# A Novel Molecular Classification Method for Glioblastoma Based on Tumor Cell Differentiation Trajectories

**DOI:** 10.1155/2023/2826815

**Published:** 2023-02-22

**Authors:** Guanghao Zhang, Xiaolong Xu, Luojiang Zhu, Sisi Li, Rundong Chen, Nan Lv, Zifu Li, Jing Wang, Qiang Li, Wang Zhou, Pengfei Yang, Jianmin Liu

**Affiliations:** ^1^Neurovascular Center, Changhai Hospital, Naval Medical University, Shanghai 200433, China; ^2^Neurosurgery Department, 922th Hospital of Joint Logistics Support Force, PLA, China

## Abstract

The latest 2021 WHO classification redefines glioblastoma (GBM) as the hierarchical reporting standard by eliminating glioblastoma, IDH-mutant and only retaining the tumor entity of “glioblastoma, IDH-wild type.” Knowing that subclassification of tumors based on molecular features is supposed to facilitate the therapeutic choice and increase the response rate in cancer patients, it is necessary to carry out molecular classification of the newly defined GBM. Although differentiation trajectory inference based on single-cell sequencing (scRNA-seq) data holds great promise for identifying cell heterogeneity, it has not been used in the study of GBM molecular classification. Single-cell transcriptome sequencing data from 10 GBM samples were used to identify molecular classification based on differentiation trajectories. The expressions of identified features were validated by public bulk RNA-sequencing data. Clinical feasibility of the classification system was examined in tissue samples by immunohistochemical (IHC) staining and immunofluorescence, and their clinical significance was investigated in public cohorts and clinical samples with complete clinical follow-up information. By analyzing scRNA-seq data of 10 GBM samples, four differentiation trajectories from the glioblastoma stem cell-like (GSCL) cluster were identified, based on which malignant cells were classified into five characteristic subclusters. Each cluster exhibited different potential drug sensitivities, pathways, functions, and transcriptional modules. The classification model was further examined in TCGA and CGGA datasets. According to the different abundance of five characteristic cell clusters, the patients were classified into five groups which we named Ac-G, Class-G, Neo-G, Opc-G, and Undiff-G groups. It was found that the Undiff-G group exhibited the worst overall survival (OS) in both TCGA and CGGA cohorts. In addition, the classification model was verified by IHC staining in 137 GBM samples to further clarify the difference in OS between the five groups. Furthermore, the novel biomarkers of glioblastoma stem cells (GSCs) were also described. In summary, we identified five classifications of GBM and found that they exhibited distinct drug sensitivities and different prognoses, suggesting that the new grouping system may be able to provide important prognostic information and have certain guiding significance for the treatment of GBM, and identified the GSCL cluster in GBM tissues and described its characteristic program, which may help develop new potential therapeutic targets for GSCs in GBM.

## 1. Introduction

Glioblastoma (GBM) is the most common brain primary malignant tumor derived from the neuroepithelial tissue with an annual incidence of 23.79 per million worldwide [1]. Since 2005, the combination of temozolomide and radiation therapy (also known as the “Stupp protocol”) has greatly improved the life expectancy of GBM patients, but the mean survival time remains at a low level of less than 2 years, and the 5-year survival rate is only 5.8% [2–4]. Recently, several targeted drugs such as erlotinib, perhexiline, and salinomycin have been found to work in some GBM patients, but toxic effects led to dose reduction or treatment interruption in a greater proportion of the patients [5]. Given the understanding that the isocitrate dehydrogenase (IDH) mutation status is a hallmark of molecular alteration to determine prognosis, previous WHO classification of tumors of the central nervous system (CNS) divided GBM into “IDH-wild type” and “IDH-mutant” [6]. The former is the most common type of adult glioma, accounting for more than 90% of all GBM patients [7]. Based on the unique biological behavior and molecular phenotype, the latest 2021 WHO classification redefines GBM as the hierarchical reporting standard by eliminating “glioblastoma, IDH-mutant” and only retaining the tumor entity of “glioblastoma, IDH-wild type” and classifies the latter as a category of “adult-type diffuse glioma” [8]. However, this classification does not lead to any significant change in the treatment strategy of GBM [9], signifying a great unmet need.

Over the past decade, extensive heterogeneity of GBM has been exhibited by increasing information derived from high-throughput sequencing data [10]. For instance, epithelial growth factor receptor (EGFR), platelet-derived growth factor receptor (PDGFR), and APO-1 have been used to detect expression alterations in some GBMs [11–13]. Although targeted agents have achieved success in early clinical studies, they have not brought significant benefits in phase II/III clinical trials [12, 14, 15]. Arduous efforts have been made to benefit more patients, but some patients with GBM still exhibited resistance to radiotherapy and chemotherapy [16, 17]. This calls for improving patient stratification according to tumor molecular classification and refining possible biomarkers as predictors of their respective treatment responses for the sake of developing biomarker-driven personalized treatment programs.

Molecular classification according to high-dimensional omics characteristics can reduce the complexity of tumors and help develop personalized treatment strategies [18, 19]. Molecular classification systems of GBM have been reported in several studies [20]. Philips et al. discovered three molecular subclasses of high-grade astrocytoma with significant prognostic value and named them proneural (PN), proliferative (Prolif), and mesenchymal (Mes) by recognizing the dominant features of the gene list that characterize each subclass. Later, Verhaak et al. identified four subtypes based on gene expression profiles and termed them as proneural, neural, mesenchymal, and classical [21]. However, these GBM molecular classification systems do not seem to have improved the prognosis and chemotherapy sensibility of patients [22, 23]. The possible reason is that they did not consider the mutation status of IDH and failed to capture the heterogeneity of tumor cell components by analysis based on the bulk GBM tissue. Despite the recent change in the definition of GBM, no appropriate molecular classification model based on the newly defined primary GBM is available, primarily because it is difficult to accurately define the differentiation process and tumor cell characteristics of GBM due to the limitations of the research technologies available. Therefore, establishing a valuable molecular classification standard of GBM remains a challenge in current research.

According to the characteristics of neural stem cells (NSCs), GBM stem cells (GSCs), also known as GBM initiation cells, have been identified in several studies [24, 25]. This cell population has been shown to be one of the critical components contributing to divarication in evolutionary trajectory, driving factors, drug sensitivity, and recurrence and leading to the heterogeneous phenotype of GBM [26]. However, the precise characteristics of GSCs and the process of their differentiation into mature GBM tumor cells remain unclear due to the scarcity of GSCs, sorting difficulty, and sharing of many characteristic genes and antigens with normal adult NCSs and progenitor [27, 28], which hinders the study of the mechanism underlying their escape from conventional therapies and development of targeted drugs for GSCs.

The single-cell RNA sequencing (scRNA-seq) technique has enabled additional evidence that GBM cells do not exist as separate populations, but rather as a continuum along the stemness–differentiation axis [29]. Moreover, at single-cell resolution, stem-like populations in their native environments can be identified with less bias and without culture [30]. Furthermore, recent scRNA-seq research of GBM has shown that the tumor cell hierarchy produced by tumor stem cells can partly explain the heterogeneity of tumor cells [25]. Therefore, molecular classification characterized by differentiation trajectory of GBM cells may make classification-specific therapy possible.

In the present study, we analyzed the scRNA-seq data of 10 adult GBM samples from the clinical patients (2021 WHO grading criteria for glioblastoma, IDH-wild type). According to the differentiation branches of malignant cells, we divided malignant cells into six clusters and analyzed their differences in genes, pathways, functions, and transcription factors (TFs). Based on the characteristics of these clusters, we proposed a new molecular classification system. Using the bulk RNA-seq-based samples in TCGA and CGGA cohorts and our molecular classification system, we divided the patients into five groups. We hope that this new classification system and knowledge about GSCs described herein may serve as a unique reference for the precision treatment and development of therapeutic targets for GBM.

## 2. Methods

### 2.1. Patient Tissue Samples Collected in Clinical Practice

One hundred and thirty-seven IDH GBM samples were obtained from GBM patients who underwent tumor resection surgery or biopsy in Changhai Hospital (Shanghai, China) from July 2013 to September 2019. The IDH status was confirmed by immunohistochemistry and/or genetic sequencing. All the GBM tissues were IDH-wild type. Patient consent was obtained for the study, and the sample collection was under ethical approval. This study was approved by the Research Ethics Committee of the said hospital.

### 2.2. Single-Cell and Bulk RNA Sequencing Data for Bioinformatics Analysis

The Gene Expression Omnibus of 10x genomics sequencing data (GEO: GSE131928) was obtained from Neftel et al., which sorted cells by the panimmune marker CD45 and profiled primarily CD45- cells and only a more limited extent CD45+ cells to focus on malignant cells. In this series, ten tumor tissue samples from nine patients pathologically (one patient contains two tumor samples) diagnosed with GBM were chosen to explore the cellular composition. Then, malignant and nonmalignant cell (including microglia, oligodendrocytes, and T cells) types were annotated. Bulk GBM RNA sequencing data were screened from The Cancer Genome Atlas (TCGA) program data portal and Chinese Glioma Genome Atlas (CGGA) data portal, from which IDH mutation GBM was screened out. The standardized RNA-sequence FPKM and clinic files were downloaded from the TCGA and CGGA data portal on January 30, 2021, obtaining 102 and 206 samples with complete clinical follow-up information.

### 2.3. Quality Control, Batch Correction, and Clustering

Analysis of scRNA-seq data was performed in the R statistical environment (v4.1.3). The raw data of 10 samples were processed separately with the Seurat method of data cleaning. To remove low-expressed genes and low-quality cells, we kept the genes expressed in at least 3 cells and filtered the cells with more than 20% mitochondrial reads and less than 5% ribosomal reads. In addition, we deleted cells with less than 200 genes or more than 5000 genes and doublets that were detected with DoubletFinder (https://github.com/chris-mcginnis-ucsf/DoubletFinder). Then, we used the *NormalizeData ()* function to normalize the count data with the LogNormalize method selected and the *FindVariableFeatures ()* function to screen out 2000 variable genes for principal component analysis (PCA). We passed the Seurat object consisting of the 9 data to *RunHarmony ()* function, which is supported by Harmony (https://github.com/immuno-genomics/harmony), and the “plot_convergence” parameter was set as TRUE to integrate the batch effects. *FindNeighbors ()* constructed a shared nearest neighbor graph (SNN) with Harmony reduction and 50 dimension inputs. The same parameters were also used in the formation of the Uniform Manifold Approximation and Projection (UMAP). The classification of all the cells was manually labeled according to the characteristics of expression. We used the dimensionality reduction and cell clustering method provided by Monocle3 downstream to reanalyze the tumor cells and the CSC-like subgroup distinguished from the tumor separately. Similarly, the batch effect from samples was eliminated by running the align_cds() function.

### 2.4. Calculation and Display of Differential Genes

We used the *FindAllMarkers ()* and *FindMarkers ()* functions of the scran package to perform a Wilcoxon test between pairs of cell clusters to find the genes specifically expressed in each cluster. For endovascular cells and Clara cell populations subdivided by Monocle3, we mapped the grouping information of these cell subgroups back to the Seurat object and calculated the differential genes for the Seurat object that rewrites the grouping information. According to the results of the calculation, the ggplot2 and heatmap packages were used to visually display the heat, violin, and bubble maps.

### 2.5. Pathway Enrichment

To assess gene expression signatures and pathway activation, GSVA was performed using gene sets of C2 and C5 collection obtained from the molecular signature database to assess the activation level of the relative pathway in each cell and visualize it through the heatmap.

### 2.6. Regulon Activity Analysis

pySCENIC (V1.22) algorithm combined Arboreto package GRNBoost2 method and cisTarget human motif database (V9) was used to build the gene regulatory network (GRN) in all cells. Raw expression data and labeled clusters were extracted from the Seurat data and Monocle3 data. Filtration was performed with default parameters of pySCENIC pipeline, and GRN was computed by using the grnboost2 method. Enriched motifs were identified by cisTarget databases containing hg38_refseq-r80__10kb_up_and_down_tss.mc9nr.feather and hg38_refseq-r80_10kb_up_and_down_tss.mc9nr.feather and the transcription factor motif annotation database (v9). All cells were finally scored by AUCell function to show regulon activities, and the similarity score was calculated for the regulons in each cluster and transferred to the specific score based on the Jensen–Shannon divergence.

### 2.7. Cell Differentiation Trajectory Analysis

Monocle3 (V0.2.3.0) algorithm was used to order cells along the trajectories based on the pseudotime in malignant cells. The expression matrix of the mesenchymal cells derived from the Seurat object was passed to Monocle3. The *new_cell_data_set ()* function was used to create a cds object and perform dimensionality reduction, cell clustering, and differentiation trajectory inference.

### 2.8. Chromosome Copy Number Variation Analysis

The inferCNV (V1.6.0) method and the recommended parameters for 10x data were used to illustrate the diverse patterns of chromosome copy number variation in malignant cell clusters, using nonmalignant cells as the reference.

### 2.9. Validation in External RNA-seq Data

CIBERSORTx tool (https://cibersortx.stanford.edu) was used to detect the relative abundance of cell types defined by our single-cell data in bulk RNA-seq database. Before being loaded into CIBERSORTx analysis, the downloaded TCGA and CGGA data were normalized. The patients were divided into five groups after calculating the Euclidean distance and clustering with hclust method. The Kaplan-Meier survival curves of different groups in the data set were drawn using the survival package. The OS rate from the diagnosis to death or the last follow-up was calculated.

### 2.10. Immunohistochemistry (IHC) and Immunofluorescence

The sample was fixed with 4% paraformaldehyde, dehydrated through a graded series of ethanol, paraffin-embedded, and sliced into 4 *μ*m sections. IHC staining for ALDOA (ab252953, Abcam, USA), MGST1 (ab131059, Abcam), ANXA1 (ab214486, Abcam), MRC2 (ab224113, Abcam), RAB34 (ab262930, Abcam), SPRY1 (ab111532, Abcam), SEMA6D (ab191169, Abcam), TUBB2A (ab170931, Abcam), TERF2IP (ab14404, Abcam), NFIB (ab186738, Abcam), PTPRZ1 (ab181131, Abcam), MARCKSL1 (ab184546, Abcam), OLIG2 (ab109186, Abcam), FABP5 (ab255276, Abcam), TIMP1 (ab21926, Abcam), and CRYAB (ab281561, Abcam) was carried out using the standard histological procedures described in the manual for Histostain-Plus (DAB) kit (Mingrui Biotech, China). The staining degree of each protein was calculated by using the ImageJ software. Immunofluorescence staining was performed with BRCA1 (ab213929, Abcam), C16orf59 (ab96760, Abcam), CASC5 (ab70537, Abcam), CCNE2 (ab40890, Abcam), CHAF1A (ab126625, Abcam), FBXO5 (PA5-83055, Thermo Fisher), TIMELESS (ab109512, Abcam), MCM2 (ab108935, Abcam), and NCAPH (PA5-64393, Thermo Fisher). DAPI was used as a counterstain to label individual cell nuclei. Sections were examined under a fluorescent microscope (Olympus BX53).

### 2.11. Prognostic Model Based on Clinical Features and Classifications

Univariate Cox proportional hazard regression analysis was performed using survival package for clinical features (gender, age, tumor size, radiotherapy, chemotherapy, surgery type, and classification) with a *p* value < 0.05 as cutoff. Based on the selected features, a multivariate Cox model was built, and a nomogram chart was drawn using rms package.

## 3. Result

### 3.1. Single-Cell Transcriptomic Profiles of All Single Cells

**Figure 1:**
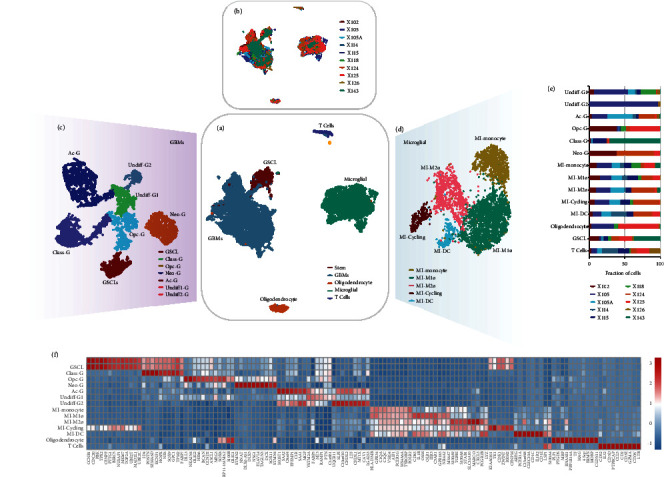
Single-cell atlas of GBM samples. (a) UMAP plot of GBM transcriptomes, color-coded for 5 phenotypes identified by graph-based clustering. (b) UMAP plot color-coded for each GBM sample. (c) UMAP plot of GBM tumor cell cluster color-coded for 7 phenotypes. (d) UMAP plot of microglial cells color-coded for 5 phenotypes. (e) Reproducible cell subset distributions across samples. Fractions of cells in each cluster derived from all samples are shown. (f) The heatmap of the expression of subset-specific markers across cell subsets.

After quality filtration, a total of 11917 cells available were obtained for subsequent analysis. Following principal component analysis (PCA), the top 50 principal components were chosen for Uniform Manifold Approximation and Projection (UMAP) analysis for dimension reduction. Nonmalignant cells were cataloged into three distinct cell lineages annotated with a list of marker genes published previously. In malignant cells, GSCs were identified via CD133 and SOX2 using as markers of glioblastoma stem cells. As a result, 810 GSCs, 6298 glioblastoma cells, 4164 microglia, 425 oligodendrocytes, and 220 T cells were identified (Figure 1(a) and Supplementary Figure 1A-D). The distribution of the cells from each sample in the UMAP is shown in Figure 1(b). Clustering analysis was further performed in each cell type. GBM stem cells and tumor cells were further subdivided into 6 clusters, which were named glioblastoma stem cell-like (GSCL), Class-G, Opc-G, Neo-G, Ac-G, and Undiff-G (including Undiff-G1 and Undiff-G2) (Figure 1(c)), while tumor-associated microglia and macrophages (TAM) fell into five clusters: MI-monocyte, MI-M1 macrophage, MI-M2 macrophage, MI-Cycling, and MI-dendritic cell (MI-DC) (Figure 1(d)). Each cluster included cells from multiple samples, showing a clear difference in distribution between each of these samples (Figure 1(e)). In addition, the heatmap indicated that each cluster exhibited a distinct gene expression pattern (Figure 1(f) and Supplementary Table 1). To confirm the reliability of the cluster method, correlation analysis was performed, showing that clusters from the same cell lineage had higher similarities than those from others (Supplementary Figure 1E).

### 3.2. The Distinct Transcriptome Program in Malignant Cells

**Figure 2:**
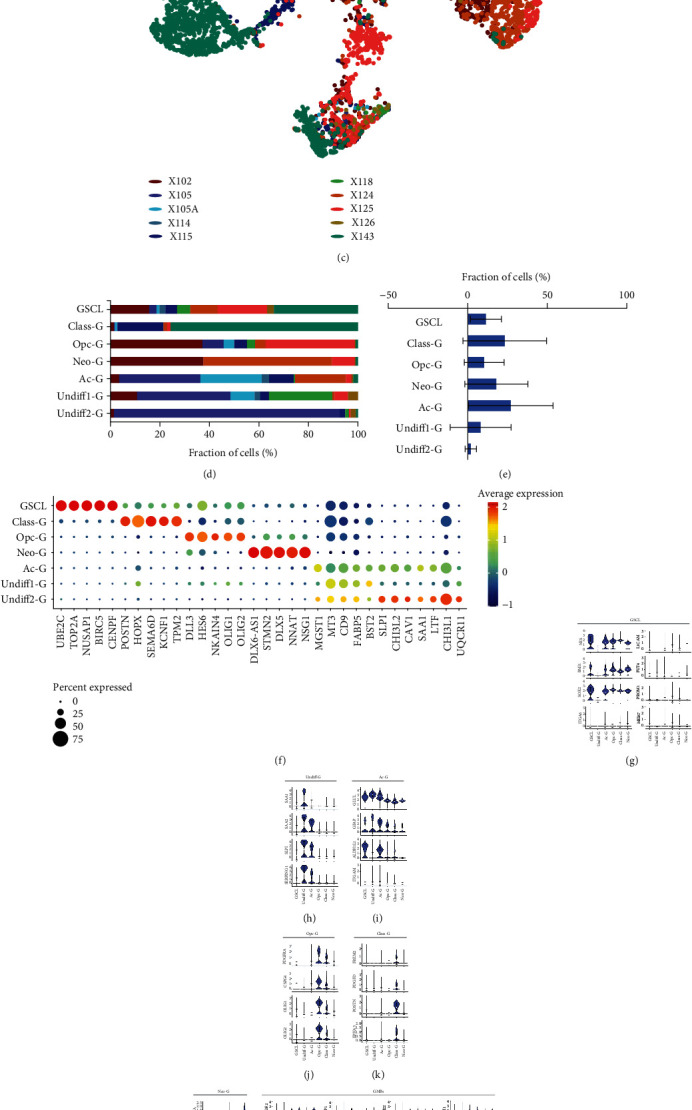
Differences in cell composition and gene expression in GBM samples. (a) UMAP plots of tumor cells by Monocle3 method. (b) Pseudotime trajectory of tumor cells. (c) UMAP plot of tumor cells color-coded for each sample. (d) Tumor cell subset distributions across samples. (e) Differences in cell proportion of GBM samples in each tumor cluster. (f) Dot-plot heatmap of the most significant genes of each cluster in tumor cells. (g–l) Violin plots showing different expressions of GSCL, Undiff-G, Ac-G, Opc-G, Class-G, and Neo-G markers in each tumor cluster. (m) Violin plots showing previously reported markers expressed in GBM are distributed in GSCL and malignant cells. (n–s) Differences in gene expressions between GSCL and malignant cells clusters. ^∗^ means *p* < 0.05.

Monocle3 orders single-cell transcriptomes in a pseudotemporal way to reveal the similarity of tumor subclusters with developmental lineages, which can be employed to identify differentiation trajectories of cancer stem cells developing in various directions [31]. By employing Monocle3 algorithm, GBM tumor cell clusters, including a GSCL cluster and four branches with three clusters in three branches and three clusters in one branch, were acquired (Figure 2(a)). In addition to GSCL, we subdivided malignant cells into five subtypes, which we named classical-like (Class-G), astrocyte-like (Ac-G), oligodendrocyte progenitor-like (Opc-G), neuron-like (Neo-G), and undifferentiated-type (Undiff-G). The pseudotime trajectory analysis demonstrated that the GSCL cluster was the beginning of each branch during cell differentiation (Figure 2(b)).  Figure 2(c) shows that tumor cells from different samples were uniformly enriched in every branch except the Undiff-G2 subcluster, indicating the intratumor heterogeneity of GBM samples. Interestingly, comparison of one cluster with the other showed high enrichment of Undiff-G2 in one sample (Figure 2(d)). Barplot of cell proportion analysis showed that the proportion of Undiff-G2 subcluster at the end of the stemness–differentiation axis was relatively rare when as compared with the other subclusters (Figure 2(e)). Besides, the abundance of every cluster was highly discrete in this system, indicating that there was extensive intertumor heterogeneity in these samples. However, the results about cluster abundance in GBM need to be interpreted with caution, for only 10 samples were included in this analysis.

The top five most significant markers of each tumor cluster are shown in Figure 2(f) and Supplementary Table 2, demonstrating that the selected markers were well capable of characterizing each subcluster. As shown in Figure 2(m), the previously reported markers expressed in GBM were distributed in CSCL and malignant cells in varying degrees. In addition, certain specific marker genes for stem, glial, and neuronal cells were also able to characterize these clusters (Figures 2(g)–2(l)). For instance, the stem cell markers were highly expressed in GSCL cluster and partly expressed in Class-G, Opc-G, Neo-G, and Ac-G subclusters, suggesting that tumor cells in branch-Class-G/Opc-G/Neo-G/Ac-G seemed to have stronger stemness than those in branch-Undiff-G. Other clusters included Ac-G cluster expressing astrocyte markers, Opc-G cluster expressing oligodendrocyte progenitor cell markers, Class-G cluster expressing canonical genes regulating invasion and proliferation such as POSTN and PDGFD, Neo-G cluster expressing neuron markers, and Undiff-G cluster expressing embryonic development markers. The results of difference analysis of the transcriptional profiles between GSCL and other clusters are shown in Figures 2(n)–2(s) and Supplementary Tables 3-7, indicating that the overexpression of these genes was involved in cell cycle, stemness, and proliferation in GSCL cells, such as MKI67, CCNA2, CDKN3, and TOP2A. Based on these findings, we suppose that that GSCL might be a precursor cell of other clusters, given the ability of GBM stem cells to confer phenotypic and functional diversity to malignant cells.

### 3.3. Identification and Characterization of GBM Stem Cells

**Figure 3:**
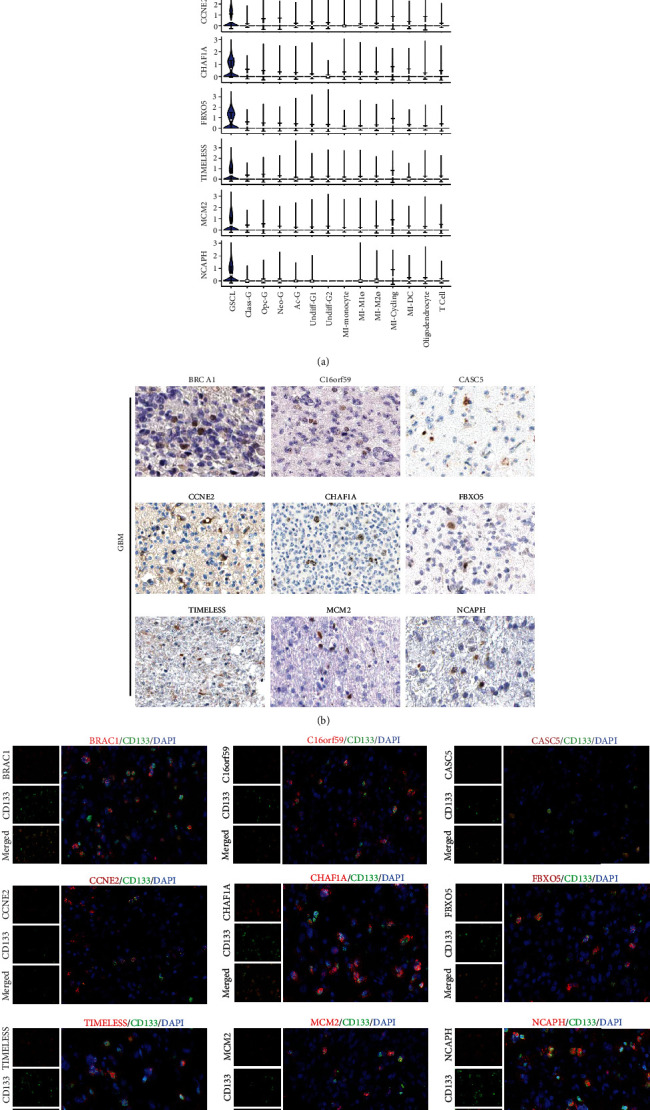
The molecular features of GBM stem cell. (a) Violin plots showing selected marker of GSCL. (b) IHC staining of BRCA1, C16orf59, CASC5, CCNF2, CHAF1A, FBXO5, TIMELESS, MCM2, and NCAPH in GBM samples. (c) Immunofluorescence colocalization of selected markers (red) and CD133 (green).

**Figure 4:**
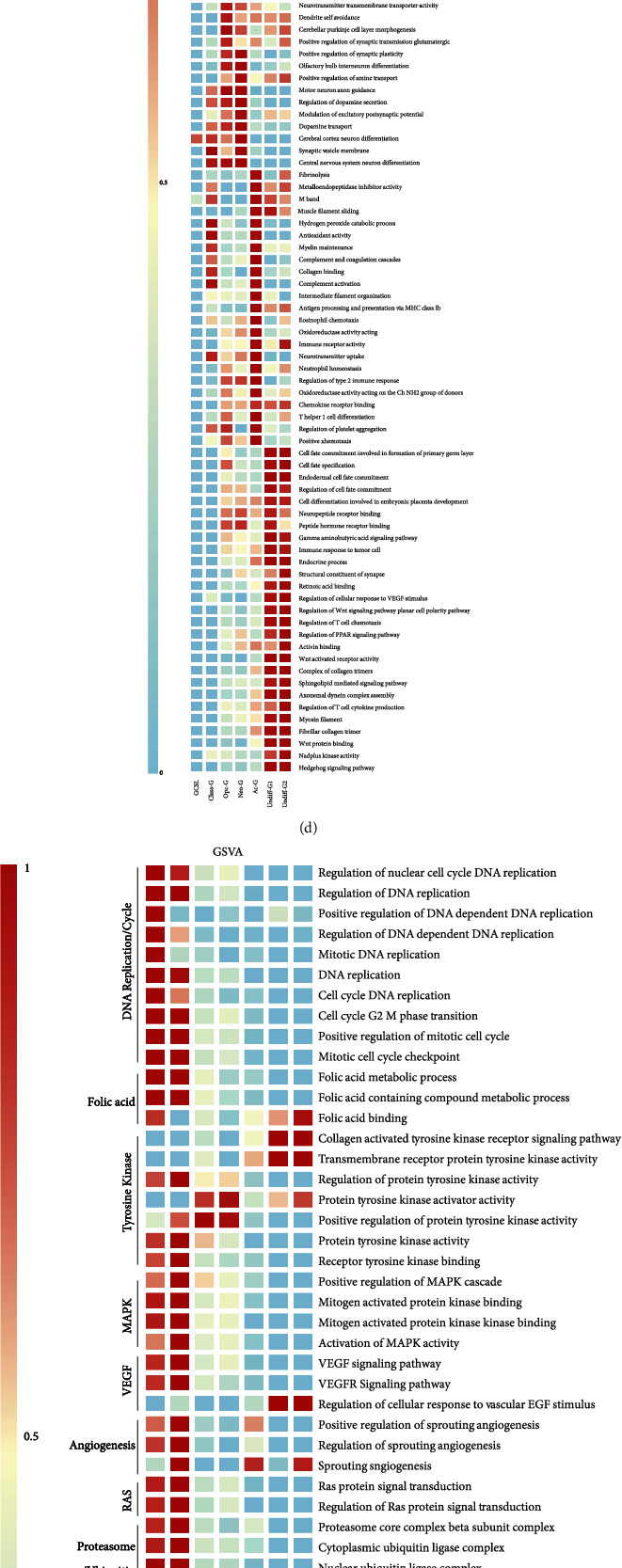
Transcriptional distinguishment between the four differentiation trajectories of tumor cells. (a) Differences in total GO pathway enrichment between the seven tumor clusters. (b) Differences in total transcripts between the seven tumor clusters. (c) Differences in the proportion of cell cycle phases in GSCL and malignant cell clusters. (d) Heatmap showing differences in activation of pathways between the seven tumor clusters. (e) Heatmap showing differences in activation of pathways related to targeted therapies in Class-G/Opc-G/Neo-G/Ac-G/Undiff-G clusters calculated by GSVA method. (f) Heatmaps of differences in single-cell copy number between the tumor clusters and other cell clusters. (g) TF activity in the GSCL/Undiff-G/Ac-G/Opc-G/Class-G/Neo-G clusters. The top 7 activated TFs were marked in each cluster.

Knowing that identification of endogenous GSCs is crucial for the targeting and prognosis of GBM, GSCs should be well correlated with progression and relapse of the disease [32]. In addition, CSCs have not been effectively characterized because of their plasticity [33]. We therefore further explored biomarkers of the GSCL cluster and found that they could be characterized by several distinct gene expression patterns with the known markers (Figures 3(a) and 3(b)). High expression of these biomarkers and their colocalization with the GSC marker, CD133, were further confirmed by immunofluorescence experiments (Figure 3(c)). By focusing on the specific pathway activation among GSCL cluster, we found that GSCL exhibited stronger pluripotentiality than the other clusters (Figure 4(d)). Transcriptional factor (TF) analysis highlighted the relative activation of EZH2 and CTCF in GSCL cells (Figure 4(g)), and their activation was found to be correlated with tumor cell stemness, proliferation, and drug resistance [34, 35]. These results suggested that GSCL has distinct transcriptional profiles from other malignant cell clusters, which may help the identification of GSCs.

### 3.4. Specific Molecular Features of the Four Differentiation Branches of Malignant Cells

ssGSEA method was performed to further analyze the signature of the clusters in the stemness–differentiation axis. Analysis of the mean value of GO enrichment signature and RNA expression level revealed that GSCL and Class-G had high average pathway expression and gene expression, suggesting that these two clusters were rich in functional abundance; Opc-G, Neo-G, and Ac-G underwent a gradual decrease; and the expression of Undiff-G was the lowest (Figures 4(a) and 4(b)). We further investigated the distribution of cell cycle stages within each cluster and found that the number of cells in GSCL and Undiff-G was relatively more enriched in the G2/M stage, compared with the remaining clusters (Figure 4(c)). These results demonstrated that GSCL had enriched functional activities and extensive proliferative phenotypes, then differentiated in different directions with various enrichment functions. Noticeably, Undiff-G cluster displayed extremely low functional enrichment. This may suggest a specific dedifferentiation phenomenon related to the drug resistance of GBM [36]. We further visualized the specific pathway activation among GSCL and malignant cell clusters and observed that DNA synthesis and metabolism-related pathways were enriched in GSCL, classic pathways of glioma were enriched in Class-G, inflammatory chemotaxis-related pathways were enriched in Opc-G, dopamine transmitter synthesis-related pathways were enriched in Neo-G, embryonic development-related pathways were enriched in Unidiff-G, and energy metabolism-related pathways were enriched in Ac-G (Figure 4(d) and Supplementary Table 8). Moreover, we integrated the targeting pathway of current clinical chemotherapeutic agents with our clustering system. As shown in Figure 4(e), for Class-G, a variety of agents had potential therapeutic effects, and for OPC-G and Neo-G, few drugs were able to target these clusters accurately, except for some tyrosine kinase receptor inhibitors. It was found in our study that Ac-G classification had the strongest resistance to chemotherapy drugs. Mifamurtide is one of the chemotherapeutic drugs currently used in osteosarcoma, mainly via activation of monocytes and macrophages to play an antitumor effect [37]. We speculate that Ac-G is sensitive to mifamurtide, while the activity of Wnt pathway, hedgehog pathway, and Hippo pathway in Undiff-G is increased. Therefore, drugs targeting these three pathways may have better effects on Undiff-G cluster.

Chromosome copy number variation (CNV) is one of the main reasons for the diversity of gene expression profiles in cancer cells [38]. According to the calculation results of CNV based on scRNA-data, we noticed that clusters within the same differentiation branch possessed similar CNVs, while clusters within different differentiation branches had diverse CNVs (Figure 4(f)). The CNV changes of Class-G were mainly focused on the gains of Chr20. OPC-G mainly showed the gains of Chr2 and Chr8. Neo-G presented with the gains of Chr7 and the losses of Chr15. Ac-G showed the losses of Chr22 and the gains of Chr1 and Chr16. Undiff-G presented with the gains of Chr11 and Chr12. These results revealed the heterogeneity among the differentiation branches of GBM samples.

Subsequently, we used pySCENIC algorithm to investigate the differences in activity of transcriptional regulatory modules in an individual cell and screened out top eight potential TFs with the highest AUC score (Figure 4(g)). The activity of transcription modules in each cluster is consistent with our annotation of GBM cell cluster differentiation direction and function, which further verifies the accuracy of our classification system. This result underscored the relative activation of EZH2, CTCF, and HOXB7 in GSCL cluster, knowing that their activation was associated with DNA damage repair [39], cell cycle regulation [40], chromatin looping mediation [41], and stemness maintenance [42]. In addition, we also found activation and expression of SOX9, E2F4, and KLF10 in Ac-G cluster, which was associated with GBM tumorigenesis, proliferation, and invasion; activation of transcriptive modules (including PRRX2 and HXC9) in Class-G cluster, which was related to immune response regulation [43] and radiotherapy resistance [44]; and activation of CD59 and FOXC2 in Neo-G cluster, which was associated with immunosuppression [45], pathological angiogenesis, and neovascularization [46]. Importantly, chemotherapy resistance-related transcriptional programs, such as MAFK and MEIS1, were activated in Undiff-G cluster [47, 48]. Whether this result is related to Undiff-G's dedifferentiation phenotype needs further investigation.

### 3.5. GBM Classification Based on the Differentiation Branches of Malignant Cells

**Figure 5:**
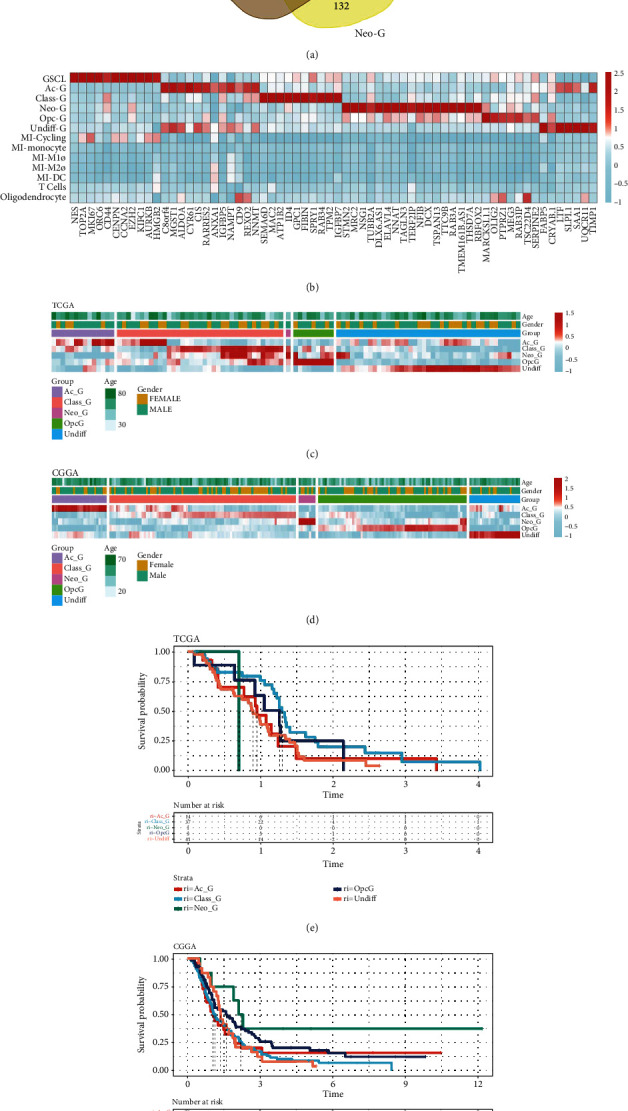
Classification of GBM samples from TCGA and CGGA datasets based on the expression profiles of the five clusters of malignant cells. (a) Venn diagram showing the similarities and differences of the calculated markers of Ac-G/Class-G/Neo-G/Opc-G/Undiff-G clusters. (b) Heatmap showing the expressions of the selected 64 marker genes of Ac-G/Class-G/Neo-G/Opc-G/Undiff-G in all 13 clusters. (c, d) Heatmap showing the abundance of the 5 clusters in the five clustered groups of TCGA and CGGA samples. (e, f) The Kaplan-Meier curves of OS for the five clustered groups of TCGA and CGGA patients.

Given the differences in transcriptional profiles, CNV, and transcriptional regulation modules of the malignant cells, we classified GBM samples into five categories based on the relative abundance of the differentiation branches of malignant cells. By calculating and overlapping the specific genes characterizing these clusters, we found that there were limited intersections between these genes (Figure 5(a)). Then, we selected 64 most specific markers to characterize CSCL/Ac-G/Class-G/Neo-G/Opc-G/Undiff-G clusters, including 11 markers in CSCL, 12 in Ac-G, 10 in Class-G, 17 in Neo-G, 7 in Opc-G, and 7 in Undiff-G (Figure 5(b)), suggesting that the markers of CSCL/Ac-G/Class-G/Neo-G/Opc-G/Undiff-G could be used to identify the differentiation branch in the GBM samples. Next, we used the CIBERSORTx deconvolution algorithm to calculate the relative abundance of the five clusters of malignant cells in 216 GBM samples in CGGA and 102 samples in TCGA dataset. By calculating the Euclidean distance between the samples and clustering with hclust method, we divided the patients from TCGA and CGGA cohorts into C1, C2, C3, C4, and C5 groups. It was found that five groups had good clustering results (Figures 5(c) and 5(d)). Samples in the C1, C2, C3, C4, and C5 groups mainly expressed Ac-G character-related genes, Class-G character-related genes, Neo-G character-related genes, Opc-G character-related genes, and Undiff-G character-related genes, respectively. The 102 samples in TCGA cohort included 14 Ac-G, 37 Class-G, 1 Neo-G, and 41 Undiff-G. The 216 samples in CGGA cohort included 26 Ac-G, 88 Class-G, 8 Neo-G, 70 Opc-G, and 24 Undiff-G. The survival curve showed that the prognosis of patients in the Undiff-G and Ac-G groups was the worst, which was confirmed in both cohorts. In addition, the prognosis was relatively good in the Neo-G group of CGGA cohort (Figures 5(e) and 5(f)). As there was only one case in the Neo-G group in TCGA cohort, it was excluded from analysis.

### 3.6. Verification of the Classification System in Clinical GBM Samples

**Figure 6:**
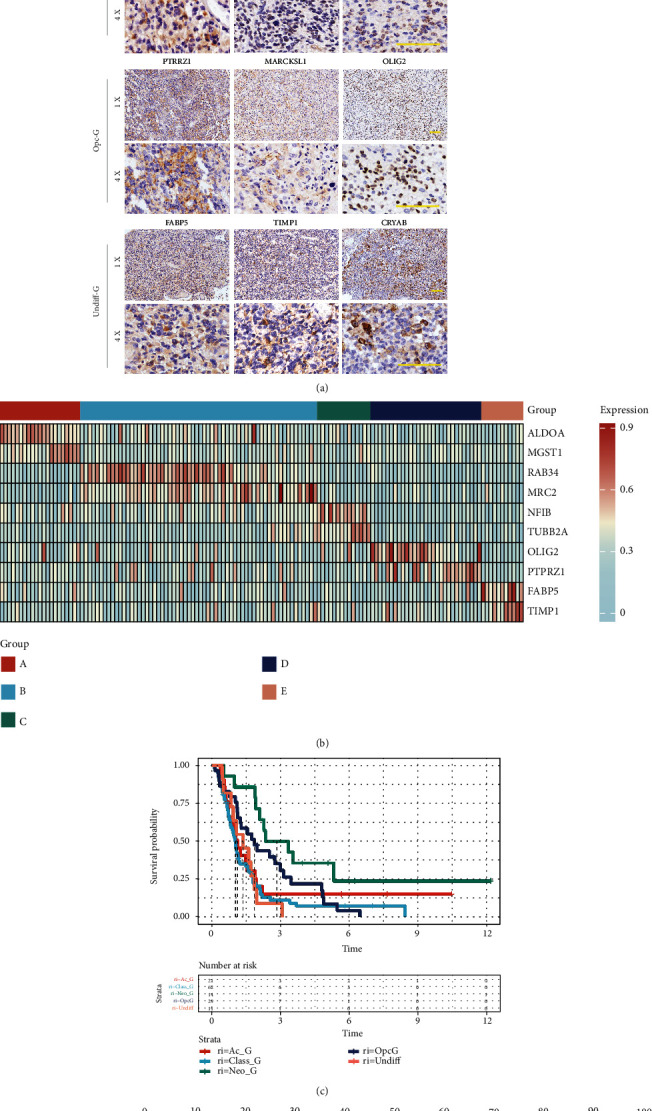
Group classification of GBM samples based on IHC staining. (a) IHC staining of sixteen gene makers selected from Ac-G/Class-G/Neo-G/Opc-G/Undiff-G clusters. (b) Heatmap showing the expressions of the selected ten gene markers in each GBM sample. (c) The Kaplan-Meier curves of GBM patients for the five groups. (d) Nomogram predicting patients' 1-, 3-, and 5-year overall survival.

To further examine the clinical significance of the classification system in GBM samples, we performed IHC staining of sixteen gene makers selected from Ac-G/Class-G/Neo-G/Opc-G/Undiff-G clusters in 137 GBM samples (Ac-G: ALDOA, MGST1, and ANXA1; Class-G: MRC2, RAB34, SPRY1, and SEMA6D; Neo-G: TUBB2A, TERF2IP, and NFIB; Opc-G: PTPRZ1, MARCKSL1, and OLIG2; and Undiff-G: FABP5, TIMP1, and CRYAB) (Figure 6(a)). According to the most highly expressed marker of each sample, GBM patients were divided into five groups. It was found that group A/B/C/D/E highly expressed the markers of Ac-G/Class-G/Neo/G/Opc-G/Undiff-G clusters, respectively, parallels to group Ac-G/Class-G/Neo/G/Opc-G/Undiff-G in TCGA and CGGA cohorts (Figure 6(b)). In addition, K-M curves showed that the GBM patients in group E (Undiff-G) exhibited significantly worse OS than those in the other four groups (Figure 6(c)), similar with the result of survival analysis in TCGA and CGGA cohorts. These results suggested that the classification of GBM samples could be performed through the IHC staining of the samples, which may help the prognostic evaluation of GBM patients. We integrated IHC staining result and clinical data and divided the clinical samples based on pathological information. The statistical information is shown in Supplementary Table 9. It was found that the correlation between the five groups of classification made by pathological data and various clinical indicators was not statistically significant, but it was closely related to the survival status of patients. Through univariate Cox regression analysis of the clinical data of the patients, including age, postoperative radiotherapy, postoperative chemotherapy, surgery type, and IHC classification (Supplementary Table 10), we screened out four statistically significant variables according to the *p* < 0.05 standard. Subsequent multivariate analysis indicated that the four factors were significantly correlated with OS (Supplementary Table 10). These results indicated that the classification characterized by the ten markers was an independent prognostic factor. To establish a clinically applicable method for predicting the prognosis of GBM patients, we established a prognostic nomogram to predict the survival probability at 1, 3, and 5 years based on clinical samples (Figure 6(d)).

### 3.7. Distribution Change and Molecular Features of Tumor-Associated Microglia and Macrophages in GBM

TAM account for about 30% of the total GBM tissue, playing crucial roles in tumor immunity and tumor interactions of GBM [49]. Five clusters (monocyte, M1*Φ*, M2*Φ*, cycling, and DC) were identified in this cell lineage (Supplementary Figure 2A). The distribution of the cells from each sample in the UMAP is shown in Supplementary Figure 2B. Each cluster was composed of cells from multiple samples (Supplementary Figure 2C). The top 10 significant markers of each TAM cluster are shown in Supplementary Figure 2D and Supplementary Table 2. Further analysis showed each TAM cluster could be characterized by a distinct gene expression pattern with the known markers (Supplementary Figure 2F), such as CD14 and CD6A in monocytes, TNF and IL1B in M1*Φ*, CD163 and MRC1 in M2*Φ*, and CCL17, CD1C, and FCGRA in DC. Despite the fact that microglial activation can also be classified as M1 or M2 polarization, microglia exhibit more heterogeneous phenotypes than peripheral macrophages due to brain-specific regional variation and pathological conditions [50]. Notably, we identified a group of cells with active self-renewal and proliferation named MI-cycling with eight percent in total TAM cells (Supplementary Figure 2E), outside the conventional M1, M2, and DC classifications, which expressed high levels of Ki67 and CDK1, suggesting that the TAM system in the GBM brain was highly activated, though its effect on tumors needs further study. GSVA analysis was performed to detect the function of microglia (Supplementary Figure 2G). Consistent with the known roles of each cluster, the tumor-resistant pathways were activated in M1*Φ*, and pathways with tumor-promoting capabilities involving immunosuppression, angiogenesis, and neovascularization were activated in M2*Φ*. In addition, pathways related to the regulation of DNA replication, chromatin remodeling at centromere, and DNA strand elongation were activated in MI-cycling, suggesting that MI-cycling may play a role in promoting proliferation and tumor progression in GBM.

### 3.8. Ligand-Receptor-Mediated Intercellular Interactions in the GBM Microenvironment

**Figure 7:**
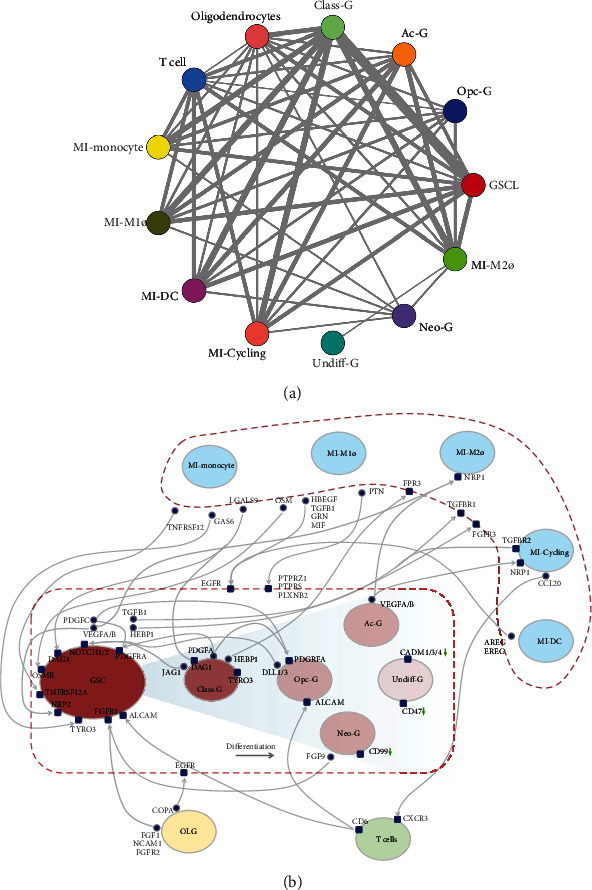
Ligand-receptor interactions between the clusters in GBM samples. (a) Cell-cell interaction networks estimated in GBM samples. (b) Schematic representation of differences in the microenvironment among CSCL/Ac-G/Class-G/Opc-G/Neo-G/Undiff-G cell clusters in GBM samples.

CellPhoneDB analysis was performed to detect interactions between the clusters. The intercellular interactions in the GBM microenvironment are displayed in Figure 7(a). It was found that except for a small amount of interaction between Undiff-G and MI-M2, the other four clusters of malignant cells had relatively abundant interaction with microglia, T cells, and oligodendrocytes and that the GSCL/malignant cell clusters displayed a strong capability of proliferation (Figure 7(b)). For instance, oligodendrocyte secreted COPA that bound to EGFR on GSCL/malignant cells, and there was a PTN-PTPRZ1/PTPRS/PLXNB2 interaction between myeloid and GSCL/malignant cells. In addition, the communications between GSCL/malignant cell clusters and other subsets were obviously different, such as FGF1/NCAM1/FGFR2-FGFR1 interactions between oligodendrocyte and GSCL cell, PDGFA-PDGFRA interaction between Class-G and GSCL cell, self-interaction of PDGFC-PDGFRA interaction in GSCL, and PDGFC-PDGFRA interaction between GSCL and Opc-G cells. Moreover, CSCL expressed high levels of Notch receptors that interacted with ligands secreted by autocrine and Class-G and Opc-G clusters, which may play a positive role in proliferation and stemness maintenance. Furthermore, LGALS9 secreted from TAM facilitated tumor migration and maintenance of extracellular matrix homeostasis by binding to DAG1 on GSCL and Class-G cells. Interestingly, in addition to the interaction shared with other malignant cells, Undiff-G exhibited a relatively lower expression level of IGSF4 (CADM1) which enabled cytotoxic T cells to recognize tumor cells [51], suggesting the existence of an immunosuppressive phenotype in this cluster that was associated with poor prognosis in our cohort. Otherwise, Neo-G lacked growth factor receptor and low expression of CD99 related to GBM invasion [52], suggesting that the malignancy of Neo-G may be lower than that of other clusters. This may be the reason why the Neo-G group has a relatively good prognosis in our cohort. These results indicated that the tumor microenvironment was various in GBM issues, which might provide cues for individualized targeted therapy of the five classifications.

## 4. Discussion

With the molecular background of GBM becoming clearer and clearer, the latest GBM diagnostic criteria not only break the conventional histological characterization of GBM but also introduce more molecular diagnostic information. Due to the extensive heterogeneity of GBM, various molecular features of GBM have different biological behaviors and clinical outcomes [53]. Molecular classification and diagnosis are helpful to judge the prognosis of patients more accurately and give precise individualized treatment [54, 55]. In 2010, TCGA project proposed a new classification of GBM into proneural, neural, classical, and mesenchyma [21]. Based on CGGA project, another study identified three subtypes in this classification system except the “classical” subtype [56]. Furthermore, a transcriptome-based alternative classification of GBM with three distinct molecular subtypes labeled invasive, mitotic, and intermediate was proposed [57]. However, sequencing based on the bulk tumor tissue may cause artifacts given the presence of nontumor cells. Due to the limitations of earlier sequencing techniques, it was a formidable challenge to characterize different GBM subtypes accurately and establish a reliable molecular classification system^21(p1)^. Meanwhile, since the definition of GBM has undergone several changes, it is not appropriate to use previous molecular classification to annotate the new version of GBM. In addition, given the effect of age on GBM molecular classification, adult GBM and pediatric GBM have been listed separately in many studies [58, 59]. In this study, we created a novel molecular classification system based on the transcriptional characteristics of GBM defined by 2021 WHO classification (fifth edition) in adults, which should be helpful to provide more clinical options for diagnosis and targeted therapy. There is an obvious trend to use scRNA-seq technique to classify tumors [60, 61]. Studies have shown that malignant cells from different samples have various differentiation trajectories with diverse molecular features [19, 55], which indicates that GBM can be classified according to the differentiation trajectories of malignant cells. Pseudotime estimation from single-cell expression level allows for recovering differentiation information from the static profile of an individual cell [62]. In our present study, we characterized tumor cells from GBM samples based on the features of the differentiation branches in stem and malignant cells and divided GBM samples into five subgroups based on the differentiation branches. Then, we analyzed the potential drug sensitivity of each subtype and revealed the phenomena and causes of inter- and intratumor heterogeneity.

Transcriptional regulation is an important manner of gene expression [63]. The five GBM clusters exhibited distinct transcriptional statuses, suggesting that the transcriptional regulatory modules in the GBM clusters were different. Consistent with the known roles of each cluster, our results identified SOX9 as the characteristic transcriptional module of Ac-G cluster. It was reported that SOX9 induces generation of astrocytes in human inducible pluripotent stem cell-derived neural model [64]. In addition, we found that the TF related to regulation of oligodendrocyte development were activated in Opc-G cluster [65]. Meanwhile, TFs, such as TCF7L2 and MEF2A, which regulated the differentiation of neural stem cells into neurons, were activated in Neo-G [66, 67]. In addition, TF related to GBM malignant behavior were activated in Class-G and stemness maintenance in Undiff-G cluster. These findings may provide a fresh perspective on the GBM transcriptional program.

GSCs are considered to be a group of cells with the characteristics of hierarchical arrangement and dynamic regulation, which are at the top of the lineage development, showing stem cell-like regeneration ability, and can reproduce the functional phenotype of primary GBM cells and even differentiate into more heterogeneous GBM cell clusters [68]. The roles and mechanisms of some molecular markers have been reported in GSCs [69]. However, since GSCs share common molecular markers with normal adult neural stem cells and progenitor cells, the current controversy over GSCs lies in the ambiguity of their definition and identification [70]. In our study, we identified GSC clusters based on pseudotime analysis, known markers, and functional analysis of individual cells and demonstrated the role of GSCs in maintaining GBM heterogeneity. The results obtained may provide a theoretical basis for further prospective identification and reclustering of GSCs.

Based on the relative abundance of the five clusters, we classified the GBM samples into five groups in TCGA and CGGA cohorts and performed survival analysis. Samples in groups 1, 3, and 5 in TCGA and CGGA cohorts were characterized by the features of Neo-G, OpcG, and Class-G, respectively. We observed that although the survival analysis of the five groups of patients in the two cohorts was not statistically significant, it showed a consistent trend. In addition, patients in the Neo-G group exhibited better prognoses, while the prognosis of the Undiff-G and Ac-G groups was poor. Combined with drug sensitivity analysis, we found that although Neo-G had a relatively good prognosis, it was difficult to benefit from drug treatment, and Class-G was sensitive to a variety of drugs, suggesting that the combination of drugs may offer greater benefits, and Undiff-G and AC-g had poor prognosis and presented strong resistance to temozolomide and other chemotherapy drugs, suggesting a low benefit to the commonly used treatment.

There are some limitations in our study. First, the scRNA-seq data were obtained from only nine GBM samples. More results of scRNA-seq from GBM samples are required to further verify and improve the classification of GBM samples. In addition, although we applied three independent cohorts to analyze and verify the molecular classification system, larger cohort studies are required to provide more accurate classification results. Finally, well-designed clinical trials are needed to verify the efficacy of the potential drugs in each subtype and confirm our hypothesis.

In conclusion, we constructed a novel classification system of GBM samples based on stem cell and tumor cell differentiation branches and verified it by bulk RNA-seq data and IHC. We identified five subtypes of GBM and found that they exhibited distinct drug sensitivities and different prognoses, suggesting that the new grouping system may be able to provide important prognostic information and have certain guiding significance for the treatment of GBM. Furthermore, we identified the GSC cluster in GBM tissues and described its transcriptional program, which may help develop new potential therapeutic targets for GSCs in GBM.

## Data Availability

All data generated during this study are included in this published article and its supplementary files.

## Supplementary Materials

Supplementary 1 Supplementary Figure 1: Validation cohorts of the single-cell atlas. (A-D) The split UMAP plots show cells color-coded for expression of indicated marker genes. (E) Grouping of the cell clusters by similarity. Heatmap showing the Pearson correlation coefficients between the log-averaged expression profiles of clusters.

Supplementary 2 Supplementary Figure 2: Activation of tumor-associated macrophages (TAMs) in GBM. (A) UMAP plots of TAM using Seurat method. (B) UMAP plot of TAM color-coded for each GBM sample. (C) TAM cell cluster distributions across samples. (D) Dot-plot heatmap of the most significant genes of each cluster in TAM cells. (E) Differences in cell proportion of GBM samples in each TAM cluster. (F) Violin plots showing different expressions of monocyte, M1*Φ*, M2*Φ*, cycling, and DC markers in each TAM cluster.

Supplementary 3 Supplementary Table 1: differentially expressed genes (DEGs) in identified clusters.

Supplementary 4 Supplementary Table 2: different gene list of tumor cell clusters.

Supplementary 5 Supplementary Table 3: differentially expressed genes (DEGs) in GSCL and Class-G.

Supplementary 6 Supplementary Table 4: differentially expressed genes (DEGs) in GSCL and Opc-G.

Supplementary 7 Supplementary Table 5: differentially expressed genes (DEGs) in GSCL and Neo-G.

Supplementary 8 Supplementary Table 6: differentially expressed genes (DEGs) in GSCL and Ac-G.

Supplementary 9 Supplementary Table 7: differentially expressed genes (DEGs) in GSCL and Undiff-G.

Supplementary 10 Supplementary Table 8: different pathway list of the 6 clusters in tumor cell.

Supplementary 11 Supplementary Table 9: clinical characteristics of 137 human IDH-mut GBM samples.

Supplementary 12 Supplementary Table 10: Univariate and multivariate Cox proportional hazards analyses of clinicopathological variables and 5 classifications in 137 human IDH-mut GBM samples.
