# Supplementary figures and images for: A Novel Molecular Classification Method for Glioblastoma Based on Tumor Cell Differentiation Trajectories
Source: Stem Cells Int. 2023 Feb 22;2023:2826815. doi: 10.1155/2023/2826815 (PMC10643041; doi:10.1155/2023/2826815)

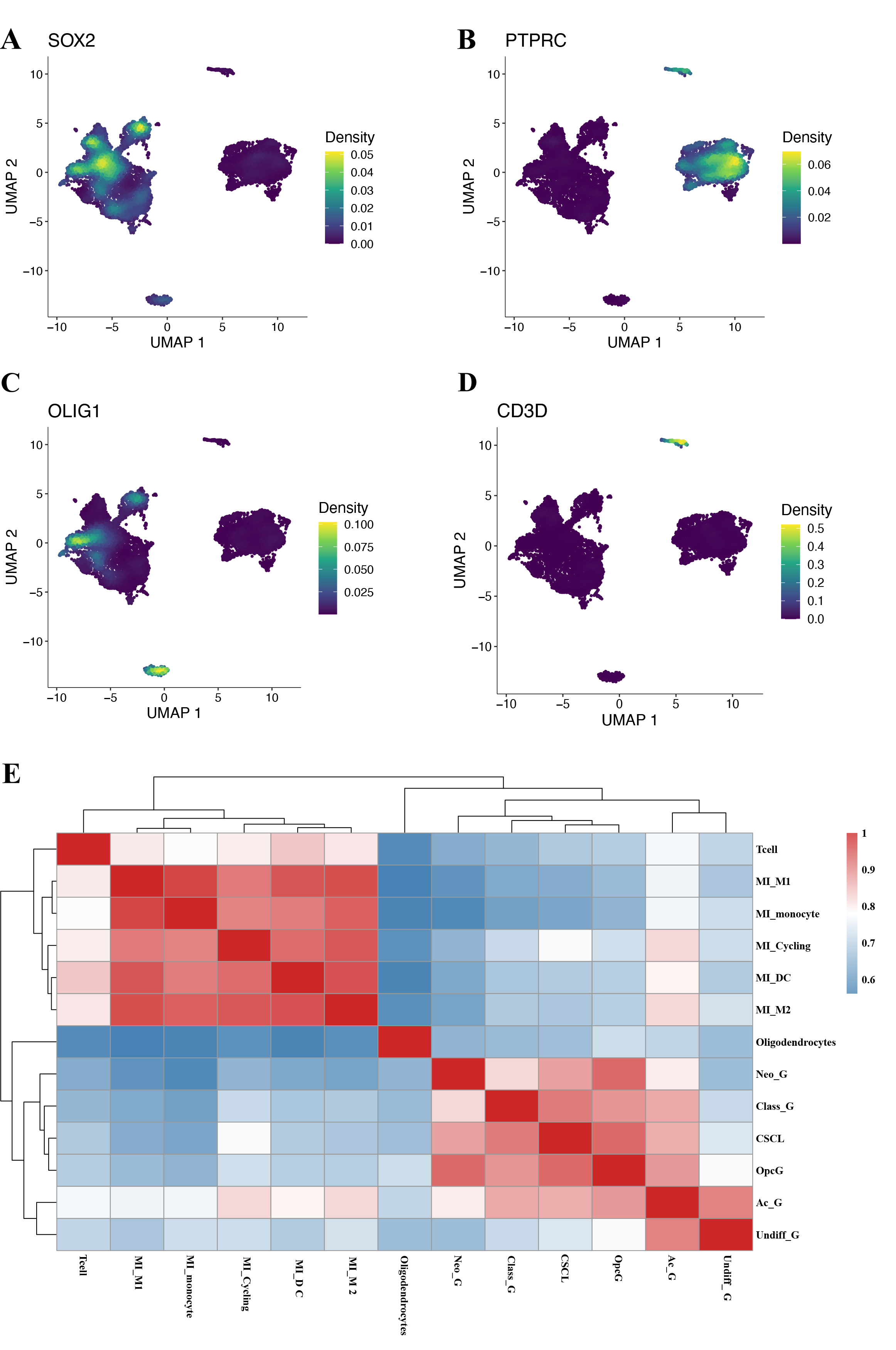

Supplement: Supplementary 1 — Supplementary Figure 1: Validation cohorts of the single-cell atlas. (A-D) The split UMAP plots show cells color-coded for expression of indicated marker genes. (E) Grouping of the cell clusters by similarity. Heatmap showing the Pearson correlation coefficients between the log-averaged expression profiles of clusters. [file 2826815.f1.png]

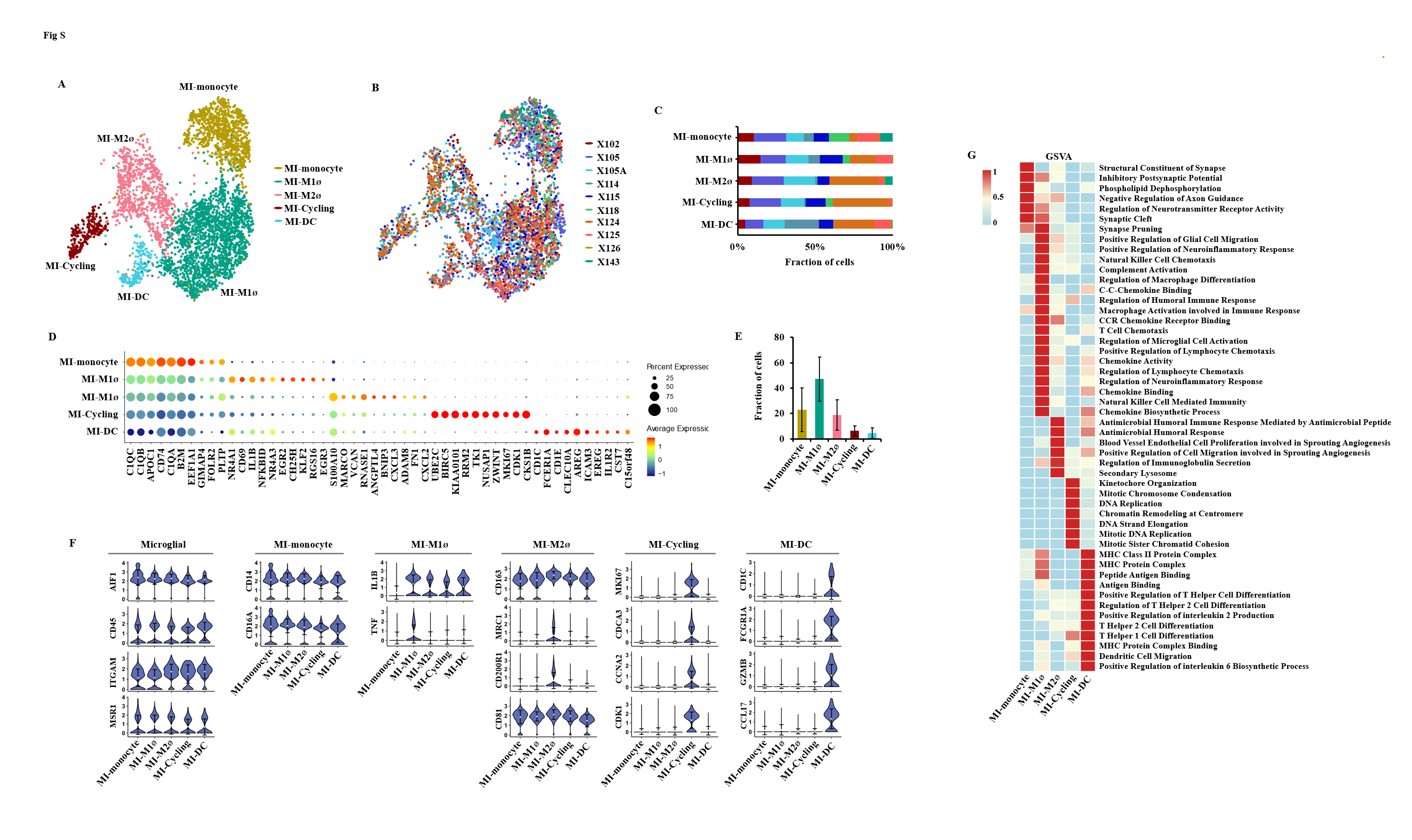

Supplement: Supplementary 2 — Supplementary Figure 2: Activation of tumor-associated macrophages (TAMs) in GBM. (A) UMAP plots of TAM using Seurat method. (B) UMAP plot of TAM color-coded for each GBM sample. (C) TAM cell cluster distributions across samples. (D) Dot-plot heatmap of the most significant genes of each cluster in TAM cells. (E) Differences in cell proportion of GBM samples in each TAM cluster. (F) Violin plots showing different expressions of monocyte, M1Φ, M2Φ, cycling, and DC markers in each TAM cluster. [file 2826815.f2.png]
