# Supplementary material for: A Novel Molecular Classification Method for Glioblastoma Based on Tumor Cell Differentiation Trajectories
Source: Stem Cells Int. 2023 Feb 22;2023:2826815. doi: 10.1155/2023/2826815 (PMC10643041; doi:10.1155/2023/2826815)
Supplement: Supplementary 4 — Supplementary Table 2: different gene list of tumor cell clusters. [file 2826815.f4.pdf]

	p_val	avg_logF(	pct.1	pct.2	p_val_adj	cluster	gene
1	3.18E-68	0.96734	0.963	0.996	4.75E-64	Undiff-G2	MALAT1
2	1.52E-62	1.570013	0.941	0.8	2.27E-58	Undiff-G2	SEC61G
3	6.76E-62	1.151372	0.956	0.994	1.01E-57	Undiff-G2	FTL
4	7.71E-62	1.582528	0.934	0.785	1.15E-57	Undiff-G2	MT2A
5	7.86E-62	1.868105	0.89	0.524	1.17E-57	Undiff-G2	CHI3L1
6	1.35E-61	1.048791	0.971	0.997	2.02E-57	Undiff-G2	TMSB4X
7	3.08E-60	1.039806	0.971	0.993	4.60E-56	Undiff-G2	ACTB
8	1.13E-55	1.201921	0.934	0.986	1.69E-51	Undiff-G2	GAPDH
9	1.95E-53	1.414593	0.904	0.784	2.91E-49	Undiff-G2	CLU
10	5.81E-49	1.007381	0.919	0.971	8.69E-45	Undiff-G2	B2M
11	2.09E-46	1.144315	0.897	0.966	3.12E-42	Undiff-G2	TMSB10
12	4.82E-42	1.137912	0.868	0.827	7.20E-38	Undiff-G2	EEF1A1
13	3.82E-41	1.807194	0.794	0.578	5.71E-37	Undiff-G2	MT1X
14	4.99E-34	1.902137	0.463	0.144	7.46E-30	Undiff-G2	SLPI
15	2.56E-33	1.905187	0.5	0.175	3.83E-29	Undiff-G2	CHI3L2
16	2.62E-33	0.988373	0.846	0.95	3.92E-29	Undiff-G2	ACTG1
17	1.91E-30	1.741473	0.699	0.483	2.86E-26	Undiff-G2	LGALS3
18	3.44E-30	1.620559	0.39	0.11	5.15E-26	Undiff-G2	UQCR11
19	6.87E-27	1.804149	0.426	0.146	1.03E-22	Undiff-G2	LTF
20	4.55E-25	1.015052	0.794	0.976	6.80E-21	Undiff-G2	EIF1
21	1.80E-22	1.470588	0.699	0.623	2.68E-18	Undiff-G2	TIMP1
22	1.26E-21	1.596021	0.243	0.058	1.89E-17	Undiff-G2	PSMA2
23	2.46E-21	1.041132	0.757	0.927	3.67E-17	Undiff-G2	CD63
24	6.96E-20	1.880217	0.346	0.123	1.04E-15	Undiff-G2	CAV1
25	8.75E-20	0.877461	0.772	0.97	1.31E-15	Undiff-G2	PTMA
26	8.92E-20	1.241972	0.706	0.769	1.33E-15	Undiff-G2	LDHA
27	2.24E-19	1.007235	0.743	0.898	3.35E-15	Undiff-G2	HLA-A
28	1.12E-18	0.826089	0.75	0.974	1.67E-14	Undiff-G2	H3F3B
29	2.38E-18	1.638391	0.331	0.115	3.56E-14	Undiff-G2	SAA1
30	1.32E-14	1.55218	0.279	0.1	1.97E-10	Undiff-G2	MGP
31	2.32E-14	0.955488	0.713	0.961	3.47E-10	Undiff-G2	FAU
32	4.28E-14	0.446973	0.176	0.648	6.40E-10	Undiff-G2	SSBP1
33	5.19E-14	1.21842	0.654	0.707	7.75E-10	Undiff-G2	LGALS1
34	7.65E-14	0.898326	0.713	0.954	1.14E-09	Undiff-G2	UBC
35	4.58E-13	1.007962	0.684	0.855	6.84E-09	Undiff-G2	HLA-B
36	1.19E-12	1.279445	0.625	0.647	1.78E-08	Undiff-G2	S100A6
37	4.99E-12	0.466987	0.147	0.556	7.46E-08	Undiff-G2	PRDX4
38	1.18E-11	0.471388	0.191	0.637	1.76E-07	Undiff-G2	BUD31
39	2.64E-11	0.463518	0.132	0.509	3.94E-07	Undiff-G2	GTF2A2
40	3.24E-11	0.451624	0.199	0.64	4.84E-07	Undiff-G2	VDAC1
41	3.81E-11	0.850448	0.691	0.946	5.69E-07	Undiff-G2	GNB2L1
42	1.06E-10	0.431865	0.176	0.584	1.58E-06	Undiff-G2	TMCO1
43	1.32E-10	1.362557	0.551	0.5	1.98E-06	Undiff-G2	CRYAB
44	1.83E-10	0.839705	0.691	0.956	2.74E-06	Undiff-G2	TPT1
45	6.59E-10	0.438353	0.272	0.771	9.84E-06	Undiff-G2	SSR4
46	6.91E-10	0.443128	0.162	0.535	1.03E-05	Undiff-G2	SSR3
47	8.57E-10	0.493424	0.199	0.597	1.28E-05	Undiff-G2	EIF2S2
48	8.96E-10	0.478106	0.243	0.697	1.34E-05	Undiff-G2	TMED9
49	9.05E-10	0.535739	0.206	0.629	1.35E-05	Undiff-G2	ARF4
50	1.03E-09	0.560827	0.199	0.6	1.54E-05	Undiff-G2	NDUFV2

51	1.21E-09	0.48655	0.176	0.55	1.81E-05	Undiff-G2EIF4H
52	1.30E-09	0.646888	0.206	0.628	1.94E-05	Undiff-G2RAB1A
53	1.30E-09	0.474135	0.257	0.733	1.95E-05	Undiff-G2PSMB1
54	1.81E-09	0.503189	0.199	0.6	2.71E-05	Undiff-G2TMEM14C
55	2.58E-09	0.495831	0.176	0.547	3.86E-05	Undiff-G2BZW1
56	3.32E-09	0.864881	0.676	0.945	4.96E-05	Undiff-G2MYL6
57	3.97E-09	0.43496	0.088	0.374	5.93E-05	Undiff-G2PPHLN1
58	5.38E-09	0.44364	0.272	0.738	8.03E-05	Undiff-G2SRSF3
59	8.38E-09	0.442988	0.265	0.719	0.000125	Undiff-G2SON
60	9.68E-09	0.529315	0.162	0.511	0.000145	Undiff-G2PGAM1
61	1.09E-08	0.505041	0.191	0.568	0.000163	Undiff-G2ARPC1A
62	1.09E-08	0.478971	0.243	0.67	0.000163	Undiff-G2BANF1
63	1.24E-08	0.466716	0.206	0.585	0.000185	Undiff-G2ERGIC3
64	1.28E-08	0.455267	0.125	0.429	0.000192	Undiff-G2IAH1
65	1.45E-08	0.485456	0.272	0.738	0.000217	Undiff-G2SPCS2
66	1.78E-08	0.4642	0.243	0.675	0.000266	Undiff-G2TXN
67	1.86E-08	0.452849	0.287	0.779	0.000278	Undiff-G2COX8A
68	2.06E-08	0.534617	0.118	0.412	0.000308	Undiff-G2MESDC2
69	2.09E-08	0.437947	0.147	0.469	0.000313	Undiff-G2RBP1
70	2.19E-08	0.472933	0.162	0.492	0.000327	Undiff-G2MED10
71	2.89E-08	0.489477	0.14	0.446	0.000432	Undiff-G2TMED4
72	3.16E-08	0.456685	0.096	0.368	0.000472	Undiff-G2ACTR10
73	3.16E-08	0.449699	0.213	0.594	0.000472	Undiff-G2SAT2
74	3.28E-08	0.472693	0.096	0.368	0.000489	Undiff-G2PDZD11
75	3.88E-08	0.519438	0.176	0.514	0.000581	Undiff-G2SMIM7
76	5.30E-08	1.475265	0.287	0.155	0.000792	Undiff-G2AC009501.4
77	5.37E-08	0.447784	0.118	0.398	0.000802	Undiff-G2HBB
78	5.37E-08	1.598935	0.206	0.088	0.000802	Undiff-G2C10orf10
79	5.55E-08	0.547893	0.14	0.442	0.000829	Undiff-G2IP6K2
80	6.04E-08	0.437249	0.309	0.806	0.000902	Undiff-G2HNRNPDL
81	6.70E-08	0.978281	0.632	0.842	0.001001	Undiff-G2FOS
82	6.72E-08	0.553674	0.199	0.564	0.001005	Undiff-G2C7orf73
83	7.44E-08	1.357529	0.272	0.141	0.001112	Undiff-G2MGST1
84	7.59E-08	0.453983	0.176	0.511	0.001135	Undiff-G2OSTC
85	7.60E-08	0.462456	0.14	0.439	0.001136	Undiff-G2PRKCSH
86	7.82E-08	0.495424	0.294	0.756	0.001169	Undiff-G2CNBP
87	8.05E-08	0.525092	0.206	0.562	0.001202	Undiff-G2TAF7
88	8.60E-08	1.032002	0.64	0.855	0.001285	Undiff-G2SPP1
89	8.66E-08	0.497953	0.25	0.66	0.001295	Undiff-G2PHPT1
90	8.70E-08	0.994072	0.632	0.848	0.0013	Undiff-G2DBI
91	9.55E-08	0.481174	0.118	0.397	0.001427	Undiff-G2GGCT
92	1.13E-07	0.529598	0.272	0.723	0.001688	Undiff-G2ERH
93	1.16E-07	1.51517	0.456	0.384	0.001727	Undiff-G2MT1E
94	1.21E-07	0.537876	0.14	0.435	0.001809	Undiff-G2DDIT3
95	1.24E-07	0.495386	0.294	0.768	0.001854	Undiff-G2ATP6V0E1
96	1.31E-07	0.500133	0.162	0.474	0.001956	Undiff-G2SNHG8
97	1.70E-07	0.516132	0.221	0.595	0.002535	Undiff-G2SLC3A2
98	1.83E-07	0.485048	0.118	0.387	0.002731	Undiff-G2SCCPDH
99	1.86E-07	0.44797	0.11	0.373	0.002781	Undiff-G2ILK
100	1.95E-07	0.571385	0.265	0.682	0.002915	Undiff-G2PSMD8
101	2.00E-07	0.45353	0.279	0.714	0.002987	Undiff-G2TRMT112

102	2.30E-07	0.513667	0.081	0.319	0.003441	Undiff-G2C14orf119
103	2.58E-07	0.59299	0.206	0.561	0.003857	Undiff-G2NDUFC1
104	2.64E-07	0.509799	0.199	0.542	0.003948	Undiff-G2RHOC
105	3.12E-07	0.514136	0.132	0.413	0.004663	Undiff-G2SYPL1
106	3.28E-07	0.444462	0.081	0.313	0.004901	Undiff-G2CHMP2B
107	3.31E-07	0.73666	0.169	0.478	0.004953	Undiff-G2COPS8
108	3.42E-07	0.571703	0.199	0.546	0.005108	Undiff-G2C12orf57
109	3.57E-07	0.53249	0.147	0.437	0.005329	Undiff-G2SPTSSA
110	3.68E-07	1.550234	0.375	0.271	0.005493	Undiff-G2IGFBP5
111	3.86E-07	0.607233	0.228	0.591	0.005767	Undiff-G2REEP5
112	4.03E-07	0.530973	0.169	0.481	0.006028	Undiff-G2UBE2L6
113	4.42E-07	1.12086	0.125	0.042	0.006598	Undiff-G2NDUFA7
114	5.01E-07	0.436188	0.074	0.295	0.007493	Undiff-G2RRP7A
115	5.07E-07	0.57229	0.235	0.618	0.007574	Undiff-G2SBDS
116	6.23E-07	0.532983	0.176	0.481	0.009306	Undiff-G2NAA38
117	7.00E-07	0.469953	0.096	0.329	0.010467	Undiff-G2ARL16
118	7.09E-07	0.496462	0.066	0.279	0.010591	Undiff-G2NDUFAF2
119	7.14E-07	0.60091	0.287	0.712	0.010671	Undiff-G2PSMB3
120	7.52E-07	1.273923	0.14	0.051	0.011238	Undiff-G2CTD-2090I13.1
121	7.81E-07	0.468594	0.257	0.653	0.011676	Undiff-G2GNG5
122	8.71E-07	0.54419	0.132	0.398	0.013017	Undiff-G2ZNF667-AS1
123	9.94E-07	0.46394	0.059	0.262	0.014852	Undiff-G2BRIX1
124	9.94E-07	0.711063	0.14	0.413	0.01486	Undiff-G2CALU
125	1.15E-06	0.469674	0.103	0.336	0.017219	Undiff-G2PTS
126	1.15E-06	0.483021	0.066	0.274	0.01723	Undiff-G2TWISTNB
127	1.29E-06	0.540446	0.015	0.001	0.019211	Undiff-G2HBD
128	1.34E-06	0.493251	0.14	0.406	0.020098	Undiff-G2FAM162A
129	1.40E-06	0.469215	0.096	0.323	0.020978	Undiff-G2CFLAR
130	1.42E-06	0.455475	0.331	0.819	0.021192	Undiff-G2NDUFS5
131	1.56E-06	0.579723	0.191	0.504	0.023263	Undiff-G2TMEM205
132	1.70E-06	1.043231	0.596	0.775	0.025466	Undiff-G2PKM
133	2.24E-06	0.467949	0.066	0.266	0.033408	Undiff-G2YIPF5
134	2.61E-06	0.525549	0.294	0.708	0.038943	Undiff-G2FKBP1A
135	2.91E-06	0.701844	0.132	0.386	0.04346	Undiff-G2FBL
136	3.04E-06	0.44739	0.309	0.737	0.045503	Undiff-G2SRI
137	3.09E-06	0.509287	0.103	0.326	0.046175	Undiff-G2COX11
138	3.12E-06	0.496919	0.081	0.288	0.046554	Undiff-G2TMEM222
139	3.12E-06	0.470158	0.331	0.809	0.046596	Undiff-G2PSMA7
140	3.24E-06	0.454069	0.169	0.453	0.04837	Undiff-G2TXNIP
141	3.29E-06	1.431919	0.456	0.437	0.049217	Undiff-G2NAMPT
142	3.32E-06	0.708343	0.221	0.549	0.049655	Undiff-G2PDCD5
143	3.84E-06	0.533105	0.081	0.287	0.057347	Undiff-G2AGTRAP
144	3.89E-06	0.695091	0.265	0.656	0.058117	Undiff-G2PDIA6
145	4.39E-06	0.469547	0.059	0.245	0.065579	Undiff-G2NUP62
146	4.71E-06	0.555242	0.257	0.627	0.070452	Undiff-G2DSTN
147	5.19E-06	0.515026	0.074	0.27	0.077498	Undiff-G2POP4
148	5.50E-06	0.471359	0.074	0.268	0.082224	Undiff-G2CDC26
149	6.52E-06	1.145296	0.103	0.034	0.09745	Undiff-G2SMIM3
150	6.53E-06	0.504382	0.074	0.267	0.097621	Undiff-G2CCND3
151	6.96E-06	1.0659	0.551	0.66	0.104073	Undiff-G2S100A11
152	7.59E-06	0.458319	0.331	0.779	0.113428	Undiff-G2TUBB

153	7.63E-06	0.447741	0.346	0.815	0.113954	Undiff-G2PRDX1
154	9.09E-06	0.48369	0.081	0.275	0.135856	Undiff-G2LLPH
155	9.23E-06	1.46613	0.228	0.126	0.137926	Undiff-G2MT1G
156	9.83E-06	0.611813	0.11	0.33	0.14692	Undiff-G2CADM1
157	1.01E-05	0.483114	0.088	0.289	0.15088	Undiff-G2ZFAND2A
158	1.06E-05	0.647857	0.169	0.431	0.158083	Undiff-G2POLD2
159	1.07E-05	0.545399	0.147	0.395	0.16042	Undiff-G2IFI16
160	1.09E-05	0.570204	0.132	0.364	0.16234	Undiff-G2UROD
161	1.10E-05	0.504923	0.331	0.788	0.164633	Undiff-G2OST4
162	1.15E-05	0.443857	0.081	0.274	0.172339	Undiff-G2CCNG1
163	1.25E-05	0.461797	0.11	0.325	0.187026	Undiff-G2REXO2
164	1.44E-05	0.447829	0.059	0.229	0.215457	Undiff-G2PPP1R8
165	1.57E-05	1.477478	0.191	0.097	0.234029	Undiff-G2SNRPN
166	1.59E-05	0.516748	0.338	0.817	0.237558	Undiff-G2NDUFB2
167	1.68E-05	1.231347	0.485	0.497	0.251516	Undiff-G2MT3
168	1.78E-05	0.514125	0.184	0.457	0.265891	Undiff-G2LGALS3BP
169	1.82E-05	0.458344	0.375	0.874	0.271766	Undiff-G2SRP14
170	1.88E-05	1.359648	0.316	0.221	0.28048	Undiff-G2EFEMP1
171	1.90E-05	1.012919	0.537	0.625	0.283776	Undiff-G2GPM6B
172	1.91E-05	0.514841	0.096	0.291	0.285892	Undiff-G2COPB1
173	2.25E-05	1.399513	0.309	0.217	0.335513	Undiff-G2C1R
174	2.69E-05	0.459956	0.066	0.237	0.402674	Undiff-G2CTTN
175	3.12E-05	0.483217	0.096	0.288	0.466746	Undiff-G2SP100
176	3.19E-05	0.466121	0.059	0.221	0.476701	Undiff-G2PEMT
177	3.40E-05	0.702677	0.294	0.7	0.508785	Undiff-G2MGST3
178	3.45E-05	0.732883	0.213	0.511	0.515915	Undiff-G2WBP5
179	3.58E-05	0.551826	0.331	0.779	0.53555	Undiff-G2LAPTM4A
180	3.61E-05	0.53961	0.125	0.336	0.539579	Undiff-G2USE1
181	4.30E-05	0.623928	0.331	0.765	0.642215	Undiff-G2RAN
182	4.36E-05	0.798681	0.184	0.45	0.651635	Undiff-G2PSMA3
183	4.60E-05	1.447837	0.125	0.052	0.688117	Undiff-G2PI3
184	4.66E-05	0.503214	0.051	0.204	0.696191	Undiff-G2RCHY1
185	4.87E-05	0.476357	0.184	0.444	0.727175	Undiff-G2RGS2
186	4.92E-05	0.933837	0.596	0.85	0.735043	Undiff-G2ALDOA
187	5.00E-05	0.729406	0.162	0.398	0.747159	Undiff-G2AK2
188	5.44E-05	0.431725	0.037	0.176	0.812301	Undiff-G2C11orf74
189	5.53E-05	0.610043	0.103	0.291	0.826343	Undiff-G2ENO2
190	5.53E-05	1.230685	0.316	0.23	0.826729	Undiff-G2SERPING1
191	5.64E-05	0.488032	0.081	0.251	0.8434	Undiff-G2FUNDC1
192	5.85E-05	0.44095	0.103	0.288	0.87353	Undiff-G2GADD45A
193	6.07E-05	0.69293	0.088	0.264	0.907018	Undiff-G2IFT43
194	6.57E-05	0.432015	0.044	0.185	0.981549	Undiff-G2NUP54
195	7.62E-05	0.852782	0.154	0.386	1	Undiff-G2DNAJB9
196	7.63E-05	0.457004	0.081	0.248	1	Undiff-G2SRSF10
197	8.94E-05	0.491015	0.125	0.321	1	Undiff-G2ZNF302
198	8.95E-05	1.197481	0.485	0.549	1	Undiff-G2S100A10
199	0.000105	0.56968	0.029	0.005	1	Undiff-G2ASB3
200	0.000122	0.482701	0.382	0.876	1	Undiff-G2NPM1
201	0.000147	0.452356	0.059	0.2	1	Undiff-G2DDX6
202	0.000159	0.47826	0.066	0.215	1	Undiff-G2PIGH
203	0.000167	0.44526	0.037	0.163	1	Undiff-G2GSTZ1

204	0.000173	0.97517	0.081	0.029	1	Undiff-G2MT1A
205	0.000174	1.055101	0.118	0.05	1	Undiff-G2SAA2
206	0.000186	0.508447	0.206	0.468	1	Undiff-G2A2M
207	0.000196	0.46738	0.074	0.224	1	Undiff-G2PPP2R2B
208	0.000202	0.780337	0.294	0.654	1	Undiff-G2C4orf3
209	0.00024	0.59837	0.081	0.234	1	Undiff-G2NIT2
210	0.000241	0.562095	0.338	0.75	1	Undiff-G2BSG
211	0.000248	0.905654	0.515	0.623	1	Undiff-G2PTN
212	0.000275	0.488989	0.051	0.182	1	Undiff-G2ARV1
213	0.000286	1.028668	0.125	0.057	1	Undiff-G2TMBIM4
214	0.000289	0.581826	0.037	0.008	1	Undiff-G2RP11-284F21.8
215	0.000323	0.495641	0.25	0.535	1	Undiff-G2S100B
216	0.000334	0.471594	0.044	0.167	1	Undiff-G2GPATCH2
217	0.000373	0.46771	0.029	0.006	1	Undiff-G2RP11-242D8.1
218	0.000375	0.659977	0.287	0.621	1	Undiff-G2TMED10
219	0.000394	0.776364	0.228	0.499	1	Undiff-G2PSENEN
220	0.00043	0.609634	0.029	0.006	1	Undiff-G2SERPINA3
221	0.000507	0.530641	0.044	0.162	1	Undiff-G2PIAS1
222	0.000565	0.614961	0.206	0.436	1	Undiff-G2PMP22
223	0.000577	0.787331	0.154	0.352	1	Undiff-G2PLP2
224	0.000579	0.781306	0.037	0.009	1	Undiff-G2PAGR1
225	0.000582	0.499682	0.059	0.185	1	Undiff-G2ARL14EP
226	0.000632	0.601021	0.059	0.184	1	Undiff-G2C1orf27
227	0.000639	0.807586	0.147	0.336	1	Undiff-G2LMAN1
228	0.000649	0.775557	0.096	0.247	1	Undiff-G2RBM7
229	0.000676	0.703218	0.213	0.465	1	Undiff-G2GABARAP
230	0.000684	0.954468	0.147	0.078	1	Undiff-G2ATXN8OS
231	0.000694	1.10144	0.441	0.473	1	Undiff-G2GAP43
232	0.000736	0.649184	0.338	0.75	1	Undiff-G2PRDX5
233	0.000809	0.443555	0.11	0.264	1	Undiff-G2H1F0
234	0.000862	1.022042	0.096	0.042	1	Undiff-G2CADPS
235	0.000876	0.513447	0.044	0.155	1	Undiff-G2UBXN2B
236	0.000906	0.689787	0.353	0.751	1	Undiff-G2WDR83OS
237	0.000985	0.511397	0.044	0.153	1	Undiff-G2TIMM21
238	0.001031	0.805611	0.051	0.016	1	Undiff-G2LINC00869
239	0.001068	0.798832	0.176	0.38	1	Undiff-G2BLVRB
240	0.001147	0.851578	0.088	0.037	1	Undiff-G2IER3IP1
241	0.001219	0.578469	0.243	0.501	1	Undiff-G2MDK
242	0.001231	0.600456	0.228	0.477	1	Undiff-G2CNN3
243	0.001464	0.498907	0.404	0.89	1	Undiff-G2PFDN5
244	0.001573	0.48509	0.037	0.134	1	Undiff-G2ZNF720
245	0.001582	0.488951	0.066	0.184	1	Undiff-G2EMP1
246	0.001586	1.459877	0.25	0.186	1	Undiff-G2ADM
247	0.00164	0.65181	0.074	0.196	1	Undiff-G2HIST1H1C
248	0.001716	0.660022	0.074	0.195	1	Undiff-G2MYADM
249	0.001745	0.651475	0.14	0.31	1	Undiff-G2MT1F
250	0.001869	0.630238	0.081	0.205	1	Undiff-G2ARMC8
251	0.001884	0.903242	0.574	0.871	1	Undiff-G2TPI1
252	0.002099	0.663083	0.331	0.7	1	Undiff-G2CALR
253	0.002353	0.555326	0.096	0.227	1	Undiff-G2PSMA6
254	0.002423	0.49515	0.066	0.176	1	Undiff-G2NIP7

255	0.002436	0.598925	0.257	0.522	1	Undiff-G2	TAGLN2
256	0.00245	0.954513	0.132	0.073	1	Undiff-G2	MTRNR2L10
257	0.002526	0.768067	0.11	0.252	1	Undiff-G2	SMOX
258	0.002534	0.448777	0.044	0.141	1	Undiff-G2	SLC2A1
259	0.00254	0.564852	0.066	0.178	1	Undiff-G2	PIGU
260	0.002676	0.584625	0.059	0.165	1	Undiff-G2	GMPPA
261	0.00276	0.639641	0.338	0.691	1	Undiff-G2	C19orf53
262	0.002861	1.556732	0.279	0.23	1	Undiff-G2	HILPDA
263	0.003015	0.605754	0.096	0.222	1	Undiff-G2	VEGFA
264	0.003039	0.528988	0.404	0.893	1	Undiff-G2	COX7C
265	0.003094	0.488102	0.397	0.858	1	Undiff-G2	TMA7
266	0.003138	0.465345	0.074	0.185	1	Undiff-G2	AK4
267	0.003291	0.561326	0.059	0.161	1	Undiff-G2	C1orf174
268	0.003337	0.886335	0.118	0.261	1	Undiff-G2	UPP1
269	0.003435	0.577553	0.397	0.853	1	Undiff-G2	COX6B1
270	0.003577	0.543103	0.066	0.17	1	Undiff-G2	TIPARP
271	0.003696	0.481487	0.029	0.111	1	Undiff-G2	ZFYVE19
272	0.003868	0.835886	0.5	0.627	1	Undiff-G2	HLA-DRA
273	0.004049	0.454296	0.022	0.005	1	Undiff-G2	ZNF8
274	0.004146	0.771112	0.191	0.388	1	Undiff-G2	GLRX
275	0.004149	0.809222	0.154	0.32	1	Undiff-G2	SERTAD1
276	0.004492	0.453896	0.044	0.133	1	Undiff-G2	ZNF429
277	0.004581	0.477408	0.022	0.005	1	Undiff-G2	APOBEC3A
278	0.004672	0.52586	0.088	0.203	1	Undiff-G2	HES1
279	0.004961	0.523302	0.044	0.131	1	Undiff-G2	CREBRF
280	0.005021	0.742699	0.11	0.241	1	Undiff-G2	LHFP
281	0.005186	0.502036	0.015	0.002	1	Undiff-G2	SEC14L4
282	0.005186	0.487738	0.015	0.002	1	Undiff-G2	CCL26
283	0.005231	1.150411	0.382	0.389	1	Undiff-G2	FABP5
284	0.005518	0.81965	0.228	0.455	1	Undiff-G2	EMP3
285	0.005762	0.432024	0.272	0.529	1	Undiff-G2	HLA-DRB1
286	0.005793	0.872435	0.051	0.019	1	Undiff-G2	TSLP
287	0.005904	0.952301	0.191	0.378	1	Undiff-G2	RAB13
288	0.006112	0.544442	0.404	0.828	1	Undiff-G2	GSTP1
289	0.006149	0.45282	0.037	0.117	1	Undiff-G2	DNALI1
290	0.006163	0.682754	0.066	0.165	1	Undiff-G2	STEAP3
291	0.007086	0.575372	0.404	0.849	1	Undiff-G2	GPX4
292	0.007128	0.511313	0.426	0.917	1	Undiff-G2	COX4I1
293	0.007426	0.905308	0.331	0.662	1	Undiff-G2	HSPA5
294	0.00752	0.442689	0.044	0.126	1	Undiff-G2	IQCG
295	0.008208	0.495884	0.169	0.328	1	Undiff-G2	SEPP1
296	0.008746	0.553552	0.051	0.136	1	Undiff-G2	PIK3R3
297	0.008866	0.558678	0.029	0.1	1	Undiff-G2	HRH1
298	0.009422	0.511391	0.022	0.005	1	Undiff-G2	GS1-18A18.2
299	0.009658	0.801232	0.096	0.206	1	Undiff-G2	INSIG2
300	0.00994	0.443731	0.213	0.396	1	Undiff-G2	HLA-DRB5
301	0	1.951144	0.433	0.057	0	Ac-G	SAA1
302	0	1.733389	0.496	0.083	0	Ac-G	LTF
303	0	1.732148	0.488	0.083	0	Ac-G	SLPI
304	0	1.72185	0.577	0.06	0	Ac-G	MGST1
305	0	1.719219	0.592	0.1	0	Ac-G	CHI3L2

306	0	1.681798	0.423	0.041	0	Ac-G	MGP
307	0	1.55461	0.614	0.157	0	Ac-G	C8orf4
308	0	1.537289	0.23	0.017	0	Ac-G	SAA2
309	0	1.523599	0.577	0.154	0	Ac-G	EFEMP1
310	0	1.495285	0.582	0.148	0	Ac-G	C1R
311	0	1.474012	0.845	0.468	0	Ac-G	CHI3L1
312	0	1.452206	0.489	0.056	0	Ac-G	CAV1
313	0	1.392692	0.483	0.102	0	Ac-G	C1S
314	0	1.353045	0.548	0.17	0	Ac-G	SERPING1
315	0	1.284982	0.461	0.089	0	Ac-G	RARRES2
316	0	1.225577	0.831	0.437	0	Ac-G	CRYAB
317	0	1.17355	0.844	0.53	0	Ac-G	MT1X
318	0	1.122243	0.786	0.428	0	Ac-G	LGALS3
319	0	1.068348	0.858	0.579	0	Ac-G	TIMP1
320	0	1.049687	0.969	0.75	0	Ac-G	CLU
321	0	0.939207	0.959	0.771	0	Ac-G	SEC61G
322	0	0.923746	0.948	0.756	0	Ac-G	MT2A
323	0	0.553032	0.986	0.985	0	Ac-G	GAPDH
324	0	0.517055	0.99	0.995	0	Ac-G	FTL
325	########	1.101967	0.433	0.119	########	Ac-G	CYR61
326	########	0.558845	0.975	0.944	########	Ac-G	ACTG1
327	########	0.940465	0.84	0.609	########	Ac-G	S100A6
328	########	0.728259	0.246	0.03	########	Ac-G	BGN
329	########	0.863553	0.316	0.058	########	Ac-G	CADM3
330	########	1.046715	0.754	0.496	########	Ac-G	SOD2
331	########	0.491035	0.992	0.993	########	Ac-G	ACTB
332	########	0.975015	0.645	0.335	########	Ac-G	MT1E
333	########	1.189215	0.392	0.115	########	Ac-G	NNMT
334	########	0.71238	0.212	0.025	########	Ac-G	IL13RA2
335	########	0.543031	0.175	0.014	########	Ac-G	CA9
336	########	0.976151	0.64	0.336	########	Ac-G	CD44
337	########	0.407009	0.996	0.995	########	Ac-G	MALAT1
338	########	0.818112	0.333	0.079	########	Ac-G	NTRK2
339	########	1.169778	0.645	0.345	########	Ac-G	OCIAD2
340	########	0.870361	0.692	0.351	########	Ac-G	ANXA1
341	########	0.973283	0.683	0.39	########	Ac-G	NAMPT
342	########	0.655687	0.193	0.021	########	Ac-G	PIFO
343	########	0.759485	0.257	0.045	########	Ac-G	PRSS23
344	########	1.085875	0.474	0.176	########	Ac-G	SPOCD1
345	########	0.841974	0.741	0.511	########	Ac-G	S100A10
346	########	0.68007	0.249	0.043	########	Ac-G	SYTL2
347	########	0.971292	0.275	0.054	########	Ac-G	C10orf10
348	########	0.938156	0.586	0.262	########	Ac-G	S100A16
349	########	0.639084	0.871	0.755	########	Ac-G	PKM
350	########	0.544979	0.165	0.015	########	Ac-G	HSPB8
351	########	0.95238	0.713	0.427	########	Ac-G	GAP43
352	########	0.638818	0.854	0.752	########	Ac-G	LDHA
353	########	0.484181	0.877	0.842	########	Ac-G	ALDOA
354	########	0.511878	0.132	0.008	########	Ac-G	CP
355	########	1.101504	0.228	0.04	########	Ac-G	TAGLN
356	########	0.972731	0.615	0.348	########	Ac-G	SPARC

357	########	0.845846	0.296	0.069	########	Ac-G	VSTM2A
358	########	1.0161	0.573	0.308	########	Ac-G	SPARCL1
359	########	1.119242	0.549	0.263	########	Ac-G	CSRP2
360	########	0.538918	0.941	0.922	########	Ac-G	CD63
361	########	0.999002	0.505	0.227	########	Ac-G	IGFBP5
362	########	0.971667	0.475	0.206	########	Ac-G	MT1M
363	########	0.645381	0.268	0.063	########	Ac-G	SLC39A14
364	########	0.716826	0.823	0.586	########	Ac-G	GPM6B
365	########	0.495999	0.899	0.86	########	Ac-G	SKP1
366	########	0.782006	0.78	0.6	########	Ac-G	PRDX6
367	########	0.786778	0.722	0.454	########	Ac-G	MT3
368	########	0.582778	0.153	0.017	########	Ac-G	GALNT15
369	########	0.707301	0.682	0.435	########	Ac-G	CNN3
370	########	0.472724	0.972	0.964	########	Ac-G	TMSB10
371	########	0.85305	0.333	0.106	########	Ac-G	CXCL14
372	########	0.915347	0.11	0.007	########	Ac-G	HP
373	########	0.609317	0.229	0.049	########	Ac-G	GJA1
374	########	0.571688	0.187	0.031	########	Ac-G	AZGP1
375	########	0.761237	0.296	0.09	########	Ac-G	AEBP1
376	########	0.939371	0.301	0.091	########	Ac-G	DIRAS3
377	########	0.9598	0.621	0.379	########	Ac-G	RAMP1
378	########	0.414165	0.968	0.971	########	Ac-G	B2M
379	########	0.772486	0.295	0.091	########	Ac-G	SH3BGR
380	########	0.93499	0.424	0.194	########	Ac-G	HILPDA
381	########	0.720836	0.809	0.586	########	Ac-G	PTN
382	########	0.44513	0.878	0.865	########	Ac-G	TPI1
383	########	0.76241	0.206	0.047	########	Ac-G	VCAM1
384	########	0.652109	0.307	0.1	########	Ac-G	RASD1
385	########	0.638924	0.737	0.534	########	Ac-G	CD9
386	########	0.716141	0.533	0.284	########	Ac-G	NUPR1
387	########	0.719474	0.254	0.071	########	Ac-G	ANGPTL4
388	########	1.108547	0.136	0.019	########	Ac-G	PLA2G2A
389	########	0.759873	0.141	0.021	########	Ac-G	CA3
390	########	0.589052	0.128	0.017	########	Ac-G	FOLR1
391	########	0.727923	0.323	0.112	########	Ac-G	SGCE
392	########	0.634179	0.203	0.048	########	Ac-G	PDLIM4
393	########	0.704121	0.657	0.471	########	Ac-G	TMEM205
394	########	0.723546	0.586	0.396	########	Ac-G	CALD1
395	########	0.862644	0.335	0.122	########	Ac-G	AC009501.4
396	########	1.247798	0.332	0.134	########	Ac-G	SLN
397	########	0.541461	0.245	0.073	########	Ac-G	IL32
398	########	0.456388	0.853	0.787	########	Ac-G	ENO1
399	########	0.53171	0.801	0.689	########	Ac-G	LGALS1
400	########	0.440239	0.761	0.649	########	Ac-G	VIM
401	########	0.683814	0.696	0.483	########	Ac-G	ANXA2
402	########	0.424715	0.906	0.895	########	Ac-G	NDUFA4
403	########	0.771171	0.497	0.289	########	Ac-G	REXO2
404	########	0.491689	0.173	0.04	########	Ac-G	CFI
405	########	0.72195	0.343	0.148	########	Ac-G	TMEM45A
406	########	0.43463	0.136	0.024	########	Ac-G	CLCF1
407	########	0.389964	0.086	0.007	########	Ac-G	TSLP

408	########	0.432341	0.921	0.946	########	Ac-G	MYL6
409	########	0.947428	0.519	0.316	########	Ac-G	GFAP
410	########	0.710248	0.234	0.073	########	Ac-G	MAOB
411	########	0.650498	0.318	0.123	########	Ac-G	GBP1
412	########	0.403534	0.109	0.014	########	Ac-G	MT1A
413	########	0.395074	0.837	0.841	########	Ac-G	TMBIM6
414	########	0.943505	0.156	0.033	########	Ac-G	PI3
415	########	0.603033	0.671	0.49	########	Ac-G	TAGLN2
416	########	0.674428	0.333	0.14	########	Ac-G	MYC
417	########	0.468021	0.8	0.751	########	Ac-G	SOD1
418	########	0.577356	0.258	0.089	########	Ac-G	PDLIM3
419	########	0.6715	0.305	0.125	########	Ac-G	FBXO32
420	########	0.421086	0.102	0.013	########	Ac-G	ASS1
421	########	0.714525	0.348	0.156	########	Ac-G	ADM
422	########	0.568055	0.176	0.045	########	Ac-G	IGFBP6
423	########	0.556133	0.78	0.624	2.65E-99	Ac-G	NGFRAP1
424	########	0.769393	0.416	0.228	3.65E-99	Ac-G	NRN1
425	########	0.784143	0.415	0.228	8.66E-97	Ac-G	DTNA
426	########	0.404976	0.992	0.997	2.93E-96	Ac-G	TMSB4X
427	########	0.679506	0.489	0.299	1.01E-95	Ac-G	CYSTM1
428	########	0.583124	0.746	0.622	1.17E-95	Ac-G	CD99
429	2.18E-99	0.426008	0.082	0.008	3.25E-95	Ac-G	PRKCG
430	3.38E-99	0.516836	0.137	0.028	5.04E-95	Ac-G	FRZB
431	3.05E-97	0.574312	0.648	0.481	4.55E-93	Ac-G	WBP5
432	5.01E-96	0.726655	0.474	0.296	7.49E-92	Ac-G	CETN2
433	9.23E-94	0.49089	0.135	0.029	1.38E-89	Ac-G	LOX
434	6.48E-92	0.563238	0.266	0.1	9.68E-88	Ac-G	MT1G
435	1.12E-91	0.552326	0.606	0.423	1.68E-87	Ac-G	EMP3
436	5.55E-91	0.778213	0.452	0.272	8.29E-87	Ac-G	S100A13
437	4.18E-90	0.456728	0.781	0.738	6.25E-86	Ac-G	PRDX5
438	5.03E-90	0.728371	0.236	0.087	7.52E-86	Ac-G	TNFAIP6
439	1.16E-89	0.508743	0.812	0.781	1.74E-85	Ac-G	GLUL
440	6.54E-89	0.608833	0.1	0.016	9.78E-85	Ac-G	MMP7
441	6.72E-87	0.448333	0.791	0.721	1.00E-82	Ac-G	SRI
442	1.19E-86	0.412485	0.801	0.769	1.78E-82	Ac-G	LAPTM4A
443	2.09E-85	0.453237	0.698	0.598	3.12E-81	Ac-G	SBDS
444	2.22E-85	0.735307	0.546	0.375	3.31E-81	Ac-G	VAMP5
445	5.01E-84	0.69698	0.218	0.079	7.49E-80	Ac-G	TIMP4
446	1.45E-83	0.482179	0.313	0.13	2.16E-79	Ac-G	APOD
447	2.03E-83	0.427634	0.772	0.751	3.04E-79	Ac-G	PGK1
448	4.47E-83	0.779686	0.345	0.182	6.68E-79	Ac-G	TNC
449	4.52E-83	0.577855	0.324	0.156	6.75E-79	Ac-G	EMP1
450	7.66E-83	0.575511	0.312	0.143	1.14E-78	Ac-G	NDRG1
451	2.14E-82	0.420782	0.16	0.045	3.20E-78	Ac-G	EGLN3
452	4.97E-82	0.486535	0.862	0.843	7.43E-78	Ac-G	DBI
453	5.10E-82	0.41509	0.842	0.856	7.62E-78	Ac-G	TOMM7
454	5.44E-82	0.810448	0.366	0.198	8.13E-78	Ac-G	AGT
455	4.49E-81	0.394174	0.846	0.83	6.71E-77	Ac-G	UBL5
456	5.72E-80	0.716913	0.61	0.486	8.54E-76	Ac-G	IFITM3
457	2.55E-79	0.477495	0.656	0.539	3.81E-75	Ac-G	TAF7
458	5.93E-79	0.433625	0.773	0.717	8.87E-75	Ac-G	SHFM1

459	8.89E-79	0.418719	0.105	0.021	1.33E-74	Ac-G	PCSK1
460	3.12E-78	0.742401	0.389	0.215	4.66E-74	Ac-G	RARRES3
461	5.80E-78	0.508489	0.212	0.076	8.66E-74	Ac-G	GEM
462	1.77E-77	0.490953	0.218	0.084	2.64E-73	Ac-G	EPAS1
463	1.72E-73	0.505905	0.672	0.514	2.58E-69	Ac-G	MAP1B
464	4.51E-73	0.587359	0.495	0.294	6.74E-69	Ac-G	SEPP1
465	8.10E-73	0.618207	0.347	0.196	1.21E-68	Ac-G	VEGFA
466	8.12E-73	0.600636	0.421	0.26	1.21E-68	Ac-G	GADD45A
467	1.03E-72	0.41041	0.74	0.687	1.54E-68	Ac-G	LAMTOR5
468	1.24E-72	0.398835	0.821	0.824	1.86E-68	Ac-G	GSTP1
469	2.08E-72	0.562122	0.212	0.08	3.10E-68	Ac-G	SERPINE1
470	2.65E-70	0.5548	0.316	0.162	3.96E-66	Ac-G	GBE1
471	7.20E-70	0.528178	0.262	0.12	1.08E-65	Ac-G	BDH2
472	7.74E-70	0.546629	0.472	0.312	1.16E-65	Ac-G	CLIC4
473	1.04E-69	0.565312	0.365	0.215	1.55E-65	Ac-G	SLC16A1
474	1.45E-69	0.452242	0.159	0.05	2.17E-65	Ac-G	FAS
475	2.34E-67	0.562413	0.281	0.144	3.50E-63	Ac-G	PLOD2
476	2.51E-67	0.536826	0.515	0.372	3.75E-63	Ac-G	ZNF667-AS1
477	3.06E-67	0.451584	0.187	0.07	4.58E-63	Ac-G	RDH10
478	9.28E-67	0.457577	0.717	0.661	1.39E-62	Ac-G	TXN
479	1.54E-66	0.669829	0.288	0.144	2.30E-62	Ac-G	AQP4
480	1.93E-66	0.575196	0.475	0.326	2.88E-62	Ac-G	PLP2
481	7.45E-66	0.539994	0.253	0.12	1.11E-61	Ac-G	SPOCK2
482	8.48E-66	0.406374	0.748	0.702	1.27E-61	Ac-G	TRMT112
483	1.24E-65	0.588141	0.341	0.182	1.86E-61	Ac-G	GBP2
484	8.16E-65	0.432628	0.694	0.651	1.22E-60	Ac-G	HSPA5
485	3.72E-64	0.622741	0.31	0.172	5.56E-60	Ac-G	AKAP12
486	9.96E-64	0.42088	0.705	0.607	1.49E-59	Ac-G	DSTN
487	1.39E-63	0.539296	0.618	0.462	2.07E-59	Ac-G	GADD45B
488	1.52E-63	0.632401	0.51	0.406	2.28E-59	Ac-G	ITM2C
489	1.74E-63	0.404877	0.841	0.84	2.59E-59	Ac-G	FOS
490	1.92E-63	0.549702	0.246	0.119	2.87E-59	Ac-G	CA12
491	3.27E-63	0.505167	0.2	0.081	4.89E-59	Ac-G	ABCC3
492	3.61E-62	1.024593	0.513	0.366	5.40E-58	Ac-G	FABP5
493	2.52E-61	0.470148	0.631	0.52	3.77E-57	Ac-G	RHOC
494	5.57E-61	0.448369	0.774	0.692	8.32E-57	Ac-G	PRDX2
495	8.45E-61	0.490381	0.529	0.387	1.26E-56	Ac-G	SYPL1
496	5.50E-60	0.432019	0.685	0.653	8.22E-56	Ac-G	HSPB1
497	2.56E-57	0.414844	0.695	0.62	3.83E-53	Ac-G	BUD31
498	4.00E-57	0.542842	0.498	0.354	5.98E-53	Ac-G	BLVRB
499	9.32E-57	0.408631	0.051	0.006	1.39E-52	Ac-G	OSR1
500	1.56E-56	0.53886	0.367	0.228	2.33E-52	Ac-G	SMOX
501	5.04E-56	0.489171	0.135	0.045	7.54E-52	Ac-G	SRPX2
502	9.74E-56	0.577527	0.14	0.045	1.46E-51	Ac-G	PSMA2
503	6.90E-55	0.656481	0.543	0.438	1.03E-50	Ac-G	PDPN
504	1.68E-54	0.663755	0.204	0.091	2.52E-50	Ac-G	PLA2G5
505	1.77E-54	0.512829	0.53	0.415	2.64E-50	Ac-G	SPTSSA
506	1.88E-54	0.498763	0.351	0.223	2.81E-50	Ac-G	TPST1
507	6.52E-54	0.43409	0.7	0.651	9.74E-50	Ac-G	CHMP2A
508	1.16E-53	0.498764	0.621	0.538	1.73E-49	Ac-G	PRDX4
509	7.93E-53	0.534259	0.363	0.231	1.18E-48	Ac-G	IFT57

510	3.47E-52	0.513639	0.539	0.438	5.18E-48	Ac-G	PON2
511	1.19E-51	0.576559	0.426	0.296	1.77E-47	Ac-G	PKIG
512	4.64E-51	0.529765	0.335	0.202	6.94E-47	Ac-G	BET1
513	1.91E-50	0.492409	0.425	0.296	2.85E-46	Ac-G	ARL1
514	9.73E-50	0.681664	0.212	0.095	1.45E-45	Ac-G	UQCR11
515	1.48E-49	0.45192	0.572	0.472	2.21E-45	Ac-G	MED10
516	6.16E-49	0.396921	0.773	0.741	9.20E-45	Ac-G	WDR83OS
517	6.46E-49	0.498816	0.258	0.136	9.65E-45	Ac-G	FN1
518	4.46E-48	0.395846	0.212	0.098	6.66E-44	Ac-G	PRUNE2
519	2.57E-47	0.400993	0.287	0.16	3.85E-43	Ac-G	SRPX
520	6.78E-47	0.525914	0.358	0.234	1.01E-42	Ac-G	TRIP6
521	1.12E-46	0.504384	0.408	0.288	1.68E-42	Ac-G	MT1F
522	1.29E-46	0.467591	0.22	0.11	1.93E-42	Ac-G	EFNA1
523	1.62E-46	0.482412	0.342	0.216	2.42E-42	Ac-G	COL6A1
524	4.76E-46	0.470323	0.098	0.029	7.12E-42	Ac-G	FBLN5
525	5.20E-46	0.458248	0.103	0.031	7.77E-42	Ac-G	NDUFA7
526	7.58E-46	0.836436	0.366	0.251	1.13E-41	Ac-G	ID3
527	8.67E-46	0.502402	0.269	0.152	1.30E-41	Ac-G	TMEM38B
528	1.18E-45	0.776928	0.366	0.254	1.76E-41	Ac-G	RCAN1
529	1.68E-45	0.467542	0.231	0.122	2.51E-41	Ac-G	SLC2A1
530	3.09E-45	0.491705	0.167	0.07	4.62E-41	Ac-G	CXCL2
531	3.80E-45	0.87789	0.328	0.211	5.68E-41	Ac-G	CCL2
532	7.11E-45	0.450173	0.184	0.086	1.06E-40	Ac-G	SDC4
533	1.40E-44	0.441766	0.148	0.061	2.09E-40	Ac-G	TRIB3
534	1.53E-43	0.476221	0.135	0.053	2.29E-39	Ac-G	FAM181A
535	9.49E-43	0.427946	0.285	0.173	1.42E-38	Ac-G	PTRF
536	7.98E-42	0.444805	0.617	0.528	1.19E-37	Ac-G	C12orf57
537	1.40E-41	0.634222	0.254	0.138	2.10E-37	Ac-G	F13A1
538	3.47E-41	0.514482	0.196	0.1	5.18E-37	Ac-G	ROM1
539	4.62E-41	0.439802	0.488	0.395	6.90E-37	Ac-G	CALU
540	9.03E-40	0.477711	0.271	0.149	1.35E-35	Ac-G	PTGDS
541	1.56E-39	0.401504	0.624	0.545	2.32E-35	Ac-G	POLR2I
542	2.27E-39	0.476729	0.509	0.413	3.39E-35	Ac-G	POLD2
543	3.14E-39	0.505391	0.566	0.472	4.69E-35	Ac-G	CD151
544	4.76E-39	0.595789	0.115	0.044	7.11E-35	Ac-G	CLDN10
545	5.89E-39	0.460821	0.587	0.52	8.81E-35	Ac-G	SSR3
546	1.52E-38	0.517312	0.334	0.22	2.27E-34	Ac-G	CDO1
547	3.12E-38	0.448962	0.691	0.653	4.66E-34	Ac-G	S100A11
548	1.29E-37	0.432459	0.601	0.534	1.92E-33	Ac-G	PDCD5
549	8.81E-36	0.438327	0.489	0.373	1.32E-31	Ac-G	IFI16
550	1.17E-35	0.552142	0.274	0.174	1.75E-31	Ac-G	TUBB6
551	1.91E-35	0.463286	0.304	0.203	2.85E-31	Ac-G	FKBP10
552	2.37E-35	0.406379	0.541	0.462	3.55E-31	Ac-G	COPS8
553	8.04E-35	0.45619	0.346	0.245	1.20E-30	Ac-G	ACTN1
554	2.48E-34	0.394716	0.228	0.13	3.71E-30	Ac-G	IMMP2L
555	1.73E-33	0.424528	0.082	0.026	2.58E-29	Ac-G	SMIM3
556	2.34E-33	0.537756	0.358	0.259	3.49E-29	Ac-G	SSBP2
557	2.48E-33	0.4086	0.492	0.406	3.70E-29	Ac-G	IMPDH2
558	7.64E-33	0.431744	0.19	0.101	1.14E-28	Ac-G	EMP2
559	1.10E-32	0.407945	0.534	0.422	1.65E-28	Ac-G	UCHL1
560	1.29E-32	0.559599	0.171	0.086	1.92E-28	Ac-G	APOL2

561	3.43E-32	0.421772	0.262	0.169	5.13E-28	Ac-G	AK4
562	4.42E-32	0.550903	0.583	0.482	6.61E-28	Ac-G	MDK
563	5.43E-32	0.42045	0.467	0.367	8.12E-28	Ac-G	DNAJB9
564	7.90E-32	0.431628	0.135	0.062	1.18E-27	Ac-G	GDF15
565	9.72E-31	0.393781	0.098	0.038	1.45E-26	Ac-G	PGAM2
566	1.97E-30	0.42671	0.585	0.552	2.94E-26	Ac-G	CD59
567	2.24E-30	0.53721	0.449	0.362	3.35E-26	Ac-G	RAB13
568	2.96E-30	0.672958	0.211	0.128	4.43E-26	Ac-G	BBOX1
569	6.12E-30	0.575415	0.266	0.175	9.14E-26	Ac-G	PPIC
570	7.56E-30	0.395082	0.164	0.087	1.13E-25	Ac-G	HRH1
571	1.18E-29	0.567547	0.434	0.343	1.76E-25	Ac-G	PMP2
572	1.49E-29	0.402404	0.366	0.274	2.22E-25	Ac-G	ENO2
573	1.93E-29	0.401772	0.115	0.047	2.89E-25	Ac-G	TMBIM4
574	1.09E-28	0.435135	0.395	0.298	1.63E-24	Ac-G	TSPAN7
575	2.10E-28	0.453541	0.473	0.389	3.14E-24	Ac-G	FAM162A
576	7.69E-27	0.41125	0.399	0.302	1.15E-22	Ac-G	SERTAD1
577	5.72E-25	0.401484	0.609	0.573	8.55E-21	Ac-G	PSMB2
578	2.03E-24	0.506994	0.178	0.108	3.04E-20	Ac-G	PTX3
579	6.97E-24	0.39731	0.457	0.384	1.04E-19	Ac-G	AK2
580	1.22E-23	0.457945	0.158	0.087	1.83E-19	Ac-G	SNRPN
581	1.87E-23	0.398309	0.428	0.361	2.80E-19	Ac-G	TMEM9
582	3.53E-23	0.426713	0.253	0.177	5.27E-19	Ac-G	DOK5
583	1.08E-22	0.507027	0.269	0.19	1.61E-18	Ac-G	IGFBP3
584	1.75E-22	0.42741	0.254	0.177	2.62E-18	Ac-G	UAP1
585	3.22E-22	0.403318	0.231	0.155	4.82E-18	Ac-G	TWSG1
586	8.01E-22	0.396643	0.509	0.443	1.20E-17	Ac-G	LGALS3BP
587	2.00E-21	0.39076	0.425	0.349	2.99E-17	Ac-G	UROD
588	7.06E-20	0.457723	0.44	0.385	1.06E-15	Ac-G	GGCT
589	4.62E-19	0.396137	0.209	0.142	6.90E-15	Ac-G	RFX4
590	7.72E-19	0.391932	0.239	0.17	1.15E-14	Ac-G	KIF9
591	4.50E-18	0.46215	0.393	0.333	6.73E-14	Ac-G	CISD1
592	6.02E-18	0.498134	0.34	0.274	9.00E-14	Ac-G	AGTRAP
593	6.42E-18	0.416959	0.22	0.153	9.60E-14	Ac-G	STEAP3
594	1.13E-15	0.410281	0.27	0.213	1.69E-11	Ac-G	EFEMP2
595	1.92E-15	0.449784	0.377	0.325	2.88E-11	Ac-G	AHCYL1
596	1.04E-14	0.415112	0.497	0.473	1.55E-10	Ac-G	UBE2L6
597	1.17E-14	0.393103	0.262	0.199	1.75E-10	Ac-G	HEPN1
598	1.49E-14	0.466731	0.479	0.462	2.23E-10	Ac-G	A2M
599	4.03E-14	0.408256	0.344	0.289	6.02E-10	Ac-G	TMED1
600	2.34E-09	0.393554	0.372	0.338	3.50E-05	Ac-G	BLVRA
601	########	0.534464	0.991	0.996	########	CSCL	MALAT1
602	########	0.585858	0.991	0.985	########	CSCL	GAPDH
603	########	0.931288	0.827	0.611	########	CSCL	PTN
604	########	1.203533	0.752	0.484	########	CSCL	MT3
605	########	0.535905	0.916	0.944	########	CSCL	GNB2L1
606	########	0.732344	0.867	0.845	########	CSCL	DBI
607	########	0.399031	0.977	0.998	########	CSCL	TMSB4X
608	########	0.419022	0.92	0.951	########	CSCL	ACTG1
609	########	0.5114	0.946	0.972	########	CSCL	B2M
610	########	0.803682	0.848	0.784	########	CSCL	MT2A
611	########	0.391846	0.911	0.974	########	CSCL	H3F3B

612	########	0.405399	0.956	0.996	########	CSCL	FTL
613	########	0.550039	0.864	0.808	########	CSCL	TUBA1A
614	########	0.720171	0.794	0.615	########	CSCL	GPM6B
615	########	0.694528	0.838	0.782	5.58E-98	CSCL	CLU
616	2.99E-97	0.766753	0.845	0.799	4.47E-93	CSCL	SEC61G
617	2.31E-96	0.463644	0.892	0.961	3.46E-92	CSCL	FAU
618	8.58E-95	0.504169	0.857	0.898	1.28E-90	CSCL	NDUFA4
619	2.76E-91	0.759138	0.771	0.643	4.12E-87	CSCL	NGFRAP1
620	6.47E-89	0.376295	0.895	0.971	9.67E-85	CSCL	PTMA
621	6.15E-88	0.52101	0.866	0.928	9.19E-84	CSCL	CD63
622	1.27E-87	0.282721	0.934	0.996	1.89E-83	CSCL	ACTB
623	9.75E-87	0.584649	0.82	0.828	1.46E-82	CSCL	EEF1A1
624	4.64E-84	0.367202	0.899	0.978	6.94E-80	CSCL	EIF1
625	3.40E-79	0.710163	0.757	0.731	5.09E-75	CSCL	SRI
626	7.71E-79	0.386061	0.862	0.955	1.15E-74	CSCL	UBC
627	3.74E-77	0.398881	0.899	0.969	5.59E-73	CSCL	TMSB10
628	4.79E-74	0.41007	0.873	0.957	7.17E-70	CSCL	TPT1
629	3.10E-67	0.505422	0.798	0.87	4.64E-63	CSCL	SKP1
630	8.08E-67	0.91756	0.613	0.408	1.21E-62	CSCL	RAMP1
631	4.23E-66	0.315293	0.857	0.955	6.32E-62	CSCL	CFL1
632	7.02E-66	0.659523	0.75	0.783	1.05E-61	CSCL	LDHB
633	1.48E-64	0.889858	0.658	0.535	2.21E-60	CSCL	C1orf61
634	5.81E-64	0.422095	0.799	0.894	8.69E-60	CSCL	HSP90AA1
635	6.10E-58	0.449801	0.791	0.881	9.12E-54	CSCL	PPIA
636	6.84E-57	0.639133	0.716	0.705	1.02E-52	CSCL	PRDX2
637	1.90E-56	0.853621	0.312	0.103	2.85E-52	CSCL	UQCR11
638	1.03E-51	0.770278	0.66	0.562	1.53E-47	CSCL	CD9
639	9.91E-51	0.461887	0.766	0.873	1.48E-46	CSCL	TPI1
640	2.17E-50	0.401488	0.789	0.902	3.25E-46	CSCL	HLA-A
641	3.81E-50	0.408869	0.78	0.905	5.70E-46	CSCL	BTF3
642	6.40E-49	0.430028	0.77	0.893	9.56E-45	CSCL	COX7C
643	1.26E-46	0.887268	0.613	0.527	1.88E-42	CSCL	S100B
644	5.77E-46	0.295106	0.815	0.939	8.62E-42	CSCL	CALM2
645	9.67E-46	0.730048	0.157	0.037	1.45E-41	CSCL	NDUFA7
646	2.33E-45	0.457064	0.742	0.853	3.48E-41	CSCL	ALDOA
647	3.42E-44	0.530228	0.724	0.818	5.10E-40	CSCL	NDUFS5
648	5.78E-44	0.350148	0.785	0.933	8.63E-40	CSCL	NACA
649	1.28E-43	0.301151	0.815	0.949	1.92E-39	CSCL	MYL6
650	2.01E-43	0.334062	0.77	0.887	3.00E-39	CSCL	CHCHD2
651	1.43E-41	0.389467	0.742	0.873	2.14E-37	CSCL	CIRBP
652	1.52E-41	0.376019	0.742	0.874	2.26E-37	CSCL	HSPA8
653	1.77E-41	0.377711	0.766	0.919	2.64E-37	CSCL	COX4I1
654	1.76E-39	0.313413	0.768	0.919	2.63E-35	CSCL	UBB
655	2.10E-37	0.362505	0.742	0.886	3.14E-33	CSCL	HNRNPA1
656	4.96E-37	0.364639	0.717	0.854	7.41E-33	CSCL	HNRNPA2B1
657	1.82E-36	0.437603	0.712	0.839	2.72E-32	CSCL	COX6C
658	1.31E-35	0.6562	0.607	0.587	1.96E-31	CSCL	CKB
659	1.03E-33	0.43863	0.684	0.753	1.54E-29	CSCL	H3F3A
660	1.11E-33	0.878584	0.445	0.301	1.66E-29	CSCL	MEG3
661	8.09E-33	0.481943	0.696	0.802	1.21E-28	CSCL	ENO1
662	6.97E-32	0.852972	0.237	0.099	1.04E-27	CSCL	VSTM2A

663	4.26E-30	0.42018	0.688	0.812	6.36E-26	CSCL	PEBP1
664	2.69E-29	0.795019	0.525	0.465	4.02E-25	CSCL	FXYD6
665	2.37E-28	0.328831	0.728	0.87	3.54E-24	CSCL	HMGB1
666	8.79E-28	0.440589	0.688	0.856	1.31E-23	CSCL	COX6B1
667	1.07E-27	0.958165	0.485	0.384	1.59E-23	CSCL	FABP5
668	1.56E-27	0.400924	0.686	0.837	2.33E-23	CSCL	HINT1
669	7.11E-27	0.587584	0.62	0.624	1.06E-22	CSCL	TIMP1
670	7.86E-27	0.421579	0.684	0.84	1.17E-22	CSCL	UBL5
671	4.47E-26	0.346957	0.695	0.845	6.68E-22	CSCL	DYNLL1
672	2.20E-25	0.565031	0.182	0.068	3.29E-21	CSCL	MTRNR2L10
673	3.37E-25	0.387712	0.695	0.862	5.04E-21	CSCL	TOMM7
674	3.84E-25	0.350132	0.681	0.855	5.73E-21	CSCL	COX6A1
675	7.50E-25	0.560255	0.159	0.055	1.12E-20	CSCL	PSMA2
676	8.12E-25	0.32639	0.696	0.857	1.21E-20	CSCL	COX5B
677	3.46E-24	0.623725	0.56	0.497	5.18E-20	CSCL	CRYAB
678	8.80E-24	0.433553	0.635	0.751	1.32E-19	CSCL	BSG
679	2.23E-21	0.329459	0.716	0.86	3.33E-17	CSCL	HLA-B
680	3.51E-21	0.669189	0.576	0.58	5.24E-17	CSCL	MT1X
681	4.69E-21	0.503778	0.621	0.735	7.01E-17	CSCL	ANXA5
682	5.74E-21	0.342064	0.691	0.88	8.58E-17	CSCL	ATP5G2
683	1.29E-20	0.759901	0.461	0.388	1.92E-16	CSCL	FABP7
684	4.07E-20	0.540815	0.108	0.034	6.08E-16	CSCL	IER3IP1
685	5.75E-20	0.420486	0.665	0.817	8.60E-16	CSCL	PRDX1
686	3.39E-19	0.307062	0.695	0.894	5.07E-15	CSCL	PFDN5
687	6.35E-19	0.36346	0.634	0.799	9.50E-15	CSCL	HNRNPK
688	6.13E-18	0.38293	0.642	0.78	9.17E-14	CSCL	PKM
689	1.36E-17	0.392613	0.639	0.82	2.04E-13	CSCL	NDUFB2
690	1.42E-17	0.323072	0.674	0.862	2.12E-13	CSCL	TMA7
691	1.47E-17	0.284391	0.686	0.879	2.19E-13	CSCL	NPM1
692	2.17E-17	0.48999	0.134	0.052	3.24E-13	CSCL	NDUFB8
693	3.57E-17	0.378868	0.62	0.767	5.33E-13	CSCL	SUB1
694	4.15E-17	0.371941	0.653	0.832	6.20E-13	CSCL	GSTP1
695	5.52E-17	0.382331	0.632	0.782	8.24E-13	CSCL	NDUFA13
696	1.56E-16	0.915411	0.354	0.278	2.33E-12	CSCL	BCAN
697	2.12E-16	0.447474	0.611	0.776	3.18E-12	CSCL	PARK7
698	5.67E-16	0.465121	0.595	0.712	8.47E-12	CSCL	7-Sep
699	6.71E-16	0.636283	0.501	0.485	1.00E-11	CSCL	LGALS3
700	9.06E-16	0.482856	0.606	0.758	1.35E-11	CSCL	SNRPD2
701	1.25E-15	0.415978	0.166	0.074	1.87E-11	CSCL	ATXN8OS
702	2.60E-15	0.444635	0.604	0.767	3.89E-11	CSCL	SOD1
703	2.83E-15	0.347847	0.644	0.841	4.24E-11	CSCL	EEF2
704	4.01E-15	0.336051	0.667	0.848	6.00E-11	CSCL	FOS
705	4.08E-15	0.363066	0.63	0.81	6.10E-11	CSCL	UQCRQ
706	1.55E-14	0.62064	0.255	0.154	2.32E-10	CSCL	APOD
707	1.97E-14	0.689475	0.475	0.474	2.95E-10	CSCL	TSC22D4
708	2.57E-14	0.492673	0.482	0.486	3.84E-10	CSCL	NOVA1
709	4.92E-14	0.674577	0.421	0.391	7.35E-10	CSCL	PTPRZ1
710	5.54E-14	0.3878	0.553	0.561	8.27E-10	CSCL	TUBB2B
711	6.59E-14	0.664568	0.18	0.094	9.85E-10	CSCL	SNRPN
712	1.10E-13	0.508646	0.574	0.65	1.65E-09	CSCL	S100A6
713	3.10E-13	0.373118	0.054	0.015	4.63E-09	CSCL	LINC00869

714	8.10E-13	0.447901	0.581	0.731	1.21E-08	CSCL	NDUFB7
715	2.46E-12	0.419007	0.588	0.671	3.67E-08	CSCL	VIM
716	2.49E-12	0.727419	0.363	0.312	3.72E-08	CSCL	S100A16
717	2.71E-12	0.301383	0.642	0.833	4.04E-08	CSCL	SUMO2
718	3.67E-12	0.383867	0.6	0.782	5.48E-08	CSCL	COPE
719	3.91E-12	0.330855	0.621	0.819	5.84E-08	CSCL	ATP5J2
720	1.67E-11	0.449656	0.616	0.798	2.49E-07	CSCL	CST3
721	2.61E-11	0.29275	0.64	0.813	3.90E-07	CSCL	HLA-C
722	2.65E-11	0.734191	0.384	0.353	3.96E-07	CSCL	BEX1
723	1.01E-10	0.30186	0.623	0.836	1.52E-06	CSCL	TCEB2
724	1.15E-10	0.28448	0.079	0.201	1.71E-06	CSCL	HBA2
725	4.71E-10	0.594657	0.471	0.521	7.04E-06	CSCL	TAGLN2
726	1.01E-09	0.415556	0.562	0.722	1.51E-05	CSCL	TBCA
727	1.05E-09	0.715695	0.394	0.381	1.56E-05	CSCL	IGFBP2
728	1.13E-09	0.380901	0.597	0.784	1.69E-05	CSCL	CALM1
729	1.19E-09	0.27749	0.63	0.873	1.79E-05	CSCL	ATP5L
730	1.22E-09	0.347665	0.586	0.788	1.83E-05	CSCL	ATP5B
731	1.48E-09	0.61748	0.403	0.391	2.21E-05	CSCL	SPARC
732	1.75E-09	0.46567	0.471	0.512	2.62E-05	CSCL	GPM6A
733	1.83E-09	0.37938	0.579	0.769	2.74E-05	CSCL	RAN
734	1.12E-08	0.578951	0.122	0.065	0.000167	CSCL	FAM195B
735	1.15E-08	0.519399	0.492	0.551	0.000172	CSCL	S100A10
736	1.54E-08	0.594054	0.373	0.349	0.000231	CSCL	SPARCL1
737	1.79E-08	0.293612	0.19	0.373	0.000268	CSCL	PDZD11
738	5.87E-08	0.546598	0.112	0.06	0.000877	CSCL	ALDH1L1
739	6.82E-08	0.318106	0.175	0.339	0.001019	CSCL	RFXANK
740	7.19E-08	0.295098	0.162	0.316	0.001074	CSCL	KIF21A
741	9.53E-08	0.455162	0.459	0.521	0.001425	CSCL	TSC22D1
742	1.05E-07	0.570267	0.063	0.027	0.00157	CSCL	C6orf15
743	1.91E-07	0.317454	0.175	0.332	0.002853	CSCL	PEF1
744	2.66E-07	0.640203	0.321	0.289	0.00398	CSCL	HOPX
745	2.99E-07	0.280239	0.194	0.363	0.004469	CSCL	ISOC2
746	3.04E-07	0.741308	0.307	0.27	0.004546	CSCL	IGFBP5
747	3.49E-07	0.285041	0.194	0.36	0.005218	CSCL	WDR61
748	3.96E-07	0.290442	0.244	0.451	0.005912	CSCL	SUCLG1
749	4.40E-07	0.287713	0.131	0.257	0.006579	CSCL	SHISA4
750	6.00E-07	0.363992	0.558	0.764	0.008961	CSCL	ATP5J
751	6.70E-07	0.294685	0.147	0.278	0.010015	CSCL	GSTM3
752	9.70E-07	0.31755	0.094	0.049	0.014496	CSCL	NHSL2
753	1.04E-06	0.314293	0.145	0.276	0.015534	CSCL	TRIB2
754	1.11E-06	0.289445	0.188	0.349	0.016592	CSCL	MANBAL
755	1.11E-06	0.359794	0.047	0.019	0.016659	CSCL	PRKCG
756	1.25E-06	0.353638	0.571	0.771	0.018705	CSCL	EIF3K
757	1.36E-06	0.347503	0.569	0.794	0.020283	CSCL	UQCRH
758	1.61E-06	0.784953	0.157	0.107	0.024108	CSCL	PLA2G5
759	1.72E-06	0.300546	0.243	0.44	0.025761	CSCL	PAFAH1B3
760	1.76E-06	0.307437	0.161	0.298	0.026291	CSCL	TRIM44
761	1.90E-06	0.400963	0.532	0.706	0.028446	CSCL	HNRNPC
762	2.34E-06	0.311757	0.176	0.323	0.035026	CSCL	PAICS
763	2.67E-06	0.321464	0.239	0.429	0.039975	CSCL	IMPDH2
764	2.84E-06	0.309835	0.099	0.197	0.042439	CSCL	ARHGAP12

765	2.96E-06	0.389256	0.447	0.474	0.044297	CSCL	GAP43
766	3.11E-06	0.493085	0.243	0.189	0.046545	CSCL	MTRNR2L8
767	3.15E-06	0.594188	0.38	0.385	0.047099	CSCL	MT1E
768	4.18E-06	0.365104	0.55	0.739	0.062492	CSCL	ANAPC11
769	4.29E-06	0.495251	0.195	0.14	0.06413	CSCL	MGST1
770	5.47E-06	0.651348	0.101	0.06	0.081735	CSCL	ATP1A2
771	6.28E-06	0.366579	0.208	0.368	0.093891	CSCL	ISG15
772	6.74E-06	0.411617	0.534	0.695	0.10071	CSCL	C19orf53
773	7.32E-06	0.318222	0.169	0.305	0.109415	CSCL	C14orf1
774	7.92E-06	0.519296	0.202	0.145	0.118402	CSCL	SLPI
775	7.96E-06	0.579309	0.127	0.082	0.118891	CSCL	GPR37L1
776	8.11E-06	0.279535	0.141	0.256	0.121214	CSCL	VCAN
777	8.50E-06	0.296824	0.068	0.033	0.126991	CSCL	SMIM3
778	8.80E-06	0.29054	0.106	0.203	0.131433	CSCL	ZEB1
779	9.20E-06	0.28485	0.288	0.509	0.137447	CSCL	CAMLG
780	1.11E-05	0.336943	0.553	0.761	0.166306	CSCL	CNBP
781	1.12E-05	0.281065	0.237	0.416	0.166812	CSCL	PNN
782	1.20E-05	0.573758	0.257	0.216	0.178819	CSCL	C1R
783	1.63E-05	0.343922	0.557	0.758	0.243775	CSCL	NEDD8
784	1.82E-05	0.283037	0.237	0.411	0.271354	CSCL	FAM162A
785	1.89E-05	0.329566	0.26	0.458	0.283018	CSCL	PEA15
786	2.42E-05	0.349438	0.105	0.195	0.361183	CSCL	LINC00665
787	2.91E-05	0.319356	0.115	0.21	0.434161	CSCL	RGMA
788	3.10E-05	0.375794	0.19	0.332	0.462952	CSCL	C16orf45
789	3.21E-05	0.484249	0.475	0.531	0.480346	CSCL	CHI3L1
790	4.11E-05	0.291107	0.309	0.544	0.614472	CSCL	RWDD1
791	4.12E-05	0.467838	0.497	0.659	0.615803	CSCL	GNAS
792	4.59E-05	0.292686	0.079	0.152	0.68566	CSCL	FBXO17
793	5.31E-05	0.686125	0.202	0.165	0.793967	CSCL	AQP4
794	5.91E-05	0.387784	0.176	0.304	0.883907	CSCL	FAM229B
795	7.05E-05	0.338676	0.195	0.332	1	CSCL	CETN2
796	7.72E-05	0.312841	0.148	0.257	1	CSCL	HEY1
797	8.27E-05	0.638482	0.262	0.229	1	CSCL	C8orf4
798	8.33E-05	0.285967	0.579	0.778	1	CSCL	LDHA
799	9.17E-05	0.278667	0.098	0.178	1	CSCL	RNF180
800	9.21E-05	0.279423	0.22	0.371	1	CSCL	FHL1
801	9.83E-05	0.296007	0.279	0.472	1	CSCL	PNKD
802	9.95E-05	0.385035	0.545	0.757	1	CSCL	WDR83OS
803	0.000109	0.379031	0.168	0.285	1	CSCL	SDR39U1
804	0.000112	0.277504	0.297	0.509	1	CSCL	PSMC4
805	0.000115	0.501089	0.344	0.358	1	CSCL	PMP2
806	0.000126	0.303324	0.117	0.204	1	CSCL	FAM46A
807	0.000135	0.296232	0.209	0.353	1	CSCL	POLR2J3
808	0.000136	0.341829	0.194	0.327	1	CSCL	DCAF13
809	0.000139	0.609706	0.342	0.358	1	CSCL	NDRG2
810	0.000146	0.511629	0.059	0.032	1	CSCL	MIR7-3HG
811	0.000147	0.411776	0.237	0.196	1	CSCL	MTRNR2L12
812	0.000149	0.350608	0.251	0.417	1	CSCL	IFI6
813	0.000155	0.583428	0.365	0.394	1	CSCL	OCIAD2
814	0.000157	0.625172	0.188	0.153	1	CSCL	ALDOC
815	0.000159	0.283932	0.108	0.19	1	CSCL	LINC00662

816	0.000171	0.300795	0.264	0.442	1	CSCL	SPTSSA
817	0.000185	0.514396	0.346	0.349	1	CSCL	GFAP
818	0.000189	0.347769	0.124	0.214	1	CSCL	RP11-14N7.2
819	0.000228	0.292189	0.096	0.17	1	CSCL	RND2
820	0.000239	0.285699	0.253	0.424	1	CSCL	RHOBTB3
821	0.000254	0.367023	0.206	0.343	1	CSCL	QTRT1
822	0.000265	0.323964	0.532	0.72	1	CSCL	EID1
823	0.000266	0.306129	0.541	0.756	1	CSCL	GABARAPL2
824	0.000277	0.321168	0.176	0.291	1	CSCL	GADD45A
825	0.000309	0.426026	0.372	0.381	1	CSCL	ZNF90
826	0.000312	0.294624	0.073	0.135	1	CSCL	KLHL4
827	0.000339	0.434654	0.096	0.063	1	CSCL	BGN
828	0.000347	0.314446	0.08	0.144	1	CSCL	BCAT2
829	0.000348	0.421094	0.433	0.49	1	CSCL	GADD45B
830	0.000356	0.437996	0.417	0.477	1	CSCL	CNN3
831	0.000366	0.363497	0.195	0.323	1	CSCL	PKIG
832	0.000398	0.310312	0.147	0.244	1	CSCL	TRIM9
833	0.000398	0.302305	0.192	0.313	1	CSCL	MT1F
834	0.000403	0.289995	0.332	0.572	1	CSCL	SDHC
835	0.000409	0.339747	0.052	0.028	1	CSCL	MT1A
836	0.000416	0.308407	0.291	0.487	1	CSCL	UBE2L6
837	0.000446	0.449317	0.162	0.123	1	CSCL	CAV1
838	0.000483	0.357743	0.126	0.211	1	CSCL	C1orf21
839	0.000484	0.351057	0.215	0.356	1	CSCL	MAGED1
840	0.000501	0.29473	0.538	0.742	1	CSCL	HSPE1
841	0.000505	0.401429	0.126	0.209	1	CSCL	CDC42EP4
842	0.000519	0.294361	0.15	0.247	1	CSCL	CREB5
843	0.000532	0.354042	0.209	0.34	1	CSCL	USE1
844	0.000611	0.736383	0.277	0.276	1	CSCL	TTYH1
845	0.000615	0.286437	0.066	0.121	1	CSCL	CAMK2B
846	0.000627	0.319178	0.264	0.438	1	CSCL	C7orf55
847	0.000653	0.386638	0.12	0.201	1	CSCL	PCDH17
848	0.00068	0.306587	0.129	0.212	1	CSCL	TNC
849	0.00068	0.303063	0.173	0.277	1	CSCL	RCAN1
850	0.000687	0.33226	0.119	0.197	1	CSCL	COL9A3
851	0.000702	0.367239	0.157	0.257	1	CSCL	ETV1
852	0.000717	0.458616	0.49	0.667	1	CSCL	CHMP2A
853	0.000808	0.336035	0.131	0.215	1	CSCL	EFHC1
854	0.000874	0.368618	0.302	0.51	1	CSCL	HSD17B10
855	0.00103	0.369403	0.489	0.636	1	CSCL	PRDX6
856	0.001031	0.576123	0.209	0.183	1	CSCL	OLIG1
857	0.001162	0.343933	0.124	0.202	1	CSCL	MYO10
858	0.001187	0.643522	0.272	0.269	1	CSCL	SCG3
859	0.001252	0.337166	0.337	0.574	1	CSCL	NDUFA12
860	0.001349	0.365738	0.155	0.249	1	CSCL	CD82
861	0.001513	0.428733	0.19	0.304	1	CSCL	TMED1
862	0.001656	0.329341	0.049	0.028	1	CSCL	NPPA
863	0.001683	0.300082	0.532	0.756	1	CSCL	PRDX5
864	0.00178	0.298675	0.087	0.145	1	CSCL	HHLA3
865	0.001872	0.31715	0.175	0.274	1	CSCL	SCG2
866	0.001895	0.354838	0.243	0.382	1	CSCL	RAB13

867	0.001912	0.294062	0.077	0.131	1	CSCL	DDAH1
868	0.002069	0.339565	0.101	0.165	1	CSCL	EXOG
869	0.002299	0.370844	0.215	0.339	1	CSCL	AHCYL1
870	0.002398	0.496428	0.133	0.212	1	CSCL	NMB
871	0.002416	0.285368	0.337	0.555	1	CSCL	PDCD5
872	0.002489	0.347992	0.129	0.203	1	CSCL	U2AF1L4
873	0.002597	0.357005	0.045	0.026	1	CSCL	PPP1R1C
874	0.002854	0.313904	0.171	0.266	1	CSCL	THY1
875	0.002892	0.361098	0.129	0.204	1	CSCL	HRSP12
876	0.002952	0.479182	0.403	0.487	1	CSCL	BST2
877	0.002975	0.386594	0.239	0.379	1	CSCL	TMEM9
878	0.003522	0.455898	0.176	0.148	1	CSCL	RARRES2
879	0.003658	0.299341	0.29	0.468	1	CSCL	ATP6V0E2
880	0.003897	0.322052	0.209	0.326	1	CSCL	CRIP2
881	0.003904	0.294535	0.152	0.235	1	CSCL	DSEL
882	0.00393	0.312639	0.157	0.243	1	CSCL	ATP1B2
883	0.003974	0.289513	0.534	0.768	1	CSCL	ATP6V1G1
884	0.004087	0.472012	0.115	0.088	1	CSCL	TMEM100
885	0.00415	0.302011	0.044	0.025	1	CSCL	C2orf74
886	0.004293	0.279368	0.101	0.159	1	CSCL	TPPP3
887	0.004344	0.33367	0.302	0.492	1	CSCL	FKBP3
888	0.005079	0.313479	0.483	0.65	1	CSCL	CD99
889	0.005213	0.41943	0.131	0.101	1	CSCL	MGP
890	0.005366	0.29481	0.105	0.163	1	CSCL	LPL
891	0.005494	0.440817	0.365	0.44	1	CSCL	SOX2
892	0.005544	0.31997	0.277	0.436	1	CSCL	POLD2
893	0.005851	0.312921	0.515	0.737	1	CSCL	SHFM1
894	0.006232	0.434933	0.475	0.657	1	CSCL	POLR2J
895	0.006294	0.476123	0.155	0.234	1	CSCL	HILPDA
896	0.006501	0.333954	0.108	0.168	1	CSCL	DPP6
897	0.007071	0.312982	0.066	0.109	1	CSCL	RDH5
898	0.007875	0.513831	0.382	0.458	1	CSCL	PON2
899	0.008246	0.304088	0.08	0.127	1	CSCL	EPSTI1
900	0.008583	0.366798	0.323	0.52	1	CSCL	SMIM7
2101	0	2.036785	0.838	0.043	0	Neo-G	DLX6-AS1
2102	0	2.013176	0.984	0.089	0	Neo-G	STMN2
2103	0	1.84312	0.865	0.056	0	Neo-G	DLX5
2104	0	1.805289	0.87	0.113	0	Neo-G	NNAT
2105	0	1.631422	0.879	0.091	0	Neo-G	NSG1
2106	0	1.531945	0.869	0.094	0	Neo-G	ELAVL4
2107	0	1.52202	0.848	0.11	0	Neo-G	TAGLN3
2108	0	1.498095	0.962	0.228	0	Neo-G	STMN4
2109	0	1.474861	0.814	0.134	0	Neo-G	DCX
2110	0	1.440814	0.866	0.207	0	Neo-G	SOX11
2111	0	1.419427	0.634	0.045	0	Neo-G	DLX2
2112	0	1.395417	0.951	0.317	0	Neo-G	NFIB
2113	0	1.376866	0.671	0.08	0	Neo-G	THSD7A
2114	0	1.363376	0.632	0.028	0	Neo-G	KLHL35
2115	0	1.353701	0.6	0.031	0	Neo-G	CELF4
2116	0	1.349263	0.86	0.198	0	Neo-G	CRMP1
2117	0	1.346341	0.609	0.038	0	Neo-G	SYT1

2118	0	1.332681	0.998	0.387	0	Neo-G	SOX4
2119	0	1.311801	0.61	0.056	0	Neo-G	DLX1
2120	0	1.302745	0.692	0.148	0	Neo-G	MEIS2
2121	0	1.298242	0.686	0.051	0	Neo-G	TTC9B
2122	0	1.281421	0.71	0.104	0	Neo-G	GPC2
2123	0	1.263259	0.697	0.1	0	Neo-G	RAB3A
2124	0	1.248092	0.987	0.396	0	Neo-G	MLLT11
2125	0	1.246254	0.877	0.231	0	Neo-G	TSPAN13
2126	0	1.210203	0.854	0.251	0	Neo-G	DBN1
2127	0	1.20051	0.523	0.032	0	Neo-G	LY6H
2128	0	1.191546	0.638	0.084	0	Neo-G	PAK3
2129	0	1.179335	0.706	0.162	0	Neo-G	RBFOX2
2130	0	1.177647	0.761	0.234	0	Neo-G	TMEM161B-AS1
2131	0	1.166218	0.647	0.094	0	Neo-G	LBH
2132	0	1.160803	0.78	0.235	0	Neo-G	ATAT1
2133	0	1.148643	0.647	0.112	0	Neo-G	MIAT
2134	0	1.141015	0.657	0.13	0	Neo-G	TMSB15A
2135	0	1.13985	0.651	0.104	0	Neo-G	NRXN1
2136	0	1.12608	0.982	0.424	0	Neo-G	TUBB2A
2137	0	1.125582	0.595	0.12	0	Neo-G	ARL4D
2138	0	1.124502	0.559	0.048	0	Neo-G	DYNC1I1
2139	0	1.124377	0.746	0.219	0	Neo-G	KIF5C
2140	0	1.123346	0.976	0.362	0	Neo-G	PCSK1N
2141	0	1.119244	0.691	0.191	0	Neo-G	RND3
2142	0	1.109209	0.745	0.26	0	Neo-G	CXADR
2143	0	1.1064	0.801	0.257	0	Neo-G	PODXL2
2144	0	1.105799	0.581	0.065	0	Neo-G	ATP1A3
2145	0	1.10416	0.816	0.256	0	Neo-G	ELAVL3
2146	0	1.09535	0.899	0.293	0	Neo-G	APLP1
2147	0	1.091609	0.511	0.024	0	Neo-G	ACTL6B
2148	0	1.084699	0.742	0.223	0	Neo-G	NREP
2149	0	1.084521	0.77	0.222	0	Neo-G	DNER
2150	0	1.078245	0.626	0.085	0	Neo-G	SNAP25
2151	0	1.075965	0.791	0.233	0	Neo-G	GSTA4
2152	0	1.07498	0.826	0.203	0	Neo-G	SCG3
2153	0	1.065927	0.52	0.039	0	Neo-G	CELF5
2154	0	1.06415	1	0.497	0	Neo-G	MARCKSL1
2155	0	1.064108	0.969	0.468	0	Neo-G	TERF2IP
2156	0	1.062935	0.678	0.155	0	Neo-G	ZC2HC1A
2157	0	1.058102	0.531	0.092	0	Neo-G	ASIC4
2158	0	1.057604	0.964	0.377	0	Neo-G	UCHL1
2159	0	1.055144	0.446	0.012	0	Neo-G	MYT1L
2160	0	1.054799	0.587	0.14	0	Neo-G	FNBP1L
2161	0	1.013201	0.676	0.119	0	Neo-G	TCEAL2
2162	0	1.01251	0.777	0.194	0	Neo-G	BEX2
2163	0	1.007124	0.958	0.477	0	Neo-G	TCF4
2164	0	1.003805	0.753	0.231	0	Neo-G	MAPT
2165	0	0.994147	0.406	0.033	0	Neo-G	GNG3
2166	0	0.98651	0.991	0.485	0	Neo-G	MAP1B
2167	0	0.986113	0.999	0.508	0	Neo-G	TUBB2B
2168	0	0.970242	0.468	0.087	0	Neo-G	CDCA7

2169	0	0.968506	0.796	0.291	0	Neo-G	MAP2
2170	0	0.967204	0.449	0.034	0	Neo-G	ELAVL2
2171	0	0.964395	0.462	0.049	0	Neo-G	PKIA
2172	0	0.964245	0.464	0.068	0	Neo-G	CD200
2173	0	0.963158	0.564	0.136	0	Neo-G	MEX3A
2174	0	0.955319	0.435	0.021	0	Neo-G	CELF3
2175	0	0.952956	0.352	0.032	0	Neo-G	RPRM
2176	0	0.950512	0.709	0.281	0	Neo-G	LINC00461
2177	0	0.941165	0.46	0.056	0	Neo-G	CSRNP3
2178	0	0.939037	0.43	0.043	0	Neo-G	HOXC9
2179	0	0.931032	0.422	0.029	0	Neo-G	SCN3B
2180	0	0.918934	0.46	0.057	0	Neo-G	GDAP1L1
2181	0	0.918516	0.415	0.048	0	Neo-G	DUSP26
2182	0	0.917867	0.853	0.295	0	Neo-G	BEX1
2183	0	0.914987	0.513	0.072	0	Neo-G	TCEAL5
2184	0	0.913251	0.751	0.254	0	Neo-G	TCEAL7
2185	0	0.912929	0.999	0.593	0	Neo-G	STMN1
2186	0	0.907663	0.935	0.472	0	Neo-G	TTC3
2187	0	0.906824	0.471	0.081	0	Neo-G	AC004540.4
2188	0	0.89981	0.491	0.091	0	Neo-G	SYP
2189	0	0.896494	0.773	0.316	0	Neo-G	KLC1
2190	0	0.892563	0.346	0.021	0	Neo-G	DLX6
2191	0	0.887991	0.475	0.078	0	Neo-G	ATCAY
2192	0	0.887119	0.925	0.411	0	Neo-G	RBP1
2193	0	0.884832	0.519	0.095	0	Neo-G	SLC38A1
2194	0	0.883453	0.98	0.454	0	Neo-G	GPM6A
2195	0	0.873553	0.419	0.055	0	Neo-G	CDK5R1
2196	0	0.873036	0.368	0.034	0	Neo-G	LINC01102
2197	0	0.857557	0.316	0.003	0	Neo-G	SLC32A1
2198	0	0.849555	0.393	0.033	0	Neo-G	SRRM3
2199	0	0.84664	0.677	0.203	0	Neo-G	GADD45G
2200	0	0.845424	0.433	0.07	0	Neo-G	KLRC2
2201	0	0.827248	0.394	0.057	0	Neo-G	FAM57B
2202	0	0.820525	0.808	0.383	0	Neo-G	PHF14
2203	0	0.817549	0.41	0.048	0	Neo-G	CHGB
2204	0	0.811694	0.412	0.063	0	Neo-G	HOXA7
2205	0	0.808522	0.392	0.056	0	Neo-G	TMEM35
2206	0	0.797997	0.361	0.035	0	Neo-G	VGF
2207	0	0.78914	0.86	0.419	0	Neo-G	UBE2E3
2208	0	0.787276	0.813	0.385	0	Neo-G	PAFAH1B3
2209	0	0.784308	0.408	0.06	0	Neo-G	RUNDC3A
2210	0	0.781135	0.286	0.008	0	Neo-G	PDZRN4
2211	0	0.778392	0.286	0.011	0	Neo-G	SP9
2212	0	0.775686	0.375	0.057	0	Neo-G	LINC00632
2213	0	0.774961	0.339	0.035	0	Neo-G	STX1A
2214	0	0.774121	0.288	0.008	0	Neo-G	FABP6
2215	0	0.773727	0.357	0.05	0	Neo-G	REEP1
2216	0	0.769982	0.342	0.027	0	Neo-G	SMIM18
2217	0	0.762788	0.915	0.414	0	Neo-G	FXYD6
2218	0	0.761964	0.822	0.41	0	Neo-G	HDAC2
2219	0	0.760268	0.389	0.06	0	Neo-G	BEX5

2220	0	0.754897	0.306	0.01	0	Neo-G	INA
2221	0	0.75444	0.333	0.042	0	Neo-G	SBK1
2222	0	0.752338	0.951	0.511	0	Neo-G	PTMS
2223	0	0.746101	0.266	0.012	0	Neo-G	ARX
2224	0	0.72699	0.295	0.013	0	Neo-G	SYT5
2225	0	0.723262	0.328	0.041	0	Neo-G	HOXA2
2226	0	0.70756	0.288	0.03	0	Neo-G	ERBB4
2227	0	0.705614	0.298	0.028	0	Neo-G	C1QTNF4
2228	0	0.694725	0.98	0.608	0	Neo-G	YWHAQ
2229	0	0.681648	0.291	0.017	0	Neo-G	PAK7
2230	0	0.67675	0.987	0.656	0	Neo-G	BTG1
2231	0	0.674915	0.285	0.022	0	Neo-G	SORBS2
2232	0	0.67461	0.258	0.015	0	Neo-G	SLCO5A1
2233	0	0.671556	0.276	0.023	0	Neo-G	FGF9
2234	0	0.662602	0.93	0.469	0	Neo-G	TSC22D1
2235	0	0.66029	0.966	0.559	0	Neo-G	MARCKS
2236	0	0.636318	0.248	0.018	0	Neo-G	KIAA1107
2237	0	0.635392	0.227	0.007	0	Neo-G	SRRM4
2238	0	0.622219	0.984	0.642	0	Neo-G	HN1
2239	########	0.736789	0.349	0.049	########	Neo-G	SPOCK1
2240	########	0.87457	0.721	0.293	########	Neo-G	NFIX
2241	########	0.602961	0.241	0.019	########	Neo-G	ONECUT2
2242	########	0.655235	0.278	0.029	########	Neo-G	HOXA3
2243	########	0.77154	0.815	0.345	########	Neo-G	CPE
2244	########	0.716444	0.342	0.048	########	Neo-G	NCAN
2245	########	0.808581	0.481	0.107	########	Neo-G	NKX2-2
2246	########	0.675857	0.284	0.032	########	Neo-G	EFNA3
2247	########	0.73104	0.302	0.039	########	Neo-G	INADL
2248	########	0.795031	0.732	0.331	########	Neo-G	CBX1
2249	########	0.88337	0.59	0.193	########	Neo-G	KIF3A
2250	########	0.702129	0.87	0.466	########	Neo-G	FSCN1
2251	########	0.779458	0.379	0.068	########	Neo-G	NAP1L3
2252	########	0.732775	0.326	0.045	########	Neo-G	RIT2
2253	########	0.653216	0.889	0.5	########	Neo-G	KMT2E
2254	########	0.782551	0.749	0.352	########	Neo-G	YWHAG
2255	########	0.637578	0.888	0.509	########	Neo-G	ZNF428
2256	########	0.818352	0.7	0.284	########	Neo-G	DPYSL3
2257	########	0.825135	0.582	0.186	########	Neo-G	SNN
2258	########	0.866175	0.529	0.156	########	Neo-G	MAPK10
2259	########	0.783285	0.47	0.114	########	Neo-G	GDAP1
2260	########	0.660203	0.301	0.042	########	Neo-G	PRKAR2B
2261	########	0.804789	0.528	0.139	########	Neo-G	HOTAIRM1
2262	########	0.802952	0.518	0.142	########	Neo-G	CCNG2
2263	########	0.718745	0.771	0.373	########	Neo-G	SRPK2
2264	########	0.713178	0.426	0.09	########	Neo-G	KIF1A
2265	########	0.607864	0.876	0.521	########	Neo-G	CBX3
2266	########	0.786746	0.625	0.22	########	Neo-G	ERV3-1
2267	########	0.752204	0.43	0.091	########	Neo-G	SHD
2268	########	0.653196	0.791	0.389	########	Neo-G	SLC25A4
2269	########	0.760785	0.747	0.371	########	Neo-G	MKRN1
2270	########	0.822874	0.6	0.216	########	Neo-G	KLHDC8A

2271	########	0.760659	0.704	0.297	########	Neo-G	NCAM1
2272	########	0.805567	0.628	0.241	########	Neo-G	KLHL7
2273	########	0.765522	0.695	0.278	########	Neo-G	CAMK2N1
2274	########	0.794087	0.65	0.269	########	Neo-G	RUFY3
2275	########	0.663944	0.822	0.394	########	Neo-G	PFN2
2276	########	0.618336	0.271	0.035	########	Neo-G	HOXA5
2277	########	0.783634	0.715	0.339	########	Neo-G	WHSC1L1
2278	########	0.774702	0.307	0.051	########	Neo-G	PLXNA4
2279	########	0.795541	0.645	0.252	########	Neo-G	CHD7
2280	########	0.797133	0.47	0.13	########	Neo-G	KLHL23
2281	########	0.729841	0.733	0.35	########	Neo-G	PSIP1
2282	########	0.776363	0.577	0.204	########	Neo-G	DAAM1
2283	########	0.759463	0.651	0.268	########	Neo-G	KIF21A
2284	########	0.729713	0.676	0.279	########	Neo-G	MAGEH1
2285	########	0.764945	0.465	0.129	########	Neo-G	ZNF821
2286	########	0.721707	0.711	0.331	########	Neo-G	MAP1LC3A
2287	########	0.642349	0.797	0.455	########	Neo-G	KHDRBS1
2288	########	0.679843	0.776	0.414	########	Neo-G	AKAP9
2289	########	0.774319	0.439	0.118	########	Neo-G	BCL7A
2290	########	0.673499	0.765	0.411	########	Neo-G	ATRX
2291	########	0.78399	0.448	0.126	########	Neo-G	NDRG4
2292	########	0.838446	0.412	0.099	########	Neo-G	RGMB
2293	########	0.755552	0.426	0.111	########	Neo-G	ZNF711
2294	########	0.632915	0.695	0.286	########	Neo-G	SCG5
2295	########	0.601918	0.157	0.009	########	Neo-G	NHLH1
2296	########	0.813282	0.487	0.153	########	Neo-G	TMSB15B
2297	########	0.722259	0.35	0.072	########	Neo-G	PCDHB10
2298	########	0.692016	0.7	0.319	########	Neo-G	RSBN1L
2299	########	0.730503	0.602	0.23	########	Neo-G	C3orf14
2300	########	0.79201	0.627	0.27	########	Neo-G	MYCBP2
2301	########	0.757115	0.716	0.345	########	Neo-G	NFIA
2302	########	0.69534	0.38	0.086	########	Neo-G	NRXN2
2303	########	0.780553	0.583	0.227	########	Neo-G	CCDC112
2304	########	0.65231	0.246	0.031	########	Neo-G	CA8
2305	########	0.640986	0.769	0.406	########	Neo-G	PTOV1
2306	########	0.679263	0.278	0.045	########	Neo-G	3-Sep
2307	########	0.702325	0.687	0.304	########	Neo-G	NLRP1
2308	########	0.604964	0.806	0.433	########	Neo-G	RCN2
2309	########	0.735306	0.557	0.202	########	Neo-G	THRA
2310	########	0.696011	0.476	0.143	########	Neo-G	ASNS
2311	########	0.629947	0.327	0.062	########	Neo-G	C14orf132
2312	########	0.7077	0.653	0.292	########	Neo-G	NPDC1
2313	########	0.70334	0.616	0.247	########	Neo-G	HMGB3
2314	########	0.707599	0.309	0.062	########	Neo-G	TOX
2315	########	0.685658	0.555	0.2	########	Neo-G	CDO1
2316	########	0.737264	0.591	0.243	########	Neo-G	SEZ6L2
2317	########	0.757098	0.442	0.133	########	Neo-G	CDKN2D
2318	########	0.724567	0.463	0.147	########	Neo-G	MAPRE3
2319	########	0.751661	0.513	0.179	########	Neo-G	GRIA2
2320	########	0.663514	0.32	0.066	########	Neo-G	GSE1
2321	########	0.645798	0.307	0.062	########	Neo-G	FAXC

2322	########	0.655317	0.712	0.353	########	Neo-G	ZBTB20
2323	########	0.711587	0.592	0.257	########	Neo-G	BLCAP
2324	########	0.70064	0.256	0.042	########	Neo-G	NXPH4
2325	########	0.63581	0.313	0.065	########	Neo-G	CCDC136
2326	########	0.781459	0.519	0.198	########	Neo-G	ACOT7
2327	########	0.654127	0.262	0.044	########	Neo-G	TOX3
2328	########	0.666702	0.384	0.103	########	Neo-G	HSDL1
2329	########	0.746767	0.414	0.12	########	Neo-G	RP3-525N10.2
2330	########	0.93179	0.504	0.198	########	Neo-G	ENC1
2331	########	0.756241	0.566	0.228	########	Neo-G	CENPV
2332	########	0.717384	0.489	0.169	########	Neo-G	MAGI1
2333	########	0.658417	0.335	0.074	########	Neo-G	PEG10
2334	########	0.64923	0.551	0.198	########	Neo-G	ATP1B1
2335	########	0.622994	0.35	0.085	########	Neo-G	NEDD4L
2336	########	0.695981	0.513	0.189	########	Neo-G	TBCC
2337	########	0.642521	0.876	0.439	########	Neo-G	NOVA1
2338	########	0.62378	0.516	0.174	########	Neo-G	PTPRN2
2339	########	0.697228	0.467	0.162	########	Neo-G	PJA1
2340	########	0.651335	0.313	0.071	########	Neo-G	KIAA1549
2341	########	0.698265	0.39	0.107	########	Neo-G	PLK2
2342	########	0.6996	0.493	0.173	########	Neo-G	KLF7
2343	########	0.623496	0.574	0.236	########	Neo-G	BZW2
2344	########	0.716583	0.353	0.093	########	Neo-G	INSM1
2345	########	0.686342	0.561	0.236	########	Neo-G	KIDINS220
2346	########	0.805528	0.621	0.345	########	Neo-G	BASP1
2347	########	0.619694	0.416	0.112	########	Neo-G	DLL3
2348	########	0.639304	0.276	0.056	########	Neo-G	TIAM2
2349	########	0.665716	0.356	0.096	########	Neo-G	STXBP1
2350	########	0.639076	0.417	0.127	########	Neo-G	ANKRD46
2351	########	0.720826	0.503	0.176	########	Neo-G	ARC
2352	########	0.623632	0.639	0.314	########	Neo-G	DYNC1LI2
2353	########	0.626723	0.65	0.335	########	Neo-G	SMARCA4
2354	########	0.763698	0.393	0.122	########	Neo-G	ATP6V1G2
2355	########	0.638968	0.691	0.356	########	Neo-G	H1FX
2356	########	0.600677	0.774	0.449	########	Neo-G	LMO4
2357	########	0.696475	0.357	0.102	########	Neo-G	STARD4-AS1
2358	########	0.628496	0.606	0.289	########	Neo-G	GOLM1
2359	########	0.650952	0.475	0.166	########	Neo-G	CLASP2
2360	########	0.695395	0.441	0.157	########	Neo-G	TRIM36
2361	########	0.647782	0.581	0.266	########	Neo-G	NT5C3A
2362	########	0.643501	0.39	0.116	########	Neo-G	SRGAP3
2363	########	0.637451	0.376	0.112	########	Neo-G	BEND5
2364	########	0.680615	0.396	0.129	########	Neo-G	MYH10
2365	########	0.608249	0.241	0.044	########	Neo-G	NKX2-5
2366	########	0.653733	0.572	0.244	########	Neo-G	KCNQ1OT1
2367	########	0.6206	0.483	0.181	########	Neo-G	ARMCX1
2368	########	0.633085	0.692	0.348	########	Neo-G	ARL4C
2369	########	0.628352	0.665	0.353	########	Neo-G	CXXC5
2370	########	0.61521	0.291	0.07	########	Neo-G	STOX2
2371	########	0.612858	0.38	0.119	########	Neo-G	DPF1
2372	########	0.647933	0.463	0.178	########	Neo-G	ZNF3

2373	########	0.60615	0.605	0.307	########	Neo-G	MAP4
2374	########	0.64082	0.582	0.281	########	Neo-G	CSNK1E
2375	########	0.671704	0.523	0.233	########	Neo-G	TMEM59L
2376	########	0.629923	0.229	0.044	########	Neo-G	HRK
2377	########	0.683947	0.386	0.12	########	Neo-G	B4GALNT1
2378	########	0.663569	0.485	0.203	########	Neo-G	ZSCAN18
2379	########	0.640928	0.471	0.19	########	Neo-G	RSBN1
2380	########	0.631916	0.518	0.234	########	Neo-G	SPATS2
2381	########	0.652338	0.541	0.259	########	Neo-G	PNMA1
2382	########	0.655894	0.531	0.248	########	Neo-G	BRD3
2383	########	0.650475	0.518	0.234	########	Neo-G	CEP170
2384	########	0.651428	0.448	0.18	########	Neo-G	KDM1A
2385	########	0.610021	0.297	0.078	########	Neo-G	RPS6KL1
2386	########	0.634983	0.407	0.144	########	Neo-G	LSAMP
2387	########	0.604758	0.442	0.177	########	Neo-G	KIFAP3
2388	########	0.654055	0.375	0.134	########	Neo-G	DPYSL4
2389	########	0.64868	0.475	0.214	########	Neo-G	KIF2A
2390	########	0.6173	0.426	0.173	########	Neo-G	DCLK2
2391	########	0.610603	0.526	0.238	########	Neo-G	SCG2
2392	########	0.644769	0.346	0.113	########	Neo-G	C11orf96
2393	########	0.633378	0.406	0.162	########	Neo-G	PGAP1
2394	########	0.691215	0.412	0.177	########	Neo-G	NDN
2395	########	0.607768	0.379	0.143	########	Neo-G	PPP2R5B
2396	########	0.644371	0.499	0.236	########	Neo-G	PTPRS
2397	########	0.613884	0.363	0.145	########	Neo-G	PROX1
2398	########	0.669719	0.382	0.163	########	Neo-G	ROBO1
2399	3.18E-90	0.640941	0.285	0.103	4.75E-86	Neo-G	KIF5A
2400	5.60E-70	0.749753	0.45	0.291	8.37E-66	Neo-G	MEG3
3001	0	1.332915	0.667	0.109	0	Opc-G	DLL3
3002	0	1.294482	0.792	0.26	0	Opc-G	HES6
3003	0	1.099339	0.498	0.075	0	Opc-G	NKAIN4
3004	0	0.948055	0.449	0.058	0	Opc-G	VIPR2
3005	0	0.944096	0.418	0.048	0	Opc-G	DLL1
3006	########	1.082536	0.659	0.157	########	Opc-G	ASCL1
3007	########	0.7006	0.26	0.016	########	Opc-G	CA10
3008	########	1.226304	0.62	0.154	########	Opc-G	OLIG2
3009	########	0.622862	0.252	0.016	########	Opc-G	RP11-676J15.1
3010	########	0.890027	0.44	0.066	########	Opc-G	TMEM100
3011	########	0.852667	0.361	0.043	########	Opc-G	NEU4
3012	########	1.160528	0.616	0.155	########	Opc-G	OLIG1
3013	########	0.958427	0.515	0.101	########	Opc-G	SHD
3014	########	1.171555	0.728	0.252	########	Opc-G	BCAN
3015	########	1.052566	0.591	0.153	########	Opc-G	SOX8
3016	########	0.991498	0.552	0.126	########	Opc-G	GLCCI1
3017	########	0.787634	0.319	0.037	########	Opc-G	TNR
3018	########	0.810718	0.919	0.437	########	Opc-G	FXYD6
3019	########	0.806628	0.916	0.457	########	Opc-G	NOVA1
3020	########	0.878588	0.519	0.113	########	Opc-G	ASIC4
3021	########	1.012628	0.745	0.306	########	Opc-G	CCND2
3022	########	0.923505	0.393	0.068	########	Opc-G	BEST3
3023	########	0.8574	0.79	0.374	########	Opc-G	SCD5

3024	########	0.758019	0.918	0.526	########	Opc-G	MARCKSL1
3025	########	0.822986	0.807	0.364	########	Opc-G	PTPRZ1
3026	########	0.988087	0.529	0.145	########	Opc-G	RP11-161M6.2
3027	########	0.960608	0.607	0.206	########	Opc-G	DSEL
3028	########	0.736194	0.276	0.035	########	Opc-G	FERMT1
3029	########	0.868622	0.861	0.424	########	Opc-G	SOX4
3030	########	0.66912	0.288	0.039	########	Opc-G	CCER2
3031	########	0.981601	0.635	0.226	########	Opc-G	RAB3IP
3032	########	0.829561	0.418	0.09	########	Opc-G	CSPG5
3033	########	0.707863	0.382	0.077	########	Opc-G	CACNA1A
3034	########	0.741058	0.438	0.106	########	Opc-G	RFTN2
3035	########	0.867046	0.444	0.116	########	Opc-G	RP11-849I19.1
3036	########	0.497095	0.163	0.012	########	Opc-G	GSX1
3037	########	0.631278	0.963	0.534	########	Opc-G	TUBB2B
3038	########	0.581508	0.19	0.018	########	Opc-G	NPPA
3039	########	0.77087	0.728	0.279	########	Opc-G	MEG3
3040	########	0.616332	0.907	0.483	########	Opc-G	GPM6A
3041	########	0.890564	0.408	0.103	########	Opc-G	LHFPL3
3042	########	0.47218	0.977	0.716	########	Opc-G	SRI
3043	########	0.616089	0.886	0.508	########	Opc-G	S100B
3044	########	0.754293	0.741	0.37	########	Opc-G	MEST
3045	########	0.592352	0.38	0.08	########	Opc-G	CMTM5
3046	########	0.65842	0.858	0.432	########	Opc-G	MLLT11
3047	########	0.764856	0.704	0.324	########	Opc-G	SCRG1
3048	########	0.68052	0.421	0.102	########	Opc-G	AC004540.4
3049	########	0.72732	0.317	0.061	########	Opc-G	FXYD7
3050	########	0.711239	0.385	0.09	########	Opc-G	KLRC2
3051	########	0.670919	0.794	0.416	########	Opc-G	PFN2
3052	########	0.583381	0.94	0.564	########	Opc-G	CKB
3053	########	0.725486	0.367	0.089	########	Opc-G	LRRN1
3054	########	0.704314	0.254	0.041	########	Opc-G	RASL11B
3055	########	0.749427	0.671	0.316	########	Opc-G	LAPTM4B
3056	########	0.755686	0.631	0.245	########	Opc-G	SCG3
3057	########	0.624329	0.875	0.519	########	Opc-G	C1orf61
3058	########	0.795877	0.343	0.079	########	Opc-G	AGAP2-AS1
3059	########	0.723735	0.683	0.322	########	Opc-G	MAP2
3060	########	0.65293	0.527	0.153	########	Opc-G	ELAVL4
3061	########	0.666529	0.733	0.394	########	Opc-G	RHOBTB3
3062	########	0.771193	0.588	0.242	########	Opc-G	CENPV
3063	########	0.702518	0.728	0.329	########	Opc-G	BEX1
3064	########	0.773769	0.353	0.085	########	Opc-G	CDKN2A
3065	########	0.683819	0.483	0.147	########	Opc-G	GPC2
3066	########	0.523306	0.169	0.019	########	Opc-G	AC114730.3
3067	########	0.629943	0.293	0.061	########	Opc-G	LMO1
3068	########	0.959492	0.535	0.233	########	Opc-G	ETV1
3069	########	0.611602	0.268	0.053	########	Opc-G	ARHGEF25
3070	########	0.695149	0.639	0.293	########	Opc-G	PODXL2
3071	########	0.680635	0.467	0.142	########	Opc-G	NRXN1
3072	########	0.444729	0.202	0.029	########	Opc-G	MASP1
3073	########	0.599067	0.757	0.407	########	Opc-G	C7orf55
3074	########	0.713118	0.625	0.277	########	Opc-G	SERPINE2

3075	########	0.539099	0.242	0.044	########	Opc-G	AGAP2
3076	########	0.514798	0.843	0.474	########	Opc-G	MDK
3077	########	0.53016	0.301	0.064	########	Opc-G	OMG
3078	########	0.639426	0.789	0.478	########	Opc-G	EGR1
3079	########	0.593105	0.788	0.413	########	Opc-G	SOX2
3080	########	0.621037	0.786	0.403	########	Opc-G	PCSK1N
3081	########	0.497669	0.181	0.024	########	Opc-G	SMOC1
3082	########	0.655142	0.607	0.246	########	Opc-G	CRMP1
3083	########	0.819859	0.409	0.131	########	Opc-G	B4GALNT1
3084	########	0.688641	0.602	0.274	########	Opc-G	CHD7
3085	########	0.619614	0.359	0.094	########	Opc-G	NTM
3086	########	0.577517	0.279	0.062	########	Opc-G	DSCAM
3087	########	0.595379	0.74	0.407	########	Opc-G	PHF14
3088	########	0.512569	0.866	0.495	########	Opc-G	TSC22D1
3089	########	0.532169	0.208	0.034	########	Opc-G	RP5-1177M21.1
3090	########	0.757595	0.507	0.191	########	Opc-G	ARC
3091	########	0.895928	0.534	0.238	########	Opc-G	DTX3
3092	########	0.610054	0.381	0.108	########	Opc-G	IFITM10
3093	########	0.70747	0.497	0.19	########	Opc-G	FAM181B
3094	########	0.524485	0.286	0.063	########	Opc-G	HOXA10
3095	########	0.518773	0.224	0.04	########	Opc-G	BTBD17
3096	########	0.621537	0.724	0.362	########	Opc-G	NFIB
3097	########	0.613334	0.653	0.284	########	Opc-G	STMN4
3098	########	0.449521	0.199	0.034	1.63E-98	Opc-G	ZDHHC22
3099	########	0.57437	0.254	0.055	3.67E-98	Opc-G	RP11-277P12.20
3100	########	0.653339	0.591	0.256	4.84E-98	Opc-G	SOX11
3101	########	0.924781	0.393	0.138	7.92E-98	Opc-G	PDGFRA
3102	########	0.552736	0.782	0.453	1.77E-97	Opc-G	TSC22D4
3103	########	0.654019	0.576	0.259	2.42E-97	Opc-G	KCNQ1OT1
3104	########	0.52949	0.789	0.416	6.45E-97	Opc-G	UCHL1
3105	########	0.700781	0.348	0.107	1.00E-96	Opc-G	C1QL1
3106	########	0.57591	0.323	0.085	3.56E-96	Opc-G	GDAP1L1
3107	########	0.572752	0.365	0.104	7.06E-96	Opc-G	ATP1A3
3108	########	0.649354	0.66	0.384	7.66E-96	Opc-G	ODC1
3109	########	0.504537	0.278	0.064	7.74E-96	Opc-G	PHACTR3
3110	########	0.572021	0.408	0.129	9.56E-96	Opc-G	NKX2-2
3111	3.26E-99	0.59628	0.622	0.293	4.88E-95	Opc-G	TSPAN7
3112	4.23E-99	0.430648	0.183	0.029	6.32E-95	Opc-G	MYT1
3113	1.02E-98	0.426716	0.182	0.029	1.52E-94	Opc-G	CNTN1
3114	1.60E-98	0.620546	0.614	0.296	2.39E-94	Opc-G	DBN1
3115	1.48E-96	0.464162	0.795	0.531	2.22E-92	Opc-G	POLR2F
3116	2.27E-96	0.56384	0.692	0.364	3.39E-92	Opc-G	NFIA
3117	3.40E-96	0.658041	0.56	0.233	5.09E-92	Opc-G	GADD45G
3118	1.51E-95	0.638982	0.774	0.535	2.25E-91	Opc-G	CCT2
3119	2.42E-95	0.627609	0.535	0.243	3.62E-91	Opc-G	THY1
3120	3.32E-95	0.644851	0.49	0.205	4.96E-91	Opc-G	KCNQ2
3121	2.51E-93	0.846748	0.389	0.142	3.75E-89	Opc-G	9-Mar
3122	3.82E-93	0.667484	0.478	0.204	5.71E-89	Opc-G	ACAP3
3123	8.73E-92	0.438317	0.228	0.048	1.30E-87	Opc-G	JPH4
3124	9.85E-92	0.57176	0.309	0.09	1.47E-87	Opc-G	FAM212B
3125	3.68E-90	0.562384	0.633	0.321	5.49E-86	Opc-G	NCAM1

3126	4.40E-90	0.560099	0.627	0.331	6.58E-86	Opc-G	MAGED1
3127	7.97E-90	0.567541	0.675	0.372	1.19E-85	Opc-G	ZBTB20
3128	9.01E-90	0.568783	0.316	0.093	1.35E-85	Opc-G	NELL2
3129	1.14E-89	0.601104	0.452	0.172	1.70E-85	Opc-G	CADM2
3130	1.45E-89	0.628469	0.365	0.123	2.16E-85	Opc-G	SEZ6L
3131	1.85E-89	0.586775	0.34	0.109	2.77E-85	Opc-G	TSPAN12
3132	3.90E-89	0.712384	0.697	0.477	5.83E-85	Opc-G	CDK4
3133	1.07E-88	0.535083	0.741	0.493	1.60E-84	Opc-G	FSCN1
3134	7.91E-88	0.462327	0.252	0.059	1.18E-83	Opc-G	HOXA2
3135	1.21E-87	0.691024	0.522	0.26	1.81E-83	Opc-G	TRAF4
3136	2.05E-87	0.424152	0.835	0.587	3.07E-83	Opc-G	MARCKS
3137	2.50E-87	0.471963	0.27	0.069	3.73E-83	Opc-G	AMOTL2
3138	4.57E-87	0.708928	0.365	0.125	6.82E-83	Opc-G	MBD6
3139	7.13E-87	0.497855	0.732	0.448	1.07E-82	Opc-G	UBE2E3
3140	3.59E-86	0.441722	0.394	0.129	5.37E-82	Opc-G	SGCE
3141	4.57E-86	0.60029	0.42	0.163	6.83E-82	Opc-G	CLIP2
3142	2.04E-85	0.605251	0.6	0.289	3.04E-81	Opc-G	EGFR
3143	2.18E-85	0.553227	0.551	0.236	3.25E-81	Opc-G	BEX2
3144	2.41E-85	0.488742	0.239	0.055	3.61E-81	Opc-G	TOX3
3145	6.47E-85	0.555411	0.575	0.273	9.67E-81	Opc-G	GSTA4
3146	2.30E-84	0.615494	0.548	0.273	3.44E-80	Opc-G	SLC22A17
3147	2.50E-84	0.640296	0.412	0.164	3.74E-80	Opc-G	ZNF462
3148	4.85E-84	0.552677	0.28	0.078	7.25E-80	Opc-G	AVIL
3149	4.80E-83	0.46796	0.264	0.067	7.17E-79	Opc-G	NCAN
3150	5.91E-83	0.653825	0.352	0.123	8.84E-79	Opc-G	NXPH1
3151	5.05E-82	0.423245	0.781	0.544	7.54E-78	Opc-G	CBX3
3152	1.84E-81	0.597831	0.58	0.316	2.74E-77	Opc-G	SGCB
3153	4.34E-81	0.513655	0.663	0.337	6.49E-77	Opc-G	NDRG2
3154	1.71E-80	0.617127	0.336	0.115	2.56E-76	Opc-G	TNK2
3155	8.57E-80	0.51382	0.307	0.092	1.28E-75	Opc-G	RAB33A
3156	1.12E-79	0.586722	0.576	0.303	1.67E-75	Opc-G	CRIP2
3157	2.33E-79	0.473025	0.701	0.45	3.48E-75	Opc-G	TSPAN3
3158	3.50E-79	0.559774	0.433	0.169	5.24E-75	Opc-G	PHYHIPL
3159	5.28E-79	0.57573	0.417	0.166	7.89E-75	Opc-G	MEX3A
3160	9.94E-79	0.474414	0.23	0.056	1.49E-74	Opc-G	CRB1
3161	1.12E-78	0.543053	0.27	0.078	1.68E-74	Opc-G	MDFI
3162	1.20E-78	0.577864	0.562	0.296	1.80E-74	Opc-G	CSNK1E
3163	7.34E-78	0.625439	0.355	0.13	1.10E-73	Opc-G	SOX6
3164	9.43E-78	0.61351	0.452	0.167	1.41E-73	Opc-G	STMN2
3165	4.85E-77	0.580657	0.562	0.29	7.25E-73	Opc-G	PIK3R1
3166	9.13E-77	0.543511	0.586	0.303	1.36E-72	Opc-G	MAGEH1
3167	1.02E-76	0.670489	0.36	0.136	1.52E-72	Opc-G	METTL1
3168	1.47E-76	0.511854	0.671	0.398	2.20E-72	Opc-G	SRPK2
3169	4.36E-76	0.70207	0.509	0.264	6.51E-72	Opc-G	TSFM
3170	5.14E-76	0.575303	0.38	0.147	7.69E-72	Opc-G	ABAT
3171	1.06E-74	0.550677	0.228	0.059	1.58E-70	Opc-G	LNX1
3172	1.09E-74	0.592216	0.321	0.109	1.62E-70	Opc-G	KIF5A
3173	1.19E-74	0.590053	0.45	0.199	1.77E-70	Opc-G	GRIA2
3174	1.51E-74	0.553737	0.509	0.253	2.25E-70	Opc-G	CADM4
3175	4.35E-74	0.475498	0.252	0.069	6.50E-70	Opc-G	XRCC6BP1
3176	7.97E-74	0.512144	0.481	0.216	1.19E-69	Opc-G	RIC3

3177	1.29E-73	0.570091	0.591	0.322	1.92E-69	Opc-G	NFIX
3178	1.37E-73	0.572529	0.511	0.246	2.05E-69	Opc-G	MCM7
3179	1.48E-73	0.427778	0.772	0.526	2.22E-69	Opc-G	KMT2E
3180	1.87E-73	0.765921	0.452	0.215	2.80E-69	Opc-G	NUP107
3181	3.57E-73	0.493722	0.426	0.164	5.34E-69	Opc-G	HOTAIRM1
3182	4.87E-73	0.458532	0.244	0.067	7.28E-69	Opc-G	CXXC4
3183	1.34E-72	0.496599	0.587	0.281	2.01E-68	Opc-G	TSPAN13
3184	1.98E-72	0.522327	0.347	0.122	2.96E-68	Opc-G	CTTNBP2
3185	1.99E-72	0.544266	0.421	0.162	2.97E-68	Opc-G	TCEAL2
3186	4.09E-72	0.522557	0.336	0.121	6.11E-68	Opc-G	CNTFR
3187	1.61E-71	0.587716	0.429	0.188	2.40E-67	Opc-G	MAGI1
3188	3.10E-71	0.475319	0.291	0.093	4.63E-67	Opc-G	MAGI2
3189	8.09E-71	0.536278	0.247	0.071	1.21E-66	Opc-G	AC009506.1
3190	1.10E-70	0.490936	0.401	0.153	1.64E-66	Opc-G	MIAT
3191	1.16E-70	0.556849	0.41	0.175	1.73E-66	Opc-G	BCHE
3192	3.37E-70	0.430662	0.74	0.475	5.04E-66	Opc-G	RPAIN
3193	7.27E-70	0.488257	0.263	0.081	1.09E-65	Opc-G	ANTXR1
3194	8.00E-70	0.432645	0.218	0.057	1.20E-65	Opc-G	NTRK3
3195	1.58E-69	0.671646	0.495	0.258	2.36E-65	Opc-G	RP11-620J15.3
3196	2.26E-69	0.636462	0.297	0.102	3.37E-65	Opc-G	FRS2
3197	2.47E-69	0.523344	0.529	0.275	3.69E-65	Opc-G	TCF12
3198	3.01E-69	0.690389	0.405	0.176	4.50E-65	Opc-G	SLC35E3
3199	5.14E-68	0.549528	0.335	0.128	7.68E-64	Opc-G	PCDHB16
3200	1.62E-67	0.522772	0.412	0.176	2.42E-63	Opc-G	ST3GAL5
3201	4.97E-67	0.501547	0.343	0.126	7.42E-63	Opc-G	C8orf46
3202	5.61E-67	0.529301	0.429	0.193	8.38E-63	Opc-G	ZNF3
3203	6.65E-67	0.540633	0.356	0.137	9.94E-63	Opc-G	RP3-525N10.2
3204	4.84E-65	0.525669	0.412	0.171	7.23E-61	Opc-G	TMSB15A
3205	5.98E-65	0.566301	0.537	0.312	8.93E-61	Opc-G	LIMA1
3206	6.87E-65	0.530448	0.469	0.225	1.03E-60	Opc-G	THRA
3207	1.27E-64	0.468663	0.578	0.313	1.90E-60	Opc-G	SCG5
3208	4.11E-64	0.533689	0.441	0.209	6.15E-60	Opc-G	HIPK2
3209	5.04E-64	0.482629	0.235	0.069	7.53E-60	Opc-G	CNPY1
3210	9.73E-64	0.497294	0.548	0.318	1.45E-59	Opc-G	FYN
3211	2.14E-63	0.46963	0.274	0.09	3.19E-59	Opc-G	RPS6KL1
3212	4.28E-63	0.579793	0.663	0.501	6.40E-59	Opc-G	DCTN2
3213	4.46E-63	0.476141	0.544	0.263	6.66E-59	Opc-G	DNER
3214	5.22E-63	0.426005	0.193	0.049	7.80E-59	Opc-G	SIX1
3215	5.92E-63	0.52863	0.308	0.113	8.85E-59	Opc-G	METAP1D
3216	8.12E-63	0.422741	0.688	0.432	1.21E-58	Opc-G	MYL6B
3217	9.10E-63	0.677685	0.499	0.282	1.36E-58	Opc-G	TSPAN31
3218	9.78E-63	0.468984	0.56	0.324	1.46E-58	Opc-G	DDR1
3219	1.26E-62	0.60251	0.416	0.195	1.89E-58	Opc-G	CDK6
3220	1.68E-62	0.48804	0.444	0.19	2.51E-58	Opc-G	DCX
3221	1.31E-61	0.481102	0.57	0.31	1.95E-57	Opc-G	LINC00461
3222	3.67E-61	0.623439	0.327	0.129	5.48E-57	Opc-G	SEZ6
3223	5.92E-61	0.561474	0.325	0.125	8.84E-57	Opc-G	C11orf96
3224	1.36E-60	0.509358	0.478	0.248	2.03E-56	Opc-G	PCBP4
3225	3.00E-60	0.565826	0.319	0.12	4.48E-56	Opc-G	RGMB
3226	1.64E-59	0.528388	0.482	0.264	2.45E-55	Opc-G	REPIN1
3227	2.99E-59	0.43334	0.25	0.076	4.47E-55	Opc-G	DUSP26

3228	5.36E-59	0.471221	0.414	0.174	8.01E-55	Opc-G	TAGLN3
3229	5.84E-59	0.496469	0.608	0.377	8.73E-55	Opc-G	H1FX
3230	2.39E-58	0.460927	0.583	0.345	3.57E-54	Opc-G	RSBN1L
3231	3.57E-58	0.509558	0.272	0.098	5.33E-54	Opc-G	TMEM121
3232	9.54E-58	0.511391	0.24	0.078	1.43E-53	Opc-G	FAM84B
3233	3.58E-57	0.432889	0.228	0.07	5.35E-53	Opc-G	AC004540.5
3234	4.35E-57	0.480862	0.36	0.153	6.51E-53	Opc-G	KLHL23
3235	1.30E-56	0.425175	0.635	0.402	1.95E-52	Opc-G	AES
3236	3.50E-56	0.512371	0.275	0.099	5.23E-52	Opc-G	PIP4K2C
3237	6.80E-56	0.456235	0.335	0.13	1.02E-51	Opc-G	PAK3
3238	7.28E-56	0.427681	0.165	0.04	1.09E-51	Opc-G	FGF12
3239	1.34E-55	0.42366	0.659	0.439	2.00E-51	Opc-G	PGRMC1
3240	1.74E-55	0.465702	0.214	0.065	2.59E-51	Opc-G	LRRC17
3241	3.67E-55	0.445234	0.623	0.406	5.48E-51	Opc-G	MIDN
3242	8.69E-55	0.464803	0.28	0.105	1.30E-50	Opc-G	APBB2
3243	1.20E-54	0.566086	0.493	0.29	1.79E-50	Opc-G	FIP1L1
3244	1.28E-54	0.508731	0.348	0.153	1.91E-50	Opc-G	DPP6
3245	4.31E-54	0.537312	0.454	0.238	6.44E-50	Opc-G	HEY1
3246	1.05E-53	0.66288	0.444	0.245	1.57E-49	Opc-G	YEATS4
3247	2.07E-53	0.478124	0.489	0.268	3.10E-49	Opc-G	KLHL7
3248	2.33E-52	0.536227	0.378	0.184	3.49E-48	Opc-G	LINC01003
3249	3.84E-52	0.47278	0.503	0.272	5.74E-48	Opc-G	MAPT
3250	1.10E-51	0.488522	0.465	0.25	1.64E-47	Opc-G	TMEM59L
3251	1.54E-51	0.525687	0.378	0.186	2.30E-47	Opc-G	ZEB1
3252	1.55E-51	0.482201	0.258	0.097	2.32E-47	Opc-G	MMP16
3253	1.55E-51	0.425645	0.291	0.109	2.32E-47	Opc-G	ATCAY
3254	3.82E-51	0.533874	0.347	0.16	5.71E-47	Opc-G	BMP7
3255	5.96E-50	0.439083	0.171	0.046	8.90E-46	Opc-G	CA8
3256	4.42E-49	0.429042	0.477	0.259	6.60E-45	Opc-G	BZW2
3257	1.09E-48	0.625242	0.388	0.2	1.63E-44	Opc-G	MDM2
3258	1.80E-48	0.550233	0.3	0.131	2.69E-44	Opc-G	USP46
3259	3.40E-48	0.428727	0.286	0.114	5.08E-44	Opc-G	NCALD
3260	3.60E-48	0.428296	0.437	0.23	5.38E-44	Opc-G	TMEM98
3261	4.69E-48	0.483794	0.398	0.205	7.02E-44	Opc-G	PBX1
3262	5.42E-48	0.423178	0.519	0.31	8.10E-44	Opc-G	GOLM1
3263	5.60E-48	0.456626	0.38	0.184	8.37E-44	Opc-G	MAPK10
3264	7.63E-48	0.436944	0.483	0.265	1.14E-43	Opc-G	NREP
3265	1.09E-47	0.606667	0.39	0.181	1.63E-43	Opc-G	NNAT
3266	2.94E-46	0.427445	0.465	0.25	4.39E-42	Opc-G	ERV3-1
3267	4.95E-46	0.425984	0.493	0.279	7.40E-42	Opc-G	ATAT1
3268	9.49E-46	0.48444	0.444	0.231	1.42E-41	Opc-G	RND3
3269	1.16E-45	0.440446	0.459	0.262	1.74E-41	Opc-G	NRCAM
3270	3.02E-45	0.459566	0.279	0.108	4.51E-41	Opc-G	TTC9B
3271	3.46E-45	0.49685	0.222	0.077	5.17E-41	Opc-G	FBLN1
3272	7.07E-45	0.448058	0.547	0.339	1.06E-40	Opc-G	BAALC
3273	9.55E-45	0.435823	0.327	0.147	1.43E-40	Opc-G	SNTG1
3274	1.45E-44	0.470402	0.288	0.126	2.16E-40	Opc-G	WSCD1
3275	3.26E-44	0.49652	0.649	0.529	4.88E-40	Opc-G	OS9
3276	3.37E-44	0.481901	0.365	0.187	5.04E-40	Opc-G	MYO10
3277	5.43E-44	0.613045	0.438	0.269	8.12E-40	Opc-G	CTDSP2
3278	2.14E-42	0.444993	0.351	0.171	3.19E-38	Opc-G	SYNE2

3279	3.20E-41	0.422757	0.268	0.114	4.78E-37	Opc-G	AC004158.3
3280	3.90E-41	0.444143	0.274	0.12	5.82E-37	Opc-G	CPXM1
3281	4.35E-41	0.431575	0.499	0.289	6.50E-37	Opc-G	METRN
3282	4.59E-41	0.430152	0.384	0.178	6.86E-37	Opc-G	MTRNR2L1
3283	4.88E-41	0.43011	0.499	0.312	7.30E-37	Opc-G	BAZ2B
3284	5.57E-41	0.461073	0.444	0.252	8.32E-37	Opc-G	PTPRS
3285	7.64E-41	0.441781	0.17	0.055	1.14E-36	Opc-G	RP11-698N11.2
3286	9.30E-41	0.422782	0.405	0.223	1.39E-36	Opc-G	MKLN1
3287	1.54E-40	0.44226	0.254	0.107	2.30E-36	Opc-G	ZIC1
3288	9.63E-39	0.430639	0.254	0.109	1.44E-34	Opc-G	NLGN3
3289	1.38E-37	0.43253	0.437	0.259	2.06E-33	Opc-G	TRIB2
3290	2.22E-37	0.430536	0.402	0.235	3.32E-33	Opc-G	ITGB8
3291	3.01E-37	0.442151	0.446	0.263	4.49E-33	Opc-G	KIF5C
3292	6.37E-36	0.429977	0.321	0.167	9.52E-32	Opc-G	BCAR1
3293	1.31E-35	0.433854	0.254	0.112	1.96E-31	Opc-G	INSM1
3294	2.67E-35	0.46852	0.38	0.221	3.99E-31	Opc-G	RP11-553L6.5
3295	9.06E-35	0.43242	0.319	0.165	1.35E-30	Opc-G	RTKN
3296	2.30E-34	0.438054	0.393	0.232	3.43E-30	Opc-G	CREB5
3297	7.28E-34	0.441803	0.307	0.157	1.09E-29	Opc-G	AASDH
3298	1.12E-33	0.463482	0.284	0.14	1.67E-29	Opc-G	APC2
3299	8.19E-28	0.439915	0.396	0.258	1.22E-23	Opc-G	POLR2B
3300	2.19E-24	0.425425	0.179	0.079	3.27E-20	Opc-G	CASP9
3301	0	1.293274	0.958	0.183	0	Class-G	HOPX
3302	0	1.251146	0.739	0.04	0	Class-G	POSTN
3303	0	1.17982	0.951	0.178	0	Class-G	NES
3304	0	1.156994	0.863	0.12	0	Class-G	ID4
3305	0	1.114532	0.935	0.175	0	Class-G	SOX9
3306	0	1.091745	0.882	0.143	0	Class-G	GPC1
3307	0	1.085898	0.811	0.061	0	Class-G	SEMA6D
3308	0	1.083739	0.988	0.295	0	Class-G	FABP7
3309	0	1.067891	0.716	0.037	0	Class-G	KCNF1
3310	0	1.065348	0.787	0.072	0	Class-G	TPM2
3311	0	1.003196	0.796	0.097	0	Class-G	COL9A3
3312	0	0.995161	0.979	0.286	0	Class-G	IGFBP2
3313	0	0.987681	0.717	0.12	0	Class-G	IGFBP3
3314	0	0.983265	0.798	0.114	0	Class-G	FJX1
3315	0	0.968769	0.877	0.179	0	Class-G	IGFBP7
3316	0	0.968194	0.859	0.122	0	Class-G	POU3F2
3317	0	0.966813	0.866	0.181	0	Class-G	TTYH1
3318	0	0.953681	0.943	0.2	0	Class-G	METRN
3319	0	0.933045	0.902	0.133	0	Class-G	ATP1B2
3320	0	0.917326	0.947	0.206	0	Class-G	EGFR
3321	0	0.914434	0.885	0.137	0	Class-G	SPRY1
3322	0	0.911692	0.83	0.102	0	Class-G	LYPD1
3323	0	0.901653	0.735	0.097	0	Class-G	RP11-395G23.3
3324	0	0.895986	0.814	0.108	0	Class-G	RGMA
3325	0	0.89292	0.741	0.077	0	Class-G	WWTR1
3326	0	0.890984	0.768	0.068	0	Class-G	NPAS3
3327	0	0.886199	0.711	0.083	0	Class-G	COL6A2
3328	0	0.885679	0.776	0.106	0	Class-G	F3
3329	0	0.885435	0.846	0.206	0	Class-G	TNFRSF12A

3330	0	0.860511	0.794	0.077	0	Class-G	CNIH3
3331	0	0.857127	0.886	0.129	0	Class-G	FIBIN
3332	0	0.846681	0.734	0.124	0	Class-G	PMEPA1
3333	0	0.834972	0.733	0.107	0	Class-G	EDNRB
3334	0	0.8333	0.837	0.139	0	Class-G	COL6A1
3335	0	0.831694	0.72	0.074	0	Class-G	SOCS2
3336	0	0.829905	0.687	0.073	0	Class-G	CITED1
3337	0	0.82629	0.87	0.172	0	Class-G	SCG2
3338	0	0.815405	0.84	0.127	0	Class-G	SPRY2
3339	0	0.802805	0.62	0.061	0	Class-G	LHX2
3340	0	0.801911	0.944	0.315	0	Class-G	PHLDA1
3341	0	0.801681	0.69	0.065	0	Class-G	FAM84A
3342	0	0.796015	0.665	0.047	0	Class-G	ARSJ
3343	0	0.792379	0.892	0.17	0	Class-G	TRIB2
3344	0	0.791625	0.953	0.262	0	Class-G	PMP2
3345	0	0.78891	0.972	0.299	0	Class-G	PTPRZ1
3346	0	0.779748	0.733	0.076	0	Class-G	JAG1
3347	0	0.775746	0.859	0.153	0	Class-G	CAMK2D
3348	0	0.775453	0.922	0.211	0	Class-G	MAP2K2
3349	0	0.774586	0.776	0.102	0	Class-G	PRR7
3350	0	0.773001	0.699	0.055	0	Class-G	ITGA7
3351	0	0.771254	0.722	0.099	0	Class-G	SMAD1
3352	0	0.764836	0.665	0.064	0	Class-G	TMEM158
3353	0	0.763235	0.841	0.143	0	Class-G	LHFP
3354	0	0.761202	0.902	0.194	0	Class-G	CDH2
3355	0	0.760181	0.625	0.075	0	Class-G	DCBLD2
3356	0	0.756989	0.737	0.084	0	Class-G	MXRA7
3357	0	0.747	0.672	0.051	0	Class-G	COL4A2
3358	0	0.744698	0.944	0.268	0	Class-G	PTPRA
3359	0	0.744023	0.865	0.206	0	Class-G	NEK6
3360	0	0.742215	0.84	0.119	0	Class-G	METTL7B
3361	0	0.742212	0.453	0.022	0	Class-G	CBLN4
3362	0	0.739113	0.698	0.131	0	Class-G	FAM181B
3363	0	0.729898	0.882	0.195	0	Class-G	RAB34
3364	0	0.7296	0.788	0.112	0	Class-G	SEMA6A
3365	0	0.727998	0.75	0.116	0	Class-G	B4GALT5
3366	0	0.72587	0.639	0.088	0	Class-G	C21orf62
3367	0	0.725629	0.674	0.075	0	Class-G	CHL1
3368	0	0.725506	0.609	0.039	0	Class-G	COL4A1
3369	0	0.722535	0.922	0.235	0	Class-G	NPDC1
3370	0	0.720947	0.939	0.261	0	Class-G	GCSH
3371	0	0.712006	0.686	0.09	0	Class-G	RFFL
3372	0	0.711619	0.628	0.064	0	Class-G	CHST2
3373	0	0.711137	0.816	0.147	0	Class-G	CCDC106
3374	0	0.704713	0.726	0.087	0	Class-G	TLE1
3375	0	0.703961	0.803	0.12	0	Class-G	SERPINH1
3376	0	0.703107	0.814	0.134	0	Class-G	PLS3
3377	0	0.696781	0.545	0.032	0	Class-G	FZD7
3378	0	0.694443	0.915	0.25	0	Class-G	PJA2
3379	0	0.690338	0.791	0.165	0	Class-G	HEY1
3380	0	0.688168	0.634	0.057	0	Class-G	PIPOX

3381	0	0.686906	0.927	0.232	0	Class-G	MSI2
3382	0	0.686871	0.702	0.1	0	Class-G	DHCR7
3383	0	0.685707	0.981	0.349	0	Class-G	SOX2
3384	0	0.680749	0.651	0.122	0	Class-G	HES4
3385	0	0.673406	0.839	0.179	0	Class-G	TSPAN5
3386	0	0.673096	0.56	0.036	0	Class-G	LGR4
3387	0	0.669974	0.865	0.208	0	Class-G	ZNF580
3388	0	0.667047	0.682	0.132	0	Class-G	NMB
3389	0	0.665458	0.921	0.261	0	Class-G	BAALC
3390	0	0.665056	0.802	0.154	0	Class-G	EPN2
3391	0	0.662487	0.944	0.213	0	Class-G	S100A16
3392	0	0.662388	0.575	0.083	0	Class-G	GAS1
3393	0	0.660336	0.744	0.132	0	Class-G	SEPN1
3394	0	0.66022	0.756	0.136	0	Class-G	KHDRBS3
3395	0	0.66	0.496	0.049	0	Class-G	SLC4A4
3396	0	0.659286	0.824	0.145	0	Class-G	TRIM9
3397	0	0.658854	0.688	0.095	0	Class-G	PRRX1
3398	0	0.655596	0.85	0.223	0	Class-G	CHPT1
3399	0	0.644175	0.646	0.083	0	Class-G	SACS
3400	0	0.644073	0.8	0.17	0	Class-G	KLHDC8A
3401	0	0.643968	0.918	0.289	0	Class-G	TCEA2
3402	0	0.643333	0.717	0.095	0	Class-G	MRC2
3403	0	0.641362	0.8	0.126	0	Class-G	FKBP10
3404	0	0.640595	0.907	0.255	0	Class-G	NLRP1
3405	0	0.640012	0.64	0.075	0	Class-G	MLC1
3406	0	0.639277	0.747	0.1	0	Class-G	PPIC
3407	0	0.639097	0.521	0.03	0	Class-G	CPNE4
3408	0	0.63758	0.717	0.144	0	Class-G	KCNQ2
3409	0	0.633461	0.638	0.091	0	Class-G	CTXN1
3410	0	0.631903	0.805	0.17	0	Class-G	FLNA
3411	0	0.625101	0.519	0.039	0	Class-G	EFNB2
3412	0	0.624884	0.66	0.084	0	Class-G	OSBPL6
3413	0	0.619908	0.799	0.156	0	Class-G	ITGB8
3414	0	0.617794	0.993	0.405	0	Class-G	NOVA1
3415	0	0.617605	0.847	0.233	0	Class-G	MMP24-AS1
3416	0	0.61699	0.898	0.234	0	Class-G	LIMA1
3417	0	0.616663	0.984	0.392	0	Class-G	CNN3
3418	0	0.61652	0.77	0.133	0	Class-G	WLS
3419	0	0.615838	0.745	0.138	0	Class-G	EFEMP2
3420	0	0.613047	0.914	0.298	0	Class-G	DST
3421	0	0.61184	0.582	0.042	0	Class-G	ACSS3
3422	0	0.611621	0.728	0.128	0	Class-G	BICD1
3423	0	0.609234	0.582	0.061	0	Class-G	NPTXR
3424	0	0.608536	0.602	0.065	0	Class-G	DPF3
3425	0	0.607114	0.44	0.075	0	Class-G	ID1
3426	0	0.60652	0.606	0.112	0	Class-G	SRPX
3427	0	0.60613	0.695	0.107	0	Class-G	TJP1
3428	0	0.605558	0.569	0.058	0	Class-G	EVA1B
3429	0	0.603942	0.907	0.271	0	Class-G	GATM
3430	0	0.603884	0.458	0.017	0	Class-G	EPHA3
3431	0	0.602097	0.637	0.082	0	Class-G	ZFHX4

3432	0	0.601285	0.777	0.197	0	Class-G	FAM210B
3433	0	0.598784	0.589	0.067	0	Class-G	RP11-71N10.1
3434	0	0.597384	0.908	0.232	0	Class-G	CD320
3435	0	0.596365	0.862	0.214	0	Class-G	RRBP1
3436	0	0.596016	0.737	0.153	0	Class-G	TM7SF2
3437	0	0.591924	0.357	0.028	0	Class-G	RP3-428L16.2
3438	0	0.590822	0.676	0.094	0	Class-G	GRIA3
3439	0	0.589933	0.626	0.117	0	Class-G	HMGCS1
3440	0	0.589372	0.889	0.262	0	Class-G	9-Sep
3441	0	0.587413	0.838	0.195	0	Class-G	FZD3
3442	0	0.587307	0.585	0.057	0	Class-G	PLEKHA4
3443	0	0.586065	0.8	0.179	0	Class-G	CHPF
3444	0	0.585784	0.727	0.126	0	Class-G	FERMT2
3445	0	0.585362	0.763	0.151	0	Class-G	TLK1
3446	0	0.58275	0.585	0.102	0	Class-G	LMO2
3447	0	0.578949	0.859	0.271	0	Class-G	PPP1R14B
3448	0	0.578876	0.841	0.157	0	Class-G	ETV1
3449	0	0.576806	0.616	0.102	0	Class-G	EHBP1
3450	0	0.576284	0.828	0.189	0	Class-G	SSBP4
3451	0	0.575741	0.672	0.134	0	Class-G	RNF19A
3452	0	0.573492	0.708	0.108	0	Class-G	PTRF
3453	0	0.573445	0.697	0.104	0	Class-G	NAT14
3454	0	0.572657	0.86	0.227	0	Class-G	STMN3
3455	0	0.572225	0.591	0.07	0	Class-G	BTBD3
3456	0	0.572198	0.47	0.021	0	Class-G	PDGFD
3457	0	0.571266	0.716	0.126	0	Class-G	SPRED1
3458	0	0.569951	0.767	0.158	0	Class-G	ABHD17A
3459	0	0.569413	0.608	0.093	0	Class-G	HOMER1
3460	0	0.567637	0.841	0.239	0	Class-G	CAMK2N1
3461	0	0.567394	0.669	0.102	0	Class-G	DAG1
3462	0	0.565163	0.683	0.129	0	Class-G	PRKCA
3463	0	0.563416	0.508	0.04	0	Class-G	SFRP1
3464	0	0.562061	0.868	0.257	0	Class-G	COPRS
3465	0	0.561826	0.829	0.204	0	Class-G	TCF12
3466	0	0.561737	0.626	0.115	0	Class-G	ROBO1
3467	0	0.560661	0.91	0.247	0	Class-G	DDR1
3468	0	0.559555	0.65	0.127	0	Class-G	AKAP7
3469	0	0.559349	0.658	0.132	0	Class-G	MSMO1
3470	0	0.558458	0.497	0.053	0	Class-G	ZNF385D
3471	0	0.557838	0.886	0.244	0	Class-G	FYN
3472	0	0.556671	0.487	0.042	0	Class-G	ELOVL2
3473	0	0.555416	0.469	0.045	0	Class-G	FGFBP3
3474	0	0.554016	0.87	0.236	0	Class-G	VGLL4
3475	0	0.553862	0.88	0.29	0	Class-G	MAP1LC3A
3476	0	0.551751	0.726	0.15	0	Class-G	HYAL2
3477	0	0.551154	0.713	0.17	0	Class-G	PDLIM7
3478	0	0.55019	0.472	0.023	0	Class-G	TLCD1
3479	0	0.54916	0.789	0.188	0	Class-G	FMNL2
3480	0	0.548695	0.716	0.126	0	Class-G	REEP2
3481	0	0.548478	0.931	0.347	0	Class-G	C17orf89
3482	0	0.547205	0.699	0.125	0	Class-G	TMEM54

3483	0	0.547108	0.83	0.224	0	Class-G	SLC35B2
3484	0	0.547006	0.715	0.146	0	Class-G	AGT
3485	0	0.546114	0.802	0.243	0	Class-G	CTDNEP1
3486	0	0.545503	0.515	0.079	0	Class-G	SLC1A2
3487	0	0.54489	0.843	0.217	0	Class-G	SPECC1
3488	0	0.544734	0.535	0.072	0	Class-G	WSCD1
3489	0	0.544215	0.931	0.299	0	Class-G	CXXC5
3490	0	0.544126	0.848	0.246	0	Class-G	ECI2
3491	0	0.543886	0.729	0.147	0	Class-G	OGFOD3
3492	0	0.543213	0.71	0.15	0	Class-G	NRP2
3493	0	0.54241	0.775	0.174	0	Class-G	SH3GL1
3494	0	0.541961	0.626	0.113	0	Class-G	TRIM47
3495	0	0.541797	0.818	0.172	0	Class-G	PCDH9
3496	0	0.541778	0.756	0.12	0	Class-G	TNC
3497	0	0.539709	0.645	0.106	0	Class-G	LDLRAD3
3498	0	0.539389	0.823	0.22	0	Class-G	PPP2CB
3499	0	0.538647	0.836	0.224	0	Class-G	KIF21A
3500	0	0.537896	0.496	0.048	0	Class-G	KCNE4
3501	0	0.537382	0.785	0.172	0	Class-G	RBMS1
3502	0	0.536798	0.603	0.1	0	Class-G	NETO2
3503	0	0.533951	0.578	0.068	0	Class-G	SLC25A23
3504	0	0.533802	0.592	0.073	0	Class-G	COLGALT2
3505	0	0.533772	0.649	0.115	0	Class-G	DOK5
3506	0	0.532608	0.811	0.206	0	Class-G	CRLS1
3507	0	0.531555	0.848	0.224	0	Class-G	11-Sep
3508	0	0.528411	0.874	0.261	0	Class-G	SLC39A3
3509	0	0.527789	0.567	0.092	0	Class-G	WEE1
3510	0	0.525798	0.86	0.281	0	Class-G	ATP2B1
3511	0	0.52561	0.485	0.044	0	Class-G	IGSF10
3512	0	0.525176	0.635	0.093	0	Class-G	GCAT
3513	0	0.524886	0.517	0.038	0	Class-G	RHOJ
3514	0	0.523652	0.912	0.276	0	Class-G	FHL1
3515	0	0.523277	0.856	0.243	0	Class-G	MACF1
3516	0	0.522936	0.93	0.304	0	Class-G	SYT11
3517	0	0.522314	0.809	0.188	0	Class-G	GIPC1
3518	0	0.519796	0.475	0.047	0	Class-G	TMEM132B
3519	0	0.519377	0.447	0.042	0	Class-G	SOX21
3520	0	0.519317	0.732	0.159	0	Class-G	BCAT1
3521	0	0.518625	0.658	0.131	0	Class-G	DKK3
3522	0	0.51742	0.498	0.052	0	Class-G	CHST7
3523	0	0.517402	0.783	0.196	0	Class-G	TRAF4
3524	0	0.517225	0.554	0.065	0	Class-G	CERS1
3525	0	0.516516	0.703	0.149	0	Class-G	HEBP2
3526	0	0.515022	0.412	0.038	0	Class-G	LMCD1
3527	0	0.514973	0.579	0.092	0	Class-G	HIVEP3
3528	0	0.513914	0.835	0.218	0	Class-G	C5orf24
3529	0	0.51382	0.773	0.177	0	Class-G	OXLD1
3530	0	0.513641	0.483	0.053	0	Class-G	SLC35F2
3531	0	0.512981	0.583	0.074	0	Class-G	GNG12
3532	0	0.512593	0.515	0.053	0	Class-G	LAMA5
3533	0	0.512275	0.893	0.292	0	Class-G	TSEN34

3534	0	0.512202	0.668	0.125	0	Class-G	SPATC1L
3535	0	0.511877	0.634	0.124	0	Class-G	AKAP12
3536	0	0.51186	0.579	0.078	0	Class-G	DMWD
3537	0	0.510888	0.497	0.045	0	Class-G	LAMC1
3538	0	0.505183	0.46	0.04	0	Class-G	THSD1
3539	0	0.504692	0.48	0.068	0	Class-G	SERINC2
3540	0	0.50443	0.733	0.168	0	Class-G	HOMER3
3541	0	0.504186	0.76	0.181	0	Class-G	ACTN1
3542	0	0.5039	0.592	0.078	0	Class-G	VWA1
3543	0	0.503573	0.491	0.048	0	Class-G	TNFRSF19
3544	0	0.503538	0.446	0.034	0	Class-G	PITX1
3545	0	0.503478	0.566	0.08	0	Class-G	TNIK
3546	0	0.502812	0.413	0.029	0	Class-G	LOXL1
3547	0	0.502282	0.867	0.235	0	Class-G	GOLM1
3548	0	0.502098	0.579	0.082	0	Class-G	TEAD1
3549	0	0.501668	0.826	0.224	0	Class-G	GAMT
3550	0	0.501323	0.949	0.359	0	Class-G	NFIC
3551	0	0.501154	0.768	0.191	0	Class-G	ENAH
3552	0	0.500092	0.412	0.022	0	Class-G	ACSBG1
3553	0	0.500048	0.763	0.17	0	Class-G	REEP3
3554	0	0.499974	0.441	0.029	0	Class-G	ELMOD1
3555	0	0.499341	0.577	0.084	0	Class-G	TMEM131
3556	0	0.494976	0.712	0.152	0	Class-G	CASC4
3557	0	0.494715	0.69	0.125	0	Class-G	CERS4
3558	0	0.493517	0.699	0.146	0	Class-G	CHN1
3559	0	0.493169	0.743	0.166	0	Class-G	RAB22A
3560	0	0.491924	0.58	0.092	0	Class-G	FOXG1
3561	0	0.491924	0.683	0.132	0	Class-G	SV2A
3562	0	0.491454	0.878	0.259	0	Class-G	NELFCD
3563	0	0.491037	0.549	0.106	0	Class-G	AQP4
3564	0	0.490872	0.814	0.219	0	Class-G	KIF1B
3565	0	0.490242	0.741	0.177	0	Class-G	SQLE
3566	0	0.490063	0.865	0.239	0	Class-G	C16orf45
3567	0	0.490044	0.43	0.032	0	Class-G	ILDR2
3568	0	0.489354	0.857	0.273	0	Class-G	POLDIP2
3569	0	0.48922	0.767	0.177	0	Class-G	TMEM203
3570	0	0.487749	0.74	0.175	0	Class-G	TP53I13
3571	0	0.486552	0.6	0.095	0	Class-G	PCYT2
3572	0	0.485508	0.714	0.141	0	Class-G	ECI1
3573	0	0.484661	0.554	0.08	0	Class-G	SCARA3
3574	0	0.483326	0.511	0.062	0	Class-G	BEND6
3575	0	0.482943	0.572	0.086	0	Class-G	ZNF771
3576	0	0.482811	0.84	0.269	0	Class-G	ACSL3
3577	0	0.481161	0.8	0.197	0	Class-G	SPTBN1
3578	0	0.480846	0.503	0.062	0	Class-G	CNKSR3
3579	0	0.480695	0.797	0.186	0	Class-G	PSRC1
3580	0	0.480567	0.423	0.024	0	Class-G	FAM69C
3581	0	0.478872	0.654	0.132	0	Class-G	REST
3582	0	0.478655	0.737	0.17	0	Class-G	MAPK1
3583	0	0.477744	0.391	0.021	0	Class-G	TRPM3
3584	0	0.476198	0.553	0.103	0	Class-G	IL1RAP

3585	0	0.475407	0.786	0.202	0	Class-G	TSR3
3586	0	0.475287	0.398	0.028	0	Class-G	ADAMTS9
3587	0	0.47513	0.745	0.185	0	Class-G	PCBP4
3588	0	0.474918	0.642	0.11	0	Class-G	AK4
3589	0	0.474678	0.664	0.127	0	Class-G	CDC25B
3590	0	0.474576	0.611	0.1	0	Class-G	NRSN2
3591	0	0.474122	0.433	0.032	0	Class-G	ABHD17C
3592	########	0.496671	0.985	0.501	########	Class-G	GADD45GIP1
3593	########	0.53084	0.992	0.615	########	Class-G	VIM
3594	########	0.501837	0.572	0.146	########	Class-G	CDC42EP4
3595	########	0.492882	0.913	0.31	########	Class-G	MEST
3596	########	0.489693	0.905	0.303	########	Class-G	CHCHD10
3597	########	0.539089	0.995	0.523	########	Class-G	CKB
3598	########	0.477474	0.9	0.331	########	Class-G	SDF4
3599	########	0.473765	0.964	0.439	########	Class-G	PPP1CB
3600	########	0.507437	0.901	0.351	########	Class-G	PCSK1N
3601	0	1.700525	0.833	0.076	0	Undiff-G1	UBE2C
3602	0	1.636268	0.785	0.046	0	Undiff-G1	TOP2A
3603	0	1.566521	0.802	0.031	0	Undiff-G1	PBK
3604	0	1.564516	0.772	0.063	0	Undiff-G1	CENPF
3605	0	1.549912	0.814	0.064	0	Undiff-G1	NUSAP1
3606	0	1.537733	0.809	0.065	0	Undiff-G1	BIRC5
3607	0	1.437942	0.717	0.033	0	Undiff-G1	FAM64A
3608	0	1.391973	0.835	0.087	0	Undiff-G1	UBE2T
3609	0	1.386021	0.831	0.196	0	Undiff-G1	PTTG1
3610	0	1.331772	0.69	0.049	0	Undiff-G1	TPX2
3611	0	1.302954	0.665	0.06	0	Undiff-G1	CDKN3
3612	0	1.296543	0.717	0.062	0	Undiff-G1	CDK1
3613	0	1.270329	0.859	0.174	0	Undiff-G1	SMC4
3614	0	1.269536	0.769	0.082	0	Undiff-G1	KIAA0101
3615	0	1.263597	0.814	0.089	0	Undiff-G1	MAD2L1
3616	0	1.24089	0.599	0.061	0	Undiff-G1	CCNB1
3617	0	1.22959	0.772	0.068	0	Undiff-G1	CENPU
3618	0	1.221984	0.647	0.031	0	Undiff-G1	RRM2
3619	0	1.220349	0.702	0.052	0	Undiff-G1	PRC1
3620	0	1.215538	0.669	0.027	0	Undiff-G1	NUF2
3621	0	1.21053	0.802	0.125	0	Undiff-G1	TYMS
3622	0	1.208959	0.543	0.04	0	Undiff-G1	CDC20
3623	0	1.199131	0.698	0.021	0	Undiff-G1	SGOL1
3624	0	1.178352	0.637	0.031	0	Undiff-G1	MKI67
3625	0	1.142594	0.56	0.033	0	Undiff-G1	CCNB2
3626	0	1.110852	0.62	0.03	0	Undiff-G1	SPC25
3627	0	1.102844	0.58	0.019	0	Undiff-G1	AURKB
3628	0	1.098732	0.633	0.059	0	Undiff-G1	SGOL2
3629	0	1.087681	0.601	0.026	0	Undiff-G1	CCNA2
3630	0	1.086637	0.783	0.188	0	Undiff-G1	H2AFX
3631	0	1.084694	0.71	0.066	0	Undiff-G1	CENPK
3632	0	1.079895	0.614	0.027	0	Undiff-G1	GTSE1
3633	0	1.065784	0.7	0.06	0	Undiff-G1	ZWINT
3634	0	1.059761	0.711	0.139	0	Undiff-G1	CKAP2
3635	0	1.054636	0.535	0.02	0	Undiff-G1	ASPM

3636	0	1.053543	0.517	0.029	0	Undiff-G1CENPE
3637	0	1.028114	0.504	0.024	0	Undiff-G1CENPA
3638	0	1.016791	0.509	0.026	0	Undiff-G1CDCA3
3639	0	1.014309	0.574	0.016	0	Undiff-G1CKAP2L
3640	0	1.004231	0.458	0.032	0	Undiff-G1PLK1
3641	0	1.001006	0.502	0.054	0	Undiff-G1AURKA
3642	0	0.998239	0.78	0.168	0	Undiff-G1SMC2
3643	0	0.997062	0.59	0.033	0	Undiff-G1MXD3
3644	0	0.988768	0.525	0.013	0	Undiff-G1HJURP
3645	0	0.984292	0.53	0.02	0	Undiff-G1NDC80
3646	0	0.979663	0.696	0.139	0	Undiff-G1CCDC34
3647	0	0.969703	0.977	0.467	0	Undiff-G1HMGB2
3648	0	0.966338	0.636	0.058	0	Undiff-G1CENPN
3649	0	0.962576	0.593	0.029	0	Undiff-G1KIFC1
3650	0	0.961921	0.615	0.045	0	Undiff-G1CENPM
3651	0	0.958703	0.641	0.061	0	Undiff-G1ORC6
3652	0	0.95605	0.71	0.094	0	Undiff-G1CENPH
3653	0	0.952443	0.707	0.118	0	Undiff-G1LMNB1
3654	0	0.928867	0.774	0.18	0	Undiff-G1RNASEH2A
3655	0	0.924233	0.573	0.034	0	Undiff-G1ASF1B
3656	0	0.923401	0.822	0.231	0	Undiff-G1GGH
3657	0	0.898022	0.568	0.023	0	Undiff-G1FOXM1
3658	0	0.894503	0.66	0.125	0	Undiff-G1DHFR
3659	0	0.892731	0.456	0.015	0	Undiff-G1CDCA8
3660	0	0.884782	0.512	0.023	0	Undiff-G1ECT2
3661	0	0.881941	0.496	0.018	0	Undiff-G1KIF2C
3662	0	0.879939	0.688	0.124	0	Undiff-G1EZH2
3663	0	0.879919	0.573	0.055	0	Undiff-G1RACGAP1
3664	0	0.879663	0.552	0.073	0	Undiff-G1FBXO5
3665	0	0.879594	0.517	0.024	0	Undiff-G1SPC24
3666	0	0.878242	0.541	0.077	0	Undiff-G1GINS2
3667	0	0.86921	0.57	0.049	0	Undiff-G1TACC3
3668	0	0.861651	0.491	0.024	0	Undiff-G1MYBL2
3669	0	0.859018	0.611	0.094	0	Undiff-G1DBF4
3670	0	0.853572	0.523	0.021	0	Undiff-G1CDCA5
3671	0	0.839085	0.506	0.016	0	Undiff-G1NCAPG
3672	0	0.838983	0.515	0.021	0	Undiff-G1MELK
3673	0	0.836514	0.43	0.012	0	Undiff-G1DLGAP5
3674	0	0.825999	0.731	0.164	0	Undiff-G1TMPO
3675	0	0.823353	0.436	0.022	0	Undiff-G1TROAP
3676	0	0.815057	0.615	0.092	0	Undiff-G1CENPW
3677	0	0.812116	0.475	0.042	0	Undiff-G1CLSPN
3678	0	0.809589	0.53	0.042	0	Undiff-G1RAD51AP1
3679	0	0.808186	0.456	0.017	0	Undiff-G1KIF23
3680	0	0.801397	0.405	0.012	0	Undiff-G1NEK2
3681	0	0.798718	0.595	0.113	0	Undiff-G1FEN1
3682	0	0.797722	0.4	0.015	0	Undiff-G1FAM83D
3683	0	0.797409	0.533	0.047	0	Undiff-G1TK1
3684	0	0.793725	0.469	0.041	0	Undiff-G1CDT1
3685	0	0.792431	0.52	0.054	0	Undiff-G1ATAD2
3686	0	0.79236	0.516	0.029	0	Undiff-G1C21orf58

3687	0	0.783095	0.421	0.018	0	Undiff-G1BUB1
3688	0	0.772596	0.494	0.034	0	Undiff-G1POC1A
3689	0	0.76792	0.462	0.021	0	Undiff-G1SPAG5
3690	0	0.759601	0.47	0.057	0	Undiff-G1KNSTRN
3691	0	0.758233	0.462	0.013	0	Undiff-G1ESCO2
3692	0	0.748606	0.537	0.065	0	Undiff-G1RFC3
3693	0	0.747749	0.52	0.067	0	Undiff-G1CDCA4
3694	0	0.73479	0.462	0.021	0	Undiff-G1PKMYT1
3695	0	0.724209	0.394	0.019	0	Undiff-G1HMMR
3696	0	0.721852	0.422	0.014	0	Undiff-G1NCAPH
3697	0	0.716676	0.414	0.015	0	Undiff-G1DEPDC1
3698	0	0.703523	0.46	0.044	0	Undiff-G1PRR11
3699	0	0.69936	0.423	0.021	0	Undiff-G1OIP5
3700	0	0.699136	0.507	0.069	0	Undiff-G1CHAF1A
3701	0	0.696231	0.414	0.014	0	Undiff-G1KIF4A
3702	0	0.695725	0.383	0.04	0	Undiff-G1E2F1
3703	0	0.692611	0.456	0.024	0	Undiff-G1MND1
3704	0	0.691218	0.428	0.013	0	Undiff-G1KIF15
3705	0	0.684356	0.463	0.051	0	Undiff-G1PSMC3IP
3706	0	0.683751	0.467	0.03	0	Undiff-G1SHCBP1
3707	0	0.681693	0.401	0.018	0	Undiff-G1ARHGAP11A
3708	0	0.659667	0.51	0.08	0	Undiff-G1DSN1
3709	0	0.658648	0.399	0.023	0	Undiff-G1XRCC2
3710	0	0.65861	0.388	0.018	0	Undiff-G1CDC45
3711	0	0.654558	0.453	0.057	0	Undiff-G1MNS1
3712	0	0.654532	0.39	0.012	0	Undiff-G1CDCA2
3713	0	0.653511	0.386	0.036	0	Undiff-G1SAPCD2
3714	0	0.648198	0.381	0.011	0	Undiff-G1SKA1
3715	0	0.646411	0.283	0.011	0	Undiff-G1NMU
3716	0	0.638715	0.453	0.026	0	Undiff-G1TRIP13
3717	0	0.638444	0.457	0.058	0	Undiff-G1ATAD5
3718	0	0.636714	0.419	0.015	0	Undiff-G1SKA3
3719	0	0.633151	0.451	0.06	0	Undiff-G1CHEK1
3720	0	0.632286	0.342	0.008	0	Undiff-G1KIF20A
3721	0	0.632253	0.423	0.027	0	Undiff-G1FANCI
3722	0	0.631059	0.406	0.031	0	Undiff-G1KIAA1524
3723	0	0.609479	0.306	0.01	0	Undiff-G1TTK
3724	0	0.597614	0.367	0.017	0	Undiff-G1DEPDC1B
3725	0	0.595747	0.41	0.038	0	Undiff-G1GINS1
3726	0	0.594809	0.385	0.012	0	Undiff-G1CASC5
3727	0	0.592886	0.443	0.042	0	Undiff-G1NCAPG2
3728	0	0.590666	0.375	0.016	0	Undiff-G1RTKN2
3729	0	0.582112	0.354	0.012	0	Undiff-G1KIF14
3730	0	0.580354	0.372	0.034	0	Undiff-G1TCF19
3731	0	0.567485	0.399	0.044	0	Undiff-G1TIMELESS
3732	0	0.553433	0.377	0.028	0	Undiff-G1PARPBP
3733	0	0.552457	0.356	0.023	0	Undiff-G1ARHGAP11B
3734	0	0.547519	0.415	0.04	0	Undiff-G1BRCA1
3735	0	0.543326	0.354	0.031	0	Undiff-G1C16orf59
3736	0	0.543237	0.301	0.006	0	Undiff-G1CDC25C
3737	0	0.538082	0.341	0.016	0	Undiff-G1DIAPH3

3738	0	0.52985	0.337	0.021	0	Undiff-G1CCNF
3739	0	0.514292	0.325	0.009	0	Undiff-G1BUB1B
3740	0	0.512049	0.306	0.022	0	Undiff-G1CDC6
3741	0	0.508389	0.264	0.014	0	Undiff-G1DTL
3742	0	0.500521	0.34	0.031	0	Undiff-G1BRCA2
3743	0	0.49826	0.319	0.023	0	Undiff-G1FANCD2
3744	0	0.497519	0.348	0.029	0	Undiff-G1CENPO
3745	0	0.490208	0.283	0.015	0	Undiff-G1RAD51
3746	########	0.509456	0.269	0.018	########	Undiff-G1HIST1H1D
3747	########	0.732507	0.67	0.15	########	Undiff-G1GMNN
3748	########	0.530059	0.379	0.044	########	Undiff-G1FANCA
3749	########	0.598512	0.46	0.067	########	Undiff-G1LRR1
3750	########	0.91654	0.823	0.247	########	Undiff-G1HMGB3
3751	########	0.483528	0.4	0.049	########	Undiff-G1C14orf80
3752	########	0.878551	0.842	0.301	########	Undiff-G1DTYMK
3753	########	0.693276	0.48	0.077	########	Undiff-G1MCM4
3754	########	0.535278	0.386	0.047	########	Undiff-G1SPDL1
3755	########	0.506751	0.251	0.017	########	Undiff-G1FAM111B
3756	########	0.643149	0.432	0.061	########	Undiff-G1CEP135
3757	########	0.592708	0.454	0.065	########	Undiff-G1KIF20B
3758	########	1.086426	0.812	0.319	########	Undiff-G1UBE2S
3759	########	0.605857	0.504	0.085	########	Undiff-G1MIS18A
3760	########	0.648612	0.521	0.096	########	Undiff-G1LMNB2
3761	########	0.521233	0.32	0.032	########	Undiff-G1APOLD1
3762	########	0.499909	0.417	0.058	########	Undiff-G1ZWILCH
3763	########	0.628186	0.381	0.049	########	Undiff-G1HELLS
3764	########	0.759191	0.643	0.154	########	Undiff-G1CRNDE
3765	########	0.718805	0.61	0.133	########	Undiff-G1RFC4
3766	########	0.572274	0.349	0.041	########	Undiff-G1WDR76
3767	########	0.702587	0.583	0.124	########	Undiff-G1BARD1
3768	########	0.672415	0.579	0.121	########	Undiff-G1ITGB3BP
3769	########	0.78368	0.677	0.183	########	Undiff-G1WDR34
3770	########	0.534255	0.433	0.066	########	Undiff-G1HAUS8
3771	########	0.864156	0.738	0.233	########	Undiff-G1CKS1B
3772	########	0.638628	0.541	0.108	########	Undiff-G1PHF19
3773	########	0.586879	0.472	0.081	########	Undiff-G1VRK1
3774	########	0.575464	0.37	0.051	########	Undiff-G1MCM2
3775	########	0.646358	0.679	0.17	########	Undiff-G1KIF22
3776	########	1.039777	0.851	0.402	########	Undiff-G1HIST1H4C
3777	########	0.769346	0.704	0.203	########	Undiff-G1C19orf48
3778	########	0.570697	0.416	0.068	########	Undiff-G1CDK2
3779	########	0.693603	0.667	0.175	########	Undiff-G1ASRGL1
3780	########	0.714075	0.673	0.173	########	Undiff-G1PHGDH
3781	########	0.645579	0.596	0.143	########	Undiff-G1TEX30
3782	########	0.86181	0.846	0.339	########	Undiff-G1CKS2
3783	########	0.685411	0.635	0.154	########	Undiff-G1TMSB15A
3784	########	0.684982	0.626	0.162	########	Undiff-G1RRM1
3785	########	0.627189	0.599	0.151	########	Undiff-G1TMEM97
3786	########	0.835056	0.725	0.229	########	Undiff-G1MCM7
3787	########	0.507593	0.414	0.072	########	Undiff-G1RFC5
3788	########	0.68987	0.594	0.153	########	Undiff-G1GPSM2

3789	########	0.628324	0.599	0.155	########	Undiff-G1ACYP1
3790	########	0.794704	0.783	0.29	########	Undiff-G1PCNA
3791	########	0.545749	0.581	0.14	########	Undiff-G1DNAJC9
3792	########	0.776558	0.714	0.243	########	Undiff-G1ANP32E
3793	########	0.606992	0.595	0.152	########	Undiff-G1SYNE2
3794	########	0.547223	0.98	0.725	########	Undiff-G1H2AFZ
3795	########	0.813303	0.737	0.258	########	Undiff-G1CDKN2C
3796	########	0.580199	0.549	0.133	########	Undiff-G1WHSC1
3797	########	0.720245	0.87	0.441	########	Undiff-G1DEK
3798	########	0.579262	0.468	0.098	########	Undiff-G1GNG4
3799	########	0.500607	0.368	0.063	########	Undiff-G1PAX6
3800	########	0.565718	0.611	0.164	########	Undiff-G1TMEM237
3801	########	0.830141	0.752	0.26	########	Undiff-G1HES6
3802	########	0.694601	0.925	0.5	########	Undiff-G1H2AFV
3803	########	0.718741	0.706	0.231	########	Undiff-G1MZT1
3804	########	0.547891	0.285	0.038	########	Undiff-G1PIF1
3805	########	0.719115	0.594	0.159	########	Undiff-G1ASCL1
3806	########	0.649428	0.706	0.235	########	Undiff-G1USP1
3807	########	0.487521	0.353	0.058	########	Undiff-G1LIG1
3808	########	0.590776	0.527	0.129	########	Undiff-G1CDCA7L
3809	########	0.679349	0.707	0.232	########	Undiff-G1CENPV
3810	########	0.515046	0.451	0.093	########	Undiff-G1FAM111A
3811	########	0.640708	0.509	0.124	########	Undiff-G1MCM3
3812	########	0.50986	0.525	0.129	########	Undiff-G1MGME1
3813	########	0.633135	0.702	0.244	########	Undiff-G1TMEM106C
3814	########	0.571588	0.546	0.146	########	Undiff-G1NT5DC2
3815	########	0.626454	0.842	0.323	########	Undiff-G1NUDT1
3816	########	0.577176	0.586	0.159	########	Undiff-G1TMSB15B
3817	########	0.574057	0.6	0.174	########	Undiff-G1RHNO1
3818	########	0.783922	0.795	0.331	########	Undiff-G1KPNA2
3819	########	0.696497	0.505	0.129	########	Undiff-G1PDGFRA
3820	########	0.737652	0.568	0.174	########	Undiff-G1CDC25B
3821	########	0.561149	0.52	0.134	########	Undiff-G1DRAXIN
3822	########	0.535504	0.302	0.047	########	Undiff-G1CCNE2
3823	########	0.685295	0.735	0.284	########	Undiff-G1SKA2
3824	########	0.593502	0.647	0.212	########	Undiff-G1TUBG1
3825	########	0.516731	0.51	0.132	########	Undiff-G1WEE1
3826	########	0.604147	0.806	0.317	########	Undiff-G1RPA3
3827	########	0.676894	0.702	0.25	########	Undiff-G1SAE1
3828	########	0.639499	0.638	0.202	########	Undiff-G1DSEL
3829	########	0.689435	0.658	0.222	########	Undiff-G1ETV1
3830	########	0.557893	0.528	0.146	########	Undiff-G1BMP7
3831	########	0.642005	0.806	0.341	########	Undiff-G1RAD21
3832	########	0.653073	0.71	0.264	########	Undiff-G1DNMT1
3833	########	0.517004	0.531	0.146	########	Undiff-G1HIRIP3
3834	########	0.481787	0.491	0.126	########	Undiff-G1ZNF738
3835	########	0.673829	0.895	0.403	########	Undiff-G1SOX2
3836	########	0.558298	0.527	0.153	########	Undiff-G1SAC3D1
3837	########	0.549418	0.62	0.202	########	Undiff-G1CSE1L
3838	########	0.563561	0.536	0.158	########	Undiff-G1EMC9
3839	########	0.541812	0.494	0.138	########	Undiff-G1CKAP5

3840	########	0.515916	0.627	0.207	########	Undiff-G1CCDC14
3841	########	0.558481	0.51	0.141	########	Undiff-G1CDKN2D
3842	########	0.596637	0.819	0.369	########	Undiff-G1MAD2L2
3843	########	0.648669	0.817	0.36	########	Undiff-G1PSIP1
3844	########	0.618803	0.968	0.56	########	Undiff-G1CKB
3845	########	0.700219	0.723	0.279	########	Undiff-G1EGFR
3846	########	0.640119	0.806	0.372	########	Undiff-G1ODC1
3847	########	0.557597	0.602	0.204	########	Undiff-G1PXMP2
3848	########	0.5765	0.995	0.61	########	Undiff-G1STMN1
3849	########	0.484538	0.493	0.139	########	Undiff-G1SOGA1
3850	########	0.54489	0.337	0.068	########	Undiff-G1FBLN1
3851	########	0.609079	0.931	0.558	########	Undiff-G1RANBP1
3852	########	0.596415	0.463	0.128	########	Undiff-G1GAS1
3853	########	0.543718	0.767	0.322	########	Undiff-G1CBX5
3854	########	0.525198	0.904	0.541	########	Undiff-G1LSM4
3855	########	0.593928	0.591	0.197	########	Undiff-G1POU3F2
3856	########	0.603929	0.841	0.359	########	Undiff-G1PTPRZ1
3857	########	0.487518	0.533	0.166	########	Undiff-G1RAC3
3858	########	0.506905	0.49	0.146	########	Undiff-G1NETO2
3859	########	0.47921	0.452	0.133	########	Undiff-G1ALYREF
3860	########	0.592468	0.872	0.471	########	Undiff-G1TUBB4B
3861	########	0.535127	0.884	0.617	########	Undiff-G1HMGN2
3862	########	0.680889	0.579	0.204	########	Undiff-G1NUP107
3863	########	0.615974	0.819	0.35	########	Undiff-G1IGFBP2
3864	########	0.564347	0.552	0.185	########	Undiff-G1FAM181B
3865	########	0.635051	0.441	0.127	########	Undiff-G1B4GALNT1
3866	########	0.500041	0.674	0.261	########	Undiff-G1ARL6IP6
3867	########	0.4873	0.88	0.486	########	Undiff-G1LSM5
3868	########	0.51151	0.501	0.16	########	Undiff-G1OLIG2
3869	########	0.599615	0.651	0.255	########	Undiff-G1BCAN
3870	########	0.516506	0.728	0.304	########	Undiff-G1SGCB
3871	########	0.511475	0.694	0.288	########	Undiff-G1PAICS
3872	########	0.515784	0.749	0.334	########	Undiff-G1SSRP1
3873	########	0.502501	0.815	0.387	########	Undiff-G1NASP
3874	########	0.48859	0.605	0.224	########	Undiff-G1RFC2
3875	########	0.496503	0.774	0.35	########	Undiff-G1BTG3
3876	########	0.547876	0.819	0.435	########	Undiff-G1DUT
3877	########	0.504323	0.381	0.105	########	Undiff-G1TSPAN12
3878	########	0.677225	0.599	0.233	########	Undiff-G1YEATS4
3879	########	0.509317	0.826	0.386	########	Undiff-G1RHOBTB3
3880	########	0.51699	0.786	0.37	########	Undiff-G1DDX39A
3881	########	0.560381	0.307	0.072	########	Undiff-G1BEST3
3882	########	0.489423	0.828	0.402	########	Undiff-G1PAFAH1B3
3883	########	0.58752	0.612	0.248	########	Undiff-G1RP11-620J15.3
3884	########	0.527217	0.705	0.306	########	Undiff-G1CCND2
3885	########	0.490138	0.778	0.395	########	Undiff-G1HNRNPD
3886	########	0.4953	0.616	0.256	########	Undiff-G1HDGF
3887	########	0.490252	0.973	0.531	3.55E-99	Undiff-G1TUBB2B
3888	8.57E-99	0.498269	0.772	0.366	1.28E-94	Undiff-G1MEST
3889	1.18E-98	0.511066	0.749	0.365	1.77E-94	Undiff-G1H1FX
3890	1.59E-98	0.496979	0.902	0.436	2.38E-94	Undiff-G1FXYD6

3891	5.08E-98	0.484511	0.591	0.247	7.59E-94	Undiff-G1PSRC1
3892	1.58E-97	0.488499	0.638	0.28	2.36E-93	Undiff-G1GLRX5
3893	3.88E-96	0.502495	0.637	0.259	5.80E-92	Undiff-G1NES
3894	8.23E-94	0.507169	0.348	0.106	1.23E-89	Undiff-G1LHFPL3
3895	2.04E-93	0.51115	0.612	0.256	3.05E-89	Undiff-G1SOX9
3896	1.68E-91	0.564621	0.837	0.466	2.51E-87	Undiff-G1CDK4
3897	1.56E-89	0.520197	0.752	0.365	2.33E-85	Undiff-G1FABP7
3898	2.23E-86	0.497433	0.486	0.189	3.33E-82	Undiff-G1CDK6
3899	5.63E-64	0.481188	0.428	0.176	8.41E-60	Undiff-G1COL9A3
3900	1.28E-60	0.48787	0.258	0.084	1.91E-56	Undiff-G1AGAP2-AS1
