# Supplementary material for: A Novel Molecular Classification Method for Glioblastoma Based on Tumor Cell Differentiation Trajectories
Source: Stem Cells Int. 2023 Feb 22;2023:2826815. doi: 10.1155/2023/2826815 (PMC10643041; doi:10.1155/2023/2826815)
Supplement: Supplementary 5 — Supplementary Table 3: differentially expressed genes (DEGs) in GSCL and Class-G. [file 2826815.f5.pdf]

	p_val	avg_logFC	pct.1	pct.2	p_val_adj
C4orf48	0	0.94689	0.916	0.084	0
RALY	0	0.908995	0.887	0.074	0
PHLDA1	0	0.879545	0.92	0.126	0
NEK6	0	0.821559	0.835	0.067	0
SOX9	0	0.813803	0.911	0.118	0
TOP1	0	0.767065	0.859	0.068	0
C17orf89	0	0.762968	0.907	0.092	0
CHCHD10	0	0.756375	0.882	0.082	0
SDF4	0	0.750932	0.875	0.085	0
RRBP1	0	0.725679	0.838	0.075	0
C20orf24	0	0.721349	0.884	0.092	0
COPRS	0	0.712443	0.838	0.072	0
PTPRA	0	0.706394	0.926	0.111	0
MAP2K2	0	0.701282	0.893	0.096	0
CCDC85B	0	0.697618	0.86	0.084	0
NES	0	0.684629	0.934	0.15	0
TMEM160	0	0.682269	0.876	0.084	0
GAMT	0	0.680506	0.8	0.048	0
EMC6	0	0.680415	0.89	0.101	0
DST	0	0.677544	0.891	0.123	0
AP1S2	0	0.671544	0.916	0.135	0
LAMP1	0	0.667056	0.898	0.109	0
TNFRSF1A	0	0.660833	0.811	0.075	0
PPM1G	0	0.650596	0.84	0.077	0
TCEA2	0	0.647917	0.895	0.123	0
POLDIP2	0	0.646073	0.83	0.077	0
NPDC1	0	0.64445	0.901	0.115	0
MARCKS	0	0.640488	0.948	0.188	0
CHMP4B	0	0.638451	0.894	0.128	0
ABHD12	0	0.634406	0.815	0.067	0
METRN	0	0.632235	0.92	0.142	0
KTN1	0	0.629153	0.921	0.14	0
MAP1LC3A	0	0.624823	0.848	0.108	0
PTMS	0	0.618609	0.957	0.21	0
ARGLU1	0	0.616264	0.906	0.128	0
TSEN34	0	0.615371	0.866	0.092	0
RBCK1	0	0.613127	0.789	0.063	0
CDH2	0	0.612578	0.881	0.104	0
ZNF580	0	0.609738	0.836	0.077	0
RAB34	0	0.601055	0.86	0.099	0
KIF5B	0	0.597016	0.887	0.118	0
FEZ2	0	0.596134	0.856	0.096	0
DDX17	0	0.596055	0.835	0.096	0
GNAI2	0	0.59593	0.85	0.091	0
RCN2	0	0.591929	0.935	0.149	0
SLC39A3	0	0.585726	0.843	0.082	0
RDX	0	0.58169	0.935	0.161	0
PAIP1	0	0.5802	0.848	0.091	0
NME3	0	0.579605	0.879	0.121	0
STUB1	0	0.57941	0.878	0.103	0

TMEM165	0	0.575328	0.818	0.089	0
COMMD7	0	0.575207	0.824	0.079	0
SNX5	0	0.573514	0.863	0.104	0
TPM4	0	0.573456	0.884	0.149	0
NCOR1	0	0.571673	0.913	0.14	0
STAU1	0	0.568799	0.831	0.082	0
SPRY1	0	0.5683	0.86	0.125	0
VGLL4	0	0.56805	0.844	0.097	0
QKI	0	0.561681	0.933	0.15	0
CD47	0	0.561186	0.807	0.074	0
UBE2S	0	0.55993	0.799	0.068	0
TSPAN5	0	0.557108	0.815	0.082	0
MTDH	0	0.556817	0.926	0.166	0
NLRP1	0	0.55657	0.888	0.128	0
SCAND1	0	0.552163	0.939	0.183	0
MLEC	0	0.551603	0.858	0.087	0
HNRNPD	0	0.548238	0.82	0.084	0
PGLS	0	0.54658	0.903	0.133	0
C19orf24	0	0.546402	0.833	0.082	0
PJA2	0	0.54578	0.891	0.121	0
UBE2J2	0	0.544085	0.776	0.062	0
AP2B1	0	0.539473	0.812	0.085	0
SRSF9	0	0.534587	0.947	0.169	0
SMARCA4	0	0.53322	0.849	0.099	0
SKA2	0	0.531933	0.795	0.072	0
ADAM9	0	0.531653	0.826	0.087	0
DTD1	0	0.52634	0.815	0.097	0
CAMK2D	0	0.526159	0.835	0.099	0
C20orf27	0	0.523391	0.79	0.087	0
NAA10	0	0.520418	0.828	0.085	0
CD320	0	0.519273	0.892	0.113	0
BPTF	0	0.51783	0.79	0.07	0
SMARCB1	0	0.515842	0.849	0.103	0
STMN3	0	0.511763	0.835	0.111	0
NME4	0	0.509348	0.831	0.104	0
SSBP4	0	0.507173	0.801	0.074	0
TMEM259	0	0.501737	0.813	0.091	0
GCSH	0	0.49898	0.913	0.125	0
LHFP	0	0.491539	0.821	0.101	0
NDFIP1	0	0.489116	0.955	0.183	0
GATM	0	0.487409	0.901	0.145	0
C6orf62	0	0.487166	0.822	0.096	0
HMG20B	0	0.486523	0.827	0.099	0
BRI3	0	0.484115	0.857	0.125	0
DEK	0	0.472616	0.819	0.091	0
HIST1H4C	0	0.469767	0.825	0.113	0
ETV1	0	0.468323	0.818	0.094	0
PTOV1	0	0.46741	0.902	0.14	0
ATP1B2	0	0.46603	0.885	0.15	0
RER1	0	0.465832	0.888	0.128	0
RAB31	0	0.465392	0.875	0.137	0

FYN	0	0.465018	0.862	0.118	0
AKR7A2	0	0.463739	0.752	0.062	0
MTCH1	0	0.460237	0.905	0.14	0
DDR1	0	0.454443	0.892	0.135	0
AP3D1	0	0.454111	0.804	0.094	0
GNB1	0	0.453005	0.85	0.106	0
TTC3	0	0.451712	0.918	0.171	0
SLC25A39	0	0.450653	0.797	0.085	0
CCDC88A	0	0.450557	0.778	0.082	0
P4HB	0	0.448651	0.939	0.19	0
MACF1	0	0.445263	0.832	0.115	0
SRSF2	0	0.444678	0.935	0.183	0
PRELID1	0	0.444035	0.921	0.162	0
NFIC	0	0.443449	0.925	0.166	0
HP1BP3	0	0.442485	0.874	0.126	0
NELFCD	0	0.440037	0.853	0.118	0
RTF1	0	0.439689	0.832	0.111	0
SYT11	0	0.43835	0.914	0.15	0
SERPINH1	0	0.432462	0.772	0.08	0
SNF8	0	0.431954	0.835	0.113	0
C1QBP	0	0.431616	0.929	0.176	0
CSNK2A1	0	0.431094	0.778	0.082	0
DUT	0	0.429893	0.869	0.12	0
DNPH1	0	0.429576	0.853	0.113	0
GSPT1	0	0.428057	0.756	0.072	0
MZT2B	0	0.427926	0.938	0.203	0
PFDN4	0	0.427331	0.787	0.085	0
GTF3A	0	0.426012	0.853	0.108	0
ATP2B1	0	0.424446	0.837	0.13	0
GRINA	0	0.424267	0.778	0.084	0
PTP4A2	0	0.422892	0.908	0.164	0
RTN4	0	0.420051	0.955	0.236	0
9-Sep	0	0.419242	0.863	0.13	0
ENOPH1	0	0.412559	0.834	0.096	0
FSCN1	0	0.410674	0.835	0.123	0
CLTC	0	0.409743	0.79	0.099	0
DGCR6L	0	0.406739	0.815	0.096	0
CNP	0	0.406243	0.855	0.108	0
PRAF2	0	0.40583	0.793	0.094	0
CTSA	0	0.397244	0.833	0.097	0
ECI2	0	0.397081	0.821	0.125	0
EIF3J	0	0.393447	0.817	0.097	0
DPYSL3	0	0.39343	0.809	0.118	0
MATR3	0	0.392612	0.89	0.138	0
CLTB	0	0.392262	0.843	0.108	0
PPP1CB	0	0.391775	0.942	0.21	0
DLGAP4	0	0.390681	0.747	0.075	0
DPP7	0	0.389867	0.828	0.116	0
AHCY	0	0.389744	0.775	0.091	0
C19orf60	0	0.389549	0.863	0.132	0
PYURF	0	0.38918	0.922	0.168	0

CSNK1A1	0	0.388665	0.948	0.203	0
BRD4	0	0.388449	0.799	0.092	0
GIPC1	0	0.387065	0.783	0.097	0
APLP2	0	0.386658	0.95	0.198	0
EPN1	0	0.386033	0.77	0.082	0
LINC00657	0	0.382182	0.838	0.109	0
UBE2D2	0	0.380946	0.941	0.178	0
C9orf16	0	0.380263	0.91	0.2	0
PGRMC1	0	0.380003	0.794	0.103	0
PIGT	0	0.379894	0.847	0.116	0
MTPN	0	0.378322	0.825	0.089	0
TRIB2	0	0.377434	0.871	0.135	0
MAD2L2	0	0.377083	0.841	0.121	0
METTL9	0	0.376449	0.853	0.12	0
JTB	0	0.375819	0.936	0.183	0
HINT2	0	0.37449	0.772	0.092	0
SRRM2	0	0.373084	0.917	0.176	0
HNRNPM	0	0.371085	0.875	0.138	0
SLC25A11	0	0.37058	0.834	0.113	0
ARL6IP4	0	0.369061	0.938	0.209	0
GADD45GIF	0	0.366284	0.965	0.272	0
SRSF4	0	0.36316	0.769	0.087	0
LYPLA1	0	0.362056	0.854	0.116	0
DDAH2	0	0.361193	0.791	0.118	0
SPATS2L	0	0.359409	0.824	0.12	0
GRHPR	0	0.359124	0.843	0.118	0
ZNF428	0	0.358908	0.967	0.232	0
GBAS	0	0.358186	0.774	0.091	0
TTC19	0	0.357514	0.807	0.106	0
SNRNP70	0	0.357376	0.861	0.138	0
SH3GLB1	0	0.357367	0.809	0.125	0
IRS2	0	0.357043	0.828	0.132	0
LAGE3	0	0.355432	0.81	0.108	0
MVB12A	0	0.35354	0.791	0.115	0
C1orf122	0	0.352152	0.941	0.217	0
LAPTM4B	0	0.351864	0.838	0.12	0
SSNA1	0	0.351735	0.832	0.115	0
C19orf43	0	0.351398	0.964	0.248	0
ABI2	0	0.347642	0.87	0.145	0
RNF187	0	0.347435	0.869	0.138	0
YWHAB	0	0.34706	0.966	0.268	0
GNB2	0	0.344558	0.916	0.19	0
ALKBH7	0	0.342223	0.911	0.162	0
ITPA	0	0.341847	0.851	0.13	0
ESF1	0	0.340282	0.825	0.118	0
KPNB1	0	0.339652	0.825	0.128	0
DDRGK1	0	0.338012	0.866	0.144	0
MAF1	0	0.336505	0.765	0.099	0
KRT10	0	0.336244	0.925	0.209	0
CCDC47	0	0.335466	0.812	0.12	0
MPG	0	0.334484	0.806	0.101	0

HMGN1	0	0.33185	0.926	0.214	0
KDELR2	0	0.330704	0.879	0.147	0
NENF	0	0.329706	0.907	0.171	0
SPG21	0	0.328537	0.758	0.077	0
APMAP	0	0.32826	0.856	0.121	0
MUM1	0	0.324479	0.841	0.137	0
CMPK1	0	0.324252	0.771	0.096	0
TXNL4A	0	0.32411	0.862	0.126	0
FEZ1	0	0.322941	0.945	0.212	0
GOLM1	0	0.322633	0.852	0.128	0
EGFR	0	0.322543	0.929	0.215	0
TUBB2A	0	0.32226	0.929	0.236	0
KHDRBS1	0	0.320351	0.844	0.135	0
EXOC7	0	0.318157	0.778	0.104	0
HIF1A	0	0.316309	0.883	0.144	0
EI24	0	0.313952	0.88	0.149	0
SOX2	0	0.313884	0.969	0.287	0
CALM3	0	0.313875	0.898	0.164	0
CXXC5	0	0.311541	0.909	0.186	0
H2AFY	0	0.311209	0.91	0.179	0
COX16	0	0.309994	0.879	0.156	0
UBXN6	0	0.309799	0.749	0.087	0
PTTG1IP	0	0.309546	0.845	0.138	0
ARHGDIA	0	0.309368	0.905	0.168	0
PPP4C	0	0.309314	0.893	0.176	0
FHL1	0	0.308125	0.897	0.171	0
CLPTM1	0	0.307722	0.772	0.097	0
LSM2	0	0.306405	0.842	0.128	0
FLOT1	0	0.303854	0.878	0.157	0
ELAVL1	0	0.303693	0.767	0.104	0
PPP1CC	0	0.302779	0.873	0.147	0
C16orf13	0	0.302316	0.879	0.152	0
PARP1	0	0.299967	0.874	0.138	0
SRM	0	0.299379	0.861	0.133	0
GTF2I	0	0.298862	0.876	0.162	0
UBE2I	0	0.297669	0.887	0.162	0
DNTTIP1	0	0.296596	0.774	0.108	0
ARL5A	0	0.296578	0.828	0.128	0
TOMM40	0	0.296225	0.775	0.103	0
SQSTM1	0	0.295495	0.902	0.2	0
HDAC2	0	0.29501	0.766	0.099	0
THRAP3	0	0.293566	0.831	0.13	0
C21orf59	0	0.291774	0.74	0.089	0
CCNI	0	0.290271	0.961	0.28	0
NFE2L2	0	0.289829	0.84	0.142	0
ACADVL	0	0.28968	0.856	0.138	0
B3GAT3	0	0.288664	0.823	0.132	0
PRPF40A	0	0.288077	0.821	0.123	0
ARID4B	0	0.287893	0.772	0.113	0
RNH1	0	0.287186	0.881	0.188	0
LSM14A	0	0.287014	0.823	0.126	0

PRKAR1A	0	0.286011	0.846	0.142	0
CAMTA1	0	0.285977	0.895	0.156	0
ZNF593	0	0.284622	0.784	0.096	0
GPS1	0	0.283819	0.74	0.094	0
ATP6AP1	0	0.282931	0.847	0.137	0
SFT2D1	0	0.28227	0.838	0.137	0
TPR	0	0.282019	0.822	0.126	0
TERF2IP	0	0.280544	0.849	0.145	0
GRN	0	0.279793	0.847	0.161	0
SFPQ	0	0.27834	0.906	0.188	0
LMO4	0	0.278148	0.939	0.231	0
NUTF2	0	0.276216	0.831	0.115	0
MARCKSL1	0	0.275166	0.972	0.297	0
NOP56	0	0.27429	0.854	0.14	0
11-Sep	0	0.273596	0.826	0.133	0
DDT	0	0.27357	0.913	0.191	0
TIMM10	0	0.272716	0.774	0.106	0
ILF3	0	0.271543	0.843	0.144	0
SPTBN1	0	0.269961	0.784	0.118	0
NSA2	0	0.269006	0.8	0.113	0
PFN1	0	0.26862	0.98	0.362	0
SERPINB6	0	0.268271	0.822	0.13	0
RAB10	0	0.267523	0.875	0.142	0
PDIA3	0	0.2665	0.899	0.183	0
ERLEC1	0	0.263945	0.813	0.123	0
COMT	0	0.263072	0.939	0.203	0
BRD2	0	0.263006	0.776	0.118	0
UBE2E3	0	0.262881	0.836	0.144	0
PIN1	0	0.259665	0.947	0.221	0
WDR45B	0	0.25933	0.843	0.137	0
IDS	0	0.258042	0.829	0.166	0
VAMP3	0	0.257385	0.731	0.085	0
MKKS	0	0.255319	0.838	0.152	0
CHD9	0	0.255073	0.851	0.145	0
TADA3	0	0.24907	0.832	0.147	0
SLC39A1	0	0.247728	0.759	0.104	0
PRPF6	0	0.247645	0.793	0.109	0
XRN2	0	0.247007	0.847	0.135	0
UBE2L3	0	0.243663	0.907	0.169	0
ATRX	0	0.242464	0.796	0.142	0
MZT2A	0	0.242222	0.794	0.128	0
ECHS1	0	0.241537	0.812	0.121	0
NOVA1	0	0.240399	0.985	0.393	0
CLPP	0	0.239647	0.883	0.156	0
MAP1LC3B	0	0.238235	0.854	0.152	0
HDLBP	0	0.237639	0.747	0.104	0
DHX36	0	0.236377	0.82	0.132	0
SERP1	0	0.23599	0.875	0.168	0
NAP1L4	0	0.234881	0.827	0.132	0
FIBIN	0	0.23436	0.872	0.174	0
NOL7	0	0.232979	0.799	0.125	0

RAD23B	0	0.232089	0.782	0.109	0
STOML2	0	0.230888	0.863	0.156	0
ATP5D	0	0.229676	0.967	0.294	0
TSPAN3	0	0.228186	0.935	0.2	0
PRRC2C	0	0.227475	0.869	0.154	0
SF1	0	0.226986	0.854	0.156	0
BAALC	0	0.226612	0.904	0.207	0
RNF114	0	0.22565	0.836	0.133	0
MAGEF1	0	0.225191	0.831	0.147	0
BBX	0	0.224521	0.847	0.138	0
BTG1	0	0.222629	0.914	0.239	0
SGTA	0	0.221008	0.737	0.097	0
LIMA1	0	0.220638	0.872	0.176	0
LMNA	0	0.220479	0.853	0.174	0
PLEKHJ1	0	0.219772	0.818	0.14	0
FAM3C	0	0.21896	0.86	0.169	0
ATP6V0E2	0	0.217908	0.951	0.234	0
PTPRZ1	0	0.21671	0.969	0.342	0
RHOBTB3	0	0.214417	0.897	0.197	0
EIF4G2	0	0.213554	0.955	0.284	0
GGNBP2	0	0.213424	0.742	0.111	0
RTFDC1	0	0.21318	0.909	0.183	0
LSM4	0	0.212012	0.926	0.243	0
SCAF11	0	0.211719	0.769	0.113	0
APP	0	0.209258	0.964	0.268	0
CLTA	0	0.209201	0.917	0.197	0
SCD5	0	0.208276	0.855	0.188	0
ARPP19	0	0.207206	0.787	0.125	0
FARSA	0	0.207069	0.755	0.118	0
YBX3	0	0.206757	0.845	0.186	0
DCXR	0	0.206398	0.867	0.176	0
ACTN4	0	0.201986	0.82	0.157	0
SAP30BP	0	0.201214	0.764	0.115	0
EIF3F	0	0.201149	0.91	0.193	0
APOA1BP	0	0.201147	0.805	0.123	0
DNAJB11	0	0.200425	0.745	0.106	0
SET	0	0.200045	0.931	0.224	0
STX10	0	0.198678	0.8	0.125	0
DYNC1I2	0	0.197253	0.85	0.159	0
LYRM2	0	0.189561	0.733	0.106	0
CYC1	0	0.189384	0.925	0.203	0
WDR1	0	0.189158	0.752	0.126	0
RPS19BP1	0	0.188859	0.902	0.19	0
GLTSCR2	0	0.188355	0.878	0.162	0
SDF2	0	0.188268	0.782	0.116	0
CBX3	0	0.185875	0.884	0.195	0
ATG3	0	0.185661	0.737	0.091	0
SMARCE1	0	0.1852	0.742	0.12	0
PRKRA	0	0.184146	0.764	0.132	0
TMEM9B	0	0.183234	0.89	0.188	0
PCBP1	0	0.180529	0.884	0.169	0

TCEA1	0	0.180225	0.93	0.234	0
DNASE2	0	0.179852	0.766	0.128	0
GTF2F2	0	0.179094	0.758	0.121	0
PDCD6	0	0.178806	0.902	0.193	0
PRPF31	0	0.178702	0.859	0.171	0
VCP	0	0.178339	0.773	0.13	0
PSMD11	0	0.177124	0.761	0.116	0
TCF4	0	0.177092	0.942	0.274	0
NUDT5	0	0.176121	0.68	0.075	0
ITM2B	0	0.175381	0.979	0.32	0
SNRPB	0	0.174437	0.928	0.234	0
ZSWIM7	0	0.173422	0.758	0.113	0
CNDP2	0	0.173402	0.769	0.118	0
ERI3	0	0.172567	0.78	0.13	0
NCL	0	0.171545	0.914	0.205	0
NSFL1C	0	0.171254	0.811	0.138	0
DDA1	0	0.171088	0.765	0.121	0
MAPRE1	0	0.169121	0.854	0.152	0
BAD	0	0.168264	0.86	0.169	0
ZNF207	0	0.168039	0.806	0.137	0
C8orf59	0	0.167353	0.818	0.132	0
ARF3	0	0.166493	0.766	0.12	0
AES	0	0.166113	0.864	0.179	0
MIDN	0	0.163891	0.764	0.144	0
LARS	0	0.163492	0.758	0.125	0
SDF2L1	0	0.162785	0.748	0.108	0
HNRNPU	0	0.159496	0.894	0.191	0
GLO1	0	0.158266	0.846	0.156	0
PSMF1	0	0.157408	0.849	0.144	0
UBA2	0	0.157185	0.731	0.113	0
ATXN10	0	0.157128	0.745	0.116	0
RSF1	0	0.156421	0.78	0.133	0
G6PC3	0	0.156103	0.797	0.149	0
SF3B1	0	0.155721	0.753	0.121	0
PABPN1	0	0.155444	0.802	0.138	0
HNRNPF	0	0.155069	0.855	0.164	0
ZNF706	0	0.15391	0.913	0.243	0
FAM127A	0	0.153823	0.886	0.2	0
U2AF1	0	0.152453	0.923	0.227	0
TFG	0	0.152433	0.813	0.162	0
TXN2	0	0.151914	0.837	0.15	0
APRT	0	0.150318	0.859	0.195	0
CCDC124	0	0.150188	0.897	0.2	0
YBX1	0	0.149673	0.976	0.407	0
AUP1	0	0.147998	0.837	0.164	0
ZBTB20	0	0.147771	0.844	0.186	0
GUK1	0	0.147739	0.969	0.323	0
YWHAQ	0	0.147211	0.969	0.304	0
SNX3	0	0.144505	0.986	0.344	0
ITM2C	0	0.144057	0.849	0.183	0
AURKAIP1	0	0.14376	0.957	0.27	0

RAB6A	0	0.143127	0.751	0.13	0
RCN1	0	0.142415	0.844	0.174	0
ISCU	0	0.141197	0.831	0.164	0
PPP1CA	0	0.141033	0.88	0.198	0
CRIP2	0	0.139327	0.823	0.174	0
ADRM1	0	0.1387	0.877	0.197	0
PPP2CA	0	0.138531	0.837	0.152	0
FAM192A	0	0.138471	0.742	0.115	0
CANX	0	0.138361	0.941	0.244	0
DDX49	0	0.137751	0.727	0.116	0
TRIM9	0	0.137077	0.805	0.162	0
SNX17	0	0.136251	0.818	0.159	0
CTNNA1	0	0.136132	0.817	0.162	0
COX5A	0	0.134844	0.947	0.244	0
RAB14	0	0.13393	0.797	0.132	0
SSR1	0	0.133577	0.809	0.152	0
MAPK1IP1L	0	0.132291	0.79	0.138	0
BOLA3	0	0.132035	0.749	0.125	0
EWSR1	0	0.130118	0.806	0.142	0
SCCPDH	0	0.12935	0.78	0.14	0
RPN2	0	0.129165	0.953	0.268	0
RAC1	0	0.128765	0.973	0.364	0
UBA1	0	0.128513	0.717	0.12	0
CHURC1	0	0.127168	0.751	0.13	0
SLC35B1	0	0.127014	0.831	0.169	0
MEST	0	0.12506	0.898	0.248	0
MSI2	0	0.124276	0.905	0.239	0
CCDC167	0	0.123715	0.747	0.125	0
FIBP	0	0.121955	0.88	0.203	0
FLYWCH2	0	0.120408	0.734	0.123	0
HNRNPA0	0	0.119939	0.925	0.219	0
NOP10	0	0.119605	0.905	0.248	0
KLHDC3	0	0.119244	0.712	0.108	0
NDUFAF3	0	0.118997	0.831	0.164	0
C7orf50	0	0.1168	0.848	0.178	0
DNAJC7	0	0.116505	0.817	0.156	0
WASF2	0	0.116127	0.81	0.152	0
SRA1	0	0.115613	0.804	0.138	0
RNF167	0	0.115346	0.843	0.174	0
RAP1B	0	0.115022	0.815	0.15	0
RAB11B	0	0.114387	0.876	0.198	0
DPM2	0	0.114221	0.735	0.116	0
FAM200B	0	0.113551	0.796	0.137	0
XRCC5	0	0.11011	0.85	0.181	0
USP16	0	0.109965	0.748	0.12	0
ANP32A	0	0.109837	0.731	0.111	0
RAB9A	0	0.108226	0.752	0.118	0
TOMM22	0	0.107007	0.825	0.144	0
PHB2	0	0.107006	0.813	0.147	0
IGFBP2	0	0.105229	0.96	0.378	0
N4BP2L2	0	0.105047	0.894	0.221	0

PSMD2	0	0.104285	0.816	0.152	0
UQCRFS1	0	0.103818	0.93	0.227	0
VAPA	0	0.103327	0.948	0.262	0
C16orf45	0	0.10086	0.84	0.173	0
DECR1	0	-0.1001	0.763	0.193	0
YIPF3	0	-0.10069	0.907	0.251	0
IK	0	-0.10087	0.698	0.152	0
POLR2H	0	-0.10118	0.652	0.128	0
PMP22	0	-0.10178	0.933	0.354	0
DCTN2	0	-0.10253	0.822	0.203	0
PAPOLA	0	-0.10276	0.852	0.195	0
HSPB11	0	-0.10294	0.671	0.126	0
MESDC2	0	-0.10301	0.803	0.19	0
TRIAP1	0	-0.10312	0.709	0.13	0
EIF2S2	0	-0.10442	0.949	0.338	0
RBX1	0	-0.10537	0.946	0.318	0
COA1	0	-0.10545	0.718	0.166	0
TRAPPC2L	0	-0.10559	0.755	0.173	0
TAF9	0	-0.1057	0.872	0.217	0
VPS25	0	-0.10675	0.693	0.133	0
TRAM1	0	-0.1071	0.731	0.154	0
CCT5	0	-0.1077	0.775	0.188	0
TUBA1C	0	-0.10791	0.729	0.179	0
UBXN1	0	-0.1084	0.878	0.253	0
DDX5	0	-0.1087	0.983	0.47	0
TMEM50A	0	-0.10873	0.878	0.236	0
C1orf123	0	-0.10882	0.708	0.147	0
IAH1	0	-0.10961	0.785	0.185	0
TMEM11	0	-0.10962	0.665	0.123	0
PDHB	0	-0.10996	0.726	0.169	0
DDX24	0	-0.11041	0.817	0.186	0
RAB7A	0	-0.11191	0.938	0.289	0
VAMP5	0	-0.11245	0.823	0.227	0
NDUFA8	0	-0.11274	0.88	0.232	0
TEX264	0	-0.11336	0.769	0.178	0
ARL2	0	-0.11371	0.72	0.147	0
TMEM14B	0	-0.11414	0.819	0.214	0
CHCHD5	0	-0.11417	0.755	0.174	0
DCTN3	0	-0.11471	0.839	0.205	0
TMX2	0	-0.11546	0.767	0.178	0
SPAG7	0	-0.11819	0.855	0.236	0
PET100	0	-0.11823	0.88	0.265	0
SEC11C	0	-0.11837	0.83	0.214	0
CNIH4	0	-0.11848	0.709	0.162	0
PAFAH1B3	0	-0.11869	0.852	0.224	0
GGPS1	0	-0.11952	0.699	0.152	0
POLR2E	0	-0.11987	0.911	0.27	0
SRSF5	0	-0.12087	0.911	0.292	0
CLNS1A	0	-0.12164	0.853	0.224	0
RTN3	0	-0.12256	0.923	0.318	0
ASNA1	0	-0.12256	0.925	0.282	0

FAM32A	0	-0.12518	0.861	0.226	0
LAMTOR2	0	-0.12519	0.909	0.26	0
ROMO1	0	-0.12559	0.932	0.368	0
EIF5	0	-0.12631	0.943	0.328	0
ISOC2	0	-0.12815	0.793	0.197	0
DCTPP1	0	-0.12982	0.684	0.142	0
C14orf2	0	-0.13057	0.966	0.39	0
MEA1	0	-0.13111	0.817	0.197	0
WSB1	0	-0.1312	0.91	0.311	0
TCP1	0	-0.13159	0.808	0.207	0
WBP2	0	-0.1316	0.807	0.214	0
CAPZB	0	-0.13166	0.897	0.272	0
PSMC6	0	-0.13214	0.727	0.183	0
ASAH1	0	-0.13274	0.831	0.229	0
SUPT4H1	0	-0.13294	0.813	0.188	0
POLR2J3	0	-0.13398	0.78	0.203	0
ATP6V1D	0	-0.13544	0.72	0.14	0
PPIG	0	-0.13548	0.835	0.203	0
UGP2	0	-0.13565	0.774	0.198	0
NSL1	0	-0.13577	0.66	0.145	0
DCTN6	0	-0.13594	0.786	0.193	0
PDIA6	0	-0.13618	0.955	0.34	0
EIF4H	0	-0.13707	0.883	0.251	0
TMEM208	0	-0.13792	0.835	0.215	0
CD151	0	-0.13806	0.936	0.338	0
NME1	0	-0.14001	0.952	0.369	0
SNRPC	0	-0.14006	0.868	0.207	0
STX8	0	-0.14067	0.671	0.142	0
TM2D1	0	-0.1407	0.787	0.21	0
FUS	0	-0.14116	0.937	0.33	0
CCT6A	0	-0.14169	0.876	0.27	0
DNAJC15	0	-0.14376	0.695	0.144	0
CHCHD3	0	-0.1443	0.715	0.161	0
SEC13	0	-0.14565	0.704	0.149	0
FDPS	0	-0.14567	0.841	0.231	0
C7orf55	0	-0.14624	0.878	0.246	0
GOLGA7	0	-0.14711	0.852	0.222	0
PA2G4	0	-0.14859	0.872	0.236	0
C9orf78	0	-0.14953	0.753	0.171	0
MLLT11	0	-0.15002	0.794	0.227	0
PSMA4	0	-0.15004	0.881	0.258	0
PNKD	0	-0.15238	0.891	0.26	0
SPARC	0	-0.1534	0.951	0.374	0
CACYBP	0	-0.15362	0.79	0.191	0
C1orf43	0	-0.15364	0.936	0.316	0
PDZD11	0	-0.15494	0.783	0.2	0
RPN1	0	-0.1551	0.662	0.145	0
PRKCSH	0	-0.15651	0.786	0.224	0
TIMMDC1	0	-0.15693	0.78	0.195	0
RBBP7	0	-0.15699	0.821	0.224	0
SLIRP	0	-0.15841	0.955	0.378	0

GTF2A2	0	-0.15973	0.865	0.234	0
CCDC12	0	-0.16017	0.756	0.203	0
PSMB4	0	-0.16072	0.805	0.209	0
LMAN2	0	-0.16079	0.917	0.292	0
VPS29	0	-0.16114	0.81	0.207	0
QTRT1	0	-0.16218	0.772	0.21	0
EIF1AX	0	-0.16358	0.907	0.277	0
TALDO1	0	-0.16427	0.812	0.21	0
DPM1	0	-0.16576	0.766	0.181	0
GGCT	0	-0.16656	0.767	0.193	0
NIFK	0	-0.16689	0.739	0.173	0
SRP19	0	-0.1678	0.711	0.156	0
RSRC2	0	-0.16844	0.823	0.195	0
MMADHC	0	-0.16893	0.743	0.176	0
ATOX1	0	-0.1717	0.94	0.335	0
PEF1	0	-0.17192	0.706	0.157	0
DERL2	0	-0.17395	0.69	0.142	0
S100A16	0	-0.17442	0.934	0.376	0
GPX1	0	-0.17462	0.956	0.379	0
BTF3L4	0	-0.17487	0.814	0.207	0
AP2S1	0	-0.17535	0.928	0.321	0
UQCRC2	0	-0.17664	0.806	0.207	0
NDUFS8	0	-0.1767	0.944	0.335	0
NUCKS1	0	-0.17707	0.964	0.364	0
PSMB8	0	-0.17783	0.8	0.207	0
CALD1	0	-0.1799	0.897	0.294	0
ERCC1	0	-0.1822	0.807	0.217	0
CWC15	0	-0.18261	0.846	0.212	0
EIF3G	0	-0.18282	0.948	0.359	0
TMEM256	0	-0.18314	0.725	0.169	0
ILF3-AS1	0	-0.18399	0.699	0.178	0
MPHOSPH8	0	-0.18513	0.732	0.164	0
METAP2	0	-0.18558	0.866	0.256	0
CYB5B	0	-0.18638	0.659	0.147	0
RHEB	0	-0.18642	0.927	0.318	0
SRRM1	0	-0.18694	0.829	0.221	0
MPLKIP	0	-0.188	0.813	0.203	0
PSMC1	0	-0.18877	0.795	0.207	0
TXNDC12	0	-0.18936	0.723	0.183	0
SF3B5	0	-0.19019	0.915	0.304	0
PPA1	0	-0.19084	0.684	0.164	0
ZDHHC4	0	-0.19161	0.72	0.168	0
UQCR10	0	-0.19408	0.96	0.426	0
TMED1	0	-0.19435	0.769	0.195	0
BCAP31	0	-0.19443	0.9	0.263	0
ATP5H	0	-0.19611	0.942	0.345	0
AP2M1	0	-0.19809	0.955	0.415	0
LINC00493	0	-0.19869	0.913	0.28	0
PABPC1	0	-0.19894	0.889	0.316	0
POLR2L	0	-0.19937	0.971	0.421	0
ZFP36L1	0	-0.19951	0.813	0.275	0

POLR1D	0	-0.1996	0.681	0.142	0
MPC2	0	-0.20079	0.818	0.234	0
DDX18	0	-0.20163	0.815	0.227	0
CCT7	0	-0.20209	0.873	0.27	0
C14orf119	0	-0.20235	0.635	0.126	0
MOB4	0	-0.20408	0.706	0.171	0
TIMM17A	0	-0.20416	0.736	0.152	0
RNF7	0	-0.20547	0.872	0.275	0
FXYD6	0	-0.20551	0.965	0.424	0
FAM103A1	0	-0.20563	0.614	0.118	0
FIS1	0	-0.20574	0.94	0.335	0
VDAC3	0	-0.20584	0.706	0.188	0
CST3	0	-0.20685	0.996	0.569	0
SRSF7	0	-0.20744	0.872	0.267	0
DNAJC8	0	-0.20868	0.867	0.25	0
MED4	0	-0.20912	0.736	0.179	0
EID1	0	-0.21074	0.98	0.485	0
HAX1	0	-0.21146	0.819	0.224	0
DYNLRB1	0	-0.21222	0.963	0.414	0
POLD2	0	-0.21288	0.866	0.275	0
NDUFB5	0	-0.21349	0.896	0.299	0
VBP1	0	-0.21367	0.841	0.236	0
ZCRB1	0	-0.21402	0.879	0.25	0
SNRPD1	0	-0.21478	0.901	0.279	0
PNISR	0	-0.21502	0.938	0.357	0
POLR2G	0	-0.21566	0.901	0.303	0
LSM5	0	-0.21579	0.835	0.219	0
PCMT1	0	-0.21623	0.848	0.231	0
TRA2B	0	-0.21658	0.836	0.248	0
SUMO1	0	-0.2166	0.935	0.34	0
CCNL1	0	-0.2168	0.724	0.203	0
BZW1	0	-0.21717	0.89	0.277	0
PEA15	0	-0.22008	0.827	0.253	0
AHSA1	0	-0.22098	0.667	0.159	0
LRPAP1	0	-0.22228	0.867	0.287	0
EIF5B	0	-0.22255	0.841	0.241	0
CALM1	0	-0.22264	0.99	0.537	0
HSPB1	0	-0.22455	0.942	0.393	0
EMP3	0	-0.2247	0.801	0.256	0
TRAPPC4	0	-0.22516	0.787	0.195	0
SNRPD3	0	-0.22535	0.8	0.215	0
VKORC1	0	-0.22582	0.806	0.219	0
PSMC3	0	-0.22615	0.876	0.294	0
SNRPB2	0	-0.22779	0.918	0.325	0
TMEM14C	0	-0.22832	0.867	0.251	0
LSM3	0	-0.23043	0.878	0.27	0
RHOC	0	-0.23088	0.961	0.407	0
PSAP	0	-0.23138	0.965	0.4	0
LSM1	0	-0.23183	0.812	0.229	0
ALG5	0	-0.23207	0.67	0.159	0
AKR1A1	0	-0.23251	0.722	0.181	0

NSMCE1	0	-0.23258	0.739	0.198	0
COX8A	0	-0.23285	0.981	0.499	0
ARPC1A	0	-0.23379	0.903	0.303	0
NDUFA11	0	-0.23435	0.978	0.475	0
DHPS	0	-0.23982	0.798	0.236	0
GNG5	0	-0.2402	0.972	0.467	0
DNAJC19	0	-0.24022	0.762	0.215	0
C11orf58	0	-0.2409	0.954	0.398	0
EMC3	0	-0.24091	0.777	0.215	0
NDUFV2	0	-0.2414	0.916	0.32	0
FAM133B	0	-0.24144	0.786	0.207	0
PSMA3	0	-0.24189	0.774	0.205	0
THYN1	0	-0.24194	0.822	0.25	0
TBCB	0	-0.24245	0.971	0.417	0
UBE2V2	0	-0.24262	0.866	0.244	0
UFC1	0	-0.24276	0.898	0.28	0
RNF181	0	-0.24337	0.839	0.238	0
COA3	0	-0.24396	0.836	0.253	0
TCEAL8	0	-0.24645	0.742	0.207	0
ACTR10	0	-0.24678	0.677	0.159	0
TCEB1	0	-0.24693	0.913	0.313	0
TRAPPC1	0	-0.24707	0.878	0.304	0
UROD	0	-0.25011	0.649	0.157	0
NDUFA9	0	-0.25048	0.698	0.191	0
CUTA	0	-0.25117	0.958	0.386	0
CCT3	0	-0.25276	0.91	0.333	0
PSMD7	0	-0.25279	0.832	0.234	0
SUB1	0	-0.25311	0.993	0.576	0
ARPC3	0	-0.25366	0.948	0.39	0
PIN4	0	-0.2538	0.824	0.227	0
NHP2	0	-0.25439	0.938	0.345	0
BLOC1S1	0	-0.25464	0.908	0.32	0
C19orf70	0	-0.25534	0.949	0.395	0
ATP6V0B	0	-0.25601	0.955	0.39	0
SH3BGRL	0	-0.25757	0.838	0.265	0
PLP2	0	-0.25892	0.687	0.179	0
PRMT1	0	-0.26015	0.924	0.34	0
ESD	0	-0.26019	0.852	0.243	0
FAM96B	0	-0.26083	0.938	0.368	0
ATPIF1	0	-0.26099	0.928	0.35	0
CIB1	0	-0.26154	0.791	0.229	0
ATP5G3	0	-0.26343	0.963	0.441	0
CHMP5	0	-0.26389	0.774	0.229	0
CLDND1	0	-0.26453	0.73	0.197	0
COPZ1	0	-0.26475	0.792	0.229	0
SELT	0	-0.26534	0.925	0.32	0
PDPN	0	-0.26735	0.874	0.316	0
NUDC	0	-0.26825	0.92	0.326	0
MEAF6	0	-0.26884	0.804	0.229	0
DARS	0	-0.27022	0.721	0.19	0
UBE2B	0	-0.27142	0.764	0.203	0

CAPZA2	0	-0.27214	0.887	0.306	0
NDUFB3	0	-0.27236	0.908	0.299	0
ILF2	0	-0.27242	0.868	0.292	0
SUCLG1	0	-0.27292	0.772	0.219	0
MYEOV2	0	-0.27297	0.949	0.395	0
EIF3I	0	-0.27517	0.926	0.345	0
15-Sep	0	-0.27577	0.943	0.366	0
C7orf73	0	-0.27712	0.889	0.318	0
FKBP3	0	-0.2778	0.874	0.282	0
DAZAP2	0	-0.27801	0.843	0.255	0
CCT4	0	-0.27801	0.828	0.262	0
S100A13	0	-0.27869	0.781	0.239	0
DGUOK	0	-0.28116	0.84	0.253	0
PSMA7	0	-0.28337	0.982	0.573	0
PSMG2	0	-0.28351	0.698	0.176	0
MAGED2	0	-0.28517	0.819	0.277	0
RNF5	0	-0.28572	0.63	0.152	0
PRDX3	0	-0.28768	0.715	0.2	0
COX14	0	-0.28818	0.767	0.198	0
COX17	0	-0.28845	0.827	0.248	0
PSMD14	0	-0.28972	0.806	0.231	0
CD99	0	-0.29053	0.97	0.496	0
NDUFS2	0	-0.29075	0.872	0.309	0
HSPD1	0	-0.29143	0.929	0.345	0
KMT2E	0	-0.29251	0.828	0.256	0
SNRPE	0	-0.29327	0.923	0.34	0
NDUFB9	0	-0.29356	0.967	0.417	0
POLR2C	0	-0.29888	0.655	0.178	0
ENY2	0	-0.29908	0.875	0.299	0
TMEM9	0	-0.29908	0.783	0.229	0
NAP1L1	0	-0.29947	0.931	0.378	0
TSG101	0	-0.30056	0.687	0.191	0
RSL1D1	0	-0.30112	0.792	0.217	0
HSD17B10	0	-0.30221	0.865	0.277	0
FCGRT	0	-0.30405	0.665	0.183	0
ATP5G1	0	-0.30418	0.947	0.364	0
SNRPF	0	-0.30436	0.873	0.289	0
ATF4	0	-0.30555	0.856	0.279	0
VDAC1	0	-0.30582	0.96	0.39	0
GPX4	0	-0.30641	0.986	0.598	0
HSP90B1	0	-0.30654	0.925	0.366	0
CFDP1	0	-0.30998	0.793	0.227	0
RABAC1	0	-0.31048	0.979	0.436	0
OSTC	0	-0.31141	0.818	0.253	0
SRP9	0	-0.31187	0.975	0.453	0
RHOA	0	-0.31194	0.963	0.398	0
PRR13	0	-0.31228	0.78	0.214	0
RAB11A	0	-0.31378	0.793	0.224	0
MED28	0	-0.31398	0.641	0.159	0
CD59	0	-0.31471	0.871	0.292	0
PCNP	0	-0.31633	0.882	0.306	0

MORF4L2	0	-0.3184	0.929	0.366	0
MAGOH	0	-0.31849	0.683	0.174	0
CTSB	0	-0.32047	0.911	0.374	0
MORF4L1	0	-0.32054	0.965	0.446	0
SLC25A3	0	-0.32177	0.952	0.438	0
CSNK2B	0	-0.32336	0.885	0.309	0
MRFAP1	0	-0.32412	0.955	0.415	0
COX7A2	0	-0.32555	0.985	0.561	0
UBE2D3	0	-0.32595	0.933	0.354	0
RWDD1	0	-0.32633	0.853	0.284	0
POMP	0	-0.32663	0.952	0.417	0
XRCC6	0	-0.32691	0.822	0.285	0
SH3BGRL3	0	-0.32862	0.948	0.462	0
GPI	0	-0.32943	0.71	0.229	0
NDUFC1	0	-0.32982	0.922	0.366	0
NDUFB11	0	-0.3313	0.967	0.434	0
VPS28	0	-0.33131	0.893	0.308	0
HSPA9	0	-0.33356	0.813	0.289	0
FTH1	0	-0.33374	0.997	0.757	0
EEF2	0	-0.33444	0.993	0.632	0
TUBB	0	-0.33504	0.967	0.523	0
GHITM	0	-0.33507	0.726	0.215	0
SNRPG	0	-0.33643	0.958	0.419	0
BRK1	0	-0.33715	0.978	0.496	0
NDUFB6	0	-0.33789	0.751	0.219	0
UBE2L6	0	-0.33829	0.841	0.277	0
GSTK1	0	-0.34157	0.849	0.279	0
WDR61	0	-0.34186	0.69	0.191	0
MED10	0	-0.34324	0.828	0.258	0
COMMD1	0	-0.34342	0.591	0.161	0
NDUFB1	0	-0.34436	0.923	0.378	0
KRTCAP2	0	-0.34466	0.804	0.291	0
DNAJA1	0	-0.34471	0.785	0.255	0
WRB	0	-0.34527	0.744	0.219	0
PAIP2	0	-0.34536	0.886	0.337	0
NEAT1	0	-0.3461	0.969	0.629	0
POLR3GL	0	-0.34775	0.653	0.174	0
PSMB9	0	-0.34788	0.75	0.207	0
SRSF3	0	-0.3487	0.95	0.414	0
TCEAL4	0	-0.34905	0.91	0.357	0
SPTSSA	0	-0.34953	0.855	0.289	0
C6orf48	0	-0.35059	0.924	0.366	0
DPY30	0	-0.35212	0.809	0.256	0
PRDX4	0	-0.35299	0.896	0.337	0
REEP5	0	-0.35419	0.844	0.277	0
ARL6IP5	0	-0.35533	0.885	0.321	0
VDAC2	0	-0.35569	0.91	0.356	0
ERP29	0	-0.35606	0.875	0.308	0
PSMB6	0	-0.35649	0.952	0.448	0
EEF1B2	0	-0.35708	0.93	0.403	0
SIVA1	0	-0.35996	0.824	0.27	0

SEC62	0	-0.36071	0.948	0.386	0
OCIAD1	0	-0.36113	0.933	0.393	0
TOMM20	0	-0.36343	0.856	0.28	0
MDH2	0	-0.36605	0.946	0.415	0
AK2	0	-0.36608	0.78	0.212	0
ACP1	0	-0.36703	0.824	0.263	0
CSTB	0	-0.36803	0.935	0.391	0
FKBP2	0	-0.36815	0.938	0.386	0
HSPE1	0	-0.36843	0.982	0.526	0
COPS6	0	-0.36904	0.909	0.368	0
TXNL1	0	-0.3703	0.791	0.241	0
SRP14	0	-0.37095	0.991	0.61	0
PHPT1	0	-0.37127	0.968	0.46	0
EAPP	0	-0.37288	0.668	0.188	0
YPEL5	0	-0.37314	0.629	0.179	0
SON	0	-0.3735	0.957	0.39	0
CCT2	0	-0.37396	0.864	0.328	0
SEPW1	0	-0.37684	0.968	0.467	0
TMEM205	0	-0.37819	0.926	0.39	0
RBM8A	0	-0.3791	0.902	0.352	0
C14orf166	0	-0.37932	0.887	0.335	0
PSMA1	0	-0.37945	0.897	0.362	0
NDUFB4	0	-0.3798	0.956	0.444	0
CAMLG	0	-0.3821	0.825	0.27	0
EDF1	0	-0.38219	0.977	0.52	0
OST4	0	-0.38489	0.967	0.511	0
ATP5I	0	-0.38495	0.972	0.521	0
RAB1A	0	-0.38668	0.859	0.284	0
SSR3	0	-0.3874	0.807	0.238	0
GABARAPL2	0	-0.38798	0.988	0.497	0
LAP3	0	-0.38845	0.79	0.287	0
SSBP1	0	-0.38958	0.908	0.349	0
UXT	0	-0.39005	0.815	0.27	0
ATP5E	0	-0.39039	0.992	0.735	0
BSG	0	-0.39054	0.993	0.597	0
ARL6IP1	0	-0.391	0.92	0.4	0
ANAPC11	0	-0.39209	0.974	0.544	0
PSMC4	0	-0.39327	0.837	0.308	0
SAT2	0	-0.39339	0.914	0.369	0
TECR	0	-0.3935	0.941	0.407	0
AP1S1	0	-0.3944	0.798	0.268	0
NDUFB7	0	-0.39509	0.987	0.579	0
IFITM3	0	-0.39536	0.908	0.395	0
HIGD2A	0	-0.39708	0.929	0.386	0
HMGN2	0	-0.39734	0.947	0.477	0
SARS	0	-0.39991	0.806	0.287	0
ARF1	0	-0.39991	0.916	0.378	0
SYPL1	0	-0.40112	0.689	0.212	0
TMCO1	0	-0.40231	0.901	0.35	0
NDUFA12	0	-0.40442	0.913	0.32	0
WBSCR22	0	-0.40551	0.774	0.267	0

ATP5A1	0	-0.4069	0.963	0.45	0
PRDX5	0	-0.40715	0.979	0.518	0
HLA-C	0	-0.4078	0.988	0.631	0
C4orf3	0	-0.40978	0.928	0.376	0
EIF4A1	0	-0.41126	0.988	0.574	0
PSMD8	0	-0.41542	0.958	0.479	0
SDHC	0	-0.41683	0.898	0.345	0
USE1	0	-0.4177	0.693	0.217	0
HSP90AB1	0	-0.41836	0.988	0.573	0
SUMO2	0	-0.41916	0.983	0.605	0
CETN2	0	-0.42018	0.644	0.203	0
MDH1	0	-0.42187	0.825	0.308	0
COX7A2L	0	-0.42242	0.879	0.342	0
STMN1	0	-0.42251	0.907	0.388	0
TBCA	0	-0.423	0.985	0.581	0
ANAPC16	0	-0.4245	0.764	0.231	0
EIF3H	0	-0.42532	0.882	0.333	0
NDUFA6	0	-0.42559	0.932	0.403	0
TMED9	0	-0.4258	0.961	0.456	0
PSMB1	0	-0.42841	0.958	0.444	0
CCT8	0	-0.42875	0.77	0.275	0
NDUFA1	0	-0.42898	0.962	0.47	0
PSMB3	0	-0.43425	0.935	0.434	0
PSMB7	0	-0.43431	0.932	0.409	0
APEX1	0	-0.43484	0.822	0.303	0
COPS8	0	-0.435	0.832	0.27	0
MYL12B	0	-0.43505	0.936	0.424	0
PRDX6	0	-0.4351	0.952	0.504	0
FUNDC2	0	-0.43594	0.66	0.209	0
HSBP1	0	-0.43669	0.963	0.455	0
SMIM7	0	-0.43695	0.889	0.342	0
BCAS2	0	-0.43855	0.646	0.221	0
GPM6B	0	-0.43921	0.998	0.725	0
NDUFS4	0	-0.43986	0.873	0.318	0
PSMB2	0	-0.44027	0.909	0.362	0
DNAJB6	0	-0.4403	0.901	0.361	0
SERF2	0	-0.44084	0.988	0.691	0
BNIP3L	0	-0.44242	0.845	0.303	0
EIF3E	0	-0.4426	0.897	0.357	0
SSR4	0	-0.44502	0.971	0.482	0
NDUFC2	0	-0.44897	0.97	0.477	0
SSR2	0	-0.44978	0.951	0.443	0
ZNHIT1	0	-0.45069	0.95	0.446	0
ATP5J	0	-0.45107	0.98	0.535	0
SEC11A	0	-0.45207	0.905	0.342	0
ATRAID	0	-0.45208	0.919	0.385	0
SBDS	0	-0.45254	0.931	0.393	0
PSMB5	0	-0.45487	0.939	0.424	0
SYF2	0	-0.45504	0.797	0.267	0
PTGES3	0	-0.45505	0.961	0.474	0
BANF1	0	-0.45578	0.957	0.45	0

COX6C	0	-0.4561	0.995	0.66	0
H3F3A	0	-0.45693	0.965	0.65	0
NEDD8	0	-0.45792	0.967	0.533	0
COMMD6	0	-0.45888	0.958	0.489	0
ECH1	0	-0.4605	0.903	0.376	0
PDCD5	0	-0.46056	0.909	0.368	0
NDUFA13	0	-0.46285	0.988	0.593	0
SEC61B	0	-0.46343	0.966	0.503	0
TCEB2	0	-0.4638	0.982	0.626	0
ATP5B	0	-0.46396	0.982	0.557	0
TXN	0	-0.46459	0.952	0.46	0
TMED10	0	-0.46976	0.93	0.417	0
POLR2I	0	-0.47248	0.933	0.402	0
PFDN2	0	-0.47288	0.9	0.352	0
SSB	0	-0.4743	0.878	0.354	0
PPIB	0	-0.47752	0.968	0.477	0
PSME2	0	-0.47837	0.847	0.332	0
TUBA1A	0	-0.48056	0.999	0.768	0
IP6K2	0	-0.48131	0.806	0.304	0
C19orf53	0	-0.48148	0.979	0.571	0
SLC25A6	0	-0.48286	0.985	0.632	0
LAPTM4A	0	-0.48557	0.958	0.482	0
PSENEN	0	-0.48685	0.797	0.306	0
HNRNPA2B1	0	-0.48731	0.995	0.663	0
IMPDH2	0	-0.48921	0.704	0.255	0
COX5B	0	-0.48976	0.995	0.682	0
POLR2J	0	-0.49098	0.959	0.458	0
MT1E	0	-0.49701	0.863	0.417	0
LAMTOR4	0	-0.49762	0.964	0.48	0
ZNF667-AS	0	-0.49805	0.859	0.326	0
SLC3A2	0	-0.49868	0.889	0.386	0
TMEM258	0	-0.50211	0.957	0.48	0
PSME1	0	-0.50256	0.88	0.364	0
EIF3M	0	-0.50345	0.781	0.308	0
TUBA1B	0	-0.50386	0.99	0.701	0
UQCRQ	0	-0.50396	0.985	0.622	0
PKM	0	-0.50465	0.982	0.658	0
SLC25A5	0	-0.50606	0.941	0.484	0
ATP5O	0	-0.50859	0.967	0.499	0
SNHG8	0	-0.51131	0.699	0.243	0
ZFAS1	0	-0.5127	0.955	0.526	0
HNRNPK	0	-0.5174	0.987	0.598	0
SDCBP	0	-0.5178	0.914	0.438	0
ATP5F1	0	-0.52266	0.917	0.403	0
COPE	0	-0.52342	0.983	0.631	0
HMGB1	0	-0.52659	0.997	0.691	0
ATP5J2	0	-0.52702	0.985	0.591	0
COX6A1	0	-0.53117	0.994	0.656	0
HNRNPDL	0	-0.5317	0.977	0.544	0
PARK7	0	-0.53768	0.98	0.603	0
HSPA5	0	-0.54132	0.905	0.422	0

LAMTOR5	0	-0.54148	0.97	0.537	0
ARF4	0	-0.54399	0.854	0.371	0
CHMP2A	0	-0.54471	0.96	0.48	0
PEBP1	0	-0.5451	0.996	0.656	0
HNRNPA1	0	-0.5453	0.994	0.696	0
C12orf57	0	-0.54728	0.906	0.412	0
ATP6V1G1	0	-0.5475	0.967	0.526	0
EIF4A2	0	-0.54781	0.944	0.491	0
SELK	0	-0.55009	0.945	0.446	0
ERH	0	-0.55031	0.961	0.509	0
TMA7	0	-0.55466	0.99	0.703	0
TAGLN2	0	-0.55629	0.936	0.497	0
EEF1D	0	-0.56624	0.983	0.583	0
PRDX1	0	-0.56629	0.983	0.614	0
ATP5EP2	0	-0.56801	0.713	0.301	0
ATP6V1F	0	-0.56822	0.958	0.492	0
SHFM1	0	-0.57162	0.963	0.528	0
CIRBP	0	-0.5729	0.997	0.718	0
PTN	0	-0.57295	0.997	0.776	0
SPCS1	0	-0.5762	0.952	0.497	0
HINT1	0	-0.5765	0.989	0.694	0
CYCS	0	-0.57656	0.968	0.504	0
H2AFZ	0	-0.57786	0.932	0.497	0
TMEM59	0	-0.57805	0.94	0.48	0
S100A11	0	-0.57853	0.798	0.378	0
CFL1	0	-0.57922	0.998	0.798	0
NAA38	0	-0.57932	0.708	0.272	0
NDUFB2	0	-0.57987	0.984	0.586	0
UQCRH	0	-0.58056	0.974	0.588	0
PRDX2	0	-0.58203	0.988	0.68	0
FBL	0	-0.58245	0.658	0.243	0
ATP5G2	0	-0.58425	0.992	0.651	0
BUD31	0	-0.586	0.919	0.439	0
OAZ1	0	-0.58691	0.996	0.754	0
CHCHD2	0	-0.58784	0.997	0.747	0
BLVRB	0	-0.58858	0.643	0.212	0
HNRNPC	0	-0.58919	0.969	0.538	0
FAM162A	0	-0.59042	0.724	0.282	0
7-Sep	0	-0.59078	0.96	0.59	0
MYL6	0	-0.59116	0.997	0.793	0
NDUFS5	0	-0.59401	0.989	0.685	0
ATP6V0E1	0	-0.59532	0.948	0.492	0
TAF7	0	-0.59739	0.895	0.436	0
HLA-A	0	-0.59884	0.995	0.798	0
TMBIM6	0	-0.60828	0.989	0.626	0
GABARAP	0	-0.60961	0.618	0.244	0
DYNLL1	0	-0.61831	0.989	0.706	0
WBP5	0	-0.61951	0.872	0.385	0
TRMT112	0	-0.62088	0.945	0.497	0
EIF1B	0	-0.62456	0.816	0.347	0
SPCS2	0	-0.62833	0.961	0.528	0

TMSB4X	0	-0.63166	0.999	0.973	0
ATP5L	0	-0.63196	0.987	0.639	0
GSTP1	0	-0.63697	0.989	0.644	0
UBB	0	-0.63785	0.995	0.779	0
SRI	0	-0.63823	0.988	0.687	0
UBL5	0	-0.63898	0.989	0.733	0
HLA-B	0	-0.63905	0.989	0.713	0
RAN	0	-0.64187	0.971	0.588	0
DAD1	0	-0.64354	0.933	0.482	0
HSPA8	0	-0.65054	0.99	0.716	0
SAT1	0	-0.65542	0.911	0.501	0
NDUFA5	0	-0.65668	0.877	0.436	0
ANXA5	0	-0.65673	0.983	0.68	0
ACTB	0	-0.66232	1	0.92	0
COX6B1	0	-0.6647	0.992	0.708	0
PFDN5	0	-0.66616	0.986	0.685	0
NGFRAP1	0	-0.66716	0.99	0.699	0
ENO1	0	-0.66776	0.987	0.764	0
CALM2	0	-0.67512	0.999	0.769	0
PPIA	0	-0.67617	0.991	0.747	0
LGALS3	0	-0.6801	0.936	0.583	0
ACTG1	0	-0.6843	0.999	0.909	0
COX7C	0	-0.68652	0.996	0.718	0
EIF3K	0	-0.69167	0.955	0.552	0
S100A6	0	-0.69239	0.933	0.605	0
SNRPD2	0	-0.69374	0.974	0.597	0
TPI1	0	-0.69461	0.995	0.786	0
NDUFA4	0	-0.69712	0.999	0.821	0
NPC2	0	-0.69776	0.762	0.345	0
H3F3B	0	-0.70141	0.999	0.889	0
ALDOA	0	-0.7021	0.995	0.805	0
CD63	0	-0.70365	0.997	0.856	0
CLU	0	-0.70786	0.997	0.896	0
SOD1	0	-0.70823	0.971	0.624	0
MALAT1	0	-0.70884	1	0.979	0
HSP90AA1	0	-0.71007	0.995	0.754	0
TIMP1	0	-0.71793	0.973	0.68	0
TOMM7	0	-0.72301	0.99	0.696	0
WDR83OS	0	-0.72497	0.97	0.6	0
NRN1	0	-0.73986	0.627	0.26	0
DBI	0	-0.74685	0.998	0.817	0
S100A10	0	-0.75367	0.885	0.533	0
CNBP	0	-0.75476	0.959	0.583	0
SEC61G	0	-0.75795	0.994	0.935	0
MGST3	0	-0.76452	0.894	0.468	0
NACA	0	-0.76804	0.996	0.797	0
B2M	0	-0.76848	0.998	0.947	0
LDHB	0	-0.77481	0.985	0.706	0
BTF3	0	-0.77743	0.993	0.774	0
EEF1A1	0	-0.77982	0.973	0.851	0
NPM1	0	-0.78135	0.983	0.715	0

LDHA	0	-0.78699	0.951	0.66	0
SAP18	0	-0.78906	0.958	0.576	0
TMSB10	0	-0.79156	0.996	0.918	0
COX4I1	0	-0.79545	0.996	0.764	0
SKP1	0	-0.80438	0.998	0.791	0
GNB2L1	0	-0.80506	1	0.906	0
PGK1	0	-0.83173	0.917	0.545	0
TPT1	0	-0.84126	0.994	0.889	0
GLUL	0	-0.87036	0.895	0.535	0
DNAJB1	0	-0.87871	0.813	0.443	0
PTMA	0	-0.88478	0.998	0.887	0
GAPDH	0	-0.90062	1	0.99	0
FAU	0	-0.92283	0.997	0.903	0
EIF1	0	-0.93958	0.999	0.918	0
MT3	0	-0.96443	0.954	0.802	0
FTL	0	-0.96775	0.999	0.974	0
UBC	0	-0.97475	0.992	0.86	0
MT2A	0	-1.05598	0.998	0.944	0
NBEAL1	0	-1.12888	0.747	0.494	0
MT1X	0	-1.16144	0.933	0.634	0
FOS	0	-1.25756	0.8	0.704	0
CRYAB	0	-1.29072	0.745	0.617	0
ACSL3	4.9406564	0.562234	0.816	0.084	7.38331701145159e-320
FZD3	4.9406564	0.523167	0.81	0.094	7.38331701145159e-320
PHF14	4.9406564	0.299718	0.809	0.13	7.38331701145159e-320
PSMD12	4.9406564	0.210047	0.745	0.109	7.38331701145159e-320
CNOT7	4.9406564	0.159511	0.69	0.094	7.38331701145159e-320
ARPC5L	9.881312S	0.532493	0.778	0.07	1.47666340229032e-319
DTNA	9.881312S	-0.30873	0.704	0.198	1.47666340229032e-319
DCAF7	1.482196S	0.253821	0.723	0.089	2.21499510343548e-319
ANKRD12	1.482196S	-0.21536	0.683	0.185	2.21499510343548e-319
PCSK1N	1.976262E	0.872106	0.878	0.113	2.95332680458064e-319
DENR	3.458459E	0.289714	0.734	0.089	5.16832190801611e-319
JUNB	3.458459E	-0.30009	0.879	0.374	5.16832190801611e-319
HSPBP1	4.9406564	0.334179	0.762	0.094	7.38331701145159e-319
C5orf24	6.916919C	0.595874	0.807	0.072	1.03366438160322e-318
ITGB1	7.9050503	0.724345	0.82	0.079	1.18133072183225e-318
BLOC1S2	1.0375378	-0.10119	0.626	0.121	1.55049657240483e-318
NRBP1	1.581010C	0.301553	0.733	0.089	2.36266144366451e-318
SYAP1	2.5691413	0.140359	0.702	0.103	3.83932484595483e-318
RNF10	3.409052S	0.270693	0.753	0.101	5.0944887379016e-318
AGTRAP	4.644217C	-0.2074	0.596	0.121	6.94031799076449e-318
TSSC4	4.8418433	0.118689	0.711	0.111	7.23565067122256e-318
ODC1	5.829974E	0.529293	0.821	0.103	8.71231407351287e-318
YY1	6.8675124	0.433227	0.752	0.077	1.02628106459177e-317
UFM1	9.7330932	-0.1383	0.658	0.144	1.45451345125596e-317
FKBP10	1.3438585	0.31098	0.774	0.115	2.00826222711483e-317
USP22	1.4920782	0.455872	0.766	0.08	2.22976173745838e-317
CSNK1D	1.674882E	0.384835	0.749	0.085	2.50294446688209e-317
IFRD1	2.766767E	-0.33011	0.676	0.209	4.13465752641289e-317
METTL23	4.0068723	0.500916	0.769	0.077	5.98787009628724e-317

SPRY2	4.1106261	0.576714	0.81	0.087	6.14291975352772e-317
CAPNS1	4.3576589	0.451227	0.761	0.082	6.5120856041003e-317
HNRNPUL1	5.2815617	0.175035	0.717	0.099	7.89276588524175e-317
SLC39A6	6.2153458	0.36585	0.766	0.092	9.2882128004061e-317
DTYMK	1.0355615	0.322348	0.734	0.084	1.54754324560025e-316
SEC63	1.5938557	0.207005	0.718	0.092	2.38185806789428e-316
COA5	1.8962239	0.126045	0.719	0.115	2.83371706899512e-316
COMMD3	2.8675570	-0.10986	0.624	0.128	4.2852771934465e-316
AKIRIN1	4.0819703	0.418863	0.769	0.084	6.1000965148613e-316
TCF12	4.4861160	0.428415	0.806	0.101	6.70405184639804e-316
SETD5	5.8808633	0.394279	0.796	0.096	8.78836223873083e-316
PTBP1	6.0226602	0.262277	0.721	0.089	9.00026343695949e-316
DCAF13	6.2598117	-0.26996	0.626	0.161	9.35466265350916e-316
PPP2CB	1.2453912	0.603153	0.796	0.079	1.8611127190766e-315
TIMM9	1.9801657	-0.23686	0.587	0.121	2.95915962501968e-315
LINC00461	5.7979097	0.312246	0.815	0.135	8.66439634610855e-315
CBX1	5.8661896	0.159964	0.692	0.096	8.76643378720681e-315
TPGS2	8.0899791	0.132849	0.671	0.097	1.20896647740612e-314
EIF4A3	9.8038434	0.311919	0.751	0.092	1.46508636121636e-314
TUSC2	1.0099739	0.390924	0.739	0.074	1.50930504679795e-314
CPNE3	2.0577142	0.142387	0.717	0.118	3.07504816883143e-314
TPD52L2	2.1554651	0.200004	0.689	0.087	3.221127095903e-314
SYNCRIP	2.5035541	0.281837	0.749	0.096	3.74131131262781e-314
TM2D3	3.1128309	-0.33744	0.632	0.166	4.651814583163e-314
RALBP1	5.5965582	0.299742	0.714	0.079	8.36349664462386e-314
KIF21A	6.7542676	0.251429	0.82	0.144	1.00935776366982e-313
RBM17	7.9079109	0.630555	0.766	0.048	1.18175821588722e-313
TPGS1	1.0827735	0.458655	0.753	0.065	1.61809674629828e-313
BCLAF1	1.0923588	0.386469	0.779	0.101	1.6324211196322e-313
SURF4	1.7543549	0.256848	0.742	0.099	2.62170807433809e-313
CCDC59	3.7830917	-0.18877	0.597	0.118	5.65345235056565e-313
PSMC2	3.8723575	-0.19072	0.723	0.195	5.78685116900176e-313
SERINC3	4.9799119	0.392174	0.781	0.103	7.44198041843438e-313
SNHG7	5.0591976	0.354776	0.8	0.113	7.56046493650735e-313
ZNF24	5.7348857	-0.19812	0.634	0.152	8.57021327764707e-313
NARF	7.4278026	0.304597	0.739	0.097	1.11001083282844e-312
PTRHD1	1.0952914	0.138399	0.683	0.097	1.63680356127752e-312
HNRNPAB	1.5194666	0.459422	0.766	0.077	2.27069100764502e-312
PAFAH1B1	1.7485439	0.536275	0.783	0.07	2.6130241122019e-312
FAM50A	3.9205048	0.426105	0.778	0.099	5.85880247927639e-312
SREK1IP1	4.0010001	-0.12999	0.616	0.121	5.97909458102011e-312
SPOP	4.5334195	0.124853	0.689	0.106	6.77474223379346e-312
ECSIT	4.5436023	-0.11793	0.66	0.138	6.78995939782041e-312
ACBD6	7.6751232	0.252719	0.685	0.07	1.14697041832397e-311
REXO2	8.3398471	-0.35918	0.64	0.186	1.24630675411008e-311
JAGN1	1.0365018	0.116533	0.689	0.108	1.54894841634392e-311
LYPD1	1.7069733	0.567708	0.807	0.084	2.55090103053389e-311
NUDT16L1	2.1641313	0.265174	0.724	0.085	3.23407794338996e-311
SDHD	2.8955576	-0.29506	0.638	0.178	4.3271213156774e-311
OCIAD2	2.9485360	-0.40766	0.839	0.376	4.4062923212752e-311
CRK	3.7663044	0.473701	0.772	0.084	5.62836543415722e-311

ADD1	7.1519947	0.165056	0.703	0.096	1.06879410205393e-310
DPYSL2	8.0849589	0.271876	0.772	0.115	1.20821626373291e-310
CYB5R3	1.0271913	0.261709	0.727	0.099	1.53503479613198e-310
WDR18	1.8701334	0.454993	0.742	0.062	2.79472743974896e-310
CDC26	1.9606831	-0.18097	0.575	0.106	2.93004492829023e-310
KEAP1	2.0996259	0.42749	0.76	0.079	3.13768101390253e-310
VPS35	2.5076290	0.104989	0.709	0.109	3.74740078602615e-310
ACAA1	3.1825869	0.417225	0.747	0.074	4.75605788841655e-310
RAB13	6.5052129	-0.45911	0.712	0.246	9.72139016733037e-310
SHISA5	6.9559391	0.149205	0.683	0.108	0.00E+00
GRSF1	7.3692166	0.219549	0.69	0.089	0.00E+00
SPP1	9.0230685	-1.13996	0.765	0.638	0.00E+00
SCG5	2.0049826	-0.40918	0.68	0.236	0.00E+00
SCNM1	2.2941541	-0.15697	0.653	0.149	0.00E+00
BID	2.9646652	0.205488	0.715	0.101	0.00E+00
HLA-E	3.8519746	-0.19535	0.815	0.248	0.00E+00
SOD2	7.1818174	-0.96026	0.629	0.328	0.00E+00
PPP1R14B	1.0544112	0.726637	0.828	0.085	0.00E+00
ENO2	1.5043621	-0.53081	0.558	0.191	########
HNRNPH2	1.7508589	-0.20164	0.668	0.178	########
FAM104A	1.9652411	0.315062	0.676	0.063	########
TMEM134	3.5379555	0.474761	0.757	0.079	########
CRLS1	4.0349338	0.63771	0.78	0.056	########
IDH1	4.3719530	0.160892	0.749	0.128	########
JOSD2	5.2100126	0.460708	0.76	0.082	########
C12orf76	5.4215826	0.105689	0.696	0.111	########
MDK	2.3487949	0.154934	0.91	0.304	########
CTSL	4.1767860	-0.352	0.668	0.217	########
UBL3	4.3735220	0.355213	0.758	0.091	########
CCDC28B	7.1734727	0.124694	0.67	0.096	########
NFIX	7.3994046	0.487928	0.8	0.099	########
USP8	1.3045217	0.179523	0.674	0.085	########
A1BG	1.3829981	0.40654	0.742	0.089	########
ISG15	1.5254659	-0.20136	0.737	0.188	########
RBM42	2.0378651	0.287298	0.771	0.115	########
SUGT1	2.0718382	-0.12927	0.626	0.133	########
RAB4A	2.4120994	0.364167	0.75	0.091	########
DDX39A	2.9300232	-0.39003	0.616	0.195	########
SSRP1	3.3899737	0.348847	0.749	0.092	########
GLRX5	3.8109780	0.478755	0.735	0.055	########
DYNC1H1	5.5652342	0.261934	0.752	0.108	########
ACTR2	5.9579757	0.403677	0.733	0.077	########
PCNA	7.5433128	-0.13741	0.635	0.126	########
LRRC59	7.6420560	0.480884	0.761	0.068	########
MAP2	0.00E+00	0.411857	0.81	0.115	########
MMP24-AS1	0.00E+00	0.857653	0.812	0.053	########
PQBP1	0.00E+00	-0.22099	0.628	0.15	########
ATF6B	0.00E+00	0.207611	0.757	0.128	########
PRKDC	0.00E+00	0.272747	0.709	0.08	########
COPS7A	0.00E+00	0.143827	0.659	0.092	########
CNIH1	0.00E+00	-0.33787	0.652	0.193	########

CHPT1	0.00E+00	0.620297	0.823	0.092	########
GOLGA4	0.00E+00	0.1333	0.686	0.109	########
YIF1B	0.00E+00	0.207767	0.716	0.103	########
CXCL16	0.00E+00	0.353122	0.709	0.062	########
DHRS7	0.00E+00	-0.24433	0.652	0.173	########
SMIM12	0.00E+00	0.231392	0.669	0.074	########
SLC35B2	0.00E+00	0.746415	0.806	0.06	########
FAM96A	0.00E+00	-0.22417	0.58	0.135	########
CYTH2	0.00E+00	0.268334	0.706	0.085	########
PRPSAP1	0.00E+00	0.479037	0.749	0.075	########
PMP2	########	0.313239	0.941	0.294	########
PMF1	########	-0.13064	0.633	0.132	########
NUDT3	########	0.318575	0.733	0.087	########
SPECC1	########	0.726562	0.824	0.068	########
TAF12	########	-0.2801	0.587	0.152	########
IDI1	########	0.470634	0.757	0.072	########
PSMD3	########	0.387959	0.747	0.091	########
SPAG16	########	-0.16829	0.639	0.152	########
YWHAH	########	0.42875	0.756	0.084	########
POU3F2	########	0.808174	0.829	0.067	########
SCARB2	########	0.380505	0.728	0.08	########
SMIM20	########	-0.10882	0.622	0.123	########
TMEM203	########	0.470205	0.742	0.06	########
EPN2	########	0.47743	0.786	0.08	########
ZFR	########	0.248048	0.753	0.113	########
SLTM	########	0.184259	0.704	0.092	########
PPP1R12A	########	0.297248	0.759	0.109	########
C9orf142	########	0.443661	0.745	0.068	########
HERPUD1	########	-0.59546	0.648	0.263	########
CYB5D2	########	0.277787	0.698	0.085	########
NUCB2	########	-0.23335	0.604	0.147	########
TNRC6B	########	0.471074	0.816	0.113	########
DYNC1LI2	########	0.18252	0.708	0.097	########
RNF13	########	-0.12498	0.701	0.174	########
ZRANB2	########	0.138476	0.732	0.13	########
PRPF38B	########	0.143899	0.715	0.123	########
PSRC1	########	0.273911	0.778	0.128	########
C19orf25	########	0.4506	0.76	0.08	########
CLIC4	########	0.137055	0.789	0.176	########
ZFAND3	########	0.442493	0.704	0.053	########
HDGF	########	0.271722	0.68	0.068	########
FAM213A	########	-0.19116	0.674	0.174	########
COQ9	########	0.113528	0.671	0.101	########
CAPZA1	########	0.475922	0.74	0.065	########
NDUFAF2	########	-0.25618	0.575	0.128	########
ZYX	########	0.437124	0.803	0.115	########
ZFAND5	########	0.171698	0.726	0.125	########
CCZ1	########	0.229571	0.727	0.111	########
GSTO1	########	-0.3598	0.576	0.15	########
RFC1	########	0.289841	0.681	0.068	########
GPAA1	########	0.59183	0.779	0.067	########

GPC1	########	0.840744	0.848	0.099	########
SPAG9	########	0.535343	0.799	0.096	########
SEZ6L2	########	0.27908	0.714	0.108	########
P4HTM	########	0.430347	0.752	0.084	########
CCDC25	########	-0.1656	0.587	0.115	########
TUBB4B	########	-0.10713	0.717	0.19	########
SHISA4	########	0.150266	0.72	0.116	########
PDCD2	########	0.399322	0.695	0.058	########
CNIH3	########	0.589579	0.766	0.062	########
KLC1	########	0.324225	0.756	0.104	########
EIF4EBP1	########	-0.10473	0.689	0.164	########
IDH2	########	0.405586	0.784	0.103	########
PNRC1	########	0.18186	0.752	0.137	########
TRAF4	########	0.613966	0.761	0.051	########
WIPI2	########	0.371537	0.743	0.089	########
TAPBP	########	0.33249	0.715	0.08	########
CAPRIN1	########	0.481056	0.746	0.07	########
MTFR1L	########	0.198238	0.682	0.092	########
KIF1B	########	0.449734	0.8	0.115	########
S100B	########	-0.84312	0.836	0.545	########
TSR3	########	0.636615	0.759	0.051	########
DCUN1D5	########	0.196095	0.659	0.084	########
PRCC	########	0.172673	0.668	0.091	########
VTI1B	########	0.315751	0.692	0.085	########
CEBPB	########	0.249751	0.796	0.145	########
C8orf33	########	0.129416	0.69	0.108	########
NRBF2	########	0.448733	0.741	0.074	########
TNC	########	0.103581	0.736	0.142	########
TIMM22	########	0.29752	0.686	0.068	########
SH3GL1	########	0.507547	0.745	0.063	########
SEMA6A	########	0.543642	0.762	0.067	########
ACYP2	########	-0.13239	0.669	0.161	########
WDR13	########	0.113096	0.693	0.121	########
FABP7	########	0.111784	0.98	0.472	########
PTPMT1	########	0.417447	0.711	0.063	########
RAE1	########	0.176255	0.658	0.082	########
CTDNEP1	########	0.857421	0.77	0.038	########
GRB2	########	0.52097	0.741	0.065	########
PLEKHB2	########	0.230999	0.676	0.085	########
PHF20	########	0.233444	0.689	0.092	########
FDFT1	########	0.222413	0.738	0.109	########
UCHL1	########	-0.62154	0.669	0.32	########
SEPHS2	########	0.432961	0.716	0.068	########
IFT57	########	-0.12137	0.625	0.14	########
YIPF4	########	0.215125	0.693	0.103	########
ANP32B	########	0.232443	0.717	0.104	########
COL6A1	########	0.556799	0.806	0.104	########
SNRPA1	########	-0.28644	0.566	0.13	########
NTPCR	########	-0.13047	0.62	0.125	########
EMC10	########	0.276646	0.77	0.123	########
ABHD17A	########	0.648555	0.735	0.041	########

LINC00152	########	-0.10484	0.689	0.168	########
CHD4	########	0.386463	0.715	0.074	########
CHPF	########	0.371189	0.774	0.111	########
CAMK2N1	########	0.504388	0.817	0.12	########
MED31	########	0.103974	0.621	0.08	########
OSER1	########	-0.33453	0.54	0.125	########
TFPT	########	0.437732	0.723	0.079	########
CCDC106	########	0.739326	0.786	0.051	########
IGBP1	########	-0.1528	0.619	0.132	########
SCG2	########	0.32285	0.843	0.181	########
WLS	########	0.373047	0.752	0.094	########
NXT1	########	0.314927	0.682	0.072	########
C22orf39	########	0.405803	0.692	0.062	########
KHDRBS3	########	0.369579	0.731	0.085	########
APPBP2	########	0.112895	0.595	0.068	########
MAP7D1	########	0.605281	0.734	0.041	########
VMA21	########	0.294535	0.66	0.058	########
NT5C	########	0.451873	0.741	0.077	########
POMGNT1	########	0.520363	0.748	0.06	########
CPE	########	0.212612	0.825	0.185	########
HAGH	########	0.4973	0.734	0.065	########
SMIM14	########	-0.31417	0.562	0.147	########
IFNGR2	########	0.441759	0.727	0.074	########
EBPL	########	0.448947	0.712	0.063	########
BRD7	########	0.486104	0.705	0.051	########
GID8	########	0.304141	0.651	0.053	########
SERINC1	########	-0.1008	0.652	0.147	########
PKIB	########	0.231418	0.745	0.125	########
SCAMP3	########	-0.14624	0.606	0.121	########
SMARCA5	########	0.574444	0.756	0.063	########
TAF1D	########	-0.19384	0.605	0.15	########
ISCA2	########	0.184724	0.65	0.092	########
PIGF	########	-0.21098	0.57	0.133	########
UBE2M	########	0.495902	0.685	0.044	########
BCAT1	########	0.213685	0.713	0.109	########
ALKBH5	########	0.619567	0.768	0.065	########
NUDT22	########	0.167637	0.626	0.07	########
ARL1	########	-0.20267	0.614	0.157	########
CREM	########	0.29788	0.67	0.068	########
CHMP2B	########	-0.11753	0.57	0.089	########
NT5C3B	########	0.570439	0.755	0.07	########
FLNA	########	0.729419	0.78	0.056	########
LSM6	########	-0.27639	0.591	0.157	########
SDR39U1	########	-0.24119	0.595	0.147	########
SLC35C2	########	0.34144	0.704	0.084	########
ADAR	########	0.319521	0.723	0.085	########
SUGP2	########	0.14827	0.663	0.097	########
RPA3	########	-0.41733	0.535	0.152	########
C1orf61	########	-0.36285	0.968	0.585	########
HMOX2	########	-0.18331	0.569	0.113	########
PPP2R2A	########	-0.11241	0.585	0.121	########

ZKSCAN1	########	0.212156	0.687	0.092	########
MXRA7	########	0.535498	0.712	0.055	########
TLK1	########	0.521401	0.742	0.062	########
GNAI3	########	0.120225	0.673	0.103	########
RAB22A	########	0.500312	0.714	0.05	########
GLRX3	########	-0.16712	0.541	0.099	########
POLD4	########	0.185442	0.687	0.108	########
LGALS1	########	-0.32512	0.953	0.598	########
CDS2	########	0.444482	0.706	0.062	########
FNTA	########	0.334196	0.715	0.084	########
ASH1L	########	0.163298	0.693	0.111	########
STARD7	########	0.286641	0.699	0.085	########
CBX5	########	0.157402	0.712	0.116	########
FERMT2	########	0.366389	0.708	0.074	########
CCND2	########	0.177978	0.751	0.133	########
TWF1	########	0.2261	0.666	0.082	########
CCDC53	########	-0.24069	0.565	0.132	########
TMEM183A	########	0.398433	0.707	0.07	########
PNMA1	########	0.517533	0.743	0.07	########
PLS3	########	0.617974	0.788	0.079	########
ZFAND2A	########	-0.57543	0.467	0.147	########
NAT14	########	0.433612	0.678	0.05	########
WDR33	########	-0.11855	0.579	0.099	########
ARL4A	########	-0.30875	0.73	0.255	########
CCDC107	########	0.231481	0.693	0.099	########
RPA2	########	-0.28762	0.55	0.144	########
GADD45A	########	-0.20682	0.745	0.222	########
MARK3	########	0.346513	0.717	0.089	########
OXLD1	########	0.58362	0.743	0.062	########
ABCF1	########	0.322755	0.671	0.065	########
NECAP2	########	-0.10972	0.591	0.096	########
REEP3	########	0.679899	0.736	0.032	########
CHCHD1	########	0.10638	0.571	0.058	########
IFT52	########	0.208405	0.623	0.07	########
MT1F	########	-0.13461	0.673	0.174	########
BOD1	########	0.344935	0.704	0.077	########
RNF11	########	0.327014	0.684	0.068	########
CMC1	########	0.111927	0.632	0.094	########
PFKL	########	0.328745	0.699	0.082	########
MFSD10	########	0.459023	0.7	0.056	########
OS9	########	-0.1622	0.751	0.207	########
HEY1	########	0.33735	0.774	0.123	########
EIF2AK2	########	0.277059	0.671	0.075	########
C17orf62	########	0.619108	0.763	0.072	########
CORO1B	########	0.43775	0.678	0.053	########
GINM1	########	0.422258	0.701	0.065	########
FAM136A	########	0.140504	0.616	0.077	########
KNOP1	########	0.201167	0.658	0.085	########
SERTAD1	########	-0.7286	0.507	0.217	########
TAOK3	########	0.170323	0.715	0.123	########
UFSP2	########	-0.21574	0.525	0.109	########

RALA	########	0.107995	0.645	0.089	########
PRPF4B	########	0.374578	0.698	0.075	########
CAPN2	########	0.353043	0.701	0.08	########
ITGB8	########	0.582647	0.778	0.072	########
NEMF	########	0.180304	0.667	0.094	########
SRSF6	########	0.356811	0.704	0.074	########
BRIX1	########	-0.33844	0.536	0.138	########
RUFY3	########	0.272944	0.693	0.094	########
EIF4G1	########	0.220193	0.636	0.08	########
RBMS1	########	0.621675	0.759	0.06	########
TMEM123	########	0.380112	0.72	0.085	########
NAMPT	########	-1.01942	0.475	0.311	########
CSTF3	########	0.132302	0.652	0.097	########
ATP1B3	########	0.355249	0.714	0.084	########
ITFG1	########	0.374303	0.708	0.079	########
BRMS1	########	0.251589	0.653	0.08	########
RAD21	########	0.11515	0.658	0.109	########
TRIP6	########	0.129104	0.668	0.12	########
DHX29	########	0.195593	0.675	0.084	########
CCDC109B	########	0.833655	0.799	0.06	########
ASNSD1	########	-0.25404	0.516	0.121	########
ECI1	########	0.514337	0.683	0.038	########
PTRF	########	0.215281	0.677	0.082	########
6-Mar	########	0.295194	0.736	0.104	########
WTAP	########	0.157792	0.704	0.118	########
AMD1	########	-0.21032	0.579	0.142	########
PPIC	########	0.600006	0.73	0.051	########
ADSL	########	0.173307	0.616	0.072	########
SMIM19	########	0.164158	0.614	0.082	########
HOPX	########	0.319299	0.947	0.349	########
CAST	########	0.194392	0.672	0.092	########
DNAJB2	########	0.181499	0.686	0.099	########
LLPH	########	0.11456	0.6	0.074	########
SLC39A7	########	0.131234	0.666	0.101	########
RGMA	########	0.395506	0.794	0.123	########
PMPCB	########	-0.35822	0.572	0.171	########
GLG1	########	0.410487	0.712	0.072	########
YAF2	########	-0.17707	0.644	0.161	########
TAOK1	########	0.505773	0.723	0.065	########
CCDC130	########	0.261771	0.644	0.075	########
ADIPOR1	########	0.547742	0.721	0.05	########
HMGB2	########	-0.59591	0.568	0.219	########
KLHDC8A	########	0.456389	0.781	0.101	########
IGFBP7	########	0.208134	0.853	0.219	########
IL6ST	########	0.483381	0.706	0.056	########
GSS	########	0.407932	0.665	0.056	########
SEMA6D	########	1.009025	0.781	0.026	########
ANKRD11	########	0.33422	0.712	0.089	########
GTF2F1	########	0.146686	0.652	0.092	########
PSIP1	########	0.304655	0.728	0.103	########
CLSTN1	########	0.206764	0.694	0.103	########

ERAL1	########	0.13395	0.597	0.084	########
TIMM44	########	0.266197	0.705	0.106	########
HRAS	########	0.63115	0.731	0.046	########
RNF126	########	0.540288	0.715	0.053	########
CMTM6	########	0.169094	0.629	0.074	########
POLR3H	########	0.32017	0.643	0.06	########
TSNAX	########	-0.11534	0.583	0.109	########
AKT1S1	########	0.372359	0.691	0.067	########
LEPROTL1	########	0.277904	0.684	0.087	########
FRG1	########	-0.17456	0.545	0.111	########
GSTM3	########	-0.21882	0.552	0.125	########
PPP4R2	########	0.236173	0.61	0.062	########
PCM1	########	0.326713	0.698	0.085	########
PEMT	########	0.179636	0.641	0.085	########
H1FX	########	0.565481	0.769	0.079	########
TIMP2	########	0.473532	0.714	0.063	########
TPM1	########	0.361321	0.712	0.089	########
COPS4	########	-0.23964	0.558	0.137	########
MPDU1	########	0.187124	0.638	0.085	########
JMJD1C	########	0.538716	0.732	0.062	########
MLST8	########	0.262578	0.63	0.063	########
RP11-1094	########	0.138085	0.631	0.084	########
EIF2AK1	########	0.454716	0.707	0.063	########
NCBP2-AS2	########	0.594671	0.73	0.055	########
STK17A	########	0.44042	0.724	0.077	########
OSBPL8	########	0.520076	0.702	0.053	########
GPRC5B	########	0.258086	0.689	0.094	########
ENAH	########	0.601956	0.738	0.056	########
RIC8A	########	0.181855	0.624	0.075	########
SURF1	########	0.556043	0.73	0.06	########
CHI3L1	########	-1.00744	0.833	0.583	########
NUDT21	########	-0.19501	0.493	0.092	########
ZMAT5	########	0.137646	0.585	0.065	########
REEP2	########	0.475323	0.691	0.048	########
PSMD6	########	-0.1473	0.6	0.133	########
DDIT3	########	-0.4259	0.642	0.222	########
SCAMP4	########	0.321673	0.68	0.077	########
SMIM4	########	0.136896	0.629	0.092	########
GANAB	########	0.387447	0.683	0.068	########
IER2	########	-0.21776	0.735	0.234	########
ACTN1	########	0.529083	0.737	0.074	########
ZFYVE21	########	0.172815	0.724	0.12	########
UBE2K	########	0.206119	0.611	0.067	########
BNIP3	########	-0.41511	0.557	0.161	########
ABCD4	########	0.191631	0.636	0.084	########
CDC34	########	0.34327	0.657	0.068	########
COQ4	########	0.215118	0.651	0.089	########
ADI1	########	0.187966	0.645	0.089	########
MBOAT7	########	0.55436	0.687	0.038	########
CTSF	########	0.235059	0.661	0.077	########
ARHGAP21	########	0.390756	0.719	0.085	########

PGD	########	0.20696	0.627	0.072	########
ID4	########	0.915176	0.833	0.085	########
TCTEX1D2	########	-0.18391	0.526	0.109	########
WDR41	########	-0.15867	0.557	0.116	########
DUSP14	########	0.136904	0.548	0.053	########
AKAP8L	########	0.315043	0.67	0.07	########
GORASP2	########	0.172168	0.617	0.072	########
MCRS1	########	0.29148	0.658	0.074	########
MAP4	########	0.166166	0.665	0.108	########
BCL7C	########	0.62465	0.701	0.036	########
ITGAV	########	0.717414	0.749	0.046	########
CCNH	########	-0.21062	0.542	0.13	########
TAF11	########	0.126757	0.582	0.075	########
DEF8	########	0.275708	0.63	0.065	########
NPAS3	########	0.611119	0.747	0.06	########
MSN	########	0.293403	0.681	0.084	########
FMNL2	########	0.921519	0.762	0.026	########
SCFD1	########	-0.25342	0.531	0.125	########
CHCHD6	########	0.15815	0.582	0.07	########
ACOT8	########	0.153574	0.592	0.067	########
ICT1	########	0.328012	0.632	0.056	########
C11orf49	########	0.155695	0.629	0.085	########
CSNK1E	########	0.27889	0.694	0.103	########
GLT8D1	########	0.332492	0.677	0.075	########
NRCAM	########	0.305638	0.713	0.108	########
TTYH1	########	0.162066	0.86	0.243	########
TMED3	########	0.383413	0.67	0.067	########
PAK2	########	0.474334	0.707	0.068	########
CDKN2C	########	-0.43484	0.624	0.224	########
FGD5-AS1	########	0.369954	0.66	0.06	########
RMDN3	########	0.352941	0.654	0.065	########
TMEM248	########	0.230727	0.607	0.058	########
ARF6	########	0.184405	0.645	0.089	########
ZNF148	########	0.277433	0.645	0.063	########
HARS	########	0.154834	0.627	0.091	########
DLD	########	-0.16358	0.6	0.145	########
SEC14L1	########	0.170786	0.702	0.108	########
FYTTD1	########	0.197197	0.608	0.062	########
UBE3A	########	0.509196	0.704	0.062	########
SPNS1	########	0.626508	0.722	0.046	########
NAA50	########	0.176553	0.6	0.074	########
CCDC82	########	0.240032	0.647	0.079	########
PIGX	########	0.442627	0.691	0.062	########
PTP4A1	########	0.372319	0.698	0.079	########
CCS	########	-0.14445	0.544	0.113	########
TM7SF2	########	0.627378	0.722	0.048	########
KLHDC2	########	0.338172	0.661	0.063	########
CHN1	########	0.249255	0.679	0.084	########
TMEM141	########	0.164896	0.576	0.063	########
ARPC1B	########	0.304255	0.677	0.089	########
DNPEP	########	0.301998	0.636	0.067	########

TGOLN2	########	0.305601	0.635	0.07	########
CD9	########	-1.21728	0.672	0.66	########
DHCR7	########	0.37353	0.682	0.068	########
FAM49B	########	0.329705	0.646	0.062	########
SEC23B	########	0.336863	0.66	0.062	########
RBBP6	########	0.322811	0.677	0.079	########
G3BP1	########	0.128454	0.651	0.109	########
UBE2F	########	0.161602	0.594	0.072	########
MLF1	########	-0.14053	0.541	0.106	########
MSRB1	########	0.170834	0.57	0.067	########
OGFR	########	0.619359	0.727	0.044	########
EMC8	########	0.347921	0.637	0.056	########
THOC2	########	0.183445	0.655	0.099	########
MCUR1	########	0.536417	0.682	0.048	########
HEXB	########	0.342599	0.668	0.07	########
CYHR1	########	0.255785	0.667	0.089	########
ZFP36L2	########	0.103435	0.8	0.214	########
DAP	########	0.497345	0.661	0.041	########
TMEM54	########	0.491959	0.67	0.044	########
SMOX	########	-0.15099	0.608	0.138	########
SV2A	########	0.389237	0.653	0.06	########
DDX27	########	0.294766	0.638	0.068	########
ARL8A	########	0.270411	0.609	0.065	########
PARL	########	0.147762	0.586	0.072	########
SPATS2	########	0.17442	0.623	0.089	########
JUND	########	0.103286	0.687	0.135	########
LARP1	########	0.341675	0.646	0.063	########
ALDH9A1	########	-0.12019	0.608	0.133	########
HSPH1	########	0.102395	0.604	0.084	########
CHD6	########	0.247216	0.634	0.07	########
DFFA	########	0.165901	0.583	0.067	########
TMEM251	########	0.270568	0.603	0.056	########
CELF1	########	0.301822	0.638	0.065	########
AFF4	########	0.28034	0.673	0.096	########
SEPN1	########	0.600865	0.715	0.051	########
TMEM30A	########	0.332866	0.687	0.087	########
MRC2	########	0.627431	0.697	0.034	########
COTL1	########	0.241005	0.655	0.077	########
EFEMP2	########	0.655348	0.717	0.048	########
OXR1	########	0.360163	0.648	0.062	########
EIF3A	########	0.272003	0.635	0.072	########
ANAPC13	########	-0.34721	0.542	0.15	########
ZDHHC12	########	0.302235	0.575	0.044	########
TSTA3	########	0.12363	0.588	0.072	########
GSN	########	-0.10776	0.596	0.126	########
MTHFD2L	########	0.343583	0.641	0.065	########
IFI27L2	########	-0.45614	0.616	0.239	########
WDR34	########	0.191947	0.587	0.065	########
PXMP2	########	0.291748	0.625	0.058	########
DHRS4L2	########	0.233142	0.607	0.068	########
AKIRIN2	########	0.361986	0.675	0.072	########

SLC22A17	########	0.403471	0.68	0.068	########
PTGES2	########	0.414361	0.655	0.053	########
WHSC1L1	########	0.585625	0.725	0.058	########
LSM12	########	0.64673	0.712	0.038	########
TCTN1	########	0.129648	0.588	0.082	########
MPST	########	0.541557	0.688	0.044	########
DHX9	########	0.159907	0.598	0.075	########
CDKN2AIPN	########	0.144814	0.585	0.079	########
URI1	########	0.443823	0.659	0.051	########
HILPDA	########	-1.03558	0.364	0.193	########
JAG1	########	0.447961	0.707	0.072	########
SLC44A1	########	0.270305	0.689	0.094	########
SHARPIN	########	0.403967	0.642	0.05	########
B4GALT5	########	0.590583	0.719	0.056	########
NEIL2	########	0.128499	0.576	0.072	########
POP4	########	-0.15435	0.532	0.108	########
C19orf66	########	0.353835	0.668	0.07	########
PRR7	########	0.859265	0.749	0.027	########
LIMS1	########	0.411582	0.73	0.104	########
PON2	########	-0.34944	0.797	0.342	########
PCBP4	########	0.640902	0.719	0.051	########
SMAP1	########	0.379171	0.645	0.053	########
ARL4C	########	0.572844	0.749	0.085	########
TTC14	########	0.141114	0.612	0.091	########
BICD1	########	0.613216	0.709	0.044	########
MAPK1	########	0.656918	0.712	0.043	########
NPM3	########	-0.18161	0.469	0.092	########
SURF2	########	0.139186	0.567	0.065	########
DOK5	########	0.199196	0.628	0.08	########
LINC00665	########	-0.20721	0.525	0.126	########
TAF15	########	0.141838	0.597	0.072	########
KIAA1715	########	0.240754	0.624	0.08	########
UBE2G2	########	0.155796	0.591	0.07	########
CD44	########	-0.3432	0.636	0.231	########
TGIF1	########	0.144784	0.667	0.118	########
HSPA4	########	0.168949	0.586	0.065	########
NR1H2	########	0.334107	0.67	0.074	########
EGLN2	########	0.530197	0.692	0.053	########
NMT1	########	0.469622	0.699	0.072	########
DCTD	########	0.170622	0.586	0.075	########
RSPRY1	########	0.151386	0.6	0.075	########
PITHD1	########	0.313878	0.634	0.068	########
HDAC1	########	0.284968	0.586	0.048	########
NUFIP2	########	0.505091	0.659	0.041	########
SDC3	########	-0.18404	0.614	0.15	########
SRSF10	########	-0.42764	0.477	0.149	########
VAPB	########	0.373049	0.614	0.046	########
TPM2	########	0.797308	0.755	0.056	########
FJX1	########	0.806142	0.771	0.062	########
TXNDC9	########	0.135994	0.538	0.058	########
ABHD14A	########	0.40579	0.642	0.05	########

ATP6V0A1	########	-0.16162	0.515	0.115	########
ZSCAN16-A	########	0.19649	0.618	0.084	########
NGDN	########	-0.43099	0.504	0.147	########
NUPR1	########	-0.77347	0.516	0.234	########
ELOVL5	########	0.467659	0.686	0.062	########
TNFRSF12A	########	0.612616	0.818	0.142	########
CHMP1A	########	0.20256	0.579	0.062	########
ZNF644	########	0.27702	0.654	0.079	########
PSMD9	########	-0.32495	0.499	0.138	########
ABHD2	########	0.418423	0.69	0.072	########
EBP	########	0.142731	0.579	0.07	########
PHF20L1	########	0.449336	0.68	0.063	########
MOCS2	########	-0.10575	0.582	0.135	########
MOSPD3	########	0.147784	0.579	0.079	########
ACAT1	########	-0.40588	0.547	0.186	########
TMUB2	########	0.13861	0.569	0.072	########
ELAVL3	########	0.349546	0.677	0.085	########
GCAT	########	0.363545	0.61	0.044	########
ARFRP1	########	0.309598	0.622	0.056	########
HOMER3	########	0.667553	0.704	0.039	########
TCEAL7	########	-0.21362	0.542	0.121	########
OGFOD3	########	0.698149	0.701	0.031	########
DNMT1	########	0.351488	0.645	0.06	########
ETV5	########	0.36368	0.641	0.056	########
IFNAR1	########	0.224608	0.599	0.07	########
UBE2H	########	0.288339	0.635	0.068	########
EMG1	########	-0.18861	0.473	0.091	########
FUNDC1	########	-0.25891	0.503	0.118	########
RBM6	########	0.251785	0.651	0.084	########
PPCS	########	-0.18513	0.515	0.115	########
VPS4A	########	0.277776	0.633	0.072	########
SQLE	########	0.561168	0.725	0.065	########
SNAP29	########	0.206373	0.559	0.055	########
LPP	########	0.422926	0.647	0.055	########
TUBG1	########	0.24252	0.563	0.05	########
PDCD7	########	0.275017	0.595	0.053	########
RTCB	########	0.283877	0.624	0.062	########
BAG6	########	0.332496	0.636	0.065	########
MAX	########	-0.10243	0.514	0.094	########
EXOSC8	########	-0.25201	0.514	0.13	########
AATF	########	0.180279	0.583	0.07	########
RING1	########	0.24475	0.597	0.065	########
TLE1	########	0.513897	0.702	0.06	########
SLBP	########	0.264459	0.62	0.07	########
F3	########	0.510529	0.762	0.108	########
DAG1	########	0.429588	0.651	0.048	########
XPO1	########	0.175752	0.582	0.072	########
FAM210B	########	0.872672	0.749	0.039	########
TMEM199	########	0.203228	0.584	0.065	########
ACP2	########	0.10405	0.547	0.074	########
RASSF2	########	0.425198	0.682	0.067	########

SMARCD3	########	0.385033	0.664	0.067	########
ARMC10	########	0.372603	0.651	0.06	########
TJP1	########	0.518016	0.668	0.043	########
CTTN	########	0.232423	0.652	0.079	########
NREP	########	0.341398	0.653	0.072	########
CCNL2	########	0.331328	0.692	0.087	########
PAFAH1B2	########	0.430008	0.675	0.06	########
FBXO7	########	0.378241	0.614	0.044	########
STK25	########	0.529464	0.687	0.051	########
YIPF5	########	-0.19263	0.51	0.106	########
RRP15	########	0.108607	0.557	0.056	########
C1R	########	-0.73145	0.513	0.251	########
PDCD10	########	0.108391	0.548	0.065	########
CCDC115	########	-0.1923	0.485	0.106	########
MAZ	########	0.43301	0.642	0.051	########
MAPKAP1	########	0.39422	0.642	0.058	########
PYGB	########	0.335475	0.644	0.065	########
SAFB2	########	0.322637	0.685	0.089	########
SLC35A4	########	0.216737	0.561	0.048	########
CMTM3	########	0.570905	0.694	0.05	########
HSD11B1L	########	0.376401	0.641	0.056	########
TAP1	########	0.140928	0.693	0.116	########
BBOX1	########	-0.1269	0.548	0.115	########
FKBP4	########	0.212713	0.623	0.082	########
HSF1	########	0.256699	0.606	0.063	########
YME1L1	########	0.210445	0.573	0.056	########
SLC25A37	########	-0.11445	0.576	0.138	########
EDEM2	########	0.153816	0.57	0.07	########
RABL6	########	0.545273	0.657	0.032	########
SPATC1L	########	0.501575	0.639	0.039	########
STX4	########	-0.13016	0.513	0.106	########
APPL1	########	0.330008	0.622	0.058	########
DMAP1	########	-0.10704	0.535	0.101	########
BLOC1S4	########	0.483548	0.647	0.039	########
CERS4	########	0.546067	0.676	0.041	########
SLC16A1	########	-0.2346	0.53	0.132	########
RABEP1	########	0.615918	0.677	0.032	########
DEAF1	########	0.385407	0.64	0.055	########
CRYL1	########	0.238479	0.612	0.074	########
TDG	########	0.173282	0.609	0.085	########
TP53I13	########	0.734083	0.711	0.034	########
LRRFIP1	########	0.158812	0.596	0.079	########
EEA1	########	0.396328	0.626	0.055	########
IQGAP1	########	0.217155	0.602	0.065	########
FAM89B	########	0.642524	0.679	0.029	########
SLC52A2	########	0.575767	0.686	0.048	########
MED11	########	0.243485	0.59	0.063	########
ZNF226	########	-0.1646	0.504	0.109	########
EXOSC4	########	0.412804	0.638	0.05	########
HMGA1	########	0.661345	0.718	0.053	########
CHD2	########	0.154203	0.573	0.075	########

RNF145	########	0.472038	0.686	0.067	########
MT1M	########	-0.53393	0.617	0.241	########
GON4L	########	0.110959	0.551	0.068	########
CORO1C	########	-0.10151	0.612	0.154	########
APLP1	########	0.338155	0.676	0.089	########
RNF24	########	0.376283	0.651	0.067	########
ATAT1	########	0.393496	0.64	0.058	########
TSEN15	########	0.116888	0.552	0.067	########
SORT1	########	0.370917	0.621	0.048	########
NEU1	########	0.200205	0.585	0.063	########
TSC22D4	########	-0.50691	0.82	0.421	########
FAM92A1	########	0.273084	0.582	0.055	########
LYPLA2	########	0.159671	0.532	0.06	########
GRIA3	########	0.339843	0.658	0.074	########
HOOK3	########	0.220374	0.589	0.065	########
RANBP3	########	0.218472	0.552	0.055	########
ARHGAP12	########	0.174393	0.583	0.067	########
MMAB	########	0.163075	0.58	0.077	########
EIF3B	########	0.228066	0.581	0.065	########
ADPRHL2	########	0.240744	0.609	0.068	########
PRPF8	########	0.216358	0.597	0.079	########
ASPH	########	0.293053	0.646	0.074	########
CASC4	########	0.573689	0.685	0.046	########
RAMP1	########	-0.72412	0.803	0.538	########
DDX42	########	0.302615	0.584	0.053	########
COL9A3	########	0.514776	0.772	0.109	########
ATP2A2	########	0.197158	0.617	0.092	########
GOLIM4	########	0.420366	0.733	0.101	########
PRRX1	########	0.439935	0.665	0.063	########
SLC25A1	########	0.371601	0.629	0.053	########
FUBP3	########	0.334386	0.654	0.065	########
DAPK3	########	0.398898	0.66	0.067	########
MAN1B1	########	0.287698	0.61	0.056	########
TMEM98	########	0.416627	0.633	0.062	########
NTHL1	########	0.113854	0.544	0.072	########
ABT1	########	-0.11519	0.492	0.089	########
G6PD	########	0.401236	0.617	0.044	########
ACIN1	########	0.170825	0.601	0.079	########
MAP1A	########	0.220355	0.606	0.07	########
CKAP4	########	0.422628	0.65	0.067	########
THY1	########	0.226317	0.635	0.091	########
CLCN3	########	0.277595	0.635	0.077	########
CD74	########	-1.50382	0.444	0.629	########
LINC01003	########	0.419133	0.623	0.044	########
TSSC1	########	0.166485	0.547	0.063	########
RP11-345J	########	0.402993	0.6	0.038	########
NDFIP2	########	0.148291	0.567	0.074	########
LRIF1	########	-0.17623	0.465	0.091	########
ACAT2	########	0.350859	0.645	0.068	########
PRPF19	########	0.146308	0.546	0.065	########
KCNQ2	########	0.627593	0.693	0.058	########

TRIM28	########	0.174657	0.6	0.087	########
THAP7	########	0.150324	0.568	0.074	########
MAN1A2	########	0.395974	0.623	0.046	########
FAM173A	########	0.451102	0.654	0.055	########
ENDOV	########	0.173685	0.595	0.079	########
TACC1	########	0.505219	0.665	0.05	########
SPTAN1	########	0.223345	0.635	0.091	########
HEBP2	########	0.710471	0.674	0.024	########
DHX30	########	0.401029	0.655	0.062	########
A2M	########	-0.45386	0.532	0.171	########
BLVRA	########	-0.49651	0.474	0.162	########
ITGA7	########	0.605831	0.677	0.039	########
FTSJ2	########	-0.10236	0.475	0.089	########
RNF213	########	0.397607	0.633	0.051	########
ATXN2	########	0.202363	0.582	0.07	########
DUSP6	########	0.21979	0.639	0.092	########
AZI2	########	-0.10765	0.503	0.087	########
UBE2G1	########	0.314387	0.589	0.051	########
WNK1	########	0.252119	0.568	0.055	########
ATPAF2	########	0.31399	0.588	0.051	########
NDUFS1	########	0.353796	0.604	0.051	########
JKAMP	########	0.303339	0.604	0.067	########
PPP2R5C	########	0.148	0.546	0.063	########
INTS10	########	0.180616	0.61	0.085	########
ZNF32	########	0.252098	0.555	0.046	########
HYAL2	########	0.716271	0.695	0.031	########
RNF130	########	0.554721	0.677	0.048	########
KATNBL1	########	0.33517	0.583	0.043	########
DDX52	########	0.438286	0.646	0.053	########
NMRAL1	########	-0.20811	0.472	0.106	########
SRP68	########	0.193156	0.557	0.062	########
TINF2	########	0.243692	0.578	0.056	########
APBA2	########	0.400746	0.652	0.065	########
IFT27	########	0.366275	0.616	0.055	########
GOSR1	########	0.133384	0.58	0.084	########
PHF23	########	0.393356	0.625	0.058	########
UTP18	########	0.16496	0.565	0.068	########
TPP1	########	0.134519	0.549	0.075	########
RAD50	########	0.166558	0.582	0.085	########
CRELD2	########	0.613778	0.667	0.034	########
COMMD10	########	-0.22284	0.488	0.108	########
KRR1	########	0.191447	0.565	0.06	########
NOL12	########	0.146081	0.539	0.062	########
SRPRB	########	0.172325	0.588	0.065	########
TNPO1	########	0.280453	0.633	0.08	########
TMEM170A	########	0.2239	0.595	0.074	########
PRKAR2A	########	0.238598	0.577	0.07	########
CADM4	########	0.454996	0.677	0.062	########
C8orf4	########	-0.97115	0.443	0.265	########
YTHDF1	########	0.368036	0.578	0.039	########
PPIL1	########	-0.11342	0.44	0.07	########

SOX4	########	0.250519	0.84	0.231	########
ANP32E	########	0.166612	0.598	0.075	########
COL4A2	########	0.517139	0.647	0.039	########
BCKDK	########	0.409742	0.633	0.06	########
DBN1	########	0.510829	0.668	0.053	########
MFGE8	########	0.347525	0.623	0.062	########
TAF10	########	0.616259	0.652	0.027	########
SFXN5	########	0.112165	0.575	0.087	########
VPS26B	########	0.359969	0.585	0.046	########
CDC25B	########	0.321293	0.639	0.074	########
PSMG1	########	-0.11365	0.475	0.085	########
EFTUD2	########	0.425583	0.638	0.051	########
CDV3	########	0.528102	0.647	0.039	########
EMD	########	0.291902	0.616	0.068	########
LONP1	########	0.328938	0.611	0.055	########
GSTA4	########	-0.19344	0.522	0.128	########
NIPA2	########	0.328899	0.602	0.051	########
FLOT2	########	0.210819	0.522	0.044	########
COPA	########	0.202429	0.625	0.089	########
PHIP	########	0.183662	0.582	0.077	########
PBDC1	########	-0.24382	0.475	0.103	########
YTHDC1	########	0.266528	0.603	0.065	########
SNX27	########	0.393602	0.612	0.05	########
GALNT2	########	0.452906	0.639	0.051	########
SPCS3	########	0.2475	0.562	0.056	########
FAM134A	########	0.433918	0.663	0.067	########
C7orf55-L	########	0.159589	0.602	0.087	########
PDCD6IP	########	0.139306	0.571	0.082	########
PDLIM7	########	0.722284	0.692	0.041	########
TMEM223	########	0.285344	0.589	0.058	########
MRTO4	########	0.114824	0.563	0.075	########
SOCS2	########	0.601123	0.701	0.06	########
MAP2K1	########	0.176903	0.529	0.053	########
CC2D1A	########	0.331087	0.594	0.05	########
FAM63B	########	0.283852	0.583	0.062	########
CIAO1	########	0.286622	0.569	0.05	########
GOPC	########	0.135906	0.565	0.08	########
GDE1	########	0.352777	0.594	0.043	########
ACLY	########	0.478623	0.638	0.048	########
ASPSCR1	########	0.423935	0.641	0.056	########
PROSC	########	0.186244	0.56	0.063	########
GPR137	########	0.392043	0.604	0.041	########
TPD52	########	-0.19058	0.499	0.113	########
ABCE1	########	0.184922	0.573	0.068	########
WDR45	########	-0.1204	0.504	0.097	########
NOP58	########	-0.28758	0.482	0.121	########
TWISTNB	########	-0.15494	0.504	0.085	########
NF1	########	0.340624	0.604	0.056	########
MAML2	########	0.476781	0.642	0.05	########
BSDC1	########	0.143286	0.615	0.096	########
SNAPC2	########	0.316041	0.589	0.053	########

BIRC2	########	0.249059	0.591	0.065	########
WAC	########	0.447817	0.629	0.05	########
OARD1	########	-0.18186	0.467	0.096	########
LARP6	########	0.454702	0.617	0.043	########
TSR1	########	0.184755	0.528	0.056	########
ELP5	########	0.337164	0.606	0.06	########
CDIPT	########	0.275528	0.607	0.07	########
TXNIP	########	-0.12578	0.622	0.169	########
BAG1	########	0.114398	0.57	0.077	########
ID2	########	-0.67366	0.624	0.323	########
TMEM158	########	0.475743	0.639	0.051	########
ERGIC1	########	0.114074	0.613	0.103	########
XIAP	########	0.310733	0.586	0.055	########
WSB2	########	0.371377	0.598	0.044	########
STK4	########	0.114424	0.544	0.07	########
KPNA3	########	0.222716	0.545	0.055	########
FDX1	########	0.508438	0.644	0.043	########
PRRC2A	########	0.443739	0.633	0.053	########
MLC1	########	0.334293	0.619	0.068	########
IVD	########	0.270472	0.578	0.056	########
WIPF2	########	0.298962	0.56	0.044	########
TMEM59L	########	0.212933	0.663	0.113	########
PODXL2	########	0.577411	0.695	0.06	########
ZCCHC11	########	0.163518	0.563	0.07	########
WDR82	########	0.180047	0.553	0.068	########
AGGF1	########	0.3668	0.613	0.055	########
UBE2J1	########	0.542127	0.677	0.055	########
ZNF302	########	-0.17427	0.596	0.173	########
TTLL7	########	0.191519	0.611	0.096	########
BAZ1A	########	0.353428	0.623	0.074	########
SUPT16H	########	-0.23026	0.481	0.106	########
CECR5	########	0.529477	0.633	0.038	########
PDXK	########	0.122121	0.528	0.065	########
TWF2	########	0.18412	0.569	0.068	########
SOCS6	########	0.339692	0.613	0.058	########
EZR	########	0.223483	0.573	0.068	########
PPP2R4	########	0.268769	0.581	0.063	########
NBPF1	########	0.513896	0.641	0.039	########
EPB41L4A-	########	-0.3472	0.472	0.123	########
ZNF106	########	0.240568	0.566	0.062	########
GTPBP6	########	0.288387	0.58	0.051	########
YARS	########	-0.16006	0.478	0.096	########
TMEM161B-	########	-0.10554	0.544	0.12	########
NKIRAS2	########	0.169704	0.556	0.065	########
NUDT1	########	-0.39933	0.468	0.128	########
SUPT5H	########	0.127201	0.553	0.082	########
BET1	########	-0.34671	0.421	0.13	########
LYPLAL1	########	-0.14454	0.466	0.092	########
SLC25A36	########	0.273898	0.59	0.07	########
ZNF830	########	-0.13583	0.454	0.077	########
FAM195A	########	0.358834	0.598	0.051	########

CDK16	########	0.408693	0.626	0.06	########
AKIP1	########	0.210493	0.569	0.07	########
GGA1	########	0.141652	0.545	0.077	########
TM9SF2	########	0.269016	0.59	0.065	########
YKT6	########	0.121852	0.535	0.068	########
KHSRP	########	0.491117	0.636	0.043	########
DUSP12	########	-0.17146	0.462	0.096	########
RCAN1	########	-0.1917	0.619	0.173	########
C6orf89	########	0.217447	0.52	0.048	########
SAFB	########	0.265751	0.601	0.068	########
WWTR1	########	0.869762	0.712	0.032	########
ARRDC3	########	0.160831	0.576	0.087	########
ORAI2	########	0.400773	0.633	0.063	########
RTN1	########	-0.49388	0.339	0.096	########
UBTF	########	0.311126	0.582	0.051	########
PKN1	########	0.562692	0.655	0.038	########
UBALD2	########	0.32438	0.629	0.07	########
DDX41	########	0.25704	0.554	0.051	########
DCAF8	########	0.224155	0.561	0.062	########
MYO9B	########	0.259337	0.612	0.077	########
GCC2	########	0.144147	0.525	0.067	########
STAT1	########	0.194658	0.634	0.091	########
MLX	########	0.198621	0.569	0.074	########
RPF2	########	-0.11876	0.477	0.087	########
HDAC3	########	0.218148	0.551	0.062	########
ANKIB1	########	0.239338	0.535	0.044	########
GOLT1B	########	-0.24602	0.424	0.084	########
C16orf91	########	0.124799	0.475	0.046	########
RBMX2	########	-0.13431	0.494	0.104	########
NQO2	########	0.160125	0.52	0.055	########
PIPOX	########	0.262577	0.617	0.075	########
CCDC14	########	0.173549	0.558	0.067	########
RHOG	########	0.142046	0.493	0.05	########
THAP9-AS1	########	-0.10686	0.487	0.08	########
MEG3	########	-0.66955	0.629	0.378	########
USF2	########	0.312316	0.622	0.068	########
SLC25A33	########	0.405391	0.597	0.043	########
RFK	########	0.270539	0.561	0.053	########
THOP1	########	0.425061	0.578	0.032	########
SIL1	########	0.104556	0.54	0.08	########
SHMT2	########	-0.23487	0.508	0.126	########
HAT1	########	-0.19514	0.499	0.115	########
MSMO1	########	0.346938	0.649	0.075	########
FNDC4	########	0.529188	0.62	0.034	########
ARSJ	########	0.45419	0.639	0.058	########
CCDC50	########	0.326413	0.577	0.044	########
DERL1	########	-0.10024	0.5	0.099	########
FAM174A	########	0.227331	0.565	0.062	########
TM9SF4	########	0.176342	0.54	0.065	########
NRP2	########	0.619202	0.681	0.05	########
ERP44	########	-0.12838	0.51	0.111	########

RPRD1A	########	0.131842	0.534	0.067	########
DEXI	########	0.492547	0.617	0.034	########
BAZ1B	########	0.528241	0.655	0.044	########
EIF2B1	########	0.192349	0.58	0.077	########
SF3A2	########	0.37183	0.619	0.063	########
CDK5	########	0.13131	0.515	0.058	########
ZNF292	########	0.366497	0.598	0.055	########
SUPT6H	########	0.309973	0.54	0.043	########
GNL1	########	0.408237	0.613	0.05	########
RAB4B	########	0.101957	0.538	0.068	########
7-Mar	########	0.134353	0.516	0.063	########
PMEPA1	########	0.975494	0.706	0.026	########
ALDH3A2	########	0.286951	0.634	0.082	########
AK3	########	0.129023	0.532	0.068	########
RBM27	########	0.403058	0.596	0.043	########
LRRC41	########	0.110122	0.545	0.084	########
SPRED1	########	0.741111	0.687	0.027	########
PLOD1	########	0.246636	0.544	0.06	########
STRADB	########	0.555048	0.641	0.034	########
RP11-620J	########	-0.37405	0.475	0.142	########
RPUSD3	########	0.195658	0.52	0.051	########
CCM2	########	0.262455	0.566	0.058	########
SLC30A9	########	0.321629	0.591	0.056	########
ZFHX4	########	0.451695	0.62	0.048	########
WDR46	########	0.174069	0.533	0.056	########
CGGBP1	########	0.168083	0.558	0.065	########
CAND1	########	0.12587	0.528	0.074	########
IMPAD1	########	0.275743	0.543	0.044	########
PHC2	########	0.291226	0.564	0.053	########
ZNF90	########	-1.51354	0.272	0.427	########
SLC25A23	########	0.357464	0.557	0.041	########
MAGEH1	########	-0.15934	0.525	0.123	########
GNAQ	########	0.295372	0.557	0.044	########
G3BP2	########	0.19907	0.526	0.048	########
RIT1	########	0.204383	0.532	0.048	########
DHRSX	########	0.322486	0.573	0.051	########
NIPBL	########	0.208479	0.532	0.056	########
HMBS	########	0.347952	0.581	0.043	########
RWDD4	########	0.240784	0.55	0.06	########
SMAD1	########	0.732637	0.696	0.046	########
POSTN	########	0.900508	0.714	0.046	########
LRP10	########	0.196248	0.56	0.07	########
IPO5	########	0.274778	0.561	0.055	########
RND2	########	0.184462	0.547	0.063	########
AAMDC	########	-0.32035	0.411	0.097	########
ELOVL1	########	0.175014	0.534	0.06	########
TRPT1	########	0.222151	0.576	0.074	########
8-Sep	########	0.222551	0.568	0.065	########
FADS1	########	0.289085	0.573	0.058	########
TSPAN7	########	-0.37413	0.614	0.234	########
TRIM69	########	0.210668	0.553	0.063	########

RP11-553L	########	0.337019	0.619	0.072	########
CHD1	########	0.435731	0.632	0.06	########
CEBPD	########	-0.23365	0.697	0.251	########
UBAP2L	########	0.100663	0.512	0.067	########
U2AF2	########	0.377112	0.605	0.053	########
TMX4	########	0.517683	0.635	0.043	########
BLOC1S6	########	0.195712	0.503	0.044	########
PPP1R10	########	0.123345	0.515	0.063	########
KLF6	########	-0.56015	0.55	0.255	########
TRAP1	########	0.373014	0.605	0.051	########
CD276	########	0.390949	0.583	0.046	########
GLTP	########	0.103754	0.529	0.067	########
KLHL7	########	0.140939	0.519	0.067	########
CHL1	########	0.661886	0.649	0.034	########
NHLRC3	########	-0.12756	0.489	0.104	########
CRBN	########	-0.18405	0.532	0.125	########
BCAP29	########	-0.20998	0.548	0.149	########
C15orf40	########	-0.16015	0.411	0.075	########
PANK2	########	0.427417	0.598	0.043	########
GNPTG	########	0.272086	0.55	0.05	########
DAXX	########	0.258943	0.536	0.051	########
BTG2	########	-0.52676	0.54	0.226	########
TMEM33	########	0.343383	0.589	0.055	########
TCEAL1	########	-0.13227	0.489	0.106	########
LINC00662	########	-0.2269	0.447	0.103	########
AC010642.	########	0.203235	0.528	0.051	########
PDLIM5	########	0.41551	0.604	0.048	########
PRRC2B	########	0.391029	0.594	0.044	########
GPX7	########	0.135707	0.513	0.06	########
KIF5C	########	0.543896	0.634	0.043	########
ROCK1	########	0.331367	0.576	0.051	########
USP10	########	0.205493	0.535	0.055	########
TMCO3	########	0.17653	0.512	0.051	########
NDN	########	0.594783	0.626	0.026	########
CPNE2	########	0.138501	0.572	0.074	########
COIL	########	0.113948	0.5	0.058	########
ALG3	########	0.251229	0.533	0.048	########
SBNO1	########	0.203553	0.557	0.072	########
TP53RK	########	0.446135	0.594	0.038	########
PREX1	########	0.233891	0.534	0.046	########
FAM84A	########	0.54417	0.663	0.055	########
COMMD2	########	0.108743	0.561	0.085	########
MIF4GD	########	0.121114	0.494	0.055	########
FARSB	########	-0.20711	0.452	0.109	########
DNAJC3	########	0.191422	0.576	0.072	########
SERPINE2	########	0.281232	0.716	0.13	########
FN3KRP	########	0.142099	0.502	0.06	########
PCMTD2	########	0.408543	0.619	0.055	########
SIRT7	########	0.419921	0.587	0.039	########
CISD3	########	0.502277	0.574	0.022	########
LTBP3	########	0.587349	0.642	0.032	########

DPF2	########	0.179103	0.557	0.07	########
SCRN1	########	0.217058	0.575	0.074	########
ANKRD17	########	0.336068	0.568	0.046	########
CITED1	########	0.356636	0.663	0.089	########
PES1	########	0.273559	0.509	0.036	########
EDNRB	########	0.73164	0.717	0.062	########
CKS2	########	-0.25584	0.447	0.115	########
TMEM107	########	-0.17166	0.406	0.084	########
TMEM45A	########	-0.71168	0.352	0.149	########
FGFR1	########	0.173171	0.541	0.062	########
BECN1	########	0.304475	0.538	0.041	########
NCSTN	########	0.139327	0.578	0.087	########
ERBB2IP	########	0.605557	0.663	0.043	########
PSD3	########	0.474786	0.627	0.046	########
GPATCH4	########	0.135897	0.512	0.063	########
KCNF1	########	0.954261	0.684	0.019	########
LENG8	########	0.351677	0.616	0.068	########
NFYC	########	-0.24271	0.422	0.101	########
CCDC137	########	0.56131	0.628	0.034	########
EXOG	########	-0.11236	0.432	0.079	########
PTRH1	########	0.377795	0.544	0.031	########
TM7SF3	########	0.395681	0.586	0.044	########
FBXW5	########	0.42854	0.63	0.062	########
SCD	########	0.169142	0.576	0.079	########
ARFIP2	########	0.221156	0.564	0.075	########
TMBIM1	########	-0.1279	0.412	0.07	########
LITAF	########	-0.53246	0.44	0.171	########
STAG2	########	0.228992	0.56	0.065	########
BRE	########	-0.22229	0.413	0.092	########
REPIN1	########	0.463173	0.622	0.046	########
SMPD1	########	0.618407	0.652	0.034	########
AKAP13	########	0.238935	0.589	0.072	########
COPS2	########	0.139085	0.466	0.051	########
AIMP2	########	-0.15998	0.454	0.096	########
C3orf14	########	-0.3141	0.439	0.123	########
PYCR1	########	0.32418	0.582	0.058	########
DKK3	########	0.427291	0.647	0.065	########
AGPAT1	########	0.317166	0.579	0.056	########
SIPA1L1	########	0.289234	0.529	0.043	########
SMARCD1	########	0.378445	0.574	0.044	########
RFFL	########	0.742754	0.657	0.027	########
GALNT11	########	0.201713	0.532	0.06	########
USB1	########	0.18062	0.488	0.05	########
PSME3	########	0.196587	0.509	0.051	########
RNF34	########	0.106074	0.497	0.06	########
BEX1	########	-0.79161	0.529	0.345	########
RAB32	########	0.592177	0.592	0.015	########
RAB21	########	0.33341	0.528	0.036	########
KPNA6	########	0.210755	0.545	0.06	########
CTNNBIP1	########	0.506226	0.587	0.026	########
MYCBP2	########	0.422338	0.582	0.039	########

PAK1IP1	########	-0.17809	0.437	0.089	########
FAM228B	########	0.145443	0.497	0.058	########
DNAJB9	########	-0.99226	0.412	0.256	########
NSD1	########	0.162061	0.513	0.065	########
ZEB1	########	0.290395	0.594	0.068	########
OSBPL6	########	0.574192	0.641	0.032	########
ARHGAP5	########	0.202486	0.551	0.07	########
HMGXB4	########	0.234016	0.511	0.048	########
STIM2	########	0.409416	0.584	0.043	########
RP11-395G	########	0.87294	0.704	0.039	########
RBM26	########	0.41186	0.592	0.039	########
TMUB1	########	0.4599	0.602	0.039	########
UBAC1	########	0.359596	0.529	0.029	########
BAZ2B	########	0.294613	0.62	0.08	########
NCOA4	########	0.159021	0.491	0.048	########
SEC31A	########	0.136143	0.518	0.07	########
GAA	########	0.350512	0.571	0.05	########
PSMG4	########	0.351062	0.566	0.051	########
PET117	########	0.225193	0.524	0.056	########
UBE2D1	########	0.39289	0.549	0.031	########
HAUS1	########	-0.13884	0.441	0.084	########
ARHGEF12	########	0.258366	0.524	0.046	########
CFLAR	########	-0.22857	0.468	0.113	########
MITD1	########	-0.14195	0.475	0.104	########
SSFA2	########	0.26949	0.577	0.058	########
C19orf48	########	0.121196	0.542	0.079	########
TRPC4AP	########	0.443115	0.602	0.043	########
DNM1L	########	0.152711	0.523	0.072	########
SNX4	########	0.135975	0.498	0.065	########
ABRACL	########	0.381649	0.556	0.032	########
CERS2	########	0.231338	0.52	0.048	########
TBC1D20	########	0.431786	0.591	0.039	########
TMEM87A	########	0.250623	0.542	0.055	########
HECTD1	########	0.355129	0.555	0.041	########
PRKRIP1	########	0.225362	0.531	0.058	########
CXADR	########	0.582435	0.661	0.055	########
FAM120A	########	0.444468	0.572	0.031	########
PPIH	########	-0.1708	0.397	0.07	########
TACO1	########	0.503537	0.592	0.031	########
ASB8	########	-0.12872	0.431	0.082	########
PAAF1	########	-0.24909	0.439	0.111	########
UBAC2	########	0.103485	0.493	0.063	########
UBE2R2	########	0.369098	0.538	0.034	########
KDM5A	########	0.246053	0.547	0.056	########
CRYZL1	########	-0.17962	0.459	0.109	########
TSPAN31	########	-0.13881	0.503	0.115	########
CSRP1	########	0.332596	0.537	0.034	########
NCKAP1	########	0.451126	0.611	0.05	########
CDC5L	########	0.247872	0.539	0.055	########
TOLLIP	########	0.30191	0.554	0.053	########
HMGB3	########	-0.11391	0.453	0.087	########

RPP25L	########	0.21335	0.518	0.048	########
PGRMC2	########	0.133254	0.497	0.056	########
SDHAF1	########	0.19138	0.478	0.041	########
FAM172A	########	0.176556	0.512	0.062	########
BDP1	########	0.185255	0.523	0.058	########
RPA1	########	0.192855	0.526	0.053	########
HRSP12	########	-0.18314	0.452	0.103	########
C18orf21	########	0.235593	0.486	0.036	########
RNF19A	########	0.568124	0.646	0.046	########
TOR1AIP2	########	0.336056	0.597	0.063	########
FAM207A	########	0.344451	0.535	0.036	########
PCYOX1	########	0.25388	0.516	0.051	########
ERF	########	0.188983	0.555	0.068	########
PLSCR1	########	-0.24488	0.459	0.118	########
TMEM115	########	0.252964	0.546	0.056	########
KCMF1	########	0.327	0.52	0.036	########
LINC0096S	########	-0.19756	0.434	0.096	########
VEGFA	########	-0.43283	0.464	0.157	########
ACTR1A	########	0.126993	0.471	0.05	########
CLIP1	########	0.117467	0.529	0.077	########
C6orf226	########	0.461787	0.59	0.038	########
2-Mar	########	0.466549	0.563	0.026	########
ZMYND8	########	0.191397	0.548	0.072	########
SDAD1	########	0.194254	0.514	0.05	########
KDSR	########	0.250397	0.545	0.063	########
WDR60	########	0.108331	0.526	0.077	########
RAB3GAP2	########	0.214516	0.535	0.062	########
NINJ1	########	0.310385	0.556	0.053	########
AZIN1	########	0.129341	0.554	0.089	########
LDLRAD3	########	0.530339	0.626	0.041	########
TNRC6A	########	0.174216	0.538	0.072	########
GSTM4	########	0.127204	0.507	0.068	########
F2R	########	0.250517	0.55	0.058	########
MRAS	########	0.343295	0.531	0.034	########
IFRD2	########	0.248268	0.567	0.072	########
COL6A2	########	0.76971	0.684	0.053	########
HGS	########	0.373435	0.556	0.043	########
CUX1	########	0.346441	0.564	0.051	########
MTHFD1	########	0.110109	0.516	0.079	########
BAIAP2	########	0.360624	0.6	0.062	########
GOT2	########	0.194723	0.484	0.048	########
TSFM	########	-0.15616	0.487	0.108	########
RNF180	########	0.204463	0.573	0.08	########
UBE2Q1	########	0.324521	0.512	0.032	########
RNASEH1	########	0.324427	0.54	0.043	########
RHOT1	########	0.507182	0.605	0.039	########
SCPEP1	########	-0.10233	0.424	0.074	########
PTCD3	########	0.290032	0.544	0.051	########
TRIM2	########	0.261541	0.559	0.06	########
POLR2B	########	0.172528	0.512	0.053	########
IGFBP5	########	-0.84504	0.58	0.361	########

CELF2	########	0.277152	0.583	0.068	########
PLEKHA4	########	0.322555	0.566	0.051	########
MPHOSPH10	########	-0.10927	0.468	0.085	########
ENDOG	########	0.221577	0.477	0.043	########
PEX10	########	0.479456	0.6	0.039	########
SUN1	########	-0.2173	0.432	0.111	########
RAF1	########	0.315562	0.571	0.056	########
AGO3	########	0.104214	0.477	0.06	########
FNDC3A	########	0.377523	0.567	0.048	########
ATG4B	########	0.234477	0.552	0.06	########
ARL14EP	########	-0.15774	0.411	0.077	########
PHYH	########	0.188965	0.491	0.05	########
ASAP1	########	0.419021	0.586	0.044	########
UROS	########	0.53718	0.597	0.029	########
DGKZ	########	0.435155	0.595	0.046	########
PRKCA	########	0.771471	0.659	0.024	########
NMB	########	0.100752	0.664	0.133	########
TRIP11	########	0.350617	0.558	0.05	########
INO80E	########	0.348105	0.557	0.046	########
FNBP4	########	0.304417	0.571	0.063	########
PTPN1	########	0.524243	0.577	0.024	########
ACOT13	########	-0.10024	0.453	0.072	########
SPG7	########	0.222654	0.536	0.053	########
NRSN2	########	0.539753	0.586	0.024	########
GNB4	########	0.335376	0.519	0.034	########
APEH	########	0.267054	0.494	0.034	########
FBXO9	########	0.422015	0.577	0.038	########
SLC35E1	########	0.18351	0.461	0.046	########
MECR	########	0.238727	0.498	0.044	########
AKAP7	########	0.40578	0.627	0.072	########
EIF2B5	########	0.163122	0.475	0.048	########
TMEM101	########	0.206487	0.495	0.051	########
PUM1	########	0.248851	0.55	0.053	########
DNM2	########	0.243065	0.509	0.048	########
BCL7B	########	0.198275	0.522	0.062	########
POLE3	########	-0.11876	0.423	0.074	########
C21orf62	########	0.495237	0.619	0.06	########
REST	########	0.558181	0.63	0.043	########
CBY1	########	0.110669	0.471	0.05	########
RBM10	########	0.171851	0.488	0.051	########
TOR1A	########	0.258954	0.509	0.05	########
ING2	########	0.100997	0.435	0.044	########
GNG12	########	0.353653	0.564	0.043	########
ABI1	########	0.204104	0.48	0.041	########
CAMK1	########	0.549044	0.61	0.039	########
PPP2R5E	########	0.415502	0.561	0.038	########
CLN5	########	0.252224	0.516	0.046	########
PLA2G12A	########	0.2422	0.558	0.068	########
ALKBH4	########	0.137223	0.486	0.06	########
MYEF2	########	0.482772	0.607	0.041	########
CTNND1	########	0.232544	0.515	0.051	########

PLEKHA3	########	0.356803	0.557	0.041	########
TBL1XR1	########	0.311894	0.564	0.056	########
DESI2	########	0.44933	0.585	0.036	########
C1orf50	########	0.151403	0.49	0.053	########
HIBADH	########	0.267218	0.524	0.051	########
USP48	########	0.218794	0.529	0.056	########
TMEM161A	########	0.129567	0.512	0.062	########
CTBP1	########	0.308609	0.531	0.043	########
SUPT20H	########	0.257593	0.532	0.055	########
NR3C1	########	0.169754	0.535	0.072	########
CCNG1	########	-0.31627	0.404	0.113	########
SLC44A2	########	0.230079	0.561	0.07	########
C1orf35	########	0.319145	0.548	0.05	########
SPARCL1	########	-0.25307	0.758	0.332	########
CRELD1	########	0.164051	0.488	0.053	########
WASL	########	0.210376	0.51	0.058	########
ZNF524	########	0.418433	0.55	0.029	########
CBX6	########	0.542605	0.664	0.063	########
SUCLG2	########	0.165655	0.529	0.067	########
CHD7	########	0.209688	0.566	0.077	########
UPF3B	########	-0.2094	0.419	0.096	########
SMARCC2	########	0.160963	0.541	0.08	########
RNASEH1-A	########	0.270556	0.517	0.048	########
ABCD3	########	0.330564	0.561	0.05	########
MAP9	########	0.262229	0.544	0.065	########
TMEM60	########	-0.10196	0.426	0.07	########
USP5	########	0.15377	0.494	0.056	########
USP14	########	0.256711	0.526	0.051	########
TOP2B	########	0.367458	0.547	0.039	########
STX16	########	0.179007	0.516	0.063	########
ANAPC10	########	-0.11415	0.403	0.075	########
ZNF511	########	0.363434	0.518	0.031	########
IRF2BPL	########	0.356545	0.581	0.055	########
FAM3A	########	0.360278	0.544	0.041	########
CHP1	########	0.250469	0.528	0.051	########
EPC1	########	0.400261	0.573	0.043	########
DCLK2	########	0.259102	0.553	0.063	########
MYO6	########	0.20199	0.576	0.084	########
GRAMD1A	########	0.353194	0.572	0.055	########
PTTG1	########	0.107123	0.489	0.062	########
HMGCS1	########	0.441492	0.616	0.056	########
CDC40	########	0.142668	0.453	0.044	########
SCYL1	########	0.36728	0.535	0.039	########
PI4KB	########	0.105879	0.527	0.074	########
PRKACB	########	0.195671	0.495	0.056	########
RIOK3	########	0.191965	0.483	0.05	########
BHLHE40	########	-0.20189	0.405	0.062	########
FAM181B	########	0.717373	0.673	0.053	########
SORBS3	########	0.31963	0.532	0.041	########
DSEL	########	0.209653	0.574	0.084	########
SCO2	########	0.361533	0.488	0.021	########

MAEA	########	0.206719	0.502	0.056	########
DHRS7B	########	0.107672	0.471	0.063	########
KIF2A	########	0.446668	0.57	0.034	########
TRIM27	########	0.252628	0.499	0.046	########
EXTL2	########	0.222233	0.495	0.046	########
LRRC58	########	0.315806	0.545	0.048	########
EHBP1	########	0.506383	0.593	0.041	########
GPR137B	########	0.280632	0.525	0.044	########
ZDHHC24	########	0.44301	0.533	0.022	########
ACTR6	########	0.125718	0.483	0.056	########
UBL4A	########	0.233079	0.504	0.041	########
TOX4	########	-0.23474	0.461	0.123	########
PDDC1	########	0.170654	0.479	0.055	########
MAPK3	########	0.113535	0.468	0.056	########
UBXN2A	########	0.244354	0.495	0.046	########
CCDC174	########	-0.14726	0.414	0.077	########
TGFB1I1	########	0.266498	0.515	0.046	########
CTDSP2	########	0.272261	0.569	0.068	########
HPS4	########	0.183538	0.494	0.058	########
GPATCH2L	########	0.28842	0.541	0.06	########
SMARCC1	########	0.49897	0.588	0.032	########
IWS1	########	0.18069	0.481	0.044	########
GSK3A	########	0.301512	0.541	0.05	########
NLN	########	0.41676	0.553	0.036	########
IPO9	########	0.179155	0.559	0.082	########
SUPT7L	########	0.108611	0.466	0.058	########
RARS2	########	0.183659	0.477	0.046	########
HS2ST1	########	0.44742	0.569	0.038	########
HEXIM1	########	0.305539	0.537	0.05	########
PURA	########	0.317118	0.541	0.05	########
IARS2	########	0.23701	0.484	0.044	########
NACC1	########	0.322154	0.523	0.039	########
NR2C2AP	########	-0.12413	0.426	0.08	########
TRAPPC2P1	########	-0.44888	0.377	0.13	########
GLIPR1	########	0.157139	0.504	0.056	########
CHIC2	########	0.294259	0.52	0.044	########
BRD3	########	0.382537	0.604	0.06	########
RB1CC1	########	0.422252	0.582	0.05	########
IFI35	########	-0.15587	0.438	0.094	########
TXNRD1	########	0.309244	0.54	0.048	########
SIKE1	########	0.100177	0.459	0.06	########
RNFT1	########	0.390695	0.53	0.032	########
CD82	########	-0.15467	0.528	0.128	########
SCARA3	########	0.254605	0.538	0.06	########
ARMC1	########	0.179728	0.502	0.062	########
MOAP1	########	0.103462	0.464	0.058	########
ZNF358	########	0.431315	0.554	0.031	########
UQCC1	########	0.182303	0.499	0.055	########
EPHX1	########	0.318969	0.563	0.053	########
STARD3	########	0.319089	0.541	0.05	########
PLOD3	########	0.154699	0.509	0.058	########

TRIM37	########	0.346308	0.497	0.026	########
TMEM132A	########	0.37602	0.539	0.038	########
NCOR2	########	0.326681	0.53	0.044	########
MAGT1	########	0.15112	0.487	0.055	########
BEX4	########	-0.7039	0.439	0.251	########
RRP1	########	0.195727	0.511	0.062	########
KANSL1-AS	########	0.304757	0.516	0.043	########
IST1	########	0.138157	0.507	0.063	########
C15orf61	########	0.167269	0.453	0.043	########
PLEKHO1	########	0.399058	0.556	0.041	########
EBAG9	########	0.149431	0.482	0.051	########
HOMER1	########	0.507722	0.581	0.031	########
PBX1	########	0.25499	0.565	0.075	########
SENP6	########	0.321444	0.522	0.043	########
B4GALT7	########	0.421027	0.538	0.029	########
EPS15L1	########	0.181315	0.469	0.046	########
TOB1	########	0.102054	0.498	0.067	########
TMEM189	########	0.54008	0.567	0.019	########
IGFBP3	########	0.662563	0.684	0.082	########
CSAD	########	0.433641	0.554	0.034	########
ARID1A	########	0.233191	0.515	0.058	########
SECISBP2L	########	0.261959	0.469	0.032	########
KLF3	########	0.331742	0.519	0.043	########
KRAS	########	0.309264	0.522	0.044	########
DPCD	########	-0.16548	0.384	0.062	########
SMAD5	########	0.112437	0.463	0.058	########
ENTPD6	########	0.452201	0.573	0.039	########
TLN1	########	0.37335	0.561	0.046	########
ZFAND2B	########	0.165721	0.491	0.058	########
C7orf49	########	0.220717	0.479	0.043	########
CGRRF1	########	-0.17828	0.379	0.077	########
RELA	########	0.215932	0.473	0.041	########
SMC3	########	0.192804	0.515	0.063	########
ASXL1	########	0.244876	0.49	0.05	########
SYNRG	########	0.320129	0.511	0.031	########
SAMM50	########	0.225051	0.48	0.043	########
BFAR	########	0.194223	0.491	0.053	########
CEP57	########	0.142831	0.478	0.062	########
DSCR3	########	-0.22085	0.333	0.065	########
RPS6KB2	########	0.15566	0.494	0.065	########
SPPL3	########	0.232465	0.489	0.046	########
PTPRE	########	0.438286	0.558	0.032	########
HES4	########	0.680964	0.635	0.039	########
ZNF91	########	0.190299	0.496	0.063	########
CSE1L	########	0.279058	0.506	0.039	########
ENC1	########	0.694752	0.638	0.039	########
CUL5	########	0.455011	0.548	0.027	########
ACSS3	########	0.425339	0.556	0.038	########
SIRT6	########	0.19267	0.496	0.056	########
MGAT2	########	0.189157	0.455	0.041	########
IPO7	########	0.320147	0.528	0.043	########

DYNC2LI1	########	-0.15885	0.392	0.08	########
KMT2A	########	0.332702	0.533	0.044	########
COLGALT2	########	0.454318	0.569	0.036	########
FOXG1	########	0.385645	0.559	0.048	########
KMT2C	########	0.208524	0.535	0.074	########
VPS51	########	0.255756	0.535	0.058	########
SUZ12	########	0.478058	0.551	0.026	########
CREB1	########	0.172384	0.472	0.053	########
TTPAL	########	0.116456	0.425	0.044	########
CCSER2	########	0.181026	0.469	0.044	########
MAPK8IP1	########	0.191975	0.516	0.058	########
TFAM	########	0.250851	0.487	0.043	########
ELP2	########	-0.23303	0.405	0.103	########
NAA15	########	0.113697	0.471	0.062	########
CENPV	########	0.661186	0.626	0.038	########
NCLN	########	0.334828	0.498	0.029	########
MICU2	########	0.153159	0.481	0.055	########
EXOSC1	########	-0.10608	0.359	0.058	########
DAZAP1	########	0.219465	0.488	0.05	########
PTPRS	########	0.512199	0.657	0.068	########
TUBGCP2	########	0.305967	0.5	0.029	########
SLC30A5	########	0.285074	0.519	0.044	########
TRIM47	########	0.430502	0.603	0.058	########
GTF2H2	########	0.171115	0.492	0.063	########
CIZ1	########	0.235841	0.496	0.05	########
CTD-2336C	########	0.310065	0.51	0.038	########
ERV3-1	########	-0.27166	0.405	0.113	########
OXA1L	########	-0.33406	0.421	0.123	########
ATP6V1C1	########	0.117829	0.471	0.062	########
RERE	########	0.22097	0.508	0.058	########
GLRX	########	-0.62183	0.331	0.132	########
TMED7	########	0.321489	0.57	0.065	########
AFG3L2	########	0.343798	0.521	0.038	########
RRP7A	########	0.128919	0.457	0.044	########
KIAA1033	########	0.1864	0.488	0.055	########
NT5DC2	########	0.430508	0.539	0.031	########
NDUFAF6	########	0.221277	0.456	0.034	########
ATF6	########	0.287706	0.497	0.044	########
MTA1	########	0.39488	0.581	0.055	########
AC093673.	########	0.271938	0.542	0.067	########
FAM53C	########	-0.2535	0.37	0.096	########
NDRG4	########	0.103519	0.455	0.058	########
ANK2	########	0.449599	0.57	0.041	########
ARMC8	########	-0.31192	0.371	0.092	########
CHMP6	########	0.360738	0.524	0.034	########
USP34	########	0.397425	0.553	0.046	########
RNF170	########	0.114548	0.44	0.05	########
GGH	########	0.163987	0.538	0.077	########
SECISBP2	########	0.165834	0.531	0.079	########
KDM1A	########	0.198983	0.452	0.036	########
FABP5	########	-1.68383	0.292	0.501	########

PIGS	########	0.374701	0.523	0.038	########
FBXW2	########	0.312036	0.514	0.043	########
IRF2	########	-0.10209	0.42	0.085	########
CDC42BPA	########	0.134238	0.491	0.065	########
CBS	########	0.197989	0.55	0.079	########
STK16	########	0.167176	0.434	0.041	########
U2AF1L4	########	-0.36576	0.422	0.14	########
MED21	########	-0.13328	0.415	0.084	########
TMEM206	########	0.319141	0.527	0.044	########
PDE4B	########	0.201973	0.572	0.087	########
PDHX	########	0.155825	0.44	0.043	########
CAT	########	-0.13259	0.411	0.085	########
EMP1	########	-0.22915	0.538	0.178	########
PPP6C	########	0.157672	0.49	0.058	########
JAK1	########	0.377741	0.538	0.039	########
TTYH3	########	0.413925	0.564	0.046	########
PAM	########	0.482017	0.562	0.031	########
RHOJ	########	0.288015	0.502	0.034	########
RIC3	########	-0.59208	0.422	0.185	########
KCTD20	########	0.236677	0.477	0.043	########
COQ5	########	0.115468	0.449	0.051	########
TMEM41B	########	0.128934	0.462	0.058	########
COL4A1	########	0.574103	0.587	0.027	########
PKN2	########	0.323594	0.538	0.044	########
ACOT7	########	0.205895	0.464	0.039	########
N6AMT2	########	-0.13402	0.353	0.06	########
RRAGC	########	0.106221	0.447	0.053	########
SNX21	########	0.326446	0.491	0.024	########
MED13	########	0.27441	0.491	0.041	########
RITA1	########	0.110189	0.405	0.038	########
PHACTR4	########	0.107645	0.46	0.063	########
DPF3	########	0.514905	0.585	0.039	########
NFE2L1	########	0.259769	0.488	0.043	########
RNF115	########	0.26183	0.529	0.056	########
ZNF638	########	0.2233	0.517	0.065	########
KIAA0319L	########	0.148555	0.458	0.05	########
SPOCD1	########	0.124526	0.646	0.166	########
GGT7	########	0.437581	0.559	0.036	########
PTPN12	########	0.253944	0.494	0.046	########
ZNF131	########	0.180972	0.49	0.058	########
DNAJB14	########	0.178588	0.463	0.048	########
SRR	########	0.15897	0.437	0.044	########
MBD3	########	0.422189	0.54	0.032	########
VOPP1	########	0.114549	0.462	0.06	########
ENDOD1	########	0.406955	0.547	0.038	########
DNAJC4	########	0.257731	0.479	0.039	########
RBM23	########	-0.24691	0.424	0.121	########
PPP1R15A	########	-0.36708	0.495	0.179	########
SACS	########	0.732543	0.619	0.017	########
CCDC66	########	0.50876	0.584	0.036	########
GTF2E2	########	0.421583	0.529	0.031	########

TNFAIP1	########	0.508115	0.572	0.032	########
FUCA2	########	0.17988	0.462	0.046	########
GMPPA	########	-0.10375	0.37	0.067	########
PREPL	########	0.111857	0.472	0.07	########
UNC119	########	0.40844	0.532	0.031	########
C1orf27	########	-0.2014	0.392	0.077	########
HSCB	########	0.116165	0.417	0.039	########
DR1	########	0.215198	0.494	0.053	########
RBM4	########	0.205582	0.493	0.048	########
SLC27A5	########	-0.31201	0.352	0.092	########
GALNT1	########	0.666286	0.592	0.015	########
PINK1	########	0.163827	0.52	0.067	########
EVI5	########	0.433887	0.53	0.026	########
ALDH6A1	########	0.216405	0.548	0.077	########
TMEM181	########	0.467361	0.537	0.022	########
ACAD8	########	0.259694	0.512	0.048	########
RIF1	########	0.300435	0.49	0.034	########
DLG1	########	0.366806	0.56	0.058	########
OSTM1	########	0.210195	0.444	0.034	########
ADNP	########	0.372837	0.535	0.039	########
CRNDE	########	0.240526	0.523	0.062	########
UHMK1	########	0.33307	0.496	0.031	########
SNX1	########	0.150913	0.454	0.048	########
ARHGEF2	########	0.111412	0.555	0.106	########
CRYZ	########	-0.14088	0.365	0.07	########
CRCP	########	0.107134	0.436	0.053	########
KIAA0907	########	-0.11327	0.413	0.089	########
C1S	########	-0.60473	0.349	0.15	########
IDH3A	########	0.124995	0.44	0.046	########
RRP8	########	0.135437	0.406	0.038	########
ZNF83	########	0.308895	0.547	0.06	########
SHKBP1	########	0.458477	0.53	0.021	########
MAPK10	########	0.171104	0.456	0.051	########
TMEM218	########	0.225652	0.482	0.048	########
MBOAT2	########	0.101906	0.525	0.082	########
ZNF414	########	0.50872	0.576	0.029	########
CDO1	########	-0.33731	0.412	0.121	########
PHLDA3	########	0.20142	0.505	0.06	########
PPFIA1	########	0.359686	0.518	0.041	########
KLHL4	########	0.170191	0.522	0.068	########
CLINT1	########	0.248368	0.474	0.039	########
TRAPPC6A	########	-0.14689	0.436	0.094	########
STT3B	########	0.246677	0.481	0.05	########
VCAN	########	0.189435	0.51	0.06	########
TRAF7	########	0.311334	0.487	0.036	########
FAM134B	########	0.36271	0.534	0.043	########
NFKBIL1	########	0.259615	0.498	0.05	########
SLC4A1AP	########	0.133123	0.428	0.039	########
CUL3	########	0.265306	0.469	0.041	########
RDH14	########	0.106411	0.433	0.056	########
SCRG1	########	-0.17026	0.59	0.19	########

BTBD3	########	0.520873	0.573	0.031	########
SFSWAP	########	0.177744	0.434	0.038	########
CLEC11A	########	0.42433	0.563	0.048	########
VPS37B	########	0.285634	0.511	0.046	########
FAHD2A	########	0.116171	0.425	0.05	########
TEAD1	########	0.521192	0.557	0.026	########
PABPC4	########	0.267745	0.51	0.048	########
POR	########	0.129099	0.506	0.084	########
REXO4	########	0.263444	0.488	0.043	########
PALLD	########	0.494053	0.557	0.036	########
CHST2	########	0.710445	0.601	0.017	########
TNPO2	########	0.176844	0.497	0.063	########
SMAD2	########	0.204016	0.453	0.043	########
CERS1	########	0.313496	0.541	0.053	########
ZNF771	########	0.482907	0.55	0.027	########
PPFIBP1	########	0.305548	0.484	0.034	########
SALL1	########	0.347491	0.507	0.039	########
NEDD9	########	0.154597	0.537	0.089	########
PGAP1	########	0.236709	0.472	0.044	########
STXBP3	########	0.138402	0.46	0.055	########
RFX4	########	-0.19431	0.447	0.125	########
TMX3	########	0.163394	0.437	0.046	########
EIF1AY	########	0.599945	0.564	0.019	########
FHL3	########	0.238848	0.459	0.034	########
DTNBP1	########	0.172681	0.427	0.041	########
ZNF664	########	0.18016	0.418	0.036	########
POLDIP3	########	0.219369	0.466	0.036	########
VWA1	########	0.570174	0.574	0.024	########
GALK1	########	0.481522	0.563	0.036	########
RBFOX2	########	0.249968	0.462	0.041	########
UBE2Q2	########	0.404146	0.538	0.039	########
RP11-51J9	########	-0.32428	0.332	0.08	########
BROX	########	0.385288	0.541	0.039	########
WEE1	########	0.36229	0.549	0.051	########
DNAJC5	########	0.452359	0.539	0.029	########
ACBD5	########	0.39182	0.531	0.038	########
CTNNB1	########	0.369806	0.62	0.082	########
SEC14L2	########	0.217647	0.497	0.051	########
ARL8B	########	0.18863	0.437	0.039	########
AKT2	########	0.12999	0.518	0.077	########
PRR14	########	0.234837	0.483	0.048	########
GPN3	########	-0.14834	0.398	0.08	########
RBM38	########	0.198711	0.487	0.056	########
TEN1	########	0.582092	0.547	0.01	########
RP11-345F	########	0.284321	0.509	0.05	########
RNF40	########	0.268613	0.484	0.043	########
INSIG2	########	-0.35028	0.345	0.097	########
MVP	########	0.230087	0.471	0.044	########
GRWD1	########	0.168012	0.434	0.039	########
PTRH2	########	-0.20477	0.317	0.063	########
SCRN2	########	0.123522	0.453	0.058	########

DALRD3	########	0.341448	0.474	0.027	########
PDK2	########	0.238097	0.449	0.038	########
ARRB2	########	0.108841	0.426	0.044	########
FSD1	########	0.211534	0.458	0.044	########
ZSCAN18	########	0.138688	0.456	0.056	########
CTNND2	########	0.141328	0.491	0.067	########
KCTD5	########	0.205987	0.456	0.046	########
SIGMAR1	########	0.287231	0.483	0.036	########
PPP3CA	########	0.369601	0.561	0.055	########
PLOD2	########	0.246266	0.515	0.06	########
TMEM38B	########	-0.3102	0.32	0.087	########
MGAT1	########	0.290686	0.482	0.036	########
ZNF444	########	0.231193	0.473	0.039	########
R3HDM4	########	0.445034	0.555	0.036	########
PRADC1	########	0.34472	0.5	0.038	########
ARHGEF26	########	0.147899	0.478	0.062	########
ZNF576	########	-0.13301	0.387	0.08	########
C19orf52	########	0.417006	0.519	0.026	########
PSMA6	########	-0.49562	0.345	0.14	########
ZHX1	########	0.350583	0.543	0.048	########
PCYT2	########	0.615078	0.58	0.019	########
PCED1A	########	0.303986	0.467	0.029	########
SNAP23	########	0.148055	0.458	0.053	########
CTA-29F11	########	-0.23897	0.362	0.072	########
CRTAP	########	0.220974	0.478	0.048	########
KCTD3	########	0.241514	0.458	0.038	########
TANC2	########	0.48116	0.559	0.031	########
ACBD3	########	0.185271	0.438	0.041	########
CHRNB1	########	-0.12088	0.393	0.079	########
VEZF1	########	0.322625	0.526	0.05	########
TCEB3	########	0.209784	0.44	0.038	########
TMEM129	########	0.362863	0.542	0.044	########
FAM120AOS	########	0.191606	0.433	0.036	########
GAR1	########	0.164011	0.451	0.041	########
NIP7	########	-0.10302	0.374	0.058	########
FARP1	########	0.313292	0.499	0.038	########
SYT17	########	0.308445	0.517	0.046	########
CAPN1	########	0.193262	0.478	0.06	########
MTRNR2L12	########	-1.154	0.239	0.263	########
ADAM10	########	0.224971	0.428	0.036	########
SUCO	########	0.221658	0.46	0.041	########
TRMT61A	########	0.114963	0.398	0.041	########
NPTXR	########	0.582931	0.561	0.021	########
ZDHHC9	########	0.1462	0.425	0.044	########
HHLA3	########	-0.22212	0.327	0.072	########
ALYREF	########	0.523215	0.538	0.019	########
LUZP1	########	0.246536	0.485	0.05	########
SAP30	########	0.376831	0.49	0.029	########
C11orf1	########	-0.33715	0.289	0.075	########
ADPGK	########	0.185579	0.441	0.043	########
MFSD12	########	0.545703	0.554	0.021	########

PPIL3	########	0.229331	0.462	0.043	########
WDR83	########	0.190602	0.468	0.05	########
CTXN1	########	0.800351	0.607	0.01	########
IBTK	########	0.176911	0.424	0.043	########
GTF2B	########	-0.25178	0.365	0.096	########
TNIP2	########	0.279193	0.436	0.024	########
CS	########	0.246754	0.431	0.027	########
PCGF2	########	0.339305	0.499	0.032	########
DNAJC10	########	0.283861	0.486	0.044	########
LIMD2	########	0.602063	0.545	0.012	########
ROCK2	########	0.398238	0.509	0.029	########
MGEA5	########	0.107551	0.458	0.068	########
PCIF1	########	0.201996	0.441	0.041	########
APC	########	0.466003	0.553	0.038	########
ATN1	########	0.382314	0.519	0.039	########
GATAD1	########	0.264992	0.508	0.062	########
SNTG1	########	-0.45983	0.357	0.142	########
DVL2	########	0.377244	0.523	0.043	########
CLPTM1L	########	0.429117	0.515	0.027	########
PHF3	########	0.223329	0.485	0.051	########
DNAJA3	########	0.121169	0.434	0.046	########
FOXO3	########	0.181902	0.499	0.067	########
USO1	########	0.117969	0.44	0.062	########
MSRA	########	-0.3053	0.377	0.109	########
VPS16	########	0.320865	0.48	0.029	########
NME7	########	-0.1884	0.371	0.082	########
KIDINS22C	########	0.235898	0.503	0.062	########
CERS5	########	0.104863	0.467	0.068	########
DUSP4	########	0.237268	0.519	0.063	########
NETO2	########	0.601945	0.579	0.024	########
C20orf96	########	0.329092	0.479	0.029	########
TM9SF3	########	0.379759	0.503	0.031	########
ARCN1	########	0.28561	0.46	0.027	########
RBM4B	########	0.109162	0.447	0.06	########
LRRC47	########	0.259518	0.507	0.051	########
SLC36A4	########	0.180972	0.458	0.05	########
MTRNR2L8	########	-1.23756	0.222	0.289	########
CCDC112	########	0.4927	0.533	0.022	########
TMEM80	########	0.307792	0.49	0.041	########
API5	########	0.177014	0.487	0.055	########
DCAF11	########	0.218049	0.462	0.046	########
GAPVD1	########	0.122497	0.404	0.034	########
MOB2	########	0.132485	0.423	0.048	########
ATXN3	########	-0.29602	0.324	0.091	########
BAZ2A	########	0.198955	0.468	0.055	########
VPS13C	########	0.201058	0.437	0.043	########
DCAKD	########	0.300794	0.46	0.027	########
TMF1	########	0.146655	0.439	0.046	########
ECHDC2	########	0.305015	0.522	0.056	########
GPBP1L1	########	0.202781	0.462	0.05	########
ROGDI	########	0.401283	0.493	0.024	########

ASCC2	########	0.193244	0.443	0.043	########
MPZL1	########	0.187446	0.439	0.041	########
MBIP	########	-0.19451	0.36	0.072	########
AMFR	########	0.237659	0.471	0.043	########
MUS81	########	0.247107	0.462	0.043	########
PITPNA	########	0.593428	0.555	0.017	########
LRWD1	########	0.246678	0.448	0.032	########
TTF1	########	0.166329	0.456	0.056	########
WIZ	########	0.180508	0.406	0.029	########
PNKP	########	0.20641	0.458	0.051	########
GNS	########	0.206313	0.446	0.043	########
YPEL3	########	0.134206	0.464	0.07	########
C16orf87	########	0.103585	0.417	0.05	########
TXLNA	########	0.291357	0.452	0.029	########
SNRNP35	########	-0.13222	0.377	0.079	########
RAB3IP	########	-0.14399	0.523	0.164	########
QSOX1	########	0.247394	0.475	0.041	########
SLC35A5	########	0.166577	0.439	0.044	########
PPP1R18	########	0.207228	0.436	0.036	########
ALKBH3	########	-0.15087	0.299	0.056	########
ZDHHC3	########	0.236829	0.439	0.029	########
MGRN1	########	0.388538	0.529	0.039	########
BEST1	########	-0.32534	0.336	0.074	########
TMEM131	########	0.552473	0.559	0.024	########
SHC1	########	0.236054	0.44	0.034	########
NR2F1	########	0.405668	0.532	0.041	########
CHTF8	########	0.209027	0.403	0.026	########
MTMR2	########	0.365171	0.474	0.024	########
PGM3	########	0.187352	0.458	0.053	########
RMDN1	########	0.187671	0.437	0.043	########
EIF2AK4	########	0.415767	0.508	0.027	########
RP11-390F	########	0.303501	0.484	0.039	########
PRKAA1	########	0.272533	0.464	0.039	########
PBRM1	########	0.239208	0.47	0.05	########
DOLK	########	0.108261	0.4	0.048	########
HIVEP3	########	0.53379	0.549	0.027	########
HNRNPUL2	########	0.1899	0.402	0.027	########
PFKM	########	0.233002	0.489	0.053	########
DIMT1	########	0.195335	0.447	0.051	########
TRADD	########	0.270976	0.415	0.022	########
INO80C	########	0.170553	0.399	0.029	########
EFCAB14	########	0.403765	0.503	0.029	########
ZHX3	########	0.123044	0.459	0.062	########
DUSP3	########	0.180314	0.468	0.056	########
PPM1B	########	0.104491	0.505	0.087	########
CCPG1	########	0.19002	0.465	0.055	########
CEP350	########	0.258489	0.45	0.036	########
CLK1	########	-0.39281	0.439	0.173	########
TRIM24	########	0.459638	0.535	0.032	########
ROBO1	########	0.856886	0.601	0.007	########
C12orf73	########	0.142908	0.427	0.041	########

ANAPC7	########	0.140877	0.441	0.051	########
TP53TG1	########	-0.26347	0.359	0.094	########
FZD7	########	0.592626	0.523	0.012	########
TRMT1	########	0.140554	0.493	0.074	########
MTRF1L	########	0.480118	0.548	0.031	########
HERC2	########	0.267009	0.493	0.053	########
NR2F6	########	0.449853	0.51	0.024	########
IQGAP2	########	0.385002	0.509	0.038	########
BNIP2	########	0.366679	0.484	0.029	########
TPST1	########	0.255398	0.504	0.055	########
NFS1	########	0.301951	0.452	0.026	########
DCTN4	########	0.16874	0.439	0.048	########
CTNNAL1	########	0.102015	0.406	0.051	########
HEPN1	########	0.114507	0.628	0.14	########
SOGA1	########	0.285603	0.478	0.043	########
ZNF608	########	0.406086	0.53	0.038	########
CEP290	########	0.224727	0.466	0.051	########
MDM4	########	0.153602	0.52	0.082	########
DPP8	########	0.195369	0.44	0.039	########
RBFA	########	0.223821	0.445	0.039	########
UBA5	########	0.178493	0.419	0.036	########
AP4M1	########	-0.18543	0.396	0.103	########
LETM1	########	0.38918	0.524	0.039	########
PVRL2	########	0.359104	0.504	0.038	########
RRP9	########	0.107908	0.423	0.043	########
RNF14	########	0.262236	0.453	0.041	########
CDC16	########	0.2818	0.46	0.034	########
LHX2	########	0.781426	0.59	0.017	########
HCFC1R1	########	-0.19513	0.406	0.094	########
RAC3	########	0.587724	0.548	0.019	########
WSCD1	########	0.247948	0.522	0.07	########
TFDP1	########	0.349047	0.477	0.027	########
TNIP1	########	0.250186	0.452	0.034	########
ACTR1B	########	0.157254	0.442	0.051	########
CANT1	########	0.307266	0.482	0.036	########
MIER1	########	0.238085	0.461	0.038	########
EGR1	########	-0.64569	0.517	0.304	########
ATXN7L3B	########	0.12384	0.417	0.048	########
NDRG3	########	0.303925	0.477	0.038	########
RARRES3	########	-0.68729	0.377	0.176	########
ST6GALNAC	########	0.128385	0.41	0.043	########
BST2	########	-0.91311	0.557	0.426	########
HTRA2	########	0.164028	0.449	0.05	########
EXOC5	########	0.216495	0.443	0.039	########
LMO2	########	0.420145	0.572	0.065	########
LTV1	########	0.145957	0.405	0.034	########
GAS2L1	########	0.423849	0.522	0.034	########
KPNA4	########	0.261973	0.433	0.029	########
HELZ	########	0.246189	0.477	0.051	########
APIP	########	-0.10843	0.359	0.065	########
RCHY1	########	-0.24329	0.364	0.085	########

KAT7	########	0.206133	0.43	0.038	########
SLC27A1	########	0.216483	0.495	0.055	########
HBS1L	########	0.174886	0.425	0.044	########
ELMO2	########	0.113724	0.374	0.036	########
IFI16	########	-0.46173	0.434	0.193	########
ZBTB7A	########	0.37922	0.504	0.032	########
ATXN2L	########	0.18554	0.439	0.043	########
DPH5	########	-0.10165	0.358	0.065	########
BCAR1	########	0.233261	0.441	0.041	########
PNPLA2	########	0.397079	0.504	0.032	########
KLHL42	########	0.186108	0.452	0.051	########
CEP164	########	0.214605	0.429	0.034	########
RPAP2	########	0.207647	0.449	0.05	########
B3GALT6	########	0.408422	0.507	0.029	########
EIF4G3	########	0.233888	0.465	0.043	########
GFER	########	0.403949	0.493	0.024	########
METTL2A	########	0.235037	0.439	0.036	########
MPRIP	########	0.186374	0.422	0.041	########
ADAM17	########	0.474905	0.551	0.038	########
MTMR4	########	0.299119	0.453	0.026	########
DMWD	########	0.53873	0.559	0.029	########
RGS17	########	0.351562	0.504	0.044	########
WARS	########	-0.13491	0.483	0.108	########
DGCR6	########	0.263686	0.495	0.048	########
MAFG	########	0.274353	0.485	0.039	########
TMEM97	########	0.196854	0.438	0.039	########
GOLGB1	########	0.205843	0.474	0.053	########
SLC2A4RG	########	0.324689	0.467	0.032	########
RFC2	########	-0.201	0.362	0.082	########
HSPA14	########	0.120939	0.359	0.034	########
KIFAP3	########	0.250754	0.451	0.039	########
SMARCA1	########	0.196635	0.425	0.038	########
DTWD1	########	0.234678	0.41	0.026	########
RNF26	########	0.203159	0.423	0.034	########
PHLDB1	########	0.109172	0.383	0.041	########
NPRL3	########	0.223659	0.46	0.053	########
UXS1	########	0.325132	0.466	0.031	########
NADK	########	0.361949	0.483	0.026	########
BLMH	########	0.231472	0.444	0.038	########
OSBPL2	########	0.161145	0.396	0.032	########
APBB1	########	0.244528	0.449	0.039	########
RAB8B	########	0.125961	0.4	0.039	########
MTG2	########	0.369793	0.468	0.024	########
NFKBIA	########	-0.20063	0.506	0.149	########
BMS1	########	0.13332	0.395	0.036	########
FAF1	########	0.21425	0.443	0.039	########
SNAPC1	########	-0.11633	0.354	0.058	########
TARBP2	########	0.248763	0.418	0.029	########
PQLC3	########	0.338678	0.458	0.027	########
THAP11	########	0.426966	0.513	0.029	########
IFT81	########	0.28871	0.463	0.036	########

HSPA13	########	-0.14219	0.401	0.085	########
NOL8	########	0.273517	0.484	0.048	########
FAM219B	########	0.224087	0.447	0.043	########
LRRFIP2	########	0.208388	0.434	0.034	########
BCAN	########	-0.12293	0.692	0.248	########
DRAM2	########	-0.16489	0.38	0.08	########
BMP7	########	0.244936	0.518	0.063	########
TMEM18	########	-0.14681	0.377	0.079	########
NOTCH2	########	0.451806	0.504	0.022	########
FNBP1	########	0.384929	0.52	0.041	########
TSC22D3	########	-0.33848	0.401	0.125	########
FNDC3B	########	0.354322	0.491	0.031	########
GSK3B	########	0.223594	0.442	0.043	########
LYSMD2	########	0.278696	0.449	0.031	########
ABCF2	########	0.146402	0.462	0.06	########
USP39	########	0.189419	0.417	0.036	########
DDX54	########	0.25906	0.483	0.046	########
FAM222B	########	0.266481	0.411	0.026	########
FAIM2	########	-0.16347	0.378	0.085	########
CASP6	########	0.297058	0.44	0.029	########
CRNKL1	########	0.282671	0.425	0.024	########
FAHD1	########	0.184536	0.432	0.044	########
TTC37	########	0.382481	0.504	0.038	########
UBE2D4	########	0.127217	0.431	0.048	########
EML4	########	0.213455	0.43	0.036	########
FAM104B	########	0.258553	0.457	0.039	########
SDSL	########	0.232323	0.432	0.034	########
FUZ	########	0.153357	0.434	0.048	########
SCO1	########	0.41629	0.492	0.026	########
USP9X	########	0.289007	0.466	0.038	########
CEP170	########	0.331378	0.482	0.036	########
DNAAF2	########	0.250405	0.428	0.031	########
RMND5B	########	0.13901	0.39	0.036	########
DCBLD2	########	0.796608	0.597	0.021	########
ZNFX1	########	0.104752	0.395	0.048	########
DDX19B	########	0.167858	0.398	0.039	########
GTF2H3	########	0.193062	0.43	0.041	########
SMCHD1	########	0.364992	0.479	0.029	########
RNF157	########	0.229809	0.46	0.044	########
BOLA1	########	0.178468	0.405	0.034	########
BRWD1	########	0.352726	0.508	0.039	########
LONP2	########	0.192658	0.485	0.065	########
PHKB	########	0.201253	0.443	0.048	########
TUG1	########	0.365971	0.472	0.027	########
ANKS3	########	0.256282	0.465	0.048	########
NF2	########	0.310875	0.443	0.026	########
DCAF5	########	0.107004	0.371	0.038	########
HGSNAT	########	0.307717	0.472	0.032	########
BASP1	########	0.682196	0.564	0.019	########
RNF139	########	0.358261	0.491	0.034	########
TNIK	########	0.521345	0.549	0.032	########

TSTD1	########	-0.23877	0.39	0.111	########
C11orf74	########	0.110031	0.389	0.036	########
TSPYL1	########	0.247172	0.455	0.041	########
PLEC	########	0.272157	0.441	0.036	########
RNF8	########	0.249618	0.422	0.031	########
SAC3D1	########	0.290744	0.437	0.026	########
MAT2B	########	-0.24615	0.319	0.079	########
XPOT	########	0.134459	0.457	0.063	########
CSNK1G2	########	0.5006	0.509	0.015	########
ARHGEF6	########	0.339287	0.451	0.024	########
DYNLL2	########	0.269265	0.442	0.034	########
TIPIN	########	-0.48877	0.245	0.08	########
ARL13B	########	-0.17093	0.326	0.055	########
SNX7	########	0.206107	0.454	0.05	########
VASP	########	0.241652	0.418	0.027	########
ATXN1	########	0.218903	0.428	0.039	########
ELAC2	########	0.249537	0.43	0.031	########
FRA10AC1	########	0.287135	0.445	0.027	########
XXYLT1	########	0.151813	0.387	0.027	########
C8orf82	########	0.397446	0.496	0.031	########
HIST1H1C	########	-0.37191	0.336	0.082	########
FBXW9	########	0.295028	0.418	0.026	########
LIX1L	########	0.335878	0.436	0.021	########
RNASEH2B	########	0.197069	0.422	0.039	########
FKBP9	########	0.342665	0.522	0.051	########
SLAIN1	########	0.523129	0.518	0.019	########
OCEL1	########	0.145971	0.399	0.039	########
FEM1C	########	0.140629	0.421	0.05	########
EXOSC5	########	-0.32637	0.345	0.104	########
OSBPL1A	########	0.247169	0.474	0.044	########
SPOCK2	########	0.257252	0.49	0.053	########
ABHD14B	########	0.200857	0.415	0.036	########
SRRD	########	0.369458	0.44	0.014	########
HLTF	########	0.231919	0.43	0.031	########
PPAT	########	0.408046	0.475	0.022	########
VAT1	########	0.261528	0.434	0.034	########
CEND1	########	0.111264	0.461	0.067	########
HNRNPLL	########	0.108328	0.396	0.041	########
DNAJC1	########	0.247477	0.436	0.039	########
RGL2	########	0.22003	0.453	0.05	########
ADORA1	########	0.18055	0.474	0.062	########
IGSF8	########	0.234638	0.497	0.06	########
MRS2	########	0.124075	0.415	0.046	########
CTDSP1	########	0.215855	0.459	0.051	########
CARS2	########	0.267967	0.406	0.024	########
RGS6	########	0.149825	0.45	0.056	########
TPST2	########	0.218737	0.43	0.034	########
BCAS4	########	-0.15714	0.283	0.051	########
MAPK6	########	0.205129	0.456	0.053	########
BCAM	########	0.257656	0.437	0.034	########
LAMA5	########	0.453734	0.496	0.022	########

ARMCX1	########	-0.26363	0.348	0.092	########
DFNA5	########	-0.14835	0.32	0.068	########
GTPBP8	########	-0.15149	0.273	0.044	########
BTBD10	########	0.248322	0.409	0.031	########
ARFGAP1	########	0.201427	0.43	0.046	########
CES2	########	0.422269	0.484	0.022	########
PIP5K1A	########	0.133247	0.395	0.043	########
DNAL4	########	0.279741	0.427	0.029	########
ANXA6	########	0.166603	0.409	0.043	########
RRS1	########	0.227591	0.421	0.032	########
ZNF330	########	-0.35229	0.318	0.091	########
RSAD1	########	0.337828	0.467	0.029	########
CDK6	########	0.193442	0.509	0.075	########
ATRN	########	0.410355	0.468	0.012	########
C1QB	########	-0.91259	0.281	0.246	########
CPD	########	0.336697	0.472	0.032	########
POMGNT2	########	0.190116	0.412	0.038	########
DPYSL5	########	0.407173	0.489	0.029	########
SGK1	########	-0.14659	0.393	0.091	########
EXTL3	########	0.341	0.478	0.032	########
OGFOD1	########	0.14523	0.4	0.034	########
LASP1	########	0.1287	0.4	0.044	########
ACAP3	########	0.24597	0.483	0.058	########
NCOA6	########	0.392735	0.476	0.026	########
E2F6	########	0.302734	0.442	0.027	########
COMMD8	########	-0.19302	0.337	0.082	########
LMF2	########	0.258643	0.462	0.044	########
RBM14	########	0.205654	0.408	0.034	########
MFSD1	########	0.150928	0.402	0.044	########
ZC3H11A	########	0.179923	0.433	0.048	########
CAMSAP2	########	0.272116	0.448	0.034	########
SERTAD3	########	-0.27543	0.325	0.079	########
ZNF506	########	0.17359	0.403	0.038	########
TCF3	########	0.342081	0.449	0.024	########
SGSM3	########	0.221962	0.466	0.055	########
EPB41L2	########	0.385002	0.482	0.031	########
UBE2Z	########	0.214056	0.421	0.041	########
DOHH	########	0.3477	0.471	0.031	########
C17orf58	########	0.413846	0.481	0.027	########
MVD	########	0.260123	0.458	0.036	########
TPMT	########	-0.19461	0.297	0.06	########
TCERG1	########	0.188428	0.429	0.048	########
TOMM34	########	0.153371	0.385	0.032	########
PXDC1	########	0.321797	0.458	0.032	########
MAP2K7	########	0.277724	0.459	0.043	########
SLC35E2B	########	0.280898	0.446	0.038	########
CTC-444N2	########	0.114076	0.427	0.065	########
SC5D	########	-0.1145	0.393	0.079	########
RP9	########	0.104744	0.401	0.051	########
DZIP1	########	0.277159	0.434	0.034	########
ATP2C1	########	0.204773	0.409	0.038	########

AKAP11	########	0.274051	0.422	0.026	########
DDX6	########	-0.25446	0.344	0.08	########
FBXO11	########	0.162122	0.399	0.041	########
SNX14	########	0.166969	0.414	0.043	########
MTMR14	########	0.2488	0.432	0.036	########
FBXW11	########	0.410254	0.487	0.026	########
ZNF430	########	-0.2004	0.339	0.084	########
CDC42SE2	########	0.173118	0.4	0.039	########
GAS1	########	0.376606	0.553	0.067	########
C21orf2	########	0.338554	0.479	0.039	########
HSD17B11	########	-0.32993	0.332	0.097	########
SUDS3	########	0.450341	0.512	0.029	########
ATG4D	########	0.34882	0.459	0.027	########
PHYKPL	########	0.230234	0.446	0.046	########
DDX23	########	0.157817	0.384	0.036	########
RNASET2	########	0.283399	0.466	0.043	########
ANKFY1	########	0.211	0.385	0.024	########
SP3	########	0.160338	0.416	0.05	########
PPAN	########	0.119423	0.408	0.051	########
TRIM16	########	0.271288	0.415	0.026	########
SNTA1	########	0.234982	0.392	0.021	########
HCCS	########	0.124291	0.378	0.036	########
TPRA1	########	0.310409	0.438	0.026	########
WAC-AS1	########	0.10946	0.37	0.038	########
CHST7	########	0.383114	0.485	0.029	########
SPATA6	########	0.362115	0.52	0.051	########
SREBF2	########	0.347707	0.452	0.027	########
TAF9B	########	0.138802	0.386	0.039	########
CPNE4	########	0.579638	0.5	0.01	########
OBSL1	########	0.165191	0.458	0.07	########
SIGIRR	########	0.458624	0.469	0.017	########
CASP3	########	0.198288	0.439	0.051	########
CEP63	########	0.232835	0.406	0.027	########
TSPAN17	########	0.290758	0.421	0.027	########
NUDT16	########	0.134818	0.384	0.038	########
NNMT	########	-0.37836	0.408	0.13	########
TLK2	########	0.209388	0.377	0.022	########
ITGB3BP	########	-0.12443	0.319	0.058	########
GLRX2	########	0.218689	0.409	0.032	########
SEL1L	########	0.13147	0.386	0.034	########
LGR4	########	0.684898	0.541	0.007	########
C19orf12	########	0.259858	0.422	0.031	########
CSNK1G3	########	0.312979	0.438	0.026	########
SPRED2	########	0.279678	0.443	0.036	########
GRIA2	########	0.159656	0.499	0.084	########
NDUFAF5	########	0.193818	0.396	0.032	########
CDH11	########	0.167319	0.407	0.044	########
KCNE4	########	0.326032	0.477	0.041	########
SLC35F1	########	0.402256	0.497	0.031	########
GBE1	########	-0.25217	0.326	0.082	########
SRFBP1	########	0.194195	0.417	0.043	########

TRIP12	########	0.322937	0.434	0.021	########
GCLM	########	0.15296	0.438	0.055	########
TBC1D7	########	0.126928	0.379	0.039	########
SMC5	########	0.226095	0.439	0.039	########
MBTPS1	########	0.209391	0.395	0.036	########
RP11-386G	########	-0.33213	0.252	0.068	########
MED16	########	0.426496	0.49	0.024	########
ANKRD54	########	0.213643	0.407	0.031	########
ST3GAL4	########	0.211889	0.41	0.031	########
DUS1L	########	0.433519	0.483	0.019	########
CHCHD7	########	-0.24448	0.313	0.077	########
TNRC6C	########	0.15843	0.394	0.038	########
NARFL	########	0.118714	0.388	0.048	########
SLMAP	########	0.116202	0.365	0.041	########
PAXBP1	########	0.460899	0.516	0.032	########
EVA1B	########	0.68693	0.543	0.005	########
HDDC3	########	0.123184	0.38	0.048	########
SIAH1	########	0.141047	0.408	0.051	########
C9orf114	########	0.108516	0.394	0.044	########
WDR43	########	0.208621	0.435	0.046	########
NTAN1	########	0.391397	0.469	0.022	########
DIS3	########	-0.10123	0.336	0.051	########
PRKACA	########	0.327298	0.422	0.021	########
AC009501.	########	-0.91916	0.201	0.185	########
TMEM237	########	0.157155	0.429	0.051	########
RAB11B-AS	########	0.334149	0.444	0.024	########
KANSL1	########	0.278148	0.437	0.036	########
AQP4	########	-0.16603	0.55	0.193	########
FAF2	########	0.246879	0.441	0.046	########
GMNN	########	-0.14211	0.359	0.08	########
FADS3	########	0.167987	0.398	0.041	########
SYNGR2	########	0.394983	0.45	0.019	########
PICALM	########	0.279991	0.44	0.031	########
UCKL1	########	0.307765	0.449	0.032	########
AP3B1	########	0.276661	0.432	0.031	########
GALE	########	0.170747	0.39	0.038	########
ASMTL	########	0.153547	0.405	0.041	########
BPHL	########	0.134984	0.386	0.041	########
CBR4	########	0.36261	0.463	0.031	########
FAM118B	########	0.169418	0.379	0.031	########
ZNF322	########	0.314143	0.419	0.021	########
ZBED5	########	0.174931	0.392	0.031	########
TSKU	########	0.158426	0.361	0.024	########
SART1	########	0.356184	0.449	0.024	########
PGP	########	0.510682	0.519	0.019	########
ZNF544	########	-0.13321	0.333	0.07	########
PSPC1	########	0.184062	0.411	0.043	########
TRABD	########	0.403065	0.465	0.019	########
BMPR2	########	0.292308	0.438	0.031	########
SLC35F6	########	0.404709	0.482	0.029	########
ACOX1	########	0.477352	0.481	0.014	########

PIK3C2A	########	0.284911	0.451	0.036	########
UBALD1	########	0.356224	0.451	0.027	########
PPP1R37	########	0.179142	0.395	0.036	########
REV3L	########	0.169455	0.46	0.063	########
BRD9	########	0.291197	0.446	0.036	########
RBM15B	########	0.378429	0.444	0.019	########
ZNF688	########	0.280465	0.431	0.031	########
DHFR	########	0.271971	0.415	0.026	########
LYST	########	0.318908	0.461	0.036	########
MLH1	########	-0.1054	0.3	0.05	########
PDS5B	########	0.237656	0.443	0.046	########
NIPSNAP1	########	0.357152	0.44	0.022	########
L3HYPDH	########	0.456878	0.491	0.022	########
PGPEP1	########	0.22793	0.428	0.039	########
INTS12	########	-0.19245	0.335	0.084	########
PTPN9	########	0.195749	0.4	0.032	########
ANKRD13D	########	0.212071	0.393	0.031	########
C1orf174	########	0.107369	0.353	0.031	########
YES1	########	0.340354	0.466	0.032	########
PARVA	########	0.177685	0.374	0.029	########
JAKMIP2	########	0.254193	0.448	0.041	########
MCC	########	0.362074	0.484	0.032	########
STRN3	########	0.202908	0.412	0.032	########
GLYR1	########	0.177457	0.408	0.041	########
RRP36	########	0.576174	0.5	0.007	########
BIRC6	########	0.107296	0.376	0.046	########
ADO	########	0.261565	0.43	0.031	########
TOR3A	########	0.155847	0.384	0.036	########
SETX	########	0.307991	0.434	0.027	########
CDIP1	########	0.174278	0.408	0.046	########
POFUT1	########	0.333095	0.447	0.029	########
ZNF12	########	0.106877	0.335	0.038	########
BTBD1	########	0.270092	0.422	0.031	########
PREB	########	0.248599	0.443	0.039	########
LAMC1	########	0.428747	0.48	0.024	########
LRIG1	########	0.13324	0.392	0.048	########
NIT1	########	0.110657	0.387	0.043	########
EHMT2	########	0.306866	0.476	0.046	########
APOE	########	-0.12482	0.693	0.263	########
FAM217B	########	0.346485	0.444	0.027	########
CITED2	########	-0.10721	0.378	0.084	########
GRIPAP1	########	0.203351	0.441	0.051	########
SH3BP5	########	0.580141	0.504	0.015	########
RHBDD3	########	0.266588	0.425	0.036	########
ORAI1	########	0.443078	0.463	0.014	########
SLC38A2	########	-0.28023	0.351	0.113	########
ZNF37A	########	0.283053	0.41	0.024	########
ADSS	########	0.143214	0.398	0.044	########
QRICH1	########	0.128854	0.357	0.034	########
SH2B1	########	0.206413	0.399	0.038	########
CRAT	########	0.340332	0.446	0.026	########

NPLOC4	########	0.230658	0.398	0.027	########
PITPNB	########	0.173568	0.408	0.044	########
MDP1	########	-0.10041	0.277	0.05	########
EFNA1	########	-0.45232	0.28	0.111	########
FAM210A	########	0.108774	0.339	0.031	########
ETNK1	########	0.281995	0.456	0.043	########
EPDR1	########	0.25653	0.426	0.034	########
ZMYM2	########	0.229754	0.425	0.039	########
WIPI1	########	0.152142	0.383	0.038	########
IQCK	########	0.110965	0.411	0.051	########
MIEF1	########	0.205874	0.43	0.048	########
ZMYM4	########	0.294085	0.408	0.024	########
POLR2M	########	0.323793	0.449	0.031	########
NQO1	########	0.113179	0.361	0.036	########
FBXO18	########	0.268459	0.408	0.026	########
HUWE1	########	0.24302	0.432	0.041	########
TMEM63B	########	0.325406	0.437	0.024	########
CASK	########	0.10608	0.361	0.044	########
ELMOD3	########	0.191693	0.399	0.039	########
DAAM1	########	0.240746	0.471	0.065	########
GSKIP	########	0.115935	0.355	0.034	########
TRIM13	########	0.189715	0.449	0.063	########
ELOVL2	########	0.366328	0.475	0.036	########
PLA2G5	########	-0.5039	0.381	0.159	########
FKBP5	########	-0.38098	0.367	0.137	########
NECAP1	########	-0.1823	0.342	0.084	########
SCAMP1	########	0.134202	0.408	0.055	########
ARHGAP35	########	0.398825	0.465	0.024	########
CYP27A1	########	0.133199	0.375	0.038	########
SLC25A38	########	-0.1842	0.299	0.053	########
MCOLN1	########	0.275697	0.438	0.041	########
CDK12	########	0.256663	0.41	0.029	########
YRDC	########	0.16781	0.393	0.043	########
MIA3	########	0.224569	0.433	0.044	########
MMP2	########	0.384003	0.49	0.038	########
MPI	########	-0.12427	0.28	0.048	########
SCAF1	########	0.345241	0.433	0.021	########
NADSYN1	########	0.132814	0.357	0.026	########
H2AFJ	########	0.43757	0.454	0.014	########
RUFY1	########	0.124528	0.378	0.039	########
MIB2	########	0.29334	0.457	0.036	########
FBXO32	########	-0.37696	0.335	0.116	########
PROX1	########	0.301262	0.458	0.039	########
RNPEP	########	0.252327	0.398	0.027	########
FRYL	########	0.425971	0.488	0.031	########
NOTCH2NL	########	0.457689	0.506	0.029	########
PELP1	########	0.191243	0.373	0.024	########
CCNB1IP1	########	-0.29155	0.336	0.106	########
PLXNB2	########	0.293917	0.409	0.024	########
SLC38A10	########	0.135964	0.39	0.043	########
TLCD1	########	0.420566	0.459	0.017	########

MYADM	########	-0.17678	0.314	0.062	########
BATF3	########	0.242635	0.389	0.022	########
ENOSF1	########	0.505924	0.468	0.009	########
PURB	########	0.148753	0.39	0.043	########
PRPS1	########	-0.18619	0.349	0.079	########
SULF2	########	0.422659	0.479	0.032	########
CMBL	########	0.109408	0.373	0.038	########
VARS	########	0.163007	0.36	0.027	########
PNPLA6	########	0.250449	0.404	0.031	########
KPNA1	########	0.221106	0.399	0.031	########
PTPN13	########	0.26441	0.402	0.024	########
ANKLE2	########	0.248914	0.422	0.036	########
RP11-410L	########	0.295379	0.441	0.036	########
CKAP5	########	0.241256	0.378	0.024	########
SP100	########	-0.58327	0.249	0.13	########
PDGFC	########	0.331284	0.455	0.034	########
DESI1	########	0.383023	0.452	0.022	########
LINC00094	########	0.259526	0.405	0.027	########
ENHO	########	0.452988	0.487	0.029	########
ZNF579	########	0.322861	0.426	0.024	########
MSL1	########	0.120995	0.382	0.048	########
BTBD7	########	0.171571	0.371	0.034	########
LYAR	########	-0.10876	0.289	0.051	########
PDPK1	########	0.314629	0.444	0.026	########
EPM2AIP1	########	0.146971	0.412	0.053	########
GABARAPL1	########	-0.21416	0.352	0.091	########
ICMT	########	0.325423	0.424	0.021	########
ARFIP1	########	0.12524	0.375	0.046	########
DENND5A	########	0.185483	0.403	0.039	########
DVL3	########	0.307889	0.451	0.038	########
RANBP2	########	0.227399	0.397	0.029	########
RGS16	########	-0.2992	0.442	0.14	########
RPP14	########	0.228154	0.42	0.036	########
MEIS3	########	0.251209	0.378	0.026	########
ANKRD40	########	0.36745	0.472	0.034	########
CHKA	########	0.132941	0.392	0.041	########
TBCC	########	0.138475	0.381	0.039	########
DPH7	########	0.289284	0.429	0.034	########
ZNF43	########	0.171047	0.394	0.041	########
NAB2	########	0.286083	0.435	0.034	########
JAM3	########	0.190332	0.409	0.041	########
UBE2W	########	0.227085	0.426	0.043	########
SCYL2	########	0.301124	0.414	0.026	########
THAP1	########	-0.10289	0.313	0.051	########
LIPA	########	0.238414	0.443	0.044	########
PARP9	########	0.243805	0.446	0.044	########
KCTD6	########	0.194858	0.37	0.027	########
UBLCP1	########	0.287684	0.413	0.026	########
MGARP	########	0.245509	0.384	0.027	########
CNOT8	########	0.103008	0.342	0.032	########
SFRP1	########	0.529817	0.487	0.014	########

MAPKAPK5	########	0.398747	0.474	0.031	########
NUDT2	########	0.298785	0.424	0.026	########
GCA	########	-0.40143	0.275	0.096	########
ZNF721	########	0.133216	0.372	0.043	########
DHCR24	########	0.350631	0.419	0.021	########
ZNF439	########	0.278734	0.421	0.031	########
TFDP2	########	0.201805	0.411	0.034	########
ZMYND11	########	0.414275	0.458	0.019	########
USP3	########	0.173877	0.384	0.034	########
IQCG	########	-0.18693	0.3	0.058	########
SLC9A3R2	########	0.244345	0.393	0.027	########
PDS5A	########	0.187841	0.37	0.031	########
TMOD3	########	0.242322	0.391	0.027	########
EIF3J-AS1	########	0.158257	0.377	0.038	########
RPIA	########	0.138232	0.362	0.036	########
PAPD4	########	0.186499	0.398	0.039	########
RNF220	########	0.135312	0.351	0.032	########
ATF2	########	-0.10064	0.319	0.062	########
ZC3HAV1	########	0.22857	0.396	0.032	########
SLC25A17	########	0.162402	0.388	0.036	########
PTAR1	########	0.338085	0.443	0.029	########
UBAP1	########	0.102607	0.38	0.039	########
MSRB2	########	0.290918	0.434	0.032	########
GMPS	########	0.37126	0.447	0.024	########
COX19	########	0.146906	0.398	0.05	########
FAM179B	########	0.125179	0.367	0.036	########
TBC1D9B	########	0.145591	0.382	0.041	########
ZNF462	########	0.200436	0.443	0.05	########
TRAPPC12	########	0.262018	0.437	0.039	########
MBNL1	########	0.297132	0.42	0.031	########
DCTN1	########	0.175728	0.407	0.044	########
CXCR4	########	-0.21203	0.403	0.106	########
TAF13	########	0.351235	0.427	0.022	########
CERCAM	########	0.275354	0.402	0.026	########
TLE4	########	0.281671	0.441	0.043	########
EVL	########	0.198943	0.433	0.055	########
SDC2	########	0.170539	0.411	0.046	########
WRAP73	########	0.23141	0.392	0.036	########
TNFRSF21	########	0.44241	0.486	0.029	########
DRAXIN	########	0.22066	0.399	0.036	########
ZNF800	########	0.255414	0.434	0.041	########
SOX11	########	0.228735	0.48	0.068	########
ZCCHC3	########	0.252982	0.364	0.017	########
IMPACT	########	0.157691	0.374	0.038	########
XPC	########	0.107589	0.371	0.048	########
GOLGA5	########	0.117204	0.375	0.041	########
CNOT3	########	0.391679	0.462	0.024	########
MRGBP	########	0.539467	0.507	0.015	########
CPSF2	########	0.213955	0.374	0.032	########
EGLN1	########	0.18731	0.419	0.048	########
LTA4H	########	-0.24156	0.305	0.077	########

LRCH3	########	0.131313	0.349	0.036	########
USP33	########	0.178565	0.396	0.041	########
ZNF827	########	0.263599	0.407	0.034	########
ATP6V1A	########	0.308722	0.431	0.031	########
BTN3A2	########	-0.20461	0.378	0.099	########
EFNB2	########	0.630826	0.499	0.007	########
MAP3K2	########	0.310411	0.424	0.031	########
KANSL2	########	-0.13404	0.316	0.07	########
ILDR2	########	0.332436	0.417	0.024	########
POLR1C	########	-0.23374	0.291	0.072	########
R3HDM2	########	0.536443	0.508	0.021	########
CASP4	########	-0.25726	0.285	0.077	########
DDX19A	########	0.131508	0.382	0.038	########
STK19	########	0.206314	0.367	0.029	########
FAM168B	########	0.270296	0.416	0.032	########
STX18	########	0.105329	0.353	0.038	########
RUSC1	########	0.226091	0.361	0.021	########
MED24	########	0.147208	0.373	0.036	########
SRD5A1	########	0.196298	0.331	0.017	########
GADD45B	########	-1.28606	0.356	0.462	########
RB1	########	0.296606	0.439	0.038	########
EXT2	########	0.325615	0.434	0.029	########
LETMD1	########	-0.1912	0.283	0.07	########
TMEM69	########	-0.22545	0.296	0.08	########
AC004951.	########	0.289015	0.41	0.027	########
AP5M1	########	0.174059	0.371	0.041	########
ASPHD1	########	0.241879	0.416	0.039	########
COLGALT1	########	0.198908	0.402	0.039	########
MAP7D3	########	0.171172	0.387	0.038	########
HBP1	########	-0.27314	0.335	0.097	########
CDK5RAP1	########	0.261174	0.407	0.032	########
SEPP1	########	-0.98002	0.347	0.337	########
CDKN1B	########	0.131343	0.421	0.062	########
AAGAB	########	0.103361	0.316	0.027	########
EXOSC3	########	0.206287	0.378	0.029	########
NCOA1	########	0.268313	0.419	0.038	########
PITPNC1	########	0.468092	0.463	0.019	########
LAMB1	########	0.226655	0.382	0.027	########
NMI	########	-0.28983	0.302	0.084	########
RAP1GDS1	########	0.102516	0.367	0.036	########
TMEM192	########	-0.10693	0.311	0.072	########
CHST10	########	0.249434	0.424	0.041	########
PSEN1	########	0.141518	0.358	0.039	########
INSIG1	########	0.119903	0.411	0.056	########
SLC50A1	########	0.147088	0.341	0.031	########
PUM2	########	0.184418	0.383	0.039	########
PYGL	########	-0.23319	0.294	0.072	########
PTGFRN	########	0.204335	0.387	0.032	########
SMG6	########	0.317701	0.421	0.029	########
CMTR2	########	-0.117	0.296	0.062	########
RGS19	########	0.403365	0.448	0.017	########

SRGAP1	########	0.151475	0.41	0.051	########
TEX261	########	0.243003	0.401	0.032	########
PARP14	########	0.1282	0.384	0.043	########
ZNF426	########	-0.13584	0.297	0.062	########
E2F4	########	0.258106	0.407	0.031	########
BMI1	########	0.299835	0.392	0.019	########
PLCG1	########	0.183015	0.374	0.036	########
KCTD17	########	0.23359	0.374	0.024	########
ANO6	########	0.159545	0.337	0.024	########
RAB35	########	0.15858	0.349	0.031	########
CLDN12	########	-0.11032	0.354	0.08	########
FPGS	########	0.257201	0.391	0.024	########
CABIN1	########	0.196789	0.372	0.032	########
MAP1S	########	0.29163	0.398	0.021	########
GATAD2B	########	0.135512	0.378	0.044	########
SMG1	########	0.191817	0.37	0.036	########
TNFRSF19	########	0.45587	0.479	0.026	########
WNK3	########	0.126966	0.383	0.039	########
IGSF10	########	0.408916	0.466	0.029	########
LIMCH1	########	0.153202	0.425	0.056	########
TBL2	########	0.183355	0.399	0.044	########
NLGN1	########	0.323005	0.443	0.039	########
HABP4	########	0.30007	0.408	0.026	########
HMBOX1	########	-0.24056	0.327	0.092	########
SLC25A29	########	0.375312	0.45	0.027	########
LEO1	########	0.115232	0.381	0.046	########
TBC1D23	########	0.172709	0.389	0.041	########
SLC39A10	########	0.202729	0.39	0.034	########
CASC3	########	0.18756	0.405	0.046	########
PTDSS2	########	0.458862	0.477	0.021	########
SKIL	########	0.230165	0.415	0.036	########
ATMIN	########	0.199975	0.336	0.017	########
CTU2	########	0.185948	0.365	0.029	########
FGFBP3	########	0.485551	0.455	0.015	########
PHF12	########	0.121966	0.347	0.036	########
FAM216A	########	0.154906	0.347	0.031	########
BEND6	########	0.585768	0.496	0.007	########
C6orf47	########	0.249517	0.369	0.019	########
GPR155	########	0.195053	0.395	0.044	########
TPT1-AS1	########	0.12767	0.357	0.039	########
HDAC5	########	0.229491	0.402	0.038	########
TMEM132B	########	0.431426	0.464	0.029	########
MTIF2	########	0.168855	0.35	0.026	########
SLC2A1	########	-0.16577	0.321	0.065	########
LINC00685	########	0.126653	0.376	0.055	########
ATP9A	########	0.149337	0.323	0.029	########
CNIH2	########	0.574127	0.49	0.012	########
FTSJ3	########	0.244438	0.395	0.027	########
SLC4A7	########	0.378705	0.472	0.039	########
IL17RC	########	0.379076	0.445	0.022	########
SNX13	########	-0.20527	0.297	0.07	########

IL10RB	########	0.241234	0.388	0.029	########
WDR26	########	0.212912	0.395	0.038	########
KDM4B	########	0.111069	0.347	0.041	########
CREBBP	########	0.184494	0.376	0.032	########
DPY19L1	########	0.179723	0.395	0.043	########
KIN	########	0.185672	0.359	0.027	########
MOB3A	########	0.210132	0.377	0.029	########
DCAF10	########	0.264508	0.41	0.031	########
STK40	########	0.177821	0.361	0.031	########
IFNAR2	########	0.536021	0.488	0.014	########
RP4-665J2	########	0.294509	0.428	0.038	########
FNBP1L	########	0.305341	0.427	0.034	########
ASAP2	########	0.254616	0.374	0.022	########
GUSB	########	-0.22223	0.312	0.087	########
SOX12	########	0.455803	0.449	0.014	########
GPCPD1	########	0.388896	0.437	0.022	########
CCDC102B	########	0.125157	0.404	0.056	########
AHI1	########	0.202445	0.422	0.055	########
CDK10	########	-0.15992	0.328	0.084	########
VPS53	########	0.261571	0.409	0.034	########
RECQL	########	0.142706	0.344	0.034	########
C2orf69	########	0.239715	0.404	0.038	########
HLA-DRA	########	-1.36153	0.271	0.426	########
RUNX1	########	0.408355	0.46	0.031	########
SRGAP2	########	0.348623	0.436	0.029	########
KANK2	########	0.131671	0.365	0.038	########
SLC4A4	########	0.101238	0.501	0.101	########
HN1L	########	0.18883	0.359	0.029	########
RRAGD	########	0.327883	0.407	0.022	########
R3HDM1	########	0.170189	0.365	0.034	########
OLIG2	########	0.124814	0.46	0.082	########
SYNC	########	0.198614	0.405	0.038	########
MBD1	########	0.17563	0.377	0.041	########
RPP38	########	0.292633	0.39	0.019	########
CTR9	########	0.142214	0.358	0.029	########
SOX6	########	0.286957	0.424	0.039	########
RP11-390E	########	-0.15778	0.289	0.067	########
DDAH1	########	0.137081	0.428	0.062	########
SEPHS1	########	0.156867	0.318	0.022	########
TRIM36	########	0.325637	0.408	0.026	########
PTPN18	########	0.183499	0.343	0.026	########
SLC12A9	########	0.266568	0.384	0.024	########
SNX9	########	0.23658	0.377	0.026	########
HELZ2	########	0.122115	0.38	0.05	########
NAV1	########	0.296014	0.409	0.031	########
RNF113A	########	-0.11031	0.28	0.05	########
RP11-505K	########	0.239536	0.346	0.017	########
VAMP7	########	0.127752	0.33	0.032	########
AKT1	########	0.192668	0.389	0.038	########
OPTN	########	0.178651	0.362	0.031	########
GAN	########	0.268732	0.434	0.05	########

TSC2	########	0.106642	0.353	0.046	########
RRAS	########	0.3716	0.4	0.015	########
CLCC1	########	0.183113	0.371	0.032	########
APC2	########	0.317608	0.451	0.041	########
ZNF385D	########	0.559375	0.478	0.017	########
STK3	########	-0.12552	0.279	0.056	########
LDLR	########	0.16976	0.401	0.043	########
POU3F3	########	0.192107	0.375	0.038	########
CLN8	########	-0.17477	0.285	0.063	########
IL1RAP	########	0.604765	0.531	0.036	########
C6orf120	########	0.414753	0.441	0.015	########
ITGA3	########	0.338232	0.414	0.026	########
DCLK1	########	0.42004	0.46	0.031	########
LAMB2	########	0.17435	0.376	0.036	########
EID2	########	0.203262	0.33	0.019	########
RALGAPA2	########	0.265209	0.38	0.027	########
OTUD5	########	0.18966	0.35	0.024	########
TSPAN4	########	0.34204	0.412	0.022	########
TOB2	########	0.245449	0.426	0.043	########
SLC35F2	########	0.498665	0.465	0.017	########
OAT	########	-0.38754	0.294	0.104	########
SMARCAD1	########	0.110424	0.299	0.026	########
MLLT6	########	0.181426	0.345	0.032	########
ADCK4	########	0.174871	0.381	0.041	########
ANKRD35	########	0.334106	0.406	0.017	########
ZNF770	########	0.260353	0.387	0.026	########
NOL10	########	0.183826	0.367	0.031	########
RANBP9	########	0.220639	0.379	0.032	########
DHRS11	########	0.475008	0.448	0.01	########
POM121	########	0.1316	0.346	0.036	########
ADIPOR2	########	0.122541	0.349	0.036	########
SGSM2	########	0.262582	0.387	0.029	########
DIAPH1	########	0.298297	0.4	0.022	########
NFAT5	########	0.128633	0.353	0.043	########
XAB2	########	0.135237	0.403	0.056	########
POU2F1	########	0.174138	0.371	0.034	########
C11orf68	########	0.257298	0.377	0.021	########
9-Mar	########	0.147312	0.371	0.039	########
NUS1	########	0.12446	0.362	0.039	########
SYNE2	########	0.111783	0.346	0.044	########
PSEN2	########	0.223211	0.361	0.024	########
UGDH	########	-0.11704	0.283	0.06	########
KIF3B	########	0.498729	0.463	0.012	########
FKBP1B	########	0.313781	0.377	0.012	########
SPPL2A	########	0.151031	0.354	0.038	########
SLC35F5	########	0.167459	0.364	0.034	########
PQLC2	########	0.232937	0.371	0.027	########
TGFB1	########	0.577177	0.485	0.009	########
FAM69C	########	0.394847	0.412	0.014	########
SLC25A25	########	0.159523	0.389	0.046	########
FSTL1	########	0.373297	0.426	0.022	########

PHF19	########	-0.10476	0.329	0.065	########
THAP4	########	0.356013	0.412	0.019	########
TFE3	########	0.123539	0.316	0.027	########
MESP1	########	0.458903	0.471	0.021	########
SF3B3	########	0.122695	0.379	0.053	########
DGCR2	########	0.178232	0.384	0.044	########
CACNG8	########	0.201626	0.349	0.024	########
STMN4	########	-0.18383	0.422	0.132	########
CLK4	########	-0.14712	0.314	0.079	########
KLHDC10	########	0.19792	0.361	0.034	########
SUMF1	########	0.18309	0.33	0.026	########
CLASP1	########	0.327205	0.434	0.038	########
TNKS	########	0.373797	0.433	0.026	########
UBR5	########	0.152667	0.343	0.032	########
IER5	########	0.11074	0.355	0.039	########
CCNDBP1	########	0.148733	0.34	0.032	########
NELFB	########	0.354441	0.423	0.022	########
LPIN2	########	0.249243	0.352	0.015	########
PDXDC1	########	0.154763	0.356	0.034	########
UBE2O	########	0.306913	0.402	0.026	########
SMC6	########	0.273068	0.392	0.029	########
CDC27	########	0.372122	0.412	0.017	########
TMEM184B	########	0.201188	0.375	0.032	########
ZNF160	########	0.198956	0.36	0.032	########
FAM110B	########	0.233924	0.403	0.038	########
YIPF2	########	-0.17068	0.261	0.06	########
SLC26A11	########	0.248164	0.361	0.022	########
MXD4	########	0.400393	0.431	0.019	########
HSPA1B	########	-0.66248	0.417	0.222	########
SDCCAG3	########	0.168723	0.326	0.024	########
KDM5B	########	0.347574	0.422	0.027	########
KCTD12	########	0.157527	0.344	0.029	########
BCL2L12	########	0.263108	0.374	0.019	########
RIN1	########	0.224805	0.372	0.027	########
GTF3C1	########	0.168412	0.333	0.026	########
SENP2	########	0.109389	0.322	0.032	########
CYP20A1	########	-0.27004	0.288	0.084	########
RSBN1	########	0.244808	0.403	0.034	########
RXRB	########	0.143237	0.345	0.031	########
C1QA	########	-0.62605	0.267	0.161	########
ATM	########	0.164129	0.378	0.046	########
KIAA2013	########	0.411512	0.432	0.019	########
ZFP91	########	0.224925	0.348	0.021	########
GAK	########	0.173602	0.342	0.027	########
OSGEP	########	-0.16381	0.324	0.075	########
SCG3	########	-0.48601	0.43	0.207	########
AP5S1	########	0.222236	0.37	0.026	########
HSPA1A	########	-0.24462	0.494	0.157	########
C16orf72	########	0.202229	0.343	0.022	########
DIDO1	########	0.533981	0.49	0.017	########
ZNRF1	########	0.466731	0.43	0.009	########

SRRT	########	0.138485	0.374	0.048	########
CCDC58	########	0.348354	0.425	0.026	########
ANXA1	########	-1.14261	0.277	0.323	########
MAU2	########	0.150735	0.362	0.038	########
NUBP1	########	0.243675	0.399	0.034	########
DOCK7	########	0.24625	0.401	0.038	########
BOD1L1	########	0.112626	0.322	0.038	########
IGF2BP3	########	0.305812	0.393	0.022	########
SCAP	########	0.308619	0.402	0.024	########
RBM12	########	0.145906	0.322	0.031	########
MRI1	########	0.256738	0.392	0.031	########
MGAT4B	########	0.455099	0.422	0.005	########
CDC42EP1	########	0.493596	0.466	0.021	########
TPPP3	########	-0.26557	0.32	0.082	########
GALNS	########	0.376491	0.422	0.021	########
NTN1	########	0.348331	0.395	0.019	########
SLC30A7	########	0.265344	0.387	0.027	########
FBXL3	########	0.144755	0.362	0.041	########
CDC42BPB	########	0.329194	0.384	0.015	########
RP11-798M	########	-0.21296	0.267	0.067	########
ZNF451	########	0.134824	0.361	0.039	########
MAPRE3	########	0.145661	0.347	0.034	########
ST6GALNAC	########	0.212495	0.368	0.024	########
AIFM1	########	0.187756	0.338	0.022	########
LIPE-AS1	########	0.267781	0.376	0.024	########
SRPK1	########	0.122439	0.349	0.031	########
AGPAT3	########	0.304654	0.391	0.022	########
MMD	########	0.307013	0.362	0.012	########
FZR1	########	0.328329	0.427	0.031	########
HIPK2	########	0.196836	0.384	0.039	########
ABCA1	########	0.448899	0.503	0.055	########
FAM208A	########	0.397059	0.418	0.017	########
VPS26A	########	-0.10306	0.294	0.06	########
B9D1	########	0.117382	0.338	0.034	########
LRRC4B	########	0.395408	0.421	0.015	########
ZNF720	########	0.127428	0.349	0.039	########
REV1	########	0.161394	0.374	0.039	########
ITPR2	########	0.205884	0.346	0.026	########
FLII	########	0.24528	0.373	0.022	########
PRMT5	########	-0.17296	0.32	0.082	########
ARNT2	########	0.349147	0.41	0.017	########
RFT1	########	0.167762	0.335	0.026	########
UNC93B1	########	0.179238	0.329	0.021	########
PRRT2	########	0.144022	0.325	0.032	########
NFATC2IP	########	0.220425	0.365	0.027	########
CTB-3102C	########	0.307443	0.398	0.026	########
BPNT1	########	-0.11858	0.258	0.051	########
SLC6A8	########	0.117765	0.332	0.034	########
ADM	########	-0.89718	0.253	0.152	########
STOM	########	-0.20577	0.25	0.06	########
PPM1A	########	0.31749	0.394	0.019	########

NUFIP1	########	-0.10365	0.278	0.043	########
SMC2	########	0.178342	0.332	0.026	########
TBCD	########	0.291555	0.374	0.017	########
ASTN1	########	0.282503	0.392	0.027	########
UBQLN2	########	0.312127	0.415	0.029	########
CAPG	########	-0.19211	0.295	0.084	########
CDYL	########	0.534932	0.453	0.005	########
HINT3	########	0.156382	0.374	0.044	########
RAD17	########	0.114794	0.319	0.026	########
IREB2	########	0.323344	0.403	0.024	########
CHPF2	########	0.18678	0.351	0.032	########
TYMS	########	0.217682	0.372	0.032	########
DHX33	########	0.216074	0.361	0.026	########
SIRPA	########	0.439255	0.46	0.026	########
DHX40	########	0.392725	0.43	0.021	########
HIST1H2AC	########	-0.20017	0.329	0.082	########
PCGF5	########	0.101226	0.36	0.043	########
ZNF639	########	0.296119	0.383	0.021	########
GNA12	########	0.216457	0.387	0.039	########
SPATA13	########	0.23155	0.392	0.039	########
ESCO1	########	0.235767	0.362	0.024	########
FBXO3	########	0.20251	0.381	0.041	########
ATF1	########	-0.13359	0.268	0.053	########
RPS6KB1	########	0.240564	0.371	0.022	########
RPS6KA3	########	0.200577	0.336	0.026	########
NRP1	########	0.335697	0.388	0.021	########
SMAD4	########	0.251975	0.37	0.029	########
STK39	########	0.31966	0.388	0.019	########
C11orf24	########	0.2433	0.358	0.024	########
TMSB15B	########	0.177753	0.357	0.032	########
CTCF	########	0.326382	0.386	0.015	########
FADD	########	0.429656	0.424	0.012	########
NBN	########	0.258141	0.38	0.027	########
SAP30L	########	0.264148	0.342	0.014	########
RFWD2	########	0.256213	0.374	0.024	########
RNF20	########	0.126584	0.339	0.038	########
KIAA0232	########	0.34892	0.41	0.021	########
HAGHL	########	0.214141	0.34	0.024	########
TULP3	########	0.116837	0.35	0.044	########
YY1AP1	########	0.150708	0.357	0.031	########
PUS7L	########	0.14614	0.358	0.036	########
EIF2B4	########	0.162287	0.37	0.039	########
GPT2	########	0.157818	0.38	0.048	########
PTPN11	########	0.287952	0.398	0.031	########
RUFY2	########	0.338278	0.4	0.024	########
TMEM243	########	-0.35272	0.26	0.092	########
SNTB2	########	0.16193	0.316	0.026	########
MYL5	########	0.186754	0.324	0.024	########
ZFAND1	########	-0.13571	0.28	0.067	########
PQLC1	########	0.289415	0.362	0.017	########
PPP1R35	########	0.371829	0.427	0.026	########

XRRA1	########	0.228583	0.395	0.041	########
PTX3	########	0.392759	0.433	0.029	########
PFKFB3	########	0.195387	0.401	0.053	########
CNPY4	########	0.130634	0.318	0.031	########
PDGFRA	########	0.636313	0.478	0.012	########
ADAL	########	0.305908	0.392	0.021	########
AGAP1	########	0.14896	0.337	0.032	########
CNKSR3	########	0.563554	0.489	0.019	########
AGFG1	########	0.271843	0.358	0.019	########
CHERP	########	0.253271	0.376	0.029	########
IMMP2L	########	-0.14748	0.269	0.058	########
ABCB8	########	0.200446	0.341	0.026	########
DIRAS3	########	-0.57085	0.327	0.162	########
PHGDH	########	-0.32615	0.393	0.145	########
FAIM	########	0.212948	0.37	0.036	########
CKAP2	########	0.265765	0.352	0.019	########
LEMD2	########	0.296408	0.393	0.026	########
VAC14	########	0.259212	0.358	0.017	########
SLC25A46	########	0.11211	0.341	0.041	########
STEAP3	########	0.179276	0.39	0.05	########
RBM28	########	0.130121	0.354	0.039	########
WDR12	########	0.177225	0.363	0.038	########
SH3RF1	########	0.192687	0.355	0.032	########
FAM129A	########	0.369731	0.409	0.024	########
SIAH2	########	0.271424	0.378	0.026	########
MED15	########	0.21142	0.367	0.034	########
OPA1	########	0.232502	0.336	0.019	########
MCM7	########	-0.1169	0.334	0.079	########
ZNF880	########	0.151276	0.37	0.046	########
TOM1L2	########	0.19551	0.353	0.032	########
DLC1	########	0.11218	0.357	0.046	########
RNF2	########	0.275615	0.378	0.024	########
ATG13	########	0.10972	0.295	0.024	########
LAMP5	########	0.367313	0.412	0.022	########
5-Mar	########	0.160755	0.358	0.032	########
GATAD2A	########	0.387836	0.412	0.015	########
HK1	########	0.106542	0.342	0.041	########
TCAIM	########	0.122485	0.298	0.026	########
MEX3A	########	0.210626	0.36	0.034	########
FBRS	########	0.102184	0.292	0.029	########
LSS	########	0.10549	0.347	0.043	########
ZNF7	########	0.108027	0.339	0.05	########
SOX21	########	0.373531	0.431	0.031	########
TRPM3	########	0.1793	0.369	0.038	########
LRPPRC	########	0.291593	0.408	0.032	########
ACAP2	########	0.192111	0.354	0.031	########
PAK4	########	0.12847	0.32	0.031	########
SPIN1	########	0.221202	0.368	0.032	########
CD68	########	-0.45098	0.219	0.089	########
GPS2	########	0.131658	0.336	0.041	########
NECAB3	########	0.363102	0.401	0.017	########

IRAK1	########	0.174463	0.333	0.024	########
MAP4K5	########	0.191466	0.357	0.034	########
ATP13A1	########	0.29913	0.414	0.034	########
ZC3H18	########	0.326417	0.384	0.017	########
FUCA1	########	0.337976	0.386	0.017	########
FAM126A	########	0.16345	0.355	0.038	########
HCFC1	########	0.261199	0.357	0.022	########
H2AFX	########	0.273165	0.377	0.024	########
NBPF10	########	0.564862	0.484	0.017	########
WHSC1	########	0.138348	0.332	0.038	########
TXLNG	########	-0.1083	0.266	0.043	########
PDE4DIP	########	-0.33636	0.377	0.147	########
KCNMA1	########	0.106369	0.316	0.026	########
EMC1	########	0.178044	0.349	0.036	########
STX17	########	0.186676	0.348	0.029	########
TANC1	########	0.336477	0.399	0.026	########
DNAJC21	########	0.276567	0.361	0.017	########
UBR4	########	0.191268	0.359	0.038	########
NNT	########	0.112415	0.344	0.041	########
CDK11A	########	0.172427	0.378	0.051	########
GBP2	########	-0.40192	0.273	0.094	########
RHOQ	########	0.502712	0.451	0.014	########
B3GALNT1	########	0.232051	0.389	0.038	########
BOC	########	0.356724	0.399	0.019	########
SAMD8	########	0.233116	0.381	0.032	########
OPA3	########	0.126026	0.288	0.027	########
LRRC37B	########	0.199648	0.343	0.029	########
SETD3	########	0.206598	0.33	0.026	########
DCAF6	########	0.19212	0.341	0.031	########
LBR	########	0.11721	0.347	0.053	########
RPP21	########	-0.18958	0.244	0.058	########
HS6ST1	########	0.391943	0.405	0.021	########
PIAS2	########	0.165585	0.33	0.027	########
MLLT4	########	0.27178	0.37	0.024	########
SLC35B4	########	0.144361	0.348	0.041	########
MACROD1	########	0.299613	0.37	0.021	########
PTK2B	########	0.151611	0.33	0.029	########
DPYSL4	########	0.352681	0.395	0.021	########
ZFHX3	########	0.274323	0.376	0.026	########
CHAMP1	########	0.306391	0.362	0.014	########
C5orf28	########	-0.1266	0.283	0.07	########
SMYD2	########	0.107851	0.358	0.044	########
RIOK1	########	-0.106	0.264	0.046	########
TNFSF12	########	0.104897	0.314	0.031	########
SLC4A2	########	0.202113	0.346	0.029	########
POM121C	########	0.136045	0.319	0.034	########
ZNF280D	########	0.176559	0.314	0.021	########
LIMK1	########	0.144786	0.318	0.032	########
DDIT4	########	-0.56069	0.405	0.198	########
UBN1	########	0.411114	0.418	0.017	########
METTL16	########	0.156953	0.316	0.027	########

EHMT1	########	0.22354	0.349	0.027	########
ATF3	########	-0.44269	0.318	0.13	########
PPP2R5A	########	0.280311	0.384	0.026	########
TEAD2	########	0.323704	0.365	0.014	########
ANKRD26	########	0.252075	0.362	0.026	########
SNRPN	########	-1.15234	0.142	0.202	########
UTP23	########	0.108655	0.317	0.032	########
ZFP36	########	-1.06303	0.384	0.431	########
DNAJC30	########	0.157446	0.354	0.041	########
ATAD3A	########	0.287653	0.397	0.031	########
KIF22	########	-0.33462	0.258	0.084	########
ADCK3	########	0.165881	0.349	0.039	########
C20orf194	########	0.195758	0.35	0.036	########
RNMTL1	########	0.30899	0.372	0.019	########
ZFP64	########	0.15505	0.273	0.015	########
THOC3	########	0.278115	0.379	0.027	########
CCDC6	########	0.275311	0.357	0.017	########
GS1-124K5	########	-0.1281	0.264	0.051	########
PACS2	########	0.119002	0.294	0.026	########
TRIP10	########	0.11968	0.308	0.034	########
ZNF521	########	0.140519	0.315	0.026	########
B4GALT4	########	0.124722	0.313	0.029	########
DPP9	########	0.146089	0.335	0.032	########
ARHGEF1	########	0.161553	0.374	0.05	########
AC004158.	########	0.123582	0.33	0.036	########
KLHL20	########	0.159433	0.348	0.036	########
PRPF18	########	0.151878	0.313	0.027	########
KLHL5	########	0.35101	0.393	0.015	########
ACSBG1	########	0.228274	0.4	0.043	########
C1orf56	########	0.117387	0.394	0.056	########
THSD1	########	0.464133	0.444	0.021	########
RILPL2	########	0.167576	0.306	0.021	########
REEP6	########	0.272837	0.351	0.019	########
PHF21A	########	0.159239	0.321	0.026	########
LSM14B	########	0.250693	0.348	0.019	########
SOX8	########	0.189796	0.367	0.039	########
TCF7L2	########	0.238812	0.352	0.022	########
PCDHB16	########	0.275639	0.388	0.034	########
RALGAPB	########	0.40041	0.406	0.012	########
SLC12A2	########	0.170594	0.361	0.039	########
PXDN	########	0.182607	0.348	0.027	########
TMEM198	########	0.271833	0.368	0.024	########
HMGXB3	########	0.274723	0.37	0.026	########
CTBP2	########	0.137075	0.324	0.031	########
MAP3K11	########	0.195512	0.341	0.031	########
CXCL14	########	-0.3167	0.327	0.113	########
SPEN	########	0.204245	0.351	0.034	########
NRBP2	########	0.399328	0.428	0.024	########
CDCA7L	########	-0.10127	0.313	0.065	########
KDM4A	########	0.142509	0.305	0.029	########
PEX26	########	0.141869	0.317	0.034	########

DHRS13	########	0.349968	0.387	0.019	########
NCAPH2	########	0.121248	0.291	0.027	########
NOC4L	########	0.244858	0.351	0.024	########
PSMB10	########	-0.13924	0.302	0.075	########
XPO6	########	0.153302	0.316	0.022	########
BAP1	########	0.171074	0.335	0.034	########
ZNF577	########	0.176437	0.327	0.026	########
PHF11	########	0.134703	0.285	0.021	########
HEXDC	########	0.286813	0.349	0.015	########
PIAS4	########	0.222464	0.348	0.024	########
IRF1	########	-0.40076	0.345	0.138	########
VEGFB	########	0.209597	0.352	0.029	########
SH3PXD2B	########	0.457005	0.427	0.014	########
APBB2	########	-0.10269	0.279	0.056	########
PDGFD	########	0.565015	0.455	0.007	########
MEN1	########	0.220105	0.34	0.022	########
ZYG11B	########	0.271407	0.376	0.026	########
PRKAB1	########	0.125709	0.312	0.032	########
ZNF528	########	0.154649	0.33	0.029	########
UBTD2	########	0.148651	0.291	0.017	########
EML3	########	0.316594	0.367	0.015	########
DENND5B	########	0.149613	0.293	0.019	########
PACSIN2	########	0.1869	0.353	0.036	########
BHLHE41	########	0.241727	0.393	0.048	########
SLC20A1	########	0.301046	0.393	0.027	########
VHL	########	0.276514	0.371	0.026	########
ABHD17C	########	0.453117	0.418	0.012	########
SLC9A3R1	########	0.291013	0.368	0.022	########
FAM102A	########	0.144059	0.294	0.026	########
AHSA2	########	0.139179	0.334	0.039	########
LY96	########	0.156787	0.327	0.026	########
SLC25A22	########	0.100959	0.3	0.031	########
GPATCH11	########	0.204833	0.326	0.024	########
NSMCE4A	########	0.161167	0.311	0.022	########
CD99L2	########	0.161068	0.328	0.027	########
CNOT11	########	0.303526	0.349	0.012	########
BNIP1	########	-0.17963	0.267	0.051	########
MIER2	########	0.112775	0.314	0.034	########
PIK3C3	########	0.176276	0.31	0.024	########
ZNF821	########	0.143771	0.302	0.027	########
PXN	########	0.361937	0.401	0.026	########
EDEM3	########	0.19205	0.345	0.031	########
METAP1D	########	-0.10201	0.29	0.065	########
SORBS1	########	0.374963	0.391	0.019	########
SS18	########	-0.20983	0.22	0.06	########
NISCH	########	0.146158	0.317	0.032	########
HTT	########	0.203028	0.318	0.022	########
ZFP90	########	0.227003	0.331	0.026	########
C1orf216	########	0.169083	0.324	0.029	########
SNAPC3	########	0.11566	0.345	0.039	########
RP11-66N1	########	0.147775	0.324	0.029	########

CDK2AP1	########	0.269291	0.341	0.015	########
MCAT	########	0.36149	0.381	0.014	########
TMEM175	########	0.217437	0.355	0.032	########
ABHD13	########	0.177225	0.317	0.022	########
PCOLCE2	########	-0.24077	0.21	0.056	########
FECH	########	0.185922	0.309	0.019	########
STRN4	########	0.256081	0.339	0.019	########
PALM	########	0.234526	0.338	0.024	########
TMEM184C	########	0.139359	0.286	0.022	########
RP11-111M	########	-0.10411	0.264	0.053	########
SLC33A1	########	0.140346	0.31	0.031	########
CWC22	########	0.112787	0.291	0.026	########
ZNF561	########	0.159714	0.323	0.034	########
SERINC2	########	0.662241	0.463	0.005	########
KAT2A	########	0.238514	0.434	0.062	########
TASP1	########	0.202658	0.318	0.024	########
SLC4A3	########	0.211848	0.336	0.026	########
SOBP	########	0.205771	0.361	0.034	########
GNA11	########	0.257219	0.356	0.024	########
KIAA0355	########	0.106014	0.344	0.051	########
CACNG7	########	0.19884	0.322	0.026	########
POLRMT	########	0.289508	0.405	0.039	########
STX6	########	0.201338	0.339	0.029	########
SLC39A13	########	0.290996	0.378	0.026	########
ACAA2	########	-0.30272	0.231	0.072	########
KCTD13	########	0.230954	0.346	0.026	########
RABL2B	########	0.146535	0.307	0.031	########
FAM57A	########	0.324612	0.371	0.017	########
ARMC7	########	0.156212	0.276	0.014	########
ZNF281	########	0.132928	0.317	0.036	########
SLC23A2	########	0.222233	0.332	0.024	########
RABGAP1	########	0.216744	0.329	0.021	########
DENND2A	########	0.168848	0.354	0.034	########
OSMR	########	0.193615	0.332	0.027	########
NACA2	########	-0.99465	0.133	0.171	########
SEH1L	########	0.12442	0.299	0.029	########
PARD6A	########	0.14303	0.276	0.017	########
RRP1B	########	0.367556	0.387	0.015	########
POLR2D	########	0.116178	0.314	0.031	########
SIK3	########	0.267489	0.351	0.019	########
ASCC3	########	0.193398	0.33	0.029	########
QRSL1	########	0.2015	0.324	0.022	########
ELMOD1	########	0.471368	0.428	0.014	########
RBM33	########	0.146889	0.328	0.029	########
FTO	########	0.220088	0.339	0.024	########
KDM2A	########	0.109136	0.301	0.027	########
SATB2	########	0.166126	0.317	0.027	########
PIP4K2B	########	0.194778	0.329	0.027	########
ZNF121	########	0.174847	0.314	0.026	########
ACACA	########	0.267233	0.324	0.014	########
NACC2	########	0.396299	0.383	0.01	########

COL4A3BP	########	0.116579	0.32	0.036	########
RP5-940J5	########	-0.72114	0.195	0.133	########
FAM168A	########	0.453913	0.431	0.017	########
KCNMB4	########	0.287162	0.358	0.021	########
LPGAT1	########	0.15449	0.299	0.021	########
ITCH	########	0.163508	0.308	0.029	########
FOXP1	########	-0.90594	0.185	0.209	########
AGRN	########	0.216088	0.329	0.026	########
FAM58A	########	0.345258	0.374	0.014	########
TRIM65	########	0.13285	0.269	0.019	########
PPP4R1	########	0.130187	0.264	0.019	########
MTHFD2	########	-0.40003	0.27	0.111	########
ASH2L	########	0.153135	0.333	0.032	########
ALG2	########	0.243457	0.357	0.026	########
FER	########	0.214974	0.319	0.019	########
BACE1	########	0.128543	0.291	0.027	########
CIC	########	0.194407	0.345	0.034	########
SAMD1	########	0.244264	0.345	0.022	########
IKBKB	########	0.135814	0.277	0.021	########
MAP2K3	########	0.507582	0.432	0.012	########
TBK1	########	0.180103	0.324	0.029	########
MAPK7	########	0.228874	0.327	0.017	########
PDP1	########	0.397257	0.398	0.024	########
PRR3	########	0.128726	0.263	0.017	########
ANKRD39	########	0.268207	0.356	0.027	########
GRK6	########	0.212404	0.317	0.019	########
HEXIM2	########	0.110067	0.282	0.026	########
RUNDC1	########	0.21137	0.336	0.026	########
ZNF652	########	0.189992	0.342	0.032	########
SPIN2B	########	0.1485	0.309	0.026	########
CEP104	########	0.251827	0.34	0.022	########
ARFGEF1	########	0.473315	0.415	0.009	########
TMEM255A	########	0.203765	0.352	0.034	########
PCYT1A	########	0.129568	0.32	0.032	########
C9orf116	########	0.22629	0.302	0.014	########
CCDC80	########	-0.14552	0.403	0.12	########
C5orf51	########	0.236958	0.31	0.012	########
PTK7	########	0.310102	0.363	0.019	########
TP53I3	########	0.276683	0.354	0.022	########
SDC4	########	-0.12271	0.254	0.041	########
PRKRIR	########	0.272955	0.333	0.017	########
RPUSD1	########	0.242037	0.326	0.021	########
ISYNA1	########	0.185235	0.308	0.024	########
RAB3GAP1	########	0.103608	0.285	0.027	########
EP400	########	0.178734	0.308	0.024	########
CAPRIN2	########	0.381913	0.406	0.024	########
CERS6	########	0.124381	0.256	0.015	########
PPP2R5B	########	0.282435	0.388	0.029	########
SESN3	########	0.305794	0.373	0.021	########
BARD1	########	0.235413	0.351	0.026	########
UBXN11	########	-0.23435	0.247	0.079	########

PANK3	########	0.188432	0.32	0.021	########
TMEM178A	########	0.15704	0.33	0.031	########
FAR1	########	0.150969	0.305	0.022	########
DDX39B	########	0.154717	0.28	0.021	########
IVNS1ABP	########	0.215827	0.343	0.026	########
SENP5	########	0.209946	0.335	0.027	########
AFTPH	########	0.11428	0.298	0.031	########
ANKRD52	########	0.152509	0.277	0.019	########
IRF7	########	0.225459	0.335	0.026	########
SLC10A4	########	0.424321	0.396	0.014	########
YDJC	########	0.317553	0.374	0.021	########
ADA	########	0.389322	0.371	0.009	########
ST8SIA1	########	0.315168	0.389	0.029	########
SLC43A2	########	0.216968	0.339	0.031	########
TMEM245	########	0.108237	0.314	0.044	########
INPPL1	########	0.360425	0.374	0.014	########
DIRAS1	########	0.124817	0.322	0.038	########
ASCL1	########	0.169249	0.394	0.062	########
AAED1	########	0.266862	0.358	0.022	########
ECE1	########	-0.14368	0.248	0.062	########
YTHDF3	########	0.141654	0.293	0.029	########
NSMF	########	0.214494	0.335	0.027	########
SLN	########	-0.20959	0.435	0.13	########
NPHP3	########	0.179221	0.332	0.038	########
KLHL8	########	0.104452	0.31	0.041	########
NLGN4X	########	0.191752	0.349	0.036	########
AC007246.	########	0.215985	0.334	0.029	########
TSPAN12	########	0.102113	0.357	0.053	########
CUEDC1	########	0.274913	0.33	0.015	########
TOR2A	########	0.117984	0.283	0.027	########
CACUL1	########	0.264124	0.355	0.026	########
SMURF2	########	0.206764	0.322	0.026	########
DCK	########	0.384048	0.395	0.015	########
QSER1	########	0.301492	0.351	0.015	########
SEZ6L	########	0.155941	0.402	0.067	########
DUS3L	########	0.132385	0.318	0.039	########
FGF1	########	0.12237	0.354	0.046	########
LACTB2	########	-0.12898	0.286	0.056	########
CTD-3184A	########	0.417888	0.403	0.012	########
TCHP	########	0.12681	0.307	0.034	########
PITX1	########	0.512569	0.428	0.009	########
ERC1	########	0.181012	0.317	0.027	########
FGGY	########	-0.2295	0.194	0.048	########
RP11-539L	########	-0.18862	0.22	0.048	########
PUSL1	########	0.37888	0.378	0.01	########
RBBP9	########	0.344164	0.367	0.012	########
XPR1	########	0.14493	0.307	0.027	########
TK2	########	0.187977	0.317	0.021	########
ABHD8	########	0.347258	0.383	0.019	########
CUL2	########	0.215361	0.306	0.019	########
SSX2IP	########	0.40061	0.386	0.01	########

GSPT2	########	0.313064	0.371	0.022	########
CASP1	########	-0.29313	0.262	0.08	########
YLPM1	########	0.122917	0.285	0.027	########
RFTN1	########	0.422756	0.396	0.014	########
CYR61	########	-0.57138	0.308	0.168	########
TMEM209	########	0.231679	0.323	0.019	########
UQCR11	########	-1.97324	0.012	0.369	########
SMARCD2	########	0.149289	0.271	0.014	########
ATL3	########	0.194578	0.299	0.017	########
EFR3A	########	0.343279	0.375	0.015	########
POGLUT1	########	0.218671	0.324	0.021	########
UBTD1	########	0.330091	0.373	0.019	########
PIGQ	########	0.187078	0.3	0.021	########
H2AFY2	########	0.439743	0.389	0.003	########
METTL13	########	0.153101	0.301	0.027	########
PIAS3	########	0.107777	0.27	0.024	########
ROBO2	########	0.343542	0.385	0.022	########
ARL9	########	0.442661	0.393	0.012	########
OSBP	########	0.12828	0.269	0.019	########
PPFIA3	########	0.229895	0.321	0.019	########
PEX14	########	0.103512	0.275	0.027	########
TRIM16L	########	0.171321	0.27	0.014	########
ZNF260	########	0.25551	0.329	0.015	########
COQ2	########	0.239266	0.303	0.012	########
SNX12	########	0.104872	0.297	0.031	########
SAV1	########	0.137559	0.276	0.024	########
ENKUR	########	-0.21605	0.255	0.075	########
SLC2A3	########	-0.38023	0.238	0.096	########
EPHA3	########	0.61319	0.442	0.002	########
RNF185	########	0.138793	0.277	0.022	########
WBSCR16	########	0.224029	0.32	0.021	########
CREG1	########	0.288397	0.336	0.015	########
TMEM136	########	0.113243	0.284	0.026	########
COG2	########	0.159364	0.313	0.029	########
TEFM	########	0.155563	0.303	0.026	########
FAM131A	########	0.208963	0.335	0.029	########
ZC3H7A	########	0.224074	0.317	0.022	########
CDC37L1	########	-0.13087	0.226	0.05	########
CCDC92	########	0.158342	0.291	0.022	########
ETV6	########	0.234999	0.345	0.026	########
CCND1	########	0.106232	0.378	0.063	########
ANKZF1	########	0.111361	0.327	0.043	########
FAM122A	########	0.148219	0.29	0.024	########
TRMU	########	0.182231	0.33	0.032	########
CHST11	########	0.163992	0.315	0.027	########
C16orf52	########	0.247012	0.313	0.017	########
MFSD3	########	0.354916	0.375	0.014	########
CCZ1B	########	0.242797	0.326	0.021	########
AP1G1	########	0.198199	0.321	0.021	########
FOSL2	########	0.325596	0.401	0.038	########
EGR2	########	0.161345	0.361	0.05	########

CEP85L	########	0.133097	0.306	0.029	########
CYB5R4	########	0.269265	0.33	0.014	########
SLC35D2	########	0.361214	0.369	0.01	########
LRRC23	########	0.201006	0.313	0.021	########
PPP6R3	########	0.132715	0.309	0.034	########
NCK2	########	0.462149	0.394	0.005	########
TTYH2	########	0.413696	0.385	0.01	########
SIPA1L2	########	0.235214	0.348	0.027	########
FOXJ3	########	0.225291	0.333	0.027	########
F8A1	########	0.441939	0.414	0.014	########
CTSC	########	-0.25202	0.235	0.068	########
DUSP15	########	0.450741	0.403	0.009	########
GLI4	########	0.122526	0.292	0.029	########
AKAP1	########	0.132207	0.296	0.027	########
MAK16	########	0.140888	0.266	0.017	########
SETD2	########	0.297408	0.36	0.022	########
UBE4A	########	0.288289	0.347	0.021	########
REPS1	########	0.292167	0.365	0.024	########
HERC4	########	0.183156	0.318	0.031	########
OGDH	########	0.100138	0.279	0.027	########
SLAIN2	########	0.330687	0.349	0.01	########
ATP13A2	########	0.261143	0.365	0.027	########
MKS1	########	0.135788	0.292	0.026	########
NBAS	########	0.213215	0.31	0.015	########
WIPF1	########	0.243993	0.339	0.024	########
OTUD6B	########	0.161483	0.311	0.031	########
BIVM	########	0.212107	0.305	0.017	########
SMAP2	########	0.148024	0.272	0.019	########
PPP6R2	########	0.339525	0.376	0.017	########
EFEMP1	########	-0.49407	0.387	0.193	########
MLLT10	########	0.154571	0.292	0.022	########
GMPR	########	0.127431	0.308	0.036	########
LRRCC1	########	0.174936	0.32	0.027	########
COG1	########	0.16698	0.292	0.026	########
MYNN	########	-0.18534	0.203	0.044	########
TYRO3	########	0.364203	0.386	0.022	########
SH3KBP1	########	0.166756	0.319	0.029	########
NXPH1	########	0.255675	0.376	0.041	########
CBLN4	########	0.450536	0.443	0.043	########
FAM189B	########	0.154524	0.292	0.024	########
FBLIM1	########	-0.14521	0.241	0.063	########
SSBP3	########	0.172571	0.312	0.029	########
KRI1	########	0.199132	0.301	0.021	########
FADS2	########	0.358052	0.388	0.022	########
USP7	########	0.223295	0.313	0.021	########
ST3GAL2	########	0.216168	0.316	0.024	########
GIT1	########	0.230582	0.308	0.014	########
CDK13	########	0.150938	0.292	0.026	########
CWF19L2	########	0.104099	0.317	0.041	########
CNOT1	########	0.146473	0.298	0.027	########
HPCAL1	########	0.253641	0.317	0.014	########

TTLL1	########	0.139148	0.283	0.021	########
PRKD3	########	0.210483	0.34	0.034	########
SMAGP	########	0.124341	0.261	0.019	########
POMT1	########	0.18996	0.354	0.041	########
PARVB	########	0.225171	0.303	0.015	########
SYDE1	########	0.167652	0.294	0.021	########
SLC22A18	########	0.17856	0.302	0.021	########
AGPAT2	########	0.461858	0.413	0.012	########
RIMKLB	########	0.255115	0.35	0.027	########
NAA35	########	0.183195	0.31	0.026	########
JOSD1	########	0.334286	0.368	0.019	########
SKI	########	0.189069	0.316	0.029	########
ATG10	########	0.147583	0.302	0.027	########
VCL	########	0.282802	0.345	0.021	########
DBX2	########	0.219107	0.348	0.034	########
C2orf68	########	0.143679	0.29	0.026	########
ANGEL2	########	0.111302	0.293	0.029	########
NRIP1	########	0.436334	0.389	0.01	########
FAM221A	########	-0.39865	0.264	0.108	########
ZNF263	########	0.116768	0.275	0.026	########
VAMP4	########	-0.17624	0.229	0.063	########
BEX2	########	-0.83352	0.222	0.197	########
CSTF2T	########	0.163009	0.282	0.017	########
TSEN54	########	0.135035	0.291	0.026	########
TREX1	########	0.235359	0.332	0.021	########
CENPB	########	0.248399	0.318	0.014	########
FUT8	########	0.230794	0.314	0.019	########
SLC40A1	########	0.265685	0.326	0.015	########
HPRT1	########	-0.26253	0.201	0.063	########
HS6ST2	########	0.364055	0.352	0.009	########
GNPNAT1	########	0.212248	0.314	0.017	########
AUTS2	########	0.305562	0.346	0.019	########
IER5L	########	0.253366	0.337	0.024	########
HERPUD2	########	0.213034	0.327	0.024	########
LRRC27	########	0.140345	0.263	0.017	########
PXN-AS1	########	0.12486	0.254	0.019	########
ZCCHC7	########	0.165841	0.314	0.031	########
CORO2B	########	0.380293	0.387	0.019	########
MED1	########	0.220167	0.313	0.019	########
PIGK	########	-0.12488	0.253	0.056	########
NOTCH3	########	0.237097	0.318	0.019	########
NCOA3	########	0.289401	0.345	0.021	########
LHFPL3	########	0.227603	0.328	0.026	########
CRKL	########	0.181781	0.323	0.029	########
STK32A	########	0.122021	0.272	0.022	########
FAM199X	########	0.27866	0.345	0.019	########
TRAFD1	########	0.115088	0.292	0.031	########
RP11-262H	########	0.183505	0.32	0.027	########
EXD2	########	0.199624	0.304	0.024	########
GTF2A1	########	0.182096	0.311	0.026	########
ZNF217	########	0.269944	0.323	0.014	########

KLHDC8B	########	0.102295	0.263	0.022	########
FGF14	########	0.168434	0.308	0.029	########
MYH10	########	0.164024	0.323	0.034	########
PACSIN3	########	0.254478	0.315	0.015	########
ZFP14	########	0.119105	0.277	0.027	########
EIF4EBP2	########	0.1096	0.27	0.027	########
WRNIP1	########	0.24369	0.305	0.012	########
CMIP	########	0.102514	0.274	0.026	########
LIN7B	########	0.261655	0.335	0.022	########
TRPS1	########	0.222027	0.343	0.031	########
ENTPD3-AS	########	-0.13306	0.179	0.034	########
IGF2R	########	0.22088	0.301	0.017	########
MAN1C1	########	0.256913	0.316	0.014	########
FEM1B	########	0.155967	0.285	0.026	########
MAPK12	########	0.300088	0.314	0.009	########
C17orf10C	########	0.330041	0.339	0.01	########
STX2	########	0.25397	0.317	0.015	########
PHF10	########	0.104682	0.251	0.022	########
SPG11	########	0.137443	0.286	0.026	########
ATP13A3	########	0.304808	0.343	0.015	########
ZNF354A	########	0.125372	0.273	0.021	########
SMCR5	########	0.14008	0.257	0.021	########
NAB1	########	0.26721	0.324	0.017	########
PBX3	########	0.187365	0.289	0.019	########
MGST2	########	-0.12467	0.232	0.046	########
MOGS	########	0.230478	0.322	0.022	########
SLC35A2	########	0.277689	0.358	0.027	########
FASN	########	0.430145	0.395	0.012	########
TMEM108	########	0.202292	0.317	0.027	########
SYNM	########	0.315736	0.355	0.017	########
SPHK2	########	0.145341	0.31	0.034	########
KIAA1109	########	0.16969	0.293	0.022	########
NOC3L	########	0.126787	0.263	0.019	########
CRTC3	########	0.227132	0.311	0.021	########
RHBDF1	########	0.136372	0.263	0.014	########
SHPRH	########	0.152268	0.296	0.026	########
SFT2D2	########	0.256085	0.314	0.017	########
RYK	########	0.194044	0.286	0.015	########
ELK1	########	0.198301	0.278	0.012	########
MSI1	########	0.257917	0.383	0.041	########
ORAI3	########	0.157754	0.278	0.019	########
TMOD2	########	0.225517	0.322	0.024	########
CAB39	########	0.236907	0.332	0.024	########
PGS1	########	0.243929	0.316	0.021	########
CRTC1	########	0.103292	0.277	0.027	########
SOCS4	########	0.145339	0.262	0.019	########
GPR107	########	0.150466	0.281	0.021	########
NFKBIE	########	0.157307	0.26	0.014	########
MPP5	########	0.247843	0.305	0.014	########
SESTD1	########	0.103992	0.283	0.026	########
ZNF136	########	-0.2103	0.217	0.048	########

DPP4	########	0.45194	0.385	0.005	########
ETV4	########	0.145462	0.342	0.039	########
TMEM214	########	0.169079	0.293	0.022	########
FAM213B	########	0.150278	0.286	0.027	########
CBFA2T2	########	0.247425	0.331	0.027	########
SPA17	########	-0.17933	0.193	0.044	########
NDE1	########	0.120406	0.283	0.026	########
USP46	########	0.302517	0.334	0.014	########
TMEM121	########	0.266354	0.329	0.019	########
DMRTA2	########	0.244661	0.341	0.029	########
WDR70	########	0.115968	0.281	0.029	########
MYH9	########	0.261261	0.324	0.019	########
HINFP	########	-0.14594	0.226	0.055	########
CLCN7	########	0.40431	0.382	0.012	########
PLEKHF2	########	0.127452	0.273	0.022	########
CTU1	########	0.200478	0.304	0.021	########
MAP2K4	########	0.195939	0.291	0.021	########
ABCF3	########	0.203477	0.325	0.022	########
QTRTD1	########	0.107275	0.274	0.024	########
RGS3	########	0.270438	0.332	0.019	########
ISG20	########	-0.48923	0.19	0.096	########
CBWD1	########	-0.28526	0.227	0.087	########
EHD2	########	0.289679	0.352	0.022	########
CELSR2	########	0.260675	0.323	0.017	########
ZBTB4	########	0.220932	0.321	0.022	########
CTC-524C5	########	0.253775	0.304	0.012	########
NLGN2	########	0.176011	0.279	0.017	########
C6orf136	########	0.120149	0.269	0.022	########
HMGCR	########	0.208325	0.338	0.032	########
SEMA5A	########	0.124752	0.362	0.055	########
FAM98B	########	0.129697	0.274	0.022	########
TRIM4	########	0.20341	0.315	0.029	########
DEDD	########	0.209769	0.3	0.021	########
APTX	########	0.150678	0.296	0.027	########
C9orf69	########	0.28704	0.324	0.012	########
SNX10	########	0.37946	0.37	0.019	########
CCDC71	########	0.205006	0.279	0.014	########
CDKN2D	########	0.133441	0.285	0.027	########
NPEPL1	########	0.100169	0.3	0.039	########
PAQR4	########	0.284939	0.329	0.015	########
H6PD	########	0.131365	0.275	0.021	########
KATNB1	########	0.251814	0.33	0.024	########
ANKRD9	########	0.270847	0.302	0.009	########
TULP4	########	0.21546	0.308	0.021	########
CASP7	########	0.158333	0.296	0.027	########
CIB2	########	0.395664	0.355	0.003	########
KIAA0100	########	0.195307	0.276	0.014	########
SLC5A6	########	0.147161	0.295	0.029	########
SH3BGR	########	-0.37172	0.199	0.075	########
LONRF2	########	0.363135	0.38	0.029	########
FAM160A2	########	0.258358	0.328	0.024	########

ESRRA	########	0.195763	0.3	0.022	########
ZFYVE19	########	0.200809	0.285	0.015	########
TNKS1BP1	########	0.196875	0.296	0.017	########
SIRT3	########	0.150614	0.301	0.034	########
GOT1	########	-0.33155	0.191	0.068	########
TBC1D10B	########	0.209499	0.287	0.014	########
CUL4A	########	0.281155	0.342	0.022	########
RIOK2	########	-0.10055	0.223	0.044	########
GPSM2	########	0.126424	0.361	0.058	########
PRKCI	########	0.219591	0.299	0.015	########
PLXNA1	########	0.265824	0.317	0.017	########
LACTB	########	0.121108	0.25	0.019	########
UBFD1	########	0.223053	0.303	0.019	########
DLEU1	########	0.153512	0.285	0.022	########
TBC1D16	########	0.249194	0.312	0.017	########
DUSP1	########	-0.682	0.343	0.243	########
DPH6	########	0.118363	0.289	0.031	########
USP32	########	0.122712	0.262	0.024	########
E4F1	########	0.148308	0.289	0.024	########
MIR210HG	########	0.134014	0.304	0.038	########
DNM1	########	0.248953	0.301	0.012	########
LSAMP	########	-0.31285	0.288	0.113	########
ZC3H6	########	0.160163	0.288	0.029	########
LMCD1	########	0.500609	0.402	0.015	########
ZNF205	########	0.22176	0.31	0.019	########
HCG18	########	0.241127	0.301	0.014	########
CDK9	########	0.155595	0.283	0.024	########
ZBED1	########	0.198412	0.299	0.019	########
HPS3	########	0.161673	0.28	0.021	########
EYA4	########	0.267421	0.309	0.012	########
LINC00511	########	0.227123	0.349	0.032	########
CDH6	########	0.27014	0.349	0.026	########
LZTS1	########	0.108775	0.247	0.021	########
SPIRE1	########	0.215428	0.323	0.026	########
ABHD11	########	0.194919	0.323	0.032	########
TMTC3	########	0.300834	0.339	0.017	########
RASSF1	########	-0.10493	0.229	0.043	########
ZNF568	########	-0.18536	0.217	0.055	########
DMTF1	########	0.155004	0.311	0.036	########
GLA	########	-0.12468	0.209	0.046	########
LRP6	########	0.212283	0.301	0.021	########
TOMM70A	########	0.290229	0.323	0.014	########
CTDSPL2	########	0.147285	0.292	0.021	########
RP11-294J	########	0.256649	0.348	0.031	########
C5orf42	########	0.189647	0.303	0.026	########
GNB5	########	0.324026	0.351	0.017	########
SRD5A3	########	0.181738	0.293	0.021	########
MFSD5	########	0.284066	0.307	0.009	########
MPHOSPH9	########	0.100046	0.288	0.038	########
IPO13	########	0.148843	0.255	0.019	########
HMG20A	########	0.152565	0.286	0.024	########

RP13-1032	########	0.271524	0.294	0.01	########
MIR22HG	########	-0.23033	0.181	0.039	########
PIP5K1C	########	0.184477	0.295	0.024	########
ZFHX4-AS1	########	0.101113	0.242	0.024	########
TMEM185A	########	0.155968	0.263	0.019	########
NUMBL	########	0.323465	0.349	0.015	########
TEAD4	########	0.152306	0.255	0.015	########
GDAP1	########	-0.2935	0.231	0.068	########
BYSL	########	0.148459	0.267	0.017	########
GCFC2	########	0.191655	0.293	0.021	########
RAB40B	########	0.233301	0.286	0.012	########
SIN3B	########	0.116037	0.283	0.032	########
SLC38A6	########	-0.10373	0.207	0.039	########
RNPEPL1	########	0.236814	0.301	0.017	########
DXO	########	0.17009	0.305	0.029	########
GIT2	########	0.158098	0.275	0.022	########
RASSF8	########	0.280827	0.342	0.024	########
ACSF3	########	0.182733	0.299	0.027	########
CHST3	########	0.254053	0.298	0.012	########
ZFC3H1	########	0.218363	0.305	0.019	########
POGK	########	0.332295	0.337	0.01	########
PLEKHH3	########	0.188076	0.291	0.019	########
GPR162	########	0.182762	0.3	0.022	########
METTL3	########	-0.10407	0.282	0.067	########
FAM76B	########	0.221903	0.301	0.019	########
MCM3AP	########	0.181333	0.298	0.022	########
NADK2	########	0.269069	0.337	0.022	########
PCOLCE	########	-0.13586	0.204	0.041	########
HOMER2	########	0.110826	0.253	0.019	########
KDM2B	########	0.214517	0.291	0.015	########
CBL	########	0.20466	0.286	0.017	########
EEF1G	########	-0.33612	0.15	0.063	########
ABHD5	########	-0.1885	0.19	0.044	########
BCL7A	########	0.239249	0.307	0.019	########
LOXL1	########	0.512293	0.398	0.005	########
ABHD3	########	0.17042	0.315	0.032	########
MAP6	########	0.285473	0.309	0.009	########
CEP78	########	0.191226	0.275	0.015	########
AEBP1	########	-0.1531	0.241	0.058	########
ACOX2	########	0.131493	0.24	0.014	########
ZDHHC7	########	0.232856	0.313	0.024	########
EPAS1	########	-0.13253	0.263	0.058	########
NIN	########	0.202891	0.289	0.019	########
ANKRD13C	########	0.180733	0.295	0.026	########
ZBTB22	########	0.159276	0.273	0.019	########
RP11-698N	########	-0.17336	0.217	0.05	########
GPALPP1	########	0.15576	0.302	0.034	########
NUP88	########	0.187788	0.288	0.019	########
KAT8	########	0.111636	0.286	0.038	########
RASGRP1	########	0.345741	0.339	0.012	########
DPY19L4	########	0.124141	0.273	0.026	########

APBA3	########	0.117268	0.275	0.027	########
DCAF15	########	0.195445	0.294	0.021	########
ASNS	########	-0.25996	0.259	0.085	########
STXBP1	########	0.109326	0.257	0.029	########
RP11-421L	########	-0.11718	0.199	0.036	########
ELOVL6	########	0.282762	0.314	0.014	########
RFNG	########	0.199993	0.306	0.026	########
ACSS2	########	0.213506	0.273	0.014	########
KLHL12	########	0.128048	0.254	0.021	########
STK32C	########	0.148768	0.277	0.017	########
SNHG10	########	0.130911	0.285	0.029	########
POLR3E	########	0.152954	0.279	0.026	########
IRF2BP1	########	0.30143	0.333	0.017	########
GAB1	########	0.221341	0.316	0.026	########
PACS1	########	0.156952	0.265	0.019	########
CALCOCO1	########	0.140297	0.307	0.039	########
KDM5C	########	0.191698	0.299	0.024	########
IKBKG	########	0.267479	0.325	0.019	########
ATP6V0A2	########	0.210795	0.276	0.014	########
TP53BP2	########	0.126	0.294	0.036	########
MEX3C	########	0.242865	0.327	0.027	########
FN1	########	-0.14432	0.255	0.055	########
BBS7	########	0.152513	0.267	0.022	########
PARN	########	0.132905	0.268	0.024	########
KLF13	########	0.223278	0.302	0.021	########
ITSN2	########	0.274425	0.318	0.014	########
RP11-161M	########	0.190237	0.313	0.027	########
MYBBP1A	########	0.147796	0.253	0.019	########
CCNY	########	0.188809	0.272	0.015	########
TP53BP1	########	0.257745	0.315	0.019	########
INTS3	########	0.235942	0.334	0.031	########
SUN2	########	0.154681	0.302	0.032	########
RPGRIP1L	########	-0.11752	0.19	0.029	########
MEIS1	########	0.128654	0.297	0.034	########
MAP2K5	########	0.213788	0.285	0.015	########
KLHL13	########	0.315574	0.321	0.009	########
ITPK1	########	0.140606	0.302	0.034	########
MTFMT	########	0.228087	0.286	0.014	########
TRIM25	########	0.23315	0.294	0.017	########
TAF2	########	0.125863	0.248	0.022	########
INPP5K	########	0.15798	0.27	0.019	########
ZNF768	########	0.26092	0.304	0.015	########
CTBS	########	0.26238	0.313	0.017	########
CHST8	########	0.281495	0.33	0.021	########
IL13RA1	########	0.339016	0.332	0.009	########
AKAP17A	########	0.281751	0.324	0.017	########
CREB3L1	########	0.275008	0.302	0.012	########
VPS33A	########	0.137264	0.266	0.022	########
HOXD11	########	0.197081	0.291	0.019	########
RBL2	########	0.142412	0.293	0.031	########
SLC2A8	########	0.256807	0.312	0.015	########

PLK3	########	0.132088	0.295	0.043	########
ADCK2	########	0.236489	0.305	0.015	########
CPT2	########	0.1453	0.278	0.027	########
PCGF3	########	0.179358	0.264	0.017	########
DIP2B	########	0.219804	0.291	0.019	########
DHODH	########	-0.13752	0.211	0.038	########
LINC00936	########	0.117226	0.26	0.026	########
EPB41L1	########	0.317582	0.317	0.009	########
PRRT3	########	0.138482	0.233	0.014	########
NKAIN3	########	0.38724	0.364	0.019	########
SLC35E3	########	-0.15289	0.261	0.072	########
SPATA2L	########	0.184581	0.277	0.019	########
CPXM1	########	0.326946	0.339	0.019	########
IRF9	########	-0.23323	0.244	0.068	########
B4GALT2	########	0.13594	0.25	0.021	########
TFB1M	########	0.153983	0.252	0.017	########
RP11-25K1	########	0.311722	0.327	0.014	########
TTI1	########	0.152812	0.267	0.022	########
ZNF692	########	0.121961	0.277	0.032	########
LPCAT1	########	0.216732	0.336	0.038	########
UPF1	########	0.182202	0.272	0.017	########
PMS1	########	0.137822	0.286	0.027	########
RRN3	########	-0.13245	0.227	0.055	########
ID1	########	0.610181	0.424	0.027	########
PML	########	0.137745	0.268	0.022	########
SLC35A3	########	0.104398	0.275	0.034	########
C7orf26	########	0.162331	0.276	0.022	########
SCRIB	########	0.205164	0.303	0.024	########
PRPS2	########	0.171147	0.263	0.015	########
STAM	########	0.119153	0.235	0.015	########
COL22A1	########	0.40031	0.355	0.014	########
FAM161A	########	0.114928	0.26	0.026	########
EVA1C	########	0.174209	0.288	0.024	########
N4BP2	########	0.181883	0.286	0.022	########
HAUS2	########	0.217665	0.266	0.01	1.18E-99
TMEM47	########	0.174446	0.292	0.026	1.19E-99
NBL1	########	0.379519	0.358	0.012	1.20E-99
ULK3	########	0.190616	0.313	0.031	1.31E-99
ALG12	########	0.225541	0.276	0.014	2.08E-99
B9D2	########	0.101611	0.242	0.024	2.35E-99
ZNF667	########	-0.12665	0.208	0.055	2.87E-99
CACNB4	########	0.182961	0.272	0.015	2.97E-99
GALC	########	0.152275	0.263	0.021	3.09E-99
LRRC4C	########	-0.22524	0.2	0.056	4.12E-99
LPIN1	########	0.108285	0.273	0.034	4.22E-99
SLC39A9	########	0.226302	0.273	0.014	4.53E-99
DOK1	########	0.224036	0.285	0.017	5.42E-99
NUDT15	########	-0.11149	0.207	0.041	7.38E-99
PWWP2A	########	0.13978	0.277	0.029	8.41E-99
SAYSD1	########	0.16366	0.261	0.017	8.52E-99
FAM120C	########	0.138974	0.232	0.014	1.05E-98

TMEM57	########	0.249084	0.305	0.021	1.07E-98
MDGA1	########	0.418186	0.358	0.003	1.08E-98
MSANTD3	########	0.160883	0.258	0.021	1.21E-98
BTBD2	########	0.276584	0.312	0.015	1.21E-98
EPS15	########	0.225138	0.313	0.026	1.30E-98
CTNS	########	0.116393	0.228	0.014	1.31E-98
LIMD1	########	0.213787	0.283	0.021	1.38E-98
GSG1L	########	-0.17476	0.211	0.05	1.66E-98
ZNF704	########	0.191774	0.302	0.031	1.76E-98
GJC1	########	0.1807	0.254	0.017	1.80E-98
DCLRE1C	########	0.208271	0.308	0.021	1.81E-98
CRADD	########	0.194176	0.283	0.021	1.91E-98
SMG7	########	0.191363	0.269	0.017	1.98E-98
ALG1	########	0.171073	0.261	0.017	2.14E-98
ELP3	########	0.103909	0.257	0.026	2.56E-98
FIGN	########	0.199121	0.295	0.027	3.00E-98
VPS8	########	0.161144	0.267	0.022	3.03E-98
SERPING1	########	-1.16966	0.113	0.186	3.48E-98
BRF1	########	0.141609	0.269	0.022	3.55E-98
DCHS1	########	0.232672	0.289	0.014	3.60E-98
IFI44	########	-0.18707	0.249	0.07	4.63E-98
UBQLN4	########	0.117656	0.256	0.024	4.72E-98
CUL4B	########	0.12228	0.245	0.017	4.93E-98
TM4SF1	########	0.122895	0.278	0.027	5.61E-98
BIN1	########	0.288086	0.327	0.017	7.38E-98
LDOC1L	########	0.256542	0.295	0.012	7.39E-98
MFI2-AS1	########	0.14925	0.25	0.017	7.43E-98
LRRC16A	########	0.35738	0.359	0.019	7.69E-98
NOA1	########	0.255773	0.294	0.012	7.80E-98
FAM134C	########	0.104916	0.258	0.029	8.57E-98
ABR	########	0.190777	0.307	0.032	8.95E-98
MEOX2	########	-0.11883	0.28	0.072	9.13E-98
GFAP	########	-0.80527	0.356	0.304	9.92E-98
DNAJB4	########	-0.18856	0.182	0.048	9.98E-98
MFN1	########	0.124109	0.262	0.017	9.98E-98
TNFAIP6	########	-0.58385	0.232	0.123	1.08E-97
SOX5	########	0.156227	0.261	0.022	1.09E-97
SLC2A10	########	0.258713	0.298	0.014	1.20E-97
ANKRD37	########	-0.10022	0.204	0.038	1.20E-97
KIF3C	########	0.144349	0.262	0.026	1.38E-97
PHYHIPL	########	-0.32311	0.321	0.15	1.41E-97
MIB1	########	0.196518	0.279	0.017	1.43E-97
KLC2	########	0.306654	0.323	0.015	1.73E-97
EPHA4	########	0.231575	0.296	0.022	1.80E-97
FXN	########	0.207377	0.272	0.014	2.23E-97
TYK2	########	0.222118	0.323	0.036	2.91E-97
KLHL36	########	0.191229	0.255	0.012	3.14E-97
LONRF1	########	0.2745	0.309	0.015	3.77E-97
GZF1	########	0.165348	0.251	0.015	3.95E-97
TAB2	########	0.254587	0.294	0.012	3.96E-97
GLS	########	0.26969	0.312	0.019	4.12E-97

FBXW7	########	0.258776	0.316	0.021	4.22E-97
MICALL2	########	0.104519	0.241	0.022	4.33E-97
RALGAPA1	########	0.186035	0.295	0.026	4.54E-97
RRAS2	########	0.19476	0.266	0.015	4.69E-97
TSPAN14	########	0.23649	0.288	0.015	5.16E-97
NAA16	########	0.157608	0.279	0.027	5.72E-97
WDR59	########	0.188266	0.285	0.024	5.84E-97
METTL6	########	0.151929	0.273	0.024	6.48E-97
MYC	########	-0.53465	0.227	0.126	6.55E-97
ZNF516	########	0.110238	0.265	0.031	7.60E-97
TYW5	########	-0.12019	0.2	0.053	8.20E-97
4-Sep	########	0.341817	0.324	0.01	9.18E-97
ELK4	########	0.208021	0.282	0.019	1.27E-96
RCE1	########	0.199985	0.288	0.024	1.39E-96
ADAM19	########	0.359387	0.351	0.019	1.41E-96
NDRG2	########	-0.68704	0.415	0.326	1.47E-96
MIS18A	########	-0.10738	0.178	0.038	1.55E-96
ZNF337	########	0.168771	0.25	0.015	1.62E-96
RASA1	########	0.244231	0.301	0.019	1.63E-96
FBXL15	########	0.207912	0.296	0.021	1.98E-96
C12orf75	########	0.447512	0.364	0.003	2.63E-96
CHSY1	########	0.170359	0.251	0.015	2.72E-96
RP11-472N	########	0.302988	0.302	0.012	2.77E-96
DIS3L	########	0.22243	0.276	0.014	2.81E-96
SIDT2	########	-0.15012	0.184	0.039	2.81E-96
NOP2	########	0.104083	0.251	0.022	2.84E-96
TUBGCP4	########	0.18272	0.303	0.032	4.41E-96
UBXN7	########	0.141146	0.263	0.026	4.72E-96
TMOD1	########	-0.17348	0.362	0.123	5.43E-96
MTHFD1L	########	0.230206	0.272	0.009	5.74E-96
ZNF672	########	0.340166	0.348	0.017	5.89E-96
ADAT1	########	0.122184	0.249	0.019	6.62E-96
LMBR1	########	0.22467	0.286	0.017	6.94E-96
ARNTL	########	0.266819	0.314	0.019	7.08E-96
TENM1	########	0.158679	0.287	0.029	7.38E-96
ZNF48	########	0.260467	0.287	0.01	7.79E-96
CTSO	########	0.190251	0.275	0.019	8.12E-96
GNA13	########	0.139577	0.242	0.019	9.30E-96
PTEN	########	0.106976	0.224	0.017	9.90E-96
TTL	########	0.115645	0.252	0.029	1.06E-95
RP11-140K	########	0.105434	0.225	0.019	1.21E-95
TBC1D1	########	0.156554	0.251	0.017	1.24E-95
DHX16	1.09E-99	0.150421	0.257	0.019	1.63E-95
YTHDC2	1.19E-99	0.193138	0.279	0.019	1.78E-95
RBBP5	1.22E-99	0.10105	0.272	0.038	1.82E-95
SPRY4	1.61E-99	0.130518	0.299	0.036	2.41E-95
BTN2A1	2.07E-99	0.170614	0.279	0.024	3.09E-95
CDH4	2.40E-99	0.309312	0.324	0.017	3.58E-95
VCAM1	2.77E-99	-0.34266	0.203	0.051	4.14E-95
RRAGB	3.28E-99	0.145138	0.272	0.026	4.90E-95
POMT2	3.32E-99	0.144588	0.255	0.022	4.96E-95

D2HGDH	3.38E-99	0.253456	0.293	0.015	5.05E-95
ARID1B	3.79E-99	0.25064	0.293	0.015	5.66E-95
ACTR8	4.70E-99	0.105529	0.234	0.024	7.02E-95
TXNRD2	5.54E-99	0.353469	0.34	0.012	8.28E-95
FRMD8	5.67E-99	0.238443	0.303	0.022	8.47E-95
FAM69B	6.27E-99	0.202581	0.252	0.009	9.36E-95
OTUD4	6.41E-99	0.362746	0.329	0.007	9.57E-95
NAPEPLD	6.56E-99	0.11169	0.247	0.024	9.80E-95
MTF1	7.02E-99	0.114254	0.255	0.026	1.05E-94
NAGLU	8.82E-99	0.168626	0.246	0.017	1.32E-94
CCDC22	1.28E-98	0.266749	0.307	0.019	1.91E-94
RAB40C	1.31E-98	0.109616	0.242	0.024	1.96E-94
PHTF1	1.39E-98	0.333379	0.326	0.012	2.08E-94
SHROOM3	1.44E-98	0.110265	0.225	0.015	2.15E-94
FLYWCH1	1.52E-98	0.156558	0.256	0.021	2.27E-94
PHLPP1	1.74E-98	0.101981	0.267	0.029	2.60E-94
SSH2	2.32E-98	0.162967	0.27	0.022	3.46E-94
GAD1	2.74E-98	0.419108	0.372	0.024	4.09E-94
ACTR3B	2.92E-98	0.188823	0.277	0.022	4.36E-94
MAP3K4	3.11E-98	0.360176	0.334	0.009	4.65E-94
ATF7IP	3.33E-98	0.116028	0.247	0.024	4.98E-94
AC093323.	3.54E-98	0.25861	0.289	0.014	5.29E-94
NME6	3.66E-98	-0.28246	0.163	0.05	5.47E-94
PRR14L	3.67E-98	0.268826	0.288	0.01	5.48E-94
NMNAT1	3.68E-98	0.148709	0.251	0.021	5.50E-94
ATP11A	4.60E-98	0.242782	0.273	0.01	6.88E-94
LLGL1	4.82E-98	0.232075	0.288	0.017	7.20E-94
EFS	4.82E-98	0.150599	0.272	0.024	7.20E-94
ZNF599	5.27E-98	0.132056	0.264	0.027	7.87E-94
MCMBP	5.49E-98	0.136851	0.25	0.021	8.20E-94
MYO19	5.56E-98	0.13031	0.228	0.015	8.31E-94
HEG1	6.11E-98	0.247035	0.282	0.012	9.13E-94
KLHL23	6.33E-98	0.241805	0.291	0.015	9.46E-94
EXD3	6.60E-98	0.145471	0.257	0.024	9.87E-94
STAG1	7.83E-98	0.106831	0.256	0.029	1.17E-93
TFCP2	8.00E-98	0.247621	0.299	0.019	1.20E-93
NANOS3	8.65E-98	0.15218	0.241	0.015	1.29E-93
NUDT14	9.36E-98	0.225129	0.275	0.014	1.40E-93
MINK1	9.40E-98	0.151916	0.255	0.021	1.40E-93
WDR5	9.62E-98	0.165261	0.269	0.024	1.44E-93
UEVLD	1.07E-97	0.138622	0.239	0.017	1.60E-93
SH3BP2	1.23E-97	0.135429	0.242	0.019	1.85E-93
MVK	1.24E-97	0.14855	0.264	0.021	1.85E-93
MCEE	1.25E-97	-0.14342	0.204	0.046	1.87E-93
ATP2B4	1.54E-97	0.14176	0.255	0.022	2.30E-93
PEX7	1.59E-97	0.163625	0.246	0.015	2.37E-93
IMPDH1	1.60E-97	0.310737	0.316	0.012	2.39E-93
FAM91A1	1.70E-97	0.286613	0.305	0.012	2.53E-93
NUDT19	1.79E-97	0.14049	0.266	0.022	2.67E-93
GBA2	1.86E-97	0.113029	0.258	0.026	2.78E-93
RABEP2	1.92E-97	0.23342	0.291	0.017	2.87E-93

LRP12	1.93E-97	0.147471	0.244	0.019	2.89E-93
NDRG1	1.98E-97	-0.91124	0.145	0.138	2.95E-93
TUBE1	2.22E-97	-0.12347	0.197	0.041	3.32E-93
PNMAL1	2.30E-97	0.204981	0.269	0.014	3.44E-93
LMBR1L	2.65E-97	0.167397	0.276	0.026	3.97E-93
CSRP2BP	2.70E-97	0.162521	0.25	0.017	4.03E-93
ARL10	2.86E-97	0.146997	0.253	0.022	4.28E-93
IZUMO4	3.04E-97	0.164675	0.283	0.026	4.54E-93
ZFX	3.53E-97	0.100107	0.263	0.029	5.28E-93
PEX11G	3.78E-97	0.249899	0.272	0.01	5.66E-93
STYXL1	3.88E-97	-0.21254	0.153	0.043	5.80E-93
MMGT1	4.25E-97	0.125227	0.249	0.021	6.35E-93
RP11-448A	4.37E-97	0.149833	0.289	0.034	6.52E-93
ZNF649	4.84E-97	0.227693	0.267	0.012	7.23E-93
ALKBH6	5.81E-97	0.193421	0.288	0.026	8.69E-93
NCDN	7.97E-97	0.20393	0.27	0.014	1.19E-92
KDM3A	9.05E-97	0.143356	0.253	0.026	1.35E-92
STRBP	9.57E-97	0.313657	0.309	0.007	1.43E-92
L3MBTL2	1.14E-96	0.21481	0.273	0.015	1.71E-92
LRRC8D	1.17E-96	0.199092	0.253	0.012	1.75E-92
SLC35C1	1.21E-96	0.13357	0.216	0.012	1.81E-92
ARAP3	1.27E-96	0.174401	0.251	0.015	1.89E-92
ZNF277	1.32E-96	-0.12934	0.239	0.055	1.98E-92
ANKRD46	1.46E-96	-0.16343	0.209	0.056	2.18E-92
TNKS2	1.56E-96	0.197304	0.274	0.017	2.33E-92
P4HA2	1.58E-96	0.150092	0.25	0.021	2.36E-92
STK11	1.65E-96	0.275343	0.304	0.017	2.47E-92
DHX38	1.66E-96	0.195923	0.268	0.017	2.49E-92
ZCCHC24	1.80E-96	0.118448	0.247	0.024	2.69E-92
ZBED3	1.82E-96	0.215255	0.273	0.017	2.73E-92
RDH13	1.90E-96	0.143523	0.248	0.026	2.85E-92
GUCD1	1.92E-96	0.267415	0.307	0.015	2.87E-92
UCK2	1.97E-96	0.134154	0.248	0.022	2.94E-92
CCDC93	2.07E-96	0.108588	0.256	0.029	3.10E-92
TICAM1	2.07E-96	0.134451	0.238	0.015	3.10E-92
MDFI	2.13E-96	0.232901	0.277	0.015	3.18E-92
SLC37A4	2.29E-96	0.180333	0.267	0.017	3.42E-92
TELO2	2.50E-96	0.247064	0.301	0.021	3.74E-92
ZSCAN9	2.94E-96	-0.11984	0.169	0.027	4.39E-92
HLA-DRB1	2.96E-96	-1.01081	0.201	0.255	4.42E-92
CNR1	3.50E-96	0.186406	0.293	0.029	5.24E-92
C16orf58	3.64E-96	0.12865	0.259	0.024	5.44E-92
C1QC	3.65E-96	-0.58954	0.19	0.125	5.45E-92
UCHL5	3.74E-96	0.130369	0.253	0.029	5.58E-92
SGPL1	4.36E-96	0.187231	0.267	0.019	6.51E-92
MARK2	4.43E-96	0.237045	0.279	0.014	6.61E-92
SYCE1L	4.45E-96	0.325	0.311	0.01	6.65E-92
TBCK	4.46E-96	0.164687	0.242	0.019	6.67E-92
NUP50	5.99E-96	0.214953	0.263	0.012	8.96E-92
OAF	6.41E-96	0.301654	0.313	0.014	9.58E-92
N4BP2L1	6.74E-96	-0.16525	0.219	0.056	1.01E-91

NUDT8	7.45E-96	0.275181	0.286	0.01	1.11E-91
MYPOP	7.66E-96	0.141845	0.255	0.022	1.14E-91
LINC0052€	9.37E-96	0.155298	0.245	0.017	1.40E-91
TET1	1.00E-95	0.405018	0.346	0.009	1.50E-91
PKD2	1.09E-95	0.198822	0.255	0.015	1.63E-91
SLC35E4	1.09E-95	0.237072	0.255	0.005	1.63E-91
CLUH	1.21E-95	0.153112	0.223	0.01	1.81E-91
INO80	1.25E-95	0.110718	0.222	0.019	1.86E-91
FOSB	1.47E-95	-0.62291	0.215	0.154	2.20E-91
AGPS	1.64E-95	0.125024	0.223	0.014	2.45E-91
ZNHIT6	1.66E-95	0.138799	0.251	0.022	2.48E-91
MSH6	1.73E-95	0.164003	0.255	0.019	2.58E-91
SPAST	1.73E-95	0.282521	0.293	0.01	2.59E-91
C1QL1	1.78E-95	0.373832	0.345	0.022	2.65E-91
PWAR6	1.86E-95	0.140038	0.241	0.019	2.78E-91
HUS1	2.04E-95	-0.14422	0.191	0.043	3.05E-91
AMDHD2	2.10E-95	0.21765	0.272	0.015	3.13E-91
MPND	2.30E-95	0.357861	0.32	0.003	3.44E-91
SETD7	2.53E-95	0.114877	0.244	0.026	3.78E-91
ZBTB44	2.59E-95	0.15375	0.251	0.022	3.87E-91
KIAA1429	2.75E-95	0.100871	0.238	0.024	4.11E-91
HLA-DPA1	2.84E-95	-1.04592	0.215	0.27	4.24E-91
TMPO	3.13E-95	0.138319	0.258	0.026	4.68E-91
STK24	3.21E-95	0.126485	0.234	0.015	4.79E-91
FOXRED2	3.84E-95	0.23063	0.27	0.012	5.74E-91
PABPC1L	4.53E-95	0.166488	0.28	0.026	6.77E-91
SHOX2	4.92E-95	0.254226	0.277	0.01	7.35E-91
TEX2	5.27E-95	0.207934	0.27	0.017	7.87E-91
PAX6	5.89E-95	0.181105	0.288	0.026	8.81E-91
DIABLO	6.62E-95	-0.15987	0.161	0.038	9.89E-91
NOTCH1	7.03E-95	0.244852	0.305	0.024	1.05E-90
INSR	7.19E-95	0.105718	0.286	0.043	1.07E-90
ZNF609	7.95E-95	0.181297	0.249	0.015	1.19E-90
PTCD1	7.97E-95	-0.1966	0.164	0.046	1.19E-90
ETFDH	8.23E-95	-0.2143	0.197	0.063	1.23E-90
MEGF8	8.40E-95	0.184443	0.293	0.031	1.25E-90
FAM171A1	9.88E-95	0.195472	0.25	0.012	1.48E-90
APITD1	1.04E-94	0.121556	0.237	0.019	1.55E-90
MAPK8IP3	1.26E-94	0.149194	0.241	0.017	1.88E-90
BAIAP2-AS	1.27E-94	0.20967	0.263	0.014	1.89E-90
STARD4-AS	1.28E-94	0.354005	0.336	0.019	1.92E-90
MTFR1	1.41E-94	0.21137	0.257	0.012	2.10E-90
RP11-212F	1.56E-94	0.146261	0.234	0.019	2.33E-90
DTX3L	2.15E-94	0.103033	0.219	0.015	3.21E-90
RAB3D	2.62E-94	0.144843	0.223	0.017	3.92E-90
BCKDHB	2.89E-94	0.155184	0.248	0.022	4.31E-90
NMT2	2.96E-94	0.117595	0.234	0.021	4.43E-90
FBXO5	3.33E-94	0.120789	0.225	0.012	4.97E-90
MMP16	3.33E-94	0.372896	0.331	0.01	4.98E-90
MAPKAPK3	3.48E-94	0.294185	0.294	0.007	5.20E-90
RP11-379H	3.99E-94	0.139738	0.242	0.019	5.96E-90

ORC5	4.28E-94	-0.18193	0.169	0.036	6.40E-90
RAD54L2	4.39E-94	0.170363	0.263	0.021	6.56E-90
GTPBP3	4.92E-94	0.104135	0.275	0.038	7.35E-90
C1GALT1	5.18E-94	0.244395	0.305	0.022	7.74E-90
DCX	5.35E-94	0.398387	0.343	0.021	8.00E-90
CPNE5	5.75E-94	0.250262	0.277	0.014	8.60E-90
WDR36	6.46E-94	0.130921	0.214	0.017	9.65E-90
SLC48A1	8.09E-94	0.171564	0.264	0.021	1.21E-89
COPG2	8.97E-94	0.163806	0.245	0.014	1.34E-89
HDAC9	9.33E-94	0.113752	0.236	0.026	1.39E-89
N6AMT1	9.43E-94	0.159515	0.232	0.012	1.41E-89
CACFD1	9.53E-94	0.154238	0.229	0.014	1.42E-89
CLN6	1.07E-93	0.121074	0.235	0.022	1.60E-89
MFN2	1.16E-93	0.16038	0.262	0.022	1.73E-89
MON1A	1.29E-93	0.166044	0.244	0.015	1.93E-89
SETDB1	1.37E-93	-0.11475	0.16	0.032	2.04E-89
ZNF532	1.46E-93	0.273344	0.293	0.014	2.18E-89
FAM181A	1.63E-93	-0.18711	0.208	0.056	2.44E-89
CCP110	1.64E-93	-0.12431	0.187	0.05	2.45E-89
SH3GLB2	1.97E-93	0.331126	0.315	0.01	2.94E-89
ZNF232	2.00E-93	0.119684	0.216	0.015	2.98E-89
LMNB2	2.00E-93	0.280734	0.293	0.012	3.00E-89
CLYBL	2.08E-93	-0.14006	0.156	0.034	3.10E-89
PI4KA	2.64E-93	0.204328	0.258	0.012	3.95E-89
KLF10	2.73E-93	-0.18228	0.225	0.068	4.08E-89
GBP1	2.89E-93	-0.61043	0.296	0.168	4.32E-89
JMY	3.03E-93	0.323411	0.317	0.015	4.52E-89
LMF1	3.37E-93	0.219488	0.277	0.017	5.03E-89
WDR55	3.50E-93	0.129519	0.234	0.017	5.22E-89
RP11-126K	3.55E-93	0.14426	0.238	0.019	5.30E-89
MYO1C	3.99E-93	0.223603	0.279	0.021	5.96E-89
GLIS3	4.11E-93	0.265493	0.294	0.017	6.14E-89
MPDZ	4.13E-93	0.23063	0.274	0.014	6.18E-89
ZNF362	4.53E-93	0.211174	0.261	0.015	6.76E-89
C10orf10	4.61E-93	-0.84224	0.113	0.106	6.89E-89
TMCC1	5.50E-93	0.380142	0.331	0.007	8.21E-89
C9orf85	7.91E-93	0.166433	0.238	0.012	1.18E-88
BOLA2B	8.03E-93	-0.16767	0.167	0.039	1.20E-88
GALNT10	8.88E-93	0.125721	0.251	0.026	1.33E-88
CAPN7	8.99E-93	0.169264	0.262	0.022	1.34E-88
IGF2BP2	9.08E-93	0.234947	0.301	0.026	1.36E-88
GNB1L	1.03E-92	0.112656	0.226	0.019	1.54E-88
ADAMTS6	1.28E-92	0.266506	0.298	0.019	1.91E-88
PCDH19	1.44E-92	0.227908	0.257	0.009	2.15E-88
CBX4	1.58E-92	0.110086	0.238	0.026	2.36E-88
GPX3	1.86E-92	-0.10398	0.193	0.036	2.77E-88
CEP57L1	1.97E-92	0.214754	0.279	0.019	2.94E-88
DGCR8	2.07E-92	0.133363	0.243	0.024	3.10E-88
NRDE2	2.25E-92	-0.14038	0.198	0.046	3.36E-88
CSPG5	2.44E-92	-0.13096	0.315	0.094	3.65E-88
DIP2C	2.97E-92	0.115022	0.22	0.019	4.43E-88

RFX5	3.10E-92	0.13885	0.23	0.019	4.63E-88
SARNP	3.12E-92	-0.29344	0.172	0.063	4.66E-88
CDKN1A	3.21E-92	-0.49552	0.248	0.135	4.80E-88
ABHD6	4.66E-92	0.166588	0.245	0.017	6.96E-88
DDX3Y	4.67E-92	0.184006	0.234	0.01	6.98E-88
VWA5A	5.20E-92	-0.16564	0.187	0.053	7.77E-88
PCDH10	5.41E-92	0.188124	0.255	0.019	8.09E-88
PTPN14	8.42E-92	0.208214	0.275	0.021	1.26E-87
ASUN	9.32E-92	0.153336	0.255	0.022	1.39E-87
DCBLD1	1.01E-91	0.165657	0.264	0.022	1.51E-87
MAPK14	1.15E-91	0.256952	0.283	0.014	1.72E-87
HBB	1.34E-91	-1.0457	0.219	0.226	2.00E-87
KIF1C	1.38E-91	0.170433	0.245	0.017	2.06E-87
FAM208B	1.49E-91	0.309515	0.285	0.003	2.23E-87
ARSA	1.55E-91	0.167204	0.244	0.015	2.31E-87
NSUN2	1.60E-91	0.229497	0.283	0.017	2.39E-87
TYW1	1.71E-91	0.117159	0.244	0.022	2.56E-87
RIC8B	1.77E-91	0.107575	0.243	0.027	2.65E-87
1-Sep	2.18E-91	0.186925	0.252	0.017	3.26E-87
ELK3	2.20E-91	0.169713	0.237	0.012	3.28E-87
H1F0	2.34E-91	-0.16549	0.294	0.104	3.50E-87
ZNF436	2.35E-91	0.408641	0.347	0.014	3.51E-87
RWDD2B	2.64E-91	0.15984	0.242	0.015	3.95E-87
CAPN5	2.64E-91	0.14213	0.239	0.022	3.95E-87
TRAF6	2.75E-91	0.101002	0.218	0.015	4.11E-87
SEC16A	3.26E-91	0.230084	0.283	0.021	4.86E-87
PSKH1	3.44E-91	0.193388	0.231	0.007	5.14E-87
TAF3	3.86E-91	0.174873	0.256	0.019	5.76E-87
THAP2	4.85E-91	0.137462	0.247	0.022	7.25E-87
MAP4K3	4.89E-91	0.250331	0.276	0.012	7.31E-87
CNTFR	5.36E-91	0.228857	0.287	0.024	8.01E-87
FN3K	6.08E-91	0.152017	0.251	0.024	9.08E-87
NUP35	6.66E-91	-0.12017	0.19	0.043	9.96E-87
ZNF587	6.95E-91	0.138893	0.25	0.026	1.04E-86
TOM1L1	8.10E-91	0.209712	0.254	0.012	1.21E-86
CNOT6	8.15E-91	0.110344	0.201	0.012	1.22E-86
KBTBD2	8.57E-91	0.126788	0.236	0.019	1.28E-86
RPRD1B	9.73E-91	0.206062	0.26	0.015	1.45E-86
TMEM39B	1.18E-90	0.115468	0.247	0.024	1.77E-86
ARRDC1	1.35E-90	0.147716	0.245	0.019	2.02E-86
UTP14C	1.36E-90	0.209684	0.258	0.014	2.04E-86
CTTNBP2NL	1.43E-90	0.248931	0.283	0.015	2.14E-86
ZNF584	1.64E-90	0.263468	0.288	0.01	2.46E-86
TOP1MT	1.81E-90	0.165206	0.229	0.01	2.70E-86
PM20D2	2.05E-90	0.275587	0.29	0.01	3.07E-86
PROS1	2.47E-90	0.179693	0.242	0.012	3.69E-86
C17orf80	2.52E-90	0.213328	0.269	0.017	3.76E-86
10-Sep	2.52E-90	0.185369	0.258	0.019	3.77E-86
ODF2	2.72E-90	0.180325	0.274	0.024	4.07E-86
RP5-1085F	3.12E-90	0.162027	0.234	0.015	4.67E-86
ZNF16	3.26E-90	-0.10403	0.178	0.036	4.88E-86

COL16A1	3.54E-90	0.176289	0.326	0.046	5.29E-86
FAM8A1	3.65E-90	0.109402	0.229	0.021	5.46E-86
PRMT6	4.42E-90	0.172017	0.265	0.021	6.60E-86
MAD1L1	4.64E-90	0.158966	0.241	0.019	6.94E-86
ASB16-AS1	4.66E-90	0.208844	0.244	0.01	6.96E-86
SLC7A1	4.92E-90	0.103744	0.227	0.022	7.35E-86
AGBL5	5.73E-90	0.323008	0.298	0.005	8.56E-86
MESDC1	5.96E-90	0.231212	0.261	0.01	8.91E-86
FREM2	6.96E-90	0.435723	0.345	0.005	1.04E-85
SAMD4A	7.36E-90	0.159759	0.264	0.026	1.10E-85
PI4K2B	7.49E-90	0.167844	0.244	0.015	1.12E-85
ZNF605	8.88E-90	-0.18566	0.172	0.06	1.33E-85
FGFRL1	9.02E-90	0.254776	0.272	0.01	1.35E-85
TAZ	1.03E-89	0.183328	0.268	0.021	1.54E-85
TBC1D22A	1.09E-89	0.195089	0.225	0.007	1.62E-85
RP3-428L1	1.09E-89	0.643094	0.34	0	1.63E-85
RNF169	1.12E-89	0.207371	0.25	0.012	1.68E-85
ZNF582-AS	1.15E-89	-0.40257	0.133	0.063	1.71E-85
LHFPL2	1.19E-89	0.112727	0.221	0.015	1.78E-85
PTP4A3	1.86E-89	0.219346	0.288	0.026	2.78E-85
METTL7A	1.87E-89	-0.289	0.308	0.132	2.80E-85
SP1	1.92E-89	0.153726	0.242	0.019	2.88E-85
TTC28	2.00E-89	0.287912	0.298	0.015	2.98E-85
TAGLN3	2.12E-89	-0.15467	0.203	0.055	3.17E-85
LZTS2	2.37E-89	0.146793	0.228	0.015	3.55E-85
RNF111	2.81E-89	0.217682	0.247	0.009	4.20E-85
SYP	3.08E-89	-0.16518	0.196	0.058	4.61E-85
TECPR1	3.62E-89	0.272595	0.283	0.012	5.42E-85
JRKL	3.70E-89	0.158715	0.259	0.021	5.52E-85
CTB-113I2	4.26E-89	0.187045	0.235	0.009	6.37E-85
PPP2R3B	4.76E-89	0.170798	0.226	0.007	7.11E-85
SLC22A23	5.31E-89	0.189092	0.238	0.012	7.94E-85
USP38	5.46E-89	0.101298	0.206	0.017	8.17E-85
PLXNA3	5.47E-89	-0.11633	0.171	0.036	8.17E-85
HDAC10	5.62E-89	0.258891	0.261	0.005	8.40E-85
ATXN1L	6.65E-89	0.145863	0.213	0.012	9.93E-85
PPP1R9B	6.67E-89	0.28361	0.284	0.01	9.96E-85
PCNX	6.90E-89	0.120526	0.228	0.022	1.03E-84
SPATA20	7.00E-89	0.14417	0.247	0.022	1.05E-84
CNKSR2	7.60E-89	0.223946	0.26	0.015	1.14E-84
TRAK1	7.60E-89	0.163203	0.25	0.021	1.14E-84
PLEKHA2	7.89E-89	0.139884	0.249	0.026	1.18E-84
MYO1E	7.90E-89	0.133063	0.21	0.009	1.18E-84
DBNDD1	8.16E-89	0.206872	0.247	0.014	1.22E-84
PLCB1	8.22E-89	0.204723	0.248	0.014	1.23E-84
B3GALT1	9.84E-89	0.178476	0.247	0.017	1.47E-84
PLAU	1.00E-88	0.384604	0.327	0.017	1.49E-84
MTR	1.13E-88	0.170564	0.255	0.019	1.69E-84
BCAS3	1.22E-88	0.143871	0.214	0.01	1.82E-84
NLK	1.47E-88	0.270027	0.279	0.01	2.19E-84
DHX8	1.72E-88	0.326556	0.307	0.009	2.57E-84

TRIM56	1.78E-88	-0.33421	0.159	0.055	2.66E-84
RBBP8	1.78E-88	0.142561	0.206	0.01	2.66E-84
ZBTB10	1.95E-88	0.118926	0.225	0.019	2.91E-84
ERCC6L2	2.41E-88	0.16634	0.245	0.017	3.60E-84
GMDS	2.54E-88	0.147994	0.233	0.017	3.80E-84
CREBRF	3.00E-88	-0.25514	0.168	0.036	4.48E-84
ICAM3	3.21E-88	0.431212	0.336	0	4.80E-84
RNF168	3.62E-88	0.181231	0.261	0.021	5.42E-84
FAT1	3.69E-88	0.292154	0.291	0.012	5.52E-84
RPS6KA2	3.88E-88	0.133247	0.259	0.032	5.80E-84
PORCN	4.00E-88	0.214656	0.267	0.015	5.98E-84
TMEM216	4.18E-88	-0.13435	0.178	0.041	6.24E-84
ZNF513	4.18E-88	0.117177	0.219	0.019	6.25E-84
EMID1	4.23E-88	0.274761	0.279	0.014	6.32E-84
CRIM1	4.71E-88	0.156974	0.246	0.017	7.04E-84
NHSL1	4.76E-88	0.142572	0.255	0.027	7.11E-84
PDIA5	5.30E-88	0.188993	0.232	0.012	7.93E-84
DNAJC14	5.37E-88	0.100614	0.214	0.021	8.03E-84
CAHM	6.20E-88	0.221681	0.259	0.012	9.27E-84
KIAA2026	6.41E-88	0.102648	0.236	0.027	9.58E-84
MCHR1	6.56E-88	0.356255	0.303	0.01	9.81E-84
MAP3K1	9.81E-88	0.269521	0.293	0.019	1.47E-83
AP5Z1	1.20E-87	0.175458	0.258	0.021	1.79E-83
RHOU	1.38E-87	0.125343	0.289	0.05	2.07E-83
FRMD3	1.63E-87	-0.39185	0.144	0.072	2.44E-83
EZH2	1.94E-87	0.101856	0.223	0.022	2.90E-83
ACAD9	2.96E-87	0.116629	0.227	0.021	4.43E-83
PAG1	3.04E-87	0.106133	0.241	0.027	4.54E-83
ZNHIT2	3.69E-87	0.159633	0.253	0.019	5.51E-83
FAM78B	4.11E-87	0.109971	0.178	0.007	6.14E-83
FAM219A	4.60E-87	0.141154	0.229	0.017	6.88E-83
IL32	5.10E-87	-0.13828	0.184	0.032	7.62E-83
TMEM8B	5.57E-87	0.163162	0.224	0.01	8.32E-83
PCGF1	5.78E-87	-0.12861	0.173	0.038	8.64E-83
ANAPC2	7.14E-87	0.242937	0.29	0.021	1.07E-82
CAPN10	8.74E-87	0.150565	0.242	0.021	1.31E-82
N4BP1	8.87E-87	0.200816	0.241	0.012	1.33E-82
TESK1	8.93E-87	0.179843	0.234	0.012	1.33E-82
ZSWIM6	9.15E-87	0.298282	0.296	0.012	1.37E-82
TAF6L	9.73E-87	0.263327	0.273	0.012	1.45E-82
NOMO1	1.03E-86	0.194799	0.256	0.017	1.54E-82
CRISPLD1	1.63E-86	0.135001	0.241	0.022	2.43E-82
CHRAC1	1.69E-86	0.140887	0.241	0.021	2.52E-82
NXT2	1.87E-86	0.140846	0.226	0.017	2.80E-82
CHI3L2	2.33E-86	-1.57272	0.069	0.193	3.48E-82
ZNF174	2.35E-86	0.121547	0.207	0.014	3.51E-82
PAN3	2.75E-86	0.151797	0.245	0.027	4.11E-82
MED12L	3.16E-86	0.295889	0.294	0.014	4.72E-82
DYRK1B	3.18E-86	0.148447	0.228	0.017	4.75E-82
NRM	3.18E-86	0.101805	0.191	0.01	4.75E-82
LRRC14	3.25E-86	0.130542	0.231	0.021	4.86E-82

TDRD3	3.30E-86	0.172015	0.253	0.021	4.93E-82
FANCF	3.38E-86	0.10892	0.231	0.022	5.06E-82
SERINC5	3.60E-86	0.186484	0.236	0.014	5.38E-82
ANXA11	3.70E-86	0.246891	0.263	0.009	5.53E-82
COL5A2	4.02E-86	0.321596	0.297	0.012	6.01E-82
C22orf29	4.73E-86	0.124477	0.215	0.015	7.07E-82
DGAT1	4.90E-86	0.240911	0.261	0.009	7.32E-82
NEO1	5.51E-86	0.165047	0.224	0.012	8.24E-82
VPS13A	5.65E-86	0.166204	0.229	0.015	8.44E-82
TRIOBP	6.52E-86	0.260821	0.258	0.007	9.74E-82
PPP2R2D	6.53E-86	0.253389	0.251	0.005	9.76E-82
GOLGA8A	6.58E-86	0.217906	0.257	0.014	9.84E-82
CA11	8.04E-86	0.130957	0.272	0.039	1.20E-81
TP53INP2	8.27E-86	0.184513	0.231	0.01	1.24E-81
IQCE	8.94E-86	0.229668	0.27	0.017	1.34E-81
E2F5	1.08E-85	0.35035	0.299	0.002	1.61E-81
DOT1L	1.17E-85	0.212821	0.26	0.017	1.74E-81
KTI12	1.54E-85	0.217607	0.264	0.014	2.30E-81
TRIM14	1.62E-85	0.176928	0.238	0.015	2.42E-81
NFKBIZ	1.65E-85	-0.11421	0.21	0.058	2.47E-81
ASXL2	1.71E-85	0.213354	0.254	0.014	2.55E-81
MSH2	1.88E-85	0.144569	0.236	0.021	2.81E-81
COG5	1.92E-85	0.113975	0.233	0.024	2.87E-81
AC091729.	2.15E-85	0.210344	0.259	0.017	3.21E-81
BACH1	2.16E-85	0.103971	0.207	0.021	3.23E-81
UTRN	2.29E-85	0.181251	0.255	0.021	3.43E-81
CTPS1	2.34E-85	0.100484	0.229	0.022	3.49E-81
OBFC1	2.63E-85	0.177209	0.218	0.007	3.93E-81
ZNF334	2.90E-85	0.148374	0.229	0.019	4.33E-81
SLC16A3	3.24E-85	0.332215	0.308	0.022	4.84E-81
ICK	3.26E-85	0.126403	0.211	0.015	4.87E-81
SLC38A7	3.38E-85	0.121612	0.229	0.022	5.05E-81
MEMO1	3.42E-85	0.13598	0.227	0.022	5.12E-81
FAM214B	3.46E-85	0.315448	0.29	0.007	5.17E-81
FBXL17	3.65E-85	0.273931	0.272	0.009	5.46E-81
ABCA3	3.70E-85	0.142281	0.239	0.021	5.53E-81
ZNF395	4.20E-85	-0.12675	0.153	0.032	6.28E-81
DDHD1	5.27E-85	0.120763	0.231	0.022	7.88E-81
ZNF512B	5.41E-85	0.321309	0.302	0.01	8.09E-81
PLCD3	5.57E-85	0.366442	0.309	0.007	8.32E-81
GUF1	6.03E-85	0.219502	0.258	0.012	9.01E-81
RMND5A	6.39E-85	0.13665	0.244	0.029	9.54E-81
ARSD	6.49E-85	0.106248	0.214	0.022	9.70E-81
TAOK2	6.50E-85	0.252317	0.264	0.01	9.71E-81
SEC24B	6.62E-85	0.1476	0.22	0.012	9.89E-81
EPC2	7.40E-85	0.134412	0.219	0.021	1.11E-80
ALCAM	7.97E-85	0.275333	0.286	0.019	1.19E-80
UBE3C	9.01E-85	0.189906	0.26	0.022	1.35E-80
NR2C1	1.06E-84	0.152873	0.233	0.017	1.59E-80
VPS9D1	1.15E-84	0.104554	0.214	0.015	1.72E-80
PYGO2	1.21E-84	0.145584	0.22	0.015	1.80E-80

CAAP1	1.37E-84	0.159356	0.217	0.012	2.04E-80
FILIP1L	1.49E-84	0.370391	0.311	0.014	2.23E-80
ZDHHC15	1.62E-84	0.187161	0.229	0.01	2.42E-80
KIAA0930	1.64E-84	0.150084	0.209	0.01	2.46E-80
RYBP	1.77E-84	0.1392	0.219	0.017	2.65E-80
ESYT1	1.89E-84	0.135297	0.219	0.019	2.83E-80
WDR3	1.90E-84	0.219189	0.26	0.017	2.84E-80
FAT3	2.07E-84	0.174775	0.255	0.022	3.09E-80
C3orf70	2.41E-84	0.217979	0.273	0.022	3.61E-80
TIMP4	2.48E-84	-0.28457	0.265	0.099	3.70E-80
NR1D1	2.68E-84	-0.23801	0.151	0.041	4.01E-80
SNCAIP	2.74E-84	0.18107	0.264	0.024	4.09E-80
TBL1X	3.02E-84	0.175101	0.231	0.009	4.52E-80
MSH3	3.10E-84	0.342123	0.29	0.005	4.63E-80
KATNAL1	3.40E-84	0.256913	0.272	0.012	5.08E-80
ZNF714	3.88E-84	-0.14422	0.188	0.058	5.79E-80
ST3GAL3	3.97E-84	0.201985	0.244	0.012	5.93E-80
PLEKHG1	4.05E-84	0.284069	0.279	0.009	6.06E-80
SRCAP	4.41E-84	0.220727	0.251	0.012	6.59E-80
PARD3	4.53E-84	0.25732	0.265	0.009	6.77E-80
TMEM8A	4.84E-84	0.200913	0.242	0.01	7.23E-80
LRFN4	5.99E-84	0.290716	0.284	0.005	8.96E-80
PCNXL2	6.51E-84	0.145826	0.241	0.021	9.73E-80
RP11-115C	6.70E-84	0.162696	0.238	0.019	1.00E-79
PODXL	6.74E-84	0.263282	0.26	0.01	1.01E-79
RNF166	7.15E-84	0.128639	0.234	0.024	1.07E-79
TGIF2	7.23E-84	0.352267	0.31	0.005	1.08E-79
TMEM19	1.03E-83	0.184502	0.256	0.019	1.54E-79
MOSPD2	1.05E-83	0.122444	0.217	0.019	1.58E-79
GALNT7	1.26E-83	0.218645	0.249	0.014	1.89E-79
ZDHHC21	1.51E-83	0.216556	0.253	0.012	2.26E-79
MAPK11	1.69E-83	0.197073	0.238	0.012	2.53E-79
RBM19	1.74E-83	0.148716	0.221	0.015	2.59E-79
MAN2A2	1.80E-83	0.107575	0.197	0.014	2.69E-79
TYMP	1.91E-83	0.126099	0.215	0.019	2.85E-79
NEK8	1.91E-83	0.148462	0.212	0.012	2.86E-79
STARD10	2.07E-83	0.121611	0.217	0.017	3.09E-79
ENTHD2	2.11E-83	0.10498	0.206	0.019	3.16E-79
SASH1	2.86E-83	0.106258	0.233	0.027	4.28E-79
VSTM2A	3.08E-83	-1.14898	0.102	0.258	4.61E-79
RAD51D	3.23E-83	0.109248	0.21	0.017	4.82E-79
DIRC2	3.24E-83	0.167005	0.219	0.012	4.84E-79
EMP2	3.24E-83	-0.25461	0.165	0.053	4.85E-79
HAUS8	3.33E-83	-0.11466	0.152	0.027	4.97E-79
KIAA0430	3.71E-83	0.124796	0.226	0.024	5.55E-79
RP1-79C4.	4.03E-83	0.179057	0.232	0.014	6.02E-79
ANKMY2	4.20E-83	-0.20306	0.174	0.05	6.28E-79
SERPINE1	4.38E-83	-0.59393	0.146	0.091	6.54E-79
PIANP	4.89E-83	0.282544	0.276	0.01	7.31E-79
SLC35D1	6.00E-83	0.183164	0.229	0.012	8.97E-79
EML1	6.26E-83	0.288095	0.275	0.009	9.35E-79

PPP1R14C	6.47E-83	0.166061	0.233	0.015	9.66E-79
XIST	7.03E-83	-0.76303	0.178	0.202	1.05E-78
CNOT6L	7.24E-83	0.180958	0.234	0.015	1.08E-78
MIAT	7.65E-83	0.101849	0.269	0.044	1.14E-78
SRGAP3	8.13E-83	0.122574	0.244	0.031	1.21E-78
EEFSEC	8.68E-83	0.167522	0.216	0.01	1.30E-78
NR1D2	9.03E-83	0.151312	0.226	0.017	1.35E-78
SOX21-AS1	9.26E-83	0.239442	0.279	0.022	1.38E-78
EYA2	9.45E-83	0.209188	0.272	0.024	1.41E-78
TMEM260	1.26E-82	0.213023	0.248	0.014	1.88E-78
UBR3	1.69E-82	0.121337	0.209	0.017	2.53E-78
ENKD1	1.93E-82	0.141008	0.21	0.015	2.89E-78
PRKD2	1.98E-82	0.141133	0.221	0.014	2.96E-78
VPS11	2.02E-82	0.163972	0.236	0.019	3.02E-78
APOL2	2.10E-82	-0.24936	0.204	0.062	3.14E-78
C18orf8	2.43E-82	0.166439	0.254	0.021	3.62E-78
POLG	4.21E-82	0.196282	0.238	0.012	6.28E-78
EP300	4.50E-82	0.122172	0.206	0.015	6.73E-78
TMEM104	4.74E-82	0.105982	0.199	0.015	7.08E-78
NAA30	4.78E-82	0.163944	0.227	0.014	7.15E-78
TRAPPC11	5.11E-82	0.159384	0.223	0.014	7.63E-78
HEY2	5.22E-82	0.256658	0.269	0.014	7.80E-78
RP11-977G	5.28E-82	0.237299	0.25	0.01	7.89E-78
TMTC4	7.46E-82	0.10084	0.22	0.026	1.11E-77
FKBP7	7.96E-82	-0.18865	0.144	0.039	1.19E-77
TTLL4	9.35E-82	0.106101	0.219	0.024	1.40E-77
SCAMP5	9.77E-82	0.208151	0.248	0.014	1.46E-77
FBXO28	1.04E-81	0.159747	0.217	0.014	1.55E-77
PARP4	1.04E-81	0.154848	0.205	0.01	1.55E-77
PDSS2	1.29E-81	0.117222	0.223	0.021	1.92E-77
COG8	1.75E-81	0.145715	0.234	0.024	2.62E-77
HECTD4	2.01E-81	0.111828	0.208	0.022	3.00E-77
ZNF708	2.43E-81	0.155723	0.241	0.024	3.64E-77
XPNPEP3	2.53E-81	0.124688	0.227	0.022	3.78E-77
SHC4	2.75E-81	0.20481	0.24	0.014	4.11E-77
FAM135A	2.80E-81	0.103051	0.207	0.021	4.18E-77
RP11-400F	3.27E-81	0.284203	0.27	0.007	4.88E-77
FBN2	3.37E-81	0.224719	0.245	0.012	5.04E-77
CD302	3.66E-81	0.155652	0.219	0.014	5.47E-77
IRGQ	3.74E-81	0.179479	0.241	0.017	5.60E-77
YTHDF3-AS	4.33E-81	0.182598	0.228	0.01	6.47E-77
MT1G	4.55E-81	-0.84138	0.122	0.126	6.79E-77
MAFG-AS1	4.58E-81	0.324729	0.294	0.007	6.84E-77
WDR75	4.97E-81	0.109446	0.235	0.029	7.43E-77
PLD2	5.50E-81	0.122524	0.191	0.01	8.22E-77
ZNF606	6.19E-81	0.145653	0.241	0.024	9.24E-77
ZNF431	6.22E-81	-0.17023	0.144	0.05	9.30E-77
RGAG4	6.81E-81	0.274529	0.267	0.009	1.02E-76
THBS3	7.60E-81	0.15116	0.217	0.015	1.14E-76
AP4S1	8.01E-81	-0.15835	0.118	0.024	1.20E-76
PLAUR	8.81E-81	-0.43349	0.135	0.068	1.32E-76

CAMSAP1	1.02E-80	0.101358	0.176	0.01	1.53E-76
ZNF668	1.12E-80	0.203017	0.228	0.01	1.68E-76
LZTFL1	1.13E-80	-0.2066	0.153	0.051	1.68E-76
PLCE1	1.17E-80	0.104299	0.216	0.022	1.75E-76
SETD6	1.21E-80	0.146993	0.233	0.022	1.81E-76
MAPK9	1.79E-80	0.14687	0.209	0.014	2.67E-76
ZNF211	2.09E-80	-0.1582	0.18	0.039	3.13E-76
STYX	2.71E-80	0.152326	0.212	0.01	4.05E-76
UBE2E2	2.82E-80	-0.18498	0.178	0.055	4.22E-76
TBC1D25	3.05E-80	0.116115	0.201	0.017	4.56E-76
KDELR3	3.90E-80	0.250178	0.262	0.012	5.83E-76
RP11-849I	3.97E-80	0.206774	0.285	0.032	5.94E-76
HSD17B1	4.56E-80	0.267175	0.254	0.005	6.82E-76
CPEB2	4.75E-80	0.117677	0.204	0.019	7.10E-76
CDK19	6.19E-80	0.130999	0.222	0.021	9.25E-76
SLC16A4	6.55E-80	0.200524	0.239	0.014	9.78E-76
TAB1	6.86E-80	0.14132	0.244	0.029	1.02E-75
B3GNT5	7.40E-80	0.235427	0.251	0.01	1.11E-75
TARBP1	7.71E-80	0.139103	0.217	0.017	1.15E-75
ZNRF3	8.54E-80	0.213513	0.228	0.009	1.28E-75
THEM6	9.59E-80	0.164488	0.229	0.015	1.43E-75
HYI	9.75E-80	0.139177	0.238	0.026	1.46E-75
CSK	1.06E-79	0.1678	0.204	0.007	1.58E-75
DHX57	1.19E-79	0.125465	0.217	0.024	1.78E-75
POLR1A	1.26E-79	0.115232	0.189	0.012	1.88E-75
EFNB3	1.35E-79	0.183956	0.222	0.009	2.01E-75
KLHDC9	1.37E-79	0.148593	0.228	0.019	2.04E-75
CTH	1.45E-79	-0.19904	0.156	0.043	2.16E-75
PTPN23	1.45E-79	0.147427	0.208	0.01	2.17E-75
DYRK1A	1.65E-79	0.143942	0.21	0.014	2.47E-75
ZNF33A	2.09E-79	0.11151	0.197	0.015	3.12E-75
FAM171B	2.43E-79	0.11327	0.232	0.027	3.63E-75
PDE4A	2.57E-79	0.126016	0.209	0.017	3.83E-75
RPTOR	2.79E-79	0.114981	0.19	0.015	4.18E-75
MARK4	2.95E-79	0.14563	0.216	0.017	4.41E-75
TMEM176B	2.99E-79	-0.53026	0.171	0.101	4.47E-75
ASPHD2	3.07E-79	0.250872	0.255	0.007	4.59E-75
MORC3	3.61E-79	0.148891	0.201	0.014	5.39E-75
NPB	3.95E-79	0.233613	0.236	0.007	5.90E-75
ZDHHC16	4.84E-79	0.328156	0.291	0.005	7.24E-75
SLC11A1	5.15E-79	-0.21857	0.12	0.043	7.70E-75
NAGPA	5.50E-79	0.256213	0.266	0.012	8.22E-75
USP42	6.56E-79	0.207112	0.241	0.014	9.80E-75
CTC-338M1	7.94E-79	-0.13887	0.172	0.039	1.19E-74
SLC43A3	8.53E-79	-0.14407	0.157	0.036	1.27E-74
NOVA2	8.73E-79	0.129601	0.202	0.015	1.30E-74
MYO5A	9.73E-79	0.313615	0.289	0.009	1.45E-74
MTRNR2L1C	1.03E-78	-1.19501	0.024	0.209	1.53E-74
TRAF2	1.13E-78	0.135823	0.203	0.012	1.69E-74
HSF2	1.18E-78	-0.16764	0.15	0.038	1.76E-74
ANGPTL2	1.22E-78	-0.28422	0.183	0.084	1.82E-74

DDHD2	1.30E-78	0.175922	0.238	0.021	1.94E-74
ARFGEF2	1.31E-78	0.192257	0.221	0.007	1.96E-74
RNF103	1.34E-78	0.12596	0.211	0.021	2.00E-74
AMOTL1	1.41E-78	0.225261	0.24	0.009	2.11E-74
RELB	1.44E-78	0.323001	0.273	0.002	2.15E-74
CHRNA9	1.45E-78	0.194594	0.247	0.019	2.17E-74
FAM76A	1.47E-78	0.178052	0.246	0.022	2.20E-74
SLC2A13	1.60E-78	0.258964	0.272	0.019	2.40E-74
PRKAR1B	1.66E-78	0.137877	0.238	0.022	2.48E-74
NOP14	1.67E-78	0.222921	0.239	0.01	2.49E-74
6-Sep	1.92E-78	0.283176	0.261	0.005	2.87E-74
TRIM22	2.04E-78	-0.15617	0.185	0.053	3.05E-74
MED13L	2.11E-78	0.139749	0.226	0.024	3.15E-74
ITGA5	2.23E-78	-0.13376	0.146	0.034	3.33E-74
GTF3C4	2.25E-78	0.166442	0.21	0.012	3.36E-74
RC3H2	2.67E-78	0.279931	0.257	0.005	3.98E-74
PTPN4	2.76E-78	0.175917	0.236	0.019	4.12E-74
DHRS12	3.27E-78	0.183387	0.222	0.012	4.89E-74
ANTXR1	4.56E-78	0.154572	0.222	0.017	6.81E-74
TJAP1	4.89E-78	0.105396	0.202	0.015	7.31E-74
TSPYL2	5.20E-78	-0.15699	0.14	0.029	7.78E-74
SERAC1	6.45E-78	-0.27851	0.125	0.027	9.64E-74
CENPQ	6.52E-78	0.126058	0.215	0.017	9.74E-74
SPATA2	7.34E-78	0.124864	0.193	0.007	1.10E-73
KCNJ16	7.49E-78	0.20037	0.244	0.019	1.12E-73
INSM1	7.74E-78	0.246417	0.248	0.012	1.16E-73
TRIM7	8.15E-78	0.204134	0.223	0.005	1.22E-73
NME2	8.86E-78	0.2373	0.241	0.007	1.32E-73
RFX1	9.06E-78	0.245894	0.261	0.012	1.35E-73
FRMD5	1.05E-77	0.312394	0.273	0.003	1.57E-73
MFAP3L	1.13E-77	0.165354	0.213	0.01	1.69E-73
MINPP1	1.21E-77	0.147306	0.24	0.026	1.80E-73
THOC5	1.23E-77	0.133723	0.201	0.017	1.84E-73
VAV3	1.33E-77	0.195328	0.256	0.026	1.99E-73
CBWD5	1.45E-77	0.106058	0.223	0.026	2.17E-73
BOK	1.82E-77	0.376866	0.3	0	2.71E-73
APOC1	2.16E-77	-0.57892	0.168	0.128	3.23E-73
POT1	2.41E-77	-0.14565	0.153	0.041	3.61E-73
PHACTR2	2.79E-77	0.361484	0.292	0.002	4.17E-73
ZZEF1	2.99E-77	0.220236	0.239	0.012	4.47E-73
CD2AP	3.25E-77	0.178405	0.223	0.012	4.86E-73
UTP14A	3.34E-77	0.151812	0.213	0.014	5.00E-73
SMG5	3.55E-77	0.115188	0.219	0.022	5.31E-73
ARC	3.63E-77	-0.24203	0.314	0.14	5.42E-73
SS18L1	3.96E-77	0.288051	0.263	0.007	5.92E-73
FAM195B	4.66E-77	-0.93474	0.083	0.147	6.96E-73
TTBK2	4.90E-77	0.120489	0.198	0.017	7.32E-73
ANGPTL1	5.05E-77	0.13676	0.198	0.012	7.54E-73
BRINP2	5.07E-77	0.346094	0.28	0.01	7.58E-73
ADAM15	6.84E-77	0.116364	0.215	0.022	1.02E-72
CBX8	7.96E-77	0.155583	0.217	0.017	1.19E-72

HOXD-AS2	1.09E-76	0.152571	0.191	0.007	1.63E-72
CHST14	1.10E-76	0.334212	0.291	0.007	1.64E-72
ZMYM3	1.33E-76	0.186237	0.228	0.015	1.99E-72
RDH10	1.40E-76	0.204864	0.266	0.029	2.09E-72
SHC3	1.44E-76	0.15347	0.214	0.017	2.15E-72
GKAP1	1.46E-76	0.127292	0.21	0.019	2.19E-72
MIR4458HG	1.50E-76	-0.57852	0.196	0.183	2.24E-72
ALKBH1	1.58E-76	-0.13317	0.125	0.027	2.36E-72
CDK17	1.74E-76	0.214625	0.261	0.024	2.61E-72
AEBP2	2.02E-76	0.237962	0.254	0.014	3.01E-72
PCSK5	2.14E-76	0.111038	0.22	0.026	3.20E-72
OSGEPL1	2.27E-76	-0.1073	0.135	0.031	3.40E-72
PIGZ	2.44E-76	0.243343	0.238	0.005	3.65E-72
FGF2	2.45E-76	0.141036	0.2	0.01	3.65E-72
UBE4B	3.07E-76	0.154671	0.236	0.022	4.59E-72
ZNF25	3.18E-76	0.151587	0.225	0.019	4.75E-72
RP11-81A2	3.44E-76	0.223366	0.23	0.007	5.13E-72
SEC61A2	3.72E-76	0.140458	0.215	0.014	5.56E-72
RNF38	3.81E-76	0.148595	0.201	0.01	5.69E-72
TSC22D2	3.87E-76	0.100841	0.201	0.021	5.79E-72
OSGIN2	4.09E-76	0.154678	0.206	0.012	6.11E-72
ATXN7	4.37E-76	0.188572	0.247	0.019	6.53E-72
SCMH1	4.37E-76	0.144571	0.213	0.015	6.53E-72
PLK2	4.65E-76	-0.10729	0.157	0.032	6.95E-72
MLYCD	4.81E-76	0.124589	0.189	0.012	7.19E-72
IPO8	6.38E-76	0.133503	0.21	0.019	9.53E-72
MSTO1	7.24E-76	0.209691	0.24	0.014	1.08E-71
FASTKD3	7.88E-76	0.166386	0.217	0.014	1.18E-71
ZNF558	8.08E-76	0.241415	0.253	0.014	1.21E-71
SLC41A1	1.15E-75	0.112975	0.21	0.021	1.73E-71
SLC25A44	1.39E-75	0.201394	0.223	0.009	2.07E-71
UBE2C	1.39E-75	0.15456	0.2	0.01	2.08E-71
VPS39	2.26E-75	0.151845	0.203	0.014	3.38E-71
PAQR3	2.83E-75	0.220495	0.233	0.01	4.23E-71
LARP4B	3.63E-75	0.193966	0.224	0.009	5.43E-71
ARID2	3.79E-75	0.105651	0.225	0.029	5.66E-71
ZNF787	4.74E-75	0.305105	0.272	0.003	7.09E-71
VAX2	5.35E-75	0.12953	0.187	0.01	8.00E-71
DNAJC3-AS	6.35E-75	-0.11676	0.13	0.032	9.48E-71
ZBTB25	6.46E-75	0.184439	0.221	0.012	9.66E-71
RALGDS	6.58E-75	0.147764	0.242	0.027	9.83E-71
COA7	6.85E-75	-0.20553	0.132	0.036	1.02E-70
TTC26	6.95E-75	-0.19294	0.103	0.029	1.04E-70
COPZ2	7.25E-75	0.146723	0.193	0.01	1.08E-70
PIGG	7.84E-75	0.18913	0.225	0.014	1.17E-70
SOS1	8.27E-75	0.142047	0.214	0.019	1.24E-70
PXMP4	8.39E-75	0.121175	0.181	0.01	1.25E-70
BCL3	9.12E-75	0.154731	0.2	0.01	1.36E-70
LRRTM3	9.16E-75	0.308035	0.258	0.005	1.37E-70
NID1	9.69E-75	0.26453	0.254	0.007	1.45E-70
THAP6	1.05E-74	0.116208	0.198	0.015	1.57E-70

F12	1.08E-74	0.347734	0.288	0.009	1.61E-70
SOS2	1.34E-74	0.249776	0.247	0.01	2.01E-70
STAT5B	1.43E-74	0.100128	0.193	0.017	2.13E-70
PRMT7	1.51E-74	0.105092	0.22	0.027	2.26E-70
DDX28	1.73E-74	0.110769	0.198	0.019	2.59E-70
TLN2	2.94E-74	0.129668	0.207	0.017	4.40E-70
ERAP2	3.33E-74	0.145337	0.244	0.031	4.97E-70
ZNF598	3.39E-74	0.20579	0.21	0.005	5.06E-70
FAM193A	3.45E-74	0.155887	0.219	0.017	5.16E-70
SPHK1	4.60E-74	0.363087	0.283	0.005	6.87E-70
SLC16A2	4.60E-74	0.191604	0.218	0.01	6.88E-70
KAZN	4.67E-74	0.117236	0.211	0.019	6.98E-70
PLEKHH2	4.88E-74	0.233729	0.25	0.017	7.29E-70
XAF1	5.20E-74	-0.26961	0.241	0.097	7.77E-70
ZMYND19	6.96E-74	0.220907	0.223	0.003	1.04E-69
HIST2H2AC	6.96E-74	0.142498	0.179	0.005	1.04E-69
C9orf3	8.13E-74	-0.3712	0.135	0.07	1.22E-69
NR2E1	8.75E-74	0.257181	0.242	0.007	1.31E-69
ZNF629	9.14E-74	0.239357	0.243	0.01	1.37E-69
RRM2B	9.23E-74	0.10378	0.197	0.022	1.38E-69
TRAPPC2	1.05E-73	0.124368	0.212	0.022	1.58E-69
TOX	1.16E-73	0.21741	0.238	0.012	1.74E-69
AGO2	1.22E-73	0.114594	0.181	0.01	1.82E-69
ITGA6	1.23E-73	0.143606	0.19	0.009	1.84E-69
LRTOMT	1.38E-73	0.116545	0.203	0.015	2.07E-69
DHRS3	1.45E-73	-0.2586	0.16	0.055	2.17E-69
TLE2	1.51E-73	0.200713	0.261	0.027	2.26E-69
JMJD4	1.52E-73	0.104118	0.184	0.015	2.28E-69
RCOR1	1.53E-73	0.144516	0.2	0.014	2.29E-69
LRRC45	1.72E-73	0.283621	0.269	0.009	2.57E-69
PIGO	2.21E-73	0.122789	0.206	0.017	3.30E-69
PPP3CB	2.22E-73	0.132685	0.211	0.021	3.32E-69
FBXO45	2.29E-73	0.160142	0.191	0.007	3.42E-69
SNHG12	2.43E-73	-0.26202	0.123	0.044	3.63E-69
FLRT3	2.47E-73	0.121445	0.223	0.026	3.69E-69
RNFT2	2.78E-73	0.176224	0.191	0.005	4.15E-69
YPEL1	3.12E-73	0.29137	0.266	0.009	4.66E-69
KIAA1958	3.38E-73	0.177811	0.229	0.019	5.05E-69
MAML1	3.51E-73	0.171053	0.206	0.01	5.24E-69
RAB11FIP2	3.57E-73	0.14773	0.181	0.007	5.33E-69
WDFY3	3.57E-73	0.157276	0.204	0.015	5.34E-69
CXorf23	4.38E-73	0.121037	0.181	0.01	6.54E-69
SRSF12	4.67E-73	0.173585	0.209	0.01	6.98E-69
PRUNE2	5.24E-73	-0.26483	0.187	0.08	7.82E-69
MGST1	6.58E-73	-1.34737	0.041	0.188	9.84E-69
CTHRC1	7.54E-73	0.432003	0.29	0.003	1.13E-68
PDLIM3	8.11E-73	-0.37488	0.185	0.103	1.21E-68
SLC30A1	8.20E-73	0.239561	0.236	0.005	1.22E-68
DUSP5	9.17E-73	0.146927	0.198	0.012	1.37E-68
IFT80	9.63E-73	0.142351	0.21	0.022	1.44E-68
CLOCK	9.66E-73	0.206364	0.25	0.024	1.44E-68

NAT10	1.02E-72	0.170401	0.216	0.017	1.53E-68
EHD1	1.04E-72	0.184084	0.234	0.019	1.55E-68
ALDOC	1.17E-72	-0.46511	0.276	0.183	1.75E-68
MMP15	1.31E-72	0.253563	0.237	0.005	1.96E-68
VCPIP1	1.61E-72	0.27075	0.251	0.007	2.40E-68
SGTB	1.62E-72	0.181746	0.222	0.015	2.42E-68
FAM120B	2.01E-72	0.139836	0.203	0.012	3.00E-68
SLC25A32	2.14E-72	0.12652	0.197	0.015	3.19E-68
HMOX1	2.37E-72	-0.38868	0.13	0.051	3.55E-68
MNS1	2.64E-72	0.164255	0.209	0.014	3.94E-68
WWP1	2.68E-72	0.175498	0.206	0.01	4.00E-68
TRIM5	3.15E-72	0.100704	0.166	0.01	4.71E-68
ALAD	3.16E-72	0.119901	0.198	0.014	4.72E-68
ARHGAP17	3.27E-72	0.187636	0.204	0.007	4.88E-68
LRRN2	3.29E-72	0.260064	0.244	0.009	4.92E-68
PRDM4	3.42E-72	0.161502	0.206	0.012	5.11E-68
NAAA	4.11E-72	0.216396	0.219	0.005	6.14E-68
OSBPL10	4.22E-72	0.228662	0.216	0.003	6.30E-68
TAF1C	4.58E-72	0.15489	0.212	0.015	6.84E-68
PIK3CA	4.58E-72	0.139211	0.207	0.019	6.84E-68
SYBU	4.68E-72	0.224577	0.258	0.024	6.99E-68
CCDC57	6.17E-72	0.121664	0.175	0.009	9.23E-68
C5orf63	6.23E-72	-0.10528	0.147	0.032	9.31E-68
LPCAT4	6.25E-72	0.209356	0.218	0.007	9.33E-68
KLF16	6.96E-72	0.144971	0.187	0.01	1.04E-67
KAT2B	7.44E-72	0.115958	0.202	0.015	1.11E-67
CMTR1	7.68E-72	0.102584	0.188	0.017	1.15E-67
ZNF445	8.97E-72	-0.13145	0.125	0.036	1.34E-67
ATXN7L3	1.01E-71	0.143609	0.181	0.009	1.51E-67
ZC3H4	1.03E-71	0.232842	0.252	0.019	1.55E-67
DNAJC16	1.10E-71	0.206622	0.236	0.012	1.64E-67
KIAA1328	1.14E-71	-0.12484	0.128	0.032	1.70E-67
POFUT2	1.19E-71	0.137607	0.2	0.015	1.77E-67
AP3M2	1.23E-71	0.170776	0.217	0.01	1.84E-67
TADA2A	1.31E-71	0.169878	0.207	0.01	1.96E-67
BMPR1A	1.35E-71	0.119341	0.204	0.019	2.01E-67
RGL3	1.89E-71	0.139059	0.194	0.012	2.82E-67
TTLL3	1.92E-71	0.124751	0.195	0.015	2.87E-67
ZNF775	1.94E-71	0.270136	0.247	0.007	2.90E-67
NMRK1	2.15E-71	-0.12984	0.148	0.034	3.21E-67
DONSON	2.17E-71	0.211244	0.214	0.005	3.24E-67
CERK	2.39E-71	0.222602	0.229	0.007	3.57E-67
C3	2.72E-71	-1.03845	0.077	0.168	4.07E-67
VPS18	2.75E-71	0.138038	0.197	0.012	4.11E-67
THBS4	2.97E-71	0.176394	0.219	0.015	4.44E-67
GTPBP1	3.00E-71	0.150462	0.2	0.015	4.48E-67
AHCTF1	3.03E-71	0.138055	0.211	0.019	4.53E-67
SMIM13	3.20E-71	0.233955	0.236	0.01	4.78E-67
GID4	3.21E-71	0.108887	0.169	0.009	4.80E-67
UNC119B	3.33E-71	0.158962	0.212	0.014	4.97E-67
NCBP1	3.55E-71	0.179487	0.202	0.007	5.30E-67

ZBTB45	4.03E-71	0.111137	0.182	0.012	6.02E-67
NCS1	4.84E-71	0.130382	0.18	0.01	7.23E-67
ZNF234	4.92E-71	0.158487	0.196	0.01	7.35E-67
ABCA2	5.26E-71	0.211123	0.245	0.017	7.85E-67
RAP2B	5.97E-71	0.231796	0.236	0.01	8.92E-67
PTPRN2	6.34E-71	-0.44371	0.163	0.092	9.48E-67
SSH1	6.35E-71	0.132476	0.187	0.012	9.49E-67
MSANTD2	7.87E-71	0.108844	0.178	0.014	1.18E-66
PGBD1	8.24E-71	0.158192	0.196	0.01	1.23E-66
RPS6KA4	8.52E-71	0.242129	0.236	0.009	1.27E-66
CARD16	9.12E-71	-0.39844	0.225	0.116	1.36E-66
COX18	1.18E-70	0.108024	0.195	0.019	1.77E-66
ALS2	1.20E-70	0.134761	0.178	0.009	1.80E-66
MTMR3	1.25E-70	0.136644	0.197	0.014	1.87E-66
GORASP1	1.35E-70	0.122442	0.215	0.026	2.01E-66
ZNF320	1.57E-70	0.135115	0.173	0.007	2.35E-66
PPP1R12C	1.60E-70	0.231281	0.238	0.012	2.39E-66
MAN2A1	2.07E-70	0.193777	0.213	0.01	3.09E-66
EPSTI1	2.23E-70	-0.49239	0.095	0.056	3.33E-66
FKTN	2.39E-70	0.161172	0.197	0.01	3.58E-66
FAM111A	2.44E-70	0.100888	0.185	0.017	3.64E-66
PIP4K2A	2.60E-70	0.153265	0.207	0.015	3.89E-66
ETV2	2.80E-70	0.113935	0.165	0.005	4.19E-66
NEK11	3.43E-70	-0.10096	0.116	0.026	5.13E-66
PCDH8	3.77E-70	0.104974	0.181	0.014	5.63E-66
SLPI	4.17E-70	-1.67134	0.033	0.209	6.23E-66
SENP7	4.17E-70	0.143323	0.207	0.019	6.24E-66
C14orf37	4.24E-70	0.225171	0.227	0.01	6.33E-66
C14orf79	4.34E-70	0.119396	0.173	0.01	6.49E-66
ITGA2	5.33E-70	0.358355	0.267	0.007	7.96E-66
TARSL2	5.38E-70	0.215076	0.254	0.026	8.04E-66
PRKCZ	5.67E-70	0.136716	0.197	0.015	8.47E-66
RNF44	6.14E-70	0.150936	0.198	0.014	9.18E-66
CCNYL1	6.17E-70	0.11276	0.166	0.012	9.22E-66
SHB	7.17E-70	0.213374	0.22	0.009	1.07E-65
RGS2	7.80E-70	-0.86399	0.103	0.13	1.17E-65
SIX5	8.12E-70	0.13966	0.182	0.007	1.21E-65
SGSH	8.97E-70	0.164341	0.203	0.012	1.34E-65
DIXDC1	9.18E-70	0.224636	0.22	0.007	1.37E-65
MVB12B	9.93E-70	0.102102	0.178	0.014	1.48E-65
BMP1	1.05E-69	0.124802	0.18	0.012	1.57E-65
RP11-119E	1.23E-69	0.13339	0.191	0.014	1.84E-65
ADNP2	1.24E-69	0.131015	0.182	0.01	1.85E-65
C12orf4	1.38E-69	0.111069	0.201	0.021	2.06E-65
RAB6B	1.62E-69	0.128265	0.181	0.012	2.42E-65
RAB30	1.68E-69	0.181879	0.209	0.012	2.52E-65
AFAP1	1.71E-69	0.19664	0.216	0.012	2.55E-65
SLC25A20	2.04E-69	-0.14976	0.118	0.027	3.06E-65
PHRF1	2.20E-69	0.212714	0.22	0.009	3.29E-65
MORC4	2.78E-69	0.178413	0.219	0.014	4.15E-65
UTP20	2.81E-69	0.182759	0.203	0.009	4.21E-65

RBAK-RBAK	2.94E-69	-0.15754	0.156	0.046	4.39E-65
GABBR1	2.98E-69	0.122709	0.203	0.021	4.45E-65
RFX3	3.19E-69	0.22199	0.225	0.012	4.77E-65
ETAA1	3.50E-69	0.160535	0.191	0.009	5.23E-65
TGFB2	3.86E-69	0.103487	0.207	0.026	5.76E-65
PLP1	3.91E-69	-0.16314	0.209	0.058	5.85E-65
TBC1D24	4.07E-69	0.190123	0.196	0.005	6.08E-65
PTPRG	4.51E-69	0.155594	0.217	0.019	6.73E-65
ZNF35	4.76E-69	0.100043	0.157	0.009	7.11E-65
ABLIM1	5.20E-69	0.116762	0.197	0.019	7.76E-65
TRIM23	6.56E-69	0.108483	0.178	0.017	9.80E-65
PCBD2	7.25E-69	0.198241	0.214	0.01	1.08E-64
DICER1-AS	7.29E-69	-0.14802	0.12	0.027	1.09E-64
KMT2E-AS1	7.75E-69	-0.16691	0.156	0.039	1.16E-64
ATXN8OS	8.36E-69	-1.29445	0.008	0.215	1.25E-64
PTBP3	1.20E-68	0.147618	0.197	0.014	1.79E-64
ICAM1	1.27E-68	-0.11105	0.189	0.046	1.90E-64
EID2B	1.34E-68	0.120468	0.217	0.024	2.00E-64
ZNF865	1.36E-68	0.220071	0.227	0.01	2.03E-64
LIG3	1.47E-68	0.124302	0.192	0.017	2.19E-64
KHK	1.50E-68	0.15783	0.207	0.015	2.25E-64
CAV1	1.54E-68	-1.4076	0.048	0.212	2.30E-64
PDCD4	1.60E-68	-0.13865	0.141	0.038	2.39E-64
SYTL2	1.77E-68	-0.29998	0.119	0.058	2.65E-64
TRIM11	1.79E-68	0.114337	0.183	0.015	2.67E-64
STRN	1.79E-68	0.140466	0.172	0.007	2.68E-64
PRMT3	1.82E-68	0.163598	0.217	0.019	2.72E-64
TOPBP1	1.89E-68	0.19238	0.219	0.012	2.82E-64
C1QTNF6	2.16E-68	0.172617	0.186	0.007	3.23E-64
BCL9	2.17E-68	0.135641	0.178	0.01	3.25E-64
TSTD3	2.29E-68	0.12987	0.178	0.009	3.43E-64
MAD2L1	2.38E-68	-0.14105	0.115	0.027	3.55E-64
IFFO1	2.55E-68	0.200661	0.236	0.022	3.81E-64
NPW	2.58E-68	0.148332	0.184	0.009	3.85E-64
ENOX2	3.44E-68	0.118523	0.175	0.01	5.15E-64
TMEM64	3.45E-68	0.165799	0.208	0.017	5.16E-64
AUH	3.83E-68	0.109607	0.173	0.014	5.72E-64
HAS2	4.18E-68	0.10132	0.193	0.017	6.24E-64
MAPK8	4.41E-68	0.214105	0.214	0.005	6.58E-64
SESN2	4.43E-68	-0.11233	0.137	0.036	6.61E-64
EXOC6B	5.13E-68	0.104918	0.163	0.01	7.67E-64
SPICE1	5.17E-68	-0.19937	0.111	0.027	7.73E-64
TRIM52	5.59E-68	0.115172	0.187	0.015	8.36E-64
RILP	5.61E-68	0.254917	0.234	0.005	8.38E-64
SLC29A4	6.11E-68	0.121578	0.19	0.015	9.13E-64
TBC1D13	6.60E-68	0.141864	0.181	0.009	9.86E-64
CHDH	9.59E-68	0.113439	0.209	0.024	1.43E-63
TRRAP	9.88E-68	0.170694	0.2	0.012	1.48E-63
NUP133	9.91E-68	0.148925	0.217	0.022	1.48E-63
PATZ1	1.04E-67	0.22798	0.229	0.01	1.55E-63
NDST1	1.15E-67	0.273938	0.248	0.005	1.72E-63

TYROBP	1.30E-67	-0.47638	0.137	0.092	1.94E-63
C17orf51	1.59E-67	0.187091	0.201	0.01	2.37E-63
ADAM12	1.71E-67	0.219332	0.216	0.01	2.55E-63
RIPK1	2.17E-67	0.10583	0.178	0.017	3.24E-63
DUS4L	2.18E-67	0.100025	0.178	0.015	3.26E-63
SLX4IP	2.51E-67	0.158202	0.188	0.01	3.76E-63
ACO1	2.54E-67	0.104394	0.196	0.019	3.80E-63
KIF13A	2.64E-67	0.124718	0.168	0.009	3.94E-63
GRIA1	2.97E-67	0.268735	0.239	0.01	4.43E-63
ALDH5A1	3.04E-67	0.214959	0.218	0.009	4.54E-63
KCTD1	3.10E-67	0.141873	0.19	0.014	4.64E-63
FAM206A	3.12E-67	0.126934	0.188	0.01	4.67E-63
CD200	3.41E-67	0.123569	0.181	0.012	5.09E-63
PCDHB2	3.48E-67	0.271369	0.248	0.012	5.20E-63
LTBP1	3.50E-67	0.276097	0.234	0.007	5.22E-63
USP13	3.64E-67	0.160878	0.203	0.017	5.44E-63
AC159540.	3.74E-67	0.115158	0.221	0.032	5.58E-63
ZNF703	3.91E-67	0.186807	0.209	0.012	5.84E-63
SMO	4.36E-67	0.174821	0.19	0.007	6.51E-63
TUBD1	4.40E-67	0.123575	0.204	0.022	6.57E-63
LINC00339	4.65E-67	-0.11194	0.122	0.024	6.94E-63
HERC1	6.50E-67	0.138524	0.193	0.012	9.71E-63
SNAP25	7.18E-67	-0.60412	0.095	0.101	1.07E-62
HEATR6	7.71E-67	0.130724	0.182	0.01	1.15E-62
ABHD10	8.38E-67	0.112907	0.175	0.014	1.25E-62
SRF	1.21E-66	0.167511	0.2	0.012	1.81E-62
LRRC24	1.24E-66	0.10371	0.162	0.01	1.86E-62
C3orf58	1.44E-66	0.184666	0.213	0.015	2.15E-62
CBX2	1.55E-66	0.123548	0.18	0.012	2.32E-62
FAM173B	1.68E-66	0.129764	0.18	0.012	2.52E-62
ANO10	1.73E-66	0.104961	0.162	0.009	2.58E-62
DOCK10	2.07E-66	0.2227	0.22	0.01	3.10E-62
NCKIPSD	2.64E-66	0.169791	0.194	0.007	3.94E-62
TMEM37	2.73E-66	-0.15035	0.08	0.022	4.07E-62
E2F3	3.26E-66	0.2278	0.22	0.007	4.86E-62
CENPK	3.61E-66	0.145772	0.18	0.01	5.39E-62
SREBF1	4.02E-66	0.155003	0.204	0.019	6.00E-62
RAI1	4.05E-66	0.234075	0.214	0.003	6.05E-62
AP006621.	4.05E-66	0.13895	0.182	0.014	6.05E-62
CREB3L2	4.66E-66	0.147757	0.205	0.019	6.97E-62
KLF12	4.84E-66	0.152193	0.201	0.021	7.24E-62
DDB2	5.18E-66	-0.18392	0.134	0.039	7.73E-62
HLA-DPB1	6.41E-66	-0.74492	0.149	0.162	9.59E-62
HBA2	7.02E-66	-0.86296	0.131	0.097	1.05E-61
PDZD8	7.54E-66	0.109098	0.189	0.019	1.13E-61
FAM86C1	7.75E-66	0.104292	0.158	0.009	1.16E-61
HARS2	8.04E-66	0.138134	0.19	0.014	1.20E-61
ACVR2A	8.20E-66	0.210574	0.204	0.005	1.23E-61
RGS20	8.21E-66	0.160624	0.192	0.012	1.23E-61
PCYOX1L	8.91E-66	0.155604	0.2	0.012	1.33E-61
SARM1	9.25E-66	0.211576	0.212	0.009	1.38E-61

ZSCAN30	1.03E-65	0.162166	0.191	0.009	1.54E-61
SMPDL3A	1.11E-65	0.230895	0.232	0.012	1.66E-61
PPTC7	1.24E-65	0.170728	0.194	0.009	1.86E-61
USP19	1.32E-65	0.131026	0.187	0.014	1.97E-61
ZNF34	1.43E-65	-0.19038	0.11	0.031	2.13E-61
CTD-3065J	1.49E-65	0.157346	0.178	0.005	2.23E-61
TXNDC11	2.01E-65	0.110391	0.18	0.015	3.01E-61
ALDH16A1	2.21E-65	0.179002	0.205	0.014	3.30E-61
MED18	2.75E-65	0.13498	0.173	0.007	4.11E-61
NUDT12	2.84E-65	0.17214	0.205	0.012	4.24E-61
AVEN	3.66E-65	0.14042	0.172	0.005	5.47E-61
UBE2T	4.05E-65	0.14793	0.174	0.007	6.06E-61
FBXO33	5.17E-65	0.10833	0.165	0.01	7.72E-61
SPTLC2	5.26E-65	0.182887	0.199	0.01	7.87E-61
C16orf46	5.29E-65	0.109425	0.171	0.012	7.90E-61
CAD	5.59E-65	0.144184	0.171	0.007	8.35E-61
FBXL6	5.68E-65	0.175396	0.197	0.012	8.49E-61
NEDD1	5.81E-65	0.161892	0.19	0.01	8.68E-61
MIPEP	6.35E-65	0.124194	0.19	0.012	9.50E-61
SH2B2	6.39E-65	0.214836	0.221	0.014	9.55E-61
LINC00672	6.65E-65	-0.13248	0.104	0.021	9.94E-61
TRAPPC10	6.99E-65	0.211092	0.21	0.007	1.04E-60
PLXNA4	7.12E-65	0.152768	0.194	0.015	1.06E-60
RETSAT	7.61E-65	0.233887	0.231	0.009	1.14E-60
GNL3L	8.27E-65	0.101512	0.179	0.017	1.24E-60
CXorf56	8.59E-65	0.141059	0.185	0.01	1.28E-60
IFIT2	8.78E-65	-0.11994	0.152	0.032	1.31E-60
RBM12B	9.47E-65	0.191525	0.206	0.01	1.42E-60
RGMB	9.99E-65	-0.14821	0.151	0.053	1.49E-60
ITPRIPL2	1.19E-64	0.200319	0.198	0.007	1.77E-60
FUT11	1.19E-64	0.190749	0.195	0.007	1.78E-60
DROSHA	1.47E-64	0.155665	0.201	0.014	2.20E-60
KIAA1211L	1.58E-64	0.240476	0.222	0.007	2.37E-60
GYS1	1.65E-64	0.143768	0.187	0.012	2.47E-60
FCER1G	1.87E-64	-0.25993	0.144	0.063	2.79E-60
TTC23	1.93E-64	0.151912	0.187	0.012	2.89E-60
KIFC2	2.04E-64	0.168754	0.201	0.014	3.05E-60
FBLN7	2.16E-64	0.183175	0.194	0.007	3.23E-60
FAM160B2	2.17E-64	0.129677	0.179	0.015	3.24E-60
AGAP3	2.23E-64	0.160262	0.188	0.009	3.34E-60
KLHL2	2.35E-64	0.161538	0.188	0.01	3.51E-60
NIPAL3	2.68E-64	0.165916	0.185	0.009	4.00E-60
RP11-395A	2.73E-64	0.245766	0.22	0.003	4.08E-60
TAPBPL	2.76E-64	-0.11012	0.176	0.041	4.12E-60
MOXD1	2.78E-64	0.30771	0.231	0.005	4.16E-60
PRICKLE2	2.95E-64	0.187587	0.193	0.007	4.40E-60
HAUS7	3.06E-64	-0.13787	0.124	0.034	4.58E-60
ZNF318	4.05E-64	0.120253	0.174	0.01	6.06E-60
EXOSC6	4.85E-64	0.106191	0.168	0.012	7.24E-60
ZC3H3	5.22E-64	0.165294	0.187	0.009	7.80E-60
SEMA3E	5.41E-64	0.271887	0.233	0.014	8.09E-60

RHPN2	5.79E-64	-0.12161	0.129	0.031	8.65E-60
MARK1	5.90E-64	0.266615	0.244	0.012	8.81E-60
KLHL9	5.96E-64	0.226057	0.216	0.009	8.91E-60
TM9SF1	6.01E-64	0.137098	0.179	0.01	8.98E-60
IFIT3	6.26E-64	-0.11675	0.193	0.051	9.36E-60
PINLYP	6.49E-64	-0.21116	0.109	0.036	9.69E-60
LCORL	6.79E-64	0.175676	0.196	0.009	1.01E-59
PARP11	6.87E-64	0.174219	0.194	0.01	1.03E-59
SLC2A6	7.30E-64	0.167503	0.179	0.005	1.09E-59
QPRT	7.35E-64	-0.24175	0.122	0.044	1.10E-59
ACSF2	1.28E-63	0.133907	0.164	0.007	1.92E-59
FAHD2B	1.31E-63	0.15602	0.197	0.014	1.95E-59
LRP5	1.33E-63	0.14587	0.185	0.01	1.98E-59
PRKD1	1.36E-63	0.170982	0.206	0.014	2.03E-59
PPARA	1.37E-63	0.152528	0.176	0.009	2.05E-59
TNFRSF11E	1.40E-63	-0.12702	0.118	0.031	2.08E-59
SLC19A1	1.53E-63	0.139409	0.168	0.007	2.29E-59
PITRM1	1.66E-63	0.11082	0.168	0.01	2.47E-59
EBF4	1.68E-63	0.223129	0.216	0.007	2.51E-59
RIMKLA	1.88E-63	0.193933	0.207	0.01	2.81E-59
RP11-538F	1.88E-63	0.114231	0.188	0.019	2.81E-59
SGK223	2.33E-63	0.292444	0.241	0.003	3.48E-59
ARHGAP5-A	2.37E-63	-0.11495	0.097	0.021	3.54E-59
CENPBD1	2.56E-63	0.104419	0.157	0.01	3.83E-59
XKR4	2.57E-63	-0.12614	0.115	0.036	3.85E-59
SIRT5	3.39E-63	0.107008	0.18	0.019	5.06E-59
CACTIN	3.40E-63	0.231582	0.219	0.005	5.08E-59
ARHGAP20	3.80E-63	0.282409	0.239	0.009	5.68E-59
PIEZO1	3.97E-63	0.170658	0.188	0.009	5.93E-59
CRLF3	4.76E-63	0.167459	0.195	0.012	7.11E-59
GLRB	5.16E-63	0.12071	0.188	0.017	7.71E-59
LOX	5.71E-63	-0.29023	0.081	0.032	8.54E-59
PUS1	5.77E-63	0.117173	0.182	0.015	8.62E-59
ACVR1	6.22E-63	0.157271	0.176	0.007	9.30E-59
MTG1	6.78E-63	0.135115	0.17	0.009	1.01E-58
TMEM87B	7.11E-63	0.109583	0.16	0.01	1.06E-58
LRIG2	8.32E-63	0.145857	0.193	0.014	1.24E-58
HNRNPU-AS	9.49E-63	0.109665	0.19	0.022	1.42E-58
MEX3D	9.64E-63	0.198774	0.196	0.005	1.44E-58
PTGS1	1.30E-62	0.17564	0.201	0.012	1.94E-58
HIP1R	1.55E-62	0.112141	0.212	0.029	2.32E-58
PCDHGA10	1.87E-62	0.199326	0.204	0.007	2.79E-58
PANK4	2.32E-62	0.107238	0.181	0.019	3.47E-58
PREP	2.65E-62	0.156546	0.175	0.007	3.97E-58
C11orf96	3.06E-62	0.101835	0.223	0.034	4.58E-58
PHF13	3.22E-62	0.108103	0.165	0.012	4.82E-58
TRAM2	3.47E-62	0.139887	0.179	0.01	5.19E-58
STK35	3.68E-62	0.146535	0.181	0.007	5.50E-58
DUS2	4.65E-62	0.144164	0.182	0.01	6.95E-58
FAM175A	4.67E-62	0.101582	0.166	0.015	6.98E-58
SELO	4.72E-62	0.174898	0.176	0.003	7.06E-58

1-Mar	4.78E-62	0.180376	0.194	0.009	7.15E-58
CHAF1A	4.95E-62	0.149198	0.198	0.019	7.40E-58
TNFRSF10E	5.20E-62	-0.21755	0.116	0.038	7.78E-58
TGFBR2	5.69E-62	0.16847	0.184	0.007	8.50E-58
GPSM1	5.70E-62	0.146545	0.18	0.01	8.52E-58
FGF14-AS2	5.74E-62	0.14376	0.18	0.01	8.58E-58
UBASH3B	5.76E-62	0.277324	0.236	0.003	8.61E-58
SLC4A8	6.51E-62	0.161716	0.21	0.021	9.73E-58
MIR497HG	8.78E-62	-0.28559	0.131	0.056	1.31E-57
TACC2	1.14E-61	0.101202	0.175	0.015	1.70E-57
ACBD4	1.15E-61	0.15198	0.178	0.01	1.72E-57
LIMK2	1.19E-61	0.170411	0.186	0.007	1.78E-57
LRRN1	1.22E-61	0.17335	0.209	0.015	1.83E-57
TUBGCP3	1.28E-61	0.108599	0.184	0.021	1.91E-57
FIZ1	1.32E-61	0.130191	0.153	0.003	1.98E-57
KLHL25	1.60E-61	0.215938	0.199	0.005	2.39E-57
GEMIN4	1.95E-61	0.123088	0.157	0.007	2.91E-57
GPR75-ASE	2.06E-61	0.206306	0.219	0.015	3.08E-57
USP40	2.26E-61	0.173266	0.191	0.009	3.37E-57
RNF208	2.31E-61	0.192386	0.194	0.007	3.45E-57
GAL3ST3	2.73E-61	0.194163	0.187	0.005	4.08E-57
FAN1	2.77E-61	0.101251	0.166	0.015	4.14E-57
SPSB4	2.79E-61	0.227839	0.214	0.007	4.18E-57
LBH	3.32E-61	-0.14801	0.114	0.039	4.95E-57
ARHGEF18	5.37E-61	0.149795	0.193	0.014	8.02E-57
GPATCH3	5.61E-61	0.175591	0.186	0.007	8.39E-57
TBX2	6.01E-61	0.260541	0.227	0.01	8.98E-57
LRRC3B	6.15E-61	0.264925	0.204	0.003	9.20E-57
ABHD17B	6.28E-61	0.138651	0.19	0.014	9.38E-57
MAFF	6.46E-61	-0.16787	0.136	0.048	9.65E-57
SEC24A	6.79E-61	0.177528	0.195	0.01	1.01E-56
AGO1	6.86E-61	0.237992	0.212	0.005	1.02E-56
DCAF4	9.07E-61	0.120712	0.153	0.005	1.36E-56
FBN1	1.01E-60	0.122371	0.153	0.005	1.51E-56
PDE8B	1.12E-60	0.197622	0.205	0.012	1.67E-56
ZSCAN2	1.13E-60	-0.11469	0.075	0.022	1.70E-56
PHC1	1.14E-60	0.196948	0.209	0.014	1.70E-56
GNG10	1.14E-60	-0.13346	0.089	0.027	1.71E-56
UBR1	1.19E-60	0.224338	0.22	0.012	1.79E-56
2-Mar	1.20E-60	0.125915	0.176	0.012	1.80E-56
GEM	1.52E-60	-0.40423	0.103	0.053	2.27E-56
SLC16A1-A	1.75E-60	-0.2161	0.107	0.041	2.62E-56
MTO1	1.80E-60	-0.10868	0.122	0.029	2.68E-56
ZNF273	1.83E-60	-0.21814	0.115	0.046	2.73E-56
GPR37L1	2.15E-60	-0.15618	0.272	0.121	3.21E-56
HSDL1	2.35E-60	0.202664	0.206	0.009	3.51E-56
TET2	3.35E-60	0.112533	0.169	0.015	5.01E-56
ZSCAN5A	3.78E-60	-0.11874	0.112	0.029	5.64E-56
MCPH1	3.78E-60	0.132673	0.17	0.014	5.65E-56
LMLN	4.76E-60	0.122559	0.162	0.007	7.12E-56
REXO1	5.64E-60	0.223495	0.218	0.009	8.43E-56

STK38	5.85E-60	0.106932	0.159	0.01	8.75E-56
MDC1	6.64E-60	0.12684	0.174	0.014	9.92E-56
CLCF1	6.72E-60	-0.22789	0.085	0.026	1.00E-55
KANSL3	6.94E-60	0.105782	0.156	0.014	1.04E-55
RPS6KL1	7.08E-60	0.111675	0.175	0.012	1.06E-55
VLDLR	7.69E-60	0.102529	0.147	0.009	1.15E-55
NPAS1	8.04E-60	0.145361	0.162	0.005	1.20E-55
EMILIN1	8.50E-60	0.166267	0.186	0.01	1.27E-55
ZNF496	8.84E-60	0.172156	0.187	0.007	1.32E-55
CEP97	8.91E-60	0.105329	0.162	0.012	1.33E-55
RAB2B	1.20E-59	0.122871	0.169	0.014	1.80E-55
PROSER1	1.24E-59	0.192245	0.196	0.009	1.85E-55
PLIN2	1.24E-59	-0.84665	0.086	0.108	1.85E-55
MTA2	1.27E-59	0.172999	0.173	0.003	1.90E-55
PER1	1.29E-59	-0.13501	0.171	0.056	1.93E-55
VSIG10	1.55E-59	0.148925	0.193	0.017	2.31E-55
ALDH18A1	1.61E-59	0.122087	0.17	0.01	2.40E-55
NT5E	1.63E-59	0.119114	0.173	0.014	2.44E-55
DOCK1	2.27E-59	0.132279	0.175	0.014	3.39E-55
EMILIN2	2.35E-59	0.131551	0.162	0.009	3.51E-55
MORN4	2.93E-59	0.139168	0.174	0.01	4.38E-55
ULK2	3.36E-59	0.187562	0.188	0.007	5.02E-55
SLC46A1	3.98E-59	0.125574	0.157	0.007	5.95E-55
S1PR3	4.23E-59	-0.1518	0.116	0.038	6.32E-55
HLA-DMA	4.33E-59	-0.64701	0.104	0.111	6.48E-55
HTATIP2	4.96E-59	0.307285	0.235	0	7.41E-55
ZNF248	5.06E-59	0.118787	0.162	0.014	7.56E-55
B3GNTL1	5.45E-59	0.191416	0.18	0.002	8.14E-55
SLK	5.76E-59	0.135477	0.173	0.012	8.61E-55
CMTM8	6.14E-59	0.291926	0.241	0.002	9.18E-55
MFHAS1	6.18E-59	0.124051	0.165	0.009	9.24E-55
PRSS23	6.71E-59	-0.15733	0.122	0.036	1.00E-54
P2RY1	7.07E-59	0.237454	0.222	0.012	1.06E-54
FBXO34	7.34E-59	0.15114	0.185	0.017	1.10E-54
TAF4	7.53E-59	0.1149	0.143	0.005	1.13E-54
FAM129B	8.03E-59	0.19955	0.199	0.012	1.20E-54
SEL1L3	8.37E-59	0.216571	0.198	0.007	1.25E-54
PSMA2	8.98E-59	-1.291	0.011	0.205	1.34E-54
SLC47A1	9.23E-59	0.116219	0.158	0.012	1.38E-54
FAM69A	1.12E-58	0.218152	0.2	0.005	1.67E-54
CGREF1	1.39E-58	0.105409	0.164	0.012	2.07E-54
CTD-2017D	1.57E-58	0.122995	0.161	0.009	2.35E-54
STON2	1.71E-58	0.173662	0.178	0.007	2.56E-54
KCTD2	1.73E-58	0.194964	0.193	0.005	2.59E-54
HSPA4L	1.92E-58	-0.1378	0.133	0.038	2.86E-54
ZNF557	2.14E-58	-0.13902	0.094	0.031	3.20E-54
CCDC152	2.17E-58	-0.24104	0.097	0.039	3.25E-54
SDCBP2	2.22E-58	-0.12578	0.127	0.031	3.31E-54
ZNF592	2.50E-58	0.131647	0.172	0.014	3.73E-54
FAM13A	2.63E-58	-0.11454	0.126	0.027	3.93E-54
CENPC	2.91E-58	0.118391	0.179	0.021	4.35E-54

UVRAG	4.32E-58	0.134595	0.171	0.014	6.46E-54
TAGLN	4.42E-58	-0.58516	0.077	0.051	6.60E-54
VAV2	4.45E-58	0.175945	0.176	0.005	6.65E-54
ABTB1	4.63E-58	-0.13837	0.11	0.032	6.92E-54
SCARF2	4.86E-58	0.199226	0.193	0.005	7.26E-54
CYB561D1	4.90E-58	-0.1504	0.092	0.022	7.33E-54
SEMA4B	5.32E-58	0.125727	0.172	0.014	7.94E-54
C9orf72	5.93E-58	-0.14812	0.126	0.039	8.87E-54
PDCD11	6.82E-58	0.110012	0.16	0.01	1.02E-53
CD83	7.37E-58	-0.12454	0.125	0.026	1.10E-53
CCDC102A	7.45E-58	0.201127	0.198	0.005	1.11E-53
MANEA	7.91E-58	0.284753	0.231	0.002	1.18E-53
MAST3	1.15E-57	0.121047	0.162	0.009	1.71E-53
GRIK1	1.19E-57	0.202911	0.184	0.007	1.78E-53
CDKN3	1.33E-57	0.105491	0.141	0.005	1.99E-53
C22orf46	1.44E-57	-0.1315	0.097	0.029	2.15E-53
DHX32	1.48E-57	0.255793	0.224	0.005	2.21E-53
VASH1	1.56E-57	0.104753	0.163	0.014	2.34E-53
CCL3	1.83E-57	-0.34969	0.141	0.085	2.74E-53
INTS2	1.99E-57	0.184967	0.184	0.005	2.97E-53
PLCB3	2.07E-57	0.154678	0.187	0.015	3.09E-53
C8orf44	2.30E-57	0.153313	0.183	0.012	3.44E-53
IFI27	2.45E-57	-0.49067	0.189	0.111	3.66E-53
ARHGEF10	2.63E-57	-0.11191	0.115	0.029	3.94E-53
BLOC1S3	2.65E-57	0.137139	0.165	0.009	3.95E-53
OGFRL1	2.67E-57	0.245096	0.214	0.003	3.99E-53
OSBP2	2.85E-57	0.111619	0.153	0.01	4.26E-53
THRB	2.90E-57	0.134843	0.156	0.007	4.33E-53
SMYD5	4.21E-57	0.121118	0.169	0.014	6.29E-53
PSORS1C1	4.46E-57	-0.10417	0.151	0.036	6.67E-53
AMMECR1	4.52E-57	0.17575	0.191	0.009	6.76E-53
MAMLD1	5.83E-57	0.151277	0.169	0.009	8.71E-53
SAMD10	5.95E-57	0.216524	0.197	0.005	8.90E-53
CCDC157	6.06E-57	0.198561	0.184	0.003	9.05E-53
KIF5A	7.41E-57	0.176484	0.203	0.017	1.11E-52
TMEM116	7.55E-57	-0.10621	0.119	0.031	1.13E-52
HLA-DRB5	8.07E-57	-0.84463	0.089	0.125	1.21E-52
NCOA2	8.32E-57	0.107175	0.149	0.01	1.24E-52
CTSS	1.12E-56	-0.18395	0.115	0.036	1.68E-52
FKRP	1.40E-56	0.119462	0.167	0.014	2.09E-52
ARL5B	1.52E-56	0.198224	0.187	0.005	2.27E-52
IGDCC3	1.79E-56	0.280196	0.226	0.003	2.68E-52
NDUFA7	1.84E-56	-1.10372	0.011	0.179	2.74E-52
STOX2	1.93E-56	0.210166	0.194	0.005	2.89E-52
APOM	2.08E-56	-0.13415	0.108	0.032	3.11E-52
TGFBR1	2.09E-56	0.151339	0.173	0.012	3.13E-52
ACAD10	2.27E-56	0.162992	0.188	0.012	3.40E-52
KIAA1549	2.92E-56	0.156616	0.166	0.009	4.36E-52
ATP1B1	3.01E-56	-0.21207	0.14	0.05	4.50E-52
CCDC117	3.07E-56	0.112921	0.139	0.005	4.59E-52
C1RL	3.42E-56	-0.1946	0.094	0.034	5.12E-52

LIN37	3.95E-56	-0.21605	0.084	0.027	5.90E-52
PCDHGC3	4.67E-56	0.171992	0.174	0.005	6.98E-52
OAS1	4.71E-56	-0.34172	0.19	0.101	7.03E-52
HPCA	4.75E-56	-0.12439	0.1	0.029	7.09E-52
TTLL12	5.19E-56	0.194613	0.187	0.007	7.75E-52
IFI44L	6.05E-56	-0.38245	0.201	0.111	9.05E-52
EPHB2	6.16E-56	0.158165	0.173	0.01	9.21E-52
CPT1A	6.24E-56	0.184801	0.191	0.01	9.32E-52
ZNF687	8.03E-56	0.175448	0.178	0.007	1.20E-51
SRGN	9.37E-56	-0.66814	0.099	0.138	1.40E-51
MAP3K7CL	1.06E-55	-0.11769	0.105	0.021	1.59E-51
PPIL6	1.12E-55	-0.10859	0.102	0.024	1.68E-51
FBXO30	1.38E-55	0.168422	0.169	0.003	2.06E-51
NUP153	1.47E-55	0.103011	0.153	0.012	2.20E-51
IL11RA	1.55E-55	0.166297	0.179	0.012	2.32E-51
RP11-195F	1.59E-55	-0.18618	0.127	0.044	2.37E-51
SFT2D3	1.65E-55	0.186985	0.181	0.003	2.46E-51
DYRK2	1.72E-55	0.10332	0.146	0.012	2.56E-51
ANKRD16	1.96E-55	0.254114	0.218	0.002	2.93E-51
FXYD5	2.09E-55	-0.45393	0.096	0.065	3.12E-51
OLFML3	2.09E-55	0.168969	0.182	0.012	3.12E-51
EVI5L	2.63E-55	0.163702	0.169	0.007	3.92E-51
LINC0091C	2.91E-55	0.104573	0.154	0.012	4.35E-51
HIPK3	3.42E-55	0.137649	0.171	0.012	5.11E-51
ADD2	3.52E-55	0.168886	0.175	0.009	5.26E-51
ALDH1L1	3.66E-55	-0.43466	0.159	0.12	5.48E-51
NR1H3	3.89E-55	-0.12038	0.09	0.021	5.82E-51
TTC33	3.91E-55	-0.17329	0.108	0.034	5.85E-51
SIPA1	4.90E-55	0.262479	0.219	0.003	7.32E-51
TXLNB	8.67E-55	0.178776	0.175	0.007	1.30E-50
ZNF853	8.88E-55	0.163309	0.166	0.007	1.33E-50
GBP3	9.21E-55	-0.252	0.152	0.06	1.38E-50
EPHB3	9.34E-55	0.100227	0.142	0.009	1.40E-50
FURIN	1.02E-54	0.109015	0.141	0.009	1.53E-50
NPL	1.14E-54	-0.10164	0.13	0.038	1.70E-50
ZNF548	1.29E-54	-0.1873	0.095	0.034	1.93E-50
HRASLS	1.30E-54	0.117643	0.157	0.009	1.94E-50
ICA1	1.39E-54	0.150328	0.159	0.005	2.08E-50
CDR2L	1.50E-54	0.191157	0.186	0.005	2.24E-50
BAMBI	1.51E-54	0.219366	0.185	0.005	2.25E-50
TOP3B	1.64E-54	0.106455	0.146	0.012	2.44E-50
ELOVL4	1.68E-54	0.158274	0.171	0.009	2.51E-50
LINC-PINT	1.69E-54	-0.13267	0.152	0.051	2.52E-50
CUL9	2.43E-54	0.125867	0.149	0.005	3.63E-50
PAPD5	2.91E-54	0.127746	0.165	0.01	4.36E-50
DSN1	2.96E-54	0.223831	0.194	0.007	4.42E-50
ATCAY	3.21E-54	-0.10459	0.122	0.032	4.79E-50
C14orf93	3.46E-54	-0.12009	0.095	0.024	5.18E-50
PLEKHM1	3.57E-54	0.185318	0.185	0.007	5.34E-50
CTDSPL	3.72E-54	0.105303	0.138	0.007	5.56E-50
PIBF1	3.93E-54	0.139968	0.175	0.017	5.87E-50

C1QTNF3	4.10E-54	-0.1644	0.107	0.029	6.12E-50
LTBP4	4.88E-54	0.103109	0.153	0.015	7.29E-50
SNX29	8.45E-54	0.105167	0.151	0.012	1.26E-49
KIAA1549L	9.06E-54	0.16371	0.166	0.007	1.35E-49
SLC45A1	9.65E-54	0.169851	0.169	0.007	1.44E-49
EPT1	1.00E-53	0.133491	0.146	0.005	1.50E-49
ATP5L2	1.04E-53	-0.4582	0.083	0.067	1.56E-49
USP30	1.05E-53	0.110382	0.146	0.01	1.56E-49
GDF15	1.20E-53	-0.33965	0.195	0.096	1.79E-49
FAXC	1.39E-53	0.108379	0.152	0.012	2.08E-49
MANSC1	1.47E-53	0.107361	0.146	0.01	2.19E-49
PC	1.92E-53	0.145916	0.176	0.012	2.87E-49
SLC25A24	1.93E-53	0.175118	0.174	0.007	2.89E-49
MFAP3	2.14E-53	0.179009	0.175	0.005	3.20E-49
CSTF2	2.22E-53	0.107998	0.147	0.01	3.32E-49
CNGA3	2.44E-53	0.114263	0.153	0.012	3.64E-49
IQSEC1	2.66E-53	0.150233	0.163	0.005	3.98E-49
CDK18	2.87E-53	-0.14251	0.146	0.048	4.28E-49
ZBTB33	2.98E-53	0.119328	0.159	0.014	4.46E-49
KIAA1456	3.24E-53	0.236892	0.197	0.005	4.84E-49
SOX15	3.82E-53	-0.25312	0.114	0.051	5.71E-49
MFSD6	4.76E-53	0.138873	0.159	0.009	7.12E-49
LTF	7.53E-53	-1.4235	0.011	0.162	1.12E-48
IGDCC4	9.10E-53	0.244823	0.206	0.003	1.36E-48
NPAS2	9.62E-53	0.177162	0.181	0.01	1.44E-48
C2CD2	1.02E-52	0.112178	0.137	0.007	1.52E-48
ITPKC	1.50E-52	0.194769	0.176	0.003	2.24E-48
GPNMB	1.52E-52	-0.31415	0.083	0.044	2.27E-48
RP5-1136G	1.72E-52	0.118972	0.132	0.003	2.57E-48
GDPD1	2.16E-52	0.212747	0.189	0.005	3.23E-48
ZNF689	3.05E-52	0.173907	0.189	0.015	4.55E-48
LCA5	3.40E-52	0.140982	0.15	0.007	5.08E-48
EGLN3	3.48E-52	-0.25821	0.078	0.036	5.21E-48
ZNF346	4.13E-52	0.126706	0.157	0.01	6.17E-48
PCNXL3	4.41E-52	0.155084	0.153	0.003	6.59E-48
CYBA	4.67E-52	-0.16034	0.112	0.036	6.97E-48
SYNJ1	4.99E-52	0.123905	0.153	0.009	7.46E-48
ENTPD5	5.12E-52	0.137567	0.164	0.01	7.66E-48
PEX12	6.19E-52	0.174421	0.173	0.007	9.25E-48
SLC27A4	6.35E-52	0.163869	0.157	0.002	9.49E-48
SBNO2	6.60E-52	0.146773	0.166	0.01	9.87E-48
LAYN	7.28E-52	0.266097	0.209	0.002	1.09E-47
INO80B	7.74E-52	0.201277	0.189	0.009	1.16E-47
CACNA1A	8.69E-52	-0.25234	0.083	0.05	1.30E-47
ZNF74	9.19E-52	0.107246	0.145	0.012	1.37E-47
WIPF3	9.32E-52	0.220629	0.19	0.007	1.39E-47
HNMT	9.34E-52	-0.11021	0.123	0.031	1.40E-47
LMO3	9.34E-52	-0.24329	0.132	0.068	1.40E-47
DENND4B	9.78E-52	0.142437	0.154	0.007	1.46E-47
RP11-315A	1.03E-51	-0.15374	0.089	0.027	1.54E-47
PYCARD	1.21E-51	0.14952	0.163	0.012	1.80E-47

RAB24	1.29E-51	0.101275	0.135	0.007	1.93E-47
RP11-455F	1.50E-51	-0.13431	0.086	0.024	2.24E-47
WBP1L	1.56E-51	0.109721	0.157	0.014	2.33E-47
NLE1	1.58E-51	0.197193	0.187	0.005	2.35E-47
TRIM3	1.58E-51	0.111117	0.145	0.01	2.36E-47
C1orf159	2.26E-51	0.123195	0.136	0.003	3.37E-47
DDX20	2.40E-51	0.159617	0.165	0.007	3.59E-47
SFRP4	3.74E-51	-0.12822	0.138	0.039	5.60E-47
TUBGCP5	4.06E-51	0.163271	0.169	0.007	6.07E-47
ADORA2B	4.24E-51	0.17884	0.172	0.005	6.34E-47
PDE7A	4.43E-51	0.114052	0.143	0.007	6.62E-47
CDK8	4.57E-51	0.155635	0.167	0.007	6.83E-47
BRPF1	5.12E-51	0.103722	0.138	0.009	7.65E-47
SLC39A11	6.08E-51	0.169401	0.165	0.003	9.09E-47
CXXC4	7.30E-51	0.16152	0.165	0.009	1.09E-46
GK	8.20E-51	0.103927	0.144	0.01	1.23E-46
FBLN5	8.52E-51	-0.15744	0.101	0.029	1.27E-46
CHN2	9.72E-51	0.108953	0.135	0.009	1.45E-46
GPR161	1.21E-50	0.211877	0.19	0.005	1.82E-46
SYNJ2	1.23E-50	0.153301	0.169	0.01	1.83E-46
WRN	1.24E-50	0.116564	0.138	0.007	1.85E-46
PI3	1.40E-50	-0.63518	0.07	0.043	2.09E-46
PNRC2	1.49E-50	-0.42301	0.07	0.046	2.23E-46
FRMD4B	1.55E-50	-0.12623	0.118	0.034	2.32E-46
ZNF747	1.64E-50	0.120653	0.138	0.005	2.45E-46
RP11-182L	2.29E-50	0.170615	0.171	0.009	3.42E-46
APOD	2.50E-50	-1.06923	0.078	0.207	3.74E-46
XYLT2	2.74E-50	0.171125	0.171	0.01	4.10E-46
NAT8L	3.01E-50	0.133551	0.156	0.01	4.50E-46
TRIM8	3.61E-50	0.26782	0.206	0.002	5.40E-46
IPPK	3.80E-50	0.118924	0.153	0.012	5.67E-46
AQR	3.82E-50	0.13246	0.157	0.01	5.71E-46
MDFIC	3.93E-50	0.131882	0.141	0.003	5.88E-46
RP11-490M	4.36E-50	0.137652	0.153	0.009	6.52E-46
FBXL19	4.60E-50	0.113164	0.134	0.005	6.87E-46
RP11-544A	5.21E-50	0.104125	0.206	0.041	7.79E-46
EHBP1L1	6.17E-50	0.135784	0.14	0.002	9.22E-46
PTGDS	6.22E-50	-0.72225	0.122	0.12	9.29E-46
CYP2U1	6.72E-50	0.13579	0.15	0.009	1.00E-45
ZNF717	7.08E-50	0.108887	0.125	0.003	1.06E-45
OXTR	7.71E-50	0.257933	0.205	0.014	1.15E-45
TMC7	8.37E-50	0.120719	0.146	0.009	1.25E-45
ZNF682	9.23E-50	0.103392	0.154	0.012	1.38E-45
STK32B	9.65E-50	0.254877	0.205	0.005	1.44E-45
MCM4	1.04E-49	0.184312	0.178	0.01	1.56E-45
PARG	1.20E-49	0.120513	0.137	0.005	1.80E-45
TTTY15	1.24E-49	0.138202	0.146	0.005	1.85E-45
PCDHB14	1.41E-49	-0.10943	0.152	0.053	2.11E-45
MOB1B	1.49E-49	0.18418	0.167	0.003	2.22E-45
FBRSL1	1.76E-49	0.131149	0.149	0.005	2.63E-45
ZNF574	1.82E-49	0.185896	0.184	0.012	2.72E-45

B3GNT9	2.18E-49	0.172928	0.172	0.005	3.26E-45
EXT1	2.43E-49	0.171564	0.169	0.005	3.64E-45
WDR44	2.44E-49	0.102327	0.129	0.007	3.65E-45
ZDHHC14	2.59E-49	0.117643	0.153	0.01	3.87E-45
TCFL5	2.70E-49	0.177547	0.172	0.005	4.03E-45
SLC22A4	3.79E-49	0.285177	0.209	0.002	5.66E-45
SH3BP1	4.00E-49	0.166541	0.163	0.005	5.97E-45
PDLIM4	4.59E-49	-0.25316	0.144	0.068	6.87E-45
INF2	4.87E-49	0.120824	0.15	0.012	7.27E-45
PPP2R5D	6.48E-49	0.178621	0.176	0.007	9.68E-45
MAEL	6.65E-49	0.165922	0.165	0.007	9.94E-45
MGP	9.23E-49	-1.28723	0.024	0.128	1.38E-44
VPS54	1.01E-48	0.124623	0.141	0.007	1.51E-44
FAM86B1	1.20E-48	0.155112	0.159	0.009	1.79E-44
CYP3A5	1.24E-48	0.132031	0.153	0.01	1.85E-44
ZNF615	1.26E-48	0.116842	0.143	0.01	1.88E-44
TMEM63A	1.36E-48	0.101887	0.154	0.017	2.03E-44
HAUS6	1.50E-48	0.131038	0.155	0.01	2.24E-44
ATP1A2	1.51E-48	-0.2404	0.2	0.113	2.26E-44
AIF1	1.53E-48	-0.20287	0.09	0.039	2.29E-44
PLEKHF1	2.18E-48	0.131514	0.16	0.012	3.26E-44
TRIM26	2.43E-48	0.139005	0.156	0.01	3.63E-44
B3GNT2	2.51E-48	0.218366	0.179	0.007	3.75E-44
MMS19	2.68E-48	0.16811	0.172	0.01	4.00E-44
LPAR1	2.81E-48	0.101927	0.122	0.005	4.20E-44
GPD1L	2.88E-48	0.176167	0.16	0.003	4.30E-44
MED23	3.74E-48	0.134148	0.155	0.012	5.59E-44
RP11-73E1	4.12E-48	0.137981	0.143	0.007	6.16E-44
C11orf71	4.32E-48	-0.14759	0.081	0.026	6.45E-44
AC002456.	4.57E-48	-0.14727	0.108	0.031	6.82E-44
ANGPT2	4.97E-48	-0.23758	0.103	0.043	7.43E-44
ZNF653	5.24E-48	0.112178	0.129	0.005	7.83E-44
ERAP1	5.51E-48	0.134379	0.159	0.01	8.24E-44
SCYL3	5.96E-48	0.107336	0.138	0.01	8.91E-44
OTP	6.05E-48	0.260541	0.199	0.002	9.04E-44
PANX1	6.55E-48	0.121114	0.143	0.009	9.79E-44
CACNB3	7.30E-48	0.143039	0.153	0.009	1.09E-43
KDM1B	9.37E-48	0.137037	0.149	0.007	1.40E-43
SHROOM1	1.05E-47	0.182026	0.168	0.007	1.57E-43
MAF	1.06E-47	-0.31934	0.084	0.048	1.59E-43
SLC8B1	1.23E-47	0.117089	0.151	0.012	1.83E-43
TMEM176A	1.24E-47	-0.30699	0.113	0.056	1.85E-43
MAGI3	1.26E-47	0.229389	0.193	0.003	1.88E-43
LIN7A	1.26E-47	0.209651	0.186	0.007	1.88E-43
MBP	1.30E-47	-0.67773	0.06	0.07	1.94E-43
SDC1	1.40E-47	0.147793	0.154	0.007	2.09E-43
IL17RB	1.46E-47	-0.17858	0.107	0.041	2.18E-43
NR2F2	1.71E-47	-0.17578	0.107	0.044	2.55E-43
GSR	1.74E-47	0.141398	0.135	0.002	2.60E-43
GPAM	1.76E-47	0.133294	0.147	0.005	2.63E-43
MPP2	1.83E-47	0.159438	0.155	0.005	2.74E-43

LINC01137	1.95E-47	0.146885	0.147	0.005	2.92E-43
TMEM117	1.97E-47	0.10694	0.129	0.005	2.94E-43
FUOM	2.05E-47	0.13784	0.14	0.003	3.06E-43
KIAA1551	2.24E-47	0.111467	0.139	0.009	3.34E-43
C9orf40	2.39E-47	0.14125	0.142	0.003	3.57E-43
CEP135	2.40E-47	0.133015	0.151	0.009	3.59E-43
ALKBH8	2.76E-47	0.190219	0.173	0.005	4.13E-43
AHNAK2	3.38E-47	0.253479	0.191	0.007	5.05E-43
ZNF143	3.61E-47	0.160255	0.162	0.007	5.40E-43
ENGASE	4.44E-47	0.137892	0.154	0.009	6.64E-43
DTNB	4.44E-47	0.240006	0.191	0	6.64E-43
VARS2	5.86E-47	0.154683	0.163	0.012	8.75E-43
CTD-2587M	6.29E-47	0.105019	0.116	0.002	9.39E-43
PEX6	6.30E-47	0.124533	0.156	0.015	9.42E-43
ADAM23	6.33E-47	0.175777	0.171	0.009	9.45E-43
MORC2	7.08E-47	0.154093	0.152	0.005	1.06E-42
PGAM5	8.66E-47	0.19775	0.172	0.003	1.29E-42
FAM43A	9.10E-47	0.266064	0.19	0	1.36E-42
RASAL2	9.24E-47	0.141262	0.144	0.005	1.38E-42
PAXIP1	1.18E-46	-0.1082	0.098	0.021	1.76E-42
CRB2	1.41E-46	0.213819	0.175	0.01	2.10E-42
LAPTM5	1.51E-46	-0.3893	0.088	0.063	2.26E-42
KLF15	1.94E-46	-0.11163	0.121	0.043	2.90E-42
RCBTB1	2.00E-46	0.124666	0.146	0.009	3.00E-42
EPOR	2.20E-46	0.139813	0.15	0.009	3.28E-42
CLCN2	2.28E-46	0.117946	0.135	0.007	3.41E-42
RASD1	2.44E-46	-0.53084	0.127	0.121	3.65E-42
IL27RA	2.62E-46	0.131812	0.144	0.007	3.91E-42
SEMA3A	2.66E-46	0.240059	0.188	0	3.98E-42
YJEFN3	2.68E-46	0.18702	0.172	0.007	4.01E-42
FHL2	2.75E-46	0.201519	0.182	0.012	4.12E-42
RHPN1	3.11E-46	0.165779	0.156	0.003	4.64E-42
ZFP1	3.13E-46	0.151722	0.152	0.007	4.67E-42
GLIS2	3.14E-46	0.100031	0.122	0.005	4.69E-42
CLDN10	3.83E-46	-0.33068	0.113	0.053	5.73E-42
DZANK1	4.37E-46	0.143976	0.143	0.005	6.53E-42
SCAF8	4.69E-46	0.209354	0.178	0.007	7.01E-42
SNAI2	5.24E-46	0.157863	0.151	0.007	7.83E-42
DIO2	5.59E-46	-0.21272	0.13	0.056	8.36E-42
TAPT1-AS1	5.73E-46	0.218067	0.18	0.003	8.56E-42
FAS	7.68E-46	-0.2643	0.076	0.034	1.15E-41
SLC9A1	8.95E-46	0.117957	0.134	0.005	1.34E-41
SVIP	9.23E-46	0.279701	0.181	0.007	1.38E-41
SH3BP5L	9.46E-46	0.126228	0.14	0.009	1.41E-41
ELL2	1.03E-45	-0.22327	0.086	0.043	1.53E-41
CHRDL1	1.04E-45	0.140317	0.135	0.002	1.55E-41
GSTCD	1.22E-45	0.100159	0.127	0.007	1.82E-41
NDUFB8	1.30E-45	-1.09171	0.009	0.164	1.95E-41
SIX1	1.51E-45	0.11013	0.133	0.007	2.25E-41
ANKS1A	1.71E-45	0.145213	0.143	0.005	2.56E-41
RAB33A	1.81E-45	-0.28558	0.067	0.031	2.71E-41

ZFP30	2.03E-45	0.137225	0.143	0.007	3.04E-41
C7orf43	2.17E-45	0.15408	0.145	0.003	3.24E-41
SYNGR3	2.21E-45	0.137663	0.136	0.003	3.30E-41
KIF1A	2.52E-45	-0.14159	0.103	0.038	3.77E-41
ZBTB41	2.58E-45	0.103691	0.128	0.007	3.85E-41
PIFO	2.63E-45	-0.50545	0.062	0.062	3.93E-41
B4GALNT4	3.19E-45	0.118624	0.132	0.005	4.76E-41
WWC2	3.23E-45	0.125684	0.143	0.01	4.83E-41
LRP8	3.30E-45	0.102017	0.127	0.007	4.93E-41
CHKB	3.54E-45	0.116279	0.123	0.003	5.29E-41
ZDHHC1	3.77E-45	0.198932	0.173	0.002	5.64E-41
PRIM1	3.89E-45	0.112488	0.147	0.012	5.81E-41
DENND1B	4.12E-45	0.134856	0.147	0.007	6.16E-41
EPS8L1	4.20E-45	0.250907	0.182	0.002	6.28E-41
ARHGAP18	4.37E-45	-0.11164	0.108	0.027	6.53E-41
ST6GALNAC	5.15E-45	0.173595	0.15	0.005	7.69E-41
SLC16A9	5.34E-45	0.159693	0.149	0.003	7.99E-41
MUC12	5.43E-45	-0.28118	0.082	0.038	8.11E-41
WWC3	8.59E-45	0.10731	0.133	0.007	1.28E-40
AREL1	8.85E-45	0.167292	0.152	0.003	1.32E-40
PITPNM1	9.40E-45	0.219074	0.182	0	1.40E-40
IER3IP1	1.05E-44	-1.04094	0.005	0.132	1.56E-40
USP53	1.09E-44	0.100329	0.137	0.012	1.63E-40
UTP15	1.14E-44	0.111864	0.138	0.01	1.70E-40
TMEM164	1.14E-44	0.120107	0.13	0.005	1.70E-40
ABCC10	1.19E-44	0.110554	0.137	0.01	1.78E-40
STX1A	1.35E-44	-0.12694	0.063	0.021	2.02E-40
STK11IP	1.70E-44	0.101633	0.122	0.009	2.54E-40
SBF1	2.29E-44	0.164079	0.155	0.003	3.42E-40
NIPA1	3.23E-44	0.117234	0.133	0.007	4.83E-40
NR4A1	3.40E-44	-0.34649	0.087	0.058	5.08E-40
RP4-639F2	3.49E-44	0.152878	0.152	0.005	5.22E-40
SHC2	3.59E-44	0.123303	0.14	0.009	5.36E-40
TUBA4A	3.92E-44	-0.32412	0.07	0.034	5.85E-40
ZNF660	4.09E-44	0.138957	0.15	0.007	6.11E-40
AMH	4.87E-44	0.167489	0.154	0.005	7.28E-40
TPX2	4.92E-44	0.115611	0.129	0.005	7.36E-40
KLHDC4	6.18E-44	0.164398	0.154	0.005	9.24E-40
TAF1A	6.39E-44	-0.17611	0.075	0.022	9.55E-40
ATAD2	7.05E-44	0.133227	0.134	0.005	1.05E-39
GJB2	7.15E-44	0.217481	0.171	0.01	1.07E-39
MSL2	7.69E-44	0.11597	0.128	0.007	1.15E-39
GJA1	8.28E-44	-0.37834	0.141	0.099	1.24E-39
ZFYVE9	8.78E-44	0.129471	0.147	0.012	1.31E-39
ASB13	9.30E-44	0.197815	0.17	0.003	1.39E-39
LRFN3	9.92E-44	0.134925	0.138	0.007	1.48E-39
AC079922.	1.07E-43	0.108632	0.119	0.005	1.60E-39
SPAG1	1.18E-43	0.151733	0.143	0.003	1.77E-39
EFNA4	1.26E-43	0.125975	0.133	0.003	1.89E-39
ZNF628	1.70E-43	0.102271	0.127	0.009	2.55E-39
GALNT18	1.80E-43	0.102083	0.119	0.007	2.69E-39

ALMS1	1.94E-43	0.106152	0.143	0.014	2.90E-39
BEST3	1.96E-43	-0.16762	0.118	0.044	2.93E-39
TRAF3IP1	2.34E-43	0.12541	0.133	0.007	3.49E-39
IKZF4	2.56E-43	0.117377	0.13	0.005	3.83E-39
PPP1R14A	2.75E-43	-0.20206	0.1	0.041	4.10E-39
WWP2	3.13E-43	0.113921	0.133	0.009	4.68E-39
KDM5D	3.25E-43	0.211524	0.176	0	4.85E-39
SPAG4	3.58E-43	-0.22744	0.055	0.019	5.35E-39
NCAPD2	3.93E-43	0.121255	0.123	0.005	5.88E-39
TPCN1	3.94E-43	0.12044	0.15	0.015	5.89E-39
PFKFB4	4.29E-43	-0.12159	0.068	0.019	6.42E-39
OPN3	4.57E-43	0.102071	0.121	0.005	6.83E-39
IFIT1	5.25E-43	-0.11409	0.118	0.032	7.85E-39
SQRDL	5.54E-43	-0.13506	0.072	0.022	8.27E-39
ZNF837	5.92E-43	0.122747	0.134	0.007	8.85E-39
RASSF7	6.05E-43	0.192334	0.163	0.002	9.04E-39
HVCN1	6.35E-43	-0.14283	0.087	0.029	9.48E-39
GRIK3	7.31E-43	0.143892	0.143	0.009	1.09E-38
DDX31	7.72E-43	0.139381	0.143	0.007	1.15E-38
C16orf70	8.53E-43	0.107121	0.124	0.005	1.27E-38
PTCH1	1.42E-42	0.138984	0.139	0.009	2.12E-38
GAS2L3	1.42E-42	0.20382	0.162	0.003	2.13E-38
BBS10	1.53E-42	0.19024	0.156	0.003	2.28E-38
RAB26	1.65E-42	0.117686	0.122	0.003	2.47E-38
DVL1	1.81E-42	0.145152	0.14	0.003	2.70E-38
TMEM145	2.63E-42	0.100212	0.157	0.021	3.93E-38
SPATA5L1	3.38E-42	0.133003	0.143	0.01	5.06E-38
RUNDC3A	3.44E-42	-0.12811	0.083	0.031	5.14E-38
ASAP3	3.52E-42	0.156056	0.144	0.003	5.26E-38
MMP11	3.83E-42	0.236809	0.172	0	5.72E-38
ANTXR2	4.77E-42	0.108351	0.116	0.003	7.12E-38
PRKX	5.15E-42	0.148441	0.134	0.003	7.70E-38
NHSL2	5.48E-42	-0.88366	0.008	0.115	8.18E-38
BANP	6.37E-42	0.170657	0.153	0.005	9.52E-38
RBM47	8.84E-42	0.131182	0.143	0.01	1.32E-37
MTRNR2L1	8.85E-42	-0.86461	0.059	0.174	1.32E-37
TRAPPC8	8.92E-42	0.199028	0.169	0.003	1.33E-37
TNS1	9.06E-42	-0.12046	0.084	0.027	1.35E-37
DNMBP	1.08E-41	0.119713	0.127	0.005	1.61E-37
AADAT	1.52E-41	0.16597	0.148	0.002	2.28E-37
CACHD1	1.56E-41	0.100993	0.13	0.009	2.34E-37
SPDL1	1.58E-41	0.108468	0.122	0.007	2.36E-37
EMX2	1.84E-41	-0.14124	0.079	0.034	2.75E-37
RTTN	2.14E-41	0.132521	0.14	0.01	3.20E-37
TMEM220	2.26E-41	0.132403	0.123	0.002	3.37E-37
KIAA1919	2.84E-41	0.101041	0.118	0.007	4.24E-37
LDLRAP1	4.46E-41	0.148739	0.143	0.003	6.66E-37
BTN3A3	5.67E-41	0.117675	0.134	0.01	8.47E-37
LIPE	5.76E-41	0.103315	0.122	0.003	8.61E-37
MBTD1	5.96E-41	0.170317	0.147	0.003	8.91E-37
ANKRD13B	6.96E-41	0.147271	0.138	0.005	1.04E-36

RBL1	7.81E-41	0.139011	0.135	0.005	1.17E-36
GADD45G	7.92E-41	-0.27585	0.127	0.075	1.18E-36
RFWD3	9.95E-41	0.115086	0.134	0.01	1.49E-36
CCL4	1.17E-40	-0.26724	0.11	0.063	1.75E-36
RP11-22P6	1.58E-40	-0.10535	0.081	0.022	2.36E-36
AQP11	1.73E-40	0.125064	0.128	0.003	2.58E-36
TCF20	1.77E-40	0.113001	0.131	0.009	2.65E-36
RP11-680F	1.81E-40	0.2125	0.165	0	2.71E-36
FBXO46	2.19E-40	0.159012	0.141	0.002	3.27E-36
CEP112	2.35E-40	0.168098	0.151	0.005	3.51E-36
IRAK1BP1	2.53E-40	0.103559	0.119	0.005	3.78E-36
SAA1	2.59E-40	-1.36561	0.016	0.121	3.87E-36
IL33	3.13E-40	0.181583	0.143	0.005	4.67E-36
MCF2L	4.65E-40	0.110326	0.134	0.012	6.95E-36
ZBED4	4.97E-40	0.102078	0.118	0.007	7.42E-36
PREX2	5.75E-40	0.110106	0.118	0.005	8.59E-36
MAPKBP1	5.76E-40	0.120709	0.119	0.002	8.61E-36
TMEM201	5.78E-40	0.186311	0.158	0.005	8.64E-36
PPP1R3D	7.18E-40	0.118687	0.127	0.009	1.07E-35
EPB41L4A	7.25E-40	0.125497	0.124	0.005	1.08E-35
THEM4	8.96E-40	0.107062	0.123	0.009	1.34E-35
MCF2L-AS1	9.97E-40	0.128598	0.123	0.002	1.49E-35
CCDC149	1.10E-39	0.104931	0.112	0.003	1.64E-35
TMEM91	1.10E-39	0.133801	0.131	0.005	1.65E-35
KITLG	1.18E-39	0.125453	0.132	0.007	1.76E-35
MFAP4	1.19E-39	-0.11436	0.102	0.026	1.77E-35
IL1B	1.45E-39	-0.1505	0.078	0.031	2.16E-35
PGAM2	1.62E-39	-0.21999	0.074	0.027	2.42E-35
AHCYL2	1.83E-39	0.171018	0.153	0.01	2.73E-35
STXBP4	2.27E-39	0.125914	0.121	0.003	3.39E-35
EPG5	2.76E-39	0.103676	0.126	0.01	4.12E-35
FLNB	3.53E-39	0.129785	0.123	0.002	5.28E-35
ZBTB16	4.01E-39	-0.46748	0.102	0.101	5.99E-35
CTD-2325M	5.27E-39	0.122744	0.124	0.005	7.88E-35
PAPD7	5.68E-39	0.120583	0.115	0.002	8.49E-35
APAF1	8.40E-39	0.101914	0.116	0.007	1.25E-34
TCEAL2	1.01E-38	-0.7658	0.057	0.137	1.51E-34
NEK3	1.04E-38	0.113885	0.124	0.007	1.55E-34
MORN3	1.19E-38	-0.1735	0.052	0.021	1.77E-34
RP11-375N	1.44E-38	0.158045	0.138	0.005	2.16E-34
HOXD9	1.87E-38	0.13244	0.131	0.003	2.79E-34
WDR89	1.93E-38	0.136701	0.135	0.005	2.88E-34
CTD-3131K	2.04E-38	-0.16684	0.07	0.021	3.04E-34
PARD3B	2.15E-38	0.143536	0.128	0.003	3.22E-34
PARP8	2.30E-38	0.124443	0.119	0.003	3.44E-34
SLC25A43	2.42E-38	0.106095	0.124	0.009	3.61E-34
CD14	2.44E-38	-0.40456	0.08	0.074	3.65E-34
SYNDIG1	2.66E-38	0.161672	0.135	0.005	3.98E-34
ATAD2B	3.16E-38	0.138168	0.128	0.005	4.72E-34
NKX2-2	4.05E-38	-0.11533	0.127	0.048	6.05E-34
SLC7A5	4.08E-38	-0.14999	0.087	0.038	6.10E-34

CRABP2	5.83E-38	-0.27021	0.054	0.034	8.72E-34
GAL3ST4	5.97E-38	-0.16754	0.071	0.032	8.92E-34
RGS1	7.09E-38	-0.39921	0.093	0.085	1.06E-33
TMEM65	7.83E-38	0.144763	0.133	0.005	1.17E-33
FBXW8	9.20E-38	0.103011	0.115	0.007	1.37E-33
CMTM7	9.45E-38	0.21462	0.154	0	1.41E-33
KSR1	1.04E-37	0.131183	0.125	0.005	1.55E-33
PIF1	1.21E-37	0.142814	0.133	0.003	1.81E-33
PDE9A	1.23E-37	0.109972	0.141	0.017	1.83E-33
RARRES2	1.32E-37	-0.8308	0.061	0.133	1.97E-33
RIN2	1.34E-37	0.156813	0.153	0.01	2.00E-33
REEP1	1.53E-37	0.124242	0.122	0.005	2.29E-33
OSBPL5	1.76E-37	0.144817	0.137	0.007	2.62E-33
FHIT	1.88E-37	-0.24996	0.073	0.038	2.80E-33
FGFR3	2.04E-37	0.109166	0.143	0.021	3.05E-33
RP11-15A1	2.05E-37	0.164969	0.15	0.012	3.07E-33
WDR86	2.07E-37	0.141096	0.13	0.003	3.09E-33
UBE2V1	2.16E-37	0.180989	0.152	0.003	3.23E-33
PLLP	2.19E-37	-0.23448	0.124	0.056	3.27E-33
SOD3	3.05E-37	0.232245	0.143	0.002	4.55E-33
NRXN1	3.12E-37	-0.16142	0.106	0.043	4.67E-33
CTD-2090I	4.11E-37	-0.76275	0.008	0.089	6.15E-33
SNIP1	4.38E-37	0.112109	0.121	0.009	6.54E-33
CELSR1	4.60E-37	0.124407	0.125	0.003	6.88E-33
PAQR7	4.85E-37	0.119769	0.124	0.007	7.25E-33
MAST4	5.06E-37	-0.20049	0.105	0.051	7.56E-33
SLC30A6	5.78E-37	0.138391	0.131	0.005	8.64E-33
CTPS2	6.21E-37	0.106728	0.117	0.007	9.28E-33
ATAD5	6.29E-37	0.109595	0.128	0.01	9.40E-33
AGAP2-AS1	7.57E-37	0.101113	0.14	0.017	1.13E-32
DSCC1	1.58E-36	0.115828	0.128	0.009	2.36E-32
CTC-444N2	1.92E-36	0.11202	0.115	0.003	2.87E-32
HSPA2	1.99E-36	-0.44697	0.086	0.08	2.97E-32
STAG3	2.05E-36	-0.24211	0.05	0.038	3.06E-32
RCAN3	2.11E-36	0.191642	0.149	0	3.15E-32
THAP7-AS1	2.87E-36	0.13719	0.127	0.003	4.28E-32
ZNF625	3.05E-36	0.123573	0.131	0.009	4.56E-32
EFNA2	3.09E-36	0.184362	0.145	0.003	4.62E-32
PARD6B	3.13E-36	0.106294	0.119	0.009	4.67E-32
PAQR8	3.25E-36	0.106474	0.134	0.015	4.86E-32
C2CD4A	4.19E-36	0.252328	0.148	0	6.26E-32
NUAK1	4.85E-36	0.127436	0.118	0.003	7.25E-32
DOPEY2	5.09E-36	0.105683	0.118	0.009	7.61E-32
S100A9	5.12E-36	-0.14802	0.064	0.026	7.65E-32
H3F3C	6.20E-36	-0.54133	0.029	0.079	9.27E-32
IDUA	9.31E-36	0.140249	0.13	0.005	1.39E-31
CACNG4	1.05E-35	0.115754	0.115	0.007	1.57E-31
TMEM170B	1.17E-35	0.187605	0.146	0	1.75E-31
NSG1	1.18E-35	-0.27167	0.092	0.051	1.76E-31
GFOD1	1.28E-35	0.150433	0.138	0.007	1.91E-31
NTRK2	1.85E-35	-0.45688	0.122	0.116	2.76E-31

TIMELESS	1.99E-35	0.126393	0.123	0.005	2.97E-31
FOLR1	2.51E-35	-0.19516	0.049	0.021	3.75E-31
TBC1D12	2.60E-35	0.193457	0.149	0.002	3.89E-31
GLIPR1L2	2.63E-35	-0.12407	0.072	0.019	3.93E-31
AC023590.	2.72E-35	-0.1438	0.068	0.029	4.07E-31
CTB-5506.	3.28E-35	0.195168	0.144	0	4.91E-31
SMAD3	3.60E-35	0.117051	0.113	0.005	5.38E-31
SGCE	4.91E-35	-0.70879	0.054	0.12	7.34E-31
ALOX5AP	5.55E-35	-0.41538	0.061	0.067	8.30E-31
KCNN2	6.44E-35	0.107533	0.113	0.009	9.62E-31
RNF217	6.51E-35	0.187832	0.143	0	9.73E-31
CHGB	6.60E-35	-0.18076	0.071	0.031	9.86E-31
AC004540.	7.20E-35	-0.10302	0.093	0.032	1.08E-30
SGMS1	8.51E-35	0.15679	0.136	0.005	1.27E-30
DAB2IP	9.03E-35	0.126498	0.121	0.005	1.35E-30
ZNF441	9.87E-35	0.107731	0.119	0.009	1.47E-30
ARG2	1.12E-34	-0.19883	0.048	0.022	1.67E-30
SCHIP1	1.22E-34	0.127816	0.125	0.009	1.82E-30
MAP3K3	1.52E-34	0.140352	0.125	0.002	2.27E-30
PCSK1	1.83E-34	-0.10051	0.078	0.022	2.73E-30
SCN1B	2.01E-34	0.144934	0.13	0.002	3.01E-30
C19orf47	2.24E-34	0.108228	0.111	0.007	3.34E-30
KHDC1	2.93E-34	-0.12286	0.09	0.032	4.38E-30
PKP4	3.26E-34	0.116003	0.112	0.003	4.87E-30
TRAM2-AS1	4.39E-34	0.127186	0.118	0.003	6.57E-30
PPM1L	4.53E-34	0.146643	0.125	0.002	6.77E-30
MAP3K5	5.19E-34	0.104397	0.105	0.003	7.76E-30
TTC21A	5.64E-34	0.114978	0.126	0.01	8.43E-30
BMP2K	5.68E-34	0.129289	0.124	0.007	8.49E-30
FOXP4	5.82E-34	0.150649	0.132	0.002	8.70E-30
B3GAT2	5.96E-34	0.166288	0.139	0.007	8.90E-30
CMTM4	6.87E-34	0.166857	0.136	0.002	1.03E-29
C6orf15	7.55E-34	-0.28701	0.118	0.067	1.13E-29
KIAA1161	7.90E-34	0.109897	0.102	0.002	1.18E-29
PLEKHA8	9.49E-34	0.145244	0.131	0.005	1.42E-29
ANGPTL4	1.01E-33	-0.55732	0.054	0.074	1.51E-29
MAFB	1.11E-33	-0.35229	0.061	0.053	1.66E-29
RP11-479J	1.22E-33	-0.11795	0.048	0.017	1.83E-29
PRKAG2-AS	1.55E-33	-0.19667	0.072	0.039	2.32E-29
DCAF17	1.63E-33	0.13375	0.115	0.002	2.44E-29
GIMAP2	1.81E-33	-0.15433	0.054	0.019	2.71E-29
MRM1	1.97E-33	0.17098	0.137	0	2.95E-29
RP13-270F	1.98E-33	0.103398	0.1	0.002	2.95E-29
PTGES	2.08E-33	-0.16153	0.04	0.017	3.11E-29
FCHO2	2.66E-33	0.114141	0.115	0.007	3.98E-29
KCNJ8	3.12E-33	0.104797	0.099	0.002	4.66E-29
SLC1A4	3.15E-33	0.133503	0.118	0.003	4.71E-29
RIT2	3.19E-33	-0.51122	0.057	0.072	4.77E-29
RP11-152N	3.23E-33	-0.11657	0.053	0.015	4.83E-29
RENBP	3.65E-33	-0.25489	0.062	0.038	5.46E-29
ARHGAP23	4.12E-33	0.182623	0.149	0.005	6.15E-29

SALL3	5.34E-33	0.106859	0.101	0.003	7.98E-29
C2	5.72E-33	-0.29041	0.04	0.034	8.54E-29
KIFC1	6.21E-33	-0.10607	0.046	0.012	9.28E-29
CDC6	6.35E-33	-0.13068	0.052	0.012	9.49E-29
USP21	8.31E-33	0.103215	0.112	0.009	1.24E-28
ZC3H12A	9.12E-33	-0.25535	0.048	0.026	1.36E-28
LA16c-431	9.64E-33	-0.1013	0.052	0.021	1.44E-28
PAOX	9.92E-33	0.162036	0.135	0.002	1.48E-28
G0S2	1.09E-32	-0.46036	0.08	0.068	1.63E-28
TMCC3	1.19E-32	0.133092	0.109	0.002	1.78E-28
SLCO4A1	1.36E-32	-0.18707	0.045	0.024	2.04E-28
HEATR3	1.39E-32	0.108931	0.105	0.003	2.07E-28
CTD-3185F	1.45E-32	0.123421	0.119	0.003	2.17E-28
NLRC5	1.47E-32	0.104354	0.112	0.009	2.20E-28
NEURL4	1.57E-32	0.133919	0.116	0.002	2.35E-28
TNFAIP3	1.96E-32	-0.22667	0.055	0.027	2.93E-28
PPP1R1C	2.43E-32	-0.25091	0.084	0.046	3.63E-28
BRCA1	2.59E-32	0.100515	0.106	0.007	3.86E-28
VAMP8	3.10E-32	-0.10818	0.069	0.022	4.63E-28
KLF4	3.31E-32	-0.18869	0.086	0.043	4.94E-28
CLDND2	4.25E-32	0.131692	0.119	0.005	6.36E-28
SLC38A1	4.30E-32	-0.22306	0.086	0.055	6.43E-28
ARSB	4.82E-32	0.133082	0.118	0.003	7.20E-28
LRP11	4.83E-32	0.136615	0.125	0.005	7.22E-28
PSPN	5.20E-32	0.111862	0.103	0.002	7.77E-28
RELT	5.75E-32	0.125996	0.112	0.003	8.59E-28
ARRDC4	6.07E-32	0.10454	0.106	0.007	9.07E-28
FGF7	7.32E-32	-0.33622	0.042	0.044	1.09E-27
LDOC1	7.57E-32	-0.26057	0.075	0.055	1.13E-27
IPO9-AS1	8.13E-32	0.140738	0.122	0.003	1.21E-27
GRAMD1C	8.44E-32	0.172489	0.138	0.009	1.26E-27
ABHD15	8.82E-32	0.103958	0.106	0.005	1.32E-27
ZBTB9	9.73E-32	0.105391	0.097	0.002	1.45E-27
CYB5R2	1.12E-31	-0.13722	0.065	0.024	1.68E-27
PDK3	1.16E-31	0.100352	0.099	0.003	1.73E-27
ARHGDIB	1.35E-31	-0.18415	0.06	0.031	2.01E-27
RPH3A	1.37E-31	-0.10748	0.091	0.038	2.05E-27
HID1	1.49E-31	0.119611	0.107	0.002	2.23E-27
AMMECR1L	1.56E-31	0.101283	0.107	0.007	2.33E-27
ZNF415	1.64E-31	-0.15425	0.056	0.022	2.46E-27
USP2	1.91E-31	0.102361	0.109	0.009	2.85E-27
BEX5	2.35E-31	-0.69992	0.026	0.094	3.52E-27
BIRC3	2.42E-31	-0.28956	0.061	0.021	3.62E-27
ASIC4	2.45E-31	-0.195	0.111	0.06	3.67E-27
HCST	2.83E-31	-0.10918	0.048	0.019	4.23E-27
RP11-314A	3.20E-31	0.133999	0.116	0.002	4.78E-27
F13A1	3.27E-31	-0.83258	0.014	0.094	4.89E-27
RP1-122K4	3.49E-31	-0.11069	0.033	0.012	5.22E-27
HMGA2	3.69E-31	0.217417	0.135	0.005	5.51E-27
RP3-402G1	3.77E-31	0.135915	0.123	0.005	5.63E-27
MAP1LC3B2	4.98E-31	-0.13963	0.066	0.029	7.43E-27

RP11-127E	5.08E-31	0.111276	0.106	0.005	7.59E-27
SCML1	7.27E-31	-0.21016	0.071	0.041	1.09E-26
KIAA0040	7.94E-31	-0.16336	0.072	0.032	1.19E-26
SHQ1	1.15E-30	0.128882	0.113	0.002	1.72E-26
C4orf47	1.17E-30	-0.13488	0.056	0.021	1.75E-26
SMPD2	1.58E-30	0.114274	0.113	0.005	2.36E-26
RP11-499F	1.69E-30	0.143103	0.119	0.003	2.53E-26
SMURF1	1.83E-30	0.115935	0.108	0.003	2.74E-26
MAP3K10	2.32E-30	0.134685	0.12	0.005	3.47E-26
TEAD3	2.51E-30	0.141634	0.124	0.002	3.75E-26
CNN2	3.01E-30	0.108768	0.105	0.005	4.50E-26
BGN	3.15E-30	-0.69034	0.03	0.082	4.71E-26
UNC13B	3.37E-30	0.121458	0.107	0.003	5.03E-26
SLC25A45	4.34E-30	-0.10286	0.052	0.019	6.49E-26
HECA	4.95E-30	0.104566	0.102	0.005	7.39E-26
ACTA2	5.69E-30	-0.10239	0.068	0.017	8.50E-26
ZNF575	5.73E-30	0.106627	0.102	0.003	8.56E-26
S100A4	7.28E-30	-0.2936	0.055	0.032	1.09E-25
ZNF280B	7.77E-30	0.113834	0.112	0.009	1.16E-25
SMIM3	1.01E-29	-0.94053	0.006	0.106	1.51E-25
RP11-420L	1.12E-29	0.119284	0.102	0.002	1.68E-25
MT1A	1.34E-29	-0.53326	0.023	0.067	2.01E-25
NKAIN4	1.60E-29	-0.1498	0.102	0.038	2.40E-25
NDUFA4L2	1.81E-29	-0.37747	0.046	0.043	2.71E-25
LINC00882	1.92E-29	-0.20428	0.051	0.024	2.87E-25
C19orf44	2.54E-29	0.150507	0.124	0.003	3.79E-25
CMYA5	3.68E-29	-0.1414	0.079	0.029	5.50E-25
ZWINT	4.11E-29	0.110711	0.097	0.002	6.14E-25
SLC26A6	4.94E-29	0.138915	0.116	0.002	7.39E-25
XKR6	5.80E-29	0.10916	0.103	0.005	8.67E-25
TCEAL5	6.15E-29	-0.55125	0.043	0.07	9.19E-25
GPR137C	6.73E-29	0.105988	0.1	0.005	1.01E-24
EID3	6.80E-29	-0.15623	0.052	0.022	1.02E-24
MGAT5	7.77E-29	0.110933	0.106	0.003	1.16E-24
SLC38A9	9.87E-29	0.134852	0.116	0.005	1.47E-24
MYO1F	1.14E-28	-0.15162	0.049	0.021	1.70E-24
FOXD1	1.51E-28	0.145716	0.118	0.002	2.26E-24
RTP4	1.66E-28	-0.1531	0.055	0.019	2.48E-24
DOK4	1.68E-28	0.106114	0.101	0.003	2.50E-24
MARVELD1	1.87E-28	0.148519	0.117	0	2.79E-24
ZNF84	2.17E-28	-0.16102	0.034	0.022	3.24E-24
PIM1	2.27E-28	-0.21186	0.064	0.034	3.39E-24
ANKS1B	2.81E-28	-0.18017	0.066	0.031	4.21E-24
PRDM5	3.24E-28	0.131258	0.104	0.002	4.84E-24
GBP4	3.62E-28	-0.22602	0.113	0.062	5.41E-24
LST1	4.61E-28	-0.1505	0.058	0.021	6.89E-24
TMBIM4	5.08E-28	-0.80029	0.007	0.101	7.59E-24
PTPRN	6.22E-28	-0.26733	0.062	0.044	9.30E-24
MYH7B	6.29E-28	-0.13586	0.032	0.021	9.39E-24
RORB	7.40E-28	-0.18479	0.046	0.031	1.11E-23
FKBP1C	7.96E-28	-0.12108	0.038	0.012	1.19E-23

CEACAM1	8.97E-28	-0.11129	0.054	0.021	1.34E-23
PALB2	9.89E-28	0.14156	0.114	0	1.48E-23
ATP11C	1.03E-27	0.112296	0.102	0.003	1.54E-23
PHLDB3	1.14E-27	0.140618	0.114	0.002	1.70E-23
PPP1R3C	1.22E-27	-0.17163	0.051	0.022	1.82E-23
ZNF442	1.28E-27	-0.12258	0.042	0.014	1.92E-23
LUZP2	1.75E-27	0.167215	0.118	0.015	2.62E-23
ZNF180	2.13E-27	0.112395	0.106	0.005	3.18E-23
AF127936.	2.68E-27	0.13898	0.112	0	4.01E-23
C21orf58	2.74E-27	0.109698	0.102	0.003	4.10E-23
MS4A7	2.77E-27	-0.19238	0.049	0.031	4.14E-23
OCRL	3.02E-27	0.132799	0.112	0.002	4.51E-23
SP4	4.25E-27	0.106546	0.106	0.009	6.35E-23
LGALS9	5.20E-27	-0.10752	0.068	0.022	7.77E-23
GDPD2	6.55E-27	0.131147	0.109	0.005	9.78E-23
RP1-43E13	1.01E-26	-0.17351	0.042	0.022	1.52E-22
SGPP1	1.16E-26	0.12977	0.104	0.002	1.74E-22
HERC6	1.47E-26	0.117539	0.097	0.003	2.20E-22
CCNO	1.48E-26	0.138662	0.117	0.002	2.22E-22
ZFY	1.63E-26	0.116614	0.102	0.002	2.43E-22
GRB14	1.80E-26	0.136806	0.108	0.002	2.69E-22
LINC01114	2.18E-26	-0.20353	0.048	0.031	3.25E-22
ZBTB47	2.74E-26	0.13401	0.105	0.002	4.10E-22
CHML	3.19E-26	0.113302	0.097	0.002	4.76E-22
C5AR1	3.21E-26	-0.19297	0.039	0.024	4.80E-22
LRFN1	3.35E-26	0.111249	0.104	0.007	5.00E-22
KIAA1614	3.39E-26	0.12548	0.106	0.003	5.06E-22
ZNF154	4.42E-26	-0.11065	0.045	0.019	6.60E-22
HOTAIRM1	4.46E-26	-0.29074	0.084	0.063	6.67E-22
FAM71E1	4.60E-26	-0.18692	0.047	0.022	6.87E-22
RSPH1	8.75E-26	-0.28479	0.039	0.034	1.31E-21
MIR181A2H	1.01E-25	0.103846	0.09	0.003	1.50E-21
COL28A1	1.03E-25	0.176688	0.116	0.012	1.53E-21
ZNF365	1.30E-25	0.10842	0.098	0.003	1.94E-21
LINC00115	1.30E-25	-0.22907	0.043	0.027	1.94E-21
MGMT	1.32E-25	-0.35885	0.039	0.051	1.97E-21
BTAF1	1.53E-25	0.110439	0.099	0.005	2.29E-21
ZNF784	1.57E-25	0.115632	0.099	0.003	2.35E-21
ELMO1	1.78E-25	-0.45178	0.041	0.07	2.66E-21
IL6	2.68E-25	-0.17099	0.034	0.015	4.01E-21
ERICH2	2.75E-25	0.101982	0.09	0.002	4.11E-21
GUCY1A2	3.68E-25	0.140686	0.109	0.009	5.50E-21
WFDC2	3.76E-25	-0.15821	0.039	0.017	5.62E-21
POPDC3	3.85E-25	0.144172	0.103	0	5.75E-21
RN7SL832P	3.95E-25	-0.11043	0.038	0.017	5.90E-21
RP11-49I1	3.99E-25	-0.12665	0.034	0.014	5.96E-21
GDAP1L1	5.71E-25	-0.27275	0.053	0.044	8.53E-21
CYB5RL	8.32E-25	0.110845	0.096	0.002	1.24E-20
SYTL4	9.84E-25	-0.1261	0.055	0.027	1.47E-20
AZGP1	1.05E-24	-0.35982	0.063	0.053	1.57E-20
NMNAT3	1.07E-24	-0.1721	0.057	0.031	1.60E-20

CRISPLD2	1.08E-24	-0.16302	0.061	0.027	1.61E-20
ADCY8	1.30E-24	-0.16809	0.048	0.017	1.94E-20
HLA-DQA1	1.59E-24	-0.45181	0.03	0.044	2.38E-20
AIFM2	2.25E-24	0.118995	0.105	0.003	3.36E-20
NTM	2.50E-24	-0.15901	0.092	0.051	3.73E-20
HLA-DQB1	3.49E-24	-0.19986	0.048	0.036	5.21E-20
CTNNA2	5.69E-24	-0.21562	0.048	0.039	8.50E-20
HOXC10	5.75E-24	-0.15413	0.06	0.039	8.60E-20
ZNF286B	5.95E-24	0.113925	0.097	0.003	8.89E-20
GRID2	8.09E-24	0.139066	0.106	0.003	1.21E-19
WDR81	8.87E-24	0.105873	0.094	0.003	1.33E-19
C7orf61	9.24E-24	-0.15585	0.052	0.027	1.38E-19
DAPL1	1.03E-23	-0.16251	0.052	0.024	1.54E-19
HSD17B14	1.25E-23	-0.16137	0.062	0.031	1.87E-19
CDKN2B-AS	1.26E-23	0.106195	0.093	0.002	1.88E-19
SLC6A6	1.43E-23	-0.30857	0.026	0.034	2.13E-19
TMEM100	1.45E-23	-0.30043	0.083	0.062	2.16E-19
GAL	1.73E-23	0.141697	0.096	0.005	2.58E-19
AC084219.	2.05E-23	-0.11443	0.04	0.024	3.06E-19
HSPA6	2.92E-23	-0.71834	0.019	0.056	4.36E-19
ABCC4	3.83E-23	0.118884	0.095	0	5.73E-19
STEAP1	5.28E-23	-0.30991	0.046	0.038	7.89E-19
TUBB4A	7.34E-23	-0.18273	0.049	0.024	1.10E-18
EFHD1	8.37E-23	-0.14972	0.047	0.027	1.25E-18
TREM2	1.07E-22	-0.2847	0.039	0.039	1.60E-18
DPYD	1.24E-22	-0.217	0.04	0.027	1.86E-18
SNX25	1.42E-22	0.1189	0.093	0	2.12E-18
CADM3	1.45E-22	-0.44542	0.024	0.048	2.16E-18
CDH13	1.85E-22	-0.35009	0.046	0.051	2.76E-18
CHSY3	1.97E-22	0.112906	0.092	0	2.94E-18
BBS5	1.97E-22	0.115933	0.092	0	2.94E-18
LRRC69	2.01E-22	-0.16682	0.04	0.021	3.01E-18
RP11-658F	2.72E-22	0.121444	0.092	0	4.07E-18
CIT	3.09E-22	-0.1316	0.036	0.017	4.62E-18
FBXL2	3.43E-22	-0.10151	0.032	0.014	5.12E-18
CLEC2D	3.44E-22	-0.1746	0.028	0.024	5.13E-18
TBCEL	4.67E-22	0.118398	0.097	0.002	6.98E-18
RP11-834C	5.07E-22	-0.25397	0.041	0.038	7.57E-18
IFI30	5.30E-22	-0.17381	0.033	0.021	7.91E-18
GNG11	6.46E-22	-0.39554	0.046	0.043	9.66E-18
ZNF665	8.71E-22	-0.15839	0.036	0.027	1.30E-17
IGSF21	9.82E-22	-0.21879	0.048	0.036	1.47E-17
TRIQK	1.30E-21	-0.10655	0.042	0.017	1.94E-17
RP1-17K7.	1.93E-21	-0.50119	0.01	0.068	2.89E-17
PRKCE	2.67E-21	0.113923	0.087	0	3.98E-17
HOXA7	4.04E-21	-0.21195	0.061	0.043	6.03E-17
FAM107A	5.40E-21	-0.11452	0.068	0.029	8.07E-17
HAS3	6.90E-21	0.106275	0.075	0.002	1.03E-16
TF	6.99E-21	-0.12039	0.029	0.015	1.04E-16
FCGR2A	9.56E-21	-0.15984	0.039	0.024	1.43E-16
ZNF107	1.00E-20	0.105325	0.08	0.002	1.50E-16

FZD5	1.05E-20	0.105559	0.086	0.002	1.57E-16
TMEM144	2.41E-20	-0.14938	0.025	0.015	3.60E-16
ETV7	2.48E-20	-0.1337	0.043	0.017	3.70E-16
RIIAD1	2.54E-20	-0.10668	0.031	0.012	3.80E-16
CXCL2	2.68E-20	-0.34266	0.027	0.031	4.00E-16
ACOT11	3.01E-20	-0.14079	0.044	0.021	4.49E-16
SPOCK1	3.03E-20	-0.10134	0.064	0.031	4.52E-16
ELAVL4	4.67E-20	-0.40335	0.028	0.06	6.98E-16
PRKCG	5.52E-20	-0.52812	0.007	0.056	8.25E-16
TSLP	6.30E-20	-0.15436	0.019	0.012	9.41E-16
ANG	6.37E-20	-0.39231	0.026	0.034	9.52E-16
CD37	6.52E-20	-0.16023	0.027	0.024	9.74E-16
LINC00869	6.68E-20	-0.55247	0.004	0.058	9.98E-16
RNASE1	7.02E-20	-0.3231	0.037	0.041	1.05E-15
OMG	7.91E-20	-0.18284	0.051	0.036	1.18E-15
VSIG4	9.13E-20	-0.2765	0.039	0.038	1.36E-15
MICALL1	9.45E-20	0.100958	0.081	0	1.41E-15
HLA-DMB	1.00E-19	-0.10595	0.043	0.017	1.49E-15
PTPRK	1.06E-19	-0.14768	0.039	0.026	1.59E-15
MIR7-3HG	1.22E-19	-0.65139	0.003	0.06	1.82E-15
RPS6KA5	1.22E-19	-0.18232	0.035	0.027	1.83E-15
HIST1H4E	1.68E-19	-0.35016	0.025	0.024	2.51E-15
SSTR2	2.56E-19	-0.12676	0.033	0.017	3.82E-15
RP11-138I	2.58E-19	-0.2232	0.026	0.022	3.85E-15
RP3-460G2	3.08E-19	-0.4895	0.024	0.067	4.61E-15
CD70	3.44E-19	-0.25714	0.023	0.017	5.14E-15
DGKQ	3.44E-19	0.104319	0.078	0	5.14E-15
MASTL	3.44E-19	0.104524	0.078	0	5.14E-15
OSR2	3.44E-19	-0.14252	0.041	0.019	5.15E-15
VGF	3.72E-19	-0.13995	0.068	0.038	5.56E-15
AGMO	4.81E-19	-0.1483	0.034	0.021	7.19E-15
CA3	5.49E-19	-0.37206	0.026	0.029	8.20E-15
DUOX1	8.06E-19	0.117503	0.082	0.002	1.20E-14
SKAP2	8.70E-19	-0.43047	0.034	0.07	1.30E-14
LEPR	9.06E-19	0.100119	0.077	0	1.35E-14
SGIP1	9.15E-19	-0.13292	0.059	0.031	1.37E-14
MAOA	9.20E-19	-0.18074	0.037	0.021	1.38E-14
PPM1N	9.36E-19	-0.17439	0.029	0.024	1.40E-14
ELFN1	1.25E-18	0.110126	0.076	0	1.87E-14
MAMSTR	1.25E-18	0.102498	0.076	0	1.87E-14
C14orf132	1.67E-18	-0.18143	0.051	0.041	2.49E-14
NR2F2-AS1	2.06E-18	-0.11853	0.032	0.021	3.07E-14
ATP1A3	2.14E-18	-0.12759	0.051	0.032	3.20E-14
LPP-AS2	2.98E-18	0.102163	0.082	0.002	4.45E-14
DISP2	3.31E-18	-0.13201	0.017	0.015	4.95E-14
LAIR1	7.13E-18	-0.13312	0.032	0.026	1.07E-13
CH25H	8.25E-18	-0.3937	0.017	0.036	1.23E-13
IGFLR1	9.32E-18	-0.1958	0.023	0.026	1.39E-13
IL6R	1.03E-17	-0.13202	0.024	0.024	1.54E-13
FGF22	1.62E-17	-0.12059	0.026	0.015	2.42E-13
MASP1	2.16E-17	-0.20588	0.039	0.036	3.23E-13

SERPINB1	2.39E-17	-0.44434	0.02	0.039	3.57E-13
TREM1	4.66E-17	-0.54453	0.008	0.05	6.96E-13
CLEC2B	4.73E-17	-0.35121	0.029	0.038	7.06E-13
TNFSF10	5.49E-17	-0.13913	0.035	0.019	8.21E-13
CA9	6.10E-17	-0.31396	0.027	0.043	9.12E-13
PLA2G2A	6.48E-17	-0.44288	0.03	0.034	9.68E-13
APLN	9.97E-17	-0.18959	0.025	0.019	1.49E-12
FCGBP	1.13E-16	-0.2423	0.029	0.038	1.69E-12
SAA2	1.16E-16	-0.79735	0.007	0.048	1.73E-12
GDPGP1	1.33E-16	-0.11005	0.018	0.01	1.98E-12
RP5-821D1	1.60E-16	-0.10879	0.029	0.01	2.39E-12
ESRRG	1.67E-16	-0.1336	0.031	0.019	2.49E-12
HIF3A	1.78E-16	-0.11198	0.048	0.026	2.66E-12
ANLN	1.89E-16	-0.11658	0.027	0.012	2.82E-12
TNFSF13B	2.40E-16	-0.18574	0.036	0.026	3.58E-12
C2orf74	2.75E-16	-0.37512	0.018	0.036	4.11E-12
RP11-46H1	3.30E-16	-0.35588	0.01	0.048	4.93E-12
CSF1R	3.65E-16	-0.13132	0.027	0.022	5.45E-12
STK17B	4.04E-16	-0.13077	0.032	0.021	6.03E-12
DUSP23	4.55E-16	-0.24389	0.036	0.032	6.80E-12
AC005618.	4.83E-16	-0.16982	0.034	0.029	7.22E-12
FABP3	5.32E-16	-0.30804	0.029	0.022	7.95E-12
PEG10	5.52E-16	-0.35502	0.037	0.051	8.25E-12
GPR19	6.30E-16	-0.14044	0.036	0.021	9.42E-12
FRZB	6.39E-16	-0.12664	0.032	0.012	9.55E-12
SELPLG	7.34E-16	-0.14684	0.025	0.017	1.10E-11
UNC79	7.87E-16	-0.14606	0.019	0.019	1.18E-11
PRRG1	1.18E-15	-0.11849	0.029	0.012	1.76E-11
AC114730.	1.85E-15	-0.10163	0.051	0.022	2.76E-11
RND1	2.30E-15	-0.12151	0.042	0.026	3.43E-11
AKNAD1	2.41E-15	-0.11271	0.027	0.015	3.60E-11
TGFBR3	2.66E-15	-0.14478	0.018	0.012	3.98E-11
ASS1	3.75E-15	-0.19351	0.025	0.021	5.60E-11
C8orf48	4.13E-15	-0.10221	0.021	0.01	6.18E-11
HPS1	4.23E-15	-0.17193	0.025	0.026	6.32E-11
FAM198B	7.00E-15	-0.15769	0.021	0.017	1.05E-10
ALDH1L1-A	1.02E-14	-0.11079	0.037	0.022	1.52E-10
ENTPD1	1.26E-14	-0.1629	0.023	0.021	1.89E-10
SNX15	2.50E-14	-0.11792	0.018	0.015	3.74E-10
SLC7A11	4.89E-14	-0.1253	0.03	0.027	7.31E-10
ADHFE1	5.67E-14	-0.10921	0.031	0.017	8.48E-10
GPR183	6.19E-14	-0.17064	0.02	0.022	9.24E-10
CMTM5	7.74E-14	-0.2888	0.038	0.048	1.16E-09
SERPINA1	9.55E-14	-0.42282	0.026	0.039	1.43E-09
KB-1980E6	1.48E-13	-0.11398	0.025	0.014	2.22E-09
CTD-2020K	1.54E-13	-0.16416	0.015	0.024	2.31E-09
RP11-727F	1.85E-13	-0.10856	0.029	0.017	2.76E-09
CXCL3	2.79E-13	-0.44722	0.012	0.034	4.17E-09
MS4A6A	2.90E-13	-0.13503	0.029	0.022	4.34E-09
HIST1H2BN	3.08E-13	-0.14221	0.017	0.014	4.60E-09
RNF144B	4.52E-13	-0.22407	0.012	0.021	6.75E-09

C1orf194	4.67E-13	-0.19615	0.014	0.017	6.98E-09
DGCR5	4.71E-13	-0.11676	0.026	0.021	7.04E-09
TSIX	5.59E-13	-0.20445	0.018	0.029	8.35E-09
KCNJ3	5.62E-13	-0.36523	0.007	0.043	8.40E-09
HSD11B1	6.14E-13	-0.17992	0.015	0.014	9.18E-09
APOL4	6.32E-13	-0.15349	0.027	0.019	9.45E-09
PNPLA4	7.29E-13	-0.29902	0.012	0.029	1.09E-08
RPP25	9.87E-13	-0.17768	0.009	0.012	1.47E-08
HSD17B6	1.03E-12	-0.1034	0.041	0.021	1.54E-08
MMP7	1.09E-12	-0.31668	0.015	0.026	1.63E-08
HOXB7	1.18E-12	-0.1641	0.026	0.021	1.76E-08
SCN3B	1.20E-12	-0.16959	0.029	0.029	1.79E-08
CD163	1.44E-12	-0.1753	0.023	0.024	2.14E-08
RP11-1143	2.14E-12	-0.16412	0.023	0.017	3.20E-08
NAA60	2.47E-12	-0.37271	0.008	0.036	3.70E-08
MORN5	2.78E-12	-0.13719	0.015	0.012	4.15E-08
PGAM4	3.54E-12	-0.15465	0.011	0.01	5.30E-08
AC010987.	3.69E-12	-0.12451	0.012	0.009	5.51E-08
CCER2	3.73E-12	-0.20411	0.026	0.034	5.57E-08
NPTX1	4.34E-12	-0.27242	0.007	0.021	6.49E-08
CCBE1	4.99E-12	-0.22259	0.007	0.026	7.45E-08
GNG3	5.18E-12	-0.16782	0.031	0.021	7.75E-08
CADPS	5.54E-12	-0.23351	0.018	0.027	8.28E-08
RPRM	5.84E-12	-0.13107	0.024	0.019	8.72E-08
STC2	5.94E-12	-0.16958	0.032	0.031	8.87E-08
ABHD1	7.90E-12	-0.20285	0.012	0.019	1.18E-07
IL1R1	8.65E-12	-0.11157	0.016	0.012	1.29E-07
PAIP2B	1.07E-11	-0.11424	0.015	0.01	1.60E-07
TFR2	1.15E-11	-0.16093	0.015	0.019	1.72E-07
PAWR	1.17E-11	-0.14134	0.016	0.017	1.75E-07
RP11-284F	1.21E-11	-0.27356	0.002	0.034	1.81E-07
GALNT15	1.37E-11	-0.18365	0.02	0.027	2.05E-07
MTRNR2L3	1.50E-11	-0.18902	0.014	0.026	2.25E-07
CPPED1	1.65E-11	-0.11988	0.012	0.012	2.47E-07
CCL5	2.29E-11	-0.1578	0.017	0.015	3.42E-07
FAM218A	2.67E-11	-0.11702	0.026	0.01	3.99E-07
SLC5A3	2.78E-11	-0.16308	0.007	0.017	4.16E-07
TTC9B	2.87E-11	-0.11214	0.046	0.029	4.29E-07
GREM1	3.94E-11	-0.29127	0.002	0.034	5.89E-07
BCL2L15	6.51E-11	-0.2432	0.006	0.026	9.72E-07
FXYD1	6.66E-11	-0.15729	0.029	0.026	9.95E-07
AC114803.	7.33E-11	-0.14322	0.011	0.015	1.10E-06
RP11-1246	7.96E-11	-0.15532	0.018	0.014	1.19E-06
NUDT7	8.01E-11	-0.14813	0.022	0.019	1.20E-06
LRRC61	8.63E-11	-0.11042	0.02	0.014	1.29E-06
FCGR1A	9.79E-11	-0.14963	0.013	0.014	1.46E-06
RNASE4	1.00E-10	-0.29026	0.005	0.027	1.50E-06
ARHGAP24	1.21E-10	-0.22815	0.014	0.031	1.80E-06
ACTL6B	1.33E-10	-0.22702	0.012	0.031	1.99E-06
SRRM3	1.33E-10	-0.10074	0.034	0.022	1.99E-06
AC015936.	1.46E-10	-0.11085	0.023	0.019	2.18E-06

C11orf70	2.15E-10	-0.17748	0.014	0.019	3.21E-06
EPHB6	3.00E-10	-0.125	0.014	0.017	4.48E-06
DMKN	3.37E-10	-0.17899	0.007	0.021	5.03E-06
TCEAL6	5.88E-10	-0.2827	0.015	0.027	8.78E-06
HSPB8	6.94E-10	-0.15952	0.017	0.021	1.04E-05
ASPA	7.06E-10	-0.14608	0.012	0.015	1.06E-05
IL13RA2	7.31E-10	-0.25598	0.017	0.032	1.09E-05
AC006946.	7.80E-10	-0.15942	0.01	0.014	1.17E-05
ZNF600	9.84E-10	-0.10726	0.015	0.012	1.47E-05
RP5-1177M	1.15E-09	-0.16415	0.011	0.026	1.71E-05
TSHZ2	1.29E-09	-0.29481	0.014	0.034	1.93E-05
RP11-299L	1.76E-09	-0.11794	0.011	0.015	2.63E-05
FAM132B	1.87E-09	-0.1177	0.011	0.014	2.79E-05
FGF12	2.13E-09	-0.10364	0.034	0.024	3.19E-05
S100A1	4.07E-09	-0.26712	0.009	0.017	6.08E-05
SYT4	4.18E-09	-0.16118	0.005	0.015	6.25E-05
C16orf89	4.58E-09	-0.10069	0.011	0.009	6.85E-05
FXYD7	4.60E-09	-0.1178	0.04	0.032	6.87E-05
RP11-437L	4.79E-09	-0.10832	0.012	0.012	7.16E-05
STMN2	7.39E-09	-0.32621	0.027	0.053	0.00011
LRAT	8.20E-09	-0.10556	0.015	0.012	0.000123
KRBOX1	8.46E-09	-0.31878	0.002	0.032	0.000126
DHRS4-AS1	1.07E-08	-0.26645	0.001	0.027	0.000159
CDIPT-AS1	1.18E-08	-0.228	0.009	0.021	0.000177
KLHL35	1.27E-08	-0.15941	0.008	0.017	0.00019
RP11-11N9	1.48E-08	-0.22556	0.012	0.019	0.000222
COLEC12	1.65E-08	-0.17158	0.007	0.019	0.000247
CLDN11	1.87E-08	-0.19099	0.009	0.014	0.00028
FPR1	1.89E-08	-0.24123	0.01	0.029	0.000282
CCL26	1.95E-08	-0.16189	0.007	0.005	0.000291
IGF2	1.95E-08	-0.29427	0.007	0.019	0.000291
DEPTOR	2.08E-08	-0.13741	0.009	0.009	0.000311
SELENBP1	2.10E-08	-0.19076	0.012	0.015	0.000314
NPPA	2.16E-08	-0.30692	0.008	0.032	0.000323
RBFOX3	2.65E-08	-0.18177	0.002	0.019	0.000395
LRRC2	2.83E-08	-0.2001	0.002	0.024	0.000424
NNAT	3.31E-08	-0.31777	0.03	0.053	0.000495
PHACTR3	3.42E-08	-0.12848	0.019	0.021	0.000511
DYNLRB2	3.49E-08	-0.15994	0.007	0.015	0.000522
NEGR1	3.83E-08	-0.17836	0.005	0.022	0.000572
HLA-DQA2	4.08E-08	-0.29654	0.001	0.021	0.000609
ZC2HC1C	4.75E-08	-0.10108	0.008	0.012	0.000711
LINC00632	5.47E-08	-0.11456	0.018	0.019	0.000818
CHST6	5.79E-08	-0.10392	0.007	0.007	0.000866
GPR89B	7.12E-08	-0.21271	0.001	0.021	0.001064
SLIT1	7.89E-08	-0.14159	0.007	0.014	0.001179
CDKN2A	8.55E-08	-0.27181	0.012	0.024	0.001278
C5AR2	8.73E-08	-0.19326	0.002	0.019	0.001305
TMEM232	9.88E-08	-0.11931	0.01	0.009	0.001476
SULT1C2	1.17E-07	-0.11571	0.004	0.014	0.001752
MSMP	1.20E-07	-0.10837	0.019	0.01	0.001798

MUC20	1.34E-07	-0.1193	0.015	0.015	0.001999
PRPH	1.41E-07	-0.10635	0.01	0.012	0.002106
C12orf54	1.49E-07	-0.1674	0.003	0.019	0.002229
HOXA4	1.52E-07	-0.14688	0.008	0.012	0.002274
SLC7A8	1.62E-07	-0.14255	0.009	0.021	0.002419
LUC7L2	1.73E-07	-0.24698	0.004	0.027	0.002581
SYNPO2L	1.85E-07	-0.16116	0.008	0.021	0.002762
HOXB-AS1	2.20E-07	-0.16486	0.01	0.019	0.003284
TNR	3.14E-07	-0.10681	0.046	0.036	0.004688
ASB3	3.19E-07	-0.25424	0.001	0.021	0.004764
EVI2A	3.22E-07	-0.18633	0.01	0.024	0.004814
HSPB2	3.26E-07	-0.15399	0.005	0.015	0.004875
MYCBPAP	4.55E-07	-0.11311	0.012	0.014	0.006793
LCP1	5.79E-07	-0.14082	0.008	0.007	0.008646
CRIP1	6.02E-07	-0.17412	0.005	0.012	0.008999
NPPC	6.89E-07	-0.12517	0.011	0.015	0.010296
CLN3	8.41E-07	-0.22816	0.005	0.022	0.012567
EIF4EBP3	8.46E-07	-0.16471	0.001	0.021	0.012645
CHIT1	9.10E-07	-0.21414	0.004	0.014	0.013605
SDS	9.25E-07	-0.12057	0.008	0.01	0.01382
ACE	1.06E-06	-0.10133	0.007	0.01	0.01591
FCGR2B	1.08E-06	-0.12163	0.007	0.012	0.016106
RCSD1	1.16E-06	-0.12221	0.007	0.014	0.017325
RNU12	1.32E-06	-0.11039	0.007	0.012	0.019773
OSR1	1.55E-06	-0.14202	0.005	0.01	0.023108
PATL2	1.63E-06	-0.11746	0.005	0.012	0.024309
SCN9A	1.64E-06	-0.13939	0.011	0.017	0.024439
HOXA-AS2	1.71E-06	-0.19621	0.011	0.022	0.025611
MYO15A	1.76E-06	-0.12244	0.005	0.014	0.026241
RAB3C	1.89E-06	-0.16875	0.008	0.021	0.028226
MYCNOS	3.41E-06	-0.12481	0.006	0.012	0.050948
THBS1	3.91E-06	-0.14043	0.01	0.015	0.058363
DDIT4L	4.31E-06	-0.2161	0.002	0.019	0.064462
CCL4L2	4.44E-06	-0.16446	0.003	0.017	0.066373
CRABP1	5.70E-06	-0.18876	0.002	0.012	0.085126
ZNF8	6.02E-06	-0.17636	0	0.014	0.089998
SCNN1A	6.65E-06	-0.16778	0.015	0.029	0.09931
HP	8.55E-06	-0.28859	0.001	0.015	0.127772
PCP4	9.03E-06	-0.17842	0.007	0.014	0.134956
HIST1H2BG	9.59E-06	-0.14176	0.001	0.012	0.143288
CA5A	9.63E-06	-0.14533	0.001	0.015	0.143853
C8orf34	9.90E-06	-0.1008	0.004	0.012	0.148009
AL592528.	1.10E-05	-0.12459	0.005	0.009	0.164506
GPRC5A	1.10E-05	-0.1715	0.001	0.015	0.165115
RP11-383M	1.17E-05	-0.11617	0.002	0.012	0.175004
RAMP3	1.45E-05	-0.13672	0.009	0.014	0.21657
GLRA3	1.46E-05	-0.14045	0.002	0.015	0.217573
FOLR2	1.56E-05	-0.11176	0.008	0.009	0.232569
THTPA	1.57E-05	-0.16095	0.008	0.015	0.233934
SLC16A10	1.57E-05	-0.10807	0.005	0.012	0.234953
RP11-242D	1.69E-05	-0.15284	0.005	0.015	0.251851

MYH14	1.70E-05	-0.14163	0.004	0.012	0.253872
RP11-306G	2.05E-05	-0.15176	0.001	0.015	0.306171
GNLY	2.12E-05	-0.16086	0.004	0.012	0.317173
SLC16A7	2.36E-05	-0.10392	0.005	0.01	0.351936
RP11-469H	2.87E-05	-0.11698	0.007	0.009	0.429157
CNDP1	3.55E-05	-0.10156	0.005	0.007	0.530885
GS1-18A18	3.63E-05	-0.19259	0.015	0.015	0.54266
TRIM72	4.34E-05	-0.14851	0.001	0.017	0.648195
CAMP	4.72E-05	-0.16934	0.001	0.009	0.704869
HCG11	6.22E-05	-0.13455	0.004	0.01	0.930194
SERPINA3	7.97E-05	-0.14542	0.001	0.012	1
AC015849.	8.31E-05	-0.11657	0.004	0.012	1
C8orf22	8.57E-05	-0.14807	0.001	0.012	1
MARCO	8.72E-05	-0.15533	0.001	0.014	1
EDN1	0.000128	-0.11651	0.005	0.009	1
LINC00637	0.000152	-0.10119	0.002	0.01	1
NAP1L6	0.000154	-0.10839	0.004	0.01	1
LINC01138	0.000168	-0.11329	0.003	0.01	1
NPFFR1	0.000211	-0.10043	0.003	0.007	1
FAM180A	0.000218	-0.13509	0.003	0.01	1
PDZK1IP1	0.000236	-0.15437	0.003	0.009	1
PLCG2	0.000266	-0.16482	0.004	0.015	1
C8orf86	0.000308	-0.11953	0.001	0.009	1
LINC0032C	0.000364	-0.12865	0.004	0.012	1
SAA4	0.000502	-0.1016	0.002	0.005	1
RP11-495F	0.000523	-0.12423	0.001	0.009	1
CTD-2369F	0.000538	-0.12417	0.002	0.012	1
CNR2	0.000544	-0.10059	0.002	0.01	1
RP11-522E	0.00079	-0.11287	0.001	0.009	1
SLITRK4	0.000801	-0.11208	0.001	0.01	1
COL1A2	0.000873	-0.10728	0.002	0.009	1
GJC3	0.001011	-0.13077	0.005	0.014	1
PLEKHG4B	0.001127	-0.10397	0.001	0.01	1
WIF1	0.001146	-0.15026	0.001	0.007	1
BCAS1	0.001191	-0.11636	0.011	0.015	1
CCL3L3	0.00128	-0.11029	0.001	0.01	1
HIST2H2BF	0.001494	-0.11505	0.004	0.007	1
RP11-89N1	0.001795	-0.11466	0	0.007	1
CSMD3	0.002466	-0.13384	0.007	0.012	1
HIST3H2A	0.002769	-0.11547	0.002	0.009	1
ACSM5	0.00355	-0.12088	0.004	0.01	1
MSC	0.004182	-0.17509	0.002	0.005	1
STC1	0.006632	-0.11024	0.001	0.005	1
FAM110C	0.009747	-0.15034	0.001	0.007	1
RP11-264E	0.034333	-0.10051	0	0.003	1
