# Supplementary material for: A Novel Molecular Classification Method for Glioblastoma Based on Tumor Cell Differentiation Trajectories
Source: Stem Cells Int. 2023 Feb 22;2023:2826815. doi: 10.1155/2023/2826815 (PMC10643041; doi:10.1155/2023/2826815)
Supplement: Supplementary 6 — Supplementary Table 4: differentially expressed genes (DEGs) in GSCL and Opc-G. [file 2826815.f6.pdf]

	p_val	avg_logFC	pct.1	pct.2	p_val_adj
SEC61G	4.1828271	-1.47794	0.791	0.935	6.25081696417212e-311
MT2A	########	-1.62515	0.605	0.944	########
GAPDH	########	-0.83641	0.996	0.99	########
CLU	########	-1.47282	0.648	0.896	########
B2M	########	-0.99892	0.951	0.947	########
CD63	########	-1.04021	0.862	0.856	########
FTL	########	-0.79598	0.982	0.974	########
TPI1	########	-0.84174	0.839	0.786	########
MT3	########	-1.39239	0.568	0.802	########
EIF1	########	-0.70466	0.983	0.918	########
HES6	########	1.424899	0.875	0.097	########
ENO1	########	-0.94373	0.757	0.764	########
TMSB4X	########	-0.64681	0.99	0.973	########
NDUFB2	########	-0.40612	0.87	0.586	########
PCSK1N	########	1.123635	0.85	0.113	########
HLA-A	########	-1.16139	0.654	0.798	########
PTN	########	-0.79343	0.843	0.776	########
FAU	########	-0.67224	0.979	0.903	########
SKP1	########	-0.57578	0.952	0.791	########
DLL3	########	1.706159	0.765	0.029	########
ALDOA	########	-1.01299	0.745	0.805	########
UBL5	########	-0.73364	0.845	0.733	########
MYL6	########	-0.57629	0.941	0.793	########
ITM2B	########	0.330185	0.905	0.32	########
SOX4	########	1.114038	0.954	0.231	########
OAZ1	########	-0.59881	0.9	0.754	########
HLA-B	########	-1.1153	0.629	0.713	########
TIMP1	########	-1.81897	0.295	0.68	########
PARK7	########	-0.51828	0.837	0.603	########
TMSB10	########	-0.6681	0.969	0.918	########
MARCKS	########	0.96631	0.902	0.188	########
ATP5E	########	-0.52395	0.913	0.735	########
NDUFA4	########	-0.45828	0.977	0.821	########
UBC	########	-0.57038	0.973	0.86	########
PRDX2	########	-0.40599	0.94	0.68	########
UQCRQ	########	-0.38897	0.892	0.622	########
SNRPD2	########	-0.56191	0.836	0.597	########
HN1	########	0.376282	0.873	0.287	########
HSBP1	########	-0.24742	0.831	0.455	########
NDUFS5	########	-0.49909	0.909	0.685	########
PEBP1	########	-0.41126	0.925	0.656	########
TPT1	########	-0.64928	0.96	0.889	########
HNRNPA0	########	0.557526	0.843	0.219	########
COX7C	########	-0.42971	0.947	0.718	########
MARCKSL1	########	0.853373	0.969	0.297	########
ACTG1	########	-0.51759	0.99	0.909	########
MLLT11	########	0.656032	0.874	0.227	########
RAC1	########	0.479773	0.953	0.364	########
LAMTOR4	########	-0.28677	0.821	0.48	########
PRDX1	########	-0.49472	0.86	0.614	########

COX6A1	########	-0.31397	0.94	0.656	########
MALAT1	########	-0.42968	0.997	0.979	########
COX6C	########	-0.33101	0.935	0.66	########
GNB2L1	########	-0.56162	0.987	0.906	########
SERF2	########	-0.56947	0.87	0.691	########
CD9	########	-1.27319	0.472	0.66	########
COX6B1	########	-0.57486	0.884	0.708	########
ATP5J2	########	-0.21561	0.925	0.591	########
GSTP1	########	-0.46965	0.871	0.644	########
DYNLL1	########	-0.5044	0.901	0.706	########
CIRBP	########	-0.36984	0.943	0.718	########
TCEB2	########	-0.34002	0.91	0.626	########
MYEOV2	########	-0.16552	0.793	0.395	########
BTG1	########	0.673151	0.884	0.239	########
BTF3	########	-0.48942	0.957	0.774	########
COX4I1	########	-0.56671	0.923	0.764	########
TOMM7	########	-0.47987	0.914	0.696	########
NPM1	########	-0.42506	0.94	0.715	########
CST3	########	-0.45438	0.861	0.569	########
TMBIM6	########	-0.58233	0.823	0.626	########
HINT1	########	-0.51483	0.899	0.694	########
BRK1	########	-0.33522	0.813	0.496	########
CYCS	########	-0.36764	0.814	0.504	########
GPM6B	########	-0.43087	0.93	0.725	########
CD74	########	-1.06885	0.586	0.629	########
TMA7	########	-0.51057	0.887	0.703	########
NGFRAP1	########	-0.39164	0.952	0.699	########
BSG	########	-0.36064	0.874	0.597	########
VIM	########	-1.55302	0.333	0.641	########
ATP5L	########	-0.4529	0.883	0.639	########
SUMO2	########	-0.2012	0.939	0.605	########
SRP9	########	-0.25666	0.792	0.453	########
ANAPC11	########	-0.4151	0.827	0.544	########
PFN2	########	0.676881	0.852	0.209	########
H2AFZ	########	-0.28006	0.848	0.497	########
ERH	########	-0.27463	0.858	0.509	########
DBI	########	-0.53646	0.966	0.817	########
SSR2	########	-0.17014	0.831	0.443	########
PFDN5	########	-0.48585	0.9	0.685	########
YBX1	########	0.303536	0.941	0.407	########
COX5B	########	-0.32904	0.931	0.682	########
RTN4	########	0.754204	0.888	0.236	########
CFL1	########	-0.35727	0.986	0.798	########
COPE	########	-0.66657	0.745	0.631	########
ATP5G3	########	-0.11171	0.857	0.441	########
PKM	########	-0.90328	0.668	0.658	########
POLR2J	########	-0.28302	0.797	0.458	########
HSPA8	########	-0.35009	0.945	0.716	########
NDUFA13	########	-0.42077	0.836	0.593	########
CALM1	########	-0.17743	0.908	0.537	########
NACA	########	-0.4628	0.966	0.797	########

CBX3	########	0.716347	0.836	0.195	########
EDF1	########	-0.32651	0.831	0.52	########
SOD1	########	-0.67695	0.763	0.624	########
RAN	########	-0.39731	0.865	0.588	########
H3F3B	########	-0.42652	0.995	0.889	########
HLA-C	########	-1.16202	0.506	0.631	########
SRP14	########	-0.17649	0.958	0.61	########
PPIA	########	-0.42088	0.943	0.747	########
SPCS2	########	-0.51481	0.763	0.528	########
HMGB1	########	-0.31998	0.961	0.691	########
SPCS1	########	-0.53614	0.73	0.497	########
UQCRH	########	-0.45358	0.84	0.588	########
C1QBP	########	0.513845	0.766	0.176	########
EIF3K	########	-0.50872	0.779	0.552	########
ATP5O	########	-0.2756	0.839	0.499	########
COX7A2	########	-0.20535	0.908	0.561	########
NDUFB7	########	-0.51113	0.806	0.579	########
YWHAQ	########	0.438483	0.878	0.304	########
CCNI	########	0.447208	0.861	0.28	########
LDHA	########	-1.17467	0.553	0.66	########
COMMD6	########	-0.35132	0.802	0.489	########
ATP5J	########	-0.3539	0.835	0.535	########
DNAJB6	########	0.159922	0.857	0.361	########
HMGN1	########	0.522027	0.798	0.214	########
ANXA5	########	-0.94857	0.646	0.68	########
CRYAB	########	-1.38644	0.454	0.617	########
KMT2E	########	0.357274	0.817	0.256	########
GABARAPL2	########	-0.31898	0.814	0.497	########
POLR2L	########	-0.35628	0.717	0.421	########
NDUFC2	########	-0.28077	0.815	0.477	########
LDHB	########	-0.34466	0.958	0.706	########
OST4	########	-0.28769	0.834	0.511	########
WDR83OS	########	-0.68078	0.758	0.6	########
PGK1	########	-0.81105	0.657	0.545	########
FIS1	########	0.146909	0.823	0.335	########
ZNHIT1	########	-0.23254	0.797	0.446	########
STMN1	########	0.398424	0.948	0.388	########
SEC61B	########	-0.48914	0.754	0.503	########
UBE2E3	########	0.689043	0.743	0.144	########
ATP5G2	########	-0.38493	0.9	0.651	########
SAP18	########	-0.57937	0.782	0.576	########
COX8A	########	-0.33033	0.808	0.499	########
BUD31	########	-0.25668	0.792	0.439	########
HNRNPA2B1	########	-0.18615	0.954	0.663	########
MT1X	########	-1.4105	0.476	0.634	########
ATP5B	########	-0.2652	0.874	0.557	########
POLR2F	########	0.45949	0.828	0.248	########
ATP6V1G1	########	-0.36653	0.824	0.526	########
PHF14	########	0.86069	0.772	0.13	########
PSMB6	########	-0.29828	0.791	0.448	########
CNPY2	########	0.212741	0.792	0.287	########

EID1	########	-0.14835	0.86	0.485	########
RBM39	########	-0.15034	0.745	0.34	########
SEC62	########	-0.10458	0.797	0.386	########
SLC25A3	########	-0.12564	0.834	0.438	########
NEAT1	########	-1.18589	0.446	0.629	########
EIF4A2	########	-0.16363	0.853	0.491	########
ATP6V1F	########	-0.29884	0.819	0.492	########
SPP1	########	-0.76742	0.774	0.638	########
SRSF9	########	0.538496	0.75	0.169	########
BANF1	########	-0.42909	0.714	0.45	########
CHI3L1	########	-2.36837	0.126	0.583	########
C12orf57	########	-0.30565	0.735	0.412	########
PSMB1	########	-0.30822	0.791	0.444	########
DBN1	########	1.123508	0.689	0.053	########
GPX1	########	-0.13893	0.775	0.379	########
EEF2	########	-0.20644	0.935	0.632	########
CRMP1	########	0.930397	0.653	0.062	########
GPM6A	########	0.335073	0.908	0.374	########
TSC22D1	########	0.226797	0.883	0.391	########
MIF	########	0.108556	0.774	0.299	########
AP2M1	########	-0.19779	0.77	0.415	########
UQCR10	########	-0.17316	0.806	0.426	########
LGALS3	########	-2.0123	0.161	0.583	########
TTC3	########	0.743094	0.792	0.171	########
CHMP2A	########	-0.52496	0.709	0.48	########
ASCL1	########	1.159226	0.698	0.062	########
2-Sep	########	0.296606	0.796	0.27	########
PPIB	########	-0.49809	0.731	0.477	########
PFDN2	########	-0.25171	0.711	0.352	########
GLUL	########	-0.98994	0.577	0.535	########
ATP5I	########	-0.26927	0.822	0.521	########
ZNF428	########	0.480724	0.802	0.232	########
CNBP	########	-0.46556	0.824	0.583	########
UCHL1	########	0.147314	0.787	0.32	########
HNRNPC	########	-0.40347	0.808	0.538	########
SELK	########	-0.33497	0.766	0.446	########
C19orf53	########	-0.75812	0.691	0.571	########
RPAIN	########	0.339026	0.753	0.219	########
S100B	########	-0.29708	0.85	0.545	########
NDUFA1	########	-0.53135	0.678	0.47	########
SOX2	########	0.389057	0.824	0.287	########
U2AF1	########	0.38262	0.774	0.227	########
PCBP1	########	0.550206	0.745	0.169	########
ARF5	########	0.495365	0.72	0.176	########
LAMTOR5	########	-0.61339	0.713	0.537	########
CALM2	########	-0.34438	0.969	0.769	########
FSCN1	########	1.032846	0.775	0.123	########
ILF2	########	0.139521	0.769	0.292	########
SSR4	########	-0.24321	0.832	0.482	########
SHFM1	########	-0.29076	0.848	0.528	########
PTGES3	########	-0.33511	0.771	0.474	########

OCIAD1	########	-0.11041	0.766	0.393	########
CCT6A	########	0.317328	0.796	0.27	########
SNRPG	########	-0.27367	0.743	0.419	########
TRMT112	########	-0.66481	0.657	0.497	########
VDAC2	########	-0.20977	0.698	0.356	########
WBP5	########	-0.3359	0.71	0.385	########
MDK	########	0.352421	0.831	0.304	########
TMEM59	########	-0.36841	0.765	0.48	########
XRCC5	########	0.514532	0.744	0.181	########
DDX5	########	0.160632	0.93	0.47	########
S100A6	########	-1.25223	0.468	0.605	########
C19orf43	########	0.404855	0.8	0.248	########
RTN3	########	0.388614	0.867	0.318	########
EEF1B2	########	-0.15091	0.784	0.403	########
NDUFA11	########	-0.2606	0.804	0.475	########
EIF3L	########	0.42651	0.756	0.215	########
SSBP1	########	-0.10564	0.745	0.349	########
TMEM258	########	-0.39898	0.744	0.48	########
GNAS	########	-0.2487	0.802	0.462	########
YWHAB	########	0.334215	0.801	0.268	########
TBCA	########	-0.36702	0.843	0.581	########
C7orf73	########	-0.19988	0.651	0.318	########
ROMO1	########	-0.20115	0.707	0.368	########
NDFIP1	########	0.550371	0.749	0.183	########
SBDS	########	-0.23721	0.741	0.393	########
FXYD6	########	0.217708	0.919	0.424	########
SEPW1	########	-0.30312	0.776	0.467	########
PSMB5	########	-0.28201	0.748	0.424	########
DNAJA1	########	0.190869	0.737	0.255	########
C11orf58	########	-0.16791	0.759	0.398	########
C7orf55	########	0.197604	0.732	0.246	########
GLTSCR2	########	0.526718	0.718	0.162	########
CCND2	########	0.943397	0.767	0.133	########
GPX4	########	-0.48807	0.808	0.598	########
HNRNPK	########	-0.14719	0.925	0.598	########
ZFAS1	########	-0.43019	0.791	0.526	########
VAMP2	########	0.18453	0.834	0.347	########
RAB1A	########	-0.13301	0.684	0.284	########
MAP2	########	0.890981	0.731	0.115	########
SCD5	########	0.764773	0.798	0.188	########
UQCRB	########	0.287955	0.81	0.275	########
UBE2D3	########	-0.23408	0.7	0.354	########
7-Sep	########	-0.33995	0.831	0.59	########
CHCHD2	########	-0.29839	0.961	0.747	########
SLC25A5	########	-0.49078	0.692	0.484	########
POMP	########	-0.39036	0.714	0.417	########
MORF4L1	########	-0.4376	0.7	0.446	########
SRSF2	########	0.543999	0.748	0.183	########
MFF	########	0.341753	0.713	0.197	########
PSMB3	########	-0.5605	0.658	0.434	########
EIF4H	########	0.154576	0.718	0.251	########

NDUFB9	########	-0.10023	0.814	0.417	########
MZT2B	########	0.532159	0.765	0.203	########
HDAC2	########	0.715841	0.663	0.099	########
TERF2IP	########	0.710292	0.74	0.145	########
EEF1D	########	-0.25867	0.899	0.583	########
ARF4	########	-0.30937	0.684	0.371	########
EIF1B	########	-0.12319	0.732	0.347	########
PODXL2	########	1.145704	0.679	0.06	########
DAD1	########	-0.45934	0.731	0.482	########
NDUFA5	########	-0.23822	0.759	0.436	########
LAPTM4A	########	-0.45557	0.713	0.482	########
SNRPE	########	-0.12465	0.713	0.34	########
UBE2I	########	0.540598	0.713	0.162	########
LAPTM4B	########	0.747335	0.7	0.12	########
ATP6V0E1	########	-0.76133	0.609	0.492	########
SLC25A6	########	-0.15889	0.941	0.632	########
NOVA1	########	0.50703	0.94	0.393	########
FTH1	########	-0.27759	0.98	0.757	########
ATRAID	########	-0.32592	0.675	0.385	########
WSB1	########	0.192557	0.788	0.311	########
SCAND1	########	0.534592	0.737	0.183	########
CUTA	########	-0.14067	0.757	0.386	########
RAB7A	########	-0.15279	0.642	0.289	########
DYNLRB1	########	-0.33559	0.706	0.414	########
TCP1	########	0.229629	0.681	0.207	########
GNB2	########	0.537946	0.743	0.19	########
NEDD8	########	-0.45778	0.761	0.533	########
RHOA	########	-0.36794	0.684	0.398	########
C7orf50	########	0.492941	0.7	0.178	########
SET	########	0.342905	0.736	0.224	########
NFIB	########	0.817377	0.77	0.162	########
SFPQ	########	0.582201	0.748	0.188	########
PRDX5	########	-0.47375	0.732	0.518	########
PDIA6	########	-0.20321	0.697	0.34	########
PGRMC1	########	0.804713	0.679	0.103	########
PDAP1	########	0.207323	0.728	0.253	########
NBEAL1	########	-0.71901	0.597	0.494	########
CALM3	########	0.543409	0.713	0.164	########
NDUFA3	########	-0.19074	0.689	0.374	########
KHDRBS1	########	0.628925	0.683	0.135	########
UBB	########	-0.38783	0.923	0.779	########
SCRG1	########	0.620814	0.752	0.19	########
SLIRP	########	-0.16787	0.723	0.378	########
PSMB7	########	-0.16791	0.763	0.409	########
EIF4G2	########	0.210487	0.761	0.284	########
ODC1	########	1.008491	0.715	0.103	########
SOX8	########	1.283096	0.636	0.039	########
HIST1H4C	########	0.693708	0.664	0.113	########
NDUFB3	########	-0.3481	0.564	0.299	########
FKBP1A	########	-0.25013	0.676	0.359	########
PSMD8	########	-0.38649	0.74	0.479	########

SRI	########	-0.16206	0.975	0.687	########
ACTB	########	-0.41927	0.995	0.92	########
PCBP2	########	0.174656	0.762	0.296	########
SSB	########	-0.16184	0.717	0.354	########
TUBA1B	########	-0.36992	0.912	0.701	########
SH3BGRL3	########	-0.73718	0.585	0.462	########
PRELID1	########	0.387781	0.667	0.162	########
APP	########	0.130662	0.714	0.268	########
APEX1	########	0.188362	0.782	0.303	########
ATP5A1	########	-0.35589	0.715	0.45	########
UBE2D2	########	0.377271	0.674	0.178	########
SRSF5	########	0.150537	0.749	0.292	########
TMED9	########	-0.51341	0.685	0.456	########
RHOBTB3	########	0.558313	0.75	0.197	########
IGFBP2	########	-0.40788	0.629	0.378	########
PSMA7	########	-0.29187	0.86	0.573	########
NDUFS6	########	-0.16008	0.701	0.35	########
C4orf3	########	-0.5893	0.566	0.376	########
ARF1	########	-0.33699	0.646	0.378	########
HNRNPM	########	0.674052	0.704	0.138	########
RABAC1	########	-0.34669	0.714	0.436	########
TCF4	########	0.440208	0.806	0.274	########
OLIG2	########	1.182324	0.694	0.082	########
C14orf2	########	-0.20008	0.732	0.39	########
MGST3	########	-0.61414	0.623	0.468	########
15-Sep	########	-0.48494	0.579	0.366	########
ATP6V0B	########	-0.20245	0.735	0.39	########
RBMX	########	0.268179	0.739	0.258	########
ATXN10	########	0.476578	0.609	0.116	########
CNN3	########	-0.52543	0.583	0.403	########
SF3B5	########	-0.18511	0.645	0.304	########
RBM8A	########	-0.25975	0.674	0.352	########
RAB2A	########	0.314037	0.737	0.227	########
RCN2	########	0.655123	0.715	0.149	########
PHPT1	########	-0.64083	0.618	0.46	########
CSNK1A1	########	0.257727	0.654	0.203	########
ATP6V0E2	########	0.208013	0.691	0.234	########
ATP5F1	########	-0.42327	0.644	0.403	########
RP11-161M	########	1.345257	0.605	0.027	########
H2AFV	########	0.356996	0.67	0.183	########
RNPS1	########	0.264298	0.711	0.234	########
FAM96B	########	-0.18877	0.709	0.368	########
KRT10	########	0.350467	0.7	0.209	########
ATP5G1	########	-0.19435	0.706	0.364	########
PRDX6	########	-1.0526	0.443	0.504	########
MYL12B	########	-0.55268	0.629	0.424	########
EIF3F	########	0.418293	0.706	0.193	########
TXN	########	-0.46226	0.679	0.46	########
SHD	########	1.131676	0.571	0.026	########
NDUFA12	########	-0.39008	0.592	0.32	########
RBPJ	########	0.692199	0.654	0.109	########

NDUFB1	########	-0.36266	0.618	0.378	########
BTF3L4	########	0.166007	0.644	0.207	########
PYURF	########	0.332779	0.642	0.168	########
H2AFY	########	0.510558	0.707	0.179	########
ATP5H	########	-0.37961	0.618	0.345	########
RAB5C	########	-0.1547	0.618	0.267	########
HNRNPU	########	0.270116	0.641	0.191	########
PUF60	########	0.228732	0.723	0.251	########
SRPK2	########	0.657675	0.688	0.137	########
GNG5	########	-0.79746	0.518	0.467	########
POLR2I	########	-0.52933	0.596	0.402	########
TIMM13	########	-0.17669	0.728	0.379	########
PTMA	########	-0.37866	0.993	0.887	########
DCTN3	########	0.12708	0.631	0.205	########
BLOC1S1	########	-0.19514	0.65	0.32	########
PTMS	########	0.640651	0.795	0.21	########
ATF4	########	-0.13278	0.628	0.279	########
PSAP	########	-0.34537	0.679	0.4	########
CSNK2B	########	-0.24268	0.618	0.309	########
GLCCI1	########	1.132062	0.61	0.043	########
CENPV	########	1.149942	0.606	0.038	########
MKRN1	########	0.591864	0.599	0.094	########
PABPC1	########	0.280261	0.818	0.316	########
IER2	########	0.674967	0.817	0.234	########
DDX18	########	0.147889	0.671	0.227	########
TUBB2B	########	0.327094	0.951	0.472	########
AKR1B1	########	0.342513	0.65	0.174	########
SAT1	########	-0.90826	0.54	0.501	########
STRAP	########	0.151107	0.719	0.27	########
DSTN	########	-0.22865	0.7	0.376	########
TBCB	########	-0.18862	0.757	0.417	########
TSPAN3	########	0.357399	0.674	0.2	########
CHD7	########	0.95552	0.654	0.077	########
NDUFA6	########	-0.49292	0.602	0.403	########
HSP90AA1	########	-0.20865	0.978	0.754	########
DSEL	########	0.96623	0.654	0.084	########
SNRPB2	########	-0.30308	0.623	0.325	########
COX7B	########	-0.17359	0.658	0.32	########
SELT	########	-0.2611	0.609	0.32	########
EIF5B	########	0.126093	0.671	0.241	########
TAF7	########	-0.31793	0.726	0.436	########
ERGIC3	########	-0.12865	0.672	0.318	########
KDELR2	########	0.477811	0.638	0.147	########
HNRNPAB	########	0.748663	0.598	0.077	########
CYC1	########	0.275067	0.681	0.203	########
PRMT1	########	-0.14305	0.693	0.34	########
ZC3H15	########	0.254882	0.642	0.179	########
PPP2R1A	########	0.17686	0.681	0.238	########
TUBB	########	0.14527	0.953	0.523	########
CLTA	########	0.283782	0.667	0.197	########
PAIP2	########	-0.17309	0.662	0.337	########

MORF4L2	########	-0.18822	0.691	0.366	########
CAMLG	########	-0.13632	0.618	0.27	########
SRRM2	########	0.548135	0.713	0.176	########
LMO4	########	0.328797	0.707	0.231	########
ZNF90	########	-0.84348	0.456	0.427	########
RSF1	########	0.522301	0.646	0.133	########
SAT2	########	-0.36705	0.649	0.369	########
PHB	########	-0.21344	0.631	0.318	########
NCL	########	0.262646	0.667	0.205	########
GTF3C6	########	0.198357	0.629	0.195	########
COX7A2L	########	-0.31616	0.624	0.342	########
MATR3	########	0.593429	0.659	0.138	########
TSPAN13	########	0.897505	0.609	0.07	########
RAB10	########	0.258264	0.56	0.142	########
ARPC3	########	-0.27013	0.683	0.39	########
RBP1	########	0.149325	0.717	0.284	########
PDCD5	########	-0.41923	0.616	0.368	########
MAGED1	########	0.359545	0.614	0.156	########
MEST	########	0.449626	0.756	0.248	########
NFIA	########	0.338341	0.689	0.221	########
PARP1	########	0.387185	0.601	0.138	########
HNRNPA1	########	-0.14148	0.971	0.696	########
PAFAH1B3	########	0.128544	0.636	0.224	########
PTOV1	########	0.553008	0.655	0.14	########
ACP1	########	-0.19074	0.573	0.263	########
EMC10	########	0.508165	0.612	0.123	########
CCT5	########	0.157584	0.603	0.188	########
VDAC1	########	-0.48008	0.59	0.39	########
YWHAG	########	0.441526	0.563	0.111	########
SOX11	########	1.09694	0.641	0.068	########
SMDT1	########	0.288183	0.694	0.219	########
RHOB	########	0.366453	0.762	0.268	########
SF1	########	0.449437	0.648	0.156	########
QKI	########	0.520637	0.649	0.15	########
MTDH	########	0.601574	0.705	0.166	########
IP6K2	########	-0.16126	0.637	0.304	########
MAD2L2	########	0.522673	0.603	0.121	########
TOMM22	########	0.245562	0.571	0.144	########
DPY30	########	-0.14583	0.576	0.256	########
C14orf166	########	-0.30366	0.614	0.335	########
H1FX	########	0.945792	0.636	0.079	########
NUCKS1	########	-0.21937	0.67	0.364	########
HSP90B1	########	-0.37878	0.62	0.366	########
AP2S1	########	-0.30768	0.59	0.321	########
CALR	########	-0.25795	0.684	0.393	########
HNRNPA3	########	0.119189	0.661	0.239	########
TCEB1	########	-0.16266	0.668	0.313	########
HNRNPR	########	0.185055	0.635	0.214	########
SLC3A2	########	-0.55406	0.56	0.386	########
EIF3I	########	-0.25559	0.64	0.345	########
NAA38	########	-0.29164	0.533	0.272	########

NDUFS8	########	-0.29294	0.62	0.335	########
TRAF4	########	0.897461	0.554	0.051	########
NDUFC1	########	-0.55041	0.537	0.366	########
SMARCB1	########	0.646045	0.606	0.103	########
RAMP1	########	-0.57548	0.657	0.538	########
C9orf16	########	0.235687	0.633	0.2	########
EIF3G	########	-0.21883	0.662	0.359	########
NUDC	########	-0.31739	0.588	0.326	########
EMC4	########	0.149816	0.599	0.186	########
HLA-DRA	########	-1.1982	0.33	0.426	########
S100A10	########	-1.6887	0.164	0.533	########
SNF8	########	0.596826	0.611	0.113	########
UFC1	########	-0.18492	0.59	0.28	########
EEF1A1	########	-0.81949	0.684	0.851	########
FAM213A	########	0.168979	0.563	0.174	########
MIDN	########	0.607868	0.653	0.144	########
ZNF207	########	0.290268	0.562	0.137	########
GTF2A2	########	-0.23836	0.512	0.234	########
GPC2	########	1.13689	0.544	0.022	########
SDHC	########	-0.58956	0.485	0.345	########
BLVRA	########	0.119546	0.547	0.162	########
YWHAH	########	0.776016	0.601	0.084	########
NOP10	########	-0.12443	0.554	0.248	########
PPA1	########	0.218612	0.589	0.164	########
MDH1	########	-0.26097	0.579	0.308	########
RAB11B	########	0.22501	0.614	0.198	########
UBXN4	########	0.124972	0.638	0.222	########
AP1S1	########	-0.12687	0.594	0.268	########
SDCBP	########	-0.52105	0.603	0.438	########
NCAM1	########	0.607619	0.651	0.138	########
NONO	########	0.275477	0.615	0.183	########
NDUFB4	########	-0.4281	0.664	0.444	########
COX16	########	0.34855	0.598	0.156	########
SEC11A	########	-0.69447	0.455	0.342	########
EIF2S2	########	-0.40811	0.577	0.338	########
ARHGDIA	########	0.105329	0.541	0.168	########
NRXN1	########	0.929085	0.537	0.043	########
TMED10	########	-0.6534	0.532	0.417	########
TMEM160	########	0.688747	0.581	0.084	########
CAPZB	########	-0.19209	0.568	0.272	########
NKAIN4	########	1.113968	0.549	0.038	########
FABP5	########	-1.38765	0.336	0.501	########
HSPA5	########	-0.61017	0.594	0.422	########
NAP1L1	########	-0.30331	0.649	0.378	########
PIK3R1	########	0.783907	0.601	0.087	########
UBXN1	########	0.167169	0.692	0.253	########
PSMC5	########	-0.11266	0.593	0.263	########
STMN4	########	0.807485	0.674	0.132	########
CSDE1	########	0.18882	0.645	0.222	########
TOMM20	########	-0.3609	0.537	0.28	########
ELAVL4	########	0.930968	0.577	0.06	########

EWSR1	########	0.414253	0.599	0.142	########
FKBP8	########	-0.21603	0.625	0.33	########
SNRNP70	########	0.541644	0.628	0.138	########
ZBTB20	########	0.495687	0.691	0.186	########
RSRC2	########	0.110799	0.606	0.195	########
PPP2CA	########	0.321853	0.589	0.152	########
FAM127A	########	0.245686	0.628	0.2	########
PPM1G	########	0.775596	0.589	0.077	########
OLA1	########	0.14207	0.601	0.203	########
MEAF6	########	-0.17027	0.52	0.229	########
ARL5A	########	0.532507	0.615	0.128	########
HIGD2A	########	-0.5531	0.545	0.386	########
YTHDF2	########	0.251014	0.573	0.159	########
TMEM230	########	-0.29331	0.627	0.361	########
CWC15	########	-0.13516	0.525	0.212	########
GDI1	########	0.197764	0.657	0.227	########
VIPR2	########	1.196027	0.512	0.012	########
NCBP2	########	0.194838	0.607	0.195	########
CCDC88A	########	0.717325	0.572	0.082	########
EGR1	########	0.451638	0.813	0.304	########
GSTA4	########	0.475633	0.592	0.128	########
NDUFA2	########	-0.19094	0.59	0.291	########
NDUFS4	########	-0.403	0.546	0.318	########
NAP1L4	########	0.386024	0.571	0.132	########
COPS8	########	-0.20817	0.597	0.27	########
PDPN	########	-0.49792	0.48	0.316	########
ASNA1	########	-0.3558	0.511	0.282	########
LGALS1	########	-1.14651	0.428	0.598	########
SYT11	########	0.322712	0.57	0.15	########
ENY2	########	-0.15167	0.623	0.299	########
BZW1	########	-0.11812	0.599	0.277	########
RSBN1L	########	0.629319	0.612	0.113	########
DHX36	########	0.431948	0.585	0.132	########
TECR	########	-0.96471	0.382	0.407	########
ZKSCAN1	########	0.563688	0.557	0.092	########
JTB	########	0.319849	0.636	0.183	########
THY1	########	0.64973	0.56	0.091	########
GADD45G	########	1.075979	0.622	0.075	########
KCNQ1OT1	########	0.754068	0.641	0.111	########
SF3B2	########	0.106994	0.597	0.214	########
DRAP1	########	-0.33391	0.605	0.362	########
ZCRB1	########	-0.33817	0.505	0.25	########
LSM3	########	-0.1684	0.584	0.27	########
NDUFS2	########	-0.16444	0.602	0.309	########
LINC00493	########	-0.35356	0.546	0.28	########
CDC37	########	-0.15766	0.538	0.248	########
METTL9	########	0.431418	0.568	0.12	########
GAP43	########	-0.91363	0.433	0.443	########
ARL2BP	########	0.239535	0.545	0.149	########
CALD1	########	-0.10325	0.623	0.294	########
C1orf61	########	-0.19835	0.869	0.585	########

TRA2B	########	0.171981	0.67	0.248	########
PSMA1	########	-0.3606	0.594	0.362	########
FKBP3	########	-0.23606	0.558	0.282	########
TUBB2A	########	0.155512	0.629	0.236	########
KIF5B	########	0.594214	0.61	0.118	########
HSD17B10	########	-0.21841	0.567	0.277	########
FEZ1	########	0.19965	0.616	0.212	########
NDUFS7	########	-0.25546	0.605	0.32	########
PHB2	########	0.238221	0.551	0.147	########
NDUFV2	########	-0.3264	0.564	0.32	########
PSMB2	########	-0.41247	0.585	0.362	########
PNRC1	########	0.415387	0.575	0.137	########
PON2	########	-0.30676	0.579	0.342	########
CTSB	########	-0.73267	0.454	0.374	########
HSPA9	########	-0.3665	0.501	0.289	########
LAMTOR1	########	-0.13476	0.616	0.289	########
TCEAL7	########	0.325103	0.537	0.121	########
SLC22A17	########	0.844227	0.568	0.068	########
PDCD6	########	0.165429	0.584	0.193	########
MAGEH1	########	0.589992	0.616	0.123	########
ZNF667-AS	########	-0.40209	0.546	0.326	########
SRP72	########	0.492978	0.62	0.144	########
HNRNPD	########	0.643373	0.546	0.084	########
AES	########	0.359487	0.631	0.179	########
ATPIF1	########	-0.38756	0.56	0.35	########
POLR2E	########	-0.14327	0.571	0.27	########
ARL6IP4	########	0.140088	0.593	0.209	########
POLR2G	########	-0.15438	0.612	0.303	########
PRPF40A	########	0.450763	0.573	0.123	########
C4orf48	########	0.737766	0.571	0.084	########
SMS	########	-0.45516	0.466	0.296	########
LUC7L3	########	0.239074	0.681	0.234	########
MCM7	########	0.749505	0.56	0.079	########
FAM3C	########	0.370844	0.597	0.169	########
COA3	########	-0.15204	0.553	0.253	########
CHD9	########	0.430312	0.594	0.145	########
ESD	########	-0.29982	0.495	0.243	########
PCNP	########	-0.27119	0.56	0.306	########
UBE2L3	########	0.145356	0.55	0.169	########
PSMC2	########	0.121045	0.577	0.195	########
ECH1	########	-0.53037	0.529	0.376	########
SRM	########	0.371055	0.558	0.133	########
PRDX4	########	-0.34569	0.572	0.337	########
SRSF11	########	0.143956	0.638	0.238	########
FYN	########	0.553504	0.583	0.118	########
SMIM7	########	-0.48946	0.531	0.342	########
HNRNPH3	########	0.373256	0.575	0.14	########
OLIG1	########	1.057916	0.668	0.113	########
KLHL7	########	0.704404	0.52	0.067	########
TMCO1	########	-0.33655	0.597	0.35	########
KRTCAP2	########	-0.30088	0.514	0.291	########

LSM4	########	0.140065	0.637	0.243	########
NASP	########	0.333929	0.536	0.13	########
PSMA4	########	-0.15456	0.555	0.258	########
AKAP9	########	0.52291	0.631	0.147	########
MED10	########	-0.31071	0.508	0.258	########
PSMD2	########	0.135815	0.514	0.152	########
ATP1B3	########	0.643075	0.547	0.084	########
ORMDL1	########	0.116475	0.559	0.185	########
SERPINE2	########	0.631274	0.625	0.13	########
ARMCX3	########	0.198742	0.577	0.186	########
TSC22D4	########	-0.14091	0.735	0.421	########
JUN	########	0.138198	0.813	0.405	########
NDUFB5	########	-0.18829	0.581	0.299	########
DTD1	########	0.612314	0.566	0.097	########
SCG3	########	0.294918	0.64	0.207	########
FAM181B	########	0.853975	0.527	0.053	########
KTN1	########	0.452379	0.594	0.14	########
RBM25	########	0.343732	0.568	0.138	########
LSM2	########	0.213448	0.502	0.128	########
PCMT1	########	-0.15332	0.538	0.231	########
LSM1	########	-0.11471	0.541	0.229	########
NFIX	########	0.797221	0.611	0.099	########
DEK	########	0.520264	0.519	0.091	########
RDX	########	0.386689	0.597	0.161	########
CBX1	########	0.495039	0.529	0.096	########
GBAS	########	0.439985	0.505	0.091	########
C19orf70	########	-0.63051	0.503	0.395	########
MCL1	########	0.170454	0.586	0.203	########
HMGB3	########	0.440391	0.49	0.087	1.37E-99
CSTB	########	-0.55794	0.532	0.391	1.67E-99
LINC00461	########	0.353891	0.547	0.135	2.11E-99
SCP2	########	-0.11655	0.588	0.277	2.19E-99
POP7	########	0.289231	0.538	0.135	2.47E-99
DNAJC8	########	-0.12549	0.57	0.25	2.47E-99
C16orf45	########	0.132613	0.538	0.173	3.69E-99
TRAPPC1	########	-0.439	0.473	0.304	4.46E-99
TTYH1	########	-0.44373	0.417	0.243	5.89E-99
MTCH1	########	0.293781	0.545	0.14	6.23E-99
BCAS2	########	-0.14729	0.505	0.221	6.27E-99
DCX	########	1.083829	0.495	0.021	1.00E-98
ENOPH1	########	0.415377	0.502	0.096	1.07E-98
CBX5	########	0.50265	0.571	0.116	1.17E-98
CADM4	########	0.774928	0.531	0.062	1.46E-98
CLTB	########	0.384722	0.502	0.108	1.77E-98
TCF12	########	0.56092	0.553	0.101	1.83E-98
SMARCA4	########	0.627512	0.571	0.099	1.83E-98
BNIP3L	########	-0.28502	0.571	0.303	1.84E-98
GADD45B	########	-1.18909	0.326	0.462	1.99E-98
CACYBP	########	0.146919	0.572	0.191	2.19E-98
TMEM14B	########	-0.1254	0.507	0.214	2.32E-98
ATAT1	########	0.721	0.507	0.058	2.55E-98

TAGLN2	########	-1.5807	0.14	0.497	2.66E-98
TMEM14C	########	-0.26663	0.512	0.251	2.84E-98
RDH11	########	0.278254	0.549	0.147	3.61E-98
NREP	########	0.620148	0.503	0.072	4.01E-98
PSMD7	########	-0.23543	0.505	0.234	1.03E-97
YIPF3	########	-0.16763	0.534	0.251	1.21E-97
USMG5	########	-0.14861	0.554	0.26	1.41E-97
APMAP	########	0.362662	0.534	0.121	1.66E-97
STUB1	########	0.531913	0.554	0.103	1.75E-97
CPE	########	0.161368	0.557	0.185	1.94E-97
C1orf43	########	-0.2704	0.572	0.316	2.30E-97
C17orf89	########	0.517867	0.523	0.092	3.00E-97
RWDD1	########	-0.29614	0.52	0.284	3.29E-97
HMGN2	########	-0.55199	0.559	0.477	7.90E-97
MAP1LC3B	########	0.371762	0.581	0.152	9.69E-97
TMEM147	########	-0.15086	0.611	0.311	1.66E-96
PPP1CB	########	-0.11061	0.503	0.21	2.12E-96
PNN	########	0.202101	0.585	0.193	2.44E-96
PSMC3	########	-0.12499	0.593	0.294	4.38E-96
RHOC	########	-0.79452	0.424	0.407	4.47E-96
ATRX	########	0.465892	0.586	0.142	5.51E-96
CNP	########	0.41352	0.512	0.108	6.13E-96
PRRC2C	########	0.223657	0.538	0.154	7.40E-96
CLK1	########	0.477096	0.649	0.173	7.90E-96
DNAJC7	########	0.311621	0.572	0.156	1.06E-95
MINOS1	########	-0.3666	0.49	0.27	1.07E-95
ARL4C	########	0.684595	0.542	0.085	1.44E-95
ASIC4	1.28E-99	0.950622	0.549	0.06	1.91E-95
PPP1R14B	1.62E-99	0.70936	0.555	0.085	2.43E-95
PAPOLA	1.73E-99	-0.10816	0.499	0.195	2.59E-95
KPNB1	3.06E-99	0.44259	0.551	0.128	4.57E-95
DYNC1LI2	3.12E-99	0.479294	0.521	0.097	4.66E-95
SMARCE1	4.62E-99	0.411298	0.533	0.12	6.90E-95
GOLGA7	7.05E-99	-0.25276	0.462	0.222	1.05E-94
PPP1CA	8.20E-99	-0.13166	0.462	0.198	1.23E-94
AUP1	9.77E-99	0.238175	0.553	0.164	1.46E-94
BZW2	1.01E-98	0.552375	0.493	0.079	1.51E-94
SIVA1	1.07E-98	-0.48897	0.437	0.27	1.59E-94
EIF6	1.10E-98	-0.13897	0.531	0.243	1.64E-94
LMAN2	1.40E-98	-0.3524	0.516	0.292	2.09E-94
HADHA	1.87E-98	0.26495	0.515	0.135	2.79E-94
PSIP1	2.43E-98	0.610029	0.563	0.103	3.63E-94
BCAN	4.29E-98	0.575562	0.745	0.248	6.41E-94
REPIN1	4.71E-98	0.824713	0.501	0.046	7.04E-94
SYF2	5.01E-98	-0.25265	0.529	0.267	7.49E-94
RALY	5.29E-98	0.632736	0.511	0.074	7.91E-94
CD99	7.30E-98	-0.94987	0.398	0.496	1.09E-93
BASP1	8.89E-98	1.145019	0.481	0.019	1.33E-93
KCNQ2	1.02E-97	0.722937	0.498	0.058	1.52E-93
GTF3A	1.25E-97	0.280971	0.469	0.108	1.87E-93
PSMC4	1.91E-97	-0.38287	0.498	0.308	2.85E-93

CHMP5	2.02E-97	-0.10445	0.508	0.229	3.02E-93
DDAH2	2.47E-97	0.326453	0.499	0.118	3.69E-93
GOLM1	2.66E-97	0.282759	0.508	0.128	3.97E-93
PPP1CC	2.73E-97	0.310632	0.538	0.147	4.07E-93
POLD2	3.07E-97	-0.17638	0.554	0.275	4.59E-93
DDR1	3.78E-97	0.449395	0.559	0.135	5.65E-93
CCZ1	6.51E-97	0.517456	0.538	0.111	9.73E-93
LHFPL3	8.81E-97	0.988575	0.458	0.026	1.32E-92
SPARC	9.45E-97	-1.00768	0.311	0.374	1.41E-92
COA1	1.76E-96	0.156119	0.523	0.166	2.63E-92
SERP1	2.36E-96	0.300574	0.568	0.168	3.53E-92
TMEM50A	2.57E-96	-0.29451	0.462	0.236	3.85E-92
OAZ2	2.58E-96	-0.21269	0.453	0.214	3.85E-92
CCT8	2.99E-96	-0.15897	0.562	0.275	4.46E-92
METAP2	5.16E-96	-0.21335	0.523	0.256	7.71E-92
TUBB4B	5.27E-96	0.138363	0.553	0.19	7.87E-92
WIPI2	8.20E-96	0.590124	0.531	0.089	1.23E-91
TMED4	8.20E-96	0.196133	0.566	0.188	1.23E-91
BTG2	2.23E-95	0.140758	0.598	0.226	3.33E-91
TMSB15A	2.24E-95	0.811487	0.434	0.026	3.35E-91
TMEM165	2.50E-95	0.888954	0.586	0.089	3.74E-91
RTF1	2.90E-95	0.593536	0.57	0.111	4.33E-91
CSNK1E	3.60E-95	0.732278	0.581	0.103	5.38E-91
MTPN	4.19E-95	0.335932	0.462	0.089	6.26E-91
BCAP31	4.34E-95	-0.2952	0.501	0.263	6.49E-91
BAZ2B	4.57E-95	0.795058	0.557	0.08	6.82E-91
PEA15	6.36E-95	-0.30406	0.464	0.253	9.50E-91
STAT3	6.45E-95	0.278684	0.571	0.178	9.64E-91
BRD2	7.11E-95	0.425985	0.525	0.118	1.06E-90
PCBP4	1.08E-94	0.763978	0.49	0.051	1.61E-90
TSN	1.12E-94	0.240627	0.515	0.137	1.67E-90
DNAJB9	1.13E-94	-0.519	0.46	0.256	1.70E-90
B4GALNT1	1.25E-94	1.209952	0.477	0.022	1.86E-90
HIPK2	1.55E-94	0.848894	0.477	0.039	2.31E-90
ARPC5	1.84E-94	-0.25448	0.515	0.268	2.75E-90
EIF3M	2.07E-94	-0.27624	0.547	0.308	3.09E-90
NME4	2.28E-94	0.4422	0.506	0.104	3.40E-90
CCDC167	2.63E-94	0.276804	0.495	0.125	3.92E-90
MUM1	2.73E-94	0.457443	0.568	0.137	4.07E-90
RAB31	2.91E-94	0.433437	0.553	0.137	4.35E-90
EIF3D	4.31E-94	0.149648	0.523	0.174	6.44E-90
ID2	4.58E-94	-0.34755	0.553	0.323	6.84E-90
ATOX1	4.94E-94	-0.48467	0.493	0.335	7.38E-90
ZFAND5	6.11E-94	0.427297	0.536	0.125	9.13E-90
TXN2	6.32E-94	0.199857	0.518	0.15	9.44E-90
EI24	6.85E-94	0.217548	0.514	0.149	1.02E-89
TPRKB	7.05E-94	0.288535	0.45	0.094	1.05E-89
TXNDC17	9.19E-94	-0.5529	0.428	0.308	1.37E-89
YIPF4	9.56E-94	0.520765	0.519	0.103	1.43E-89
NUBP2	9.76E-94	0.201734	0.492	0.142	1.46E-89
ATP6V0D1	1.50E-93	0.173735	0.485	0.135	2.24E-89

PDGFRA	1.59E-93	1.31667	0.454	0.012	2.37E-89
SARS	2.10E-93	-0.47463	0.458	0.287	3.14E-89
PTPRZ1	2.67E-93	0.313672	0.785	0.342	3.98E-89
TXNL1	2.69E-93	-0.33408	0.471	0.241	4.02E-89
SPAG7	4.35E-93	-0.20141	0.476	0.236	6.50E-89
TMEM205	4.50E-93	-0.93699	0.338	0.39	6.73E-89
GFAP	6.51E-93	-0.49437	0.475	0.304	9.73E-89
FOS	6.60E-93	-0.37654	0.86	0.704	9.86E-89
MPC2	8.14E-93	-0.15801	0.49	0.234	1.22E-88
CDKN2A	8.15E-93	0.90527	0.404	0.024	1.22E-88
MEX3A	1.23E-92	0.792855	0.451	0.034	1.84E-88
FHL1	1.25E-92	0.146414	0.518	0.171	1.86E-88
GPBP1	1.78E-92	0.11316	0.521	0.176	2.66E-88
CXXC5	1.96E-92	0.146546	0.54	0.186	2.93E-88
ACAP3	2.48E-92	0.892533	0.524	0.058	3.71E-88
CFDP1	2.98E-92	-0.25675	0.462	0.227	4.46E-88
ERV3-1	3.05E-92	0.383213	0.497	0.113	4.56E-88
CDKN2C	3.34E-92	-0.19794	0.468	0.224	5.00E-88
SGCB	5.05E-92	0.403142	0.598	0.169	7.55E-88
COPS5	7.09E-92	0.154426	0.508	0.159	1.06E-87
HSPB1	9.59E-92	-0.16038	0.688	0.393	1.43E-87
ILF3	1.33E-91	0.309775	0.536	0.144	1.99E-87
DLL1	1.34E-91	1.085273	0.464	0.021	2.01E-87
LAMTOR2	1.38E-91	-0.32739	0.477	0.26	2.06E-87
EGFR	1.53E-91	0.19331	0.606	0.215	2.28E-87
RFTN2	1.80E-91	0.76176	0.459	0.039	2.69E-87
IGFBP5	1.83E-91	-0.9007	0.377	0.361	2.74E-87
PNKD	1.98E-91	-0.1575	0.545	0.26	2.95E-87
UQCRC1	2.20E-91	-0.10815	0.481	0.207	3.29E-87
TMEM98	2.35E-91	0.601232	0.458	0.062	3.51E-87
MPV17	2.50E-91	0.111034	0.437	0.125	3.73E-87
EIF4EBP1	4.99E-91	0.187068	0.524	0.164	7.45E-87
NDRG2	5.10E-91	-0.18002	0.598	0.326	7.62E-87
DNAJB11	6.97E-91	0.274384	0.459	0.106	1.04E-86
PPT1	7.11E-91	0.10934	0.495	0.162	1.06E-86
UXT	7.77E-91	-0.31024	0.486	0.27	1.16E-86
RP11-849I	8.85E-91	0.961585	0.471	0.032	1.32E-86
DDX46	1.04E-90	0.143952	0.516	0.161	1.56E-86
STARD3NL	1.37E-90	0.301792	0.499	0.135	2.05E-86
LIMA1	1.45E-90	0.307578	0.56	0.176	2.16E-86
ZFP36	1.66E-90	-1.05619	0.316	0.431	2.48E-86
CHID1	1.95E-90	-0.23341	0.456	0.231	2.92E-86
ZFAND2A	2.26E-90	-0.16386	0.391	0.147	3.38E-86
ETV1	2.26E-90	0.985502	0.584	0.094	3.38E-86
VCP	3.29E-90	0.258709	0.49	0.13	4.92E-86
SIRT2	3.58E-90	0.265774	0.557	0.171	5.35E-86
C16orf13	3.68E-90	0.218139	0.508	0.152	5.49E-86
XRN2	3.74E-90	0.155926	0.468	0.135	5.59E-86
TSG101	5.09E-90	-0.14576	0.445	0.191	7.60E-86
TCEA2	6.81E-90	0.415795	0.525	0.123	1.02E-85
KIF5C	1.72E-89	0.856352	0.477	0.043	2.57E-85

RNF7	1.88E-89	-0.3321	0.484	0.275	2.81E-85
MT1E	2.11E-89	-1.54083	0.151	0.417	3.15E-85
PABPN1	2.17E-89	0.236186	0.495	0.138	3.24E-85
SYNCRIP	2.21E-89	0.341095	0.458	0.096	3.31E-85
TERF1	2.50E-89	0.274064	0.455	0.111	3.74E-85
DNER	2.64E-89	0.629956	0.541	0.104	3.94E-85
ANAPC5	3.56E-89	0.131187	0.467	0.145	5.32E-85
CNOT7	3.65E-89	0.376466	0.467	0.094	5.46E-85
REEP5	4.11E-89	-0.41506	0.468	0.277	6.14E-85
SNHG8	4.38E-89	-0.25005	0.507	0.243	6.55E-85
TMEM208	4.44E-89	-0.21623	0.446	0.215	6.64E-85
SEC63	4.45E-89	0.342773	0.45	0.092	6.65E-85
UBE2S	4.46E-89	0.464541	0.441	0.068	6.67E-85
UBA2	4.73E-89	0.21099	0.445	0.113	7.07E-85
MAGOH	5.02E-89	-0.19137	0.42	0.174	7.50E-85
PSME2	5.58E-89	-0.75858	0.372	0.332	8.33E-85
NT5C3B	6.39E-89	0.707992	0.498	0.07	9.55E-85
HNRNPUL1	6.60E-89	0.315856	0.45	0.099	9.86E-85
CPSF6	7.86E-89	0.605315	0.527	0.099	1.17E-84
CD151	9.01E-89	-0.72684	0.38	0.338	1.35E-84
ITM2C	9.74E-89	0.255084	0.554	0.183	1.46E-84
ERP29	1.68E-88	-0.40667	0.482	0.308	2.51E-84
AKIRIN2	1.80E-88	0.635337	0.492	0.072	2.68E-84
HNRNPH1	1.85E-88	0.26258	0.571	0.193	2.76E-84
MEA1	2.02E-88	-0.31038	0.403	0.197	3.02E-84
DDX17	2.09E-88	0.560093	0.512	0.096	3.12E-84
SUMO3	2.16E-88	0.331112	0.424	0.084	3.23E-84
RAB3IP	2.90E-88	0.766629	0.662	0.164	4.34E-84
C7orf55-L	2.95E-88	0.546764	0.494	0.087	4.41E-84
ATP5EP2	3.54E-88	-0.57553	0.377	0.301	5.29E-84
PAIP1	5.19E-88	0.527639	0.499	0.091	7.75E-84
NOC2L	5.19E-88	0.236428	0.447	0.111	7.75E-84
KDELR1	5.55E-88	-0.10022	0.495	0.224	8.29E-84
EIF4E	9.76E-88	-0.1839	0.447	0.195	1.46E-83
APH1A	1.08E-87	-0.1012	0.468	0.21	1.61E-83
VPS29	1.08E-87	-0.24415	0.43	0.207	1.62E-83
PSMD11	1.40E-87	0.193449	0.447	0.116	2.09E-83
UBE2B	2.22E-87	-0.25643	0.428	0.203	3.32E-83
GCSH	2.81E-87	0.101896	0.427	0.125	4.20E-83
RNF181	3.24E-87	-0.33503	0.45	0.238	4.84E-83
NCOR1	3.75E-87	0.365716	0.537	0.14	5.61E-83
YPEL5	3.97E-87	-0.16862	0.42	0.179	5.94E-83
GNAI2	5.14E-87	0.451003	0.467	0.091	7.68E-83
NDUFAF3	5.28E-87	0.183021	0.518	0.164	7.89E-83
PSMG3	6.04E-87	0.200437	0.449	0.118	9.02E-83
MAP4K4	6.11E-87	0.469146	0.414	0.065	9.13E-83
S100A11	6.29E-87	-1.59027	0.137	0.378	9.41E-83
KXD1	6.73E-87	-0.31225	0.441	0.244	1.01E-82
ZEB2	7.31E-87	0.57263	0.446	0.065	1.09E-82
PRPF38B	7.54E-87	0.436958	0.515	0.123	1.13E-82
MIAT	9.04E-87	0.767771	0.454	0.044	1.35E-82

DEGS1	9.33E-87	0.196041	0.492	0.14	1.39E-82
EMC6	1.46E-86	0.402091	0.477	0.101	2.18E-82
TOP1	1.51E-86	0.61208	0.472	0.068	2.26E-82
CCNL1	1.63E-86	0.12053	0.541	0.203	2.44E-82
RALA	1.66E-86	0.350936	0.449	0.089	2.47E-82
AC004540.	1.99E-86	0.89615	0.453	0.032	2.98E-82
HOTAIRM1	2.50E-86	0.570961	0.437	0.063	3.74E-82
ANP32B	2.67E-86	0.473812	0.502	0.104	3.98E-82
RAD23A	2.90E-86	-0.2908	0.499	0.292	4.33E-82
KLC1	2.91E-86	0.450875	0.492	0.104	4.35E-82
FDPS	3.13E-86	-0.3072	0.432	0.231	4.68E-82
EIF4A3	3.78E-86	0.293683	0.434	0.092	5.65E-82
DYNC1I2	4.86E-86	0.288273	0.521	0.159	7.27E-82
PSMB4	5.24E-86	-0.29907	0.399	0.209	7.84E-82
NUTF2	5.26E-86	0.225351	0.454	0.115	7.86E-82
ISCU	5.73E-86	0.158476	0.495	0.164	8.56E-82
PNMA1	6.81E-86	0.605614	0.472	0.07	1.02E-81
DLGAP4	7.18E-86	0.297618	0.399	0.075	1.07E-81
C1D	9.22E-86	0.212736	0.397	0.087	1.38E-81
LAMP1	1.13E-85	0.390601	0.49	0.109	1.69E-81
SRSF1	1.26E-85	0.126586	0.475	0.156	1.88E-81
PGLS	1.52E-85	0.311037	0.498	0.133	2.28E-81
RAB6A	1.53E-85	0.245585	0.479	0.13	2.29E-81
WHSC1L1	1.55E-85	0.82746	0.501	0.058	2.31E-81
ZNF3	1.98E-85	0.692056	0.468	0.058	2.95E-81
HSPA1A	2.03E-85	0.763814	0.638	0.157	3.03E-81
VIMP	2.12E-85	-0.31881	0.372	0.195	3.17E-81
NLRP1	2.16E-85	0.253974	0.467	0.128	3.22E-81
MZT2A	2.84E-85	0.320141	0.489	0.128	4.24E-81
SDHB	2.89E-85	-0.12143	0.401	0.156	4.32E-81
C1orf122	3.24E-85	0.132498	0.563	0.217	4.84E-81
RAB14	3.28E-85	0.158407	0.454	0.132	4.90E-81
TRA2A	3.36E-85	0.349549	0.493	0.121	5.03E-81
YIPF6	3.49E-85	0.264559	0.476	0.115	5.22E-81
OSTC	3.97E-85	-0.48935	0.388	0.253	5.94E-81
SF3B1	4.04E-85	0.404571	0.514	0.121	6.03E-81
POLR2H	5.68E-85	0.141532	0.436	0.128	8.49E-81
ARGLU1	5.78E-85	0.440555	0.525	0.128	8.64E-81
DTX3	7.42E-85	1.067844	0.575	0.087	1.11E-80
PTPRA	7.96E-85	0.230099	0.44	0.111	1.19E-80
C20orf24	9.03E-85	0.462837	0.48	0.092	1.35E-80
METTL5	9.47E-85	0.127741	0.43	0.125	1.42E-80
BRI3	9.79E-85	0.137879	0.432	0.125	1.46E-80
BBX	9.88E-85	0.180886	0.469	0.138	1.48E-80
MAF1	1.08E-84	0.531308	0.503	0.099	1.62E-80
PSMA3	1.22E-84	-0.15619	0.462	0.205	1.83E-80
CCDC85B	1.26E-84	0.467423	0.464	0.084	1.89E-80
SNRPD3	1.46E-84	-0.2382	0.437	0.215	2.18E-80
ARL6IP5	1.63E-84	-0.23496	0.571	0.321	2.43E-80
MMP2	1.69E-84	0.689476	0.414	0.038	2.52E-80
GRHPR	2.07E-84	0.349144	0.482	0.118	3.10E-80

NAE1	2.24E-84	0.317946	0.499	0.13	3.34E-80
PPHLN1	2.25E-84	0.139729	0.411	0.106	3.36E-80
TXNL4A	2.27E-84	0.139793	0.437	0.126	3.40E-80
C8orf33	2.48E-84	0.32531	0.468	0.108	3.70E-80
OCIAD2	2.90E-84	-1.54726	0.116	0.376	4.34E-80
HIGD1A	3.40E-84	-0.14004	0.449	0.2	5.08E-80
BTG3	4.12E-84	0.189668	0.451	0.132	6.16E-80
BPTF	9.85E-84	0.483049	0.433	0.07	1.47E-79
EPB41L4A-	1.10E-83	0.1597	0.451	0.123	1.64E-79
PGAM1	1.40E-83	-0.35575	0.397	0.222	2.10E-79
HERPUD1	1.58E-83	-0.31567	0.477	0.263	2.37E-79
DBNL	1.86E-83	0.350868	0.459	0.104	2.78E-79
TSFM	2.77E-83	0.71352	0.54	0.108	4.14E-79
DNAJB1	3.01E-83	-0.36494	0.661	0.443	4.49E-79
LEPROTL1	3.31E-83	0.494361	0.472	0.087	4.95E-79
DCTN2	4.98E-83	0.682611	0.685	0.203	7.45E-79
THYN1	5.93E-83	-0.24474	0.463	0.25	8.85E-79
IFI27L2	7.09E-83	-0.13554	0.497	0.239	1.06E-78
SCARB2	8.05E-83	0.433985	0.438	0.08	1.20E-78
TMEM100	9.81E-83	0.706974	0.464	0.062	1.47E-78
DPYSL2	1.38E-82	0.310291	0.467	0.115	2.06E-78
CITED2	1.42E-82	0.486293	0.456	0.084	2.12E-78
MAPT	1.61E-82	0.361517	0.514	0.135	2.40E-78
WDR61	1.92E-82	-0.40239	0.334	0.191	2.88E-78
APLP1	1.93E-82	0.663922	0.51	0.089	2.89E-78
HNRNPH2	1.99E-82	-0.10394	0.421	0.178	2.97E-78
ELAVL3	2.67E-82	0.685689	0.506	0.085	3.99E-78
ARID4B	3.37E-82	0.417638	0.49	0.113	5.04E-78
COTL1	3.85E-82	0.320691	0.388	0.077	5.75E-78
PTP4A1	4.28E-82	0.592621	0.479	0.079	6.40E-78
BAG1	5.39E-82	0.408063	0.425	0.077	8.06E-78
IFITM3	6.03E-82	-1.74028	0.066	0.395	9.01E-78
RUFY3	6.15E-82	0.46544	0.469	0.094	9.19E-78
FABP7	7.26E-82	-0.55281	0.534	0.472	1.09E-77
MAGI1	7.57E-82	0.601797	0.464	0.074	1.13E-77
RBFOX2	8.12E-82	0.696933	0.419	0.041	1.21E-77
LRRN1	8.56E-82	0.863642	0.39	0.015	1.28E-77
MANBAL	9.13E-82	-0.11924	0.42	0.179	1.36E-77
ABI2	9.24E-82	0.228139	0.475	0.145	1.38E-77
PSMD3	1.19E-81	0.494027	0.473	0.091	1.78E-77
HAX1	1.66E-81	-0.37104	0.401	0.224	2.48E-77
RBBP7	1.93E-81	-0.19796	0.443	0.224	2.88E-77
ENSA	2.01E-81	-0.34354	0.388	0.217	3.00E-77
TPR	2.09E-81	0.260344	0.467	0.126	3.13E-77
ARHGEF2	2.24E-81	0.465772	0.488	0.106	3.35E-77
NSA2	2.29E-81	0.17582	0.424	0.113	3.42E-77
ZNF462	2.39E-81	0.749551	0.45	0.05	3.58E-77
COX17	2.57E-81	-0.4523	0.381	0.248	3.83E-77
MBP	2.95E-81	0.226926	0.365	0.07	4.42E-77
MAT2A	2.96E-81	0.328461	0.473	0.121	4.43E-77
NDUFA8	4.75E-81	-0.22952	0.455	0.232	7.10E-77

TAGLN3	4.78E-81	0.674134	0.441	0.055	7.14E-77
DLD	5.44E-81	0.123293	0.45	0.145	8.13E-77
CMTM6	5.75E-81	0.259965	0.369	0.074	8.60E-77
GABARAP	5.90E-81	-0.48351	0.369	0.244	8.81E-77
FKBP2	6.53E-81	-0.79876	0.388	0.386	9.75E-77
ADI1	6.60E-81	0.3658	0.424	0.089	9.87E-77
PDZD11	6.72E-81	-0.36002	0.358	0.2	1.00E-76
RAB11A	7.25E-81	-0.453	0.371	0.224	1.08E-76
CLIP2	7.69E-81	0.646501	0.427	0.05	1.15E-76
LARS	1.02E-80	0.136693	0.425	0.125	1.52E-76
PSME1	1.04E-80	-0.84005	0.345	0.364	1.55E-76
RNF145	1.19E-80	0.64843	0.466	0.067	1.78E-76
CRIPT	1.21E-80	0.212221	0.45	0.128	1.80E-76
GRINA	1.41E-80	0.530261	0.463	0.084	2.11E-76
MAP1LC3A	1.65E-80	0.244553	0.42	0.108	2.47E-76
GTF2I	1.65E-80	0.537458	0.583	0.162	2.47E-76
NOP56	1.75E-80	0.182716	0.463	0.14	2.62E-76
CHCHD5	1.77E-80	-0.28476	0.345	0.174	2.64E-76
NOSIP	1.79E-80	-0.2461	0.404	0.202	2.67E-76
NSFL1C	2.37E-80	0.112697	0.438	0.138	3.54E-76
SLC25A11	2.90E-80	0.207035	0.428	0.113	4.33E-76
NPC2	3.27E-80	-1.06355	0.269	0.345	4.88E-76
VDAC3	5.08E-80	0.152252	0.52	0.188	7.59E-76
HMGB2	5.65E-80	-0.27023	0.447	0.219	8.44E-76
MPC1	6.42E-80	-0.46454	0.388	0.253	9.60E-76
TRIB2	6.59E-80	0.138336	0.43	0.135	9.85E-76
HEY1	6.99E-80	0.256485	0.441	0.123	1.04E-75
ELOF1	7.78E-80	-0.29046	0.424	0.229	1.16E-75
MAPK10	1.02E-79	0.573119	0.407	0.051	1.53E-75
CADM2	1.21E-79	0.682377	0.475	0.072	1.81E-75
KLHL23	1.69E-79	0.804503	0.386	0.015	2.53E-75
TM2D3	1.70E-79	-0.22581	0.38	0.166	2.54E-75
MOSPD3	1.73E-79	0.349864	0.398	0.079	2.59E-75
KLHDC2	1.74E-79	0.509722	0.428	0.063	2.61E-75
KLHDC3	1.83E-79	0.114631	0.388	0.108	2.73E-75
FEZ2	1.97E-79	0.495771	0.481	0.096	2.94E-75
GNB1	2.43E-79	0.308619	0.446	0.106	3.63E-75
NRCAM	2.48E-79	0.472811	0.481	0.108	3.71E-75
UQCC2	2.57E-79	-0.1557	0.412	0.173	3.84E-75
CYB5R3	2.58E-79	0.298544	0.421	0.099	3.85E-75
RND3	2.61E-79	0.821994	0.489	0.067	3.90E-75
DPM1	2.71E-79	-0.25356	0.378	0.181	4.05E-75
ACTR10	2.89E-79	-0.15352	0.399	0.159	4.32E-75
SPTSSA	2.95E-79	-0.56052	0.382	0.289	4.41E-75
HP1BP3	2.98E-79	0.301562	0.472	0.126	4.45E-75
LYPLA1	3.29E-79	0.197812	0.428	0.116	4.91E-75
CDK4	3.93E-79	0.664525	0.733	0.25	5.87E-75
ZDHHC4	4.13E-79	-0.20004	0.375	0.168	6.17E-75
TMEM106B	4.15E-79	0.352293	0.475	0.12	6.21E-75
FIP1L1	4.45E-79	0.508756	0.503	0.111	6.65E-75
TNRC6B	4.62E-79	0.485532	0.501	0.113	6.91E-75

MTSS1	5.43E-79	0.799625	0.389	0.021	8.11E-75
ERCC1	5.56E-79	-0.14772	0.467	0.217	8.31E-75
GSTK1	5.61E-79	-0.60231	0.363	0.279	8.38E-75
SLC1A3	5.69E-79	-0.78649	0.293	0.289	8.51E-75
ZNHIT3	5.82E-79	0.161799	0.454	0.135	8.70E-75
MSI2	6.72E-79	-0.25765	0.427	0.239	1.00E-74
LRPAP1	6.96E-79	-0.37443	0.44	0.287	1.04E-74
EIF2AK1	7.11E-79	0.568773	0.432	0.063	1.06E-74
C14orf1	7.79E-79	-0.23647	0.33	0.154	1.16E-74
PPP4C	8.28E-79	0.124821	0.484	0.176	1.24E-74
9-Mar	1.02E-78	1.013476	0.442	0.039	1.52E-74
TMEM123	1.15E-78	0.521909	0.455	0.085	1.72E-74
CSPG5	1.27E-78	0.322634	0.411	0.094	1.90E-74
CRIP2	1.72E-78	0.327994	0.549	0.174	2.57E-74
WBP2	1.85E-78	-0.26704	0.401	0.214	2.76E-74
TXNIP	2.53E-78	0.318999	0.537	0.169	3.78E-74
UBE2L6	2.57E-78	-0.67341	0.325	0.277	3.84E-74
DHX9	2.94E-78	0.369131	0.402	0.075	4.40E-74
NT5C3A	3.13E-78	0.373926	0.416	0.08	4.68E-74
CD59	3.87E-78	-0.77652	0.303	0.292	5.78E-74
THRA	3.96E-78	0.616088	0.473	0.08	5.91E-74
SNRNP200	4.10E-78	0.335459	0.424	0.091	6.12E-74
S100A16	4.30E-78	-1.57811	0.073	0.376	6.42E-74
BST2	5.06E-78	-1.69722	0.055	0.426	7.57E-74
PLEKHB2	6.42E-78	0.293166	0.391	0.085	9.59E-74
DTYMK	6.44E-78	0.321452	0.407	0.084	9.63E-74
SRSF6	9.19E-78	0.513643	0.442	0.074	1.37E-73
GGCT	1.15E-77	-0.26725	0.377	0.193	1.72E-73
EAPP	1.22E-77	-0.13448	0.434	0.188	1.82E-73
NEU4	1.26E-77	1.010755	0.401	0.017	1.88E-73
BABAM1	1.28E-77	-0.18335	0.391	0.19	1.92E-73
SIAH1	1.31E-77	0.699093	0.442	0.051	1.96E-73
DAZAP2	1.34E-77	-0.48699	0.376	0.255	2.00E-73
MKLN1	1.60E-77	0.427603	0.438	0.082	2.39E-73
ZFAND6	1.71E-77	-0.43101	0.355	0.221	2.55E-73
TRAPPC4	1.79E-77	-0.23055	0.41	0.195	2.67E-73
USP22	1.87E-77	0.397215	0.411	0.08	2.79E-73
EXOC7	2.00E-77	0.34706	0.44	0.104	2.99E-73
EIF4B	2.15E-77	0.268389	0.424	0.101	3.22E-73
BCL7B	2.93E-77	0.534133	0.417	0.062	4.38E-73
NAMPT	2.97E-77	-1.22827	0.177	0.311	4.44E-73
PTPRS	3.37E-77	0.7524	0.477	0.068	5.04E-73
SSR3	3.48E-77	-0.70179	0.304	0.238	5.21E-73
MAP7D1	3.63E-77	0.55076	0.381	0.041	5.43E-73
SLC39A6	3.89E-77	0.370397	0.429	0.092	5.81E-73
RPA3	3.93E-77	-0.19896	0.355	0.152	5.88E-73
CYTH2	4.27E-77	0.308917	0.399	0.085	6.38E-73
KARS	5.98E-77	0.229198	0.438	0.121	8.94E-73
MED4	7.07E-77	-0.1756	0.399	0.179	1.06E-72
PHIP	8.31E-77	0.503727	0.438	0.077	1.24E-72
PRKCSH	8.98E-77	-0.18005	0.44	0.224	1.34E-72

CCT2	9.81E-77	0.415551	0.778	0.328	1.47E-72
NOL7	1.15E-76	0.159745	0.436	0.125	1.71E-72
DNAJC19	1.15E-76	-0.2054	0.421	0.215	1.72E-72
GRB2	1.16E-76	0.474015	0.41	0.065	1.73E-72
OS9	1.16E-76	0.711404	0.685	0.207	1.73E-72
HILPDA	1.24E-76	-0.79268	0.268	0.193	1.85E-72
GRIA2	1.48E-76	0.65991	0.489	0.084	2.21E-72
NARS	1.50E-76	-0.18426	0.407	0.188	2.25E-72
FBL	1.58E-76	-0.4174	0.412	0.243	2.36E-72
KLRC2	1.67E-76	1.040003	0.408	0.019	2.49E-72
RP11-14N7	2.06E-76	0.133495	0.364	0.096	3.08E-72
NRN1	2.46E-76	-0.84761	0.291	0.26	3.68E-72
HMGA1	4.25E-76	0.420311	0.367	0.053	6.36E-72
SOD2	4.40E-76	-1.36662	0.19	0.328	6.57E-72
CKS2	4.73E-76	0.314576	0.449	0.115	7.07E-72
CDH2	4.74E-76	0.325275	0.437	0.104	7.08E-72
TIA1	6.85E-76	0.411774	0.425	0.084	1.02E-71
CHD4	7.71E-76	0.43009	0.406	0.074	1.15E-71
IFITM10	8.13E-76	0.902631	0.402	0.022	1.21E-71
FLOT1	8.81E-76	-0.10633	0.39	0.157	1.32E-71
MPDU1	9.00E-76	0.37037	0.41	0.085	1.34E-71
THSD7A	9.49E-76	0.788217	0.375	0.021	1.42E-71
ITPA	1.02E-75	0.192539	0.442	0.13	1.53E-71
PRPF6	1.15E-75	0.209229	0.417	0.109	1.72E-71
COX14	1.20E-75	-0.41701	0.343	0.198	1.79E-71
MPST	1.25E-75	0.708739	0.42	0.044	1.87E-71
ST3GAL5	1.81E-75	0.577427	0.412	0.056	2.71E-71
METRN	1.89E-75	0.332553	0.495	0.142	2.82E-71
6-Mar	1.95E-75	0.396389	0.456	0.104	2.91E-71
NSRP1	2.45E-75	-0.10339	0.384	0.15	3.66E-71
HSPH1	3.35E-75	0.40387	0.416	0.084	5.01E-71
KMT2C	3.52E-75	0.545282	0.437	0.074	5.26E-71
APOPT1	3.86E-75	-0.17667	0.382	0.176	5.77E-71
HSPA1B	4.74E-75	0.21371	0.571	0.222	7.09E-71
PSMA5	5.30E-75	-0.19945	0.378	0.176	7.92E-71
STMN2	5.55E-75	1.15094	0.476	0.053	8.30E-71
FBXO21	6.47E-75	0.356676	0.477	0.123	9.66E-71
C19orf60	7.97E-75	0.14726	0.428	0.132	1.19E-70
VCAN	9.26E-75	0.638704	0.428	0.06	1.38E-70
VGLL4	1.05E-74	0.379008	0.433	0.097	1.57E-70
DNAJC2	1.05E-74	0.254388	0.401	0.101	1.57E-70
EMC7	1.11E-74	-0.15597	0.398	0.176	1.66E-70
DPYSL3	1.20E-74	0.30068	0.446	0.118	1.80E-70
MAGEF1	1.31E-74	0.18292	0.454	0.147	1.96E-70
SKA2	1.50E-74	0.357926	0.385	0.072	2.25E-70
NKX2-2	1.54E-74	0.652746	0.415	0.048	2.31E-70
CHMP3	2.25E-74	0.125986	0.394	0.121	3.36E-70
LINC01003	2.26E-74	0.566982	0.382	0.044	3.38E-70
SMARCA5	2.72E-74	0.55436	0.42	0.063	4.06E-70
AK1	2.83E-74	0.296128	0.436	0.115	4.23E-70
ARPC4	3.00E-74	-0.16651	0.372	0.159	4.48E-70

ZNF326	3.01E-74	0.332682	0.384	0.077	4.49E-70
CAP1	3.32E-74	-0.1806	0.39	0.169	4.95E-70
NKTR	4.02E-74	0.156479	0.441	0.149	6.01E-70
TSPAN31	5.39E-74	0.789771	0.544	0.115	8.05E-70
RP11-620J	5.71E-74	0.496084	0.524	0.142	8.54E-70
RBM23	7.16E-74	0.133185	0.395	0.121	1.07E-69
CTDSP2	7.66E-74	0.951468	0.493	0.068	1.15E-69
ENAH	8.03E-74	0.51554	0.394	0.056	1.20E-69
CACNA1A	8.78E-74	0.738782	0.427	0.05	1.31E-69
STK25	8.99E-74	0.638986	0.416	0.051	1.34E-69
SMARCC1	8.99E-74	0.707287	0.394	0.032	1.34E-69
MDM2	1.24E-73	0.872701	0.44	0.053	1.86E-69
POLR2B	1.34E-73	0.727429	0.432	0.053	2.00E-69
ZNF821	1.73E-73	0.623423	0.36	0.027	2.59E-69
TKT	1.76E-73	-0.17066	0.411	0.202	2.63E-69
SEZ6L	1.76E-73	0.447507	0.384	0.067	2.63E-69
NAA10	1.88E-73	0.399032	0.43	0.085	2.81E-69
CNOT4	1.90E-73	0.39692	0.416	0.08	2.84E-69
SSRP1	1.95E-73	0.391282	0.434	0.092	2.91E-69
UGP2	2.00E-73	-0.41395	0.316	0.198	2.99E-69
STMN3	2.02E-73	0.414397	0.466	0.111	3.03E-69
ARL4A	2.08E-73	-0.54566	0.339	0.255	3.11E-69
SEC13	2.32E-73	-0.13158	0.375	0.149	3.47E-69
MBD6	2.40E-73	1.01326	0.417	0.029	3.59E-69
LSM14A	2.67E-73	0.204993	0.432	0.126	4.00E-69
SNX6	2.78E-73	-0.13528	0.378	0.159	4.16E-69
AFF4	2.80E-73	0.543075	0.472	0.096	4.18E-69
SLC39A3	2.81E-73	0.318092	0.39	0.082	4.20E-69
USP16	2.82E-73	0.12708	0.404	0.12	4.21E-69
ACTR2	3.18E-73	0.363339	0.39	0.077	4.76E-69
YEATS4	3.27E-73	0.764123	0.468	0.077	4.89E-69
MIEN1	3.77E-73	-0.16115	0.454	0.229	5.64E-69
IDH2	3.93E-73	0.344756	0.433	0.103	5.87E-69
PMVK	4.45E-73	-0.17392	0.38	0.179	6.65E-69
ARPP19	4.93E-73	0.192137	0.425	0.125	7.36E-69
BCHE	5.04E-73	0.203776	0.397	0.109	7.53E-69
CORO1C	5.28E-73	0.328187	0.511	0.154	7.89E-69
NDUFA9	5.46E-73	-0.10041	0.429	0.191	8.16E-69
CIR1	5.56E-73	0.235007	0.408	0.101	8.30E-69
ZNF677	5.76E-73	0.385655	0.363	0.055	8.60E-69
UBE2R2	6.20E-73	0.604704	0.364	0.034	9.27E-69
GARS	6.44E-73	0.369456	0.401	0.087	9.63E-69
ITFG1	6.56E-73	0.448351	0.415	0.079	9.81E-69
MTX2	7.63E-73	0.28285	0.424	0.111	1.14E-68
ZFP36L1	8.49E-73	-0.40573	0.403	0.275	1.27E-68
ERI3	9.06E-73	0.218718	0.445	0.13	1.35E-68
TWISTNB	9.38E-73	0.118016	0.372	0.085	1.40E-68
GSPT1	1.01E-72	0.371825	0.388	0.072	1.50E-68
BAZ1A	1.03E-72	0.687037	0.464	0.074	1.55E-68
PAICS	1.10E-72	0.121227	0.437	0.145	1.64E-68
BEST3	1.15E-72	0.86839	0.425	0.044	1.72E-68

ZC3H13	1.16E-72	0.175974	0.377	0.092	1.74E-68
TMEM59L	1.33E-72	0.363008	0.453	0.113	1.99E-68
GPAA1	1.49E-72	0.578982	0.427	0.067	2.22E-68
PEPD	1.70E-72	-0.14068	0.39	0.171	2.54E-68
C12orf10	1.72E-72	-0.11495	0.341	0.123	2.58E-68
RBM42	1.80E-72	0.282397	0.434	0.115	2.70E-68
AGAP2-AS1	2.28E-72	1.081369	0.389	0.017	3.41E-68
ANXA6	2.46E-72	0.541696	0.363	0.043	3.68E-68
CKLF	2.55E-72	-0.24998	0.337	0.159	3.82E-68
TNK2	2.87E-72	0.764675	0.372	0.027	4.29E-68
PSENEN	2.87E-72	-0.69677	0.32	0.306	4.29E-68
CD320	3.46E-72	0.25115	0.424	0.113	5.16E-68
GPS1	3.98E-72	0.246089	0.39	0.094	5.94E-68
BPGM	4.02E-72	0.246188	0.373	0.085	6.00E-68
PRAF2	4.42E-72	0.325065	0.411	0.094	6.61E-68
BCLAF1	6.25E-72	0.433091	0.455	0.101	9.34E-68
GLO1	8.43E-72	-0.1126	0.372	0.156	1.26E-67
PNPLA8	8.71E-72	0.212601	0.388	0.109	1.30E-67
AKIRIN1	1.08E-71	0.320517	0.39	0.084	1.62E-67
PDHB	1.09E-71	-0.14875	0.38	0.169	1.63E-67
ANKRD12	1.63E-71	-0.1307	0.406	0.185	2.44E-67
VPS35	1.69E-71	0.212871	0.421	0.109	2.53E-67
CCDC115	2.10E-71	0.12033	0.367	0.106	3.13E-67
YY1	2.21E-71	0.354145	0.385	0.077	3.30E-67
TIMM50	2.35E-71	0.251263	0.432	0.116	3.51E-67
CNTFR	2.55E-71	0.724735	0.372	0.024	3.81E-67
TTC19	2.57E-71	0.21583	0.407	0.106	3.84E-67
PPM1B	3.04E-71	0.440827	0.429	0.087	4.54E-67
ITGB8	3.27E-71	0.505141	0.416	0.072	4.89E-67
RPA2	3.77E-71	-0.2327	0.302	0.144	5.63E-67
POLR2C	3.83E-71	-0.14563	0.402	0.178	5.73E-67
EMC3	4.90E-71	-0.26795	0.408	0.215	7.33E-67
CDK6	4.92E-71	0.632637	0.443	0.075	7.35E-67
IDH1	5.44E-71	0.111053	0.394	0.128	8.13E-67
PRKAR1A	6.71E-71	0.128523	0.421	0.142	1.00E-66
NARF	6.77E-71	0.148379	0.372	0.097	1.01E-66
DUT	8.03E-71	0.143498	0.398	0.12	1.20E-66
DECR1	8.73E-71	0.113859	0.49	0.193	1.30E-66
SETD5	8.75E-71	0.373894	0.433	0.096	1.31E-66
GTF2H5	1.01E-70	-0.22248	0.341	0.154	1.52E-66
RNF167	1.13E-70	-0.17584	0.388	0.174	1.68E-66
NUP107	1.23E-70	0.924003	0.518	0.091	1.84E-66
VPS51	1.34E-70	0.477986	0.382	0.058	2.01E-66
ING3	1.40E-70	0.412504	0.349	0.053	2.09E-66
WDR18	1.64E-70	0.316758	0.347	0.062	2.44E-66
HAT1	1.65E-70	0.115264	0.385	0.115	2.47E-66
ZEB1	1.68E-70	0.54334	0.42	0.068	2.51E-66
GHITM	1.77E-70	-0.37027	0.365	0.215	2.65E-66
ARFGAP2	1.83E-70	0.27315	0.391	0.092	2.74E-66
HTATSF1	1.86E-70	0.35211	0.381	0.075	2.78E-66
NUPL2	2.29E-70	0.360018	0.421	0.099	3.43E-66

ETFB	2.84E-70	-0.3057	0.381	0.212	4.25E-66
CADM1	3.17E-70	0.18576	0.395	0.103	4.73E-66
SDF4	4.00E-70	0.365351	0.404	0.085	5.98E-66
YIF1A	4.20E-70	-0.22562	0.398	0.212	6.28E-66
ALG5	4.62E-70	-0.24787	0.329	0.159	6.90E-66
DPM3	4.69E-70	-0.18539	0.368	0.178	7.01E-66
NUDCD2	4.80E-70	-0.15398	0.386	0.178	7.17E-66
ZFP36L2	5.62E-70	-0.12091	0.424	0.214	8.40E-66
GLRX5	5.75E-70	0.530859	0.393	0.055	8.59E-66
CCDC47	6.21E-70	0.182354	0.411	0.12	9.28E-66
PSMG2	6.43E-70	-0.30751	0.338	0.176	9.60E-66
RAD21	6.60E-70	0.297516	0.425	0.109	9.86E-66
PFDN4	7.35E-70	0.351532	0.399	0.085	1.10E-65
ELAVL1	8.02E-70	0.286143	0.412	0.104	1.20E-65
ISOC2	9.80E-70	-0.45204	0.299	0.197	1.46E-65
RUVBL1	9.81E-70	0.222592	0.356	0.085	1.47E-65
UBE2H	1.03E-69	0.378771	0.373	0.068	1.54E-65
C6orf62	1.08E-69	0.289387	0.399	0.096	1.61E-65
PLP1	1.17E-69	0.724234	0.425	0.058	1.75E-65
SEPP1	1.26E-69	-1.11939	0.179	0.337	1.88E-65
PRKRA	1.28E-69	0.174953	0.424	0.132	1.91E-65
NGDN	1.39E-69	-0.11671	0.381	0.147	2.08E-65
NINJ1	1.45E-69	0.474109	0.362	0.053	2.17E-65
ATG12	1.58E-69	0.104333	0.385	0.121	2.36E-65
STARD7	1.96E-69	0.30563	0.38	0.085	2.93E-65
RAF1	2.21E-69	0.56105	0.397	0.056	3.31E-65
F2R	2.33E-69	0.298301	0.326	0.058	3.48E-65
BOD1	2.40E-69	0.461216	0.415	0.077	3.58E-65
LAP3	2.58E-69	-0.67572	0.312	0.287	3.86E-65
ZCCHC17	2.86E-69	0.117824	0.373	0.115	4.28E-65
PDCD2	2.93E-69	0.290525	0.334	0.058	4.38E-65
LITAF	3.02E-69	-0.47045	0.273	0.171	4.52E-65
SRSF4	3.32E-69	0.268201	0.384	0.087	4.96E-65
SOX9	3.48E-69	0.22818	0.406	0.118	5.20E-65
METTL1	3.62E-69	0.668676	0.391	0.05	5.41E-65
FAM110B	4.08E-69	0.689584	0.386	0.038	6.10E-65
ATP6V1D	4.40E-69	-0.21381	0.346	0.14	6.58E-65
DYNC1H1	6.73E-69	0.338161	0.43	0.108	1.01E-64
SPARCL1	7.07E-69	-0.44615	0.459	0.332	1.06E-64
PELI1	7.27E-69	0.648681	0.347	0.027	1.09E-64
UBE2J2	7.78E-69	0.329386	0.349	0.062	1.16E-64
EXOC1	7.99E-69	0.651024	0.419	0.062	1.19E-64
ESF1	8.66E-69	0.136429	0.388	0.118	1.29E-64
ATP1A3	9.02E-69	0.793377	0.391	0.032	1.35E-64
CLDND1	9.36E-69	-0.21796	0.391	0.197	1.40E-64
ACAT1	9.46E-69	-0.24389	0.354	0.186	1.41E-64
CHIC2	9.84E-69	0.659335	0.38	0.044	1.47E-64
LINC0011€	1.18E-68	-0.17414	0.351	0.166	1.77E-64
U2SURP	1.22E-68	0.162573	0.41	0.123	1.83E-64
SCAF11	1.23E-68	0.185225	0.407	0.113	1.83E-64
RP3-525N1	1.27E-68	0.546974	0.382	0.055	1.89E-64

ANAPC16	1.42E-68	-0.62325	0.306	0.231	2.12E-64
FKBP4	1.48E-68	0.274381	0.367	0.082	2.22E-64
NAA20	1.53E-68	-0.33673	0.378	0.209	2.29E-64
KDM1A	1.56E-68	0.487584	0.334	0.036	2.33E-64
HOXA10	1.61E-68	0.782806	0.329	0.007	2.41E-64
ING4	1.63E-68	0.280191	0.388	0.096	2.43E-64
TPGS2	1.66E-68	0.129088	0.352	0.097	2.48E-64
PIGT	1.76E-68	-0.11065	0.311	0.116	2.63E-64
CLIP3	1.86E-68	0.194354	0.346	0.079	2.78E-64
BAZ1B	1.87E-68	0.655853	0.395	0.044	2.79E-64
ARL2	1.93E-68	-0.21439	0.336	0.147	2.89E-64
CUEDC2	2.14E-68	0.158962	0.459	0.161	3.19E-64
SAP30BP	2.54E-68	0.112164	0.376	0.115	3.80E-64
TMEM167A	3.00E-68	-0.12994	0.352	0.15	4.48E-64
CFL2	3.20E-68	-0.22351	0.368	0.178	4.79E-64
CAPRIN1	3.43E-68	0.495708	0.41	0.07	5.12E-64
RAB33A	3.59E-68	0.508475	0.32	0.031	5.36E-64
AKAP8L	4.01E-68	0.351492	0.373	0.07	5.99E-64
ATF6B	4.03E-68	0.276356	0.443	0.128	6.02E-64
AP2B1	4.17E-68	0.322025	0.382	0.085	6.24E-64
C21orf59	4.78E-68	0.19795	0.352	0.089	7.14E-64
IMMT	4.81E-68	0.107335	0.334	0.085	7.19E-64
PIP4K2C	6.49E-68	0.73947	0.316	0.014	9.69E-64
STOML2	6.55E-68	-0.1065	0.363	0.156	9.78E-64
COA5	8.01E-68	0.118436	0.378	0.115	1.20E-63
LSM10	8.38E-68	-0.35485	0.313	0.173	1.25E-63
SMARCD3	9.85E-68	0.417921	0.373	0.067	1.47E-63
FAM212B	1.08E-67	0.53659	0.339	0.038	1.61E-63
COMMD7	1.27E-67	0.384591	0.389	0.079	1.90E-63
ITGB1BP1	1.40E-67	-0.17204	0.365	0.168	2.08E-63
ACAA1	1.43E-67	0.324814	0.365	0.074	2.13E-63
KLF6	1.56E-67	-0.44627	0.356	0.255	2.33E-63
PAFAH1B2	1.74E-67	0.475823	0.385	0.06	2.60E-63
HNRNPA1L2	1.79E-67	0.398214	0.352	0.055	2.68E-63
CREB1	1.93E-67	0.473705	0.358	0.053	2.89E-63
SLC44A1	1.94E-67	0.500683	0.445	0.094	2.90E-63
SOX6	2.38E-67	0.758099	0.385	0.039	3.56E-63
BOLA3	2.77E-67	-0.11313	0.313	0.125	4.14E-63
TIMM17A	3.35E-67	-0.28725	0.328	0.152	5.00E-63
RTCB	4.49E-67	0.394333	0.364	0.062	6.71E-63
ARC	4.70E-67	0.506861	0.512	0.14	7.02E-63
APBA2	5.05E-67	0.436843	0.368	0.065	7.54E-63
FLAD1	5.10E-67	0.213081	0.342	0.077	7.62E-63
PLEKHJ1	5.22E-67	0.102753	0.41	0.14	7.80E-63
PSMD1	5.51E-67	0.175992	0.364	0.099	8.23E-63
DDIT3	5.78E-67	0.209269	0.564	0.222	8.65E-63
POLE4	6.15E-67	0.142959	0.312	0.077	9.18E-63
DDX56	6.46E-67	0.183695	0.345	0.087	9.65E-63
ZNF22	7.33E-67	0.252551	0.397	0.104	1.10E-62
NELFCD	7.48E-67	0.192114	0.402	0.118	1.12E-62
GGNBP2	7.85E-67	0.196758	0.391	0.111	1.17E-62

AKR1A1	8.89E-67	-0.21651	0.373	0.181	1.33E-62
KIDINS22C	9.17E-67	0.571065	0.402	0.062	1.37E-62
SMIM15	9.83E-67	-0.14863	0.296	0.132	1.47E-62
C1QL1	1.09E-66	0.860855	0.363	0.022	1.63E-62
TRIM13	1.13E-66	0.5739	0.404	0.063	1.69E-62
FZD3	1.20E-66	0.370553	0.408	0.094	1.80E-62
SNHG9	1.33E-66	-0.18522	0.346	0.185	1.99E-62
TMEM9	1.42E-66	-0.34728	0.372	0.229	2.12E-62
ABAT	1.44E-66	0.506148	0.381	0.062	2.16E-62
SPOP	1.50E-66	0.141579	0.362	0.106	2.24E-62
PARL	1.70E-66	0.339065	0.364	0.072	2.55E-62
PRCC	1.89E-66	0.170971	0.351	0.091	2.83E-62
GLTP	1.98E-66	0.334413	0.358	0.067	2.96E-62
CMTM5	2.03E-66	0.709324	0.395	0.048	3.04E-62
RGS16	2.11E-66	-0.1836	0.333	0.14	3.15E-62
UBQLN2	2.22E-66	0.648483	0.354	0.029	3.32E-62
STAU1	3.06E-66	0.276072	0.365	0.082	4.58E-62
MAP4	3.37E-66	0.220669	0.384	0.108	5.03E-62
PRKDC	3.53E-66	0.243269	0.356	0.08	5.27E-62
JUND	4.34E-66	0.196683	0.415	0.135	6.48E-62
PPP1R11	4.80E-66	-0.13683	0.324	0.13	7.17E-62
TSPAN12	5.01E-66	0.417166	0.342	0.053	7.48E-62
SF3A3	5.16E-66	0.117056	0.313	0.07	7.71E-62
ECSIT	5.58E-66	-0.11724	0.338	0.138	8.34E-62
IMPDH2	5.76E-66	-0.41656	0.378	0.255	8.60E-62
C1QB	5.87E-66	-0.43193	0.346	0.246	8.77E-62
NCOR2	5.99E-66	0.563514	0.356	0.044	8.96E-62
PTP4A2	6.42E-66	-0.16208	0.354	0.164	9.59E-62
RAD23B	7.73E-66	0.206714	0.402	0.109	1.16E-61
TSEN34	7.82E-66	0.208925	0.36	0.092	1.17E-61
CIAPIN1	8.38E-66	0.321462	0.368	0.077	1.25E-61
ALDH7A1	8.43E-66	-0.19288	0.362	0.179	1.26E-61
PTPMT1	1.01E-65	0.302827	0.328	0.063	1.51E-61
MOAP1	1.02E-65	0.416732	0.356	0.058	1.52E-61
HOPX	1.03E-65	-1.46481	0.082	0.349	1.53E-61
TCEAL2	1.14E-65	0.132069	0.403	0.137	1.71E-61
EIF3J	1.18E-65	0.142084	0.35	0.097	1.77E-61
TOMM40	1.26E-65	0.157017	0.369	0.103	1.88E-61
GNG2	1.27E-65	0.548206	0.343	0.038	1.90E-61
NTM	1.29E-65	0.674292	0.393	0.051	1.93E-61
CMPK1	1.43E-65	0.270729	0.389	0.096	2.14E-61
PIN4	1.45E-65	-0.34447	0.378	0.227	2.16E-61
HSF1	1.52E-65	0.398243	0.364	0.063	2.27E-61
SLC25A39	1.71E-65	0.102914	0.319	0.085	2.55E-61
NRBP1	1.73E-65	0.166857	0.341	0.089	2.58E-61
PPP1R10	1.85E-65	0.412535	0.36	0.063	2.77E-61
FNTA	1.92E-65	0.35778	0.385	0.084	2.87E-61
ZSCAN18	2.10E-65	0.490343	0.371	0.056	3.15E-61
CDC34	2.15E-65	0.374587	0.363	0.068	3.22E-61
BAD	2.21E-65	-0.15193	0.358	0.169	3.31E-61
EZR	2.22E-65	0.517132	0.386	0.068	3.32E-61

CLSTN1	2.33E-65	0.148117	0.358	0.103	3.49E-61
SLC35E3	2.38E-65	0.860109	0.454	0.072	3.56E-61
TM2D1	2.40E-65	-0.28916	0.365	0.21	3.59E-61
FAM49B	3.32E-65	0.456838	0.373	0.062	4.96E-61
ZNF92	3.51E-65	0.51512	0.354	0.046	5.24E-61
LRP1	3.62E-65	-0.22591	0.311	0.152	5.41E-61
PRNP	4.34E-65	-0.13621	0.391	0.188	6.49E-61
CLIC1	4.53E-65	-1.08014	0.172	0.297	6.78E-61
MLEC	4.83E-65	0.301937	0.377	0.087	7.22E-61
GDE1	5.00E-65	0.308606	0.293	0.043	7.47E-61
PRPSAP2	5.15E-65	0.241702	0.354	0.085	7.70E-61
YTHDC1	5.69E-65	0.43421	0.377	0.065	8.50E-61
PBX1	6.06E-65	0.505256	0.406	0.075	9.05E-61
PMP22	6.07E-65	-1.27528	0.133	0.354	9.07E-61
DDB1	6.18E-65	0.230388	0.356	0.08	9.23E-61
SDHD	6.42E-65	-0.26782	0.334	0.178	9.59E-61
PAPSS1	6.80E-65	0.296046	0.373	0.087	1.02E-60
BRD3	7.03E-65	0.599779	0.403	0.06	1.05E-60
SQSTM1	7.39E-65	-0.32139	0.346	0.2	1.10E-60
MRPS23	7.64E-65	-0.15242	0.338	0.149	1.14E-60
TOP2B	8.24E-65	0.623421	0.363	0.039	1.23E-60
CCDC112	8.46E-65	0.713246	0.338	0.022	1.26E-60
UBE2F	8.86E-65	0.281498	0.345	0.072	1.32E-60
PCMTD2	8.91E-65	0.598344	0.39	0.055	1.33E-60
USE1	9.20E-65	-0.3667	0.365	0.217	1.37E-60
FAM89B	9.90E-65	0.527455	0.322	0.029	1.48E-60
ARID4A	1.01E-64	0.408663	0.416	0.092	1.50E-60
ZNF680	1.03E-64	0.34109	0.347	0.07	1.54E-60
MPHOSPH6	1.24E-64	0.184026	0.35	0.089	1.86E-60
VEZT	1.48E-64	0.220184	0.338	0.082	2.21E-60
EIF3B	1.96E-64	0.384125	0.355	0.065	2.92E-60
KIF5A	2.39E-64	0.930306	0.358	0.017	3.57E-60
SPAG9	2.39E-64	0.490988	0.44	0.096	3.57E-60
C8orf46	2.40E-64	0.5016	0.33	0.041	3.59E-60
RSPRY1	2.49E-64	0.203258	0.326	0.075	3.72E-60
SYNE2	2.60E-64	0.617522	0.375	0.044	3.88E-60
ARMC10	2.81E-64	0.423355	0.365	0.06	4.20E-60
ARMC1	2.88E-64	0.505715	0.377	0.062	4.31E-60
PHLDA1	3.23E-64	0.268629	0.427	0.126	4.82E-60
UQCR11	3.84E-64	-1.72985	0.03	0.369	5.74E-60
JMJD1C	4.03E-64	0.535584	0.384	0.062	6.02E-60
SPSB3	4.96E-64	0.388311	0.39	0.085	7.42E-60
TRAPPC3	4.97E-64	-0.45559	0.302	0.203	7.42E-60
KIF1B	5.31E-64	0.2903	0.41	0.115	7.94E-60
GRSF1	6.35E-64	0.192285	0.349	0.089	9.49E-60
RGCC	6.75E-64	0.381015	0.372	0.075	1.01E-59
DGCR6L	9.09E-64	0.169129	0.352	0.096	1.36E-59
CECR5	1.01E-63	0.63673	0.358	0.038	1.52E-59
NXPH1	1.06E-63	0.724428	0.376	0.041	1.59E-59
PRR13	1.09E-63	-0.7117	0.244	0.214	1.63E-59
NXT1	1.14E-63	0.251429	0.334	0.072	1.70E-59

KPNA2	1.16E-63	0.25768	0.346	0.08	1.74E-59
CISD2	1.25E-63	-0.12301	0.329	0.137	1.87E-59
GNAI3	1.36E-63	0.150414	0.356	0.103	2.03E-59
ASRGL1	1.43E-63	0.546262	0.341	0.038	2.13E-59
GPATCH8	1.43E-63	0.183142	0.389	0.116	2.14E-59
DCAF7	1.53E-63	0.193365	0.351	0.089	2.29E-59
PAK2	1.58E-63	0.455006	0.375	0.068	2.36E-59
RBM6	1.62E-63	0.457747	0.412	0.084	2.42E-59
CNIH1	1.72E-63	-0.33371	0.337	0.193	2.57E-59
TRAPPC2L	1.79E-63	-0.20864	0.356	0.173	2.68E-59
PRKRIP1	1.98E-63	0.47894	0.363	0.058	2.96E-59
BCAR1	2.27E-63	0.537443	0.341	0.041	3.39E-59
RP11-1094	2.36E-63	0.142839	0.326	0.084	3.52E-59
GABARAPL1	2.82E-63	0.144064	0.338	0.091	4.22E-59
LYRM5	3.00E-63	-0.11752	0.364	0.166	4.48E-59
USP46	3.06E-63	0.820702	0.326	0.014	4.57E-59
CREB5	3.25E-63	0.169718	0.391	0.128	4.85E-59
SLTM	3.56E-63	0.138757	0.351	0.092	5.32E-59
H3F3A	3.91E-63	-0.43092	0.676	0.65	5.84E-59
NSMCE1	3.94E-63	-0.18813	0.378	0.198	5.88E-59
CLASP2	4.24E-63	0.600675	0.36	0.043	6.34E-59
MAP1A	4.43E-63	0.450178	0.382	0.07	6.63E-59
MRFAP1L1	4.45E-63	0.291984	0.322	0.065	6.64E-59
PCDHB16	4.47E-63	0.636463	0.352	0.034	6.68E-59
TMEM87A	4.85E-63	0.441993	0.347	0.055	7.25E-59
CCND1	5.04E-63	0.548646	0.381	0.063	7.54E-59
QTRT1	5.56E-63	-0.22928	0.38	0.21	8.31E-59
TMSB15B	5.69E-63	0.55156	0.328	0.032	8.51E-59
GPI	6.98E-63	-0.58803	0.265	0.229	1.04E-58
MTF2	7.09E-63	0.281376	0.325	0.068	1.06E-58
RBM17	7.46E-63	0.507975	0.346	0.048	1.11E-58
MYO10	7.96E-63	0.179585	0.377	0.109	1.19E-58
ILF3-AS1	8.05E-63	-0.11951	0.389	0.178	1.20E-58
NUCB1	9.37E-63	-0.16813	0.365	0.178	1.40E-58
ARL8A	1.06E-62	0.368297	0.346	0.065	1.58E-58
DCTPP1	1.10E-62	-0.13942	0.332	0.142	1.65E-58
NTMT1	1.12E-62	-0.11761	0.299	0.113	1.67E-58
IRF2BPL	1.17E-62	0.450123	0.355	0.055	1.75E-58
SNHG7	1.25E-62	0.191793	0.382	0.113	1.87E-58
TMEM161B-	1.33E-62	0.150681	0.378	0.12	1.99E-58
CUX1	1.80E-62	0.499343	0.354	0.051	2.69E-58
PSMC1	2.30E-62	-0.43705	0.322	0.207	3.44E-58
ELOVL5	2.47E-62	0.383816	0.343	0.062	3.69E-58
C7orf26	2.86E-62	0.573691	0.312	0.022	4.27E-58
C16orf87	3.25E-62	0.440779	0.338	0.05	4.86E-58
TSSC1	3.44E-62	0.28216	0.313	0.063	5.14E-58
ECHS1	3.50E-62	-0.1002	0.315	0.121	5.23E-58
ARMCX1	4.34E-62	0.178071	0.355	0.092	6.49E-58
SMARCD1	4.41E-62	0.482791	0.332	0.044	6.59E-58
ZNF24	4.67E-62	-0.17645	0.33	0.152	6.98E-58
EIF1AY	4.73E-62	0.761738	0.336	0.019	7.07E-58

ARPC5L	4.74E-62	0.292021	0.338	0.07	7.08E-58
DARS	4.86E-62	-0.3104	0.341	0.19	7.26E-58
UBL3	5.07E-62	0.158396	0.337	0.091	7.58E-58
POLDIP2	5.11E-62	0.367021	0.369	0.077	7.64E-58
ZMIZ1	5.95E-62	0.518207	0.285	0.017	8.89E-58
SKAP2	6.08E-62	0.243062	0.313	0.07	9.08E-58
TMX1	6.14E-62	-0.25869	0.32	0.181	9.18E-58
ALKBH7	6.24E-62	-0.13259	0.354	0.162	9.33E-58
TGOLN2	6.45E-62	0.310468	0.334	0.07	9.64E-58
SRSF10	6.60E-62	-0.15888	0.339	0.149	9.87E-58
IFT52	8.65E-62	0.200843	0.3	0.07	1.29E-57
KRIT1	8.86E-62	0.210158	0.333	0.075	1.32E-57
RFC1	1.08E-61	0.161082	0.299	0.068	1.62E-57
CLPP	1.14E-61	-0.11322	0.351	0.156	1.70E-57
EPC1	1.34E-61	0.562983	0.358	0.043	2.00E-57
NEMF	1.35E-61	0.290371	0.382	0.094	2.02E-57
PAK3	1.60E-61	0.734584	0.356	0.031	2.40E-57
MED19	1.72E-61	0.140292	0.324	0.084	2.57E-57
KRCC1	1.73E-61	0.277858	0.338	0.077	2.59E-57
NDUFS1	2.00E-61	0.393841	0.321	0.051	2.99E-57
CMSS1	3.03E-61	0.118508	0.349	0.109	4.53E-57
GORASP2	3.06E-61	0.187071	0.313	0.072	4.57E-57
RAB9A	3.11E-61	-0.12961	0.3	0.118	4.65E-57
BRIX1	3.29E-61	-0.17784	0.322	0.138	4.92E-57
ATP6V1H	3.33E-61	0.164044	0.354	0.092	4.97E-57
RNF11	3.42E-61	0.247917	0.324	0.068	5.12E-57
HNRNPL	3.66E-61	0.30877	0.398	0.099	5.48E-57
SURF1	4.15E-61	0.451452	0.36	0.06	6.21E-57
TBPL1	4.37E-61	0.227808	0.282	0.055	6.52E-57
UBE2E2	4.53E-61	0.475537	0.354	0.055	6.77E-57
CTTNBP2	4.61E-61	0.732888	0.371	0.038	6.90E-57
TAOK3	4.68E-61	0.240353	0.41	0.123	6.99E-57
SRRT	4.73E-61	0.547729	0.359	0.048	7.07E-57
PHGDH	5.57E-61	-0.12112	0.326	0.145	8.32E-57
CHMP1A	5.81E-61	0.242742	0.307	0.062	8.68E-57
DDX39A	6.07E-61	-0.19667	0.375	0.195	9.08E-57
WDR6	6.36E-61	0.208015	0.356	0.099	9.51E-57
DST	6.92E-61	0.311621	0.425	0.123	1.03E-56
LGALS3BP	9.55E-61	-0.76029	0.259	0.289	1.43E-56
DENR	9.95E-61	0.130509	0.325	0.089	1.49E-56
NPDC1	1.09E-60	0.296425	0.415	0.115	1.62E-56
CSNK2A1	1.11E-60	0.29207	0.35	0.082	1.66E-56
ZNF711	1.12E-60	0.579872	0.337	0.034	1.68E-56
SLC16A1	1.15E-60	-0.16289	0.306	0.132	1.72E-56
ALKBH4	1.17E-60	0.344466	0.324	0.06	1.75E-56
AMOTL2	1.17E-60	0.550799	0.291	0.017	1.76E-56
CHMP4B	1.18E-60	0.159981	0.394	0.128	1.77E-56
TMEM219	1.40E-60	-0.27792	0.338	0.188	2.09E-56
AVIL	1.44E-60	0.667723	0.324	0.026	2.16E-56
IFI16	1.57E-60	-0.39916	0.3	0.193	2.34E-56
DNAJC15	1.58E-60	-0.40168	0.256	0.144	2.37E-56

PSPC1	1.61E-60	0.448032	0.317	0.043	2.41E-56
LSM6	1.67E-60	-0.16831	0.326	0.157	2.50E-56
SMARCC2	1.77E-60	0.449708	0.394	0.08	2.65E-56
ZNF580	1.91E-60	0.114644	0.293	0.077	2.85E-56
PEF1	1.97E-60	-0.29688	0.289	0.157	2.95E-56
FUBP1	1.99E-60	0.318265	0.328	0.063	2.97E-56
ARL6IP6	2.25E-60	0.212363	0.347	0.094	3.37E-56
COMMD2	2.26E-60	0.384953	0.39	0.085	3.37E-56
C21orf33	2.55E-60	-0.12598	0.324	0.142	3.81E-56
TAF6	2.57E-60	0.160256	0.293	0.067	3.84E-56
SNRPA	2.64E-60	0.25918	0.332	0.074	3.95E-56
HTRA1	2.71E-60	-0.28332	0.368	0.221	4.05E-56
GOLIM4	2.98E-60	0.388161	0.406	0.101	4.46E-56
FXR1	3.00E-60	0.15903	0.359	0.109	4.49E-56
EZH2	3.03E-60	0.607964	0.319	0.022	4.53E-56
TMEM248	3.26E-60	0.166549	0.283	0.058	4.88E-56
ACIN1	3.27E-60	0.292267	0.351	0.079	4.88E-56
ACTR1A	3.31E-60	0.392608	0.32	0.05	4.95E-56
HOXA2	3.31E-60	0.723513	0.285	0.003	4.95E-56
TMEM206	3.37E-60	0.506271	0.336	0.044	5.04E-56
PPP1R15A	3.52E-60	-0.21986	0.329	0.179	5.25E-56
ILKAP	3.52E-60	0.375353	0.336	0.056	5.26E-56
CAPNS1	3.65E-60	0.227955	0.334	0.082	5.45E-56
ASF1A	3.70E-60	0.299929	0.347	0.084	5.53E-56
ALCAM	3.78E-60	0.785057	0.334	0.019	5.64E-56
KNOP1	3.81E-60	0.224383	0.343	0.085	5.69E-56
YBX3	4.13E-60	-0.25825	0.319	0.186	6.17E-56
MT1M	4.35E-60	-0.81589	0.241	0.241	6.50E-56
OXR1	4.66E-60	0.310625	0.317	0.062	6.96E-56
AAK1	5.54E-60	0.345936	0.371	0.089	8.28E-56
G6PC3	8.45E-60	-0.17017	0.317	0.149	1.26E-55
DDIT4	9.60E-60	-0.27591	0.375	0.198	1.43E-55
PHF20L1	9.70E-60	0.548642	0.382	0.063	1.45E-55
TPM3	9.86E-60	-0.30794	0.338	0.197	1.47E-55
SPATS2	1.01E-59	0.346497	0.371	0.089	1.50E-55
PTBP1	1.02E-59	0.125651	0.317	0.089	1.53E-55
TUSC2	1.08E-59	0.251501	0.33	0.074	1.62E-55
AMD1	1.10E-59	-0.16649	0.312	0.142	1.64E-55
MAML2	1.14E-59	0.482157	0.338	0.05	1.70E-55
C9orf142	1.26E-59	0.311352	0.334	0.068	1.88E-55
HSD17B12	1.33E-59	-0.19774	0.345	0.181	1.99E-55
RASL11B	1.61E-59	0.939669	0.304	0.007	2.41E-55
ATCAY	1.70E-59	0.527823	0.313	0.032	2.54E-55
SLC35B2	1.73E-59	0.418447	0.345	0.06	2.58E-55
TXNDC12	1.79E-59	-0.34045	0.312	0.183	2.68E-55
ORC4	1.86E-59	0.28382	0.302	0.058	2.79E-55
FNDC4	2.47E-59	0.437666	0.296	0.034	3.70E-55
PPP2R5E	2.55E-59	0.489318	0.316	0.038	3.80E-55
MDFI	2.94E-59	0.644406	0.29	0.015	4.40E-55
HIP1	3.05E-59	0.231317	0.332	0.082	4.55E-55
DPP6	3.08E-59	0.297991	0.343	0.082	4.60E-55

ATP2A2	3.17E-59	0.247599	0.35	0.092	4.74E-55
BMP7	4.01E-59	0.465666	0.363	0.063	5.99E-55
MAFB	4.06E-59	0.289884	0.289	0.053	6.07E-55
CTDNEP1	4.23E-59	0.408323	0.295	0.038	6.32E-55
CBR4	4.46E-59	0.665971	0.339	0.031	6.67E-55
METTL2B	4.60E-59	0.264297	0.294	0.053	6.87E-55
JKAMP	4.76E-59	0.461086	0.359	0.067	7.11E-55
DYNC1LI1	4.79E-59	0.230963	0.333	0.077	7.16E-55
EBNA1BP2	4.87E-59	-0.23687	0.312	0.156	7.28E-55
PFKL	5.22E-59	0.152352	0.309	0.082	7.80E-55
RBM5	6.08E-59	0.274101	0.377	0.096	9.09E-55
LRRC59	6.65E-59	0.272181	0.341	0.068	9.95E-55
ACADVL	6.75E-59	-0.14851	0.308	0.138	1.01E-54
NDUFAF2	6.89E-59	-0.15814	0.303	0.128	1.03E-54
TIMMDC1	7.15E-59	-0.31598	0.32	0.195	1.07E-54
RPS6KL1	7.44E-59	0.647636	0.294	0.012	1.11E-54
DHRSX	7.90E-59	0.469368	0.336	0.051	1.18E-54
VPS37B	8.09E-59	0.475467	0.332	0.046	1.21E-54
G3BP2	8.85E-59	0.278485	0.283	0.048	1.32E-54
CCL3	9.16E-59	0.257592	0.345	0.085	1.37E-54
FOXO3	1.12E-58	0.342857	0.332	0.067	1.68E-54
BCL7A	1.33E-58	0.638024	0.307	0.019	1.99E-54
VEZF1	1.52E-58	0.459089	0.338	0.05	2.27E-54
PITHD1	1.70E-58	0.304671	0.329	0.068	2.54E-54
TM2D2	1.71E-58	-0.10956	0.299	0.128	2.56E-54
VKORC1	1.88E-58	-0.42295	0.336	0.219	2.81E-54
DPYSL4	2.09E-58	0.567296	0.298	0.021	3.12E-54
GDAP1L1	2.16E-58	0.54762	0.337	0.044	3.23E-54
ZIC1	2.19E-58	0.285001	0.282	0.053	3.27E-54
SDF2L1	2.22E-58	-0.23557	0.254	0.108	3.32E-54
7-Mar	2.23E-58	0.309826	0.315	0.063	3.33E-54
TM4SF1	2.28E-58	0.470617	0.278	0.027	3.40E-54
RP11-553L	2.97E-58	0.577184	0.397	0.072	4.44E-54
TUBB4A	3.26E-58	0.48069	0.283	0.024	4.88E-54
HMOX2	3.27E-58	-0.22966	0.256	0.113	4.89E-54
PPP1R12A	3.35E-58	0.209662	0.372	0.109	5.00E-54
RCC2	3.42E-58	0.263555	0.248	0.032	5.11E-54
CDC5L	3.49E-58	0.407583	0.334	0.055	5.21E-54
TPGS1	3.91E-58	0.35028	0.342	0.065	5.85E-54
G3BP1	3.92E-58	0.115382	0.337	0.109	5.85E-54
BRWD1	4.22E-58	0.54746	0.336	0.039	6.30E-54
CCDC107	4.63E-58	0.18639	0.35	0.099	6.92E-54
PGD	4.88E-58	0.23339	0.313	0.072	7.30E-54
GRAMD1A	5.19E-58	0.519541	0.358	0.055	7.76E-54
ARHGEF25	5.32E-58	0.692688	0.293	0.014	7.95E-54
ZNF292	6.37E-58	0.530301	0.358	0.055	9.51E-54
RFXANK	6.43E-58	-0.53921	0.252	0.183	9.62E-54
NUDT4	6.55E-58	0.141117	0.267	0.055	9.79E-54
COPS4	6.59E-58	-0.11303	0.319	0.137	9.85E-54
TNRC6C	6.92E-58	0.395656	0.289	0.038	1.03E-53
DNAAF2	7.23E-58	0.466268	0.291	0.031	1.08E-53

CDCA7L	7.46E-58	0.262187	0.313	0.065	1.11E-53
MED28	8.65E-58	-0.22419	0.332	0.159	1.29E-53
PDRG1	9.10E-58	-0.14077	0.274	0.108	1.36E-53
TIMP2	1.01E-57	0.364409	0.334	0.063	1.51E-53
NSL1	1.03E-57	-0.14377	0.321	0.145	1.55E-53
COX19	1.10E-57	0.453565	0.322	0.05	1.65E-53
SNN	1.11E-57	0.48537	0.338	0.051	1.65E-53
DCP1A	1.39E-57	0.298356	0.326	0.065	2.08E-53
NUDT3	1.46E-57	0.258374	0.352	0.087	2.18E-53
RBM4	1.51E-57	0.501884	0.337	0.048	2.26E-53
ZNF638	1.53E-57	0.527593	0.377	0.065	2.28E-53
MED30	1.53E-57	0.458434	0.341	0.058	2.29E-53
AP3S1	1.61E-57	-0.10839	0.329	0.14	2.41E-53
GGPS1	1.72E-57	-0.20179	0.316	0.152	2.56E-53
YKT6	1.81E-57	0.15037	0.283	0.068	2.70E-53
APOA1BP	2.02E-57	0.119798	0.368	0.123	3.02E-53
TTC1	2.17E-57	-0.21214	0.303	0.144	3.24E-53
PHYHIPL	2.18E-57	0.179301	0.421	0.15	3.26E-53
ORAI2	2.20E-57	0.409428	0.337	0.063	3.29E-53
ZNF655	2.24E-57	0.340477	0.337	0.067	3.34E-53
PPP2CB	2.48E-57	0.301629	0.334	0.079	3.71E-53
CA10	2.60E-57	0.741863	0.285	0.009	3.89E-53
LRRC4C	2.70E-57	0.199637	0.268	0.056	4.03E-53
ZNF146	3.23E-57	0.227408	0.336	0.089	4.82E-53
KMT2A	3.27E-57	0.519137	0.333	0.044	4.89E-53
SND1	3.70E-57	0.224439	0.282	0.058	5.54E-53
YBEY	3.78E-57	0.106328	0.307	0.084	5.65E-53
C22orf39	4.01E-57	0.29515	0.307	0.062	5.99E-53
PRR4	4.81E-57	0.319916	0.346	0.074	7.19E-53
ANXA1	4.87E-57	-1.4069	0.077	0.323	7.28E-53
RP9	5.53E-57	0.421774	0.321	0.051	8.26E-53
VOPP1	5.91E-57	0.184685	0.269	0.06	8.83E-53
SPTAN1	5.93E-57	0.269025	0.35	0.091	8.87E-53
CPXM1	6.35E-57	0.666892	0.307	0.019	9.49E-53
MARK3	6.41E-57	0.295887	0.36	0.089	9.58E-53
RBM22	6.51E-57	0.151368	0.346	0.103	9.72E-53
DHX29	6.54E-57	0.170688	0.337	0.084	9.77E-53
AGAP2	6.79E-57	0.749946	0.274	0.002	1.01E-52
EIF2B1	6.80E-57	0.376145	0.363	0.077	1.02E-52
TAF15	7.19E-57	0.173449	0.304	0.072	1.07E-52
GAMT	7.56E-57	0.226322	0.272	0.048	1.13E-52
IWS1	7.85E-57	0.375479	0.304	0.044	1.17E-52
TRAP1	9.23E-57	0.447738	0.329	0.051	1.38E-52
ZNF791	9.38E-57	0.161112	0.381	0.128	1.40E-52
ZNF131	9.39E-57	0.402993	0.329	0.058	1.40E-52
TM9SF2	1.01E-56	0.306316	0.315	0.065	1.51E-52
DCLK2	1.01E-56	0.268669	0.306	0.063	1.51E-52
TBCC	1.09E-56	0.518067	0.325	0.039	1.62E-52
FAM60A	1.17E-56	0.449056	0.32	0.046	1.75E-52
TMEM45A	1.25E-56	-0.56231	0.226	0.149	1.87E-52
R3HCC1	1.34E-56	0.18568	0.33	0.089	2.01E-52

TMEM256	1.37E-56	-0.17546	0.345	0.169	2.05E-52
TMEM259	1.40E-56	0.290517	0.359	0.091	2.09E-52
RPN1	1.56E-56	-0.20322	0.299	0.145	2.34E-52
MAGI2	1.62E-56	0.540591	0.315	0.032	2.42E-52
BRD7	1.77E-56	0.381591	0.312	0.051	2.65E-52
C11orf96	1.83E-56	0.700183	0.338	0.034	2.74E-52
LRWD1	1.86E-56	0.299916	0.25	0.032	2.78E-52
ZNF75A	1.92E-56	0.323303	0.334	0.072	2.87E-52
HBB	2.09E-56	-0.76483	0.303	0.226	3.12E-52
RGS2	2.12E-56	-0.17802	0.307	0.13	3.17E-52
C14orf119	2.18E-56	-0.23118	0.272	0.126	3.26E-52
NSUN5	2.18E-56	0.157219	0.286	0.072	3.26E-52
PTGES2	2.36E-56	0.375625	0.319	0.053	3.53E-52
SEZ6	2.44E-56	0.706454	0.369	0.05	3.64E-52
C5orf24	2.46E-56	0.312794	0.336	0.072	3.67E-52
UROD	2.66E-56	-0.31054	0.29	0.157	3.98E-52
NKIRAS2	2.97E-56	0.258835	0.307	0.065	4.44E-52
RP11-676J	3.33E-56	0.745632	0.287	0.005	4.97E-52
CELF1	3.40E-56	0.263674	0.306	0.065	5.08E-52
TCEAL5	4.28E-56	0.101389	0.276	0.07	6.40E-52
CHD6	4.46E-56	0.289122	0.324	0.07	6.67E-52
FYTTD1	4.70E-56	0.134831	0.273	0.062	7.03E-52
CUL1	4.88E-56	0.1717	0.285	0.068	7.29E-52
RARRES2	5.35E-56	-0.18353	0.293	0.133	7.99E-52
ADSL	5.87E-56	0.134477	0.287	0.072	8.77E-52
IPO9	6.58E-56	0.277299	0.343	0.082	9.83E-52
TMEM179B	7.84E-56	-0.31305	0.274	0.15	1.17E-51
ABCD4	8.05E-56	0.175623	0.315	0.084	1.20E-51
BAG6	8.87E-56	0.345803	0.332	0.065	1.33E-51
HAGH	8.88E-56	0.437393	0.347	0.065	1.33E-51
SAFB	1.07E-55	0.35141	0.336	0.068	1.59E-51
DSCAM	1.07E-55	0.518411	0.304	0.034	1.60E-51
NUCB2	1.13E-55	-0.23311	0.286	0.147	1.69E-51
APRT	1.24E-55	-0.19243	0.362	0.195	1.85E-51
C1QA	1.28E-55	-0.27053	0.281	0.161	1.91E-51
TIMM9	1.55E-55	-0.1638	0.291	0.121	2.32E-51
ZC2HC1A	1.59E-55	0.446613	0.322	0.055	2.37E-51
STX10	1.59E-55	-0.1308	0.291	0.125	2.38E-51
WDR1	1.62E-55	-0.12026	0.294	0.126	2.43E-51
CDKN1B	1.94E-55	0.362296	0.328	0.062	2.90E-51
HLA-DPA1	2.05E-55	-1.03884	0.176	0.27	3.06E-51
PLRG1	2.11E-55	0.172945	0.277	0.063	3.16E-51
OMG	2.28E-55	0.590344	0.315	0.036	3.41E-51
CDC42EP4	2.38E-55	-0.34173	0.234	0.132	3.56E-51
VPS4A	2.42E-55	0.271431	0.325	0.072	3.62E-51
HOXB2	2.51E-55	0.662769	0.278	0.009	3.75E-51
ALDH9A1	2.62E-55	-0.14151	0.308	0.133	3.92E-51
SATB1	2.72E-55	0.582274	0.306	0.026	4.07E-51
GDAP1	2.73E-55	0.158769	0.295	0.068	4.07E-51
BID	2.75E-55	0.132388	0.333	0.101	4.11E-51
PAQR4	2.78E-55	0.581709	0.274	0.015	4.15E-51

TAF11	3.03E-55	0.187238	0.303	0.075	4.53E-51
VMP1	3.05E-55	-0.46682	0.294	0.215	4.55E-51
TRIM24	3.20E-55	0.500509	0.303	0.032	4.78E-51
GRN	3.24E-55	-0.19053	0.309	0.161	4.84E-51
CRK	3.41E-55	0.283816	0.345	0.084	5.09E-51
RTKN	3.47E-55	0.223351	0.317	0.082	5.19E-51
SPG7	3.86E-55	0.320508	0.302	0.053	5.76E-51
NOA1	3.90E-55	0.648833	0.274	0.012	5.83E-51
KIF2A	4.05E-55	0.527081	0.304	0.034	6.05E-51
DLX5	4.17E-55	1.023906	0.316	0.015	6.23E-51
NCAN	4.27E-55	0.617173	0.285	0.017	6.37E-51
RNF216	4.62E-55	0.167649	0.322	0.097	6.91E-51
WDR82	4.92E-55	0.242539	0.295	0.068	7.35E-51
EBPL	4.93E-55	0.44201	0.347	0.063	7.37E-51
TSR3	4.96E-55	0.494241	0.338	0.051	7.42E-51
HSPBP1	5.00E-55	0.127885	0.322	0.094	7.47E-51
XRCC6BP1	5.25E-55	0.644233	0.283	0.015	7.85E-51
SAFB2	5.42E-55	0.357874	0.371	0.089	8.10E-51
UPF3B	5.46E-55	0.148391	0.333	0.096	8.16E-51
C8orf4	5.50E-55	-1.0958	0.165	0.265	8.21E-51
SNRPA1	5.83E-55	-0.20128	0.295	0.13	8.71E-51
NNAT	6.02E-55	1.093375	0.401	0.053	9.00E-51
MPHOSPH1C	6.16E-55	0.178747	0.337	0.085	9.21E-51
HMG20B	6.42E-55	0.146055	0.33	0.099	9.59E-51
APBB2	6.42E-55	0.232718	0.274	0.056	9.60E-51
TRMT10C	6.50E-55	0.208355	0.312	0.079	9.72E-51
RRP7A	7.13E-55	0.31423	0.287	0.044	1.07E-50
NACA2	7.34E-55	-0.60257	0.208	0.171	1.10E-50
DCTN1	7.35E-55	0.449618	0.319	0.044	1.10E-50
NUB1	8.13E-55	0.297766	0.308	0.06	1.22E-50
PDCL3	8.36E-55	0.106838	0.313	0.096	1.25E-50
SMAP1	8.54E-55	0.309191	0.295	0.053	1.28E-50
RB1CC1	8.57E-55	0.555713	0.342	0.05	1.28E-50
EPRS	9.16E-55	0.124237	0.347	0.118	1.37E-50
PDCD7	9.76E-55	0.304925	0.296	0.053	1.46E-50
GATM	1.10E-54	-0.17337	0.299	0.145	1.65E-50
GPN1	1.15E-54	0.173945	0.277	0.058	1.71E-50
HNRNPLL	1.22E-54	0.258164	0.257	0.041	1.82E-50
C18orf32	1.25E-54	0.136238	0.272	0.067	1.87E-50
TPST1	1.25E-54	0.332131	0.298	0.055	1.88E-50
REV3L	1.43E-54	0.508683	0.358	0.063	2.14E-50
TRAM1	1.48E-54	-0.28607	0.295	0.154	2.22E-50
PLEKHO1	1.50E-54	0.366649	0.285	0.041	2.24E-50
PHACTR3	1.53E-54	0.567171	0.285	0.021	2.29E-50
EPN2	1.61E-54	0.166559	0.307	0.08	2.40E-50
UBTF	1.76E-54	0.388515	0.312	0.051	2.63E-50
SESTD1	1.90E-54	0.367782	0.25	0.026	2.85E-50
PRPSAP1	1.92E-54	0.256341	0.321	0.075	2.88E-50
SCFD1	2.12E-54	-0.12157	0.299	0.125	3.17E-50
AMZ2	2.51E-54	-0.28937	0.307	0.178	3.75E-50
CNDP2	2.62E-54	-0.18374	0.263	0.118	3.92E-50

OSER1	2.65E-54	-0.21524	0.289	0.125	3.95E-50
CDKN2AIP	2.99E-54	0.312598	0.239	0.026	4.47E-50
AHCY	3.03E-54	0.123516	0.309	0.091	4.53E-50
RICTOR	3.08E-54	0.556579	0.358	0.058	4.60E-50
POU3F3	3.19E-54	0.415193	0.285	0.038	4.77E-50
RNF187	3.27E-54	0.12417	0.382	0.138	4.89E-50
UBE2A	3.29E-54	-0.29051	0.3	0.169	4.92E-50
FERMT1	3.56E-54	0.845491	0.313	0.019	5.32E-50
LANCL2	3.61E-54	0.125164	0.291	0.079	5.39E-50
POU3F2	3.76E-54	0.287245	0.315	0.067	5.61E-50
RAP1A	4.39E-54	-0.24922	0.294	0.147	6.56E-50
SLC37A3	4.41E-54	0.434281	0.291	0.038	6.59E-50
ARRDC3	4.81E-54	0.241237	0.324	0.087	7.19E-50
RSBN1	5.03E-54	0.477911	0.302	0.034	7.52E-50
IGFBP7	5.26E-54	-1.06089	0.131	0.219	7.86E-50
UBXN2A	6.13E-54	0.332138	0.274	0.046	9.15E-50
HOOK3	6.40E-54	0.322207	0.317	0.065	9.57E-50
URM1	6.85E-54	-0.22731	0.299	0.159	1.02E-49
FBXO11	7.89E-54	0.4628	0.307	0.041	1.18E-49
SF3A2	8.02E-54	0.359355	0.322	0.063	1.20E-49
FDX1L	8.12E-54	-0.12001	0.272	0.116	1.21E-49
C1orf35	8.57E-54	0.443205	0.319	0.05	1.28E-49
HLA-DRB1	9.29E-54	-1.03863	0.148	0.255	1.39E-49
CNIH4	1.01E-53	-0.17318	0.324	0.162	1.50E-49
AZIN1	1.12E-53	0.350083	0.362	0.089	1.67E-49
LRRC47	1.21E-53	0.369402	0.309	0.051	1.81E-49
FBXO7	1.36E-53	0.318045	0.277	0.044	2.03E-49
LBR	1.40E-53	0.495968	0.334	0.053	2.09E-49
TNR	1.40E-53	0.691778	0.332	0.036	2.10E-49
RIF1	1.53E-53	0.358896	0.261	0.034	2.29E-49
FRS2	1.59E-53	0.774442	0.322	0.029	2.38E-49
TIMM22	1.63E-53	0.227775	0.308	0.068	2.44E-49
RNF130	1.78E-53	0.569956	0.337	0.048	2.67E-49
CSRP2	1.92E-53	-0.87635	0.174	0.232	2.87E-49
RAB13	1.94E-53	-0.90979	0.204	0.246	2.90E-49
TXNDC9	2.11E-53	0.223363	0.281	0.058	3.16E-49
PTCD3	2.16E-53	0.458529	0.325	0.051	3.22E-49
MIR4458HG	2.16E-53	-0.3912	0.273	0.183	3.23E-49
ACAT2	2.28E-53	0.114712	0.263	0.068	3.41E-49
CXADR	2.37E-53	0.482051	0.328	0.055	3.54E-49
CEP170	2.51E-53	0.48636	0.302	0.036	3.75E-49
ANKIB1	2.51E-53	0.174947	0.239	0.044	3.75E-49
CCNG2	2.58E-53	0.629015	0.308	0.027	3.86E-49
ARID1A	2.70E-53	0.335917	0.308	0.058	4.03E-49
CXXC4	3.05E-53	0.595281	0.261	0.009	4.56E-49
NELL2	3.20E-53	0.522654	0.312	0.041	4.79E-49
PCSK7	3.62E-53	0.185742	0.368	0.123	5.41E-49
ATP1A1	4.02E-53	-0.20183	0.336	0.183	6.01E-49
HRAS	4.61E-53	0.412583	0.303	0.046	6.89E-49
CDK5	4.90E-53	0.247354	0.289	0.058	7.33E-49
ACOT7	5.68E-53	0.384944	0.276	0.039	8.49E-49

SYPL1	5.91E-53	-0.6639	0.237	0.212	8.84E-49
IFT20	6.08E-53	-0.20332	0.296	0.154	9.09E-49
NSD1	6.46E-53	0.338812	0.311	0.065	9.66E-49
CDK5RAP3	7.12E-53	-0.13365	0.328	0.162	1.06E-48
HEPACAM	7.28E-53	-0.19609	0.286	0.142	1.09E-48
DCUN1D5	8.10E-53	0.14613	0.303	0.084	1.21E-48
CRLS1	8.16E-53	0.444999	0.328	0.056	1.22E-48
PGAP1	8.46E-53	0.360469	0.278	0.044	1.26E-48
UBE3A	8.47E-53	0.410195	0.329	0.062	1.27E-48
CORO7	8.48E-53	0.529709	0.256	0.014	1.27E-48
HTRA2	9.07E-53	0.461222	0.321	0.05	1.35E-48
LBH	9.91E-53	0.470946	0.293	0.039	1.48E-48
BSDC1	1.07E-52	0.168811	0.325	0.096	1.59E-48
ATIC	1.09E-52	0.26049	0.3	0.065	1.63E-48
PKIG	1.11E-52	-0.33134	0.294	0.183	1.65E-48
DNAJA2	1.16E-52	0.12338	0.316	0.099	1.73E-48
CPM	1.25E-52	0.856553	0.306	0.019	1.86E-48
TM7SF2	1.46E-52	0.335508	0.285	0.048	2.18E-48
MEIS2	1.49E-52	0.391413	0.307	0.053	2.22E-48
WDR60	1.63E-52	0.358042	0.352	0.077	2.44E-48
NSG1	1.74E-52	0.483355	0.308	0.051	2.60E-48
MMP16	1.90E-52	0.719293	0.282	0.01	2.85E-48
SLC38A1	1.97E-52	0.327032	0.286	0.055	2.94E-48
KIAA1715	2.10E-52	0.19199	0.296	0.08	3.15E-48
AIF1L	2.24E-52	-0.14713	0.228	0.089	3.35E-48
ANKS1B	2.51E-52	0.350123	0.243	0.031	3.74E-48
MARS	2.52E-52	0.205386	0.309	0.082	3.76E-48
ACSL3	2.62E-52	0.103731	0.291	0.084	3.92E-48
ZFAND3	2.69E-52	0.128169	0.241	0.053	4.01E-48
ACYP2	2.95E-52	-0.22824	0.291	0.161	4.40E-48
FGD5-AS1	3.22E-52	0.252982	0.277	0.06	4.81E-48
AC004158.	3.40E-52	0.479087	0.291	0.036	5.08E-48
TMEM121	3.72E-52	0.622336	0.285	0.019	5.56E-48
TMPO	3.77E-52	0.515535	0.283	0.026	5.63E-48
DRAXIN	4.00E-52	0.393112	0.273	0.036	5.98E-48
TTC14	4.07E-52	0.161314	0.311	0.091	6.08E-48
DYNLL2	4.18E-52	0.397212	0.27	0.034	6.25E-48
MLLT4	5.78E-52	0.629966	0.298	0.024	8.64E-48
BLOC1S6	5.98E-52	0.249995	0.255	0.044	8.94E-48
ZNF281	6.39E-52	0.484785	0.289	0.036	9.55E-48
CHST11	7.05E-52	0.368447	0.243	0.027	1.05E-47
LMNA	7.51E-52	-0.19589	0.319	0.174	1.12E-47
FOXP1	8.73E-52	-0.5843	0.224	0.209	1.30E-47
ABCF1	9.65E-52	0.195632	0.282	0.065	1.44E-47
MRTO4	9.70E-52	0.216748	0.308	0.075	1.45E-47
DDX52	1.03E-51	0.405809	0.313	0.053	1.54E-47
RBBP6	1.07E-51	0.365511	0.349	0.079	1.60E-47
BCS1L	1.13E-51	0.277654	0.273	0.051	1.70E-47
GTF2IRD2E	1.27E-51	0.361212	0.286	0.046	1.90E-47
ANTXR1	1.36E-51	0.586159	0.277	0.017	2.03E-47
HMBOX1	1.38E-51	0.143014	0.316	0.092	2.07E-47

ZNF91	1.43E-51	0.382794	0.322	0.063	2.14E-47
PLGRKT	1.59E-51	0.132657	0.26	0.065	2.38E-47
THOC2	1.67E-51	0.235631	0.342	0.099	2.50E-47
TSNAX	1.72E-51	-0.11976	0.277	0.109	2.57E-47
TMEM183A	1.82E-51	0.145683	0.272	0.07	2.72E-47
PCNA	1.91E-51	-0.11344	0.293	0.126	2.85E-47
DNMT1	1.94E-51	0.30725	0.298	0.06	2.90E-47
SMIM14	1.98E-51	-0.33537	0.263	0.147	2.96E-47
ARIH2	2.17E-51	0.161722	0.285	0.074	3.24E-47
NRXN2	2.17E-51	0.47575	0.302	0.039	3.24E-47
SDHAF2	2.19E-51	0.162373	0.293	0.074	3.28E-47
MYEF2	2.43E-51	0.441524	0.299	0.041	3.64E-47
IFNGR2	2.45E-51	0.108495	0.272	0.074	3.66E-47
1-Mar	2.63E-51	0.673626	0.272	0.01	3.92E-47
SLC39A7	2.99E-51	0.105876	0.329	0.101	4.46E-47
LARP7	3.04E-51	0.116829	0.343	0.123	4.54E-47
EPM2AIP1	3.05E-51	0.403618	0.308	0.053	4.56E-47
ATG4B	3.10E-51	0.335314	0.313	0.06	4.64E-47
SDC3	3.12E-51	-0.54408	0.198	0.15	4.66E-47
DDX41	3.16E-51	0.17284	0.246	0.051	4.72E-47
H1F0	3.30E-51	0.455327	0.41	0.104	4.93E-47
FUBP3	3.43E-51	0.264296	0.306	0.065	5.12E-47
INSM1	3.57E-51	0.703057	0.277	0.012	5.34E-47
XPO1	3.60E-51	0.206847	0.295	0.072	5.38E-47
GLRX3	3.84E-51	-0.1884	0.242	0.099	5.74E-47
WSCD1	3.86E-51	0.244684	0.291	0.07	5.76E-47
TMEM30A	4.33E-51	0.31453	0.343	0.087	6.47E-47
MTRNR2L8	4.72E-51	-1.22689	0.082	0.289	7.05E-47
ECI2	5.24E-51	-0.18403	0.267	0.125	7.83E-47
PRDX3	5.26E-51	-0.36596	0.309	0.2	7.87E-47
EXOSC4	5.84E-51	0.491461	0.319	0.05	8.73E-47
LAMP2	5.97E-51	-0.47689	0.259	0.198	8.92E-47
GOPC	6.30E-51	0.323556	0.341	0.08	9.41E-47
VPS41	6.84E-51	0.17386	0.247	0.053	1.02E-46
LUC7L	8.10E-51	0.224785	0.309	0.079	1.21E-46
TRIM33	8.16E-51	0.271202	0.254	0.044	1.22E-46
LMO1	8.20E-51	0.713	0.3	0.022	1.23E-46
TAX1BP3	8.86E-51	-0.11282	0.283	0.116	1.32E-46
PCM1	8.97E-51	0.255304	0.329	0.085	1.34E-46
RP11-792A	1.00E-50	0.167256	0.311	0.091	1.50E-46
AASDH	1.09E-50	0.445434	0.32	0.056	1.64E-46
SEZ6L2	1.16E-50	0.253708	0.351	0.108	1.73E-46
KCTD5	1.16E-50	0.347563	0.28	0.046	1.74E-46
IFT27	1.19E-50	0.367104	0.3	0.055	1.78E-46
RNF24	1.26E-50	0.246556	0.295	0.067	1.88E-46
FAM210B	1.38E-50	0.639687	0.324	0.039	2.07E-46
CCDC50	1.42E-50	0.333688	0.281	0.044	2.12E-46
SUCLA2	1.44E-50	-0.12537	0.283	0.13	2.16E-46
NT5C	1.63E-50	0.282648	0.32	0.077	2.43E-46
BIN1	1.63E-50	0.609424	0.28	0.017	2.44E-46
PAFAH1B1	1.94E-50	0.277034	0.317	0.07	2.90E-46

ANKRD17	2.03E-50	0.34101	0.276	0.046	3.04E-46
COPA	2.11E-50	0.209569	0.316	0.089	3.15E-46
MORN2	2.13E-50	-0.21435	0.241	0.111	3.18E-46
TFDP2	2.17E-50	0.405772	0.269	0.034	3.25E-46
ATP6V1B2	2.25E-50	0.163646	0.302	0.087	3.36E-46
C19orf25	2.32E-50	0.234331	0.311	0.08	3.47E-46
SNX22	2.35E-50	0.5764	0.241	0.01	3.51E-46
CCDC14	2.39E-50	0.286261	0.307	0.067	3.56E-46
GNAQ	2.75E-50	0.201386	0.239	0.044	4.10E-46
ING2	2.88E-50	0.254412	0.246	0.044	4.31E-46
SURF2	3.09E-50	0.130401	0.265	0.065	4.62E-46
UBE2G2	3.10E-50	0.201964	0.293	0.07	4.63E-46
CPNE1	3.25E-50	-0.14945	0.276	0.13	4.86E-46
TEX264	3.43E-50	-0.34935	0.283	0.178	5.13E-46
NCALD	3.48E-50	0.291235	0.283	0.062	5.20E-46
LNX1	3.70E-50	0.741493	0.267	0.01	5.53E-46
NFU1	4.03E-50	0.108083	0.304	0.101	6.02E-46
CELF5	4.27E-50	0.675066	0.261	0.005	6.38E-46
MESDC2	4.31E-50	-0.605	0.221	0.19	6.44E-46
SPECC1	4.32E-50	0.413542	0.337	0.068	6.45E-46
MAD1L1	4.66E-50	0.566896	0.272	0.019	6.97E-46
FADS1	4.75E-50	0.288524	0.286	0.058	7.10E-46
SECISBP2	4.99E-50	0.373949	0.343	0.079	7.45E-46
PEG10	5.01E-50	0.193338	0.248	0.051	7.49E-46
CBWD1	5.88E-50	0.11064	0.282	0.087	8.79E-46
NUP160	6.25E-50	0.25879	0.257	0.05	9.34E-46
KPNA3	6.73E-50	0.147079	0.242	0.055	1.01E-45
HARS	7.61E-50	0.182429	0.313	0.091	1.14E-45
KLF7	7.64E-50	0.377311	0.286	0.05	1.14E-45
TMEM33	7.71E-50	0.26831	0.277	0.055	1.15E-45
USF2	8.30E-50	0.209954	0.282	0.068	1.24E-45
TTYH3	8.75E-50	0.370952	0.286	0.046	1.31E-45
CCM2	1.03E-49	0.203525	0.267	0.058	1.55E-45
RBM10	1.10E-49	0.263018	0.265	0.051	1.64E-45
LMBR1L	1.13E-49	0.572487	0.289	0.026	1.69E-45
FAM162A	1.25E-49	-0.92081	0.199	0.282	1.87E-45
RNASEH1	1.37E-49	0.392477	0.285	0.043	2.05E-45
RING1	1.39E-49	0.183015	0.273	0.065	2.07E-45
RABL6	1.43E-49	0.377987	0.257	0.032	2.14E-45
CCDC53	1.48E-49	-0.18406	0.277	0.132	2.21E-45
BLOC1S4	1.66E-49	0.381137	0.276	0.039	2.48E-45
HYAL2	1.82E-49	0.475412	0.281	0.031	2.72E-45
COQ7	1.83E-49	0.298826	0.277	0.056	2.73E-45
USP10	1.99E-49	0.244132	0.272	0.055	2.98E-45
MYCBP2	2.00E-49	0.406503	0.276	0.039	2.99E-45
AC004951.	2.16E-49	0.461156	0.268	0.027	3.23E-45
SREK1	2.26E-49	-0.13148	0.307	0.144	3.38E-45
ZC3H8	2.58E-49	0.401839	0.272	0.038	3.85E-45
SRP68	2.92E-49	0.139837	0.252	0.062	4.36E-45
CCL4	3.04E-49	0.286532	0.302	0.063	4.54E-45
CPEB4	3.04E-49	0.201078	0.278	0.072	4.55E-45

FAM134A	3.05E-49	0.392198	0.328	0.067	4.56E-45
TIPIN	3.07E-49	-0.23155	0.204	0.08	4.58E-45
NUDT11	3.21E-49	0.28657	0.248	0.038	4.80E-45
AATF	3.34E-49	0.280781	0.307	0.07	4.99E-45
TRIM9	3.50E-49	-0.18627	0.3	0.162	5.23E-45
PCGF2	3.54E-49	0.411356	0.27	0.032	5.29E-45
11-Sep	3.72E-49	-0.19923	0.27	0.133	5.56E-45
CCER2	3.74E-49	0.563565	0.298	0.034	5.58E-45
ACBD6	4.25E-49	0.128468	0.269	0.07	6.35E-45
SERTAD1	4.77E-49	-0.86948	0.187	0.217	7.12E-45
RP11-332H	5.70E-49	0.365221	0.257	0.036	8.52E-45
PJA1	5.71E-49	0.340864	0.286	0.051	8.53E-45
KHSRP	6.10E-49	0.344197	0.269	0.043	9.11E-45
C19orf48	6.58E-49	0.217787	0.307	0.079	9.83E-45
NABP2	6.74E-49	-0.25366	0.254	0.132	1.01E-44
PSMD9	6.85E-49	-0.30864	0.243	0.138	1.02E-44
MLST8	7.39E-49	0.186452	0.267	0.063	1.10E-44
ALDH2	8.67E-49	0.353925	0.32	0.072	1.30E-44
C1R	8.70E-49	-1.3973	0.029	0.251	1.30E-44
ALKBH2	8.71E-49	0.107688	0.289	0.085	1.30E-44
LIMD2	9.15E-49	0.500948	0.233	0.012	1.37E-44
CTSL	1.08E-48	-0.67366	0.205	0.217	1.61E-44
KATNBL1	1.16E-48	0.273783	0.254	0.043	1.73E-44
MXD4	1.28E-48	0.575123	0.27	0.019	1.91E-44
DHX15	1.31E-48	0.239316	0.3	0.075	1.95E-44
WDR46	1.33E-48	0.145567	0.247	0.056	1.99E-44
UBE2G1	1.42E-48	0.276551	0.267	0.051	2.12E-44
LRRTM2	1.42E-48	0.279898	0.23	0.034	2.12E-44
TANK	1.49E-48	-0.23578	0.237	0.128	2.22E-44
ALKBH5	1.50E-48	0.223498	0.283	0.065	2.24E-44
CISD1	1.56E-48	-0.29083	0.281	0.162	2.33E-44
PJA2	1.66E-48	-0.21971	0.248	0.121	2.48E-44
U2AF2	1.87E-48	0.31131	0.281	0.053	2.80E-44
CETN2	1.95E-48	-0.68445	0.202	0.203	2.92E-44
EMC8	1.98E-48	0.275233	0.277	0.056	2.95E-44
TMUB1	2.14E-48	0.406726	0.278	0.039	3.20E-44
MTMR9	2.17E-48	0.384411	0.287	0.05	3.24E-44
ZNF32	2.22E-48	0.233739	0.244	0.046	3.32E-44
B3GAT3	2.24E-48	-0.1519	0.289	0.132	3.34E-44
TMEM54	2.29E-48	0.194808	0.231	0.044	3.42E-44
BRD9	2.44E-48	0.416488	0.274	0.036	3.65E-44
GNPTG	2.46E-48	0.402145	0.291	0.05	3.67E-44
NKAIN3	2.46E-48	0.504439	0.254	0.019	3.68E-44
RP11-277F	2.83E-48	0.640876	0.27	0.017	4.22E-44
FXYD7	3.03E-48	0.728191	0.306	0.032	4.53E-44
LRRC17	3.13E-48	0.256045	0.224	0.039	4.67E-44
APOD	3.24E-48	-0.18821	0.364	0.207	4.84E-44
PTBP2	3.86E-48	0.460076	0.252	0.017	5.77E-44
CPSF4	4.00E-48	0.253027	0.268	0.058	5.98E-44
CASC15	4.24E-48	0.522901	0.263	0.024	6.34E-44
TOX3	4.64E-48	0.625165	0.265	0.015	6.94E-44

PLOD3	4.66E-48	0.14314	0.251	0.058	6.97E-44
RMND5B	4.69E-48	0.208329	0.215	0.036	7.01E-44
H2AFX	4.83E-48	0.473541	0.257	0.024	7.21E-44
EMP3	4.97E-48	-1.18705	0.078	0.256	7.43E-44
ENHO	5.19E-48	0.554572	0.283	0.029	7.75E-44
PSMB9	5.50E-48	-0.81944	0.172	0.207	8.21E-44
DNPH1	5.60E-48	0.102389	0.319	0.113	8.37E-44
CELF2	6.05E-48	0.399877	0.33	0.068	9.05E-44
UBE2D4	6.40E-48	0.192065	0.243	0.048	9.56E-44
NCBP2-AS2	7.26E-48	0.514265	0.332	0.055	1.08E-43
ADAR	7.88E-48	0.152555	0.299	0.085	1.18E-43
KIAA1549	8.39E-48	0.5801	0.248	0.009	1.25E-43
LONP2	9.43E-48	0.386663	0.319	0.065	1.41E-43
CHST12	9.94E-48	0.183788	0.317	0.094	1.49E-43
BTBD17	1.03E-47	0.594028	0.239	0.01	1.54E-43
PRPF8	1.04E-47	0.194008	0.29	0.079	1.55E-43
RNF139	1.09E-47	0.531913	0.29	0.034	1.62E-43
BMPR2	1.22E-47	0.459164	0.27	0.031	1.83E-43
IGSF21	1.25E-47	0.487776	0.276	0.036	1.87E-43
TRIM36	1.28E-47	0.486271	0.259	0.026	1.92E-43
FANCL	1.56E-47	0.258798	0.256	0.044	2.33E-43
CD164	1.74E-47	-0.56623	0.213	0.197	2.60E-43
ZBED5	1.75E-47	0.375044	0.252	0.031	2.61E-43
DYNC1I1	1.78E-47	0.717377	0.25	0.007	2.67E-43
MPPED2	1.85E-47	0.466805	0.222	0.009	2.77E-43
MCRS1	1.87E-47	0.264918	0.302	0.074	2.79E-43
DFFA	1.94E-47	0.125492	0.257	0.067	2.90E-43
MED17	2.01E-47	0.239639	0.268	0.056	3.01E-43
EBAG9	2.46E-47	0.251895	0.257	0.051	3.68E-43
NIN	2.55E-47	0.504152	0.25	0.019	3.81E-43
LINC00665	2.56E-47	-0.30793	0.212	0.126	3.83E-43
ABCF2	2.57E-47	0.30647	0.29	0.06	3.84E-43
RAD50	2.75E-47	0.162897	0.286	0.085	4.11E-43
CHD2	2.90E-47	0.11209	0.259	0.075	4.33E-43
ZNF195	2.90E-47	0.40961	0.278	0.043	4.34E-43
TSPYL1	2.92E-47	0.346162	0.261	0.041	4.36E-43
KDM6B	3.14E-47	0.484714	0.255	0.024	4.69E-43
RQCD1	3.22E-47	0.205311	0.237	0.046	4.81E-43
CYR61	3.37E-47	-0.81523	0.122	0.168	5.04E-43
WASF2	3.58E-47	-0.31174	0.264	0.152	5.35E-43
RAB8A	3.59E-47	-0.20831	0.233	0.108	5.36E-43
PAAF1	3.75E-47	-0.12648	0.252	0.111	5.60E-43
MLX	3.95E-47	0.139147	0.263	0.074	5.90E-43
ACTN4	4.25E-47	-0.19415	0.296	0.157	6.36E-43
PSME3	4.31E-47	0.211492	0.241	0.051	6.45E-43
PPP4R2	4.41E-47	0.212161	0.273	0.062	6.59E-43
GPANK1	4.43E-47	0.111645	0.246	0.062	6.62E-43
ZMYM2	4.51E-47	0.417853	0.277	0.039	6.74E-43
RAC3	4.53E-47	0.573222	0.263	0.019	6.78E-43
TSPAN14	4.83E-47	0.483103	0.237	0.015	7.21E-43
USP47	5.05E-47	0.159941	0.289	0.077	7.54E-43

AK2	5.32E-47	-0.65735	0.254	0.212	7.96E-43
ROCK1	5.44E-47	0.284498	0.27	0.051	8.13E-43
RNF126	5.86E-47	0.391678	0.296	0.053	8.76E-43
NLGN3	6.05E-47	0.225643	0.25	0.05	9.05E-43
EFEMP1	6.45E-47	-0.73056	0.181	0.193	9.63E-43
CHMP2B	6.46E-47	-0.17471	0.234	0.089	9.65E-43
NTHL1	6.54E-47	0.105969	0.248	0.072	9.77E-43
SLAIN1	6.68E-47	0.521139	0.252	0.019	9.98E-43
PCDH9	6.75E-47	-0.66566	0.19	0.195	1.01E-42
TRIM2	7.44E-47	0.276677	0.281	0.06	1.11E-42
CIAO1	8.63E-47	0.234143	0.25	0.05	1.29E-42
AKAP7	8.72E-47	0.257381	0.295	0.072	1.30E-42
NTRK3	9.18E-47	0.299734	0.228	0.029	1.37E-42
SMOX	9.57E-47	-0.384	0.222	0.138	1.43E-42
TMEM222	1.01E-46	-0.17059	0.269	0.125	1.51E-42
TNPO1	1.15E-46	0.159633	0.286	0.08	1.72E-42
DRG1	1.15E-46	0.1932	0.282	0.07	1.72E-42
EHMT2	1.17E-46	0.548527	0.319	0.046	1.74E-42
HMGXB4	1.18E-46	0.265388	0.255	0.048	1.77E-42
COPB1	1.21E-46	-0.18791	0.242	0.113	1.80E-42
GOLT1B	1.27E-46	-0.24575	0.2	0.084	1.90E-42
DEF8	1.34E-46	0.152723	0.257	0.065	2.01E-42
TDG	1.36E-46	0.168517	0.298	0.085	2.03E-42
GGA1	1.38E-46	0.215153	0.289	0.077	2.06E-42
TP53I3	1.42E-46	0.509713	0.259	0.022	2.12E-42
PID1	1.43E-46	0.448239	0.267	0.034	2.14E-42
CDC42SE1	1.47E-46	0.180291	0.295	0.082	2.20E-42
WDR77	1.53E-46	0.191026	0.238	0.05	2.29E-42
ZCCHC11	1.66E-46	0.147956	0.268	0.07	2.47E-42
KHDRBS3	1.67E-46	0.118307	0.287	0.085	2.49E-42
RBM15B	1.77E-46	0.529132	0.252	0.019	2.64E-42
SRGAP1	1.89E-46	0.19901	0.239	0.051	2.83E-42
MYL12A	1.94E-46	-0.62978	0.189	0.152	2.90E-42
PHF20	2.04E-46	0.121506	0.296	0.092	3.05E-42
DPF2	2.06E-46	0.181258	0.281	0.07	3.07E-42
TRIM28	2.08E-46	0.164159	0.291	0.087	3.10E-42
MT1F	2.20E-46	-0.41835	0.238	0.174	3.29E-42
PKIB	2.34E-46	-0.40448	0.195	0.125	3.50E-42
ATG3	2.40E-46	-0.11124	0.234	0.091	3.58E-42
GGH	2.42E-46	0.209036	0.29	0.077	3.62E-42
IFT43	2.69E-46	-0.14776	0.26	0.123	4.01E-42
TLK1	2.74E-46	0.235432	0.272	0.062	4.09E-42
SHARPIN	2.90E-46	0.33498	0.269	0.05	4.33E-42
METAP1D	3.06E-46	0.357137	0.308	0.065	4.57E-42
ABRACL	3.14E-46	0.212398	0.222	0.032	4.69E-42
C16orf91	3.15E-46	0.257227	0.247	0.046	4.71E-42
PNPT1	3.17E-46	0.238443	0.286	0.072	4.74E-42
ZBTB1	3.30E-46	0.247164	0.221	0.034	4.93E-42
FAM126B	3.37E-46	0.190635	0.234	0.048	5.03E-42
GLYR1	3.43E-46	0.286307	0.244	0.041	5.13E-42
LRRC4B	3.47E-46	0.466963	0.237	0.015	5.19E-42

KAT7	3.59E-46	0.347086	0.252	0.038	5.36E-42
CYHR1	3.74E-46	0.284391	0.325	0.089	5.59E-42
PRKAG1	3.82E-46	0.141241	0.3	0.094	5.71E-42
RNASEH1-A	3.90E-46	0.293433	0.255	0.048	5.82E-42
DERL2	3.91E-46	-0.43356	0.226	0.142	5.84E-42
TRAPPC2P1	3.92E-46	-0.32476	0.226	0.13	5.86E-42
FAM50A	4.01E-46	0.141267	0.304	0.099	5.99E-42
MTIF3	4.59E-46	-0.20651	0.246	0.12	6.86E-42
EXOC4	4.77E-46	0.23438	0.259	0.05	7.13E-42
ARMC8	4.93E-46	-0.11623	0.244	0.092	7.36E-42
COPRS	5.26E-46	0.193152	0.276	0.072	7.86E-42
SAMM50	5.33E-46	0.329307	0.254	0.043	7.96E-42
YIF1B	5.42E-46	-0.11259	0.231	0.103	8.10E-42
HOXA7	5.54E-46	0.325075	0.255	0.043	8.27E-42
REXO2	5.61E-46	-0.74725	0.174	0.186	8.38E-42
RNF146	5.79E-46	0.132211	0.278	0.082	8.66E-42
PXMP2	5.89E-46	0.174743	0.248	0.058	8.81E-42
BEND5	6.18E-46	0.219359	0.251	0.05	9.23E-42
SMIM19	6.50E-46	0.15279	0.278	0.082	9.71E-42
RERE	6.85E-46	0.264894	0.269	0.058	1.02E-41
PCDHB10	7.46E-46	0.510874	0.238	0.015	1.12E-41
PLIN3	7.93E-46	-0.58683	0.192	0.183	1.19E-41
MEIS3	9.35E-46	0.31693	0.215	0.026	1.40E-41
MICU2	9.87E-46	0.189334	0.244	0.055	1.47E-41
GART	1.03E-45	0.241508	0.263	0.056	1.54E-41
MFGE8	1.04E-45	0.114635	0.234	0.062	1.56E-41
AIG1	1.11E-45	-0.29193	0.216	0.12	1.65E-41
DHX30	1.15E-45	0.313514	0.29	0.062	1.71E-41
ANKRD46	1.22E-45	0.346716	0.287	0.056	1.82E-41
UCP2	1.28E-45	0.402717	0.211	0.017	1.91E-41
CIB1	1.37E-45	-0.8837	0.15	0.229	2.05E-41
AC004540.	1.43E-45	0.598415	0.242	0.012	2.14E-41
NECAP2	1.51E-45	-0.23044	0.23	0.096	2.25E-41
AK3	1.52E-45	0.120489	0.251	0.068	2.28E-41
CD47	1.80E-45	0.119316	0.257	0.074	2.68E-41
TINF2	1.87E-45	0.106118	0.229	0.056	2.80E-41
RTCA	1.91E-45	0.103794	0.248	0.07	2.85E-41
SCAMP3	1.93E-45	-0.22688	0.251	0.121	2.88E-41
SHISA4	1.93E-45	-0.11875	0.255	0.116	2.88E-41
EIF2AK2	1.97E-45	0.184923	0.283	0.075	2.94E-41
APPL1	2.03E-45	0.198338	0.25	0.058	3.04E-41
LINC00511	2.06E-45	0.463443	0.269	0.032	3.08E-41
CYB5B	2.35E-45	-0.36898	0.231	0.147	3.51E-41
TAF12	2.40E-45	-0.3895	0.228	0.152	3.59E-41
CCAR1	2.56E-45	0.265187	0.291	0.067	3.83E-41
CD44	2.60E-45	-1.3729	0.025	0.231	3.89E-41
HLA-E	2.61E-45	-0.98095	0.14	0.248	3.89E-41
CNOT8	2.98E-45	0.243988	0.215	0.032	4.45E-41
TOPORS	2.99E-45	0.409155	0.247	0.031	4.47E-41
CEBPD	3.22E-45	-0.60641	0.241	0.251	4.82E-41
TTC9B	3.33E-45	0.650548	0.286	0.029	4.97E-41

MAPK8IP1	3.33E-45	0.202373	0.256	0.058	4.97E-41
ZHX1	3.39E-45	0.404237	0.287	0.048	5.07E-41
ZNF883	3.58E-45	0.221654	0.23	0.044	5.34E-41
SLC39A1	3.60E-45	-0.12652	0.239	0.104	5.38E-41
ZNF738	3.91E-45	0.166097	0.237	0.056	5.84E-41
POLR3GL	3.91E-45	-0.49003	0.237	0.174	5.84E-41
RGMB	3.92E-45	0.591954	0.32	0.053	5.86E-41
MCFD2	4.09E-45	-0.13669	0.242	0.099	6.11E-41
ZNF254	4.09E-45	0.284519	0.238	0.041	6.12E-41
UFM1	4.25E-45	-0.25414	0.267	0.144	6.36E-41
MNAT1	4.26E-45	0.252414	0.287	0.067	6.37E-41
PRPF19	4.33E-45	0.194904	0.265	0.065	6.47E-41
SBNO1	4.39E-45	0.263401	0.291	0.072	6.56E-41
C2orf68	4.44E-45	0.393192	0.235	0.026	6.63E-41
DUSP26	4.86E-45	0.638029	0.273	0.024	7.26E-41
TOR1A	4.96E-45	0.248974	0.247	0.05	7.41E-41
C7orf49	5.01E-45	0.218351	0.226	0.043	7.48E-41
NUP62	6.13E-45	0.18945	0.264	0.063	9.17E-41
EIF2B4	6.52E-45	0.400791	0.268	0.039	9.74E-41
SLC52A2	6.56E-45	0.415621	0.285	0.048	9.81E-41
PDE6D	8.01E-45	0.352291	0.273	0.05	1.20E-40
OLFM1	8.05E-45	0.334151	0.285	0.06	1.20E-40
CHERP	8.11E-45	0.429318	0.254	0.029	1.21E-40
BLOC1S2	8.89E-45	-0.18041	0.251	0.121	1.33E-40
METTL21A	9.56E-45	0.209078	0.247	0.048	1.43E-40
TMEM50B	1.00E-44	-0.23696	0.242	0.137	1.50E-40
SYT17	1.05E-44	0.301493	0.255	0.046	1.57E-40
AC009506.	1.08E-44	0.556688	0.257	0.024	1.62E-40
ZBED1	1.09E-44	0.447152	0.231	0.019	1.63E-40
C2orf80	1.13E-44	0.265183	0.239	0.046	1.70E-40
TOB1	1.15E-44	0.133742	0.256	0.067	1.72E-40
GADD45A	1.20E-44	-0.86757	0.166	0.222	1.79E-40
CGGBP1	1.23E-44	0.122816	0.25	0.065	1.84E-40
FBXL3	1.28E-44	0.300366	0.242	0.041	1.92E-40
ELP2	1.38E-44	-0.15956	0.229	0.103	2.06E-40
SMIM20	1.50E-44	-0.25598	0.238	0.123	2.24E-40
POMGNT1	1.53E-44	0.217307	0.267	0.06	2.28E-40
SMC6	1.55E-44	0.502505	0.264	0.029	2.32E-40
TMEM97	1.64E-44	0.312656	0.239	0.039	2.45E-40
EFS	1.66E-44	0.532947	0.263	0.024	2.48E-40
ABCE1	1.79E-44	0.142768	0.259	0.068	2.68E-40
DNTTIP2	2.00E-44	0.236317	0.28	0.07	2.99E-40
ENO2	2.10E-44	-0.52784	0.244	0.191	3.14E-40
CYFIP2	2.11E-44	0.360477	0.225	0.027	3.15E-40
RASSF2	2.11E-44	0.19271	0.265	0.067	3.15E-40
VPS26B	2.15E-44	0.308515	0.251	0.046	3.21E-40
CORO1B	2.25E-44	0.229601	0.251	0.053	3.36E-40
MTX1	2.26E-44	-0.27219	0.181	0.082	3.37E-40
GNG4	2.39E-44	0.267761	0.221	0.034	3.57E-40
NR3C1	2.62E-44	0.31804	0.299	0.072	3.91E-40
UBA3	2.86E-44	0.159386	0.273	0.079	4.28E-40

ARHGEF7	2.91E-44	0.294477	0.26	0.053	4.36E-40
POGZ	2.99E-44	0.138748	0.268	0.074	4.46E-40
SNAP25	2.99E-44	-0.12913	0.22	0.101	4.47E-40
GLT8D1	3.09E-44	0.124059	0.264	0.075	4.62E-40
RBCK1	3.28E-44	0.137541	0.247	0.063	4.90E-40
TMEM55B	3.33E-44	0.290274	0.248	0.044	4.97E-40
FRYL	3.41E-44	0.566051	0.283	0.031	5.10E-40
PRRC2B	3.53E-44	0.330773	0.257	0.044	5.27E-40
RABEPK	3.58E-44	-0.14005	0.199	0.077	5.34E-40
CUL3	3.59E-44	0.38015	0.256	0.041	5.37E-40
MEX3B	3.77E-44	0.553944	0.218	0.005	5.64E-40
FLYWCH2	4.36E-44	-0.11132	0.268	0.123	6.51E-40
YARS	4.38E-44	-0.2481	0.203	0.096	6.54E-40
C1QC	4.57E-44	-0.26853	0.226	0.125	6.83E-40
TOMM5	4.83E-44	-0.10511	0.166	0.053	7.22E-40
RNF165	5.61E-44	0.478319	0.23	0.015	8.39E-40
FCGRT	5.67E-44	-0.7129	0.163	0.183	8.47E-40
MED24	5.71E-44	0.315473	0.241	0.036	8.53E-40
ACTR6	5.76E-44	0.246497	0.257	0.056	8.61E-40
FOXN3	6.31E-44	0.227996	0.251	0.056	9.43E-40
HOXB7	6.44E-44	0.357314	0.209	0.021	9.63E-40
SLC30A9	6.45E-44	0.30368	0.273	0.056	9.63E-40
SWI5	6.56E-44	-0.33051	0.192	0.103	9.80E-40
SEPHS2	6.77E-44	0.147563	0.251	0.068	1.01E-39
VTI1B	7.39E-44	0.198962	0.289	0.085	1.10E-39
DFNA5	7.41E-44	0.102364	0.242	0.068	1.11E-39
PHC2	7.42E-44	0.217375	0.244	0.053	1.11E-39
SRPRB	7.57E-44	0.137732	0.264	0.065	1.13E-39
SMOC1	7.60E-44	0.798396	0.22	0.002	1.14E-39
EIF3A	8.13E-44	0.240229	0.286	0.072	1.21E-39
ASAP1	8.25E-44	0.40561	0.277	0.044	1.23E-39
KCNH2	8.47E-44	0.439399	0.225	0.017	1.27E-39
PBRM1	8.73E-44	0.37686	0.278	0.05	1.30E-39
MOB3B	8.93E-44	0.517135	0.242	0.017	1.33E-39
BRE	9.66E-44	-0.18608	0.202	0.092	1.44E-39
GOSR1	9.84E-44	0.105178	0.269	0.084	1.47E-39
PABPC4	1.02E-43	0.26709	0.252	0.048	1.53E-39
CSRNP3	1.16E-43	0.53589	0.229	0.012	1.73E-39
CLCN3	1.20E-43	0.204206	0.283	0.077	1.80E-39
GOT2	1.24E-43	0.263317	0.242	0.048	1.85E-39
PSMA6	1.35E-43	-0.32326	0.228	0.14	2.02E-39
RAB18	1.37E-43	-0.11192	0.298	0.147	2.05E-39
SLBP	1.40E-43	0.228922	0.281	0.07	2.10E-39
SUPT5H	1.43E-43	0.183476	0.283	0.082	2.14E-39
ID3	1.64E-43	-0.56495	0.177	0.126	2.45E-39
PUM1	1.85E-43	0.287704	0.276	0.053	2.76E-39
SERPINB6	1.90E-43	-0.18318	0.26	0.13	2.83E-39
PRDM2	2.01E-43	0.298409	0.229	0.034	3.00E-39
FAM96A	2.01E-43	-0.3162	0.225	0.135	3.00E-39
LRIF1	2.08E-43	-0.1194	0.231	0.091	3.11E-39
GTF2IRD2	2.19E-43	0.28328	0.242	0.048	3.28E-39

CCDC136	2.50E-43	0.348349	0.216	0.021	3.74E-39
TMEM35	2.55E-43	0.458379	0.241	0.024	3.81E-39
TMA16	2.65E-43	0.143584	0.256	0.072	3.96E-39
POM121	2.68E-43	0.307815	0.234	0.036	4.00E-39
SPATC1L	3.39E-43	0.323052	0.246	0.039	5.07E-39
ZNF277	3.50E-43	0.259049	0.264	0.055	5.23E-39
PFKM	3.53E-43	0.318131	0.27	0.053	5.28E-39
CTNNB1	3.81E-43	0.302914	0.313	0.082	5.70E-39
GNAI1	3.82E-43	0.5883	0.222	0.005	5.71E-39
ZNF138	4.04E-43	0.46445	0.28	0.041	6.04E-39
INTS10	4.08E-43	0.113058	0.269	0.085	6.10E-39
CAV1	4.54E-43	-1.35658	0.057	0.212	6.78E-39
LRP6	4.95E-43	0.463989	0.237	0.021	7.40E-39
BFAR	5.14E-43	0.264467	0.257	0.053	7.68E-39
APC	5.34E-43	0.482173	0.274	0.038	7.99E-39
ANXA2	5.37E-43	-1.24855	0.098	0.287	8.03E-39
TRIM27	5.72E-43	0.291719	0.244	0.046	8.55E-39
NUPR1	5.79E-43	-1.25309	0.086	0.234	8.66E-39
LMNB1	5.89E-43	0.442174	0.229	0.021	8.80E-39
GLG1	6.19E-43	0.107175	0.248	0.072	9.25E-39
FNBP4	6.21E-43	0.31054	0.282	0.063	9.28E-39
UGDH	6.42E-43	0.179327	0.242	0.06	9.60E-39
PPP1R8	6.48E-43	-0.15752	0.189	0.068	9.68E-39
IRF1	6.51E-43	-0.11217	0.286	0.138	9.73E-39
NUDCD3	6.94E-43	0.246861	0.27	0.065	1.04E-38
FMNL2	6.94E-43	0.560798	0.264	0.026	1.04E-38
PLTP	7.17E-43	-0.78112	0.131	0.19	1.07E-38
ANAPC7	7.45E-43	0.163489	0.222	0.051	1.11E-38
CRELD2	7.64E-43	0.386565	0.246	0.034	1.14E-38
PES1	7.82E-43	0.264092	0.221	0.036	1.17E-38
RAP1B	8.04E-43	-0.4904	0.19	0.15	1.20E-38
8-Sep	8.27E-43	0.217597	0.264	0.065	1.24E-38
DLX2	8.44E-43	0.707719	0.25	0.014	1.26E-38
CMTM3	9.29E-43	0.293962	0.255	0.05	1.39E-38
TANC2	9.70E-43	0.404405	0.254	0.031	1.45E-38
SCNM1	9.92E-43	-0.22698	0.27	0.149	1.48E-38
SAR1A	1.02E-42	-0.1197	0.217	0.087	1.53E-38
CLIC4	1.03E-42	-0.36179	0.254	0.176	1.54E-38
BET1	1.03E-42	-0.24991	0.222	0.13	1.54E-38
NIPSNAP1	1.03E-42	0.423868	0.229	0.022	1.54E-38
C17orf62	1.07E-42	0.190361	0.265	0.072	1.61E-38
ILK	1.08E-42	-0.58807	0.183	0.154	1.61E-38
CSTF2T	1.11E-42	0.327393	0.198	0.017	1.66E-38
CTSA	1.15E-42	-0.26943	0.213	0.097	1.73E-38
CAHM	1.16E-42	0.521022	0.224	0.012	1.74E-38
CTNNBIP1	1.21E-42	0.315008	0.213	0.026	1.81E-38
RBM14	1.22E-42	0.292332	0.226	0.034	1.82E-38
PPP1R14A	1.26E-42	0.381823	0.247	0.041	1.88E-38
CNPY1	1.34E-42	0.528145	0.246	0.021	2.01E-38
SIAH2	1.36E-42	0.489907	0.254	0.026	2.03E-38
SLC12A9	1.46E-42	0.377533	0.221	0.024	2.18E-38

SERP2	1.61E-42	0.445632	0.231	0.021	2.40E-38
TRMT61B	1.63E-42	0.128068	0.238	0.06	2.43E-38
ELAVL2	1.66E-42	0.509681	0.212	0.005	2.47E-38
TMED1	1.68E-42	-0.66577	0.187	0.195	2.51E-38
FAM173A	1.68E-42	0.323631	0.273	0.055	2.51E-38
VMA21	1.69E-42	0.106479	0.226	0.058	2.52E-38
EVL	1.74E-42	0.45466	0.302	0.055	2.60E-38
PILRB	1.88E-42	0.245264	0.261	0.06	2.82E-38
HLA-DPB1	2.05E-42	-0.65131	0.155	0.162	3.07E-38
G0S2	2.06E-42	-0.14741	0.186	0.068	3.08E-38
POP4	2.08E-42	-0.15865	0.237	0.108	3.10E-38
CHORDC1	2.09E-42	0.114674	0.228	0.06	3.12E-38
NMRAL1	2.10E-42	-0.26145	0.203	0.106	3.14E-38
PYCR1	2.14E-42	0.336683	0.281	0.058	3.20E-38
LTV1	2.19E-42	0.22184	0.208	0.034	3.27E-38
PRRC2A	2.22E-42	0.385423	0.278	0.053	3.32E-38
MGRN1	2.23E-42	0.389787	0.264	0.039	3.33E-38
DUSP10	2.56E-42	0.273307	0.27	0.063	3.82E-38
METTL21B	2.56E-42	0.322621	0.194	0.017	3.83E-38
USP42	2.57E-42	0.519203	0.234	0.014	3.84E-38
YAE1D1	2.96E-42	0.355208	0.281	0.06	4.43E-38
PGM2L1	3.11E-42	-0.26934	0.264	0.159	4.64E-38
MTHFD2L	3.27E-42	0.200892	0.251	0.065	4.88E-38
SMC3	3.42E-42	0.418987	0.308	0.063	5.11E-38
IPO5	3.47E-42	0.21408	0.242	0.055	5.19E-38
PPAT	3.57E-42	0.458368	0.23	0.022	5.33E-38
PHKB	3.77E-42	0.244705	0.229	0.048	5.63E-38
HECTD1	3.98E-42	0.367991	0.256	0.041	5.94E-38
MIF4GD	4.00E-42	0.15465	0.224	0.055	5.97E-38
KDM5A	4.08E-42	0.295818	0.27	0.056	6.10E-38
PHACTR4	4.17E-42	0.228162	0.263	0.063	6.22E-38
APBB1	4.24E-42	0.300108	0.231	0.039	6.33E-38
NF1	4.26E-42	0.289623	0.263	0.056	6.37E-38
TMEM203	4.45E-42	0.138825	0.241	0.06	6.65E-38
HIP1R	4.70E-42	0.514274	0.267	0.029	7.03E-38
APC2	4.78E-42	0.468386	0.272	0.041	7.15E-38
TSR2	5.21E-42	0.153619	0.278	0.085	7.78E-38
SYT1	6.34E-42	0.568706	0.222	0.009	9.48E-38
MTMR4	6.35E-42	0.316812	0.213	0.026	9.48E-38
WNK1	6.76E-42	0.163689	0.23	0.055	1.01E-37
PRADC1	7.08E-42	0.260912	0.218	0.038	1.06E-37
B4GALT3	7.12E-42	0.165599	0.243	0.062	1.06E-37
ZCCHC10	7.36E-42	0.153314	0.27	0.082	1.10E-37
SNX1	8.27E-42	0.167163	0.222	0.048	1.24E-37
ECI1	8.53E-42	0.200497	0.211	0.038	1.28E-37
NLGN2	8.68E-42	0.41086	0.211	0.017	1.30E-37
LINC00844	8.79E-42	0.3488	0.269	0.056	1.31E-37
VARS	9.59E-42	0.311899	0.215	0.027	1.43E-37
HDAC3	9.70E-42	0.189241	0.247	0.062	1.45E-37
MRPS25	9.86E-42	-0.1251	0.222	0.096	1.47E-37
CPSF3	1.07E-41	0.297086	0.237	0.038	1.60E-37

REC8	1.09E-41	0.421452	0.22	0.021	1.64E-37
MDM4	1.11E-41	0.281666	0.311	0.082	1.66E-37
MPZL1	1.16E-41	0.373487	0.259	0.041	1.73E-37
IRF2BP2	1.27E-41	0.141269	0.302	0.108	1.90E-37
CCS	1.29E-41	-0.21333	0.22	0.113	1.92E-37
DDA1	1.37E-41	-0.19998	0.233	0.121	2.05E-37
NBPF1	1.38E-41	0.257513	0.228	0.039	2.06E-37
FDFT1	1.48E-41	-0.10205	0.248	0.109	2.21E-37
GTPBP6	1.53E-41	0.108745	0.218	0.051	2.29E-37
GRIA3	1.59E-41	0.178029	0.267	0.074	2.38E-37
CAPZA1	1.73E-41	0.11717	0.235	0.065	2.58E-37
PTPRD	1.74E-41	0.548291	0.22	0.009	2.60E-37
CNPPD1	1.78E-41	0.209082	0.237	0.05	2.66E-37
PKIA	1.96E-41	0.431035	0.218	0.017	2.93E-37
CHTF8	1.96E-41	0.232392	0.191	0.026	2.93E-37
ZNF493	2.01E-41	0.225378	0.255	0.06	3.00E-37
ABHD12	2.12E-41	0.149323	0.256	0.067	3.17E-37
SNRPN	2.12E-41	-1.02697	0.095	0.202	3.17E-37
SCAMP1	2.48E-41	0.27385	0.255	0.055	3.70E-37
FAHD2A	2.57E-41	0.191693	0.225	0.05	3.85E-37
VAMP5	2.67E-41	-1.24002	0.06	0.227	3.99E-37
LDLRAD3	2.73E-41	0.351936	0.25	0.041	4.08E-37
ROGDI	2.80E-41	0.281161	0.2	0.024	4.18E-37
EXOSC7	2.81E-41	0.20077	0.246	0.056	4.21E-37
UBAP2L	2.97E-41	0.112797	0.234	0.067	4.44E-37
SCN3A	3.21E-41	0.604232	0.242	0.014	4.80E-37
SYMPK	3.33E-41	-0.21913	0.231	0.13	4.98E-37
RBM28	3.35E-41	0.381154	0.259	0.039	5.01E-37
NAT14	3.57E-41	0.125693	0.209	0.05	5.33E-37
ZNF43	3.67E-41	0.230194	0.216	0.041	5.48E-37
FUNDC1	3.83E-41	-0.15624	0.252	0.118	5.72E-37
LRCH3	4.03E-41	0.356962	0.235	0.036	6.02E-37
PLEKHA3	4.64E-41	0.37125	0.255	0.041	6.93E-37
XPA	4.65E-41	0.157417	0.231	0.058	6.95E-37
TXNRD1	4.71E-41	0.279446	0.241	0.048	7.04E-37
GPATCH4	4.94E-41	0.175952	0.248	0.063	7.39E-37
OXA1L	5.68E-41	-0.25484	0.235	0.123	8.49E-37
NFYB	5.81E-41	-0.13349	0.185	0.074	8.68E-37
PDE4DIP	5.92E-41	-0.33571	0.229	0.147	8.85E-37
TFAP2B	6.26E-41	0.5646	0.192	0	9.36E-37
POLR2A	6.29E-41	0.104742	0.211	0.048	9.39E-37
ZMAT3	6.32E-41	0.116236	0.243	0.074	9.45E-37
PAF1	6.46E-41	0.109847	0.218	0.056	9.65E-37
SIX1	6.74E-41	0.519359	0.205	0.007	1.01E-36
SZRD1	6.86E-41	-0.26492	0.182	0.085	1.03E-36
FIBIN	7.48E-41	-0.39373	0.234	0.174	1.12E-36
S100A13	7.58E-41	-1.16786	0.074	0.239	1.13E-36
LINC0111€	7.80E-41	0.3418	0.247	0.043	1.17E-36
FJX1	7.80E-41	0.127271	0.233	0.062	1.17E-36
TRPC4AP	8.20E-41	0.287542	0.238	0.043	1.23E-36
M6PR	8.45E-41	-0.1265	0.221	0.091	1.26E-36

ARL9	8.56E-41	0.55408	0.222	0.012	1.28E-36
EXOC5	8.74E-41	0.273048	0.222	0.039	1.31E-36
NIPBL	8.85E-41	0.19374	0.233	0.056	1.32E-36
ARRB2	9.69E-41	0.215275	0.225	0.044	1.45E-36
GPR155	9.71E-41	0.323235	0.243	0.044	1.45E-36
FDX1	9.81E-41	0.368811	0.257	0.043	1.47E-36
SLC35B4	1.07E-40	0.414766	0.263	0.041	1.59E-36
C3	1.15E-40	-0.78242	0.134	0.168	1.72E-36
ATP1B1	1.22E-40	0.209534	0.231	0.05	1.82E-36
SYNC	1.22E-40	0.303198	0.237	0.038	1.82E-36
HOXC9	1.27E-40	0.542621	0.212	0.005	1.90E-36
WDR13	1.28E-40	-0.14432	0.241	0.121	1.91E-36
DRG2	1.31E-40	-0.13245	0.182	0.077	1.96E-36
EMG1	1.39E-40	-0.25968	0.182	0.091	2.07E-36
SUN2	1.48E-40	0.421648	0.246	0.032	2.21E-36
GPATCH2L	1.49E-40	0.302979	0.269	0.06	2.23E-36
A2M	1.64E-40	-0.96636	0.101	0.171	2.46E-36
COA6	1.69E-40	-0.14607	0.213	0.087	2.53E-36
WSB2	1.74E-40	0.171587	0.211	0.044	2.60E-36
CTNNAL1	1.77E-40	0.161422	0.217	0.051	2.64E-36
NMT2	1.81E-40	0.44822	0.225	0.021	2.71E-36
ZDHHC22	1.84E-40	0.404299	0.216	0.019	2.74E-36
BUB3	1.85E-40	0.15398	0.286	0.094	2.76E-36
BECN1	1.90E-40	0.145064	0.194	0.041	2.84E-36
TMED3	1.94E-40	0.118113	0.234	0.067	2.90E-36
COQ10B	1.95E-40	-0.10635	0.224	0.096	2.92E-36
WASL	1.99E-40	0.178237	0.229	0.058	2.98E-36
UBLCP1	2.01E-40	0.321195	0.207	0.026	3.01E-36
TOLLIP	2.05E-40	0.22828	0.235	0.053	3.07E-36
NPRL3	2.12E-40	0.345101	0.267	0.053	3.16E-36
PBDC1	2.31E-40	-0.27104	0.202	0.103	3.46E-36
ATP2C1	2.33E-40	0.27386	0.216	0.038	3.48E-36
FAM84B	2.44E-40	0.369806	0.254	0.046	3.64E-36
SPNS1	2.45E-40	0.349556	0.259	0.046	3.66E-36
DGCR2	2.55E-40	0.417986	0.269	0.044	3.81E-36
MYT1	3.03E-40	0.59408	0.209	0.005	4.52E-36
MFNG	3.11E-40	0.5404	0.221	0.012	4.64E-36
PAXIP1-AS	3.13E-40	0.27133	0.243	0.051	4.67E-36
ARHGAP12	3.24E-40	0.156224	0.248	0.067	4.84E-36
SP3	3.30E-40	0.329899	0.259	0.05	4.93E-36
RAB4A	3.31E-40	0.112216	0.273	0.091	4.94E-36
CERS6	3.45E-40	0.297334	0.178	0.015	5.16E-36
SLC43A2	3.51E-40	0.391444	0.234	0.031	5.25E-36
GSTO1	3.52E-40	-0.56829	0.178	0.15	5.26E-36
FARP1	3.71E-40	0.242608	0.218	0.038	5.55E-36
RUNDC3A	3.78E-40	0.364711	0.221	0.031	5.64E-36
CDC26	3.90E-40	-0.44457	0.165	0.106	5.83E-36
USP5	4.04E-40	0.188526	0.241	0.056	6.04E-36
EYA1	4.22E-40	0.506127	0.202	0.005	6.31E-36
CNRIP1	4.36E-40	0.343036	0.256	0.05	6.52E-36
ZMYND8	4.41E-40	0.167988	0.256	0.072	6.59E-36

RMDN3	5.29E-40	0.110665	0.23	0.065	7.91E-36
KIF3A	5.41E-40	0.257446	0.267	0.067	8.08E-36
SUPT20H	5.52E-40	0.234646	0.243	0.055	8.25E-36
JPH4	5.76E-40	0.536241	0.229	0.014	8.60E-36
COQ9	6.08E-40	-0.13687	0.222	0.101	9.09E-36
ZNF714	6.19E-40	0.280575	0.257	0.058	9.26E-36
ARID5B	6.55E-40	-0.29216	0.231	0.132	9.79E-36
ANGPTL2	6.68E-40	0.154006	0.265	0.084	9.99E-36
RXRB	7.23E-40	0.252309	0.205	0.031	1.08E-35
CHI3L2	7.46E-40	-1.64774	0.014	0.193	1.11E-35
SLPI	7.82E-40	-1.6837	0.018	0.209	1.17E-35
TP53	8.99E-40	0.273948	0.208	0.031	1.34E-35
SF3B3	9.02E-40	0.302458	0.252	0.053	1.35E-35
CTCF	9.29E-40	0.450867	0.216	0.015	1.39E-35
SALL3	9.32E-40	0.429626	0.183	0.003	1.39E-35
OSBPL8	9.40E-40	0.171701	0.222	0.053	1.40E-35
SLC25A36	1.07E-39	0.311841	0.283	0.07	1.60E-35
CCNG1	1.10E-39	-0.25525	0.198	0.113	1.64E-35
CCNB1IP1	1.10E-39	-0.14141	0.216	0.106	1.64E-35
GMCL1	1.13E-39	0.356189	0.238	0.038	1.69E-35
LYRM7	1.16E-39	0.224193	0.281	0.084	1.73E-35
C3orf70	1.18E-39	0.406903	0.215	0.022	1.77E-35
EEA1	1.20E-39	0.109036	0.209	0.055	1.80E-35
CYB5A	1.23E-39	-0.30042	0.211	0.126	1.84E-35
PREB	1.27E-39	0.361097	0.251	0.039	1.90E-35
CMC2	1.27E-39	-0.19719	0.222	0.104	1.90E-35
PRKAR1B	1.28E-39	0.429834	0.226	0.022	1.92E-35
NCKAP5L	1.31E-39	0.24752	0.192	0.029	1.96E-35
RABGGTB	1.41E-39	0.118165	0.257	0.077	2.10E-35
ADAM22	1.62E-39	0.436038	0.208	0.015	2.42E-35
NAA15	1.72E-39	0.191912	0.248	0.062	2.57E-35
SPPL3	1.75E-39	0.311763	0.244	0.046	2.61E-35
ABT1	1.77E-39	-0.1629	0.203	0.089	2.65E-35
LINC00632	1.89E-39	0.5291	0.229	0.019	2.82E-35
ELN	1.92E-39	0.105152	0.207	0.055	2.86E-35
BDP1	1.92E-39	0.236329	0.254	0.058	2.87E-35
SART1	2.03E-39	0.390229	0.22	0.024	3.03E-35
RHOT1	2.26E-39	0.32578	0.238	0.039	3.38E-35
GALNT11	2.40E-39	0.106347	0.22	0.06	3.59E-35
GPSM2	2.41E-39	0.381977	0.287	0.058	3.61E-35
BRD8	2.52E-39	0.107396	0.209	0.053	3.77E-35
RNF41	2.53E-39	0.241813	0.205	0.036	3.78E-35
SRGAP3	2.59E-39	0.42233	0.239	0.031	3.88E-35
CSE1L	2.72E-39	0.279192	0.229	0.039	4.06E-35
TRIO	2.73E-39	-0.30553	0.215	0.128	4.08E-35
ME2	2.86E-39	0.188025	0.202	0.039	4.28E-35
MTRNR2L12	2.92E-39	-1.00959	0.117	0.263	4.36E-35
RPP14	3.03E-39	0.2907	0.224	0.036	4.53E-35
UFSP2	3.07E-39	-0.19798	0.23	0.109	4.59E-35
COMMD5	3.14E-39	-0.17704	0.213	0.096	4.69E-35
RP11-390E	3.18E-39	0.174491	0.244	0.067	4.76E-35

ATF3	3.22E-39	-0.44888	0.174	0.13	4.82E-35
CCDC66	3.37E-39	0.396279	0.246	0.036	5.04E-35
TMEM106C	3.38E-39	-0.23344	0.189	0.087	5.06E-35
RBM4B	3.43E-39	0.213462	0.25	0.06	5.12E-35
APOO	3.44E-39	-0.25127	0.182	0.097	5.15E-35
PIK3R3	3.54E-39	0.162729	0.212	0.043	5.29E-35
PPP1R9A	3.79E-39	0.586772	0.224	0.01	5.67E-35
SCFD2	3.80E-39	0.406479	0.216	0.024	5.68E-35
FOXN2	4.03E-39	0.290185	0.211	0.031	6.02E-35
RPF1	4.07E-39	-0.12233	0.241	0.109	6.09E-35
OAT	4.09E-39	-0.18652	0.22	0.104	6.11E-35
ZBTB17	4.10E-39	0.261553	0.195	0.027	6.12E-35
ZNF397	4.17E-39	0.131472	0.226	0.062	6.24E-35
ZSCAN21	4.21E-39	0.351615	0.202	0.021	6.29E-35
SUZ12	4.40E-39	0.375236	0.217	0.026	6.57E-35
GATAD1	4.56E-39	0.31339	0.268	0.062	6.81E-35
RNF220	4.99E-39	0.218401	0.198	0.032	7.46E-35
IST1	5.76E-39	0.150602	0.244	0.063	8.61E-35
ZNF148	6.22E-39	0.125981	0.238	0.063	9.29E-35
RWDD4	6.26E-39	0.204724	0.241	0.06	9.35E-35
SAP30	6.43E-39	0.39121	0.218	0.029	9.61E-35
TMEM218	7.33E-39	0.275704	0.239	0.048	1.10E-34
ARFRP1	7.67E-39	0.121247	0.228	0.056	1.15E-34
RSU1	7.86E-39	-0.17956	0.194	0.085	1.18E-34
TGS1	7.92E-39	0.161132	0.213	0.046	1.18E-34
THAP9-AS1	8.01E-39	-0.15208	0.208	0.08	1.20E-34
RNASEH2B	8.18E-39	0.386667	0.243	0.039	1.22E-34
PIGU	8.38E-39	-0.11242	0.178	0.067	1.25E-34
RPA1	8.68E-39	0.118914	0.215	0.053	1.30E-34
CRCP	8.86E-39	0.113871	0.205	0.053	1.32E-34
CNPY3	9.65E-39	0.180427	0.234	0.06	1.44E-34
E2F4	1.03E-38	0.310788	0.218	0.031	1.54E-34
ARHGEF9	1.07E-38	0.287419	0.215	0.038	1.60E-34
KPNA4	1.11E-38	0.208141	0.185	0.029	1.66E-34
PTPN12	1.12E-38	0.204568	0.218	0.046	1.67E-34
TNPO3	1.15E-38	0.301573	0.228	0.039	1.72E-34
UBE2Q2	1.16E-38	0.344116	0.241	0.039	1.73E-34
MIR181A1H	1.22E-38	0.389914	0.209	0.019	1.82E-34
BCL2L1	1.24E-38	-0.36933	0.161	0.113	1.85E-34
DAAM1	1.25E-38	0.473121	0.309	0.065	1.87E-34
SCAMP4	1.25E-38	0.114424	0.25	0.077	1.87E-34
GOLGA2	1.26E-38	-0.14791	0.186	0.08	1.89E-34
IFT57	1.36E-38	-0.42768	0.186	0.14	2.03E-34
SERPING1	1.39E-38	-1.15896	0.057	0.186	2.08E-34
ISG20L2	1.45E-38	0.15891	0.23	0.055	2.17E-34
CEP68	1.56E-38	0.269082	0.244	0.055	2.32E-34
RGMA	1.59E-38	-0.20821	0.231	0.123	2.37E-34
NLGN1	1.60E-38	0.436847	0.254	0.039	2.39E-34
SCAMP5	1.65E-38	0.458438	0.212	0.014	2.47E-34
PDCL	1.81E-38	0.128748	0.218	0.055	2.70E-34
ZNF439	1.93E-38	0.368568	0.229	0.031	2.88E-34

RFK	2.01E-38	0.19622	0.226	0.053	3.00E-34
C6orf1	2.12E-38	-0.43886	0.177	0.135	3.17E-34
TMEM57	2.15E-38	0.55234	0.242	0.021	3.22E-34
EFNA1	2.25E-38	-0.29894	0.187	0.111	3.36E-34
PCDHB7	2.31E-38	0.504103	0.226	0.015	3.45E-34
LMAN1	2.35E-38	-0.4446	0.182	0.123	3.52E-34
TRAF7	2.37E-38	0.181508	0.192	0.036	3.54E-34
KPNA6	2.38E-38	0.21228	0.252	0.06	3.56E-34
DDX27	2.41E-38	0.140028	0.242	0.068	3.60E-34
ZCCHC24	2.52E-38	0.288702	0.191	0.024	3.77E-34
BRAT1	2.58E-38	0.205867	0.239	0.058	3.86E-34
DTNA	2.68E-38	-0.81485	0.147	0.198	4.00E-34
STAG2	2.68E-38	0.140293	0.233	0.065	4.00E-34
ADD2	2.77E-38	0.445193	0.199	0.009	4.14E-34
PPP2R4	2.93E-38	0.136342	0.229	0.063	4.38E-34
DPF1	2.94E-38	0.262865	0.26	0.06	4.39E-34
MAPRE3	2.99E-38	0.315332	0.222	0.034	4.46E-34
MFSD10	3.03E-38	0.169657	0.225	0.056	4.52E-34
GANAB	3.15E-38	0.14222	0.239	0.068	4.70E-34
PPP2R5C	3.34E-38	0.10661	0.228	0.063	4.99E-34
PNO1	3.44E-38	0.144703	0.228	0.06	5.15E-34
JAM3	3.54E-38	0.270042	0.222	0.041	5.29E-34
ING5	3.61E-38	0.307209	0.205	0.027	5.40E-34
NDUFAF6	3.65E-38	0.24398	0.2	0.034	5.45E-34
SMIM4	3.68E-38	-0.20369	0.183	0.092	5.50E-34
ZFP82	3.69E-38	0.223793	0.187	0.032	5.51E-34
ORMDL3	3.71E-38	0.215237	0.2	0.039	5.55E-34
LPCAT1	3.74E-38	0.440234	0.25	0.038	5.59E-34
WDR45	3.94E-38	-0.19592	0.205	0.097	5.89E-34
SAR1B	3.95E-38	-0.21523	0.222	0.116	5.90E-34
TXNDC15	4.12E-38	-0.13417	0.237	0.12	6.15E-34
C14orf37	4.15E-38	0.538496	0.213	0.01	6.20E-34
USB1	4.22E-38	0.119558	0.196	0.05	6.30E-34
CHD3	4.49E-38	0.399576	0.199	0.014	6.70E-34
MOCS2	4.50E-38	-0.19819	0.241	0.135	6.73E-34
CA2	4.84E-38	-1.17035	0.035	0.231	7.23E-34
TRAPPC12	4.86E-38	0.325805	0.233	0.039	7.26E-34
PPP3CA	5.24E-38	0.246146	0.244	0.055	7.83E-34
SQLE	5.29E-38	0.360208	0.282	0.065	7.90E-34
XIAP	5.48E-38	0.115952	0.208	0.055	8.19E-34
DAP	5.73E-38	0.114678	0.181	0.041	8.57E-34
CNTN1	5.83E-38	0.530837	0.202	0.009	8.72E-34
DUSP6	5.88E-38	-0.17697	0.19	0.092	8.78E-34
NUFIP2	6.24E-38	0.147221	0.192	0.041	9.32E-34
KBTBD6	6.41E-38	0.23004	0.196	0.031	9.57E-34
HEXA	6.61E-38	-0.28137	0.204	0.12	9.88E-34
SLC9A3R1	6.94E-38	0.567097	0.238	0.022	1.04E-33
CCDC126	7.16E-38	0.382653	0.228	0.032	1.07E-33
ZNF512	7.35E-38	0.286093	0.216	0.034	1.10E-33
ZNF775	7.91E-38	0.516298	0.204	0.007	1.18E-33
ATP2B1	8.10E-38	-0.21642	0.233	0.13	1.21E-33

MAP2K7	8.12E-38	0.345964	0.244	0.043	1.21E-33
IVNS1ABP	8.15E-38	0.415561	0.222	0.026	1.22E-33
SCYL1	8.24E-38	0.237697	0.209	0.039	1.23E-33
RRP1	8.32E-38	0.145323	0.229	0.062	1.24E-33
ADIPOR1	8.48E-38	0.234586	0.234	0.05	1.27E-33
RNF44	8.67E-38	0.529748	0.216	0.014	1.30E-33
ZNF394	8.84E-38	0.236961	0.194	0.031	1.32E-33
TUBG1	8.85E-38	0.158031	0.213	0.05	1.32E-33
MED29	9.70E-38	-0.15755	0.207	0.096	1.45E-33
DNM3	1.04E-37	0.512324	0.212	0.012	1.55E-33
RND2	1.12E-37	0.133615	0.226	0.063	1.67E-33
RP11-262H	1.17E-37	0.383861	0.221	0.027	1.74E-33
SKIL	1.19E-37	0.314937	0.23	0.036	1.77E-33
SLC29A4	1.20E-37	0.43739	0.207	0.015	1.79E-33
ABHD14A	1.25E-37	0.192188	0.22	0.05	1.87E-33
RCAN1	1.34E-37	-0.61397	0.17	0.173	2.00E-33
RBM27	1.40E-37	0.21259	0.212	0.043	2.10E-33
NBR1	1.41E-37	0.124859	0.215	0.056	2.10E-33
MSH6	1.46E-37	0.321257	0.19	0.019	2.19E-33
VPS45	1.52E-37	0.166704	0.22	0.051	2.27E-33
MASP1	1.55E-37	0.258988	0.203	0.036	2.31E-33
ARMCX6	1.57E-37	-0.22587	0.191	0.089	2.34E-33
PLIN2	1.60E-37	-0.59326	0.14	0.108	2.39E-33
EFNB1	1.64E-37	0.378688	0.192	0.015	2.45E-33
UBALD2	1.79E-37	0.156191	0.242	0.07	2.67E-33
NUDT5	1.81E-37	-0.1097	0.195	0.075	2.71E-33
MAD2L1BP	1.83E-37	0.132467	0.204	0.044	2.73E-33
STRIP1	1.85E-37	0.287754	0.182	0.022	2.77E-33
STARD4-AS	1.85E-37	0.515166	0.231	0.019	2.77E-33
ZNF268	1.87E-37	0.288445	0.213	0.036	2.80E-33
UCK1	1.91E-37	0.109063	0.216	0.058	2.86E-33
AKT1S1	1.99E-37	0.104189	0.23	0.067	2.98E-33
CD276	2.03E-37	0.168515	0.205	0.046	3.03E-33
ATXN7L3B	2.12E-37	0.135163	0.202	0.048	3.17E-33
PPP2R2B	2.23E-37	-0.35315	0.229	0.15	3.34E-33
PPA2	2.44E-37	-0.20284	0.212	0.113	3.65E-33
SOBP	2.52E-37	0.353004	0.226	0.034	3.76E-33
MGEA5	2.55E-37	0.246009	0.26	0.068	3.81E-33
NUP35	2.60E-37	0.14488	0.191	0.043	3.88E-33
SNX4	2.61E-37	0.16644	0.231	0.065	3.90E-33
RHOT2	2.69E-37	0.193209	0.261	0.075	4.02E-33
MGST1	2.75E-37	-1.33012	0.027	0.188	4.11E-33
ELP5	2.78E-37	0.183796	0.23	0.06	4.15E-33
PPCS	3.13E-37	-0.48678	0.143	0.115	4.68E-33
C11orf74	3.17E-37	0.112379	0.172	0.036	4.73E-33
INSIG2	3.18E-37	-0.24803	0.189	0.097	4.76E-33
FSD1	3.20E-37	0.257765	0.222	0.044	4.79E-33
KDM5B	3.21E-37	0.402074	0.224	0.027	4.80E-33
PTK2	3.34E-37	0.155618	0.229	0.058	4.99E-33
MMD	3.39E-37	0.316671	0.17	0.012	5.07E-33
CHST10	3.51E-37	0.275314	0.222	0.041	5.24E-33

GNG7	3.77E-37	-0.52321	0.153	0.14	5.63E-33
MYH10	3.78E-37	0.410573	0.242	0.034	5.65E-33
EXOSC5	3.82E-37	-0.19927	0.204	0.104	5.71E-33
ZFPL1	4.01E-37	-0.15708	0.203	0.091	6.00E-33
ADNP	4.13E-37	0.281879	0.222	0.039	6.17E-33
SFSWAP	4.22E-37	0.211378	0.202	0.038	6.30E-33
MCM5	4.26E-37	0.305205	0.173	0.019	6.36E-33
SKIV2L2	4.48E-37	0.186685	0.233	0.06	6.69E-33
FAM174A	4.49E-37	0.132353	0.218	0.062	6.70E-33
CDIP1	4.70E-37	0.270271	0.221	0.046	7.02E-33
NME7	5.64E-37	-0.15204	0.181	0.082	8.42E-33
NTPCR	5.76E-37	-0.15714	0.254	0.125	8.61E-33
MITD1	6.30E-37	-0.15494	0.218	0.104	9.42E-33
KRAS	6.38E-37	0.347878	0.243	0.044	9.53E-33
CYSTM1	6.42E-37	-0.96385	0.096	0.191	9.60E-33
PTGDS	6.64E-37	-0.31664	0.221	0.12	9.92E-33
WDR70	7.70E-37	0.350456	0.218	0.029	1.15E-32
POR	7.71E-37	0.213477	0.272	0.084	1.15E-32
RP11-345F	8.03E-37	0.255694	0.233	0.05	1.20E-32
MAP4K5	8.04E-37	0.338402	0.22	0.034	1.20E-32
CRB1	8.79E-37	0.551806	0.247	0.026	1.31E-32
SMC2	8.85E-37	0.324278	0.194	0.026	1.32E-32
CRNDE	9.23E-37	0.179377	0.231	0.062	1.38E-32
RIMKLB	9.95E-37	0.381812	0.218	0.027	1.49E-32
BIRC6	1.02E-36	0.234212	0.217	0.046	1.52E-32
GATAD2B	1.14E-36	0.272683	0.23	0.044	1.71E-32
COMMD10	1.15E-36	-0.4251	0.152	0.108	1.72E-32
FADS2	1.16E-36	0.483199	0.228	0.022	1.73E-32
CCSER2	1.18E-36	0.180348	0.207	0.044	1.76E-32
IGF2BP3	1.19E-36	0.269318	0.181	0.022	1.77E-32
NETO2	1.24E-36	0.372814	0.207	0.024	1.85E-32
EXTL2	1.32E-36	0.133333	0.194	0.046	1.97E-32
TCERG1	1.33E-36	0.404788	0.264	0.048	1.99E-32
C2orf69	1.38E-36	0.375847	0.238	0.038	2.06E-32
EFHC1	1.44E-36	-0.25957	0.202	0.116	2.16E-32
CCAR2	1.61E-36	0.262872	0.209	0.034	2.40E-32
ICA1	1.62E-36	0.463281	0.186	0.005	2.41E-32
ATL1	1.64E-36	0.230974	0.194	0.034	2.44E-32
DNM1L	1.72E-36	0.123428	0.235	0.072	2.58E-32
ACTR1B	1.75E-36	0.149593	0.205	0.051	2.62E-32
SRGN	2.03E-36	-0.22696	0.224	0.138	3.03E-32
MBTPS1	2.06E-36	0.250875	0.199	0.036	3.08E-32
PSMB8	2.11E-36	-0.73999	0.166	0.207	3.15E-32
DEAF1	2.15E-36	0.171567	0.224	0.055	3.21E-32
EIF4G3	2.17E-36	0.284396	0.233	0.043	3.24E-32
FTX	2.18E-36	0.317681	0.226	0.041	3.26E-32
PPP6C	2.31E-36	0.197468	0.237	0.058	3.45E-32
RFC4	2.36E-36	0.292196	0.224	0.043	3.52E-32
REEP2	2.40E-36	0.148821	0.205	0.048	3.59E-32
CASC3	2.42E-36	0.366939	0.254	0.046	3.62E-32
CHD1	2.52E-36	0.171752	0.222	0.06	3.76E-32

COL9A2	2.61E-36	0.443813	0.209	0.021	3.91E-32
TBL1XR1	2.71E-36	0.20749	0.231	0.056	4.05E-32
TYROBP	2.78E-36	-0.15401	0.196	0.092	4.16E-32
PROX1	2.86E-36	0.234222	0.208	0.039	4.27E-32
TAF10	2.91E-36	0.211176	0.182	0.027	4.34E-32
B4GALT7	2.92E-36	0.227883	0.186	0.029	4.36E-32
SNX27	2.96E-36	0.132971	0.198	0.05	4.42E-32
CS	2.99E-36	0.177092	0.169	0.027	4.47E-32
PIGX	3.19E-36	0.211959	0.246	0.062	4.77E-32
ASPH	3.24E-36	0.107048	0.237	0.074	4.84E-32
GTF2H2	3.34E-36	0.143462	0.221	0.063	4.99E-32
BLVRB	3.40E-36	-1.16264	0.068	0.212	5.07E-32
POMGNT2	3.55E-36	0.206574	0.196	0.038	5.31E-32
SEMA5A	3.68E-36	0.102569	0.208	0.055	5.50E-32
DHX33	3.69E-36	0.345995	0.207	0.026	5.51E-32
CALCOCO1	3.69E-36	0.268794	0.213	0.039	5.52E-32
STX6	4.32E-36	0.366725	0.217	0.029	6.46E-32
MED27	4.55E-36	0.228495	0.203	0.036	6.81E-32
PCSK2	4.66E-36	0.60088	0.17	0	6.97E-32
B3GALT2	4.74E-36	0.413345	0.179	0.009	7.09E-32
SH3BP4	5.05E-36	0.301662	0.199	0.027	7.55E-32
PURA	5.13E-36	0.260001	0.23	0.05	7.66E-32
ETHE1	5.16E-36	-0.35473	0.155	0.104	7.71E-32
TJP1	5.28E-36	0.113149	0.189	0.043	7.90E-32
NOTCH1	5.32E-36	0.412055	0.217	0.024	7.95E-32
C14orf132	5.36E-36	0.409253	0.241	0.041	8.01E-32
PURB	5.39E-36	0.257886	0.217	0.043	8.05E-32
NOL10	5.82E-36	0.258889	0.195	0.031	8.70E-32
SRPX	5.91E-36	-0.99464	0.061	0.171	8.83E-32
HLTF	6.08E-36	0.337056	0.212	0.031	9.09E-32
LPIN1	6.58E-36	0.392298	0.23	0.034	9.83E-32
CDC40	6.80E-36	0.183312	0.207	0.044	1.02E-31
TOR3A	7.42E-36	0.194625	0.191	0.036	1.11E-31
STX5	7.44E-36	-0.12942	0.215	0.091	1.11E-31
GPT2	7.80E-36	0.177799	0.2	0.048	1.17E-31
AAMDC	8.85E-36	-0.44116	0.138	0.097	1.32E-31
MIA3	9.07E-36	0.273435	0.224	0.044	1.36E-31
WDR41	9.46E-36	-0.39221	0.168	0.116	1.41E-31
VSTM2A	9.53E-36	-0.96898	0.095	0.258	1.42E-31
GSK3B	9.91E-36	0.31332	0.229	0.043	1.48E-31
GNL1	9.93E-36	0.307527	0.247	0.05	1.48E-31
DR1	1.01E-35	0.181998	0.221	0.053	1.51E-31
GSX1	1.02E-35	0.559388	0.177	0.002	1.52E-31
5-Mar	1.05E-35	0.30804	0.218	0.032	1.57E-31
ZC3H11A	1.08E-35	0.157589	0.205	0.048	1.61E-31
FAIM	1.17E-35	0.289395	0.208	0.036	1.74E-31
MSI1	1.18E-35	0.348935	0.239	0.041	1.76E-31
NMB	1.22E-35	-0.82786	0.088	0.133	1.83E-31
AGT	1.29E-35	-0.91292	0.095	0.193	1.93E-31
GAS1	1.32E-35	0.144229	0.225	0.067	1.97E-31
SPATS2L	1.33E-35	-0.36764	0.179	0.12	1.98E-31

TMEM237	1.34E-35	0.273687	0.238	0.051	2.01E-31
PDLIM5	1.37E-35	0.220265	0.216	0.048	2.04E-31
SMIM18	1.45E-35	0.511749	0.178	0.003	2.17E-31
CKAP4	1.47E-35	0.239366	0.252	0.067	2.20E-31
UGT8	1.48E-35	0.460106	0.172	0.003	2.21E-31
ARFIP2	1.48E-35	0.144786	0.241	0.075	2.21E-31
CBX6	1.55E-35	0.390566	0.282	0.063	2.32E-31
ANKH	1.56E-35	0.254729	0.17	0.021	2.33E-31
IDS	1.62E-35	-0.55814	0.153	0.166	2.42E-31
LRRC58	1.76E-35	0.218508	0.218	0.048	2.63E-31
SPEN	1.79E-35	0.338389	0.218	0.034	2.67E-31
ENC1	1.82E-35	0.387785	0.228	0.039	2.72E-31
TEAD2	1.84E-35	0.330445	0.17	0.014	2.75E-31
WASF1	1.86E-35	0.335115	0.177	0.015	2.78E-31
ABHD17B	1.90E-35	0.472864	0.205	0.014	2.85E-31
ZFYVE21	1.97E-35	-0.30517	0.192	0.12	2.95E-31
CTBP1	2.07E-35	0.206286	0.204	0.043	3.10E-31
NAV2	2.11E-35	0.215524	0.248	0.07	3.15E-31
BPHL	2.22E-35	0.248397	0.211	0.041	3.32E-31
PRPF4B	2.23E-35	0.12749	0.244	0.075	3.34E-31
SOX5	2.41E-35	0.306433	0.186	0.022	3.59E-31
TAF9B	2.42E-35	0.179916	0.187	0.039	3.61E-31
ARL1	2.50E-35	-0.41917	0.198	0.157	3.73E-31
CDKN1C	2.56E-35	0.483516	0.204	0.017	3.82E-31
MID1IP1	2.59E-35	0.243132	0.247	0.065	3.86E-31
ZNF414	2.61E-35	0.389994	0.225	0.029	3.90E-31
HINT3	2.65E-35	0.268531	0.22	0.044	3.95E-31
KLF10	2.80E-35	0.20277	0.241	0.068	4.18E-31
TCTEX1D2	2.85E-35	-0.27519	0.183	0.109	4.27E-31
PSMD5-AS1	2.93E-35	0.133054	0.237	0.072	4.38E-31
GYG1	2.95E-35	-0.13767	0.161	0.068	4.42E-31
UBE2M	3.10E-35	0.125459	0.182	0.044	4.63E-31
KANSL1	3.24E-35	0.306081	0.215	0.036	4.85E-31
POU4F1	3.30E-35	0.506409	0.166	0	4.93E-31
DDX19B	3.32E-35	0.220287	0.195	0.039	4.96E-31
FNBP1L	3.63E-35	0.335733	0.215	0.034	5.42E-31
RNF115	3.94E-35	0.237239	0.239	0.056	5.89E-31
RP11-1275	3.99E-35	0.510375	0.199	0.009	5.96E-31
RAD1	4.44E-35	0.157806	0.228	0.065	6.64E-31
ZC3H18	4.80E-35	0.366038	0.191	0.017	7.17E-31
SPOCD1	4.97E-35	-1.14507	0.008	0.166	7.43E-31
ZNF451	5.03E-35	0.211101	0.202	0.039	7.51E-31
MAEA	5.46E-35	0.178143	0.217	0.056	8.16E-31
USP39	5.92E-35	0.212373	0.191	0.036	8.85E-31
PPM1A	6.62E-35	0.354273	0.191	0.019	9.89E-31
KLHL9	6.74E-35	0.441939	0.191	0.009	1.01E-30
ARL14EP	7.03E-35	-0.17029	0.178	0.077	1.05E-30
B3GALNT1	7.04E-35	0.250518	0.203	0.038	1.05E-30
FAM20C	7.14E-35	-0.29614	0.153	0.091	1.07E-30
LHFP	7.17E-35	-0.25743	0.181	0.101	1.07E-30
MED31	7.40E-35	-0.11974	0.194	0.08	1.11E-30

HOXA9	7.74E-35	0.464329	0.179	0.003	1.16E-30
ETF1	7.98E-35	-0.20504	0.192	0.094	1.19E-30
TMEM178A	8.02E-35	0.100196	0.159	0.031	1.20E-30
HBA2	8.09E-35	-0.68716	0.159	0.097	1.21E-30
DNAJA3	8.25E-35	0.133272	0.196	0.046	1.23E-30
RALBP1	8.28E-35	-0.16214	0.173	0.079	1.24E-30
SIRT7	8.37E-35	0.269547	0.213	0.039	1.25E-30
MTRNR2L1C	8.40E-35	-1.11034	0.025	0.209	1.25E-30
PLA2G16	9.02E-35	-0.70172	0.156	0.168	1.35E-30
FKBP10	9.03E-35	-0.13901	0.216	0.115	1.35E-30
INPP1	9.11E-35	0.196677	0.203	0.043	1.36E-30
GFER	9.36E-35	0.304558	0.194	0.024	1.40E-30
POU2F1	9.59E-35	0.299151	0.209	0.034	1.43E-30
ATN1	9.91E-35	0.259711	0.207	0.039	1.48E-30
TTC32	1.03E-34	0.132092	0.202	0.051	1.54E-30
RPF2	1.05E-34	-0.10168	0.213	0.087	1.56E-30
TLE3	1.12E-34	0.430308	0.213	0.022	1.68E-30
MOB2	1.17E-34	0.110945	0.183	0.048	1.75E-30
ASCC2	1.20E-34	0.21078	0.204	0.043	1.79E-30
LTA4H	1.31E-34	-0.12394	0.181	0.077	1.96E-30
FERMT2	1.35E-34	-0.10958	0.179	0.074	2.01E-30
DDX23	1.39E-34	0.195385	0.185	0.036	2.08E-30
TMEM243	1.40E-34	-0.22817	0.174	0.092	2.09E-30
CHAMP1	1.58E-34	0.351524	0.174	0.014	2.37E-30
NCKAP1	1.66E-34	0.320532	0.242	0.05	2.48E-30
TAOK1	1.69E-34	0.174146	0.239	0.065	2.53E-30
VPS36	1.74E-34	0.122241	0.204	0.058	2.61E-30
VASH1	1.76E-34	0.392122	0.186	0.014	2.62E-30
HDAC9	1.86E-34	0.339417	0.199	0.026	2.78E-30
ZC3H7A	1.86E-34	0.379833	0.199	0.022	2.79E-30
CRISPLD1	1.87E-34	0.316213	0.179	0.022	2.79E-30
PSD3	1.90E-34	0.231822	0.218	0.046	2.83E-30
RABEP1	1.93E-34	0.285612	0.2	0.032	2.89E-30
VAMP3	2.11E-34	-0.31233	0.142	0.085	3.15E-30
SMARCA1	2.19E-34	0.19653	0.191	0.038	3.27E-30
LY6E	2.30E-34	0.166575	0.3	0.118	3.44E-30
DDX42	2.40E-34	0.180717	0.212	0.053	3.59E-30
RHOU	2.50E-34	0.40784	0.251	0.05	3.74E-30
LTBP3	2.53E-34	0.338548	0.216	0.032	3.78E-30
NPM3	2.69E-34	-0.3069	0.153	0.092	4.02E-30
WBSCR16	3.03E-34	0.290519	0.185	0.021	4.53E-30
GAR1	3.06E-34	0.154476	0.192	0.041	4.58E-30
EIF2S3	3.44E-34	-0.10866	0.209	0.091	5.14E-30
DMTF1	3.47E-34	0.323724	0.217	0.036	5.18E-30
POLDIP3	3.60E-34	0.211146	0.198	0.036	5.38E-30
DAZAP1	3.63E-34	0.157505	0.202	0.05	5.42E-30
CARKD	4.02E-34	0.359102	0.211	0.029	6.01E-30
ZFHX4	4.11E-34	0.205228	0.204	0.048	6.14E-30
COQ5	4.12E-34	0.156702	0.205	0.051	6.15E-30
PLP2	4.24E-34	-0.96499	0.083	0.179	6.34E-30
CDK2AP2	4.27E-34	-0.36803	0.165	0.111	6.38E-30

TUG1	4.57E-34	0.36433	0.207	0.027	6.84E-30
LANCL1	4.59E-34	0.257525	0.183	0.027	6.86E-30
ASIC1	4.67E-34	0.383066	0.183	0.015	6.99E-30
TECPR1	4.83E-34	0.447495	0.19	0.012	7.22E-30
MCF2L	4.88E-34	0.447078	0.199	0.012	7.29E-30
RPAP2	5.10E-34	0.263924	0.22	0.05	7.62E-30
ARHGAP21	5.94E-34	0.123733	0.251	0.085	8.88E-30
SDHAF1	6.34E-34	0.167585	0.187	0.041	9.47E-30
ROBO1	6.41E-34	0.477286	0.19	0.007	9.58E-30
COPG2	6.44E-34	0.314739	0.164	0.014	9.63E-30
KRBOX4	7.05E-34	0.192524	0.178	0.032	1.05E-29
NIT2	7.25E-34	-0.12543	0.173	0.077	1.08E-29
EN1	7.43E-34	0.401925	0.204	0.019	1.11E-29
PITX2	7.86E-34	0.43978	0.187	0.009	1.17E-29
KAT6A	7.90E-34	0.235843	0.204	0.043	1.18E-29
FAM103A1	7.92E-34	-0.43438	0.178	0.118	1.18E-29
ACAD8	7.92E-34	0.137289	0.192	0.048	1.18E-29
DDX6	7.99E-34	-0.1006	0.209	0.08	1.19E-29
AC009501.	8.47E-34	-0.89709	0.078	0.185	1.27E-29
TENM3	8.50E-34	0.437446	0.16	0	1.27E-29
MEF2C	9.69E-34	0.161424	0.179	0.039	1.45E-29
RBM26	9.74E-34	0.271965	0.216	0.039	1.46E-29
SULF2	1.02E-33	0.399129	0.22	0.032	1.52E-29
TMEM69	1.02E-33	-0.1007	0.183	0.08	1.52E-29
FOXK1	1.05E-33	0.140127	0.179	0.043	1.57E-29
PPIF	1.06E-33	0.212812	0.177	0.027	1.58E-29
SRPK1	1.07E-33	0.265829	0.202	0.031	1.60E-29
CPNE2	1.09E-33	-0.23596	0.151	0.074	1.63E-29
PTRH1	1.15E-33	0.144268	0.161	0.031	1.73E-29
METTL2A	1.29E-33	0.153449	0.177	0.036	1.93E-29
THOP1	1.29E-33	0.165762	0.176	0.032	1.93E-29
SPCS3	1.33E-33	0.123476	0.203	0.056	1.99E-29
BEST1	1.38E-33	-0.33291	0.148	0.074	2.06E-29
LGMN	1.40E-33	-0.34397	0.156	0.096	2.09E-29
USP8	1.42E-33	-0.19665	0.176	0.085	2.13E-29
HSDL1	1.45E-33	0.479506	0.187	0.009	2.16E-29
DCAF10	1.62E-33	0.303391	0.203	0.031	2.42E-29
PELP1	1.63E-33	0.193973	0.164	0.024	2.43E-29
PXDN	1.63E-33	0.210744	0.178	0.027	2.44E-29
PMM1	1.63E-33	-0.1807	0.186	0.087	2.44E-29
UBE2J1	1.66E-33	0.235765	0.228	0.055	2.48E-29
UBA5	1.67E-33	0.136171	0.17	0.036	2.49E-29
MCOLN1	1.70E-33	0.237013	0.196	0.041	2.55E-29
GLRX	1.73E-33	-0.62769	0.137	0.132	2.58E-29
GSK3A	1.79E-33	0.227558	0.222	0.05	2.67E-29
NOC4L	1.86E-33	0.312819	0.189	0.024	2.77E-29
C6orf47	1.97E-33	0.212994	0.156	0.019	2.94E-29
MAPK6	2.05E-33	0.147401	0.205	0.053	3.06E-29
CYFIP1	2.06E-33	-0.12375	0.156	0.056	3.07E-29
TSPYL4	2.13E-33	0.133497	0.147	0.022	3.18E-29
DLX1	2.15E-33	0.607576	0.2	0.012	3.21E-29

SENP2	2.16E-33	0.234155	0.189	0.032	3.23E-29
COMMD8	2.34E-33	-0.14271	0.173	0.082	3.49E-29
CASP2	2.46E-33	0.248218	0.172	0.021	3.67E-29
PAG1	2.55E-33	0.241053	0.177	0.027	3.81E-29
KIF1A	2.57E-33	0.192838	0.185	0.038	3.84E-29
POLR3K	2.65E-33	-0.17532	0.166	0.079	3.96E-29
FBLN1	2.68E-33	0.518455	0.228	0.032	4.00E-29
IPO7	2.79E-33	0.188153	0.199	0.043	4.17E-29
ZBTB8A	2.83E-33	0.174444	0.187	0.043	4.23E-29
SIRT6	2.84E-33	0.119312	0.202	0.056	4.24E-29
AGPAT1	3.09E-33	0.184469	0.221	0.056	4.61E-29
DOLPP1	3.27E-33	0.151744	0.164	0.031	4.89E-29
NPPA	3.65E-33	0.37806	0.208	0.032	5.45E-29
INO80E	3.65E-33	0.278791	0.215	0.046	5.45E-29
TMEM55A	3.70E-33	-0.13644	0.189	0.087	5.52E-29
RP11-212F	3.83E-33	0.376949	0.187	0.019	5.73E-29
TIPRL	3.98E-33	-0.13091	0.183	0.082	5.94E-29
NAV1	3.98E-33	0.237386	0.181	0.031	5.95E-29
RP11-488C	3.99E-33	0.3838	0.19	0.017	5.96E-29
GEMIN6	4.08E-33	0.161152	0.194	0.048	6.09E-29
GCA	4.13E-33	-0.31045	0.152	0.096	6.17E-29
ACLY	4.24E-33	0.223507	0.205	0.048	6.33E-29
UBN2	4.29E-33	0.219199	0.173	0.031	6.41E-29
POMZP3	4.71E-33	0.402026	0.185	0.012	7.04E-29
SYNRG	4.96E-33	0.135311	0.169	0.031	7.41E-29
UTP23	5.09E-33	0.299507	0.196	0.032	7.61E-29
TMEFF2	5.10E-33	0.565164	0.194	0.009	7.62E-29
RRM1	5.47E-33	0.24744	0.218	0.048	8.17E-29
DGKB	5.49E-33	0.459944	0.177	0.009	8.21E-29
SCAMP2	5.52E-33	-0.20601	0.173	0.089	8.26E-29
AMER2	5.55E-33	0.311051	0.209	0.038	8.29E-29
CKS1B	5.80E-33	-0.2076	0.144	0.07	8.67E-29
PEX10	5.82E-33	0.254318	0.203	0.039	8.70E-29
RP11-472N	5.86E-33	0.27056	0.144	0.012	8.76E-29
ZNF330	6.09E-33	-0.27613	0.172	0.091	9.10E-29
ZNF664	6.11E-33	0.178456	0.178	0.036	9.13E-29
SUDS3	6.27E-33	0.408458	0.22	0.029	9.37E-29
WDR54	6.41E-33	-0.35731	0.172	0.125	9.58E-29
ZNF444	6.55E-33	0.152976	0.187	0.039	9.79E-29
SLC25A13	7.07E-33	0.289606	0.192	0.031	1.06E-28
DBF4	7.24E-33	0.26694	0.172	0.019	1.08E-28
SHMT2	7.29E-33	-0.37434	0.179	0.126	1.09E-28
PDPK1	7.49E-33	0.316435	0.198	0.026	1.12E-28
AP2A2	7.53E-33	0.138129	0.208	0.06	1.13E-28
HMBS	7.65E-33	0.162423	0.192	0.043	1.14E-28
NCOA1	7.68E-33	0.27172	0.199	0.038	1.15E-28
FAM200A	7.71E-33	0.281832	0.181	0.024	1.15E-28
PCCB	7.87E-33	0.137336	0.213	0.063	1.18E-28
RRAGC	7.97E-33	0.105262	0.195	0.053	1.19E-28
VANGL2	8.05E-33	0.197954	0.159	0.024	1.20E-28
CCZ1B	8.26E-33	0.433947	0.196	0.021	1.23E-28

ITGAV	8.40E-33	0.189339	0.202	0.046	1.26E-28
CLOCK	8.46E-33	0.608636	0.234	0.024	1.26E-28
LRP4	9.87E-33	0.284726	0.17	0.021	1.47E-28
FAM46A	1.00E-32	-0.10687	0.23	0.115	1.50E-28
EGLN2	1.03E-32	0.177174	0.211	0.053	1.54E-28
TULP4	1.14E-32	0.300146	0.181	0.021	1.71E-28
CDC16	1.18E-32	0.207334	0.178	0.034	1.77E-28
GSPT2	1.21E-32	0.338349	0.187	0.022	1.82E-28
BCL7C	1.22E-32	0.193621	0.182	0.036	1.83E-28
TRIM37	1.24E-32	0.10754	0.144	0.026	1.85E-28
CDIPT	1.27E-32	0.21037	0.246	0.07	1.90E-28
ZNF800	1.34E-32	0.224151	0.207	0.041	2.00E-28
ZC3H6	1.34E-32	0.450926	0.217	0.029	2.01E-28
TMEM199	1.39E-32	0.149972	0.224	0.065	2.07E-28
IARS2	1.40E-32	0.156326	0.185	0.044	2.09E-28
DOK5	1.42E-32	-0.31804	0.142	0.08	2.12E-28
RAB22A	1.44E-32	0.12476	0.195	0.05	2.16E-28
TCF7	1.45E-32	0.238383	0.218	0.05	2.16E-28
USP14	1.55E-32	0.161901	0.207	0.051	2.32E-28
THOC3	1.67E-32	0.204976	0.168	0.027	2.49E-28
TGIF1	1.67E-32	-0.22124	0.199	0.118	2.50E-28
TOB2	1.72E-32	0.344667	0.231	0.043	2.57E-28
TMED7	1.74E-32	0.271664	0.25	0.065	2.61E-28
FAHD1	1.76E-32	0.242271	0.205	0.044	2.63E-28
FAM127B	1.76E-32	-0.28885	0.205	0.144	2.64E-28
HEBP2	1.80E-32	0.238869	0.169	0.024	2.68E-28
KCMF1	1.89E-32	0.190933	0.181	0.036	2.82E-28
PXDC1	1.94E-32	0.241035	0.186	0.032	2.90E-28
PSMA2	2.01E-32	-1.20952	0.018	0.205	3.01E-28
ACBD3	2.05E-32	0.138031	0.179	0.041	3.06E-28
CTXN1	2.06E-32	0.490254	0.192	0.01	3.08E-28
FAM207A	2.18E-32	0.2099	0.183	0.036	3.25E-28
CHGB	2.19E-32	0.284457	0.186	0.031	3.27E-28
ATXN8OS	2.34E-32	-1.15147	0.023	0.215	3.50E-28
CUL5	2.35E-32	0.379056	0.204	0.027	3.52E-28
TM7SF3	2.59E-32	0.120055	0.181	0.044	3.86E-28
ZNF219	2.68E-32	0.322731	0.186	0.022	4.01E-28
CAT	2.91E-32	-0.1087	0.189	0.085	4.35E-28
PALLD	2.98E-32	0.266612	0.194	0.036	4.45E-28
LRPPRC	3.00E-32	0.36324	0.216	0.032	4.49E-28
PUS3	3.23E-32	0.251558	0.176	0.027	4.83E-28
CENPT	3.36E-32	0.244076	0.237	0.062	5.02E-28
RPUSD1	3.38E-32	0.295814	0.17	0.021	5.05E-28
UBTD2	3.40E-32	0.233267	0.152	0.017	5.08E-28
RP11-315A	3.49E-32	0.162406	0.152	0.027	5.22E-28
HERPUD2	3.68E-32	0.315887	0.189	0.024	5.50E-28
HIBADH	3.73E-32	0.147101	0.189	0.051	5.58E-28
WAC	3.77E-32	0.263491	0.222	0.05	5.63E-28
AQP4	3.86E-32	-1.00267	0.059	0.193	5.76E-28
RGS3	3.91E-32	0.275594	0.165	0.019	5.85E-28
RP11-51J9	3.98E-32	-0.17498	0.176	0.08	5.95E-28

BLMH	4.19E-32	0.174731	0.181	0.038	6.26E-28
CFLAR	4.34E-32	-0.3228	0.179	0.113	6.49E-28
MLF1	4.39E-32	-0.4004	0.146	0.106	6.57E-28
QRICH1	4.42E-32	0.181213	0.173	0.034	6.60E-28
TMEM38B	4.43E-32	-0.37937	0.12	0.087	6.61E-28
TLE2	4.45E-32	0.346398	0.2	0.027	6.66E-28
FKBP7	4.57E-32	0.189098	0.187	0.039	6.83E-28
MOB1A	4.64E-32	-0.11164	0.166	0.07	6.93E-28
MSN	4.75E-32	-0.11948	0.178	0.084	7.09E-28
RBAK-RBAK	4.88E-32	0.147514	0.19	0.046	7.29E-28
SSH2	5.13E-32	0.383859	0.194	0.022	7.67E-28
ZNF503	5.29E-32	0.313286	0.195	0.031	7.91E-28
DENND2A	5.60E-32	0.169376	0.178	0.034	8.36E-28
LTF	6.00E-32	-1.41414	0.012	0.162	8.96E-28
FAM3A	6.11E-32	0.17885	0.182	0.041	9.13E-28
RAB1B	6.23E-32	-0.19471	0.148	0.077	9.30E-28
PLA2G12A	6.75E-32	0.124762	0.218	0.068	1.01E-27
ZDHHC3	6.91E-32	0.124106	0.16	0.029	1.03E-27
UBE2D1	6.92E-32	0.224957	0.173	0.031	1.03E-27
RRP1B	7.28E-32	0.361375	0.178	0.015	1.09E-27
NUP93	7.30E-32	0.115109	0.183	0.046	1.09E-27
CES2	7.53E-32	0.337425	0.183	0.022	1.12E-27
BNIP2	7.62E-32	0.291111	0.187	0.029	1.14E-27
RCHY1	7.66E-32	-0.13548	0.19	0.085	1.14E-27
DENND5A	8.12E-32	0.207992	0.194	0.039	1.21E-27
MAPRE2	8.28E-32	0.19234	0.181	0.034	1.24E-27
C12orf43	8.69E-32	0.161712	0.187	0.046	1.30E-27
KIAA1143	8.69E-32	-0.13438	0.147	0.06	1.30E-27
CAND1	8.79E-32	0.141986	0.225	0.074	1.31E-27
KCTD17	8.80E-32	0.235027	0.166	0.024	1.32E-27
RBM33	9.20E-32	0.250888	0.189	0.029	1.37E-27
PRKD3	9.26E-32	0.319008	0.207	0.034	1.38E-27
RP5-1177M	9.28E-32	0.547529	0.218	0.026	1.39E-27
CAB39	9.42E-32	0.337265	0.192	0.024	1.41E-27
DDX54	9.71E-32	0.129456	0.186	0.046	1.45E-27
SLC25A17	1.01E-31	0.235147	0.192	0.036	1.50E-27
RRS1	1.05E-31	0.136634	0.16	0.032	1.57E-27
RP5-940J5	1.06E-31	-0.76275	0.083	0.133	1.59E-27
GTF3C2	1.12E-31	0.161907	0.156	0.026	1.67E-27
KDM7A	1.13E-31	0.309351	0.143	0.005	1.69E-27
MAP3K1	1.16E-31	0.336947	0.181	0.019	1.73E-27
KLHL13	1.18E-31	0.336123	0.156	0.009	1.76E-27
SORBS3	1.19E-31	0.162802	0.187	0.041	1.78E-27
TEX261	1.27E-31	0.249907	0.185	0.032	1.90E-27
BAMBI	1.30E-31	0.424198	0.161	0.005	1.94E-27
RP11-386G	1.32E-31	-0.28984	0.124	0.068	1.98E-27
COL9A3	1.33E-31	-0.26247	0.186	0.109	1.99E-27
HPCAL1	1.35E-31	0.259454	0.148	0.014	2.02E-27
TAPBP	1.37E-31	-0.15327	0.168	0.08	2.04E-27
TUSC1	1.48E-31	0.371192	0.156	0.003	2.21E-27
ABHD6	1.62E-31	0.418471	0.185	0.017	2.42E-27

DEDD2	1.63E-31	0.146118	0.169	0.036	2.43E-27
LIMS1	1.65E-31	-0.15932	0.194	0.104	2.47E-27
ZNF226	1.75E-31	-0.38174	0.151	0.109	2.62E-27
METTL7B	1.75E-31	-1.19107	0.034	0.231	2.62E-27
FAM171B	1.80E-31	0.345089	0.198	0.027	2.68E-27
PPIL3	1.80E-31	0.167162	0.189	0.043	2.69E-27
BYSL	1.81E-31	0.246496	0.156	0.017	2.70E-27
WDR12	2.05E-31	0.351902	0.216	0.038	3.07E-27
RFX4	2.09E-31	-0.26039	0.187	0.125	3.13E-27
PIGC	2.14E-31	-0.16212	0.169	0.072	3.19E-27
HSPB11	2.19E-31	-0.36279	0.19	0.126	3.28E-27
NVL	2.20E-31	0.218632	0.174	0.031	3.28E-27
ALDOC	2.24E-31	-0.60196	0.153	0.183	3.35E-27
STK16	2.51E-31	0.117576	0.168	0.041	3.75E-27
HSD11B1L	2.52E-31	0.193066	0.217	0.056	3.76E-27
WDR43	2.56E-31	0.289114	0.224	0.046	3.82E-27
C1orf27	2.58E-31	-0.18383	0.178	0.077	3.85E-27
TPPP3	2.61E-31	-0.1885	0.169	0.082	3.90E-27
C1orf21	2.67E-31	-0.39342	0.179	0.133	3.99E-27
GPC1	2.68E-31	-0.14867	0.195	0.099	4.01E-27
STOX2	2.68E-31	0.41239	0.164	0.005	4.01E-27
CHMP7	2.74E-31	0.17322	0.194	0.046	4.09E-27
SYP	2.75E-31	0.24799	0.228	0.058	4.11E-27
SLC39A10	2.77E-31	0.205869	0.179	0.034	4.14E-27
JARID2	2.77E-31	0.111834	0.172	0.039	4.15E-27
CTU2	2.79E-31	0.191382	0.169	0.029	4.16E-27
URGCP	2.81E-31	0.23761	0.178	0.027	4.20E-27
SCG2	2.84E-31	-0.67636	0.139	0.181	4.25E-27
CEP63	2.86E-31	0.256763	0.178	0.027	4.27E-27
KBTBD7	2.99E-31	0.270904	0.166	0.022	4.46E-27
GALNT13	3.18E-31	0.510055	0.186	0.012	4.76E-27
RPIA	3.33E-31	0.204737	0.182	0.036	4.98E-27
MTA1	3.43E-31	0.301375	0.237	0.055	5.13E-27
IGSF8	3.61E-31	0.172719	0.22	0.06	5.39E-27
NDUFAF1	3.72E-31	0.140686	0.186	0.048	5.56E-27
NUDT10	3.80E-31	0.334267	0.178	0.015	5.68E-27
MEIS1	3.91E-31	0.210883	0.179	0.034	5.85E-27
RAB3GAP1	3.92E-31	0.224678	0.168	0.027	5.86E-27
SMPD1	3.93E-31	0.340917	0.211	0.034	5.88E-27
PRKCZ	3.99E-31	0.331966	0.169	0.015	5.96E-27
FAM168B	4.05E-31	0.318919	0.204	0.032	6.05E-27
GRID2	4.21E-31	0.483134	0.169	0.003	6.29E-27
NAPEPLD	4.25E-31	0.296518	0.176	0.024	6.36E-27
ANKRD39	4.27E-31	0.367128	0.198	0.027	6.38E-27
B3GAT2	4.61E-31	0.493085	0.179	0.007	6.89E-27
PRTFDC1	4.67E-31	0.212532	0.208	0.053	6.98E-27
ZMIZ2	4.85E-31	0.3021	0.172	0.021	7.24E-27
TBC1D14	4.89E-31	0.244286	0.192	0.038	7.30E-27
SOX12	4.97E-31	0.361181	0.169	0.014	7.43E-27
CHCHD7	5.14E-31	-0.1389	0.169	0.077	7.68E-27
MYC	5.24E-31	-0.42758	0.163	0.126	7.83E-27

RAB28	5.35E-31	-0.11129	0.165	0.072	7.99E-27
DDX39B	5.42E-31	0.199235	0.147	0.021	8.10E-27
SSBP2	5.47E-31	-0.54782	0.15	0.161	8.17E-27
ALDH6A1	5.64E-31	0.146554	0.233	0.077	8.42E-27
STYXL1	5.75E-31	0.158548	0.179	0.043	8.60E-27
LINC00685	6.00E-31	0.230382	0.213	0.055	8.96E-27
RNASET2	6.02E-31	0.25963	0.208	0.043	9.00E-27
POLK	6.08E-31	0.140578	0.198	0.053	9.09E-27
PGP	6.26E-31	0.380431	0.194	0.019	9.35E-27
CCDC93	6.30E-31	0.314224	0.194	0.029	9.42E-27
NAP1L5	6.77E-31	0.203792	0.209	0.051	1.01E-26
SLC25A37	6.82E-31	-0.42072	0.155	0.138	1.02E-26
SERTAD2	6.90E-31	0.154119	0.174	0.036	1.03E-26
RFXAP	7.11E-31	0.260457	0.163	0.019	1.06E-26
CENPH	7.32E-31	0.308482	0.161	0.017	1.09E-26
ADAM17	7.68E-31	0.373362	0.218	0.038	1.15E-26
CWF19L2	7.99E-31	0.213014	0.194	0.041	1.19E-26
KIAA2026	8.08E-31	0.360275	0.196	0.027	1.21E-26
HBS1L	8.22E-31	0.175325	0.183	0.044	1.23E-26
BNIP3	8.22E-31	-0.80777	0.118	0.161	1.23E-26
FBXO3	8.45E-31	0.277559	0.203	0.041	1.26E-26
PCF11	9.35E-31	0.149955	0.215	0.06	1.40E-26
PTPN4	9.80E-31	0.383012	0.19	0.019	1.46E-26
TP53TG1	1.08E-30	-0.2949	0.15	0.094	1.61E-26
SYS1	1.08E-30	-0.42205	0.124	0.091	1.62E-26
PYCR2	1.10E-30	-0.24284	0.16	0.087	1.65E-26
CLASRP	1.11E-30	0.147029	0.186	0.043	1.67E-26
ASPSCR1	1.14E-30	0.249172	0.228	0.056	1.70E-26
WHSC1	1.15E-30	0.296462	0.205	0.038	1.72E-26
UBAC1	1.24E-30	0.183581	0.161	0.029	1.86E-26
PRPS1	1.30E-30	-0.22828	0.152	0.079	1.94E-26
REST	1.31E-30	0.277471	0.209	0.043	1.96E-26
RAB21	1.32E-30	0.178921	0.17	0.036	1.97E-26
AIDA	1.32E-30	-0.21596	0.148	0.075	1.98E-26
ZNF649	1.35E-30	0.314598	0.159	0.012	2.01E-26
GAL3ST4	1.35E-30	0.206157	0.165	0.032	2.02E-26
DUSP11	1.42E-30	0.120249	0.196	0.05	2.11E-26
SMIM8	1.42E-30	0.179218	0.159	0.027	2.12E-26
NUBP1	1.45E-30	0.273247	0.191	0.034	2.16E-26
SESN3	1.47E-30	0.282708	0.17	0.021	2.19E-26
CHMP6	1.48E-30	0.151067	0.168	0.034	2.22E-26
ZNF12	1.49E-30	0.191877	0.174	0.038	2.23E-26
BRINP2	1.58E-30	0.47281	0.178	0.01	2.36E-26
SPRY2	1.58E-30	-0.13949	0.174	0.087	2.37E-26
KLHL24	1.67E-30	0.146871	0.198	0.055	2.49E-26
AKAP13	1.72E-30	-0.10568	0.163	0.072	2.56E-26
APIP	1.75E-30	-0.14344	0.15	0.065	2.61E-26
NFYC	1.80E-30	-0.21327	0.181	0.101	2.70E-26
PLXNB1	1.90E-30	0.153757	0.161	0.029	2.84E-26
TMEM68	1.93E-30	0.191441	0.199	0.053	2.89E-26
RP11-571M	1.95E-30	0.329354	0.152	0.007	2.91E-26

MED8	1.95E-30	-0.18941	0.173	0.085	2.92E-26
IFI6	1.98E-30	-0.28581	0.282	0.198	2.96E-26
VAX2	2.00E-30	0.308427	0.15	0.01	3.00E-26
EMP1	2.02E-30	-1.02841	0.021	0.178	3.02E-26
TSTD1	2.04E-30	-0.5708	0.105	0.111	3.04E-26
ERCC3	2.07E-30	0.197562	0.176	0.038	3.09E-26
NISCH	2.17E-30	0.192719	0.168	0.032	3.24E-26
SIGMAR1	2.23E-30	0.202638	0.179	0.036	3.33E-26
RARA	2.23E-30	0.309247	0.157	0.014	3.33E-26
KIFAP3	2.24E-30	0.239597	0.187	0.039	3.35E-26
TRMT2A	2.30E-30	-0.15662	0.146	0.063	3.43E-26
LYPLAL1	2.38E-30	-0.15183	0.185	0.092	3.55E-26
ATP1B2	2.39E-30	-0.49951	0.152	0.15	3.57E-26
FAXC	2.45E-30	0.451439	0.182	0.012	3.66E-26
ISY1	2.49E-30	0.120299	0.186	0.05	3.72E-26
N6AMT2	2.49E-30	-0.16154	0.133	0.06	3.72E-26
FAM98A	2.53E-30	0.169629	0.179	0.041	3.78E-26
DUSP14	2.53E-30	-0.18024	0.126	0.053	3.78E-26
TYMS	2.62E-30	0.209258	0.168	0.032	3.91E-26
CEP57	2.74E-30	0.138654	0.208	0.062	4.10E-26
HDAC5	2.77E-30	0.22854	0.189	0.038	4.13E-26
WARS	2.78E-30	-0.58715	0.1	0.108	4.16E-26
KDM4B	2.83E-30	0.212938	0.19	0.041	4.23E-26
UXS1	2.94E-30	0.227458	0.172	0.031	4.39E-26
ANXA7	2.98E-30	-0.23433	0.183	0.109	4.46E-26
DESI2	3.00E-30	0.178274	0.178	0.036	4.48E-26
DSCR3	3.19E-30	-0.21327	0.14	0.065	4.77E-26
CEBPB	3.25E-30	-0.66263	0.104	0.145	4.85E-26
BROX	3.30E-30	0.131394	0.17	0.039	4.94E-26
REV1	3.32E-30	0.233412	0.194	0.039	4.96E-26
REL	3.37E-30	0.162729	0.187	0.044	5.03E-26
OGFR	3.43E-30	0.248215	0.211	0.044	5.12E-26
TANC1	3.53E-30	0.347253	0.189	0.026	5.27E-26
DLX6	3.64E-30	0.439759	0.143	0	5.44E-26
SCAF1	3.69E-30	0.238992	0.156	0.021	5.51E-26
KDSR	3.78E-30	0.153204	0.212	0.063	5.65E-26
CHCHD10	4.14E-30	-0.14204	0.174	0.082	6.19E-26
RCOR2	4.15E-30	0.476148	0.155	0.003	6.21E-26
KCTD20	4.33E-30	0.101081	0.169	0.043	6.47E-26
ZNF354A	4.40E-30	0.185785	0.15	0.021	6.58E-26
CKAP2	4.42E-30	0.293764	0.16	0.019	6.60E-26
FBXO25	4.47E-30	0.176592	0.161	0.029	6.69E-26
USP9X	4.52E-30	0.249286	0.191	0.038	6.76E-26
SERINC2	4.82E-30	0.364898	0.163	0.005	7.20E-26
AKAP12	5.05E-30	-0.58209	0.111	0.115	7.55E-26
REXO4	5.14E-30	0.206816	0.194	0.043	7.68E-26
EML4	5.52E-30	0.20083	0.178	0.036	8.25E-26
CCDC94	5.60E-30	0.138311	0.19	0.053	8.37E-26
DUOX1	5.63E-30	0.428199	0.156	0.002	8.41E-26
COL20A1	5.68E-30	0.472479	0.165	0.007	8.49E-26
RBFA	5.93E-30	0.119692	0.164	0.039	8.86E-26

STRN4	5.97E-30	0.289241	0.165	0.019	8.92E-26
CACUL1	6.04E-30	0.373843	0.194	0.026	9.02E-26
ARPC1B	6.05E-30	-0.18495	0.172	0.089	9.05E-26
UBALD1	6.21E-30	0.269377	0.179	0.027	9.28E-26
TMCC1	6.46E-30	0.440187	0.168	0.007	9.65E-26
MICAL1	6.89E-30	0.376699	0.161	0.009	1.03E-25
CC2D1A	7.04E-30	0.165324	0.195	0.05	1.05E-25
DNAJC18	7.16E-30	0.185173	0.177	0.038	1.07E-25
LETM1	7.69E-30	0.240353	0.196	0.039	1.15E-25
PPP1R35	8.20E-30	0.414361	0.205	0.026	1.23E-25
C12orf73	8.27E-30	0.164237	0.181	0.041	1.24E-25
AUTS2	8.58E-30	0.306011	0.166	0.019	1.28E-25
MLXIP	8.64E-30	0.201468	0.195	0.046	1.29E-25
AFG3L2	9.12E-30	0.166041	0.174	0.038	1.36E-25
TUBGCP4	9.38E-30	0.261644	0.191	0.032	1.40E-25
RAP2A	9.70E-30	0.296608	0.179	0.027	1.45E-25
SEC23A	1.04E-29	-0.14159	0.173	0.085	1.55E-25
FAM122A	1.09E-29	0.211927	0.156	0.024	1.63E-25
SMC1A	1.13E-29	0.158291	0.198	0.051	1.69E-25
LYRM9	1.14E-29	-0.11686	0.156	0.072	1.71E-25
GPCPD1	1.17E-29	0.285577	0.172	0.022	1.75E-25
TBC1D7	1.18E-29	0.167893	0.174	0.039	1.76E-25
CYP20A1	1.25E-29	-0.13084	0.181	0.084	1.86E-25
GBP1	1.28E-29	-1.1837	0.022	0.168	1.91E-25
PSEN1	1.30E-29	0.200038	0.179	0.039	1.94E-25
CDK16	1.31E-29	0.107405	0.19	0.06	1.96E-25
SSBP3	1.45E-29	0.242853	0.172	0.029	2.17E-25
GRIK3	1.47E-29	0.314241	0.15	0.009	2.20E-25
DPH7	1.48E-29	0.247334	0.183	0.034	2.21E-25
FGF9	1.52E-29	0.463647	0.172	0.01	2.27E-25
CHP1	1.54E-29	-0.10939	0.139	0.051	2.31E-25
PPP6R2	1.58E-29	0.409386	0.187	0.017	2.37E-25
CYTH1	1.59E-29	0.372676	0.166	0.012	2.38E-25
CASP3	1.65E-29	0.162125	0.186	0.051	2.47E-25
KCND2	1.76E-29	0.282986	0.144	0.01	2.63E-25
SCAP	1.83E-29	0.302154	0.178	0.024	2.74E-25
RHBDD3	1.84E-29	0.239345	0.186	0.036	2.75E-25
YRDC	1.87E-29	0.272724	0.203	0.043	2.79E-25
ZNF704	1.95E-29	0.342026	0.195	0.031	2.91E-25
ZBTB16	1.95E-29	0.203018	0.27	0.101	2.91E-25
NR2C2AP	1.97E-29	-0.2396	0.144	0.08	2.95E-25
RRP9	2.01E-29	0.168947	0.195	0.043	3.00E-25
YLPM1	2.05E-29	0.263529	0.172	0.027	3.07E-25
RGS1	2.10E-29	-0.1599	0.166	0.085	3.14E-25
TTC17	2.13E-29	0.146311	0.211	0.062	3.18E-25
TMED5	2.13E-29	-0.31197	0.152	0.091	3.19E-25
NMD3	2.18E-29	-0.1137	0.202	0.106	3.25E-25
ZNF721	2.19E-29	0.196648	0.186	0.043	3.28E-25
IRF3	2.24E-29	-0.31525	0.157	0.109	3.34E-25
GPSM1	2.25E-29	0.350397	0.159	0.01	3.37E-25
HIST1H1C	2.26E-29	-0.35186	0.143	0.082	3.37E-25

CTPS1	2.33E-29	0.240201	0.161	0.022	3.47E-25
LYPD1	2.49E-29	-0.3992	0.116	0.084	3.72E-25
KLHL35	2.73E-29	0.362993	0.168	0.017	4.08E-25
SRRM3	2.89E-29	0.430906	0.191	0.022	4.33E-25
FTSJ3	2.91E-29	0.211077	0.165	0.027	4.35E-25
NEIL2	2.97E-29	-0.10001	0.166	0.072	4.44E-25
ACOT13	3.00E-29	-0.20235	0.159	0.072	4.48E-25
RARRES3	3.00E-29	-1.14582	0.03	0.176	4.48E-25
POM121C	3.30E-29	0.265857	0.186	0.034	4.93E-25
SRR	3.32E-29	0.111397	0.169	0.044	4.96E-25
ELMO1	3.36E-29	0.128581	0.208	0.07	5.02E-25
THUMPD1	3.46E-29	0.145987	0.196	0.053	5.17E-25
ATP9A	3.56E-29	0.242424	0.165	0.029	5.32E-25
SLC30A5	3.59E-29	0.201883	0.198	0.044	5.37E-25
GATS	3.77E-29	0.342719	0.17	0.015	5.64E-25
BCL11A	3.78E-29	0.344241	0.146	0.005	5.65E-25
MTRNR2L1	3.89E-29	0.313393	0.381	0.174	5.81E-25
ZNF827	3.99E-29	0.327106	0.198	0.034	5.96E-25
HCFC1	3.99E-29	0.28418	0.168	0.022	5.97E-25
USP7	4.17E-29	0.361084	0.178	0.021	6.23E-25
URI1	4.17E-29	0.218171	0.204	0.051	6.23E-25
KLHL42	4.33E-29	0.133512	0.191	0.051	6.47E-25
HPS4	4.79E-29	0.112442	0.191	0.058	7.16E-25
DOHH	4.94E-29	0.285964	0.185	0.031	7.39E-25
ANKRD54	5.14E-29	0.251023	0.181	0.031	7.68E-25
LRRC4	5.17E-29	0.419671	0.147	0.002	7.73E-25
DYNLT3	5.20E-29	-0.50754	0.103	0.097	7.77E-25
DPYSL5	5.33E-29	0.167807	0.156	0.029	7.97E-25
KLF12	5.48E-29	0.382917	0.187	0.021	8.20E-25
DHFR	5.75E-29	0.151645	0.148	0.026	8.59E-25
RAMP2	5.90E-29	0.168742	0.183	0.05	8.82E-25
ADM	6.03E-29	-1.18629	0.046	0.152	9.00E-25
TNFRSF21	6.43E-29	0.316953	0.187	0.029	9.61E-25
KDM2A	6.53E-29	0.161171	0.157	0.027	9.76E-25
FKBP5	6.67E-29	-0.21991	0.213	0.137	9.97E-25
TUBA1C	6.68E-29	-0.73516	0.129	0.179	9.98E-25
AP5Z1	6.73E-29	0.311173	0.173	0.021	1.01E-24
LENG8	7.00E-29	0.102398	0.203	0.068	1.05E-24
U2AF1L4	7.22E-29	-0.45237	0.16	0.14	1.08E-24
EIF2B5	7.34E-29	0.106288	0.174	0.048	1.10E-24
PEX5	7.35E-29	0.195005	0.147	0.022	1.10E-24
IMPAD1	7.50E-29	0.152188	0.181	0.044	1.12E-24
TFPT	7.56E-29	-0.1487	0.161	0.079	1.13E-24
VEGFA	7.74E-29	-0.84647	0.077	0.157	1.16E-24
MAFF	7.74E-29	0.277923	0.209	0.048	1.16E-24
CDKN1A	8.22E-29	-0.68513	0.094	0.135	1.23E-24
ANKRD36	8.64E-29	0.204696	0.217	0.06	1.29E-24
TMEM101	8.82E-29	0.140542	0.189	0.051	1.32E-24
CEP41	8.85E-29	0.22267	0.174	0.034	1.32E-24
ZNF85	9.40E-29	0.174063	0.134	0.017	1.41E-24
ST6GAL2	9.87E-29	0.438599	0.165	0.007	1.47E-24

SNAPC5	9.97E-29	-0.10375	0.146	0.062	1.49E-24
RGL2	1.01E-28	0.140974	0.182	0.05	1.50E-24
SC5D	1.04E-28	-0.16423	0.16	0.079	1.55E-24
LENG1	1.04E-28	-0.22527	0.159	0.08	1.55E-24
MAZ	1.14E-28	0.193821	0.196	0.051	1.70E-24
RFWD2	1.14E-28	0.248845	0.161	0.024	1.70E-24
FAM45A	1.15E-28	0.150035	0.182	0.044	1.72E-24
NUDT22	1.17E-28	-0.17794	0.146	0.07	1.75E-24
AKTIP	1.18E-28	-0.10198	0.16	0.065	1.77E-24
TRABD	1.22E-28	0.272899	0.159	0.019	1.82E-24
HERC2	1.24E-28	0.271427	0.218	0.053	1.85E-24
SLAIN2	1.29E-28	0.329265	0.15	0.01	1.92E-24
UNC119	1.33E-28	0.129021	0.155	0.031	1.99E-24
GALNT1	1.34E-28	0.329604	0.163	0.015	2.01E-24
CDK2AP1	1.36E-28	0.312659	0.16	0.015	2.03E-24
IP6K1	1.37E-28	0.108057	0.183	0.05	2.04E-24
CPT1C	1.40E-28	0.139381	0.187	0.051	2.10E-24
FKBP14	1.48E-28	0.137079	0.14	0.024	2.22E-24
DVL2	1.54E-28	0.29324	0.205	0.043	2.30E-24
DEXI	1.58E-28	0.230645	0.181	0.034	2.37E-24
NFKBIL1	1.59E-28	0.253263	0.211	0.05	2.38E-24
SLC20A1	1.66E-28	0.154745	0.152	0.027	2.48E-24
TOR1AIP2	1.89E-28	0.121357	0.204	0.063	2.82E-24
HYI	1.95E-28	0.331009	0.183	0.026	2.91E-24
GLIPR2	1.96E-28	-0.49593	0.129	0.111	2.93E-24
PHLDB1	2.06E-28	0.210889	0.183	0.041	3.07E-24
DYRK4	2.08E-28	-0.20911	0.156	0.082	3.11E-24
NEDD4L	2.18E-28	0.332694	0.169	0.017	3.26E-24
RP11-126K	2.21E-28	0.246097	0.152	0.019	3.30E-24
UROS	2.24E-28	0.293444	0.182	0.029	3.35E-24
SH2B2	2.37E-28	0.35019	0.166	0.014	3.54E-24
CHPF2	2.41E-28	0.207364	0.169	0.032	3.60E-24
BAP1	2.48E-28	0.221295	0.173	0.034	3.70E-24
ZNF766	2.52E-28	0.187312	0.169	0.036	3.77E-24
CSNK1G3	2.64E-28	0.238934	0.165	0.026	3.95E-24
TSC2	2.64E-28	0.160746	0.182	0.046	3.95E-24
RDH14	2.65E-28	0.190096	0.207	0.056	3.95E-24
SLC35F1	2.69E-28	0.217132	0.173	0.031	4.02E-24
MAPK1	2.79E-28	0.184972	0.176	0.043	4.17E-24
HOMER3	2.80E-28	0.121776	0.157	0.039	4.18E-24
OGFOD3	2.82E-28	0.203816	0.166	0.031	4.22E-24
C1orf131	2.85E-28	-0.11181	0.179	0.087	4.25E-24
SMG1	2.85E-28	0.233695	0.179	0.036	4.26E-24
FAM217B	2.89E-28	0.287659	0.174	0.027	4.32E-24
NOL4	3.03E-28	0.328523	0.152	0.009	4.53E-24
TMX4	3.05E-28	0.305828	0.209	0.043	4.56E-24
FEN1	3.68E-28	0.231418	0.147	0.021	5.51E-24
C1S	3.71E-28	-1.05193	0.021	0.15	5.54E-24
ACOX1	3.79E-28	0.276976	0.146	0.014	5.66E-24
TLE1	3.93E-28	0.117185	0.192	0.06	5.87E-24
POLR2D	3.96E-28	0.166932	0.164	0.031	5.92E-24

HOXB4	3.98E-28	0.332884	0.137	0.005	5.94E-24
ATP1A2	4.24E-28	-0.71433	0.069	0.113	6.33E-24
MCUR1	4.30E-28	0.124705	0.176	0.048	6.42E-24
TMOD1	4.84E-28	-0.53163	0.127	0.123	7.23E-24
RP11-182L	4.97E-28	0.361404	0.159	0.009	7.42E-24
EXTL3	5.02E-28	0.132912	0.156	0.032	7.50E-24
CA8	5.19E-28	0.562049	0.181	0.014	7.75E-24
PSMG1	5.19E-28	-0.20861	0.16	0.085	7.75E-24
ANKS3	5.24E-28	0.243102	0.2	0.048	7.83E-24
MCM6	5.24E-28	0.2331	0.14	0.015	7.83E-24
NR2F1	5.26E-28	0.202387	0.178	0.041	7.86E-24
PLAT	5.45E-28	-0.21524	0.157	0.085	8.14E-24
ZFHX3	5.46E-28	0.15977	0.144	0.026	8.16E-24
TTF1	5.51E-28	0.242562	0.221	0.056	8.24E-24
ZNF566	5.54E-28	0.104734	0.185	0.06	8.28E-24
CLN5	5.85E-28	0.127466	0.177	0.046	8.75E-24
HOXB8	5.90E-28	0.383992	0.133	0	8.82E-24
CBX2	5.97E-28	0.356868	0.159	0.012	8.93E-24
SLC25A29	6.21E-28	0.289017	0.177	0.027	9.27E-24
PNMAL1	6.60E-28	0.25042	0.142	0.014	9.86E-24
PSMG4	6.72E-28	0.160808	0.189	0.051	1.00E-23
AAAS	6.78E-28	0.105318	0.225	0.08	1.01E-23
CASD1	6.83E-28	0.325573	0.157	0.015	1.02E-23
SGSM3	6.85E-28	0.205144	0.208	0.055	1.02E-23
ZNF286A	6.86E-28	0.268208	0.143	0.014	1.03E-23
RMND5A	7.00E-28	0.308116	0.182	0.029	1.05E-23
LINC0059(	8.06E-28	0.378347	0.14	0.003	1.21E-23
GCFC2	8.50E-28	0.269942	0.157	0.021	1.27E-23
RANGRF	8.60E-28	-0.17229	0.153	0.074	1.28E-23
CTBP2	8.87E-28	0.14325	0.155	0.031	1.33E-23
SEC14L1	9.02E-28	-0.41515	0.14	0.108	1.35E-23
HGSNAT	9.47E-28	0.249947	0.176	0.032	1.42E-23
SMPD4	9.48E-28	0.144685	0.135	0.019	1.42E-23
CASC4	9.91E-28	0.166207	0.187	0.046	1.48E-23
TSHZ1	1.01E-27	0.18142	0.126	0.014	1.51E-23
HGS	1.01E-27	0.224065	0.187	0.043	1.52E-23
CWC22	1.04E-27	0.139755	0.138	0.026	1.56E-23
TM9SF3	1.06E-27	0.289039	0.182	0.031	1.59E-23
ZNF358	1.11E-27	0.257884	0.177	0.031	1.65E-23
AP5M1	1.12E-27	0.201041	0.177	0.041	1.67E-23
MSH2	1.13E-27	0.290813	0.165	0.021	1.68E-23
HCG18	1.13E-27	0.302769	0.15	0.014	1.69E-23
NDP	1.16E-27	-0.10854	0.137	0.058	1.73E-23
CERS4	1.17E-27	0.18395	0.178	0.041	1.75E-23
FBXW11	1.19E-27	0.291182	0.173	0.026	1.77E-23
ZNF789	1.19E-27	0.287184	0.147	0.014	1.78E-23
RANBP2	1.20E-27	0.164265	0.155	0.029	1.79E-23
PTDSS2	1.21E-27	0.36505	0.181	0.021	1.80E-23
LSM12	1.26E-27	0.134858	0.17	0.038	1.89E-23
LARP1	1.27E-27	0.122246	0.199	0.063	1.90E-23
CD68	1.29E-27	-0.41675	0.112	0.089	1.92E-23

VHL	1.37E-27	0.280764	0.172	0.026	2.05E-23
VPS16	1.38E-27	0.153793	0.153	0.029	2.06E-23
TMEM198	1.43E-27	0.256533	0.161	0.024	2.13E-23
NDUFA7	1.44E-27	-1.01704	0.02	0.179	2.15E-23
BAZ2A	1.48E-27	0.155046	0.192	0.055	2.21E-23
RBM48	1.54E-27	0.260131	0.163	0.022	2.30E-23
MAD2L1	1.57E-27	0.146295	0.143	0.027	2.35E-23
GINS2	1.59E-27	0.281684	0.165	0.026	2.37E-23
PICALM	1.62E-27	0.139506	0.152	0.031	2.42E-23
IRS2	1.67E-27	-0.34701	0.181	0.132	2.49E-23
ANKFY1	1.68E-27	0.113537	0.131	0.024	2.52E-23
AP006222.	1.71E-27	-0.3411	0.117	0.08	2.55E-23
AKAP17A	1.73E-27	0.339752	0.168	0.017	2.58E-23
ZNF300	1.74E-27	0.363861	0.16	0.01	2.60E-23
MLC1	1.76E-27	-0.14657	0.139	0.068	2.63E-23
CASK	1.78E-27	0.167707	0.176	0.044	2.66E-23
RP11-71N1	1.83E-27	-0.45963	0.124	0.123	2.74E-23
AGAP3	1.92E-27	0.326792	0.143	0.009	2.86E-23
SH3KBP1	1.92E-27	0.160355	0.151	0.029	2.88E-23
ASH2L	1.94E-27	0.253721	0.176	0.032	2.90E-23
MMP24-AS1	2.08E-27	0.105533	0.178	0.053	3.10E-23
POLR3D	2.08E-27	0.241627	0.183	0.038	3.11E-23
LINGO1	2.17E-27	0.350288	0.148	0.012	3.24E-23
ETFDH	2.32E-27	0.125603	0.204	0.063	3.46E-23
ZP3	2.42E-27	0.193633	0.137	0.021	3.62E-23
CHRAC1	2.44E-27	0.268255	0.155	0.021	3.65E-23
ZFP14	2.46E-27	0.232175	0.16	0.027	3.67E-23
INTS4	2.50E-27	0.162857	0.139	0.022	3.73E-23
CAMSAP2	2.53E-27	0.203825	0.173	0.034	3.78E-23
AKAP1	2.57E-27	0.195033	0.152	0.027	3.83E-23
NRSN2	2.60E-27	0.175724	0.146	0.024	3.88E-23
NFATC2IP	2.64E-27	0.244552	0.163	0.027	3.95E-23
SENP6	2.66E-27	0.284694	0.195	0.043	3.98E-23
ARNT2	2.69E-27	0.324626	0.165	0.017	4.03E-23
ALG8	2.71E-27	-0.15543	0.124	0.055	4.05E-23
UBE2W	2.82E-27	0.173093	0.177	0.043	4.21E-23
PDE9A	2.86E-27	0.444813	0.185	0.017	4.28E-23
FAF2	2.86E-27	0.277259	0.205	0.046	4.28E-23
TMEM5	2.90E-27	-0.13162	0.157	0.08	4.34E-23
FRMD4A	2.95E-27	0.320063	0.166	0.024	4.41E-23
DDX19A	3.15E-27	0.1577	0.172	0.038	4.71E-23
ANKRD13D	3.27E-27	0.19657	0.159	0.031	4.88E-23
TPMT	3.38E-27	-0.22865	0.122	0.06	5.05E-23
GGT7	3.38E-27	0.21254	0.174	0.036	5.05E-23
RBMXL1	3.39E-27	0.166899	0.177	0.043	5.06E-23
AMN1	3.40E-27	0.157156	0.142	0.027	5.08E-23
ARMCX5	3.43E-27	0.170912	0.137	0.019	5.12E-23
SLN	3.49E-27	-1.23554	0.008	0.13	5.21E-23
AFF3	3.63E-27	0.4516	0.156	0.007	5.42E-23
PLEKHH2	3.66E-27	0.383521	0.172	0.017	5.47E-23
GPATCH11	3.70E-27	0.347399	0.174	0.024	5.53E-23

NAB1	3.82E-27	0.267668	0.148	0.017	5.71E-23
ARVCF	4.06E-27	0.219339	0.124	0.009	6.07E-23
ZNF639	4.12E-27	0.350989	0.17	0.021	6.15E-23
TJAP1	4.12E-27	0.271315	0.151	0.015	6.16E-23
ROCK2	4.15E-27	0.164232	0.152	0.029	6.21E-23
TYW1	4.17E-27	0.289513	0.166	0.022	6.24E-23
CD200	4.20E-27	0.210128	0.127	0.012	6.27E-23
SECISBP2L	4.29E-27	0.113864	0.146	0.032	6.41E-23
HSPA2	4.34E-27	0.185876	0.229	0.08	6.48E-23
RPP38	4.45E-27	0.247712	0.148	0.019	6.65E-23
TBL2	4.65E-27	0.233284	0.189	0.044	6.95E-23
IGLON5	4.77E-27	0.381015	0.146	0.003	7.12E-23
SAC3D1	4.88E-27	0.187708	0.153	0.026	7.29E-23
TNPO2	4.99E-27	0.116182	0.202	0.063	7.46E-23
CDKAL1	5.22E-27	0.22008	0.135	0.017	7.79E-23
STXBP1	5.26E-27	0.205967	0.155	0.029	7.86E-23
AC114730.	5.37E-27	0.393083	0.17	0.022	8.03E-23
CAMSAP1	5.82E-27	0.257366	0.127	0.01	8.70E-23
CDC42SE2	5.94E-27	0.132631	0.157	0.039	8.87E-23
CDK10	6.12E-27	-0.14248	0.161	0.084	9.14E-23
MGP	6.13E-27	-1.29342	0.016	0.128	9.16E-23
ELMOD3	6.14E-27	0.164078	0.169	0.039	9.18E-23
ITFG2	6.14E-27	0.195411	0.157	0.027	9.18E-23
SPRY1	6.23E-27	-0.5335	0.108	0.125	9.31E-23
THAP4	6.28E-27	0.316821	0.161	0.019	9.39E-23
ATG4D	6.31E-27	0.246825	0.165	0.027	9.43E-23
NF2	6.59E-27	0.170265	0.147	0.026	9.84E-23
PTPN2	6.60E-27	-0.10997	0.142	0.065	9.87E-23
RAB35	6.67E-27	0.238455	0.169	0.031	9.97E-23
ZNF274	6.69E-27	0.261865	0.168	0.029	1.00E-22
POGK	6.84E-27	0.352533	0.151	0.01	1.02E-22
EPHB3	6.92E-27	0.203945	0.116	0.009	1.03E-22
TMEM181	7.09E-27	0.244542	0.155	0.022	1.06E-22
RPRM	7.25E-27	0.263342	0.148	0.019	1.08E-22
EHD1	7.41E-27	0.364788	0.176	0.019	1.11E-22
CBX4	7.77E-27	0.29547	0.169	0.026	1.16E-22
EFHD2	7.81E-27	0.262497	0.202	0.051	1.17E-22
MAPKAPK5	7.89E-27	0.355787	0.192	0.031	1.18E-22
DPF3	7.98E-27	0.206006	0.177	0.039	1.19E-22
PCYOX1	8.13E-27	0.116582	0.176	0.051	1.22E-22
CDV3	8.46E-27	0.242708	0.183	0.039	1.26E-22
CLINT1	9.56E-27	0.1134	0.159	0.039	1.43E-22
ZNF7	9.65E-27	0.285812	0.212	0.05	1.44E-22
LUZP2	9.73E-27	0.369642	0.16	0.015	1.45E-22
TRAPPC6B	9.90E-27	0.115916	0.155	0.038	1.48E-22
LMBR1	1.01E-26	0.3677	0.166	0.017	1.51E-22
ARHGEF1	1.02E-26	0.12975	0.174	0.05	1.52E-22
PWWP2A	1.02E-26	0.267948	0.17	0.029	1.52E-22
MTMR14	1.04E-26	0.1437	0.156	0.036	1.55E-22
KIF3C	1.06E-26	0.239661	0.152	0.026	1.58E-22
TMEM132A	1.12E-26	0.107201	0.15	0.038	1.68E-22

NLN	1.15E-26	0.196398	0.166	0.036	1.71E-22
NHSL1	1.16E-26	0.2245	0.159	0.027	1.73E-22
ANKZF1	1.17E-26	0.122272	0.164	0.043	1.75E-22
PDXDC1	1.18E-26	0.210844	0.172	0.034	1.76E-22
FNIP2	1.19E-26	0.19128	0.173	0.041	1.77E-22
GRIPAP1	1.28E-26	0.208754	0.2	0.051	1.91E-22
CDH11	1.32E-26	0.139141	0.168	0.044	1.97E-22
BCAT1	1.34E-26	-0.52305	0.1	0.109	2.00E-22
API5	1.36E-26	0.111076	0.189	0.055	2.03E-22
CDK5R1	1.37E-26	0.309402	0.146	0.012	2.04E-22
TCF3	1.38E-26	0.212373	0.152	0.024	2.07E-22
FAM57B	1.41E-26	0.437774	0.185	0.024	2.11E-22
VPS53	1.50E-26	0.206002	0.172	0.034	2.24E-22
KDM4A	1.50E-26	0.178869	0.153	0.029	2.24E-22
ASXL1	1.52E-26	0.236859	0.194	0.05	2.27E-22
KCNIP1	1.53E-26	-0.38964	0.108	0.082	2.28E-22
EEF1G	1.53E-26	-0.13582	0.131	0.063	2.29E-22
MCAT	1.54E-26	0.386954	0.161	0.014	2.31E-22
NOM1	1.63E-26	0.22968	0.159	0.027	2.44E-22
PMEPA1	1.65E-26	0.183608	0.148	0.026	2.46E-22
AASS	1.65E-26	0.173359	0.202	0.062	2.47E-22
DCK	1.68E-26	0.365608	0.165	0.015	2.52E-22
CSPG4	1.71E-26	0.319764	0.144	0.009	2.55E-22
3-Sep	1.76E-26	0.173208	0.124	0.015	2.63E-22
AGPS	1.79E-26	0.262835	0.14	0.014	2.68E-22
TIMM21	1.79E-26	-0.12894	0.125	0.043	2.68E-22
NSMCE4A	1.80E-26	0.189994	0.142	0.022	2.69E-22
TFRC	1.92E-26	-0.15408	0.125	0.056	2.87E-22
NCK1	1.96E-26	-0.26157	0.153	0.094	2.93E-22
WRAP73	1.98E-26	0.153277	0.152	0.036	2.96E-22
SOCS3	2.01E-26	-0.41432	0.152	0.132	3.01E-22
LEO1	2.07E-26	0.103183	0.172	0.046	3.09E-22
ZNF786	2.07E-26	0.240825	0.147	0.021	3.10E-22
TOMM70A	2.11E-26	0.284311	0.146	0.014	3.16E-22
TNFAIP6	2.12E-26	-0.76997	0.079	0.123	3.17E-22
UGGT1	2.26E-26	0.188516	0.146	0.026	3.37E-22
COL6A1	2.37E-26	-0.32329	0.143	0.104	3.55E-22
COASY	2.39E-26	-0.16903	0.146	0.077	3.57E-22
NKD1	2.52E-26	0.395328	0.134	0.002	3.77E-22
AP1B1	2.63E-26	0.167794	0.164	0.038	3.93E-22
CSAD	2.68E-26	0.206196	0.165	0.034	4.01E-22
DNAJC11	2.71E-26	0.150733	0.152	0.034	4.04E-22
PPP4R1	2.72E-26	0.138155	0.117	0.019	4.07E-22
GSN	2.80E-26	-0.66628	0.092	0.126	4.19E-22
DACT3	2.82E-26	0.277367	0.151	0.017	4.21E-22
PMS1	2.83E-26	0.127771	0.143	0.027	4.24E-22
ATAD1	2.84E-26	0.17121	0.173	0.044	4.24E-22
STARD3	2.88E-26	0.180089	0.191	0.05	4.31E-22
AK4	2.92E-26	-0.47804	0.101	0.089	4.36E-22
ZNF263	3.13E-26	0.252939	0.161	0.026	4.67E-22
ATF6	3.19E-26	0.198428	0.179	0.044	4.77E-22

ZNF212	3.26E-26	0.150105	0.138	0.024	4.87E-22
PCDHB2	3.34E-26	0.353901	0.163	0.012	4.99E-22
CARS	3.42E-26	-0.19661	0.16	0.087	5.12E-22
TIAM2	3.46E-26	0.121349	0.126	0.022	5.17E-22
PUS7L	3.52E-26	0.201462	0.174	0.036	5.27E-22
TRIM4	3.68E-26	0.265132	0.17	0.029	5.49E-22
LINC01102	3.70E-26	0.29301	0.15	0.015	5.53E-22
ELF2	3.74E-26	0.224117	0.19	0.041	5.58E-22
RNFT1	3.76E-26	0.149541	0.148	0.032	5.62E-22
STX16	3.93E-26	0.107163	0.196	0.063	5.87E-22
SYNE1	3.95E-26	0.111904	0.159	0.034	5.90E-22
E2F6	3.98E-26	0.168706	0.144	0.027	5.95E-22
EDEM2	4.35E-26	-0.17079	0.14	0.07	6.50E-22
FNDC3A	4.51E-26	0.113093	0.165	0.048	6.74E-22
SMAP2	4.58E-26	0.214096	0.139	0.019	6.84E-22
GIGYF1	4.72E-26	0.319111	0.186	0.032	7.05E-22
GSKIP	4.87E-26	0.133613	0.152	0.034	7.28E-22
ZNF100	4.90E-26	0.215931	0.147	0.021	7.32E-22
FUT8	5.18E-26	0.236909	0.138	0.019	7.75E-22
GLI4	5.58E-26	0.246978	0.164	0.029	8.35E-22
RAB40B	5.73E-26	0.276096	0.135	0.012	8.56E-22
MTSS1L	5.76E-26	0.106596	0.168	0.05	8.61E-22
NT5DC2	5.81E-26	0.150696	0.146	0.031	8.68E-22
DNAJC30	6.14E-26	0.275945	0.194	0.041	9.17E-22
EGLN1	6.14E-26	0.102152	0.17	0.048	9.17E-22
OSER1-AS1	6.37E-26	0.194974	0.172	0.036	9.52E-22
NCAPH2	6.58E-26	0.109275	0.129	0.027	9.84E-22
VAC14	6.60E-26	0.198256	0.134	0.017	9.87E-22
FAM104B	6.74E-26	0.178584	0.168	0.039	1.01E-21
RBM7	6.85E-26	-0.31335	0.139	0.091	1.02E-21
TNKS	6.85E-26	0.34886	0.178	0.026	1.02E-21
ANKLE2	7.03E-26	0.255988	0.182	0.036	1.05E-21
DMWD	7.08E-26	0.341314	0.187	0.029	1.06E-21
DLX6-AS1	7.65E-26	0.596199	0.16	0.01	1.14E-21
SSSCA1	7.65E-26	-0.1446	0.116	0.048	1.14E-21
DNAJB14	7.73E-26	0.141037	0.178	0.048	1.16E-21
C17orf58	7.85E-26	0.329969	0.174	0.027	1.17E-21
ANKRD35	8.39E-26	0.189937	0.134	0.017	1.25E-21
ABCG1	8.50E-26	0.368091	0.147	0.009	1.27E-21
ZNF253	8.97E-26	0.287531	0.151	0.019	1.34E-21
ORC6	9.30E-26	0.375337	0.144	0.01	1.39E-21
NAA16	9.67E-26	0.151092	0.142	0.027	1.45E-21
ARHGAP1	9.69E-26	0.101699	0.172	0.053	1.45E-21
CTR9	9.96E-26	0.148412	0.153	0.029	1.49E-21
HOXA5	1.00E-25	0.25864	0.137	0.014	1.49E-21
R3HDM4	1.00E-25	0.206401	0.173	0.036	1.50E-21
CYB5R1	1.01E-25	-0.29015	0.151	0.096	1.51E-21
CCDC137	1.04E-25	0.233215	0.172	0.034	1.56E-21
C12orf45	1.06E-25	-0.12228	0.142	0.063	1.59E-21
PKN2	1.06E-25	0.114642	0.169	0.044	1.59E-21
EHMT1	1.10E-25	0.214782	0.156	0.027	1.65E-21

TRAFD1	1.14E-25	0.164565	0.15	0.031	1.70E-21
SRD5A3	1.17E-25	0.237865	0.144	0.021	1.75E-21
CACFD1	1.18E-25	0.216811	0.127	0.014	1.76E-21
CTSK	1.21E-25	0.182285	0.124	0.014	1.81E-21
TNFRSF12A	1.23E-25	-0.81727	0.06	0.142	1.83E-21
OXLD1	1.24E-25	0.101943	0.191	0.062	1.86E-21
SNCAIP	1.24E-25	0.3153	0.17	0.024	1.86E-21
PAK7	1.26E-25	0.372645	0.144	0.005	1.88E-21
ST5	1.31E-25	-0.25657	0.129	0.072	1.95E-21
REEP1	1.32E-25	0.336888	0.137	0.005	1.98E-21
MIEF1	1.33E-25	0.156503	0.178	0.048	1.99E-21
RSAD1	1.33E-25	0.1699	0.151	0.029	1.99E-21
RNF2	1.34E-25	0.284209	0.164	0.024	2.00E-21
ZNF140	1.37E-25	0.217666	0.152	0.026	2.05E-21
SLC25A25	1.39E-25	0.105175	0.165	0.046	2.07E-21
STAT1	1.39E-25	-0.25565	0.147	0.091	2.08E-21
FARS2	1.44E-25	-0.10755	0.116	0.038	2.15E-21
GPKOW	1.50E-25	0.106223	0.151	0.038	2.25E-21
BTBD10	1.54E-25	0.134089	0.138	0.031	2.30E-21
LRRCC1	1.54E-25	0.176945	0.147	0.027	2.30E-21
C11orf54	1.59E-25	0.160594	0.147	0.029	2.37E-21
ARPP21	1.59E-25	0.352473	0.147	0.014	2.38E-21
ZNF544	1.63E-25	-0.19173	0.134	0.07	2.43E-21
DENND5B	1.64E-25	0.109263	0.113	0.019	2.45E-21
OR4N2	1.72E-25	0.460038	0.121	0	2.57E-21
GPR82	1.75E-25	0.286143	0.169	0.027	2.61E-21
C1QTNF4	1.83E-25	0.311656	0.143	0.01	2.74E-21
SNX14	1.86E-25	0.116561	0.164	0.043	2.79E-21
USP15	1.87E-25	0.322961	0.229	0.06	2.79E-21
PNPLA2	1.91E-25	0.204403	0.164	0.032	2.85E-21
PIGS	1.95E-25	0.178974	0.163	0.038	2.92E-21
CREBBP	2.04E-25	0.132333	0.147	0.032	3.04E-21
ZCCHC3	2.09E-25	0.182656	0.129	0.017	3.12E-21
STX18	2.10E-25	0.129442	0.155	0.038	3.13E-21
CNOT11	2.12E-25	0.254555	0.131	0.012	3.16E-21
BMI1	2.12E-25	0.227695	0.143	0.019	3.16E-21
NDRG1	2.12E-25	-0.93425	0.061	0.138	3.16E-21
USP33	2.12E-25	0.18699	0.174	0.041	3.17E-21
CNOT1	2.27E-25	0.187299	0.15	0.027	3.38E-21
CEP350	2.27E-25	0.150586	0.155	0.036	3.39E-21
GSE1	2.39E-25	0.406969	0.164	0.017	3.57E-21
SFXN5	2.42E-25	-0.30711	0.126	0.087	3.62E-21
DGCR6	2.46E-25	0.1035	0.169	0.048	3.67E-21
RNF14	2.48E-25	0.163107	0.164	0.041	3.71E-21
CASP6	2.50E-25	0.144519	0.131	0.029	3.73E-21
SAAL1	2.72E-25	0.107448	0.151	0.039	4.06E-21
PCDH15	2.72E-25	0.444534	0.134	0.003	4.07E-21
ZNF276	2.73E-25	0.197745	0.15	0.027	4.09E-21
ARID1B	2.84E-25	0.33737	0.155	0.015	4.24E-21
SMAD1	2.87E-25	0.193517	0.178	0.046	4.30E-21
TMEM169	3.02E-25	0.282196	0.146	0.015	4.51E-21

TPD52	3.09E-25	-0.34607	0.153	0.113	4.62E-21
KLHL20	3.21E-25	0.230938	0.173	0.036	4.80E-21
PLEKHB1	3.26E-25	-0.27309	0.143	0.094	4.87E-21
NELFB	3.31E-25	0.273439	0.163	0.022	4.95E-21
APTX	3.38E-25	0.246448	0.157	0.027	5.05E-21
ITSN2	3.64E-25	0.309102	0.146	0.014	5.43E-21
SMAD9	3.67E-25	0.226667	0.118	0.009	5.48E-21
NDRG3	3.67E-25	0.184608	0.165	0.038	5.49E-21
PTGR1	3.73E-25	-0.28037	0.1	0.065	5.57E-21
UBN1	4.43E-25	0.342624	0.16	0.017	6.61E-21
METTL7A	4.57E-25	-0.36188	0.164	0.132	6.83E-21
KAT6B	4.69E-25	0.292992	0.182	0.032	7.01E-21
SPHK2	4.80E-25	0.247126	0.172	0.034	7.17E-21
GPR108	4.84E-25	-0.20208	0.151	0.087	7.23E-21
CNTLN	4.97E-25	0.187373	0.142	0.024	7.42E-21
IREB2	5.24E-25	0.27108	0.161	0.024	7.83E-21
AGPAT5	5.30E-25	-0.2298	0.151	0.091	7.92E-21
R3HDM1	5.32E-25	0.119788	0.144	0.034	7.96E-21
CDH13	5.43E-25	0.144809	0.179	0.051	8.11E-21
CAMKMT	5.54E-25	0.370109	0.159	0.019	8.28E-21
CMIP	5.61E-25	0.142829	0.138	0.026	8.38E-21
PDZD4	5.70E-25	0.210992	0.117	0.01	8.52E-21
C1orf216	5.71E-25	0.234584	0.161	0.029	8.53E-21
PITPNB	5.87E-25	0.117063	0.166	0.044	8.76E-21
HOXA3	5.89E-25	0.272773	0.131	0.01	8.80E-21
PTTG1	5.91E-25	-0.16257	0.126	0.062	8.83E-21
WDR26	6.04E-25	0.126784	0.153	0.038	9.03E-21
RNF168	6.12E-25	0.277014	0.157	0.021	9.15E-21
DIRAS3	6.17E-25	-0.96866	0.035	0.162	9.21E-21
STT3B	6.21E-25	0.198782	0.185	0.05	9.27E-21
CTNNA2	6.40E-25	0.173699	0.161	0.039	9.57E-21
DIP2B	6.51E-25	0.270096	0.147	0.019	9.73E-21
BOLA1	6.53E-25	0.163381	0.15	0.034	9.76E-21
RAB3A	6.64E-25	0.196972	0.169	0.043	9.92E-21
PPP2R5B	6.92E-25	0.259193	0.172	0.029	1.03E-20
MECP2	6.97E-25	-0.11408	0.144	0.065	1.04E-20
SLC30A7	6.99E-25	0.169489	0.144	0.027	1.04E-20
CLEC11A	7.05E-25	0.354104	0.211	0.048	1.05E-20
CEP95	7.14E-25	0.204048	0.168	0.034	1.07E-20
YPEL3	7.20E-25	0.284942	0.229	0.07	1.08E-20
SVIP	7.27E-25	0.245247	0.12	0.007	1.09E-20
OTUD5	7.27E-25	0.125484	0.13	0.024	1.09E-20
MIOS	7.28E-25	0.180542	0.131	0.021	1.09E-20
ANAPC10	7.42E-25	-0.21371	0.134	0.075	1.11E-20
NNMT	7.54E-25	-1.04893	0.027	0.13	1.13E-20
TCEB3	7.76E-25	0.160044	0.16	0.038	1.16E-20
C8orf82	7.81E-25	0.298296	0.173	0.031	1.17E-20
SPIN1	7.86E-25	0.157379	0.151	0.032	1.17E-20
CHRDL1	7.99E-25	0.254959	0.107	0.002	1.19E-20
EID2	8.19E-25	0.192327	0.131	0.019	1.22E-20
EPHB1	8.88E-25	0.331675	0.139	0.009	1.33E-20

PCOLCE2	9.24E-25	-0.1924	0.108	0.056	1.38E-20
STRADB	1.01E-24	0.286861	0.178	0.034	1.51E-20
RNF8	1.02E-24	0.106794	0.137	0.031	1.53E-20
WLS	1.06E-24	-0.14483	0.174	0.094	1.59E-20
B3GAT1	1.07E-24	0.1164	0.129	0.026	1.60E-20
RALGDS	1.07E-24	0.340357	0.177	0.027	1.60E-20
RNF20	1.10E-24	0.191693	0.166	0.038	1.64E-20
POLD4	1.12E-24	-0.40976	0.124	0.108	1.67E-20
KAZN	1.14E-24	0.169311	0.127	0.019	1.70E-20
EIF3J-AS1	1.14E-24	0.137509	0.152	0.038	1.71E-20
NR2F6	1.18E-24	0.237666	0.15	0.024	1.76E-20
LMAN2L	1.20E-24	0.13546	0.143	0.034	1.79E-20
MSRB2	1.22E-24	0.412465	0.191	0.032	1.82E-20
PRR3	1.23E-24	0.172557	0.122	0.017	1.83E-20
SEMA4C	1.28E-24	0.250723	0.134	0.015	1.92E-20
MSX1	1.35E-24	0.169033	0.137	0.024	2.01E-20
PHYKPL	1.35E-24	0.14724	0.166	0.046	2.02E-20
XAB2	1.41E-24	0.136693	0.185	0.056	2.10E-20
ACTR3B	1.43E-24	0.240116	0.148	0.022	2.13E-20
LIPT1	1.49E-24	0.128182	0.114	0.017	2.23E-20
CASP9	1.53E-24	0.538238	0.19	0.026	2.28E-20
RAB11B-AS	1.63E-24	0.217009	0.147	0.024	2.43E-20
SPAST	1.68E-24	0.360492	0.146	0.01	2.51E-20
PRKCA	1.69E-24	0.259218	0.156	0.024	2.52E-20
C15orf40	1.70E-24	-0.28705	0.121	0.075	2.54E-20
MVB12B	1.79E-24	0.221615	0.127	0.014	2.68E-20
SLC1A2	1.84E-24	-0.33797	0.143	0.103	2.75E-20
MED16	1.86E-24	0.241325	0.155	0.024	2.78E-20
SLC38A7	1.88E-24	0.283955	0.161	0.022	2.82E-20
PRPF3	1.93E-24	0.10288	0.13	0.029	2.88E-20
EXT2	1.93E-24	0.228863	0.156	0.029	2.89E-20
MFAP1	2.01E-24	-0.10621	0.143	0.067	3.00E-20
CCL2	2.06E-24	-0.90793	0.039	0.08	3.08E-20
GTPBP1	2.07E-24	0.307734	0.147	0.015	3.09E-20
DCPS	2.07E-24	-0.19046	0.127	0.063	3.10E-20
TMEM132B	2.17E-24	0.26044	0.164	0.029	3.24E-20
TFB2M	2.17E-24	0.169767	0.126	0.019	3.24E-20
NANS	2.17E-24	-0.17512	0.152	0.082	3.25E-20
PTPN18	2.20E-24	0.178336	0.138	0.026	3.29E-20
GMPS	2.20E-24	0.23945	0.151	0.024	3.29E-20
GMPR2	2.28E-24	-0.13818	0.159	0.08	3.41E-20
D2HGDH	2.31E-24	0.335097	0.151	0.015	3.45E-20
PRKAG2	2.35E-24	0.384477	0.164	0.019	3.51E-20
CITED1	2.46E-24	-0.53845	0.072	0.089	3.67E-20
OBSL1	2.50E-24	0.113294	0.192	0.07	3.73E-20
P2RX7	2.52E-24	0.270307	0.139	0.014	3.76E-20
IMPDH1	2.53E-24	0.267242	0.137	0.012	3.77E-20
DCHS1	2.81E-24	0.203505	0.126	0.014	4.20E-20
AC113189.	2.82E-24	0.163268	0.135	0.027	4.22E-20
CAP2	3.07E-24	0.16473	0.107	0.009	4.59E-20
LINC00152	3.07E-24	-0.98285	0.031	0.168	4.59E-20

BCR	3.14E-24	0.327277	0.159	0.024	4.70E-20
WDR47	3.18E-24	0.218877	0.134	0.019	4.75E-20
TCTN1	3.33E-24	-0.10563	0.157	0.082	4.98E-20
PATZ1	3.35E-24	0.339176	0.146	0.01	5.00E-20
CDKL3	3.40E-24	0.125317	0.137	0.031	5.08E-20
SORBS2	3.43E-24	0.293021	0.121	0.007	5.12E-20
SARNP	3.55E-24	-0.1259	0.137	0.063	5.31E-20
ARL13B	3.58E-24	-0.30085	0.098	0.055	5.35E-20
TEAD1	3.58E-24	0.262061	0.156	0.026	5.35E-20
PLSCR1	3.60E-24	-0.65329	0.073	0.118	5.39E-20
TFAP2A	3.64E-24	0.366978	0.127	0.002	5.44E-20
GLB1	3.68E-24	-0.18871	0.144	0.079	5.50E-20
KDM2B	3.81E-24	0.262357	0.138	0.015	5.70E-20
ZBTB10	3.81E-24	0.264341	0.146	0.019	5.70E-20
MEN1	3.92E-24	0.15852	0.135	0.022	5.86E-20
ZCCHC7	3.98E-24	0.25258	0.166	0.031	5.95E-20
FBXW7	4.05E-24	0.351149	0.168	0.021	6.05E-20
DHX57	4.05E-24	0.357979	0.173	0.024	6.05E-20
YY1AP1	4.11E-24	0.11084	0.146	0.031	6.14E-20
TNFAIP1	4.18E-24	0.204349	0.161	0.032	6.25E-20
ZFPM2	4.19E-24	0.460668	0.139	0.005	6.26E-20
SEC22C	4.30E-24	-0.12891	0.155	0.068	6.42E-20
ADAM9	4.31E-24	-0.13747	0.163	0.087	6.44E-20
ABHD11	4.50E-24	0.268781	0.17	0.032	6.73E-20
AGTRAP	4.69E-24	-0.8004	0.052	0.121	7.00E-20
DUS3L	4.71E-24	0.161184	0.161	0.039	7.04E-20
SPRTN	4.80E-24	0.106622	0.152	0.038	7.17E-20
DCLK1	4.82E-24	0.149495	0.143	0.031	7.20E-20
NSUN6	4.87E-24	0.303724	0.164	0.024	7.28E-20
RCBTB2	4.99E-24	0.218267	0.142	0.022	7.45E-20
UBXN7	5.00E-24	0.252637	0.159	0.026	7.48E-20
BARD1	5.16E-24	0.235207	0.15	0.026	7.70E-20
C19orf52	5.49E-24	0.186765	0.139	0.026	8.21E-20
RHOG	6.07E-24	-0.15721	0.108	0.05	9.07E-20
PREP	6.33E-24	0.319939	0.133	0.007	9.47E-20
CACNG7	6.34E-24	0.214768	0.15	0.026	9.47E-20
ATAD3A	6.41E-24	0.253306	0.17	0.031	9.58E-20
F3	6.85E-24	-0.42445	0.113	0.108	1.02E-19
MED1	6.90E-24	0.220861	0.139	0.019	1.03E-19
LRRC8B	7.04E-24	0.164326	0.121	0.017	1.05E-19
RNF219	7.06E-24	0.167647	0.137	0.027	1.06E-19
FTO	7.30E-24	0.224675	0.146	0.024	1.09E-19
C7orf43	7.39E-24	0.278978	0.117	0.003	1.10E-19
TP53RK	7.47E-24	0.1378	0.155	0.038	1.12E-19
PIGP	7.83E-24	-0.10948	0.134	0.06	1.17E-19
GPATCH2	7.91E-24	-0.13604	0.143	0.062	1.18E-19
C7orf60	7.95E-24	0.154478	0.126	0.017	1.19E-19
RAD17	8.04E-24	0.111197	0.131	0.026	1.20E-19
CCNDBP1	8.63E-24	0.137739	0.139	0.032	1.29E-19
GABBR1	8.64E-24	0.310417	0.153	0.021	1.29E-19
CLIP1	8.81E-24	-0.21592	0.129	0.077	1.32E-19

NOMO1	9.04E-24	0.223064	0.131	0.017	1.35E-19
COG5	9.06E-24	0.186143	0.139	0.024	1.35E-19
GAK	9.07E-24	0.127384	0.13	0.027	1.35E-19
PIGM	9.66E-24	0.136118	0.135	0.029	1.44E-19
DDAH1	9.95E-24	-0.1655	0.121	0.062	1.49E-19
CDCA7	1.05E-23	0.200216	0.16	0.038	1.58E-19
ATF7	1.06E-23	-0.20279	0.099	0.044	1.59E-19
CAST	1.06E-23	-0.34943	0.126	0.092	1.59E-19
GPR89A	1.06E-23	0.106954	0.152	0.046	1.59E-19
KIF22	1.14E-23	-0.18735	0.157	0.084	1.71E-19
SAA1	1.17E-23	-1.37276	0.01	0.121	1.75E-19
HLA-DMA	1.18E-23	-0.53549	0.098	0.111	1.76E-19
TMEM80	1.18E-23	0.12616	0.151	0.041	1.76E-19
ZNF362	1.26E-23	0.200503	0.124	0.015	1.88E-19
ETNK1	1.31E-23	0.197002	0.173	0.043	1.96E-19
ST6GAL1	1.34E-23	0.342885	0.131	0.005	2.01E-19
POMT1	1.36E-23	0.21623	0.176	0.041	2.03E-19
PLA2G5	1.39E-23	-1.06795	0.018	0.159	2.08E-19
TRIM16	1.41E-23	0.141577	0.127	0.026	2.11E-19
TFAM	1.46E-23	0.101288	0.151	0.043	2.18E-19
CRNKL1	1.50E-23	0.111359	0.117	0.024	2.24E-19
TMEM184B	1.56E-23	0.1886	0.159	0.032	2.33E-19
ZBTB7A	1.68E-23	0.16019	0.151	0.032	2.52E-19
TSEN2	1.78E-23	0.167351	0.166	0.043	2.66E-19
C11orf1	1.85E-23	-0.40511	0.088	0.075	2.76E-19
BRF1	1.89E-23	0.211849	0.143	0.022	2.82E-19
TRIM69	1.95E-23	-0.17869	0.116	0.063	2.92E-19
ZNF205	1.97E-23	0.286403	0.151	0.019	2.94E-19
CEP135	2.04E-23	0.352644	0.135	0.009	3.05E-19
NSUN2	2.08E-23	0.31496	0.152	0.017	3.10E-19
TP53I13	2.11E-23	0.235515	0.164	0.034	3.15E-19
FLNA	2.15E-23	0.103856	0.172	0.056	3.21E-19
TARBP2	2.17E-23	0.106209	0.127	0.029	3.24E-19
MCM4	2.19E-23	0.360343	0.144	0.01	3.28E-19
DAGLB	2.26E-23	0.294894	0.139	0.012	3.38E-19
ZNF583	2.35E-23	0.122255	0.129	0.026	3.51E-19
CERK	2.40E-23	0.314997	0.131	0.007	3.58E-19
TCAIM	2.48E-23	0.116503	0.124	0.026	3.70E-19
PDE7A	2.52E-23	0.285487	0.121	0.007	3.77E-19
MSL1	2.57E-23	0.149546	0.172	0.048	3.83E-19
C16orf72	2.62E-23	0.139332	0.124	0.022	3.91E-19
MLLT3	2.66E-23	0.185244	0.134	0.024	3.97E-19
PAXBP1	2.69E-23	0.271848	0.17	0.032	4.03E-19
DVL3	2.70E-23	0.246907	0.177	0.038	4.04E-19
SLC2A6	2.71E-23	0.23441	0.109	0.005	4.05E-19
NR2F2	2.72E-23	-0.10292	0.109	0.044	4.07E-19
NUP88	2.83E-23	0.210012	0.134	0.019	4.23E-19
FAM19A5	2.87E-23	0.300506	0.144	0.017	4.29E-19
KLHDC10	2.96E-23	0.145845	0.146	0.034	4.43E-19
SCD	3.10E-23	-0.29392	0.125	0.079	4.64E-19
MICU1	3.11E-23	0.143707	0.137	0.031	4.65E-19

TTC37	3.12E-23	0.223377	0.166	0.038	4.67E-19
TM9SF4	3.18E-23	-0.10989	0.137	0.065	4.76E-19
BIVM	3.19E-23	0.211306	0.126	0.017	4.77E-19
HOXB3	3.20E-23	0.281467	0.129	0.009	4.78E-19
CRKL	3.27E-23	0.125208	0.138	0.029	4.88E-19
NCK2	3.40E-23	0.294269	0.117	0.005	5.09E-19
FBXL15	3.45E-23	0.327226	0.159	0.021	5.16E-19
GRK4	3.50E-23	0.246613	0.15	0.024	5.23E-19
OPHN1	3.52E-23	0.407602	0.182	0.029	5.26E-19
ABCB8	3.58E-23	0.156869	0.134	0.026	5.35E-19
GPS2	3.68E-23	0.191539	0.161	0.041	5.50E-19
TTL	3.69E-23	0.208359	0.148	0.029	5.51E-19
AGAP1	3.72E-23	0.103751	0.134	0.032	5.55E-19
MAF	3.72E-23	-0.19003	0.095	0.048	5.56E-19
ZADH2	3.76E-23	0.118768	0.117	0.021	5.62E-19
MTRF1L	3.78E-23	0.22545	0.157	0.031	5.65E-19
SMCR5	3.79E-23	0.165245	0.12	0.021	5.67E-19
LIPE-AS1	3.81E-23	0.176925	0.133	0.024	5.70E-19
USP34	3.82E-23	0.296754	0.195	0.046	5.71E-19
PTCH1	3.88E-23	0.363932	0.139	0.009	5.80E-19
ZSCAN16	3.94E-23	0.223778	0.14	0.024	5.89E-19
RP11-798M	3.94E-23	-0.21763	0.116	0.067	5.89E-19
FGF12	3.95E-23	0.443787	0.17	0.024	5.91E-19
TPT1-AS1	3.97E-23	0.199552	0.165	0.039	5.94E-19
KLF13	4.00E-23	0.285263	0.147	0.021	5.98E-19
EPHA4	4.08E-23	0.225215	0.137	0.022	6.10E-19
GPR1	4.26E-23	0.344488	0.118	0.002	6.36E-19
SRGAP2	4.31E-23	0.2103	0.153	0.029	6.44E-19
SCO2	4.37E-23	0.122089	0.116	0.021	6.53E-19
TIMP4	4.44E-23	-0.60669	0.068	0.099	6.63E-19
AFTPH	4.60E-23	0.137117	0.138	0.031	6.88E-19
ITGB1	4.61E-23	-0.27049	0.126	0.079	6.88E-19
FBXO32	4.67E-23	-0.52564	0.107	0.116	6.97E-19
ID4	4.71E-23	-0.18211	0.146	0.085	7.04E-19
SLITRK1	4.75E-23	0.290817	0.109	0	7.10E-19
TMEM107	4.90E-23	-0.31429	0.113	0.084	7.32E-19
FLYWCH1	5.38E-23	0.170151	0.126	0.021	8.05E-19
THAP11	5.71E-23	0.247143	0.159	0.029	8.53E-19
B4GALT5	5.79E-23	-0.13423	0.116	0.056	8.66E-19
ZFP91	5.86E-23	0.151282	0.124	0.021	8.76E-19
FIGN	5.91E-23	0.178075	0.14	0.027	8.83E-19
FXYD5	5.95E-23	-0.25519	0.109	0.065	8.90E-19
RAD54L2	5.98E-23	0.300713	0.152	0.021	8.94E-19
HUWE1	6.23E-23	0.266435	0.183	0.041	9.32E-19
ZFP90	6.35E-23	0.248502	0.146	0.026	9.49E-19
XKR4	6.41E-23	0.107704	0.131	0.036	9.58E-19
KCTD13	6.45E-23	0.217521	0.138	0.026	9.63E-19
POLM	6.58E-23	0.17594	0.138	0.027	9.83E-19
ZBED3	6.62E-23	0.306316	0.146	0.017	9.90E-19
TRIP12	7.00E-23	0.169741	0.13	0.021	1.05E-18
LRRC37B	7.32E-23	0.119793	0.135	0.029	1.09E-18

SAMD14	7.78E-23	0.232635	0.118	0.01	1.16E-18
TRAPPC2	7.87E-23	0.256732	0.143	0.022	1.18E-18
HELZ	8.26E-23	0.156931	0.178	0.051	1.23E-18
NOL8	8.30E-23	0.133124	0.168	0.048	1.24E-18
RP11-410L	8.48E-23	0.210422	0.155	0.036	1.27E-18
NAA35	8.51E-23	0.19162	0.139	0.026	1.27E-18
YTHDF3	8.84E-23	0.201383	0.143	0.029	1.32E-18
HHLA3	8.89E-23	-0.40295	0.074	0.072	1.33E-18
MTG2	8.98E-23	0.131445	0.125	0.024	1.34E-18
CDK11A	9.05E-23	0.204733	0.185	0.051	1.35E-18
ZNF37A	9.33E-23	0.258815	0.146	0.024	1.39E-18
ALG14	9.75E-23	0.100349	0.179	0.062	1.46E-18
C19orf12	9.76E-23	0.127287	0.133	0.031	1.46E-18
UCKL1	9.87E-23	0.153672	0.144	0.032	1.47E-18
FAM195B	1.00E-22	-0.84495	0.048	0.147	1.50E-18
HOOK2	1.02E-22	-0.19434	0.17	0.101	1.52E-18
CTSC	1.02E-22	-0.35061	0.082	0.068	1.52E-18
PHLDA3	1.04E-22	-0.16586	0.114	0.06	1.55E-18
EPB41L2	1.06E-22	0.177203	0.146	0.031	1.58E-18
TMEM161A	1.06E-22	-0.18006	0.121	0.062	1.59E-18
UIMC1	1.10E-22	0.130258	0.137	0.031	1.65E-18
RBM12	1.11E-22	0.129487	0.134	0.031	1.66E-18
ALKBH3	1.13E-22	-0.14349	0.116	0.056	1.68E-18
ZNF770	1.13E-22	0.143623	0.129	0.026	1.69E-18
NARS2	1.16E-22	0.181492	0.143	0.031	1.73E-18
MAPK7	1.22E-22	0.161601	0.121	0.017	1.82E-18
TTPAL	1.23E-22	-0.11266	0.104	0.044	1.83E-18
NAP1L3	1.24E-22	0.270861	0.15	0.022	1.85E-18
TOPORS-AS	1.27E-22	-0.10593	0.109	0.046	1.89E-18
UBQLN4	1.30E-22	0.139265	0.127	0.024	1.94E-18
C12orf75	1.30E-22	0.302437	0.12	0.003	1.95E-18
TACO1	1.31E-22	0.181085	0.148	0.031	1.95E-18
RIT2	1.32E-22	-0.48784	0.06	0.072	1.97E-18
NQO2	1.33E-22	-0.16454	0.109	0.055	1.99E-18
LRRC1	1.39E-22	0.231108	0.13	0.019	2.08E-18
PTRH2	1.44E-22	-0.18607	0.125	0.063	2.15E-18
SBK1	1.45E-22	0.356508	0.138	0.01	2.17E-18
XIST	1.46E-22	-0.83521	0.083	0.202	2.18E-18
THAP3	1.47E-22	-0.12238	0.12	0.043	2.20E-18
TTC28	1.49E-22	0.342875	0.15	0.015	2.23E-18
AFAP1L2	1.55E-22	0.238669	0.101	0.002	2.31E-18
ATP13A2	1.56E-22	0.185053	0.147	0.027	2.33E-18
SRFBP1	1.58E-22	0.129488	0.152	0.043	2.36E-18
PARP2	1.58E-22	0.184604	0.164	0.041	2.37E-18
C5orf30	1.64E-22	0.108103	0.122	0.027	2.45E-18
WDR73	1.65E-22	-0.12418	0.131	0.065	2.47E-18
GRIK2	1.68E-22	0.300042	0.138	0.014	2.50E-18
POLR2M	1.69E-22	0.168685	0.143	0.031	2.53E-18
CAPN10	1.70E-22	0.26505	0.144	0.021	2.54E-18
RRP36	1.75E-22	0.306691	0.121	0.007	2.61E-18
YIPF1	1.82E-22	-0.304	0.1	0.067	2.71E-18

ZNF582-AS	1.83E-22	-0.30917	0.088	0.063	2.73E-18
ATXN2L	1.83E-22	0.122274	0.159	0.043	2.74E-18
MBD3	1.89E-22	0.200995	0.152	0.032	2.82E-18
SLC35F6	1.95E-22	0.181054	0.146	0.029	2.92E-18
KBTBD2	1.96E-22	0.229474	0.138	0.019	2.93E-18
SCAF4	1.98E-22	0.126179	0.159	0.048	2.96E-18
NSDHL	1.98E-22	-0.20174	0.083	0.038	2.97E-18
ALDH18A1	1.99E-22	0.262021	0.122	0.01	2.98E-18
RPUSD4	2.30E-22	0.127237	0.122	0.022	3.44E-18
OTUD6B	2.36E-22	0.24201	0.156	0.031	3.53E-18
MMGT1	2.68E-22	0.129011	0.118	0.021	4.01E-18
NLGN4X	2.70E-22	0.122727	0.142	0.036	4.04E-18
CERS5	2.72E-22	0.114986	0.191	0.068	4.07E-18
ATG13	2.87E-22	0.13	0.122	0.024	4.29E-18
EXOC3	2.89E-22	0.117961	0.124	0.026	4.32E-18
ZNF260	2.94E-22	0.184623	0.117	0.015	4.39E-18
LIAS	2.95E-22	0.182332	0.127	0.019	4.41E-18
GBE1	3.04E-22	-0.20709	0.142	0.082	4.54E-18
CENPU	3.09E-22	0.276867	0.118	0.009	4.61E-18
MEOX2	3.09E-22	-0.2177	0.12	0.072	4.62E-18
SERTAD3	3.14E-22	-0.27916	0.131	0.079	4.69E-18
RP11-1114	3.27E-22	0.184777	0.148	0.031	4.89E-18
DCUN1D4	3.32E-22	0.181647	0.146	0.032	4.95E-18
SMURF2	3.39E-22	0.202753	0.139	0.026	5.07E-18
SHPRH	3.40E-22	0.17324	0.135	0.026	5.08E-18
C5orf28	3.41E-22	-0.17553	0.113	0.07	5.09E-18
RNF166	3.56E-22	0.249343	0.148	0.024	5.32E-18
NDUFB8	3.68E-22	-0.96803	0.021	0.164	5.51E-18
GTF2IRD1	3.72E-22	0.118371	0.13	0.029	5.56E-18
ISG15	4.13E-22	-0.54962	0.164	0.188	6.17E-18
PAX3	4.24E-22	0.283211	0.116	0.003	6.34E-18
MXD1	4.24E-22	0.257071	0.148	0.019	6.34E-18
PEX26	4.38E-22	0.159907	0.143	0.034	6.55E-18
ATP2B4	4.43E-22	0.244116	0.143	0.022	6.62E-18
RP11-398K	4.50E-22	0.130543	0.118	0.022	6.72E-18
CBFA2T2	4.69E-22	0.252856	0.152	0.027	7.00E-18
AGK	4.82E-22	0.12312	0.13	0.029	7.21E-18
MOGS	4.91E-22	0.194421	0.134	0.022	7.34E-18
MAT2B	4.92E-22	-0.17128	0.146	0.079	7.35E-18
TSC22D2	4.95E-22	0.18193	0.131	0.021	7.40E-18
SLC41A3	5.04E-22	-0.12796	0.122	0.058	7.53E-18
PLK2	5.20E-22	0.158498	0.146	0.032	7.77E-18
EGR3	5.32E-22	0.259438	0.125	0.014	7.95E-18
VWA9	5.43E-22	-0.10794	0.12	0.053	8.12E-18
CUEDC1	5.44E-22	0.168431	0.116	0.015	8.14E-18
ARHGAP35	5.46E-22	0.1718	0.134	0.024	8.17E-18
RALGAPA1	5.49E-22	0.238575	0.147	0.026	8.20E-18
DCLRE1C	5.56E-22	0.26015	0.15	0.021	8.30E-18
INSR	5.65E-22	0.175234	0.16	0.043	8.44E-18
RCBTB1	5.66E-22	0.250495	0.118	0.009	8.47E-18
OPA1	5.73E-22	0.155016	0.114	0.019	8.56E-18

TMEM39B	5.74E-22	0.179073	0.134	0.024	8.58E-18
XPC	5.89E-22	0.104704	0.153	0.048	8.80E-18
ROBO2	5.93E-22	0.246069	0.143	0.022	8.87E-18
LAPTM5	5.98E-22	-0.20365	0.111	0.063	8.94E-18
MAP1S	6.13E-22	0.136315	0.117	0.021	9.16E-18
PRR14	6.48E-22	0.162744	0.169	0.048	9.69E-18
STX4	6.70E-22	-0.38773	0.12	0.106	1.00E-17
TAP1	6.77E-22	-0.48592	0.114	0.116	1.01E-17
DYNC2LI1	6.84E-22	-0.14863	0.15	0.08	1.02E-17
MT1G	6.96E-22	-0.87367	0.048	0.126	1.04E-17
ADAT1	7.00E-22	0.206431	0.129	0.019	1.05E-17
PRKAR2B	7.34E-22	0.303093	0.124	0.007	1.10E-17
CNIH2	7.45E-22	0.391411	0.14	0.012	1.11E-17
CDK5RAP1	8.04E-22	0.154823	0.138	0.032	1.20E-17
SH2B1	8.05E-22	0.158298	0.144	0.038	1.20E-17
ILVBL	8.58E-22	-0.37838	0.111	0.097	1.28E-17
TRMT5	8.62E-22	0.136804	0.121	0.024	1.29E-17
ZNF398	8.70E-22	0.112558	0.105	0.017	1.30E-17
EPHB2	8.92E-22	0.326666	0.135	0.01	1.33E-17
METTL15	8.95E-22	-0.10509	0.108	0.05	1.34E-17
CSRNP1	9.00E-22	0.132943	0.127	0.027	1.34E-17
CAMK1	9.07E-22	-0.10924	0.103	0.039	1.36E-17
ERRFI1	9.36E-22	-0.42887	0.094	0.089	1.40E-17
NHSL2	9.58E-22	-0.87514	0.005	0.115	1.43E-17
ZNF669	9.61E-22	0.238613	0.137	0.022	1.44E-17
KIAA0895L	9.71E-22	0.13821	0.127	0.026	1.45E-17
ARRDC4	9.73E-22	0.328853	0.129	0.007	1.45E-17
SLC26A10	9.80E-22	0.26278	0.121	0.01	1.47E-17
COLGALT2	1.00E-21	0.197667	0.155	0.036	1.49E-17
KCTD16	1.01E-21	0.265134	0.103	0.003	1.51E-17
NR0B1	1.01E-21	0.230923	0.098	0.002	1.51E-17
TBC1D17	1.04E-21	0.152011	0.174	0.051	1.56E-17
ARL15	1.04E-21	0.270239	0.113	0.005	1.56E-17
ZNF581	1.10E-21	-0.21966	0.12	0.067	1.65E-17
ZNF573	1.11E-21	0.150663	0.133	0.029	1.66E-17
FNBP1	1.17E-21	0.125421	0.15	0.041	1.74E-17
AGO1	1.20E-21	0.328947	0.122	0.005	1.79E-17
CNOT6	1.20E-21	0.118775	0.095	0.012	1.79E-17
CACNG4	1.21E-21	0.281945	0.117	0.007	1.81E-17
TMEM209	1.22E-21	0.151075	0.117	0.019	1.82E-17
PAK1	1.22E-21	0.141363	0.116	0.019	1.82E-17
KLHL4	1.24E-21	-0.21055	0.118	0.068	1.85E-17
C10orf10	1.25E-21	-0.97242	0.013	0.106	1.87E-17
MTMR2	1.25E-21	0.145952	0.124	0.024	1.87E-17
CPD	1.27E-21	0.102186	0.133	0.032	1.90E-17
SNRK	1.29E-21	0.287216	0.148	0.022	1.93E-17
PIK3C3	1.30E-21	0.141885	0.12	0.024	1.95E-17
ZC4H2	1.34E-21	0.341659	0.122	0.005	2.00E-17
INPP5K	1.35E-21	0.190398	0.121	0.019	2.01E-17
COQ6	1.35E-21	0.122947	0.139	0.034	2.02E-17
ING1	1.37E-21	0.112287	0.131	0.034	2.05E-17

TRO	1.38E-21	0.13141	0.111	0.017	2.07E-17
NFX1	1.46E-21	0.227322	0.14	0.024	2.19E-17
CAMK2D	1.53E-21	-0.32742	0.127	0.099	2.28E-17
IFNAR2	1.57E-21	0.335975	0.143	0.014	2.35E-17
QRSL1	1.65E-21	0.202816	0.133	0.022	2.46E-17
ZNF117	1.68E-21	0.251646	0.146	0.026	2.51E-17
YES1	1.69E-21	0.132338	0.139	0.032	2.53E-17
SPIN2B	1.70E-21	0.108569	0.122	0.026	2.53E-17
ZNF74	1.72E-21	0.262803	0.127	0.012	2.57E-17
TELO2	1.73E-21	0.271089	0.143	0.021	2.59E-17
LRRC8D	1.79E-21	0.230397	0.117	0.012	2.68E-17
SENP5	1.80E-21	0.144522	0.13	0.027	2.69E-17
CEP78	1.84E-21	0.148089	0.109	0.015	2.74E-17
SOX15	1.86E-21	-0.1313	0.105	0.051	2.78E-17
INTS1	1.88E-21	0.173625	0.125	0.021	2.81E-17
TRMU	1.88E-21	0.280461	0.166	0.032	2.81E-17
ZMYND11	1.88E-21	0.222524	0.13	0.019	2.81E-17
TMEM141	1.90E-21	-0.27961	0.099	0.063	2.84E-17
VRK1	1.98E-21	0.141471	0.126	0.026	2.96E-17
ZNF232	2.01E-21	0.241368	0.125	0.015	3.00E-17
FAM189B	2.14E-21	0.15262	0.127	0.024	3.19E-17
RTN1	2.16E-21	-0.14711	0.165	0.096	3.23E-17
ZNF280D	2.22E-21	0.149862	0.12	0.021	3.32E-17
TRIM41	2.26E-21	0.18802	0.14	0.029	3.38E-17
PKN1	2.27E-21	0.195983	0.16	0.038	3.39E-17
CREG1	2.29E-21	0.236203	0.125	0.015	3.42E-17
ANP32E	2.34E-21	-0.15999	0.148	0.075	3.50E-17
ATP10B	2.41E-21	0.303997	0.111	0.003	3.60E-17
AQP1	2.46E-21	0.206127	0.113	0.012	3.68E-17
SLITRK2	2.47E-21	0.288547	0.12	0.007	3.68E-17
NAA30	2.58E-21	0.255826	0.126	0.014	3.85E-17
XPR1	2.59E-21	0.126193	0.126	0.027	3.87E-17
DNAJC5	2.61E-21	0.21484	0.148	0.029	3.90E-17
IRF2	2.68E-21	-0.25714	0.124	0.085	4.00E-17
FAM131B	2.68E-21	0.195301	0.133	0.021	4.00E-17
VPS9D1	2.71E-21	0.159783	0.114	0.015	4.05E-17
CREBZF	2.74E-21	0.171307	0.164	0.044	4.10E-17
THUMPD2	2.80E-21	0.216226	0.143	0.026	4.18E-17
RHNO1	2.86E-21	-0.17187	0.114	0.06	4.28E-17
PRMT6	2.88E-21	0.228327	0.139	0.021	4.31E-17
RNMTL1	2.89E-21	0.27238	0.137	0.019	4.31E-17
ELAC1	3.07E-21	-0.1086	0.104	0.043	4.59E-17
NMI	3.12E-21	-0.50379	0.074	0.084	4.66E-17
GNA12	3.17E-21	0.14886	0.147	0.039	4.74E-17
STAM	3.28E-21	0.181796	0.114	0.015	4.90E-17
STRBP	3.38E-21	0.301125	0.12	0.007	5.05E-17
PIGQ	3.42E-21	0.161443	0.12	0.021	5.10E-17
MICAL3	3.49E-21	0.155801	0.121	0.024	5.22E-17
HTT	3.57E-21	0.136028	0.116	0.022	5.33E-17
RND1	3.61E-21	0.18567	0.129	0.026	5.40E-17
ZFYVE16	3.65E-21	0.129741	0.164	0.053	5.45E-17

ZCCHC9	3.81E-21	-0.19481	0.121	0.067	5.70E-17
RCL1	3.83E-21	0.101631	0.094	0.01	5.72E-17
DHX38	3.86E-21	0.20235	0.122	0.017	5.77E-17
JAZF1	3.90E-21	0.192419	0.12	0.015	5.83E-17
ARID5A	3.94E-21	-0.24684	0.129	0.079	5.88E-17
HLA-DRB5	4.09E-21	-0.90577	0.036	0.125	6.11E-17
RNFT2	4.18E-21	0.240779	0.101	0.005	6.25E-17
ZNF652	4.29E-21	0.137301	0.14	0.032	6.42E-17
2-Mar	4.33E-21	0.108447	0.12	0.026	6.47E-17
TRPS1	4.35E-21	0.236235	0.153	0.031	6.50E-17
LRRTM1	4.44E-21	0.306397	0.117	0.003	6.64E-17
MICU3	4.45E-21	0.263724	0.133	0.017	6.65E-17
DIAPH1	4.49E-21	0.234017	0.138	0.022	6.71E-17
IQCE	4.72E-21	0.245004	0.129	0.017	7.06E-17
REEP3	4.72E-21	0.107092	0.135	0.032	7.06E-17
TPRA1	4.78E-21	0.175352	0.133	0.026	7.14E-17
CNOT3	4.78E-21	0.207274	0.14	0.024	7.14E-17
CKAP5	5.08E-21	0.104415	0.111	0.024	7.60E-17
PCGF3	5.27E-21	0.16762	0.109	0.017	7.87E-17
PARN	5.32E-21	0.147109	0.122	0.024	7.95E-17
ARL4D	5.36E-21	0.142248	0.17	0.051	8.01E-17
AHDC1	5.52E-21	0.108956	0.153	0.046	8.26E-17
GABPB1	5.69E-21	0.113002	0.109	0.019	8.50E-17
ZNF804A	5.85E-21	0.344826	0.109	0.002	8.74E-17
C11orf24	5.89E-21	0.143746	0.121	0.024	8.80E-17
IGDCC3	5.95E-21	0.331796	0.118	0.003	8.89E-17
BTN2A1	5.95E-21	0.181883	0.13	0.024	8.89E-17
CCDC142	5.99E-21	0.303473	0.146	0.019	8.94E-17
SNX8	6.03E-21	0.169956	0.137	0.029	9.01E-17
ELP3	6.27E-21	0.196633	0.139	0.026	9.36E-17
CBWD5	6.77E-21	0.156521	0.126	0.026	1.01E-16
RNF38	6.81E-21	0.288702	0.122	0.01	1.02E-16
SLC10A4	7.05E-21	0.270456	0.127	0.014	1.05E-16
ATP13A1	7.11E-21	0.228995	0.16	0.034	1.06E-16
FBXO28	7.21E-21	0.29338	0.13	0.014	1.08E-16
RAB34	7.40E-21	-0.64326	0.062	0.099	1.11E-16
PPM1D	7.56E-21	0.141025	0.117	0.021	1.13E-16
CDK12	7.63E-21	0.163336	0.135	0.029	1.14E-16
CTC-524C5	7.85E-21	0.201766	0.109	0.012	1.17E-16
NFATC3	8.09E-21	0.186867	0.146	0.031	1.21E-16
ANTXR2	8.24E-21	0.243354	0.099	0.003	1.23E-16
SLC16A9	8.25E-21	0.296256	0.108	0.003	1.23E-16
PUM2	8.28E-21	0.207959	0.16	0.039	1.24E-16
AC093673.	8.29E-21	-0.2036	0.1	0.067	1.24E-16
MBNL1	8.45E-21	0.108406	0.125	0.031	1.26E-16
RP11-395A	8.54E-21	0.261981	0.107	0.003	1.28E-16
FAM58A	8.75E-21	0.277374	0.127	0.014	1.31E-16
PXK	8.87E-21	0.211261	0.135	0.024	1.33E-16
LHX2	8.93E-21	0.159332	0.112	0.017	1.33E-16
AC016700.	9.01E-21	0.322461	0.121	0.01	1.35E-16
HECTD4	9.03E-21	0.221599	0.131	0.022	1.35E-16

KIAA1429	9.03E-21	0.312358	0.15	0.024	1.35E-16
GCNT2	9.29E-21	0.278547	0.109	0.003	1.39E-16
TTLL1	9.34E-21	0.145013	0.114	0.021	1.40E-16
LACTB2	9.63E-21	-0.16271	0.117	0.056	1.44E-16
PGS1	1.02E-20	0.1946	0.124	0.021	1.52E-16
FCER1G	1.03E-20	-0.10508	0.125	0.063	1.54E-16
SPSB4	1.03E-20	0.31103	0.124	0.007	1.54E-16
ZKSCAN5	1.04E-20	0.221974	0.116	0.012	1.56E-16
FBXO44	1.06E-20	-0.11934	0.118	0.06	1.59E-16
CYP3A5	1.11E-20	0.327771	0.13	0.01	1.65E-16
MCMBP	1.15E-20	0.183874	0.122	0.021	1.72E-16
GPR17	1.16E-20	0.402197	0.112	0.003	1.73E-16
RP11-231C	1.16E-20	0.317426	0.111	0.005	1.73E-16
TP53INP2	1.17E-20	0.174523	0.101	0.01	1.74E-16
SART3	1.22E-20	0.19502	0.15	0.032	1.82E-16
ABCC5	1.22E-20	0.220066	0.155	0.034	1.83E-16
RINT1	1.22E-20	0.138378	0.131	0.032	1.83E-16
TNC	1.26E-20	-0.857	0.036	0.142	1.88E-16
TRRAP	1.27E-20	0.235893	0.118	0.012	1.90E-16
MAFG	1.27E-20	0.12586	0.153	0.039	1.90E-16
ZNF682	1.29E-20	0.263867	0.125	0.012	1.93E-16
GRAMD3	1.35E-20	-0.14759	0.138	0.082	2.01E-16
E4F1	1.35E-20	0.17206	0.129	0.024	2.02E-16
MINA	1.36E-20	-0.10715	0.104	0.046	2.03E-16
GPR162	1.37E-20	0.247889	0.139	0.022	2.04E-16
JAG1	1.37E-20	-0.14445	0.134	0.072	2.04E-16
FASN	1.41E-20	0.302769	0.13	0.012	2.10E-16
FAM161A	1.51E-20	0.254607	0.143	0.026	2.25E-16
CA14	1.55E-20	0.179457	0.118	0.024	2.32E-16
CALCRL	1.68E-20	0.372391	0.134	0.01	2.51E-16
PDS5A	1.72E-20	0.112848	0.127	0.031	2.57E-16
ZNF121	1.74E-20	0.190885	0.133	0.026	2.59E-16
PIAS4	1.79E-20	0.171719	0.129	0.024	2.68E-16
FIGNL1	1.83E-20	0.152453	0.1	0.012	2.73E-16
NUDT2	1.85E-20	0.159665	0.13	0.026	2.76E-16
TRIM26	1.90E-20	0.301162	0.126	0.01	2.85E-16
HN1L	1.97E-20	0.145203	0.129	0.029	2.94E-16
DDX50	1.97E-20	0.101074	0.135	0.039	2.94E-16
CCNT2	1.99E-20	0.177837	0.134	0.027	2.97E-16
EPS8	2.18E-20	0.160198	0.13	0.029	3.26E-16
ELMSAN1	2.24E-20	0.120577	0.127	0.027	3.34E-16
RNF150	2.38E-20	0.275157	0.099	0.002	3.55E-16
HOXC-AS1	2.50E-20	0.217671	0.113	0.012	3.74E-16
MKRN3	2.52E-20	0.161927	0.129	0.027	3.76E-16
ZNF579	2.52E-20	0.199218	0.135	0.024	3.77E-16
CELF3	2.53E-20	0.315645	0.117	0.007	3.79E-16
ZDHHC12	2.54E-20	-0.14335	0.092	0.044	3.80E-16
ZYG11B	2.56E-20	0.212408	0.14	0.026	3.82E-16
RB1	2.60E-20	0.115461	0.137	0.038	3.89E-16
NME6	2.63E-20	-0.15488	0.107	0.05	3.93E-16
PGM5	2.64E-20	0.271115	0.1	0.002	3.94E-16

TRIP11	2.64E-20	0.12238	0.156	0.05	3.95E-16
KATNB1	2.66E-20	0.261938	0.146	0.024	3.97E-16
SLC2A3	2.66E-20	-0.37102	0.105	0.096	3.98E-16
CD14	2.69E-20	-0.22048	0.108	0.074	4.02E-16
MSMO1	2.77E-20	-0.21081	0.125	0.075	4.14E-16
ZNF692	2.79E-20	0.161669	0.135	0.032	4.17E-16
CDC42BPB	2.82E-20	0.153367	0.104	0.015	4.21E-16
CRLF3	2.86E-20	0.2636	0.122	0.012	4.27E-16
DDX3Y	2.87E-20	0.234546	0.114	0.01	4.29E-16
RNF25	2.96E-20	0.170571	0.124	0.024	4.43E-16
KLHL22	2.99E-20	0.170248	0.125	0.022	4.47E-16
MAP3K11	2.99E-20	0.187341	0.143	0.031	4.47E-16
GNAO1	3.00E-20	0.218776	0.131	0.024	4.49E-16
RASSF4	3.01E-20	-0.26325	0.147	0.101	4.49E-16
EFNA2	3.03E-20	0.30411	0.113	0.003	4.53E-16
MARK2	3.24E-20	0.248521	0.124	0.014	4.84E-16
SPRYD7	3.26E-20	-0.18189	0.117	0.067	4.86E-16
AKT1	3.30E-20	0.204257	0.157	0.038	4.93E-16
BRAP	3.33E-20	0.138151	0.124	0.026	4.98E-16
GTF2E2	3.39E-20	0.196875	0.14	0.031	5.06E-16
TTTY15	3.44E-20	0.300895	0.111	0.005	5.14E-16
ZNF384	3.58E-20	0.211584	0.109	0.01	5.35E-16
NSMAF	3.60E-20	0.137488	0.109	0.019	5.38E-16
DHCR7	3.78E-20	-0.41107	0.075	0.068	5.65E-16
EXOSC3	3.85E-20	0.142796	0.129	0.029	5.75E-16
ELOVL4	4.00E-20	0.264766	0.114	0.009	5.98E-16
C6orf226	4.02E-20	0.108427	0.131	0.038	6.01E-16
SKIV2L	4.05E-20	0.101254	0.105	0.021	6.06E-16
RAB3GAP2	4.07E-20	-0.13821	0.12	0.062	6.09E-16
CTTNBP2NL	4.18E-20	0.193668	0.118	0.015	6.25E-16
KLHL12	4.24E-20	0.140375	0.112	0.021	6.34E-16
AC005618.	4.25E-20	0.154461	0.127	0.029	6.35E-16
CRAT	4.32E-20	0.154708	0.127	0.026	6.46E-16
PINX1	4.32E-20	0.236513	0.129	0.021	6.46E-16
ZNF433	4.33E-20	0.163145	0.096	0.01	6.47E-16
HMGCL	4.39E-20	-0.28301	0.13	0.103	6.56E-16
GDPD1	4.72E-20	0.325547	0.117	0.005	7.06E-16
ARL10	4.85E-20	0.169101	0.121	0.022	7.25E-16
CHRNB1	4.86E-20	-0.26532	0.111	0.079	7.26E-16
SCO1	4.89E-20	0.163402	0.126	0.026	7.31E-16
FAM107B	4.99E-20	0.281054	0.12	0.012	7.45E-16
BOD1L1	5.12E-20	0.124017	0.137	0.038	7.65E-16
PPP3CC	5.15E-20	0.174681	0.148	0.038	7.70E-16
CDT1	5.24E-20	0.16807	0.099	0.012	7.83E-16
PIANP	5.26E-20	0.32278	0.126	0.01	7.86E-16
MKNK2	5.30E-20	-0.12469	0.122	0.062	7.91E-16
ZFP62	5.79E-20	0.155634	0.117	0.022	8.65E-16
CBWD2	5.88E-20	-0.17045	0.105	0.055	8.78E-16
ATP5L2	5.96E-20	-0.25635	0.101	0.067	8.91E-16
DXO	6.02E-20	0.159654	0.13	0.029	9.00E-16
ZBTB18	6.23E-20	0.31559	0.116	0.007	9.31E-16

TRIP6	6.30E-20	-0.47048	0.096	0.12	9.42E-16
C4orf46	6.51E-20	0.138338	0.101	0.015	9.73E-16
UBE2Z	6.54E-20	0.103565	0.138	0.041	9.77E-16
ABCF3	6.58E-20	0.216036	0.135	0.022	9.84E-16
EEFSEC	6.74E-20	0.2615	0.111	0.01	1.01E-15
TSEN54	6.78E-20	0.142617	0.124	0.026	1.01E-15
TIMM10B	6.81E-20	-0.19563	0.113	0.068	1.02E-15
C14orf80	6.83E-20	0.265126	0.105	0.007	1.02E-15
GTF3C3	6.88E-20	0.161809	0.12	0.021	1.03E-15
LDLRAD4	7.01E-20	0.199856	0.113	0.014	1.05E-15
RFC3	7.24E-20	0.145809	0.099	0.014	1.08E-15
CADM3	7.61E-20	-0.24787	0.082	0.048	1.14E-15
DDX55	8.01E-20	0.131495	0.142	0.036	1.20E-15
CSK	8.03E-20	0.176044	0.096	0.007	1.20E-15
PRKRIR	8.11E-20	0.148096	0.108	0.017	1.21E-15
BBS2	9.12E-20	-0.1131	0.164	0.097	1.36E-15
TMEM178B	9.34E-20	0.245835	0.117	0.012	1.40E-15
SAMD8	9.49E-20	0.155843	0.142	0.032	1.42E-15
COG8	9.50E-20	0.2436	0.137	0.024	1.42E-15
GPR161	9.59E-20	0.306652	0.117	0.005	1.43E-15
GOT1	9.83E-20	-0.31639	0.091	0.068	1.47E-15
CDC42EP1	9.90E-20	0.1655	0.121	0.021	1.48E-15
AGFG1	1.01E-19	0.167189	0.114	0.019	1.51E-15
MTURN	1.02E-19	0.236257	0.109	0.009	1.52E-15
DEDD	1.03E-19	0.124084	0.108	0.021	1.54E-15
FAM109A	1.04E-19	0.181526	0.098	0.009	1.56E-15
RP11-115C	1.04E-19	0.238085	0.126	0.019	1.56E-15
NOVA2	1.08E-19	0.200777	0.114	0.015	1.61E-15
WRNIP1	1.12E-19	0.184362	0.108	0.012	1.67E-15
FAM84A	1.23E-19	-0.10951	0.114	0.055	1.83E-15
DNALI1	1.23E-19	-0.14196	0.088	0.039	1.83E-15
PEX1	1.24E-19	0.116041	0.088	0.012	1.85E-15
XPO7	1.24E-19	0.158854	0.111	0.019	1.86E-15
ATOH8	1.25E-19	0.384876	0.138	0.014	1.86E-15
C3orf17	1.29E-19	-0.18979	0.103	0.058	1.93E-15
CXCR4	1.30E-19	-0.66313	0.064	0.106	1.94E-15
PPWD1	1.30E-19	0.109453	0.126	0.032	1.95E-15
HOXC6	1.32E-19	0.158306	0.091	0.009	1.97E-15
CDH10	1.36E-19	0.284013	0.118	0.01	2.04E-15
C21orf2	1.38E-19	0.113623	0.137	0.039	2.07E-15
ARID2	1.39E-19	0.201022	0.14	0.029	2.08E-15
ACVR1B	1.41E-19	0.21567	0.107	0.01	2.11E-15
AKAP11	1.41E-19	0.112223	0.118	0.026	2.11E-15
PXN-AS1	1.44E-19	0.143239	0.109	0.019	2.16E-15
ELFN1	1.46E-19	0.246578	0.092	0	2.19E-15
ERBB2IP	1.50E-19	0.136813	0.151	0.043	2.24E-15
C5orf38	1.54E-19	0.322745	0.12	0.009	2.29E-15
NBN	1.56E-19	0.148294	0.122	0.027	2.33E-15
RNF103	1.59E-19	0.296575	0.14	0.021	2.38E-15
LETMD1	1.67E-19	-0.14111	0.126	0.07	2.50E-15
MSANTD4	1.67E-19	-0.16743	0.103	0.051	2.50E-15

LCORL	1.72E-19	0.246154	0.111	0.009	2.57E-15
ZNF514	1.73E-19	0.117448	0.113	0.024	2.58E-15
VWA1	1.81E-19	0.133265	0.114	0.024	2.70E-15
CHEK1	1.81E-19	0.104218	0.09	0.014	2.71E-15
ZNF703	1.86E-19	0.138339	0.096	0.012	2.78E-15
RP11-660L	1.89E-19	-0.14675	0.118	0.055	2.82E-15
MTHFS	1.89E-19	-0.18255	0.095	0.058	2.83E-15
ZNF282	1.94E-19	0.161052	0.098	0.01	2.90E-15
MEMO1	1.97E-19	0.205745	0.126	0.022	2.94E-15
MTAP	1.98E-19	0.176764	0.121	0.022	2.96E-15
PHF21A	2.01E-19	0.156763	0.127	0.026	3.00E-15
CAAP1	2.02E-19	0.120294	0.088	0.012	3.02E-15
TBC1D24	2.05E-19	0.22098	0.095	0.005	3.06E-15
AFAP1	2.07E-19	0.263155	0.121	0.012	3.09E-15
CDK19	2.07E-19	0.241944	0.134	0.021	3.09E-15
PDS5B	2.07E-19	0.125202	0.151	0.046	3.10E-15
GTDC1	2.10E-19	0.172946	0.116	0.021	3.13E-15
CHADL	2.10E-19	0.299511	0.117	0.007	3.14E-15
CLDN10	2.16E-19	-0.34005	0.079	0.053	3.22E-15
CDKN2D	2.21E-19	0.175375	0.13	0.027	3.30E-15
ATXN3	2.23E-19	-0.251	0.133	0.091	3.34E-15
SRBD1	2.32E-19	0.143869	0.092	0.01	3.46E-15
MAP3K2	2.32E-19	0.11977	0.124	0.031	3.46E-15
CTB-3102C	2.35E-19	0.198007	0.129	0.026	3.51E-15
HPCA	2.37E-19	0.170333	0.125	0.029	3.53E-15
GPR75-ASE	2.38E-19	0.311667	0.134	0.015	3.55E-15
DZIP3	2.41E-19	0.16666	0.13	0.029	3.59E-15
KIF9	2.45E-19	-0.29278	0.105	0.082	3.66E-15
JAK1	2.57E-19	0.121797	0.14	0.039	3.84E-15
TRAPPC11	2.57E-19	0.15451	0.104	0.014	3.85E-15
SNX12	2.66E-19	0.133434	0.129	0.031	3.97E-15
IVD	2.67E-19	-0.10618	0.117	0.056	3.98E-15
HOXC11	2.70E-19	0.25483	0.091	0	4.04E-15
PRKX	2.83E-19	0.257356	0.101	0.003	4.22E-15
TMEM145	2.84E-19	0.200712	0.127	0.021	4.24E-15
TSPAN11	2.89E-19	0.159755	0.09	0.009	4.32E-15
C7orf61	2.93E-19	0.193292	0.126	0.027	4.38E-15
NDUFAF7	2.98E-19	0.183455	0.127	0.026	4.45E-15
PLAUR	3.08E-19	-0.38211	0.078	0.068	4.61E-15
MAN1B1	3.10E-19	-0.12836	0.124	0.056	4.63E-15
GMPPA	3.16E-19	-0.32668	0.079	0.067	4.72E-15
NUP50	3.16E-19	0.220957	0.113	0.012	4.72E-15
TTTY14	3.18E-19	0.246303	0.096	0.003	4.75E-15
ZNF519	3.19E-19	0.171294	0.098	0.01	4.76E-15
IRF2BP1	3.32E-19	0.218511	0.124	0.017	4.97E-15
MBD1	3.52E-19	0.143373	0.146	0.041	5.25E-15
SLC35A3	3.54E-19	0.112796	0.131	0.034	5.29E-15
FAM98B	3.55E-19	0.163578	0.121	0.022	5.31E-15
ZNF420	3.63E-19	0.139177	0.143	0.039	5.42E-15
TOM1	3.66E-19	0.116421	0.121	0.027	5.47E-15
CDC73	3.67E-19	-0.18887	0.118	0.07	5.48E-15

ZNF197	3.69E-19	0.121839	0.105	0.021	5.51E-15
FAM134C	3.84E-19	0.147991	0.127	0.029	5.74E-15
RC3H1	3.86E-19	0.113643	0.109	0.022	5.77E-15
MLLT10	3.91E-19	0.170139	0.118	0.022	5.84E-15
ARHGEF26	3.96E-19	-0.18413	0.107	0.062	5.92E-15
CPLX1	3.97E-19	0.380409	0.131	0.014	5.93E-15
TMEM175	4.09E-19	0.206001	0.143	0.032	6.11E-15
ZNF713	4.32E-19	0.221734	0.108	0.01	6.45E-15
VAT1	4.32E-19	0.122129	0.129	0.034	6.46E-15
CRTC3	4.38E-19	0.152621	0.111	0.021	6.54E-15
CUL4A	4.40E-19	0.267758	0.139	0.022	6.58E-15
KLHL8	4.41E-19	0.102238	0.134	0.041	6.58E-15
ORC3	4.52E-19	0.105271	0.113	0.024	6.76E-15
RUNDC1	4.62E-19	0.151365	0.124	0.026	6.90E-15
DUS4L	4.87E-19	0.19394	0.107	0.015	7.28E-15
ZNF143	4.90E-19	0.274416	0.111	0.007	7.33E-15
CRY1	5.07E-19	-0.13281	0.121	0.062	7.57E-15
NCOA5	5.15E-19	0.109565	0.103	0.017	7.69E-15
ZDHHC6	5.23E-19	-0.15098	0.1	0.051	7.82E-15
GTF2H2C	5.27E-19	0.129131	0.124	0.029	7.88E-15
VPS37D	5.31E-19	0.291265	0.12	0.009	7.94E-15
AC084219.	5.32E-19	0.126792	0.111	0.024	7.95E-15
UBE4A	5.36E-19	0.178945	0.118	0.021	8.01E-15
CDK17	5.51E-19	0.241179	0.138	0.024	8.24E-15
RASAL2	5.59E-19	0.246323	0.1	0.005	8.35E-15
MAPK8	5.70E-19	0.243025	0.099	0.005	8.52E-15
SNAPC1	6.21E-19	-0.12269	0.131	0.058	9.28E-15
PRMT7	6.28E-19	0.192857	0.134	0.027	9.38E-15
SRRD	6.76E-19	0.183784	0.104	0.014	1.01E-14
TTC5	6.76E-19	0.190584	0.133	0.029	1.01E-14
MCM2	7.06E-19	0.210781	0.107	0.012	1.06E-14
PITPNA	7.09E-19	0.204669	0.12	0.017	1.06E-14
ENTPD4	7.16E-19	0.115065	0.107	0.021	1.07E-14
VCPKMT	7.39E-19	0.238994	0.118	0.015	1.10E-14
PI4KA	7.45E-19	0.150097	0.098	0.012	1.11E-14
POLR1C	7.50E-19	-0.15234	0.133	0.072	1.12E-14
LMF1	7.66E-19	0.232919	0.122	0.017	1.14E-14
ZNF107	7.92E-19	0.27512	0.099	0.002	1.18E-14
PTPN1	7.94E-19	0.138528	0.114	0.024	1.19E-14
AGPAT4	8.10E-19	0.105848	0.103	0.022	1.21E-14
DNASE2	8.12E-19	-0.45354	0.122	0.128	1.21E-14
KAT8	8.14E-19	0.184971	0.144	0.038	1.22E-14
FAM220A	8.21E-19	0.23635	0.112	0.012	1.23E-14
LDOC1L	8.23E-19	0.236951	0.113	0.012	1.23E-14
RP11-448A	8.54E-19	0.248465	0.153	0.034	1.28E-14
ATG14	8.86E-19	0.127343	0.107	0.019	1.32E-14
GPALPP1	8.86E-19	0.214267	0.15	0.034	1.32E-14
MYLIP	9.12E-19	0.193102	0.118	0.021	1.36E-14
FAM216A	9.32E-19	0.156683	0.126	0.031	1.39E-14
TOP1MT	9.37E-19	0.180231	0.095	0.01	1.40E-14
HYPK	9.90E-19	0.135143	0.108	0.019	1.48E-14

C17orf96	9.97E-19	0.160483	0.095	0.01	1.49E-14
KIAA1211	1.01E-18	0.29529	0.107	0.003	1.50E-14
MYO5A	1.02E-18	0.289889	0.12	0.009	1.53E-14
ACVR2A	1.06E-18	0.178441	0.088	0.005	1.58E-14
TCF7L1	1.06E-18	0.164071	0.095	0.009	1.59E-14
FLOT2	1.08E-18	-0.12225	0.098	0.044	1.61E-14
ULK3	1.11E-18	0.165803	0.137	0.031	1.66E-14
ZNF708	1.11E-18	0.18202	0.122	0.024	1.67E-14
ZDHHC21	1.16E-18	0.209208	0.109	0.012	1.73E-14
ATF7IP	1.16E-18	0.216518	0.127	0.024	1.74E-14
SP100	1.19E-18	-0.76152	0.038	0.13	1.78E-14
PSD2	1.23E-18	0.191016	0.118	0.021	1.84E-14
B3GALNT2	1.26E-18	0.132827	0.127	0.032	1.89E-14
DMD	1.27E-18	-0.13435	0.1	0.048	1.90E-14
ATF1	1.28E-18	-0.18129	0.096	0.053	1.91E-14
SYT6	1.29E-18	0.179449	0.094	0.009	1.94E-14
CCDC80	1.30E-18	-0.68335	0.051	0.12	1.94E-14
MED13L	1.33E-18	0.212122	0.127	0.024	1.99E-14
BGN	1.36E-18	-0.51948	0.068	0.082	2.03E-14
PSRC1	1.39E-18	-0.45267	0.116	0.128	2.08E-14
FBXO22	1.39E-18	-0.22088	0.107	0.063	2.08E-14
ZBTB44	1.42E-18	0.24505	0.13	0.022	2.12E-14
SCAI	1.44E-18	0.18693	0.125	0.024	2.15E-14
HIRIP3	1.46E-18	0.113881	0.146	0.044	2.18E-14
NOLC1	1.53E-18	0.126866	0.135	0.036	2.28E-14
KIN	1.53E-18	0.151813	0.122	0.027	2.28E-14
JADE1	1.56E-18	0.246126	0.155	0.036	2.34E-14
MTFR1	1.66E-18	0.227377	0.105	0.012	2.48E-14
COG1	1.72E-18	0.130267	0.113	0.026	2.57E-14
IER3IP1	1.78E-18	-0.92057	0.016	0.132	2.66E-14
MPI	1.80E-18	-0.17139	0.096	0.048	2.70E-14
UGCG	1.83E-18	-0.15874	0.091	0.048	2.74E-14
DOCK10	1.87E-18	0.320673	0.12	0.01	2.79E-14
SETD2	1.88E-18	0.205335	0.125	0.022	2.81E-14
RABGAP1	1.93E-18	0.169825	0.117	0.021	2.88E-14
F13A1	2.00E-18	-0.83785	0.01	0.094	2.98E-14
RMI1	2.02E-18	0.103755	0.092	0.017	3.02E-14
ANAPC2	2.06E-18	0.261978	0.134	0.021	3.07E-14
PSMB10	2.10E-18	-0.17272	0.118	0.075	3.13E-14
ZCCHC8	2.11E-18	0.11851	0.101	0.019	3.15E-14
PREX1	2.12E-18	-0.33751	0.059	0.046	3.17E-14
NIPSNAP3A	2.13E-18	-0.21797	0.079	0.044	3.18E-14
PPP1R15B	2.14E-18	0.184204	0.124	0.024	3.19E-14
SEMA6B	2.16E-18	0.233909	0.104	0.009	3.23E-14
ZNRF1	2.17E-18	0.182585	0.091	0.009	3.25E-14
C1GALT1	2.18E-18	0.238958	0.135	0.022	3.26E-14
FEM1C	2.19E-18	-0.15694	0.095	0.05	3.27E-14
NXN	2.20E-18	0.316133	0.1	0.002	3.28E-14
ACHE	2.21E-18	0.202204	0.129	0.024	3.30E-14
HIST1H2AC	2.22E-18	-0.20486	0.137	0.082	3.31E-14
METTL13	2.26E-18	0.12265	0.118	0.027	3.38E-14

POLR1A	2.28E-18	0.168171	0.1	0.012	3.41E-14
DIDO1	2.31E-18	0.310503	0.138	0.017	3.45E-14
ALOX5AP	2.32E-18	-0.19754	0.103	0.067	3.47E-14
TIGD1	2.51E-18	0.19637	0.111	0.015	3.75E-14
PPP3R1	2.52E-18	0.108212	0.1	0.017	3.76E-14
RAB2B	2.55E-18	0.195696	0.108	0.014	3.81E-14
SREBF2	2.65E-18	0.148867	0.121	0.027	3.96E-14
CXCL16	2.82E-18	-0.31171	0.088	0.062	4.22E-14
CHGA	2.83E-18	0.334794	0.099	0.002	4.23E-14
WNT5A	2.83E-18	0.225263	0.086	0.002	4.23E-14
ARHGAP33	2.93E-18	0.25267	0.114	0.012	4.37E-14
FAM208A	2.93E-18	0.200068	0.112	0.017	4.38E-14
BTN3A2	2.93E-18	-0.62304	0.048	0.099	4.38E-14
POLR3A	2.99E-18	0.140716	0.103	0.014	4.47E-14
SLC5A6	3.11E-18	0.104476	0.117	0.029	4.64E-14
ASXL3	3.13E-18	0.305464	0.086	0	4.67E-14
PDP1	3.15E-18	0.142836	0.111	0.024	4.70E-14
AP3M2	3.18E-18	0.223246	0.108	0.01	4.75E-14
TMEM196	3.18E-18	0.254677	0.1	0.003	4.75E-14
RDH5	3.19E-18	-0.25079	0.09	0.062	4.77E-14
AGRN	3.35E-18	0.112397	0.111	0.026	5.00E-14
COQ2	3.36E-18	0.162818	0.098	0.012	5.03E-14
WDYHV1	3.46E-18	-0.13048	0.091	0.041	5.17E-14
FXN	3.46E-18	0.175225	0.103	0.014	5.18E-14
CACNB4	3.51E-18	0.158604	0.105	0.015	5.25E-14
C16orf58	3.55E-18	0.169589	0.122	0.024	5.31E-14
DNAJC3	3.56E-18	-0.21469	0.122	0.072	5.32E-14
ZNF513	3.57E-18	0.161127	0.112	0.019	5.33E-14
C17orf49	3.59E-18	-0.13017	0.107	0.058	5.36E-14
LRRC23	3.59E-18	0.117347	0.105	0.021	5.36E-14
TPP1	3.59E-18	-0.23572	0.108	0.075	5.37E-14
CDH24	3.67E-18	0.265895	0.088	0.002	5.49E-14
EPHB6	3.70E-18	0.227229	0.113	0.017	5.53E-14
PTPRF	3.75E-18	-0.17292	0.103	0.063	5.61E-14
NOVA1-AS1	3.80E-18	0.204082	0.099	0.01	5.68E-14
MAPK3	4.04E-18	-0.10898	0.113	0.056	6.03E-14
CELF4	4.10E-18	0.207347	0.108	0.012	6.13E-14
TMEM176B	4.11E-18	-0.57709	0.068	0.101	6.14E-14
JMY	4.14E-18	0.212698	0.114	0.015	6.19E-14
SETD9	4.15E-18	-0.1297	0.101	0.051	6.21E-14
ST3GAL2	4.20E-18	0.151793	0.117	0.024	6.27E-14
MLH3	4.25E-18	0.129853	0.129	0.032	6.36E-14
LINC-PINT	4.29E-18	0.142736	0.156	0.051	6.42E-14
IPO13	4.38E-18	0.1576	0.107	0.019	6.54E-14
ZNF688	4.41E-18	0.184629	0.134	0.031	6.59E-14
CAV2	4.43E-18	0.21184	0.113	0.015	6.62E-14
CDC25B	4.45E-18	-0.27366	0.098	0.074	6.65E-14
DNASE1	4.54E-18	0.1666	0.125	0.026	6.79E-14
UPP1	4.60E-18	-0.5156	0.061	0.063	6.88E-14
MAP2K5	4.67E-18	0.187021	0.108	0.015	6.97E-14
GNG11	4.73E-18	-0.26334	0.081	0.043	7.07E-14

ZFP1	4.87E-18	0.307527	0.111	0.007	7.28E-14
CCNE2	4.95E-18	0.334673	0.122	0.012	7.40E-14
ZNF576	5.00E-18	-0.34281	0.095	0.08	7.47E-14
SPR	5.05E-18	0.173058	0.094	0.009	7.55E-14
PEX11B	5.09E-18	-0.15592	0.096	0.046	7.60E-14
DHX40	5.13E-18	0.223401	0.126	0.021	7.66E-14
TRIP4	5.14E-18	0.110519	0.111	0.027	7.68E-14
SNX10	5.16E-18	0.134522	0.105	0.019	7.71E-14
MAK16	5.18E-18	0.135755	0.098	0.017	7.74E-14
KDM3B	5.24E-18	0.141807	0.116	0.024	7.84E-14
HES1	5.39E-18	-0.62289	0.061	0.104	8.05E-14
CDK13	5.42E-18	0.138737	0.111	0.026	8.09E-14
HSPA6	5.45E-18	-0.36065	0.087	0.056	8.14E-14
ASAH2B	5.76E-18	0.240298	0.085	0	8.61E-14
SLC30A3	5.76E-18	0.225261	0.085	0	8.61E-14
SYBU	5.88E-18	0.266469	0.138	0.024	8.78E-14
NEURL1B	5.95E-18	0.238415	0.09	0.002	8.88E-14
KLHL18	5.95E-18	0.103071	0.109	0.026	8.89E-14
UBE3C	5.96E-18	0.196258	0.124	0.022	8.91E-14
ZNF674-AS	6.21E-18	0.103394	0.094	0.019	9.27E-14
BANP	6.23E-18	0.281916	0.104	0.005	9.31E-14
SMAD4	6.44E-18	0.146762	0.121	0.029	9.62E-14
PHTF2	6.45E-18	0.168921	0.091	0.009	9.63E-14
KCNJ10	6.47E-18	0.228874	0.114	0.014	9.67E-14
ACO1	6.60E-18	0.175136	0.114	0.019	9.86E-14
C12orf29	6.70E-18	-0.10734	0.113	0.053	1.00E-13
ARAF	6.82E-18	-0.15295	0.12	0.07	1.02E-13
KAT2B	6.84E-18	0.106314	0.094	0.015	1.02E-13
ZNF71	7.29E-18	0.140401	0.101	0.017	1.09E-13
GAL3ST3	7.35E-18	0.248691	0.096	0.005	1.10E-13
PCDH19	7.44E-18	0.213124	0.096	0.009	1.11E-13
CACNA2D1	7.87E-18	0.287301	0.098	0.003	1.18E-13
NCOA6	7.89E-18	0.141173	0.116	0.026	1.18E-13
SNHG10	7.94E-18	0.150978	0.125	0.029	1.19E-13
AHI1	8.10E-18	0.136295	0.159	0.055	1.21E-13
DIS3L	8.19E-18	0.107552	0.086	0.014	1.22E-13
N4BP2	8.60E-18	0.144368	0.112	0.022	1.29E-13
C20orf96	8.63E-18	0.11349	0.117	0.029	1.29E-13
USF1	8.70E-18	0.299678	0.116	0.014	1.30E-13
C14orf142	8.74E-18	-0.21786	0.082	0.053	1.31E-13
CREM	9.45E-18	-0.17879	0.116	0.068	1.41E-13
C1orf53	9.58E-18	-0.12485	0.069	0.032	1.43E-13
ZHX3	1.00E-17	-0.13292	0.116	0.062	1.50E-13
PELO	1.01E-17	-0.14256	0.085	0.032	1.51E-13
MMAB	1.10E-17	-0.27248	0.101	0.077	1.64E-13
LPAR6	1.10E-17	-0.13929	0.087	0.046	1.65E-13
CLEC2B	1.11E-17	-0.18687	0.079	0.038	1.66E-13
BEX5	1.13E-17	-0.43617	0.082	0.094	1.69E-13
SCRN3	1.19E-17	0.138232	0.13	0.032	1.77E-13
KDM5C	1.23E-17	0.151395	0.117	0.024	1.83E-13
PTPRK	1.25E-17	0.147087	0.117	0.026	1.87E-13

TAF2	1.25E-17	0.179188	0.113	0.022	1.87E-13
ZNF322	1.27E-17	0.137915	0.109	0.021	1.90E-13
NSMF	1.29E-17	0.15816	0.122	0.027	1.93E-13
RP11-166C	1.32E-17	0.193929	0.104	0.012	1.97E-13
CXCL14	1.33E-17	-0.80672	0.018	0.113	1.99E-13
DNAJC14	1.39E-17	0.141142	0.109	0.021	2.08E-13
IFI27	1.40E-17	-0.8868	0.022	0.111	2.09E-13
GUCD1	1.41E-17	0.254279	0.12	0.015	2.11E-13
MLLT6	1.55E-17	0.133401	0.125	0.032	2.31E-13
ANKRD13B	1.58E-17	0.282319	0.103	0.005	2.37E-13
SLC11A1	1.63E-17	-0.12166	0.078	0.043	2.44E-13
KIRREL3	1.65E-17	0.261309	0.104	0.007	2.46E-13
NT5E	1.69E-17	0.125331	0.094	0.014	2.53E-13
POLR3E	1.69E-17	0.105781	0.104	0.026	2.53E-13
TEN1	1.71E-17	0.185495	0.094	0.01	2.56E-13
C2orf49	1.75E-17	-0.14813	0.09	0.043	2.61E-13
RASD1	1.78E-17	-0.2131	0.161	0.121	2.66E-13
IFT81	1.80E-17	0.136579	0.131	0.036	2.69E-13
SCMH1	1.91E-17	0.140359	0.099	0.015	2.85E-13
GSX2	1.95E-17	0.287904	0.082	0	2.92E-13
LNX1-AS1	1.95E-17	0.287536	0.082	0	2.92E-13
TAPT1-AS1	2.01E-17	0.335661	0.101	0.003	3.01E-13
BCAT2	2.02E-17	-0.39755	0.09	0.084	3.01E-13
FAM219A	2.05E-17	0.109544	0.094	0.017	3.06E-13
OPCML	2.12E-17	0.21173	0.091	0.007	3.16E-13
SENP7	2.12E-17	0.272908	0.13	0.019	3.17E-13
NEDD1	2.17E-17	0.180435	0.096	0.01	3.24E-13
MATN2	2.17E-17	-0.11016	0.095	0.043	3.24E-13
CENPQ	2.19E-17	0.118756	0.099	0.017	3.28E-13
ASTN1	2.20E-17	0.118287	0.114	0.027	3.29E-13
LEMD2	2.25E-17	0.155666	0.121	0.026	3.37E-13
CLCN7	2.27E-17	0.269893	0.114	0.012	3.40E-13
ZNF174	2.33E-17	0.146272	0.095	0.014	3.49E-13
ZBTB2	2.35E-17	0.247014	0.125	0.021	3.51E-13
TGFBR1	2.35E-17	0.283892	0.118	0.012	3.51E-13
PDXK	2.35E-17	-0.2167	0.109	0.065	3.51E-13
GBP2	2.37E-17	-0.76471	0.016	0.094	3.54E-13
MON1B	2.48E-17	0.116934	0.107	0.022	3.70E-13
STAG1	2.49E-17	0.177204	0.129	0.029	3.72E-13
SNX24	2.49E-17	-0.25118	0.068	0.044	3.72E-13
SLC8A3	2.50E-17	0.281317	0.086	0.002	3.73E-13
CORO2B	2.50E-17	0.122833	0.101	0.019	3.74E-13
ZNF136	2.54E-17	-0.20612	0.092	0.048	3.80E-13
ALAD	2.58E-17	0.147141	0.099	0.014	3.85E-13
GCLC	2.63E-17	0.133047	0.092	0.015	3.92E-13
CTD-2017D	2.65E-17	0.197176	0.094	0.009	3.96E-13
MORC3	2.72E-17	0.210853	0.105	0.014	4.07E-13
TUBGCP3	2.73E-17	0.235305	0.125	0.021	4.08E-13
NPHP3	2.78E-17	0.133818	0.133	0.038	4.15E-13
ST7L	2.79E-17	-0.11277	0.087	0.039	4.18E-13
UCHL5	2.82E-17	0.182184	0.129	0.029	4.22E-13

KRI1	2.86E-17	0.152237	0.105	0.021	4.27E-13
HERC1	2.87E-17	0.203296	0.103	0.012	4.29E-13
CCDC159	2.89E-17	-0.2245	0.105	0.075	4.32E-13
GFM2	2.91E-17	0.125691	0.108	0.022	4.35E-13
FBXO9	2.91E-17	0.140156	0.137	0.038	4.35E-13
SETBP1	2.93E-17	0.108527	0.1	0.022	4.37E-13
L3MBTL2	2.96E-17	0.195783	0.107	0.015	4.42E-13
L1CAM	3.00E-17	0.310936	0.1	0.007	4.48E-13
TYRO3	3.05E-17	0.217734	0.124	0.022	4.55E-13
FAM155A	3.13E-17	0.240241	0.094	0.003	4.68E-13
ZNF200	3.20E-17	0.177156	0.094	0.01	4.78E-13
LEAP2	3.34E-17	0.148872	0.109	0.021	4.99E-13
BTBD2	3.43E-17	0.208023	0.107	0.015	5.12E-13
LRCH4	3.45E-17	0.137366	0.144	0.043	5.16E-13
MIB1	3.64E-17	0.117176	0.099	0.017	5.43E-13
PLOD2	3.65E-17	-0.17301	0.098	0.06	5.45E-13
HELLS	3.74E-17	0.21511	0.108	0.015	5.59E-13
RP11-108M	3.86E-17	0.180288	0.096	0.01	5.76E-13
DNAJB5	3.97E-17	0.165819	0.095	0.012	5.94E-13
SCNN1A	3.97E-17	0.432661	0.152	0.029	5.94E-13
ZNF781	3.98E-17	0.24158	0.107	0.012	5.94E-13
OAS1	4.03E-17	-0.78299	0.025	0.101	6.02E-13
CCDC88C	4.28E-17	0.191383	0.086	0.005	6.39E-13
CALCOCO2	4.54E-17	-0.14605	0.116	0.063	6.78E-13
TNFRSF1A	4.55E-17	-0.29455	0.09	0.075	6.80E-13
RAB24	4.76E-17	0.17247	0.088	0.007	7.12E-13
C5orf45	4.83E-17	-0.20047	0.122	0.074	7.22E-13
UBR5	4.83E-17	0.125665	0.121	0.032	7.22E-13
RBM12B	4.85E-17	0.252377	0.109	0.01	7.25E-13
UBR3	4.88E-17	0.204547	0.108	0.017	7.29E-13
ATXN1L	5.18E-17	0.148021	0.091	0.012	7.74E-13
AGO2	5.22E-17	0.197321	0.098	0.01	7.81E-13
SCPEP1	5.25E-17	-0.37131	0.085	0.074	7.85E-13
FAM76B	5.35E-17	0.21753	0.117	0.019	7.99E-13
ZHX2	5.39E-17	0.208798	0.116	0.019	8.05E-13
SH3BGR	5.51E-17	-0.34243	0.086	0.075	8.23E-13
POLB	5.55E-17	0.185886	0.112	0.021	8.30E-13
FOXRED2	5.59E-17	0.197287	0.1	0.012	8.35E-13
SHKBP1	5.75E-17	0.186532	0.113	0.021	8.60E-13
SLC4A7	5.98E-17	0.118411	0.134	0.039	8.93E-13
COL11A1	6.00E-17	0.122931	0.078	0.009	8.97E-13
LINC0052€	6.14E-17	0.201486	0.109	0.017	9.18E-13
NCBP1	6.22E-17	0.166489	0.087	0.007	9.30E-13
NTN4	6.22E-17	0.26398	0.105	0.007	9.30E-13
RBBP5	6.36E-17	0.137049	0.133	0.038	9.50E-13
CENPK	6.55E-17	0.216338	0.096	0.01	9.79E-13
CRTAC1	6.59E-17	0.279874	0.079	0	9.86E-13
WDR92	6.61E-17	0.161572	0.113	0.024	9.88E-13
ASB16-AS1	6.62E-17	0.184854	0.094	0.01	9.90E-13
DENND1B	6.68E-17	0.214484	0.098	0.007	9.98E-13
STRIP2	6.75E-17	0.187711	0.075	0.002	1.01E-12

TRIM11	6.77E-17	0.202892	0.109	0.015	1.01E-12
DAPL1	6.77E-17	0.247674	0.12	0.024	1.01E-12
TTC27	7.04E-17	0.129129	0.103	0.021	1.05E-12
FASTKD5	7.05E-17	0.112995	0.122	0.034	1.05E-12
MFSD3	7.06E-17	0.310897	0.118	0.014	1.05E-12
CARF	7.14E-17	0.105741	0.111	0.027	1.07E-12
THAP2	7.24E-17	0.176757	0.116	0.022	1.08E-12
SMO	7.66E-17	0.220816	0.094	0.007	1.14E-12
UBR4	7.73E-17	0.161789	0.134	0.038	1.15E-12
MFN2	7.90E-17	0.108179	0.101	0.022	1.18E-12
SERAC1	8.14E-17	-0.25327	0.065	0.027	1.22E-12
B4GALT2	8.34E-17	0.11276	0.098	0.021	1.25E-12
PLEKHH3	8.49E-17	0.112879	0.1	0.019	1.27E-12
DTD2	8.91E-17	0.226845	0.114	0.019	1.33E-12
FARP2	9.29E-17	0.115053	0.098	0.017	1.39E-12
ANKRD44	9.44E-17	0.236671	0.113	0.017	1.41E-12
ASXL2	9.51E-17	0.230672	0.109	0.014	1.42E-12
SLC25A44	9.58E-17	0.179914	0.091	0.009	1.43E-12
TBC1D16	9.69E-17	0.187659	0.108	0.017	1.45E-12
CCDC34	1.01E-16	-0.15918	0.096	0.051	1.51E-12
TMEM185B	1.01E-16	0.149397	0.091	0.012	1.51E-12
C9orf85	1.02E-16	0.149885	0.09	0.012	1.52E-12
LPAR4	1.02E-16	0.216202	0.085	0.003	1.53E-12
GULP1	1.03E-16	0.154445	0.079	0.005	1.54E-12
SACS	1.04E-16	0.259482	0.12	0.017	1.56E-12
DCBLD1	1.09E-16	0.148446	0.113	0.022	1.63E-12
LLGL1	1.11E-16	0.144816	0.1	0.017	1.66E-12
ICK	1.11E-16	0.153604	0.096	0.015	1.66E-12
CTD-2090I	1.14E-16	-0.70996	0.016	0.089	1.70E-12
GSTM4	1.16E-16	-0.28183	0.081	0.068	1.73E-12
TBCD	1.16E-16	0.14811	0.101	0.017	1.74E-12
MMP14	1.18E-16	-0.31447	0.066	0.056	1.77E-12
COG2	1.19E-16	0.128885	0.116	0.029	1.78E-12
TBX5	1.21E-16	0.211067	0.078	0	1.81E-12
STX2	1.21E-16	0.179832	0.103	0.015	1.81E-12
PTPN11	1.21E-16	0.174793	0.131	0.031	1.82E-12
MARK4	1.25E-16	0.209414	0.113	0.017	1.87E-12
ZNF454	1.26E-16	0.203469	0.081	0.003	1.88E-12
ORAOV1	1.26E-16	0.105637	0.099	0.021	1.88E-12
PYGL	1.27E-16	-0.4737	0.053	0.072	1.89E-12
TXNRD2	1.29E-16	0.250338	0.111	0.012	1.93E-12
NBAS	1.31E-16	0.157783	0.096	0.015	1.96E-12
GPRASP2	1.32E-16	0.239925	0.105	0.01	1.97E-12
BBOX1	1.33E-16	-0.75894	0.027	0.115	1.98E-12
ZNF184	1.42E-16	0.199069	0.085	0.005	2.13E-12
HIST1H4J	1.48E-16	0.144347	0.085	0.007	2.21E-12
GSG1L	1.49E-16	-0.10586	0.1	0.05	2.23E-12
RRP8	1.52E-16	-0.12769	0.078	0.038	2.27E-12
PGAM2	1.59E-16	-0.11594	0.066	0.027	2.38E-12
HAUS2	1.60E-16	0.133554	0.081	0.01	2.40E-12
ACTL6B	1.62E-16	0.121074	0.108	0.031	2.43E-12

SERPINE1	1.63E-16	-0.80967	0.008	0.091	2.43E-12
FBXL18	1.63E-16	0.180907	0.094	0.012	2.44E-12
AP1G1	1.65E-16	0.13764	0.109	0.021	2.46E-12
ATG10	1.66E-16	0.12443	0.109	0.027	2.48E-12
MESP1	1.67E-16	0.255526	0.127	0.021	2.49E-12
CASP1	1.72E-16	-0.68851	0.009	0.08	2.57E-12
RGP1	1.77E-16	0.164079	0.117	0.024	2.65E-12
SMYD5	1.78E-16	0.190825	0.104	0.014	2.66E-12
PVRL1	1.80E-16	0.215835	0.107	0.014	2.69E-12
CARD16	1.82E-16	-0.80205	0.023	0.116	2.73E-12
NRBF2	1.87E-16	-0.30519	0.1	0.074	2.80E-12
BOC	1.91E-16	0.244666	0.114	0.019	2.85E-12
RNF182	1.91E-16	0.231636	0.09	0.003	2.86E-12
TACC2	1.93E-16	0.169729	0.101	0.015	2.88E-12
PCMTD1	1.95E-16	0.200061	0.138	0.034	2.91E-12
SERGEF	1.99E-16	-0.14512	0.09	0.044	2.97E-12
PWP2	1.99E-16	0.163092	0.12	0.027	2.97E-12
TMTC4	2.00E-16	0.123479	0.109	0.026	2.98E-12
SBF1	2.02E-16	0.259203	0.09	0.003	3.02E-12
SMIM3	2.03E-16	-0.8936	0.009	0.106	3.04E-12
TGFB1I1	2.05E-16	-0.15259	0.086	0.046	3.06E-12
TMEM184C	2.06E-16	0.110821	0.101	0.022	3.07E-12
PRDM8	2.06E-16	0.252451	0.088	0.002	3.08E-12
ITGB3BP	2.08E-16	-0.2119	0.094	0.058	3.11E-12
MFSD12	2.18E-16	0.101885	0.096	0.021	3.25E-12
DTNB	2.22E-16	0.225531	0.077	0	3.32E-12
NPTXR	2.26E-16	0.151842	0.105	0.021	3.38E-12
RANGAP1	2.27E-16	0.125897	0.087	0.014	3.40E-12
TRAK1	2.37E-16	0.155492	0.109	0.021	3.54E-12
HDAC4	2.40E-16	0.286929	0.101	0.007	3.59E-12
PRKCI	2.44E-16	0.11391	0.094	0.015	3.65E-12
TFAP4	2.46E-16	0.182713	0.1	0.012	3.67E-12
GCLM	2.47E-16	-0.21661	0.081	0.055	3.68E-12
AADAT	2.54E-16	0.248359	0.086	0.002	3.79E-12
SLC12A5	2.56E-16	0.209222	0.094	0.01	3.82E-12
UHRF2	2.58E-16	0.156169	0.088	0.01	3.85E-12
FRA10AC1	2.59E-16	0.112296	0.112	0.027	3.88E-12
SORT1	2.66E-16	-0.17128	0.09	0.048	3.97E-12
MEGF11	2.67E-16	0.216713	0.099	0.01	3.99E-12
ZNF620	2.69E-16	0.159666	0.095	0.014	4.03E-12
PEAK1	2.70E-16	-0.12072	0.066	0.026	4.03E-12
HRASLS	2.70E-16	0.295014	0.099	0.009	4.04E-12
EPC2	2.83E-16	0.193078	0.112	0.021	4.23E-12
MFSD5	2.83E-16	0.194763	0.086	0.009	4.23E-12
DLG2	2.88E-16	0.202787	0.096	0.009	4.31E-12
FBRSL1	3.43E-16	0.259668	0.092	0.005	5.12E-12
PISD	3.43E-16	0.155134	0.107	0.017	5.13E-12
IFIT5	3.48E-16	0.129269	0.111	0.026	5.21E-12
PUSL1	3.71E-16	0.215245	0.098	0.01	5.55E-12
GFPT1	3.89E-16	0.233012	0.112	0.015	5.81E-12
ANGEL1	3.90E-16	0.139411	0.092	0.015	5.83E-12

MTPAP	3.92E-16	0.157707	0.105	0.017	5.86E-12
FZR1	4.00E-16	0.152666	0.125	0.031	5.98E-12
ZNF236	4.01E-16	0.239247	0.109	0.015	5.99E-12
ZNF746	4.02E-16	0.150894	0.1	0.017	6.01E-12
TPM2	4.04E-16	-0.31233	0.066	0.056	6.04E-12
TMOD2	4.06E-16	0.117811	0.104	0.024	6.07E-12
NFE2L3	4.08E-16	0.222462	0.075	0	6.10E-12
ZNF134	4.12E-16	0.103755	0.101	0.022	6.15E-12
ACOT9	4.31E-16	-0.16291	0.086	0.048	6.44E-12
TERF2	4.33E-16	0.105697	0.092	0.017	6.48E-12
RP11-713F	4.34E-16	0.17624	0.101	0.015	6.48E-12
FAM222A	4.37E-16	0.252363	0.087	0.003	6.53E-12
NEDD9	4.39E-16	-0.29921	0.105	0.089	6.56E-12
PITPNC1	4.43E-16	0.174433	0.104	0.019	6.62E-12
ZNF532	4.43E-16	0.170161	0.1	0.014	6.62E-12
ATG16L1	4.45E-16	0.114581	0.081	0.01	6.65E-12
ONECUT2	4.49E-16	0.238191	0.09	0.005	6.71E-12
QSER1	4.55E-16	0.16608	0.099	0.015	6.80E-12
AGBL5	4.61E-16	0.226842	0.088	0.005	6.89E-12
FBXO33	4.87E-16	0.150411	0.088	0.01	7.28E-12
GAB1	5.07E-16	0.111526	0.109	0.026	7.58E-12
CDK8	5.24E-16	0.265325	0.098	0.007	7.83E-12
LRP12	5.33E-16	0.154619	0.1	0.019	7.96E-12
INHBB	5.39E-16	0.168413	0.113	0.022	8.05E-12
TAF3	5.45E-16	0.176288	0.111	0.019	8.14E-12
SLC27A5	5.49E-16	-0.42696	0.088	0.092	8.20E-12
CLASP1	5.52E-16	0.181071	0.139	0.038	8.25E-12
MAPK8IP2	5.88E-16	0.188077	0.087	0.01	8.79E-12
TMEM150A	5.90E-16	-0.11997	0.09	0.046	8.81E-12
PCOLCE	5.95E-16	-0.16946	0.075	0.041	8.90E-12
GPATCH3	6.06E-16	0.188868	0.088	0.007	9.06E-12
BCL11B	6.09E-16	0.252776	0.092	0.007	9.10E-12
DACT1	6.16E-16	0.291172	0.104	0.007	9.21E-12
MAP1LC3B2	6.18E-16	0.115211	0.107	0.029	9.23E-12
ERCC5	6.21E-16	0.204634	0.104	0.015	9.27E-12
USP49	6.32E-16	0.204103	0.086	0.007	9.45E-12
NDST1	7.01E-16	0.224019	0.094	0.005	1.05E-11
WDR76	7.03E-16	0.228196	0.086	0.005	1.05E-11
XAF1	7.33E-16	-0.72687	0.018	0.097	1.10E-11
UBE2T	7.40E-16	0.232604	0.088	0.007	1.11E-11
TMEM42	7.44E-16	-0.15175	0.096	0.053	1.11E-11
ZNF331	7.47E-16	-0.10606	0.104	0.05	1.12E-11
DNAJA4	7.53E-16	0.25155	0.098	0.007	1.12E-11
LRRC24	7.73E-16	0.179147	0.087	0.01	1.16E-11
SOWAHC	7.89E-16	0.11388	0.098	0.021	1.18E-11
MBNL2	8.20E-16	0.107772	0.09	0.017	1.23E-11
METTL14	8.31E-16	0.131298	0.111	0.027	1.24E-11
PIK3IP1	8.66E-16	0.180396	0.113	0.022	1.29E-11
DGCR8	8.89E-16	0.137992	0.107	0.024	1.33E-11
TIPARP	8.94E-16	-0.14191	0.1	0.05	1.34E-11
RCE1	8.97E-16	0.201266	0.12	0.024	1.34E-11

FAM118A	9.42E-16	0.178511	0.1	0.017	1.41E-11
PAQR6	9.52E-16	0.122346	0.109	0.027	1.42E-11
INTS8	9.88E-16	0.201023	0.105	0.017	1.48E-11
NCKAP5	1.01E-15	0.224574	0.107	0.014	1.50E-11
HEBP1	1.01E-15	-0.22391	0.07	0.043	1.50E-11
WDR25	1.05E-15	-0.19521	0.062	0.034	1.57E-11
SGPL1	1.07E-15	0.144667	0.1	0.019	1.61E-11
ETS2	1.09E-15	0.114041	0.092	0.019	1.63E-11
EMILIN3	1.11E-15	0.165133	0.099	0.019	1.67E-11
ADAMTS10	1.17E-15	0.191099	0.082	0.005	1.75E-11
MSH3	1.19E-15	0.176771	0.079	0.005	1.78E-11
COL9A1	1.19E-15	0.160336	0.072	0.003	1.78E-11
SETD6	1.22E-15	0.257487	0.127	0.022	1.83E-11
B4GALNT4	1.24E-15	0.226975	0.085	0.005	1.85E-11
GAD1	1.30E-15	0.135262	0.108	0.024	1.95E-11
NPY	1.34E-15	0.367108	0.094	0.005	2.00E-11
DCTN5	1.34E-15	-0.1101	0.072	0.034	2.00E-11
SLC4A4	1.38E-15	-0.69544	0.021	0.101	2.06E-11
C16orf52	1.40E-15	0.161102	0.098	0.017	2.09E-11
TARSL2	1.42E-15	0.23398	0.13	0.026	2.12E-11
RP11-127E	1.46E-15	0.173691	0.091	0.012	2.18E-11
VPS8	1.54E-15	0.164196	0.107	0.022	2.30E-11
MFN1	1.56E-15	0.137349	0.099	0.017	2.33E-11
ZNF423	1.58E-15	0.252856	0.086	0.003	2.36E-11
PLOD1	1.58E-15	-0.18441	0.087	0.06	2.36E-11
SLC37A1	1.60E-15	0.191655	0.09	0.007	2.39E-11
CDK9	1.60E-15	0.170079	0.114	0.024	2.40E-11
TMEM2	1.60E-15	0.290003	0.107	0.01	2.40E-11
KIAA2013	1.65E-15	0.187095	0.105	0.019	2.47E-11
XPOT	1.66E-15	-0.16352	0.101	0.063	2.48E-11
ACTN1	1.67E-15	-0.26852	0.096	0.074	2.49E-11
NBPF10	1.67E-15	0.288106	0.124	0.017	2.50E-11
SETD3	1.68E-15	0.116361	0.104	0.026	2.51E-11
PDLIM4	1.69E-15	-0.45332	0.043	0.068	2.52E-11
RYBP	1.73E-15	0.134443	0.095	0.017	2.58E-11
DSN1	1.76E-15	0.251095	0.094	0.007	2.64E-11
MAPK8IP3	1.77E-15	0.142456	0.098	0.017	2.65E-11
LRP5	1.78E-15	0.142088	0.085	0.01	2.67E-11
ISG20	1.79E-15	-0.68829	0.018	0.096	2.67E-11
CPVL	1.85E-15	-0.1098	0.087	0.046	2.77E-11
ACACA	1.87E-15	0.175453	0.092	0.014	2.80E-11
RIC8B	1.87E-15	0.110785	0.108	0.027	2.80E-11
MON2	1.89E-15	0.175208	0.129	0.034	2.82E-11
ZNF250	1.95E-15	0.120363	0.101	0.024	2.91E-11
CHFR	1.95E-15	-0.11751	0.092	0.053	2.92E-11
THAP6	1.99E-15	0.145081	0.09	0.015	2.98E-11
UTP14C	2.03E-15	0.145574	0.092	0.014	3.04E-11
KIAA0196	2.06E-15	0.15336	0.113	0.026	3.08E-11
DESI1	2.10E-15	0.138903	0.105	0.022	3.13E-11
MPDZ	2.23E-15	0.213003	0.103	0.014	3.33E-11
PPP2R3B	2.34E-15	0.133859	0.078	0.007	3.50E-11

IGFBP3	2.37E-15	-0.8421	0.016	0.082	3.54E-11
ICA1L	2.39E-15	0.153306	0.134	0.041	3.58E-11
CDC27	2.42E-15	0.18496	0.101	0.017	3.62E-11
NR2C1	2.44E-15	0.148208	0.098	0.017	3.64E-11
LRIG1	2.58E-15	-0.12589	0.086	0.048	3.85E-11
LPL	2.68E-15	-0.58645	0.059	0.113	4.01E-11
GTPBP8	2.74E-15	-0.2241	0.074	0.044	4.09E-11
TMEM179	2.79E-15	0.23665	0.083	0.002	4.17E-11
FKBP11	2.79E-15	-0.21591	0.079	0.048	4.17E-11
SERPINH1	2.81E-15	-0.40542	0.078	0.08	4.19E-11
S100PBP	2.82E-15	-0.11939	0.085	0.043	4.21E-11
ALYREF	2.83E-15	0.224509	0.111	0.019	4.22E-11
TNRC18	2.95E-15	0.19082	0.104	0.017	4.41E-11
GATAD2A	3.03E-15	0.20114	0.101	0.015	4.53E-11
PRDM4	3.10E-15	0.18996	0.098	0.012	4.63E-11
SMCHD1	3.12E-15	0.124762	0.112	0.029	4.66E-11
SFT2D3	3.17E-15	0.248304	0.086	0.003	4.73E-11
TTC8	3.22E-15	-0.18086	0.074	0.044	4.81E-11
CNOT6L	3.27E-15	0.214782	0.104	0.015	4.89E-11
PRR7	3.27E-15	0.190515	0.122	0.027	4.89E-11
RAP2B	3.29E-15	0.196595	0.094	0.01	4.91E-11
EPSTI1	3.33E-15	-0.28166	0.082	0.056	4.98E-11
SS18L1	3.33E-15	0.233799	0.092	0.007	4.98E-11
SULT1C4	3.51E-15	0.134455	0.075	0.009	5.25E-11
PPP2R5D	3.54E-15	0.243075	0.096	0.007	5.30E-11
UST	3.58E-15	0.149508	0.072	0.005	5.35E-11
RAPGEF2	3.58E-15	0.162448	0.074	0.005	5.35E-11
WRN	3.66E-15	0.140583	0.073	0.007	5.46E-11
KIAA0930	3.66E-15	0.147753	0.082	0.01	5.47E-11
PIBF1	3.77E-15	0.293462	0.121	0.017	5.63E-11
FAM171A1	3.78E-15	0.158918	0.087	0.012	5.65E-11
PROSER1	3.78E-15	0.242209	0.098	0.009	5.65E-11
STK32B	3.79E-15	0.318142	0.096	0.005	5.67E-11
TRIM8	3.85E-15	0.247654	0.083	0.002	5.75E-11
DOLK	3.90E-15	-0.12703	0.086	0.048	5.83E-11
LMNB2	3.94E-15	0.165585	0.091	0.012	5.89E-11
SAV1	3.97E-15	0.124076	0.104	0.024	5.93E-11
IL17D	3.97E-15	0.111154	0.075	0.01	5.94E-11
ADNP2	3.99E-15	0.153773	0.086	0.01	5.96E-11
WDR3	4.03E-15	0.261362	0.114	0.017	6.02E-11
PHC1	4.07E-15	0.264937	0.112	0.014	6.08E-11
PSMC3IP	4.08E-15	0.187251	0.086	0.009	6.09E-11
HOTAIR	4.19E-15	0.176232	0.087	0.009	6.26E-11
1-Mar	4.21E-15	0.228236	0.094	0.009	6.29E-11
CAPN7	4.21E-15	0.186678	0.112	0.022	6.30E-11
FASTKD2	4.34E-15	0.135139	0.117	0.029	6.48E-11
HPRT1	4.41E-15	-0.2085	0.092	0.063	6.59E-11
CPSF1	4.42E-15	0.131819	0.105	0.026	6.60E-11
WDR86	4.44E-15	0.254563	0.086	0.003	6.63E-11
GNA11	4.57E-15	0.112308	0.1	0.024	6.83E-11
SLC7A6	4.61E-15	0.139609	0.094	0.015	6.89E-11

ZDHHC14	4.63E-15	0.139716	0.081	0.01	6.92E-11
SLC2A1	4.73E-15	-0.31937	0.087	0.065	7.08E-11
FAM110A	4.77E-15	0.213969	0.088	0.009	7.13E-11
PGM2	5.08E-15	-0.13116	0.082	0.041	7.59E-11
TSTD2	5.36E-15	0.157991	0.101	0.021	8.00E-11
CDYL	5.39E-15	0.180474	0.079	0.005	8.06E-11
MAU2	5.51E-15	0.108741	0.125	0.038	8.23E-11
PROM1	5.54E-15	0.269736	0.092	0.005	8.28E-11
TEFM	5.75E-15	0.102424	0.101	0.026	8.59E-11
SIL1	6.06E-15	-0.24161	0.107	0.08	9.06E-11
METTL6	6.17E-15	0.109283	0.1	0.024	9.22E-11
RP11-379H	6.22E-15	0.146606	0.098	0.019	9.29E-11
MESDC1	6.29E-15	0.199057	0.092	0.01	9.40E-11
IRGQ	6.42E-15	0.201032	0.107	0.017	9.60E-11
TTI2	6.47E-15	0.104095	0.094	0.021	9.66E-11
ZC3H3	6.48E-15	0.192138	0.087	0.009	9.69E-11
GPNMB	6.76E-15	-0.25232	0.059	0.044	1.01E-10
DHX16	6.87E-15	0.115338	0.096	0.019	1.03E-10
C6orf120	7.14E-15	0.119981	0.088	0.015	1.07E-10
CENPW	7.15E-15	0.106048	0.077	0.012	1.07E-10
HSDL2	7.20E-15	-0.33531	0.057	0.051	1.08E-10
BMPR1A	7.32E-15	0.11696	0.094	0.019	1.09E-10
ZWILCH	7.33E-15	0.137986	0.077	0.009	1.10E-10
PPFIA2	7.39E-15	0.238773	0.096	0.009	1.10E-10
HOXB5	7.41E-15	0.220994	0.079	0.002	1.11E-10
GNB1L	7.49E-15	0.146755	0.098	0.019	1.12E-10
ADAL	7.95E-15	0.126763	0.1	0.021	1.19E-10
TFDP1	7.95E-15	0.103204	0.103	0.027	1.19E-10
CCDC22	8.01E-15	0.204226	0.109	0.019	1.20E-10
ZNF536	8.14E-15	0.183329	0.07	0.002	1.22E-10
CARS2	8.43E-15	0.100341	0.095	0.024	1.26E-10
DDHD1	8.47E-15	0.124482	0.098	0.022	1.27E-10
PPP1R16A	8.55E-15	-0.11144	0.12	0.07	1.28E-10
WDR5	8.74E-15	0.124733	0.103	0.024	1.31E-10
RFX5	8.80E-15	0.12498	0.09	0.019	1.31E-10
FASTKD1	8.91E-15	0.146102	0.103	0.021	1.33E-10
ZNF675	9.22E-15	0.125908	0.091	0.017	1.38E-10
LINC01117	9.43E-15	0.245265	0.087	0.005	1.41E-10
ANKRD13A	9.44E-15	0.230471	0.113	0.019	1.41E-10
ARID3A	9.50E-15	0.127712	0.103	0.026	1.42E-10
LHX1	9.71E-15	0.313187	0.098	0.009	1.45E-10
TAF1C	1.01E-14	0.167312	0.096	0.015	1.51E-10
GAS2L1	1.03E-14	0.27795	0.139	0.034	1.54E-10
IQCK	1.05E-14	-0.10385	0.103	0.051	1.56E-10
MYCN	1.05E-14	0.300162	0.098	0.007	1.57E-10
TAZ	1.08E-14	0.172992	0.107	0.021	1.62E-10
ASUN	1.10E-14	0.17757	0.108	0.022	1.65E-10
C19orf68	1.15E-14	0.162172	0.081	0.007	1.72E-10
LOXL1	1.15E-14	0.213688	0.086	0.005	1.72E-10
ZNF222	1.17E-14	0.122244	0.087	0.015	1.75E-10
ZNF500	1.18E-14	0.117527	0.094	0.019	1.77E-10

H2AFY2	1.21E-14	0.207386	0.075	0.003	1.81E-10
TTC21B	1.25E-14	0.243164	0.09	0.007	1.87E-10
GPR19	1.27E-14	0.111281	0.094	0.021	1.90E-10
MMS22L	1.28E-14	0.113768	0.099	0.024	1.92E-10
CLHC1	1.29E-14	0.23123	0.12	0.024	1.93E-10
CA12	1.30E-14	-0.58532	0.018	0.07	1.94E-10
PLAA	1.30E-14	0.106705	0.077	0.012	1.95E-10
RP13-1032	1.31E-14	0.1591	0.079	0.01	1.96E-10
MTA2	1.35E-14	0.180178	0.072	0.003	2.02E-10
GNB5	1.39E-14	0.217967	0.107	0.017	2.08E-10
LRP10	1.40E-14	-0.26004	0.085	0.07	2.09E-10
SETMAR	1.52E-14	0.163935	0.088	0.014	2.28E-10
EBF1	1.54E-14	0.234513	0.068	0	2.30E-10
HOXA10-AS	1.54E-14	0.20906	0.068	0	2.30E-10
CENPJ	1.59E-14	0.14214	0.088	0.015	2.38E-10
DGAT1	1.64E-14	0.203838	0.087	0.009	2.45E-10
ALDH1L1	1.68E-14	-0.6429	0.048	0.12	2.51E-10
CSTF2	1.74E-14	0.195958	0.09	0.01	2.59E-10
GDF15	1.74E-14	-0.75282	0.018	0.096	2.60E-10
FOXRED1	1.79E-14	0.138959	0.105	0.026	2.67E-10
EPHX1	1.80E-14	-0.14031	0.095	0.053	2.69E-10
TMCC3	1.80E-14	0.175377	0.068	0.002	2.69E-10
MGAT4A	1.86E-14	0.129469	0.098	0.022	2.78E-10
FAM69B	1.93E-14	0.123695	0.073	0.009	2.88E-10
HEXDC	1.94E-14	0.183022	0.094	0.015	2.90E-10
DROSHA	1.94E-14	0.188792	0.103	0.014	2.91E-10
PHTF1	1.96E-14	0.233147	0.1	0.012	2.93E-10
CYP27B1	2.03E-14	0.196483	0.081	0.003	3.04E-10
SPRED1	2.04E-14	0.104901	0.107	0.027	3.05E-10
CUL2	2.19E-14	0.12638	0.088	0.019	3.27E-10
AC093323.	2.22E-14	0.188046	0.095	0.014	3.32E-10
GIT1	2.25E-14	0.112813	0.082	0.014	3.36E-10
HAUS6	2.32E-14	0.162234	0.087	0.01	3.47E-10
EIF1AD	2.34E-14	-0.10121	0.085	0.043	3.49E-10
FBLIM1	2.50E-14	-0.23463	0.073	0.063	3.74E-10
THOC5	2.52E-14	0.184835	0.1	0.017	3.76E-10
ATAD2B	2.74E-14	0.20426	0.082	0.005	4.10E-10
PRUNE2	2.75E-14	-0.28072	0.09	0.08	4.10E-10
XRRA1	2.93E-14	0.1654	0.135	0.041	4.38E-10
ZFP37	2.95E-14	0.140443	0.087	0.015	4.40E-10
TTC26	2.97E-14	-0.16595	0.052	0.029	4.44E-10
RIN2	3.01E-14	0.247669	0.101	0.01	4.49E-10
YPEL1	3.14E-14	0.253857	0.098	0.009	4.70E-10
DPCD	3.15E-14	-0.37791	0.073	0.062	4.70E-10
P4HA1	3.15E-14	-0.44227	0.062	0.082	4.71E-10
NCAM2	3.17E-14	0.114423	0.105	0.027	4.74E-10
KCTD12	3.27E-14	-0.17242	0.056	0.029	4.89E-10
ZNF736	3.42E-14	0.102888	0.088	0.021	5.11E-10
C17orf10C	3.54E-14	0.216852	0.09	0.01	5.29E-10
TGFBRAP1	3.58E-14	0.122319	0.077	0.012	5.36E-10
PTPRO	3.59E-14	0.200118	0.088	0.007	5.36E-10

KLC2	3.62E-14	0.172528	0.095	0.015	5.41E-10
SERINC5	3.79E-14	0.190471	0.091	0.014	5.66E-10
UBFD1	3.79E-14	0.1172	0.088	0.019	5.66E-10
TYW5	3.79E-14	-0.10482	0.091	0.053	5.67E-10
EPB41	4.01E-14	0.254575	0.086	0.005	5.99E-10
SLC27A1	4.08E-14	-0.3515	0.052	0.055	6.09E-10
TRIM52	4.25E-14	0.1058	0.083	0.015	6.36E-10
OXSM	4.31E-14	-0.10818	0.068	0.027	6.44E-10
PANK3	4.45E-14	0.103608	0.094	0.021	6.65E-10
FRMD3	4.49E-14	-0.58611	0.005	0.072	6.72E-10
SRSF12	4.52E-14	0.19075	0.086	0.01	6.75E-10
R3HDM2	4.52E-14	0.260155	0.116	0.021	6.76E-10
LINC00237	4.71E-14	0.160967	0.078	0.007	7.04E-10
ERBB3	4.72E-14	0.253171	0.087	0.007	7.05E-10
PNRC2	4.80E-14	-0.27248	0.07	0.046	7.17E-10
HECW1	4.88E-14	0.223016	0.081	0.003	7.30E-10
C17orf51	4.96E-14	0.216465	0.092	0.01	7.41E-10
LAMA5	4.98E-14	0.12494	0.095	0.022	7.44E-10
TENM2	4.99E-14	0.123009	0.062	0.003	7.46E-10
SYNDIG1	5.00E-14	0.197864	0.081	0.005	7.47E-10
ATP6V0A2	5.14E-14	0.192061	0.092	0.014	7.68E-10
SLC16A14	5.15E-14	0.177267	0.065	0	7.70E-10
RAB8B	5.23E-14	-0.13118	0.079	0.039	7.81E-10
SUOX	5.34E-14	0.152079	0.085	0.014	7.99E-10
GBA	5.35E-14	-0.14907	0.064	0.032	7.99E-10
BRSK2	5.36E-14	0.253776	0.092	0.007	8.01E-10
PRIM2	5.51E-14	0.183051	0.073	0.003	8.24E-10
ZNF346	5.53E-14	0.213853	0.092	0.01	8.26E-10
EIF2AK4	5.54E-14	0.103926	0.103	0.027	8.28E-10
H3F3C	5.97E-14	-0.4305	0.046	0.079	8.92E-10
SOS1	6.00E-14	0.175286	0.101	0.019	8.96E-10
INO80B	6.03E-14	0.228085	0.094	0.009	9.02E-10
ZSWIM6	6.15E-14	0.233258	0.1	0.012	9.18E-10
CYB5R4	6.24E-14	0.170465	0.09	0.014	9.33E-10
FAM212B-A	6.34E-14	0.237656	0.085	0.007	9.48E-10
NR4A2	6.39E-14	0.143536	0.087	0.014	9.55E-10
ZNF609	6.43E-14	0.146828	0.09	0.015	9.61E-10
TTI1	6.47E-14	0.15738	0.101	0.022	9.67E-10
LZTR1	6.66E-14	0.140652	0.086	0.014	9.95E-10
FLCN	6.69E-14	-0.15378	0.096	0.056	1.00E-09
UBE4B	7.00E-14	0.242067	0.116	0.022	1.05E-09
TREM2	7.19E-14	-0.1185	0.074	0.039	1.07E-09
MAP2K4	7.30E-14	0.15938	0.099	0.021	1.09E-09
DHX8	7.33E-14	0.216095	0.09	0.009	1.10E-09
SMG7	7.38E-14	0.153206	0.092	0.017	1.10E-09
RRAGB	7.40E-14	0.101953	0.098	0.026	1.11E-09
FLII	7.56E-14	0.106766	0.096	0.022	1.13E-09
SLC39A13	7.61E-14	0.181445	0.113	0.026	1.14E-09
SIPA1L1	7.67E-14	-0.16907	0.07	0.043	1.15E-09
KTI12	7.77E-14	0.175701	0.092	0.014	1.16E-09
GPR37L1	7.84E-14	-0.46339	0.096	0.121	1.17E-09

MAST1	7.99E-14	0.186662	0.088	0.012	1.19E-09
EN2	8.04E-14	0.234845	0.074	0.002	1.20E-09
PAN3	8.13E-14	0.136866	0.105	0.027	1.22E-09
AC002454.	8.17E-14	0.125889	0.073	0.012	1.22E-09
ETV6	8.34E-14	0.144369	0.108	0.026	1.25E-09
HK1	8.60E-14	-0.10183	0.081	0.041	1.29E-09
SCRIB	8.63E-14	0.200664	0.112	0.024	1.29E-09
ZNF48	8.67E-14	0.182632	0.085	0.01	1.30E-09
CTPS2	9.02E-14	0.167437	0.078	0.007	1.35E-09
ZNF598	9.12E-14	0.155583	0.072	0.005	1.36E-09
RP3-428L1	9.41E-14	0.174908	0.064	0	1.41E-09
FN1	9.43E-14	-0.3261	0.066	0.055	1.41E-09
ANKRD6	9.61E-14	0.229882	0.095	0.012	1.44E-09
EMID1	1.00E-13	0.172453	0.088	0.014	1.50E-09
ZNF668	1.01E-13	0.215377	0.09	0.01	1.51E-09
FANCA	1.01E-13	0.16849	0.078	0.009	1.51E-09
POLR3C	1.01E-13	0.131594	0.092	0.019	1.51E-09
ZNF853	1.04E-13	0.164507	0.077	0.007	1.55E-09
GCC1	1.06E-13	0.227197	0.101	0.014	1.58E-09
FAM120B	1.07E-13	0.128556	0.083	0.012	1.60E-09
EXD2	1.08E-13	0.143889	0.103	0.024	1.61E-09
DSCAML1	1.10E-13	0.22658	0.085	0.005	1.65E-09
AC074289.	1.13E-13	0.203795	0.075	0.005	1.69E-09
NME2	1.13E-13	0.189091	0.078	0.007	1.69E-09
PRKD2	1.14E-13	0.107442	0.081	0.014	1.70E-09
NACAD	1.14E-13	0.147632	0.103	0.026	1.71E-09
PIK3CA	1.16E-13	0.191541	0.1	0.019	1.73E-09
HDAC11	1.17E-13	0.125347	0.07	0.009	1.75E-09
C16orf59	1.18E-13	0.182738	0.077	0.003	1.77E-09
STK38	1.19E-13	0.12884	0.074	0.01	1.78E-09
RAI1	1.21E-13	0.233887	0.077	0.003	1.81E-09
ZNF320	1.26E-13	0.134068	0.069	0.007	1.88E-09
ELOVL6	1.26E-13	0.151522	0.087	0.014	1.89E-09
INTS9	1.27E-13	0.111006	0.066	0.007	1.90E-09
PPIC	1.32E-13	-0.19331	0.077	0.051	1.97E-09
MYADM	1.33E-13	-0.37408	0.066	0.062	1.99E-09
ST3GAL4	1.35E-13	-0.1433	0.059	0.031	2.01E-09
TNFRSF10E	1.36E-13	-0.20008	0.065	0.038	2.03E-09
OGFRL1	1.38E-13	0.230099	0.079	0.003	2.06E-09
PHOSPHO2	1.38E-13	0.165649	0.087	0.014	2.07E-09
CEP97	1.39E-13	0.161774	0.083	0.012	2.08E-09
HMOX1	1.42E-13	-0.45083	0.044	0.051	2.12E-09
KIAA1614	1.42E-13	0.227038	0.081	0.003	2.12E-09
PC	1.44E-13	0.159968	0.086	0.012	2.16E-09
GPHN	1.45E-13	0.149858	0.108	0.026	2.17E-09
ELOVL1	1.47E-13	-0.34645	0.059	0.06	2.19E-09
AGA	1.50E-13	-0.19238	0.081	0.055	2.24E-09
FXYD1	1.52E-13	0.18518	0.103	0.026	2.28E-09
NBL1	1.54E-13	0.213345	0.096	0.012	2.30E-09
STAT5B	1.54E-13	0.110919	0.087	0.017	2.30E-09
FLVCR1	1.56E-13	0.104514	0.092	0.021	2.34E-09

GJA1	1.57E-13	-0.57703	0.051	0.099	2.34E-09
DBR1	1.61E-13	0.151581	0.095	0.017	2.41E-09
RP11-436K	1.63E-13	0.240368	0.086	0.007	2.43E-09
DUSP8	1.67E-13	0.178914	0.075	0.005	2.49E-09
C2orf42	1.68E-13	0.15338	0.082	0.012	2.51E-09
CRYZ	1.69E-13	-0.36434	0.069	0.07	2.52E-09
MMP15	1.69E-13	0.187613	0.074	0.005	2.52E-09
LRRN2	1.69E-13	0.160604	0.079	0.009	2.53E-09
ENTPD3-AS	1.71E-13	-0.13282	0.062	0.034	2.55E-09
KDM5D	1.72E-13	0.256218	0.062	0	2.57E-09
AMPH	1.72E-13	0.167662	0.062	0	2.57E-09
ZNF345	1.74E-13	0.114295	0.075	0.014	2.61E-09
SLC2A8	1.79E-13	0.132097	0.086	0.015	2.67E-09
ZNF771	1.80E-13	0.103728	0.1	0.027	2.69E-09
NLGN4Y	1.84E-13	0.168935	0.073	0.003	2.75E-09
RBBP9	1.87E-13	0.142578	0.083	0.012	2.79E-09
YDJC	1.88E-13	0.201041	0.107	0.021	2.81E-09
LCLAT1	1.91E-13	0.107102	0.069	0.009	2.86E-09
MANSC1	1.93E-13	0.133059	0.075	0.01	2.88E-09
TMEM19	1.98E-13	0.185067	0.1	0.019	2.95E-09
XRCC2	1.99E-13	0.227339	0.074	0.002	2.97E-09
ABHD5	1.99E-13	-0.24866	0.064	0.044	2.98E-09
CCDC121	2.13E-13	0.118809	0.065	0.007	3.19E-09
HHIP-AS1	2.14E-13	0.168632	0.07	0.003	3.20E-09
ATAD2	2.14E-13	0.179783	0.075	0.005	3.20E-09
GUF1	2.16E-13	0.141509	0.083	0.012	3.23E-09
CACNB3	2.17E-13	0.174404	0.083	0.009	3.24E-09
KCTD15	2.18E-13	0.123714	0.086	0.017	3.25E-09
MBTPS2	2.18E-13	0.159083	0.094	0.019	3.26E-09
SCLT1	2.20E-13	0.110942	0.068	0.007	3.29E-09
TOPBP1	2.21E-13	0.236832	0.096	0.012	3.30E-09
SLC25A32	2.26E-13	0.165445	0.092	0.015	3.37E-09
SFT2D2	2.26E-13	0.103878	0.081	0.017	3.38E-09
METAP1	2.32E-13	-0.10204	0.07	0.029	3.47E-09
ACAD10	2.33E-13	0.194485	0.092	0.012	3.48E-09
LIPE	2.34E-13	0.153632	0.068	0.003	3.50E-09
UTP14A	2.42E-13	0.113364	0.079	0.014	3.61E-09
C17orf80	2.43E-13	0.157348	0.098	0.017	3.62E-09
IFI44L	2.46E-13	-0.71713	0.036	0.111	3.68E-09
RASA1	2.47E-13	0.118379	0.088	0.019	3.70E-09
PFKP	2.50E-13	-0.19042	0.094	0.062	3.73E-09
SPTBN2	2.55E-13	0.17547	0.081	0.007	3.80E-09
HOXD3	2.58E-13	0.226938	0.073	0.002	3.86E-09
MGST2	2.59E-13	-0.26104	0.059	0.046	3.87E-09
TAPT1	2.61E-13	0.152229	0.085	0.015	3.90E-09
SLC37A4	2.74E-13	0.151979	0.091	0.017	4.09E-09
CTD-2339F	2.79E-13	0.336603	0.073	0.002	4.17E-09
GPD2	2.89E-13	0.10531	0.085	0.019	4.31E-09
TRUB1	2.93E-13	0.144412	0.086	0.014	4.39E-09
SWAP70	3.01E-13	-0.27214	0.07	0.051	4.50E-09
NKAIN1	3.13E-13	0.19439	0.061	0	4.68E-09

INA	3.13E-13	0.191736	0.061	0	4.68E-09
AC073283.	3.14E-13	0.162364	0.077	0.01	4.69E-09
RP11-111F	3.17E-13	0.131797	0.079	0.01	4.73E-09
ZIK1	3.27E-13	0.200393	0.092	0.014	4.88E-09
SOX10	3.28E-13	0.164886	0.066	0.005	4.90E-09
ADCK2	3.30E-13	0.10008	0.083	0.015	4.93E-09
GPATCH1	3.39E-13	0.127615	0.098	0.022	5.07E-09
REPS1	3.40E-13	0.104438	0.096	0.024	5.08E-09
NEUROD1	3.45E-13	0.148626	0.074	0.009	5.16E-09
KCNMB2	3.52E-13	0.151583	0.079	0.012	5.26E-09
DUSP4	3.52E-13	-0.32076	0.062	0.063	5.27E-09
ZNHIT6	3.53E-13	0.110818	0.094	0.022	5.28E-09
SLC27A3	3.62E-13	-0.2346	0.07	0.05	5.42E-09
POLG	3.63E-13	0.152483	0.085	0.012	5.42E-09
HNRNPU-AS	3.64E-13	0.171755	0.104	0.022	5.44E-09
GZF1	3.65E-13	0.167965	0.09	0.015	5.46E-09
IFI35	3.65E-13	-0.65727	0.018	0.094	5.46E-09
PM20D2	3.73E-13	0.126119	0.078	0.01	5.57E-09
WDR59	3.73E-13	0.189592	0.109	0.024	5.58E-09
TFCP2	3.80E-13	0.158266	0.096	0.019	5.68E-09
GKAP1	3.97E-13	0.129197	0.088	0.019	5.94E-09
IL17RB	3.97E-13	-0.18461	0.062	0.041	5.94E-09
NEK6	4.00E-13	-0.37715	0.061	0.067	5.98E-09
GBP4	4.10E-13	-0.37407	0.055	0.062	6.12E-09
E2F3	4.13E-13	0.183237	0.079	0.007	6.17E-09
PIPOX	4.33E-13	-0.36269	0.074	0.075	6.48E-09
RP1-17K7.	4.36E-13	-0.3884	0.04	0.068	6.52E-09
ZDBF2	4.47E-13	0.129541	0.065	0.005	6.68E-09
ZDHHC15	4.57E-13	0.110783	0.07	0.01	6.82E-09
CSMD1	4.61E-13	0.160891	0.07	0.005	6.90E-09
C11orf95	4.67E-13	0.212703	0.088	0.01	6.98E-09
ZBTB11	4.69E-13	0.104013	0.072	0.01	7.00E-09
FBXO30	4.72E-13	0.133042	0.064	0.003	7.05E-09
YTHDC2	4.76E-13	0.155137	0.095	0.019	7.11E-09
SUCLG2	4.79E-13	-0.32629	0.066	0.067	7.16E-09
KANSL1-AS	4.81E-13	-0.15731	0.065	0.043	7.19E-09
LRFN4	4.82E-13	0.16146	0.075	0.005	7.20E-09
MSANTD3	4.89E-13	0.167676	0.098	0.021	7.31E-09
CNKSR3	4.96E-13	0.226704	0.109	0.019	7.41E-09
MPND	5.20E-13	0.162788	0.068	0.003	7.76E-09
NEO1	5.50E-13	0.100573	0.074	0.012	8.22E-09
EPB41L4A	5.60E-13	0.162449	0.07	0.005	8.37E-09
ARSD	5.61E-13	0.123963	0.091	0.022	8.39E-09
USP37	5.67E-13	0.148461	0.085	0.014	8.47E-09
ACTR8	5.71E-13	0.136409	0.096	0.024	8.53E-09
NUP133	5.81E-13	0.110709	0.095	0.022	8.68E-09
MAML1	5.90E-13	0.156622	0.079	0.01	8.82E-09
STAT2	5.90E-13	-0.14099	0.087	0.055	8.82E-09
SEMA6C	6.09E-13	0.178848	0.073	0.003	9.10E-09
CEP104	6.13E-13	0.105493	0.088	0.022	9.16E-09
MLYCD	6.35E-13	0.171124	0.083	0.012	9.49E-09

OTUD4	6.36E-13	0.14092	0.072	0.007	9.50E-09
DIP2C	6.38E-13	0.164598	0.094	0.019	9.53E-09
BMP1	6.79E-13	0.165767	0.083	0.012	1.02E-08
RRBP1	6.98E-13	-0.27553	0.081	0.075	1.04E-08
COCH	7.10E-13	0.101429	0.074	0.014	1.06E-08
DDX28	7.23E-13	0.145851	0.094	0.019	1.08E-08
RP11-259K	7.31E-13	0.243799	0.079	0.005	1.09E-08
DES	7.44E-13	0.241284	0.107	0.019	1.11E-08
IFFO1	7.48E-13	0.120134	0.091	0.022	1.12E-08
SOS2	7.54E-13	0.157317	0.081	0.01	1.13E-08
RAB3B	7.68E-13	0.164798	0.075	0.009	1.15E-08
TMBIM1	8.01E-13	-0.55344	0.029	0.07	1.20E-08
ZC3H4	8.01E-13	0.199131	0.103	0.019	1.20E-08
PLEKHA2	8.14E-13	0.136487	0.103	0.026	1.22E-08
GLIS2	8.16E-13	0.138707	0.064	0.005	1.22E-08
PANK4	8.22E-13	0.123109	0.088	0.019	1.23E-08
ABHD10	8.54E-13	0.102072	0.074	0.014	1.28E-08
ERBB4	8.72E-13	0.138268	0.075	0.009	1.30E-08
EMILIN2	9.07E-13	0.10688	0.068	0.009	1.36E-08
ALG1	9.14E-13	0.151789	0.087	0.017	1.37E-08
MFI2-AS1	9.25E-13	0.109065	0.082	0.017	1.38E-08
ALG12	9.35E-13	0.110819	0.075	0.014	1.40E-08
JMJD4	9.53E-13	0.113774	0.083	0.015	1.42E-08
GPD1	9.54E-13	0.306141	0.086	0.009	1.43E-08
ZFHX2	9.63E-13	0.19761	0.064	0.002	1.44E-08
GTF3C4	1.02E-12	0.183135	0.085	0.012	1.52E-08
AQR	1.05E-12	0.196787	0.085	0.01	1.57E-08
PDLIM1	1.06E-12	-0.14463	0.061	0.029	1.58E-08
SCARA3	1.07E-12	-0.20055	0.082	0.06	1.60E-08
KIF3B	1.07E-12	0.200422	0.088	0.012	1.60E-08
PHF19	1.10E-12	-0.34872	0.064	0.065	1.65E-08
PIP5K1C	1.11E-12	0.109613	0.094	0.024	1.66E-08
TIMP3	1.12E-12	-0.54453	0.053	0.103	1.67E-08
ATRNL1	1.15E-12	0.220773	0.082	0.007	1.72E-08
PTPRG	1.16E-12	0.213918	0.103	0.019	1.73E-08
MANEA	1.16E-12	0.226196	0.07	0.002	1.73E-08
KIAA0232	1.17E-12	0.179355	0.099	0.021	1.75E-08
NEK3	1.18E-12	0.221541	0.082	0.007	1.76E-08
IRF9	1.19E-12	-0.22252	0.099	0.068	1.77E-08
ABCA5	1.20E-12	-0.10084	0.073	0.036	1.80E-08
USP19	1.20E-12	0.169868	0.086	0.014	1.80E-08
ZDHHC7	1.24E-12	0.197439	0.108	0.024	1.85E-08
SARM1	1.28E-12	0.232724	0.086	0.009	1.91E-08
GLS	1.29E-12	0.215259	0.101	0.019	1.93E-08
MSL2	1.34E-12	0.196589	0.079	0.007	2.01E-08
PRIM1	1.38E-12	0.216613	0.09	0.012	2.06E-08
CAPG	1.39E-12	-0.33177	0.074	0.084	2.08E-08
ZDHHC16	1.42E-12	0.23037	0.079	0.005	2.12E-08
DUSP3	1.44E-12	-0.18835	0.083	0.056	2.15E-08
FAM19A2	1.46E-12	0.157916	0.069	0.005	2.18E-08
FAM193A	1.47E-12	0.123499	0.085	0.017	2.20E-08

CCDC117	1.49E-12	0.169963	0.07	0.005	2.23E-08
MAP4K3	1.50E-12	0.159968	0.083	0.012	2.24E-08
COLGALT1	1.51E-12	-0.12349	0.073	0.039	2.26E-08
CTA-384D8	1.52E-12	0.142654	0.06	0.002	2.27E-08
RC3H2	1.57E-12	0.201196	0.073	0.005	2.35E-08
BCL9	1.64E-12	0.109948	0.073	0.01	2.45E-08
SHF	1.71E-12	0.122296	0.075	0.012	2.55E-08
HOXD8	1.72E-12	0.203665	0.07	0.002	2.58E-08
ZNF829	1.76E-12	0.134564	0.07	0.009	2.62E-08
MT1A	1.80E-12	-0.55016	0.007	0.067	2.69E-08
PGBD1	1.83E-12	0.188682	0.082	0.01	2.74E-08
GLRB	1.84E-12	0.159363	0.091	0.017	2.74E-08
ATP13A3	1.89E-12	0.136472	0.086	0.015	2.83E-08
APOL2	1.92E-12	-0.47351	0.039	0.062	2.88E-08
CLGN	1.95E-12	0.157528	0.072	0.009	2.92E-08
ZNF34	2.00E-12	-0.12158	0.066	0.031	2.99E-08
PTGR2	2.00E-12	0.150147	0.087	0.019	2.99E-08
NUMBL	2.07E-12	0.133963	0.085	0.015	3.09E-08
SLC20A2	2.08E-12	-0.28475	0.07	0.065	3.10E-08
ABCA3	2.09E-12	0.108425	0.09	0.021	3.13E-08
MAPK9	2.14E-12	0.152687	0.083	0.014	3.20E-08
FAM181A	2.18E-12	-0.37633	0.048	0.056	3.25E-08
AMDHD2	2.18E-12	0.11385	0.081	0.015	3.25E-08
SMYD4	2.22E-12	-0.11039	0.088	0.053	3.31E-08
TOMM40L	2.37E-12	0.101526	0.073	0.014	3.54E-08
RP11-445F	2.37E-12	0.24374	0.078	0.009	3.55E-08
SOX21-AS1	2.38E-12	0.177714	0.103	0.022	3.55E-08
WDR75	2.39E-12	0.144691	0.109	0.029	3.58E-08
MEF2A	2.49E-12	-0.14053	0.072	0.041	3.73E-08
GYPC	2.57E-12	-0.41702	0.035	0.053	3.84E-08
FOXD1	2.58E-12	0.216951	0.069	0.002	3.85E-08
NAT16	2.64E-12	0.196556	0.073	0.005	3.95E-08
ZNF667	2.70E-12	-0.10142	0.095	0.055	4.03E-08
BRPF1	2.72E-12	0.222005	0.083	0.009	4.07E-08
CTIF	2.73E-12	0.106194	0.068	0.01	4.08E-08
MAST4	2.93E-12	-0.19589	0.073	0.051	4.38E-08
DLEU1	2.94E-12	0.100264	0.087	0.022	4.39E-08
RNF122	2.94E-12	0.215186	0.066	0.002	4.39E-08
MTHFSD	2.97E-12	0.121897	0.077	0.014	4.43E-08
BRAF	3.01E-12	0.131259	0.091	0.021	4.49E-08
SLC39A9	3.03E-12	0.143201	0.078	0.014	4.52E-08
ODF2	3.13E-12	0.107334	0.095	0.024	4.67E-08
C9orf9	3.32E-12	-0.16093	0.074	0.05	4.97E-08
4-Sep	3.36E-12	0.149661	0.075	0.01	5.02E-08
MAGT1	3.42E-12	-0.27765	0.062	0.055	5.11E-08
SIK1	3.44E-12	0.136149	0.072	0.01	5.15E-08
CBX8	3.50E-12	0.143187	0.087	0.017	5.22E-08
CEP57L1	3.51E-12	0.140352	0.09	0.019	5.25E-08
CSRP1	3.55E-12	-0.23342	0.051	0.034	5.30E-08
TMBIM4	3.59E-12	-0.70203	0.017	0.101	5.36E-08
ME3	3.62E-12	0.182521	0.07	0.005	5.40E-08

PTRF	3.62E-12	-0.61994	0.034	0.082	5.41E-08
MFSD6	3.69E-12	0.148454	0.073	0.009	5.52E-08
RP11-400F	3.72E-12	0.165636	0.074	0.007	5.56E-08
DGKG	3.79E-12	0.194422	0.101	0.024	5.66E-08
RP11-725F	3.82E-12	0.187762	0.062	0.003	5.70E-08
CBLB	3.86E-12	0.125612	0.088	0.021	5.77E-08
SLCO5A1	3.89E-12	0.1959	0.073	0.005	5.81E-08
PHRF1	3.93E-12	0.181974	0.078	0.009	5.88E-08
TBC1D10B	3.95E-12	0.106218	0.07	0.014	5.90E-08
TTLL12	3.97E-12	0.17611	0.074	0.007	5.93E-08
SLC24A3	3.99E-12	0.151604	0.077	0.01	5.97E-08
CECR6	4.05E-12	0.206084	0.068	0.003	6.05E-08
ZDHHC13	4.08E-12	0.106817	0.075	0.015	6.09E-08
AUH	4.12E-12	0.105217	0.073	0.014	6.15E-08
FBXL17	4.15E-12	0.175676	0.075	0.009	6.20E-08
ST3GAL3	4.24E-12	0.136986	0.075	0.012	6.34E-08
VCPIP1	4.42E-12	0.209562	0.079	0.007	6.60E-08
GRB14	4.43E-12	0.175443	0.066	0.002	6.61E-08
SMPD3	4.50E-12	0.158422	0.062	0.003	6.72E-08
ZNF569	4.59E-12	0.128238	0.082	0.015	6.86E-08
TUBB6	4.70E-12	-0.53684	0.021	0.068	7.02E-08
NOS1AP	4.92E-12	-0.25234	0.051	0.036	7.35E-08
TMEM246	4.95E-12	0.267428	0.079	0.005	7.40E-08
FAM160B2	4.98E-12	0.180254	0.088	0.015	7.45E-08
SEC24B	5.00E-12	0.139762	0.074	0.012	7.48E-08
AKAP6	5.06E-12	-0.14692	0.085	0.053	7.56E-08
HID1	5.11E-12	0.201758	0.064	0.002	7.64E-08
RHOJ	5.12E-12	-0.15609	0.068	0.034	7.65E-08
HDAC10	5.12E-12	0.12527	0.062	0.005	7.66E-08
DDHD2	5.14E-12	0.199574	0.1	0.021	7.68E-08
SENP3	5.36E-12	0.129529	0.073	0.01	8.00E-08
FAM89A	5.39E-12	0.145085	0.061	0.003	8.06E-08
USP40	5.48E-12	0.16547	0.073	0.009	8.18E-08
GALNS	5.53E-12	0.166991	0.096	0.021	8.26E-08
PKP4	5.53E-12	0.189714	0.068	0.003	8.27E-08
SNX29	5.55E-12	0.182448	0.086	0.012	8.29E-08
ANXA4	5.57E-12	-0.27557	0.073	0.06	8.32E-08
ZNF286B	5.63E-12	0.190519	0.073	0.003	8.41E-08
DDX20	5.63E-12	0.139908	0.066	0.007	8.42E-08
SOCS2	5.77E-12	-0.18363	0.079	0.06	8.62E-08
XPNPEP3	5.94E-12	0.133697	0.095	0.022	8.88E-08
LIN52	5.97E-12	0.117346	0.075	0.014	8.92E-08
C9orf64	6.16E-12	0.123963	0.087	0.021	9.21E-08
RFX3	6.22E-12	0.172003	0.082	0.012	9.29E-08
ZNF496	6.29E-12	0.166478	0.072	0.007	9.41E-08
TMEM170B	6.30E-12	0.17249	0.055	0	9.42E-08
CDH8	6.30E-12	0.158944	0.055	0	9.42E-08
TUT1	6.41E-12	0.157477	0.082	0.014	9.58E-08
AC091729.	6.66E-12	0.195037	0.092	0.017	9.96E-08
CCDC152	6.77E-12	-0.10485	0.072	0.039	1.01E-07
PQLC1	6.82E-12	0.12858	0.082	0.017	1.02E-07

GPR137C	6.91E-12	0.203868	0.074	0.005	1.03E-07
ZNF574	7.02E-12	0.248757	0.095	0.012	1.05E-07
AP4S1	7.06E-12	-0.13438	0.052	0.024	1.05E-07
RBM38	7.13E-12	-0.11347	0.094	0.056	1.07E-07
PIGG	7.27E-12	0.161942	0.085	0.014	1.09E-07
CXorf56	7.31E-12	0.114373	0.07	0.01	1.09E-07
ZBED6	7.42E-12	0.117459	0.086	0.021	1.11E-07
UTP20	7.51E-12	0.145106	0.069	0.009	1.12E-07
SP4	7.58E-12	0.194452	0.083	0.009	1.13E-07
FCHSD2	7.59E-12	0.115221	0.082	0.017	1.13E-07
ZBTB25	7.84E-12	0.129859	0.074	0.012	1.17E-07
CSPP1	8.00E-12	0.110695	0.109	0.038	1.20E-07
PPP1R9B	8.08E-12	0.200682	0.083	0.01	1.21E-07
KIF13A	8.23E-12	0.176645	0.073	0.009	1.23E-07
EVI5L	8.31E-12	0.179732	0.072	0.007	1.24E-07
FDXR	8.40E-12	-0.12427	0.059	0.034	1.25E-07
KLHDC4	8.83E-12	0.239023	0.075	0.005	1.32E-07
MON1A	9.02E-12	0.123635	0.081	0.015	1.35E-07
CLEC16A	9.15E-12	0.139178	0.07	0.009	1.37E-07
CHAC2	9.25E-12	0.106884	0.059	0.007	1.38E-07
IL11RA	9.25E-12	0.158112	0.077	0.012	1.38E-07
RALGAPB	9.34E-12	0.149965	0.078	0.012	1.40E-07
SAA2	9.59E-12	-0.8236	0	0.048	1.43E-07
YPEL4	9.88E-12	0.212088	0.074	0.007	1.48E-07
POLRMT	9.88E-12	0.103368	0.113	0.039	1.48E-07
RUFY2	9.97E-12	0.135966	0.092	0.024	1.49E-07
FAM117B	1.00E-11	0.162782	0.074	0.009	1.50E-07
SLC38A6	1.01E-11	-0.1746	0.06	0.039	1.51E-07
RP11-127E	1.01E-11	0.125614	0.064	0.005	1.52E-07
MBTD1	1.04E-11	0.183348	0.068	0.003	1.56E-07
MAP6	1.05E-11	0.151101	0.069	0.009	1.56E-07
FADD	1.05E-11	0.171837	0.081	0.012	1.57E-07
CTH	1.06E-11	-0.2605	0.052	0.043	1.58E-07
FAM208B	1.10E-11	0.154231	0.061	0.003	1.64E-07
YIPF2	1.11E-11	-0.25095	0.073	0.06	1.65E-07
TADA2A	1.13E-11	0.124817	0.072	0.01	1.69E-07
MCPH1	1.13E-11	0.205616	0.09	0.014	1.69E-07
GPR153	1.14E-11	0.185809	0.064	0.003	1.70E-07
TIMELESS	1.16E-11	0.178971	0.072	0.005	1.74E-07
PIM1	1.19E-11	-0.10065	0.072	0.034	1.77E-07
CLSTN3	1.21E-11	0.118257	0.083	0.019	1.81E-07
BAIAP2	1.21E-11	-0.10323	0.107	0.062	1.81E-07
SGK3	1.31E-11	0.130447	0.064	0.005	1.95E-07
SLC35D2	1.32E-11	0.147398	0.074	0.01	1.97E-07
USP13	1.32E-11	0.171941	0.09	0.017	1.97E-07
ZNF672	1.32E-11	0.209273	0.099	0.017	1.97E-07
DDB2	1.35E-11	-0.19877	0.053	0.039	2.02E-07
DHRS11	1.36E-11	0.106104	0.068	0.01	2.03E-07
VAMP4	1.38E-11	-0.20444	0.083	0.063	2.07E-07
KALRN	1.39E-11	-0.22218	0.06	0.046	2.08E-07
TMEM158	1.41E-11	-0.31578	0.048	0.051	2.11E-07

USP36	1.45E-11	-0.13119	0.073	0.043	2.17E-07
TFB1M	1.46E-11	0.127309	0.081	0.017	2.18E-07
RP11-73E1	1.48E-11	0.217095	0.075	0.007	2.22E-07
ARL5B	1.51E-11	0.1884	0.072	0.005	2.25E-07
ZKSCAN8	1.52E-11	0.125529	0.069	0.009	2.26E-07
FHOD3	1.61E-11	0.161244	0.064	0.003	2.41E-07
SEC16A	1.62E-11	0.164541	0.095	0.021	2.42E-07
RAB9B	1.67E-11	0.181111	0.083	0.014	2.49E-07
BEND6	1.68E-11	0.146139	0.069	0.007	2.50E-07
NACC2	1.70E-11	0.121418	0.068	0.01	2.54E-07
MOB1B	1.70E-11	0.126764	0.059	0.003	2.54E-07
MYCL	1.73E-11	0.152638	0.062	0.003	2.58E-07
POU2F2	1.75E-11	0.196279	0.077	0.009	2.62E-07
ZBTB38	1.76E-11	-0.25788	0.061	0.055	2.63E-07
BCDIN3D	1.82E-11	0.146327	0.074	0.012	2.71E-07
KIF20B	1.82E-11	0.110504	0.07	0.012	2.73E-07
NCKIPSD	1.84E-11	0.139344	0.064	0.007	2.75E-07
RNF112	1.91E-11	0.172213	0.066	0.003	2.86E-07
RP11-356C	1.95E-11	0.172791	0.075	0.007	2.91E-07
SMYD2	1.99E-11	-0.10611	0.083	0.044	2.98E-07
EBF4	2.04E-11	0.157564	0.072	0.007	3.05E-07
GAB2	2.05E-11	0.117747	0.072	0.012	3.06E-07
NCAPG2	2.06E-11	0.105492	0.074	0.015	3.08E-07
SIX6	2.09E-11	0.156919	0.052	0	3.12E-07
ABTB1	2.11E-11	-0.13303	0.056	0.032	3.15E-07
TMEM53	2.12E-11	-0.29318	0.043	0.048	3.17E-07
LL22NC03-	2.15E-11	0.171403	0.082	0.01	3.21E-07
ANKRD26	2.16E-11	0.114476	0.092	0.026	3.23E-07
SS18	2.19E-11	-0.31321	0.052	0.06	3.27E-07
RP4-798A1	2.23E-11	0.145441	0.081	0.015	3.34E-07
RTN2	2.26E-11	0.184422	0.069	0.007	3.38E-07
MCAM	2.30E-11	0.105237	0.075	0.015	3.44E-07
SEC61A2	2.32E-11	0.142698	0.079	0.014	3.46E-07
GPR85	2.34E-11	0.111606	0.055	0.003	3.50E-07
ARHGAP17	2.36E-11	0.146748	0.066	0.007	3.52E-07
EPB41L5	2.38E-11	0.142706	0.066	0.007	3.55E-07
SREBF1	2.38E-11	0.222074	0.098	0.019	3.56E-07
DPAGT1	2.45E-11	-0.22719	0.064	0.05	3.67E-07
ANGPTL4	2.46E-11	-0.60525	0.016	0.074	3.68E-07
CCDC58	2.47E-11	0.179103	0.104	0.026	3.69E-07
LIN7B	2.48E-11	0.112403	0.086	0.022	3.71E-07
IQCG	2.54E-11	-0.35532	0.057	0.058	3.79E-07
GFOD1	2.59E-11	0.233669	0.079	0.007	3.88E-07
PCNX	2.60E-11	0.136866	0.09	0.022	3.88E-07
MARK1	2.61E-11	0.203817	0.088	0.012	3.90E-07
AREL1	2.61E-11	0.168486	0.064	0.003	3.90E-07
CTSS	2.62E-11	-0.2319	0.046	0.036	3.91E-07
5-Sep	2.69E-11	0.18484	0.072	0.005	4.03E-07
ZNF605	2.76E-11	-0.13647	0.085	0.06	4.12E-07
DIXDC1	2.79E-11	0.192882	0.072	0.007	4.17E-07
MEX3D	2.80E-11	0.142455	0.064	0.005	4.18E-07

ATP8A1	2.82E-11	0.185192	0.06	0.002	4.21E-07
TMEM67	2.92E-11	-0.21244	0.056	0.039	4.36E-07
EFCAB7	2.93E-11	0.162013	0.095	0.022	4.38E-07
PRMT3	2.94E-11	0.191505	0.096	0.019	4.40E-07
ATG2A	2.95E-11	0.122244	0.074	0.015	4.41E-07
LIMK2	3.15E-11	0.166919	0.07	0.007	4.70E-07
ZNF628	3.18E-11	0.131273	0.069	0.009	4.75E-07
PLEKHA5	3.21E-11	0.175219	0.085	0.017	4.79E-07
TP53BP1	3.22E-11	0.167274	0.091	0.019	4.81E-07
ELP4	3.23E-11	-0.17132	0.065	0.041	4.82E-07
MORC2	3.25E-11	0.175808	0.07	0.005	4.86E-07
ERCC6L2	3.27E-11	0.140031	0.085	0.017	4.89E-07
GNL3L	3.38E-11	0.109852	0.079	0.017	5.05E-07
CYBRD1	3.41E-11	-0.32493	0.06	0.063	5.10E-07
SLC2A13	3.58E-11	0.175377	0.094	0.019	5.35E-07
UBE2O	3.60E-11	0.172857	0.101	0.026	5.38E-07
MORC2-AS1	3.62E-11	0.123113	0.07	0.012	5.41E-07
RGL1	3.64E-11	-0.11249	0.052	0.022	5.44E-07
SLC35G2	3.64E-11	-0.29204	0.073	0.068	5.44E-07
RHPN1	3.72E-11	0.187781	0.064	0.003	5.56E-07
E2F1	3.74E-11	0.130665	0.073	0.012	5.59E-07
ARFGEF1	3.74E-11	0.164716	0.072	0.009	5.59E-07
RNF213	3.74E-11	-0.16337	0.081	0.051	5.59E-07
BMP2	3.76E-11	0.222149	0.072	0.005	5.61E-07
EVI2A	3.78E-11	0.300088	0.108	0.024	5.64E-07
MYT1L	3.80E-11	0.163173	0.051	0	5.68E-07
OPN3	3.86E-11	0.124328	0.061	0.005	5.77E-07
F8A1	3.96E-11	0.19342	0.088	0.014	5.92E-07
MCM3AP	4.07E-11	0.10449	0.09	0.022	6.08E-07
UNC13D	4.11E-11	0.108654	0.056	0.005	6.15E-07
GRIN2B	4.42E-11	0.151561	0.06	0.002	6.61E-07
ZNF614	4.47E-11	0.112871	0.077	0.017	6.68E-07
PRR14L	4.50E-11	0.134824	0.07	0.01	6.73E-07
STC2	4.52E-11	0.252471	0.113	0.031	6.76E-07
LCOR	4.55E-11	0.107757	0.072	0.015	6.80E-07
UCK2	4.58E-11	0.104156	0.087	0.022	6.85E-07
COL4A4	4.61E-11	0.167484	0.062	0.003	6.90E-07
CASP4	4.67E-11	-0.56528	0.016	0.077	6.98E-07
LONRF1	4.76E-11	0.184887	0.085	0.015	7.12E-07
AP3B2	4.99E-11	0.128282	0.06	0.005	7.46E-07
TBCK	5.01E-11	0.143701	0.085	0.019	7.48E-07
TDRD3	5.01E-11	0.111828	0.085	0.021	7.48E-07
WDR36	5.02E-11	0.128991	0.079	0.017	7.50E-07
VASH2	5.02E-11	0.135836	0.056	0.003	7.50E-07
PCDH11X	5.03E-11	0.223254	0.081	0.01	7.52E-07
OTP	5.25E-11	0.220357	0.062	0.002	7.85E-07
DBP	5.31E-11	-0.11009	0.06	0.029	7.93E-07
STK3	5.45E-11	-0.29178	0.057	0.056	8.14E-07
RP11-401F	5.45E-11	0.150324	0.056	0.002	8.14E-07
ZWINT	5.55E-11	0.189464	0.059	0.002	8.30E-07
SLC25A24	5.58E-11	0.134979	0.064	0.007	8.33E-07

CHST14	5.58E-11	0.188857	0.075	0.007	8.33E-07
SAMD10	5.58E-11	0.187341	0.066	0.005	8.35E-07
GOLGA3	5.66E-11	-0.18689	0.083	0.051	8.45E-07
GNG12	5.75E-11	-0.22092	0.059	0.043	8.59E-07
GREB1	6.10E-11	0.205704	0.07	0.005	9.12E-07
SOAT1	6.16E-11	-0.10788	0.062	0.032	9.21E-07
FBXO4	6.35E-11	-0.1952	0.059	0.046	9.49E-07
MAP3K4	6.44E-11	0.17422	0.074	0.009	9.62E-07
GNE	6.45E-11	0.119597	0.064	0.009	9.64E-07
KANSL3	6.55E-11	0.137228	0.077	0.014	9.79E-07
STOM	6.78E-11	-0.43392	0.034	0.06	1.01E-06
PLCXD1	6.81E-11	0.123611	0.069	0.01	1.02E-06
NMNAT2	6.90E-11	0.187844	0.049	0	1.03E-06
RP11-658F	6.90E-11	0.151822	0.049	0	1.03E-06
SCRT2	6.90E-11	0.173772	0.049	0	1.03E-06
FA2H	6.90E-11	0.136654	0.049	0	1.03E-06
NOMO3	6.93E-11	0.161838	0.061	0.003	1.04E-06
BHLHE40	6.95E-11	-0.69864	0.014	0.062	1.04E-06
TUBD1	6.95E-11	0.155725	0.092	0.022	1.04E-06
ZNF621	7.18E-11	0.109935	0.073	0.014	1.07E-06
DUS2	7.35E-11	0.161779	0.074	0.01	1.10E-06
CELSR3	7.49E-11	0.174412	0.066	0.007	1.12E-06
ID1	7.50E-11	0.12489	0.094	0.027	1.12E-06
TRIM56	7.54E-11	-0.41485	0.043	0.055	1.13E-06
C21orf62	7.54E-11	-0.35301	0.055	0.06	1.13E-06
LINC00643	7.59E-11	0.183175	0.074	0.01	1.13E-06
ALDH16A1	7.65E-11	0.113938	0.073	0.014	1.14E-06
SEC14L2	7.77E-11	-0.14626	0.087	0.051	1.16E-06
CD58	7.98E-11	-0.29322	0.048	0.048	1.19E-06
ADORA1	8.03E-11	-0.36274	0.038	0.062	1.20E-06
AGO4	8.11E-11	0.187372	0.068	0.005	1.21E-06
MPV17L	8.46E-11	-0.14979	0.061	0.038	1.26E-06
RP11-138A	8.57E-11	0.209682	0.125	0.039	1.28E-06
TAF5L	8.70E-11	0.120211	0.061	0.007	1.30E-06
AK9	8.77E-11	0.154849	0.061	0.005	1.31E-06
FAM173B	8.78E-11	0.141	0.074	0.012	1.31E-06
NRBP2	8.84E-11	0.149123	0.092	0.024	1.32E-06
WASF3	8.84E-11	-0.25769	0.053	0.048	1.32E-06
SCML1	9.08E-11	-0.15455	0.062	0.041	1.36E-06
TMEM255A	9.08E-11	-0.10961	0.062	0.034	1.36E-06
EGR2	9.18E-11	-0.15337	0.073	0.05	1.37E-06
OCEL1	9.24E-11	-0.17756	0.056	0.039	1.38E-06
PLEKHA8	9.30E-11	0.165502	0.07	0.005	1.39E-06
DGKI	9.34E-11	0.139027	0.066	0.009	1.40E-06
PAPD5	9.45E-11	0.168657	0.077	0.01	1.41E-06
EEF1A2	9.58E-11	0.160849	0.066	0.009	1.43E-06
SLC9B2	9.79E-11	0.120843	0.07	0.012	1.46E-06
ZMYND19	9.91E-11	0.116192	0.053	0.003	1.48E-06
TP53INP1	9.93E-11	0.192944	0.065	0.003	1.48E-06
CYP2U1	9.94E-11	0.138532	0.065	0.009	1.49E-06
AHCYL2	1.01E-10	0.163193	0.077	0.01	1.50E-06

TNFSF12	1.01E-10	-0.12574	0.059	0.031	1.51E-06
ST8SIA1	1.02E-10	0.101624	0.092	0.029	1.52E-06
SRF	1.03E-10	0.158768	0.078	0.012	1.54E-06
SOX1	1.04E-10	0.141673	0.065	0.009	1.56E-06
PDCD11	1.05E-10	0.105212	0.065	0.01	1.58E-06
PGAM5	1.07E-10	0.158953	0.062	0.003	1.60E-06
POFUT2	1.08E-10	0.113268	0.074	0.015	1.61E-06
PPTC7	1.08E-10	0.102106	0.064	0.009	1.62E-06
PGAP2	1.11E-10	-0.12184	0.078	0.044	1.66E-06
RP4-635E1	1.11E-10	0.187705	0.086	0.015	1.66E-06
LRRK2	1.13E-10	0.149672	0.075	0.012	1.69E-06
CERS1	1.13E-10	-0.15523	0.079	0.053	1.70E-06
CAND2	1.15E-10	0.200867	0.074	0.007	1.72E-06
PAQR3	1.15E-10	0.138444	0.07	0.01	1.72E-06
FGD3	1.15E-10	0.117485	0.053	0.002	1.72E-06
VPS39	1.20E-10	0.117233	0.07	0.014	1.79E-06
PTPRN	1.21E-10	-0.10384	0.074	0.044	1.81E-06
C17orf97	1.23E-10	0.143247	0.081	0.017	1.84E-06
CAMK2B	1.24E-10	-0.43816	0.046	0.072	1.86E-06
ZKSCAN7	1.25E-10	0.155241	0.048	0	1.88E-06
DEPDC7	1.25E-10	0.152785	0.048	0	1.88E-06
SUPV3L1	1.26E-10	0.160579	0.068	0.007	1.88E-06
CLSPN	1.31E-10	0.152769	0.066	0.009	1.96E-06
GEM	1.32E-10	-0.40742	0.042	0.053	1.97E-06
ZNF35	1.37E-10	0.104197	0.06	0.009	2.04E-06
PCDHB14	1.37E-10	-0.2195	0.064	0.053	2.04E-06
LIN37	1.39E-10	-0.19204	0.043	0.027	2.08E-06
INF2	1.43E-10	0.129436	0.072	0.012	2.14E-06
TCF20	1.46E-10	0.21725	0.078	0.009	2.19E-06
TMEM220	1.52E-10	0.103269	0.048	0.002	2.27E-06
SLC27A4	1.53E-10	0.120743	0.049	0.002	2.29E-06
TMEM176A	1.57E-10	-0.35649	0.046	0.056	2.34E-06
RP1-223E5	1.57E-10	0.184614	0.07	0.009	2.35E-06
CTSO	1.61E-10	0.146636	0.083	0.019	2.41E-06
PHKG2	1.65E-10	-0.12639	0.074	0.044	2.46E-06
ZNF507	1.67E-10	0.109414	0.07	0.014	2.49E-06
CCDC102B	1.68E-10	-0.16005	0.082	0.056	2.51E-06
10-Sep	1.70E-10	0.119509	0.081	0.019	2.54E-06
SLC19A1	1.70E-10	0.111116	0.057	0.007	2.54E-06
RP1-40E16	1.71E-10	0.159048	0.059	0.002	2.55E-06
HIC2	1.71E-10	0.185251	0.069	0.005	2.55E-06
MAPT-AS1	1.74E-10	0.233241	0.086	0.015	2.61E-06
PHF13	1.77E-10	0.115374	0.069	0.012	2.65E-06
ZNF529	1.77E-10	0.236556	0.085	0.012	2.65E-06
NFKBIZ	1.77E-10	-0.19592	0.07	0.058	2.65E-06
AEBP2	1.81E-10	0.124615	0.074	0.014	2.71E-06
RP11-69E1	1.84E-10	0.184492	0.072	0.009	2.75E-06
NOP14	1.84E-10	0.139572	0.07	0.01	2.75E-06
TXNDC11	1.88E-10	0.106238	0.074	0.015	2.81E-06
USP20	1.88E-10	0.142524	0.075	0.012	2.81E-06
KIAA1324L	1.93E-10	0.131193	0.049	0.002	2.88E-06

RBL1	2.00E-10	0.14786	0.062	0.005	2.99E-06
VCAM1	2.01E-10	-0.67269	0.001	0.051	3.01E-06
EID2B	2.02E-10	0.121797	0.092	0.024	3.02E-06
TMEM260	2.06E-10	0.127048	0.074	0.014	3.08E-06
NACC1	2.10E-10	-0.11152	0.07	0.039	3.13E-06
MIR181A2H	2.13E-10	0.160792	0.061	0.003	3.18E-06
PCP4	2.17E-10	0.113131	0.064	0.014	3.25E-06
PNPLA4	2.21E-10	-0.18904	0.043	0.029	3.31E-06
LRIG2	2.25E-10	0.134453	0.077	0.014	3.37E-06
RAI2	2.28E-10	0.143402	0.047	0	3.41E-06
VSTM2B	2.28E-10	0.151368	0.047	0	3.41E-06
TAGLN	2.32E-10	-0.65215	0.014	0.051	3.47E-06
CNIH3	2.38E-10	-0.36687	0.047	0.062	3.55E-06
RP11-637A	2.39E-10	0.138677	0.055	0.003	3.57E-06
USPL1	2.45E-10	0.117787	0.074	0.017	3.66E-06
LRP1B	2.45E-10	0.12033	0.07	0.012	3.67E-06
ZNF615	2.46E-10	0.177401	0.073	0.01	3.67E-06
MXRA7	2.55E-10	-0.26589	0.06	0.055	3.81E-06
NEK9	2.55E-10	0.137032	0.079	0.017	3.82E-06
BCAS4	2.66E-10	-0.37047	0.039	0.051	3.97E-06
GNS	2.67E-10	-0.15678	0.064	0.043	3.99E-06
TUB	2.69E-10	0.144182	0.055	0.002	4.02E-06
CXorf23	2.71E-10	0.132257	0.064	0.01	4.05E-06
MRGBP	2.72E-10	0.163665	0.085	0.015	4.06E-06
HAUS8	2.73E-10	-0.10595	0.057	0.027	4.08E-06
VPS54	2.82E-10	0.134845	0.064	0.007	4.22E-06
C6orf15	2.89E-10	-0.65395	0.008	0.067	4.32E-06
ZNFX1	3.01E-10	-0.15171	0.073	0.048	4.49E-06
SFI1	3.06E-10	0.135551	0.068	0.01	4.57E-06
FKTN	3.17E-10	0.127717	0.066	0.01	4.73E-06
DLC1	3.18E-10	-0.27789	0.044	0.046	4.76E-06
ECE1	3.30E-10	-0.32068	0.046	0.062	4.94E-06
PLEKHG2	3.33E-10	0.102739	0.062	0.012	4.98E-06
PCYT2	3.37E-10	0.131129	0.082	0.019	5.04E-06
SIDT2	3.39E-10	-0.22265	0.049	0.039	5.07E-06
USP30	3.40E-10	0.127692	0.068	0.01	5.09E-06
CDK5RAP2	3.43E-10	-0.12716	0.06	0.032	5.13E-06
KITLG	3.46E-10	0.170694	0.066	0.007	5.18E-06
ITM2A	3.55E-10	0.30905	0.075	0.009	5.31E-06
TRIP10	3.61E-10	-0.10493	0.057	0.034	5.39E-06
DCAF17	3.77E-10	0.157619	0.053	0.002	5.64E-06
RP11-536C	3.78E-10	0.163869	0.049	0.002	5.64E-06
SRCAP	3.78E-10	0.12421	0.069	0.012	5.65E-06
PTP4A3	3.79E-10	0.138715	0.09	0.026	5.66E-06
CNTRL	3.80E-10	0.135208	0.095	0.026	5.68E-06
CCDC30	3.86E-10	-0.1638	0.039	0.027	5.77E-06
RAPGEF4	3.94E-10	0.198314	0.066	0.005	5.88E-06
PHF1	3.96E-10	-0.11227	0.081	0.051	5.91E-06
ZNF211	4.03E-10	-0.20803	0.064	0.039	6.02E-06
PUS1	4.12E-10	0.113295	0.075	0.015	6.16E-06
FANCE	4.14E-10	0.122723	0.046	0	6.19E-06

LINC00574	4.14E-10	0.149438	0.046	0	6.19E-06
SIX4	4.14E-10	0.147522	0.046	0	6.19E-06
DUSP9	4.14E-10	0.212573	0.046	0	6.19E-06
RP11-119E	4.17E-10	0.15427	0.075	0.014	6.23E-06
CREB3L2	4.30E-10	0.104232	0.077	0.019	6.42E-06
VIM-AS1	4.34E-10	-0.201	0.035	0.024	6.48E-06
DHRS3	4.37E-10	-0.50454	0.01	0.055	6.53E-06
MSANTD2	4.42E-10	0.114769	0.07	0.014	6.60E-06
ADAMTS6	4.51E-10	0.101319	0.074	0.019	6.74E-06
SLC12A4	4.55E-10	-0.10021	0.046	0.021	6.80E-06
C2orf74	4.66E-10	-0.30356	0.033	0.036	6.96E-06
KIAA1147	4.74E-10	0.13644	0.075	0.015	7.09E-06
RFX1	4.79E-10	0.145674	0.075	0.012	7.16E-06
TBC1D12	4.86E-10	0.188125	0.057	0.002	7.26E-06
ZNF865	4.86E-10	0.105466	0.065	0.01	7.26E-06
SCRT1	4.92E-10	0.185207	0.064	0.005	7.35E-06
PLS3	4.97E-10	-0.3504	0.061	0.079	7.42E-06
RP11-499F	5.07E-10	0.172304	0.061	0.003	7.57E-06
LRRTM3	5.14E-10	0.118891	0.056	0.005	7.68E-06
ZNF678	5.31E-10	0.169542	0.072	0.009	7.94E-06
MKL2	5.48E-10	-0.14169	0.049	0.027	8.19E-06
ZZEF1	5.55E-10	0.12272	0.069	0.012	8.30E-06
CTD-2035E	5.57E-10	0.183685	0.07	0.007	8.33E-06
JOSD1	5.58E-10	0.172444	0.088	0.019	8.34E-06
RALGPS1	5.70E-10	0.131435	0.07	0.012	8.51E-06
CDK5R2	5.75E-10	0.163651	0.059	0.007	8.60E-06
TTC13	5.78E-10	0.122429	0.062	0.009	8.64E-06
ATAD5	5.80E-10	0.162433	0.073	0.01	8.66E-06
CHKB	5.80E-10	0.153752	0.055	0.003	8.67E-06
RFNG	5.97E-10	0.128379	0.091	0.026	8.92E-06
VWCE	6.03E-10	0.133839	0.064	0.009	9.00E-06
SH3BP5L	6.03E-10	0.116459	0.062	0.009	9.02E-06
GUCY1A2	6.66E-10	0.20403	0.074	0.009	9.95E-06
TMEM17	6.76E-10	0.126891	0.065	0.012	1.01E-05
TTBK2	6.85E-10	0.115442	0.075	0.017	1.02E-05
SP110	6.91E-10	-0.17139	0.064	0.048	1.03E-05
PPP2R2D	6.98E-10	0.140386	0.056	0.005	1.04E-05
IQGAP1	7.45E-10	-0.49728	0.022	0.065	1.11E-05
STEAP3	7.47E-10	-0.26561	0.049	0.05	1.12E-05
GMIP	7.52E-10	0.142108	0.044	0	1.12E-05
PRKCE	7.52E-10	0.138859	0.044	0	1.12E-05
DRP2	7.52E-10	0.153135	0.044	0	1.12E-05
MAPK14	7.86E-10	0.134965	0.073	0.014	1.18E-05
MIR7-3HG	7.87E-10	-0.51357	0.029	0.06	1.18E-05
OGFOD2	7.95E-10	-0.11513	0.052	0.027	1.19E-05
RGR	7.96E-10	0.20157	0.062	0.007	1.19E-05
SDC1	8.00E-10	0.145033	0.062	0.007	1.20E-05
HOXD10	8.02E-10	0.109984	0.065	0.01	1.20E-05
TAF6L	8.12E-10	0.137895	0.07	0.012	1.21E-05
USP9Y	8.24E-10	0.157069	0.055	0.003	1.23E-05
EP400NL	8.27E-10	0.108476	0.059	0.009	1.24E-05

TBKBP1	8.33E-10	0.112603	0.062	0.009	1.24E-05
CHML	8.39E-10	0.134802	0.053	0.002	1.25E-05
PARP11	8.49E-10	0.142785	0.069	0.01	1.27E-05
NLE1	8.55E-10	0.147813	0.061	0.005	1.28E-05
EPT1	8.64E-10	0.129048	0.057	0.005	1.29E-05
NEURL4	8.77E-10	0.149848	0.052	0.002	1.31E-05
FAM91A1	8.83E-10	0.163014	0.073	0.012	1.32E-05
NADSYN1	9.16E-10	-0.11768	0.056	0.026	1.37E-05
PDGFA	9.20E-10	-0.21015	0.072	0.058	1.37E-05
CDC45	9.27E-10	0.114463	0.046	0.002	1.39E-05
SLC30A1	9.47E-10	0.152982	0.059	0.005	1.41E-05
TMPRSS5	9.49E-10	0.143476	0.069	0.012	1.42E-05
ZBTB33	9.90E-10	0.150443	0.075	0.014	1.48E-05
SPATA6	1.02E-09	-0.14992	0.072	0.051	1.53E-05
NCS1	1.03E-09	0.128857	0.064	0.01	1.53E-05
TRIM22	1.03E-09	-0.32022	0.042	0.053	1.54E-05
ZNF724P	1.03E-09	0.170823	0.068	0.007	1.54E-05
NAA40	1.05E-09	0.114074	0.074	0.019	1.57E-05
SFXN3	1.07E-09	-0.19655	0.059	0.043	1.60E-05
BBS10	1.08E-09	0.152801	0.055	0.003	1.61E-05
AKT3	1.08E-09	0.100153	0.075	0.021	1.62E-05
ACSF3	1.11E-09	0.110153	0.088	0.027	1.67E-05
LIN9	1.12E-09	0.138556	0.061	0.005	1.68E-05
NMRK1	1.14E-09	-0.11421	0.065	0.034	1.70E-05
CABP1	1.14E-09	0.105404	0.052	0.005	1.70E-05
PCDH7	1.14E-09	0.123348	0.047	0.002	1.71E-05
TRAPPC10	1.27E-09	0.128235	0.06	0.007	1.90E-05
SPEG	1.28E-09	-0.11617	0.064	0.036	1.91E-05
PPP3CB	1.29E-09	0.163076	0.087	0.021	1.92E-05
NEK7	1.30E-09	0.133651	0.081	0.019	1.94E-05
PLEKHM1	1.31E-09	0.153401	0.065	0.007	1.96E-05
LSS	1.32E-09	-0.19674	0.053	0.043	1.97E-05
RP11-588G	1.32E-09	0.152003	0.051	0.002	1.97E-05
TMPO-AS1	1.33E-09	0.208803	0.053	0.002	1.99E-05
TSPAN33	1.36E-09	0.133833	0.043	0	2.04E-05
BACH2	1.36E-09	0.131685	0.043	0	2.04E-05
ZNF625	1.37E-09	0.170492	0.072	0.009	2.04E-05
CHAF1A	1.43E-09	0.122106	0.079	0.019	2.14E-05
WWP2	1.43E-09	0.15553	0.068	0.009	2.14E-05
ZNF610	1.47E-09	0.1414	0.051	0.002	2.19E-05
GRAMD4	1.51E-09	0.144741	0.065	0.009	2.26E-05
SULF1	1.60E-09	0.178656	0.062	0.007	2.39E-05
AC009948.	1.63E-09	0.145767	0.061	0.007	2.44E-05
YJEFN3	1.64E-09	0.150083	0.065	0.007	2.45E-05
VWA5A	1.65E-09	-0.27733	0.042	0.053	2.47E-05
SH3GLB2	1.73E-09	0.166312	0.07	0.01	2.58E-05
TCEAL6	1.75E-09	-0.13061	0.049	0.027	2.61E-05
TXNDC16	1.76E-09	0.102097	0.064	0.012	2.63E-05
EBLN2	1.77E-09	0.144434	0.069	0.012	2.65E-05
UBXN11	1.81E-09	-0.40991	0.038	0.079	2.71E-05
PPM1L	1.84E-09	0.132612	0.051	0.002	2.75E-05

RAB11FIP2	1.87E-09	0.119562	0.053	0.007	2.80E-05
CCDC28A	1.88E-09	-0.15065	0.061	0.039	2.81E-05
DIO2	1.89E-09	-0.49165	0.017	0.056	2.83E-05
MSTO1	1.94E-09	0.125882	0.072	0.014	2.90E-05
LMO2	2.03E-09	-0.40478	0.029	0.065	3.04E-05
TPST2	2.12E-09	-0.20068	0.049	0.034	3.16E-05
SPTLC2	2.18E-09	0.156067	0.066	0.01	3.26E-05
TAOK2	2.22E-09	0.127979	0.064	0.01	3.31E-05
MTHFR	2.35E-09	0.11556	0.074	0.017	3.52E-05
SGMS1	2.41E-09	0.183938	0.064	0.005	3.60E-05
CCNJ	2.42E-09	0.12762	0.052	0.003	3.61E-05
C16orf70	2.43E-09	0.128982	0.055	0.005	3.63E-05
MANEAL	2.43E-09	0.108865	0.062	0.01	3.64E-05
ATP6V0E2-	2.44E-09	0.113951	0.052	0.005	3.65E-05
ZNF367	2.48E-09	0.149389	0.042	0	3.70E-05
FOXP2	2.54E-09	0.114403	0.051	0.003	3.80E-05
SNHG15	2.55E-09	-0.36325	0.068	0.075	3.81E-05
MAL	2.57E-09	0.108232	0.046	0.002	3.85E-05
C15orf41	2.61E-09	0.121386	0.061	0.01	3.90E-05
S100A9	2.63E-09	-0.11739	0.042	0.026	3.93E-05
DLGAP1-AS	2.69E-09	-0.11335	0.049	0.029	4.02E-05
UTY	2.73E-09	0.173509	0.053	0.002	4.08E-05
POLR1B	2.78E-09	0.149043	0.069	0.01	4.16E-05
MBLAC1	2.85E-09	0.181666	0.052	0.002	4.25E-05
RP11-357K	2.87E-09	0.132925	0.053	0.007	4.29E-05
ATXN7	2.90E-09	0.168958	0.086	0.019	4.33E-05
RAB30	2.93E-09	0.126987	0.066	0.012	4.38E-05
AMMECR1L	2.93E-09	0.162505	0.064	0.007	4.38E-05
ECHDC2	2.98E-09	-0.28475	0.039	0.056	4.45E-05
RP11-195F	2.98E-09	-0.18109	0.061	0.044	4.46E-05
RASL10A	2.98E-09	0.265496	0.082	0.015	4.46E-05
NANOS1	3.00E-09	0.143585	0.051	0.005	4.49E-05
PEX6	3.06E-09	0.16397	0.078	0.015	4.57E-05
ZCCHC14	3.07E-09	0.109069	0.057	0.007	4.59E-05
SEMA7A	3.10E-09	0.110856	0.052	0.005	4.64E-05
UBR1	3.21E-09	0.149849	0.073	0.012	4.80E-05
EFHD1	3.30E-09	0.160029	0.091	0.027	4.93E-05
PARP8	3.30E-09	0.137664	0.052	0.003	4.93E-05
SMURF1	3.35E-09	0.133882	0.055	0.003	5.01E-05
CACTIN	3.36E-09	0.129882	0.056	0.005	5.03E-05
TEF	3.37E-09	0.147767	0.055	0.003	5.03E-05
DPY19L1	3.38E-09	-0.17585	0.056	0.043	5.06E-05
RAD51AP1	3.41E-09	0.101934	0.052	0.007	5.09E-05
PSMG3-AS1	3.47E-09	0.12354	0.065	0.012	5.19E-05
EMP2	3.54E-09	-0.37239	0.036	0.053	5.29E-05
CPQ	3.55E-09	-0.26726	0.043	0.051	5.31E-05
FYB	3.64E-09	0.171294	0.057	0.005	5.44E-05
FNDC3B	3.65E-09	-0.12332	0.057	0.031	5.45E-05
MGAT4B	3.70E-09	0.105241	0.048	0.005	5.52E-05
MAP2K3	3.82E-09	0.113097	0.065	0.012	5.70E-05
ASGR1	3.90E-09	0.116483	0.061	0.009	5.83E-05

CITF22-92	3.98E-09	0.238368	0.059	0.003	5.95E-05
KCTD2	4.08E-09	0.100016	0.052	0.005	6.09E-05
MFAP3	4.16E-09	0.164147	0.06	0.005	6.22E-05
PHF21B	4.22E-09	0.163832	0.072	0.012	6.30E-05
ANKRD53	4.49E-09	0.130359	0.04	0	6.71E-05
RP11-473I	4.51E-09	0.116991	0.064	0.012	6.74E-05
MFSD2A	4.62E-09	-0.18959	0.051	0.038	6.91E-05
RASGRP2	4.66E-09	0.13537	0.049	0.002	6.97E-05
IKZF4	4.70E-09	0.160235	0.059	0.005	7.03E-05
LINC01122	4.77E-09	0.12069	0.057	0.009	7.13E-05
AGTPBP1	4.80E-09	0.144742	0.052	0.002	7.17E-05
RP11-110G	4.82E-09	0.115205	0.052	0.005	7.20E-05
KCTD1	4.86E-09	0.110019	0.066	0.014	7.27E-05
HOXD1	4.89E-09	0.132359	0.051	0.002	7.30E-05
GLIPR1	4.97E-09	-0.53872	0.017	0.056	7.43E-05
PLCL1	5.02E-09	0.177361	0.056	0.003	7.51E-05
KIAA1328	5.07E-09	-0.1019	0.059	0.032	7.58E-05
HEATR3	5.08E-09	0.115394	0.051	0.003	7.59E-05
MACROD1	5.24E-09	0.12077	0.077	0.021	7.84E-05
ELOVL2	5.35E-09	-0.16555	0.051	0.036	8.00E-05
ZFP30	5.41E-09	0.105715	0.055	0.007	8.08E-05
CXorf40A	5.42E-09	-0.11827	0.057	0.034	8.09E-05
RP11-11N9	5.44E-09	-0.12008	0.038	0.019	8.12E-05
HELZ2	5.48E-09	-0.14872	0.07	0.05	8.19E-05
C12orf4	5.54E-09	0.110876	0.078	0.021	8.28E-05
PBXIP1	5.74E-09	-0.40823	0.03	0.062	8.57E-05
PPP1R1C	5.82E-09	-0.26073	0.049	0.046	8.70E-05
HOXD9	5.83E-09	0.110999	0.051	0.003	8.71E-05
AC005076.	5.84E-09	0.112543	0.059	0.01	8.73E-05
NINL	5.91E-09	0.146207	0.061	0.005	8.84E-05
SDCCAG3	5.95E-09	-0.10313	0.044	0.024	8.90E-05
NFXL1	6.07E-09	0.100606	0.066	0.014	9.07E-05
FGF7	6.22E-09	-0.2945	0.031	0.044	9.30E-05
ZNF611	6.24E-09	0.130986	0.072	0.015	9.32E-05
CDR2L	6.31E-09	0.146162	0.057	0.005	9.42E-05
TNFSF13B	6.36E-09	-0.15111	0.039	0.026	9.50E-05
TFE3	6.40E-09	-0.1136	0.048	0.027	9.56E-05
CHRNA5	6.60E-09	0.118559	0.051	0.003	9.86E-05
WDFY3	6.84E-09	0.11164	0.068	0.015	0.000102
C1orf159	6.96E-09	0.115884	0.046	0.003	0.000104
CLSTN2	7.07E-09	0.113574	0.057	0.009	0.000106
TNFRSF11E	7.21E-09	-0.13699	0.048	0.031	0.000108
HDDC3	7.29E-09	-0.12643	0.068	0.048	0.000109
CENPC	7.47E-09	0.148046	0.083	0.021	0.000112
ZNF334	7.51E-09	0.16221	0.079	0.019	0.000112
TRIM23	7.58E-09	0.137662	0.073	0.017	0.000113
UTP15	7.86E-09	0.115436	0.061	0.01	0.000117
NAGA	7.94E-09	-0.1148	0.038	0.021	0.000119
ZNF512B	8.01E-09	0.154975	0.069	0.01	0.00012
LPAR2	8.15E-09	0.118651	0.039	0	0.000122
SEMA3A	8.15E-09	0.110289	0.039	0	0.000122

MYOT	8.15E-09	0.13616	0.039	0	0.000122
TUFT1	8.15E-09	0.126513	0.039	0	0.000122
ZFP69B	8.15E-09	0.117443	0.039	0	0.000122
TET1	8.24E-09	0.157878	0.064	0.009	0.000123
POLE2	8.28E-09	0.148464	0.059	0.007	0.000124
KIF19	8.46E-09	0.205127	0.049	0.002	0.000126
PIFO	8.48E-09	-0.53419	0.009	0.062	0.000127
SPATA5L1	8.50E-09	0.164907	0.069	0.01	0.000127
KDELC2	8.51E-09	-0.14441	0.042	0.026	0.000127
COX18	8.61E-09	0.10508	0.074	0.019	0.000129
PDE6A	8.69E-09	0.112949	0.052	0.005	0.00013
HRH1	8.97E-09	-0.43388	0.005	0.048	0.000134
DLGAP1	9.35E-09	0.153328	0.047	0.002	0.00014
FBXO45	9.35E-09	0.102873	0.052	0.007	0.00014
ACVR2B	9.35E-09	0.135014	0.059	0.007	0.00014
SDC2	9.36E-09	-0.26151	0.047	0.046	0.00014
CDKN2B	9.36E-09	0.106558	0.07	0.017	0.00014
BHLHB9	9.47E-09	0.127736	0.052	0.005	0.000141
ADAMTS9	9.56E-09	-0.42835	0.016	0.053	0.000143
BCL9L	9.93E-09	0.122681	0.055	0.007	0.000148
ROM1	9.98E-09	-0.38994	0.026	0.056	0.000149
CPLX2	1.00E-08	0.143076	0.047	0.002	0.00015
SLC44A5	1.05E-08	0.111356	0.048	0.003	0.000157
PCNXL2	1.05E-08	0.137499	0.081	0.021	0.000157
SLC35A2	1.06E-08	0.11889	0.087	0.027	0.000158
SEC24D	1.06E-08	-0.18907	0.038	0.031	0.000158
ZNF395	1.11E-08	-0.1591	0.048	0.032	0.000166
UVRAG	1.13E-08	0.159948	0.073	0.014	0.000169
PIGZ	1.15E-08	0.109076	0.049	0.005	0.000172
SCYL3	1.15E-08	0.129257	0.062	0.01	0.000172
LIFR-AS1	1.16E-08	0.171452	0.068	0.012	0.000174
SEMA5B	1.16E-08	0.136961	0.056	0.007	0.000174
ZNF660	1.17E-08	0.131654	0.06	0.007	0.000174
CTD-3184A	1.17E-08	0.156152	0.069	0.012	0.000175
RAPGEF1	1.19E-08	-0.1784	0.038	0.026	0.000178
ZBED4	1.26E-08	0.134811	0.057	0.007	0.000188
GBP3	1.30E-08	-0.51092	0.009	0.06	0.000195
DYRK2	1.33E-08	0.123977	0.064	0.012	0.000199
COL6A2	1.35E-08	-0.3866	0.021	0.053	0.000201
LDLR	1.35E-08	-0.32644	0.031	0.043	0.000202
SNX32	1.41E-08	0.151297	0.056	0.005	0.00021
SPDL1	1.41E-08	0.120562	0.056	0.007	0.000211
PINLYP	1.41E-08	-0.22944	0.04	0.036	0.000211
KLHL2	1.42E-08	0.100871	0.059	0.01	0.000213
HDHD3	1.48E-08	-0.18637	0.047	0.039	0.000221
NYAP1	1.48E-08	0.127926	0.038	0	0.000221
RP11-342K	1.48E-08	0.136228	0.038	0	0.000221
TPRN	1.48E-08	0.118248	0.038	0	0.000221
FUT9	1.48E-08	0.113691	0.038	0	0.000221
CNNM3	1.48E-08	0.126723	0.038	0	0.000221
EZH1	1.49E-08	-0.19354	0.055	0.044	0.000223

RP11-15A1	1.54E-08	0.123327	0.066	0.012	0.00023
CENPBD1	1.61E-08	0.107447	0.056	0.01	0.00024
HCFC2	1.62E-08	-0.17506	0.036	0.027	0.000242
RBAK	1.64E-08	0.105044	0.065	0.014	0.000245
ANKHD1	1.66E-08	0.141701	0.081	0.022	0.000248
CCDC149	1.69E-08	0.112329	0.044	0.003	0.000252
UBL7-AS1	1.75E-08	0.112013	0.057	0.009	0.000262
UNKL	1.77E-08	0.167039	0.07	0.012	0.000264
MYBPC1	1.84E-08	-0.3963	0.01	0.039	0.000275
FCGR3A	1.95E-08	0.138675	0.061	0.01	0.000292
VPS13B	1.96E-08	0.107615	0.066	0.017	0.000292
KCNJ3	1.99E-08	-0.20112	0.049	0.043	0.000297
LINC00324	2.04E-08	0.157406	0.048	0.002	0.000305
DOK4	2.06E-08	0.156297	0.052	0.003	0.000308
DSCC1	2.12E-08	0.181538	0.065	0.009	0.000317
BOLA2B	2.12E-08	-0.22825	0.044	0.039	0.000318
PCDHB5	2.24E-08	0.202687	0.053	0.003	0.000334
SERPINB1	2.25E-08	-0.4227	0.02	0.039	0.000336
NRG1	2.25E-08	0.181994	0.062	0.007	0.000336
TMEM150C	2.31E-08	0.11218	0.044	0.003	0.000345
RFWD3	2.37E-08	0.112492	0.06	0.01	0.000355
DMXL2	2.52E-08	0.111579	0.073	0.019	0.000377
LINC00869	2.56E-08	-0.50829	0.008	0.058	0.000383
NQO1	2.57E-08	-0.32068	0.021	0.036	0.000384
MAP7D3	2.58E-08	-0.16654	0.052	0.038	0.000385
PANX1	2.67E-08	0.144639	0.059	0.009	0.0004
FAM149A	2.68E-08	0.148615	0.036	0	0.0004
WNK2	2.68E-08	0.103685	0.036	0	0.0004
TIGD3	2.68E-08	0.105525	0.036	0	0.0004
AC006994.	2.68E-08	0.103474	0.036	0	0.0004
ZNF699	2.70E-08	0.165594	0.056	0.005	0.000403
S100A4	2.73E-08	-0.23526	0.04	0.032	0.000408
DUSP15	2.76E-08	0.120775	0.057	0.009	0.000412
NDUFA4L2	2.86E-08	-0.2978	0.038	0.043	0.000427
ACVR1	2.88E-08	0.12193	0.052	0.007	0.000431
DAPK1	2.91E-08	0.110389	0.046	0.003	0.000435
CYP27C1	2.96E-08	0.15486	0.052	0.005	0.000443
BDH2	2.97E-08	-0.41464	0.026	0.063	0.000444
PLD6	3.01E-08	0.141226	0.047	0.002	0.00045
ZNF787	3.03E-08	0.136558	0.049	0.003	0.000453
IPPK	3.25E-08	0.103387	0.061	0.012	0.000486
IQSEC1	3.34E-08	0.102552	0.047	0.005	0.000499
SLC16A1-A	3.39E-08	-0.27486	0.031	0.041	0.000507
ARSJ	3.48E-08	-0.42855	0.008	0.058	0.000521
ZBTB37	3.49E-08	0.128134	0.07	0.015	0.000521
TGIF2	3.59E-08	0.144368	0.055	0.005	0.000536
CTHRC1	3.64E-08	0.13364	0.049	0.003	0.000543
ENKUR	3.67E-08	-0.41737	0.036	0.075	0.000548
ZNF629	3.80E-08	0.114893	0.06	0.01	0.000568
PTCHD2	3.93E-08	0.136531	0.051	0.005	0.000587
SMN2	3.96E-08	0.10096	0.057	0.01	0.000591

PRKCG	3.98E-08	-0.49251	0.008	0.056	0.000594
GPX3	4.04E-08	-0.33861	0.017	0.036	0.000604
KCNN2	4.08E-08	0.119574	0.055	0.009	0.00061
VARS2	4.19E-08	0.126357	0.064	0.012	0.000626
BCAS1	4.45E-08	0.195686	0.072	0.015	0.000665
ZNF415	4.50E-08	-0.12337	0.042	0.022	0.000672
PI3	4.54E-08	-0.71016	0.007	0.043	0.000678
HR	4.57E-08	0.152897	0.048	0.003	0.000683
GAS7	4.62E-08	-0.22784	0.049	0.044	0.00069
PLCL2	4.81E-08	0.122658	0.046	0.002	0.000719
LZTFL1	4.85E-08	-0.23816	0.051	0.051	0.000725
ACKR3	4.86E-08	0.107709	0.035	0	0.000726
AL645728.	4.86E-08	0.126019	0.035	0	0.000726
NAP1L2	4.86E-08	0.13647	0.035	0	0.000726
CACNG2	4.86E-08	0.152418	0.035	0	0.000726
RP11-867G	4.86E-08	0.114623	0.035	0	0.000726
SNAP91	4.86E-08	0.146051	0.035	0	0.000726
LTBP4	4.88E-08	0.19159	0.074	0.015	0.000729
SSPN	4.92E-08	-0.17066	0.055	0.044	0.000735
ZCCHC6	4.93E-08	-0.10025	0.051	0.032	0.000736
ZNF618	4.99E-08	0.123835	0.046	0.002	0.000746
MIR22HG	5.05E-08	-0.3429	0.033	0.039	0.000755
FBXO34	5.13E-08	0.133788	0.073	0.017	0.000766
C19orf47	5.22E-08	0.117144	0.053	0.007	0.00078
GABRB3	5.32E-08	0.147034	0.052	0.005	0.000795
GOLGA8A	5.41E-08	0.124172	0.065	0.014	0.000808
PAN2	5.48E-08	0.11073	0.069	0.019	0.000819
EGLN3	5.52E-08	-0.27055	0.027	0.036	0.000825
CTC-239J1	5.57E-08	0.166761	0.048	0.003	0.000832
ZNF687	5.59E-08	0.118991	0.052	0.007	0.000835
TMOD3	5.64E-08	-0.10032	0.048	0.027	0.000843
SPA17	5.65E-08	-0.20844	0.052	0.044	0.000844
SLC38A9	5.71E-08	0.181446	0.056	0.005	0.000853
LRRC45	5.81E-08	0.150085	0.062	0.009	0.000869
ZSCAN5A	5.82E-08	-0.14676	0.039	0.029	0.000869
ANGPT2	6.05E-08	-0.24345	0.047	0.043	0.000905
ADAM23	6.21E-08	0.170184	0.062	0.009	0.000928
EYA3	6.29E-08	-0.122	0.043	0.024	0.000941
MINPP1	6.30E-08	0.113504	0.082	0.026	0.000941
NRP2	6.55E-08	-0.31168	0.033	0.05	0.000979
PTK2B	6.75E-08	-0.2562	0.026	0.029	0.001009
ZNF784	6.98E-08	0.132175	0.049	0.003	0.001043
PKMYT1	7.20E-08	0.134777	0.046	0.002	0.001076
FBXL6	7.27E-08	0.119523	0.061	0.012	0.001086
ASB13	7.31E-08	0.139145	0.049	0.003	0.001092
U73166.2	7.35E-08	0.173191	0.062	0.009	0.001099
PQLC3	7.36E-08	-0.13506	0.039	0.027	0.0011
PLEKHG3	7.52E-08	0.116266	0.052	0.005	0.001124
FGGY	7.56E-08	-0.30804	0.038	0.048	0.001129
RP11-118F	7.70E-08	0.173132	0.051	0.005	0.001151
GNG10	7.71E-08	-0.13433	0.038	0.027	0.001152

SCAF8	7.71E-08	0.138319	0.057	0.007	0.001153
ENOX1	7.83E-08	0.117115	0.044	0.002	0.00117
SLC30A6	7.89E-08	0.158351	0.053	0.005	0.001179
LRRC20	8.24E-08	0.118279	0.059	0.012	0.001232
SIM2	8.28E-08	0.148249	0.046	0.002	0.001237
IL32	8.29E-08	-0.40443	0.012	0.032	0.001238
REXO1	8.47E-08	0.161042	0.062	0.009	0.001266
ZNF696	8.57E-08	0.110001	0.051	0.007	0.00128
B3GNT7	8.81E-08	0.105015	0.034	0	0.001316
MATN4	8.81E-08	0.141473	0.034	0	0.001316
GS1-124K5	8.81E-08	0.121973	0.034	0	0.001316
FGD6	8.81E-08	0.102958	0.034	0	0.001316
AZGP1	9.05E-08	-0.37941	0.031	0.053	0.001352
TM9SF1	9.07E-08	0.100313	0.056	0.01	0.001356
ZFY	9.14E-08	0.125812	0.044	0.002	0.001365
SYTL2	9.14E-08	-0.32899	0.038	0.058	0.001366
RP3-460G2	9.31E-08	-0.38782	0.035	0.067	0.001391
RP11-727F	9.39E-08	0.10193	0.053	0.009	0.001404
LRP8	9.54E-08	0.101028	0.048	0.007	0.001426
ZNF768	9.85E-08	0.102134	0.062	0.015	0.001472
TREM1	1.02E-07	-0.48398	0.018	0.05	0.001518
LZTS1	1.02E-07	-0.16391	0.026	0.021	0.001522
LYST	1.07E-07	-0.15114	0.046	0.036	0.0016
C1QTNF3	1.08E-07	-0.28228	0.023	0.029	0.001616
APOM	1.09E-07	-0.11513	0.048	0.032	0.001636
CXCL2	1.10E-07	-0.36705	0.01	0.031	0.001643
TRIP13	1.14E-07	0.125905	0.049	0.005	0.001705
SP9	1.17E-07	0.132462	0.042	0.002	0.001749
RP11-46H1	1.23E-07	-0.35459	0.005	0.048	0.00184
EPAS1	1.26E-07	-0.43406	0.023	0.058	0.001881
MAGI3	1.28E-07	0.134242	0.049	0.003	0.00191
HLA-DQA1	1.34E-07	-0.446	0.016	0.044	0.001996
NAAA	1.34E-07	0.11736	0.047	0.005	0.002004
RHPN2	1.36E-07	-0.27541	0.018	0.031	0.002029
ARSK	1.36E-07	-0.12561	0.04	0.027	0.002031
PFKFB3	1.41E-07	-0.16536	0.061	0.053	0.002112
NRF1	1.43E-07	0.119285	0.043	0.002	0.002132
CTD-3138E	1.46E-07	-0.23621	0.025	0.027	0.002175
SIAE	1.52E-07	-0.16266	0.042	0.036	0.002266
ARHGAP22	1.57E-07	0.125686	0.043	0.002	0.00234
PPP1R1B	1.60E-07	0.101595	0.033	0	0.002385
VSTM2L	1.60E-07	0.151556	0.033	0	0.002385
HTATIP2	1.60E-07	0.107601	0.033	0	0.002385
TAPBPL	1.60E-07	-0.27127	0.039	0.041	0.002396
TRAPPC8	1.65E-07	0.124314	0.048	0.003	0.002467
HSF2BP	1.69E-07	0.13875	0.04	0.002	0.002521
NKX2-5	1.74E-07	0.105354	0.064	0.021	0.002607
C10orf2	1.78E-07	0.12091	0.048	0.005	0.002659
FAM107A	1.79E-07	-0.20528	0.033	0.029	0.00267
GMPR	1.83E-07	-0.2017	0.034	0.036	0.002728
STK10	1.87E-07	0.104346	0.047	0.005	0.002802

CEP250	1.89E-07	-0.20987	0.034	0.032	0.002825
RP11-480C	2.00E-07	0.118552	0.057	0.01	0.002996
PRSS27	2.01E-07	0.11355	0.046	0.005	0.003009
AC023590.	2.07E-07	-0.12054	0.042	0.029	0.003096
KIF13B	2.09E-07	-0.12012	0.038	0.019	0.003129
SLC25A18	2.12E-07	-0.21627	0.069	0.062	0.003168
TTC9	2.15E-07	0.105392	0.043	0.003	0.003208
COL4A3	2.30E-07	0.119441	0.048	0.005	0.003431
NFASC	2.30E-07	-0.18806	0.061	0.05	0.003438
RIMS1	2.32E-07	0.109712	0.046	0.003	0.003461
HBEGF	2.35E-07	-0.10298	0.06	0.039	0.003514
LRCH2	2.43E-07	0.116796	0.047	0.005	0.003629
PIAS3	2.43E-07	-0.11016	0.04	0.024	0.003629
FRS3	2.44E-07	0.135687	0.042	0.002	0.003654
NR1D1	2.50E-07	-0.40093	0.016	0.041	0.003731
BAHD1	2.54E-07	0.108229	0.049	0.007	0.003789
HARS2	2.68E-07	0.103525	0.06	0.014	0.004005
CDC6	2.80E-07	-0.10431	0.031	0.012	0.004188
RALYL	2.89E-07	0.13117	0.031	0	0.004323
KIAA0922	2.94E-07	0.117944	0.047	0.005	0.004401
NFKB1	3.01E-07	-0.13368	0.036	0.022	0.004505
OPTN	3.19E-07	-0.13003	0.047	0.031	0.004766
RPH3A	3.20E-07	-0.13764	0.049	0.038	0.004784
LAMA4	3.28E-07	-0.29206	0.025	0.038	0.004902
RASGEF1C	3.37E-07	0.10503	0.035	0.002	0.005042
RNASE1	3.54E-07	-0.23526	0.044	0.041	0.005297
LCA5	3.75E-07	0.129336	0.049	0.007	0.005605
BHLHE41	4.20E-07	-0.32793	0.012	0.048	0.006277
URB2	4.21E-07	0.116149	0.048	0.007	0.006299
ZNF684	4.34E-07	0.141503	0.062	0.014	0.006487
ELL2	4.43E-07	-0.19968	0.044	0.043	0.006623
NUAK1	4.54E-07	0.131656	0.043	0.003	0.006782
CXCL3	4.66E-07	-0.44691	0.01	0.034	0.006959
MPP2	4.66E-07	0.100748	0.046	0.005	0.006968
ATP11C	4.75E-07	0.17566	0.047	0.003	0.007094
FCHO2	4.91E-07	0.10425	0.049	0.007	0.007341
PLA2G2A	4.96E-07	-0.50621	0.005	0.034	0.007419
SERPINA1	5.02E-07	-0.38138	0.033	0.039	0.007503
KLHL32	5.24E-07	0.130237	0.03	0	0.007836
KIF26A	5.24E-07	0.125458	0.03	0	0.007836
RIPPLY2	5.29E-07	0.138	0.052	0.009	0.007902
FREM2	5.36E-07	0.101591	0.047	0.005	0.008015
ZFP28	5.56E-07	0.105963	0.051	0.009	0.008311
ZNF248	5.68E-07	0.151965	0.064	0.014	0.008487
SGPP1	5.75E-07	0.115001	0.039	0.002	0.008594
THEM4	5.78E-07	0.129823	0.053	0.009	0.008632
GSDMD	5.89E-07	-0.39915	0.016	0.048	0.008809
ZNF141	5.92E-07	0.126362	0.055	0.009	0.008845
TSPAN15	6.03E-07	0.113955	0.039	0.002	0.009009
RHOF	6.19E-07	0.130159	0.052	0.007	0.009252
ANKAR	6.42E-07	0.115746	0.048	0.007	0.009589

RP11-214N	6.85E-07	0.153471	0.044	0.003	0.010236
OSTF1	6.93E-07	-0.13302	0.036	0.022	0.010361
SDC4	7.34E-07	-0.44614	0.007	0.041	0.01097
XKR8	7.73E-07	-0.149	0.033	0.027	0.011551
CH25H	7.78E-07	-0.37432	0.014	0.036	0.011632
CHST9	7.98E-07	0.131332	0.07	0.022	0.011918
FGF1	8.19E-07	-0.40049	0.013	0.046	0.012236
RGS6	8.20E-07	-0.4144	0.014	0.056	0.012247
ZBTB5	8.20E-07	0.134537	0.043	0.003	0.012253
IGDCC4	8.22E-07	0.104133	0.043	0.003	0.012277
ATXN7L1	8.23E-07	0.101505	0.047	0.005	0.012304
IPO9-AS1	8.46E-07	0.116905	0.043	0.003	0.012648
IL1RAPL1	8.49E-07	0.114065	0.038	0.002	0.012693
S1PR3	8.57E-07	-0.23188	0.026	0.038	0.0128
LOX	8.58E-07	-0.3888	0.003	0.032	0.012826
CLCF1	8.71E-07	-0.33275	0	0.026	0.013014
MMP7	8.71E-07	-0.35165	0	0.026	0.013014
PRSS23	8.77E-07	-0.28582	0.02	0.036	0.013107
CTA-292E1	9.23E-07	0.123489	0.046	0.005	0.013791
SNIP1	9.24E-07	0.104869	0.051	0.009	0.013816
KANK2	9.43E-07	-0.22074	0.04	0.038	0.014094
RP3-508I1	9.49E-07	-0.33517	0.009	0.041	0.014178
PURG	9.50E-07	0.101472	0.029	0	0.014204
CTD-2162K	9.50E-07	0.116101	0.029	0	0.014204
PIK3C2B	9.58E-07	0.122296	0.046	0.005	0.014317
RILPL2	9.65E-07	-0.13371	0.03	0.021	0.014425
HLA-DQB1	9.95E-07	-0.17315	0.035	0.036	0.014875
IGSF9B	1.00E-06	0.160102	0.044	0.003	0.014992
AC002456.	1.01E-06	-0.17642	0.039	0.031	0.015052
RFX2	1.01E-06	-0.12846	0.057	0.039	0.015064
CD163	1.01E-06	-0.12841	0.033	0.024	0.015075
IRAK1BP1	1.04E-06	0.121689	0.044	0.005	0.015532
RUSC2	1.04E-06	0.109421	0.046	0.005	0.015547
FSD1L	1.06E-06	0.105584	0.047	0.007	0.015889
MGAT5	1.07E-06	0.114964	0.043	0.003	0.015993
MAOB	1.10E-06	-0.17956	0.046	0.044	0.016417
ATXN7L2	1.12E-06	0.14229	0.044	0.003	0.016696
SWSAP1	1.23E-06	-0.12727	0.036	0.024	0.018456
PHLDB3	1.25E-06	0.138099	0.039	0.002	0.018662
TRIB1	1.27E-06	-0.10505	0.046	0.031	0.019026
RP11-490M	1.29E-06	0.110057	0.049	0.009	0.019326
SLC1A4	1.30E-06	0.163031	0.043	0.003	0.019498
IBA57	1.34E-06	0.114952	0.053	0.01	0.019967
LRFN3	1.37E-06	0.107881	0.047	0.007	0.020472
CTGF	1.38E-06	0.162026	0.055	0.01	0.020582
ANG	1.39E-06	-0.32183	0.03	0.034	0.020779
LINC01152	1.40E-06	-0.10125	0.025	0.014	0.020912
RARRES1	1.42E-06	0.109658	0.034	0.002	0.02119
FABP6	1.42E-06	0.158333	0.046	0.005	0.0212
PPP1R3F	1.46E-06	0.131091	0.047	0.005	0.021762
CLYBL	1.48E-06	-0.25489	0.026	0.034	0.02208

FAM71E1	1.49E-06	-0.16775	0.029	0.022	0.022327
OLR1	1.52E-06	0.115309	0.043	0.005	0.02271
LRRC26	1.53E-06	0.102136	0.044	0.007	0.022891
C2	1.55E-06	-0.33662	0.005	0.034	0.023203
CDK18	1.61E-06	-0.23482	0.046	0.048	0.024095
CYB561D1	1.63E-06	-0.17627	0.027	0.022	0.024329
NR1H3	1.63E-06	-0.13035	0.031	0.021	0.024338
ATP6V1B1	1.65E-06	0.13282	0.048	0.005	0.024602
HOXA6	1.72E-06	0.104305	0.027	0	0.02575
LINC00339	1.74E-06	-0.15661	0.031	0.024	0.026035
MUC12	1.77E-06	-0.28973	0.029	0.038	0.026415
WIPI1	1.78E-06	-0.21428	0.034	0.038	0.026582
ZFP41	1.79E-06	0.139055	0.042	0.003	0.026687
TET3	1.80E-06	0.139091	0.051	0.007	0.026905
TMEM241	1.82E-06	0.114161	0.042	0.003	0.027272
ABCA8	1.92E-06	-0.38011	0.012	0.053	0.028642
FBXO46	1.95E-06	0.102774	0.035	0.002	0.029069
AC093901.	1.98E-06	0.126561	0.043	0.003	0.029545
KIAA0556	2.01E-06	0.140713	0.038	0.002	0.030036
AC068535.	2.03E-06	0.112492	0.056	0.014	0.030379
IGFBP6	2.06E-06	-0.11598	0.035	0.022	0.030782
ATE1	2.09E-06	0.100575	0.047	0.009	0.031187
SHC1	2.12E-06	-0.25181	0.029	0.034	0.031723
SPOCK2	2.16E-06	-0.35546	0.022	0.053	0.032303
CDCA5	2.20E-06	0.100242	0.036	0.003	0.032837
SAMD9L	2.23E-06	-0.36779	0.01	0.031	0.033317
GUCY1B3	2.28E-06	0.14304	0.043	0.003	0.034067
MRC2	2.33E-06	-0.28576	0.021	0.034	0.03487
FAH	2.41E-06	-0.3588	0.014	0.036	0.036077
TRIB3	2.42E-06	-0.29053	0.027	0.044	0.036133
SLC10A3	2.46E-06	-0.12946	0.03	0.022	0.03677
CBLN4	2.46E-06	-0.3522	0.007	0.043	0.036833
C22orf46	2.48E-06	-0.10505	0.044	0.029	0.037128
OXNAD1	2.51E-06	-0.121	0.03	0.021	0.037446
HUNK	2.55E-06	0.121108	0.036	0.002	0.038112
RUNX1T1	2.55E-06	0.146013	0.053	0.01	0.038162
PRCD	2.63E-06	0.131675	0.048	0.009	0.039308
SRPX2	2.69E-06	-0.3239	0.001	0.032	0.040126
F12	2.69E-06	0.123446	0.051	0.009	0.040242
ANKRD16	2.74E-06	0.104813	0.036	0.002	0.041006
NAA60	2.85E-06	-0.3749	0.003	0.036	0.04258
PLCB1	3.03E-06	0.16694	0.06	0.014	0.045294
LINC01023	3.10E-06	-0.13856	0.046	0.027	0.046284
RP11-138I	3.11E-06	-0.14595	0.031	0.022	0.046454
ARHGEF11	3.20E-06	0.135792	0.048	0.007	0.047863
ARAP2	3.30E-06	-0.14785	0.038	0.031	0.049338
LAMA2	3.30E-06	-0.23737	0.001	0.031	0.049368
TMEM216	3.35E-06	-0.25813	0.03	0.041	0.050109
KANSL1L	3.36E-06	0.110054	0.043	0.005	0.050161
LGALS9	3.37E-06	-0.14434	0.031	0.022	0.05035
ZNF180	3.38E-06	0.120276	0.046	0.005	0.050581

TRAPPC12-	3.41E-06	0.108532	0.036	0.002	0.050911
C5AR1	3.44E-06	-0.16055	0.027	0.024	0.051441
RALGPS2	3.44E-06	0.10111	0.039	0.003	0.051479
HAP1	3.49E-06	-0.28073	0.02	0.05	0.052195
PPP5D1	3.53E-06	-0.1001	0.038	0.024	0.052747
NCOA2	3.54E-06	0.101322	0.049	0.01	0.052901
LIMS2	3.63E-06	0.107861	0.035	0.002	0.054281
LRFN1	3.66E-06	0.125212	0.049	0.007	0.054732
CAPS	3.76E-06	-0.17819	0.033	0.027	0.05615
PPP1R3B	3.76E-06	-0.1426	0.035	0.021	0.056209
TMED8	3.87E-06	-0.10143	0.033	0.024	0.057873
FCGBP	4.15E-06	-0.11426	0.051	0.038	0.06201
ZEB2-AS1	4.16E-06	0.122893	0.051	0.01	0.062124
ALKBH8	4.27E-06	0.106823	0.043	0.005	0.063756
CDK20	4.33E-06	-0.11258	0.04	0.027	0.064674
GIMAP2	4.45E-06	-0.17553	0.021	0.019	0.066511
THSD4	4.56E-06	-0.12439	0.047	0.032	0.068168
TMEM201	4.65E-06	0.119257	0.044	0.005	0.069563
FAM124A	4.69E-06	0.10287	0.044	0.007	0.070149
MTMR11	4.88E-06	-0.3912	0.007	0.036	0.07287
MED23	5.02E-06	0.120074	0.056	0.012	0.075067
ADAMTS9-A	5.05E-06	-0.15874	0	0.022	0.075479
CLN3	5.05E-06	-0.24668	0	0.022	0.075479
IQGAP2	5.09E-06	-0.14653	0.044	0.038	0.076
KCNMB3	5.21E-06	0.10236	0.036	0.003	0.077842
ZBTB21	5.42E-06	-0.16953	0.034	0.031	0.080969
TPTEP1	5.49E-06	0.30708	0.046	0.005	0.082058
MSTN	5.53E-06	0.119679	0.039	0.005	0.082595
HMGN3-AS1	5.67E-06	0.102404	0.025	0	0.084685
PDE10A	5.67E-06	0.111026	0.025	0	0.084685
ANKRD42	5.68E-06	-0.18967	0.025	0.021	0.084885
RORA	5.75E-06	-0.10016	0.064	0.041	0.085972
GALM	6.06E-06	-0.19316	0.038	0.036	0.090618
PCGF5	6.11E-06	-0.24214	0.04	0.043	0.091242
ZNF689	6.22E-06	0.130119	0.061	0.015	0.092982
IFIT2	6.23E-06	-0.23712	0.034	0.032	0.093098
SDSL	6.28E-06	-0.18052	0.035	0.034	0.093838
SFRP4	6.29E-06	-0.27309	0.029	0.039	0.094028
TTN-AS1	6.42E-06	0.112777	0.039	0.003	0.095867
GPRC5C	6.51E-06	-0.33966	0.025	0.046	0.097228
KISS1	6.68E-06	0.194461	0.035	0.002	0.099827
TMCC2	6.69E-06	0.156181	0.04	0.003	0.100016
USP6NL	6.75E-06	-0.13946	0.036	0.034	0.100912
NEK11	7.08E-06	-0.14552	0.029	0.026	0.105874
RP11-347F	7.24E-06	0.154993	0.057	0.012	0.108231
RP11-191L	7.33E-06	0.121538	0.048	0.01	0.109542
ARHGAP23	7.75E-06	0.102575	0.044	0.005	0.115858
PARP14	7.79E-06	-0.32542	0.014	0.043	0.116373
USP21	7.99E-06	0.147261	0.051	0.009	0.11934
ARHGAP26	8.09E-06	-0.24017	0.023	0.031	0.120922
RUNX1	8.18E-06	-0.16487	0.026	0.031	0.122192

ZNF84	8.25E-06	-0.11556	0.027	0.022	0.123273
DDR2	8.27E-06	-0.123	0.03	0.021	0.123593
ZNF25	8.50E-06	0.145133	0.066	0.019	0.127095
ICAM1	8.51E-06	-0.34203	0.022	0.046	0.127122
DDX31	8.57E-06	0.109426	0.046	0.007	0.128024
POSTN	8.69E-06	-0.40471	0.013	0.046	0.129804
ULK1	8.71E-06	0.126033	0.057	0.014	0.130196
TIFA	8.78E-06	-0.17354	0.02	0.019	0.131136
SMIM13	8.83E-06	0.120399	0.051	0.01	0.131881
FOXP4	9.10E-06	0.108195	0.034	0.002	0.135925
TMSB4Y	9.55E-06	0.111503	0.034	0.002	0.142706
RP11-263K	9.62E-06	-0.29501	0.016	0.038	0.143836
C1RL	9.72E-06	-0.29714	0.007	0.034	0.145263
RENBP	9.76E-06	-0.25407	0.029	0.038	0.145916
REPS2	1.03E-05	0.121977	0.023	0	0.153652
AMH	1.06E-05	0.118452	0.042	0.005	0.15804
ARHGAP5-A	1.08E-05	-0.11706	0.031	0.021	0.160733
SYDE1	1.10E-05	-0.10424	0.034	0.021	0.163905
SLCO4A1	1.10E-05	-0.18137	0.022	0.024	0.163943
RSPH1	1.10E-05	-0.32357	0.003	0.034	0.164155
ITPR2	1.11E-05	-0.22572	0.018	0.026	0.166258
GPR158	1.11E-05	0.11215	0.056	0.015	0.166625
RP13-188A	1.15E-05	0.113685	0.047	0.009	0.172221
COL4A2	1.15E-05	-0.31916	0.013	0.039	0.172273
DDX25	1.20E-05	0.117727	0.048	0.01	0.179706
DMKN	1.22E-05	-0.19062	0	0.021	0.182033
FRAT2	1.23E-05	0.108535	0.051	0.012	0.184329
CRABP2	1.26E-05	-0.2977	0.014	0.034	0.187989
PCSK5	1.28E-05	-0.10469	0.034	0.026	0.190733
DHCR24	1.30E-05	-0.14073	0.025	0.021	0.193755
UACA	1.35E-05	-0.11503	0.027	0.019	0.202129
MMP17	1.37E-05	0.107015	0.033	0.002	0.204587
SORL1	1.37E-05	-0.15798	0.046	0.046	0.205045
FBLN5	1.50E-05	-0.21478	0.027	0.029	0.223476
ITGA7	1.54E-05	-0.19177	0.036	0.039	0.229875
HCN3	1.65E-05	0.119904	0.036	0.003	0.245972
MIR210HG	1.65E-05	-0.21624	0.025	0.038	0.24709
ABHD14B	1.69E-05	-0.18826	0.038	0.036	0.252622
RP3-402G1	1.72E-05	0.109343	0.042	0.005	0.257274
IFIT3	1.77E-05	-0.23076	0.051	0.051	0.263871
ITGA5	1.78E-05	-0.28264	0.013	0.034	0.265363
FAM114A1	1.83E-05	-0.24174	0.021	0.029	0.273649
TNFAIP3	1.85E-05	-0.19845	0.03	0.027	0.276103
HVCN1	1.85E-05	-0.22649	0.017	0.029	0.27712
GAD2	1.87E-05	0.114349	0.022	0	0.27892
HLA-DQA2	1.89E-05	-0.28432	0.007	0.021	0.281827
RP11-5301	1.90E-05	-0.11219	0.027	0.014	0.284133
SERPINF2	1.94E-05	0.140722	0.033	0.002	0.289499
RPGRIP1L	2.00E-05	-0.17491	0.042	0.029	0.298821
SELENBP1	2.01E-05	-0.17394	0.016	0.015	0.300258
AFAP1L1	2.05E-05	-0.1704	0.025	0.027	0.306511

RP11-390F	2.06E-05	-0.21873	0.034	0.039	0.307148
INTU	2.07E-05	-0.1677	0.033	0.034	0.30888
ACSS3	2.17E-05	-0.25837	0.014	0.038	0.323887
ARHGAP24	2.28E-05	0.103648	0.072	0.031	0.340631
ADCK5	2.29E-05	0.107653	0.048	0.01	0.341828
CNR1	2.37E-05	-0.17649	0.025	0.029	0.354191
ADCY8	2.40E-05	-0.15103	0.027	0.017	0.358377
ZDHHC2	2.44E-05	0.13582	0.053	0.012	0.364499
SYT16	2.53E-05	0.105577	0.033	0.003	0.377377
PLEKHA4	2.58E-05	-0.32337	0.026	0.051	0.385836
SIGIRR	2.72E-05	-0.12198	0.023	0.017	0.406036
IL13RA2	2.74E-05	-0.2386	0.021	0.032	0.409521
RDH10	2.75E-05	-0.19742	0.02	0.029	0.411279
C6orf141	2.94E-05	-0.20143	0	0.019	0.439619
TMEM231	3.00E-05	-0.15258	0.034	0.032	0.448117
ENTPD1	3.06E-05	-0.13489	0.023	0.021	0.457584
DPYD	3.10E-05	-0.20632	0.022	0.027	0.463544
CA3	3.16E-05	-0.39891	0.007	0.029	0.472234
S100A1	3.18E-05	-0.14433	0.027	0.017	0.474762
KLHL15	3.24E-05	0.102459	0.031	0.002	0.483946
RP11-849H	3.33E-05	0.108686	0.04	0.007	0.497035
KCNE4	3.35E-05	-0.22716	0.033	0.041	0.500905
HSD17B8	3.37E-05	-0.2628	0.022	0.036	0.503797
LRRTM4	3.49E-05	0.102432	0.034	0.003	0.521872
HIVEP3	3.52E-05	-0.11731	0.034	0.027	0.525511
ARHGAP18	3.56E-05	-0.26588	0.013	0.027	0.532629
SLC31A2	3.57E-05	-0.1293	0.027	0.019	0.53317
TNFRSF14	3.59E-05	-0.17333	0.023	0.024	0.535746
PHKA2	3.66E-05	0.129646	0.043	0.007	0.547648
LCAT	3.67E-05	-0.17937	0.023	0.022	0.548851
FAM122C	3.71E-05	0.124301	0.04	0.005	0.55423
FAS	3.82E-05	-0.34124	0.008	0.034	0.570362
MROH1	3.86E-05	-0.10844	0.021	0.015	0.577439
FRMD4B	3.99E-05	-0.25386	0.02	0.034	0.595604
ZNF441	4.12E-05	0.106701	0.044	0.009	0.615236
ETV4	4.12E-05	-0.30505	0.021	0.039	0.615289
ZNF665	4.17E-05	-0.15473	0.022	0.027	0.623168
TUBA4A	4.19E-05	-0.37967	0.013	0.034	0.62644
DDX60L	4.20E-05	-0.10062	0.026	0.015	0.62739
RP11-498F	4.24E-05	0.103027	0.035	0.003	0.634161
SNTA1	4.78E-05	-0.18193	0.021	0.021	0.713732
HACE1	4.93E-05	0.127827	0.042	0.007	0.736483
XXbac-BPG	5.27E-05	-0.14289	0.018	0.017	0.786876
PEX11A	5.38E-05	-0.15741	0.021	0.022	0.804548
PNPO	5.47E-05	-0.10846	0.023	0.017	0.817901
RP1-313I6	5.57E-05	-0.13114	0.026	0.021	0.832314
PDZD2	5.82E-05	-0.19272	0.034	0.039	0.869218
PLSCR4	6.07E-05	-0.18683	0.016	0.026	0.906811
SLC6A6	6.26E-05	-0.2677	0.018	0.034	0.934849
EVA1C	6.66E-05	-0.1582	0.026	0.024	0.99578
PTGES	7.12E-05	-0.21348	0	0.017	1

RP11-479J	7.12E-05	-0.17293	0	0.017	1
UNC93B1	7.16E-05	-0.19526	0.016	0.021	1
KCNMA1	7.23E-05	-0.22643	0.025	0.026	1
RAB20	7.50E-05	-0.17631	0.022	0.027	1
WWTR1	7.52E-05	-0.20892	0.021	0.032	1
PTX3	7.71E-05	-0.24781	0.01	0.029	1
RP11-355E	7.88E-05	0.107936	0.036	0.005	1
BIRC3	7.99E-05	-0.38714	0.009	0.021	1
TMEM144	8.00E-05	-0.11425	0.021	0.015	1
PTHLH	8.03E-05	-0.25345	0.007	0.032	1
ZNF546	8.05E-05	0.103451	0.038	0.005	1
RP11-468E	8.12E-05	-0.11837	0.035	0.027	1
LGI1	8.37E-05	-0.12211	0.031	0.027	1
KIAA1107	8.41E-05	0.101381	0.039	0.007	1
LINC0011	8.46E-05	-0.19829	0.029	0.027	1
ZC3H12A	8.59E-05	-0.28864	0.01	0.026	1
FPGT	8.64E-05	-0.17072	0.03	0.027	1
PPIL6	8.67E-05	-0.16618	0.022	0.024	1
DUSP23	8.71E-05	-0.24149	0.021	0.032	1
SERF1A	9.02E-05	-0.11024	0.02	0.017	1
PDE8A	9.02E-05	-0.20694	0.014	0.031	1
CLDND2	9.11E-05	0.105917	0.036	0.005	1
GALNT15	9.12E-05	-0.21313	0.007	0.027	1
CA9	9.12E-05	-0.31015	0.012	0.043	1
HCST	9.28E-05	-0.10453	0.022	0.019	1
SPTSSB	9.40E-05	-0.28071	0.003	0.026	1
KCNN3	9.43E-05	-0.12554	0.017	0.014	1
MAP3K10	9.43E-05	0.105931	0.038	0.005	1
BATF3	9.82E-05	-0.27154	0.007	0.022	1
PHYHD1	0.0001	0.115888	0.04	0.009	1
ITGA3	0.000101	-0.22206	0.009	0.026	1
C1orf54	0.000103	-0.30832	0.008	0.032	1
ACSBG1	0.000104	-0.30539	0.013	0.043	1
HIST1H4E	0.000105	-0.29672	0.021	0.024	1
ITPKB	0.000106	-0.23318	0.008	0.024	1
ZNF700	0.000106	-0.12042	0.026	0.026	1
COL16A1	0.000114	-0.27378	0.022	0.046	1
RNASE4	0.000115	-0.27549	0.007	0.027	1
CSF1	0.000116	-0.25291	0.027	0.039	1
TMEM37	0.000116	-0.23602	0.004	0.022	1
SCN2A	0.000118	0.102021	0.034	0.005	1
H6PD	0.00012	-0.12553	0.027	0.021	1
KRBOX1	0.000124	-0.289	0.004	0.032	1
CLDN5	0.000124	-0.18085	0.023	0.024	1
SLC16A3	0.000133	-0.1178	0.026	0.022	1
MYCBP	0.000137	-0.1135	0.03	0.022	1
MGARP	0.000139	-0.17031	0.02	0.027	1
KIAA0040	0.000139	-0.24746	0.014	0.032	1
AHNAK	0.000144	-0.19877	0.018	0.021	1
FKBP1C	0.000146	-0.12282	0.017	0.012	1
IFI30	0.000147	-0.20962	0.009	0.021	1

LUC7L2	0.000149	-0.24675	0.003	0.027	1
GREM1	0.000151	-0.25934	0.008	0.034	1
XKR6	0.000151	0.100577	0.035	0.005	1
PPP1R3C	0.000157	-0.20177	0.017	0.022	1
NMNAT3	0.000159	-0.19027	0.025	0.031	1
SLC6A9	0.000159	-0.18294	0.023	0.029	1
NPTX1	0.000163	-0.26224	0.007	0.021	1
SELPLG	0.000164	-0.11019	0.022	0.017	1
APOL6	0.000167	-0.296	0.008	0.031	1
GPRC5A	0.000173	-0.17472	0	0.015	1
RP11-306G	0.000173	-0.15374	0	0.015	1
CA5A	0.000173	-0.14831	0	0.015	1
TRPM3	0.000178	-0.28472	0.016	0.038	1
AC007405.	0.000182	-0.20166	0.017	0.024	1
BLM	0.000191	-0.17681	0.043	0.05	1
WNT3	0.000195	-0.18212	0.018	0.022	1
RP11-1143	0.000195	-0.10203	0.029	0.017	1
PPARD	0.000201	-0.1072	0.036	0.027	1
DMRTA2	0.00022	-0.12323	0.031	0.029	1
DHX32	0.000221	0.100474	0.035	0.005	1
SRRM4	0.000221	0.113845	0.031	0.003	1
SPICE1	0.000224	-0.29047	0.014	0.027	1
STEAP1	0.000224	-0.3383	0.01	0.038	1
ZFYVE9	0.000226	0.102337	0.046	0.012	1
RTP4	0.000238	-0.22627	0.01	0.019	1
WDFY2	0.000238	-0.13393	0.03	0.026	1
APLN	0.000252	-0.23433	0.001	0.019	1
CCDC88B	0.000255	-0.1635	0.02	0.022	1
MVP	0.000255	-0.29182	0.022	0.044	1
CRISPLD2	0.000258	-0.18391	0.027	0.027	1
RP5-1068E	0.000263	-0.10444	0.03	0.021	1
PTCHD1	0.000266	-0.22952	0.001	0.021	1
PCDH1	0.000268	-0.15454	0.035	0.032	1
RMRP	0.000269	0.104864	0.027	0.003	1
LGR6	0.000272	-0.237	0.004	0.026	1
HES7	0.000272	0.101061	0.026	0.002	1
SPAG4	0.000274	-0.28504	0.005	0.019	1
TSHR	0.000284	0.169739	0.026	0.002	1
EID3	0.000284	-0.15353	0.023	0.022	1
OAS3	0.000285	-0.20154	0.017	0.024	1
CTC-479C5	0.000286	-0.12317	0.021	0.021	1
CCL5	0.000286	-0.12644	0.021	0.015	1
RAB32	0.000289	-0.11371	0.022	0.015	1
SLC7A11	0.000301	-0.16485	0.008	0.027	1
DBX2	0.000303	-0.24424	0.012	0.034	1
RP11-783K	0.000317	-0.21058	0.014	0.022	1
LINC00882	0.00033	-0.20963	0.018	0.024	1
GLIPR1L2	0.000331	-0.12724	0.027	0.019	1
SLC39A14	0.000343	-0.25139	0.021	0.038	1
CD163L1	0.000349	0.104964	0.042	0.01	1
SDCBP2	0.00035	-0.25693	0.016	0.031	1

KCNF1	0.000362	-0.11448	0.023	0.019	1
DMRTA1	0.000382	-0.15629	0.001	0.022	1
FAM129A	0.000384	-0.2121	0.007	0.024	1
PGAM4	0.000401	-0.14959	0.009	0.01	1
TFR2	0.000403	-0.13404	0.017	0.019	1
TSLP	0.000417	-0.15198	0.01	0.012	1
CFH	0.00042	-0.13758	0	0.014	1
ADAMTS3	0.00042	-0.13878	0	0.014	1
ADAM19	0.000422	-0.17697	0.013	0.019	1
RPGR	0.000431	-0.12738	0.026	0.022	1
SYTL4	0.000433	-0.12407	0.026	0.027	1
ANO6	0.000435	-0.15074	0.026	0.024	1
OSR2	0.000435	-0.15106	0.02	0.019	1
PPP2R5A	0.000436	-0.14086	0.026	0.026	1
BCAR3	0.00046	-0.2506	0.008	0.031	1
GPX8	0.000463	-0.29721	0.004	0.027	1
DUSP18	0.00048	-0.10472	0.026	0.026	1
PSORS1C1	0.000488	-0.30471	0.01	0.036	1
EIF4EBP3	0.00049	-0.16355	0.001	0.021	1
CLEC2D	0.000497	-0.17805	0.012	0.024	1
ABHD1	0.000516	-0.21706	0.003	0.019	1
RP11-284F	0.000534	-0.22897	0.01	0.034	1
MAP3K7CL	0.000542	-0.21593	0.014	0.021	1
SLC39A8	0.000551	-0.1351	0.013	0.015	1
CACNG8	0.000568	-0.15854	0.023	0.024	1
FOLR1	0.00057	-0.25737	0.003	0.021	1
ENDOD1	0.000583	-0.16882	0.034	0.038	1
LY96	0.000586	-0.23258	0.016	0.026	1
CYB561	0.000626	-0.1679	0.022	0.026	1
B9D2	0.00063	-0.10018	0.027	0.024	1
FPR1	0.000651	-0.19104	0.02	0.029	1
FOSL2	0.000654	-0.15864	0.031	0.038	1
NPHP1	0.000654	-0.17765	0.018	0.026	1
C1orf226	0.000658	-0.21679	0.018	0.027	1
APOBEC3G	0.000683	-0.20904	0.016	0.029	1
CCDC163P	0.000693	-0.12857	0.021	0.021	1
CHRM3	0.000714	-0.19474	0.012	0.022	1
IFITM2	0.000742	-0.26722	0.022	0.039	1
CASP7	0.00081	-0.20378	0.014	0.027	1
ACP6	0.000815	-0.11529	0.026	0.024	1
DDIT4L	0.000825	-0.2168	0.003	0.019	1
SLC25A45	0.000826	-0.10458	0.021	0.019	1
CHST8	0.000873	-0.15967	0.021	0.021	1
SLC39A4	0.000878	-0.12245	0.018	0.017	1
C1orf194	0.00089	-0.2123	0.003	0.017	1
CCBE1	0.000905	-0.20455	0.007	0.026	1
HP	0.000907	-0.291	0.001	0.015	1
HSD17B6	0.000994	-0.13855	0.018	0.021	1
IL1R1	0.001026	-0.13147	0	0.012	1
MYBPH	0.001026	-0.11772	0	0.012	1
RP1-122K4	0.001026	-0.15002	0	0.012	1

NPNT	0.001088	-0.23078	0.008	0.024	1
BAG3	0.001097	-0.13337	0.02	0.027	1
ANGPTL1	0.001113	-0.103	0.017	0.012	1
RIN1	0.00114	-0.23856	0.005	0.027	1
FAM69C	0.001145	-0.13066	0.012	0.014	1
PHF11	0.001172	-0.17412	0.013	0.021	1
AGMO	0.001172	-0.13638	0.02	0.021	1
PIR	0.001262	-0.18048	0.01	0.021	1
ARSG	0.001323	-0.14277	0.022	0.024	1
BCL2L12	0.001333	-0.15177	0.018	0.019	1
IL1RAP	0.001339	-0.16907	0.012	0.036	1
RHBDF1	0.001342	-0.15178	0.013	0.014	1
HOMER2	0.001386	-0.14654	0.02	0.019	1
COLEC12	0.001503	-0.17476	0.001	0.019	1
FAXDC2	0.001504	-0.1129	0.033	0.029	1
OSBPL3	0.001513	-0.18395	0.016	0.024	1
PCSK1	0.001526	-0.2053	0.005	0.022	1
COL4A1	0.001555	-0.18597	0.017	0.027	1
ASS1	0.00159	-0.129	0.025	0.021	1
SHROOM3	0.001607	-0.17976	0.005	0.015	1
MORN3	0.001614	-0.21932	0.005	0.021	1
MIR155HG	0.001621	-0.19613	0.001	0.019	1
PPM1N	0.001731	-0.18899	0.008	0.024	1
LOXL2	0.001743	-0.12398	0.01	0.019	1
CD70	0.001782	-0.28376	0.003	0.017	1
LAMB2	0.001791	-0.25254	0.014	0.036	1
PLA2G6	0.00186	0.104506	0.039	0.01	1
CMYA5	0.001864	-0.25797	0.008	0.029	1
TFCP2L1	0.001914	-0.19277	0.001	0.017	1
STX3	0.001965	-0.12316	0.02	0.021	1
NOTCH3	0.001992	-0.16091	0.009	0.019	1
ZNF442	0.002017	-0.12032	0.017	0.014	1
STK33	0.002067	-0.13565	0.036	0.034	1
ASB3	0.002158	-0.24026	0.003	0.021	1
IL6	0.002293	-0.21725	0.001	0.015	1
IGFLR1	0.002371	-0.18336	0.01	0.026	1
CTSH	0.002376	-0.14941	0.02	0.026	1
PARVB	0.002379	-0.10724	0.016	0.015	1
SLC5A3	0.002403	-0.1647	0.001	0.017	1
SNX15	0.00241	-0.10027	0.014	0.015	1
LFNG	0.002449	-0.15625	0.021	0.026	1
CNR2	0.00252	-0.10516	0	0.01	1
CCL3L3	0.00252	-0.11233	0	0.01	1
LINC00637	0.00252	-0.10367	0	0.01	1
MT1H	0.00252	-0.10778	0	0.01	1
MLLT1	0.00252	-0.10004	0	0.01	1
MAOA	0.002526	-0.21809	0.009	0.021	1
AP001258.	0.002533	-0.10936	0.016	0.014	1
RCSD1	0.002537	-0.10439	0.012	0.014	1
PFKFB4	0.002541	-0.16713	0.013	0.019	1
KCTD14	0.002572	-0.13057	0.008	0.01	1

ACAP1	0.002671	-0.13901	0.01	0.017	1
TLCD1	0.002738	-0.13688	0.017	0.017	1
APOLD1	0.002741	-0.10886	0.023	0.026	1
TSIX	0.002762	-0.19599	0.009	0.029	1
HPS1	0.002784	-0.17707	0.01	0.026	1
CTD-2020K	0.002833	-0.14422	0.013	0.024	1
LHFPL2	0.002924	-0.12898	0.016	0.015	1
CTD-3131K	0.002924	-0.18415	0.02	0.021	1
GNG3	0.002937	-0.1042	0.027	0.021	1
CDIPT-AS1	0.003076	-0.2258	0.003	0.021	1
AC007161.	0.003088	0.104745	0.027	0.005	1
C5AR2	0.003127	-0.18127	0.004	0.019	1
ALDH1L1-A	0.003133	-0.15726	0.012	0.022	1
IZUMO4	0.003169	-0.18685	0.013	0.026	1
PARP9	0.003197	-0.21326	0.031	0.044	1
BCL2L15	0.003255	-0.20454	0.01	0.026	1
LRRC2	0.003515	-0.15346	0.013	0.024	1
ACOT11	0.003532	-0.18345	0.01	0.021	1
SULT1C2	0.003644	-0.11939	0.001	0.014	1
C11orf71	0.00367	-0.19263	0.013	0.026	1
METTL25	0.003714	-0.12535	0.012	0.014	1
MOV10	0.003741	-0.1278	0.022	0.026	1
SP140L	0.003744	-0.19874	0.013	0.031	1
TRAF6	0.003823	-0.10843	0.02	0.015	1
MX1	0.003825	-0.19827	0.022	0.032	1
CNTNAP3B	0.003856	-0.12024	0.017	0.022	1
MTFP1	0.004036	-0.22908	0.017	0.029	1
EYA4	0.004038	-0.10079	0.014	0.012	1
FZD7	0.004078	-0.16426	0.004	0.012	1
HSD3B7	0.004302	-0.19821	0.007	0.022	1
NOS2	0.004375	-0.20835	0.007	0.019	1
FABP3	0.004446	-0.31898	0.008	0.022	1
ABCC3	0.004478	-0.18859	0.013	0.031	1
DNAH9	0.004479	-0.16814	0.005	0.021	1
KRT7	0.004509	-0.26391	0.007	0.026	1
SCN9A	0.004614	-0.1055	0.017	0.017	1
SDS	0.004769	-0.11372	0.009	0.01	1
DHRS4-AS1	0.004912	-0.2257	0.007	0.027	1
P4HA2	0.00509	-0.16565	0.007	0.021	1
RP11-315I	0.005297	-0.10835	0.01	0.01	1
RP11-49I1	0.005318	-0.17189	0.003	0.014	1
MAFG-AS1	0.005456	0.103294	0.03	0.007	1
CALHM2	0.005523	-0.14971	0.005	0.019	1
SLC22A18	0.005558	-0.15956	0.016	0.021	1
CHIT1	0.005682	-0.20294	0.005	0.014	1
MTRNR2L3	0.005688	-0.17778	0.007	0.026	1
HSPB8	0.005811	-0.16918	0.008	0.021	1
HSD11B1	0.005987	-0.1968	0.001	0.014	1
C12orf54	0.006154	-0.15294	0.005	0.019	1
C8orf86	0.006239	-0.12011	0	0.009	1
RP11-522E	0.006239	-0.11366	0	0.009	1

AC010987.	0.006239	-0.13974	0	0.009	1
C16orf89	0.006239	-0.11763	0	0.009	1
COL5A1	0.006239	-0.10566	0	0.009	1
RP11-495F	0.006239	-0.12494	0	0.009	1
PDZK1IP1	0.006239	-0.15785	0	0.009	1
AL592528.	0.006239	-0.13233	0	0.009	1
TLR3	0.006239	-0.11461	0	0.009	1
MUC1	0.006239	-0.10112	0	0.009	1
FGF22	0.006305	-0.14117	0.007	0.015	1
TRIM72	0.006454	-0.14358	0.003	0.017	1
LYN	0.006484	-0.13487	0.017	0.017	1
CFI	0.006759	-0.13234	0.003	0.015	1
ITGB4	0.006818	-0.17259	0.013	0.021	1
PARP10	0.006837	-0.10353	0.023	0.019	1
ADSSL1	0.006996	-0.15515	0.012	0.021	1
IRAK2	0.007014	-0.15025	0.014	0.019	1
MSMP	0.007046	-0.10451	0.013	0.01	1
FGFR3	0.007117	-0.14274	0.014	0.021	1
SQRDL	0.007154	-0.18953	0.01	0.022	1
CHRNA9	0.007154	-0.19083	0.004	0.019	1
TMCO4	0.007273	-0.12943	0.001	0.015	1
ABCB9	0.007407	-0.11688	0.016	0.019	1
AXL	0.00761	-0.16403	0.012	0.024	1
ASPA	0.007745	-0.15871	0.003	0.015	1
OSMR	0.007747	-0.17124	0.012	0.027	1
CRIP1	0.00776	-0.12629	0.013	0.012	1
SYNPO2L	0.008393	-0.11141	0.017	0.021	1
MORN5	0.008661	-0.15791	0.001	0.012	1
COL18A1	0.008851	-0.1704	0.012	0.022	1
ETV7	0.009159	-0.19162	0.003	0.017	1
PCDH9-AS1	0.009173	-0.10167	0.013	0.017	1
RASSF8	0.009305	-0.1344	0.016	0.024	1
MICALL2	0.009375	-0.1119	0.022	0.022	1
ACTA2	0.009427	-0.1828	0.008	0.017	1
HLA-F	0.009805	-0.16216	0.01	0.021	1
GPR89B	0.009892	-0.1818	0.007	0.021	1
MAN1C1	0.010184	-0.12618	0.008	0.014	1
FAM198B	0.010235	-0.15991	0.009	0.017	1
ZNF8	0.010397	-0.16957	0.001	0.014	1
FCGR2A	0.010538	-0.15549	0.016	0.024	1
RAMP3	0.010628	-0.102	0.016	0.014	1
HERC5	0.010781	-0.10382	0.025	0.022	1
ZDHHC23	0.010929	-0.1453	0.009	0.015	1
AC114803.	0.011151	-0.14751	0.003	0.015	1
THBS1	0.011418	-0.12704	0.01	0.015	1
C4orf47	0.011438	-0.12574	0.018	0.021	1
AMT	0.011559	-0.1265	0.012	0.014	1
FLT3LG	0.011652	-0.12342	0.016	0.021	1
IL6R	0.012136	-0.12195	0.012	0.024	1
SPATA17	0.012221	-0.15901	0.004	0.014	1
SERPINA3	0.012316	-0.1448	0.001	0.012	1

RNF144B	0.012484	-0.19844	0.008	0.021	1
POLN	0.012556	-0.10423	0.013	0.014	1
PPARGC1A	0.012838	-0.12584	0.005	0.014	1
ZNF556	0.013692	-0.11524	0.001	0.012	1
CHST2	0.014039	-0.1156	0.014	0.017	1
MARVELD3	0.014193	-0.17502	0.008	0.024	1
MPZ	0.014281	-0.11681	0.008	0.01	1
SSTR2	0.014344	-0.13342	0.01	0.017	1
PLCG2	0.014702	-0.16896	0.003	0.015	1
PHLDA2	0.014856	-0.15192	0.005	0.019	1
HSPB2	0.014901	-0.14662	0.004	0.015	1
WIF1	0.01562	-0.15102	0	0.007	1
FAM110C	0.01562	-0.15259	0	0.007	1
RP11-383M	0.015701	-0.11525	0.001	0.012	1
EHD2	0.015751	-0.14782	0.012	0.022	1
AC006946.	0.015781	-0.16165	0.001	0.014	1
GJC3	0.016073	-0.14094	0.003	0.014	1
YAP1	0.016667	-0.13388	0.003	0.015	1
SLIT1	0.016984	-0.11385	0.01	0.014	1
ERAP2	0.017127	-0.15125	0.016	0.031	1
CCL4L2	0.017136	-0.1527	0.004	0.017	1
VWA3B	0.018034	-0.15811	0.013	0.029	1
HYKK	0.018177	-0.10459	0.001	0.012	1
IGF2	0.018199	-0.24355	0.009	0.019	1
OTX1	0.018442	-0.10788	0.01	0.015	1
ARHGEF4	0.018461	-0.11194	0.016	0.019	1
SLC44A3	0.019996	-0.15041	0.005	0.014	1
THTPA	0.020257	-0.16882	0.003	0.015	1
SCN7A	0.020269	-0.10622	0.001	0.012	1
RIIAD1	0.020577	-0.12508	0.008	0.012	1
WWC1	0.020604	-0.1197	0.003	0.014	1
C8orf34	0.020732	-0.10194	0.001	0.012	1
EPHA1-AS1	0.020765	-0.11345	0.009	0.009	1
SPRED3	0.020903	-0.11296	0.007	0.01	1
CALB1	0.021555	-0.13424	0.005	0.012	1
ACOX2	0.022897	-0.12187	0.01	0.014	1
RP3-325F2	0.022915	-0.11286	0.004	0.014	1
RGL3	0.023104	-0.10824	0.003	0.012	1
TMEM232	0.023428	-0.1105	0.008	0.009	1
CLMN	0.023527	-0.13332	0.007	0.021	1
COL23A1	0.02395	-0.14079	0.004	0.015	1
GS1-18A18	0.024083	-0.23468	0.005	0.015	1
COL22A1	0.024267	-0.11796	0.007	0.014	1
HIST1H2BC	0.024382	-0.12982	0.004	0.012	1
BATF2	0.024739	-0.18115	0.007	0.024	1
RNASEK-C1	0.02503	-0.10056	0.001	0.012	1
TNFSF10	0.026563	-0.16936	0.005	0.019	1
PROCR	0.026932	-0.13518	0.003	0.012	1
ZNF264	0.028235	-0.11014	0.012	0.017	1
USP6	0.028294	-0.10345	0.003	0.012	1
CD34	0.028935	-0.10452	0.001	0.01	1

TNFAIP2	0.029124	-0.12757	0.004	0.01	1
MYLK	0.029417	-0.12856	0.007	0.012	1
TGFBR3	0.030674	-0.14089	0.007	0.012	1
LINC01138	0.030719	-0.11536	0.001	0.01	1
SMIM10	0.031135	-0.12838	0.01	0.014	1
ADCY2	0.031851	-0.1295	0.013	0.022	1
TNXB	0.0323	-0.10852	0.007	0.015	1
GDPGP1	0.032679	-0.12435	0.003	0.01	1
GBP7	0.033012	-0.1003	0.001	0.012	1
RGS20	0.03342	-0.10297	0.001	0.012	1
MILR1	0.033888	-0.10334	0.005	0.009	1
PLAU	0.034254	-0.10355	0.01	0.017	1
AC015936.	0.034591	-0.12885	0.008	0.019	1
SLC6A11	0.035084	-0.1328	0.009	0.014	1
RP11-326C	0.035444	-0.11094	0.003	0.014	1
RASGRP1	0.036462	-0.10794	0.005	0.012	1
RP11-244H	0.037675	-0.10579	0.003	0.012	1
OAS2	0.038129	-0.12367	0.004	0.01	1
FAM180A	0.038346	-0.13773	0.001	0.01	1
ZNF600	0.038362	-0.11432	0.007	0.012	1
ATP13A4	0.038829	-0.10142	0.013	0.014	1
RP11-1246	0.03891	-0.15164	0.009	0.014	1
TRIM38	0.038936	-0.13089	0.005	0.017	1
ALDH3B1	0.03959	-0.10507	0.009	0.014	1
HIST1H2BF	0.03979	-0.10678	0	0.005	1
SAA4	0.03979	-0.10498	0	0.005	1
CCL26	0.03979	-0.17007	0	0.005	1
SLC7A8	0.039938	-0.12114	0.008	0.021	1
CHST6	0.041626	-0.10617	0.004	0.007	1
NEBL	0.042214	-0.1151	0.012	0.021	1
CAMP	0.042685	-0.16552	0.001	0.009	1
RP11-242D	0.043728	-0.13658	0.007	0.015	1
CYB5R2	0.043731	-0.17101	0.009	0.024	1
RP11-160C	0.044392	-0.11451	0.004	0.01	1
SYNM	0.045153	-0.13213	0.007	0.017	1
DCN	0.045171	-0.10853	0.001	0.01	1
SYT4	0.045319	-0.13778	0.005	0.015	1
NPTX2	0.046023	-0.10322	0.009	0.014	1
C11orf70	0.047577	-0.11105	0.016	0.019	1
PTPRT	0.048064	-0.11254	0.008	0.01	1
LSR	0.049636	-0.10554	0.012	0.017	1
TMEM71	0.050099	-0.1157	0.001	0.01	1
MET	0.050989	-0.11058	0.007	0.009	1
DYNLRB2	0.051478	-0.14177	0.005	0.015	1
MSC	0.051888	-0.17707	0.003	0.005	1
TEAD4	0.052368	-0.13386	0.004	0.015	1
C1QTNF9B-	0.052504	-0.11992	0.004	0.014	1
SLFN12	0.054466	-0.10109	0.003	0.012	1
LRRC69	0.054577	-0.15232	0.016	0.021	1
MMP9	0.058526	-0.116	0.001	0.009	1
GJB2	0.061127	-0.107	0.005	0.01	1

CAPS2	0.063388	-0.14034	0.005	0.015	1
RRAS	0.064453	-0.11837	0.008	0.015	1
MARCO	0.068936	-0.131	0.007	0.014	1
RP11-367J	0.07232	-0.11195	0.004	0.01	1
INCA1	0.075918	-0.13546	0.003	0.01	1
ALG1L	0.077822	-0.11062	0.007	0.009	1
ACSM5	0.080542	-0.10043	0.009	0.01	1
PAXIP1-AS	0.080975	-0.11841	0.001	0.009	1
GGACT	0.08255	-0.10127	0.001	0.009	1
C8orf48	0.083735	-0.1046	0.007	0.01	1
OGDHL	0.085364	-0.10495	0.003	0.01	1
ECM2	0.087193	-0.11359	0.004	0.014	1
RP11-437L	0.090381	-0.11028	0.004	0.012	1
ADAMTS18	0.09644	-0.10592	0.005	0.017	1
MMP19	0.097888	-0.10983	0.004	0.012	1
PTGS1	0.102442	-0.1023	0.008	0.012	1
CTD-2369F	0.103894	-0.10549	0.003	0.012	1
RP11-264E	0.104413	-0.10051	0	0.003	1
GNLY	0.111798	-0.12874	0.007	0.012	1
RP11-745C	0.114287	-0.11563	0.005	0.01	1
MYO1E	0.115591	-0.10275	0.008	0.009	1
RP11-395E	0.11621	-0.10326	0.012	0.017	1
HCG11	0.124546	-0.11766	0.005	0.01	1
MYCBPAP	0.137402	-0.10023	0.008	0.014	1
AQP3	0.146252	-0.11093	0.001	0.005	1
GPR158-AS	0.208199	-0.10695	0.005	0.01	1
