# Supplementary material for: A Novel Molecular Classification Method for Glioblastoma Based on Tumor Cell Differentiation Trajectories
Source: Stem Cells Int. 2023 Feb 22;2023:2826815. doi: 10.1155/2023/2826815 (PMC10643041; doi:10.1155/2023/2826815)
Supplement: Supplementary 7 — Supplementary Table 5: differentially expressed genes (DEGs) in GSCL and Neo-G. [file 2826815.f7.pdf]

	p_val	avg_logFC	pct.1	pct.2	p_val_adj
STMN2	0	1.64234	0.984	0.221	0
STMN4	0	1.292063	0.962	0.373	0
TERF2IP	0	0.937158	0.969	0.719	0
MAP1B	0	0.765441	0.991	0.836	0
HMGB2	0	-1.36915	0.351	0.977	0
PBK	0	-1.60435	0.019	0.802	0
BIRC5	0	-1.71183	0.021	0.809	0
NUSAP1	0	-1.73776	0.013	0.814	0
UBE2C	0	-1.85455	0.031	0.833	0
NSG1	1.9762625	1.446257	0.879	0.196	2.95332680458064e-319
DLX6-AS1	5.9475622	1.864895	0.838	0.114	8.88803701838542e-316
MLLT11	1.2117454	0.794433	0.987	0.798	1.81083233022862e-315
TOP2A	4.5236551	-1.67298	0.028	0.785	6.76015028905105e-314
KIAA0101	########	-1.49875	0.029	0.769	########
FAM64A	########	-1.49392	0.009	0.717	########
SOX4	########	0.718099	0.998	0.832	########
TYMS	########	-1.38656	0.053	0.802	########
PTTG1	########	-1.68926	0.084	0.831	########
UCHL1	########	0.889604	0.964	0.673	########
CDK1	########	-1.46781	0.015	0.717	########
CENPF	########	-1.62524	0.046	0.772	########
SGOL1	########	-1.23885	0.009	0.698	########
MARCKSL1	########	0.514496	1	0.906	########
BTG1	########	0.714529	0.987	0.831	########
ZWINT	########	-1.22822	0.013	0.7	########
CENPK	########	-1.20863	0.02	0.71	########
TPX2	########	-1.42371	0.016	0.69	########
NUF2	########	-1.26439	0.01	0.669	########
RRM2	########	-1.33703	0.005	0.647	########
APLP1	########	0.882779	0.899	0.633	########
DLX5	########	1.470843	0.865	0.185	########
SMC4	########	-1.3207	0.134	0.859	########
TUBB2A	########	0.633212	0.982	0.83	########
CENPU	########	-1.22857	0.069	0.772	########
MKI67	########	-1.26706	0.009	0.637	########
GTSE1	########	-1.15186	0.005	0.614	########
CENPM	########	-1.09107	0.006	0.615	########
PRC1	########	-1.26903	0.04	0.702	########
NFIB	########	0.715717	0.951	0.79	########
DCX	########	1.040833	0.814	0.388	########
ATAT1	########	0.903085	0.78	0.494	########
TCF4	########	0.566894	0.958	0.864	########
CXADR	########	1.025294	0.745	0.42	########
TTC3	########	0.618766	0.935	0.805	########
CCNA2	########	-1.14021	0.009	0.601	########
CENPN	########	-1.09087	0.02	0.636	########
KIFC1	########	-1.04037	0.007	0.593	########
MAD2L1	########	-1.18601	0.124	0.814	########
UBE2T	########	-1.22998	0.148	0.835	########
CRMP1	########	0.878048	0.86	0.515	########

AURKB	########	-1.15409	0.005	0.58	########
CDKN3	########	-1.36462	0.036	0.665	########
ASF1B	########	-1.02179	0.004	0.573	########
NNAT	########	1.24769	0.87	0.264	########
TAGLN3	########	1.082745	0.848	0.349	########
RAB3A	########	1.258699	0.697	0.143	########
CCNB2	########	-1.24818	0.005	0.56	########
CKAP2L	########	-1.03177	0.009	0.574	########
PCSK1N	########	0.638168	0.976	0.784	########
MT2A	########	-1.34831	0.381	0.889	########
MT3	########	-1.49646	0.122	0.768	########
FOXM1	########	-0.94193	0.009	0.568	########
SGOL2	########	-1.15022	0.035	0.633	########
TACC3	########	-1.00228	0.013	0.57	########
TUBB2B	########	0.372839	0.999	0.973	########
PHF19	########	-0.94605	0.006	0.541	########
KIF5C	########	0.83087	0.746	0.499	########
ELAVL4	########	1.020084	0.869	0.312	########
RNASEH2A	########	-1.09402	0.13	0.774	########
NDC80	########	-1.03031	0.005	0.53	########
CDC20	########	-1.30284	0.009	0.543	########
HJURP	########	-1.00906	0.005	0.525	########
ASPM	########	-1.09217	0.007	0.535	########
GPM6A	########	0.536311	0.98	0.865	########
MELK	########	-0.88858	0.003	0.515	########
GNG5	########	-1.21296	0.174	0.798	########
MXD3	########	-1.01446	0.027	0.59	########
H2AFX	########	-1.12881	0.159	0.783	########
PHGDH	########	-1.0924	0.067	0.673	########
KLHL35	########	1.360516	0.632	0.046	########
SOX11	########	0.794897	0.866	0.628	########
TK1	########	-0.96864	0.01	0.533	########
TSPAN13	########	0.839907	0.877	0.51	########
GSTA4	########	0.816356	0.791	0.504	########
CCNB1	########	-1.33287	0.035	0.599	########
CENPA	########	-1.08396	0.004	0.504	########
DNAJB6	########	0.522489	0.961	0.848	########
TMEM161B-	########	0.856265	0.761	0.489	########
RAD51AP1	########	-0.91229	0.012	0.53	########
NCAPG	########	-0.86674	0.006	0.506	########
CDCA5	########	-0.87568	0.012	0.523	########
BASP1	########	0.74221	0.621	0.491	########
ECT2	########	-0.92105	0.009	0.512	########
MYBL2	########	-0.91277	0.004	0.491	########
GMNN	########	-0.9226	0.079	0.67	########
KIF2C	########	-0.91697	0.005	0.496	########
SPC25	########	-1.05093	0.05	0.62	########
MAPT	########	0.891141	0.753	0.384	########
RTN4	########	0.436712	0.984	0.888	########
DBN1	########	0.690735	0.854	0.644	########
SMC2	########	-0.99644	0.158	0.78	########

TUBA1A	########	0.329015	1	0.989	########
POC1A	########	-0.84596	0.007	0.494	########
EGFR	########	-1.22162	0.126	0.723	########
CELF4	########	1.255118	0.6	0.09	########
ORC6	########	-0.93062	0.069	0.641	########
BEX2	########	1.09693	0.777	0.263	########
DHFR	########	-0.96941	0.082	0.66	########
AKAP9	########	0.68262	0.776	0.605	########
SPC24	########	-0.88374	0.018	0.517	########
RBFOX2	########	0.877425	0.706	0.39	########
ESCO2	########	-0.78751	0.004	0.462	########
MEIS2	########	0.918963	0.692	0.404	########
C4orf48	########	0.657978	0.831	0.641	########
SPAG5	########	-0.81651	0.005	0.462	########
SHCBP1	########	-0.76485	0.006	0.467	########
CKS2	########	-1.07227	0.298	0.846	########
CKS1B	########	-1.09918	0.159	0.738	########
CENPH	########	-0.85622	0.124	0.71	########
CDCA3	########	-1.04117	0.02	0.509	########
KIF23	########	-0.84549	0.005	0.456	########
SLC1A3	########	-1.04131	0.056	0.595	########
SPARC	########	-1.06485	0.068	0.616	########
CENPW	########	-0.89854	0.068	0.615	########
MAGED2	########	0.626973	0.841	0.683	########
VAMP2	########	0.489721	0.931	0.844	########
C21orf58	########	-0.76215	0.027	0.516	########
AURKA	########	-1.09481	0.02	0.502	########
MEG3	########	0.586716	0.45	0.528	########
CENPE	########	-1.06776	0.026	0.517	########
MIS18BP1	########	-0.80703	0.016	0.486	########
LBH	########	1.09063	0.647	0.18	########
CDCA8	########	-0.91533	0.008	0.456	########
FAM111A	########	-0.79901	0.008	0.451	########
ELAVL3	########	0.6749	0.816	0.605	########
DTYMK	########	-0.91142	0.284	0.842	########
PLK1	########	-1.07257	0.01	0.458	########
TROAP	########	-0.8771	0.005	0.436	########
PTPRZ1	########	-0.97177	0.295	0.841	########
LINC00461	########	0.627497	0.709	0.612	########
YWHAG	########	0.603485	0.749	0.6	########
GATM	########	-0.85797	0.037	0.53	########
C19orf48	########	-0.91501	0.135	0.704	########
PKMYT1	########	-0.75198	0.013	0.462	########
H2AFZ	########	-0.50788	0.863	0.98	########
DEPDC1	########	-0.74931	0.002	0.414	########
RAB2A	########	0.536536	0.9	0.775	########
PMP22	########	-1.08319	0.05	0.557	########
SKA3	########	-0.66707	0.003	0.419	########
RAMP1	########	-0.96354	0.145	0.709	########
DLX2	########	1.200331	0.634	0.131	########
OIP5	########	-0.73427	0.005	0.423	########

KLC1	########	0.664256	0.773	0.584	########
LY6H	########	1.223622	0.523	0.038	########
ATAD2	########	-0.82117	0.036	0.52	########
ARL4D	########	0.994634	0.595	0.257	########
KMT2E	########	0.532799	0.889	0.767	########
NEK2	########	-0.83004	0.002	0.405	########
FANCI	########	-0.68364	0.006	0.423	########
DLGAP5	########	-0.85155	0.007	0.43	########
BLCAP	########	0.736947	0.592	0.384	########
MYCBP2	########	0.791302	0.627	0.391	########
NREP	########	0.610307	0.742	0.585	########
THSD7A	########	1.031787	0.671	0.249	########
VIM	########	-1.24187	0.326	0.722	########
MIAT	########	1.034338	0.647	0.209	########
KIF15	########	-0.70504	0.008	0.428	########
BUB1	########	-0.8084	0.006	0.421	########
ZFP36L1	########	-0.99564	0.084	0.609	########
MKRN1	########	0.573391	0.747	0.631	########
TTYH1	########	-1.02807	0.064	0.573	########
TCEAL7	########	0.771812	0.751	0.498	########
TRIP13	########	-0.66812	0.016	0.453	########
EIF4G2	########	0.426528	0.921	0.849	########
CDT1	########	-0.83391	0.021	0.469	########
DNER	########	0.835835	0.77	0.432	########
SPARCL1	########	-1.07429	0.084	0.604	########
ARHGAP11A	########	-0.7228	0.003	0.401	########
PODXL2	########	0.585834	0.801	0.641	########
PCNA	########	-0.98872	0.231	0.783	########
PRR11	########	-0.75565	0.021	0.46	########
DBF4	########	-0.87143	0.091	0.611	########
PAK3	########	0.961355	0.638	0.233	########
MND1	########	-0.71364	0.02	0.456	########
HOPX	########	-1.39147	0.039	0.465	########
RACGAP1	########	-0.84884	0.071	0.573	########
IGFBP7	########	-1.18105	0.044	0.516	########
SKA1	########	-0.67306	0.001	0.381	########
KIF4A	########	-0.70758	0.008	0.414	########
RUFY3	########	0.66186	0.65	0.469	########
CSRP2	########	-0.93917	0.13	0.668	########
NCAPH	########	-0.72877	0.01	0.422	########
WEE1	########	-0.70159	0.044	0.51	########
SCG5	########	0.894399	0.695	0.322	########
CCDC34	########	-0.90199	0.152	0.696	########
ANP32E	########	-0.94051	0.169	0.714	########
CDCA4	########	-0.78394	0.048	0.52	########
LRR1	########	-0.73624	0.024	0.46	########
TTC9B	########	1.018368	0.686	0.153	########
CDC45	########	-0.69836	0.003	0.388	########
MNS1	########	-0.70629	0.024	0.453	########
CASC5	########	-0.6193	0.003	0.385	########
ATRX	########	0.537532	0.765	0.657	########

TUBA1B	########	-0.45072	0.954	1	########
NDFIP1	########	0.458152	0.853	0.781	########
MGME1	########	-0.69656	0.056	0.525	########
SYT1	########	1.120764	0.609	0.126	########
CRNDE	########	-0.68734	0.134	0.643	########
ITGB3BP	########	-0.75152	0.083	0.579	########
HMMR	########	-0.77255	0.005	0.394	########
MAP1LC3B	########	0.539605	0.78	0.664	########
CDCA2	########	-0.66289	0.005	0.39	########
DDAH2	########	0.546029	0.781	0.673	########
DSN1	########	-0.72905	0.049	0.51	########
CDC25B	########	-0.95106	0.076	0.568	########
TUBB6	########	-0.81764	0.013	0.42	########
CDK2	########	-0.70272	0.014	0.416	########
REST	########	-0.66913	0.016	0.425	########
DLX1	########	1.016993	0.61	0.201	########
OLIG1	########	-0.97056	0.033	0.474	########
RBP1	########	0.481018	0.925	0.832	########
FNBP1L	########	0.747573	0.587	0.348	########
PON2	########	-0.90035	0.154	0.684	########
ETV1	########	-0.91953	0.136	0.658	########
RPA3	########	-0.82907	0.266	0.806	########
METTL7B	########	-0.89075	0.004	0.374	########
PMP2	########	-1.00876	0.162	0.677	########
FBXO5	########	-0.85004	0.072	0.552	########
CHAF1A	########	-0.7171	0.053	0.507	########
PDGFRA	########	-0.90148	0.05	0.505	########
FAM83D	########	-0.80311	0.011	0.4	########
ATP6V1G1	########	0.446715	0.927	0.84	########
EDNRB	########	-0.76699	0.012	0.401	########
ZC2HC1A	########	0.806654	0.678	0.335	########
BCAN	########	-1.11323	0.142	0.651	########
CD9	########	-0.96267	0.112	0.615	########
PAICS	########	-0.80639	0.17	0.694	########
DBI	########	-0.655	0.696	0.957	########
S100A16	########	-1.08588	0.041	0.48	########
SLC25A4	########	0.529256	0.791	0.662	########
NDUFA5	########	0.450509	0.864	0.788	########
CKAP2	########	-0.88894	0.186	0.711	########
CLSPN	########	-0.81795	0.039	0.475	########
ZFP36L2	########	-0.79982	0.113	0.61	########
RFC3	########	-0.70507	0.074	0.537	########
RAB34	########	-0.79673	0.021	0.426	########
ANKRD12	########	0.55826	0.633	0.522	########
TMX1	########	-0.65007	0.131	0.619	########
COMMD4	########	-0.6936	0.138	0.637	########
GINS1	########	-0.60816	0.02	0.41	########
MFGE8	########	-0.65676	0.06	0.504	########
GPC2	########	0.836099	0.71	0.354	########
KIF20A	########	-0.65255	0.001	0.342	########
CKLF	########	-0.76429	0.236	0.762	########

BMP7	########	-0.6502	0.079	0.528	########
RAB13	########	-0.83997	0.061	0.51	########
SCG3	########	0.684942	0.826	0.494	########
CCNI	########	0.390384	0.959	0.872	########
SERPINH1	########	-0.61022	0.018	0.391	########
MAP2	########	0.46344	0.796	0.712	########
MAP1LC3A	########	0.535023	0.711	0.583	########
GINS2	########	-0.86425	0.078	0.541	########
MYT1L	########	1.028082	0.446	0.025	########
PHF14	########	0.44934	0.808	0.76	########
TPM2	########	-0.8598	0.016	0.395	########
HAUS8	########	-0.6229	0.034	0.433	########
BEX1	########	0.626786	0.853	0.578	########
RAB1A	########	0.435945	0.8	0.735	########
KIF3A	########	0.717179	0.59	0.358	########
BRCA1	########	-0.58884	0.025	0.415	########
NDN	########	0.575293	0.412	0.33	########
WDR34	########	-0.71861	0.173	0.677	########
RTN3	########	0.40322	0.913	0.851	########
PDPN	########	-0.67309	0.176	0.662	########
ARHGAP11E	########	-0.61104	0.006	0.356	########
ANXA5	########	-0.80557	0.343	0.837	########
BCL2L12	########	-0.63764	0.012	0.372	########
SERINC1	########	0.576063	0.625	0.491	########
HTRA1	########	-0.80637	0.128	0.61	########
CENPQ	########	-0.55432	0.035	0.425	########
DEK	########	-0.69983	0.463	0.87	########
RAB6A	########	0.481279	0.625	0.568	########
DPYSL3	########	0.584414	0.7	0.578	########
MT1E	########	-0.91172	0.055	0.483	########
MAGEH1	########	0.666398	0.676	0.463	########
SNX5	########	-0.80171	0.137	0.622	########
MT1M	########	-0.70718	0.046	0.458	########
KIF14	########	-0.58908	0.009	0.354	########
SPRY2	########	-0.7743	0.045	0.458	########
JAM2	########	-0.5333	0.104	0.528	########
MCM3	########	-0.76957	0.071	0.509	########
METRN	########	-0.78149	0.161	0.652	########
PSMB9	########	-0.78576	0.056	0.472	########
DNAJB9	########	0.769441	0.643	0.323	########
DIAPH3	########	-0.56487	0.005	0.341	########
CD200	########	0.871243	0.464	0.146	########
LGALS1	########	-0.9348	0.408	0.767	########
RTKN2	########	-0.58529	0.016	0.375	########
ENC1	########	0.489302	0.504	0.52	########
IFITM3	########	-0.8381	0.029	0.416	########
HEPN1	########	-0.69104	0.015	0.374	########
ANLN	########	-0.55993	0.016	0.37	########
MAP4	########	0.538404	0.605	0.51	########
CAMLG	########	0.410865	0.701	0.683	########
DSEL	########	-0.74871	0.156	0.638	########

CEP135	########	-0.68814	0.038	0.432	########
ACTL6B	########	0.978531	0.511	0.078	########
E2F1	########	-0.74556	0.018	0.383	########
FEN1	########	-0.76116	0.125	0.595	########
DDX5	########	0.319507	0.98	0.919	########
SNN	########	0.647239	0.582	0.39	########
KIF20B	########	-0.66708	0.051	0.454	########
OLIG2	########	-0.83105	0.071	0.501	########
NCAPD2	########	-0.53981	0.016	0.365	########
ID2	########	0.601004	0.747	0.595	########
CHMP5	########	0.447412	0.706	0.647	########
ARSJ	########	-0.49673	0.002	0.315	########
RND3	########	0.821311	0.691	0.395	########
CDC42	########	0.427407	0.804	0.73	########
SNAP25	########	0.870704	0.626	0.219	########
BUB1B	########	-0.5204	0.005	0.325	########
HMGB1	########	-0.33816	0.958	0.991	########
BRCA2	########	-0.56772	0.009	0.34	########
C14orf80	########	-0.47062	0.04	0.4	########
SPRY1	########	-0.84767	0.045	0.443	########
PTRF	########	-0.56948	0.009	0.336	########
COL4A2	########	-0.55758	0.009	0.342	########
IFRD1	########	0.498438	0.736	0.626	########
TCF19	########	-0.61853	0.019	0.372	########
GAS1	########	-0.77524	0.055	0.463	########
MRC2	########	-0.44652	0.003	0.312	########
RSF1	########	0.388992	0.672	0.677	########
BNIP3L	########	0.528018	0.761	0.663	########
KIF22	########	-0.60103	0.207	0.679	########
LMNB2	########	-0.54698	0.102	0.521	########
KLF7	########	0.775687	0.493	0.24	########
YWHAQ	########	0.305622	0.98	0.916	########
SNRPB	########	-0.63291	0.535	0.894	########
TLE1	########	-0.56368	0.035	0.401	########
RGMA	########	-0.64323	0.053	0.448	########
TEX30	########	-0.62868	0.143	0.596	########
VRK1	########	-0.62476	0.067	0.472	########
WLS	########	-0.5953	0.042	0.417	########
DUT	########	-0.73074	0.353	0.819	########
POSTN	########	-0.78342	0.007	0.327	########
FKBP10	########	-0.54081	0.049	0.423	########
PAX6	########	-0.5387	0.025	0.368	########
TIMELESS	########	-0.5616	0.035	0.399	########
ANP32B	########	-0.67257	0.327	0.81	########
PSAT1	########	-0.67302	0.082	0.5	########
AK1	########	0.5108	0.628	0.54	########
F3	########	-0.66162	0.026	0.384	########
PDGFC	########	-0.49722	0.012	0.336	########
NCAPG2	########	-0.54082	0.057	0.443	########
KNSTRN	########	-0.73215	0.064	0.47	########
BEX4	########	0.719979	0.738	0.409	########

IP6K2	########	0.441075	0.751	0.688	########
BARD1	########	-0.57491	0.143	0.583	########
DYNC1I1	########	0.94786	0.559	0.128	########
WWTR1	########	-0.56378	0.01	0.336	########
SAPCD2	########	-0.66724	0.027	0.386	########
CDC25C	########	-0.55054	0.002	0.301	########
UBE2V2	########	0.371637	0.805	0.775	########
KIDINS22C	########	0.613708	0.561	0.389	########
CCDC112	########	0.531747	0.583	0.477	########
CEP55	########	-0.50154	0.004	0.304	########
SCARA3	########	-0.47914	0.057	0.421	########
ARMCX3	########	0.514317	0.658	0.551	########
ID3	########	-0.79684	0.057	0.454	########
TMEM97	########	-0.56899	0.156	0.599	########
MALAT1	########	0.231161	0.998	0.988	########
MLC1	########	-0.53436	0.008	0.321	########
TTK	########	-0.62637	0.004	0.306	########
GABARAPL2	########	0.365526	0.914	0.857	1.31E-99
S100A13	########	-0.78885	0.035	0.402	1.32E-99
CDC6	########	-0.56025	0.005	0.306	1.83E-99
ZFAND5	########	0.462883	0.662	0.59	2.92E-99
ATP6V0B	########	0.372558	0.888	0.828	3.26E-99
IKBIP	########	-0.53133	0.101	0.502	3.51E-99
TJP1	########	-0.41482	0.07	0.428	6.94E-99
TP53I13	########	-0.58124	0.098	0.507	7.25E-99
NDE1	########	-0.50834	0.017	0.347	7.51E-99
TNFRSF1A	########	-0.59847	0.055	0.432	1.04E-98
DNAJC9	########	-0.51006	0.155	0.581	1.56E-98
PARPBP	########	-0.54592	0.028	0.377	2.83E-98
PLIN3	########	-0.7309	0.06	0.453	3.85E-98
PLD3	########	0.372835	0.608	0.6	4.00E-98
EIF4A2	########	0.353759	0.892	0.847	4.36E-98
NMB	########	-0.69326	0.035	0.398	5.91E-98
NFIC	########	-0.70883	0.282	0.753	6.30E-98
ITGB8	########	-0.5019	0.106	0.505	6.57E-98
CPE	########	0.522387	0.815	0.663	8.28E-98
CELF3	########	0.89167	0.435	0.047	1.07E-97
LINC00152	########	-0.67061	0.029	0.38	1.16E-97
TMSB10	########	0.281468	0.998	0.974	1.48E-97
ALYREF	########	-0.51564	0.075	0.452	1.50E-97
TIMP2	########	-0.48784	0.104	0.496	2.30E-97
COQ2	########	-0.45244	0.068	0.43	2.31E-97
EIF1AY	########	-0.39997	0.093	0.449	2.93E-97
HES1	########	-0.66833	0.016	0.343	3.35E-97
XRCC2	########	-0.61328	0.037	0.399	3.76E-97
CLTB	########	0.44471	0.684	0.619	6.04E-97
REEP3	########	-0.53198	0.058	0.425	6.83E-97
RBBP8	########	-0.52669	0.027	0.367	7.60E-97
RSBN1L	########	0.473747	0.7	0.611	1.34E-96
RPA1	########	-0.43274	0.173	0.579	1.66E-96
TSPAN12	########	-0.68332	0.031	0.381	1.70E-96

MCM4	########	-0.64646	0.079	0.48	2.29E-96
KIF18B	########	-0.452	0.004	0.295	3.40E-96
ATP1B1	########	0.746073	0.551	0.296	3.83E-96
YTHDF2	########	0.367911	0.699	0.683	3.99E-96
MCM10	########	-0.47471	0.003	0.29	7.21E-96
RAD51	########	-0.53026	0.002	0.283	8.57E-96
AHCY	########	-0.59257	0.152	0.586	1.07E-95
LGALS3	########	-0.92982	0.073	0.47	1.10E-95
KIAA1524	########	-0.59696	0.042	0.406	1.32E-95
MTPN	1.03E-99	0.336525	0.651	0.653	1.54E-95
TCEAL4	1.18E-99	0.471177	0.823	0.728	1.77E-95
CDKN2C	1.24E-99	-0.68599	0.272	0.737	1.85E-95
TNFAIP6	1.49E-99	-0.73796	0.004	0.294	2.23E-95
DEPDC1B	1.84E-99	-0.5454	0.029	0.367	2.75E-95
RNASEH2C	1.96E-99	-0.58767	0.168	0.605	2.92E-95
STMN1	2.42E-99	0.240227	0.999	0.995	3.62E-95
SAT2	3.46E-99	0.396019	0.727	0.689	5.17E-95
NR3C1	3.56E-99	-0.4569	0.072	0.432	5.32E-95
TNC	3.68E-99	-0.55061	0.021	0.344	5.49E-95
ARID4B	4.40E-99	0.447051	0.665	0.599	6.57E-95
BSDC1	9.37E-99	0.487732	0.501	0.425	1.40E-94
CENPO	9.89E-99	-0.49609	0.023	0.348	1.48E-94
CD82	1.22E-98	-0.72467	0.049	0.42	1.83E-94
GNG12	3.04E-98	-0.43872	0.016	0.325	4.54E-94
ATP6V1H	6.05E-98	0.595501	0.55	0.398	9.05E-94
SCN3B	7.33E-98	0.914945	0.422	0.047	1.09E-93
MCM5	7.52E-98	-0.70602	0.035	0.388	1.12E-93
VAMP5	8.50E-98	-0.64674	0.045	0.405	1.27E-93
MIS18A	1.75E-97	-0.54704	0.104	0.504	2.62E-93
HERPUD1	2.03E-97	0.549227	0.638	0.494	3.03E-93
HSP90AB1	2.34E-97	0.285802	0.98	0.933	3.50E-93
COL6A2	3.59E-97	-0.63051	0.016	0.332	5.37E-93
FANCA	8.36E-97	-0.52915	0.037	0.379	1.25E-92
FANCD2	9.57E-97	-0.54059	0.012	0.319	1.43E-92
TMPO	1.23E-96	-0.55698	0.276	0.731	1.84E-92
COL4A1	1.70E-96	-0.47318	0.004	0.286	2.55E-92
TXN	2.15E-96	0.390355	0.888	0.817	3.21E-92
CTNNAL1	2.58E-96	-0.52534	0.071	0.433	3.85E-92
TSC22D1	2.63E-96	0.346544	0.93	0.854	3.94E-92
SPDL1	2.87E-96	-0.53558	0.04	0.386	4.28E-92
PTBP1	3.41E-96	-0.50764	0.224	0.659	5.09E-92
PRDX6	5.57E-96	-0.77082	0.304	0.751	8.32E-92
REEP4	6.91E-96	-0.55411	0.044	0.391	1.03E-91
SPOCD1	1.61E-95	-0.65331	0.008	0.301	2.41E-91
CLIC1	1.64E-95	-0.88476	0.194	0.631	2.46E-91
RAB11B	3.06E-95	0.37166	0.764	0.73	4.58E-91
CITED2	6.92E-95	0.737436	0.636	0.365	1.03E-90
ELOVL2	7.34E-95	-0.50259	0.015	0.321	1.10E-90
WDR76	1.16E-94	-0.61142	0.024	0.349	1.73E-90
ZWILCH	1.41E-94	-0.46144	0.068	0.417	2.11E-90
FSCN1	1.43E-94	0.397215	0.87	0.774	2.13E-90

RBMS1	2.19E-94	-0.58103	0.04	0.39	3.27E-90
SEZ6L2	2.60E-94	0.536382	0.591	0.473	3.88E-90
SIVA1	3.47E-94	-0.65234	0.305	0.762	5.19E-90
SLC39A1	3.65E-94	-0.47319	0.15	0.552	5.46E-90
PRMT2	3.98E-94	0.409203	0.617	0.584	5.94E-90
EMP3	4.11E-94	-0.75393	0.067	0.447	6.14E-90
BCAS2	6.27E-94	0.441221	0.686	0.602	9.36E-90
WHSC1L1	6.67E-94	0.432884	0.715	0.664	9.97E-90
6-Mar	8.71E-94	0.43547	0.596	0.551	1.30E-89
NLRP1	8.91E-94	0.453898	0.687	0.6	1.33E-89
MCM8	9.01E-94	-0.43286	0.016	0.312	1.35E-89
HAT1	1.15E-93	-0.55335	0.244	0.684	1.72E-89
ZFHX4	1.21E-93	-0.28622	0.071	0.365	1.81E-89
CDO1	1.50E-93	0.589235	0.555	0.407	2.24E-89
NPDC1	1.69E-93	0.442628	0.653	0.567	2.53E-89
TMEM237	2.10E-93	-0.41823	0.207	0.611	3.14E-89
PIPOX	2.79E-93	-0.56803	0.02	0.332	4.16E-89
TMEM106C	3.27E-93	-0.66197	0.249	0.702	4.89E-89
CREB5	3.54E-93	-0.48204	0.128	0.511	5.29E-89
RMDN3	3.61E-93	-0.45931	0.093	0.459	5.39E-89
ATP6V0D1	4.24E-93	0.422982	0.619	0.569	6.33E-89
SPSB3	5.67E-93	0.516389	0.537	0.422	8.48E-89
YPEL5	6.68E-93	0.574269	0.562	0.401	9.99E-89
CBR3	7.88E-93	-0.47896	0.053	0.388	1.18E-88
HMGXB4	1.02E-92	-0.45081	0.192	0.596	1.53E-88
IL6ST	1.08E-92	-0.41964	0.079	0.428	1.61E-88
LMNB1	1.24E-92	-0.60222	0.259	0.707	1.86E-88
FIBIN	1.24E-92	-0.6355	0.056	0.406	1.86E-88
SOX21	2.53E-92	-0.41518	0.007	0.283	3.78E-88
GRB2	2.67E-92	0.339734	0.585	0.59	3.98E-88
NOTCH1	2.68E-92	-0.47088	0.025	0.332	4.01E-88
CALU	3.55E-92	-0.56828	0.298	0.747	5.31E-88
LYPD1	3.59E-92	-0.41035	0.064	0.385	5.36E-88
ARF4	5.47E-92	0.37411	0.797	0.764	8.18E-88
CELF5	6.68E-92	0.835956	0.52	0.151	9.98E-88
PLP2	6.84E-92	-0.6778	0.09	0.478	1.02E-87
CMTM3	7.34E-92	-0.39009	0.108	0.457	1.10E-87
NCAPH2	8.50E-92	-0.45423	0.1	0.469	1.27E-87
HELLS	8.90E-92	-0.62545	0.042	0.381	1.33E-87
PPA1	1.00E-91	0.423269	0.663	0.599	1.49E-87
SRPK2	1.30E-91	0.359534	0.771	0.725	1.94E-87
MAPRE3	2.08E-91	0.67111	0.463	0.235	3.10E-87
SPRED1	2.27E-91	-0.4701	0.03	0.344	3.40E-87
KIF18A	2.67E-91	-0.43078	0.01	0.291	4.00E-87
VAMP3	2.81E-91	-0.46969	0.075	0.426	4.20E-87
SYF2	2.86E-91	0.316471	0.617	0.64	4.28E-87
RFC4	4.56E-91	-0.59031	0.182	0.61	6.81E-87
RRBP1	4.95E-91	-0.603	0.068	0.432	7.40E-87
WASF2	1.08E-90	-0.58612	0.13	0.532	1.61E-86
CDC42EP1	1.09E-90	-0.39034	0.017	0.299	1.62E-86
SLAIN1	1.24E-90	0.48108	0.441	0.378	1.86E-86

ADAM9	2.55E-90	-0.47709	0.102	0.467	3.80E-86
TGIF1	2.63E-90	-0.62461	0.118	0.516	3.94E-86
CEP170	2.65E-90	0.475843	0.518	0.433	3.95E-86
ATAD5	2.86E-90	-0.52134	0.089	0.457	4.27E-86
NOTCH2	3.00E-90	-0.43158	0.013	0.296	4.49E-86
EMC9	4.18E-90	-0.55876	0.139	0.536	6.25E-86
CHEK1	4.50E-90	-0.57583	0.079	0.451	6.72E-86
PNRC1	4.86E-90	0.540738	0.645	0.514	7.27E-86
RB1CC1	6.10E-90	0.453914	0.513	0.448	9.11E-86
NCBP2	6.14E-90	0.291825	0.654	0.689	9.18E-86
KLHL7	6.75E-90	0.468491	0.628	0.528	1.01E-85
AP1S1	9.34E-90	0.37783	0.731	0.695	1.40E-85
PKIB	1.15E-89	-0.64753	0.063	0.425	1.72E-85
GTF2I	1.15E-89	0.270246	0.639	0.686	1.72E-85
PPIC	1.23E-89	-0.43354	0.023	0.322	1.84E-85
ZSCAN18	1.60E-89	0.552511	0.485	0.347	2.40E-85
TMED5	1.84E-89	-0.40668	0.096	0.44	2.74E-85
KIF11	2.09E-89	-0.45897	0.008	0.281	3.12E-85
DGCR6L	2.65E-89	-0.41036	0.2	0.589	3.96E-85
APP	2.95E-89	0.425247	0.872	0.786	4.41E-85
RAB32	2.97E-89	-0.35478	0.023	0.298	4.43E-85
PSRC1	3.17E-89	-0.69336	0.167	0.591	4.74E-85
SLC32A1	3.28E-89	0.850516	0.316	0.007	4.91E-85
LMBR1L	3.72E-89	0.623084	0.424	0.251	5.56E-85
WDR62	4.24E-89	-0.47395	0.009	0.29	6.34E-85
RP11-390F	4.63E-89	-0.36602	0.016	0.283	6.92E-85
MTX2	4.93E-89	0.423902	0.604	0.551	7.37E-85
EPB41L2	5.81E-89	-0.46073	0.068	0.41	8.68E-85
SPATA6	6.33E-89	-0.43062	0.024	0.32	9.46E-85
ARMCX1	7.45E-89	0.704201	0.483	0.24	1.11E-84
CLK1	7.90E-89	0.580959	0.705	0.501	1.18E-84
CCDC18	8.52E-89	-0.54013	0.042	0.377	1.27E-84
H2AFV	2.00E-88	-0.53466	0.625	0.925	2.98E-84
NFIX	2.24E-88	0.416707	0.721	0.674	3.35E-84
ABHD3	2.24E-88	-0.52544	0.071	0.422	3.35E-84
PRRX1	2.26E-88	-0.45439	0.058	0.381	3.38E-84
MPST	2.40E-88	-0.2942	0.22	0.58	3.59E-84
RCC1	2.78E-88	-0.49267	0.057	0.399	4.16E-84
UBE2B	3.02E-88	0.279446	0.578	0.631	4.51E-84
HN1	3.56E-88	0.283918	0.984	0.914	5.33E-84
WIPI2	4.04E-88	0.304843	0.572	0.614	6.03E-84
NT5DC2	4.90E-88	-0.43411	0.164	0.546	7.32E-84
RRAGA	5.30E-88	0.446981	0.589	0.51	7.92E-84
POU3F2	5.30E-88	-0.56512	0.181	0.591	7.93E-84
ECI1	6.70E-88	-0.47466	0.134	0.512	1.00E-83
GGH	9.49E-88	-0.56719	0.389	0.822	1.42E-83
JAG1	1.09E-87	-0.5405	0.049	0.383	1.63E-83
ZDHHC12	1.17E-87	-0.42431	0.037	0.346	1.75E-83
MYO10	1.21E-87	-0.57608	0.079	0.443	1.81E-83
TMEM59L	1.24E-87	0.496212	0.523	0.438	1.86E-83
TLK1	1.24E-87	-0.51645	0.13	0.52	1.86E-83

WSCD1	1.27E-87	-0.51235	0.03	0.341	1.89E-83
NUDT1	1.45E-87	-0.51972	0.415	0.842	2.16E-83
RP11-71N1	1.51E-87	-0.49145	0.014	0.3	2.26E-83
ATP1A3	1.54E-87	0.785255	0.581	0.219	2.29E-83
SORBS3	2.00E-87	-0.40584	0.079	0.416	2.99E-83
RFC5	2.20E-87	-0.50245	0.068	0.414	3.28E-83
NCAM1	2.75E-87	0.355561	0.704	0.686	4.11E-83
BRD3	3.45E-87	0.408885	0.531	0.494	5.15E-83
ERV3-1	3.62E-87	0.57526	0.625	0.446	5.41E-83
MARCKS	5.10E-87	0.322744	0.966	0.87	7.63E-83
APOLD1	5.47E-87	-0.56157	0.021	0.32	8.18E-83
RAB18	5.54E-87	0.293481	0.456	0.502	8.28E-83
ANP32A	5.78E-87	-0.4417	0.244	0.651	8.64E-83
PTPRN2	6.92E-87	0.7436	0.516	0.231	1.03E-82
NDRG4	7.36E-87	0.721738	0.448	0.183	1.10E-82
AGTRAP	7.51E-87	-0.4198	0.02	0.309	1.12E-82
WBP11	8.65E-87	-0.17258	0.28	0.593	1.29E-82
HIST2H2AC	9.34E-87	-0.48813	0.017	0.304	1.40E-82
TMEM106B	9.99E-87	0.4154	0.526	0.493	1.49E-82
CDK5R1	1.22E-86	0.731272	0.419	0.153	1.82E-82
TAX1BP1	1.22E-86	0.318808	0.687	0.691	1.83E-82
DYNC1LI2	1.31E-86	0.392555	0.639	0.59	1.96E-82
MCM2	1.43E-86	-0.58515	0.042	0.37	2.14E-82
MOB4	1.65E-86	0.267644	0.56	0.622	2.47E-82
TBCC	1.71E-86	0.554621	0.513	0.368	2.55E-82
ASAH1	1.74E-86	0.338606	0.61	0.64	2.59E-82
PLK4	1.84E-86	-0.41771	0.005	0.26	2.76E-82
SEPN1	1.98E-86	-0.42273	0.071	0.4	2.96E-82
EXO1	1.99E-86	-0.41673	0.001	0.242	2.98E-82
DHRS7	2.31E-86	0.470393	0.584	0.523	3.45E-82
GCAT	2.37E-86	-0.46707	0.077	0.426	3.54E-82
TAGLN2	2.50E-86	-0.91734	0.14	0.544	3.73E-82
CDCA7L	2.84E-86	-0.53941	0.14	0.527	4.25E-82
ACTN4	3.41E-86	-0.47369	0.183	0.575	5.09E-82
ACSS3	3.55E-86	-0.409	0.002	0.248	5.30E-82
SPINT2	3.75E-86	0.817973	0.346	0.037	5.61E-82
DTL	4.00E-86	-0.53554	0.005	0.264	5.98E-82
MAPK10	4.01E-86	0.636494	0.529	0.33	5.99E-82
CAMK2N1	4.94E-86	0.569164	0.695	0.499	7.38E-82
SLC16A1	5.03E-86	-0.47433	0.112	0.479	7.52E-82
MZT1	6.22E-86	-0.61039	0.273	0.706	9.29E-82
NPAS3	7.49E-86	-0.45178	0.028	0.321	1.12E-81
TMEM14A	7.62E-86	0.492236	0.577	0.475	1.14E-81
ATP6V0A1	7.80E-86	0.550906	0.486	0.356	1.17E-81
CA2	8.12E-86	-0.57229	0.013	0.296	1.21E-81
CEP152	9.85E-86	-0.42886	0.005	0.26	1.47E-81
EVA1B	1.13E-85	-0.34478	0.009	0.259	1.69E-81
LUC7L3	1.15E-85	0.308493	0.786	0.794	1.72E-81
POLE4	1.52E-85	-0.52972	0.107	0.477	2.28E-81
CD99	1.64E-85	-0.6734	0.241	0.674	2.45E-81
RANGAP1	1.72E-85	-0.39152	0.071	0.39	2.56E-81

FBL	1.86E-85	-0.47589	0.24	0.646	2.78E-81
BOLA3	1.97E-85	-0.47472	0.21	0.61	2.95E-81
TMEM54	2.36E-85	-0.37312	0.172	0.531	3.53E-81
MAPK1	2.42E-85	-0.38697	0.104	0.441	3.61E-81
TCF12	2.62E-85	-0.49272	0.214	0.615	3.92E-81
MVB12A	2.77E-85	-0.4043	0.17	0.541	4.14E-81
PPP2R5B	3.09E-85	0.571217	0.379	0.232	4.62E-81
HSD11B1L	3.72E-85	0.458565	0.388	0.332	5.56E-81
KIFAP3	3.98E-85	0.600973	0.442	0.26	5.94E-81
LHX2	4.27E-85	-0.50397	0.076	0.42	6.38E-81
PKM	5.30E-85	-0.59259	0.571	0.878	7.92E-81
RAD54L	7.89E-85	-0.38683	0	0.231	1.18E-80
DPM3	9.60E-85	-0.3276	0.181	0.535	1.43E-80
DCXR	9.83E-85	-0.5919	0.317	0.751	1.47E-80
STX12	1.55E-84	0.439219	0.54	0.477	2.31E-80
SYP	2.40E-84	0.76996	0.491	0.198	3.59E-80
RHNO1	3.45E-84	-0.56881	0.189	0.6	5.15E-80
KIAA1715	3.87E-84	0.300595	0.397	0.477	5.79E-80
TMEM189	4.87E-84	-0.36581	0.066	0.375	7.27E-80
PAK2	5.80E-84	0.251069	0.476	0.549	8.67E-80
RHOJ	6.84E-84	-0.38957	0.005	0.258	1.02E-79
VPS28	7.87E-84	0.366934	0.793	0.746	1.18E-79
SLC2A4RG	8.23E-84	-0.47798	0.049	0.375	1.23E-79
HDAC1	8.86E-84	-0.36167	0.095	0.416	1.32E-79
PNMA1	1.21E-83	0.351146	0.541	0.544	1.80E-79
PSMC3IP	1.38E-83	-0.50425	0.105	0.463	2.06E-79
CAPZA2	1.42E-83	0.280173	0.806	0.816	2.13E-79
DLGAP4	1.53E-83	0.367774	0.5	0.479	2.28E-79
CNIH4	1.57E-83	-0.5502	0.242	0.658	2.34E-79
GJC1	2.79E-83	-0.38658	0.025	0.306	4.17E-79
TMEM123	3.03E-83	-0.31166	0.215	0.563	4.53E-79
C7orf49	3.11E-83	-0.3667	0.121	0.457	4.65E-79
H2AFJ	3.75E-83	-0.42239	0.029	0.311	5.60E-79
EIF1B	4.08E-83	0.323269	0.799	0.78	6.10E-79
CBX1	4.45E-83	0.27917	0.732	0.752	6.64E-79
UBE2E3	5.63E-83	0.339873	0.86	0.811	8.41E-79
KDM5B	6.41E-83	0.512685	0.353	0.257	9.58E-79
NUDT5	9.30E-83	-0.37594	0.15	0.499	1.39E-78
GRINA	9.65E-83	0.344072	0.462	0.499	1.44E-78
ENOSF1	1.03E-82	-0.3235	0.012	0.264	1.54E-78
RPAIN	1.13E-82	0.32019	0.816	0.799	1.68E-78
TSC22D4	1.27E-82	-0.54937	0.263	0.672	1.89E-78
TM4SF1	1.27E-82	-0.54231	0.029	0.332	1.90E-78
NCAPD3	1.46E-82	-0.4209	0.057	0.37	2.18E-78
TCEAL5	1.51E-82	0.804106	0.513	0.154	2.25E-78
MBD3	1.57E-82	-0.31722	0.096	0.404	2.35E-78
CIB1	1.67E-82	-0.51005	0.129	0.501	2.50E-78
HDLBP	1.70E-82	-0.29727	0.222	0.569	2.54E-78
TUBB4B	1.80E-82	-0.64511	0.524	0.872	2.69E-78
HIST1H1D	1.92E-82	-0.54671	0.009	0.269	2.87E-78
HS2ST1	2.24E-82	-0.41323	0.065	0.383	3.35E-78

UBC	2.38E-82	0.231703	0.991	0.97	3.55E-78
GLO1	2.43E-82	-0.53326	0.306	0.732	3.63E-78
ASIC4	2.59E-82	0.85905	0.531	0.215	3.87E-78
CD47	3.29E-82	-0.49243	0.204	0.602	4.91E-78
MPHOSPH8	3.80E-82	0.39475	0.588	0.553	5.67E-78
SALL1	4.04E-82	-0.39992	0.033	0.316	6.04E-78
STX1A	6.17E-82	0.775903	0.339	0.058	9.21E-78
PTGR1	6.86E-82	-0.44871	0.036	0.337	1.03E-77
RNASEH2B	7.19E-82	-0.4216	0.232	0.621	1.08E-77
IQGAP2	8.67E-82	-0.36449	0.016	0.267	1.30E-77
NEK6	9.04E-82	-0.69521	0.071	0.422	1.35E-77
DHX36	1.12E-81	0.247128	0.611	0.677	1.67E-77
GPSM2	1.15E-81	-0.58142	0.189	0.594	1.72E-77
PJA1	1.22E-81	0.505375	0.467	0.353	1.82E-77
SNRPD3	1.24E-81	-0.45493	0.313	0.719	1.85E-77
TMEM98	1.24E-81	-0.34706	0.238	0.606	1.85E-77
DCPS	1.33E-81	-0.40673	0.082	0.402	1.99E-77
LGR4	1.37E-81	-0.3778	0.013	0.273	2.04E-77
KDM1A	1.70E-81	0.344556	0.448	0.463	2.54E-77
FAM96A	1.77E-81	-0.39014	0.136	0.48	2.65E-77
BBOX1	1.80E-81	-0.42389	0.005	0.248	2.70E-77
CTSA	1.84E-81	-0.50083	0.131	0.499	2.75E-77
TMEM251	1.87E-81	-0.28007	0.148	0.462	2.80E-77
CAMK2D	2.14E-81	-0.46311	0.101	0.446	3.19E-77
ZNF292	2.22E-81	0.353226	0.468	0.477	3.31E-77
ITGA7	2.50E-81	-0.37297	0.035	0.317	3.74E-77
MAGT1	2.51E-81	-0.396	0.02	0.295	3.75E-77
CSNK1E	3.03E-81	0.396458	0.582	0.568	4.52E-77
EMP2	3.05E-81	-0.4938	0.029	0.321	4.55E-77
IQGAP1	3.16E-81	-0.38405	0.034	0.316	4.72E-77
PEBP1	3.16E-81	0.262568	0.98	0.932	4.72E-77
DNAJA1	3.18E-81	0.351132	0.861	0.796	4.76E-77
DNPH1	3.44E-81	-0.39136	0.151	0.496	5.14E-77
SH3BP5	3.58E-81	0.47214	0.331	0.251	5.36E-77
CCDC28B	4.64E-81	0.467687	0.408	0.347	6.94E-77
TRIOBP	4.89E-81	-0.33859	0.032	0.299	7.30E-77
BRD7	5.39E-81	-0.32016	0.24	0.596	8.06E-77
NDUFS2	6.02E-81	0.320212	0.716	0.694	8.99E-77
EME1	6.80E-81	-0.38471	0.009	0.265	1.02E-76
ACYP1	6.87E-81	-0.40981	0.222	0.599	1.03E-76
SUZ12	8.66E-81	-0.43032	0.149	0.51	1.29E-76
CD81	8.81E-81	-0.12953	0.163	0.422	1.32E-76
COLGALT2	9.25E-81	-0.40032	0.048	0.344	1.38E-76
WRB	9.36E-81	0.351417	0.676	0.665	1.40E-76
UBE2D1	1.02E-80	0.298757	0.324	0.378	1.52E-76
RNF11	1.21E-80	0.453748	0.515	0.441	1.81E-76
BZW2	1.27E-80	0.532829	0.574	0.4	1.90E-76
ZKSCAN1	1.33E-80	0.351577	0.508	0.504	1.98E-76
TNFRSF12A	1.34E-80	-0.89058	0.079	0.425	2.00E-76
TGFB1	1.67E-80	-0.32504	0.013	0.265	2.50E-76
HOTAIRM1	2.02E-80	0.791376	0.528	0.225	3.02E-76

COL9A3	2.25E-80	-0.78835	0.076	0.428	3.36E-76
AK2	2.59E-80	-0.41781	0.2	0.57	3.87E-76
RNFT1	2.65E-80	-0.28402	0.089	0.373	3.96E-76
MPLKIP	2.73E-80	0.334936	0.671	0.673	4.08E-76
ZNF524	2.79E-80	-0.3588	0.038	0.32	4.17E-76
RSBN1	2.85E-80	0.45882	0.471	0.391	4.26E-76
SMIM4	3.67E-80	-0.38928	0.112	0.443	5.49E-76
UFD1L	4.02E-80	-0.45026	0.227	0.616	6.01E-76
TRIP6	4.09E-80	-0.4176	0.049	0.347	6.10E-76
STRAP	4.09E-80	0.292727	0.811	0.794	6.11E-76
RFK	4.36E-80	0.305596	0.391	0.42	6.51E-76
PREX1	4.54E-80	-0.37537	0.019	0.283	6.78E-76
BOD1	4.58E-80	0.246055	0.539	0.606	6.85E-76
PSMB8	4.73E-80	-0.53568	0.121	0.478	7.07E-76
STAT1	4.94E-80	-0.49808	0.086	0.428	7.38E-76
BEX5	5.35E-80	0.880673	0.389	0.051	8.00E-76
RALA	5.36E-80	0.260513	0.574	0.63	8.01E-76
CCNF	5.45E-80	-0.47198	0.038	0.337	8.15E-76
DAAM1	5.99E-80	0.524238	0.577	0.432	8.95E-76
DPYSL2	6.64E-80	0.296345	0.593	0.611	9.92E-76
TRIM9	7.33E-80	-0.4955	0.104	0.454	1.10E-75
THOP1	7.91E-80	-0.37712	0.178	0.535	1.18E-75
COMMD3	8.27E-80	0.345548	0.478	0.494	1.24E-75
NSL1	9.97E-80	-0.32543	0.263	0.627	1.49E-75
FABP7	1.02E-79	-0.7117	0.459	0.752	1.53E-75
EMP1	1.18E-79	-0.59878	0.014	0.283	1.76E-75
PAIP2	1.49E-79	0.303835	0.77	0.764	2.23E-75
B2M	1.58E-79	-0.48294	0.853	0.968	2.36E-75
RFFL	1.64E-79	-0.45931	0.047	0.354	2.45E-75
TXNDC12	1.72E-79	-0.44136	0.241	0.633	2.56E-75
HDAC2	1.76E-79	0.317931	0.822	0.789	2.64E-75
RASSF2	1.77E-79	-0.34092	0.087	0.391	2.64E-75
SNHG9	2.03E-79	-0.31734	0.207	0.551	3.03E-75
FLNA	2.07E-79	-0.47019	0.106	0.449	3.09E-75
CHMP1A	2.26E-79	0.150394	0.345	0.484	3.37E-75
PLXNB2	2.39E-79	-0.2521	0.058	0.317	3.58E-75
APC	2.43E-79	0.448055	0.422	0.357	3.63E-75
DCTN6	2.54E-79	0.265183	0.544	0.616	3.79E-75
CELF1	2.98E-79	0.304164	0.464	0.493	4.45E-75
CCDC90B	3.26E-79	0.358809	0.632	0.609	4.88E-75
LINC0011б	3.29E-79	-0.29273	0.227	0.567	4.91E-75
DUSP1	3.45E-79	0.59128	0.745	0.48	5.15E-75
MAP7D3	4.47E-79	-0.34	0.006	0.249	6.67E-75
LHFP	4.91E-79	-0.49439	0.145	0.51	7.34E-75
CHCHD5	5.02E-79	-0.28991	0.26	0.606	7.50E-75
MEAF6	5.58E-79	0.267357	0.642	0.688	8.34E-75
CHGB	5.99E-79	0.807147	0.41	0.085	8.95E-75
RNF24	6.10E-79	0.361031	0.469	0.464	9.12E-75
FAM111B	6.63E-79	-0.5314	0.006	0.251	9.91E-75
NME4	6.91E-79	-0.43673	0.296	0.695	1.03E-74
10-Sep	8.09E-79	-0.35505	0.069	0.365	1.21E-74

GAS2L1	8.85E-79	-0.34146	0.068	0.359	1.32E-74
KIF2A	9.41E-79	0.355671	0.475	0.483	1.41E-74
PIF1	1.02E-78	-0.60414	0.016	0.285	1.52E-74
HSDL2	1.11E-78	-0.42455	0.038	0.333	1.66E-74
ARF5	1.27E-78	0.311896	0.739	0.738	1.90E-74
PLSCR1	1.30E-78	-0.42877	0.05	0.351	1.94E-74
CPNE4	1.31E-78	-0.40042	0.009	0.259	1.96E-74
GRAMD1A	1.31E-78	0.375447	0.446	0.425	1.96E-74
YWHAZ	1.41E-78	0.309254	0.722	0.707	2.11E-74
CUTA	1.41E-78	0.316299	0.854	0.814	2.11E-74
TIMP1	1.55E-78	-0.85567	0.272	0.628	2.31E-74
RBM38	1.82E-78	-0.32394	0.063	0.344	2.72E-74
C22orf39	2.23E-78	-0.301	0.215	0.553	3.33E-74
ZMAT2	2.42E-78	0.310547	0.54	0.575	3.61E-74
SPOCK1	2.47E-78	0.828425	0.349	0.04	3.69E-74
CHPT1	2.50E-78	-0.49362	0.163	0.536	3.73E-74
TMEM141	3.09E-78	-0.35327	0.072	0.367	4.62E-74
SYDE1	3.26E-78	-0.30465	0.005	0.231	4.88E-74
PGP	3.34E-78	-0.31242	0.236	0.583	5.00E-74
TSG101	4.30E-78	0.353138	0.579	0.558	6.43E-74
FZD7	4.94E-78	-0.36115	0.004	0.237	7.39E-74
SEC61G	5.42E-78	-0.58037	0.819	0.923	8.11E-74
PSMD3	5.55E-78	0.224087	0.586	0.658	8.29E-74
TMEM158	6.70E-78	-0.53594	0.039	0.342	1.00E-73
ILDR2	6.78E-78	-0.31768	0.008	0.249	1.01E-73
ELOVL5	7.83E-78	-0.3437	0.241	0.6	1.17E-73
NT5C3A	8.27E-78	0.303397	0.581	0.602	1.24E-73
KHSRP	1.09E-77	-0.26499	0.218	0.542	1.63E-73
NT5C3B	1.26E-77	0.208937	0.574	0.652	1.88E-73
IER2	1.30E-77	0.394594	0.899	0.778	1.94E-73
NKIRAS2	1.31E-77	0.192272	0.391	0.5	1.96E-73
GOLM1	1.39E-77	0.249421	0.606	0.657	2.08E-73
ITFG1	1.43E-77	0.305435	0.524	0.553	2.13E-73
STAG2	2.05E-77	-0.32429	0.148	0.472	3.06E-73
SDHD	2.15E-77	-0.27659	0.218	0.549	3.21E-73
MOSPD3	2.26E-77	0.376093	0.473	0.454	3.38E-73
AKAP12	2.40E-77	-0.46357	0.086	0.414	3.59E-73
VPS35	2.69E-77	0.309138	0.592	0.611	4.01E-73
ETV4	2.69E-77	-0.41249	0.015	0.274	4.03E-73
HIST1H1C	2.71E-77	-0.50618	0.049	0.344	4.06E-73
NRM	2.72E-77	-0.38231	0.044	0.325	4.07E-73
XPO1	2.91E-77	-0.34837	0.248	0.611	4.34E-73
LPL	2.96E-77	-0.4348	0.008	0.251	4.42E-73
ASXL1	3.08E-77	-0.2265	0.142	0.426	4.61E-73
MXRA7	3.25E-77	-0.3387	0.054	0.331	4.85E-73
RAB9A	3.69E-77	-0.23833	0.171	0.475	5.51E-73
SRPX	3.76E-77	-0.71264	0.038	0.342	5.61E-73
PAFAH1B2	3.98E-77	0.226271	0.478	0.546	5.94E-73
NIPA2	4.05E-77	-0.33869	0.173	0.512	6.05E-73
CMAS	4.28E-77	0.303839	0.401	0.432	6.39E-73
SNX1	5.48E-77	-0.29104	0.065	0.337	8.19E-73

KATNBL1	5.61E-77	-0.15921	0.202	0.472	8.38E-73
GNG3	6.36E-77	0.863351	0.406	0.069	9.50E-73
CMC2	6.39E-77	-0.3905	0.318	0.705	9.55E-73
SLC44A2	6.56E-77	-0.33769	0.139	0.459	9.80E-73
NRXN1	6.68E-77	0.689449	0.651	0.326	9.98E-73
RANGRF	7.36E-77	-0.39542	0.119	0.449	1.10E-72
SS18L2	7.37E-77	0.190818	0.419	0.53	1.10E-72
EBAG9	7.49E-77	0.365093	0.423	0.428	1.12E-72
C16orf59	7.67E-77	-0.47148	0.049	0.354	1.15E-72
FAM127A	7.96E-77	0.324917	0.65	0.681	1.19E-72
C12orf75	1.06E-76	-0.45552	0.146	0.501	1.59E-72
DUSP26	1.10E-76	0.865556	0.415	0.091	1.64E-72
RFXANK	1.15E-76	-0.41855	0.142	0.486	1.71E-72
DAG1	1.16E-76	-0.38172	0.093	0.407	1.74E-72
SUCLG2	1.17E-76	-0.40027	0.023	0.284	1.75E-72
PRADC1	1.33E-76	-0.29807	0.207	0.536	1.99E-72
TLN1	1.34E-76	-0.30605	0.06	0.335	2.00E-72
SH3GL1	1.41E-76	-0.35773	0.17	0.51	2.10E-72
YBX3	1.47E-76	-0.51132	0.207	0.594	2.20E-72
AP3S1	1.64E-76	0.253125	0.543	0.605	2.45E-72
DGUOK	1.99E-76	0.255347	0.735	0.767	2.97E-72
ITGAV	2.02E-76	-0.40357	0.09	0.406	3.02E-72
PROX1	2.07E-76	0.446813	0.363	0.314	3.10E-72
NCKAP1	2.10E-76	-0.11759	0.211	0.467	3.13E-72
ADSL	2.28E-76	-0.2278	0.186	0.481	3.41E-72
ZNHIT3	2.36E-76	0.276965	0.543	0.596	3.52E-72
SNRPA	2.39E-76	-0.34312	0.225	0.574	3.57E-72
FBXO11	2.61E-76	0.41781	0.397	0.349	3.90E-72
NETO2	2.63E-76	-0.46285	0.136	0.49	3.94E-72
MAP7D1	2.78E-76	0.305061	0.479	0.509	4.15E-72
MTA1	2.83E-76	-0.11228	0.189	0.438	4.24E-72
TNFRSF19	2.84E-76	-0.34629	0.023	0.28	4.24E-72
SEC63	2.98E-76	0.259405	0.551	0.609	4.46E-72
GNB4	3.19E-76	-0.31514	0.085	0.375	4.76E-72
PRCP	3.54E-76	-0.46494	0.218	0.598	5.30E-72
NOTCH2NL	3.56E-76	-0.38419	0.033	0.304	5.33E-72
ERI1	4.23E-76	-0.34233	0.057	0.343	6.31E-72
HEPACAM	5.10E-76	-0.54262	0.022	0.295	7.63E-72
PDCD6	5.17E-76	0.211476	0.631	0.717	7.73E-72
ORC4	5.49E-76	0.423342	0.465	0.404	8.20E-72
CRYZL1	5.74E-76	0.493908	0.432	0.333	8.58E-72
QKI	5.76E-76	-0.46178	0.334	0.737	8.60E-72
IFI6	5.87E-76	-0.63452	0.137	0.502	8.78E-72
MIS12	6.35E-76	-0.38175	0.092	0.402	9.50E-72
ING4	7.06E-76	0.494383	0.511	0.386	1.06E-71
CENPL	8.05E-76	-0.39801	0.031	0.304	1.20E-71
TCEAL2	8.50E-76	0.687055	0.676	0.309	1.27E-71
AAK1	8.94E-76	0.262533	0.402	0.457	1.34E-71
TCP1	9.08E-76	0.241488	0.805	0.822	1.36E-71
CFL1	1.08E-75	0.199261	0.998	0.964	1.61E-71
DNAJC1	1.15E-75	-0.33641	0.072	0.357	1.72E-71

TOX	1.16E-75	0.62073	0.309	0.138	1.73E-71
EZH2	1.17E-75	-0.44049	0.297	0.688	1.76E-71
SCOC	1.19E-75	0.285398	0.605	0.643	1.78E-71
MTHFD1	1.19E-75	-0.48136	0.11	0.451	1.78E-71
ISCA1	1.21E-75	0.248893	0.4	0.468	1.81E-71
NUP37	1.22E-75	-0.36491	0.067	0.359	1.82E-71
THRA	1.43E-75	0.540724	0.557	0.386	2.14E-71
PRPSAP2	1.50E-75	0.315834	0.478	0.494	2.24E-71
MLST8	1.57E-75	-0.13486	0.222	0.488	2.35E-71
YIPF4	1.71E-75	0.201176	0.467	0.565	2.56E-71
FUCA2	1.75E-75	-0.26908	0.081	0.347	2.61E-71
LAMC1	1.84E-75	-0.30191	0.016	0.253	2.74E-71
SFT2D1	1.86E-75	-0.34288	0.23	0.584	2.78E-71
TOP2B	1.87E-75	0.335436	0.427	0.432	2.79E-71
MTMR9	2.01E-75	0.420955	0.405	0.356	3.01E-71
COX7A2L	2.21E-75	0.352886	0.755	0.712	3.30E-71
FAM46A	2.42E-75	-0.24067	0.11	0.378	3.62E-71
CSRNP3	2.53E-75	0.734701	0.46	0.162	3.79E-71
IPO7	3.00E-75	-0.25857	0.156	0.458	4.49E-71
PLXNA4	3.29E-75	0.723136	0.307	0.096	4.91E-71
MEX3A	3.46E-75	0.461874	0.564	0.465	5.18E-71
SEC11A	3.54E-75	-0.51337	0.313	0.716	5.29E-71
MT1X	3.93E-75	-0.64662	0.216	0.614	5.88E-71
IGFBP2	4.51E-75	-0.63235	0.465	0.819	6.73E-71
SLC25A36	4.64E-75	0.388238	0.436	0.405	6.93E-71
SF3A2	4.77E-75	-0.2625	0.269	0.6	7.13E-71
KLHL4	4.95E-75	-0.40891	0.032	0.306	7.40E-71
CNOT7	5.55E-75	0.226281	0.548	0.637	8.29E-71
MAML2	6.44E-75	-0.33876	0.104	0.407	9.62E-71
NOL12	6.48E-75	-0.11098	0.209	0.467	9.69E-71
G6PC3	7.01E-75	-0.26691	0.24	0.56	1.05E-70
C9orf78	7.17E-75	0.169886	0.539	0.652	1.07E-70
CSDE1	8.36E-75	0.25015	0.763	0.774	1.25E-70
TMEM256	9.54E-75	-0.30494	0.237	0.577	1.43E-70
FBXO21	1.01E-74	0.372215	0.561	0.521	1.51E-70
DHRS7B	1.21E-74	-0.22429	0.134	0.412	1.81E-70
TMED1	1.35E-74	-0.42154	0.126	0.46	2.02E-70
EFNB2	1.39E-74	-0.33579	0.015	0.262	2.08E-70
PEX2	1.59E-74	-0.2732	0.17	0.478	2.37E-70
NRGN	1.65E-74	-0.31945	0.016	0.252	2.46E-70
BTF3L4	1.75E-74	0.275153	0.67	0.694	2.61E-70
SDF2L1	1.77E-74	-0.59653	0.149	0.519	2.64E-70
ETV5	1.98E-74	-0.30015	0.145	0.456	2.96E-70
CTNNA1	2.13E-74	0.204791	0.463	0.558	3.18E-70
PPIH	2.28E-74	-0.41224	0.177	0.528	3.41E-70
FIS1	2.32E-74	0.322506	0.796	0.788	3.46E-70
ANKRD13D	2.50E-74	0.382925	0.321	0.295	3.73E-70
ENO1	2.83E-74	-0.55818	0.599	0.895	4.23E-70
KIF1B	3.81E-74	0.15442	0.41	0.549	5.70E-70
TTC14	4.15E-74	0.253741	0.337	0.411	6.20E-70
TRAPPC4	4.26E-74	0.307171	0.625	0.62	6.37E-70

PCDH17	4.71E-74	-0.4238	0.119	0.451	7.04E-70
TRABD	5.49E-74	-0.22571	0.112	0.377	8.21E-70
GINM1	5.55E-74	-0.2926	0.175	0.491	8.30E-70
ALCAM	5.60E-74	-0.27556	0.066	0.32	8.37E-70
ROBO1	6.14E-74	0.314324	0.382	0.433	9.17E-70
EIF4A1	7.28E-74	0.246102	0.977	0.937	1.09E-69
NCLN	7.40E-74	-0.20744	0.094	0.349	1.11E-69
MTHFD2L	8.09E-74	-0.2416	0.148	0.433	1.21E-69
FERMT2	8.96E-74	-0.30864	0.138	0.444	1.34E-69
LIMS1	1.02E-73	-0.42391	0.162	0.509	1.52E-69
POLA2	1.24E-73	-0.40695	0.029	0.295	1.86E-69
FUBP3	1.27E-73	-0.22509	0.169	0.457	1.90E-69
UFM1	1.34E-73	0.223791	0.39	0.474	2.01E-69
ZC3H13	1.38E-73	0.340612	0.501	0.502	2.07E-69
SYPL1	1.46E-73	-0.32891	0.174	0.494	2.18E-69
UBE2S	1.56E-73	-0.63687	0.554	0.812	2.33E-69
CLIC4	1.85E-73	-0.451	0.221	0.589	2.77E-69
GPM6B	2.17E-73	-0.43722	0.764	0.937	3.24E-69
GPATCH8	2.39E-73	0.245433	0.463	0.533	3.58E-69
NCOR2	2.42E-73	-0.18188	0.157	0.419	3.62E-69
C1QL1	2.49E-73	-0.38562	0.071	0.357	3.72E-69
SSFA2	2.62E-73	-0.37873	0.073	0.369	3.92E-69
NES	2.84E-73	-0.68627	0.249	0.637	4.25E-69
GABARAPL1	3.41E-73	0.640004	0.45	0.22	5.09E-69
MOB1A	3.50E-73	-0.29122	0.139	0.44	5.23E-69
TMEM206	3.67E-73	0.200476	0.311	0.41	5.48E-69
ANXA2	3.81E-73	-0.89784	0.128	0.477	5.69E-69
SYT5	4.05E-73	0.721449	0.295	0.021	6.05E-69
ISCA2	4.07E-73	-0.28233	0.171	0.478	6.09E-69
APOBEC3B	4.31E-73	-0.40028	0.018	0.259	6.44E-69
DNASE2	4.60E-73	-0.2986	0.1	0.384	6.88E-69
TUBG1	4.73E-73	-0.43726	0.265	0.647	7.07E-69
CASC4	4.95E-73	-0.24179	0.148	0.436	7.40E-69
TRAF7	5.06E-73	-0.29192	0.115	0.407	7.56E-69
DDX1	5.42E-73	0.168839	0.487	0.601	8.10E-69
UBQLN2	7.07E-73	0.426006	0.421	0.363	1.06E-68
NFE2L2	7.65E-73	-0.26944	0.277	0.606	1.14E-68
CADM1	8.33E-73	0.352538	0.47	0.477	1.24E-68
RNF26	8.59E-73	-0.37794	0.12	0.437	1.28E-68
RMI2	1.03E-72	-0.44178	0.013	0.258	1.53E-68
DAZAP1	1.09E-72	-0.33377	0.213	0.551	1.62E-68
COMT	1.10E-72	-0.27439	0.325	0.668	1.64E-68
CBFB	1.17E-72	-0.3455	0.141	0.459	1.75E-68
TRIM28	1.34E-72	-0.26135	0.257	0.583	2.01E-68
DDX39A	1.39E-72	-0.5815	0.389	0.786	2.08E-68
ARL8A	1.42E-72	0.320403	0.405	0.427	2.12E-68
TRIM2	1.44E-72	0.332071	0.407	0.412	2.15E-68
SMARCE1	1.50E-72	0.232069	0.594	0.652	2.24E-68
AKR7A2	1.77E-72	-0.30969	0.218	0.548	2.64E-68
ERF	2.00E-72	-0.34914	0.06	0.332	2.98E-68
GSK3B	2.13E-72	0.436257	0.377	0.317	3.18E-68

PKIA	2.28E-72	0.7449	0.462	0.162	3.40E-68
PHF20L1	2.43E-72	0.256941	0.462	0.526	3.63E-68
MMS22L	2.50E-72	-0.46345	0.046	0.333	3.74E-68
AIF1L	2.65E-72	-0.64994	0.028	0.304	3.96E-68
CCDC47	2.73E-72	-0.32667	0.291	0.643	4.07E-68
DNMT1	2.78E-72	-0.50154	0.315	0.71	4.16E-68
SDC1	3.06E-72	-0.34257	0.027	0.268	4.58E-68
ARHGAP21	3.26E-72	-0.25479	0.173	0.469	4.87E-68
HNRNPA0	3.38E-72	0.259451	0.928	0.894	5.05E-68
MATR3	3.39E-72	0.295072	0.78	0.764	5.06E-68
YIPF3	3.40E-72	0.253828	0.617	0.652	5.09E-68
NTAN1	3.50E-72	-0.16619	0.13	0.377	5.24E-68
AKIP1	3.57E-72	0.110418	0.31	0.456	5.33E-68
UBXN2A	3.79E-72	-0.19301	0.194	0.473	5.67E-68
EXOC7	3.93E-72	0.291993	0.527	0.548	5.87E-68
LMF2	3.93E-72	-0.35286	0.098	0.399	5.88E-68
ANKIB1	4.34E-72	0.288293	0.335	0.365	6.48E-68
RNF10	4.37E-72	0.225854	0.441	0.51	6.53E-68
ZNF91	4.73E-72	0.392568	0.416	0.38	7.07E-68
IPO5	4.75E-72	-0.26252	0.194	0.502	7.10E-68
N4BP2L2	5.37E-72	0.182866	0.56	0.657	8.02E-68
BORA	5.64E-72	-0.41532	0.005	0.228	8.43E-68
SPECC1	5.79E-72	-0.39439	0.251	0.616	8.65E-68
DHRSX	5.81E-72	-0.21491	0.201	0.493	8.69E-68
RAB31	6.11E-72	-0.332	0.253	0.6	9.14E-68
TMEM57	6.64E-72	0.487507	0.396	0.291	9.92E-68
EXOSC8	7.36E-72	-0.37053	0.324	0.691	1.10E-67
MED11	7.46E-72	-0.13614	0.137	0.369	1.12E-67
NMU	7.94E-72	-0.60432	0.021	0.283	1.19E-67
ZNF821	8.04E-72	0.57653	0.465	0.288	1.20E-67
LINC00998	8.05E-72	-0.30672	0.223	0.553	1.20E-67
EMC10	8.17E-72	0.234967	0.607	0.659	1.22E-67
SPTSSA	9.62E-72	-0.47092	0.253	0.631	1.44E-67
U2SURP	9.86E-72	0.193601	0.54	0.628	1.47E-67
RNF213	1.02E-71	-0.31006	0.037	0.285	1.52E-67
PPP2R5C	1.04E-71	-0.31241	0.183	0.499	1.55E-67
ZNF511	1.05E-71	-0.26129	0.085	0.343	1.57E-67
RFC1	1.08E-71	-0.31476	0.244	0.584	1.62E-67
GNAI3	1.16E-71	0.238788	0.511	0.573	1.74E-67
OLA1	1.29E-71	0.243809	0.752	0.784	1.93E-67
SKP1	1.36E-71	0.229687	0.973	0.936	2.04E-67
PSMA3	1.38E-71	-0.20561	0.372	0.691	2.06E-67
DNAJB2	1.56E-71	0.268315	0.425	0.477	2.33E-67
SZRD1	1.63E-71	-0.24575	0.15	0.428	2.44E-67
RFWD3	2.00E-71	-0.39214	0.028	0.285	2.98E-67
FRG1	2.18E-71	0.194995	0.413	0.504	3.25E-67
PLOD1	2.18E-71	-0.23109	0.062	0.302	3.26E-67
NUP50	2.50E-71	-0.35574	0.071	0.359	3.73E-67
ZNF638	2.77E-71	0.235748	0.372	0.443	4.14E-67
CSE1L	2.91E-71	-0.38552	0.259	0.62	4.36E-67
CALCOCO2	2.96E-71	-0.20297	0.064	0.296	4.43E-67

PITPNA-AS	3.33E-71	-0.19884	0.176	0.454	4.98E-67
NFIA	3.53E-71	0.279475	0.716	0.757	5.27E-67
ARHGAP5	3.93E-71	-0.24544	0.101	0.363	5.87E-67
SORT1	3.97E-71	-0.27583	0.117	0.401	5.93E-67
TMEM134	4.32E-71	-0.23488	0.155	0.432	6.46E-67
ARX	4.43E-71	0.741648	0.266	0.02	6.62E-67
CENPP	4.92E-71	-0.44038	0.024	0.28	7.36E-67
BAG6	5.04E-71	0.168344	0.43	0.535	7.54E-67
RABL6	5.07E-71	-0.20869	0.242	0.533	7.57E-67
ZCCHC11	5.25E-71	0.193398	0.338	0.437	7.85E-67
ABHD2	5.96E-71	-0.40478	0.091	0.4	8.91E-67
IFI27L1	6.35E-71	-0.25789	0.171	0.464	9.49E-67
VGF	6.50E-71	0.74741	0.361	0.079	9.71E-67
LAMP2	6.50E-71	-0.40846	0.152	0.486	9.72E-67
FSTL1	7.02E-71	-0.34488	0.027	0.277	1.05E-66
BAZ1A	7.03E-71	-0.15156	0.293	0.577	1.05E-66
CERS2	7.64E-71	-0.2541	0.087	0.349	1.14E-66
SPCS3	7.80E-71	-0.28181	0.154	0.451	1.17E-66
KLHDC2	7.91E-71	0.167966	0.392	0.517	1.18E-66
HIST1H4C	8.06E-71	-0.5992	0.668	0.851	1.20E-66
NAPA	8.38E-71	0.185419	0.43	0.527	1.25E-66
C8orf4	9.06E-71	-0.50754	0.031	0.298	1.35E-66
DAP	9.44E-71	-0.28428	0.181	0.491	1.41E-66
SPOP	1.00E-70	0.252635	0.43	0.483	1.50E-66
BLOC1S4	1.00E-70	0.186319	0.324	0.433	1.50E-66
TEN1	1.02E-70	-0.23749	0.069	0.316	1.53E-66
POLE3	1.07E-70	-0.31767	0.215	0.541	1.60E-66
SV2A	1.10E-70	0.253154	0.371	0.423	1.64E-66
GSS	1.16E-70	-0.33787	0.167	0.486	1.74E-66
LEPROTL1	1.20E-70	0.139645	0.454	0.586	1.80E-66
FAM207A	1.21E-70	-0.25207	0.086	0.347	1.82E-66
PNISR	1.27E-70	0.286238	0.84	0.83	1.90E-66
AKR1B1	1.31E-70	0.257327	0.731	0.748	1.96E-66
ATP1B2	1.33E-70	-0.50914	0.078	0.394	1.98E-66
TRAIP	1.44E-70	-0.33735	0.035	0.289	2.15E-66
GINS4	1.49E-70	-0.37294	0.026	0.273	2.22E-66
RNF130	1.64E-70	-0.2268	0.214	0.507	2.46E-66
CD58	1.79E-70	-0.37113	0.018	0.259	2.68E-66
PNPLA8	1.94E-70	0.344231	0.541	0.511	2.91E-66
MYO9B	1.98E-70	-0.16239	0.148	0.391	2.95E-66
RHBDD2	2.04E-70	0.270531	0.67	0.694	3.05E-66
IRF2BP2	2.04E-70	0.360958	0.404	0.385	3.05E-66
NT5C	2.16E-70	-0.18228	0.209	0.485	3.22E-66
WDR54	2.32E-70	-0.31576	0.211	0.533	3.47E-66
MAP4K4	2.33E-70	0.227797	0.391	0.478	3.48E-66
FREM2	2.36E-70	-0.29376	0.009	0.227	3.53E-66
PLK2	2.57E-70	0.757471	0.39	0.147	3.84E-66
DNM1L	2.61E-70	0.258649	0.371	0.415	3.89E-66
KCNF1	2.67E-70	-0.52611	0.03	0.301	3.99E-66
DESI2	2.82E-70	-0.32792	0.152	0.464	4.21E-66
OSBPL8	2.92E-70	0.224242	0.452	0.515	4.37E-66

CUL5	3.34E-70	0.171816	0.299	0.406	5.00E-66
C6orf62	3.37E-70	0.184542	0.5	0.595	5.04E-66
SPP1	3.65E-70	0.375626	0.874	0.753	5.45E-66
IARS	3.78E-70	-0.27249	0.156	0.452	5.65E-66
TOMM5	3.85E-70	-0.23429	0.134	0.407	5.75E-66
VCP	3.95E-70	0.30055	0.606	0.601	5.90E-66
YME1L1	4.02E-70	-0.17507	0.137	0.389	6.01E-66
DYNLL2	4.06E-70	0.122604	0.342	0.47	6.06E-66
GID8	4.24E-70	-0.2228	0.15	0.422	6.34E-66
MAPK8IP1	4.41E-70	0.413926	0.35	0.293	6.59E-66
ZEB1	4.41E-70	-0.44081	0.163	0.509	6.59E-66
OTUB1	4.66E-70	0.216286	0.425	0.511	6.97E-66
RNF139	4.95E-70	0.342796	0.379	0.39	7.40E-66
AASDHPPT	5.11E-70	0.270338	0.428	0.468	7.64E-66
MGST3	5.40E-70	0.273789	0.782	0.783	8.07E-66
PPFIBP1	5.50E-70	0.43636	0.302	0.242	8.22E-66
CDK16	6.34E-70	-0.19106	0.249	0.536	9.48E-66
STARD7	7.12E-70	-0.31167	0.24	0.574	1.06E-65
EFNA3	7.58E-70	0.708848	0.284	0.033	1.13E-65
EIF3J	7.65E-70	-0.2993	0.265	0.594	1.14E-65
CEBPB	7.69E-70	-0.52279	0.092	0.414	1.15E-65
ERICH1	7.74E-70	0.484137	0.345	0.289	1.16E-65
C3orf14	9.00E-70	0.465729	0.602	0.478	1.34E-65
VEGFA	9.60E-70	-0.42678	0.046	0.32	1.43E-65
TACC1	9.76E-70	-0.38492	0.18	0.514	1.46E-65
CXCL16	9.77E-70	-0.35366	0.101	0.39	1.46E-65
PPP1R15A	1.07E-69	0.51654	0.556	0.407	1.60E-65
U2AF2	1.12E-69	-0.20023	0.236	0.527	1.68E-65
SARS	1.19E-69	0.261526	0.559	0.591	1.78E-65
CHD7	1.28E-69	0.394359	0.645	0.585	1.91E-65
PRRC2B	1.35E-69	0.255338	0.338	0.396	2.01E-65
HNRNPH2	1.40E-69	0.30187	0.48	0.491	2.09E-65
PLA2G16	1.50E-69	-0.20083	0.121	0.374	2.24E-65
CNIH3	1.52E-69	-0.47587	0.055	0.348	2.28E-65
CNIH1	1.53E-69	-0.30095	0.214	0.537	2.29E-65
CTDSP1	1.54E-69	-0.29345	0.034	0.27	2.30E-65
GRN	1.55E-69	-0.28909	0.211	0.523	2.31E-65
VMA21	1.58E-69	-0.16316	0.239	0.507	2.37E-65
CELF2	1.64E-69	0.109698	0.332	0.472	2.45E-65
MTFR2	1.77E-69	-0.38258	0.002	0.205	2.64E-65
GDI1	1.83E-69	0.229472	0.761	0.79	2.73E-65
G3BP2	1.97E-69	0.336218	0.418	0.406	2.95E-65
KMT2A	2.23E-69	0.223552	0.445	0.517	3.33E-65
KIAA1143	2.26E-69	-0.22886	0.146	0.415	3.38E-65
FTL	2.30E-69	0.19224	1	0.981	3.43E-65
ILVBL	2.41E-69	-0.38087	0.16	0.484	3.60E-65
CHST2	2.60E-69	-0.35704	0.03	0.284	3.88E-65
ENDOV	2.76E-69	0.228822	0.319	0.404	4.13E-65
CLN6	2.85E-69	-0.37386	0.053	0.323	4.26E-65
DUSP6	2.91E-69	-0.47795	0.108	0.435	4.35E-65
FAM120A	3.55E-69	-0.32462	0.068	0.342	5.31E-65

MRFAP1L1	3.68E-69	0.310607	0.368	0.39	5.50E-65
SLC39A6	3.97E-69	0.176262	0.535	0.626	5.94E-65
OGFOD3	4.19E-69	-0.24941	0.137	0.409	6.27E-65
HNRNPUL2	4.53E-69	-0.31432	0.099	0.386	6.78E-65
SFXN5	4.81E-69	-0.28353	0.102	0.375	7.19E-65
CMTM6	5.03E-69	-0.25012	0.24	0.549	7.52E-65
PFDN4	5.16E-69	0.23324	0.547	0.604	7.72E-65
ATF6B	5.19E-69	0.163929	0.46	0.581	7.76E-65
RUNDC3A	5.38E-69	0.710793	0.408	0.114	8.04E-65
IL1RAP	5.38E-69	-0.36182	0.016	0.249	8.05E-65
HN1L	5.38E-69	-0.16242	0.178	0.435	8.05E-65
GPRC5B	5.58E-69	-0.25659	0.122	0.391	8.33E-65
ALDH2	6.40E-69	0.541809	0.461	0.3	9.57E-65
R3HCC1	6.73E-69	0.178732	0.412	0.509	1.01E-64
AGT	6.75E-69	-0.54813	0.046	0.326	1.01E-64
PYCR2	7.03E-69	-0.34531	0.107	0.402	1.05E-64
HADHB	7.03E-69	-0.29075	0.304	0.637	1.05E-64
M6PR	7.17E-69	-0.18115	0.172	0.427	1.07E-64
LINC01102	7.21E-69	0.770776	0.368	0.098	1.08E-64
NABP2	7.29E-69	-0.32594	0.238	0.573	1.09E-64
SRRM3	7.64E-69	0.75984	0.393	0.072	1.14E-64
FKBP4	8.19E-69	0.284552	0.528	0.552	1.22E-64
HOOK3	8.27E-69	0.214418	0.374	0.464	1.24E-64
EEF1D	1.04E-68	-0.48339	0.64	0.911	1.55E-64
GPN3	1.09E-68	-0.12966	0.227	0.474	1.63E-64
FNDC4	1.11E-68	0.20252	0.295	0.393	1.65E-64
CTTN	1.15E-68	0.151536	0.304	0.411	1.72E-64
KRAS	1.24E-68	0.274593	0.395	0.433	1.86E-64
DOK5	1.37E-68	-0.12245	0.116	0.33	2.05E-64
DCUN1D5	1.42E-68	0.12569	0.389	0.514	2.12E-64
SOGA1	1.43E-68	-0.29077	0.187	0.493	2.14E-64
TGFB1I1	1.69E-68	-0.2773	0.04	0.277	2.53E-64
DPP4	1.71E-68	-0.24577	0.003	0.204	2.56E-64
PMF1	1.82E-68	-0.30842	0.249	0.578	2.71E-64
RP11-472N	1.99E-68	-0.34375	0.005	0.216	2.97E-64
USP22	2.00E-68	0.16416	0.481	0.593	2.99E-64
AP2A1	2.01E-68	-0.10644	0.23	0.467	3.01E-64
PLA2G12A	2.06E-68	0.318134	0.401	0.401	3.07E-64
CREM	2.09E-68	-0.47197	0.101	0.415	3.12E-64
TSSC1	2.32E-68	0.250436	0.408	0.468	3.47E-64
PEX10	2.36E-68	-0.16281	0.135	0.377	3.53E-64
ORAI1	2.61E-68	-0.28021	0.022	0.247	3.89E-64
NUFIP2	2.64E-68	-0.19844	0.153	0.411	3.95E-64
TTC19	2.69E-68	0.187256	0.506	0.594	4.02E-64
COPA	2.71E-68	0.223564	0.381	0.462	4.06E-64
EMD	2.80E-68	-0.21284	0.24	0.526	4.18E-64
SLN	2.81E-68	-0.61607	0.005	0.221	4.20E-64
ZNF367	3.00E-68	-0.44427	0.01	0.238	4.49E-64
TTLL7	3.01E-68	0.197241	0.373	0.464	4.50E-64
FAM49B	3.17E-68	0.324707	0.533	0.527	4.74E-64
PANK2	3.35E-68	-0.24978	0.137	0.411	5.01E-64

SPAG9	3.40E-68	0.143854	0.494	0.623	5.08E-64
TAF10	3.69E-68	-0.22387	0.143	0.41	5.52E-64
GGCT	3.80E-68	-0.4057	0.329	0.699	5.68E-64
PHC2	3.92E-68	-0.10336	0.228	0.467	5.87E-64
GTPBP6	4.10E-68	-0.20317	0.146	0.409	6.12E-64
TIMM10	4.41E-68	-0.45595	0.256	0.628	6.59E-64
SEPHS2	4.77E-68	0.23338	0.364	0.43	7.13E-64
RBM18	4.80E-68	0.276101	0.304	0.332	7.17E-64
SLC38A1	4.96E-68	0.661419	0.519	0.223	7.41E-64
EPC1	5.32E-68	0.140713	0.367	0.485	7.95E-64
NEDD1	5.58E-68	-0.31944	0.052	0.309	8.34E-64
PDRG1	6.20E-68	0.183679	0.394	0.493	9.27E-64
FABP6	6.41E-68	0.740103	0.288	0.026	9.57E-64
ATP6V1F	6.92E-68	0.283046	0.88	0.852	1.03E-63
CUX1	7.05E-68	-0.10741	0.24	0.491	1.05E-63
SSX2IP	7.05E-68	-0.23447	0.053	0.288	1.05E-63
BLM	7.07E-68	-0.35029	0.017	0.246	1.06E-63
ELAVL1	7.35E-68	-0.3319	0.313	0.664	1.10E-63
C21orf62	7.51E-68	-0.472	0.039	0.311	1.12E-63
TMEM107	8.43E-68	-0.31221	0.149	0.449	1.26E-63
ARF6	8.51E-68	-0.23802	0.245	0.547	1.27E-63
SMIM14	8.82E-68	0.328394	0.489	0.485	1.32E-63
TANC1	8.86E-68	-0.31953	0.023	0.253	1.32E-63
ALDH7A1	8.90E-68	-0.32123	0.263	0.598	1.33E-63
TLE4	9.49E-68	-0.33222	0.038	0.284	1.42E-63
CPNE3	9.55E-68	0.307439	0.449	0.459	1.43E-63
RPP25L	9.62E-68	-0.28501	0.037	0.274	1.44E-63
CNPY3	9.75E-68	-0.20831	0.195	0.481	1.46E-63
TACO1	1.03E-67	-0.2332	0.102	0.351	1.54E-63
PTP4A1	1.05E-67	0.185317	0.576	0.663	1.57E-63
SCAMP2	1.11E-67	-0.24252	0.097	0.356	1.66E-63
COMMD9	1.15E-67	0.359384	0.357	0.348	1.72E-63
DPM2	1.22E-67	-0.24391	0.306	0.622	1.82E-63
POFUT1	1.30E-67	-0.31635	0.041	0.291	1.95E-63
GANAB	1.34E-67	-0.27893	0.226	0.537	2.00E-63
CDK5RAP2	1.35E-67	-0.34913	0.085	0.363	2.02E-63
RHOA	1.42E-67	-0.48012	0.498	0.857	2.12E-63
GDAP1L1	1.42E-67	0.769435	0.46	0.14	2.13E-63
PPP2R3C	1.61E-67	-0.26094	0.26	0.572	2.41E-63
GRSF1	1.67E-67	0.139245	0.405	0.528	2.49E-63
ZNF43	1.85E-67	-0.26798	0.155	0.44	2.77E-63
RBM6	1.89E-67	0.270245	0.474	0.507	2.82E-63
MED10	2.07E-67	0.248718	0.635	0.67	3.09E-63
KMT2C	2.19E-67	0.193687	0.358	0.454	3.27E-63
COX19	2.23E-67	0.285861	0.375	0.404	3.33E-63
LIG1	2.24E-67	-0.46611	0.062	0.353	3.34E-63
LYRM2	2.27E-67	-0.28291	0.255	0.57	3.39E-63
FIGN	2.72E-67	-0.32821	0.027	0.262	4.07E-63
ARL6IP5	2.75E-67	0.336582	0.59	0.565	4.11E-63
EIF3D	2.79E-67	-0.17416	0.355	0.652	4.17E-63
ARID4A	2.86E-67	0.365009	0.493	0.465	4.28E-63

ALG3	2.90E-67	-0.22152	0.091	0.338	4.34E-63
POLD4	2.93E-67	-0.12122	0.102	0.31	4.39E-63
PLEKHB2	3.21E-67	0.130345	0.431	0.546	4.79E-63
ARAF	3.25E-67	-0.24713	0.08	0.326	4.85E-63
SEC31A	3.78E-67	0.129272	0.223	0.333	5.64E-63
IK	3.79E-67	0.195144	0.499	0.584	5.67E-63
SLC4A2	4.03E-67	-0.28222	0.053	0.304	6.02E-63
FAM181B	4.07E-67	-0.50568	0.196	0.552	6.07E-63
NAP1L3	4.17E-67	0.720317	0.379	0.105	6.23E-63
TXN2	4.19E-67	-0.12858	0.38	0.66	6.26E-63
DNAJA2	4.35E-67	0.206313	0.411	0.48	6.51E-63
PRKDC	4.60E-67	-0.315	0.291	0.63	6.87E-63
UBQLN1	4.63E-67	0.215981	0.379	0.451	6.92E-63
AC010642.	4.80E-67	-0.10539	0.191	0.426	7.17E-63
ITGB1BP1	4.96E-67	-0.37185	0.317	0.678	7.41E-63
STK17A	4.97E-67	-0.35625	0.185	0.507	7.42E-63
NAGK	5.25E-67	0.12549	0.374	0.51	7.84E-63
UBE2H	6.44E-67	0.412353	0.438	0.375	9.62E-63
CHD4	6.65E-67	0.188488	0.452	0.544	9.94E-63
HDGF	6.71E-67	-0.47485	0.247	0.616	1.00E-62
IRF3	7.16E-67	-0.26983	0.126	0.402	1.07E-62
GSPT2	8.84E-67	0.48242	0.334	0.24	1.32E-62
THAP7	9.37E-67	-0.23753	0.161	0.438	1.40E-62
CNOT4	9.43E-67	0.329578	0.455	0.459	1.41E-62
MAZ	9.55E-67	-0.3333	0.233	0.563	1.43E-62
ASH1L	9.81E-67	0.181333	0.396	0.484	1.47E-62
OXR1	1.22E-66	0.220239	0.33	0.409	1.82E-62
AGPAT2	1.24E-66	-0.25625	0.018	0.222	1.85E-62
ZNF530	1.26E-66	-0.34088	0.041	0.289	1.88E-62
ARF3	1.31E-66	0.183837	0.414	0.515	1.95E-62
HADH	1.33E-66	-0.27293	0.17	0.462	1.98E-62
ATXN7L3B	1.35E-66	0.345311	0.339	0.326	2.02E-62
SLU7	1.38E-66	0.36961	0.43	0.407	2.07E-62
CHCHD10	1.45E-66	-0.43293	0.124	0.441	2.16E-62
USP47	1.45E-66	0.274708	0.402	0.435	2.16E-62
DYNC1H1	1.60E-66	0.280313	0.482	0.526	2.39E-62
PSMD2	1.64E-66	0.207014	0.664	0.715	2.45E-62
IDH2	1.93E-66	-0.43829	0.331	0.705	2.89E-62
EPHX1	1.94E-66	-0.17424	0.121	0.354	2.90E-62
CLCN3	2.03E-66	0.185193	0.342	0.433	3.03E-62
CCZ1	2.06E-66	0.176612	0.525	0.61	3.07E-62
SEC62	2.08E-66	0.29622	0.84	0.802	3.11E-62
VDAC3	2.14E-66	0.315854	0.769	0.737	3.19E-62
KRI1	2.43E-66	-0.26902	0.054	0.296	3.63E-62
MAP9	2.45E-66	0.373982	0.34	0.31	3.67E-62
DYNC1LI1	2.47E-66	0.398113	0.48	0.416	3.69E-62
SIRPA	3.00E-66	-0.24516	0.03	0.251	4.49E-62
C12orf76	3.14E-66	0.259596	0.353	0.391	4.69E-62
CLASP2	3.39E-66	0.530865	0.475	0.32	5.07E-62
CCDC115	3.43E-66	0.477889	0.45	0.359	5.12E-62
METTL7A	3.61E-66	-0.51323	0.04	0.304	5.40E-62

ABRACL	3.72E-66	0.319483	0.351	0.372	5.56E-62
SWI5	3.86E-66	-0.21154	0.14	0.401	5.78E-62
NDUFB1	3.87E-66	-0.40288	0.422	0.79	5.78E-62
SRP19	3.93E-66	0.133007	0.465	0.593	5.87E-62
CHST7	4.07E-66	-0.31999	0.01	0.228	6.08E-62
RHOG	4.27E-66	-0.24563	0.075	0.32	6.38E-62
ACTL6A	4.50E-66	-0.2728	0.258	0.572	6.72E-62
PKN1	4.70E-66	-0.21884	0.171	0.442	7.03E-62
NOC2L	5.66E-66	0.130226	0.424	0.563	8.46E-62
GNL1	5.75E-66	-0.19575	0.249	0.528	8.59E-62
CCDC12	5.75E-66	0.191599	0.549	0.651	8.59E-62
MAF1	5.90E-66	0.210081	0.552	0.622	8.82E-62
8-Sep	6.12E-66	-0.28067	0.118	0.393	9.14E-62
PAFAH1B1	6.76E-66	0.172585	0.471	0.562	1.01E-61
DONSON	6.85E-66	-0.33412	0.051	0.307	1.02E-61
CTBP1	7.35E-66	0.120644	0.279	0.402	1.10E-61
HSDL1	7.45E-66	0.522377	0.384	0.238	1.11E-61
COA6	7.52E-66	-0.21701	0.198	0.477	1.12E-61
WTAP	7.65E-66	0.160391	0.537	0.635	1.14E-61
SLC22A17	8.37E-66	0.286641	0.492	0.544	1.25E-61
ACTN1	8.70E-66	-0.37075	0.112	0.405	1.30E-61
MAGOH	9.06E-66	-0.13071	0.372	0.647	1.35E-61
DDX24	9.11E-66	0.259204	0.575	0.615	1.36E-61
GALNT2	9.28E-66	-0.23825	0.091	0.34	1.39E-61
IFNAR2	1.04E-65	-0.15278	0.127	0.36	1.56E-61
PLEKHA4	1.11E-65	-0.3149	0.015	0.235	1.66E-61
COA4	1.37E-65	-0.22281	0.329	0.635	2.05E-61
FAM129A	1.42E-65	-0.30212	0.007	0.214	2.13E-61
ZRANB2	1.45E-65	0.132093	0.381	0.501	2.17E-61
RRP7A	1.49E-65	-0.23189	0.24	0.537	2.22E-61
TRAF3IP2	1.58E-65	-0.38404	0.034	0.28	2.36E-61
MOAP1	1.63E-65	0.400334	0.375	0.332	2.43E-61
CDK5	1.70E-65	0.227138	0.357	0.426	2.54E-61
RAD51C	1.74E-65	-0.32495	0.286	0.621	2.60E-61
INA	1.79E-65	0.700218	0.306	0.035	2.67E-61
ALG8	1.82E-65	-0.28149	0.093	0.357	2.72E-61
PLEKHA3	1.89E-65	0.200349	0.328	0.417	2.82E-61
MMADHC	1.91E-65	0.235615	0.566	0.623	2.86E-61
UBR7	1.98E-65	-0.39558	0.068	0.34	2.96E-61
NRBF2	2.02E-65	-0.34407	0.112	0.398	3.01E-61
FBLN7	2.02E-65	-0.31239	0.013	0.225	3.02E-61
SMARCC2	2.07E-65	0.258018	0.448	0.488	3.09E-61
ATAD3A	2.15E-65	-0.18706	0.149	0.399	3.21E-61
SAE1	2.22E-65	-0.53335	0.322	0.702	3.32E-61
POU2F1	2.42E-65	0.435238	0.35	0.293	3.62E-61
TFAM	2.61E-65	-0.20737	0.15	0.41	3.91E-61
CFDP1	2.97E-65	0.119415	0.571	0.715	4.44E-61
TMED4	3.04E-65	0.231961	0.615	0.657	4.55E-61
CYBRD1	3.29E-65	-0.28858	0.028	0.251	4.91E-61
NSRP1	3.60E-65	-0.27578	0.321	0.638	5.37E-61
CISD3	3.62E-65	-0.27403	0.071	0.315	5.40E-61

ATP6V1G2	3.62E-65	0.643449	0.393	0.215	5.41E-61
FOXRED2	3.74E-65	-0.30218	0.051	0.296	5.59E-61
CCNL2	3.96E-65	0.274338	0.383	0.42	5.92E-61
NFYB	4.01E-65	-0.24462	0.178	0.46	5.99E-61
KHDRBS3	4.27E-65	-0.21607	0.189	0.462	6.38E-61
ARHGEF6	4.33E-65	-0.29542	0.009	0.217	6.47E-61
AFF4	4.73E-65	0.207889	0.441	0.526	7.06E-61
BCLAF1	4.75E-65	0.154121	0.525	0.627	7.10E-61
PLTP	4.97E-65	-0.41558	0.092	0.385	7.43E-61
LRRCC1	5.26E-65	-0.38736	0.108	0.405	7.87E-61
CHN1	5.30E-65	-0.11445	0.299	0.556	7.93E-61
MYCBP	5.71E-65	-0.27362	0.008	0.212	8.53E-61
TFDP1	5.97E-65	-0.33319	0.172	0.483	8.92E-61
PUM1	6.52E-65	0.21221	0.312	0.375	9.75E-61
TRIM36	6.70E-65	0.285019	0.441	0.467	1.00E-60
MGAT2	7.08E-65	-0.20945	0.119	0.369	1.06E-60
RBM4B	7.12E-65	0.36421	0.357	0.333	1.06E-60
FDXR	7.17E-65	-0.24234	0.07	0.309	1.07E-60
RRP36	7.25E-65	-0.12829	0.124	0.343	1.08E-60
MGRN1	7.39E-65	0.222226	0.255	0.325	1.11E-60
MAT2A	7.80E-65	-0.14895	0.334	0.606	1.17E-60
SDC4	7.94E-65	-0.30337	0.004	0.2	1.19E-60
ATP6V1D	8.03E-65	0.200269	0.433	0.526	1.20E-60
ID1	8.06E-65	-0.3711	0.048	0.298	1.20E-60
MPV17	8.31E-65	-0.15176	0.275	0.542	1.24E-60
INADL	8.72E-65	0.685211	0.302	0.081	1.30E-60
RPRM	9.58E-65	0.832653	0.352	0.078	1.43E-60
USP8	9.60E-65	-0.21961	0.132	0.389	1.43E-60
FEZ1	9.81E-65	0.22933	0.758	0.784	1.47E-60
BCL7A	1.01E-64	0.529491	0.439	0.289	1.51E-60
PFKP	1.03E-64	-0.20539	0.065	0.291	1.54E-60
EFEMP2	1.12E-64	-0.19607	0.184	0.443	1.67E-60
OAZ2	1.24E-64	0.167702	0.573	0.651	1.85E-60
PRKCSH	1.24E-64	-0.35556	0.335	0.684	1.86E-60
C17orf58	1.37E-64	0.136205	0.305	0.427	2.05E-60
KCNQ2	1.39E-64	-0.16881	0.251	0.521	2.08E-60
PSMG2	1.42E-64	-0.16793	0.339	0.621	2.12E-60
TSPAN4	1.56E-64	-0.23935	0.064	0.299	2.33E-60
TIMM50	1.56E-64	-0.30967	0.252	0.572	2.34E-60
DNAJC7	1.65E-64	0.168571	0.606	0.696	2.46E-60
PITX1	1.73E-64	-0.20519	0.032	0.232	2.58E-60
MLX	1.78E-64	0.162678	0.32	0.433	2.66E-60
ACOT7	1.80E-64	0.36475	0.519	0.514	2.69E-60
RERE	1.88E-64	0.270486	0.353	0.393	2.81E-60
TMEM170A	1.88E-64	-0.2475	0.15	0.42	2.81E-60
MED4	1.88E-64	0.14423	0.484	0.601	2.82E-60
RBCK1	1.89E-64	-0.20332	0.244	0.528	2.82E-60
EVL	2.04E-64	0.388296	0.351	0.333	3.05E-60
FNDC3B	2.09E-64	-0.25459	0.03	0.251	3.12E-60
BCL7C	2.32E-64	-0.44122	0.222	0.574	3.47E-60
SDHAF2	2.44E-64	0.147818	0.307	0.423	3.65E-60

CHMP1B	2.44E-64	0.356265	0.449	0.401	3.65E-60
PRR14	2.48E-64	0.1121	0.239	0.364	3.71E-60
VOPP1	2.57E-64	0.13292	0.36	0.48	3.83E-60
TSEN15	2.61E-64	-0.16846	0.235	0.499	3.90E-60
CRLS1	2.62E-64	-0.22903	0.238	0.531	3.91E-60
CHEK2	2.65E-64	-0.3553	0.01	0.221	3.96E-60
SRGAP1	2.65E-64	0.502737	0.404	0.281	3.96E-60
AUTS2	2.71E-64	0.343741	0.266	0.283	4.05E-60
CD44	2.82E-64	-0.41575	0.036	0.285	4.21E-60
EBPL	2.86E-64	0.217645	0.428	0.517	4.27E-60
TMUB2	2.89E-64	0.157815	0.319	0.419	4.32E-60
CCNG2	2.97E-64	0.524469	0.518	0.356	4.43E-60
PARL	3.04E-64	0.125882	0.364	0.496	4.54E-60
CYB5D2	3.12E-64	-0.12368	0.197	0.433	4.67E-60
C8orf88	3.13E-64	-0.2804	0.009	0.214	4.68E-60
GLOD4	3.28E-64	0.14689	0.558	0.673	4.91E-60
TM2D2	3.36E-64	0.314003	0.375	0.405	5.03E-60
FBXW2	3.46E-64	-0.22839	0.131	0.39	5.17E-60
DMRTA2	3.74E-64	-0.28728	0.015	0.227	5.59E-60
CITED1	3.87E-64	-0.43925	0.057	0.333	5.78E-60
TMEM87A	4.10E-64	-0.12729	0.225	0.468	6.12E-60
IDI1	4.30E-64	-0.28335	0.207	0.507	6.42E-60
ATXN10	4.75E-64	0.232694	0.648	0.7	7.10E-60
HDDC2	5.12E-64	0.128539	0.477	0.614	7.65E-60
PSMD13	5.29E-64	0.184128	0.504	0.59	7.90E-60
RPN1	6.19E-64	-0.15531	0.307	0.578	9.25E-60
CTDSPL2	6.21E-64	-0.30269	0.082	0.347	9.27E-60
TGIF2	6.23E-64	-0.30721	0.008	0.209	9.31E-60
CTNNBL1	6.31E-64	-0.1796	0.288	0.565	9.43E-60
PTS	6.66E-64	0.266725	0.546	0.564	9.95E-60
EFTUD2	7.08E-64	-0.16291	0.171	0.414	1.06E-59
AKAP8L	7.54E-64	0.174281	0.346	0.438	1.13E-59
C4orf27	7.56E-64	0.17174	0.556	0.649	1.13E-59
MPHOSPH9	7.65E-64	-0.27519	0.131	0.4	1.14E-59
MORC4	8.17E-64	-0.36561	0.012	0.226	1.22E-59
MASTL	8.34E-64	-0.41182	0.009	0.22	1.25E-59
SENP6	8.49E-64	0.122037	0.257	0.375	1.27E-59
UBA1	8.81E-64	0.120066	0.393	0.52	1.32E-59
ICMT	9.34E-64	-0.32391	0.065	0.325	1.40E-59
PPP2R1A	9.49E-64	0.226507	0.743	0.764	1.42E-59
BTBD3	9.53E-64	-0.26097	0.051	0.285	1.42E-59
ZBTB20	9.62E-64	0.267099	0.712	0.731	1.44E-59
UQCC2	9.73E-64	-0.46684	0.322	0.694	1.45E-59
SMARCA5	1.06E-63	0.141957	0.501	0.619	1.58E-59
CAST	1.09E-63	-0.34282	0.082	0.356	1.63E-59
BCKDK	1.11E-63	-0.13661	0.212	0.451	1.66E-59
SIL1	1.13E-63	-0.16491	0.079	0.29	1.69E-59
DHRS11	1.19E-63	-0.22086	0.061	0.285	1.78E-59
CCND1	1.21E-63	-0.36375	0.104	0.379	1.80E-59
TWSG1	1.22E-63	-0.22296	0.07	0.302	1.82E-59
NELFE	1.25E-63	-0.31308	0.342	0.681	1.87E-59

COMMD2	1.30E-63	0.224538	0.472	0.532	1.94E-59
COX11	1.33E-63	0.141137	0.378	0.496	1.98E-59
USP11	1.33E-63	0.275737	0.622	0.617	1.99E-59
CNP	1.39E-63	-0.30901	0.329	0.665	2.08E-59
DLD	1.43E-63	0.300861	0.496	0.5	2.13E-59
ANKRD9	1.48E-63	-0.31062	0.013	0.228	2.22E-59
SEC11C	1.48E-63	0.202729	0.631	0.679	2.22E-59
SOX9	1.51E-63	-0.62258	0.243	0.612	2.26E-59
WBP4	1.53E-63	0.113919	0.379	0.519	2.28E-59
SBNO1	1.59E-63	0.181829	0.416	0.505	2.38E-59
RB1	1.61E-63	-0.18683	0.114	0.349	2.40E-59
BOC	1.63E-63	-0.22293	0.03	0.228	2.44E-59
NOL3	1.75E-63	0.563457	0.269	0.156	2.61E-59
TMEM255A	1.85E-63	-0.21007	0.027	0.223	2.76E-59
LHFPL3	1.98E-63	-0.63261	0.06	0.348	2.96E-59
TAP1	2.02E-63	-0.46247	0.096	0.388	3.03E-59
H3F3B	2.12E-63	0.1674	1	0.981	3.18E-59
PTPRE	2.25E-63	-0.2196	0.047	0.262	3.36E-59
ERAL1	2.31E-63	-0.11639	0.259	0.5	3.45E-59
RBPJ	2.44E-63	0.29582	0.61	0.605	3.65E-59
CEP78	2.58E-63	-0.25103	0.157	0.43	3.85E-59
CNOT2	2.77E-63	0.170319	0.362	0.464	4.15E-59
TYRO3	2.78E-63	-0.2994	0.038	0.272	4.15E-59
PXMP2	2.78E-63	-0.32617	0.273	0.602	4.15E-59
MSN	2.81E-63	-0.12337	0.185	0.416	4.20E-59
PTAR1	3.07E-63	-0.27585	0.063	0.306	4.59E-59
SPATS2L	3.31E-63	-0.52574	0.192	0.546	4.94E-59
CCDC14	3.50E-63	-0.19388	0.336	0.627	5.23E-59
FOPNL	3.54E-63	-0.22861	0.161	0.433	5.29E-59
AGPAT5	3.60E-63	-0.30415	0.123	0.402	5.38E-59
ERBB2IP	3.62E-63	-0.19424	0.135	0.378	5.41E-59
PRDX5	3.68E-63	0.322914	0.863	0.799	5.50E-59
NMT1	3.74E-63	-0.3156	0.146	0.436	5.59E-59
DHCR24	3.78E-63	-0.32043	0.009	0.217	5.64E-59
TUSC2	3.83E-63	0.14689	0.422	0.532	5.72E-59
DTD1	3.87E-63	0.312435	0.697	0.673	5.78E-59
DNM2	3.91E-63	-0.20999	0.119	0.365	5.84E-59
HIF1A	3.95E-63	-0.38758	0.305	0.657	5.90E-59
FRYL	4.14E-63	-0.129	0.174	0.409	6.18E-59
TMEM126B	4.16E-63	-0.16565	0.251	0.515	6.22E-59
FUZ	4.33E-63	-0.32258	0.119	0.4	6.47E-59
SAFB	4.42E-63	0.15214	0.42	0.528	6.61E-59
RRM1	4.74E-63	-0.41782	0.273	0.626	7.09E-59
PDLIM5	4.80E-63	-0.20013	0.156	0.41	7.17E-59
PRKRIP1	4.95E-63	0.139801	0.346	0.467	7.40E-59
SAFB2	4.98E-63	0.134915	0.432	0.554	7.45E-59
LPP	5.14E-63	-0.30587	0.072	0.327	7.68E-59
CLPTM1L	5.19E-63	-0.17966	0.089	0.311	7.76E-59
CDS2	5.25E-63	-0.10204	0.171	0.386	7.84E-59
SNRPA1	5.55E-63	-0.16866	0.317	0.593	8.30E-59
FTH1	5.60E-63	0.169555	1	0.981	8.37E-59

SHMT1	5.61E-63	-0.23576	0.048	0.268	8.38E-59
PLOD2	6.68E-63	-0.31752	0.052	0.299	9.98E-59
GPR108	6.75E-63	-0.17605	0.11	0.34	1.01E-58
TCF3	6.77E-63	-0.16571	0.118	0.342	1.01E-58
ENAH	6.95E-63	0.189745	0.493	0.578	1.04E-58
VEZT	7.06E-63	0.298598	0.361	0.394	1.06E-58
RSU1	7.57E-63	-0.13532	0.105	0.317	1.13E-58
MPDU1	7.58E-63	-0.14161	0.302	0.564	1.13E-58
ARIH2	7.61E-63	0.101698	0.319	0.447	1.14E-58
STIL	7.72E-63	-0.32429	0.003	0.195	1.15E-58
ATXN2L	7.73E-63	-0.10016	0.152	0.359	1.16E-58
ADD1	8.08E-63	0.142568	0.362	0.478	1.21E-58
SERINC3	8.15E-63	0.165295	0.411	0.501	1.22E-58
ATPAF2	8.34E-63	-0.12213	0.132	0.344	1.25E-58
ATXN2	8.76E-63	0.175291	0.284	0.369	1.31E-58
THOC3	8.93E-63	-0.11246	0.181	0.401	1.33E-58
HIRIP3	9.01E-63	-0.4022	0.2	0.531	1.35E-58
DUSP4	9.74E-63	-0.35533	0.046	0.295	1.46E-58
TDG	9.84E-63	0.251145	0.422	0.459	1.47E-58
ASNSD1	1.02E-62	0.159378	0.417	0.519	1.53E-58
LSM1	1.04E-62	0.201529	0.57	0.66	1.56E-58
C12orf10	1.05E-62	0.178096	0.371	0.46	1.56E-58
TMA16	1.09E-62	-0.23274	0.134	0.391	1.62E-58
NUB1	1.09E-62	0.275387	0.345	0.379	1.63E-58
ASPH	1.14E-62	-0.24265	0.249	0.542	1.71E-58
FAM89B	1.14E-62	0.205192	0.394	0.468	1.71E-58
DYNC1I2	1.19E-62	0.194784	0.612	0.667	1.78E-58
SCD	1.19E-62	-0.31789	0.043	0.284	1.78E-58
VWA1	1.19E-62	-0.27335	0.039	0.263	1.78E-58
VAPB	1.25E-62	-0.25376	0.166	0.442	1.86E-58
ATP2A2	1.28E-62	-0.12858	0.247	0.496	1.92E-58
FAM177A1	1.32E-62	0.137467	0.451	0.583	1.98E-58
IFT27	1.33E-62	0.124569	0.324	0.458	1.99E-58
ATP6AP2	1.36E-62	0.234722	0.676	0.714	2.04E-58
MYH10	1.40E-62	0.314284	0.396	0.389	2.09E-58
CYTH2	1.44E-62	0.234785	0.596	0.635	2.15E-58
CDV3	1.46E-62	-0.23221	0.159	0.423	2.18E-58
TIMM17A	1.58E-62	0.106122	0.43	0.567	2.36E-58
CAND1	1.62E-62	0.242131	0.357	0.419	2.42E-58
BRMS1	1.67E-62	-0.11219	0.266	0.509	2.50E-58
PGAP1	1.76E-62	0.250208	0.406	0.449	2.64E-58
CCNH	1.80E-62	0.264533	0.493	0.536	2.69E-58
RECQL	1.88E-62	-0.2461	0.118	0.373	2.81E-58
SLC30A9	2.02E-62	0.182119	0.375	0.462	3.01E-58
THUMPD3	2.04E-62	-0.26662	0.135	0.406	3.05E-58
PRRC2A	2.23E-62	-0.1541	0.25	0.506	3.33E-58
MAPKAP1	2.28E-62	-0.17785	0.188	0.443	3.40E-58
NUDT4	2.37E-62	-0.25293	0.148	0.416	3.54E-58
FZR1	2.50E-62	-0.2838	0.118	0.386	3.73E-58
GRHPR	2.61E-62	-0.26954	0.302	0.612	3.91E-58
CST3	2.62E-62	-0.51121	0.705	0.925	3.92E-58

ABHD12	2.67E-62	-0.42019	0.235	0.58	3.99E-58
UBL3	2.70E-62	-0.1242	0.236	0.475	4.03E-58
MEOX2	2.72E-62	-0.36948	0.035	0.273	4.07E-58
CEBPD	2.75E-62	-0.58215	0.095	0.396	4.11E-58
ADA	2.82E-62	-0.29325	0.005	0.196	4.22E-58
KANK2	2.85E-62	-0.32694	0.013	0.221	4.27E-58
BRIX1	2.91E-62	-0.24456	0.261	0.552	4.35E-58
ATP6V1B2	2.99E-62	0.327457	0.427	0.419	4.47E-58
NKTR	3.10E-62	0.19393	0.497	0.581	4.63E-58
CMSS1	3.15E-62	-0.14182	0.34	0.602	4.71E-58
DERL2	3.26E-62	-0.13515	0.255	0.507	4.87E-58
SP9	3.27E-62	0.721358	0.286	0.041	4.89E-58
RBM4	3.29E-62	0.247235	0.383	0.432	4.92E-58
DCLK1	3.32E-62	0.464604	0.314	0.216	4.96E-58
IFNAR1	3.38E-62	-0.2417	0.178	0.454	5.06E-58
SLC9A3R1	3.42E-62	-0.24422	0.076	0.309	5.10E-58
CBY1	3.53E-62	-0.14991	0.148	0.375	5.27E-58
ASNS	3.69E-62	0.519659	0.476	0.299	5.51E-58
MIF4GD	3.69E-62	-0.3413	0.101	0.378	5.52E-58
RPA2	3.87E-62	-0.32626	0.305	0.635	5.79E-58
REPIN1	3.96E-62	0.152805	0.387	0.493	5.92E-58
KIF1A	4.26E-62	0.656063	0.426	0.175	6.37E-58
ACTR10	4.48E-62	0.217344	0.466	0.536	6.69E-58
CRIP2	4.52E-62	0.229838	0.502	0.604	6.76E-58
HIGD1A	4.63E-62	-0.36138	0.297	0.636	6.92E-58
NR1H2	4.90E-62	-0.12654	0.166	0.389	7.33E-58
GOLGA4	4.97E-62	0.128905	0.369	0.48	7.43E-58
GON4L	5.10E-62	0.175427	0.266	0.358	7.62E-58
GSTO1	5.27E-62	-0.35765	0.153	0.452	7.88E-58
PRKAR1A	5.35E-62	0.156252	0.539	0.627	7.99E-58
MNAT1	5.38E-62	-0.13141	0.183	0.415	8.04E-58
CCZ1B	5.47E-62	0.315171	0.295	0.301	8.18E-58
CCDC88A	5.56E-62	0.188871	0.627	0.701	8.31E-58
CXXC5	5.95E-62	0.281755	0.665	0.68	8.89E-58
SLC20A1	6.00E-62	-0.3791	0.112	0.4	8.96E-58
MAPKAPK5-	6.27E-62	0.326124	0.335	0.344	9.37E-58
TBPL1	6.58E-62	0.369802	0.398	0.363	9.83E-58
GSTZ1	6.83E-62	-0.22986	0.068	0.286	1.02E-57
PSME1	6.97E-62	-0.39736	0.285	0.627	1.04E-57
ARHGAP12	7.13E-62	-0.30368	0.079	0.333	1.07E-57
GNB1	7.73E-62	0.126745	0.547	0.664	1.16E-57
CHD2	7.84E-62	0.17208	0.306	0.4	1.17E-57
GNA11	8.89E-62	-0.1185	0.093	0.29	1.33E-57
SP3	9.17E-62	0.214735	0.357	0.433	1.37E-57
CHMP4A	9.35E-62	-0.17098	0.328	0.604	1.40E-57
SF3B1	9.58E-62	0.170701	0.562	0.654	1.43E-57
ZNF428	9.83E-62	0.240941	0.888	0.868	1.47E-57
KDM5A	1.04E-61	0.221083	0.316	0.38	1.55E-57
RUVBL2	1.04E-61	-0.35343	0.335	0.675	1.55E-57
BNIP2	1.08E-61	-0.18696	0.134	0.37	1.61E-57
IFRD2	1.11E-61	-0.23759	0.126	0.383	1.66E-57

MPZL1	1.12E-61	0.306844	0.333	0.344	1.68E-57
TSN	1.13E-61	0.20393	0.58	0.643	1.69E-57
ENO2	1.22E-61	0.397773	0.574	0.472	1.82E-57
TAPBP	1.22E-61	-0.2187	0.146	0.399	1.82E-57
RSPRY1	1.23E-61	0.170474	0.367	0.462	1.83E-57
MCUR1	1.24E-61	-0.34005	0.205	0.519	1.85E-57
CDYL	1.25E-61	-0.2588	0.045	0.268	1.87E-57
PPP2R4	1.27E-61	-0.12989	0.181	0.411	1.90E-57
SNX21	1.33E-61	-0.24791	0.061	0.291	1.98E-57
RPE	1.40E-61	-0.1802	0.092	0.31	2.08E-57
B3GALNT1	1.44E-61	0.370839	0.391	0.341	2.16E-57
GHITM	1.52E-61	0.147535	0.464	0.577	2.28E-57
CNOT1	1.53E-61	-0.13429	0.121	0.341	2.28E-57
MED16	1.54E-61	-0.12004	0.122	0.333	2.29E-57
CTDSP2	1.63E-61	-0.40434	0.181	0.495	2.43E-57
ARL16	1.67E-61	0.146664	0.461	0.573	2.50E-57
WSB1	1.71E-61	0.295291	0.803	0.775	2.56E-57
BAZ2A	1.76E-61	0.201714	0.243	0.31	2.63E-57
SCRN1	1.78E-61	-0.23723	0.101	0.347	2.65E-57
NUDT16L1	1.79E-61	0.10926	0.335	0.462	2.67E-57
PPP1R2	1.82E-61	0.227606	0.437	0.491	2.72E-57
SPATS2	1.82E-61	0.207207	0.518	0.584	2.72E-57
NUP62	1.83E-61	-0.14248	0.271	0.526	2.74E-57
LRRC58	1.88E-61	-0.10058	0.196	0.411	2.81E-57
BCAT1	1.91E-61	-0.3626	0.118	0.402	2.85E-57
PAFAH1B3	2.00E-61	0.219506	0.813	0.828	2.98E-57
MRAS	2.07E-61	0.151971	0.214	0.31	3.09E-57
FN3KRP	2.19E-61	-0.20965	0.207	0.473	3.27E-57
ARL1	2.28E-61	-0.17169	0.201	0.454	3.41E-57
PQBP1	2.34E-61	0.169197	0.397	0.494	3.50E-57
C14orf142	2.36E-61	-0.26666	0.079	0.325	3.52E-57
MXD4	2.56E-61	0.510761	0.269	0.157	3.83E-57
MYH9	2.61E-61	-0.2484	0.027	0.231	3.90E-57
UBE2R2	2.68E-61	0.143716	0.405	0.515	4.00E-57
DNAAF2	2.68E-61	0.279225	0.277	0.315	4.01E-57
SIAH2	2.74E-61	0.246601	0.34	0.388	4.10E-57
DPYSL4	2.78E-61	0.309083	0.375	0.383	4.16E-57
MAEA	2.80E-61	0.197542	0.311	0.385	4.18E-57
PHF6	2.92E-61	0.213098	0.305	0.377	4.36E-57
NPRL3	2.92E-61	0.117276	0.231	0.352	4.36E-57
SIPA1L1	2.94E-61	-0.17131	0.084	0.298	4.40E-57
CRELD2	3.10E-61	-0.2009	0.203	0.467	4.64E-57
MESDC2	3.22E-61	-0.36393	0.203	0.523	4.82E-57
OSBPL6	3.38E-61	-0.18198	0.148	0.388	5.05E-57
TXLNA	3.49E-61	-0.23143	0.086	0.325	5.21E-57
STT3B	3.51E-61	-0.16552	0.151	0.385	5.24E-57
ITGA2	3.56E-61	-0.3418	0.005	0.2	5.32E-57
RNF126	3.66E-61	-0.10574	0.341	0.589	5.47E-57
TMEM259	3.90E-61	-0.16414	0.294	0.559	5.83E-57
SUMF2	4.05E-61	-0.1455	0.277	0.533	6.05E-57
KEAP1	4.12E-61	-0.32154	0.211	0.52	6.15E-57

TM2D3	4.16E-61	0.368498	0.452	0.417	6.21E-57
YTHDF1	4.29E-61	-0.16325	0.151	0.389	6.41E-57
SLC35A4	4.34E-61	-0.22159	0.129	0.38	6.49E-57
CSTF1	4.54E-61	-0.23351	0.089	0.331	6.79E-57
RGS6	4.62E-61	-0.28419	0.004	0.19	6.91E-57
FKBP5	4.89E-61	-0.36452	0.085	0.347	7.30E-57
CYHR1	4.93E-61	0.137076	0.279	0.4	7.37E-57
AAMDC	4.98E-61	-0.42584	0.067	0.335	7.45E-57
SDHB	5.23E-61	-0.17787	0.314	0.589	7.81E-57
QSER1	5.26E-61	-0.11566	0.148	0.359	7.87E-57
PCBP4	5.50E-61	0.175028	0.409	0.512	8.22E-57
MPHOSPH6	5.65E-61	0.150568	0.357	0.468	8.45E-57
WHSC1	5.66E-61	-0.2424	0.255	0.549	8.46E-57
CNTRL	5.74E-61	-0.27281	0.087	0.34	8.58E-57
SPNS1	5.93E-61	0.120824	0.309	0.423	8.86E-57
RRAGD	5.93E-61	-0.25785	0.057	0.29	8.86E-57
ITGAE	6.11E-61	-0.36768	0.358	0.707	9.14E-57
HRAS	6.21E-61	0.1744	0.497	0.583	9.29E-57
NACC1	6.36E-61	-0.2345	0.074	0.305	9.51E-57
APPL1	6.50E-61	-0.15067	0.261	0.516	9.72E-57
ELP6	6.54E-61	-0.17767	0.216	0.473	9.78E-57
GPBP1	6.57E-61	0.193473	0.631	0.693	9.82E-57
MGEA5	6.68E-61	0.352412	0.364	0.335	9.98E-57
SOCS6	6.76E-61	-0.2768	0.09	0.341	1.01E-56
UBXN6	6.81E-61	0.100884	0.288	0.419	1.02E-56
RBBP7	7.23E-61	-0.41586	0.408	0.768	1.08E-56
LMNA	7.58E-61	-0.60738	0.219	0.578	1.13E-56
ZNF667-AS	7.87E-61	0.219761	0.621	0.672	1.18E-56
BCL7B	8.57E-61	0.1177	0.452	0.58	1.28E-56
PTGFRN	8.66E-61	-0.31426	0.027	0.253	1.29E-56
CWC27	8.69E-61	0.186445	0.4	0.49	1.30E-56
PRR13	8.77E-61	-0.32086	0.224	0.53	1.31E-56
SNAP23	8.85E-61	-0.32039	0.039	0.274	1.32E-56
PDHX	8.86E-61	0.20248	0.201	0.253	1.32E-56
COQ10B	9.13E-61	0.232713	0.302	0.351	1.36E-56
APOA1BP	1.00E-60	-0.30029	0.308	0.631	1.50E-56
CNOT3	1.05E-60	-0.1271	0.159	0.378	1.58E-56
LAMA5	1.10E-60	-0.16152	0.063	0.259	1.64E-56
POLR2A	1.12E-60	-0.11225	0.193	0.417	1.68E-56
RPF1	1.13E-60	-0.21084	0.185	0.448	1.69E-56
RNF187	1.16E-60	0.305839	0.543	0.568	1.73E-56
ATP6AP1	1.18E-60	0.206222	0.487	0.565	1.76E-56
FAM32A	1.19E-60	0.199083	0.618	0.684	1.78E-56
ZBTB7A	1.27E-60	-0.1018	0.13	0.328	1.90E-56
FAM92A1	1.37E-60	0.203423	0.368	0.446	2.05E-56
ARMC1	1.40E-60	0.167795	0.447	0.552	2.09E-56
DDX21	1.46E-60	-0.12489	0.176	0.402	2.19E-56
FYTTD1	1.52E-60	0.102989	0.352	0.484	2.27E-56
NECAP2	1.59E-60	-0.16446	0.154	0.39	2.38E-56
TWF1	1.62E-60	-0.11522	0.244	0.47	2.43E-56
DCTN1	1.67E-60	0.259128	0.334	0.365	2.50E-56

RAB1B	1.77E-60	-0.14458	0.148	0.373	2.65E-56
TBL1XR1	1.82E-60	0.115858	0.333	0.452	2.72E-56
DCP2	1.88E-60	-0.2912	0.124	0.394	2.80E-56
DCP1A	2.13E-60	0.331583	0.378	0.372	3.18E-56
NECAP1	2.15E-60	0.478476	0.412	0.295	3.21E-56
ZNF738	2.18E-60	-0.21605	0.218	0.491	3.26E-56
PNKD	2.24E-60	-0.24361	0.411	0.726	3.34E-56
TCF25	2.35E-60	0.149234	0.617	0.725	3.51E-56
BCAP29	2.37E-60	-0.22133	0.29	0.574	3.54E-56
FSD1	2.38E-60	0.196623	0.313	0.381	3.55E-56
CKAP5	2.44E-60	-0.40309	0.174	0.494	3.64E-56
ARHGEF26	2.47E-60	-0.34649	0.054	0.3	3.69E-56
UBXN1	2.51E-60	0.234071	0.687	0.723	3.75E-56
MAFG	2.55E-60	0.12284	0.215	0.336	3.81E-56
ARID5A	2.89E-60	-0.22482	0.101	0.337	4.32E-56
TMEM128	2.96E-60	0.134279	0.355	0.464	4.42E-56
ROGDI	3.00E-60	0.338231	0.302	0.281	4.48E-56
MAGED1	3.03E-60	0.208261	0.584	0.635	4.52E-56
ILK	3.04E-60	-0.27566	0.202	0.49	4.54E-56
VGLL4	3.04E-60	-0.11448	0.332	0.58	4.55E-56
DYNLT1	3.33E-60	0.272726	0.871	0.838	4.98E-56
STAU2	3.49E-60	0.392034	0.317	0.265	5.22E-56
RFC2	3.52E-60	-0.48852	0.252	0.605	5.25E-56
DNTTIP2	3.58E-60	0.229165	0.372	0.43	5.34E-56
APBB2	3.64E-60	-0.38731	0.072	0.337	5.44E-56
PRPF40A	3.67E-60	0.106074	0.588	0.721	5.49E-56
EXOC5	3.80E-60	-0.13642	0.185	0.415	5.68E-56
GSTM3	3.97E-60	0.328644	0.467	0.442	5.94E-56
HNRNPUL1	4.00E-60	-0.28924	0.401	0.725	5.97E-56
ADAM17	4.07E-60	-0.1391	0.159	0.386	6.08E-56
SNAPIN	4.15E-60	-0.17129	0.349	0.626	6.20E-56
ZC3H15	4.19E-60	0.235004	0.694	0.726	6.26E-56
CHMP7	4.34E-60	0.334656	0.318	0.3	6.48E-56
WDR5	4.40E-60	-0.10866	0.124	0.322	6.58E-56
BUB3	4.55E-60	-0.40703	0.302	0.648	6.81E-56
GALNT1	4.89E-60	-0.22194	0.122	0.369	7.30E-56
COA3	4.92E-60	0.17652	0.596	0.691	7.36E-56
APC2	4.92E-60	0.557961	0.306	0.163	7.36E-56
CAPRIN2	5.08E-60	-0.20144	0.058	0.268	7.58E-56
ACTR3	5.16E-60	0.214207	0.665	0.7	7.71E-56
6-Sep	5.22E-60	0.472939	0.218	0.101	7.81E-56
ALDH6A1	5.60E-60	-0.2392	0.104	0.347	8.37E-56
PDZRN4	5.71E-60	0.70339	0.286	0.035	8.53E-56
NRBP1	5.78E-60	-0.10333	0.324	0.56	8.63E-56
WDR82	5.78E-60	0.253092	0.429	0.453	8.64E-56
ATCAY	5.79E-60	0.622585	0.475	0.221	8.65E-56
DPP7	5.91E-60	-0.23206	0.227	0.502	8.84E-56
HMOX2	6.02E-60	0.22055	0.324	0.39	9.00E-56
YWHAH	6.28E-60	0.260118	0.809	0.81	9.39E-56
RUNX1	6.33E-60	-0.28023	0.017	0.225	9.45E-56
HSPA14	6.40E-60	-0.24163	0.101	0.347	9.56E-56

TRMU	6.49E-60	-0.14316	0.13	0.353	9.69E-56
MAP2K7	6.55E-60	0.16585	0.262	0.354	9.78E-56
7-Mar	6.55E-60	0.114779	0.334	0.457	9.79E-56
ENDOD1	6.60E-60	-0.30148	0.024	0.241	9.87E-56
C1orf123	6.66E-60	-0.17863	0.342	0.619	9.96E-56
DERL1	6.78E-60	0.23508	0.357	0.409	1.01E-55
RHOT2	6.82E-60	0.135777	0.313	0.421	1.02E-55
URM1	6.92E-60	-0.11992	0.266	0.507	1.03E-55
MAPK1IP1L	7.13E-60	-0.21388	0.328	0.616	1.07E-55
FAM120AOS	7.22E-60	-0.24989	0.117	0.374	1.08E-55
PREPL	7.77E-60	0.227195	0.25	0.305	1.16E-55
HGSNAT	8.18E-60	0.329765	0.295	0.3	1.22E-55
METTL4	8.50E-60	-0.14801	0.06	0.248	1.27E-55
SMARCD1	8.55E-60	0.106382	0.326	0.456	1.28E-55
PTTG1IP	8.76E-60	0.157551	0.452	0.546	1.31E-55
RBM25	8.80E-60	0.14673	0.576	0.69	1.31E-55
ANKRD46	9.23E-60	0.537124	0.417	0.252	1.38E-55
TM7SF3	9.56E-60	-0.21592	0.192	0.458	1.43E-55
ZFYVE21	9.58E-60	-0.24807	0.138	0.393	1.43E-55
MAP3K13	9.85E-60	0.157778	0.541	0.633	1.47E-55
UNC119	9.91E-60	0.153214	0.272	0.367	1.48E-55
ARFGAP2	1.01E-59	0.269507	0.423	0.454	1.51E-55
DAXX	1.02E-59	-0.25499	0.179	0.452	1.52E-55
STARD3NL	1.02E-59	0.195792	0.59	0.647	1.53E-55
FAT1	1.03E-59	-0.23666	0.011	0.194	1.53E-55
PLAT	1.05E-59	-0.38875	0.031	0.268	1.57E-55
RGMB	1.06E-59	0.675636	0.412	0.22	1.58E-55
CFLAR	1.11E-59	-0.28019	0.123	0.383	1.67E-55
TMEM5	1.13E-59	0.254968	0.288	0.325	1.69E-55
SKA2	1.16E-59	-0.49562	0.37	0.735	1.74E-55
EIF3H	1.19E-59	0.276704	0.754	0.765	1.78E-55
HLA-E	1.20E-59	-0.67109	0.135	0.462	1.80E-55
HTATSF1	1.34E-59	0.21458	0.474	0.531	2.01E-55
TSPYL1	1.37E-59	0.314937	0.347	0.332	2.05E-55
SPTAN1	1.37E-59	0.289594	0.428	0.438	2.05E-55
SRGAP3	1.39E-59	0.626217	0.39	0.181	2.08E-55
FAM57A	1.40E-59	-0.20476	0.049	0.258	2.10E-55
DCAF15	1.44E-59	-0.23532	0.095	0.331	2.15E-55
CD276	1.51E-59	-0.17711	0.166	0.407	2.26E-55
E2F6	1.54E-59	-0.15298	0.108	0.32	2.30E-55
ELOVL1	1.64E-59	-0.32059	0.049	0.29	2.46E-55
GALNT11	1.69E-59	0.297263	0.374	0.367	2.53E-55
C1QTNF4	1.74E-59	0.666154	0.298	0.054	2.60E-55
DR1	1.75E-59	-0.1481	0.236	0.48	2.61E-55
TPM1	1.75E-59	-0.26902	0.187	0.467	2.62E-55
SFRP1	1.79E-59	-0.3441	0.031	0.259	2.67E-55
PRDX3	1.88E-59	-0.2101	0.295	0.573	2.80E-55
SLC25A33	1.93E-59	-0.11954	0.206	0.431	2.88E-55
DLG1	1.94E-59	-0.22088	0.082	0.311	2.90E-55
TUBB4A	2.02E-59	0.692961	0.386	0.101	3.01E-55
TRIM24	2.05E-59	0.13433	0.339	0.451	3.06E-55

MBOAT7	2.12E-59	-0.30928	0.126	0.398	3.17E-55
DNAJC15	2.13E-59	-0.24687	0.243	0.526	3.18E-55
CDH11	2.20E-59	-0.26879	0.046	0.263	3.28E-55
PITPNC1	2.20E-59	-0.20002	0.078	0.296	3.28E-55
DPY19L1	2.20E-59	-0.24665	0.02	0.214	3.29E-55
FLAD1	2.21E-59	0.230943	0.334	0.389	3.30E-55
RAB5A	2.29E-59	0.219721	0.529	0.584	3.42E-55
CDKN1B	2.30E-59	0.173138	0.452	0.536	3.44E-55
EXTL3	2.32E-59	-0.16264	0.101	0.315	3.47E-55
AKT1S1	2.33E-59	-0.14394	0.179	0.406	3.48E-55
TMEM9B	2.35E-59	0.119048	0.523	0.642	3.52E-55
RGS19	2.36E-59	-0.29865	0.051	0.288	3.52E-55
UBL7-AS1	2.40E-59	-0.30787	0.03	0.251	3.59E-55
GDAP1	2.42E-59	0.608752	0.47	0.221	3.62E-55
GNAQ	2.49E-59	0.212438	0.388	0.437	3.73E-55
MIDN	2.55E-59	0.243187	0.6	0.643	3.80E-55
SHMT2	2.57E-59	-0.41001	0.118	0.411	3.84E-55
LENG1	2.63E-59	-0.28676	0.121	0.381	3.93E-55
RP11-553L	2.71E-59	0.199824	0.389	0.475	4.04E-55
SAR1B	2.80E-59	-0.16389	0.221	0.469	4.18E-55
NTPCR	2.82E-59	-0.19151	0.182	0.435	4.21E-55
USP34	2.85E-59	0.126824	0.254	0.36	4.25E-55
STAMBP	2.86E-59	-0.13435	0.252	0.494	4.28E-55
COL22A1	2.88E-59	-0.251	0.002	0.175	4.31E-55
TMX3	2.93E-59	-0.20422	0.091	0.319	4.38E-55
KLHDC8A	2.97E-59	0.319374	0.6	0.567	4.44E-55
TP53RK	3.09E-59	-0.14544	0.154	0.377	4.62E-55
ATP6V1A	3.26E-59	0.429975	0.328	0.231	4.87E-55
TMEM138	3.29E-59	-0.18258	0.12	0.346	4.92E-55
ABHD17A	3.31E-59	-0.26725	0.155	0.422	4.95E-55
CD164	3.33E-59	-0.30112	0.211	0.505	4.97E-55
SMC1A	3.36E-59	-0.26753	0.241	0.533	5.02E-55
RPAP2	3.51E-59	0.281127	0.316	0.335	5.25E-55
PPME1	3.82E-59	0.140805	0.288	0.39	5.71E-55
SIGMAR1	4.15E-59	-0.25182	0.138	0.401	6.20E-55
ADD3	4.17E-59	-0.31232	0.036	0.259	6.22E-55
NTHL1	4.47E-59	-0.20372	0.186	0.441	6.68E-55
MRGBP	4.49E-59	-0.15408	0.126	0.346	6.71E-55
RALY	4.79E-59	-0.42527	0.443	0.798	7.15E-55
GRIA3	4.79E-59	-0.22451	0.115	0.358	7.16E-55
REEP1	5.29E-59	0.588834	0.357	0.148	7.90E-55
CHCHD6	5.59E-59	0.183877	0.328	0.415	8.36E-55
HARS	5.65E-59	0.213834	0.401	0.459	8.44E-55
UQCRC2	5.75E-59	0.26314	0.584	0.595	8.59E-55
ANAPC16	5.95E-59	-0.21672	0.275	0.552	8.90E-55
LSM4	6.13E-59	-0.41505	0.668	0.904	9.16E-55
VPS26B	6.28E-59	0.186187	0.367	0.438	9.39E-55
SMAD5	6.46E-59	-0.26903	0.126	0.388	9.66E-55
NDUFAF4	6.52E-59	0.291883	0.324	0.338	9.74E-55
XPOT	6.56E-59	-0.15851	0.103	0.315	9.81E-55
OSTC	6.81E-59	-0.27579	0.339	0.651	1.02E-54

EAPP	7.40E-59	0.240137	0.42	0.488	1.11E-54
FAM122B	7.59E-59	-0.31548	0.062	0.305	1.13E-54
POLDIP3	7.62E-59	-0.1162	0.192	0.416	1.14E-54
DERA	7.63E-59	-0.31381	0.16	0.443	1.14E-54
HMBS	7.88E-59	-0.11193	0.167	0.379	1.18E-54
PRPF19	7.96E-59	-0.20028	0.212	0.473	1.19E-54
PSMC2	8.06E-59	0.149196	0.569	0.658	1.21E-54
SLC25A23	8.39E-59	-0.1337	0.071	0.262	1.25E-54
GSDMD	8.46E-59	-0.25188	0.008	0.198	1.26E-54
YIF1B	8.46E-59	-0.1245	0.276	0.517	1.26E-54
PPT1	8.56E-59	-0.26236	0.306	0.614	1.28E-54
WNK1	8.62E-59	-0.10932	0.248	0.473	1.29E-54
PAK4	8.79E-59	-0.29733	0.101	0.36	1.31E-54
PFKL	8.88E-59	-0.138	0.267	0.51	1.33E-54
KIAA2013	9.07E-59	-0.1378	0.074	0.265	1.35E-54
BRWD1	9.97E-59	0.325543	0.407	0.388	1.49E-54
GOSR1	9.98E-59	0.155417	0.37	0.465	1.49E-54
PPAT	1.01E-58	-0.43464	0.113	0.411	1.51E-54
PRTFDC1	1.03E-58	-0.27248	0.124	0.38	1.53E-54
PFDN1	1.05E-58	0.150844	0.544	0.633	1.56E-54
NELFB	1.05E-58	-0.10947	0.141	0.351	1.57E-54
C5orf24	1.06E-58	-0.19623	0.293	0.569	1.58E-54
TNPO2	1.07E-58	-0.10398	0.203	0.42	1.60E-54
WDR60	1.09E-58	0.310968	0.397	0.4	1.62E-54
SDC3	1.10E-58	-0.23236	0.106	0.348	1.64E-54
PPP2R2A	1.11E-58	0.160105	0.41	0.51	1.66E-54
NUDCD2	1.12E-58	-0.39661	0.345	0.695	1.67E-54
PRKCA	1.12E-58	-0.11232	0.167	0.377	1.67E-54
CTSD	1.15E-58	-0.1081	0.342	0.588	1.71E-54
YPEL1	1.15E-58	0.114504	0.181	0.293	1.72E-54
CIAPIN1	1.16E-58	0.107551	0.381	0.51	1.74E-54
HCCS	1.22E-58	-0.21733	0.088	0.312	1.82E-54
SSR3	1.23E-58	-0.35262	0.299	0.628	1.84E-54
TRPT1	1.32E-58	-0.23619	0.154	0.41	1.97E-54
GLT8D1	1.32E-58	-0.14462	0.282	0.532	1.98E-54
TMEM199	1.38E-58	0.112708	0.298	0.422	2.06E-54
CRCP	1.54E-58	0.263954	0.307	0.332	2.31E-54
ZSCAN16-A	1.55E-58	-0.29595	0.162	0.441	2.31E-54
MTFR1L	1.57E-58	0.181565	0.376	0.451	2.34E-54
AP2A2	1.60E-58	0.260416	0.307	0.336	2.39E-54
POLR2D	1.63E-58	-0.21339	0.152	0.401	2.43E-54
LDLR	1.64E-58	-0.34245	0.015	0.222	2.45E-54
SNX14	1.65E-58	0.183655	0.282	0.356	2.47E-54
TTC1	1.67E-58	0.140314	0.375	0.483	2.49E-54
TRNAU1AP	1.67E-58	0.295279	0.32	0.336	2.50E-54
RSL1D1	1.69E-58	0.12115	0.537	0.658	2.53E-54
TMEM209	1.83E-58	-0.24019	0.087	0.32	2.74E-54
CEP57L1	1.88E-58	-0.19858	0.13	0.364	2.81E-54
TMEM179B	1.89E-58	-0.26608	0.207	0.49	2.83E-54
FAM127B	1.89E-58	0.368642	0.43	0.385	2.83E-54
RAD18	1.92E-58	-0.36699	0.041	0.28	2.87E-54

PSME2	1.93E-58	-0.52195	0.367	0.727	2.88E-54
ENOPH1	1.96E-58	-0.22051	0.43	0.731	2.93E-54
STXBP1	1.96E-58	0.503413	0.356	0.22	2.94E-54
ZNF32	2.00E-58	0.159995	0.266	0.365	2.99E-54
ENHO	2.12E-58	0.121231	0.271	0.391	3.16E-54
INTS10	2.17E-58	0.10091	0.322	0.451	3.25E-54
ZNF106	2.18E-58	-0.17044	0.148	0.377	3.26E-54
ASAP2	2.18E-58	-0.2215	0.089	0.32	3.26E-54
ACOT9	2.22E-58	-0.16512	0.056	0.251	3.32E-54
EXOSC10	2.23E-58	-0.24306	0.096	0.332	3.34E-54
COPS5	2.27E-58	0.117661	0.526	0.66	3.40E-54
TARS	2.30E-58	-0.10833	0.201	0.422	3.43E-54
ZC3HAV1	2.37E-58	-0.29088	0.035	0.253	3.54E-54
C1orf52	2.47E-58	0.220515	0.416	0.465	3.70E-54
JOSD2	2.49E-58	-0.11471	0.214	0.438	3.73E-54
NPEPPS	2.58E-58	0.229873	0.324	0.369	3.85E-54
DDB1	2.62E-58	0.146282	0.393	0.486	3.92E-54
PHF23	2.67E-58	0.144057	0.265	0.359	3.99E-54
PARP9	2.80E-58	-0.29665	0.008	0.194	4.18E-54
SNTA1	2.84E-58	-0.24117	0.016	0.212	4.24E-54
C6orf1	2.86E-58	0.157988	0.324	0.43	4.28E-54
PWP1	2.88E-58	0.196438	0.43	0.494	4.30E-54
NXPH4	2.92E-58	0.619749	0.256	0.117	4.36E-54
NQO1	2.93E-58	-0.27453	0.019	0.22	4.38E-54
DBNL	2.95E-58	0.128968	0.434	0.547	4.41E-54
KIF9	3.03E-58	-0.25519	0.102	0.348	4.53E-54
SF3B3	3.03E-58	-0.1526	0.218	0.459	4.53E-54
ADPGK	3.04E-58	-0.14223	0.104	0.31	4.54E-54
E2F8	3.06E-58	-0.30322	0.001	0.17	4.57E-54
STX10	3.09E-58	-0.2916	0.276	0.58	4.62E-54
CYB5B	3.09E-58	-0.15821	0.291	0.548	4.62E-54
TMSB15B	3.25E-58	0.161996	0.487	0.586	4.86E-54
RBM17	3.36E-58	-0.16562	0.347	0.62	5.03E-54
NBPF10	3.46E-58	-0.3177	0.037	0.264	5.17E-54
DCLRE1C	3.48E-58	-0.21459	0.147	0.395	5.21E-54
LONP1	3.78E-58	-0.10064	0.185	0.4	5.65E-54
MZT2A	3.97E-58	-0.22032	0.45	0.756	5.93E-54
HAUS1	4.03E-58	-0.3314	0.245	0.552	6.03E-54
SLC35B4	4.06E-58	0.354409	0.369	0.338	6.07E-54
BEND5	4.16E-58	0.411125	0.376	0.309	6.21E-54
EPN2	4.61E-58	-0.15187	0.223	0.462	6.89E-54
FAM229B	4.67E-58	0.234725	0.456	0.515	6.98E-54
KCNE4	4.70E-58	-0.2539	0.033	0.238	7.03E-54
SEMA6D	4.89E-58	-0.4099	0.079	0.352	7.30E-54
CPSF2	4.95E-58	-0.19779	0.056	0.263	7.40E-54
LZIC	5.01E-58	-0.20001	0.124	0.362	7.49E-54
ELAVL2	5.75E-58	0.666525	0.449	0.165	8.59E-54
DEXI	5.85E-58	0.230549	0.284	0.332	8.74E-54
METTL9	6.24E-58	0.26275	0.708	0.714	9.33E-54
CMC1	6.52E-58	-0.22738	0.179	0.44	9.75E-54
NUDT3	6.61E-58	0.104254	0.463	0.583	9.88E-54

CCHCR1	7.00E-58	-0.23064	0.064	0.284	1.05E-53
ANAPC13	7.01E-58	0.278671	0.529	0.525	1.05E-53
AKT1	7.21E-58	-0.1296	0.1	0.296	1.08E-53
MUM1	7.60E-58	0.187508	0.513	0.59	1.14E-53
DCLK2	7.75E-58	0.372121	0.426	0.378	1.16E-53
HEXA	8.50E-58	-0.13952	0.162	0.375	1.27E-53
SIK3	8.52E-58	-0.22225	0.038	0.241	1.27E-53
OXLD1	9.06E-58	-0.24542	0.159	0.42	1.35E-53
STARD4-AS	9.10E-58	0.47527	0.357	0.249	1.36E-53
GOLIM4	9.41E-58	-0.33691	0.27	0.59	1.41E-53
MLLT4	9.62E-58	0.314986	0.334	0.348	1.44E-53
CHAMP1	9.91E-58	-0.19578	0.122	0.356	1.48E-53
EFCAB11	1.01E-57	-0.31201	0.022	0.232	1.50E-53
WDR6	1.04E-57	0.365542	0.369	0.335	1.55E-53
TECR	1.05E-57	-0.53558	0.396	0.747	1.56E-53
NQO2	1.05E-57	0.116927	0.262	0.388	1.56E-53
RAP1A	1.06E-57	-0.20661	0.239	0.5	1.59E-53
STK39	1.15E-57	-0.22205	0.052	0.258	1.71E-53
KDELR1	1.15E-57	-0.25206	0.391	0.698	1.72E-53
SOCS2	1.19E-57	-0.41324	0.093	0.36	1.77E-53
BLVRA	1.22E-57	0.302625	0.549	0.558	1.83E-53
PLEKHB1	1.27E-57	-0.33334	0.059	0.302	1.90E-53
LRP10	1.38E-57	-0.26197	0.047	0.265	2.07E-53
GIPC1	1.43E-57	-0.24592	0.217	0.491	2.14E-53
UACA	1.45E-57	-0.23435	0.014	0.2	2.16E-53
DEAF1	1.46E-57	0.162518	0.318	0.41	2.18E-53
EZR	1.52E-57	0.151922	0.452	0.562	2.28E-53
G3BP1	1.55E-57	-0.19981	0.266	0.533	2.32E-53
NUDT21	1.63E-57	-0.10939	0.221	0.442	2.43E-53
UBE2A	1.65E-57	-0.1186	0.34	0.583	2.46E-53
CCT8	1.68E-57	0.173704	0.684	0.738	2.52E-53
SF3A1	1.72E-57	-0.20844	0.112	0.343	2.58E-53
JADE1	1.90E-57	-0.30966	0.14	0.409	2.85E-53
JOSD1	1.91E-57	-0.11141	0.088	0.274	2.85E-53
ZNF131	2.00E-57	0.235271	0.402	0.454	2.99E-53
BPHL	2.00E-57	0.357742	0.323	0.3	2.99E-53
ZNF791	2.06E-57	0.291823	0.415	0.432	3.07E-53
HOOK2	2.09E-57	0.302323	0.339	0.356	3.13E-53
RNMT	2.25E-57	0.226028	0.364	0.406	3.37E-53
ARHGEF2	2.26E-57	0.132623	0.385	0.504	3.38E-53
SEPHS1	2.37E-57	-0.2264	0.077	0.301	3.53E-53
BDP1	2.40E-57	0.188422	0.383	0.459	3.59E-53
LSAMP	2.45E-57	0.532394	0.407	0.257	3.66E-53
RP3-525N1	2.49E-57	0.563464	0.414	0.27	3.72E-53
TAF12	2.62E-57	-0.20798	0.266	0.537	3.92E-53
NEMF	2.78E-57	0.140961	0.368	0.481	4.16E-53
ABHD17C	2.79E-57	-0.22166	0.02	0.207	4.17E-53
SYT11	2.96E-57	0.188877	0.576	0.647	4.42E-53
DNTTIP1	2.97E-57	-0.24027	0.294	0.584	4.44E-53
IVD	3.07E-57	-0.19829	0.079	0.295	4.59E-53
ARID5B	3.11E-57	0.175568	0.257	0.344	4.64E-53

TMSB15A	3.17E-57	0.315382	0.657	0.635	4.74E-53
KHDRBS1	3.22E-57	0.173876	0.797	0.84	4.80E-53
PIK3R3	3.27E-57	0.334431	0.352	0.331	4.89E-53
MSRA	3.40E-57	0.262898	0.408	0.446	5.07E-53
RTF1	3.52E-57	0.162017	0.621	0.712	5.26E-53
UBTD1	3.54E-57	-0.24371	0.016	0.202	5.29E-53
ZNF302	3.62E-57	0.148529	0.471	0.568	5.41E-53
ZNF580	3.63E-57	-0.22092	0.247	0.522	5.42E-53
UBE2G1	3.77E-57	0.19191	0.354	0.431	5.64E-53
AP1S2	3.79E-57	0.266668	0.708	0.723	5.67E-53
HINT2	3.97E-57	-0.43014	0.266	0.606	5.93E-53
TNIP2	4.19E-57	-0.17539	0.084	0.291	6.26E-53
ABCD3	4.19E-57	-0.20459	0.187	0.438	6.26E-53
MEIS3	4.20E-57	0.447144	0.314	0.226	6.27E-53
SMAD1	4.42E-57	-0.21326	0.159	0.411	6.60E-53
JAGN1	4.52E-57	-0.10074	0.279	0.509	6.75E-53
TIMM9	4.67E-57	-0.16918	0.237	0.486	6.97E-53
LDLRAD3	4.76E-57	-0.13505	0.147	0.359	7.11E-53
COLGALT1	4.86E-57	-0.12711	0.08	0.264	7.27E-53
RP11-698N	4.90E-57	-0.32493	0.006	0.193	7.33E-53
DCBLD2	4.91E-57	-0.34819	0.079	0.338	7.33E-53
C11orf96	5.08E-57	0.409683	0.346	0.306	7.60E-53
STT3A	5.63E-57	-0.1082	0.125	0.317	8.42E-53
EPN1	5.73E-57	-0.17738	0.299	0.558	8.57E-53
SAC3D1	5.81E-57	-0.29652	0.233	0.527	8.68E-53
HOXC9	5.90E-57	0.691814	0.43	0.151	8.82E-53
HLTF	5.95E-57	0.118381	0.291	0.406	8.89E-53
APBB1	6.06E-57	0.299352	0.328	0.322	9.05E-53
PET117	6.22E-57	-0.1037	0.149	0.353	9.29E-53
FBXL5	6.23E-57	0.106643	0.315	0.436	9.31E-53
ACLY	6.27E-57	-0.10474	0.19	0.401	9.37E-53
NXT1	6.87E-57	0.10199	0.444	0.573	1.03E-52
EPM2AIP1	7.18E-57	0.242005	0.332	0.372	1.07E-52
NME3	7.29E-57	0.116838	0.358	0.478	1.09E-52
LSM14A	7.30E-57	-0.19423	0.401	0.681	1.09E-52
NUDT2	7.51E-57	-0.13228	0.139	0.352	1.12E-52
NIFK	7.79E-57	-0.10048	0.361	0.599	1.16E-52
MIEN1	8.10E-57	0.212137	0.583	0.642	1.21E-52
SSR1	8.21E-57	-0.15072	0.35	0.601	1.23E-52
GCLM	8.23E-57	-0.13516	0.119	0.325	1.23E-52
PARP14	8.43E-57	-0.25859	0.003	0.177	1.26E-52
KIAA1191	8.46E-57	0.176704	0.378	0.46	1.26E-52
C16orf87	8.56E-57	0.113362	0.329	0.453	1.28E-52
SUPT4H1	8.84E-57	0.109156	0.489	0.614	1.32E-52
VTA1	8.90E-57	0.127501	0.252	0.356	1.33E-52
CEP112	1.02E-56	-0.24592	0.015	0.207	1.53E-52
CADM4	1.06E-56	0.188954	0.445	0.517	1.58E-52
COL6A1	1.06E-56	-0.39166	0.119	0.402	1.59E-52
PTOV1	1.15E-56	0.219519	0.769	0.789	1.72E-52
CHAF1B	1.16E-56	-0.35733	0.023	0.237	1.73E-52
YIPF6	1.22E-56	0.152719	0.377	0.486	1.83E-52

SCCPDH	1.27E-56	-0.22753	0.368	0.659	1.90E-52
CUL1	1.31E-56	0.245887	0.386	0.417	1.96E-52
PBRM1	1.33E-56	0.205009	0.436	0.486	1.98E-52
SPG20	1.48E-56	-0.27036	0.13	0.386	2.21E-52
ZCCHC24	1.49E-56	-0.29665	0.006	0.185	2.22E-52
DFFA	1.59E-56	-0.16005	0.176	0.404	2.37E-52
ACTR1A	1.65E-56	0.262622	0.399	0.414	2.46E-52
GLIPR1	1.70E-56	-0.30766	0.042	0.262	2.54E-52
MLEC	1.79E-56	-0.20611	0.364	0.648	2.68E-52
TAF11	1.88E-56	0.182326	0.405	0.486	2.82E-52
EI24	1.89E-56	0.122966	0.643	0.737	2.82E-52
EXOSC3	1.96E-56	-0.19269	0.132	0.363	2.92E-52
AES	2.00E-56	0.204848	0.686	0.749	2.98E-52
UBE2Q1	2.00E-56	-0.10994	0.124	0.315	2.98E-52
TMEM60	2.07E-56	-0.15712	0.24	0.484	3.09E-52
ELN	2.15E-56	-0.31957	0.098	0.344	3.21E-52
ING5	2.35E-56	-0.14784	0.118	0.33	3.51E-52
YTHDC1	2.41E-56	0.19755	0.467	0.53	3.60E-52
BAG1	2.47E-56	0.302115	0.437	0.43	3.70E-52
SNW1	2.48E-56	-0.12127	0.344	0.59	3.71E-52
CCDC137	2.51E-56	-0.24105	0.17	0.43	3.75E-52
COA1	2.56E-56	-0.18312	0.435	0.719	3.83E-52
NFATC2IP	2.68E-56	-0.19245	0.159	0.396	4.01E-52
HPCAL1	2.74E-56	0.440362	0.328	0.238	4.09E-52
PTPRS	2.87E-56	0.277431	0.499	0.542	4.29E-52
TRIM44	2.96E-56	0.143462	0.39	0.493	4.42E-52
MED17	3.13E-56	0.349653	0.366	0.325	4.68E-52
ZCCHC9	3.17E-56	-0.18345	0.064	0.262	4.74E-52
C19orf52	3.20E-56	-0.14698	0.161	0.38	4.78E-52
CRAT	3.23E-56	-0.15913	0.142	0.362	4.82E-52
FAM84A	3.26E-56	-0.33861	0.071	0.319	4.88E-52
TAF7	3.27E-56	0.181483	0.754	0.794	4.88E-52
EWSR1	3.29E-56	-0.14532	0.518	0.786	4.91E-52
RNF146	3.43E-56	0.365261	0.39	0.348	5.13E-52
ASCL1	3.52E-56	-0.30036	0.295	0.594	5.26E-52
NUDT22	3.53E-56	-0.2006	0.131	0.362	5.27E-52
ZDHHC24	3.54E-56	-0.21936	0.055	0.263	5.29E-52
CNRIP1	3.55E-56	0.314415	0.427	0.431	5.31E-52
VIMP	3.56E-56	-0.23827	0.319	0.611	5.32E-52
GARS	3.60E-56	0.254841	0.478	0.495	5.38E-52
EIF2S1	3.73E-56	-0.10377	0.329	0.568	5.57E-52
APITD1	3.87E-56	-0.28296	0.045	0.264	5.78E-52
POU3F3	3.92E-56	0.267941	0.338	0.365	5.86E-52
ARL6IP6	3.97E-56	-0.23765	0.377	0.674	5.93E-52
CHCHD3	3.98E-56	-0.26296	0.409	0.716	5.95E-52
HMG20B	4.28E-56	-0.31413	0.251	0.556	6.39E-52
ZNF714	4.31E-56	-0.29458	0.109	0.353	6.45E-52
SHISA4	4.40E-56	0.118372	0.257	0.368	6.57E-52
ORAI2	4.43E-56	0.175942	0.357	0.436	6.62E-52
PPM1A	4.45E-56	0.178634	0.236	0.316	6.66E-52
SRRM1	4.46E-56	0.12999	0.609	0.715	6.66E-52

SCAF11	4.47E-56	-0.14607	0.332	0.585	6.67E-52
NSA2	4.55E-56	-0.16432	0.334	0.598	6.79E-52
PDLIM1	4.56E-56	-0.27966	0.024	0.23	6.81E-52
PAM	4.68E-56	-0.16506	0.085	0.286	6.99E-52
FLOT2	4.76E-56	-0.14165	0.101	0.304	7.12E-52
REXO4	4.94E-56	0.126423	0.246	0.349	7.37E-52
PXDC1	5.08E-56	-0.167	0.079	0.279	7.60E-52
LIMA1	5.24E-56	-0.29786	0.372	0.689	7.84E-52
SCO1	5.31E-56	-0.12524	0.134	0.337	7.93E-52
MRPS25	5.32E-56	-0.10027	0.196	0.407	7.95E-52
SYNE2	5.48E-56	-0.1398	0.345	0.595	8.19E-52
COPS4	5.53E-56	0.275219	0.482	0.48	8.26E-52
SRSF10	5.80E-56	-0.27727	0.283	0.573	8.67E-52
FADS1	5.90E-56	-0.18129	0.225	0.477	8.81E-52
NSD1	5.99E-56	0.163903	0.332	0.419	8.95E-52
RUNDC1	6.27E-56	0.341686	0.269	0.23	9.37E-52
NEAT1	6.35E-56	-0.62327	0.328	0.669	9.49E-52
NSFL1C	6.35E-56	0.19863	0.537	0.604	9.50E-52
ZC3H14	6.41E-56	-0.13919	0.194	0.42	9.57E-52
G6PD	6.47E-56	-0.22375	0.121	0.359	9.67E-52
NOTCH3	6.49E-56	-0.22981	0.002	0.17	9.70E-52
CHRAC1	6.52E-56	-0.27437	0.085	0.319	9.75E-52
IGFBP4	6.93E-56	-0.35586	0.017	0.219	1.04E-51
H2AFY2	6.93E-56	-0.15001	0.045	0.217	1.04E-51
PRKRIR	6.96E-56	-0.14675	0.087	0.28	1.04E-51
MFSD12	7.48E-56	-0.23028	0.074	0.296	1.12E-51
FAM134A	7.98E-56	0.121466	0.39	0.504	1.19E-51
RNF157	8.01E-56	-0.12024	0.164	0.374	1.20E-51
CPNE2	8.05E-56	-0.4394	0.108	0.391	1.20E-51
FAM57B	8.44E-56	0.629073	0.394	0.157	1.26E-51
IRAK1	8.54E-56	-0.24927	0.06	0.279	1.28E-51
PHF1	8.58E-56	-0.15448	0.133	0.348	1.28E-51
EFNB3	8.64E-56	0.512551	0.247	0.099	1.29E-51
HAUS2	8.71E-56	-0.25168	0.052	0.268	1.30E-51
ARL2	8.80E-56	-0.23183	0.307	0.591	1.31E-51
PYCR1	9.36E-56	-0.17725	0.185	0.421	1.40E-51
ST3GAL4	9.41E-56	-0.26669	0.099	0.335	1.41E-51
TOPORS-AS	1.01E-55	-0.16338	0.075	0.269	1.51E-51
FAM228B	1.05E-55	0.312453	0.349	0.344	1.56E-51
STXBP3	1.06E-55	-0.32705	0.053	0.285	1.58E-51
ACYP2	1.10E-55	0.128686	0.29	0.411	1.64E-51
IGSF8	1.13E-55	0.403048	0.317	0.256	1.69E-51
ISOC2	1.14E-55	-0.35394	0.21	0.512	1.70E-51
CCDC25	1.20E-55	-0.11101	0.304	0.537	1.79E-51
FARSA	1.21E-55	-0.19826	0.286	0.553	1.80E-51
CDC42EP3	1.23E-55	0.575462	0.348	0.158	1.83E-51
SRGAP2	1.25E-55	-0.26177	0.112	0.358	1.87E-51
SMARCA4	1.26E-55	0.13096	0.65	0.748	1.88E-51
HSPH1	1.37E-55	0.120855	0.5	0.61	2.05E-51
NAT14	1.39E-55	-0.18957	0.159	0.394	2.08E-51
CIZ1	1.41E-55	-0.18704	0.158	0.395	2.11E-51

RP11-798M	1.42E-55	-0.15184	0.132	0.34	2.13E-51
GOLGB1	1.45E-55	0.277356	0.303	0.319	2.16E-51
GADD45G	1.45E-55	0.580907	0.677	0.364	2.17E-51
TMEM18	1.45E-55	-0.27932	0.147	0.409	2.17E-51
ZBTB38	1.46E-55	0.359168	0.266	0.219	2.18E-51
DDT	1.49E-55	-0.31138	0.397	0.721	2.22E-51
RNF114	1.56E-55	-0.3334	0.311	0.631	2.33E-51
POP5	1.57E-55	-0.15635	0.278	0.523	2.35E-51
LAP3	1.58E-55	-0.37606	0.223	0.528	2.36E-51
NUP85	1.62E-55	-0.28948	0.086	0.33	2.42E-51
CD2BP2	1.73E-55	0.109917	0.329	0.457	2.59E-51
KIF3B	1.75E-55	0.175053	0.189	0.251	2.61E-51
PCYT2	1.83E-55	-0.1225	0.112	0.307	2.74E-51
RFT1	1.87E-55	-0.23398	0.068	0.285	2.79E-51
DNAJC3	1.91E-55	-0.13392	0.17	0.383	2.85E-51
RAMP2	1.93E-55	0.413894	0.302	0.264	2.89E-51
FAM103A1	2.06E-55	-0.22078	0.17	0.411	3.08E-51
E2F5	2.21E-55	-0.14243	0.027	0.193	3.31E-51
TM9SF3	2.23E-55	-0.16893	0.108	0.319	3.33E-51
CAPN2	2.28E-55	-0.18905	0.2	0.442	3.41E-51
NDC1	2.44E-55	-0.28605	0.032	0.242	3.65E-51
A1BG	2.48E-55	-0.19227	0.167	0.401	3.71E-51
GMPPA	2.58E-55	-0.12421	0.093	0.28	3.85E-51
SNX4	2.63E-55	0.301715	0.345	0.346	3.93E-51
PRAF2	2.69E-55	0.163803	0.426	0.522	4.01E-51
KXD1	2.79E-55	-0.35047	0.322	0.652	4.17E-51
CTXN1	2.93E-55	0.185964	0.277	0.349	4.38E-51
HRK	2.93E-55	0.590104	0.229	0.084	4.39E-51
SEC22C	2.96E-55	-0.20314	0.207	0.458	4.43E-51
CIR1	3.02E-55	0.247043	0.399	0.426	4.51E-51
PNN	3.03E-55	-0.17343	0.447	0.72	4.53E-51
MSI2	3.07E-55	-0.32148	0.306	0.619	4.59E-51
MACROD1	3.45E-55	-0.17505	0.099	0.307	5.16E-51
COPS7A	3.54E-55	-0.12794	0.215	0.441	5.28E-51
NTN1	3.80E-55	-0.18319	0.033	0.212	5.67E-51
USP13	3.92E-55	-0.24344	0.071	0.295	5.86E-51
CLPTM1	4.14E-55	-0.24138	0.279	0.562	6.19E-51
IDH3G	4.53E-55	0.127285	0.416	0.537	6.77E-51
CLIP3	4.64E-55	0.499327	0.368	0.244	6.94E-51
HCFC1R1	5.13E-55	0.47484	0.452	0.337	7.66E-51
DIDO1	5.16E-55	-0.13245	0.13	0.338	7.71E-51
ALKBH2	5.40E-55	-0.20305	0.176	0.422	8.07E-51
SASS6	5.45E-55	-0.24513	0.058	0.274	8.15E-51
AP3B1	5.48E-55	-0.23192	0.09	0.314	8.18E-51
AK4	5.53E-55	-0.39551	0.053	0.298	8.26E-51
CAPNS1	5.66E-55	-0.1698	0.247	0.499	8.45E-51
TMEM70	5.78E-55	0.224602	0.425	0.479	8.64E-51
CHST12	6.26E-55	0.299917	0.386	0.389	9.35E-51
LYAR	6.35E-55	-0.14673	0.157	0.377	9.49E-51
MAGOHB	6.37E-55	-0.26253	0.296	0.583	9.52E-51
NEURL1B	6.52E-55	-0.34928	0.03	0.246	9.74E-51

PAK7	6.89E-55	0.623864	0.291	0.046	1.03E-50
RP11-25K1	7.08E-55	0.442485	0.255	0.189	1.06E-50
RNF4	7.28E-55	-0.19099	0.167	0.406	1.09E-50
KRR1	7.39E-55	-0.15995	0.21	0.447	1.10E-50
PEMT	7.74E-55	-0.16698	0.185	0.417	1.16E-50
NUCKS1	7.76E-55	-0.38537	0.73	0.94	1.16E-50
RRAS	8.03E-55	-0.19899	0.003	0.156	1.20E-50
APH1A	8.29E-55	-0.24628	0.407	0.707	1.24E-50
HEY1	8.36E-55	-0.42883	0.229	0.549	1.25E-50
SIAH1	8.66E-55	0.274731	0.445	0.47	1.29E-50
ZNF3	8.92E-55	0.349138	0.463	0.425	1.33E-50
C1orf174	9.10E-55	-0.15562	0.137	0.351	1.36E-50
CTSF	9.37E-55	0.215113	0.334	0.389	1.40E-50
MBNL1	9.75E-55	-0.23678	0.083	0.31	1.46E-50
ODF2L	9.82E-55	0.430466	0.34	0.254	1.47E-50
RBM42	1.00E-54	-0.1967	0.392	0.669	1.49E-50
SUPT16H	1.05E-54	-0.18791	0.299	0.559	1.57E-50
YRDC	1.10E-54	0.197814	0.252	0.32	1.64E-50
TM7SF2	1.16E-54	0.394454	0.334	0.281	1.73E-50
HINT3	1.17E-54	0.212991	0.305	0.351	1.76E-50
HLA-B	1.20E-54	-0.64823	0.482	0.796	1.79E-50
HSPB11	1.24E-54	-0.27835	0.32	0.619	1.85E-50
BRD4	1.25E-54	-0.13951	0.324	0.575	1.86E-50
CHTF18	1.34E-54	-0.27531	0.051	0.272	2.00E-50
PRPF38A	1.36E-54	-0.11443	0.306	0.541	2.03E-50
GRIA2	1.36E-54	0.43403	0.513	0.407	2.03E-50
NAA38	1.39E-54	-0.26029	0.449	0.757	2.08E-50
CANT1	1.42E-54	-0.13936	0.177	0.393	2.11E-50
GPBP1L1	1.42E-54	-0.17943	0.103	0.309	2.12E-50
WIZ	1.45E-54	-0.12359	0.095	0.281	2.17E-50
PDIA4	1.51E-54	-0.26643	0.403	0.707	2.26E-50
RUFY2	1.56E-54	0.330152	0.287	0.263	2.32E-50
SUCLG1	1.56E-54	0.117872	0.528	0.648	2.33E-50
QDPR	1.56E-54	-0.12782	0.301	0.538	2.33E-50
TRMT61A	1.60E-54	-0.21722	0.063	0.274	2.39E-50
SLC25A1	1.67E-54	-0.25416	0.142	0.391	2.49E-50
SLC35B2	1.71E-54	-0.22254	0.315	0.594	2.56E-50
PAPOLA	1.74E-54	-0.2132	0.434	0.72	2.60E-50
ARHGEF12	1.76E-54	0.114219	0.203	0.3	2.62E-50
PDE4DIP	1.80E-54	0.528142	0.416	0.231	2.69E-50
UBA6	1.92E-54	0.163555	0.246	0.328	2.88E-50
RDH14	1.94E-54	0.117518	0.239	0.347	2.90E-50
FAM104B	1.95E-54	-0.23434	0.142	0.388	2.91E-50
ACBD5	2.01E-54	-0.24298	0.055	0.267	3.00E-50
EXOSC6	2.01E-54	-0.22545	0.022	0.204	3.01E-50
COMMD1	2.09E-54	0.17473	0.384	0.484	3.13E-50
RNF145	2.11E-54	0.123661	0.521	0.627	3.15E-50
ERCC1	2.19E-54	-0.215	0.351	0.631	3.28E-50
GPAA1	2.32E-54	-0.16674	0.399	0.665	3.46E-50
TMEM9	2.38E-54	0.142147	0.456	0.547	3.56E-50
DTWD1	2.38E-54	-0.14128	0.075	0.26	3.56E-50

FAM104A	2.39E-54	-0.12948	0.259	0.486	3.58E-50
ATP1B3	2.39E-54	0.163025	0.641	0.717	3.58E-50
CCDC107	2.41E-54	0.147745	0.452	0.557	3.60E-50
RP4-665J2	2.48E-54	0.298338	0.153	0.19	3.71E-50
NIF3L1	2.49E-54	-0.26221	0.115	0.359	3.71E-50
ATG12	2.54E-54	0.115364	0.418	0.535	3.80E-50
SYNGR2	2.67E-54	-0.12487	0.051	0.219	3.98E-50
GPI	2.70E-54	-0.18389	0.209	0.451	4.03E-50
FNDC3A	2.76E-54	-0.12321	0.145	0.346	4.12E-50
WRAP73	2.78E-54	-0.13819	0.112	0.311	4.16E-50
C9orf142	2.82E-54	-0.19816	0.398	0.683	4.22E-50
CASP6	2.95E-54	0.21772	0.254	0.311	4.42E-50
RCC2	3.00E-54	-0.17391	0.133	0.354	4.49E-50
SAP30L	3.02E-54	-0.30817	0.06	0.294	4.52E-50
TRIP10	3.13E-54	-0.23428	0.019	0.204	4.67E-50
ZCCHC10	3.45E-54	0.174282	0.294	0.368	5.16E-50
MYEF2	3.54E-54	-0.12587	0.26	0.488	5.28E-50
COMMD10	3.61E-54	-0.12584	0.146	0.353	5.39E-50
MTF2	3.67E-54	0.250022	0.439	0.472	5.49E-50
SECISBP2	3.76E-54	0.309489	0.372	0.364	5.62E-50
CDIPT	3.92E-54	0.242006	0.361	0.396	5.86E-50
ZNF90	3.96E-54	-0.31435	0.169	0.435	5.92E-50
TIMM23	4.05E-54	-0.10438	0.109	0.296	6.06E-50
STMN3	4.05E-54	0.110847	0.563	0.675	6.06E-50
LMCD1	4.08E-54	-0.26128	0.008	0.186	6.09E-50
SMC5	4.15E-54	-0.15037	0.172	0.388	6.20E-50
DDX41	4.23E-54	0.117478	0.283	0.388	6.32E-50
TMEM101	4.44E-54	-0.16903	0.098	0.305	6.64E-50
TMEM38B	4.56E-54	-0.25973	0.08	0.309	6.81E-50
MPC2	4.76E-54	0.100513	0.581	0.698	7.11E-50
CORO2B	4.86E-54	-0.24208	0.054	0.264	7.27E-50
FAM72D	4.88E-54	-0.26602	0.002	0.163	7.30E-50
A2M	5.04E-54	-0.35142	0.079	0.326	7.53E-50
RBM15B	5.16E-54	0.132091	0.272	0.359	7.70E-50
SF1	5.35E-54	0.153239	0.675	0.753	8.00E-50
CMIP	5.37E-54	0.498086	0.296	0.174	8.02E-50
PIGP	5.44E-54	-0.13117	0.095	0.289	8.13E-50
MGAT1	5.61E-54	-0.16883	0.126	0.344	8.38E-50
RIF1	5.72E-54	-0.1455	0.284	0.522	8.54E-50
NR2F6	6.24E-54	0.133112	0.214	0.311	9.32E-50
ENDOG	6.38E-54	0.151553	0.203	0.293	9.53E-50
PURA	6.41E-54	0.119668	0.279	0.379	9.58E-50
DDX11	6.53E-54	-0.29705	0.033	0.24	9.76E-50
HSBP1	6.55E-54	0.228216	0.892	0.874	9.79E-50
ZNF397	6.57E-54	0.302372	0.282	0.272	9.82E-50
CHL1	7.58E-54	-0.29595	0.084	0.323	1.13E-49
HERC2	7.77E-54	-0.12632	0.174	0.385	1.16E-49
RAB10	8.02E-54	0.11237	0.579	0.688	1.20E-49
LRRC61	8.29E-54	0.617839	0.281	0.043	1.24E-49
ZNF711	8.62E-54	0.426412	0.426	0.322	1.29E-49
PRPS2	8.73E-54	-0.27977	0.046	0.264	1.30E-49

RNASEH1-A	8.75E-54	0.125307	0.241	0.36	1.31E-49
ZMYM2	9.38E-54	0.312942	0.269	0.264	1.40E-49
ITM2C	9.38E-54	-0.19082	0.374	0.646	1.40E-49
DHX29	9.39E-54	0.172072	0.434	0.516	1.40E-49
CS	9.60E-54	-0.19432	0.123	0.349	1.43E-49
SRSF1	9.81E-54	-0.12167	0.493	0.743	1.47E-49
PIGS	9.85E-54	-0.12025	0.117	0.31	1.47E-49
DLX6	9.99E-54	0.717843	0.346	0.086	1.49E-49
TNRC6C	1.01E-53	0.254542	0.323	0.347	1.51E-49
IL17RC	1.03E-53	-0.19415	0.025	0.204	1.54E-49
LCORL	1.04E-53	-0.13138	0.104	0.296	1.55E-49
STAG1	1.09E-53	-0.16857	0.132	0.346	1.63E-49
PIGT	1.10E-53	-0.33817	0.277	0.586	1.64E-49
MCC	1.12E-53	-0.20111	0.076	0.283	1.67E-49
TBCD	1.13E-53	-0.10313	0.141	0.336	1.69E-49
FUNDC1	1.15E-53	0.166291	0.304	0.393	1.71E-49
KAT7	1.18E-53	-0.25865	0.113	0.356	1.76E-49
FNBP1	1.24E-53	0.153017	0.225	0.305	1.85E-49
GTF2B	1.24E-53	0.367908	0.362	0.302	1.85E-49
CPD	1.32E-53	-0.10989	0.096	0.275	1.97E-49
SEC14L1	1.33E-53	-0.26501	0.152	0.404	1.98E-49
ROCK2	1.34E-53	-0.11353	0.139	0.341	2.01E-49
USE1	1.36E-53	0.114628	0.357	0.479	2.03E-49
ZMAT3	1.41E-53	-0.27074	0.092	0.325	2.10E-49
POLD2	1.47E-53	-0.33931	0.448	0.775	2.20E-49
DESI1	1.52E-53	-0.16355	0.065	0.252	2.27E-49
NHLRC3	1.56E-53	0.158512	0.357	0.448	2.33E-49
SAP30	1.59E-53	-0.28608	0.249	0.535	2.38E-49
TMOD3	1.60E-53	-0.14781	0.062	0.246	2.38E-49
TAF1D	1.62E-53	0.147762	0.328	0.422	2.42E-49
CCDC150	1.64E-53	-0.28798	0.009	0.188	2.45E-49
PRPF6	1.72E-53	0.103079	0.472	0.598	2.57E-49
FAM212B	1.92E-53	-0.16725	0.101	0.304	2.87E-49
CTCF	1.98E-53	-0.12385	0.227	0.443	2.96E-49
CIB2	1.98E-53	-0.23877	0.058	0.27	2.96E-49
AQP4	2.00E-53	-0.51835	0.015	0.211	2.99E-49
ACTB	2.05E-53	0.138511	1	0.996	3.06E-49
SNRNP40	2.05E-53	-0.24174	0.352	0.64	3.06E-49
HMGN2	2.05E-53	-0.503	0.63	0.884	3.07E-49
KAT5	2.07E-53	0.250631	0.339	0.363	3.09E-49
CLDND1	2.11E-53	-0.13437	0.357	0.602	3.16E-49
E2F7	2.15E-53	-0.21836	0.001	0.153	3.22E-49
RHOQ	2.20E-53	-0.14705	0.049	0.226	3.29E-49
CCDC106	2.22E-53	-0.24268	0.162	0.412	3.32E-49
KDELR3	2.24E-53	-0.18509	0	0.148	3.34E-49
GPX7	2.27E-53	-0.24033	0.125	0.364	3.40E-49
FAM173A	2.31E-53	0.17918	0.312	0.386	3.45E-49
BCAR1	2.36E-53	0.164442	0.281	0.359	3.52E-49
CDCA7	2.42E-53	0.511597	0.468	0.331	3.62E-49
IFI35	2.49E-53	-0.27741	0.016	0.204	3.72E-49
PKN2	2.53E-53	-0.1219	0.189	0.4	3.78E-49

TMEM131	2.59E-53	-0.18691	0.093	0.301	3.87E-49
C1R	2.61E-53	-0.43193	0.021	0.227	3.90E-49
BROX	2.65E-53	-0.16333	0.138	0.348	3.95E-49
DIMT1	2.73E-53	-0.14646	0.132	0.335	4.07E-49
PPP1R11	2.83E-53	0.193692	0.407	0.467	4.22E-49
OSTM1	2.96E-53	0.125575	0.243	0.333	4.42E-49
PDPK1	3.05E-53	0.140331	0.278	0.373	4.55E-49
TSNAX	3.13E-53	0.190287	0.367	0.435	4.67E-49
HSPBP1	3.18E-53	-0.11086	0.315	0.542	4.75E-49
SPIN1	3.18E-53	0.231219	0.28	0.312	4.75E-49
TEAD1	3.25E-53	-0.16862	0.14	0.36	4.86E-49
IMMP1L	3.39E-53	-0.10457	0.311	0.536	5.07E-49
C11orf49	3.62E-53	0.118531	0.321	0.428	5.41E-49
ODF2	3.62E-53	-0.34442	0.137	0.405	5.41E-49
SUV39H2	3.77E-53	-0.27158	0.051	0.265	5.63E-49
BMPR2	3.82E-53	0.245745	0.285	0.314	5.71E-49
CAPZA1	3.94E-53	-0.1165	0.295	0.521	5.89E-49
VPS4B	3.97E-53	0.103716	0.242	0.349	5.93E-49
COTL1	4.06E-53	0.255255	0.542	0.554	6.06E-49
C11orf24	4.17E-53	-0.20173	0.078	0.285	6.23E-49
MAP2K1	4.24E-53	-0.2432	0.119	0.359	6.33E-49
DUSP12	4.34E-53	0.225393	0.388	0.433	6.48E-49
ASB8	4.42E-53	0.34973	0.374	0.326	6.61E-49
DLST	4.46E-53	-0.10618	0.108	0.29	6.67E-49
SDF4	4.52E-53	-0.2469	0.341	0.633	6.75E-49
KIAA1107	4.55E-53	0.62208	0.248	0.03	6.80E-49
DMWD	4.58E-53	-0.10593	0.132	0.325	6.85E-49
TDP1	4.61E-53	-0.24218	0.043	0.242	6.89E-49
AIMP1	4.65E-53	-0.12583	0.302	0.538	6.94E-49
TBC1D9B	4.82E-53	-0.1144	0.079	0.256	7.20E-49
C5orf34	4.88E-53	-0.35931	0.01	0.191	7.29E-49
NUP93	4.96E-53	-0.18574	0.181	0.415	7.42E-49
SHC1	5.08E-53	-0.22086	0.02	0.202	7.59E-49
SACS	5.69E-53	-0.20821	0.136	0.364	8.51E-49
TNRC6A	5.71E-53	0.167126	0.278	0.353	8.53E-49
DDAH1	5.81E-53	-0.2572	0.097	0.331	8.69E-49
WDR41	6.18E-53	-0.20855	0.168	0.404	9.24E-49
DDOST	6.21E-53	-0.13391	0.386	0.632	9.28E-49
BPGM	6.47E-53	0.236826	0.427	0.457	9.67E-49
PLAU	6.51E-53	-0.37062	0.014	0.206	9.73E-49
TRMT10C	6.79E-53	0.173349	0.361	0.442	1.01E-48
NAA10	6.84E-53	-0.17781	0.413	0.683	1.02E-48
SNAPC1	6.86E-53	-0.29298	0.097	0.337	1.03E-48
FANCG	7.45E-53	-0.28795	0.077	0.311	1.11E-48
LUC7L	7.84E-53	0.120502	0.361	0.472	1.17E-48
SCLT1	8.26E-53	-0.27193	0.06	0.283	1.23E-48
TLCD1	8.32E-53	-0.2471	0.001	0.156	1.24E-48
CERS4	8.61E-53	-0.13175	0.111	0.302	1.29E-48
MTHFD1L	8.92E-53	-0.15839	0.046	0.22	1.33E-48
RBM5	9.14E-53	0.276035	0.43	0.435	1.37E-48
3-Sep	9.59E-53	0.543546	0.278	0.138	1.43E-48

GLRX	9.72E-53	0.519366	0.357	0.204	1.45E-48
PRKACA	1.07E-52	-0.16237	0.104	0.307	1.60E-48
SNHG7	1.10E-52	-0.13634	0.326	0.56	1.65E-48
GEMIN6	1.19E-52	-0.20498	0.129	0.356	1.78E-48
UXT	1.20E-52	-0.11116	0.438	0.684	1.79E-48
IFNGR2	1.22E-52	-0.11992	0.276	0.501	1.82E-48
MEPCE	1.24E-52	-0.18914	0.091	0.302	1.85E-48
NCOA1	1.29E-52	0.157123	0.208	0.293	1.92E-48
LMO2	1.30E-52	-0.30624	0.027	0.228	1.95E-48
LINC00969	1.30E-52	0.214778	0.354	0.4	1.95E-48
GIGYF2	1.32E-52	0.152902	0.235	0.311	1.97E-48
PPP1R12C	1.32E-52	-0.12488	0.043	0.207	1.98E-48
PRIM2	1.34E-52	-0.35793	0.042	0.262	2.00E-48
GNG2	1.41E-52	0.460048	0.421	0.305	2.11E-48
PPP2R3B	1.52E-52	-0.20734	0.059	0.258	2.28E-48
NFATC3	1.52E-52	-0.1151	0.123	0.317	2.28E-48
RAB22A	1.61E-52	-0.18879	0.199	0.441	2.40E-48
BTG3	1.66E-52	-0.28573	0.464	0.774	2.48E-48
EYA2	1.68E-52	-0.23507	0.032	0.227	2.52E-48
VAT1	1.73E-52	0.388548	0.262	0.205	2.58E-48
L3HYPDH	1.78E-52	-0.24487	0.017	0.2	2.67E-48
EHMT2	1.82E-52	0.238298	0.35	0.391	2.72E-48
RPIA	1.83E-52	0.123365	0.225	0.317	2.73E-48
TMF1	1.83E-52	0.208288	0.278	0.323	2.74E-48
TMEM248	1.83E-52	-0.13578	0.203	0.425	2.74E-48
CCS	1.85E-52	0.163446	0.206	0.286	2.76E-48
KBTBD2	1.90E-52	-0.20244	0.122	0.343	2.84E-48
TRA2A	1.93E-52	0.195839	0.488	0.543	2.89E-48
DTNA	1.94E-52	-0.25902	0.12	0.358	2.89E-48
NRCAM	1.94E-52	-0.12827	0.332	0.574	2.90E-48
SREK1	1.99E-52	-0.12457	0.335	0.568	2.97E-48
FBXO3	2.11E-52	0.277744	0.302	0.3	3.15E-48
ZBTB8OS	2.25E-52	-0.20013	0.244	0.499	3.36E-48
RNF5	2.29E-52	0.108513	0.547	0.659	3.42E-48
CABIN1	2.31E-52	-0.15557	0.067	0.256	3.45E-48
PUF60	2.35E-52	0.202856	0.726	0.763	3.51E-48
BHLHE40	2.52E-52	-0.23731	0.011	0.179	3.77E-48
UBE2M	2.60E-52	-0.16668	0.22	0.453	3.89E-48
USP1	2.74E-52	-0.35327	0.378	0.706	4.10E-48
PET100	2.88E-52	-0.13388	0.46	0.71	4.30E-48
LINC00662	2.92E-52	0.328451	0.311	0.3	4.37E-48
FAM174A	2.94E-52	0.210413	0.25	0.306	4.40E-48
POLD3	2.99E-52	-0.37827	0.094	0.354	4.47E-48
CTNND1	3.25E-52	0.126535	0.223	0.312	4.86E-48
ATP6V1C1	3.38E-52	0.110572	0.207	0.31	5.05E-48
XIST	3.39E-52	0.730378	0.434	0.146	5.06E-48
RRS1	3.45E-52	-0.10939	0.085	0.258	5.16E-48
SPSB4	3.50E-52	-0.15883	0.058	0.242	5.23E-48
RAB14	3.59E-52	0.155098	0.521	0.6	5.36E-48
LYPLA2	3.60E-52	0.103017	0.214	0.315	5.38E-48
ERI2	3.66E-52	-0.21063	0.026	0.201	5.48E-48

MPP5	3.67E-52	-0.21967	0.028	0.211	5.48E-48
METTL23	3.70E-52	-0.18773	0.261	0.514	5.52E-48
TNFAIP8L1	3.90E-52	-0.36341	0.038	0.258	5.83E-48
TRMT1	3.91E-52	-0.11125	0.133	0.321	5.85E-48
PSENEN	3.96E-52	-0.10826	0.345	0.575	5.92E-48
ZMYND11	4.01E-52	-0.1285	0.121	0.314	6.00E-48
PDE4B	4.10E-52	-0.26735	0.107	0.338	6.12E-48
PAPSS1	4.10E-52	0.341715	0.452	0.409	6.13E-48
SRPK1	4.21E-52	0.123732	0.318	0.426	6.30E-48
CNPY2	4.31E-52	0.20765	0.731	0.767	6.44E-48
ATP5SL	4.33E-52	-0.13687	0.215	0.435	6.47E-48
CDKN1A	4.38E-52	-0.39237	0.088	0.332	6.54E-48
LRPAP1	4.55E-52	-0.23367	0.364	0.649	6.80E-48
GRK6	4.58E-52	-0.14301	0.144	0.352	6.84E-48
DHRS4L2	4.58E-52	-0.1545	0.119	0.319	6.84E-48
SUGP2	4.59E-52	-0.22534	0.322	0.596	6.86E-48
GRIPAP1	4.63E-52	0.180777	0.237	0.299	6.92E-48
BCAM	4.91E-52	-0.22782	0.035	0.223	7.34E-48
MSI1	5.00E-52	0.2214	0.263	0.307	7.47E-48
FUT8	5.05E-52	-0.13681	0.09	0.277	7.55E-48
RASSF8	5.42E-52	-0.24899	0.016	0.199	8.09E-48
UBE2E1	5.79E-52	-0.11258	0.394	0.626	8.66E-48
ASRGL1	5.99E-52	-0.19146	0.396	0.667	8.95E-48
LYRM7	6.02E-52	0.161863	0.324	0.395	8.99E-48
OSER1	6.20E-52	0.221648	0.375	0.419	9.26E-48
HIST1H1B	6.62E-52	-0.30214	0.002	0.162	9.90E-48
C1orf21	6.64E-52	-0.15575	0.208	0.438	9.92E-48
MED24	6.67E-52	0.187359	0.279	0.331	9.97E-48
PPIL1	6.89E-52	-0.1483	0.205	0.427	1.03E-47
SPPL2A	6.97E-52	-0.27385	0.044	0.248	1.04E-47
DKC1	7.17E-52	-0.15942	0.299	0.542	1.07E-47
DKK3	7.63E-52	0.124019	0.3	0.4	1.14E-47
FAIM2	7.65E-52	0.469777	0.383	0.26	1.14E-47
ACTR6	7.94E-52	0.30415	0.393	0.372	1.19E-47
TOLLIP	8.02E-52	0.1703	0.288	0.36	1.20E-47
MFAP1	8.24E-52	-0.20775	0.134	0.363	1.23E-47
GAS2L3	8.65E-52	-0.33076	0.098	0.347	1.29E-47
CIT	8.66E-52	-0.26034	0.036	0.236	1.29E-47
RECQL4	8.83E-52	-0.33445	0.026	0.232	1.32E-47
C7orf26	8.84E-52	0.29995	0.321	0.309	1.32E-47
MOCS2	9.14E-52	-0.23542	0.225	0.48	1.37E-47
PPM1B	9.30E-52	0.115116	0.423	0.533	1.39E-47
WIPF1	9.64E-52	-0.20626	0.049	0.246	1.44E-47
GMPS	9.72E-52	-0.17695	0.17	0.398	1.45E-47
UNK	9.79E-52	-0.17308	0.101	0.301	1.46E-47
NUP54	1.04E-51	-0.10146	0.209	0.414	1.55E-47
MED19	1.06E-51	0.217716	0.481	0.517	1.58E-47
TMED10	1.09E-51	-0.24247	0.382	0.674	1.63E-47
MCL1	1.14E-51	0.198505	0.57	0.631	1.71E-47
MIR7-3HG	1.18E-51	0.631234	0.215	0.011	1.76E-47
INPPL1	1.18E-51	-0.1676	0.016	0.172	1.76E-47

RAB8B	1.18E-51	-0.20567	0.048	0.241	1.77E-47
FZD3	1.23E-51	0.110584	0.466	0.577	1.84E-47
SH2B1	1.23E-51	0.169589	0.195	0.257	1.84E-47
BLOC1S2	1.25E-51	0.124083	0.346	0.438	1.87E-47
EBP	1.29E-51	-0.13279	0.233	0.458	1.93E-47
C8orf59	1.29E-51	-0.21385	0.364	0.646	1.93E-47
ELF2	1.32E-51	0.168141	0.244	0.311	1.97E-47
SH3PXD2B	1.36E-51	-0.16937	0.042	0.221	2.03E-47
KCNQ1OT1	1.37E-51	0.466791	0.572	0.426	2.04E-47
ANXA7	1.44E-51	-0.21323	0.138	0.36	2.15E-47
POLR3K	1.46E-51	-0.15743	0.233	0.463	2.19E-47
PRR4	1.48E-51	0.243711	0.406	0.441	2.21E-47
ATP6V1E1	1.49E-51	0.135269	0.411	0.527	2.23E-47
MSRB1	1.56E-51	-0.10018	0.173	0.363	2.33E-47
EFCAB14	1.57E-51	-0.19359	0.071	0.269	2.34E-47
ANAPC10	1.57E-51	-0.11633	0.141	0.337	2.35E-47
TROVE2	1.57E-51	0.113547	0.588	0.685	2.35E-47
TMEM55A	1.57E-51	0.181806	0.253	0.319	2.35E-47
CCDC6	1.58E-51	-0.10397	0.053	0.21	2.36E-47
MMP14	1.60E-51	-0.23789	0.042	0.228	2.40E-47
IARS2	1.62E-51	-0.11166	0.178	0.378	2.41E-47
RP11-111M	1.62E-51	-0.24604	0.068	0.277	2.42E-47
DRAM2	1.63E-51	-0.11042	0.146	0.34	2.44E-47
IFI27L2	1.76E-51	0.146742	0.39	0.507	2.63E-47
TTF2	1.83E-51	-0.25465	0.038	0.236	2.73E-47
METTL12	1.91E-51	-0.23279	0.076	0.288	2.85E-47
ZNF195	1.93E-51	0.360905	0.325	0.274	2.88E-47
HNRNPF	1.97E-51	-0.38294	0.374	0.705	2.94E-47
CHORDC1	2.00E-51	0.180556	0.259	0.32	2.99E-47
CARD8	2.03E-51	-0.23142	0.044	0.237	3.03E-47
NUDCD3	2.03E-51	0.315052	0.332	0.317	3.04E-47
CDC27	2.04E-51	-0.18055	0.154	0.37	3.04E-47
NAA50	2.08E-51	-0.10182	0.269	0.477	3.10E-47
KIAA0232	2.11E-51	0.286596	0.254	0.247	3.15E-47
UTRN	2.13E-51	-0.24241	0.01	0.185	3.18E-47
PRKRA	2.16E-51	0.147352	0.496	0.594	3.22E-47
CHAC2	2.46E-51	-0.29942	0.018	0.21	3.68E-47
HIST1H2BH	2.51E-51	-0.34926	0.009	0.184	3.75E-47
ACBD3	2.61E-51	0.149245	0.211	0.288	3.91E-47
UBE2W	2.64E-51	0.103322	0.201	0.305	3.95E-47
CCDC15	2.69E-51	-0.3102	0.03	0.232	4.03E-47
ADAM10	2.73E-51	-0.12675	0.069	0.242	4.07E-47
TPST1	2.86E-51	0.176131	0.342	0.401	4.28E-47
ABI1	2.89E-51	-0.17014	0.097	0.296	4.32E-47
TRADD	3.10E-51	-0.1311	0.03	0.183	4.63E-47
NACC2	3.19E-51	-0.22573	0.024	0.207	4.77E-47
CXXC1	3.24E-51	-0.13995	0.174	0.384	4.85E-47
TMEM161A	3.26E-51	-0.16537	0.097	0.295	4.87E-47
COPS8	3.32E-51	0.121224	0.664	0.767	4.96E-47
SNX17	3.33E-51	-0.12747	0.376	0.619	4.97E-47
JAKMIP2	3.33E-51	0.307194	0.288	0.274	4.98E-47

SBK1	3.35E-51	0.577914	0.333	0.14	5.01E-47
BTG2	3.37E-51	0.360304	0.511	0.449	5.04E-47
WRAP53	3.44E-51	-0.2004	0.093	0.304	5.14E-47
EPB41L4A-	3.55E-51	0.331015	0.39	0.37	5.30E-47
SH3GLB1	3.68E-51	-0.15426	0.321	0.564	5.49E-47
CBS	3.73E-51	-0.22839	0.137	0.37	5.57E-47
CBR1	3.88E-51	-0.13769	0.422	0.677	5.80E-47
PCNXL4	4.09E-51	-0.11736	0.103	0.285	6.11E-47
IGSF21	4.12E-51	0.685032	0.414	0.141	6.15E-47
PGM2L1	4.13E-51	0.378822	0.495	0.427	6.17E-47
FXYD6	4.13E-51	0.193316	0.915	0.902	6.17E-47
KIF21A	4.22E-51	0.245396	0.651	0.652	6.31E-47
FEZ2	4.35E-51	0.181259	0.539	0.604	6.49E-47
NDUFB6	4.38E-51	0.104597	0.624	0.731	6.55E-47
FOXO3	4.50E-51	0.246152	0.364	0.381	6.72E-47
SNX7	4.55E-51	-0.16959	0.126	0.335	6.80E-47
BHLHE41	4.58E-51	-0.27691	0.006	0.173	6.85E-47
CETN2	4.63E-51	0.253857	0.384	0.409	6.92E-47
C9orf114	4.81E-51	-0.22937	0.086	0.302	7.19E-47
KLHL24	5.03E-51	0.543216	0.315	0.146	7.52E-47
PTMS	5.05E-51	0.267091	0.951	0.856	7.55E-47
B4GALT3	5.36E-51	0.139377	0.29	0.37	8.02E-47
CAMTA1	5.36E-51	-0.36028	0.382	0.707	8.02E-47
PGD	5.45E-51	-0.14928	0.239	0.469	8.14E-47
HMGCL	5.71E-51	-0.13784	0.096	0.284	8.53E-47
TIAM2	5.94E-51	0.57995	0.276	0.105	8.88E-47
BAD	6.10E-51	0.114204	0.52	0.626	9.12E-47
HSPA13	6.20E-51	0.107332	0.285	0.402	9.26E-47
ZNF771	6.22E-51	-0.20782	0.128	0.353	9.29E-47
CHST14	7.09E-51	-0.21866	0.025	0.205	1.06E-46
SMAD2	7.18E-51	-0.12724	0.149	0.352	1.07E-46
NUP88	7.41E-51	-0.13713	0.159	0.365	1.11E-46
FGFR1	7.44E-51	-0.11621	0.164	0.363	1.11E-46
SCPEP1	7.49E-51	-0.10053	0.053	0.207	1.12E-46
ARL13B	7.64E-51	-0.21122	0.127	0.351	1.14E-46
PDXK	7.72E-51	-0.10952	0.154	0.347	1.15E-46
GRWD1	7.78E-51	-0.13058	0.122	0.317	1.16E-46
ARMC6	8.54E-51	-0.14542	0.172	0.384	1.28E-46
LSM2	8.67E-51	-0.26033	0.439	0.743	1.30E-46
PSMD9	8.80E-51	-0.14385	0.189	0.399	1.32E-46
SPHK1	8.84E-51	-0.25608	0.006	0.173	1.32E-46
S100A11	9.06E-51	-0.5612	0.165	0.474	1.35E-46
ZFP90	9.14E-51	0.254685	0.247	0.277	1.37E-46
DPP8	9.38E-51	-0.11119	0.101	0.28	1.40E-46
LOXL1	9.54E-51	-0.22418	0.01	0.178	1.43E-46
RP11-849I	9.77E-51	-0.37507	0.138	0.407	1.46E-46
ATRAID	9.78E-51	-0.16151	0.525	0.79	1.46E-46
WDHD1	9.90E-51	-0.3132	0.024	0.216	1.48E-46
POLR1E	1.03E-50	-0.1172	0.104	0.284	1.55E-46
PARP2	1.05E-50	-0.27712	0.142	0.391	1.57E-46
RNF8	1.06E-50	0.115892	0.233	0.322	1.58E-46

C7orf73	1.06E-50	0.184048	0.695	0.746	1.58E-46
SETD9	1.08E-50	-0.16963	0.079	0.272	1.61E-46
CHST3	1.09E-50	-0.18905	0.012	0.174	1.62E-46
C18orf32	1.10E-50	0.144483	0.24	0.315	1.64E-46
NNT	1.14E-50	-0.20793	0.124	0.341	1.70E-46
AKAP17A	1.15E-50	0.157932	0.235	0.302	1.72E-46
F8A1	1.16E-50	-0.12844	0.106	0.294	1.73E-46
GPR155	1.16E-50	0.430869	0.298	0.212	1.74E-46
CYFIP1	1.18E-50	-0.25987	0.072	0.288	1.77E-46
GAMT	1.20E-50	-0.26838	0.279	0.562	1.80E-46
NLGN1	1.22E-50	-0.1038	0.181	0.377	1.83E-46
GGPS1	1.30E-50	0.13348	0.406	0.502	1.94E-46
FDPS	1.31E-50	-0.2982	0.426	0.73	1.95E-46
PDDC1	1.31E-50	-0.1123	0.124	0.31	1.96E-46
UBE2J1	1.35E-50	-0.13556	0.207	0.425	2.02E-46
YES1	1.35E-50	-0.11109	0.132	0.316	2.02E-46
C1orf109	1.44E-50	-0.17628	0.115	0.325	2.15E-46
TRAPPC12	1.49E-50	0.200701	0.244	0.295	2.22E-46
GNPTAB	1.52E-50	0.357697	0.211	0.168	2.27E-46
S100PBP	1.55E-50	-0.12224	0.072	0.243	2.32E-46
XIAP	1.58E-50	-0.15234	0.173	0.385	2.37E-46
MAD2L2	1.60E-50	-0.33006	0.502	0.819	2.40E-46
SRA1	1.73E-50	-0.2257	0.313	0.588	2.59E-46
LIMD1	1.79E-50	-0.21931	0.031	0.214	2.67E-46
PTK7	1.92E-50	-0.10774	0.083	0.256	2.87E-46
SUPT6H	1.99E-50	-0.13332	0.107	0.293	2.97E-46
HIST1H1E	2.00E-50	-0.25701	0.014	0.188	2.98E-46
BABAM1	2.01E-50	-0.1853	0.383	0.647	3.00E-46
PAK1IP1	2.05E-50	0.155448	0.28	0.368	3.06E-46
ECHDC2	2.08E-50	-0.23676	0.065	0.272	3.11E-46
ITSN1	2.11E-50	0.386155	0.302	0.226	3.15E-46
POU2F2	2.11E-50	0.620253	0.276	0.063	3.16E-46
ARF1	2.12E-50	0.134829	0.739	0.804	3.17E-46
GFOD2	2.16E-50	0.348296	0.3	0.254	3.22E-46
CYB561D2	2.17E-50	-0.10153	0.124	0.306	3.24E-46
GEMIN2	2.24E-50	-0.1654	0.105	0.304	3.35E-46
PPFIA1	2.26E-50	-0.11981	0.123	0.307	3.38E-46
FAM172A	2.28E-50	0.187995	0.266	0.321	3.41E-46
ATL3	2.30E-50	-0.24305	0.028	0.216	3.43E-46
PFDN2	2.34E-50	0.212815	0.818	0.817	3.50E-46
DPYSL5	2.46E-50	0.305358	0.341	0.33	3.67E-46
MEA1	2.49E-50	-0.22599	0.408	0.696	3.73E-46
SBDS	2.52E-50	0.135507	0.72	0.802	3.77E-46
WDR61	2.70E-50	-0.25019	0.253	0.519	4.04E-46
PCED1A	2.72E-50	-0.10007	0.085	0.252	4.07E-46
TOB1	2.87E-50	0.109516	0.268	0.378	4.29E-46
SF3B2	2.93E-50	0.139264	0.699	0.777	4.38E-46
GLRX5	2.93E-50	-0.26918	0.342	0.638	4.38E-46
TEAD2	2.94E-50	-0.14547	0.068	0.24	4.39E-46
SHPRH	3.03E-50	0.243647	0.205	0.225	4.53E-46
DPM1	3.05E-50	-0.15461	0.383	0.631	4.55E-46

ARHGAP35	3.23E-50	-0.15663	0.134	0.34	4.83E-46
SSRP1	3.28E-50	-0.28839	0.444	0.749	4.90E-46
NDUFA8	3.29E-50	0.140523	0.588	0.674	4.91E-46
LBR	3.29E-50	-0.27009	0.286	0.569	4.91E-46
APEH	3.50E-50	-0.13854	0.124	0.316	5.23E-46
IQGAP3	3.54E-50	-0.29417	0.002	0.153	5.29E-46
PGM1	3.63E-50	-0.13816	0.237	0.458	5.43E-46
CENPJ	3.67E-50	-0.10651	0.137	0.325	5.48E-46
TCEAL8	3.79E-50	0.156614	0.468	0.551	5.66E-46
FCGRT	3.97E-50	-0.2591	0.115	0.344	5.93E-46
AKIRIN2	3.99E-50	0.162876	0.526	0.61	5.97E-46
DDX6	4.23E-50	0.290046	0.29	0.283	6.32E-46
SYS1	4.35E-50	-0.12872	0.133	0.326	6.51E-46
ZNF117	4.36E-50	0.447127	0.28	0.174	6.52E-46
MTHFD2	4.62E-50	-0.32102	0.163	0.427	6.90E-46
SREBF2	4.80E-50	-0.17817	0.057	0.243	7.17E-46
BCHE	4.90E-50	-0.35068	0.172	0.444	7.32E-46
SMTN	5.16E-50	-0.30703	0.023	0.214	7.71E-46
TRIM16	5.39E-50	0.149886	0.178	0.241	8.06E-46
C7orf50	5.59E-50	0.125096	0.72	0.799	8.36E-46
PLOD3	5.81E-50	-0.19761	0.137	0.358	8.68E-46
ATM	5.97E-50	-0.10099	0.111	0.286	8.93E-46
C15orf40	5.98E-50	-0.19704	0.076	0.274	8.93E-46
PINK1	6.02E-50	0.314329	0.288	0.265	9.00E-46
PRKD3	6.48E-50	-0.13519	0.128	0.322	9.68E-46
CCDC22	6.55E-50	-0.1171	0.064	0.225	9.79E-46
NID1	7.40E-50	-0.19342	0.003	0.154	1.11E-45
ZNF608	7.46E-50	0.209959	0.245	0.283	1.11E-45
HEBP2	7.59E-50	-0.11181	0.197	0.402	1.13E-45
DHCR7	7.72E-50	-0.29425	0.088	0.32	1.15E-45
ARFGAP3	7.85E-50	-0.13894	0.185	0.395	1.17E-45
IL17RB	8.36E-50	-0.40056	0.015	0.201	1.25E-45
NCAN	8.46E-50	0.652604	0.342	0.084	1.26E-45
KDM6B	9.00E-50	0.525363	0.295	0.148	1.34E-45
NUPL2	9.33E-50	0.133289	0.39	0.48	1.39E-45
SWAP70	9.63E-50	-0.21787	0.064	0.263	1.44E-45
SNAP47	9.95E-50	0.158226	0.299	0.381	1.49E-45
PRKAG1	9.98E-50	0.116383	0.317	0.422	1.49E-45
TRAPPC1	1.03E-49	0.169637	0.606	0.673	1.54E-45
ZNF85	1.04E-49	-0.19185	0.097	0.302	1.55E-45
CNTLN	1.04E-49	-0.33699	0.094	0.337	1.56E-45
CDK4	1.04E-49	-0.45868	0.525	0.837	1.56E-45
TPM4	1.06E-49	-0.4694	0.423	0.744	1.58E-45
DPP9	1.08E-49	-0.22814	0.068	0.273	1.61E-45
FKBP3	1.09E-49	-0.16406	0.504	0.77	1.62E-45
ITGB1	1.09E-49	-0.53913	0.191	0.505	1.63E-45
MAP6	1.10E-49	0.379357	0.24	0.175	1.65E-45
DNAJC10	1.16E-49	-0.11498	0.132	0.32	1.74E-45
MDM2	1.24E-49	-0.38429	0.18	0.459	1.86E-45
ACD	1.28E-49	0.102825	0.258	0.368	1.91E-45
RBM14	1.28E-49	-0.12301	0.223	0.438	1.91E-45

STK24	1.33E-49	-0.18224	0.043	0.223	1.98E-45
NFS1	1.33E-49	-0.1441	0.077	0.262	1.99E-45
EMG1	1.37E-49	-0.13328	0.165	0.367	2.05E-45
ICT1	1.50E-49	-0.18759	0.189	0.417	2.25E-45
C9orf16	1.55E-49	0.22201	0.75	0.76	2.31E-45
CCDC80	1.57E-49	-0.38144	0.029	0.232	2.35E-45
TMEM132A	1.61E-49	0.136266	0.226	0.305	2.40E-45
RCOR1	1.70E-49	-0.16048	0.023	0.178	2.54E-45
IREB2	1.76E-49	-0.12963	0.096	0.277	2.62E-45
LGALS3BP	1.81E-49	-0.4751	0.203	0.51	2.71E-45
WNK3	1.85E-49	0.302608	0.24	0.241	2.76E-45
NEDD4L	1.88E-49	0.508176	0.35	0.193	2.81E-45
SPG7	1.97E-49	0.106516	0.279	0.388	2.94E-45
RBL1	1.98E-49	-0.24385	0.029	0.217	2.96E-45
BLMH	1.98E-49	-0.16384	0.187	0.41	2.96E-45
DGKZ	2.00E-49	-0.12923	0.153	0.349	2.99E-45
CINP	2.03E-49	-0.15585	0.163	0.372	3.03E-45
SPTBN1	2.03E-49	0.105396	0.407	0.521	3.03E-45
RAB4B	2.05E-49	0.244197	0.266	0.294	3.06E-45
HEG1	2.08E-49	-0.17063	0.024	0.188	3.11E-45
TSKU	2.17E-49	-0.18084	0.044	0.223	3.24E-45
ZNF322	2.18E-49	0.192135	0.203	0.241	3.26E-45
EPHA3	2.27E-49	-0.20791	0.004	0.162	3.39E-45
ANKS3	2.47E-49	0.171955	0.258	0.33	3.69E-45
IRS2	2.48E-49	-0.18328	0.212	0.448	3.70E-45
DUS1L	2.49E-49	-0.17656	0.078	0.27	3.72E-45
EHMT1	2.59E-49	0.123965	0.191	0.273	3.88E-45
CHD6	2.62E-49	0.196241	0.359	0.412	3.91E-45
SMOX	2.79E-49	-0.17241	0.155	0.369	4.18E-45
WBP2	2.81E-49	0.107437	0.423	0.527	4.20E-45
RARS2	2.81E-49	0.121872	0.253	0.341	4.20E-45
LONP2	2.85E-49	0.237296	0.324	0.352	4.25E-45
ST5	2.86E-49	-0.12524	0.086	0.26	4.28E-45
MTHFS	2.87E-49	-0.13116	0.07	0.243	4.29E-45
NBEAL1	2.87E-49	-0.32971	0.324	0.625	4.30E-45
P4HA1	2.90E-49	-0.20234	0.053	0.241	4.34E-45
ZNHIT1	2.98E-49	0.172453	0.767	0.812	4.45E-45
ERBB4	3.07E-49	0.616401	0.288	0.072	4.58E-45
TBCB	3.13E-49	0.244195	0.883	0.864	4.68E-45
RTKN	3.24E-49	-0.11651	0.121	0.302	4.85E-45
DBF4B	3.24E-49	-0.23222	0.039	0.231	4.85E-45
CD59	3.39E-49	0.149043	0.549	0.626	5.06E-45
FAR1	3.40E-49	-0.11395	0.076	0.24	5.08E-45
AZIN1	3.47E-49	0.240587	0.46	0.48	5.18E-45
XPR1	3.55E-49	0.323334	0.24	0.201	5.31E-45
TPMT	3.56E-49	-0.1909	0.076	0.268	5.32E-45
TYK2	3.77E-49	-0.14188	0.046	0.21	5.63E-45
GINS3	3.77E-49	-0.30084	0.014	0.193	5.63E-45
ZNF83	3.82E-49	-0.1558	0.214	0.432	5.70E-45
HDHD2	3.85E-49	-0.11914	0.243	0.451	5.75E-45
CSNK1G3	3.85E-49	0.130359	0.241	0.322	5.76E-45

MTMR4	4.12E-49	0.248045	0.281	0.306	6.16E-45
TMEM245	4.14E-49	-0.13616	0.116	0.306	6.19E-45
TRIB2	4.14E-49	-0.31862	0.316	0.619	6.19E-45
FAHD1	4.15E-49	0.253139	0.259	0.288	6.21E-45
MOSPD1	4.22E-49	0.244699	0.196	0.214	6.31E-45
ANXA6	4.25E-49	-0.22972	0.144	0.374	6.36E-45
HNRNPK	4.42E-49	0.188693	0.949	0.938	6.60E-45
CDH6	4.47E-49	-0.21381	0.007	0.163	6.68E-45
EIF4E	4.52E-49	0.100049	0.599	0.698	6.75E-45
SSH2	4.70E-49	-0.16007	0.138	0.343	7.02E-45
POP7	4.75E-49	0.178807	0.699	0.759	7.10E-45
TOPBP1	4.78E-49	-0.18287	0.108	0.314	7.14E-45
GPC1	4.78E-49	-0.53438	0.179	0.488	7.14E-45
NASP	4.86E-49	-0.32658	0.508	0.815	7.26E-45
GPATCH4	4.88E-49	-0.23845	0.119	0.343	7.30E-45
REXO2	4.94E-49	-0.22036	0.14	0.367	7.39E-45
BECN1	5.01E-49	-0.10496	0.156	0.337	7.49E-45
CREB1	5.06E-49	0.160882	0.375	0.447	7.56E-45
USP3	5.07E-49	-0.1078	0.126	0.309	7.57E-45
LAPTM4B	5.27E-49	0.118454	0.581	0.7	7.88E-45
SRRM4	5.36E-49	0.608871	0.227	0.02	8.00E-45
VPS13C	5.40E-49	-0.11822	0.075	0.241	8.07E-45
MAGEF1	5.48E-49	0.153682	0.605	0.675	8.19E-45
MCM6	5.61E-49	-0.46939	0.077	0.331	8.39E-45
KATNA1	5.64E-49	-0.16315	0.106	0.302	8.43E-45
ZCCHC7	5.70E-49	0.186234	0.207	0.258	8.52E-45
SRSF6	5.79E-49	-0.13221	0.383	0.615	8.65E-45
DNAJC21	5.85E-49	-0.17325	0.064	0.252	8.74E-45
NUP160	6.43E-49	0.286923	0.313	0.317	9.62E-45
NFU1	6.49E-49	0.16705	0.411	0.483	9.69E-45
PRKX	6.82E-49	0.414798	0.276	0.177	1.02E-44
TMPO-AS1	6.89E-49	-0.21111	0.027	0.194	1.03E-44
GPR180	6.91E-49	-0.18714	0.035	0.211	1.03E-44
COMMD7	7.09E-49	0.14903	0.519	0.607	1.06E-44
NUP35	7.29E-49	-0.27654	0.122	0.359	1.09E-44
KDELC2	7.32E-49	-0.27814	0.042	0.241	1.09E-44
CCDC136	7.54E-49	0.562278	0.313	0.126	1.13E-44
TMBIM1	7.66E-49	-0.2057	0.019	0.185	1.14E-44
RIT2	8.13E-49	0.680054	0.326	0.096	1.22E-44
GYG1	8.16E-49	-0.10085	0.13	0.305	1.22E-44
C7orf55	8.29E-49	0.112345	0.626	0.744	1.24E-44
GTF3C5	8.60E-49	-0.13616	0.212	0.43	1.29E-44
PNPLA2	8.79E-49	-0.14228	0.115	0.307	1.31E-44
LY6E	9.13E-49	0.105087	0.363	0.493	1.36E-44
NRXN2	9.44E-49	0.548515	0.38	0.19	1.41E-44
EFR3A	9.53E-49	-0.11657	0.045	0.202	1.42E-44
ELOVL4	9.61E-49	0.514208	0.258	0.1	1.44E-44
SNUPN	9.68E-49	-0.17391	0.101	0.3	1.45E-44
NDUFA6	9.83E-49	-0.34195	0.506	0.817	1.47E-44
REEP2	9.95E-49	0.148639	0.311	0.395	1.49E-44
FBXL3	1.03E-48	0.103865	0.213	0.317	1.54E-44

RIMKLB	1.07E-48	0.293837	0.324	0.32	1.60E-44
CSNK1G2	1.09E-48	-0.23901	0.083	0.295	1.63E-44
XBP1	1.12E-48	-0.14007	0.189	0.398	1.67E-44
RNF13	1.13E-48	-0.10096	0.265	0.467	1.69E-44
C4orf46	1.14E-48	-0.18164	0.104	0.305	1.71E-44
MYL6B	1.17E-48	0.209671	0.745	0.765	1.76E-44
RAD1	1.17E-48	-0.16178	0.258	0.483	1.76E-44
PCMT1	1.19E-48	0.145959	0.699	0.767	1.79E-44
UBALD1	1.23E-48	0.171823	0.211	0.279	1.84E-44
TOMM40	1.24E-48	-0.14433	0.39	0.631	1.86E-44
PXN	1.26E-48	-0.18619	0.044	0.225	1.88E-44
RNASET2	1.27E-48	0.254681	0.266	0.284	1.90E-44
ZNF680	1.30E-48	-0.23949	0.242	0.501	1.95E-44
PCBD2	1.36E-48	-0.27596	0.025	0.209	2.03E-44
GOPC	1.36E-48	0.218875	0.403	0.442	2.04E-44
CCT6A	1.38E-48	0.188824	0.841	0.842	2.06E-44
EFHC1	1.55E-48	0.10797	0.225	0.332	2.32E-44
SLC12A2	1.60E-48	-0.19338	0.079	0.277	2.39E-44
NSUN5	1.61E-48	0.136803	0.313	0.401	2.41E-44
POR	1.69E-48	0.261833	0.363	0.368	2.53E-44
ALKBH4	1.85E-48	0.152987	0.31	0.388	2.77E-44
SLC47A1	1.92E-48	-0.19907	0.005	0.156	2.87E-44
IMPDH2	1.96E-48	-0.20304	0.351	0.616	2.93E-44
LAMTOR3	1.98E-48	0.144266	0.279	0.359	2.96E-44
RRAGC	2.10E-48	0.219665	0.305	0.344	3.14E-44
NDRG3	2.12E-48	0.181041	0.243	0.302	3.17E-44
KBTBD6	2.18E-48	0.271021	0.296	0.291	3.26E-44
CERS6	2.22E-48	0.346578	0.306	0.254	3.32E-44
VIM-AS1	2.27E-48	-0.22117	0.019	0.191	3.39E-44
ANXA2R	2.34E-48	0.120198	0.257	0.374	3.50E-44
PSMC1	2.37E-48	-0.32988	0.302	0.6	3.54E-44
PNKP	2.37E-48	-0.11843	0.121	0.31	3.54E-44
CRYZ	2.46E-48	-0.23544	0.031	0.21	3.67E-44
STRN4	2.68E-48	-0.12394	0.131	0.326	4.01E-44
TFPT	2.73E-48	-0.16443	0.189	0.41	4.08E-44
SORBS2	2.74E-48	0.604571	0.285	0.062	4.10E-44
ATP6V0E2	2.75E-48	0.178562	0.767	0.79	4.11E-44
FANCB	2.75E-48	-0.24091	0.008	0.17	4.12E-44
MORF4L2	2.91E-48	0.193074	0.862	0.857	4.34E-44
IP6K1	2.95E-48	0.131744	0.202	0.283	4.41E-44
GBA	2.95E-48	-0.25597	0.053	0.258	4.42E-44
SCP2	3.02E-48	-0.2218	0.445	0.722	4.51E-44
IL13RA1	3.05E-48	-0.15436	0.018	0.17	4.55E-44
ARPC1B	3.25E-48	-0.18827	0.203	0.432	4.85E-44
MGAT4B	3.35E-48	-0.11731	0.038	0.188	5.01E-44
MPHOSPH1C	3.49E-48	0.200378	0.363	0.417	5.22E-44
DDX50	3.50E-48	-0.10204	0.126	0.305	5.23E-44
TAOK3	3.53E-48	0.22079	0.438	0.475	5.27E-44
DCTN3	3.55E-48	0.211693	0.808	0.802	5.31E-44
SHKBP1	3.60E-48	-0.15206	0.097	0.285	5.38E-44
COQ7	3.62E-48	0.18372	0.321	0.388	5.40E-44

BIN1	3.62E-48	0.388646	0.337	0.256	5.41E-44
MANF	3.82E-48	-0.138	0.436	0.68	5.70E-44
PCDH9	3.84E-48	-0.25379	0.173	0.412	5.73E-44
FKBP1B	3.96E-48	0.355351	0.233	0.194	5.92E-44
CDC73	4.43E-48	-0.13003	0.106	0.29	6.61E-44
HAUS6	4.59E-48	-0.16781	0.092	0.28	6.85E-44
DNAJC8	4.99E-48	0.1274	0.698	0.77	7.45E-44
SNRNP25	5.02E-48	-0.16797	0.408	0.665	7.50E-44
SOAT1	5.11E-48	-0.19198	0.042	0.219	7.64E-44
EDEM2	5.14E-48	-0.14394	0.112	0.304	7.68E-44
NOSIP	5.20E-48	-0.14086	0.422	0.665	7.77E-44
PRKAR2B	5.37E-48	0.547258	0.301	0.117	8.03E-44
SERPINE2	5.42E-48	-0.3562	0.318	0.625	8.11E-44
RNF168	5.56E-48	-0.11663	0.178	0.374	8.31E-44
PPP1R16A	6.48E-48	0.195968	0.246	0.284	9.68E-44
LINC01003	6.60E-48	0.182253	0.243	0.314	9.86E-44
DZIP1	6.78E-48	-0.12691	0.083	0.259	1.01E-43
STK11	7.03E-48	-0.19281	0.046	0.226	1.05E-43
MLF1	7.04E-48	0.153059	0.29	0.367	1.05E-43
CES2	7.11E-48	0.186113	0.237	0.289	1.06E-43
WBP5	7.17E-48	0.189417	0.72	0.777	1.07E-43
RAD50	7.18E-48	0.134408	0.345	0.425	1.07E-43
GNPNAT1	7.22E-48	-0.22462	0.047	0.236	1.08E-43
NFE2L1	7.28E-48	-0.14109	0.127	0.323	1.09E-43
GBE1	7.31E-48	-0.32079	0.063	0.278	1.09E-43
SAMD1	7.37E-48	-0.18037	0.075	0.263	1.10E-43
CRTAP	7.46E-48	-0.25224	0.091	0.31	1.12E-43
PIGU	8.02E-48	-0.18687	0.142	0.349	1.20E-43
ENSA	8.34E-48	-0.35355	0.378	0.691	1.25E-43
ATG3	8.50E-48	-0.15476	0.307	0.543	1.27E-43
PPP5C	8.53E-48	-0.23523	0.185	0.427	1.27E-43
TMEM100	8.56E-48	-0.52933	0.042	0.264	1.28E-43
ANKRD35	8.85E-48	-0.14448	0.1	0.285	1.32E-43
CLIP1	9.00E-48	0.165966	0.246	0.302	1.35E-43
ZEB2	9.32E-48	0.210177	0.382	0.433	1.39E-43
MYO6	9.33E-48	0.293701	0.396	0.369	1.39E-43
SLCO5A1	9.43E-48	0.607803	0.258	0.049	1.41E-43
PSPH	9.47E-48	-0.10131	0.198	0.388	1.41E-43
MEX3C	9.57E-48	-0.13883	0.067	0.237	1.43E-43
DHTKD1	9.64E-48	-0.14966	0.078	0.257	1.44E-43
GALK1	9.82E-48	-0.23288	0.076	0.279	1.47E-43
DFNA5	1.04E-47	0.460587	0.339	0.221	1.56E-43
FADS2	1.04E-47	-0.13404	0.159	0.359	1.56E-43
BAZ2B	1.05E-47	0.230114	0.506	0.533	1.58E-43
SLC25A37	1.08E-47	-0.20856	0.11	0.321	1.62E-43
BOD1L1	1.08E-47	0.24285	0.231	0.253	1.62E-43
PRKACB	1.09E-47	0.135434	0.225	0.306	1.63E-43
SLC39A3	1.13E-47	-0.25822	0.344	0.628	1.68E-43
RAB5B	1.13E-47	0.17526	0.223	0.29	1.68E-43
AC004381.	1.15E-47	-0.38515	0.029	0.228	1.71E-43
HEXIM1	1.15E-47	-0.21766	0.095	0.302	1.72E-43

GOLGA2	1.16E-47	0.118954	0.222	0.306	1.74E-43
RANBP1	1.17E-47	-0.39197	0.745	0.931	1.75E-43
PIK3C2A	1.22E-47	-0.12503	0.086	0.26	1.82E-43
CAPN1	1.28E-47	0.316417	0.308	0.275	1.91E-43
MBD4	1.34E-47	-0.15407	0.204	0.42	2.00E-43
E2F2	1.36E-47	-0.29373	0.002	0.146	2.04E-43
ALDH3A2	1.46E-47	-0.19344	0.219	0.457	2.18E-43
CDK2AP2	1.46E-47	-0.18703	0.235	0.47	2.18E-43
PLRG1	1.48E-47	0.14449	0.313	0.391	2.22E-43
TMTC3	1.50E-47	-0.15337	0.051	0.215	2.24E-43
NIPSNAP1	1.58E-47	0.309847	0.286	0.275	2.36E-43
FGFRL1	1.60E-47	-0.17466	0.002	0.138	2.40E-43
LRRC59	1.60E-47	-0.14474	0.375	0.611	2.40E-43
TIMM21	1.66E-47	-0.21248	0.105	0.315	2.48E-43
STOX2	1.67E-47	0.508262	0.291	0.148	2.50E-43
CWC15	1.68E-47	0.186693	0.617	0.664	2.50E-43
GSE1	1.72E-47	0.531596	0.32	0.172	2.57E-43
AUP1	1.85E-47	-0.11479	0.474	0.712	2.76E-43
PACSIN3	1.93E-47	-0.19696	0.004	0.151	2.88E-43
GPX8	1.98E-47	-0.18473	0.002	0.147	2.96E-43
CLCC1	2.00E-47	-0.12374	0.102	0.278	2.98E-43
MSRB2	2.04E-47	0.23908	0.251	0.288	3.05E-43
GADD45A	2.21E-47	-0.42901	0.176	0.463	3.31E-43
NSMCE4A	2.22E-47	-0.23027	0.116	0.337	3.32E-43
AIDA	2.33E-47	-0.137	0.155	0.351	3.48E-43
RPP14	2.33E-47	0.177912	0.257	0.314	3.48E-43
RGS12	2.33E-47	0.113717	0.344	0.446	3.48E-43
LMO7	2.35E-47	-0.31381	0.022	0.205	3.52E-43
NADK	2.37E-47	-0.1934	0.086	0.281	3.54E-43
CACUL1	2.38E-47	0.167899	0.217	0.285	3.56E-43
ACAP3	2.68E-47	0.248309	0.405	0.433	4.01E-43
TOP3A	2.76E-47	-0.16169	0.103	0.295	4.12E-43
KAT6A	2.78E-47	0.20762	0.244	0.293	4.15E-43
STIM2	2.86E-47	-0.19276	0.127	0.34	4.28E-43
ANKRD52	2.88E-47	-0.11267	0.044	0.193	4.30E-43
NGLY1	3.04E-47	-0.1577	0.175	0.385	4.54E-43
AZI2	3.04E-47	0.13093	0.344	0.436	4.54E-43
FIP1L1	3.08E-47	-0.15134	0.372	0.612	4.61E-43
XAB2	3.31E-47	-0.11127	0.16	0.346	4.94E-43
PRIM1	3.37E-47	-0.3047	0.108	0.342	5.04E-43
ANK2	3.46E-47	0.114356	0.231	0.323	5.17E-43
OXCT1	3.66E-47	-0.30503	0.159	0.412	5.47E-43
ARC	3.68E-47	0.429252	0.503	0.401	5.50E-43
ARNT2	3.69E-47	-0.21899	0.081	0.284	5.52E-43
RAE1	3.81E-47	-0.1123	0.333	0.548	5.70E-43
COPG1	3.85E-47	0.224007	0.29	0.31	5.76E-43
C12orf43	3.94E-47	0.113711	0.217	0.309	5.89E-43
FAHD2A	4.22E-47	-0.10591	0.173	0.358	6.31E-43
MESP1	4.41E-47	-0.13396	0.152	0.348	6.60E-43
GTF2A1	4.57E-47	-0.15026	0.071	0.248	6.82E-43
POLRMT	4.83E-47	-0.12383	0.081	0.252	7.22E-43

XPA	5.05E-47	0.168179	0.288	0.348	7.54E-43
MTCH2	5.16E-47	-0.11351	0.43	0.658	7.71E-43
VPS53	5.18E-47	0.255858	0.215	0.23	7.74E-43
RHOB	5.53E-47	0.254362	0.719	0.738	8.26E-43
YWHAB	5.91E-47	0.229186	0.917	0.869	8.83E-43
NDUFB5	5.93E-47	-0.16484	0.489	0.742	8.86E-43
STRBP	5.94E-47	0.280393	0.289	0.277	8.87E-43
AKT2	6.01E-47	-0.1105	0.171	0.357	8.98E-43
PHF12	6.30E-47	-0.10352	0.079	0.241	9.41E-43
SDSL	6.47E-47	-0.11208	0.053	0.206	9.66E-43
RAP1B	6.52E-47	-0.28219	0.273	0.546	9.74E-43
MTMR2	6.54E-47	-0.17243	0.123	0.323	9.77E-43
GATAD2A	6.80E-47	-0.22085	0.07	0.26	1.02E-42
ERP44	6.84E-47	0.115567	0.342	0.435	1.02E-42
RNF180	7.06E-47	-0.11256	0.189	0.38	1.05E-42
OTUD4	7.39E-47	-0.1102	0.072	0.231	1.10E-42
GABPB1	7.50E-47	-0.13925	0.105	0.285	1.12E-42
TXNIP	8.31E-47	-0.30171	0.321	0.602	1.24E-42
NPM3	8.31E-47	-0.16807	0.119	0.311	1.24E-42
PPP2R5A	8.46E-47	-0.21063	0.009	0.17	1.26E-42
THAP10	8.46E-47	-0.22128	0.024	0.191	1.26E-42
TULP4	8.79E-47	0.2627	0.251	0.246	1.31E-42
ARL4C	9.17E-47	0.380283	0.692	0.627	1.37E-42
CHD3	9.26E-47	0.479408	0.323	0.195	1.38E-42
VPS37A	9.39E-47	0.108967	0.227	0.322	1.40E-42
MAPRE2	9.53E-47	0.336127	0.284	0.23	1.42E-42
SPRY4	1.00E-46	-0.18379	0.016	0.175	1.50E-42
AKAP13	1.03E-46	-0.17296	0.127	0.326	1.54E-42
AIG1	1.05E-46	-0.16816	0.223	0.449	1.56E-42
IGBP1	1.06E-46	0.222927	0.374	0.416	1.59E-42
ANKRD40	1.11E-46	-0.19293	0.1	0.299	1.66E-42
LARP6	1.13E-46	-0.15867	0.163	0.364	1.69E-42
XRCC1	1.14E-46	-0.26755	0.104	0.328	1.70E-42
C14orf119	1.15E-46	-0.12439	0.235	0.442	1.71E-42
YIPF5	1.15E-46	0.229525	0.312	0.336	1.72E-42
HOXA7	1.20E-46	0.560943	0.412	0.199	1.79E-42
DNALI1	1.22E-46	-0.25517	0.069	0.275	1.83E-42
ARL14EP	1.23E-46	0.139662	0.216	0.295	1.84E-42
RBBP9	1.30E-46	-0.17392	0.064	0.244	1.95E-42
CHIC2	1.37E-46	-0.19185	0.252	0.489	2.05E-42
PLA2G5	1.37E-46	-0.37336	0	0.13	2.05E-42
DOLK	1.43E-46	-0.13849	0.068	0.238	2.13E-42
TMED2	1.49E-46	0.167948	0.746	0.775	2.23E-42
MAX	1.55E-46	0.179847	0.252	0.317	2.31E-42
UGP2	1.61E-46	-0.2998	0.411	0.706	2.41E-42
CCSER2	1.64E-46	0.249396	0.307	0.316	2.45E-42
AC004540.	1.65E-46	0.528798	0.471	0.278	2.46E-42
REV1	1.80E-46	0.105521	0.276	0.372	2.69E-42
RAD21	1.82E-46	-0.3522	0.5	0.806	2.72E-42
FAM53C	1.86E-46	0.269895	0.279	0.288	2.77E-42
UBALD2	1.88E-46	-0.3211	0.234	0.511	2.81E-42

CENPT	1.88E-46	-0.13981	0.269	0.486	2.81E-42
CCDC174	1.91E-46	0.169843	0.222	0.294	2.85E-42
CTHRC1	1.94E-46	-0.27822	0.024	0.211	2.89E-42
CYB5R1	1.94E-46	0.128127	0.196	0.275	2.90E-42
TMEM35	1.95E-46	0.548911	0.392	0.189	2.92E-42
ZNF281	1.99E-46	0.368188	0.364	0.294	2.98E-42
CRKL	2.12E-46	-0.14183	0.082	0.258	3.17E-42
SMIM7	2.12E-46	-0.12676	0.499	0.728	3.17E-42
MBIP	2.24E-46	0.183746	0.266	0.323	3.35E-42
AAAS	2.33E-46	-0.20547	0.2	0.433	3.48E-42
UPF3B	2.37E-46	0.208784	0.43	0.467	3.54E-42
CLINT1	2.41E-46	-0.11391	0.166	0.354	3.60E-42
KIF1C	2.45E-46	-0.24911	0.02	0.194	3.66E-42
ZNF451	2.51E-46	0.103278	0.214	0.307	3.75E-42
XRCC4	2.53E-46	-0.12487	0.081	0.248	3.78E-42
CHCHD1	2.57E-46	-0.14526	0.178	0.379	3.84E-42
PCDHB10	2.61E-46	0.533916	0.35	0.184	3.90E-42
HES6	2.70E-46	-0.53447	0.435	0.752	4.04E-42
TIPRL	2.94E-46	0.160856	0.353	0.412	4.39E-42
MTFR1	2.95E-46	-0.10849	0.075	0.235	4.41E-42
GDI2	2.96E-46	-0.18052	0.365	0.614	4.42E-42
ZNF100	3.04E-46	-0.19275	0.055	0.232	4.55E-42
FDFT1	3.15E-46	-0.2288	0.237	0.481	4.71E-42
AMFR	3.17E-46	-0.10086	0.115	0.289	4.74E-42
IGFBP3	3.35E-46	-0.65444	0.092	0.344	5.00E-42
HM13	3.35E-46	-0.14882	0.35	0.581	5.01E-42
TMEM261	3.38E-46	-0.12383	0.297	0.511	5.04E-42
PSMF1	3.45E-46	-0.12206	0.367	0.595	5.15E-42
CAMK1	3.70E-46	-0.10237	0.167	0.349	5.53E-42
HCFC1	3.74E-46	-0.13482	0.137	0.326	5.59E-42
SLC38A10	3.85E-46	-0.11841	0.097	0.27	5.75E-42
PES1	3.86E-46	-0.12882	0.146	0.34	5.77E-42
CAHM	3.95E-46	-0.21236	0.055	0.233	5.90E-42
TPR	3.99E-46	-0.16335	0.473	0.725	5.96E-42
SNX8	4.21E-46	-0.16775	0.064	0.242	6.29E-42
UBAC1	4.39E-46	-0.10799	0.191	0.383	6.56E-42
KLC2	4.54E-46	0.267008	0.234	0.23	6.78E-42
ATP6V0A2	4.54E-46	-0.16362	0.077	0.256	6.79E-42
PGRMC1	4.56E-46	0.144122	0.681	0.749	6.81E-42
YBEY	4.79E-46	0.161031	0.26	0.331	7.16E-42
VPS41	4.96E-46	0.259983	0.328	0.321	7.41E-42
CNKSR3	4.96E-46	-0.20943	0.058	0.247	7.42E-42
DHRS13	5.42E-46	-0.22286	0.063	0.258	8.10E-42
NCSTN	5.53E-46	-0.11403	0.187	0.377	8.26E-42
PI4K2B	6.03E-46	-0.19239	0.035	0.206	9.01E-42
ACTG1	6.17E-46	0.138634	0.998	0.981	9.22E-42
LIFR	6.48E-46	-0.31713	0.028	0.214	9.68E-42
ARHGEF7	6.49E-46	0.374496	0.338	0.264	9.70E-42
MIA3	6.49E-46	0.146216	0.258	0.323	9.70E-42
RIN1	6.58E-46	-0.22349	0.008	0.163	9.84E-42
EFHD2	7.07E-46	-0.13679	0.09	0.265	1.06E-41

SPATA33	7.30E-46	-0.18388	0.186	0.409	1.09E-41
MAP2K2	7.65E-46	-0.32995	0.26	0.546	1.14E-41
AKTIP	8.43E-46	0.120351	0.182	0.263	1.26E-41
UBE2N	9.15E-46	0.141973	0.687	0.747	1.37E-41
ECHS1	9.73E-46	-0.1184	0.357	0.581	1.45E-41
MOXD1	9.86E-46	-0.23503	0.011	0.167	1.47E-41
CCNL1	9.91E-46	0.131155	0.581	0.663	1.48E-41
GLIS3	1.18E-45	-0.22444	0.007	0.16	1.76E-41
MOB2	1.20E-45	0.123164	0.221	0.302	1.80E-41
ACADM	1.22E-45	0.100018	0.434	0.538	1.83E-41
NUDT11	1.27E-45	0.360625	0.328	0.268	1.90E-41
LAMP1	1.29E-45	-0.15568	0.427	0.674	1.93E-41
CCDC53	1.30E-45	0.156237	0.393	0.472	1.94E-41
NOL8	1.30E-45	-0.11448	0.161	0.344	1.95E-41
LIN7B	1.32E-45	0.278862	0.222	0.231	1.97E-41
C1orf216	1.32E-45	0.384549	0.252	0.173	1.97E-41
NPRL2	1.38E-45	0.266307	0.227	0.237	2.06E-41
ERC1	1.42E-45	0.307769	0.221	0.199	2.12E-41
CBFA2T2	1.44E-45	0.267027	0.215	0.223	2.16E-41
PBX3	1.54E-45	0.125296	0.159	0.238	2.31E-41
SPRYD7	1.55E-45	0.107214	0.207	0.286	2.32E-41
RFWD2	1.56E-45	0.139304	0.239	0.311	2.34E-41
GUCD1	1.71E-45	-0.19858	0.069	0.256	2.55E-41
SOCS4	1.71E-45	-0.13542	0.053	0.212	2.56E-41
CENPB	1.73E-45	-0.13572	0.07	0.237	2.58E-41
BST2	1.91E-45	-0.72579	0.031	0.232	2.85E-41
MDC1	1.91E-45	-0.19835	0.042	0.216	2.85E-41
SPA17	1.96E-45	-0.23512	0.06	0.249	2.93E-41
SLC35D2	2.00E-45	-0.20114	0.032	0.202	2.99E-41
JAK1	2.00E-45	0.203404	0.271	0.315	2.99E-41
RAC1	2.10E-45	0.168253	0.987	0.941	3.14E-41
LRRC4B	2.11E-45	0.142777	0.212	0.285	3.16E-41
DTNBP1	2.15E-45	0.185555	0.27	0.32	3.22E-41
ITPK1	2.17E-45	-0.21933	0.046	0.228	3.24E-41
NEUROD2	2.18E-45	0.607464	0.187	0.014	3.25E-41
DDRGK1	2.25E-45	-0.10756	0.357	0.574	3.36E-41
ZHX1	2.26E-45	0.239497	0.295	0.319	3.37E-41
NLN	2.34E-45	0.169565	0.228	0.28	3.50E-41
GPANK1	2.35E-45	-0.18624	0.181	0.402	3.51E-41
RBMXL1	2.40E-45	-0.16599	0.158	0.363	3.58E-41
SNRPG	2.75E-45	-0.35563	0.7	0.907	4.10E-41
CTDNEP1	2.81E-45	-0.16887	0.32	0.557	4.19E-41
SPRED2	2.83E-45	-0.22722	0.053	0.243	4.23E-41
CLUAP1	2.85E-45	0.202609	0.239	0.285	4.26E-41
CAMSAP2	2.92E-45	0.115686	0.22	0.296	4.36E-41
NAP1L5	2.96E-45	0.491924	0.333	0.165	4.42E-41
SLC25A22	3.02E-45	0.209177	0.233	0.258	4.52E-41
CDC23	3.03E-45	-0.22343	0.105	0.31	4.53E-41
CCNK	3.05E-45	-0.13153	0.064	0.228	4.56E-41
TRIM7	3.17E-45	-0.13542	0.013	0.147	4.74E-41
FOSL2	3.19E-45	-0.14354	0.049	0.204	4.76E-41

CHD1L	3.46E-45	-0.11162	0.082	0.242	5.17E-41
C19orf60	3.55E-45	0.135673	0.495	0.591	5.30E-41
ICAM3	3.67E-45	-0.16357	0.009	0.151	5.48E-41
DIRAS1	3.75E-45	0.318786	0.244	0.207	5.60E-41
CHP1	3.83E-45	-0.15472	0.1	0.279	5.73E-41
NUCB1	4.00E-45	-0.1023	0.311	0.514	5.98E-41
TBC1D1	4.04E-45	-0.1355	0.065	0.231	6.03E-41
HDAC9	4.29E-45	0.510075	0.294	0.141	6.41E-41
SERTAD1	4.41E-45	-0.2978	0.09	0.306	6.59E-41
BTBD1	4.49E-45	-0.12832	0.086	0.254	6.71E-41
C14orf37	4.51E-45	0.398717	0.26	0.184	6.74E-41
DDR1	4.96E-45	-0.16607	0.39	0.637	7.41E-41
NDUFV1	5.15E-45	-0.13683	0.5	0.735	7.70E-41
TSR2	5.19E-45	-0.11709	0.259	0.464	7.76E-41
SRSF5	5.26E-45	0.159434	0.748	0.802	7.86E-41
AGPAT3	5.41E-45	-0.17163	0.073	0.253	8.09E-41
GPR161	5.41E-45	0.378511	0.255	0.172	8.09E-41
MYL12A	5.66E-45	-0.37022	0.196	0.477	8.45E-41
DOT1L	6.04E-45	-0.17064	0.054	0.225	9.03E-41
CERK	6.31E-45	0.248934	0.216	0.23	9.43E-41
HOMER2	6.89E-45	-0.14894	0.023	0.172	1.03E-40
RQCD1	7.22E-45	-0.10526	0.224	0.419	1.08E-40
PCCB	7.32E-45	-0.25476	0.102	0.314	1.09E-40
CBX5	7.89E-45	-0.22832	0.493	0.767	1.18E-40
AP3D1	7.92E-45	-0.1393	0.331	0.552	1.18E-40
ATG5	8.10E-45	0.137601	0.251	0.325	1.21E-40
SOBP	8.18E-45	0.338048	0.299	0.262	1.22E-40
NUP107	8.22E-45	-0.46104	0.277	0.579	1.23E-40
VASP	8.37E-45	-0.10909	0.102	0.268	1.25E-40
SLC39A10	8.57E-45	0.232151	0.241	0.267	1.28E-40
GNG4	8.66E-45	-0.20809	0.23	0.468	1.29E-40
ARFIP2	8.93E-45	0.103308	0.284	0.378	1.33E-40
QTRT1	9.06E-45	-0.12399	0.303	0.514	1.35E-40
PFN2	9.30E-45	0.269285	0.822	0.783	1.39E-40
TIAL1	9.58E-45	-0.13502	0.161	0.354	1.43E-40
GOSR2	9.75E-45	0.133311	0.22	0.304	1.46E-40
SQSTM1	9.86E-45	-0.18016	0.329	0.574	1.47E-40
CERS1	1.02E-44	-0.20977	0.065	0.253	1.53E-40
ZBED5	1.03E-44	0.163259	0.301	0.36	1.54E-40
ZNF439	1.05E-44	0.269209	0.288	0.29	1.57E-40
SLC7A1	1.08E-44	-0.12253	0.049	0.2	1.61E-40
CBX4	1.10E-44	0.292384	0.229	0.225	1.64E-40
SLC35A5	1.13E-44	0.133585	0.224	0.304	1.70E-40
VKORC1	1.14E-44	-0.15903	0.387	0.63	1.70E-40
MED30	1.15E-44	0.140426	0.412	0.494	1.72E-40
RCAN1	1.20E-44	-0.35227	0.156	0.409	1.79E-40
PAF1	1.20E-44	-0.1672	0.212	0.425	1.79E-40
ACTR1B	1.26E-44	0.144621	0.269	0.343	1.88E-40
DPY19L4	1.30E-44	-0.12169	0.069	0.228	1.94E-40
ABHD8	1.34E-44	0.168239	0.165	0.209	2.00E-40
MAP3K2	1.36E-44	0.119756	0.189	0.274	2.03E-40

UBE2L3	1.36E-44	-0.27841	0.467	0.756	2.03E-40
FBXO22	1.46E-44	-0.1513	0.08	0.253	2.18E-40
GSN	1.48E-44	-0.29596	0.104	0.327	2.21E-40
HEY2	1.53E-44	-0.22461	0.009	0.163	2.29E-40
IFT57	1.53E-44	-0.11867	0.24	0.436	2.29E-40
TTC17	1.54E-44	0.120976	0.255	0.336	2.29E-40
OCIAD1	1.57E-44	0.177644	0.777	0.806	2.34E-40
HSD17B10	1.57E-44	-0.16352	0.487	0.735	2.35E-40
GNAO1	1.61E-44	0.593757	0.319	0.094	2.41E-40
S100A10	1.67E-44	-0.49889	0.241	0.542	2.49E-40
PARVB	1.67E-44	-0.16037	0.027	0.181	2.50E-40
TSC22D2	1.68E-44	0.313533	0.248	0.22	2.51E-40
MLH1	1.70E-44	-0.1539	0.172	0.365	2.54E-40
IRF2	1.71E-44	-0.1767	0.082	0.262	2.55E-40
TBC1D14	1.79E-44	0.332777	0.278	0.232	2.67E-40
NAV1	1.88E-44	0.121275	0.267	0.356	2.82E-40
KIAA1549	1.98E-44	0.38458	0.313	0.237	2.96E-40
RTTN	1.98E-44	-0.18419	0.007	0.152	2.96E-40
FAM60A	2.01E-44	0.207165	0.291	0.332	3.00E-40
ADK	2.04E-44	-0.17263	0.108	0.298	3.05E-40
IGSF10	2.15E-44	-0.20888	0.051	0.231	3.22E-40
GMPR2	2.18E-44	-0.1693	0.101	0.289	3.25E-40
DDX3Y	2.18E-44	-0.12431	0.046	0.194	3.26E-40
METTL2B	2.23E-44	0.207749	0.345	0.379	3.34E-40
RFX2	2.25E-44	-0.24701	0.042	0.226	3.36E-40
NKX2-5	2.29E-44	0.614581	0.241	0.064	3.42E-40
SMARCA1	2.42E-44	0.157822	0.306	0.362	3.62E-40
KATNB1	2.50E-44	0.119842	0.181	0.256	3.74E-40
ZFHX3	2.57E-44	0.121387	0.174	0.262	3.83E-40
STOML2	2.60E-44	-0.18096	0.405	0.656	3.89E-40
HS6ST2	2.69E-44	-0.19318	0.003	0.143	4.02E-40
DNM1	2.90E-44	0.104466	0.105	0.165	4.34E-40
RNF216	3.05E-44	0.234885	0.338	0.352	4.55E-40
RAB8A	3.05E-44	-0.21736	0.272	0.517	4.56E-40
DCAKD	3.14E-44	-0.15003	0.143	0.338	4.70E-40
LDHA	3.22E-44	-0.4333	0.606	0.865	4.81E-40
FRMD8	3.25E-44	-0.15328	0.059	0.225	4.86E-40
NOP56	3.25E-44	-0.2851	0.459	0.751	4.86E-40
TAF15	3.26E-44	-0.19498	0.323	0.564	4.87E-40
TP73	3.31E-44	-0.19026	0.029	0.18	4.94E-40
SLC35F2	3.33E-44	-0.10232	0.069	0.221	4.97E-40
PDCL	3.50E-44	0.135771	0.271	0.342	5.23E-40
TMEM109	3.53E-44	-0.1071	0.067	0.217	5.28E-40
TCEAL1	3.55E-44	0.285037	0.306	0.305	5.30E-40
TPT1-AS1	3.58E-44	0.194114	0.207	0.247	5.36E-40
SERPINB6	3.82E-44	-0.24742	0.284	0.541	5.70E-40
C12orf73	3.84E-44	0.197119	0.222	0.256	5.74E-40
KIAA2026	3.84E-44	0.352807	0.268	0.209	5.74E-40
METTL21A	3.93E-44	-0.15547	0.177	0.375	5.88E-40
ZCCHC12	4.18E-44	0.512459	0.186	0.012	6.24E-40
PCMTD2	4.25E-44	0.105421	0.317	0.419	6.35E-40

DLEU1	4.28E-44	-0.21198	0.059	0.243	6.39E-40
TADA3	4.46E-44	-0.1269	0.409	0.636	6.66E-40
THUMPD1	4.69E-44	0.188131	0.306	0.343	7.01E-40
PTPN13	4.82E-44	-0.15625	0.026	0.172	7.20E-40
CHRNA5	4.82E-44	-0.21465	0.024	0.186	7.20E-40
SNRPB2	4.87E-44	0.181632	0.74	0.772	7.28E-40
PI4KB	5.00E-44	0.122379	0.226	0.305	7.47E-40
KIAA1586	5.14E-44	0.195256	0.232	0.269	7.68E-40
HRH1	5.21E-44	-0.16896	0.009	0.153	7.79E-40
MBOAT2	5.37E-44	-0.11596	0.242	0.438	8.03E-40
TRPM3	5.47E-44	-0.19725	0.002	0.138	8.18E-40
TMEM19	5.91E-44	-0.17125	0.117	0.306	8.84E-40
PTPN9	5.97E-44	-0.14818	0.067	0.236	8.92E-40
UQCRC1	6.44E-44	-0.18007	0.498	0.747	9.63E-40
SEC14L2	6.52E-44	-0.19891	0.037	0.202	9.75E-40
KIAA1033	6.86E-44	-0.19852	0.094	0.291	1.02E-39
KCTD13	7.21E-44	0.342368	0.313	0.267	1.08E-39
TPD52	7.29E-44	-0.10431	0.181	0.358	1.09E-39
RABGAP1	7.83E-44	0.104334	0.152	0.233	1.17E-39
TCF7L2	8.19E-44	-0.25997	0.023	0.193	1.22E-39
CLDN10	8.28E-44	-0.44409	0.003	0.143	1.24E-39
LMAN2	8.44E-44	-0.18309	0.488	0.743	1.26E-39
TRIM47	8.52E-44	-0.21969	0.112	0.322	1.27E-39
TPST2	8.64E-44	-0.1443	0.03	0.175	1.29E-39
NFKBIB	8.64E-44	-0.17982	0.109	0.304	1.29E-39
TULP3	8.65E-44	-0.13027	0.135	0.316	1.29E-39
NONO	9.10E-44	-0.20232	0.552	0.807	1.36E-39
ZNF48	9.12E-44	0.172486	0.184	0.228	1.36E-39
G2E3	9.17E-44	-0.30494	0.121	0.346	1.37E-39
MBTPS1	9.25E-44	0.117297	0.205	0.284	1.38E-39
KLF6	9.27E-44	-0.25948	0.258	0.505	1.39E-39
STRN3	9.28E-44	-0.17134	0.089	0.273	1.39E-39
WDR47	9.32E-44	0.382961	0.264	0.18	1.39E-39
SPAST	9.85E-44	0.221298	0.2	0.223	1.47E-39
LPAR6	1.01E-43	-0.20469	0.017	0.169	1.50E-39
CBX2	1.01E-43	-0.23578	0.08	0.277	1.51E-39
C16orf72	1.04E-43	0.112493	0.137	0.209	1.55E-39
EFCAB2	1.07E-43	-0.27928	0.064	0.259	1.59E-39
FAM217B	1.08E-43	0.246301	0.238	0.244	1.61E-39
USP6NL	1.09E-43	-0.11838	0.027	0.165	1.63E-39
GPATCH11	1.12E-43	-0.18797	0.115	0.312	1.68E-39
NME7	1.13E-43	0.151284	0.219	0.293	1.69E-39
JAM3	1.22E-43	-0.19575	0.111	0.31	1.82E-39
GAS7	1.28E-43	-0.37391	0.02	0.194	1.92E-39
AFAP1	1.33E-43	0.276562	0.189	0.185	1.99E-39
TWISTNB	1.41E-43	0.120774	0.31	0.406	2.10E-39
PTPRN	1.44E-43	0.559422	0.258	0.059	2.15E-39
MAGI1	1.49E-43	0.261068	0.489	0.493	2.22E-39
SMARCD2	1.67E-43	-0.18693	0.018	0.172	2.49E-39
PPM1K	1.71E-43	0.23566	0.32	0.331	2.56E-39
ST7	1.71E-43	0.434349	0.297	0.191	2.56E-39

TMEM242	1.78E-43	0.119517	0.268	0.353	2.66E-39
CPSF3	1.79E-43	-0.10893	0.214	0.407	2.68E-39
LAMB2	1.81E-43	-0.16963	0.005	0.142	2.70E-39
MDGA1	1.94E-43	-0.11489	0.046	0.189	2.90E-39
WDR45B	1.95E-43	-0.12366	0.408	0.631	2.91E-39
RPP38	1.96E-43	-0.20225	0.073	0.259	2.93E-39
SORD	2.11E-43	-0.23175	0.027	0.198	3.16E-39
RHOC	2.15E-43	-0.46193	0.31	0.621	3.22E-39
WRNIP1	2.23E-43	-0.10102	0.115	0.281	3.34E-39
RP11-161M	2.28E-43	0.372318	0.371	0.319	3.41E-39
FUCA1	2.36E-43	0.104055	0.12	0.178	3.52E-39
XRCC3	2.44E-43	-0.22407	0.016	0.17	3.64E-39
C19orf24	2.49E-43	-0.1908	0.296	0.537	3.72E-39
CDH4	2.61E-43	0.281617	0.218	0.202	3.89E-39
KLHL12	2.66E-43	0.208094	0.189	0.21	3.97E-39
OSMR	2.72E-43	-0.16757	0.008	0.147	4.06E-39
LITAF	2.78E-43	-0.1116	0.229	0.421	4.16E-39
UMPS	2.86E-43	-0.15748	0.122	0.305	4.28E-39
FGF14	2.93E-43	0.281466	0.167	0.156	4.38E-39
PCNT	3.14E-43	-0.13131	0.075	0.24	4.69E-39
C10orf35	3.16E-43	0.568768	0.25	0.041	4.72E-39
IFT20	3.33E-43	0.127754	0.36	0.443	4.97E-39
TSPAN6	3.33E-43	-0.13121	0.119	0.295	4.98E-39
ALKBH3	3.33E-43	0.141693	0.149	0.212	4.98E-39
SLC9A3R2	3.40E-43	0.233715	0.163	0.177	5.09E-39
ZNF724P	3.47E-43	-0.29736	0.033	0.216	5.19E-39
LRIG1	3.70E-43	-0.16794	0.038	0.195	5.54E-39
USP33	3.73E-43	0.205228	0.264	0.29	5.57E-39
ACSBG1	3.75E-43	-0.2107	0.002	0.132	5.61E-39
CHMP4B	3.99E-43	-0.31817	0.318	0.607	5.96E-39
NDUFAF3	4.03E-43	-0.12342	0.489	0.717	6.02E-39
UFL1	4.05E-43	-0.10741	0.142	0.316	6.05E-39
STON2	4.06E-43	-0.20022	0.006	0.148	6.07E-39
XPNPEP3	4.10E-43	-0.15099	0.051	0.207	6.13E-39
CDC7	4.20E-43	-0.27536	0.042	0.227	6.28E-39
LRRC41	4.24E-43	0.184303	0.258	0.302	6.33E-39
2-Sep	4.43E-43	-0.31102	0.581	0.86	6.62E-39
CD320	4.85E-43	-0.25893	0.397	0.675	7.24E-39
SUV39H1	4.90E-43	-0.22182	0.024	0.186	7.32E-39
MANEA	5.04E-43	-0.14838	0.038	0.194	7.54E-39
PQLC3	5.30E-43	-0.10907	0.046	0.186	7.92E-39
MDFI	5.51E-43	-0.26137	0.038	0.211	8.23E-39
TMEM129	5.91E-43	-0.17482	0.112	0.3	8.82E-39
PRPF31	6.00E-43	-0.2178	0.369	0.622	8.97E-39
LSM12	6.15E-43	-0.21318	0.251	0.486	9.18E-39
CA12	6.18E-43	-0.26113	0.02	0.18	9.24E-39
SLC37A4	6.64E-43	-0.12912	0.072	0.235	9.92E-39
ZNF354A	6.76E-43	0.241762	0.207	0.214	1.01E-38
ATG4C	7.54E-43	-0.2679	0.067	0.258	1.13E-38
ABI2	8.08E-43	-0.20424	0.474	0.736	1.21E-38
NIPSNAP3A	8.51E-43	0.171509	0.194	0.235	1.27E-38

CASP7	8.61E-43	-0.2715	0.005	0.146	1.29E-38
ZADH2	8.70E-43	0.130508	0.181	0.246	1.30E-38
LGMN	8.86E-43	-0.22686	0.143	0.354	1.32E-38
PTX3	9.15E-43	-0.16005	0.053	0.216	1.37E-38
ANO6	9.46E-43	-0.19732	0.013	0.159	1.41E-38
NPHP1	9.83E-43	-0.15574	0.02	0.17	1.47E-38
CCL2	1.01E-42	-0.36653	0.047	0.247	1.51E-38
RGS17	1.01E-42	0.269255	0.321	0.317	1.51E-38
PDGFD	1.02E-42	-0.16759	0.006	0.143	1.52E-38
GLIPR2	1.03E-42	-0.27222	0.116	0.331	1.54E-38
POLR3GL	1.12E-42	0.108494	0.364	0.454	1.67E-38
GATAD2B	1.13E-42	0.255191	0.257	0.263	1.68E-38
VWA9	1.13E-42	-0.1805	0.083	0.265	1.69E-38
HIPK2	1.14E-42	0.148508	0.314	0.385	1.70E-38
NCOA3	1.15E-42	-0.14272	0.071	0.231	1.72E-38
LINC00094	1.18E-42	0.237216	0.191	0.188	1.76E-38
PHACTR2	1.22E-42	-0.15353	0.003	0.137	1.82E-38
UQCRB	1.28E-42	0.177524	0.826	0.83	1.91E-38
BNIP3	1.34E-42	-0.25701	0.124	0.335	2.00E-38
SLC7A6OS	1.39E-42	0.16859	0.175	0.222	2.07E-38
DDX56	1.39E-42	0.103953	0.365	0.456	2.07E-38
LINC00511	1.43E-42	-0.22846	0.089	0.285	2.13E-38
ATF4	1.44E-42	-0.1424	0.497	0.731	2.15E-38
BTBD10	1.46E-42	0.120491	0.276	0.353	2.19E-38
HEXB	1.47E-42	-0.33125	0.227	0.49	2.20E-38
RPUSD1	1.49E-42	0.111461	0.174	0.253	2.23E-38
PPIF	1.51E-42	-0.27399	0.135	0.367	2.26E-38
B4GALT5	1.52E-42	-0.15299	0.18	0.38	2.27E-38
POLR2L	1.74E-42	-0.38207	0.571	0.847	2.60E-38
FAM162A	1.78E-42	-0.27167	0.185	0.417	2.66E-38
YAE1D1	1.79E-42	0.326913	0.322	0.291	2.68E-38
SLC43A3	1.82E-42	-0.19095	0.104	0.299	2.72E-38
DCAF10	1.90E-42	0.19166	0.262	0.306	2.83E-38
MAT2B	1.97E-42	-0.14513	0.112	0.288	2.95E-38
RAB30-AS1	2.02E-42	-0.11081	0.137	0.31	3.02E-38
INO80C	2.07E-42	-0.14798	0.135	0.32	3.09E-38
COPRS	2.13E-42	-0.315	0.336	0.625	3.19E-38
FAM118B	2.22E-42	0.142614	0.189	0.258	3.31E-38
GUSB	2.23E-42	-0.15976	0.213	0.421	3.33E-38
NR2C2AP	2.27E-42	-0.20013	0.158	0.37	3.39E-38
ABHD14B	2.34E-42	-0.17621	0.009	0.149	3.50E-38
SUN2	2.36E-42	-0.13158	0.218	0.414	3.53E-38
LAMP5	2.44E-42	0.184087	0.182	0.235	3.64E-38
DEDD2	2.45E-42	0.419055	0.292	0.188	3.66E-38
UBAP1	2.50E-42	0.150437	0.185	0.242	3.73E-38
DYRK4	2.67E-42	-0.12033	0.124	0.296	3.99E-38
ENKUR	2.69E-42	-0.20293	0.008	0.146	4.01E-38
VMP1	2.79E-42	-0.21762	0.347	0.598	4.17E-38
AGA	2.79E-42	-0.1353	0.06	0.214	4.17E-38
PDHA1	2.79E-42	-0.14668	0.353	0.568	4.18E-38
GDF15	2.84E-42	-0.43408	0.007	0.154	4.24E-38

TMEM59	2.95E-42	0.168073	0.839	0.854	4.41E-38
ACAT1	3.13E-42	0.217711	0.412	0.43	4.68E-38
PIEZO1	3.14E-42	-0.21788	0.003	0.136	4.70E-38
DDX54	3.21E-42	-0.13165	0.158	0.342	4.79E-38
DNAJA3	3.29E-42	-0.12183	0.174	0.357	4.91E-38
EIF2S3	3.50E-42	-0.16452	0.184	0.388	5.22E-38
PTPRA	3.51E-42	-0.23061	0.384	0.648	5.25E-38
ZYG11B	3.77E-42	0.196186	0.221	0.253	5.63E-38
SPATA13	3.90E-42	-0.13153	0.053	0.206	5.82E-38
FKBP8	3.96E-42	0.154615	0.701	0.754	5.92E-38
OLFM2	4.17E-42	0.439831	0.384	0.265	6.24E-38
CHMP2A	4.26E-42	0.108299	0.682	0.763	6.37E-38
FHL3	4.35E-42	-0.12824	0.066	0.223	6.50E-38
CCDC86	4.62E-42	-0.12285	0.057	0.212	6.91E-38
MPC1	4.72E-42	-0.26988	0.416	0.696	7.05E-38
SLC25A19	4.72E-42	-0.19247	0.044	0.209	7.05E-38
CCDC8	4.85E-42	-0.15315	0.013	0.148	7.25E-38
DNAJB14	5.01E-42	0.107716	0.206	0.284	7.49E-38
PIK3R1	5.18E-42	0.337994	0.49	0.422	7.74E-38
PPP6R3	5.24E-42	-0.13546	0.076	0.241	7.83E-38
CBX8	5.28E-42	0.231425	0.145	0.156	7.88E-38
PHYKPL	5.47E-42	0.131545	0.169	0.236	8.18E-38
NUP188	5.80E-42	-0.19176	0.057	0.226	8.66E-38
PPP1CA	5.93E-42	-0.30637	0.43	0.722	8.86E-38
SERGEF	5.97E-42	0.129503	0.157	0.22	8.93E-38
TMEM51	5.99E-42	-0.19587	0.024	0.175	8.95E-38
FAF2	6.02E-42	0.109054	0.263	0.352	8.99E-38
ZNF677	6.08E-42	0.237714	0.325	0.333	9.09E-38
CLVS1	6.11E-42	0.538087	0.219	0.026	9.13E-38
RFX4	6.24E-42	-0.30667	0.102	0.317	9.32E-38
TMEM214	6.28E-42	-0.13369	0.067	0.228	9.38E-38
FUBP1	6.28E-42	0.237611	0.43	0.43	9.39E-38
ATP9A	6.37E-42	0.212738	0.199	0.22	9.52E-38
SSB	6.56E-42	0.127278	0.731	0.801	9.80E-38
GAN	6.73E-42	0.16129	0.199	0.246	1.01E-37
RP11-262H	6.80E-42	0.159205	0.196	0.253	1.02E-37
STX6	6.82E-42	0.202497	0.228	0.269	1.02E-37
TBL1X	7.01E-42	-0.11423	0.02	0.148	1.05E-37
ISYNA1	7.06E-42	0.178523	0.247	0.285	1.05E-37
SATB2	7.60E-42	-0.12482	0.03	0.17	1.14E-37
ARHGAP33	8.01E-42	-0.10478	0.174	0.351	1.20E-37
MTSS1	8.03E-42	0.419719	0.46	0.333	1.20E-37
AHCTF1	8.38E-42	-0.11133	0.059	0.204	1.25E-37
PPCDC	8.73E-42	-0.16826	0.02	0.164	1.30E-37
KDM2B	8.75E-42	0.170224	0.223	0.267	1.31E-37
MKS1	8.95E-42	-0.16461	0.061	0.228	1.34E-37
POLE2	9.16E-42	-0.21204	0.034	0.19	1.37E-37
COX7A2	9.41E-42	0.185708	0.965	0.928	1.41E-37
CNOT10	9.48E-42	-0.1243	0.126	0.296	1.42E-37
TEAD4	9.75E-42	-0.15958	0.002	0.13	1.46E-37
PEAK1	9.90E-42	-0.18006	0.018	0.165	1.48E-37

CTC-260E6	9.91E-42	-0.22559	0.017	0.173	1.48E-37
NIN	1.03E-41	-0.15678	0.117	0.299	1.54E-37
JPH4	1.04E-41	0.582379	0.254	0.051	1.56E-37
LYRM1	1.09E-41	0.102867	0.252	0.338	1.63E-37
DHPS	1.10E-41	0.171333	0.71	0.757	1.65E-37
ARL4A	1.13E-41	-0.39009	0.273	0.553	1.69E-37
NYAP1	1.17E-41	0.525195	0.213	0.03	1.74E-37
PARP1	1.17E-41	-0.29956	0.518	0.802	1.75E-37
ZHX3	1.20E-41	-0.25351	0.071	0.262	1.80E-37
EXOSC1	1.22E-41	-0.20107	0.104	0.291	1.83E-37
LAMA4	1.24E-41	-0.201	0.009	0.143	1.86E-37
PDXDC1	1.28E-41	0.150856	0.238	0.299	1.91E-37
PRPF4	1.28E-41	-0.14742	0.184	0.379	1.92E-37
PLEKHH2	1.33E-41	-0.23187	0.024	0.181	1.99E-37
SPEN	1.34E-41	0.103052	0.199	0.279	2.00E-37
PCGF5	1.34E-41	-0.17896	0.047	0.207	2.01E-37
RBMX	1.35E-41	-0.34818	0.656	0.901	2.02E-37
ZFAND1	1.37E-41	0.165437	0.178	0.235	2.05E-37
CDK2AP1	1.45E-41	-0.14287	0.069	0.231	2.17E-37
HES4	1.48E-41	-0.29923	0.185	0.432	2.21E-37
DNAJC30	1.50E-41	0.198141	0.212	0.249	2.25E-37
C20orf27	1.55E-41	-0.21261	0.263	0.5	2.32E-37
PITPNB	1.56E-41	-0.13824	0.149	0.332	2.33E-37
PFKM	1.61E-41	0.237577	0.256	0.263	2.41E-37
ADAL	1.78E-41	-0.13556	0.077	0.24	2.66E-37
TMEM165	1.86E-41	-0.2001	0.439	0.689	2.77E-37
SLC25A39	1.90E-41	-0.13378	0.344	0.562	2.84E-37
RARRES2	1.92E-41	-0.62449	0.06	0.277	2.88E-37
TMEM55B	2.05E-41	0.108576	0.239	0.33	3.07E-37
HERPUD2	2.24E-41	0.234863	0.245	0.257	3.34E-37
KAT6B	2.35E-41	0.391111	0.289	0.19	3.51E-37
TMEM230	2.35E-41	-0.31579	0.49	0.78	3.51E-37
MDH2	2.42E-41	0.173719	0.845	0.849	3.61E-37
TOM1L1	2.48E-41	-0.18165	0.005	0.138	3.71E-37
NISCH	2.49E-41	0.112554	0.156	0.228	3.72E-37
MVP	2.60E-41	-0.11231	0.02	0.144	3.88E-37
CRBN	2.63E-41	0.251772	0.355	0.363	3.93E-37
IGF2BP2	2.73E-41	-0.16317	0.043	0.198	4.08E-37
SSSCA1	2.75E-41	-0.14499	0.1	0.273	4.11E-37
SCAMP1	2.76E-41	0.22599	0.354	0.372	4.12E-37
GRK4	2.81E-41	0.377064	0.24	0.169	4.20E-37
HAUS7	2.85E-41	-0.12499	0.075	0.232	4.26E-37
SYNM	2.88E-41	-0.16778	0.005	0.135	4.31E-37
MPND	2.97E-41	-0.11147	0.056	0.204	4.43E-37
HTT	3.20E-41	0.10967	0.13	0.205	4.78E-37
ZC3H6	3.24E-41	0.272751	0.272	0.273	4.84E-37
SMPD4	3.51E-41	-0.14136	0.109	0.283	5.24E-37
DIXDC1	3.51E-41	0.38112	0.21	0.133	5.24E-37
WDR1	3.59E-41	-0.19477	0.312	0.549	5.37E-37
CCNG1	3.63E-41	-0.20734	0.198	0.41	5.43E-37
ITGA3	3.67E-41	-0.16117	0.054	0.215	5.48E-37

EYA4	4.06E-41	-0.15317	0.001	0.122	6.07E-37
ATP13A2	4.07E-41	0.233398	0.218	0.227	6.08E-37
KLF10	4.16E-41	0.360246	0.35	0.3	6.22E-37
PPP3CB	4.18E-41	0.250562	0.183	0.18	6.24E-37
SSBP4	4.22E-41	-0.11677	0.266	0.47	6.30E-37
CRISPLD1	4.24E-41	-0.32723	0.06	0.258	6.33E-37
LRP4	4.47E-41	-0.23832	0.013	0.159	6.68E-37
PRKCZ	4.50E-41	0.446842	0.272	0.132	6.73E-37
KLRC2	4.51E-41	0.578505	0.433	0.198	6.74E-37
SERINC2	4.56E-41	-0.1738	0.054	0.219	6.81E-37
BCAT2	4.72E-41	-0.12784	0.064	0.216	7.06E-37
ASPHD1	4.75E-41	0.283393	0.223	0.212	7.09E-37
LRRC8A	4.93E-41	0.103357	0.243	0.326	7.37E-37
EPHA4	5.12E-41	-0.23359	0.031	0.198	7.65E-37
ARV1	5.42E-41	0.113168	0.224	0.302	8.09E-37
POLR2M	5.43E-41	-0.20249	0.111	0.304	8.11E-37
SMIM18	5.48E-41	0.559232	0.342	0.116	8.19E-37
RNF170	5.48E-41	0.172771	0.205	0.252	8.20E-37
EPOR	5.98E-41	-0.18035	0.026	0.174	8.94E-37
PEA15	6.31E-41	-0.16388	0.423	0.651	9.44E-37
TRAF2	6.53E-41	-0.10126	0.075	0.225	9.76E-37
ANKMY2	6.94E-41	0.31382	0.277	0.227	1.04E-36
SERP2	6.98E-41	0.491009	0.308	0.16	1.04E-36
CEP44	7.23E-41	-0.11675	0.059	0.207	1.08E-36
CDK12	7.25E-41	0.101656	0.192	0.275	1.08E-36
C1orf112	7.50E-41	-0.21513	0.027	0.181	1.12E-36
RIOK1	7.51E-41	0.1291	0.203	0.27	1.12E-36
KALRN	7.60E-41	0.289863	0.185	0.159	1.14E-36
CNTFR	7.95E-41	0.164596	0.308	0.359	1.19E-36
CPEB4	8.57E-41	0.345533	0.35	0.29	1.28E-36
TBC1D17	1.00E-40	0.301184	0.208	0.177	1.50E-36
PPA2	1.02E-40	-0.1202	0.243	0.435	1.52E-36
HERC5	1.07E-40	-0.21904	0.01	0.152	1.60E-36
COL5A2	1.11E-40	-0.19303	0.009	0.147	1.66E-36
CHRNB1	1.12E-40	0.330994	0.211	0.167	1.68E-36
ATF2	1.21E-40	0.212989	0.247	0.278	1.81E-36
GPD1	1.22E-40	0.574285	0.216	0.036	1.82E-36
BLVRB	1.23E-40	-0.10901	0.131	0.296	1.83E-36
ZNF726	1.32E-40	-0.27096	0.027	0.194	1.97E-36
SNHG10	1.40E-40	-0.11855	0.079	0.232	2.08E-36
DCAF6	1.42E-40	0.139548	0.14	0.195	2.12E-36
CKB	1.42E-40	-0.27159	0.918	0.968	2.12E-36
NUDT15	1.45E-40	-0.15418	0.125	0.307	2.17E-36
TAF13	1.51E-40	-0.1673	0.075	0.246	2.26E-36
CLEC11A	1.53E-40	0.251457	0.256	0.281	2.29E-36
ACAT2	1.54E-40	-0.15356	0.395	0.622	2.30E-36
DDB2	1.56E-40	-0.18912	0.029	0.183	2.34E-36
CCPG1	1.66E-40	-0.14003	0.124	0.299	2.49E-36
AP006222.	1.73E-40	-0.12522	0.118	0.283	2.59E-36
DICER1	1.73E-40	0.141673	0.174	0.238	2.59E-36
ISG20L2	1.78E-40	0.132662	0.269	0.342	2.66E-36

CTTNBP2NL	1.78E-40	0.225665	0.203	0.211	2.66E-36
EAF1	1.84E-40	0.1139	0.123	0.179	2.75E-36
AP2M1	1.87E-40	0.162053	0.859	0.862	2.79E-36
BRI3BP	2.00E-40	-0.19499	0.077	0.256	2.99E-36
MGARP	2.00E-40	-0.10946	0.031	0.165	2.99E-36
C14orf166	2.01E-40	-0.28198	0.485	0.765	3.00E-36
PRDX2	2.04E-40	0.176365	0.962	0.932	3.05E-36
SLC5A6	2.05E-40	-0.11884	0.08	0.238	3.07E-36
TCEB1	2.12E-40	0.183311	0.803	0.823	3.17E-36
SLC25A5	2.13E-40	-0.36959	0.651	0.896	3.18E-36
ITM2B	2.15E-40	0.197684	0.919	0.895	3.21E-36
AGBL5	2.15E-40	-0.13434	0.061	0.219	3.21E-36
KIN	2.17E-40	0.102347	0.143	0.222	3.24E-36
TMEM25	2.30E-40	0.281341	0.198	0.186	3.44E-36
RPS6KL1	2.37E-40	0.451781	0.297	0.18	3.55E-36
P4HB	2.42E-40	-0.40187	0.483	0.769	3.61E-36
EIF2B5	2.50E-40	0.125338	0.229	0.296	3.74E-36
CBX3	2.63E-40	0.171049	0.876	0.879	3.94E-36
TBRG1	2.79E-40	0.139668	0.258	0.326	4.17E-36
FAM126B	2.87E-40	0.300298	0.252	0.217	4.28E-36
TIPIN	2.91E-40	-0.22104	0.078	0.262	4.35E-36
SLC35F5	2.93E-40	-0.117	0.068	0.214	4.37E-36
AEN	2.96E-40	-0.28067	0.053	0.242	4.42E-36
GFM1	3.05E-40	0.148452	0.215	0.281	4.56E-36
ZNF12	3.10E-40	0.117876	0.189	0.262	4.64E-36
ANKRD36	3.21E-40	0.132495	0.291	0.36	4.79E-36
CFL2	3.53E-40	-0.11464	0.343	0.546	5.28E-36
IFI16	3.55E-40	-0.43665	0.142	0.396	5.31E-36
SS18L1	3.74E-40	0.179051	0.159	0.198	5.60E-36
TRIM69	3.79E-40	-0.21874	0.156	0.365	5.67E-36
CAB39	3.89E-40	0.121439	0.186	0.252	5.82E-36
AC009506.	3.98E-40	-0.33139	0.059	0.253	5.95E-36
ZNF138	4.00E-40	0.105464	0.307	0.393	5.98E-36
SNRNP35	4.02E-40	0.166521	0.272	0.33	6.01E-36
NRP2	4.08E-40	-0.17267	0.133	0.311	6.09E-36
AMN1	4.20E-40	0.33298	0.266	0.219	6.28E-36
ARPC3	4.34E-40	0.189021	0.812	0.817	6.49E-36
ELP2	4.61E-40	0.212655	0.263	0.281	6.90E-36
GAB1	5.09E-40	-0.19749	0.049	0.214	7.60E-36
KIF3C	5.16E-40	0.365771	0.241	0.18	7.71E-36
TMEM68	5.38E-40	0.113438	0.255	0.341	8.04E-36
SLC35F1	5.38E-40	-0.11253	0.124	0.283	8.05E-36
ZDHHC6	5.43E-40	-0.16909	0.105	0.28	8.12E-36
ARL10	5.49E-40	0.218462	0.177	0.185	8.20E-36
ADAMTS6	5.55E-40	-0.26027	0.016	0.17	8.29E-36
KIAA0586	5.59E-40	-0.17404	0.062	0.226	8.36E-36
ITCH	5.76E-40	-0.13283	0.048	0.194	8.61E-36
GEN1	6.21E-40	-0.19411	0.018	0.164	9.28E-36
PARD3	6.34E-40	-0.1244	0.038	0.179	9.47E-36
NAPB	6.90E-40	0.472955	0.236	0.099	1.03E-35
LRP12	7.69E-40	0.366284	0.218	0.149	1.15E-35

JAKMIP1	7.76E-40	0.487501	0.198	0.02	1.16E-35
SP110	7.82E-40	-0.25812	0.016	0.165	1.17E-35
LRRC40	8.05E-40	0.273562	0.312	0.288	1.20E-35
BUD31	8.11E-40	0.176407	0.841	0.853	1.21E-35
FTO	8.16E-40	0.111812	0.189	0.263	1.22E-35
UBA3	8.20E-40	0.108167	0.288	0.372	1.23E-35
EID2	8.36E-40	0.146459	0.183	0.243	1.25E-35
EMID1	8.42E-40	-0.10069	0.055	0.193	1.26E-35
MAPKAPK3	8.56E-40	-0.11688	0.026	0.154	1.28E-35
NDP	8.73E-40	-0.18822	0.041	0.198	1.30E-35
EIF1	9.49E-40	0.132879	0.995	0.983	1.42E-35
PKP4	1.03E-39	-0.17875	0.044	0.204	1.53E-35
SMIM13	1.03E-39	-0.11078	0.049	0.189	1.54E-35
MIER1	1.04E-39	-0.13107	0.112	0.279	1.56E-35
AUNIP	1.09E-39	-0.25891	0.003	0.13	1.63E-35
CD302	1.15E-39	-0.16163	0.016	0.152	1.72E-35
PDP1	1.20E-39	0.155937	0.167	0.21	1.79E-35
OSER1-AS1	1.39E-39	0.14416	0.194	0.251	2.08E-35
SESTD1	1.42E-39	0.215876	0.343	0.365	2.12E-35
CDC37	1.44E-39	-0.16399	0.505	0.747	2.14E-35
SMG9	1.47E-39	-0.1359	0.094	0.256	2.20E-35
POC1B	1.53E-39	-0.18721	0.044	0.205	2.29E-35
C6orf203	1.54E-39	0.142071	0.233	0.291	2.30E-35
PSMG1	1.56E-39	-0.15324	0.191	0.385	2.33E-35
TRAPPC2P1	1.64E-39	0.276907	0.317	0.296	2.45E-35
TOP1	1.64E-39	-0.25606	0.394	0.667	2.45E-35
FBXW7	1.66E-39	0.18725	0.218	0.262	2.48E-35
EML3	1.73E-39	-0.13015	0.024	0.154	2.58E-35
RARS	1.76E-39	0.115665	0.288	0.36	2.62E-35
C6orf48	1.81E-39	-0.30116	0.573	0.842	2.70E-35
LRP1	1.85E-39	-0.15173	0.194	0.39	2.76E-35
CCND2	1.87E-39	-0.31717	0.427	0.705	2.79E-35
RXRB	1.95E-39	0.197947	0.234	0.252	2.91E-35
SAP18	1.95E-39	0.155648	0.859	0.878	2.91E-35
SLC2A8	2.01E-39	-0.11036	0.097	0.252	3.00E-35
NECAB3	2.15E-39	-0.15874	0.043	0.195	3.21E-35
MAP3K4	2.17E-39	-0.15098	0.049	0.201	3.25E-35
POLR2E	2.24E-39	-0.24558	0.504	0.772	3.34E-35
NUDT6	2.44E-39	-0.11232	0.057	0.198	3.64E-35
BAMBI	2.52E-39	-0.20671	0.039	0.196	3.76E-35
ECI2	2.59E-39	-0.28379	0.285	0.543	3.86E-35
POLR2F	2.79E-39	-0.20669	0.562	0.819	4.17E-35
FBXO44	2.80E-39	0.209212	0.163	0.19	4.19E-35
FAM168A	3.03E-39	-0.13144	0.075	0.228	4.54E-35
HECTD4	3.05E-39	0.237275	0.175	0.188	4.55E-35
DDA1	3.05E-39	-0.13173	0.319	0.527	4.55E-35
C12orf45	3.07E-39	-0.1059	0.152	0.325	4.59E-35
GCSH	3.12E-39	-0.35119	0.364	0.653	4.66E-35
SAV1	3.16E-39	-0.14606	0.068	0.223	4.72E-35
TMEM243	3.16E-39	-0.25949	0.086	0.283	4.72E-35
DPP3	3.27E-39	0.21671	0.237	0.257	4.89E-35

MDFIC	3.42E-39	-0.16405	0.002	0.123	5.11E-35
FAM184B	3.48E-39	-0.17375	0.038	0.191	5.20E-35
ZYX	3.64E-39	-0.29879	0.248	0.507	5.43E-35
DLC1	3.75E-39	-0.21616	0.009	0.149	5.60E-35
MAN1C1	3.80E-39	-0.1434	0.002	0.116	5.68E-35
FAM69C	3.84E-39	-0.15793	0.002	0.121	5.74E-35
PCDH10	4.41E-39	-0.17523	0.005	0.13	6.59E-35
YY1AP1	4.50E-39	0.115038	0.227	0.294	6.72E-35
ZNF462	4.53E-39	0.147761	0.3	0.362	6.78E-35
ANKRA2	4.59E-39	0.146234	0.187	0.243	6.86E-35
CEP68	4.64E-39	0.231926	0.211	0.228	6.93E-35
SLC38A2	5.03E-39	0.127623	0.288	0.362	7.52E-35
MAP3K1	5.04E-39	-0.21052	0.031	0.189	7.54E-35
NDUFC1	5.10E-39	-0.26068	0.467	0.74	7.62E-35
TOPORS	5.10E-39	0.21079	0.297	0.312	7.63E-35
ZNF217	5.29E-39	-0.13688	0.02	0.156	7.90E-35
SYT4	5.42E-39	0.479739	0.167	0.01	8.10E-35
FAM131A	5.44E-39	0.260224	0.214	0.202	8.12E-35
SNX10	5.50E-39	0.219351	0.24	0.243	8.22E-35
BDH2	6.03E-39	-0.16448	0.007	0.132	9.01E-35
FAM118A	6.50E-39	0.426867	0.266	0.151	9.71E-35
TOMM34	6.60E-39	-0.17205	0.101	0.274	9.86E-35
MED25	7.16E-39	-0.28062	0.083	0.281	1.07E-34
UBL7	7.50E-39	-0.12439	0.173	0.349	1.12E-34
C11orf58	7.63E-39	0.167967	0.829	0.84	1.14E-34
ATG10	8.07E-39	0.200387	0.222	0.258	1.21E-34
LINC00632	8.43E-39	0.593398	0.375	0.137	1.26E-34
USP42	8.59E-39	0.188761	0.221	0.251	1.28E-34
C19orf53	8.97E-39	-0.39518	0.562	0.821	1.34E-34
SLC26A2	9.38E-39	-0.15601	0.049	0.204	1.40E-34
METTL10	9.39E-39	-0.17364	0.085	0.253	1.40E-34
USP18	9.63E-39	-0.23247	0.007	0.144	1.44E-34
GAD1	9.94E-39	0.335576	0.284	0.23	1.49E-34
MED21	1.02E-38	0.113425	0.321	0.402	1.52E-34
TBC1D31	1.04E-38	-0.25882	0.036	0.205	1.56E-34
RAP1GDS1	1.06E-38	0.155464	0.214	0.262	1.58E-34
TMEM47	1.07E-38	-0.23677	0.03	0.193	1.60E-34
LACTB2	1.08E-38	-0.26645	0.035	0.199	1.61E-34
IBTK	1.09E-38	0.11866	0.176	0.238	1.62E-34
SAMD8	1.13E-38	-0.12042	0.068	0.217	1.69E-34
RAB35	1.17E-38	0.112977	0.185	0.257	1.74E-34
NKAIN3	1.18E-38	0.236856	0.272	0.283	1.77E-34
UGGT1	1.19E-38	-0.12166	0.1	0.262	1.77E-34
CNPY4	1.19E-38	-0.15259	0.073	0.231	1.78E-34
LRRC16A	1.20E-38	-0.16404	0.023	0.162	1.79E-34
IPO8	1.22E-38	-0.12938	0.033	0.17	1.82E-34
POLK	1.23E-38	0.149586	0.233	0.293	1.84E-34
TAF8	1.24E-38	-0.14403	0.037	0.177	1.86E-34
B3GAT3	1.26E-38	-0.1652	0.341	0.567	1.88E-34
ZNF775	1.27E-38	0.127084	0.16	0.225	1.89E-34
RAB7A	1.34E-38	0.114579	0.707	0.774	2.01E-34

PIM3	1.35E-38	-0.19313	0.128	0.319	2.01E-34
CYSTM1	1.38E-38	-0.10725	0.194	0.372	2.06E-34
GCFC2	1.39E-38	-0.15006	0.102	0.272	2.07E-34
SOX6	1.39E-38	0.166703	0.193	0.247	2.08E-34
DIS3L	1.47E-38	-0.11642	0.093	0.246	2.20E-34
ABHD17B	1.48E-38	0.318979	0.276	0.226	2.21E-34
RNF166	1.49E-38	0.133839	0.184	0.241	2.23E-34
TRIO	1.71E-38	-0.18803	0.137	0.325	2.55E-34
TDP2	1.86E-38	0.253105	0.237	0.23	2.78E-34
APEX2	1.92E-38	-0.18208	0.031	0.178	2.88E-34
ADAMTS9	2.01E-38	-0.20551	0.01	0.144	3.01E-34
SP1	2.28E-38	-0.14562	0.028	0.172	3.40E-34
SMURF2	2.43E-38	0.102599	0.148	0.215	3.63E-34
NMT2	2.47E-38	-0.13281	0.103	0.267	3.69E-34
NRIP1	2.59E-38	-0.10824	0.074	0.216	3.87E-34
CADM3	2.63E-38	0.521782	0.236	0.048	3.93E-34
TRIM33	2.68E-38	0.159297	0.294	0.338	4.00E-34
OPTN	2.73E-38	0.234671	0.193	0.19	4.08E-34
UGGT2	2.79E-38	-0.15228	0.053	0.206	4.16E-34
GSTCD	2.85E-38	-0.12244	0.031	0.164	4.26E-34
PXMP4	2.90E-38	-0.20132	0.028	0.173	4.33E-34
ANKRD39	2.99E-38	0.105037	0.205	0.286	4.47E-34
RASSF8-AS	3.22E-38	0.134887	0.218	0.286	4.82E-34
SGCB	3.30E-38	-0.18139	0.495	0.728	4.93E-34
TSPAN7	3.37E-38	0.185672	0.52	0.557	5.03E-34
FOXN3	3.85E-38	0.168288	0.246	0.286	5.76E-34
NMI	4.01E-38	-0.22408	0.021	0.17	5.99E-34
TCF7	4.03E-38	0.244174	0.225	0.219	6.03E-34
NBN	4.04E-38	-0.14386	0.111	0.28	6.04E-34
RNPEPL1	4.07E-38	-0.12468	0.042	0.181	6.08E-34
PPP1R14B	4.29E-38	0.132551	0.54	0.626	6.42E-34
RASGRP1	4.41E-38	-0.17985	0.023	0.17	6.59E-34
KNTC1	4.73E-38	-0.25455	0.013	0.156	7.07E-34
STX4	5.08E-38	-0.10062	0.131	0.286	7.59E-34
NKX2-2	5.51E-38	0.531162	0.481	0.253	8.23E-34
TUBA1C	5.89E-38	-0.5531	0.326	0.616	8.81E-34
KLHL23	6.04E-38	0.238876	0.47	0.459	9.03E-34
CEP89	6.06E-38	-0.12123	0.175	0.344	9.05E-34
ZNF704	6.19E-38	0.225967	0.307	0.319	9.25E-34
LPCAT1	6.35E-38	0.126188	0.285	0.352	9.49E-34
ELAC2	6.41E-38	-0.15962	0.12	0.296	9.58E-34
BBS2	6.49E-38	-0.22454	0.107	0.298	9.70E-34
CBL	6.66E-38	-0.11332	0.069	0.215	9.96E-34
ELMO1	6.82E-38	0.566232	0.321	0.106	1.02E-33
PRPS1	7.31E-38	-0.22271	0.144	0.346	1.09E-33
CNTROB	7.44E-38	-0.18262	0.085	0.257	1.11E-33
ZFAND6	7.57E-38	-0.27773	0.353	0.607	1.13E-33
TMEM127	7.58E-38	0.174892	0.152	0.196	1.13E-33
PDAP1	7.61E-38	0.123805	0.761	0.821	1.14E-33
ALDH16A1	7.99E-38	-0.17001	0.054	0.209	1.19E-33
SCN2A	8.22E-38	0.489432	0.183	0.017	1.23E-33

ZFAS1	8.38E-38	-0.32171	0.596	0.856	1.25E-33
OAF	8.44E-38	-0.14771	0.002	0.114	1.26E-33
CALCOCO1	8.58E-38	0.352952	0.241	0.173	1.28E-33
CSNK2B	9.07E-38	-0.10891	0.605	0.81	1.36E-33
NUP214	9.07E-38	-0.11013	0.094	0.244	1.36E-33
EIF4EBP1	9.50E-38	-0.16588	0.417	0.648	1.42E-33
STK3	9.67E-38	-0.15151	0.028	0.165	1.45E-33
ZNF140	9.92E-38	0.248817	0.192	0.195	1.48E-33
SLC25A46	9.95E-38	0.200451	0.178	0.201	1.49E-33
NDEL1	1.01E-37	0.256646	0.194	0.189	1.51E-33
LINC00493	1.02E-37	-0.14689	0.463	0.696	1.52E-33
CDK13	1.02E-37	0.121472	0.159	0.221	1.53E-33
SLC27A1	1.04E-37	-0.12709	0.068	0.221	1.56E-33
CANX	1.05E-37	-0.24604	0.456	0.715	1.58E-33
NAA35	1.07E-37	0.14052	0.219	0.27	1.60E-33
NCOR1	1.09E-37	0.131995	0.668	0.728	1.62E-33
HMBOX1	1.10E-37	0.328014	0.346	0.29	1.65E-33
PABPC1L	1.13E-37	-0.15143	0.027	0.167	1.69E-33
SND1	1.16E-37	0.164618	0.295	0.337	1.73E-33
CREBZF	1.17E-37	0.100756	0.196	0.268	1.75E-33
SEMA5A	1.18E-37	-0.24935	0.036	0.2	1.77E-33
DENND2A	1.23E-37	-0.16399	0.075	0.238	1.84E-33
PRMT1	1.24E-37	0.119987	0.736	0.805	1.85E-33
CLPP	1.26E-37	-0.28001	0.383	0.648	1.88E-33
SKP2	1.26E-37	-0.19297	0.038	0.189	1.89E-33
YARS	1.27E-37	-0.1065	0.203	0.374	1.90E-33
PLCG1	1.38E-37	-0.13741	0.08	0.235	2.06E-33
DYNC2LI1	1.38E-37	0.174713	0.204	0.242	2.07E-33
GNG7	1.48E-37	-0.29267	0.104	0.311	2.21E-33
CDKN2D	1.51E-37	0.134049	0.442	0.51	2.25E-33
HEXDC	1.61E-37	0.138161	0.123	0.173	2.40E-33
CDKN2AIP	1.61E-37	0.199794	0.275	0.298	2.41E-33
TMEM192	1.79E-37	0.394392	0.291	0.183	2.68E-33
TEX2	1.80E-37	-0.1276	0.024	0.153	2.69E-33
ZNF493	1.83E-37	0.248817	0.255	0.26	2.74E-33
TTPAL	1.92E-37	0.17291	0.194	0.228	2.87E-33
HSD17B11	1.95E-37	-0.26982	0.161	0.378	2.92E-33
EXOSC9	2.12E-37	-0.23238	0.165	0.369	3.16E-33
RYK	2.15E-37	-0.10215	0.062	0.199	3.21E-33
ATP5G1	2.16E-37	0.135362	0.751	0.805	3.22E-33
CHRDL1	2.27E-37	-0.24018	0.014	0.157	3.40E-33
ADORA1	2.29E-37	-0.14129	0.036	0.175	3.42E-33
MLXIP	2.29E-37	0.166575	0.224	0.269	3.43E-33
DNAJC4	2.43E-37	0.171216	0.178	0.227	3.63E-33
RCN2	2.47E-37	0.192906	0.806	0.801	3.69E-33
PYGL	2.67E-37	-0.19246	0.013	0.147	3.98E-33
N6AMT2	2.68E-37	0.108684	0.161	0.232	4.00E-33
SOX21-AS1	2.70E-37	-0.19718	0.01	0.143	4.04E-33
HOXA5	2.70E-37	0.540011	0.271	0.086	4.04E-33
ZIC1	2.76E-37	-0.30739	0.111	0.322	4.13E-33
MAPRE1	2.86E-37	-0.15992	0.516	0.741	4.27E-33

RP11-192H	2.90E-37	0.223672	0.224	0.223	4.33E-33
HAUS5	2.91E-37	-0.21209	0.055	0.223	4.35E-33
CCT7	2.92E-37	0.103102	0.718	0.788	4.36E-33
NIT2	2.99E-37	-0.13679	0.166	0.341	4.47E-33
IRX3	3.12E-37	-0.14549	0	0.104	4.67E-33
LEMD1	3.46E-37	0.467501	0.185	0.042	5.18E-33
C18orf54	3.51E-37	-0.19662	0.013	0.148	5.25E-33
EMILIN3	3.60E-37	-0.29796	0.009	0.141	5.38E-33
PGAM5	3.62E-37	-0.12172	0.032	0.163	5.41E-33
GTF2A2	3.77E-37	-0.13378	0.513	0.733	5.63E-33
IGF2BP3	4.01E-37	-0.16573	0.112	0.289	6.00E-33
BEST3	4.09E-37	-0.49536	0.09	0.307	6.11E-33
LMO4	4.42E-37	0.243256	0.774	0.78	6.61E-33
MIB1	4.66E-37	-0.11149	0.078	0.222	6.97E-33
BATF3	4.81E-37	-0.19855	0.01	0.144	7.18E-33
PPIA	5.14E-37	-0.25097	0.914	0.983	7.68E-33
TSPAN3	5.20E-37	-0.26419	0.5	0.765	7.77E-33
KLHDC10	5.33E-37	0.108	0.191	0.256	7.97E-33
MTOR	5.56E-37	-0.12263	0.044	0.183	8.32E-33
KTN1	5.89E-37	-0.1617	0.519	0.759	8.80E-33
PGK1	6.34E-37	0.101537	0.727	0.804	9.48E-33
HSD17B4	6.38E-37	0.135539	0.226	0.288	9.53E-33
NLGN3	6.39E-37	-0.16324	0.112	0.284	9.56E-33
IGF2R	6.42E-37	-0.12469	0.031	0.163	9.59E-33
CCNB1IP1	6.95E-37	-0.13358	0.167	0.338	1.04E-32
BCL3	7.02E-37	-0.14028	0.02	0.148	1.05E-32
PRMT5	7.05E-37	-0.21813	0.103	0.286	1.05E-32
ZNF883	7.13E-37	0.107632	0.212	0.28	1.07E-32
EFEMP1	7.35E-37	-0.29719	0.06	0.241	1.10E-32
IFT80	7.84E-37	-0.14144	0.041	0.179	1.17E-32
MAPK8	8.04E-37	0.264423	0.213	0.189	1.20E-32
PLK3	8.41E-37	0.24476	0.2	0.191	1.26E-32
KLHL13	8.77E-37	0.147875	0.196	0.246	1.31E-32
MEIS1	9.25E-37	-0.2001	0.072	0.24	1.38E-32
ZFAND2A	9.42E-37	0.155367	0.391	0.452	1.41E-32
COX17	9.60E-37	-0.2449	0.427	0.681	1.43E-32
CRELD1	9.77E-37	0.142532	0.153	0.205	1.46E-32
SYCE1L	1.00E-36	-0.1316	0.022	0.153	1.50E-32
POLR2B	1.01E-36	0.123378	0.438	0.51	1.51E-32
NT5C2	1.04E-36	0.187714	0.158	0.179	1.55E-32
OAS3	1.06E-36	-0.1762	0.013	0.138	1.59E-32
TRMT13	1.08E-36	0.127508	0.189	0.249	1.61E-32
CEP192	1.10E-36	-0.15844	0.025	0.16	1.64E-32
TMTC4	1.13E-36	0.326958	0.211	0.177	1.69E-32
PGAM1	1.15E-36	-0.33184	0.407	0.684	1.72E-32
HNRNPA1L2	1.16E-36	-0.25347	0.197	0.421	1.73E-32
PHF10	1.17E-36	-0.12118	0.048	0.185	1.74E-32
HILPDA	1.17E-36	-0.13163	0.261	0.452	1.75E-32
CCDC77	1.22E-36	-0.16433	0.068	0.223	1.82E-32
CSRP1	1.26E-36	-0.19739	0.104	0.284	1.88E-32
TMEM198	1.29E-36	0.27325	0.228	0.201	1.92E-32

SNAI2	1.53E-36	-0.1754	0.007	0.128	2.29E-32
DPAGT1	1.56E-36	-0.1287	0.079	0.228	2.34E-32
RP5-1085F	1.58E-36	0.12955	0.147	0.193	2.37E-32
OXA1L	1.60E-36	-0.16705	0.145	0.326	2.38E-32
TSC22D3	1.81E-36	0.298954	0.443	0.374	2.71E-32
MED15	1.83E-36	-0.10737	0.084	0.231	2.74E-32
AMER2	1.86E-36	0.501332	0.321	0.138	2.78E-32
PLCB3	1.94E-36	-0.12605	0.009	0.125	2.90E-32
DBX2	1.98E-36	-0.20703	0.003	0.121	2.96E-32
SSBP3	2.01E-36	0.135791	0.218	0.273	3.01E-32
CORO1A	2.03E-36	0.297891	0.24	0.199	3.03E-32
KIAA0895L	2.09E-36	0.434998	0.275	0.144	3.12E-32
ZNF880	2.12E-36	0.184779	0.212	0.246	3.16E-32
ETV6	2.12E-36	-0.1065	0.06	0.195	3.17E-32
TAB2	2.24E-36	-0.10686	0.057	0.193	3.35E-32
DNAJB1	2.34E-36	-0.11738	0.56	0.773	3.50E-32
CLN8	2.35E-36	0.287906	0.253	0.215	3.52E-32
KPNA2	2.45E-36	-0.44182	0.526	0.795	3.66E-32
POLD1	2.54E-36	-0.20059	0.038	0.191	3.79E-32
CTNNB1	2.67E-36	-0.18261	0.293	0.509	4.00E-32
MKKS	2.68E-36	-0.28847	0.396	0.669	4.00E-32
KCNMB4	2.83E-36	0.127461	0.149	0.2	4.22E-32
STX7	2.87E-36	0.336995	0.271	0.216	4.29E-32
PEX19	2.88E-36	-0.11007	0.127	0.28	4.31E-32
FAM96B	3.27E-36	0.142588	0.775	0.816	4.89E-32
TRAPPC6A	3.29E-36	0.147435	0.185	0.249	4.92E-32
NUP210	3.29E-36	-0.12696	0.036	0.168	4.92E-32
RIC3	3.38E-36	0.345943	0.452	0.364	5.04E-32
SEL1L	3.52E-36	-0.11283	0.081	0.223	5.25E-32
GPR82	3.53E-36	0.351821	0.193	0.123	5.28E-32
LYRM9	3.69E-36	0.164935	0.19	0.233	5.51E-32
THSD1	3.69E-36	-0.15891	0.05	0.201	5.52E-32
WHAMM	3.71E-36	-0.14448	0.053	0.198	5.54E-32
SGK1	3.76E-36	-0.23632	0.094	0.277	5.62E-32
ZZZ3	4.04E-36	0.106657	0.13	0.191	6.04E-32
FHL2	4.05E-36	-0.29611	0.013	0.149	6.05E-32
PMEPA1	4.13E-36	-0.30789	0.181	0.415	6.17E-32
CNOT11	4.46E-36	-0.13193	0.085	0.24	6.67E-32
CPVL	4.47E-36	-0.11652	0.042	0.17	6.67E-32
PPIP5K2	4.47E-36	0.160898	0.175	0.209	6.69E-32
KLHL20	4.63E-36	0.163601	0.185	0.227	6.91E-32
E2F3	4.64E-36	-0.17423	0.072	0.23	6.94E-32
NAPEPLD	4.69E-36	-0.18167	0.074	0.235	7.02E-32
RAD23A	4.73E-36	-0.35656	0.54	0.806	7.06E-32
FOXO1	4.75E-36	-0.24097	0.019	0.159	7.09E-32
DUSP8	4.90E-36	0.48133	0.214	0.073	7.32E-32
SCRIB	4.97E-36	-0.14413	0.055	0.2	7.42E-32
FAXC	5.11E-36	0.41149	0.307	0.2	7.64E-32
NADSYN1	6.34E-36	-0.12888	0.044	0.184	9.48E-32
MIR155HG	6.59E-36	-0.13169	0	0.1	9.84E-32
TTYH2	6.76E-36	-0.10199	0.021	0.137	1.01E-31

LLGL1	6.82E-36	-0.12111	0.075	0.217	1.02E-31
DMTF1	6.83E-36	0.119135	0.24	0.307	1.02E-31
ANKS1B	7.00E-36	0.519025	0.337	0.149	1.05E-31
DENND5B	7.05E-36	0.193482	0.183	0.207	1.05E-31
RER1	7.13E-36	-0.21835	0.453	0.705	1.07E-31
FAM134B	7.38E-36	0.239302	0.213	0.217	1.10E-31
CTSL	7.71E-36	-0.11341	0.219	0.389	1.15E-31
HIST1H2BK	7.82E-36	-0.22552	0.073	0.246	1.17E-31
DNAL1	7.93E-36	-0.11518	0.078	0.221	1.19E-31
TRIM25	8.51E-36	-0.13413	0.013	0.136	1.27E-31
MCCC2	8.54E-36	-0.13402	0.071	0.212	1.28E-31
SLIRP	8.54E-36	-0.28321	0.566	0.826	1.28E-31
ATP13A3	8.60E-36	-0.13989	0.079	0.231	1.28E-31
OXNAD1	8.62E-36	-0.15487	0.034	0.172	1.29E-31
PPCS	8.91E-36	0.110548	0.21	0.275	1.33E-31
C17orf53	9.09E-36	-0.18818	0.012	0.14	1.36E-31
HPS3	9.27E-36	-0.10502	0.061	0.196	1.38E-31
YPEL3	9.37E-36	0.202987	0.261	0.293	1.40E-31
ENKD1	9.75E-36	-0.10743	0.057	0.194	1.46E-31
SMKR1	1.01E-35	0.478509	0.223	0.077	1.51E-31
ZFYVE19	1.02E-35	-0.11937	0.05	0.184	1.52E-31
WDYHV1	1.07E-35	0.107659	0.167	0.238	1.59E-31
LSM5	1.07E-35	-0.3216	0.642	0.88	1.60E-31
OAT	1.07E-35	0.24987	0.262	0.26	1.60E-31
SAAL1	1.11E-35	-0.19832	0.139	0.326	1.66E-31
YEATS4	1.16E-35	-0.43962	0.327	0.599	1.73E-31
XRCC5	1.18E-35	0.144109	0.822	0.84	1.76E-31
CLIP2	1.21E-35	0.167853	0.295	0.346	1.80E-31
NHLH1	1.26E-35	0.587727	0.157	0.02	1.89E-31
ELMOD1	1.34E-35	-0.13799	0.027	0.159	2.00E-31
CTPS1	1.39E-35	-0.15477	0.161	0.341	2.07E-31
TATDN3	1.42E-35	0.157478	0.189	0.233	2.12E-31
TMEM43	1.48E-35	0.10879	0.196	0.263	2.20E-31
FLYWCH1	1.54E-35	0.136816	0.143	0.181	2.30E-31
SPATA2L	1.54E-35	0.212226	0.155	0.148	2.30E-31
RABL2B	1.55E-35	0.11343	0.142	0.195	2.32E-31
ZSCAN21	1.76E-35	0.169575	0.187	0.215	2.62E-31
ZFR2	1.86E-35	0.47353	0.203	0.028	2.77E-31
UTP23	1.94E-35	0.126998	0.196	0.253	2.90E-31
NAGA	1.99E-35	-0.13618	0.028	0.159	2.98E-31
ELOVL6	2.00E-35	0.111181	0.132	0.189	2.98E-31
CSPG5	2.01E-35	-0.433	0.053	0.243	3.01E-31
ST13	2.09E-35	-0.13956	0.612	0.828	3.12E-31
PLCB1	2.12E-35	0.253995	0.202	0.189	3.17E-31
GNB1L	2.15E-35	-0.10639	0.067	0.201	3.22E-31
NEO1	2.22E-35	-0.11287	0.034	0.158	3.32E-31
NXT2	2.25E-35	-0.21564	0.027	0.178	3.37E-31
BAX	2.26E-35	-0.11129	0.501	0.705	3.37E-31
CREG1	2.34E-35	0.1686	0.165	0.2	3.50E-31
ZNF692	2.57E-35	0.194435	0.186	0.206	3.84E-31
RNF41	2.81E-35	0.177413	0.222	0.252	4.19E-31

NEDD9	2.96E-35	-0.15746	0.104	0.267	4.42E-31
ZNF205	3.06E-35	0.181455	0.178	0.199	4.57E-31
C1orf50	3.24E-35	0.188228	0.212	0.237	4.84E-31
IFT122	3.51E-35	-0.17323	0.024	0.164	5.25E-31
AMDHD2	3.62E-35	-0.1058	0.05	0.184	5.41E-31
ZNF273	3.62E-35	-0.24548	0.141	0.34	5.42E-31
CYTH1	3.78E-35	0.335509	0.214	0.163	5.65E-31
NENF	4.57E-35	-0.16073	0.529	0.757	6.82E-31
ELK3	5.07E-35	-0.14334	0.005	0.12	7.58E-31
DTX3L	5.16E-35	-0.14567	0.005	0.119	7.72E-31
GUK1	5.43E-35	0.175671	0.848	0.846	8.11E-31
SFT2D2	5.48E-35	-0.13531	0.063	0.206	8.19E-31
ZNF254	5.63E-35	0.203946	0.253	0.268	8.41E-31
RDH10	5.63E-35	-0.2078	0.016	0.149	8.42E-31
CEP85L	5.74E-35	0.189361	0.173	0.189	8.58E-31
EGLN1	6.01E-35	0.142439	0.214	0.263	8.99E-31
DPF1	6.09E-35	0.146535	0.38	0.422	9.09E-31
AAGAB	7.02E-35	-0.13313	0.096	0.251	1.05E-30
ST18	7.32E-35	0.422963	0.15	0.007	1.09E-30
CADM2	7.54E-35	0.328901	0.405	0.346	1.13E-30
FAM101B	7.86E-35	-0.14993	0.013	0.135	1.17E-30
CENPC	7.99E-35	-0.12147	0.128	0.281	1.19E-30
MIR210HG	8.31E-35	-0.13062	0.025	0.146	1.24E-30
RABL2A	8.98E-35	0.358559	0.17	0.101	1.34E-30
ADORA2B	9.17E-35	-0.10534	0.004	0.105	1.37E-30
DGCR2	9.27E-35	-0.13264	0.137	0.301	1.39E-30
GRAMD3	9.93E-35	-0.24663	0.013	0.146	1.48E-30
ZNF667	1.00E-34	0.325827	0.18	0.121	1.50E-30
C19orf12	1.05E-34	0.163331	0.18	0.209	1.56E-30
CDK8	1.09E-34	0.220206	0.148	0.147	1.63E-30
NSMF	1.12E-34	0.214645	0.178	0.178	1.68E-30
PUSL1	1.15E-34	-0.13655	0.076	0.227	1.71E-30
FANCL	1.16E-34	-0.21215	0.183	0.385	1.73E-30
PHF11	1.19E-34	-0.14057	0.011	0.13	1.77E-30
RP1-79C4.	1.19E-34	-0.16446	0.015	0.137	1.78E-30
ZNF268	1.23E-34	0.19053	0.196	0.227	1.84E-30
GTF2IRD2E	1.26E-34	0.348236	0.284	0.209	1.89E-30
LRRC57	1.29E-34	-0.13064	0.043	0.177	1.93E-30
RP11-51J9	1.34E-34	0.119582	0.15	0.214	2.00E-30
CTBS	1.35E-34	-0.10158	0.027	0.144	2.01E-30
RP13-1032	1.46E-34	-0.14872	0.059	0.204	2.18E-30
PTPN14	1.46E-34	-0.12582	0.013	0.131	2.18E-30
IQCE	1.47E-34	0.233004	0.225	0.222	2.20E-30
CXCR4	1.47E-34	-0.30516	0.102	0.293	2.20E-30
PLEKHG1	1.61E-34	-0.12301	0.008	0.121	2.40E-30
TRNT1	1.75E-34	-0.15775	0.086	0.242	2.61E-30
SP100	1.75E-34	-0.27157	0.023	0.163	2.62E-30
ARID1B	1.85E-34	0.185306	0.212	0.235	2.77E-30
DARS2	1.87E-34	-0.15134	0.011	0.132	2.80E-30
SH3RF1	1.87E-34	0.105605	0.149	0.201	2.80E-30
NR2E1	2.01E-34	-0.15361	0.011	0.133	3.00E-30

CDIP1	2.06E-34	0.149252	0.218	0.264	3.07E-30
ZNF512	2.14E-34	0.315973	0.307	0.247	3.20E-30
ZNF577	2.26E-34	-0.20485	0.053	0.211	3.38E-30
INCENP	2.26E-34	-0.15792	0.026	0.16	3.38E-30
CDC42EP4	2.30E-34	-0.20437	0.231	0.43	3.44E-30
FBXL15	2.35E-34	0.299544	0.207	0.163	3.51E-30
ZNF429	2.41E-34	0.224974	0.226	0.236	3.60E-30
GBP1	2.49E-34	-0.34248	0.008	0.133	3.72E-30
HIGD2A	2.51E-34	-0.11795	0.46	0.664	3.75E-30
RGS2	2.54E-34	0.480463	0.42	0.227	3.80E-30
KLHL9	2.65E-34	0.233033	0.21	0.204	3.96E-30
ABCC5	2.84E-34	0.316011	0.182	0.128	4.25E-30
5-Mar	2.90E-34	0.127706	0.252	0.298	4.34E-30
IRF1	2.96E-34	-0.2885	0.111	0.306	4.43E-30
CCDC102A	2.98E-34	-0.137	0.001	0.104	4.45E-30
BIVM	3.11E-34	0.214645	0.203	0.217	4.65E-30
PKD2	3.30E-34	-0.11165	0.057	0.188	4.93E-30
AP4M1	3.47E-34	0.203038	0.344	0.357	5.18E-30
TRAF3IP2-	3.47E-34	-0.12622	0.081	0.226	5.19E-30
TMEM201	3.50E-34	-0.14049	0.027	0.156	5.23E-30
HMGN5	3.63E-34	-0.30406	0.047	0.216	5.43E-30
HOMER1	3.76E-34	-0.10475	0.192	0.354	5.61E-30
ARHGEF39	3.82E-34	-0.17744	0.01	0.128	5.71E-30
RAI2	3.82E-34	0.423268	0.149	0.009	5.71E-30
MTMR6	4.03E-34	0.134968	0.186	0.233	6.03E-30
TNKS1BP1	4.04E-34	-0.11081	0.022	0.138	6.03E-30
KDM4B	4.10E-34	0.124083	0.176	0.235	6.13E-30
CAPG	4.34E-34	-0.13168	0.084	0.227	6.49E-30
GAPDH	4.49E-34	-0.18604	0.995	0.996	6.71E-30
CDK9	4.57E-34	0.164241	0.162	0.191	6.82E-30
TCTN3	4.60E-34	-0.13651	0.089	0.238	6.88E-30
GALNT7	5.04E-34	-0.13645	0.032	0.16	7.53E-30
P2RX4	5.08E-34	-0.12081	0.049	0.18	7.59E-30
BAG3	5.11E-34	-0.132	0.013	0.127	7.64E-30
PCOLCE	5.13E-34	0.149741	0.126	0.169	7.67E-30
NME2	5.14E-34	-0.11293	0.032	0.156	7.68E-30
SNRPC	5.30E-34	-0.12282	0.555	0.76	7.92E-30
CCDC93	5.32E-34	0.193646	0.217	0.238	7.96E-30
SLC22A4	5.58E-34	-0.15106	0.009	0.125	8.34E-30
CHSY1	5.70E-34	-0.1406	0.027	0.158	8.51E-30
PRMT3	5.81E-34	-0.10416	0.075	0.206	8.68E-30
QSOX1	6.08E-34	0.131195	0.189	0.236	9.09E-30
RPS6KA4	6.10E-34	-0.11287	0.046	0.177	9.12E-30
RP11-977G	6.32E-34	-0.11363	0.023	0.14	9.44E-30
RAB9B	6.74E-34	0.455548	0.229	0.081	1.01E-29
INSIG1	7.37E-34	-0.17688	0.138	0.311	1.10E-29
COMMD6	7.61E-34	0.202115	0.841	0.823	1.14E-29
NOP10	7.97E-34	-0.22117	0.48	0.728	1.19E-29
CCDC91	8.00E-34	0.270074	0.268	0.242	1.20E-29
C1orf198	8.08E-34	0.276438	0.183	0.153	1.21E-29
FRMD4A	8.23E-34	0.455316	0.287	0.153	1.23E-29

THAP2	9.22E-34	0.232938	0.17	0.167	1.38E-29
RGP1	9.41E-34	0.172577	0.156	0.183	1.41E-29
TMCC1	1.01E-33	0.166141	0.156	0.186	1.50E-29
FAM219A	1.02E-33	0.153215	0.156	0.188	1.52E-29
TP53INP2	1.04E-33	0.117484	0.114	0.165	1.55E-29
YAP1	1.04E-33	-0.1306	0	0.094	1.56E-29
TSPYL4	1.04E-33	0.335989	0.22	0.163	1.56E-29
EEFSEC	1.08E-33	0.142475	0.152	0.196	1.61E-29
EGR2	1.11E-33	-0.14752	0.066	0.209	1.65E-29
EMC6	1.13E-33	-0.16645	0.456	0.683	1.69E-29
PDE7A	1.17E-33	0.153801	0.163	0.21	1.76E-29
SORBS1	1.23E-33	-0.16839	0.057	0.207	1.83E-29
ABR	1.33E-33	0.163934	0.134	0.158	1.98E-29
TRIM13	1.45E-33	0.21885	0.386	0.388	2.16E-29
HCG18	1.45E-33	0.201903	0.165	0.18	2.17E-29
ARHGEF3	1.49E-33	0.168889	0.108	0.13	2.23E-29
SMAP2	1.53E-33	0.193037	0.17	0.185	2.28E-29
RCHY1	1.54E-33	0.201773	0.284	0.298	2.30E-29
LMBRD1	1.55E-33	0.129648	0.159	0.212	2.32E-29
SH3BP4	1.57E-33	-0.16751	0.057	0.199	2.35E-29
ZNF266	1.61E-33	-0.11715	0.081	0.216	2.41E-29
CHST11	1.76E-33	-0.15636	0.115	0.272	2.63E-29
PLCD3	1.79E-33	-0.14573	0.01	0.132	2.67E-29
FAM199X	1.80E-33	-0.1078	0.057	0.184	2.69E-29
4-Sep	1.92E-33	0.236712	0.163	0.157	2.87E-29
MTA3	1.93E-33	-0.11828	0.042	0.169	2.88E-29
MSX1	1.99E-33	-0.15979	0.03	0.162	2.97E-29
CYR61	2.01E-33	-0.36844	0.053	0.228	3.01E-29
UBR5	2.04E-33	0.107959	0.156	0.219	3.05E-29
PPP1CB	2.10E-33	-0.36205	0.468	0.737	3.14E-29
SLC29A1	2.33E-33	-0.18281	0.019	0.151	3.49E-29
AFTPH	2.39E-33	0.131473	0.233	0.284	3.57E-29
TET1	2.40E-33	0.151834	0.121	0.153	3.59E-29
RILPL2	2.41E-33	-0.10869	0.021	0.133	3.60E-29
9-Sep	2.53E-33	-0.23316	0.371	0.612	3.78E-29
RAB40B	2.75E-33	0.309274	0.191	0.142	4.11E-29
RNF38	2.77E-33	0.151119	0.146	0.183	4.14E-29
DOCK1	2.82E-33	-0.16486	0.005	0.116	4.21E-29
FGF2	2.88E-33	-0.10717	0.009	0.111	4.30E-29
CBLN4	2.89E-33	-0.15458	0.015	0.123	4.32E-29
PDZRN3	2.96E-33	0.37938	0.213	0.128	4.42E-29
DALRD3	2.96E-33	0.142229	0.197	0.241	4.42E-29
PRR3	3.03E-33	0.122286	0.163	0.219	4.53E-29
ACSL3	3.10E-33	-0.13302	0.391	0.591	4.64E-29
RPP30	3.20E-33	-0.1641	0.128	0.296	4.78E-29
APBA3	3.23E-33	-0.12655	0.043	0.172	4.83E-29
SRSF12	3.29E-33	0.108184	0.151	0.214	4.92E-29
WARS	3.39E-33	-0.17653	0.106	0.269	5.06E-29
WDR48	3.74E-33	0.24511	0.255	0.232	5.59E-29
GTF3C1	3.75E-33	-0.14282	0.074	0.22	5.61E-29
ZC3H4	3.77E-33	-0.13316	0.057	0.193	5.64E-29

PCLO	3.92E-33	0.434371	0.181	0.027	5.86E-29
SOCS3	3.93E-33	-0.23142	0.147	0.332	5.87E-29
CHRNA9	4.08E-33	-0.13843	0.001	0.1	6.10E-29
ADIPOR2	4.36E-33	-0.11578	0.112	0.258	6.52E-29
BBC3	4.57E-33	0.361335	0.17	0.098	6.83E-29
SLC30A1	4.90E-33	-0.13993	0.017	0.137	7.32E-29
SMYD3	4.90E-33	0.132906	0.174	0.228	7.33E-29
PTP4A3	4.94E-33	0.321306	0.173	0.125	7.38E-29
GYPC	5.17E-33	-0.28308	0.052	0.215	7.73E-29
FZD2	5.28E-33	-0.12782	0.015	0.128	7.90E-29
PKN3	5.55E-33	-0.16775	0.003	0.11	8.29E-29
FGFR1OP	5.72E-33	-0.16733	0.075	0.227	8.55E-29
STRIP1	5.81E-33	0.317301	0.267	0.205	8.69E-29
SLC1A2	5.88E-33	-0.26905	0.096	0.279	8.78E-29
ZNF599	6.14E-33	-0.16418	0.038	0.175	9.18E-29
MLLT3	6.38E-33	0.257532	0.216	0.201	9.53E-29
ZNF232	7.46E-33	-0.10063	0.116	0.254	1.12E-28
COL16A1	7.54E-33	-0.15035	0.009	0.125	1.13E-28
KAT8	7.55E-33	0.108369	0.196	0.259	1.13E-28
CCBL2	8.49E-33	-0.13794	0.07	0.207	1.27E-28
MEF2C	8.98E-33	0.234643	0.201	0.194	1.34E-28
LINC00844	9.12E-33	-0.40561	0.031	0.19	1.36E-28
CTD-3138E	9.24E-33	-0.12267	0.021	0.138	1.38E-28
DNASE1	9.70E-33	0.288326	0.196	0.154	1.45E-28
VAX2	9.83E-33	0.301717	0.225	0.188	1.47E-28
MUTYH	9.84E-33	-0.13604	0.104	0.252	1.47E-28
ERAP2	1.00E-32	-0.15775	0.007	0.119	1.50E-28
ABL1	1.02E-32	-0.14985	0.053	0.19	1.52E-28
NARS2	1.06E-32	0.111808	0.172	0.227	1.59E-28
CARKD	1.12E-32	0.201719	0.228	0.236	1.67E-28
SRD5A3	1.14E-32	-0.14519	0.134	0.295	1.70E-28
TOX4	1.21E-32	0.115268	0.295	0.36	1.80E-28
ACAA2	1.21E-32	-0.15328	0.117	0.278	1.80E-28
REL	1.27E-32	0.13857	0.207	0.264	1.91E-28
LAMTOR5	1.30E-32	0.126946	0.765	0.806	1.94E-28
C17orf10C	1.32E-32	0.116717	0.125	0.178	1.97E-28
SMAD9	1.34E-32	0.28347	0.237	0.198	2.01E-28
SYBU	1.37E-32	0.25503	0.148	0.126	2.04E-28
PUS7L	1.38E-32	0.269862	0.241	0.214	2.06E-28
CAMK2B	1.47E-32	0.22932	0.191	0.191	2.20E-28
ZFHX4-AS1	1.53E-32	-0.14429	0.01	0.121	2.28E-28
SRD5A1	1.56E-32	0.219237	0.181	0.174	2.33E-28
CASP4	1.58E-32	-0.10196	0.012	0.115	2.36E-28
PPP1CC	1.72E-32	-0.17137	0.541	0.76	2.57E-28
ZNRF3	1.77E-32	-0.12341	0.013	0.122	2.64E-28
ERRFI1	1.80E-32	-0.22248	0.096	0.269	2.69E-28
CMTM8	1.89E-32	-0.11928	0.013	0.126	2.82E-28
SLC37A3	1.91E-32	0.322827	0.307	0.251	2.86E-28
FCF1	1.92E-32	-0.13072	0.029	0.151	2.87E-28
FAM13B	1.94E-32	0.15582	0.148	0.174	2.90E-28
CHCHD7	1.96E-32	0.107478	0.186	0.254	2.92E-28

ARL9	1.97E-32	0.15475	0.176	0.219	2.95E-28
ARHGEF40	2.02E-32	-0.11052	0.067	0.195	3.02E-28
TONSL	2.07E-32	-0.19831	0.015	0.14	3.09E-28
HSP90B1	2.14E-32	-0.32152	0.606	0.847	3.20E-28
SIX5	2.15E-32	-0.10789	0	0.09	3.22E-28
DZIP3	2.17E-32	0.229236	0.284	0.285	3.25E-28
VSIG10	2.18E-32	-0.15994	0.042	0.178	3.26E-28
CHST8	2.23E-32	-0.12509	0.028	0.146	3.33E-28
ROBO3	2.23E-32	0.333425	0.162	0.125	3.34E-28
SLC19A1	2.25E-32	-0.12434	0.035	0.159	3.37E-28
TRAFD1	2.32E-32	0.109685	0.156	0.215	3.46E-28
SH3KBP1	2.35E-32	-0.13244	0.137	0.288	3.51E-28
REM2	2.39E-32	0.384542	0.122	0.004	3.56E-28
ADAM12	2.60E-32	-0.16378	0.002	0.105	3.89E-28
COX5A	2.71E-32	-0.29599	0.575	0.822	4.05E-28
KLF9	2.79E-32	-0.17748	0.031	0.156	4.17E-28
EHD1	2.80E-32	0.168145	0.171	0.196	4.18E-28
LIN9	2.81E-32	-0.17925	0.037	0.172	4.20E-28
KDELC1	2.83E-32	-0.14589	0.024	0.148	4.23E-28
GGCX	2.88E-32	-0.12513	0.14	0.296	4.30E-28
INSM1	2.91E-32	0.249746	0.353	0.347	4.35E-28
AASS	3.06E-32	-0.13942	0.091	0.237	4.58E-28
ACTR3B	3.08E-32	0.242592	0.223	0.204	4.61E-28
PTPN4	3.17E-32	0.245834	0.214	0.202	4.73E-28
UBE2I	3.22E-32	0.123535	0.788	0.827	4.81E-28
NUP205	3.25E-32	-0.12808	0.061	0.196	4.86E-28
ORMDL3	3.27E-32	0.15384	0.201	0.236	4.89E-28
HLA-C	3.38E-32	-0.52125	0.497	0.716	5.04E-28
RYBP	3.51E-32	0.111179	0.15	0.201	5.25E-28
EIF3G	3.52E-32	-0.21227	0.528	0.768	5.26E-28
LIN54	3.54E-32	-0.19116	0.018	0.146	5.28E-28
ACVR1	3.57E-32	0.191478	0.116	0.132	5.33E-28
CEP128	3.66E-32	-0.16317	0.008	0.122	5.47E-28
CACFD1	3.72E-32	0.262116	0.189	0.164	5.56E-28
EVA1C	3.74E-32	-0.19848	0.013	0.135	5.59E-28
RAB30	3.76E-32	0.253379	0.151	0.131	5.61E-28
COG1	3.89E-32	0.107554	0.143	0.194	5.81E-28
PDIA3	4.05E-32	-0.24093	0.485	0.73	6.05E-28
NOVA2	4.19E-32	0.374256	0.223	0.121	6.27E-28
BOK	4.23E-32	-0.1048	0.012	0.117	6.32E-28
B3GNT5	4.25E-32	-0.14982	0.069	0.204	6.35E-28
SRPX2	4.55E-32	-0.13339	0.002	0.098	6.80E-28
TFPI	4.62E-32	-0.28597	0.006	0.121	6.90E-28
FANCF	4.63E-32	0.352629	0.179	0.109	6.91E-28
CMBL	4.83E-32	0.102266	0.154	0.211	7.23E-28
DIS3	4.86E-32	0.167877	0.222	0.251	7.26E-28
MAFB	4.99E-32	0.519073	0.344	0.159	7.45E-28
MFI2-AS1	5.14E-32	-0.11705	0.097	0.241	7.68E-28
HDAC5	5.27E-32	0.147234	0.174	0.207	7.87E-28
HBP1	5.43E-32	0.271013	0.245	0.217	8.12E-28
LSM3	5.50E-32	-0.26246	0.629	0.863	8.22E-28

NADK2	5.68E-32	-0.10256	0.073	0.202	8.49E-28
SLC2A10	5.77E-32	-0.12003	0.009	0.116	8.63E-28
FBN2	6.15E-32	-0.14673	0.006	0.114	9.19E-28
OSGEP	6.17E-32	-0.11303	0.164	0.314	9.22E-28
DSTYK	6.21E-32	0.164454	0.157	0.19	9.27E-28
CEP70	6.21E-32	-0.1482	0.159	0.321	9.28E-28
GLDC	6.36E-32	-0.12195	0.049	0.175	9.50E-28
PCDH19	6.41E-32	-0.16008	0.042	0.177	9.59E-28
HSPA8	6.55E-32	0.11203	0.956	0.959	9.79E-28
TUBGCP4	6.67E-32	0.115245	0.193	0.248	9.97E-28
POLE	6.99E-32	-0.15908	0.029	0.157	1.04E-27
SERF2	8.44E-32	-0.27971	0.767	0.914	1.26E-27
PSMB2	8.47E-32	-0.15543	0.588	0.791	1.27E-27
GEM	8.59E-32	-0.17251	0.022	0.147	1.28E-27
NOC3L	8.82E-32	-0.11129	0.046	0.168	1.32E-27
RAB23	9.23E-32	-0.15251	0.053	0.194	1.38E-27
ACSF2	9.65E-32	-0.10783	0.001	0.095	1.44E-27
FAM20B	9.91E-32	-0.13104	0.045	0.174	1.48E-27
TYW3	9.94E-32	-0.13285	0.078	0.219	1.49E-27
ZCRB1	1.01E-31	-0.10643	0.567	0.763	1.52E-27
TUBGCP3	1.06E-31	-0.17727	0.101	0.264	1.59E-27
RNF113A	1.14E-31	0.107171	0.145	0.2	1.70E-27
PNMA2	1.14E-31	0.100754	0.248	0.314	1.71E-27
KDM4A	1.18E-31	0.105267	0.148	0.209	1.76E-27
ANK3	1.21E-31	0.417215	0.186	0.057	1.80E-27
RASSF4	1.24E-31	-0.17574	0.07	0.219	1.85E-27
BUD13	1.24E-31	-0.16445	0.077	0.221	1.86E-27
SLC2A1	1.34E-31	-0.1237	0.098	0.238	2.00E-27
ATP2B1	1.36E-31	-0.21929	0.332	0.549	2.03E-27
KIF13A	1.38E-31	-0.10749	0.024	0.136	2.06E-27
C1QB	1.42E-31	0.309114	0.386	0.31	2.12E-27
CSNK1G1	1.64E-31	-0.10643	0.037	0.154	2.45E-27
CDK6	1.70E-31	-0.3279	0.251	0.486	2.53E-27
SLC39A9	1.75E-31	-0.12756	0.055	0.19	2.61E-27
CBWD1	1.87E-31	0.268703	0.297	0.256	2.79E-27
SLC12A4	1.88E-31	-0.11185	0.022	0.131	2.81E-27
RP1-39G22	1.90E-31	0.125281	0.184	0.244	2.84E-27
LMBR1	1.98E-31	0.173707	0.167	0.193	2.96E-27
CDKAL1	2.04E-31	0.14161	0.174	0.216	3.05E-27
DLGAP1-AS	2.09E-31	-0.16633	0.009	0.125	3.13E-27
BAALC	2.12E-31	-0.14366	0.471	0.672	3.16E-27
MSANTD4	2.19E-31	0.130044	0.191	0.227	3.27E-27
AHDC1	2.21E-31	0.243394	0.178	0.164	3.30E-27
TMEM64	2.29E-31	-0.14063	0.014	0.126	3.42E-27
DCLRE1B	2.36E-31	-0.19043	0.054	0.204	3.53E-27
DDIT3	2.39E-31	-0.10697	0.441	0.627	3.57E-27
TCEA1	2.42E-31	-0.17188	0.539	0.756	3.62E-27
TBX2	2.47E-31	-0.14592	0.011	0.122	3.70E-27
RUFY1	2.49E-31	-0.10075	0.099	0.232	3.73E-27
NUP155	2.68E-31	-0.16207	0.064	0.206	4.00E-27
TP53BP2	2.69E-31	-0.10329	0.084	0.215	4.03E-27

KLHL18	2.71E-31	0.121725	0.141	0.186	4.04E-27
S100B	2.72E-31	-0.27475	0.511	0.754	4.07E-27
KBTBD4	2.76E-31	0.194595	0.156	0.165	4.12E-27
NCEH1	2.86E-31	-0.14352	0.009	0.119	4.27E-27
EXOSC2	3.04E-31	-0.13493	0.096	0.241	4.55E-27
HYI	3.09E-31	-0.10191	0.152	0.301	4.62E-27
CREBL2	3.12E-31	0.114469	0.122	0.169	4.66E-27
UCP2	3.18E-31	-0.28609	0.104	0.29	4.76E-27
FGF9	3.21E-31	0.510011	0.276	0.099	4.79E-27
HSF2	3.23E-31	0.322695	0.336	0.257	4.83E-27
NPC1	3.26E-31	-0.10685	0.023	0.135	4.87E-27
KPNB1	3.28E-31	-0.17577	0.576	0.794	4.89E-27
FAM110B	3.37E-31	0.104867	0.267	0.336	5.03E-27
GABBR1	3.37E-31	0.291161	0.176	0.132	5.04E-27
STK32B	3.60E-31	-0.10643	0.049	0.172	5.39E-27
GSG1L	3.66E-31	-0.21992	0.015	0.135	5.47E-27
STK33	3.71E-31	-0.11103	0.036	0.151	5.54E-27
RELT	3.72E-31	-0.10687	0.039	0.157	5.56E-27
HID1	3.80E-31	0.382643	0.17	0.059	5.67E-27
SENP7	3.85E-31	0.239933	0.156	0.138	5.76E-27
TECPR1	3.92E-31	0.1655	0.188	0.211	5.86E-27
ZNF71	3.97E-31	0.220015	0.184	0.181	5.93E-27
N4BP2	4.42E-31	0.123505	0.207	0.258	6.60E-27
LTBP1	4.55E-31	-0.14235	0.009	0.121	6.80E-27
GPR162	4.56E-31	0.2883	0.187	0.154	6.82E-27
SH2B2	4.70E-31	0.263905	0.243	0.209	7.03E-27
SPATA5	4.83E-31	-0.10375	0.033	0.147	7.22E-27
ARMCX2	4.90E-31	0.2003	0.163	0.177	7.33E-27
METAP1	5.04E-31	-0.10191	0.079	0.205	7.53E-27
VPS37D	5.30E-31	0.385115	0.193	0.1	7.91E-27
TUBG2	5.36E-31	0.151174	0.127	0.16	8.01E-27
H6PD	5.41E-31	-0.11593	0.025	0.142	8.09E-27
ANGPTL1	5.76E-31	-0.21061	0.002	0.099	8.61E-27
MEF2A	5.90E-31	-0.13586	0.06	0.195	8.82E-27
ZNF107	6.44E-31	-0.20451	0.023	0.151	9.63E-27
ANKZF1	6.54E-31	0.230901	0.187	0.18	9.78E-27
PDIA5	6.58E-31	-0.10601	0.013	0.119	9.84E-27
KB-1980E6	7.33E-31	0.403968	0.123	0.011	1.10E-26
KDELR2	7.48E-31	-0.18108	0.592	0.812	1.12E-26
CLK4	7.94E-31	0.247419	0.25	0.232	1.19E-26
EFNB1	8.02E-31	0.228582	0.232	0.227	1.20E-26
PIGW	8.19E-31	-0.11382	0.035	0.151	1.22E-26
PSMC3	8.31E-31	-0.21114	0.621	0.842	1.24E-26
C19orf70	8.52E-31	-0.20987	0.437	0.667	1.27E-26
C9orf40	8.65E-31	-0.10995	0.06	0.185	1.29E-26
MDM1	8.71E-31	-0.16788	0.049	0.186	1.30E-26
MICAL1	8.81E-31	0.336455	0.219	0.152	1.32E-26
RAB3C	8.82E-31	0.453229	0.21	0.046	1.32E-26
KIAA1211L	8.91E-31	-0.12356	0.002	0.101	1.33E-26
CASC15	9.14E-31	0.472777	0.293	0.152	1.37E-26
DLAT	9.36E-31	-0.10506	0.118	0.253	1.40E-26

PRKAR1B	9.80E-31	0.354837	0.306	0.215	1.46E-26
TTLL1	1.03E-30	0.167321	0.121	0.144	1.54E-26
EID2B	1.07E-30	0.175529	0.159	0.19	1.60E-26
MON1A	1.07E-30	0.136088	0.13	0.17	1.60E-26
RP11-452F	1.09E-30	-0.10186	0.04	0.153	1.62E-26
MOV10	1.13E-30	-0.13648	0.013	0.123	1.68E-26
PHTF2	1.14E-30	-0.11405	0.118	0.26	1.70E-26
NDUFC2	1.15E-30	0.14992	0.888	0.88	1.72E-26
RP11-620J	1.18E-30	-0.2791	0.378	0.612	1.76E-26
CYFIP2	1.21E-30	0.280589	0.257	0.21	1.81E-26
DDHD2	1.24E-30	0.271241	0.2	0.17	1.85E-26
ANTXR1	1.34E-30	-0.11029	0.088	0.221	2.00E-26
ZBTB43	1.35E-30	0.13787	0.197	0.237	2.02E-26
ADM	1.37E-30	-0.32815	0.047	0.204	2.04E-26
CCDC126	1.38E-30	0.386055	0.264	0.172	2.06E-26
VARS2	1.38E-30	-0.10105	0.027	0.137	2.07E-26
ZNF559	1.41E-30	0.103764	0.207	0.263	2.11E-26
TMEM246	1.42E-30	0.457538	0.198	0.057	2.12E-26
RP11-295G	1.44E-30	-0.16139	0.003	0.104	2.15E-26
PRPSAP1	1.47E-30	-0.13801	0.434	0.638	2.19E-26
CDK5R2	1.47E-30	0.491063	0.19	0.049	2.19E-26
RHOBTB3	1.47E-30	-0.10011	0.639	0.826	2.19E-26
TSTD2	1.52E-30	0.103971	0.115	0.168	2.27E-26
RAB33A	1.54E-30	0.481582	0.298	0.136	2.30E-26
MFSD2A	1.60E-30	-0.10167	0.056	0.172	2.39E-26
RAD54B	1.60E-30	-0.1736	0.026	0.151	2.39E-26
C1orf54	1.62E-30	-0.17122	0.014	0.128	2.43E-26
MPP6	1.63E-30	-0.11924	0.075	0.209	2.44E-26
ONECUT2	1.63E-30	0.479039	0.241	0.074	2.44E-26
KANK1	1.68E-30	-0.19385	0.02	0.144	2.50E-26
C17orf89	1.83E-30	-0.21894	0.452	0.694	2.74E-26
BRSK1	1.86E-30	0.174454	0.162	0.178	2.78E-26
EPHB4	1.97E-30	-0.11672	0.004	0.1	2.95E-26
FAM131B	2.34E-30	0.213811	0.152	0.149	3.50E-26
CACNA2D3	2.38E-30	0.38123	0.13	0.006	3.55E-26
FEM1B	2.40E-30	-0.12661	0.075	0.21	3.58E-26
CSTF2T	2.43E-30	-0.14178	0.093	0.235	3.63E-26
ABCA1	2.56E-30	0.211496	0.183	0.184	3.83E-26
TAF2	2.65E-30	0.116667	0.137	0.181	3.96E-26
PSAP	2.80E-30	0.130726	0.74	0.76	4.18E-26
FANCM	2.84E-30	-0.16503	0.008	0.116	4.24E-26
RP11-83A2	2.85E-30	0.246621	0.229	0.209	4.26E-26
CYLD	2.96E-30	0.214744	0.148	0.137	4.42E-26
HAPLN3	3.02E-30	-0.10707	0.009	0.109	4.52E-26
TAF1B	3.03E-30	-0.1038	0.078	0.206	4.53E-26
RP11-395G	3.09E-30	-0.13061	0.178	0.337	4.62E-26
YTHDF3-AS	3.12E-30	-0.14762	0.023	0.141	4.67E-26
ASNA1	3.19E-30	-0.17277	0.523	0.74	4.76E-26
RNF20	3.20E-30	0.108275	0.207	0.259	4.78E-26
ST8SIA1	3.28E-30	-0.15581	0.05	0.185	4.90E-26
SLC35E4	3.29E-30	-0.13125	0.025	0.142	4.92E-26

DUS4L	3.41E-30	0.106662	0.132	0.181	5.09E-26
FAM200A	3.51E-30	0.283469	0.198	0.165	5.25E-26
CDC37L1	3.52E-30	0.222343	0.229	0.221	5.26E-26
CAPN7	3.63E-30	0.142387	0.162	0.194	5.43E-26
LINC00599	3.76E-30	0.494976	0.179	0.033	5.62E-26
EIF2S2	3.81E-30	-0.3191	0.533	0.78	5.70E-26
SLC4A3	3.89E-30	0.199438	0.138	0.144	5.81E-26
PARP4	3.90E-30	-0.13878	0.017	0.128	5.83E-26
LZTS2	3.97E-30	-0.1143	0.027	0.14	5.93E-26
CTSC	4.29E-30	-0.2297	0.053	0.202	6.41E-26
TMEFF2	4.71E-30	0.481158	0.276	0.112	7.05E-26
PPP1R14C	4.73E-30	-0.19867	0.046	0.186	7.06E-26
MTFP1	4.74E-30	-0.11716	0.013	0.12	7.08E-26
INPP5K	4.97E-30	0.130484	0.167	0.205	7.43E-26
WWC3	5.07E-30	-0.11509	0.032	0.143	7.58E-26
SMARCAD1	5.08E-30	0.177234	0.186	0.194	7.60E-26
MVB12B	5.28E-30	0.230281	0.191	0.177	7.89E-26
MZF1	5.31E-30	0.311666	0.17	0.1	7.93E-26
RRAGB	5.48E-30	0.319497	0.204	0.146	8.20E-26
MINPP1	5.50E-30	0.101689	0.127	0.179	8.22E-26
MTRNR2L1	5.82E-30	0.598912	0.333	0.174	8.70E-26
OSBPL10	6.03E-30	-0.15056	0.01	0.117	9.01E-26
C3orf58	6.04E-30	-0.15867	0.021	0.142	9.02E-26
HDDC3	6.55E-30	-0.10908	0.063	0.188	9.78E-26
MAEL	6.69E-30	-0.13151	0.003	0.098	9.99E-26
ZNF583	7.20E-30	0.260339	0.203	0.172	1.08E-25
ARL17A	7.62E-30	-0.12118	0.039	0.158	1.14E-25
FAM84B	7.68E-30	-0.14881	0.06	0.19	1.15E-25
SETBP1	7.77E-30	0.310613	0.203	0.132	1.16E-25
GRIK3	8.14E-30	-0.20782	0.026	0.157	1.22E-25
IGSF11	8.15E-30	-0.12532	0.046	0.165	1.22E-25
KCNJ2	8.15E-30	-0.19591	0.009	0.121	1.22E-25
BAK1	8.49E-30	-0.13279	0.051	0.18	1.27E-25
AC004158.	8.51E-30	0.261195	0.255	0.226	1.27E-25
POLG	8.56E-30	-0.10238	0.062	0.184	1.28E-25
SLC35E3	8.59E-30	-0.24199	0.23	0.428	1.28E-25
TRH	9.20E-30	0.34322	0.132	0.007	1.37E-25
C14orf132	9.28E-30	0.492747	0.327	0.149	1.39E-25
TMOD2	1.09E-29	0.109532	0.143	0.19	1.63E-25
KAT2B	1.13E-29	-0.17812	0.022	0.142	1.68E-25
COX20	1.14E-29	-0.12415	0.57	0.759	1.70E-25
RARRES3	1.16E-29	-0.14637	0.018	0.127	1.73E-25
DIRAS3	1.22E-29	-0.23101	0.02	0.144	1.83E-25
DYNLRB1	1.24E-29	0.145613	0.797	0.826	1.85E-25
RREB1	1.25E-29	-0.11362	0.005	0.1	1.87E-25
IFI44L	1.26E-29	-0.39564	0.002	0.099	1.88E-25
OSBPL3	1.26E-29	-0.14855	0.007	0.111	1.89E-25
CD63	1.32E-29	-0.33918	0.829	0.9	1.97E-25
GPSM1	1.36E-29	0.256182	0.165	0.153	2.03E-25
LRRC28	1.38E-29	-0.11711	0.058	0.185	2.06E-25
CDKN1C	1.40E-29	0.390453	0.174	0.102	2.09E-25

RMI1	1.64E-29	-0.15746	0.094	0.237	2.45E-25
DIS3L2	1.68E-29	0.149714	0.141	0.17	2.51E-25
DLG4	1.76E-29	0.157437	0.119	0.148	2.64E-25
MPI	1.83E-29	-0.10284	0.055	0.173	2.73E-25
ZNF669	1.86E-29	0.276766	0.214	0.17	2.78E-25
TST	2.07E-29	-0.12575	0.025	0.137	3.09E-25
PIN1	2.09E-29	-0.27534	0.61	0.84	3.12E-25
ARHGAP32	2.16E-29	0.110484	0.131	0.175	3.23E-25
WAC-AS1	2.18E-29	0.113385	0.15	0.195	3.25E-25
TTLL5	2.18E-29	-0.11831	0.037	0.149	3.26E-25
CCNE1	2.27E-29	-0.21533	0.045	0.186	3.40E-25
HMGA2	2.46E-29	-0.13956	0	0.081	3.67E-25
FNTB	2.52E-29	-0.22071	0.038	0.177	3.77E-25
HNRNPA3	2.62E-29	-0.25121	0.61	0.838	3.91E-25
FAM181A	2.62E-29	-0.16368	0.003	0.099	3.91E-25
SLC4A4	2.70E-29	-0.15743	0.043	0.168	4.03E-25
ITFG2	3.30E-29	0.103789	0.13	0.185	4.93E-25
PTN	3.42E-29	-0.27389	0.861	0.916	5.11E-25
TRAM2	3.45E-29	-0.1081	0.011	0.112	5.15E-25
EPC2	3.47E-29	0.173184	0.163	0.185	5.18E-25
EEF1A1	3.51E-29	-0.35074	0.682	0.878	5.24E-25
COX7C	3.53E-29	0.142473	0.949	0.938	5.28E-25
SYN1	3.59E-29	0.416845	0.178	0.035	5.37E-25
GLI3	3.64E-29	-0.177	0.005	0.104	5.44E-25
AEBP1	3.72E-29	-0.12962	0.065	0.194	5.56E-25
SPIN2B	3.81E-29	0.1875	0.148	0.148	5.69E-25
UNC93B1	3.86E-29	-0.10091	0.012	0.11	5.77E-25
RELB	3.88E-29	-0.11063	0.013	0.116	5.79E-25
SUMO1	3.93E-29	0.122088	0.844	0.863	5.87E-25
ZW10	4.01E-29	-0.14094	0.057	0.185	5.99E-25
CLSTN3	4.02E-29	0.375765	0.167	0.057	6.00E-25
TRMT61B	4.04E-29	0.161176	0.254	0.285	6.03E-25
NANP	4.07E-29	-0.10327	0.037	0.148	6.08E-25
FAM72A	4.18E-29	-0.16823	0.007	0.11	6.24E-25
IFT46	4.23E-29	-0.11212	0.105	0.238	6.32E-25
CA8	4.33E-29	0.537954	0.246	0.078	6.46E-25
LRRN3	4.42E-29	0.338079	0.272	0.206	6.60E-25
ARL6IP4	4.49E-29	-0.26301	0.543	0.781	6.71E-25
LRTM1	4.94E-29	0.409661	0.132	0.01	7.38E-25
ZCCHC8	5.00E-29	-0.10734	0.086	0.216	7.47E-25
FAM114A1	5.03E-29	-0.11352	0.038	0.153	7.52E-25
SDC2	5.13E-29	-0.10428	0.072	0.193	7.66E-25
ZFP14	5.25E-29	0.290605	0.205	0.162	7.85E-25
ZNF536	5.42E-29	0.441858	0.196	0.043	8.10E-25
RP3-428L1	5.75E-29	-0.11101	0.038	0.152	8.60E-25
CAD	6.12E-29	-0.15317	0.031	0.153	9.15E-25
S100A6	6.17E-29	-0.43479	0.365	0.62	9.22E-25
PRKD1	6.47E-29	-0.13487	0.023	0.135	9.67E-25
DUSP11	6.55E-29	0.137943	0.202	0.24	9.79E-25
SMCR5	6.69E-29	0.168215	0.178	0.2	9.99E-25
DCHS1	7.08E-29	0.118599	0.15	0.196	1.06E-24

C1orf61	7.40E-29	-0.32983	0.72	0.893	1.11E-24
KLHDC9	7.42E-29	0.313507	0.159	0.09	1.11E-24
SAMD14	7.50E-29	0.407996	0.229	0.11	1.12E-24
SIN3A	7.66E-29	-0.12452	0.074	0.204	1.14E-24
F12	7.86E-29	0.224053	0.145	0.132	1.17E-24
ARG2	7.88E-29	0.420911	0.204	0.083	1.18E-24
ATL1	8.22E-29	0.219281	0.246	0.246	1.23E-24
GZF1	8.73E-29	0.145857	0.109	0.135	1.30E-24
ZNF263	8.74E-29	0.166562	0.2	0.221	1.31E-24
LRP6	8.98E-29	0.150901	0.181	0.214	1.34E-24
SREBF1	9.53E-29	0.160147	0.086	0.106	1.42E-24
ETFB	1.01E-28	-0.24384	0.419	0.651	1.51E-24
HDAC7	1.05E-28	0.131006	0.115	0.147	1.56E-24
RNF44	1.08E-28	0.135518	0.154	0.194	1.62E-24
NT5E	1.11E-28	-0.23671	0.014	0.132	1.67E-24
NDUFS5	1.13E-28	0.15577	0.954	0.93	1.70E-24
STOM	1.21E-28	-0.14329	0.028	0.142	1.81E-24
POLR3C	1.27E-28	-0.12186	0.058	0.18	1.89E-24
MXI1	1.28E-28	0.352597	0.207	0.119	1.92E-24
FOXP1	1.29E-28	-0.35378	0.062	0.228	1.92E-24
ZNF236	1.46E-28	0.281108	0.165	0.127	2.18E-24
PPP1R21	1.55E-28	0.209607	0.152	0.148	2.32E-24
PSMA6	1.56E-28	-0.12793	0.159	0.299	2.33E-24
MBD2	1.57E-28	-0.12348	0.008	0.109	2.35E-24
FAH	1.66E-28	-0.13226	0.023	0.135	2.49E-24
FKBP14	1.70E-28	-0.13956	0.057	0.188	2.54E-24
RTN2	1.78E-28	0.405613	0.179	0.057	2.65E-24
ZNF394	1.83E-28	0.188936	0.161	0.174	2.74E-24
SATB1	1.92E-28	0.414005	0.277	0.156	2.87E-24
HPRT1	2.00E-28	0.196907	0.267	0.279	2.99E-24
RNF103	2.05E-28	0.15134	0.147	0.179	3.06E-24
UPP1	2.09E-28	-0.16783	0.133	0.284	3.12E-24
FBXO4	2.09E-28	-0.21114	0.046	0.185	3.13E-24
NAP1L1	2.15E-28	-0.29388	0.642	0.859	3.21E-24
RP11-498C	2.29E-28	-0.10646	0.024	0.13	3.42E-24
DNAJB5	2.47E-28	0.318475	0.222	0.154	3.69E-24
ISG15	2.51E-28	-0.25201	0.189	0.374	3.75E-24
FOXK1	2.56E-28	0.153373	0.156	0.179	3.83E-24
NGFRAP1	2.64E-28	0.148446	0.963	0.953	3.95E-24
WASF1	2.75E-28	0.332362	0.26	0.173	4.12E-24
BCL2L13	2.81E-28	-0.11458	0.089	0.22	4.20E-24
PDE4D	2.84E-28	0.223981	0.179	0.163	4.24E-24
L3MBTL2	2.87E-28	-0.10631	0.075	0.2	4.30E-24
PDCD5	3.01E-28	-0.15097	0.618	0.817	4.50E-24
SMO	3.04E-28	-0.15174	0.03	0.149	4.54E-24
ARHGAP1	3.10E-28	0.205459	0.182	0.19	4.64E-24
ZMYM5	3.16E-28	0.157951	0.197	0.219	4.73E-24
PARP6	3.17E-28	0.294561	0.22	0.16	4.73E-24
ARPC2	3.20E-28	0.151519	0.841	0.849	4.78E-24
CAMSAP1	3.36E-28	0.215677	0.168	0.17	5.02E-24
POT1	3.38E-28	-0.11345	0.077	0.199	5.05E-24

KCNMA1	3.44E-28	-0.16448	0.036	0.162	5.14E-24
SLC25A18	3.46E-28	-0.22534	0.004	0.101	5.17E-24
KIAA1109	3.56E-28	0.14369	0.141	0.172	5.32E-24
CTB-58E17	3.68E-28	-0.11684	0.02	0.126	5.50E-24
TTC13	3.71E-28	0.256747	0.168	0.132	5.54E-24
ADD2	4.05E-28	0.21571	0.244	0.235	6.05E-24
CAMKV	4.12E-28	0.348623	0.126	0.007	6.15E-24
YEATS2	4.23E-28	-0.12959	0.054	0.177	6.32E-24
RP11-294J	4.26E-28	-0.1017	0.093	0.219	6.37E-24
VAV3	4.36E-28	-0.18031	0.026	0.146	6.51E-24
NAA40	4.52E-28	0.153916	0.131	0.153	6.75E-24
RAB20	4.64E-28	-0.1033	0.013	0.107	6.93E-24
CEP76	4.87E-28	-0.11786	0.05	0.167	7.28E-24
CNPY1	4.96E-28	-0.18731	0.139	0.299	7.41E-24
LPIN1	5.07E-28	0.305672	0.238	0.186	7.58E-24
DSCC1	5.10E-28	-0.18964	0.098	0.252	7.62E-24
SEPP1	5.22E-28	-0.26488	0.034	0.168	7.81E-24
STYX	5.48E-28	-0.10387	0.026	0.127	8.19E-24
CCNE2	5.59E-28	-0.30609	0.12	0.302	8.35E-24
FAM193A	5.60E-28	0.109611	0.108	0.153	8.37E-24
INTS8	6.25E-28	0.186969	0.148	0.152	9.34E-24
FAT3	6.29E-28	-0.16089	0.03	0.152	9.40E-24
MFSD3	6.41E-28	0.16019	0.123	0.151	9.59E-24
SNRPF	6.62E-28	-0.11897	0.64	0.821	9.90E-24
ZNF280B	6.85E-28	-0.15514	0.013	0.122	1.02E-23
RPN2	7.18E-28	-0.26889	0.59	0.819	1.07E-23
FAM8A1	7.25E-28	0.172072	0.118	0.12	1.08E-23
FBXL17	7.71E-28	0.131751	0.093	0.114	1.15E-23
DNAJC12	7.76E-28	0.367433	0.159	0.074	1.16E-23
FAM65A	7.87E-28	0.130938	0.134	0.163	1.18E-23
GOLGA3	7.89E-28	0.102326	0.109	0.152	1.18E-23
SH2D3C	8.37E-28	0.341869	0.126	0.011	1.25E-23
SLC2A6	8.46E-28	0.229801	0.145	0.115	1.26E-23
ATAD1	8.47E-28	0.150544	0.233	0.257	1.27E-23
ITPR2	8.92E-28	-0.1426	0.019	0.126	1.33E-23
ELF1	9.18E-28	-0.11533	0.031	0.141	1.37E-23
ATP5J	9.43E-28	0.143479	0.875	0.88	1.41E-23
BHLHE22	9.55E-28	0.484567	0.108	0.002	1.43E-23
FAM49A	9.80E-28	0.204531	0.151	0.141	1.46E-23
EPB41L1	9.80E-28	0.163647	0.123	0.137	1.47E-23
LDOC1	1.05E-27	0.45668	0.236	0.07	1.57E-23
ACTR8	1.10E-27	0.125593	0.148	0.183	1.65E-23
BCL10	1.17E-27	0.1529	0.145	0.168	1.75E-23
TOE1	1.18E-27	-0.17296	0.068	0.204	1.77E-23
SSR4	1.26E-27	0.10215	0.756	0.815	1.88E-23
FBN1	1.26E-27	-0.10655	0.005	0.099	1.88E-23
NUP43	1.27E-27	-0.13846	0.079	0.209	1.90E-23
SNRPD1	1.28E-27	-0.2322	0.645	0.858	1.91E-23
POLB	1.34E-27	0.329919	0.19	0.122	2.00E-23
PARD6A	1.35E-27	0.124259	0.154	0.194	2.01E-23
LRRC27	1.52E-27	0.202519	0.131	0.125	2.27E-23

PAG1	1.57E-27	0.224668	0.204	0.196	2.34E-23
GORAB	1.58E-27	-0.12287	0.035	0.144	2.35E-23
ZDHHC21	1.75E-27	0.118756	0.115	0.147	2.62E-23
FAM188A	1.89E-27	0.123069	0.119	0.154	2.82E-23
FOXD1	1.95E-27	-0.1081	0.016	0.112	2.91E-23
ZNF277	1.99E-27	0.107865	0.188	0.236	2.98E-23
C1S	2.13E-27	-0.16562	0.023	0.135	3.18E-23
BTN3A2	2.16E-27	-0.17523	0.028	0.151	3.23E-23
NACAD	2.17E-27	0.302958	0.185	0.121	3.25E-23
TSTD1	2.21E-27	-0.27154	0.075	0.221	3.31E-23
UTP3	2.22E-27	0.105179	0.142	0.191	3.32E-23
ALG6	2.26E-27	-0.1434	0.09	0.227	3.37E-23
PAK1	2.29E-27	0.322503	0.236	0.165	3.42E-23
HAS2	2.41E-27	-0.12698	0.002	0.086	3.59E-23
ZNF253	2.64E-27	0.222415	0.182	0.181	3.94E-23
PPP1R9A	2.64E-27	0.457253	0.284	0.116	3.95E-23
MTMR11	2.78E-27	-0.14064	0.003	0.09	4.16E-23
CIRBP	3.10E-27	0.152761	0.952	0.94	4.63E-23
OXTR	3.19E-27	-0.13255	0.007	0.104	4.77E-23
SKAP2	3.23E-27	0.448567	0.33	0.179	4.82E-23
CPT1C	3.37E-27	0.344198	0.237	0.154	5.03E-23
TCEAL6	3.42E-27	0.428274	0.179	0.032	5.11E-23
STK17B	3.46E-27	-0.21656	0.019	0.137	5.18E-23
PSMA4	3.51E-27	-0.13764	0.592	0.781	5.24E-23
TLE2	3.59E-27	0.12799	0.17	0.207	5.36E-23
JARID2	3.90E-27	0.120381	0.14	0.178	5.83E-23
CACNB4	3.95E-27	0.175529	0.143	0.151	5.90E-23
HMGA1	3.95E-27	-0.24807	0.342	0.565	5.91E-23
SCAI	4.15E-27	0.207706	0.172	0.165	6.21E-23
NOS1AP	4.65E-27	0.262416	0.155	0.119	6.95E-23
ACSL1	4.70E-27	0.315972	0.18	0.11	7.03E-23
ZNF426	4.76E-27	0.101192	0.16	0.206	7.11E-23
MMP15	5.03E-27	0.193135	0.125	0.119	7.52E-23
GTF2IRD2	5.22E-27	0.247925	0.213	0.185	7.81E-23
RARA	5.27E-27	0.149699	0.145	0.169	7.87E-23
ZNF250	5.43E-27	0.296002	0.209	0.154	8.11E-23
ZNF668	5.93E-27	0.133893	0.134	0.159	8.86E-23
TMEM44	6.02E-27	0.223834	0.132	0.114	9.00E-23
ASUN	6.03E-27	-0.10522	0.097	0.219	9.01E-23
FAM171B	6.13E-27	0.286478	0.258	0.205	9.16E-23
BRAP	6.34E-27	0.11192	0.137	0.174	9.47E-23
PIANP	6.53E-27	0.188818	0.152	0.16	9.76E-23
KIFC2	6.65E-27	0.208834	0.134	0.125	9.94E-23
PFN1	6.79E-27	-0.23216	0.835	0.905	1.01E-22
HMGB3	7.33E-27	-0.21954	0.616	0.823	1.10E-22
PIGO	8.03E-27	-0.13294	0.019	0.12	1.20E-22
SLC27A3	8.20E-27	-0.11209	0.109	0.235	1.23E-22
CCDC74A	8.93E-27	-0.10899	0.079	0.199	1.33E-22
DNAJC22	9.25E-27	-0.13579	0.008	0.104	1.38E-22
ZNF33A	9.29E-27	0.183388	0.119	0.12	1.39E-22
ERLIN1	9.33E-27	-0.10732	0.029	0.133	1.39E-22

MED12L	9.80E-27	-0.1138	0.025	0.13	1.46E-22
NPNT	9.95E-27	-0.14638	0	0.074	1.49E-22
POLH	1.03E-26	-0.1525	0.043	0.164	1.54E-22
CREB3L1	1.07E-26	-0.1554	0.012	0.117	1.60E-22
B3GALT1	1.12E-26	-0.12442	0.019	0.12	1.68E-22
UBE3B	1.14E-26	-0.12376	0.072	0.191	1.70E-22
NBEA	1.16E-26	0.396516	0.178	0.057	1.73E-22
DOK4	1.19E-26	0.338127	0.166	0.072	1.77E-22
SLC35D1	1.24E-26	-0.10567	0.02	0.121	1.85E-22
MCM7	1.28E-26	-0.37084	0.485	0.725	1.92E-22
NKIRAS1	1.46E-26	0.138006	0.154	0.186	2.18E-22
CTB-5506.	1.48E-26	-0.13249	0.011	0.11	2.21E-22
HNRNPC	1.49E-26	-0.18848	0.712	0.899	2.23E-22
ATP5G3	1.55E-26	0.14571	0.893	0.884	2.32E-22
FOSL1	1.62E-26	-0.12075	0.003	0.09	2.42E-22
SNCB	1.63E-26	0.388372	0.144	0.03	2.43E-22
ATP5O	1.64E-26	0.115578	0.872	0.893	2.44E-22
RDX	1.71E-26	-0.23282	0.608	0.827	2.56E-22
TREX1	1.72E-26	-0.16127	0.025	0.136	2.57E-22
CPT2	1.74E-26	-0.12789	0.061	0.181	2.60E-22
ZNF789	1.75E-26	0.142548	0.155	0.178	2.62E-22
N4BP2L1	1.76E-26	0.258555	0.168	0.135	2.64E-22
NFKBIZ	1.78E-26	-0.18019	0.034	0.149	2.67E-22
CAB39L	1.85E-26	0.270088	0.162	0.114	2.77E-22
IZUMO4	1.86E-26	-0.11046	0.02	0.115	2.79E-22
FAM98C	1.88E-26	-0.10312	0.087	0.209	2.80E-22
FANCC	2.03E-26	-0.1827	0.016	0.127	3.04E-22
KCTD9	2.09E-26	-0.14957	0.038	0.151	3.12E-22
CNR1	2.15E-26	0.128445	0.109	0.128	3.21E-22
TOM1L2	2.20E-26	0.13387	0.141	0.174	3.29E-22
SLC25A14	2.24E-26	0.108906	0.161	0.202	3.35E-22
PCSK5	2.32E-26	-0.14539	0.006	0.101	3.46E-22
MICU3	2.56E-26	0.386854	0.214	0.11	3.82E-22
RRN3	2.57E-26	0.243924	0.236	0.212	3.84E-22
RP11-545E	2.58E-26	-0.10615	0.004	0.089	3.86E-22
GOLGA8A	2.64E-26	-0.11063	0.024	0.125	3.94E-22
CPLX1	2.65E-26	0.445083	0.197	0.049	3.96E-22
LIN52	2.67E-26	-0.11633	0.06	0.175	3.99E-22
PPIB	2.68E-26	-0.14241	0.61	0.801	4.01E-22
SHC4	2.76E-26	-0.10576	0.027	0.125	4.13E-22
GPR137C	2.78E-26	0.194119	0.139	0.137	4.16E-22
DPH6	3.02E-26	-0.15841	0.037	0.157	4.52E-22
GLUD1	3.04E-26	-0.25913	0.115	0.286	4.54E-22
CLOCK	3.23E-26	-0.13794	0.171	0.321	4.82E-22
SELM	3.65E-26	0.270114	0.152	0.105	5.45E-22
HOXA2	3.74E-26	0.423847	0.328	0.188	5.58E-22
ADSSL1	3.81E-26	-0.10118	0.018	0.109	5.69E-22
SPOCK2	3.84E-26	-0.18677	0.036	0.162	5.74E-22
ANKRD36C	3.90E-26	0.163569	0.31	0.32	5.83E-22
AP3M2	3.95E-26	0.19631	0.196	0.191	5.91E-22
STK35	4.09E-26	-0.11178	0.028	0.132	6.10E-22

DHX57	4.18E-26	0.120665	0.159	0.196	6.25E-22
PPP3CC	4.20E-26	0.172019	0.213	0.215	6.28E-22
TMEM205	4.39E-26	-0.12279	0.405	0.58	6.56E-22
TPI1	4.46E-26	-0.25332	0.854	0.953	6.66E-22
AL592183.	4.64E-26	0.282098	0.209	0.153	6.94E-22
PWWP2A	4.72E-26	0.181789	0.206	0.211	7.05E-22
RALGDS	4.77E-26	0.319357	0.237	0.165	7.13E-22
DBNDD1	5.03E-26	0.218541	0.121	0.111	7.51E-22
SHROOM3	5.06E-26	-0.10943	0.003	0.089	7.56E-22
CDC25A	5.19E-26	-0.20182	0.035	0.16	7.75E-22
PDLIM3	5.24E-26	-0.21148	0.07	0.21	7.83E-22
RNF165	5.45E-26	0.27399	0.207	0.156	8.14E-22
MSANTD2	5.45E-26	0.131562	0.097	0.122	8.14E-22
FNIP2	5.63E-26	0.112889	0.186	0.238	8.41E-22
LTBP4	5.90E-26	0.173388	0.09	0.095	8.82E-22
PTK2B	6.17E-26	-0.15637	0.02	0.132	9.23E-22
LRRC17	6.31E-26	-0.26496	0.06	0.206	9.43E-22
MAML1	6.67E-26	-0.16081	0.05	0.168	9.97E-22
L1CAM	6.87E-26	0.434055	0.186	0.041	1.03E-21
KTI12	7.17E-26	0.200338	0.188	0.188	1.07E-21
METTL1	7.19E-26	-0.30596	0.123	0.3	1.08E-21
C6orf15	7.32E-26	-0.44283	0	0.072	1.09E-21
PDCD2L	7.89E-26	0.170597	0.177	0.189	1.18E-21
NNMT	7.94E-26	-0.38377	0.035	0.17	1.19E-21
RICTOR	8.24E-26	0.195969	0.25	0.252	1.23E-21
SS18	8.54E-26	-0.12327	0.035	0.142	1.28E-21
VANGL2	8.64E-26	0.112474	0.129	0.165	1.29E-21
KCNJ16	8.70E-26	-0.18319	0.023	0.137	1.30E-21
MEMO1	8.84E-26	0.132394	0.151	0.175	1.32E-21
MTRNR2L12	8.95E-26	-0.27879	0.041	0.18	1.34E-21
GAP43	9.22E-26	0.288554	0.8	0.638	1.38E-21
TMEM79	9.57E-26	-0.16656	0.052	0.18	1.43E-21
IGFBP5	1.10E-25	-0.26921	0.194	0.379	1.64E-21
EMILIN1	1.10E-25	-0.14924	0.014	0.12	1.64E-21
SCD5	1.10E-25	-0.1938	0.571	0.775	1.65E-21
ANKRD37	1.16E-25	0.126995	0.121	0.157	1.74E-21
PPFIA2	1.18E-25	0.403227	0.183	0.042	1.76E-21
C12orf4	1.25E-25	-0.10229	0.077	0.194	1.87E-21
ELP4	1.25E-25	-0.13992	0.042	0.153	1.87E-21
UBAP2	1.26E-25	0.164944	0.148	0.16	1.88E-21
PLEKHA5	1.29E-25	0.100066	0.095	0.14	1.93E-21
APOL6	1.29E-25	-0.1154	0.007	0.09	1.93E-21
STX3	1.31E-25	0.306242	0.118	0.03	1.96E-21
AARSD1	1.33E-25	0.163341	0.141	0.162	1.99E-21
PCGF3	1.38E-25	0.143064	0.152	0.179	2.07E-21
SAMD9L	1.38E-25	-0.18255	0.002	0.086	2.07E-21
ZNF566	1.38E-25	0.181602	0.203	0.207	2.07E-21
AC009005.	1.42E-25	-0.12398	0.002	0.083	2.12E-21
HNRNPD	1.43E-25	-0.1547	0.579	0.778	2.14E-21
RP11-263K	1.46E-25	-0.11491	0.006	0.095	2.18E-21
SEC61B	1.47E-25	0.151904	0.84	0.831	2.19E-21

COX8A	1.47E-25	-0.24184	0.8	0.931	2.20E-21
KPNA5	1.53E-25	0.299641	0.19	0.136	2.28E-21
ADAM19	1.56E-25	-0.14875	0.045	0.162	2.33E-21
PCDHB2	1.57E-25	0.152867	0.143	0.158	2.34E-21
PPP2R3A	1.63E-25	0.246511	0.126	0.088	2.44E-21
SETMAR	1.64E-25	0.198462	0.115	0.107	2.45E-21
CEP72	1.64E-25	-0.23317	0.02	0.132	2.46E-21
ZFP1	1.66E-25	0.195436	0.167	0.156	2.47E-21
ZUFSP	1.75E-25	0.170868	0.128	0.14	2.61E-21
CEP85	1.79E-25	-0.13348	0.02	0.12	2.68E-21
CPLX2	1.80E-25	0.379894	0.152	0.022	2.69E-21
TMEM116	1.87E-25	-0.11457	0.061	0.173	2.79E-21
COPZ2	1.98E-25	-0.10558	0	0.07	2.96E-21
SCFD2	2.04E-25	-0.20068	0.082	0.227	3.05E-21
AMPD2	2.14E-25	0.16038	0.122	0.13	3.20E-21
NLK	2.26E-25	-0.10253	0.046	0.152	3.38E-21
WDR90	2.33E-25	-0.12531	0.029	0.136	3.48E-21
GS1-124K5	2.33E-25	0.129089	0.151	0.194	3.49E-21
FLVCR1-AS	2.38E-25	-0.1405	0.016	0.115	3.55E-21
LRRC24	2.40E-25	0.335906	0.203	0.117	3.59E-21
YJEFN3	2.42E-25	0.297951	0.148	0.09	3.62E-21
MFAP3L	2.56E-25	-0.10397	0.005	0.09	3.83E-21
NCS1	2.57E-25	0.110873	0.097	0.131	3.83E-21
FMNL3	2.65E-25	-0.12284	0.02	0.12	3.97E-21
SMG5	2.69E-25	-0.13421	0.051	0.17	4.02E-21
SMPDL3A	2.76E-25	0.152811	0.093	0.101	4.13E-21
C11orf1	2.81E-25	0.116925	0.148	0.188	4.20E-21
RP11-386G	2.82E-25	-0.12129	0.093	0.215	4.22E-21
KIAA0930	2.83E-25	0.211041	0.131	0.123	4.22E-21
USP15	2.96E-25	0.127788	0.251	0.293	4.43E-21
DPCD	2.99E-25	-0.1165	0.105	0.228	4.46E-21
SRP14	3.29E-25	0.145249	0.954	0.93	4.92E-21
FAM43A	3.29E-25	-0.11071	0.002	0.083	4.92E-21
9-Mar	3.34E-25	-0.17244	0.214	0.381	4.99E-21
SLC29A4	3.36E-25	0.264939	0.181	0.143	5.02E-21
FGF1	3.37E-25	-0.10928	0.001	0.075	5.04E-21
RNF219	3.40E-25	0.138795	0.25	0.28	5.08E-21
EPHB2	3.43E-25	0.110736	0.13	0.164	5.12E-21
MBOAT1	3.43E-25	-0.1222	0.005	0.091	5.13E-21
NDUFA2	3.55E-25	-0.10103	0.581	0.758	5.30E-21
GFOD1	3.71E-25	0.320547	0.167	0.098	5.55E-21
PDGFA	3.79E-25	-0.29763	0.042	0.175	5.67E-21
SMS	3.97E-25	-0.20269	0.581	0.789	5.93E-21
NDUFS6	4.00E-25	-0.24612	0.651	0.856	5.98E-21
MATN2	5.12E-25	-0.17876	0.013	0.115	7.65E-21
GAL	5.16E-25	0.371799	0.098	0.067	7.70E-21
DUOX1	5.26E-25	0.377279	0.219	0.111	7.86E-21
ZNF793	5.45E-25	0.12951	0.193	0.221	8.14E-21
TMED9	5.58E-25	-0.14304	0.623	0.805	8.34E-21
PIBF1	5.60E-25	0.119651	0.153	0.188	8.37E-21
ZKSCAN5	6.06E-25	0.126855	0.127	0.154	9.06E-21

COBL	6.39E-25	0.253317	0.121	0.096	9.55E-21
CBLB	6.40E-25	0.217254	0.112	0.098	9.56E-21
NMNAT2	6.45E-25	0.336473	0.131	0.015	9.64E-21
SETD6	6.97E-25	0.138694	0.155	0.18	1.04E-20
YPEL4	7.14E-25	0.389641	0.141	0.02	1.07E-20
TMOD1	8.10E-25	0.306517	0.228	0.158	1.21E-20
CXXC4	8.46E-25	0.190752	0.184	0.196	1.26E-20
GPR37L1	8.54E-25	-0.36715	0.014	0.122	1.28E-20
CD163L1	8.64E-25	0.408195	0.135	0.022	1.29E-20
METTL17	8.85E-25	-0.10133	0.09	0.205	1.32E-20
CPT1A	9.01E-25	-0.12276	0.019	0.115	1.35E-20
TMEM150C	9.26E-25	0.36949	0.174	0.059	1.38E-20
PCMTD1	9.66E-25	0.300599	0.205	0.144	1.44E-20
NUDT14	1.01E-24	0.162631	0.124	0.144	1.51E-20
ERCC6L	1.02E-24	-0.1513	0.001	0.077	1.53E-20
MZT2B	1.07E-24	-0.2621	0.723	0.877	1.60E-20
ZDHHC17	1.08E-24	0.111969	0.118	0.144	1.62E-20
C1QA	1.10E-24	0.205924	0.312	0.294	1.65E-20
GCA	1.12E-24	0.151348	0.215	0.249	1.67E-20
C20orf194	1.16E-24	0.103858	0.13	0.17	1.74E-20
HIP1R	1.26E-24	0.244001	0.233	0.196	1.88E-20
RIC8B	1.29E-24	0.101748	0.148	0.188	1.93E-20
KCNH2	1.30E-24	0.408313	0.238	0.1	1.94E-20
PYCRL	1.35E-24	-0.15261	0.038	0.149	2.01E-20
GJB2	1.45E-24	-0.15001	0	0.068	2.17E-20
KRBOX4	1.50E-24	0.182761	0.189	0.193	2.24E-20
GALC	1.54E-24	-0.11024	0.02	0.114	2.30E-20
ZBTB22	1.60E-24	0.151726	0.104	0.112	2.39E-20
NKX2-1	1.62E-24	0.334923	0.119	0.01	2.42E-20
ZBTB8A	1.63E-24	0.107437	0.207	0.249	2.43E-20
ZNF300	1.63E-24	0.235163	0.218	0.194	2.44E-20
HOXD-AS2	1.64E-24	-0.13207	0.024	0.126	2.45E-20
IFITM10	1.64E-24	0.430761	0.215	0.105	2.45E-20
HMGN3	1.69E-24	-0.12297	0.658	0.835	2.53E-20
ACSS1	1.72E-24	-0.11321	0.053	0.157	2.56E-20
SH3BP5L	1.78E-24	0.1695	0.108	0.106	2.66E-20
TIMP4	1.81E-24	-0.35364	0.015	0.123	2.70E-20
SRSF7	1.87E-24	-0.1637	0.648	0.84	2.79E-20
ROMO1	1.95E-24	-0.1904	0.592	0.802	2.92E-20
C2CD4A	1.97E-24	-0.103	0.001	0.075	2.95E-20
IRF9	2.00E-24	-0.19729	0.033	0.148	2.98E-20
USP28	2.01E-24	-0.13636	0.04	0.149	3.00E-20
AVEN	2.09E-24	-0.11077	0.012	0.101	3.12E-20
NDUFA3	2.31E-24	-0.20037	0.603	0.809	3.45E-20
WIPF3	2.77E-24	0.254201	0.152	0.099	4.14E-20
LRRC49	2.92E-24	0.163196	0.13	0.14	4.37E-20
ZNF568	2.96E-24	0.170489	0.158	0.165	4.42E-20
FIG4	3.07E-24	0.208283	0.166	0.151	4.59E-20
UBN2	3.19E-24	0.165852	0.194	0.201	4.76E-20
LINC0091C	3.32E-24	0.103506	0.135	0.169	4.96E-20
SCN3A	3.33E-24	0.409181	0.263	0.125	4.97E-20

NDUFB11	3.37E-24	0.120591	0.891	0.902	5.03E-20
PHLPP1	3.69E-24	-0.16305	0.042	0.158	5.52E-20
SMIM10	3.72E-24	-0.12176	0.011	0.099	5.57E-20
XAF1	3.92E-24	-0.30489	0.004	0.089	5.86E-20
EMILIN2	3.99E-24	-0.14116	0.005	0.091	5.97E-20
CACNB3	4.02E-24	0.195927	0.156	0.147	6.00E-20
FAM98A	4.35E-24	0.117976	0.205	0.241	6.49E-20
BCL11A	4.58E-24	0.370897	0.179	0.079	6.85E-20
ERGIC3	4.59E-24	-0.21684	0.565	0.777	6.86E-20
TJP2	4.77E-24	-0.129	0.009	0.098	7.12E-20
ARHGAP19	4.81E-24	-0.11765	0.002	0.079	7.19E-20
CALB2	5.22E-24	0.384246	0.121	0.014	7.80E-20
DNM3	5.27E-24	0.238981	0.203	0.165	7.88E-20
AGAP3	5.28E-24	0.146751	0.17	0.194	7.89E-20
MCPH1	5.47E-24	0.108247	0.117	0.143	8.18E-20
SPEG	5.78E-24	0.211131	0.127	0.1	8.64E-20
CTA-384D8	5.92E-24	-0.13848	0.022	0.121	8.85E-20
HRASLS	6.10E-24	-0.10754	0.035	0.135	9.12E-20
INSR	6.25E-24	0.172787	0.201	0.206	9.34E-20
RP11-445F	6.35E-24	0.432137	0.135	0.057	9.49E-20
CORO7	6.77E-24	0.167857	0.178	0.189	1.01E-19
KIF5A	6.92E-24	0.385139	0.285	0.226	1.03E-19
ZNF286A	7.24E-24	0.220159	0.15	0.126	1.08E-19
ARNTL	7.28E-24	-0.13387	0.054	0.167	1.09E-19
NELL2	7.57E-24	0.104509	0.217	0.272	1.13E-19
C5orf30	7.59E-24	0.173479	0.158	0.157	1.13E-19
ARAP2	7.90E-24	-0.12337	0.018	0.111	1.18E-19
GPC4	8.05E-24	-0.15231	0.01	0.101	1.20E-19
CTTNBP2	8.07E-24	0.24512	0.25	0.23	1.21E-19
LINC00685	8.39E-24	0.174616	0.198	0.207	1.25E-19
AC004540.	8.80E-24	0.399018	0.249	0.151	1.31E-19
PHB	9.68E-24	-0.16031	0.577	0.773	1.45E-19
RDM1	9.70E-24	-0.13474	0.002	0.08	1.45E-19
CDKL3	1.03E-23	0.154604	0.156	0.173	1.54E-19
CEP19	1.11E-23	0.180543	0.17	0.172	1.67E-19
MINOS1	1.21E-23	-0.2476	0.56	0.775	1.80E-19
NOTCH4	1.23E-23	0.222195	0.13	0.117	1.84E-19
NDUFV2	1.29E-23	-0.15763	0.57	0.751	1.93E-19
CALR	1.30E-23	-0.27946	0.617	0.82	1.95E-19
CNN3	1.41E-23	-0.26659	0.615	0.822	2.11E-19
ZNF101	1.46E-23	-0.12644	0.02	0.121	2.18E-19
ANXA11	1.53E-23	-0.17803	0.016	0.115	2.29E-19
PDIA6	1.60E-23	-0.22272	0.65	0.849	2.39E-19
HSPB1	1.65E-23	-0.15539	0.597	0.794	2.47E-19
HDHD3	1.69E-23	0.210194	0.13	0.101	2.52E-19
CBWD5	1.70E-23	0.10278	0.15	0.188	2.53E-19
RP11-315A	1.72E-23	-0.16007	0.01	0.1	2.57E-19
PTPRK	1.77E-23	0.40965	0.156	0.058	2.64E-19
ATP1A2	1.77E-23	-0.35881	0.005	0.093	2.65E-19
GPRASP2	1.88E-23	0.252551	0.18	0.146	2.81E-19
PABPC1	1.96E-23	-0.11745	0.683	0.849	2.93E-19

IGDCC3	2.11E-23	0.167455	0.123	0.128	3.15E-19
IMMP2L	2.18E-23	0.255192	0.193	0.165	3.26E-19
PFDN5	2.25E-23	0.129539	0.903	0.906	3.37E-19
JUP	2.33E-23	0.297636	0.107	0.01	3.48E-19
KIAA0226L	2.37E-23	-0.2619	0.026	0.143	3.54E-19
SOX2	2.42E-23	-0.22496	0.715	0.895	3.61E-19
CD151	2.43E-23	-0.24299	0.387	0.605	3.63E-19
DACT3	2.45E-23	0.28277	0.156	0.095	3.67E-19
KLF16	2.53E-23	-0.1033	0.016	0.105	3.78E-19
SERBP1	2.65E-23	-0.12157	0.675	0.846	3.96E-19
PTBP2	2.71E-23	0.323234	0.299	0.199	4.05E-19
GPR85	2.71E-23	0.363626	0.137	0.023	4.06E-19
SEMA6C	3.04E-23	0.299711	0.12	0.041	4.55E-19
MRE11A	3.05E-23	-0.18048	0.037	0.151	4.57E-19
ARSK	3.54E-23	-0.13502	0.024	0.12	5.29E-19
ETAA1	3.57E-23	-0.17447	0.019	0.12	5.33E-19
DLGAP1	3.60E-23	0.354207	0.181	0.072	5.38E-19
PTP4A2	3.74E-23	-0.15272	0.496	0.683	5.59E-19
LIN7A	4.76E-23	-0.12874	0.011	0.101	7.11E-19
ZNF606	4.82E-23	0.116465	0.122	0.151	7.21E-19
ARNTL2	4.90E-23	-0.10416	0.024	0.122	7.33E-19
SAMHD1	5.22E-23	-0.11023	0.072	0.184	7.80E-19
FAM160B2	5.57E-23	0.10418	0.082	0.111	8.33E-19
MCF2L	5.67E-23	0.3395	0.183	0.099	8.47E-19
XRCC6BP1	6.04E-23	-0.32072	0.087	0.243	9.03E-19
ZNF708	6.16E-23	0.145371	0.156	0.168	9.21E-19
RBX1	6.30E-23	-0.123	0.648	0.825	9.42E-19
UBA6-AS1	6.39E-23	0.110549	0.128	0.163	9.54E-19
GRIA4	7.01E-23	0.182267	0.24	0.24	1.05E-18
AGPAT4	7.27E-23	0.105318	0.145	0.175	1.09E-18
CMPK2	7.46E-23	0.259792	0.119	0.065	1.11E-18
GNG11	7.79E-23	-0.2338	0.005	0.09	1.16E-18
FN1	7.95E-23	-0.1121	0.056	0.164	1.19E-18
SYNE1	8.23E-23	0.185588	0.203	0.202	1.23E-18
WASF3	8.26E-23	-0.10788	0.046	0.143	1.23E-18
AP5Z1	8.85E-23	0.14143	0.16	0.184	1.32E-18
ACTA2	9.14E-23	-0.10867	0.006	0.088	1.37E-18
TCF7L1	9.71E-23	-0.11242	0.005	0.084	1.45E-18
PRMT6	1.01E-22	-0.10125	0.086	0.196	1.52E-18
ZMIZ2	1.03E-22	0.162621	0.159	0.169	1.55E-18
FKBP2	1.04E-22	-0.1393	0.463	0.643	1.56E-18
CHI3L1	1.10E-22	-0.53482	0.294	0.506	1.64E-18
HOXA3	1.10E-22	0.394093	0.278	0.143	1.64E-18
TSFM	1.13E-22	-0.24005	0.318	0.509	1.68E-18
C14orf2	1.17E-22	-0.2089	0.658	0.851	1.74E-18
ETFDH	1.35E-22	0.111781	0.182	0.219	2.02E-18
RTN1	1.44E-22	-0.31906	0.097	0.252	2.16E-18
IDS	1.69E-22	-0.20688	0.292	0.474	2.53E-18
GAL3ST3	1.79E-22	0.22401	0.13	0.105	2.68E-18
PCDHB3	1.85E-22	0.259864	0.114	0.057	2.77E-18
TMEM187	1.95E-22	-0.11362	0.037	0.136	2.91E-18

SALL3	2.20E-22	-0.20649	0.028	0.14	3.29E-18
ICA1	2.34E-22	0.279211	0.207	0.138	3.49E-18
SLC16A14	2.40E-22	-0.11523	0.043	0.148	3.58E-18
TIFA	2.54E-22	-0.12907	0.018	0.111	3.80E-18
PA2G4	2.77E-22	-0.19439	0.608	0.804	4.13E-18
TNFRSF11E	2.92E-22	-0.11447	0.005	0.084	4.36E-18
FRS2	3.09E-22	-0.17163	0.111	0.241	4.61E-18
ORAOV1	3.26E-22	0.140074	0.13	0.144	4.87E-18
FKBP7	3.45E-22	0.223491	0.176	0.167	5.16E-18
SH2D4A	3.45E-22	-0.1094	0.001	0.069	5.16E-18
MYL6	3.49E-22	0.123874	0.987	0.964	5.22E-18
PDIK1L	3.50E-22	0.146474	0.127	0.132	5.23E-18
RGAG4	3.60E-22	0.100509	0.086	0.11	5.38E-18
MBD6	3.90E-22	-0.22747	0.146	0.298	5.83E-18
SHFM1	3.99E-22	0.118881	0.868	0.884	5.96E-18
NME1-NME2	4.26E-22	-0.13794	0.039	0.146	6.37E-18
FAM127C	4.58E-22	0.126828	0.124	0.149	6.85E-18
TBC1D24	5.48E-22	0.111948	0.123	0.147	8.19E-18
COX4I1	6.19E-22	0.121339	0.95	0.941	9.25E-18
PAPD5	6.28E-22	0.110751	0.111	0.138	9.38E-18
ZNF324	6.39E-22	0.169009	0.134	0.135	9.54E-18
FBXO32	6.64E-22	0.201214	0.107	0.102	9.92E-18
MCAM	6.75E-22	-0.12199	0.029	0.123	1.01E-17
PWAR6	6.77E-22	0.271475	0.152	0.086	1.01E-17
RCAN2	6.93E-22	0.294758	0.123	0.015	1.04E-17
PREP	7.15E-22	0.212755	0.165	0.143	1.07E-17
CYP3A5	7.26E-22	0.129683	0.097	0.12	1.09E-17
GAD2	7.53E-22	0.493333	0.117	0.014	1.13E-17
RPS6KA2	7.64E-22	0.232469	0.146	0.115	1.14E-17
KLHL28	7.68E-22	0.192047	0.17	0.169	1.15E-17
STARD9	8.06E-22	-0.11673	0.02	0.111	1.20E-17
MNT	8.19E-22	0.129171	0.104	0.115	1.22E-17
SC5D	8.63E-22	0.117983	0.184	0.21	1.29E-17
IDNK	9.02E-22	0.231909	0.122	0.101	1.35E-17
RND1	9.13E-22	0.358606	0.209	0.104	1.36E-17
TRIM56	9.34E-22	-0.13927	0.006	0.088	1.40E-17
SH3BGR	9.38E-22	0.180577	0.145	0.144	1.40E-17
NCDN	1.02E-21	0.178096	0.121	0.106	1.52E-17
TARBP1	1.05E-21	0.110885	0.091	0.107	1.57E-17
RAB6B	1.07E-21	0.232434	0.125	0.081	1.60E-17
MORC2	1.17E-21	0.145927	0.108	0.109	1.75E-17
CASKIN2	1.19E-21	-0.10211	0.02	0.105	1.78E-17
SERPINI1	1.24E-21	0.312634	0.144	0.054	1.86E-17
KCNJ10	1.35E-21	-0.12035	0.006	0.08	2.01E-17
XKR4	1.38E-21	0.365686	0.189	0.079	2.06E-17
BCL11B	1.39E-21	0.387942	0.174	0.057	2.08E-17
JUNB	1.40E-21	0.137203	0.773	0.767	2.10E-17
FRMD6	1.43E-21	-0.158	0.006	0.086	2.14E-17
PSMB5	1.43E-21	-0.11981	0.687	0.847	2.14E-17
TTC23	1.50E-21	-0.16378	0.01	0.096	2.24E-17
ZNF420	1.57E-21	0.190175	0.19	0.183	2.35E-17

SYNGR3	1.82E-21	0.276936	0.112	0.046	2.72E-17
RP11-127E	1.98E-21	0.230495	0.14	0.096	2.95E-17
FSD1L	1.98E-21	0.261569	0.156	0.102	2.96E-17
ZNF274	2.22E-21	0.108007	0.197	0.23	3.32E-17
C1QC	2.30E-21	0.287979	0.276	0.205	3.44E-17
C1QBP	2.37E-21	0.109937	0.776	0.802	3.55E-17
PLEKHA8	2.38E-21	0.159714	0.141	0.138	3.56E-17
RP11-563K	2.51E-21	0.10657	0.135	0.177	3.75E-17
ZNF853	2.53E-21	-0.10274	0.041	0.138	3.78E-17
S1PR3	2.97E-21	-0.1159	0.01	0.091	4.43E-17
LDLRAP1	2.97E-21	-0.11592	0.002	0.072	4.43E-17
LDHB	3.02E-21	-0.22751	0.849	0.967	4.52E-17
ZNF786	3.15E-21	0.113246	0.126	0.149	4.70E-17
C11orf95	3.23E-21	0.268677	0.129	0.073	4.83E-17
JUN	3.38E-21	0.10709	0.857	0.883	5.05E-17
DDR2	3.49E-21	-0.10919	0.014	0.099	5.22E-17
ACVR1B	3.71E-21	0.283477	0.191	0.125	5.54E-17
NEDD4	3.84E-21	-0.106	0.004	0.075	5.74E-17
SYTL4	3.89E-21	-0.14223	0.003	0.073	5.81E-17
SLC16A9	3.95E-21	-0.10173	0.051	0.147	5.91E-17
NPM2	4.09E-21	0.303745	0.097	0.007	6.12E-17
ZNF592	4.12E-21	-0.1025	0.021	0.11	6.16E-17
PSMC5	4.78E-21	-0.18865	0.577	0.772	7.14E-17
C9orf72	4.93E-21	0.302289	0.22	0.147	7.37E-17
FASTKD2	5.29E-21	0.12451	0.14	0.158	7.90E-17
DDX25	5.33E-21	0.338807	0.17	0.065	7.96E-17
TRIM5	5.55E-21	-0.11091	0.005	0.078	8.30E-17
ZNF454	5.58E-21	0.258447	0.145	0.08	8.34E-17
PICK1	5.70E-21	0.22011	0.115	0.094	8.52E-17
MITF	5.73E-21	-0.13711	0.005	0.078	8.56E-17
RANBP6	5.74E-21	0.228496	0.144	0.107	8.57E-17
PPP1R12B	5.90E-21	0.108317	0.101	0.121	8.81E-17
SLC35A1	6.35E-21	0.165397	0.194	0.198	9.49E-17
TMEM163	6.72E-21	0.310268	0.135	0.028	1.00E-16
AGO1	6.96E-21	0.107647	0.115	0.144	1.04E-16
KLHDC4	7.11E-21	-0.12318	0.046	0.143	1.06E-16
TECPR2	7.18E-21	-0.10377	0.027	0.115	1.07E-16
GATS	7.20E-21	0.302994	0.185	0.102	1.08E-16
FGF14-AS2	7.83E-21	-0.15073	0.023	0.12	1.17E-16
SCG2	8.10E-21	0.290135	0.526	0.443	1.21E-16
AGAP2-AS1	8.11E-21	-0.27025	0.114	0.258	1.21E-16
SAMD13	8.69E-21	-0.13139	0.004	0.077	1.30E-16
ACVR2A	8.87E-21	0.147402	0.126	0.127	1.33E-16
SYNPR	8.91E-21	0.35266	0.091	0.005	1.33E-16
UVRAG	9.12E-21	0.112822	0.102	0.126	1.36E-16
PLSCR4	9.34E-21	-0.11159	0.002	0.072	1.40E-16
SCRT1	9.48E-21	0.348644	0.134	0.027	1.42E-16
ARHGEF9	9.86E-21	0.242375	0.235	0.193	1.47E-16
ARSD	9.86E-21	0.193292	0.101	0.094	1.47E-16
PTCH1	1.02E-20	-0.10758	0.043	0.135	1.52E-16
FAM220A	1.07E-20	0.185003	0.141	0.128	1.60E-16

HEATR5A	1.07E-20	-0.11891	0.019	0.106	1.60E-16
SLC1A5	1.11E-20	-0.15962	0.02	0.117	1.66E-16
GDPD1	1.21E-20	0.258072	0.144	0.091	1.81E-16
ZNF620	1.27E-20	0.11631	0.146	0.165	1.90E-16
ZNF74	1.50E-20	0.181154	0.12	0.119	2.25E-16
RASA4	1.52E-20	0.288144	0.133	0.049	2.28E-16
MTDH	1.55E-20	0.161157	0.793	0.786	2.32E-16
GALM	1.55E-20	-0.12626	0.017	0.105	2.32E-16
KIF5B	1.57E-20	-0.23086	0.607	0.802	2.35E-16
SMARCA2	1.62E-20	0.193098	0.18	0.162	2.42E-16
NPB	1.70E-20	0.181467	0.125	0.125	2.54E-16
C15orf41	1.80E-20	0.264231	0.139	0.095	2.69E-16
ZNF574	1.96E-20	0.167406	0.174	0.169	2.92E-16
CLCN4	2.01E-20	0.289923	0.129	0.046	3.00E-16
ENOX2	2.04E-20	-0.13618	0.026	0.121	3.05E-16
MTRNR2L8	2.17E-20	-0.17715	0.042	0.149	3.24E-16
MAFF	2.49E-20	0.262104	0.21	0.164	3.72E-16
TBC1D25	2.50E-20	0.133754	0.115	0.135	3.74E-16
ZFYVE27	2.57E-20	0.20255	0.128	0.105	3.85E-16
C7orf60	2.75E-20	0.216004	0.156	0.133	4.11E-16
HSF4	2.75E-20	0.277295	0.093	0.02	4.11E-16
SMPD3	2.90E-20	0.364037	0.159	0.041	4.34E-16
CASP1	2.95E-20	-0.23961	0.012	0.1	4.41E-16
TBCK	3.13E-20	0.116966	0.112	0.136	4.67E-16
PHLDA2	3.28E-20	-0.13049	0.007	0.083	4.90E-16
RBM15	3.31E-20	-0.12958	0.038	0.135	4.94E-16
PROS1	3.42E-20	-0.10623	0.028	0.117	5.11E-16
LTA4H	3.64E-20	0.10258	0.174	0.205	5.44E-16
H3F3A	3.86E-20	-0.13933	0.681	0.838	5.76E-16
MAP3K7CL	3.89E-20	-0.139	0.004	0.075	5.81E-16
ASGR1	3.98E-20	0.239802	0.114	0.073	5.95E-16
TSHZ1	4.02E-20	0.175866	0.17	0.164	6.01E-16
RAB27A	4.02E-20	-0.1137	0.017	0.098	6.01E-16
SNX13	4.04E-20	-0.12831	0.128	0.247	6.03E-16
GPATCH1	4.06E-20	0.124884	0.12	0.132	6.07E-16
NRG2	4.08E-20	0.114255	0.075	0.098	6.09E-16
RAB39B	4.17E-20	0.202713	0.152	0.135	6.24E-16
ABCA8	4.23E-20	-0.12962	0.001	0.063	6.32E-16
PCNXL2	4.25E-20	0.111059	0.09	0.112	6.34E-16
ATP10B	4.37E-20	-0.15382	0.001	0.063	6.53E-16
EEF1B2	4.39E-20	-0.19756	0.723	0.89	6.57E-16
IFIT1	4.52E-20	-0.19368	0.002	0.07	6.75E-16
TMEM145	4.81E-20	0.244507	0.178	0.132	7.19E-16
NDUFB4	5.38E-20	0.117725	0.77	0.788	8.05E-16
NCKAP5L	6.34E-20	0.241305	0.149	0.11	9.47E-16
KLC4	6.38E-20	0.1641	0.138	0.133	9.53E-16
COG6	6.55E-20	0.130783	0.115	0.123	9.79E-16
RP11-676J	6.57E-20	-0.26419	0.028	0.135	9.81E-16
1-Mar	6.57E-20	0.396121	0.292	0.168	9.82E-16
SOX5	7.60E-20	0.204742	0.181	0.159	1.14E-15
VASH1	7.64E-20	0.123546	0.109	0.136	1.14E-15

EFNA2	7.66E-20	0.106193	0.107	0.135	1.14E-15
GRAMD1C	8.01E-20	-0.11483	0.005	0.077	1.20E-15
TOX3	8.02E-20	0.382499	0.262	0.172	1.20E-15
FTX	8.87E-20	0.312868	0.199	0.123	1.33E-15
ZNF675	9.58E-20	0.115264	0.112	0.133	1.43E-15
IRGQ	9.59E-20	0.116346	0.137	0.156	1.43E-15
SAMD10	1.00E-19	0.133724	0.102	0.107	1.50E-15
SSPN	1.00E-19	-0.10109	0.024	0.107	1.50E-15
PER3	1.02E-19	-0.1185	0.009	0.086	1.53E-15
CASP9	1.07E-19	-0.16036	0.042	0.142	1.59E-15
NPW	1.09E-19	-0.13309	0.011	0.089	1.63E-15
LHX1	1.11E-19	0.308478	0.115	0.068	1.67E-15
CHN2	1.23E-19	0.262392	0.145	0.081	1.84E-15
HPCA	1.25E-19	0.349557	0.205	0.123	1.86E-15
FBXO43	1.32E-19	-0.12369	0.007	0.081	1.97E-15
C1orf106	1.33E-19	-0.1084	0.006	0.075	1.99E-15
EIF5AL1	1.34E-19	-0.15428	0.091	0.212	2.01E-15
RBAK-RBAK	1.39E-19	0.195117	0.189	0.184	2.08E-15
XRN1	1.40E-19	0.119175	0.115	0.135	2.10E-15
WDSUB1	1.52E-19	0.195119	0.13	0.117	2.28E-15
DGCR5	1.54E-19	0.347533	0.123	0.027	2.31E-15
TCF20	1.55E-19	0.100064	0.09	0.121	2.32E-15
TP53I11	1.59E-19	0.138264	0.084	0.086	2.37E-15
SNRPD2	1.59E-19	-0.20692	0.735	0.896	2.38E-15
PNRC2	1.60E-19	-0.21472	0.018	0.11	2.39E-15
ULBP2	1.62E-19	0.233748	0.105	0.058	2.42E-15
PEG3	1.63E-19	0.333673	0.181	0.078	2.43E-15
ATF7IP	1.68E-19	0.144484	0.188	0.194	2.51E-15
NXPH1	1.72E-19	0.156079	0.274	0.284	2.58E-15
PPP1R1A	1.75E-19	0.28914	0.078	0.002	2.62E-15
ARHGAP10	1.78E-19	-0.10261	0.007	0.079	2.66E-15
ALAD	1.88E-19	0.145124	0.111	0.127	2.81E-15
ZNF627	2.05E-19	0.107238	0.119	0.144	3.07E-15
SVOP	2.06E-19	0.262441	0.094	0.009	3.08E-15
THBS4	2.14E-19	-0.10582	0.016	0.099	3.20E-15
EPHB3	2.30E-19	-0.11545	0.028	0.117	3.44E-15
NCALD	2.44E-19	0.177346	0.223	0.225	3.65E-15
CDKN2A	2.54E-19	-0.4954	0.083	0.217	3.79E-15
ENTPD3-AS	2.68E-19	-0.11595	0.043	0.132	4.01E-15
ARMC5	2.74E-19	0.120177	0.086	0.096	4.09E-15
GBP3	2.74E-19	-0.2062	0.002	0.064	4.10E-15
DENND1B	2.96E-19	0.188448	0.153	0.143	4.42E-15
MPP1	3.00E-19	0.194857	0.148	0.131	4.49E-15
MYT1	3.15E-19	0.350169	0.163	0.056	4.70E-15
PER1	3.18E-19	-0.16346	0.07	0.181	4.75E-15
NME6	3.21E-19	0.111172	0.152	0.172	4.79E-15
UBR3	3.28E-19	0.119133	0.098	0.117	4.90E-15
ARVCF	3.39E-19	0.149316	0.108	0.109	5.07E-15
HIST2H2BE	3.50E-19	0.293208	0.176	0.094	5.24E-15
LMO3	3.76E-19	0.412401	0.231	0.122	5.62E-15
SFR1	4.28E-19	-0.17559	0.024	0.117	6.40E-15

RINT1	4.32E-19	0.13357	0.163	0.168	6.46E-15
NMNAT1	4.52E-19	-0.10567	0.036	0.123	6.76E-15
MEX3B	4.72E-19	0.2438	0.208	0.17	7.06E-15
KCNMB2	4.86E-19	0.364415	0.16	0.051	7.27E-15
PEX5	4.88E-19	0.20719	0.162	0.138	7.30E-15
RALYL	4.89E-19	0.362606	0.152	0.037	7.31E-15
MIEF2	4.96E-19	0.162406	0.107	0.094	7.41E-15
NRSN1	4.99E-19	0.26514	0.196	0.138	7.45E-15
CAMKK2	5.31E-19	0.272352	0.149	0.089	7.93E-15
SLC1A6	5.33E-19	0.267587	0.101	0.011	7.97E-15
TSEN2	5.62E-19	0.104888	0.131	0.162	8.40E-15
ARSE	5.67E-19	-0.13079	0	0.052	8.47E-15
PANX1	5.76E-19	0.104266	0.09	0.109	8.60E-15
LEAP2	5.76E-19	0.143537	0.118	0.123	8.61E-15
APOE	5.87E-19	0.135639	0.724	0.67	8.78E-15
ANXA1	6.02E-19	-0.56488	0.068	0.198	9.00E-15
PVRL1	6.54E-19	0.173579	0.102	0.096	9.77E-15
PDK1	6.81E-19	0.136503	0.111	0.123	1.02E-14
URGCP	6.83E-19	0.133518	0.204	0.216	1.02E-14
RAI14	7.01E-19	0.164842	0.132	0.135	1.05E-14
FAM107B	7.03E-19	0.161461	0.169	0.17	1.05E-14
BRF2	7.07E-19	0.120551	0.131	0.146	1.06E-14
MGAT5	7.31E-19	0.12167	0.086	0.104	1.09E-14
TMEM196	8.58E-19	0.328476	0.145	0.042	1.28E-14
GIGYF1	8.75E-19	0.132097	0.164	0.18	1.31E-14
GAS6	8.80E-19	0.112958	0.117	0.136	1.32E-14
LRCH2	8.86E-19	0.320643	0.145	0.04	1.32E-14
SCML4	8.89E-19	0.239829	0.089	0.006	1.33E-14
GABRG2	9.34E-19	0.228086	0.079	0.004	1.40E-14
SNRPE	9.53E-19	-0.13231	0.723	0.873	1.42E-14
PLCXD1	9.64E-19	-0.10302	0.055	0.147	1.44E-14
PHLDA1	1.02E-18	-0.32012	0.414	0.617	1.53E-14
COX7B	1.03E-18	-0.14916	0.677	0.837	1.53E-14
RAN	1.03E-18	-0.20605	0.873	0.947	1.54E-14
TMEM150A	1.05E-18	0.123218	0.079	0.09	1.57E-14
HSPB6	1.07E-18	-0.13467	0.025	0.109	1.60E-14
ABHD4	1.15E-18	-0.17449	0.016	0.101	1.71E-14
GPR153	1.22E-18	0.268844	0.112	0.067	1.82E-14
AVIL	1.22E-18	-0.17816	0.119	0.242	1.83E-14
SLC37A1	1.29E-18	0.30725	0.15	0.057	1.93E-14
ADCYAP1	1.30E-18	0.309371	0.099	0.014	1.95E-14
GNAI1	1.40E-18	0.338297	0.273	0.159	2.09E-14
FBXL7	1.49E-18	-0.11218	0.013	0.091	2.23E-14
FRRS1L	1.53E-18	0.275129	0.112	0.026	2.28E-14
TSPYL2	1.55E-18	0.216526	0.187	0.154	2.31E-14
ANAPC11	1.61E-18	-0.19816	0.826	0.917	2.41E-14
PTTG2	1.63E-18	-0.15214	0.001	0.058	2.44E-14
ZNF585A	1.70E-18	0.110909	0.101	0.116	2.55E-14
SPATA20	1.75E-18	0.140726	0.091	0.096	2.62E-14
ANKS6	1.93E-18	-0.11077	0.009	0.083	2.88E-14
MPP2	2.27E-18	0.222385	0.137	0.094	3.39E-14

SOX13	2.28E-18	-0.10321	0.007	0.074	3.41E-14
ZNF713	2.38E-18	0.157939	0.12	0.119	3.56E-14
PCBP2	2.39E-18	-0.13394	0.715	0.873	3.58E-14
KCNA5	2.43E-18	0.247065	0.084	0.006	3.63E-14
PGGT1B	2.43E-18	-0.13421	0.057	0.157	3.64E-14
DMTN	2.57E-18	0.247352	0.091	0.014	3.84E-14
NKD1	2.61E-18	-0.14314	0.019	0.1	3.89E-14
FAM214B	2.67E-18	0.19529	0.128	0.1	3.99E-14
ZNF14	2.92E-18	0.143886	0.091	0.089	4.37E-14
MPP3	3.20E-18	0.288454	0.125	0.042	4.79E-14
KIF21B	3.25E-18	0.307508	0.123	0.028	4.85E-14
MAST1	3.51E-18	0.245134	0.138	0.094	5.24E-14
PRKAG2	3.54E-18	0.295889	0.149	0.089	5.30E-14
ZBTB11	3.73E-18	0.118937	0.104	0.117	5.57E-14
CHGA	3.91E-18	0.34348	0.147	0.036	5.84E-14
COL28A1	4.07E-18	-0.12721	0	0.049	6.08E-14
MAOB	4.08E-18	0.272062	0.178	0.115	6.10E-14
GBA2	4.57E-18	0.122707	0.11	0.123	6.83E-14
AC008060.	4.66E-18	0.311237	0.111	0.02	6.97E-14
SNX3	4.80E-18	-0.21668	0.714	0.869	7.17E-14
PNP	4.88E-18	-0.17189	0.024	0.115	7.30E-14
RWDD2A	4.90E-18	0.161452	0.101	0.093	7.33E-14
RGN	5.12E-18	0.301087	0.137	0.064	7.65E-14
MADD	5.41E-18	0.114414	0.078	0.091	8.08E-14
CACNA1A	6.04E-18	0.307279	0.247	0.2	9.03E-14
LGALS8	6.12E-18	0.141322	0.153	0.153	9.15E-14
ZNF408	6.33E-18	0.169582	0.13	0.119	9.46E-14
AUH	6.96E-18	0.110657	0.081	0.098	1.04E-13
CYP2R1	7.19E-18	0.137007	0.089	0.094	1.07E-13
RCOR2	8.34E-18	0.279735	0.138	0.083	1.25E-13
NPAS1	8.86E-18	0.142148	0.056	0.053	1.32E-13
TMEM176B	8.90E-18	0.291862	0.164	0.091	1.33E-13
RSPH3	8.93E-18	0.16342	0.104	0.09	1.33E-13
CLP1	9.34E-18	0.12784	0.104	0.114	1.40E-13
OLFM3	9.37E-18	0.275162	0.072	0.002	1.40E-13
TSGA10	9.48E-18	0.156248	0.097	0.094	1.42E-13
JMY	9.73E-18	0.157993	0.14	0.123	1.45E-13
RP13-650J	1.07E-17	-0.12161	0.005	0.073	1.60E-13
SNRK	1.27E-17	0.155345	0.149	0.151	1.90E-13
TLE3	1.33E-17	0.21151	0.191	0.165	1.98E-13
ARHGEF4	1.37E-17	0.147659	0.087	0.088	2.04E-13
PTPRD	1.39E-17	0.224185	0.162	0.127	2.08E-13
LRRK2	1.43E-17	-0.19209	0.009	0.083	2.13E-13
CCNT2	1.43E-17	0.122392	0.137	0.153	2.14E-13
BRAF	1.46E-17	0.10541	0.104	0.125	2.18E-13
FAM155A	1.49E-17	0.319426	0.158	0.047	2.23E-13
LINC00205	1.53E-17	0.210156	0.096	0.049	2.28E-13
SERPINE1	1.68E-17	-0.2627	0.014	0.096	2.51E-13
LRRC1	1.71E-17	0.128288	0.157	0.173	2.55E-13
MBTD1	1.72E-17	0.102144	0.084	0.102	2.57E-13
SRGN	1.73E-17	0.357814	0.31	0.16	2.58E-13

METTL22	1.73E-17	0.122534	0.101	0.109	2.59E-13
ZNF25	1.80E-17	0.106793	0.104	0.12	2.70E-13
FAM13A	2.08E-17	0.114173	0.093	0.11	3.10E-13
DNAJC6	2.11E-17	0.13536	0.074	0.073	3.16E-13
TMEM178B	2.13E-17	0.217737	0.131	0.099	3.18E-13
SCGN	2.16E-17	0.331081	0.09	0.009	3.23E-13
TRO	2.20E-17	0.258748	0.149	0.086	3.29E-13
RNF175	2.22E-17	0.312337	0.152	0.046	3.32E-13
GARNL3	2.28E-17	0.231238	0.088	0.04	3.41E-13
PHPT1	2.30E-17	-0.15906	0.69	0.848	3.44E-13
CCER2	2.34E-17	0.380129	0.191	0.078	3.49E-13
SGK3	2.46E-17	-0.11572	0.009	0.078	3.67E-13
TRIM22	2.59E-17	-0.11726	0.017	0.095	3.87E-13
FBXL16	2.60E-17	0.138179	0.103	0.105	3.88E-13
IFIT5	2.62E-17	0.134748	0.11	0.114	3.91E-13
PDE9A	2.64E-17	0.14666	0.127	0.136	3.95E-13
RP5-940J5	2.83E-17	-0.16129	0.066	0.169	4.22E-13
SLC25A42	2.87E-17	0.107205	0.079	0.096	4.30E-13
PLLP	2.91E-17	-0.20223	0.011	0.086	4.34E-13
FOSB	3.04E-17	0.221941	0.321	0.28	4.54E-13
PRRT1	3.05E-17	0.144093	0.079	0.083	4.55E-13
GPR75-ASB	3.06E-17	-0.10163	0.093	0.186	4.58E-13
RAB3D	3.07E-17	0.121875	0.086	0.089	4.59E-13
FBLIM1	3.29E-17	0.143518	0.119	0.119	4.92E-13
MTUS1	3.35E-17	0.253342	0.225	0.152	5.00E-13
LGALSL	3.45E-17	0.129599	0.118	0.128	5.16E-13
CD3EAP	3.85E-17	-0.10875	0.073	0.17	5.76E-13
PPP1R3E	4.14E-17	0.11061	0.084	0.107	6.19E-13
TRAPPC9	4.22E-17	0.11901	0.096	0.111	6.31E-13
HBB	4.78E-17	0.210816	0.5	0.332	7.15E-13
TP53	4.97E-17	-0.1123	0.122	0.227	7.43E-13
TRIM52	5.17E-17	0.108469	0.104	0.123	7.72E-13
LANCL2	5.33E-17	0.212663	0.333	0.281	7.97E-13
GCC1	5.56E-17	0.210155	0.137	0.102	8.31E-13
SPSB1	5.62E-17	-0.11228	0.016	0.089	8.40E-13
DGKG	5.69E-17	-0.13796	0.009	0.078	8.50E-13
CREBRF	5.75E-17	0.260803	0.164	0.104	8.60E-13
GLI4	5.77E-17	0.159146	0.169	0.17	8.62E-13
TACC2	6.04E-17	0.108658	0.102	0.114	9.02E-13
YWHAE	6.41E-17	0.102465	0.969	0.946	9.57E-13
RECQL5	6.68E-17	-0.10181	0.031	0.111	9.98E-13
STYXL1	7.38E-17	0.207711	0.175	0.163	1.10E-12
PROM1	7.75E-17	-0.16875	0.01	0.084	1.16E-12
BACE2	8.00E-17	-0.11078	0.006	0.072	1.20E-12
RBM48	8.39E-17	0.167001	0.139	0.138	1.25E-12
HOXA6	8.44E-17	0.297333	0.134	0.035	1.26E-12
SASH1	8.63E-17	-0.13177	0.023	0.104	1.29E-12
FEV	8.98E-17	0.37097	0.08	0.007	1.34E-12
RAMP3	9.51E-17	0.301748	0.086	0.009	1.42E-12
UPRT	1.03E-16	0.220592	0.122	0.079	1.54E-12
MOB3B	1.08E-16	0.217619	0.165	0.142	1.61E-12

NRXN3	1.09E-16	0.289173	0.115	0.023	1.63E-12
FGGY	1.23E-16	-0.13168	0.031	0.116	1.83E-12
SLC38A7	1.25E-16	0.12054	0.133	0.144	1.87E-12
MYC	1.31E-16	-0.11952	0.092	0.185	1.96E-12
RAPGEF5	1.43E-16	0.285227	0.109	0.022	2.13E-12
MFAP4	1.52E-16	-0.22367	0.041	0.146	2.28E-12
CDS1	1.54E-16	0.195179	0.064	0.001	2.30E-12
UBE2E2	1.55E-16	0.264782	0.351	0.285	2.32E-12
APOC1	1.65E-16	0.183726	0.327	0.302	2.46E-12
CTSV	1.69E-16	-0.11316	0.006	0.072	2.52E-12
THEM4	1.70E-16	0.172481	0.12	0.098	2.53E-12
NKRF	1.73E-16	0.230581	0.146	0.095	2.58E-12
PRDM8	1.75E-16	0.306315	0.125	0.047	2.61E-12
RBFOX3	1.78E-16	0.307047	0.082	0.011	2.67E-12
RP5-1024C	1.91E-16	-0.1372	0.007	0.073	2.85E-12
MIR503HG	1.99E-16	-0.13391	0.005	0.067	2.97E-12
ERC2	2.00E-16	0.254122	0.103	0.025	2.98E-12
SCAMP5	2.26E-16	0.231821	0.199	0.152	3.37E-12
METTL21B	2.27E-16	-0.14597	0.033	0.121	3.40E-12
FGD4	2.29E-16	0.279024	0.148	0.07	3.43E-12
ZNF398	2.30E-16	0.103659	0.094	0.112	3.44E-12
CCL3	2.30E-16	0.24721	0.297	0.247	3.44E-12
ZSCAN16	2.44E-16	0.170131	0.148	0.14	3.65E-12
AQP11	2.51E-16	0.131184	0.069	0.078	3.76E-12
C8orf46	2.58E-16	0.277478	0.319	0.236	3.85E-12
KIAA1324L	2.66E-16	0.298501	0.104	0.026	3.98E-12
BANF1	2.74E-16	-0.18416	0.72	0.873	4.09E-12
ALDH1L1	2.87E-16	-0.24191	0.003	0.062	4.29E-12
CA11	2.93E-16	0.134721	0.088	0.094	4.37E-12
SVIP	2.97E-16	-0.27508	0.118	0.26	4.44E-12
DHRS12	3.09E-16	0.117001	0.095	0.102	4.61E-12
PLXNA3	3.33E-16	0.220565	0.106	0.065	4.97E-12
SGIP1	3.63E-16	0.310131	0.141	0.044	5.42E-12
SCN9A	3.96E-16	0.303462	0.127	0.036	5.92E-12
CADPS	4.05E-16	0.281525	0.106	0.021	6.05E-12
ASIC1	4.26E-16	0.297317	0.147	0.067	6.36E-12
TMEM169	4.29E-16	0.186397	0.138	0.116	6.41E-12
AC005076.	4.30E-16	-0.11819	0.03	0.105	6.42E-12
IFI27	4.33E-16	-0.37192	0.013	0.09	6.47E-12
EDF1	4.37E-16	0.104806	0.878	0.886	6.53E-12
PTPN5	4.71E-16	0.238668	0.093	0.014	7.03E-12
LPAR2	5.32E-16	0.25643	0.107	0.033	7.95E-12
OAS1	5.72E-16	-0.30085	0.005	0.068	8.55E-12
KBTBD7	5.75E-16	0.204636	0.175	0.14	8.59E-12
DOCK4	6.13E-16	0.16803	0.116	0.1	9.17E-12
EGR3	6.78E-16	0.262074	0.136	0.086	1.01E-11
TENM3	6.82E-16	-0.22157	0.024	0.114	1.02E-11
EFHD1	6.85E-16	-0.1566	0.032	0.114	1.02E-11
TXNDC16	7.38E-16	0.117748	0.111	0.114	1.10E-11
AACS	7.86E-16	0.135731	0.103	0.104	1.18E-11
CARD16	8.12E-16	-0.30619	0.01	0.081	1.21E-11

SLITRK2	8.46E-16	-0.18755	0.021	0.105	1.26E-11
GBP2	8.51E-16	-0.15914	0.013	0.085	1.27E-11
ZDHHC13	8.53E-16	0.256081	0.183	0.115	1.27E-11
FOS	8.55E-16	0.105164	0.844	0.791	1.28E-11
CTC-534A2	8.74E-16	0.106176	0.086	0.099	1.31E-11
GRID2	9.01E-16	-0.15765	0.035	0.122	1.35E-11
RASAL2	9.38E-16	0.127472	0.129	0.13	1.40E-11
EPSTI1	9.41E-16	-0.28266	0.016	0.096	1.41E-11
SNX29	9.47E-16	0.130835	0.106	0.109	1.42E-11
SFXN3	1.01E-15	0.115007	0.063	0.065	1.50E-11
GNAZ	1.02E-15	0.159285	0.125	0.125	1.52E-11
MORC2-AS1	1.17E-15	0.226956	0.117	0.074	1.75E-11
LRRC73	1.24E-15	0.133058	0.074	0.062	1.85E-11
MAP1LC3B2	1.35E-15	0.273634	0.126	0.073	2.02E-11
ZNF654	1.37E-15	0.105848	0.073	0.081	2.04E-11
ZNF595	1.50E-15	0.110159	0.126	0.141	2.25E-11
JAZF1	1.57E-15	0.141606	0.115	0.116	2.34E-11
PEX1	1.59E-15	0.135409	0.13	0.126	2.37E-11
AIFM2	1.71E-15	-0.10375	0.006	0.067	2.55E-11
MTAP	1.74E-15	-0.16937	0.022	0.1	2.60E-11
CD74	1.79E-15	0.154677	0.596	0.527	2.68E-11
RIPPLY2	1.81E-15	0.150269	0.086	0.079	2.70E-11
MB21D2	1.84E-15	0.235804	0.108	0.049	2.75E-11
GADD45GIP	1.97E-15	-0.16286	0.628	0.794	2.95E-11
VPS8	1.98E-15	0.124754	0.121	0.13	2.96E-11
CXorf40A	2.01E-15	0.130398	0.097	0.095	3.00E-11
RABGAP1L	2.04E-15	0.193858	0.108	0.073	3.05E-11
PELI1	2.06E-15	0.146326	0.241	0.243	3.08E-11
MTURN	2.11E-15	0.223441	0.18	0.128	3.16E-11
IGFBP6	2.47E-15	0.173321	0.08	0.07	3.69E-11
GADD45B	2.50E-15	-0.32927	0.197	0.348	3.73E-11
ATAD2B	2.56E-15	0.124451	0.097	0.099	3.83E-11
GSX1	2.58E-15	-0.19747	0.016	0.091	3.86E-11
RHOBTB2	2.61E-15	0.121263	0.084	0.085	3.91E-11
AC079922.	2.67E-15	-0.13337	0.02	0.094	3.99E-11
FAM117B	2.72E-15	0.278586	0.133	0.048	4.07E-11
NAT16	3.20E-15	0.263925	0.113	0.033	4.78E-11
SNX30	3.38E-15	0.127832	0.071	0.073	5.06E-11
TRUB1	3.41E-15	0.172109	0.093	0.072	5.10E-11
ARHGEF10	3.59E-15	0.100106	0.093	0.11	5.36E-11
ZNF350	3.78E-15	0.157425	0.116	0.104	5.65E-11
RGS11	3.82E-15	0.244179	0.101	0.031	5.71E-11
RELL2	3.87E-15	0.182045	0.089	0.051	5.78E-11
AC002454.	4.00E-15	-0.16623	0.013	0.08	5.97E-11
ST8SIA4	4.09E-15	0.17189	0.086	0.053	6.11E-11
HARS2	4.41E-15	0.145125	0.132	0.128	6.59E-11
LRRC26	4.51E-15	0.306755	0.126	0.033	6.73E-11
HAUS4	5.09E-15	-0.13874	0.012	0.08	7.61E-11
TBC1D19	5.34E-15	0.187689	0.112	0.085	7.98E-11
TAC3	5.41E-15	0.350047	0.13	0.032	8.09E-11
PLAA	5.58E-15	0.145915	0.153	0.148	8.34E-11

SCRT2	5.66E-15	0.292416	0.116	0.027	8.45E-11
PTGS1	6.67E-15	-0.11152	0.009	0.074	9.96E-11
TRMT10B	6.68E-15	0.103288	0.13	0.151	9.99E-11
C19orf54	6.83E-15	-0.10447	0.019	0.088	1.02E-10
RAB33B	6.97E-15	0.126303	0.088	0.091	1.04E-10
CASD1	7.29E-15	0.205525	0.174	0.14	1.09E-10
APOL2	7.46E-15	-0.11272	0.025	0.099	1.11E-10
EFNA1	7.47E-15	0.141006	0.109	0.109	1.12E-10
KIAA1522	7.58E-15	0.129854	0.07	0.06	1.13E-10
HOXD3	7.94E-15	0.265582	0.123	0.044	1.19E-10
ZNF781	8.78E-15	0.107307	0.084	0.094	1.31E-10
MORN4	9.00E-15	0.115504	0.079	0.079	1.34E-10
DMD	9.22E-15	-0.10516	0.068	0.157	1.38E-10
SFRP2	9.45E-15	-0.13162	0.002	0.051	1.41E-10
SPIRE2	9.63E-15	0.156024	0.11	0.095	1.44E-10
PARK7	9.64E-15	-0.19511	0.786	0.906	1.44E-10
PHACTR3	1.03E-14	0.319833	0.264	0.138	1.54E-10
CCDC170	1.04E-14	0.246392	0.101	0.026	1.55E-10
DLL1	1.08E-14	-0.23968	0.089	0.207	1.62E-10
STRIP2	1.11E-14	-0.1144	0.013	0.081	1.65E-10
CA10	1.11E-14	-0.15281	0.032	0.11	1.65E-10
SRSF2	1.13E-14	-0.17896	0.745	0.878	1.70E-10
FCER1G	1.16E-14	0.114805	0.165	0.175	1.74E-10
NXN	1.25E-14	0.298083	0.14	0.06	1.86E-10
FAM109A	1.30E-14	0.187821	0.104	0.073	1.95E-10
LINC00237	1.31E-14	0.264514	0.131	0.047	1.96E-10
OPHN1	1.40E-14	0.189919	0.137	0.117	2.09E-10
SLC7A5	1.42E-14	0.123059	0.09	0.095	2.12E-10
PPP1R1C	1.47E-14	-0.12557	0.003	0.056	2.19E-10
COX5B	1.51E-14	0.108734	0.931	0.916	2.26E-10
PSD2	1.59E-14	0.297868	0.145	0.054	2.38E-10
ZNF385A	1.60E-14	0.103304	0.084	0.094	2.39E-10
SNX32	1.60E-14	0.219765	0.091	0.021	2.39E-10
ST8SIA3	1.69E-14	0.250685	0.105	0.022	2.53E-10
PTPRO	1.78E-14	0.273886	0.14	0.047	2.67E-10
THUMPD2	1.81E-14	-0.10942	0.095	0.185	2.71E-10
WDR86	1.96E-14	0.255885	0.104	0.054	2.93E-10
ZFP36	2.22E-14	-0.37894	0.178	0.327	3.32E-10
MB21D1	2.32E-14	-0.11409	0.007	0.064	3.46E-10
RUNX1T1	2.39E-14	0.183067	0.076	0.052	3.57E-10
HOXC6	2.47E-14	0.300643	0.134	0.047	3.69E-10
FHOD3	2.52E-14	0.102002	0.091	0.102	3.76E-10
PPP2R2C	2.58E-14	0.167378	0.056	0.001	3.85E-10
PCDHB15	3.11E-14	0.231993	0.09	0.016	4.64E-10
VWA5A	3.20E-14	0.118783	0.084	0.083	4.78E-10
RP11-108M	3.28E-14	-0.11688	0.028	0.1	4.90E-10
SLC17A6	3.33E-14	0.249721	0.097	0.02	4.98E-10
KLHL21	3.56E-14	-0.11133	0.011	0.074	5.33E-10
RP6-65G23	3.68E-14	0.241461	0.079	0.015	5.51E-10
SLC25A12	3.73E-14	0.159634	0.116	0.104	5.57E-10
C7orf43	3.80E-14	0.11928	0.108	0.115	5.67E-10

ZNF879	3.87E-14	0.107242	0.069	0.073	5.79E-10
APBB3	4.00E-14	0.174333	0.106	0.079	5.98E-10
CYBA	4.13E-14	0.233511	0.194	0.135	6.17E-10
NACA2	4.16E-14	-0.13976	0.074	0.165	6.22E-10
ZNF623	4.18E-14	0.113965	0.081	0.086	6.25E-10
PRKAB2	4.21E-14	0.10965	0.086	0.094	6.29E-10
USP49	4.56E-14	0.139391	0.109	0.101	6.81E-10
CRB1	4.75E-14	0.152058	0.147	0.148	7.10E-10
FNDC5	5.43E-14	0.233252	0.085	0.015	8.11E-10
CCL4	5.75E-14	0.162028	0.247	0.228	8.59E-10
DACT1	6.16E-14	0.271147	0.112	0.044	9.21E-10
SOD3	6.44E-14	-0.13256	0.008	0.065	9.63E-10
PCP4	6.54E-14	0.385186	0.159	0.057	9.77E-10
DAGLB	6.71E-14	0.137144	0.118	0.112	1.00E-09
ANKRD13B	6.73E-14	0.119652	0.098	0.102	1.01E-09
HDX	6.87E-14	0.171787	0.096	0.068	1.03E-09
ACSL4	7.35E-14	0.138746	0.083	0.08	1.10E-09
RP4-798A1	7.57E-14	0.10305	0.077	0.094	1.13E-09
SCRG1	7.70E-14	-0.18784	0.487	0.642	1.15E-09
FBXL2	7.72E-14	0.214163	0.095	0.036	1.15E-09
HBEGF	8.16E-14	0.190574	0.12	0.094	1.22E-09
MAGEE1	8.28E-14	0.213067	0.096	0.019	1.24E-09
TFAP2A	8.33E-14	0.238414	0.146	0.107	1.24E-09
SAMD11	9.09E-14	0.208959	0.07	0.01	1.36E-09
C2CD2L	9.60E-14	0.144581	0.076	0.063	1.43E-09
RBFOX1	1.00E-13	0.223868	0.058	0.004	1.50E-09
RPRML	1.07E-13	-0.11377	0.007	0.062	1.61E-09
ME3	1.13E-13	0.189902	0.101	0.078	1.69E-09
HECW1	1.13E-13	0.275934	0.129	0.038	1.69E-09
SAMD9	1.15E-13	-0.12903	0.016	0.081	1.72E-09
C5orf63	1.17E-13	0.108519	0.104	0.117	1.76E-09
SLC16A1-A	1.21E-13	-0.19964	0.031	0.112	1.81E-09
CLU	1.24E-13	-0.29611	0.765	0.828	1.86E-09
AP2S1	1.26E-13	-0.10194	0.705	0.83	1.89E-09
ARMCX5	1.28E-13	0.163243	0.144	0.128	1.92E-09
MYBPC1	1.52E-13	-0.17369	0.003	0.053	2.27E-09
EN1	1.62E-13	0.255605	0.169	0.107	2.42E-09
MYOM2	1.64E-13	0.176566	0.078	0.054	2.46E-09
EPS8L1	1.68E-13	0.126808	0.071	0.063	2.51E-09
RAPGEF4	1.75E-13	0.230693	0.119	0.059	2.62E-09
ARID3A	1.84E-13	0.108771	0.101	0.107	2.74E-09
EFCAB7	1.84E-13	0.126619	0.128	0.132	2.75E-09
SH3BGRL3	1.86E-13	0.113685	0.701	0.705	2.77E-09
SERPINF1	2.11E-13	0.129624	0.064	0.07	3.16E-09
ZFP37	2.22E-13	0.142526	0.133	0.11	3.31E-09
PLXNA2	2.30E-13	0.123099	0.079	0.08	3.44E-09
SLFN11	2.47E-13	-0.11962	0.006	0.06	3.69E-09
CABYR	2.50E-13	0.175781	0.082	0.053	3.73E-09
RAB11FIP4	2.70E-13	0.246189	0.098	0.037	4.03E-09
RCL1	2.74E-13	0.112458	0.111	0.114	4.10E-09
TMTC2	2.78E-13	0.221725	0.102	0.059	4.16E-09

DHFRL1	2.95E-13	-0.113	0.043	0.115	4.41E-09
ZNF804A	3.05E-13	0.279492	0.139	0.057	4.55E-09
P2RX7	3.21E-13	-0.15237	0.035	0.116	4.79E-09
PAPOLG	4.18E-13	0.131114	0.096	0.102	6.24E-09
RP11-834C	4.22E-13	0.288078	0.162	0.074	6.31E-09
NOS2	4.24E-13	-0.1423	0.002	0.046	6.33E-09
PEX6	4.25E-13	0.141955	0.092	0.083	6.34E-09
CAMK2G	4.31E-13	0.134147	0.135	0.126	6.43E-09
TXNDC17	4.37E-13	-0.14278	0.562	0.712	6.53E-09
ZBTB8B	4.40E-13	0.160779	0.11	0.083	6.57E-09
BHLHB9	4.44E-13	0.212057	0.109	0.063	6.64E-09
TMEM151A	4.47E-13	0.192231	0.07	0.017	6.68E-09
MGAT5B	4.51E-13	0.11518	0.073	0.075	6.74E-09
CCDC157	4.56E-13	0.129619	0.078	0.065	6.82E-09
5-Sep	4.56E-13	0.138846	0.083	0.078	6.82E-09
AFF3	4.76E-13	0.23839	0.175	0.117	7.11E-09
QPRT	4.77E-13	-0.13585	0.063	0.146	7.12E-09
CTSH	4.94E-13	-0.10182	0.027	0.095	7.39E-09
KIAA0895	5.26E-13	0.193888	0.101	0.058	7.87E-09
ZNF548	5.46E-13	0.136387	0.148	0.14	8.16E-09
EEF1G	5.99E-13	-0.11071	0.038	0.111	8.95E-09
PIP5KL1	7.53E-13	0.140709	0.056	0.033	1.13E-08
TAF1A	7.77E-13	0.120016	0.123	0.12	1.16E-08
FAM65B	8.09E-13	0.132716	0.068	0.056	1.21E-08
RALGPS1	8.60E-13	0.126688	0.12	0.115	1.29E-08
EEF1A2	9.43E-13	0.105347	0.08	0.093	1.41E-08
FXYD5	9.60E-13	-0.11704	0.057	0.132	1.43E-08
RP11-22P6	9.80E-13	0.163301	0.084	0.072	1.46E-08
PGM5	9.89E-13	-0.10452	0.002	0.044	1.48E-08
NKAIN1	1.04E-12	0.227642	0.083	0.019	1.55E-08
CCDC24	1.06E-12	0.121179	0.08	0.081	1.58E-08
CPM	1.07E-12	-0.27882	0.049	0.144	1.60E-08
CACNA1E	1.20E-12	0.159779	0.057	0.004	1.80E-08
TSPAN18	1.22E-12	0.22727	0.092	0.033	1.82E-08
SHD	1.24E-12	0.254638	0.43	0.351	1.86E-08
RHEB	1.44E-12	-0.16922	0.821	0.926	2.15E-08
STC1	1.46E-12	0.279859	0.126	0.044	2.18E-08
BMPER	1.47E-12	0.130857	0.069	0.062	2.20E-08
PRCD	1.50E-12	0.313906	0.123	0.046	2.24E-08
ARHGEF11	1.61E-12	0.113341	0.068	0.072	2.41E-08
ATP2B4	1.68E-12	0.123951	0.086	0.086	2.51E-08
NTRK2	1.72E-12	-0.32035	0.053	0.149	2.57E-08
MAGI2	1.79E-12	0.149566	0.199	0.183	2.68E-08
DYRK1B	1.80E-12	0.180686	0.085	0.057	2.69E-08
4-Mar	1.85E-12	0.195183	0.053	0.005	2.77E-08
RCBTB2	1.91E-12	0.134562	0.207	0.195	2.86E-08
CELSR3	1.95E-12	0.241059	0.12	0.059	2.91E-08
C9orf3	2.06E-12	0.140568	0.159	0.158	3.08E-08
RP11-69E1	2.07E-12	0.191189	0.101	0.064	3.09E-08
RP11-629C	2.11E-12	-0.13562	0.012	0.07	3.15E-08
ZSCAN9	2.24E-12	0.136548	0.146	0.133	3.35E-08

EFNA5	2.29E-12	0.211327	0.062	0.011	3.42E-08
PDZD4	2.29E-12	0.155013	0.139	0.128	3.43E-08
SRI	2.45E-12	-0.18054	0.868	0.946	3.66E-08
RP11-196G	2.48E-12	-0.11683	0.01	0.068	3.71E-08
FAM66C	2.81E-12	0.170015	0.091	0.064	4.20E-08
TRIM59	2.82E-12	-0.14605	0.009	0.067	4.21E-08
UCN	2.90E-12	0.199678	0.09	0.031	4.34E-08
BTBD17	3.08E-12	0.279821	0.152	0.083	4.60E-08
USF1	3.21E-12	0.214685	0.118	0.089	4.80E-08
VASH2	3.34E-12	0.233785	0.104	0.043	4.99E-08
IGFBPL1	3.36E-12	0.222177	0.088	0.021	5.02E-08
MYLIP	3.36E-12	0.15997	0.086	0.074	5.02E-08
RUSC2	3.74E-12	0.185158	0.093	0.052	5.59E-08
BBS9	3.75E-12	0.151087	0.09	0.074	5.60E-08
PCSK2	3.88E-12	-0.13496	0.054	0.128	5.79E-08
THSD4	4.05E-12	-0.17633	0.002	0.046	6.05E-08
NOL4	4.86E-12	0.190448	0.218	0.177	7.26E-08
RP11-195F	4.89E-12	0.143865	0.18	0.165	7.30E-08
NUDT10	4.93E-12	0.154768	0.145	0.132	7.37E-08
RP11-490M	5.14E-12	0.140273	0.082	0.068	7.68E-08
COL20A1	5.48E-12	-0.12763	0.004	0.051	8.19E-08
C16orf70	5.57E-12	0.113832	0.082	0.078	8.33E-08
TLDC1	5.69E-12	0.13437	0.073	0.058	8.50E-08
ZNF135	6.27E-12	0.197209	0.108	0.059	9.38E-08
ZCCHC18	7.20E-12	0.200038	0.092	0.046	1.08E-07
NFX1	7.48E-12	0.111333	0.119	0.117	1.12E-07
EGR1	7.80E-12	0.140352	0.726	0.659	1.17E-07
CD14	8.33E-12	0.186983	0.142	0.11	1.24E-07
FRY	8.55E-12	0.216772	0.089	0.026	1.28E-07
CREB3L4	9.00E-12	0.148464	0.091	0.079	1.35E-07
ATP8A2	9.17E-12	0.157346	0.057	0.005	1.37E-07
BOLA3-AS1	9.80E-12	0.104729	0.071	0.077	1.46E-07
NUAK1	9.89E-12	0.17334	0.098	0.075	1.48E-07
MAPK8IP2	9.92E-12	0.214137	0.109	0.068	1.48E-07
KDM7A	1.03E-11	0.23819	0.124	0.041	1.54E-07
KCNK12	1.07E-11	0.196662	0.065	0.021	1.60E-07
SLC17A5	1.10E-11	0.121909	0.092	0.085	1.64E-07
CGREF1	1.10E-11	0.105803	0.056	0.06	1.64E-07
ZNF618	1.14E-11	0.20455	0.102	0.053	1.71E-07
GALT	1.16E-11	0.184256	0.106	0.067	1.74E-07
TYROBP	1.28E-11	0.126265	0.161	0.151	1.91E-07
RASGRP2	1.29E-11	0.179272	0.075	0.038	1.92E-07
AIF1	1.35E-11	0.157764	0.134	0.117	2.01E-07
PCAT6	1.35E-11	0.187248	0.075	0.028	2.01E-07
AURKAIP1	1.44E-11	-0.10038	0.694	0.817	2.15E-07
CTC-503J8	1.66E-11	0.158612	0.071	0.041	2.49E-07
CDK20	1.73E-11	0.149933	0.088	0.068	2.59E-07
GTF2IRD1	2.31E-11	0.110055	0.132	0.133	3.45E-07
MYOT	2.36E-11	-0.11725	0.003	0.044	3.53E-07
RNF182	2.43E-11	0.105481	0.138	0.141	3.63E-07
DUSP16	2.44E-11	0.121315	0.06	0.053	3.65E-07

TRIM46	2.46E-11	0.206212	0.077	0.022	3.68E-07
ABLIM1	2.53E-11	-0.1107	0.024	0.089	3.78E-07
FRAT2	2.70E-11	0.176791	0.093	0.064	4.03E-07
ZNF573	2.80E-11	0.183145	0.117	0.084	4.18E-07
RGCC	2.80E-11	-0.29937	0.095	0.204	4.19E-07
XKR6	2.81E-11	0.152099	0.065	0.048	4.20E-07
CTNNA2	2.83E-11	0.276403	0.181	0.105	4.22E-07
DHRS3	2.92E-11	-0.12227	0.012	0.067	4.36E-07
RAB3B	2.96E-11	0.163763	0.093	0.077	4.43E-07
LINGO1	2.99E-11	0.19083	0.104	0.073	4.47E-07
IGSF3	3.65E-11	0.122533	0.095	0.089	5.46E-07
HAPLN4	3.84E-11	0.180109	0.071	0.021	5.74E-07
PPIL6	3.86E-11	0.142701	0.078	0.06	5.77E-07
RNF112	3.88E-11	0.157643	0.105	0.079	5.79E-07
AP001372.	3.95E-11	0.10992	0.092	0.088	5.90E-07
TUSC1	4.29E-11	0.181555	0.189	0.148	6.41E-07
CTSZ	4.30E-11	0.139623	0.075	0.072	6.43E-07
FXYD7	4.31E-11	0.337582	0.24	0.141	6.45E-07
SNAP91	4.45E-11	0.216638	0.081	0.016	6.65E-07
RP11-111M	4.65E-11	0.110608	0.084	0.085	6.95E-07
ACHE	4.75E-11	0.180362	0.086	0.067	7.10E-07
RP11-166F	4.90E-11	0.143295	0.063	0.036	7.33E-07
AGTPBP1	4.95E-11	0.141284	0.079	0.072	7.39E-07
PRPF40B	5.03E-11	0.125476	0.108	0.098	7.52E-07
PLCB4	5.52E-11	0.181919	0.075	0.02	8.25E-07
UNC13A	5.66E-11	0.138729	0.079	0.057	8.45E-07
LINC00839	5.76E-11	0.19031	0.083	0.021	8.62E-07
BCAR3	5.80E-11	-0.10424	0.009	0.057	8.67E-07
APOD	5.91E-11	-0.16714	0.056	0.132	8.84E-07
ALDOC	6.01E-11	0.24315	0.152	0.102	8.97E-07
C4orf36	6.59E-11	-0.10886	0.031	0.09	9.85E-07
FAM71E1	6.67E-11	0.210143	0.097	0.031	9.97E-07
GRIN2B	7.92E-11	0.208448	0.092	0.025	1.18E-06
NEUROD1	8.22E-11	0.295198	0.174	0.075	1.23E-06
ZNF540	8.24E-11	0.16287	0.057	0.009	1.23E-06
PDZD7	8.74E-11	0.144638	0.047	0.004	1.31E-06
TP53INP1	8.91E-11	0.102687	0.062	0.063	1.33E-06
TIGD3	9.63E-11	0.154832	0.083	0.049	1.44E-06
TCP11L1	9.77E-11	-0.10617	0.035	0.098	1.46E-06
ZNF850	9.79E-11	-0.10518	0.011	0.062	1.46E-06
KHDC1	1.01E-10	0.138949	0.143	0.135	1.51E-06
HSPA2	1.06E-10	-0.12722	0.086	0.158	1.58E-06
ZFP28	1.06E-10	0.157811	0.099	0.073	1.58E-06
PRKCDBP	1.10E-10	-0.11407	0.005	0.048	1.65E-06
DDIT4	1.13E-10	-0.16297	0.213	0.312	1.69E-06
EPHB1	1.16E-10	0.20482	0.128	0.081	1.73E-06
DIRAS2	1.17E-10	-0.11241	0.093	0.173	1.76E-06
NMRK1	1.29E-10	0.110259	0.09	0.086	1.93E-06
ATP8A1	1.30E-10	0.119348	0.066	0.054	1.94E-06
BACH2	1.46E-10	0.149794	0.079	0.053	2.18E-06
FBXL20	1.58E-10	0.130039	0.075	0.059	2.37E-06

PGAM2	2.15E-10	0.226639	0.098	0.037	3.21E-06
PPP1R3F	2.33E-10	0.204832	0.098	0.053	3.48E-06
DDX60	2.34E-10	-0.10486	0.008	0.054	3.49E-06
AMOTL2	2.47E-10	0.104396	0.162	0.177	3.68E-06
PIR	2.51E-10	-0.10756	0.024	0.084	3.75E-06
RP5-1177M	2.51E-10	0.31308	0.168	0.098	3.76E-06
HIST1H2BJ	2.99E-10	-0.26659	0.005	0.049	4.47E-06
ZNF641	3.00E-10	0.11457	0.064	0.053	4.48E-06
FBXO16	3.13E-10	0.170386	0.098	0.058	4.67E-06
PDLIM4	3.13E-10	-0.19518	0.018	0.078	4.68E-06
MYH7	3.30E-10	0.182518	0.051	0.007	4.94E-06
LINC-PINT	3.33E-10	0.160351	0.09	0.073	4.98E-06
LDLRAD4	3.43E-10	0.107397	0.116	0.119	5.13E-06
TRIQK	3.52E-10	0.195137	0.097	0.058	5.26E-06
RP11-686C	3.73E-10	0.13469	0.064	0.058	5.58E-06
RP11-1391	4.23E-10	0.104748	0.067	0.057	6.33E-06
PURG	4.36E-10	0.120971	0.057	0.044	6.51E-06
PNCK	4.51E-10	0.177233	0.076	0.019	6.75E-06
CNTN2	4.66E-10	0.183902	0.074	0.016	6.96E-06
ZFP69B	4.69E-10	0.16369	0.081	0.048	7.01E-06
CLEC2B	5.03E-10	-0.13269	0.026	0.088	7.51E-06
VIP	5.07E-10	0.161857	0.031	0	7.58E-06
SRCIN1	5.23E-10	0.188414	0.078	0.028	7.81E-06
CLMN	5.52E-10	-0.1076	0.009	0.054	8.25E-06
PCDHB7	5.53E-10	0.195414	0.207	0.163	8.26E-06
GPRIN1	5.71E-10	0.11553	0.106	0.106	8.54E-06
PEG10	5.72E-10	0.272613	0.335	0.242	8.55E-06
GPR173	5.93E-10	0.151547	0.078	0.057	8.87E-06
NR4A2	6.19E-10	0.191598	0.123	0.083	9.24E-06
WNT5A	6.39E-10	-0.12107	0.02	0.074	9.54E-06
GPR19	6.77E-10	0.252414	0.161	0.089	1.01E-05
TBC1D9	7.22E-10	0.134972	0.059	0.037	1.08E-05
ZNF527	7.23E-10	0.129803	0.089	0.069	1.08E-05
ARSF	7.29E-10	0.164912	0.057	0.007	1.09E-05
PDE2A	7.75E-10	0.129158	0.043	0.002	1.16E-05
RP11-2E11	7.76E-10	0.155056	0.093	0.063	1.16E-05
RADIL	8.13E-10	0.162548	0.071	0.037	1.21E-05
CLDN15	8.21E-10	0.110266	0.101	0.098	1.23E-05
CHST9	8.35E-10	-0.10768	0.026	0.083	1.25E-05
RIMS3	9.10E-10	0.15833	0.053	0.006	1.36E-05
KLF15	1.21E-09	-0.11953	0.013	0.062	1.80E-05
YPEL2	1.23E-09	0.183405	0.077	0.03	1.84E-05
KCTD16	1.28E-09	-0.12966	0.011	0.06	1.91E-05
GNAS	1.32E-09	-0.16028	0.805	0.893	1.97E-05
ZNF596	1.36E-09	0.154196	0.075	0.053	2.04E-05
CPEB3	1.38E-09	0.149459	0.052	0.007	2.07E-05
ADAMTS10	1.43E-09	0.193608	0.108	0.06	2.14E-05
B4GALNT4	1.46E-09	0.170785	0.103	0.075	2.18E-05
ASAH2B	1.50E-09	0.145194	0.09	0.065	2.24E-05
CAP2	1.50E-09	0.112065	0.111	0.104	2.25E-05
C3orf18	1.54E-09	0.137911	0.066	0.043	2.31E-05

TSHZ2	1.56E-09	0.239799	0.124	0.058	2.33E-05
ETS2	1.64E-09	0.14175	0.086	0.074	2.45E-05
RP11-713F	1.67E-09	0.234654	0.09	0.033	2.50E-05
BRSK2	1.90E-09	0.181392	0.106	0.069	2.84E-05
RHBDL1	2.12E-09	0.153476	0.06	0.026	3.16E-05
TOMM40L	2.25E-09	0.100768	0.082	0.08	3.36E-05
TSHZ3	2.30E-09	0.198877	0.085	0.041	3.44E-05
DOPEY1	2.38E-09	0.139525	0.075	0.053	3.55E-05
DLG2	2.40E-09	0.16098	0.106	0.074	3.59E-05
ZNF502	2.55E-09	0.102876	0.078	0.075	3.81E-05
ZC4H2	2.56E-09	0.180067	0.167	0.126	3.82E-05
HOXD8	2.72E-09	0.172517	0.104	0.058	4.07E-05
WNT6	2.98E-09	0.158365	0.044	0.004	4.46E-05
BSG	3.15E-09	-0.14141	0.834	0.915	4.71E-05
ALOX5AP	3.42E-09	0.20309	0.134	0.084	5.10E-05
NCAM2	3.45E-09	0.139499	0.127	0.111	5.16E-05
CACNG2	3.56E-09	0.224602	0.086	0.025	5.32E-05
PPDPF	3.64E-09	-0.10172	0.884	0.916	5.44E-05
MDK	3.77E-09	0.118921	0.874	0.867	5.63E-05
HEATR5B	3.81E-09	0.113711	0.066	0.06	5.69E-05
ESRRG	3.91E-09	0.221085	0.109	0.043	5.84E-05
SEMA6B	3.97E-09	0.169132	0.09	0.056	5.94E-05
LOX	3.98E-09	-0.14843	0.016	0.068	5.94E-05
HYAL3	4.26E-09	0.10521	0.04	0.03	6.37E-05
ZNF554	4.27E-09	0.134662	0.079	0.049	6.39E-05
RASD1	4.53E-09	0.239676	0.197	0.131	6.77E-05
TUFT1	4.62E-09	0.130468	0.071	0.048	6.91E-05
CSDC2	4.87E-09	0.177594	0.074	0.02	7.28E-05
LRRTM4	4.89E-09	0.182454	0.069	0.016	7.31E-05
LMTK3	4.89E-09	0.128568	0.042	0.02	7.31E-05
TBC1D22B	4.91E-09	0.102737	0.074	0.068	7.34E-05
LHX9	4.98E-09	0.199492	0.082	0.026	7.44E-05
ZNF783	4.98E-09	0.142637	0.079	0.057	7.45E-05
SCNN1A	5.42E-09	0.329416	0.14	0.074	8.10E-05
TMEM176A	5.70E-09	0.118599	0.082	0.074	8.51E-05
SRSF3	5.73E-09	-0.11362	0.861	0.943	8.57E-05
GMIP	6.18E-09	0.131774	0.058	0.035	9.23E-05
ZMYM6	6.23E-09	-0.11097	0.043	0.099	9.31E-05
SCML1	6.33E-09	-0.10832	0.087	0.158	9.46E-05
APOL4	7.06E-09	0.196781	0.113	0.064	0.000105
KCNQ3	8.04E-09	0.209496	0.082	0.025	0.00012
FAM212B-A	8.83E-09	0.147325	0.073	0.051	0.000132
FRS3	8.86E-09	0.119642	0.064	0.046	0.000132
UQCR11	9.47E-09	-0.26084	0	0.022	0.000141
NPY	9.54E-09	-0.19126	0.027	0.088	0.000143
GLIPR1L2	1.01E-08	0.124845	0.053	0.036	0.000151
DTX1	1.06E-08	0.188401	0.084	0.041	0.000158
CYGB	1.07E-08	0.139611	0.04	0.005	0.00016
AMBRA1	1.08E-08	0.114333	0.056	0.042	0.000162
MCM3AP-AS	1.18E-08	0.205863	0.093	0.046	0.000176
ASXL3	1.18E-08	0.180231	0.116	0.07	0.000177

ARHGAP24	1.19E-08	0.221139	0.083	0.027	0.000178
GABRB1	1.21E-08	0.156922	0.071	0.017	0.000181
TMEM130	1.29E-08	0.11375	0.043	0.009	0.000193
RNF150	1.29E-08	0.16551	0.091	0.057	0.000193
MKNK1	1.37E-08	0.112711	0.085	0.077	0.000204
SLC9B2	1.37E-08	0.109966	0.094	0.081	0.000205
HOXC10	1.44E-08	0.212242	0.153	0.101	0.000215
CAMK4	1.48E-08	0.176403	0.106	0.058	0.000221
PRPH	1.49E-08	0.159075	0.045	0.01	0.000222
ALDOA	1.56E-08	-0.18011	0.816	0.89	0.000233
DAB1	1.56E-08	0.115071	0.041	0.004	0.000233
SPTBN4	1.62E-08	0.157423	0.058	0.027	0.000242
COMTD1	1.63E-08	0.11225	0.064	0.053	0.000244
C10orf88	1.65E-08	0.103493	0.065	0.054	0.000247
SVEP1	1.67E-08	0.167338	0.059	0.017	0.00025
RP11-455F	1.71E-08	0.106215	0.068	0.063	0.000256
ZNF589	1.81E-08	0.132881	0.09	0.069	0.00027
CRY2	1.82E-08	0.126676	0.074	0.056	0.000273
TMEM179	1.87E-08	0.187601	0.128	0.081	0.000279
ST8SIA5	1.89E-08	0.140701	0.118	0.096	0.000282
EPB41	1.90E-08	0.132827	0.136	0.119	0.000284
GJA1	1.91E-08	-0.12213	0.011	0.054	0.000285
SH3RF3	1.94E-08	0.128462	0.06	0.04	0.00029
RP11-357K	1.96E-08	0.207267	0.064	0.016	0.000293
SH3GL2	1.97E-08	0.192001	0.1	0.037	0.000294
VWC2L	1.97E-08	0.145763	0.046	0.009	0.000295
PITX2	2.16E-08	-0.15481	0.047	0.116	0.000324
MAFK	2.17E-08	0.113625	0.052	0.031	0.000325
ABCG1	2.18E-08	0.210038	0.125	0.059	0.000326
SIK1	2.24E-08	0.119285	0.064	0.056	0.000335
PLP1	2.28E-08	-0.11274	0.147	0.217	0.00034
PGBD5	2.29E-08	0.145952	0.057	0.035	0.000342
ZNF124	2.38E-08	0.14873	0.092	0.063	0.000355
ST6GAL1	2.46E-08	0.19325	0.082	0.044	0.000367
ANKRD44	2.70E-08	0.137597	0.108	0.085	0.000403
HNMT	2.71E-08	-0.11607	0.053	0.115	0.000405
IFIT2	2.80E-08	-0.10083	0.007	0.046	0.000418
MAP3K9	2.84E-08	0.150196	0.057	0.015	0.000424
S100A8	2.92E-08	0.139267	0.06	0.036	0.000437
ABCC8	3.37E-08	0.150808	0.053	0.011	0.000503
PTCHD2	3.40E-08	0.19386	0.068	0.023	0.000509
ZNF222	3.48E-08	0.14026	0.105	0.074	0.000521
RGS1	3.56E-08	0.144278	0.174	0.146	0.000531
SPON2	3.61E-08	0.136476	0.048	0.012	0.000539
CDKL2	3.85E-08	0.10622	0.037	0.002	0.000576
ANKRD65	3.97E-08	-0.1585	0.008	0.046	0.000593
FAM13C	4.00E-08	0.128564	0.071	0.052	0.000598
SLC12A5	4.16E-08	0.166575	0.125	0.088	0.000621
PRSS27	4.93E-08	0.131694	0.068	0.053	0.000736
CTC-239J1	4.95E-08	0.146838	0.06	0.046	0.00074
NAP1L2	5.78E-08	0.117654	0.046	0.006	0.000864

AC005618.	5.81E-08	0.192427	0.13	0.084	0.000869
NEU4	5.87E-08	-0.21401	0.042	0.109	0.000877
DOCK11	5.88E-08	0.157912	0.066	0.027	0.000879
ANKH	5.99E-08	0.121754	0.152	0.137	0.000895
PDE7B	6.12E-08	-0.11257	0.002	0.032	0.000915
NEGR1	6.37E-08	0.198215	0.09	0.036	0.000953
ZGLP1	6.49E-08	0.124692	0.066	0.043	0.000969
HNRNPA1	6.88E-08	-0.11854	0.94	0.98	0.001028
HOXA10	7.30E-08	0.230026	0.27	0.195	0.001091
EN2	7.38E-08	0.177964	0.095	0.059	0.001102
TMBIM6	7.81E-08	-0.10473	0.803	0.893	0.001167
PKNOX2	7.94E-08	0.139731	0.062	0.025	0.001187
CROCC	8.03E-08	0.125114	0.068	0.052	0.001201
ZNF582-AS	8.07E-08	0.153889	0.113	0.086	0.001206
GABRB3	8.14E-08	0.178657	0.067	0.019	0.001217
MURC	8.35E-08	0.160099	0.081	0.048	0.001248
IGLON5	8.67E-08	0.199295	0.146	0.085	0.001295
NT5DC3	8.91E-08	0.11211	0.068	0.046	0.001332
CSGALNACT	9.08E-08	0.106311	0.065	0.058	0.001357
SLC39A8	9.77E-08	-0.13119	0.006	0.042	0.00146
HLA-DMA	1.02E-07	0.115344	0.115	0.107	0.001519
HOXB3	1.04E-07	0.111705	0.099	0.102	0.001555
NAV3	1.19E-07	0.133015	0.053	0.019	0.001781
MXD1	1.20E-07	0.11002	0.116	0.104	0.001798
ITM2A	1.21E-07	0.267154	0.124	0.056	0.001804
VAMP8	1.25E-07	0.119083	0.062	0.049	0.001872
PIP5K1B	1.28E-07	0.146929	0.054	0.014	0.001914
RIMS1	1.34E-07	0.173488	0.083	0.035	0.002
VWA3B	1.35E-07	0.103496	0.048	0.036	0.002015
LYG2	1.40E-07	0.145539	0.063	0.027	0.002093
PIK3IP1	1.49E-07	0.163736	0.064	0.026	0.002232
HLA-DRB1	1.50E-07	0.105703	0.189	0.172	0.002237
CAMKK1	1.61E-07	-0.11055	0.024	0.074	0.002399
FAM50B	1.61E-07	0.172451	0.079	0.026	0.0024
HOXB5	1.69E-07	0.197381	0.07	0.036	0.002521
CTIF	1.81E-07	0.128867	0.089	0.065	0.002703
PHF21B	1.81E-07	0.111266	0.072	0.063	0.002708
PRKAG2-AS	1.82E-07	0.173344	0.115	0.088	0.002715
FAM133A	1.82E-07	0.169841	0.071	0.025	0.002722
FERMT1	1.93E-07	-0.10191	0.046	0.101	0.002888
KCNN2	1.96E-07	0.135704	0.076	0.057	0.002922
HCN3	2.29E-07	0.132082	0.078	0.053	0.003425
LINC01122	2.31E-07	0.188191	0.086	0.031	0.003447
SEMA4A	2.61E-07	0.125618	0.047	0.011	0.003899
KCNC1	2.61E-07	0.158894	0.079	0.036	0.003904
TNFSF13B	2.66E-07	-0.10817	0.018	0.062	0.003972
COCH	2.72E-07	0.119596	0.105	0.093	0.004063
LINC00884	2.97E-07	0.126559	0.044	0.014	0.004443
PCYT1B	3.07E-07	0.13774	0.07	0.042	0.004592
GABBR2	3.22E-07	0.13343	0.062	0.025	0.004809
DGKB	3.24E-07	-0.12094	0.052	0.112	0.004836

TRPC1	3.48E-07	0.104328	0.054	0.041	0.005207
RP11-527D	3.59E-07	0.154585	0.049	0.011	0.005372
KCNN1	3.67E-07	0.13013	0.046	0.016	0.005478
VSTM2B	3.73E-07	0.109488	0.045	0.007	0.005572
GPLD1	3.75E-07	0.127377	0.055	0.035	0.005603
RGS14	3.75E-07	0.126766	0.056	0.019	0.005605
ARHGDIG	3.83E-07	0.124701	0.051	0.021	0.005724
WNT7A	3.92E-07	0.144002	0.053	0.021	0.005853
LRP1B	3.97E-07	0.104624	0.048	0.037	0.005932
MROH8	4.08E-07	-0.10218	0.036	0.084	0.006096
DSCAML1	4.58E-07	0.170059	0.077	0.035	0.006842
CACNA1C	4.71E-07	0.134157	0.05	0.01	0.007037
UNC79	4.89E-07	0.135271	0.054	0.026	0.007301
HAND2	5.00E-07	0.149969	0.066	0.026	0.007479
SSTR2	5.64E-07	0.147782	0.047	0.021	0.008425
MDGA2	5.67E-07	0.141182	0.048	0.01	0.008475
DLL3	6.00E-07	0.210257	0.416	0.316	0.008962
HHIP	6.29E-07	-0.10063	0.008	0.042	0.009397
ALX3	6.39E-07	0.16771	0.09	0.041	0.009552
RP11-480C	6.95E-07	0.14357	0.075	0.037	0.010393
HOXA-AS2	7.42E-07	0.192061	0.131	0.077	0.011085
FMNL1	7.72E-07	0.132352	0.059	0.025	0.011538
MYCL	7.77E-07	0.143215	0.056	0.017	0.011619
ADCYAP1R1	8.48E-07	0.123323	0.075	0.047	0.01267
ABLIM2	8.63E-07	0.143627	0.071	0.038	0.012895
MT1G	8.65E-07	-0.12623	0.022	0.065	0.012926
TNFRSF25	8.79E-07	0.114509	0.04	0.006	0.01313
SERPING1	9.71E-07	0.134704	0.092	0.065	0.014511
PCDHB14	1.03E-06	0.140997	0.08	0.049	0.015366
GFAP	1.16E-06	-0.20144	0.255	0.354	0.017332
KCNJ6	1.23E-06	0.137509	0.04	0.01	0.018406
ZFP69	1.25E-06	0.123403	0.049	0.014	0.018627
SMOC1	1.28E-06	-0.15757	0.028	0.077	0.019152
LRRC4	1.47E-06	0.15066	0.124	0.094	0.021961
CACNA2D2	1.82E-06	0.140894	0.044	0.009	0.027169
NDRG2	1.82E-06	-0.11127	0.312	0.385	0.027239
ZXDA	1.93E-06	0.108397	0.046	0.026	0.028813
FCGBP	1.97E-06	0.135853	0.071	0.051	0.029502
CACNB1	2.01E-06	0.124781	0.06	0.023	0.030015
LINC00882	2.06E-06	0.12077	0.058	0.032	0.030764
VMO1	2.12E-06	0.106876	0.058	0.04	0.031741
L3MBTL3	2.18E-06	0.121743	0.049	0.016	0.032607
LA16c-444	2.41E-06	0.104155	0.029	0.002	0.035953
LYZ	2.50E-06	0.125418	0.073	0.048	0.037289
GABRA2	2.50E-06	0.122185	0.049	0.011	0.037397
SPOCK3	2.61E-06	0.129229	0.029	0.004	0.038951
ZNF518B	2.62E-06	0.119591	0.057	0.021	0.039154
MAPT-AS1	2.71E-06	0.185805	0.06	0.019	0.040446
HOXC8	2.74E-06	0.183697	0.107	0.062	0.040879
C19orf81	2.77E-06	0.186133	0.092	0.043	0.041455
TUB	2.82E-06	0.115273	0.048	0.03	0.042119

FGD3	2.85E-06	0.118883	0.047	0.012	0.042529
SLCO3A1	3.00E-06	0.117954	0.045	0.011	0.044873
FBXO41	3.06E-06	0.13393	0.049	0.021	0.045723
LINC01117	3.06E-06	0.103676	0.06	0.053	0.045793
RGS7	3.13E-06	0.143808	0.056	0.023	0.046759
LRRTM1	3.22E-06	0.155867	0.09	0.052	0.048128
TFR2	3.43E-06	0.102736	0.053	0.041	0.051315
HNRNPA2B1	3.53E-06	-0.11137	0.958	0.974	0.052733
BTBD8	3.59E-06	0.107477	0.042	0.017	0.053656
EPHX2	3.64E-06	0.11831	0.053	0.028	0.054444
MYCNOS	3.72E-06	0.165108	0.06	0.026	0.055574
CLSTN2	3.72E-06	0.165814	0.078	0.033	0.055628
FPR1	3.88E-06	0.118111	0.046	0.014	0.057985
KIAA1211	3.98E-06	0.108022	0.084	0.073	0.059485
KIF26A	4.03E-06	0.143312	0.05	0.015	0.06027
SRC	4.07E-06	0.129098	0.091	0.057	0.060861
MAP2K6	4.98E-06	0.102451	0.055	0.036	0.074364
ZNF737	5.07E-06	0.146842	0.083	0.04	0.075761
LAPTM5	5.24E-06	0.170343	0.159	0.096	0.078306
PSMA2	5.47E-06	-0.17175	0.001	0.02	0.081723
HYDIN	5.64E-06	0.114869	0.047	0.016	0.084271
RP11-266J	6.20E-06	0.105319	0.041	0.014	0.092713
MAB21L1	6.31E-06	0.142877	0.059	0.028	0.09434
FCGR3A	6.66E-06	0.129678	0.077	0.048	0.099468
RNF122	6.68E-06	0.143085	0.07	0.04	0.099836
WNK2	6.85E-06	0.136864	0.069	0.031	0.102428
HINT1	6.91E-06	-0.10792	0.922	0.963	0.103251
ARRB1	7.81E-06	0.109485	0.038	0.011	0.116725
FAM26F	7.85E-06	0.101713	0.034	0.006	0.117369
SIM2	8.05E-06	0.155393	0.068	0.025	0.120367
EBF1	8.72E-06	0.15412	0.073	0.041	0.130285
RP13-188A	8.87E-06	0.140784	0.051	0.017	0.132544
CD247	9.07E-06	0.145328	0.046	0.02	0.135475
PNMAL2	9.80E-06	0.109521	0.041	0.019	0.146476
CD163	1.06E-05	0.130288	0.064	0.036	0.159123
NYAP2	1.10E-05	0.134419	0.049	0.014	0.164556
C11orf63	1.11E-05	0.121885	0.058	0.021	0.165296
STX1B	1.11E-05	0.142866	0.065	0.028	0.165735
RAMP2-AS1	1.11E-05	0.1172	0.045	0.02	0.165912
RP11-259K	1.12E-05	0.156054	0.071	0.03	0.167347
WDR66	1.18E-05	0.102892	0.042	0.019	0.176801
NOVA1	1.36E-05	0.13425	0.876	0.879	0.203599
MMP24	1.38E-05	0.124041	0.063	0.036	0.205631
ARL15	1.49E-05	0.108287	0.082	0.065	0.222633
ZSWIM5	1.60E-05	0.12268	0.041	0.012	0.239551
RP11-725F	1.78E-05	0.149839	0.078	0.048	0.265437
OPCML	1.79E-05	0.134303	0.059	0.021	0.268016
NFE2L3	1.83E-05	0.130704	0.075	0.044	0.272812
CTGF	1.93E-05	0.134451	0.045	0.028	0.288572
WFDC2	2.25E-05	0.106417	0.081	0.067	0.33653
ASPDH	2.27E-05	0.114179	0.038	0.009	0.33849

NDUFB8	2.44E-05	-0.10524	0	0.012	0.364745
BSN	2.47E-05	0.129199	0.054	0.021	0.369406
RHCE	2.52E-05	0.120187	0.044	0.012	0.376478
HLA-G	2.54E-05	0.116115	0.043	0.02	0.379452
PLIN2	2.75E-05	0.139329	0.154	0.115	0.410862
CAV1	2.78E-05	-0.23522	0.023	0.063	0.415808
PSD	2.81E-05	0.106036	0.061	0.038	0.420556
ADORA3	2.90E-05	0.113379	0.059	0.037	0.433967
PLCL1	3.08E-05	0.121442	0.064	0.03	0.460373
SLC6A11	3.20E-05	0.103442	0.046	0.016	0.478643
DIO2	3.38E-05	-0.11048	0.01	0.04	0.505064
S100A1	3.51E-05	-0.10731	0.002	0.022	0.524025
SUSD4	3.54E-05	0.14468	0.08	0.048	0.529089
AC068535.	3.64E-05	0.174016	0.083	0.049	0.543647
PIRT	3.67E-05	0.10839	0.031	0.006	0.548755
CD55	4.32E-05	0.109762	0.039	0.015	0.644862
HOXC-AS1	4.38E-05	0.142476	0.077	0.051	0.654499
CACNA2D1	5.06E-05	0.155271	0.114	0.079	0.755424
SYT6	5.48E-05	0.126962	0.096	0.067	0.818949
HOXA4	5.61E-05	0.103543	0.071	0.028	0.83905
NDUFA4L2	5.77E-05	0.131687	0.092	0.044	0.862513
STXBP5L	6.00E-05	0.116848	0.053	0.023	0.896376
RP11-545I	6.94E-05	0.106147	0.045	0.027	1
NR2F2	7.04E-05	-0.11925	0.039	0.084	1
HSD17B14	7.19E-05	0.130356	0.063	0.037	1
VAT1L	7.39E-05	0.134055	0.064	0.027	1
NMNAT3	7.90E-05	0.121992	0.082	0.044	1
HOXA9	8.30E-05	0.126765	0.121	0.104	1
HOXB-AS1	8.33E-05	0.108985	0.064	0.054	1
S100A4	9.03E-05	0.125668	0.089	0.051	1
CDKL5	9.09E-05	0.102466	0.038	0.015	1
STC2	9.12E-05	0.141137	0.075	0.06	1
DHRS2	9.50E-05	-0.17924	0.001	0.016	1
ANKRD53	0.000101	0.124681	0.057	0.026	1
NALCN	0.000115	0.104996	0.044	0.017	1
ARPP21	0.00014	0.138244	0.095	0.067	1
ZFPM2	0.000143	0.121779	0.088	0.073	1
SLC16A7	0.000164	0.1044	0.048	0.02	1
RP11-436K	0.000169	0.114199	0.096	0.07	1
GPR183	0.00017	0.138669	0.083	0.044	1
CPPED1	0.000181	0.103027	0.038	0.014	1
AC114730.	0.000185	-0.11415	0.02	0.053	1
JPH3	0.000276	0.100289	0.048	0.017	1
GPX4	0.000316	-0.10149	0.88	0.917	1
PLCL2	0.00032	0.101745	0.06	0.032	1
SERPINA1	0.000331	0.117431	0.057	0.027	1
NHLH2	0.000332	0.135763	0.051	0.023	1
MGST1	0.00034	-0.13098	0.02	0.052	1
GALNT13	0.000466	0.124771	0.098	0.079	1
VSIG4	0.000503	0.103241	0.092	0.057	1
GBP4	0.000648	-0.12829	0.027	0.062	1

AC073283.	0.000688	0.108722	0.064	0.04	1
THNSL2	0.000722	0.119199	0.039	0.015	1
HOXB2	0.000765	0.131076	0.236	0.207	1
IGF2	0.000957	-0.15929	0.002	0.016	1
FGF12	0.000969	0.150314	0.132	0.093	1
NKAIN4	0.001069	-0.13643	0.154	0.216	1
NANOS1	0.001172	0.112756	0.059	0.035	1
GUCY1B3	0.00122	0.111006	0.079	0.06	1
ZBTB16	0.002765	0.114007	0.2	0.174	1
H3F3C	0.003253	-0.1118	0.024	0.048	1
NTN4	0.004964	0.102111	0.064	0.035	1
SST	0.005989	0.108192	0.016	0.002	1
HSPA6	0.009834	0.157019	0.086	0.063	1
C10orf10	0.053278	-0.19805	0.02	0.037	1
FABP5	0.199555	-0.12456	0.357	0.338	1
HLA-A	0.299448	-0.11218	0.816	0.841	1
