# Supplementary material for: A Novel Molecular Classification Method for Glioblastoma Based on Tumor Cell Differentiation Trajectories
Source: Stem Cells Int. 2023 Feb 22;2023:2826815. doi: 10.1155/2023/2826815 (PMC10643041; doi:10.1155/2023/2826815)
Supplement: Supplementary 8 — Supplementary Table 6: differentially expressed genes (DEGs) in GSCL and Ac-G. [file 2826815.f8.pdf]

	p_val	avg_logFC	pct.1	pct.2	p_val_adj
ZWINT	0	-1.22286	0.021	0.7	0
NUF2	0	-1.25918	0.012	0.669	0
SGOL1	0	-1.25999	0.005	0.698	0
TYMS	0	-1.32476	0.079	0.802	0
CENPU	0	-1.36516	0.031	0.772	0
CDK1	0	-1.4535	0.025	0.717	0
UBE2T	0	-1.46367	0.054	0.835	0
KIAA0101	0	-1.46976	0.034	0.769	0
FAM64A	0	-1.49497	0.01	0.717	0
PBK	0	-1.62211	0.017	0.802	0
CENPF	0	-1.63856	0.034	0.772	0
BIRC5	0	-1.68635	0.025	0.809	0
NUSAP1	0	-1.68695	0.032	0.814	0
TOP2A	0	-1.71731	0.02	0.785	0
UBE2C	0	-1.81904	0.039	0.833	0
SMC4	1.5316035	-1.35777	0.136	0.859	2.28882827354999e-318
MKI67	2.2145998	-1.28005	0.007	0.637	3.30949801721306e-315
RRM2	7.0188447	-1.32401	0.009	0.647	1.04889616459785e-314
HMGB2	2.1034561	-1.19379	0.36	0.977	3.14340492081735e-310
TPX2	2.1332280	-1.38167	0.032	0.69	3.18789596367352e-310
PRC1	########	-1.26222	0.033	0.702	########
CENPK	########	-1.15737	0.039	0.71	########
MAD2L1	########	-1.21196	0.098	0.814	########
HIST1H4C	########	-1.51388	0.203	0.851	########
SPC25	########	-1.15708	0.011	0.62	########
CHI3L1	########	1.963068	0.845	0.506	########
CCNA2	########	-1.1424	0.008	0.601	########
STMN1	########	-1.03895	0.426	0.995	########
UBE2S	########	-1.42973	0.166	0.812	########
GTSE1	########	-1.11967	0.015	0.614	########
HMGB3	########	-1.23198	0.126	0.823	########
CENPM	########	-1.06347	0.015	0.615	########
AURKB	########	-1.15938	0.007	0.58	########
FTL	########	0.887242	0.99	0.981	########
LMNB1	########	-1.14129	0.055	0.707	########
MT1X	########	1.560649	0.844	0.614	########
CKAP2L	########	-1.04417	0.008	0.574	########
CDKN3	########	-1.34532	0.04	0.665	########
FOXM1	########	-0.9456	0.007	0.568	########
KIFC1	########	-1.00104	0.014	0.593	########
CRYAB	########	1.710304	0.831	0.506	########
MXD3	########	-1.06067	0.016	0.59	########
SOD2	########	1.687139	0.754	0.443	########
CENPV	########	-1.1241	0.074	0.707	########
PTTG1	########	-1.35919	0.192	0.831	########
HJURP	########	-1.01924	0.004	0.525	########
CKAP2	########	-1.19564	0.079	0.711	########
CENPN	########	-1.03871	0.038	0.636	########
CCNB2	########	-1.21971	0.013	0.56	########
TACC3	########	-0.98667	0.015	0.57	########

TMPO	########	-1.04741	0.086	0.731	########
H2AFX	########	-1.16582	0.162	0.783	########
ASPM	########	-1.09055	0.009	0.535	########
NDC80	########	-1.02483	0.009	0.53	########
EZH2	########	-0.99522	0.072	0.688	########
CDC20	########	-1.28699	0.016	0.543	########
CDCA5	########	-0.88458	0.009	0.523	########
SGOL2	########	-1.12511	0.052	0.633	########
ASF1B	########	-0.95349	0.024	0.573	########
CENPW	########	-0.9598	0.041	0.615	########
ORC6	########	-0.9876	0.052	0.641	########
NCAPG	########	-0.86513	0.007	0.506	########
TK1	########	-0.95469	0.014	0.533	########
KIF2C	########	-0.92294	0.005	0.496	########
SPC24	########	-0.92024	0.01	0.517	########
C21orf58	########	-0.83975	0.011	0.516	########
CENPH	########	-0.93973	0.093	0.71	########
CDCA3	########	-1.05722	0.009	0.509	########
SMC2	########	-1.03983	0.151	0.78	########
CENPE	########	-1.11521	0.013	0.517	########
MELK	########	-0.85715	0.012	0.515	########
RAD51AP1	########	-0.90011	0.016	0.53	########
TMSB15A	########	-1.02738	0.056	0.635	########
MYBL2	########	-0.90215	0.007	0.491	########
CLU	########	1.157008	0.969	0.828	########
CKS2	########	-1.12174	0.228	0.846	########
HES6	########	-1.25903	0.135	0.752	########
DHFR	########	-0.99392	0.073	0.66	########
NUDT1	########	-0.86067	0.21	0.842	########
CENPA	########	-1.0544	0.015	0.504	########
NAMPT	########	1.685599	0.683	0.293	########
DEK	########	-0.96895	0.311	0.87	########
ESCO2	########	-0.786	0.005	0.462	########
ECT2	########	-0.87656	0.018	0.512	########
GGH	########	-0.92038	0.199	0.822	########
RNASEH2A	########	-0.87719	0.156	0.774	########
MARCKSL1	########	-0.94937	0.382	0.906	########
GLUL	########	1.122213	0.812	0.732	########
CHI3L2	########	2.294984	0.592	0.059	########
SHCBP1	########	-0.7561	0.009	0.467	########
PKMYT1	########	-0.77076	0.008	0.462	########
CKB	########	-0.84436	0.455	0.968	########
RACGAP1	########	-0.90208	0.045	0.573	########
KIF23	########	-0.83405	0.008	0.456	########
GINS2	########	-1.0162	0.036	0.541	########
DLGAP5	########	-0.86399	0.003	0.43	########
CDCA8	########	-0.92248	0.008	0.456	########
H2AFV	########	-0.8597	0.381	0.925	########
ASCL1	########	-1.0267	0.057	0.594	########
AURKA	########	-1.09201	0.022	0.502	########
MARCKS	########	-0.93303	0.3	0.87	########

MND1	########	-0.74093	0.01	0.456	########
CCNB1	########	-1.13936	0.064	0.599	########
NCAPH	########	-0.75642	0.004	0.422	########
SKA3	########	-0.66356	0.004	0.419	########
SPAG5	########	-0.79153	0.013	0.462	########
TROAP	########	-0.87003	0.007	0.436	########
S100A6	########	1.274052	0.84	0.62	########
FAM181B	########	-0.97765	0.048	0.552	########
KIF15	########	-0.70751	0.007	0.428	########
S100A10	########	1.131129	0.741	0.542	########
POC1A	########	-0.79302	0.026	0.494	########
MGST1	########	1.973136	0.577	0.052	########
CD44	########	1.512141	0.64	0.285	########
PRR11	########	-0.77511	0.017	0.46	########
H1FX	########	-0.95199	0.173	0.749	########
OIP5	########	-0.73168	0.007	0.423	########
DTYMK	########	-0.8194	0.264	0.842	########
SKA2	########	-0.88912	0.166	0.735	########
SPP1	########	1.137098	0.856	0.753	########
BUB1	########	-0.81359	0.008	0.421	########
FXYD6	########	-0.79693	0.331	0.902	########
KIF4A	########	-0.71234	0.007	0.414	########
DEPDC1	########	-0.73577	0.007	0.414	########
TRIP13	########	-0.65126	0.018	0.453	########
CCDC34	########	-0.88756	0.135	0.696	########
CDT1	########	-0.84049	0.022	0.469	########
PLK1	########	-1.04218	0.019	0.458	########
NEK2	########	-0.82124	0.005	0.405	########
CLSPN	########	-0.86653	0.025	0.475	########
NT5DC2	########	-0.74546	0.054	0.546	########
HNRNPD	########	-0.87763	0.215	0.778	########
TCF12	########	-0.83245	0.088	0.615	########
FANCI	########	-0.67962	0.011	0.423	########
ODC1	########	-0.91186	0.253	0.806	########
ARHGAP11A	########	-0.7164	0.006	0.401	########
ATAD2	########	-0.81651	0.044	0.52	########
MIS18BP1	########	-0.75176	0.031	0.486	########
PGK1	########	0.746329	0.772	0.804	########
C8orf4	########	1.56299	0.614	0.298	########
CHAF1A	########	-0.75739	0.04	0.507	########
CASC5	########	-0.62003	0.004	0.385	########
CD74	########	1.333077	0.805	0.527	########
SERPING1	########	1.978002	0.548	0.065	########
RANBP1	########	-0.80373	0.418	0.931	########
ETV1	########	-0.92012	0.12	0.658	########
CDC45	########	-0.69733	0.005	0.388	########
CDCA2	########	-0.67144	0.006	0.39	########
SKA1	########	-0.66835	0.004	0.381	########
YWHAH	########	-0.79662	0.245	0.81	########
TMSB15B	########	-0.79666	0.08	0.586	########
ASRGL1	########	-0.82663	0.129	0.667	########

HMMR	########	-0.76542	0.007	0.394	########
ATP6V0E1	########	0.836885	0.746	0.726	########
EIF1	########	0.616316	0.948	0.983	########
NPC2	########	1.281206	0.683	0.489	########
DNMT1	########	-0.83242	0.161	0.71	########
UBC	########	0.586054	0.924	0.97	########
FBXO5	########	-0.87504	0.066	0.552	########
PDGFRA	########	-0.94094	0.045	0.505	########
LGALS3	########	1.453795	0.786	0.47	########
ANXA1	########	1.44101	0.692	0.198	########
MT2A	########	0.874688	0.948	0.889	########
RFC3	########	-0.7263	0.062	0.537	########
C19orf48	########	-0.80698	0.161	0.704	########
TRAF4	########	-0.84055	0.096	0.602	########
SMARCA4	########	-0.73091	0.197	0.748	########
NETO2	########	-0.75302	0.041	0.49	########
RTKN2	########	-0.61702	0.006	0.375	########
SOX4	########	-0.95381	0.292	0.832	########
TIMP1	########	1.27405	0.858	0.628	########
DRAXIN	########	-0.71079	0.057	0.52	########
FAM83D	########	-0.80236	0.011	0.4	########
OLIG1	########	-0.94406	0.036	0.474	########
CDKN2C	########	-0.7755	0.196	0.737	########
MAD2L2	########	-0.68753	0.268	0.819	########
S100A11	########	1.357588	0.691	0.474	########
PHF19	########	-0.70357	0.069	0.541	########
TIMELESS	########	-0.62908	0.014	0.399	########
EIF1AY	########	-0.6256	0.031	0.449	########
KHDRBS1	########	-0.74306	0.295	0.84	########
C1R	########	1.570086	0.582	0.227	########
TCF4	########	-0.73782	0.331	0.864	########
KIF20A	########	-0.64994	0.002	0.342	########
LTF	########	2.270432	0.496	0.032	########
SAT1	########	1.032921	0.781	0.707	########
MAP2	########	-0.70313	0.181	0.712	########
DBF4	########	-0.76262	0.109	0.611	########
C14orf80	########	-0.57794	0.015	0.4	########
RALY	########	-0.79476	0.265	0.798	########
SOX8	########	-0.79	0.036	0.46	########
KIAA1524	########	-0.6656	0.017	0.406	########
UBE2E3	########	-0.67211	0.269	0.811	########
LMNB2	########	-0.71548	0.061	0.521	########
MEX3A	########	-0.7085	0.038	0.465	########
XRCC2	########	-0.68153	0.015	0.399	########
SLPI	########	2.333678	0.488	0.037	########
KIF20B	########	-0.71606	0.036	0.454	########
NASP	########	-0.71582	0.276	0.815	########
NCAPG2	########	-0.6226	0.032	0.443	########
CDCA4	########	-0.72297	0.064	0.52	########
RAD21	########	-0.72669	0.27	0.806	########
HLA-DRA	########	1.541978	0.702	0.335	########

POU3F2	########	-0.83616	0.102	0.591	########
GINS1	########	-0.635	0.02	0.41	########
EFEMP1	########	1.671002	0.577	0.241	########
TMEM160	########	-0.82354	0.243	0.76	########
HNRNPAB	########	-0.74265	0.225	0.758	########
PAFAH1B3	########	-0.68033	0.294	0.828	########
FEN1	########	-0.79369	0.105	0.595	########
SYNE2	########	-0.66201	0.109	0.595	########
LRR1	########	-0.63196	0.042	0.46	########
KNSTRN	########	-0.7994	0.043	0.47	########
PSIP1	########	-0.76327	0.284	0.817	########
KIF22	########	-0.58999	0.169	0.679	########
BUB1B	########	-0.53121	0.001	0.325	########
CCDC14	########	-0.56378	0.135	0.627	########
WHSC1	########	-0.69879	0.08	0.549	########
PGP	########	-0.69771	0.1	0.583	########
ALDOA	########	0.606174	0.877	0.89	########
E2F1	########	-0.75836	0.014	0.383	########
YBX1	########	-0.6369	0.539	0.959	########
KIF14	########	-0.59986	0.007	0.354	########
KLHDC8A	########	-0.78445	0.091	0.567	########
HN1	########	-0.69975	0.44	0.914	########
ZWILCH	########	-0.57754	0.027	0.417	########
FSCN1	########	-0.79177	0.246	0.774	########
FTH1	########	0.267873	0.833	0.981	########
C12orf75	########	-0.75617	0.059	0.501	########
OLIG2	########	-0.89258	0.059	0.501	########
ARHGAP11E	########	-0.60091	0.008	0.356	########
SAE1	########	-0.79898	0.184	0.702	########
SKP1	########	0.643119	0.899	0.936	########
NREP	########	-0.66299	0.109	0.585	########
TMBIM6	########	0.592874	0.837	0.893	########
NFIB	########	-0.73955	0.263	0.79	########
AES	########	-0.67919	0.226	0.749	########
MCM7	########	-0.88379	0.207	0.725	########
SOX11	########	-0.781	0.134	0.628	########
SOX2	########	-0.7131	0.396	0.895	########
VRK1	########	-0.65763	0.051	0.472	########
SAPCD2	########	-0.70361	0.018	0.386	########
ITM2C	########	0.548838	0.51	0.646	########
ITGB3BP	########	-0.66202	0.107	0.579	########
ACYP1	########	-0.59133	0.125	0.599	########
HSPA5	########	0.676538	0.694	0.71	########
BRCA1	########	-0.55554	0.032	0.415	########
DEPDC1B	########	-0.60471	0.013	0.367	########
CD9	########	0.954472	0.737	0.615	########
RNASEH2B	########	-0.70467	0.132	0.621	########
SOGA1	########	-0.66004	0.061	0.493	########
ANLN	########	-0.54752	0.015	0.37	########
RAC3	########	-0.66685	0.081	0.533	########
DNAJC9	########	-0.5914	0.114	0.581	########

CD63	########	0.792575	0.941	0.9	########
PODXL2	########	-0.75113	0.148	0.641	########
GAS2L3	########	-0.57326	0.01	0.347	########
BARD1	########	-0.65539	0.114	0.583	########
CHD7	########	-0.69678	0.114	0.585	########
KPNA2	########	-0.79171	0.286	0.795	########
CKS1B	########	-0.73423	0.228	0.738	########
PARPBP	########	-0.56886	0.019	0.377	########
PCSK1N	########	-0.61659	0.282	0.784	########
CBX5	########	-0.56894	0.258	0.767	########
CBX1	########	-0.60841	0.243	0.752	########
PSMC3IP	########	-0.67128	0.051	0.463	########
CEP55	########	-0.51532	0.002	0.304	########
DBN1	########	-0.66027	0.157	0.644	########
GAPDH	########	0.592907	0.986	0.996	########
LDHA	########	0.718507	0.854	0.865	########
HAUS8	########	-0.57595	0.045	0.433	########
SFPQ	########	-0.68347	0.358	0.854	########
DSEL	########	-0.7501	0.152	0.638	########
PRDX5	########	0.68947	0.781	0.799	########
CDC25C	########	-0.55387	0.002	0.301	########
ATAD5	########	-0.66159	0.049	0.457	########
HMGN1	########	-0.63345	0.478	0.923	########
GLRX5	########	-0.76003	0.153	0.638	########
KIF18B	########	-0.45361	0.002	0.295	########
NCAPD2	########	-0.52499	0.019	0.365	########
CEP135	########	-0.69786	0.04	0.432	########
RHOBTB3	########	-0.61024	0.321	0.826	########
IFITM3	########	1.266113	0.61	0.416	########
DSN1	########	-0.66324	0.076	0.51	########
CSTB	########	0.658257	0.672	0.691	########
FAU	########	0.544891	0.912	0.969	########
CRNDE	########	-0.67488	0.158	0.643	########
GMNN	########	-0.50224	0.196	0.67	########
DUT	########	-0.73153	0.325	0.819	########
RHNO1	########	-0.48445	0.143	0.6	########
SAA1	########	2.368165	0.433	0.026	########
HMGXB4	########	-0.60075	0.135	0.596	########
A2M	########	1.295822	0.479	0.326	########
CCNF	########	-0.57191	0.01	0.337	########
RAC1	########	-0.55996	0.484	0.941	########
MCM4	########	-0.73282	0.063	0.48	########
TTK	########	-0.62534	0.004	0.306	########
FANCA	########	-0.58749	0.023	0.379	########
EGFR	########	-0.84967	0.23	0.723	########
TRIB2	########	-0.66307	0.146	0.619	########
CHEK1	########	-0.60726	0.053	0.451	########
PRDX1	########	0.552348	0.795	0.868	########
MNS1	########	-0.61344	0.054	0.453	########
ANP32E	########	-0.77285	0.221	0.714	########
MCM10	########	-0.47975	0.002	0.29	########

SQSTM1	########	0.519925	0.555	0.574	########
H2AFY	########	-0.65024	0.401	0.878	########
ANP32B	########	-0.66881	0.313	0.81	########
C16orf59	########	-0.57712	0.016	0.354	########
SRM	########	-0.6859	0.18	0.663	########
CCND2	########	-0.76346	0.213	0.705	########
HNRNPUL1	########	-0.59677	0.234	0.725	########
CCDC88A	########	-0.56179	0.215	0.701	########
GNG4	########	-0.65019	0.062	0.468	########
B2M	########	0.651971	0.968	0.968	########
LHX2	########	-0.65484	0.04	0.42	########
C1S	########	1.525454	0.483	0.135	########
SET	########	-0.60328	0.367	0.853	########
MAGI1	########	-0.6309	0.079	0.493	########
NUPR1	########	1.264542	0.533	0.196	########
RFC4	########	-0.66447	0.146	0.61	########
BRD7	########	-0.62811	0.136	0.596	########
TMEM97	########	-0.58064	0.143	0.599	########
NFIX	########	-0.65123	0.193	0.674	########
ZNF738	########	-0.54008	0.084	0.491	########
LAPTM4A	########	0.553161	0.801	0.872	########
MT1E	########	1.056609	0.645	0.483	########
TOMM7	########	0.616017	0.842	0.915	########
PTMS	########	-0.70283	0.402	0.856	########
NCAPH2	########	-0.56685	0.066	0.469	########
WDR34	########	-0.68578	0.195	0.677	########
PPM1G	########	-0.65895	0.276	0.767	########
NFIA	########	-0.51401	0.276	0.757	########
PARP1	########	-0.65148	0.313	0.802	########
SBDS	########	0.479555	0.698	0.802	########
H2AFZ	########	-0.50709	0.669	0.98	########
PHIP	########	-0.56561	0.203	0.674	########
DIAPH3	########	-0.53627	0.016	0.341	########
CCDC167	########	-0.52939	0.271	0.757	########
HLA-B	########	0.764369	0.867	0.796	########
LAMTOR5	########	0.560439	0.74	0.806	########
TRIM36	########	-0.57621	0.066	0.467	########
MPST	########	-0.54374	0.138	0.58	########
USP1	########	-0.58299	0.225	0.706	########
CAV1	########	1.570547	0.489	0.063	########
ARF4	########	0.501726	0.671	0.764	########
TCF19	########	-0.602	0.025	0.372	########
MGP	########	1.942439	0.423	0.028	########
SOD1	########	0.584388	0.8	0.863	########
BTG1	########	-0.26524	0.429	0.831	########
TMEM59	########	0.51105	0.764	0.854	########
SMARCB1	########	-0.60948	0.244	0.728	########
CALD1	########	0.408434	0.586	0.746	########
WTAP	########	0.230923	0.428	0.635	########
PTPRZ1	########	-0.64651	0.363	0.841	########
HELLS	########	-0.66664	0.029	0.381	########

PTOV1	########	-0.58731	0.304	0.789	########
PCNA	########	-0.66477	0.298	0.783	########
BMP7	########	-0.6529	0.099	0.528	########
CPXM1	########	-0.53108	0.036	0.389	########
CSE1L	########	-0.57887	0.16	0.62	########
ROBO1	########	-0.58471	0.051	0.433	########
RABL6	########	-0.61666	0.102	0.533	########
LSM4	########	-0.58546	0.467	0.904	########
SRSF2	########	-0.62504	0.425	0.878	########
TUBB2B	########	-0.43163	0.578	0.973	########
SAC3D1	########	-0.65469	0.099	0.527	########
GAS1	########	-0.74716	0.065	0.463	########
CENPO	########	-0.49834	0.02	0.348	########
PKM	########	0.686108	0.871	0.878	########
DCX	########	-0.68395	0.034	0.388	########
INSM1	########	-0.63246	0.019	0.347	########
SHD	########	-0.76079	0.02	0.351	########
ZFP36	########	1.21481	0.631	0.327	########
MCM2	########	-0.63956	0.027	0.37	########
CHMP2A	########	0.634681	0.7	0.763	########
TUBG1	########	-0.52339	0.188	0.647	########
APBA2	########	-0.51378	0.073	0.465	########
RCN2	########	-0.63239	0.322	0.801	########
ALYREF	########	-0.59547	0.062	0.452	########
RRM1	########	-0.65529	0.167	0.626	########
ENC1	########	-0.61028	0.1	0.52	########
HLA-DRB1	########	1.510561	0.524	0.172	########
PSAP	########	0.553323	0.711	0.76	########
BCAN	########	-0.8109	0.187	0.651	########
BNIP3L	########	0.541572	0.59	0.663	########
LAPTM4B	########	-0.61357	0.229	0.7	########
CDKN2D	########	-0.67791	0.094	0.51	########
CTSB	########	0.879005	0.716	0.689	########
MGME1	########	-0.48908	0.111	0.525	########
XPO1	########	-0.50529	0.17	0.611	########
DAD1	########	0.556818	0.746	0.82	########
CALM3	########	-0.58136	0.429	0.883	########
WDR76	########	-0.60784	0.022	0.349	########
JMJD1C	########	-0.50063	0.123	0.533	########
CMC2	########	-0.43356	0.246	0.705	########
EMC9	########	-0.56017	0.122	0.536	########
CDK6	########	-0.74055	0.083	0.486	########
MIS18A	########	-0.55074	0.098	0.504	########
KIF21A	########	-0.68834	0.191	0.652	########
TEX30	########	-0.53861	0.157	0.596	########
GADD45B	########	1.188513	0.618	0.348	########
KIF5B	########	-0.65518	0.338	0.802	########
EWSR1	########	-0.37517	0.332	0.786	########
MGST3	########	0.531506	0.684	0.783	########
MZT1	########	-0.62689	0.238	0.706	########
GPSM2	########	-0.64433	0.151	0.594	########

C9orf142	########	-0.58271	0.219	0.683	########
TMEM205	########	0.848203	0.657	0.58	########
ENHO	########	-0.46787	0.045	0.391	########
TRMT112	########	0.597611	0.748	0.802	########
KIF11	########	-0.465	0.006	0.281	########
SSR4	########	0.56791	0.724	0.815	########
FANCD2	########	-0.53083	0.015	0.319	########
TMEM98	########	-0.4286	0.179	0.606	########
CSNK1E	########	-0.48265	0.146	0.568	########
ATP6V1F	########	0.502427	0.744	0.852	########
MAP1LC3B	########	0.209581	0.485	0.664	########
DAZAP1	########	-0.54293	0.129	0.551	########
SF3A2	########	-0.41918	0.182	0.6	########
FAM210B	########	-0.67989	0.143	0.581	########
IGFBP2	########	-0.58301	0.353	0.819	########
POSTN	########	-0.74214	0.017	0.327	########
RAD51	########	-0.50676	0.007	0.283	########
HNRNPM	########	-0.59154	0.377	0.84	########
TFDP1	########	-0.60568	0.084	0.483	########
HNRNPU	########	-0.49384	0.36	0.821	########
PPIB	########	0.554424	0.709	0.801	########
ILF3	########	-0.48343	0.3	0.763	########
CEP78	########	-0.52604	0.06	0.43	########
NACA	########	0.425557	0.877	0.962	########
IDH2	########	-0.61713	0.24	0.705	########
CD59	########	0.70596	0.585	0.626	########
TRIM28	########	-0.37869	0.174	0.583	########
C4orf3	########	0.644617	0.647	0.704	########
TSPAN5	########	-0.68717	0.149	0.589	########
RBMX	########	-0.46315	0.462	0.901	########
SSRP1	########	-0.54718	0.285	0.749	########
NCAM1	########	-0.4773	0.239	0.686	########
COQ2	########	-0.53973	0.059	0.43	########
SCD5	########	-0.52522	0.32	0.775	########
PLEKHO1	########	-0.46731	0.117	0.507	########
NONO	########	-0.50654	0.347	0.807	########
MCM5	########	-0.63412	0.04	0.388	########
TMED9	########	0.458646	0.7	0.805	########
SAP30	########	-0.63129	0.115	0.535	########
ARL6IP5	########	0.689358	0.598	0.565	########
SPARC	########	0.805788	0.615	0.616	########
HDAC2	########	-0.56525	0.325	0.789	########
ECI1	########	-0.61807	0.102	0.512	########
KCNQ2	########	-0.4461	0.121	0.521	########
MCM3	########	-0.71383	0.101	0.509	########
TPI1	########	0.494825	0.878	0.953	########
CKAP5	########	-0.59511	0.095	0.494	########
SUZ12	########	-0.57684	0.104	0.51	########
MZT2B	########	-0.61342	0.455	0.877	########
CTSD	########	0.567652	0.511	0.588	########
PRDX6	########	0.812722	0.78	0.751	########

PHF14	########	-0.41825	0.322	0.76	########
WDR62	########	-0.47231	0.009	0.29	########
MEST	########	-0.62766	0.31	0.772	########
COMMD4	########	-0.33376	0.224	0.637	########
ZEB1	########	-0.60802	0.105	0.509	########
KPNB1	########	-0.48065	0.338	0.794	########
KIF1B	########	-0.39231	0.15	0.549	########
HNRNPA3	########	-0.42495	0.386	0.838	########
JUNB	########	0.205395	0.587	0.767	########
PABPN1	########	-0.31885	0.213	0.625	########
ANP32A	########	-0.25946	0.232	0.651	########
METRN	########	-0.69464	0.203	0.652	########
ATP5D	########	-0.59885	0.397	0.847	########
C22orf39	########	-0.58537	0.133	0.553	########
MYL6	########	0.602897	0.921	0.964	########
CDC25B	########	-0.70159	0.142	0.568	########
APOE	########	0.373669	0.528	0.67	########
MCM8	########	-0.42766	0.018	0.312	########
TMEM237	########	-0.38906	0.198	0.611	########
SPECC1	########	-0.48351	0.182	0.616	########
OAZ1	########	0.395657	0.876	0.965	########
TIA1	########	-0.43738	0.152	0.557	########
NMU	########	-0.65259	0.008	0.283	########
SEZ6	########	-0.7213	0.034	0.365	########
BAZ1A	########	-0.50502	0.158	0.577	########
CNIH2	########	-0.5379	0.034	0.365	########
CCDC18	########	-0.55206	0.04	0.377	########
ABI2	########	-0.50539	0.283	0.736	########
SPDL1	########	-0.50936	0.048	0.386	########
CNP	########	-0.48083	0.229	0.665	########
15-Sep	########	0.618324	0.694	0.707	########
SUGP2	########	-0.52163	0.173	0.596	########
COL9A3	########	-0.82833	0.063	0.428	########
NDUFA1	########	0.460272	0.688	0.789	########
PRKDC	########	-0.56842	0.191	0.63	########
FAM111A	########	-0.61784	0.074	0.451	########
CENPQ	########	-0.44763	0.069	0.425	########
KHSRP	########	-0.41773	0.147	0.542	########
TMSB10	########	0.647232	0.972	0.974	########
ARHGAP33	########	-0.48571	0.034	0.351	########
BCL7C	########	-0.61854	0.15	0.574	########
NBPF1	########	-0.5224	0.125	0.53	########
ACAT2	########	-0.5587	0.19	0.622	########
PHGDH	########	-0.46158	0.25	0.673	########
GOLM1	########	-0.3707	0.245	0.657	########
USP22	########	-0.42159	0.179	0.593	########
PXMP2	########	-0.57941	0.172	0.602	########
ELOVL2	########	-0.44795	0.025	0.321	########
RAD51C	########	-0.41146	0.204	0.621	########
B4GALNT1	########	-0.83003	0.07	0.441	########
UBE2B	########	0.263045	0.464	0.631	########

SPARCL1	########	0.704549	0.573	0.604	########
SRSF9	########	-0.58804	0.382	0.827	########
TUBB	########	-0.35993	0.698	0.995	########
QKI	########	-0.54806	0.285	0.737	########
ARHGAP21	########	-0.48781	0.096	0.469	########
SAP18	########	0.439444	0.764	0.878	########
SEPP1	########	1.46408	0.495	0.168	########
ACTG1	########	0.592499	0.975	0.981	########
BTG3	########	-0.43906	0.335	0.774	########
CAPZA2	########	0.277645	0.611	0.816	########
CBX3	########	-0.51026	0.448	0.879	########
GNB2	########	-0.47731	0.398	0.838	########
SDCBP	########	0.519923	0.684	0.742	########
PLK4	########	-0.39228	0.006	0.26	########
UBTF	########	-0.49186	0.126	0.521	########
RANGAP1	########	-0.46423	0.053	0.39	########
PDCD6	########	0.180582	0.497	0.717	########
ITGAE	########	-0.35369	0.276	0.707	########
DTL	########	-0.53215	0.006	0.264	########
COX4I1	########	0.488534	0.861	0.941	########
HIST2H2AC	########	-0.49994	0.017	0.304	########
TRAIP	########	-0.3991	0.015	0.289	########
KIF18A	########	-0.42468	0.014	0.291	########
TPGS2	########	-0.31696	0.271	0.679	########
NAP1L4	########	-0.4575	0.303	0.747	########
ELAVL1	########	-0.53194	0.227	0.664	########
PCM1	########	-0.47796	0.226	0.656	########
ENAH	########	-0.50912	0.164	0.578	########
RNF145	########	-0.52323	0.197	0.627	########
BRD2	########	-0.13064	0.332	0.684	########
CUX1	########	-0.39954	0.12	0.491	########
MPHOSPH9	########	-0.47707	0.059	0.4	########
HLA-A	########	0.681204	0.889	0.841	########
UQCRB	########	-0.18556	0.46	0.83	########
KLHL23	########	-0.65145	0.082	0.459	########
PTPRS	########	-0.47165	0.144	0.542	########
SNRPB	########	-0.5002	0.481	0.894	########
TPT1	########	0.568406	0.924	0.959	########
THOP1	########	-0.4772	0.138	0.535	########
EXO1	########	-0.41546	0.002	0.242	########
BAZ1B	########	-0.48963	0.225	0.653	########
EIF4A2	########	0.3353	0.704	0.847	########
SGCB	########	-0.41141	0.296	0.728	########
ELAVL3	########	-0.57351	0.18	0.605	########
SMARCC1	########	-0.49574	0.143	0.547	########
RPAIN	########	-0.31938	0.379	0.799	########
RABAC1	########	0.415893	0.659	0.799	1.59E-99
PTTG1IP	########	0.299333	0.434	0.546	1.81E-99
RPA3	########	-0.32905	0.382	0.806	1.88E-99
CDC6	########	-0.52123	0.018	0.306	1.93E-99
KDM1A	########	-0.42303	0.097	0.463	3.15E-99

RNF157	########	-0.38078	0.054	0.374	3.21E-99
FAM89B	########	-0.52528	0.09	0.468	4.87E-99
ZNHIT1	########	0.427535	0.691	0.812	5.78E-99
NUP107	########	-0.78604	0.159	0.579	6.26E-99
ATXN10	########	-0.35379	0.281	0.7	1.15E-98
REEP4	########	-0.54041	0.054	0.391	1.18E-98
NCAPD3	########	-0.44498	0.049	0.37	1.70E-98
SSR3	########	0.67419	0.587	0.628	1.90E-98
APOBEC3B	########	-0.46253	0.006	0.259	1.90E-98
SPCS1	########	0.460818	0.728	0.84	2.07E-98
PFDN5	########	0.456199	0.813	0.906	2.35E-98
ATP6V0E2	########	-0.41559	0.357	0.79	3.22E-98
GPM6A	########	-0.16676	0.471	0.865	3.45E-98
VEZF1	########	-0.44127	0.144	0.535	3.72E-98
HIST1H1D	########	-0.55314	0.008	0.269	3.88E-98
PTBP1	########	-0.41269	0.243	0.659	4.04E-98
GNB1	########	-0.48284	0.236	0.664	4.57E-98
CYR61	########	0.98155	0.433	0.228	5.98E-98
NES	########	-0.66705	0.206	0.637	8.81E-98
SPCS2	########	0.432418	0.756	0.872	9.77E-98
MMP2	########	-0.45626	0.041	0.347	1.02E-97
SLC25A11	########	-0.3924	0.274	0.699	1.10E-97
FOS	########	0.711881	0.841	0.791	1.17E-97
HP1BP3	########	-0.55898	0.316	0.759	1.28E-97
C3	########	1.303808	0.438	0.125	1.41E-97
SRSF1	########	-0.26611	0.338	0.743	1.58E-97
WEE1	########	-0.38975	0.139	0.51	1.81E-97
ACTB	########	0.567746	0.992	0.996	1.81E-97
RCC1	########	-0.47689	0.061	0.399	2.44E-97
HERPUD1	########	0.744565	0.549	0.494	2.45E-97
FJX1	########	-0.52256	0.074	0.431	3.24E-97
TPRKB	########	-0.29938	0.29	0.7	3.47E-97
ALDH7A1	########	-0.26803	0.212	0.598	3.52E-97
SPATS2	########	-0.41314	0.182	0.584	4.10E-97
NEAT1	########	0.68546	0.741	0.669	5.70E-97
IPO9	########	-0.384	0.158	0.549	6.74E-97
CDCA7L	########	-0.50872	0.132	0.527	7.03E-97
RAB1A	########	0.309527	0.583	0.735	8.28E-97
ZFP36L2	########	0.123104	0.391	0.61	8.44E-97
CHD9	########	-0.4361	0.313	0.744	1.06E-96
RCN1	########	0.108685	0.434	0.62	1.19E-96
LAMTOR4	########	0.385055	0.687	0.848	1.26E-96
LBR	########	-0.57615	0.158	0.569	1.38E-96
KIF5C	########	-0.48768	0.117	0.499	1.43E-96
HSPB1	########	0.416355	0.685	0.794	1.86E-96
LIMA1	########	-0.5441	0.253	0.689	1.90E-96
GPC2	########	-0.6414	0.037	0.354	2.04E-96
RAD54L	########	-0.38495	0.002	0.231	2.37E-96
CKLF	########	-0.32088	0.341	0.762	2.47E-96
BLVRB	########	1.099148	0.498	0.296	3.07E-96
PTP4A1	########	-0.37798	0.261	0.663	3.25E-96

PAQR4	########	-0.60965	0.06	0.406	3.61E-96
TAF7	########	0.345125	0.656	0.794	3.67E-96
SPSB4	########	-0.36482	0.004	0.242	6.33E-96
CARHSP1	########	-0.19375	0.389	0.788	6.67E-96
PPP1R14B	########	-0.54777	0.202	0.626	7.63E-96
UBALD2	########	-0.63176	0.12	0.511	8.82E-96
FBLN1	########	-0.66778	0.033	0.337	9.20E-96
BPTF	########	-0.53778	0.166	0.578	9.67E-96
MAP1B	########	0.349529	0.672	0.836	1.01E-95
MZT2A	########	-0.3922	0.328	0.756	1.02E-95
SEC62	########	0.370711	0.65	0.802	1.23E-95
PMEPA1	########	-0.6216	0.065	0.415	1.27E-95
CYC1	########	-0.43099	0.406	0.825	1.38E-95
HILPDA	1.02E-99	0.747518	0.424	0.452	1.52E-95
KCNF1	1.15E-99	-0.56292	0.019	0.301	1.72E-95
ACOT7	1.21E-99	-0.44834	0.134	0.514	1.81E-95
ATP6AP2	1.21E-99	0.432921	0.608	0.714	1.82E-95
TUBB4B	1.26E-99	-0.46651	0.452	0.872	1.89E-95
STMN3	1.32E-99	-0.3982	0.254	0.675	1.97E-95
ZBTB20	1.91E-99	-0.34504	0.323	0.731	2.86E-95
FYN	1.96E-99	-0.44931	0.233	0.653	2.92E-95
ZNF428	2.39E-99	-0.51246	0.449	0.868	3.57E-95
WDR83OS	2.53E-99	0.610534	0.773	0.828	3.78E-95
SRRM2	2.67E-99	-0.47951	0.362	0.795	4.00E-95
VAMP5	2.72E-99	1.091527	0.546	0.405	4.07E-95
C7orf50	3.22E-99	-0.18713	0.431	0.799	4.81E-95
MFGE8	3.62E-99	-0.35909	0.147	0.504	5.41E-95
RMI2	4.21E-99	-0.47127	0.007	0.258	6.29E-95
GLTSCR2	5.30E-99	-0.27877	0.353	0.733	7.92E-95
PPP2R5C	6.05E-99	-0.36067	0.134	0.499	9.04E-95
DPF1	6.21E-99	-0.44297	0.079	0.422	9.28E-95
RP11-161M	6.87E-99	-0.56261	0.025	0.319	1.03E-94
PNN	6.97E-99	-0.37967	0.301	0.72	1.04E-94
MPPED2	9.30E-99	-0.54428	0.026	0.32	1.39E-94
APLP1	9.71E-99	-0.38682	0.237	0.633	1.45E-94
SEZ6L	1.28E-98	-0.5445	0.017	0.286	1.92E-94
PSPC1	1.46E-98	-0.49991	0.129	0.512	2.18E-94
C2orf69	1.67E-98	-0.49321	0.139	0.528	2.50E-94
NFE2L2	1.83E-98	0.168857	0.428	0.606	2.74E-94
KMT2A	2.16E-98	-0.40725	0.142	0.517	3.23E-94
HLA-DRB5	2.29E-98	1.506559	0.383	0.07	3.43E-94
CTCF	2.60E-98	-0.50696	0.085	0.443	3.89E-94
TXN	2.79E-98	0.486662	0.717	0.817	4.17E-94
EXOSC8	2.98E-98	-0.32932	0.283	0.691	4.46E-94
SLC35B2	3.04E-98	-0.50059	0.183	0.594	4.55E-94
CTSL	3.27E-98	0.947546	0.561	0.389	4.88E-94
WHSC1L1	3.45E-98	-0.51268	0.241	0.664	5.16E-94
RTF1	3.84E-98	-0.38455	0.297	0.712	5.73E-94
CNN3	3.91E-98	0.33587	0.682	0.822	5.84E-94
HNRNPA0	4.12E-98	-0.41788	0.491	0.894	6.16E-94
ENO1	4.91E-98	0.515695	0.853	0.895	7.34E-94

PPP2R5E	5.62E-98	-0.43165	0.14	0.52	8.40E-94
TTC3	5.67E-98	-0.34495	0.405	0.805	8.47E-94
SYNCRIP	5.99E-98	-0.3813	0.277	0.683	8.95E-94
CYSTM1	6.79E-98	0.851271	0.489	0.372	1.01E-93
SEC61G	7.85E-98	0.783264	0.959	0.923	1.17E-93
SNRNP70	8.27E-98	-0.37644	0.346	0.753	1.24E-93
SNRPA	8.67E-98	-0.40959	0.184	0.574	1.30E-93
SMC3	8.78E-98	-0.44615	0.223	0.635	1.31E-93
CDC34	1.04E-97	-0.46371	0.127	0.5	1.55E-93
DGCR6L	1.06E-97	-0.35823	0.203	0.589	1.59E-93
TIMP2	1.07E-97	-0.23842	0.169	0.496	1.60E-93
LAMP1	1.15E-97	-0.19326	0.341	0.674	1.72E-93
YEATS4	1.53E-97	-0.78419	0.183	0.599	2.29E-93
CTNNBIP1	1.85E-97	-0.50314	0.094	0.459	2.77E-93
CPNE2	1.88E-97	-0.57944	0.057	0.391	2.81E-93
PRDX4	1.90E-97	0.546518	0.621	0.721	2.84E-93
CIB2	2.04E-97	-0.34756	0.015	0.27	3.05E-93
WSCD1	2.47E-97	-0.48761	0.038	0.341	3.70E-93
PLD3	2.80E-97	0.48521	0.504	0.6	4.19E-93
UBA2	3.57E-97	-0.33135	0.3	0.694	5.33E-93
C4orf27	4.17E-97	-0.19813	0.268	0.649	6.22E-93
CD99	4.62E-97	0.816565	0.746	0.674	6.90E-93
REEP5	4.74E-97	0.55151	0.596	0.628	7.09E-93
TRA2B	6.31E-97	-0.32214	0.414	0.826	9.43E-93
HIRIP3	9.25E-97	-0.46659	0.148	0.531	1.38E-92
EMP3	9.29E-97	0.845586	0.606	0.447	1.39E-92
KLC1	9.54E-97	-0.44616	0.19	0.584	1.43E-92
DHRSX	9.54E-97	-0.42547	0.128	0.493	1.43E-92
GNB2L1	1.08E-96	0.381366	0.888	0.979	1.62E-92
GABARAPL2	1.12E-96	0.407196	0.723	0.857	1.68E-92
DDX17	1.37E-96	-0.44389	0.24	0.648	2.04E-92
GLCCI1	1.37E-96	-0.66857	0.058	0.393	2.05E-92
CEP152	1.41E-96	-0.41926	0.009	0.26	2.11E-92
STRA13	1.53E-96	-0.24479	0.371	0.769	2.29E-92
HMGN2	1.73E-96	-0.54139	0.515	0.884	2.59E-92
GPS1	1.74E-96	-0.38082	0.194	0.589	2.60E-92
ATRAID	1.83E-96	0.414565	0.621	0.79	2.74E-92
CCNE2	1.88E-96	-0.63964	0.021	0.302	2.81E-92
NT5C3B	2.54E-96	-0.32645	0.268	0.652	3.79E-92
PRPSAP1	3.31E-96	-0.57541	0.216	0.638	4.95E-92
HLA-DPA1	3.43E-96	1.301435	0.521	0.2	5.13E-92
ATP5EP2	3.47E-96	0.587432	0.383	0.514	5.18E-92
ASAH1	3.71E-96	0.42163	0.498	0.64	5.55E-92
NOL12	4.10E-96	-0.2846	0.123	0.467	6.12E-92
PDPN	4.21E-96	0.616893	0.543	0.662	6.29E-92
SLC10A4	4.66E-96	-0.39419	0.013	0.272	6.97E-92
TSR3	4.87E-96	-0.47009	0.135	0.517	7.28E-92
CAMLG	5.05E-96	0.310695	0.508	0.683	7.54E-92
CORO1C	5.28E-96	-0.35007	0.238	0.638	7.90E-92
MYEF2	5.94E-96	-0.56779	0.111	0.488	8.88E-92
TPST1	6.19E-96	0.378203	0.351	0.401	9.25E-92

MED10	6.29E-96	0.483825	0.572	0.67	9.40E-92
KDELR2	6.32E-96	-0.12131	0.498	0.812	9.44E-92
ZNF43	9.07E-96	-0.45918	0.092	0.44	1.36E-91
CNBP	1.01E-95	0.374484	0.751	0.891	1.50E-91
CDK2	1.01E-95	-0.51227	0.074	0.416	1.51E-91
TTYH3	1.02E-95	-0.38662	0.106	0.451	1.52E-91
ATP6V1G1	1.09E-95	0.442117	0.718	0.84	1.63E-91
COX7C	1.33E-95	0.413862	0.834	0.938	1.98E-91
EME1	1.40E-95	-0.38026	0.012	0.265	2.09E-91
SNRPD3	1.44E-95	-0.31718	0.311	0.719	2.15E-91
TMED2	1.55E-95	0.120489	0.546	0.775	2.32E-91
HSPA9	1.61E-95	0.305394	0.517	0.679	2.40E-91
PIF1	1.86E-95	-0.60702	0.017	0.285	2.78E-91
NDUFA5	2.44E-95	0.362421	0.649	0.788	3.65E-91
TNRC6B	2.49E-95	-0.33495	0.199	0.567	3.73E-91
PDS5B	2.54E-95	-0.40362	0.098	0.442	3.79E-91
SLAIN1	2.98E-95	-0.47115	0.055	0.378	4.45E-91
SAT2	3.10E-95	0.55625	0.615	0.689	4.63E-91
NUP62	3.12E-95	-0.35763	0.155	0.526	4.67E-91
C4orf48	3.53E-95	-0.43556	0.252	0.641	5.27E-91
ETV5	4.41E-95	-0.4756	0.099	0.456	6.59E-91
SH3BGRL3	5.21E-95	0.752011	0.706	0.705	7.78E-91
GRK6	5.27E-95	-0.44013	0.042	0.352	7.87E-91
CPE	5.43E-95	0.15317	0.435	0.663	8.11E-91
CDCA7	6.47E-95	-0.53447	0.035	0.331	9.67E-91
SUPT16H	7.50E-95	-0.39848	0.178	0.559	1.12E-90
TAX1BP1	7.84E-95	0.183431	0.493	0.691	1.17E-90
GOLIM4	1.06E-94	-0.42815	0.201	0.59	1.59E-90
PCMT1	1.07E-94	0.174245	0.545	0.767	1.61E-90
DYNLL2	1.10E-94	-0.41294	0.113	0.47	1.64E-90
FAM111B	1.16E-94	-0.52713	0.008	0.251	1.73E-90
HNRNPH3	1.29E-94	-0.40876	0.336	0.756	1.93E-90
ARF1	1.46E-94	0.29631	0.629	0.804	2.19E-90
NENF	1.53E-94	-0.37325	0.358	0.757	2.29E-90
SLC3A2	1.67E-94	0.318835	0.583	0.757	2.49E-90
TMEM54	2.74E-94	-0.44578	0.151	0.531	4.09E-90
COLGALT2	2.96E-94	-0.39946	0.049	0.344	4.42E-90
PCBP4	3.06E-94	-0.46985	0.136	0.512	4.57E-90
NUCKS1	3.34E-94	-0.43389	0.578	0.94	5.00E-90
RBM17	3.96E-94	-0.46731	0.216	0.62	5.92E-90
HSP90B1	4.49E-94	0.144807	0.616	0.847	6.71E-90
VEGFA	4.60E-94	0.594185	0.347	0.32	6.87E-90
COA3	4.99E-94	0.278341	0.466	0.691	7.45E-90
GDI1	5.15E-94	-0.32552	0.388	0.79	7.69E-90
RBM25	7.55E-94	-0.30448	0.296	0.69	1.13E-89
QSER1	7.61E-94	-0.37076	0.052	0.359	1.14E-89
TUBB2A	8.39E-94	-0.44119	0.416	0.83	1.25E-89
RSF1	8.55E-94	-0.20717	0.312	0.677	1.28E-89
LIG1	1.05E-93	-0.55353	0.043	0.353	1.57E-89
RARRES3	1.20E-93	1.332676	0.389	0.127	1.79E-89
CTDNEP1	1.21E-93	-0.53046	0.16	0.557	1.81E-89

TMED10	1.30E-93	0.692245	0.655	0.674	1.94E-89
SETD5	1.42E-93	-0.42866	0.172	0.553	2.12E-89
MTA1	1.56E-93	-0.44329	0.094	0.438	2.34E-89
KLHDC3	1.70E-93	-0.34961	0.241	0.642	2.53E-89
FANCG	2.00E-93	-0.40024	0.031	0.311	2.99E-89
COPS3	2.01E-93	-0.16158	0.314	0.674	3.00E-89
PGAP1	2.05E-93	-0.43915	0.101	0.449	3.06E-89
RNF126	2.12E-93	-0.40847	0.203	0.589	3.17E-89
XRCC5	2.17E-93	-0.34759	0.435	0.84	3.25E-89
SNF8	2.20E-93	-0.35285	0.319	0.719	3.28E-89
WBP11	2.33E-93	-0.19471	0.249	0.593	3.48E-89
MTCH1	2.38E-93	-0.19721	0.431	0.774	3.55E-89
ZNF90	2.44E-93	0.779371	0.44	0.435	3.65E-89
STK25	2.49E-93	-0.41955	0.192	0.581	3.73E-89
PRRC2A	2.69E-93	-0.40113	0.141	0.506	4.03E-89
DCLRE1C	2.84E-93	-0.4324	0.069	0.395	4.24E-89
ENOPH1	3.08E-93	-0.33698	0.338	0.731	4.61E-89
TCEA2	3.12E-93	-0.50452	0.287	0.709	4.67E-89
PRADC1	3.69E-93	-0.3521	0.173	0.536	5.51E-89
RDX	3.79E-93	-0.33975	0.435	0.827	5.67E-89
SMC1A	4.02E-93	-0.49972	0.15	0.533	6.01E-89
TOMM40	4.17E-93	-0.43584	0.235	0.631	6.23E-89
TERF2IP	5.16E-93	-0.1179	0.406	0.719	7.71E-89
DTD1	5.51E-93	-0.43856	0.263	0.673	8.23E-89
ANAPC5	5.81E-93	-0.18443	0.323	0.672	8.68E-89
POP7	6.67E-93	-0.21015	0.375	0.759	9.96E-89
TRIM24	6.83E-93	-0.45724	0.099	0.451	1.02E-88
ARL6IP6	6.99E-93	-0.43678	0.265	0.674	1.04E-88
9-Mar	7.41E-93	-0.70462	0.056	0.381	1.11E-88
GPAA1	8.68E-93	-0.29242	0.288	0.665	1.30E-88
MALAT1	9.23E-93	0.448219	0.996	0.988	1.38E-88
RFC5	9.74E-93	-0.46071	0.078	0.414	1.45E-88
SNRNP25	1.02E-92	-0.29154	0.272	0.665	1.52E-88
STUB1	1.09E-92	-0.40407	0.325	0.728	1.63E-88
DLL3	1.21E-92	-0.80777	0.031	0.316	1.81E-88
CYTH2	1.23E-92	-0.45911	0.231	0.635	1.83E-88
RSRC2	1.26E-92	-0.12064	0.405	0.747	1.89E-88
YY1	1.34E-92	-0.32226	0.23	0.61	2.01E-88
RSL1D1	1.45E-92	0.217874	0.449	0.658	2.16E-88
NAA10	1.97E-92	-0.34978	0.286	0.683	2.94E-88
NRM	2.16E-92	-0.44603	0.034	0.325	3.23E-88
CLN6	2.41E-92	-0.45166	0.034	0.323	3.61E-88
CUTA	2.49E-92	0.273614	0.652	0.814	3.73E-88
NEURL1B	3.64E-92	-0.43371	0.008	0.246	5.44E-88
SCP2	3.75E-92	0.412709	0.58	0.722	5.60E-88
NOVA1	3.83E-92	-0.52054	0.495	0.879	5.72E-88
ASXL1	4.00E-92	-0.41065	0.092	0.426	5.98E-88
CLSTN1	4.61E-92	-0.23711	0.165	0.509	6.89E-88
UBE2L6	4.98E-92	0.660144	0.497	0.56	7.44E-88
GINS4	5.02E-92	-0.39756	0.018	0.273	7.50E-88
CXXC5	5.15E-92	-0.4784	0.268	0.68	7.70E-88

RFC2	5.70E-92	-0.47787	0.207	0.605	8.52E-88
CETN2	7.74E-92	0.798011	0.474	0.409	1.16E-87
AKIRIN2	7.76E-92	-0.45707	0.216	0.61	1.16E-87
CDH2	7.83E-92	-0.48294	0.22	0.626	1.17E-87
CCDC112	7.92E-92	-0.4353	0.122	0.477	1.18E-87
SLC39A3	8.84E-92	-0.53636	0.218	0.628	1.32E-87
PRIM1	9.05E-92	-0.50253	0.042	0.342	1.35E-87
UBE3A	9.10E-92	-0.41094	0.172	0.548	1.36E-87
RASSF2	1.33E-91	-0.38423	0.073	0.391	1.99E-87
SPAG9	1.88E-91	-0.4219	0.228	0.623	2.82E-87
PEBP1	1.95E-91	0.341202	0.801	0.932	2.91E-87
SOX9	1.99E-91	-0.68576	0.207	0.612	2.98E-87
CHN1	2.26E-91	-0.4618	0.174	0.556	3.37E-87
REXO2	2.26E-91	0.92743	0.497	0.367	3.38E-87
SRSF10	2.27E-91	-0.28053	0.21	0.573	3.39E-87
CLTA	2.27E-91	-0.29914	0.454	0.822	3.40E-87
RPA1	2.32E-91	-0.3878	0.199	0.579	3.47E-87
ACIN1	2.71E-91	-0.34478	0.15	0.501	4.05E-87
C17orf89	2.77E-91	-0.54475	0.275	0.694	4.14E-87
SNRPD2	3.05E-91	0.370489	0.763	0.896	4.55E-87
ZNF326	3.19E-91	-0.38008	0.172	0.542	4.76E-87
SRPK2	4.12E-91	-0.30848	0.342	0.725	6.15E-87
HAUS1	4.75E-91	-0.37381	0.18	0.552	7.10E-87
F2R	6.11E-91	-0.40394	0.088	0.417	9.14E-87
POLA2	6.92E-91	-0.42598	0.024	0.295	1.03E-86
RHBDD2	7.76E-91	0.180852	0.481	0.694	1.16E-86
MAP2K2	8.80E-91	-0.58012	0.158	0.546	1.31E-86
SYF2	9.28E-91	0.395443	0.49	0.64	1.39E-86
NPM1	1.01E-90	0.364778	0.821	0.938	1.51E-86
FOXRED2	1.16E-90	-0.35416	0.031	0.296	1.73E-86
FARP1	1.54E-90	-0.37237	0.089	0.41	2.31E-86
MAGED2	1.80E-90	0.262688	0.503	0.683	2.69E-86
CACYBP	2.02E-90	-0.21673	0.44	0.814	3.02E-86
MAZ	2.03E-90	-0.44886	0.181	0.563	3.03E-86
TMEM106C	2.10E-90	-0.33517	0.299	0.702	3.14E-86
FAM127A	2.21E-90	0.127986	0.441	0.681	3.30E-86
AP1S1	2.25E-90	0.311337	0.501	0.695	3.36E-86
SH3BGRL	2.57E-90	0.273178	0.476	0.702	3.83E-86
LSM14A	2.66E-90	-0.20913	0.334	0.681	3.98E-86
ERI3	2.78E-90	-0.12648	0.316	0.654	4.15E-86
NME4	3.20E-90	-0.28452	0.327	0.695	4.78E-86
COX20	3.38E-90	-0.15743	0.388	0.759	5.05E-86
FIS1	3.43E-90	0.165754	0.534	0.788	5.12E-86
CSNK1A1	4.36E-90	-0.25435	0.428	0.786	6.52E-86
DNAJC7	5.63E-90	-0.14298	0.339	0.696	8.42E-86
EMC10	6.19E-90	-0.27005	0.298	0.659	9.25E-86
CCM2	6.56E-90	-0.3325	0.193	0.552	9.81E-86
CCSAP	7.06E-90	-0.45274	0.036	0.323	1.06E-85
BASP1	7.95E-90	-0.64602	0.121	0.491	1.19E-85
IPO5	8.94E-90	-0.37239	0.146	0.502	1.34E-85
COA1	9.43E-90	-0.13202	0.359	0.719	1.41E-85

RUVBL2	9.44E-90	-0.40976	0.276	0.675	1.41E-85
UBL5	1.00E-89	0.510195	0.846	0.896	1.49E-85
PIGX	1.04E-89	-0.46492	0.189	0.575	1.55E-85
NOTCH1	1.14E-89	-0.43517	0.042	0.332	1.70E-85
MAP4K4	1.15E-89	-0.28218	0.146	0.478	1.72E-85
PSENEN	1.19E-89	0.652436	0.525	0.575	1.77E-85
GJC1	1.52E-89	-0.40614	0.029	0.306	2.27E-85
SEMA6D	1.57E-89	-0.43678	0.05	0.352	2.35E-85
EIF4A3	1.79E-89	-0.23529	0.32	0.669	2.67E-85
DDAH2	1.96E-89	-0.10137	0.336	0.673	2.93E-85
MT1M	1.99E-89	0.784554	0.475	0.458	2.97E-85
LINC00461	1.99E-89	-0.25412	0.254	0.612	2.97E-85
KIF2A	2.12E-89	-0.42351	0.127	0.483	3.16E-85
LIMD2	3.11E-89	-0.38854	0.043	0.331	4.65E-85
C2orf80	3.14E-89	-0.46735	0.074	0.398	4.69E-85
DNAJB11	3.44E-89	-0.12728	0.34	0.681	5.15E-85
MDH2	3.47E-89	0.26168	0.657	0.849	5.18E-85
EIF4A1	3.51E-89	0.249153	0.795	0.937	5.25E-85
ZNF667-AS	3.54E-89	0.329226	0.515	0.672	5.29E-85
RTN4	3.54E-89	-0.33187	0.518	0.888	5.29E-85
PTPRA	3.58E-89	-0.48814	0.243	0.648	5.36E-85
ZNF704	4.11E-89	-0.47171	0.035	0.319	6.15E-85
CSTF3	4.54E-89	-0.2719	0.221	0.584	6.78E-85
NUP50	4.73E-89	-0.39806	0.057	0.359	7.08E-85
MLLT11	4.95E-89	-0.30733	0.411	0.798	7.40E-85
ASAP2	4.96E-89	-0.38225	0.038	0.32	7.42E-85
KRTCAP2	4.97E-89	0.433618	0.559	0.672	7.43E-85
MMS22L	5.08E-89	-0.45058	0.044	0.333	7.59E-85
TXN2	5.53E-89	-0.24298	0.289	0.66	8.26E-85
CHMP5	5.92E-89	0.36089	0.523	0.647	8.84E-85
SSB	5.95E-89	0.277972	0.611	0.801	8.89E-85
C7orf55	7.01E-89	-0.15915	0.378	0.744	1.05E-84
S100B	7.38E-89	0.21343	0.499	0.754	1.10E-84
LUC7L3	7.56E-89	-0.14728	0.452	0.794	1.13E-84
RP11-1094	7.63E-89	-0.3124	0.154	0.498	1.14E-84
NAV1	7.83E-89	-0.43225	0.053	0.356	1.17E-84
USP13	8.04E-89	-0.403	0.026	0.295	1.20E-84
PNMA1	8.73E-89	-0.42018	0.173	0.544	1.30E-84
PCNP	8.76E-89	0.245465	0.566	0.754	1.31E-84
TBL1XR1	1.13E-88	-0.41384	0.109	0.452	1.69E-84
LYPLA1	1.17E-88	-0.39988	0.251	0.643	1.75E-84
CEP57	1.24E-88	-0.31444	0.176	0.526	1.85E-84
UFD1L	1.24E-88	-0.34249	0.235	0.616	1.86E-84
NUBP2	1.45E-88	-0.12734	0.328	0.67	2.16E-84
FAM136A	1.53E-88	-0.22489	0.188	0.528	2.28E-84
SELT	1.60E-88	0.441987	0.612	0.714	2.39E-84
SMARCE1	1.68E-88	-0.16555	0.292	0.652	2.51E-84
EIF3I	1.88E-88	0.288316	0.627	0.804	2.80E-84
MATR3	1.91E-88	-0.352	0.379	0.764	2.85E-84
DHX36	1.96E-88	-0.25355	0.315	0.677	2.93E-84
CAPRIN1	2.01E-88	-0.40253	0.203	0.58	3.01E-84

RFC1	2.25E-88	-0.30559	0.214	0.584	3.36E-84
CHTF18	2.67E-88	-0.38175	0.019	0.272	3.98E-84
ARPP19	2.72E-88	-0.22619	0.342	0.719	4.06E-84
PHACTR4	2.98E-88	-0.34498	0.124	0.452	4.45E-84
TMEM259	3.19E-88	-0.32688	0.208	0.559	4.77E-84
C8orf33	3.94E-88	-0.19677	0.264	0.607	5.89E-84
ARPC3	3.98E-88	0.397381	0.683	0.817	5.94E-84
FEZ1	4.33E-88	-0.32283	0.399	0.784	6.47E-84
ARF6	4.41E-88	-0.40018	0.18	0.547	6.59E-84
PAX6	5.49E-88	-0.43034	0.064	0.368	8.20E-84
BLOC1S1	5.57E-88	0.241404	0.525	0.783	8.33E-84
DEF8	5.78E-88	-0.36163	0.157	0.506	8.64E-84
SF1	6.42E-88	-0.23741	0.388	0.753	9.59E-84
PON2	6.96E-88	0.346871	0.539	0.684	1.04E-83
RAMP1	6.97E-88	0.670939	0.621	0.709	1.04E-83
MAGOHB	7.89E-88	-0.29952	0.217	0.583	1.18E-83
NDUFS5	9.67E-88	0.291527	0.789	0.93	1.44E-83
MIDN	1.13E-87	-0.38218	0.26	0.643	1.69E-83
HAGH	1.24E-87	-0.43673	0.225	0.611	1.86E-83
SLC22A17	1.28E-87	-0.30573	0.196	0.544	1.91E-83
CBX2	1.68E-87	-0.41752	0.021	0.277	2.51E-83
NUTF2	1.78E-87	-0.14449	0.332	0.69	2.65E-83
PDIA4	1.91E-87	0.196797	0.483	0.707	2.85E-83
TRAPPC1	2.31E-87	0.533039	0.579	0.673	3.45E-83
TPR	2.73E-87	-0.3684	0.33	0.725	4.08E-83
BID	2.83E-87	-0.18694	0.169	0.477	4.23E-83
GRB2	2.89E-87	-0.33734	0.226	0.59	4.32E-83
ABHD12	2.93E-87	-0.56882	0.188	0.58	4.38E-83
NARS	3.29E-87	0.312442	0.467	0.621	4.92E-83
ARL4C	3.49E-87	-0.44564	0.237	0.627	5.22E-83
HNRNPR	3.52E-87	-0.33689	0.455	0.847	5.27E-83
PRPF40A	3.53E-87	-0.19775	0.358	0.721	5.28E-83
YPEL1	3.83E-87	-0.33841	0.029	0.293	5.72E-83
WIPI2	3.93E-87	-0.24526	0.276	0.614	5.87E-83
PCBP1	4.06E-87	-0.34286	0.403	0.793	6.07E-83
COPS8	4.08E-87	0.202114	0.541	0.767	6.10E-83
CPNE4	4.60E-87	-0.36448	0.016	0.259	6.87E-83
RGS12	4.68E-87	-0.38955	0.115	0.446	6.99E-83
LSM5	4.94E-87	-0.28412	0.503	0.88	7.38E-83
LGR4	5.58E-87	-0.37556	0.019	0.273	8.34E-83
REEP3	6.67E-87	-0.46062	0.091	0.425	9.97E-83
TRIOBP	7.29E-87	-0.35188	0.036	0.299	1.09E-82
BRCA2	7.52E-87	-0.45534	0.045	0.34	1.12E-82
FAM200B	7.68E-87	-0.1268	0.299	0.644	1.15E-82
TMEM251	7.87E-87	-0.15653	0.15	0.462	1.18E-82
FAM212B	8.36E-87	-0.40627	0.031	0.304	1.25E-82
NELFE	8.92E-87	-0.10735	0.338	0.681	1.33E-82
ID3	9.03E-87	0.73281	0.366	0.454	1.35E-82
FAM96B	9.05E-87	0.327094	0.667	0.816	1.35E-82
TTYH1	1.00E-86	-0.52971	0.196	0.573	1.50E-82
DDX18	1.10E-86	0.149303	0.46	0.72	1.65E-82

TCEAL4	1.25E-86	0.32217	0.533	0.728	1.86E-82
GSPT1	1.27E-86	-0.39723	0.241	0.623	1.90E-82
PPP2R3C	1.30E-86	-0.22557	0.22	0.572	1.94E-82
CIB1	1.31E-86	0.63495	0.5	0.501	1.96E-82
GTF2I	1.39E-86	-0.43454	0.292	0.686	2.08E-82
CNPY2	1.60E-86	0.146202	0.506	0.767	2.40E-82
DCTN3	1.65E-86	-0.27625	0.426	0.802	2.46E-82
SEC11C	1.68E-86	0.235226	0.483	0.679	2.51E-82
NCOR2	1.73E-86	-0.3984	0.094	0.419	2.59E-82
ILF2	1.75E-86	-0.26436	0.539	0.902	2.62E-82
MTPN	1.80E-86	-0.1004	0.353	0.653	2.68E-82
SPOCD1	1.82E-86	1.089713	0.474	0.301	2.71E-82
N4BP2L2	2.03E-86	0.119581	0.443	0.657	3.03E-82
TMCO1	2.15E-86	0.473443	0.629	0.723	3.22E-82
WSB2	2.19E-86	-0.39396	0.122	0.463	3.27E-82
PRPF38B	2.26E-86	-0.28549	0.307	0.668	3.38E-82
CDKN2AIPN	2.40E-86	-0.20593	0.194	0.537	3.58E-82
BOD1	3.42E-86	-0.2312	0.263	0.606	5.12E-82
MUM1	3.45E-86	-0.30272	0.227	0.59	5.16E-82
NLGN1	3.85E-86	-0.44702	0.066	0.377	5.75E-82
DPYSL4	4.08E-86	-0.44112	0.069	0.383	6.09E-82
UPF3A	4.68E-86	-0.17188	0.312	0.662	6.99E-82
NUDT3	4.82E-86	-0.38641	0.208	0.583	7.21E-82
DHRS11	4.88E-86	-0.31836	0.031	0.285	7.30E-82
ERP29	5.64E-86	0.362849	0.568	0.714	8.43E-82
CNOT7	6.37E-86	-0.18614	0.293	0.637	9.52E-82
GNAS	6.62E-86	-0.44511	0.518	0.893	9.89E-82
SSBP1	7.80E-86	0.265929	0.636	0.847	1.17E-81
CBR1	8.65E-86	0.219018	0.476	0.677	1.29E-81
DDX39A	9.33E-86	-0.28089	0.404	0.786	1.39E-81
PIK3R3	9.43E-86	-0.22633	0.06	0.331	1.41E-81
KRT10	1.04E-85	-0.20483	0.478	0.811	1.56E-81
FDPS	1.06E-85	-0.24227	0.357	0.73	1.58E-81
BAZ2B	1.17E-85	-0.44333	0.166	0.533	1.75E-81
CDK16	1.20E-85	-0.34607	0.182	0.536	1.79E-81
VPS37B	1.33E-85	-0.39305	0.108	0.441	1.99E-81
CHCHD3	1.39E-85	-0.22041	0.351	0.716	2.07E-81
POLR2F	1.43E-85	-0.2551	0.438	0.819	2.14E-81
SMARCD1	1.75E-85	-0.34163	0.123	0.456	2.62E-81
RNF181	1.78E-85	0.457338	0.521	0.642	2.66E-81
UQCC2	1.80E-85	-0.13797	0.338	0.694	2.69E-81
ATP5G1	1.83E-85	0.269051	0.605	0.805	2.74E-81
RP11-553L	2.02E-85	-0.36172	0.141	0.475	3.02E-81
PAIP1	2.40E-85	-0.43417	0.285	0.678	3.58E-81
PSMA3	2.48E-85	0.223754	0.435	0.691	3.71E-81
UBXN2A	2.57E-85	-0.34816	0.139	0.473	3.84E-81
11-Sep	2.59E-85	-0.44841	0.229	0.616	3.87E-81
SACS	2.66E-85	-0.43022	0.062	0.364	3.97E-81
HMGN3	2.74E-85	-0.22445	0.462	0.835	4.10E-81
AHCY	2.74E-85	-0.32996	0.217	0.586	4.10E-81
ANKRD11	3.05E-85	-0.33496	0.195	0.541	4.56E-81

PPP1CC	3.13E-85	-0.43449	0.361	0.76	4.68E-81
GCAT	3.41E-85	-0.44965	0.094	0.426	5.09E-81
SSH2	3.60E-85	-0.39474	0.055	0.343	5.39E-81
MAML2	3.87E-85	-0.33634	0.095	0.407	5.78E-81
DTNA	3.95E-85	0.823298	0.415	0.358	5.91E-81
GFAP	4.04E-85	1.155293	0.519	0.354	6.04E-81
MYL6B	4.17E-85	-0.17984	0.407	0.765	6.23E-81
ACAP3	4.27E-85	-0.48948	0.096	0.433	6.39E-81
ARHGEF2	4.41E-85	-0.25243	0.185	0.504	6.59E-81
SCARA3	4.70E-85	-0.28731	0.115	0.421	7.02E-81
DHX9	4.73E-85	-0.33885	0.18	0.533	7.06E-81
ARL5A	4.90E-85	-0.2764	0.33	0.695	7.33E-81
NRN1	5.14E-85	0.654986	0.416	0.433	7.68E-81
BUB3	5.95E-85	-0.40345	0.262	0.648	8.89E-81
NDUFB2	6.66E-85	0.418806	0.786	0.905	9.95E-81
AP1S2	7.46E-85	-0.47193	0.322	0.723	1.11E-80
ATP1B3	7.55E-85	-0.28655	0.353	0.717	1.13E-80
CADM4	7.76E-85	-0.38534	0.167	0.517	1.16E-80
CNTFR	8.73E-85	-0.48281	0.056	0.359	1.31E-80
C8orf59	9.37E-85	-0.21383	0.305	0.646	1.40E-80
FRYL	1.01E-84	-0.4086	0.087	0.409	1.51E-80
IGFBP5	1.03E-84	0.942142	0.505	0.379	1.53E-80
PDCD2	1.05E-84	-0.47247	0.178	0.548	1.56E-80
MTFR2	1.06E-84	-0.37963	0.003	0.205	1.58E-80
DCK	1.07E-84	-0.44778	0.128	0.472	1.60E-80
NLRP1	1.09E-84	-0.27537	0.248	0.6	1.64E-80
RERE	1.14E-84	-0.31563	0.091	0.393	1.71E-80
WBP5	1.17E-84	0.401458	0.648	0.777	1.75E-80
KTN1	1.21E-84	-0.40313	0.361	0.759	1.80E-80
HAT1	1.30E-84	-0.3007	0.307	0.684	1.94E-80
RFWD3	1.31E-84	-0.39062	0.028	0.285	1.95E-80
ARMC1	1.51E-84	-0.33524	0.201	0.552	2.26E-80
CENPL	1.54E-84	-0.34997	0.039	0.304	2.30E-80
ZCRB1	1.63E-84	0.114294	0.48	0.763	2.44E-80
PBRM1	1.65E-84	-0.42842	0.137	0.486	2.46E-80
ANAPC15	1.69E-84	-0.21463	0.156	0.478	2.52E-80
DDR1	1.87E-84	-0.2419	0.286	0.637	2.80E-80
SCARB2	1.91E-84	-0.23702	0.246	0.574	2.86E-80
PSMA4	2.11E-84	-0.18119	0.417	0.781	3.15E-80
FMNL2	2.16E-84	-0.40084	0.097	0.421	3.22E-80
FREM2	2.26E-84	-0.30353	0.009	0.227	3.38E-80
ZFAND5	2.27E-84	-0.19164	0.271	0.59	3.39E-80
C9orf16	2.55E-84	-0.17349	0.409	0.76	3.81E-80
IMMP1L	2.64E-84	-0.2459	0.191	0.536	3.95E-80
GSTK1	2.70E-84	0.695963	0.48	0.504	4.04E-80
PGD	2.76E-84	-0.34607	0.136	0.469	4.12E-80
HMGA1	2.80E-84	-0.52633	0.185	0.565	4.19E-80
ATRX	3.03E-84	-0.30046	0.294	0.657	4.53E-80
PHPT1	3.22E-84	0.207378	0.643	0.848	4.81E-80
ARID1A	3.35E-84	-0.43301	0.131	0.478	5.01E-80
MAGOH	3.60E-84	0.172269	0.411	0.647	5.38E-80

RIF1	3.64E-84	-0.43777	0.158	0.522	5.44E-80
UBE2L3	4.01E-84	-0.24209	0.38	0.756	5.99E-80
EIF3K	4.67E-84	0.392665	0.679	0.835	6.98E-80
CDK4	4.81E-84	-0.52069	0.446	0.837	7.19E-80
CEP170	4.83E-84	-0.46827	0.1	0.433	7.21E-80
CRIP2	5.29E-84	-0.13063	0.281	0.604	7.90E-80
NDUFB5	5.73E-84	0.194544	0.509	0.742	8.57E-80
TOP1	6.18E-84	-0.42944	0.277	0.667	9.23E-80
NDUFA12	6.55E-84	0.154734	0.532	0.788	9.78E-80
EIF1B	6.70E-84	0.301725	0.597	0.78	1.00E-79
COPS6	6.83E-84	0.132831	0.608	0.859	1.02E-79
GSS	6.99E-84	-0.31005	0.151	0.486	1.04E-79
C1QBP	7.17E-84	-0.36156	0.425	0.802	1.07E-79
SLC44A2	7.89E-84	-0.28952	0.139	0.459	1.18E-79
LSM2	8.13E-84	-0.10752	0.405	0.743	1.21E-79
CENPP	8.34E-84	-0.38064	0.03	0.28	1.25E-79
2-Sep	9.00E-84	-0.26537	0.5	0.86	1.35E-79
HADHB	9.04E-84	0.143335	0.415	0.637	1.35E-79
JUN	1.02E-83	-0.11025	0.606	0.883	1.53E-79
FOXG1	1.04E-83	-0.37653	0.086	0.391	1.55E-79
NAA38	1.16E-83	0.134305	0.456	0.757	1.74E-79
POLR3K	1.28E-83	-0.28221	0.138	0.463	1.91E-79
TRAM1	1.32E-83	0.402851	0.45	0.516	1.97E-79
COMMD6	1.32E-83	0.444731	0.726	0.823	1.98E-79
GTF2H5	1.35E-83	0.201759	0.378	0.575	2.02E-79
COPE	1.41E-83	0.317911	0.759	0.889	2.11E-79
GGCT	1.49E-83	0.203634	0.44	0.699	2.23E-79
GOLGA7	1.74E-83	0.18709	0.467	0.68	2.59E-79
DENR	1.89E-83	-0.28228	0.24	0.594	2.82E-79
VPS28	2.02E-83	0.19257	0.504	0.746	3.02E-79
TAF10	2.04E-83	-0.38743	0.091	0.41	3.06E-79
C7orf73	2.09E-83	0.393661	0.581	0.746	3.12E-79
HN1L	2.10E-83	-0.32979	0.112	0.435	3.14E-79
POMP	2.19E-83	0.291529	0.676	0.838	3.28E-79
RAF1	2.21E-83	-0.36523	0.188	0.546	3.30E-79
TMEM14C	2.21E-83	0.27742	0.544	0.715	3.31E-79
ATP6V0B	2.41E-83	0.251433	0.659	0.828	3.60E-79
MBD3	2.62E-83	-0.32423	0.097	0.404	3.92E-79
ILKAP	2.73E-83	-0.35491	0.162	0.507	4.07E-79
SYPL1	2.85E-83	0.666735	0.529	0.494	4.26E-79
DCAF7	2.92E-83	-0.44323	0.23	0.615	4.36E-79
OLA1	3.01E-83	-0.10744	0.441	0.784	4.49E-79
PAIP2	3.01E-83	0.212267	0.578	0.764	4.50E-79
NIPA2	3.12E-83	-0.30318	0.176	0.512	4.67E-79
LEPROTL1	3.26E-83	-0.18167	0.247	0.586	4.88E-79
DGUOK	3.77E-83	0.164221	0.515	0.767	5.63E-79
WRB	3.85E-83	0.249639	0.451	0.665	5.75E-79
PAICS	3.85E-83	-0.36072	0.302	0.694	5.75E-79
HRAS	3.98E-83	-0.45107	0.204	0.583	5.95E-79
HDGF	4.13E-83	-0.47171	0.229	0.616	6.18E-79
EIF3H	4.38E-83	0.26605	0.587	0.765	6.55E-79

AKIRIN1	4.53E-83	-0.21342	0.282	0.607	6.77E-79
C9orf78	4.69E-83	0.108416	0.409	0.652	7.01E-79
MCRS1	4.92E-83	-0.26529	0.193	0.537	7.36E-79
NDUFB3	5.99E-83	0.17222	0.493	0.743	8.95E-79
PLEKHJ1	6.48E-83	-0.17855	0.292	0.633	9.69E-79
C16orf45	6.67E-83	-0.20576	0.28	0.632	9.97E-79
DBF4B	6.68E-83	-0.29956	0.011	0.231	9.98E-79
RFFL	6.83E-83	-0.43911	0.056	0.354	1.02E-78
HSBP1	7.85E-83	0.257578	0.699	0.874	1.17E-78
KNOP1	8.03E-83	-0.28839	0.209	0.558	1.20E-78
SIAH1	8.44E-83	-0.35395	0.142	0.47	1.26E-78
CHD4	8.92E-83	-0.29576	0.199	0.544	1.33E-78
PPP2R1A	9.19E-83	-0.20231	0.418	0.764	1.37E-78
PPT1	9.32E-83	0.217825	0.433	0.614	1.39E-78
MAPRE1	9.50E-83	-0.30348	0.362	0.741	1.42E-78
ADSL	9.78E-83	-0.2576	0.158	0.481	1.46E-78
U2AF2	1.05E-82	-0.15928	0.211	0.527	1.57E-78
EXOC5	1.14E-82	-0.38099	0.099	0.415	1.71E-78
NFKBIA	1.14E-82	0.616051	0.483	0.419	1.71E-78
NCL	1.21E-82	-0.33322	0.434	0.811	1.81E-78
MPDU1	1.29E-82	-0.23856	0.226	0.564	1.93E-78
GPX1	1.37E-82	0.375717	0.69	0.84	2.05E-78
FKBP8	1.38E-82	0.361349	0.62	0.754	2.07E-78
POU3F3	1.42E-82	-0.37529	0.071	0.365	2.12E-78
GSTP1	1.58E-82	0.435922	0.821	0.923	2.37E-78
RAD1	1.75E-82	-0.36674	0.147	0.483	2.61E-78
EIF3E	1.81E-82	0.149856	0.523	0.746	2.71E-78
TTC19	1.84E-82	-0.24208	0.261	0.594	2.75E-78
NTHL1	2.05E-82	-0.29066	0.122	0.441	3.07E-78
MIF	2.07E-82	0.205463	0.615	0.795	3.09E-78
SLTM	2.35E-82	-0.20233	0.239	0.565	3.52E-78
PSD3	2.77E-82	-0.31334	0.089	0.393	4.14E-78
PPP2CA	2.88E-82	-0.30264	0.368	0.735	4.30E-78
HNRNPL	3.11E-82	-0.28506	0.166	0.489	4.65E-78
FIP1L1	3.16E-82	-0.3501	0.247	0.612	4.72E-78
CASC4	3.20E-82	-0.34476	0.115	0.436	4.78E-78
MASTL	3.39E-82	-0.42027	0.007	0.22	5.06E-78
DCP2	3.51E-82	-0.41583	0.083	0.394	5.25E-78
MPC1	3.52E-82	0.153651	0.462	0.696	5.26E-78
ADNP	3.81E-82	-0.36869	0.1	0.411	5.70E-78
SSR2	3.89E-82	0.132648	0.636	0.869	5.81E-78
TMEM165	4.10E-82	-0.46588	0.3	0.689	6.13E-78
HSPB11	4.14E-82	-0.33332	0.254	0.619	6.19E-78
TMEM9B	4.22E-82	0.244636	0.46	0.642	6.30E-78
FUS	5.16E-82	-0.25865	0.543	0.898	7.72E-78
BTF3	5.47E-82	0.368113	0.854	0.954	8.18E-78
ARGLU1	6.27E-82	-0.28095	0.361	0.714	9.36E-78
MEAF6	6.31E-82	0.209626	0.478	0.688	9.43E-78
MAT2A	6.37E-82	-0.1707	0.283	0.606	9.53E-78
DHPS	6.67E-82	-0.13258	0.415	0.757	9.96E-78
CPSF6	6.84E-82	-0.33862	0.236	0.604	1.02E-77

RAB7A	7.52E-82	0.22912	0.609	0.774	1.12E-77
SRRT	8.79E-82	-0.38285	0.181	0.532	1.31E-77
ILDR2	8.82E-82	-0.28074	0.02	0.249	1.32E-77
NCOR1	1.12E-81	-0.43644	0.335	0.728	1.67E-77
PSMC3	1.18E-81	-0.10994	0.497	0.842	1.76E-77
CCT5	1.18E-81	-0.15676	0.447	0.791	1.77E-77
GANAB	1.25E-81	-0.33207	0.192	0.537	1.87E-77
PPP2R3B	1.33E-81	-0.32719	0.02	0.258	1.99E-77
ZNF680	1.40E-81	-0.40403	0.155	0.501	2.09E-77
RNF5	1.48E-81	-0.21451	0.308	0.659	2.20E-77
LSM3	1.48E-81	-0.1852	0.508	0.863	2.22E-77
LHFPL3	1.49E-81	-0.64752	0.053	0.348	2.23E-77
UQCRFS1	1.51E-81	-0.37306	0.437	0.812	2.26E-77
FGD5-AS1	1.55E-81	-0.37249	0.224	0.583	2.32E-77
COX7A2L	1.66E-81	0.376449	0.578	0.712	2.48E-77
TAF15	1.68E-81	-0.3396	0.203	0.564	2.51E-77
NDE1	1.77E-81	-0.40964	0.056	0.347	2.65E-77
EIF5B	1.83E-81	0.151651	0.51	0.767	2.74E-77
XRN2	2.16E-81	-0.29432	0.361	0.731	3.23E-77
NSL1	2.19E-81	-0.12107	0.303	0.627	3.27E-77
NSRP1	2.61E-81	-0.12817	0.317	0.638	3.90E-77
CBR3	2.67E-81	-0.34189	0.094	0.388	3.98E-77
EIF2AK1	2.75E-81	-0.31902	0.284	0.635	4.11E-77
FUNDC2	2.79E-81	0.109757	0.386	0.643	4.17E-77
PNRC1	2.79E-81	0.191848	0.372	0.514	4.17E-77
PPIH	2.95E-81	-0.21068	0.201	0.528	4.41E-77
RRP7A	2.98E-81	-0.2131	0.203	0.537	4.45E-77
BUD31	3.03E-81	0.302375	0.695	0.853	4.53E-77
SCLT1	3.13E-81	-0.35485	0.03	0.283	4.68E-77
APOLD1	3.47E-81	-0.49457	0.043	0.32	5.19E-77
SNRNP200	3.48E-81	-0.28552	0.22	0.568	5.19E-77
PCGF2	3.71E-81	-0.28114	0.098	0.386	5.54E-77
PSMB1	4.02E-81	0.359579	0.713	0.853	6.01E-77
OSBPL6	4.20E-81	-0.33608	0.089	0.388	6.27E-77
TMEM258	4.41E-81	0.327605	0.696	0.847	6.59E-77
TSPAN3	4.49E-81	-0.33251	0.387	0.765	6.70E-77
PPP1R10	4.54E-81	-0.28341	0.142	0.456	6.78E-77
SMARCA5	4.95E-81	-0.384	0.251	0.619	7.40E-77
TJP1	5.67E-81	-0.25563	0.126	0.428	8.47E-77
IP6K2	5.93E-81	0.169151	0.452	0.688	8.87E-77
RNH1	6.11E-81	0.340695	0.506	0.627	9.13E-77
TAGLN3	6.15E-81	-0.62509	0.055	0.349	9.19E-77
RRP36	6.44E-81	-0.33568	0.059	0.343	9.62E-77
IFRD1	7.06E-81	0.316739	0.512	0.626	1.06E-76
MARK3	7.20E-81	-0.26	0.194	0.505	1.08E-76
RNPS1	7.52E-81	-0.23819	0.476	0.831	1.12E-76
FKBP1A	8.35E-81	0.35382	0.648	0.81	1.25E-76
CTNNA1	8.48E-81	0.170367	0.391	0.558	1.27E-76
ACBD6	8.57E-81	-0.26165	0.195	0.537	1.28E-76
SERF2	9.28E-81	0.474802	0.837	0.914	1.39E-76
UBE2J2	1.00E-80	-0.31866	0.236	0.58	1.50E-76

KATNBL1	1.01E-80	-0.27647	0.147	0.472	1.51E-76
POLDIP2	1.06E-80	-0.41388	0.243	0.619	1.58E-76
SHFM1	1.13E-80	0.397662	0.773	0.884	1.69E-76
ABHD17A	1.17E-80	-0.41285	0.101	0.422	1.75E-76
ATP2A2	1.23E-80	-0.26821	0.177	0.496	1.84E-76
TP53I13	1.25E-80	-0.52058	0.147	0.507	1.86E-76
SOCS3	1.39E-80	0.485018	0.342	0.332	2.08E-76
ELOVL5	1.61E-80	-0.17697	0.268	0.6	2.40E-76
LSM1	1.62E-80	0.198993	0.445	0.66	2.42E-76
RBBP4	1.66E-80	-0.25199	0.362	0.728	2.49E-76
UBE2R2	1.89E-80	-0.40377	0.164	0.515	2.82E-76
CEP57L1	1.90E-80	-0.33445	0.071	0.364	2.84E-76
GLOD4	1.90E-80	-0.10431	0.343	0.673	2.84E-76
AKAP12	1.94E-80	0.339168	0.31	0.414	2.90E-76
NBEAL1	2.27E-80	0.774857	0.648	0.625	3.40E-76
CXCL14	2.34E-80	1.090713	0.333	0.101	3.49E-76
LAMTOR1	2.40E-80	0.34738	0.563	0.727	3.58E-76
FZD3	2.50E-80	-0.27468	0.231	0.577	3.74E-76
C16orf87	2.53E-80	-0.36202	0.13	0.453	3.79E-76
KMT2E	2.53E-80	-0.15781	0.432	0.767	3.79E-76
PGRMC1	2.85E-80	-0.18874	0.427	0.749	4.26E-76
SON	2.99E-80	0.249527	0.651	0.848	4.46E-76
FSD1	3.00E-80	-0.36445	0.082	0.381	4.48E-76
WBSCR22	3.04E-80	0.14717	0.483	0.728	4.55E-76
UBE2E1	3.15E-80	0.279526	0.459	0.626	4.70E-76
UBE2I	3.46E-80	-0.21296	0.469	0.827	5.17E-76
TLK1	4.09E-80	-0.40998	0.168	0.52	6.11E-76
DESI2	4.12E-80	-0.37986	0.135	0.464	6.16E-76
PDIA6	4.13E-80	0.223777	0.659	0.849	6.17E-76
CELF2	4.53E-80	-0.22387	0.165	0.472	6.76E-76
ENOSF1	4.55E-80	-0.25808	0.029	0.264	6.80E-76
ROBO2	4.64E-80	-0.39241	0.029	0.284	6.94E-76
HSD17B12	4.84E-80	0.247803	0.417	0.568	7.24E-76
PGLS	4.91E-80	-0.23192	0.348	0.686	7.34E-76
IMPDH2	5.30E-80	0.386855	0.492	0.616	7.93E-76
GALNT1	5.34E-80	-0.35663	0.077	0.369	7.98E-76
NNMT	5.62E-80	1.172293	0.392	0.17	8.40E-76
CSNK2A1	5.74E-80	-0.25849	0.251	0.593	8.58E-76
C3orf70	5.96E-80	-0.4567	0.037	0.301	8.91E-76
IFI27L1	6.22E-80	-0.19009	0.165	0.464	9.30E-76
LMAN2	6.34E-80	0.29645	0.564	0.743	9.48E-76
PSMD1	6.69E-80	-0.30068	0.251	0.605	1.00E-75
PSMD3	6.73E-80	-0.16862	0.347	0.658	1.01E-75
RNF26	7.07E-80	-0.38282	0.111	0.437	1.06E-75
NDUFV1	7.18E-80	-0.11025	0.41	0.735	1.07E-75
PRELID1	7.32E-80	-0.24834	0.433	0.767	1.09E-75
AIF1L	7.63E-80	-0.5943	0.036	0.304	1.14E-75
PLA2G16	8.31E-80	0.777064	0.437	0.374	1.24E-75
SEC61B	9.48E-80	0.337725	0.708	0.831	1.42E-75
DCTPP1	9.84E-80	-0.18869	0.253	0.586	1.47E-75
SRSF6	9.98E-80	-0.31118	0.26	0.615	1.49E-75

NDUFA13	1.09E-79	0.521178	0.769	0.862	1.63E-75
TMEM50A	1.14E-79	0.444298	0.567	0.689	1.70E-75
SRSF5	1.15E-79	0.194411	0.599	0.802	1.72E-75
METTL9	1.16E-79	-0.3012	0.361	0.714	1.73E-75
TMEM208	1.16E-79	0.319369	0.491	0.651	1.74E-75
ANXA2R	1.26E-79	-0.26433	0.094	0.374	1.89E-75
EIF3F	1.27E-79	-0.18654	0.448	0.767	1.90E-75
IRF2BPL	1.31E-79	-0.41035	0.154	0.494	1.96E-75
AKAP8L	1.33E-79	-0.12524	0.181	0.438	1.99E-75
SARS	1.33E-79	0.301573	0.448	0.591	1.99E-75
NOP56	1.33E-79	-0.23871	0.38	0.751	1.99E-75
NDUFA4	1.44E-79	0.434847	0.906	0.975	2.15E-75
BLMH	1.50E-79	-0.3065	0.105	0.41	2.24E-75
OSBPL8	1.52E-79	-0.31898	0.183	0.515	2.28E-75
TFDP2	1.57E-79	-0.41411	0.106	0.426	2.35E-75
C4orf46	1.62E-79	-0.34809	0.045	0.305	2.42E-75
SQLE	1.64E-79	-0.37306	0.187	0.537	2.44E-75
TBCB	1.66E-79	0.113086	0.612	0.864	2.48E-75
HNRNPA1L2	1.67E-79	-0.49146	0.097	0.421	2.49E-75
SNRNP40	1.69E-79	-0.10076	0.317	0.64	2.53E-75
OSTC	2.02E-79	0.45313	0.53	0.651	3.01E-75
CCL2	2.05E-79	1.222899	0.328	0.247	3.06E-75
PHC2	2.37E-79	-0.15574	0.176	0.467	3.54E-75
RP11-472N	2.60E-79	-0.30594	0.012	0.216	3.89E-75
R3HDM1	2.73E-79	-0.31158	0.057	0.331	4.09E-75
EI24	2.75E-79	-0.2177	0.398	0.737	4.11E-75
TMX4	3.02E-79	-0.37074	0.183	0.532	4.51E-75
HSPH1	3.23E-79	-0.18787	0.284	0.61	4.83E-75
ADI1	3.28E-79	-0.10401	0.323	0.614	4.91E-75
EBP	3.65E-79	-0.17053	0.156	0.458	5.46E-75
RDH11	3.80E-79	-0.14435	0.288	0.609	5.67E-75
HNRNPUL2	3.91E-79	-0.35382	0.087	0.386	5.85E-75
RHEB	4.00E-79	-0.30357	0.594	0.926	5.97E-75
HYAL2	4.12E-79	-0.31918	0.128	0.437	6.15E-75
LDHB	4.15E-79	0.136262	0.768	0.967	6.20E-75
TAX1BP3	4.36E-79	0.390155	0.346	0.464	6.52E-75
ARL9	4.49E-79	-0.33749	0.009	0.219	6.71E-75
LMO4	4.62E-79	-0.37254	0.396	0.78	6.91E-75
CENPJ	4.81E-79	-0.38284	0.05	0.325	7.19E-75
TOP2B	5.21E-79	-0.33568	0.121	0.432	7.78E-75
DPYSL5	5.27E-79	-0.438	0.049	0.33	7.88E-75
EIF3L	5.83E-79	-0.1391	0.402	0.744	8.72E-75
CLNS1A	5.86E-79	0.179608	0.461	0.686	8.76E-75
OCIAD1	6.00E-79	0.280208	0.621	0.806	8.97E-75
POLR2J	6.13E-79	0.219109	0.664	0.878	9.16E-75
GPBP1	6.25E-79	-0.12653	0.378	0.693	9.34E-75
CHID1	6.45E-79	0.148818	0.416	0.675	9.64E-75
ECH1	6.90E-79	0.371921	0.555	0.704	1.03E-74
RSBN1L	7.02E-79	-0.17361	0.282	0.611	1.05E-74
SRR	7.52E-79	-0.24057	0.106	0.39	1.12E-74
CREB5	7.55E-79	-0.35525	0.178	0.511	1.13E-74

TMEM206	7.64E-79	-0.27752	0.113	0.41	1.14E-74
POLR2G	8.55E-79	0.274701	0.544	0.759	1.28E-74
ID2	8.80E-79	0.485695	0.539	0.595	1.32E-74
APP	8.96E-79	-0.12329	0.497	0.786	1.34E-74
7-Sep	9.12E-79	0.112622	0.724	0.933	1.36E-74
CCAR1	9.34E-79	-0.31907	0.166	0.495	1.40E-74
NCK2	9.44E-79	-0.28994	0.033	0.279	1.41E-74
SNHG7	1.01E-78	-0.10082	0.29	0.56	1.50E-74
BCLAF1	1.02E-78	-0.25031	0.281	0.627	1.53E-74
UBE2D3	1.03E-78	0.331116	0.678	0.802	1.54E-74
U2SURP	1.05E-78	-0.14532	0.304	0.628	1.57E-74
MTF2	1.28E-78	-0.30246	0.157	0.472	1.91E-74
MYL12B	1.32E-78	0.446349	0.693	0.804	1.97E-74
EMC3	1.50E-78	0.267967	0.427	0.616	2.25E-74
CTDSPL2	1.52E-78	-0.35857	0.066	0.347	2.27E-74
COMMD2	1.60E-78	-0.13448	0.228	0.532	2.40E-74
IPO7	1.63E-78	-0.29337	0.148	0.458	2.43E-74
PABPC4	1.68E-78	-0.28399	0.168	0.48	2.51E-74
SRSF4	1.70E-78	-0.3183	0.27	0.625	2.54E-74
U2AF1	1.82E-78	-0.3245	0.517	0.875	2.72E-74
LINC0011€	2.07E-78	0.253983	0.368	0.567	3.09E-74
SRPK1	2.19E-78	-0.38338	0.111	0.426	3.28E-74
MAGED1	2.29E-78	-0.14972	0.307	0.635	3.43E-74
YIF1A	2.40E-78	0.511296	0.47	0.527	3.58E-74
EPC1	2.50E-78	-0.29619	0.163	0.485	3.74E-74
BCL7B	2.52E-78	-0.28944	0.237	0.58	3.76E-74
MAPK1	2.54E-78	-0.37517	0.122	0.441	3.80E-74
SORT1	2.78E-78	-0.31186	0.105	0.401	4.16E-74
SGTA	2.98E-78	-0.22422	0.261	0.581	4.46E-74
MAPK1IP1L	3.00E-78	-0.12823	0.289	0.616	4.49E-74
CCND1	3.13E-78	-0.47511	0.083	0.379	4.67E-74
LDLRAD3	3.23E-78	-0.24254	0.082	0.359	4.82E-74
AK1	3.25E-78	0.130841	0.301	0.54	4.85E-74
LAMP2	4.01E-78	0.624768	0.501	0.486	5.99E-74
JAG1	4.38E-78	-0.41481	0.086	0.383	6.55E-74
PFDN4	4.93E-78	-0.30367	0.254	0.604	7.37E-74
ITGB8	5.31E-78	-0.2679	0.184	0.505	7.94E-74
POLD2	5.72E-78	0.121732	0.509	0.775	8.54E-74
SYT11	5.76E-78	-0.24835	0.297	0.647	8.60E-74
ATP5J2	5.76E-78	0.320847	0.781	0.922	8.61E-74
RBM38	6.56E-78	-0.32197	0.07	0.344	9.81E-74
PDCD7	6.62E-78	-0.37322	0.123	0.446	9.90E-74
MAF1	6.75E-78	-0.31605	0.275	0.622	1.01E-73
RUVBL1	6.90E-78	-0.10528	0.239	0.554	1.03E-73
OST4	7.15E-78	0.414715	0.734	0.852	1.07E-73
FN3KRP	7.37E-78	-0.17972	0.167	0.473	1.10E-73
BCAP31	7.42E-78	0.441872	0.562	0.662	1.11E-73
DDX24	7.54E-78	0.200221	0.402	0.615	1.13E-73
THAP11	7.68E-78	-0.33291	0.099	0.4	1.15E-73
MPLKIP	7.86E-78	0.123799	0.428	0.673	1.17E-73
SRSF7	7.93E-78	-0.13989	0.504	0.84	1.19E-73

OAZ2	8.14E-78	0.211515	0.427	0.651	1.22E-73
PSMB2	8.39E-78	0.326889	0.609	0.791	1.25E-73
PIN4	8.52E-78	0.240681	0.422	0.631	1.27E-73
GCSH	8.55E-78	-0.45317	0.276	0.653	1.28E-73
UFC1	9.06E-78	0.186034	0.484	0.712	1.35E-73
FCGRT	9.07E-78	0.814878	0.452	0.344	1.36E-73
RAB11A	9.41E-78	0.228585	0.428	0.637	1.41E-73
PSMD8	1.04E-77	0.280918	0.689	0.873	1.56E-73
NHP2	1.05E-77	0.143406	0.561	0.798	1.56E-73
RBM26	1.19E-77	-0.29261	0.117	0.414	1.77E-73
AP3S1	1.39E-77	0.136411	0.398	0.605	2.08E-73
IKBIP	1.43E-77	-0.18466	0.194	0.502	2.14E-73
DNAJB6	1.46E-77	0.189572	0.654	0.848	2.18E-73
COX16	1.58E-77	-0.10505	0.403	0.716	2.36E-73
YWHAQ	1.68E-77	-0.38169	0.575	0.916	2.52E-73
WNK1	1.69E-77	-0.21926	0.168	0.473	2.52E-73
YIPF3	1.76E-77	0.281678	0.48	0.652	2.64E-73
HNRNPC	1.88E-77	0.13134	0.677	0.899	2.82E-73
RAD23B	1.92E-77	-0.17542	0.318	0.625	2.87E-73
PARP2	1.93E-77	-0.38375	0.097	0.391	2.89E-73
CTNNBL1	1.99E-77	-0.19744	0.24	0.565	2.98E-73
POLD4	2.03E-77	0.80185	0.364	0.31	3.04E-73
ZNF580	2.22E-77	-0.38099	0.182	0.522	3.32E-73
NGFRAP1	2.41E-77	0.295299	0.78	0.953	3.60E-73
POLR2A	2.51E-77	-0.23208	0.131	0.417	3.76E-73
RAE1	2.58E-77	-0.26566	0.221	0.548	3.85E-73
RHOA	2.58E-77	0.286874	0.697	0.857	3.85E-73
RP11-71N1	2.63E-77	-0.28531	0.056	0.3	3.92E-73
VIMP	2.65E-77	0.258625	0.435	0.611	3.97E-73
DCTN6	2.73E-77	0.142453	0.392	0.616	4.08E-73
CORO2B	2.82E-77	-0.30862	0.031	0.264	4.22E-73
PRPF8	2.85E-77	-0.21954	0.172	0.465	4.26E-73
NPTXR	2.94E-77	-0.2928	0.037	0.275	4.40E-73
EPN1	3.07E-77	-0.36715	0.208	0.558	4.58E-73
SBNO1	3.11E-77	-0.11686	0.222	0.505	4.65E-73
PSMA5	3.53E-77	0.137886	0.411	0.64	5.28E-73
CAP1	3.76E-77	0.212099	0.497	0.657	5.62E-73
EIF3D	3.83E-77	-0.13506	0.339	0.652	5.73E-73
SDHD	3.93E-77	0.364044	0.426	0.549	5.88E-73
CECR5	4.07E-77	-0.33273	0.145	0.47	6.08E-73
IFT27	4.13E-77	-0.23594	0.157	0.458	6.18E-73
TMEM14B	4.33E-77	0.127187	0.463	0.72	6.47E-73
AMD1	4.47E-77	0.328865	0.39	0.516	6.68E-73
CENPT	4.48E-77	-0.43149	0.147	0.486	6.70E-73
PRKCA	4.60E-77	-0.36406	0.083	0.377	6.87E-73
NELFB	4.68E-77	-0.27388	0.083	0.351	6.99E-73
SF3B1	4.70E-77	-0.22476	0.322	0.654	7.03E-73
SSNA1	4.71E-77	-0.2189	0.322	0.662	7.04E-73
MANF	4.72E-77	0.156584	0.46	0.68	7.05E-73
PAK2	4.92E-77	-0.34522	0.208	0.549	7.35E-73
PPP2CB	4.97E-77	-0.336	0.205	0.552	7.42E-73

NCKAP1	4.97E-77	-0.25938	0.16	0.467	7.42E-73
SELK	5.44E-77	0.24461	0.683	0.862	8.13E-73
PDE6D	5.44E-77	-0.31946	0.114	0.411	8.13E-73
NT5C3A	5.57E-77	-0.21096	0.28	0.602	8.33E-73
POLDIP3	5.62E-77	-0.27483	0.12	0.416	8.40E-73
OLFM1	5.76E-77	-0.38799	0.15	0.477	8.61E-73
MPC2	6.04E-77	0.35362	0.558	0.698	9.02E-73
LAMTOR2	6.28E-77	0.317947	0.514	0.691	9.38E-73
SAFB	6.39E-77	-0.19953	0.22	0.528	9.55E-73
UCHL1	6.43E-77	0.345109	0.534	0.673	9.61E-73
ATAD3A	6.76E-77	-0.29702	0.108	0.399	1.01E-72
SLC39A6	7.31E-77	-0.26762	0.278	0.626	1.09E-72
TIMM22	7.42E-77	-0.20589	0.185	0.5	1.11E-72
ANXA5	7.47E-77	0.385686	0.753	0.837	1.12E-72
BRD3	7.66E-77	-0.41379	0.156	0.494	1.14E-72
SNRPE	7.66E-77	-0.12593	0.548	0.873	1.15E-72
FAM192A	8.38E-77	-0.21787	0.264	0.598	1.25E-72
DDX52	8.57E-77	-0.258	0.161	0.473	1.28E-72
COX6B1	8.81E-77	0.374036	0.805	0.906	1.32E-72
TOMM22	9.45E-77	-0.17201	0.331	0.675	1.41E-72
ARID5B	9.69E-77	0.673975	0.38	0.344	1.45E-72
TRMU	1.08E-76	-0.26618	0.08	0.353	1.61E-72
C5orf24	1.08E-76	-0.29639	0.23	0.569	1.61E-72
EXTL3	1.10E-76	-0.28004	0.058	0.315	1.64E-72
MLEC	1.10E-76	-0.2685	0.305	0.648	1.64E-72
APMAP	1.13E-76	-0.15938	0.35	0.678	1.68E-72
TIMM50	1.26E-76	-0.23343	0.248	0.572	1.88E-72
VCP	1.27E-76	-0.18953	0.305	0.601	1.90E-72
UXT	1.31E-76	0.330924	0.506	0.684	1.96E-72
GRHPR	1.32E-76	-0.29964	0.265	0.612	1.97E-72
ROCK1	1.34E-76	-0.31457	0.173	0.498	2.01E-72
C1orf35	1.47E-76	-0.22985	0.213	0.525	2.19E-72
C7orf55-L	1.49E-76	-0.32137	0.301	0.654	2.23E-72
SMARCC2	1.59E-76	-0.27009	0.172	0.488	2.37E-72
CCT6A	1.59E-76	-0.1091	0.534	0.842	2.38E-72
CALR	1.71E-76	0.32595	0.683	0.82	2.55E-72
SLC25A3	1.85E-76	0.213136	0.66	0.868	2.77E-72
HSD17B10	1.85E-76	0.236947	0.514	0.735	2.77E-72
ING2	1.92E-76	-0.3175	0.179	0.504	2.87E-72
CTPS1	1.98E-76	-0.40445	0.06	0.341	2.95E-72
CHPF	2.18E-76	0.124232	0.299	0.463	3.26E-72
GAP43	2.19E-76	0.829409	0.713	0.638	3.27E-72
RSRC1	2.23E-76	-0.20205	0.25	0.585	3.33E-72
ERI1	2.24E-76	-0.16008	0.097	0.343	3.35E-72
TMED4	2.27E-76	0.198157	0.449	0.657	3.39E-72
TARDBP	2.29E-76	-0.20651	0.228	0.541	3.42E-72
COMMD7	2.32E-76	-0.39863	0.247	0.607	3.47E-72
CSRP2	2.35E-76	0.651821	0.549	0.668	3.52E-72
ERAL1	2.40E-76	-0.16687	0.194	0.5	3.59E-72
EPN2	2.41E-76	-0.35987	0.138	0.462	3.60E-72
HOMER1	2.42E-76	-0.44706	0.064	0.354	3.61E-72

DR1	2.47E-76	-0.35536	0.154	0.48	3.69E-72
HLA-C	2.54E-76	0.606924	0.809	0.716	3.80E-72
TNPO2	2.58E-76	-0.20945	0.141	0.42	3.86E-72
FDX1	2.59E-76	-0.25107	0.2	0.514	3.87E-72
TMEM147	2.65E-76	0.309966	0.586	0.725	3.96E-72
NKIRAS2	2.79E-76	-0.2074	0.191	0.5	4.16E-72
JADE1	2.87E-76	-0.39117	0.104	0.409	4.28E-72
TGOLN2	2.98E-76	-0.19873	0.221	0.528	4.46E-72
PIPOX	3.13E-76	-0.4388	0.057	0.332	4.67E-72
AC009501.	3.23E-76	1.310045	0.335	0.115	4.82E-72
ASF1A	3.36E-76	-0.37424	0.162	0.498	5.02E-72
RWDD1	3.46E-76	0.205176	0.516	0.72	5.17E-72
CTXN1	3.65E-76	-0.37742	0.065	0.349	5.46E-72
ELP6	3.74E-76	-0.18906	0.169	0.473	5.60E-72
MAGEF1	3.99E-76	-0.27338	0.33	0.675	5.96E-72
SDHC	4.16E-76	0.432782	0.558	0.669	6.21E-72
GNL1	4.29E-76	-0.25713	0.203	0.528	6.41E-72
DDX46	4.29E-76	-0.22751	0.334	0.681	6.42E-72
HTRA1	4.44E-76	-0.34227	0.27	0.61	6.63E-72
CDC5L	4.67E-76	-0.36915	0.214	0.56	6.98E-72
NIN	5.71E-76	-0.403	0.04	0.299	8.53E-72
TAF11	5.85E-76	-0.21996	0.178	0.486	8.75E-72
TKT	6.22E-76	0.34477	0.479	0.598	9.30E-72
ATP5G2	6.24E-76	0.359341	0.789	0.914	9.33E-72
SLC2A4RG	6.48E-76	-0.39172	0.08	0.375	9.68E-72
MLST8	7.72E-76	-0.18818	0.19	0.488	1.15E-71
ATP5F1	8.11E-76	0.343275	0.639	0.773	1.21E-71
PRRC2C	8.14E-76	-0.16526	0.361	0.693	1.22E-71
BEST3	8.83E-76	-0.67941	0.041	0.307	1.32E-71
GTF3A	9.95E-76	-0.29805	0.35	0.699	1.49E-71
DPM3	1.24E-75	0.13129	0.308	0.535	1.85E-71
ARL1	1.47E-75	0.513756	0.425	0.454	2.20E-71
MTHFD1	1.52E-75	-0.37367	0.129	0.451	2.27E-71
POLR2I	1.61E-75	0.311561	0.624	0.791	2.41E-71
CCDC85B	1.74E-75	-0.34279	0.256	0.604	2.60E-71
ABHD2	1.79E-75	-0.22421	0.123	0.4	2.68E-71
SAP30L	1.80E-75	-0.41478	0.037	0.294	2.69E-71
EBPL	1.90E-75	-0.41146	0.179	0.517	2.84E-71
SLC25A4	1.91E-75	0.14347	0.44	0.662	2.85E-71
C19orf70	2.09E-75	0.44629	0.584	0.667	3.12E-71
ARPC5L	2.46E-75	-0.24079	0.312	0.644	3.68E-71
SEC13	2.49E-75	0.17384	0.343	0.574	3.73E-71
COX5A	2.55E-75	-0.32068	0.467	0.822	3.81E-71
NARF	2.55E-75	-0.15725	0.266	0.579	3.81E-71
TCEB1	2.70E-75	0.122233	0.579	0.823	4.04E-71
PKN1	3.36E-75	-0.35563	0.129	0.442	5.03E-71
STIL	3.55E-75	-0.29418	0.006	0.195	5.31E-71
NELFCD	3.79E-75	-0.28932	0.282	0.631	5.66E-71
EIF3B	3.80E-75	-0.17305	0.195	0.485	5.68E-71
ITGA7	4.01E-75	-0.25734	0.068	0.317	6.00E-71
CMSS1	4.01E-75	-0.12833	0.291	0.602	6.00E-71

MDK	4.08E-75	0.170445	0.583	0.867	6.09E-71
GRIA3	4.09E-75	-0.25739	0.095	0.358	6.11E-71
MVB12A	4.47E-75	-0.14552	0.24	0.541	6.68E-71
ARPC5	4.59E-75	0.272089	0.596	0.793	6.86E-71
ZNF414	4.86E-75	-0.36372	0.089	0.383	7.26E-71
ZNF138	4.89E-75	-0.37038	0.097	0.393	7.31E-71
DCAF15	4.99E-75	-0.32027	0.065	0.331	7.45E-71
MUS81	5.13E-75	-0.26207	0.12	0.402	7.66E-71
SOX12	5.64E-75	-0.2871	0.038	0.278	8.44E-71
SMAD1	6.07E-75	-0.27994	0.119	0.411	9.07E-71
POLR2C	6.75E-75	0.139047	0.385	0.595	1.01E-70
EIF3M	6.79E-75	0.298473	0.513	0.659	1.01E-70
RANBP3	6.88E-75	-0.26381	0.152	0.447	1.03E-70
SDF4	7.72E-75	-0.34483	0.288	0.633	1.15E-70
DDX42	7.83E-75	-0.20397	0.169	0.449	1.17E-70
YWHAG	7.96E-75	-0.17259	0.29	0.6	1.19E-70
THYN1	8.41E-75	0.176641	0.41	0.647	1.26E-70
ARID4A	8.55E-75	-0.32759	0.15	0.465	1.28E-70
NUDC	8.65E-75	0.126487	0.597	0.812	1.29E-70
VMA21	8.67E-75	-0.26498	0.192	0.507	1.30E-70
NXT1	8.73E-75	-0.13847	0.282	0.573	1.31E-70
RAD18	8.88E-75	-0.36327	0.036	0.28	1.33E-70
GNAQ	8.93E-75	-0.3715	0.122	0.437	1.33E-70
FAM110A	9.19E-75	-0.3747	0.02	0.249	1.37E-70
SF3B5	1.03E-74	0.270575	0.58	0.765	1.54E-70
KBTBD2	1.12E-74	-0.35583	0.066	0.343	1.67E-70
STARD7	1.13E-74	-0.32661	0.236	0.574	1.68E-70
ACSL3	1.26E-74	-0.48411	0.225	0.591	1.89E-70
GPM6B	1.31E-74	0.46462	0.823	0.937	1.95E-70
PPAT	1.31E-74	-0.45442	0.099	0.411	1.95E-70
ISCU	1.33E-74	0.11835	0.469	0.689	1.99E-70
MT1F	1.43E-74	0.658988	0.408	0.34	2.14E-70
SUPT20H	1.56E-74	-0.26347	0.134	0.433	2.33E-70
PSMD7	1.61E-74	0.202862	0.473	0.699	2.40E-70
ZIC1	1.65E-74	-0.45103	0.052	0.322	2.46E-70
OCIAD2	1.73E-74	1.041238	0.645	0.495	2.59E-70
HPS4	1.74E-74	-0.16846	0.102	0.351	2.60E-70
PHF20L1	1.76E-74	-0.26469	0.216	0.526	2.63E-70
PITPNA-AS	1.77E-74	-0.11324	0.176	0.454	2.64E-70
NDUFS8	1.90E-74	0.292703	0.606	0.804	2.84E-70
AC004381.	1.96E-74	-0.44336	0.014	0.228	2.93E-70
OGFOD3	2.10E-74	-0.3454	0.109	0.409	3.13E-70
TXNL1	2.17E-74	0.313433	0.509	0.667	3.25E-70
POLR2B	2.26E-74	-0.43124	0.17	0.51	3.38E-70
RTCB	2.29E-74	-0.24622	0.199	0.507	3.42E-70
ARL8A	2.29E-74	-0.22012	0.14	0.427	3.43E-70
HOOK3	2.34E-74	-0.26969	0.161	0.464	3.49E-70
TAF1D	2.40E-74	0.414445	0.354	0.422	3.59E-70
NPDC1	2.47E-74	-0.39051	0.225	0.567	3.70E-70
MKRN1	2.61E-74	-0.22604	0.309	0.631	3.90E-70
SF3B3	2.62E-74	-0.32771	0.145	0.459	3.92E-70

SLC16A1	2.70E-74	0.288811	0.365	0.479	4.03E-70
PIN1	2.88E-74	-0.29158	0.478	0.84	4.31E-70
BLVRA	2.91E-74	0.326147	0.372	0.558	4.35E-70
RECQL4	3.09E-74	-0.36786	0.015	0.232	4.62E-70
UFM1	3.19E-74	0.225409	0.33	0.474	4.76E-70
TMEM203	3.23E-74	-0.38846	0.159	0.491	4.83E-70
DNAJC19	3.25E-74	0.231463	0.408	0.621	4.86E-70
DDOST	3.32E-74	0.279503	0.446	0.632	4.96E-70
CNPY1	3.37E-74	-0.51439	0.04	0.299	5.04E-70
ARHGAP35	3.38E-74	-0.37194	0.063	0.34	5.05E-70
CMPK1	3.44E-74	-0.17934	0.308	0.607	5.14E-70
PSMD2	3.48E-74	-0.13529	0.402	0.715	5.21E-70
NIPBL	3.71E-74	-0.35277	0.131	0.441	5.55E-70
GPATCH8	4.06E-74	-0.166	0.232	0.533	6.07E-70
GHITM	4.11E-74	0.42503	0.473	0.577	6.14E-70
TIMM10	4.19E-74	-0.28351	0.272	0.628	6.26E-70
CIT	4.25E-74	-0.31357	0.02	0.236	6.35E-70
CCZ1	4.49E-74	-0.24858	0.286	0.61	6.70E-70
PLTP	4.57E-74	0.836046	0.463	0.385	6.83E-70
PAFAH1B2	4.78E-74	-0.25902	0.234	0.546	7.14E-70
RCC2	4.88E-74	-0.31	0.079	0.354	7.30E-70
SEMA6A	5.01E-74	-0.36375	0.109	0.414	7.49E-70
SRGAP2	5.12E-74	-0.39899	0.071	0.358	7.66E-70
TMEM183A	5.16E-74	-0.24177	0.211	0.517	7.71E-70
LCORL	5.36E-74	-0.30027	0.051	0.296	8.01E-70
GBAS	5.55E-74	-0.16737	0.348	0.641	8.29E-70
AP2B1	5.77E-74	-0.23921	0.264	0.589	8.63E-70
MSH2	5.94E-74	-0.41241	0.077	0.364	8.88E-70
TMEM9	5.97E-74	0.364364	0.428	0.547	8.92E-70
ZFAND2B	6.04E-74	-0.13815	0.143	0.402	9.03E-70
TRAF7	6.37E-74	-0.29211	0.111	0.407	9.51E-70
SUV39H2	6.47E-74	-0.30106	0.036	0.265	9.67E-70
SCOC	6.73E-74	0.154101	0.436	0.643	1.01E-69
TRAPPC4	7.36E-74	0.272873	0.463	0.62	1.10E-69
ATF6B	7.46E-74	-0.16439	0.272	0.581	1.11E-69
PSMC4	7.49E-74	0.275356	0.482	0.652	1.12E-69
NLGN3	8.97E-74	-0.3538	0.04	0.284	1.34E-69
FADS1	1.03E-73	-0.3539	0.16	0.477	1.54E-69
IAH1	1.05E-73	0.155224	0.384	0.599	1.57E-69
DARS	1.07E-73	0.391636	0.448	0.523	1.60E-69
FKBP2	1.12E-73	0.533505	0.599	0.643	1.67E-69
IER2	1.12E-73	-0.37662	0.426	0.778	1.68E-69
DYNC1H1	1.13E-73	-0.25456	0.212	0.526	1.69E-69
HDDC2	1.18E-73	0.123946	0.365	0.614	1.76E-69
CHMP3	1.22E-73	-0.12159	0.268	0.574	1.83E-69
WAC	1.26E-73	-0.27966	0.147	0.448	1.88E-69
NAT14	1.45E-73	-0.2601	0.117	0.394	2.17E-69
RALA	1.48E-73	-0.13053	0.329	0.63	2.22E-69
ABHD3	1.51E-73	-0.39709	0.118	0.422	2.26E-69
CAPZB	1.57E-73	0.259803	0.551	0.741	2.34E-69
GAMT	1.69E-73	-0.42673	0.21	0.562	2.52E-69

RNF168	1.69E-73	-0.36765	0.086	0.374	2.52E-69
APRT	1.72E-73	0.569712	0.462	0.51	2.57E-69
TERF1	1.74E-73	-0.25427	0.267	0.604	2.59E-69
ZFHX4	1.76E-73	-0.30249	0.09	0.365	2.63E-69
PSMC2	1.83E-73	0.245075	0.474	0.658	2.74E-69
DYNC1LI2	1.85E-73	-0.20812	0.274	0.59	2.76E-69
SPPL3	1.86E-73	-0.29322	0.102	0.379	2.77E-69
PSMB3	1.87E-73	0.400328	0.701	0.842	2.79E-69
ATP5O	1.94E-73	0.208652	0.689	0.893	2.90E-69
MTDH	2.03E-73	-0.37233	0.423	0.786	3.04E-69
SFRP1	2.07E-73	-0.2992	0.036	0.259	3.09E-69
SP3	2.08E-73	-0.32285	0.132	0.433	3.10E-69
CANX	2.17E-73	0.186892	0.542	0.715	3.24E-69
PSMB4	2.17E-73	0.217816	0.4	0.612	3.24E-69
NDUFS4	2.30E-73	0.209323	0.503	0.71	3.44E-69
BSG	2.42E-73	0.302844	0.776	0.915	3.62E-69
ARMC10	2.57E-73	-0.20716	0.241	0.526	3.84E-69
CD276	2.57E-73	-0.11614	0.145	0.407	3.85E-69
TMEM256	2.72E-73	0.119902	0.338	0.577	4.06E-69
PITHD1	2.77E-73	-0.2754	0.29	0.619	4.14E-69
RQCD1	2.80E-73	-0.32169	0.122	0.419	4.18E-69
HSP90AB1	2.80E-73	0.127296	0.755	0.933	4.19E-69
ATP1A1	3.14E-73	0.245422	0.437	0.552	4.69E-69
POLE3	3.31E-73	-0.12622	0.255	0.541	4.95E-69
GGNBP2	3.38E-73	-0.12634	0.334	0.62	5.05E-69
PRDX3	3.39E-73	0.429363	0.454	0.573	5.07E-69
HIF1A	3.40E-73	-0.23649	0.313	0.657	5.09E-69
PPM1B	3.62E-73	-0.31873	0.209	0.533	5.41E-69
CDKN1B	3.85E-73	-0.36033	0.204	0.536	5.76E-69
SPTSSA	4.01E-73	0.409239	0.53	0.631	6.00E-69
EIF3J	4.07E-73	-0.14714	0.29	0.594	6.08E-69
TIMM17A	4.42E-73	0.323875	0.406	0.567	6.60E-69
ATP5J	4.70E-73	0.284519	0.716	0.88	7.03E-69
MIIP	4.82E-73	-0.1928	0.126	0.381	7.20E-69
HIVEP3	5.36E-73	-0.33165	0.043	0.294	8.01E-69
STRN4	5.40E-73	-0.31906	0.063	0.326	8.07E-69
CRMP1	5.54E-73	-0.48352	0.172	0.515	8.29E-69
ACLY	5.57E-73	-0.21606	0.129	0.401	8.32E-69
PSMA1	6.21E-73	0.434957	0.627	0.731	9.29E-69
RANGRF	6.28E-73	-0.14571	0.161	0.449	9.38E-69
MMP16	6.38E-73	-0.3851	0.028	0.265	9.54E-69
BAALC	7.19E-73	-0.22146	0.356	0.672	1.07E-68
PDCD5	7.25E-73	0.216729	0.601	0.817	1.08E-68
CNTRL	7.69E-73	-0.35104	0.068	0.34	1.15E-68
TACC1	7.72E-73	-0.38532	0.178	0.514	1.15E-68
TM2D1	8.07E-73	0.283669	0.385	0.554	1.21E-68
CRLS1	8.74E-73	-0.38476	0.195	0.531	1.31E-68
NOL11	8.90E-73	-0.1462	0.17	0.436	1.33E-68
SUPT4H1	9.55E-73	0.200597	0.412	0.614	1.43E-68
SLC35B1	9.77E-73	-0.12567	0.329	0.641	1.46E-68
DHX30	1.03E-72	-0.13347	0.189	0.479	1.54E-68

DNAJB9	1.07E-72	0.819774	0.467	0.323	1.60E-68
PSRC1	1.11E-72	-0.38295	0.238	0.591	1.65E-68
EIF5	1.15E-72	-0.1914	0.551	0.868	1.72E-68
SLC39A1	1.16E-72	0.21559	0.372	0.552	1.73E-68
PPA1	1.26E-72	0.329378	0.454	0.599	1.89E-68
NFIC	1.29E-72	-0.31911	0.388	0.753	1.93E-68
TOMM20	1.38E-72	0.142734	0.448	0.657	2.06E-68
RBM10	1.45E-72	-0.31585	0.148	0.458	2.17E-68
HIST1H2BH	1.55E-72	-0.36837	0.004	0.184	2.32E-68
ANKRD12	1.55E-72	0.246637	0.362	0.522	2.32E-68
EZR	1.57E-72	-0.24674	0.237	0.562	2.35E-68
LARS	1.63E-72	-0.14818	0.296	0.583	2.43E-68
ZNF714	1.84E-72	-0.413	0.076	0.353	2.75E-68
SFT2D1	1.87E-72	0.191727	0.391	0.584	2.79E-68
DCPS	1.90E-72	-0.20069	0.131	0.402	2.85E-68
IFI27L2	1.94E-72	0.507653	0.427	0.507	2.89E-68
GTPBP6	2.12E-72	-0.29577	0.119	0.409	3.17E-68
HNRNPLL	2.18E-72	-0.38805	0.129	0.442	3.26E-68
NRGN	2.23E-72	-0.26556	0.034	0.252	3.33E-68
AAMP	2.35E-72	0.197111	0.438	0.627	3.51E-68
UBE2M	2.42E-72	-0.31842	0.145	0.453	3.62E-68
RBBP8	2.52E-72	-0.38529	0.079	0.367	3.76E-68
TBC1D7	2.65E-72	-0.25951	0.084	0.347	3.97E-68
SH3GL1	2.66E-72	-0.30855	0.191	0.51	3.98E-68
AKR7A2	2.92E-72	-0.31791	0.229	0.548	4.37E-68
SNRNP27	3.15E-72	-0.13241	0.303	0.611	4.71E-68
SORBS3	3.43E-72	-0.33531	0.12	0.416	5.12E-68
DCXR	3.79E-72	-0.26293	0.396	0.751	5.67E-68
PHF20	3.87E-72	-0.1198	0.242	0.533	5.78E-68
OGFR	3.87E-72	-0.25688	0.161	0.454	5.78E-68
RP11-390F	4.05E-72	-0.27349	0.047	0.283	6.05E-68
KMT2C	4.13E-72	-0.27411	0.159	0.454	6.18E-68
CBX6	4.29E-72	-0.26466	0.157	0.448	6.42E-68
RABEP1	4.33E-72	-0.40803	0.146	0.47	6.48E-68
RBBP6	4.42E-72	-0.13765	0.241	0.519	6.60E-68
RCAN1	4.49E-72	0.695417	0.366	0.409	6.71E-68
ENY2	4.58E-72	0.14166	0.54	0.786	6.84E-68
RALGAPA2	4.62E-72	-0.2897	0.036	0.265	6.91E-68
ST3GAL4	4.91E-72	-0.4379	0.06	0.335	7.34E-68
RBFOX2	5.12E-72	-0.37229	0.098	0.39	7.65E-68
COX5B	5.27E-72	0.336769	0.812	0.916	7.88E-68
ARL3	5.47E-72	0.129242	0.344	0.563	8.18E-68
CADM3	5.50E-72	0.995328	0.316	0.048	8.22E-68
PSMB6	5.58E-72	0.265406	0.672	0.852	8.33E-68
SLC25A1	5.60E-72	-0.41861	0.094	0.391	8.37E-68
ABCF1	5.67E-72	-0.29005	0.226	0.548	8.48E-68
DBNL	5.74E-72	-0.1198	0.253	0.547	8.57E-68
STAU1	5.85E-72	-0.19809	0.299	0.612	8.74E-68
TCEA1	5.86E-72	-0.10568	0.457	0.756	8.76E-68
DVL3	6.08E-72	-0.27768	0.085	0.354	9.09E-68
RPN1	6.16E-72	0.290116	0.406	0.578	9.21E-68

SMIM7	6.20E-72	0.261401	0.556	0.728	9.27E-68
UBE2D2	6.32E-72	-0.27172	0.473	0.815	9.45E-68
POLR2D	6.33E-72	-0.33005	0.106	0.401	9.46E-68
BRD4	6.56E-72	-0.18474	0.261	0.575	9.81E-68
FAM104A	6.59E-72	-0.23961	0.179	0.486	9.84E-68
DUSP6	6.87E-72	-0.11532	0.177	0.435	1.03E-67
CCDC59	6.88E-72	0.149528	0.288	0.491	1.03E-67
KHDRBS3	7.00E-72	-0.3028	0.155	0.462	1.05E-67
ZMAT2	7.11E-72	0.198994	0.387	0.575	1.06E-67
EFEMP2	7.32E-72	0.19521	0.27	0.443	1.09E-67
SNRPD1	7.37E-72	-0.11425	0.541	0.858	1.10E-67
DPM1	7.43E-72	0.125814	0.394	0.631	1.11E-67
SPRED1	7.47E-72	-0.37906	0.069	0.344	1.12E-67
TMEM176B	7.51E-72	0.951898	0.335	0.091	1.12E-67
MTHFD2L	7.58E-72	-0.17458	0.15	0.433	1.13E-67
DKC1	7.60E-72	-0.23417	0.22	0.542	1.14E-67
MYL12A	7.71E-72	0.577295	0.455	0.477	1.15E-67
JTB	8.48E-72	-0.16421	0.491	0.791	1.27E-67
NDUFA8	9.40E-72	0.167671	0.464	0.674	1.40E-67
ACTR3	1.00E-71	0.118217	0.48	0.7	1.50E-67
ZNF146	1.01E-71	-0.12568	0.213	0.481	1.51E-67
SUN2	1.04E-71	-0.42889	0.109	0.414	1.55E-67
GBP2	1.04E-71	1.016515	0.341	0.085	1.55E-67
KLF6	1.06E-71	0.482414	0.462	0.505	1.59E-67
LRPAP1	1.15E-71	0.256815	0.481	0.649	1.72E-67
RBM42	1.16E-71	-0.18813	0.363	0.669	1.73E-67
CIRBP	1.26E-71	0.291605	0.811	0.94	1.88E-67
EEF1D	1.28E-71	0.324138	0.782	0.911	1.91E-67
TOPBP1	1.28E-71	-0.34949	0.057	0.314	1.91E-67
ARL2	1.32E-71	0.325572	0.435	0.591	1.98E-67
WDR18	1.35E-71	-0.3294	0.196	0.521	2.01E-67
SRP72	1.41E-71	-0.18095	0.383	0.714	2.11E-67
CDC42BPA	1.43E-71	-0.38149	0.137	0.448	2.13E-67
NUDT2	1.48E-71	-0.27177	0.087	0.352	2.21E-67
G2E3	1.55E-71	-0.43996	0.071	0.346	2.32E-67
TXNRD1	1.67E-71	-0.23532	0.176	0.469	2.50E-67
ZNF367	2.05E-71	-0.42588	0.019	0.238	3.07E-67
ARID4B	2.07E-71	-0.22574	0.281	0.599	3.09E-67
CUEDC1	2.20E-71	-0.25778	0.042	0.284	3.29E-67
NUCB2	2.47E-71	0.101083	0.39	0.619	3.70E-67
LRRTM2	2.56E-71	-0.36421	0.028	0.252	3.83E-67
GSK3A	2.75E-71	-0.29175	0.119	0.401	4.11E-67
LYRM2	2.88E-71	-0.24328	0.255	0.57	4.30E-67
TCF3	3.01E-71	-0.31565	0.073	0.342	4.50E-67
TDG	3.03E-71	-0.16322	0.18	0.459	4.53E-67
SV2A	3.09E-71	-0.27206	0.133	0.423	4.62E-67
GTF3C5	3.13E-71	-0.28364	0.138	0.43	4.68E-67
YTHDC1	3.16E-71	-0.31479	0.212	0.53	4.72E-67
ATP6V0D1	3.27E-71	0.142785	0.364	0.569	4.89E-67
UBE2K	3.33E-71	-0.11258	0.224	0.502	4.98E-67
AMZ2	3.59E-71	0.13086	0.341	0.589	5.36E-67

NAE1	3.68E-71	-0.14668	0.341	0.663	5.51E-67
SPRY2	3.71E-71	-0.47635	0.134	0.458	5.55E-67
ACTL6A	3.98E-71	-0.23474	0.246	0.572	5.95E-67
RNASEH1	4.12E-71	-0.18064	0.177	0.46	6.16E-67
RBX1	4.45E-71	-0.12524	0.502	0.825	6.65E-67
PCIF1	4.59E-71	-0.17039	0.069	0.307	6.87E-67
USP16	4.82E-71	-0.11543	0.292	0.588	7.20E-67
NDUFB9	5.23E-71	0.196495	0.665	0.86	7.82E-67
TTLL7	5.32E-71	-0.26323	0.167	0.464	7.95E-67
THAP7	5.49E-71	-0.18232	0.164	0.438	8.20E-67
NAB1	5.56E-71	-0.33816	0.055	0.314	8.30E-67
DEAF1	5.71E-71	-0.3177	0.118	0.41	8.54E-67
PSMG2	5.82E-71	0.131686	0.39	0.621	8.70E-67
TTL	5.98E-71	-0.31283	0.059	0.312	8.94E-67
SCAND1	6.24E-71	-0.37234	0.436	0.794	9.32E-67
EIF1AX	6.29E-71	-0.1024	0.499	0.798	9.40E-67
HERC2	6.61E-71	-0.26865	0.111	0.385	9.88E-67
RBMS1	6.69E-71	-0.32019	0.104	0.39	9.99E-67
IMP4	6.76E-71	0.171678	0.352	0.54	1.01E-66
C19orf25	7.17E-71	-0.22193	0.221	0.515	1.07E-66
ITFG1	7.41E-71	-0.15575	0.255	0.553	1.11E-66
RBM4	8.29E-71	-0.27089	0.137	0.432	1.24E-66
WDHD1	8.40E-71	-0.35776	0.013	0.216	1.26E-66
ARMC6	8.42E-71	-0.30814	0.102	0.384	1.26E-66
AP2S1	8.46E-71	-0.2516	0.487	0.83	1.26E-66
ZEB2	8.65E-71	-0.3506	0.13	0.433	1.29E-66
PRTFDC1	9.58E-71	-0.26062	0.109	0.38	1.43E-66
TRABD	9.65E-71	-0.26748	0.105	0.377	1.44E-66
MTSS1	1.00E-70	-0.53669	0.059	0.333	1.50E-66
TCERG1	1.02E-70	-0.3252	0.154	0.463	1.52E-66
ATAT1	1.19E-70	-0.27706	0.179	0.494	1.77E-66
C12orf57	1.29E-70	0.217952	0.617	0.828	1.93E-66
G6PC3	1.31E-70	0.116476	0.341	0.56	1.96E-66
ARNT2	1.32E-70	-0.29059	0.049	0.284	1.97E-66
BRK1	1.38E-70	0.256362	0.713	0.873	2.07E-66
TAOK1	1.39E-70	-0.31582	0.148	0.449	2.08E-66
HADH	1.47E-70	-0.1869	0.169	0.462	2.20E-66
STX8	1.49E-70	0.281306	0.33	0.506	2.23E-66
RNF7	1.50E-70	0.340873	0.504	0.649	2.24E-66
SMCHD1	1.52E-70	-0.35406	0.121	0.421	2.27E-66
PDGFC	1.75E-70	-0.28968	0.075	0.336	2.62E-66
FABP7	1.82E-70	-0.4705	0.386	0.752	2.72E-66
ASAP1	1.83E-70	-0.35278	0.126	0.43	2.74E-66
YWHAB	1.91E-70	-0.31069	0.526	0.869	2.86E-66
SSBP4	1.93E-70	-0.42122	0.146	0.47	2.88E-66
FEZ2	1.96E-70	-0.23197	0.302	0.604	2.93E-66
TTF1	2.11E-70	-0.31082	0.172	0.484	3.15E-66
UQCRH	2.13E-70	0.129825	0.739	0.927	3.18E-66
AP3D1	2.15E-70	-0.36523	0.223	0.552	3.21E-66
NDUFC2	2.21E-70	0.28363	0.715	0.88	3.31E-66
NKTR	2.23E-70	-0.12327	0.303	0.581	3.33E-66

RHOJ	2.25E-70	-0.19722	0.038	0.258	3.36E-66
RAB3IP	2.25E-70	-0.59866	0.183	0.53	3.37E-66
YIPF4	2.29E-70	-0.10476	0.295	0.565	3.42E-66
EMD	2.30E-70	-0.13967	0.238	0.526	3.44E-66
MYO10	2.37E-70	-0.37407	0.136	0.443	3.54E-66
MDH1	2.42E-70	0.136536	0.552	0.779	3.62E-66
UBE2Q2	2.66E-70	-0.26631	0.112	0.389	3.97E-66
SRI	2.66E-70	0.256387	0.791	0.946	3.98E-66
FARSA	2.82E-70	-0.1005	0.275	0.553	4.22E-66
VMP1	2.84E-70	0.295847	0.472	0.598	4.24E-66
SLC25A33	2.86E-70	-0.25244	0.151	0.431	4.28E-66
DAZAP2	3.01E-70	0.306409	0.461	0.586	4.50E-66
CUL3	3.05E-70	-0.29624	0.119	0.4	4.56E-66
SIGMAR1	3.12E-70	-0.35906	0.106	0.401	4.67E-66
FBXO32	3.22E-70	0.894354	0.305	0.102	4.81E-66
TRAP1	3.93E-70	-0.25927	0.218	0.527	5.87E-66
CDK5	4.16E-70	-0.14324	0.156	0.426	6.22E-66
FAM50A	4.23E-70	-0.38094	0.216	0.553	6.33E-66
VPS4A	4.68E-70	-0.26863	0.248	0.569	6.99E-66
CLTB	4.72E-70	-0.29419	0.289	0.619	7.06E-66
HECTD1	4.95E-70	-0.23717	0.14	0.415	7.40E-66
MED28	5.20E-70	0.288069	0.331	0.49	7.76E-66
DEGS1	5.57E-70	0.193366	0.445	0.62	8.33E-66
SRP19	6.01E-70	0.207066	0.382	0.593	8.98E-66
PCBP2	6.20E-70	-0.23212	0.545	0.873	9.26E-66
RER1	6.48E-70	-0.12208	0.408	0.705	9.68E-66
APOA1BP	6.66E-70	-0.1516	0.326	0.631	9.96E-66
WIZ	7.14E-70	-0.20827	0.055	0.281	1.07E-65
PRDX2	7.73E-70	0.293752	0.774	0.932	1.15E-65
SEPHS2	8.59E-70	0.161899	0.3	0.43	1.28E-65
SOX21	8.92E-70	-0.32561	0.042	0.283	1.33E-65
EDF1	9.55E-70	0.161006	0.688	0.886	1.43E-65
9-Sep	9.89E-70	-0.30485	0.277	0.612	1.48E-65
PMP2	9.91E-70	0.181828	0.434	0.677	1.48E-65
APPL1	1.00E-69	-0.27666	0.207	0.516	1.50E-65
RBM6	1.01E-69	-0.23048	0.206	0.507	1.50E-65
POLR2J3	1.01E-69	-0.17586	0.297	0.621	1.50E-65
TBCD	1.03E-69	-0.32258	0.069	0.336	1.53E-65
RAB5A	1.03E-69	0.151814	0.397	0.584	1.54E-65
OXCT1	1.04E-69	-0.346	0.118	0.412	1.56E-65
MAP7D1	1.05E-69	-0.32548	0.189	0.509	1.57E-65
NIF3L1	1.09E-69	-0.18585	0.108	0.359	1.63E-65
TMX2	1.14E-69	0.213571	0.416	0.6	1.71E-65
HNRNPH1	1.19E-69	-0.31199	0.376	0.727	1.78E-65
PANK2	1.21E-69	-0.33627	0.114	0.411	1.81E-65
TSPO	1.25E-69	0.40943	0.422	0.531	1.87E-65
PSMD13	1.30E-69	0.190595	0.406	0.59	1.95E-65
ZNF724P	1.32E-69	-0.32879	0.016	0.216	1.97E-65
RMDN3	1.34E-69	-0.27938	0.158	0.459	2.01E-65
USF2	1.40E-69	-0.29887	0.154	0.451	2.09E-65
NIFK	1.42E-69	0.109396	0.366	0.599	2.13E-65

ZNF292	1.52E-69	-0.29081	0.173	0.477	2.27E-65
TSSC1	1.52E-69	-0.15342	0.189	0.468	2.27E-65
GGA1	1.56E-69	-0.15198	0.143	0.414	2.33E-65
TSEN15	1.60E-69	-0.19209	0.206	0.499	2.40E-65
IMPAD1	1.65E-69	-0.25307	0.102	0.367	2.47E-65
SEC11A	1.66E-69	0.25681	0.503	0.716	2.48E-65
PRPSAP2	1.98E-69	-0.10988	0.219	0.494	2.96E-65
VDAC1	1.99E-69	0.309246	0.652	0.781	2.98E-65
KPNA3	2.09E-69	-0.3314	0.186	0.501	3.13E-65
PRR13	2.12E-69	0.577965	0.506	0.53	3.17E-65
GRIA2	2.30E-69	-0.45323	0.106	0.407	3.44E-65
ZNF22	2.34E-69	-0.16129	0.209	0.498	3.50E-65
PSMB7	3.00E-69	0.122629	0.629	0.852	4.49E-65
DGKZ	3.02E-69	-0.37978	0.074	0.349	4.52E-65
JKAMP	3.07E-69	-0.13121	0.18	0.459	4.59E-65
SAA2	3.10E-69	1.723605	0.23	0.006	4.64E-65
MACF1	3.29E-69	-0.27746	0.192	0.505	4.92E-65
COPRS	3.30E-69	-0.39713	0.273	0.625	4.94E-65
TUG1	3.51E-69	-0.31245	0.099	0.373	5.25E-65
POLR2L	3.57E-69	0.21374	0.644	0.847	5.33E-65
LRRC47	3.65E-69	-0.28823	0.155	0.451	5.45E-65
TPGS1	3.73E-69	-0.34123	0.168	0.479	5.58E-65
STAG2	4.02E-69	-0.25809	0.174	0.472	6.00E-65
PLOD2	4.11E-69	0.403725	0.281	0.299	6.14E-65
CNOT1	4.22E-69	-0.23039	0.094	0.341	6.31E-65
ASPSCR1	4.22E-69	-0.28159	0.154	0.443	6.31E-65
XXYLT1	4.24E-69	-0.34923	0.049	0.298	6.33E-65
STAG1	4.39E-69	-0.25215	0.087	0.346	6.55E-65
SLBP	4.48E-69	-0.44011	0.241	0.593	6.70E-65
NSD1	4.84E-69	-0.28533	0.131	0.419	7.23E-65
DDX49	4.87E-69	-0.11019	0.224	0.493	7.28E-65
ABAT	4.91E-69	-0.41261	0.086	0.367	7.33E-65
C19orf43	5.08E-69	-0.28284	0.566	0.891	7.60E-65
CEBPD	5.36E-69	0.410079	0.402	0.396	8.01E-65
COL4A2	5.64E-69	-0.34328	0.078	0.342	8.43E-65
RAB34	6.02E-69	-0.1864	0.158	0.426	9.00E-65
HLTF	6.06E-69	-0.31366	0.12	0.406	9.05E-65
PTCD3	6.13E-69	-0.21132	0.181	0.479	9.16E-65
CCT8	6.19E-69	0.153093	0.541	0.738	9.26E-65
MCUR1	6.21E-69	-0.41365	0.185	0.519	9.27E-65
SLC44A1	6.28E-69	-0.11333	0.248	0.5	9.39E-65
PPP1R35	6.79E-69	-0.40516	0.088	0.38	1.01E-64
SSX2IP	7.34E-69	-0.24805	0.054	0.288	1.10E-64
ANKRD9	7.52E-69	-0.29271	0.02	0.228	1.12E-64
DAG1	8.22E-69	-0.25655	0.133	0.407	1.23E-64
TXNDC12	8.35E-69	0.169412	0.448	0.633	1.25E-64
PDHA1	8.47E-69	-0.2387	0.252	0.568	1.27E-64
VPS26B	8.68E-69	-0.35395	0.134	0.438	1.30E-64
CHPT1	8.87E-69	-0.33316	0.213	0.536	1.33E-64
GNL3	9.60E-69	0.261622	0.37	0.526	1.43E-64
MIS12	1.07E-68	-0.27474	0.129	0.402	1.60E-64

TXNIP	1.08E-68	0.170755	0.422	0.602	1.62E-64
TPM4	1.13E-68	-0.12509	0.434	0.744	1.68E-64
GRSF1	1.19E-68	-0.22898	0.224	0.528	1.78E-64
HIST1H1B	1.20E-68	-0.29761	0.002	0.162	1.80E-64
RNF4	1.21E-68	-0.20098	0.136	0.406	1.80E-64
ZNF358	1.23E-68	-0.30672	0.081	0.344	1.83E-64
AATF	1.23E-68	-0.18165	0.237	0.531	1.84E-64
DSTN	1.32E-68	0.308109	0.705	0.827	1.97E-64
PMVK	1.35E-68	0.199972	0.398	0.601	2.02E-64
ZCCHC11	1.36E-68	-0.17099	0.167	0.437	2.03E-64
COX14	1.37E-68	0.416264	0.424	0.53	2.04E-64
KCMF1	1.38E-68	-0.25206	0.13	0.412	2.06E-64
PRKD3	1.38E-68	-0.26929	0.073	0.322	2.07E-64
PSME1	1.49E-68	0.512357	0.562	0.627	2.23E-64
CHAMP1	1.69E-68	-0.35205	0.081	0.356	2.53E-64
GIPC1	1.77E-68	-0.23678	0.193	0.491	2.65E-64
LRRC58	1.80E-68	-0.20933	0.142	0.411	2.70E-64
NUFIP2	1.94E-68	-0.22566	0.139	0.411	2.89E-64
CTDSP2	1.97E-68	-0.43836	0.169	0.495	2.94E-64
WBP4	2.11E-68	0.110155	0.288	0.519	3.16E-64
EPB41L2	2.17E-68	-0.32342	0.115	0.41	3.25E-64
CD320	2.30E-68	-0.28797	0.339	0.675	3.44E-64
PAFAH1B1	2.45E-68	-0.22388	0.258	0.562	3.66E-64
NT5C	2.63E-68	-0.26658	0.187	0.485	3.92E-64
MYO9B	2.68E-68	-0.19516	0.133	0.391	4.00E-64
CAMTA1	2.85E-68	-0.26756	0.364	0.707	4.26E-64
KAT7	2.94E-68	-0.34139	0.084	0.356	4.39E-64
PRIM2	2.95E-68	-0.35841	0.034	0.262	4.41E-64
THRAP3	2.96E-68	-0.15947	0.348	0.646	4.42E-64
SRSF3	3.04E-68	-0.17787	0.65	0.943	4.54E-64
LRRN1	3.04E-68	-0.50602	0.042	0.29	4.54E-64
EIF3A	3.21E-68	-0.26851	0.206	0.512	4.79E-64
NDUFA6	3.39E-68	0.143465	0.571	0.817	5.07E-64
PLGRKT	4.04E-68	-0.10277	0.203	0.477	6.04E-64
ARHGDIA	4.08E-68	-0.13619	0.43	0.742	6.09E-64
INO80E	4.30E-68	-0.2304	0.158	0.446	6.42E-64
SURF1	4.30E-68	-0.19402	0.223	0.506	6.43E-64
BTBD3	4.39E-68	-0.25069	0.051	0.285	6.56E-64
ATP5A1	4.47E-68	0.224801	0.64	0.831	6.68E-64
NUP88	4.66E-68	-0.29979	0.092	0.365	6.96E-64
SDHAF1	4.72E-68	-0.1892	0.129	0.378	7.05E-64
MAP1LC3A	5.33E-68	-0.1666	0.301	0.583	7.97E-64
GNB4	5.50E-68	-0.30308	0.096	0.375	8.22E-64
LUC7L	5.66E-68	-0.19339	0.188	0.472	8.46E-64
SCG3	5.71E-68	-0.40238	0.177	0.494	8.53E-64
ABCD3	5.75E-68	-0.27343	0.15	0.438	8.59E-64
MPHOSPH8	6.14E-68	0.103948	0.344	0.553	9.18E-64
GAS2L1	6.18E-68	-0.31682	0.093	0.359	9.24E-64
CS	6.37E-68	-0.28713	0.089	0.349	9.52E-64
STK17A	6.59E-68	-0.20803	0.222	0.507	9.85E-64
AFF4	6.78E-68	-0.27999	0.219	0.526	1.01E-63

ROMO1	6.92E-68	0.1402	0.579	0.802	1.03E-63
RHOC	7.00E-68	0.548193	0.631	0.621	1.05E-63
RNFT1	7.08E-68	-0.20605	0.107	0.373	1.06E-63
ZFAND3	7.71E-68	-0.25363	0.181	0.477	1.15E-63
SEPHS1	8.01E-68	-0.28086	0.059	0.301	1.20E-63
CNOT3	8.48E-68	-0.22908	0.119	0.378	1.27E-63
ANAPC16	8.68E-68	0.51107	0.463	0.552	1.30E-63
PRR4	8.90E-68	-0.30004	0.146	0.441	1.33E-63
C11orf31	9.03E-68	0.190721	0.572	0.783	1.35E-63
PRKACA	9.74E-68	-0.252	0.067	0.307	1.46E-63
ACTR2	9.80E-68	-0.19102	0.274	0.579	1.46E-63
HEY1	1.01E-67	-0.39569	0.22	0.549	1.51E-63
LMAN1	1.04E-67	0.370972	0.338	0.441	1.56E-63
NDUFB4	1.04E-67	0.342397	0.673	0.788	1.56E-63
NDRG1	1.05E-67	1.004322	0.312	0.074	1.57E-63
WBP2	1.08E-67	0.338185	0.445	0.527	1.61E-63
C7orf49	1.11E-67	-0.19836	0.178	0.457	1.65E-63
PSME3	1.12E-67	-0.13992	0.167	0.443	1.67E-63
CDK5RAP2	1.13E-67	-0.16531	0.117	0.363	1.69E-63
POLR2E	1.18E-67	-0.11749	0.46	0.772	1.76E-63
ARSJ	1.24E-67	-0.20013	0.08	0.315	1.86E-63
GATM	1.26E-67	-0.20367	0.242	0.53	1.88E-63
SREK1IP1	1.27E-67	0.185269	0.322	0.532	1.89E-63
CIAO1	1.33E-67	-0.1652	0.169	0.437	1.99E-63
SAMM50	1.39E-67	-0.18169	0.126	0.386	2.08E-63
THOC3	1.44E-67	-0.1577	0.148	0.401	2.15E-63
NSA2	1.54E-67	-0.14716	0.293	0.598	2.30E-63
REST	1.56E-67	-0.40537	0.122	0.425	2.34E-63
TANC2	1.69E-67	-0.27727	0.075	0.326	2.52E-63
EXOC7	1.75E-67	-0.18843	0.241	0.548	2.61E-63
NFATC2IP	1.81E-67	-0.32525	0.113	0.396	2.70E-63
CCNI	1.82E-67	-0.33012	0.541	0.872	2.72E-63
WDR82	1.91E-67	-0.33744	0.151	0.453	2.86E-63
H2AFY2	1.97E-67	-0.25215	0.018	0.217	2.94E-63
DNAJC15	2.07E-67	0.32478	0.355	0.526	3.09E-63
CHMP4A	2.10E-67	0.120374	0.365	0.604	3.14E-63
STX10	2.16E-67	-0.18462	0.276	0.58	3.22E-63
THOC2	2.28E-67	-0.22095	0.248	0.548	3.40E-63
TFAM	2.33E-67	-0.16517	0.143	0.41	3.48E-63
NDUFS2	2.35E-67	0.241211	0.517	0.694	3.51E-63
SOCS6	2.40E-67	-0.27604	0.084	0.341	3.58E-63
AP2A1	2.40E-67	-0.24638	0.177	0.467	3.59E-63
TAF6	2.44E-67	-0.21953	0.221	0.511	3.65E-63
PRNP	2.53E-67	0.272067	0.471	0.599	3.79E-63
E2F8	2.54E-67	-0.29024	0.004	0.17	3.80E-63
RHOT1	2.69E-67	-0.28691	0.155	0.447	4.02E-63
RBM15B	2.77E-67	-0.33382	0.087	0.359	4.13E-63
SUMF2	2.92E-67	-0.10529	0.265	0.533	4.36E-63
STK4	3.01E-67	-0.16533	0.149	0.409	4.50E-63
HCFC1	3.01E-67	-0.28073	0.074	0.326	4.50E-63
UBL7-AS1	3.19E-67	-0.31558	0.031	0.251	4.76E-63

NUDT5	3.21E-67	-0.16376	0.206	0.499	4.80E-63
CGGBP1	3.22E-67	-0.24451	0.206	0.505	4.80E-63
EFTUD2	3.84E-67	-0.20567	0.139	0.414	5.74E-63
LAGE3	4.03E-67	-0.12015	0.328	0.61	6.03E-63
PA2G4	4.27E-67	-0.20986	0.479	0.804	6.38E-63
C17orf58	4.35E-67	-0.26384	0.141	0.427	6.50E-63
VTI1B	4.37E-67	-0.14549	0.226	0.512	6.54E-63
CHIC2	4.50E-67	-0.30586	0.185	0.489	6.72E-63
DIAPH1	4.61E-67	-0.30451	0.062	0.31	6.88E-63
BORA	4.74E-67	-0.37376	0.02	0.228	7.08E-63
TLE1	4.84E-67	-0.28125	0.127	0.401	7.23E-63
TMEM181	5.07E-67	-0.30663	0.075	0.335	7.58E-63
TRIM37	5.18E-67	-0.32181	0.096	0.373	7.74E-63
TRIM44	5.19E-67	-0.20357	0.212	0.493	7.76E-63
BRI3	5.51E-67	-0.29408	0.297	0.627	8.24E-63
CWC15	5.76E-67	0.154407	0.431	0.664	8.61E-63
DFFA	5.88E-67	-0.18492	0.136	0.404	8.79E-63
MED4	5.95E-67	0.196884	0.394	0.601	8.90E-63
MT3	5.96E-67	0.551063	0.722	0.768	8.90E-63
EVA1B	6.29E-67	-0.32297	0.031	0.259	9.41E-63
SOCS2	6.50E-67	-0.49085	0.082	0.36	9.71E-63
CRELD2	6.86E-67	-0.29536	0.171	0.467	1.03E-62
IFNGR2	7.01E-67	-0.26619	0.206	0.501	1.05E-62
C5orf15	7.02E-67	0.136707	0.309	0.479	1.05E-62
RBM14	7.31E-67	-0.29721	0.149	0.438	1.09E-62
UNC50	7.68E-67	0.196141	0.369	0.538	1.15E-62
VAPB	7.84E-67	-0.2522	0.162	0.442	1.17E-62
MED13	7.95E-67	-0.22063	0.08	0.321	1.19E-62
EIF2AK2	8.06E-67	-0.23014	0.221	0.522	1.20E-62
DAP	8.57E-67	-0.23117	0.203	0.491	1.28E-62
ATP5H	8.69E-67	0.261297	0.6	0.783	1.30E-62
RB1	8.88E-67	-0.24304	0.099	0.349	1.33E-62
8-Sep	8.99E-67	-0.27758	0.122	0.393	1.34E-62
NNT	9.15E-67	-0.34532	0.078	0.341	1.37E-62
SASS6	9.33E-67	-0.27338	0.047	0.274	1.39E-62
TDP1	9.67E-67	-0.24146	0.038	0.242	1.45E-62
SAMD4B	9.88E-67	-0.11656	0.176	0.432	1.48E-62
CYCS	9.92E-67	0.158866	0.738	0.931	1.48E-62
TGFB1	1.01E-66	-0.16451	0.064	0.265	1.51E-62
ZNF638	1.02E-66	-0.13705	0.188	0.443	1.52E-62
PTP4A2	1.13E-66	-0.36051	0.335	0.683	1.68E-62
ZKSCAN1	1.13E-66	-0.14855	0.224	0.504	1.69E-62
BAG5	1.17E-66	-0.11311	0.148	0.385	1.75E-62
TTC1	1.17E-66	0.329779	0.374	0.483	1.75E-62
IQGAP3	1.20E-66	-0.29665	0.001	0.153	1.80E-62
ZMYND11	1.21E-66	-0.27018	0.069	0.314	1.81E-62
RAB5C	1.21E-66	0.160977	0.543	0.757	1.82E-62
HSPBP1	1.35E-66	-0.12129	0.269	0.542	2.02E-62
DAXX	1.42E-66	-0.15534	0.193	0.452	2.12E-62
LARP1	1.49E-66	-0.12692	0.188	0.431	2.22E-62
FAM72D	1.71E-66	-0.26552	0.002	0.163	2.56E-62

SEC22C	1.77E-66	-0.23781	0.176	0.458	2.65E-62
FBXW2	1.78E-66	-0.23887	0.13	0.39	2.65E-62
HIST1H1E	1.81E-66	-0.26396	0.01	0.188	2.71E-62
GABARAP	1.86E-66	0.624804	0.399	0.417	2.78E-62
TIMMDC1	1.93E-66	0.25639	0.397	0.547	2.88E-62
PTPN1	1.98E-66	-0.16294	0.115	0.352	2.97E-62
APBB2	2.04E-66	-0.3656	0.074	0.337	3.05E-62
CLPTM1L	2.14E-66	-0.27112	0.068	0.311	3.20E-62
TOP3A	2.17E-66	-0.30909	0.054	0.295	3.24E-62
ARPC1A	2.23E-66	0.100691	0.592	0.837	3.33E-62
GDE1	2.24E-66	-0.20551	0.185	0.47	3.34E-62
AGPAT1	2.36E-66	-0.17746	0.16	0.428	3.53E-62
PRCP	2.42E-66	-0.13262	0.309	0.598	3.61E-62
LRRC59	2.46E-66	-0.14322	0.32	0.611	3.68E-62
DHRS7	2.64E-66	0.280122	0.363	0.523	3.95E-62
BMI1	2.64E-66	-0.20889	0.07	0.307	3.95E-62
TFG	2.79E-66	0.10044	0.438	0.64	4.17E-62
FAM229B	2.94E-66	0.103883	0.316	0.515	4.39E-62
CNPY3	3.20E-66	-0.12103	0.213	0.481	4.77E-62
RAB18	3.75E-66	0.250214	0.358	0.502	5.61E-62
ALDH3A2	3.76E-66	-0.1974	0.172	0.457	5.62E-62
NNT-AS1	3.76E-66	-0.24142	0.173	0.457	5.62E-62
BCL2L12	3.81E-66	-0.41299	0.09	0.372	5.69E-62
ATP5I	3.84E-66	0.19908	0.693	0.869	5.74E-62
REV1	4.00E-66	-0.30352	0.101	0.372	5.98E-62
VPS25	4.09E-66	0.217805	0.315	0.505	6.12E-62
LRWD1	4.18E-66	-0.22338	0.152	0.419	6.25E-62
CTBP1	4.32E-66	-0.22469	0.136	0.402	6.45E-62
FAM96A	4.34E-66	0.103866	0.257	0.48	6.49E-62
ZNF85	4.48E-66	-0.33022	0.057	0.302	6.69E-62
ARL6IP4	4.91E-66	-0.28077	0.449	0.781	7.34E-62
EMC7	4.97E-66	0.16294	0.388	0.595	7.42E-62
OTUB1	4.99E-66	0.19965	0.333	0.511	7.46E-62
SHARPIN	5.19E-66	-0.27431	0.177	0.469	7.76E-62
TMEM179B	5.46E-66	0.353304	0.384	0.49	8.16E-62
SCAMP4	5.70E-66	-0.21379	0.195	0.489	8.51E-62
PLXNB2	6.30E-66	-0.15174	0.09	0.317	9.42E-62
PRKRIR	6.51E-66	-0.26165	0.05	0.28	9.73E-62
POLD3	6.69E-66	-0.41196	0.08	0.354	1.00E-61
CIAPIN1	6.98E-66	-0.16519	0.218	0.51	1.04E-61
SYNRG	7.34E-66	-0.19069	0.11	0.362	1.10E-61
HTATSF1	7.37E-66	-0.2652	0.224	0.531	1.10E-61
COMMD1	7.41E-66	0.21889	0.295	0.484	1.11E-61
BCAS2	7.52E-66	0.232515	0.422	0.602	1.12E-61
DNTTIP1	7.80E-66	-0.15733	0.286	0.584	1.17E-61
EXOG	7.95E-66	-0.2894	0.107	0.372	1.19E-61
BAG6	8.71E-66	-0.13183	0.259	0.535	1.30E-61
CHURC1	8.92E-66	0.314639	0.357	0.5	1.33E-61
PROX1	9.00E-66	-0.31037	0.066	0.314	1.35E-61
PSAT1	9.49E-66	-0.16666	0.244	0.5	1.42E-61
FKBP4	9.57E-66	-0.18359	0.266	0.552	1.43E-61

CCNG2	9.95E-66	-0.42479	0.084	0.356	1.49E-61
PCDH17	1.01E-65	-0.30826	0.158	0.451	1.52E-61
DNM2	1.06E-65	-0.16536	0.123	0.365	1.58E-61
ATP5B	1.13E-65	0.161037	0.734	0.917	1.69E-61
GLIPR1	1.13E-65	0.807894	0.334	0.262	1.69E-61
KIAA1715	1.17E-65	-0.15518	0.202	0.477	1.74E-61
DOK5	1.22E-65	0.356813	0.253	0.33	1.82E-61
COTL1	1.25E-65	-0.30918	0.246	0.554	1.87E-61
IDH1	1.27E-65	-0.23181	0.26	0.572	1.90E-61
CCDC82	1.43E-65	-0.17171	0.218	0.512	2.14E-61
C19orf53	1.54E-65	0.426047	0.722	0.821	2.29E-61
NPAS3	1.66E-65	-0.35329	0.065	0.321	2.48E-61
AUTS2	1.68E-65	-0.30597	0.047	0.283	2.50E-61
STAMBP	1.81E-65	-0.10496	0.222	0.494	2.70E-61
DYNC1I2	1.81E-65	-0.16666	0.362	0.667	2.71E-61
PKIG	1.95E-65	0.384013	0.426	0.533	2.92E-61
VKORC1	2.00E-65	0.276716	0.475	0.63	2.99E-61
SCAF11	2.00E-65	-0.12377	0.303	0.585	3.00E-61
CKAP4	2.19E-65	-0.2328	0.171	0.446	3.28E-61
EMC8	2.24E-65	-0.2959	0.218	0.527	3.34E-61
WRAP53	2.35E-65	-0.21549	0.072	0.304	3.51E-61
MSL1	2.39E-65	-0.2258	0.092	0.336	3.57E-61
RNF114	2.47E-65	-0.1011	0.344	0.631	3.68E-61
F8A1	2.54E-65	-0.2988	0.052	0.294	3.80E-61
SALL1	2.56E-65	-0.34451	0.062	0.316	3.83E-61
SPTBN1	2.60E-65	-0.21328	0.229	0.521	3.88E-61
PRRC2B	2.70E-65	-0.21223	0.135	0.396	4.04E-61
F13A1	2.76E-65	1.289213	0.254	0.02	4.12E-61
DRAP1	3.16E-65	0.133758	0.608	0.796	4.72E-61
SRRD	3.22E-65	-0.23893	0.049	0.274	4.82E-61
FARSB	3.25E-65	-0.10685	0.224	0.495	4.86E-61
AKR1A1	3.33E-65	0.192993	0.363	0.54	4.98E-61
SPAG16	3.37E-65	0.130926	0.351	0.537	5.04E-61
MDM2	3.47E-65	-0.52432	0.147	0.459	5.19E-61
CAPZA1	3.48E-65	-0.23127	0.23	0.521	5.20E-61
PRR7	3.49E-65	-0.36759	0.102	0.384	5.21E-61
ANXA2	3.65E-65	0.876616	0.696	0.477	5.46E-61
REPIN1	3.65E-65	-0.24426	0.207	0.493	5.46E-61
ZNF131	3.70E-65	-0.29572	0.161	0.454	5.53E-61
H3F3B	3.78E-65	0.361138	0.935	0.981	5.65E-61
SGSM3	3.81E-65	-0.26552	0.104	0.363	5.69E-61
EIF4E	3.97E-65	-0.11069	0.403	0.698	5.93E-61
PNPLA8	4.15E-65	0.253559	0.383	0.511	6.19E-61
ELOF1	4.25E-65	0.184957	0.45	0.649	6.35E-61
RASSF1	4.33E-65	-0.19206	0.129	0.373	6.48E-61
TRAPPC3	4.36E-65	0.317687	0.433	0.569	6.51E-61
TNC	4.41E-65	0.657598	0.345	0.344	6.59E-61
HSF1	4.42E-65	-0.19446	0.212	0.493	6.60E-61
CBFB	4.50E-65	-0.201	0.183	0.459	6.73E-61
GSN	4.51E-65	0.666214	0.391	0.327	6.73E-61
DIDO1	4.84E-65	-0.26003	0.089	0.338	7.23E-61

AK2	5.16E-65	0.403535	0.457	0.57	7.72E-61
ZFP36L1	5.35E-65	0.502391	0.561	0.609	8.00E-61
TRIM2	5.41E-65	-0.1436	0.178	0.412	8.09E-61
TRAPPC2L	5.59E-65	0.253796	0.381	0.543	8.35E-61
SAMD1	5.59E-65	-0.2659	0.044	0.263	8.35E-61
CLDND1	5.82E-65	0.176363	0.416	0.602	8.70E-61
HINT2	5.88E-65	-0.36075	0.27	0.606	8.78E-61
CD164	6.14E-65	0.364165	0.451	0.505	9.18E-61
RGS19	6.24E-65	-0.28477	0.055	0.288	9.32E-61
GNAI2	6.61E-65	-0.32303	0.248	0.569	9.88E-61
USP11	6.73E-65	-0.148	0.325	0.617	1.01E-60
NDUFS1	7.48E-65	-0.23274	0.203	0.49	1.12E-60
SNCAIP	7.82E-65	-0.32383	0.037	0.26	1.17E-60
AP2M1	8.75E-65	0.157757	0.664	0.862	1.31E-60
ARRB2	8.97E-65	-0.22446	0.147	0.425	1.34E-60
DECR1	9.06E-65	0.279652	0.391	0.575	1.35E-60
RBPJ	9.20E-65	-0.19847	0.308	0.605	1.38E-60
IGF2BP3	9.23E-65	-0.33285	0.051	0.289	1.38E-60
ZBTB7A	9.33E-65	-0.27804	0.084	0.328	1.39E-60
GFER	9.74E-65	-0.36868	0.117	0.405	1.46E-60
CRK	9.75E-65	-0.25112	0.255	0.557	1.46E-60
RAB22A	1.03E-64	-0.31257	0.149	0.441	1.53E-60
MBOAT2	1.03E-64	-0.3564	0.144	0.438	1.54E-60
FAM49B	1.08E-64	-0.20316	0.237	0.527	1.61E-60
C6orf1	1.10E-64	0.436408	0.363	0.43	1.65E-60
NCBP2-AS2	1.12E-64	-0.2241	0.237	0.523	1.68E-60
ZNF148	1.20E-64	-0.22298	0.16	0.436	1.79E-60
FUT8	1.27E-64	-0.18091	0.066	0.277	1.89E-60
EAPP	1.27E-64	0.365542	0.356	0.488	1.89E-60
ZDHHC4	1.28E-64	0.196889	0.373	0.564	1.91E-60
CMTM3	1.30E-64	-0.14933	0.202	0.457	1.95E-60
RBL1	1.31E-64	-0.27558	0.02	0.217	1.96E-60
PBX1	1.56E-64	-0.35742	0.16	0.464	2.33E-60
NGDN	1.69E-64	0.127619	0.262	0.453	2.53E-60
DRG1	1.71E-64	-0.11825	0.191	0.451	2.55E-60
SMARCD3	1.73E-64	-0.26036	0.183	0.47	2.59E-60
MDGA1	1.76E-64	-0.22068	0.013	0.189	2.63E-60
IER5	2.00E-64	-0.21259	0.111	0.354	2.99E-60
POLR3H	2.00E-64	-0.12868	0.146	0.404	3.00E-60
DNAJB1	2.05E-64	0.361175	0.608	0.773	3.06E-60
CASP2	2.07E-64	-0.33824	0.062	0.311	3.10E-60
SIK3	2.10E-64	-0.21114	0.039	0.241	3.14E-60
ATP2B1	2.47E-64	-0.36206	0.229	0.549	3.70E-60
WDR61	2.50E-64	0.235735	0.341	0.519	3.74E-60
CDK2AP1	2.55E-64	-0.27767	0.025	0.231	3.81E-60
TMEM230	2.61E-64	0.259241	0.61	0.78	3.90E-60
ATP6V1D	2.65E-64	0.271803	0.365	0.526	3.96E-60
SPATA33	2.71E-64	-0.23574	0.139	0.409	4.05E-60
CCDC15	2.72E-64	-0.29264	0.027	0.232	4.06E-60
AKAP7	2.80E-64	-0.33824	0.091	0.358	4.19E-60
AEBP1	2.84E-64	0.67952	0.296	0.194	4.24E-60

POMGNT1	2.87E-64	-0.2723	0.173	0.464	4.29E-60
SREK1	2.97E-64	-0.10521	0.303	0.568	4.43E-60
ZNF302	2.97E-64	0.22656	0.369	0.568	4.45E-60
MSI2	3.02E-64	-0.22695	0.299	0.619	4.52E-60
CHAF1B	3.04E-64	-0.34552	0.027	0.237	4.54E-60
HSPA8	3.12E-64	0.249944	0.834	0.959	4.66E-60
AC004540.	3.52E-64	-0.4689	0.043	0.278	5.25E-60
MKLN1	3.71E-64	-0.19525	0.173	0.442	5.54E-60
TXNL4A	3.94E-64	-0.23405	0.315	0.64	5.89E-60
CCDC66	3.96E-64	-0.13775	0.186	0.437	5.91E-60
AGTRAP	4.06E-64	0.802655	0.34	0.309	6.06E-60
NXPH1	4.26E-64	-0.49369	0.044	0.284	6.37E-60
ACTR10	4.32E-64	0.235354	0.355	0.536	6.46E-60
TMEM131	4.68E-64	-0.26932	0.063	0.301	7.00E-60
MIEN1	4.68E-64	0.16457	0.429	0.642	7.00E-60
APOO	4.83E-64	-0.14758	0.204	0.478	7.22E-60
CHST10	5.32E-64	-0.20349	0.082	0.315	7.95E-60
ANKRD17	5.41E-64	-0.33478	0.124	0.405	8.09E-60
CCDC150	5.52E-64	-0.27763	0.011	0.188	8.26E-60
CEBPB	5.55E-64	0.18209	0.275	0.414	8.30E-60
CELF1	5.74E-64	-0.13356	0.233	0.493	8.58E-60
ZFAS1	6.01E-64	0.208221	0.664	0.856	8.98E-60
ANKLE2	6.17E-64	-0.20953	0.137	0.395	9.22E-60
ABCD4	6.25E-64	-0.10095	0.164	0.402	9.34E-60
DMWD	6.38E-64	-0.31096	0.072	0.325	9.54E-60
AHCYL1	6.64E-64	0.354684	0.377	0.526	9.92E-60
COL4A1	7.50E-64	-0.30813	0.055	0.286	1.12E-59
S100A13	7.61E-64	0.755793	0.452	0.402	1.14E-59
MYEOV2	8.39E-64	0.20171	0.62	0.836	1.25E-59
NCOA6	8.62E-64	-0.31973	0.063	0.307	1.29E-59
ZMYND8	8.66E-64	-0.30912	0.12	0.394	1.29E-59
CNIH1	8.82E-64	0.264647	0.368	0.537	1.32E-59
NCLN	8.85E-64	-0.23947	0.1	0.349	1.32E-59
ZNF273	8.87E-64	-0.36604	0.08	0.34	1.33E-59
FUBP3	8.98E-64	-0.18861	0.192	0.457	1.34E-59
TNKS	9.08E-64	-0.2634	0.09	0.336	1.36E-59
FADS2	9.50E-64	-0.24989	0.104	0.359	1.42E-59
CCDC137	9.55E-64	-0.29719	0.147	0.43	1.43E-59
TCEB2	9.66E-64	0.305693	0.795	0.911	1.44E-59
CFL1	1.09E-63	0.392813	0.908	0.964	1.62E-59
NR2F6	1.11E-63	-0.22941	0.08	0.311	1.65E-59
MRPS23	1.13E-63	0.132554	0.335	0.563	1.68E-59
TP73	1.14E-63	-0.28037	0.01	0.18	1.70E-59
CCT2	1.22E-63	-0.26665	0.511	0.833	1.82E-59
SH3GLB1	1.25E-63	0.284867	0.431	0.564	1.86E-59
TEAD1	1.25E-63	-0.35326	0.089	0.36	1.87E-59
CCP110	1.28E-63	-0.33946	0.092	0.359	1.92E-59
TEAD2	1.29E-63	-0.31299	0.027	0.24	1.92E-59
PPP1R15A	1.29E-63	0.362656	0.364	0.407	1.93E-59
EFHD2	1.32E-63	-0.31331	0.042	0.265	1.97E-59
ATPIF1	1.42E-63	0.212796	0.589	0.756	2.12E-59

CLCN3	1.46E-63	-0.14243	0.185	0.433	2.18E-59
CDC42SE1	1.47E-63	-0.18245	0.166	0.422	2.20E-59
EHMT2	1.51E-63	-0.32416	0.116	0.391	2.26E-59
VIPR2	1.56E-63	-0.61278	0.03	0.247	2.33E-59
MRGBP	1.59E-63	-0.2918	0.092	0.346	2.38E-59
ASPH	1.86E-63	-0.13624	0.282	0.542	2.78E-59
E2F2	1.92E-63	-0.29777	0.001	0.146	2.87E-59
ZNF664	1.96E-63	-0.21305	0.097	0.331	2.93E-59
APEX1	2.12E-63	0.131196	0.457	0.694	3.16E-59
CIZ1	2.21E-63	-0.23667	0.133	0.395	3.30E-59
TNPO1	2.22E-63	-0.22389	0.198	0.477	3.31E-59
PHLDA1	2.24E-63	-0.52175	0.27	0.617	3.35E-59
ACTN4	2.27E-63	-0.11509	0.311	0.575	3.38E-59
HM13	2.27E-63	0.141687	0.412	0.581	3.39E-59
EGLN2	2.28E-63	-0.21833	0.191	0.474	3.40E-59
CALM1	2.32E-63	-0.22178	0.656	0.94	3.47E-59
ME2	2.38E-63	-0.27288	0.116	0.384	3.55E-59
PREX1	2.61E-63	-0.27694	0.055	0.283	3.91E-59
IDI1	2.72E-63	-0.19138	0.22	0.507	4.07E-59
NME3	2.74E-63	0.166927	0.315	0.478	4.09E-59
MKKS	2.75E-63	-0.23865	0.335	0.669	4.11E-59
PDS5A	3.06E-63	-0.2093	0.112	0.359	4.57E-59
STT3B	3.06E-63	-0.24578	0.123	0.385	4.58E-59
ZC3H18	3.24E-63	-0.29214	0.096	0.359	4.85E-59
PITPNC1	3.33E-63	-0.2559	0.066	0.296	4.97E-59
RP11-698N	3.34E-63	-0.19869	0.023	0.193	4.99E-59
SLC52A2	3.44E-63	-0.2041	0.222	0.495	5.15E-59
SMAP1	3.52E-63	-0.21448	0.225	0.506	5.26E-59
CLPTM1	3.80E-63	-0.13907	0.297	0.562	5.68E-59
STIM2	3.87E-63	-0.27012	0.086	0.34	5.79E-59
ODF2	3.91E-63	-0.39079	0.118	0.405	5.85E-59
LRRC45	4.37E-63	-0.29368	0.031	0.246	6.54E-59
SAR1B	4.52E-63	0.253126	0.347	0.469	6.76E-59
KEAP1	4.90E-63	-0.1591	0.251	0.52	7.32E-59
ZC3H13	4.94E-63	-0.10547	0.242	0.502	7.38E-59
JOSD1	5.17E-63	-0.25483	0.051	0.274	7.73E-59
LINC00493	5.41E-63	0.244233	0.522	0.696	8.09E-59
TSEN34	5.55E-63	-0.21242	0.344	0.632	8.29E-59
COX6A1	5.71E-63	0.318169	0.827	0.931	8.54E-59
LMO1	5.89E-63	-0.56675	0.03	0.248	8.81E-59
DLGAP4	6.13E-63	-0.13713	0.209	0.479	9.15E-59
TMPO-AS1	6.18E-63	-0.30346	0.013	0.194	9.24E-59
EVL	6.50E-63	-0.23383	0.095	0.333	9.72E-59
HES4	6.73E-63	-0.43711	0.134	0.432	1.01E-58
AAAS	6.92E-63	-0.25447	0.159	0.433	1.03E-58
CCDC107	7.09E-63	-0.12114	0.295	0.557	1.06E-58
SMS	7.24E-63	0.126107	0.565	0.789	1.08E-58
VPS51	7.31E-63	-0.31882	0.153	0.438	1.09E-58
NDUFC1	7.33E-63	0.315964	0.591	0.74	1.09E-58
UNC119	7.44E-63	-0.23417	0.114	0.367	1.11E-58
STAT1	7.67E-63	-0.31903	0.143	0.428	1.15E-58

MCM6	8.28E-63	-0.49632	0.068	0.331	1.24E-58
GIT1	8.69E-63	-0.22968	0.059	0.28	1.30E-58
RGMA	8.72E-63	-0.26432	0.171	0.448	1.30E-58
TPM2	8.91E-63	-0.49312	0.107	0.395	1.33E-58
CREB1	9.00E-63	-0.27311	0.16	0.447	1.35E-58
TMEM261	9.28E-63	0.109056	0.312	0.511	1.39E-58
TMEM45A	1.01E-62	0.649422	0.343	0.298	1.51E-58
NF2	1.04E-62	-0.21295	0.077	0.309	1.55E-58
DPP4	1.05E-62	-0.11513	0.034	0.204	1.57E-58
CISD1	1.13E-62	0.438361	0.393	0.504	1.68E-58
ERGIC3	1.14E-62	0.139014	0.553	0.777	1.70E-58
C1QL1	1.15E-62	-0.43394	0.085	0.357	1.72E-58
DYNLL1	1.19E-62	0.273851	0.848	0.964	1.78E-58
ZNF649	1.19E-62	-0.3013	0.04	0.262	1.78E-58
ELAVL4	1.22E-62	-0.67855	0.061	0.312	1.83E-58
RNF10	1.24E-62	-0.10337	0.264	0.51	1.85E-58
IMMT	1.24E-62	-0.20474	0.232	0.525	1.85E-58
HSPA14	1.28E-62	-0.22112	0.104	0.347	1.92E-58
KCTD17	1.29E-62	-0.29644	0.066	0.306	1.93E-58
EXTL2	1.29E-62	-0.22852	0.126	0.385	1.93E-58
ATOX1	1.45E-62	0.397796	0.591	0.71	2.16E-58
CHD1	1.49E-62	-0.31307	0.168	0.463	2.23E-58
GRAMD1A	1.50E-62	-0.28783	0.145	0.425	2.25E-58
URM1	1.59E-62	0.192323	0.339	0.507	2.37E-58
PAK4	1.65E-62	-0.27475	0.104	0.36	2.47E-58
ZFR	1.68E-62	-0.26299	0.247	0.548	2.52E-58
BEND5	1.70E-62	-0.10421	0.098	0.309	2.54E-58
SUB1	1.78E-62	-0.13433	0.689	0.956	2.66E-58
NUP54	1.79E-62	-0.15097	0.169	0.414	2.68E-58
FZR1	1.83E-62	-0.35948	0.11	0.386	2.73E-58
DDX3Y	1.85E-62	-0.26349	0.014	0.194	2.77E-58
RAB9A	1.99E-62	0.134587	0.278	0.475	2.98E-58
COX6C	2.07E-62	0.311491	0.795	0.923	3.09E-58
ADM	2.22E-62	0.865588	0.348	0.204	3.31E-58
TXLNA	2.24E-62	-0.23885	0.085	0.325	3.34E-58
ARIH2	2.26E-62	-0.11994	0.197	0.447	3.38E-58
CD151	2.31E-62	0.591676	0.566	0.605	3.46E-58
PCMTD2	2.37E-62	-0.34983	0.132	0.419	3.55E-58
DERL2	2.42E-62	0.389963	0.396	0.507	3.62E-58
MCOLN1	2.50E-62	-0.18161	0.149	0.394	3.74E-58
7-Mar	2.82E-62	-0.18005	0.19	0.457	4.21E-58
CSTF1	3.06E-62	-0.18631	0.098	0.331	4.58E-58
YTHDF1	3.30E-62	-0.14658	0.149	0.389	4.93E-58
TNFRSF19	3.41E-62	-0.23167	0.063	0.28	5.09E-58
GLT8D1	3.50E-62	-0.11882	0.277	0.532	5.23E-58
ATXN2L	3.51E-62	-0.18673	0.124	0.359	5.24E-58
KPNA6	3.52E-62	-0.13398	0.18	0.426	5.26E-58
NF1	3.53E-62	-0.23959	0.132	0.396	5.27E-58
TOMM5	3.54E-62	-0.16224	0.154	0.407	5.29E-58
ANKRD54	3.60E-62	-0.1688	0.095	0.326	5.38E-58
SEZ6L2	3.72E-62	-0.11413	0.225	0.473	5.56E-58

USP48	4.04E-62	-0.2167	0.17	0.426	6.04E-58
TECR	4.37E-62	0.251714	0.579	0.747	6.54E-58
UCP2	4.72E-62	-0.44713	0.051	0.29	7.05E-58
LGALS3BP	4.75E-62	0.579061	0.509	0.51	7.09E-58
LSM14B	4.90E-62	-0.30639	0.042	0.265	7.33E-58
TSPAN12	5.46E-62	-0.48481	0.1	0.381	8.16E-58
ZNF83	5.59E-62	-0.25117	0.159	0.432	8.36E-58
ICMT	5.61E-62	-0.36388	0.068	0.325	8.38E-58
C20orf24	6.11E-62	-0.33457	0.364	0.7	9.14E-58
LRRCC1	6.26E-62	-0.1546	0.159	0.405	9.35E-58
FN1	6.49E-62	0.700771	0.258	0.164	9.70E-58
SDHA	6.95E-62	-0.10587	0.271	0.517	1.04E-57
6-Mar	7.52E-62	-0.12315	0.271	0.551	1.12E-57
CDC7	7.57E-62	-0.3249	0.027	0.227	1.13E-57
NDC1	7.76E-62	-0.2634	0.039	0.242	1.16E-57
AC009506.	7.87E-62	-0.37441	0.037	0.253	1.18E-57
HIP1	8.28E-62	-0.17076	0.106	0.336	1.24E-57
NRSN2	8.51E-62	-0.18302	0.124	0.369	1.27E-57
MEOX2	8.89E-62	-0.29129	0.051	0.273	1.33E-57
DUS1L	8.97E-62	-0.27695	0.046	0.27	1.34E-57
GAD1	9.50E-62	-0.23091	0.044	0.23	1.42E-57
AAK1	9.73E-62	-0.2435	0.181	0.457	1.45E-57
NKAIN3	1.00E-61	-0.28651	0.06	0.283	1.50E-57
UBE2Q1	1.05E-61	-0.18593	0.086	0.315	1.57E-57
TMEM128	1.07E-61	0.108484	0.28	0.464	1.60E-57
RNF180	1.12E-61	-0.27922	0.12	0.38	1.67E-57
EDNRB	1.14E-61	-0.25649	0.137	0.401	1.70E-57
CHRNA5	1.18E-61	-0.26591	0.011	0.186	1.77E-57
SRP14	1.21E-61	0.222239	0.771	0.93	1.81E-57
COPZ1	1.22E-61	0.261231	0.496	0.656	1.82E-57
ETV4	1.26E-61	-0.29023	0.05	0.274	1.88E-57
PLP2	1.26E-61	0.538791	0.475	0.478	1.89E-57
ACTR1A	1.28E-61	-0.20933	0.15	0.414	1.92E-57
IL6ST	1.32E-61	-0.11104	0.191	0.428	1.98E-57
TOR1A	1.33E-61	-0.17818	0.191	0.462	1.99E-57
PHF6	1.35E-61	-0.28039	0.111	0.377	2.01E-57
SNRPG	1.37E-61	-0.13647	0.62	0.907	2.05E-57
MRFAP1	1.42E-61	0.115169	0.681	0.885	2.13E-57
KRAS	1.46E-61	-0.29272	0.155	0.433	2.18E-57
PAF1	1.47E-61	-0.19179	0.164	0.425	2.19E-57
BAD	1.47E-61	0.161457	0.44	0.626	2.19E-57
ALCAM	1.48E-61	-0.27234	0.078	0.32	2.21E-57
METTL4	1.63E-61	-0.14603	0.056	0.248	2.44E-57
CNTLN	1.65E-61	-0.38356	0.078	0.337	2.47E-57
UQCRQ	1.67E-61	0.264866	0.768	0.915	2.50E-57
FAM84A	1.90E-61	-0.26828	0.085	0.319	2.84E-57
HMOX2	1.96E-61	0.438436	0.298	0.39	2.93E-57
ING5	1.98E-61	-0.26328	0.083	0.33	2.95E-57
TMEM126A	2.00E-61	0.150461	0.279	0.483	2.99E-57
ISCA1	2.02E-61	0.119907	0.283	0.468	3.02E-57
FAHD2A	2.02E-61	-0.21231	0.11	0.358	3.02E-57

ZNF530	2.07E-61	-0.28812	0.062	0.289	3.09E-57
POGZ	2.13E-61	-0.28794	0.128	0.4	3.19E-57
DVL2	2.23E-61	-0.20392	0.126	0.381	3.34E-57
GLRX	2.36E-61	1.008807	0.345	0.204	3.53E-57
BLOC1S4	2.39E-61	-0.24336	0.161	0.433	3.57E-57
USP3	2.41E-61	-0.19006	0.086	0.309	3.60E-57
COQ7	2.42E-61	-0.23795	0.132	0.388	3.62E-57
TMED3	2.56E-61	0.200059	0.266	0.401	3.83E-57
C11orf58	2.63E-61	0.174585	0.658	0.84	3.94E-57
WRNIP1	2.90E-61	-0.26205	0.054	0.281	4.33E-57
PPP5C	3.02E-61	-0.27914	0.146	0.427	4.51E-57
MORF4L1	3.03E-61	0.229226	0.711	0.846	4.53E-57
SLC25A39	3.12E-61	-0.24395	0.268	0.562	4.67E-57
SDC1	3.20E-61	-0.34124	0.042	0.268	4.78E-57
TMEM219	3.29E-61	0.298337	0.436	0.573	4.92E-57
ELP5	3.49E-61	-0.13173	0.241	0.514	5.21E-57
NSMCE1	3.49E-61	0.290086	0.397	0.543	5.21E-57
AIMP1	3.59E-61	0.236792	0.37	0.538	5.37E-57
PITPNA	3.66E-61	-0.2118	0.096	0.326	5.47E-57
SURF2	3.83E-61	-0.24916	0.164	0.444	5.73E-57
AFG3L2	3.90E-61	-0.18109	0.153	0.398	5.83E-57
SRA1	3.94E-61	-0.11417	0.301	0.588	5.89E-57
URI1	3.96E-61	-0.22211	0.16	0.423	5.92E-57
SLN	4.35E-61	1.241869	0.332	0.221	6.50E-57
ZNF644	4.40E-61	-0.19007	0.205	0.469	6.57E-57
ID4	4.85E-61	-0.38365	0.138	0.421	7.24E-57
C1orf43	4.88E-61	0.200192	0.58	0.726	7.29E-57
SIAH2	4.93E-61	-0.21894	0.141	0.388	7.36E-57
FAM162A	5.66E-61	0.670198	0.473	0.417	8.45E-57
TMEM14A	5.70E-61	0.301594	0.349	0.475	8.52E-57
C20orf96	5.99E-61	-0.16103	0.102	0.325	8.95E-57
TRIM27	6.60E-61	-0.2032	0.137	0.384	9.86E-57
TMEM189	6.69E-61	-0.28482	0.111	0.375	1.00E-56
SNN	7.04E-61	-0.27185	0.13	0.39	1.05E-56
KIAA2013	7.14E-61	-0.16748	0.068	0.265	1.07E-56
BCHE	7.55E-61	-0.30886	0.161	0.444	1.13E-56
PRKRIP1	7.71E-61	-0.12189	0.204	0.467	1.15E-56
IFT43	8.90E-61	0.150923	0.261	0.444	1.33E-56
ASNA1	8.99E-61	0.152958	0.538	0.74	1.34E-56
BRD9	9.20E-61	-0.31899	0.121	0.395	1.37E-56
FXR1	9.26E-61	-0.22565	0.259	0.554	1.38E-56
GSTA4	9.77E-61	-0.1214	0.227	0.504	1.46E-56
KLHL7	1.03E-60	-0.29326	0.23	0.528	1.53E-56
LMF2	1.03E-60	-0.23097	0.13	0.399	1.54E-56
CYB5B	1.03E-60	0.156271	0.363	0.548	1.54E-56
TRIP12	1.07E-60	-0.1331	0.092	0.315	1.60E-56
SCNM1	1.09E-60	0.132545	0.287	0.477	1.62E-56
UBN1	1.31E-60	-0.28582	0.106	0.364	1.96E-56
ATP6V1E1	1.33E-60	0.231907	0.357	0.527	1.99E-56
DBI	1.33E-60	0.44339	0.862	0.957	1.99E-56
DYNLRB1	1.33E-60	0.21138	0.647	0.826	1.99E-56

HIGD2A	1.34E-60	0.338419	0.525	0.664	2.01E-56
UBE2G2	1.39E-60	-0.16849	0.196	0.459	2.08E-56
UBR7	1.39E-60	-0.25572	0.097	0.34	2.08E-56
ITPA	1.52E-60	0.106605	0.356	0.591	2.28E-56
ILK	1.54E-60	0.339382	0.351	0.49	2.30E-56
SLC30A9	1.55E-60	-0.17987	0.193	0.462	2.31E-56
LRRC37B	1.55E-60	-0.27431	0.056	0.284	2.31E-56
BOC	1.57E-60	-0.23649	0.032	0.228	2.35E-56
CPSF3	1.58E-60	-0.26576	0.137	0.407	2.36E-56
KDM2B	1.60E-60	-0.29714	0.046	0.267	2.39E-56
CDC27	1.80E-60	-0.32442	0.104	0.37	2.68E-56
DLL1	1.80E-60	-0.47533	0.017	0.207	2.69E-56
TSC22D4	2.04E-60	0.170136	0.476	0.672	3.06E-56
SUDS3	2.13E-60	-0.26203	0.134	0.395	3.18E-56
MMD	2.24E-60	-0.15349	0.167	0.419	3.34E-56
TSG101	2.45E-60	0.248418	0.397	0.558	3.66E-56
NTPCR	3.06E-60	0.140281	0.242	0.435	4.58E-56
RWDD4	3.08E-60	-0.21827	0.181	0.447	4.61E-56
C8orf88	3.32E-60	-0.1924	0.027	0.214	4.96E-56
PEPD	3.36E-60	0.36163	0.37	0.499	5.03E-56
ADAT1	3.39E-60	-0.23498	0.058	0.277	5.07E-56
NTAN1	3.41E-60	-0.19905	0.123	0.377	5.10E-56
ZNF92	3.55E-60	-0.33694	0.126	0.399	5.30E-56
RP11-849I	3.63E-60	-0.47577	0.119	0.407	5.42E-56
C10orf10	3.66E-60	1.047114	0.275	0.037	5.47E-56
CLIC4	3.73E-60	0.282253	0.472	0.589	5.58E-56
PITX1	3.91E-60	-0.25114	0.031	0.232	5.84E-56
CLASP1	4.15E-60	-0.20832	0.075	0.293	6.20E-56
MAPK6	4.19E-60	-0.1763	0.12	0.347	6.26E-56
METTL23	4.23E-60	-0.10039	0.252	0.514	6.33E-56
FDX1L	4.32E-60	0.141308	0.24	0.43	6.46E-56
TCEAL8	4.54E-60	0.26397	0.39	0.551	6.79E-56
COX11	4.58E-60	0.133983	0.315	0.496	6.84E-56
RNMT	4.71E-60	0.154685	0.274	0.406	7.05E-56
TAGLN	4.72E-60	1.250877	0.228	0.038	7.05E-56
TMEM33	4.99E-60	-0.10293	0.21	0.452	7.46E-56
UNK	5.07E-60	-0.162	0.089	0.301	7.58E-56
OXR1	5.09E-60	-0.16912	0.157	0.409	7.60E-56
C12orf10	5.17E-60	0.238341	0.302	0.46	7.72E-56
MDC1	5.35E-60	-0.26091	0.026	0.216	8.00E-56
PRKAR2A	5.43E-60	-0.10732	0.205	0.444	8.11E-56
ZNRF1	5.45E-60	-0.26923	0.058	0.285	8.15E-56
DCLK2	6.08E-60	-0.28944	0.117	0.378	9.09E-56
MFSD12	6.24E-60	-0.22261	0.072	0.296	9.33E-56
TRPC4AP	6.25E-60	-0.2001	0.173	0.427	9.35E-56
MED1	6.75E-60	-0.23157	0.065	0.286	1.01E-55
ISCA2	6.84E-60	0.136388	0.269	0.478	1.02E-55
ACAA1	7.19E-60	-0.13492	0.229	0.468	1.07E-55
MORN2	7.45E-60	0.24204	0.315	0.467	1.11E-55
RNF2	7.47E-60	-0.12311	0.093	0.307	1.12E-55
TMEM222	8.04E-60	0.133965	0.297	0.474	1.20E-55

CXXC1	8.07E-60	-0.12236	0.146	0.384	1.21E-55
IFNAR2	8.19E-60	-0.18985	0.124	0.36	1.22E-55
SMIM4	8.59E-60	-0.10603	0.195	0.443	1.28E-55
ATN1	9.17E-60	-0.15898	0.126	0.362	1.37E-55
USE1	9.40E-60	0.341656	0.341	0.479	1.40E-55
PCSK7	1.07E-59	-0.15902	0.206	0.463	1.60E-55
PHF23	1.13E-59	-0.1153	0.129	0.359	1.69E-55
PSMG4	1.14E-59	-0.21159	0.132	0.383	1.70E-55
CLPP	1.16E-59	-0.23799	0.341	0.648	1.74E-55
SAP30BP	1.17E-59	0.117741	0.342	0.542	1.75E-55
CERK	1.23E-59	-0.28952	0.028	0.23	1.83E-55
GPI	1.26E-59	0.323597	0.361	0.451	1.89E-55
PQBP1	1.33E-59	0.162499	0.306	0.494	1.98E-55
SLC20A1	1.34E-59	-0.2647	0.137	0.4	2.01E-55
FAM173A	1.38E-59	-0.28127	0.127	0.386	2.07E-55
ADD1	1.39E-59	-0.12942	0.228	0.478	2.08E-55
SNX21	1.43E-59	-0.19986	0.078	0.291	2.13E-55
DHX15	1.48E-59	-0.13246	0.212	0.468	2.22E-55
XBP1	1.52E-59	0.204194	0.29	0.398	2.27E-55
EIF3G	1.75E-59	0.160669	0.569	0.768	2.62E-55
BCAT1	1.81E-59	-0.22984	0.149	0.402	2.71E-55
HIPK2	1.86E-59	-0.29533	0.122	0.385	2.78E-55
CPSF2	1.95E-59	-0.12564	0.073	0.263	2.92E-55
NEDD1	2.02E-59	-0.25413	0.077	0.309	3.01E-55
COIL	2.03E-59	-0.17685	0.142	0.393	3.03E-55
C1orf174	2.05E-59	-0.11596	0.132	0.351	3.06E-55
MAFG	2.08E-59	-0.193	0.109	0.336	3.11E-55
DUSP4	2.08E-59	-0.1211	0.103	0.295	3.11E-55
SLC25A6	2.10E-59	0.112376	0.717	0.927	3.14E-55
NUP93	2.19E-59	-0.25262	0.142	0.415	3.27E-55
NEMF	2.19E-59	-0.13241	0.23	0.481	3.27E-55
MTMR2	2.20E-59	-0.25055	0.087	0.323	3.28E-55
TYRO3	2.23E-59	-0.23825	0.058	0.272	3.34E-55
ZNF511	2.26E-59	-0.3007	0.09	0.343	3.37E-55
GNA11	2.31E-59	-0.21203	0.07	0.29	3.45E-55
PMM1	2.31E-59	0.263642	0.26	0.396	3.45E-55
VPS16	2.31E-59	-0.13603	0.118	0.343	3.46E-55
ATP5SL	2.52E-59	-0.17987	0.179	0.435	3.76E-55
ATP5L	2.54E-59	0.242688	0.778	0.92	3.79E-55
FNBP4	2.55E-59	-0.20966	0.201	0.459	3.80E-55
SERINC1	2.66E-59	0.223184	0.369	0.491	3.97E-55
AURKAIP1	2.67E-59	-0.12816	0.535	0.817	3.99E-55
AGPAT5	2.91E-59	0.130275	0.228	0.402	4.36E-55
TBC1D31	2.94E-59	-0.31402	0.018	0.205	4.40E-55
MESP1	3.05E-59	-0.37639	0.088	0.348	4.56E-55
HEBP2	3.20E-59	-0.31815	0.127	0.402	4.79E-55
CNRIP1	3.26E-59	-0.21623	0.167	0.431	4.87E-55
TAGLN2	3.31E-59	0.650872	0.671	0.544	4.95E-55
ANK2	3.31E-59	-0.24145	0.092	0.323	4.95E-55
ZNF32	3.32E-59	-0.18004	0.127	0.365	4.96E-55
NUP35	3.33E-59	-0.15588	0.12	0.359	4.98E-55

FAM103A1	3.35E-59	0.170991	0.223	0.411	5.01E-55
ERBB2IP	3.81E-59	-0.11404	0.15	0.378	5.70E-55
TM7SF3	4.59E-59	-0.34456	0.168	0.458	6.86E-55
NDUFV2	4.67E-59	0.224003	0.576	0.751	6.98E-55
AKT1	4.79E-59	-0.18217	0.083	0.296	7.16E-55
CCDC50	5.07E-59	-0.20841	0.192	0.456	7.57E-55
C15orf61	5.20E-59	-0.16508	0.132	0.358	7.77E-55
EFS	5.33E-59	-0.24849	0.055	0.264	7.97E-55
SUGT1	5.45E-59	0.124724	0.351	0.536	8.14E-55
SPTAN1	5.68E-59	-0.12321	0.193	0.438	8.50E-55
CDK2AP2	5.88E-59	0.192212	0.324	0.47	8.79E-55
RAB13	6.10E-59	0.552905	0.449	0.51	9.12E-55
IFT81	6.24E-59	-0.28589	0.142	0.41	9.33E-55
LRRC4B	6.30E-59	-0.27812	0.059	0.285	9.42E-55
PLEKHA3	6.99E-59	-0.20275	0.168	0.417	1.04E-54
HUWE1	7.13E-59	-0.24626	0.096	0.331	1.07E-54
FAM134A	7.17E-59	-0.26773	0.22	0.504	1.07E-54
INIP	7.59E-59	-0.1804	0.155	0.42	1.13E-54
SERTAD1	8.34E-59	0.762263	0.399	0.306	1.25E-54
DCTN2	9.87E-59	-0.28636	0.438	0.757	1.48E-54
CEP41	1.00E-58	-0.2826	0.114	0.369	1.49E-54
TMEM19	1.01E-58	-0.27984	0.076	0.306	1.51E-54
ALDH9A1	1.04E-58	0.321037	0.386	0.462	1.56E-54
PKN2	1.14E-58	-0.19972	0.154	0.4	1.70E-54
DYNLT3	1.19E-58	0.452029	0.365	0.374	1.78E-54
BLM	1.20E-58	-0.23583	0.044	0.246	1.79E-54
DDX23	1.37E-58	-0.22747	0.124	0.375	2.05E-54
PTGES2	1.45E-58	-0.26659	0.168	0.44	2.16E-54
NDUFA11	1.46E-58	0.217108	0.675	0.862	2.18E-54
EFNB2	1.47E-58	-0.26454	0.048	0.262	2.20E-54
PURA	1.51E-58	-0.33289	0.112	0.379	2.26E-54
CLIP2	1.51E-58	-0.33111	0.091	0.346	2.26E-54
DDIT3	1.52E-58	0.131771	0.44	0.627	2.28E-54
RUFY3	1.55E-58	-0.1792	0.217	0.469	2.32E-54
ZNF100	1.60E-58	-0.29495	0.031	0.232	2.39E-54
IL32	1.76E-58	0.784723	0.245	0.088	2.63E-54
SERTAD2	1.79E-58	-0.17054	0.068	0.275	2.67E-54
UBE2A	1.79E-58	0.185318	0.422	0.583	2.68E-54
DCAF11	1.84E-58	-0.11088	0.119	0.34	2.75E-54
CXADR	1.91E-58	-0.17249	0.172	0.42	2.86E-54
SEC14L1	1.97E-58	-0.11127	0.159	0.404	2.95E-54
NUMA1	2.16E-58	-0.17816	0.115	0.342	3.23E-54
CBS	2.35E-58	-0.28881	0.121	0.37	3.51E-54
ANKRD13D	2.44E-58	-0.18844	0.084	0.295	3.65E-54
SLC39A14	2.48E-58	0.684485	0.268	0.088	3.70E-54
FAM120AOS	2.71E-58	-0.21485	0.13	0.374	4.05E-54
SLC43A2	2.79E-58	-0.22309	0.097	0.32	4.16E-54
ARHGAP12	2.80E-58	-0.30509	0.087	0.333	4.18E-54
GOT2	3.13E-58	-0.21557	0.133	0.381	4.68E-54
KRI1	3.40E-58	-0.19121	0.084	0.296	5.07E-54
HMBS	3.62E-58	-0.13866	0.139	0.379	5.41E-54

TMEM106B	3.65E-58	0.178111	0.328	0.493	5.46E-54
MINOS1	3.75E-58	0.155044	0.557	0.775	5.60E-54
NUCB1	3.90E-58	0.130926	0.349	0.514	5.84E-54
NSMCE4A	3.91E-58	-0.35904	0.085	0.337	5.84E-54
ORAI1	4.01E-58	-0.27172	0.037	0.247	6.00E-54
10-Sep	4.38E-58	-0.35755	0.1	0.365	6.54E-54
RP11-111M	4.54E-58	-0.18949	0.066	0.277	6.78E-54
CUL5	5.39E-58	-0.18819	0.162	0.406	8.06E-54
ATG3	5.48E-58	-0.13637	0.282	0.543	8.19E-54
CEP350	5.87E-58	-0.23793	0.104	0.34	8.78E-54
NEDD8	6.16E-58	0.303711	0.683	0.848	9.20E-54
MAPKAPK5	6.52E-58	-0.19279	0.133	0.372	9.75E-54
FUZ	6.53E-58	-0.20087	0.157	0.4	9.75E-54
EIF4G2	6.54E-58	-0.19334	0.552	0.849	9.77E-54
FBXO7	6.64E-58	-0.18973	0.193	0.448	9.93E-54
TMUB1	6.74E-58	-0.22082	0.211	0.477	1.01E-53
SNX27	6.75E-58	-0.21338	0.156	0.405	1.01E-53
ATG4D	7.15E-58	-0.27321	0.118	0.37	1.07E-53
ZNF3	7.31E-58	-0.28799	0.151	0.425	1.09E-53
STK39	8.09E-58	-0.26392	0.048	0.258	1.21E-53
EXOSC3	8.21E-58	-0.27009	0.112	0.363	1.23E-53
TEN1	8.31E-58	-0.16788	0.098	0.316	1.24E-53
NDUFAF6	8.48E-58	-0.27095	0.126	0.384	1.27E-53
TP53RK	8.49E-58	-0.16598	0.149	0.377	1.27E-53
DAPK3	8.70E-58	-0.11842	0.224	0.453	1.30E-53
TCHP	8.76E-58	-0.24626	0.072	0.295	1.31E-53
ZNF106	8.85E-58	-0.1077	0.15	0.377	1.32E-53
PALLD	9.00E-58	-0.127	0.187	0.417	1.34E-53
HINT1	9.27E-58	0.272904	0.84	0.963	1.39E-53
SESTD1	1.00E-57	-0.36584	0.101	0.365	1.50E-53
HLA-DPB1	1.03E-57	0.955485	0.413	0.202	1.55E-53
ENO2	1.06E-57	0.237171	0.366	0.472	1.58E-53
KLF13	1.06E-57	-0.26587	0.033	0.233	1.58E-53
UBAC1	1.06E-57	-0.27343	0.12	0.383	1.59E-53
KLHL4	1.08E-57	-0.15318	0.086	0.306	1.61E-53
CCHCR1	1.16E-57	-0.25246	0.065	0.284	1.73E-53
LINC00657	1.21E-57	-0.13865	0.264	0.509	1.81E-53
AIG1	1.25E-57	0.282442	0.305	0.449	1.86E-53
FNBP1L	1.32E-57	-0.35895	0.091	0.348	1.97E-53
CDKN2A	1.33E-57	-0.70653	0.023	0.217	1.98E-53
ARMCX3	1.33E-57	0.188313	0.394	0.551	1.99E-53
MIF4GD	1.38E-57	-0.1723	0.139	0.378	2.06E-53
RB1CC1	1.40E-57	-0.11414	0.213	0.448	2.10E-53
YWHAE	1.41E-57	-0.13434	0.693	0.946	2.11E-53
ROCK2	1.55E-57	-0.2352	0.1	0.341	2.32E-53
LYRM5	1.57E-57	0.265469	0.335	0.468	2.35E-53
UBE2O	1.61E-57	-0.20314	0.053	0.249	2.40E-53
PSMD6	1.76E-57	0.127511	0.358	0.57	2.63E-53
CDO1	1.84E-57	0.417648	0.334	0.407	2.74E-53
USP39	2.07E-57	-0.17895	0.152	0.383	3.09E-53
LZIC	2.24E-57	-0.13277	0.134	0.362	3.35E-53

SHISA5	2.29E-57	0.216926	0.321	0.447	3.42E-53
RP9	2.38E-57	-0.14256	0.17	0.405	3.55E-53
RHBDD3	2.40E-57	-0.16753	0.095	0.307	3.59E-53
RSU1	2.72E-57	0.292745	0.232	0.317	4.06E-53
DHCR24	2.73E-57	-0.10098	0.054	0.217	4.08E-53
SRP54	2.88E-57	0.269772	0.305	0.414	4.31E-53
TMED5	3.09E-57	0.2714	0.33	0.44	4.62E-53
HMGB1	3.13E-57	-0.17089	0.806	0.991	4.68E-53
UCK1	3.17E-57	-0.23579	0.134	0.386	4.73E-53
RP11-277F	3.24E-57	-0.40059	0.02	0.206	4.84E-53
C14orf166	3.36E-57	0.119747	0.545	0.765	5.03E-53
DUSP1	3.37E-57	0.281931	0.417	0.48	5.03E-53
DENND2A	3.56E-57	-0.23912	0.042	0.238	5.32E-53
MTIF3	3.83E-57	0.305373	0.312	0.441	5.73E-53
MPND	3.92E-57	-0.22735	0.021	0.204	5.86E-53
SSBP2	4.04E-57	0.405851	0.358	0.457	6.03E-53
ZNF91	4.13E-57	-0.114	0.156	0.38	6.17E-53
WDR1	4.36E-57	0.130534	0.359	0.549	6.52E-53
PPP2R5D	4.58E-57	-0.21885	0.046	0.241	6.84E-53
EFCAB11	4.92E-57	-0.24706	0.038	0.232	7.35E-53
TMEM50B	4.94E-57	0.354073	0.347	0.422	7.38E-53
HTRA2	5.02E-57	-0.17994	0.173	0.428	7.50E-53
CANT1	5.16E-57	-0.21034	0.141	0.393	7.72E-53
GMPS	5.28E-57	-0.24303	0.141	0.398	7.89E-53
TTC14	5.47E-57	0.153818	0.249	0.411	8.18E-53
ZNF232	5.50E-57	-0.26703	0.048	0.254	8.21E-53
MED30	5.54E-57	-0.25879	0.211	0.494	8.28E-53
UGGT1	5.61E-57	-0.17832	0.062	0.262	8.39E-53
SNHG8	5.68E-57	0.565735	0.428	0.451	8.49E-53
CCNE1	6.12E-57	-0.32587	0.013	0.186	9.14E-53
BICD1	6.60E-57	-0.29809	0.145	0.41	9.87E-53
ZCCHC3	6.73E-57	-0.26486	0.046	0.257	1.01E-52
DNAAF2	6.92E-57	-0.1363	0.106	0.315	1.03E-52
ZNF593	6.92E-57	0.156039	0.275	0.459	1.03E-52
MED16	6.95E-57	-0.18662	0.112	0.333	1.04E-52
SMOX	7.11E-57	0.527345	0.367	0.369	1.06E-52
ADO	7.25E-57	-0.23714	0.053	0.262	1.08E-52
CA12	7.60E-57	0.511079	0.246	0.18	1.14E-52
GADD45A	7.84E-57	0.404217	0.421	0.463	1.17E-52
ZNF639	7.84E-57	-0.25654	0.082	0.312	1.17E-52
RAP1A	8.04E-57	0.307473	0.397	0.5	1.20E-52
MCFD2	8.31E-57	0.182792	0.274	0.417	1.24E-52
KLHL13	9.10E-57	-0.30149	0.038	0.246	1.36E-52
DDX21	9.13E-57	0.111209	0.243	0.402	1.36E-52
HLA-DQA1	1.01E-56	1.160225	0.213	0.026	1.51E-52
TAF9B	1.01E-56	-0.2538	0.107	0.344	1.51E-52
DNAJC3	1.16E-56	0.222587	0.278	0.383	1.73E-52
ZNF524	1.17E-56	-0.17568	0.103	0.32	1.75E-52
MLH1	1.20E-56	-0.21466	0.121	0.365	1.79E-52
BMPR2	1.21E-56	-0.27325	0.081	0.314	1.80E-52
ARFRP1	1.24E-56	-0.17034	0.182	0.431	1.85E-52

RNF11	1.27E-56	-0.14877	0.202	0.441	1.90E-52
RBM5	1.41E-56	-0.16897	0.19	0.435	2.11E-52
EXOSC10	1.45E-56	-0.19579	0.107	0.332	2.17E-52
UBE2G1	1.49E-56	-0.18965	0.181	0.431	2.22E-52
FKBP10	1.49E-56	0.240059	0.304	0.423	2.23E-52
PDPK1	1.62E-56	-0.27146	0.121	0.373	2.42E-52
CPSF3L	1.62E-56	0.15437	0.266	0.438	2.43E-52
BDP1	1.67E-56	-0.20803	0.197	0.459	2.50E-52
KIAA1549	1.68E-56	-0.3297	0.034	0.237	2.51E-52
TMEM121	1.69E-56	-0.3504	0.038	0.248	2.53E-52
FAM110B	1.72E-56	-0.27638	0.095	0.336	2.57E-52
FAM92A1	1.76E-56	-0.15327	0.183	0.446	2.63E-52
ARFGAP2	1.85E-56	-0.14369	0.205	0.454	2.77E-52
SPATC1L	1.86E-56	-0.29369	0.112	0.367	2.78E-52
CAHM	1.87E-56	-0.35042	0.032	0.233	2.79E-52
CDH4	1.88E-56	-0.26964	0.022	0.202	2.81E-52
B4GALT7	1.92E-56	-0.14305	0.137	0.367	2.87E-52
RNF13	1.93E-56	0.355261	0.4	0.467	2.89E-52
CBR4	1.93E-56	-0.24649	0.116	0.357	2.89E-52
CDC42EP1	1.95E-56	-0.22916	0.079	0.299	2.91E-52
FAM168B	2.00E-56	-0.15831	0.119	0.34	2.98E-52
NQO2	2.10E-56	0.173136	0.235	0.388	3.14E-52
CDYL	2.11E-56	-0.27197	0.053	0.268	3.15E-52
SH3GLB2	2.14E-56	-0.20671	0.047	0.254	3.19E-52
GPCPD1	2.18E-56	-0.20021	0.09	0.315	3.26E-52
SCAF4	2.20E-56	-0.22935	0.087	0.309	3.29E-52
HOMER2	2.23E-56	-0.11945	0.027	0.172	3.33E-52
RRP1B	2.24E-56	-0.22166	0.103	0.341	3.35E-52
ENTPD6	2.26E-56	-0.17731	0.127	0.364	3.37E-52
ZNF711	2.33E-56	-0.39553	0.076	0.322	3.48E-52
SPRY1	2.41E-56	-0.38965	0.164	0.443	3.60E-52
NDUFB7	2.47E-56	0.274964	0.724	0.869	3.69E-52
DLD	2.53E-56	0.309241	0.386	0.5	3.78E-52
LAP3	2.58E-56	0.396984	0.421	0.528	3.85E-52
MEPCE	2.77E-56	-0.20328	0.084	0.302	4.14E-52
DCAF16	2.90E-56	-0.20043	0.09	0.31	4.34E-52
SDF2L1	2.91E-56	-0.25176	0.25	0.519	4.36E-52
TRIM69	2.92E-56	-0.17399	0.13	0.365	4.37E-52
MYH10	3.02E-56	-0.31406	0.124	0.389	4.52E-52
CNTROB	3.19E-56	-0.28738	0.048	0.257	4.77E-52
UQCRC2	3.26E-56	0.212436	0.436	0.595	4.87E-52
EIF4G1	3.30E-56	-0.10753	0.23	0.464	4.94E-52
VSTM2A	3.37E-56	0.95592	0.296	0.079	5.03E-52
STX18	3.47E-56	-0.12298	0.102	0.319	5.18E-52
ANAPC7	3.67E-56	-0.17664	0.123	0.36	5.49E-52
HMG20B	3.68E-56	-0.32213	0.254	0.556	5.49E-52
ATPAF2	3.83E-56	-0.1147	0.126	0.344	5.72E-52
AGAP1	3.87E-56	-0.19918	0.072	0.279	5.79E-52
XRCC3	3.91E-56	-0.23166	0.011	0.17	5.84E-52
SNAP29	3.96E-56	-0.18892	0.12	0.351	5.92E-52
ASB16-AS1	4.00E-56	-0.21087	0.053	0.247	5.98E-52

LSM12	4.08E-56	-0.28888	0.196	0.486	6.09E-52
PFKL	4.37E-56	-0.10147	0.277	0.51	6.53E-52
PCBD1	4.57E-56	0.187925	0.281	0.437	6.82E-52
PRDM2	4.61E-56	-0.20384	0.068	0.275	6.89E-52
PPP2R2D	4.87E-56	-0.22337	0.04	0.231	7.28E-52
NFATC3	5.24E-56	-0.2025	0.096	0.317	7.83E-52
PEX10	5.33E-56	-0.11688	0.156	0.377	7.97E-52
LAMA5	5.47E-56	-0.2164	0.057	0.259	8.18E-52
C19orf52	6.01E-56	-0.24299	0.133	0.38	8.99E-52
HNRNPH2	6.55E-56	0.268653	0.369	0.491	9.79E-52
MFSD10	6.59E-56	0.164424	0.248	0.407	9.85E-52
KCTD20	6.79E-56	-0.24317	0.108	0.352	1.02E-51
P4HTM	6.95E-56	-0.10713	0.23	0.449	1.04E-51
KIAA0586	6.95E-56	-0.25891	0.034	0.226	1.04E-51
MGAT2	6.99E-56	-0.19986	0.135	0.369	1.04E-51
CD68	7.31E-56	0.756072	0.317	0.165	1.09E-51
DKK3	7.36E-56	-0.16309	0.173	0.4	1.10E-51
UBL3	7.74E-56	-0.10573	0.239	0.475	1.16E-51
POFUT1	8.19E-56	-0.18204	0.077	0.291	1.22E-51
TSR1	8.24E-56	-0.1048	0.161	0.398	1.23E-51
NUDCD2	8.28E-56	-0.11043	0.404	0.695	1.24E-51
SCAF1	8.60E-56	-0.1971	0.095	0.306	1.29E-51
PPDPF	8.86E-56	-0.15957	0.639	0.916	1.32E-51
KXD1	9.53E-56	0.234811	0.479	0.652	1.42E-51
PTX3	9.76E-56	0.426599	0.178	0.216	1.46E-51
STX12	1.01E-55	0.273588	0.356	0.477	1.51E-51
TRIP6	1.03E-55	0.540687	0.358	0.347	1.54E-51
GSTO1	1.04E-55	0.296752	0.341	0.452	1.55E-51
ING1	1.04E-55	-0.18968	0.107	0.338	1.56E-51
TNRC6C	1.06E-55	-0.2212	0.114	0.347	1.59E-51
FDFT1	1.11E-55	-0.15137	0.223	0.481	1.66E-51
MEIS1	1.23E-55	-0.30803	0.038	0.24	1.83E-51
NTN1	1.23E-55	-0.14041	0.042	0.212	1.84E-51
MTMR4	1.23E-55	-0.22802	0.087	0.306	1.85E-51
DCTN1	1.29E-55	-0.18853	0.133	0.365	1.92E-51
RBMXL1	1.31E-55	-0.25307	0.124	0.363	1.95E-51
TAPBP	1.36E-55	0.108318	0.258	0.399	2.03E-51
DONSON	1.39E-55	-0.29715	0.075	0.307	2.08E-51
CCDC106	1.52E-55	-0.23638	0.157	0.412	2.27E-51
GRN	1.56E-55	0.201567	0.365	0.523	2.32E-51
C8orf82	1.57E-55	-0.28036	0.091	0.327	2.35E-51
TMEM132A	1.59E-55	-0.17425	0.096	0.305	2.38E-51
ERI2	1.71E-55	-0.21151	0.028	0.201	2.55E-51
CYB5A	1.82E-55	0.411182	0.355	0.457	2.72E-51
AGT	1.82E-55	0.7357	0.366	0.326	2.72E-51
CXXC4	1.85E-55	-0.30447	0.02	0.196	2.76E-51
PCNT	1.87E-55	-0.24178	0.043	0.24	2.80E-51
OSER1	1.94E-55	0.19523	0.255	0.419	2.91E-51
RRP1	1.96E-55	-0.11773	0.159	0.401	2.93E-51
TCEAL7	2.02E-55	0.125255	0.293	0.498	3.02E-51
PPP4R2	2.14E-55	-0.17612	0.206	0.463	3.20E-51

GOPC	2.17E-55	-0.21899	0.188	0.442	3.24E-51
NUDT4	2.17E-55	-0.16786	0.175	0.416	3.25E-51
SDHAF2	2.23E-55	0.126838	0.252	0.423	3.33E-51
EMC6	2.23E-55	-0.18001	0.393	0.683	3.34E-51
CEP63	2.23E-55	-0.22928	0.14	0.381	3.34E-51
UPF3B	2.26E-55	-0.18039	0.21	0.467	3.38E-51
GOLT1B	2.41E-55	0.235081	0.273	0.381	3.60E-51
PRSS23	2.43E-55	0.738891	0.257	0.091	3.63E-51
GPATCH2L	2.49E-55	-0.14631	0.139	0.359	3.72E-51
SMAD4	2.50E-55	-0.19245	0.075	0.283	3.74E-51
APC	2.60E-55	-0.19765	0.13	0.357	3.88E-51
UTP18	2.61E-55	-0.16096	0.21	0.458	3.90E-51
PYGB	2.62E-55	-0.12702	0.169	0.399	3.92E-51
DNM1L	2.72E-55	-0.16494	0.169	0.415	4.07E-51
RMI1	2.77E-55	-0.35015	0.036	0.237	4.13E-51
FLYWCH2	2.83E-55	0.17978	0.308	0.475	4.22E-51
CSNK1G2	2.88E-55	-0.30028	0.069	0.295	4.31E-51
CD81	2.90E-55	0.108784	0.248	0.422	4.34E-51
BTG2	2.95E-55	0.302213	0.335	0.449	4.41E-51
AKT2	3.00E-55	-0.19911	0.125	0.357	4.48E-51
EPHA3	3.20E-55	-0.18714	0.01	0.162	4.78E-51
REV3L	3.29E-55	-0.29879	0.168	0.436	4.92E-51
AP5M1	3.45E-55	-0.17744	0.108	0.33	5.15E-51
ZSCAN16-A	3.68E-55	-0.16787	0.186	0.441	5.50E-51
PNPT1	3.76E-55	-0.14698	0.154	0.384	5.62E-51
CLTC	3.83E-55	-0.19806	0.229	0.494	5.72E-51
SMAD2	3.88E-55	-0.11186	0.138	0.352	5.79E-51
ERP44	3.91E-55	0.234727	0.331	0.435	5.84E-51
POMGNT2	3.98E-55	-0.18665	0.115	0.343	5.95E-51
PEF1	4.18E-55	0.101851	0.316	0.512	6.24E-51
SIRT7	4.28E-55	-0.16912	0.139	0.367	6.40E-51
SPATA6	4.30E-55	-0.23286	0.091	0.32	6.43E-51
ATXN2	4.34E-55	-0.11349	0.152	0.369	6.49E-51
CTTN	4.46E-55	0.191237	0.279	0.411	6.66E-51
LRRFIP2	4.59E-55	-0.11793	0.17	0.389	6.86E-51
KCTD5	4.61E-55	-0.27287	0.134	0.385	6.88E-51
VEZT	4.66E-55	-0.15676	0.155	0.394	6.96E-51
DSCC1	4.86E-55	-0.33757	0.043	0.252	7.27E-51
SF3A1	4.87E-55	-0.23573	0.106	0.343	7.27E-51
SSFA2	5.10E-55	-0.16418	0.146	0.369	7.62E-51
SNX5	5.11E-55	-0.29946	0.305	0.622	7.64E-51
FANCB	5.87E-55	-0.21133	0.014	0.17	8.77E-51
RFXANK	5.94E-55	0.161458	0.303	0.486	8.88E-51
FBXW11	6.21E-55	-0.13598	0.131	0.343	9.28E-51
NAA20	6.52E-55	0.180004	0.47	0.651	9.74E-51
ANGPTL4	7.38E-55	0.941125	0.254	0.033	1.10E-50
TMEM59L	7.62E-55	-0.10346	0.223	0.438	1.14E-50
POLE2	7.70E-55	-0.25376	0.023	0.19	1.15E-50
MBD6	8.13E-55	-0.52886	0.063	0.298	1.21E-50
ZNF771	8.16E-55	-0.27559	0.108	0.353	1.22E-50
CPNE3	8.47E-55	0.234271	0.327	0.459	1.27E-50

CHCHD10	8.73E-55	-0.29244	0.175	0.441	1.31E-50
DNAJC5	9.00E-55	-0.23325	0.103	0.332	1.35E-50
PCYT2	9.47E-55	-0.13268	0.106	0.307	1.42E-50
CHERP	9.57E-55	-0.14384	0.152	0.37	1.43E-50
RBM12	9.76E-55	-0.23748	0.066	0.278	1.46E-50
PAK3	1.09E-54	-0.34284	0.035	0.233	1.63E-50
TUBA1B	1.13E-54	-0.13536	0.842	1	1.68E-50
PHLDB1	1.16E-54	-0.12711	0.062	0.241	1.73E-50
CAPRIN2	1.21E-54	-0.20596	0.066	0.268	1.81E-50
SREBF2	1.22E-54	-0.19138	0.054	0.243	1.82E-50
SLU7	1.22E-54	0.248894	0.301	0.407	1.82E-50
LSM7	1.25E-54	-0.10988	0.579	0.859	1.86E-50
BLOC1S6	1.29E-54	-0.21116	0.169	0.415	1.92E-50
TSPAN14	1.31E-54	-0.24827	0.052	0.254	1.96E-50
UROS	1.31E-54	-0.24876	0.133	0.375	1.96E-50
TM2D2	1.32E-54	0.528827	0.372	0.405	1.97E-50
ANKIB1	1.33E-54	-0.20241	0.134	0.365	1.99E-50
PUM1	1.33E-54	-0.11031	0.16	0.375	1.99E-50
ZNF362	1.34E-54	-0.20903	0.031	0.207	2.01E-50
C1orf52	1.39E-54	0.204414	0.322	0.465	2.08E-50
FH	1.42E-54	-0.15504	0.2	0.453	2.12E-50
APPBP2	1.43E-54	-0.10116	0.157	0.377	2.14E-50
WDR5	1.45E-54	-0.17589	0.108	0.322	2.17E-50
ERH	1.51E-54	0.164996	0.726	0.914	2.26E-50
ZNF726	1.54E-54	-0.25619	0.023	0.194	2.31E-50
SLC25A37	1.62E-54	0.51144	0.322	0.321	2.42E-50
DHX33	1.62E-54	-0.24399	0.074	0.291	2.42E-50
PCDH19	1.64E-54	-0.25175	0.014	0.177	2.45E-50
LIMD1	1.64E-54	-0.20564	0.033	0.214	2.45E-50
DDX11	1.75E-54	-0.30229	0.039	0.24	2.61E-50
NUP85	1.76E-54	-0.2204	0.102	0.33	2.63E-50
TNFAIP1	1.78E-54	-0.16749	0.122	0.348	2.66E-50
CCDC53	1.79E-54	0.226355	0.327	0.472	2.67E-50
ANAPC13	1.82E-54	0.150641	0.369	0.525	2.72E-50
C19orf24	2.08E-54	-0.15397	0.278	0.537	3.10E-50
RGS2	2.08E-54	1.017987	0.422	0.227	3.11E-50
CDV3	2.14E-54	-0.20518	0.175	0.423	3.19E-50
IQGAP2	2.21E-54	-0.24363	0.062	0.267	3.30E-50
PPP1R12C	2.44E-54	-0.12728	0.042	0.207	3.64E-50
CFL2	2.48E-54	0.240276	0.406	0.546	3.71E-50
TEX264	2.52E-54	0.418469	0.393	0.478	3.77E-50
RNF34	2.52E-54	-0.12141	0.167	0.4	3.77E-50
PRPF31	2.53E-54	-0.10895	0.358	0.622	3.79E-50
SMC6	2.59E-54	-0.31292	0.106	0.357	3.87E-50
IWS1	2.65E-54	-0.19693	0.161	0.414	3.96E-50
MFSD11	2.65E-54	-0.1136	0.135	0.363	3.96E-50
CSAD	2.68E-54	-0.23344	0.097	0.328	4.00E-50
NUDT15	2.70E-54	-0.20085	0.094	0.307	4.04E-50
SERPINB6	2.82E-54	0.226562	0.398	0.541	4.22E-50
MTMR9	2.93E-54	-0.20728	0.124	0.356	4.37E-50
TNFRSF1A	2.96E-54	0.186307	0.347	0.432	4.43E-50

ACYP2	2.98E-54	0.370121	0.31	0.411	4.46E-50
CDH24	3.00E-54	-0.26084	0.01	0.169	4.48E-50
TSPAN7	3.07E-54	0.19848	0.395	0.557	4.58E-50
SMIM20	3.08E-54	0.273151	0.329	0.446	4.61E-50
PRPF4B	3.17E-54	-0.13313	0.195	0.44	4.74E-50
LY6E	3.19E-54	0.217607	0.336	0.493	4.77E-50
MAP3K7	3.60E-54	-0.10731	0.156	0.372	5.37E-50
CNOT2	3.61E-54	0.146828	0.292	0.464	5.39E-50
MLLT4	3.61E-54	-0.32627	0.101	0.348	5.40E-50
CC2D1A	3.94E-54	-0.22873	0.126	0.363	5.89E-50
PDE4B	3.95E-54	-0.15137	0.129	0.338	5.91E-50
EPHX1	4.12E-54	0.257901	0.238	0.354	6.16E-50
TMEM132B	4.67E-54	-0.26231	0.018	0.19	6.98E-50
DZIP1	5.54E-54	-0.19736	0.057	0.259	8.29E-50
ACAT1	5.58E-54	0.469747	0.424	0.43	8.34E-50
TWSG1	5.66E-54	0.352266	0.231	0.302	8.46E-50
PKNOX1	5.81E-54	-0.21082	0.083	0.296	8.69E-50
SLC25A25	5.84E-54	-0.19651	0.136	0.363	8.73E-50
ZNF75A	5.85E-54	-0.15697	0.201	0.446	8.74E-50
JAGN1	6.15E-54	0.228691	0.341	0.509	9.19E-50
NBL1	6.50E-54	-0.20336	0.086	0.298	9.71E-50
TLK2	6.58E-54	-0.18326	0.072	0.277	9.84E-50
DCAF13	6.98E-54	0.162206	0.365	0.52	1.04E-49
USP34	7.08E-54	-0.16793	0.135	0.36	1.06E-49
CRAT	7.42E-54	-0.24461	0.123	0.362	1.11E-49
RARRES2	7.91E-54	0.922336	0.461	0.277	1.18E-49
CARS2	8.30E-54	-0.17023	0.076	0.278	1.24E-49
LAMC1	8.34E-54	-0.13714	0.069	0.253	1.25E-49
ALKBH4	8.83E-54	-0.14359	0.16	0.388	1.32E-49
ICT1	9.37E-54	-0.15123	0.17	0.417	1.40E-49
CDC25A	9.70E-54	-0.30024	0.008	0.16	1.45E-49
XRCC1	1.02E-53	-0.23391	0.103	0.328	1.52E-49
IARS2	1.02E-53	-0.14155	0.152	0.378	1.53E-49
SEC31A	1.03E-53	0.124921	0.213	0.333	1.54E-49
BCS1L	1.04E-53	-0.17254	0.138	0.368	1.56E-49
MAN1A2	1.05E-53	-0.21111	0.141	0.377	1.57E-49
TRMT6	1.11E-53	-0.13022	0.154	0.385	1.66E-49
PFDN6	1.15E-53	0.124784	0.334	0.535	1.71E-49
CHMP2B	1.18E-53	0.272475	0.305	0.416	1.76E-49
TMEM178A	1.19E-53	-0.32078	0.05	0.259	1.77E-49
VKORC1L1	1.22E-53	-0.20591	0.101	0.309	1.83E-49
GLYR1	1.25E-53	-0.19384	0.154	0.39	1.88E-49
RP11-620J	1.28E-53	-0.35371	0.302	0.612	1.91E-49
RAB4A	1.28E-53	-0.2433	0.262	0.542	1.91E-49
TGIF2	1.33E-53	-0.26852	0.026	0.209	1.98E-49
LEPROT	1.38E-53	0.165923	0.311	0.469	2.06E-49
B3GALT6	1.41E-53	-0.24626	0.061	0.265	2.10E-49
ERF	1.52E-53	-0.21926	0.114	0.332	2.27E-49
B4GALT5	1.52E-53	-0.19329	0.136	0.38	2.28E-49
GLTP	1.53E-53	-0.15344	0.215	0.454	2.29E-49
GTF2E2	1.86E-53	-0.21826	0.122	0.353	2.77E-49

PKP4	1.96E-53	-0.2533	0.024	0.204	2.92E-49
XIAP	1.97E-53	-0.20154	0.149	0.385	2.94E-49
AIMP2	2.01E-53	0.100158	0.259	0.442	3.01E-49
DPP6	2.11E-53	-0.24687	0.117	0.351	3.15E-49
EMID1	2.16E-53	-0.2057	0.023	0.193	3.22E-49
HELZ	2.43E-53	-0.16582	0.113	0.331	3.63E-49
ZNF462	2.76E-53	-0.26211	0.112	0.362	4.13E-49
ATP6V0A2	2.82E-53	-0.24448	0.051	0.256	4.21E-49
TACO1	2.97E-53	-0.25268	0.114	0.351	4.43E-49
ZC3H14	3.00E-53	-0.12188	0.186	0.42	4.48E-49
TTF2	3.12E-53	-0.24118	0.041	0.236	4.66E-49
HSPE1	3.15E-53	0.111304	0.714	0.902	4.71E-49
BLOC1S2	3.22E-53	0.226895	0.293	0.438	4.81E-49
RBBP9	3.28E-53	-0.24586	0.046	0.244	4.90E-49
H2AFJ	3.29E-53	-0.33083	0.079	0.311	4.92E-49
TMA7	3.33E-53	0.312104	0.815	0.914	4.98E-49
IFI16	3.37E-53	0.681703	0.489	0.396	5.03E-49
BCL7A	3.52E-53	-0.31086	0.069	0.289	5.26E-49
RTKN	3.72E-53	-0.1308	0.093	0.302	5.56E-49
JUND	3.80E-53	-0.1209	0.286	0.526	5.68E-49
CSNK1G3	3.89E-53	-0.27771	0.092	0.322	5.81E-49
DNASE2	3.89E-53	0.301771	0.284	0.384	5.82E-49
NAA50	3.90E-53	-0.15862	0.23	0.477	5.83E-49
FAM122B	4.01E-53	-0.283	0.081	0.305	5.99E-49
SPIN1	4.31E-53	-0.19515	0.099	0.312	6.45E-49
IFT57	4.69E-53	0.355755	0.363	0.436	7.01E-49
RCOR1	4.74E-53	-0.18173	0.022	0.178	7.08E-49
SETD2	4.79E-53	-0.20479	0.092	0.302	7.16E-49
MSN	5.02E-53	0.180611	0.285	0.416	7.50E-49
SUPT6H	5.32E-53	-0.18384	0.084	0.293	7.95E-49
KLHL42	5.36E-53	-0.28701	0.109	0.348	8.01E-49
SART1	5.86E-53	-0.24832	0.137	0.38	8.76E-49
CHEK2	5.91E-53	-0.24094	0.037	0.221	8.82E-49
LYRM7	6.08E-53	-0.18602	0.161	0.395	9.08E-49
ENDOG	6.09E-53	-0.19581	0.085	0.293	9.09E-49
STARD4-AS	6.13E-53	-0.30654	0.046	0.249	9.17E-49
EXOSC9	6.47E-53	-0.31922	0.12	0.369	9.67E-49
SMAD5	6.53E-53	-0.12456	0.157	0.388	9.77E-49
PAXBP1	6.81E-53	-0.19172	0.111	0.331	1.02E-48
CREM	6.85E-53	-0.31316	0.154	0.415	1.02E-48
NDUFAF5	7.27E-53	-0.17545	0.09	0.298	1.09E-48
CTNNB1	7.29E-53	-0.11774	0.262	0.509	1.09E-48
BST2	7.93E-53	0.862832	0.461	0.232	1.18E-48
TM2D3	7.97E-53	0.282021	0.319	0.417	1.19E-48
G3BP2	8.93E-53	-0.16459	0.176	0.406	1.33E-48
GEMIN2	9.51E-53	-0.15365	0.098	0.304	1.42E-48
SCRN1	9.91E-53	-0.16016	0.13	0.347	1.48E-48
CEP97	1.00E-52	-0.22539	0.046	0.236	1.50E-48
ZNF682	1.06E-52	-0.31107	0.05	0.26	1.59E-48
WDR74	1.08E-52	0.179083	0.23	0.374	1.62E-48
SENP6	1.09E-52	-0.14502	0.159	0.375	1.62E-48

PTDSS2	1.15E-52	-0.24937	0.092	0.317	1.72E-48
ICA1L	1.28E-52	-0.21704	0.085	0.302	1.91E-48
RP11-262H	1.34E-52	-0.21667	0.057	0.253	2.00E-48
TRIM33	1.35E-52	-0.26992	0.107	0.338	2.02E-48
SNTG1	1.36E-52	0.175045	0.168	0.33	2.03E-48
COPB1	1.36E-52	0.109453	0.274	0.433	2.03E-48
ZNF579	1.40E-52	-0.20174	0.048	0.237	2.09E-48
TMSB4X	1.41E-52	0.506385	0.992	0.994	2.11E-48
BNIP2	1.42E-52	-0.12787	0.159	0.37	2.12E-48
GEN1	1.42E-52	-0.20875	0.014	0.164	2.12E-48
PSPH	1.48E-52	-0.12778	0.17	0.388	2.21E-48
E2F5	1.48E-52	-0.13234	0.03	0.193	2.21E-48
IGSF10	1.58E-52	-0.23343	0.045	0.231	2.37E-48
MAD1L1	1.59E-52	-0.23001	0.093	0.314	2.38E-48
COPS7B	1.64E-52	-0.10376	0.157	0.38	2.45E-48
MTG2	1.64E-52	-0.15902	0.093	0.304	2.45E-48
USP47	1.78E-52	-0.12886	0.205	0.435	2.66E-48
DOHH	1.89E-52	-0.17402	0.152	0.377	2.83E-48
TMEM70	1.95E-52	0.104565	0.322	0.479	2.91E-48
POLR3GL	1.97E-52	0.109388	0.282	0.454	2.94E-48
SMTN	2.01E-52	-0.28502	0.03	0.214	3.00E-48
SFXN5	2.12E-52	0.156749	0.218	0.375	3.17E-48
PSME2	2.15E-52	0.176386	0.522	0.727	3.21E-48
TAOK3	2.15E-52	-0.12051	0.239	0.475	3.21E-48
TMED1	2.43E-52	0.363098	0.344	0.46	3.64E-48
FAM60A	2.44E-52	-0.25583	0.1	0.332	3.64E-48
MYCBP2	2.63E-52	-0.31145	0.134	0.391	3.92E-48
ATF6	2.66E-52	-0.10832	0.172	0.378	3.98E-48
NCOA1	2.93E-52	-0.1171	0.099	0.293	4.38E-48
NACC1	3.17E-52	-0.15883	0.097	0.305	4.74E-48
ATF3	3.20E-52	0.560719	0.334	0.228	4.79E-48
ZC3H8	3.32E-52	-0.20569	0.111	0.34	4.96E-48
IFI6	3.42E-52	0.290498	0.421	0.502	5.11E-48
MFAP1	3.48E-52	-0.11706	0.149	0.363	5.20E-48
TBC1D20	3.59E-52	-0.15265	0.128	0.335	5.37E-48
FAM120A	3.83E-52	-0.1646	0.122	0.342	5.73E-48
YBX3	4.29E-52	-0.10501	0.356	0.594	6.41E-48
USP5	4.61E-52	-0.22057	0.184	0.44	6.89E-48
EMILIN3	4.76E-52	-0.32074	0.004	0.141	7.11E-48
CTBP2	4.80E-52	-0.25756	0.072	0.29	7.17E-48
UBAP2L	4.93E-52	-0.16167	0.158	0.38	7.36E-48
MDM4	4.93E-52	-0.21661	0.171	0.414	7.37E-48
CASP4	4.95E-52	0.804133	0.255	0.115	7.40E-48
ZNF827	4.95E-52	-0.27703	0.056	0.267	7.40E-48
FBXO11	5.19E-52	-0.22393	0.12	0.349	7.75E-48
TMCO3	5.25E-52	-0.10572	0.186	0.396	7.85E-48
NRBF2	5.50E-52	-0.10536	0.173	0.398	8.21E-48
SETD9	5.53E-52	-0.1167	0.079	0.272	8.27E-48
FZD7	5.64E-52	-0.21807	0.046	0.237	8.43E-48
E2F7	5.78E-52	-0.1912	0.009	0.153	8.63E-48
KANSL1	5.83E-52	-0.26911	0.088	0.31	8.72E-48

PPIE	5.98E-52	0.116029	0.28	0.442	8.93E-48
EML4	6.00E-52	-0.19743	0.09	0.302	8.96E-48
SCAMP2	6.71E-52	0.226279	0.221	0.356	1.00E-47
DAAM1	7.37E-52	-0.13955	0.194	0.432	1.10E-47
SPRED2	7.53E-52	-0.21811	0.055	0.243	1.12E-47
YES1	7.66E-52	-0.24175	0.095	0.316	1.14E-47
G6PD	7.69E-52	-0.12789	0.138	0.359	1.15E-47
STX16	8.02E-52	-0.11476	0.173	0.383	1.20E-47
PARK7	8.47E-52	0.241181	0.745	0.906	1.27E-47
FAM20C	8.48E-52	0.403723	0.274	0.304	1.27E-47
GABPB1	9.25E-52	-0.20498	0.077	0.285	1.38E-47
DHRS13	1.01E-51	-0.18223	0.07	0.258	1.52E-47
CEP70	1.06E-51	-0.31508	0.088	0.321	1.59E-47
RP11-332H	1.14E-51	-0.22818	0.106	0.332	1.71E-47
FANCL	1.26E-51	-0.25116	0.137	0.385	1.89E-47
HAUS2	1.28E-51	-0.24247	0.061	0.268	1.91E-47
STK24	1.32E-51	-0.15406	0.049	0.223	1.98E-47
HABP4	1.33E-51	-0.23635	0.077	0.29	1.99E-47
ITGAV	1.38E-51	-0.1709	0.172	0.406	2.06E-47
YPEL5	1.40E-51	0.482945	0.409	0.401	2.10E-47
TGS1	1.42E-51	-0.15977	0.129	0.359	2.13E-47
NEU1	1.50E-51	0.178813	0.247	0.364	2.24E-47
PDDC1	1.76E-51	-0.11402	0.11	0.31	2.64E-47
MYC	1.89E-51	0.757258	0.333	0.185	2.83E-47
ZNF821	1.95E-51	-0.27745	0.072	0.288	2.92E-47
TUBGCP2	2.00E-51	-0.16172	0.108	0.322	2.98E-47
UACA	2.02E-51	-0.17715	0.032	0.2	3.03E-47
MAP3K1	2.17E-51	-0.24568	0.022	0.189	3.24E-47
ATPAF1	2.35E-51	-0.309	0.181	0.442	3.51E-47
CASP3	2.35E-51	-0.24001	0.146	0.386	3.51E-47
DLST	2.36E-51	-0.11017	0.099	0.29	3.53E-47
PPIF	2.41E-51	-0.26445	0.124	0.367	3.60E-47
APITD1	2.44E-51	-0.13746	0.073	0.264	3.64E-47
CAST	2.47E-51	0.288519	0.308	0.356	3.69E-47
C12orf76	2.49E-51	0.168463	0.231	0.391	3.72E-47
BANF1	2.68E-51	0.128304	0.677	0.873	4.00E-47
SLC36A4	2.70E-51	-0.18724	0.175	0.404	4.03E-47
CHD6	2.87E-51	-0.20336	0.168	0.412	4.29E-47
ABHD17C	2.88E-51	-0.17324	0.038	0.207	4.31E-47
DLG1	2.96E-51	-0.16564	0.106	0.311	4.42E-47
HIST1H1C	3.07E-51	0.189117	0.181	0.344	4.59E-47
KLHL5	3.22E-51	-0.18372	0.075	0.274	4.81E-47
CIR1	3.22E-51	0.208415	0.301	0.426	4.81E-47
PLA2G12A	3.33E-51	-0.14908	0.184	0.401	4.98E-47
MESDC2	3.34E-51	0.368237	0.398	0.523	4.99E-47
NMT2	3.41E-51	-0.25685	0.06	0.267	5.09E-47
TSR2	3.45E-51	-0.12609	0.218	0.464	5.16E-47
FAM98B	3.46E-51	-0.201	0.045	0.226	5.18E-47
TMEM209	3.52E-51	-0.1988	0.1	0.32	5.25E-47
POLR3F	3.62E-51	-0.15593	0.119	0.323	5.41E-47
CLASP2	3.80E-51	-0.33384	0.09	0.32	5.68E-47

GNB5	3.86E-51	-0.24054	0.075	0.281	5.76E-47
SLC25A29	4.16E-51	-0.22441	0.096	0.305	6.22E-47
COX17	4.18E-51	0.117654	0.469	0.681	6.25E-47
MBOAT7	4.30E-51	-0.21535	0.16	0.398	6.42E-47
NR3C1	4.32E-51	-0.16123	0.2	0.432	6.46E-47
WDR77	4.52E-51	-0.13785	0.167	0.394	6.76E-47
CEP89	4.63E-51	-0.15933	0.129	0.344	6.92E-47
QTRT1	4.78E-51	0.2197	0.367	0.514	7.14E-47
KLHL8	4.84E-51	-0.18167	0.088	0.283	7.24E-47
ZDHHC3	4.98E-51	-0.122	0.139	0.348	7.44E-47
TBCA	5.00E-51	0.159719	0.727	0.9	7.47E-47
METTL7B	5.41E-51	0.35719	0.218	0.374	8.09E-47
SMC5	5.51E-51	-0.17791	0.161	0.388	8.23E-47
ARID2	5.61E-51	-0.27114	0.06	0.267	8.39E-47
MLF1	5.72E-51	0.288167	0.29	0.367	8.54E-47
MTX1	5.91E-51	0.119586	0.196	0.36	8.82E-47
EXOSC6	6.02E-51	-0.15117	0.037	0.204	8.99E-47
M6PR	6.08E-51	0.114776	0.276	0.427	9.09E-47
TSKU	6.09E-51	-0.21518	0.04	0.223	9.10E-47
TMEM158	6.26E-51	-0.2751	0.106	0.342	9.35E-47
MED27	6.38E-51	-0.2073	0.11	0.328	9.53E-47
SRP68	6.99E-51	-0.11513	0.208	0.431	1.04E-46
DOT1L	7.06E-51	-0.18042	0.051	0.225	1.06E-46
ADAM17	7.14E-51	-0.11589	0.175	0.386	1.07E-46
TBCC	7.26E-51	-0.23933	0.138	0.368	1.09E-46
KIDINS22C	7.42E-51	-0.1679	0.168	0.389	1.11E-46
PDCL3	7.47E-51	0.115292	0.249	0.444	1.12E-46
RBMX2	7.52E-51	-0.1199	0.204	0.431	1.12E-46
PHTF2	7.61E-51	-0.23916	0.064	0.26	1.14E-46
SH3BGR	7.72E-51	0.785278	0.295	0.144	1.15E-46
ASH2L	8.11E-51	-0.1889	0.136	0.363	1.21E-46
DLEU1	8.45E-51	-0.13648	0.061	0.243	1.26E-46
REEP2	8.64E-51	-0.20407	0.166	0.395	1.29E-46
ZMYM4	8.67E-51	-0.21976	0.056	0.252	1.30E-46
TANK	9.47E-51	0.245352	0.331	0.448	1.41E-46
FABP5	9.52E-51	1.314348	0.513	0.338	1.42E-46
MLF2	9.88E-51	0.100655	0.639	0.823	1.48E-46
FKBP9	1.04E-50	0.105305	0.213	0.341	1.55E-46
PPM1D	1.11E-50	-0.27368	0.067	0.279	1.66E-46
UBE2J1	1.13E-50	-0.13814	0.205	0.425	1.69E-46
DCHS1	1.16E-50	-0.2416	0.026	0.196	1.73E-46
PNKP	1.16E-50	-0.15992	0.109	0.31	1.73E-46
CTHRC1	1.16E-50	-0.27148	0.029	0.211	1.74E-46
GTPBP1	1.26E-50	-0.25152	0.049	0.24	1.88E-46
BRI3BP	1.31E-50	-0.24835	0.056	0.256	1.95E-46
CASP6	1.42E-50	-0.13808	0.1	0.311	2.12E-46
CHRAC1	1.45E-50	-0.21703	0.107	0.319	2.17E-46
RASL11B	1.59E-50	-0.50436	0.014	0.174	2.37E-46
GGT7	1.60E-50	-0.15325	0.117	0.33	2.39E-46
SYCE1L	1.79E-50	-0.12502	0.018	0.153	2.67E-46
YLPM1	1.80E-50	-0.20178	0.063	0.258	2.69E-46

MAP1S	1.82E-50	-0.12315	0.117	0.31	2.72E-46
AP3B1	1.85E-50	-0.20393	0.096	0.314	2.76E-46
AHI1	1.91E-50	-0.11174	0.218	0.448	2.86E-46
CYHR1	1.97E-50	0.185117	0.285	0.4	2.94E-46
GADD45GIF	1.99E-50	-0.23239	0.506	0.794	2.98E-46
RP3-525N1	2.12E-50	-0.24111	0.072	0.27	3.17E-46
CHCHD2	2.12E-50	0.24353	0.869	0.97	3.17E-46
USP6NL	2.14E-50	-0.10784	0.026	0.165	3.20E-46
UBTD2	2.18E-50	-0.23328	0.059	0.259	3.26E-46
RP11-676J	2.24E-50	-0.34692	0.004	0.135	3.35E-46
GSK3B	2.45E-50	-0.20942	0.103	0.317	3.66E-46
SRPRB	2.48E-50	0.148881	0.263	0.41	3.70E-46
NFYC	2.58E-50	-0.11268	0.159	0.379	3.86E-46
DCAKD	2.59E-50	-0.12395	0.112	0.338	3.88E-46
MEX3D	2.72E-50	-0.20159	0.025	0.19	4.07E-46
SLC2A1	2.76E-50	0.371836	0.231	0.238	4.12E-46
WIPF1	2.78E-50	-0.13754	0.067	0.246	4.15E-46
TSPAN13	2.83E-50	-0.38587	0.222	0.51	4.22E-46
TXNDC15	2.91E-50	0.310696	0.326	0.419	4.35E-46
SNX10	2.94E-50	0.224517	0.196	0.243	4.39E-46
SMIM14	3.06E-50	0.272828	0.359	0.485	4.58E-46
RSBN1	3.25E-50	-0.11443	0.183	0.391	4.85E-46
LIX1L	3.26E-50	-0.25582	0.069	0.275	4.87E-46
POGK	3.29E-50	-0.17347	0.075	0.272	4.92E-46
ARHGEF12	3.51E-50	-0.13156	0.11	0.3	5.24E-46
SCD	3.53E-50	0.101459	0.16	0.284	5.27E-46
KPNA4	3.75E-50	-0.15087	0.122	0.332	5.60E-46
PPP1R11	4.36E-50	0.136183	0.302	0.467	6.52E-46
MARK2	4.46E-50	-0.20498	0.068	0.267	6.67E-46
E2F4	4.46E-50	-0.13068	0.158	0.358	6.67E-46
ATP6V1B2	4.54E-50	0.14739	0.279	0.419	6.78E-46
RASGRP1	4.63E-50	-0.18095	0.019	0.17	6.92E-46
MRPS25	4.64E-50	0.218516	0.254	0.407	6.93E-46
WRAP73	4.82E-50	-0.10546	0.118	0.311	7.20E-46
MED24	5.47E-50	-0.21938	0.111	0.331	8.18E-46
EPB41L4A-	5.75E-50	0.293737	0.293	0.37	8.59E-46
COPS4	6.37E-50	0.133787	0.325	0.48	9.52E-46
NDUFAF2	6.41E-50	0.282846	0.324	0.414	9.58E-46
UROD	6.47E-50	0.504133	0.425	0.431	9.67E-46
OPA1	6.56E-50	-0.22793	0.065	0.258	9.80E-46
ETF1	6.83E-50	0.261425	0.302	0.42	1.02E-45
UAP1	7.09E-50	0.36446	0.254	0.323	1.06E-45
NUDT22	7.17E-50	0.189171	0.21	0.362	1.07E-45
GPX4	7.20E-50	0.306913	0.803	0.917	1.08E-45
FOXN3	7.60E-50	-0.1885	0.091	0.286	1.14E-45
CNOT10	8.03E-50	-0.18539	0.093	0.296	1.20E-45
PCDH9	8.41E-50	0.234342	0.267	0.412	1.26E-45
VCAM1	8.43E-50	0.872719	0.206	0.042	1.26E-45
TBC1D9B	8.72E-50	-0.10639	0.084	0.256	1.30E-45
LZTS1	8.75E-50	-0.1202	0.026	0.158	1.31E-45
PELP1	9.83E-50	-0.19759	0.117	0.328	1.47E-45

TMEM245	9.95E-50	-0.20926	0.099	0.306	1.49E-45
SLC47A1	1.01E-49	-0.16001	0.014	0.156	1.50E-45
PPFIA3	1.01E-49	-0.21586	0.041	0.22	1.51E-45
CHMP4B	1.04E-49	-0.16448	0.348	0.607	1.55E-45
ERV3-1	1.07E-49	-0.1286	0.213	0.446	1.59E-45
CERS6	1.08E-49	-0.24139	0.059	0.254	1.61E-45
CHRDL1	1.08E-49	-0.26593	0.009	0.157	1.61E-45
COPB2	1.08E-49	0.249735	0.327	0.44	1.62E-45
HYI	1.09E-49	-0.26081	0.089	0.301	1.64E-45
EIF4G3	1.12E-49	-0.23711	0.084	0.301	1.67E-45
RRAGD	1.17E-49	-0.178	0.091	0.29	1.74E-45
NUP37	1.18E-49	-0.14191	0.136	0.359	1.76E-45
FASN	1.22E-49	-0.20712	0.053	0.238	1.83E-45
PTGFRN	1.25E-49	-0.10954	0.079	0.253	1.87E-45
SUV39H1	1.37E-49	-0.22292	0.025	0.186	2.05E-45
SCO1	1.42E-49	-0.13512	0.136	0.337	2.12E-45
E2F3	1.42E-49	-0.32005	0.037	0.23	2.12E-45
VWA1	1.43E-49	-0.26113	0.06	0.263	2.13E-45
SHISA4	1.46E-49	0.115127	0.217	0.368	2.18E-45
MBNL1	1.50E-49	-0.16034	0.11	0.31	2.24E-45
ZBED5	1.61E-49	-0.24078	0.129	0.36	2.40E-45
CALM2	1.62E-49	0.21779	0.888	0.989	2.43E-45
ACTN1	1.65E-49	0.333623	0.346	0.405	2.46E-45
PCDHB16	1.67E-49	-0.29968	0.086	0.311	2.50E-45
DMRTA2	1.72E-49	-0.16055	0.057	0.227	2.56E-45
FUBP1	1.75E-49	-0.26925	0.176	0.43	2.61E-45
CASC3	1.78E-49	-0.23209	0.141	0.372	2.66E-45
PHF12	1.82E-49	-0.18144	0.058	0.241	2.73E-45
BCAR1	1.85E-49	-0.14876	0.146	0.359	2.76E-45
TREM1	1.88E-49	1.039328	0.208	0.02	2.82E-45
KCTD3	1.91E-49	-0.18892	0.108	0.314	2.85E-45
CCS	1.93E-49	0.454986	0.268	0.286	2.88E-45
TANC1	1.99E-49	-0.23034	0.059	0.253	2.98E-45
ILVBL	2.00E-49	-0.12167	0.241	0.484	2.98E-45
PDE7A	2.18E-49	-0.23373	0.036	0.21	3.25E-45
INCENP	2.23E-49	-0.20222	0.013	0.16	3.33E-45
MAP2K7	2.30E-49	-0.14237	0.145	0.354	3.44E-45
SYTL2	2.40E-49	0.692789	0.249	0.064	3.58E-45
CCDC92	2.41E-49	-0.10016	0.051	0.209	3.60E-45
LARP6	2.49E-49	-0.12565	0.164	0.364	3.72E-45
PDLIM7	2.56E-49	-0.12476	0.18	0.4	3.82E-45
MSH6	2.62E-49	-0.37522	0.098	0.34	3.92E-45
PGM3	2.67E-49	0.1118	0.226	0.333	4.00E-45
FBLN7	2.78E-49	-0.27923	0.036	0.225	4.15E-45
FUNDC1	2.82E-49	0.244213	0.254	0.393	4.22E-45
LMO7	3.17E-49	-0.32425	0.027	0.205	4.73E-45
REXO4	3.28E-49	-0.16999	0.139	0.349	4.90E-45
CPSF4	3.30E-49	-0.13332	0.201	0.422	4.93E-45
BDH2	3.33E-49	0.692779	0.262	0.132	4.97E-45
KIAA1586	3.33E-49	-0.1611	0.079	0.269	4.98E-45
GINS3	3.49E-49	-0.23911	0.028	0.193	5.22E-45

TNFAIP8L1	3.93E-49	-0.25817	0.063	0.258	5.87E-45
PES1	3.99E-49	-0.2238	0.117	0.34	5.97E-45
PTAR1	4.20E-49	-0.24207	0.091	0.306	6.28E-45
CRLF3	4.35E-49	-0.13996	0.046	0.215	6.50E-45
DIRAS3	4.92E-49	0.987602	0.301	0.144	7.35E-45
PTDSS1	4.99E-49	-0.14195	0.139	0.353	7.46E-45
ZFHX3	5.04E-49	-0.14369	0.083	0.262	7.53E-45
FAM161A	5.28E-49	-0.22075	0.109	0.327	7.89E-45
COL6A1	5.33E-49	0.319511	0.342	0.402	7.96E-45
LGALS1	5.92E-49	0.505616	0.801	0.767	8.85E-45
NDFIP2	5.97E-49	0.107251	0.194	0.349	8.92E-45
SLC35E3	6.04E-49	-0.50786	0.156	0.428	9.03E-45
CAMK2D	6.15E-49	-0.14654	0.211	0.446	9.20E-45
SOCS4	6.15E-49	-0.15311	0.046	0.212	9.20E-45
FBXO18	6.26E-49	-0.13302	0.073	0.249	9.35E-45
MEX3B	6.36E-49	-0.30545	0.015	0.17	9.50E-45
AK3	6.66E-49	-0.12036	0.201	0.414	9.95E-45
NOTCH2	6.76E-49	-0.14417	0.104	0.296	1.01E-44
CREBBP	8.00E-49	-0.23895	0.079	0.285	1.20E-44
YRDC	8.17E-49	-0.15164	0.121	0.32	1.22E-44
GMFB	8.45E-49	-0.10743	0.144	0.347	1.26E-44
SSBP3	8.48E-49	-0.24759	0.07	0.273	1.27E-44
GRIK3	8.70E-49	-0.24106	0.013	0.157	1.30E-44
SLC4A7	8.79E-49	-0.22802	0.121	0.344	1.31E-44
TMEM184B	8.80E-49	-0.22748	0.072	0.275	1.31E-44
ABCB8	8.93E-49	-0.13968	0.09	0.28	1.34E-44
VAMP3	9.38E-49	0.262129	0.304	0.426	1.40E-44
ZFAND6	9.39E-49	0.158642	0.426	0.607	1.40E-44
PCNXL4	9.75E-49	-0.15139	0.084	0.285	1.46E-44
POLD1	1.04E-48	-0.22813	0.027	0.191	1.55E-44
PLEKHB1	1.05E-48	0.271551	0.225	0.302	1.57E-44
SLC25A26	1.10E-48	0.232051	0.23	0.36	1.65E-44
AC093323.	1.15E-48	-0.19523	0.062	0.253	1.72E-44
PHKB	1.18E-48	-0.23284	0.112	0.333	1.76E-44
TTI1	1.25E-48	-0.17097	0.071	0.262	1.86E-44
ATP6V1H	1.31E-48	0.188327	0.271	0.398	1.96E-44
MT1G	1.32E-48	0.984321	0.266	0.065	1.97E-44
HOXA2	1.32E-48	-0.37526	0.02	0.188	1.97E-44
SECISBP2L	1.37E-48	-0.11054	0.096	0.273	2.04E-44
ZNF451	1.41E-48	-0.12403	0.109	0.307	2.10E-44
ZC3H7A	1.49E-48	-0.25619	0.103	0.321	2.22E-44
BAP1	1.52E-48	-0.16367	0.097	0.305	2.27E-44
BET1	1.58E-48	0.531726	0.335	0.325	2.36E-44
CABIN1	1.59E-48	-0.10137	0.086	0.256	2.37E-44
CLOCK	1.61E-48	-0.35663	0.091	0.321	2.40E-44
LINC01003	1.66E-48	-0.28995	0.09	0.314	2.48E-44
PUM2	1.72E-48	-0.20743	0.103	0.31	2.57E-44
C9orf40	1.78E-48	-0.24442	0.02	0.185	2.65E-44
HEG1	1.81E-48	-0.15159	0.03	0.188	2.70E-44
DGAT1	1.82E-48	-0.21591	0.03	0.2	2.73E-44
ID1	1.83E-48	-0.14326	0.111	0.298	2.74E-44

SLC12A2	1.97E-48	-0.1782	0.085	0.277	2.94E-44
MITD1	2.01E-48	0.245655	0.269	0.377	3.01E-44
SNX2	2.02E-48	0.176237	0.332	0.475	3.02E-44
CASK	2.05E-48	-0.15665	0.075	0.259	3.07E-44
CDK5RAP1	2.17E-48	-0.17757	0.108	0.315	3.24E-44
KNTC1	2.23E-48	-0.27457	0.01	0.156	3.33E-44
SNX24	2.24E-48	-0.1352	0.072	0.248	3.35E-44
SLC25A23	2.25E-48	-0.13194	0.078	0.262	3.37E-44
USP46	2.28E-48	-0.36098	0.084	0.311	3.41E-44
LETM1	2.29E-48	-0.18169	0.12	0.338	3.42E-44
CNOT8	2.37E-48	-0.15337	0.133	0.341	3.54E-44
HINT3	2.59E-48	-0.14347	0.147	0.351	3.86E-44
ZFYVE21	2.70E-48	0.122591	0.246	0.393	4.04E-44
MFI2-AS1	2.73E-48	-0.31301	0.045	0.241	4.08E-44
CBL	2.77E-48	-0.1802	0.043	0.215	4.14E-44
CHST14	2.78E-48	-0.19555	0.033	0.205	4.15E-44
MAPK7	2.79E-48	-0.15407	0.097	0.295	4.17E-44
CHST3	2.87E-48	-0.12183	0.031	0.174	4.28E-44
COG4	2.88E-48	-0.21265	0.133	0.356	4.30E-44
POLR1E	2.88E-48	-0.15907	0.091	0.284	4.30E-44
NNAT	2.94E-48	-0.68663	0.123	0.264	4.39E-44
N4BP2	3.37E-48	-0.27907	0.06	0.258	5.04E-44
CFLAR	3.65E-48	0.262703	0.287	0.383	5.45E-44
ARPC1B	3.70E-48	0.282105	0.312	0.432	5.54E-44
RMND5B	3.73E-48	-0.16267	0.118	0.33	5.57E-44
FBXW5	3.80E-48	-0.16973	0.196	0.414	5.68E-44
DST	4.05E-48	-0.21822	0.275	0.537	6.05E-44
MLC1	4.57E-48	-0.11854	0.127	0.321	6.83E-44
LSM10	4.58E-48	0.232022	0.375	0.531	6.84E-44
RECQL	4.59E-48	-0.17049	0.156	0.373	6.86E-44
NDN	4.74E-48	-0.11322	0.146	0.33	7.08E-44
ZNF439	4.88E-48	-0.21726	0.09	0.29	7.30E-44
C5orf34	4.96E-48	-0.30785	0.024	0.191	7.41E-44
NDRG3	5.12E-48	-0.15992	0.097	0.302	7.66E-44
IFT20	5.24E-48	0.233194	0.332	0.443	7.83E-44
CPD	5.75E-48	0.17671	0.171	0.275	8.59E-44
IRS2	6.26E-48	0.175503	0.322	0.448	9.36E-44
HAUS3	6.27E-48	-0.21042	0.077	0.267	9.37E-44
CYB5R1	6.29E-48	0.437414	0.229	0.275	9.40E-44
POM121	7.10E-48	-0.19159	0.119	0.33	1.06E-43
RNMTL1	7.29E-48	-0.17159	0.091	0.291	1.09E-43
MTIF2	8.04E-48	-0.11328	0.099	0.285	1.20E-43
MEIS2	8.30E-48	-0.27519	0.156	0.404	1.24E-43
R3HDM4	8.74E-48	-0.19306	0.138	0.356	1.31E-43
METTL2A	8.86E-48	-0.14005	0.135	0.342	1.32E-43
BGN	9.16E-48	0.788311	0.246	0.044	1.37E-43
AKT1S1	9.19E-48	-0.1175	0.2	0.406	1.37E-43
STRBP	9.21E-48	-0.28776	0.067	0.277	1.38E-43
CNIH3	9.66E-48	-0.14096	0.143	0.348	1.44E-43
NCBP1	9.81E-48	-0.16729	0.047	0.22	1.47E-43
MGAT1	1.00E-47	0.128601	0.234	0.344	1.50E-43

ANKFY1	1.01E-47	-0.12067	0.087	0.26	1.50E-43
CDK19	1.06E-47	-0.20439	0.063	0.244	1.59E-43
SPATS2L	1.06E-47	-0.35631	0.253	0.546	1.59E-43
SH3PXD2B	1.08E-47	-0.10056	0.061	0.221	1.61E-43
CRYL1	1.11E-47	0.32493	0.288	0.359	1.65E-43
SMG1	1.13E-47	-0.11799	0.108	0.291	1.69E-43
BEX1	1.13E-47	-0.12229	0.34	0.578	1.69E-43
MAPKAPK5-	1.14E-47	0.2296	0.221	0.344	1.71E-43
MACROD1	1.17E-47	-0.2339	0.092	0.307	1.75E-43
FAM127B	1.22E-47	0.394133	0.354	0.385	1.82E-43
CDC16	1.23E-47	-0.10768	0.17	0.364	1.84E-43
AGBL5	1.24E-47	-0.1676	0.049	0.219	1.85E-43
PPM1A	1.30E-47	-0.23149	0.1	0.316	1.94E-43
CTC-260E6	1.32E-47	-0.22691	0.019	0.173	1.98E-43
POLE	1.34E-47	-0.22498	0.012	0.157	2.00E-43
SNAPC5	1.35E-47	0.126076	0.198	0.344	2.02E-43
PTK7	1.56E-47	-0.18632	0.069	0.256	2.33E-43
SESN3	1.58E-47	-0.18211	0.073	0.259	2.36E-43
SHMT1	1.59E-47	-0.16451	0.08	0.268	2.38E-43
MAD2L1BP	1.61E-47	-0.16884	0.123	0.325	2.41E-43
AC004158.	1.73E-47	-0.17694	0.051	0.226	2.59E-43
ZBED1	1.74E-47	-0.28983	0.085	0.304	2.60E-43
TTC28	1.75E-47	-0.14944	0.056	0.23	2.61E-43
CYB561D2	1.79E-47	0.130611	0.158	0.306	2.67E-43
PLSCR1	1.83E-47	0.41884	0.293	0.351	2.74E-43
RAB30-AS1	1.89E-47	-0.12957	0.112	0.31	2.83E-43
IFI35	1.95E-47	0.627811	0.261	0.204	2.91E-43
GPANK1	1.98E-47	-0.24812	0.16	0.402	2.96E-43
SYNC	2.06E-47	-0.25237	0.159	0.386	3.08E-43
NR2F1	2.24E-47	-0.12369	0.163	0.357	3.35E-43
ATP13A1	2.28E-47	-0.12077	0.111	0.301	3.41E-43
ALG12	2.28E-47	-0.12505	0.034	0.188	3.41E-43
MED31	2.38E-47	0.121023	0.224	0.377	3.56E-43
RNFT2	2.50E-47	-0.25993	0.022	0.185	3.74E-43
LIN9	2.50E-47	-0.25306	0.017	0.172	3.74E-43
KBTBD6	2.53E-47	-0.20103	0.088	0.291	3.79E-43
RHOG	2.57E-47	0.21119	0.207	0.32	3.84E-43
RPS6KL1	2.58E-47	-0.30314	0.019	0.18	3.86E-43
PSMA7	2.68E-47	0.142524	0.758	0.923	4.01E-43
PIGC	2.93E-47	0.150736	0.206	0.351	4.38E-43
SWI5	3.02E-47	0.287193	0.282	0.401	4.51E-43
TOMM70A	3.21E-47	-0.18749	0.08	0.275	4.80E-43
CUL1	3.33E-47	-0.11898	0.2	0.417	4.97E-43
SLC39A10	3.37E-47	-0.15307	0.086	0.267	5.04E-43
SHROOM2	3.50E-47	-0.18013	0.015	0.153	5.23E-43
DPF3	3.52E-47	-0.10787	0.065	0.232	5.27E-43
ZADH2	3.85E-47	-0.22034	0.055	0.246	5.75E-43
STRN3	3.90E-47	-0.11524	0.094	0.273	5.83E-43
MEX3C	3.94E-47	-0.13114	0.073	0.237	5.88E-43
AGAP2	3.98E-47	-0.32453	0.007	0.143	5.94E-43
EEF1A1	4.28E-47	0.439405	0.803	0.878	6.39E-43

VHL	4.32E-47	-0.17734	0.115	0.316	6.46E-43
SMARCA1	4.40E-47	-0.20927	0.14	0.362	6.58E-43
ANAPC10	4.51E-47	0.111571	0.173	0.337	6.74E-43
PPP6R2	4.54E-47	-0.20806	0.064	0.252	6.79E-43
MCC	4.60E-47	-0.18775	0.09	0.283	6.87E-43
GALNT2	4.70E-47	0.111865	0.197	0.34	7.02E-43
PTGR1	4.81E-47	0.229039	0.212	0.337	7.18E-43
CHAC2	4.85E-47	-0.15833	0.043	0.21	7.25E-43
TRIM7	5.01E-47	-0.15994	0.01	0.147	7.48E-43
COQ10B	5.06E-47	0.294188	0.269	0.351	7.55E-43
AGO3	5.12E-47	-0.14903	0.138	0.336	7.65E-43
AGPAT3	5.18E-47	-0.21013	0.066	0.253	7.74E-43
MCAT	5.50E-47	-0.17177	0.079	0.267	8.22E-43
ELMO2	5.52E-47	-0.1817	0.09	0.289	8.24E-43
SLC35F1	5.72E-47	-0.25154	0.077	0.283	8.55E-43
MAP4K5	5.74E-47	-0.18248	0.101	0.295	8.57E-43
SH3BP5	5.75E-47	-0.11826	0.084	0.251	8.59E-43
UCKL1	5.78E-47	-0.15231	0.119	0.316	8.63E-43
RANBP2	5.83E-47	-0.1199	0.104	0.29	8.72E-43
GNG7	6.24E-47	-0.18175	0.11	0.311	9.33E-43
SNX8	6.62E-47	-0.10824	0.069	0.242	9.89E-43
USP14	6.85E-47	-0.12713	0.222	0.432	1.02E-42
HIBADH	7.90E-47	0.325791	0.25	0.314	1.18E-42
CAMSAP2	8.25E-47	-0.20619	0.093	0.296	1.23E-42
PQLC1	8.53E-47	-0.19328	0.051	0.232	1.28E-42
SCG5	9.29E-47	0.664987	0.369	0.322	1.39E-42
LEMD2	9.36E-47	-0.16576	0.098	0.291	1.40E-42
MED8	1.02E-46	0.181141	0.234	0.362	1.52E-42
TSNAX	1.04E-46	0.182973	0.294	0.435	1.55E-42
COL6A2	1.05E-46	0.166722	0.22	0.332	1.56E-42
ADD2	1.07E-46	-0.24391	0.05	0.235	1.60E-42
MAPK8IP1	1.08E-46	-0.11624	0.107	0.293	1.62E-42
NAV2	1.11E-46	-0.23745	0.091	0.291	1.66E-42
FAM168A	1.16E-46	-0.20576	0.051	0.228	1.73E-42
FAM76B	1.24E-46	-0.21247	0.071	0.257	1.85E-42
TXNDC17	1.24E-46	0.178215	0.525	0.712	1.85E-42
LRCH3	1.61E-46	-0.17294	0.085	0.27	2.41E-42
UQCR11	1.82E-46	1.444807	0.212	0.022	2.73E-42
KRIT1	1.87E-46	-0.14023	0.205	0.427	2.80E-42
ZNF655	1.92E-46	-0.13309	0.154	0.369	2.87E-42
NELL2	1.95E-46	-0.365	0.064	0.272	2.92E-42
IRAK1	1.96E-46	-0.19323	0.084	0.279	2.92E-42
PLCG1	1.98E-46	-0.2145	0.055	0.235	2.95E-42
AMH	2.03E-46	-0.23692	0.037	0.211	3.04E-42
SNX14	2.04E-46	-0.13023	0.152	0.356	3.05E-42
TMX3	2.08E-46	-0.10066	0.127	0.319	3.11E-42
POU2F1	2.11E-46	-0.20642	0.089	0.293	3.15E-42
PMPCB	2.27E-46	0.152394	0.345	0.514	3.40E-42
KIAA1841	2.32E-46	-0.23488	0.037	0.21	3.47E-42
USP7	2.37E-46	-0.14986	0.083	0.267	3.55E-42
AP5S1	2.44E-46	-0.11926	0.042	0.199	3.65E-42

MAPK10	2.47E-46	-0.20858	0.115	0.33	3.69E-42
ELF2	2.51E-46	-0.12017	0.126	0.311	3.75E-42
DLX1	2.56E-46	-0.35734	0.03	0.201	3.83E-42
MESDC1	2.57E-46	-0.24064	0.039	0.217	3.84E-42
EMP2	2.59E-46	0.181929	0.19	0.321	3.87E-42
FTSJ1	2.78E-46	-0.11728	0.101	0.28	4.15E-42
CAPG	2.92E-46	0.675566	0.304	0.227	4.37E-42
POMZP3	3.45E-46	-0.31239	0.048	0.243	5.15E-42
POP4	3.93E-46	0.202578	0.243	0.385	5.87E-42
EMG1	3.98E-46	0.289197	0.27	0.367	5.95E-42
EYA2	4.05E-46	-0.12686	0.063	0.227	6.05E-42
ZBTB17	4.10E-46	-0.11038	0.061	0.221	6.13E-42
GRWD1	4.34E-46	-0.15659	0.117	0.317	6.49E-42
PPP4R1	4.39E-46	-0.18081	0.076	0.265	6.57E-42
AUNIP	4.57E-46	-0.25702	0.005	0.13	6.83E-42
DERL1	4.74E-46	0.234947	0.298	0.409	7.08E-42
LYSMD2	4.86E-46	-0.22088	0.061	0.251	7.27E-42
GPALPP1	5.43E-46	-0.1005	0.09	0.267	8.11E-42
GNG2	5.80E-46	-0.2202	0.098	0.305	8.66E-42
YDJC	5.99E-46	-0.19368	0.071	0.256	8.95E-42
C21orf2	6.20E-46	-0.17055	0.123	0.325	9.26E-42
C18orf32	6.58E-46	0.133082	0.196	0.315	9.83E-42
GBP1	7.26E-46	0.781663	0.318	0.133	1.08E-41
DPP8	7.27E-46	-0.14586	0.095	0.28	1.09E-41
GNG5	7.36E-46	0.251871	0.677	0.798	1.10E-41
GBE1	7.43E-46	0.491639	0.316	0.278	1.11E-41
BEST1	7.56E-46	0.337438	0.242	0.285	1.13E-41
EXOSC7	8.59E-46	0.178203	0.232	0.374	1.28E-41
CHTF8	8.78E-46	-0.10454	0.135	0.326	1.31E-41
RRAGC	9.19E-46	0.131119	0.237	0.344	1.37E-41
SHMT2	9.30E-46	0.126734	0.26	0.411	1.39E-41
SYDE1	1.08E-45	-0.12893	0.066	0.231	1.62E-41
MYO5A	1.09E-45	-0.15604	0.034	0.186	1.63E-41
WDR13	1.11E-45	0.203763	0.29	0.411	1.67E-41
QRICH1	1.12E-45	-0.15844	0.098	0.286	1.68E-41
CARD8	1.12E-45	-0.16286	0.06	0.237	1.68E-41
TUBB6	1.31E-45	0.162522	0.274	0.42	1.96E-41
ISOC2	1.39E-45	0.159833	0.359	0.512	2.08E-41
SH3KBP1	1.48E-45	-0.30747	0.078	0.288	2.21E-41
RTTN	1.49E-45	-0.16542	0.015	0.152	2.23E-41
MED29	1.51E-45	0.152451	0.239	0.385	2.26E-41
SLAIN2	1.54E-45	-0.17955	0.079	0.268	2.30E-41
KIF3B	1.55E-45	-0.15605	0.076	0.251	2.31E-41
TPP1	1.69E-45	0.380345	0.218	0.251	2.52E-41
PCED1A	1.72E-45	-0.10647	0.086	0.252	2.57E-41
TCTN1	1.78E-45	0.240835	0.255	0.365	2.66E-41
FAM57A	1.81E-45	-0.13323	0.084	0.258	2.70E-41
HSPA1A	1.84E-45	-0.47527	0.296	0.584	2.75E-41
SLC2A3	1.87E-45	0.614175	0.279	0.178	2.79E-41
NRXN1	1.96E-45	-0.52143	0.096	0.326	2.93E-41
PBX3	1.99E-45	-0.19797	0.06	0.238	2.98E-41

TRPM3	2.04E-45	-0.14378	0.015	0.138	3.05E-41
AP1B1	2.13E-45	-0.13914	0.078	0.249	3.18E-41
LAMTOR3	2.14E-45	0.24305	0.272	0.359	3.20E-41
CALCOCO2	2.15E-45	0.229732	0.219	0.296	3.21E-41
PTPN9	2.22E-45	-0.15575	0.062	0.236	3.32E-41
NAT9	2.23E-45	0.113261	0.211	0.346	3.34E-41
ATRN	2.26E-45	-0.12256	0.062	0.216	3.38E-41
ADAL	2.31E-45	-0.13758	0.072	0.24	3.45E-41
ALG3	2.38E-45	0.201979	0.208	0.338	3.56E-41
DCBLD2	2.50E-45	-0.26731	0.115	0.338	3.74E-41
SIL1	2.51E-45	0.352979	0.237	0.29	3.75E-41
C1QB	2.54E-45	0.709666	0.444	0.31	3.80E-41
SPG7	2.56E-45	-0.17507	0.173	0.388	3.83E-41
PRPF4	2.60E-45	-0.12957	0.165	0.379	3.89E-41
RNF139	2.77E-45	-0.15981	0.172	0.39	4.14E-41
CENPB	2.77E-45	-0.17218	0.061	0.237	4.14E-41
HGSNAT	2.77E-45	-0.18526	0.1	0.3	4.14E-41
FAF1	2.80E-45	-0.2037	0.135	0.352	4.18E-41
MTRNR2L12	2.84E-45	0.95364	0.228	0.18	4.25E-41
DPH7	2.94E-45	-0.11298	0.157	0.341	4.39E-41
GSKIP	2.97E-45	-0.11992	0.107	0.29	4.44E-41
NUP210	3.03E-45	-0.179	0.021	0.168	4.53E-41
PIGP	3.46E-45	0.266302	0.197	0.289	5.18E-41
DUSP14	3.63E-45	0.241541	0.224	0.335	5.42E-41
C18orf54	3.71E-45	-0.21883	0.01	0.148	5.54E-41
EID2	3.77E-45	-0.22506	0.057	0.243	5.63E-41
RASD1	3.98E-45	0.69806	0.307	0.131	5.95E-41
SOBP	4.03E-45	-0.22616	0.072	0.262	6.02E-41
HEXA	4.09E-45	0.247342	0.249	0.375	6.11E-41
PRKACB	4.16E-45	-0.10609	0.123	0.306	6.22E-41
CHMP1B	4.28E-45	0.328043	0.35	0.401	6.39E-41
COMMD10	4.39E-45	0.197834	0.215	0.353	6.55E-41
SRD5A3	4.48E-45	-0.23619	0.095	0.295	6.70E-41
SPRY4	4.73E-45	-0.1049	0.037	0.175	7.07E-41
P4HA1	5.12E-45	0.361357	0.234	0.241	7.65E-41
STOM	5.24E-45	0.665311	0.236	0.142	7.83E-41
RDH14	5.47E-45	-0.12952	0.154	0.347	8.17E-41
OTP	5.60E-45	-0.19468	0.005	0.125	8.37E-41
STX2	6.19E-45	-0.22474	0.065	0.254	9.25E-41
ING3	6.26E-45	-0.22549	0.157	0.394	9.36E-41
ZNF609	6.51E-45	-0.17866	0.024	0.173	9.73E-41
EIF2AK4	6.56E-45	-0.14041	0.118	0.31	9.80E-41
TMEM38B	6.56E-45	0.405566	0.269	0.309	9.81E-41
ELN	6.74E-45	-0.10336	0.138	0.344	1.01E-40
PDXDC1	6.80E-45	-0.1232	0.121	0.299	1.02E-40
KAT6A	6.89E-45	-0.20643	0.095	0.293	1.03E-40
NOL3	7.05E-45	0.521937	0.23	0.156	1.05E-40
PSMD9	7.07E-45	0.213837	0.288	0.399	1.06E-40
TWF2	7.21E-45	0.175561	0.222	0.348	1.08E-40
ATP9A	7.80E-45	-0.16554	0.053	0.22	1.17E-40
C11orf96	7.90E-45	-0.17521	0.114	0.306	1.18E-40

SYS1	7.94E-45	0.136293	0.197	0.326	1.19E-40
PIK3C2A	8.04E-45	-0.16001	0.084	0.26	1.20E-40
GTF2A1	8.20E-45	-0.15564	0.074	0.248	1.23E-40
TBC1D10B	8.28E-45	-0.18608	0.048	0.215	1.24E-40
S100A16	8.47E-45	0.676808	0.586	0.48	1.27E-40
TUBGCP3	8.56E-45	-0.28863	0.065	0.264	1.28E-40
ZFP91	8.66E-45	-0.18707	0.087	0.274	1.29E-40
PPCS	8.83E-45	0.535287	0.325	0.275	1.32E-40
NOTCH3	9.06E-45	-0.10928	0.033	0.17	1.35E-40
GMCL1	9.08E-45	-0.19038	0.15	0.365	1.36E-40
ARID1B	9.36E-45	-0.23278	0.056	0.235	1.40E-40
CAMK2N1	9.40E-45	-0.26328	0.248	0.499	1.40E-40
LRP1	1.02E-44	0.23332	0.28	0.39	1.52E-40
ZFAND2A	1.04E-44	0.204546	0.299	0.452	1.55E-40
MSI1	1.08E-44	-0.22954	0.1	0.307	1.62E-40
YARS	1.10E-44	0.102386	0.246	0.374	1.64E-40
DESI1	1.12E-44	-0.10628	0.086	0.252	1.68E-40
CERS1	1.13E-44	-0.12081	0.083	0.253	1.69E-40
C15orf40	1.17E-44	0.165962	0.154	0.274	1.75E-40
TMEM161B-	1.25E-44	-0.13243	0.253	0.489	1.86E-40
UCHL3	1.29E-44	0.149143	0.232	0.379	1.93E-40
ST3GAL2	1.37E-44	-0.13958	0.059	0.223	2.04E-40
AC004951.	1.38E-44	-0.22575	0.106	0.315	2.06E-40
PPP3CA	1.39E-44	-0.12326	0.187	0.385	2.08E-40
THSD1	1.44E-44	-0.19254	0.037	0.201	2.15E-40
CXCL2	1.52E-44	0.850525	0.167	0.011	2.27E-40
ZNF48	1.58E-44	-0.18373	0.058	0.228	2.36E-40
ARHGEF6	1.65E-44	-0.14159	0.054	0.217	2.47E-40
DUS3L	1.66E-44	-0.17113	0.11	0.299	2.48E-40
CCAR2	1.72E-44	-0.1545	0.119	0.33	2.57E-40
BTBD1	1.85E-44	-0.14207	0.083	0.254	2.77E-40
POR	1.95E-44	0.248825	0.295	0.368	2.92E-40
SYT17	2.07E-44	-0.15235	0.088	0.265	3.10E-40
CARM1	2.19E-44	-0.13787	0.045	0.193	3.27E-40
FAM58A	2.22E-44	-0.14683	0.089	0.27	3.32E-40
MPRIP	2.29E-44	-0.10463	0.121	0.305	3.42E-40
GEMIN7	2.31E-44	0.115485	0.144	0.273	3.46E-40
C1orf112	2.32E-44	-0.23837	0.025	0.181	3.46E-40
USP9X	2.36E-44	-0.10361	0.133	0.316	3.53E-40
RNPEPL1	2.42E-44	-0.15326	0.031	0.181	3.62E-40
HEPN1	2.43E-44	0.277485	0.262	0.374	3.63E-40
CCZ1B	2.62E-44	-0.10844	0.117	0.301	3.92E-40
PITPNB	2.76E-44	-0.15725	0.123	0.332	4.12E-40
MEN1	2.76E-44	-0.14562	0.092	0.264	4.13E-40
HAUS5	2.84E-44	-0.17853	0.057	0.223	4.24E-40
PRKAG1	2.89E-44	0.129238	0.265	0.422	4.32E-40
TESK1	2.96E-44	-0.13769	0.04	0.185	4.43E-40
TMEM260	2.96E-44	-0.13341	0.029	0.167	4.43E-40
RP11-345J	2.97E-44	-0.16886	0.18	0.393	4.44E-40
LRRC16A	3.02E-44	-0.15714	0.025	0.162	4.51E-40
HS6ST2	3.07E-44	-0.16112	0.013	0.143	4.59E-40

ATP1A3	3.40E-44	-0.43026	0.038	0.219	5.08E-40
AKAP17A	3.41E-44	-0.17826	0.109	0.302	5.10E-40
IGBP1	3.45E-44	0.146463	0.27	0.416	5.16E-40
ABHD14A	3.51E-44	-0.15972	0.129	0.336	5.25E-40
PSMB9	3.74E-44	0.362476	0.377	0.472	5.60E-40
SMPD4	4.02E-44	-0.15893	0.096	0.283	6.01E-40
LDOC1L	4.04E-44	-0.24458	0.037	0.209	6.04E-40
HMGCS1	4.11E-44	-0.10264	0.143	0.34	6.14E-40
DHTKD1	4.21E-44	-0.1689	0.074	0.257	6.29E-40
ZFP90	4.24E-44	-0.19912	0.085	0.277	6.33E-40
ZDHHC22	4.29E-44	-0.22808	0.007	0.13	6.41E-40
EFHC1	4.37E-44	0.275511	0.231	0.332	6.53E-40
RAD17	4.45E-44	-0.12593	0.108	0.283	6.65E-40
TBC1D1	4.65E-44	-0.20757	0.054	0.231	6.95E-40
DNPEP	4.70E-44	0.125728	0.241	0.347	7.03E-40
SLC35D2	4.78E-44	-0.20627	0.037	0.202	7.14E-40
API5	4.91E-44	-0.11479	0.164	0.363	7.33E-40
FBXO9	5.06E-44	-0.17227	0.145	0.349	7.56E-40
DGCR8	5.09E-44	-0.18073	0.059	0.228	7.60E-40
SCPEP1	5.24E-44	0.571636	0.257	0.207	7.83E-40
GTF2H1	5.31E-44	0.139092	0.186	0.311	7.94E-40
UCHL5	5.83E-44	-0.1573	0.136	0.338	8.71E-40
NAB2	6.07E-44	-0.11202	0.099	0.265	9.07E-40
ZHX3	6.90E-44	-0.27796	0.069	0.262	1.03E-39
PTPN18	7.07E-44	-0.15534	0.094	0.277	1.06E-39
HSD11B1L	7.14E-44	-0.12915	0.14	0.332	1.07E-39
SPOCK2	7.42E-44	0.553493	0.253	0.162	1.11E-39
AKAP11	7.48E-44	-0.14343	0.091	0.273	1.12E-39
GUCD1	7.63E-44	-0.20551	0.074	0.256	1.14E-39
PATZ1	7.95E-44	-0.21107	0.035	0.201	1.19E-39
PGBD1	8.15E-44	-0.19373	0.049	0.22	1.22E-39
CIC	8.46E-44	-0.15444	0.079	0.248	1.26E-39
DIS3L	8.52E-44	-0.24387	0.06	0.246	1.27E-39
NARFL	8.87E-44	-0.11106	0.11	0.294	1.33E-39
PAK1IP1	8.91E-44	0.179762	0.25	0.368	1.33E-39
ABCC3	8.98E-44	0.68512	0.2	0.053	1.34E-39
CACNG7	9.33E-44	-0.17044	0.032	0.185	1.39E-39
DPP9	9.41E-44	-0.19359	0.088	0.273	1.41E-39
TWISTNB	9.77E-44	0.19211	0.277	0.406	1.46E-39
SCFD1	1.00E-43	0.234588	0.259	0.36	1.50E-39
SPRTN	1.02E-43	-0.14653	0.099	0.278	1.53E-39
ITPK1	1.12E-43	-0.19267	0.053	0.228	1.67E-39
ZNF770	1.13E-43	-0.11448	0.094	0.26	1.69E-39
PXDC1	1.18E-43	-0.18885	0.09	0.279	1.77E-39
C14orf119	1.19E-43	0.143044	0.295	0.442	1.78E-39
PLRG1	1.22E-43	0.149331	0.259	0.391	1.82E-39
TPPP3	1.44E-43	0.457018	0.233	0.198	2.14E-39
RAB11B-AS	1.49E-43	-0.18375	0.065	0.243	2.23E-39
ARHGEF26	1.50E-43	-0.22646	0.1	0.3	2.24E-39
GYG1	1.66E-43	0.369723	0.258	0.305	2.48E-39
KDM5A	1.68E-43	-0.12753	0.185	0.38	2.51E-39

BBOX1	1.69E-43	0.556087	0.211	0.248	2.52E-39
SETD3	1.96E-43	-0.13755	0.091	0.263	2.94E-39
HDAC6	2.00E-43	-0.2277	0.079	0.272	2.99E-39
MYO19	2.02E-43	-0.13872	0.054	0.211	3.01E-39
GOLPH3	2.02E-43	-0.20087	0.117	0.322	3.02E-39
ZNF519	2.05E-43	-0.24824	0.02	0.163	3.07E-39
DIP2B	2.10E-43	-0.14488	0.051	0.202	3.14E-39
HBS1L	2.13E-43	-0.1638	0.166	0.373	3.18E-39
DHX8	2.14E-43	-0.1289	0.075	0.235	3.20E-39
EHBP1	2.19E-43	-0.10599	0.14	0.32	3.27E-39
TRIM47	2.19E-43	0.179771	0.216	0.322	3.28E-39
LITAF	2.27E-43	0.298268	0.336	0.421	3.39E-39
HOMER3	2.36E-43	-0.16312	0.153	0.359	3.52E-39
ZBTB18	2.36E-43	-0.2316	0.013	0.146	3.53E-39
NUB1	2.44E-43	0.133965	0.26	0.379	3.65E-39
ASCC2	2.59E-43	-0.15844	0.134	0.33	3.87E-39
DNMT3A	2.70E-43	-0.21239	0.042	0.209	4.03E-39
PTK2	2.74E-43	-0.13446	0.132	0.319	4.10E-39
PREPL	2.80E-43	0.131877	0.188	0.305	4.19E-39
CCDC109B	2.84E-43	-0.35138	0.253	0.528	4.24E-39
ARFIP2	2.90E-43	0.128229	0.255	0.378	4.33E-39
PPP1R9B	2.90E-43	-0.14576	0.041	0.189	4.34E-39
ARID5A	2.94E-43	0.220737	0.226	0.337	4.40E-39
MKS1	2.95E-43	-0.10323	0.07	0.228	4.41E-39
SRSF12	2.95E-43	-0.15361	0.048	0.214	4.41E-39
ADA	3.02E-43	-0.22272	0.033	0.196	4.51E-39
ZNF775	3.18E-43	-0.21796	0.049	0.225	4.75E-39
PDXK	3.25E-43	0.124365	0.206	0.347	4.86E-39
PHTF1	3.54E-43	-0.15245	0.079	0.257	5.28E-39
GTF3C2	3.91E-43	-0.14626	0.057	0.223	5.84E-39
RP11-126K	3.94E-43	-0.19795	0.038	0.195	5.89E-39
LEO1	4.03E-43	-0.13787	0.108	0.295	6.02E-39
PAM	4.21E-43	0.199284	0.214	0.286	6.29E-39
ATP1B2	4.34E-43	0.108599	0.257	0.394	6.49E-39
ROGDI	4.85E-43	-0.19626	0.092	0.281	7.25E-39
BRD8	5.02E-43	-0.19444	0.165	0.379	7.50E-39
PJA2	5.08E-43	-0.1272	0.28	0.51	7.60E-39
STK11	5.16E-43	-0.19413	0.056	0.226	7.71E-39
MUTYH	5.49E-43	-0.22484	0.071	0.252	8.21E-39
BCL2L1	5.69E-43	0.196391	0.22	0.323	8.50E-39
VIM	5.76E-43	0.312476	0.761	0.722	8.61E-39
DNAJC1	5.96E-43	-0.19647	0.15	0.357	8.91E-39
RBM18	6.36E-43	0.121316	0.205	0.332	9.50E-39
ARL14EP	7.08E-43	0.128318	0.164	0.295	1.06E-38
ETHE1	7.18E-43	0.382892	0.253	0.298	1.07E-38
DNAL4	7.28E-43	-0.16244	0.083	0.262	1.09E-38
EHMT1	7.60E-43	-0.15851	0.09	0.273	1.14E-38
KLHL22	8.10E-43	-0.17567	0.058	0.221	1.21E-38
MVD	8.17E-43	-0.17914	0.11	0.305	1.22E-38
TMEM5	8.20E-43	0.123106	0.2	0.325	1.22E-38
RP11-395G	8.35E-43	-0.23161	0.129	0.337	1.25E-38

SMCR5	8.42E-43	-0.19895	0.041	0.2	1.26E-38
C9orf69	8.54E-43	-0.19226	0.044	0.21	1.28E-38
WDR45	8.67E-43	0.309947	0.233	0.315	1.30E-38
TMEM63B	8.87E-43	-0.10764	0.085	0.251	1.33E-38
CCDC6	9.15E-43	-0.10182	0.063	0.21	1.37E-38
PCID2	9.22E-43	0.137949	0.207	0.33	1.38E-38
CXCL16	9.77E-43	0.144805	0.235	0.39	1.46E-38
ZNF436	9.81E-43	-0.19193	0.037	0.193	1.47E-38
CCDC77	9.91E-43	-0.2514	0.049	0.223	1.48E-38
RRP15	1.02E-42	0.112574	0.197	0.354	1.53E-38
PLIN2	1.06E-42	0.9996	0.306	0.115	1.59E-38
PPIC	1.08E-42	0.465521	0.266	0.322	1.61E-38
WARS	1.11E-42	0.349766	0.255	0.269	1.66E-38
ELOVL1	1.15E-42	0.120821	0.149	0.29	1.72E-38
SKIL	1.19E-42	-0.27865	0.117	0.337	1.78E-38
PIM3	1.20E-42	-0.21402	0.116	0.319	1.80E-38
GSTCD	1.21E-42	-0.16856	0.022	0.164	1.81E-38
TRAPPC11	1.23E-42	-0.18313	0.042	0.2	1.83E-38
COMMD9	1.24E-42	0.143051	0.222	0.348	1.85E-38
TMEM201	1.28E-42	-0.17442	0.018	0.156	1.92E-38
CACNA1A	1.30E-42	-0.28138	0.038	0.2	1.94E-38
MTRNR2L8	1.30E-42	0.846762	0.221	0.149	1.95E-38
UBQLN2	1.40E-42	-0.14783	0.165	0.363	2.09E-38
LAMP5	1.41E-42	-0.23383	0.055	0.235	2.10E-38
TLE4	1.44E-42	-0.13358	0.109	0.284	2.16E-38
NADK	1.50E-42	-0.13368	0.099	0.281	2.24E-38
PHACTR2	1.60E-42	-0.13641	0.011	0.137	2.39E-38
SKIV2L2	1.61E-42	-0.21321	0.129	0.337	2.41E-38
PXDN	1.75E-42	-0.14687	0.077	0.247	2.61E-38
SEC61A2	1.79E-42	-0.12933	0.043	0.19	2.67E-38
NRIP1	2.02E-42	-0.18215	0.05	0.216	3.02E-38
C17orf53	2.04E-42	-0.18768	0.012	0.14	3.05E-38
ELAVL2	2.09E-42	-0.33612	0.018	0.165	3.13E-38
PTRF	2.16E-42	0.252677	0.285	0.336	3.23E-38
FBXL3	2.21E-42	-0.12259	0.138	0.317	3.30E-38
SKP2	2.24E-42	-0.19729	0.034	0.189	3.35E-38
IFNGR1	2.29E-42	0.31478	0.31	0.352	3.43E-38
TAF4	2.37E-42	-0.14643	0.029	0.169	3.55E-38
GAR1	2.48E-42	0.19123	0.228	0.353	3.70E-38
APAF1	2.57E-42	-0.19136	0.025	0.172	3.83E-38
RFWD2	2.59E-42	-0.21797	0.108	0.311	3.87E-38
ATP5E	2.69E-42	0.275158	0.845	0.933	4.02E-38
CHST11	2.71E-42	-0.25737	0.079	0.272	4.05E-38
ANKS3	3.01E-42	-0.11276	0.144	0.33	4.50E-38
C17orf80	3.11E-42	-0.16576	0.052	0.215	4.64E-38
FOXN2	3.13E-42	-0.2025	0.055	0.226	4.68E-38
PPFIA1	3.21E-42	-0.13677	0.124	0.307	4.80E-38
U2AF1L4	3.85E-42	0.274799	0.194	0.289	5.76E-38
MTHFD1L	3.93E-42	-0.11417	0.063	0.22	5.87E-38
FZD2	4.05E-42	-0.1383	0.009	0.128	6.05E-38
EBF4	4.12E-42	-0.13719	0.025	0.165	6.16E-38

CCDC138	4.39E-42	-0.20849	0.031	0.186	6.57E-38
NCK1	4.59E-42	0.162046	0.228	0.356	6.86E-38
RAP2A	4.63E-42	-0.14237	0.078	0.248	6.92E-38
BEND6	4.69E-42	-0.17382	0.079	0.263	7.01E-38
NLGN4X	5.08E-42	-0.2454	0.074	0.26	7.60E-38
PEAK1	5.11E-42	-0.15751	0.027	0.165	7.64E-38
TMEM39B	5.13E-42	-0.10469	0.088	0.263	7.66E-38
UFSP2	5.18E-42	0.123555	0.216	0.358	7.74E-38
CDH6	5.43E-42	-0.14461	0.028	0.163	8.12E-38
TRIM13	5.84E-42	-0.28792	0.158	0.388	8.73E-38
DPY19L4	6.37E-42	-0.14614	0.068	0.228	9.51E-38
CENPC	6.55E-42	-0.21743	0.091	0.281	9.79E-38
CTNNAL1	6.75E-42	-0.20362	0.203	0.433	1.01E-37
UBE2H	6.93E-42	-0.14804	0.175	0.375	1.04E-37
GPR108	7.04E-42	0.226453	0.242	0.34	1.05E-37
TPD52	7.17E-42	0.20981	0.262	0.358	1.07E-37
FAT1	7.42E-42	-0.16028	0.041	0.194	1.11E-37
EML1	7.49E-42	-0.10108	0.042	0.178	1.12E-37
EPAS1	7.49E-42	0.577248	0.218	0.09	1.12E-37
POM121C	7.70E-42	-0.14898	0.109	0.29	1.15E-37
FBXO45	8.19E-42	-0.17661	0.034	0.184	1.22E-37
CEP192	9.53E-42	-0.16194	0.024	0.16	1.42E-37
SEC23A	9.60E-42	0.1033	0.183	0.307	1.43E-37
LRRC17	9.79E-42	-0.28573	0.042	0.206	1.46E-37
KAT5	1.00E-41	0.123324	0.226	0.363	1.50E-37
PTN	1.03E-41	0.379736	0.809	0.916	1.55E-37
CA14	1.04E-41	-0.30111	0.009	0.136	1.55E-37
CCND3	1.20E-41	0.235388	0.221	0.342	1.79E-37
NUP155	1.20E-41	-0.20311	0.046	0.206	1.80E-37
GTDC1	1.21E-41	-0.20404	0.053	0.225	1.81E-37
DTNBP1	1.21E-41	-0.10609	0.144	0.32	1.81E-37
SMYD2	1.26E-41	-0.14982	0.1	0.283	1.88E-37
GAK	1.28E-41	-0.14357	0.08	0.248	1.92E-37
KATNB1	1.28E-41	-0.21871	0.07	0.256	1.92E-37
CYB5R4	1.29E-41	-0.13088	0.066	0.228	1.93E-37
SELM	1.35E-41	0.53016	0.222	0.105	2.01E-37
NANS	1.45E-41	0.300607	0.306	0.364	2.17E-37
NEIL2	1.47E-41	0.223471	0.217	0.31	2.20E-37
STEAP3	1.50E-41	0.5783	0.22	0.181	2.24E-37
RPP38	1.50E-41	-0.18331	0.077	0.259	2.24E-37
LLPH	1.55E-41	0.169894	0.262	0.386	2.32E-37
UBL4A	1.57E-41	-0.12414	0.14	0.333	2.34E-37
RMND5A	1.65E-41	-0.12815	0.079	0.237	2.46E-37
COQ5	1.72E-41	0.105983	0.197	0.342	2.58E-37
CBX4	1.73E-41	-0.19058	0.06	0.225	2.59E-37
LRP10	1.79E-41	0.28304	0.227	0.265	2.67E-37
HGS	1.85E-41	-0.10593	0.186	0.379	2.76E-37
KLHDC10	2.02E-41	-0.16036	0.087	0.256	3.03E-37
PDLIM2	2.03E-41	0.360919	0.269	0.312	3.04E-37
SS18L1	2.06E-41	-0.12792	0.053	0.198	3.08E-37
PPIL4	2.08E-41	0.121757	0.235	0.374	3.11E-37

CADM2	2.18E-41	-0.33042	0.125	0.346	3.26E-37
TRIO	2.18E-41	-0.21835	0.126	0.325	3.26E-37
GCFC2	2.29E-41	-0.15327	0.102	0.272	3.42E-37
NOB1	2.30E-41	0.171702	0.204	0.299	3.44E-37
TOR3A	2.34E-41	-0.14144	0.162	0.356	3.49E-37
ZNF93	2.34E-41	-0.19579	0.043	0.202	3.50E-37
PGM2L1	2.35E-41	0.205627	0.298	0.427	3.51E-37
MKNK2	2.38E-41	0.179803	0.16	0.236	3.56E-37
GATAD2B	2.45E-41	-0.18128	0.086	0.263	3.66E-37
SPG20	2.58E-41	0.122238	0.272	0.386	3.85E-37
KLF3	2.58E-41	-0.14994	0.118	0.299	3.86E-37
DTNB	2.61E-41	-0.19919	0.035	0.191	3.89E-37
B3GNT5	2.66E-41	-0.23434	0.045	0.204	3.98E-37
AP001347.	2.67E-41	-0.22983	0.005	0.121	3.99E-37
PALM	2.69E-41	-0.20092	0.075	0.252	4.03E-37
IMPDH1	2.79E-41	-0.13012	0.096	0.264	4.18E-37
SPEN	2.94E-41	-0.13235	0.106	0.279	4.40E-37
OXA1L	3.14E-41	0.250935	0.219	0.326	4.69E-37
ATP6V1C1	3.17E-41	0.154794	0.198	0.31	4.74E-37
PCSK2	3.34E-41	-0.33415	0.008	0.128	4.99E-37
NOP16	3.42E-41	0.195988	0.224	0.349	5.11E-37
FBXW7	3.44E-41	-0.20344	0.08	0.262	5.14E-37
STX6	3.48E-41	-0.18038	0.09	0.269	5.20E-37
TM9SF3	3.55E-41	-0.139	0.137	0.319	5.31E-37
RBM7	3.66E-41	0.33837	0.289	0.311	5.47E-37
CXCR4	3.92E-41	0.369568	0.244	0.293	5.86E-37
TMEM176A	4.16E-41	0.670221	0.227	0.074	6.21E-37
RFT1	4.18E-41	-0.13477	0.103	0.285	6.25E-37
GAB1	4.30E-41	-0.2264	0.046	0.214	6.43E-37
HES1	4.37E-41	0.151464	0.236	0.343	6.52E-37
KIAA1958	4.40E-41	-0.19762	0.029	0.178	6.57E-37
CA10	4.42E-41	-0.25426	0.004	0.11	6.60E-37
NIT2	4.68E-41	0.266894	0.232	0.341	6.99E-37
CNKSR3	4.79E-41	-0.13961	0.074	0.247	7.16E-37
PAXIP1-AS	4.83E-41	-0.17946	0.161	0.368	7.22E-37
RRBP1	5.21E-41	-0.22725	0.211	0.432	7.79E-37
RCE1	5.36E-41	-0.1177	0.097	0.26	8.01E-37
TRAF3IP2	5.42E-41	-0.16479	0.101	0.28	8.10E-37
NOTCH2NL	5.44E-41	-0.10911	0.133	0.304	8.12E-37
B3GALNT1	5.63E-41	-0.14199	0.136	0.341	8.42E-37
FBXO34	5.64E-41	-0.16828	0.049	0.204	8.43E-37
EXOC4	5.85E-41	-0.17693	0.134	0.331	8.75E-37
GALNT15	6.07E-41	0.654949	0.153	0.007	9.08E-37
APOD	6.41E-41	0.954325	0.313	0.132	9.58E-37
SLC9A3R1	6.45E-41	-0.23554	0.109	0.309	9.64E-37
EPOR	6.60E-41	-0.16177	0.033	0.174	9.87E-37
RABGGTB	6.68E-41	0.228023	0.279	0.335	9.98E-37
DRAM2	6.98E-41	0.197971	0.248	0.34	1.04E-36
PLS3	6.99E-41	0.13446	0.245	0.395	1.04E-36
TXNRD2	7.10E-41	-0.16459	0.066	0.226	1.06E-36
RP5-940J5	7.32E-41	0.611725	0.247	0.169	1.09E-36

ACSS3	7.37E-41	-0.11473	0.087	0.248	1.10E-36
ZNF625	7.44E-41	-0.17051	0.033	0.172	1.11E-36
CRKL	7.52E-41	-0.16194	0.085	0.258	1.12E-36
GPATCH11	7.57E-41	-0.24259	0.107	0.312	1.13E-36
CCDC127	7.65E-41	0.110112	0.131	0.244	1.14E-36
PYGL	7.80E-41	0.530871	0.196	0.147	1.17E-36
MAP3K11	7.99E-41	-0.14364	0.102	0.288	1.19E-36
C11orf84	8.19E-41	-0.14384	0.017	0.148	1.22E-36
R3HDM2	8.30E-41	-0.11992	0.092	0.272	1.24E-36
PARD3	9.08E-41	-0.11876	0.039	0.179	1.36E-36
PDCL	9.32E-41	-0.16574	0.146	0.342	1.39E-36
FAM217B	9.36E-41	-0.1992	0.072	0.244	1.40E-36
RPF2	1.00E-40	0.29924	0.266	0.357	1.50E-36
GRIA4	1.02E-40	-0.2993	0.058	0.24	1.52E-36
CEP112	1.03E-40	-0.12847	0.055	0.207	1.53E-36
COLGALT1	1.04E-40	-0.13824	0.09	0.264	1.55E-36
RNF220	1.07E-40	-0.16024	0.078	0.249	1.60E-36
ZNF652	1.10E-40	-0.15439	0.081	0.253	1.64E-36
XRCC6BP1	1.11E-40	-0.42656	0.055	0.243	1.65E-36
C7orf26	1.11E-40	-0.19849	0.115	0.309	1.66E-36
METTL10	1.17E-40	-0.15115	0.083	0.253	1.75E-36
ARFGEF1	1.21E-40	-0.13606	0.061	0.216	1.81E-36
PIP4K2B	1.23E-40	-0.13153	0.059	0.21	1.83E-36
AGPAT2	1.25E-40	-0.11814	0.076	0.222	1.87E-36
MGEA5	1.28E-40	-0.11953	0.155	0.335	1.91E-36
MIB1	1.43E-40	-0.16321	0.062	0.222	2.14E-36
ANXA7	1.44E-40	0.349292	0.316	0.36	2.15E-36
PIFO	1.44E-40	0.685865	0.193	0.026	2.16E-36
LINC00667	1.46E-40	0.167648	0.235	0.359	2.18E-36
BAIAP2	1.51E-40	0.175677	0.189	0.281	2.26E-36
CACUL1	1.53E-40	-0.1352	0.109	0.285	2.28E-36
CSPG5	1.56E-40	-0.41493	0.056	0.243	2.33E-36
SLC25A22	1.57E-40	-0.12793	0.089	0.258	2.34E-36
NID1	1.60E-40	-0.12236	0.025	0.154	2.39E-36
CA2	1.60E-40	0.501772	0.263	0.296	2.39E-36
NACC2	1.61E-40	-0.14287	0.057	0.207	2.41E-36
DDAH1	1.64E-40	-0.19769	0.13	0.331	2.46E-36
KATNAL1	1.66E-40	-0.11823	0.061	0.221	2.48E-36
RFX3	1.70E-40	-0.14722	0.052	0.2	2.53E-36
RBM28	1.71E-40	-0.15014	0.138	0.331	2.55E-36
TRAF3IP2-	1.73E-40	-0.18354	0.057	0.226	2.58E-36
SCAMP3	1.84E-40	0.150083	0.292	0.41	2.75E-36
BUD13	1.87E-40	-0.20952	0.057	0.221	2.79E-36
GPR180	1.94E-40	-0.18301	0.049	0.211	2.90E-36
CEP128	1.98E-40	-0.162	0.008	0.122	2.96E-36
RP11-563K	2.10E-40	-0.22187	0.03	0.177	3.14E-36
ANKRD36	2.20E-40	-0.2604	0.145	0.36	3.29E-36
MAX	2.22E-40	0.240289	0.237	0.317	3.31E-36
SPHK2	2.24E-40	-0.18131	0.085	0.263	3.35E-36
ANKRD39	2.41E-40	-0.15215	0.101	0.286	3.60E-36
NOC4L	2.57E-40	-0.1995	0.126	0.317	3.85E-36

FIGN	2.59E-40	-0.17111	0.085	0.262	3.87E-36
TBRG1	2.65E-40	0.1214	0.206	0.326	3.96E-36
KIF9	2.71E-40	0.212259	0.239	0.348	4.05E-36
TMEM242	2.74E-40	0.130741	0.214	0.353	4.09E-36
SLC35F2	3.07E-40	-0.11345	0.061	0.221	4.59E-36
C6orf47	3.08E-40	-0.13683	0.1	0.273	4.60E-36
TTTY15	3.16E-40	-0.16481	0.009	0.121	4.72E-36
IRF2BP1	3.51E-40	-0.151	0.074	0.24	5.24E-36
KCNAB3	3.65E-40	-0.20354	0.012	0.136	5.45E-36
RAD54B	3.94E-40	-0.19337	0.019	0.151	5.89E-36
MANEA	4.04E-40	-0.10747	0.045	0.194	6.04E-36
RP13-1032	4.13E-40	-0.20493	0.043	0.204	6.17E-36
FAM184B	4.17E-40	0.101245	0.092	0.191	6.23E-36
RIMKLB	4.40E-40	-0.20234	0.12	0.32	6.57E-36
DCLRE1B	4.69E-40	-0.20351	0.045	0.204	7.02E-36
ANKRD26	4.80E-40	-0.16862	0.08	0.256	7.17E-36
SLC25A19	5.03E-40	-0.1451	0.06	0.209	7.52E-36
TONSL	5.12E-40	-0.19329	0.013	0.14	7.66E-36
SMURF2	5.14E-40	-0.16533	0.056	0.215	7.69E-36
ATM	5.24E-40	-0.10867	0.118	0.286	7.83E-36
ATP2C1	5.45E-40	-0.11328	0.173	0.353	8.15E-36
KDM2A	5.55E-40	-0.10933	0.075	0.226	8.30E-36
PURB	5.84E-40	-0.12201	0.134	0.311	8.72E-36
FAXC	6.22E-40	-0.27054	0.038	0.2	9.30E-36
ZBTB8A	6.35E-40	-0.20698	0.075	0.249	9.49E-36
CDKN1A	6.44E-40	0.382631	0.33	0.332	9.63E-36
BRWD1	6.47E-40	-0.21758	0.172	0.388	9.67E-36
INPP1	6.78E-40	-0.1827	0.112	0.298	1.01E-35
C11orf68	6.99E-40	-0.11741	0.067	0.216	1.04E-35
NIPSNAP1	7.24E-40	-0.17473	0.091	0.275	1.08E-35
MSANTD3	7.55E-40	-0.15992	0.057	0.221	1.13E-35
KLF12	8.06E-40	-0.22629	0.042	0.206	1.20E-35
LINC00662	8.28E-40	0.134408	0.191	0.3	1.24E-35
CTU2	8.40E-40	-0.15672	0.11	0.289	1.26E-35
COPG2	8.48E-40	-0.22015	0.084	0.28	1.27E-35
MAPK9	8.89E-40	-0.17886	0.037	0.189	1.33E-35
THSD7A	9.44E-40	-0.40672	0.06	0.249	1.41E-35
TULP4	9.52E-40	-0.16146	0.081	0.246	1.42E-35
FOXO3	9.55E-40	-0.12909	0.187	0.381	1.43E-35
FAT3	9.60E-40	-0.19805	0.018	0.152	1.44E-35
SPATA5	9.67E-40	-0.11044	0.027	0.147	1.44E-35
ANTXR1	1.01E-39	-0.26663	0.049	0.221	1.51E-35
CARS	1.02E-39	0.240057	0.277	0.348	1.52E-35
TMEM68	1.05E-39	-0.1104	0.162	0.341	1.57E-35
YIPF5	1.05E-39	0.325373	0.301	0.336	1.57E-35
NCOA3	1.18E-39	-0.21706	0.061	0.231	1.77E-35
STK32B	1.26E-39	-0.1517	0.034	0.172	1.89E-35
NECAB3	1.32E-39	-0.18486	0.04	0.195	1.98E-35
ZNF672	1.46E-39	-0.17383	0.084	0.258	2.18E-35
TEX261	1.48E-39	-0.20521	0.129	0.327	2.22E-35
HSPB8	1.49E-39	0.596466	0.165	0.012	2.22E-35

CDC42BPB	1.50E-39	-0.16505	0.053	0.209	2.24E-35
NUP188	1.54E-39	-0.20024	0.059	0.226	2.30E-35
ZNF280D	1.69E-39	-0.1773	0.045	0.196	2.52E-35
DNAJC21	1.71E-39	-0.18655	0.077	0.252	2.55E-35
UCK2	1.71E-39	-0.10355	0.049	0.186	2.56E-35
GMPPA	1.73E-39	0.158861	0.167	0.28	2.58E-35
EIF2S3	1.79E-39	0.121491	0.271	0.388	2.67E-35
CCDC115	1.83E-39	0.289327	0.289	0.359	2.74E-35
CEP72	1.98E-39	-0.26915	0.01	0.132	2.95E-35
THAP10	2.01E-39	-0.15501	0.044	0.191	3.00E-35
LINC00152	2.02E-39	0.269815	0.27	0.38	3.02E-35
CCNK	2.04E-39	-0.11752	0.076	0.228	3.05E-35
WNK3	2.07E-39	-0.14513	0.078	0.241	3.09E-35
FBF1	2.13E-39	-0.15047	0.023	0.153	3.18E-35
DENND5B	2.37E-39	-0.17709	0.049	0.207	3.54E-35
NPM3	2.39E-39	0.171373	0.195	0.311	3.58E-35
ZNF76	2.43E-39	-0.15766	0.068	0.231	3.63E-35
CEP44	2.52E-39	-0.11766	0.06	0.207	3.77E-35
MTHFS	2.62E-39	0.246707	0.158	0.243	3.91E-35
TMEM51	2.65E-39	-0.21829	0.027	0.175	3.97E-35
ZNF37A	2.81E-39	-0.14467	0.086	0.257	4.20E-35
KCTD13	2.85E-39	-0.27943	0.077	0.267	4.26E-35
RP11-398K	3.09E-39	-0.1927	0.045	0.199	4.62E-35
UPP1	3.30E-39	0.465914	0.296	0.284	4.92E-35
SHOX2	3.32E-39	-0.17167	0.034	0.184	4.96E-35
CDC42EP4	3.40E-39	-0.15481	0.225	0.43	5.09E-35
ZNF12	3.49E-39	-0.14678	0.092	0.262	5.21E-35
WDR46	3.76E-39	0.141357	0.223	0.354	5.62E-35
SPAST	4.08E-39	-0.22737	0.052	0.223	6.10E-35
CHMP7	4.26E-39	-0.12313	0.121	0.3	6.36E-35
EP400	4.31E-39	-0.16133	0.073	0.238	6.45E-35
PXN-AS1	4.33E-39	-0.14812	0.041	0.19	6.47E-35
LPCAT3	4.41E-39	0.188682	0.142	0.263	6.59E-35
RP5-1085F	4.55E-39	-0.11783	0.056	0.193	6.80E-35
PILRB	4.61E-39	-0.1981	0.139	0.333	6.89E-35
GALNS	4.67E-39	-0.17004	0.084	0.26	6.97E-35
ACBD5	4.68E-39	-0.1571	0.091	0.267	7.00E-35
LOXL1	5.47E-39	-0.15764	0.036	0.178	8.17E-35
ZFYVE16	5.59E-39	-0.14717	0.099	0.272	8.35E-35
PIAS4	5.77E-39	-0.19163	0.105	0.288	8.62E-35
RELT	5.96E-39	-0.15822	0.024	0.157	8.90E-35
PPP1R16A	6.03E-39	0.173025	0.214	0.284	9.01E-35
TSPAN6	6.21E-39	0.202804	0.194	0.295	9.28E-35
SLC8A1	6.55E-39	-0.20004	0.048	0.2	9.79E-35
ULK3	6.64E-39	-0.13003	0.092	0.26	9.93E-35
CHST7	6.71E-39	-0.16547	0.065	0.228	1.00E-34
PHYHIPL	6.78E-39	-0.25378	0.139	0.351	1.01E-34
MGAT4B	6.98E-39	-0.10552	0.052	0.188	1.04E-34
GATAD2A	7.19E-39	-0.17455	0.087	0.26	1.07E-34
ZNF107	7.61E-39	-0.22697	0.019	0.151	1.14E-34
KDM3B	7.74E-39	-0.17392	0.054	0.209	1.16E-34

MORC4	7.80E-39	-0.28804	0.051	0.226	1.17E-34
TMEM55A	8.12E-39	0.221251	0.251	0.319	1.21E-34
SNHG15	8.59E-39	0.314893	0.213	0.288	1.28E-34
DNAJA3	8.60E-39	-0.115	0.179	0.357	1.29E-34
DCAF8	8.70E-39	0.130584	0.176	0.285	1.30E-34
EXOSC2	8.80E-39	-0.11266	0.081	0.241	1.32E-34
CAT	8.84E-39	0.496888	0.295	0.278	1.32E-34
LPCAT1	9.46E-39	-0.21098	0.152	0.352	1.41E-34
TMEM35	9.65E-39	-0.31548	0.032	0.189	1.44E-34
RAI1	9.95E-39	-0.1474	0.029	0.163	1.49E-34
ITGB1	1.02E-38	-0.20673	0.259	0.505	1.52E-34
ARHGEF39	1.05E-38	-0.1841	0.011	0.128	1.58E-34
PDCD6IP	1.07E-38	0.197085	0.249	0.358	1.60E-34
ELAC2	1.14E-38	-0.1631	0.112	0.296	1.70E-34
PEX26	1.17E-38	-0.10525	0.062	0.206	1.75E-34
RUFY2	1.18E-38	-0.14133	0.099	0.263	1.76E-34
ARAP3	1.19E-38	-0.15142	0.023	0.146	1.79E-34
FGFBP3	1.26E-38	-0.17886	0.027	0.165	1.88E-34
MTRF1L	1.31E-38	-0.13107	0.162	0.344	1.95E-34
MTOR	1.38E-38	-0.11995	0.041	0.183	2.06E-34
SCAP	1.38E-38	-0.13177	0.107	0.289	2.06E-34
CRYZL1	1.42E-38	0.216038	0.244	0.333	2.12E-34
PLEKHF2	1.53E-38	-0.16071	0.078	0.244	2.29E-34
MVP	1.56E-38	0.526824	0.202	0.144	2.34E-34
ALDH16A1	1.62E-38	-0.19665	0.052	0.209	2.42E-34
PIK3R1	1.69E-38	-0.31581	0.187	0.422	2.52E-34
BRAT1	1.70E-38	-0.2331	0.158	0.37	2.54E-34
DCAF10	1.72E-38	-0.10852	0.142	0.306	2.57E-34
TCOF1	1.74E-38	-0.16241	0.108	0.289	2.60E-34
AGFG1	1.87E-38	-0.1583	0.07	0.237	2.79E-34
GLB1	1.89E-38	0.218279	0.211	0.272	2.83E-34
SLC27A4	1.90E-38	-0.15019	0.018	0.141	2.83E-34
CMTM8	1.90E-38	-0.11735	0.014	0.126	2.84E-34
NUP205	1.99E-38	-0.17819	0.049	0.196	2.97E-34
CEP164	2.18E-38	-0.14601	0.122	0.301	3.25E-34
SMAD9	2.20E-38	-0.17972	0.05	0.198	3.29E-34
TAF3	2.30E-38	-0.17182	0.075	0.243	3.44E-34
ZNF581	2.33E-38	0.2333	0.143	0.206	3.48E-34
FAF2	2.39E-38	-0.12012	0.167	0.352	3.57E-34
TRRAP	2.42E-38	-0.1362	0.06	0.206	3.61E-34
C21orf62	2.46E-38	0.178385	0.209	0.311	3.67E-34
CELSR2	2.53E-38	-0.12476	0.04	0.178	3.78E-34
DMTF1	2.55E-38	-0.15283	0.127	0.307	3.81E-34
EDEM2	2.57E-38	0.206	0.195	0.304	3.84E-34
SLC35B4	2.63E-38	-0.12206	0.156	0.338	3.93E-34
RALGAPB	2.74E-38	-0.1581	0.065	0.221	4.09E-34
ALDH2	2.87E-38	0.264263	0.275	0.3	4.29E-34
NPAT	3.27E-38	-0.12816	0.061	0.215	4.88E-34
SLC4A8	3.27E-38	-0.16627	0.056	0.209	4.89E-34
TUBA1A	3.45E-38	0.139201	0.859	0.989	5.16E-34
NQO1	3.46E-38	0.221294	0.136	0.22	5.18E-34

C5orf45	3.53E-38	0.175035	0.161	0.241	5.27E-34
PARVB	3.55E-38	-0.13048	0.044	0.181	5.31E-34
ZNF768	3.83E-38	-0.13379	0.066	0.216	5.72E-34
CLASRP	3.92E-38	-0.11524	0.126	0.29	5.86E-34
C1QA	4.51E-38	0.560356	0.371	0.294	6.75E-34
C2orf68	4.58E-38	-0.10933	0.099	0.265	6.84E-34
MLLT10	4.58E-38	-0.11741	0.079	0.238	6.84E-34
TMEM57	5.10E-38	-0.1582	0.118	0.291	7.63E-34
FAM20B	5.20E-38	-0.16226	0.034	0.174	7.77E-34
GLA	5.23E-38	-0.19612	0.095	0.274	7.82E-34
STX4	5.77E-38	0.368619	0.265	0.286	8.62E-34
NUP214	6.18E-38	-0.15707	0.083	0.244	9.23E-34
PMS1	6.19E-38	-0.15047	0.087	0.249	9.25E-34
MAP6D1	6.43E-38	-0.2269	0.028	0.177	9.62E-34
C19orf66	6.44E-38	0.203628	0.244	0.326	9.63E-34
MAP9	6.57E-38	0.128955	0.206	0.31	9.83E-34
DHRS4L2	6.58E-38	0.256026	0.221	0.319	9.83E-34
TSPAN17	6.66E-38	-0.16878	0.09	0.262	9.95E-34
PHC1	6.72E-38	-0.18316	0.051	0.204	1.00E-33
ZNF280B	6.99E-38	-0.13809	0.011	0.122	1.04E-33
ADAMTS6	7.22E-38	-0.21185	0.027	0.17	1.08E-33
ZBTB1	7.45E-38	-0.18864	0.091	0.27	1.11E-33
NTRK2	8.05E-38	0.660663	0.333	0.149	1.20E-33
RPUSD1	8.57E-38	-0.19008	0.078	0.253	1.28E-33
UMPS	8.98E-38	-0.12472	0.129	0.305	1.34E-33
POLR2M	9.01E-38	-0.19995	0.117	0.304	1.35E-33
CEP290	9.23E-38	-0.14253	0.13	0.304	1.38E-33
DPH3	9.25E-38	0.259942	0.239	0.332	1.38E-33
MAP3K4	9.61E-38	-0.14691	0.054	0.201	1.44E-33
GOLGB1	9.64E-38	0.146785	0.226	0.319	1.44E-33
CLN5	1.00E-37	0.155701	0.195	0.279	1.50E-33
TRAF2	1.01E-37	-0.15279	0.069	0.225	1.51E-33
TP53TG1	1.02E-37	0.524526	0.242	0.226	1.52E-33
CNOT6	1.04E-37	-0.19395	0.03	0.172	1.55E-33
RTN1	1.05E-37	-0.36433	0.069	0.252	1.57E-33
NRBP2	1.06E-37	-0.15013	0.068	0.222	1.58E-33
ZCCHC9	1.15E-37	0.112132	0.155	0.262	1.72E-33
GTPBP3	1.18E-37	-0.10796	0.104	0.262	1.76E-33
MSH3	1.18E-37	-0.1381	0.067	0.221	1.76E-33
CAMK1D	1.21E-37	-0.18407	0.03	0.168	1.80E-33
MDFI	1.22E-37	-0.22879	0.052	0.211	1.82E-33
RND2	1.31E-37	0.214038	0.162	0.24	1.95E-33
SDC2	1.33E-37	0.458955	0.257	0.193	1.99E-33
ICK	1.42E-37	-0.17263	0.042	0.189	2.12E-33
ZBTB44	1.48E-37	-0.1202	0.086	0.242	2.21E-33
POC1B	1.60E-37	-0.12713	0.06	0.205	2.40E-33
UBXN7	1.67E-37	-0.11522	0.075	0.231	2.50E-33
VARS	1.69E-37	-0.15558	0.087	0.254	2.53E-33
CCNG1	1.70E-37	0.219502	0.317	0.41	2.54E-33
OTUD4	1.87E-37	-0.21327	0.059	0.231	2.79E-33
FAM3A	1.97E-37	0.177425	0.207	0.322	2.95E-33

ZNF322	1.99E-37	-0.11077	0.086	0.241	2.97E-33
PDHX	2.02E-37	0.240987	0.183	0.253	3.02E-33
GSDMD	2.04E-37	0.232027	0.136	0.198	3.04E-33
FPGS	2.10E-37	-0.14281	0.079	0.247	3.14E-33
ASPHD2	2.12E-37	-0.12874	0.022	0.148	3.17E-33
NAPEPLD	2.13E-37	-0.127	0.082	0.235	3.18E-33
ZMYND19	2.17E-37	-0.19563	0.025	0.164	3.25E-33
IKBKG	2.18E-37	-0.16769	0.064	0.225	3.26E-33
SLC16A14	2.20E-37	-0.20051	0.018	0.148	3.28E-33
CAD	2.23E-37	-0.14232	0.031	0.153	3.33E-33
ZDHHC9	2.35E-37	0.424736	0.228	0.227	3.51E-33
ATMIN	2.38E-37	-0.14017	0.063	0.212	3.56E-33
B3GALNT2	2.42E-37	-0.14382	0.06	0.206	3.62E-33
CETN3	2.48E-37	-0.12086	0.131	0.311	3.70E-33
HECTD4	2.50E-37	-0.13443	0.045	0.188	3.74E-33
FAM222B	2.51E-37	-0.12939	0.05	0.196	3.75E-33
NDUFAF4	2.52E-37	0.241058	0.235	0.338	3.76E-33
AP2A2	2.53E-37	-0.10393	0.167	0.336	3.78E-33
SMARCD2	2.53E-37	-0.12768	0.036	0.172	3.79E-33
MVB12B	2.68E-37	-0.15523	0.037	0.177	4.01E-33
MOGS	2.75E-37	-0.12393	0.074	0.22	4.12E-33
CYBRD1	2.76E-37	0.355646	0.22	0.251	4.13E-33
ZCCHC8	2.86E-37	-0.15558	0.06	0.216	4.27E-33
PDSS1	2.90E-37	-0.1428	0.022	0.149	4.33E-33
PIGQ	3.04E-37	-0.16102	0.08	0.246	4.54E-33
TRAPPC2P1	3.05E-37	0.397679	0.28	0.296	4.55E-33
HAUS6	3.09E-37	-0.2053	0.095	0.28	4.61E-33
RP11-25K1	3.13E-37	-0.16577	0.044	0.189	4.68E-33
ZNF444	3.14E-37	-0.13808	0.115	0.281	4.70E-33
PRR14L	3.38E-37	-0.12621	0.038	0.173	5.06E-33
KDELC2	3.41E-37	-0.17411	0.072	0.241	5.10E-33
MICU1	3.43E-37	-0.12256	0.082	0.24	5.12E-33
GLIPR2	3.49E-37	0.151283	0.24	0.331	5.22E-33
C1QTNF2	3.54E-37	-0.23823	0.013	0.136	5.28E-33
TTC37	3.64E-37	-0.13474	0.128	0.305	5.44E-33
DDX54	3.82E-37	-0.15781	0.155	0.342	5.71E-33
POLG	3.91E-37	-0.14237	0.048	0.184	5.85E-33
DHX40	3.92E-37	-0.15081	0.117	0.291	5.85E-33
UBB	4.14E-37	0.170216	0.843	0.965	6.18E-33
NFS1	4.36E-37	-0.10425	0.102	0.262	6.51E-33
EXOC1	4.42E-37	-0.16794	0.212	0.422	6.61E-33
NRSN1	4.52E-37	-0.17626	0.02	0.138	6.75E-33
PCBD2	4.59E-37	-0.1704	0.051	0.209	6.86E-33
SDCCAG3	4.75E-37	-0.15059	0.049	0.198	7.10E-33
RP3-428L1	4.85E-37	-0.1644	0.023	0.152	7.25E-33
LINC00665	4.92E-37	0.130411	0.236	0.347	7.36E-33
FAM53C	5.20E-37	0.129503	0.179	0.288	7.77E-33
ARHGEF7	5.47E-37	-0.20637	0.09	0.264	8.17E-33
CDKN2AIP	5.52E-37	-0.1063	0.125	0.298	8.25E-33
SNHG10	5.52E-37	-0.15787	0.073	0.232	8.25E-33
TERF2	5.64E-37	-0.16121	0.049	0.199	8.43E-33

YEATS2	5.86E-37	-0.16249	0.039	0.177	8.76E-33
COPG1	6.17E-37	0.171144	0.236	0.31	9.22E-33
GRPEL1	6.44E-37	0.112725	0.243	0.378	9.62E-33
GJA1	6.46E-37	0.657964	0.229	0.054	9.65E-33
AFTPH	7.26E-37	-0.1388	0.115	0.284	1.09E-32
SEH1L	7.32E-37	-0.10333	0.076	0.23	1.09E-32
TMBIM1	7.56E-37	0.528188	0.232	0.185	1.13E-32
AVIL	7.59E-37	-0.39081	0.062	0.242	1.13E-32
AFAP1	7.62E-37	-0.14025	0.048	0.185	1.14E-32
DHCR7	8.10E-37	-0.15067	0.135	0.32	1.21E-32
FBXO22	8.51E-37	0.128543	0.136	0.253	1.27E-32
SOS2	9.49E-37	-0.13171	0.033	0.162	1.42E-32
NEK1	9.71E-37	-0.14005	0.102	0.274	1.45E-32
LRP4	9.75E-37	-0.14876	0.034	0.159	1.46E-32
CTTNBP2	1.02E-36	-0.29254	0.056	0.23	1.52E-32
SCMH1	1.02E-36	-0.16821	0.058	0.211	1.52E-32
ZNF787	1.02E-36	-0.11305	0.042	0.17	1.53E-32
RARS	1.06E-36	0.141227	0.25	0.36	1.58E-32
TLE2	1.10E-36	-0.26264	0.047	0.207	1.64E-32
FGFR1OP2	1.19E-36	0.159909	0.313	0.428	1.78E-32
DGCR2	1.23E-36	-0.15753	0.12	0.301	1.84E-32
BRF1	1.27E-36	-0.16594	0.053	0.201	1.90E-32
NVL	1.29E-36	-0.22725	0.082	0.254	1.93E-32
EPHB2	1.31E-36	-0.21851	0.026	0.164	1.96E-32
TMEM101	1.32E-36	0.139095	0.185	0.305	1.98E-32
NME7	1.33E-36	0.195336	0.206	0.293	1.99E-32
SLC38A2	1.37E-36	0.115675	0.252	0.362	2.05E-32
SAV1	1.46E-36	-0.16542	0.064	0.223	2.18E-32
AK4	1.46E-36	0.2758	0.262	0.298	2.18E-32
ZNF513	1.56E-36	-0.12468	0.026	0.144	2.33E-32
NAA30	1.85E-36	-0.11648	0.043	0.174	2.77E-32
ZNF266	1.86E-36	-0.16521	0.068	0.216	2.78E-32
LMBR1L	1.88E-36	-0.11375	0.094	0.251	2.81E-32
CRNKL1	1.88E-36	-0.10336	0.12	0.277	2.81E-32
PHF13	1.91E-36	-0.15342	0.049	0.189	2.86E-32
PIP5K1C	1.92E-36	-0.15593	0.053	0.202	2.87E-32
NMRAL1	2.04E-36	0.222498	0.231	0.336	3.04E-32
CCDC174	2.06E-36	0.1406	0.187	0.294	3.08E-32
ZSWIM6	2.25E-36	-0.15967	0.045	0.188	3.37E-32
GALK1	2.30E-36	0.167796	0.169	0.279	3.43E-32
RNF165	2.44E-36	-0.21193	0.023	0.156	3.64E-32
MCMBP	2.58E-36	-0.13857	0.11	0.273	3.86E-32
GNB1L	2.66E-36	-0.15791	0.056	0.201	3.97E-32
MAPK8	2.67E-36	-0.17992	0.046	0.189	4.00E-32
PPP6R3	2.70E-36	-0.10069	0.089	0.241	4.03E-32
EFNB1	2.74E-36	-0.22293	0.063	0.227	4.10E-32
CDC23	2.85E-36	-0.14976	0.129	0.31	4.25E-32
ECHDC2	2.87E-36	0.270628	0.218	0.272	4.29E-32
LLGL1	2.93E-36	-0.15811	0.062	0.217	4.37E-32
GLDC	2.99E-36	-0.14428	0.04	0.175	4.47E-32
B3GAT1	3.37E-36	-0.20393	0.032	0.175	5.04E-32

RNF219	3.44E-36	-0.23515	0.097	0.28	5.15E-32
TRAPPC12	3.45E-36	-0.13095	0.125	0.295	5.15E-32
PTGDS	3.69E-36	1.045311	0.271	0.123	5.52E-32
XPO6	3.70E-36	-0.13476	0.065	0.216	5.52E-32
PSMB8	3.76E-36	0.234414	0.364	0.478	5.62E-32
PACS1	3.79E-36	-0.10408	0.051	0.183	5.66E-32
ADAM19	3.83E-36	-0.22696	0.025	0.162	5.73E-32
BNIP3	3.85E-36	0.287628	0.263	0.335	5.75E-32
MAFG-AS1	4.02E-36	-0.11789	0.039	0.174	6.01E-32
ITGA2	4.22E-36	-0.22125	0.044	0.2	6.30E-32
PI4K2B	4.36E-36	-0.16647	0.054	0.206	6.51E-32
HLA-DQA2	4.75E-36	0.879263	0.128	0.005	7.10E-32
UBE2D4	4.91E-36	-0.11367	0.171	0.342	7.33E-32
CNOT11	4.99E-36	-0.21322	0.069	0.24	7.46E-32
EGR1	5.02E-36	-0.24677	0.414	0.659	7.50E-32
SIKE1	5.05E-36	0.139026	0.193	0.296	7.54E-32
GPR137C	5.10E-36	-0.15316	0.02	0.137	7.62E-32
TELO2	5.35E-36	-0.15218	0.09	0.262	7.99E-32
ADCK2	5.36E-36	-0.14295	0.056	0.204	8.01E-32
RP11-538F	6.07E-36	-0.18272	0.054	0.212	9.08E-32
EIF3J-AS1	6.74E-36	0.102077	0.118	0.227	1.01E-31
TOX3	7.64E-36	-0.33451	0.028	0.172	1.14E-31
GSX1	7.92E-36	-0.25703	0.002	0.091	1.18E-31
IGDCC3	8.41E-36	-0.17493	0.014	0.128	1.26E-31
RP11-212F	8.49E-36	-0.18737	0.045	0.188	1.27E-31
CD58	9.05E-36	0.112091	0.155	0.259	1.35E-31
MED25	9.05E-36	-0.24277	0.099	0.281	1.35E-31
RFX4	9.13E-36	0.144053	0.209	0.317	1.36E-31
SCRIB	9.68E-36	-0.18422	0.051	0.2	1.45E-31
DDB2	9.98E-36	0.145239	0.106	0.183	1.49E-31
CTBS	1.01E-35	0.24003	0.134	0.144	1.50E-31
WWC3	1.03E-35	-0.14799	0.021	0.143	1.54E-31
TCEAL1	1.09E-35	0.35355	0.266	0.305	1.64E-31
FOXRED1	1.10E-35	-0.1545	0.086	0.246	1.64E-31
ATP13A2	1.11E-35	-0.18024	0.069	0.227	1.66E-31
ARHGEF40	1.17E-35	-0.14832	0.053	0.195	1.75E-31
RP11-192H	1.17E-35	-0.14902	0.072	0.223	1.75E-31
RAP2B	1.33E-35	-0.11583	0.086	0.246	1.99E-31
PBDC1	1.34E-35	0.166529	0.257	0.372	2.00E-31
MAP3K2	1.35E-35	-0.11989	0.114	0.274	2.02E-31
PKD2	1.37E-35	-0.13527	0.049	0.188	2.04E-31
FANCC	1.39E-35	-0.17008	0.014	0.127	2.07E-31
CD82	1.39E-35	-0.18317	0.21	0.42	2.08E-31
LIMK1	1.45E-35	-0.12884	0.062	0.209	2.16E-31
FADS3	1.47E-35	0.223411	0.197	0.216	2.20E-31
SFMBT1	1.51E-35	-0.18474	0.05	0.2	2.25E-31
AP006222.	1.55E-35	0.130603	0.159	0.283	2.32E-31
NLGN2	1.58E-35	-0.20042	0.052	0.207	2.36E-31
ALMS1	1.60E-35	-0.17047	0.021	0.149	2.39E-31
CUL2	1.65E-35	-0.14148	0.068	0.216	2.46E-31
REXO1	1.68E-35	-0.11792	0.033	0.154	2.51E-31

TMEM129	1.80E-35	-0.19875	0.119	0.3	2.70E-31
TFCP2	1.92E-35	-0.13746	0.076	0.226	2.87E-31
AMOTL1	1.97E-35	-0.15789	0.04	0.181	2.94E-31
ANXA6	2.02E-35	-0.11862	0.185	0.374	3.02E-31
BCCIP	2.02E-35	0.14258	0.169	0.283	3.03E-31
TP53BP2	2.24E-35	-0.15163	0.062	0.215	3.34E-31
TMOD2	2.27E-35	-0.11501	0.054	0.19	3.39E-31
SETX	2.35E-35	-0.14643	0.098	0.262	3.51E-31
DUSP10	2.35E-35	-0.16278	0.114	0.277	3.52E-31
FNIP2	2.40E-35	-0.14557	0.087	0.238	3.58E-31
IKBKB	2.42E-35	-0.11164	0.06	0.207	3.62E-31
MAP4K3	2.50E-35	-0.12489	0.04	0.165	3.74E-31
ACSF3	2.55E-35	-0.15003	0.057	0.205	3.80E-31
LINC00511	2.56E-35	-0.11087	0.131	0.285	3.82E-31
CHD3	2.57E-35	-0.24425	0.044	0.195	3.84E-31
SH3BP4	2.61E-35	-0.20499	0.047	0.199	3.90E-31
PNO1	2.62E-35	0.151493	0.251	0.351	3.91E-31
ZNF195	2.66E-35	-0.14448	0.105	0.274	3.97E-31
USP42	2.76E-35	-0.17131	0.089	0.251	4.13E-31
PTPRF	2.79E-35	0.141885	0.142	0.211	4.17E-31
QRSL1	2.83E-35	-0.13773	0.092	0.259	4.24E-31
HMGCL	2.87E-35	0.25442	0.213	0.284	4.29E-31
ASL	3.06E-35	0.346414	0.189	0.223	4.57E-31
ETV2	3.06E-35	-0.12506	0.016	0.117	4.58E-31
AAMDC	3.11E-35	0.199362	0.236	0.335	4.65E-31
TBC1D5	3.13E-35	-0.12036	0.094	0.247	4.68E-31
CAMSAP1	3.24E-35	-0.20377	0.032	0.17	4.84E-31
GFOD2	3.28E-35	-0.1117	0.102	0.254	4.91E-31
SORD	3.31E-35	-0.11274	0.053	0.198	4.95E-31
COASY	3.33E-35	0.122171	0.191	0.291	4.97E-31
TP53INP2	3.39E-35	-0.11107	0.041	0.165	5.06E-31
MMP14	3.43E-35	0.233372	0.18	0.228	5.13E-31
TPP2	3.58E-35	-0.11324	0.085	0.227	5.35E-31
DDX6	3.63E-35	0.104255	0.154	0.283	5.43E-31
GTF3C4	3.90E-35	-0.14319	0.042	0.174	5.83E-31
COMMD8	3.91E-35	0.138403	0.167	0.27	5.84E-31
IGFBP3	3.98E-35	0.29193	0.269	0.344	5.95E-31
ASIC4	4.02E-35	-0.37763	0.05	0.215	6.01E-31
SCAMP1	4.12E-35	-0.19507	0.172	0.372	6.15E-31
MID1IP1	4.19E-35	0.261136	0.244	0.306	6.26E-31
CSRP2BP	4.21E-35	-0.1844	0.04	0.183	6.29E-31
SLC35E2B	4.64E-35	-0.14348	0.089	0.249	6.93E-31
DYRK4	4.81E-35	0.187797	0.213	0.296	7.18E-31
NIPSNAP3A	4.87E-35	0.170064	0.158	0.235	7.28E-31
NAA16	4.97E-35	-0.16698	0.102	0.275	7.43E-31
OSBPL1A	5.05E-35	-0.18524	0.124	0.306	7.55E-31
C16orf52	5.15E-35	-0.15552	0.056	0.206	7.70E-31
MEIS3	5.39E-35	-0.13188	0.072	0.226	8.05E-31
HRSP12	5.41E-35	0.320462	0.243	0.29	8.09E-31
SLC1A2	5.60E-35	-0.10966	0.118	0.279	8.36E-31
MAPK11	5.63E-35	-0.17934	0.019	0.147	8.41E-31

CPVL	5.64E-35	0.364394	0.136	0.17	8.43E-31
NSF	5.98E-35	-0.11278	0.092	0.247	8.93E-31
TENM3	6.19E-35	-0.26954	0.007	0.114	9.25E-31
ZDHHC24	6.37E-35	-0.12851	0.102	0.263	9.52E-31
SSSCA1	6.44E-35	0.175245	0.17	0.273	9.62E-31
ARHGEF25	6.66E-35	-0.30436	0.031	0.174	9.96E-31
CELF5	7.35E-35	-0.29276	0.021	0.151	1.10E-30
ZYG11B	7.44E-35	-0.13684	0.095	0.253	1.11E-30
FAM69C	7.72E-35	-0.12017	0.014	0.121	1.15E-30
PGAM5	7.87E-35	-0.11123	0.036	0.163	1.18E-30
TIMM10B	8.12E-35	0.209102	0.17	0.268	1.21E-30
SLC2A8	8.60E-35	-0.11637	0.094	0.252	1.28E-30
CTB-3102C	9.03E-35	-0.21317	0.073	0.233	1.35E-30
ZBTB10	9.09E-35	-0.19851	0.057	0.214	1.36E-30
AKAP1	9.23E-35	-0.12845	0.097	0.249	1.38E-30
GTF2B	9.39E-35	0.281391	0.246	0.302	1.40E-30
SFI1	9.48E-35	-0.19447	0.027	0.16	1.42E-30
TET1	9.63E-35	-0.11171	0.034	0.153	1.44E-30
GTF3C1	9.89E-35	-0.12865	0.071	0.22	1.48E-30
LYRM1	9.96E-35	0.134733	0.203	0.338	1.49E-30
ENKD1	1.01E-34	-0.15447	0.051	0.194	1.51E-30
HMGCR	1.03E-34	-0.10846	0.073	0.212	1.53E-30
MAST2	1.03E-34	-0.17709	0.046	0.188	1.53E-30
EFCAB2	1.03E-34	-0.15884	0.096	0.259	1.55E-30
NBN	1.08E-34	-0.16626	0.108	0.28	1.61E-30
YTHDC2	1.12E-34	-0.11583	0.076	0.216	1.67E-30
NCOA5	1.13E-34	-0.16549	0.052	0.196	1.68E-30
LIN7B	1.14E-34	-0.12346	0.084	0.231	1.70E-30
ZC3H4	1.23E-34	-0.20535	0.046	0.193	1.83E-30
PPP3CB	1.23E-34	-0.11914	0.05	0.18	1.84E-30
MOB3A	1.28E-34	-0.10322	0.086	0.235	1.91E-30
ST5	1.33E-34	0.168389	0.177	0.26	1.98E-30
BRSK1	1.34E-34	-0.1443	0.041	0.178	2.01E-30
TADA2A	1.39E-34	-0.17785	0.046	0.189	2.08E-30
RP11-498C	1.41E-34	-0.13365	0.017	0.13	2.11E-30
PPIL2	1.49E-34	-0.11728	0.071	0.215	2.22E-30
ACOT13	1.49E-34	0.312515	0.228	0.288	2.23E-30
TIPARP	1.50E-34	0.396283	0.21	0.158	2.23E-30
LIPE	1.53E-34	-0.18758	0.029	0.162	2.29E-30
RP11-1114	1.61E-34	-0.16426	0.045	0.178	2.41E-30
MLLT3	1.63E-34	-0.14292	0.059	0.201	2.43E-30
CSRP1	1.64E-34	0.382526	0.237	0.284	2.45E-30
FAM208A	1.79E-34	-0.152	0.098	0.26	2.67E-30
GSTM4	1.88E-34	0.133118	0.145	0.24	2.81E-30
SALL3	1.95E-34	-0.2418	0.017	0.14	2.92E-30
TSFM	1.99E-34	-0.24946	0.275	0.509	2.97E-30
USO1	2.17E-34	0.120829	0.197	0.301	3.25E-30
DNAJC11	2.23E-34	-0.10558	0.062	0.204	3.33E-30
FAM101B	2.23E-34	-0.16144	0.016	0.135	3.33E-30
SLC50A1	2.23E-34	0.256815	0.127	0.18	3.34E-30
PTPN2	2.24E-34	0.185962	0.193	0.274	3.35E-30

KIAA1211L	2.30E-34	-0.10198	0.008	0.101	3.44E-30
CORO7	2.42E-34	-0.19722	0.043	0.189	3.62E-30
FOSB	2.43E-34	0.530662	0.345	0.28	3.64E-30
CSK	2.50E-34	-0.15853	0.032	0.16	3.74E-30
GMEB1	2.56E-34	-0.15385	0.069	0.211	3.83E-30
RRAS	2.64E-34	0.121482	0.098	0.156	3.95E-30
TFAP2B	2.75E-34	-0.25992	0.006	0.106	4.11E-30
PTMA	2.99E-34	0.225893	0.911	0.985	4.47E-30
SP1	3.01E-34	-0.11234	0.044	0.172	4.50E-30
DDX39B	3.26E-34	-0.17586	0.06	0.206	4.88E-30
KCNMA1	3.30E-34	-0.15866	0.035	0.162	4.93E-30
KIAA0232	3.37E-34	-0.13988	0.091	0.247	5.04E-30
PLXNA1	3.37E-34	-0.12322	0.042	0.175	5.04E-30
OTUD6B	3.50E-34	-0.14706	0.099	0.256	5.23E-30
CAB39	3.61E-34	-0.14921	0.094	0.252	5.40E-30
SUCLG2	3.67E-34	0.222545	0.219	0.284	5.48E-30
DYRK1A	3.90E-34	-0.16093	0.049	0.186	5.82E-30
KDELC1	3.90E-34	-0.15063	0.025	0.148	5.83E-30
AQP4	3.94E-34	0.597207	0.288	0.211	5.89E-30
ARL10	4.20E-34	-0.10942	0.054	0.185	6.28E-30
PHF10	4.33E-34	-0.12688	0.055	0.185	6.48E-30
HIP1R	4.65E-34	-0.23946	0.046	0.196	6.95E-30
ARHGEF1	4.79E-34	-0.13012	0.107	0.26	7.16E-30
DNAJC18	4.83E-34	-0.2074	0.085	0.249	7.22E-30
DZIP3	5.10E-34	-0.19306	0.111	0.285	7.62E-30
PCOLCE	5.29E-34	0.247541	0.097	0.169	7.91E-30
KIF3C	5.44E-34	-0.16667	0.042	0.18	8.13E-30
GPR137B	5.58E-34	0.167871	0.175	0.23	8.34E-30
DGCR14	5.74E-34	-0.13542	0.086	0.236	8.57E-30
FBXO28	5.87E-34	-0.11131	0.066	0.201	8.77E-30
CCPG1	5.94E-34	0.101905	0.193	0.299	8.88E-30
PI4KA	6.14E-34	-0.14983	0.045	0.183	9.18E-30
SRBD1	6.15E-34	-0.15609	0.05	0.185	9.19E-30
UBE2Z	6.57E-34	-0.10761	0.13	0.293	9.82E-30
FAM189B	6.60E-34	-0.14461	0.059	0.201	9.86E-30
FAM219B	6.91E-34	-0.11938	0.091	0.236	1.03E-29
SERGEF	7.38E-34	0.307815	0.201	0.22	1.10E-29
DHX57	7.64E-34	-0.12707	0.064	0.196	1.14E-29
LINC01116	7.66E-34	-0.14494	0.137	0.307	1.14E-29
ROM1	7.67E-34	0.594234	0.196	0.127	1.15E-29
TNPO3	7.81E-34	-0.20703	0.137	0.323	1.17E-29
PLCB1	8.08E-34	-0.20743	0.043	0.189	1.21E-29
HEY2	8.31E-34	-0.12529	0.041	0.163	1.24E-29
MAPK14	8.34E-34	-0.10819	0.067	0.195	1.25E-29
PCGF6	8.51E-34	-0.1443	0.059	0.201	1.27E-29
PKN3	8.90E-34	-0.15414	0.008	0.11	1.33E-29
KCNQ1OT1	9.05E-34	-0.25581	0.213	0.426	1.35E-29
THOC5	9.61E-34	-0.10473	0.042	0.17	1.44E-29
TRAK1	9.85E-34	-0.1414	0.035	0.163	1.47E-29
TSEN54	9.92E-34	-0.14549	0.076	0.225	1.48E-29
RND3	9.92E-34	-0.11775	0.214	0.395	1.48E-29

SDC4	1.01E-33	0.358049	0.184	0.2	1.51E-29
YPEL3	1.04E-33	-0.10757	0.135	0.293	1.55E-29
INSIG2	1.05E-33	0.365408	0.261	0.284	1.58E-29
VAX2	1.21E-33	-0.23058	0.039	0.188	1.81E-29
SLC39A9	1.22E-33	-0.12413	0.058	0.19	1.82E-29
ZFHX4-AS1	1.26E-33	-0.15471	0.011	0.121	1.89E-29
FMR1	1.32E-33	-0.11527	0.066	0.209	1.98E-29
MAOB	1.37E-33	0.686529	0.234	0.115	2.04E-29
ORMDL2	1.42E-33	0.362663	0.237	0.27	2.11E-29
MYO6	1.48E-33	-0.12574	0.2	0.369	2.21E-29
CNOT6L	1.55E-33	-0.10488	0.05	0.175	2.32E-29
ACAA2	1.62E-33	0.297474	0.197	0.278	2.42E-29
TMCC1	1.64E-33	-0.14486	0.047	0.186	2.45E-29
NDP	1.65E-33	0.173394	0.113	0.198	2.46E-29
CPQ	1.66E-33	0.517565	0.173	0.107	2.49E-29
RNF166	1.68E-33	-0.16591	0.082	0.241	2.50E-29
FER	1.74E-33	-0.19374	0.044	0.19	2.60E-29
TNFRSF21	1.74E-33	-0.1586	0.085	0.236	2.60E-29
PLEKHH3	1.88E-33	-0.13832	0.044	0.178	2.81E-29
KDM4B	1.93E-33	-0.16208	0.082	0.235	2.88E-29
MAPK3	2.05E-33	0.198065	0.17	0.253	3.06E-29
TECPR1	2.05E-33	-0.12494	0.067	0.211	3.06E-29
GEM	2.09E-33	0.565121	0.212	0.147	3.12E-29
MFSD6	2.16E-33	-0.14236	0.032	0.158	3.23E-29
ACACA	2.20E-33	-0.13693	0.052	0.185	3.28E-29
TIMP3	2.38E-33	0.365979	0.203	0.164	3.56E-29
ZNF226	2.39E-33	0.387204	0.246	0.258	3.57E-29
NOA1	2.41E-33	-0.263	0.098	0.28	3.60E-29
LIG3	2.60E-33	-0.15127	0.031	0.159	3.89E-29
ALG2	2.61E-33	0.165968	0.179	0.23	3.90E-29
FAM72A	2.69E-33	-0.16819	0.008	0.11	4.03E-29
LRFN4	2.69E-33	-0.16223	0.026	0.152	4.03E-29
BRCC3	2.71E-33	-0.11976	0.081	0.225	4.05E-29
MED14	2.78E-33	-0.13838	0.036	0.158	4.16E-29
RASSF4	2.79E-33	0.391093	0.224	0.219	4.17E-29
ZC3H6	2.89E-33	-0.11825	0.119	0.273	4.32E-29
CLIP1	2.91E-33	0.145261	0.196	0.302	4.34E-29
UBQLN4	3.03E-33	-0.22488	0.074	0.235	4.52E-29
CEP76	3.03E-33	-0.10989	0.051	0.167	4.53E-29
PTPN4	3.06E-33	-0.18621	0.057	0.202	4.58E-29
ZNF608	3.07E-33	-0.10017	0.127	0.283	4.58E-29
HLA-E	3.08E-33	0.253303	0.434	0.462	4.60E-29
TAF6L	3.09E-33	-0.14683	0.048	0.191	4.62E-29
HDAC8	3.14E-33	-0.1466	0.06	0.201	4.69E-29
MIER1	3.17E-33	-0.13522	0.118	0.279	4.74E-29
MARK4	3.59E-33	-0.15594	0.032	0.16	5.37E-29
MDFIC	3.65E-33	-0.11798	0.017	0.123	5.46E-29
CSTF2T	3.78E-33	-0.15048	0.076	0.235	5.64E-29
ARL17A	3.89E-33	-0.1485	0.032	0.158	5.82E-29
FAM171A1	4.03E-33	-0.1752	0.025	0.153	6.03E-29
FAM57B	4.18E-33	-0.26181	0.027	0.157	6.25E-29

ZNF521	4.41E-33	-0.11774	0.051	0.183	6.58E-29
EED	4.60E-33	-0.14022	0.088	0.244	6.87E-29
ARHGAP23	4.60E-33	-0.11059	0.018	0.123	6.87E-29
CDKAL1	4.64E-33	-0.20423	0.061	0.216	6.94E-29
TSC22D3	4.79E-33	0.275311	0.297	0.374	7.16E-29
TCF7L2	4.89E-33	-0.11065	0.062	0.193	7.31E-29
PCYOX1L	5.04E-33	-0.16294	0.024	0.146	7.54E-29
RYBP	5.16E-33	-0.14873	0.06	0.201	7.71E-29
TAOK2	5.17E-33	-0.13265	0.064	0.2	7.73E-29
TMTC3	5.20E-33	-0.13228	0.069	0.215	7.76E-29
FEM1B	5.24E-33	-0.1286	0.073	0.21	7.83E-29
NUFIP1	5.46E-33	-0.14054	0.102	0.258	8.16E-29
KIF1C	5.67E-33	-0.14791	0.057	0.194	8.48E-29
ADD3	5.68E-33	0.192692	0.173	0.259	8.49E-29
TOX	5.89E-33	-0.11263	0.027	0.138	8.80E-29
CLIC1	6.13E-33	0.356401	0.578	0.631	9.16E-29
KDM4A	6.61E-33	-0.11826	0.07	0.209	9.87E-29
C1orf56	6.77E-33	-0.10764	0.126	0.286	1.01E-28
NSUN6	7.46E-33	-0.17449	0.044	0.181	1.12E-28
ALG13	7.77E-33	0.186731	0.193	0.262	1.16E-28
TUBGCP4	7.80E-33	-0.14865	0.092	0.248	1.17E-28
CDK5R1	7.92E-33	-0.16548	0.032	0.153	1.18E-28
FAM122A	8.27E-33	-0.18569	0.091	0.252	1.24E-28
ERICH1	8.59E-33	0.103092	0.174	0.289	1.28E-28
LIPE-AS1	8.74E-33	-0.12562	0.057	0.193	1.31E-28
SBK1	9.60E-33	-0.20003	0.021	0.14	1.43E-28
RNASET2	9.73E-33	0.183399	0.236	0.284	1.45E-28
PRKX	9.78E-33	-0.20747	0.036	0.177	1.46E-28
POC5	1.05E-32	-0.14014	0.047	0.174	1.57E-28
SOX6	1.05E-32	-0.23191	0.079	0.247	1.57E-28
PI3	1.06E-32	1.043069	0.156	0.046	1.59E-28
CFI	1.07E-32	0.486553	0.173	0.073	1.60E-28
BAMBI	1.09E-32	-0.22793	0.046	0.196	1.62E-28
SMARCAD1	1.11E-32	-0.19635	0.05	0.194	1.66E-28
SERTAD3	1.17E-32	0.366687	0.176	0.217	1.76E-28
VAV3	1.21E-32	-0.18255	0.026	0.146	1.81E-28
MMP15	1.25E-32	-0.1306	0.015	0.119	1.86E-28
ADIPOR2	1.33E-32	-0.16119	0.099	0.258	1.99E-28
HP	1.34E-32	0.994729	0.11	0.001	2.01E-28
YIPF1	1.37E-32	0.183733	0.148	0.232	2.05E-28
PSMA6	1.37E-32	0.283881	0.245	0.299	2.05E-28
NOL4	1.38E-32	-0.26751	0.036	0.177	2.06E-28
NPRL2	1.38E-32	0.234269	0.189	0.237	2.07E-28
OS9	1.50E-32	-0.23738	0.425	0.653	2.25E-28
VANGL2	1.51E-32	-0.1195	0.045	0.165	2.25E-28
SCG2	1.54E-32	-0.2529	0.22	0.443	2.30E-28
NKD1	1.57E-32	-0.20382	0.005	0.1	2.34E-28
EIF4EBP2	1.59E-32	-0.15202	0.076	0.22	2.38E-28
NLK	1.60E-32	-0.17779	0.028	0.152	2.39E-28
TNK2	1.61E-32	-0.20616	0.065	0.22	2.41E-28
TAB2	1.71E-32	-0.10443	0.065	0.193	2.55E-28

CTSC	1.78E-32	0.533476	0.219	0.202	2.65E-28
CPOX	1.82E-32	-0.1179	0.033	0.151	2.71E-28
PELI1	1.82E-32	-0.25732	0.077	0.243	2.72E-28
KIAA1328	2.04E-32	-0.12139	0.048	0.172	3.05E-28
STMN2	2.05E-32	-0.56046	0.059	0.221	3.07E-28
AGAP2-AS1	2.06E-32	-0.48888	0.08	0.258	3.08E-28
ATCAY	2.10E-32	-0.30012	0.061	0.221	3.14E-28
SLC25A13	2.10E-32	-0.1308	0.083	0.235	3.15E-28
MIAT	2.12E-32	-0.17346	0.069	0.209	3.16E-28
ZMIZ1	2.13E-32	-0.22153	0.059	0.211	3.19E-28
NLE1	2.18E-32	-0.12147	0.04	0.16	3.25E-28
LPGAT1	2.22E-32	-0.14091	0.078	0.228	3.32E-28
PHYH	2.34E-32	0.131638	0.16	0.246	3.50E-28
RP11-448A	2.37E-32	-0.10839	0.059	0.183	3.54E-28
ATL1	2.54E-32	-0.10453	0.097	0.246	3.80E-28
GEMIN4	2.74E-32	-0.11687	0.04	0.159	4.09E-28
ARL5B	2.79E-32	-0.13889	0.039	0.165	4.16E-28
PABPC1L	2.83E-32	-0.10712	0.045	0.167	4.23E-28
N4BP1	2.90E-32	-0.14718	0.048	0.181	4.33E-28
PRKCI	2.99E-32	-0.1125	0.083	0.217	4.46E-28
TRIB3	3.03E-32	0.413521	0.148	0.093	4.53E-28
CBFA2T2	3.13E-32	-0.10999	0.087	0.223	4.68E-28
AZGP1	3.29E-32	0.603071	0.187	0.038	4.91E-28
ZBTB38	3.31E-32	0.307095	0.188	0.219	4.95E-28
ZDHHC16	3.38E-32	-0.10123	0.067	0.2	5.06E-28
SMIM13	3.40E-32	-0.14954	0.053	0.189	5.09E-28
ST3GAL3	3.66E-32	-0.17952	0.066	0.215	5.48E-28
SSH1	3.80E-32	-0.13288	0.03	0.146	5.68E-28
C1orf159	3.85E-32	-0.16827	0.031	0.16	5.75E-28
RDH10	3.94E-32	0.374119	0.187	0.149	5.89E-28
SLC25A44	4.31E-32	-0.12508	0.067	0.209	6.44E-28
TOX4	4.34E-32	0.130247	0.26	0.36	6.48E-28
LPL	4.35E-32	0.224584	0.168	0.251	6.50E-28
FIGNL1	4.43E-32	-0.25716	0.031	0.167	6.61E-28
PDLIM4	4.50E-32	0.573981	0.203	0.078	6.72E-28
ISG15	4.59E-32	0.109033	0.264	0.374	6.86E-28
MARK1	4.60E-32	-0.15872	0.033	0.16	6.87E-28
LRRC41	4.92E-32	0.243843	0.238	0.302	7.36E-28
LYPLAL1	5.19E-32	0.276522	0.245	0.288	7.76E-28
EFNA1	5.26E-32	0.602041	0.22	0.109	7.87E-28
RPS6KA4	5.88E-32	-0.1294	0.048	0.177	8.79E-28
KLRC2	6.30E-32	-0.39549	0.047	0.198	9.41E-28
GNPDA1	6.89E-32	0.254655	0.209	0.278	1.03E-27
MAGI3	7.24E-32	-0.15574	0.023	0.143	1.08E-27
TLCD1	7.64E-32	-0.16343	0.029	0.156	1.14E-27
FAM84B	7.69E-32	-0.22934	0.046	0.19	1.15E-27
LIN54	7.98E-32	-0.14415	0.031	0.146	1.19E-27
MTA2	8.17E-32	-0.18039	0.032	0.162	1.22E-27
TOB2	8.27E-32	0.129157	0.166	0.237	1.24E-27
ATG4C	8.48E-32	-0.15716	0.102	0.258	1.27E-27
NEDD4L	8.61E-32	-0.11964	0.06	0.193	1.29E-27

VAT1	8.67E-32	0.243854	0.179	0.205	1.30E-27
UGDH	8.70E-32	0.250742	0.227	0.288	1.30E-27
RFTN2	8.72E-32	-0.30635	0.077	0.251	1.30E-27
PNMAL1	8.86E-32	-0.22611	0.056	0.205	1.32E-27
CWF19L2	9.66E-32	0.121439	0.176	0.263	1.44E-27
SGK1	1.02E-31	0.203766	0.225	0.277	1.53E-27
ARV1	1.14E-31	0.177757	0.217	0.302	1.70E-27
CA9	1.19E-31	0.507112	0.175	0.027	1.78E-27
PPP2R2B	1.21E-31	0.222819	0.234	0.326	1.80E-27
PCNX	1.21E-31	-0.13507	0.045	0.17	1.81E-27
RGS16	1.24E-31	0.321401	0.262	0.328	1.86E-27
IGFBP6	1.27E-31	0.585854	0.176	0.07	1.89E-27
POLR1A	1.28E-31	-0.11864	0.024	0.133	1.92E-27
TMEM79	1.29E-31	-0.10419	0.054	0.18	1.93E-27
C17orf10C	1.32E-31	-0.1021	0.052	0.178	1.97E-27
MAML1	1.33E-31	-0.19616	0.039	0.168	1.98E-27
FOLR1	1.35E-31	0.642243	0.128	0.012	2.01E-27
SAP130	1.37E-31	-0.12246	0.031	0.147	2.05E-27
HCFC1R1	1.39E-31	0.117435	0.218	0.337	2.08E-27
NOM1	1.45E-31	-0.16953	0.109	0.273	2.17E-27
GPC1	1.74E-31	-0.2265	0.262	0.488	2.61E-27
LARP4B	1.76E-31	-0.11835	0.041	0.167	2.62E-27
PARD6G	1.95E-31	-0.11611	0.01	0.105	2.92E-27
FOXD1	2.00E-31	-0.14982	0.01	0.112	2.98E-27
PLA2G2A	2.08E-31	1.191985	0.136	0.028	3.10E-27
XPNPEP3	2.10E-31	-0.12251	0.071	0.207	3.13E-27
3-Sep	2.19E-31	-0.17457	0.022	0.138	3.27E-27
HEPACAM	2.34E-31	0.19297	0.201	0.295	3.50E-27
NT5M	2.40E-31	-0.1634	0.021	0.136	3.58E-27
SARS2	2.43E-31	-0.12713	0.034	0.148	3.63E-27
ZNF300	2.48E-31	-0.22251	0.05	0.194	3.71E-27
AGPS	2.49E-31	-0.16157	0.065	0.212	3.73E-27
C17orf51	2.57E-31	-0.11325	0.042	0.167	3.84E-27
SMIM18	2.62E-31	-0.27371	0.011	0.116	3.91E-27
B9D1	2.67E-31	0.187373	0.118	0.21	3.99E-27
ZNF337	2.81E-31	-0.12011	0.042	0.165	4.20E-27
KIAA0141	2.87E-31	-0.11868	0.06	0.189	4.29E-27
SPECC1L	2.98E-31	-0.12262	0.025	0.135	4.46E-27
NBPF10	3.07E-31	-0.1604	0.108	0.264	4.58E-27
VSIG10	3.10E-31	-0.10096	0.053	0.178	4.63E-27
FGFR1OP	3.26E-31	-0.10989	0.079	0.227	4.88E-27
DIP2C	3.28E-31	-0.12228	0.042	0.163	4.90E-27
SCAPER	3.71E-31	-0.19127	0.076	0.225	5.55E-27
DCUN1D4	3.73E-31	-0.11713	0.093	0.233	5.58E-27
EFNA2	3.78E-31	-0.18274	0.019	0.135	5.65E-27
AADAT	3.82E-31	-0.14029	0.025	0.137	5.71E-27
OSMR	4.06E-31	0.272644	0.151	0.147	6.07E-27
LIN52	4.09E-31	-0.15369	0.045	0.175	6.11E-27
CAPN10	4.36E-31	-0.13858	0.048	0.177	6.52E-27
ANKRD36C	4.47E-31	-0.25576	0.136	0.32	6.67E-27
LRP3	4.48E-31	-0.13511	0.027	0.138	6.70E-27

CACNG4	4.60E-31	-0.17918	0.025	0.143	6.88E-27
PLEKHH2	4.61E-31	-0.15431	0.048	0.181	6.89E-27
CHKA	4.80E-31	-0.20934	0.112	0.28	7.17E-27
ZMYM1	5.19E-31	-0.13772	0.033	0.157	7.76E-27
AHCTF1	6.03E-31	-0.11604	0.072	0.204	9.01E-27
GALNT7	6.10E-31	-0.10221	0.048	0.16	9.12E-27
ANXA4	6.22E-31	0.532056	0.218	0.136	9.29E-27
SPG11	6.30E-31	-0.11122	0.055	0.189	9.41E-27
C1orf50	6.40E-31	0.121933	0.139	0.237	9.57E-27
HRH1	6.53E-31	0.394844	0.164	0.153	9.76E-27
REL	6.92E-31	-0.11925	0.111	0.264	1.03E-26
ZNF865	7.16E-31	-0.13133	0.036	0.156	1.07E-26
APPL2	7.78E-31	0.123821	0.155	0.26	1.16E-26
PRRT2	8.24E-31	-0.12113	0.082	0.219	1.23E-26
TMTC4	8.42E-31	-0.18311	0.044	0.177	1.26E-26
IGFBP4	8.58E-31	-0.13693	0.077	0.219	1.28E-26
SDSL	9.07E-31	0.211074	0.131	0.206	1.36E-26
PITX2	9.09E-31	-0.23841	0.013	0.116	1.36E-26
YTHDF3-AS	9.43E-31	-0.11553	0.031	0.141	1.41E-26
METTL8	9.43E-31	-0.14326	0.084	0.23	1.41E-26
NUDT16	9.47E-31	0.145723	0.13	0.21	1.41E-26
AKTIP	9.79E-31	0.242406	0.204	0.263	1.46E-26
WDR90	1.02E-30	-0.12864	0.026	0.136	1.52E-26
MAT2B	1.04E-30	0.106201	0.182	0.288	1.55E-26
MDP1	1.10E-30	0.176118	0.112	0.188	1.64E-26
ZNF628	1.11E-30	-0.14318	0.018	0.128	1.66E-26
FKBPL	1.11E-30	-0.11422	0.063	0.199	1.66E-26
FBXO42	1.12E-30	-0.10979	0.064	0.185	1.67E-26
KCNJ16	1.16E-30	-0.18582	0.022	0.137	1.74E-26
PPP3R1	1.24E-30	-0.19285	0.071	0.222	1.85E-26
FLVCR1	1.27E-30	-0.15965	0.051	0.18	1.90E-26
DCBLD1	1.36E-30	-0.15687	0.064	0.202	2.03E-26
SLC16A2	1.37E-30	-0.12845	0.024	0.133	2.05E-26
PHRF1	1.39E-30	-0.14673	0.043	0.168	2.07E-26
ELP2	1.51E-30	0.170455	0.216	0.281	2.25E-26
ZC2HC1A	1.57E-30	-0.17922	0.156	0.335	2.34E-26
SCFD2	1.75E-30	-0.26382	0.068	0.227	2.62E-26
OTUD7B	1.82E-30	0.149041	0.12	0.168	2.72E-26
ATXN8OS	1.85E-30	0.87281	0.142	0.015	2.77E-26
NHSL1	1.93E-30	-0.15358	0.063	0.2	2.89E-26
GPR161	1.94E-30	-0.18143	0.039	0.172	2.90E-26
PRMT6	1.94E-30	-0.14315	0.064	0.196	2.90E-26
TRIM26	1.99E-30	-0.13634	0.069	0.211	2.98E-26
SOWAHC	1.99E-30	-0.14467	0.042	0.168	2.98E-26
ZNF281	2.05E-30	-0.21733	0.12	0.294	3.06E-26
IPO8	2.11E-30	-0.11422	0.041	0.17	3.15E-26
DNAJC4	2.35E-30	0.23354	0.177	0.227	3.52E-26
MAPK12	2.44E-30	-0.13616	0.033	0.158	3.64E-26
SCYL2	2.46E-30	-0.12488	0.082	0.22	3.67E-26
RAB23	2.49E-30	-0.1377	0.061	0.194	3.72E-26
WDR73	2.52E-30	0.107877	0.132	0.228	3.77E-26

THRA	2.69E-30	-0.11639	0.21	0.386	4.02E-26
ZFAND1	2.80E-30	0.11535	0.152	0.235	4.19E-26
PFAS	2.93E-30	-0.11259	0.017	0.115	4.38E-26
LRRC8D	2.99E-30	-0.14761	0.035	0.154	4.47E-26
HLA-DMA	3.11E-30	0.716053	0.246	0.107	4.65E-26
BCL3	3.15E-30	-0.10919	0.037	0.148	4.71E-26
C17orf96	3.30E-30	-0.15152	0.031	0.149	4.93E-26
ZNF330	3.49E-30	0.258457	0.212	0.256	5.21E-26
DIRAS2	3.87E-30	-0.26064	0.039	0.173	5.79E-26
RNF169	3.90E-30	-0.1028	0.051	0.169	5.83E-26
PROSER1	4.00E-30	-0.12212	0.041	0.16	5.97E-26
DTX3	4.15E-30	-0.40697	0.21	0.432	6.20E-26
NMD3	4.28E-30	0.141312	0.242	0.325	6.39E-26
PTRH2	4.40E-30	0.214351	0.156	0.204	6.57E-26
BCL9	4.60E-30	-0.10388	0.019	0.119	6.88E-26
SHC1	4.64E-30	0.176266	0.13	0.202	6.93E-26
DYNC2LI1	4.83E-30	0.359372	0.228	0.242	7.22E-26
SNRPN	4.93E-30	0.781423	0.158	0.078	7.37E-26
CSRNP2	4.96E-30	-0.16934	0.057	0.195	7.42E-26
ADAM22	5.05E-30	-0.2739	0.03	0.159	7.55E-26
RBM12B	5.37E-30	-0.11404	0.051	0.172	8.02E-26
PRMT7	5.86E-30	-0.17209	0.073	0.215	8.75E-26
HSD17B4	5.95E-30	0.112459	0.196	0.288	8.90E-26
DHRS1	6.27E-30	0.250272	0.134	0.191	9.37E-26
INTS1	6.31E-30	-0.13358	0.054	0.188	9.44E-26
EMC2	6.55E-30	0.234377	0.231	0.311	9.79E-26
RNF214	6.62E-30	-0.16964	0.069	0.214	9.89E-26
SLC27A5	6.63E-30	0.209428	0.143	0.212	9.91E-26
INTS7	6.68E-30	-0.15817	0.046	0.179	9.98E-26
TMEM43	6.71E-30	0.196636	0.22	0.263	1.00E-25
RLIM	7.08E-30	-0.13255	0.073	0.206	1.06E-25
ADORA1	7.14E-30	0.196845	0.132	0.175	1.07E-25
XIST	7.49E-30	0.514811	0.357	0.146	1.12E-25
ORC3	7.55E-30	-0.14676	0.097	0.247	1.13E-25
ZNF253	7.88E-30	-0.17742	0.049	0.181	1.18E-25
DUSP23	8.56E-30	0.606116	0.201	0.049	1.28E-25
CRBN	8.56E-30	0.117221	0.267	0.363	1.28E-25
SP100	8.68E-30	0.627134	0.253	0.163	1.30E-25
ZW10	9.08E-30	-0.15306	0.054	0.185	1.36E-25
FMNL3	9.16E-30	-0.1371	0.017	0.12	1.37E-25
EMP1	9.16E-30	0.415652	0.324	0.283	1.37E-25
DCLK1	9.39E-30	0.157051	0.146	0.216	1.40E-25
FANCM	9.43E-30	-0.14247	0.016	0.116	1.41E-25
FRS2	1.01E-29	-0.3167	0.076	0.241	1.52E-25
CDR2L	1.07E-29	-0.1159	0.029	0.141	1.60E-25
PFKM	1.13E-29	0.151265	0.183	0.263	1.69E-25
AGA	1.14E-29	0.350037	0.196	0.214	1.71E-25
SND1	1.19E-29	0.165192	0.241	0.337	1.78E-25
MFSD2A	1.23E-29	0.327061	0.177	0.172	1.84E-25
MFSD1	1.26E-29	0.211719	0.173	0.225	1.88E-25
FBN2	1.28E-29	-0.11125	0.017	0.114	1.91E-25

PXMP4	1.33E-29	-0.18745	0.042	0.173	1.99E-25
GPX3	1.34E-29	0.377086	0.125	0.121	2.01E-25
CACFD1	1.36E-29	-0.12073	0.046	0.164	2.03E-25
PEX13	1.36E-29	0.122637	0.173	0.284	2.03E-25
PLIN3	1.40E-29	0.138491	0.333	0.453	2.10E-25
ABCA1	1.56E-29	0.281428	0.21	0.184	2.32E-25
ST6GAL2	1.57E-29	-0.21937	0.005	0.093	2.34E-25
NFKBIZ	1.66E-29	0.424752	0.204	0.149	2.48E-25
PLEKHG2	1.70E-29	-0.13385	0.019	0.117	2.54E-25
VASH1	1.79E-29	-0.13802	0.025	0.136	2.68E-25
CH25H	1.80E-29	0.738295	0.153	0.023	2.70E-25
PLAU	1.81E-29	-0.10631	0.082	0.206	2.71E-25
L3MBTL2	1.95E-29	-0.16959	0.06	0.2	2.91E-25
HOXD-AS2	1.99E-29	-0.17359	0.017	0.126	2.98E-25
MBOAT1	2.05E-29	-0.11965	0.007	0.091	3.06E-25
C1QC	2.12E-29	0.666091	0.313	0.205	3.17E-25
ZNF286A	2.30E-29	-0.14174	0.022	0.126	3.44E-25
NOL9	2.35E-29	-0.12191	0.071	0.2	3.51E-25
SRPX2	2.46E-29	0.466132	0.135	0.098	3.68E-25
XPO7	2.60E-29	-0.1655	0.071	0.215	3.89E-25
ZBTB2	2.64E-29	-0.14253	0.056	0.184	3.95E-25
ATXN1L	2.85E-29	-0.11127	0.051	0.169	4.26E-25
ALKBH3	2.91E-29	0.209486	0.15	0.212	4.34E-25
GPSM1	3.36E-29	-0.16031	0.033	0.153	5.02E-25
VAMP7	3.51E-29	0.138303	0.159	0.241	5.24E-25
RP11-119E	3.53E-29	-0.14135	0.032	0.151	5.27E-25
PLAUR	3.59E-29	0.528452	0.251	0.162	5.36E-25
FRMD4A	3.62E-29	-0.17989	0.031	0.153	5.41E-25
ASXL2	3.76E-29	-0.10446	0.059	0.178	5.61E-25
ADNP2	3.78E-29	-0.1284	0.06	0.183	5.64E-25
DLAT	4.10E-29	-0.14229	0.107	0.253	6.13E-25
WIPI1	4.26E-29	0.258621	0.132	0.126	6.37E-25
HOXB2	4.44E-29	-0.35918	0.056	0.207	6.64E-25
RP11-400F	4.56E-29	-0.10625	0.042	0.154	6.81E-25
PMP22	4.59E-29	0.321704	0.509	0.557	6.86E-25
OAT	4.60E-29	0.376637	0.273	0.26	6.87E-25
FGF14	4.87E-29	-0.11007	0.045	0.156	7.28E-25
MIR181A1F	4.92E-29	-0.20298	0.039	0.169	7.35E-25
ANKRA2	4.93E-29	0.15256	0.164	0.243	7.37E-25
KIAA0196	5.11E-29	-0.11302	0.066	0.19	7.64E-25
FAIM	5.31E-29	0.138805	0.155	0.252	7.93E-25
ZNF512B	5.38E-29	-0.10348	0.035	0.142	8.04E-25
IQCG	5.80E-29	0.368914	0.188	0.185	8.67E-25
PAPD4	6.59E-29	0.171747	0.157	0.217	9.85E-25
CHCHD7	6.75E-29	0.308419	0.22	0.254	1.01E-24
ABHD17B	6.79E-29	-0.18102	0.08	0.226	1.02E-24
ZNF598	6.86E-29	-0.13228	0.051	0.173	1.03E-24
TSC22D2	6.87E-29	-0.10187	0.092	0.22	1.03E-24
RNF6	7.13E-29	0.139352	0.139	0.207	1.06E-24
NUP43	7.58E-29	-0.17174	0.069	0.209	1.13E-24
5-Mar	7.64E-29	-0.10483	0.152	0.298	1.14E-24

ATF1	7.91E-29	0.151867	0.158	0.225	1.18E-24
CP	8.02E-29	0.50286	0.132	0.019	1.20E-24
DHX38	8.18E-29	-0.10044	0.084	0.209	1.22E-24
ICAM1	8.39E-29	0.494633	0.159	0.058	1.25E-24
GPD2	8.44E-29	-0.16131	0.058	0.195	1.26E-24
SPPL2A	9.32E-29	-0.16211	0.096	0.248	1.39E-24
TBC1D14	9.46E-29	-0.11665	0.093	0.232	1.41E-24
TBX2	9.70E-29	-0.14564	0.017	0.122	1.45E-24
CLCF1	9.78E-29	0.469372	0.136	0.037	1.46E-24
CHML	1.02E-28	-0.1363	0.015	0.115	1.52E-24
STARD9	1.10E-28	-0.14774	0.013	0.111	1.65E-24
SERPINE1	1.11E-28	0.648249	0.212	0.096	1.65E-24
GRIK2	1.11E-28	-0.15779	0.011	0.104	1.66E-24
ERCC6L	1.20E-28	-0.13911	0.003	0.077	1.79E-24
CYFIP2	1.20E-28	-0.18972	0.068	0.21	1.79E-24
FBXO43	1.21E-28	-0.13916	0.003	0.081	1.80E-24
TRAPPC6A	1.21E-28	0.40954	0.246	0.249	1.81E-24
LRP6	1.32E-28	-0.20111	0.068	0.214	1.98E-24
ASNS	1.34E-28	0.121738	0.226	0.299	2.00E-24
CBLN4	1.38E-28	-0.10726	0.031	0.123	2.07E-24
DUSP3	1.46E-28	0.209205	0.143	0.195	2.18E-24
EAF1	1.47E-28	0.262394	0.125	0.179	2.20E-24
ZNF577	1.47E-28	-0.19141	0.069	0.211	2.20E-24
FAM131B	1.56E-28	-0.11077	0.04	0.149	2.33E-24
RP11-182L	1.66E-28	-0.13203	0.036	0.146	2.48E-24
HUNK	1.69E-28	-0.11743	0.016	0.11	2.52E-24
RGP1	1.70E-28	-0.16214	0.051	0.183	2.55E-24
LMAN2L	1.81E-28	-0.13185	0.118	0.263	2.70E-24
TTC9B	1.83E-28	-0.3834	0.029	0.153	2.74E-24
CLUAP1	1.91E-28	0.189776	0.197	0.285	2.85E-24
CYTH1	1.92E-28	-0.14722	0.042	0.163	2.87E-24
DPAGT1	1.92E-28	0.144244	0.15	0.228	2.87E-24
EEFSEC	1.93E-28	-0.10425	0.072	0.196	2.89E-24
BHLHE40	1.94E-28	0.358909	0.162	0.179	2.90E-24
GOLGA3	2.05E-28	0.156104	0.114	0.152	3.07E-24
LMO2	2.12E-28	-0.11693	0.089	0.228	3.17E-24
PLA2G5	2.15E-28	0.65224	0.204	0.13	3.21E-24
SLC19A1	2.23E-28	-0.1121	0.042	0.159	3.33E-24
ABCA3	2.32E-28	-0.14743	0.056	0.189	3.47E-24
STRN	2.33E-28	-0.10152	0.025	0.127	3.48E-24
DNM3	2.40E-28	-0.17093	0.041	0.165	3.59E-24
GRID2	2.52E-28	-0.24438	0.016	0.122	3.77E-24
METAP1	2.53E-28	-0.10983	0.07	0.205	3.79E-24
DUSP15	2.58E-28	-0.13195	0.042	0.16	3.85E-24
CHD1L	2.65E-28	0.131201	0.156	0.242	3.97E-24
PACS2	2.66E-28	-0.1695	0.063	0.196	3.98E-24
DIRC2	2.67E-28	-0.12007	0.032	0.142	3.99E-24
HSDL1	2.82E-28	-0.1299	0.094	0.238	4.21E-24
NARS2	2.83E-28	-0.12465	0.092	0.227	4.22E-24
CHST8	2.96E-28	-0.14036	0.031	0.146	4.42E-24
GUF1	2.98E-28	-0.10686	0.062	0.18	4.45E-24

KCTD15	3.09E-28	-0.15436	0.03	0.143	4.62E-24
LCA5	3.27E-28	-0.11149	0.032	0.14	4.88E-24
ZNF689	3.32E-28	-0.13641	0.038	0.151	4.96E-24
IL17RB	3.51E-28	-0.12908	0.073	0.201	5.24E-24
BCOR	3.52E-28	-0.17687	0.024	0.136	5.26E-24
PNMA2	3.53E-28	-0.10351	0.156	0.314	5.28E-24
SCLY	3.56E-28	-0.1288	0.018	0.12	5.32E-24
PARD6A	4.10E-28	-0.16402	0.06	0.194	6.12E-24
CPSF1	4.74E-28	-0.17619	0.057	0.188	7.08E-24
TMSB4Y	4.97E-28	-0.12098	0.004	0.083	7.42E-24
MFN2	4.98E-28	-0.11082	0.078	0.204	7.45E-24
PDZD4	5.24E-28	-0.20096	0.019	0.128	7.83E-24
ECE1	5.37E-28	0.12106	0.115	0.18	8.03E-24
POU4F1	5.55E-28	-0.19666	0.004	0.085	8.30E-24
GKAP1	5.67E-28	-0.19501	0.069	0.211	8.48E-24
TMEM169	5.70E-28	-0.15212	0.016	0.116	8.52E-24
RIT1	6.21E-28	0.153362	0.17	0.242	9.27E-24
ZNF611	6.21E-28	-0.15039	0.037	0.151	9.28E-24
BRE	6.24E-28	0.158406	0.214	0.296	9.32E-24
BAG4	6.28E-28	-0.11831	0.075	0.205	9.38E-24
OSTF1	6.44E-28	0.126387	0.104	0.2	9.62E-24
IGSF11	6.58E-28	-0.15606	0.043	0.165	9.83E-24
ZNF620	6.69E-28	-0.14446	0.043	0.165	1.00E-23
RNF113A	6.78E-28	0.125724	0.123	0.2	1.01E-23
RP1-79C4.	6.95E-28	-0.13249	0.03	0.137	1.04E-23
STXBP1	7.41E-28	-0.13733	0.084	0.22	1.11E-23
TOPORS	7.44E-28	-0.17042	0.152	0.312	1.11E-23
AGAP3	7.62E-28	-0.2093	0.055	0.194	1.14E-23
POLA1	7.78E-28	-0.15053	0.015	0.114	1.16E-23
TOE1	9.29E-28	-0.17084	0.068	0.204	1.39E-23
SLC16A3	9.62E-28	0.310734	0.162	0.13	1.44E-23
ALG6	9.80E-28	-0.1117	0.091	0.227	1.46E-23
KLHL9	1.03E-27	-0.13373	0.072	0.204	1.54E-23
MICAL1	1.04E-27	-0.16203	0.037	0.152	1.55E-23
KAT2B	1.10E-27	-0.13012	0.034	0.142	1.64E-23
C2	1.16E-27	0.554551	0.141	0.026	1.74E-23
CRISPLD1	1.23E-27	-0.1819	0.109	0.258	1.83E-23
CPNE5	1.25E-27	-0.11673	0.01	0.095	1.87E-23
CTA-384D8	1.30E-27	-0.16573	0.018	0.121	1.94E-23
SLC12A9	1.32E-27	-0.16704	0.1	0.248	1.97E-23
MPP6	1.33E-27	-0.1387	0.074	0.209	1.99E-23
KCTD1	1.46E-27	-0.16577	0.054	0.179	2.18E-23
EYA4	1.52E-27	-0.10088	0.023	0.122	2.27E-23
ARNTL2	1.61E-27	-0.12874	0.019	0.122	2.40E-23
CAPN1	1.61E-27	0.147475	0.191	0.275	2.40E-23
FOSL2	1.62E-27	0.126644	0.153	0.204	2.42E-23
DLX5	1.64E-27	-0.43537	0.046	0.185	2.45E-23
LRRC8B	1.64E-27	-0.15203	0.053	0.183	2.46E-23
PLCXD1	1.64E-27	-0.17923	0.032	0.147	2.46E-23
AGRN	1.69E-27	-0.15385	0.046	0.167	2.53E-23
SIX1	1.78E-27	-0.18552	0.037	0.158	2.67E-23

RNF44	1.86E-27	-0.13577	0.064	0.194	2.78E-23
FBXO8	1.89E-27	0.156535	0.176	0.238	2.82E-23
TGFBRAP1	1.95E-27	-0.10648	0.025	0.128	2.91E-23
PIANP	1.95E-27	-0.15481	0.041	0.16	2.92E-23
TMEM243	2.12E-27	0.207615	0.218	0.283	3.17E-23
LANCL2	2.16E-27	-0.33222	0.11	0.281	3.23E-23
SRGAP3	2.24E-27	-0.18333	0.053	0.181	3.35E-23
RGS3	2.87E-27	-0.15077	0.071	0.199	4.29E-23
PFKFB3	2.89E-27	0.133762	0.13	0.173	4.32E-23
ERCC3	2.94E-27	-0.14038	0.114	0.268	4.40E-23
PHLPP1	2.97E-27	-0.13449	0.044	0.158	4.44E-23
DUSP28	3.03E-27	-0.15453	0.048	0.169	4.53E-23
FBXO44	3.08E-27	0.158334	0.126	0.19	4.60E-23
OAS3	3.13E-27	-0.10314	0.033	0.138	4.67E-23
PIGZ	3.18E-27	-0.11102	0.035	0.14	4.76E-23
ZDHHC7	3.41E-27	-0.12598	0.097	0.233	5.09E-23
AQR	3.49E-27	-0.12165	0.034	0.14	5.21E-23
USP18	3.50E-27	-0.13971	0.034	0.144	5.23E-23
LRRN2	3.66E-27	-0.12729	0.023	0.125	5.47E-23
ASMTL	3.79E-27	0.104533	0.146	0.228	5.66E-23
HPCAL1	3.84E-27	-0.11601	0.106	0.238	5.75E-23
HOXA9	4.08E-27	-0.24521	0.01	0.104	6.09E-23
ISG20	4.29E-27	0.561594	0.151	0.067	6.40E-23
CSF1	4.71E-27	0.277203	0.154	0.122	7.04E-23
ZFP1	4.86E-27	-0.14936	0.039	0.156	7.27E-23
DDIT4	4.88E-27	0.239655	0.286	0.312	7.29E-23
CACNB3	5.19E-27	-0.1546	0.034	0.147	7.76E-23
EIF4ENIF1	5.32E-27	-0.1226	0.044	0.159	7.95E-23
HIST1H2BK	5.48E-27	0.101647	0.164	0.246	8.20E-23
TRIM41	5.91E-27	-0.15378	0.073	0.204	8.84E-23
APOC1	6.16E-27	0.636777	0.372	0.302	9.20E-23
TNFAIP6	6.34E-27	0.301084	0.236	0.294	9.48E-23
CCNDBP1	6.38E-27	0.128527	0.137	0.206	9.54E-23
TAP1	6.51E-27	-0.15494	0.219	0.388	9.73E-23
ADCK3	6.81E-27	0.148167	0.133	0.189	1.02E-22
ZNF629	6.82E-27	-0.12329	0.034	0.138	1.02E-22
SULF2	6.85E-27	-0.15392	0.105	0.252	1.02E-22
ZDHHC15	7.06E-27	-0.11106	0.031	0.141	1.06E-22
SEC16A	7.25E-27	-0.10101	0.084	0.206	1.08E-22
AVEN	7.56E-27	-0.12755	0.01	0.101	1.13E-22
KPTN	7.65E-27	-0.17614	0.046	0.172	1.14E-22
ZP3	8.04E-27	-0.15881	0.055	0.185	1.20E-22
PPP1R14C	8.39E-27	-0.24271	0.05	0.186	1.25E-22
MAP6	8.46E-27	-0.11342	0.056	0.175	1.26E-22
NKAIN4	8.46E-27	-0.34414	0.066	0.216	1.26E-22
SPIN4	8.69E-27	-0.16041	0.01	0.101	1.30E-22
IRF1	8.74E-27	0.184071	0.232	0.306	1.31E-22
ZNF318	8.80E-27	-0.1335	0.029	0.133	1.32E-22
IGSF21	9.02E-27	-0.24982	0.03	0.141	1.35E-22
RRP8	9.07E-27	0.130167	0.124	0.206	1.36E-22
AGL	9.50E-27	-0.11043	0.053	0.164	1.42E-22

MEGF9	9.50E-27	-0.11596	0.047	0.163	1.42E-22
OSBPL10	1.01E-26	-0.12312	0.021	0.117	1.51E-22
BBS2	1.04E-26	0.239224	0.234	0.298	1.56E-22
RCHY1	1.09E-26	0.14404	0.217	0.298	1.62E-22
RP11-66N1	1.11E-26	-0.10959	0.041	0.151	1.66E-22
TASP1	1.12E-26	0.161519	0.092	0.167	1.68E-22
UBXN11	1.15E-26	0.269068	0.181	0.199	1.72E-22
RTN4IP1	1.18E-26	-0.10568	0.032	0.131	1.77E-22
SIMC1	1.19E-26	-0.15201	0.038	0.157	1.77E-22
NCALD	1.19E-26	-0.13074	0.094	0.225	1.78E-22
HHLA3	1.24E-26	0.339553	0.189	0.219	1.85E-22
ARFGEF2	1.25E-26	-0.11151	0.032	0.14	1.87E-22
ZNF184	1.28E-26	-0.18418	0.03	0.143	1.92E-22
USP28	1.35E-26	-0.12401	0.042	0.149	2.02E-22
ZNF844	1.38E-26	-0.10394	0.052	0.157	2.06E-22
KAT6B	1.40E-26	-0.12405	0.068	0.19	2.09E-22
CCDC97	1.43E-26	-0.1465	0.068	0.196	2.13E-22
AC093673.	1.43E-26	0.185628	0.211	0.263	2.14E-22
SLC35E4	1.48E-26	-0.12264	0.033	0.142	2.21E-22
NPHP1	1.50E-26	0.135533	0.096	0.17	2.23E-22
RNF20	1.52E-26	-0.10834	0.119	0.259	2.27E-22
SAMHD1	1.54E-26	-0.14403	0.06	0.184	2.30E-22
SMO	1.58E-26	-0.12793	0.038	0.149	2.35E-22
LINC0091C	1.62E-26	-0.12835	0.055	0.169	2.43E-22
UBR1	1.72E-26	-0.14865	0.044	0.163	2.57E-22
LYRM9	1.72E-26	0.24842	0.168	0.233	2.57E-22
CAPN5	1.79E-26	-0.10918	0.054	0.164	2.68E-22
CD46	1.84E-26	0.134266	0.178	0.263	2.74E-22
C19orf57	1.84E-26	-0.1432	0.02	0.119	2.75E-22
SLC16A9	1.88E-26	-0.16927	0.033	0.147	2.81E-22
C19orf12	1.91E-26	0.116763	0.125	0.209	2.85E-22
TNRC18	2.00E-26	-0.14212	0.036	0.146	3.00E-22
PIK3R2	2.06E-26	-0.11421	0.024	0.116	3.07E-22
BIVM	2.26E-26	-0.16688	0.079	0.217	3.38E-22
FKTN	2.42E-26	-0.1086	0.041	0.149	3.62E-22
TRNT1	2.48E-26	0.161488	0.15	0.242	3.71E-22
USP37	2.50E-26	-0.12854	0.051	0.167	3.73E-22
TMEM198	2.67E-26	-0.12156	0.073	0.201	4.00E-22
FUCA1	2.69E-26	0.176995	0.128	0.178	4.02E-22
SAYSD1	2.91E-26	0.1007	0.127	0.207	4.35E-22
B3GALT1	3.16E-26	-0.13566	0.02	0.12	4.72E-22
HOXB3	3.20E-26	-0.16433	0.016	0.102	4.79E-22
RHOU	3.37E-26	-0.22875	0.07	0.212	5.04E-22
ZBED3	3.52E-26	-0.12368	0.038	0.151	5.26E-22
LRRC14	3.64E-26	-0.15557	0.043	0.159	5.45E-22
IMMP2L	3.84E-26	0.445838	0.228	0.165	5.74E-22
HIC2	3.95E-26	-0.12162	0.013	0.102	5.90E-22
RP11-386G	3.98E-26	0.170489	0.137	0.215	5.95E-22
OPTN	4.00E-26	0.133822	0.149	0.19	5.98E-22
ZCCHC24	4.01E-26	-0.12574	0.063	0.185	5.99E-22
IFI44	4.03E-26	-0.13778	0.103	0.244	6.02E-22

ZNF71	4.11E-26	-0.11147	0.067	0.181	6.14E-22
RP11-73E1	4.40E-26	-0.14521	0.017	0.112	6.58E-22
GSE1	4.78E-26	-0.16205	0.052	0.172	7.15E-22
ATP6V1G2	4.93E-26	-0.12631	0.087	0.215	7.36E-22
FAS	5.10E-26	0.538336	0.159	0.068	7.62E-22
LZTS2	5.47E-26	-0.11693	0.035	0.14	8.17E-22
EPHB3	5.49E-26	-0.14699	0.019	0.117	8.20E-22
SEMA7A	5.92E-26	-0.10156	0.011	0.094	8.84E-22
KIAA1456	6.68E-26	-0.10098	0.018	0.11	9.99E-22
PPP1R14A	6.69E-26	-0.15168	0.057	0.172	1.00E-21
PIP4K2C	6.87E-26	-0.27493	0.078	0.23	1.03E-21
DSCAM	7.18E-26	-0.25414	0.055	0.193	1.07E-21
SPIRE1	7.30E-26	-0.10192	0.069	0.186	1.09E-21
LMBRD1	7.98E-26	0.348743	0.187	0.212	1.19E-21
TADA1	8.01E-26	-0.10611	0.04	0.141	1.20E-21
USP15	8.25E-26	-0.10436	0.156	0.293	1.23E-21
IRGQ	8.40E-26	-0.153	0.041	0.156	1.26E-21
PSMA2	8.64E-26	0.904556	0.14	0.02	1.29E-21
MAPK8IP3	9.30E-26	-0.11828	0.043	0.149	1.39E-21
LRP11	9.65E-26	-0.12262	0.033	0.138	1.44E-21
AKAP5	9.72E-26	-0.10563	0.013	0.094	1.45E-21
LYST	1.00E-25	0.237559	0.147	0.152	1.49E-21
PDE4DIP	1.04E-25	0.414913	0.287	0.231	1.56E-21
SFT2D3	1.05E-25	-0.11762	0.024	0.122	1.57E-21
SIPA1	1.05E-25	-0.10068	0.027	0.126	1.57E-21
PDE9A	1.08E-25	-0.14562	0.029	0.136	1.61E-21
NDEL1	1.12E-25	0.184323	0.141	0.189	1.68E-21
RAD54L2	1.12E-25	-0.11437	0.078	0.2	1.68E-21
UGT8	1.20E-25	-0.20517	0.008	0.091	1.79E-21
C1RL	1.21E-25	0.458695	0.12	0.021	1.81E-21
TMBIM4	1.22E-25	0.879201	0.115	0.01	1.83E-21
FAM114A1	1.39E-25	0.376542	0.194	0.153	2.08E-21
HOXA10	1.46E-25	-0.35971	0.06	0.195	2.18E-21
STK3	1.52E-25	0.226505	0.139	0.165	2.27E-21
MYL5	1.52E-25	0.184844	0.089	0.136	2.28E-21
RP11-395A	1.54E-25	-0.11299	0.045	0.156	2.31E-21
FGF9	1.57E-25	-0.19384	0.012	0.099	2.34E-21
ZNF16	1.61E-25	-0.10502	0.046	0.154	2.41E-21
ZNF853	1.62E-25	-0.14453	0.031	0.138	2.42E-21
ZNF713	1.66E-25	-0.10982	0.027	0.119	2.48E-21
ENTPD5	1.67E-25	-0.10632	0.027	0.125	2.50E-21
SCO2	1.81E-25	0.10633	0.112	0.198	2.71E-21
RP11-345F	1.89E-25	-0.17609	0.123	0.277	2.82E-21
FBRSL1	2.00E-25	-0.11491	0.02	0.115	2.99E-21
PBXIP1	2.04E-25	0.445248	0.152	0.101	3.05E-21
TMEM42	2.17E-25	0.239954	0.172	0.201	3.25E-21
ZNF512	2.19E-25	-0.15132	0.104	0.247	3.27E-21
CRB1	2.26E-25	-0.17043	0.038	0.148	3.37E-21
CEP85	2.27E-25	-0.14575	0.021	0.12	3.40E-21
CRYZ	2.42E-25	0.235084	0.161	0.21	3.62E-21
SELO	2.59E-25	-0.11336	0.028	0.128	3.87E-21

PLEKHA2	2.64E-25	-0.1532	0.045	0.162	3.94E-21
LETMD1	2.69E-25	0.150651	0.145	0.209	4.02E-21
SARM1	2.77E-25	-0.13398	0.024	0.125	4.14E-21
NLGN4Y	2.89E-25	-0.13152	0.007	0.088	4.32E-21
BIN3	2.93E-25	0.133542	0.17	0.259	4.38E-21
BAG3	2.94E-25	0.234526	0.146	0.127	4.39E-21
FAIM2	2.95E-25	0.21287	0.19	0.26	4.42E-21
LAMB2	3.20E-25	0.158511	0.106	0.142	4.78E-21
GPX8	3.22E-25	0.130535	0.084	0.147	4.82E-21
ALKBH6	3.28E-25	-0.14511	0.064	0.188	4.90E-21
REC8	3.36E-25	-0.24712	0.039	0.16	5.02E-21
TMEM44	3.41E-25	-0.10082	0.022	0.114	5.10E-21
CRELD1	3.49E-25	0.212648	0.159	0.205	5.21E-21
FOXP1	3.53E-25	0.63892	0.254	0.228	5.27E-21
PTCH1	3.59E-25	-0.14776	0.029	0.135	5.36E-21
RPP21	3.61E-25	0.11356	0.138	0.214	5.40E-21
KIAA1614	3.69E-25	-0.15424	0.011	0.099	5.51E-21
ALDOC	3.75E-25	0.564753	0.236	0.102	5.61E-21
SCARF2	3.91E-25	-0.10622	0.018	0.111	5.84E-21
TMEM179	3.98E-25	-0.16919	0.005	0.081	5.95E-21
ZSCAN29	4.04E-25	-0.11371	0.016	0.105	6.04E-21
ARMCX1	4.11E-25	0.259017	0.22	0.24	6.14E-21
ANKRD13A	4.27E-25	-0.12076	0.059	0.177	6.39E-21
SLC35G2	4.28E-25	0.289833	0.167	0.211	6.39E-21
LRRC4	4.31E-25	-0.18987	0.009	0.094	6.45E-21
POLR3C	4.59E-25	-0.11077	0.061	0.18	6.85E-21
EPDR1	4.82E-25	0.128125	0.164	0.23	7.20E-21
PCOLCE2	5.50E-25	0.259445	0.134	0.133	8.22E-21
RIC3	5.85E-25	0.225473	0.288	0.364	8.75E-21
STK35	5.92E-25	-0.10425	0.032	0.132	8.85E-21
CHRNB1	6.03E-25	0.336316	0.157	0.167	9.02E-21
TYW3	6.20E-25	0.108596	0.131	0.219	9.26E-21
KIFAP3	6.56E-25	0.235004	0.215	0.26	9.81E-21
DPCD	6.60E-25	0.228859	0.187	0.228	9.86E-21
PAG1	7.20E-25	-0.13624	0.073	0.196	1.08E-20
CD14	7.35E-25	0.529364	0.227	0.11	1.10E-20
NPPA	7.40E-25	-0.26155	0.004	0.075	1.11E-20
HSF2	7.47E-25	-0.15726	0.112	0.257	1.12E-20
CAMK2N2	7.68E-25	-0.16703	0.009	0.093	1.15E-20
ZNF660	8.39E-25	-0.10894	0.03	0.128	1.25E-20
ATXN3	8.52E-25	0.109521	0.127	0.202	1.27E-20
HEBP1	9.74E-25	0.240777	0.188	0.202	1.45E-20
SYP	1.01E-24	-0.13912	0.074	0.198	1.52E-20
C14orf37	1.05E-24	-0.14829	0.062	0.184	1.56E-20
EGLN3	1.07E-24	0.459306	0.16	0.068	1.60E-20
FAM107B	1.16E-24	-0.23792	0.044	0.17	1.74E-20
TRIM11	1.17E-24	-0.11561	0.077	0.196	1.75E-20
FAM184A	1.18E-24	-0.15927	0.034	0.147	1.77E-20
TSHZ1	1.22E-24	-0.19798	0.043	0.164	1.82E-20
1-Mar	1.33E-24	-0.23962	0.046	0.168	1.98E-20
FXYD7	1.35E-24	-0.33949	0.029	0.141	2.02E-20

RALGAPA1	1.38E-24	-0.15127	0.08	0.206	2.06E-20
CRTC1	1.43E-24	-0.11893	0.059	0.169	2.14E-20
AC002454.	1.46E-24	-0.20298	0.005	0.08	2.18E-20
STOML1	1.49E-24	0.177331	0.103	0.179	2.22E-20
MPI	1.53E-24	0.177055	0.121	0.173	2.28E-20
TGFBR1	1.56E-24	-0.16662	0.046	0.164	2.33E-20
STOX2	1.59E-24	-0.15935	0.038	0.148	2.37E-20
TSPAN31	1.66E-24	-0.14366	0.255	0.43	2.48E-20
CROT	1.68E-24	0.301549	0.176	0.209	2.51E-20
RP11-977G	1.79E-24	-0.10792	0.033	0.13	2.67E-20
ZNF74	1.79E-24	-0.11819	0.025	0.119	2.67E-20
ARHGEF9	1.99E-24	-0.16895	0.065	0.193	2.98E-20
PIEZO1	2.26E-24	-0.11435	0.032	0.136	3.38E-20
TMEM109	2.35E-24	0.148346	0.139	0.217	3.50E-20
RP4-665J2	2.36E-24	0.112169	0.124	0.19	3.52E-20
DNMT3B	2.46E-24	-0.11142	0.004	0.072	3.67E-20
KLF10	2.55E-24	0.179373	0.225	0.3	3.81E-20
MAEL	2.68E-24	-0.11631	0.013	0.098	4.01E-20
METTL1	2.74E-24	-0.22673	0.138	0.3	4.10E-20
NUDT8	2.78E-24	-0.12872	0.039	0.151	4.16E-20
FANCE	2.86E-24	-0.12149	0.013	0.098	4.28E-20
LRRC23	2.91E-24	0.122125	0.143	0.22	4.35E-20
AASS	2.93E-24	-0.115	0.106	0.237	4.38E-20
ADAMTS9	3.09E-24	-0.10061	0.047	0.144	4.62E-20
ZNF346	3.18E-24	-0.14438	0.042	0.153	4.75E-20
PCGF5	3.37E-24	0.211486	0.16	0.207	5.04E-20
ERCC6L2	3.64E-24	-0.14736	0.059	0.184	5.44E-20
SERPINB1	3.68E-24	0.558379	0.134	0.025	5.50E-20
RP11-108M	3.77E-24	-0.19205	0.012	0.1	5.63E-20
RARA	3.80E-24	-0.10841	0.057	0.169	5.68E-20
GPRIN1	4.00E-24	-0.1595	0.015	0.106	5.98E-20
PHACTR3	4.14E-24	-0.23513	0.031	0.138	6.19E-20
OCEL1	4.17E-24	0.309291	0.16	0.157	6.23E-20
GADD45G	4.35E-24	-0.33987	0.18	0.364	6.50E-20
STK32C	4.49E-24	-0.16045	0.043	0.157	6.71E-20
GAS7	4.53E-24	-0.21297	0.067	0.194	6.76E-20
PLEKHA5	4.63E-24	-0.11707	0.037	0.14	6.92E-20
DBX2	4.66E-24	-0.13005	0.029	0.121	6.96E-20
KCNJ2	4.88E-24	-0.15332	0.024	0.121	7.29E-20
PRUNE2	4.99E-24	0.543244	0.212	0.088	7.46E-20
AC009005.	5.25E-24	-0.10364	0.008	0.083	7.84E-20
KLHL25	5.74E-24	-0.12655	0.03	0.132	8.58E-20
NOVA1-AS1	5.80E-24	-0.12464	0.013	0.098	8.67E-20
NDRG2	6.02E-24	0.301566	0.345	0.385	8.99E-20
HIST1H2AC	6.18E-24	0.544244	0.212	0.078	9.23E-20
ZNF133	6.20E-24	-0.10076	0.05	0.156	9.27E-20
PARP9	6.48E-24	0.166875	0.148	0.194	9.68E-20
ZNF335	7.30E-24	-0.11317	0.054	0.162	1.09E-19
ZNF766	7.40E-24	-0.12023	0.11	0.236	1.11E-19
SHF	7.71E-24	-0.12833	0.013	0.094	1.15E-19
TP53BP1	7.91E-24	-0.13266	0.062	0.18	1.18E-19

INF2	8.22E-24	-0.12622	0.028	0.127	1.23E-19
RP5-1177M	8.35E-24	-0.25093	0.011	0.098	1.25E-19
RFXAP	8.53E-24	-0.13634	0.09	0.217	1.27E-19
RECK	9.13E-24	-0.12484	0.043	0.147	1.36E-19
OSGEPL1	1.02E-23	-0.10068	0.053	0.159	1.52E-19
CLEC11A	1.03E-23	-0.14387	0.136	0.281	1.53E-19
PTBP2	1.08E-23	-0.23722	0.063	0.199	1.61E-19
STMN4	1.10E-23	-0.36323	0.186	0.373	1.64E-19
ZC3H3	1.14E-23	-0.10959	0.059	0.165	1.70E-19
SOX5	1.26E-23	-0.11436	0.044	0.159	1.88E-19
FBXL16	1.26E-23	-0.11502	0.018	0.105	1.89E-19
FAH	1.34E-23	0.251666	0.106	0.135	2.01E-19
CUTC	1.42E-23	0.147403	0.107	0.167	2.12E-19
MOB3B	1.45E-23	-0.16981	0.034	0.142	2.17E-19
MORC3	1.56E-23	-0.11943	0.063	0.175	2.33E-19
CSPG4	1.64E-23	-0.11923	0.011	0.09	2.45E-19
PSEN2	1.71E-23	0.216074	0.105	0.151	2.56E-19
CTPS2	1.74E-23	-0.11741	0.027	0.123	2.60E-19
LINC00094	1.79E-23	0.112462	0.142	0.188	2.67E-19
FAM69B	1.79E-23	-0.10629	0.021	0.111	2.68E-19
SLC35A2	1.82E-23	0.250057	0.179	0.22	2.72E-19
NUP133	1.82E-23	-0.17584	0.073	0.201	2.72E-19
ATF7IP	1.82E-23	-0.19214	0.065	0.194	2.72E-19
LY96	1.96E-23	0.207707	0.118	0.107	2.94E-19
TAF5L	2.00E-23	-0.11163	0.021	0.111	2.98E-19
MID1	2.02E-23	-0.10151	0.05	0.149	3.03E-19
CMBL	2.05E-23	0.125098	0.154	0.211	3.06E-19
4-Sep	2.22E-23	0.123692	0.097	0.157	3.32E-19
TMOD1	2.23E-23	0.347059	0.214	0.158	3.33E-19
AP3M2	2.25E-23	-0.13474	0.072	0.191	3.36E-19
ITSN1	2.59E-23	-0.10964	0.107	0.226	3.87E-19
TMEM69	2.59E-23	0.125878	0.168	0.247	3.87E-19
LRRC20	2.72E-23	-0.14212	0.024	0.119	4.07E-19
RP11-1275	2.90E-23	-0.14967	0.041	0.147	4.33E-19
GRK4	2.91E-23	-0.10224	0.061	0.169	4.34E-19
ZBTB33	3.02E-23	-0.13394	0.039	0.144	4.51E-19
DLGAP1-AS	3.05E-23	0.255204	0.106	0.125	4.56E-19
SESN2	3.11E-23	0.289632	0.149	0.11	4.64E-19
TMEM53	3.23E-23	0.217461	0.104	0.151	4.83E-19
NOP14	3.67E-23	-0.11428	0.056	0.165	5.48E-19
PIK3CA	3.72E-23	-0.10743	0.061	0.165	5.56E-19
CLK4	3.97E-23	0.121133	0.165	0.232	5.94E-19
DDIT4L	4.19E-23	0.330012	0.08	0.001	6.27E-19
ANGPTL1	4.27E-23	-0.17603	0.014	0.099	6.38E-19
HBP1	4.34E-23	0.257249	0.218	0.217	6.49E-19
SLC22A4	4.60E-23	-0.11427	0.028	0.125	6.88E-19
HOXB4	4.61E-23	-0.15737	0.011	0.084	6.89E-19
PLEKHG3	4.94E-23	-0.14169	0.005	0.077	7.39E-19
CABLES2	4.95E-23	-0.12076	0.014	0.1	7.40E-19
CHPF2	4.96E-23	0.11854	0.165	0.227	7.41E-19
CAND2	5.61E-23	-0.10336	0.01	0.081	8.38E-19

PHKG2	5.92E-23	0.221611	0.13	0.174	8.85E-19
TP53	6.01E-23	-0.13136	0.092	0.227	8.98E-19
DDX20	6.17E-23	-0.1199	0.059	0.168	9.22E-19
POLH	6.21E-23	-0.11628	0.057	0.164	9.27E-19
CCDC117	6.22E-23	-0.10248	0.036	0.131	9.30E-19
CSRNP3	7.06E-23	-0.22865	0.043	0.162	1.06E-18
DPYD	7.47E-23	0.401126	0.135	0.025	1.12E-18
CCDC88C	7.64E-23	-0.1339	0.01	0.09	1.14E-18
MIR22HG	8.00E-23	0.545579	0.15	0.054	1.19E-18
TENM1	9.08E-23	-0.13896	0.035	0.131	1.36E-18
GRB10	9.53E-23	0.136446	0.1	0.132	1.42E-18
HLA-DQB1	9.69E-23	0.47683	0.136	0.04	1.45E-18
SNX11	1.06E-22	-0.11566	0.059	0.173	1.59E-18
TMEM8B	1.07E-22	-0.10614	0.034	0.128	1.59E-18
MSL2	1.10E-22	-0.10297	0.04	0.131	1.64E-18
CCDC159	1.14E-22	0.287185	0.137	0.154	1.70E-18
CTD-2017D	1.14E-22	-0.10298	0.029	0.117	1.70E-18
C5orf28	1.16E-22	0.269995	0.166	0.158	1.74E-18
H1FX-AS1	1.21E-22	-0.12141	0.015	0.094	1.80E-18
MGST2	1.25E-22	0.418816	0.139	0.135	1.87E-18
RABL2B	1.32E-22	0.115337	0.121	0.195	1.97E-18
MT1A	1.43E-22	0.4763	0.109	0.012	2.14E-18
ERLIN2	1.45E-22	0.162334	0.132	0.19	2.17E-18
RP11-263K	1.46E-22	0.26842	0.114	0.095	2.18E-18
B3GALT2	1.50E-22	-0.19208	0.011	0.093	2.24E-18
STRIP1	1.63E-22	-0.14752	0.083	0.205	2.44E-18
RFX7	1.80E-22	-0.11178	0.016	0.098	2.69E-18
SMYD3	1.86E-22	0.1729	0.157	0.228	2.78E-18
RCBTB1	1.87E-22	-0.15415	0.036	0.137	2.79E-18
SRGN	1.87E-22	0.579384	0.312	0.16	2.80E-18
BCR	1.89E-22	-0.14159	0.036	0.137	2.82E-18
CCDC74A	2.02E-22	-0.10478	0.084	0.199	3.02E-18
EEF1A2	2.05E-22	-0.16891	0.011	0.093	3.07E-18
MVK	2.07E-22	-0.12622	0.048	0.146	3.09E-18
ENKUR	2.11E-22	0.2826	0.117	0.146	3.15E-18
LNPEP	2.15E-22	-0.10507	0.073	0.179	3.22E-18
KIF5A	2.20E-22	-0.29456	0.083	0.226	3.29E-18
GGA2	2.25E-22	-0.10922	0.069	0.175	3.36E-18
CDC37L1	2.29E-22	0.125213	0.164	0.221	3.42E-18
EPC2	2.31E-22	-0.11081	0.072	0.185	3.46E-18
KCND3	2.82E-22	-0.10509	0.028	0.112	4.22E-18
SLC38A6	2.89E-22	0.195106	0.113	0.14	4.32E-18
NACA2	3.24E-22	0.368079	0.178	0.165	4.85E-18
CREBL2	3.27E-22	0.156802	0.123	0.169	4.89E-18
PEX11B	3.55E-22	0.198209	0.125	0.168	5.30E-18
SGCE	3.72E-22	0.467662	0.323	0.305	5.56E-18
ZNF736	3.73E-22	-0.14443	0.028	0.123	5.58E-18
MAN1C1	3.73E-22	0.120768	0.076	0.116	5.58E-18
MYADM	3.86E-22	0.165292	0.169	0.21	5.76E-18
ZNF433	3.96E-22	-0.10554	0.034	0.126	5.91E-18
BRPF1	4.01E-22	-0.11986	0.043	0.146	6.00E-18

KIRREL3	4.20E-22	-0.13708	0.004	0.067	6.27E-18
SLC48A1	4.21E-22	0.136924	0.126	0.184	6.30E-18
SH2B2	4.23E-22	-0.1964	0.075	0.209	6.32E-18
C19orf47	4.26E-22	-0.11617	0.027	0.111	6.37E-18
PRR12	4.29E-22	-0.11387	0.018	0.104	6.41E-18
TAB1	4.72E-22	-0.11172	0.045	0.147	7.05E-18
ANKRD37	4.72E-22	0.149956	0.115	0.157	7.06E-18
GRAMD3	4.92E-22	0.37138	0.193	0.146	7.36E-18
PHF7	4.99E-22	-0.10445	0.034	0.13	7.46E-18
PLP1	5.05E-22	-0.10739	0.097	0.217	7.54E-18
CTSH	5.26E-22	0.443549	0.153	0.095	7.86E-18
ELFN1	5.39E-22	-0.1061	0.005	0.07	8.05E-18
NRG2	5.45E-22	-0.13554	0.014	0.098	8.15E-18
RDM1	5.78E-22	-0.12121	0.008	0.08	8.64E-18
LRRC1	5.86E-22	-0.19519	0.053	0.173	8.75E-18
GPR1	5.98E-22	-0.16694	0.014	0.098	8.94E-18
DYNC1I1	6.01E-22	-0.28482	0.028	0.128	8.99E-18
MMP7	6.07E-22	0.677107	0.1	0.014	9.07E-18
EXOSC5	6.12E-22	0.210208	0.18	0.233	9.15E-18
C11orf1	6.17E-22	0.377836	0.182	0.188	9.21E-18
NEK3	7.07E-22	-0.11544	0.034	0.13	1.06E-17
TMEM37	7.40E-22	0.540611	0.091	0.026	1.11E-17
NPY	7.42E-22	-0.24313	0.013	0.088	1.11E-17
KLHDC4	8.30E-22	-0.1322	0.043	0.143	1.24E-17
PKIA	8.80E-22	-0.22586	0.046	0.162	1.32E-17
REEP1	9.50E-22	-0.20207	0.038	0.148	1.42E-17
PELO	9.60E-22	0.13006	0.133	0.183	1.44E-17
ATP9B	9.98E-22	-0.11066	0.039	0.132	1.49E-17
HMOX1	1.10E-21	0.523883	0.174	0.095	1.64E-17
CDH10	1.14E-21	-0.16768	0.01	0.088	1.71E-17
TUBG2	1.17E-21	0.223971	0.134	0.16	1.75E-17
CCDC152	1.17E-21	0.109524	0.057	0.106	1.75E-17
METTL15	1.18E-21	0.103026	0.13	0.181	1.76E-17
C1orf106	1.18E-21	-0.11637	0.006	0.075	1.76E-17
GPC6	1.22E-21	0.136356	0.076	0.127	1.82E-17
ZMIZ2	1.22E-21	-0.10674	0.061	0.169	1.82E-17
ERBB2	1.22E-21	0.122324	0.06	0.109	1.83E-17
C9orf116	1.27E-21	0.137178	0.104	0.142	1.90E-17
HMGN5	1.29E-21	-0.18022	0.085	0.216	1.93E-17
ZIK1	1.30E-21	-0.11647	0.052	0.151	1.94E-17
TFAP2A	1.31E-21	-0.18915	0.018	0.107	1.96E-17
ZNF114	1.32E-21	-0.10368	0.009	0.077	1.97E-17
ZNF568	1.36E-21	0.120091	0.119	0.165	2.04E-17
MIR210HG	1.39E-21	0.165759	0.112	0.146	2.08E-17
HDAC10	1.54E-21	-0.1103	0.038	0.135	2.31E-17
DHRS3	1.55E-21	0.475164	0.168	0.067	2.31E-17
PRKAR1B	1.69E-21	-0.13332	0.09	0.215	2.53E-17
TBC1D17	1.80E-21	0.125857	0.144	0.177	2.69E-17
PARP11	1.81E-21	-0.10228	0.045	0.147	2.70E-17
CBWD1	1.85E-21	0.221658	0.227	0.256	2.76E-17
BEX2	2.01E-21	0.445693	0.329	0.263	3.00E-17

ZNF181	2.06E-21	0.108343	0.115	0.17	3.07E-17
CCBL2	2.07E-21	0.100005	0.142	0.207	3.10E-17
USP19	2.07E-21	-0.11908	0.047	0.148	3.10E-17
TFE3	2.11E-21	0.186461	0.111	0.138	3.16E-17
ALDH18A1	2.16E-21	-0.13086	0.073	0.185	3.23E-17
CCDC61	2.18E-21	-0.1069	0.014	0.089	3.26E-17
RRNAD1	2.24E-21	0.102366	0.117	0.181	3.35E-17
BEX4	2.31E-21	0.361278	0.428	0.409	3.46E-17
TMEM150A	2.43E-21	0.388125	0.138	0.09	3.63E-17
GDAP1	2.68E-21	-0.19317	0.091	0.221	4.00E-17
TMEM145	2.73E-21	-0.11254	0.041	0.132	4.07E-17
HYLS1	2.75E-21	-0.25794	0.027	0.126	4.11E-17
KIAA1429	3.17E-21	-0.10781	0.084	0.2	4.74E-17
HOXA3	3.34E-21	-0.23102	0.037	0.143	4.99E-17
PTK2B	3.44E-21	0.140668	0.079	0.132	5.14E-17
CLN8	3.46E-21	0.10538	0.139	0.215	5.16E-17
TCF20	3.59E-21	-0.1096	0.03	0.121	5.37E-17
AFF3	3.85E-21	-0.20673	0.023	0.117	5.75E-17
HPCA	4.01E-21	-0.21044	0.028	0.123	5.99E-17
UTP14C	4.30E-21	-0.12808	0.053	0.159	6.43E-17
WASF1	4.44E-21	-0.123	0.065	0.173	6.63E-17
SLC24A3	4.91E-21	-0.11277	0.009	0.08	7.33E-17
GFM2	5.35E-21	-0.10361	0.078	0.181	8.00E-17
ACTR3B	5.46E-21	-0.10368	0.094	0.204	8.16E-17
KIAA0100	5.66E-21	-0.11873	0.049	0.153	8.46E-17
ARHGEF3	5.79E-21	0.119144	0.087	0.13	8.65E-17
AHNAK	5.98E-21	0.326256	0.134	0.094	8.94E-17
PLK3	5.98E-21	0.135266	0.148	0.191	8.94E-17
ARMCX2	6.16E-21	0.345771	0.172	0.177	9.21E-17
KIAA1211	6.67E-21	-0.14104	0.006	0.073	9.96E-17
C8orf46	6.82E-21	-0.24043	0.099	0.236	1.02E-16
GGA3	6.89E-21	-0.10018	0.049	0.147	1.03E-16
NDUFA7	7.06E-21	0.699407	0.103	0.012	1.06E-16
SPIRE2	7.08E-21	-0.10604	0.018	0.095	1.06E-16
MIR155HG	7.31E-21	0.168517	0.063	0.1	1.09E-16
RCBTB2	7.57E-21	-0.15514	0.077	0.195	1.13E-16
TBC1D15	7.65E-21	0.145879	0.18	0.215	1.14E-16
DOCK10	8.01E-21	-0.11091	0.026	0.11	1.20E-16
PDLIM3	8.10E-21	0.417532	0.258	0.21	1.21E-16
CASD1	8.31E-21	-0.16266	0.037	0.14	1.24E-16
ELF1	8.32E-21	0.165719	0.121	0.141	1.24E-16
ITGA5	9.04E-21	0.244355	0.087	0.069	1.35E-16
ZNF627	9.15E-21	-0.10369	0.046	0.144	1.37E-16
GDF15	9.53E-21	0.219814	0.135	0.154	1.42E-16
IGLON5	1.03E-20	-0.16663	0.01	0.085	1.54E-16
KITLG	1.03E-20	-0.1413	0.019	0.105	1.54E-16
RP11-390E	1.03E-20	0.132653	0.135	0.216	1.55E-16
TIMP4	1.04E-20	0.602593	0.218	0.123	1.56E-16
DHX34	1.07E-20	-0.12858	0.037	0.128	1.60E-16
DEDD2	1.11E-20	0.171214	0.164	0.188	1.66E-16
AMOTL2	1.21E-20	-0.17925	0.057	0.177	1.80E-16

TMEM100	1.23E-20	-0.11623	0.13	0.264	1.83E-16
ASXL3	1.24E-20	-0.13519	0.006	0.07	1.85E-16
FKBP11	1.24E-20	0.257295	0.131	0.158	1.86E-16
PEG10	1.26E-20	-0.31364	0.097	0.242	1.88E-16
RP5-1136G	1.37E-20	-0.11443	0.024	0.111	2.04E-16
RNF182	1.38E-20	-0.1722	0.037	0.141	2.06E-16
SLC6A8	1.66E-20	0.128986	0.113	0.146	2.48E-16
CTD-2035E	1.67E-20	-0.13501	0.018	0.099	2.50E-16
ABHD14B	1.74E-20	0.288293	0.138	0.149	2.60E-16
NFASC	1.75E-20	0.409363	0.152	0.084	2.62E-16
BIRC3	1.76E-20	0.494531	0.115	0.032	2.63E-16
C12orf49	1.82E-20	-0.10245	0.065	0.175	2.73E-16
PTPRD	1.92E-20	-0.14941	0.033	0.127	2.87E-16
RP11-488C	2.28E-20	-0.15092	0.026	0.114	3.41E-16
NMI	2.42E-20	0.343211	0.188	0.17	3.62E-16
PHF2	2.43E-20	-0.1502	0.037	0.132	3.63E-16
DENND6A	2.68E-20	-0.12815	0.031	0.121	4.00E-16
KDM5D	2.79E-20	-0.10734	0.008	0.078	4.16E-16
CASP9	2.85E-20	-0.1461	0.044	0.142	4.27E-16
PTTG2	2.88E-20	-0.13851	0.003	0.058	4.31E-16
SLITRK1	2.98E-20	-0.12519	0.003	0.062	4.45E-16
DDHD1	3.22E-20	-0.10847	0.051	0.149	4.81E-16
AGO1	3.23E-20	-0.11399	0.048	0.144	4.83E-16
CCDC151	3.32E-20	0.307064	0.089	0.052	4.96E-16
SIAE	3.43E-20	0.100019	0.08	0.147	5.13E-16
AASDH	3.48E-20	-0.10495	0.158	0.29	5.20E-16
TMED8	3.61E-20	-0.11514	0.034	0.127	5.40E-16
AC159540.	3.66E-20	-0.11479	0.067	0.173	5.47E-16
ANGPTL2	3.70E-20	0.189504	0.127	0.153	5.53E-16
UTP3	3.91E-20	0.173135	0.146	0.191	5.84E-16
KRT7	4.03E-20	0.326671	0.07	0.057	6.03E-16
ANKRD49	4.26E-20	0.113583	0.113	0.158	6.37E-16
CA3	4.38E-20	0.730474	0.141	0.049	6.54E-16
MAST1	4.68E-20	-0.10357	0.018	0.094	7.00E-16
CFB	4.71E-20	0.385551	0.09	0.01	7.04E-16
SPAG4	4.72E-20	0.432666	0.115	0.03	7.05E-16
ACTR5	4.76E-20	-0.10239	0.039	0.131	7.11E-16
CARD16	4.86E-20	0.56407	0.212	0.081	7.27E-16
DFNA5	4.93E-20	0.242632	0.197	0.221	7.36E-16
LINGO1	4.95E-20	-0.1148	0.007	0.073	7.39E-16
RBM15	5.11E-20	-0.10614	0.044	0.135	7.64E-16
ZSCAN16	5.11E-20	-0.16302	0.039	0.14	7.64E-16
ADSSL1	5.34E-20	0.122431	0.084	0.109	7.98E-16
MYLK	5.66E-20	0.212483	0.064	0.078	8.46E-16
FCHSD2	5.70E-20	-0.1199	0.037	0.133	8.52E-16
MTRNR2L1C	5.86E-20	0.586191	0.107	0.016	8.75E-16
DGKB	6.11E-20	-0.19881	0.024	0.112	9.13E-16
MCAM	6.28E-20	-0.14374	0.031	0.123	9.38E-16
HSD17B7	6.35E-20	0.236356	0.115	0.137	9.49E-16
LDLRAD4	6.71E-20	-0.10831	0.031	0.119	1.00E-15
MINA	6.82E-20	0.135498	0.139	0.188	1.02E-15

TSLP	7.05E-20	0.416115	0.086	0.011	1.05E-15
SATB1	7.08E-20	-0.17345	0.047	0.156	1.06E-15
MRE11A	7.43E-20	-0.11102	0.059	0.151	1.11E-15
RAB20	7.62E-20	0.192728	0.094	0.107	1.14E-15
DUSP11	7.65E-20	0.126108	0.177	0.24	1.14E-15
FRZB	7.77E-20	0.495915	0.137	0.06	1.16E-15
TDP2	8.38E-20	0.145651	0.161	0.23	1.25E-15
LIPA	8.77E-20	0.257758	0.145	0.149	1.31E-15
OPN3	9.19E-20	-0.11171	0.033	0.121	1.37E-15
EHD2	9.73E-20	0.27334	0.13	0.079	1.45E-15
TACC2	1.02E-19	-0.13659	0.026	0.114	1.52E-15
IL13RA2	1.04E-19	0.541857	0.212	0.086	1.55E-15
BHLHE41	1.04E-19	0.142397	0.141	0.173	1.56E-15
C9orf9	1.16E-19	0.177236	0.137	0.168	1.73E-15
AC016700.	1.16E-19	-0.11854	0.028	0.112	1.74E-15
TCTA	1.17E-19	0.188748	0.169	0.23	1.75E-15
TRIB1	1.18E-19	0.341267	0.153	0.073	1.77E-15
MIOS	1.31E-19	-0.13873	0.076	0.191	1.96E-15
COQ3	1.33E-19	-0.13447	0.048	0.149	1.99E-15
RP11-196G	1.35E-19	-0.1198	0.006	0.068	2.01E-15
PDIA5	1.35E-19	0.203269	0.09	0.119	2.01E-15
CADPS	1.41E-19	0.447608	0.114	0.021	2.11E-15
ATOH8	1.43E-19	-0.15305	0.023	0.106	2.13E-15
CTD-2090I	1.48E-19	0.585219	0.089	0.009	2.21E-15
FAM181A	1.49E-19	0.447018	0.135	0.099	2.23E-15
CDK7	1.82E-19	0.130024	0.114	0.165	2.72E-15
RP5-1024C	1.84E-19	-0.12672	0.008	0.073	2.75E-15
TRMT11	1.93E-19	0.154995	0.165	0.205	2.88E-15
GMEB2	1.98E-19	-0.11825	0.032	0.12	2.96E-15
CYLD	2.37E-19	0.158313	0.115	0.137	3.54E-15
RP11-571M	2.38E-19	-0.13392	0.016	0.091	3.56E-15
CEPT1	2.82E-19	0.121715	0.17	0.223	4.21E-15
SC5D	2.99E-19	0.123928	0.155	0.21	4.47E-15
NRXN2	3.05E-19	-0.17634	0.074	0.19	4.56E-15
MAGI2	3.08E-19	-0.15789	0.071	0.183	4.60E-15
BACE1	3.35E-19	-0.11546	0.085	0.196	5.01E-15
SP4	3.48E-19	-0.10224	0.038	0.122	5.20E-15
ZNF274	3.53E-19	-0.10089	0.114	0.23	5.27E-15
CTSV	3.58E-19	-0.10109	0.008	0.072	5.35E-15
PDZRN3	3.68E-19	-0.1492	0.034	0.128	5.50E-15
TGFBI	4.03E-19	0.361476	0.122	0.026	6.03E-15
APOL2	4.10E-19	0.659489	0.171	0.099	6.13E-15
C15orf57	4.19E-19	0.116833	0.088	0.159	6.25E-15
SYVN1	4.35E-19	0.108125	0.112	0.151	6.49E-15
RALGPS1	4.56E-19	-0.14103	0.029	0.115	6.81E-15
ICA1	4.80E-19	-0.15956	0.04	0.138	7.18E-15
HSD17B8	4.98E-19	0.171517	0.087	0.115	7.44E-15
SMIM3	4.99E-19	0.705794	0.082	0.006	7.46E-15
ELL2	5.16E-19	0.374491	0.157	0.069	7.70E-15
MTMR11	5.37E-19	0.29465	0.122	0.09	8.03E-15
RP11-445F	5.99E-19	-0.11886	0.004	0.057	8.95E-15

ARVCF	6.02E-19	-0.11887	0.025	0.109	9.00E-15
MKL2	6.05E-19	0.124875	0.097	0.123	9.05E-15
BCL6	6.26E-19	0.322903	0.167	0.102	9.35E-15
N4BP2L1	6.37E-19	0.272694	0.149	0.135	9.51E-15
WIPF3	6.40E-19	-0.11596	0.02	0.099	9.56E-15
VAV2	6.42E-19	-0.11717	0.03	0.117	9.60E-15
GCA	6.58E-19	0.137627	0.201	0.249	9.83E-15
APH1B	7.07E-19	0.147838	0.092	0.122	1.06E-14
SEMA4C	7.33E-19	-0.15595	0.055	0.162	1.09E-14
FBLIM1	7.60E-19	0.179805	0.09	0.119	1.14E-14
HRASLS	8.61E-19	-0.14126	0.038	0.135	1.29E-14
SNRK	8.64E-19	-0.11809	0.053	0.151	1.29E-14
LZTFL1	8.93E-19	0.31841	0.152	0.106	1.33E-14
CXCL3	9.11E-19	0.514631	0.121	0.025	1.36E-14
C5orf30	9.18E-19	-0.12779	0.057	0.157	1.37E-14
PRKCG	9.49E-19	0.459053	0.082	0.01	1.42E-14
SCAMP5	1.01E-18	-0.14189	0.049	0.152	1.51E-14
NFKBIE	1.07E-18	-0.10217	0.07	0.172	1.60E-14
PASK	1.11E-18	-0.10983	0.007	0.069	1.66E-14
HSD3B7	1.15E-18	0.345273	0.104	0.021	1.72E-14
USP49	1.20E-18	-0.10714	0.025	0.101	1.79E-14
MCF2L	1.23E-18	-0.14538	0.019	0.099	1.84E-14
B3GAT2	1.38E-18	-0.1825	0.063	0.177	2.06E-14
ZNF829	1.41E-18	-0.11506	0.039	0.127	2.11E-14
NEU4	1.44E-18	-0.28987	0.024	0.109	2.15E-14
PHF11	1.48E-18	0.111847	0.089	0.13	2.21E-14
LIFR	1.51E-18	-0.1118	0.105	0.214	2.26E-14
BTN3A2	1.54E-18	0.286255	0.13	0.151	2.30E-14
ZNF395	1.65E-18	0.137894	0.113	0.137	2.46E-14
C9orf85	1.67E-18	-0.1092	0.06	0.158	2.50E-14
SSPN	1.73E-18	0.316426	0.154	0.107	2.58E-14
FAM195B	1.75E-18	0.440281	0.095	0.068	2.61E-14
NKX2-2	1.80E-18	-0.25041	0.114	0.253	2.69E-14
ZC4H2	1.82E-18	-0.21787	0.031	0.126	2.73E-14
ABHD5	1.84E-18	0.184337	0.117	0.149	2.76E-14
SEMA3A	1.97E-18	-0.10438	0.037	0.125	2.94E-14
RP11-318A	2.00E-18	-0.10217	0.004	0.059	2.99E-14
BRINP2	2.03E-18	-0.14023	0.031	0.121	3.04E-14
TMEM216	2.10E-18	0.310691	0.101	0.104	3.14E-14
LHX1	2.18E-18	-0.11227	0.008	0.068	3.26E-14
GS1-124K5	2.21E-18	0.162099	0.134	0.194	3.31E-14
PSMB10	2.32E-18	0.294064	0.166	0.162	3.47E-14
PARP6	2.38E-18	-0.10717	0.064	0.16	3.56E-14
AC004540.	2.43E-18	-0.18858	0.049	0.151	3.63E-14
RBM41	2.46E-18	-0.15745	0.063	0.165	3.67E-14
YIPF2	2.65E-18	0.422897	0.167	0.138	3.95E-14
RP11-437L	2.87E-18	0.361117	0.056	0	4.29E-14
3-Mar	2.87E-18	0.315838	0.135	0.11	4.29E-14
AC079922.	2.97E-18	-0.12304	0.019	0.094	4.43E-14
FBXL7	3.19E-18	-0.10266	0.018	0.091	4.77E-14
STRIP2	3.22E-18	-0.12019	0.012	0.081	4.81E-14

GNAZ	3.63E-18	-0.11494	0.037	0.125	5.42E-14
RSPH1	3.75E-18	0.318747	0.091	0.012	5.61E-14
ST8SIA5	3.75E-18	-0.13013	0.024	0.096	5.61E-14
NGRN	3.90E-18	0.118723	0.091	0.137	5.82E-14
ATRIP	3.93E-18	-0.10108	0.025	0.1	5.87E-14
ZNF394	4.29E-18	0.111246	0.125	0.174	6.41E-14
NHSL2	4.53E-18	0.513315	0.086	0.01	6.77E-14
PORCN	4.97E-18	0.113609	0.071	0.119	7.42E-14
EPB41	5.85E-18	-0.16809	0.03	0.119	8.75E-14
CAAP1	6.02E-18	-0.10721	0.077	0.179	8.99E-14
ZNF480	6.72E-18	-0.10082	0.057	0.149	1.00E-13
HOXA7	7.27E-18	-0.10715	0.091	0.199	1.09E-13
ZMYM5	7.34E-18	0.105677	0.156	0.219	1.10E-13
DNAJC25	7.85E-18	0.127486	0.109	0.133	1.17E-13
GDAP1L1	8.26E-18	-0.13736	0.05	0.14	1.23E-13
VWA5A	8.31E-18	0.347026	0.145	0.083	1.24E-13
C6orf141	8.53E-18	0.357915	0.091	0.028	1.27E-13
C6orf15	9.32E-18	-0.4037	0.008	0.072	1.39E-13
INO80B	9.46E-18	-0.14496	0.041	0.137	1.41E-13
FAM171B	9.92E-18	-0.12706	0.094	0.205	1.48E-13
ANKRD13B	9.93E-18	-0.11756	0.023	0.102	1.48E-13
ACTA2	1.02E-17	0.338541	0.115	0.088	1.52E-13
LOX	1.12E-17	0.406437	0.135	0.068	1.68E-13
RILPL2	1.21E-17	0.199651	0.109	0.133	1.81E-13
MYCN	1.21E-17	-0.11371	0.006	0.063	1.81E-13
AC011043.	1.31E-17	-0.10976	0.075	0.172	1.95E-13
PROS1	1.34E-17	0.139288	0.104	0.117	2.01E-13
THUMPD2	1.54E-17	-0.1328	0.083	0.185	2.30E-13
LSS	1.54E-17	0.104034	0.078	0.122	2.30E-13
TMCC3	1.59E-17	-0.11382	0.015	0.084	2.38E-13
CPT1A	1.66E-17	-0.10902	0.033	0.115	2.47E-13
TBK1	1.90E-17	0.117514	0.135	0.185	2.84E-13
MFNG	2.05E-17	-0.16966	0.01	0.074	3.07E-13
MANEAL	2.07E-17	-0.13049	0.021	0.101	3.09E-13
DEPDC7	2.09E-17	-0.13276	0.009	0.073	3.12E-13
APLN	2.21E-17	0.336056	0.119	0.031	3.30E-13
GPNMB	2.22E-17	0.491544	0.132	0.062	3.32E-13
ROBO3	2.44E-17	0.241638	0.129	0.125	3.65E-13
STX7	2.50E-17	0.136187	0.159	0.216	3.74E-13
LL22NC03-	2.62E-17	-0.11783	0.016	0.086	3.91E-13
TNR	2.93E-17	-0.21471	0.025	0.106	4.38E-13
FBLN5	2.96E-17	0.488872	0.098	0.03	4.43E-13
SLC44A3	3.10E-17	0.367488	0.084	0.019	4.63E-13
LTA4H	3.23E-17	0.245232	0.215	0.205	4.83E-13
TXNDC16	3.36E-17	-0.10736	0.037	0.114	5.01E-13
RGS1	3.37E-17	0.436545	0.228	0.146	5.04E-13
IL6	3.43E-17	0.401723	0.089	0.015	5.13E-13
TYROBP	3.57E-17	0.425239	0.207	0.151	5.34E-13
DNAJB4	3.63E-17	0.130176	0.145	0.196	5.43E-13
RPS6KC1	4.36E-17	-0.13515	0.044	0.135	6.51E-13
LACTB2	4.40E-17	0.259877	0.192	0.199	6.57E-13

DUOX1	4.45E-17	-0.15094	0.029	0.111	6.65E-13
GJB2	4.48E-17	0.33638	0.103	0.068	6.69E-13
RGMB	4.74E-17	-0.19913	0.096	0.22	7.08E-13
AC015936.	5.00E-17	0.346381	0.074	0.01	7.48E-13
CLDN10	5.06E-17	0.322147	0.115	0.143	7.56E-13
TVP23B	5.09E-17	0.107014	0.122	0.16	7.61E-13
CAPS	5.15E-17	0.224341	0.114	0.095	7.70E-13
SETD7	5.27E-17	0.158298	0.095	0.141	7.88E-13
OPHN1	5.30E-17	-0.13728	0.036	0.117	7.92E-13
P4HA2	5.34E-17	0.142007	0.092	0.121	7.98E-13
RAB33A	5.36E-17	-0.16653	0.043	0.136	8.01E-13
PTPRN2	5.62E-17	0.101403	0.164	0.231	8.39E-13
ANKRD50	5.90E-17	-0.10286	0.029	0.101	8.82E-13
HAP1	6.22E-17	0.293369	0.124	0.075	9.29E-13
ZNF286B	6.47E-17	-0.10279	0.017	0.086	9.66E-13
SPTSSB	6.85E-17	0.328741	0.073	0.053	1.02E-12
TRAK2	6.95E-17	-0.11448	0.049	0.133	1.04E-12
SREBF1	6.96E-17	0.120838	0.089	0.106	1.04E-12
EYA1	7.14E-17	-0.16615	0.016	0.089	1.07E-12
GABARAPL1	7.50E-17	0.235254	0.2	0.22	1.12E-12
ARHGAP1	8.26E-17	0.172925	0.149	0.19	1.23E-12
ASS1	8.42E-17	0.41894	0.102	0.021	1.26E-12
FAM134C	8.81E-17	-0.1025	0.083	0.184	1.32E-12
GLI4	8.96E-17	-0.1839	0.062	0.17	1.34E-12
UNG	9.65E-17	-0.10202	0.135	0.263	1.44E-12
NUDT10	1.02E-16	-0.12222	0.045	0.132	1.52E-12
COL9A2	1.24E-16	-0.10186	0.043	0.121	1.85E-12
SNHG12	1.24E-16	0.290182	0.154	0.149	1.85E-12
HDHD3	1.26E-16	0.341215	0.135	0.101	1.88E-12
STAT2	1.29E-16	0.122764	0.104	0.141	1.93E-12
SETBP1	1.32E-16	-0.10072	0.045	0.132	1.98E-12
SERPINA1	1.34E-16	0.38614	0.111	0.027	2.01E-12
TNFRSF10E	1.37E-16	0.239805	0.129	0.111	2.05E-12
DNAJB5	1.41E-16	-0.11106	0.063	0.154	2.10E-12
GPR75-ASE	1.45E-16	-0.13107	0.088	0.186	2.17E-12
CCDC28A	1.46E-16	0.239351	0.149	0.146	2.18E-12
TDRD3	1.48E-16	-0.11493	0.073	0.167	2.20E-12
TTTY14	1.51E-16	-0.13624	0.005	0.058	2.26E-12
CCDC142	1.55E-16	-0.10649	0.045	0.132	2.32E-12
POFUT2	1.61E-16	0.114193	0.12	0.149	2.41E-12
IER3IP1	1.69E-16	0.589835	0.086	0.011	2.53E-12
DLX2	1.94E-16	-0.24935	0.038	0.131	2.90E-12
RGCC	2.00E-16	-0.30889	0.084	0.204	2.99E-12
TEX26	2.02E-16	0.262202	0.091	0.014	3.02E-12
RCOR2	2.03E-16	-0.12969	0.016	0.083	3.03E-12
SLC25A24	2.13E-16	-0.13385	0.048	0.142	3.18E-12
SPATA20	2.32E-16	0.290961	0.129	0.096	3.47E-12
NEUROD1	2.32E-16	-0.20553	0.011	0.075	3.47E-12
LRP5	2.48E-16	-0.10731	0.029	0.107	3.71E-12
KBTBD3	2.76E-16	0.104357	0.097	0.137	4.13E-12
FHOD3	2.78E-16	-0.11845	0.026	0.102	4.16E-12

PCSK1	2.91E-16	0.403377	0.105	0.035	4.35E-12
ADCY6	2.94E-16	-0.1024	0.029	0.102	4.40E-12
CTSO	3.41E-16	0.128673	0.109	0.126	5.10E-12
PRKAR2B	3.66E-16	-0.13474	0.034	0.117	5.47E-12
ZNF615	4.36E-16	-0.1244	0.032	0.109	6.52E-12
RP11-195F	4.62E-16	0.229866	0.164	0.165	6.91E-12
RPH3A	4.88E-16	-0.1401	0.008	0.065	7.29E-12
SEC24D	5.13E-16	0.161497	0.1	0.105	7.66E-12
SYBU	5.24E-16	0.106785	0.091	0.126	7.83E-12
PODXL	5.40E-16	0.103978	0.086	0.125	8.07E-12
ABCA8	5.64E-16	0.317271	0.097	0.063	8.44E-12
SVIP	5.99E-16	-0.21509	0.131	0.26	8.95E-12
BACE2	6.09E-16	0.266005	0.141	0.072	9.10E-12
ANGPT2	6.11E-16	0.294852	0.079	0.057	9.13E-12
HRCT1	6.82E-16	0.123778	0.052	0.078	1.02E-11
SERP2	7.00E-16	-0.1033	0.07	0.16	1.05E-11
FGF1	7.20E-16	0.262531	0.099	0.075	1.08E-11
RNF149	7.41E-16	0.211807	0.17	0.14	1.11E-11
PCDHB10	7.57E-16	-0.18963	0.073	0.184	1.13E-11
SORL1	8.86E-16	0.123022	0.083	0.111	1.32E-11
GLI3	8.97E-16	-0.1022	0.03	0.104	1.34E-11
TMEM170B	9.04E-16	-0.10428	0.024	0.096	1.35E-11
ZRANB3	9.08E-16	-0.10036	0.015	0.08	1.36E-11
RP11-146F	9.21E-16	-0.11591	0.024	0.098	1.38E-11
PLLP	9.83E-16	-0.18678	0.017	0.086	1.47E-11
CYB561	1.04E-15	0.156491	0.104	0.127	1.55E-11
ZHX2	1.05E-15	-0.14451	0.03	0.111	1.57E-11
FAM127C	1.11E-15	0.201956	0.115	0.149	1.66E-11
SLC6A6	1.16E-15	0.297897	0.08	0.019	1.74E-11
SLC16A1-A	1.22E-15	-0.10194	0.041	0.112	1.83E-11
LINC01094	1.30E-15	0.428428	0.084	0.019	1.95E-11
DLX6	1.41E-15	-0.19303	0.017	0.086	2.10E-11
S1PR3	1.47E-15	0.119236	0.069	0.091	2.20E-11
C1orf54	1.51E-15	0.181371	0.099	0.128	2.25E-11
C1orf198	1.51E-15	0.10887	0.118	0.153	2.26E-11
GNG10	1.55E-15	0.18708	0.077	0.074	2.32E-11
FICD	1.85E-15	0.140267	0.116	0.137	2.77E-11
TMEM2	2.03E-15	-0.11535	0.03	0.105	3.04E-11
BBC3	2.16E-15	0.162817	0.102	0.098	3.22E-11
RP4-569M2	2.27E-15	0.100906	0.028	0.063	3.39E-11
MYL9	2.42E-15	0.366396	0.089	0.027	3.62E-11
CAMKK1	2.44E-15	-0.13862	0.012	0.074	3.64E-11
SLC1A5	2.49E-15	0.103573	0.099	0.117	3.72E-11
ANKS1B	2.50E-15	-0.13547	0.059	0.149	3.74E-11
AMER2	2.62E-15	-0.15379	0.05	0.138	3.92E-11
TUBA4A	2.79E-15	0.329278	0.117	0.041	4.17E-11
TRIM56	2.95E-15	0.308101	0.13	0.088	4.41E-11
RRAGB	3.08E-15	0.114814	0.1	0.146	4.60E-11
SFRP4	3.08E-15	0.381121	0.107	0.069	4.61E-11
POLR1C	3.46E-15	0.191065	0.149	0.177	5.17E-11
BLZF1	3.46E-15	0.123259	0.147	0.179	5.17E-11

TTC8	3.53E-15	0.100598	0.096	0.151	5.28E-11
VPS54	3.54E-15	-0.11016	0.038	0.116	5.29E-11
GAS6	3.66E-15	0.165559	0.146	0.136	5.47E-11
BTBD17	3.78E-15	-0.16532	0.016	0.083	5.65E-11
ANKRD28	3.80E-15	0.256522	0.146	0.125	5.68E-11
TSPYL2	4.21E-15	0.178363	0.146	0.154	6.29E-11
LRRC2	4.53E-15	0.270485	0.069	0.007	6.77E-11
ABCC5	5.01E-15	0.129361	0.081	0.128	7.49E-11
SPTLC1	5.14E-15	0.111949	0.087	0.128	7.67E-11
DENND1B	5.23E-15	-0.13105	0.053	0.143	7.82E-11
PDGFA	5.42E-15	-0.19544	0.074	0.175	8.10E-11
ZNF212	5.58E-15	-0.11466	0.064	0.154	8.34E-11
RALB	5.84E-15	0.116847	0.115	0.175	8.73E-11
NMRK1	5.95E-15	0.317943	0.138	0.086	8.89E-11
SLC22A18	6.39E-15	0.224239	0.086	0.078	9.55E-11
BCL11B	8.58E-15	-0.10199	0.008	0.057	1.28E-10
LNX1	8.65E-15	-0.20882	0.051	0.146	1.29E-10
ZNF385A	8.68E-15	0.182153	0.088	0.094	1.30E-10
PROCR	9.25E-15	0.284616	0.072	0.04	1.38E-10
TMEM117	1.07E-14	-0.10266	0.022	0.089	1.60E-10
ANG	1.14E-14	0.360106	0.124	0.057	1.70E-10
KIAA0895L	1.16E-14	-0.12405	0.057	0.144	1.73E-10
GCNT2	1.22E-14	-0.11441	0.015	0.078	1.82E-10
HK2	1.23E-14	0.14913	0.079	0.077	1.83E-10
SPEG	1.26E-14	0.130603	0.08	0.1	1.88E-10
ENPP5	1.36E-14	0.305261	0.071	0.007	2.04E-10
FAM107A	1.39E-14	0.361654	0.114	0.042	2.08E-10
QSOX2	1.45E-14	-0.10084	0.037	0.106	2.16E-10
IFITM2	1.47E-14	0.310681	0.147	0.065	2.19E-10
DNAJC27	1.48E-14	-0.13841	0.031	0.102	2.21E-10
MXRA8	1.51E-14	0.267172	0.086	0.027	2.26E-10
TFRC	1.59E-14	0.146386	0.147	0.165	2.38E-10
SCN3A	1.66E-14	-0.1751	0.039	0.125	2.48E-10
PAX3	1.66E-14	-0.10381	0.006	0.054	2.48E-10
SH3BP2	1.85E-14	0.212798	0.115	0.106	2.77E-10
GM2A	1.99E-14	0.143751	0.08	0.091	2.98E-10
SPSB1	2.20E-14	0.248371	0.118	0.089	3.29E-10
THBS1	2.26E-14	0.289512	0.086	0.017	3.37E-10
TMEM192	2.36E-14	0.208253	0.168	0.183	3.52E-10
VASN	2.54E-14	0.224433	0.076	0.014	3.79E-10
RAMP3	2.64E-14	0.301383	0.072	0.009	3.94E-10
ITPKB	2.72E-14	0.153549	0.073	0.072	4.07E-10
ZFP37	2.74E-14	-0.10957	0.04	0.11	4.10E-10
CAMKMT	2.76E-14	-0.11848	0.057	0.146	4.12E-10
TCN2	3.19E-14	0.19876	0.071	0.074	4.77E-10
CASC15	3.19E-14	-0.13565	0.062	0.152	4.77E-10
ATP5L2	3.27E-14	0.208966	0.085	0.106	4.88E-10
SAA4	3.41E-14	0.397894	0.05	0.001	5.10E-10
KLRG1	3.71E-14	-0.10245	0.017	0.077	5.54E-10
OXSM	3.87E-14	0.180754	0.094	0.101	5.79E-10
WDR55	4.04E-14	0.116911	0.103	0.133	6.03E-10

SLC39A4	4.45E-14	0.263597	0.074	0.033	6.65E-10
PTPRN	4.61E-14	0.307691	0.136	0.059	6.89E-10
SARNP	4.63E-14	0.184015	0.125	0.164	6.92E-10
HOXC9	4.80E-14	-0.20567	0.056	0.151	7.17E-10
PDK1	4.81E-14	0.112246	0.101	0.123	7.20E-10
LAPTM5	5.10E-14	0.410232	0.197	0.096	7.63E-10
MCEE	5.56E-14	0.100137	0.115	0.149	8.30E-10
PCDHB7	5.67E-14	-0.1805	0.064	0.163	8.48E-10
EPS8	6.07E-14	-0.15822	0.114	0.231	9.07E-10
AC005076.	6.35E-14	-0.13846	0.032	0.105	9.49E-10
AMPH	6.51E-14	-0.12656	0.015	0.077	9.73E-10
FILIP1L	6.55E-14	0.109468	0.088	0.104	9.79E-10
HHIP-AS1	6.57E-14	-0.10608	0.009	0.062	9.82E-10
TRIM59	7.31E-14	-0.12457	0.011	0.067	1.09E-09
AMPD2	7.87E-14	0.117167	0.107	0.13	1.18E-09
IRAK4	8.05E-14	0.114085	0.091	0.116	1.20E-09
MYT1	8.11E-14	-0.10347	0.007	0.056	1.21E-09
SP110	9.21E-14	0.11313	0.135	0.165	1.38E-09
PGAM2	9.92E-14	0.43541	0.098	0.037	1.48E-09
DDR2	1.01E-13	0.199924	0.103	0.099	1.50E-09
CCL4	1.12E-13	-0.15217	0.118	0.228	1.68E-09
LINC01023	1.15E-13	0.111679	0.056	0.083	1.71E-09
PREP	1.15E-13	-0.10329	0.061	0.143	1.72E-09
SMOC1	1.15E-13	-0.18211	0.015	0.077	1.72E-09
RCAN2	1.35E-13	0.260957	0.084	0.015	2.01E-09
LUCAT1	1.40E-13	0.207593	0.059	0.016	2.09E-09
TMEM38A	1.43E-13	0.185392	0.1	0.074	2.13E-09
RP11-118F	1.44E-13	-0.10865	0.004	0.043	2.15E-09
NDUFB8	1.62E-13	0.552566	0.079	0.012	2.43E-09
TCP11L1	1.66E-13	-0.13147	0.027	0.098	2.48E-09
TNFAIP3	1.69E-13	0.329042	0.112	0.049	2.53E-09
CLP1	1.71E-13	0.102477	0.082	0.114	2.56E-09
CLEC2B	1.85E-13	0.428131	0.108	0.088	2.76E-09
ASIC1	2.00E-13	-0.10357	0.013	0.067	2.99E-09
RRM2B	2.03E-13	0.148534	0.103	0.109	3.04E-09
PDZK1IP1	2.06E-13	0.228956	0.053	0.002	3.08E-09
LINC-PINT	2.37E-13	0.310668	0.136	0.073	3.54E-09
MXI1	2.40E-13	0.235097	0.15	0.119	3.59E-09
AC002456.	3.21E-13	0.178937	0.063	0.089	4.80E-09
DHRS12	3.25E-13	0.17013	0.085	0.102	4.86E-09
RINT1	3.52E-13	0.118692	0.147	0.168	5.25E-09
GPRC5A	3.66E-13	0.201831	0.048	0.002	5.47E-09
HAS2	3.93E-13	0.257261	0.092	0.086	5.88E-09
DLX6-AS1	4.01E-13	-0.21924	0.036	0.114	5.99E-09
KCNIP1	4.13E-13	-0.15698	0.059	0.144	6.17E-09
TUBB4A	4.13E-13	-0.16644	0.03	0.101	6.18E-09
RP11-479J	4.60E-13	0.252772	0.048	0.014	6.88E-09
NCOA7	4.83E-13	0.144532	0.114	0.132	7.22E-09
PCGF1	5.26E-13	0.135837	0.125	0.149	7.85E-09
RIOK2	5.38E-13	0.113003	0.14	0.185	8.04E-09
IDNK	5.98E-13	0.289507	0.123	0.101	8.94E-09

SLC30A3	6.05E-13	-0.11537	0.01	0.062	9.04E-09
SQRDL	6.16E-13	0.304577	0.101	0.052	9.20E-09
HSPB2	6.26E-13	0.259554	0.061	0.007	9.35E-09
MAFB	6.64E-13	0.319187	0.203	0.159	9.92E-09
SYT6	6.74E-13	-0.12582	0.012	0.067	1.01E-08
MYBPH	6.74E-13	0.217905	0.062	0.01	1.01E-08
CTSS	6.76E-13	0.396393	0.136	0.083	1.01E-08
MUC12	6.98E-13	0.335717	0.095	0.051	1.04E-08
LIN37	7.15E-13	0.109492	0.05	0.067	1.07E-08
C11orf70	7.30E-13	0.256282	0.073	0.011	1.09E-08
FXYD5	8.47E-13	0.186181	0.118	0.132	1.27E-08
SLC12A5	8.60E-13	-0.1646	0.022	0.088	1.28E-08
SPRYD4	8.75E-13	0.10404	0.059	0.098	1.31E-08
MIR497HG	9.13E-13	0.162262	0.115	0.149	1.37E-08
CASP1	9.43E-13	0.365889	0.164	0.1	1.41E-08
COL8A1	9.62E-13	0.330701	0.08	0.015	1.44E-08
NABP1	9.65E-13	0.36466	0.106	0.036	1.44E-08
COL20A1	9.72E-13	-0.1129	0.006	0.051	1.45E-08
CPM	9.72E-13	-0.27148	0.059	0.144	1.45E-08
SFXN3	1.06E-12	0.19495	0.077	0.065	1.59E-08
P2RX7	1.14E-12	-0.1165	0.04	0.116	1.71E-08
HDAC4	1.14E-12	-0.11084	0.027	0.091	1.71E-08
ZFPM2	1.19E-12	-0.17904	0.015	0.073	1.78E-08
TOM1	1.20E-12	0.100423	0.094	0.125	1.79E-08
COL9A1	1.24E-12	-0.10593	0.006	0.051	1.85E-08
ATP6V0E2-	1.31E-12	-0.10318	0.018	0.074	1.96E-08
KCND2	1.33E-12	-0.13565	0.031	0.1	1.99E-08
TLE3	1.37E-12	-0.10387	0.083	0.165	2.05E-08
LIX1	1.39E-12	0.31703	0.068	0.012	2.08E-08
TTC21B	1.41E-12	-0.13099	0.039	0.114	2.11E-08
SP140L	1.52E-12	0.28774	0.089	0.062	2.27E-08
TAPBPL	1.53E-12	0.34116	0.143	0.09	2.28E-08
CACNA2D1	1.58E-12	-0.15581	0.021	0.079	2.36E-08
SPRYD3	1.69E-12	0.15746	0.097	0.1	2.53E-08
LY6H	1.93E-12	0.278523	0.106	0.038	2.89E-08
FAM19A5	2.16E-12	-0.12548	0.036	0.109	3.22E-08
CLYBL	2.55E-12	0.171617	0.075	0.078	3.82E-08
SLC47A2	2.59E-12	0.237358	0.063	0.042	3.88E-08
SLC25A18	2.80E-12	0.291421	0.107	0.101	4.18E-08
RP11-783K	2.90E-12	0.313778	0.075	0.059	4.33E-08
PACRG	3.40E-12	0.335553	0.088	0.032	5.08E-08
RPRML	3.54E-12	-0.11694	0.011	0.062	5.29E-08
STYXL1	3.55E-12	0.180025	0.143	0.163	5.31E-08
XKR4	3.63E-12	-0.11437	0.02	0.079	5.42E-08
MIR4458HG	3.80E-12	0.398526	0.258	0.185	5.68E-08
C1orf53	3.88E-12	0.22405	0.099	0.112	5.79E-08
ORAI3	3.94E-12	0.201924	0.113	0.089	5.88E-08
DMKN	4.07E-12	0.271715	0.043	0.001	6.08E-08
ZNF211	4.10E-12	0.101387	0.081	0.107	6.13E-08
GBP3	4.19E-12	0.31166	0.145	0.064	6.27E-08
USF1	4.21E-12	-0.13572	0.025	0.089	6.29E-08

IL33	4.58E-12	0.281476	0.104	0.058	6.85E-08
IL1R1	4.62E-12	0.207116	0.052	0.014	6.90E-08
SLC39A8	4.66E-12	0.227114	0.119	0.042	6.96E-08
CCER2	5.39E-12	-0.15998	0.018	0.078	8.05E-08
CRB2	5.42E-12	0.39731	0.079	0.035	8.10E-08
FPR1	5.45E-12	0.279819	0.066	0.014	8.14E-08
ANKRD6	5.55E-12	-0.11957	0.042	0.115	8.29E-08
FRMD3	5.55E-12	0.272746	0.118	0.059	8.30E-08
CD83	7.27E-12	0.189378	0.12	0.151	1.09E-07
TGM2	7.29E-12	0.217709	0.071	0.035	1.09E-07
TGDS	7.61E-12	0.141012	0.111	0.123	1.14E-07
MARCO	8.90E-12	0.310602	0.059	0.007	1.33E-07
MAOA	9.17E-12	0.28362	0.073	0.016	1.37E-07
THBS3	9.85E-12	0.113147	0.059	0.059	1.47E-07
ARHGAP29	1.04E-11	0.218599	0.095	0.044	1.55E-07
RP3-460G2	1.12E-11	0.465822	0.098	0.052	1.68E-07
TFB2M	1.14E-11	0.153682	0.111	0.136	1.71E-07
FCGBP	1.16E-11	0.302666	0.113	0.051	1.74E-07
KLHL24	1.39E-11	0.165427	0.14	0.146	2.07E-07
ZRSR2	1.47E-11	0.145872	0.127	0.13	2.20E-07
SLC16A4	1.47E-11	0.118545	0.091	0.104	2.20E-07
APOBEC3C	1.54E-11	0.153863	0.074	0.104	2.30E-07
CTGF	1.55E-11	0.312013	0.078	0.028	2.32E-07
ACTL6B	1.56E-11	-0.11712	0.022	0.078	2.33E-07
FAM13A	1.59E-11	0.197718	0.118	0.11	2.37E-07
GDPD2	1.61E-11	0.219728	0.075	0.042	2.41E-07
NXN	1.69E-11	-0.11288	0.011	0.06	2.52E-07
CCDC170	1.78E-11	0.192906	0.092	0.026	2.66E-07
HVCN1	1.78E-11	0.132124	0.056	0.064	2.66E-07
HNMT	1.90E-11	0.415721	0.163	0.115	2.84E-07
ADCY2	2.00E-11	0.18428	0.064	0.035	2.99E-07
DYNLRB2	2.04E-11	0.198399	0.046	0.002	3.04E-07
DMXL2	2.29E-11	-0.10055	0.036	0.1	3.42E-07
HBEGF	2.44E-11	0.128469	0.094	0.094	3.64E-07
CLGN	2.74E-11	-0.17526	0.044	0.121	4.09E-07
ARHGEF10	2.84E-11	0.123636	0.089	0.11	4.25E-07
TSHZ2	2.94E-11	0.316289	0.129	0.058	4.39E-07
PLSCR4	2.95E-11	0.190094	0.085	0.072	4.41E-07
RASL10A	3.00E-11	-0.11787	0.011	0.06	4.49E-07
RP11-436K	3.12E-11	-0.12391	0.017	0.07	4.67E-07
SERPINB8	3.13E-11	0.111643	0.084	0.095	4.68E-07
ASTE1	3.39E-11	0.100027	0.062	0.096	5.06E-07
FANCF	3.56E-11	0.110993	0.093	0.109	5.32E-07
KLHDC9	3.65E-11	0.140246	0.087	0.09	5.46E-07
LRRC24	3.69E-11	-0.13423	0.043	0.117	5.51E-07
CCDC88B	3.69E-11	0.294447	0.09	0.051	5.52E-07
AQP1	3.74E-11	0.186858	0.055	0.064	5.59E-07
PCMTD1	3.82E-11	0.128425	0.13	0.144	5.71E-07
C16orf89	3.98E-11	0.246618	0.046	0.004	5.94E-07
NR1D1	4.09E-11	0.163623	0.082	0.073	6.11E-07
SMIM10	4.34E-11	0.132799	0.077	0.099	6.49E-07

FGGY	4.82E-11	0.144362	0.098	0.116	7.20E-07
MICU3	4.89E-11	-0.10573	0.042	0.11	7.31E-07
EEF1G	4.90E-11	0.152814	0.079	0.111	7.32E-07
EDARADD	5.45E-11	0.206209	0.057	0.025	8.14E-07
LINC00844	5.63E-11	-0.10047	0.105	0.19	8.42E-07
PLEKHF1	6.48E-11	0.119822	0.062	0.051	9.68E-07
RENBP	6.72E-11	0.222232	0.071	0.022	1.00E-06
FERMT1	7.26E-11	-0.18062	0.033	0.101	1.08E-06
RP1-17K7.	7.33E-11	0.453927	0.102	0.04	1.10E-06
PRTN3	7.41E-11	0.13382	0.032	0	1.11E-06
HGF	9.46E-11	0.158276	0.043	0.002	1.41E-06
FCER1G	9.82E-11	0.224385	0.172	0.175	1.47E-06
CCDC181	1.03E-10	0.152142	0.148	0.16	1.54E-06
HIST2H2BE	1.04E-10	0.245507	0.11	0.094	1.55E-06
ANKRD42	1.09E-10	0.113195	0.077	0.079	1.63E-06
ECM2	1.18E-10	0.238586	0.062	0.01	1.76E-06
ARHGAP5-A	1.22E-10	0.131298	0.042	0.049	1.82E-06
MAP3K7CL	1.22E-10	0.21461	0.095	0.075	1.82E-06
HLA-DMB	1.31E-10	0.268068	0.1	0.042	1.96E-06
TRIM22	1.41E-10	0.114532	0.085	0.095	2.11E-06
HSD11B1	1.60E-10	0.297273	0.054	0.015	2.39E-06
MTURN	1.69E-10	-0.12358	0.055	0.128	2.53E-06
PINLYP	1.82E-10	0.145589	0.083	0.104	2.72E-06
CTSK	1.87E-10	0.265692	0.111	0.078	2.80E-06
RNF144B	2.06E-10	0.181558	0.042	0.002	3.08E-06
RGN	2.07E-10	0.250746	0.1	0.064	3.09E-06
TGFB2	2.07E-10	0.197009	0.117	0.075	3.10E-06
FAM149A	2.17E-10	-0.11936	0.007	0.047	3.24E-06
ALOX5AP	2.17E-10	0.33355	0.154	0.084	3.24E-06
LINC00869	2.40E-10	0.319297	0.042	0.002	3.58E-06
MMP19	2.42E-10	0.217963	0.062	0.014	3.61E-06
C14orf132	2.76E-10	-0.10999	0.072	0.149	4.13E-06
ATP6AP1L	2.98E-10	0.134956	0.068	0.073	4.45E-06
SERPINA3	3.24E-10	0.245386	0.03	0	4.84E-06
ATP10B	3.50E-10	-0.11683	0.014	0.063	5.23E-06
CSRNP1	3.60E-10	0.163871	0.133	0.121	5.39E-06
STAP2	3.88E-10	0.132565	0.048	0.051	5.79E-06
GGT5	3.95E-10	0.146426	0.056	0.012	5.90E-06
CTH	4.13E-10	0.223832	0.094	0.06	6.16E-06
CXorf40A	4.15E-10	0.136254	0.085	0.095	6.20E-06
STX3	4.20E-10	0.17734	0.068	0.03	6.28E-06
HSPB6	4.29E-10	0.156124	0.122	0.109	6.40E-06
MYOT	4.32E-10	-0.11581	0.006	0.044	6.45E-06
CMTM5	4.41E-10	-0.15223	0.074	0.156	6.59E-06
FGFBP2	5.08E-10	0.173133	0.036	0.001	7.59E-06
CXCL12	5.14E-10	0.227628	0.057	0.011	7.68E-06
OLFML3	5.96E-10	0.149102	0.068	0.063	8.90E-06
CHIT1	6.10E-10	0.357541	0.039	0.002	9.12E-06
TNFRSF14	6.21E-10	0.223922	0.082	0.046	9.27E-06
CYBA	6.77E-10	0.199626	0.165	0.135	1.01E-05
TNFAIP2	6.77E-10	0.217415	0.06	0.021	1.01E-05

ALDH1L1	7.19E-10	0.317204	0.143	0.062	1.07E-05
PTGES	7.79E-10	0.243424	0.064	0.021	1.16E-05
PPP1R3C	8.08E-10	0.231896	0.072	0.025	1.21E-05
TVP23A	8.36E-10	0.24142	0.074	0.021	1.25E-05
PRSS35	8.85E-10	0.151628	0.059	0.01	1.32E-05
PPP1R9A	8.87E-10	-0.16964	0.046	0.116	1.33E-05
NCAN	9.56E-10	-0.13694	0.027	0.084	1.43E-05
ARG2	9.61E-10	0.116849	0.067	0.083	1.44E-05
COL5A1	9.62E-10	0.155798	0.053	0.009	1.44E-05
VSIG4	9.63E-10	0.347595	0.121	0.057	1.44E-05
CREBRF	9.77E-10	0.188644	0.12	0.104	1.46E-05
MAPK8IP2	1.07E-09	-0.10381	0.018	0.068	1.59E-05
NTM	1.09E-09	-0.22582	0.106	0.2	1.62E-05
SLFN5	1.09E-09	0.202116	0.069	0.038	1.63E-05
GSTM5	1.13E-09	0.199255	0.036	0.001	1.69E-05
HOPX	1.14E-09	-0.17781	0.347	0.465	1.70E-05
ARHGAP24	1.25E-09	0.259039	0.087	0.027	1.87E-05
NR4A1	1.34E-09	0.161051	0.154	0.151	2.00E-05
SYT1	1.40E-09	-0.1669	0.053	0.126	2.09E-05
IL18BP	1.49E-09	0.131697	0.037	0.026	2.22E-05
LDOC1	1.54E-09	0.36319	0.147	0.07	2.30E-05
COLEC12	1.72E-09	0.185494	0.06	0.012	2.58E-05
PPIL6	1.79E-09	0.200213	0.098	0.06	2.67E-05
HBB	1.81E-09	0.229945	0.417	0.332	2.70E-05
LRRK2	1.89E-09	-0.12227	0.027	0.083	2.82E-05
RABL2A	1.91E-09	0.137943	0.084	0.101	2.85E-05
HLA-DOA	1.91E-09	0.171352	0.044	0.005	2.85E-05
INCA1	2.03E-09	0.100533	0.031	0.041	3.04E-05
G0S2	2.09E-09	0.405096	0.124	0.138	3.12E-05
IL6R	2.19E-09	0.134289	0.051	0.009	3.28E-05
ZNF350	2.26E-09	0.115805	0.101	0.104	3.38E-05
MAST4	2.29E-09	0.16811	0.084	0.079	3.42E-05
SELENBP1	2.31E-09	0.219921	0.053	0.01	3.46E-05
LCAT	2.32E-09	0.127462	0.041	0.052	3.46E-05
ZNF419	2.48E-09	0.151663	0.087	0.083	3.71E-05
NIM1K	2.51E-09	0.228577	0.122	0.089	3.75E-05
PDCD4	2.84E-09	0.135453	0.1	0.106	4.24E-05
APOBEC3G	2.86E-09	0.165493	0.071	0.07	4.28E-05
RP11-326C	3.01E-09	0.183301	0.038	0.002	4.50E-05
TF	3.06E-09	0.249225	0.098	0.053	4.57E-05
SLITRK2	3.11E-09	-0.114	0.042	0.105	4.65E-05
C5AR1	3.24E-09	0.197038	0.077	0.031	4.84E-05
LOXL2	3.29E-09	0.191819	0.076	0.052	4.91E-05
CCL3	3.30E-09	0.175566	0.219	0.247	4.93E-05
H1F0	3.33E-09	0.126587	0.265	0.291	4.98E-05
RNASE4	3.34E-09	0.239868	0.058	0.014	5.00E-05
PEX12	3.53E-09	0.124704	0.073	0.083	5.27E-05
RCN3	3.69E-09	0.152677	0.086	0.062	5.51E-05
CYB5R2	3.71E-09	0.177393	0.037	0.035	5.54E-05
SAMD14	4.21E-09	-0.10478	0.045	0.11	6.29E-05
PLK2	4.40E-09	0.13632	0.127	0.147	6.57E-05

CRABP1	4.45E-09	-0.20525	0.002	0.03	6.65E-05
HHIP	5.14E-09	-0.10552	0.007	0.042	7.68E-05
SLC15A4	5.18E-09	0.178257	0.078	0.056	7.74E-05
MS4A7	5.30E-09	0.193251	0.075	0.063	7.92E-05
TTC26	5.42E-09	0.103357	0.079	0.106	8.09E-05
HLA-G	5.50E-09	0.173794	0.048	0.02	8.21E-05
OSR1	5.64E-09	0.397057	0.051	0.009	8.43E-05
RP11-160C	5.79E-09	0.105828	0.032	0.043	8.65E-05
EEPD1	5.81E-09	0.178198	0.085	0.052	8.69E-05
OSGIN1	5.86E-09	0.166449	0.057	0.023	8.76E-05
ONECUT2	5.89E-09	-0.11669	0.022	0.074	8.80E-05
KLF4	6.00E-09	0.186078	0.115	0.091	8.97E-05
MYOF	6.61E-09	0.160887	0.05	0.011	9.88E-05
THSD4	6.86E-09	-0.16039	0.008	0.046	0.000102
C4orf47	7.01E-09	0.222017	0.086	0.047	0.000105
CKMT2	7.21E-09	0.137797	0.032	0.001	0.000108
KCTD11	7.53E-09	0.113786	0.069	0.063	0.000113
RCCD1	8.34E-09	-0.11832	0.039	0.098	0.000125
ATP2B4	8.96E-09	0.139665	0.097	0.086	0.000134
FSTL3	9.12E-09	0.191826	0.08	0.043	0.000136
TNFAIP8L3	1.01E-08	0.129252	0.032	0.001	0.000151
RP11-536C	1.01E-08	-0.11113	0.004	0.033	0.000151
ST8SIA4	1.11E-08	0.141648	0.055	0.053	0.000166
ITGB4	1.12E-08	0.196287	0.064	0.044	0.000168
GOLPH3L	1.13E-08	0.121639	0.11	0.123	0.000168
ASIP	1.33E-08	0.141431	0.043	0.017	0.000199
MICA	1.39E-08	0.130325	0.064	0.043	0.000208
PIR	1.45E-08	0.137468	0.076	0.084	0.000217
SDCBP2	1.45E-08	0.174395	0.082	0.054	0.000217
ZC3H12A	1.69E-08	0.204276	0.086	0.035	0.000253
TRIM38	1.83E-08	0.11804	0.042	0.049	0.000274
NOS2	1.85E-08	-0.11658	0.009	0.046	0.000276
RAB6B	1.87E-08	0.113076	0.085	0.081	0.000279
ALDH3B1	1.92E-08	0.223661	0.063	0.023	0.000287
ZNF550	2.13E-08	0.135797	0.079	0.077	0.000318
ZNF582-AS	2.15E-08	0.297093	0.135	0.086	0.000322
WDR19	2.23E-08	0.121038	0.066	0.068	0.000334
DUSP22	2.63E-08	0.123789	0.085	0.091	0.000394
PTPRU	2.64E-08	0.165261	0.04	0.019	0.000394
NKD2	2.81E-08	0.100906	0.042	0.005	0.000421
HPS1	2.94E-08	0.198958	0.062	0.019	0.00044
HSF4	3.10E-08	0.130791	0.053	0.02	0.000464
PNPLA4	3.37E-08	0.283631	0.078	0.028	0.000503
CLIC2	3.54E-08	0.21169	0.048	0.012	0.000529
RHPN2	3.57E-08	0.176171	0.069	0.06	0.000533
TNFSF13B	3.66E-08	0.254104	0.115	0.062	0.000547
TREM2	3.76E-08	0.259438	0.092	0.051	0.000562
PSORS1C1	4.14E-08	0.176938	0.054	0.046	0.000619
RP11-395E	4.34E-08	0.210426	0.052	0.027	0.000649
ACBD4	4.60E-08	0.122892	0.049	0.052	0.000687
GFPT2	4.62E-08	0.230191	0.11	0.083	0.00069

NOV	4.82E-08	0.126314	0.043	0.006	0.000721
NKX2-5	5.15E-08	0.169607	0.085	0.064	0.00077
RORA	5.16E-08	0.114071	0.099	0.088	0.000772
PHYHD1	5.23E-08	0.189422	0.053	0.011	0.000782
CCDC3	5.23E-08	0.119956	0.042	0.011	0.000782
APLNR	6.15E-08	0.202322	0.042	0.006	0.000918
C9orf3	6.60E-08	0.121709	0.138	0.158	0.000986
LSR	6.66E-08	0.102582	0.048	0.051	0.000995
GLIPR1L2	6.84E-08	0.195202	0.069	0.036	0.001021
SERPINF1	6.93E-08	0.10186	0.048	0.07	0.001036
CTD-2270L	6.98E-08	0.172025	0.057	0.06	0.001043
ADAMTS9-A	7.79E-08	0.141496	0.042	0.017	0.001164
NEBL	7.87E-08	0.117304	0.052	0.011	0.001177
BEX5	7.99E-08	0.309223	0.11	0.051	0.001194
EFHD1	8.14E-08	-0.10909	0.055	0.114	0.001217
RSPH3	8.51E-08	0.109701	0.078	0.09	0.001272
ERICH2	9.50E-08	0.135742	0.063	0.051	0.00142
PLXNA3	9.59E-08	0.109618	0.052	0.065	0.001433
EPHX2	9.85E-08	0.249273	0.072	0.028	0.001473
MFAP4	9.95E-08	0.101912	0.1	0.146	0.001486
PLA2G4C	1.12E-07	0.145619	0.067	0.028	0.00168
SCN9A	1.17E-07	0.247941	0.089	0.036	0.001748
LRRN4CL	1.22E-07	0.126717	0.031	0.002	0.001825
KCNN3	1.27E-07	0.172076	0.06	0.023	0.001898
RNASE1	1.27E-07	0.299494	0.082	0.043	0.001901
ZNF557	1.30E-07	0.112759	0.041	0.067	0.001936
AC114803.	1.50E-07	0.163463	0.037	0.006	0.002239
KIAA0040	1.52E-07	0.196461	0.088	0.035	0.002279
GREM1	1.59E-07	0.335835	0.037	0.005	0.00237
ZNF503	1.60E-07	-0.1339	0.085	0.157	0.002398
THEMIS2	1.65E-07	0.107183	0.049	0.057	0.00247
CAMP	1.69E-07	0.220907	0.021	0	0.00253
MX1	1.75E-07	0.213406	0.097	0.053	0.002618
HSBP1L1	1.79E-07	0.145189	0.08	0.035	0.002672
CD163	1.84E-07	0.239797	0.081	0.036	0.002752
MAP1LC3B2	2.00E-07	0.118875	0.072	0.073	0.002984
PCDH11X	2.00E-07	-0.14163	0.029	0.075	0.002995
HMGN4	2.11E-07	0.115693	0.044	0.037	0.00316
S100A4	2.35E-07	0.319464	0.1	0.051	0.003506
RNPC3	2.49E-07	0.107611	0.104	0.101	0.003728
SLC7A5	2.51E-07	0.116622	0.096	0.095	0.003747
GNAI1	2.54E-07	-0.16368	0.086	0.159	0.003803
GPR183	2.61E-07	0.229336	0.099	0.044	0.003896
STAB1	2.87E-07	0.125961	0.034	0.016	0.004288
HLA-F	2.94E-07	0.193705	0.088	0.047	0.004396
IL1B	3.01E-07	0.207629	0.107	0.073	0.004494
C11orf71	3.03E-07	0.26879	0.041	0.02	0.00453
CD163L1	3.14E-07	0.141541	0.068	0.022	0.0047
LRRC61	3.42E-07	0.204241	0.098	0.043	0.005109
HSD17B14	3.42E-07	0.202277	0.089	0.037	0.005109
NME5	3.49E-07	0.131474	0.07	0.072	0.005211

KCTD16	3.84E-07	-0.10241	0.019	0.06	0.005745
CDK20	3.88E-07	0.143617	0.089	0.068	0.005797
SBF2-AS1	3.91E-07	0.102544	0.045	0.041	0.005848
PRKAG2	4.19E-07	0.127815	0.079	0.089	0.006255
HRH2	4.35E-07	0.102397	0.037	0.005	0.006496
WNT5A	4.59E-07	-0.12391	0.027	0.074	0.006856
LINC01088	5.22E-07	0.183239	0.031	0.005	0.007801
ZMYND12	5.58E-07	0.150715	0.027	0.001	0.008345
CYP19A1	5.92E-07	0.176308	0.03	0.005	0.008852
EVA1A	6.14E-07	0.137031	0.055	0.021	0.009181
RMDN2	6.54E-07	0.144837	0.046	0.048	0.009766
SLC25A20	6.82E-07	0.138426	0.065	0.084	0.010193
FAM180A	6.86E-07	0.122968	0.03	0.002	0.010253
RBKS	6.88E-07	0.133821	0.045	0.022	0.010284
RP11-806H	7.81E-07	0.152155	0.051	0.032	0.011668
BCO2	7.95E-07	0.147602	0.05	0.022	0.011887
ECE2	8.53E-07	0.134359	0.063	0.056	0.012753
LSP1	8.62E-07	0.156675	0.053	0.021	0.012888
FGF12	8.77E-07	-0.16013	0.042	0.093	0.013108
CTSZ	8.83E-07	0.114832	0.085	0.072	0.013202
FGF7	9.12E-07	0.233643	0.047	0.016	0.013635
S100A9	9.37E-07	0.22401	0.074	0.067	0.014004
EMX2	9.80E-07	0.121785	0.053	0.048	0.014641
MR1	1.06E-06	0.156371	0.071	0.052	0.015823
C12orf54	1.07E-06	0.116247	0.018	0	0.015971
TNFSF10	1.16E-06	0.274634	0.05	0.017	0.017384
LINC01152	1.18E-06	0.189048	0.042	0.035	0.017637
CMYA5	1.20E-06	0.150103	0.072	0.037	0.017893
OSR2	1.31E-06	0.19285	0.043	0.028	0.019554
IGFBP7-AS	1.32E-06	0.21198	0.076	0.051	0.019668
LINC00882	1.40E-06	0.226602	0.062	0.032	0.020918
STEAP1	1.47E-06	0.263462	0.074	0.038	0.02191
NEK11	1.58E-06	0.177446	0.093	0.068	0.023622
SYNPO	1.62E-06	0.165607	0.066	0.026	0.024231
SKAP2	1.63E-06	0.148077	0.168	0.179	0.024395
HCLS1	1.69E-06	0.165164	0.061	0.019	0.025304
MBP	1.74E-06	-0.13225	0.1	0.173	0.026029
ZNF295-AS	1.78E-06	0.123193	0.025	0.002	0.026536
TLDC1	1.97E-06	0.139443	0.052	0.058	0.02942
MORN5	2.07E-06	0.128986	0.043	0.01	0.030984
VAMP8	2.12E-06	0.153205	0.062	0.049	0.031667
HSD17B6	2.14E-06	0.112697	0.032	0.04	0.032055
ZBTB11-AS	2.32E-06	0.105774	0.031	0.037	0.034694
FAM198B	2.51E-06	0.185232	0.053	0.023	0.037583
AIF1	2.63E-06	0.20871	0.138	0.117	0.03924
DYRK3	2.66E-06	0.105327	0.053	0.036	0.039682
SMKR1	2.67E-06	0.114298	0.087	0.077	0.039929
RBFOX3	2.74E-06	0.187165	0.033	0.011	0.04091
CEBPA	2.87E-06	0.123528	0.039	0.014	0.042943
GSAP	2.99E-06	0.16464	0.068	0.056	0.044671
CCL7	3.08E-06	0.176357	0.023	0.006	0.046088

MLIP	3.10E-06	0.134873	0.05	0.014	0.046254
PIGV	3.29E-06	0.102781	0.068	0.077	0.04921
ETS2	3.45E-06	0.139926	0.096	0.074	0.051625
CFH	3.50E-06	0.132057	0.042	0.011	0.052342
PTGS2	3.53E-06	0.132396	0.041	0.01	0.052798
GUCY1B3	3.62E-06	-0.11326	0.022	0.06	0.054137
CYP1B1	3.75E-06	0.120228	0.037	0.007	0.056076
VWA3B	3.78E-06	0.105893	0.052	0.036	0.056458
CA8	3.94E-06	-0.12779	0.032	0.078	0.058866
C1QTNF1	4.11E-06	0.165788	0.073	0.041	0.061422
MKNK1	4.42E-06	0.104718	0.064	0.077	0.066086
KISS1R	4.55E-06	0.112315	0.041	0.016	0.067934
PPP1R13L	4.64E-06	0.124651	0.039	0.04	0.069385
ELMO1	4.68E-06	0.255932	0.136	0.106	0.069882
C1orf194	4.99E-06	0.155625	0.054	0.016	0.074541
TNXB	5.01E-06	0.106775	0.039	0.011	0.074804
AKR1C3	5.39E-06	0.136428	0.029	0.004	0.080574
SLC7A8	5.41E-06	0.126631	0.033	0.009	0.080837
TMEFF2	5.56E-06	-0.11446	0.058	0.112	0.083019
LAMA2	5.71E-06	0.184228	0.046	0.012	0.085309
LGALS9	5.82E-06	0.141063	0.056	0.038	0.087012
EIF4EBP3	5.89E-06	0.16063	0.023	0.001	0.088039
PLXDC2	5.97E-06	0.16114	0.045	0.021	0.089167
IGFLR1	6.38E-06	0.154664	0.04	0.035	0.095352
CD37	6.59E-06	0.152441	0.047	0.032	0.09852
PLCD1	6.64E-06	0.141514	0.068	0.047	0.099278
COX7A1	6.77E-06	0.110894	0.016	0	0.10113
UNC5B	6.87E-06	0.101044	0.052	0.033	0.1026
FCGR3A	7.13E-06	0.218767	0.083	0.048	0.106534
FBXO2	7.24E-06	0.102841	0.034	0.006	0.108267
ITGB2	7.30E-06	0.17026	0.064	0.063	0.109044
SLC9A9	7.32E-06	0.145107	0.058	0.026	0.109356
GCNT1	8.12E-06	0.117618	0.031	0.009	0.121304
TCTEX1D1	8.68E-06	0.137754	0.038	0.009	0.129753
MT1H	8.99E-06	0.149952	0.028	0.006	0.134318
C10orf107	9.40E-06	0.13588	0.048	0.041	0.1405
PALMD	1.05E-05	0.148977	0.057	0.019	0.156981
UPRT	1.11E-05	0.110623	0.085	0.079	0.16627
CHST9	1.17E-05	-0.10756	0.037	0.083	0.174799
IFI30	1.18E-05	0.141253	0.034	0.02	0.176353
EGOT	1.20E-05	0.118417	0.022	0.002	0.179176
C1QTNF3	1.37E-05	-0.16467	0.017	0.051	0.20408
GALNT13	1.48E-05	-0.12261	0.037	0.079	0.220506
DNAH11	1.50E-05	0.146347	0.064	0.027	0.224176
SLCO4A1	1.57E-05	0.109345	0.066	0.06	0.235309
C2orf74	1.90E-05	0.218355	0.038	0.022	0.283714
TDRP	1.92E-05	0.103074	0.044	0.02	0.286514
APOL3	1.94E-05	0.116445	0.04	0.014	0.289815
C1orf167	2.34E-05	0.134257	0.026	0.004	0.349034
SPRED3	2.49E-05	0.130222	0.026	0.02	0.371974
RP11-367J	2.57E-05	0.130116	0.027	0.009	0.384487

PIK3IP1	2.60E-05	0.147756	0.051	0.026	0.387853
RP11-92G1	2.74E-05	0.179562	0.041	0.011	0.410041
H3F3C	2.75E-05	0.199928	0.069	0.048	0.41148
LCA5L	2.81E-05	0.109206	0.045	0.02	0.420486
LST1	2.84E-05	0.150495	0.069	0.041	0.424745
CDH13	3.00E-05	-0.11652	0.053	0.101	0.447629
NDUFA4L2	3.06E-05	0.164112	0.083	0.044	0.457205
RIT2	3.39E-05	0.16458	0.096	0.096	0.506024
ENTPD1	3.44E-05	0.128547	0.041	0.022	0.514257
RP11-138A	3.47E-05	0.140136	0.085	0.067	0.518924
TMEM163	3.50E-05	0.114318	0.065	0.028	0.522307
HS3ST1	3.50E-05	0.121984	0.052	0.044	0.522564
RTP4	3.64E-05	0.164119	0.048	0.017	0.543904
SLC11A1	3.73E-05	0.118142	0.073	0.067	0.557052
AC104653.	4.01E-05	0.125821	0.018	0.004	0.599879
RP11-745C	4.11E-05	0.158578	0.028	0.007	0.614619
CA4	4.16E-05	0.195362	0.018	0.001	0.621147
AC007228.	4.19E-05	0.130635	0.039	0.023	0.625828
CBX7	4.30E-05	0.105993	0.054	0.063	0.643194
SNCA	4.32E-05	0.135688	0.024	0.004	0.645783
C10orf11	4.36E-05	0.12827	0.055	0.021	0.652152
EPSTI1	4.50E-05	-0.14155	0.052	0.096	0.672616
C1orf162	4.60E-05	0.174068	0.062	0.028	0.687174
IL34	4.68E-05	0.101369	0.028	0.007	0.699692
CD70	4.90E-05	0.161037	0.037	0.011	0.732439
CNTNAP3B	5.25E-05	0.144515	0.062	0.053	0.78424
CCL20	5.65E-05	0.152972	0.034	0.01	0.844715
FBXL2	5.75E-05	0.135235	0.061	0.036	0.859471
PAPPA2	5.88E-05	0.117945	0.03	0.006	0.878385
ADCYAP1R1	5.89E-05	0.102135	0.054	0.047	0.880203
MSRB3	5.89E-05	0.130982	0.046	0.022	0.880339
RP11-22N1	6.00E-05	0.120087	0.04	0.028	0.895897
IQCA1	6.06E-05	0.124805	0.045	0.014	0.906276
MYBPC1	6.35E-05	0.220483	0.084	0.053	0.948913
CD53	6.43E-05	0.166293	0.052	0.022	0.961261
SAMD9L	6.58E-05	0.112647	0.088	0.086	0.983876
SLC7A11	6.65E-05	0.105964	0.058	0.025	0.99406
SELPLG	6.81E-05	0.133001	0.034	0.017	1
ARHGDIB	7.35E-05	0.175745	0.096	0.07	1
TCEAL5	7.46E-05	0.102467	0.142	0.154	1
RP11-446N	7.94E-05	0.118171	0.021	0.006	1
LY86	8.50E-05	0.134391	0.033	0.028	1
IER3	8.53E-05	0.108368	0.088	0.084	1
DENND2D	9.60E-05	0.144352	0.035	0.012	1
FAM183A	9.61E-05	0.120875	0.028	0.006	1
DHRS2	9.68E-05	-0.17175	0.002	0.016	1
NPTX1	0.0001	0.115273	0.022	0.005	1
TDO2	0.000101	0.102387	0.022	0.004	1
C12orf66	0.000102	0.120637	0.05	0.031	1
PPM1N	0.000104	0.121422	0.034	0.017	1
METTL20	0.000104	0.118055	0.044	0.047	1

FHIT	0.000104	0.142705	0.096	0.08	1
PCDHB14	0.000105	0.125096	0.079	0.049	1
RP1-122K4	0.000108	0.115214	0.016	0.005	1
DNAH9	0.000115	0.12554	0.062	0.036	1
AXL	0.000117	0.160394	0.06	0.037	1
CCDC122	0.000125	0.107036	0.04	0.021	1
WDR78	0.000127	0.122876	0.042	0.028	1
CSGALNACT	0.000131	0.12937	0.05	0.058	1
SNHG18	0.000138	0.137142	0.056	0.027	1
ALPK1	0.000139	0.113548	0.028	0.02	1
GIMAP2	0.000149	0.119092	0.042	0.035	1
RP11-434H	0.000151	0.100823	0.036	0.03	1
RP11-834C	0.000151	0.179589	0.114	0.074	1
RRP12	0.000153	0.116356	0.036	0.04	1
ACY1	0.000153	0.12373	0.028	0.019	1
AC018647.	0.00016	0.114166	0.049	0.025	1
FXYD1	0.000161	0.147943	0.053	0.037	1
CDIPT-AS1	0.000167	0.134407	0.027	0.006	1
TEX29	0.000188	0.132285	0.024	0.006	1
RP11-499E	0.000192	0.142135	0.073	0.057	1
ACOT11	0.000194	0.116844	0.033	0.019	1
HSPA2	0.0002	0.199671	0.162	0.158	1
APOL4	0.000213	0.106097	0.064	0.064	1
RP3-325F2	0.000223	0.111055	0.028	0.01	1
PDK4	0.000224	0.137059	0.062	0.03	1
LYN	0.000229	0.114561	0.056	0.056	1
MASP1	0.000229	0.120797	0.07	0.069	1
RASA4	0.000249	0.114566	0.054	0.049	1
RP11-11N9	0.000254	0.209369	0.069	0.035	1
GPR37	0.000279	0.130851	0.074	0.062	1
CSF1R	0.000296	0.13135	0.036	0.023	1
TMEM144	0.000318	0.139537	0.039	0.019	1
DEPTOR	0.000322	0.100844	0.02	0.007	1
UNC79	0.000327	0.108073	0.033	0.026	1
TMEM255B	0.000328	0.125944	0.028	0.011	1
LINC0032C	0.000363	0.132309	0.022	0.005	1
CLN3	0.000377	0.210993	0.022	0.005	1
SLC38A5	0.000383	0.104855	0.023	0.006	1
SLC14A1	0.000392	0.135017	0.027	0.01	1
SULT1A1	0.000394	0.140744	0.033	0.016	1
CCL5	0.000398	0.100927	0.031	0.016	1
PDGFRL	0.00043	0.127931	0.038	0.02	1
CLEC18B	0.000441	0.131752	0.033	0.02	1
DAB2	0.000446	0.130639	0.062	0.033	1
MAPK4	0.000452	0.100387	0.048	0.042	1
TNS1	0.000477	0.107585	0.048	0.043	1
SLC16A7	0.000497	0.108764	0.047	0.02	1
MS4A6A	0.000545	0.156467	0.075	0.058	1
EFNA3	0.000548	0.100445	0.054	0.033	1
TNFAIP8	0.000585	0.146973	0.042	0.027	1
CCK	0.000592	0.115899	0.019	0.002	1

HIST1H2BD	0.000651	0.135622	0.053	0.031	1
RP11-138A	0.000666	0.113615	0.11	0.094	1
HIST1H2BJ	0.000753	-0.18312	0.021	0.049	1
SMIM5	0.000763	0.118044	0.022	0.006	1
FABP3	0.000946	0.129311	0.032	0.01	1
LINC00632	0.000978	-0.10789	0.092	0.137	1
ETV7	0.001035	0.108276	0.025	0.011	1
SUSD4	0.001127	0.139862	0.071	0.048	1
TMEM44-AS	0.001169	0.101287	0.038	0.03	1
RP11-46H1	0.001171	0.136713	0.033	0.012	1
FCGR2A	0.001185	0.17051	0.052	0.03	1
TUBB3	0.001242	0.115786	0.008	0	1
MGMT	0.001276	0.211776	0.098	0.064	1
AC015987.	0.001318	0.108518	0.023	0.007	1
RP11-244H	0.001372	0.106866	0.021	0.007	1
NAA60	0.001442	0.175053	0.025	0.006	1
AC069363.	0.001521	0.179098	0.039	0.019	1
LRRC69	0.001528	0.110079	0.033	0.036	1
MILR1	0.001653	0.147773	0.038	0.023	1
GLDN	0.001931	0.12641	0.021	0.005	1
AC006946.	0.002043	0.10305	0.017	0.004	1
C3AR1	0.002233	0.122113	0.033	0.012	1
C10orf35	0.00226	0.141543	0.072	0.041	1
ACSM5	0.002308	0.122885	0.019	0.004	1
CALB1	0.002328	0.222655	0.03	0.011	1
LINC00623	0.002361	0.177626	0.022	0.009	1
MAF	0.002599	0.128984	0.068	0.067	1
FAM50B	0.002867	0.130993	0.053	0.026	1
FST	0.002886	0.111166	0.038	0.025	1
C10orf54	0.003172	0.113633	0.068	0.04	1
FAM178B	0.0032	0.102203	0.019	0.005	1
ZNF665	0.003412	0.115014	0.038	0.022	1
S100A8	0.003452	0.16409	0.043	0.036	1
AMT	0.003733	0.108383	0.046	0.04	1
YPEL4	0.004081	0.115642	0.043	0.02	1
TSIX	0.004159	0.116723	0.031	0.02	1
LINC00987	0.004691	0.10677	0.014	0.004	1
STEAP2	0.004947	0.133217	0.034	0.021	1
MS4A4A	0.005264	0.135124	0.046	0.025	1
CLDN11	0.005287	0.200949	0.031	0.021	1
IFI27	0.005313	0.152936	0.118	0.09	1
ANKRD65	0.005589	-0.104	0.024	0.046	1
SGIP1	0.005669	0.118392	0.055	0.044	1
LRAT	0.005732	0.114043	0.028	0.025	1
LUC7L2	0.006701	0.114891	0.015	0.002	1
LINC01114	0.006879	0.125233	0.07	0.052	1
THTPA	0.007311	0.124201	0.019	0.005	1
FLNC	0.008195	0.100581	0.059	0.049	1
HCST	0.00863	0.123628	0.043	0.028	1
GNG11	0.008984	0.134575	0.084	0.09	1
NUDT7	0.009975	0.110433	0.053	0.035	1

MSC	0.01253	0.12488	0.018	0.011	1
EAF1-AS1	0.01504	0.109916	0.02	0.011	1
MIR7-3HG	0.018101	0.106619	0.022	0.011	1
RP11-284F	0.023702	0.113077	0.02	0.007	1
HSPA6	0.025611	0.211165	0.074	0.063	1
KRBOX1	0.030784	0.133325	0.013	0.004	1
NMNAT3	0.033196	0.101401	0.06	0.044	1
ZBTB16	0.048086	0.13746	0.199	0.174	1
STC2	0.050683	0.101784	0.079	0.06	1
FOLR2	0.05756	0.12173	0.028	0.023	1
IFI44L	0.110566	-0.10357	0.076	0.099	1
PCP4	0.15449	0.121483	0.059	0.057	1
