# Supplementary material for: A Novel Molecular Classification Method for Glioblastoma Based on Tumor Cell Differentiation Trajectories
Source: Stem Cells Int. 2023 Feb 22;2023:2826815. doi: 10.1155/2023/2826815 (PMC10643041; doi:10.1155/2023/2826815)
Supplement: Supplementary 9 — Supplementary Table 7: differentially expressed genes (DEGs) in GSCL and Undiff-G. [file 2826815.f9.pdf]

	p_val	avg_logFC	pct.1	pct.2	p_val_adj
FTL	########	0.855064	0.956	0.981	########
MALAT1	########	0.619855	0.991	0.988	########
UBE2T	########	-1.54966	0.01	0.835	########
UBE2C	########	-1.86966	0.01	0.833	########
NUSAP1	########	-1.67432	0.01	0.814	########
FAU	########	0.72561	0.892	0.969	########
HMGB2	########	-1.18078	0.187	0.977	########
ATP6V1G1	########	0.571737	0.534	0.84	########
LAMTOR4	########	0.527485	0.513	0.848	########
PBK	########	-1.47489	0.016	0.802	########
SKP1	########	0.719564	0.798	0.936	########
MAD2L1	########	-1.24951	0.03	0.814	########
SMC4	########	-1.21794	0.063	0.859	########
SOD1	########	0.625973	0.604	0.863	########
NUDT1	########	-0.49446	0.138	0.842	########
GABARAPL2	########	0.524424	0.541	0.857	########
TOP2A	########	-1.70301	0.007	0.785	########
TOMM7	########	0.64552	0.695	0.915	########
NDUFA1	########	0.565595	0.473	0.789	########
ATP6V1F	########	0.484144	0.482	0.852	########
LAMTOR5	########	0.584446	0.496	0.806	########
NHP2	########	0.331539	0.373	0.798	########
DEK	########	-0.98403	0.113	0.87	########
BIRC5	########	-1.61729	0.021	0.809	########
ATP5G1	########	0.417532	0.412	0.805	########
MYEOV2	########	0.210523	0.377	0.836	########
BANF1	########	0.225275	0.442	0.873	########
NDUFB2	########	0.54648	0.639	0.905	########
EDF1	########	0.369434	0.517	0.886	########
H2AFV	########	-0.75718	0.192	0.925	########
LSM3	########	-0.16056	0.267	0.863	########
ATP5J	########	0.460537	0.558	0.88	########
CENPF	########	-1.70872	0.005	0.772	########
TYMS	########	-1.20758	0.037	0.802	########
PSMB6	########	0.372491	0.459	0.852	########
POLR2J	########	0.348674	0.475	0.878	########
WDR83OS	########	0.651661	0.545	0.828	########
OST4	########	0.455976	0.524	0.852	########
COMMD6	########	0.486434	0.478	0.823	########
ATP5I	########	0.387681	0.511	0.869	########
NDUFB3	########	0.328162	0.305	0.743	########
CENPU	########	-1.31091	0.012	0.772	########
CNBP	########	0.461281	0.553	0.891	########
DTYMK	########	-0.9027	0.103	0.842	########
PTTG1	########	-1.50897	0.047	0.831	########
SNRPB2	########	0.194043	0.309	0.772	########
SELK	########	0.203144	0.4	0.862	########
PRMT1	########	0.292366	0.366	0.805	########
NDUFA13	########	0.583092	0.632	0.862	########
ATP6V0E1	########	0.735391	0.473	0.726	########

PRDX5	########	0.605042	0.532	0.799	########
TRMT112	########	0.484959	0.445	0.802	########
SPCS1	########	0.473212	0.496	0.84	########
CSNK2B	########	0.117203	0.29	0.81	########
CHMP2A	########	0.715312	0.49	0.763	########
LSM5	########	-0.45287	0.209	0.88	########
COX6A1	########	0.444482	0.681	0.931	########
ATP5L	########	0.443383	0.63	0.92	########
ATP5G2	########	0.575032	0.691	0.914	########
TMEM258	########	0.413893	0.489	0.847	########
CIRBP	########	0.542592	0.742	0.94	########
SEC62	########	0.364927	0.419	0.802	########
UBL5	########	0.587437	0.684	0.896	########
SMC2	########	-1.06846	0.042	0.78	########
SNRPD1	########	-0.14322	0.277	0.858	########
SNRPD2	########	0.534504	0.606	0.896	########
PFDN5	########	0.63721	0.695	0.906	########
KIAA0101	########	-1.41108	0.016	0.769	########
COX4I1	########	0.644069	0.766	0.941	########
FAM96B	########	0.242157	0.36	0.816	########
ATP5O	########	0.348416	0.508	0.893	########
NDUFS8	########	0.253621	0.358	0.804	########
NDUFC2	########	0.376513	0.518	0.88	########
MGST3	########	0.621527	0.476	0.783	########
COX6B1	########	0.610492	0.688	0.906	########
ATRAID	########	0.453345	0.412	0.79	########
SSB	########	0.365826	0.384	0.801	########
UQCRH	########	0.347527	0.569	0.927	########
COX7C	########	0.650596	0.77	0.938	########
EIF1	########	0.647822	0.899	0.983	########
PARK7	########	0.458437	0.611	0.906	########
VBP1	########	-0.18592	0.222	0.825	########
HSBP1	########	0.383168	0.492	0.874	########
C4orf3	########	0.549982	0.36	0.704	########
ERH	########	0.229764	0.504	0.914	########
SAP18	########	0.437779	0.524	0.878	########
ZNHIT1	########	0.498824	0.466	0.812	########
POLR2I	########	0.399214	0.387	0.791	########
NDUFA6	########	0.279839	0.386	0.817	########
BUD31	########	0.163904	0.386	0.853	########
NDUFB9	########	0.305421	0.468	0.86	########
PSMB3	########	0.328837	0.431	0.842	########
PRDX4	########	0.356052	0.326	0.721	########
CNPY2	########	0.235021	0.328	0.767	########
ATP6V0B	########	0.288455	0.421	0.828	########
TIMM8B	########	0.182622	0.295	0.754	########
CYC1	########	-0.14295	0.248	0.825	########
LINC00493	########	0.195238	0.244	0.696	########
SEC61B	########	0.391716	0.475	0.831	########
DAD1	########	0.517484	0.476	0.82	########
SPCS2	########	0.425515	0.518	0.872	########

RHOA	########	0.224301	0.417	0.857	########
BRK1	########	0.367821	0.515	0.873	########
CLTA	########	-0.18297	0.225	0.822	########
BTF3	########	0.574653	0.78	0.954	########
AP2M1	########	0.226158	0.447	0.862	########
POLR2L	########	0.318213	0.455	0.847	########
U2AF1	########	-0.28028	0.274	0.875	########
UQCRQ	########	0.433135	0.63	0.915	########
PEBP1	########	0.509483	0.688	0.932	########
TMBIM6	########	0.488419	0.623	0.893	########
NDUFS5	########	0.585095	0.724	0.93	########
GGH	########	-0.86283	0.098	0.822	########
ASNA1	########	0.13513	0.262	0.74	########
COPS6	########	0.186739	0.391	0.859	########
BLOC1S1	########	0.295307	0.36	0.783	########
PYURF	########	-0.1761	0.215	0.775	########
SRP14	########	0.390844	0.64	0.93	########
ARPC3	########	0.303428	0.419	0.817	########
PPIB	########	0.509078	0.468	0.801	########
SHFM1	########	0.343039	0.515	0.884	########
SERF2	########	0.555441	0.703	0.914	########
NACA	########	0.553408	0.785	0.962	########
ROMO1	########	0.213684	0.372	0.802	########
DYNLRB1	########	0.240256	0.405	0.826	########
TCEB2	########	0.397703	0.623	0.911	########
MRFAP1	########	0.117084	0.431	0.885	########
NDUFB7	########	0.492493	0.581	0.869	########
MT1X	########	1.318041	0.576	0.614	########
TMA7	########	0.461077	0.674	0.914	########
KDELR2	########	-0.48192	0.164	0.812	########
RBX1	########	0.162454	0.36	0.825	########
UBE2I	########	-0.39383	0.197	0.827	########
ILF2	########	-0.29102	0.286	0.902	########
ATP5J2	########	0.413418	0.621	0.922	########
FKBP8	########	0.282639	0.344	0.754	########
FKBP1A	########	0.233501	0.393	0.81	########
ZNF428	########	-0.12462	0.318	0.868	########
SSBP1	########	0.228079	0.387	0.847	########
PRDX1	########	0.690556	0.665	0.868	########
LSM2	########	-0.31245	0.147	0.743	########
SSR2	########	0.197644	0.431	0.869	########
TMEM59	########	0.395084	0.482	0.854	########
NDUFA12	########	0.305568	0.337	0.788	########
GNB2L1	########	0.674312	0.916	0.979	########
C12orf57	########	0.290936	0.401	0.828	########
RPA3	########	-0.53238	0.131	0.806	########
PCNP	########	0.14682	0.283	0.754	########
SAT2	########	0.55429	0.36	0.689	########
ANP32B	########	-0.72036	0.11	0.81	########
CUTA	########	0.270521	0.398	0.814	########
PAFAH1B3	########	-0.16357	0.243	0.828	########

SON	########	0.198451	0.408	0.848	########
HNRNPR	########	-0.31628	0.239	0.847	########
MYL12B	########	0.325461	0.4	0.804	########
STRA13	########	-0.26071	0.169	0.769	########
PDAP1	########	-0.10746	0.265	0.821	########
PGK1	########	0.62539	0.49	0.804	########
TBCB	########	0.236086	0.435	0.864	########
KRT10	########	-0.28173	0.23	0.811	########
H2AFX	########	-1.21685	0.045	0.783	########
RWDD1	########	0.339522	0.309	0.72	########
GPX1	########	0.180901	0.396	0.84	########
SSR4	########	0.509033	0.487	0.815	########
GSTP1	########	0.463365	0.653	0.923	########
H2AFY	########	-0.54488	0.215	0.878	########
SNRPB	########	-0.47599	0.248	0.894	########
SRSF2	########	-0.57672	0.215	0.878	########
SRP9	########	0.119661	0.475	0.919	########
C14orf2	########	0.15553	0.389	0.851	########
HMGN1	########	-0.58416	0.267	0.923	########
PRDX2	########	0.529363	0.716	0.932	########
TTC3	########	-0.19487	0.229	0.805	########
PIN1	########	-0.30953	0.237	0.84	########
COX6C	########	0.513616	0.712	0.923	########
CCT3	########	0.187868	0.366	0.827	########
PSMA3	########	0.139259	0.213	0.691	########
BSG	########	0.401123	0.635	0.915	########
RBMX	########	-0.32708	0.286	0.901	########
ZFAS1	########	0.297772	0.462	0.856	########
UQCR10	########	0.214302	0.443	0.86	########
PSMD8	########	0.236788	0.443	0.873	########
TPT1	########	0.667526	0.873	0.959	########
HNRNPA0	########	-0.36264	0.271	0.894	########
PSMB5	########	0.28278	0.417	0.847	########
RPAIN	########	-0.10712	0.237	0.799	########
MAD2L2	########	-0.67508	0.136	0.819	########
ENY2	########	0.151805	0.318	0.786	########
CYCS	########	0.138254	0.483	0.931	########
HMGB3	########	-0.81066	0.108	0.823	########
EEF1B2	########	0.149735	0.445	0.89	########
DUT	########	-0.77434	0.12	0.819	########
TBCA	########	0.353706	0.562	0.9	########
HNRNPM	########	-0.53204	0.175	0.84	########
KHDRBS1	########	-0.56973	0.164	0.84	########
CAPZA2	########	0.160633	0.335	0.816	########
RNASEH2A	########	-0.85059	0.075	0.774	########
C19orf43	########	-0.29667	0.297	0.891	########
NDUFA11	########	0.345521	0.51	0.862	########
TPI1	########	0.569972	0.766	0.953	########
RANBP1	########	-0.37773	0.298	0.931	########
HNRNPC	########	0.33958	0.532	0.899	########
CLU	########	1.015751	0.838	0.828	########

PSMB1	########	0.300971	0.452	0.853	########
SRSF5	########	0.14919	0.333	0.802	########
OCIAD1	########	0.357255	0.433	0.806	########
15-Sep	########	0.440114	0.353	0.707	########
SLC25A3	########	0.238364	0.45	0.868	########
RHEB	########	-0.35478	0.318	0.926	########
MDH2	########	0.277588	0.438	0.849	########
PARP1	########	-0.38874	0.175	0.802	########
SF3B2	########	-0.14399	0.222	0.777	########
CKLF	########	-0.33488	0.152	0.762	########
RABAC1	########	0.481489	0.457	0.799	########
CALM3	########	-0.63839	0.195	0.883	########
HSD17B10	########	0.317116	0.302	0.735	########
COPE	########	0.472015	0.6	0.889	########
C11orf58	########	0.245369	0.447	0.84	########
NONO	########	-0.20646	0.215	0.807	########
SEC11A	########	0.379541	0.319	0.716	########
DBI	########	0.734477	0.867	0.957	########
GNB2	########	-0.46197	0.202	0.838	########
RBM8A	########	0.114505	0.344	0.822	########
PA2G4	########	-0.1812	0.227	0.804	########
H3F3B	########	0.530411	0.911	0.981	########
RCN2	########	-0.36354	0.199	0.801	########
GPM6B	########	0.57405	0.794	0.937	########
USMG5	########	0.195678	0.277	0.728	########
GLUL	########	0.836277	0.508	0.732	########
NAA38	########	0.164672	0.265	0.757	########
RNPS1	########	-0.19504	0.264	0.831	########
SLC25A6	########	0.36642	0.635	0.927	########
TMEM14C	########	0.145725	0.253	0.715	########
ARHGDIA	########	-0.20498	0.188	0.742	########
HNRNPA3	########	-0.23859	0.25	0.838	########
COX7A2	########	0.24066	0.567	0.928	########
SF3B5	########	0.103857	0.286	0.765	########
CACYBP	########	-0.26431	0.211	0.814	########
ACTG1	########	0.541503	0.92	0.981	########
FAM64A	########	-1.49073	0.005	0.717	########
RTN4	########	-0.319	0.309	0.888	########
ST13	########	0.133205	0.36	0.828	########
PNISR	########	0.230936	0.405	0.83	########
YWHAH	########	-0.65152	0.136	0.81	########
MORF4L1	########	0.189757	0.417	0.846	########
HNRNPU	########	-0.25571	0.225	0.821	########
HNRNPK	########	0.334498	0.634	0.938	########
COA3	########	0.300641	0.269	0.691	########
PPIG	########	-0.16732	0.202	0.777	########
NDUFA4	########	0.564833	0.857	0.975	########
POP7	########	-0.49395	0.122	0.759	########
TIMM13	########	0.336262	0.405	0.772	########
SEPW1	########	0.423361	0.483	0.841	########
TCP1	########	-0.35195	0.204	0.822	########

COX5B	########	0.481298	0.696	0.916	########
SLC25A5	########	0.263837	0.483	0.896	########
CENPK	########	-1.16653	0.01	0.71	########
PHPT1	########	0.313104	0.447	0.848	########
CSNK1A1	########	-0.31135	0.204	0.786	########
LSM4	########	-0.59582	0.239	0.904	########
ATP5G3	########	0.226426	0.468	0.884	########
RNF7	########	0.363487	0.265	0.649	########
PHB	########	0.193939	0.34	0.773	########
ATP5A1	########	0.416302	0.468	0.831	########
NDUFB11	########	0.18977	0.48	0.902	########
RAD21	########	-0.71424	0.119	0.806	########
DRAP1	########	0.114353	0.349	0.796	########
HNRNPAB	########	-0.64497	0.101	0.758	########
FKBP3	########	0.169482	0.302	0.77	########
RALY	########	-0.90309	0.094	0.798	########
POMP	########	0.186535	0.389	0.838	########
LMAN2	########	0.20465	0.298	0.743	########
NASP	########	-0.49687	0.159	0.815	########
YWHAB	########	-0.13366	0.346	0.869	########
FIS1	########	0.330604	0.382	0.788	########
PFDN2	########	0.104526	0.332	0.817	########
RDX	########	-0.4942	0.19	0.827	########
C7orf50	########	-0.21405	0.22	0.799	########
ATOX1	########	0.384837	0.344	0.71	########
GAPDH	########	0.700238	0.991	0.996	########
NEDD8	########	0.487388	0.557	0.848	########
NDUFB4	########	0.379285	0.433	0.788	########
CSTB	########	0.506338	0.361	0.691	########
C7orf73	########	0.295593	0.337	0.746	########
ARF1	########	0.106017	0.33	0.804	########
C11orf31	########	0.175554	0.372	0.783	########
EIF5B	########	0.206543	0.3	0.767	########
CAMLG	########	0.384854	0.288	0.683	########
EIF3K	########	0.610283	0.571	0.835	########
ATP5C1	########	0.105362	0.262	0.733	########
SBDS	########	0.245721	0.366	0.802	########
PPP1CA	########	-0.10399	0.202	0.722	########
EI24	########	-0.24063	0.166	0.737	########
ATP5H	########	0.259485	0.353	0.783	########
NGFRAP1	########	0.557967	0.771	0.953	########
CDK1	########	-1.41404	0.01	0.717	########
TXN	########	0.415764	0.469	0.817	########
NDUFA3	########	0.189952	0.391	0.809	########
XRN2	########	-0.26449	0.15	0.731	########
SRI	########	0.556646	0.757	0.946	########
SGOL1	########	-1.26451	0.002	0.698	########
NDUFC1	########	0.367421	0.358	0.74	########
SMARCA4	########	-0.42618	0.136	0.748	########
SRRM2	########	-0.23279	0.229	0.795	########
EEF1D	########	0.433233	0.611	0.911	########

NDUFB1	########	0.313461	0.389	0.79	########
HINT1	########	0.350102	0.686	0.963	########
SRSF9	########	-0.36047	0.229	0.827	########
UBC	########	0.644027	0.862	0.97	########
RTFDC1	########	-0.1522	0.18	0.74	########
JTB	########	-0.27766	0.206	0.791	########
EIF4H	########	-0.18148	0.239	0.8	########
PDCD5	########	0.137211	0.337	0.817	########
PCBP1	########	-0.24647	0.213	0.793	########
C1QBP	########	-0.27432	0.222	0.802	########
C14orf166	########	0.20116	0.316	0.765	########
SET	########	-0.27795	0.267	0.853	########
COX5A	########	-0.11005	0.269	0.822	########
EID1	########	0.290727	0.532	0.895	########
CLNS1A	########	0.238942	0.25	0.686	########
COX7A2L	########	0.387904	0.337	0.712	########
HDAC2	########	-0.56963	0.133	0.789	########
AURKAIP1	########	-0.12344	0.279	0.817	########
POLR2G	########	0.295829	0.344	0.759	########
SAT1	########	0.741058	0.461	0.707	########
VAMP2	########	0.111544	0.375	0.844	########
SELT	########	0.421482	0.354	0.714	########
PSMD7	########	0.101167	0.218	0.699	########
C6orf48	########	0.123785	0.375	0.842	########
NAP1L4	########	-0.33674	0.155	0.747	########
CCT4	########	-0.16024	0.243	0.822	########
CENPH	########	-1.04953	0.017	0.71	########
PKM	########	0.542042	0.642	0.878	########
HNRNPUL1	########	-0.39617	0.12	0.725	########
EIF3G	########	0.189272	0.332	0.768	########
LAPTM4A	########	0.273797	0.471	0.872	########
METTL9	########	-0.25867	0.152	0.714	########
PUF60	########	0.115806	0.298	0.763	########
MTCH1	########	-0.45942	0.15	0.774	########
MZT2A	########	-0.41607	0.141	0.756	########
NME1	########	0.101853	0.401	0.864	########
ZWINT	########	-1.23924	0.005	0.7	########
METAP2	########	0.176954	0.253	0.711	########
TMED9	########	0.350712	0.433	0.805	########
NDFIP1	########	-0.13751	0.243	0.781	########
COA4	########	0.109678	0.178	0.635	########
MED10	########	0.259389	0.246	0.67	########
VPS28	########	0.430944	0.379	0.746	########
CBX3	########	-0.44904	0.248	0.879	########
DPY30	########	0.199548	0.271	0.74	########
TRAPPC1	########	0.412204	0.323	0.673	########
RHOBTB3	########	-0.19524	0.253	0.826	########
ITGAE	########	-0.3016	0.129	0.707	########
EIF3E	########	0.503077	0.394	0.746	########
ECH1	########	0.534439	0.377	0.704	########
CD63	########	0.850906	0.866	0.9	########

COA1	########	-0.14227	0.173	0.719	########
UBE2E3	########	-0.40431	0.187	0.811	########
TSC22D1	########	0.207434	0.459	0.854	########
TMED10	########	0.597332	0.396	0.674	########
NDUFB5	########	0.219056	0.295	0.742	########
CCDC167	########	-0.38197	0.152	0.757	########
TMSB10	########	0.642735	0.899	0.974	########
PRPF40A	########	-0.30592	0.141	0.721	########
SKA2	########	-0.68468	0.087	0.735	########
HIGD2A	########	0.576844	0.379	0.664	########
ANXA5	########	0.622472	0.621	0.837	########
LAMTOR2	########	0.272422	0.264	0.691	########
TCEAL4	########	0.428619	0.363	0.728	########
EWSR1	########	-0.35167	0.168	0.786	########
CCNI	########	-0.22201	0.328	0.872	########
PTOV1	########	-0.39703	0.178	0.789	########
SH3BGRL3	########	0.663863	0.462	0.705	########
RNF181	########	0.304137	0.23	0.642	########
EIF3F	########	-0.1409	0.215	0.767	########
PET100	########	0.20229	0.283	0.71	########
UBXN1	########	0.113035	0.257	0.723	########
TECR	########	0.381858	0.387	0.747	########
ATP5B	########	0.347961	0.586	0.917	########
LDHB	########	0.531528	0.75	0.967	########
PSMA1	########	0.393429	0.368	0.731	########
C8orf59	########	-0.10425	0.136	0.646	########
NENF	########	-0.27687	0.183	0.757	########
SLIRP	########	0.147754	0.384	0.826	########
PRC1	########	-1.25918	0.012	0.702	########
NUCKS1	########	-0.23674	0.382	0.94	########
UQCRC1	########	-0.13994	0.195	0.747	########
CBX1	########	-0.43397	0.134	0.752	########
HNRNPD	########	-0.60125	0.141	0.778	########
B2M	########	0.7968	0.946	0.968	########
HAX1	########	0.135583	0.204	0.66	########
SCP2	########	0.299344	0.314	0.722	########
WBP5	########	0.328975	0.368	0.777	########
EIF4A2	########	0.408047	0.499	0.847	########
KTN1	########	-0.38114	0.171	0.759	########
NDUFV2	########	0.210007	0.325	0.751	########
PGLS	########	-0.20584	0.154	0.686	########
ARPP19	########	-0.16947	0.171	0.719	########
EEF1A1	########	0.714054	0.82	0.878	########
COPS8	########	0.111065	0.272	0.767	########
ENO1	########	0.600737	0.696	0.895	########
SFPQ	########	-0.47536	0.225	0.854	########
MYL6	########	0.538252	0.815	0.964	########
TMPO	########	-0.99268	0.04	0.731	########
ERGIC3	########	0.135941	0.333	0.777	########
NUTF2	########	-0.21287	0.133	0.69	########
PSMB7	########	0.268066	0.443	0.852	########

OSTC	########	0.255313	0.239	0.651	########
SOX2	########	-0.19598	0.365	0.895	########
TRA2B	########	-0.19208	0.255	0.826	########
PSMD2	########	-0.16744	0.175	0.715	########
PRELID1	########	-0.33193	0.183	0.767	########
UBE2L3	########	-0.23268	0.182	0.756	########
NDUFA5	########	0.435452	0.415	0.788	########
UQCRFS1	########	-0.16439	0.246	0.812	########
TMEM106C	########	-0.48742	0.099	0.702	########
KIF5B	########	-0.53105	0.169	0.802	########
NOP56	########	-0.35956	0.154	0.751	########
C17orf89	########	-0.39187	0.129	0.694	########
MZT2B	########	-0.49107	0.258	0.877	########
MEAF6	########	0.181657	0.234	0.688	########
CMC2	########	-0.52231	0.091	0.705	########
RAD23A	########	-0.15951	0.267	0.806	########
ATPIF1	########	0.272082	0.347	0.756	########
RTF1	########	-0.28144	0.148	0.712	########
KPNB1	########	-0.54639	0.152	0.794	########
NUF2	########	-1.30208	0	0.669	########
SMIM7	########	0.348051	0.323	0.728	########
C19orf70	########	0.586903	0.393	0.667	########
FEZ1	########	-0.11237	0.25	0.784	########
CWC15	########	0.174655	0.227	0.664	########
PSMG2	########	0.154786	0.175	0.621	########
UQCRB	########	0.171166	0.363	0.83	########
SDHC	########	0.499757	0.332	0.669	########
SRSF7	########	-0.15714	0.288	0.84	########
ENOPH1	########	-0.41424	0.133	0.731	########
PSMB2	########	0.288985	0.372	0.791	########
ATP5F1	########	0.408317	0.41	0.773	########
MTPN	########	-0.18226	0.122	0.653	########
CFL1	########	0.482666	0.857	0.964	########
CALU	########	-0.23303	0.183	0.747	########
NCL	########	-0.24336	0.239	0.811	########
YWHAQ	########	-0.34129	0.335	0.916	########
TOMM22	########	-0.10821	0.161	0.675	########
LSM1	########	0.245441	0.241	0.66	########
GLOD4	########	-0.12245	0.154	0.673	########
TUBB4B	########	-0.70681	0.192	0.872	########
ILF3	########	-0.38389	0.157	0.763	########
UBE2S	########	-1.21228	0.089	0.812	########
STUB1	########	-0.32576	0.154	0.728	########
CKS1B	########	-0.93808	0.068	0.738	########
UBE2D2	########	-0.39767	0.216	0.815	########
COMT	########	0.12534	0.223	0.668	########
PPM1G	########	-0.69697	0.106	0.767	########
OAZ1	########	0.408529	0.736	0.965	########
TPX2	########	-1.38009	0.009	0.69	########
ERP29	########	0.300637	0.302	0.714	########
ARF4	########	0.281484	0.34	0.764	########

ALDOA	########	0.64692	0.742	0.89	########
TPR	########	-0.27289	0.157	0.725	########
MT2A	########	0.915263	0.848	0.889	########
EIF5	########	-0.14214	0.333	0.868	########
COX16	########	-0.16771	0.178	0.716	########
MAPRE1	########	-0.34873	0.152	0.741	########
CCT5	########	-0.25158	0.202	0.791	########
HNRNPDL	########	0.184457	0.565	0.937	########
RSL1D1	########	0.179513	0.208	0.658	########
CKS2	########	-1.06314	0.126	0.846	########
SRSF3	########	-0.13109	0.433	0.943	########
CAMTA1	########	-0.21037	0.173	0.707	########
GLO1	########	-0.29824	0.159	0.732	########
HSP90AB1	########	0.287768	0.602	0.933	########
EIF3H	########	0.277125	0.337	0.765	########
TMEM230	########	0.288275	0.401	0.78	########
AES	########	-0.15199	0.202	0.749	########
AKR1B1	########	-0.12091	0.215	0.748	########
GPM6A	########	0.231567	0.471	0.865	########
NFIC	########	-0.35236	0.169	0.753	########
CARHSP1	########	-0.23503	0.206	0.788	########
TMCO1	########	0.421726	0.366	0.723	########
GCSH	########	-0.13723	0.154	0.653	########
HNRNPH3	########	-0.39577	0.159	0.756	########
UXT	########	0.32721	0.279	0.684	########
PAIP2	########	0.263397	0.346	0.764	########
DCTN3	########	-0.15185	0.243	0.802	########
CRYAB	########	1.394409	0.56	0.506	########
SSRP1	########	-0.60082	0.108	0.749	########
ZNF667-AS	########	0.401758	0.3	0.672	########
C19orf53	########	0.499432	0.534	0.821	########
MIEN1	########	0.199317	0.223	0.642	########
CCT6A	########	-0.15797	0.297	0.842	########
LAMTOR1	########	0.246551	0.319	0.727	########
PRRC2C	########	-0.13211	0.175	0.693	########
SMARCB1	########	-0.45063	0.131	0.728	########
EEF2	########	0.353855	0.644	0.936	########
PCNA	########	-0.72592	0.11	0.783	########
BTG3	########	-0.66853	0.119	0.774	########
TCF4	########	-0.13775	0.333	0.864	########
SUB1	########	0.210783	0.62	0.956	########
SRSF1	########	-0.27007	0.173	0.743	########
NDUFS2	########	0.372481	0.326	0.694	########
GDI1	########	-0.12757	0.243	0.79	########
SEC11C	########	0.132671	0.225	0.679	########
ABI2	########	-0.38583	0.15	0.736	########
MDH1	########	0.104725	0.3	0.779	########
RAB10	########	-0.22909	0.138	0.688	########
TXNDC17	########	0.179064	0.295	0.712	########
AUP1	########	-0.17852	0.178	0.712	########
SLC25A11	########	-0.32875	0.127	0.699	########

TMEM160	########	-0.6943	0.112	0.76	########
CCDC34	########	-0.92049	0.042	0.696	########
CHID1	########	0.222388	0.251	0.675	########
NDUFA8	########	0.21463	0.234	0.674	########
ADRM1	########	-0.18393	0.195	0.733	########
PSIP1	########	-0.72275	0.14	0.817	########
TMEM208	########	0.271882	0.237	0.651	########
C1orf43	########	0.107701	0.269	0.726	########
MARCKS	########	-0.27397	0.312	0.87	########
COX17	########	0.19058	0.255	0.681	########
WBSCR22	########	0.218311	0.297	0.728	########
DYNLL1	########	0.32602	0.695	0.964	########
DPM2	########	-0.12078	0.12	0.622	########
CD99	########	0.64906	0.483	0.674	########
EIF4E	########	-0.11024	0.178	0.698	########
USP1	########	-0.70572	0.07	0.706	########
PPP4C	########	-0.34831	0.152	0.722	########
CKAP2	########	-1.19865	0.026	0.711	########
APMAP	########	-0.18104	0.157	0.678	########
CDKN3	########	-1.41946	0.003	0.665	########
PABPC1	########	0.108627	0.384	0.849	########
ANAPC11	########	0.209824	0.55	0.917	########
RPN2	########	-0.11357	0.297	0.819	########
ODC1	########	-0.66487	0.155	0.806	########
COX20	########	-0.15036	0.208	0.759	########
KPNA2	########	-1.0728	0.089	0.795	########
BRD2	########	-0.32026	0.126	0.684	########
EIF4A1	########	0.289498	0.628	0.937	########
FAM32A	########	0.108808	0.213	0.684	########
MPC1	########	0.132026	0.244	0.696	########
RAN	########	0.173782	0.579	0.947	########
PNKD	########	0.186594	0.279	0.726	########
TMEM205	########	0.718117	0.361	0.58	########
C9orf142	########	-0.54771	0.08	0.683	########
WSB1	########	0.242389	0.377	0.775	########
NPM1	########	0.438399	0.686	0.938	########
SDCBP	########	0.420781	0.412	0.742	########
HSPE1	########	0.216865	0.538	0.902	########
SNRNP25	########	-0.22355	0.129	0.665	########
SNRNP70	########	-0.38017	0.166	0.753	########
AP1S1	########	0.258631	0.265	0.695	########
TPRKB	########	-0.44883	0.099	0.7	########
OAZ2	########	0.208085	0.23	0.651	########
UFC1	########	0.286677	0.297	0.712	########
SMS	########	-0.11102	0.269	0.789	########
CD151	########	0.548966	0.342	0.605	########
PIN4	########	0.303995	0.241	0.631	########
LMNB1	########	-1.0208	0.033	0.707	########
NUBP2	########	-0.22249	0.138	0.67	########
MATR3	########	-0.36573	0.169	0.764	########
SIVA1	########	-0.11182	0.234	0.762	########

NCOR1	########	-0.28838	0.175	0.728	########
PCSK1N	########	-0.34332	0.227	0.784	########
EIF4G2	########	-0.21884	0.318	0.849	########
XRCC5	########	-0.38101	0.227	0.84	########
SF1	########	-0.30441	0.185	0.753	########
NAA10	########	-0.4533	0.094	0.683	########
ATP6AP2	########	0.219295	0.311	0.714	########
CSDE1	########	-0.18226	0.225	0.774	########
RER1	########	-0.41918	0.129	0.705	########
EIF4A3	########	-0.353	0.103	0.669	########
NAA20	########	0.121597	0.208	0.651	########
SSNA1	########	-0.35163	0.11	0.662	########
GNB1	########	-0.33196	0.113	0.664	########
DCXR	########	-0.3478	0.18	0.751	########
NELFE	########	-0.22838	0.141	0.681	########
PSMB4	########	0.248615	0.201	0.612	########
PSMD4	########	0.116591	0.277	0.737	########
WDR34	########	-0.66493	0.066	0.677	########
PRDX6	########	0.5532	0.489	0.751	########
UGP2	########	-0.14995	0.185	0.706	########
WRB	########	0.220796	0.236	0.665	########
SNF8	########	-0.27361	0.162	0.719	########
RAC1	########	-0.14748	0.459	0.941	########
PPP1CC	########	-0.39814	0.168	0.76	########
ISCU	########	-0.14523	0.171	0.689	########
VDAC1	########	0.2261	0.372	0.781	########
GNAS	########	0.196631	0.497	0.893	########
GADD45GIF	########	-0.11858	0.283	0.794	########
TMEM147	########	0.22065	0.321	0.725	########
SERP1	########	-0.20262	0.19	0.736	########
TMEM50A	########	0.13839	0.237	0.689	########
CHCHD5	########	0.110949	0.171	0.606	########
CBX5	########	-0.38157	0.166	0.767	########
P4HB	########	-0.27398	0.218	0.769	########
EIF1B	########	0.238354	0.339	0.78	########
ARL5A	########	-0.10743	0.182	0.695	########
AP1S2	########	-0.37948	0.147	0.723	########
PAPOLA	########	-0.13269	0.19	0.72	########
CALD1	########	0.224671	0.318	0.746	########
DPM3	########	0.313717	0.188	0.535	########
CNP	########	-0.13645	0.15	0.665	########
QKI	########	-0.18831	0.211	0.737	########
ARL6IP4	########	-0.30093	0.216	0.781	########
LSM14A	########	-0.3222	0.122	0.681	########
SPP1	########	0.945638	0.637	0.753	########
TAF7	########	0.330743	0.412	0.794	########
BCAP31	########	0.332841	0.281	0.662	########
RAB11A	########	0.249549	0.218	0.637	########
PGRMC1	########	-0.33081	0.171	0.749	########
FXYD6	########	0.284112	0.525	0.902	########
BTF3L4	########	0.130156	0.243	0.694	########

PPP2CA	########	-0.21738	0.18	0.735	########
PTN	########	0.67554	0.827	0.916	########
BZW1	########	-0.12618	0.257	0.788	########
NBEAL1	########	0.857737	0.447	0.625	########
DDX39A	########	-0.42198	0.175	0.786	########
RNF5	########	-0.11838	0.143	0.659	########
ELOF1	########	0.146438	0.223	0.649	########
ARGLU1	########	-0.29137	0.166	0.714	########
C16orf13	########	-0.25774	0.145	0.686	########
CTSB	########	0.444833	0.37	0.689	########
PSAP	########	0.396795	0.438	0.76	########
CNN3	########	0.193366	0.417	0.822	########
PTBP1	########	-0.4393	0.087	0.659	########
TOP1	########	-0.45312	0.105	0.667	########
NPC2	########	0.869887	0.311	0.489	########
NDUFS4	########	0.409637	0.34	0.71	########
SYF2	########	0.312616	0.251	0.64	########
SCAND1	########	-0.29132	0.234	0.794	########
LDHA	########	0.484066	0.579	0.865	########
BOLA3	########	-0.14087	0.126	0.61	########
TCEA2	########	-0.38419	0.145	0.709	########
MTDH	########	-0.34964	0.223	0.786	########
HIST1H4C	########	-1.23997	0.136	0.851	########
APLP2	########	-0.1745	0.223	0.737	########
LAPTM4B	########	-0.17182	0.173	0.7	########
7-Sep	########	0.309647	0.595	0.933	########
THYN1	########	0.330325	0.269	0.647	########
PAIP1	########	-0.43314	0.108	0.678	########
DNMT1	########	-0.77863	0.065	0.71	########
PLD3	########	0.25675	0.22	0.6	########
YIPF3	########	0.183598	0.232	0.652	########
S100B	########	0.864597	0.613	0.754	########
COPS3	########	-0.29418	0.12	0.674	########
C4orf27	########	-0.20015	0.113	0.649	########
GTF3A	########	-0.4041	0.127	0.699	########
PDIA3	########	-0.17708	0.199	0.73	########
VDAC3	########	-0.16578	0.194	0.737	########
CD9	########	1.165764	0.66	0.615	########
MLEC	########	-0.32605	0.105	0.648	########
HSPA8	########	0.411877	0.742	0.959	########
RRM2	########	-1.34589	0.002	0.647	########
CD59	########	0.492782	0.304	0.626	########
DDT	########	-0.18231	0.19	0.721	########
BTG1	########	-0.15677	0.295	0.831	########
MKKS	########	-0.2605	0.15	0.669	########
FAM200B	########	-0.11119	0.138	0.644	########
SSR3	########	0.114069	0.188	0.628	########
NDUFS7	########	0.279329	0.358	0.74	########
RAB1A	########	0.154558	0.291	0.735	########
RSRC2	########	-0.14489	0.202	0.747	########
PDZD11	########	0.222476	0.19	0.594	########

SLC3A2	########	0.14773	0.328	0.757	########
ACTR3	########	-0.12335	0.188	0.7	########
TOMM20	########	0.218955	0.251	0.657	########
MAP2	########	-0.22414	0.176	0.712	########
CCT8	########	0.101008	0.265	0.738	########
MT3	########	1.037298	0.752	0.768	########
HIGD1A	########	0.168515	0.222	0.636	########
PLEKHJ1	########	-0.12968	0.14	0.633	########
LAMP1	########	-0.27395	0.148	0.674	########
ATP5E	########	0.426962	0.729	0.933	########
STMN3	########	-0.30983	0.134	0.675	########
KLHDC3	########	-0.31667	0.101	0.642	########
IP6K2	########	0.358373	0.304	0.688	########
ASAH1	########	0.264612	0.248	0.64	########
CHCHD3	########	-0.28217	0.152	0.716	########
HN1	########	-0.32675	0.349	0.914	########
TCEA1	########	-0.24208	0.208	0.756	########
ANP32E	########	-0.81337	0.072	0.714	########
TXNL1	########	0.114454	0.209	0.667	########
CCDC124	########	-0.11438	0.202	0.716	########
SUCLG1	########	0.273335	0.244	0.648	########
IMPDH2	########	0.359597	0.239	0.616	########
TUBG1	########	-0.67282	0.049	0.647	########
KIF22	########	-0.5378	0.086	0.679	########
PSMA7	########	0.194667	0.572	0.923	########
HDDC2	########	0.161635	0.194	0.614	########
KRTCAP2	########	0.278	0.279	0.672	########
HNRNPF	########	-0.21494	0.171	0.705	########
NME4	########	-0.32997	0.134	0.695	########
RSRC1	########	-0.19249	0.094	0.585	########
MKI67	########	-1.24465	0.003	0.637	########
MAGED2	########	0.331548	0.305	0.683	########
STIP1	########	-0.26803	0.11	0.638	########
HP1BP3	########	-0.56977	0.141	0.759	########
DHX36	########	-0.10218	0.169	0.677	########
SH3BGRL	########	0.323013	0.307	0.702	########
C1orf61	########	0.504573	0.658	0.893	########
ESD	########	0.148915	0.239	0.688	########
SUPT4H1	########	0.17461	0.187	0.614	########
LGALS3	########	1.213197	0.501	0.47	########
POLDIP2	########	-0.36535	0.099	0.619	########
APOPT1	########	0.137292	0.194	0.621	########
CBR1	########	0.201197	0.267	0.677	########
DNAJB11	########	-0.389	0.112	0.681	########
TUBB2A	########	-0.31189	0.264	0.83	########
CCDC88A	########	-0.34932	0.14	0.701	########
NDUFAF3	########	-0.15623	0.183	0.717	########
ITGB1BP1	########	-0.21236	0.157	0.678	########
STMN1	########	-0.36204	0.468	0.995	########
SARS	########	0.347521	0.23	0.591	########
EMC7	########	0.20457	0.192	0.595	########

DTD1	########	-0.32287	0.133	0.673	########
YY1	########	-0.23898	0.108	0.61	########
DCTN6	########	0.250969	0.216	0.616	########
TRAPPC3	########	0.233952	0.188	0.569	########
CALM1	########	0.227338	0.597	0.94	########
ERI3	########	-0.12746	0.145	0.654	########
CENPN	########	-1.0536	0.007	0.636	########
PTP4A2	########	-0.33998	0.143	0.683	########
PHF14	########	-0.21507	0.208	0.76	########
ETFB	########	0.123689	0.229	0.651	########
CCDC47	########	-0.2218	0.133	0.643	########
PTPRA	########	-0.2145	0.148	0.648	########
CHD9	########	-0.19065	0.202	0.744	########
DENR	########	-0.22535	0.094	0.594	########
N4BP2L2	########	0.172955	0.248	0.657	########
RHOC	########	0.604277	0.417	0.621	########
DHFR	########	-0.89298	0.033	0.66	########
PSME1	########	0.568066	0.333	0.627	########
S100A6	########	1.034587	0.574	0.62	########
TSEN34	########	-0.2722	0.119	0.632	########
SPARC	########	0.644747	0.403	0.616	########
EMC6	########	-0.35831	0.143	0.683	########
STOML2	########	-0.13699	0.159	0.656	########
VKORC1	########	0.15031	0.222	0.63	########
PSMD3	########	-0.37574	0.103	0.658	########
GPBP1	########	-0.11891	0.19	0.693	########
PSMC4	########	0.392717	0.297	0.652	########
ATXN10	########	-0.24271	0.166	0.7	########
YIF1A	########	0.413271	0.227	0.527	########
ARPC5L	########	-0.51054	0.08	0.644	########
KMT2E	########	0.108724	0.307	0.767	########
PSENEN	########	0.58733	0.3	0.575	########
CIB1	########	0.449704	0.208	0.501	########
EMC3	########	0.242525	0.218	0.616	########
CST3	########	0.422404	0.616	0.925	########
EXOSC8	########	-0.2847	0.136	0.691	########
TMEM219	########	0.332692	0.23	0.573	########
TPM4	########	-0.51099	0.155	0.744	########
NUDCD2	########	-0.18386	0.175	0.695	########
PFN1	########	-0.13673	0.417	0.905	########
SRM	########	-0.1159	0.166	0.663	########
NOL7	########	-0.19441	0.141	0.656	########
ATP1B3	########	-0.51005	0.119	0.717	########
FKBP2	########	0.54635	0.372	0.643	########
CCDC12	########	0.156118	0.225	0.651	########
SAE1	########	-0.66086	0.084	0.702	########
CD320	########	-0.32696	0.134	0.675	########
ELAVL1	########	-0.35394	0.124	0.664	########
CHTOP	########	-0.25332	0.122	0.652	########
GSTK1	########	0.697376	0.281	0.504	########
C19orf48	########	-0.71394	0.079	0.704	########

DNAJC19	########	0.183886	0.206	0.621	########
LMO4	########	-0.12672	0.283	0.78	########
TADA3	########	-0.12821	0.152	0.636	########
NDUFA10	########	0.181573	0.206	0.62	########
WBP11	########	-0.30905	0.08	0.593	########
MED4	########	0.154113	0.183	0.601	########
UBE2B	########	0.123491	0.187	0.631	########
TPGS2	########	-0.41282	0.112	0.679	########
COMMD4	########	-0.33762	0.101	0.637	########
RBBP4	########	-0.10743	0.213	0.728	########
SDF4	########	-0.42287	0.092	0.633	########
THRAP3	########	-0.20584	0.14	0.646	########
THOC7	########	-0.20585	0.113	0.625	########
CNOT7	########	-0.30895	0.099	0.637	########
PPP1CB	########	-0.18088	0.22	0.737	########
LINC0011€	########	0.173778	0.171	0.567	########
TOMM40	########	-0.22126	0.119	0.631	########
UBE2L6	########	0.615621	0.291	0.56	########
ARL6IP5	########	0.741406	0.361	0.565	########
SNRNP27	########	-0.15666	0.122	0.611	########
EZH2	########	-0.89163	0.045	0.688	########
UFD1L	########	-0.32617	0.087	0.616	########
DGCR6L	########	-0.15375	0.11	0.589	########
PSMD11	########	-0.25424	0.117	0.633	########
TIMP1	########	1.023184	0.62	0.628	########
FYN	########	-0.10971	0.166	0.653	########
ZNF207	########	-0.24611	0.169	0.701	########
ZBTB20	########	-0.10358	0.225	0.731	########
IDH2	########	-0.51716	0.115	0.705	########
UBA2	########	-0.43385	0.113	0.694	########
CHMP5	########	0.197516	0.225	0.647	########
SCCPDH	########	-0.18476	0.145	0.659	########
RSF1	########	-0.14886	0.171	0.677	########
GMNN	########	-0.66361	0.068	0.67	########
ATP5EP2	########	0.573499	0.271	0.514	########
PCM1	########	-0.44797	0.096	0.656	########
TSPO	########	0.393145	0.223	0.531	########
FUNDC2	########	0.16265	0.208	0.643	########
TRIB2	########	-0.12661	0.145	0.619	########
CLDND1	########	0.139883	0.187	0.602	########
FSCN1	########	-0.39035	0.192	0.774	########
HSPB11	########	-0.10901	0.126	0.619	########
LYPLA1	########	-0.18535	0.136	0.643	########
PPA1	########	0.209337	0.195	0.599	########
TROVE2	########	0.102904	0.23	0.685	########
PDPN	########	0.400185	0.325	0.662	########
GPS1	########	-0.1712	0.113	0.589	########
CENPV	########	-0.94588	0.063	0.707	########
LAGE3	########	-0.16818	0.119	0.61	########
BRI3	########	-0.17613	0.143	0.627	########
GPX4	########	0.180995	0.565	0.917	########

MANF	########	-0.2374	0.161	0.68	########
TRAPPC4	########	0.122288	0.192	0.62	########
BRD4	########	-0.27961	0.092	0.575	########
UBB	########	0.356248	0.768	0.965	########
RNF114	########	-0.36208	0.103	0.631	########
BNIP3L	########	0.316125	0.29	0.663	########
SLC39A6	########	-0.20563	0.133	0.626	########
SLC25A4	########	0.122707	0.232	0.662	########
DDAH2	########	-0.28002	0.134	0.673	########
MAGEF1	########	-0.15708	0.173	0.675	########
SGOL2	########	-1.17036	0.01	0.633	########
PFDN4	########	-0.33787	0.086	0.604	########
GSPT1	########	-0.40155	0.087	0.623	########
SRSF4	########	-0.32818	0.103	0.625	########
DDX17	########	-0.19689	0.15	0.648	########
TMEM256	########	0.197342	0.175	0.577	########
SNRNP40	########	-0.24675	0.113	0.64	########
HLA-A	########	0.759938	0.789	0.841	########
PAICS	########	-0.17277	0.176	0.694	########
CAPRIN1	########	-0.31604	0.079	0.58	########
PSME2	########	0.255888	0.325	0.727	########
HSPB1	########	0.233168	0.403	0.794	########
GTSE1	########	-1.1614	0.002	0.614	########
SLC39A1	########	-0.10302	0.115	0.552	########
SMARCE1	########	-0.33997	0.112	0.652	########
TERF2IP	########	-0.15671	0.197	0.719	########
PRR13	########	0.402285	0.206	0.53	########
AKIRIN1	########	-0.36707	0.077	0.607	########
EIF3J	########	-0.27282	0.099	0.594	########
CNIH4	########	-0.10079	0.168	0.658	########
UBE2E1	########	0.241582	0.23	0.626	########
FAM192A	########	-0.15776	0.117	0.598	########
HSPA9	########	0.116612	0.237	0.679	########
TKT	########	0.174076	0.199	0.598	########
RAD23B	########	-0.17316	0.126	0.625	########
RAD51C	########	-0.30641	0.103	0.621	########
GNG5	########	0.303632	0.461	0.798	########
ORC6	########	-0.99885	0.019	0.641	########
SYNCRIP	########	-0.47012	0.108	0.683	########
TIMM17A	########	0.10384	0.147	0.567	########
NELFCD	########	-0.25706	0.126	0.631	########
C20orf24	########	-0.6733	0.101	0.7	########
PRKDC	########	-0.40804	0.098	0.63	########
CCDC14	########	-0.46906	0.079	0.627	########
NSA2	########	-0.14476	0.129	0.598	########
NT5C3B	########	-0.52532	0.084	0.652	########
RHOB	########	0.121572	0.304	0.738	########
SDHD	########	0.287546	0.192	0.549	########
U2SURP	########	-0.14506	0.134	0.628	########
MAPK1IP1L	########	-0.10963	0.145	0.616	########
HADHB	########	0.124727	0.206	0.637	########

BRD7	########	-0.37104	0.084	0.596	########
ACAT2	########	-0.55495	0.061	0.622	########
ZMAT2	########	0.196628	0.183	0.575	########
SEC61A1	########	-0.13404	0.117	0.599	########
DYNC1I2	########	-0.20662	0.161	0.667	########
EIF3M	########	0.39691	0.309	0.659	########
LRPAP1	########	0.297691	0.293	0.649	########
IGFBP2	########	0.10693	0.394	0.819	########
CHI3L1	########	1.376049	0.475	0.506	########
NIFK	########	0.120283	0.173	0.599	########
FLOT1	########	-0.18691	0.159	0.643	########
APOA1BP	########	-0.1884	0.136	0.631	########
SPC25	########	-1.10161	0.01	0.62	########
TM2D1	########	0.223943	0.183	0.554	########
MSI2	########	0.165811	0.237	0.619	########
HDLBP	########	-0.12489	0.11	0.569	########
BAALC	########	0.110524	0.243	0.672	########
CENPW	########	-0.99863	0.012	0.615	########
SGTA	########	-0.27489	0.086	0.581	########
STAU1	########	-0.43861	0.084	0.612	########
DDX24	########	0.169643	0.201	0.615	########
GTF2H5	########	0.152001	0.164	0.575	########
ANP32A	########	-0.28411	0.122	0.651	########
S100A11	########	0.90496	0.34	0.474	########
PPIA	########	0.380109	0.791	0.983	########
CENPM	########	-1.04793	0.007	0.615	########
PSMC2	########	0.110752	0.22	0.658	########
STX10	########	-0.12167	0.124	0.58	########
CLTB	########	-0.25601	0.117	0.619	########
GBAS	########	-0.25711	0.117	0.641	########
H1FX	########	-0.72938	0.122	0.749	########
FBL	########	0.158196	0.218	0.646	########
SUMO2	########	0.180323	0.642	0.957	########
DNAJB1	########	0.385767	0.407	0.773	########
GRHPR	########	-0.10076	0.14	0.612	########
MED28	########	0.318088	0.161	0.49	########
PHIP	########	-0.45183	0.11	0.674	########
CCDC85B	########	-0.25784	0.115	0.604	########
11-Sep	########	-0.17095	0.129	0.616	########
LSM10	########	0.222278	0.164	0.531	########
PEA15	########	0.245372	0.26	0.651	########
JUN	########	0.100772	0.459	0.883	########
ERCC1	########	0.229661	0.232	0.631	########
ITM2C	########	0.173777	0.248	0.646	########
FAM3C	########	-0.15533	0.211	0.721	########
WTAP	########	-0.17776	0.138	0.635	########
TIMMDC1	########	0.189173	0.171	0.547	########
PTP4A1	########	-0.49255	0.094	0.663	########
TIMM10	########	-0.35441	0.11	0.628	########
ARL3	########	0.137138	0.162	0.563	########
UBE2J2	########	-0.40379	0.063	0.58	########

PTMS	########	-0.43071	0.283	0.856	########
CMSS1	########	-0.25766	0.105	0.602	########
C4orf48	########	-0.34945	0.126	0.641	########
PSMC1	########	0.161227	0.199	0.6	########
ACTB	########	0.440815	0.934	0.996	########
CHMP4B	########	-0.24854	0.129	0.607	########
REEP5	########	0.378088	0.283	0.628	########
MZT1	########	-0.77214	0.084	0.706	########
CD74	########	1.199111	0.621	0.527	########
APEX1	########	0.377665	0.342	0.694	########
PRKRA	########	-0.1702	0.127	0.594	########
NAE1	########	-0.25986	0.143	0.663	########
BAZ1B	########	-0.6253	0.07	0.653	########
SEC63	########	-0.2466	0.105	0.609	########
GPAA1	########	-0.46713	0.112	0.665	########
HIF1A	########	-0.25015	0.157	0.657	########
MAP1LC3B	########	-0.16638	0.166	0.664	########
HSD17B12	########	0.123088	0.169	0.568	########
CLPP	########	-0.13496	0.168	0.648	########
RNASEH2B	########	-0.6633	0.045	0.621	########
RALBP1	########	-0.16975	0.082	0.527	########
PIH1D1	########	0.153449	0.171	0.564	########
ANAPC16	########	0.349184	0.201	0.552	########
C8orf33	########	-0.17679	0.126	0.607	########
AK2	########	0.262364	0.19	0.57	########
RBM42	########	-0.33732	0.129	0.669	########
TUBA1A	########	0.37623	0.864	0.989	########
SLC39A3	########	-0.37495	0.112	0.628	########
ZDHHC4	########	0.203679	0.185	0.564	########
RHNO1	########	-0.53336	0.059	0.6	########
USP22	########	-0.30232	0.103	0.593	########
HAT1	########	-0.34106	0.131	0.684	########
EMC10	########	-0.10714	0.173	0.659	########
TMEM11	########	-0.11499	0.127	0.594	########
URM1	########	0.187032	0.145	0.507	########
GHITM	########	0.284795	0.208	0.577	########
SNX6	########	0.194741	0.175	0.56	########
IFITM3	########	1.15046	0.396	0.416	########
IAH1	########	0.131015	0.19	0.599	########
CHCHD2	########	0.317297	0.77	0.97	########
RBM17	########	-0.48913	0.08	0.62	########
BOD1	########	-0.38343	0.087	0.606	########
TMEM237	########	-0.54072	0.056	0.611	########
CISD2	########	-0.12877	0.134	0.606	########
TTC19	########	-0.18152	0.119	0.594	########
HSP90AA1	########	0.335915	0.799	0.986	########
WDR45B	########	-0.2941	0.129	0.631	########
TSG101	########	0.21959	0.187	0.558	########
XPO1	########	-0.36558	0.087	0.611	########
SRP72	########	-0.26606	0.173	0.714	########
KIF21A	########	-0.1207	0.162	0.652	########

WIPI2	########	-0.34808	0.094	0.614	########
PXMP2	########	-0.50653	0.063	0.602	########
VPS25	########	0.154677	0.126	0.505	########
CCNA2	########	-1.13321	0.005	0.601	########
NFIB	########	-0.26227	0.239	0.79	########
MXD3	########	-0.99853	0.007	0.59	########
COPRS	########	-0.56164	0.08	0.625	########
CTNNA1	########	0.136697	0.176	0.558	########
UNC50	########	0.158072	0.162	0.538	########
WHSC1L1	########	-0.49825	0.101	0.664	########
TEX30	########	-0.56203	0.052	0.596	########
SMARCA5	########	-0.45194	0.086	0.619	########
NSMCE1	########	0.327184	0.206	0.543	########
CDH2	########	-0.31667	0.12	0.626	########
CTSD	########	0.247151	0.218	0.588	########
NUCB2	########	-0.14935	0.133	0.619	########
TMEM98	########	-0.31097	0.112	0.606	########
YBX1	########	-0.23344	0.478	0.959	########
SLC35B2	########	-0.48627	0.075	0.594	########
NARF	########	-0.34712	0.089	0.579	########
C14orf1	########	0.224776	0.169	0.523	########
AHCY	########	-0.26358	0.103	0.586	########
NFIX	########	-0.30391	0.154	0.674	########
ZNF580	########	-0.18734	0.084	0.522	########
LEPROTL1	########	-0.19663	0.106	0.586	########
PRPF38B	########	-0.18465	0.161	0.668	########
SRPK2	########	-0.26266	0.183	0.725	########
SPECC1	########	-0.45473	0.091	0.616	########
DBF4	########	-0.99878	0.017	0.611	########
MAP1LC3A	########	-0.19176	0.124	0.583	########
RFXANK	########	0.342555	0.175	0.486	########
EIF2AK1	########	-0.51809	0.079	0.635	########
ASRGL1	########	-0.76461	0.063	0.667	########
PHAX	########	-0.14427	0.105	0.564	########
KIFC1	########	-0.84643	0.019	0.593	########
CYB5B	########	0.118902	0.148	0.548	########
COMMD7	########	-0.37708	0.094	0.607	########
CSNK2A1	########	-0.30399	0.099	0.593	########
EIF2S1	########	-0.23524	0.091	0.568	########
CMPK1	########	-0.30447	0.105	0.607	########
TMEM97	########	-0.55379	0.054	0.599	########
SLTM	########	-0.18025	0.098	0.565	########
PRPSAP1	########	-0.55597	0.089	0.638	########
RNF126	########	-0.57507	0.052	0.589	########
KARS	########	-0.25171	0.119	0.614	########
SNRPA	########	-0.27232	0.084	0.574	########
SPAG9	########	-0.21269	0.143	0.623	########
PRPF6	########	-0.19996	0.122	0.598	########
RAMP1	########	0.789052	0.613	0.709	########
HLA-B	########	0.781118	0.716	0.796	########
PTS	########	-0.23414	0.089	0.564	########

SPTSSA	########	0.282678	0.264	0.631	########
PTMA	########	0.445195	0.895	0.985	########
CCDC90B	########	0.130118	0.201	0.609	########
ARL4C	########	-0.4389	0.113	0.627	########
GLRX5	########	-0.51386	0.087	0.638	########
HNRNPA1	########	0.266818	0.742	0.98	########
SF3B1	########	-0.16376	0.161	0.654	########
PEPD	########	0.285456	0.169	0.499	########
LRRC59	########	-0.57003	0.068	0.611	########
TMSB15A	########	-0.89314	0.037	0.635	########
RFC4	########	-0.69247	0.044	0.61	########
MAGED1	########	0.122054	0.215	0.635	########
FAM229B	########	0.294828	0.176	0.515	########
MYL12A	########	0.195438	0.143	0.477	########
SPG21	########	-0.17792	0.08	0.525	########
HSPA5	########	0.384182	0.386	0.71	########
GRB2	########	-0.38406	0.082	0.59	########
SOD2	########	1.089525	0.33	0.443	########
HNRNPH1	########	-0.24471	0.206	0.727	########
RPA1	########	-0.42461	0.061	0.579	########
CRIPT	########	0.109406	0.131	0.509	########
PPDPF	########	-0.12113	0.478	0.916	########
PRKAR1A	########	-0.13201	0.162	0.627	########
HSPH1	########	-0.37568	0.087	0.61	########
PPP1R14B	########	-0.27522	0.138	0.626	########
COX14	########	0.319696	0.19	0.53	########
SEC61G	########	0.780187	0.845	0.923	########
MESDC2	########	0.248087	0.188	0.523	########
NEAT1	########	0.731202	0.581	0.669	########
DAZAP2	########	0.261031	0.223	0.586	########
DNTTIP1	########	-0.24368	0.113	0.584	########
APRT	########	0.408874	0.225	0.51	########
HMGXB4	########	-0.50414	0.061	0.596	########
ERLEC1	########	-0.10089	0.122	0.573	########
PQBP1	########	0.220866	0.141	0.494	########
CYTH2	########	-0.40823	0.098	0.635	########
ARL6IP6	########	-0.55556	0.099	0.674	########
ZFAND6	########	0.175341	0.22	0.607	########
PRDX3	########	0.24807	0.195	0.573	########
YWHAG	########	-0.1658	0.124	0.6	########
ANKRD12	########	0.173313	0.162	0.522	########
RPA2	########	-0.2041	0.136	0.635	########
TMSB4X	########	0.554269	0.977	0.994	########
TCF12	########	-0.11671	0.154	0.615	########
9-Sep	########	-0.26741	0.133	0.612	########
NUDT5	########	-0.1027	0.082	0.499	########
KNOP1	########	-0.12446	0.106	0.558	########
WBP4	########	0.157963	0.136	0.519	########
PIGT	########	-0.2215	0.113	0.586	########
SLC25A39	########	-0.26397	0.094	0.562	########
CSE1L	########	-0.6208	0.056	0.62	########

DCUN1D5	########	-0.13908	0.087	0.514	########
MCM7	########	-0.78255	0.105	0.725	########
GNAI3	########	-0.21222	0.098	0.573	########
RALA	########	-0.39606	0.099	0.63	########
VPS4A	########	-0.4468	0.058	0.569	########
BPTF	########	-0.23125	0.108	0.578	########
TERF1	########	-0.12519	0.138	0.604	########
MAF1	########	-0.29126	0.115	0.622	########
HRAS	########	-0.57748	0.051	0.583	########
GTF2I	########	-0.10346	0.209	0.686	########
CDK4	########	-0.32958	0.279	0.837	########
CCNB1	########	-1.31875	0.01	0.599	########
MAT2A	########	-0.14327	0.138	0.606	########
CRNDE	########	-0.66639	0.066	0.643	########
SF3B4	########	-0.13147	0.113	0.564	########
ACSL3	########	-0.27239	0.113	0.591	########
S100A10	########	0.968736	0.492	0.542	########
VMP1	########	0.162992	0.215	0.598	########
UQCRC2	########	0.181763	0.204	0.595	########
AP2B1	########	-0.35745	0.096	0.589	########
CTNNBL1	########	-0.1983	0.096	0.565	########
HINT2	########	-0.31783	0.113	0.606	########
LSM6	########	0.146027	0.166	0.557	########
NLRP1	########	-0.12473	0.15	0.6	########
PHF5A	########	-0.11309	0.126	0.578	########
SMC3	########	-0.52592	0.08	0.635	########
ASF1B	########	-0.94172	0.007	0.573	########
SLC1A3	########	0.375147	0.3	0.595	########
ITGB3BP	########	-0.39768	0.068	0.579	########
HNRNPA2B1	########	0.20353	0.717	0.974	########
HAGH	########	-0.54296	0.072	0.611	########
RNF145	########	-0.38158	0.115	0.627	########
H2AFZ	########	-0.32268	0.49	0.98	########
CSTF3	########	-0.2409	0.099	0.584	########
KHSRP	########	-0.45606	0.049	0.542	########
VGLL4	########	-0.21157	0.122	0.58	########
STARD3NL	########	-0.17342	0.164	0.647	########
TMEM165	########	-0.38485	0.148	0.689	########
ACTR2	########	-0.28964	0.099	0.579	########
SCARB2	########	-0.15022	0.119	0.574	########
ATRX	########	-0.1089	0.185	0.657	########
CUEDC2	########	0.154389	0.195	0.584	########
CKAP2L	########	-1.05142	0.002	0.574	########
ARMCX3	########	0.316329	0.222	0.551	########
AURKB	########	-1.1628	0.003	0.58	########
BCAS2	########	0.200703	0.213	0.602	########
CNDP2	########	0.110909	0.127	0.498	########
IDH3G	########	0.192752	0.168	0.537	########
ATP6V1D	########	0.101055	0.134	0.526	########
B3GAT3	########	-0.10546	0.134	0.567	########
FEN1	########	-0.87495	0.024	0.595	########

ACBD6	########	-0.15382	0.091	0.537	########
SQSTM1	########	0.185021	0.202	0.574	########
PTPMT1	########	-0.22659	0.084	0.536	########
OTUB1	########	0.207926	0.154	0.511	########
C5orf24	########	-0.39782	0.075	0.569	########
CCNB2	########	-1.25941	0	0.56	########
AKR1A1	########	0.171417	0.161	0.54	########
ARF3	########	0.11533	0.147	0.515	########
ACTR10	########	0.223575	0.176	0.536	########
LYRM2	########	-0.1705	0.108	0.57	########
ALDH7A1	########	0.127046	0.19	0.598	########
LMAN1	########	0.21301	0.117	0.441	########
SF3A2	########	-0.5364	0.07	0.6	########
RUVBL2	########	-0.35902	0.133	0.675	########
SCAF11	########	-0.15053	0.133	0.585	########
MKRN1	########	-0.26968	0.122	0.631	########
C19orf24	########	-0.34794	0.072	0.537	########
SUGP2	########	-0.29407	0.101	0.596	########
ZFAND5	########	-0.10092	0.145	0.59	########
SGCB	########	-0.11706	0.227	0.728	########
FOXM1	########	-0.92095	0.005	0.568	########
CHMP3	########	-0.12722	0.124	0.574	########
NT5DC2	########	-0.59225	0.035	0.546	########
TMEM9	########	0.405862	0.239	0.547	########
ENAH	########	-0.40383	0.08	0.578	########
ACYP1	########	-0.57684	0.058	0.599	########
NES	########	-0.2041	0.175	0.637	########
PKIG	########	0.268107	0.195	0.533	########
ELOVL5	########	-0.40059	0.087	0.6	########
NRBP1	########	-0.22996	0.098	0.56	########
DCAF7	########	-0.35218	0.105	0.615	########
ARL6IP1	########	-0.22859	0.407	0.916	########
EPN1	########	-0.24192	0.098	0.558	########
CCZ1	########	-0.21861	0.136	0.61	########
SS18L2	########	-0.17082	0.087	0.53	########
ISOC2	########	0.280783	0.194	0.512	########
TMEM54	########	-0.45839	0.045	0.531	########
PGP	########	-0.82716	0.017	0.583	########
PPP2R3C	########	-0.19117	0.106	0.572	########
NT5C3A	########	-0.31588	0.101	0.602	########
LARP7	########	-0.21644	0.115	0.59	########
SRSF6	########	-0.32727	0.112	0.615	########
GGNBP2	########	-0.27962	0.126	0.62	########
CTDNEP1	########	-0.46295	0.065	0.557	########
SNX5	########	-0.40643	0.119	0.622	########
IDI1	########	-0.15166	0.079	0.507	########
EIF4EBP1	########	-0.2494	0.15	0.648	########
APLP1	########	-0.26654	0.14	0.633	########
TACC3	########	-0.94972	0.009	0.57	########
CD47	########	-0.29777	0.112	0.602	########
PIGX	########	-0.39926	0.073	0.575	########

AKAP9	########	0.105029	0.204	0.605	########
RAB18	########	0.235591	0.164	0.502	########
DPYSL2	########	-0.18164	0.134	0.611	########
BLVRA	########	0.163661	0.173	0.558	########
CYB5R3	########	-0.15318	0.12	0.562	########
TMEM222	########	0.169939	0.127	0.474	########
CORO1C	########	-0.10974	0.171	0.638	########
FAM136A	########	-0.22734	0.079	0.528	########
CRIP2	########	0.111951	0.209	0.604	########
C6orf62	########	-0.34268	0.103	0.595	########
SNHG7	########	-0.11445	0.122	0.56	########
FIP1L1	########	-0.27544	0.117	0.612	########
TSPAN5	########	-0.29993	0.105	0.589	########
MPST	########	-0.46697	0.065	0.58	########
RFC1	########	-0.30576	0.092	0.584	########
BCL7B	########	-0.31181	0.092	0.58	########
DHRS7	########	0.276581	0.208	0.523	########
LAP3	########	0.587419	0.288	0.528	########
TRIM28	########	-0.35493	0.086	0.583	########
METRN	########	-0.20469	0.178	0.652	########
CHN1	########	-0.22097	0.089	0.556	########
AHCYL1	########	0.341686	0.215	0.526	########
MPG	########	0.13968	0.127	0.468	########
RFC2	########	-0.53369	0.068	0.605	########
PSMD1	########	-0.20349	0.124	0.605	########
BUB3	########	-0.52545	0.096	0.648	########
STX8	########	0.281657	0.171	0.506	########
CCM2	########	-0.35797	0.07	0.552	########
PHF20	########	-0.16979	0.098	0.533	########
TMEM123	########	-0.19981	0.108	0.563	########
ATP1A1	########	0.243634	0.215	0.552	########
FEZ2	########	-0.22512	0.134	0.604	########
CHURC1	########	0.169481	0.159	0.5	########
GRINA	########	0.121383	0.143	0.499	########
HDGF	########	-0.55181	0.075	0.616	########
BCLAF1	########	-0.27403	0.138	0.627	########
DNAJC9	########	-0.63233	0.042	0.581	########
TMEM183A	########	-0.43227	0.042	0.517	########
C20orf27	########	-0.19215	0.086	0.5	########
ERAL1	########	-0.12136	0.089	0.5	########
MVB12A	########	-0.19702	0.099	0.541	########
CCDC82	########	-0.18001	0.094	0.512	########
STARD7	########	-0.15985	0.12	0.574	########
SH3GLB1	########	-0.19093	0.113	0.564	########
CCDC107	########	-0.20471	0.099	0.557	########
RRM1	########	-0.73312	0.056	0.626	########
PON2	########	0.417678	0.382	0.684	########
CRLS1	########	-0.29435	0.075	0.531	########
ZFP36L1	########	0.248552	0.281	0.609	########
GNAI2	########	-0.17389	0.129	0.569	########
MPHOSPH8	########	0.210271	0.178	0.553	########

EMC8	########	-0.25544	0.075	0.527	########
THOP1	########	-0.66307	0.019	0.535	########
NUDT3	########	-0.24145	0.113	0.583	########
IMMP1L	########	-0.19521	0.089	0.536	########
TRAF4	########	-0.3347	0.112	0.602	########
DDRGK1	########	-0.13599	0.136	0.574	########
MTIF3	########	0.118775	0.092	0.441	########
GANAB	########	-0.2698	0.084	0.537	########
RACGAP1	########	-0.95027	0.012	0.573	########
ILK	########	0.196463	0.145	0.49	########
NOP58	########	-0.33468	0.096	0.599	########
TTC1	########	0.208374	0.138	0.483	########
SIRT2	########	0.294945	0.197	0.542	########
FKBP4	########	-0.34494	0.072	0.552	########
ARID4B	########	-0.13019	0.15	0.599	########
DECR1	########	0.243359	0.222	0.575	########
RAB14	########	-0.15955	0.141	0.6	########
SUMO3	########	-0.19001	0.124	0.591	########
PAK2	########	-0.32893	0.082	0.549	########
TSEN15	########	-0.17766	0.072	0.499	########
SYNE2	########	-0.68082	0.049	0.595	########
UBE3A	########	-0.40849	0.07	0.548	########
BARD1	########	-0.75562	0.033	0.583	########
CCND2	########	-0.25561	0.187	0.705	########
ADI1	########	-0.29115	0.117	0.614	########
CREB3	########	-0.12924	0.086	0.504	########
ACTL6A	########	-0.34634	0.08	0.572	########
TMEM179B	########	0.230356	0.155	0.49	########
PPIH	########	-0.29403	0.066	0.528	########
TSC22D4	########	0.656301	0.475	0.672	########
AKIRIN2	########	-0.46349	0.087	0.61	########
PHGDH	########	-0.29813	0.152	0.673	########
C7orf55-L	########	-0.37577	0.129	0.654	########
PITHD1	########	-0.5724	0.073	0.619	########
EZR	########	-0.32327	0.087	0.562	########
RABL6	########	-0.39085	0.063	0.533	########
CDKN2AIPN	########	-0.27385	0.077	0.537	########
DCAF13	########	0.262962	0.194	0.52	########
JUNB	########	0.288313	0.424	0.767	########
RSBN1L	########	-0.19555	0.134	0.611	########
UCHL1	########	0.363191	0.358	0.673	########
CDC123	########	0.105946	0.12	0.483	########
DARS	########	0.258266	0.182	0.523	########
NXT1	########	-0.48133	0.068	0.573	########
PPP2CB	########	-0.38977	0.08	0.552	########
TMED1	########	0.436028	0.19	0.46	########
TRAPPC2L	########	0.13381	0.159	0.543	########
FGD5-AS1	########	-0.41912	0.082	0.583	########
MPDU1	########	-0.12807	0.126	0.564	########
ECI1	########	-0.44524	0.045	0.512	########
QDPR	########	0.147399	0.162	0.538	########

HMG20B	########	-0.27192	0.103	0.556	########
SUPT16H	########	-0.10071	0.117	0.559	########
AKR7A2	########	-0.26215	0.084	0.548	########
NPDC1	########	-0.11017	0.14	0.567	########
CDKN2C	########	-0.30289	0.216	0.737	########
TIMM22	########	-0.16378	0.08	0.5	########
COMMD3	########	0.215861	0.154	0.494	########
ETV1	########	-0.27802	0.157	0.658	########
PSMD12	########	-0.29329	0.092	0.557	########
NIPA2	########	-0.34115	0.056	0.512	########
SYAP1	########	-0.10499	0.087	0.489	########
GORASP2	########	-0.22749	0.082	0.521	########
PAFAH1B1	########	-0.38302	0.086	0.562	########
ATG3	########	-0.22739	0.089	0.543	########
PMP2	########	0.200305	0.344	0.677	########
UBE2K	########	-0.17759	0.077	0.502	########
FTH1	########	0.343226	0.806	0.981	########
WDR61	########	0.314658	0.194	0.519	########
GINM1	########	-0.20272	0.075	0.491	########
CCNL1	########	-0.12062	0.194	0.663	########
DCTN2	########	-0.23305	0.236	0.757	########
VPS35	########	-0.18577	0.14	0.611	########
DTNA	########	0.840852	0.222	0.358	########
NREP	########	-0.29656	0.103	0.585	########
GRN	########	0.141094	0.175	0.523	########
FNTA	########	-0.33791	0.094	0.585	########
PAFAH1B2	########	-0.28679	0.084	0.546	########
UTP11L	########	-0.10661	0.092	0.507	########
C21orf59	########	-0.21707	0.092	0.533	########
LYRM5	########	0.344761	0.176	0.468	########
SMIM20	########	0.183806	0.12	0.446	########
DBN1	########	-0.43584	0.117	0.644	########
COMMD1	########	0.308391	0.175	0.484	########
SYPL1	########	0.330778	0.19	0.494	########
MAZ	########	-0.53192	0.052	0.563	########
DAZAP1	########	-0.47644	0.052	0.551	########
GAMT	########	-0.53471	0.058	0.562	########
KLC1	########	-0.19535	0.124	0.584	########
EIF2AK2	########	-0.11899	0.099	0.522	########
TMSB15B	########	-0.60909	0.052	0.586	########
SLBP	########	-0.58435	0.063	0.593	########
RAC3	########	-0.55194	0.042	0.533	########
CNIH1	########	0.193758	0.175	0.537	########
CALM2	########	0.324395	0.815	0.989	########
C12orf10	########	0.233881	0.134	0.46	########
RAP1A	########	0.130358	0.143	0.5	########
6-Mar	########	-0.12329	0.115	0.551	########
RBCK1	########	-0.34015	0.07	0.528	########
PPP2R5E	########	-0.44637	0.049	0.52	########
DLGAP4	########	-0.13045	0.079	0.479	########
TSR3	########	-0.26888	0.082	0.517	########

MLST8	########	-0.12999	0.079	0.488	########
TAF15	########	-0.42526	0.073	0.564	########
LCMT1	########	-0.20793	0.068	0.495	########
CFL2	########	0.126785	0.168	0.546	########
RAE1	########	-0.28612	0.084	0.548	########
CHD4	########	-0.24814	0.098	0.544	########
RUVBL1	########	-0.2244	0.099	0.554	########
TK1	########	-0.94118	0.003	0.533	########
ZFAND3	########	-0.11891	0.08	0.477	########
PEF1	########	0.256061	0.175	0.512	########
TUBB	########	-0.26391	0.56	0.995	########
NDUFA9	########	0.176451	0.204	0.579	########
TMEM259	########	-0.3103	0.089	0.559	########
IFNGR2	########	-0.24669	0.073	0.501	########
WDR1	########	-0.13066	0.127	0.549	########
SPATS2	########	-0.28298	0.112	0.584	########
VAMP5	########	0.739355	0.241	0.405	########
SH3GL1	########	-0.3082	0.066	0.51	########
BCL7C	########	-0.81701	0.031	0.574	########
IFT52	########	-0.21159	0.079	0.507	########
TSSC4	########	-0.24712	0.098	0.556	########
YIF1B	########	-0.14488	0.096	0.517	########
MAGOHB	########	-0.45459	0.073	0.583	########
TIMM9	########	0.126991	0.129	0.486	########
GNL1	########	-0.38794	0.065	0.528	########
STK25	########	-0.5302	0.066	0.581	########
CEBPZ	########	-0.18433	0.082	0.515	########
PODXL2	########	-0.4252	0.12	0.641	########
CMTM6	########	-0.19361	0.105	0.549	########
PJA2	########	-0.15872	0.106	0.51	########
ASPM	########	-1.09622	0.002	0.535	########
STX12	########	0.193418	0.15	0.477	########
SNRNP200	########	-0.27753	0.096	0.568	########
MAP2K2	########	-0.3605	0.087	0.546	########
USE1	########	0.444808	0.209	0.479	########
EGFR	########	-0.13274	0.257	0.723	########
BAZ1A	########	-0.33575	0.099	0.577	########
NKIRAS2	########	-0.16622	0.077	0.5	########
PPP1R12A	########	-0.10239	0.12	0.541	########
HJURP	########	-1.02929	0	0.525	########
HNRNPH2	########	0.298526	0.19	0.491	########
FAM210B	########	-0.71575	0.049	0.581	########
PDHB	########	0.243968	0.185	0.533	########
CISD1	########	0.225351	0.168	0.504	########
CDK16	########	-0.35387	0.072	0.536	########
TMX4	########	-0.55061	0.042	0.532	########
SPATS2L	########	-0.23952	0.11	0.546	########
CSNK1E	########	-0.14759	0.133	0.568	########
MCRS1	########	-0.32003	0.073	0.537	########
ATP6V1E1	########	0.190693	0.175	0.527	########
FZD3	########	-0.29013	0.108	0.577	########

FAM96A	########	0.146058	0.133	0.48	########
ANKRD11	########	-0.25142	0.094	0.541	########
FBXO5	########	-0.87939	0.016	0.552	########
TUSC2	########	-0.23678	0.087	0.532	########
C22orf39	########	-0.42872	0.066	0.553	########
POU3F2	########	-0.5525	0.08	0.591	########
PDCD2	########	-0.37176	0.072	0.548	########
BAG6	########	-0.26117	0.086	0.535	########
UBE2R2	########	-0.36952	0.058	0.515	########
TRAP1	########	-0.36099	0.063	0.527	########
DDX27	########	-0.14931	0.07	0.459	########
C19orf25	########	-0.23048	0.08	0.515	########
ELP5	########	-0.44264	0.047	0.514	########
MT1M	########	0.645026	0.239	0.458	########
PHF19	########	-0.45722	0.054	0.541	########
POLE3	########	-0.26757	0.086	0.541	########
TAX1BP3	########	0.17079	0.126	0.464	########
WDR18	########	-0.23823	0.08	0.521	########
DKC1	########	-0.22061	0.094	0.542	########
NBPF1	########	-0.45818	0.049	0.53	########
NCBP2-AS2	########	-0.31797	0.07	0.523	########
C6orf1	########	0.342431	0.152	0.43	########
NME3	########	0.12291	0.145	0.478	########
ARMC10	########	-0.27192	0.075	0.526	########
STK17A	########	-0.21052	0.084	0.507	########
ZNF24	########	0.173301	0.148	0.494	########
GPN3	########	-0.13261	0.075	0.474	########
ABHD12	########	-0.48913	0.082	0.58	########
MTX2	########	-0.19044	0.106	0.551	########
HES6	########	-0.3365	0.218	0.752	########
C1orf35	########	-0.39262	0.056	0.525	########
THOC2	########	-0.13714	0.12	0.548	########
SMIM19	########	-0.11587	0.099	0.505	########
METTL23	########	-0.29091	0.077	0.514	########
HAUS1	########	-0.30983	0.08	0.552	########
TIA1	########	-0.15059	0.113	0.557	########
MCUR1	########	-0.52379	0.042	0.519	########
CDC20	########	-1.28996	0.005	0.543	########
DNPH1	########	0.109253	0.145	0.496	########
ID2	########	0.443403	0.3	0.595	########
GSS	########	-0.26709	0.063	0.486	########
SSBP4	########	-0.1724	0.079	0.47	########
TJP1	########	-0.25862	0.04	0.428	########
LAMP2	########	0.334484	0.213	0.486	########
RAD51AP1	########	-0.91276	0.003	0.53	########
MORN2	########	0.141846	0.115	0.467	########
ADSL	########	-0.13198	0.082	0.481	########
WBP2	########	0.351598	0.223	0.527	########
ABCF1	########	-0.38145	0.072	0.548	########
RP11-1094	########	-0.10649	0.089	0.498	########
PLEKHB2	########	-0.16711	0.108	0.546	########

PHF20L1	########	-0.24576	0.089	0.526	########
TEX264	########	0.334014	0.185	0.478	########
NUP62	########	-0.32245	0.066	0.526	########
SF3A3	########	-0.23826	0.079	0.522	########
ASPH	########	-0.33771	0.077	0.542	########
GTF2F2	########	0.125562	0.134	0.478	########
CRK	########	-0.27101	0.108	0.557	########
RRP7A	########	-0.48284	0.052	0.537	########
GLT8D1	########	-0.30152	0.079	0.532	########
VEZF1	########	-0.36374	0.068	0.535	########
KEAP1	########	-0.22536	0.091	0.52	########
MAP7D1	########	-0.32542	0.068	0.509	########
RFC3	########	-0.80453	0.012	0.537	########
WDR33	########	-0.13769	0.089	0.511	########
GATM	########	0.114158	0.173	0.53	########
AATF	########	-0.31898	0.075	0.531	########
PDHA1	########	-0.15963	0.12	0.568	########
SAFB2	########	-0.28358	0.098	0.554	########
DEF8	########	-0.27689	0.065	0.506	########
EFEMP2	########	-0.23944	0.054	0.443	########
FAM104A	########	-0.34455	0.056	0.486	########
ACYP2	########	0.423519	0.168	0.411	########
ILF3-AS1	########	0.14089	0.162	0.522	########
GOLIM4	########	-0.26576	0.126	0.59	########
IMMT	########	-0.24864	0.082	0.525	########
NDC80	########	-1.02331	0.003	0.53	########
FTSJ2	########	-0.15293	0.086	0.501	########
SERINC3	########	-0.13205	0.101	0.501	########
ZNF90	########	0.9113	0.372	0.435	########
CHMP1A	########	-0.19752	0.07	0.484	########
LIMS1	########	-0.18088	0.101	0.509	########
KLHDC8A	########	-0.25849	0.108	0.567	########
SUCLA2	########	0.143292	0.154	0.501	########
ZNF326	########	-0.32677	0.079	0.542	########
DRG2	########	0.13454	0.087	0.381	########
MRTO4	########	-0.14518	0.077	0.478	########
EAPP	########	0.338662	0.183	0.488	########
DDX42	########	-0.19067	0.058	0.449	########
ITFG1	########	-0.24497	0.105	0.553	########
TAOK1	########	-0.16832	0.072	0.449	########
SMAP1	########	-0.27439	0.065	0.506	########
ACO2	########	0.153364	0.173	0.522	########
PNMA1	########	-0.25588	0.101	0.544	########
EIF3A	########	-0.27904	0.072	0.512	########
KLHDC2	########	-0.16842	0.094	0.517	########
OCIAD2	########	0.722607	0.365	0.495	########
ARMC1	########	-0.33539	0.084	0.552	########
OSBPL8	########	-0.40038	0.059	0.515	########
ILKAP	########	-0.26913	0.068	0.507	########
NOL12	########	-0.22609	0.061	0.467	########
DHX9	########	-0.13103	0.11	0.533	########

SDR39U1	########	0.252393	0.168	0.491	########
COTL1	########	-0.33628	0.084	0.554	########
KPNA3	########	-0.27763	0.066	0.501	########
C17orf62	########	-0.29672	0.079	0.499	########
SRSF10	########	-0.13674	0.131	0.573	########
VMA21	########	-0.23774	0.077	0.507	########
MT1E	########	0.872734	0.38	0.483	########
APPL1	########	-0.36326	0.063	0.516	########
ELAVL3	########	-0.36159	0.12	0.605	########
FXR1	########	-0.13331	0.127	0.554	########
HMGA1	########	-0.56789	0.066	0.565	########
PRCC	########	-0.12605	0.113	0.536	########
CAPZA1	########	-0.32563	0.075	0.521	########
FAM50A	########	-0.32191	0.096	0.553	########
DHX30	########	-0.18287	0.073	0.479	########
LEPROT	########	0.207086	0.147	0.469	########
PPP1R11	########	0.142767	0.133	0.467	########
ECT2	########	-0.90897	0.002	0.512	########
ACIN1	########	-0.10992	0.096	0.501	########
NRCAM	########	-0.1408	0.147	0.574	########
PRADC1	########	-0.51361	0.049	0.536	########
HILPDA	########	0.48169	0.155	0.452	########
TMEM261	########	0.146675	0.152	0.511	########
TM2D2	########	0.269471	0.136	0.405	########
CBFB	########	-0.24842	0.056	0.459	########
TMEM203	########	-0.24198	0.072	0.491	########
ATAD2	########	-0.89878	0.003	0.52	########
SAP30	########	-0.72267	0.028	0.535	########
DNAJC2	########	-0.1782	0.112	0.553	########
MAPKAP1	########	-0.10939	0.079	0.443	########
WEE1	########	-0.47874	0.044	0.51	########
EBPL	########	-0.17481	0.098	0.517	########
IER2	########	-0.21556	0.277	0.778	########
RAB8A	########	-0.13273	0.096	0.517	########
SQLE	########	-0.30435	0.087	0.537	########
RIC8A	########	-0.20132	0.087	0.516	########
TMEM106B	########	0.104703	0.148	0.493	########
ZFR	########	-0.17278	0.112	0.548	########
HSPBP1	########	-0.21698	0.101	0.542	########
TCEAL8	########	0.257357	0.211	0.551	########
RBBP6	########	-0.25171	0.086	0.519	########
TGOLN2	########	-0.27016	0.089	0.528	########
AP3D1	########	-0.29367	0.098	0.552	########
ANAPC13	########	0.167077	0.159	0.525	########
NCAPG	########	-0.88763	0	0.506	########
CSNK1D	########	-0.21812	0.072	0.475	########
ROCK1	########	-0.31798	0.059	0.498	########
WHSC1	########	-0.55552	0.051	0.549	########
BRMS1	########	-0.2879	0.072	0.509	########
C21orf58	########	-0.83147	0.003	0.516	########
NUCB1	########	0.195995	0.178	0.514	########

SPTBN1	########	-0.10139	0.115	0.521	########
PARL	########	-0.12643	0.094	0.496	########
TRIM44	########	0.195748	0.161	0.493	########
ARF6	########	-0.25202	0.096	0.547	########
CDCA3	########	-1.05911	0.002	0.509	########
CPSF6	########	-0.18331	0.147	0.604	########
IPO9	########	-0.36661	0.077	0.549	########
PRPF19	########	-0.17647	0.075	0.473	########
FOS	########	0.701145	0.667	0.791	########
ALKBH5	########	-0.4487	0.056	0.515	########
PCBP4	########	-0.30018	0.079	0.512	########
POLR3K	########	-0.13698	0.073	0.463	########
MELK	########	-0.8061	0.009	0.515	########
TNRC6B	########	-0.13321	0.143	0.567	########
SPC24	########	-0.92515	0.003	0.517	########
LGALS3BP	########	0.55104	0.307	0.51	########
AP2A1	########	-0.1715	0.072	0.467	########
SBNO1	########	-0.27041	0.073	0.505	########
CAPNS1	########	-0.13357	0.101	0.499	########
COA6	########	-0.13025	0.082	0.477	########
C2orf69	########	-0.61912	0.031	0.528	########
PHC2	########	-0.16444	0.077	0.467	########
PPP1R2	########	-0.12007	0.098	0.491	########
DDA1	########	-0.11885	0.119	0.527	########
COMMD2	########	-0.15852	0.108	0.532	########
A2M	########	0.777731	0.19	0.326	########
EMD	########	-0.45795	0.056	0.526	########
ARL1	########	0.188735	0.138	0.454	########
USP8	########	0.133913	0.089	0.389	########
DSEL	########	-0.30221	0.152	0.638	########
CDCA5	########	-0.84652	0.009	0.523	########
HDAC1	########	-0.10574	0.059	0.416	########
HLA-C	########	0.686187	0.64	0.716	########
CHPT1	########	-0.23608	0.099	0.536	########
CDC5L	########	-0.47104	0.07	0.56	########
RAF1	########	-0.39526	0.075	0.546	########
PLEKHO1	########	-0.43842	0.047	0.507	########
SURF1	########	-0.30897	0.066	0.506	########
COQ4	########	-0.10179	0.091	0.468	########
TLK1	########	-0.35242	0.072	0.52	########
THY1	########	0.116466	0.171	0.521	########
CENPE	########	-1.11838	0.003	0.517	########
RAB4A	########	-0.20044	0.113	0.542	########
FDX1	########	-0.38216	0.061	0.514	########
ZNF292	########	-0.15208	0.084	0.477	########
NT5C	########	-0.16639	0.089	0.485	########
PRRC2A	########	-0.30492	0.08	0.506	########
PMP22	########	0.401491	0.311	0.557	########
IFI27L2	########	0.521016	0.271	0.507	########
PITPNA-AS	########	-0.15676	0.063	0.454	########
KATNBL1	########	-0.28273	0.059	0.472	########

TTYH1	########	0.376035	0.277	0.573	########
YIPF6	########	0.151998	0.152	0.486	########
TAF11	########	-0.1935	0.077	0.486	########
SLC52A2	########	-0.34024	0.065	0.495	########
FAM49B	########	-0.20262	0.098	0.527	########
RNF10	########	-0.13611	0.098	0.51	########
GGPS1	########	0.100917	0.145	0.502	########
QTRT1	########	0.306124	0.206	0.514	########
LMNB2	########	-0.7712	0.014	0.521	########
EPC1	########	-0.27868	0.065	0.485	########
NAMPT	########	1.097607	0.276	0.293	########
SUZ12	########	-0.50191	0.045	0.51	########
EIF3B	########	-0.23502	0.075	0.485	########
SAFB	########	-0.405	0.063	0.528	########
CD2BP2	########	0.111907	0.113	0.457	########
PFKL	########	-0.19293	0.096	0.51	########
G3BP1	########	-0.11909	0.122	0.533	########
TAF6	########	-0.22535	0.082	0.511	########
SAC3D1	########	-0.63408	0.033	0.527	########
MEG3	########	0.762735	0.445	0.528	########
GABARAP	########	0.571082	0.232	0.417	########
PSRC1	########	-0.30952	0.129	0.591	########
SMC1A	########	-0.43474	0.061	0.533	########
HSF1	########	-0.2431	0.079	0.493	########
SMARCC1	########	-0.49818	0.056	0.547	########
CIAPIN1	########	-0.15572	0.094	0.51	########
DLD	########	0.137783	0.159	0.5	########
GPSM2	########	-0.53233	0.086	0.594	########
CCNC	########	-0.13644	0.087	0.486	########
TAGLN2	########	0.728984	0.471	0.544	########
RANGRF	########	-0.13185	0.068	0.449	########
FAM92A1	########	-0.19034	0.063	0.446	########
CADM4	########	-0.14197	0.105	0.517	########
TP53I13	########	-0.5747	0.044	0.507	########
SHISA4	########	0.330498	0.131	0.368	########
DRAXIN	########	-0.51273	0.051	0.52	########
MIS18A	########	-0.55773	0.033	0.504	########
CDC25B	########	-0.65253	0.063	0.568	########
KMT2A	########	-0.34639	0.068	0.517	########
FDX1L	########	0.117366	0.11	0.43	########
NFYB	########	-0.2241	0.059	0.46	########
AKIP1	########	-0.12446	0.077	0.456	########
CD164	########	0.141724	0.175	0.505	########
ING2	########	-0.34147	0.063	0.504	########
MTX1	########	0.15981	0.073	0.36	########
WSB2	########	-0.32527	0.047	0.463	########
RMDN3	########	-0.21315	0.066	0.459	########
CHD7	########	-0.17954	0.14	0.585	########
PPP2R5C	########	-0.25045	0.072	0.499	########
JMJD1C	########	-0.22335	0.108	0.533	########
U2AF2	########	-0.36134	0.068	0.527	########

RTCB	########	-0.2627	0.082	0.507	########
BLOC1S2	########	0.121423	0.11	0.438	########
IKBIP	########	-0.54582	0.037	0.502	########
IPO5	########	-0.27318	0.073	0.502	########
YTHDC1	########	-0.28892	0.086	0.53	########
HIRIP3	########	-0.54867	0.044	0.531	########
ADIPOR1	########	-0.3334	0.058	0.488	########
ACOT7	########	-0.40348	0.058	0.514	########
CENPA	########	-1.09124	0.002	0.504	########
CYB5A	########	0.163356	0.131	0.457	########
RAB13	########	0.458424	0.243	0.51	########
TSPAN7	########	0.374121	0.277	0.557	########
PRPF8	########	-0.18401	0.073	0.465	########
HMOX2	########	0.337586	0.122	0.39	########
NINJ1	########	-0.2711	0.065	0.48	########
RIF1	########	-0.44437	0.056	0.522	########
ECHDC1	########	-0.11049	0.098	0.494	########
PRPSAP2	########	-0.1173	0.099	0.494	########
TWF1	########	-0.14437	0.082	0.47	########
SOGA1	########	-0.45679	0.049	0.493	########
PLP2	########	0.128113	0.147	0.478	########
CDC34	########	-0.26899	0.073	0.5	########
CDCA4	########	-0.82158	0.014	0.52	########
NTPCR	########	0.248395	0.134	0.435	########
RSPRY1	########	-0.20565	0.065	0.462	########
SOX9	########	-0.43635	0.131	0.612	########
WNK1	########	-0.29793	0.056	0.473	########
H3F3A	########	0.394419	0.684	0.838	########
CTNNBIP1	########	-0.42963	0.031	0.459	########
GDE1	########	-0.28294	0.059	0.47	########
SOX11	########	-0.43647	0.127	0.628	########
ACAA1	########	-0.12696	0.082	0.468	########
SORT1	########	-0.14729	0.047	0.401	########
CETN2	########	0.549169	0.195	0.409	########
CEP57	########	-0.40583	0.068	0.526	########
MFGE8	########	-0.28672	0.079	0.504	########
GPI	########	0.427764	0.206	0.451	########
TSTA3	########	-0.18841	0.066	0.459	########
HDAC3	########	-0.15575	0.068	0.446	########
NDUFS1	########	-0.22131	0.079	0.49	########
AIG1	########	0.209003	0.143	0.449	########
PABPC4	########	-0.30239	0.056	0.48	########
KLHL7	########	-0.26435	0.096	0.528	########
ARL4A	########	0.349219	0.262	0.553	########
SERINC1	########	0.167132	0.175	0.491	########
TIMP2	########	-0.30467	0.068	0.496	########
GSTO1	########	0.297176	0.15	0.452	########
FYTTD1	########	-0.23931	0.068	0.484	########
TRA2A	########	-0.13767	0.127	0.543	########
TMEM14A	########	0.145243	0.138	0.475	########
DNASE2	########	0.258963	0.12	0.384	########

GINS2	########	-0.8552	0.028	0.541	########
ATG4B	########	-0.24455	0.066	0.473	########
MYBL2	########	-0.90555	0.002	0.491	########
MED19	########	-0.14597	0.105	0.517	########
NRN1	########	0.581533	0.209	0.433	########
RANBP3	########	-0.20418	0.059	0.447	########
RSU1	########	0.346356	0.094	0.317	########
PPM1B	########	-0.19995	0.108	0.533	########
HTRA1	########	0.164855	0.244	0.61	########
HTATSF1	########	-0.2795	0.087	0.531	########
TFDP1	########	-0.52182	0.03	0.483	########
SEPHS2	########	-0.14679	0.065	0.43	########
SERPINE2	########	-0.1521	0.18	0.625	########
GSTA4	########	0.168274	0.166	0.504	########
PSMB8	########	0.221144	0.166	0.478	########
TMEM87A	########	-0.11872	0.087	0.468	########
CCDC109B	########	-0.63335	0.058	0.528	########
CD81	########	0.307894	0.15	0.422	########
DAP	########	-0.47001	0.04	0.491	########
PMPCB	########	0.246327	0.194	0.514	########
PALLD	########	-0.22497	0.056	0.417	########
CHAF1A	########	-0.81263	0.009	0.507	########
CNPY3	########	-0.19514	0.077	0.481	########
CKAP5	########	-0.66699	0.023	0.494	########
UBTF	########	-0.34561	0.072	0.521	########
TMEM30A	########	-0.11852	0.113	0.502	########
CKAP4	########	-0.21266	0.07	0.446	########
ADAM9	########	-0.11288	0.099	0.467	########
IFT43	########	0.13944	0.122	0.444	########
EMC9	########	-0.59408	0.052	0.536	########
KRR1	########	-0.12992	0.072	0.447	########
PLGRKT	########	-0.23642	0.065	0.477	########
CDC26	########	0.125323	0.079	0.383	########
MGME1	########	-0.52241	0.047	0.525	########
PKN1	########	-0.46495	0.023	0.442	########
SPARCL1	########	0.493483	0.373	0.604	########
C12orf75	########	-0.84251	0.007	0.501	########
SRRT	########	-0.40526	0.072	0.532	########
SYMPK	########	0.181877	0.112	0.399	########
PRKAR2A	########	-0.17893	0.072	0.444	########
APOE	########	0.288116	0.33	0.67	########
MARK3	########	-0.11622	0.112	0.505	########
MOB1A	########	-0.1403	0.068	0.44	########
BMP7	########	-0.30816	0.086	0.528	########
TTC14	########	0.167539	0.122	0.411	########
LBR	########	-0.56732	0.072	0.569	########
EIF1AY	########	-0.34793	0.045	0.449	########
POLR3GL	########	0.325319	0.171	0.454	########
WAC	########	-0.24146	0.059	0.448	########
RP11-14N7	########	0.22493	0.124	0.398	########
SMIM14	########	0.116047	0.134	0.485	########

FN3KRP	########	-0.26108	0.065	0.473	########
ILVBL	########	-0.12597	0.096	0.484	########
KIF2C	########	-0.91155	0.003	0.496	########
FADS1	########	-0.27146	0.066	0.477	########
FUBP3	########	-0.14154	0.077	0.457	########
TMEM60	########	-0.29168	0.063	0.484	########
DSN1	########	-0.80091	0.012	0.51	########
ALDH3A2	########	-0.13834	0.094	0.457	########
UBL3	########	-0.16184	0.082	0.475	########
TMEM251	########	-0.24376	0.063	0.462	########
KDM1A	########	-0.28991	0.056	0.463	########
DHX29	########	-0.24829	0.089	0.516	########
SV2A	########	-0.13858	0.066	0.423	########
TGIF1	########	-0.27729	0.096	0.516	########
CDKN1B	########	-0.42591	0.07	0.536	########
TM2D3	########	0.39144	0.164	0.417	########
KCNQ2	########	-0.37386	0.08	0.521	########
CCDC130	########	-0.12256	0.073	0.427	########
KLF6	########	0.406421	0.251	0.505	########
SHARPIN	########	-0.21635	0.073	0.469	########
DHRS7B	########	-0.13409	0.061	0.412	########
HADH	########	-0.16694	0.075	0.462	########
AGTRAP	########	0.474014	0.117	0.309	########
FARSB	########	-0.11837	0.099	0.495	########
TMEM50B	########	0.175738	0.129	0.422	########
ITGB8	########	-0.15953	0.106	0.505	########
STAG2	########	-0.17419	0.082	0.472	########
SWI5	########	0.233369	0.105	0.401	########
RNF24	########	-0.19127	0.073	0.464	########
ARRDC3	########	-0.27045	0.073	0.486	########
POC1A	########	-0.82947	0.005	0.494	########
TTF1	########	-0.31718	0.063	0.484	########
CCDC53	########	0.165939	0.143	0.472	########
UBE2M	########	-0.37528	0.045	0.453	########
PTGES2	########	-0.20548	0.061	0.44	########
LSM12	########	-0.56511	0.03	0.486	########
C21orf33	########	0.146888	0.138	0.473	########
BLVRB	########	0.773657	0.178	0.296	########
ENC1	########	-0.57699	0.051	0.52	########
TACC1	########	-0.57765	0.04	0.514	########
HOOK3	########	-0.1465	0.082	0.464	########
CAPN2	########	-0.13097	0.079	0.442	########
ZEB1	########	-0.13375	0.106	0.509	########
EGLN2	########	-0.35519	0.054	0.474	########
S100A16	########	0.638892	0.363	0.48	########
FAM103A1	########	0.145908	0.094	0.411	########
TMEM128	########	-0.12646	0.091	0.464	########
RTCA	########	-0.13045	0.068	0.437	########
EIF2B1	########	-0.21191	0.089	0.504	########
DDB1	########	-0.12557	0.094	0.486	########
AP1M1	########	-0.10413	0.075	0.433	########

DAXX	########	-0.282	0.054	0.452	########
SCAMP4	########	-0.18876	0.091	0.489	########
DYNLL2	########	-0.45044	0.033	0.47	########
DESI2	########	-0.41628	0.038	0.464	########
KIF2A	########	-0.36272	0.056	0.483	########
CCDC50	########	-0.29747	0.047	0.456	########
PSPC1	########	-0.46732	0.052	0.512	########
TMEM199	########	-0.12742	0.061	0.422	########
GNG4	########	-0.456	0.035	0.468	########
ATAT1	########	-0.15773	0.098	0.494	########
AURKA	########	-1.08137	0.009	0.502	########
USP10	########	-0.15406	0.07	0.447	########
OXLD1	########	-0.19002	0.061	0.42	########
TSSC1	########	-0.19825	0.075	0.468	########
ASH1L	########	-0.12097	0.098	0.484	########
KIF5C	########	-0.28156	0.086	0.499	########
KIAA1715	########	-0.24242	0.075	0.477	########
CDKN2D	########	-0.74065	0.023	0.51	########
TOMM5	########	-0.21206	0.044	0.407	########
NGDN	########	0.188071	0.147	0.453	########
FAM89B	########	-0.40772	0.047	0.468	########
NDUFAF2	########	0.221916	0.122	0.414	########
DHRSX	########	-0.24809	0.08	0.493	########
ABHD17A	########	-0.27126	0.049	0.422	########
ZNF146	########	-0.14193	0.096	0.481	########
CELF1	########	-0.24247	0.08	0.493	########
CDCA7L	########	-0.42461	0.068	0.527	########
UTP18	########	-0.26225	0.061	0.458	########
ZYX	########	-0.12202	0.127	0.507	########
BDP1	########	-0.22437	0.066	0.459	########
PPP4R2	########	-0.21954	0.072	0.463	########
AKAP8L	########	-0.17586	0.063	0.438	########
GOSR1	########	-0.16847	0.079	0.465	########
IFI27L1	########	-0.10157	0.086	0.464	########
IPO7	########	-0.33812	0.045	0.458	########
CYHR1	########	0.175377	0.122	0.4	########
RNF13	########	0.369796	0.213	0.467	########
MIS18BP1	########	-0.76333	0.01	0.486	########
SMARCD1	########	-0.32247	0.051	0.456	########
ZCCHC11	########	-0.15816	0.072	0.437	########
IFNAR1	########	-0.23644	0.065	0.454	########
ATP2A2	########	-0.16984	0.101	0.496	########
JKAMP	########	-0.13967	0.091	0.459	########
EBP	########	-0.12854	0.079	0.458	########
TPM1	########	-0.12156	0.096	0.467	########
LITAF	########	0.322519	0.159	0.421	########
AC010642.	########	-0.26996	0.042	0.426	########
DDX52	########	-0.30191	0.072	0.473	########
RBM26	########	-0.17683	0.058	0.414	########
FABP5	########	1.425689	0.485	0.338	########
FH	########	-0.134	0.079	0.453	########

BCKDK	########	-0.25379	0.066	0.451	########
CGGBP1	########	-0.30949	0.075	0.505	########
ABCE1	########	-0.2086	0.066	0.447	########
SPCS3	########	-0.25828	0.059	0.451	########
IRF2BPL	########	-0.32332	0.079	0.494	########
AFF4	########	-0.20166	0.113	0.526	########
SLC25A33	########	-0.32987	0.037	0.431	########
BEX1	########	0.541296	0.384	0.578	########
GLRX3	########	-0.19186	0.073	0.462	########
REPIN1	########	-0.28424	0.077	0.493	########
CYSTM1	########	0.428593	0.145	0.372	########
TMUB1	########	-0.35468	0.056	0.477	########
OGFR	########	-0.35073	0.047	0.454	########
CUX1	########	-0.27534	0.075	0.491	########
UBQLN1	########	-0.26168	0.058	0.451	########
RBM10	########	-0.30291	0.054	0.458	########
SEC22C	########	-0.25588	0.061	0.458	########
CCDC112	########	-0.42521	0.047	0.477	########
MMP24-AS1	########	-0.17785	0.063	0.405	########
DHX15	########	-0.2068	0.077	0.468	########
CASC4	########	-0.31143	0.047	0.436	########
SLC39A7	########	-0.1433	0.105	0.493	########
UBE2D1	########	-0.18757	0.04	0.378	########
GSTM3	########	0.25499	0.147	0.442	########
PRKRIP1	########	-0.25979	0.065	0.467	########
IL6ST	########	-0.19664	0.063	0.428	########
C14orf119	########	0.169192	0.122	0.442	########
ASF1A	########	-0.26646	0.086	0.498	########
C7orf49	########	-0.32379	0.049	0.457	########
C12orf76	########	0.242964	0.119	0.391	########
IFT27	########	-0.34393	0.052	0.458	########
RP11-345J	########	-0.26594	0.035	0.393	########
LUC7L	########	-0.15688	0.084	0.472	########
SPAG5	########	-0.82898	0	0.462	########
UBXN2A	########	-0.4065	0.049	0.473	########
ZNF738	########	-0.34401	0.063	0.491	########
PLTP	########	0.439265	0.194	0.385	########
SHCBP1	########	-0.76329	0.002	0.467	########
BCAN	########	0.300582	0.354	0.651	########
ITGB1	########	-0.38611	0.086	0.505	########
FABP7	########	0.231764	0.461	0.752	########
CIAO1	########	-0.19088	0.063	0.437	########
VCAN	########	0.169503	0.141	0.457	########
NCKAP1	########	-0.27668	0.065	0.467	########
YPEL5	########	0.424696	0.182	0.401	########
SRP68	########	-0.13454	0.072	0.431	########
SDF2L1	########	-0.16794	0.105	0.519	########
HLA-DRA	########	1.178453	0.41	0.335	########
CDK5	########	-0.14319	0.059	0.426	########
P4HTM	########	-0.12286	0.089	0.449	########
SORBS3	########	-0.23578	0.047	0.416	########

ABCD4	########	0.105952	0.103	0.402	########
SURF2	########	-0.16066	0.072	0.444	########
TXNDC9	########	-0.16113	0.068	0.442	########
TAF1D	########	0.150468	0.119	0.422	########
TXNRD1	########	-0.37729	0.052	0.469	########
ARID1A	########	-0.35098	0.059	0.478	########
PNPLA8	########	-0.28293	0.091	0.511	########
TBL1XR1	########	-0.30198	0.058	0.452	########
LYPD1	########	0.123422	0.094	0.385	########
TMEM70	########	-0.18148	0.084	0.479	########
FAM134A	########	-0.29272	0.086	0.504	########
CMTM3	########	-0.32895	0.056	0.457	########
PPP2R4	########	-0.12771	0.065	0.411	########
S100A13	########	0.646908	0.239	0.402	########
PRR11	########	-0.78443	0.003	0.46	########
YTHDF1	########	-0.21516	0.042	0.389	########
GAP43	########	0.471332	0.447	0.638	########
FJX1	########	-0.20675	0.066	0.431	########
PLEKHA3	########	-0.16409	0.059	0.417	########
NUFIP2	########	-0.26656	0.044	0.411	########
HSPA4	########	-0.18228	0.073	0.458	########
JMJD6	########	-0.33707	0.049	0.452	########
TDG	########	-0.13086	0.089	0.459	########
MTHFD2L	########	-0.12205	0.08	0.433	########
NCAPH2	########	-0.55492	0.024	0.469	########
AFG3L2	########	-0.18883	0.047	0.398	########
CD44	########	0.852597	0.225	0.285	########
TUBA1C	########	-0.3834	0.15	0.616	########
BIRC2	########	-0.1142	0.065	0.405	########
STK4	########	-0.16422	0.054	0.409	########
EIF4G1	########	-0.17911	0.082	0.464	########
CLSPN	########	-0.88666	0.007	0.475	########
IARS	########	-0.16089	0.086	0.452	########
NIPBL	########	-0.17513	0.073	0.441	########
HYAL2	########	-0.36195	0.042	0.437	########
MAGI1	########	-0.21128	0.092	0.493	########
VPS37B	########	-0.21559	0.066	0.441	########
UBE2G2	########	-0.11304	0.086	0.459	########
TNPO1	########	-0.20105	0.084	0.477	########
GADD45A	########	0.23089	0.176	0.463	########
C16orf87	########	-0.24426	0.065	0.453	########
ETV5	########	-0.28161	0.061	0.456	########
RRAGA	########	-0.10107	0.126	0.51	########
LRWD1	########	-0.21987	0.051	0.419	########
BLOC1S4	########	-0.28053	0.051	0.433	########
ACTN1	########	-0.2277	0.063	0.405	########
PTPRS	########	-0.17582	0.127	0.542	########
PHACTR4	########	-0.18945	0.075	0.452	########
COMMD10	########	0.269881	0.103	0.353	########
RABEP1	########	-0.45032	0.045	0.47	########
C1orf21	########	0.126221	0.126	0.438	########

TANK	########	0.122855	0.134	0.448	########
VOPP1	########	-0.30889	0.068	0.48	########
CRELD2	########	-0.46195	0.042	0.467	########
NAA50	########	-0.22464	0.082	0.477	########
TRIP13	########	-0.67391	0.003	0.453	########
CXCL16	########	-0.1079	0.061	0.39	########
PSME3	########	-0.25842	0.061	0.443	########
RWDD4	########	-0.17977	0.075	0.447	########
PKIB	########	0.120677	0.119	0.425	########
LRR1	########	-0.67845	0.01	0.46	########
PHLDA1	########	-0.37008	0.164	0.617	########
MCM3	########	-0.53475	0.054	0.509	########
RAB22A	########	-0.2835	0.054	0.441	########
ARFRP1	########	-0.13336	0.07	0.431	########
ESCO2	########	-0.78968	0.002	0.462	########
PLA2G16	########	0.436431	0.159	0.374	########
CDV3	########	-0.32002	0.045	0.423	########
ABCD3	########	-0.23104	0.058	0.438	########
AZIN1	########	-0.11384	0.106	0.48	########
RNASEH1	########	-0.37468	0.047	0.46	########
THAP9-AS1	########	-0.15272	0.077	0.443	########
TOR1A	########	-0.34217	0.054	0.462	########
PDCD7	########	-0.26046	0.059	0.446	########
SHISA5	########	0.131142	0.141	0.447	########
SLC30A5	########	-0.15544	0.052	0.389	########
UBE2J1	########	-0.18598	0.066	0.425	########
USP48	########	-0.22443	0.052	0.426	########
CENPT	########	-0.31696	0.073	0.486	########
THAP7	########	-0.17184	0.07	0.438	########
RP11-553L	########	-0.11416	0.113	0.475	########
YKT6	########	-0.12062	0.059	0.406	########
IGBP1	########	0.226258	0.133	0.416	########
RHOT1	########	-0.37854	0.042	0.447	########
TM7SF3	########	-0.34993	0.051	0.458	########
ASCL1	########	-0.41094	0.129	0.594	########
REXO2	########	0.462644	0.162	0.367	########
DAG1	########	-0.17284	0.056	0.407	########
VRK1	########	-0.5692	0.03	0.472	########
PRPF4B	########	-0.12863	0.086	0.44	########
C16orf91	########	-0.16079	0.058	0.411	########
RGS12	########	0.106145	0.127	0.446	########
IFT20	########	0.126174	0.14	0.443	########
LAMTOR3	########	0.151332	0.096	0.359	########
PYCR1	########	-0.15725	0.07	0.421	########
TM9SF2	########	-0.21061	0.084	0.478	########
RNF130	########	-0.35997	0.08	0.507	########
SLC30A9	########	-0.26191	0.066	0.462	########
GNAQ	########	-0.25417	0.058	0.437	########
PSMB9	########	0.389953	0.211	0.472	########
MCM4	########	-0.789	0.014	0.48	########
SEC23B	########	-0.15241	0.065	0.422	########

LRP1	########	0.423124	0.182	0.39	########
SNX27	########	-0.16849	0.056	0.405	########
FUNDC1	########	0.185218	0.112	0.393	########
SUPT20H	########	-0.1656	0.07	0.433	########
GID8	########	-0.26444	0.051	0.422	########
CTSF	########	0.147198	0.101	0.389	########
CDT1	########	-0.84828	0.007	0.469	########
EBAG9	########	-0.1989	0.058	0.428	########
PCDH9	########	0.436428	0.194	0.412	########
DRG1	########	-0.17552	0.075	0.451	########
MTF2	########	-0.25932	0.073	0.472	########
KRAS	########	-0.24597	0.058	0.433	########
MYEF2	########	-0.40855	0.058	0.488	########
ALYREF	########	-0.554	0.028	0.452	########
CTSL	########	0.611858	0.222	0.389	########
SMARCC2	########	-0.16836	0.105	0.488	########
PGM2L1	########	0.206004	0.159	0.427	########
FAM181B	########	-0.41643	0.105	0.552	########
UBALD2	########	-0.48657	0.068	0.511	########
CTTN	########	-0.12051	0.07	0.411	########
NMB	########	0.322402	0.133	0.398	########
SNHG8	########	0.405065	0.209	0.451	########
SCRG1	########	0.166828	0.302	0.642	########
VAPB	########	-0.44278	0.031	0.442	########
PLIN3	########	0.288427	0.199	0.453	########
PBRM1	########	-0.44411	0.052	0.486	########
DOK5	########	0.121982	0.075	0.33	########
TMEM33	########	-0.23924	0.072	0.452	########
FOPNL	########	-0.17819	0.068	0.433	########
RNF26	########	-0.40567	0.035	0.437	########
HERPUD1	########	0.33126	0.234	0.494	########
LONP1	########	-0.15811	0.052	0.4	########
AAK1	########	-0.12715	0.101	0.457	########
CAMK2N1	########	-0.19729	0.108	0.499	########
POLR2B	########	-0.3408	0.08	0.51	########
MNS1	########	-0.66833	0.012	0.453	########
MLX	########	-0.19228	0.066	0.433	########
PDLIM5	########	-0.13619	0.066	0.41	########
ARMCX6	########	0.192359	0.089	0.338	########
NETO2	########	-0.56475	0.04	0.49	########
BAZ2B	########	-0.15735	0.138	0.533	########
CDCA8	########	-0.93503	0.002	0.456	########
EMG1	########	0.166246	0.091	0.367	########
MYO9B	########	-0.1037	0.072	0.391	########
TRPC4AP	########	-0.31921	0.044	0.427	########
SPATC1L	########	-0.1548	0.045	0.367	1.08E-99
PKMYT1	########	-0.78401	0.003	0.462	1.12E-99
NTHL1	########	-0.21518	0.068	0.441	1.19E-99
GADD45B	########	1.154124	0.433	0.348	1.33E-99
F2R	########	-0.10262	0.075	0.417	1.38E-99
CTBP1	########	-0.13768	0.061	0.402	1.44E-99

INO80E	########	-0.3279	0.054	0.446	1.54E-99
SMOX	########	0.320265	0.124	0.369	1.69E-99
TFAM	########	-0.21074	0.054	0.41	1.75E-99
HEXB	########	-0.32981	0.079	0.49	1.76E-99
PTPN1	########	-0.2132	0.03	0.352	1.81E-99
USP5	########	-0.19981	0.066	0.44	1.96E-99
MTFR1L	########	-0.14434	0.087	0.451	2.13E-99
COQ2	########	-0.48713	0.019	0.43	2.49E-99
CD276	########	-0.14592	0.065	0.407	2.54E-99
MED30	########	-0.30761	0.079	0.494	2.68E-99
CDO1	########	0.248887	0.133	0.407	2.98E-99
MND1	########	-0.70807	0.007	0.456	3.22E-99
SEPN1	########	-0.21706	0.051	0.4	3.34E-99
DCK	########	-0.60944	0.026	0.472	3.93E-99
SRPK1	########	-0.31495	0.04	0.426	4.50E-99
NFKBIL1	########	-0.13765	0.054	0.381	4.54E-99
GNB4	########	-0.12154	0.045	0.375	4.75E-99
SPATA33	########	-0.14176	0.061	0.409	4.94E-99
GTPBP6	########	-0.21921	0.049	0.409	5.05E-99
FLNA	########	-0.30368	0.072	0.449	5.24E-99
ICT1	########	-0.19963	0.059	0.417	5.24E-99
PLSCR1	########	0.311738	0.12	0.351	5.33E-99
TTYH3	########	-0.32473	0.059	0.451	5.93E-99
HECTD1	########	-0.16264	0.063	0.415	7.45E-99
7-Mar	########	-0.19822	0.079	0.457	7.65E-99
TRIM24	########	-0.39849	0.042	0.451	8.60E-99
ACAT1	########	0.298939	0.168	0.43	8.79E-99
CHCHD10	########	-0.17078	0.084	0.441	8.97E-99
SLC44A2	########	-0.23065	0.077	0.459	9.01E-99
ABCF2	########	-0.11314	0.073	0.411	9.13E-99
POLDIP3	########	-0.28559	0.045	0.416	1.02E-98
NRBF2	########	-0.20839	0.054	0.398	1.10E-98
DAPK3	########	-0.3317	0.054	0.453	1.17E-98
FMNL2	########	-0.29018	0.052	0.421	1.26E-98
PPP5C	########	-0.10014	0.077	0.427	1.68E-98
EMP3	########	0.500534	0.251	0.447	1.68E-98
PPID	########	-0.12621	0.054	0.391	1.77E-98
CUL5	########	-0.20798	0.051	0.406	1.91E-98
KIF23	########	-0.83662	0.003	0.456	2.23E-98
TSR1	########	-0.21469	0.045	0.398	2.34E-98
EEA1	########	-0.19488	0.059	0.419	2.47E-98
CDC42BPA	########	-0.20923	0.072	0.448	2.52E-98
POLD4	########	0.363638	0.117	0.31	2.99E-98
CECR5	########	-0.37959	0.061	0.47	3.29E-98
RNF4	########	-0.24062	0.047	0.406	3.41E-98
RBM14	########	-0.32002	0.049	0.438	3.97E-98
TRIM36	########	-0.5896	0.026	0.467	4.13E-98
HIBCH	########	0.195236	0.138	0.41	4.21E-98
USP47	########	-0.10113	0.084	0.435	4.79E-98
ID3	########	0.210957	0.15	0.454	5.16E-98
JOSD2	########	-0.11917	0.089	0.438	5.43E-98

TSR2	########	-0.12266	0.098	0.464	5.56E-98
IFT81	########	-0.28444	0.042	0.41	5.58E-98
ATP1B2	########	0.26088	0.157	0.394	6.67E-98
KCMF1	########	-0.28762	0.044	0.412	7.20E-98
URI1	########	-0.22184	0.059	0.423	7.95E-98
PTCD3	########	-0.3189	0.07	0.479	8.88E-98
FBXO7	########	-0.2617	0.061	0.448	9.22E-98
SP3	########	-0.2624	0.059	0.433	9.37E-98
BPGM	########	-0.10477	0.094	0.457	1.07E-97
SCG5	########	0.879323	0.284	0.322	1.38E-97
ARRB2	########	-0.18486	0.066	0.425	1.46E-97
POLE4	########	-0.22094	0.084	0.477	1.51E-97
BRD3	########	-0.26736	0.091	0.494	1.55E-97
POMGNT1	########	-0.18258	0.086	0.464	1.77E-97
SCARA3	########	-0.17816	0.066	0.421	2.60E-97
ITGAV	########	-0.17813	0.066	0.406	3.20E-97
PSMC3IP	########	-0.73217	0.012	0.463	3.46E-97
DNTTIP2	########	-0.13061	0.079	0.43	3.58E-97
PLK1	########	-1.02351	0.007	0.458	3.72E-97
ZNF644	########	-0.19497	0.089	0.469	3.95E-97
YEATS4	########	-0.62926	0.103	0.599	5.16E-97
CENPQ	########	-0.49306	0.017	0.425	5.17E-97
KNSTRN	########	-0.83396	0.014	0.47	5.22E-97
USF2	########	-0.1892	0.075	0.451	5.36E-97
TMEM218	########	-0.22987	0.054	0.417	5.44E-97
DDIT3	########	-0.13548	0.202	0.627	7.62E-97
UBE2G1	########	-0.22443	0.066	0.431	8.81E-97
TMEM115	########	-0.15048	0.056	0.388	8.85E-97
HNRNPUL2	########	-0.23926	0.037	0.386	9.63E-97
FCGRT	########	0.622274	0.194	0.344	9.99E-97
KMT2C	########	-0.20787	0.082	0.454	1.07E-96
SF3B3	########	-0.28205	0.065	0.459	1.09E-96
TNC	########	0.341052	0.129	0.344	1.21E-96
RCAN1	########	0.37752	0.173	0.409	1.28E-96
TINF2	########	-0.17487	0.058	0.407	1.32E-96
STX5	########	-0.16004	0.068	0.43	1.54E-96
HTRA2	########	-0.20169	0.072	0.428	1.56E-96
TLE1	########	-0.26825	0.045	0.401	1.58E-96
SIGMAR1	########	-0.15984	0.061	0.401	1.63E-96
TAF10	########	-0.38775	0.031	0.41	1.73E-96
RP11-620J	########	-0.29368	0.164	0.612	1.80E-96
MAPK1	########	-0.33898	0.054	0.441	1.97E-96
ZNF830	########	-0.1176	0.063	0.39	2.06E-96
REEP2	########	-0.12497	0.061	0.395	2.08E-96
CCDC66	########	-0.37658	0.042	0.437	2.15E-96
ATAD5	########	-0.69954	0.012	0.457	2.73E-96
GRAMD1A	########	-0.13227	0.082	0.425	3.12E-96
MGAT2	########	-0.19217	0.04	0.369	3.14E-96
SYS1	########	0.202879	0.08	0.326	3.17E-96
PDS5B	########	-0.28227	0.058	0.442	3.46E-96
FAM111A	########	-0.6833	0.014	0.451	3.58E-96

CEP78	########	-0.51889	0.016	0.43	3.93E-96
GTF3C5	########	-0.22598	0.059	0.43	4.39E-96
REEP3	########	-0.27902	0.054	0.425	4.48E-96
SPNS1	########	-0.30007	0.047	0.423	4.49E-96
CCDC28B	########	0.22381	0.099	0.347	4.76E-96
DLGAP5	########	-0.87126	0	0.43	4.91E-96
POLR2A	########	-0.2287	0.058	0.417	5.23E-96
TCERG1	########	-0.41826	0.047	0.463	6.32E-96
ZNF148	########	-0.16412	0.075	0.436	7.10E-96
RFK	########	-0.15855	0.072	0.42	8.35E-96
PAF1	########	-0.15663	0.068	0.425	8.96E-96
TRAF7	########	-0.30173	0.038	0.407	9.67E-96
MTHFD1	########	-0.25323	0.072	0.451	1.04E-95
TMCO3	########	-0.15051	0.059	0.396	1.08E-95
MAGEH1	########	0.167287	0.168	0.463	1.09E-95
KIF15	########	-0.72687	0	0.428	1.10E-95
FARP1	########	-0.20501	0.058	0.41	1.15E-95
PDCD10	########	-0.1117	0.063	0.394	1.20E-95
GARS	########	-0.24522	0.098	0.495	1.36E-95
TP53RK	########	-0.24918	0.035	0.377	1.42E-95
YME1L1	########	-0.11642	0.058	0.389	1.44E-95
DNAJC3	########	-0.12703	0.052	0.383	1.47E-95
ADPRHL2	1.03E-99	-0.19773	0.065	0.426	1.54E-95
DERA	1.18E-99	-0.1344	0.087	0.443	1.76E-95
C8orf4	1.20E-99	1.074546	0.262	0.298	1.79E-95
SEMA6A	1.29E-99	-0.15276	0.07	0.414	1.93E-95
SMCHD1	1.30E-99	-0.41266	0.03	0.421	1.95E-95
MAN1A2	1.34E-99	-0.12334	0.047	0.377	2.00E-95
WDR82	1.66E-99	-0.21205	0.082	0.453	2.48E-95
GOPC	1.81E-99	-0.13218	0.086	0.442	2.70E-95
TNFRSF1A	1.97E-99	-0.24633	0.073	0.432	2.94E-95
HNRNPLL	2.02E-99	-0.23254	0.063	0.442	3.02E-95
GART	2.08E-99	-0.12302	0.061	0.398	3.10E-95
CCDC106	2.08E-99	-0.26735	0.052	0.412	3.11E-95
DR1	2.32E-99	-0.3507	0.065	0.48	3.47E-95
SMAD1	2.48E-99	-0.30246	0.051	0.411	3.71E-95
ABHD2	3.09E-99	-0.13838	0.068	0.4	4.61E-95
USP14	3.34E-99	-0.31461	0.051	0.432	4.99E-95
PDLIM7	3.74E-99	-0.30988	0.045	0.4	5.58E-95
AGPAT1	4.37E-99	-0.22394	0.068	0.428	6.53E-95
WDR77	4.60E-99	-0.12387	0.059	0.394	6.87E-95
SLC25A36	4.75E-99	-0.12632	0.07	0.405	7.09E-95
ZFYVE21	4.86E-99	0.184423	0.12	0.393	7.27E-95
POP4	4.99E-99	0.177127	0.108	0.385	7.46E-95
SDC3	5.18E-99	0.439188	0.148	0.348	7.74E-95
NUDT4	5.42E-99	-0.19798	0.058	0.416	8.09E-95
FNDC4	6.45E-99	-0.27386	0.04	0.393	9.63E-95
ENHO	6.58E-99	-0.22689	0.051	0.391	9.84E-95
SRR	7.30E-99	-0.12642	0.063	0.39	1.09E-94
TMEM248	7.34E-99	-0.15441	0.073	0.425	1.10E-94
BLOC1S6	7.48E-99	-0.2214	0.056	0.415	1.12E-94

AHI1	7.91E-99	-0.35374	0.054	0.448	1.18E-94
KIAA1143	8.21E-99	-0.31756	0.038	0.415	1.23E-94
MPHOSPH1C	8.38E-99	-0.10579	0.075	0.417	1.25E-94
TROAP	1.05E-98	-0.84898	0.003	0.436	1.57E-94
NMT1	1.11E-98	-0.23044	0.068	0.436	1.66E-94
GPN1	1.13E-98	-0.1169	0.056	0.384	1.68E-94
SIAH1	1.18E-98	-0.25254	0.08	0.47	1.77E-94
UFSP2	1.20E-98	0.247646	0.108	0.358	1.80E-94
ATP6V1B2	1.48E-98	-0.13113	0.077	0.419	2.20E-94
LZIC	1.62E-98	-0.1114	0.045	0.362	2.42E-94
EFTUD2	1.68E-98	-0.19819	0.058	0.414	2.51E-94
PGAP1	1.86E-98	-0.26086	0.072	0.449	2.78E-94
IFI6	1.97E-98	0.448206	0.251	0.502	2.95E-94
HEXA	2.03E-98	0.198742	0.124	0.375	3.03E-94
LRRFIP2	2.03E-98	-0.30949	0.026	0.389	3.03E-94
ZSCAN16-A	2.04E-98	-0.14216	0.084	0.441	3.05E-94
ASPSCR1	2.05E-98	-0.21643	0.073	0.443	3.06E-94
HLTF	2.14E-98	-0.27818	0.042	0.406	3.20E-94
CCDC137	2.25E-98	-0.39094	0.042	0.43	3.37E-94
TMEM59L	2.40E-98	0.102577	0.145	0.438	3.59E-94
PANK2	2.46E-98	-0.33045	0.04	0.411	3.68E-94
HAUS8	2.52E-98	-0.42955	0.031	0.433	3.77E-94
SRPRB	3.15E-98	-0.15645	0.066	0.41	4.71E-94
UROD	3.27E-98	0.271037	0.157	0.431	4.89E-94
DDX41	3.61E-98	-0.21368	0.047	0.388	5.40E-94
SCAMP3	3.83E-98	0.206951	0.131	0.41	5.73E-94
RBM4	3.87E-98	-0.16762	0.073	0.432	5.78E-94
FAM162A	3.90E-98	0.572254	0.237	0.417	5.84E-94
AGGF1	3.97E-98	-0.14649	0.051	0.378	5.93E-94
NFYC	4.03E-98	0.109773	0.096	0.379	6.03E-94
ZNF131	4.28E-98	-0.23731	0.077	0.454	6.39E-94
GPC1	5.02E-98	-0.2907	0.106	0.488	7.50E-94
CHIC2	5.50E-98	-0.4372	0.063	0.489	8.21E-94
ZNF680	5.94E-98	-0.28961	0.096	0.501	8.88E-94
GSK3A	6.18E-98	-0.22815	0.051	0.401	9.24E-94
RAD1	6.26E-98	-0.27674	0.082	0.483	9.36E-94
CEBPG	6.88E-98	-0.22878	0.066	0.435	1.03E-93
PYGB	6.96E-98	-0.16124	0.065	0.399	1.04E-93
SPRY2	7.29E-98	-0.29036	0.08	0.458	1.09E-93
MBD4	8.25E-98	-0.12458	0.073	0.42	1.23E-93
CBY1	8.46E-98	-0.13908	0.047	0.375	1.26E-93
BUB1	9.11E-98	-0.83094	0	0.421	1.36E-93
ACD	1.05E-97	-0.1268	0.054	0.368	1.57E-93
HMGN2	1.10E-97	-0.21923	0.461	0.884	1.64E-93
RRBP1	1.20E-97	-0.28779	0.07	0.432	1.79E-93
USP39	1.32E-97	-0.25785	0.037	0.383	1.98E-93
RBM27	1.50E-97	-0.33377	0.04	0.405	2.24E-93
AKT1S1	1.51E-97	-0.17658	0.058	0.406	2.25E-93
PGRMC2	1.84E-97	-0.13746	0.063	0.399	2.74E-93
CBX6	2.03E-97	-0.1588	0.094	0.448	3.03E-93
OLFM1	2.22E-97	-0.28195	0.084	0.477	3.32E-93

COIL	2.50E-97	-0.20932	0.045	0.393	3.74E-93
PRRC2B	2.67E-97	-0.18978	0.059	0.396	4.00E-93
DEAF1	2.79E-97	-0.18979	0.059	0.41	4.18E-93
TRUB2	2.94E-97	0.139976	0.092	0.352	4.40E-93
THOC3	3.18E-97	-0.3906	0.026	0.401	4.76E-93
BTG2	3.46E-97	0.497887	0.257	0.449	5.17E-93
MLF1	3.52E-97	0.149028	0.103	0.367	5.26E-93
G6PD	3.55E-97	-0.15854	0.04	0.359	5.30E-93
TOP2B	3.61E-97	-0.2198	0.068	0.432	5.39E-93
CHEK1	3.73E-97	-0.6501	0.019	0.451	5.57E-93
RNF157	4.19E-97	-0.11255	0.054	0.374	6.26E-93
SKA3	4.50E-97	-0.67921	0	0.419	6.72E-93
ZFAND2A	5.87E-97	0.169087	0.145	0.452	8.77E-93
ME2	6.47E-97	-0.24805	0.038	0.384	9.66E-93
PUM1	6.61E-97	-0.10211	0.065	0.375	9.88E-93
ACLY	6.88E-97	-0.2077	0.059	0.401	1.03E-92
CTNNAL1	7.05E-97	-0.38717	0.049	0.433	1.05E-92
RRP1	8.03E-97	-0.2166	0.054	0.401	1.20E-92
PIGC	8.48E-97	0.129952	0.079	0.351	1.27E-92
STX16	1.06E-96	-0.11443	0.061	0.383	1.58E-92
NCAPG2	1.34E-96	-0.56233	0.023	0.443	2.00E-92
OGFOD3	1.41E-96	-0.35084	0.04	0.409	2.10E-92
MUS81	1.42E-96	-0.29421	0.042	0.402	2.13E-92
LINC00152	1.45E-96	0.229348	0.131	0.38	2.17E-92
ATP6V1H	1.61E-96	0.107896	0.105	0.398	2.40E-92
NEK6	1.80E-96	-0.3245	0.068	0.422	2.69E-92
SERPINH1	2.23E-96	-0.10145	0.068	0.391	3.33E-92
RQCD1	2.39E-96	-0.2806	0.051	0.419	3.57E-92
GPR137	2.42E-96	-0.23714	0.042	0.384	3.62E-92
VPS26B	2.48E-96	-0.32865	0.054	0.438	3.70E-92
VTA1	2.58E-96	-0.12781	0.051	0.356	3.86E-92
CTCF	2.59E-96	-0.41868	0.044	0.443	3.88E-92
FSD1	2.67E-96	-0.17509	0.052	0.381	4.00E-92
ATF6	2.71E-96	-0.17862	0.049	0.378	4.05E-92
ZNF791	2.78E-96	0.11254	0.143	0.432	4.15E-92
MBD3	2.78E-96	-0.32478	0.04	0.404	4.15E-92
CCNL2	2.83E-96	-0.10726	0.087	0.42	4.23E-92
ROBO1	3.00E-96	-0.58786	0.017	0.433	4.48E-92
LRRC47	3.06E-96	-0.35238	0.054	0.451	4.57E-92
CRMP1	3.42E-96	-0.29344	0.113	0.515	5.11E-92
CHMP1B	3.83E-96	0.112894	0.117	0.401	5.72E-92
GINS1	3.94E-96	-0.54367	0.012	0.41	5.89E-92
CHD1	4.05E-96	-0.41694	0.056	0.463	6.05E-92
HN1L	4.37E-96	-0.50309	0.024	0.435	6.54E-92
FNBP4	4.79E-96	-0.27554	0.077	0.459	7.16E-92
ASXL1	4.92E-96	-0.2704	0.061	0.426	7.35E-92
KIF20B	4.94E-96	-0.71078	0.017	0.454	7.38E-92
GCAT	5.16E-96	-0.22697	0.061	0.426	7.72E-92
NQO2	5.30E-96	-0.17848	0.052	0.388	7.92E-92
VPS51	5.49E-96	-0.17498	0.08	0.438	8.21E-92
TRIM37	6.21E-96	-0.26035	0.031	0.373	9.28E-92

PSD3	6.35E-96	-0.16229	0.059	0.393	9.49E-92
NTAN1	6.53E-96	-0.37252	0.023	0.377	9.76E-92
G3BP2	6.54E-96	-0.26115	0.051	0.406	9.78E-92
ZFAND2B	6.57E-96	-0.20997	0.054	0.402	9.82E-92
PTPN12	6.71E-96	-0.17023	0.065	0.396	1.00E-91
NSD1	7.40E-96	-0.1114	0.086	0.419	1.11E-91
SLC25A26	7.56E-96	0.117632	0.098	0.36	1.13E-91
TNPO2	8.02E-96	-0.15377	0.073	0.42	1.20E-91
EFHC1	8.24E-96	0.350745	0.131	0.332	1.23E-91
RECQL	8.34E-96	-0.25224	0.035	0.373	1.25E-91
ZNF43	8.97E-96	-0.29412	0.061	0.44	1.34E-91
C17orf58	9.77E-96	-0.42949	0.035	0.427	1.46E-91
DEPDC1	1.08E-95	-0.75468	0	0.414	1.61E-91
KIF4A	1.08E-95	-0.73412	0	0.414	1.61E-91
ISG15	1.18E-95	0.636721	0.208	0.374	1.77E-91
SCYL1	1.19E-95	-0.24956	0.042	0.383	1.77E-91
FANCI	1.20E-95	-0.57337	0.014	0.423	1.79E-91
FBXW2	1.25E-95	-0.27224	0.04	0.39	1.87E-91
EPB41L2	1.28E-95	-0.38616	0.035	0.41	1.91E-91
B4GALT5	1.28E-95	-0.25408	0.045	0.38	1.92E-91
PDGFRA	1.30E-95	-0.5695	0.065	0.505	1.95E-91
RB1CC1	1.33E-95	-0.3002	0.07	0.448	1.99E-91
THAP11	1.59E-95	-0.34362	0.033	0.4	2.37E-91
NSUN5	1.65E-95	-0.13115	0.066	0.401	2.47E-91
LHX2	1.67E-95	-0.60142	0.017	0.42	2.50E-91
CIZ1	1.74E-95	-0.16314	0.059	0.395	2.60E-91
OIP5	2.10E-95	-0.74062	0.002	0.423	3.14E-91
ZNF358	2.37E-95	-0.16721	0.04	0.344	3.55E-91
NAT14	2.59E-95	-0.19165	0.056	0.394	3.87E-91
CNRIP1	2.60E-95	-0.21451	0.072	0.431	3.89E-91
SCFD1	2.67E-95	0.24235	0.122	0.36	3.99E-91
NUP93	2.72E-95	-0.30276	0.045	0.415	4.07E-91
MAP3K7	3.07E-95	-0.10503	0.056	0.372	4.59E-91
KIF9	3.79E-95	0.115099	0.086	0.348	5.67E-91
BASP1	3.92E-95	-0.69076	0.042	0.491	5.86E-91
ORAI2	4.20E-95	-0.22018	0.075	0.436	6.28E-91
NCLN	4.47E-95	-0.19399	0.031	0.349	6.68E-91
SMPD1	5.30E-95	-0.23339	0.052	0.389	7.91E-91
NCAPH	5.30E-95	-0.75257	0.002	0.422	7.93E-91
TOR1AIP2	5.67E-95	-0.155	0.077	0.417	8.48E-91
ATP6V0A1	5.78E-95	0.273326	0.126	0.356	8.64E-91
SART1	7.64E-95	-0.29606	0.033	0.38	1.14E-90
MEX3A	7.94E-95	-0.38764	0.066	0.465	1.19E-90
CPSF4	8.06E-95	-0.21564	0.07	0.422	1.20E-90
IGFBP5	1.13E-94	0.876338	0.307	0.379	1.70E-90
NOB1	1.15E-94	0.229745	0.073	0.299	1.72E-90
BICD1	1.18E-94	-0.30654	0.047	0.41	1.77E-90
PCMTD2	1.22E-94	-0.13315	0.084	0.419	1.82E-90
FAM46A	1.24E-94	0.175728	0.117	0.378	1.85E-90
CEP350	1.47E-94	-0.19586	0.031	0.34	2.19E-90
FGFR1OP2	1.48E-94	-0.14069	0.079	0.428	2.21E-90

HS2ST1	1.55E-94	-0.18107	0.054	0.383	2.32E-90
SPOCD1	1.78E-94	0.575437	0.175	0.301	2.65E-90
WASL	1.94E-94	-0.14012	0.058	0.374	2.90E-90
NCOR2	2.20E-94	-0.19381	0.07	0.419	3.30E-90
NDUFAF6	2.26E-94	-0.19012	0.049	0.384	3.37E-90
BAG5	2.26E-94	-0.14056	0.052	0.385	3.37E-90
PROSC	2.58E-94	-0.1599	0.063	0.398	3.85E-90
LINC00667	2.75E-94	0.251523	0.12	0.359	4.11E-90
COX19	2.78E-94	-0.14727	0.072	0.404	4.16E-90
FOXG1	2.85E-94	-0.2534	0.051	0.391	4.26E-90
PEX16	3.06E-94	0.211526	0.08	0.311	4.57E-90
ENO2	3.10E-94	0.136007	0.157	0.472	4.63E-90
IWS1	3.41E-94	-0.22768	0.059	0.414	5.09E-90
CCNG1	3.57E-94	0.147415	0.126	0.41	5.34E-90
CXXC1	3.95E-94	-0.18513	0.045	0.384	5.90E-90
CREB1	4.94E-94	-0.14922	0.096	0.447	7.39E-90
FBXW5	5.06E-94	-0.2687	0.052	0.414	7.57E-90
DNM2	5.45E-94	-0.16462	0.047	0.365	8.14E-90
MBOAT7	6.07E-94	-0.2938	0.04	0.398	9.07E-90
LYPLA2	7.28E-94	0.112283	0.073	0.315	1.09E-89
GOLT1B	8.33E-94	-0.10314	0.063	0.381	1.25E-89
VPS16	9.00E-94	-0.13548	0.04	0.343	1.34E-89
PBDC1	9.26E-94	0.175215	0.099	0.372	1.38E-89
CINP	1.06E-93	0.137441	0.101	0.372	1.58E-89
HSPA14	1.08E-93	-0.24315	0.026	0.347	1.61E-89
UBAC1	1.10E-93	-0.24493	0.042	0.383	1.64E-89
NR3C1	1.26E-93	-0.17507	0.082	0.432	1.89E-89
CCS	1.55E-93	0.510931	0.14	0.286	2.32E-89
HEBP2	1.57E-93	-0.48768	0.021	0.402	2.35E-89
HES4	1.87E-93	-0.52097	0.031	0.432	2.79E-89
PCGF2	2.18E-93	-0.29458	0.038	0.386	3.26E-89
MTA1	2.23E-93	-0.26924	0.07	0.438	3.34E-89
REV3L	2.45E-93	-0.15016	0.091	0.436	3.66E-89
IFRD2	2.80E-93	-0.18179	0.059	0.383	4.19E-89
ASAP1	3.25E-93	-0.22138	0.073	0.43	4.86E-89
MIS12	3.32E-93	-0.2006	0.058	0.402	4.96E-89
GFER	3.42E-93	-0.36837	0.037	0.405	5.11E-89
SEPP1	3.44E-93	1.250569	0.314	0.168	5.14E-89
DENND5A	3.51E-93	-0.1162	0.038	0.326	5.24E-89
ZEB2	3.55E-93	-0.1335	0.096	0.433	5.31E-89
ATAD3A	4.24E-93	-0.32553	0.035	0.399	6.34E-89
STRADB	4.64E-93	-0.27349	0.042	0.389	6.94E-89
NUP107	5.87E-93	-0.40336	0.134	0.579	8.77E-89
RHOT2	5.96E-93	-0.15304	0.08	0.421	8.91E-89
BFAR	7.08E-93	-0.19789	0.058	0.391	1.06E-88
GSN	7.61E-93	0.355913	0.124	0.327	1.14E-88
CDK5RAP2	7.69E-93	-0.28572	0.026	0.363	1.15E-88
SIL1	9.63E-93	0.187215	0.077	0.29	1.44E-88
SMUG1	1.07E-92	0.126791	0.079	0.322	1.59E-88
ARID5B	1.09E-92	0.313235	0.12	0.344	1.63E-88
KCTD20	1.15E-92	-0.10812	0.052	0.352	1.71E-88

CDIPT	1.68E-92	-0.10393	0.07	0.396	2.51E-88
PKN2	1.70E-92	-0.27158	0.047	0.4	2.54E-88
GFAP	1.82E-92	0.916463	0.346	0.354	2.71E-88
CDK2	2.22E-92	-0.554	0.019	0.416	3.31E-88
CCDC115	2.27E-92	0.217773	0.113	0.359	3.39E-88
PLA2G12A	2.51E-92	-0.17799	0.063	0.401	3.75E-88
GAS1	2.78E-92	-0.36928	0.073	0.463	4.15E-88
GMPS	3.06E-92	-0.42901	0.024	0.398	4.57E-88
HOMER3	3.23E-92	-0.24504	0.04	0.359	4.83E-88
BCAR1	3.27E-92	-0.16257	0.051	0.359	4.88E-88
ZWILCH	3.30E-92	-0.5719	0.014	0.417	4.93E-88
COL4A2	3.49E-92	-0.25243	0.03	0.342	5.21E-88
FIBIN	3.64E-92	0.347805	0.194	0.406	5.44E-88
TMEM5	3.64E-92	0.137318	0.084	0.325	5.44E-88
RAD50	3.67E-92	-0.11836	0.089	0.425	5.48E-88
CTNNB1	3.92E-92	-0.26489	0.112	0.509	5.85E-88
LGALS1	4.86E-92	0.306494	0.539	0.767	7.26E-88
LRRC58	5.00E-92	-0.30188	0.049	0.411	7.47E-88
KLHL23	6.02E-92	-0.55804	0.044	0.459	9.00E-88
LINC00665	6.41E-92	0.234473	0.105	0.347	9.58E-88
SUDS3	7.67E-92	-0.28917	0.045	0.395	1.15E-87
BRD9	7.79E-92	-0.2295	0.054	0.395	1.16E-87
PET117	7.96E-92	-0.14227	0.051	0.353	1.19E-87
FAM173A	8.05E-92	-0.19005	0.059	0.386	1.20E-87
PSMD9	8.70E-92	0.130363	0.131	0.399	1.30E-87
ZNF83	8.77E-92	-0.27832	0.063	0.432	1.31E-87
WDR45	9.91E-92	0.331019	0.112	0.315	1.48E-87
ERBB2IP	1.10E-91	-0.22725	0.045	0.378	1.64E-87
HOOK2	1.33E-91	0.242067	0.117	0.356	1.99E-87
CDC42EP4	1.57E-91	0.128697	0.126	0.43	2.35E-87
TRIP6	1.58E-91	0.252929	0.122	0.347	2.37E-87
MCOLN1	1.63E-91	-0.33104	0.037	0.394	2.43E-87
PDE6D	1.68E-91	-0.21902	0.063	0.411	2.52E-87
CEP170	1.77E-91	-0.34228	0.052	0.433	2.64E-87
CNOT3	1.86E-91	-0.3163	0.031	0.378	2.78E-87
KIAA1524	2.12E-91	-0.6446	0.007	0.406	3.17E-87
CANT1	2.22E-91	-0.32478	0.04	0.393	3.32E-87
TUBB6	2.70E-91	-0.1859	0.075	0.42	4.03E-87
ARHGAP12	2.71E-91	0.231493	0.099	0.333	4.05E-87
ADNP	2.84E-91	-0.34502	0.045	0.411	4.25E-87
EPB41L4A-	3.16E-91	0.247005	0.127	0.37	4.72E-87
GAR1	3.53E-91	-0.16667	0.042	0.353	5.27E-87
HLA-E	3.55E-91	0.411874	0.236	0.462	5.31E-87
SNAPC2	3.68E-91	-0.11543	0.044	0.34	5.50E-87
UBAP2L	4.43E-91	-0.16153	0.056	0.38	6.62E-87
SCG3	4.48E-91	0.398416	0.272	0.494	6.69E-87
CEP135	5.32E-91	-0.66828	0.017	0.432	7.95E-87
PURA	5.67E-91	-0.158	0.063	0.379	8.47E-87
BLMH	6.35E-91	-0.37202	0.038	0.41	9.50E-87
CUL3	6.60E-91	-0.22634	0.061	0.4	9.86E-87
WWTR1	6.88E-91	-0.25094	0.031	0.336	1.03E-86

SLC2A4RG	7.31E-91	-0.36618	0.024	0.375	1.09E-86
HERC2	7.49E-91	-0.11944	0.07	0.385	1.12E-86
TMEM189	7.68E-91	-0.37132	0.024	0.375	1.15E-86
FAM120AOS	8.08E-91	-0.25382	0.037	0.374	1.21E-86
ATN1	8.27E-91	-0.15894	0.052	0.362	1.24E-86
TRIM27	8.50E-91	-0.14723	0.063	0.384	1.27E-86
METTL7B	8.67E-91	0.529686	0.208	0.374	1.30E-86
BCL2L1	8.69E-91	0.304431	0.115	0.323	1.30E-86
STARD3	8.83E-91	-0.24167	0.049	0.39	1.32E-86
TLN1	9.00E-91	-0.13036	0.04	0.335	1.34E-86
CPSF3	1.09E-90	-0.29462	0.045	0.407	1.63E-86
TFDP2	1.23E-90	-0.28996	0.061	0.426	1.83E-86
SLC36A4	1.26E-90	-0.3027	0.047	0.404	1.89E-86
PEX10	1.29E-90	-0.19215	0.052	0.377	1.93E-86
ANAPC7	1.42E-90	-0.12472	0.056	0.36	2.12E-86
ETHE1	1.54E-90	0.33736	0.103	0.298	2.30E-86
ADAM17	1.54E-90	-0.23723	0.056	0.386	2.31E-86
TCF3	1.63E-90	-0.23176	0.031	0.342	2.43E-86
PPP3CA	1.78E-90	-0.13177	0.066	0.385	2.67E-86
PLXNB2	1.88E-90	-0.18482	0.033	0.317	2.81E-86
CD82	1.94E-90	0.180551	0.155	0.42	2.89E-86
GUSB	2.27E-90	-0.12644	0.084	0.421	3.39E-86
B4GALT7	2.44E-90	-0.23636	0.04	0.367	3.65E-86
XIAP	2.61E-90	-0.18255	0.056	0.385	3.90E-86
SLC20A1	2.64E-90	-0.44226	0.031	0.4	3.95E-86
EPHX1	2.89E-90	-0.11383	0.054	0.354	4.32E-86
SLC25A1	2.99E-90	-0.20542	0.054	0.391	4.47E-86
UXS1	3.05E-90	-0.16729	0.047	0.356	4.56E-86
ANKRD17	3.37E-90	-0.15235	0.072	0.405	5.04E-86
FAM20C	3.81E-90	0.228149	0.091	0.304	5.69E-86
C1R	4.14E-90	1.053974	0.257	0.227	6.19E-86
SIRT7	4.40E-90	-0.2466	0.04	0.367	6.58E-86
C14orf80	4.61E-90	-0.47188	0.017	0.4	6.89E-86
CRYZL1	4.79E-90	0.250462	0.117	0.333	7.16E-86
IGFBP7	4.91E-90	0.227002	0.232	0.516	7.34E-86
BRCA1	5.03E-90	-0.58924	0.01	0.415	7.52E-86
GALE	5.13E-90	-0.12717	0.028	0.294	7.67E-86
POLR2D	5.68E-90	-0.37884	0.033	0.401	8.48E-86
RAB5B	5.69E-90	0.231058	0.092	0.29	8.51E-86
GRK6	6.14E-90	-0.37496	0.016	0.352	9.17E-86
DDX23	6.79E-90	-0.27333	0.038	0.375	1.02E-85
FAM104B	6.90E-90	-0.25253	0.049	0.388	1.03E-85
ABHD3	7.02E-90	-0.53558	0.026	0.422	1.05E-85
MT1F	7.61E-90	0.540246	0.192	0.34	1.14E-85
RBMS1	7.69E-90	-0.18301	0.065	0.39	1.15E-85
NEK2	7.91E-90	-0.82708	0.002	0.405	1.18E-85
PPP1R15A	8.55E-90	0.201197	0.155	0.407	1.28E-85
ACP2	8.84E-90	0.140656	0.087	0.333	1.32E-85
GPX7	8.88E-90	-0.10852	0.056	0.364	1.33E-85
IMPAD1	9.74E-90	-0.1365	0.054	0.367	1.46E-85
PPCS	9.81E-90	0.420872	0.112	0.275	1.47E-85

OXCT1	1.05E-89	-0.21631	0.063	0.412	1.57E-85
PMEPA1	1.13E-89	-0.50134	0.038	0.415	1.69E-85
RNF138	1.16E-89	-0.17573	0.044	0.353	1.73E-85
FRYL	1.21E-89	-0.31489	0.052	0.409	1.80E-85
RNFT1	1.40E-89	-0.32517	0.03	0.373	2.10E-85
MAEA	1.45E-89	-0.15231	0.066	0.385	2.17E-85
NUP54	1.54E-89	-0.32073	0.051	0.414	2.31E-85
TRMT6	1.65E-89	-0.2614	0.045	0.385	2.47E-85
CRAT	1.66E-89	-0.21268	0.044	0.362	2.48E-85
SNX14	1.70E-89	-0.13416	0.052	0.356	2.54E-85
CEBPB	1.90E-89	-0.12117	0.094	0.414	2.84E-85
EXTL2	1.95E-89	-0.16652	0.059	0.385	2.91E-85
ABRACL	2.15E-89	-0.25597	0.042	0.372	3.21E-85
FUZ	2.18E-89	-0.22239	0.056	0.4	3.26E-85
RANGAP1	2.37E-89	-0.53533	0.009	0.39	3.55E-85
PPIL3	2.81E-89	-0.30668	0.038	0.386	4.20E-85
CDCA2	3.05E-89	-0.6852	0	0.39	4.56E-85
DVL2	3.19E-89	-0.3088	0.04	0.381	4.77E-85
PSMG4	3.25E-89	-0.1715	0.059	0.383	4.85E-85
MFSD11	3.71E-89	-0.15678	0.049	0.363	5.54E-85
FAM120A	3.84E-89	-0.2316	0.031	0.342	5.73E-85
FAM127B	4.28E-89	0.192933	0.129	0.385	6.40E-85
RP9	4.61E-89	-0.24456	0.061	0.405	6.89E-85
BET1	4.82E-89	0.233933	0.11	0.325	7.20E-85
IST1	4.87E-89	-0.26419	0.061	0.407	7.28E-85
REST	5.12E-89	-0.40173	0.045	0.425	7.66E-85
ERGIC1	6.00E-89	0.102328	0.098	0.364	8.97E-85
RCC1	6.74E-89	-0.46439	0.019	0.399	1.01E-84
CBR3	7.56E-89	-0.39725	0.03	0.388	1.13E-84
REEP4	7.77E-89	-0.59419	0.01	0.391	1.16E-84
FAM207A	7.97E-89	-0.19474	0.038	0.347	1.19E-84
MMD	8.02E-89	-0.52708	0.024	0.419	1.20E-84
ZNF511	8.81E-89	-0.25872	0.031	0.343	1.32E-84
LARP6	9.41E-89	-0.16699	0.052	0.364	1.41E-84
UBE2Q2	1.01E-88	-0.33984	0.035	0.389	1.51E-84
C6orf89	1.15E-88	-0.17239	0.054	0.368	1.72E-84
C15orf61	1.24E-88	-0.12539	0.049	0.358	1.86E-84
EHBP1	1.31E-88	-0.17472	0.033	0.32	1.95E-84
DCTN1	1.42E-88	-0.11915	0.061	0.365	2.12E-84
CDC45	1.42E-88	-0.70925	0	0.388	2.13E-84
NFATC2IP	1.49E-88	-0.36405	0.037	0.396	2.23E-84
ROCK2	1.62E-88	-0.19211	0.038	0.341	2.42E-84
RRP36	1.79E-88	-0.35634	0.014	0.343	2.67E-84
LTBP3	1.94E-88	-0.15207	0.061	0.373	2.90E-84
DCP2	2.22E-88	-0.35773	0.035	0.394	3.32E-84
FAM83D	2.49E-88	-0.82652	0.002	0.4	3.73E-84
STT3B	2.65E-88	-0.1802	0.063	0.385	3.96E-84
PRPF4	2.69E-88	-0.2074	0.051	0.379	4.03E-84
SLAIN1	2.81E-88	-0.30652	0.04	0.378	4.19E-84
HEPN1	2.91E-88	0.404979	0.182	0.374	4.34E-84
TGS1	3.01E-88	-0.12091	0.052	0.359	4.49E-84

TEAD1	3.55E-88	-0.26528	0.042	0.36	5.31E-84
GOT2	3.59E-88	-0.16332	0.058	0.381	5.37E-84
CNIH3	3.75E-88	-0.11761	0.058	0.348	5.60E-84
WDR43	4.08E-88	-0.24243	0.037	0.365	6.09E-84
ARSJ	4.24E-88	-0.12879	0.04	0.315	6.34E-84
HMMR	4.54E-88	-0.74407	0.003	0.394	6.79E-84
RFFL	4.56E-88	-0.31358	0.033	0.354	6.82E-84
MPHOSPH9	6.10E-88	-0.3528	0.04	0.4	9.12E-84
PIGP	6.30E-88	0.197761	0.07	0.289	9.42E-84
CASC5	6.61E-88	-0.62971	0	0.385	9.88E-84
EXOC5	6.90E-88	-0.22607	0.066	0.415	1.03E-83
TBC1D7	6.96E-88	-0.14491	0.045	0.347	1.04E-83
UTP6	7.27E-88	-0.11457	0.054	0.352	1.09E-83
RPP25L	7.37E-88	0.136162	0.058	0.274	1.10E-83
NFKBIA	8.71E-88	0.244599	0.168	0.419	1.30E-83
CXADR	9.70E-88	-0.25617	0.07	0.42	1.45E-83
AP006222.	1.34E-87	0.159373	0.063	0.283	2.01E-83
METTL2A	1.52E-87	-0.19302	0.038	0.342	2.26E-83
LYAR	1.54E-87	-0.2154	0.047	0.377	2.30E-83
IER5	1.56E-87	-0.20927	0.04	0.354	2.34E-83
PRR14	1.96E-87	-0.17971	0.054	0.364	2.92E-83
RNASEH1-A	2.05E-87	-0.21838	0.044	0.36	3.06E-83
TBC1D20	2.29E-87	-0.21066	0.03	0.335	3.42E-83
REXO4	2.54E-87	-0.11876	0.054	0.349	3.79E-83
SOX8	2.69E-87	-0.20121	0.103	0.46	4.02E-83
MSL1	2.83E-87	-0.10429	0.049	0.336	4.22E-83
C19orf52	2.90E-87	-0.34341	0.031	0.38	4.33E-83
TRPT1	2.91E-87	-0.16983	0.072	0.41	4.35E-83
ANKLE2	3.15E-87	-0.35359	0.037	0.395	4.71E-83
ARMC6	3.30E-87	-0.11347	0.068	0.384	4.94E-83
NMRAL1	3.31E-87	0.190127	0.101	0.336	4.95E-83
OSTM1	3.89E-87	-0.17629	0.035	0.333	5.81E-83
DCAF11	3.89E-87	-0.15932	0.042	0.34	5.81E-83
QSER1	4.12E-87	-0.37338	0.017	0.359	6.15E-83
PREB	5.11E-87	-0.15639	0.056	0.365	7.63E-83
IVNS1ABP	5.56E-87	-0.27656	0.042	0.373	8.31E-83
ERI1	7.31E-87	-0.18796	0.04	0.343	1.09E-82
WIPF2	7.31E-87	-0.16982	0.047	0.346	1.09E-82
PLS3	7.44E-87	-0.14797	0.075	0.395	1.11E-82
NUP37	8.34E-87	-0.21044	0.04	0.359	1.25E-82
OSBPL6	9.11E-87	-0.35271	0.037	0.388	1.36E-82
AAAS	1.16E-86	-0.18025	0.087	0.433	1.73E-82
CS	1.16E-86	-0.26164	0.028	0.349	1.73E-82
ANLN	1.24E-86	-0.51811	0.007	0.37	1.86E-82
BCS1L	1.37E-86	-0.12171	0.061	0.368	2.05E-82
TMED7	1.43E-86	-0.13804	0.08	0.402	2.14E-82
GEMIN6	1.47E-86	-0.17936	0.051	0.356	2.20E-82
UROS	1.67E-86	-0.274	0.042	0.375	2.49E-82
KAT7	1.74E-86	-0.15363	0.051	0.356	2.60E-82
ATG4D	1.74E-86	-0.30231	0.037	0.37	2.60E-82
DVL3	1.82E-86	-0.24417	0.033	0.354	2.72E-82

FOXO3	1.95E-86	-0.14801	0.066	0.381	2.91E-82
TACO1	1.99E-86	-0.31023	0.026	0.351	2.98E-82
EHMT2	2.28E-86	-0.16263	0.075	0.391	3.41E-82
SMAD2	2.41E-86	-0.10804	0.056	0.352	3.60E-82
PPAT	2.44E-86	-0.36674	0.045	0.411	3.64E-82
LRRC8A	3.05E-86	0.114854	0.087	0.326	4.56E-82
IARS2	3.11E-86	-0.22863	0.049	0.378	4.65E-82
KPNA4	3.38E-86	-0.2273	0.03	0.332	5.04E-82
CEP63	3.48E-86	-0.30726	0.038	0.381	5.20E-82
ORC4	3.80E-86	-0.25909	0.059	0.404	5.68E-82
BCL2L12	4.43E-86	-0.55951	0.01	0.372	6.62E-82
ANXA7	4.55E-86	0.131724	0.106	0.36	6.80E-82
GLIPR1	4.58E-86	0.115268	0.058	0.262	6.85E-82
NGLY1	4.74E-86	-0.2127	0.054	0.385	7.08E-82
XRCC2	4.76E-86	-0.6858	0.005	0.399	7.12E-82
SAMM50	4.87E-86	-0.20812	0.061	0.386	7.28E-82
ZDHHC3	5.07E-86	-0.28609	0.024	0.348	7.58E-82
LETM1	5.24E-86	-0.1449	0.045	0.338	7.83E-82
ARL14EP	6.56E-86	0.130974	0.072	0.295	9.80E-82
WDR46	6.61E-86	-0.16129	0.049	0.354	9.89E-82
ARHGAP11A	6.75E-86	-0.70613	0.005	0.401	1.01E-81
ZFP36	6.84E-86	1.07186	0.41	0.327	1.02E-81
U2AF1L4	6.97E-86	0.468275	0.129	0.289	1.04E-81
PCF11	7.47E-86	-0.10817	0.063	0.354	1.12E-81
RNF115	7.49E-86	-0.22626	0.068	0.409	1.12E-81
TRABD	7.81E-86	-0.31186	0.037	0.377	1.17E-81
SMC5	8.28E-86	-0.2753	0.049	0.388	1.24E-81
APC	8.72E-86	-0.14804	0.058	0.357	1.30E-81
LRRCC1	9.11E-86	-0.38845	0.042	0.405	1.36E-81
CDK6	9.50E-86	-0.31288	0.103	0.486	1.42E-81
LDLRAD3	9.56E-86	-0.13474	0.059	0.359	1.43E-81
KCTD5	1.05E-85	-0.17938	0.063	0.385	1.58E-81
EFEMP1	1.06E-85	0.902699	0.225	0.241	1.58E-81
UNC119	1.09E-85	-0.28365	0.037	0.367	1.63E-81
MED13	1.09E-85	-0.17918	0.035	0.321	1.64E-81
PITPNA	1.14E-85	-0.2812	0.024	0.326	1.70E-81
KDSR	1.20E-85	-0.1591	0.068	0.378	1.80E-81
SNTG1	1.24E-85	0.455588	0.171	0.33	1.85E-81
RBM38	1.29E-85	-0.2015	0.038	0.344	1.93E-81
NUP50	1.31E-85	-0.36896	0.019	0.359	1.96E-81
TMEM55A	1.51E-85	0.212237	0.094	0.319	2.25E-81
DPP6	1.53E-85	0.13611	0.108	0.351	2.28E-81
NRSN2	1.65E-85	-0.30182	0.035	0.369	2.47E-81
MYH10	1.70E-85	-0.27044	0.054	0.389	2.53E-81
GLYR1	1.73E-85	-0.26403	0.051	0.39	2.58E-81
NPM3	2.00E-85	0.201672	0.087	0.311	3.00E-81
RFC5	2.04E-85	-0.50138	0.028	0.414	3.05E-81
ZNF430	2.17E-85	-0.16634	0.077	0.404	3.25E-81
SIAH2	2.17E-85	-0.32453	0.042	0.388	3.25E-81
EPM2AIP1	2.28E-85	-0.11521	0.068	0.372	3.41E-81
CAMK1	2.37E-85	-0.1839	0.044	0.349	3.55E-81

COQ7	2.43E-85	-0.11461	0.073	0.388	3.63E-81
C2orf80	2.76E-85	-0.3125	0.054	0.398	4.13E-81
STIM2	3.14E-85	-0.12348	0.051	0.34	4.70E-81
NDN	3.14E-85	-0.18954	0.03	0.33	4.70E-81
RAB32	3.47E-85	-0.19973	0.023	0.298	5.18E-81
MYCBP2	4.01E-85	-0.17458	0.068	0.391	5.99E-81
MAP2K7	4.01E-85	-0.1301	0.061	0.354	6.00E-81
UCK1	4.06E-85	-0.11515	0.073	0.386	6.07E-81
DGKZ	4.16E-85	-0.22273	0.04	0.349	6.22E-81
CASP3	4.27E-85	-0.31448	0.045	0.386	6.38E-81
CDC27	4.34E-85	-0.45316	0.019	0.37	6.48E-81
SAPCD2	4.36E-85	-0.51361	0.021	0.386	6.51E-81
DNAAF2	4.86E-85	-0.10163	0.049	0.315	7.26E-81
TIMELESS	5.14E-85	-0.62636	0.009	0.399	7.69E-81
RNF139	5.29E-85	-0.2094	0.063	0.39	7.91E-81
DEXI	5.47E-85	-0.11965	0.047	0.332	8.17E-81
HRSP12	6.29E-85	0.421636	0.129	0.29	9.40E-81
DCAKD	6.62E-85	-0.22469	0.031	0.338	9.90E-81
ID4	6.98E-85	-0.22954	0.084	0.421	1.04E-80
TOR3A	7.36E-85	-0.19058	0.047	0.356	1.10E-80
USP34	9.94E-85	-0.18306	0.051	0.36	1.49E-80
HGS	1.06E-84	-0.19584	0.061	0.379	1.58E-80
RGL2	1.12E-84	-0.10269	0.054	0.33	1.67E-80
GNL2	1.13E-84	-0.18583	0.056	0.374	1.69E-80
RBM15B	1.15E-84	-0.25493	0.038	0.359	1.72E-80
SNRNP35	1.31E-84	0.144641	0.091	0.33	1.96E-80
HCCS	1.33E-84	-0.10674	0.037	0.312	1.98E-80
CNOT1	1.37E-84	-0.28861	0.024	0.341	2.05E-80
ZBED5	1.44E-84	-0.13357	0.058	0.36	2.15E-80
PPP1R35	1.50E-84	-0.36755	0.035	0.38	2.24E-80
FBXO9	1.71E-84	-0.1749	0.042	0.349	2.56E-80
SRGAP2	1.73E-84	-0.1895	0.047	0.358	2.58E-80
ZNF414	1.85E-84	-0.32636	0.037	0.383	2.76E-80
PAK4	2.01E-84	-0.32862	0.028	0.36	3.00E-80
E2F4	2.22E-84	-0.2784	0.031	0.358	3.31E-80
ARFIP2	2.26E-84	-0.14121	0.07	0.378	3.38E-80
POMGNT2	2.52E-84	-0.11664	0.051	0.343	3.76E-80
TMEM80	2.60E-84	-0.10626	0.042	0.312	3.89E-80
NCAPD3	2.80E-84	-0.47066	0.012	0.37	4.18E-80
CHAMP1	2.81E-84	-0.28995	0.031	0.356	4.20E-80
TCF19	2.86E-84	-0.67919	0	0.372	4.28E-80
ODF2	2.88E-84	-0.50524	0.03	0.405	4.30E-80
UBAC2	3.03E-84	-0.20301	0.052	0.368	4.52E-80
FGFR1	3.09E-84	-0.18611	0.054	0.363	4.62E-80
SNN	3.46E-84	-0.24172	0.056	0.39	5.18E-80
FAHD2A	3.54E-84	-0.14133	0.056	0.358	5.29E-80
R3HDM1	3.58E-84	-0.17458	0.04	0.331	5.35E-80
GMPPA	3.87E-84	0.126392	0.061	0.28	5.79E-80
UBA5	4.06E-84	-0.13341	0.052	0.352	6.06E-80
RASSF1	4.93E-84	-0.28213	0.04	0.373	7.37E-80
RTKN	5.09E-84	0.195195	0.091	0.302	7.60E-80

SYNRG	5.30E-84	-0.26164	0.037	0.362	7.92E-80
PDS5A	5.47E-84	-0.33099	0.028	0.359	8.17E-80
HSDL2	5.66E-84	-0.153	0.037	0.333	8.46E-80
PAXIP1-AS	5.93E-84	-0.1199	0.068	0.368	8.87E-80
TARBP2	6.48E-84	-0.11293	0.035	0.299	9.68E-80
TSPAN13	6.83E-84	-0.44117	0.103	0.51	1.02E-79
RARS2	7.15E-84	-0.2478	0.031	0.341	1.07E-79
GALNT1	7.24E-84	-0.38315	0.026	0.369	1.08E-79
PDLIM2	8.61E-84	0.237058	0.101	0.312	1.29E-79
FBXW11	1.00E-83	-0.27502	0.031	0.343	1.50E-79
MAX	1.04E-83	0.204695	0.096	0.317	1.56E-79
NFATC3	1.08E-83	-0.16601	0.035	0.317	1.62E-79
YIPF5	1.15E-83	0.121974	0.094	0.336	1.71E-79
RBFOX2	1.16E-83	-0.13768	0.082	0.39	1.74E-79
SKA1	1.17E-83	-0.66675	0.002	0.381	1.75E-79
FAF1	1.22E-83	-0.24628	0.035	0.352	1.83E-79
PPFIA1	1.25E-83	-0.12985	0.042	0.307	1.87E-79
GALNT11	1.27E-83	-0.1395	0.058	0.367	1.90E-79
FAM168B	1.41E-83	-0.23226	0.035	0.34	2.10E-79
BEX4	1.44E-83	0.51677	0.269	0.409	2.15E-79
C18orf32	1.49E-83	0.13279	0.086	0.315	2.23E-79
COQ5	1.50E-83	-0.10653	0.052	0.342	2.24E-79
TPD52	1.52E-83	0.136936	0.099	0.358	2.28E-79
RBBP8	1.61E-83	-0.4904	0.012	0.367	2.40E-79
TOB1	1.70E-83	-0.11751	0.075	0.378	2.55E-79
SNX1	1.78E-83	-0.10934	0.052	0.337	2.66E-79
FBXO11	1.90E-83	-0.16122	0.052	0.349	2.84E-79
ATXN2L	1.94E-83	-0.25633	0.042	0.359	2.90E-79
TMEM158	2.04E-83	-0.19942	0.047	0.342	3.05E-79
SPDL1	2.27E-83	-0.60609	0.007	0.386	3.39E-79
TNFAIP1	2.36E-83	-0.22132	0.038	0.348	3.52E-79
GTF2H2	2.50E-83	-0.12671	0.065	0.364	3.73E-79
EDNRB	2.53E-83	-0.23695	0.07	0.401	3.79E-79
ITGA7	2.55E-83	-0.17199	0.037	0.317	3.81E-79
SCPEP1	2.58E-83	0.304771	0.07	0.207	3.86E-79
VEGFA	2.65E-83	0.299156	0.12	0.32	3.97E-79
ENTPD6	2.66E-83	-0.26767	0.042	0.364	3.97E-79
CHERP	2.66E-83	-0.20715	0.056	0.37	3.97E-79
MTMR2	3.13E-83	-0.22244	0.03	0.323	4.67E-79
SUN2	3.47E-83	-0.44596	0.042	0.414	5.18E-79
ZC3H11A	4.13E-83	-0.19643	0.04	0.343	6.17E-79
REV1	4.36E-83	-0.20633	0.052	0.372	6.51E-79
DCAF8	5.10E-83	0.134866	0.073	0.285	7.62E-79
E2F6	5.70E-83	-0.2475	0.023	0.32	8.52E-79
TMEM45A	5.98E-83	0.479173	0.136	0.298	8.94E-79
SMARCA1	6.35E-83	-0.16733	0.054	0.362	9.48E-79
ZNF664	6.82E-83	-0.12555	0.049	0.331	1.02E-78
HMCES	6.84E-83	-0.12205	0.051	0.341	1.02E-78
CA2	6.97E-83	0.649588	0.195	0.296	1.04E-78
RABIF	8.49E-83	-0.11598	0.042	0.326	1.27E-78
PLEKHB1	8.50E-83	0.273858	0.099	0.302	1.27E-78

NUP85	8.99E-83	-0.11384	0.047	0.33	1.34E-78
DCLRE1C	9.31E-83	-0.43267	0.028	0.395	1.39E-78
CTDSP2	1.16E-82	-0.29946	0.112	0.495	1.73E-78
PAQR4	1.23E-82	-0.50109	0.035	0.406	1.83E-78
MTRNR2L8	1.40E-82	1.12711	0.243	0.149	2.09E-78
LMF2	1.41E-82	-0.39235	0.04	0.399	2.10E-78
PCIF1	1.48E-82	-0.11757	0.038	0.307	2.22E-78
CDC42SE2	1.66E-82	-0.11074	0.049	0.321	2.48E-78
UBLCP1	2.05E-82	-0.22278	0.035	0.336	3.06E-78
FAM228B	2.20E-82	-0.11483	0.052	0.344	3.28E-78
EGR1	2.63E-82	0.110494	0.356	0.659	3.93E-78
GCDH	2.71E-82	0.100694	0.089	0.333	4.05E-78
SF3A1	2.77E-82	-0.14109	0.047	0.343	4.14E-78
CHMP6	3.42E-82	-0.13468	0.037	0.315	5.12E-78
RRP1B	3.57E-82	-0.30853	0.024	0.341	5.34E-78
RRAGC	3.76E-82	-0.10649	0.052	0.344	5.62E-78
SACS	4.45E-82	-0.40949	0.023	0.364	6.65E-78
FNDC3A	4.79E-82	-0.1593	0.051	0.346	7.16E-78
BEST1	4.79E-82	0.326509	0.084	0.285	7.16E-78
USB1	5.16E-82	-0.24109	0.033	0.338	7.72E-78
RIC3	5.22E-82	0.500871	0.213	0.364	7.80E-78
SMC6	5.32E-82	-0.26751	0.044	0.357	7.95E-78
ZNF3	5.42E-82	-0.17344	0.094	0.425	8.10E-78
NDE1	5.50E-82	-0.31276	0.024	0.347	8.22E-78
HIP1	5.70E-82	0.13097	0.098	0.336	8.52E-78
RP11-792A	5.95E-82	0.106724	0.103	0.352	8.89E-78
POM121	6.17E-82	-0.17566	0.04	0.33	9.21E-78
BTBD10	6.47E-82	-0.29826	0.033	0.353	9.67E-78
PRR7	6.69E-82	-0.44252	0.028	0.384	1.00E-77
DEPDC1B	6.79E-82	-0.62286	0.002	0.367	1.01E-77
CYB5R1	6.93E-82	0.397104	0.112	0.275	1.04E-77
SLC25A37	6.94E-82	0.239449	0.119	0.321	1.04E-77
TMEM223	7.01E-82	-0.10585	0.058	0.343	1.05E-77
AAMDC	7.26E-82	0.202125	0.099	0.335	1.09E-77
GCLM	7.75E-82	-0.11	0.047	0.325	1.16E-77
NUP88	8.05E-82	-0.39227	0.023	0.365	1.20E-77
NIF3L1	8.21E-82	-0.22414	0.042	0.359	1.23E-77
ZCCHC9	8.82E-82	0.100803	0.054	0.262	1.32E-77
MDM4	9.12E-82	-0.12833	0.096	0.414	1.36E-77
AGT	9.30E-82	0.607785	0.209	0.326	1.39E-77
STAG1	9.73E-82	-0.21562	0.042	0.346	1.45E-77
HNRNPA1L2	9.83E-82	-0.16291	0.084	0.421	1.47E-77
FANCL	1.04E-81	-0.25024	0.058	0.385	1.56E-77
BNIP2	1.14E-81	-0.27841	0.045	0.37	1.70E-77
PRTFDC1	1.24E-81	-0.19676	0.063	0.38	1.85E-77
E2F1	1.38E-81	-0.73534	0.007	0.383	2.06E-77
PDPK1	1.44E-81	-0.27837	0.042	0.373	2.14E-77
TPM2	1.59E-81	-0.48775	0.042	0.395	2.37E-77
NCAPD2	1.67E-81	-0.55798	0.003	0.365	2.49E-77
UBN1	1.74E-81	-0.47401	0.01	0.364	2.59E-77
ANKRD54	1.82E-81	-0.16217	0.042	0.326	2.71E-77

FDXR	1.86E-81	-0.18369	0.03	0.309	2.78E-77
HBS1L	2.11E-81	-0.17096	0.063	0.373	3.16E-77
NELFB	2.11E-81	-0.34582	0.021	0.351	3.16E-77
C1QB	2.19E-81	0.67808	0.25	0.31	3.27E-77
DPH5	2.51E-81	0.218269	0.075	0.259	3.75E-77
CCNB1IP1	2.60E-81	0.11012	0.099	0.338	3.89E-77
RSBN1	2.65E-81	-0.35195	0.045	0.391	3.96E-77
RNF14	2.79E-81	-0.28069	0.038	0.362	4.17E-77
XBP1	2.82E-81	-0.23153	0.066	0.398	4.21E-77
NDRG2	3.13E-81	0.754328	0.342	0.385	4.67E-77
PPIF	3.22E-81	-0.39031	0.026	0.367	4.82E-77
BECN1	3.79E-81	-0.22009	0.035	0.337	5.66E-77
OLIG1	3.86E-81	0.182395	0.209	0.474	5.77E-77
WDR6	3.98E-81	0.149504	0.11	0.335	5.95E-77
TEN1	4.09E-81	-0.28929	0.017	0.316	6.12E-77
RNF168	4.16E-81	-0.43582	0.021	0.374	6.22E-77
DAAM1	4.56E-81	-0.17446	0.101	0.432	6.81E-77
TIMM10B	4.68E-81	0.17778	0.072	0.268	7.00E-77
EXOSC3	4.72E-81	-0.38515	0.024	0.363	7.06E-77
DPYSL4	4.73E-81	-0.37911	0.037	0.383	7.07E-77
HOMER1	4.74E-81	-0.26913	0.04	0.354	7.09E-77
RGS16	4.90E-81	0.408969	0.143	0.328	7.33E-77
SYNC	5.09E-81	-0.2249	0.058	0.386	7.61E-77
SERTAD1	5.26E-81	0.509402	0.157	0.306	7.86E-77
HIPK2	5.68E-81	-0.20706	0.07	0.385	8.49E-77
UAP1	5.91E-81	-0.16214	0.04	0.323	8.84E-77
UBL4A	6.06E-81	-0.22391	0.037	0.333	9.06E-77
KIAA0319L	6.14E-81	0.115736	0.075	0.3	9.18E-77
DCAF16	6.27E-81	-0.23123	0.024	0.31	9.37E-77
DNAJA3	6.36E-81	-0.14827	0.054	0.357	9.50E-77
KLHL42	6.42E-81	-0.11623	0.061	0.348	9.59E-77
GNG12	6.47E-81	-0.2169	0.03	0.325	9.66E-77
TRIP12	6.59E-81	-0.23856	0.021	0.315	9.84E-77
SNAPC1	7.03E-81	-0.16116	0.042	0.337	1.05E-76
PGM3	7.57E-81	-0.15442	0.049	0.333	1.13E-76
API5	8.20E-81	-0.12082	0.068	0.363	1.23E-76
RTKN2	8.33E-81	-0.56855	0.009	0.375	1.24E-76
EMP2	8.63E-81	-0.12557	0.045	0.321	1.29E-76
MAFG	9.43E-81	-0.2203	0.033	0.336	1.41E-76
NUDT2	9.74E-81	-0.30806	0.028	0.352	1.45E-76
RELA	9.79E-81	-0.10713	0.038	0.305	1.46E-76
LGMN	9.93E-81	0.208575	0.113	0.354	1.48E-76
PARPBP	9.98E-81	-0.61812	0.003	0.377	1.49E-76
DNAJC1	1.12E-80	-0.30068	0.035	0.357	1.68E-76
DPF1	1.16E-80	-0.22919	0.079	0.422	1.73E-76
CNTRL	1.20E-80	-0.34054	0.019	0.34	1.80E-76
SALL1	1.27E-80	-0.13477	0.049	0.316	1.90E-76
C1QL1	1.27E-80	-0.16002	0.061	0.357	1.90E-76
SGSM3	1.35E-80	-0.17009	0.058	0.363	2.02E-76
BRWD1	1.37E-80	-0.2381	0.059	0.388	2.04E-76
CPXM1	1.41E-80	-0.36169	0.044	0.389	2.11E-76

LRRC41	1.43E-80	0.117554	0.084	0.302	2.13E-76
ZNF524	1.58E-80	-0.28239	0.019	0.32	2.37E-76
C1orf174	1.63E-80	-0.28957	0.031	0.351	2.43E-76
FANCA	1.74E-80	-0.58588	0.009	0.379	2.60E-76
MAPKAPK5	1.85E-80	-0.32656	0.037	0.372	2.76E-76
UBL7	1.86E-80	-0.11626	0.058	0.349	2.78E-76
HSPA1B	2.15E-80	0.132543	0.239	0.541	3.22E-76
POLR3F	2.17E-80	-0.1651	0.037	0.323	3.24E-76
EXOSC9	2.40E-80	-0.29671	0.04	0.369	3.58E-76
STX4	2.47E-80	0.23491	0.092	0.286	3.69E-76
EXT2	2.54E-80	-0.17625	0.038	0.315	3.79E-76
COMMD8	3.13E-80	0.13754	0.07	0.27	4.68E-76
10-Sep	3.22E-80	-0.4337	0.019	0.365	4.82E-76
HCFC1R1	3.35E-80	0.151127	0.101	0.337	5.00E-76
TNKS	3.42E-80	-0.27635	0.03	0.336	5.11E-76
DNAJB14	3.60E-80	0.100349	0.068	0.284	5.38E-76
NACC1	3.68E-80	-0.16715	0.033	0.305	5.49E-76
GRWD1	3.86E-80	-0.11406	0.038	0.317	5.77E-76
NANS	4.03E-80	-0.17291	0.058	0.364	6.03E-76
NR2F1	4.12E-80	-0.23523	0.049	0.357	6.16E-76
UBQLN2	4.13E-80	-0.21045	0.054	0.363	6.18E-76
TMEM242	4.64E-80	-0.10008	0.066	0.353	6.93E-76
RAB4B	4.86E-80	0.123933	0.073	0.294	7.26E-76
CHL1	4.87E-80	-0.16742	0.045	0.323	7.28E-76
ZNF138	5.24E-80	-0.31639	0.054	0.393	7.83E-76
FUBP1	5.42E-80	-0.29256	0.077	0.43	8.10E-76
VPS41	5.56E-80	-0.10265	0.052	0.321	8.32E-76
SLC4A7	7.00E-80	-0.21996	0.044	0.344	1.05E-75
ASH2L	7.11E-80	-0.30645	0.033	0.363	1.06E-75
THAP4	7.44E-80	-0.31873	0.026	0.343	1.11E-75
PAAF1	9.76E-80	0.22379	0.11	0.326	1.46E-75
GTF2B	9.93E-80	0.142113	0.086	0.302	1.48E-75
METTL2B	1.06E-79	-0.21262	0.059	0.379	1.59E-75
POLD3	1.12E-79	-0.45405	0.017	0.354	1.68E-75
HSPA1A	1.17E-79	-0.1191	0.209	0.584	1.74E-75
RPAP2	1.21E-79	-0.13363	0.056	0.335	1.81E-75
PES1	1.27E-79	-0.20556	0.038	0.34	1.89E-75
INO80C	1.55E-79	-0.1871	0.033	0.32	2.31E-75
RBM7	1.56E-79	0.123418	0.087	0.311	2.33E-75
RP11-71N1	1.59E-79	0.298902	0.112	0.3	2.37E-75
TAP1	1.78E-79	0.155144	0.129	0.388	2.66E-75
HSD17B11	1.99E-79	0.10035	0.124	0.378	2.98E-75
MTMR14	2.14E-79	-0.17316	0.037	0.317	3.19E-75
MSMO1	2.16E-79	0.110905	0.077	0.311	3.22E-75
GTPBP4	2.18E-79	-0.11827	0.066	0.353	3.25E-75
SIRT6	2.37E-79	-0.12192	0.052	0.338	3.55E-75
RNF40	2.40E-79	-0.11465	0.042	0.31	3.58E-75
RB1	3.09E-79	-0.27933	0.037	0.349	4.61E-75
MED16	3.24E-79	-0.26482	0.03	0.333	4.84E-75
CDK11A	3.44E-79	-0.23664	0.047	0.348	5.14E-75
ST3GAL4	3.69E-79	-0.25342	0.033	0.335	5.51E-75

SLC25A17	3.89E-79	-0.1096	0.047	0.328	5.81E-75
ATG5	3.97E-79	-0.15425	0.045	0.325	5.93E-75
MCM2	4.28E-79	-0.61684	0.01	0.37	6.40E-75
GPT2	4.37E-79	-0.29609	0.037	0.347	6.52E-75
CNOT8	4.40E-79	-0.2191	0.037	0.341	6.58E-75
BMI1	4.70E-79	-0.17818	0.031	0.307	7.02E-75
GYG1	4.75E-79	0.114073	0.08	0.305	7.10E-75
C20orf96	5.18E-79	-0.15189	0.04	0.325	7.74E-75
ZNF451	5.31E-79	-0.1044	0.047	0.307	7.94E-75
DOHH	5.64E-79	-0.45478	0.021	0.377	8.43E-75
FZR1	5.93E-79	-0.43241	0.03	0.386	8.86E-75
RNF19A	6.24E-79	-0.28334	0.04	0.347	9.33E-75
POU3F3	6.33E-79	-0.23175	0.052	0.365	9.46E-75
IFI16	6.94E-79	0.269241	0.178	0.396	1.04E-74
HINT3	7.52E-79	-0.20514	0.047	0.351	1.12E-74
RNF8	8.03E-79	-0.20548	0.033	0.322	1.20E-74
CDC16	8.78E-79	-0.29706	0.037	0.364	1.31E-74
MCM5	8.88E-79	-0.56161	0.026	0.388	1.33E-74
IFNAR2	9.31E-79	-0.3965	0.019	0.36	1.39E-74
CEP89	1.05E-78	-0.18856	0.045	0.344	1.57E-74
LINC00662	1.09E-78	0.266244	0.108	0.3	1.63E-74
BST2	1.15E-78	1.221358	0.403	0.232	1.72E-74
GRIA2	1.19E-78	0.116869	0.152	0.407	1.77E-74
COLGALT2	1.24E-78	-0.1358	0.054	0.344	1.85E-74
TMF1	1.40E-78	-0.14608	0.045	0.323	2.09E-74
ARHGAP35	1.46E-78	-0.34383	0.024	0.34	2.18E-74
PRKCA	1.50E-78	-0.39938	0.033	0.377	2.24E-74
DUSP1	1.53E-78	0.347682	0.271	0.48	2.28E-74
SEPHS1	1.57E-78	-0.19623	0.026	0.301	2.34E-74
NLGN1	1.63E-78	-0.25289	0.059	0.377	2.44E-74
EXTL3	1.64E-78	-0.13785	0.04	0.315	2.45E-74
UBE2D4	1.68E-78	-0.12294	0.054	0.342	2.51E-74
PIK3R3	1.77E-78	-0.16673	0.045	0.331	2.64E-74
YRDC	1.95E-78	-0.16753	0.04	0.32	2.92E-74
TRIM69	2.09E-78	-0.19638	0.058	0.365	3.12E-74
ICMT	2.16E-78	-0.30006	0.023	0.325	3.22E-74
CLINT1	2.33E-78	-0.20413	0.052	0.354	3.48E-74
PHF1	2.35E-78	-0.26927	0.035	0.348	3.51E-74
PI4KB	2.40E-78	0.172171	0.086	0.305	3.59E-74
BNIP3	2.51E-78	0.213353	0.108	0.335	3.75E-74
DDX6	2.53E-78	0.121766	0.073	0.283	3.79E-74
MRGBP	2.93E-78	-0.42048	0.014	0.346	4.37E-74
TRMU	3.02E-78	-0.24509	0.044	0.353	4.51E-74
SEMA6D	3.32E-78	-0.44184	0.021	0.352	4.97E-74
C12orf29	4.34E-78	-0.13419	0.051	0.328	6.49E-74
NME7	5.10E-78	0.127528	0.079	0.293	7.63E-74
EPS15L1	5.28E-78	-0.14756	0.038	0.312	7.89E-74
CHST2	5.51E-78	-0.29365	0.012	0.284	8.23E-74
SNX7	5.78E-78	-0.12098	0.051	0.335	8.64E-74
GAS2L1	6.01E-78	-0.23944	0.051	0.359	8.98E-74
NR2F6	6.25E-78	-0.2684	0.021	0.311	9.33E-74

ASAP2	6.32E-78	-0.27223	0.024	0.32	9.45E-74
LTV1	6.92E-78	-0.10055	0.045	0.307	1.03E-73
DYRK4	7.06E-78	0.235724	0.094	0.296	1.06E-73
ACBD3	7.14E-78	-0.10542	0.033	0.288	1.07E-73
CCP110	7.75E-78	-0.25516	0.044	0.359	1.16E-73
TXLNA	8.19E-78	-0.22716	0.033	0.325	1.22E-73
PPP2R2B	8.38E-78	0.433944	0.157	0.326	1.25E-73
COL6A2	8.52E-78	-0.16126	0.059	0.332	1.27E-73
SCD	9.62E-78	0.129723	0.072	0.284	1.44E-73
TBCD	1.01E-77	-0.30083	0.024	0.336	1.51E-73
PDGFC	1.03E-77	-0.29341	0.031	0.336	1.53E-73
MDM2	1.03E-77	-0.34865	0.091	0.459	1.54E-73
GNPDA1	1.04E-77	0.136879	0.072	0.278	1.56E-73
TBCC	1.06E-77	-0.24547	0.054	0.368	1.59E-73
ING3	1.07E-77	-0.27212	0.065	0.394	1.61E-73
EMC2	1.08E-77	0.182184	0.091	0.311	1.61E-73
INTS12	1.08E-77	0.109853	0.077	0.301	1.62E-73
CASC3	1.12E-77	-0.28821	0.045	0.372	1.67E-73
GTF2E2	1.12E-77	-0.38022	0.023	0.353	1.67E-73
GEMIN2	1.21E-77	-0.11873	0.04	0.304	1.81E-73
TCEB3	1.22E-77	-0.1889	0.033	0.305	1.82E-73
CCND1	1.23E-77	-0.10829	0.087	0.379	1.84E-73
SPRED1	1.42E-77	-0.31073	0.033	0.344	2.11E-73
MTHFS	1.43E-77	0.16915	0.065	0.243	2.14E-73
RGS17	1.45E-77	-0.19566	0.038	0.317	2.17E-73
BROX	1.46E-77	-0.34941	0.028	0.348	2.18E-73
C15orf40	1.47E-77	0.162387	0.066	0.274	2.20E-73
RPIA	1.56E-77	-0.16794	0.038	0.317	2.34E-73
TSPAN6	1.59E-77	0.293785	0.108	0.295	2.37E-73
PKNOX1	1.67E-77	-0.13224	0.037	0.296	2.49E-73
SFSWAP	1.73E-77	-0.21311	0.033	0.325	2.59E-73
JADE1	1.74E-77	-0.5072	0.037	0.409	2.61E-73
TANC2	1.95E-77	-0.1317	0.049	0.326	2.91E-73
ANKRD13D	2.00E-77	-0.10107	0.042	0.295	2.98E-73
CEP41	2.00E-77	-0.22867	0.054	0.369	2.99E-73
TSFM	2.12E-77	-0.21235	0.145	0.509	3.17E-73
CNIH2	2.30E-77	-0.47634	0.021	0.365	3.44E-73
UBR7	2.35E-77	-0.23538	0.037	0.34	3.52E-73
PAX6	2.40E-77	-0.46331	0.023	0.368	3.59E-73
TUG1	2.47E-77	-0.28931	0.045	0.373	3.69E-73
TTC9C	2.69E-77	-0.29912	0.033	0.343	4.02E-73
MMP2	2.71E-77	-0.12509	0.063	0.347	4.05E-73
TNRC6C	2.76E-77	-0.20495	0.051	0.347	4.12E-73
MEIS2	2.87E-77	-0.19691	0.082	0.404	4.28E-73
USP9X	2.87E-77	-0.11761	0.049	0.316	4.29E-73
ZNF639	3.15E-77	-0.24396	0.026	0.312	4.70E-73
PVRL2	3.15E-77	-0.13485	0.035	0.294	4.70E-73
MTMR9	3.29E-77	-0.16337	0.061	0.356	4.91E-73
RBMXL1	3.32E-77	-0.34732	0.035	0.363	4.96E-73
EIF2AK4	3.53E-77	-0.19166	0.033	0.31	5.28E-73
DIMT1	3.63E-77	-0.23162	0.038	0.335	5.42E-73

EXOC1	4.03E-77	-0.20655	0.091	0.422	6.03E-73
ANXA6	4.63E-77	-0.13013	0.077	0.374	6.92E-73
MTRNR2L12	5.35E-77	1.034533	0.237	0.18	8.00E-73
LIG1	5.52E-77	-0.51591	0.014	0.353	8.24E-73
SPATA6	6.04E-77	-0.16516	0.042	0.32	9.02E-73
DIAPH1	6.09E-77	-0.19719	0.035	0.31	9.10E-73
C16orf59	6.31E-77	-0.607	0.002	0.354	9.43E-73
CSTF1	7.11E-77	-0.2774	0.026	0.331	1.06E-72
TM4SF1	7.11E-77	-0.17046	0.051	0.332	1.06E-72
NF2	7.38E-77	-0.18814	0.031	0.309	1.10E-72
BRE	7.56E-77	0.169332	0.086	0.296	1.13E-72
NSMCE4A	7.73E-77	-0.28965	0.03	0.337	1.15E-72
GNG7	9.72E-77	0.367082	0.133	0.311	1.45E-72
MGAT1	1.03E-76	-0.26944	0.033	0.344	1.53E-72
CEP57L1	1.14E-76	-0.48699	0.016	0.364	1.71E-72
CCSAP	1.26E-76	-0.23864	0.031	0.323	1.88E-72
BBOX1	1.27E-76	0.452093	0.112	0.248	1.89E-72
NUP35	1.32E-76	-0.20765	0.056	0.359	1.98E-72
XXYLT1	1.43E-76	-0.1675	0.026	0.298	2.14E-72
MED17	1.43E-76	-0.12626	0.054	0.325	2.14E-72
TMEM181	1.47E-76	-0.24317	0.033	0.335	2.20E-72
KIF20A	1.60E-76	-0.65421	0	0.342	2.39E-72
ST5	1.64E-76	0.258024	0.08	0.26	2.45E-72
RP11-390F	1.68E-76	-0.10565	0.031	0.283	2.51E-72
PREPL	1.68E-76	-0.11342	0.045	0.305	2.51E-72
MBIP	1.75E-76	0.105101	0.094	0.323	2.61E-72
DIDO1	1.77E-76	-0.31004	0.03	0.338	2.64E-72
CCAR2	1.78E-76	-0.18438	0.044	0.33	2.66E-72
MED24	1.80E-76	-0.14641	0.049	0.331	2.69E-72
MTRF1L	2.00E-76	-0.34653	0.028	0.344	2.99E-72
CLPTM1L	2.00E-76	-0.19461	0.033	0.311	3.00E-72
DHX33	2.04E-76	-0.11405	0.042	0.291	3.05E-72
CAT	2.08E-76	0.253304	0.099	0.278	3.10E-72
NRM	2.12E-76	-0.33915	0.017	0.325	3.18E-72
CEBPD	2.14E-76	0.459868	0.229	0.396	3.20E-72
SESTD1	2.14E-76	-0.35323	0.038	0.365	3.20E-72
CTXN1	2.28E-76	-0.40496	0.021	0.349	3.41E-72
SKIL	2.32E-76	-0.18304	0.044	0.337	3.46E-72
DTNBP1	2.44E-76	-0.13451	0.047	0.32	3.65E-72
ZC3H18	2.65E-76	-0.36499	0.03	0.359	3.96E-72
TCEAL1	3.06E-76	0.291616	0.124	0.305	4.57E-72
NXF1	3.25E-76	-0.11818	0.054	0.338	4.85E-72
CTPS1	3.40E-76	-0.38773	0.021	0.341	5.07E-72
ACOT13	3.56E-76	0.185964	0.073	0.288	5.32E-72
SCO1	3.69E-76	-0.23689	0.038	0.337	5.51E-72
XAB2	3.86E-76	-0.22614	0.044	0.346	5.76E-72
TLK2	4.45E-76	-0.16719	0.024	0.277	6.65E-72
SLC25A25	4.47E-76	-0.2488	0.056	0.363	6.68E-72
CTNND2	4.56E-76	0.102932	0.089	0.319	6.81E-72
SRRD	4.86E-76	-0.13667	0.026	0.274	7.27E-72
LIMD2	4.92E-76	-0.3061	0.026	0.331	7.35E-72

MSH2	5.00E-76	-0.45962	0.023	0.364	7.47E-72
CISD3	5.16E-76	-0.17172	0.038	0.315	7.72E-72
TRIO	5.38E-76	0.34146	0.133	0.325	8.04E-72
BRD8	5.71E-76	-0.16374	0.073	0.379	8.53E-72
CSNK1G3	5.92E-76	-0.20671	0.037	0.322	8.85E-72
UBE2Q1	5.99E-76	-0.25812	0.026	0.315	8.95E-72
GLIPR2	6.23E-76	0.274682	0.113	0.331	9.31E-72
DPH3	6.44E-76	-0.21034	0.044	0.332	9.63E-72
CASP6	6.79E-76	-0.20837	0.035	0.311	1.01E-71
BRCA2	6.88E-76	-0.59735	0	0.34	1.03E-71
CENPJ	7.11E-76	-0.32473	0.021	0.325	1.06E-71
SCAF1	8.80E-76	-0.17864	0.031	0.306	1.32E-71
GSK3B	8.84E-76	-0.12952	0.051	0.317	1.32E-71
ING5	9.12E-76	-0.18576	0.044	0.33	1.36E-71
FAM3A	9.25E-76	-0.15392	0.049	0.322	1.38E-71
METTL4	1.09E-75	-0.11928	0.019	0.248	1.62E-71
FNBP1L	1.12E-75	-0.34523	0.03	0.348	1.67E-71
LRP10	1.14E-75	0.173709	0.075	0.265	1.70E-71
TUBGCP2	1.16E-75	-0.2005	0.033	0.322	1.73E-71
GAS2L3	1.22E-75	-0.59815	0.002	0.347	1.82E-71
CSAD	1.36E-75	-0.18687	0.045	0.328	2.04E-71
TNIP2	1.40E-75	-0.17525	0.028	0.291	2.09E-71
ACTR1B	1.45E-75	-0.12066	0.059	0.343	2.17E-71
DCAF15	1.45E-75	-0.4004	0.012	0.331	2.17E-71
VPS37A	1.45E-75	-0.13722	0.047	0.322	2.17E-71
ZNF92	1.61E-75	-0.29319	0.068	0.399	2.41E-71
C1orf109	1.64E-75	-0.19551	0.037	0.325	2.46E-71
SCAMP1	1.67E-75	-0.19824	0.065	0.372	2.49E-71
C8orf82	1.86E-75	-0.23524	0.037	0.327	2.79E-71
FADS2	2.13E-75	-0.32861	0.04	0.359	3.18E-71
CDK5RAP1	2.15E-75	-0.24799	0.03	0.315	3.22E-71
FKBP9	2.29E-75	-0.13622	0.063	0.341	3.42E-71
DNAJC10	2.52E-75	-0.12692	0.052	0.32	3.76E-71
PPP1R18	2.67E-75	-0.21965	0.031	0.316	3.99E-71
KIF14	2.89E-75	-0.56536	0.007	0.354	4.32E-71
R3HDM4	2.94E-75	-0.29443	0.038	0.356	4.40E-71
NFE2L1	3.19E-75	-0.19836	0.038	0.323	4.77E-71
FNBP1	3.49E-75	-0.1477	0.04	0.305	5.21E-71
ARHGAP33	3.69E-75	-0.47377	0.014	0.351	5.51E-71
ZMYND11	3.74E-75	-0.26775	0.023	0.314	5.58E-71
MOB2	3.97E-75	-0.14062	0.038	0.302	5.93E-71
CRBN	4.61E-75	0.11277	0.119	0.363	6.89E-71
C9orf114	4.67E-75	-0.12721	0.04	0.302	6.98E-71
CHTF8	4.91E-75	-0.22515	0.035	0.326	7.34E-71
TRIM13	5.24E-75	-0.19129	0.084	0.388	7.83E-71
CCL2	5.35E-75	0.601463	0.091	0.247	8.00E-71
STRN4	5.89E-75	-0.3469	0.017	0.326	8.79E-71
RAB3GAP2	5.94E-75	-0.13981	0.045	0.322	8.87E-71
SNX21	6.45E-75	-0.1513	0.031	0.291	9.63E-71
HELLS	6.47E-75	-0.60279	0.023	0.381	9.66E-71
TPP1	6.95E-75	0.29195	0.092	0.251	1.04E-70

BTBD3	7.44E-75	-0.16923	0.028	0.285	1.11E-70
PHLDB1	7.62E-75	0.174135	0.063	0.241	1.14E-70
PCYT2	9.03E-75	-0.25842	0.023	0.307	1.35E-70
HEPACAM	9.43E-75	0.511337	0.175	0.295	1.41E-70
RPE	9.91E-75	-0.11637	0.051	0.31	1.48E-70
RNF2	1.02E-74	-0.1843	0.035	0.307	1.53E-70
CHST10	1.04E-74	-0.13192	0.052	0.315	1.55E-70
KBTBD2	1.05E-74	-0.29559	0.035	0.343	1.57E-70
TSC22D3	1.14E-74	0.118118	0.127	0.374	1.71E-70
PARP2	1.16E-74	-0.35053	0.054	0.391	1.74E-70
UHMK1	1.16E-74	-0.11191	0.042	0.296	1.74E-70
KCTD17	1.22E-74	-0.26133	0.021	0.306	1.83E-70
NOL8	1.25E-74	-0.18735	0.051	0.344	1.87E-70
ZNF771	1.32E-74	-0.37129	0.03	0.353	1.97E-70
DDX54	1.33E-74	-0.24753	0.042	0.342	1.99E-70
DPH7	1.43E-74	-0.32428	0.028	0.341	2.14E-70
GTF2H1	1.56E-74	-0.11215	0.047	0.311	2.32E-70
NNT	1.57E-74	-0.23609	0.042	0.341	2.35E-70
METTL21A	1.59E-74	-0.2967	0.051	0.375	2.37E-70
ZBTB7A	1.62E-74	-0.29462	0.028	0.328	2.42E-70
PELP1	1.82E-74	-0.28255	0.028	0.328	2.72E-70
PINK1	2.04E-74	0.115892	0.058	0.265	3.05E-70
ZNRF1	2.13E-74	-0.27531	0.014	0.285	3.19E-70
CCDC174	2.14E-74	0.1669	0.079	0.294	3.19E-70
DNAJC5	2.36E-74	-0.27814	0.035	0.332	3.52E-70
RANBP9	2.36E-74	-0.12753	0.035	0.283	3.52E-70
CTDSPL2	2.81E-74	-0.43372	0.012	0.347	4.20E-70
VPS45	3.16E-74	-0.1368	0.063	0.348	4.73E-70
MAPT	3.30E-74	0.156373	0.15	0.384	4.93E-70
CCDC18	3.45E-74	-0.5553	0.017	0.377	5.15E-70
HMGCL	3.83E-74	0.241174	0.101	0.284	5.73E-70
WDR5	4.54E-74	-0.25971	0.028	0.322	6.78E-70
NCK2	4.55E-74	-0.23337	0.017	0.279	6.80E-70
RANBP2	4.57E-74	-0.15187	0.03	0.29	6.83E-70
ZBED1	4.67E-74	-0.19402	0.035	0.304	6.98E-70
TSPAN4	4.92E-74	-0.23463	0.024	0.299	7.35E-70
DNAJB9	5.46E-74	0.449687	0.19	0.323	8.16E-70
UCHL5	5.51E-74	-0.40228	0.017	0.338	8.23E-70
PNPLA2	5.74E-74	-0.22343	0.033	0.307	8.57E-70
PUM2	5.78E-74	-0.16835	0.04	0.31	8.64E-70
TNIP1	5.79E-74	-0.15894	0.035	0.298	8.66E-70
PITPNB	6.81E-74	-0.21433	0.042	0.332	1.02E-69
GLB1	6.89E-74	0.233438	0.092	0.272	1.03E-69
COL9A3	7.05E-74	-0.12655	0.119	0.428	1.05E-69
DDX19B	7.63E-74	-0.25933	0.037	0.328	1.14E-69
MCM8	8.37E-74	-0.34794	0.012	0.312	1.25E-69
PITPNC1	9.41E-74	-0.25662	0.023	0.296	1.41E-69
ASCC2	9.76E-74	-0.20724	0.042	0.33	1.46E-69
GPCPD1	9.85E-74	-0.29765	0.023	0.315	1.47E-69
SSH2	1.17E-73	-0.3311	0.035	0.343	1.74E-69
SLC35F6	1.21E-73	-0.14997	0.04	0.298	1.80E-69

MBNL1	1.21E-73	-0.2043	0.037	0.31	1.81E-69
SLC43A2	1.23E-73	-0.20566	0.045	0.32	1.84E-69
RMND5B	1.44E-73	-0.22356	0.038	0.33	2.15E-69
LYPLAL1	1.54E-73	0.269153	0.105	0.288	2.31E-69
SH3YL1	1.97E-73	0.116147	0.068	0.29	2.95E-69
DIAPH3	1.99E-73	-0.57019	0.002	0.341	2.97E-69
RP11-849I	2.02E-73	-0.40476	0.063	0.407	3.01E-69
POLR1E	2.02E-73	-0.12316	0.031	0.284	3.02E-69
SND1	2.19E-73	-0.12834	0.058	0.337	3.27E-69
CPSF7	2.19E-73	-0.15709	0.059	0.343	3.28E-69
AKTIP	2.30E-73	0.20811	0.079	0.263	3.44E-69
HABP4	2.65E-73	-0.1802	0.03	0.29	3.96E-69
TPRA1	2.96E-73	-0.13879	0.033	0.288	4.43E-69
HSD17B4	3.13E-73	0.156914	0.086	0.288	4.67E-69
JAK1	3.30E-73	-0.20203	0.035	0.315	4.94E-69
NAV1	3.38E-73	-0.28113	0.044	0.356	5.05E-69
ENOSF1	3.83E-73	-0.24426	0.009	0.264	5.73E-69
CLIP2	4.97E-73	-0.10103	0.075	0.346	7.43E-69
YIPF1	5.13E-73	0.178204	0.054	0.232	7.66E-69
TAF9B	5.46E-73	-0.25445	0.04	0.344	8.17E-69
STX18	6.23E-73	-0.18949	0.04	0.319	9.31E-69
AP5M1	6.67E-73	-0.21877	0.044	0.33	9.96E-69
CENPO	6.68E-73	-0.52982	0.007	0.348	9.98E-69
DPYSL5	6.77E-73	-0.2768	0.035	0.33	1.01E-68
ING1	7.08E-73	-0.28086	0.037	0.338	1.06E-68
ALCAM	7.56E-73	-0.10115	0.061	0.32	1.13E-68
FAM195A	9.00E-73	-0.11447	0.045	0.306	1.35E-68
ELP2	9.59E-73	0.167673	0.087	0.281	1.43E-68
ERICH1	1.02E-72	0.117805	0.073	0.289	1.52E-68
C11orf24	1.13E-72	-0.24877	0.019	0.285	1.68E-68
RP11-395G	1.13E-72	-0.37926	0.03	0.337	1.69E-68
MGRN1	1.16E-72	-0.17926	0.054	0.325	1.73E-68
ARHGAP11E	1.24E-72	-0.56558	0.01	0.356	1.86E-68
MAPK6	1.27E-72	-0.27212	0.04	0.347	1.90E-68
CCNF	1.30E-72	-0.54746	0.005	0.337	1.95E-68
TMEM68	1.32E-72	-0.17767	0.054	0.341	1.98E-68
SETD2	1.36E-72	-0.24028	0.026	0.302	2.03E-68
UCKL1	1.72E-72	-0.21617	0.037	0.316	2.57E-68
EXOC4	1.73E-72	-0.14495	0.056	0.331	2.59E-68
GGT7	1.74E-72	-0.14698	0.054	0.33	2.60E-68
HLA-DRB1	1.75E-72	1.078579	0.265	0.172	2.62E-68
MECP2	1.79E-72	0.116952	0.07	0.283	2.68E-68
KBTBD6	1.92E-72	-0.10954	0.044	0.291	2.87E-68
HELZ	2.02E-72	-0.11714	0.061	0.331	3.02E-68
DCBLD2	2.25E-72	-0.44272	0.019	0.338	3.36E-68
PAXBP1	2.36E-72	-0.23412	0.044	0.331	3.52E-68
NUDT15	2.44E-72	-0.17632	0.035	0.307	3.65E-68
FZD7	2.55E-72	-0.16094	0.017	0.237	3.81E-68
GMPR2	2.78E-72	0.17466	0.086	0.289	4.15E-68
RHBDD3	2.94E-72	-0.16011	0.042	0.307	4.39E-68
ELAC2	2.94E-72	-0.20737	0.026	0.296	4.40E-68

SLC35B4	3.04E-72	-0.17702	0.056	0.338	4.55E-68
EIF2B3	3.18E-72	-0.12173	0.059	0.335	4.76E-68
RFWD2	3.30E-72	-0.18856	0.04	0.311	4.94E-68
FKBP5	3.54E-72	0.195333	0.131	0.347	5.29E-68
AP3B1	3.65E-72	-0.17384	0.04	0.314	5.45E-68
TOMM70A	3.87E-72	-0.20862	0.023	0.275	5.78E-68
PICALM	3.91E-72	-0.16848	0.033	0.3	5.84E-68
MLLT4	3.91E-72	-0.18609	0.061	0.348	5.84E-68
C4orf46	3.94E-72	-0.30673	0.017	0.305	5.90E-68
TMEM41B	4.05E-72	-0.14126	0.045	0.306	6.05E-68
BUB1B	4.07E-72	-0.53419	0	0.325	6.08E-68
DUSP4	4.16E-72	-0.10481	0.044	0.295	6.22E-68
MED27	4.42E-72	-0.22038	0.038	0.328	6.61E-68
PHKB	4.50E-72	-0.19423	0.049	0.333	6.72E-68
HIVEP3	4.68E-72	-0.26337	0.021	0.294	6.99E-68
OXA1L	6.84E-72	0.21609	0.113	0.326	1.02E-67
RND2	7.27E-72	0.329421	0.096	0.24	1.09E-67
C6orf47	7.37E-72	-0.19343	0.019	0.273	1.10E-67
HCFC1	7.51E-72	-0.3123	0.028	0.326	1.12E-67
TSPYL1	7.72E-72	-0.1303	0.063	0.332	1.15E-67
TOPBP1	7.75E-72	-0.38981	0.012	0.314	1.16E-67
MTG2	8.20E-72	-0.25492	0.024	0.304	1.22E-67
OGG1	8.92E-72	-0.14255	0.047	0.317	1.33E-67
KRI1	8.93E-72	-0.23532	0.023	0.296	1.33E-67
WARS	9.12E-72	0.336982	0.098	0.269	1.36E-67
SAP30L	9.65E-72	-0.3144	0.016	0.294	1.44E-67
TIMM21	9.88E-72	-0.19185	0.037	0.315	1.48E-67
ZC3H7A	1.18E-71	-0.2891	0.03	0.321	1.76E-67
LPL	1.41E-71	0.357712	0.105	0.251	2.10E-67
BEND5	1.48E-71	-0.11982	0.047	0.309	2.21E-67
FAF2	2.18E-71	-0.29259	0.045	0.352	3.26E-67
ANXA2	2.28E-71	0.225215	0.265	0.477	3.41E-67
ACAP2	2.28E-71	-0.11866	0.037	0.283	3.41E-67
EIF2B4	2.60E-71	-0.20968	0.049	0.344	3.89E-67
ZHX1	2.60E-71	-0.13473	0.051	0.319	3.89E-67
RCHY1	2.69E-71	0.108761	0.084	0.298	4.03E-67
MAPK7	2.71E-71	-0.22665	0.023	0.295	4.05E-67
VKORC1L1	2.83E-71	-0.217	0.037	0.309	4.23E-67
CDC42EP1	2.96E-71	-0.21531	0.03	0.299	4.42E-67
GSTM4	2.96E-71	0.181649	0.068	0.24	4.43E-67
FAM57A	3.19E-71	-0.16345	0.017	0.258	4.77E-67
CRTAP	3.23E-71	-0.12405	0.045	0.31	4.83E-67
G2E3	3.56E-71	-0.45009	0.024	0.346	5.32E-67
NDRG3	3.83E-71	-0.13668	0.044	0.302	5.72E-67
SLC4A1AP	4.09E-71	-0.19657	0.033	0.302	6.12E-67
IRAK1	4.67E-71	-0.13818	0.028	0.279	6.98E-67
CITED2	5.32E-71	-0.12011	0.089	0.365	7.96E-67
CYBRD1	5.47E-71	0.160799	0.066	0.251	8.18E-67
MED1	5.86E-71	-0.22375	0.021	0.286	8.75E-67
CSNK1G2	5.91E-71	-0.28208	0.019	0.295	8.83E-67
GMCL1	6.85E-71	-0.38703	0.038	0.365	1.02E-66

TRAPPC2P1	7.18E-71	0.182835	0.094	0.296	1.07E-66
GSKIP	7.19E-71	-0.1328	0.038	0.29	1.07E-66
N6AMT2	7.88E-71	0.227445	0.063	0.232	1.18E-66
SOCS2	8.78E-71	-0.36241	0.047	0.36	1.31E-66
NRP2	9.06E-71	-0.27184	0.03	0.311	1.35E-66
SMAD4	9.07E-71	-0.23463	0.021	0.283	1.35E-66
LEMD2	9.13E-71	-0.25875	0.017	0.291	1.36E-66
SSX2IP	9.73E-71	-0.28094	0.014	0.288	1.45E-66
ZNF397	1.08E-70	0.100984	0.079	0.272	1.62E-66
GPBP1L1	1.15E-70	-0.17334	0.042	0.309	1.72E-66
IFI35	1.17E-70	0.444517	0.089	0.204	1.75E-66
EXOSC10	1.23E-70	-0.16939	0.054	0.332	1.84E-66
H2AFJ	1.28E-70	-0.35034	0.017	0.311	1.91E-66
GNA11	1.37E-70	-0.25944	0.019	0.29	2.05E-66
NOL10	1.43E-70	-0.2137	0.042	0.331	2.14E-66
PDXDC1	1.47E-70	-0.16878	0.033	0.299	2.19E-66
PKD1	1.66E-70	0.130126	0.065	0.242	2.48E-66
MFSD12	1.71E-70	-0.27195	0.021	0.296	2.56E-66
ID1	1.83E-70	-0.13352	0.04	0.298	2.74E-66
RFX4	1.96E-70	0.314048	0.148	0.317	2.93E-66
PRPS2	2.05E-70	-0.17349	0.023	0.264	3.06E-66
CTBP2	2.08E-70	-0.1606	0.033	0.29	3.12E-66
ANKRD40	2.40E-70	-0.22275	0.03	0.299	3.59E-66
FTSJ1	2.90E-70	-0.12616	0.033	0.28	4.33E-66
PPM1A	2.99E-70	-0.35734	0.017	0.316	4.46E-66
BRAT1	3.41E-70	-0.15401	0.077	0.37	5.10E-66
ATP2C1	3.42E-70	-0.19177	0.061	0.353	5.12E-66
MAP1S	3.71E-70	-0.31037	0.019	0.31	5.55E-66
DCX	3.74E-70	-0.33454	0.068	0.388	5.58E-66
YES1	3.76E-70	-0.25168	0.033	0.316	5.62E-66
ANKRD35	4.08E-70	-0.18069	0.028	0.285	6.10E-66
XRCC1	4.08E-70	-0.21785	0.044	0.328	6.10E-66
FAM53C	4.21E-70	0.146922	0.087	0.288	6.29E-66
PRKACA	4.28E-70	-0.27112	0.024	0.307	6.39E-66
TTC37	4.59E-70	-0.13463	0.049	0.305	6.86E-66
SCAF4	5.63E-70	-0.16078	0.045	0.309	8.42E-66
WDR76	6.20E-70	-0.56722	0.016	0.349	9.26E-66
MAD1L1	6.59E-70	-0.18538	0.045	0.314	9.85E-66
PXDC1	7.52E-70	-0.12345	0.035	0.279	1.12E-65
MVD	7.94E-70	-0.18527	0.035	0.305	1.19E-65
C5orf45	8.34E-70	0.24875	0.079	0.241	1.25E-65
NUBP1	8.53E-70	-0.2232	0.04	0.323	1.27E-65
PCID2	8.67E-70	-0.20105	0.047	0.33	1.30E-65
ZNF800	9.62E-70	-0.23195	0.038	0.323	1.44E-65
NEDD1	9.62E-70	-0.36351	0.014	0.309	1.44E-65
MIA3	1.03E-69	-0.14465	0.056	0.323	1.55E-65
FANCG	1.20E-69	-0.41112	0.009	0.311	1.79E-65
TRMT13	1.33E-69	0.116714	0.063	0.249	1.98E-65
DMWD	1.33E-69	-0.22644	0.044	0.325	1.99E-65
CNTFR	1.33E-69	-0.28892	0.054	0.359	1.99E-65
NAB1	1.51E-69	-0.28426	0.03	0.314	2.26E-65

PRKD3	2.10E-69	-0.21694	0.047	0.322	3.14E-65
ZNF85	2.12E-69	-0.30527	0.019	0.302	3.17E-65
APBB1	2.14E-69	-0.14369	0.059	0.322	3.19E-65
DOCK7	2.20E-69	-0.10167	0.03	0.251	3.29E-65
VHL	2.21E-69	-0.21961	0.04	0.316	3.31E-65
RFT1	2.40E-69	-0.15766	0.031	0.285	3.58E-65
GOLGB1	2.59E-69	-0.21283	0.042	0.319	3.88E-65
NRAS	2.61E-69	-0.20464	0.044	0.321	3.91E-65
UBE2W	2.76E-69	-0.16209	0.042	0.305	4.12E-65
ELOVL2	2.76E-69	-0.18829	0.044	0.321	4.12E-65
CASP2	2.77E-69	-0.27854	0.028	0.311	4.14E-65
MESP1	2.88E-69	-0.38462	0.03	0.348	4.30E-65
WBSCR16	2.91E-69	-0.18184	0.026	0.279	4.35E-65
ATP13A1	2.97E-69	-0.19735	0.035	0.301	4.44E-65
RARRES3	3.05E-69	1.10225	0.178	0.127	4.56E-65
C1QA	3.32E-69	0.392853	0.155	0.294	4.96E-65
SHKBP1	3.38E-69	-0.18249	0.03	0.285	5.05E-65
SLC27A1	3.85E-69	0.149806	0.051	0.221	5.76E-65
SLC43A3	4.12E-69	-0.14859	0.044	0.299	6.16E-65
NCOA1	5.07E-69	-0.11889	0.049	0.293	7.58E-65
STRN3	5.11E-69	-0.17378	0.024	0.273	7.64E-65
RNPEP	5.74E-69	-0.19021	0.03	0.286	8.58E-65
CLN6	6.08E-69	-0.37602	0.021	0.323	9.08E-65
USP3	6.74E-69	-0.25461	0.028	0.309	1.01E-64
TMEM55B	6.89E-69	-0.16667	0.052	0.33	1.03E-64
MAD2L1BP	7.62E-69	-0.23078	0.044	0.325	1.14E-64
TCHP	7.69E-69	-0.13428	0.042	0.295	1.15E-64
OSBPL1A	7.72E-69	-0.16734	0.042	0.306	1.15E-64
DYNC2LI1	8.07E-69	0.184992	0.065	0.242	1.21E-64
POFUT1	8.35E-69	-0.21022	0.028	0.291	1.25E-64
AC004951.	8.91E-69	-0.22996	0.044	0.315	1.33E-64
ZNF626	9.62E-69	0.110192	0.059	0.244	1.44E-64
SAAL1	9.66E-69	-0.25649	0.038	0.326	1.44E-64
NBL1	9.86E-69	-0.32115	0.016	0.298	1.47E-64
CHRAC1	1.01E-68	-0.23308	0.038	0.319	1.51E-64
DONSON	1.06E-68	-0.42307	0.007	0.307	1.58E-64
TNFRSF12A	1.06E-68	-0.27351	0.113	0.425	1.59E-64
CAMSAP2	1.08E-68	-0.12549	0.049	0.296	1.61E-64
ANK2	1.17E-68	-0.24811	0.04	0.323	1.75E-64
MEPCE	1.27E-68	-0.12849	0.045	0.302	1.90E-64
MACROD1	1.38E-68	-0.29824	0.024	0.307	2.06E-64
WRAP53	1.56E-68	-0.20245	0.035	0.304	2.33E-64
IGFBP3	1.59E-68	-0.19736	0.073	0.344	2.38E-64
GJC1	1.70E-68	-0.32664	0.017	0.306	2.54E-64
MCM6	1.72E-68	-0.51153	0.017	0.331	2.58E-64
ICA1L	1.79E-68	-0.16868	0.047	0.302	2.67E-64
B4GALNT1	1.88E-68	-0.5821	0.068	0.441	2.80E-64
PHYHIPL	2.04E-68	0.322462	0.183	0.351	3.04E-64
SCAP	2.23E-68	-0.21226	0.03	0.289	3.33E-64
PLAT	2.37E-68	0.219322	0.086	0.268	3.54E-64
NOC4L	2.69E-68	-0.31634	0.028	0.317	4.01E-64

C21orf2	3.05E-68	-0.22677	0.042	0.325	4.55E-64
WRAP73	3.20E-68	-0.2231	0.038	0.311	4.78E-64
AGAP1	3.23E-68	-0.18163	0.03	0.279	4.83E-64
COL4A1	3.36E-68	-0.25349	0.024	0.286	5.02E-64
CES2	3.45E-68	-0.16141	0.035	0.289	5.15E-64
GPATCH11	3.45E-68	-0.1802	0.044	0.312	5.15E-64
ZNF576	3.52E-68	0.172306	0.07	0.257	5.26E-64
TMEM209	3.53E-68	-0.34525	0.021	0.32	5.28E-64
ZNF711	4.34E-68	-0.15451	0.056	0.322	6.49E-64
LRRC37B	5.16E-68	-0.20633	0.024	0.284	7.72E-64
TMEM131	5.41E-68	-0.28996	0.023	0.301	8.08E-64
TSPAN12	5.94E-68	-0.16384	0.091	0.381	8.88E-64
PPP2R3B	6.17E-68	-0.22951	0.01	0.258	9.22E-64
NCOA6	6.75E-68	-0.29304	0.024	0.307	1.01E-63
C11orf96	7.61E-68	-0.16861	0.052	0.306	1.14E-63
TEX261	7.88E-68	-0.27621	0.038	0.327	1.18E-63
SPIN1	7.90E-68	-0.31032	0.026	0.312	1.18E-63
COLGALT1	8.00E-68	-0.12368	0.028	0.264	1.20E-63
DNAJC4	8.01E-68	0.118066	0.052	0.227	1.20E-63
APOC1	8.34E-68	0.36495	0.159	0.302	1.25E-63
NOTCH2NL	8.36E-68	-0.25648	0.03	0.304	1.25E-63
SOX21	8.67E-68	-0.16468	0.035	0.283	1.30E-63
B3GALNT1	8.82E-68	-0.27367	0.044	0.341	1.32E-63
MIB2	9.06E-68	-0.14457	0.03	0.262	1.35E-63
FAIM2	9.30E-68	0.320927	0.106	0.26	1.39E-63
TULP3	9.61E-68	-0.2519	0.031	0.316	1.44E-63
TM7SF2	9.62E-68	0.110816	0.079	0.281	1.44E-63
PRIM1	1.00E-67	-0.49309	0.019	0.342	1.50E-63
MMS22L	1.01E-67	-0.3298	0.035	0.333	1.51E-63
KLHL8	1.08E-67	-0.13119	0.038	0.283	1.61E-63
MAP3K2	1.14E-67	-0.152	0.033	0.274	1.70E-63
TIPIN	1.21E-67	0.222772	0.077	0.262	1.81E-63
RIMKLB	1.48E-67	-0.20503	0.047	0.32	2.21E-63
IQGAP2	1.62E-67	-0.13392	0.033	0.267	2.42E-63
TTK	1.75E-67	-0.63588	0	0.306	2.61E-63
PTAR1	1.76E-67	-0.25279	0.03	0.306	2.63E-63
LINC0111€	1.86E-67	-0.10151	0.059	0.307	2.78E-63
C8orf76	1.88E-67	-0.14759	0.038	0.29	2.81E-63
FOXRED2	1.91E-67	-0.321	0.019	0.296	2.86E-63
ZNF273	2.07E-67	-0.27389	0.049	0.34	3.09E-63
PRMT5	2.30E-67	0.114135	0.08	0.286	3.44E-63
HEXIM1	2.39E-67	-0.10241	0.056	0.302	3.57E-63
BMPR2	2.49E-67	-0.13486	0.054	0.314	3.72E-63
PCDHB16	2.52E-67	-0.2398	0.044	0.311	3.76E-63
CDC23	2.68E-67	-0.21571	0.038	0.31	4.01E-63
SLC35E1	2.80E-67	-0.11997	0.04	0.279	4.18E-63
FAM122B	2.99E-67	-0.27539	0.026	0.305	4.46E-63
BBS2	3.10E-67	0.182143	0.106	0.298	4.63E-63
CEP164	3.15E-67	-0.12981	0.047	0.301	4.71E-63
RPP14	3.23E-67	-0.22613	0.038	0.314	4.83E-63
CWC25	3.62E-67	-0.25256	0.052	0.346	5.41E-63

ZMYM4	3.68E-67	-0.12445	0.026	0.252	5.50E-63
NADK	3.80E-67	-0.16708	0.028	0.281	5.68E-63
PTDSS2	3.80E-67	-0.2398	0.038	0.317	5.68E-63
ISYNA1	3.98E-67	-0.20543	0.03	0.285	5.94E-63
KAT6A	4.69E-67	-0.11604	0.045	0.293	7.02E-63
CDC6	5.19E-67	-0.4686	0.009	0.306	7.75E-63
PILRB	5.36E-67	-0.14165	0.066	0.333	8.01E-63
FAM161A	5.54E-67	-0.36276	0.028	0.327	8.27E-63
RP11-161M	5.83E-67	0.2124	0.122	0.319	8.71E-63
P4HA1	5.90E-67	0.172711	0.072	0.241	8.82E-63
CEP55	7.14E-67	-0.51883	0	0.304	1.07E-62
SMG1	8.93E-67	-0.14854	0.044	0.291	1.33E-62
PNMA2	9.13E-67	-0.15958	0.051	0.314	1.36E-62
ACBD5	1.01E-66	-0.1524	0.033	0.267	1.51E-62
LPCAT1	1.02E-66	-0.2162	0.066	0.352	1.52E-62
CCNG2	1.31E-66	-0.35821	0.049	0.356	1.96E-62
SLC9A3R1	1.37E-66	-0.15256	0.049	0.309	2.04E-62
MAP3K11	1.37E-66	-0.13792	0.038	0.288	2.05E-62
ARC	1.49E-66	0.102878	0.166	0.401	2.23E-62
EMP1	1.62E-66	0.411522	0.147	0.283	2.42E-62
ZNF581	1.70E-66	0.244049	0.056	0.206	2.54E-62
CETN3	1.83E-66	-0.24206	0.037	0.311	2.74E-62
BPHL	1.89E-66	-0.11753	0.054	0.3	2.82E-62
POLR2M	1.98E-66	-0.23805	0.033	0.304	2.95E-62
CCHCR1	1.98E-66	-0.28632	0.014	0.284	2.96E-62
DNALI1	2.00E-66	-0.10098	0.037	0.275	2.99E-62
ACOX1	2.09E-66	-0.15636	0.03	0.27	3.12E-62
EFCAB14	2.47E-66	-0.13049	0.035	0.269	3.70E-62
KPNA1	2.50E-66	-0.16337	0.03	0.278	3.73E-62
GCA	2.56E-66	0.252959	0.091	0.249	3.83E-62
ATM	2.61E-66	-0.13865	0.042	0.286	3.90E-62
LCORL	2.66E-66	-0.31119	0.017	0.296	3.98E-62
CDC25C	2.91E-66	-0.5596	0	0.301	4.34E-62
NDUFAF5	3.19E-66	-0.23154	0.03	0.298	4.77E-62
CEP70	3.22E-66	-0.34849	0.03	0.321	4.81E-62
RBM12	3.28E-66	-0.14189	0.037	0.278	4.91E-62
DUS3L	3.52E-66	-0.20234	0.037	0.299	5.26E-62
KLHL5	3.57E-66	-0.21826	0.019	0.274	5.33E-62
TTI1	3.59E-66	-0.15763	0.026	0.262	5.37E-62
CCZ1B	3.80E-66	-0.22198	0.037	0.301	5.67E-62
STK39	3.87E-66	-0.15972	0.024	0.258	5.79E-62
BOLA1	3.91E-66	-0.15127	0.038	0.289	5.85E-62
CD46	4.14E-66	0.118553	0.075	0.263	6.19E-62
RP11-660L	4.86E-66	0.109665	0.049	0.227	7.27E-62
TNFRSF19	4.88E-66	-0.2021	0.031	0.28	7.29E-62
NPTXR	4.88E-66	-0.18881	0.028	0.275	7.30E-62
FANCD2	5.20E-66	-0.50319	0.009	0.319	7.77E-62
CREBBP	5.56E-66	-0.11719	0.044	0.285	8.31E-62
TRAIP	5.57E-66	-0.35824	0.009	0.289	8.33E-62
NUDCD1	5.60E-66	-0.1518	0.023	0.252	8.36E-62
HIST2H2AC	5.81E-66	-0.51298	0.003	0.304	8.68E-62

ZFAND1	5.81E-66	0.143583	0.068	0.235	8.68E-62
ZFP91	5.99E-66	-0.15645	0.031	0.274	8.96E-62
ANKFY1	6.03E-66	-0.10802	0.031	0.26	9.01E-62
SOCS3	6.48E-66	0.19667	0.124	0.332	9.68E-62
PTRH2	7.46E-66	0.14089	0.045	0.204	1.11E-61
YPEL1	7.55E-66	-0.30955	0.016	0.293	1.13E-61
TTL	7.67E-66	-0.36029	0.019	0.312	1.15E-61
TMOD3	8.13E-66	-0.12437	0.023	0.246	1.22E-61
ARCN1	8.69E-66	-0.23343	0.023	0.281	1.30E-61
FUT8	8.97E-66	-0.20374	0.03	0.277	1.34E-61
MBD1	9.09E-66	-0.14831	0.04	0.288	1.36E-61
INPP1	9.20E-66	-0.11953	0.047	0.298	1.37E-61
ORAI1	9.24E-66	-0.17978	0.019	0.247	1.38E-61
ZCCHC3	1.00E-65	-0.15666	0.023	0.257	1.50E-61
MSH6	1.11E-65	-0.43198	0.028	0.34	1.66E-61
ZADH2	1.14E-65	-0.14152	0.023	0.246	1.70E-61
CUEDC1	1.17E-65	-0.30895	0.012	0.284	1.74E-61
MTMR4	1.23E-65	-0.20413	0.037	0.306	1.84E-61
TSPAN17	1.36E-65	-0.12388	0.033	0.262	2.03E-61
FSTL1	1.36E-65	-0.2134	0.026	0.277	2.04E-61
PTPN18	1.43E-65	-0.15695	0.033	0.277	2.13E-61
ZNF530	1.48E-65	-0.39694	0.007	0.289	2.22E-61
RP11-332H	1.53E-65	-0.32504	0.037	0.332	2.28E-61
HHLA3	1.78E-65	0.354685	0.087	0.219	2.66E-61
TRIOBP	1.87E-65	-0.40454	0.007	0.299	2.79E-61
AGA	2.02E-65	0.222732	0.066	0.214	3.01E-61
ALKBH3	2.34E-65	0.179728	0.054	0.212	3.49E-61
MSI1	2.37E-65	-0.13987	0.052	0.307	3.54E-61
UBE2Z	2.64E-65	-0.19249	0.037	0.293	3.94E-61
PPP4R1	2.68E-65	-0.21001	0.023	0.265	4.00E-61
APOD	2.68E-65	1.14713	0.255	0.132	4.01E-61
SMG6	2.72E-65	-0.13431	0.026	0.247	4.06E-61
KIF18A	2.73E-65	-0.42749	0.003	0.291	4.08E-61
TOP3A	2.84E-65	-0.21847	0.035	0.295	4.24E-61
RUSC1	2.95E-65	-0.18045	0.023	0.259	4.41E-61
NARFL	3.27E-65	-0.1447	0.042	0.294	4.88E-61
NIN	3.57E-65	-0.23715	0.035	0.299	5.34E-61
CTU2	3.62E-65	-0.1316	0.044	0.289	5.41E-61
CNOT10	3.72E-65	-0.11023	0.052	0.296	5.55E-61
RRAGD	3.81E-65	-0.29621	0.017	0.29	5.69E-61
SASS6	3.84E-65	-0.26768	0.014	0.274	5.73E-61
DCAF10	3.85E-65	-0.16037	0.049	0.306	5.76E-61
POSTN	4.06E-65	-0.31878	0.045	0.327	6.07E-61
THOC1	4.59E-65	-0.19923	0.037	0.298	6.86E-61
TRAPPC12	4.60E-65	-0.14993	0.044	0.295	6.87E-61
HAUS2	4.67E-65	-0.27731	0.014	0.268	6.99E-61
GOLGA5	5.42E-65	-0.11832	0.035	0.265	8.10E-61
PTPRF	5.81E-65	0.217811	0.068	0.211	8.68E-61
CGRRF1	6.09E-65	0.121402	0.08	0.279	9.10E-61
ZNF439	6.68E-65	-0.16504	0.04	0.29	9.98E-61
CCDC58	6.91E-65	-0.15929	0.035	0.28	1.03E-60

NOTCH1	7.19E-65	-0.32114	0.037	0.332	1.08E-60
MLLT6	7.59E-65	-0.10907	0.031	0.242	1.13E-60
BAP1	8.07E-65	-0.28889	0.026	0.305	1.21E-60
TOPORS	8.74E-65	-0.19005	0.051	0.312	1.31E-60
FADD	8.97E-65	-0.20931	0.021	0.264	1.34E-60
CLASP1	9.64E-65	-0.16583	0.042	0.293	1.44E-60
ADAL	1.00E-64	-0.14194	0.021	0.24	1.50E-60
CPNE4	1.05E-64	-0.27835	0.012	0.259	1.56E-60
ADAT1	1.14E-64	-0.19884	0.03	0.277	1.71E-60
SOX12	1.18E-64	-0.27962	0.016	0.278	1.76E-60
MCC	1.21E-64	-0.15309	0.033	0.283	1.80E-60
EHMT1	1.28E-64	-0.1678	0.031	0.273	1.91E-60
ZNF562	1.30E-64	0.170857	0.07	0.233	1.94E-60
RALGAPA2	1.39E-64	-0.146	0.031	0.265	2.07E-60
CENPL	1.40E-64	-0.36758	0.016	0.304	2.09E-60
RNMTL1	1.44E-64	-0.2928	0.021	0.291	2.15E-60
SGK1	1.53E-64	0.250211	0.099	0.277	2.29E-60
SRPX	1.61E-64	0.298093	0.152	0.342	2.41E-60
NOTCH2	1.64E-64	-0.25813	0.028	0.296	2.45E-60
SUCO	1.75E-64	-0.21723	0.031	0.293	2.62E-60
ZNF608	1.83E-64	-0.20455	0.031	0.283	2.73E-60
CHD1L	1.83E-64	-0.10155	0.024	0.242	2.74E-60
LYRM9	1.99E-64	0.21293	0.075	0.233	2.98E-60
TLE4	2.19E-64	-0.15916	0.04	0.284	3.28E-60
ZNF704	2.40E-64	-0.24688	0.045	0.319	3.59E-60
TMEM63B	2.43E-64	-0.10251	0.03	0.251	3.63E-60
ETNK1	2.46E-64	-0.11932	0.058	0.305	3.67E-60
ZNF559	2.46E-64	0.180137	0.089	0.263	3.68E-60
ABHD11	2.48E-64	-0.28095	0.035	0.31	3.71E-60
MEN1	2.60E-64	-0.16371	0.024	0.264	3.89E-60
C7orf26	2.64E-64	-0.24088	0.038	0.309	3.94E-60
LRRC40	2.95E-64	-0.18581	0.038	0.288	4.41E-60
INSM1	2.99E-64	-0.48079	0.031	0.347	4.47E-60
TMEM57	3.49E-64	-0.15392	0.045	0.291	5.21E-60
ZFP90	3.71E-64	-0.15496	0.04	0.277	5.55E-60
ROBO2	3.79E-64	-0.24428	0.028	0.284	5.67E-60
ZDHHC6	3.86E-64	-0.16987	0.035	0.28	5.76E-60
IGSF8	4.00E-64	0.182918	0.082	0.256	5.98E-60
OAT	4.04E-64	0.260219	0.103	0.26	6.03E-60
AKAP17A	4.05E-64	-0.30062	0.024	0.302	6.05E-60
LIX1L	4.10E-64	-0.19077	0.028	0.275	6.13E-60
BCAT2	4.18E-64	0.348467	0.08	0.216	6.24E-60
CAPRIN2	4.28E-64	-0.19928	0.028	0.268	6.40E-60
CPSF2	4.40E-64	-0.16062	0.03	0.263	6.58E-60
C11orf1	4.42E-64	0.347453	0.068	0.188	6.61E-60
UBE2O	4.59E-64	-0.14316	0.026	0.249	6.86E-60
POGK	5.08E-64	-0.2375	0.019	0.272	7.59E-60
KCNF1	5.47E-64	-0.4365	0.017	0.301	8.17E-60
TMEM19	5.57E-64	-0.28017	0.033	0.306	8.33E-60
GIT1	5.79E-64	-0.24138	0.021	0.28	8.65E-60
SENP5	5.88E-64	-0.17904	0.03	0.267	8.79E-60

CNTLN	6.38E-64	-0.37338	0.035	0.337	9.54E-60
F8A1	6.99E-64	-0.32625	0.016	0.294	1.04E-59
EID2	7.06E-64	-0.19046	0.016	0.243	1.05E-59
STEAP3	7.35E-64	0.194397	0.052	0.181	1.10E-59
KANSL1	7.80E-64	-0.25792	0.037	0.31	1.17E-59
ZNF714	8.99E-64	-0.23291	0.065	0.353	1.34E-59
SOX6	9.36E-64	0.226113	0.096	0.247	1.40E-59
IRF1	9.41E-64	0.223329	0.124	0.306	1.41E-59
FAM129A	1.00E-63	-0.13444	0.017	0.214	1.50E-59
MARK2	1.04E-63	-0.19871	0.023	0.267	1.55E-59
SH2B1	1.04E-63	-0.11997	0.035	0.257	1.55E-59
TAGLN3	1.07E-63	-0.13021	0.086	0.349	1.60E-59
ADORA1	1.21E-63	0.226742	0.056	0.175	1.81E-59
KIF18B	1.26E-63	-0.4471	0.002	0.295	1.88E-59
ZBTB38	1.37E-63	0.126845	0.056	0.219	2.05E-59
IRF2	1.38E-63	0.165309	0.077	0.262	2.06E-59
LINC00511	1.46E-63	-0.11773	0.047	0.285	2.18E-59
ZC3HAV1	1.47E-63	-0.13165	0.03	0.253	2.19E-59
SYDE1	1.51E-63	-0.13924	0.016	0.231	2.25E-59
WRNIP1	1.75E-63	-0.31371	0.012	0.281	2.61E-59
GNPDA2	1.83E-63	-0.11628	0.049	0.284	2.74E-59
SIGIRR	1.92E-63	-0.1243	0.017	0.219	2.87E-59
POLA2	2.16E-63	-0.40702	0.01	0.295	3.23E-59
DICER1	2.22E-63	0.111464	0.068	0.238	3.32E-59
C1S	2.63E-63	0.895888	0.162	0.135	3.94E-59
SHMT1	2.78E-63	-0.20596	0.023	0.268	4.16E-59
TNPO3	3.06E-63	-0.26953	0.044	0.323	4.57E-59
FIGN	3.11E-63	-0.15566	0.035	0.262	4.65E-59
SLC35E3	3.20E-63	-0.23344	0.117	0.428	4.78E-59
HMBOX1	3.22E-63	0.204968	0.106	0.29	4.82E-59
TTC32	3.60E-63	-0.1249	0.049	0.291	5.39E-59
TYW1	3.93E-63	-0.15471	0.026	0.254	5.87E-59
CIB2	4.35E-63	-0.36342	0.003	0.27	6.49E-59
REPS1	4.70E-63	-0.24637	0.017	0.269	7.02E-59
JOSD1	4.85E-63	-0.23814	0.021	0.274	7.25E-59
ANKRD26	4.86E-63	-0.1363	0.031	0.256	7.26E-59
ZNF770	4.97E-63	-0.15819	0.03	0.26	7.43E-59
USP7	5.35E-63	-0.15615	0.035	0.267	8.00E-59
NUPR1	5.89E-63	0.879466	0.182	0.196	8.80E-59
GLRX	6.16E-63	0.599709	0.127	0.204	9.20E-59
BEX2	6.41E-63	0.604208	0.202	0.263	9.58E-59
ZFHX3	6.70E-63	-0.16897	0.026	0.262	1.00E-58
LEO1	6.93E-63	-0.11485	0.049	0.295	1.04E-58
MCMBP	7.84E-63	-0.12072	0.044	0.273	1.17E-58
RAD18	7.87E-63	-0.32124	0.014	0.28	1.18E-58
RNF219	8.70E-63	-0.15095	0.037	0.28	1.30E-58
PRKRIR	9.40E-63	-0.28815	0.017	0.28	1.40E-58
TMEM243	9.45E-63	0.103535	0.082	0.283	1.41E-58
SLC25A29	9.63E-63	-0.33519	0.024	0.305	1.44E-58
ROGDI	1.18E-62	-0.14114	0.038	0.281	1.76E-58
FAM118B	1.26E-62	-0.10752	0.031	0.258	1.88E-58

TGFB1	1.26E-62	-0.23667	0.019	0.265	1.89E-58
KDM2B	1.29E-62	-0.21327	0.024	0.267	1.93E-58
DMTF1	1.30E-62	-0.18598	0.051	0.307	1.94E-58
COPG2	1.35E-62	-0.19696	0.033	0.28	2.01E-58
CDYL	1.38E-62	-0.27048	0.016	0.268	2.06E-58
MPPED2	1.41E-62	-0.46161	0.021	0.32	2.11E-58
RP11-111M	1.47E-62	-0.16213	0.038	0.277	2.20E-58
GBE1	1.49E-62	0.104893	0.08	0.278	2.23E-58
TMEM245	1.50E-62	-0.18051	0.051	0.306	2.24E-58
DNAJC21	1.50E-62	-0.14681	0.024	0.252	2.25E-58
SLC31A1	1.50E-62	-0.11067	0.031	0.252	2.25E-58
WDR73	1.66E-62	0.109313	0.059	0.228	2.47E-58
FAM58A	1.88E-62	-0.23551	0.019	0.27	2.81E-58
BYSL	1.90E-62	-0.13535	0.021	0.244	2.84E-58
PSMA6	1.98E-62	0.169797	0.112	0.299	2.96E-58
THOC6	2.27E-62	-0.12089	0.03	0.252	3.39E-58
RAB11B-AS	2.32E-62	-0.10415	0.03	0.243	3.46E-58
POM121C	2.33E-62	-0.22274	0.033	0.29	3.49E-58
SERTAD3	2.44E-62	0.289145	0.072	0.217	3.64E-58
SIRPA	2.66E-62	-0.19605	0.017	0.251	3.98E-58
RGS19	2.80E-62	-0.34822	0.01	0.288	4.18E-58
SLC27A5	2.89E-62	0.4189	0.094	0.212	4.32E-58
GABPB1	2.90E-62	-0.30378	0.019	0.285	4.33E-58
SLC35F1	2.90E-62	-0.18266	0.035	0.283	4.34E-58
CHTF18	2.98E-62	-0.30303	0.012	0.272	4.45E-58
TMEM43	3.20E-62	0.117479	0.08	0.263	4.79E-58
SLC5A6	3.27E-62	-0.11384	0.024	0.238	4.89E-58
NAA16	3.76E-62	-0.23407	0.026	0.275	5.62E-58
ZNF330	4.24E-62	0.102855	0.066	0.256	6.34E-58
GLCCI1	4.42E-62	-0.17165	0.11	0.393	6.60E-58
TP53TG1	4.43E-62	0.397212	0.103	0.226	6.62E-58
ABCB8	4.83E-62	-0.19576	0.031	0.28	7.22E-58
UBTD2	4.98E-62	-0.1997	0.021	0.259	7.44E-58
SNCAIP	5.48E-62	-0.11927	0.04	0.26	8.19E-58
YY1AP1	6.00E-62	-0.15471	0.044	0.294	8.96E-58
GAK	6.23E-62	-0.15451	0.024	0.248	9.31E-58
SCLT1	6.36E-62	-0.32092	0.014	0.283	9.50E-58
SLC12A2	6.87E-62	-0.20233	0.03	0.277	1.03E-57
LRPPRC	7.06E-62	-0.28416	0.03	0.301	1.06E-57
AUTS2	7.82E-62	-0.26051	0.024	0.283	1.17E-57
PIM3	8.26E-62	-0.41845	0.023	0.319	1.23E-57
ZIC1	9.08E-62	-0.19568	0.061	0.322	1.36E-57
STRBP	9.66E-62	-0.25268	0.023	0.277	1.44E-57
NMU	9.69E-62	-0.67953	0	0.283	1.45E-57
WDR62	9.71E-62	-0.44807	0.005	0.29	1.45E-57
ZNF254	1.07E-61	-0.12318	0.04	0.268	1.59E-57
GNPTAB	1.20E-61	0.174438	0.04	0.168	1.80E-57
LAMC1	1.21E-61	-0.16649	0.024	0.253	1.81E-57
TOMM34	1.34E-61	-0.19717	0.028	0.274	2.01E-57
RAD9A	1.50E-61	-0.18067	0.038	0.283	2.24E-57
TRAF3IP2	1.52E-61	-0.14932	0.044	0.28	2.26E-57

9-Mar	1.66E-61	-0.34654	0.075	0.381	2.48E-57
CLASP2	1.69E-61	-0.11086	0.07	0.32	2.52E-57
IREB2	1.77E-61	-0.20208	0.031	0.277	2.64E-57
PXDN	1.79E-61	-0.11652	0.028	0.247	2.68E-57
MCM10	1.81E-61	-0.47339	0.002	0.29	2.70E-57
SH3KBP1	1.83E-61	-0.24483	0.028	0.288	2.73E-57
SLC10A4	1.98E-61	-0.28885	0.017	0.272	2.96E-57
ZC3H6	2.05E-61	-0.17775	0.04	0.273	3.07E-57
NEDD9	2.14E-61	0.133402	0.082	0.267	3.19E-57
R3HDM2	2.24E-61	-0.2186	0.026	0.272	3.35E-57
SRD5A3	2.30E-61	-0.36869	0.017	0.295	3.44E-57
RPP30	2.73E-61	-0.17966	0.044	0.296	4.09E-57
SENP2	2.95E-61	-0.10262	0.035	0.251	4.41E-57
NBN	3.11E-61	-0.18035	0.037	0.28	4.65E-57
GRIA4	3.25E-61	0.272146	0.092	0.24	4.86E-57
SLC30A7	3.41E-61	-0.1194	0.038	0.259	5.10E-57
WIPF1	3.92E-61	-0.11161	0.03	0.246	5.86E-57
GALNS	4.28E-61	-0.22788	0.021	0.26	6.40E-57
KIAA2013	4.30E-61	-0.23889	0.021	0.265	6.42E-57
MMP16	4.32E-61	-0.13983	0.042	0.265	6.45E-57
CHST11	4.59E-61	-0.13648	0.042	0.272	6.86E-57
DUS1L	4.62E-61	-0.22088	0.023	0.27	6.90E-57
TRAPPC6A	4.72E-61	0.328477	0.101	0.249	7.05E-57
NAA35	4.72E-61	-0.22598	0.026	0.27	7.05E-57
LSM14B	4.88E-61	-0.21929	0.023	0.265	7.30E-57
NIPSNAP1	5.15E-61	-0.18675	0.033	0.275	7.70E-57
MTSS1	5.35E-61	-0.29818	0.054	0.333	7.99E-57
STX6	5.63E-61	-0.16476	0.033	0.269	8.42E-57
CCDC80	5.79E-61	0.415657	0.115	0.232	8.65E-57
PRDM2	6.04E-61	-0.12744	0.042	0.275	9.03E-57
EGLN1	7.27E-61	-0.11358	0.035	0.263	1.09E-56
CENPP	7.57E-61	-0.45483	0.005	0.28	1.13E-56
VWA1	7.99E-61	-0.19989	0.026	0.263	1.19E-56
SSBP3	8.07E-61	-0.1966	0.033	0.273	1.21E-56
CDKN2AIP	8.24E-61	-0.26741	0.033	0.298	1.23E-56
EFS	8.76E-61	-0.10327	0.044	0.264	1.31E-56
USP13	9.04E-61	-0.35395	0.017	0.295	1.35E-56
SERTAD2	9.10E-61	-0.20367	0.033	0.275	1.36E-56
C3orf70	9.11E-61	-0.25704	0.038	0.301	1.36E-56
RASSF4	9.65E-61	0.517699	0.131	0.219	1.44E-56
METTL7A	1.01E-60	0.25895	0.133	0.304	1.50E-56
STX2	1.01E-60	-0.203	0.021	0.254	1.50E-56
DHRS11	1.02E-60	-0.38634	0.005	0.285	1.52E-56
ZNF12	1.05E-60	-0.14171	0.035	0.262	1.57E-56
SGCE	1.24E-60	0.177702	0.145	0.305	1.86E-56
GLUD1	1.36E-60	0.191949	0.11	0.286	2.04E-56
SLC35G2	1.40E-60	0.173637	0.068	0.211	2.09E-56
YLPM1	1.43E-60	-0.15239	0.033	0.258	2.14E-56
ZNF821	1.44E-60	-0.17655	0.047	0.288	2.15E-56
CTR9	1.45E-60	-0.19372	0.038	0.286	2.17E-56
ATXN3	1.47E-60	0.31611	0.084	0.202	2.20E-56

SLC1A2	1.56E-60	0.286657	0.112	0.279	2.33E-56
TMEM129	1.88E-60	-0.15274	0.049	0.3	2.80E-56
SPRED2	1.97E-60	-0.10592	0.03	0.243	2.94E-56
SMPD4	2.21E-60	-0.2694	0.023	0.283	3.30E-56
CRNKL1	2.34E-60	-0.16996	0.035	0.277	3.50E-56
EMC1	2.40E-60	-0.14966	0.03	0.254	3.59E-56
APOLD1	2.71E-60	-0.49844	0.019	0.32	4.04E-56
DDIT4	2.88E-60	0.503687	0.206	0.312	4.30E-56
SFRP1	2.90E-60	-0.30416	0.01	0.259	4.33E-56
SREBF2	2.98E-60	-0.12127	0.031	0.243	4.45E-56
2-Mar	3.16E-60	-0.12946	0.028	0.246	4.72E-56
CHCHD4	3.25E-60	-0.10536	0.024	0.236	4.86E-56
C3	3.33E-60	1.007703	0.194	0.125	4.97E-56
CDCA7	3.35E-60	-0.38665	0.042	0.331	5.01E-56
HDAC6	3.40E-60	-0.19372	0.03	0.272	5.08E-56
ATP6V0A2	3.68E-60	-0.19746	0.023	0.256	5.50E-56
EVA1B	3.86E-60	-0.35923	0.002	0.259	5.76E-56
PTK7	4.41E-60	-0.25179	0.014	0.256	6.59E-56
NDP	4.46E-60	0.259601	0.068	0.198	6.66E-56
SAMD1	4.49E-60	-0.23667	0.023	0.263	6.70E-56
UTP23	4.99E-60	-0.12894	0.033	0.253	7.46E-56
ZNF226	5.06E-60	0.259502	0.096	0.258	7.55E-56
CBX2	5.29E-60	-0.40099	0.009	0.277	7.90E-56
TMEM69	5.48E-60	0.186652	0.086	0.247	8.18E-56
PIGQ	5.60E-60	-0.14386	0.026	0.246	8.38E-56
ZC2HC1A	6.00E-60	-0.15881	0.075	0.335	8.97E-56
DHX40	6.01E-60	-0.30359	0.024	0.291	8.98E-56
HOPX	6.95E-60	0.31583	0.321	0.465	1.04E-55
SIK3	7.08E-60	-0.17595	0.019	0.241	1.06E-55
UBALD1	7.10E-60	-0.15606	0.04	0.279	1.06E-55
TCOF1	7.22E-60	-0.1652	0.044	0.289	1.08E-55
ANKRD46	7.29E-60	0.113649	0.075	0.252	1.09E-55
SH3BP5	7.60E-60	-0.22187	0.019	0.251	1.14E-55
LAS1L	7.83E-60	-0.13053	0.031	0.263	1.17E-55
PIAS4	7.86E-60	-0.21784	0.033	0.288	1.17E-55
RPP38	8.33E-60	-0.14662	0.031	0.259	1.24E-55
SUV39H2	9.75E-60	-0.25186	0.019	0.265	1.46E-55
PPM1D	9.92E-60	-0.29769	0.019	0.279	1.48E-55
VIM	1.10E-59	0.351631	0.588	0.722	1.64E-55
GNB5	1.17E-59	-0.33275	0.017	0.281	1.75E-55
SETD3	1.18E-59	-0.17598	0.031	0.263	1.77E-55
DESI1	1.25E-59	-0.15322	0.028	0.252	1.87E-55
AC093323.	1.28E-59	-0.21613	0.019	0.253	1.92E-55
MOB3A	1.33E-59	-0.12133	0.026	0.235	1.98E-55
LGR4	1.37E-59	-0.34262	0.01	0.273	2.05E-55
RND3	1.41E-59	-0.27642	0.096	0.395	2.11E-55
ZNF429	1.54E-59	0.17181	0.073	0.236	2.29E-55
BRI3BP	1.58E-59	-0.2514	0.016	0.256	2.36E-55
FTO	1.61E-59	-0.16136	0.033	0.263	2.41E-55
LARP1B	1.64E-59	-0.22425	0.024	0.265	2.45E-55
TMEM67	1.65E-59	0.217314	0.052	0.185	2.46E-55

IL1RAP	1.66E-59	-0.20245	0.03	0.249	2.49E-55
DLEU1	1.83E-59	-0.13805	0.026	0.243	2.73E-55
BCL7A	1.86E-59	-0.23548	0.04	0.289	2.79E-55
TMBIM1	2.17E-59	0.227883	0.056	0.185	3.24E-55
RTN1	2.26E-59	0.549867	0.143	0.252	3.37E-55
NKAIN3	2.26E-59	-0.21788	0.04	0.283	3.38E-55
EFNB2	2.33E-59	-0.29923	0.01	0.262	3.48E-55
CORO2B	2.36E-59	-0.24206	0.023	0.264	3.53E-55
GPALPP1	2.40E-59	-0.17428	0.033	0.267	3.59E-55
HLA-DPA1	2.40E-59	0.946085	0.257	0.2	3.59E-55
ZNF277	2.43E-59	0.136126	0.066	0.236	3.63E-55
MYCBP	2.44E-59	-0.10402	0.017	0.212	3.65E-55
GTF2A1	2.46E-59	-0.1747	0.023	0.248	3.68E-55
MED25	2.55E-59	-0.1364	0.044	0.281	3.81E-55
PPP6R2	2.64E-59	-0.13746	0.031	0.252	3.94E-55
ARID2	3.36E-59	-0.1264	0.044	0.267	5.03E-55
AC009501.	3.62E-59	0.894712	0.18	0.115	5.41E-55
IGF2BP3	3.66E-59	-0.23181	0.037	0.289	5.47E-55
HYI	3.80E-59	-0.29577	0.033	0.301	5.69E-55
TMEM42	4.26E-59	0.227393	0.061	0.201	6.37E-55
ACSS3	5.45E-59	-0.15498	0.028	0.248	8.14E-55
GINS4	5.52E-59	-0.34532	0.012	0.273	8.26E-55
ARMCX1	5.68E-59	0.277626	0.106	0.24	8.48E-55
UNC45A	6.01E-59	0.120681	0.068	0.248	8.98E-55
GNPNAT1	6.74E-59	-0.13569	0.023	0.236	1.01E-54
ELMOD3	7.58E-59	-0.17027	0.033	0.26	1.13E-54
OLFM2	8.45E-59	0.333322	0.136	0.265	1.26E-54
CIT	9.09E-59	-0.15767	0.023	0.236	1.36E-54
SLAIN2	9.78E-59	-0.27017	0.019	0.268	1.46E-54
RCE1	9.86E-59	-0.22908	0.023	0.26	1.47E-54
USP46	9.98E-59	-0.3536	0.035	0.311	1.49E-54
KDELC2	1.02E-58	-0.20955	0.017	0.241	1.52E-54
RP11-51J9	1.08E-58	0.282203	0.084	0.214	1.61E-54
SH3PXD2B	1.16E-58	-0.1391	0.016	0.221	1.73E-54
CBWD1	1.19E-58	0.207743	0.101	0.256	1.78E-54
PCNT	1.22E-58	-0.10801	0.028	0.24	1.82E-54
MLXIP	1.22E-58	-0.1215	0.045	0.269	1.82E-54
IMPDH1	1.23E-58	-0.27682	0.016	0.264	1.83E-54
LRRC4B	1.27E-58	-0.28369	0.023	0.285	1.90E-54
UQCR11	1.32E-58	1.699738	0.312	0.022	1.98E-54
CUL4A	1.33E-58	-0.10275	0.033	0.242	1.98E-54
CHKA	1.34E-58	-0.14871	0.044	0.28	2.00E-54
EFR3A	1.51E-58	-0.11112	0.016	0.202	2.26E-54
KIF3B	1.55E-58	-0.2169	0.019	0.251	2.32E-54
HRH1	1.82E-58	0.237761	0.044	0.153	2.71E-54
5-Mar	1.93E-58	-0.22219	0.042	0.298	2.89E-54
CLIP3	2.04E-58	0.229818	0.087	0.244	3.05E-54
OPA1	2.10E-58	-0.24006	0.019	0.258	3.13E-54
LYSMD2	2.28E-58	-0.11077	0.031	0.251	3.41E-54
SPRTN	2.29E-58	-0.15543	0.045	0.278	3.42E-54
SPEN	2.29E-58	-0.13853	0.051	0.279	3.43E-54

DZIP3	2.36E-58	-0.20308	0.04	0.285	3.53E-54
FEM1C	2.46E-58	-0.10584	0.035	0.248	3.68E-54
RPS6KB1	2.56E-58	-0.15611	0.023	0.236	3.83E-54
APITD1	2.69E-58	-0.30377	0.012	0.264	4.03E-54
B3GALT6	2.74E-58	-0.23046	0.021	0.265	4.09E-54
AAGAB	3.36E-58	-0.11322	0.033	0.251	5.02E-54
AFTPH	3.56E-58	-0.24557	0.03	0.284	5.32E-54
ZFP82	3.70E-58	-0.13846	0.037	0.252	5.53E-54
OTUD6B	4.07E-58	-0.1494	0.035	0.256	6.08E-54
ZNF331	4.32E-58	-0.32903	0.028	0.301	6.45E-54
AGFG1	5.02E-58	-0.1554	0.024	0.237	7.50E-54
DHCR24	5.03E-58	-0.10636	0.023	0.217	7.51E-54
PHTF1	5.09E-58	-0.24928	0.017	0.257	7.60E-54
ZNF814	5.48E-58	0.133264	0.056	0.212	8.20E-54
LSAMP	5.64E-58	0.399772	0.147	0.257	8.43E-54
HAUS6	5.69E-58	-0.37631	0.01	0.28	8.50E-54
VAC14	5.73E-58	-0.21034	0.017	0.248	8.57E-54
FBXW9	6.32E-58	-0.11239	0.026	0.225	9.45E-54
CAPG	6.48E-58	0.173698	0.077	0.227	9.68E-54
RAB3GAP1	6.87E-58	-0.15031	0.031	0.247	1.03E-53
RPUSD1	8.17E-58	-0.16489	0.031	0.253	1.22E-53
C6orf136	8.69E-58	-0.12065	0.021	0.226	1.30E-53
TPST2	8.78E-58	0.128251	0.035	0.175	1.31E-53
GCFC2	8.87E-58	-0.2015	0.031	0.272	1.33E-53
CHCHD7	9.35E-58	0.132361	0.077	0.254	1.40E-53
MYH9	9.64E-58	-0.17318	0.021	0.231	1.44E-53
GATAD2A	1.06E-57	-0.21999	0.024	0.26	1.59E-53
RFWD3	1.12E-57	-0.45807	0.005	0.285	1.68E-53
KLF10	1.21E-57	-0.13993	0.065	0.3	1.81E-53
ZNF827	1.28E-57	-0.13184	0.042	0.267	1.91E-53
SDC1	1.29E-57	-0.37923	0.007	0.268	1.93E-53
ERCC3	1.30E-57	-0.16429	0.038	0.268	1.94E-53
CENPC	1.30E-57	-0.37763	0.016	0.281	1.95E-53
POLRMT	1.36E-57	-0.1617	0.028	0.252	2.03E-53
EME1	1.36E-57	-0.4139	0	0.265	2.03E-53
PQLC1	1.52E-57	-0.11251	0.026	0.232	2.27E-53
RGS6	1.68E-57	0.187968	0.051	0.19	2.51E-53
SPHK2	1.72E-57	-0.13641	0.04	0.263	2.57E-53
MTFR1	1.72E-57	-0.17798	0.019	0.235	2.57E-53
H2AFY2	1.90E-57	-0.19079	0.012	0.217	2.84E-53
BTBD1	1.92E-57	-0.17936	0.031	0.254	2.87E-53
KIF11	2.23E-57	-0.42052	0.009	0.281	3.33E-53
TYRO3	2.31E-57	-0.18858	0.037	0.272	3.45E-53
SH3GLB2	2.32E-57	-0.27198	0.012	0.254	3.46E-53
CYB5R4	2.32E-57	-0.13397	0.023	0.228	3.47E-53
TAF13	2.48E-57	-0.17085	0.026	0.246	3.71E-53
PIF1	2.53E-57	-0.63778	0.003	0.285	3.79E-53
SHD	2.82E-57	-0.31495	0.079	0.351	4.21E-53
TRAF2	3.04E-57	-0.17951	0.014	0.225	4.54E-53
RBBP9	3.15E-57	-0.22154	0.016	0.244	4.71E-53
TULP4	3.29E-57	-0.18305	0.024	0.246	4.91E-53

KDM5B	3.34E-57	-0.14918	0.037	0.257	4.99E-53
FAM208A	3.44E-57	-0.26509	0.019	0.26	5.14E-53
APTX	3.64E-57	-0.18887	0.023	0.246	5.44E-53
RUNX1	3.85E-57	-0.11012	0.028	0.225	5.75E-53
RP3-525N1	3.87E-57	0.123056	0.098	0.27	5.78E-53
CLOCK	3.95E-57	-0.37642	0.037	0.321	5.91E-53
NRGN	4.16E-57	-0.31699	0.007	0.252	6.21E-53
GPC2	4.16E-57	-0.33661	0.07	0.354	6.22E-53
FOXP1	4.18E-57	0.616185	0.199	0.228	6.24E-53
CCDC22	4.62E-57	-0.10943	0.028	0.225	6.91E-53
C1GALT1	4.71E-57	-0.19532	0.037	0.274	7.04E-53
SPA17	4.76E-57	-0.12088	0.033	0.249	7.11E-53
RUFY2	4.92E-57	-0.27427	0.017	0.263	7.35E-53
TMEM178A	5.35E-57	-0.11924	0.038	0.259	8.00E-53
SERPING1	5.72E-57	1.287004	0.213	0.065	8.54E-53
CERS6	7.01E-57	-0.20032	0.024	0.254	1.05E-52
AEBP1	7.04E-57	0.305621	0.082	0.194	1.05E-52
EXOSC5	7.41E-57	0.264224	0.091	0.233	1.11E-52
CROT	7.93E-57	0.134473	0.052	0.209	1.18E-52
NLGN4X	8.00E-57	-0.1273	0.038	0.26	1.20E-52
RDH5	8.01E-57	0.27169	0.066	0.188	1.20E-52
SEZ6L	8.27E-57	0.126879	0.103	0.286	1.24E-52
ORC3	8.63E-57	-0.16565	0.026	0.247	1.29E-52
MMP14	9.95E-57	0.111571	0.061	0.228	1.49E-52
MED7	1.03E-56	-0.10791	0.033	0.243	1.54E-52
SYNGR2	1.05E-56	-0.11838	0.024	0.219	1.57E-52
DHRS1	1.06E-56	0.198149	0.058	0.191	1.58E-52
TMEM39B	1.11E-56	-0.21507	0.026	0.263	1.66E-52
BTN2A1	1.21E-56	-0.13983	0.031	0.247	1.80E-52
ULK3	1.21E-56	-0.10184	0.042	0.26	1.81E-52
RAD51	1.24E-56	-0.4893	0.007	0.283	1.86E-52
PBX3	1.37E-56	-0.19792	0.019	0.238	2.04E-52
DNAJC11	1.40E-56	0.112948	0.047	0.204	2.10E-52
ATG4C	1.51E-56	-0.20306	0.028	0.258	2.25E-52
NUFIP1	1.61E-56	-0.12582	0.035	0.258	2.41E-52
CHI3L2	1.68E-56	1.494652	0.197	0.059	2.51E-52
LAMP5	1.75E-56	-0.12724	0.026	0.235	2.62E-52
ZNF649	1.85E-56	-0.27629	0.017	0.262	2.77E-52
KATNB1	1.91E-56	-0.18523	0.03	0.256	2.86E-52
SNX8	1.95E-56	-0.15111	0.028	0.242	2.91E-52
DHRS13	1.99E-56	-0.29444	0.012	0.258	2.97E-52
PLK4	2.02E-56	-0.43517	0	0.26	3.02E-52
DTX3	2.06E-56	-0.30245	0.12	0.432	3.08E-52
ZDHHC16	2.20E-56	-0.15403	0.01	0.2	3.29E-52
KLF13	2.36E-56	-0.10386	0.03	0.233	3.52E-52
UBL7-AS1	2.47E-56	-0.24581	0.017	0.251	3.69E-52
CENPB	2.52E-56	-0.19396	0.017	0.237	3.77E-52
PHTF2	2.58E-56	-0.34675	0.01	0.26	3.85E-52
PGS1	2.58E-56	-0.13333	0.033	0.24	3.86E-52
SLN	2.62E-56	0.651945	0.143	0.221	3.92E-52
NOM1	2.65E-56	-0.3117	0.021	0.273	3.95E-52

BLM	2.74E-56	-0.12431	0.037	0.246	4.10E-52
CYR61	2.90E-56	0.527715	0.143	0.228	4.33E-52
KLHDC10	3.05E-56	-0.13061	0.037	0.256	4.55E-52
PRKAB1	3.15E-56	-0.15173	0.033	0.253	4.70E-52
HAUS3	3.22E-56	-0.24061	0.03	0.267	4.82E-52
CNTROB	3.61E-56	-0.27959	0.016	0.257	5.40E-52
RP11-386G	3.67E-56	0.209194	0.07	0.215	5.49E-52
TTF2	3.69E-56	-0.24121	0.014	0.236	5.52E-52
PATL1	4.16E-56	-0.13892	0.017	0.205	6.22E-52
CAB39	4.37E-56	-0.15267	0.033	0.252	6.53E-52
UBXN2B	4.40E-56	-0.13248	0.031	0.243	6.57E-52
CRKL	4.82E-56	-0.19566	0.028	0.258	7.21E-52
IFI44	4.91E-56	0.133009	0.077	0.244	7.33E-52
UGGT1	5.24E-56	-0.20394	0.031	0.262	7.83E-52
SESN3	5.42E-56	-0.11112	0.042	0.259	8.10E-52
LHFPL3	5.57E-56	-0.32256	0.07	0.348	8.33E-52
GTPBP3	6.30E-56	-0.1685	0.031	0.262	9.42E-52
FRMD8	7.25E-56	-0.14372	0.023	0.225	1.08E-51
REL	7.40E-56	-0.12409	0.047	0.264	1.11E-51
MTMR6	7.65E-56	-0.15155	0.026	0.233	1.14E-51
TELO2	8.49E-56	-0.20243	0.031	0.262	1.27E-51
MIR4458HG	8.79E-56	0.668769	0.173	0.185	1.31E-51
BEND6	9.13E-56	-0.31613	0.012	0.263	1.36E-51
ZYG11B	9.43E-56	-0.1948	0.026	0.253	1.41E-51
DPP4	9.45E-56	-0.19389	0.005	0.204	1.41E-51
TNFAIP6	1.08E-55	0.230367	0.115	0.294	1.62E-51
DMRTA2	1.15E-55	-0.15659	0.021	0.227	1.72E-51
TMEM127	1.65E-55	0.103165	0.049	0.196	2.47E-51
HEBP1	1.78E-55	0.147894	0.052	0.202	2.65E-51
FASN	2.00E-55	-0.2004	0.019	0.238	2.99E-51
DALRD3	2.04E-55	-0.19076	0.023	0.241	3.05E-51
MPI	2.09E-55	0.187957	0.044	0.173	3.12E-51
SART3	2.11E-55	-0.12235	0.037	0.247	3.16E-51
CCDC86	2.19E-55	-0.1067	0.021	0.212	3.27E-51
HIST1H1D	2.21E-55	-0.54201	0.003	0.269	3.30E-51
MLLT10	2.28E-55	-0.12352	0.031	0.238	3.41E-51
DENND5B	2.50E-55	-0.10667	0.019	0.207	3.73E-51
CNPY1	3.10E-55	-0.44435	0.026	0.299	4.64E-51
UCP2	3.92E-55	-0.44818	0.019	0.29	5.86E-51
DDX10	4.38E-55	-0.13063	0.028	0.235	6.54E-51
N4BP2	4.60E-55	-0.24986	0.023	0.258	6.88E-51
HERPUD2	5.02E-55	-0.18192	0.031	0.257	7.51E-51
RABGAP1	5.08E-55	-0.14147	0.026	0.233	7.59E-51
MCAT	5.14E-55	-0.26464	0.023	0.267	7.68E-51
C17orf75	5.52E-55	-0.12626	0.024	0.221	8.25E-51
C6orf120	5.67E-55	-0.12228	0.019	0.209	8.47E-51
CD99L2	6.45E-55	-0.13144	0.021	0.223	9.63E-51
PALM	7.60E-55	-0.17101	0.03	0.252	1.14E-50
YTHDC2	7.77E-55	-0.10126	0.023	0.216	1.16E-50
ANKRD39	8.02E-55	-0.31695	0.028	0.286	1.20E-50
PMS1	8.32E-55	-0.14911	0.03	0.249	1.24E-50

ASB16-AS1	9.61E-55	-0.25252	0.016	0.247	1.44E-50
GAN	1.08E-54	-0.10465	0.042	0.246	1.62E-50
STK24	1.14E-54	-0.11672	0.023	0.223	1.70E-50
SNRNP48	1.16E-54	-0.13174	0.031	0.241	1.73E-50
SEZ6	1.18E-54	-0.27527	0.086	0.365	1.76E-50
LRRTM2	1.19E-54	-0.13153	0.038	0.252	1.77E-50
POMT1	1.20E-54	-0.13009	0.03	0.236	1.79E-50
IGSF10	1.21E-54	-0.13602	0.026	0.231	1.80E-50
DTL	1.28E-54	-0.51102	0.003	0.264	1.91E-50
FOXRED1	1.31E-54	-0.10277	0.037	0.246	1.95E-50
FBLN1	1.34E-54	-0.46673	0.049	0.337	2.01E-50
C18orf8	1.44E-54	-0.23518	0.014	0.237	2.15E-50
METTL10	1.54E-54	-0.18127	0.03	0.253	2.30E-50
C8orf88	1.57E-54	-0.20739	0.007	0.214	2.34E-50
MSANTD3	1.65E-54	-0.13234	0.023	0.221	2.47E-50
CRYZ	1.68E-54	0.167666	0.065	0.21	2.51E-50
SPSB4	1.70E-54	-0.1956	0.023	0.242	2.55E-50
YDJC	1.76E-54	-0.26177	0.017	0.256	2.63E-50
CEP152	1.88E-54	-0.41372	0.003	0.26	2.81E-50
ZNF232	2.13E-54	-0.21235	0.026	0.254	3.19E-50
CRELD1	2.17E-54	0.115037	0.051	0.205	3.24E-50
FAM126B	2.44E-54	0.11581	0.065	0.217	3.64E-50
FAM217B	2.53E-54	-0.15079	0.033	0.244	3.77E-50
TBC1D1	2.85E-54	-0.17274	0.021	0.231	4.26E-50
NRP1	2.95E-54	-0.21285	0.017	0.231	4.41E-50
UNG	3.12E-54	-0.12323	0.049	0.263	4.66E-50
IBTK	3.83E-54	-0.10889	0.035	0.238	5.72E-50
CHAF1B	4.60E-54	-0.30535	0.01	0.237	6.87E-50
ZNF682	5.04E-54	-0.31821	0.017	0.26	7.54E-50
EIF4EBP2	5.33E-54	-0.11222	0.026	0.22	7.96E-50
SNX10	5.60E-54	-0.21703	0.024	0.243	8.37E-50
DHX8	5.93E-54	-0.19665	0.019	0.235	8.87E-50
AGBL5	6.30E-54	-0.21537	0.01	0.219	9.41E-50
MFSD5	6.98E-54	-0.1039	0.016	0.191	1.04E-49
LETMD1	7.83E-54	0.140916	0.058	0.209	1.17E-49
PUSL1	8.11E-54	-0.21155	0.016	0.227	1.21E-49
CNPY4	8.19E-54	-0.17319	0.024	0.231	1.22E-49
CD68	8.20E-54	0.478188	0.099	0.165	1.23E-49
NPAT	8.65E-54	-0.10062	0.03	0.215	1.29E-49
EFCAB11	9.09E-54	-0.20758	0.014	0.232	1.36E-49
PPP2R5B	9.14E-54	-0.13702	0.026	0.232	1.37E-49
E4F1	9.78E-54	-0.10087	0.033	0.232	1.46E-49
PPFIA3	1.16E-53	-0.18659	0.014	0.22	1.74E-49
ZNF48	1.20E-53	-0.19319	0.016	0.228	1.79E-49
RUNDC1	1.20E-53	-0.1088	0.031	0.23	1.80E-49
RMI2	1.23E-53	-0.38931	0.01	0.258	1.84E-49
CDADC1	1.24E-53	-0.15045	0.024	0.231	1.86E-49
CNKSR3	1.26E-53	-0.20879	0.026	0.247	1.89E-49
UBXN7	1.29E-53	-0.11391	0.037	0.231	1.93E-49
RAP2B	1.30E-53	-0.29812	0.009	0.246	1.94E-49
MGAT4B	1.34E-53	-0.11536	0.01	0.188	2.01E-49

TNIK	1.41E-53	-0.11964	0.033	0.236	2.10E-49
MAN2C1	1.47E-53	0.197742	0.054	0.178	2.20E-49
APOBEC3B	1.48E-53	-0.43312	0.005	0.259	2.21E-49
IQCG	1.48E-53	0.204594	0.052	0.185	2.21E-49
PTPRN2	1.48E-53	0.287906	0.103	0.231	2.21E-49
BRCC3	1.55E-53	-0.1422	0.023	0.225	2.32E-49
TEAD2	1.57E-53	-0.23431	0.017	0.24	2.34E-49
BCAS4	1.58E-53	0.158091	0.047	0.177	2.37E-49
AP1G1	1.66E-53	-0.20212	0.024	0.247	2.48E-49
BIN1	1.67E-53	-0.11515	0.045	0.256	2.49E-49
GLA	1.76E-53	-0.14271	0.047	0.274	2.63E-49
LRRC45	1.78E-53	-0.29606	0.009	0.246	2.66E-49
PDE4DIP	1.79E-53	0.410685	0.131	0.231	2.67E-49
UBE3C	1.91E-53	-0.15453	0.024	0.225	2.85E-49
RNF166	1.93E-53	-0.17894	0.026	0.241	2.88E-49
SVIP	1.97E-53	-0.57139	0.003	0.26	2.95E-49
FAM111B	1.99E-53	-0.48302	0.005	0.251	2.97E-49
MEX3C	1.99E-53	-0.20432	0.017	0.237	2.98E-49
LMAN2L	2.22E-53	-0.15137	0.042	0.263	3.31E-49
AGPAT3	2.34E-53	-0.18981	0.028	0.253	3.50E-49
EPDR1	2.40E-53	-0.10073	0.035	0.23	3.59E-49
UBE4A	2.45E-53	-0.1854	0.03	0.248	3.66E-49
MTHFD1L	2.56E-53	-0.23973	0.007	0.22	3.82E-49
LONRF1	2.58E-53	-0.16386	0.023	0.225	3.85E-49
USP42	2.63E-53	-0.22462	0.026	0.251	3.92E-49
SC5D	2.86E-53	0.28413	0.087	0.21	4.27E-49
ABCF3	3.01E-53	-0.15167	0.031	0.244	4.50E-49
NT5C2	3.03E-53	0.112501	0.044	0.179	4.53E-49
GLRX2	3.16E-53	-0.26341	0.021	0.258	4.72E-49
LPGAT1	3.22E-53	-0.14387	0.023	0.228	4.81E-49
DFNA5	3.28E-53	0.196914	0.079	0.221	4.91E-49
FAM122A	3.44E-53	-0.28063	0.017	0.252	5.14E-49
NACC2	3.62E-53	-0.1205	0.019	0.207	5.40E-49
LIMCH1	3.90E-53	0.116002	0.072	0.232	5.83E-49
TUBGCP3	4.16E-53	-0.27706	0.026	0.264	6.22E-49
MPP5	4.33E-53	-0.15287	0.016	0.211	6.47E-49
ZBTB44	4.44E-53	-0.14085	0.035	0.242	6.63E-49
PGBD1	4.60E-53	-0.18132	0.017	0.22	6.88E-49
GUCD1	4.81E-53	-0.22209	0.024	0.256	7.18E-49
MPND	5.18E-53	-0.17162	0.01	0.204	7.74E-49
CDK19	5.36E-53	-0.21021	0.024	0.244	8.01E-49
CNOT11	5.75E-53	-0.19353	0.023	0.24	8.59E-49
NOA1	6.30E-53	-0.2368	0.04	0.28	9.41E-49
NBPF10	7.09E-53	-0.22489	0.035	0.264	1.06E-48
MDP1	7.29E-53	0.136395	0.051	0.188	1.09E-48
CLN8	7.96E-53	0.145532	0.068	0.215	1.19E-48
SLC2A8	8.94E-53	-0.20675	0.028	0.252	1.34E-48
PLEKHF2	9.06E-53	-0.20752	0.021	0.244	1.35E-48
ELP3	9.33E-53	-0.16657	0.03	0.242	1.39E-48
FOSB	9.77E-53	0.340412	0.157	0.28	1.46E-48
UBXN11	9.87E-53	0.196714	0.07	0.199	1.48E-48

ORMDL3	1.11E-52	-0.1235	0.035	0.236	1.66E-48
TMEM53	1.14E-52	0.203782	0.045	0.151	1.71E-48
FAM222B	1.23E-52	-0.10732	0.019	0.196	1.83E-48
ZNF672	1.28E-52	-0.2865	0.017	0.258	1.91E-48
QRSL1	1.29E-52	-0.22198	0.028	0.259	1.92E-48
DNAL4	1.31E-52	-0.22068	0.031	0.262	1.96E-48
DDX55	1.33E-52	-0.1014	0.038	0.241	1.98E-48
AGPS	1.38E-52	-0.11484	0.023	0.212	2.06E-48
KLC2	1.47E-52	-0.23943	0.014	0.23	2.20E-48
FBXO44	1.57E-52	0.162555	0.058	0.19	2.35E-48
ZDHHC7	1.59E-52	-0.13309	0.033	0.233	2.37E-48
KIFAP3	1.70E-52	-0.17007	0.04	0.26	2.54E-48
RASA1	1.83E-52	-0.10664	0.021	0.196	2.73E-48
TP53I3	1.84E-52	-0.21382	0.033	0.264	2.74E-48
ABHD8	2.04E-52	-0.12098	0.021	0.209	3.04E-48
SLC25A19	2.07E-52	-0.12642	0.021	0.209	3.09E-48
MAP2K3	2.15E-52	-0.2541	0.016	0.241	3.22E-48
FAM76B	2.16E-52	-0.24136	0.026	0.257	3.23E-48
GSPT2	2.23E-52	-0.18116	0.028	0.24	3.33E-48
CASP4	2.24E-52	0.391048	0.066	0.115	3.35E-48
SEC14L2	2.36E-52	0.165459	0.052	0.202	3.53E-48
CARD8	2.39E-52	-0.13187	0.031	0.237	3.57E-48
FBXO28	2.48E-52	-0.10314	0.021	0.201	3.71E-48
NDC1	2.54E-52	-0.31014	0.009	0.242	3.79E-48
CHRNB1	2.75E-52	0.365883	0.08	0.167	4.11E-48
CA12	3.16E-52	0.319952	0.073	0.18	4.72E-48
SLC35A3	3.57E-52	-0.14341	0.031	0.236	5.33E-48
EXOSC6	3.62E-52	-0.14321	0.016	0.204	5.40E-48
NMI	3.78E-52	0.417766	0.079	0.17	5.65E-48
EFCAB2	3.87E-52	-0.19872	0.035	0.259	5.78E-48
NACA2	3.90E-52	0.63634	0.136	0.165	5.83E-48
OTUD7B	3.98E-52	0.118834	0.044	0.168	5.94E-48
SLC35D2	4.46E-52	-0.1301	0.017	0.202	6.66E-48
TAF3	4.68E-52	-0.28975	0.012	0.243	7.00E-48
PXN	4.76E-52	-0.16211	0.024	0.225	7.11E-48
SPPL2A	4.87E-52	-0.10823	0.042	0.248	7.28E-48
LPAR6	5.40E-52	0.128941	0.045	0.169	8.06E-48
POLR1C	5.77E-52	0.294403	0.068	0.177	8.62E-48
PITX1	5.99E-52	-0.27136	0.007	0.232	8.95E-48
TPPP3	6.24E-52	0.44868	0.101	0.198	9.32E-48
ATP13A3	7.39E-52	-0.16983	0.023	0.231	1.10E-47
CCNE2	7.54E-52	-0.55031	0.024	0.302	1.13E-47
ZNF880	7.81E-52	-0.11807	0.038	0.246	1.17E-47
GABARAPL1	8.42E-52	0.254666	0.098	0.22	1.26E-47
TSPAN14	8.86E-52	-0.15865	0.038	0.254	1.32E-47
TMEM100	9.05E-52	0.143227	0.115	0.264	1.35E-47
ARMCX2	9.96E-52	0.319609	0.08	0.177	1.49E-47
QSOX1	1.17E-51	-0.11433	0.033	0.236	1.74E-47
ANKRD9	1.17E-51	-0.30325	0.003	0.228	1.75E-47
KLHL12	1.23E-51	-0.15087	0.017	0.21	1.84E-47
TNFRSF21	1.30E-51	-0.12277	0.03	0.236	1.94E-47

EED	1.40E-51	-0.26304	0.017	0.244	2.10E-47
HPCAL1	1.49E-51	-0.17153	0.026	0.238	2.22E-47
ZNF512	1.52E-51	-0.11841	0.042	0.247	2.28E-47
AASDH	1.59E-51	-0.11974	0.065	0.29	2.37E-47
C16orf52	1.74E-51	-0.135	0.019	0.206	2.61E-47
TMEM121	1.87E-51	-0.11816	0.045	0.248	2.80E-47
HKR1	1.88E-51	0.132988	0.072	0.219	2.81E-47
CPVL	1.91E-51	0.162239	0.054	0.17	2.86E-47
MAP3K4	2.01E-51	-0.16259	0.012	0.201	3.01E-47
CBL	2.02E-51	-0.15383	0.017	0.215	3.02E-47
MYO19	2.06E-51	-0.10677	0.026	0.211	3.08E-47
TUBGCP4	2.10E-51	-0.22854	0.024	0.248	3.13E-47
CEP97	2.17E-51	-0.20136	0.023	0.236	3.24E-47
CAMK2B	2.22E-51	0.252563	0.066	0.191	3.31E-47
PRIM2	2.22E-51	-0.41423	0.01	0.262	3.32E-47
ST3GAL2	2.27E-51	-0.17637	0.019	0.223	3.40E-47
PPP3R1	2.28E-51	-0.19982	0.016	0.222	3.41E-47
ANKRD52	2.35E-51	-0.1332	0.014	0.193	3.51E-47
RFXAP	2.70E-51	-0.13983	0.024	0.217	4.04E-47
RALGAPB	2.81E-51	-0.18641	0.017	0.221	4.21E-47
TMEM25	2.82E-51	0.187086	0.063	0.186	4.22E-47
TCTA	3.14E-51	0.125289	0.07	0.23	4.70E-47
NRBP2	3.30E-51	-0.1091	0.035	0.222	4.93E-47
ALG6	3.32E-51	-0.14973	0.026	0.227	4.96E-47
LRRN1	3.50E-51	-0.30537	0.044	0.29	5.24E-47
PARVB	3.72E-51	-0.12846	0.009	0.181	5.55E-47
SKI	3.82E-51	-0.12656	0.019	0.21	5.70E-47
ITSN2	4.23E-51	-0.10088	0.026	0.216	6.33E-47
PPP2R2D	4.29E-51	-0.25507	0.01	0.231	6.41E-47
KALRN	4.32E-51	0.133437	0.04	0.159	6.46E-47
SLC4A4	4.40E-51	0.496986	0.105	0.168	6.57E-47
KLHL13	4.66E-51	-0.23427	0.021	0.246	6.96E-47
TCF7	4.70E-51	0.11283	0.066	0.219	7.03E-47
GTF3C1	5.16E-51	-0.11485	0.026	0.22	7.71E-47
RBL1	5.20E-51	-0.25483	0.009	0.217	7.77E-47
PCOLCE	5.45E-51	0.121083	0.04	0.169	8.14E-47
ANAPC2	5.53E-51	-0.13654	0.031	0.236	8.26E-47
IFT46	5.54E-51	-0.11141	0.037	0.238	8.27E-47
ABHD17C	5.71E-51	-0.20377	0.01	0.207	8.53E-47
EYA2	5.76E-51	-0.15025	0.026	0.227	8.61E-47
C17orf80	5.77E-51	-0.18459	0.014	0.215	8.62E-47
DSCC1	5.77E-51	-0.34704	0.014	0.252	8.63E-47
ATL3	6.23E-51	-0.13906	0.021	0.216	9.31E-47
PRR3	6.56E-51	-0.14502	0.023	0.219	9.80E-47
DPCD	6.56E-51	0.109123	0.061	0.228	9.81E-47
DIRAS3	6.99E-51	0.78289	0.147	0.144	1.04E-46
PPP2R5D	7.42E-51	-0.30073	0.009	0.241	1.11E-46
ECE1	7.56E-51	0.147679	0.051	0.18	1.13E-46
HELZ2	8.21E-51	0.10163	0.049	0.188	1.23E-46
FAM110A	8.24E-51	-0.29362	0.017	0.249	1.23E-46
SOCS4	9.07E-51	-0.15707	0.019	0.212	1.36E-46

TFCP2	1.11E-50	-0.16335	0.026	0.226	1.66E-46
POC1B	1.13E-50	-0.10512	0.021	0.205	1.69E-46
MON1B	1.31E-50	-0.12442	0.019	0.201	1.96E-46
BIVM	1.41E-50	-0.11715	0.026	0.217	2.11E-46
RGS2	1.58E-50	0.468191	0.136	0.227	2.36E-46
DIS3L	1.63E-50	-0.25206	0.019	0.246	2.43E-46
TMTC3	1.69E-50	-0.1981	0.016	0.215	2.52E-46
CLCN7	1.77E-50	-0.22655	0.023	0.241	2.64E-46
ZNF766	1.77E-50	-0.11416	0.037	0.236	2.65E-46
LPIN2	1.84E-50	-0.20442	0.017	0.232	2.75E-46
SNHG10	1.90E-50	-0.1733	0.026	0.232	2.83E-46
KCTD13	1.92E-50	-0.19137	0.04	0.267	2.87E-46
LLGL1	1.94E-50	-0.21087	0.016	0.217	2.90E-46
METTL16	1.99E-50	-0.11373	0.028	0.216	2.98E-46
AQP4	2.27E-50	0.709731	0.202	0.211	3.39E-46
SPAST	2.52E-50	-0.14875	0.026	0.223	3.77E-46
RMND5A	2.66E-50	-0.17974	0.031	0.237	3.97E-46
RECQL4	2.76E-50	-0.33509	0.005	0.232	4.12E-46
DNMT3A	2.80E-50	-0.10049	0.023	0.209	4.19E-46
C9orf69	2.88E-50	-0.15897	0.016	0.21	4.30E-46
ATF3	2.99E-50	0.359726	0.108	0.228	4.47E-46
PARN	3.04E-50	-0.2287	0.023	0.241	4.54E-46
KLHL22	3.06E-50	-0.14817	0.024	0.221	4.58E-46
HSF2	3.07E-50	-0.30399	0.023	0.257	4.59E-46
RFTN1	3.13E-50	-0.14567	0.016	0.198	4.68E-46
PLEKHM2	3.27E-50	0.149771	0.037	0.142	4.89E-46
SLC35E2B	3.29E-50	-0.21392	0.031	0.249	4.91E-46
ARID1B	3.40E-50	-0.22938	0.021	0.235	5.08E-46
DBF4B	3.95E-50	-0.302	0.005	0.231	5.91E-46
MFI2-AS1	4.04E-50	-0.21669	0.024	0.241	6.04E-46
ATL1	4.55E-50	-0.13674	0.042	0.246	6.80E-46
LTA4H	4.71E-50	0.203409	0.075	0.205	7.03E-46
RP11-698N	5.18E-50	0.108833	0.045	0.193	7.75E-46
GTPBP1	6.57E-50	-0.29632	0.014	0.24	9.82E-46
DDX11	6.86E-50	-0.31288	0.012	0.24	1.03E-45
VSTM2A	7.32E-50	1.093902	0.237	0.079	1.09E-45
GS1-124K5	7.33E-50	0.160542	0.061	0.194	1.10E-45
OTUD5	7.63E-50	-0.12133	0.026	0.216	1.14E-45
BTN3A2	7.63E-50	0.443814	0.084	0.151	1.14E-45
SEMA5A	7.83E-50	0.176081	0.068	0.2	1.17E-45
ZNF263	8.57E-50	-0.10963	0.03	0.221	1.28E-45
FBXW7	8.65E-50	-0.28851	0.024	0.262	1.29E-45
NOL3	9.08E-50	0.366937	0.086	0.156	1.36E-45
ARL4D	9.97E-50	-0.17312	0.042	0.257	1.49E-45
LIMD1	1.02E-49	-0.1765	0.019	0.214	1.52E-45
TESK1	1.03E-49	-0.12959	0.012	0.185	1.54E-45
EXO1	1.08E-49	-0.4107	0.002	0.242	1.61E-45
TTC28	1.08E-49	-0.19347	0.023	0.23	1.62E-45
ZNF100	1.11E-49	-0.13361	0.033	0.232	1.67E-45
STK11	1.13E-49	-0.25941	0.01	0.226	1.69E-45
SNRPN	1.19E-49	1.032861	0.18	0.078	1.77E-45

ACAD9	1.26E-49	-0.1443	0.021	0.209	1.88E-45
TUBG2	1.34E-49	0.145785	0.044	0.16	2.00E-45
RARRES2	1.39E-49	0.416469	0.176	0.277	2.08E-45
CEP104	1.51E-49	-0.10962	0.021	0.199	2.25E-45
RICTOR	1.54E-49	0.152278	0.106	0.252	2.30E-45
RAD54L	1.66E-49	-0.38683	0	0.231	2.49E-45
CBX4	1.67E-49	-0.15855	0.03	0.225	2.50E-45
ALG2	1.71E-49	-0.16809	0.028	0.23	2.55E-45
SPTY2D1	1.79E-49	-0.10371	0.026	0.21	2.68E-45
CTSC	1.85E-49	0.23282	0.073	0.202	2.76E-45
CARKD	1.85E-49	-0.15416	0.035	0.236	2.77E-45
DCUN1D4	1.86E-49	-0.12878	0.037	0.233	2.79E-45
RHOQ	2.21E-49	-0.24118	0.012	0.226	3.30E-45
TNFAIP8L1	2.24E-49	-0.34911	0.019	0.258	3.34E-45
ADCK2	2.29E-49	-0.19049	0.01	0.204	3.42E-45
ABHD17B	2.43E-49	-0.15865	0.03	0.226	3.63E-45
HBP1	2.43E-49	0.249139	0.092	0.217	3.64E-45
CDC42BPB	2.84E-49	-0.16073	0.017	0.209	4.25E-45
TECPR1	2.99E-49	-0.10269	0.031	0.211	4.47E-45
ATP6V1A	3.10E-49	-0.25073	0.016	0.231	4.63E-45
CTB-3102C	3.56E-49	-0.11939	0.038	0.233	5.31E-45
HSDL1	3.65E-49	-0.20295	0.024	0.238	5.45E-45
CERK	3.88E-49	-0.28482	0.009	0.23	5.80E-45
STMN4	3.96E-49	0.174404	0.204	0.373	5.92E-45
ZCCHC8	4.14E-49	-0.15504	0.023	0.216	6.19E-45
TDP1	4.39E-49	-0.31356	0.01	0.242	6.57E-45
PQLC2	4.43E-49	-0.12516	0.023	0.207	6.62E-45
SLC35F2	4.58E-49	-0.23415	0.014	0.221	6.84E-45
SAV1	4.67E-49	-0.19286	0.021	0.223	6.97E-45
DGAT1	5.10E-49	-0.16987	0.012	0.2	7.62E-45
MYC	5.15E-49	0.499847	0.124	0.185	7.70E-45
DNAJC18	5.59E-49	-0.13581	0.042	0.249	8.35E-45
MSH3	5.70E-49	-0.24372	0.01	0.221	8.52E-45
RNF214	5.71E-49	-0.11782	0.024	0.214	8.53E-45
FGF14	5.77E-49	0.115997	0.04	0.156	8.62E-45
NVL	5.92E-49	-0.10233	0.051	0.254	8.85E-45
NRIP1	5.94E-49	-0.24686	0.009	0.216	8.87E-45
ATF5	5.99E-49	-0.15272	0.028	0.227	8.95E-45
SLC2A3	6.32E-49	0.364087	0.092	0.178	9.44E-45
MOGS	6.42E-49	-0.20472	0.017	0.22	9.59E-45
TBC1D17	7.98E-49	0.218104	0.065	0.177	1.19E-44
CCDC93	8.05E-49	-0.18743	0.028	0.238	1.20E-44
PAK3	8.69E-49	-0.12506	0.049	0.233	1.30E-44
KIAA0586	8.69E-49	-0.1855	0.024	0.226	1.30E-44
TCEAL2	8.77E-49	0.114857	0.15	0.309	1.31E-44
NEURL1B	9.88E-49	-0.39981	0.007	0.246	1.48E-44
MANEA	9.91E-49	-0.15926	0.014	0.194	1.48E-44
FOXN2	1.00E-48	-0.13974	0.031	0.226	1.49E-44
MTF1	1.04E-48	-0.15978	0.021	0.216	1.55E-44
UGGT2	1.04E-48	-0.11228	0.024	0.206	1.55E-44
CREBL2	1.11E-48	0.166102	0.047	0.169	1.65E-44

USP15	1.19E-48	-0.13764	0.07	0.293	1.77E-44
CAHM	1.25E-48	-0.20675	0.026	0.233	1.86E-44
CTC-524C5	1.27E-48	-0.24647	0.012	0.221	1.91E-44
IKBKG	1.38E-48	-0.17772	0.021	0.225	2.07E-44
PPP1R12C	1.39E-48	-0.21816	0.007	0.207	2.08E-44
NCBP1	1.44E-48	-0.22651	0.012	0.22	2.15E-44
RP11-472N	1.53E-48	-0.20104	0.017	0.216	2.28E-44
CRLF3	1.58E-48	-0.10616	0.033	0.215	2.37E-44
LIN7B	1.67E-48	-0.24029	0.016	0.231	2.50E-44
ERI2	1.78E-48	-0.22882	0.007	0.201	2.65E-44
ZBTB43	1.79E-48	-0.12796	0.037	0.237	2.68E-44
DEDD	1.98E-48	-0.17819	0.021	0.217	2.96E-44
ZBTB10	2.13E-48	-0.1048	0.033	0.214	3.19E-44
NXPH1	2.19E-48	-0.14216	0.072	0.284	3.27E-44
IRF2BP1	2.25E-48	-0.26951	0.016	0.24	3.35E-44
RP11-390E	2.27E-48	0.152216	0.075	0.216	3.40E-44
ZNF367	2.32E-48	-0.44645	0.003	0.238	3.47E-44
METTL1	2.44E-48	-0.16043	0.073	0.3	3.65E-44
SEH1L	2.80E-48	-0.17329	0.024	0.23	4.19E-44
CTD-3138E	2.82E-48	0.109027	0.024	0.138	4.21E-44
NELL2	2.96E-48	-0.22304	0.051	0.272	4.42E-44
CRISPLD1	3.06E-48	-0.28917	0.03	0.258	4.57E-44
CEND1	3.24E-48	0.156503	0.07	0.207	4.85E-44
CDC37L1	3.38E-48	-0.10072	0.033	0.221	5.06E-44
BRMS1L	3.71E-48	-0.18649	0.026	0.232	5.54E-44
TP53INP2	3.80E-48	-0.10519	0.01	0.165	5.67E-44
ADA	4.11E-48	-0.19434	0.009	0.196	6.15E-44
MESDC1	4.29E-48	-0.21789	0.014	0.217	6.41E-44
BBS7	4.47E-48	-0.15649	0.017	0.201	6.68E-44
ARL9	4.55E-48	-0.21426	0.017	0.219	6.80E-44
EFNB1	4.72E-48	-0.16524	0.033	0.227	7.05E-44
CBFA2T2	4.76E-48	-0.18807	0.023	0.223	7.11E-44
SLC18B1	5.79E-48	0.195527	0.079	0.219	8.65E-44
METTL3	5.91E-48	0.17698	0.079	0.209	8.84E-44
HLA-DPB1	5.93E-48	0.534745	0.155	0.202	8.87E-44
RYBP	6.09E-48	-0.13244	0.021	0.201	9.09E-44
RBM33	6.21E-48	-0.19247	0.031	0.241	9.29E-44
NRDE2	6.55E-48	0.127213	0.051	0.184	9.78E-44
ICK	6.57E-48	-0.1318	0.016	0.189	9.82E-44
ZNF768	6.61E-48	-0.16669	0.021	0.216	9.88E-44
CEP44	7.38E-48	-0.1705	0.016	0.207	1.10E-43
RP5-1085F	7.45E-48	-0.11347	0.019	0.193	1.11E-43
HIST1H1E	7.81E-48	-0.18671	0.01	0.188	1.17E-43
AC009506.	8.51E-48	-0.26345	0.035	0.253	1.27E-43
NUP205	8.54E-48	-0.13354	0.019	0.196	1.28E-43
LAMB2	8.65E-48	0.202615	0.044	0.142	1.29E-43
INPP5K	9.05E-48	-0.13933	0.021	0.205	1.35E-43
FBLN7	9.09E-48	-0.32316	0.003	0.225	1.36E-43
LACTB2	9.13E-48	0.10738	0.051	0.199	1.36E-43
DIP2B	1.02E-47	-0.11797	0.024	0.202	1.52E-43
DOLPP1	1.03E-47	-0.13869	0.026	0.216	1.54E-43

FKBP1B	1.03E-47	-0.15815	0.01	0.194	1.55E-43
OXSR1	1.25E-47	-0.11087	0.021	0.199	1.87E-43
ADM	1.28E-47	0.484352	0.112	0.204	1.91E-43
ARFGEF1	1.33E-47	-0.23361	0.009	0.216	1.99E-43
CDKAL1	1.39E-47	-0.14325	0.026	0.216	2.07E-43
STK3	1.96E-47	0.155062	0.047	0.165	2.92E-43
PEX11B	1.98E-47	0.248304	0.054	0.168	2.96E-43
TIMM8A	2.15E-47	-0.11204	0.024	0.209	3.21E-43
EMID1	2.28E-47	-0.13839	0.017	0.193	3.40E-43
CRADD	2.38E-47	-0.12711	0.019	0.196	3.56E-43
MIB1	2.48E-47	-0.22446	0.016	0.222	3.71E-43
LRRC4C	2.87E-47	0.197791	0.077	0.195	4.28E-43
BDH2	2.96E-47	0.356459	0.063	0.132	4.42E-43
DCAF6	2.99E-47	-0.10921	0.023	0.195	4.47E-43
FAH	3.12E-47	0.185697	0.03	0.135	4.66E-43
SLPI	3.34E-47	1.646678	0.202	0.037	4.99E-43
ELAVL4	3.44E-47	-0.11128	0.113	0.312	5.14E-43
UBTD1	3.45E-47	-0.15827	0.017	0.202	5.15E-43
NCOA3	4.29E-47	-0.17908	0.03	0.231	6.42E-43
DOT1L	4.43E-47	-0.26087	0.012	0.225	6.63E-43
TSC22D2	5.04E-47	-0.17511	0.023	0.22	7.53E-43
HEG1	5.24E-47	-0.11715	0.014	0.188	7.84E-43
XPO7	5.63E-47	-0.15113	0.026	0.215	8.42E-43
MASTL	5.82E-47	-0.43686	0	0.22	8.70E-43
DDX3Y	6.21E-47	-0.15956	0.014	0.194	9.28E-43
TP53	6.31E-47	-0.12635	0.04	0.227	9.44E-43
ZNF93	6.41E-47	-0.20881	0.01	0.202	9.58E-43
AMOTL1	7.10E-47	-0.14331	0.014	0.181	1.06E-42
ZNF567	8.67E-47	-0.12264	0.028	0.216	1.30E-42
GTDC1	8.75E-47	-0.16626	0.03	0.225	1.31E-42
FREM2	8.83E-47	-0.25757	0.012	0.227	1.32E-42
ZNF362	8.99E-47	-0.14008	0.026	0.207	1.34E-42
TSTD1	9.37E-47	0.324569	0.105	0.221	1.40E-42
SNAP25	1.05E-46	0.173963	0.087	0.219	1.57E-42
ATP13A2	1.07E-46	-0.13655	0.03	0.227	1.60E-42
E2F3	1.11E-46	-0.33165	0.009	0.23	1.66E-42
MGST1	1.20E-46	1.268913	0.195	0.052	1.79E-42
RP11-538F	1.21E-46	-0.1543	0.024	0.212	1.81E-42
BPNT1	1.25E-46	0.148693	0.054	0.177	1.86E-42
IQCE	1.33E-46	-0.1606	0.03	0.222	1.99E-42
ZNF775	1.35E-46	-0.18966	0.03	0.225	2.02E-42
PARD6A	1.39E-46	-0.1079	0.019	0.194	2.08E-42
FAM160A2	1.64E-46	-0.13852	0.023	0.2	2.46E-42
CCDC15	1.73E-46	-0.36739	0.005	0.232	2.59E-42
OSBP	1.87E-46	-0.11295	0.016	0.178	2.79E-42
CHAC2	2.13E-46	-0.27592	0.007	0.21	3.18E-42
MTSS1L	2.17E-46	0.178966	0.059	0.16	3.24E-42
OTUD4	2.20E-46	-0.27483	0.01	0.231	3.29E-42
RPRD1B	2.27E-46	-0.21803	0.014	0.215	3.40E-42
RP5-940J5	2.53E-46	0.58397	0.122	0.169	3.78E-42
CSPG5	2.69E-46	0.298562	0.126	0.243	4.02E-42

RMI1	2.85E-46	-0.27337	0.023	0.237	4.26E-42
E2F5	3.03E-46	-0.18019	0.007	0.193	4.53E-42
ROM1	3.05E-46	0.352948	0.061	0.127	4.55E-42
METTL6	3.11E-46	-0.14	0.024	0.206	4.64E-42
UTRN	3.36E-46	-0.17002	0.009	0.185	5.02E-42
BUD13	3.36E-46	-0.21352	0.021	0.221	5.02E-42
MVP	3.39E-46	0.151025	0.038	0.144	5.06E-42
SMTN	3.44E-46	-0.281	0.009	0.214	5.15E-42
FAM168A	3.81E-46	-0.24799	0.014	0.228	5.69E-42
MYO5A	4.10E-46	-0.13593	0.017	0.186	6.13E-42
NNMT	4.17E-46	0.622515	0.124	0.17	6.23E-42
ZMIZ1	4.44E-46	-0.10526	0.033	0.211	6.63E-42
ANXA1	4.68E-46	0.893717	0.286	0.198	7.00E-42
PKD2	4.79E-46	-0.16544	0.012	0.188	7.16E-42
CTTNBP2NL	4.86E-46	-0.19346	0.017	0.211	7.26E-42
CSTF2T	5.57E-46	-0.25368	0.023	0.235	8.33E-42
RAPGEF1	6.03E-46	0.138097	0.028	0.13	9.01E-42
SORBS1	6.33E-46	-0.16484	0.019	0.207	9.46E-42
NSUN2	6.53E-46	-0.13029	0.026	0.205	9.76E-42
NECAB3	6.82E-46	-0.11909	0.021	0.195	1.02E-41
SLC25A14	7.02E-46	-0.11943	0.024	0.202	1.05E-41
POMZP3	7.51E-46	-0.33155	0.014	0.243	1.12E-41
SIN3A	7.57E-46	-0.13934	0.023	0.204	1.13E-41
HAUS5	7.75E-46	-0.2368	0.017	0.223	1.16E-41
RASGRP1	8.41E-46	-0.1318	0.009	0.17	1.26E-41
ZNF266	8.84E-46	-0.17221	0.026	0.216	1.32E-41
IL13RA1	8.97E-46	-0.14654	0.007	0.17	1.34E-41
ENKD1	1.00E-45	-0.1622	0.014	0.194	1.50E-41
ELK4	1.05E-45	-0.12378	0.021	0.195	1.56E-41
CARM1	1.08E-45	-0.13547	0.021	0.193	1.61E-41
TAB2	1.13E-45	-0.13944	0.016	0.193	1.69E-41
SHOX2	1.20E-45	-0.13643	0.014	0.184	1.80E-41
TXNRD2	1.30E-45	-0.24621	0.016	0.226	1.94E-41
PPP1R9B	1.41E-45	-0.12873	0.019	0.189	2.11E-41
AC004381.	1.63E-45	-0.41649	0.007	0.228	2.44E-41
SCFD2	1.70E-45	-0.21088	0.026	0.227	2.54E-41
KIAA1841	1.80E-45	-0.19315	0.019	0.21	2.69E-41
NR2C1	1.81E-45	-0.11781	0.023	0.195	2.70E-41
RP11-421L	1.82E-45	0.121991	0.04	0.162	2.72E-41
DHX38	2.12E-45	-0.1626	0.023	0.209	3.17E-41
YARS2	2.13E-45	-0.14376	0.019	0.196	3.18E-41
PM20D2	2.15E-45	-0.13509	0.016	0.188	3.21E-41
FBXO25	2.24E-45	-0.13347	0.03	0.211	3.35E-41
IMPA1	2.38E-45	0.100742	0.066	0.211	3.56E-41
LRRN3	2.58E-45	0.133683	0.072	0.206	3.85E-41
ENKUR	2.60E-45	0.300851	0.065	0.146	3.88E-41
B4GALT2	2.71E-45	-0.1136	0.014	0.172	4.05E-41
STK33	2.92E-45	0.119409	0.035	0.151	4.36E-41
XPNPEP3	2.98E-45	-0.13319	0.026	0.207	4.45E-41
MORC4	3.08E-45	-0.29433	0.012	0.226	4.61E-41
LDOC1L	3.25E-45	-0.21988	0.014	0.209	4.86E-41

LRRC8B	3.26E-45	-0.15693	0.012	0.183	4.88E-41
RNF38	3.28E-45	-0.16238	0.01	0.183	4.90E-41
AEN	3.34E-45	-0.24311	0.028	0.242	4.99E-41
FKBP11	3.41E-45	0.168078	0.047	0.158	5.09E-41
LRP6	3.47E-45	-0.11172	0.033	0.214	5.18E-41
RASSF8	3.58E-45	-0.13315	0.021	0.199	5.35E-41
KIF1C	3.68E-45	-0.14502	0.017	0.194	5.49E-41
FGFR1OP	3.69E-45	-0.24834	0.017	0.227	5.51E-41
DGCR8	3.75E-45	-0.19244	0.028	0.228	5.61E-41
THAP10	3.86E-45	-0.14687	0.016	0.191	5.77E-41
TERF2	3.89E-45	-0.12791	0.024	0.199	5.81E-41
UBQLN4	4.19E-45	-0.19214	0.03	0.235	6.26E-41
SCAPER	4.25E-45	-0.11359	0.037	0.225	6.35E-41
CAPN7	4.72E-45	-0.11497	0.021	0.194	7.05E-41
TOE1	4.97E-45	-0.16857	0.019	0.204	7.42E-41
CREG1	5.94E-45	-0.12684	0.024	0.2	8.88E-41
AGPAT2	6.02E-45	-0.29521	0.007	0.222	9.00E-41
ARMC9	6.07E-45	0.11273	0.042	0.154	9.06E-41
UPF1	6.13E-45	-0.10108	0.019	0.184	9.16E-41
ZNF274	6.22E-45	-0.14386	0.042	0.23	9.30E-41
SRSF12	6.46E-45	-0.21893	0.017	0.214	9.66E-41
WDHD1	6.75E-45	-0.28199	0.012	0.216	1.01E-40
TRPM3	6.77E-45	0.126515	0.03	0.138	1.01E-40
ELMSAN1	6.90E-45	-0.11925	0.026	0.204	1.03E-40
MAFG-AS1	6.96E-45	-0.1494	0.009	0.174	1.04E-40
MMGT1	6.99E-45	-0.13461	0.019	0.194	1.05E-40
PINX1	7.34E-45	-0.11775	0.028	0.201	1.10E-40
RFX3	7.90E-45	-0.16024	0.017	0.2	1.18E-40
BAG4	9.33E-45	-0.14645	0.021	0.205	1.39E-40
GMEB1	9.47E-45	-0.12674	0.028	0.211	1.42E-40
RYK	9.59E-45	-0.17993	0.014	0.199	1.43E-40
ANTXR1	1.02E-44	-0.13062	0.037	0.221	1.52E-40
PLA2G5	1.03E-44	0.85859	0.157	0.13	1.54E-40
SLC25A44	1.07E-44	-0.26289	0.009	0.209	1.59E-40
HPS3	1.07E-44	-0.17442	0.016	0.196	1.60E-40
FN1	1.18E-44	0.287468	0.066	0.164	1.77E-40
SLC20A2	1.21E-44	0.310385	0.063	0.141	1.80E-40
IGFBP4	1.29E-44	-0.24448	0.017	0.219	1.93E-40
ZNF253	1.43E-44	-0.12085	0.019	0.181	2.14E-40
GNA13	1.50E-44	-0.18275	0.009	0.184	2.25E-40
MFN2	1.54E-44	-0.10174	0.028	0.204	2.30E-40
SLC38A6	1.75E-44	0.206616	0.045	0.14	2.61E-40
BORA	1.77E-44	-0.40191	0.005	0.228	2.64E-40
LIFR	1.79E-44	0.130555	0.072	0.214	2.68E-40
PDE7A	1.84E-44	-0.2013	0.017	0.21	2.76E-40
RGS3	1.86E-44	-0.12071	0.026	0.199	2.78E-40
STOM	1.98E-44	0.303773	0.061	0.142	2.96E-40
GPD2	2.02E-44	-0.14331	0.023	0.195	3.02E-40
RP13-1032	2.07E-44	-0.2033	0.016	0.204	3.09E-40
STXBP1	2.34E-44	-0.16357	0.03	0.22	3.50E-40
FER	2.39E-44	-0.10239	0.023	0.19	3.57E-40

TMOD1	2.54E-44	0.630561	0.129	0.158	3.80E-40
AKAP6	2.58E-44	0.214468	0.052	0.157	3.85E-40
GPX3	2.73E-44	0.194111	0.035	0.121	4.08E-40
COG1	3.05E-44	-0.15144	0.017	0.194	4.56E-40
ELK1	3.19E-44	-0.17159	0.012	0.19	4.76E-40
DLC1	3.69E-44	0.13722	0.038	0.149	5.51E-40
PCGF6	3.88E-44	-0.16319	0.019	0.201	5.80E-40
FOSL2	3.98E-44	-0.11815	0.028	0.204	5.95E-40
ERC1	4.03E-44	-0.10083	0.028	0.199	6.03E-40
BBS4	4.09E-44	0.126253	0.045	0.158	6.11E-40
MAPK9	4.17E-44	-0.14106	0.017	0.189	6.23E-40
CEP112	4.32E-44	-0.24569	0.007	0.207	6.45E-40
RP11-126K	4.38E-44	-0.12756	0.024	0.195	6.54E-40
MYO1C	4.53E-44	-0.1573	0.019	0.194	6.77E-40
PHF13	4.57E-44	-0.14237	0.017	0.189	6.83E-40
LMCD1	4.63E-44	-0.15941	0.014	0.186	6.92E-40
KATNAL1	4.99E-44	-0.23057	0.017	0.221	7.45E-40
ST3GAL3	5.83E-44	-0.22647	0.017	0.215	8.71E-40
C1QC	6.09E-44	0.331892	0.117	0.205	9.10E-40
RMND1	6.53E-44	-0.17949	0.017	0.201	9.76E-40
RAI1	7.17E-44	-0.12405	0.009	0.163	1.07E-39
SCMH1	7.33E-44	-0.20584	0.019	0.211	1.10E-39
ACACA	7.51E-44	-0.13996	0.017	0.185	1.12E-39
SS18L1	7.54E-44	-0.17193	0.014	0.198	1.13E-39
RAB23	8.38E-44	-0.20277	0.01	0.194	1.25E-39
GAD1	8.40E-44	-0.29668	0.017	0.23	1.26E-39
GBP1	8.71E-44	0.814243	0.14	0.133	1.30E-39
GAB1	8.89E-44	-0.14964	0.03	0.214	1.33E-39
PDLIM3	9.90E-44	0.278362	0.115	0.21	1.48E-39
CSRNP2	9.96E-44	-0.10309	0.024	0.195	1.49E-39
NXT2	1.16E-43	-0.13394	0.014	0.178	1.73E-39
MDC1	1.18E-43	-0.2575	0.012	0.216	1.76E-39
PELI1	1.18E-43	-0.20821	0.045	0.243	1.77E-39
NUP155	1.19E-43	-0.15739	0.026	0.206	1.77E-39
CDC7	1.19E-43	-0.25508	0.019	0.227	1.78E-39
MTFR2	1.31E-43	-0.38661	0	0.205	1.95E-39
CHEK2	1.33E-43	-0.3156	0.01	0.221	1.98E-39
SP2	1.34E-43	-0.11315	0.021	0.189	2.01E-39
IGF1R	1.55E-43	0.115459	0.035	0.148	2.31E-39
MBD6	1.68E-43	-0.40981	0.051	0.298	2.52E-39
ASUN	1.77E-43	-0.17045	0.026	0.219	2.65E-39
MIR210HG	1.81E-43	0.101339	0.04	0.146	2.71E-39
FBXO34	1.84E-43	-0.17484	0.021	0.204	2.76E-39
PSMB10	1.86E-43	0.278567	0.072	0.162	2.78E-39
ZNF587	1.90E-43	-0.1108	0.021	0.186	2.83E-39
ZNF789	2.07E-43	-0.10597	0.019	0.178	3.10E-39
ALG12	2.31E-43	-0.14691	0.016	0.188	3.46E-39
ITGA2	2.34E-43	-0.28064	0.007	0.2	3.49E-39
HSD17B7	2.48E-43	0.251346	0.056	0.137	3.71E-39
KIAA1549	2.54E-43	-0.28593	0.024	0.237	3.80E-39
NME6	2.98E-43	0.183247	0.056	0.172	4.46E-39

CCDC77	3.56E-43	-0.20618	0.026	0.223	5.32E-39
NDRG4	3.86E-43	0.209308	0.072	0.183	5.77E-39
PTK2B	4.18E-43	0.105387	0.026	0.132	6.25E-39
DPY19L3	4.21E-43	0.138275	0.044	0.149	6.28E-39
HYOU1	4.60E-43	-0.13197	0.03	0.214	6.88E-39
RPUSD4	5.27E-43	-0.10999	0.019	0.18	7.87E-39
RP11-398K	5.27E-43	-0.12923	0.026	0.199	7.88E-39
PRRC1	5.36E-43	-0.12957	0.028	0.205	8.01E-39
LIPE-AS1	6.00E-43	-0.10582	0.024	0.193	8.96E-39
WHAMM	6.10E-43	-0.14611	0.019	0.198	9.11E-39
TRIM26	6.43E-43	-0.19547	0.024	0.211	9.61E-39
CDH4	6.52E-43	-0.20645	0.016	0.202	9.74E-39
PI4K2B	6.64E-43	-0.20872	0.014	0.206	9.92E-39
NUP153	6.71E-43	-0.10659	0.019	0.175	1.00E-38
ALDOC	7.34E-43	0.899471	0.188	0.102	1.10E-38
RALGAPA1	7.84E-43	-0.13363	0.028	0.206	1.17E-38
PYGL	8.09E-43	0.250445	0.056	0.147	1.21E-38
UCK2	8.62E-43	-0.10309	0.023	0.186	1.29E-38
APEX2	8.66E-43	-0.11455	0.016	0.178	1.29E-38
ASIC4	9.05E-43	0.270258	0.129	0.215	1.35E-38
RP11-25K1	9.07E-43	-0.16369	0.016	0.189	1.35E-38
INTS5	9.16E-43	-0.11417	0.019	0.183	1.37E-38
PCOLCE2	1.01E-42	0.292956	0.058	0.133	1.51E-38
ADNP2	1.06E-42	-0.16228	0.012	0.183	1.59E-38
PKP4	1.08E-42	-0.22731	0.012	0.204	1.61E-38
CTD-3184A	1.17E-42	-0.18428	0.007	0.179	1.74E-38
B3GNT5	1.19E-42	-0.2854	0.007	0.204	1.78E-38
BAMBI	1.31E-42	-0.19929	0.016	0.196	1.96E-38
CCDC159	1.49E-42	0.231517	0.065	0.154	2.23E-38
AMH	1.60E-42	-0.26321	0.01	0.211	2.38E-38
WIPI1	1.73E-42	0.104401	0.03	0.126	2.58E-38
PCBD2	1.80E-42	-0.26543	0.012	0.209	2.68E-38
C5orf28	1.81E-42	0.165123	0.052	0.158	2.71E-38
AHCTF1	1.85E-42	-0.17162	0.019	0.204	2.76E-38
FAM173B	1.95E-42	-0.10477	0.017	0.169	2.92E-38
PARD3	2.01E-42	-0.14973	0.01	0.179	3.01E-38
L3MBTL2	2.28E-42	-0.19519	0.017	0.2	3.41E-38
ZP3	2.39E-42	-0.12192	0.024	0.185	3.57E-38
STAT2	2.43E-42	0.201787	0.052	0.141	3.63E-38
HLA-DRB5	2.45E-42	0.937014	0.138	0.07	3.67E-38
SFMBT1	2.52E-42	-0.20592	0.016	0.2	3.76E-38
GDAP1	2.79E-42	0.194454	0.096	0.221	4.17E-38
COL22A1	2.99E-42	-0.1436	0.016	0.175	4.47E-38
SYP	3.05E-42	0.140345	0.075	0.198	4.56E-38
RP11-539L	3.23E-42	0.151063	0.052	0.179	4.83E-38
FAM84B	3.28E-42	0.148533	0.065	0.19	4.91E-38
PHC1	3.33E-42	-0.15933	0.026	0.204	4.97E-38
ANGPTL2	3.43E-42	0.398364	0.099	0.153	5.12E-38
GKAP1	3.71E-42	-0.26409	0.016	0.211	5.55E-38
MLLT3	3.84E-42	-0.12843	0.03	0.201	5.73E-38
DDX39B	4.09E-42	-0.11996	0.035	0.206	6.11E-38

TRIM41	4.17E-42	-0.13283	0.03	0.204	6.23E-38
METAP1D	4.53E-42	0.124839	0.08	0.205	6.76E-38
GTF2IRD2	4.93E-42	0.116096	0.068	0.185	7.37E-38
ZNF436	4.97E-42	-0.22479	0.009	0.193	7.43E-38
RNF169	5.17E-42	-0.10863	0.014	0.169	7.73E-38
CCDC74A	6.04E-42	-0.22119	0.014	0.199	9.02E-38
CTHRC1	6.05E-42	-0.3153	0.005	0.211	9.03E-38
PNMAL1	6.16E-42	-0.16556	0.023	0.205	9.21E-38
MSTO1	6.39E-42	-0.23122	0.007	0.191	9.55E-38
DCBLD1	6.44E-42	-0.1489	0.023	0.202	9.62E-38
SH3BP4	6.44E-42	-0.10832	0.035	0.199	9.63E-38
SERINC2	6.51E-42	-0.29224	0.009	0.219	9.73E-38
C12orf4	6.58E-42	-0.15829	0.017	0.194	9.83E-38
TOR2A	6.61E-42	-0.19672	0.019	0.206	9.88E-38
RLIM	7.70E-42	-0.17419	0.023	0.206	1.15E-37
CCDC138	7.83E-42	-0.20489	0.01	0.186	1.17E-37
RNPEPL1	8.08E-42	-0.1327	0.019	0.181	1.21E-37
PRDM4	8.39E-42	-0.15277	0.012	0.181	1.25E-37
FAM219A	8.46E-42	-0.14623	0.017	0.188	1.26E-37
ZNF71	8.49E-42	-0.10961	0.021	0.181	1.27E-37
SLC26A11	9.11E-42	-0.10436	0.021	0.178	1.36E-37
SLC39A9	9.64E-42	-0.17147	0.016	0.19	1.44E-37
GUF1	9.80E-42	-0.12388	0.017	0.18	1.46E-37
NHSL1	1.07E-41	-0.10006	0.033	0.2	1.59E-37
KLF12	1.09E-41	-0.19049	0.026	0.206	1.63E-37
FAXC	1.11E-41	-0.16877	0.026	0.2	1.66E-37
CRTC3	1.13E-41	-0.10711	0.026	0.19	1.69E-37
CXXC4	1.16E-41	-0.16926	0.026	0.196	1.74E-37
LOXL1	1.23E-41	-0.16752	0.01	0.178	1.83E-37
TADA2A	1.36E-41	-0.22071	0.009	0.189	2.03E-37
TAF4	1.50E-41	-0.13142	0.01	0.169	2.24E-37
BACE1	1.50E-41	-0.10436	0.031	0.196	2.25E-37
ELMOD2	1.51E-41	0.108396	0.044	0.163	2.26E-37
BCL3	1.59E-41	-0.10404	0.009	0.148	2.38E-37
TRIM11	1.63E-41	-0.16291	0.021	0.196	2.44E-37
TRRAP	1.65E-41	-0.19364	0.021	0.206	2.46E-37
ALDH16A1	1.73E-41	-0.26416	0.01	0.209	2.58E-37
PWWP2A	1.84E-41	-0.15041	0.031	0.211	2.75E-37
PPP1R14C	1.88E-41	-0.22411	0.01	0.186	2.81E-37
TGIF2	2.05E-41	-0.27743	0.009	0.209	3.07E-37
SP110	2.10E-41	0.223302	0.061	0.165	3.14E-37
STIL	2.12E-41	-0.33204	0	0.195	3.16E-37
ZNF584	2.36E-41	-0.15546	0.012	0.181	3.53E-37
TRIM7	2.40E-41	-0.12513	0.003	0.147	3.59E-37
APC2	2.53E-41	0.184694	0.066	0.163	3.79E-37
ELOVL6	2.78E-41	-0.12532	0.023	0.189	4.15E-37
FBXO45	2.90E-41	-0.22689	0.005	0.184	4.33E-37
FAT1	2.94E-41	-0.1937	0.014	0.194	4.40E-37
ZNF532	3.07E-41	-0.12826	0.019	0.179	4.59E-37
RFX5	3.36E-41	-0.10713	0.026	0.183	5.02E-37
SEC16A	3.51E-41	-0.18011	0.024	0.206	5.25E-37

MIOS	3.60E-41	-0.11971	0.024	0.191	5.37E-37
ADD2	3.68E-41	-0.34274	0.017	0.235	5.51E-37
XRCC6BP1	4.32E-41	-0.41044	0.026	0.243	6.46E-37
TBC1D31	4.69E-41	-0.35872	0.002	0.205	7.01E-37
GEMIN4	4.72E-41	-0.10006	0.014	0.159	7.05E-37
CCDC150	4.77E-41	-0.22747	0.009	0.188	7.13E-37
FAM49A	4.80E-41	0.130631	0.038	0.141	7.18E-37
WDR3	5.20E-41	-0.12339	0.023	0.189	7.78E-37
POLG	5.31E-41	-0.15102	0.016	0.184	7.94E-37
SLC4A8	5.40E-41	-0.19483	0.023	0.209	8.06E-37
DCHS1	5.46E-41	-0.12992	0.024	0.196	8.16E-37
TIMP3	5.75E-41	0.356035	0.091	0.164	8.60E-37
CHST14	5.89E-41	-0.2542	0.009	0.205	8.79E-37
SUV39H1	6.01E-41	-0.2262	0.007	0.186	8.97E-37
TUBD1	6.11E-41	-0.19766	0.021	0.206	9.13E-37
TFRC	6.27E-41	0.163122	0.052	0.165	9.37E-37
RAD54L2	6.93E-41	-0.15456	0.024	0.2	1.04E-36
CCDC102B	7.20E-41	0.303494	0.063	0.137	1.08E-36
EML1	7.85E-41	-0.14379	0.016	0.178	1.17E-36
DCLRE1B	7.99E-41	-0.25849	0.012	0.204	1.19E-36
TFIP11	8.02E-41	-0.1029	0.026	0.189	1.20E-36
AP3M2	8.20E-41	-0.16827	0.017	0.191	1.23E-36
IRF7	8.28E-41	-0.10324	0.017	0.168	1.24E-36
MAPK14	8.36E-41	-0.15945	0.017	0.195	1.25E-36
TTC8	9.55E-41	0.140238	0.042	0.151	1.43E-36
TRAPPC11	1.06E-40	-0.18451	0.019	0.2	1.58E-36
DHX16	1.11E-40	-0.16769	0.016	0.188	1.65E-36
PLP1	1.16E-40	0.380306	0.133	0.217	1.74E-36
PRKAR1B	1.16E-40	-0.12181	0.04	0.215	1.74E-36
NOTCH3	1.17E-40	-0.12636	0.01	0.17	1.75E-36
SPICE1	1.18E-40	0.112687	0.021	0.109	1.76E-36
GTF3C4	1.22E-40	-0.17133	0.01	0.174	1.82E-36
C9orf40	1.24E-40	-0.25225	0.003	0.185	1.85E-36
LMO7	1.26E-40	-0.30638	0.009	0.205	1.89E-36
FBXO32	1.27E-40	0.547042	0.089	0.102	1.90E-36
HBB	1.36E-40	0.438996	0.202	0.332	2.03E-36
DUS4L	1.40E-40	-0.13621	0.019	0.181	2.10E-36
AHDC1	1.41E-40	0.192886	0.063	0.164	2.11E-36
TAOK2	1.42E-40	-0.22574	0.012	0.2	2.12E-36
ZSWIM6	1.58E-40	-0.16512	0.017	0.188	2.36E-36
TMEM168	1.64E-40	0.120828	0.056	0.178	2.44E-36
ADAMTS9	1.66E-40	0.185991	0.044	0.144	2.48E-36
UBFD1	1.96E-40	-0.15242	0.021	0.19	2.92E-36
POLE2	2.00E-40	-0.23096	0.012	0.19	2.99E-36
CPQ	2.03E-40	0.265488	0.047	0.107	3.03E-36
C8orf46	2.06E-40	-0.20909	0.044	0.236	3.08E-36
ZBTB2	2.27E-40	-0.12222	0.024	0.184	3.39E-36
RFNG	2.28E-40	-0.11656	0.024	0.185	3.41E-36
NUP43	2.35E-40	-0.23859	0.017	0.209	3.52E-36
NKAIN4	2.52E-40	0.17577	0.108	0.216	3.77E-36
DPH2	2.58E-40	0.163047	0.051	0.158	3.86E-36

CAMSAP1	2.89E-40	-0.11249	0.019	0.17	4.32E-36
ZW10	2.94E-40	-0.16378	0.017	0.185	4.39E-36
BOLA2B	3.03E-40	0.215789	0.037	0.116	4.52E-36
FAM13B	3.19E-40	-0.12706	0.019	0.174	4.77E-36
PRMT6	3.60E-40	-0.1125	0.03	0.196	5.38E-36
CCDC71	3.62E-40	-0.15911	0.014	0.178	5.40E-36
AMDHD2	3.73E-40	-0.14931	0.016	0.184	5.57E-36
DHODH	4.18E-40	0.117714	0.037	0.147	6.25E-36
FAM127C	4.48E-40	0.100986	0.04	0.149	6.70E-36
CNOT6L	4.91E-40	-0.13526	0.017	0.175	7.34E-36
ZNF724P	4.96E-40	-0.3202	0.012	0.216	7.41E-36
SH2B2	5.15E-40	-0.24847	0.019	0.209	7.70E-36
DYRK1A	5.20E-40	-0.18059	0.014	0.186	7.77E-36
KLHL9	5.36E-40	-0.24335	0.016	0.204	8.01E-36
SARNP	5.48E-40	0.204497	0.065	0.164	8.19E-36
URGCP	6.27E-40	-0.18761	0.031	0.216	9.37E-36
TXNL4B	6.50E-40	-0.13227	0.021	0.183	9.71E-36
RAP2C	7.61E-40	-0.15375	0.01	0.169	1.14E-35
FAM20B	7.65E-40	-0.15771	0.012	0.174	1.14E-35
POLD1	8.14E-40	-0.22741	0.012	0.191	1.22E-35
MDGA1	8.99E-40	-0.23618	0.005	0.189	1.34E-35
RC3H2	9.13E-40	-0.21361	0.007	0.184	1.36E-35
GEM	9.43E-40	0.194876	0.045	0.147	1.41E-35
SMCR5	9.50E-40	-0.15355	0.026	0.2	1.42E-35
PRMT7	1.07E-39	-0.18887	0.028	0.215	1.61E-35
IL17RB	1.10E-39	-0.17515	0.026	0.201	1.64E-35
ELP4	1.20E-39	0.208371	0.054	0.153	1.80E-35
PATZ1	1.26E-39	-0.17587	0.026	0.201	1.88E-35
MIAT	1.30E-39	0.100557	0.082	0.209	1.94E-35
RNFT2	1.38E-39	-0.21626	0.01	0.185	2.07E-35
IMMP2L	1.39E-39	0.173656	0.063	0.165	2.08E-35
SP100	1.46E-39	0.370706	0.098	0.163	2.19E-35
TBL1X	1.79E-39	-0.11978	0.005	0.148	2.67E-35
AFAP1	1.80E-39	-0.18445	0.016	0.185	2.69E-35
CSRP2BP	1.84E-39	-0.1437	0.019	0.183	2.76E-35
TMEM260	1.86E-39	-0.11937	0.016	0.167	2.78E-35
NFRKB	1.98E-39	-0.17472	0.014	0.179	2.96E-35
VAX2	2.01E-39	-0.1477	0.021	0.188	3.01E-35
XRCC3	2.06E-39	-0.16255	0.01	0.17	3.08E-35
NOS1AP	2.11E-39	0.219148	0.033	0.119	3.15E-35
PLAUR	2.18E-39	0.256315	0.068	0.162	3.26E-35
THSD7A	2.46E-39	-0.15175	0.068	0.249	3.67E-35
ABCA3	2.47E-39	-0.13952	0.021	0.189	3.69E-35
RPS6KA4	2.52E-39	-0.14513	0.014	0.177	3.77E-35
CHD3	2.59E-39	-0.17413	0.023	0.195	3.88E-35
FKBPL	2.63E-39	-0.21928	0.016	0.199	3.93E-35
PRMT3	2.75E-39	-0.21928	0.019	0.206	4.10E-35
LRRC57	2.85E-39	-0.15473	0.014	0.177	4.26E-35
ZNF598	2.90E-39	-0.19388	0.007	0.173	4.33E-35
HS1BP3	2.92E-39	0.104127	0.026	0.123	4.37E-35
HINFP	3.16E-39	0.152216	0.051	0.156	4.73E-35

PIP4K2C	3.24E-39	-0.25083	0.037	0.23	4.84E-35
SKP2	3.67E-39	-0.23437	0.01	0.189	5.48E-35
ABCC5	3.94E-39	0.174741	0.044	0.128	5.89E-35
ASXL2	4.12E-39	-0.14666	0.016	0.178	6.16E-35
GRAMD3	4.12E-39	0.337469	0.08	0.146	6.16E-35
FAM199X	4.28E-39	-0.14785	0.019	0.184	6.39E-35
CAPN5	4.35E-39	-0.10993	0.017	0.164	6.51E-35
LIPA	4.78E-39	0.135339	0.045	0.149	7.15E-35
PIK3C3	4.96E-39	-0.11411	0.023	0.178	7.42E-35
SMAD9	5.50E-39	-0.19762	0.017	0.198	8.22E-35
ATL2	5.76E-39	-0.11784	0.014	0.169	8.61E-35
CXCL14	5.79E-39	0.651362	0.106	0.101	8.65E-35
DLGAP1-AS	5.99E-39	0.148825	0.035	0.125	8.94E-35
HIST1H2BH	6.14E-39	-0.38068	0	0.184	9.17E-35
ZNF569	6.78E-39	-0.2017	0.016	0.188	1.01E-34
ZNF726	6.97E-39	-0.28437	0.007	0.194	1.04E-34
PCDH19	7.14E-39	-0.12761	0.016	0.177	1.07E-34
PIP5K1C	7.24E-39	-0.12698	0.03	0.202	1.08E-34
PPP3CC	7.49E-39	-0.2013	0.031	0.215	1.12E-34
DTNB	7.61E-39	-0.23026	0.009	0.191	1.14E-34
SS18	7.79E-39	0.142482	0.044	0.142	1.16E-34
RFX1	7.82E-39	-0.15158	0.016	0.179	1.17E-34
TMEM192	8.23E-39	0.168271	0.08	0.183	1.23E-34
PCGF3	8.85E-39	-0.10163	0.024	0.179	1.32E-34
UTP14C	8.89E-39	-0.10365	0.014	0.159	1.33E-34
PRKX	9.25E-39	-0.19818	0.01	0.177	1.38E-34
VSIG10	1.09E-38	-0.12701	0.021	0.178	1.62E-34
FANCB	1.11E-38	-0.23058	0.002	0.17	1.66E-34
AKAP10	1.13E-38	-0.12997	0.017	0.172	1.69E-34
ALDH18A1	1.20E-38	-0.14452	0.023	0.185	1.80E-34
TAF6L	1.22E-38	-0.21066	0.012	0.191	1.83E-34
PDGFD	1.28E-38	-0.11856	0.007	0.143	1.91E-34
SLC19A1	1.47E-38	-0.16752	0.007	0.159	2.20E-34
FRS2	1.48E-38	-0.19722	0.049	0.241	2.21E-34
PGGT1B	1.48E-38	0.100351	0.045	0.157	2.21E-34
BEST3	1.49E-38	-0.20277	0.094	0.307	2.23E-34
FAM91A1	1.57E-38	-0.1604	0.019	0.189	2.35E-34
CORO7	1.58E-38	-0.13498	0.028	0.189	2.37E-34
KTI12	1.61E-38	-0.16852	0.019	0.188	2.41E-34
GINS3	1.67E-38	-0.25039	0.012	0.193	2.50E-34
FBF1	1.84E-38	-0.13269	0.007	0.153	2.76E-34
PPP3CB	1.90E-38	-0.13348	0.019	0.18	2.83E-34
PYGO2	1.91E-38	-0.19332	0.009	0.174	2.85E-34
PIBF1	1.99E-38	-0.15357	0.023	0.188	2.97E-34
MKL2	2.02E-38	0.121912	0.028	0.123	3.02E-34
C1orf112	2.04E-38	-0.21173	0.01	0.181	3.05E-34
NKX2-2	2.16E-38	-0.2126	0.065	0.253	3.23E-34
HNRNPU-AS	2.29E-38	-0.12536	0.031	0.195	3.43E-34
CACNG8	2.42E-38	0.135093	0.026	0.116	3.61E-34
RBM12B	2.50E-38	-0.10477	0.023	0.172	3.73E-34
IFT80	2.58E-38	-0.12401	0.023	0.179	3.85E-34

CEP76	2.70E-38	-0.168	0.01	0.167	4.04E-34
ALKBH6	2.74E-38	-0.17289	0.017	0.188	4.10E-34
N4BP2L1	2.79E-38	0.284779	0.063	0.135	4.17E-34
CDK17	2.94E-38	-0.18458	0.028	0.207	4.40E-34
AL592183.	3.12E-38	0.187538	0.065	0.153	4.66E-34
WDTC1	3.25E-38	-0.10909	0.021	0.172	4.85E-34
TMEM150A	3.58E-38	0.35588	0.054	0.09	5.35E-34
KDELR3	3.60E-38	-0.1067	0.01	0.148	5.38E-34
CDKL3	3.64E-38	-0.10471	0.024	0.173	5.43E-34
LINC00844	3.87E-38	0.181056	0.084	0.19	5.78E-34
ZMYND19	3.88E-38	-0.18303	0.007	0.164	5.79E-34
ZNF787	4.36E-38	-0.17305	0.007	0.17	6.52E-34
SOWAHC	4.65E-38	-0.14802	0.014	0.168	6.94E-34
C5orf42	4.87E-38	-0.13427	0.021	0.181	7.28E-34
HOXA2	5.09E-38	-0.21204	0.031	0.188	7.61E-34
KIF3C	5.37E-38	-0.18607	0.017	0.18	8.03E-34
CCNE1	6.40E-38	-0.33957	0.002	0.186	9.57E-34
PEG10	6.74E-38	-0.14954	0.07	0.242	1.01E-33
CHRNA5	6.77E-38	-0.2391	0.009	0.186	1.01E-33
EPS8	6.97E-38	-0.25094	0.04	0.231	1.04E-33
CPSF1	7.50E-38	-0.15849	0.024	0.188	1.12E-33
RP11-563K	7.81E-38	-0.13651	0.019	0.177	1.17E-33
RP11-977G	8.00E-38	-0.10008	0.009	0.14	1.20E-33
ANXA4	8.22E-38	0.25842	0.058	0.136	1.23E-33
ARL5B	8.32E-38	-0.16517	0.007	0.165	1.24E-33
GSTCD	8.51E-38	-0.15605	0.009	0.164	1.27E-33
RP11-212F	9.40E-38	-0.13463	0.028	0.188	1.40E-33
HSD17B8	9.44E-38	0.201531	0.037	0.115	1.41E-33
LAMA4	1.02E-37	0.124025	0.037	0.143	1.52E-33
SLC2A13	1.03E-37	-0.11584	0.019	0.172	1.55E-33
ZC3H4	1.04E-37	-0.16099	0.024	0.193	1.56E-33
EML3	1.06E-37	-0.10421	0.012	0.154	1.59E-33
MAST2	1.08E-37	-0.11309	0.028	0.188	1.62E-33
FXN	1.09E-37	-0.11028	0.017	0.169	1.63E-33
MGST2	1.10E-37	0.243769	0.052	0.135	1.64E-33
C5orf34	1.16E-37	-0.34839	0.005	0.191	1.73E-33
PLAU	1.16E-37	-0.26479	0.019	0.206	1.73E-33
CD3EAP	1.21E-37	-0.10203	0.021	0.17	1.81E-33
RIC8B	1.30E-37	-0.10234	0.03	0.188	1.94E-33
NEDD4L	1.40E-37	-0.19625	0.023	0.193	2.09E-33
SIAE	1.60E-37	0.152187	0.049	0.147	2.39E-33
MAPK8	1.74E-37	-0.23296	0.01	0.189	2.60E-33
ZNF335	1.83E-37	-0.12789	0.014	0.162	2.73E-33
TIPARP	1.94E-37	0.238058	0.066	0.158	2.90E-33
TMPO-AS1	2.10E-37	-0.29578	0.007	0.194	3.14E-33
SOS2	2.11E-37	-0.12263	0.016	0.162	3.16E-33
CSK	2.20E-37	-0.11801	0.014	0.16	3.29E-33
RNF103	2.48E-37	-0.10922	0.028	0.179	3.71E-33
N4BP1	2.55E-37	-0.19786	0.012	0.181	3.81E-33
TYMP	2.69E-37	-0.1085	0.019	0.165	4.02E-33
MEX3D	2.70E-37	-0.25547	0.005	0.19	4.04E-33

SH3BGR	3.23E-37	0.278337	0.065	0.144	4.83E-33
LARP4B	3.54E-37	-0.17736	0.007	0.167	5.28E-33
TP53BP1	3.55E-37	-0.11601	0.023	0.18	5.30E-33
PBXIP1	3.65E-37	0.299782	0.052	0.101	5.46E-33
KCTD1	3.89E-37	-0.18341	0.016	0.179	5.81E-33
MAPK12	3.95E-37	-0.11827	0.012	0.158	5.91E-33
RGCC	4.16E-37	0.275483	0.108	0.204	6.22E-33
ZNF668	5.36E-37	-0.11406	0.017	0.159	8.01E-33
GLIS3	5.72E-37	-0.11995	0.016	0.16	8.54E-33
ARL17A	5.89E-37	-0.11596	0.014	0.158	8.80E-33
STK32B	5.98E-37	-0.17939	0.01	0.172	8.94E-33
SLC47A1	6.61E-37	-0.11439	0.014	0.156	9.88E-33
SYCE1L	6.81E-37	-0.15016	0.005	0.153	1.02E-32
BRSK1	7.35E-37	-0.18125	0.016	0.178	1.10E-32
C17orf96	7.44E-37	-0.13014	0.009	0.149	1.11E-32
ATXN1L	7.82E-37	-0.22943	0.003	0.169	1.17E-32
CEP192	7.91E-37	-0.18002	0.007	0.16	1.18E-32
NOP14	9.47E-37	-0.15861	0.01	0.165	1.42E-32
DMD	9.63E-37	0.133894	0.051	0.157	1.44E-32
KIAA1958	1.08E-36	-0.18651	0.012	0.178	1.62E-32
THOC5	1.17E-36	-0.14752	0.014	0.17	1.74E-32
KDELC1	1.23E-36	-0.11074	0.009	0.148	1.84E-32
RP11-395A	1.26E-36	-0.15994	0.007	0.156	1.88E-32
COL16A1	1.50E-36	0.172843	0.04	0.125	2.24E-32
ZBTB33	1.52E-36	-0.1059	0.01	0.144	2.27E-32
KHK	1.54E-36	-0.18043	0.019	0.18	2.30E-32
THEM6	1.65E-36	-0.12059	0.017	0.165	2.46E-32
COA7	1.68E-36	0.128548	0.035	0.123	2.51E-32
LONRF2	1.76E-36	-0.15477	0.021	0.179	2.63E-32
IFT122	1.80E-36	-0.18495	0.007	0.164	2.68E-32
DLX1	2.25E-36	-0.25505	0.026	0.201	3.36E-32
PAQR3	2.28E-36	-0.17253	0.012	0.168	3.41E-32
AGAP3	2.33E-36	-0.20178	0.023	0.194	3.48E-32
YEATS2	2.41E-36	-0.1027	0.028	0.177	3.60E-32
RCBTB2	2.47E-36	-0.21726	0.024	0.195	3.70E-32
AMPD2	2.63E-36	0.173003	0.045	0.13	3.94E-32
EPC2	2.64E-36	-0.18652	0.019	0.185	3.95E-32
AVIL	2.67E-36	-0.17429	0.059	0.242	3.99E-32
GSE1	2.67E-36	-0.13427	0.023	0.172	3.99E-32
ZNF669	2.74E-36	-0.12331	0.024	0.17	4.10E-32
PTPN23	2.78E-36	-0.1463	0.007	0.153	4.16E-32
FAM175B	2.90E-36	0.113443	0.035	0.136	4.34E-32
GTPBP10	3.02E-36	0.140181	0.051	0.148	4.52E-32
MARK1	3.30E-36	-0.18607	0.007	0.16	4.93E-32
KCTD12	3.38E-36	0.172217	0.033	0.12	5.05E-32
ASPHD2	3.51E-36	-0.10974	0.009	0.148	5.25E-32
ZNF143	3.77E-36	-0.13775	0.017	0.165	5.63E-32
MFSD6	4.17E-36	-0.15397	0.009	0.158	6.23E-32
PTBP2	4.47E-36	-0.15564	0.037	0.199	6.68E-32
SPHK1	4.64E-36	-0.23949	0.003	0.173	6.94E-32
MTRNR2L1C	4.76E-36	1.034209	0.182	0.016	7.12E-32

IRAK4	4.80E-36	0.109449	0.035	0.116	7.18E-32
LIN52	5.14E-36	-0.24399	0.009	0.175	7.69E-32
LSS	5.48E-36	0.19525	0.044	0.122	8.20E-32
E2F8	5.81E-36	-0.30446	0	0.17	8.69E-32
HEY2	6.15E-36	-0.11473	0.017	0.163	9.18E-32
NUP133	6.38E-36	-0.25125	0.019	0.201	9.53E-32
AC159540.	6.78E-36	-0.1086	0.026	0.173	1.01E-31
ZNHIT2	7.06E-36	-0.13006	0.012	0.162	1.06E-31
SMIM13	7.24E-36	-0.2433	0.009	0.189	1.08E-31
ZNF300	7.30E-36	-0.28424	0.012	0.194	1.09E-31
STK32A	8.24E-36	0.1613	0.026	0.1	1.23E-31
DUSP15	8.97E-36	-0.18228	0.007	0.16	1.34E-31
LMO1	1.05E-35	-0.4171	0.038	0.248	1.57E-31
TLE2	1.08E-35	-0.18184	0.031	0.207	1.62E-31
GGA2	1.16E-35	-0.18189	0.016	0.175	1.73E-31
IFFO1	1.17E-35	-0.11543	0.023	0.164	1.75E-31
TMEM216	1.18E-35	0.139487	0.031	0.104	1.76E-31
GALNT7	1.19E-35	-0.14032	0.014	0.16	1.78E-31
VCPIP1	1.49E-35	-0.1419	0.009	0.151	2.23E-31
TMTC4	1.51E-35	-0.16572	0.017	0.177	2.25E-31
CSGALNACT	1.54E-35	-0.13212	0.016	0.162	2.30E-31
RTTN	1.59E-35	-0.14385	0.007	0.152	2.38E-31
DROSHA	1.66E-35	-0.11212	0.021	0.173	2.49E-31
CAMK1D	1.82E-35	-0.10532	0.023	0.168	2.72E-31
EBF4	1.87E-35	-0.1367	0.016	0.165	2.79E-31
ZBTB25	1.88E-35	-0.12108	0.017	0.159	2.81E-31
THUMPD2	1.92E-35	-0.12484	0.031	0.185	2.86E-31
HLA-DMA	2.05E-35	0.57084	0.112	0.107	3.06E-31
MAPKAPK3	2.11E-35	-0.14803	0.009	0.154	3.15E-31
PIM1	2.13E-35	-0.10517	0.033	0.186	3.18E-31
APAF1	2.25E-35	-0.19718	0.01	0.172	3.36E-31
ZNF865	2.31E-35	-0.1391	0.01	0.156	3.46E-31
ACSBG1	2.66E-35	0.134206	0.037	0.132	3.98E-31
PXMP4	2.68E-35	-0.23337	0.009	0.173	4.00E-31
C1orf54	2.70E-35	0.116093	0.03	0.128	4.04E-31
PIP4K2A	2.71E-35	-0.11282	0.016	0.158	4.05E-31
KANK1	2.89E-35	0.108361	0.042	0.144	4.32E-31
ANKRD13A	2.89E-35	-0.15648	0.023	0.177	4.32E-31
UNC119B	2.95E-35	-0.17764	0.01	0.167	4.41E-31
LIG3	3.02E-35	-0.15156	0.01	0.159	4.51E-31
UHRF2	3.08E-35	-0.14431	0.012	0.156	4.61E-31
NME2	3.28E-35	-0.16003	0.007	0.156	4.90E-31
ATP1A3	3.33E-35	-0.14569	0.059	0.219	4.97E-31
NUDT8	3.58E-35	-0.1357	0.012	0.151	5.35E-31
ACSS2	3.87E-35	-0.11271	0.012	0.147	5.79E-31
WASF3	4.43E-35	0.136797	0.042	0.143	6.62E-31
NLE1	4.65E-35	-0.18769	0.005	0.16	6.94E-31
HERC1	4.77E-35	-0.112	0.016	0.156	7.12E-31
BANP	4.94E-35	-0.2052	0.012	0.178	7.39E-31
TET1	6.88E-35	-0.15918	0.007	0.153	1.03E-30
NID1	8.03E-35	-0.16441	0.003	0.154	1.20E-30

LINC0091C	8.24E-35	-0.15889	0.019	0.169	1.23E-30
ARNTL	8.40E-35	-0.15176	0.014	0.167	1.25E-30
SIX1	9.11E-35	-0.12525	0.019	0.158	1.36E-30
GSG1L	9.66E-35	0.269555	0.058	0.135	1.44E-30
TP73	9.88E-35	-0.24382	0.01	0.18	1.48E-30
C1orf159	9.93E-35	-0.20813	0.005	0.16	1.48E-30
KCNIP1	1.07E-34	0.404093	0.087	0.144	1.60E-30
SIDT2	1.13E-34	0.16939	0.037	0.107	1.68E-30
CTC-260E6	1.14E-34	-0.26806	0.002	0.173	1.71E-30
CDH24	1.22E-34	-0.21687	0.009	0.169	1.82E-30
PAK1	1.30E-34	-0.12951	0.023	0.165	1.95E-30
ATCAY	1.40E-34	-0.16881	0.049	0.221	2.10E-30
DDHD2	1.42E-34	-0.14294	0.021	0.17	2.13E-30
DBR1	1.61E-34	-0.11563	0.021	0.165	2.41E-30
MIR497HG	1.67E-34	0.193841	0.065	0.149	2.50E-30
SETDB1	1.70E-34	0.110893	0.035	0.128	2.54E-30
ZNF667	1.79E-34	0.156023	0.042	0.121	2.68E-30
TYROBP	1.95E-34	0.337278	0.098	0.151	2.91E-30
ALS2	2.00E-34	-0.12748	0.009	0.144	2.99E-30
CEP19	2.08E-34	-0.10367	0.026	0.172	3.12E-30
GEN1	2.23E-34	-0.14297	0.016	0.164	3.34E-30
HMG20A	2.25E-34	-0.13717	0.019	0.172	3.37E-30
FKTN	2.27E-34	-0.11097	0.014	0.149	3.39E-30
FAM72D	2.36E-34	-0.27045	0	0.163	3.53E-30
FAM195B	2.48E-34	0.818485	0.122	0.068	3.71E-30
RP11-400F	2.80E-34	-0.14372	0.01	0.154	4.18E-30
RELT	2.84E-34	-0.18585	0.005	0.157	4.24E-30
SPATA5	2.85E-34	-0.10798	0.014	0.147	4.26E-30
TOM1L1	3.61E-34	-0.1043	0.01	0.138	5.39E-30
CDK5R1	3.86E-34	-0.1203	0.016	0.153	5.76E-30
MVB12B	4.15E-34	-0.14608	0.021	0.177	6.20E-30
EPHA3	4.37E-34	-0.2154	0	0.162	6.53E-30
HIST1H1B	4.37E-34	-0.30848	0	0.162	6.53E-30
ZMYM1	4.41E-34	-0.17101	0.007	0.157	6.59E-30
TMEM79	4.91E-34	-0.19493	0.017	0.18	7.34E-30
VPS13A	5.05E-34	-0.13301	0.014	0.156	7.54E-30
MGLL	5.24E-34	0.182956	0.066	0.168	7.83E-30
IL32	5.81E-34	0.385494	0.047	0.088	8.69E-30
CDK8	5.83E-34	-0.10157	0.012	0.147	8.72E-30
PHRF1	6.27E-34	-0.18342	0.014	0.168	9.37E-30
CDR2L	6.51E-34	-0.10928	0.01	0.141	9.74E-30
PIANP	6.80E-34	-0.13368	0.017	0.16	1.02E-29
EPOR	6.86E-34	-0.22827	0.007	0.174	1.03E-29
COL5A2	7.23E-34	-0.13079	0.01	0.147	1.08E-29
IRF9	7.29E-34	0.383933	0.084	0.148	1.09E-29
FAM107B	7.88E-34	-0.15404	0.019	0.17	1.18E-29
TMEM176B	9.70E-34	0.651152	0.105	0.091	1.45E-29
FNTB	1.07E-33	-0.13953	0.023	0.177	1.60E-29
VCL	1.24E-33	-0.13236	0.017	0.162	1.86E-29
GPR75-ASE	1.34E-33	-0.22406	0.021	0.186	2.00E-29
CPT2	1.35E-33	-0.2034	0.017	0.181	2.02E-29

CSF1	1.39E-33	0.150854	0.04	0.122	2.08E-29
LCA5	1.65E-33	-0.12212	0.009	0.14	2.47E-29
DNAJB5	1.68E-33	-0.10196	0.019	0.154	2.51E-29
MEX3B	1.86E-33	-0.18128	0.023	0.17	2.78E-29
CAAP1	2.37E-33	-0.17077	0.021	0.179	3.54E-29
ALMS1	2.51E-33	-0.15286	0.009	0.149	3.75E-29
RNGTT	2.67E-33	-0.18362	0.014	0.168	4.00E-29
POC5	2.77E-33	-0.17383	0.017	0.174	4.14E-29
ROBO3	3.49E-33	0.191766	0.054	0.125	5.21E-29
STON2	3.59E-33	-0.12704	0.012	0.148	5.37E-29
KIAA0100	3.70E-33	-0.11549	0.016	0.153	5.53E-29
SLC27A4	3.90E-33	-0.11242	0.01	0.141	5.82E-29
TTLL5	4.05E-33	-0.11215	0.016	0.149	6.06E-29
RINT1	4.16E-33	-0.12924	0.026	0.168	6.22E-29
LIPE	4.21E-33	-0.17344	0.014	0.162	6.30E-29
VIPR2	4.38E-33	-0.3089	0.061	0.247	6.55E-29
NUDT14	4.43E-33	-0.12506	0.01	0.144	6.62E-29
CD200	4.81E-33	-0.1299	0.012	0.146	7.19E-29
PBX2	4.90E-33	0.116446	0.038	0.125	7.33E-29
POLH	5.33E-33	-0.22585	0.007	0.164	7.96E-29
DDX20	5.35E-33	-0.20842	0.012	0.168	7.99E-29
C11orf80	5.58E-33	-0.11512	0.003	0.125	8.34E-29
CC2D2A	6.87E-33	0.188139	0.04	0.114	1.03E-28
ZNF625	7.31E-33	-0.22295	0.009	0.172	1.09E-28
SGTB	7.64E-33	-0.10104	0.014	0.144	1.14E-28
GMPR	7.82E-33	0.161866	0.033	0.098	1.17E-28
GABBR1	8.13E-33	0.109435	0.045	0.132	1.21E-28
AGAP2-AS1	8.19E-33	-0.44758	0.049	0.258	1.22E-28
LIN9	8.56E-33	-0.27142	0.005	0.172	1.28E-28
RPS6KL1	8.89E-33	-0.14636	0.033	0.18	1.33E-28
B3GAT2	1.00E-32	-0.17308	0.028	0.177	1.50E-28
CPOX	1.07E-32	-0.10723	0.014	0.151	1.60E-28
GPR161	1.12E-32	-0.20976	0.012	0.172	1.67E-28
TBC1D24	1.41E-32	-0.10348	0.016	0.147	2.10E-28
OGFRL1	1.45E-32	-0.16043	0.012	0.154	2.17E-28
LTF	1.59E-32	1.291069	0.162	0.032	2.37E-28
RP11-119E	1.63E-32	-0.13403	0.014	0.151	2.43E-28
MTA2	1.64E-32	-0.16036	0.016	0.162	2.44E-28
STK38	1.95E-32	-0.10853	0.014	0.144	2.91E-28
MBP	1.97E-32	0.4558	0.124	0.173	2.95E-28
REXO1	2.01E-32	-0.15736	0.01	0.154	3.01E-28
USP37	2.25E-32	-0.23565	0.009	0.167	3.37E-28
RBM19	2.31E-32	-0.1024	0.017	0.149	3.46E-28
PHACTR2	2.41E-32	-0.14269	0.002	0.137	3.60E-28
SPTLC2	2.48E-32	-0.12013	0.019	0.157	3.70E-28
SMG5	2.84E-32	-0.10045	0.026	0.17	4.24E-28
ATG16L1	2.88E-32	-0.17044	0.009	0.153	4.30E-28
CPT1C	3.01E-32	0.13713	0.063	0.154	4.50E-28
IQGAP3	3.18E-32	-0.29754	0	0.153	4.75E-28
CSTF2	3.22E-32	-0.16592	0.016	0.162	4.81E-28
NR2E1	3.42E-32	-0.10834	0.01	0.133	5.11E-28

GPSM1	3.49E-32	-0.11754	0.016	0.153	5.21E-28
EPHB2	3.91E-32	-0.18185	0.014	0.164	5.84E-28
KIF5A	5.13E-32	-0.2452	0.047	0.226	7.67E-28
HOXB2	5.15E-32	-0.42897	0.021	0.207	7.70E-28
MPV17L	5.62E-32	0.198973	0.04	0.101	8.39E-28
RASD1	6.31E-32	0.480124	0.113	0.131	9.43E-28
ZC3H3	6.85E-32	-0.17743	0.014	0.165	1.02E-27
OXSM	7.45E-32	0.133871	0.026	0.101	1.11E-27
PYCRL	7.66E-32	-0.14507	0.014	0.149	1.14E-27
ZZEF1	7.73E-32	-0.12461	0.014	0.147	1.15E-27
EFNA1	8.24E-32	0.489244	0.092	0.109	1.23E-27
HOXA10	8.41E-32	-0.37574	0.023	0.195	1.26E-27
MOXD1	9.09E-32	-0.24453	0.005	0.167	1.36E-27
MCPH1	9.26E-32	-0.11539	0.014	0.143	1.38E-27
SFI1	9.64E-32	-0.13846	0.019	0.16	1.44E-27
XIST	9.65E-32	0.603968	0.175	0.146	1.44E-27
PROSER1	1.01E-31	-0.20044	0.01	0.16	1.51E-27
MAPK11	1.07E-31	-0.12707	0.012	0.147	1.60E-27
ICAM3	1.08E-31	-0.19597	0	0.151	1.61E-27
GBP2	1.08E-31	0.655866	0.099	0.085	1.62E-27
GLRB	1.08E-31	-0.10019	0.017	0.147	1.62E-27
CHRDL1	1.11E-31	-0.21315	0.007	0.157	1.66E-27
E2F7	1.20E-31	-0.19917	0.003	0.153	1.79E-27
TRAPPC10	1.25E-31	-0.18836	0.005	0.159	1.87E-27
CAD	1.28E-31	-0.17917	0.009	0.153	1.91E-27
SEMA4C	1.29E-31	-0.10674	0.024	0.162	1.93E-27
LRRC14	1.32E-31	-0.10989	0.021	0.159	1.98E-27
ZFP62	1.33E-31	-0.10384	0.024	0.16	1.98E-27
RP11-412D	1.34E-31	0.104902	0.031	0.114	2.00E-27
CDC25A	1.42E-31	-0.26015	0.005	0.16	2.12E-27
GK	1.50E-31	-0.14325	0.012	0.149	2.24E-27
PCDHB2	1.80E-31	-0.13732	0.019	0.158	2.70E-27
NDUFA7	1.82E-31	1.010059	0.157	0.012	2.72E-27
PGAM5	1.84E-31	-0.22259	0.002	0.163	2.75E-27
FGGY	2.07E-31	0.25718	0.051	0.116	3.10E-27
MAML1	2.15E-31	-0.18349	0.017	0.168	3.21E-27
KCTD9	2.38E-31	-0.15934	0.012	0.151	3.55E-27
FBLIM1	2.40E-31	0.240551	0.061	0.119	3.59E-27
SRPX2	2.41E-31	0.202856	0.033	0.098	3.61E-27
TDRD3	2.52E-31	-0.17543	0.017	0.167	3.76E-27
MIR181A1H	2.59E-31	-0.11868	0.033	0.169	3.88E-27
SLC16A14	2.61E-31	-0.22166	0.002	0.148	3.91E-27
ZSCAN32	2.65E-31	-0.12172	0.016	0.147	3.95E-27
SLC25A32	2.83E-31	-0.16788	0.012	0.157	4.23E-27
TTC26	2.85E-31	0.120233	0.026	0.106	4.27E-27
YTHDF3-AS	3.06E-31	-0.10103	0.014	0.141	4.58E-27
E2F2	3.18E-31	-0.2681	0.002	0.146	4.76E-27
INCENP	3.24E-31	-0.22999	0.002	0.16	4.85E-27
IPO11	3.55E-31	-0.1199	0.01	0.137	5.30E-27
THAP8	3.57E-31	0.103153	0.033	0.122	5.34E-27
BDH1	3.93E-31	0.116496	0.035	0.128	5.87E-27

SLC30A1	3.97E-31	-0.15062	0.003	0.137	5.93E-27
KNTC1	4.26E-31	-0.18546	0.012	0.156	6.37E-27
AHNAK	4.27E-31	0.110518	0.023	0.094	6.38E-27
RP11-182L	4.43E-31	-0.13721	0.016	0.146	6.61E-27
SMO	4.53E-31	-0.12057	0.016	0.149	6.77E-27
G0S2	4.55E-31	0.226754	0.058	0.138	6.80E-27
CCDC117	5.32E-31	-0.12977	0.007	0.131	7.95E-27
RP11-277P	5.35E-31	-0.23571	0.037	0.206	8.00E-27
RP11-263K	5.44E-31	0.198727	0.037	0.095	8.13E-27
NTM	6.15E-31	0.112026	0.101	0.2	9.18E-27
PARP11	7.07E-31	-0.13212	0.014	0.147	1.06E-26
SLC16A2	7.35E-31	-0.12066	0.009	0.133	1.10E-26
KLF9	8.01E-31	0.103886	0.059	0.156	1.20E-26
TMEM231	8.10E-31	0.11618	0.033	0.111	1.21E-26
ZFP1	9.05E-31	-0.12408	0.021	0.156	1.35E-26
YIPF2	9.29E-31	0.122699	0.047	0.138	1.39E-26
NLK	1.05E-30	-0.14705	0.012	0.152	1.57E-26
MFHAS1	1.18E-30	-0.14861	0.007	0.14	1.76E-26
FIGNL1	1.22E-30	-0.24571	0.014	0.167	1.82E-26
KBTBD3	1.29E-30	0.100021	0.045	0.137	1.93E-26
ZNF611	1.31E-30	-0.145	0.016	0.151	1.96E-26
ZKSCAN5	1.33E-30	-0.11885	0.019	0.154	1.99E-26
VARS2	1.47E-30	-0.10031	0.014	0.137	2.19E-26
STK35	1.67E-30	-0.11808	0.007	0.132	2.49E-26
WWC3	1.75E-30	-0.14865	0.009	0.143	2.62E-26
RNF182	1.88E-30	-0.15359	0.012	0.141	2.81E-26
PLIN2	1.99E-30	0.534285	0.096	0.115	2.97E-26
SPAG1	2.03E-30	-0.12405	0.009	0.135	3.03E-26
POLE	2.12E-30	-0.21011	0.009	0.157	3.16E-26
LRFN4	2.26E-30	-0.13163	0.014	0.152	3.37E-26
MAGI3	2.28E-30	-0.16705	0.007	0.143	3.40E-26
FAM181A	2.38E-30	0.379151	0.065	0.099	3.56E-26
ZMYM3	2.97E-30	-0.12879	0.014	0.146	4.44E-26
C17orf53	3.23E-30	-0.20533	0.002	0.14	4.82E-26
ZNF512B	3.31E-30	-0.17077	0.003	0.142	4.95E-26
NTRK3	3.40E-30	0.2348	0.051	0.116	5.08E-26
ZIK1	3.56E-30	-0.2168	0.009	0.151	5.32E-26
BDNF-AS	4.47E-30	0.114879	0.03	0.099	6.68E-26
TUSC1	4.59E-30	-0.20227	0.014	0.148	6.86E-26
FIZ1	5.24E-30	-0.13066	0.005	0.127	7.83E-26
ARHGEF25	5.51E-30	-0.17413	0.031	0.174	8.24E-26
ATXN8OS	5.57E-30	0.99749	0.166	0.015	8.32E-26
MXI1	5.58E-30	0.12074	0.04	0.119	8.34E-26
TMEM201	6.11E-30	-0.18343	0.007	0.156	9.13E-26
PLXNB1	6.16E-30	0.190332	0.047	0.117	9.20E-26
STOX2	6.59E-30	-0.12199	0.023	0.148	9.85E-26
ZNF200	6.67E-30	-0.11003	0.01	0.128	9.97E-26
RIT2	6.79E-30	0.455852	0.082	0.096	1.02E-25
APOL2	6.88E-30	0.409643	0.063	0.099	1.03E-25
HDHD3	7.07E-30	0.151681	0.038	0.101	1.06E-25
ZNF519	7.32E-30	-0.22393	0.014	0.163	1.09E-25

NDST1	7.46E-30	-0.11893	0.01	0.137	1.12E-25
CACNB3	7.88E-30	-0.2044	0.007	0.147	1.18E-25
RGS1	8.24E-30	0.159524	0.07	0.146	1.23E-25
TCEAL5	8.96E-30	0.188912	0.073	0.154	1.34E-25
PIGZ	9.62E-30	-0.18837	0.003	0.14	1.44E-25
ZNRF3	9.83E-30	-0.10238	0.007	0.122	1.47E-25
RECK	9.94E-30	-0.14971	0.012	0.147	1.48E-25
SPATA2	1.10E-29	-0.15088	0.002	0.132	1.64E-25
PRSS23	1.14E-29	0.285826	0.051	0.091	1.71E-25
SCAF8	1.15E-29	-0.16661	0.007	0.143	1.71E-25
STRN	1.17E-29	-0.10637	0.007	0.127	1.74E-25
TGFBR1	1.21E-29	-0.18765	0.016	0.164	1.81E-25
ZNF689	1.34E-29	-0.14183	0.017	0.151	2.01E-25
RP3-428L1	1.41E-29	-0.22447	0.002	0.152	2.10E-25
C18orf54	1.49E-29	-0.18942	0.007	0.148	2.23E-25
UBR1	1.64E-29	-0.19709	0.014	0.163	2.45E-25
ZNF384	1.64E-29	-0.14271	0.017	0.152	2.46E-25
AP4S1	1.70E-29	0.143839	0.03	0.102	2.54E-25
ZNF703	1.73E-29	-0.11758	0.014	0.142	2.59E-25
FAM118A	1.83E-29	-0.11159	0.023	0.151	2.73E-25
USP40	1.86E-29	-0.10557	0.019	0.146	2.78E-25
TOX3	1.89E-29	-0.18763	0.033	0.172	2.83E-25
WDR36	1.90E-29	-0.14887	0.014	0.148	2.84E-25
LCLAT1	1.96E-29	-0.11808	0.01	0.135	2.93E-25
GRIK3	2.09E-29	-0.1366	0.021	0.157	3.12E-25
NEK3	2.33E-29	-0.13194	0.007	0.13	3.48E-25
EVA1C	2.67E-29	-0.11049	0.012	0.135	3.99E-25
SRGN	2.76E-29	0.392077	0.136	0.16	4.12E-25
GPR137C	3.14E-29	-0.13332	0.012	0.137	4.70E-25
TEX10	3.16E-29	-0.15732	0.017	0.154	4.72E-25
PTGDS	3.26E-29	0.644082	0.126	0.123	4.87E-25
PLAA	3.36E-29	-0.21378	0.007	0.148	5.02E-25
ZBTB18	3.42E-29	-0.2024	0.009	0.146	5.12E-25
PUS1	3.83E-29	-0.13953	0.01	0.14	5.72E-25
HOXC9	4.39E-29	-0.30545	0.009	0.151	6.56E-25
ACTA2	4.42E-29	0.124175	0.019	0.088	6.60E-25
SIMC1	4.44E-29	-0.20541	0.009	0.157	6.64E-25
TSGA10	4.49E-29	0.122058	0.031	0.094	6.71E-25
TUBGCP5	4.51E-29	-0.11945	0.012	0.137	6.75E-25
TOX	4.78E-29	-0.13222	0.009	0.138	7.14E-25
NR4A1	4.80E-29	0.141389	0.065	0.151	7.18E-25
SLC22A4	4.93E-29	-0.15205	0.002	0.125	7.37E-25
MRE11A	5.02E-29	-0.15798	0.016	0.151	7.50E-25
TXNDC11	5.17E-29	-0.10087	0.012	0.131	7.73E-25
NCKAP5L	5.19E-29	0.169927	0.042	0.11	7.76E-25
AGO1	5.29E-29	-0.1283	0.016	0.144	7.90E-25
ZNF629	6.62E-29	-0.16768	0.007	0.138	9.89E-25
PIEZO1	7.97E-29	-0.13514	0.009	0.136	1.19E-24
OSBPL10	8.18E-29	-0.11922	0.003	0.117	1.22E-24
LIMK2	8.68E-29	-0.1193	0.007	0.126	1.30E-24
MGP	1.02E-28	1.187544	0.131	0.028	1.53E-24

ISG20	1.02E-28	0.549914	0.073	0.067	1.53E-24
CEP128	1.06E-28	-0.12425	0.005	0.122	1.58E-24
SPRYD4	1.06E-28	0.155118	0.026	0.098	1.58E-24
FGF1	1.07E-28	0.329254	0.042	0.075	1.60E-24
SLC39A14	1.11E-28	0.258215	0.045	0.088	1.66E-24
NEK4	1.31E-28	-0.11332	0.021	0.147	1.96E-24
ZNF574	1.34E-28	-0.18792	0.023	0.169	2.00E-24
HDAC10	1.34E-28	-0.15458	0.007	0.135	2.00E-24
DENND1B	1.35E-28	-0.19559	0.007	0.143	2.02E-24
WDR53	1.44E-28	-0.14641	0.01	0.141	2.15E-24
MATN2	1.53E-28	0.159577	0.047	0.115	2.28E-24
TRIM22	1.62E-28	0.295601	0.052	0.095	2.43E-24
MICAL1	1.63E-28	-0.12923	0.023	0.152	2.43E-24
GALM	1.86E-28	0.196191	0.04	0.105	2.78E-24
NOL4	2.08E-28	-0.23719	0.023	0.177	3.11E-24
TNFRSF11E	2.19E-28	0.194204	0.033	0.084	3.28E-24
MSL2	2.51E-28	-0.10599	0.016	0.131	3.75E-24
SCML1	2.54E-28	-0.11715	0.031	0.158	3.79E-24
ZCCHC6	2.59E-28	-0.13795	0.019	0.153	3.87E-24
CACNG4	2.64E-28	-0.13965	0.017	0.143	3.95E-24
ATP5L2	2.67E-28	0.302143	0.061	0.106	3.99E-24
ARAP2	2.89E-28	0.115351	0.037	0.111	4.33E-24
C3orf58	2.95E-28	-0.12693	0.016	0.142	4.40E-24
KCTD2	2.96E-28	-0.13655	0.007	0.136	4.43E-24
ZNF628	3.03E-28	-0.12145	0.009	0.128	4.52E-24
SAA1	3.09E-28	1.389227	0.131	0.026	4.61E-24
1-Mar	3.10E-28	-0.14064	0.04	0.168	4.63E-24
INO80B	3.15E-28	-0.11686	0.016	0.137	4.71E-24
FBXL6	3.32E-28	-0.147	0.01	0.137	4.96E-24
GDF15	3.56E-28	0.191026	0.063	0.154	5.31E-24
SELO	3.56E-28	-0.15955	0.002	0.128	5.32E-24
MT1G	4.31E-28	0.699194	0.112	0.065	6.43E-24
FAM101B	4.40E-28	-0.17221	0.003	0.135	6.58E-24
AMMECR1	4.43E-28	-0.13614	0.005	0.127	6.62E-24
ANKRD28	4.48E-28	0.146271	0.047	0.125	6.69E-24
EPAS1	4.76E-28	0.309415	0.051	0.09	7.12E-24
WDR90	4.83E-28	-0.11963	0.016	0.136	7.22E-24
CAPS	4.99E-28	0.140865	0.028	0.095	7.46E-24
ZNF35	5.04E-28	-0.11702	0.017	0.142	7.53E-24
RP5-827C2	5.17E-28	0.169921	0.044	0.104	7.73E-24
CAMKMT	5.45E-28	-0.10046	0.024	0.146	8.14E-24
CACTIN	5.56E-28	-0.16731	0.007	0.14	8.30E-24
FTX	6.54E-28	0.175556	0.066	0.123	9.77E-24
PLCD3	7.14E-28	-0.11809	0.009	0.132	1.07E-23
GNAI1	8.06E-28	-0.16772	0.026	0.159	1.20E-23
FXYD5	8.76E-28	0.164587	0.054	0.132	1.31E-23
PAPD5	8.86E-28	-0.13774	0.012	0.138	1.32E-23
CEP72	9.29E-28	-0.3018	0	0.132	1.39E-23
TMEM176A	9.49E-28	0.442216	0.07	0.074	1.42E-23
RIMKLA	9.50E-28	-0.11047	0.007	0.119	1.42E-23
EEF1G	1.01E-27	0.226935	0.056	0.111	1.52E-23

LIN54	1.06E-27	-0.23027	0.003	0.146	1.58E-23
TNRC18	1.07E-27	-0.11055	0.021	0.146	1.60E-23
CAV1	1.07E-27	0.963138	0.162	0.063	1.60E-23
ETV2	1.11E-27	-0.11271	0.005	0.117	1.66E-23
ZNF829	1.13E-27	-0.10278	0.014	0.127	1.69E-23
CMTM5	1.18E-27	0.255666	0.098	0.156	1.77E-23
ELAVL2	1.19E-27	-0.28891	0.017	0.165	1.78E-23
SFT2D3	1.24E-27	-0.12009	0.009	0.122	1.85E-23
RBM15	1.24E-27	-0.11917	0.014	0.135	1.86E-23
GFPT1	1.27E-27	-0.23357	0.017	0.167	1.89E-23
USP28	1.29E-27	-0.22213	0.007	0.149	1.92E-23
FHL2	1.35E-27	-0.193	0.014	0.149	2.01E-23
CTPS2	1.39E-27	-0.14059	0.007	0.123	2.07E-23
PREP	1.45E-27	-0.13708	0.017	0.143	2.17E-23
CCDC152	1.50E-27	0.206214	0.045	0.106	2.24E-23
ACTR5	1.64E-27	-0.12907	0.009	0.131	2.45E-23
FZD2	1.67E-27	-0.12573	0.007	0.128	2.50E-23
SLC16A9	1.70E-27	-0.15345	0.017	0.147	2.54E-23
ARNTL2	1.78E-27	-0.13535	0.003	0.122	2.67E-23
VCPKMT	1.93E-27	-0.1432	0.017	0.146	2.88E-23
CSRNP1	1.97E-27	0.101633	0.04	0.121	2.94E-23
SPEG	2.52E-27	0.142801	0.035	0.1	3.76E-23
ZNF184	2.53E-27	-0.184	0.012	0.143	3.78E-23
RP11-486G	2.56E-27	0.145972	0.038	0.105	3.82E-23
BOK	2.58E-27	-0.1248	0.002	0.117	3.86E-23
EMILIN1	2.60E-27	-0.1112	0.007	0.12	3.89E-23
CLYBL	2.64E-27	0.243913	0.031	0.078	3.94E-23
APOL6	2.66E-27	0.159374	0.028	0.09	3.97E-23
GPRASP2	3.11E-27	-0.1523	0.017	0.146	4.65E-23
HAGHL	3.13E-27	0.130504	0.031	0.091	4.68E-23
VAV3	3.25E-27	-0.1212	0.019	0.146	4.86E-23
IDNK	3.47E-27	0.116291	0.031	0.101	5.18E-23
SLC22A18	3.50E-27	0.155643	0.023	0.078	5.23E-23
MDFIC	3.57E-27	-0.14792	0.002	0.123	5.33E-23
F12	3.60E-27	-0.1082	0.016	0.132	5.38E-23
TTC13	4.07E-27	-0.13655	0.01	0.132	6.08E-23
TBCCD1	4.77E-27	-0.15443	0.017	0.151	7.12E-23
C1QTNF2	5.31E-27	-0.18517	0.01	0.136	7.94E-23
AUNIP	5.38E-27	-0.23747	0.002	0.13	8.04E-23
SIPA1	5.38E-27	-0.13764	0.003	0.126	8.05E-23
SEMA3A	6.24E-27	-0.12025	0.009	0.125	9.33E-23
AADAT	6.64E-27	-0.19828	0.005	0.137	9.93E-23
IGSF21	6.74E-27	0.114108	0.065	0.141	1.01E-22
RP11-498C	6.86E-27	-0.16001	0.003	0.13	1.02E-22
DHX32	7.40E-27	-0.13559	0.007	0.128	1.11E-22
C7orf60	7.88E-27	-0.11556	0.017	0.133	1.18E-22
C19orf57	8.06E-27	-0.14518	0.005	0.119	1.20E-22
FAM120B	8.62E-27	-0.10385	0.017	0.14	1.29E-22
ZNF853	1.02E-26	-0.10296	0.019	0.138	1.52E-22
CELF5	1.06E-26	-0.1913	0.024	0.151	1.58E-22
MED12L	1.10E-26	-0.12797	0.01	0.13	1.64E-22

DNAJC3-AS	1.16E-26	0.2084	0.047	0.09	1.74E-22
CD14	1.27E-26	0.220554	0.056	0.11	1.90E-22
UBASH3B	1.30E-26	-0.13652	0.003	0.126	1.94E-22
TARSL2	1.37E-26	-0.12428	0.021	0.143	2.05E-22
PHF2	1.51E-26	-0.11105	0.016	0.132	2.25E-22
SORL1	1.54E-26	0.173019	0.052	0.111	2.30E-22
ARHGAP20	1.98E-26	-0.11777	0.003	0.116	2.96E-22
ZNF605	2.00E-26	0.159691	0.056	0.125	2.99E-22
MED20	2.13E-26	-0.10201	0.009	0.119	3.18E-22
ZNF107	2.20E-26	-0.22627	0.01	0.151	3.29E-22
REEP1	2.48E-26	-0.18118	0.017	0.148	3.71E-22
SLC25A24	2.58E-26	-0.20007	0.009	0.142	3.86E-22
PINLYP	2.87E-26	0.141602	0.031	0.104	4.28E-22
NFASC	3.42E-26	0.310017	0.054	0.084	5.12E-22
PLCXD1	3.67E-26	-0.18395	0.014	0.147	5.49E-22
RRAS2	3.95E-26	-0.13346	0.009	0.13	5.90E-22
SP4	4.58E-26	-0.1202	0.012	0.122	6.84E-22
ARHGEF39	4.97E-26	-0.18921	0.003	0.128	7.42E-22
WWP2	5.12E-26	-0.16614	0.005	0.133	7.65E-22
RP5-1136G	5.23E-26	-0.11315	0.005	0.111	7.82E-22
ZNF681	5.77E-26	-0.10572	0.014	0.122	8.63E-22
ATAD3B	5.79E-26	-0.11541	0.01	0.125	8.65E-22
OTP	6.11E-26	-0.17715	0.003	0.125	9.14E-22
AC002456.	7.32E-26	0.145534	0.026	0.089	1.09E-21
OPN3	7.70E-26	-0.1426	0.007	0.121	1.15E-21
LINC00672	7.87E-26	0.121565	0.026	0.086	1.18E-21
FLVCR1-AS	8.69E-26	-0.10063	0.01	0.115	1.30E-21
ANTXR2	9.27E-26	-0.11842	0.01	0.119	1.39E-21
PHF7	1.05E-25	-0.16021	0.007	0.13	1.56E-21
AP001347.	1.05E-25	-0.19095	0.003	0.121	1.58E-21
SSPN	1.10E-25	0.130932	0.038	0.107	1.65E-21
PLEKHA8	1.11E-25	-0.21535	0.003	0.138	1.66E-21
KIAA1551	1.17E-25	-0.12722	0.005	0.116	1.75E-21
NAT10	1.19E-25	-0.11637	0.019	0.142	1.78E-21
PKIA	1.20E-25	-0.16831	0.031	0.162	1.79E-21
FGF2	1.38E-25	-0.12296	0.002	0.111	2.06E-21
RP11-1275	1.53E-25	-0.12166	0.028	0.147	2.29E-21
TRAF6	1.63E-25	-0.10889	0.009	0.116	2.43E-21
CLP1	1.67E-25	-0.10987	0.009	0.114	2.50E-21
TIAM2	1.80E-25	0.101759	0.031	0.105	2.69E-21
USP31	1.86E-25	-0.16407	0.003	0.119	2.78E-21
ASAP3	1.87E-25	-0.1355	0.003	0.119	2.79E-21
KCTD15	2.09E-25	-0.15434	0.017	0.143	3.12E-21
LRP11	2.09E-25	-0.19662	0.003	0.138	3.13E-21
ZBTB16	2.11E-25	0.261919	0.126	0.174	3.15E-21
KLHL25	2.18E-25	-0.12818	0.016	0.132	3.25E-21
FANCM	2.41E-25	-0.16255	0.002	0.116	3.60E-21
RHBDF1	2.43E-25	0.12596	0.019	0.08	3.63E-21
RABL3	2.46E-25	-0.1141	0.009	0.117	3.68E-21
TIFA	2.52E-25	-0.10222	0.009	0.111	3.77E-21
STK17B	2.54E-25	-0.10379	0.023	0.137	3.80E-21

CASP1	2.56E-25	0.437158	0.079	0.1	3.82E-21
ICA1	2.60E-25	-0.17015	0.014	0.138	3.89E-21
SCAMP5	2.72E-25	-0.13509	0.026	0.152	4.06E-21
FHIT	2.99E-25	0.225655	0.038	0.08	4.47E-21
ENTPD5	3.64E-25	-0.15354	0.005	0.125	5.44E-21
BCOR	3.85E-25	-0.14472	0.014	0.136	5.75E-21
STMN2	3.87E-25	-0.12929	0.089	0.221	5.78E-21
KIFC2	4.07E-25	-0.11217	0.012	0.125	6.08E-21
KLHDC4	4.08E-25	-0.21183	0.01	0.143	6.10E-21
RELB	4.63E-25	-0.13777	0.002	0.116	6.92E-21
OMG	4.71E-25	0.254163	0.072	0.112	7.03E-21
ETAA1	5.24E-25	-0.1091	0.012	0.12	7.83E-21
TRIM8	5.74E-25	-0.17402	0.005	0.131	8.58E-21
HAS2	5.95E-25	0.139409	0.028	0.086	8.90E-21
HNMT	6.06E-25	0.185808	0.049	0.115	9.06E-21
DIEXF	6.35E-25	0.116078	0.026	0.085	9.49E-21
FAS	6.37E-25	0.200423	0.028	0.068	9.53E-21
VWA5A	6.38E-25	0.290511	0.058	0.083	9.54E-21
SPATA5L1	6.55E-25	-0.10317	0.01	0.115	9.78E-21
NNAT	7.40E-25	-0.41561	0.086	0.264	1.11E-20
CDKN2A	7.67E-25	-0.42395	0.049	0.217	1.15E-20
BCL6	8.03E-25	0.193429	0.052	0.102	1.20E-20
HBA2	8.41E-25	0.335252	0.079	0.177	1.26E-20
CCDC74B	9.64E-25	-0.15353	0.005	0.122	1.44E-20
PNRC2	9.66E-25	0.185055	0.044	0.11	1.44E-20
TCFL5	1.02E-24	-0.12278	0.002	0.109	1.52E-20
NTRK2	1.12E-24	0.401982	0.133	0.149	1.67E-20
CMTM8	1.22E-24	-0.1503	0.002	0.126	1.82E-20
HUNK	1.29E-24	-0.11898	0.005	0.11	1.93E-20
ACVR2A	1.44E-24	-0.17607	0.005	0.127	2.16E-20
AK9	1.50E-24	-0.14047	0.012	0.123	2.24E-20
PTCHD1	1.56E-24	0.143561	0.023	0.064	2.33E-20
RP5-1068E	1.58E-24	0.12082	0.017	0.067	2.37E-20
EFNA2	1.64E-24	-0.16253	0.012	0.135	2.45E-20
FGF14-AS2	1.66E-24	-0.13706	0.009	0.12	2.49E-20
CEP85	1.77E-24	-0.18773	0.002	0.12	2.64E-20
TRIM56	1.79E-24	0.260984	0.049	0.088	2.68E-20
CLGN	1.83E-24	-0.20344	0.01	0.121	2.74E-20
SPIN4	1.86E-24	-0.10367	0.005	0.101	2.78E-20
VPS18	1.98E-24	-0.1485	0.009	0.126	2.96E-20
HOXA3	2.09E-24	-0.26907	0.012	0.143	3.13E-20
SFR1	2.10E-24	-0.10252	0.014	0.117	3.14E-20
TIMP4	2.60E-24	0.455498	0.106	0.123	3.89E-20
VCAM1	2.90E-24	0.451233	0.047	0.042	4.33E-20
ORAI3	2.95E-24	0.101989	0.026	0.089	4.41E-20
SELM	2.99E-24	0.120301	0.038	0.105	4.47E-20
FAM72A	3.06E-24	-0.11918	0.007	0.11	4.58E-20
SAMD9L	3.08E-24	0.209543	0.033	0.086	4.61E-20
KANSL3	3.16E-24	-0.12542	0.014	0.126	4.72E-20
MTMR11	3.71E-24	0.120855	0.03	0.09	5.54E-20
LINC00632	3.80E-24	-0.12332	0.035	0.137	5.67E-20

ITPRIPL2	3.95E-24	-0.11257	0.003	0.109	5.90E-20
OPHN1	4.52E-24	0.162409	0.063	0.117	6.76E-20
EXT1	4.54E-24	-0.12176	0.002	0.105	6.78E-20
RCBTB1	4.58E-24	-0.14511	0.016	0.137	6.85E-20
ARHGAP23	5.02E-24	-0.1574	0.002	0.123	7.50E-20
GLIS2	5.34E-24	-0.10362	0.005	0.105	7.98E-20
SLC1A4	5.72E-24	-0.10031	0.009	0.112	8.55E-20
HSPA2	5.86E-24	0.176435	0.084	0.158	8.76E-20
CTSS	5.92E-24	0.250196	0.042	0.083	8.84E-20
IGDCC3	6.12E-24	-0.18162	0.007	0.128	9.15E-20
HARS2	6.20E-24	-0.10335	0.016	0.128	9.26E-20
CADM3	7.87E-24	0.589151	0.08	0.048	1.18E-19
EMILIN3	9.35E-24	-0.10076	0.028	0.141	1.40E-19
NIPA1	9.50E-24	-0.14148	0.005	0.122	1.42E-19
KCNJ2	9.88E-24	-0.14425	0.01	0.121	1.48E-19
RAB2B	1.05E-23	-0.10934	0.019	0.131	1.57E-19
FANCC	1.11E-23	-0.16107	0.01	0.127	1.66E-19
WIPF3	1.30E-23	-0.11888	0.003	0.099	1.95E-19
ZNF582-AS	1.33E-23	0.3423	0.059	0.086	1.99E-19
PSMA2	1.35E-23	0.970066	0.159	0.02	2.01E-19
FOXD1	1.35E-23	-0.14595	0.003	0.112	2.01E-19
NDRG1	1.38E-23	0.603648	0.087	0.074	2.07E-19
LMO3	1.41E-23	0.204067	0.07	0.122	2.11E-19
MANSC1	1.43E-23	-0.10901	0.017	0.125	2.14E-19
NMRK1	1.58E-23	0.143921	0.028	0.086	2.36E-19
RASAL2	2.06E-23	-0.17405	0.009	0.13	3.08E-19
RNPC3	2.07E-23	0.186715	0.049	0.101	3.09E-19
ZNF34	2.11E-23	0.100513	0.026	0.094	3.16E-19
CA11	2.14E-23	0.130114	0.038	0.094	3.21E-19
HSD17B1	2.28E-23	-0.1229	0.005	0.112	3.41E-19
SBK1	2.34E-23	-0.16195	0.019	0.14	3.49E-19
C4orf33	2.58E-23	-0.10681	0.009	0.107	3.86E-19
ITPKB	2.69E-23	0.152612	0.024	0.072	4.02E-19
CREBRF	2.72E-23	0.100884	0.031	0.104	4.07E-19
CCDC30	2.82E-23	0.107788	0.024	0.081	4.21E-19
C7orf43	3.04E-23	-0.15428	0.005	0.115	4.54E-19
ZNF280B	3.47E-23	-0.11863	0.014	0.122	5.18E-19
CTA-384D8	3.60E-23	-0.20621	0.002	0.121	5.38E-19
ELMO1	3.72E-23	0.349797	0.087	0.106	5.56E-19
POLA1	3.91E-23	-0.1319	0.007	0.114	5.84E-19
NCAM2	4.08E-23	0.104314	0.042	0.111	6.10E-19
PKN3	4.11E-23	-0.17814	0	0.11	6.14E-19
VAV2	4.32E-23	-0.13358	0.009	0.117	6.45E-19
CTSH	4.41E-23	0.152007	0.038	0.095	6.60E-19
MGAT5	4.58E-23	-0.12878	0.002	0.104	6.85E-19
BGN	5.05E-23	0.627654	0.096	0.044	7.54E-19
JMY	5.47E-23	-0.11944	0.014	0.123	8.18E-19
FSD1L	5.53E-23	-0.10754	0.007	0.102	8.26E-19
ICAM1	5.55E-23	0.321549	0.042	0.058	8.30E-19
MCAM	5.57E-23	-0.10241	0.019	0.123	8.33E-19
CCNYL1	5.59E-23	-0.13472	0.002	0.111	8.36E-19

ANKRD16	6.15E-23	-0.13362	0.002	0.114	9.19E-19
CEP120	6.24E-23	-0.10259	0.012	0.116	9.32E-19
FBXL16	7.41E-23	-0.10002	0.007	0.105	1.11E-18
HMOX1	7.55E-23	0.239344	0.042	0.095	1.13E-18
RCL1	7.60E-23	-0.13463	0.009	0.114	1.14E-18
AGAP2	7.71E-23	-0.20207	0.023	0.143	1.15E-18
GPR37L1	8.47E-23	0.515535	0.127	0.122	1.27E-18
IFITM10	8.71E-23	0.18419	0.058	0.105	1.30E-18
DLX5	8.85E-23	-0.28341	0.044	0.185	1.32E-18
RP11-783K	1.02E-22	0.140039	0.017	0.059	1.52E-18
F13A1	1.12E-22	0.832443	0.099	0.02	1.68E-18
RP11-73E1	1.12E-22	-0.153	0.005	0.112	1.68E-18
SLC1A5	1.13E-22	-0.10657	0.012	0.117	1.68E-18
SLC25A18	1.16E-22	0.33522	0.068	0.101	1.73E-18
TBX2	1.19E-22	-0.13992	0.009	0.122	1.78E-18
HIST2H2BE	1.28E-22	0.244009	0.047	0.094	1.91E-18
SEC23IP	1.30E-22	-0.10403	0.012	0.116	1.94E-18
MAST4	1.43E-22	0.209198	0.047	0.079	2.13E-18
SLC25A28	1.47E-22	-0.12778	0.005	0.109	2.19E-18
SALL3	1.70E-22	-0.18501	0.017	0.14	2.54E-18
TRAPPC5	1.82E-22	0.100418	0.021	0.07	2.71E-18
TTC31	1.85E-22	-0.13199	0.012	0.122	2.76E-18
HBEGF	1.86E-22	0.103716	0.031	0.094	2.79E-18
CTB-5506.	2.07E-22	-0.10972	0.009	0.11	3.09E-18
ATG16L2	2.29E-22	-0.11764	0.005	0.107	3.42E-18
NDUFB8	2.33E-22	0.938094	0.134	0.012	3.48E-18
PAPOLG	2.33E-22	-0.10304	0.009	0.102	3.48E-18
MAOB	2.37E-22	0.114331	0.047	0.115	3.55E-18
SFXN3	2.71E-22	0.152172	0.026	0.065	4.05E-18
ADCK1	2.96E-22	0.113338	0.014	0.062	4.43E-18
SGK223	3.16E-22	-0.13235	0.005	0.114	4.72E-18
HYLS1	3.50E-22	-0.27911	0.007	0.126	5.23E-18
RASL11B	4.26E-22	-0.2615	0.042	0.174	6.37E-18
TTLL12	4.48E-22	-0.15938	0.007	0.126	6.69E-18
ARRDC4	5.05E-22	-0.10492	0.021	0.122	7.54E-18
RP11-315A	5.20E-22	0.176092	0.049	0.1	7.77E-18
ACVR1B	5.59E-22	-0.16844	0.012	0.125	8.36E-18
TCF20	5.60E-22	-0.1299	0.012	0.121	8.36E-18
FRMD4B	5.61E-22	0.108024	0.03	0.089	8.38E-18
PRKAR2B	5.84E-22	-0.12138	0.016	0.117	8.72E-18
MORC2	6.60E-22	-0.1205	0.007	0.109	9.87E-18
ZNF230	6.76E-22	-0.11299	0.019	0.127	1.01E-17
SLC25A20	6.89E-22	0.102383	0.023	0.084	1.03E-17
GAL3ST4	7.01E-22	0.135788	0.045	0.095	1.05E-17
EPB41	8.43E-22	-0.15257	0.012	0.119	1.26E-17
FAM57B	8.43E-22	-0.14432	0.037	0.157	1.26E-17
ANXA11	8.61E-22	-0.11996	0.012	0.115	1.29E-17
ERMARD	8.96E-22	-0.10509	0.01	0.11	1.34E-17
AC016700.	9.83E-22	-0.11845	0.017	0.112	1.47E-17
NET1	9.90E-22	-0.10884	0.003	0.101	1.48E-17
UTP15	9.93E-22	-0.10776	0.01	0.109	1.48E-17

MTMR10	1.07E-21	0.10008	0.021	0.075	1.60E-17
IGFBP6	1.08E-21	0.195925	0.031	0.07	1.62E-17
EZH1	1.14E-21	0.164748	0.044	0.101	1.70E-17
EPSTI1	1.17E-21	0.468657	0.08	0.096	1.75E-17
LNX1	1.24E-21	-0.13717	0.035	0.146	1.85E-17
NRF1	1.39E-21	-0.15261	0	0.102	2.08E-17
KDM1B	1.41E-21	-0.12984	0.005	0.109	2.10E-17
ZCCHC14	1.55E-21	-0.13409	0.007	0.109	2.32E-17
RP11-455F	1.68E-21	0.185091	0.026	0.063	2.51E-17
NCKIPSD	1.98E-21	-0.12857	0.01	0.114	2.96E-17
RECQL5	1.99E-21	-0.12509	0.009	0.111	2.98E-17
CHML	2.18E-21	-0.17246	0.002	0.115	3.26E-17
TBC1D13	2.19E-21	-0.11568	0.01	0.114	3.27E-17
ZDHHC13	2.27E-21	-0.12387	0.016	0.115	3.39E-17
MTURN	2.30E-21	-0.16835	0.016	0.128	3.43E-17
AP4B1	2.30E-21	-0.10477	0.017	0.119	3.44E-17
SPTSSB	2.45E-21	0.21948	0.024	0.053	3.67E-17
KITLG	2.88E-21	-0.10492	0.01	0.105	4.30E-17
TMEM38A	2.96E-21	0.103794	0.024	0.074	4.43E-17
DICER1-AS	2.96E-21	0.16482	0.033	0.08	4.43E-17
SP140L	2.97E-21	0.147306	0.023	0.062	4.45E-17
PRUNE2	3.11E-21	0.378387	0.077	0.088	4.64E-17
DNAJC12	3.32E-21	0.184626	0.03	0.074	4.97E-17
GNG10	3.34E-21	0.104259	0.024	0.074	4.99E-17
MAP3K3	3.67E-21	-0.12039	0.003	0.102	5.49E-17
APOBEC3G	3.87E-21	0.17954	0.031	0.07	5.78E-17
TBC1D19	3.97E-21	0.101201	0.028	0.085	5.93E-17
MTRNR2L1	4.19E-21	0.440281	0.206	0.174	6.26E-17
GID4	4.59E-21	-0.10982	0.009	0.107	6.85E-17
AC009948.	4.67E-21	-0.11002	0.009	0.105	6.98E-17
OSBPL5	5.02E-21	-0.10435	0.009	0.105	7.50E-17
ZNF77	5.11E-21	-0.15871	0.003	0.107	7.63E-17
FHOD3	5.61E-21	-0.10885	0.01	0.102	8.39E-17
TENM3	6.25E-21	-0.19953	0.012	0.114	9.35E-17
ATP1A2	6.42E-21	0.534803	0.101	0.093	9.59E-17
GLI3	6.64E-21	-0.10842	0.01	0.104	9.93E-17
LIN37	6.66E-21	0.162329	0.026	0.067	9.96E-17
TCN2	6.96E-21	0.141121	0.031	0.074	1.04E-16
ZC4H2	7.34E-21	-0.11853	0.024	0.126	1.10E-16
PCSK2	9.11E-21	-0.22778	0.019	0.128	1.36E-16
TMEM17	9.24E-21	-0.13061	0.007	0.106	1.38E-16
RHPN2	1.03E-20	0.171216	0.026	0.06	1.53E-16
LHPP	1.10E-20	0.213176	0.042	0.08	1.64E-16
TRO	1.16E-20	0.159339	0.037	0.086	1.73E-16
ATP2B4	1.22E-20	0.162378	0.04	0.086	1.83E-16
MAEL	1.29E-20	-0.10143	0.005	0.098	1.92E-16
CLDN10	1.34E-20	0.121562	0.059	0.143	2.00E-16
HOXB3	1.41E-20	-0.13009	0.012	0.102	2.11E-16
BTBD6	1.80E-20	-0.10723	0.003	0.093	2.68E-16
HAP1	2.58E-20	0.141724	0.033	0.075	3.86E-16
ZNF526	2.61E-20	-0.10863	0.016	0.111	3.90E-16

FANCE	2.63E-20	-0.14515	0.002	0.098	3.94E-16
TMCC1-AS1	2.65E-20	0.112638	0.023	0.069	3.96E-16
AC005076.	2.70E-20	-0.15261	0.009	0.105	4.04E-16
FERMT1	3.15E-20	0.199586	0.059	0.101	4.70E-16
CHGB	3.37E-20	0.167454	0.044	0.085	5.04E-16
SPIRE2	3.46E-20	-0.10187	0.007	0.095	5.17E-16
DGKB	3.57E-20	-0.10251	0.024	0.112	5.33E-16
CTNNA2	3.78E-20	0.102969	0.049	0.105	5.65E-16
GNG11	4.03E-20	0.143613	0.038	0.09	6.02E-16
RP11-295G	4.05E-20	-0.11257	0.007	0.104	6.06E-16
PSMG3-AS1	4.52E-20	-0.10655	0.009	0.101	6.75E-16
LINC-PINT	5.50E-20	0.292635	0.058	0.073	8.22E-16
ZNF180	5.74E-20	-0.12959	0.01	0.112	8.57E-16
MAF	6.56E-20	0.291439	0.051	0.067	9.81E-16
PDPR	6.83E-20	0.108791	0.028	0.083	1.02E-15
TTC9B	6.93E-20	-0.18188	0.04	0.153	1.04E-15
ZSCAN5A	6.97E-20	0.130439	0.031	0.081	1.04E-15
TAGLN	7.23E-20	0.427733	0.045	0.038	1.08E-15
WDR89	7.58E-20	-0.14169	0.003	0.1	1.13E-15
QSOX2	7.98E-20	-0.10304	0.012	0.106	1.19E-15
TRMT2B	8.49E-20	-0.13997	0.005	0.102	1.27E-15
CAPN10-AS	8.99E-20	-0.13805	0.007	0.1	1.34E-15
C1orf226	9.11E-20	0.213059	0.024	0.052	1.36E-15
GPRIN1	9.34E-20	-0.12711	0.01	0.106	1.40E-15
LAYN	1.02E-19	-0.10491	0.003	0.096	1.52E-15
AVEN	1.03E-19	-0.12909	0.003	0.101	1.55E-15
SERPINE1	1.08E-19	0.313898	0.063	0.096	1.62E-15
PRUNE	1.12E-19	-0.13048	0.005	0.1	1.68E-15
VPS54	1.13E-19	-0.16397	0.01	0.116	1.68E-15
GRID2	1.13E-19	-0.13758	0.023	0.122	1.69E-15
SYTL2	1.14E-19	0.359943	0.063	0.064	1.71E-15
ABCA8	1.14E-19	0.396733	0.061	0.063	1.71E-15
TRAPPC8	1.30E-19	-0.10589	0.009	0.102	1.94E-15
SMIM18	1.34E-19	-0.12093	0.023	0.116	2.00E-15
FAM214B	1.38E-19	-0.1067	0.009	0.1	2.06E-15
RP11-676J	1.49E-19	-0.25367	0.019	0.135	2.22E-15
PCDHGC3	1.64E-19	-0.13069	0.003	0.098	2.46E-15
TMEM117	1.66E-19	-0.11417	0.003	0.089	2.49E-15
THEM4	2.01E-19	-0.10408	0.009	0.098	3.01E-15
RABGAP1L	2.02E-19	0.132363	0.028	0.073	3.02E-15
TFAP2A	2.06E-19	-0.13314	0.016	0.107	3.08E-15
LCAT	2.14E-19	0.16361	0.017	0.052	3.20E-15
IER3IP1	2.27E-19	0.81285	0.108	0.011	3.40E-15
NPL	2.47E-19	0.152062	0.042	0.083	3.70E-15
SLC9A6	2.58E-19	-0.1139	0.007	0.099	3.85E-15
GPATCH3	2.83E-19	-0.15098	0.003	0.102	4.22E-15
KIAA1614	3.01E-19	-0.13119	0.007	0.099	4.50E-15
ADCY6	3.06E-19	-0.13207	0.007	0.102	4.57E-15
USP49	3.29E-19	-0.13005	0.007	0.101	4.92E-15
AFF3	3.55E-19	-0.10126	0.026	0.117	5.30E-15
LIN7A	3.55E-19	-0.12104	0.007	0.101	5.31E-15

CLCN6	3.68E-19	0.114135	0.024	0.069	5.49E-15
SWSAP1	3.74E-19	0.107396	0.016	0.059	5.58E-15
DLX6-AS1	4.13E-19	-0.11998	0.023	0.114	6.17E-15
UBE2V1	4.74E-19	-0.10793	0.009	0.099	7.09E-15
IGDCC4	5.10E-19	-0.10344	0.005	0.094	7.62E-15
HECA	6.33E-19	-0.12173	0.005	0.098	9.45E-15
DYNC1I1	6.37E-19	-0.22525	0.021	0.128	9.51E-15
IFIT3	6.79E-19	0.394269	0.068	0.085	1.01E-14
TRMT1L	6.79E-19	-0.11526	0.01	0.105	1.02E-14
CARD16	6.88E-19	0.610087	0.119	0.081	1.03E-14
PI3	6.95E-19	0.577231	0.045	0.046	1.04E-14
MAST3	7.19E-19	-0.10495	0.003	0.089	1.08E-14
RP11-127E	8.21E-19	-0.11714	0.007	0.096	1.23E-14
RHBDD1	8.75E-19	0.118549	0.021	0.065	1.31E-14
CTH	8.83E-19	0.209575	0.031	0.06	1.32E-14
ZNF573	9.85E-19	0.112213	0.031	0.084	1.47E-14
MTX3	1.00E-18	-0.11153	0.003	0.091	1.50E-14
APBB3	1.08E-18	0.11647	0.028	0.079	1.61E-14
IFI44L	1.15E-18	0.517838	0.103	0.099	1.73E-14
STAG3	1.16E-18	0.210011	0.049	0.075	1.74E-14
ASTE1	1.16E-18	-0.12524	0.005	0.096	1.74E-14
TTC21B	1.22E-18	-0.12332	0.017	0.114	1.83E-14
EARS2	1.23E-18	0.106131	0.024	0.074	1.83E-14
PIM2	1.32E-18	-0.11535	0.007	0.1	1.97E-14
C19orf47	1.32E-18	-0.1285	0.012	0.111	1.98E-14
IFIT1	1.36E-18	0.369027	0.056	0.07	2.03E-14
SHF	1.39E-18	-0.10585	0.009	0.094	2.08E-14
PITX2	1.42E-18	-0.17734	0.019	0.116	2.12E-14
PALB2	1.51E-18	-0.12683	0	0.088	2.26E-14
TAPBPL	1.65E-18	0.240879	0.054	0.09	2.47E-14
PVRL3	1.66E-18	0.130757	0.023	0.062	2.48E-14
NR1D1	1.77E-18	0.129136	0.024	0.073	2.64E-14
GCC1	2.00E-18	-0.10908	0.012	0.102	2.99E-14
DUOX1	2.06E-18	-0.16865	0.012	0.111	3.07E-14
CHST9	2.16E-18	0.135588	0.037	0.083	3.22E-14
ABCC3	2.19E-18	0.212553	0.035	0.053	3.28E-14
SCN1B	2.43E-18	-0.10557	0.003	0.09	3.63E-14
ZNF785	2.44E-18	0.14474	0.028	0.072	3.64E-14
SLC11A1	2.77E-18	0.232643	0.042	0.067	4.14E-14
MOB1B	2.90E-18	-0.15823	0.002	0.094	4.34E-14
BTN3A1	2.97E-18	0.175339	0.031	0.07	4.44E-14
SLC30A6	3.17E-18	-0.12187	0.01	0.102	4.74E-14
NHSL2	3.83E-18	0.705119	0.094	0.01	5.72E-14
FAM24B	3.83E-18	-0.10046	0.005	0.088	5.73E-14
ZNF615	3.99E-18	-0.10264	0.017	0.109	5.96E-14
DYRK2	4.20E-18	-0.12476	0.007	0.098	6.28E-14
KRT7	4.24E-18	0.121455	0.017	0.057	6.33E-14
HLA-DQA1	4.36E-18	0.515136	0.054	0.026	6.52E-14
SGPP1	4.67E-18	-0.11471	0.005	0.093	6.98E-14
COL18A1	4.72E-18	0.119289	0.023	0.068	7.05E-14
H1FX-AS1	4.91E-18	-0.10764	0.009	0.094	7.34E-14

PPIL6	5.02E-18	0.132487	0.024	0.06	7.51E-14
IFI27	5.03E-18	0.609569	0.112	0.09	7.51E-14
SRP14-AS1	5.08E-18	0.10513	0.031	0.083	7.60E-14
NPNT	5.47E-18	0.122149	0.026	0.074	8.17E-14
NEK11	5.54E-18	0.125816	0.026	0.068	8.27E-14
SLC38A9	6.49E-18	-0.10144	0.014	0.101	9.70E-14
HVCN1	7.24E-18	0.133619	0.024	0.064	1.08E-13
BEX5	7.36E-18	0.583597	0.092	0.051	1.10E-13
PDZD2	7.55E-18	0.180011	0.037	0.068	1.13E-13
EFTUD1	7.67E-18	-0.10657	0.01	0.099	1.15E-13
PLLP	7.92E-18	0.295964	0.061	0.086	1.18E-13
AREL1	8.15E-18	-0.10189	0.007	0.09	1.22E-13
RCAN3	8.58E-18	-0.12147	0	0.084	1.28E-13
CYB561D1	8.79E-18	0.115201	0.017	0.054	1.31E-13
MBOAT1	1.02E-17	-0.13263	0.002	0.091	1.53E-13
XKR4	1.14E-17	0.123441	0.04	0.079	1.70E-13
DLX2	1.22E-17	-0.1877	0.026	0.131	1.82E-13
GSX1	1.22E-17	-0.16829	0.009	0.091	1.82E-13
PDLIM4	1.24E-17	0.389797	0.072	0.078	1.86E-13
ALOX5AP	1.35E-17	0.202453	0.056	0.084	2.02E-13
CLCF1	1.39E-17	0.239846	0.024	0.037	2.08E-13
ZNF557	1.53E-17	0.118336	0.028	0.067	2.28E-13
FAM43A	1.53E-17	-0.11912	0	0.083	2.28E-13
AC079922.	1.66E-17	-0.15757	0.005	0.094	2.49E-13
ATP11C	1.73E-17	-0.1266	0.003	0.091	2.59E-13
HMGA2	1.84E-17	-0.10348	0.003	0.081	2.76E-13
TNR	1.89E-17	0.120522	0.063	0.106	2.83E-13
TMEFF2	2.01E-17	-0.12443	0.024	0.112	3.01E-13
SYT1	2.16E-17	-0.19676	0.026	0.126	3.23E-13
TET3	2.18E-17	-0.10537	0.009	0.093	3.26E-13
LRRC34	2.35E-17	-0.10868	0.005	0.088	3.52E-13
EGLN3	2.49E-17	0.168214	0.028	0.068	3.72E-13
NEU4	2.84E-17	0.201353	0.079	0.109	4.24E-13
MPP2	3.28E-17	-0.10444	0.009	0.094	4.91E-13
SMOC1	3.66E-17	0.195539	0.045	0.077	5.46E-13
SAA2	3.74E-17	0.915825	0.063	0.006	5.60E-13
TMSB4Y	3.96E-17	-0.102	0.003	0.083	5.92E-13
RDM1	4.85E-17	-0.14213	0	0.08	7.24E-13
ENTPD1-AS	5.74E-17	-0.12566	0.007	0.09	8.57E-13
CDH13	6.07E-17	0.180014	0.063	0.101	9.06E-13
AP001372.	6.27E-17	-0.1039	0.007	0.088	9.36E-13
XAF1	6.71E-17	0.405583	0.077	0.089	1.00E-12
GJA1	6.71E-17	0.520768	0.08	0.054	1.00E-12
APCDD1	6.92E-17	0.132435	0.023	0.062	1.03E-12
ANGPT2	6.92E-17	0.221763	0.031	0.057	1.03E-12
CTD-2035E	6.95E-17	-0.16402	0.005	0.099	1.04E-12
TMEM170B	7.50E-17	-0.11203	0.009	0.096	1.12E-12
SUPV3L1	9.28E-17	-0.12424	0.009	0.093	1.39E-12
ECE2	9.57E-17	0.110157	0.016	0.056	1.43E-12
MANBA	1.03E-16	0.119505	0.026	0.063	1.53E-12
OAS1	1.08E-16	0.578757	0.096	0.068	1.61E-12

NR2F2	1.13E-16	0.142684	0.042	0.084	1.69E-12
ARHGAP26	1.15E-16	0.205634	0.028	0.057	1.72E-12
DHRS3	1.46E-16	0.282008	0.045	0.067	2.18E-12
RP11-108M	1.52E-16	-0.10176	0.016	0.1	2.27E-12
PPP1R1C	1.68E-16	0.312951	0.045	0.056	2.51E-12
LPCAT4	2.28E-16	-0.11798	0.009	0.093	3.41E-12
KIAA1549L	2.47E-16	-0.12081	0.003	0.086	3.69E-12
LRRC4	2.50E-16	-0.10287	0.016	0.094	3.74E-12
SLITRK2	2.64E-16	-0.11767	0.021	0.105	3.95E-12
PLXNA3	2.69E-16	0.153988	0.031	0.065	4.01E-12
ELL2	3.00E-16	0.215582	0.044	0.069	4.48E-12
FAM122C	3.01E-16	-0.12973	0.003	0.088	4.50E-12
EVI2A	3.30E-16	0.286184	0.045	0.053	4.94E-12
RGN	3.41E-16	0.142062	0.026	0.064	5.10E-12
GALT	3.67E-16	0.165028	0.035	0.067	5.48E-12
TNFRSF14	3.97E-16	0.235143	0.033	0.046	5.93E-12
MX1	4.18E-16	0.215282	0.031	0.053	6.24E-12
C2CD4A	4.84E-16	-0.1049	0	0.075	7.23E-12
PAQR6	4.86E-16	0.159748	0.04	0.074	7.27E-12
C4orf36	5.09E-16	-0.15628	0.007	0.09	7.60E-12
PIFO	6.43E-16	0.489443	0.054	0.026	9.60E-12
ARHGAP5-A	6.51E-16	0.195039	0.024	0.049	9.73E-12
SGMS1	6.66E-16	-0.10422	0.002	0.083	9.95E-12
HOXA9	6.71E-16	-0.20913	0.012	0.104	1.00E-11
SLC12A7	7.61E-16	0.115137	0.017	0.051	1.14E-11
TFAP2B	7.84E-16	-0.201	0.014	0.106	1.17E-11
DLX6	9.46E-16	-0.18341	0.007	0.086	1.41E-11
PPM1L	9.94E-16	-0.13155	0.003	0.086	1.49E-11
SDCBP2	1.00E-15	0.16547	0.024	0.054	1.49E-11
IL1B	1.05E-15	0.152637	0.04	0.073	1.56E-11
ZNF783	1.05E-15	0.186666	0.028	0.057	1.57E-11
HDAC4	1.13E-15	-0.13405	0.009	0.091	1.69E-11
IGLON5	1.29E-15	-0.10723	0.014	0.085	1.92E-11
ERCC6L	1.37E-15	-0.12989	0.002	0.077	2.05E-11
CCDC88B	1.41E-15	0.166841	0.024	0.051	2.10E-11
ZNF286B	1.59E-15	-0.10595	0.007	0.086	2.37E-11
MS4A7	1.64E-15	0.14265	0.031	0.063	2.45E-11
DUSP26	1.66E-15	0.139804	0.058	0.091	2.47E-11
DAPK1	1.71E-15	-0.10665	0.003	0.079	2.55E-11
TCP11L1	1.73E-15	-0.13987	0.012	0.098	2.59E-11
CDK18	1.86E-15	0.277287	0.049	0.065	2.78E-11
TAF5	1.92E-15	-0.10304	0.005	0.08	2.88E-11
LINC00342	2.10E-15	-0.13242	0.003	0.083	3.13E-11
INADL	2.17E-15	-0.12987	0.003	0.081	3.24E-11
SLC25A45	2.35E-15	0.12247	0.021	0.052	3.51E-11
GPR1	2.51E-15	-0.18712	0.009	0.098	3.76E-11
C7orf61	2.58E-15	0.23505	0.042	0.054	3.86E-11
TREM2	2.65E-15	0.236077	0.038	0.051	3.95E-11
LAPTM5	2.73E-15	0.177023	0.058	0.096	4.07E-11
RPH3A	2.78E-15	0.184878	0.04	0.065	4.15E-11
MGMT	2.96E-15	0.211743	0.044	0.064	4.43E-11

SERPINF1	3.12E-15	0.215486	0.045	0.07	4.66E-11
CTD-2090I	3.18E-15	0.607836	0.079	0.009	4.75E-11
PLA2G2A	3.23E-15	0.549385	0.038	0.028	4.83E-11
MIR22HG	3.89E-15	0.105987	0.023	0.054	5.81E-11
ASIC1	4.10E-15	0.160142	0.038	0.067	6.12E-11
ALDH1L1	4.21E-15	0.545876	0.112	0.062	6.29E-11
USP54	5.69E-15	0.108103	0.024	0.064	8.50E-11
EPB41L4A	7.49E-15	-0.10851	0.01	0.091	1.12E-10
PTHLH	7.66E-15	0.147893	0.023	0.049	1.14E-10
BCL11A	7.95E-15	-0.13251	0.003	0.079	1.19E-10
PTPRN	8.25E-15	0.272119	0.051	0.059	1.23E-10
GPNMB	8.72E-15	0.226662	0.037	0.062	1.30E-10
TLDC1	9.37E-15	0.10757	0.024	0.058	1.40E-10
ZMYM6	9.94E-15	-0.14798	0.014	0.099	1.49E-10
RP3-460G2	1.08E-14	0.422395	0.07	0.052	1.62E-10
FCHO2	1.18E-14	-0.12808	0.009	0.09	1.76E-10
SLC6A9	1.40E-14	0.11604	0.024	0.062	2.09E-10
TNFAIP3	1.63E-14	0.290205	0.038	0.049	2.43E-10
CA3	1.63E-14	0.364641	0.037	0.049	2.43E-10
PROCR	1.75E-14	0.11086	0.012	0.04	2.62E-10
MASP1	1.77E-14	0.168221	0.045	0.069	2.65E-10
CYB5R2	1.79E-14	0.229678	0.026	0.035	2.67E-10
RP11-138A	2.01E-14	0.215079	0.054	0.067	3.01E-10
LFNG	2.32E-14	0.130505	0.03	0.067	3.46E-10
NPY	2.33E-14	-0.21363	0.01	0.088	3.48E-10
KCNJ3	2.37E-14	0.119915	0.038	0.07	3.54E-10
PCDHB14	2.59E-14	0.291585	0.047	0.049	3.88E-10
MR1	2.61E-14	0.11386	0.021	0.052	3.90E-10
TREM1	3.16E-14	0.587263	0.059	0.02	4.72E-10
ST6GAL2	3.44E-14	-0.15033	0.012	0.093	5.14E-10
POU4F1	3.48E-14	-0.10001	0.016	0.085	5.21E-10
HEATR3	3.92E-14	-0.13075	0.002	0.077	5.87E-10
MIR7-3HG	3.97E-14	0.620532	0.059	0.011	5.94E-10
ARPP21	4.12E-14	0.216612	0.049	0.067	6.15E-10
LYN	4.23E-14	0.110371	0.019	0.056	6.32E-10
AMPH	4.37E-14	-0.16341	0.002	0.077	6.53E-10
SFRP4	4.48E-14	0.140432	0.031	0.069	6.70E-10
AP1S3	5.15E-14	-0.11301	0.002	0.074	7.69E-10
PROM1	7.13E-14	-0.10409	0.014	0.084	1.07E-09
GBP3	7.16E-14	0.36683	0.059	0.064	1.07E-09
ONECUT2	7.50E-14	-0.12506	0.007	0.074	1.12E-09
NCAN	7.87E-14	0.118792	0.051	0.084	1.18E-09
TMBIM4	7.95E-14	0.677544	0.086	0.01	1.19E-09
FRMD3	8.28E-14	0.295473	0.047	0.059	1.24E-09
XXbac-BPG	9.15E-14	0.115488	0.024	0.053	1.37E-09
CTSV	9.25E-14	-0.118	0.002	0.072	1.38E-09
ZNF468	9.72E-14	-0.11393	0.003	0.077	1.45E-09
SOD3	1.01E-13	-0.12995	0.002	0.065	1.51E-09
SUOX	1.09E-13	0.109075	0.026	0.054	1.63E-09
GLIPR1L2	1.10E-13	0.172317	0.016	0.036	1.65E-09
PLCD1	1.14E-13	0.135929	0.021	0.047	1.71E-09

GPR37	1.28E-13	0.229069	0.051	0.062	1.92E-09
AFAP1L1	1.34E-13	0.12144	0.023	0.056	2.00E-09
HSPA6	1.48E-13	0.422433	0.054	0.063	2.21E-09
RP13-650J	1.48E-13	-0.1023	0.005	0.073	2.21E-09
5-Sep	1.55E-13	-0.10259	0.007	0.078	2.32E-09
CCER2	1.59E-13	0.161509	0.058	0.078	2.37E-09
MUC12	1.62E-13	0.260015	0.04	0.051	2.42E-09
ELFN1	1.70E-13	-0.10229	0.003	0.07	2.55E-09
CAMKK1	1.75E-13	-0.1347	0.005	0.074	2.62E-09
PTPRK	1.81E-13	0.112124	0.028	0.058	2.71E-09
ZNF510	1.91E-13	-0.11303	0.003	0.073	2.85E-09
CLSTN3	2.15E-13	0.116612	0.03	0.057	3.21E-09
IFIT2	2.20E-13	0.288942	0.037	0.046	3.29E-09
FCGBP	2.46E-13	0.149992	0.035	0.051	3.67E-09
SLC30A3	2.60E-13	-0.14827	0	0.062	3.89E-09
LDLRAP1	3.23E-13	-0.11676	0.002	0.072	4.82E-09
RP11-258C	3.88E-13	-0.1394	0.003	0.075	5.79E-09
ADCY2	4.03E-13	0.156321	0.023	0.035	6.02E-09
TRMT44	4.30E-13	-0.10399	0.002	0.069	6.43E-09
C1RL	4.54E-13	0.327422	0.038	0.021	6.79E-09
DFFB	4.58E-13	0.102029	0.021	0.052	6.84E-09
PSORS1C1	5.18E-13	0.276073	0.035	0.046	7.74E-09
ARHGAP22	5.45E-13	-0.10189	0.002	0.068	8.15E-09
ARHGDIB	5.69E-13	0.112188	0.035	0.07	8.51E-09
DNMT3B	5.84E-13	-0.12054	0.002	0.072	8.73E-09
TMEM37	5.85E-13	0.236938	0.024	0.026	8.74E-09
TMEM179	7.17E-13	-0.14758	0.007	0.081	1.07E-08
WNT5A	7.52E-13	-0.13223	0.007	0.074	1.12E-08
LOX	8.25E-13	0.145086	0.03	0.068	1.23E-08
SERF1A	8.46E-13	0.111087	0.017	0.038	1.26E-08
RP11-196G	9.84E-13	-0.13313	0.002	0.068	1.47E-08
LDOC1	1.18E-12	0.132738	0.038	0.07	1.76E-08
ARHGEF11	1.19E-12	-0.10146	0.005	0.072	1.78E-08
KIAA0556	1.38E-12	-0.11015	0.007	0.074	2.06E-08
PTTG2	1.43E-12	-0.15542	0	0.058	2.14E-08
GRAMD1C	1.51E-12	-0.10336	0.007	0.077	2.26E-08
ZNF823	1.53E-12	-0.11236	0.003	0.069	2.28E-08
KCTD14	1.53E-12	0.118284	0.012	0.033	2.29E-08
AGTPBP1	1.57E-12	-0.11383	0.003	0.072	2.35E-08
ADCYAP1R1	1.71E-12	0.160861	0.026	0.047	2.55E-08
MB21D1	1.78E-12	-0.1222	0.002	0.064	2.65E-08
MORN3	1.81E-12	0.20156	0.019	0.035	2.71E-08
SMIM3	2.14E-12	0.643136	0.068	0.006	3.19E-08
DEPDC7	2.24E-12	-0.12119	0.005	0.073	3.35E-08
TSHZ2	2.30E-12	0.23344	0.045	0.058	3.44E-08
ITGB4	2.40E-12	0.120714	0.021	0.044	3.59E-08
APOL4	2.47E-12	0.115461	0.028	0.064	3.68E-08
TNFSF13B	2.54E-12	0.1368	0.03	0.062	3.79E-08
HSD17B6	2.79E-12	0.176625	0.024	0.04	4.16E-08
RARRES1	3.96E-12	-0.11127	0.005	0.07	5.92E-08
MAMSTR	4.22E-12	-0.1123	0.002	0.064	6.30E-08

RP1-17K7.	4.46E-12	0.347138	0.063	0.04	6.67E-08
GIMAP2	4.53E-12	0.182753	0.019	0.035	6.77E-08
TPTEP1	4.54E-12	0.21002	0.03	0.049	6.79E-08
RP11-499E	4.66E-12	0.107011	0.023	0.057	6.97E-08
FGF7	5.46E-12	0.350267	0.038	0.016	8.16E-08
C2orf74	5.49E-12	0.431592	0.044	0.022	8.21E-08
PPT2	5.67E-12	0.121401	0.023	0.049	8.47E-08
CNTN1	6.52E-12	0.109136	0.037	0.065	9.75E-08
NPPA	7.36E-12	0.175775	0.049	0.075	1.10E-07
HOXC11	9.09E-12	-0.11881	0.005	0.064	1.36E-07
LINC01114	9.81E-12	0.194935	0.038	0.052	1.47E-07
HLA-DQB1	1.06E-11	0.253238	0.042	0.04	1.58E-07
GBP4	1.23E-11	0.267592	0.051	0.062	1.83E-07
ANGPTL4	1.48E-11	0.536007	0.07	0.033	2.21E-07
DNAJA4	1.65E-11	-0.1272	0.012	0.079	2.46E-07
MT1A	1.65E-11	0.470059	0.052	0.012	2.46E-07
BIRC3	1.70E-11	0.229731	0.021	0.032	2.55E-07
SLC39A4	1.76E-11	0.209794	0.026	0.033	2.63E-07
MAP2K6	1.82E-11	0.116117	0.016	0.036	2.71E-07
AZGP1	2.03E-11	0.418846	0.063	0.038	3.03E-07
TBCEL	2.04E-11	-0.11318	0.002	0.063	3.05E-07
VAMP8	2.46E-11	0.100237	0.021	0.049	3.68E-07
C10orf10	2.52E-11	0.539699	0.08	0.037	3.77E-07
MYBPC1	2.61E-11	0.234273	0.038	0.053	3.90E-07
METTL20	2.77E-11	0.133121	0.023	0.047	4.14E-07
RPRML	3.13E-11	-0.11769	0.003	0.062	4.68E-07
NEUROD1	3.46E-11	-0.11486	0.014	0.075	5.17E-07
LINC01152	3.85E-11	0.117471	0.014	0.035	5.75E-07
LINC00869	4.06E-11	0.508556	0.054	0.002	6.07E-07
CXCL2	4.06E-11	0.445952	0.042	0.011	6.07E-07
ZNF850	4.32E-11	-0.11193	0.003	0.062	6.45E-07
S100A4	4.35E-11	0.156172	0.026	0.051	6.51E-07
RP11-395E	4.69E-11	0.178567	0.019	0.027	7.01E-07
FAM107A	5.16E-11	0.227614	0.033	0.042	7.70E-07
BRINP3	5.54E-11	0.183571	0.031	0.044	8.28E-07
KCTD16	5.70E-11	-0.11374	0.005	0.06	8.52E-07
FAXDC2	6.51E-11	0.169553	0.03	0.044	9.73E-07
IFITM2	6.80E-11	0.219944	0.044	0.065	1.02E-06
LAIR1	7.09E-11	0.12559	0.03	0.044	1.06E-06
HIST1H2AC	7.54E-11	0.279855	0.072	0.078	1.13E-06
NMNAT3	7.68E-11	0.17641	0.03	0.044	1.15E-06
PRKCG	8.51E-11	0.454093	0.047	0.01	1.27E-06
CAMK4	8.65E-11	-0.11722	0.002	0.058	1.29E-06
ZNF154	9.74E-11	0.133131	0.026	0.043	1.46E-06
TRIM59	9.82E-11	-0.13929	0.005	0.067	1.47E-06
STX3	1.01E-10	0.13974	0.021	0.03	1.51E-06
ARHGAP18	1.08E-10	0.160028	0.021	0.042	1.61E-06
GPRC5C	1.37E-10	0.288693	0.047	0.052	2.04E-06
STEAP1	1.95E-10	0.21529	0.026	0.038	2.91E-06
HHIP-AS1	2.06E-10	-0.12048	0.003	0.062	3.07E-06
KLF15	2.32E-10	0.109589	0.035	0.062	3.47E-06

ZNF582	2.61E-10	0.111036	0.026	0.049	3.91E-06
BBS1	2.76E-10	0.109959	0.017	0.031	4.12E-06
IGFLR1	2.90E-10	0.129573	0.021	0.035	4.33E-06
MYO1F	3.57E-10	0.202612	0.023	0.035	5.33E-06
LGALS9	3.93E-10	0.138907	0.019	0.038	5.88E-06
EHD3	4.37E-10	0.131163	0.017	0.031	6.53E-06
PGAM2	4.42E-10	0.298157	0.035	0.037	6.60E-06
ZNF415	4.66E-10	0.1462	0.026	0.048	6.97E-06
SULT1C4	4.76E-10	0.10511	0.023	0.042	7.11E-06
NXN	5.60E-10	-0.12265	0.003	0.06	8.37E-06
TNS1	5.76E-10	0.172557	0.033	0.043	8.61E-06
PCDH15	6.97E-10	0.237618	0.03	0.03	1.04E-05
GUCY1B3	7.57E-10	-0.11267	0.007	0.06	1.13E-05
UNC80	7.58E-10	0.160966	0.035	0.046	1.13E-05
DNAH9	7.81E-10	0.125467	0.019	0.036	1.17E-05
IRAK2	8.78E-10	0.125624	0.014	0.03	1.31E-05
C2	8.91E-10	0.246602	0.03	0.026	1.33E-05
AC016831.	9.43E-10	0.113899	0.026	0.048	1.41E-05
LST1	1.03E-09	0.119636	0.021	0.041	1.54E-05
DZIP1L	1.17E-09	-0.10549	0.002	0.053	1.74E-05
VWA3B	1.18E-09	0.136621	0.023	0.036	1.76E-05
NUDT7	1.18E-09	0.211416	0.031	0.035	1.76E-05
FXYD1	1.43E-09	0.187813	0.037	0.037	2.13E-05
CRABP2	1.44E-09	0.106979	0.024	0.054	2.15E-05
CMYA5	1.51E-09	0.167652	0.023	0.037	2.26E-05
CTC-479C5	1.53E-09	0.134283	0.021	0.032	2.29E-05
ZNF84	1.57E-09	0.172627	0.028	0.028	2.35E-05
RNASE1	1.81E-09	0.221169	0.037	0.043	2.70E-05
FAM71E1	1.83E-09	0.207317	0.023	0.031	2.74E-05
DUSP23	1.93E-09	0.268053	0.045	0.049	2.89E-05
DIO2	1.98E-09	0.358167	0.049	0.04	2.96E-05
RP1-43E13	2.32E-09	0.119145	0.019	0.042	3.47E-05
RP11-22N1	2.36E-09	0.14572	0.016	0.028	3.52E-05
RP11-326G	3.29E-09	0.100175	0.016	0.033	4.92E-05
CLEC2D	3.36E-09	0.119134	0.019	0.031	5.03E-05
FAM105A	3.63E-09	-0.10821	0.002	0.051	5.43E-05
SLC44A3	3.89E-09	0.160503	0.016	0.019	5.81E-05
PDE6B	4.23E-09	0.114643	0.03	0.047	6.32E-05
SPAG4	4.49E-09	0.107888	0.012	0.03	6.71E-05
AC023590.	4.96E-09	0.156799	0.03	0.036	7.41E-05
TCEAL6	6.31E-09	0.234323	0.028	0.032	9.43E-05
NEGR1	7.36E-09	0.142902	0.026	0.036	0.00011
FOLR1	8.23E-09	0.302098	0.028	0.012	0.000123
PCSK1	8.25E-09	0.157737	0.023	0.035	0.000123
AC009961.	8.37E-09	-0.11146	0.002	0.049	0.000125
HS3ST1	8.77E-09	0.113739	0.03	0.044	0.000131
TRIM21	1.01E-08	0.109557	0.014	0.03	0.000151
PACRG	1.01E-08	0.110601	0.019	0.032	0.000152
ARHGAP24	1.09E-08	0.347273	0.056	0.027	0.000162
CH25H	1.23E-08	0.395173	0.047	0.023	0.000184
TUBA4A	1.30E-08	0.103245	0.016	0.041	0.000194

AC114730.	1.32E-08	0.199746	0.044	0.053	0.000198
C6orf141	1.79E-08	0.141516	0.017	0.028	0.000268
HCST	1.82E-08	0.131672	0.021	0.028	0.000272
SERPINB1	2.32E-08	0.328994	0.035	0.025	0.000347
SCN3B	2.62E-08	0.130417	0.038	0.047	0.000392
CADPS	2.81E-08	0.266262	0.033	0.021	0.00042
RP11-11N9	3.17E-08	0.147469	0.019	0.035	0.000474
JPH4	3.32E-08	0.101401	0.038	0.051	0.000496
ACSL6	3.33E-08	0.100682	0.028	0.043	0.000497
KCNN3	3.40E-08	0.104622	0.012	0.023	0.000508
DPYD	3.47E-08	0.254095	0.037	0.025	0.000519
LY86	3.68E-08	0.114536	0.017	0.028	0.00055
SLC6A6	3.93E-08	0.245012	0.026	0.019	0.000587
MYH7B	4.05E-08	0.112429	0.019	0.027	0.000606
SGIP1	4.13E-08	0.11354	0.03	0.044	0.000617
ZFPM2	4.17E-08	-0.12585	0.017	0.073	0.000623
AGMO	4.68E-08	0.104905	0.019	0.036	0.0007
PCAT6	5.23E-08	0.104312	0.016	0.028	0.000782
FCGR2A	5.80E-08	0.161639	0.021	0.03	0.000867
SCN9A	6.00E-08	0.106328	0.021	0.036	0.000896
C11orf71	6.18E-08	0.20755	0.023	0.02	0.000924
CD37	7.02E-08	0.120554	0.023	0.032	0.001049
HLA-DQA2	8.18E-08	0.362776	0.033	0.005	0.001223
C1QTNF3	8.41E-08	0.160893	0.031	0.051	0.001258
RSPH1	9.52E-08	0.255162	0.026	0.012	0.001423
LGR6	9.95E-08	0.185968	0.023	0.026	0.001487
SLITRK3	1.11E-07	0.102527	0.016	0.028	0.001654
MYCNOS	1.11E-07	0.116558	0.016	0.026	0.001659
C6orf15	1.12E-07	0.262429	0.063	0.072	0.001672
RP11-479J	1.13E-07	0.162682	0.014	0.014	0.001681
SERPINA1	1.17E-07	0.217264	0.03	0.027	0.001745
RP11-713F	1.19E-07	0.165426	0.028	0.033	0.001774
MYOT	1.25E-07	-0.12633	0.002	0.044	0.001865
PNPLA4	1.36E-07	0.213139	0.03	0.028	0.002033
FBLN5	1.41E-07	0.204836	0.024	0.03	0.00211
H3F3C	1.44E-07	0.233412	0.058	0.048	0.002152
NDUFA4L2	1.48E-07	0.188501	0.031	0.044	0.002209
HLA-DMB	1.62E-07	0.183599	0.033	0.042	0.002422
CD163	1.70E-07	0.110214	0.021	0.036	0.002543
LINC00882	2.09E-07	0.132681	0.019	0.032	0.003131
SPOCK1	2.14E-07	0.147472	0.033	0.04	0.003191
KIAA0040	2.46E-07	0.185084	0.03	0.035	0.003679
CLDN11	2.66E-07	0.210052	0.023	0.021	0.003971
TNFAIP2	2.69E-07	0.149433	0.016	0.021	0.004023
HIF3A	3.00E-07	0.156213	0.035	0.041	0.004485
RP3-508I1	3.54E-07	0.292828	0.037	0.019	0.005285
IFI30	4.43E-07	0.109454	0.012	0.02	0.006616
LTBR	4.81E-07	0.155082	0.024	0.023	0.007188
ZNF665	5.69E-07	0.151124	0.019	0.022	0.008509
FAM149A	5.95E-07	-0.10559	0.005	0.047	0.008892
RENBP	6.39E-07	0.167878	0.019	0.022	0.009554

MTRNR2L3	6.49E-07	0.150647	0.019	0.02	0.009704
CTD-2020K	6.69E-07	0.184377	0.024	0.015	0.009993
MMP7	6.69E-07	0.339829	0.026	0.014	0.010002
ZNF404	8.06E-07	0.113464	0.016	0.028	0.012038
C5AR1	8.26E-07	0.133228	0.021	0.031	0.012349
CRISPLD2	9.05E-07	0.228482	0.033	0.03	0.013522
THSD4	1.02E-06	0.176607	0.035	0.046	0.015301
APLN	1.15E-06	0.110957	0.016	0.031	0.017225
FGF22	1.19E-06	0.128399	0.016	0.019	0.01785
PTGES	1.33E-06	0.10328	0.014	0.021	0.019845
HSD11B1	1.42E-06	0.166229	0.016	0.015	0.021148
SSTR2	1.49E-06	0.108126	0.014	0.021	0.022226
NR2F2-AS1	1.83E-06	0.153752	0.028	0.026	0.027342
OR4N2	1.94E-06	0.206508	0.028	0.021	0.029007
RP11-118F	2.01E-06	-0.10081	0.005	0.043	0.030047
CEACAM1	2.05E-06	0.138659	0.021	0.022	0.030662
SPRED3	2.34E-06	0.153829	0.016	0.02	0.035002
ACOT11	2.60E-06	0.151457	0.019	0.019	0.038842
HP	2.60E-06	0.396525	0.026	0.001	0.038922
HSD17B14	2.74E-06	0.17	0.028	0.037	0.040875
MYO16	2.93E-06	0.100089	0.016	0.026	0.043713
RP11-46H1	3.05E-06	0.270902	0.035	0.012	0.045526
TMEM144	3.21E-06	0.133505	0.014	0.019	0.047962
LRRC2	3.63E-06	0.214477	0.026	0.007	0.054295
PDGFRL	4.39E-06	0.123611	0.019	0.02	0.065662
GPR17	4.44E-06	0.100406	0.019	0.031	0.066356
IL1R1	4.78E-06	0.124745	0.012	0.014	0.071459
GALNT15	5.05E-06	0.226549	0.024	0.007	0.075461
RP11-138I	5.64E-06	0.12053	0.024	0.033	0.084222
ETV7	5.68E-06	0.195445	0.016	0.011	0.084924
OPCML	6.32E-06	0.128578	0.019	0.021	0.094377
STAB1	7.12E-06	0.123547	0.012	0.016	0.106393
ENTPD1	7.24E-06	0.10728	0.016	0.022	0.108187
AC015936.	7.75E-06	0.17789	0.023	0.01	0.115825
PPM1N	8.02E-06	0.175748	0.023	0.017	0.119786
RBFOX3	8.53E-06	0.192624	0.023	0.011	0.127404
DES	8.82E-06	0.127184	0.026	0.031	0.131826
FPR1	9.02E-06	0.202212	0.023	0.014	0.134755
FAM198B	9.16E-06	0.148221	0.019	0.023	0.136842
TSIX	9.65E-06	0.206274	0.033	0.02	0.144276
HLA-G	1.00E-05	0.137209	0.023	0.02	0.149845
BCL2L15	1.11E-05	0.200961	0.026	0.006	0.165251
RNASE4	1.18E-05	0.204968	0.03	0.014	0.176763
HPS1	1.32E-05	0.130357	0.017	0.019	0.197753
PPP1R3C	1.38E-05	0.100583	0.014	0.025	0.206249
GREM1	1.39E-05	0.274848	0.035	0.005	0.207577
CSF1R	1.40E-05	0.124426	0.019	0.023	0.208519
CCL5	1.40E-05	0.103687	0.014	0.016	0.209363
PAIP2B	1.45E-05	0.112266	0.012	0.017	0.217409
BCAS1	1.47E-05	0.257894	0.044	0.033	0.219377
CTGF	1.73E-05	0.128053	0.024	0.028	0.25813

TAC3	1.81E-05	-0.16787	0.002	0.032	0.270462
RP11-437L	1.83E-05	0.217501	0.019	0	0.272907
ALDH1L1-A	2.15E-05	0.205207	0.026	0.005	0.320636
TVP23A	2.18E-05	0.137638	0.024	0.021	0.325555
HSD3B7	2.56E-05	0.111718	0.017	0.021	0.383204
CHIT1	2.74E-05	0.303321	0.021	0.002	0.409984
METTL25	2.94E-05	0.105136	0.014	0.023	0.439141
RP4-613B2	3.63E-05	0.108497	0.014	0.017	0.541905
TNFSF10	3.74E-05	0.187182	0.023	0.017	0.558456
RTP4	3.84E-05	0.22929	0.023	0.017	0.574449
SLC6A11	4.23E-05	0.160414	0.017	0.016	0.632582
RP11-49I1	4.51E-05	0.125136	0.01	0.012	0.674135
RP11-16E1	5.80E-05	0.119698	0.014	0.021	0.866456
LGI1	6.20E-05	0.136864	0.028	0.03	0.926396
IL6	6.54E-05	0.186236	0.017	0.015	0.977862
HSPB8	7.16E-05	0.165888	0.019	0.012	1
KRBOX1	7.31E-05	0.275662	0.026	0.004	1
MARVELD3	7.31E-05	0.139989	0.019	0.017	1
MYH14	7.35E-05	0.152094	0.016	0.014	1
TMEM255B	7.91E-05	0.123776	0.01	0.011	1
S100A1	8.78E-05	0.192311	0.019	0.022	1
CFH	9.56E-05	0.111859	0.01	0.011	1
THBS1	9.68E-05	0.147624	0.019	0.017	1
RP11-284F	0.000104	0.22823	0.03	0.007	1
C10orf11	0.000108	0.10633	0.014	0.021	1
TGFBR3	0.000114	0.140918	0.014	0.016	1
TRIM72	0.000117	0.132997	0.016	0	1
ALG1L	0.000117	0.117674	0.009	0.014	1
MAPT-AS1	0.000119	0.17139	0.033	0.019	1
MUC20	0.000124	0.102886	0.014	0.02	1
PLIN5	0.000131	0.11478	0.016	0.02	1
CDKN2B	0.000136	0.13177	0.028	0.02	1
CA9	0.00014	0.168818	0.026	0.027	1
RP1-122K4	0.000145	0.168267	0.014	0.005	1
CHRM3	0.000147	0.132222	0.019	0.022	1
CSF2RA	0.00015	0.127625	0.016	0.012	1
SYNPO2L	0.000151	0.144138	0.017	0.014	1
SELPLG	0.000155	0.121054	0.017	0.017	1
EIF4EBP3	0.000167	0.180245	0.023	0.001	1
CLN3	0.000177	0.239774	0.023	0.005	1
C1orf194	0.000195	0.166814	0.017	0.016	1
SYT4	0.000243	0.160014	0.017	0.01	1
ZNF517	0.000245	0.120184	0.014	0.012	1
SLC8A3	0.000268	0.128144	0.017	0.014	1
CDIPT-AS1	0.000274	0.265516	0.024	0.006	1
DMKN	0.000316	0.176761	0.019	0.001	1
RP11-299L	0.000347	0.101755	0.017	0.017	1
DEPTOR	0.000365	0.162465	0.01	0.007	1
NAA60	0.000389	0.279265	0.031	0.006	1
COLEC12	0.000397	0.114899	0.014	0.012	1
PLCG2	0.000401	0.193437	0.017	0.004	1

GPR158-AS	0.00041	0.152444	0.014	0.01	1
TNXB	0.000502	0.106822	0.014	0.011	1
RIIAD1	0.000591	0.160197	0.014	0.005	1
HSPB2	0.000614	0.152323	0.017	0.007	1
GPR89B	0.000615	0.156749	0.017	0.01	1
SELENBP1	0.000652	0.131596	0.01	0.01	1
RP11-373N	0.000656	0.121065	0.016	0.01	1
CRIP1	0.000662	0.103564	0.01	0.019	1
CXCL3	0.000697	0.204745	0.03	0.025	1
RGR	0.000707	0.157719	0.019	0.009	1
RP13-188A	0.000744	0.110981	0.019	0.017	1
SERPINA3	0.000755	0.138399	0.012	0	1
SLITRK4	0.000755	0.135777	0.012	0	1
C11orf70	0.000904	0.18758	0.021	0.011	1
IL6R	0.001077	0.103072	0.017	0.009	1
ASPA	0.001207	0.139623	0.014	0.006	1
BATF2	0.001222	0.161029	0.023	0.02	1
LUC7L2	0.001234	0.216151	0.021	0.002	1
MT1H	0.001246	0.117348	0.016	0.006	1
PRPH	0.001302	0.106496	0.012	0.01	1
DYNLRB2	0.001356	0.179306	0.016	0.002	1
MAOA	0.001373	0.106134	0.016	0.016	1
CLDN5	0.001414	0.123714	0.016	0.02	1
CFB	0.001528	0.100556	0.012	0.01	1
DCN	0.00157	0.101629	0.009	0.007	1
CD70	0.001598	0.114027	0.01	0.011	1
GLRA3	0.002077	0.15514	0.021	0.009	1
FABP3	0.002149	0.308625	0.021	0.01	1
SLC7A8	0.002365	0.119204	0.017	0.009	1
RP11-643M	0.00239	0.11732	0.009	0.009	1
ZNF556	0.002551	0.117353	0.014	0.001	1
P2RX6	0.002555	0.116623	0.014	0.01	1
RP11-469H	0.002579	0.139978	0.01	0.007	1
TEX29	0.002716	0.153845	0.012	0.006	1
COL8A1	0.003442	0.105924	0.016	0.015	1
NEBL	0.003816	0.125728	0.019	0.011	1
HIST1H2BG	0.003901	0.131052	0.012	0.007	1
RNF144B	0.004114	0.165945	0.014	0.002	1
PTPRH	0.004649	0.106257	0.01	0.004	1
GNLY	0.00493	0.113779	0.01	0.007	1
RP11-495F	0.004998	0.14041	0.009	0	1
ITGAL	0.004998	0.102048	0.009	0	1
DHRS4-AS1	0.005332	0.14788	0.016	0.002	1
NPTX1	0.005437	0.146408	0.01	0.005	1
TFCP2L1	0.005708	0.149012	0.016	0.011	1
C5AR2	0.005902	0.177123	0.021	0.005	1
KCNA7	0.006149	0.104632	0.014	0.001	1
PTPRT	0.006268	0.124795	0.01	0.009	1
SLC5A3	0.007183	0.115861	0.012	0.001	1
BCL2L14	0.00795	0.113457	0.014	0.004	1
CA5A	0.008271	0.117064	0.014	0.002	1

CCL4L2	0.00953	0.12828	0.012	0.002	1
ECM2	0.00982	0.103178	0.012	0.01	1
ZNF490	0.01001	0.100129	0.009	0.009	1
LRRN4CL	0.010062	0.122403	0.01	0.002	1
WFDC1	0.011823	0.103707	0.01	0.007	1
AL592528.	0.012146	0.153191	0.012	0.004	1
CAMP	0.013059	0.129131	0.007	0	1
PCDH18	0.013272	0.119469	0.014	0.006	1
DHRS2	0.014692	-0.16744	0.002	0.016	1
SAA4	0.015434	0.138435	0.01	0.001	1
TLR3	0.015461	0.112815	0.009	0.004	1
KCNK6	0.016295	0.107332	0.009	0.002	1
TEKT1	0.018245	0.122334	0.012	0.004	1
RAMP3	0.020074	0.108706	0.012	0.009	1
MYO15A	0.020553	0.114964	0.012	0.002	1
LAMA2	0.022832	0.113883	0.016	0.012	1
ZMYND12	0.025961	0.145004	0.01	0.001	1
CCBE1	0.031065	0.120167	0.021	0.01	1
OSR1	0.031295	0.15264	0.016	0.009	1
RP11-306G	0.033719	0.118783	0.014	0.002	1
RP11-326C	0.038882	0.101575	0.012	0.002	1
LINC0032C	0.040963	0.139566	0.012	0.005	1
ENPP5	0.042932	0.130474	0.014	0.007	1
CTD-2369F	0.043768	0.124074	0.014	0.002	1
GS1-18A18	0.051875	0.113161	0.009	0.007	1
LXN	0.053094	0.107988	0.01	0.001	1
PRMT5-AS1	0.061404	0.107404	0.007	0.002	1
IGF2	0.063668	0.157078	0.019	0.016	1
GJC3	0.070494	0.116668	0.01	0.002	1
GPR27	0.076968	0.101099	0.009	0.004	1
PDZK1IP1	0.077912	0.108934	0.009	0.002	1
AC002451.	0.084548	0.101804	0.009	0.001	1
THTPA	0.090803	0.121855	0.012	0.005	1
ABHD1	0.09198	0.120148	0.012	0.006	1
RP11-242D	0.137456	0.108354	0.012	0.005	1
