# Supplementary material for: A Novel Molecular Classification Method for Glioblastoma Based on Tumor Cell Differentiation Trajectories
Source: Stem Cells Int. 2023 Feb 22;2023:2826815. doi: 10.1155/2023/2826815 (PMC10643041; doi:10.1155/2023/2826815)
Supplement: Supplementary 10 — Supplementary Table 8: different pathway list of the 6 clusters in tumor cell. [file 2826815.f10.pdf]

	CSCL	Class-G	Opc-G	Neo-G	Ac-G	Undiff-G1	Undiff-G2
GO-CONDEN	0.629478	0.02663	0.036356	0.063204	-0.06858	-0.06261	-0.04563
GO-MCM-CC	0.426188	########	0.188863	0.053144	-0.03604	-0.01087	-0.01635
GO-MITOTI	0.468391	0.09031	0.098213	0.004852	0.042448	0.014404	0.008347
GO-DNA-LI	0.436284	0.065282	0.098187	0.057879	0.007697	-0.07752	-0.17969
GO-MITOTI	0.678	0.311487	0.038421	0.017791	-0.12883	-0.21048	-0.20501
GO-CHROMC	0.320796	-0.02194	0.071588	0.064163	-0.09276	-0.06482	-0.04573
GO-COHESI	0.314341	0.019496	0.027226	0.013737	-0.11855	-0.14403	-0.15115
GO-CONDEN	0.330829	0.037115	0.073809	0.075335	-0.02337	-0.0486	-0.05126
GO-CENTRC	0.634833	0.361556	0.137113	0.107586	-0.08828	-0.27072	-0.32341
GO-HISTON	0.546558	0.275767	0.098696	0.098525	-0.03036	-0.09202	-0.06085
GO-REGULA	0.330699	0.664526	0.05893	0.012686	-0.01839	-0.24635	-0.31518
GO-REGULA	0.222206	0.542173	-0.07552	-0.1541	-0.00935	-0.31888	-0.35686
GO-SECONE	0.006931	0.322271	-0.12688	-0.05424	0.058081	-0.25376	-0.27855
NGO-MALIG	0.33348	0.620212	-0.18086	-0.18773	0.203572	-0.2155	-0.34825
GO-AMYLOI	0.033744	0.307313	-0.12829	-0.10466	0.119441	-0.14016	-0.09322
GO-L-ASCC	0.233664	0.506753	-0.05748	-0.0783	-0.01047	-0.25494	-0.27084
KEGG-PENT	0.272816	0.535324	-0.07157	-0.08731	0.009638	-0.31088	-0.39144
GO-FILOPC	0.264715	0.525946	-0.04177	-0.13447	-0.13911	-0.32904	-0.41198
GO-RETROG	0.294733	0.546186	0.036214	0.083145	-0.06374	-0.30054	-0.39178
GO-AXONAL	0.238191	0.480694	0.134175	0.25885	-0.02717	-0.28811	-0.42848
GO-CERAMI	0.049903	0.283111	-0.06359	-0.0786	-0.09784	-0.31136	-0.30211
GO-PHAGOC	0.073488	0.303705	-0.14653	-0.15088	-0.10504	-0.30745	-0.33139
GO-REGULA	-0.17745	0.128765	0.107503	-0.0003	1.33E-05	-0.03575	-0.08682
GO-POSITI	-0.19654	0.156503	0.174529	0.121322	0.058038	0.021119	-0.02521
GO-MONOCY	-0.20245	0.065838	0.174047	0.108244	0.121995	-0.09752	-0.13068
GO-EXCITA	0.101124	0.16149	0.23889	0.186305	-0.02548	-0.00357	-0.00779
GO-SPINAL	-0.18646	0.004445	0.13817	0.105242	-0.09523	-0.07629	-0.10024
GO-POSITI	-0.19724	0.041488	0.184866	0.161144	0.072452	0.092524	0.027224
GO-POSITI	-0.2092	0.001686	0.162128	0.153624	0.128695	-0.03332	0.005812
GO-NEURON	-0.20595	0.022024	0.23964	0.204067	0.037266	0.087424	0.089389
GO-CELL-D	-0.19561	-0.01911	0.200314	0.135012	-0.04343	0.051861	0.064465
GO-PARANC	-0.1848	0.014622	0.306959	0.275842	0.221699	0.189013	0.185214
GO-NEURON	-0.07686	0.092539	0.306401	0.284076	0.105634	0.183968	0.190531
GO-NEUROT	-0.19047	0.044923	0.213305	0.13703	0.15268	0.151715	0.152134
GO-NEUROT	-0.03281	0.11389	0.171018	0.155766	0.140661	0.128067	0.115039
GO-DENDRI	-0.13378	0.036978	0.29545	0.225178	0.240113	0.232296	0.244753
GO-CEREBE	-0.20031	-0.03724	0.239096	0.1938	0.086849	0.156807	0.189178
GO-POSITI	-0.20667	0.073388	0.18996	0.121391	0.143167	0.093049	0.157818
GO-POSITI	-0.23606	0.094577	0.287258	0.510884	0.094542	-0.01942	0.033476
GO-OLFACT	-0.16317	0.02696	0.220353	0.342327	0.015972	0.048691	0.089631
GO-POSITI	-0.16878	-0.01624	0.209951	0.298451	0.185452	0.219992	0.257109
GO-MOTOR-	-0.20559	0.043253	0.114528	0.181769	-0.1083	-0.12898	-0.15305
GO-REGULA	-0.19157	0.131576	0.163163	0.252929	0.011794	-0.0471	-0.04879
GO-MODULA	-0.21252	0.08746	0.130857	0.187734	0.035244	0.111045	0.105274
GO-DOPAMI	-0.24796	0.111081	0.142326	0.193851	0.024044	-0.00559	-0.0047
GO-CEREBR	0.195014	0.215652	0.171596	0.296039	-0.04002	-0.14654	-0.17763
GO-SYNAPT	-0.2009	0.068817	-0.00388	0.124584	-0.0663	-0.11333	-0.13051
GO-CENTRA	-0.16416	0.091735	0.099325	0.142071	-0.08283	-0.08416	-0.09777
GO-FIBRIN	-0.20327	0.012952	-0.01879	0.012005	0.298194	-0.02124	0.116081
GO-METALL	-0.16334	0.1366	-0.03961	-0.07233	0.26934	0.128651	0.159382

GO-M-BAND	0.119109	0.211142	-0.05539	-0.08182	0.2481	0.203055	0.17862
GO-MUSCLE	-0.15925	-0.04575	-0.07737	-0.00465	0.175905	0.111263	0.062465
GO-HYDROG	-0.18731	0.243116	0.042624	-0.01994	0.291616	-0.03027	-0.09393
GO-ANTIOX	-0.22184	0.2035	-0.02697	-0.02625	0.210045	-0.10443	-0.15072
GO-MYELIN	-0.3578	0.114854	-0.03298	-0.01248	0.185466	0.017799	0.017275
KEGG-COMP	-0.20149	0.025395	-0.04545	-0.0242	0.17118	-0.15669	-0.07525
GO-COLLAG	-0.20007	0.089291	-0.01972	-0.06737	0.189879	-0.02058	0.001077
GO-COMPLE	-0.22245	0.05598	-0.07616	-0.06285	0.121245	-0.20984	-0.15904
GO-INTERM	-0.19639	0.034118	0.023305	0.017507	0.215663	0.023431	-0.10668
GO-ANTIGE	-0.18388	-0.00139	-0.03737	-0.03904	0.136175	0.041308	0.055739
GO-EOSINO	-0.18024	0.143696	0.101989	0.148838	0.263373	0.057344	0.144815
GO-OXIDOR	-0.01144	0.157886	0.248244	0.265822	0.392032	0.198473	0.220789
GO-IMMUNE	-0.18372	-0.12208	0.037796	0.043491	0.142487	0.046	0.118588
GO-NEUROT	-0.19531	0.075071	0.021421	0.040075	0.114446	-0.05905	-0.08713
GO-NEUTRO	-0.20048	0.01554	0.114707	0.080131	0.199088	0.087536	0.115802
GO-REGULA	-0.18803	-0.03584	0.090512	0.097819	0.174271	-0.04789	-0.00161
GO-OXIDOR	-0.21054	0.097656	0.187794	0.154529	0.262292	0.13055	0.161246
GO-CHEMOK	-0.23105	-0.16342	0.137978	0.135083	0.197573	0.171947	0.189812
GO-T-HELP	-0.20122	0.044681	0.115747	0.064478	0.172008	0.055903	0.098036
GO-REGULA	-0.09004	0.096993	0.115512	-0.07515	0.159874	0.053256	0.032406
GO-POSITI	-0.19118	0.046704	0.086874	0.057562	0.127507	0.008299	0.022704
GO-CELL-F	-0.1884	-0.08036	0.01747	-0.03545	-0.02411	0.117131	0.115212
GO-CELL-F	-0.20485	-0.04804	0.137519	0.05676	0.04079	0.183376	0.189636
GO-ENDODE	-0.17798	-0.14332	0.047133	0.012917	0.00352	0.206831	0.192522
GO-REGULA	0.033476	-0.05221	0.118169	0.116657	0.080209	0.171079	0.150225
GO-CELL-D	-0.19169	-0.15454	0.069778	0.078342	0.09024	0.145172	0.139002
GO-NEUROF	-0.18993	-0.12479	0.340201	0.368381	0.302318	0.415349	0.336889
GO-PEPTID	-0.17884	-0.05684	0.154896	0.171191	0.084699	0.183679	0.10541
GO-GAMMA-	-0.22066	-0.23329	0.085362	0.064821	0.042712	0.222509	0.174757
GO-IMMUNE	-0.19444	-0.17199	0.157678	0.139808	0.16898	0.303226	0.265618
GO-ENDOCR	-0.20303	-0.15983	0.099302	0.10019	0.179177	0.272868	0.266235
GO-STRUCT	-0.03837	-0.14751	0.083225	0.199859	0.152825	0.2336	0.477066
GO-RETINO	-0.19541	-0.11695	0.165737	0.149719	0.251156	0.416667	0.590617
GO-REGULA	-0.20826	0.073456	-0.01923	-0.07841	0.043862	0.22456	0.344279
GO-REGULA	-0.24104	0.013225	0.124184	0.06591	0.133308	0.330971	0.439438
GO-REGULA	-0.01692	-0.11228	0.142118	0.123651	0.171962	0.329306	0.434834
GO-REGULA	-0.18413	-0.08221	0.108764	0.141218	0.077338	0.209708	0.309928
GO-ACTIVI	-0.18988	-0.07245	0.055915	0.086701	0.105796	0.098494	0.197397
GO-WNT-AC	-0.19605	-0.12111	-0.05608	-0.09351	0.038373	0.3383	0.43321
GO-COMPLE	-0.17926	-0.2397	0.168516	0.148903	0.37023	0.635475	0.730108
GO-SPHING	0.021119	-0.1926	0.173717	0.190951	0.183197	0.353187	0.447425
GO-AXONEM	-0.17619	-0.30128	0.19206	0.206737	0.386995	0.687201	0.77762
GO-REGULA	-0.21542	-0.05154	0.049138	0.038371	0.076141	0.096347	0.180648
GO-MYOSIN	-0.21944	-0.20771	0.19457	0.243258	0.276502	0.468529	0.549832
GO-FIBRIL	-0.20414	-0.26712	0.229789	0.228071	0.432044	0.661401	0.737465
GO-WNT-PR	-0.2022	-0.07747	0.033359	-0.00277	0.150367	0.360428	0.43389
GO-NADPLU	-0.2257	0.055559	0.047638	0.026831	0.021202	0.123073	0.196262
KEGG-HEDG	-0.18366	-0.17679	0.033288	0.011764	0.058435	0.332446	0.401405
