# Supplementary material for: A Novel Molecular Classification Method for Glioblastoma Based on Tumor Cell Differentiation Trajectories
Source: Stem Cells Int. 2023 Feb 22;2023:2826815. doi: 10.1155/2023/2826815 (PMC10643041; doi:10.1155/2023/2826815)
Supplement: Supplementary 11 — Supplementary Table 9: clinical characteristics of 137 human IDH-mut GBM samples. [file 2826815.f11.pdf]

Supplementary Table 9 Clinical characteristics of 137 human IDH-mut GBM samples

		**Total (n=137)**	**Ac_G (n=21)**	**Class_G (n=62)**	**Neo_G (n=14)**	**OpcG (n=29)**	**Undiff (n=11)**	**p-value**
**Event (%)**	Alive	18 (13.1)	4 (19.0)	7 (11.3)	6 (42.9)	3 (10.3)	0 (0.0)	**0.016**
	Death	119 (86.9)	17 (81.0)	55 (88.7)	8 (57.1)	26 (89.7)	11 (100.0)	
**Age (median [IQR])**		55.00 [45.00, 62.00]	47.00 [40.00, 58.00]	55.50 [45.00, 62.00]	54.50 [48.75, 59.50]	57.00 [49.00, 63.00]	50.00 [44.00, 64.00]	0.626
**Gender (%)**	Female	54 (39.4)	9 (42.9)	22 (35.5)	5 (35.7)	14 (48.3)	4 (36.4)	0.812
	Male	83 (60.6)	12 (57.1)	40 (64.5)	9 (64.3)	15 (51.7)	7 (63.6)	
**Radiotherapy (%)**	No	24 (17.5)	2 (9.5)	10 (16.1)	3 (21.4)	8 (27.6)	1 (9.1)	0.443
	Yes	113 (82.5)	19 (90.5)	52 (83.9)	11 (78.6)	21 (72.4)	10 (90.9)	
**Chemotherapy (%)**	No	23 (16.8)	4 (19.0)	11 (17.7)	1 (7.1)	6 (20.7)	1 (9.1)	0.767
	Yes	114 (83.2)	17 (81.0)	51 (82.3)	13 (92.9)	23 (79.3)	10 (90.9)	
**Size (median [IQR])**		4.78 [3.95, 5.50]	4.74 [4.20, 5.31]	4.90 [3.94, 5.58]	5.06 [4.36, 5.27]	4.73 [3.95, 5.56]	4.86 [3.92, 5.32]	0.988
**Surgery-type (%)**	Biopsy	10 (7.3)	1 (4.8)	5 (8.1)	1 (7.1)	1 (3.4)	2 (18.2)	0.077
	Sub-resection	47 (34.3)	13 (61.9)	16 (25.8)	7 (50.0)	8 (27.6)	3 (27.3)	
	Total-resection	80 (58.4)	7 (33.3)	41 (66.1)	6 (42.9)	20 (69.0)	6 (54.5)	
