# Supplementary material for: A Novel Molecular Classification Method for Glioblastoma Based on Tumor Cell Differentiation Trajectories
Source: Stem Cells Int. 2023 Feb 22;2023:2826815. doi: 10.1155/2023/2826815 (PMC10643041; doi:10.1155/2023/2826815)
Supplement: Supplementary 12 — Supplementary Table 10: Univariate and multivariate Cox proportional hazards analyses of clinicopathological variables and 5 classifications in 137 human IDH-mut GBM samples. [file 2826815.f12.pdf]

Supplementary Table 10 Univariate and multivariate Cox proportional hazards analyses of clinicopathological variables and 5 classifications in 137 human IDH-mut GBM samples

**Variables**		**Univariate cox**			**Multivariate cox**	
	**Hazard ratio**	**95% CI**	**p-value**	**Hazard ratio**	**95% CI**	**p-value**
**Gender**						
Female	-	-	-			
Male	0.88	0.78-1.65	0.516			
**Age**						
≤35	-	-	-	-	-	-
>35	0.58	1.04-2.87	0.035	1.76	1.05-2.94	0.034
**Size**						
≤3.5	-	-	-	-	-	-
>3.5	1.668	0.68-4.1	0.264			
**Radiotherapy**						
No	-	-	-	-	-	-
Yes	1.87	0.36-0.8	**0.002**	0.58	0.38-0.88	0.016
**Chemotherapy**						
No	-	-	-			
Yes	1.86	0.35-0.83	**0.005**			
**Surgery-type**						
Biopsy	-	-	-	-	-	-
Sub_section	1.34	0.95-1.88	0.091	1.18	0.83-1.69	0.351
Total_section	0.54	0.35-0.81	**<0.001**	0.57	0.38-0.87	0.017
**Group**						
Ac-G	-	-	-	-	-	-
Class_G	1.34	0.78-2.31	0.297	1.3	0.74-2.27	0.362
Neo-G	0.45	0.2-0.98	**0.031**	0.46	0.21-1.01	0.045

Opc-G	0.83	0.45-1.54	0.566	0.88	0.47-1.64	0.694
Undiff-G	1.42	0.66-3.05	0.374	1.58	0.73-3.42	0.241
