# Supplementary material for: A Novel Molecular Classification Method for Glioblastoma Based on Tumor Cell Differentiation Trajectories
Source: Stem Cells Int. 2023 Feb 22;2023:2826815. doi: 10.1155/2023/2826815 (PMC10643041; doi:10.1155/2023/2826815)
Supplement: Supplementary 3 — Supplementary Table 1: differentially expressed genes (DEGs) in identified clusters. [file 2826815.f3.pdf]

	p_val	avg_logFC	pct.1	pct.2	p_val_adj	cluster	gene
MALAT1	4.38E-68	0.964054	0.963	0.996	6.54E-64	Undiff-G	MALAT1
SEC61G	1.46E-62	1.571338	0.941	0.797	2.19E-58	Undiff-G	SEC61G
CHI3L1	5.47E-62	1.86183	0.89	0.52	8.17E-58	Undiff-G	CHI3L1
MT2A	8.38E-62	1.579659	0.934	0.782	1.25E-57	Undiff-G	MT2A
FTL	9.75E-62	1.145351	0.956	0.994	1.46E-57	Undiff-G	FTL
TMSB4X	1.78E-61	1.046026	0.971	0.997	2.66E-57	Undiff-G	TMSB4X
ACTB	4.78E-60	1.035659	0.971	0.993	7.15E-56	Undiff-G	ACTB
GAPDH	1.42E-55	1.198554	0.934	0.985	2.12E-51	Undiff-G	GAPDH
CLU	2.22E-53	1.412215	0.904	0.781	3.32E-49	Undiff-G	CLU
B2M	8.66E-49	1.002675	0.919	0.971	1.29E-44	Undiff-G	B2M
TMSB10	2.53E-46	1.140405	0.897	0.966	3.77E-42	Undiff-G	TMSB10
CCNI	2.10E-44	-1.61265	0.022	0.714	3.14E-40	Undiff-G	CCNI
ITM2B	2.72E-44	-1.07166	0.074	0.808	4.06E-40	Undiff-G	ITM2B
YBX1	2.34E-42	-1.11148	0.066	0.771	3.49E-38	Undiff-G	YBX1
EEF1A1	5.67E-42	1.134212	0.868	0.825	8.47E-38	Undiff-G	EEF1A1
MT1X	3.25E-41	1.79932	0.794	0.575	4.86E-37	Undiff-G	MT1X
RAC1	5.41E-41	-1.05258	0.059	0.751	8.09E-37	Undiff-G	RAC1
RTN4	3.60E-40	-1.41526	0.044	0.708	5.38E-36	Undiff-G	RTN4
C19orf43	6.83E-40	-1.34805	0.029	0.68	1.02E-35	Undiff-G	C19orf43
CLTA	3.33E-39	-1.49863	0	0.635	4.98E-35	Undiff-G	CLTA
HMGN1	4.42E-37	-1.18581	0.037	0.672	6.61E-33	Undiff-G	HMGN1
PFN1	7.47E-37	-0.71159	0.118	0.817	1.12E-32	Undiff-G	PFN1
HNRNPA0	7.56E-36	-1.2231	0.029	0.649	1.13E-31	Undiff-G	HNRNPA0
U2AF1	8.25E-36	-1.30541	0.022	0.638	1.23E-31	Undiff-G	U2AF1
JUNB	2.95E-35	-1.03809	0.074	0.731	4.41E-31	Undiff-G	JUNB
BTG1	5.30E-35	-1.14297	0.066	0.696	7.93E-31	Undiff-G	BTG1
SNX3	1.36E-34	-0.63161	0.074	0.724	2.03E-30	Undiff-G	SNX3
MTDH	4.51E-34	-1.29312	0.022	0.617	6.74E-30	Undiff-G	MTDH
JTB	7.73E-34	-1.272	0.022	0.616	1.15E-29	Undiff-G	JTB
SLPI	1.59E-33	1.891948	0.463	0.146	2.38E-29	Undiff-G	SLPI
YWHAQ	1.62E-33	-0.83352	0.044	0.651	2.42E-29	Undiff-G	YWHAQ
COX5A	2.04E-33	-1.18729	0.022	0.605	3.05E-29	Undiff-G	COX5A
ACTG1	3.18E-33	0.985502	0.846	0.95	4.75E-29	Undiff-G	ACTG1
MZT2B	3.98E-33	-1.30089	0.015	0.6	5.94E-29	Undiff-G	MZT2B
ATP5D	4.00E-33	-1.26139	0.029	0.621	5.97E-29	Undiff-G	ATP5D
YWHAB	4.21E-33	-0.71795	0.081	0.71	6.29E-29	Undiff-G	YWHAB
CHI3L2	6.18E-33	1.894952	0.5	0.177	9.23E-29	Undiff-G	CHI3L2
NDFIP1	2.48E-32	-1.18967	0.029	0.608	3.70E-28	Undiff-G	NDFIP1
ATP6V0B	3.10E-32	-0.40856	0.132	0.806	4.63E-28	Undiff-G	ATP6V0B
GLTSCR2	5.89E-32	-1.33797	0.007	0.57	8.80E-28	Undiff-G	GLTSCR2
EIF3F	6.36E-32	-0.89849	0.029	0.612	9.50E-28	Undiff-G	EIF3F
MARCKS	9.46E-32	-1.39723	0.037	0.604	1.41E-27	Undiff-G	MARCKS
HN1	1.00E-31	-0.65701	0.066	0.682	1.50E-27	Undiff-G	HN1
IER2	1.44E-31	-1.23506	0.037	0.622	2.16E-27	Undiff-G	IER2
UBE2D2	1.65E-31	-0.89032	0.022	0.595	2.46E-27	Undiff-G	UBE2D2
APOE	3.06E-31	-0.94488	0.088	0.693	4.57E-27	Undiff-G	APOE
SERP1	3.55E-31	-1.20539	0.037	0.61	5.30E-27	Undiff-G	SERP1
NDUFA3	7.45E-31	-0.68208	0.066	0.655	1.11E-26	Undiff-G	NDUFA3
LGALS3	1.04E-30	1.739438	0.699	0.478	1.56E-26	Undiff-G	LGALS3
MAP1LC3B	1.18E-30	-1.0051	0.015	0.572	1.77E-26	Undiff-G	MAP1LC3B

C1QBP	1.79E-30	-1.20821	0.015	0.561	2.68E-26	Undiff-G	C1QBP
ASAH1	2.16E-30	-0.61971	0.066	0.658	3.23E-26	Undiff-G	ASAH1
H2AFY	3.66E-30	-0.8835	0.044	0.614	5.47E-26	Undiff-G	H2AFY
FIS1	7.00E-30	-0.59951	0.059	0.645	1.05E-25	Undiff-G	FIS1
MIF	9.39E-30	-0.80724	0.081	0.677	1.40E-25	Undiff-G	MIF
UQCR11	9.69E-30	1.61225	0.39	0.112	1.45E-25	Undiff-G	UQCR11
YWHAH	1.16E-29	-1.22609	0.022	0.571	1.73E-25	Undiff-G	YWHAH
ARL6IP4	1.41E-29	-0.97335	0.059	0.631	2.10E-25	Undiff-G	ARL6IP4
PABPC1	6.20E-29	-0.39326	0.125	0.752	9.26E-25	Undiff-G	PABPC1
SCAND1	6.48E-29	-1.1351	0.037	0.577	9.68E-25	Undiff-G	SCAND1
UQCRFS1	7.27E-29	-0.90412	0.015	0.551	1.09E-24	Undiff-G	UQCRFS1
KDELR2	1.07E-28	-0.88235	0.029	0.575	1.60E-24	Undiff-G	KDELR2
CYC1	1.17E-28	-1.0107	0.015	0.546	1.75E-24	Undiff-G	CYC1
C4orf48	1.41E-28	-1.32118	0.007	0.532	2.11E-24	Undiff-G	C4orf48
SEPW1	2.18E-28	-0.58992	0.096	0.679	3.26E-24	Undiff-G	SEPW1
SRSF2	2.80E-28	-0.90552	0.051	0.612	4.19E-24	Undiff-G	SRSF2
VAPA	4.02E-28	-0.64291	0.074	0.64	6.01E-24	Undiff-G	VAPA
SRSF9	4.31E-28	-0.94002	0.029	0.568	6.44E-24	Undiff-G	SRSF9
RANBP1	4.35E-28	-0.92159	0.044	0.584	6.49E-24	Undiff-G	RANBP1
ATP1B3	4.72E-28	-1.07282	0.022	0.551	7.05E-24	Undiff-G	ATP1B3
CST3	5.30E-28	-0.50083	0.154	0.794	7.92E-24	Undiff-G	CST3
ZNF428	5.81E-28	-1.23888	0.022	0.551	8.68E-24	Undiff-G	ZNF428
AP2S1	6.43E-28	-0.44398	0.088	0.667	9.61E-24	Undiff-G	AP2S1
STMN1	9.00E-28	-0.81352	0.074	0.637	1.34E-23	Undiff-G	STMN1
PRELID1	1.88E-27	-1.07763	0.037	0.566	2.81E-23	Undiff-G	PRELID1
UQCRB	2.38E-27	-0.37434	0.096	0.685	3.56E-23	Undiff-G	UQCRB
KIF5B	7.74E-27	-1.15248	0.015	0.517	1.16E-22	Undiff-G	KIF5B
HNRNPM	8.34E-27	-0.98411	0.029	0.549	1.25E-22	Undiff-G	HNRNPM
EIF4G2	1.04E-26	-0.35335	0.088	0.666	1.56E-22	Undiff-G	EIF4G2
RHOB	1.13E-26	-0.76651	0.066	0.615	1.69E-22	Undiff-G	RHOB
LAMP1	1.31E-26	-1.18827	0.022	0.527	1.96E-22	Undiff-G	LAMP1
ARF5	1.50E-26	-0.92397	0.022	0.535	2.24E-22	Undiff-G	ARF5
LTF	1.99E-26	1.794555	0.426	0.148	2.97E-22	Undiff-G	LTF
DUSP1	2.67E-26	-1.03827	0.051	0.581	3.99E-22	Undiff-G	DUSP1
PCBP2	3.45E-26	-0.45538	0.096	0.669	5.16E-22	Undiff-G	PCBP2
GNB2	3.67E-26	-0.79922	0.051	0.575	5.49E-22	Undiff-G	GNB2
AKR1B1	7.37E-26	-0.36768	0.081	0.631	1.10E-21	Undiff-G	AKR1B1
CAPZA2	8.16E-26	-0.29829	0.096	0.661	1.22E-21	Undiff-G	CAPZA2
ARL5A	8.59E-26	-1.08686	0.022	0.523	1.28E-21	Undiff-G	ARL5A
FSCN1	9.90E-26	-0.99281	0.015	0.51	1.48E-21	Undiff-G	FSCN1
GTF3A	1.29E-25	-0.98054	0.015	0.51	1.92E-21	Undiff-G	GTF3A
RNASET2	1.94E-25	-1.45935	0.007	0.488	2.90E-21	Undiff-G	RNASET2
MATR3	1.96E-25	-1.13142	0.007	0.492	2.92E-21	Undiff-G	MATR3
PDIA3	1.97E-25	-0.57373	0.059	0.589	2.94E-21	Undiff-G	PDIA3
PYURF	2.26E-25	-0.82294	0.037	0.547	3.37E-21	Undiff-G	PYURF
CALM1	3.11E-25	-0.42898	0.162	0.779	4.64E-21	Undiff-G	CALM1
COTL1	3.30E-25	-0.98412	0.022	0.515	4.93E-21	Undiff-G	COTL1
ISCU	3.34E-25	-0.62877	0.051	0.572	5.00E-21	Undiff-G	ISCU
RHEB	4.84E-25	-0.32222	0.088	0.644	7.23E-21	Undiff-G	RHEB
EIF1	5.47E-25	1.009729	0.794	0.976	8.18E-21	Undiff-G	EIF1
MARCKSL1	7.06E-25	-1.23708	0.051	0.55	1.05E-20	Undiff-G	MARCKSL1

CALM3	7.43E-25	-0.60858	0.074	0.599	1.11E-20	Undiff-G	CALM3
UBXN1	7.87E-25	-0.40843	0.088	0.627	1.18E-20	Undiff-G	UBXN1
PCBP1	8.59E-25	-0.46815	0.066	0.594	1.28E-20	Undiff-G	PCBP1
AURKAIP1	9.95E-25	-0.51949	0.081	0.612	1.49E-20	Undiff-G	AURKAIP1
GADD45GIF	1.22E-24	-0.71476	0.059	0.569	1.82E-20	Undiff-G	GADD45GIP1
ARPC4	2.51E-24	-0.72092	0.029	0.519	3.75E-20	Undiff-G	ARPC4
PNRC1	2.63E-24	-0.75231	0.044	0.546	3.93E-20	Undiff-G	PNRC1
UBE2N	2.71E-24	-0.68294	0.037	0.527	4.05E-20	Undiff-G	UBE2N
PGLS	2.77E-24	-0.91938	0.029	0.516	4.14E-20	Undiff-G	PGLS
BLOC1S1	3.31E-24	-0.18881	0.103	0.654	4.95E-20	Undiff-G	BLOC1S1
ARPC5	4.42E-24	-0.21949	0.118	0.679	6.60E-20	Undiff-G	ARPC5
MTCH1	4.65E-24	-0.70713	0.044	0.54	6.96E-20	Undiff-G	MTCH1
RALY	4.82E-24	-1.13799	0	0.461	7.20E-20	Undiff-G	RALY
AP1S2	5.18E-24	-1.10314	0.029	0.506	7.74E-20	Undiff-G	AP1S2
RTFDC1	5.57E-24	-0.50239	0.044	0.544	8.33E-20	Undiff-G	RTFDC1
RAB31	7.76E-24	-1.17132	0.007	0.465	1.16E-19	Undiff-G	RAB31
FAM49B	1.07E-23	-1.00356	0.029	0.512	1.60E-19	Undiff-G	FAM49B
C7orf50	1.09E-23	-0.59912	0.029	0.513	1.62E-19	Undiff-G	C7orf50
P4HB	1.24E-23	-0.54834	0.066	0.578	1.85E-19	Undiff-G	P4HB
TPM3	1.40E-23	-0.54612	0.059	0.564	2.09E-19	Undiff-G	TPM3
SUB1	2.14E-23	-0.20952	0.169	0.763	3.19E-19	Undiff-G	SUB1
HNRNPU	2.23E-23	-0.69382	0.044	0.536	3.33E-19	Undiff-G	HNRNPU
USMG5	2.27E-23	-0.23591	0.096	0.626	3.39E-19	Undiff-G	USMG5
KRT10	2.29E-23	-0.78472	0.066	0.564	3.42E-19	Undiff-G	KRT10
COX16	2.61E-23	-0.92214	0.029	0.506	3.90E-19	Undiff-G	COX16
SNF8	2.94E-23	-0.86159	0.007	0.465	4.40E-19	Undiff-G	SNF8
PPP4C	3.04E-23	-0.57071	0.051	0.549	4.55E-19	Undiff-G	PPP4C
HMGN3	3.36E-23	-0.41057	0.088	0.615	5.02E-19	Undiff-G	HMGN3
NDUFB10	3.41E-23	-0.43101	0.11	0.646	5.10E-19	Undiff-G	NDUFB10
PARP1	3.63E-23	-0.95312	0.015	0.477	5.42E-19	Undiff-G	PARP1
PPP1R15A	4.00E-23	-1.18967	0.015	0.472	5.98E-19	Undiff-G	PPP1R15A
CTSD	4.09E-23	-0.34779	0.103	0.645	6.11E-19	Undiff-G	CTSD
TMEM160	5.63E-23	-1.01016	0.015	0.471	8.42E-19	Undiff-G	TMEM160
TIMM13	5.66E-23	-0.35364	0.081	0.597	8.45E-19	Undiff-G	TIMM13
H2AFV	5.87E-23	-0.51311	0.044	0.528	8.77E-19	Undiff-G	H2AFV
BRI3	6.59E-23	-0.99465	0.015	0.473	9.85E-19	Undiff-G	BRI3
NME1	6.87E-23	-0.36565	0.066	0.574	1.03E-18	Undiff-G	NME1
METTL9	6.98E-23	-0.80173	0.015	0.474	1.04E-18	Undiff-G	METTL9
DEK	7.89E-23	-1.06733	0.015	0.469	1.18E-18	Undiff-G	DEK
GNAS	8.08E-23	-0.43196	0.118	0.653	1.21E-18	Undiff-G	GNAS
TTC3	8.15E-23	-1.00972	0.044	0.522	1.22E-18	Undiff-G	TTC3
CSNK1A1	8.43E-23	-0.57698	0.066	0.564	1.26E-18	Undiff-G	CSNK1A1
GRB2	1.02E-22	-0.67399	0.022	0.486	1.53E-18	Undiff-G	GRB2
ZFP36L2	1.07E-22	-0.77031	0.044	0.521	1.59E-18	Undiff-G	ZFP36L2
GUK1	1.09E-22	-0.17214	0.14	0.71	1.63E-18	Undiff-G	GUK1
NDUFA11	1.34E-22	-0.20448	0.162	0.748	2.00E-18	Undiff-G	NDUFA11
PTMS	1.42E-22	-0.90506	0.074	0.559	2.12E-18	Undiff-G	PTMS
APLP2	1.46E-22	-0.47205	0.088	0.599	2.18E-18	Undiff-G	APLP2
TIMP1	1.46E-22	1.467713	0.699	0.62	2.19E-18	Undiff-G	TIMP1
NOP10	1.50E-22	-0.33571	0.088	0.612	2.24E-18	Undiff-G	NOP10
KHDRBS1	1.55E-22	-0.98834	0.029	0.491	2.31E-18	Undiff-G	KHDRBS1

FUS	1.68E-22	-0.22804	0.11	0.649	2.51E-18	Undiff-G	FUS
NFKBIA	1.74E-22	-0.75591	0.037	0.505	2.60E-18	Undiff-G	NFKBIA
C9orf16	1.78E-22	-0.27404	0.081	0.588	2.66E-18	Undiff-G	C9orf16
CKB	1.89E-22	-0.8866	0.088	0.588	2.83E-18	Undiff-G	CKB
KTN1	1.90E-22	-0.81555	0.051	0.533	2.84E-18	Undiff-G	KTN1
DYNC1I2	2.01E-22	-0.902	0.022	0.474	3.01E-18	Undiff-G	DYNC1I2
RBPJ	2.21E-22	-0.8189	0.022	0.474	3.30E-18	Undiff-G	RBPJ
SET	2.21E-22	-0.5842	0.074	0.566	3.31E-18	Undiff-G	SET
LAMTOR2	2.39E-22	-0.42152	0.088	0.593	3.58E-18	Undiff-G	LAMTOR2
AUP1	2.46E-22	-0.4517	0.051	0.528	3.68E-18	Undiff-G	AUP1
RTN3	2.75E-22	-0.16032	0.118	0.661	4.11E-18	Undiff-G	RTN3
NENF	2.83E-22	-0.79239	0.037	0.499	4.23E-18	Undiff-G	NENF
PUF60	3.44E-22	-0.43335	0.059	0.547	5.15E-18	Undiff-G	PUF60
C20orf24	3.77E-22	-0.81109	0.022	0.472	5.63E-18	Undiff-G	C20orf24
RWDD1	3.87E-22	-0.41128	0.051	0.533	5.78E-18	Undiff-G	RWDD1
UFC1	4.48E-22	-0.33947	0.074	0.567	6.69E-18	Undiff-G	UFC1
TERF2IP	4.75E-22	-0.78439	0.051	0.523	7.10E-18	Undiff-G	TERF2IP
RBX1	5.60E-22	-0.11953	0.125	0.672	8.38E-18	Undiff-G	RBX1
ANP32B	6.79E-22	-1.04197	0.015	0.455	1.01E-17	Undiff-G	ANP32B
TALDO1	6.84E-22	-0.17593	0.096	0.603	1.02E-17	Undiff-G	TALDO1
ACTR2	7.07E-22	-1.03844	0.022	0.464	1.06E-17	Undiff-G	ACTR2
STUB1	7.33E-22	-0.89521	0.022	0.466	1.09E-17	Undiff-G	STUB1
STRAP	8.29E-22	-0.1935	0.066	0.559	1.24E-17	Undiff-G	STRAP
POLR2E	9.10E-22	-0.44896	0.059	0.541	1.36E-17	Undiff-G	POLR2E
SQSTM1	9.38E-22	-0.69316	0.059	0.529	1.40E-17	Undiff-G	SQSTM1
LAPTM5	1.08E-21	-1.558	0.007	0.441	1.61E-17	Undiff-G	LAPTM5
DUT	1.17E-21	-1.03579	0.022	0.46	1.75E-17	Undiff-G	DUT
EIF3D	1.19E-21	-0.83499	0.029	0.482	1.77E-17	Undiff-G	EIF3D
FEZ2	1.33E-21	-0.93186	0.015	0.454	1.98E-17	Undiff-G	FEZ2
BCAP31	1.59E-21	-0.19915	0.103	0.624	2.37E-17	Undiff-G	BCAP31
NUTF2	1.59E-21	-0.65181	0.015	0.454	2.37E-17	Undiff-G	NUTF2
NUCKS1	1.75E-21	-0.34338	0.118	0.642	2.62E-17	Undiff-G	NUCKS1
SFPQ	1.78E-21	-0.61605	0.066	0.548	2.67E-17	Undiff-G	SFPQ
BAX	1.81E-21	-0.3102	0.081	0.571	2.71E-17	Undiff-G	BAX
ATP6V0D1	2.46E-21	-0.30258	0.051	0.516	3.68E-17	Undiff-G	ATP6V0D1
HSPA1A	2.46E-21	-1.05774	0.044	0.497	3.68E-17	Undiff-G	HSPA1A
PSMA2	2.57E-21	1.590678	0.243	0.059	3.84E-17	Undiff-G	PSMA2
CD63	2.76E-21	1.037496	0.757	0.926	4.12E-17	Undiff-G	CD63
C6orf62	2.88E-21	-0.7813	0.022	0.457	4.30E-17	Undiff-G	C6orf62
OAZ2	3.10E-21	-0.57258	0.051	0.511	4.64E-17	Undiff-G	OAZ2
ZFAND5	3.27E-21	-0.94339	0.029	0.468	4.89E-17	Undiff-G	ZFAND5
PDAP1	3.53E-21	-0.23266	0.066	0.541	5.28E-17	Undiff-G	PDAP1
ALKBH7	3.62E-21	-0.57697	0.029	0.477	5.41E-17	Undiff-G	ALKBH7
ZNF706	3.74E-21	-0.2226	0.088	0.58	5.59E-17	Undiff-G	ZNF706
UBE2L3	3.90E-21	-0.24619	0.066	0.541	5.82E-17	Undiff-G	UBE2L3
KDELR1	3.91E-21	-0.50384	0.044	0.498	5.85E-17	Undiff-G	KDELR1
PSMA5	4.76E-21	-0.71375	0.015	0.448	7.12E-17	Undiff-G	PSMA5
NDUFB6	4.79E-21	-0.51605	0.044	0.502	7.16E-17	Undiff-G	NDUFB6
RAB2A	5.06E-21	-0.19579	0.096	0.597	7.56E-17	Undiff-G	RAB2A
GNAI2	5.48E-21	-0.52056	0.037	0.488	8.19E-17	Undiff-G	GNAI2
UBXN4	5.61E-21	-0.20881	0.074	0.55	8.39E-17	Undiff-G	UBXN4

NCL	6.50E-21	-0.15449	0.074	0.56	9.71E-17	Undiff-G	NCL
OCIAD1	6.56E-21	-0.18256	0.118	0.642	9.80E-17	Undiff-G	OCIAD1
SRRM2	6.98E-21	-0.61561	0.066	0.538	1.04E-16	Undiff-G	SRRM2
HNRNPF	7.25E-21	-0.38061	0.051	0.514	1.08E-16	Undiff-G	HNRNPF
EI24	7.53E-21	-0.79472	0.015	0.438	1.13E-16	Undiff-G	EI24
CSDE1	7.98E-21	-0.11268	0.103	0.607	1.19E-16	Undiff-G	CSDE1
CXCL16	8.04E-21	-0.76343	0.022	0.458	1.20E-16	Undiff-G	CXCL16
GRN	8.15E-21	-0.43663	0.088	0.576	1.22E-16	Undiff-G	GRN
NUDC	9.40E-21	-0.19521	0.088	0.585	1.40E-16	Undiff-G	NUDC
OS9	9.87E-21	-0.34627	0.066	0.541	1.47E-16	Undiff-G	OS9
IFNGR1	1.02E-20	-0.98606	0.015	0.442	1.53E-16	Undiff-G	IFNGR1
NINJ1	1.03E-20	-0.7428	0.022	0.457	1.54E-16	Undiff-G	NINJ1
CCT6A	1.07E-20	-0.22576	0.088	0.58	1.59E-16	Undiff-G	CCT6A
ZC3H15	1.17E-20	-0.20533	0.051	0.509	1.75E-16	Undiff-G	ZC3H15
FTH1	1.21E-20	-0.36292	0.279	0.94	1.81E-16	Undiff-G	FTH1
CHMP4B	1.27E-20	-0.96156	0.029	0.459	1.89E-16	Undiff-G	CHMP4B
TCF25	1.27E-20	-0.68255	0.081	0.557	1.90E-16	Undiff-G	TCF25
EMC10	1.35E-20	-0.75414	0.022	0.455	2.02E-16	Undiff-G	EMC10
ENY2	1.40E-20	-0.15606	0.103	0.61	2.09E-16	Undiff-G	ENY2
ATP5C1	1.48E-20	-0.21793	0.096	0.592	2.22E-16	Undiff-G	ATP5C1
PTP4A1	1.50E-20	-0.99473	0	0.412	2.25E-16	Undiff-G	PTP4A1
TMEM219	1.53E-20	-0.11535	0.081	0.566	2.28E-16	Undiff-G	TMEM219
SF3B2	1.58E-20	-0.39916	0.066	0.53	2.37E-16	Undiff-G	SF3B2
PDCD6	1.63E-20	-0.28066	0.066	0.54	2.44E-16	Undiff-G	PDCD6
TMEM165	1.74E-20	-0.91398	0.029	0.461	2.60E-16	Undiff-G	TMEM165
HEXB	1.91E-20	-1.06785	0	0.41	2.86E-16	Undiff-G	HEXB
VCP	2.17E-20	-0.92277	0.015	0.437	3.24E-16	Undiff-G	VCP
TCF4	2.25E-20	-0.7341	0.066	0.528	3.36E-16	Undiff-G	TCF4
UBE2E3	2.49E-20	-0.63475	0.029	0.465	3.72E-16	Undiff-G	UBE2E3
ETFB	2.66E-20	-0.46181	0.051	0.505	3.97E-16	Undiff-G	ETFB
SSNA1	2.66E-20	-0.61499	0.022	0.451	3.98E-16	Undiff-G	SSNA1
PPP2R1A	2.87E-20	-0.26143	0.059	0.52	4.28E-16	Undiff-G	PPP2R1A
EIF3G	3.22E-20	-0.10855	0.118	0.626	4.81E-16	Undiff-G	EIF3G
EIF5	3.50E-20	-0.11104	0.132	0.656	5.23E-16	Undiff-G	EIF5
NCOR1	3.61E-20	-0.62746	0.037	0.469	5.39E-16	Undiff-G	NCOR1
PRPF40A	3.97E-20	-0.77722	0.029	0.455	5.93E-16	Undiff-G	PRPF40A
APH1A	4.03E-20	-0.47143	0.044	0.49	6.02E-16	Undiff-G	APH1A
NONO	4.05E-20	-0.71073	0.029	0.46	6.05E-16	Undiff-G	NONO
TRA2B	4.18E-20	-0.22426	0.074	0.545	6.25E-16	Undiff-G	TRA2B
HLA-E	4.79E-20	-0.3527	0.11	0.602	7.15E-16	Undiff-G	HLA-E
PPP2CA	4.93E-20	-0.65039	0.044	0.485	7.37E-16	Undiff-G	PPP2CA
PGRMC1	5.02E-20	-0.85609	0.029	0.452	7.51E-16	Undiff-G	PGRMC1
ATP6AP1	5.18E-20	-0.51954	0.051	0.498	7.74E-16	Undiff-G	ATP6AP1
RAB10	5.50E-20	-0.28175	0.051	0.501	8.21E-16	Undiff-G	RAB10
C19orf60	5.52E-20	-0.63294	0.059	0.507	8.24E-16	Undiff-G	C19orf60
NFE2L2	5.57E-20	-0.5569	0.037	0.473	8.33E-16	Undiff-G	NFE2L2
FEZ1	6.46E-20	-0.94109	0.022	0.434	9.65E-16	Undiff-G	FEZ1
PTOV1	6.49E-20	-0.92365	0.022	0.443	9.70E-16	Undiff-G	PTOV1
PPM1G	7.25E-20	-0.92884	0.015	0.423	1.08E-15	Undiff-G	PPM1G
DPY30	7.46E-20	-0.42543	0.051	0.501	1.12E-15	Undiff-G	DPY30
DCXR	8.10E-20	-0.57826	0.029	0.458	1.21E-15	Undiff-G	DCXR

LDHA	8.30E-20	1.238561	0.706	0.767	1.24E-15	Undiff-G	LDHA
PPP1CC	8.45E-20	-0.79599	0.029	0.45	1.26E-15	Undiff-G	PPP1CC
ILF2	9.46E-20	-0.26528	0.096	0.58	1.41E-15	Undiff-G	ILF2
ENSA	9.69E-20	-0.33223	0.059	0.506	1.45E-15	Undiff-G	ENSA
PTMA	9.89E-20	0.874744	0.772	0.969	1.48E-15	Undiff-G	PTMA
PNISR	1.06E-19	-0.27976	0.118	0.617	1.58E-15	Undiff-G	PNISR
PSMD2	1.08E-19	-0.45766	0.037	0.466	1.62E-15	Undiff-G	PSMD2
CLK1	1.09E-19	-0.60447	0.015	0.427	1.63E-15	Undiff-G	CLK1
DHRS7	1.17E-19	-0.2407	0.088	0.562	1.76E-15	Undiff-G	DHRS7
RAB11B	1.19E-19	-0.47179	0.059	0.509	1.78E-15	Undiff-G	RAB11B
BEX4	1.31E-19	-0.52078	0.044	0.482	1.96E-15	Undiff-G	BEX4
ASNA1	1.32E-19	-0.31673	0.066	0.525	1.97E-15	Undiff-G	ASNA1
CBX3	1.37E-19	-0.34676	0.088	0.559	2.05E-15	Undiff-G	CBX3
RNF130	1.43E-19	-0.59799	0.037	0.468	2.14E-15	Undiff-G	RNF130
CAV1	1.47E-19	1.872622	0.346	0.124	2.20E-15	Undiff-G	CAV1
DNAJB11	1.49E-19	-0.68826	0.022	0.438	2.22E-15	Undiff-G	DNAJB11
CYBA	1.59E-19	-1.39133	0.007	0.409	2.37E-15	Undiff-G	CYBA
STAT3	1.64E-19	-0.58886	0.037	0.463	2.45E-15	Undiff-G	STAT3
NDUFS2	1.66E-19	-0.20964	0.088	0.556	2.49E-15	Undiff-G	NDUFS2
PSMC3	1.88E-19	-0.25836	0.074	0.536	2.81E-15	Undiff-G	PSMC3
RAP1A	1.89E-19	-0.17166	0.066	0.521	2.82E-15	Undiff-G	RAP1A
APMAP	2.10E-19	-0.17529	0.066	0.523	3.14E-15	Undiff-G	APMAP
HP1BP3	2.13E-19	-0.81846	0.029	0.447	3.18E-15	Undiff-G	HP1BP3
OLA1	2.15E-19	-0.24192	0.051	0.493	3.22E-15	Undiff-G	OLA1
COMT	2.19E-19	-0.24431	0.088	0.553	3.27E-15	Undiff-G	COMT
SRGN	2.21E-19	-0.69385	0.059	0.504	3.30E-15	Undiff-G	SRGN
CCDC88A	2.29E-19	-0.91319	0.015	0.421	3.43E-15	Undiff-G	CCDC88A
UXT	2.39E-19	-0.12115	0.081	0.543	3.57E-15	Undiff-G	UXT
GTF3C6	2.45E-19	-0.28636	0.066	0.519	3.66E-15	Undiff-G	GTF3C6
DPYSL2	2.59E-19	-0.8752	0.022	0.428	3.87E-15	Undiff-G	DPYSL2
HLA-A	2.59E-19	1.002054	0.743	0.898	3.88E-15	Undiff-G	HLA-A
HNRNPD	2.60E-19	-0.92575	0.015	0.419	3.88E-15	Undiff-G	HNRNPD
PAIP2	2.81E-19	0.124316	0.132	0.636	4.20E-15	Undiff-G	PAIP2
AIF1	2.97E-19	-1.35672	0.015	0.415	4.43E-15	Undiff-G	AIF1
RCN2	3.34E-19	-0.6944	0.044	0.472	4.99E-15	Undiff-G	RCN2
LMO4	3.55E-19	-0.37249	0.051	0.483	5.30E-15	Undiff-G	LMO4
ARRB2	3.78E-19	-0.90934	0.007	0.405	5.65E-15	Undiff-G	ARRB2
ARPP19	3.95E-19	-0.85547	0.022	0.43	5.90E-15	Undiff-G	ARPP19
HTRA1	3.99E-19	-0.68209	0.044	0.465	5.96E-15	Undiff-G	HTRA1
PRPF38B	4.00E-19	-0.93041	0.029	0.44	5.97E-15	Undiff-G	PRPF38B
CLTB	4.23E-19	-0.66829	0.029	0.446	6.32E-15	Undiff-G	CLTB
RBMX	4.33E-19	-0.30575	0.103	0.572	6.47E-15	Undiff-G	RBMX
EMC6	5.00E-19	-0.61395	0.029	0.438	7.47E-15	Undiff-G	EMC6
CD81	5.09E-19	-0.86774	0.029	0.443	7.61E-15	Undiff-G	CD81
RER1	5.14E-19	-0.27356	0.059	0.502	7.68E-15	Undiff-G	RER1
GRINA	5.16E-19	-0.6213	0.029	0.441	7.71E-15	Undiff-G	GRINA
CKLF	5.40E-19	-0.19602	0.074	0.525	8.08E-15	Undiff-G	CKLF
SNRNP70	5.76E-19	-0.6159	0.051	0.48	8.60E-15	Undiff-G	SNRNP70
NDUFV1	5.87E-19	-0.55326	0.051	0.484	8.78E-15	Undiff-G	NDUFV1
TSPAN3	6.51E-19	-0.59756	0.044	0.464	9.72E-15	Undiff-G	TSPAN3
TMEM123	6.55E-19	-0.71623	0.007	0.401	9.79E-15	Undiff-G	TMEM123

LSM7	6.67E-19	-0.10798	0.132	0.636	9.97E-15	Undiff-G	LSM7
CD14	6.70E-19	-1.37115	0.015	0.413	1.00E-14	Undiff-G	CD14
SNX2	6.95E-19	-0.7313	0.015	0.415	1.04E-14	Undiff-G	SNX2
TUBB2A	7.13E-19	-0.54981	0.051	0.483	1.07E-14	Undiff-G	TUBB2A
CHMP1B	7.59E-19	-0.57972	0.029	0.443	1.13E-14	Undiff-G	CHMP1B
XRCC5	7.85E-19	-0.2281	0.096	0.566	1.17E-14	Undiff-G	XRCC5
HNRNPH3	8.35E-19	-0.38965	0.037	0.45	1.25E-14	Undiff-G	HNRNPH3
APP	8.54E-19	-0.57884	0.081	0.53	1.28E-14	Undiff-G	APP
RDX	9.00E-19	-0.53598	0.059	0.495	1.34E-14	Undiff-G	RDX
DDAH2	9.42E-19	-0.40916	0.059	0.494	1.41E-14	Undiff-G	DDAH2
BRD2	9.97E-19	-0.58087	0.044	0.458	1.49E-14	Undiff-G	BRD2
EIF4A3	1.02E-18	-0.50482	0.037	0.455	1.52E-14	Undiff-G	EIF4A3
CCT3	1.03E-18	-0.1108	0.103	0.583	1.55E-14	Undiff-G	CCT3
RGS1	1.09E-18	-0.91529	0.037	0.452	1.63E-14	Undiff-G	RGS1
DPP7	1.12E-18	-0.6717	0.051	0.472	1.67E-14	Undiff-G	DPP7
CREM	1.15E-18	-0.92707	0.015	0.411	1.72E-14	Undiff-G	CREM
QKI	1.18E-18	-0.5302	0.051	0.476	1.76E-14	Undiff-G	QKI
SELK	1.19E-18	0.186636	0.162	0.707	1.77E-14	Undiff-G	SELK
H3F3B	1.29E-18	0.822538	0.75	0.973	1.93E-14	Undiff-G	H3F3B
CCDC85B	1.31E-18	-0.80238	0.022	0.418	1.96E-14	Undiff-G	CCDC85B
TIMM8B	1.38E-18	-0.18319	0.088	0.549	2.06E-14	Undiff-G	TIMM8B
ATG3	1.43E-18	-0.96405	0	0.381	2.14E-14	Undiff-G	ATG3
CMPK1	1.53E-18	-0.71907	0.022	0.421	2.28E-14	Undiff-G	CMPK1
VPS29	1.53E-18	-0.11795	0.096	0.559	2.29E-14	Undiff-G	VPS29
NUCB1	1.59E-18	-0.49732	0.037	0.45	2.38E-14	Undiff-G	NUCB1
PRRC2C	1.67E-18	-0.54616	0.059	0.487	2.50E-14	Undiff-G	PRRC2C
C9orf142	1.69E-18	-0.93002	0	0.38	2.52E-14	Undiff-G	C9orf142
MTPN	1.70E-18	-0.53059	0.029	0.437	2.54E-14	Undiff-G	MTPN
PPA1	1.81E-18	-0.12758	0.081	0.534	2.70E-14	Undiff-G	PPA1
CEBPD	1.83E-18	-0.81247	0.044	0.464	2.74E-14	Undiff-G	CEBPD
XRN2	1.95E-18	-0.46305	0.044	0.462	2.92E-14	Undiff-G	XRN2
POLR2F	1.98E-18	-0.25451	0.088	0.547	2.95E-14	Undiff-G	POLR2F
NDUFB11	1.98E-18	0.104083	0.184	0.741	2.96E-14	Undiff-G	NDUFB11
TOP1	1.99E-18	-0.63698	0.007	0.393	2.98E-14	Undiff-G	TOP1
TKT	2.17E-18	-0.11257	0.074	0.514	3.24E-14	Undiff-G	TKT
VAMP2	2.27E-18	-0.13046	0.132	0.628	3.39E-14	Undiff-G	VAMP2
DDX5	2.29E-18	-0.12037	0.221	0.824	3.42E-14	Undiff-G	DDX5
NAA10	2.29E-18	-0.62059	0.029	0.43	3.43E-14	Undiff-G	NAA10
SCARB2	2.62E-18	-0.92698	0.007	0.386	3.92E-14	Undiff-G	SCARB2
MAF1	2.67E-18	-0.62218	0.029	0.433	3.98E-14	Undiff-G	MAF1
GBAS	2.67E-18	-0.7858	0.015	0.404	4.00E-14	Undiff-G	GBAS
WBP2	2.68E-18	-0.63948	0.044	0.457	4.00E-14	Undiff-G	WBP2
ODC1	2.81E-18	-0.97576	0.015	0.398	4.20E-14	Undiff-G	ODC1
MZT2A	2.83E-18	-0.66992	0.022	0.418	4.23E-14	Undiff-G	MZT2A
SMARCB1	2.85E-18	-0.75826	0.022	0.419	4.26E-14	Undiff-G	SMARCB1
WASF2	2.93E-18	-0.63152	0.037	0.443	4.38E-14	Undiff-G	WASF2
PCSK1N	2.99E-18	-1.05338	0.029	0.425	4.47E-14	Undiff-G	PCSK1N
ATF4	3.13E-18	-0.36912	0.081	0.522	4.68E-14	Undiff-G	ATF4
FNTA	3.14E-18	-0.87478	0.007	0.39	4.69E-14	Undiff-G	FNTA
TCEAL8	3.16E-18	-0.4792	0.037	0.446	4.72E-14	Undiff-G	TCEAL8
NUDT3	3.26E-18	-0.66001	0.015	0.404	4.87E-14	Undiff-G	NUDT3

XBP1	3.40E-18	-1.03876	0	0.375	5.08E-14	Undiff-G	XBP1
IDH2	3.47E-18	-0.7345	0.029	0.431	5.19E-14	Undiff-G	IDH2
H1FX	3.58E-18	-0.94071	0.007	0.389	5.36E-14	Undiff-G	H1FX
SRPK2	3.61E-18	-0.85599	0.022	0.415	5.39E-14	Undiff-G	SRPK2
WTAP	3.90E-18	-0.63409	0.029	0.425	5.84E-14	Undiff-G	WTAP
TOMM22	3.95E-18	-0.19286	0.051	0.474	5.90E-14	Undiff-G	TOMM22
COPE	4.00E-18	0.109372	0.199	0.777	5.97E-14	Undiff-G	COPE
KPNB1	4.27E-18	-0.59174	0.044	0.452	6.38E-14	Undiff-G	KPNB1
PSMD3	4.35E-18	-0.85356	0.022	0.409	6.50E-14	Undiff-G	PSMD3
SAA1	4.69E-18	1.628611	0.331	0.116	7.01E-14	Undiff-G	SAA1
RBM17	4.81E-18	-0.89856	0	0.373	7.19E-14	Undiff-G	RBM17
HNRNPAB	4.82E-18	-0.61693	0.015	0.402	7.20E-14	Undiff-G	HNRNPAB
SUMO3	5.04E-18	-0.39459	0.037	0.444	7.53E-14	Undiff-G	SUMO3
CACYBP	5.26E-18	-0.13236	0.074	0.511	7.86E-14	Undiff-G	CACYBP
ATP6V0E2	5.78E-18	-0.49386	0.044	0.457	8.64E-14	Undiff-G	ATP6V0E2
ABHD12	6.02E-18	-0.76206	0.007	0.386	9.00E-14	Undiff-G	ABHD12
SFT2D1	6.03E-18	-0.15852	0.074	0.506	9.01E-14	Undiff-G	SFT2D1
BRK1	6.15E-18	0.10117	0.206	0.787	9.20E-14	Undiff-G	BRK1
EIF3M	6.25E-18	-0.17392	0.088	0.531	9.34E-14	Undiff-G	EIF3M
FAM3C	6.33E-18	-0.51121	0.037	0.441	9.46E-14	Undiff-G	FAM3C
TMEM70	6.37E-18	-0.65437	0.015	0.4	9.51E-14	Undiff-G	TMEM70
PRKAR1A	6.76E-18	-0.23676	0.059	0.477	1.01E-13	Undiff-G	PRKAR1A
CTNNA1	6.88E-18	-0.45217	0.029	0.426	1.03E-13	Undiff-G	CTNNA1
GNB1	7.22E-18	-0.72491	0.029	0.42	1.08E-13	Undiff-G	GNB1
MAPRE1	7.52E-18	-0.16497	0.059	0.482	1.12E-13	Undiff-G	MAPRE1
ADI1	7.59E-18	-0.70543	0.022	0.411	1.13E-13	Undiff-G	ADI1
CLNS1A	7.70E-18	-0.20185	0.066	0.497	1.15E-13	Undiff-G	CLNS1A
PRMT1	8.39E-18	-0.20153	0.103	0.559	1.25E-13	Undiff-G	PRMT1
FLOT1	8.86E-18	-0.22289	0.074	0.508	1.32E-13	Undiff-G	FLOT1
HSPD1	8.96E-18	0.124026	0.132	0.62	1.34E-13	Undiff-G	HSPD1
LYPLA1	9.24E-18	-0.45101	0.022	0.411	1.38E-13	Undiff-G	LYPLA1
HADHA	1.02E-17	-0.45169	0.044	0.446	1.52E-13	Undiff-G	HADHA
IFNGR2	1.07E-17	-0.8934	0.015	0.394	1.59E-13	Undiff-G	IFNGR2
GDI1	1.09E-17	-0.22666	0.066	0.491	1.62E-13	Undiff-G	GDI1
HDAC2	1.13E-17	-0.33365	0.044	0.452	1.69E-13	Undiff-G	HDAC2
SIRT2	1.19E-17	-0.74095	0.029	0.421	1.78E-13	Undiff-G	SIRT2
MPC1	1.27E-17	-0.24609	0.066	0.493	1.90E-13	Undiff-G	MPC1
LMNA	1.30E-17	-0.77157	0.029	0.42	1.94E-13	Undiff-G	LMNA
SPAG7	1.46E-17	-0.15341	0.066	0.483	2.18E-13	Undiff-G	SPAG7
FXYD6	1.48E-17	-0.67694	0.059	0.465	2.20E-13	Undiff-G	FXYD6
TMEM9B	1.53E-17	-0.10332	0.096	0.541	2.29E-13	Undiff-G	TMEM9B
LEPROTL1	1.56E-17	-0.71727	0.015	0.392	2.33E-13	Undiff-G	LEPROTL1
DCTN6	1.57E-17	-0.40228	0.044	0.448	2.34E-13	Undiff-G	DCTN6
MIDN	1.66E-17	-0.65997	0.029	0.419	2.49E-13	Undiff-G	MIDN
ATP6V1F	1.69E-17	0.132222	0.221	0.808	2.52E-13	Undiff-G	ATP6V1F
C17orf89	1.72E-17	-0.78094	0.037	0.426	2.58E-13	Undiff-G	C17orf89
IMP3	1.81E-17	-0.9008	0	0.364	2.70E-13	Undiff-G	IMP3
MTCH2	1.85E-17	-0.39755	0.037	0.433	2.77E-13	Undiff-G	MTCH2
C16orf13	1.91E-17	-0.57368	0.059	0.465	2.85E-13	Undiff-G	C16orf13
CHCHD10	1.91E-17	-0.86468	0.015	0.385	2.85E-13	Undiff-G	CHCHD10
SGK1	1.99E-17	-0.81434	0.044	0.441	2.98E-13	Undiff-G	SGK1

SNX6	2.01E-17	-0.29668	0.037	0.434	3.00E-13	Undiff-G	SNX6
FCER1G	2.06E-17	-0.6909	0.044	0.446	3.08E-13	Undiff-G	FCER1G
ARPC1B	2.14E-17	-0.45248	0.096	0.536	3.20E-13	Undiff-G	ARPC1B
MAGOH	2.35E-17	-0.45573	0.029	0.418	3.52E-13	Undiff-G	MAGOH
PSIP1	2.39E-17	-0.88328	0.015	0.388	3.57E-13	Undiff-G	PSIP1
ARGLU1	2.42E-17	-0.48497	0.059	0.473	3.61E-13	Undiff-G	ARGLU1
TUBB4B	2.42E-17	-0.13149	0.074	0.497	3.62E-13	Undiff-G	TUBB4B
PAIP1	2.43E-17	-0.82304	0.022	0.4	3.63E-13	Undiff-G	PAIP1
EMC4	2.84E-17	-0.17764	0.066	0.487	4.24E-13	Undiff-G	EMC4
WIPI2	2.90E-17	-0.47173	0.022	0.402	4.33E-13	Undiff-G	WIPI2
PTP4A2	2.94E-17	-0.51739	0.051	0.458	4.39E-13	Undiff-G	PTP4A2
DDX46	3.18E-17	-0.43435	0.051	0.451	4.75E-13	Undiff-G	DDX46
NSA2	3.18E-17	-0.27515	0.022	0.403	4.75E-13	Undiff-G	NSA2
CCNL1	3.23E-17	-0.14704	0.088	0.53	4.83E-13	Undiff-G	CCNL1
TXN2	3.32E-17	-0.67476	0.044	0.438	4.96E-13	Undiff-G	TXN2
AES	3.35E-17	-0.57457	0.029	0.415	5.00E-13	Undiff-G	AES
CMTM6	3.60E-17	-0.33135	0.037	0.43	5.38E-13	Undiff-G	CMTM6
DDX17	3.66E-17	-0.47454	0.037	0.428	5.47E-13	Undiff-G	DDX17
ACADVL	3.79E-17	-0.44152	0.051	0.455	5.66E-13	Undiff-G	ACADVL
AKIRIN2	3.84E-17	-0.68798	0.015	0.385	5.73E-13	Undiff-G	AKIRIN2
HIST1H4C	4.05E-17	-0.48901	0.037	0.429	6.05E-13	Undiff-G	HIST1H4C
PTTG1IP	4.07E-17	-0.5241	0.059	0.461	6.08E-13	Undiff-G	PTTG1IP
YTHDF2	4.10E-17	-0.48307	0.044	0.435	6.13E-13	Undiff-G	YTHDF2
UGP2	4.17E-17	-0.11902	0.059	0.465	6.23E-13	Undiff-G	UGP2
YPEL5	4.43E-17	-0.21123	0.051	0.457	6.62E-13	Undiff-G	YPEL5
RNF13	4.54E-17	-0.15205	0.066	0.485	6.79E-13	Undiff-G	RNF13
MKRN1	4.84E-17	-0.52067	0.029	0.412	7.23E-13	Undiff-G	MKRN1
RHOG	4.85E-17	-0.54882	0.029	0.414	7.24E-13	Undiff-G	RHOG
BAG1	5.11E-17	-0.88055	0	0.356	7.64E-13	Undiff-G	BAG1
PSMC2	5.19E-17	-0.1167	0.059	0.463	7.76E-13	Undiff-G	PSMC2
POLR2G	5.22E-17	0.118973	0.11	0.572	7.79E-13	Undiff-G	POLR2G
GABARAPL2	5.48E-17	0.145768	0.199	0.749	8.19E-13	Undiff-G	GABARAPL2
RNF145	5.97E-17	-0.82837	0.015	0.382	8.93E-13	Undiff-G	RNF145
C8orf59	6.44E-17	-0.42627	0.022	0.398	9.62E-13	Undiff-G	C8orf59
GRHPR	6.48E-17	-0.60979	0.022	0.397	9.68E-13	Undiff-G	GRHPR
STOML2	6.91E-17	-0.27829	0.037	0.425	1.03E-12	Undiff-G	STOML2
CCDC107	7.08E-17	-0.79286	0.022	0.395	1.06E-12	Undiff-G	CCDC107
POLR1D	7.20E-17	-0.18587	0.037	0.42	1.08E-12	Undiff-G	POLR1D
MYL6B	7.27E-17	-0.18338	0.051	0.447	1.09E-12	Undiff-G	MYL6B
TPM4	7.31E-17	-0.33834	0.081	0.502	1.09E-12	Undiff-G	TPM4
CEBPB	7.43E-17	-0.82178	0.051	0.439	1.11E-12	Undiff-G	CEBPB
LAGE3	7.61E-17	-0.68439	0.029	0.403	1.14E-12	Undiff-G	LAGE3
LIMS1	7.68E-17	-0.40408	0.051	0.444	1.15E-12	Undiff-G	LIMS1
PFKL	7.70E-17	-0.82265	0.007	0.367	1.15E-12	Undiff-G	PFKL
EIF3J	7.73E-17	-0.50091	0.015	0.382	1.16E-12	Undiff-G	EIF3J
DTD1	7.97E-17	-0.58286	0.022	0.396	1.19E-12	Undiff-G	DTD1
SMARCA5	8.54E-17	-0.83361	0	0.353	1.28E-12	Undiff-G	SMARCA5
ATOX1	8.59E-17	0.131272	0.132	0.61	1.28E-12	Undiff-G	ATOX1
GDI2	9.12E-17	-0.16217	0.074	0.493	1.36E-12	Undiff-G	GDI2
CISD2	9.54E-17	-0.54856	0.029	0.408	1.43E-12	Undiff-G	CISD2
CNPY3	9.76E-17	-0.52384	0.037	0.417	1.46E-12	Undiff-G	CNPY3

BIN1	1.09E-16	-0.84618	0.007	0.365	1.63E-12	Undiff-G	BIN1
C1QB	1.14E-16	-0.43289	0.125	0.575	1.70E-12	Undiff-G	C1QB
BASP1	1.16E-16	-0.87382	0.015	0.377	1.73E-12	Undiff-G	BASP1
WDR45B	1.20E-16	-0.33607	0.029	0.407	1.79E-12	Undiff-G	WDR45B
CSTB	1.20E-16	0.149391	0.176	0.696	1.79E-12	Undiff-G	CSTB
EIF3H	1.24E-16	0.246003	0.162	0.675	1.85E-12	Undiff-G	EIF3H
AKIRIN1	1.25E-16	-0.82898	0.015	0.372	1.87E-12	Undiff-G	AKIRIN1
COMMD7	1.30E-16	-0.76308	0.029	0.404	1.94E-12	Undiff-G	COMMD7
CORO1A	1.31E-16	-1.03416	0.015	0.377	1.95E-12	Undiff-G	CORO1A
PSMD13	1.32E-16	-0.6725	0.037	0.418	1.97E-12	Undiff-G	PSMD13
NME4	1.36E-16	-0.78972	0.022	0.386	2.03E-12	Undiff-G	NME4
HMGA1	1.36E-16	-0.88992	0	0.349	2.03E-12	Undiff-G	HMGA1
SRSF11	1.42E-16	-0.20082	0.103	0.544	2.12E-12	Undiff-G	SRSF11
RAB6A	1.51E-16	-0.652	0.029	0.398	2.26E-12	Undiff-G	RAB6A
DNAJC7	1.55E-16	-0.47058	0.066	0.469	2.32E-12	Undiff-G	DNAJC7
PLEKHB2	1.60E-16	-0.37539	0.029	0.403	2.38E-12	Undiff-G	PLEKHB2
HMGB2	1.61E-16	-0.1238	0.081	0.499	2.41E-12	Undiff-G	HMGB2
EIF5A	1.62E-16	-0.10148	0.096	0.532	2.42E-12	Undiff-G	EIF5A
MFSD1	1.62E-16	-1.05634	0	0.348	2.42E-12	Undiff-G	MFSD1
UBA2	1.65E-16	-0.63266	0.007	0.362	2.47E-12	Undiff-G	UBA2
TYROBP	1.66E-16	-0.78587	0.066	0.464	2.48E-12	Undiff-G	TYROBP
MGAT1	1.69E-16	-0.91791	0.007	0.362	2.52E-12	Undiff-G	MGAT1
CD164	1.70E-16	-0.11765	0.081	0.495	2.54E-12	Undiff-G	CD164
CHMP4A	1.76E-16	-0.25086	0.066	0.473	2.64E-12	Undiff-G	CHMP4A
STAU1	1.82E-16	-0.79087	0.015	0.37	2.72E-12	Undiff-G	STAU1
SPG21	2.06E-16	-0.49424	0.022	0.389	3.07E-12	Undiff-G	SPG21
PAK2	2.07E-16	-0.81685	0.007	0.356	3.10E-12	Undiff-G	PAK2
ARID4B	2.11E-16	-0.39915	0.037	0.41	3.16E-12	Undiff-G	ARID4B
SRA1	2.12E-16	-0.45471	0.029	0.403	3.17E-12	Undiff-G	SRA1
PSMF1	2.13E-16	-0.26056	0.029	0.403	3.18E-12	Undiff-G	PSMF1
ATP5B	2.15E-16	0.204932	0.221	0.782	3.21E-12	Undiff-G	ATP5B
BCAS2	2.22E-16	-0.22812	0.066	0.467	3.31E-12	Undiff-G	BCAS2
SSR2	2.46E-16	0.244985	0.191	0.72	3.67E-12	Undiff-G	SSR2
LAMTOR1	2.47E-16	0.179725	0.169	0.66	3.69E-12	Undiff-G	LAMTOR1
IDH3B	2.47E-16	-0.36116	0.044	0.428	3.69E-12	Undiff-G	IDH3B
NDUFA12	2.50E-16	0.116192	0.118	0.563	3.74E-12	Undiff-G	NDUFA12
SOX4	2.55E-16	-0.84089	0.066	0.45	3.82E-12	Undiff-G	SOX4
RTF1	2.56E-16	-0.5181	0.051	0.435	3.83E-12	Undiff-G	RTF1
SNRPC	2.58E-16	0.174166	0.088	0.517	3.85E-12	Undiff-G	SNRPC
ATRX	2.64E-16	-0.5295	0.059	0.449	3.94E-12	Undiff-G	ATRX
VPS28	2.71E-16	0.267035	0.154	0.642	4.05E-12	Undiff-G	VPS28
MKKS	2.80E-16	-0.4105	0.037	0.408	4.18E-12	Undiff-G	MKKS
SUCLG1	3.31E-16	-0.36949	0.051	0.441	4.95E-12	Undiff-G	SUCLG1
NDUFB3	3.52E-16	0.118924	0.11	0.559	5.26E-12	Undiff-G	NDUFB3
ADIPOR1	3.71E-16	-0.5935	0.022	0.384	5.54E-12	Undiff-G	ADIPOR1
CCDC109B	3.76E-16	-0.90365	0.007	0.353	5.61E-12	Undiff-G	CCDC109B
SURF1	3.80E-16	-0.77264	0.007	0.356	5.67E-12	Undiff-G	SURF1
CNDP2	3.81E-16	-0.27051	0.051	0.435	5.69E-12	Undiff-G	CNDP2
ATP6V1B2	3.88E-16	-0.31015	0.037	0.413	5.80E-12	Undiff-G	ATP6V1B2
SDF4	3.98E-16	-0.68563	0.037	0.408	5.95E-12	Undiff-G	SDF4
ZYX	4.03E-16	-0.52962	0.037	0.406	6.03E-12	Undiff-G	ZYX

PFN2	4.06E-16	-0.2603	0.051	0.439	6.06E-12	Undiff-G	PFN2
ARL4C	4.20E-16	-0.51508	0.022	0.382	6.28E-12	Undiff-G	ARL4C
RNF187	4.48E-16	-0.74843	0.022	0.382	6.69E-12	Undiff-G	RNF187
SEC11A	4.52E-16	0.114875	0.132	0.596	6.76E-12	Undiff-G	SEC11A
SMARCE1	4.67E-16	-0.67687	0.022	0.383	6.98E-12	Undiff-G	SMARCE1
BBX	4.78E-16	-0.33187	0.037	0.411	7.14E-12	Undiff-G	BBX
MLEC	4.88E-16	-0.53948	0.037	0.405	7.30E-12	Undiff-G	MLEC
EIF4B	5.09E-16	-0.43621	0.022	0.382	7.60E-12	Undiff-G	EIF4B
EIF3K	5.26E-16	0.238331	0.213	0.765	7.86E-12	Undiff-G	EIF3K
SLC25A11	5.58E-16	-0.42093	0.044	0.424	8.34E-12	Undiff-G	SLC25A11
CCT7	6.11E-16	-0.18776	0.103	0.535	9.14E-12	Undiff-G	CCT7
DNPH1	6.24E-16	-0.50701	0.022	0.381	9.33E-12	Undiff-G	DNPH1
GCSH	6.43E-16	-0.86329	0.007	0.351	9.61E-12	Undiff-G	GCSH
COPRS	6.58E-16	-0.82932	0	0.338	9.84E-12	Undiff-G	COPRS
BTG3	6.68E-16	-0.58833	0.022	0.375	9.98E-12	Undiff-G	BTG3
STARD3NL	6.69E-16	-0.41785	0.037	0.402	1.00E-11	Undiff-G	STARD3NL
SNRPE	6.84E-16	0.128981	0.132	0.585	1.02E-11	Undiff-G	SNRPE
ATP6V1E1	6.87E-16	-0.18219	0.051	0.437	1.03E-11	Undiff-G	ATP6V1E1
ATP5G1	7.02E-16	0.211549	0.147	0.623	1.05E-11	Undiff-G	ATP5G1
RBM42	7.55E-16	-0.55271	0.029	0.39	1.13E-11	Undiff-G	RBM42
HIGD2A	7.58E-16	0.181779	0.147	0.624	1.13E-11	Undiff-G	HIGD2A
SF3B5	7.93E-16	0.232594	0.154	0.635	1.18E-11	Undiff-G	SF3B5
NDUFA1	9.74E-16	0.221599	0.199	0.73	1.46E-11	Undiff-G	NDUFA1
PEPD	9.96E-16	-0.25684	0.051	0.433	1.49E-11	Undiff-G	PEPD
ALDH2	1.03E-15	-0.72917	0.022	0.375	1.53E-11	Undiff-G	ALDH2
TAF9	1.03E-15	0.135232	0.088	0.505	1.54E-11	Undiff-G	TAF9
PSMC6	1.04E-15	-0.38978	0.029	0.391	1.55E-11	Undiff-G	PSMC6
LMAN2	1.09E-15	0.114166	0.125	0.579	1.62E-11	Undiff-G	LMAN2
NAPA	1.10E-15	-0.48297	0.037	0.398	1.65E-11	Undiff-G	NAPA
FHL1	1.13E-15	-0.85759	0.015	0.361	1.68E-11	Undiff-G	FHL1
CCL3	1.16E-15	-0.75441	0.051	0.426	1.73E-11	Undiff-G	CCL3
IFI27L2	1.20E-15	-0.11925	0.074	0.476	1.80E-11	Undiff-G	IFI27L2
WBSCR22	1.23E-15	0.124145	0.088	0.498	1.84E-11	Undiff-G	WBSCR22
TNFRSF1A	1.26E-15	-0.50356	0.022	0.376	1.88E-11	Undiff-G	TNFRSF1A
CD68	1.27E-15	-0.47048	0.059	0.446	1.90E-11	Undiff-G	CD68
TMEM126A	1.28E-15	-0.8783	0	0.333	1.92E-11	Undiff-G	TMEM126A
MAP1LC3A	1.32E-15	-0.77501	0.022	0.369	1.98E-11	Undiff-G	MAP1LC3A
TMEM179B	1.37E-15	-0.20868	0.044	0.418	2.05E-11	Undiff-G	TMEM179B
BCLAF1	1.39E-15	-0.32035	0.029	0.389	2.08E-11	Undiff-G	BCLAF1
NOL7	1.42E-15	-0.14981	0.059	0.443	2.13E-11	Undiff-G	NOL7
SELT	1.43E-15	0.122762	0.176	0.665	2.14E-11	Undiff-G	SELT
RGS10	1.45E-15	-1.07926	0.007	0.346	2.16E-11	Undiff-G	RGS10
AHSA1	1.47E-15	-0.25311	0.037	0.403	2.19E-11	Undiff-G	AHSA1
COX7B	1.48E-15	0.151584	0.169	0.648	2.22E-11	Undiff-G	COX7B
TRAPPC2L	1.50E-15	-0.22652	0.051	0.424	2.24E-11	Undiff-G	TRAPPC2L
DBNL	1.52E-15	-0.29306	0.051	0.43	2.27E-11	Undiff-G	DBNL
C19orf24	1.52E-15	-0.48216	0.022	0.369	2.27E-11	Undiff-G	C19orf24
DHX36	1.60E-15	-0.32157	0.051	0.43	2.39E-11	Undiff-G	DHX36
PSMG2	1.63E-15	-0.14511	0.044	0.416	2.43E-11	Undiff-G	PSMG2
TSEN34	1.67E-15	-0.6695	0.022	0.372	2.49E-11	Undiff-G	TSEN34
CCT5	1.70E-15	0.118478	0.081	0.487	2.54E-11	Undiff-G	CCT5

BTF3L4	1.81E-15	0.170267	0.096	0.517	2.71E-11	Undiff-G	BTF3L4
PHLDA1	1.88E-15	-0.71369	0.037	0.4	2.81E-11	Undiff-G	PHLDA1
GPAA1	1.94E-15	-0.49302	0.037	0.397	2.90E-11	Undiff-G	GPAA1
PCMT1	1.97E-15	0.168989	0.11	0.546	2.95E-11	Undiff-G	PCMT1
GATM	2.02E-15	-0.83102	0.015	0.356	3.01E-11	Undiff-G	GATM
ARL6IP1	2.11E-15	0.157431	0.213	0.748	3.15E-11	Undiff-G	ARL6IP1
APLP1	2.14E-15	-0.83446	0.015	0.355	3.21E-11	Undiff-G	APLP1
ATP5F1	2.15E-15	0.208051	0.162	0.649	3.22E-11	Undiff-G	ATP5F1
YBX3	2.22E-15	-0.56637	0.044	0.409	3.32E-11	Undiff-G	YBX3
ANAPC5	2.24E-15	-0.20026	0.037	0.4	3.34E-11	Undiff-G	ANAPC5
N4BP2L2	2.36E-15	-0.27821	0.103	0.521	3.53E-11	Undiff-G	N4BP2L2
CYB5R3	2.37E-15	-0.73909	0.022	0.365	3.54E-11	Undiff-G	CYB5R3
TSC22D1	2.44E-15	-0.20176	0.103	0.517	3.65E-11	Undiff-G	TSC22D1
ITGB2	2.46E-15	-1.27152	0	0.328	3.68E-11	Undiff-G	ITGB2
C6orf48	2.52E-15	0.246023	0.169	0.652	3.76E-11	Undiff-G	C6orf48
RAD23B	2.52E-15	-0.38457	0.029	0.384	3.76E-11	Undiff-G	RAD23B
SOX2	2.58E-15	-0.7843	0.059	0.434	3.86E-11	Undiff-G	SOX2
LITAF	2.65E-15	-0.2353	0.074	0.463	3.96E-11	Undiff-G	LITAF
PSMD11	2.92E-15	-0.16999	0.044	0.412	4.36E-11	Undiff-G	PSMD11
FAM50A	2.96E-15	-0.57254	0.029	0.377	4.43E-11	Undiff-G	FAM50A
PDHB	2.98E-15	-0.51719	0.022	0.367	4.46E-11	Undiff-G	PDHB
GRSF1	3.02E-15	-0.76889	0	0.326	4.51E-11	Undiff-G	GRSF1
APOC1	3.04E-15	-0.41666	0.103	0.521	4.54E-11	Undiff-G	APOC1
TAOK3	3.09E-15	-0.56503	0.037	0.39	4.61E-11	Undiff-G	TAOK3
TUBB2B	3.11E-15	-0.46272	0.132	0.56	4.64E-11	Undiff-G	TUBB2B
ARHGDIB	3.24E-15	-0.87621	0.029	0.376	4.85E-11	Undiff-G	ARHGDIB
NEDD8	3.33E-15	0.187903	0.213	0.751	4.98E-11	Undiff-G	NEDD8
MAP7D1	3.35E-15	-0.66162	0.015	0.35	5.00E-11	Undiff-G	MAP7D1
ARL2BP	3.42E-15	-0.13979	0.044	0.409	5.11E-11	Undiff-G	ARL2BP
GTF2I	3.44E-15	-0.38516	0.059	0.431	5.13E-11	Undiff-G	GTF2I
UBE2I	3.53E-15	0.260799	0.132	0.582	5.28E-11	Undiff-G	UBE2I
JMJD1C	3.64E-15	-0.80728	0.007	0.338	5.43E-11	Undiff-G	JMJD1C
TMEM59	3.65E-15	0.31542	0.228	0.792	5.46E-11	Undiff-G	TMEM59
SNRPD3	3.72E-15	-0.45129	0.051	0.418	5.56E-11	Undiff-G	SNRPD3
C21orf33	3.73E-15	-0.42378	0.022	0.367	5.57E-11	Undiff-G	C21orf33
MAGED2	3.88E-15	0.121247	0.103	0.513	5.80E-11	Undiff-G	MAGED2
ERCC1	3.90E-15	-0.13465	0.081	0.477	5.83E-11	Undiff-G	ERCC1
HIGD1A	3.98E-15	-0.13744	0.059	0.431	5.94E-11	Undiff-G	HIGD1A
NAP1L4	4.01E-15	-0.42871	0.044	0.408	6.00E-11	Undiff-G	NAP1L4
NSFL1C	4.07E-15	-0.30586	0.044	0.406	6.08E-11	Undiff-G	NSFL1C
RBM25	4.16E-15	-0.35509	0.066	0.445	6.22E-11	Undiff-G	RBM25
RHOBTB3	4.19E-15	-0.58706	0.051	0.413	6.26E-11	Undiff-G	RHOBTB3
ATP5A1	4.27E-15	0.225084	0.184	0.68	6.38E-11	Undiff-G	ATP5A1
IDH3G	4.54E-15	-0.25617	0.044	0.407	6.79E-11	Undiff-G	IDH3G
POLDIP2	4.56E-15	-0.72202	0.015	0.351	6.82E-11	Undiff-G	POLDIP2
ERI3	4.64E-15	-0.76769	0.029	0.373	6.93E-11	Undiff-G	ERI3
ALOX5AP	4.78E-15	-0.78016	0.044	0.403	7.15E-11	Undiff-G	ALOX5AP
WHSC1L1	4.79E-15	-0.68898	0.029	0.377	7.15E-11	Undiff-G	WHSC1L1
RNF181	4.80E-15	0.200021	0.125	0.56	7.18E-11	Undiff-G	RNF181
15-Sep	4.89E-15	0.1561	0.176	0.662	7.31E-11	Undiff-G	15-Sep
PIH1D1	4.91E-15	-0.15181	0.037	0.394	7.33E-11	Undiff-G	PIH1D1

CCNH	4.98E-15	-0.4214	0.051	0.416	7.44E-11	Undiff-G	CCNH
HSPH1	5.03E-15	-0.59899	0.022	0.365	7.51E-11	Undiff-G	HSPH1
NDUFV3	5.16E-15	-0.29503	0.051	0.414	7.71E-11	Undiff-G	NDUFV3
PEA15	5.31E-15	-0.22781	0.066	0.448	7.93E-11	Undiff-G	PEA15
ENOPH1	5.32E-15	-0.23553	0.037	0.393	7.95E-11	Undiff-G	ENOPH1
RALA	5.35E-15	-0.38024	0.022	0.364	8.00E-11	Undiff-G	RALA
KLC1	5.40E-15	-0.58294	0.022	0.363	8.07E-11	Undiff-G	KLC1
HNRNPUL1	5.42E-15	-0.49117	0.037	0.388	8.10E-11	Undiff-G	HNRNPUL1
NDUFB4	5.44E-15	0.168714	0.191	0.686	8.13E-11	Undiff-G	NDUFB4
GLOD4	5.47E-15	-0.1373	0.037	0.393	8.18E-11	Undiff-G	GLOD4
CANX	5.53E-15	0.177119	0.132	0.579	8.27E-11	Undiff-G	CANX
FUNDC2	5.66E-15	-0.21214	0.044	0.407	8.46E-11	Undiff-G	FUNDC2
SRSF4	5.71E-15	-0.43707	0.022	0.364	8.53E-11	Undiff-G	SRSF4
YIPF3	5.72E-15	-0.16169	0.088	0.488	8.55E-11	Undiff-G	YIPF3
LAPTM4B	5.87E-15	-0.81124	0.007	0.335	8.77E-11	Undiff-G	LAPTM4B
THRAP3	5.96E-15	-0.17642	0.066	0.442	8.90E-11	Undiff-G	THRAP3
PABPC4	6.02E-15	-0.82711	0.007	0.332	8.99E-11	Undiff-G	PABPC4
LSM14A	6.20E-15	-0.4533	0.037	0.385	9.26E-11	Undiff-G	LSM14A
SEC63	6.25E-15	-0.11254	0.037	0.392	9.34E-11	Undiff-G	SEC63
NFIB	6.27E-15	-0.78049	0.037	0.382	9.37E-11	Undiff-G	NFIB
ATP5H	6.34E-15	0.313699	0.169	0.655	9.48E-11	Undiff-G	ATP5H
UBL3	6.59E-15	-0.60441	0.022	0.358	9.84E-11	Undiff-G	UBL3
UBE2V2	6.73E-15	0.199357	0.096	0.499	1.01E-10	Undiff-G	UBE2V2
TGOLN2	6.94E-15	-0.45633	0.029	0.377	1.04E-10	Undiff-G	TGOLN2
UBE2A	8.17E-15	-0.24122	0.059	0.426	1.22E-10	Undiff-G	UBE2A
CTSS	8.67E-15	-0.97724	0.022	0.358	1.30E-10	Undiff-G	CTSS
MAFB	8.68E-15	-1.06232	0	0.318	1.30E-10	Undiff-G	MAFB
NDUFA10	8.75E-15	-0.10604	0.066	0.443	1.31E-10	Undiff-G	NDUFA10
PSME1	8.87E-15	0.157109	0.14	0.588	1.33E-10	Undiff-G	PSME1
PRMT2	9.05E-15	-0.144	0.074	0.453	1.35E-10	Undiff-G	PRMT2
CD47	9.07E-15	-0.41387	0.037	0.388	1.36E-10	Undiff-G	CD47
VSIG4	9.10E-15	-1.10043	0.029	0.369	1.36E-10	Undiff-G	VSIG4
EZR	9.16E-15	-0.70421	0.015	0.346	1.37E-10	Undiff-G	EZR
NASP	9.17E-15	-0.39975	0.051	0.413	1.37E-10	Undiff-G	NASP
LRRFIP1	9.34E-15	-0.65455	0.022	0.357	1.40E-10	Undiff-G	LRRFIP1
MS4A6A	9.44E-15	-1.06726	0.015	0.345	1.41E-10	Undiff-G	MS4A6A
CTDNEP1	9.70E-15	-0.79412	0	0.317	1.45E-10	Undiff-G	CTDNEP1
ORMDL1	9.75E-15	0.107668	0.088	0.487	1.46E-10	Undiff-G	ORMDL1
RBBP6	1.00E-14	-0.78407	0	0.317	1.50E-10	Undiff-G	RBBP6
SEPHS2	1.03E-14	-0.75729	0	0.317	1.53E-10	Undiff-G	SEPHS2
BLOC1S2	1.04E-14	-0.3742	0.029	0.372	1.55E-10	Undiff-G	BLOC1S2
FCGR3A	1.04E-14	-0.98857	0.015	0.345	1.56E-10	Undiff-G	FCGR3A
TMED2	1.07E-14	0.207365	0.154	0.617	1.61E-10	Undiff-G	TMED2
RCN1	1.09E-14	-0.49729	0.022	0.358	1.63E-10	Undiff-G	RCN1
KPNA2	1.12E-14	-0.61103	0.022	0.359	1.68E-10	Undiff-G	KPNA2
YY1	1.18E-14	-0.62687	0.015	0.344	1.76E-10	Undiff-G	YY1
ATP1A1	1.20E-14	-0.26121	0.088	0.478	1.79E-10	Undiff-G	ATP1A1
SEC11C	1.21E-14	0.159024	0.096	0.502	1.80E-10	Undiff-G	SEC11C
FIBP	1.23E-14	0.117216	0.088	0.487	1.83E-10	Undiff-G	FIBP
NDUFS3	1.24E-14	0.156388	0.096	0.494	1.86E-10	Undiff-G	NDUFS3
KLF6	1.25E-14	-0.18318	0.118	0.536	1.87E-10	Undiff-G	KLF6

FAM96B	1.27E-14	0.214885	0.184	0.663	1.90E-10	Undiff-G	FAM96B
SCAMP2	1.28E-14	-0.7141	0.037	0.378	1.91E-10	Undiff-G	SCAMP2
RHOA	1.31E-14	0.208697	0.228	0.761	1.95E-10	Undiff-G	RHOA
TIMM10	1.33E-14	-0.2607	0.022	0.358	1.98E-10	Undiff-G	TIMM10
CLTC	1.34E-14	-0.57434	0.022	0.353	2.00E-10	Undiff-G	CLTC
CBX1	1.39E-14	-0.3201	0.029	0.371	2.08E-10	Undiff-G	CBX1
UQCR10	1.41E-14	0.128469	0.221	0.748	2.11E-10	Undiff-G	UQCR10
PFDN2	1.47E-14	0.199341	0.154	0.616	2.19E-10	Undiff-G	PFDN2
PSMB8	1.49E-14	-0.14155	0.059	0.419	2.22E-10	Undiff-G	PSMB8
RBCK1	1.51E-14	-0.5566	0.015	0.341	2.25E-10	Undiff-G	RBCK1
SRM	1.54E-14	-0.59999	0.029	0.369	2.30E-10	Undiff-G	SRM
EID1	1.58E-14	0.156695	0.206	0.713	2.36E-10	Undiff-G	EID1
RNF167	1.61E-14	-0.25951	0.051	0.412	2.40E-10	Undiff-G	RNF167
ZCCHC17	1.62E-14	-0.59882	0.022	0.355	2.42E-10	Undiff-G	ZCCHC17
GNAI3	1.63E-14	-0.18476	0.044	0.397	2.43E-10	Undiff-G	GNAI3
PIN1	1.64E-14	0.150507	0.118	0.542	2.45E-10	Undiff-G	PIN1
ATP6AP2	1.66E-14	0.200293	0.176	0.662	2.48E-10	Undiff-G	ATP6AP2
DPM2	1.66E-14	-0.74991	0.022	0.35	2.48E-10	Undiff-G	DPM2
SURF4	1.77E-14	-0.49352	0.022	0.354	2.65E-10	Undiff-G	SURF4
SNRPF	1.77E-14	0.118362	0.125	0.552	2.65E-10	Undiff-G	SNRPF
HSBP1	1.97E-14	0.165345	0.206	0.721	2.94E-10	Undiff-G	HSBP1
AP2M1	2.00E-14	0.144632	0.213	0.718	2.99E-10	Undiff-G	AP2M1
CHD4	2.02E-14	-0.54325	0.007	0.326	3.02E-10	Undiff-G	CHD4
BCL7B	2.02E-14	-0.7034	0.007	0.326	3.02E-10	Undiff-G	BCL7B
PSAP	2.05E-14	0.128631	0.265	0.825	3.06E-10	Undiff-G	PSAP
CXADR	2.18E-14	-0.88201	0	0.311	3.25E-10	Undiff-G	CXADR
SNRPB2	2.23E-14	0.304008	0.132	0.569	3.34E-10	Undiff-G	SNRPB2
SNRNP25	2.25E-14	-0.35777	0.022	0.353	3.37E-10	Undiff-G	SNRNP25
NFIX	2.27E-14	-0.81299	0.015	0.336	3.39E-10	Undiff-G	NFIX
FKBP4	2.33E-14	-0.70008	0.007	0.325	3.49E-10	Undiff-G	FKBP4
RAD23A	2.40E-14	0.18234	0.14	0.572	3.58E-10	Undiff-G	RAD23A
EIF1B	2.40E-14	0.122698	0.147	0.593	3.58E-10	Undiff-G	EIF1B
TPR	2.46E-14	-0.19594	0.051	0.409	3.67E-10	Undiff-G	TPR
ROMO1	2.47E-14	0.157667	0.147	0.6	3.69E-10	Undiff-G	ROMO1
UBE2J2	2.48E-14	-0.51965	0.015	0.338	3.70E-10	Undiff-G	UBE2J2
JOSD2	2.48E-14	-0.59819	0.015	0.339	3.71E-10	Undiff-G	JOSD2
CAPG	2.49E-14	-0.53604	0.059	0.419	3.71E-10	Undiff-G	CAPG
FAU	2.51E-14	0.951005	0.713	0.961	3.75E-10	Undiff-G	FAU
GSPT1	2.59E-14	-0.56148	0.022	0.352	3.88E-10	Undiff-G	GSPT1
FAM192A	2.67E-14	-0.438	0.029	0.361	4.00E-10	Undiff-G	FAM192A
MGP	2.69E-14	1.54327	0.279	0.102	4.02E-10	Undiff-G	MGP
DTYMK	2.74E-14	-0.64702	0.015	0.333	4.10E-10	Undiff-G	DTYMK
SNHG7	2.92E-14	-0.68781	0.022	0.35	4.37E-10	Undiff-G	SNHG7
SRP19	2.99E-14	-0.12779	0.051	0.408	4.48E-10	Undiff-G	SRP19
NANS	3.01E-14	-0.4977	0.029	0.364	4.49E-10	Undiff-G	NANS
PAFAH1B3	3.04E-14	-0.40421	0.066	0.428	4.54E-10	Undiff-G	PAFAH1B3
HINT2	3.07E-14	-0.62077	0.022	0.35	4.59E-10	Undiff-G	HINT2
JUND	3.14E-14	-0.41785	0.051	0.403	4.69E-10	Undiff-G	JUND
C6orf1	3.19E-14	-0.53319	0.029	0.364	4.77E-10	Undiff-G	C6orf1
METTL23	3.19E-14	-0.67788	0.007	0.322	4.77E-10	Undiff-G	METTL23
PRKRA	3.22E-14	-0.44671	0.029	0.365	4.81E-10	Undiff-G	PRKRA

YIPF4	3.36E-14	-0.27064	0.037	0.378	5.02E-10	Undiff-G	YIPF4
PHB2	3.38E-14	0.104528	0.074	0.447	5.05E-10	Undiff-G	PHB2
HNRNPC	3.45E-14	0.26077	0.199	0.699	5.16E-10	Undiff-G	HNRNPC
CSNK1D	3.53E-14	-0.47063	0.029	0.363	5.27E-10	Undiff-G	CSNK1D
CHD9	3.55E-14	-0.11743	0.088	0.472	5.31E-10	Undiff-G	CHD9
FGFR1OP2	3.63E-14	-0.68903	0.007	0.321	5.43E-10	Undiff-G	FGFR1OP2
AFF4	3.70E-14	-0.65642	0.022	0.344	5.52E-10	Undiff-G	AFF4
CLPTM1	3.71E-14	-0.40634	0.022	0.349	5.54E-10	Undiff-G	CLPTM1
EIF1AX	3.88E-14	0.350317	0.132	0.565	5.80E-10	Undiff-G	EIF1AX
EVL	3.91E-14	-0.72159	0.029	0.356	5.85E-10	Undiff-G	EVL
ILF3	4.01E-14	-0.20466	0.059	0.416	6.00E-10	Undiff-G	ILF3
PLSCR1	4.06E-14	-0.46027	0.029	0.363	6.07E-10	Undiff-G	PLSCR1
NXT1	4.07E-14	-0.66326	0.007	0.32	6.08E-10	Undiff-G	NXT1
CSNK2A1	4.21E-14	-0.67566	0.007	0.32	6.30E-10	Undiff-G	CSNK2A1
CTNNB1	4.23E-14	-0.66883	0.029	0.359	6.32E-10	Undiff-G	CTNNB1
PRAF2	4.24E-14	-0.75785	0	0.306	6.34E-10	Undiff-G	PRAF2
CRK	4.25E-14	-0.62484	0.007	0.32	6.36E-10	Undiff-G	CRK
GPBP1	4.36E-14	-0.19527	0.081	0.453	6.52E-10	Undiff-G	GPBP1
TIMP2	4.41E-14	-0.75924	0.015	0.329	6.60E-10	Undiff-G	TIMP2
LGALS1	4.46E-14	1.21913	0.654	0.704	6.66E-10	Undiff-G	LGALS1
DYNC1H1	4.51E-14	-0.75465	0.022	0.342	6.73E-10	Undiff-G	DYNC1H1
PQBP1	4.51E-14	-0.37988	0.022	0.348	6.75E-10	Undiff-G	PQBP1
HAGH	4.55E-14	-0.50684	0.029	0.357	6.80E-10	Undiff-G	HAGH
EIF4EBP1	4.62E-14	-0.15404	0.066	0.431	6.90E-10	Undiff-G	EIF4EBP1
DYNLT1	4.65E-14	0.217254	0.169	0.634	6.95E-10	Undiff-G	DYNLT1
DLD	4.75E-14	-0.42235	0.022	0.348	7.10E-10	Undiff-G	DLD
NFIC	4.82E-14	-0.27458	0.074	0.438	7.20E-10	Undiff-G	NFIC
COA1	4.87E-14	-0.30744	0.051	0.401	7.28E-10	Undiff-G	COA1
TREM2	4.91E-14	-1.02518	0.029	0.354	7.34E-10	Undiff-G	TREM2
VAMP8	5.16E-14	-0.75108	0.022	0.347	7.70E-10	Undiff-G	VAMP8
PAPOLA	5.23E-14	0.212377	0.096	0.489	7.82E-10	Undiff-G	PAPOLA
TCEA2	5.37E-14	-0.56426	0.037	0.372	8.02E-10	Undiff-G	TCEA2
TAPBP	5.56E-14	-0.3674	0.022	0.346	8.32E-10	Undiff-G	TAPBP
TMEM50A	5.59E-14	0.272666	0.154	0.597	8.35E-10	Undiff-G	TMEM50A
TCEB1	5.59E-14	0.299157	0.169	0.628	8.35E-10	Undiff-G	TCEB1
CYTH2	5.63E-14	-0.54172	0.022	0.345	8.42E-10	Undiff-G	CYTH2
CXXC5	5.77E-14	-0.59856	0.044	0.384	8.63E-10	Undiff-G	CXXC5
ACSL3	5.82E-14	-0.63732	0.022	0.345	8.70E-10	Undiff-G	ACSL3
CMTM3	5.87E-14	-0.64589	0.015	0.327	8.78E-10	Undiff-G	CMTM3
C7orf55-L	5.93E-14	-0.70813	0.022	0.342	8.87E-10	Undiff-G	C7orf55-LUC7L2
RAB4A	5.93E-14	-0.48309	0.022	0.345	8.87E-10	Undiff-G	RAB4A
SF3B1	6.04E-14	-0.25271	0.074	0.441	9.03E-10	Undiff-G	SF3B1
SLC25A4	6.06E-14	-0.12254	0.066	0.431	9.06E-10	Undiff-G	SLC25A4
ARPC5L	6.20E-14	-0.33679	0.037	0.373	9.26E-10	Undiff-G	ARPC5L
PHF14	6.20E-14	-0.40638	0.066	0.427	9.26E-10	Undiff-G	PHF14
SSRP1	6.24E-14	-0.55602	0.029	0.358	9.32E-10	Undiff-G	SSRP1
TSPAN13	6.41E-14	-0.92531	0	0.302	9.58E-10	Undiff-G	TSPAN13
PRCP	6.47E-14	-0.28978	0.029	0.359	9.66E-10	Undiff-G	PRCP
PTBP1	6.48E-14	-0.63933	0.015	0.327	9.68E-10	Undiff-G	PTBP1
ESD	6.79E-14	0.234759	0.103	0.494	1.01E-09	Undiff-G	ESD
FGD5-AS1	6.94E-14	-0.5631	0.007	0.316	1.04E-09	Undiff-G	FGD5-AS1

SSBP1	7.08E-14	0.445862	0.176	0.644	1.06E-09	Undiff-G	SSBP1
SPAG9	7.09E-14	-0.60843	0.029	0.357	1.06E-09	Undiff-G	SPAG9
SCD5	7.10E-14	-0.46609	0.051	0.398	1.06E-09	Undiff-G	SCD5
SPCS3	7.14E-14	-0.76161	0.015	0.329	1.07E-09	Undiff-G	SPCS3
TYMP	7.15E-14	-1.06134	0.007	0.31	1.07E-09	Undiff-G	TYMP
USP16	7.16E-14	-0.49217	0.037	0.367	1.07E-09	Undiff-G	USP16
TWF2	7.17E-14	-0.32055	0.022	0.344	1.07E-09	Undiff-G	TWF2
USF2	7.24E-14	-0.47309	0.015	0.329	1.08E-09	Undiff-G	USF2
OSBPL8	7.25E-14	-0.72484	0.007	0.315	1.08E-09	Undiff-G	OSBPL8
HLA-DPB1	7.26E-14	-0.27566	0.103	0.499	1.09E-09	Undiff-G	HLA-DPB1
SMAP1	7.54E-14	-0.73248	0	0.301	1.13E-09	Undiff-G	SMAP1
ECH1	7.54E-14	0.187089	0.14	0.563	1.13E-09	Undiff-G	ECH1
SERPINB6	7.59E-14	0.15434	0.096	0.482	1.13E-09	Undiff-G	SERPINB6
SERINC3	7.78E-14	-0.37906	0.037	0.369	1.16E-09	Undiff-G	SERINC3
MYEOV2	7.90E-14	0.18061	0.191	0.679	1.18E-09	Undiff-G	MYEOV2
YWHAE	7.96E-14	0.214054	0.235	0.758	1.19E-09	Undiff-G	YWHAE
VPS4A	7.98E-14	-0.72026	0.007	0.311	1.19E-09	Undiff-G	VPS4A
NLRP1	8.13E-14	-0.48133	0.022	0.342	1.21E-09	Undiff-G	NLRP1
M6PR	8.18E-14	-0.12045	0.044	0.386	1.22E-09	Undiff-G	M6PR
CREG1	8.21E-14	-0.89567	0	0.3	1.23E-09	Undiff-G	CREG1
PET100	8.34E-14	0.130318	0.118	0.529	1.25E-09	Undiff-G	PET100
TPGS2	8.48E-14	-0.56702	0.022	0.342	1.27E-09	Undiff-G	TPGS2
GPM6A	8.55E-14	-0.22473	0.118	0.509	1.28E-09	Undiff-G	GPM6A
HMG20B	8.58E-14	-0.68559	0.022	0.34	1.28E-09	Undiff-G	HMG20B
UBC	8.67E-14	0.891802	0.713	0.953	1.30E-09	Undiff-G	UBC
ARMC10	8.67E-14	-0.68577	0	0.3	1.30E-09	Undiff-G	ARMC10
SLC39A3	8.68E-14	-0.62691	0.022	0.342	1.30E-09	Undiff-G	SLC39A3
IFI6	8.86E-14	-0.3678	0.059	0.41	1.32E-09	Undiff-G	IFI6
DDX18	9.12E-14	0.114426	0.11	0.506	1.36E-09	Undiff-G	DDX18
PRPF6	9.14E-14	-0.3146	0.029	0.357	1.37E-09	Undiff-G	PRPF6
CKS2	9.18E-14	-0.44573	0.037	0.37	1.37E-09	Undiff-G	CKS2
TPGS1	9.25E-14	-0.68857	0.007	0.313	1.38E-09	Undiff-G	TPGS1
NPDC1	9.32E-14	-0.74848	0.015	0.327	1.39E-09	Undiff-G	NPDC1
GYPC	9.62E-14	-0.93913	0.007	0.313	1.44E-09	Undiff-G	GYPC
KLHDC3	9.82E-14	-0.25942	0.037	0.366	1.47E-09	Undiff-G	KLHDC3
ACAA1	9.88E-14	-0.59574	0.029	0.353	1.48E-09	Undiff-G	ACAA1
PTPMT1	9.93E-14	-0.34866	0.029	0.356	1.48E-09	Undiff-G	PTPMT1
SMIM19	1.00E-13	-0.48356	0.015	0.327	1.50E-09	Undiff-G	SMIM19
SOCS3	1.02E-13	-0.72372	0.029	0.352	1.52E-09	Undiff-G	SOCS3
ATF6B	1.03E-13	-0.51488	0.051	0.391	1.54E-09	Undiff-G	ATF6B
SNRNP27	1.03E-13	-0.33591	0.029	0.356	1.54E-09	Undiff-G	SNRNP27
RBM39	1.04E-13	0.226617	0.184	0.66	1.56E-09	Undiff-G	RBM39
CORO1B	1.06E-13	-0.40543	0.037	0.365	1.58E-09	Undiff-G	CORO1B
CAPZA1	1.06E-13	-0.22102	0.051	0.396	1.59E-09	Undiff-G	CAPZA1
CFDP1	1.07E-13	-0.12172	0.066	0.42	1.61E-09	Undiff-G	CFDP1
CELF2	1.09E-13	-0.3516	0.051	0.387	1.63E-09	Undiff-G	CELF2
STMN4	1.10E-13	-1.13275	0.007	0.309	1.65E-09	Undiff-G	STMN4
NME3	1.13E-13	-0.27224	0.066	0.421	1.69E-09	Undiff-G	NME3
PITHD1	1.14E-13	-0.48522	0.029	0.35	1.70E-09	Undiff-G	PITHD1
TMEM256	1.14E-13	-0.10711	0.074	0.434	1.71E-09	Undiff-G	TMEM256
RDH11	1.16E-13	-0.12792	0.051	0.392	1.73E-09	Undiff-G	RDH11

PAPSS1	1.16E-13	-0.75276	0	0.298	1.73E-09	Undiff-G	PAPSS1
APEX1	1.16E-13	0.261685	0.125	0.542	1.74E-09	Undiff-G	APEX1
NUDT1	1.16E-13	-0.40718	0.029	0.354	1.74E-09	Undiff-G	NUDT1
PMF1	1.17E-13	-0.79635	0.015	0.324	1.74E-09	Undiff-G	PMF1
ITFG1	1.17E-13	-0.43573	0.029	0.352	1.75E-09	Undiff-G	ITFG1
PFDN4	1.17E-13	-0.50549	0.037	0.363	1.75E-09	Undiff-G	PFDN4
SDF2L1	1.22E-13	-0.5808	0.037	0.364	1.82E-09	Undiff-G	SDF2L1
RNF11	1.25E-13	-0.66524	0.007	0.311	1.88E-09	Undiff-G	RNF11
C14orf166	1.26E-13	0.316878	0.154	0.59	1.89E-09	Undiff-G	C14orf166
HNRNPH2	1.28E-13	-0.11388	0.059	0.404	1.91E-09	Undiff-G	HNRNPH2
SSB	1.32E-13	0.231751	0.154	0.597	1.97E-09	Undiff-G	SSB
C11orf31	1.32E-13	0.301167	0.191	0.673	1.98E-09	Undiff-G	C11orf31
TUSC2	1.34E-13	-0.55447	0.022	0.339	2.00E-09	Undiff-G	TUSC2
NSMCE1	1.38E-13	-0.12493	0.059	0.408	2.07E-09	Undiff-G	NSMCE1
GOLGA4	1.40E-13	-0.45981	0.029	0.35	2.09E-09	Undiff-G	GOLGA4
MRFAP1	1.41E-13	0.27132	0.191	0.676	2.10E-09	Undiff-G	MRFAP1
TMEM30A	1.41E-13	-0.64443	0.022	0.338	2.11E-09	Undiff-G	TMEM30A
ARF6	1.42E-13	-0.55584	0.029	0.347	2.12E-09	Undiff-G	ARF6
STMN3	1.44E-13	-0.63234	0.007	0.31	2.15E-09	Undiff-G	STMN3
AAMP	1.45E-13	-0.19465	0.074	0.431	2.17E-09	Undiff-G	AAMP
LY86	1.50E-13	-1.23079	0	0.296	2.24E-09	Undiff-G	LY86
CBX5	1.51E-13	-0.44018	0.029	0.348	2.25E-09	Undiff-G	CBX5
TMEM183A	1.53E-13	-0.67947	0	0.295	2.29E-09	Undiff-G	TMEM183A
CAPRIN1	1.58E-13	-0.50512	0.015	0.324	2.35E-09	Undiff-G	CAPRIN1
YWHAG	1.58E-13	-0.18299	0.051	0.394	2.36E-09	Undiff-G	YWHAG
PHF20	1.59E-13	-0.4945	0.015	0.324	2.38E-09	Undiff-G	PHF20
STK25	1.63E-13	-0.6986	0.007	0.306	2.44E-09	Undiff-G	STK25
U2SURP	1.74E-13	-0.496	0.044	0.373	2.59E-09	Undiff-G	U2SURP
MYL12A	1.78E-13	0.163866	0.132	0.548	2.66E-09	Undiff-G	MYL12A
ANKRD12	1.78E-13	-0.3046	0.074	0.43	2.67E-09	Undiff-G	ANKRD12
TMEM261	1.79E-13	-0.25849	0.037	0.364	2.68E-09	Undiff-G	TMEM261
MSN	1.81E-13	-0.22997	0.044	0.379	2.70E-09	Undiff-G	MSN
VTI1B	1.81E-13	-0.40391	0.029	0.35	2.70E-09	Undiff-G	VTI1B
MTFR1L	1.84E-13	-0.72159	0	0.294	2.74E-09	Undiff-G	MTFR1L
HCLS1	1.85E-13	-1.16703	0	0.294	2.77E-09	Undiff-G	HCLS1
C20orf27	1.86E-13	-0.36657	0.037	0.364	2.78E-09	Undiff-G	C20orf27
CWC15	1.87E-13	0.271136	0.103	0.492	2.80E-09	Undiff-G	CWC15
AKR7A2	1.89E-13	-0.53367	0.015	0.318	2.82E-09	Undiff-G	AKR7A2
TOMM40	1.90E-13	-0.68038	0.022	0.331	2.84E-09	Undiff-G	TOMM40
CDC42SE1	1.91E-13	-0.77956	0.007	0.307	2.86E-09	Undiff-G	CDC42SE1
TXNL4A	1.92E-13	-0.15652	0.066	0.414	2.87E-09	Undiff-G	TXNL4A
VBP1	1.93E-13	0.247106	0.103	0.491	2.89E-09	Undiff-G	VBP1
EIF4E2	1.98E-13	-0.40209	0.022	0.336	2.97E-09	Undiff-G	EIF4E2
EBPL	2.04E-13	-0.69743	0.007	0.306	3.05E-09	Undiff-G	EBPL
MS4A7	2.08E-13	-0.86702	0.022	0.336	3.11E-09	Undiff-G	MS4A7
CCDC50	2.10E-13	-0.73004	0.007	0.303	3.13E-09	Undiff-G	CCDC50
MPG	2.12E-13	-0.36976	0.029	0.349	3.17E-09	Undiff-G	MPG
COMMD3	2.13E-13	-0.41935	0.037	0.358	3.18E-09	Undiff-G	COMMD3
TCEAL4	2.18E-13	0.244815	0.147	0.576	3.26E-09	Undiff-G	TCEAL4
ELAVL1	2.20E-13	-0.42676	0.029	0.348	3.28E-09	Undiff-G	ELAVL1
C1QA	2.24E-13	-0.322	0.14	0.542	3.34E-09	Undiff-G	C1QA

ARMCX3	2.27E-13	0.125059	0.074	0.434	3.40E-09	Undiff-G	ARMCX3
TTC1	2.28E-13	-0.11654	0.044	0.377	3.40E-09	Undiff-G	TTC1
SRP72	2.30E-13	-0.10052	0.088	0.463	3.43E-09	Undiff-G	SRP72
IDH1	2.35E-13	-0.39134	0.051	0.384	3.52E-09	Undiff-G	IDH1
SHARPIN	2.36E-13	-0.7071	0.007	0.305	3.53E-09	Undiff-G	SHARPIN
DNASE2	2.41E-13	-0.46131	0.051	0.387	3.60E-09	Undiff-G	DNASE2
FKBP2	2.45E-13	0.299289	0.154	0.595	3.66E-09	Undiff-G	FKBP2
NHP2	2.49E-13	0.300994	0.154	0.585	3.73E-09	Undiff-G	NHP2
MMP24-AS1	2.51E-13	-0.65114	0.015	0.315	3.75E-09	Undiff-G	MMP24-AS1
CCL4	2.59E-13	-1.01195	0.022	0.328	3.87E-09	Undiff-G	CCL4
ATP2B1	2.64E-13	-0.36009	0.037	0.358	3.94E-09	Undiff-G	ATP2B1
SCP2	2.69E-13	0.226488	0.154	0.589	4.02E-09	Undiff-G	SCP2
SYNCRIP	2.72E-13	-0.19775	0.051	0.387	4.07E-09	Undiff-G	SYNCRIP
SARS	2.74E-13	0.136066	0.081	0.447	4.10E-09	Undiff-G	SARS
ATP5O	2.82E-13	0.354987	0.243	0.756	4.22E-09	Undiff-G	ATP5O
PSMC4	2.92E-13	0.122347	0.11	0.499	4.36E-09	Undiff-G	PSMC4
CD53	2.94E-13	-0.97879	0.015	0.319	4.39E-09	Undiff-G	CD53
PTPRA	3.01E-13	-0.67009	0.037	0.358	4.50E-09	Undiff-G	PTPRA
RSL1D1	3.07E-13	0.136421	0.11	0.501	4.58E-09	Undiff-G	RSL1D1
TTC19	3.14E-13	-0.29945	0.037	0.36	4.69E-09	Undiff-G	TTC19
DNAJC8	3.16E-13	0.29912	0.14	0.556	4.72E-09	Undiff-G	DNAJC8
RAD21	3.19E-13	-0.40152	0.044	0.37	4.77E-09	Undiff-G	RAD21
EPB41L2	3.24E-13	-0.83633	0.015	0.314	4.85E-09	Undiff-G	EPB41L2
SDHC	3.25E-13	0.300841	0.14	0.562	4.85E-09	Undiff-G	SDHC
PSMB2	3.25E-13	0.294069	0.147	0.579	4.85E-09	Undiff-G	PSMB2
DDRGK1	3.26E-13	-0.18616	0.074	0.422	4.87E-09	Undiff-G	DDRGK1
RAB5A	3.40E-13	0.102243	0.088	0.452	5.08E-09	Undiff-G	RAB5A
SYNGR2	3.42E-13	-0.66893	0.022	0.332	5.12E-09	Undiff-G	SYNGR2
PCM1	3.43E-13	-0.65057	0.022	0.327	5.12E-09	Undiff-G	PCM1
ALKBH5	3.44E-13	-0.64013	0.022	0.327	5.14E-09	Undiff-G	ALKBH5
RNH1	3.52E-13	0.243793	0.132	0.542	5.26E-09	Undiff-G	RNH1
FDPS	3.71E-13	-0.16346	0.074	0.42	5.54E-09	Undiff-G	FDPS
SLBP	3.72E-13	-0.68851	0.015	0.314	5.55E-09	Undiff-G	SLBP
CITED2	3.81E-13	-0.59114	0.037	0.356	5.70E-09	Undiff-G	CITED2
FPR1	3.85E-13	-1.23109	0	0.288	5.76E-09	Undiff-G	FPR1
PHB	3.88E-13	0.317246	0.14	0.559	5.80E-09	Undiff-G	PHB
TSC22D4	3.90E-13	-0.31851	0.103	0.474	5.83E-09	Undiff-G	TSC22D4
FXR1	3.92E-13	-0.40692	0.022	0.33	5.86E-09	Undiff-G	FXR1
MAGEF1	3.95E-13	-0.29463	0.044	0.367	5.91E-09	Undiff-G	MAGEF1
SRSF7	4.05E-13	0.257709	0.162	0.595	6.05E-09	Undiff-G	SRSF7
IRS2	4.05E-13	-0.34074	0.037	0.352	6.06E-09	Undiff-G	IRS2
SLC22A17	4.19E-13	-0.74583	0	0.287	6.26E-09	Undiff-G	SLC22A17
TSN	4.36E-13	-0.1231	0.051	0.381	6.52E-09	Undiff-G	TSN
HSP90B1	4.63E-13	0.290917	0.199	0.663	6.91E-09	Undiff-G	HSP90B1
IFITM2	4.70E-13	-1.08715	0	0.286	7.03E-09	Undiff-G	IFITM2
HLA-B	4.84E-13	1.001428	0.684	0.854	7.23E-09	Undiff-G	HLA-B
DCAF7	4.85E-13	-0.51017	0.022	0.324	7.25E-09	Undiff-G	DCAF7
NUBP2	4.87E-13	-0.13571	0.051	0.385	7.27E-09	Undiff-G	NUBP2
USP11	4.91E-13	-0.3748	0.037	0.356	7.33E-09	Undiff-G	USP11
VKORC1	5.10E-13	0.298952	0.11	0.501	7.63E-09	Undiff-G	VKORC1
LGMN	5.11E-13	-0.41305	0.037	0.354	7.64E-09	Undiff-G	LGMN

TM9SF2	5.19E-13	-0.23133	0.029	0.343	7.76E-09	Undiff-G	TM9SF2
GLRX5	5.31E-13	-0.50988	0.007	0.3	7.94E-09	Undiff-G	GLRX5
BPTF	5.33E-13	-0.49971	0.015	0.314	7.96E-09	Undiff-G	BPTF
APPL1	5.53E-13	-0.69164	0.007	0.299	8.26E-09	Undiff-G	APPL1
UQCRH	5.57E-13	0.277688	0.25	0.787	8.32E-09	Undiff-G	UQCRH
GPS1	5.64E-13	-0.36903	0.022	0.328	8.44E-09	Undiff-G	GPS1
NELFCD	5.76E-13	-0.54549	0.029	0.34	8.61E-09	Undiff-G	NELFCD
CTSH	5.82E-13	-0.87119	0.007	0.299	8.69E-09	Undiff-G	CTSH
ZNF580	5.85E-13	-0.71146	0.007	0.295	8.75E-09	Undiff-G	ZNF580
ATP6V1G1	5.92E-13	0.302401	0.243	0.76	8.84E-09	Undiff-G	ATP6V1G1
DGCR6L	5.92E-13	-0.59874	0.029	0.339	8.85E-09	Undiff-G	DGCR6L
G6PC3	5.93E-13	-0.33204	0.037	0.351	8.86E-09	Undiff-G	G6PC3
PRPF31	5.95E-13	-0.21745	0.059	0.396	8.90E-09	Undiff-G	PRPF31
TMEM134	5.99E-13	-0.40323	0.015	0.313	8.95E-09	Undiff-G	TMEM134
AK1	6.02E-13	-0.45739	0.029	0.339	9.00E-09	Undiff-G	AK1
CYB5B	6.04E-13	-0.29692	0.029	0.341	9.02E-09	Undiff-G	CYB5B
HRAS	6.13E-13	-0.66135	0.007	0.297	9.16E-09	Undiff-G	HRAS
MEST	6.20E-13	-0.40929	0.059	0.39	9.27E-09	Undiff-G	MEST
SAR1B	6.35E-13	-0.37578	0.022	0.326	9.48E-09	Undiff-G	SAR1B
CAMTA1	6.52E-13	0.281027	0.088	0.453	9.74E-09	Undiff-G	CAMTA1
TMED3	6.62E-13	-0.53429	0.015	0.311	9.90E-09	Undiff-G	TMED3
SLC52A2	6.65E-13	-0.67166	0	0.283	9.93E-09	Undiff-G	SLC52A2
STK17A	6.73E-13	-0.74154	0.007	0.297	1.01E-08	Undiff-G	STK17A
PDCD2	6.83E-13	-0.45819	0.007	0.298	1.02E-08	Undiff-G	PDCD2
LINC00657	6.86E-13	-0.32724	0.029	0.335	1.02E-08	Undiff-G	LINC00657
SNRPD2	7.00E-13	0.364122	0.235	0.754	1.05E-08	Undiff-G	SNRPD2
IFRD1	7.10E-13	0.187077	0.11	0.492	1.06E-08	Undiff-G	IFRD1
NR1H2	7.15E-13	-0.72891	0.015	0.307	1.07E-08	Undiff-G	NR1H2
SPNS1	7.16E-13	-0.67504	0.007	0.294	1.07E-08	Undiff-G	SPNS1
NDUFC2	7.22E-13	0.356016	0.235	0.759	1.08E-08	Undiff-G	NDUFC2
CUEDC2	7.23E-13	-0.21287	0.066	0.404	1.08E-08	Undiff-G	CUEDC2
DDX39A	7.28E-13	-0.12421	0.059	0.396	1.09E-08	Undiff-G	DDX39A
ITM2C	7.29E-13	-0.10158	0.074	0.421	1.09E-08	Undiff-G	ITM2C
IGFBP2	7.48E-13	-0.60728	0.051	0.378	1.12E-08	Undiff-G	IGFBP2
NT5C3B	7.64E-13	-0.27662	0.029	0.335	1.14E-08	Undiff-G	NT5C3B
POLR2J	7.74E-13	0.295653	0.191	0.649	1.16E-08	Undiff-G	POLR2J
MINOS1	7.79E-13	0.295433	0.184	0.645	1.16E-08	Undiff-G	MINOS1
ARF1	7.85E-13	0.379545	0.213	0.7	1.17E-08	Undiff-G	ARF1
CCDC12	7.90E-13	-0.16063	0.081	0.434	1.18E-08	Undiff-G	CCDC12
WNK1	7.93E-13	-0.72342	0	0.282	1.19E-08	Undiff-G	WNK1
MPST	8.13E-13	-0.64451	0.015	0.31	1.21E-08	Undiff-G	MPST
BAG6	8.32E-13	-0.30802	0.029	0.335	1.24E-08	Undiff-G	BAG6
AZIN1	8.39E-13	-0.53283	0.029	0.333	1.25E-08	Undiff-G	AZIN1
RHBDD2	8.44E-13	0.21922	0.096	0.463	1.26E-08	Undiff-G	RHBDD2
CPVL	8.46E-13	-0.44513	0.037	0.352	1.26E-08	Undiff-G	CPVL
RNF7	8.49E-13	0.11692	0.132	0.537	1.27E-08	Undiff-G	RNF7
SUGT1	8.49E-13	-0.21571	0.037	0.348	1.27E-08	Undiff-G	SUGT1
VIMP	8.56E-13	0.244066	0.096	0.46	1.28E-08	Undiff-G	VIMP
MCL1	8.68E-13	0.254964	0.169	0.603	1.30E-08	Undiff-G	MCL1
RAB7A	8.72E-13	0.301002	0.199	0.664	1.30E-08	Undiff-G	RAB7A
ERLEC1	9.02E-13	-0.17095	0.044	0.365	1.35E-08	Undiff-G	ERLEC1

SLC16A3	9.16E-13	-0.57964	0.022	0.324	1.37E-08	Undiff-G	SLC16A3
UFD1L	9.25E-13	-0.31848	0.029	0.334	1.38E-08	Undiff-G	UFD1L
HSPA9	9.39E-13	0.239733	0.11	0.492	1.40E-08	Undiff-G	HSPA9
GAA	9.53E-13	-0.54575	0.022	0.323	1.42E-08	Undiff-G	GAA
SPATS2L	9.55E-13	-0.58829	0.029	0.335	1.43E-08	Undiff-G	SPATS2L
DNAJA2	9.66E-13	-0.34027	0.029	0.333	1.44E-08	Undiff-G	DNAJA2
ZC3H13	9.67E-13	-0.12133	0.029	0.337	1.45E-08	Undiff-G	ZC3H13
OSTF1	9.67E-13	-0.78296	0.007	0.295	1.45E-08	Undiff-G	OSTF1
PDIA4	9.72E-13	0.187915	0.096	0.466	1.45E-08	Undiff-G	PDIA4
SYT11	9.72E-13	-0.1676	0.059	0.388	1.45E-08	Undiff-G	SYT11
FDX1	9.80E-13	-0.36436	0.015	0.309	1.46E-08	Undiff-G	FDX1
BID	1.01E-12	-0.19749	0.044	0.36	1.51E-08	Undiff-G	BID
CD37	1.01E-12	-0.95965	0.015	0.304	1.51E-08	Undiff-G	CD37
S100A6	1.04E-12	1.275746	0.625	0.644	1.55E-08	Undiff-G	S100A6
RTCB	1.05E-12	-0.10108	0.029	0.337	1.58E-08	Undiff-G	RTCB
MPHOSPH6	1.05E-12	-0.61121	0.007	0.294	1.58E-08	Undiff-G	MPHOSPH6
ADH5	1.05E-12	0.112282	0.059	0.393	1.58E-08	Undiff-G	ADH5
IGBP1	1.06E-12	-0.30105	0.037	0.349	1.59E-08	Undiff-G	IGBP1
TM2D1	1.08E-12	-0.1223	0.059	0.39	1.62E-08	Undiff-G	TM2D1
GNAQ	1.09E-12	-0.70004	0.007	0.29	1.62E-08	Undiff-G	GNAQ
C4orf27	1.10E-12	-0.33285	0.022	0.322	1.64E-08	Undiff-G	C4orf27
TSR3	1.11E-12	-0.65075	0	0.279	1.65E-08	Undiff-G	TSR3
CD163	1.13E-12	-1.15384	0	0.279	1.69E-08	Undiff-G	CD163
IFT20	1.14E-12	-0.39179	0.037	0.348	1.71E-08	Undiff-G	IFT20
ETF1	1.15E-12	-0.31357	0.037	0.349	1.72E-08	Undiff-G	ETF1
RGS19	1.17E-12	-0.83951	0	0.279	1.74E-08	Undiff-G	RGS19
QDPR	1.17E-12	-0.17843	0.044	0.364	1.75E-08	Undiff-G	QDPR
POMP	1.19E-12	0.288517	0.235	0.741	1.78E-08	Undiff-G	POMP
MVB12A	1.20E-12	-0.69403	0.015	0.305	1.80E-08	Undiff-G	MVB12A
ADAM9	1.22E-12	-0.36489	0.029	0.333	1.82E-08	Undiff-G	ADAM9
GOLIM4	1.23E-12	-0.57379	0.029	0.33	1.83E-08	Undiff-G	GOLIM4
RNASE6	1.23E-12	-1.14846	0	0.278	1.83E-08	Undiff-G	RNASE6
TOMM20	1.24E-12	0.305258	0.125	0.521	1.85E-08	Undiff-G	TOMM20
POLR2I	1.24E-12	0.239852	0.147	0.557	1.85E-08	Undiff-G	POLR2I
ISG15	1.25E-12	-0.46539	0.044	0.36	1.87E-08	Undiff-G	ISG15
SMIM12	1.27E-12	-0.59539	0.015	0.306	1.89E-08	Undiff-G	SMIM12
PSMD12	1.27E-12	-0.21951	0.037	0.346	1.89E-08	Undiff-G	PSMD12
DNTTIP1	1.28E-12	-0.38892	0.029	0.333	1.91E-08	Undiff-G	DNTTIP1
GAMT	1.28E-12	-0.46816	0.015	0.303	1.92E-08	Undiff-G	GAMT
PDHA1	1.31E-12	-0.43563	0.015	0.306	1.96E-08	Undiff-G	PDHA1
REL	1.32E-12	-0.76668	0.015	0.301	1.97E-08	Undiff-G	REL
ATF3	1.33E-12	-0.8308	0.015	0.305	1.99E-08	Undiff-G	ATF3
CDH2	1.34E-12	-0.72235	0.007	0.287	2.00E-08	Undiff-G	CDH2
YTHDC1	1.34E-12	-0.56799	0.015	0.305	2.00E-08	Undiff-G	YTHDC1
C10orf54	1.34E-12	-1.05681	0.007	0.291	2.01E-08	Undiff-G	C10orf54
TRAPPC4	1.36E-12	0.287608	0.088	0.449	2.04E-08	Undiff-G	TRAPPC4
ZNF331	1.36E-12	-0.62152	0.022	0.319	2.04E-08	Undiff-G	ZNF331
PPM1B	1.38E-12	-0.65766	0.015	0.304	2.06E-08	Undiff-G	PPM1B
PPP1R10	1.38E-12	-0.7369	0.007	0.291	2.06E-08	Undiff-G	PPP1R10
SGTA	1.41E-12	-0.49436	0.022	0.319	2.11E-08	Undiff-G	SGTA
DBN1	1.43E-12	-0.69357	0.022	0.315	2.14E-08	Undiff-G	DBN1

FKBP3	1.44E-12	0.131798	0.11	0.481	2.16E-08	Undiff-G	FKBP3
PPP1R14B	1.45E-12	-0.27224	0.037	0.348	2.17E-08	Undiff-G	PPP1R14B
TACC1	1.48E-12	-0.61079	0.022	0.316	2.21E-08	Undiff-G	TACC1
HCST	1.50E-12	-1.15316	0.007	0.288	2.24E-08	Undiff-G	HCST
IRF2BP2	1.52E-12	-0.44558	0.022	0.319	2.27E-08	Undiff-G	IRF2BP2
EBNA1BP2	1.52E-12	-0.13832	0.044	0.357	2.27E-08	Undiff-G	EBNA1BP2
RBBP7	1.57E-12	0.112863	0.081	0.428	2.34E-08	Undiff-G	RBBP7
NR4A1	1.62E-12	-1.00224	0	0.276	2.42E-08	Undiff-G	NR4A1
PAFAH1B2	1.63E-12	-0.63452	0.029	0.329	2.44E-08	Undiff-G	PAFAH1B2
CD2BP2	1.64E-12	-0.35267	0.029	0.332	2.45E-08	Undiff-G	CD2BP2
AMZ2	1.68E-12	-0.12657	0.059	0.384	2.51E-08	Undiff-G	AMZ2
SNRPG	1.70E-12	0.352002	0.191	0.645	2.54E-08	Undiff-G	SNRPG
WDR18	1.73E-12	-0.64935	0.007	0.289	2.58E-08	Undiff-G	WDR18
SCAF11	1.74E-12	0.104522	0.066	0.4	2.60E-08	Undiff-G	SCAF11
NAA50	1.80E-12	-0.69276	0	0.275	2.69E-08	Undiff-G	NAA50
ATP6V1D	1.81E-12	-0.11371	0.051	0.374	2.70E-08	Undiff-G	ATP6V1D
UBE2R2	1.81E-12	-0.34337	0.007	0.289	2.71E-08	Undiff-G	UBE2R2
EMC8	1.82E-12	-0.64362	0	0.275	2.71E-08	Undiff-G	EMC8
SMC3	1.85E-12	-0.69311	0.007	0.289	2.76E-08	Undiff-G	SMC3
YIF1A	1.87E-12	0.203818	0.081	0.428	2.80E-08	Undiff-G	YIF1A
EIF4A2	1.88E-12	0.179411	0.228	0.725	2.80E-08	Undiff-G	EIF4A2
ROCK1	1.90E-12	-0.6696	0.022	0.312	2.84E-08	Undiff-G	ROCK1
GTF2B	1.92E-12	-0.38661	0.015	0.303	2.86E-08	Undiff-G	GTF2B
CTSC	1.96E-12	-0.31931	0.059	0.388	2.93E-08	Undiff-G	CTSC
CDIPT	1.97E-12	-0.52294	0.007	0.289	2.94E-08	Undiff-G	CDIPT
G3BP1	1.97E-12	-0.34917	0.022	0.317	2.95E-08	Undiff-G	G3BP1
COPA	2.04E-12	-0.51615	0.022	0.316	3.05E-08	Undiff-G	COPA
SLC44A1	2.09E-12	-0.38022	0.022	0.317	3.12E-08	Undiff-G	SLC44A1
SCNM1	2.10E-12	-0.354	0.037	0.343	3.14E-08	Undiff-G	SCNM1
PHF5A	2.11E-12	-0.16863	0.029	0.331	3.16E-08	Undiff-G	PHF5A
PFDN6	2.11E-12	-0.22952	0.044	0.356	3.16E-08	Undiff-G	PFDN6
TPP1	2.12E-12	-0.46871	0.029	0.326	3.17E-08	Undiff-G	TPP1
LST1	2.15E-12	-0.7933	0.022	0.316	3.21E-08	Undiff-G	LST1
NAE1	2.19E-12	-0.15956	0.037	0.344	3.27E-08	Undiff-G	NAE1
PSMG3	2.20E-12	-0.26313	0.029	0.33	3.28E-08	Undiff-G	PSMG3
NFIA	2.27E-12	-0.37208	0.059	0.383	3.39E-08	Undiff-G	NFIA
HLA-DQB1	2.30E-12	-0.80355	0.029	0.329	3.44E-08	Undiff-G	HLA-DQB1
9-Sep	2.33E-12	-0.21624	0.037	0.344	3.48E-08	Undiff-G	9-Sep
BAZ2B	2.34E-12	-0.62994	0.029	0.324	3.50E-08	Undiff-G	BAZ2B
FXYD5	2.36E-12	-0.50631	0.037	0.344	3.53E-08	Undiff-G	FXYD5
ABCF1	2.38E-12	-0.60779	0.007	0.287	3.56E-08	Undiff-G	ABCF1
PMVK	2.40E-12	0.110066	0.088	0.441	3.58E-08	Undiff-G	PMVK
TMEM176B	2.40E-12	-0.66385	0.044	0.356	3.59E-08	Undiff-G	TMEM176B
PNMA1	2.42E-12	-0.64145	0.007	0.287	3.61E-08	Undiff-G	PNMA1
TMEM14B	2.54E-12	0.290342	0.132	0.53	3.80E-08	Undiff-G	TMEM14B
TSSC4	2.58E-12	-0.11473	0.022	0.315	3.85E-08	Undiff-G	TSSC4
NDUFA6	2.61E-12	0.308124	0.162	0.576	3.90E-08	Undiff-G	NDUFA6
ARL2	2.64E-12	0.136344	0.088	0.442	3.95E-08	Undiff-G	ARL2
LIPA	2.65E-12	-0.39064	0.037	0.338	3.96E-08	Undiff-G	LIPA
TNRC6B	2.65E-12	-0.39687	0.044	0.356	3.97E-08	Undiff-G	TNRC6B
CRELD2	2.67E-12	-0.62287	0.007	0.286	3.99E-08	Undiff-G	CRELD2

CUTA	2.68E-12	0.359403	0.206	0.687	4.00E-08	Undiff-G	CUTA
EPN1	2.81E-12	-0.23252	0.037	0.338	4.19E-08	Undiff-G	EPN1
PPP2CB	2.86E-12	-0.43161	0.015	0.3	4.27E-08	Undiff-G	PPP2CB
BAZ1A	2.89E-12	-0.41543	0.029	0.323	4.32E-08	Undiff-G	BAZ1A
ACTN4	2.92E-12	-0.48228	0.037	0.336	4.36E-08	Undiff-G	ACTN4
ITGAV	2.93E-12	-0.68944	0.007	0.282	4.38E-08	Undiff-G	ITGAV
IDI1	2.99E-12	-0.40408	0.029	0.327	4.47E-08	Undiff-G	IDI1
BNIP3L	3.01E-12	0.390817	0.169	0.604	4.50E-08	Undiff-G	BNIP3L
BRMS1	3.02E-12	-0.2635	0.022	0.314	4.51E-08	Undiff-G	BRMS1
PRPSAP1	3.09E-12	-0.52423	0.022	0.309	4.62E-08	Undiff-G	PRPSAP1
DST	3.14E-12	-0.52624	0.059	0.38	4.69E-08	Undiff-G	DST
C1D	3.29E-12	-0.4336	0.022	0.313	4.92E-08	Undiff-G	C1D
PSMB7	3.35E-12	0.332914	0.191	0.645	5.01E-08	Undiff-G	PSMB7
PRKAG1	3.38E-12	-0.66473	0.015	0.297	5.06E-08	Undiff-G	PRKAG1
FLYWCH2	3.41E-12	-0.23336	0.022	0.313	5.10E-08	Undiff-G	FLYWCH2
TXNDC12	3.45E-12	0.169995	0.088	0.436	5.15E-08	Undiff-G	TXNDC12
BUB3	3.54E-12	-0.6029	0.022	0.311	5.29E-08	Undiff-G	BUB3
MANF	3.55E-12	0.243476	0.096	0.455	5.31E-08	Undiff-G	MANF
PSMD6	3.56E-12	-0.25321	0.044	0.354	5.32E-08	Undiff-G	PSMD6
DENR	3.63E-12	-0.54832	0.029	0.324	5.42E-08	Undiff-G	DENR
KIF1B	3.64E-12	-0.64248	0.015	0.297	5.44E-08	Undiff-G	KIF1B
AHCY	3.74E-12	-0.4565	0.015	0.297	5.59E-08	Undiff-G	AHCY
TGIF1	3.81E-12	-0.24061	0.051	0.361	5.70E-08	Undiff-G	TGIF1
CCDC47	3.82E-12	0.183964	0.074	0.408	5.70E-08	Undiff-G	CCDC47
NCBP2-AS2	3.85E-12	-0.64665	0.007	0.28	5.76E-08	Undiff-G	NCBP2-AS2
MYL12B	3.91E-12	0.380759	0.235	0.733	5.85E-08	Undiff-G	MYL12B
ATAT1	3.92E-12	-0.72167	0.015	0.292	5.85E-08	Undiff-G	ATAT1
SMARCA4	3.93E-12	-0.3173	0.051	0.365	5.88E-08	Undiff-G	SMARCA4
GARS	3.93E-12	-0.6337	0.015	0.296	5.88E-08	Undiff-G	GARS
SAFB2	4.04E-12	-0.37263	0.037	0.335	6.04E-08	Undiff-G	SAFB2
DPM1	4.25E-12	0.164174	0.059	0.382	6.35E-08	Undiff-G	DPM1
NT5C	4.34E-12	-0.46619	0.029	0.323	6.48E-08	Undiff-G	NT5C
METAP2	4.37E-12	0.336281	0.11	0.474	6.52E-08	Undiff-G	METAP2
ZCRB1	4.37E-12	0.398919	0.118	0.495	6.53E-08	Undiff-G	ZCRB1
PPP1R18	4.38E-12	-0.62754	0.015	0.296	6.55E-08	Undiff-G	PPP1R18
HNRNPK	4.50E-12	0.269867	0.265	0.795	6.72E-08	Undiff-G	HNRNPK
C1QC	4.51E-12	-0.2559	0.132	0.506	6.74E-08	Undiff-G	C1QC
MEAF6	4.61E-12	0.222722	0.11	0.481	6.89E-08	Undiff-G	MEAF6
FCGR2A	4.67E-12	-0.9607	0.015	0.292	6.98E-08	Undiff-G	FCGR2A
CORO1C	4.71E-12	-0.19817	0.059	0.377	7.04E-08	Undiff-G	CORO1C
ACTR1A	4.73E-12	-0.4277	0.007	0.281	7.07E-08	Undiff-G	ACTR1A
HNRNPH1	4.75E-12	-0.10059	0.103	0.461	7.09E-08	Undiff-G	HNRNPH1
FYTTD1	4.79E-12	-0.4494	0.015	0.292	7.15E-08	Undiff-G	FYTTD1
HLA-DMB	4.80E-12	-0.6124	0.029	0.324	7.17E-08	Undiff-G	HLA-DMB
KLHDC2	4.84E-12	-0.4769	0.022	0.305	7.23E-08	Undiff-G	KLHDC2
NDUFS6	4.99E-12	0.334954	0.191	0.633	7.45E-08	Undiff-G	NDUFS6
EIF3A	4.99E-12	-0.4621	0.037	0.336	7.46E-08	Undiff-G	EIF3A
SPCS1	5.01E-12	0.418368	0.235	0.742	7.48E-08	Undiff-G	SPCS1
MYCBP2	5.29E-12	-0.44783	0.022	0.308	7.91E-08	Undiff-G	MYCBP2
HLA-DMA	5.44E-12	-0.39987	0.081	0.412	8.14E-08	Undiff-G	HLA-DMA
NSL1	5.45E-12	-0.18995	0.044	0.35	8.15E-08	Undiff-G	NSL1

RNF126	5.48E-12	-0.56788	0.015	0.294	8.20E-08	Undiff-G	RNF126
LAMP2	5.50E-12	0.11926	0.11	0.475	8.22E-08	Undiff-G	LAMP2
DDR1	5.57E-12	-0.50821	0.037	0.335	8.33E-08	Undiff-G	DDR1
HADHB	5.59E-12	0.244269	0.103	0.465	8.35E-08	Undiff-G	HADHB
SLC11A1	5.61E-12	-0.57036	0.022	0.309	8.38E-08	Undiff-G	SLC11A1
RSBN1L	5.70E-12	-0.21018	0.051	0.358	8.51E-08	Undiff-G	RSBN1L
TSPAN5	5.74E-12	-0.6795	0	0.265	8.58E-08	Undiff-G	TSPAN5
CHPT1	5.85E-12	-0.59441	0.022	0.306	8.75E-08	Undiff-G	CHPT1
RAB18	5.88E-12	0.159215	0.074	0.402	8.79E-08	Undiff-G	RAB18
PNPLA8	5.94E-12	0.153671	0.044	0.351	8.88E-08	Undiff-G	PNPLA8
FAM173A	5.95E-12	-0.60369	0.015	0.29	8.90E-08	Undiff-G	FAM173A
ZFAS1	6.00E-12	0.336542	0.221	0.703	8.97E-08	Undiff-G	ZFAS1
MAP2	6.04E-12	-0.50016	0.044	0.341	9.02E-08	Undiff-G	MAP2
ZEB2	6.08E-12	-0.54723	0.022	0.307	9.08E-08	Undiff-G	ZEB2
C19orf70	6.08E-12	0.255502	0.169	0.583	9.09E-08	Undiff-G	C19orf70
SH3GLB1	6.09E-12	0.111932	0.088	0.434	9.10E-08	Undiff-G	SH3GLB1
RRAGA	6.25E-12	0.337407	0.066	0.393	9.35E-08	Undiff-G	RRAGA
ZBTB20	6.26E-12	-0.38174	0.066	0.389	9.36E-08	Undiff-G	ZBTB20
DNAJC19	6.26E-12	0.206327	0.066	0.393	9.36E-08	Undiff-G	DNAJC19
MAGED1	6.36E-12	-0.21153	0.044	0.348	9.51E-08	Undiff-G	MAGED1
SKIL	6.37E-12	-0.54797	0.015	0.289	9.53E-08	Undiff-G	SKIL
UBE2S	6.40E-12	-0.33794	0.044	0.348	9.57E-08	Undiff-G	UBE2S
BCAP29	6.41E-12	-0.39213	0.029	0.32	9.58E-08	Undiff-G	BCAP29
SCRG1	6.41E-12	-0.33101	0.044	0.349	9.59E-08	Undiff-G	SCRG1
BEX1	6.53E-12	-0.46363	0.051	0.354	9.76E-08	Undiff-G	BEX1
ZNF22	6.60E-12	-0.53022	0.007	0.279	9.86E-08	Undiff-G	ZNF22
ITGB1BP1	6.60E-12	0.12524	0.066	0.39	9.86E-08	Undiff-G	ITGB1BP1
DYNC1LI2	6.65E-12	-0.31099	0.044	0.348	9.94E-08	Undiff-G	DYNC1LI2
CYB5A	6.68E-12	-0.11863	0.044	0.345	9.99E-08	Undiff-G	CYB5A
MAGEH1	6.88E-12	-0.19051	0.029	0.321	1.03E-07	Undiff-G	MAGEH1
CCDC112	6.95E-12	-0.65195	0	0.264	1.04E-07	Undiff-G	CCDC112
CCND2	7.00E-12	-0.49316	0.037	0.33	1.05E-07	Undiff-G	CCND2
UBE3A	7.03E-12	-0.6154	0.015	0.29	1.05E-07	Undiff-G	UBE3A
AKAP9	7.07E-12	0.100564	0.103	0.454	1.06E-07	Undiff-G	AKAP9
CHURC1	7.28E-12	0.21224	0.074	0.404	1.09E-07	Undiff-G	CHURC1
REPIN1	7.30E-12	-0.59902	0.007	0.277	1.09E-07	Undiff-G	REPIN1
INTS10	7.31E-12	-0.48319	0.022	0.305	1.09E-07	Undiff-G	INTS10
YIPF6	7.42E-12	-0.18981	0.029	0.316	1.11E-07	Undiff-G	YIPF6
PAFAH1B1	7.47E-12	-0.20122	0.059	0.371	1.12E-07	Undiff-G	PAFAH1B1
TUFM	7.58E-12	0.305611	0.191	0.638	1.13E-07	Undiff-G	TUFM
TCF12	7.73E-12	-0.68177	0.015	0.287	1.16E-07	Undiff-G	TCF12
ACADM	7.78E-12	-0.1934	0.037	0.334	1.16E-07	Undiff-G	ACADM
6-Mar	7.96E-12	-0.12116	0.051	0.36	1.19E-07	Undiff-G	6-Mar
IMMT	8.07E-12	-0.19165	0.022	0.305	1.21E-07	Undiff-G	IMMT
RABAC1	8.15E-12	0.362418	0.206	0.67	1.22E-07	Undiff-G	RABAC1
EIF2AK2	8.25E-12	-0.17902	0.022	0.305	1.23E-07	Undiff-G	EIF2AK2
H2AFJ	8.33E-12	-0.80446	0	0.262	1.24E-07	Undiff-G	H2AFJ
PTPRE	8.33E-12	-0.77753	0.007	0.273	1.25E-07	Undiff-G	PTPRE
BAZ1B	8.44E-12	-0.5339	0.022	0.301	1.26E-07	Undiff-G	BAZ1B
NUDCD2	8.44E-12	0.22544	0.074	0.404	1.26E-07	Undiff-G	NUDCD2
PRDX4	8.47E-12	0.466061	0.147	0.551	1.27E-07	Undiff-G	PRDX4

CDKN1B	8.50E-12	-0.70042	0	0.262	1.27E-07	Undiff-G	CDKN1B
PSMA1	8.56E-12	0.298623	0.184	0.616	1.28E-07	Undiff-G	PSMA1
COMMD6	8.62E-12	0.313929	0.257	0.782	1.29E-07	Undiff-G	COMMD6
AP1B1	8.67E-12	-0.79469	0	0.262	1.30E-07	Undiff-G	AP1B1
SUCLA2	8.75E-12	-0.41638	0.015	0.29	1.31E-07	Undiff-G	SUCLA2
TECR	8.81E-12	0.263148	0.147	0.547	1.32E-07	Undiff-G	TECR
PABPN1	8.94E-12	-0.22934	0.051	0.359	1.34E-07	Undiff-G	PABPN1
DNMT1	8.96E-12	-0.5785	0.015	0.289	1.34E-07	Undiff-G	DNMT1
LRRC59	8.99E-12	0.174533	0.051	0.361	1.34E-07	Undiff-G	LRRC59
GMFG	9.24E-12	-0.82299	0.022	0.301	1.38E-07	Undiff-G	GMFG
ANXA7	9.31E-12	-0.1516	0.044	0.342	1.39E-07	Undiff-G	ANXA7
NCAM1	9.32E-12	-0.55394	0.044	0.339	1.39E-07	Undiff-G	NCAM1
TMEM258	9.35E-12	0.294735	0.235	0.734	1.40E-07	Undiff-G	TMEM258
MOB1A	9.38E-12	-0.13267	0.044	0.344	1.40E-07	Undiff-G	MOB1A
DPM3	9.44E-12	-0.13416	0.051	0.356	1.41E-07	Undiff-G	DPM3
SLC39A6	9.56E-12	-0.1612	0.037	0.332	1.43E-07	Undiff-G	SLC39A6
FASTK	9.65E-12	-0.1764	0.044	0.341	1.44E-07	Undiff-G	FASTK
ARHGDIA	9.69E-12	0.159479	0.162	0.569	1.45E-07	Undiff-G	ARHGDIA
DNAJB2	9.69E-12	-0.23703	0.029	0.317	1.45E-07	Undiff-G	DNAJB2
ACTL6A	9.78E-12	-0.67104	0	0.261	1.46E-07	Undiff-G	ACTL6A
TCEAL3	1.00E-11	-0.1339	0.044	0.345	1.50E-07	Undiff-G	TCEAL3
MLX	1.02E-11	-0.32277	0.022	0.302	1.52E-07	Undiff-G	MLX
RSRC2	1.04E-11	0.221648	0.118	0.479	1.55E-07	Undiff-G	RSRC2
STX4	1.07E-11	-0.22451	0.029	0.317	1.60E-07	Undiff-G	STX4
HOOK3	1.07E-11	-0.6077	0.015	0.287	1.60E-07	Undiff-G	HOOK3
C5orf24	1.07E-11	-0.37818	0.022	0.299	1.60E-07	Undiff-G	C5orf24
CAMLG	1.08E-11	0.245762	0.125	0.498	1.61E-07	Undiff-G	CAMLG
SLTM	1.08E-11	-0.28913	0.051	0.353	1.61E-07	Undiff-G	SLTM
JKAMP	1.10E-11	-0.31292	0.022	0.303	1.65E-07	Undiff-G	JKAMP
GCA	1.11E-11	-0.5415	0.022	0.302	1.66E-07	Undiff-G	GCA
CDC123	1.11E-11	-0.16487	0.044	0.34	1.66E-07	Undiff-G	CDC123
MEF2C	1.12E-11	-0.79429	0.015	0.286	1.67E-07	Undiff-G	MEF2C
GDE1	1.15E-11	-0.26042	0.022	0.302	1.72E-07	Undiff-G	GDE1
NDUFS8	1.16E-11	0.375992	0.184	0.614	1.74E-07	Undiff-G	NDUFS8
GLO1	1.17E-11	0.112784	0.081	0.414	1.75E-07	Undiff-G	GLO1
PPP6C	1.17E-11	-0.68337	0	0.259	1.75E-07	Undiff-G	PPP6C
C1orf162	1.18E-11	-0.69908	0.037	0.325	1.76E-07	Undiff-G	C1orf162
FAM134A	1.19E-11	-0.47281	0.022	0.301	1.78E-07	Undiff-G	FAM134A
PYCARD	1.19E-11	-0.68999	0.015	0.288	1.78E-07	Undiff-G	PYCARD
CTSF	1.21E-11	-0.64392	0.007	0.273	1.81E-07	Undiff-G	CTSF
UBE2J1	1.23E-11	-0.58446	0.015	0.284	1.84E-07	Undiff-G	UBE2J1
SSBP4	1.23E-11	-0.56262	0.007	0.273	1.85E-07	Undiff-G	SSBP4
ECHDC1	1.25E-11	-0.15961	0.037	0.33	1.87E-07	Undiff-G	ECHDC1
TRAF4	1.27E-11	-0.65817	0.007	0.272	1.89E-07	Undiff-G	TRAF4
CRLS1	1.28E-11	-0.57969	0.015	0.286	1.91E-07	Undiff-G	CRLS1
PODXL2	1.28E-11	-0.55922	0.029	0.311	1.92E-07	Undiff-G	PODXL2
PHF20L1	1.29E-11	-0.49973	0.029	0.311	1.93E-07	Undiff-G	PHF20L1
NUDT22	1.30E-11	-0.5569	0.007	0.273	1.94E-07	Undiff-G	NUDT22
PSMB5	1.32E-11	0.314511	0.176	0.6	1.97E-07	Undiff-G	PSMB5
LILRB4	1.32E-11	-1.08149	0	0.258	1.97E-07	Undiff-G	LILRB4
ELAVL3	1.33E-11	-0.49257	0.029	0.314	1.99E-07	Undiff-G	ELAVL3

CNOT7	1.34E-11	0.114141	0.059	0.372	2.00E-07	Undiff-G	CNOT7
POLR2H	1.34E-11	-0.16407	0.037	0.326	2.01E-07	Undiff-G	POLR2H
KIDINS22C	1.34E-11	-0.6418	0.007	0.272	2.01E-07	Undiff-G	KIDINS220
MLF2	1.37E-11	0.326798	0.184	0.614	2.05E-07	Undiff-G	MLF2
NOVA1	1.38E-11	-0.24458	0.118	0.483	2.06E-07	Undiff-G	NOVA1
PPIE	1.42E-11	-0.37546	0.015	0.286	2.12E-07	Undiff-G	PPIE
MAP2K1	1.43E-11	-0.63513	0.007	0.272	2.13E-07	Undiff-G	MAP2K1
CYFIP1	1.43E-11	-0.50647	0.015	0.286	2.13E-07	Undiff-G	CYFIP1
LRP1	1.46E-11	-0.33174	0.051	0.354	2.17E-07	Undiff-G	LRP1
C9orf78	1.46E-11	0.171648	0.103	0.448	2.18E-07	Undiff-G	C9orf78
CD59	1.46E-11	0.31708	0.154	0.558	2.18E-07	Undiff-G	CD59
ATRAID	1.46E-11	0.419997	0.184	0.616	2.19E-07	Undiff-G	ATRAID
EXOC7	1.49E-11	-0.22342	0.051	0.355	2.22E-07	Undiff-G	EXOC7
FAM195A	1.49E-11	-0.64646	0	0.257	2.22E-07	Undiff-G	FAM195A
RPN1	1.49E-11	0.257508	0.081	0.414	2.22E-07	Undiff-G	RPN1
GIPC1	1.52E-11	-0.61887	0.007	0.271	2.27E-07	Undiff-G	GIPC1
ATP2A2	1.54E-11	-0.55195	0.015	0.282	2.30E-07	Undiff-G	ATP2A2
POP5	1.55E-11	-0.36909	0.022	0.299	2.32E-07	Undiff-G	POP5
TRAPPC3	1.55E-11	0.197326	0.096	0.441	2.32E-07	Undiff-G	TRAPPC3
TBCB	1.56E-11	0.291486	0.213	0.663	2.32E-07	Undiff-G	TBCB
PICALM	1.57E-11	-0.43336	0.015	0.284	2.34E-07	Undiff-G	PICALM
TERF1	1.57E-11	-0.2748	0.037	0.327	2.35E-07	Undiff-G	TERF1
SPECC1	1.58E-11	-0.55837	0.022	0.299	2.36E-07	Undiff-G	SPECC1
PON2	1.58E-11	0.100423	0.103	0.454	2.36E-07	Undiff-G	PON2
PIN4	1.58E-11	0.305157	0.088	0.422	2.37E-07	Undiff-G	PIN4
CSF1R	1.60E-11	-0.92598	0.007	0.271	2.39E-07	Undiff-G	CSF1R
SMS	1.60E-11	0.317849	0.169	0.577	2.40E-07	Undiff-G	SMS
CHMP2A	1.60E-11	0.294876	0.206	0.66	2.40E-07	Undiff-G	CHMP2A
KIF5C	1.61E-11	-0.62966	0.007	0.271	2.40E-07	Undiff-G	KIF5C
CCDC90B	1.61E-11	0.266347	0.081	0.413	2.41E-07	Undiff-G	CCDC90B
COPS6	1.62E-11	0.22768	0.184	0.609	2.43E-07	Undiff-G	COPS6
PPP1R12A	1.63E-11	-0.28698	0.059	0.363	2.44E-07	Undiff-G	PPP1R12A
PTGES2	1.64E-11	-0.59229	0.007	0.27	2.45E-07	Undiff-G	PTGES2
SLC35C2	1.66E-11	-0.64476	0	0.256	2.48E-07	Undiff-G	SLC35C2
AP3D1	1.69E-11	-0.4155	0.029	0.312	2.52E-07	Undiff-G	AP3D1
DHX9	1.69E-11	-0.50452	0.015	0.281	2.53E-07	Undiff-G	DHX9
ATP5L	1.69E-11	0.282845	0.301	0.866	2.53E-07	Undiff-G	ATP5L
FBXO7	1.72E-11	-0.12532	0.029	0.313	2.57E-07	Undiff-G	FBXO7
BAALC	1.72E-11	-0.35155	0.051	0.349	2.57E-07	Undiff-G	BAALC
CDC5L	1.75E-11	-0.4229	0.015	0.284	2.62E-07	Undiff-G	CDC5L
UBE2D1	1.79E-11	-0.5899	0.007	0.27	2.68E-07	Undiff-G	UBE2D1
TAF12	1.82E-11	-0.31242	0.029	0.312	2.72E-07	Undiff-G	TAF12
CMAS	1.83E-11	-0.58453	0.007	0.27	2.74E-07	Undiff-G	CMAS
BUD31	1.85E-11	0.471352	0.191	0.633	2.77E-07	Undiff-G	BUD31
NDUFB9	1.89E-11	0.358828	0.221	0.694	2.83E-07	Undiff-G	NDUFB9
MORF4L1	1.90E-11	0.37094	0.25	0.75	2.84E-07	Undiff-G	MORF4L1
HDLBP	1.91E-11	-0.13733	0.044	0.339	2.86E-07	Undiff-G	HDLBP
WBP4	1.92E-11	-0.23594	0.029	0.308	2.87E-07	Undiff-G	WBP4
MIF4GD	1.92E-11	-0.71947	0	0.255	2.88E-07	Undiff-G	MIF4GD
THYN1	1.93E-11	0.153871	0.081	0.41	2.88E-07	Undiff-G	THYN1
SERPINE2	1.93E-11	-0.61263	0.022	0.296	2.89E-07	Undiff-G	SERPINE2

LYRM2	1.94E-11	-0.43699	0.022	0.297	2.90E-07	Undiff-G	LYRM2
SAMSN1	1.94E-11	-1.04193	0	0.255	2.90E-07	Undiff-G	SAMSN1
GHITM	1.98E-11	0.378828	0.14	0.528	2.95E-07	Undiff-G	GHITM
PHIP	1.98E-11	-0.41842	0.044	0.335	2.96E-07	Undiff-G	PHIP
ASPH	1.99E-11	-0.53202	0.015	0.28	2.97E-07	Undiff-G	ASPH
RAF1	2.00E-11	-0.58917	0.015	0.28	2.98E-07	Undiff-G	RAF1
VGLL4	2.01E-11	-0.37284	0.037	0.321	3.00E-07	Undiff-G	VGLL4
11-Sep	2.05E-11	-0.5224	0.029	0.306	3.07E-07	Undiff-G	11-Sep
PAICS	2.07E-11	-0.23742	0.029	0.311	3.09E-07	Undiff-G	PAICS
FMNL2	2.07E-11	-0.63242	0.007	0.268	3.09E-07	Undiff-G	FMNL2
PLEKHO1	2.08E-11	-0.65923	0.015	0.278	3.11E-07	Undiff-G	PLEKHO1
YEATS4	2.08E-11	-0.74769	0	0.254	3.11E-07	Undiff-G	YEATS4
ANKRD10	2.10E-11	-0.37424	0.029	0.309	3.14E-07	Undiff-G	ANKRD10
EBP	2.10E-11	-0.68758	0	0.254	3.14E-07	Undiff-G	EBP
TMEM170A	2.12E-11	-0.66195	0	0.254	3.17E-07	Undiff-G	TMEM170A
FAM32A	2.17E-11	0.102134	0.11	0.456	3.24E-07	Undiff-G	FAM32A
CRMP1	2.19E-11	-0.64759	0.007	0.268	3.27E-07	Undiff-G	CRMP1
DAZAP2	2.21E-11	0.256759	0.154	0.541	3.31E-07	Undiff-G	DAZAP2
NIPA2	2.24E-11	-0.59817	0.007	0.267	3.35E-07	Undiff-G	NIPA2
PIK3R1	2.24E-11	-0.32421	0.029	0.31	3.35E-07	Undiff-G	PIK3R1
HSD17B12	2.28E-11	0.114084	0.088	0.417	3.40E-07	Undiff-G	HSD17B12
CSNK1E	2.30E-11	-0.44217	0.029	0.309	3.44E-07	Undiff-G	CSNK1E
IFNAR1	2.30E-11	-0.25242	0.022	0.293	3.44E-07	Undiff-G	IFNAR1
MAPKAP1	2.32E-11	-0.61889	0	0.253	3.47E-07	Undiff-G	MAPKAP1
PGD	2.36E-11	-0.23562	0.022	0.296	3.53E-07	Undiff-G	PGD
FARSA	2.46E-11	-0.17002	0.037	0.323	3.67E-07	Undiff-G	FARSA
NDUFB1	2.47E-11	0.214149	0.176	0.598	3.70E-07	Undiff-G	NDUFB1
TAF1D	2.49E-11	-0.28832	0.051	0.346	3.72E-07	Undiff-G	TAF1D
RBM8A	2.49E-11	0.319427	0.191	0.618	3.72E-07	Undiff-G	RBM8A
MPHOSPH8	2.50E-11	0.138564	0.081	0.403	3.73E-07	Undiff-G	MPHOSPH8
RNF114	2.53E-11	0.242003	0.088	0.419	3.79E-07	Undiff-G	RNF114
NAA20	2.55E-11	0.314439	0.132	0.501	3.82E-07	Undiff-G	NAA20
PTPRZ1	2.61E-11	-0.31102	0.074	0.388	3.90E-07	Undiff-G	PTPRZ1
EEF1B2	2.61E-11	0.410374	0.265	0.782	3.91E-07	Undiff-G	EEF1B2
C7orf55	2.64E-11	0.200838	0.096	0.427	3.94E-07	Undiff-G	C7orf55
C1orf35	2.65E-11	-0.58468	0.007	0.266	3.96E-07	Undiff-G	C1orf35
KIF21A	2.66E-11	-0.62666	0.029	0.305	3.98E-07	Undiff-G	KIF21A
HSPB11	2.66E-11	-0.23637	0.044	0.332	3.98E-07	Undiff-G	HSPB11
CADM4	2.69E-11	-0.50856	0.007	0.266	4.02E-07	Undiff-G	CADM4
VPS51	2.70E-11	-0.58131	0.007	0.266	4.03E-07	Undiff-G	VPS51
PEF1	2.71E-11	-0.2949	0.037	0.323	4.05E-07	Undiff-G	PEF1
MTIF3	2.73E-11	-0.12388	0.051	0.349	4.08E-07	Undiff-G	MTIF3
RHOT1	2.74E-11	-0.59406	0	0.252	4.10E-07	Undiff-G	RHOT1
AIMP1	2.78E-11	0.147664	0.066	0.374	4.15E-07	Undiff-G	AIMP1
TDG	2.81E-11	-0.64532	0.015	0.276	4.21E-07	Undiff-G	TDG
ATF6	2.83E-11	-0.65729	0	0.251	4.22E-07	Undiff-G	ATF6
LARP7	2.84E-11	-0.13717	0.059	0.363	4.25E-07	Undiff-G	LARP7
MPLKIP	2.89E-11	0.240316	0.103	0.449	4.31E-07	Undiff-G	MPLKIP
GPR183	2.91E-11	-0.77602	0.022	0.293	4.34E-07	Undiff-G	GPR183
VPS4B	2.91E-11	-0.65664	0	0.251	4.35E-07	Undiff-G	VPS4B
CLCN3	2.94E-11	-0.60641	0	0.251	4.39E-07	Undiff-G	CLCN3

STARD7	2.97E-11	-0.52218	0.037	0.32	4.43E-07	Undiff-G	STARD7
VOPP1	3.01E-11	-0.32289	0.022	0.294	4.49E-07	Undiff-G	VOPP1
OGFR	3.02E-11	-0.46382	0.022	0.29	4.51E-07	Undiff-G	OGFR
RFC1	3.03E-11	-0.26314	0.015	0.28	4.53E-07	Undiff-G	RFC1
SAFB	3.03E-11	-0.48241	0.022	0.292	4.53E-07	Undiff-G	SAFB
SOX11	3.05E-11	-0.80473	0.015	0.275	4.56E-07	Undiff-G	SOX11
VPS72	3.26E-11	-0.30596	0.015	0.279	4.87E-07	Undiff-G	VPS72
TLN1	3.29E-11	-0.66349	0.022	0.288	4.92E-07	Undiff-G	TLN1
ADORA3	3.35E-11	-0.99037	0.007	0.264	5.01E-07	Undiff-G	ADORA3
RNF149	3.53E-11	-0.68567	0.015	0.274	5.28E-07	Undiff-G	RNF149
RRAGC	3.54E-11	-0.63491	0	0.249	5.29E-07	Undiff-G	RRAGC
CAMK2N1	3.68E-11	-0.26001	0.037	0.32	5.50E-07	Undiff-G	CAMK2N1
RALBP1	3.70E-11	-0.23509	0.029	0.306	5.52E-07	Undiff-G	RALBP1
GRAMD1A	3.71E-11	-0.40151	0.015	0.278	5.55E-07	Undiff-G	GRAMD1A
CPSF6	3.81E-11	-0.24782	0.044	0.333	5.69E-07	Undiff-G	CPSF6
ANP32A	3.81E-11	0.33458	0.096	0.432	5.70E-07	Undiff-G	ANP32A
BOD1	3.83E-11	-0.20759	0.029	0.306	5.72E-07	Undiff-G	BOD1
SCOC	3.83E-11	0.21355	0.088	0.418	5.73E-07	Undiff-G	SCOC
SEC13	3.84E-11	0.201688	0.066	0.372	5.73E-07	Undiff-G	SEC13
HTATSF1	3.89E-11	-0.30304	0.015	0.277	5.81E-07	Undiff-G	HTATSF1
TMEM59L	3.89E-11	-0.64204	0.007	0.261	5.82E-07	Undiff-G	TMEM59L
C22orf39	3.93E-11	-0.59592	0.007	0.262	5.87E-07	Undiff-G	C22orf39
SEC61B	3.98E-11	0.386898	0.25	0.742	5.95E-07	Undiff-G	SEC61B
GNB2L1	3.98E-11	0.846719	0.691	0.945	5.95E-07	Undiff-G	GNB2L1
CNIH1	4.07E-11	0.216425	0.066	0.376	6.08E-07	Undiff-G	CNIH1
MAP3K13	4.26E-11	0.189804	0.081	0.397	6.37E-07	Undiff-G	MAP3K13
MOSPD3	4.32E-11	-0.31636	0.022	0.29	6.46E-07	Undiff-G	MOSPD3
DRAM2	4.36E-11	-0.34167	0.051	0.339	6.52E-07	Undiff-G	DRAM2
TP53I13	4.44E-11	-0.59773	0	0.248	6.63E-07	Undiff-G	TP53I13
TGFB1	4.52E-11	-0.71336	0	0.247	6.76E-07	Undiff-G	TGFB1
FCGR1A	4.63E-11	-0.94156	0.007	0.262	6.91E-07	Undiff-G	FCGR1A
GTF2A2	4.67E-11	0.464283	0.132	0.503	6.97E-07	Undiff-G	GTF2A2
FAM200B	4.68E-11	0.199927	0.066	0.37	6.99E-07	Undiff-G	FAM200B
CNP	4.71E-11	-0.27878	0.074	0.38	7.03E-07	Undiff-G	CNP
7-Sep	4.71E-11	0.222718	0.235	0.708	7.03E-07	Undiff-G	7-Sep
GANAB	4.75E-11	-0.38666	0.015	0.276	7.09E-07	Undiff-G	GANAB
AKIP1	4.77E-11	-0.15676	0.022	0.29	7.12E-07	Undiff-G	AKIP1
H2AFZ	4.78E-11	0.361414	0.25	0.745	7.15E-07	Undiff-G	H2AFZ
EGLN2	4.80E-11	-0.42005	0.022	0.286	7.17E-07	Undiff-G	EGLN2
CLPP	4.84E-11	0.190599	0.081	0.402	7.24E-07	Undiff-G	CLPP
UCP2	4.85E-11	-0.68808	0.015	0.275	7.25E-07	Undiff-G	UCP2
VDAC1	4.85E-11	0.451087	0.199	0.636	7.25E-07	Undiff-G	VDAC1
CTSL	4.92E-11	0.20312	0.147	0.528	7.35E-07	Undiff-G	CTSL
GNG7	4.96E-11	-0.33773	0.029	0.302	7.41E-07	Undiff-G	GNG7
TMED7	5.06E-11	-0.57585	0.015	0.274	7.56E-07	Undiff-G	TMED7
AKAP13	5.06E-11	-0.25671	0.044	0.329	7.56E-07	Undiff-G	AKAP13
BRD4	5.09E-11	-0.15957	0.044	0.33	7.60E-07	Undiff-G	BRD4
TBCA	5.10E-11	0.375999	0.243	0.716	7.62E-07	Undiff-G	TBCA
CTSZ	5.11E-11	-0.88962	0.007	0.259	7.63E-07	Undiff-G	CTSZ
MUM1	5.18E-11	-0.22186	0.066	0.367	7.74E-07	Undiff-G	MUM1
KEAP1	5.21E-11	-0.42261	0.015	0.275	7.79E-07	Undiff-G	KEAP1

PVRL2	5.29E-11	-0.63225	0	0.246	7.90E-07	Undiff-G	PVRL2
DDX56	5.29E-11	-0.63713	0.007	0.26	7.90E-07	Undiff-G	DDX56
TMEM33	5.30E-11	-0.26453	0.022	0.289	7.93E-07	Undiff-G	TMEM33
WDR13	5.33E-11	-0.37492	0.022	0.289	7.96E-07	Undiff-G	WDR13
KRAS	5.40E-11	-0.60162	0.007	0.257	8.06E-07	Undiff-G	KRAS
CCZ1	5.45E-11	-0.13647	0.059	0.354	8.15E-07	Undiff-G	CCZ1
NMT1	5.49E-11	-0.57521	0.015	0.271	8.20E-07	Undiff-G	NMT1
ADSL	5.55E-11	-0.42873	0.007	0.26	8.30E-07	Undiff-G	ADSL
UBAC2	5.67E-11	-0.29947	0.015	0.274	8.47E-07	Undiff-G	UBAC2
DEF8	5.72E-11	-0.46459	0.015	0.273	8.55E-07	Undiff-G	DEF8
TMEM259	5.85E-11	-0.4557	0.051	0.339	8.74E-07	Undiff-G	TMEM259
TMEM248	5.88E-11	-0.23607	0.029	0.298	8.79E-07	Undiff-G	TMEM248
CNPY2	5.90E-11	0.327281	0.169	0.573	8.81E-07	Undiff-G	CNPY2
FOXN3	6.00E-11	-0.71632	0.007	0.258	8.97E-07	Undiff-G	FOXN3
DNAJC2	6.01E-11	-0.37312	0.029	0.301	8.98E-07	Undiff-G	DNAJC2
PLAUR	6.06E-11	-0.19754	0.066	0.37	9.05E-07	Undiff-G	PLAUR
GABARAPL1	6.06E-11	-0.31263	0.029	0.301	9.06E-07	Undiff-G	GABARAPL1
BIRC2	6.13E-11	-0.40004	0.022	0.284	9.17E-07	Undiff-G	BIRC2
TMEM208	6.20E-11	0.33062	0.118	0.471	9.26E-07	Undiff-G	TMEM208
TPD52L2	6.22E-11	-0.32627	0.044	0.327	9.30E-07	Undiff-G	TPD52L2
TSTA3	6.32E-11	-0.47759	0.015	0.272	9.44E-07	Undiff-G	TSTA3
GRPEL1	6.37E-11	-0.39292	0.015	0.273	9.52E-07	Undiff-G	GRPEL1
EIF4E	6.40E-11	0.372532	0.132	0.493	9.57E-07	Undiff-G	EIF4E
7-Mar	6.44E-11	-0.60264	0.007	0.258	9.62E-07	Undiff-G	7-Mar
TMUB1	6.54E-11	-0.57189	0.015	0.269	9.77E-07	Undiff-G	TMUB1
DNER	6.67E-11	-0.61358	0.022	0.283	9.97E-07	Undiff-G	DNER
TBXAS1	6.68E-11	-0.99	0	0.244	9.98E-07	Undiff-G	TBXAS1
RTCA	6.72E-11	-0.37615	0.015	0.273	1.00E-06	Undiff-G	RTCA
RRBP1	6.74E-11	-0.4251	0.029	0.3	1.01E-06	Undiff-G	RRBP1
PSMD14	6.87E-11	0.375997	0.118	0.471	1.03E-06	Undiff-G	PSMD14
RNF139	6.87E-11	-0.50939	0.007	0.258	1.03E-06	Undiff-G	RNF139
CELF1	6.91E-11	-0.26106	0.037	0.311	1.03E-06	Undiff-G	CELF1
PRDX3	6.95E-11	0.187355	0.118	0.471	1.04E-06	Undiff-G	PRDX3
NDUFS1	7.00E-11	-0.48373	0.007	0.258	1.05E-06	Undiff-G	NDUFS1
NTMT1	7.04E-11	-0.11848	0.044	0.328	1.05E-06	Undiff-G	NTMT1
UROS	7.09E-11	-0.59916	0.007	0.255	1.06E-06	Undiff-G	UROS
RMDN3	7.15E-11	-0.59941	0	0.243	1.07E-06	Undiff-G	RMDN3
CHID1	7.17E-11	0.288929	0.103	0.441	1.07E-06	Undiff-G	CHID1
DDOST	7.18E-11	0.289655	0.118	0.469	1.07E-06	Undiff-G	DDOST
BRD7	7.50E-11	-0.56513	0.007	0.257	1.12E-06	Undiff-G	BRD7
ISCA1	7.60E-11	-0.16179	0.029	0.3	1.14E-06	Undiff-G	ISCA1
IL6ST	7.61E-11	-0.595	0.007	0.257	1.14E-06	Undiff-G	IL6ST
PDCD10	7.62E-11	-0.14681	0.022	0.286	1.14E-06	Undiff-G	PDCD10
GOLGB1	7.65E-11	-0.65043	0	0.243	1.14E-06	Undiff-G	GOLGB1
ZFR	7.71E-11	-0.23977	0.037	0.309	1.15E-06	Undiff-G	ZFR
PEX2	7.75E-11	-0.38893	0.015	0.271	1.16E-06	Undiff-G	PEX2
FAM133B	7.82E-11	0.28055	0.125	0.475	1.17E-06	Undiff-G	FAM133B
SEC14L1	7.83E-11	-0.42518	0.029	0.295	1.17E-06	Undiff-G	SEC14L1
EIF4A1	7.93E-11	0.23672	0.309	0.868	1.18E-06	Undiff-G	EIF4A1
DPYSL3	7.93E-11	-0.41668	0.044	0.325	1.18E-06	Undiff-G	DPYSL3
GLTP	8.00E-11	-0.16854	0.015	0.271	1.20E-06	Undiff-G	GLTP

TM7SF3	8.01E-11	-0.59563	0.007	0.254	1.20E-06	Undiff-G	TM7SF3
PSMD1	8.14E-11	-0.23064	0.044	0.326	1.22E-06	Undiff-G	PSMD1
GORASP2	8.20E-11	-0.13324	0.029	0.296	1.23E-06	Undiff-G	GORASP2
ACIN1	8.24E-11	-0.31863	0.015	0.268	1.23E-06	Undiff-G	ACIN1
QTRT1	8.34E-11	-0.23486	0.051	0.334	1.25E-06	Undiff-G	QTRT1
MED30	8.34E-11	-0.48164	0.007	0.256	1.25E-06	Undiff-G	MED30
SCG3	8.52E-11	-0.73552	0.015	0.269	1.27E-06	Undiff-G	SCG3
ALG5	8.52E-11	0.190844	0.066	0.369	1.27E-06	Undiff-G	ALG5
NIPBL	8.59E-11	-0.59004	0	0.242	1.28E-06	Undiff-G	NIPBL
SPI1	8.59E-11	-0.92295	0	0.242	1.28E-06	Undiff-G	SPI1
DHX29	8.63E-11	0.181621	0.037	0.313	1.29E-06	Undiff-G	DHX29
SPOP	8.77E-11	-0.16836	0.037	0.312	1.31E-06	Undiff-G	SPOP
YKT6	8.84E-11	-0.58951	0	0.241	1.32E-06	Undiff-G	YKT6
RNASEH2B	8.84E-11	-0.63243	0	0.241	1.32E-06	Undiff-G	RNASEH2B
PLGRKT	8.89E-11	-0.44321	0.015	0.27	1.33E-06	Undiff-G	PLGRKT
PJA2	8.91E-11	-0.25091	0.051	0.338	1.33E-06	Undiff-G	PJA2
ELP5	8.95E-11	-0.30405	0.015	0.27	1.34E-06	Undiff-G	ELP5
RABEP1	9.05E-11	-0.51695	0.007	0.256	1.35E-06	Undiff-G	RABEP1
RNF10	9.05E-11	-0.1459	0.051	0.336	1.35E-06	Undiff-G	RNF10
TMEM203	9.13E-11	-0.53989	0.007	0.255	1.36E-06	Undiff-G	TMEM203
GPR34	9.17E-11	-0.8097	0.015	0.27	1.37E-06	Undiff-G	GPR34
DCK	9.30E-11	-0.59529	0.015	0.269	1.39E-06	Undiff-G	DCK
WAC	9.35E-11	-0.43561	0.015	0.267	1.40E-06	Undiff-G	WAC
KLHL7	9.46E-11	-0.47987	0.022	0.282	1.41E-06	Undiff-G	KLHL7
CD83	9.47E-11	-0.43831	0.029	0.298	1.42E-06	Undiff-G	CD83
GPSM3	9.51E-11	-0.96508	0.015	0.265	1.42E-06	Undiff-G	GPSM3
DKC1	9.52E-11	-0.5788	0.015	0.268	1.42E-06	Undiff-G	DKC1
EPRS	9.66E-11	-0.36645	0.029	0.297	1.44E-06	Undiff-G	EPRS
RB1CC1	9.81E-11	-0.51868	0.022	0.28	1.47E-06	Undiff-G	RB1CC1
VAMP3	9.85E-11	-0.21604	0.059	0.352	1.47E-06	Undiff-G	VAMP3
HES6	9.85E-11	-0.69166	0.029	0.289	1.47E-06	Undiff-G	HES6
ABRACL	9.88E-11	-0.18665	0.037	0.307	1.48E-06	Undiff-G	ABRACL
SLC38A2	9.94E-11	-0.46112	0.022	0.282	1.49E-06	Undiff-G	SLC38A2
TWF1	9.96E-11	-0.47542	0.015	0.266	1.49E-06	Undiff-G	TWF1
DDX1	1.00E-10	0.11598	0.066	0.363	1.49E-06	Undiff-G	DDX1
ACBD6	1.01E-10	-0.26611	0.022	0.28	1.51E-06	Undiff-G	ACBD6
AP2A2	1.02E-10	-0.57858	0.007	0.254	1.52E-06	Undiff-G	AP2A2
ITPA	1.03E-10	0.137991	0.088	0.408	1.54E-06	Undiff-G	ITPA
TRAM1	1.04E-10	0.360642	0.103	0.437	1.56E-06	Undiff-G	TRAM1
PWP1	1.04E-10	-0.4057	0.037	0.306	1.56E-06	Undiff-G	PWP1
TOLLIP	1.05E-10	-0.40893	0.015	0.269	1.56E-06	Undiff-G	TOLLIP
RASSF4	1.06E-10	-0.21877	0.051	0.332	1.58E-06	Undiff-G	RASSF4
PRPF38A	1.06E-10	-0.3409	0.029	0.293	1.59E-06	Undiff-G	PRPF38A
MAP4	1.07E-10	-0.13514	0.051	0.338	1.59E-06	Undiff-G	MAP4
SHKBP1	1.07E-10	-0.62687	0	0.24	1.60E-06	Undiff-G	SHKBP1
TMEM50B	1.10E-10	-0.26594	0.037	0.306	1.64E-06	Undiff-G	TMEM50B
CDKN1A	1.12E-10	-0.37865	0.037	0.31	1.67E-06	Undiff-G	CDKN1A
CAST	1.12E-10	-0.23064	0.044	0.321	1.68E-06	Undiff-G	CAST
CRTAP	1.12E-10	-0.65233	0	0.239	1.68E-06	Undiff-G	CRTAP
TRPC4AP	1.15E-10	-0.52839	0.007	0.253	1.72E-06	Undiff-G	TRPC4AP
RAB20	1.16E-10	-0.86218	0	0.239	1.73E-06	Undiff-G	RAB20

SMAP2	1.17E-10	-0.74984	0	0.239	1.75E-06	Undiff-G	SMAP2
IMP4	1.18E-10	0.223016	0.066	0.362	1.77E-06	Undiff-G	IMP4
GPATCH8	1.18E-10	-0.47744	0.037	0.307	1.77E-06	Undiff-G	GPATCH8
HDAC3	1.19E-10	-0.47167	0.015	0.266	1.78E-06	Undiff-G	HDAC3
PPP2R3C	1.19E-10	-0.28162	0.037	0.309	1.78E-06	Undiff-G	PPP2R3C
HMGB3	1.20E-10	-0.26653	0.022	0.282	1.79E-06	Undiff-G	HMGB3
OLR1	1.21E-10	-0.89634	0.007	0.253	1.80E-06	Undiff-G	OLR1
SZRD1	1.21E-10	-0.2148	0.015	0.267	1.81E-06	Undiff-G	SZRD1
EPC1	1.22E-10	-0.33615	0.029	0.294	1.82E-06	Undiff-G	EPC1
NBPF1	1.23E-10	-0.56065	0	0.238	1.83E-06	Undiff-G	NBPF1
DAD1	1.24E-10	0.402909	0.243	0.726	1.85E-06	Undiff-G	DAD1
RABGGTB	1.25E-10	-0.34615	0.029	0.294	1.87E-06	Undiff-G	RABGGTB
COMMD2	1.25E-10	-0.23021	0.029	0.292	1.87E-06	Undiff-G	COMMD2
FDFT1	1.28E-10	-0.12166	0.037	0.309	1.91E-06	Undiff-G	FDFT1
TRIM28	1.28E-10	-0.53832	0.007	0.252	1.91E-06	Undiff-G	TRIM28
ABHD17A	1.29E-10	-0.57465	0	0.238	1.92E-06	Undiff-G	ABHD17A
MBD4	1.31E-10	-0.51277	0.015	0.266	1.96E-06	Undiff-G	MBD4
SPSB3	1.31E-10	-0.37401	0.037	0.307	1.96E-06	Undiff-G	SPSB3
CRYAB	1.35E-10	1.352686	0.551	0.499	2.01E-06	Undiff-G	CRYAB
USP22	1.37E-10	-0.21061	0.044	0.322	2.04E-06	Undiff-G	USP22
SHISA4	1.37E-10	-0.62852	0.007	0.249	2.05E-06	Undiff-G	SHISA4
IL13RA1	1.38E-10	-0.56695	0.007	0.252	2.06E-06	Undiff-G	IL13RA1
EXOSC8	1.41E-10	-0.38602	0.029	0.292	2.11E-06	Undiff-G	EXOSC8
LIMD2	1.41E-10	-0.57812	0.007	0.252	2.11E-06	Undiff-G	LIMD2
EIF2AK1	1.42E-10	-0.19732	0.051	0.335	2.12E-06	Undiff-G	EIF2AK1
MICU2	1.42E-10	-0.59695	0	0.237	2.12E-06	Undiff-G	MICU2
CYCS	1.44E-10	0.408949	0.25	0.739	2.16E-06	Undiff-G	CYCS
COPS7A	1.44E-10	-0.25684	0.022	0.279	2.16E-06	Undiff-G	COPS7A
PLD3	1.45E-10	0.326206	0.154	0.533	2.16E-06	Undiff-G	PLD3
MCUR1	1.45E-10	-0.53325	0.015	0.265	2.17E-06	Undiff-G	MCUR1
STK4	1.46E-10	-0.53084	0.029	0.29	2.18E-06	Undiff-G	STK4
RSRC1	1.46E-10	-0.17908	0.037	0.308	2.19E-06	Undiff-G	RSRC1
DAP	1.47E-10	-0.33028	0.022	0.279	2.20E-06	Undiff-G	DAP
TMX4	1.47E-10	-0.56822	0.007	0.248	2.20E-06	Undiff-G	TMX4
NKTR	1.51E-10	-0.3778	0.066	0.36	2.25E-06	Undiff-G	NKTR
KARS	1.56E-10	0.196846	0.074	0.377	2.33E-06	Undiff-G	KARS
LSM12	1.57E-10	-0.43093	0.015	0.262	2.34E-06	Undiff-G	LSM12
ADPRHL2	1.58E-10	-0.52911	0.015	0.264	2.36E-06	Undiff-G	ADPRHL2
ACP1	1.58E-10	0.361652	0.14	0.503	2.37E-06	Undiff-G	ACP1
MCFD2	1.63E-10	-0.37191	0.029	0.289	2.43E-06	Undiff-G	MCFD2
TMCO1	1.64E-10	0.429268	0.176	0.58	2.45E-06	Undiff-G	TMCO1
ERGIC1	1.68E-10	-0.33013	0.029	0.292	2.50E-06	Undiff-G	ERGIC1
FBXW5	1.68E-10	-0.37856	0.029	0.291	2.52E-06	Undiff-G	FBXW5
RABL6	1.71E-10	-0.53148	0.007	0.25	2.56E-06	Undiff-G	RABL6
2-Mar	1.73E-10	-0.60578	0	0.235	2.59E-06	Undiff-G	2-Mar
A1BG	1.74E-10	-0.17789	0.037	0.302	2.60E-06	Undiff-G	A1BG
BRD3	1.75E-10	-0.36625	0.022	0.277	2.61E-06	Undiff-G	BRD3
TTYH3	1.75E-10	-0.43454	0.007	0.25	2.61E-06	Undiff-G	TTYH3
UBXN6	1.82E-10	-0.30178	0.037	0.302	2.72E-06	Undiff-G	UBXN6
ERICH1	1.82E-10	-0.6133	0.022	0.275	2.72E-06	Undiff-G	ERICH1
UBALD2	1.91E-10	-0.45646	0.015	0.262	2.85E-06	Undiff-G	UBALD2

DDX49	1.91E-10	-0.4142	0.022	0.277	2.85E-06	Undiff-G	DDX49
TRABD	1.92E-10	-0.59983	0.015	0.262	2.86E-06	Undiff-G	TRABD
USP8	1.93E-10	-0.43003	0.015	0.262	2.89E-06	Undiff-G	USP8
DAZAP1	1.94E-10	-0.58914	0	0.234	2.90E-06	Undiff-G	DAZAP1
CLEC11A	1.94E-10	-0.59024	0	0.234	2.90E-06	Undiff-G	CLEC11A
NTPCR	1.96E-10	-0.17103	0.022	0.276	2.93E-06	Undiff-G	NTPCR
C19orf25	1.98E-10	-0.3794	0.037	0.302	2.96E-06	Undiff-G	C19orf25
TPT1	1.98E-10	0.835507	0.691	0.956	2.96E-06	Undiff-G	TPT1
BCKDK	1.99E-10	-0.10334	0.044	0.317	2.97E-06	Undiff-G	BCKDK
SDHAF2	2.00E-10	-0.22366	0.022	0.274	2.99E-06	Undiff-G	SDHAF2
CHMP1A	2.08E-10	-0.16482	0.022	0.276	3.11E-06	Undiff-G	CHMP1A
PSMC1	2.09E-10	0.331745	0.081	0.388	3.13E-06	Undiff-G	PSMC1
SAE1	2.12E-10	-0.30544	0.022	0.276	3.17E-06	Undiff-G	SAE1
TMEM87A	2.14E-10	-0.14316	0.022	0.276	3.21E-06	Undiff-G	TMEM87A
SNX27	2.20E-10	-0.55661	0.007	0.247	3.29E-06	Undiff-G	SNX27
ZNF106	2.22E-10	-0.56333	0.015	0.26	3.32E-06	Undiff-G	ZNF106
ARL8A	2.22E-10	-0.28653	0.022	0.275	3.32E-06	Undiff-G	ARL8A
TRAPPC6A	2.24E-10	-0.46928	0.015	0.262	3.35E-06	Undiff-G	TRAPPC6A
ARID1A	2.25E-10	-0.58542	0.007	0.245	3.36E-06	Undiff-G	ARID1A
ZFAND3	2.30E-10	-0.2257	0.022	0.276	3.44E-06	Undiff-G	ZFAND3
VDAC3	2.34E-10	0.388902	0.125	0.468	3.50E-06	Undiff-G	VDAC3
NOC2L	2.35E-10	-0.28592	0.044	0.316	3.51E-06	Undiff-G	NOC2L
TRAP1	2.37E-10	-0.49156	0.015	0.261	3.54E-06	Undiff-G	TRAP1
SIRPA	2.37E-10	-0.63997	0.007	0.246	3.54E-06	Undiff-G	SIRPA
USP1	2.40E-10	-0.24326	0.015	0.261	3.59E-06	Undiff-G	USP1
MSRB2	2.41E-10	-0.52545	0.015	0.258	3.60E-06	Undiff-G	MSRB2
CLSTN1	2.42E-10	-0.38446	0.029	0.285	3.62E-06	Undiff-G	CLSTN1
POLR2L	2.45E-10	0.397482	0.228	0.693	3.67E-06	Undiff-G	POLR2L
CPNE1	2.46E-10	-0.21542	0.059	0.342	3.68E-06	Undiff-G	CPNE1
GPR137B	2.49E-10	-0.59801	0	0.232	3.71E-06	Undiff-G	GPR137B
UBE2G1	2.53E-10	-0.40597	0.015	0.261	3.78E-06	Undiff-G	UBE2G1
SEC62	2.55E-10	0.428267	0.235	0.696	3.81E-06	Undiff-G	SEC62
LRPAP1	2.57E-10	0.298262	0.169	0.551	3.84E-06	Undiff-G	LRPAP1
RNASEH1	2.63E-10	-0.58028	0	0.232	3.93E-06	Undiff-G	RNASEH1
CHCHD3	2.67E-10	0.247583	0.074	0.372	3.99E-06	Undiff-G	CHCHD3
DHPS	2.68E-10	0.355231	0.118	0.451	4.00E-06	Undiff-G	DHPS
PIGT	2.68E-10	0.247442	0.088	0.401	4.01E-06	Undiff-G	PIGT
FUCA2	2.70E-10	-0.58078	0.007	0.246	4.03E-06	Undiff-G	FUCA2
FAM204A	2.76E-10	-0.41503	0.015	0.26	4.12E-06	Undiff-G	FAM204A
TAX1BP3	2.76E-10	0.245299	0.081	0.385	4.13E-06	Undiff-G	TAX1BP3
KPNA3	2.82E-10	-0.31224	0.015	0.258	4.21E-06	Undiff-G	KPNA3
FOSB	2.84E-10	-0.15947	0.088	0.395	4.24E-06	Undiff-G	FOSB
PLEKHJ1	2.87E-10	0.120809	0.074	0.37	4.28E-06	Undiff-G	PLEKHJ1
TFPT	2.88E-10	-0.1819	0.044	0.311	4.31E-06	Undiff-G	TFPT
ZDHHC4	2.95E-10	0.343758	0.066	0.358	4.41E-06	Undiff-G	ZDHHC4
CGGBP1	3.04E-10	-0.1985	0.022	0.272	4.55E-06	Undiff-G	CGGBP1
ROGDI	3.18E-10	-0.60399	0	0.23	4.75E-06	Undiff-G	ROGDI
PARL	3.19E-10	0.16777	0.059	0.341	4.77E-06	Undiff-G	PARL
ANAPC13	3.21E-10	0.212464	0.066	0.357	4.80E-06	Undiff-G	ANAPC13
PIGX	3.21E-10	-0.3969	0.015	0.256	4.80E-06	Undiff-G	PIGX
MZT1	3.23E-10	-0.49461	0.015	0.258	4.82E-06	Undiff-G	MZT1

IER3	3.23E-10	-0.8241	0.007	0.244	4.83E-06	Undiff-G	IER3
B3GAT3	3.30E-10	0.214099	0.081	0.382	4.93E-06	Undiff-G	B3GAT3
SYF2	3.33E-10	0.376934	0.154	0.522	4.98E-06	Undiff-G	SYF2
RB1	3.44E-10	-0.39774	0.044	0.308	5.14E-06	Undiff-G	RB1
SMARCC1	3.44E-10	-0.5029	0.015	0.257	5.14E-06	Undiff-G	SMARCC1
CUX1	3.45E-10	-0.54161	0.007	0.243	5.16E-06	Undiff-G	CUX1
GTF2F1	3.46E-10	-0.16591	0.029	0.286	5.17E-06	Undiff-G	GTF2F1
EIF3I	3.52E-10	0.413064	0.191	0.607	5.26E-06	Undiff-G	EIF3I
C3AR1	3.56E-10	-0.97026	0.007	0.241	5.33E-06	Undiff-G	C3AR1
RBM27	3.66E-10	-0.53135	0	0.229	5.47E-06	Undiff-G	RBM27
RUFY3	3.77E-10	-0.35544	0.044	0.308	5.63E-06	Undiff-G	RUFY3
SREK1IP1	3.86E-10	0.177392	0.059	0.341	5.77E-06	Undiff-G	SREK1IP1
ANP32E	4.01E-10	-0.18293	0.022	0.27	6.00E-06	Undiff-G	ANP32E
SECISBP2	4.01E-10	-0.30988	0.015	0.256	6.00E-06	Undiff-G	SECISBP2
SMARCC2	4.02E-10	-0.57397	0.022	0.266	6.00E-06	Undiff-G	SMARCC2
CISD3	4.04E-10	-0.33509	0.015	0.256	6.04E-06	Undiff-G	CISD3
NR4A2	4.06E-10	-0.80435	0.015	0.254	6.06E-06	Undiff-G	NR4A2
CHD7	4.14E-10	-0.53527	0.037	0.292	6.18E-06	Undiff-G	CHD7
UTP18	4.19E-10	-0.46496	0.022	0.267	6.26E-06	Undiff-G	UTP18
METRN	4.20E-10	-0.35194	0.037	0.297	6.27E-06	Undiff-G	METRN
GPN3	4.22E-10	-0.32057	0.015	0.256	6.30E-06	Undiff-G	GPN3
ARF3	4.33E-10	0.282926	0.081	0.381	6.47E-06	Undiff-G	ARF3
MAZ	4.33E-10	-0.44529	0.015	0.253	6.48E-06	Undiff-G	MAZ
PARK7	4.37E-10	0.392728	0.272	0.771	6.54E-06	Undiff-G	PARK7
CHD1	4.38E-10	-0.3868	0.037	0.296	6.54E-06	Undiff-G	CHD1
ANAPC11	4.39E-10	0.259283	0.257	0.732	6.56E-06	Undiff-G	ANAPC11
KIAA1033	4.40E-10	-0.52725	0.007	0.241	6.58E-06	Undiff-G	KIAA1033
NQO2	4.44E-10	-0.30009	0.015	0.256	6.63E-06	Undiff-G	NQO2
RAB32	4.45E-10	-0.535	0.007	0.241	6.64E-06	Undiff-G	RAB32
BSDC1	4.50E-10	-0.2124	0.037	0.293	6.72E-06	Undiff-G	BSDC1
LARP1	4.58E-10	-0.3712	0.015	0.255	6.85E-06	Undiff-G	LARP1
EGFR	4.61E-10	-0.4417	0.044	0.303	6.89E-06	Undiff-G	EGFR
C12orf76	4.63E-10	-0.18158	0.022	0.269	6.92E-06	Undiff-G	C12orf76
BNIP2	4.68E-10	-0.57408	0.022	0.267	7.00E-06	Undiff-G	BNIP2
PROSC	4.70E-10	-0.42969	0.015	0.255	7.02E-06	Undiff-G	PROSC
MOCS2	4.70E-10	-0.3993	0.022	0.266	7.03E-06	Undiff-G	MOCS2
EXOSC4	4.72E-10	-0.46042	0.015	0.254	7.05E-06	Undiff-G	EXOSC4
LPAR6	4.81E-10	-0.74483	0.007	0.24	7.19E-06	Undiff-G	LPAR6
HSP90AB1	4.95E-10	0.191296	0.294	0.827	7.39E-06	Undiff-G	HSP90AB1
YAF2	4.97E-10	-0.10771	0.066	0.35	7.42E-06	Undiff-G	YAF2
SLC30A9	5.02E-10	-0.17816	0.015	0.254	7.51E-06	Undiff-G	SLC30A9
ZNF638	5.02E-10	-0.44741	0.015	0.254	7.51E-06	Undiff-G	ZNF638
ZNF292	5.04E-10	-0.53303	0.022	0.267	7.53E-06	Undiff-G	ZNF292
PXMP2	5.04E-10	-0.5243	0	0.226	7.53E-06	Undiff-G	PXMP2
COX6C	5.04E-10	0.198312	0.301	0.837	7.54E-06	Undiff-G	COX6C
FYB	5.14E-10	-0.91303	0	0.225	7.68E-06	Undiff-G	FYB
DR1	5.17E-10	-0.55943	0.007	0.237	7.73E-06	Undiff-G	DR1
KATNBL1	5.24E-10	-0.42092	0.015	0.254	7.83E-06	Undiff-G	KATNBL1
IMPDH2	5.24E-10	0.24319	0.103	0.42	7.83E-06	Undiff-G	IMPDH2
AC010642.	5.28E-10	-0.55574	0	0.225	7.90E-06	Undiff-G	AC010642.1
TMEM128	5.32E-10	-0.25877	0.037	0.294	7.94E-06	Undiff-G	TMEM128

FNBP1	5.33E-10	-0.56236	0.022	0.266	7.97E-06	Undiff-G	FNBP1
ZNF32	5.33E-10	-0.55831	0	0.225	7.97E-06	Undiff-G	ZNF32
C14orf2	5.33E-10	0.383718	0.221	0.659	7.97E-06	Undiff-G	C14orf2
KAT5	5.34E-10	-0.3557	0.015	0.254	7.98E-06	Undiff-G	KAT5
AAK1	5.42E-10	-0.53013	0.029	0.28	8.09E-06	Undiff-G	AAK1
RANBP3	5.43E-10	-0.54316	0	0.225	8.12E-06	Undiff-G	RANBP3
DMAP1	5.46E-10	-0.16805	0.015	0.254	8.16E-06	Undiff-G	DMAP1
TMED5	5.49E-10	0.171149	0.044	0.309	8.20E-06	Undiff-G	TMED5
ASPSCR1	5.49E-10	-0.52478	0.007	0.239	8.20E-06	Undiff-G	ASPSCR1
ANXA5	5.49E-10	0.255186	0.265	0.732	8.21E-06	Undiff-G	ANXA5
SMPD1	5.60E-10	-0.33668	0.007	0.239	8.37E-06	Undiff-G	SMPD1
CD276	5.64E-10	-0.56091	0	0.225	8.43E-06	Undiff-G	CD276
PGM1	5.66E-10	-0.20927	0.015	0.253	8.46E-06	Undiff-G	PGM1
TRIB2	5.68E-10	-0.59582	0.022	0.266	8.49E-06	Undiff-G	TRIB2
MLST8	5.76E-10	-0.3433	0.007	0.239	8.61E-06	Undiff-G	MLST8
KCTD12	5.82E-10	-0.46705	0.007	0.239	8.69E-06	Undiff-G	KCTD12
ANKRD11	5.83E-10	-0.17493	0.051	0.319	8.71E-06	Undiff-G	ANKRD11
COPB2	5.89E-10	-0.11437	0.044	0.309	8.80E-06	Undiff-G	COPB2
MARK3	5.93E-10	-0.47247	0.037	0.29	8.86E-06	Undiff-G	MARK3
PNN	5.93E-10	0.118367	0.096	0.405	8.86E-06	Undiff-G	PNN
TNPO1	5.96E-10	-0.21516	0.029	0.281	8.90E-06	Undiff-G	TNPO1
IL1B	5.97E-10	-0.94322	0	0.224	8.92E-06	Undiff-G	IL1B
SH3GL1	6.00E-10	-0.31176	0.015	0.253	8.97E-06	Undiff-G	SH3GL1
ERV3-1	6.05E-10	-0.62194	0.022	0.263	9.04E-06	Undiff-G	ERV3-1
WDR1	6.05E-10	0.21308	0.103	0.418	9.05E-06	Undiff-G	WDR1
DARS	6.15E-10	0.243814	0.096	0.406	9.19E-06	Undiff-G	DARS
ORAI2	6.17E-10	-0.35201	0.029	0.279	9.22E-06	Undiff-G	ORAI2
NRP2	6.19E-10	-0.60785	0	0.224	9.26E-06	Undiff-G	NRP2
AP3S1	6.21E-10	0.353478	0.11	0.43	9.28E-06	Undiff-G	AP3S1
RBMX2	6.22E-10	-0.51666	0.007	0.238	9.29E-06	Undiff-G	RBMX2
LINC00493	6.22E-10	0.401347	0.162	0.534	9.30E-06	Undiff-G	LINC00493
CHD2	6.24E-10	-0.20952	0.037	0.295	9.33E-06	Undiff-G	CHD2
URI1	6.31E-10	-0.53879	0	0.224	9.43E-06	Undiff-G	URI1
FN3KRP	6.31E-10	-0.59318	0	0.224	9.43E-06	Undiff-G	FN3KRP
AHCYL1	6.37E-10	-0.10922	0.059	0.331	9.52E-06	Undiff-G	AHCYL1
KIF2A	6.39E-10	-0.29277	0.007	0.238	9.55E-06	Undiff-G	KIF2A
CWC27	6.39E-10	-0.11182	0.022	0.266	9.55E-06	Undiff-G	CWC27
SNX4	6.40E-10	-0.20151	0.015	0.252	9.57E-06	Undiff-G	SNX4
DNTTIP2	6.47E-10	-0.16128	0.037	0.29	9.67E-06	Undiff-G	DNTTIP2
ESF1	6.53E-10	-0.14561	0.066	0.349	9.75E-06	Undiff-G	ESF1
MGAT4A	6.61E-10	-0.79756	0	0.223	9.88E-06	Undiff-G	MGAT4A
ZFPL1	6.62E-10	-0.40095	0.007	0.238	9.89E-06	Undiff-G	ZFPL1
UBQLN2	6.68E-10	-0.56386	0	0.223	9.98E-06	Undiff-G	UBQLN2
KIF3A	6.68E-10	-0.59427	0.007	0.237	9.99E-06	Undiff-G	KIF3A
CCDC53	6.70E-10	0.140648	0.059	0.332	1.00E-05	Undiff-G	CCDC53
RP11-1094	6.73E-10	-0.55692	0.007	0.237	1.01E-05	Undiff-G	RP11-1094M14.11
CPM	6.76E-10	-0.76959	0.007	0.237	1.01E-05	Undiff-G	CPM
PLA2G16	6.85E-10	0.174855	0.103	0.408	1.02E-05	Undiff-G	PLA2G16
GSTA4	6.93E-10	-0.16523	0.037	0.293	1.04E-05	Undiff-G	GSTA4
RP11-345J	6.94E-10	-0.32867	0.007	0.237	1.04E-05	Undiff-G	RP11-345J4.5
ATP1B1	7.00E-10	-0.5735	0.007	0.237	1.05E-05	Undiff-G	ATP1B1

CEBPG	7.10E-10	-0.28032	0.015	0.251	1.06E-05	Undiff-G	CEBPG
FUCA1	7.13E-10	-0.66965	0	0.222	1.06E-05	Undiff-G	FUCA1
NCOA4	7.16E-10	-0.15098	0.022	0.265	1.07E-05	Undiff-G	NCOA4
CPNE3	7.22E-10	0.106275	0.059	0.334	1.08E-05	Undiff-G	CPNE3
TM9SF3	7.26E-10	-0.55959	0	0.222	1.08E-05	Undiff-G	TM9SF3
TAOK1	7.27E-10	-0.3413	0.022	0.262	1.09E-05	Undiff-G	TAOK1
FOXO3	7.30E-10	-0.37161	0.029	0.275	1.09E-05	Undiff-G	FOXO3
MRFAP1L1	7.40E-10	-0.5915	0	0.222	1.11E-05	Undiff-G	MRFAP1L1
RSPRY1	7.49E-10	-0.29833	0.015	0.251	1.12E-05	Undiff-G	RSPRY1
CEP170	7.52E-10	-0.35487	0.022	0.264	1.12E-05	Undiff-G	CEP170
KXD1	7.54E-10	0.341139	0.125	0.452	1.13E-05	Undiff-G	KXD1
EEA1	7.55E-10	-0.50039	0.015	0.248	1.13E-05	Undiff-G	EEA1
G3BP2	7.55E-10	-0.19507	0.029	0.279	1.13E-05	Undiff-G	G3BP2
HEY1	7.56E-10	-0.60632	0.015	0.25	1.13E-05	Undiff-G	HEY1
REEP3	7.64E-10	-0.3846	0.015	0.247	1.14E-05	Undiff-G	REEP3
SSR4	7.85E-10	0.433719	0.272	0.768	1.17E-05	Undiff-G	SSR4
KNOP1	7.86E-10	-0.35376	0.022	0.262	1.17E-05	Undiff-G	KNOP1
CTSA	7.86E-10	0.291926	0.096	0.403	1.17E-05	Undiff-G	CTSA
ACYP2	8.00E-10	-0.21978	0.051	0.315	1.20E-05	Undiff-G	ACYP2
ANAPC15	8.03E-10	-0.21628	0.022	0.264	1.20E-05	Undiff-G	ANAPC15
MID1IP1	8.08E-10	-0.27781	0.022	0.264	1.21E-05	Undiff-G	MID1IP1
CYB5R1	8.16E-10	-0.43326	0.022	0.261	1.22E-05	Undiff-G	CYB5R1
USP14	8.26E-10	-0.51481	0.022	0.26	1.23E-05	Undiff-G	USP14
CDC42SE2	8.27E-10	-0.61077	0	0.221	1.24E-05	Undiff-G	CDC42SE2
CHCHD5	8.43E-10	0.168238	0.081	0.376	1.26E-05	Undiff-G	CHCHD5
TRIM44	8.51E-10	-0.336	0.037	0.29	1.27E-05	Undiff-G	TRIM44
GNB4	8.53E-10	-0.38456	0.007	0.235	1.27E-05	Undiff-G	GNB4
CCDC106	8.66E-10	-0.52594	0.007	0.235	1.29E-05	Undiff-G	CCDC106
COX6A1	8.73E-10	0.321639	0.309	0.851	1.31E-05	Undiff-G	COX6A1
UBAC1	8.83E-10	-0.55005	0	0.22	1.32E-05	Undiff-G	UBAC1
MAPT	8.88E-10	-0.39027	0.037	0.288	1.33E-05	Undiff-G	MAPT
RASSF2	8.89E-10	-0.52305	0.022	0.261	1.33E-05	Undiff-G	RASSF2
YPEL3	8.90E-10	-0.60717	0.015	0.247	1.33E-05	Undiff-G	YPEL3
DCTPP1	8.91E-10	0.167923	0.059	0.333	1.33E-05	Undiff-G	DCTPP1
THEMIS2	8.91E-10	-0.70832	0.015	0.244	1.33E-05	Undiff-G	THEMIS2
ZDHHC12	8.91E-10	-0.24052	0.022	0.263	1.33E-05	Undiff-G	ZDHHC12
C11orf73	8.92E-10	0.291686	0.11	0.424	1.33E-05	Undiff-G	C11orf73
PTPN1	8.93E-10	-0.55449	0.007	0.234	1.34E-05	Undiff-G	PTPN1
OARD1	9.10E-10	-0.26777	0.007	0.235	1.36E-05	Undiff-G	OARD1
CIR1	9.32E-10	0.13017	0.059	0.332	1.39E-05	Undiff-G	CIR1
ABL2	9.44E-10	-0.61396	0.029	0.271	1.41E-05	Undiff-G	ABL2
CCDC66	9.52E-10	-0.52917	0.007	0.232	1.42E-05	Undiff-G	CCDC66
HAVCR2	9.53E-10	-0.70582	0.007	0.234	1.42E-05	Undiff-G	HAVCR2
PCNA	9.55E-10	0.151822	0.051	0.318	1.43E-05	Undiff-G	PCNA
PLEKHA3	9.65E-10	-0.47078	0.007	0.234	1.44E-05	Undiff-G	PLEKHA3
GOLM1	9.71E-10	0.227705	0.051	0.319	1.45E-05	Undiff-G	GOLM1
PPA2	9.82E-10	0.250475	0.051	0.319	1.47E-05	Undiff-G	PPA2
MTX1	9.91E-10	-0.13586	0.015	0.248	1.48E-05	Undiff-G	MTX1
DNAJC4	9.95E-10	-0.61804	0.007	0.231	1.49E-05	Undiff-G	DNAJC4
DRG1	1.00E-09	-0.20632	0.015	0.248	1.50E-05	Undiff-G	DRG1
XPO1	1.00E-09	-0.4743	0.022	0.261	1.50E-05	Undiff-G	XPO1

EEF1E1	1.01E-09	0.154462	0.051	0.318	1.51E-05	Undiff-G	EEF1E1
MMD	1.02E-09	-0.44412	0.015	0.247	1.52E-05	Undiff-G	MMD
TSPAN31	1.02E-09	-0.26446	0.044	0.298	1.52E-05	Undiff-G	TSPAN31
SEC23B	1.02E-09	-0.10957	0.029	0.273	1.52E-05	Undiff-G	SEC23B
NREP	1.03E-09	-0.26765	0.029	0.276	1.54E-05	Undiff-G	NREP
TMEM251	1.03E-09	-0.35022	0.015	0.245	1.54E-05	Undiff-G	TMEM251
CD151	1.03E-09	0.325882	0.14	0.486	1.54E-05	Undiff-G	CD151
SPINT2	1.03E-09	-0.79429	0	0.219	1.54E-05	Undiff-G	SPINT2
R3HCC1	1.04E-09	-0.19255	0.037	0.289	1.55E-05	Undiff-G	R3HCC1
OSTM1	1.04E-09	-0.55227	0.007	0.231	1.55E-05	Undiff-G	OSTM1
CENPV	1.04E-09	-0.53482	0.022	0.258	1.55E-05	Undiff-G	CENPV
SSR3	1.04E-09	0.440048	0.162	0.531	1.56E-05	Undiff-G	SSR3
CDK2AP2	1.05E-09	0.131906	0.059	0.332	1.57E-05	Undiff-G	CDK2AP2
HECTD1	1.06E-09	-0.54841	0.007	0.231	1.58E-05	Undiff-G	HECTD1
RILPL2	1.08E-09	-0.47739	0.022	0.261	1.62E-05	Undiff-G	RILPL2
UBE2M	1.09E-09	-0.49513	0.007	0.233	1.62E-05	Undiff-G	UBE2M
ANXA11	1.10E-09	-0.70156	0	0.218	1.65E-05	Undiff-G	ANXA11
ARFIP2	1.11E-09	-0.49815	0.015	0.246	1.65E-05	Undiff-G	ARFIP2
ASAP1	1.11E-09	-0.53689	0.007	0.23	1.66E-05	Undiff-G	ASAP1
DECR1	1.12E-09	0.293673	0.103	0.416	1.67E-05	Undiff-G	DECR1
MS4A4A	1.12E-09	-0.422	0.037	0.289	1.68E-05	Undiff-G	MS4A4A
FAM210B	1.13E-09	-0.47483	0.029	0.273	1.69E-05	Undiff-G	FAM210B
GUSB	1.14E-09	-0.25562	0.029	0.275	1.70E-05	Undiff-G	GUSB
ABCF2	1.14E-09	-0.55153	0	0.218	1.71E-05	Undiff-G	ABCF2
ARF4	1.15E-09	0.530912	0.206	0.627	1.72E-05	Undiff-G	ARF4
LARS	1.15E-09	0.145556	0.066	0.345	1.72E-05	Undiff-G	LARS
PKN1	1.17E-09	-0.49503	0.015	0.246	1.74E-05	Undiff-G	PKN1
POLE4	1.17E-09	-0.19565	0.037	0.288	1.76E-05	Undiff-G	POLE4
MEF2A	1.18E-09	-0.68811	0	0.218	1.76E-05	Undiff-G	MEF2A
TMED9	1.18E-09	0.474372	0.243	0.694	1.76E-05	Undiff-G	TMED9
SCAMP4	1.18E-09	-0.43944	0.029	0.271	1.76E-05	Undiff-G	SCAMP4
ARID5B	1.19E-09	0.182218	0.074	0.359	1.78E-05	Undiff-G	ARID5B
CHIC2	1.21E-09	-0.31122	0.015	0.246	1.81E-05	Undiff-G	CHIC2
MCOLN1	1.21E-09	-0.49667	0.015	0.246	1.81E-05	Undiff-G	MCOLN1
DDX27	1.22E-09	-0.21123	0.037	0.285	1.82E-05	Undiff-G	DDX27
KCNQ2	1.22E-09	-0.58701	0	0.217	1.82E-05	Undiff-G	KCNQ2
DDIT4	1.25E-09	0.180803	0.103	0.412	1.86E-05	Undiff-G	DDIT4
CTNNBIP1	1.27E-09	-0.50784	0	0.217	1.89E-05	Undiff-G	CTNNBIP1
BLOC1S4	1.29E-09	-0.10032	0.015	0.246	1.92E-05	Undiff-G	BLOC1S4
CCDC25	1.29E-09	-0.2134	0.044	0.3	1.92E-05	Undiff-G	CCDC25
CCSER2	1.29E-09	-0.55169	0.007	0.231	1.93E-05	Undiff-G	CCSER2
EIF2S2	1.30E-09	0.495413	0.199	0.592	1.94E-05	Undiff-G	EIF2S2
SIAH1	1.31E-09	-0.33242	0.007	0.231	1.95E-05	Undiff-G	SIAH1
SNX10	1.31E-09	-0.55673	0.007	0.231	1.96E-05	Undiff-G	SNX10
SCYL1	1.32E-09	-0.53602	0.007	0.229	1.97E-05	Undiff-G	SCYL1
MAPK1	1.32E-09	-0.394	0.015	0.245	1.97E-05	Undiff-G	MAPK1
COQ9	1.33E-09	-0.49408	0.015	0.245	1.98E-05	Undiff-G	COQ9
RFC2	1.35E-09	-0.40237	0.015	0.245	2.01E-05	Undiff-G	RFC2
UQCRC1	1.36E-09	0.391092	0.147	0.492	2.03E-05	Undiff-G	UQCRC1
RPA1	1.37E-09	-0.35149	0.015	0.242	2.04E-05	Undiff-G	RPA1
EAPP	1.37E-09	0.296059	0.074	0.358	2.05E-05	Undiff-G	EAPP

CHCHD1	1.37E-09	-0.13075	0.029	0.273	2.05E-05	Undiff-G	CHCHD1
ADAM17	1.38E-09	-0.34965	0.022	0.258	2.06E-05	Undiff-G	ADAM17
DCTD	1.38E-09	-0.41483	0.022	0.258	2.07E-05	Undiff-G	DCTD
LAMTOR5	1.40E-09	0.419259	0.243	0.697	2.09E-05	Undiff-G	LAMTOR5
AP1S1	1.40E-09	0.399523	0.132	0.471	2.10E-05	Undiff-G	AP1S1
CCM2	1.42E-09	-0.10302	0.037	0.287	2.12E-05	Undiff-G	CCM2
IFT52	1.43E-09	-0.10649	0.022	0.259	2.14E-05	Undiff-G	IFT52
CLN8	1.44E-09	-0.38093	0.029	0.272	2.15E-05	Undiff-G	CLN8
JAK1	1.44E-09	-0.3084	0.022	0.255	2.15E-05	Undiff-G	JAK1
SH3KBP1	1.44E-09	-0.49455	0.015	0.244	2.15E-05	Undiff-G	SH3KBP1
BZW2	1.45E-09	-0.17865	0.029	0.273	2.16E-05	Undiff-G	BZW2
CD58	1.46E-09	-0.65675	0.007	0.23	2.18E-05	Undiff-G	CD58
PDLIM7	1.48E-09	-0.52351	0.015	0.241	2.21E-05	Undiff-G	PDLIM7
STX7	1.48E-09	-0.22254	0.029	0.27	2.21E-05	Undiff-G	STX7
ATPAF2	1.48E-09	-0.54911	0	0.216	2.21E-05	Undiff-G	ATPAF2
PRKRIP1	1.49E-09	-0.40299	0.015	0.244	2.23E-05	Undiff-G	PRKRIP1
PKIB	1.50E-09	-0.19468	0.037	0.287	2.24E-05	Undiff-G	PKIB
DAPK3	1.50E-09	-0.49747	0.015	0.241	2.24E-05	Undiff-G	DAPK3
VPS37B	1.50E-09	-0.47452	0.022	0.257	2.25E-05	Undiff-G	VPS37B
RNF146	1.51E-09	-0.24094	0.022	0.258	2.26E-05	Undiff-G	RNF146
POP7	1.52E-09	0.399106	0.096	0.398	2.27E-05	Undiff-G	POP7
ACSL1	1.52E-09	-0.62721	0.007	0.23	2.28E-05	Undiff-G	ACSL1
COQ4	1.54E-09	-0.11723	0.029	0.27	2.30E-05	Undiff-G	COQ4
NDUFV2	1.54E-09	0.560227	0.199	0.596	2.31E-05	Undiff-G	NDUFV2
SBNO1	1.54E-09	-0.1722	0.029	0.268	2.31E-05	Undiff-G	SBNO1
GLIPR2	1.56E-09	-0.29766	0.029	0.271	2.33E-05	Undiff-G	GLIPR2
TSPAN14	1.59E-09	-0.63556	0	0.215	2.38E-05	Undiff-G	TSPAN14
CTDSP2	1.62E-09	-0.43866	0.037	0.279	2.41E-05	Undiff-G	CTDSP2
LIMA1	1.62E-09	-0.17605	0.059	0.321	2.41E-05	Undiff-G	LIMA1
PSMB1	1.62E-09	0.471069	0.257	0.73	2.42E-05	Undiff-G	PSMB1
GTF2H5	1.65E-09	0.308181	0.103	0.408	2.46E-05	Undiff-G	GTF2H5
JMJD6	1.65E-09	-0.11403	0.037	0.285	2.47E-05	Undiff-G	JMJD6
HSF1	1.66E-09	-0.31372	0.037	0.281	2.49E-05	Undiff-G	HSF1
GALNT11	1.67E-09	-0.38112	0.007	0.229	2.50E-05	Undiff-G	GALNT11
SAP30	1.69E-09	-0.4689	0.015	0.243	2.52E-05	Undiff-G	SAP30
CDK5	1.69E-09	-0.28494	0.015	0.241	2.52E-05	Undiff-G	CDK5
NUDT16L1	1.69E-09	-0.24705	0.037	0.284	2.52E-05	Undiff-G	NUDT16L1
RAB1A	1.69E-09	0.641456	0.206	0.625	2.52E-05	Undiff-G	RAB1A
SLC25A3	1.72E-09	0.346001	0.265	0.752	2.57E-05	Undiff-G	SLC25A3
CTD-2336C	1.73E-09	-0.56917	0.007	0.226	2.59E-05	Undiff-G	CTD-233602.1
GTPBP6	1.73E-09	-0.51537	0	0.214	2.59E-05	Undiff-G	GTPBP6
ATP5EP2	1.75E-09	0.214435	0.125	0.451	2.61E-05	Undiff-G	ATP5EP2
GLG1	1.75E-09	-0.44669	0.029	0.269	2.62E-05	Undiff-G	GLG1
C5orf15	1.76E-09	0.148737	0.059	0.327	2.63E-05	Undiff-G	C5orf15
UBE2E2	1.77E-09	-0.25804	0.015	0.243	2.65E-05	Undiff-G	UBE2E2
GSS	1.79E-09	-0.19448	0.015	0.243	2.67E-05	Undiff-G	GSS
SNN	1.79E-09	-0.53668	0.007	0.228	2.67E-05	Undiff-G	SNN
ATPAF1	1.79E-09	-0.39871	0.015	0.243	2.68E-05	Undiff-G	ATPAF1
PRPSAP2	1.80E-09	-0.20797	0.029	0.269	2.68E-05	Undiff-G	PRPSAP2
AKT1S1	1.80E-09	-0.29539	0.015	0.243	2.69E-05	Undiff-G	AKT1S1
MTF2	1.80E-09	-0.28067	0.022	0.256	2.69E-05	Undiff-G	MTF2

TPST1	1.82E-09	-0.45826	0.015	0.242	2.72E-05	Undiff-G	TPST1
VASP	1.84E-09	-0.2773	0.015	0.242	2.75E-05	Undiff-G	VASP
HSPE1	1.85E-09	0.407077	0.257	0.735	2.76E-05	Undiff-G	HSPE1
INO80E	1.85E-09	-0.39939	0.007	0.228	2.77E-05	Undiff-G	INO80E
RBM5	1.89E-09	-0.14274	0.037	0.281	2.83E-05	Undiff-G	RBM5
RAE1	1.92E-09	0.111484	0.029	0.27	2.87E-05	Undiff-G	RAE1
TXNDC17	1.93E-09	0.397723	0.169	0.529	2.88E-05	Undiff-G	TXNDC17
PRPF19	1.94E-09	-0.18928	0.022	0.256	2.90E-05	Undiff-G	PRPF19
ZC2HC1A	1.95E-09	-0.61573	0	0.213	2.92E-05	Undiff-G	ZC2HC1A
MAP2K2	1.96E-09	-0.46128	0.051	0.305	2.92E-05	Undiff-G	MAP2K2
EIF4H	1.97E-09	0.487215	0.176	0.545	2.94E-05	Undiff-G	EIF4H
TRMT10C	1.99E-09	-0.41927	0.022	0.252	2.97E-05	Undiff-G	TRMT10C
ETNK1	1.99E-09	-0.5625	0.007	0.226	2.97E-05	Undiff-G	ETNK1
KHSRP	1.99E-09	-0.46714	0.015	0.241	2.98E-05	Undiff-G	KHSRP
SPTLC2	2.02E-09	-0.72121	0	0.213	3.02E-05	Undiff-G	SPTLC2
CECR5	2.03E-09	-0.17331	0.022	0.254	3.03E-05	Undiff-G	CECR5
RAB22A	2.04E-09	-0.3902	0.015	0.241	3.04E-05	Undiff-G	RAB22A
RGCC	2.05E-09	-0.70696	0.022	0.253	3.06E-05	Undiff-G	RGCC
KMT2A	2.05E-09	-0.43185	0.015	0.239	3.06E-05	Undiff-G	KMT2A
ING4	2.08E-09	-0.22699	0.029	0.269	3.10E-05	Undiff-G	ING4
TIMM44	2.08E-09	-0.49631	0.029	0.265	3.11E-05	Undiff-G	TIMM44
IQGAP1	2.09E-09	-0.17141	0.022	0.255	3.13E-05	Undiff-G	IQGAP1
TM2D3	2.10E-09	0.104107	0.066	0.334	3.13E-05	Undiff-G	TM2D3
TRIM27	2.14E-09	-0.52849	0	0.212	3.20E-05	Undiff-G	TRIM27
PCNP	2.14E-09	0.420156	0.176	0.541	3.20E-05	Undiff-G	PCNP
EBAG9	2.14E-09	-0.15667	0.022	0.255	3.21E-05	Undiff-G	EBAG9
TBRG4	2.17E-09	-0.36764	0.015	0.24	3.24E-05	Undiff-G	TBRG4
RFK	2.19E-09	-0.45073	0.015	0.24	3.28E-05	Undiff-G	RFK
LASP1	2.20E-09	-0.57738	0	0.212	3.28E-05	Undiff-G	LASP1
RGS16	2.22E-09	-0.40678	0.037	0.278	3.31E-05	Undiff-G	RGS16
B4GALT7	2.22E-09	-0.52355	0	0.212	3.31E-05	Undiff-G	B4GALT7
SLC35F6	2.22E-09	-0.5563	0	0.212	3.31E-05	Undiff-G	SLC35F6
HYAL2	2.26E-09	-0.49366	0.007	0.225	3.37E-05	Undiff-G	HYAL2
LAIR1	2.26E-09	-0.87536	0.015	0.233	3.38E-05	Undiff-G	LAIR1
TPRKB	2.29E-09	0.319603	0.081	0.367	3.43E-05	Undiff-G	TPRKB
PUM1	2.31E-09	-0.24174	0.015	0.24	3.45E-05	Undiff-G	PUM1
TXNRD1	2.32E-09	-0.34196	0.015	0.24	3.46E-05	Undiff-G	TXNRD1
BEST1	2.32E-09	-0.44181	0.037	0.278	3.47E-05	Undiff-G	BEST1
EPN2	2.33E-09	-0.50027	0.015	0.24	3.49E-05	Undiff-G	EPN2
ILVBL	2.36E-09	-0.27	0.022	0.254	3.52E-05	Undiff-G	ILVBL
PLRG1	2.37E-09	-0.22765	0.015	0.24	3.54E-05	Undiff-G	PLRG1
ADPGK	2.38E-09	-0.29842	0.029	0.264	3.55E-05	Undiff-G	ADPGK
SUPT5H	2.38E-09	-0.37481	0.022	0.252	3.56E-05	Undiff-G	SUPT5H
DERA	2.38E-09	-0.34618	0.022	0.254	3.56E-05	Undiff-G	DERA
TOR1A	2.40E-09	-0.25508	0.029	0.264	3.58E-05	Undiff-G	TOR1A
CAND1	2.43E-09	-0.53335	0.015	0.238	3.63E-05	Undiff-G	CAND1
FAM136A	2.43E-09	-0.20717	0.029	0.265	3.63E-05	Undiff-G	FAM136A
MANBAL	2.44E-09	0.200699	0.066	0.338	3.65E-05	Undiff-G	MANBAL
C1orf52	2.45E-09	0.128777	0.051	0.31	3.66E-05	Undiff-G	C1orf52
LY96	2.46E-09	-0.47107	0.007	0.225	3.67E-05	Undiff-G	LY96
KDM6B	2.46E-09	-0.62702	0.007	0.225	3.68E-05	Undiff-G	KDM6B

GGA1	2.46E-09	-0.41157	0.044	0.292	3.68E-05	Undiff-G	GGA1
HLA-DQA1	2.46E-09	-0.26299	0.051	0.309	3.68E-05	Undiff-G	HLA-DQA1
BTG2	2.47E-09	0.293489	0.14	0.471	3.69E-05	Undiff-G	BTG2
SLC35A4	2.52E-09	-0.52159	0	0.211	3.77E-05	Undiff-G	SLC35A4
CCND1	2.53E-09	-0.66622	0.007	0.221	3.78E-05	Undiff-G	CCND1
ATP6V0A1	2.54E-09	-0.51851	0.044	0.288	3.80E-05	Undiff-G	ATP6V0A1
FAM174A	2.55E-09	-0.41566	0.007	0.225	3.81E-05	Undiff-G	FAM174A
PLA2G12A	2.57E-09	-0.47708	0.015	0.236	3.83E-05	Undiff-G	PLA2G12A
HIST1H2BK	2.57E-09	-0.63363	0	0.21	3.84E-05	Undiff-G	HIST1H2BK
CUL5	2.62E-09	-0.31244	0.007	0.225	3.92E-05	Undiff-G	CUL5
CPSF3L	2.63E-09	-0.23831	0.037	0.277	3.92E-05	Undiff-G	CPSF3L
TMEM14C	2.63E-09	0.501407	0.199	0.595	3.93E-05	Undiff-G	TMEM14C
TATDN1	2.65E-09	-0.2009	0.015	0.239	3.96E-05	Undiff-G	TATDN1
VMP1	2.70E-09	0.248001	0.14	0.476	4.03E-05	Undiff-G	VMP1
AFG3L2	2.74E-09	-0.49116	0	0.21	4.09E-05	Undiff-G	AFG3L2
P4HTM	2.75E-09	-0.47979	0.029	0.264	4.11E-05	Undiff-G	P4HTM
ARMC1	2.77E-09	-0.19412	0.029	0.267	4.14E-05	Undiff-G	ARMC1
PMP2	2.77E-09	-0.40856	0.081	0.353	4.15E-05	Undiff-G	PMP2
NES	2.79E-09	-0.2961	0.037	0.279	4.18E-05	Undiff-G	NES
TNFRSF12A	2.81E-09	-0.24412	0.044	0.29	4.20E-05	Undiff-G	TNFRSF12A
SUPT20H	2.81E-09	-0.5074	0	0.21	4.20E-05	Undiff-G	SUPT20H
CTDSP1	2.82E-09	-0.48448	0.007	0.224	4.21E-05	Undiff-G	CTDSP1
RAB1B	2.84E-09	-0.27204	0.015	0.238	4.25E-05	Undiff-G	RAB1B
PPIF	2.87E-09	-0.59486	0.007	0.223	4.29E-05	Undiff-G	PPIF
MTHFD2	2.91E-09	-0.45993	0.044	0.291	4.35E-05	Undiff-G	MTHFD2
KMT2C	2.93E-09	-0.32359	0.029	0.264	4.38E-05	Undiff-G	KMT2C
CHP1	2.94E-09	-0.39452	0.015	0.237	4.39E-05	Undiff-G	CHP1
RAB4B	2.95E-09	-0.24055	0.029	0.265	4.41E-05	Undiff-G	RAB4B
CHCHD6	2.95E-09	-0.22514	0.015	0.238	4.41E-05	Undiff-G	CHCHD6
CDK16	2.96E-09	-0.29295	0.015	0.238	4.43E-05	Undiff-G	CDK16
DHRSX	2.96E-09	-0.45708	0.015	0.235	4.43E-05	Undiff-G	DHRSX
PPP1R11	2.97E-09	0.328838	0.081	0.363	4.44E-05	Undiff-G	PPP1R11
ORC4	2.98E-09	-0.40698	0.015	0.237	4.45E-05	Undiff-G	ORC4
ZMAT5	2.98E-09	-0.20144	0.015	0.238	4.45E-05	Undiff-G	ZMAT5
CLN5	2.98E-09	-0.33872	0.007	0.224	4.45E-05	Undiff-G	CLN5
CDC34	2.98E-09	-0.24466	0.015	0.237	4.46E-05	Undiff-G	CDC34
HIPK2	3.02E-09	-0.5393	0.007	0.223	4.51E-05	Undiff-G	HIPK2
SRSF6	3.02E-09	0.240329	0.081	0.362	4.51E-05	Undiff-G	SRSF6
NFYC	3.02E-09	-0.47918	0.007	0.223	4.52E-05	Undiff-G	NFYC
PMPCA	3.02E-09	-0.45358	0.022	0.249	4.52E-05	Undiff-G	PMPCA
FKBP5	3.06E-09	-0.29536	0.059	0.32	4.57E-05	Undiff-G	FKBP5
SQLE	3.06E-09	-0.29399	0.022	0.251	4.57E-05	Undiff-G	SQLE
HDGF	3.06E-09	-0.14798	0.037	0.276	4.58E-05	Undiff-G	HDGF
TBL1XR1	3.08E-09	-0.44114	0.022	0.251	4.60E-05	Undiff-G	TBL1XR1
TMEM98	3.09E-09	-0.18917	0.015	0.238	4.62E-05	Undiff-G	TMEM98
RGS12	3.17E-09	-0.49885	0.037	0.273	4.74E-05	Undiff-G	RGS12
CDS2	3.22E-09	-0.34799	0.022	0.251	4.81E-05	Undiff-G	CDS2
CAPN1	3.22E-09	-0.30886	0.029	0.261	4.81E-05	Undiff-G	CAPN1
AGGF1	3.22E-09	-0.4942	0.007	0.222	4.81E-05	Undiff-G	AGGF1
ARL8B	3.22E-09	-0.54396	0	0.208	4.82E-05	Undiff-G	ARL8B
POLR2B	3.22E-09	-0.2224	0.029	0.265	4.82E-05	Undiff-G	POLR2B

MSRB1	3.27E-09	-0.45569	0.007	0.223	4.89E-05	Undiff-G	MSRB1
COA6	3.30E-09	0.122145	0.037	0.279	4.94E-05	Undiff-G	COA6
NABP2	3.31E-09	-0.1799	0.037	0.279	4.94E-05	Undiff-G	NABP2
GALNT1	3.31E-09	-0.49984	0	0.208	4.95E-05	Undiff-G	GALNT1
CACUL1	3.31E-09	-0.56307	0	0.208	4.95E-05	Undiff-G	CACUL1
GOPC	3.31E-09	-0.48667	0.029	0.26	4.95E-05	Undiff-G	GOPC
ASF1A	3.35E-09	-0.28949	0.015	0.237	5.00E-05	Undiff-G	ASF1A
WIF1	3.40E-09	0.33469	0.015	0.001	5.08E-05	Undiff-G	WIF1
RP11-286N	3.40E-09	0.326086	0.015	0.001	5.08E-05	Undiff-G	RP11-286N22.8
PDXK	3.41E-09	-0.15043	0.044	0.287	5.09E-05	Undiff-G	PDXK
HSPA4	3.41E-09	0.13989	0.037	0.279	5.10E-05	Undiff-G	HSPA4
MED28	3.42E-09	0.104994	0.074	0.342	5.12E-05	Undiff-G	MED28
TM7SF2	3.43E-09	-0.54611	0.015	0.233	5.12E-05	Undiff-G	TM7SF2
CBFB	3.44E-09	-0.3453	0.015	0.236	5.14E-05	Undiff-G	CBFB
H2AFX	3.49E-09	-0.54315	0.007	0.222	5.21E-05	Undiff-G	H2AFX
MYL6	3.50E-09	0.860697	0.676	0.945	5.23E-05	Undiff-G	MYL6
PDLIM5	3.50E-09	-0.4813	0.007	0.222	5.24E-05	Undiff-G	PDLIM5
SIVA1	3.51E-09	0.403994	0.147	0.489	5.24E-05	Undiff-G	SIVA1
SRGAP2	3.53E-09	-0.5524	0.015	0.235	5.27E-05	Undiff-G	SRGAP2
MOB4	3.54E-09	0.343679	0.088	0.375	5.30E-05	Undiff-G	MOB4
LBR	3.59E-09	-0.53548	0.015	0.235	5.36E-05	Undiff-G	LBR
NUDT16	3.60E-09	-0.51764	0.007	0.222	5.37E-05	Undiff-G	NUDT16
ZCCHC10	3.63E-09	-0.26233	0.015	0.236	5.42E-05	Undiff-G	ZCCHC10
FAM207A	3.66E-09	-0.51116	0	0.207	5.47E-05	Undiff-G	FAM207A
TMEM109	3.66E-09	-0.65953	0	0.207	5.47E-05	Undiff-G	TMEM109
NRBF2	3.66E-09	-0.12802	0.037	0.274	5.47E-05	Undiff-G	NRBF2
PCSK7	3.69E-09	-0.51126	0.044	0.285	5.51E-05	Undiff-G	PCSK7
GAS6	3.69E-09	-0.6616	0	0.207	5.52E-05	Undiff-G	GAS6
BICD1	3.76E-09	-0.47955	0	0.207	5.62E-05	Undiff-G	BICD1
SENP6	3.76E-09	-0.50045	0.007	0.221	5.62E-05	Undiff-G	SENP6
HEBP2	3.79E-09	-0.43542	0.007	0.221	5.66E-05	Undiff-G	HEBP2
DCX	3.79E-09	-0.68736	0	0.207	5.67E-05	Undiff-G	DCX
PPIL4	3.82E-09	-0.17665	0.022	0.249	5.71E-05	Undiff-G	PPIL4
PRPF4B	3.90E-09	-0.2056	0.037	0.276	5.83E-05	Undiff-G	PRPF4B
TMEM55B	3.92E-09	-0.51027	0.007	0.221	5.86E-05	Undiff-G	TMEM55B
SLC25A33	3.95E-09	-0.4663	0.007	0.221	5.91E-05	Undiff-G	SLC25A33
DHX30	3.96E-09	-0.25203	0.029	0.26	5.92E-05	Undiff-G	DHX30
DCTN1	3.96E-09	-0.55276	0.007	0.218	5.92E-05	Undiff-G	DCTN1
SAMHD1	4.00E-09	-0.6859	0.007	0.217	5.98E-05	Undiff-G	SAMHD1
BZW1	4.00E-09	0.496043	0.176	0.542	5.98E-05	Undiff-G	BZW1
FAM89B	4.06E-09	-0.26165	0.022	0.249	6.07E-05	Undiff-G	FAM89B
HCFC1R1	4.13E-09	-0.23561	0.044	0.285	6.18E-05	Undiff-G	HCFC1R1
NHLRC3	4.16E-09	-0.11717	0.037	0.273	6.22E-05	Undiff-G	NHLRC3
AASDHPPT	4.19E-09	0.331411	0.044	0.291	6.27E-05	Undiff-G	AASDHPPT
NUDT21	4.24E-09	0.11109	0.029	0.263	6.34E-05	Undiff-G	NUDT21
SMC1A	4.28E-09	-0.52148	0.007	0.22	6.39E-05	Undiff-G	SMC1A
ACP2	4.30E-09	-0.44986	0.022	0.246	6.43E-05	Undiff-G	ACP2
SMARCD3	4.32E-09	-0.38496	0.015	0.234	6.46E-05	Undiff-G	SMARCD3
SEZ6L2	4.34E-09	-0.14727	0.037	0.276	6.48E-05	Undiff-G	SEZ6L2
PTPRC	4.35E-09	-0.82808	0	0.205	6.49E-05	Undiff-G	PTPRC
SF3A2	4.36E-09	-0.22461	0.029	0.262	6.52E-05	Undiff-G	SF3A2

TMEM107	4.37E-09	-0.51957	0.007	0.22	6.54E-05	Undiff-G	TMEM107
VPS26B	4.39E-09	-0.44976	0.015	0.234	6.56E-05	Undiff-G	VPS26B
MOAP1	4.39E-09	-0.4874	0.007	0.22	6.57E-05	Undiff-G	MOAP1
U2AF2	4.41E-09	-0.17638	0.029	0.26	6.59E-05	Undiff-G	U2AF2
KIFAP3	4.46E-09	-0.54197	0	0.205	6.67E-05	Undiff-G	KIFAP3
LAT2	4.46E-09	-0.83432	0	0.205	6.67E-05	Undiff-G	LAT2
EVI2B	4.50E-09	-0.86641	0	0.205	6.73E-05	Undiff-G	EVI2B
FAM213A	4.52E-09	0.268574	0.096	0.386	6.76E-05	Undiff-G	FAM213A
RARS	4.55E-09	-0.23427	0.022	0.247	6.80E-05	Undiff-G	RARS
DHX15	4.61E-09	-0.43288	0.022	0.247	6.89E-05	Undiff-G	DHX15
PLK3	4.62E-09	-0.53879	0.022	0.243	6.90E-05	Undiff-G	PLK3
CSF2RA	4.67E-09	-0.88882	0	0.205	6.98E-05	Undiff-G	CSF2RA
NPM3	4.73E-09	-0.39654	0.007	0.219	7.06E-05	Undiff-G	NPM3
DNAJC15	4.73E-09	0.284273	0.103	0.393	7.07E-05	Undiff-G	DNAJC15
TMEM206	4.78E-09	-0.19308	0.015	0.233	7.14E-05	Undiff-G	TMEM206
RBFOX2	4.81E-09	-0.54726	0.007	0.218	7.19E-05	Undiff-G	RBFOX2
DERL1	4.86E-09	0.183566	0.059	0.316	7.27E-05	Undiff-G	DERL1
CCDC137	4.89E-09	-0.47619	0	0.204	7.30E-05	Undiff-G	CCDC137
MECP2	4.89E-09	-0.54553	0	0.204	7.30E-05	Undiff-G	MECP2
SLA	4.93E-09	-0.83458	0	0.204	7.37E-05	Undiff-G	SLA
BOLA3	4.98E-09	0.111157	0.074	0.339	7.44E-05	Undiff-G	BOLA3
BRWD1	5.00E-09	-0.43161	0.022	0.244	7.47E-05	Undiff-G	BRWD1
MSRA	5.04E-09	-0.32634	0.029	0.259	7.54E-05	Undiff-G	MSRA
SF3A3	5.06E-09	0.118424	0.044	0.288	7.56E-05	Undiff-G	SF3A3
VAT1	5.07E-09	-0.54067	0	0.204	7.57E-05	Undiff-G	VAT1
SOX9	5.10E-09	-0.35036	0.037	0.274	7.62E-05	Undiff-G	SOX9
BECN1	5.12E-09	-0.33067	0.015	0.232	7.65E-05	Undiff-G	BECN1
MMP2	5.13E-09	-0.55409	0.015	0.229	7.66E-05	Undiff-G	MMP2
SRP68	5.14E-09	-0.43658	0.015	0.232	7.69E-05	Undiff-G	SRP68
ARL1	5.21E-09	0.188974	0.059	0.316	7.79E-05	Undiff-G	ARL1
PSMB10	5.22E-09	-0.43519	0.029	0.258	7.79E-05	Undiff-G	PSMB10
IL10RB	5.22E-09	-0.31809	0.022	0.246	7.80E-05	Undiff-G	IL10RB
PCMTD2	5.32E-09	-0.2973	0.015	0.23	7.95E-05	Undiff-G	PCMTD2
HDHD2	5.32E-09	-0.16344	0.029	0.26	7.95E-05	Undiff-G	HDHD2
ELF1	5.33E-09	-0.19016	0.022	0.246	7.96E-05	Undiff-G	ELF1
TCEAL1	5.34E-09	-0.2751	0.029	0.258	7.98E-05	Undiff-G	TCEAL1
ACAT1	5.34E-09	0.332443	0.088	0.369	7.98E-05	Undiff-G	ACAT1
PMEPA1	5.40E-09	-0.5817	0	0.203	8.06E-05	Undiff-G	PMEPA1
PRKACB	5.46E-09	-0.49383	0.007	0.218	8.17E-05	Undiff-G	PRKACB
EIF2S3	5.48E-09	-0.10296	0.051	0.297	8.19E-05	Undiff-G	EIF2S3
PCBP4	5.49E-09	-0.37003	0.029	0.258	8.20E-05	Undiff-G	PCBP4
EFTUD2	5.52E-09	-0.49022	0.007	0.217	8.24E-05	Undiff-G	EFTUD2
PPIL3	5.53E-09	-0.52988	0.007	0.215	8.27E-05	Undiff-G	PPIL3
SYS1	5.53E-09	-0.3516	0.022	0.243	8.27E-05	Undiff-G	SYS1
SAR1A	5.54E-09	0.113785	0.051	0.301	8.28E-05	Undiff-G	SAR1A
ASRGL1	5.54E-09	-0.53936	0	0.203	8.28E-05	Undiff-G	ASRGL1
VPS36	5.54E-09	-0.56013	0	0.203	8.28E-05	Undiff-G	VPS36
USO1	5.56E-09	-0.32559	0.007	0.218	8.31E-05	Undiff-G	USO1
SORT1	5.58E-09	-0.33751	0.015	0.232	8.34E-05	Undiff-G	SORT1
UNC93B1	5.59E-09	-0.62536	0	0.203	8.36E-05	Undiff-G	UNC93B1
CTNND1	5.62E-09	-0.49352	0.007	0.217	8.40E-05	Undiff-G	CTNND1

QARS	5.71E-09	-0.28119	0.044	0.285	8.54E-05	Undiff-G	QARS
TTYH1	5.76E-09	-0.43413	0.037	0.271	8.61E-05	Undiff-G	TTYH1
SMIM20	5.81E-09	0.191134	0.074	0.343	8.68E-05	Undiff-G	SMIM20
KIAA1715	5.81E-09	-0.20938	0.029	0.258	8.69E-05	Undiff-G	KIAA1715
CSTF3	5.82E-09	-0.12919	0.037	0.27	8.70E-05	Undiff-G	CSTF3
DDX41	5.85E-09	-0.24276	0.022	0.245	8.74E-05	Undiff-G	DDX41
APOO	5.86E-09	-0.16583	0.015	0.231	8.76E-05	Undiff-G	APOO
PPHLN1	5.88E-09	0.43297	0.088	0.37	8.79E-05	Undiff-G	PPHLN1
SMARCD1	5.89E-09	-0.46332	0.007	0.217	8.80E-05	Undiff-G	SMARCD1
CAMK1	5.91E-09	-0.45501	0.015	0.228	8.83E-05	Undiff-G	CAMK1
SNRPA1	5.92E-09	0.153667	0.051	0.298	8.85E-05	Undiff-G	SNRPA1
CTNNBL1	5.98E-09	0.170438	0.051	0.299	8.94E-05	Undiff-G	CTNNBL1
RND3	5.98E-09	-0.35388	0.022	0.245	8.94E-05	Undiff-G	RND3
CHTOP	6.01E-09	0.355262	0.088	0.37	8.99E-05	Undiff-G	CHTOP
DNPEP	6.04E-09	-0.35587	0.022	0.244	9.03E-05	Undiff-G	DNPEP
GART	6.06E-09	-0.53223	0	0.202	9.06E-05	Undiff-G	GART
MRPS23	6.09E-09	0.379341	0.088	0.37	9.11E-05	Undiff-G	MRPS23
BFAR	6.13E-09	-0.25979	0.015	0.231	9.16E-05	Undiff-G	BFAR
DNM2	6.14E-09	-0.22294	0.022	0.245	9.18E-05	Undiff-G	DNM2
RNF141	6.19E-09	-0.34058	0.007	0.217	9.26E-05	Undiff-G	RNF141
ALDH9A1	6.22E-09	0.271191	0.074	0.342	9.29E-05	Undiff-G	ALDH9A1
DEAF1	6.22E-09	-0.4952	0.007	0.214	9.29E-05	Undiff-G	DEAF1
PGRMC2	6.23E-09	-0.37014	0.015	0.228	9.31E-05	Undiff-G	PGRMC2
ECI2	6.23E-09	0.124768	0.066	0.326	9.32E-05	Undiff-G	ECI2
SORL1	6.26E-09	-0.49922	0.015	0.23	9.35E-05	Undiff-G	SORL1
MYO6	6.39E-09	-0.53048	0.015	0.227	9.55E-05	Undiff-G	MYO6
PPIH	6.41E-09	-0.44563	0.015	0.23	9.57E-05	Undiff-G	PPIH
E2F4	6.41E-09	-0.47043	0.007	0.216	9.58E-05	Undiff-G	E2F4
TPM1	6.54E-09	-0.31489	0.029	0.255	9.78E-05	Undiff-G	TPM1
CD86	6.58E-09	-0.78278	0.007	0.216	9.84E-05	Undiff-G	CD86
HIBADH	6.59E-09	-0.16397	0.015	0.23	9.85E-05	Undiff-G	HIBADH
DAAM1	6.63E-09	-0.34473	0.022	0.243	9.91E-05	Undiff-G	DAAM1
S100A9	6.64E-09	-0.9593	0.015	0.226	9.92E-05	Undiff-G	S100A9
ERAL1	6.64E-09	-0.227	0.022	0.243	9.92E-05	Undiff-G	ERAL1
INSIG1	6.66E-09	-0.55394	0.015	0.229	9.96E-05	Undiff-G	INSIG1
AGPAT1	6.67E-09	-0.25022	0.015	0.229	9.97E-05	Undiff-G	AGPAT1
CREB1	6.69E-09	-0.25699	0.022	0.244	0.0001	Undiff-G	CREB1
HOMER3	6.74E-09	-0.21278	0.022	0.244	0.000101	Undiff-G	HOMER3
CCPG1	6.80E-09	-0.51237	0.007	0.215	0.000102	Undiff-G	CCPG1
ECI1	6.84E-09	-0.21105	0.007	0.216	0.000102	Undiff-G	ECI1
PDCL3	6.88E-09	0.125899	0.051	0.296	0.000103	Undiff-G	PDCL3
S100A4	6.93E-09	-0.30591	0.051	0.298	0.000104	Undiff-G	S100A4
ZNF146	6.96E-09	-0.26586	0.015	0.23	0.000104	Undiff-G	ZNF146
KLHDC8A	6.96E-09	-0.50556	0.029	0.253	0.000104	Undiff-G	KLHDC8A
ACOT7	7.00E-09	-0.44327	0.015	0.229	0.000105	Undiff-G	ACOT7
SRSF3	7.06E-09	0.444135	0.272	0.734	0.000105	Undiff-G	SRSF3
TMEM242	7.09E-09	-0.16867	0.022	0.243	0.000106	Undiff-G	TMEM242
PSME3	7.15E-09	-0.45131	0.015	0.229	0.000107	Undiff-G	PSME3
RAD51C	7.18E-09	-0.13932	0.037	0.269	0.000107	Undiff-G	RAD51C
HSD17B4	7.18E-09	-0.15939	0.022	0.244	0.000107	Undiff-G	HSD17B4
MSI2	7.20E-09	-0.13192	0.066	0.324	0.000108	Undiff-G	MSI2

RPS6KB2	7.20E-09	-0.36976	0.015	0.229	0.000108	Undiff-G	RPS6KB2
PPP1CB	7.20E-09	0.300902	0.162	0.51	0.000108	Undiff-G	PPP1CB
UBTF	7.30E-09	-0.34661	0.015	0.229	0.000109	Undiff-G	UBTF
FABP7	7.31E-09	-0.17456	0.103	0.387	0.000109	Undiff-G	FABP7
SP3	7.35E-09	-0.52214	0.007	0.212	0.00011	Undiff-G	SP3
THUMPD1	7.37E-09	-0.52002	0.007	0.214	0.00011	Undiff-G	THUMPD1
CBX6	7.40E-09	-0.1617	0.022	0.243	0.000111	Undiff-G	CBX6
SLC2A3	7.40E-09	-0.23652	0.029	0.257	0.000111	Undiff-G	SLC2A3
SNHG9	7.43E-09	-0.11501	0.059	0.307	0.000111	Undiff-G	SNHG9
AK3	7.45E-09	-0.30451	0.022	0.24	0.000111	Undiff-G	AK3
EEF1D	7.54E-09	0.266208	0.331	0.851	0.000113	Undiff-G	EEF1D
C5AR1	7.56E-09	-0.52166	0.022	0.24	0.000113	Undiff-G	C5AR1
PNPLA2	7.58E-09	-0.43472	0.007	0.215	0.000113	Undiff-G	PNPLA2
CHORDC1	7.58E-09	-0.4686	0.007	0.215	0.000113	Undiff-G	CHORDC1
NF1	7.65E-09	-0.4977	0.015	0.226	0.000114	Undiff-G	NF1
COX14	7.83E-09	0.420043	0.14	0.464	0.000117	Undiff-G	COX14
LGALS9	7.93E-09	-0.40841	0.022	0.243	0.000118	Undiff-G	LGALS9
SPPL3	7.93E-09	-0.50775	0	0.2	0.000119	Undiff-G	SPPL3
RANBP2	7.97E-09	-0.55893	0.007	0.211	0.000119	Undiff-G	RANBP2
UFL1	8.05E-09	-0.51247	0.007	0.214	0.00012	Undiff-G	UFL1
EIF4G1	8.06E-09	-0.23843	0.044	0.28	0.00012	Undiff-G	EIF4G1
VAPB	8.15E-09	-0.46519	0	0.199	0.000122	Undiff-G	VAPB
ARHGEF2	8.21E-09	-0.26753	0.051	0.292	0.000123	Undiff-G	ARHGEF2
QSOX1	8.33E-09	-0.4516	0.022	0.239	0.000125	Undiff-G	QSOX1
MGST2	8.34E-09	-0.20558	0.029	0.256	0.000125	Undiff-G	MGST2
ARIH2	8.34E-09	-0.29104	0.029	0.253	0.000125	Undiff-G	ARIH2
HTRA2	8.40E-09	-0.36581	0.015	0.227	0.000126	Undiff-G	HTRA2
THRA	8.43E-09	-0.45593	0.022	0.241	0.000126	Undiff-G	THRA
CASP3	8.45E-09	-0.51794	0	0.199	0.000126	Undiff-G	CASP3
OFD1	8.51E-09	-0.34274	0.044	0.277	0.000127	Undiff-G	OFD1
PYGB	8.58E-09	-0.47323	0.007	0.213	0.000128	Undiff-G	PYGB
NDN	8.60E-09	-0.49886	0	0.199	0.000129	Undiff-G	NDN
FNBP4	8.66E-09	-0.40477	0.029	0.254	0.000129	Undiff-G	FNBP4
ATP5I	8.67E-09	0.314516	0.279	0.757	0.00013	Undiff-G	ATP5I
TRIM13	8.68E-09	-0.52938	0.029	0.252	0.00013	Undiff-G	TRIM13
SIAH2	8.70E-09	-0.50401	0.007	0.213	0.00013	Undiff-G	SIAH2
CSE1L	8.80E-09	-0.42009	0.015	0.225	0.000131	Undiff-G	CSE1L
TMEM223	9.01E-09	-0.20023	0.015	0.227	0.000135	Undiff-G	TMEM223
TIMM17A	9.01E-09	0.393374	0.096	0.379	0.000135	Undiff-G	TIMM17A
SIGIRR	9.15E-09	-0.56786	0	0.198	0.000137	Undiff-G	SIGIRR
OXR1	9.17E-09	-0.11503	0.029	0.254	0.000137	Undiff-G	OXR1
AKAP8L	9.18E-09	-0.41759	0.029	0.252	0.000137	Undiff-G	AKAP8L
NT5C3A	9.20E-09	0.291022	0.051	0.297	0.000137	Undiff-G	NT5C3A
COQ5	9.24E-09	-0.54702	0	0.198	0.000138	Undiff-G	COQ5
FAM120A	9.32E-09	-0.41352	0.007	0.213	0.000139	Undiff-G	FAM120A
TSPYL1	9.45E-09	-0.19328	0.022	0.24	0.000141	Undiff-G	TSPYL1
EXOC1	9.46E-09	-0.28254	0.029	0.252	0.000141	Undiff-G	EXOC1
MAGOHB	9.49E-09	-0.27666	0.022	0.24	0.000142	Undiff-G	MAGOHB
WDR46	9.59E-09	-0.3722	0.022	0.238	0.000143	Undiff-G	WDR46
HPCAL1	9.59E-09	-0.43434	0.007	0.212	0.000143	Undiff-G	HPCAL1
IVNS1ABP	9.65E-09	-0.18782	0.022	0.24	0.000144	Undiff-G	IVNS1ABP

PELI1	9.76E-09	-0.62054	0.007	0.211	0.000146	Undiff-G	PELI1
MTMR14	9.80E-09	-0.46216	0.015	0.224	0.000147	Undiff-G	MTMR14
RAB9A	9.81E-09	0.249956	0.059	0.307	0.000147	Undiff-G	RAB9A
GPN1	9.81E-09	-0.25563	0.015	0.226	0.000147	Undiff-G	GPN1
RNPEP	9.83E-09	-0.36773	0.015	0.226	0.000147	Undiff-G	RNPEP
FBXL15	9.92E-09	-0.54262	0	0.198	0.000148	Undiff-G	FBXL15
LRP10	1.00E-08	-0.2463	0.029	0.254	0.00015	Undiff-G	LRP10
MPZL1	1.00E-08	-0.19223	0.029	0.251	0.00015	Undiff-G	MPZL1
THAP7	1.01E-08	-0.44524	0.007	0.212	0.00015	Undiff-G	THAP7
METTL5	1.01E-08	0.264354	0.088	0.363	0.000151	Undiff-G	METTL5
RNF34	1.02E-08	-0.49095	0	0.197	0.000152	Undiff-G	RNF34
NUFIP2	1.03E-08	-0.44941	0.022	0.239	0.000153	Undiff-G	NUFIP2
SON	1.03E-08	0.43982	0.265	0.716	0.000154	Undiff-G	SON
WSB2	1.03E-08	-0.13393	0.015	0.226	0.000154	Undiff-G	WSB2
PDE4B	1.04E-08	-0.15922	0.044	0.279	0.000155	Undiff-G	PDE4B
WDR77	1.04E-08	-0.24344	0.007	0.212	0.000156	Undiff-G	WDR77
AIG1	1.05E-08	0.285488	0.074	0.337	0.000156	Undiff-G	AIG1
TOP2B	1.06E-08	-0.19132	0.022	0.24	0.000158	Undiff-G	TOP2B
KDM5A	1.06E-08	-0.45269	0.029	0.25	0.000158	Undiff-G	KDM5A
UTP6	1.07E-08	-0.36995	0.007	0.211	0.000159	Undiff-G	UTP6
ARFGAP2	1.08E-08	-0.10305	0.051	0.295	0.000161	Undiff-G	ARFGAP2
GGPS1	1.08E-08	0.154123	0.066	0.32	0.000162	Undiff-G	GGPS1
ENAH	1.10E-08	-0.14136	0.037	0.267	0.000164	Undiff-G	ENAH
TMEM218	1.12E-08	-0.31087	0.015	0.224	0.000167	Undiff-G	TMEM218
KLF4	1.13E-08	-0.75196	0.007	0.208	0.00017	Undiff-G	KLF4
ANKIB1	1.14E-08	-0.39315	0.007	0.211	0.000171	Undiff-G	ANKIB1
CCAR1	1.15E-08	-0.24789	0.029	0.252	0.000172	Undiff-G	CCAR1
XPA	1.15E-08	-0.44784	0.015	0.224	0.000172	Undiff-G	XPA
UTP11L	1.17E-08	0.156263	0.051	0.294	0.000174	Undiff-G	UTP11L
GNL3	1.17E-08	0.324374	0.081	0.349	0.000175	Undiff-G	GNL3
BHLHE41	1.17E-08	-0.58066	0.007	0.21	0.000175	Undiff-G	BHLHE41
CAT	1.17E-08	0.131697	0.059	0.308	0.000175	Undiff-G	CAT
TMPO	1.17E-08	-0.5392	0	0.196	0.000175	Undiff-G	TMPO
ETS2	1.18E-08	-0.69628	0.007	0.209	0.000176	Undiff-G	ETS2
SLCO2B1	1.18E-08	-0.68005	0.015	0.223	0.000177	Undiff-G	SLCO2B1
ABHD14A	1.19E-08	-0.23116	0.015	0.224	0.000178	Undiff-G	ABHD14A
KCTD5	1.21E-08	-0.22492	0.007	0.21	0.000181	Undiff-G	KCTD5
ACAT2	1.21E-08	0.121984	0.044	0.28	0.000181	Undiff-G	ACAT2
SLC43A2	1.21E-08	-0.36175	0.029	0.248	0.000181	Undiff-G	SLC43A2
EIF3B	1.22E-08	-0.32177	0.029	0.249	0.000182	Undiff-G	EIF3B
DFFA	1.23E-08	-0.26884	0.015	0.222	0.000183	Undiff-G	DFFA
GPANK1	1.23E-08	-0.49926	0	0.195	0.000183	Undiff-G	GPANK1
C7orf49	1.23E-08	-0.24203	0.007	0.21	0.000184	Undiff-G	C7orf49
SPRY2	1.24E-08	-0.53521	0.015	0.22	0.000185	Undiff-G	SPRY2
KANSL1-AS	1.24E-08	-0.52707	0	0.195	0.000185	Undiff-G	KANSL1-AS1
ZMYND8	1.24E-08	-0.32344	0.007	0.21	0.000186	Undiff-G	ZMYND8
ARL3	1.24E-08	0.288898	0.081	0.349	0.000186	Undiff-G	ARL3
MNAT1	1.26E-08	-0.50693	0	0.195	0.000188	Undiff-G	MNAT1
C12orf75	1.26E-08	-0.57509	0	0.195	0.000188	Undiff-G	C12orf75
LYSMD2	1.28E-08	-0.49438	0	0.195	0.000192	Undiff-G	LYSMD2
CHPF	1.31E-08	-0.39193	0.037	0.261	0.000196	Undiff-G	CHPF

HERPUD1	1.31E-08	0.374298	0.235	0.647	0.000196	Undiff-G	HERPUD1
DEXI	1.33E-08	-0.44408	0.007	0.209	0.000199	Undiff-G	DEXI
ETFA	1.35E-08	0.395673	0.096	0.375	0.000202	Undiff-G	ETFA
PEX10	1.35E-08	-0.45591	0	0.195	0.000202	Undiff-G	PEX10
PLS3	1.36E-08	-0.26518	0.015	0.223	0.000203	Undiff-G	PLS3
ZNF326	1.37E-08	-0.34347	0.029	0.25	0.000205	Undiff-G	ZNF326
LINC0100З	1.38E-08	-0.47282	0	0.194	0.000206	Undiff-G	LINC01003
NEK6	1.40E-08	0.117652	0.051	0.292	0.000209	Undiff-G	NEK6
PLIN3	1.40E-08	0.353862	0.088	0.361	0.000209	Undiff-G	PLIN3
NUDT14	1.40E-08	-0.61055	0	0.194	0.00021	Undiff-G	NUDT14
ING2	1.41E-08	-0.38363	0.007	0.209	0.00021	Undiff-G	ING2
DNM1L	1.41E-08	-0.25794	0.022	0.234	0.000211	Undiff-G	DNM1L
MAN1A2	1.42E-08	-0.45835	0.007	0.206	0.000213	Undiff-G	MAN1A2
CCS	1.42E-08	-0.13278	0.037	0.262	0.000213	Undiff-G	CCS
LYZ	1.43E-08	-0.65013	0.007	0.208	0.000214	Undiff-G	LYZ
ZMYM2	1.44E-08	-0.52162	0.007	0.207	0.000215	Undiff-G	ZMYM2
HNRNPA1	1.44E-08	0.276205	0.346	0.883	0.000216	Undiff-G	HNRNPA1
MFSD10	1.45E-08	0.112432	0.059	0.304	0.000216	Undiff-G	MFSD10
CHD6	1.45E-08	-0.42762	0.029	0.247	0.000216	Undiff-G	CHD6
CD300A	1.45E-08	-0.80173	0	0.194	0.000217	Undiff-G	CD300A
TRIM24	1.47E-08	-0.45313	0	0.194	0.000219	Undiff-G	TRIM24
PFKFB3	1.50E-08	-0.33907	0.029	0.246	0.000224	Undiff-G	PFKFB3
SLAIN1	1.50E-08	-0.59452	0.007	0.205	0.000224	Undiff-G	SLAIN1
RSBN1	1.51E-08	-0.20845	0.015	0.219	0.000226	Undiff-G	RSBN1
BANF1	1.51E-08	0.478993	0.243	0.666	0.000226	Undiff-G	BANF1
SNRNP40	1.53E-08	0.210047	0.074	0.332	0.000229	Undiff-G	SNRNP40
PPP2R5E	1.55E-08	-0.25431	0.029	0.25	0.000231	Undiff-G	PPP2R5E
PGAM1	1.57E-08	0.530694	0.162	0.505	0.000235	Undiff-G	PGAM1
UBE2H	1.58E-08	-0.16275	0.037	0.262	0.000237	Undiff-G	UBE2H
IFI30	1.60E-08	-0.51999	0.015	0.222	0.000239	Undiff-G	IFI30
SPTAN1	1.60E-08	-0.22574	0.037	0.263	0.000239	Undiff-G	SPTAN1
TIMM50	1.62E-08	0.165059	0.066	0.313	0.000242	Undiff-G	TIMM50
FCGBP	1.62E-08	-0.70883	0.022	0.232	0.000243	Undiff-G	FCGBP
1-Mar	1.63E-08	-0.65762	0	0.193	0.000244	Undiff-G	1-Mar
LONP1	1.64E-08	-0.42635	0.007	0.207	0.000245	Undiff-G	LONP1
AZI2	1.66E-08	0.135685	0.059	0.304	0.000248	Undiff-G	AZI2
SLC30A5	1.66E-08	-0.21573	0.007	0.207	0.000249	Undiff-G	SLC30A5
TMF1	1.69E-08	-0.21583	0.015	0.221	0.000253	Undiff-G	TMF1
RPA3	1.69E-08	0.340259	0.081	0.346	0.000253	Undiff-G	RPA3
FLAD1	1.69E-08	-0.35477	0.022	0.234	0.000253	Undiff-G	FLAD1
PTCD3	1.71E-08	-0.42545	0.022	0.232	0.000256	Undiff-G	PTCD3
MAP1A	1.73E-08	-0.15819	0.022	0.232	0.000258	Undiff-G	MAP1A
PREX1	1.73E-08	-0.45665	0.015	0.22	0.000258	Undiff-G	PREX1
GSK3A	1.73E-08	-0.4594	0	0.192	0.000259	Undiff-G	GSK3A
GADD45G	1.74E-08	-0.56867	0.037	0.258	0.00026	Undiff-G	GADD45G
RAP1B	1.74E-08	0.349713	0.132	0.438	0.00026	Undiff-G	RAP1B
HNMT	1.74E-08	-0.33667	0.015	0.221	0.000261	Undiff-G	HNMT
SOCS6	1.75E-08	-0.51461	0.022	0.232	0.000261	Undiff-G	SOCS6
PRPF8	1.75E-08	-0.14249	0.037	0.259	0.000261	Undiff-G	PRPF8
DDX42	1.75E-08	-0.20755	0.015	0.221	0.000262	Undiff-G	DDX42
SURF2	1.75E-08	-0.15306	0.022	0.234	0.000262	Undiff-G	SURF2

LUC7L	1.75E-08	-0.28574	0.029	0.248	0.000262	Undiff-G	LUC7L
ZNF593	1.76E-08	0.286302	0.059	0.304	0.000264	Undiff-G	ZNF593
C6orf226	1.76E-08	-0.50329	0	0.192	0.000264	Undiff-G	C6orf226
VCAN	1.78E-08	-0.19075	0.029	0.249	0.000265	Undiff-G	VCAN
ARPC1A	1.78E-08	0.507641	0.191	0.562	0.000266	Undiff-G	ARPC1A
ZSCAN18	1.78E-08	-0.43392	0.022	0.234	0.000266	Undiff-G	ZSCAN18
ANKRD17	1.78E-08	-0.40789	0.015	0.219	0.000267	Undiff-G	ANKRD17
RPF1	1.82E-08	-0.12693	0.029	0.248	0.000272	Undiff-G	RPF1
SPCS2	1.82E-08	0.481952	0.272	0.735	0.000272	Undiff-G	SPCS2
GALNT2	1.82E-08	-0.36909	0.029	0.246	0.000272	Undiff-G	GALNT2
CHST12	1.82E-08	-0.11403	0.029	0.248	0.000272	Undiff-G	CHST12
RELA	1.83E-08	-0.29261	0.007	0.206	0.000274	Undiff-G	RELA
RAB21	1.83E-08	-0.25965	0.007	0.206	0.000274	Undiff-G	RAB21
CDKN2C	1.84E-08	-0.10943	0.051	0.285	0.000275	Undiff-G	CDKN2C
ABHD2	1.84E-08	-0.14259	0.022	0.232	0.000275	Undiff-G	ABHD2
HEBP1	1.84E-08	-0.54929	0	0.191	0.000276	Undiff-G	HEBP1
IFT27	1.86E-08	-0.2248	0.037	0.259	0.000278	Undiff-G	IFT27
EFCAB14	1.86E-08	-0.46932	0.007	0.205	0.000278	Undiff-G	EFCAB14
FAF2	1.86E-08	-0.47856	0	0.191	0.000278	Undiff-G	FAF2
NDUFB7	1.87E-08	0.413	0.272	0.725	0.000279	Undiff-G	NDUFB7
UNC119	1.87E-08	-0.28091	0.015	0.219	0.000279	Undiff-G	UNC119
EXOSC7	1.87E-08	-0.17568	0.022	0.231	0.00028	Undiff-G	EXOSC7
RAB28	1.87E-08	-0.39231	0.015	0.22	0.00028	Undiff-G	RAB28
APOC2	1.88E-08	-1.00614	0	0.191	0.00028	Undiff-G	APOC2
ERGIC3	1.89E-08	0.467988	0.206	0.58	0.000283	Undiff-G	ERGIC3
CERS2	1.90E-08	-0.22127	0.022	0.234	0.000283	Undiff-G	CERS2
TNFSF13B	1.90E-08	-0.49111	0.007	0.206	0.000284	Undiff-G	TNFSF13B
BCAT1	1.91E-08	-0.29789	0.022	0.234	0.000285	Undiff-G	BCAT1
FNDC3A	1.91E-08	-0.48508	0.015	0.217	0.000286	Undiff-G	FNDC3A
ID4	1.92E-08	-0.61351	0.015	0.218	0.000288	Undiff-G	ID4
BDP1	1.92E-08	-0.24286	0.029	0.247	0.000288	Undiff-G	BDP1
PDE6D	1.93E-08	-0.49563	0	0.191	0.000288	Undiff-G	PDE6D
ZSCAN16-A	1.93E-08	-0.36207	0.037	0.26	0.000289	Undiff-G	ZSCAN16-AS1
SPATS2	1.94E-08	-0.22136	0.037	0.261	0.00029	Undiff-G	SPATS2
ZMPSTE24	1.94E-08	-0.35522	0.015	0.22	0.00029	Undiff-G	ZMPSTE24
SWI5	1.95E-08	-0.11253	0.029	0.248	0.000291	Undiff-G	SWI5
IER5	1.95E-08	-0.55095	0.007	0.205	0.000292	Undiff-G	IER5
IAH1	1.96E-08	0.453746	0.125	0.424	0.000293	Undiff-G	IAH1
ATP5SL	1.97E-08	-0.27543	0.022	0.233	0.000294	Undiff-G	ATP5SL
THY1	1.98E-08	-0.33925	0.037	0.258	0.000296	Undiff-G	THY1
NCOR2	1.98E-08	-0.21324	0.022	0.233	0.000297	Undiff-G	NCOR2
UBA1	1.99E-08	0.305099	0.081	0.345	0.000297	Undiff-G	UBA1
ZC3H14	2.00E-08	-0.2582	0.007	0.205	0.000299	Undiff-G	ZC3H14
LRRC47	2.02E-08	-0.43683	0.015	0.219	0.000302	Undiff-G	LRRC47
COX19	2.03E-08	-0.22457	0.022	0.233	0.000303	Undiff-G	COX19
MXD4	2.03E-08	-0.49134	0	0.191	0.000304	Undiff-G	MXD4
PRADC1	2.05E-08	-0.406	0.015	0.219	0.000306	Undiff-G	PRADC1
CNOT2	2.06E-08	0.127847	0.059	0.301	0.000308	Undiff-G	CNOT2
SRRT	2.09E-08	-0.18574	0.015	0.219	0.000312	Undiff-G	SRRT
PYCR2	2.10E-08	-0.23048	0.029	0.244	0.000314	Undiff-G	PYCR2
LTA4H	2.10E-08	-0.3964	0.022	0.232	0.000314	Undiff-G	LTA4H

ZMAT3	2.13E-08	-0.44095	0.007	0.204	0.000319	Undiff-G	ZMAT3
CUL3	2.13E-08	-0.47571	0.007	0.202	0.000319	Undiff-G	CUL3
LMBRD1	2.14E-08	-0.27254	0.015	0.219	0.000319	Undiff-G	LMBRD1
POU3F2	2.14E-08	-0.50578	0.015	0.218	0.000321	Undiff-G	POU3F2
HNRNPA2B1	2.15E-08	0.293204	0.324	0.851	0.000321	Undiff-G	HNRNPA2B1
CAMK2D	2.16E-08	-0.14405	0.029	0.246	0.000322	Undiff-G	CAMK2D
ZDHHC24	2.16E-08	-0.46824	0	0.19	0.000323	Undiff-G	ZDHHC24
LRRFIP2	2.18E-08	-0.46104	0	0.19	0.000326	Undiff-G	LRRFIP2
PRRC2A	2.18E-08	-0.18465	0.029	0.245	0.000326	Undiff-G	PRRC2A
IGFBP7	2.19E-08	-0.32367	0.044	0.271	0.000327	Undiff-G	IGFBP7
IGSF8	2.21E-08	-0.51502	0.015	0.215	0.00033	Undiff-G	IGSF8
SLC12A9	2.21E-08	-0.49969	0.007	0.204	0.00033	Undiff-G	SLC12A9
REEP2	2.23E-08	-0.18694	0.007	0.204	0.000333	Undiff-G	REEP2
SV2A	2.24E-08	-0.33779	0.007	0.204	0.000335	Undiff-G	SV2A
SMCHD1	2.26E-08	-0.46239	0	0.19	0.000337	Undiff-G	SMCHD1
THOP1	2.26E-08	-0.42486	0.007	0.204	0.000338	Undiff-G	THOP1
MBOAT7	2.27E-08	-0.27269	0.029	0.245	0.000339	Undiff-G	MBOAT7
USP9X	2.28E-08	-0.3314	0.007	0.204	0.000341	Undiff-G	USP9X
CIB1	2.29E-08	0.418625	0.162	0.496	0.000342	Undiff-G	CIB1
TMEM189	2.30E-08	-0.45062	0	0.189	0.000343	Undiff-G	TMEM189
COX8A	2.30E-08	0.453149	0.287	0.776	0.000344	Undiff-G	COX8A
CARHSP1	2.31E-08	0.414529	0.125	0.426	0.000345	Undiff-G	CARHSP1
GLA	2.31E-08	-0.4463	0.015	0.218	0.000346	Undiff-G	GLA
USB1	2.32E-08	-0.23181	0.022	0.232	0.000347	Undiff-G	USB1
KHDRBS3	2.34E-08	-0.22878	0.015	0.217	0.00035	Undiff-G	KHDRBS3
RPP14	2.36E-08	-0.47052	0	0.189	0.000352	Undiff-G	RPP14
TMEM9	2.37E-08	0.253452	0.096	0.37	0.000354	Undiff-G	TMEM9
LYRM7	2.38E-08	-0.40012	0.007	0.203	0.000356	Undiff-G	LYRM7
USP10	2.39E-08	-0.10688	0.029	0.245	0.000357	Undiff-G	USP10
NAA15	2.41E-08	-0.27528	0.015	0.218	0.00036	Undiff-G	NAA15
PSME2	2.41E-08	0.429339	0.191	0.562	0.000361	Undiff-G	PSME2
BLVRA	2.42E-08	0.35209	0.081	0.343	0.000361	Undiff-G	BLVRA
DSEL	2.42E-08	-0.52789	0.022	0.227	0.000361	Undiff-G	DSEL
SREK1	2.44E-08	0.127659	0.074	0.325	0.000365	Undiff-G	SREK1
TXN	2.44E-08	0.462714	0.243	0.671	0.000365	Undiff-G	TXN
MGAT2	2.44E-08	-0.45981	0	0.189	0.000365	Undiff-G	MGAT2
FUBP3	2.46E-08	-0.37782	0.029	0.242	0.000368	Undiff-G	FUBP3
EDEM2	2.46E-08	-0.16005	0.029	0.243	0.000368	Undiff-G	EDEM2
GMPR2	2.51E-08	-0.15883	0.015	0.217	0.000375	Undiff-G	GMPR2
CISD1	2.52E-08	0.252607	0.081	0.34	0.000376	Undiff-G	CISD1
SMC4	2.53E-08	-0.50499	0.015	0.214	0.000379	Undiff-G	SMC4
ZNF644	2.54E-08	-0.18991	0.037	0.255	0.000379	Undiff-G	ZNF644
HNRNPA1L2	2.54E-08	-0.35702	0.007	0.203	0.000379	Undiff-G	HNRNPA1L2
NPL	2.56E-08	-0.52607	0.007	0.203	0.000382	Undiff-G	NPL
FOSL2	2.57E-08	-0.55873	0	0.188	0.000385	Undiff-G	FOSL2
CLINT1	2.58E-08	-0.42242	0.007	0.203	0.000385	Undiff-G	CLINT1
TMA16	2.58E-08	-0.33104	0.007	0.203	0.000386	Undiff-G	TMA16
C19orf48	2.63E-08	-0.39485	0.022	0.23	0.000393	Undiff-G	C19orf48
KLF3	2.63E-08	-0.30087	0.015	0.217	0.000393	Undiff-G	KLF3
C17orf58	2.64E-08	-0.44066	0	0.188	0.000395	Undiff-G	C17orf58
MAP3K2	2.65E-08	-0.43906	0.007	0.202	0.000396	Undiff-G	MAP3K2

NTAN1	2.66E-08	-0.47229	0.007	0.202	0.000398	Undiff-G	NTAN1
SRPRB	2.68E-08	0.111736	0.037	0.258	0.0004	Undiff-G	SRPRB
ASCL1	2.69E-08	-0.61715	0	0.188	0.000402	Undiff-G	ASCL1
DRG2	2.72E-08	-0.28772	0.007	0.202	0.000407	Undiff-G	DRG2
REST	2.74E-08	-0.4521	0.007	0.2	0.000409	Undiff-G	REST
MCM7	2.76E-08	-0.19945	0.037	0.257	0.000412	Undiff-G	MCM7
CYSTM1	2.77E-08	0.319093	0.074	0.327	0.000413	Undiff-G	CYSTM1
SEMA6A	2.79E-08	-0.4526	0.007	0.202	0.000417	Undiff-G	SEMA6A
IFNAR2	2.80E-08	-0.44253	0.007	0.202	0.000418	Undiff-G	IFNAR2
MTSS1	2.83E-08	-0.55603	0.015	0.215	0.000422	Undiff-G	MTSS1
CBWD1	2.85E-08	-0.33572	0.015	0.216	0.000426	Undiff-G	CBWD1
FIP1L1	2.87E-08	0.257519	0.059	0.299	0.000429	Undiff-G	FIP1L1
ENC1	2.89E-08	-0.3903	0.022	0.227	0.000432	Undiff-G	ENC1
ZC3H11A	2.89E-08	-0.32719	0.007	0.202	0.000432	Undiff-G	ZC3H11A
ZNF281	2.91E-08	-0.49166	0	0.187	0.000435	Undiff-G	ZNF281
ACER3	2.91E-08	-0.55924	0	0.187	0.000435	Undiff-G	ACER3
ATP6V1A	2.93E-08	-0.45566	0.007	0.201	0.000438	Undiff-G	ATP6V1A
ZBTB8OS	2.94E-08	0.199003	0.059	0.297	0.000439	Undiff-G	ZBTB8OS
RP11-553L	2.97E-08	-0.41288	0.022	0.229	0.000444	Undiff-G	RP11-553L6.5
ENTPD6	2.97E-08	-0.45627	0.007	0.201	0.000445	Undiff-G	ENTPD6
COMMD8	3.00E-08	-0.21951	0.022	0.227	0.000448	Undiff-G	COMMD8
NAGA	3.01E-08	-0.64291	0	0.187	0.00045	Undiff-G	NAGA
ABCA1	3.03E-08	-0.421	0.029	0.241	0.000453	Undiff-G	ABCA1
CHST11	3.05E-08	-0.46568	0.015	0.215	0.000456	Undiff-G	CHST11
UBQLN1	3.08E-08	0.168816	0.051	0.284	0.00046	Undiff-G	UBQLN1
ABI1	3.10E-08	-0.14913	0.029	0.243	0.000463	Undiff-G	ABI1
WDR82	3.11E-08	-0.16884	0.037	0.257	0.000464	Undiff-G	WDR82
MBNL1	3.12E-08	-0.43131	0.022	0.226	0.000467	Undiff-G	MBNL1
TMEM176A	3.12E-08	-0.26779	0.022	0.229	0.000467	Undiff-G	TMEM176A
IRF2	3.13E-08	-0.52613	0.007	0.2	0.000467	Undiff-G	IRF2
HINT3	3.13E-08	-0.33126	0.007	0.201	0.000468	Undiff-G	HINT3
BCAN	3.15E-08	-0.36692	0.051	0.278	0.000471	Undiff-G	BCAN
MESDC2	3.20E-08	0.533074	0.118	0.407	0.000478	Undiff-G	MESDC2
OLFML3	3.20E-08	-0.38339	0.022	0.229	0.000478	Undiff-G	OLFML3
ISCA2	3.21E-08	0.251062	0.051	0.284	0.00048	Undiff-G	ISCA2
EPM2AIP1	3.24E-08	-0.31686	0.007	0.2	0.000485	Undiff-G	EPM2AIP1
SMIM14	3.24E-08	0.356979	0.081	0.333	0.000485	Undiff-G	SMIM14
GOSR1	3.25E-08	0.134304	0.044	0.27	0.000486	Undiff-G	GOSR1
OXLD1	3.26E-08	-0.21397	0.037	0.255	0.000487	Undiff-G	OXLD1
HRSP12	3.29E-08	-0.39656	0.007	0.2	0.000492	Undiff-G	HRSP12
MED10	3.32E-08	0.471827	0.162	0.487	0.000496	Undiff-G	MED10
GPCPD1	3.33E-08	-0.34737	0.007	0.2	0.000497	Undiff-G	GPCPD1
WASL	3.35E-08	-0.4453	0	0.186	0.0005	Undiff-G	WASL
SMIM4	3.36E-08	0.105784	0.044	0.27	0.000502	Undiff-G	SMIM4
MAP3K8	3.38E-08	-0.74879	0	0.186	0.000504	Undiff-G	MAP3K8
TIAL1	3.38E-08	-0.22163	0.022	0.228	0.000505	Undiff-G	TIAL1
IPO7	3.42E-08	-0.32103	0.015	0.214	0.000511	Undiff-G	IPO7
LYRM1	3.43E-08	-0.51441	0	0.185	0.000513	Undiff-G	LYRM1
HDAC1	3.44E-08	-0.1629	0.022	0.228	0.000514	Undiff-G	HDAC1
CETN2	3.45E-08	0.353927	0.074	0.325	0.000515	Undiff-G	CETN2
PKN2	3.48E-08	-0.33833	0.015	0.213	0.00052	Undiff-G	PKN2

TMX1	3.49E-08	0.344891	0.118	0.406	0.000521	Undiff-G	TMX1
SAP30BP	3.49E-08	0.32764	0.096	0.365	0.000521	Undiff-G	SAP30BP
C1orf54	3.49E-08	-0.36871	0.022	0.228	0.000522	Undiff-G	C1orf54
TAF10	3.50E-08	-0.11595	0.022	0.228	0.000523	Undiff-G	TAF10
RNMT	3.52E-08	0.276923	0.066	0.306	0.000526	Undiff-G	RNMT
GSK3B	3.53E-08	-0.39537	0.007	0.2	0.000527	Undiff-G	GSK3B
INIP	3.60E-08	-0.27197	0.007	0.199	0.000538	Undiff-G	INIP
RBP1	3.60E-08	0.439277	0.147	0.463	0.000538	Undiff-G	RBP1
PSPC1	3.61E-08	-0.3783	0.015	0.213	0.000539	Undiff-G	PSPC1
EVI2A	3.61E-08	-0.57115	0.022	0.225	0.000539	Undiff-G	EVI2A
HMBS	3.62E-08	-0.45092	0.007	0.199	0.000541	Undiff-G	HMBS
SNAPC5	3.62E-08	-0.13305	0.015	0.214	0.000541	Undiff-G	SNAPC5
ZNF677	3.63E-08	-0.29187	0.007	0.199	0.000542	Undiff-G	ZNF677
ARHGEF1	3.65E-08	-0.50939	0.007	0.199	0.000545	Undiff-G	ARHGEF1
EFHD2	3.65E-08	-0.46393	0.015	0.213	0.000546	Undiff-G	EFHD2
FLNA	3.68E-08	-0.34352	0.037	0.253	0.00055	Undiff-G	FLNA
ISOC2	3.70E-08	0.386178	0.088	0.352	0.000552	Undiff-G	ISOC2
PHAX	3.73E-08	0.226888	0.066	0.308	0.000558	Undiff-G	PHAX
ABHD3	3.75E-08	-0.51344	0	0.185	0.00056	Undiff-G	ABHD3
PPM1K	3.75E-08	-0.30796	0.022	0.227	0.00056	Undiff-G	PPM1K
TFRC	3.77E-08	-0.58067	0.015	0.211	0.000563	Undiff-G	TFRC
LZIC	3.78E-08	-0.48651	0	0.184	0.000565	Undiff-G	LZIC
RNASEH2A	3.81E-08	-0.18031	0.015	0.213	0.000569	Undiff-G	RNASEH2A
PI4KB	3.84E-08	-0.43408	0.015	0.212	0.000574	Undiff-G	PI4KB
HBB	3.84E-08	0.44019	0.118	0.402	0.000574	Undiff-G	HBB
PTPN18	3.88E-08	-0.24158	0.015	0.213	0.00058	Undiff-G	PTPN18
TMEM141	3.88E-08	0.118909	0.037	0.255	0.000581	Undiff-G	TMEM141
NUP214	3.89E-08	-0.63482	0.007	0.197	0.000582	Undiff-G	NUP214
C2orf69	3.94E-08	-0.4618	0.007	0.196	0.000589	Undiff-G	C2orf69
COPS2	3.94E-08	-0.41324	0.015	0.212	0.000589	Undiff-G	COPS2
NNAT	3.95E-08	-0.91355	0.007	0.198	0.00059	Undiff-G	NNAT
PLXDC2	3.95E-08	-0.52738	0.015	0.211	0.000591	Undiff-G	PLXDC2
PANK2	3.96E-08	-0.4315	0.015	0.21	0.000592	Undiff-G	PANK2
RBMS1	3.96E-08	-0.18543	0.037	0.252	0.000592	Undiff-G	RBMS1
SPATC1L	3.96E-08	-0.42101	0.007	0.197	0.000592	Undiff-G	SPATC1L
SCAMP3	3.97E-08	0.214534	0.059	0.293	0.000593	Undiff-G	SCAMP3
CDC16	3.97E-08	-0.39777	0.007	0.198	0.000593	Undiff-G	CDC16
CSRP1	3.97E-08	-0.21338	0.029	0.241	0.000594	Undiff-G	CSRP1
OGFOD3	3.98E-08	-0.33034	0.022	0.224	0.000595	Undiff-G	OGFOD3
EPB41L4A-	3.99E-08	0.176379	0.059	0.296	0.000596	Undiff-G	EPB41L4A-AS1
ALCAM	4.01E-08	-0.51278	0.007	0.198	0.0006	Undiff-G	ALCAM
ARHGAP21	4.03E-08	-0.37389	0.037	0.253	0.000602	Undiff-G	ARHGAP21
PITPNA	4.05E-08	-0.42333	0	0.184	0.000606	Undiff-G	PITPNA
ACTR1B	4.06E-08	-0.42246	0.015	0.212	0.000607	Undiff-G	ACTR1B
STOM	4.07E-08	-0.14885	0.037	0.254	0.000608	Undiff-G	STOM
ERBB2IP	4.07E-08	-0.27462	0.022	0.224	0.000608	Undiff-G	ERBB2IP
VEZF1	4.10E-08	-0.12402	0.029	0.24	0.000612	Undiff-G	VEZF1
HELZ	4.11E-08	-0.42166	0.007	0.198	0.000614	Undiff-G	HELZ
ATP2C1	4.11E-08	-0.24499	0.022	0.226	0.000614	Undiff-G	ATP2C1
LPCAT1	4.12E-08	-0.48765	0	0.184	0.000616	Undiff-G	LPCAT1
ITSN2	4.12E-08	-0.50079	0	0.184	0.000616	Undiff-G	ITSN2

SNRNP35	4.14E-08	-0.34024	0.007	0.198	0.000618	Undiff-G	SNRNP35
8-Sep	4.14E-08	-0.47887	0.007	0.198	0.000619	Undiff-G	8-Sep
RING1	4.15E-08	-0.16947	0.044	0.264	0.00062	Undiff-G	RING1
SDCCAG8	4.17E-08	0.216503	0.081	0.332	0.000624	Undiff-G	SDCCAG8
LTBP3	4.18E-08	-0.30281	0.015	0.212	0.000624	Undiff-G	LTBP3
CNOT8	4.20E-08	-0.4762	0	0.183	0.000627	Undiff-G	CNOT8
CCNG2	4.20E-08	-0.53983	0	0.183	0.000627	Undiff-G	CCNG2
MIA3	4.20E-08	-0.25455	0.015	0.212	0.000628	Undiff-G	MIA3
AAED1	4.23E-08	-0.49176	0	0.183	0.000632	Undiff-G	AAED1
SAT2	4.25E-08	0.4457	0.213	0.59	0.000635	Undiff-G	SAT2
LY6E	4.25E-08	0.277004	0.103	0.379	0.000636	Undiff-G	LY6E
SUZ12	4.27E-08	-0.42744	0.007	0.197	0.000639	Undiff-G	SUZ12
DCAF8	4.36E-08	-0.2432	0.015	0.21	0.000651	Undiff-G	DCAF8
TOMM5	4.39E-08	-0.39905	0.015	0.211	0.000656	Undiff-G	TOMM5
ZHX1	4.45E-08	-0.37127	0.015	0.211	0.000665	Undiff-G	ZHX1
TCOF1	4.45E-08	-0.49273	0.007	0.197	0.000665	Undiff-G	TCOF1
TMED4	4.46E-08	0.488729	0.14	0.441	0.000667	Undiff-G	TMED4
RNF14	4.47E-08	-0.42911	0.007	0.197	0.000668	Undiff-G	RNF14
SIRT6	4.51E-08	-0.25843	0.007	0.197	0.000674	Undiff-G	SIRT6
LYPLA2	4.52E-08	-0.18584	0.022	0.223	0.000675	Undiff-G	LYPLA2
AXL	4.54E-08	-0.76296	0	0.183	0.000678	Undiff-G	AXL
HMGXB4	4.60E-08	-0.34676	0.022	0.225	0.000688	Undiff-G	HMGXB4
IL18	4.62E-08	-0.79257	0.007	0.195	0.00069	Undiff-G	IL18
CTBP1	4.65E-08	-0.28918	0.022	0.225	0.000696	Undiff-G	CTBP1
GNA13	4.70E-08	-0.56676	0	0.182	0.000702	Undiff-G	GNA13
GFER	4.70E-08	-0.30411	0.015	0.211	0.000702	Undiff-G	GFER
ARHGAP5	4.72E-08	-0.45248	0.007	0.196	0.000705	Undiff-G	ARHGAP5
PRRC2B	4.72E-08	-0.14464	0.029	0.239	0.000705	Undiff-G	PRRC2B
GLUD1	4.72E-08	-0.18627	0.037	0.25	0.000705	Undiff-G	GLUD1
SLC25A1	4.75E-08	-0.31947	0.007	0.197	0.00071	Undiff-G	SLC25A1
RBM4	4.77E-08	-0.41495	0.022	0.222	0.000713	Undiff-G	RBM4
FBXO11	4.79E-08	-0.44133	0.015	0.208	0.000716	Undiff-G	FBXO11
CDC25B	4.82E-08	-0.4701	0.007	0.196	0.000721	Undiff-G	CDC25B
PSD3	4.83E-08	-0.254	0.007	0.196	0.000722	Undiff-G	PSD3
GPR137	4.85E-08	-0.19904	0.015	0.211	0.000725	Undiff-G	GPR137
NUDT9	4.85E-08	-0.1452	0.022	0.225	0.000725	Undiff-G	NUDT9
WDR33	4.87E-08	0.298995	0.066	0.303	0.000727	Undiff-G	WDR33
WBP11	4.88E-08	0.275711	0.059	0.294	0.000729	Undiff-G	WBP11
TSEN15	4.89E-08	-0.39559	0.029	0.235	0.00073	Undiff-G	TSEN15
ZKSCAN1	4.98E-08	0.207264	0.074	0.319	0.000744	Undiff-G	ZKSCAN1
NR3C1	4.99E-08	-0.29117	0.037	0.251	0.000746	Undiff-G	NR3C1
PTPN12	4.99E-08	-0.18786	0.022	0.224	0.000746	Undiff-G	PTPN12
CALCOCO2	5.03E-08	-0.51419	0.007	0.196	0.000752	Undiff-G	CALCOCO2
ACTR10	5.06E-08	0.455352	0.096	0.363	0.000756	Undiff-G	ACTR10
PIGF	5.07E-08	0.227871	0.059	0.29	0.000758	Undiff-G	PIGF
ARC	5.08E-08	-0.52183	0.015	0.21	0.000759	Undiff-G	ARC
CIZ1	5.12E-08	-0.43978	0	0.181	0.000765	Undiff-G	CIZ1
AGPAT2	5.12E-08	-0.49426	0	0.181	0.000765	Undiff-G	AGPAT2
PGP	5.15E-08	-0.25904	0.022	0.221	0.000769	Undiff-G	PGP
MMP14	5.17E-08	-0.15964	0.022	0.224	0.000773	Undiff-G	MMP14
GLRX3	5.18E-08	0.315243	0.066	0.307	0.000773	Undiff-G	GLRX3

MYEF2	5.24E-08	-0.40268	0.007	0.195	0.000782	Undiff-G	MYEF2
TFDP1	5.27E-08	-0.44857	0.007	0.194	0.000788	Undiff-G	TFDP1
BRD9	5.27E-08	-0.32734	0.007	0.196	0.000788	Undiff-G	BRD9
ST3GAL5	5.32E-08	-0.54999	0.007	0.193	0.000794	Undiff-G	ST3GAL5
NGDN	5.36E-08	0.220867	0.044	0.266	0.000801	Undiff-G	NGDN
MAPRE3	5.39E-08	-0.46573	0	0.181	0.000806	Undiff-G	MAPRE3
POLR3K	5.46E-08	-0.16046	0.022	0.223	0.000816	Undiff-G	POLR3K
CDKN2AIPN	5.47E-08	0.125102	0.037	0.251	0.000817	Undiff-G	CDKN2AIPNL
DENND5A	5.49E-08	-0.48348	0	0.181	0.00082	Undiff-G	DENND5A
GNPTG	5.49E-08	-0.30449	0.044	0.26	0.00082	Undiff-G	GNPTG
APBA2	5.50E-08	-0.42334	0.015	0.209	0.000822	Undiff-G	APBA2
PHF23	5.52E-08	-0.19973	0.015	0.209	0.000824	Undiff-G	PHF23
GSTM4	5.54E-08	-0.41904	0.015	0.207	0.000827	Undiff-G	GSTM4
SNAP23	5.55E-08	-0.24485	0.037	0.247	0.00083	Undiff-G	SNAP23
NOB1	5.56E-08	-0.47609	0.007	0.195	0.00083	Undiff-G	NOB1
ZNF131	5.56E-08	-0.29134	0.029	0.234	0.000831	Undiff-G	ZNF131
PINK1	5.63E-08	-0.25112	0.015	0.209	0.000841	Undiff-G	PINK1
TMSB15A	5.63E-08	-0.57365	0	0.18	0.000842	Undiff-G	TMSB15A
AC009501.	5.63E-08	1.468214	0.287	0.155	0.000842	Undiff-G	AC009501.4
HSD11B1L	5.65E-08	-0.27835	0.015	0.207	0.000844	Undiff-G	HSD11B1L
LARP6	5.69E-08	-0.38664	0.007	0.195	0.00085	Undiff-G	LARP6
CPEB4	5.70E-08	-0.33515	0.037	0.249	0.000852	Undiff-G	CPEB4
SLC31A2	5.71E-08	-0.66856	0.007	0.195	0.000854	Undiff-G	SLC31A2
TEN1	5.73E-08	-0.42782	0	0.18	0.000856	Undiff-G	TEN1
PMPCB	5.75E-08	0.359439	0.088	0.346	0.000859	Undiff-G	PMPCB
MED11	5.79E-08	-0.418	0.022	0.22	0.000865	Undiff-G	MED11
LRRC58	5.80E-08	-0.29176	0.015	0.209	0.000867	Undiff-G	LRRC58
EPHX1	5.82E-08	-0.27633	0.029	0.234	0.000869	Undiff-G	EPHX1
PDZD11	5.82E-08	0.473994	0.096	0.362	0.00087	Undiff-G	PDZD11
MEX3A	5.83E-08	-0.49266	0	0.18	0.000871	Undiff-G	MEX3A
TRMT1	5.84E-08	-0.26972	0.044	0.26	0.000873	Undiff-G	TRMT1
MTHFD2L	5.85E-08	-0.27092	0.015	0.208	0.000875	Undiff-G	MTHFD2L
DTNBP1	5.89E-08	-0.24319	0.015	0.209	0.00088	Undiff-G	DTNBP1
SPRY1	5.90E-08	-0.30286	0.029	0.235	0.000881	Undiff-G	SPRY1
TMEM101	5.90E-08	-0.22993	0.015	0.209	0.000882	Undiff-G	TMEM101
SMIM7	5.93E-08	0.518174	0.176	0.509	0.000886	Undiff-G	SMIM7
TINF2	5.98E-08	0.226228	0.037	0.251	0.000893	Undiff-G	TINF2
EIF1AY	5.98E-08	-0.44419	0	0.18	0.000894	Undiff-G	EIF1AY
PJA1	6.00E-08	-0.34583	0.007	0.194	0.000896	Undiff-G	PJA1
DERL2	6.03E-08	0.293376	0.096	0.355	0.000901	Undiff-G	DERL2
PLOD3	6.06E-08	-0.21104	0.022	0.22	0.000906	Undiff-G	PLOD3
RHBDF2	6.09E-08	-0.70529	0	0.18	0.00091	Undiff-G	RHBDF2
HGSNAT	6.11E-08	-0.26807	0.015	0.208	0.000913	Undiff-G	HGSNAT
ATP5G2	6.12E-08	0.38582	0.346	0.875	0.000914	Undiff-G	ATP5G2
CYBB	6.15E-08	-0.6131	0.007	0.194	0.000919	Undiff-G	CYBB
NFKBIL1	6.15E-08	-0.32964	0.015	0.206	0.00092	Undiff-G	NFKBIL1
TEX264	6.16E-08	0.359373	0.118	0.399	0.000921	Undiff-G	TEX264
KLF7	6.18E-08	-0.46012	0.015	0.208	0.000923	Undiff-G	KLF7
ABI3	6.21E-08	-0.74706	0.007	0.192	0.000928	Undiff-G	ABI3
MAPK8IP1	6.24E-08	-0.48212	0.007	0.193	0.000932	Undiff-G	MAPK8IP1
PES1	6.32E-08	-0.33241	0.007	0.194	0.000944	Undiff-G	PES1

MTRF1L	6.32E-08	-0.40735	0.007	0.192	0.000945	Undiff-G	MTRF1L
SOX8	6.35E-08	-0.56198	0	0.179	0.00095	Undiff-G	SOX8
SUN2	6.36E-08	-0.46908	0.022	0.221	0.000951	Undiff-G	SUN2
APIP	6.44E-08	-0.12415	0.029	0.235	0.000962	Undiff-G	APIP
TTF1	6.46E-08	-0.45469	0.015	0.205	0.000965	Undiff-G	TTF1
WIPF1	6.46E-08	-0.18732	0.022	0.222	0.000966	Undiff-G	WIPF1
LINC00667	6.47E-08	-0.14389	0.037	0.246	0.000967	Undiff-G	LINC00667
5-Mar	6.49E-08	-0.22976	0.007	0.194	0.00097	Undiff-G	5-Mar
MFSD12	6.49E-08	-0.39004	0.007	0.193	0.000971	Undiff-G	MFSD12
TMEM60	6.52E-08	0.131241	0.037	0.25	0.000974	Undiff-G	TMEM60
GOLGA2	6.52E-08	-0.39957	0.015	0.207	0.000975	Undiff-G	GOLGA2
TAGLN3	6.61E-08	-0.64649	0.007	0.19	0.000988	Undiff-G	TAGLN3
ALG13	6.64E-08	-0.14075	0.022	0.222	0.000992	Undiff-G	ALG13
PLOD1	6.66E-08	-0.44334	0.007	0.193	0.000996	Undiff-G	PLOD1
P2RX4	6.69E-08	-0.578	0	0.179	0.001	Undiff-G	P2RX4
SLC35B4	6.69E-08	-0.39793	0.007	0.193	0.001	Undiff-G	SLC35B4
USP15	6.70E-08	-0.3537	0.037	0.245	0.001001	Undiff-G	USP15
GPRC5B	6.71E-08	-0.23897	0.022	0.221	0.001003	Undiff-G	GPRC5B
KCMF1	6.74E-08	-0.3609	0.007	0.193	0.001008	Undiff-G	KCMF1
PTPRS	6.80E-08	-0.27011	0.044	0.26	0.001016	Undiff-G	PTPRS
ZC3HAV1	6.81E-08	-0.4923	0	0.179	0.001017	Undiff-G	ZC3HAV1
ASNSD1	6.83E-08	0.262434	0.051	0.274	0.001021	Undiff-G	ASNSD1
CLIP2	6.87E-08	-0.47625	0	0.178	0.001026	Undiff-G	CLIP2
POMGNT1	6.98E-08	-0.25065	0.044	0.261	0.001043	Undiff-G	POMGNT1
CD302	6.99E-08	-0.43243	0.015	0.207	0.001045	Undiff-G	CD302
MTMR9	7.04E-08	-0.46687	0.007	0.191	0.001052	Undiff-G	MTMR9
C1orf56	7.10E-08	-0.5443	0.015	0.204	0.001061	Undiff-G	C1orf56
ETV5	7.10E-08	-0.21224	0.044	0.261	0.001061	Undiff-G	ETV5
KAT8	7.11E-08	-0.50508	0	0.178	0.001062	Undiff-G	KAT8
STT3B	7.11E-08	-0.36915	0.015	0.206	0.001062	Undiff-G	STT3B
SIL1	7.12E-08	-0.35791	0.044	0.257	0.001064	Undiff-G	SIL1
MYO9B	7.13E-08	-0.14045	0.051	0.272	0.001065	Undiff-G	MYO9B
HNRNPDL	7.13E-08	0.435659	0.309	0.803	0.001066	Undiff-G	HNRNPDL
C10orf10	7.14E-08	1.593219	0.206	0.089	0.001067	Undiff-G	C10orf10
UHMK1	7.16E-08	-0.42844	0.015	0.205	0.00107	Undiff-G	UHMK1
NFKBIB	7.17E-08	-0.47158	0	0.178	0.001071	Undiff-G	NFKBIB
ALDH3A2	7.18E-08	-0.11961	0.029	0.234	0.001073	Undiff-G	ALDH3A2
PIM3	7.19E-08	-0.36814	0.007	0.193	0.001075	Undiff-G	PIM3
HGS	7.28E-08	-0.18285	0.022	0.218	0.001088	Undiff-G	HGS
DCAF11	7.32E-08	-0.21109	0.007	0.192	0.001094	Undiff-G	DCAF11
IMMP1L	7.35E-08	-0.15205	0.029	0.234	0.001099	Undiff-G	IMMP1L
SLC25A6	7.37E-08	0.324365	0.338	0.864	0.001101	Undiff-G	SLC25A6
ILKAP	7.47E-08	-0.28629	0.029	0.231	0.001116	Undiff-G	ILKAP
FOS	7.54E-08	0.970099	0.632	0.843	0.001127	Undiff-G	FOS
PTTG1	7.58E-08	-0.13622	0.029	0.233	0.001133	Undiff-G	PTTG1
STMN2	7.61E-08	-0.9701	0.007	0.189	0.001137	Undiff-G	STMN2
RNF40	7.61E-08	-0.4231	0	0.177	0.001138	Undiff-G	RNF40
APBB1	7.64E-08	-0.24534	0.007	0.192	0.001142	Undiff-G	APBB1
MT1E	7.73E-08	1.512381	0.456	0.379	0.001156	Undiff-G	MT1E
BLMH	7.75E-08	-0.43439	0	0.177	0.001157	Undiff-G	BLMH
TNFAIP1	7.81E-08	-0.40346	0	0.177	0.001167	Undiff-G	TNFAIP1

ETV1	8.02E-08	-0.19475	0.037	0.247	0.001199	Undiff-G	ETV1
IP6K2	8.03E-08	0.545829	0.14	0.438	0.0012	Undiff-G	IP6K2
HBEGF	8.10E-08	-0.53071	0.022	0.218	0.00121	Undiff-G	HBEGF
NBR1	8.11E-08	-0.22485	0.007	0.191	0.001212	Undiff-G	NBR1
DPP3	8.14E-08	-0.45975	0.007	0.191	0.001216	Undiff-G	DPP3
FAM53C	8.16E-08	-0.19906	0.037	0.244	0.001219	Undiff-G	FAM53C
DBI	8.17E-08	0.992944	0.632	0.846	0.001221	Undiff-G	DBI
RBM10	8.18E-08	-0.40906	0.015	0.204	0.001222	Undiff-G	RBM10
MAP2K3	8.18E-08	-0.35167	0.007	0.191	0.001222	Undiff-G	MAP2K3
HERPUD2	8.22E-08	-0.46557	0	0.177	0.001229	Undiff-G	HERPUD2
SPAG16	8.23E-08	0.357313	0.066	0.299	0.00123	Undiff-G	SPAG16
TLK1	8.24E-08	-0.25091	0.029	0.23	0.001231	Undiff-G	TLK1
PBX1	8.28E-08	-0.38616	0.022	0.216	0.001238	Undiff-G	PBX1
FAM60A	8.37E-08	-0.46701	0	0.176	0.00125	Undiff-G	FAM60A
WAS	8.37E-08	-0.70026	0	0.176	0.00125	Undiff-G	WAS
NCKAP1	8.50E-08	-0.40077	0.007	0.19	0.00127	Undiff-G	NCKAP1
PCCB	8.51E-08	-0.49982	0	0.176	0.001272	Undiff-G	PCCB
FRYL	8.58E-08	-0.44788	0.007	0.19	0.001283	Undiff-G	FRYL
EHMT2	8.60E-08	-0.43326	0.015	0.202	0.001285	Undiff-G	EHMT2
SLC4A7	8.69E-08	-0.37112	0.022	0.216	0.001299	Undiff-G	SLC4A7
TNFRSF1B	8.70E-08	-0.62982	0.007	0.19	0.0013	Undiff-G	TNFRSF1B
ACLY	8.75E-08	-0.22808	0.022	0.219	0.001307	Undiff-G	ACLY
ABCD4	8.78E-08	-0.20303	0.044	0.259	0.001312	Undiff-G	ABCD4
SLC44A2	8.79E-08	-0.21631	0.015	0.205	0.001314	Undiff-G	SLC44A2
SECISBP2L	8.88E-08	-0.47169	0	0.176	0.001328	Undiff-G	SECISBP2L
GTF2E2	9.00E-08	-0.40354	0.007	0.19	0.001345	Undiff-G	GTF2E2
RNFT1	9.03E-08	-0.22041	0.015	0.204	0.001349	Undiff-G	RNFT1
FERMT2	9.03E-08	-0.17205	0.015	0.204	0.001349	Undiff-G	FERMT2
DUSP23	9.03E-08	-0.63015	0.007	0.19	0.00135	Undiff-G	DUSP23
C19orf52	9.04E-08	-0.41121	0	0.176	0.001351	Undiff-G	C19orf52
PRR7	9.13E-08	-0.34826	0.007	0.19	0.001365	Undiff-G	PRR7
STK16	9.14E-08	-0.27553	0.007	0.19	0.001366	Undiff-G	STK16
CXXC1	9.19E-08	-0.46157	0	0.176	0.001374	Undiff-G	CXXC1
RIT1	9.20E-08	-0.1235	0.022	0.216	0.001375	Undiff-G	RIT1
SPRED1	9.22E-08	-0.40343	0.015	0.203	0.001377	Undiff-G	SPRED1
IPO5	9.26E-08	-0.13991	0.022	0.218	0.001384	Undiff-G	IPO5
ZNF524	9.30E-08	-0.32161	0.007	0.19	0.00139	Undiff-G	ZNF524
PTDSS1	9.31E-08	-0.32314	0.015	0.204	0.001391	Undiff-G	PTDSS1
POLR3H	9.32E-08	-0.29317	0.022	0.217	0.001393	Undiff-G	POLR3H
PAK1IP1	9.38E-08	-0.18842	0.022	0.218	0.001402	Undiff-G	PAK1IP1
ITGB8	9.46E-08	-0.22625	0.037	0.242	0.001414	Undiff-G	ITGB8
GNPAT	9.61E-08	-0.15289	0.037	0.242	0.001436	Undiff-G	GNPAT
KCTD20	9.62E-08	-0.31733	0.007	0.19	0.001438	Undiff-G	KCTD20
CNBP	9.64E-08	0.492309	0.294	0.754	0.00144	Undiff-G	CNBP
GON4L	9.66E-08	-0.38186	0.022	0.217	0.001444	Undiff-G	GON4L
MFAP1	9.68E-08	-0.45237	0	0.175	0.001446	Undiff-G	MFAP1
UBXN2A	9.71E-08	-0.21978	0.007	0.19	0.001451	Undiff-G	UBXN2A
AMFR	9.76E-08	-0.43883	0	0.175	0.001459	Undiff-G	AMFR
SMUG1	9.80E-08	-0.2308	0.022	0.215	0.001464	Undiff-G	SMUG1
CMIP	9.85E-08	-0.49032	0	0.175	0.001471	Undiff-G	CMIP
NGLY1	9.94E-08	-0.20807	0.022	0.217	0.001486	Undiff-G	NGLY1

STX5	9.98E-08	0.118438	0.051	0.271	0.001491	Undiff-G	STX5
PTRH1	1.00E-07	-0.39928	0.007	0.189	0.001497	Undiff-G	PTRH1
SDSL	1.00E-07	-0.32349	0.007	0.189	0.001501	Undiff-G	SDSL
COIL	1.01E-07	-0.43782	0.007	0.187	0.00151	Undiff-G	COIL
IWS1	1.01E-07	-0.17674	0.022	0.215	0.001512	Undiff-G	IWS1
CERS4	1.02E-07	-0.30812	0.015	0.201	0.001519	Undiff-G	CERS4
SPP1	1.02E-07	1.022616	0.64	0.856	0.001522	Undiff-G	SPP1
TMEM237	1.02E-07	-0.42147	0.007	0.189	0.001523	Undiff-G	TMEM237
TUBGCP2	1.03E-07	-0.28697	0.022	0.215	0.001541	Undiff-G	TUBGCP2
SH3BP5	1.03E-07	-0.33328	0.015	0.203	0.001543	Undiff-G	SH3BP5
FAM92A1	1.03E-07	-0.11745	0.015	0.203	0.001544	Undiff-G	FAM92A1
C7orf73	1.04E-07	0.553465	0.199	0.559	0.001547	Undiff-G	C7orf73
ARHGAP18	1.04E-07	-0.67867	0	0.174	0.001549	Undiff-G	ARHGAP18
LMBR1L	1.05E-07	-0.50662	0.007	0.186	0.001562	Undiff-G	LMBR1L
SIGMAR1	1.05E-07	-0.40399	0	0.174	0.001575	Undiff-G	SIGMAR1
GPNMB	1.06E-07	-0.55032	0.015	0.203	0.001587	Undiff-G	GPNMB
R3HDM4	1.07E-07	-0.34159	0.015	0.203	0.001598	Undiff-G	R3HDM4
DEDD2	1.07E-07	-0.48687	0	0.174	0.001603	Undiff-G	DEDD2
SPPL2A	1.07E-07	-0.48627	0.007	0.186	0.001603	Undiff-G	SPPL2A
BCHE	1.08E-07	-0.31756	0.007	0.188	0.001607	Undiff-G	BCHE
MPP1	1.08E-07	-0.32458	0.015	0.203	0.001607	Undiff-G	MPP1
PSMB9	1.08E-07	0.378794	0.125	0.408	0.001616	Undiff-G	PSMB9
OSTC	1.08E-07	0.451576	0.176	0.507	0.001618	Undiff-G	OSTC
VEZT	1.10E-07	-0.38842	0.029	0.229	0.001638	Undiff-G	VEZT
POLR2C	1.11E-07	0.404154	0.096	0.353	0.001652	Undiff-G	POLR2C
SPG7	1.11E-07	-0.1118	0.037	0.243	0.001655	Undiff-G	SPG7
MGST1	1.11E-07	1.348858	0.272	0.143	0.001655	Undiff-G	MGST1
RAB5B	1.11E-07	-0.20438	0.022	0.216	0.001657	Undiff-G	RAB5B
SEC31A	1.12E-07	-0.15094	0.029	0.23	0.001673	Undiff-G	SEC31A
GTF3C5	1.12E-07	-0.30508	0.007	0.188	0.001677	Undiff-G	GTF3C5
LINC00665	1.12E-07	-0.24105	0.007	0.188	0.001678	Undiff-G	LINC00665
STX6	1.12E-07	-0.46683	0.015	0.199	0.001679	Undiff-G	STX6
TAF7	1.14E-07	0.522511	0.206	0.557	0.001703	Undiff-G	TAF7
NIPSNAP3A	1.14E-07	-0.27111	0.015	0.202	0.001705	Undiff-G	NIPSNAP3A
PHYHIPL	1.14E-07	-0.5852	0.007	0.187	0.001706	Undiff-G	PHYHIPL
CCDC14	1.15E-07	-0.21196	0.029	0.229	0.001714	Undiff-G	CCDC14
PHPT1	1.15E-07	0.496838	0.25	0.656	0.00172	Undiff-G	PHPT1
FAM120AOS	1.16E-07	-0.36	0.007	0.188	0.001731	Undiff-G	FAM120AOS
MAP1S	1.17E-07	-0.43071	0	0.173	0.001745	Undiff-G	MAP1S
TNFSF12	1.17E-07	-0.3525	0.007	0.188	0.00175	Undiff-G	TNFSF12
PGAP1	1.18E-07	-0.44302	0.007	0.185	0.001757	Undiff-G	PGAP1
DNAJC1	1.18E-07	-0.41561	0.007	0.187	0.001766	Undiff-G	DNAJC1
WIPI1	1.19E-07	-0.33778	0.007	0.187	0.00178	Undiff-G	WIPI1
IFI44	1.20E-07	-0.54869	0.007	0.186	0.001794	Undiff-G	IFI44
DDX54	1.20E-07	-0.21765	0.015	0.201	0.001797	Undiff-G	DDX54
PSMD9	1.21E-07	0.181895	0.059	0.283	0.001804	Undiff-G	PSMD9
RABEPK	1.21E-07	-0.16508	0.015	0.201	0.001815	Undiff-G	RABEPK
CYHR1	1.22E-07	0.105269	0.059	0.281	0.001824	Undiff-G	CYHR1
RNF166	1.23E-07	-0.42826	0.007	0.187	0.001841	Undiff-G	RNF166
PRKCSH	1.24E-07	0.463523	0.14	0.434	0.001857	Undiff-G	PRKCSH
ECSIT	1.25E-07	0.296845	0.051	0.27	0.001861	Undiff-G	ECSIT

SLC2A5	1.25E-07	-0.81267	0	0.172	0.001868	Undiff-G	SLC2A5
SERPINB1	1.25E-07	-0.12822	0.051	0.269	0.00187	Undiff-G	SERPINB1
PDRG1	1.25E-07	0.393265	0.051	0.271	0.001871	Undiff-G	PDRG1
MAPKAPK5-	1.25E-07	-0.24634	0.029	0.226	0.001872	Undiff-G	MAPKAPK5-AS1
DNAJC5	1.25E-07	-0.30738	0.022	0.214	0.001873	Undiff-G	DNAJC5
PQLC3	1.26E-07	-0.4591	0.007	0.186	0.001888	Undiff-G	PQLC3
SNX14	1.26E-07	-0.20108	0.022	0.213	0.00189	Undiff-G	SNX14
HMOX2	1.28E-07	0.301049	0.066	0.297	0.001917	Undiff-G	HMOX2
AIMP2	1.29E-07	0.205673	0.037	0.243	0.001935	Undiff-G	AIMP2
CASC4	1.30E-07	-0.26408	0.029	0.226	0.001938	Undiff-G	CASC4
RP11-792A	1.30E-07	-0.19268	0.022	0.212	0.001941	Undiff-G	RP11-792A8.4
GOLPH3	1.31E-07	-0.30909	0.007	0.186	0.001956	Undiff-G	GOLPH3
SART1	1.33E-07	-0.37586	0.015	0.2	0.00198	Undiff-G	SART1
SERPINF1	1.33E-07	-0.59639	0.029	0.227	0.001982	Undiff-G	SERPINF1
ATN1	1.33E-07	-0.41425	0.007	0.185	0.001985	Undiff-G	ATN1
SRGAP1	1.33E-07	-0.13626	0.029	0.227	0.001989	Undiff-G	SRGAP1
THOC3	1.34E-07	-0.42024	0	0.172	0.002	Undiff-G	THOC3
PAK1	1.34E-07	-0.57559	0	0.172	0.002	Undiff-G	PAK1
CAB39	1.35E-07	-0.44443	0.007	0.186	0.002014	Undiff-G	CAB39
RERE	1.36E-07	-0.38358	0.022	0.213	0.002026	Undiff-G	RERE
SNAP29	1.36E-07	-0.14688	0.029	0.227	0.002029	Undiff-G	SNAP29
ADAP2	1.36E-07	-0.58653	0.007	0.186	0.002038	Undiff-G	ADAP2
CES2	1.38E-07	-0.32104	0.007	0.186	0.002056	Undiff-G	CES2
ZNF800	1.38E-07	-0.42501	0.007	0.184	0.002057	Undiff-G	ZNF800
TRAPPC12	1.38E-07	-0.37931	0.015	0.2	0.002058	Undiff-G	TRAPPC12
ZNRD1	1.38E-07	-0.33346	0.022	0.213	0.002059	Undiff-G	ZNRD1
ALKBH4	1.38E-07	-0.22421	0.015	0.2	0.002059	Undiff-G	ALKBH4
PDK4	1.40E-07	-0.74382	0	0.171	0.002087	Undiff-G	PDK4
TRIM69	1.40E-07	-0.3614	0.015	0.2	0.002097	Undiff-G	TRIM69
SP110	1.40E-07	-0.25482	0.015	0.2	0.002098	Undiff-G	SP110
PTPRN2	1.41E-07	-0.21829	0.022	0.214	0.002108	Undiff-G	PTPRN2
MYO1F	1.41E-07	-0.45	0.007	0.186	0.002112	Undiff-G	MYO1F
SOAT1	1.41E-07	-0.2813	0.015	0.2	0.002113	Undiff-G	SOAT1
ATP6V0E1	1.41E-07	0.489322	0.294	0.766	0.002114	Undiff-G	ATP6V0E1
TTC37	1.42E-07	-0.30047	0.007	0.186	0.002129	Undiff-G	TTC37
HSP90AA1	1.43E-07	0.213479	0.36	0.894	0.002135	Undiff-G	HSP90AA1
MMAB	1.43E-07	-0.24052	0.015	0.199	0.00214	Undiff-G	MMAB
B3GALNT1	1.44E-07	-0.24419	0.007	0.186	0.002146	Undiff-G	B3GALNT1
PHC2	1.45E-07	0.237906	0.059	0.279	0.002173	Undiff-G	PHC2
CKAP2	1.47E-07	-0.47401	0	0.171	0.002196	Undiff-G	CKAP2
SWAP70	1.47E-07	-0.1816	0.029	0.224	0.002197	Undiff-G	SWAP70
SCO2	1.47E-07	-0.17202	0.022	0.213	0.002198	Undiff-G	SCO2
MAPKAPK5	1.47E-07	-0.31023	0.007	0.185	0.002202	Undiff-G	MAPKAPK5
TOB2	1.48E-07	-0.22346	0.015	0.199	0.002216	Undiff-G	TOB2
F2R	1.49E-07	-0.41196	0	0.171	0.002234	Undiff-G	F2R
MLXIP	1.50E-07	-0.25881	0.007	0.185	0.002241	Undiff-G	MLXIP
LRIF1	1.50E-07	-0.15443	0.015	0.199	0.002242	Undiff-G	LRIF1
RAC3	1.51E-07	-0.38545	0.007	0.185	0.002264	Undiff-G	RAC3
ERH	1.53E-07	0.529926	0.272	0.719	0.002281	Undiff-G	ERH
USP34	1.53E-07	-0.38169	0.022	0.212	0.002289	Undiff-G	USP34
RNF213	1.55E-07	-0.20946	0.051	0.264	0.002315	Undiff-G	RNF213

DNAJC10	1.55E-07	-0.13935	0.015	0.199	0.002321	Undiff-G	DNAJC10
TIA1	1.56E-07	-0.13672	0.044	0.25	0.002333	Undiff-G	TIA1
UPF3B	1.57E-07	0.107856	0.044	0.253	0.00234	Undiff-G	UPF3B
TRIM2	1.57E-07	-0.25502	0.029	0.226	0.002347	Undiff-G	TRIM2
LTC4S	1.59E-07	-0.69419	0	0.17	0.00237	Undiff-G	LTC4S
ACAA2	1.59E-07	-0.19934	0.022	0.212	0.002381	Undiff-G	ACAA2
FAM172A	1.59E-07	-0.23953	0.015	0.198	0.002382	Undiff-G	FAM172A
TFAM	1.60E-07	-0.44467	0.007	0.183	0.002385	Undiff-G	TFAM
SNHG8	1.60E-07	0.496544	0.162	0.472	0.002391	Undiff-G	SNHG8
JAGN1	1.60E-07	0.4026	0.081	0.323	0.002397	Undiff-G	JAGN1
BCL2L1	1.62E-07	0.113924	0.051	0.266	0.002416	Undiff-G	BCL2L1
B4GALT5	1.62E-07	-0.22657	0.015	0.199	0.00242	Undiff-G	B4GALT5
PPIL1	1.63E-07	-0.24461	0.015	0.196	0.002437	Undiff-G	PPIL1
TMEM54	1.63E-07	-0.39142	0.015	0.198	0.002443	Undiff-G	TMEM54
UPF3A	1.64E-07	0.161103	0.096	0.348	0.002446	Undiff-G	UPF3A
MRAS	1.64E-07	-0.4092	0	0.17	0.002452	Undiff-G	MRAS
FNIP2	1.65E-07	-0.31165	0.015	0.198	0.002467	Undiff-G	FNIP2
PEBP1	1.65E-07	0.403642	0.324	0.809	0.00247	Undiff-G	PEBP1
LRPPRC	1.65E-07	-0.43521	0	0.17	0.002473	Undiff-G	LRPPRC
SYMPK	1.66E-07	-0.3247	0.029	0.225	0.002474	Undiff-G	SYMPK
CPSF4	1.66E-07	-0.29048	0.015	0.198	0.002478	Undiff-G	CPSF4
NEU1	1.67E-07	0.329126	0.066	0.295	0.002493	Undiff-G	NEU1
PREB	1.67E-07	-0.14728	0.022	0.212	0.0025	Undiff-G	PREB
TGFBI	1.68E-07	-0.57842	0.022	0.207	0.002505	Undiff-G	TGFBI
ZNF511	1.68E-07	-0.36571	0.015	0.198	0.002511	Undiff-G	ZNF511
MSR1	1.70E-07	-0.73003	0.007	0.182	0.002542	Undiff-G	MSR1
AP1M1	1.71E-07	-0.24249	0.029	0.225	0.002555	Undiff-G	AP1M1
RPUSD3	1.72E-07	-0.12822	0.015	0.198	0.002568	Undiff-G	RPUSD3
TRIM47	1.72E-07	-0.4525	0.007	0.182	0.00257	Undiff-G	TRIM47
RNF8	1.74E-07	-0.41354	0	0.169	0.002602	Undiff-G	RNF8
CCDC34	1.74E-07	-0.46822	0	0.169	0.002602	Undiff-G	CCDC34
DDIT3	1.74E-07	0.536125	0.14	0.431	0.002607	Undiff-G	DDIT3
PPME1	1.77E-07	-0.33515	0.007	0.183	0.00265	Undiff-G	PPME1
CHMP6	1.77E-07	-0.23649	0.015	0.197	0.00265	Undiff-G	CHMP6
WDR34	1.78E-07	-0.36	0.022	0.209	0.00266	Undiff-G	WDR34
FAM20C	1.78E-07	0.133579	0.029	0.226	0.002661	Undiff-G	FAM20C
TUBA1C	1.78E-07	0.276054	0.118	0.383	0.002664	Undiff-G	TUBA1C
GRIPAP1	1.79E-07	-0.44078	0.015	0.195	0.002675	Undiff-G	GRIPAP1
RABIF	1.79E-07	-0.2304	0.007	0.183	0.002677	Undiff-G	RABIF
EIF2AK4	1.80E-07	-0.34636	0.007	0.183	0.002692	Undiff-G	EIF2AK4
ZFHX3	1.80E-07	-0.11397	0.015	0.197	0.002695	Undiff-G	ZFHX3
GGCT	1.81E-07	0.485453	0.118	0.39	0.002698	Undiff-G	GGCT
KPNA6	1.81E-07	0.10056	0.037	0.237	0.0027	Undiff-G	KPNA6
CDK6	1.82E-07	-0.46078	0.022	0.208	0.002727	Undiff-G	CDK6
STX16	1.84E-07	-0.29963	0.022	0.211	0.002756	Undiff-G	STX16
DUSP4	1.84E-07	-0.51368	0.007	0.181	0.002756	Undiff-G	DUSP4
C16orf45	1.85E-07	0.316095	0.081	0.321	0.002765	Undiff-G	C16orf45
NDUFAF6	1.86E-07	-0.37823	0.007	0.183	0.002785	Undiff-G	NDUFAF6
BROX	1.87E-07	-0.12538	0.037	0.237	0.002801	Undiff-G	BROX
ACOX1	1.88E-07	-0.40892	0	0.168	0.002808	Undiff-G	ACOX1
ICT1	1.88E-07	0.201176	0.037	0.239	0.002811	Undiff-G	ICT1

SRFBP1	1.88E-07	-0.313	0.007	0.183	0.002813	Undiff-G	SRFBP1
FBXW11	1.88E-07	-0.12476	0.007	0.183	0.002817	Undiff-G	FBXW11
ATAD1	1.89E-07	-0.46139	0	0.168	0.002832	Undiff-G	ATAD1
FERMT3	1.89E-07	-0.66594	0	0.168	0.002832	Undiff-G	FERMT3
LINC00659	1.90E-07	0.278875	0.007	0	0.002838	Undiff-G	LINC00659
NBL1	1.90E-07	-0.47699	0.007	0.18	0.002842	Undiff-G	NBL1
UBA6	1.92E-07	-0.22174	0.015	0.197	0.002863	Undiff-G	UBA6
DESI2	1.94E-07	-0.30078	0.015	0.196	0.002899	Undiff-G	DESI2
LACTB	1.94E-07	-0.49391	0	0.168	0.002904	Undiff-G	LACTB
LSP1	1.96E-07	-0.75877	0	0.168	0.002929	Undiff-G	LSP1
PURA	1.96E-07	-0.15552	0.022	0.21	0.002933	Undiff-G	PURA
6-Sep	1.98E-07	-0.51524	0	0.168	0.002954	Undiff-G	6-Sep
MGRN1	1.98E-07	-0.14809	0.022	0.21	0.00296	Undiff-G	MGRN1
RAB35	1.99E-07	-0.42751	0	0.168	0.002979	Undiff-G	RAB35
DCAF6	1.99E-07	-0.44302	0	0.168	0.002979	Undiff-G	DCAF6
SEC23A	2.01E-07	-0.42266	0.015	0.195	0.003006	Undiff-G	SEC23A
POLK	2.02E-07	-0.40631	0.007	0.182	0.003017	Undiff-G	POLK
SMC5	2.03E-07	-0.39688	0.007	0.182	0.003034	Undiff-G	SMC5
DOHH	2.04E-07	-0.33123	0.015	0.196	0.003047	Undiff-G	DOHH
NRP1	2.04E-07	-0.45428	0	0.167	0.003055	Undiff-G	NRP1
ATF5	2.05E-07	-0.41358	0.015	0.196	0.003066	Undiff-G	ATF5
WDR6	2.06E-07	-0.25638	0.037	0.234	0.003072	Undiff-G	WDR6
SLC36A4	2.08E-07	-0.39151	0.015	0.195	0.003103	Undiff-G	SLC36A4
COQ2	2.11E-07	-0.40144	0.007	0.182	0.003154	Undiff-G	COQ2
TMUB2	2.13E-07	-0.1994	0.051	0.262	0.003182	Undiff-G	TMUB2
FBXO9	2.15E-07	-0.3736	0.022	0.208	0.003207	Undiff-G	FBXO9
TBCC	2.15E-07	0.140768	0.029	0.224	0.003214	Undiff-G	TBCC
MDM2	2.15E-07	-0.29951	0.022	0.208	0.003218	Undiff-G	MDM2
RP9	2.16E-07	-0.41159	0.007	0.181	0.003224	Undiff-G	RP9
ENG	2.17E-07	-0.62066	0	0.167	0.003241	Undiff-G	ENG
SLC1A3	2.18E-07	0.259011	0.169	0.48	0.003254	Undiff-G	SLC1A3
TNIP2	2.19E-07	-0.40387	0	0.167	0.003269	Undiff-G	TNIP2
DUSP14	2.19E-07	-0.29455	0.007	0.181	0.003272	Undiff-G	DUSP14
PSEN1	2.19E-07	-0.27325	0.007	0.181	0.003278	Undiff-G	PSEN1
MORN2	2.20E-07	0.272326	0.051	0.264	0.003281	Undiff-G	MORN2
MGEA5	2.20E-07	-0.35839	0.037	0.235	0.003288	Undiff-G	MGEA5
PPP3CA	2.21E-07	-0.14356	0.044	0.249	0.003305	Undiff-G	PPP3CA
GNG2	2.21E-07	-0.26823	0.007	0.181	0.003306	Undiff-G	GNG2
TOPORS	2.21E-07	-0.45146	0.007	0.181	0.003309	Undiff-G	TOPORS
COG4	2.22E-07	-0.41527	0.015	0.193	0.003314	Undiff-G	COG4
ZNF358	2.22E-07	-0.38184	0	0.167	0.003324	Undiff-G	ZNF358
B4GALT3	2.24E-07	-0.2063	0.029	0.22	0.003341	Undiff-G	B4GALT3
RBM22	2.24E-07	0.416783	0.074	0.305	0.003345	Undiff-G	RBM22
KLF10	2.25E-07	-0.11745	0.037	0.235	0.003365	Undiff-G	KLF10
SUPT16H	2.25E-07	0.275033	0.044	0.251	0.003367	Undiff-G	SUPT16H
SLC3A2	2.25E-07	0.51401	0.221	0.592	0.003369	Undiff-G	SLC3A2
SRP54	2.27E-07	0.107668	0.059	0.275	0.003388	Undiff-G	SRP54
KLHL24	2.27E-07	-0.31925	0.007	0.181	0.003389	Undiff-G	KLHL24
CNRIP1	2.27E-07	-0.29494	0.022	0.206	0.003396	Undiff-G	CNRIP1
NSD1	2.28E-07	-0.10743	0.037	0.235	0.003411	Undiff-G	NSD1
ZBED5	2.28E-07	-0.32452	0.007	0.181	0.003414	Undiff-G	ZBED5

MVD	2.31E-07	-0.40947	0.007	0.18	0.003453	Undiff-G	MVD
CMTM7	2.32E-07	-0.58179	0	0.166	0.003467	Undiff-G	CMTM7
TP53RK	2.33E-07	-0.34733	0.015	0.193	0.003477	Undiff-G	TP53RK
AHI1	2.34E-07	-0.27114	0.022	0.207	0.0035	Undiff-G	AHI1
TRMT112	2.36E-07	0.449074	0.279	0.712	0.003522	Undiff-G	TRMT112
ELAVL4	2.37E-07	-0.57985	0.007	0.18	0.003536	Undiff-G	ELAVL4
ELL2	2.37E-07	-0.22221	0.015	0.195	0.00354	Undiff-G	ELL2
GALK1	2.37E-07	-0.2818	0.022	0.206	0.003548	Undiff-G	GALK1
RP11-345F	2.38E-07	-0.39179	0	0.166	0.003556	Undiff-G	RP11-345P4.9
PARVB	2.38E-07	-0.48279	0	0.166	0.003556	Undiff-G	PARVB
REXO4	2.38E-07	-0.38307	0.015	0.192	0.003556	Undiff-G	REXO4
IGFBP5	2.39E-07	1.548914	0.375	0.267	0.003565	Undiff-G	IGFBP5
FAM181B	2.43E-07	-0.4853	0.022	0.205	0.003625	Undiff-G	FAM181B
CEP63	2.43E-07	-0.26268	0.007	0.18	0.003626	Undiff-G	CEP63
ACAP2	2.43E-07	-0.41765	0.007	0.18	0.003636	Undiff-G	ACAP2
NDFIP2	2.45E-07	-0.10264	0.015	0.194	0.003657	Undiff-G	NDFIP2
GPBP1L1	2.46E-07	-0.1828	0.022	0.208	0.00368	Undiff-G	GPBP1L1
IFT57	2.47E-07	0.248609	0.044	0.25	0.003688	Undiff-G	IFT57
DYNLL2	2.48E-07	-0.2174	0.022	0.208	0.003706	Undiff-G	DYNLL2
BBIP1	2.49E-07	-0.3901	0.007	0.18	0.003714	Undiff-G	BBIP1
CSRNP1	2.49E-07	-0.52252	0.015	0.193	0.003726	Undiff-G	CSRNP1
CENPT	2.50E-07	-0.38823	0.015	0.192	0.003742	Undiff-G	CENPT
FGL2	2.51E-07	-0.70381	0.007	0.179	0.003747	Undiff-G	FGL2
OLFM2	2.52E-07	-0.27696	0.015	0.193	0.00376	Undiff-G	OLFM2
RBM14	2.53E-07	-0.27159	0.015	0.194	0.003777	Undiff-G	RBM14
UBE2Q2	2.54E-07	-0.33435	0.022	0.207	0.003794	Undiff-G	UBE2Q2
NCK1	2.54E-07	0.162939	0.044	0.246	0.003803	Undiff-G	NCK1
PSMD8	2.56E-07	0.569599	0.265	0.678	0.003822	Undiff-G	PSMD8
LDLRAD3	2.58E-07	-0.41189	0.007	0.178	0.003854	Undiff-G	LDLRAD3
TMEM167B	2.58E-07	-0.21988	0.022	0.207	0.003858	Undiff-G	TMEM167B
CLK3	2.59E-07	-0.46263	0	0.165	0.003868	Undiff-G	CLK3
PLAU	2.59E-07	-0.53507	0	0.165	0.003868	Undiff-G	PLAU
FCGR1B	2.59E-07	-0.78437	0	0.165	0.003868	Undiff-G	FCGR1B
TAF15	2.61E-07	0.170593	0.059	0.276	0.003899	Undiff-G	TAF15
TBRG1	2.61E-07	0.100717	0.044	0.247	0.003906	Undiff-G	TBRG1
NDRG3	2.62E-07	-0.20951	0.007	0.179	0.003911	Undiff-G	NDRG3
HNRNPLL	2.67E-07	-0.40012	0.015	0.193	0.003993	Undiff-G	HNRNPLL
CDC27	2.68E-07	-0.40486	0	0.165	0.004	Undiff-G	CDC27
MAP3K7	2.68E-07	-0.21497	0.022	0.207	0.004011	Undiff-G	MAP3K7
LPP	2.70E-07	-0.3338	0.029	0.219	0.004032	Undiff-G	LPP
RUNX1	2.73E-07	-0.27531	0.015	0.192	0.004075	Undiff-G	RUNX1
ACAP3	2.73E-07	-0.11122	0.029	0.218	0.004082	Undiff-G	ACAP3
TRAF7	2.75E-07	-0.21174	0.015	0.191	0.004104	Undiff-G	TRAF7
ANKRD40	2.75E-07	-0.43235	0.007	0.177	0.004106	Undiff-G	ANKRD40
SDHAF1	2.76E-07	-0.38561	0.007	0.178	0.004119	Undiff-G	SDHAF1
C19orf66	2.76E-07	-0.11143	0.051	0.258	0.004119	Undiff-G	C19orf66
FAM105A	2.77E-07	-0.65984	0	0.164	0.004137	Undiff-G	FAM105A
ANAPC10	2.77E-07	-0.31584	0.007	0.179	0.004139	Undiff-G	ANAPC10
MYH9	2.79E-07	-0.47088	0.015	0.19	0.004166	Undiff-G	MYH9
SRD5A3	2.79E-07	-0.4377	0	0.164	0.004172	Undiff-G	SRD5A3
FAM127B	2.80E-07	0.330606	0.074	0.303	0.004178	Undiff-G	FAM127B

CHN1	2.81E-07	-0.10117	0.029	0.218	0.004193	Undiff-G	CHN1
CLDND1	2.82E-07	0.353054	0.125	0.397	0.00421	Undiff-G	CLDND1
UBE2Z	2.82E-07	-0.40455	0.007	0.178	0.004211	Undiff-G	UBE2Z
PPP5C	2.82E-07	-0.40855	0.007	0.178	0.004217	Undiff-G	PPP5C
CNOT3	2.84E-07	-0.36936	0	0.164	0.004242	Undiff-G	CNOT3
NPRL3	2.84E-07	-0.34288	0.015	0.192	0.004246	Undiff-G	NPRL3
ENTPD1	2.86E-07	-0.68199	0	0.164	0.004278	Undiff-G	ENTPD1
PTPN6	2.91E-07	-0.65621	0	0.164	0.00435	Undiff-G	PTPN6
ASH2L	2.92E-07	-0.2489	0.007	0.178	0.004359	Undiff-G	ASH2L
ILF3-AS1	2.93E-07	0.14431	0.081	0.312	0.004378	Undiff-G	ILF3-AS1
ILK	2.93E-07	0.447633	0.11	0.368	0.004385	Undiff-G	ILK
TGFBR1	2.94E-07	-0.51206	0	0.164	0.004387	Undiff-G	TGFBR1
DOCK4	2.94E-07	-0.54401	0	0.164	0.004387	Undiff-G	DOCK4
SIGLEC10	2.94E-07	-0.67341	0	0.164	0.004387	Undiff-G	SIGLEC10
C1orf61	2.96E-07	0.253169	0.206	0.539	0.004424	Undiff-G	C1orf61
CYB5R4	2.97E-07	-0.28898	0.007	0.178	0.004434	Undiff-G	CYB5R4
ACOT13	3.03E-07	0.124727	0.029	0.22	0.004526	Undiff-G	ACOT13
BAZ2A	3.04E-07	-0.37119	0.015	0.192	0.004536	Undiff-G	BAZ2A
STAB1	3.04E-07	-0.72065	0	0.163	0.004537	Undiff-G	STAB1
MIER1	3.04E-07	-0.42461	0.007	0.176	0.00455	Undiff-G	MIER1
THAP11	3.04E-07	-0.1431	0.007	0.178	0.00455	Undiff-G	THAP11
CEP57	3.06E-07	-0.26838	0.037	0.232	0.004571	Undiff-G	CEP57
EIF2B4	3.06E-07	-0.25099	0.015	0.19	0.00458	Undiff-G	EIF2B4
RPIA	3.07E-07	-0.24822	0.015	0.19	0.004582	Undiff-G	RPIA
FBXL3	3.07E-07	-0.19683	0.007	0.178	0.004589	Undiff-G	FBXL3
ATP13A1	3.09E-07	-0.39388	0	0.163	0.004613	Undiff-G	ATP13A1
ATP5J2	3.09E-07	0.369779	0.324	0.813	0.004624	Undiff-G	ATP5J2
POLR2J3	3.11E-07	0.280427	0.096	0.342	0.004641	Undiff-G	POLR2J3
MTHFS	3.11E-07	-0.28708	0.015	0.191	0.004646	Undiff-G	MTHFS
ZNF688	3.11E-07	-0.35468	0.007	0.178	0.004647	Undiff-G	ZNF688
SF3B3	3.11E-07	-0.19618	0.015	0.192	0.004655	Undiff-G	SF3B3
C14orf119	3.12E-07	0.511181	0.081	0.316	0.004664	Undiff-G	C14orf119
UBE2D4	3.16E-07	-0.28587	0.015	0.192	0.00472	Undiff-G	UBE2D4
PLEK	3.16E-07	-0.69711	0.007	0.177	0.004725	Undiff-G	PLEK
GCAT	3.17E-07	-0.35588	0	0.163	0.004731	Undiff-G	GCAT
PDPK1	3.17E-07	-0.39668	0.015	0.189	0.004738	Undiff-G	PDPK1
MAGT1	3.19E-07	-0.27488	0.022	0.205	0.004772	Undiff-G	MAGT1
IRF2BPL	3.20E-07	0.135742	0.044	0.244	0.004779	Undiff-G	IRF2BPL
TYMS	3.22E-07	-0.46182	0	0.163	0.004811	Undiff-G	TYMS
IRF7	3.25E-07	-0.4682	0	0.163	0.004851	Undiff-G	IRF7
RP11-620J	3.27E-07	0.174217	0.059	0.272	0.004891	Undiff-G	RP11-620J15.3
YRDC	3.27E-07	-0.39745	0	0.163	0.004892	Undiff-G	YRDC
HAMP	3.27E-07	-0.84567	0	0.163	0.004892	Undiff-G	HAMP
XIST	3.30E-07	-0.21448	0.059	0.268	0.004938	Undiff-G	XIST
SCCPDH	3.31E-07	0.488855	0.118	0.38	0.00494	Undiff-G	SCCPDH
SMAD2	3.31E-07	-0.12176	0.015	0.191	0.004947	Undiff-G	SMAD2
ZNF581	3.32E-07	-0.46171	0.007	0.175	0.004969	Undiff-G	ZNF581
GATAD2B	3.33E-07	-0.42335	0	0.162	0.004974	Undiff-G	GATAD2B
TBL2	3.33E-07	-0.22884	0.015	0.191	0.004977	Undiff-G	TBL2
CLEC7A	3.33E-07	-0.57931	0.007	0.177	0.004977	Undiff-G	CLEC7A
SLC25A37	3.35E-07	0.123006	0.081	0.313	0.005004	Undiff-G	SLC25A37

PHYKPL	3.36E-07	-0.37034	0.022	0.204	0.005016	Undiff-G	PHYKPL
KIF9	3.37E-07	-0.4192	0.007	0.176	0.005041	Undiff-G	KIF9
CANT1	3.39E-07	-0.16084	0.007	0.177	0.005072	Undiff-G	CANT1
LYN	3.40E-07	-0.57006	0.007	0.176	0.005081	Undiff-G	LYN
EGLN1	3.41E-07	-0.30951	0.015	0.189	0.005096	Undiff-G	EGLN1
EIF4G3	3.41E-07	-0.40525	0.007	0.176	0.005096	Undiff-G	EIF4G3
FRMD4A	3.42E-07	-0.55973	0.015	0.19	0.005105	Undiff-G	FRMD4A
ISY1	3.43E-07	-0.45363	0.007	0.176	0.00512	Undiff-G	ISY1
CREBL2	3.43E-07	-0.36879	0.015	0.191	0.005123	Undiff-G	CREBL2
SELM	3.47E-07	-0.30802	0.015	0.19	0.005186	Undiff-G	SELM
PGM3	3.47E-07	-0.38201	0.007	0.176	0.005187	Undiff-G	PGM3
FNDC3B	3.49E-07	-0.41223	0.007	0.176	0.005214	Undiff-G	FNDC3B
PKIG	3.50E-07	0.367706	0.081	0.313	0.005235	Undiff-G	PKIG
SLC7A7	3.52E-07	-0.64161	0.007	0.176	0.005255	Undiff-G	SLC7A7
NMB	3.52E-07	-0.347	0.022	0.204	0.005265	Undiff-G	NMB
ZNF655	3.53E-07	-0.12774	0.044	0.242	0.005278	Undiff-G	ZNF655
ACBD3	3.54E-07	-0.1929	0.007	0.176	0.005289	Undiff-G	ACBD3
LTV1	3.57E-07	-0.42192	0.015	0.188	0.00534	Undiff-G	LTV1
CCDC130	3.60E-07	-0.30459	0.037	0.23	0.005383	Undiff-G	CCDC130
RAP1GDS1	3.65E-07	-0.42475	0.007	0.176	0.005452	Undiff-G	RAP1GDS1
GRIA2	3.65E-07	-0.39524	0.029	0.214	0.005455	Undiff-G	GRIA2
SF3A1	3.69E-07	-0.29896	0.007	0.176	0.00551	Undiff-G	SF3A1
BPHL	3.69E-07	-0.15696	0.007	0.176	0.005514	Undiff-G	BPHL
ZDHHC3	3.72E-07	-0.21078	0.022	0.202	0.005557	Undiff-G	ZDHHC3
TM9SF4	3.73E-07	-0.24446	0.015	0.19	0.005579	Undiff-G	TM9SF4
NDUFC1	3.77E-07	0.592702	0.206	0.556	0.00563	Undiff-G	NDUFC1
SULF2	3.77E-07	-0.39359	0	0.161	0.005639	Undiff-G	SULF2
CBY1	3.77E-07	-0.41618	0	0.161	0.005639	Undiff-G	CBY1
CIAO1	3.77E-07	0.135459	0.044	0.245	0.005641	Undiff-G	CIAO1
MTHFD1	3.80E-07	-0.22599	0.015	0.189	0.005684	Undiff-G	MTHFD1
THAP4	3.84E-07	-0.39644	0	0.161	0.005734	Undiff-G	THAP4
TTLL7	3.84E-07	-0.32868	0.044	0.239	0.005738	Undiff-G	TTLL7
C15orf61	3.85E-07	-0.22933	0.007	0.175	0.00575	Undiff-G	C15orf61
STAU2	3.87E-07	-0.26899	0.007	0.175	0.00579	Undiff-G	STAU2
FJX1	3.87E-07	-0.2828	0.022	0.204	0.00579	Undiff-G	FJX1
SCD	3.93E-07	-0.15181	0.029	0.214	0.005867	Undiff-G	SCD
DAG1	3.94E-07	-0.26203	0.007	0.175	0.005894	Undiff-G	DAG1
FLII	3.97E-07	-0.25671	0.015	0.189	0.005932	Undiff-G	FLII
RHOC	4.00E-07	0.510615	0.199	0.537	0.00598	Undiff-G	RHOC
NUDCD3	4.01E-07	-0.41375	0.022	0.2	0.005985	Undiff-G	NUDCD3
EDNRB	4.02E-07	-0.37767	0.015	0.189	0.006013	Undiff-G	EDNRB
MSMO1	4.04E-07	-0.13347	0.022	0.203	0.006036	Undiff-G	MSMO1
UQCRQ	4.06E-07	0.411738	0.324	0.805	0.006069	Undiff-G	UQCRQ
PAXIP1-AS	4.07E-07	-0.40913	0	0.16	0.006079	Undiff-G	PAXIP1-AS1
ZNF414	4.11E-07	-0.32026	0.015	0.187	0.006141	Undiff-G	ZNF414
NXF1	4.15E-07	-0.40057	0.022	0.2	0.006196	Undiff-G	NXF1
IRF8	4.17E-07	-0.65787	0	0.16	0.006232	Undiff-G	IRF8
UCK1	4.17E-07	-0.22163	0.015	0.187	0.006237	Undiff-G	UCK1
DNAAF2	4.18E-07	-0.23576	0.007	0.175	0.006248	Undiff-G	DNAAF2
TM6SF1	4.21E-07	-0.72292	0	0.16	0.006285	Undiff-G	TM6SF1
GAK	4.24E-07	-0.43173	0	0.16	0.006337	Undiff-G	GAK

SYPL1	4.28E-07	0.511028	0.132	0.409	0.0064	Undiff-G	SYPL1
UGCG	4.31E-07	-0.44988	0	0.16	0.006444	Undiff-G	UGCG
VARS	4.35E-07	-0.39922	0	0.16	0.006498	Undiff-G	VARS
GPX1	4.42E-07	0.368478	0.324	0.819	0.006607	Undiff-G	GPX1
CASP1	4.44E-07	-0.12129	0.037	0.228	0.006631	Undiff-G	CASP1
XIAP	4.44E-07	-0.10035	0.029	0.215	0.006639	Undiff-G	XIAP
GMPS	4.46E-07	-0.31481	0.015	0.186	0.006658	Undiff-G	GMPS
CHMP2B	4.46E-07	0.442245	0.081	0.31	0.006661	Undiff-G	CHMP2B
CLASP2	4.48E-07	-0.43044	0.022	0.199	0.006697	Undiff-G	CLASP2
NLN	4.51E-07	-0.36047	0.015	0.187	0.006738	Undiff-G	NLN
PEX16	4.51E-07	0.222674	0.037	0.23	0.006744	Undiff-G	PEX16
REEP5	4.53E-07	0.601849	0.228	0.589	0.006763	Undiff-G	REEP5
GNS	4.55E-07	-0.17815	0.022	0.201	0.006794	Undiff-G	GNS
IMPDH1	4.57E-07	-0.40341	0	0.159	0.00683	Undiff-G	IMPDH1
PRRX1	4.58E-07	-0.43056	0.007	0.173	0.006841	Undiff-G	PRRX1
TMEM97	4.58E-07	-0.21321	0.007	0.174	0.006847	Undiff-G	TMEM97
BCAR1	4.58E-07	-0.18066	0.007	0.174	0.006849	Undiff-G	BCAR1
DKK3	4.60E-07	-0.26582	0.022	0.202	0.006879	Undiff-G	DKK3
TNRC6C	4.62E-07	-0.3048	0.007	0.174	0.006897	Undiff-G	TNRC6C
ANAPC7	4.62E-07	-0.25581	0.007	0.174	0.006901	Undiff-G	ANAPC7
HSPA13	4.63E-07	-0.10716	0.022	0.202	0.006913	Undiff-G	HSPA13
ALAS1	4.64E-07	-0.34478	0.007	0.173	0.006933	Undiff-G	ALAS1
S100A8	4.67E-07	-0.60373	0.015	0.187	0.006986	Undiff-G	S100A8
FNBP1L	4.69E-07	-0.40175	0.015	0.187	0.007005	Undiff-G	FNBP1L
PDHX	4.73E-07	-0.27109	0.007	0.173	0.007074	Undiff-G	PDHX
NOP16	4.74E-07	-0.10407	0.022	0.201	0.007077	Undiff-G	NOP16
GAL3ST4	4.81E-07	-0.46133	0.007	0.173	0.007192	Undiff-G	GAL3ST4
OXA1L	4.82E-07	0.169929	0.081	0.304	0.007207	Undiff-G	OXA1L
TRIB1	4.84E-07	-0.56242	0	0.159	0.00724	Undiff-G	TRIB1
C8orf82	4.87E-07	-0.34252	0.007	0.173	0.007285	Undiff-G	C8orf82
ABHD11	4.89E-07	-0.47453	0.007	0.171	0.007311	Undiff-G	ABHD11
RBM15B	4.90E-07	-0.2344	0.007	0.173	0.00732	Undiff-G	RBM15B
MKNK2	4.91E-07	-0.33935	0.015	0.186	0.007334	Undiff-G	MKNK2
SLC38A10	4.93E-07	-0.27043	0.022	0.201	0.007361	Undiff-G	SLC38A10
RFC4	4.93E-07	-0.4383	0	0.158	0.007361	Undiff-G	RFC4
RPAP2	4.93E-07	-0.37637	0.015	0.186	0.007372	Undiff-G	RPAP2
SMAD5	4.93E-07	-0.39719	0.007	0.172	0.007372	Undiff-G	SMAD5
TOR3A	4.94E-07	-0.24726	0.029	0.212	0.007379	Undiff-G	TOR3A
AP3B1	4.96E-07	-0.40008	0.007	0.172	0.007407	Undiff-G	AP3B1
HOMER1	4.97E-07	-0.37815	0	0.158	0.007422	Undiff-G	HOMER1
TSC22D2	4.97E-07	-0.45268	0	0.158	0.007422	Undiff-G	TSC22D2
COPS8	4.99E-07	0.735599	0.169	0.473	0.007459	Undiff-G	COPS8
SOCS2	5.01E-07	-0.41857	0	0.158	0.007484	Undiff-G	SOCS2
JAM2	5.02E-07	0.144928	0.051	0.255	0.007507	Undiff-G	JAM2
C11orf49	5.03E-07	0.15293	0.044	0.239	0.007521	Undiff-G	C11orf49
PPP2R4	5.05E-07	-0.17897	0.044	0.239	0.007541	Undiff-G	PPP2R4
PTGS1	5.05E-07	-0.56109	0	0.158	0.007547	Undiff-G	PTGS1
GGH	5.06E-07	0.181153	0.059	0.265	0.007557	Undiff-G	GGH
TBC1D20	5.06E-07	-0.36711	0.015	0.185	0.007567	Undiff-G	TBC1D20
UBA5	5.07E-07	-0.28125	0.015	0.186	0.00758	Undiff-G	UBA5
CKAP5	5.09E-07	-0.39167	0	0.158	0.00761	Undiff-G	CKAP5

BIRC6	5.15E-07	-0.30944	0.015	0.186	0.007692	Undiff-G	BIRC6
KIAA0319L	5.16E-07	-0.28033	0.015	0.186	0.007707	Undiff-G	KIAA0319L
SETD3	5.18E-07	-0.41537	0	0.158	0.007737	Undiff-G	SETD3
CTBS	5.18E-07	-0.46249	0	0.158	0.007737	Undiff-G	CTBS
CFD	5.18E-07	-0.67813	0	0.158	0.007737	Undiff-G	CFD
REV3L	5.21E-07	-0.35777	0.029	0.211	0.007789	Undiff-G	REV3L
BRAT1	5.22E-07	-0.3894	0.015	0.186	0.007807	Undiff-G	BRAT1
NDUFA7	5.23E-07	1.117351	0.125	0.042	0.007821	Undiff-G	NDUFA7
NOL8	5.26E-07	-0.15969	0.015	0.186	0.007862	Undiff-G	NOL8
PPFIBP1	5.26E-07	-0.39405	0	0.158	0.007867	Undiff-G	PPFIBP1
C12orf57	5.27E-07	0.572804	0.199	0.54	0.00788	Undiff-G	C12orf57
MAP2K7	5.28E-07	-0.34897	0.022	0.198	0.007888	Undiff-G	MAP2K7
RRP1B	5.31E-07	-0.37258	0	0.158	0.007932	Undiff-G	RRP1B
PALLD	5.33E-07	-0.27496	0.022	0.2	0.007968	Undiff-G	PALLD
NEDD9	5.35E-07	-0.34568	0.037	0.223	0.007991	Undiff-G	NEDD9
MAGI1	5.35E-07	-0.35857	0.022	0.199	0.007995	Undiff-G	MAGI1
PIGS	5.35E-07	-0.21547	0.015	0.185	0.007999	Undiff-G	PIGS
NOP58	5.36E-07	0.264158	0.074	0.295	0.008005	Undiff-G	NOP58
FH	5.38E-07	0.186511	0.037	0.228	0.008034	Undiff-G	FH
CKAP4	5.46E-07	-0.2716	0.029	0.211	0.008159	Undiff-G	CKAP4
CLIP3	5.48E-07	-0.17966	0.022	0.199	0.008197	Undiff-G	CLIP3
SAC3D1	5.55E-07	-0.22393	0.007	0.172	0.00829	Undiff-G	SAC3D1
UBE2L6	5.55E-07	0.526609	0.169	0.477	0.008295	Undiff-G	UBE2L6
ACD	5.55E-07	-0.4367	0.007	0.17	0.008298	Undiff-G	ACD
PAG1	5.61E-07	-0.46671	0.007	0.171	0.008379	Undiff-G	PAG1
BAIAP2	5.68E-07	-0.1387	0.022	0.199	0.008484	Undiff-G	BAIAP2
SPTSSA	5.71E-07	0.532682	0.147	0.431	0.008527	Undiff-G	SPTSSA
SVIP	5.73E-07	-0.51545	0.007	0.171	0.008557	Undiff-G	SVIP
PEX19	5.75E-07	-0.35936	0.007	0.171	0.008589	Undiff-G	PEX19
CCDC91	5.81E-07	-0.13754	0.022	0.199	0.008683	Undiff-G	CCDC91
TRPT1	5.92E-07	0.176038	0.037	0.225	0.00885	Undiff-G	TRPT1
RP11-161M	5.95E-07	-0.51489	0.007	0.168	0.008886	Undiff-G	RP11-161M6.2
PURB	5.95E-07	-0.37936	0.007	0.171	0.008888	Undiff-G	PURB
FSD1	6.01E-07	-0.37114	0	0.156	0.008982	Undiff-G	FSD1
ABCD3	6.03E-07	-0.36296	0.022	0.197	0.009015	Undiff-G	ABCD3
TRIM33	6.04E-07	-0.3291	0.015	0.185	0.009027	Undiff-G	TRIM33
TDP2	6.14E-07	-0.22378	0.015	0.185	0.009173	Undiff-G	TDP2
PUM2	6.16E-07	-0.37508	0	0.156	0.009208	Undiff-G	PUM2
TRA2A	6.16E-07	0.304986	0.103	0.346	0.009213	Undiff-G	TRA2A
DPYSL4	6.21E-07	-0.37429	0	0.156	0.009285	Undiff-G	DPYSL4
MED24	6.23E-07	-0.36922	0.007	0.17	0.009316	Undiff-G	MED24
OGFRL1	6.24E-07	-0.23192	0.015	0.183	0.009332	Undiff-G	OGFRL1
MAP3K11	6.25E-07	-0.20011	0.015	0.184	0.009343	Undiff-G	MAP3K11
FAM58A	6.26E-07	-0.37961	0	0.156	0.009362	Undiff-G	FAM58A
DOK1	6.26E-07	-0.46377	0	0.156	0.009362	Undiff-G	DOK1
DGKZ	6.30E-07	-0.33715	0.022	0.198	0.009421	Undiff-G	DGKZ
IFRD2	6.40E-07	-0.22804	0.029	0.211	0.009557	Undiff-G	IFRD2
TPST2	6.41E-07	-0.45285	0.007	0.169	0.009586	Undiff-G	TPST2
APPBP2	6.42E-07	-0.18106	0.029	0.211	0.009596	Undiff-G	APPBP2
SLC31A1	6.45E-07	-0.44494	0.007	0.17	0.009637	Undiff-G	SLC31A1
TRIM36	6.45E-07	-0.35755	0.015	0.184	0.00964	Undiff-G	TRIM36

EGR2	6.48E-07	-0.48452	0.022	0.194	0.009684	Undiff-G	EGR2
NMI	6.50E-07	-0.16562	0.015	0.184	0.009713	Undiff-G	NMI
HK1	6.59E-07	-0.25423	0.022	0.197	0.009852	Undiff-G	HK1
IGSF6	6.64E-07	-0.69681	0	0.155	0.00992	Undiff-G	IGSF6
REV1	6.64E-07	-0.38962	0.007	0.169	0.009928	Undiff-G	REV1
STXBP3	6.68E-07	-0.21418	0.022	0.196	0.009984	Undiff-G	STXBP3
RBFA	6.68E-07	-0.38134	0.007	0.169	0.009986	Undiff-G	RBFA
ST6GALNAC	6.69E-07	-0.40569	0	0.155	0.010002	Undiff-G	ST6GALNAC6
EZH2	6.69E-07	-0.42658	0	0.155	0.010002	Undiff-G	EZH2
PLD4	6.69E-07	-0.69849	0	0.155	0.010002	Undiff-G	PLD4
HOTAIRM1	6.69E-07	-0.2191	0.015	0.184	0.010003	Undiff-G	HOTAIRM1
TRADD	6.70E-07	-0.36373	0.007	0.17	0.010007	Undiff-G	TRADD
AKAP7	6.71E-07	-0.33542	0.022	0.197	0.010035	Undiff-G	AKAP7
XPOT	6.78E-07	-0.36599	0.007	0.169	0.010137	Undiff-G	XPOT
CD69	6.80E-07	-0.78034	0	0.155	0.010169	Undiff-G	CD69
ALDH6A1	6.82E-07	-0.15664	0.029	0.209	0.010189	Undiff-G	ALDH6A1
MBOAT2	6.85E-07	-0.26963	0.015	0.184	0.010241	Undiff-G	MBOAT2
TRIM9	6.87E-07	-0.11333	0.044	0.235	0.010264	Undiff-G	TRIM9
SBDS	6.87E-07	0.569279	0.235	0.614	0.010272	Undiff-G	SBDS
UBALD1	6.91E-07	-0.36087	0.007	0.169	0.010326	Undiff-G	UBALD1
YIPF1	6.92E-07	-0.15172	0.015	0.182	0.010334	Undiff-G	YIPF1
IBTK	6.98E-07	-0.26904	0.007	0.169	0.010437	Undiff-G	IBTK
NIPSNAP1	6.99E-07	-0.19485	0.007	0.169	0.010448	Undiff-G	NIPSNAP1
SNX1	7.02E-07	0.125063	0.037	0.224	0.010487	Undiff-G	SNX1
CSNK1G3	7.03E-07	-0.35857	0	0.155	0.01051	Undiff-G	CSNK1G3
C21orf2	7.10E-07	-0.18506	0.022	0.197	0.010612	Undiff-G	C21orf2
HCK	7.11E-07	-0.42646	0.007	0.169	0.010622	Undiff-G	HCK
BTBD10	7.16E-07	-0.12711	0.015	0.183	0.010704	Undiff-G	BTBD10
TRIP12	7.18E-07	-0.38445	0.007	0.167	0.010729	Undiff-G	TRIP12
GGT7	7.19E-07	-0.37659	0.007	0.167	0.01075	Undiff-G	GGT7
SDC2	7.20E-07	-0.30302	0.022	0.194	0.010759	Undiff-G	SDC2
ELAC2	7.21E-07	-0.36913	0	0.154	0.010774	Undiff-G	ELAC2
CSF3R	7.21E-07	-0.68018	0	0.154	0.010774	Undiff-G	CSF3R
COLGALT1	7.28E-07	-0.23246	0.022	0.194	0.010875	Undiff-G	COLGALT1
ATP1B2	7.30E-07	-0.25539	0.044	0.235	0.010915	Undiff-G	ATP1B2
SUN1	7.32E-07	-0.17381	0.029	0.21	0.010937	Undiff-G	SUN1
DRAXIN	7.33E-07	-0.40557	0	0.154	0.010953	Undiff-G	DRAXIN
COQ10B	7.33E-07	0.340217	0.059	0.265	0.01096	Undiff-G	COQ10B
TTC17	7.37E-07	-0.17075	0.037	0.221	0.01101	Undiff-G	TTC17
HIVEP3	7.39E-07	-0.36631	0	0.154	0.011044	Undiff-G	HIVEP3
FAM26F	7.47E-07	-0.49896	0.007	0.169	0.011158	Undiff-G	FAM26F
DUSP12	7.48E-07	0.286146	0.044	0.238	0.011176	Undiff-G	DUSP12
TJP1	7.54E-07	-0.19052	0.015	0.182	0.011263	Undiff-G	TJP1
HLA-DPA1	7.56E-07	0.17142	0.213	0.537	0.011295	Undiff-G	HLA-DPA1
UBN1	7.58E-07	-0.34907	0.007	0.168	0.011329	Undiff-G	UBN1
PXDC1	7.64E-07	-0.3719	0	0.154	0.011414	Undiff-G	PXDC1
CCDC174	7.66E-07	-0.2402	0.022	0.195	0.011447	Undiff-G	CCDC174
CYP27A1	7.68E-07	-0.43405	0.007	0.166	0.011478	Undiff-G	CYP27A1
KPNA4	7.73E-07	-0.27084	0.015	0.182	0.011556	Undiff-G	KPNA4
GAS2L1	7.76E-07	-0.35124	0	0.154	0.011604	Undiff-G	GAS2L1
MBD1	7.89E-07	-0.37432	0	0.153	0.011797	Undiff-G	MBD1

VAMP7	7.92E-07	-0.3704	0.007	0.168	0.011832	Undiff-G	VAMP7
OLIG2	7.95E-07	-0.50957	0.015	0.18	0.011886	Undiff-G	OLIG2
TEX261	7.98E-07	-0.12353	0.007	0.168	0.011923	Undiff-G	TEX261
CCNDBP1	7.98E-07	-0.41435	0.029	0.207	0.011931	Undiff-G	CCNDBP1
ZEB1	8.01E-07	-0.33081	0.022	0.195	0.011969	Undiff-G	ZEB1
BCS1L	8.04E-07	-0.12729	0.007	0.168	0.012021	Undiff-G	BCS1L
RIOK3	8.05E-07	-0.16029	0.022	0.195	0.012036	Undiff-G	RIOK3
PAPD4	8.14E-07	-0.22671	0.015	0.181	0.012168	Undiff-G	PAPD4
TCEAL7	8.15E-07	0.415753	0.081	0.305	0.012174	Undiff-G	TCEAL7
FADS1	8.21E-07	-0.22754	0.037	0.22	0.012267	Undiff-G	FADS1
USP3	8.24E-07	-0.25603	0.007	0.168	0.012314	Undiff-G	USP3
POLE3	8.27E-07	0.175285	0.051	0.249	0.012355	Undiff-G	POLE3
FAM134B	8.28E-07	-0.3757	0.007	0.167	0.012373	Undiff-G	FAM134B
ADK	8.30E-07	-0.33437	0.007	0.167	0.012401	Undiff-G	ADK
RRP7A	8.34E-07	0.436957	0.074	0.29	0.012467	Undiff-G	RRP7A
EML4	8.36E-07	-0.30277	0.022	0.193	0.012486	Undiff-G	EML4
SCRN1	8.41E-07	-0.2475	0.015	0.181	0.012567	Undiff-G	SCRN1
FAM45A	8.44E-07	-0.10696	0.022	0.193	0.012608	Undiff-G	FAM45A
TNPO2	8.44E-07	-0.23785	0.015	0.181	0.012617	Undiff-G	TNPO2
CEBPZ	8.45E-07	0.375252	0.066	0.274	0.012623	Undiff-G	CEBPZ
RGL2	8.46E-07	-0.1821	0.029	0.206	0.012636	Undiff-G	RGL2
UCHL3	8.63E-07	0.199021	0.051	0.25	0.012896	Undiff-G	UCHL3
NR4A3	8.64E-07	-0.64469	0	0.153	0.012915	Undiff-G	NR4A3
SCIN	8.64E-07	-0.74357	0	0.153	0.012915	Undiff-G	SCIN
HPRT1	8.78E-07	0.133951	0.044	0.233	0.013115	Undiff-G	HPRT1
CRNKL1	8.79E-07	-0.35849	0	0.152	0.013129	Undiff-G	CRNKL1
PNKP	8.79E-07	-0.38887	0.015	0.18	0.013134	Undiff-G	PNKP
C18orf21	8.80E-07	-0.19909	0.007	0.167	0.013146	Undiff-G	C18orf21
RIMKLB	8.86E-07	-0.39821	0	0.152	0.013237	Undiff-G	RIMKLB
PSMB3	8.86E-07	0.59865	0.287	0.708	0.013241	Undiff-G	PSMB3
FAM104B	8.95E-07	-0.13018	0.022	0.193	0.013381	Undiff-G	FAM104B
MNDA	8.97E-07	-0.56103	0.015	0.179	0.013399	Undiff-G	MNDA
C20orf96	9.00E-07	-0.36484	0	0.152	0.013457	Undiff-G	C20orf96
ATP13A3	9.00E-07	-0.39419	0	0.152	0.013457	Undiff-G	ATP13A3
ADIPOR2	9.00E-07	-0.43544	0	0.152	0.013457	Undiff-G	ADIPOR2
ZBTB1	9.04E-07	-0.36317	0.007	0.166	0.013515	Undiff-G	ZBTB1
PTK2	9.05E-07	-0.25138	0.015	0.18	0.013519	Undiff-G	PTK2
ZNF3	9.06E-07	-0.23361	0.029	0.207	0.013538	Undiff-G	ZNF3
RFWD2	9.18E-07	-0.22614	0.007	0.166	0.013711	Undiff-G	RFWD2
CHMP7	9.18E-07	-0.15587	0.015	0.179	0.013721	Undiff-G	CHMP7
SLC9A3R1	9.23E-07	-0.39905	0	0.152	0.013793	Undiff-G	SLC9A3R1
DAB2	9.23E-07	-0.6382	0	0.152	0.013793	Undiff-G	DAB2
PROX1	9.27E-07	-0.40338	0.007	0.166	0.013849	Undiff-G	PROX1
C2orf76	9.46E-07	-0.34886	0.007	0.166	0.01414	Undiff-G	C2orf76
SERPINA1	9.47E-07	0.105129	0.081	0.302	0.014156	Undiff-G	SERPINA1
NRSN2	9.47E-07	-0.16434	0.007	0.166	0.014157	Undiff-G	NRSN2
PRKACA	9.54E-07	-0.38515	0	0.151	0.014253	Undiff-G	PRKACA
FAIM2	9.57E-07	-0.26074	0.022	0.193	0.014305	Undiff-G	FAIM2
SSBP2	9.58E-07	0.181973	0.066	0.272	0.01432	Undiff-G	SSBP2
HBS1L	9.71E-07	-0.27441	0.022	0.193	0.014511	Undiff-G	HBS1L
CPNE2	9.76E-07	-0.3748	0.015	0.179	0.014579	Undiff-G	CPNE2

TRIM37	9.78E-07	-0.3351	0	0.151	0.014609	Undiff-G	TRIM37
LMNB1	9.78E-07	-0.43113	0	0.151	0.014609	Undiff-G	LMNB1
PAQR4	9.79E-07	-0.27711	0.007	0.166	0.014636	Undiff-G	PAQR4
NAA38	9.82E-07	0.533308	0.176	0.475	0.014675	Undiff-G	NAA38
ICAM1	9.86E-07	-0.44414	0.007	0.166	0.014735	Undiff-G	ICAM1
TRNAU1AP	9.89E-07	-0.18893	0.029	0.204	0.014786	Undiff-G	TRNAU1AP
TCEB3	9.93E-07	-0.31654	0.015	0.178	0.01484	Undiff-G	TCEB3
CTD-2090I	9.98E-07	1.26912	0.14	0.052	0.01492	Undiff-G	CTD-2090I13.1
FOLR2	1.00E-06	-0.49991	0.015	0.18	0.014943	Undiff-G	FOLR2
NRXN1	1.01E-06	-0.5446	0.007	0.165	0.015037	Undiff-G	NRXN1
TMEM181	1.02E-06	-0.2866	0.007	0.165	0.015227	Undiff-G	TMEM181
CTCF	1.02E-06	-0.24202	0.007	0.165	0.015254	Undiff-G	CTCF
FAM228B	1.02E-06	-0.13983	0.037	0.217	0.015281	Undiff-G	FAM228B
ARFIP1	1.02E-06	-0.38329	0.007	0.164	0.015284	Undiff-G	ARFIP1
TCERG1	1.03E-06	-0.21409	0.029	0.206	0.015334	Undiff-G	TCERG1
TLE4	1.03E-06	-0.33397	0.007	0.165	0.015349	Undiff-G	TLE4
VHL	1.03E-06	-0.37119	0.007	0.165	0.015364	Undiff-G	VHL
DCAF10	1.03E-06	-0.13867	0.015	0.179	0.015382	Undiff-G	DCAF10
TANC2	1.03E-06	-0.35264	0.022	0.191	0.015404	Undiff-G	TANC2
FAM103A1	1.03E-06	0.237818	0.051	0.245	0.01544	Undiff-G	FAM103A1
ASXL1	1.03E-06	-0.3312	0.015	0.179	0.015445	Undiff-G	ASXL1
GNG5	1.03E-06	0.468925	0.257	0.649	0.015449	Undiff-G	GNG5
NELFB	1.04E-06	-0.33521	0	0.151	0.015472	Undiff-G	NELFB
SNAP47	1.04E-06	-0.1012	0.029	0.204	0.015492	Undiff-G	SNAP47
RP11-395G	1.04E-06	-0.16206	0.015	0.179	0.015532	Undiff-G	RP11-395G23.3
ZNF821	1.04E-06	-0.35625	0.007	0.165	0.015571	Undiff-G	ZNF821
RND2	1.05E-06	-0.19381	0.007	0.165	0.015627	Undiff-G	RND2
SDAD1	1.05E-06	0.183517	0.037	0.221	0.015637	Undiff-G	SDAD1
IQGAP2	1.05E-06	-0.39017	0	0.15	0.015728	Undiff-G	IQGAP2
ATXN1	1.05E-06	-0.35818	0.007	0.165	0.015755	Undiff-G	ATXN1
GPSM2	1.06E-06	-0.36637	0.015	0.179	0.015806	Undiff-G	GPSM2
LINC00116	1.06E-06	0.355094	0.118	0.367	0.015812	Undiff-G	LINC00116
CNPPD1	1.06E-06	0.163465	0.029	0.207	0.015863	Undiff-G	CNPPD1
SNX9	1.06E-06	-0.40529	0.022	0.191	0.015902	Undiff-G	SNX9
NDUFAF2	1.07E-06	0.497143	0.066	0.275	0.015944	Undiff-G	NDUFAF2
PHACTR4	1.07E-06	-0.16553	0.029	0.204	0.015978	Undiff-G	PHACTR4
HNRNPUL2	1.07E-06	-0.34016	0.007	0.165	0.01598	Undiff-G	HNRNPUL2
RNF20	1.07E-06	-0.38656	0	0.15	0.015988	Undiff-G	RNF20
CDC73	1.08E-06	-0.10742	0.022	0.191	0.016119	Undiff-G	CDC73
MLC1	1.08E-06	-0.38674	0	0.15	0.01612	Undiff-G	MLC1
ARHGAP24	1.08E-06	-0.6611	0	0.15	0.01612	Undiff-G	ARHGAP24
GLIPR1	1.08E-06	0.269087	0.103	0.337	0.016147	Undiff-G	GLIPR1
TNFRSF21	1.08E-06	-0.22404	0.007	0.165	0.016151	Undiff-G	TNFRSF21
RCAN1	1.08E-06	0.178032	0.066	0.27	0.016152	Undiff-G	RCAN1
FIBIN	1.08E-06	-0.14366	0.044	0.229	0.016162	Undiff-G	FIBIN
GSKIP	1.09E-06	-0.39198	0	0.15	0.016252	Undiff-G	GSKIP
SYNC	1.09E-06	-0.36657	0.015	0.178	0.01633	Undiff-G	SYNC
MEIS2	1.10E-06	-0.16935	0.029	0.205	0.016369	Undiff-G	MEIS2
SFSWAP	1.10E-06	-0.21821	0.015	0.178	0.016506	Undiff-G	SFSWAP
NUDT2	1.11E-06	-0.38217	0.015	0.177	0.01652	Undiff-G	NUDT2
PTPN11	1.11E-06	-0.35952	0	0.15	0.016521	Undiff-G	PTPN11

DNAJB12	1.12E-06	-0.47367	0	0.15	0.016793	Undiff-G	DNAJB12
CYBRD1	1.12E-06	-0.3756	0.015	0.178	0.0168	Undiff-G	CYBRD1
PRR4	1.13E-06	-0.12499	0.037	0.218	0.016889	Undiff-G	PRR4
VPS16	1.13E-06	-0.30039	0.015	0.177	0.016891	Undiff-G	VPS16
ZNF75A	1.13E-06	-0.20759	0.037	0.216	0.016941	Undiff-G	ZNF75A
TBC1D7	1.14E-06	-0.22656	0.007	0.164	0.016964	Undiff-G	TBC1D7
ZNF195	1.14E-06	-0.18351	0.007	0.164	0.017103	Undiff-G	ZNF195
RGMA	1.15E-06	-0.3821	0.029	0.202	0.017141	Undiff-G	RGMA
GMNN	1.15E-06	-0.16521	0.015	0.178	0.017142	Undiff-G	GMNN
ECHDC2	1.15E-06	-0.33343	0.022	0.19	0.01721	Undiff-G	ECHDC2
NCK2	1.15E-06	-0.40018	0	0.15	0.017211	Undiff-G	NCK2
TMEM243	1.15E-06	-0.3115	0.029	0.204	0.017243	Undiff-G	TMEM243
ETV6	1.17E-06	-0.41988	0	0.149	0.017495	Undiff-G	ETV6
DNAJC9	1.17E-06	-0.26614	0.007	0.164	0.017521	Undiff-G	DNAJC9
BRD8	1.17E-06	-0.16077	0.007	0.164	0.017531	Undiff-G	BRD8
SSH2	1.18E-06	-0.11579	0.015	0.178	0.0176	Undiff-G	SSH2
KDM5B	1.18E-06	-0.29719	0.007	0.164	0.017654	Undiff-G	KDM5B
LUZP1	1.18E-06	-0.10842	0.022	0.19	0.017691	Undiff-G	LUZP1
LPCAT2	1.20E-06	-0.57139	0	0.149	0.017929	Undiff-G	LPCAT2
MLLT4	1.20E-06	-0.32297	0.007	0.164	0.017941	Undiff-G	MLLT4
TPRA1	1.21E-06	-0.21093	0.007	0.163	0.018103	Undiff-G	TPRA1
TSR1	1.21E-06	-0.21163	0.022	0.191	0.018132	Undiff-G	TSR1
TUG1	1.22E-06	-0.28525	0.015	0.177	0.018221	Undiff-G	TUG1
ENDOG	1.22E-06	-0.33814	0	0.149	0.018225	Undiff-G	ENDOG
CSK	1.22E-06	-0.46141	0	0.149	0.018225	Undiff-G	CSK
TRIP11	1.22E-06	-0.18794	0.022	0.191	0.018252	Undiff-G	TRIP11
TOR2A	1.23E-06	-0.40928	0	0.149	0.018375	Undiff-G	TOR2A
NETO2	1.23E-06	-0.33883	0.007	0.163	0.018383	Undiff-G	NETO2
SIKE1	1.23E-06	-0.37337	0.022	0.19	0.018425	Undiff-G	SIKE1
DUSP3	1.26E-06	-0.11268	0.015	0.177	0.018761	Undiff-G	DUSP3
PLEC	1.26E-06	-0.15324	0.022	0.188	0.018807	Undiff-G	PLEC
ZDHHC7	1.26E-06	-0.37971	0	0.149	0.018831	Undiff-G	ZDHHC7
AKT1	1.27E-06	-0.39288	0	0.148	0.018985	Undiff-G	AKT1
ZC3H6	1.27E-06	-0.40665	0	0.148	0.018985	Undiff-G	ZC3H6
TMEM57	1.27E-06	-0.36435	0.015	0.176	0.01901	Undiff-G	TMEM57
FAM110B	1.27E-06	-0.36268	0.022	0.188	0.019035	Undiff-G	FAM110B
FUZ	1.28E-06	-0.31922	0.007	0.163	0.019106	Undiff-G	FUZ
LMF2	1.28E-06	-0.17269	0.029	0.203	0.019125	Undiff-G	LMF2
PIK3IP1	1.28E-06	-0.57851	0.007	0.163	0.019137	Undiff-G	PIK3IP1
HTATIP2	1.28E-06	-0.47086	0	0.148	0.019141	Undiff-G	HTATIP2
CS	1.29E-06	-0.29788	0.007	0.163	0.019274	Undiff-G	CS
TARBP2	1.30E-06	-0.3659	0	0.148	0.019456	Undiff-G	TARBP2
FAM118B	1.30E-06	-0.38831	0	0.148	0.019456	Undiff-G	FAM118B
SERPINB9	1.30E-06	-0.61489	0	0.148	0.019456	Undiff-G	SERPINB9
CDC42EP4	1.32E-06	-0.23107	0.029	0.203	0.019712	Undiff-G	CDC42EP4
ARL16	1.32E-06	0.474334	0.096	0.322	0.019734	Undiff-G	ARL16
ZMYND11	1.32E-06	-0.34332	0	0.148	0.019776	Undiff-G	ZMYND11
CYTH1	1.33E-06	-0.48663	0.007	0.16	0.019901	Undiff-G	CYTH1
LHFPL2	1.34E-06	-0.39744	0.007	0.162	0.019953	Undiff-G	LHFPL2
GGCX	1.35E-06	-0.13476	0.015	0.176	0.020169	Undiff-G	GGCX
APC	1.35E-06	-0.10531	0.037	0.215	0.020223	Undiff-G	APC

VPS13C	1.36E-06	-0.30138	0.015	0.176	0.020255	Undiff-G	VPS13C
SUDS3	1.36E-06	-0.24895	0.022	0.19	0.0203	Undiff-G	SUDS3
AAAS	1.36E-06	-0.36944	0.022	0.188	0.020302	Undiff-G	AAAS
MTMR2	1.37E-06	-0.34682	0	0.148	0.020432	Undiff-G	MTMR2
ZNF92	1.37E-06	-0.42607	0	0.148	0.020432	Undiff-G	ZNF92
NECAP2	1.37E-06	0.345302	0.081	0.294	0.020481	Undiff-G	NECAP2
IFT81	1.38E-06	-0.35358	0.007	0.161	0.020569	Undiff-G	IFT81
SCFD1	1.38E-06	0.241421	0.059	0.255	0.020624	Undiff-G	SCFD1
CRAT	1.38E-06	-0.23969	0.007	0.162	0.020627	Undiff-G	CRAT
IVD	1.38E-06	-0.19872	0.029	0.201	0.020641	Undiff-G	IVD
GPR155	1.39E-06	-0.32777	0.022	0.189	0.020719	Undiff-G	GPR155
NCF4	1.39E-06	-0.66255	0	0.148	0.020769	Undiff-G	NCF4
CDKN2D	1.41E-06	-0.40588	0.007	0.161	0.02102	Undiff-G	CDKN2D
ALYREF	1.41E-06	-0.32637	0	0.147	0.02111	Undiff-G	ALYREF
CHKA	1.41E-06	-0.1384	0.022	0.189	0.021136	Undiff-G	CHKA
SLC27A1	1.41E-06	-0.28022	0.007	0.162	0.021137	Undiff-G	SLC27A1
DCP1A	1.42E-06	0.108068	0.044	0.229	0.021186	Undiff-G	DCP1A
SAMM50	1.42E-06	0.192668	0.037	0.217	0.021238	Undiff-G	SAMM50
PCYT2	1.43E-06	-0.22502	0.007	0.162	0.021315	Undiff-G	PCYT2
EXOSC3	1.43E-06	-0.22467	0.007	0.162	0.021319	Undiff-G	EXOSC3
ZNF138	1.44E-06	-0.39172	0	0.147	0.021457	Undiff-G	ZNF138
SORBS3	1.44E-06	-0.28053	0.015	0.174	0.021528	Undiff-G	SORBS3
NFE2L1	1.44E-06	-0.27725	0.022	0.189	0.021552	Undiff-G	NFE2L1
CUL4A	1.45E-06	-0.36616	0	0.147	0.021633	Undiff-G	CUL4A
ABAT	1.45E-06	-0.37696	0.007	0.161	0.021695	Undiff-G	ABAT
RRP36	1.46E-06	-0.32232	0	0.147	0.02181	Undiff-G	RRP36
OSM	1.46E-06	-0.72863	0	0.147	0.02181	Undiff-G	OSM
TIPRL	1.46E-06	0.406952	0.066	0.268	0.021873	Undiff-G	TIPRL
SNX7	1.46E-06	-0.18311	0.015	0.175	0.021874	Undiff-G	SNX7
FLOT2	1.46E-06	-0.22243	0.022	0.188	0.021874	Undiff-G	FLOT2
TP53I3	1.47E-06	-0.40533	0	0.147	0.021988	Undiff-G	TP53I3
BOD1L1	1.47E-06	-0.36443	0.007	0.161	0.022015	Undiff-G	BOD1L1
CTXN1	1.47E-06	-0.31824	0.007	0.161	0.022028	Undiff-G	CTXN1
PHGDH	1.48E-06	-0.29138	0.029	0.202	0.022052	Undiff-G	PHGDH
HES4	1.48E-06	-0.34194	0.022	0.189	0.02208	Undiff-G	HES4
TWISTNB	1.48E-06	0.481589	0.066	0.271	0.022143	Undiff-G	TWISTNB
ZNF667-AS	1.49E-06	0.546336	0.132	0.391	0.022264	Undiff-G	ZNF667-AS1
POLR2M	1.51E-06	-0.33178	0.007	0.161	0.022557	Undiff-G	POLR2M
TBC1D14	1.52E-06	-0.27433	0.015	0.173	0.022663	Undiff-G	TBC1D14
STX18	1.52E-06	-0.27826	0.007	0.161	0.022717	Undiff-G	STX18
DTNA	1.52E-06	0.240436	0.059	0.255	0.022728	Undiff-G	DTNA
PRR14	1.52E-06	-0.13008	0.029	0.202	0.022732	Undiff-G	PRR14
ABHD8	1.53E-06	-0.34716	0	0.147	0.022902	Undiff-G	ABHD8
KIAA0930	1.53E-06	-0.4696	0	0.147	0.022902	Undiff-G	KIAA0930
RNASE2	1.54E-06	-0.71084	0.007	0.159	0.022985	Undiff-G	RNASE2
PKM	1.54E-06	1.042024	0.596	0.772	0.023007	Undiff-G	PKM
TEAD1	1.55E-06	-0.31113	0	0.146	0.023089	Undiff-G	TEAD1
MTMR6	1.56E-06	-0.40768	0	0.146	0.023278	Undiff-G	MTMR6
SEC22C	1.56E-06	0.345318	0.044	0.23	0.023294	Undiff-G	SEC22C
FHL3	1.56E-06	-0.15669	0.007	0.161	0.02332	Undiff-G	FHL3
TMEM132A	1.57E-06	-0.32468	0	0.146	0.023468	Undiff-G	TMEM132A

FAM162A	1.57E-06	0.487574	0.14	0.404	0.023524	Undiff-G	FAM162A
MAFG	1.58E-06	-0.13937	0.022	0.186	0.023612	Undiff-G	MAFG
HBD	1.58E-06	0.540291	0.015	0.001	0.023639	Undiff-G	HBD
MCMBP	1.58E-06	-0.38971	0	0.146	0.02366	Undiff-G	MCMBP
C11orf1	1.58E-06	-0.45953	0	0.146	0.02366	Undiff-G	C11orf1
AC008268.	1.61E-06	0.209071	0.015	0.001	0.024006	Undiff-G	AC008268.1
WDR83	1.61E-06	-0.35223	0.015	0.173	0.02402	Undiff-G	WDR83
CORO7	1.61E-06	-0.45097	0.007	0.16	0.024074	Undiff-G	CORO7
TRIO	1.61E-06	-0.34956	0.015	0.174	0.024082	Undiff-G	TRIO
HUWE1	1.62E-06	-0.25189	0.015	0.174	0.024158	Undiff-G	HUWE1
NDUFS5	1.62E-06	0.453151	0.331	0.816	0.024215	Undiff-G	NDUFS5
POM121	1.63E-06	-0.37977	0.007	0.159	0.024329	Undiff-G	POM121
STXBP2	1.64E-06	-0.3244	0.015	0.174	0.024491	Undiff-G	STXBP2
TMEM129	1.64E-06	-0.28586	0.015	0.174	0.024514	Undiff-G	TMEM129
VWA1	1.65E-06	-0.36659	0	0.146	0.024642	Undiff-G	VWA1
COL4A3BP	1.65E-06	-0.38485	0	0.146	0.024642	Undiff-G	COL4A3BP
TARS	1.66E-06	0.2143	0.044	0.229	0.024761	Undiff-G	TARS
COA5	1.66E-06	0.206245	0.088	0.308	0.024771	Undiff-G	COA5
BST2	1.66E-06	0.222356	0.191	0.489	0.024861	Undiff-G	BST2
METTL2A	1.66E-06	-0.10983	0.007	0.16	0.024881	Undiff-G	METTL2A
CFLAR	1.68E-06	0.465245	0.096	0.321	0.025127	Undiff-G	CFLAR
PITPNB	1.68E-06	-0.32784	0.015	0.173	0.025156	Undiff-G	PITPNB
KLHL5	1.68E-06	-0.39323	0.015	0.172	0.025168	Undiff-G	KLHL5
DLX5	1.69E-06	-0.73669	0	0.145	0.02525	Undiff-G	DLX5
CCDC28A	1.69E-06	-0.29131	0.007	0.16	0.025269	Undiff-G	CCDC28A
BCL2A1	1.69E-06	-0.61746	0.007	0.159	0.025321	Undiff-G	BCL2A1
APBB1IP	1.69E-06	-0.59428	0.007	0.158	0.025327	Undiff-G	APBB1IP
ZFYVE21	1.70E-06	0.340874	0.066	0.268	0.025436	Undiff-G	ZFYVE21
IRAK1	1.71E-06	-0.27221	0.007	0.16	0.02549	Undiff-G	IRAK1
FAM122A	1.72E-06	-0.38491	0	0.145	0.025664	Undiff-G	FAM122A
UGDH	1.72E-06	-0.17172	0.007	0.16	0.025697	Undiff-G	UGDH
SAMD8	1.73E-06	-0.37642	0	0.145	0.025873	Undiff-G	SAMD8
DPP8	1.74E-06	-0.35348	0.007	0.159	0.025946	Undiff-G	DPP8
EXT2	1.74E-06	-0.25899	0.015	0.173	0.026054	Undiff-G	EXT2
HSCB	1.75E-06	-0.25931	0.015	0.174	0.026078	Undiff-G	HSCB
PDS5A	1.75E-06	-0.17462	0.007	0.16	0.026123	Undiff-G	PDS5A
CALU	1.76E-06	0.715868	0.14	0.406	0.02625	Undiff-G	CALU
NUMB	1.76E-06	-0.35733	0.015	0.172	0.02625	Undiff-G	NUMB
GM2A	1.76E-06	-0.49267	0	0.145	0.026297	Undiff-G	GM2A
FAH	1.77E-06	-0.4092	0.007	0.159	0.026434	Undiff-G	FAH
BRIX1	1.77E-06	0.467185	0.059	0.256	0.026514	Undiff-G	BRIX1
PRKAB1	1.78E-06	-0.39663	0.007	0.159	0.026534	Undiff-G	PRKAB1
EVI5	1.79E-06	-0.25302	0.022	0.186	0.026704	Undiff-G	EVI5
TLE1	1.79E-06	-0.34592	0.015	0.171	0.026739	Undiff-G	TLE1
JOSD1	1.79E-06	-0.22931	0.007	0.159	0.026761	Undiff-G	JOSD1
USP39	1.79E-06	-0.2079	0.022	0.187	0.026765	Undiff-G	USP39
NOA1	1.80E-06	-0.37675	0	0.145	0.026946	Undiff-G	NOA1
C7orf26	1.80E-06	-0.29168	0.007	0.159	0.026963	Undiff-G	C7orf26
PPP1R35	1.81E-06	-0.32458	0.007	0.159	0.027082	Undiff-G	PPP1R35
SPATA6	1.81E-06	-0.37844	0.007	0.158	0.027093	Undiff-G	SPATA6
RNF26	1.82E-06	-0.25847	0.007	0.159	0.027161	Undiff-G	RNF26

DCBLD2	1.82E-06	-0.35155	0	0.145	0.027165	Undiff-G	DCBLD2
BRE	1.82E-06	0.295131	0.044	0.228	0.027175	Undiff-G	BRE
CBS	1.83E-06	-0.3084	0.007	0.159	0.027273	Undiff-G	CBS
KIAA2026	1.83E-06	-0.40278	0	0.145	0.027387	Undiff-G	KIAA2026
CDK11A	1.84E-06	-0.12407	0.029	0.198	0.027531	Undiff-G	CDK11A
MUT	1.85E-06	-0.18762	0.007	0.159	0.027644	Undiff-G	MUT
MAFF	1.87E-06	-0.19606	0.015	0.173	0.027925	Undiff-G	MAFF
SEMA6D	1.87E-06	-0.38861	0.007	0.159	0.027944	Undiff-G	SEMA6D
PLXNB2	1.90E-06	-0.20895	0.007	0.159	0.028324	Undiff-G	PLXNB2
SCAP	1.90E-06	-0.21165	0.007	0.159	0.028324	Undiff-G	SCAP
ZC3H18	1.90E-06	-0.34402	0.007	0.158	0.028457	Undiff-G	ZC3H18
OCEL1	1.90E-06	-0.39624	0.007	0.158	0.028459	Undiff-G	OCEL1
MAD1L1	1.91E-06	-0.38975	0.007	0.158	0.028566	Undiff-G	MAD1L1
UBAP1	1.91E-06	-0.10053	0.022	0.186	0.028592	Undiff-G	UBAP1
SLC25A17	1.92E-06	-0.27993	0.007	0.159	0.028741	Undiff-G	SLC25A17
C11orf24	1.92E-06	-0.38854	0	0.144	0.028753	Undiff-G	C11orf24
HPS4	1.93E-06	-0.37028	0.022	0.185	0.028825	Undiff-G	HPS4
ZNF83	1.93E-06	-0.1224	0.029	0.199	0.028876	Undiff-G	ZNF83
NIT1	1.94E-06	-0.18125	0.015	0.172	0.028917	Undiff-G	NIT1
SMC6	1.94E-06	-0.10412	0.007	0.158	0.029032	Undiff-G	SMC6
RPP30	1.95E-06	-0.27732	0.015	0.17	0.029199	Undiff-G	RPP30
ARHGEF6	1.96E-06	-0.3672	0	0.144	0.029223	Undiff-G	ARHGEF6
MPRIP	1.96E-06	-0.22514	0.007	0.158	0.029257	Undiff-G	MPRIP
SPTBN1	1.96E-06	0.171946	0.074	0.277	0.029288	Undiff-G	SPTBN1
FBXO3	1.97E-06	-0.31555	0.015	0.172	0.029439	Undiff-G	FBXO3
COMMD10	1.97E-06	0.271418	0.044	0.227	0.029446	Undiff-G	COMMD10
GPATCH2L	2.00E-06	-0.12745	0.044	0.225	0.029833	Undiff-G	GPATCH2L
RAB3GAP2	2.00E-06	-0.20555	0.015	0.172	0.029912	Undiff-G	RAB3GAP2
ZNF711	2.00E-06	-0.39277	0	0.144	0.029942	Undiff-G	ZNF711
MKNK1	2.02E-06	-0.28702	0.015	0.172	0.030114	Undiff-G	MKNK1
BCCIP	2.04E-06	-0.20968	0.022	0.185	0.030423	Undiff-G	BCCIP
NOC4L	2.04E-06	-0.10229	0.007	0.158	0.030477	Undiff-G	NOC4L
PSMG4	2.04E-06	0.10394	0.029	0.2	0.030481	Undiff-G	PSMG4
LENG8	2.05E-06	-0.11117	0.044	0.225	0.030638	Undiff-G	LENG8
PHF11	2.05E-06	-0.42378	0	0.143	0.030678	Undiff-G	PHF11
KIN	2.07E-06	-0.38479	0	0.143	0.030928	Undiff-G	KIN
TMSB15B	2.08E-06	-0.35298	0.022	0.183	0.031049	Undiff-G	TMSB15B
TCEB2	2.08E-06	0.423059	0.346	0.829	0.031141	Undiff-G	TCEB2
TBC1D5	2.09E-06	-0.38692	0	0.143	0.031179	Undiff-G	TBC1D5
AP5M1	2.10E-06	-0.36355	0.007	0.157	0.031432	Undiff-G	AP5M1
PTS	2.11E-06	0.474729	0.103	0.329	0.031516	Undiff-G	PTS
B3GNT5	2.12E-06	-0.45862	0	0.143	0.031688	Undiff-G	B3GNT5
ADAM28	2.14E-06	-0.6424	0	0.143	0.031946	Undiff-G	ADAM28
OLIG1	2.14E-06	-0.57472	0.022	0.182	0.031983	Undiff-G	OLIG1
COX5B	2.15E-06	0.407001	0.353	0.854	0.032119	Undiff-G	COX5B
SETD5	2.20E-06	0.124912	0.081	0.291	0.032805	Undiff-G	SETD5
AP2A1	2.21E-06	0.148871	0.051	0.238	0.032977	Undiff-G	AP2A1
LINC00662	2.21E-06	-0.1289	0.022	0.185	0.032988	Undiff-G	LINC00662
ALKBH2	2.21E-06	-0.25517	0.029	0.197	0.033027	Undiff-G	ALKBH2
ACBD5	2.22E-06	-0.3523	0.015	0.169	0.033127	Undiff-G	ACBD5
FAM217B	2.22E-06	-0.34462	0.007	0.157	0.033214	Undiff-G	FAM217B

NR2F6	2.23E-06	-0.31218	0	0.143	0.033265	Undiff-G	NR2F6
VAC14	2.23E-06	-0.3372	0	0.143	0.033265	Undiff-G	VAC14
C6orf47	2.23E-06	-0.34661	0	0.143	0.033265	Undiff-G	C6orf47
CDK9	2.23E-06	-0.35646	0	0.143	0.033265	Undiff-G	CDK9
CTTNBP2NL	2.23E-06	-0.40708	0.007	0.155	0.033295	Undiff-G	CTTNBP2NL
FTSJ2	2.23E-06	0.16106	0.051	0.239	0.033356	Undiff-G	FTSJ2
CD4	2.25E-06	-0.55003	0.007	0.157	0.033577	Undiff-G	CD4
GRIA3	2.25E-06	-0.33917	0.015	0.17	0.033671	Undiff-G	GRIA3
SOGA1	2.25E-06	-0.30003	0.007	0.157	0.033673	Undiff-G	SOGA1
TMEM205	2.25E-06	0.578314	0.191	0.499	0.033697	Undiff-G	TMEM205
FZR1	2.26E-06	-0.29121	0.007	0.157	0.0337	Undiff-G	FZR1
FUOM	2.26E-06	-0.54554	0	0.142	0.033807	Undiff-G	FUOM
GPC1	2.26E-06	0.108885	0.051	0.239	0.033842	Undiff-G	GPC1
POMGNT2	2.28E-06	-0.33433	0	0.142	0.034081	Undiff-G	POMGNT2
CLCN7	2.29E-06	-0.33846	0.007	0.156	0.034226	Undiff-G	CLCN7
DCLK2	2.30E-06	-0.15839	0.029	0.198	0.034302	Undiff-G	DCLK2
PDS5B	2.30E-06	-0.2611	0.015	0.169	0.034405	Undiff-G	PDS5B
COASY	2.30E-06	-0.28488	0.029	0.196	0.034409	Undiff-G	COASY
EHBP1	2.32E-06	-0.33621	0.015	0.169	0.034632	Undiff-G	EHBP1
ARL4D	2.33E-06	-0.34211	0.015	0.171	0.034748	Undiff-G	ARL4D
RAB8B	2.34E-06	-0.23624	0.007	0.156	0.034952	Undiff-G	RAB8B
RIPK2	2.35E-06	-0.23407	0.015	0.17	0.035127	Undiff-G	RIPK2
ZNF672	2.36E-06	-0.34995	0	0.142	0.035201	Undiff-G	ZNF672
SLC25A19	2.36E-06	-0.29919	0.015	0.17	0.035271	Undiff-G	SLC25A19
HADH	2.36E-06	0.175532	0.037	0.209	0.035272	Undiff-G	HADH
SCG5	2.36E-06	0.369906	0.103	0.331	0.035293	Undiff-G	SCG5
TMEM115	2.36E-06	0.108491	0.037	0.21	0.035328	Undiff-G	TMEM115
MAP4K5	2.37E-06	-0.30956	0.007	0.156	0.035422	Undiff-G	MAP4K5
VPS45	2.37E-06	-0.12406	0.015	0.17	0.03544	Undiff-G	VPS45
ATP13A2	2.37E-06	-0.33958	0	0.142	0.035487	Undiff-G	ATP13A2
PHKG2	2.38E-06	-0.18788	0.007	0.156	0.035527	Undiff-G	PHKG2
UBL4A	2.41E-06	0.114135	0.022	0.184	0.035945	Undiff-G	UBL4A
ACOT9	2.41E-06	-0.24563	0.022	0.184	0.036005	Undiff-G	ACOT9
PXK	2.41E-06	-0.42078	0	0.142	0.036065	Undiff-G	PXK
MAPK10	2.44E-06	-0.14092	0.029	0.197	0.036427	Undiff-G	MAPK10
F8A1	2.45E-06	-0.33319	0	0.142	0.036652	Undiff-G	F8A1
C2orf68	2.45E-06	-0.39449	0	0.142	0.036652	Undiff-G	C2orf68
NOTCH2NL	2.47E-06	-0.32871	0.015	0.168	0.036845	Undiff-G	NOTCH2NL
PPM1A	2.47E-06	-0.12087	0.022	0.184	0.036925	Undiff-G	PPM1A
MIAT	2.47E-06	-0.43472	0.015	0.169	0.036935	Undiff-G	MIAT
POU3F3	2.47E-06	-0.24993	0.007	0.156	0.036982	Undiff-G	POU3F3
BAP1	2.49E-06	-0.34847	0	0.141	0.037248	Undiff-G	BAP1
ADCK2	2.49E-06	-0.39178	0	0.141	0.037248	Undiff-G	ADCK2
GPC2	2.51E-06	-0.3748	0.015	0.17	0.03751	Undiff-G	GPC2
RPRD1A	2.52E-06	-0.16798	0.037	0.209	0.037637	Undiff-G	RPRD1A
ANKRD13D	2.52E-06	-0.32999	0.015	0.169	0.037648	Undiff-G	ANKRD13D
SLC50A1	2.52E-06	-0.26206	0.015	0.169	0.037684	Undiff-G	SLC50A1
CIAPIN1	2.52E-06	0.374759	0.066	0.262	0.037719	Undiff-G	CIAPIN1
CRCP	2.53E-06	-0.10735	0.022	0.183	0.037763	Undiff-G	CRCP
BMP7	2.53E-06	-0.36698	0.015	0.168	0.037831	Undiff-G	BMP7
LIX1L	2.55E-06	-0.32843	0	0.141	0.038161	Undiff-G	LIX1L

SCAF1	2.57E-06	-0.32462	0.007	0.155	0.038363	Undiff-G	SCAF1
LINC01094	2.57E-06	-0.41275	0.007	0.155	0.038457	Undiff-G	LINC01094
BMI1	2.57E-06	-0.32882	0	0.141	0.03847	Undiff-G	BMI1
BTBD7	2.60E-06	-0.37391	0	0.141	0.038782	Undiff-G	BTBD7
DNAJC30	2.60E-06	-0.25018	0.007	0.155	0.038814	Undiff-G	DNAJC30
SH3YL1	2.62E-06	0.11948	0.022	0.183	0.039212	Undiff-G	SH3YL1
FBXW2	2.63E-06	-0.11774	0.029	0.194	0.039357	Undiff-G	FBXW2
ZBED1	2.64E-06	-0.35326	0	0.141	0.039412	Undiff-G	ZBED1
MITD1	2.67E-06	0.189557	0.051	0.236	0.039858	Undiff-G	MITD1
FOXG1	2.68E-06	-0.30997	0.007	0.155	0.040038	Undiff-G	FOXG1
RAP2B	2.68E-06	-0.21062	0.015	0.167	0.040049	Undiff-G	RAP2B
LBH	2.68E-06	-0.21931	0.007	0.155	0.040101	Undiff-G	LBH
AGA	2.70E-06	-0.31155	0.007	0.155	0.040329	Undiff-G	AGA
C1GALT1C1	2.70E-06	-0.23359	0.007	0.155	0.040369	Undiff-G	C1GALT1C1
CEP164	2.71E-06	-0.33124	0.007	0.155	0.040526	Undiff-G	CEP164
ANKRD39	2.71E-06	-0.13887	0.015	0.169	0.040542	Undiff-G	ANKRD39
SLAIN2	2.72E-06	-0.33754	0	0.14	0.040704	Undiff-G	SLAIN2
SLC40A1	2.72E-06	-0.44378	0	0.14	0.040704	Undiff-G	SLC40A1
YIPF5	2.74E-06	0.465626	0.066	0.264	0.040961	Undiff-G	YIPF5
ZFAND2B	2.74E-06	-0.20842	0.037	0.208	0.040964	Undiff-G	ZFAND2B
PNPLA6	2.74E-06	-0.35578	0.015	0.168	0.040977	Undiff-G	PNPLA6
ZNF37A	2.75E-06	-0.34421	0	0.14	0.041033	Undiff-G	ZNF37A
SMYD3	2.77E-06	-0.41185	0	0.14	0.041365	Undiff-G	SMYD3
C1orf50	2.77E-06	-0.19341	0.022	0.182	0.041384	Undiff-G	C1orf50
CDO1	2.77E-06	0.209277	0.051	0.237	0.04142	Undiff-G	CDO1
ATAD3A	2.78E-06	-0.33013	0.007	0.153	0.041567	Undiff-G	ATAD3A
DCAKD	2.79E-06	-0.33109	0	0.14	0.041699	Undiff-G	DCAKD
ALOX5	2.79E-06	-0.5804	0	0.14	0.041699	Undiff-G	ALOX5
NDUFAF4	2.80E-06	0.309596	0.044	0.223	0.041866	Undiff-G	NDUFAF4
APTX	2.81E-06	-0.38608	0	0.14	0.042037	Undiff-G	APTX
SLC7A5	2.81E-06	-0.51463	0	0.14	0.042037	Undiff-G	SLC7A5
SETX	2.82E-06	-0.34324	0.015	0.168	0.042193	Undiff-G	SETX
STT3A	2.83E-06	-0.15561	0.015	0.168	0.042226	Undiff-G	STT3A
PLA2G15	2.84E-06	-0.45899	0	0.14	0.042377	Undiff-G	PLA2G15
IL10RA	2.84E-06	-0.57663	0	0.14	0.042377	Undiff-G	IL10RA
VRK3	2.85E-06	0.144024	0.044	0.22	0.04261	Undiff-G	VRK3
NNT-AS1	2.85E-06	0.273661	0.044	0.223	0.042627	Undiff-G	NNT-AS1
B3GALT6	2.86E-06	-0.32427	0	0.14	0.042719	Undiff-G	B3GALT6
AFTPH	2.88E-06	-0.18009	0.007	0.154	0.043104	Undiff-G	AFTPH
RALGDS	2.91E-06	-0.41622	0.007	0.154	0.043413	Undiff-G	RALGDS
HN1L	2.93E-06	-0.21623	0.007	0.154	0.043794	Undiff-G	HN1L
PTDSS2	2.94E-06	-0.31024	0.007	0.154	0.043986	Undiff-G	PTDSS2
WDR54	2.95E-06	0.287483	0.051	0.236	0.044013	Undiff-G	WDR54
RAB3A	2.95E-06	-0.32342	0.015	0.166	0.044089	Undiff-G	RAB3A
NOLC1	2.97E-06	-0.37705	0.007	0.153	0.044386	Undiff-G	NOLC1
RUSC1	2.98E-06	-0.34023	0	0.139	0.044473	Undiff-G	RUSC1
LIMK1	2.98E-06	-0.3842	0	0.139	0.044473	Undiff-G	LIMK1
TIMM23	2.99E-06	-0.30359	0.007	0.154	0.044685	Undiff-G	TIMM23
LRCH3	3.00E-06	-0.35847	0	0.139	0.044833	Undiff-G	LRCH3
UTP3	3.00E-06	-0.39062	0	0.139	0.044833	Undiff-G	UTP3
GPKOW	3.02E-06	-0.37706	0	0.139	0.045195	Undiff-G	GPKOW

DSE	3.02E-06	-0.51678	0	0.139	0.045195	Undiff-G	DSE
SLC29A1	3.05E-06	-0.5371	0	0.139	0.04556	Undiff-G	SLC29A1
SPATA13	3.05E-06	-0.2036	0.015	0.167	0.045627	Undiff-G	SPATA13
CALD1	3.07E-06	0.355902	0.154	0.423	0.045811	Undiff-G	CALD1
C6orf120	3.07E-06	-0.30689	0	0.139	0.045927	Undiff-G	C6orf120
CHD1L	3.07E-06	-0.38984	0	0.139	0.045927	Undiff-G	CHD1L
SKI	3.09E-06	-0.39588	0.007	0.153	0.046225	Undiff-G	SKI
ZNF43	3.10E-06	-0.3404	0	0.139	0.046298	Undiff-G	ZNF43
MARS	3.10E-06	0.103416	0.037	0.208	0.046387	Undiff-G	MARS
CLPTM1L	3.12E-06	-0.31692	0.007	0.153	0.046567	Undiff-G	CLPTM1L
ARAF	3.12E-06	-0.23209	0.022	0.179	0.04664	Undiff-G	ARAF
USP46	3.12E-06	-0.36979	0	0.139	0.046672	Undiff-G	USP46
GDAP1	3.13E-06	-0.15489	0.007	0.153	0.046719	Undiff-G	GDAP1
ERRFI1	3.13E-06	-0.34509	0.007	0.153	0.046752	Undiff-G	ERRFI1
SLC16A1	3.13E-06	0.223535	0.051	0.236	0.046803	Undiff-G	SLC16A1
FBXO25	3.15E-06	-0.34493	0.007	0.153	0.047045	Undiff-G	FBXO25
TEX30	3.16E-06	-0.27356	0.015	0.167	0.047263	Undiff-G	TEX30
RBM18	3.17E-06	0.259017	0.037	0.208	0.047364	Undiff-G	RBM18
C12orf45	3.17E-06	-0.19231	0.022	0.18	0.047382	Undiff-G	C12orf45
NUS1	3.18E-06	-0.10326	0.007	0.153	0.047476	Undiff-G	NUS1
KANSL1	3.19E-06	-0.17407	0.015	0.167	0.047642	Undiff-G	KANSL1
SLC18B1	3.22E-06	-0.1533	0.022	0.181	0.048169	Undiff-G	SLC18B1
MED27	3.23E-06	-0.35624	0.007	0.153	0.048295	Undiff-G	MED27
AAMDC	3.26E-06	0.109282	0.037	0.208	0.048699	Undiff-G	AAMDC
FKBP1A	3.27E-06	0.525452	0.294	0.704	0.048902	Undiff-G	FKBP1A
PILRA	3.28E-06	-0.45596	0.015	0.167	0.048949	Undiff-G	PILRA
CDK10	3.30E-06	-0.39481	0.015	0.165	0.04925	Undiff-G	CDK10
ROBO1	3.30E-06	-0.27907	0.022	0.18	0.049258	Undiff-G	ROBO1
IP6K1	3.31E-06	-0.12059	0.007	0.153	0.049409	Undiff-G	IP6K1
ZNF385A	3.33E-06	-0.45012	0	0.138	0.04977	Undiff-G	ZNF385A
NF2	3.33E-06	-0.21176	0.007	0.153	0.049797	Undiff-G	NF2
RANBP9	3.33E-06	-0.13733	0.007	0.153	0.049817	Undiff-G	RANBP9
NAMPT	3.35E-06	1.423389	0.456	0.437	0.050086	Undiff-G	NAMPT
PTAR1	3.36E-06	-0.32036	0	0.138	0.050171	Undiff-G	PTAR1
AAR2	3.36E-06	-0.34714	0	0.138	0.050171	Undiff-G	AAR2
KIAA2013	3.36E-06	-0.3333	0.007	0.152	0.050178	Undiff-G	KIAA2013
ALG8	3.38E-06	-0.12808	0.022	0.18	0.050492	Undiff-G	ALG8
GIMAP4	3.39E-06	-0.63956	0.007	0.151	0.050726	Undiff-G	GIMAP4
RFFL	3.40E-06	-0.27681	0.015	0.166	0.050825	Undiff-G	RFFL
RHBDD3	3.41E-06	-0.33878	0.007	0.151	0.050982	Undiff-G	RHBDD3
C1GALT1	3.41E-06	-0.37762	0.015	0.164	0.051017	Undiff-G	C1GALT1
FAM168B	3.41E-06	-0.35262	0.015	0.165	0.051019	Undiff-G	FAM168B
PAM	3.43E-06	-0.18159	0.015	0.166	0.051308	Undiff-G	PAM
GLRX2	3.44E-06	-0.21072	0.015	0.165	0.051335	Undiff-G	GLRX2
KRIT1	3.45E-06	-0.17825	0.044	0.219	0.051484	Undiff-G	KRIT1
OSBPL1A	3.45E-06	-0.10061	0.029	0.193	0.051496	Undiff-G	OSBPL1A
DCP2	3.50E-06	-0.14766	0.007	0.152	0.052261	Undiff-G	DCP2
C9orf72	3.52E-06	-0.32996	0.007	0.152	0.052608	Undiff-G	C9orf72
BEX2	3.52E-06	0.238229	0.066	0.259	0.052625	Undiff-G	BEX2
TBK1	3.57E-06	-0.3735	0.007	0.151	0.053365	Undiff-G	TBK1
DYNC2LI1	3.57E-06	-0.10429	0.015	0.166	0.053385	Undiff-G	DYNC2LI1

FPGS	3.59E-06	-0.34036	0.007	0.151	0.053587	Undiff-G	FPGS
PDGFRA	3.60E-06	-0.43085	0.007	0.15	0.053756	Undiff-G	PDGFRA
HEXIM1	3.60E-06	0.249056	0.044	0.22	0.053828	Undiff-G	HEXIM1
NT5DC2	3.61E-06	-0.19662	0.015	0.166	0.053963	Undiff-G	NT5DC2
BMPR2	3.63E-06	-0.19779	0.022	0.179	0.05424	Undiff-G	BMPR2
PSMA7	3.64E-06	0.470486	0.331	0.807	0.054335	Undiff-G	PSMA7
SESN3	3.64E-06	-0.26886	0.007	0.152	0.054342	Undiff-G	SESN3
NCOA6	3.67E-06	-0.29811	0	0.137	0.0548	Undiff-G	NCOA6
RBM38	3.70E-06	-0.32888	0	0.137	0.055241	Undiff-G	RBM38
PGS1	3.70E-06	-0.35822	0	0.137	0.055241	Undiff-G	PGS1
MTHFD1L	3.70E-06	-0.38427	0	0.137	0.055241	Undiff-G	MTHFD1L
RAMP2	3.70E-06	-0.38667	0	0.137	0.055241	Undiff-G	RAMP2
SNUPN	3.70E-06	-0.20067	0.015	0.164	0.055352	Undiff-G	SNUPN
IMPACT	3.74E-06	-0.13148	0.015	0.164	0.055833	Undiff-G	IMPACT
ALG2	3.75E-06	-0.13821	0.015	0.165	0.056041	Undiff-G	ALG2
MCM5	3.75E-06	-0.35186	0.007	0.151	0.056044	Undiff-G	MCM5
RNF19A	3.76E-06	0.104153	0.037	0.204	0.056195	Undiff-G	RNF19A
ASNS	3.76E-06	-0.2163	0.022	0.179	0.056259	Undiff-G	ASNS
BCL7A	3.77E-06	-0.35623	0.007	0.151	0.056381	Undiff-G	BCL7A
ZFP91	3.79E-06	-0.32789	0	0.137	0.056585	Undiff-G	ZFP91
POU2F2	3.79E-06	-0.50652	0	0.137	0.056585	Undiff-G	POU2F2
NISCH	3.80E-06	-0.29605	0.022	0.178	0.056735	Undiff-G	NISCH
GRK6	3.82E-06	-0.33549	0	0.137	0.05704	Undiff-G	GRK6
SRI	3.83E-06	0.446475	0.309	0.734	0.057251	Undiff-G	SRI
SH2B1	3.83E-06	-0.34396	0.007	0.151	0.057284	Undiff-G	SH2B1
FILIP1L	3.88E-06	-0.39887	0.007	0.151	0.058014	Undiff-G	FILIP1L
IGSF21	3.90E-06	-0.43197	0.015	0.165	0.058238	Undiff-G	IGSF21
DESI1	3.90E-06	-0.13566	0.022	0.177	0.058351	Undiff-G	DESI1
QRICH1	3.92E-06	-0.30286	0.007	0.151	0.058544	Undiff-G	QRICH1
ANKH	3.92E-06	-0.45025	0.007	0.15	0.058638	Undiff-G	ANKH
LPIN2	3.93E-06	-0.2871	0.007	0.151	0.058741	Undiff-G	LPIN2
CGRRF1	3.94E-06	-0.10859	0.015	0.165	0.058818	Undiff-G	CGRRF1
KCTD17	3.94E-06	-0.33624	0	0.136	0.058897	Undiff-G	KCTD17
PADI2	3.94E-06	-0.56328	0	0.136	0.058897	Undiff-G	PADI2
TNFAIP3	3.98E-06	-0.38492	0.015	0.165	0.059489	Undiff-G	TNFAIP3
KLHL23	3.99E-06	-0.235	0.015	0.163	0.059653	Undiff-G	KLHL23
VPS41	4.00E-06	-0.13654	0.037	0.203	0.059793	Undiff-G	VPS41
ARSA	4.00E-06	-0.44097	0	0.136	0.059847	Undiff-G	ARSA
ZNF664	4.01E-06	-0.18813	0.007	0.151	0.059877	Undiff-G	ZNF664
DYRK4	4.09E-06	0.180244	0.044	0.219	0.061092	Undiff-G	DYRK4
ARHGAP12	4.14E-06	-0.15424	0.029	0.191	0.061903	Undiff-G	ARHGAP12
DPYSL5	4.15E-06	-0.19155	0.015	0.164	0.062079	Undiff-G	DPYSL5
TNKS	4.16E-06	-0.3037	0.007	0.15	0.062229	Undiff-G	TNKS
NFATC2IP	4.17E-06	-0.26537	0.015	0.164	0.06237	Undiff-G	NFATC2IP
FTO	4.20E-06	-0.35072	0	0.136	0.06279	Undiff-G	FTO
RPUSD1	4.24E-06	-0.31607	0	0.136	0.063294	Undiff-G	RPUSD1
BTBD1	4.24E-06	-0.12199	0.007	0.15	0.06332	Undiff-G	BTBD1
TXNIP	4.24E-06	0.450976	0.169	0.449	0.06334	Undiff-G	TXNIP
NFKBIZ	4.25E-06	-0.20588	0.022	0.177	0.06352	Undiff-G	NFKBIZ
PQLC1	4.27E-06	-0.34022	0	0.136	0.063802	Undiff-G	PQLC1
NSUN2	4.27E-06	-0.34614	0	0.136	0.063802	Undiff-G	NSUN2

LEMD2	4.28E-06	-0.30592	0.007	0.15	0.06402	Undiff-G	LEMD2
SYNE2	4.33E-06	-0.15255	0.022	0.176	0.064715	Undiff-G	SYNE2
UXS1	4.37E-06	0.258769	0.037	0.205	0.065316	Undiff-G	UXS1
SLC35E1	4.40E-06	-0.15342	0.015	0.164	0.065761	Undiff-G	SLC35E1
NAAA	4.41E-06	-0.41202	0	0.135	0.065874	Undiff-G	NAAA
ALKBH3	4.41E-06	-0.39259	0.007	0.149	0.065915	Undiff-G	ALKBH3
GIGYF2	4.43E-06	-0.16151	0.022	0.177	0.066127	Undiff-G	GIGYF2
ATG4D	4.43E-06	-0.30309	0.015	0.162	0.0662	Undiff-G	ATG4D
TSPAN17	4.43E-06	-0.29448	0.007	0.149	0.06624	Undiff-G	TSPAN17
ODF2L	4.43E-06	-0.32141	0.022	0.177	0.066262	Undiff-G	ODF2L
NUPL2	4.44E-06	0.336672	0.074	0.271	0.066394	Undiff-G	NUPL2
SLC25A23	4.44E-06	-0.29204	0	0.135	0.066403	Undiff-G	SLC25A23
COL9A3	4.45E-06	-0.3289	0.029	0.188	0.066487	Undiff-G	COL9A3
MRGBP	4.46E-06	-0.30763	0.007	0.148	0.066659	Undiff-G	MRGBP
WARS	4.48E-06	-0.14408	0.037	0.203	0.066882	Undiff-G	WARS
TMEM245	4.48E-06	-0.34137	0	0.135	0.066935	Undiff-G	TMEM245
GPR65	4.51E-06	-0.57468	0	0.135	0.067472	Undiff-G	GPR65
ABT1	4.52E-06	0.321976	0.044	0.218	0.067592	Undiff-G	ABT1
APC2	4.53E-06	-0.32636	0.007	0.149	0.067673	Undiff-G	APC2
PARVG	4.57E-06	-0.37821	0.015	0.163	0.068257	Undiff-G	PARVG
IL1RAP	4.58E-06	-0.30166	0.015	0.161	0.068427	Undiff-G	IL1RAP
NDUFAF1	4.62E-06	-0.12884	0.022	0.177	0.069051	Undiff-G	NDUFAF1
NOL11	4.62E-06	0.148325	0.029	0.191	0.069104	Undiff-G	NOL11
COX11	4.63E-06	0.509353	0.103	0.321	0.069249	Undiff-G	COX11
LRRC4B	4.66E-06	-0.29845	0	0.135	0.069662	Undiff-G	LRRC4B
KLHL8	4.66E-06	-0.35493	0	0.135	0.069662	Undiff-G	KLHL8
FBL	4.69E-06	0.702477	0.132	0.38	0.070065	Undiff-G	FBL
DICER1	4.73E-06	-0.13306	0.022	0.176	0.070671	Undiff-G	DICER1
TOPORS-AS	4.74E-06	-0.1946	0.007	0.149	0.070787	Undiff-G	TOPORS-AS1
HLA-F	4.74E-06	-0.53033	0.007	0.148	0.070877	Undiff-G	HLA-F
MTUS1	4.77E-06	-0.27721	0.007	0.149	0.071311	Undiff-G	MTUS1
ACTN1	4.77E-06	0.36037	0.066	0.257	0.071328	Undiff-G	ACTN1
ARFGAP3	4.79E-06	0.393099	0.051	0.231	0.071574	Undiff-G	ARFGAP3
CABIN1	4.81E-06	-0.34287	0	0.134	0.071921	Undiff-G	CABIN1
NCOA1	4.82E-06	-0.19523	0.015	0.163	0.07202	Undiff-G	NCOA1
RCC2	4.85E-06	-0.21631	0.022	0.176	0.072413	Undiff-G	RCC2
MIS18BP1	4.88E-06	-0.43506	0.007	0.148	0.072865	Undiff-G	MIS18BP1
GFM1	4.89E-06	-0.16471	0.029	0.188	0.07304	Undiff-G	GFM1
PPP3CC	4.89E-06	-0.36517	0	0.134	0.073077	Undiff-G	PPP3CC
COQ7	4.89E-06	-0.1496	0.029	0.189	0.073138	Undiff-G	COQ7
PRKD3	4.92E-06	-0.34686	0.007	0.148	0.073554	Undiff-G	PRKD3
RASSF1	4.94E-06	-0.33197	0.015	0.16	0.073857	Undiff-G	RASSF1
BARD1	4.94E-06	-0.33084	0.007	0.148	0.073883	Undiff-G	BARD1
SSFA2	4.96E-06	-0.15742	0.029	0.188	0.074169	Undiff-G	SSFA2
AGTRAP	4.97E-06	0.529094	0.081	0.284	0.074201	Undiff-G	AGTRAP
SLC48A1	4.97E-06	-0.42146	0	0.134	0.074252	Undiff-G	SLC48A1
HIST2H2BE	4.97E-06	-0.18611	0.007	0.148	0.074252	Undiff-G	HIST2H2BE
CTNNAL1	4.97E-06	-0.21106	0.007	0.148	0.074252	Undiff-G	CTNNAL1
TACO1	4.98E-06	-0.13196	0.022	0.176	0.074413	Undiff-G	TACO1
NOTCH2	4.98E-06	-0.31316	0.007	0.148	0.074464	Undiff-G	NOTCH2
PDIA6	5.00E-06	0.694222	0.265	0.652	0.074724	Undiff-G	PDIA6

ZNF267	5.01E-06	-0.43002	0	0.134	0.074846	Undiff-G	ZNF267
SNAPC1	5.01E-06	0.163046	0.029	0.19	0.07489	Undiff-G	SNAPC1
HS2ST1	5.02E-06	0.102088	0.022	0.176	0.075006	Undiff-G	HS2ST1
PDCD5	5.03E-06	0.711314	0.221	0.543	0.075177	Undiff-G	PDCD5
RNF2	5.05E-06	-0.19134	0.007	0.148	0.075414	Undiff-G	RNF2
RHOT2	5.05E-06	0.116583	0.066	0.253	0.075528	Undiff-G	RHOT2
C1orf131	5.07E-06	-0.10668	0.037	0.202	0.075825	Undiff-G	C1orf131
DNAJB14	5.08E-06	0.137631	0.037	0.202	0.075965	Undiff-G	DNAJB14
FAIM	5.09E-06	-0.14275	0.007	0.148	0.076017	Undiff-G	FAIM
SSSCA1	5.09E-06	0.100236	0.029	0.189	0.0761	Undiff-G	SSSCA1
LDLRAD4	5.15E-06	-0.48366	0.007	0.147	0.076917	Undiff-G	LDLRAD4
KDM4B	5.17E-06	-0.33553	0	0.133	0.077271	Undiff-G	KDM4B
WRAP73	5.17E-06	-0.13933	0.007	0.148	0.077282	Undiff-G	WRAP73
CASP4	5.18E-06	0.230722	0.051	0.23	0.077337	Undiff-G	CASP4
ARHGAP35	5.19E-06	-0.25434	0.007	0.148	0.077608	Undiff-G	ARHGAP35
EHMT1	5.19E-06	-0.30721	0.007	0.148	0.077629	Undiff-G	EHMT1
NTHL1	5.25E-06	0.173928	0.029	0.189	0.07843	Undiff-G	NTHL1
MIR4458HG	5.25E-06	-0.21287	0.015	0.162	0.078497	Undiff-G	MIR4458HG
SQRDL	5.28E-06	-0.19722	0.015	0.162	0.078859	Undiff-G	SQRDL
MED16	5.28E-06	-0.19187	0.015	0.161	0.078948	Undiff-G	MED16
TRIM4	5.31E-06	-0.31442	0.007	0.148	0.079315	Undiff-G	TRIM4
NDRG4	5.34E-06	-0.11222	0.015	0.162	0.079859	Undiff-G	NDRG4
TMEM222	5.35E-06	0.499059	0.081	0.282	0.07998	Undiff-G	TMEM222
KLF2	5.38E-06	-0.55918	0	0.133	0.08041	Undiff-G	KLF2
MRS2	5.39E-06	-0.11002	0.015	0.162	0.080555	Undiff-G	MRS2
ARMCX1	5.40E-06	0.102098	0.044	0.215	0.080654	Undiff-G	ARMCX1
ARHGAP1	5.40E-06	-0.22385	0.015	0.161	0.080719	Undiff-G	ARHGAP1
MSL1	5.41E-06	-0.32326	0.015	0.16	0.080816	Undiff-G	MSL1
DYNC1LI1	5.44E-06	0.403163	0.074	0.269	0.081252	Undiff-G	DYNC1LI1
C1orf21	5.45E-06	0.273969	0.037	0.202	0.081479	Undiff-G	C1orf21
ABCG1	5.47E-06	-0.47059	0	0.133	0.0817	Undiff-G	ABCG1
CDK5RAP1	5.51E-06	-0.31485	0.015	0.16	0.082268	Undiff-G	CDK5RAP1
SC5D	5.51E-06	-0.15457	0.022	0.175	0.082315	Undiff-G	SC5D
FRA10AC1	5.52E-06	-0.28319	0.015	0.161	0.082507	Undiff-G	FRA10AC1
OSBPL9	5.53E-06	-0.12096	0.029	0.188	0.082676	Undiff-G	OSBPL9
VMO1	5.55E-06	-0.39402	0.015	0.161	0.083002	Undiff-G	VMO1
HLA-DQA2	5.59E-06	-0.62314	0.022	0.174	0.083481	Undiff-G	HLA-DQA2
DCPS	5.60E-06	-0.22781	0.022	0.173	0.083616	Undiff-G	DCPS
SRGAP3	5.62E-06	-0.34395	0.007	0.147	0.083946	Undiff-G	SRGAP3
PAXBP1	5.62E-06	-0.10008	0.015	0.161	0.084005	Undiff-G	PAXBP1
ZFP90	5.64E-06	-0.33482	0	0.133	0.084342	Undiff-G	ZFP90
RPS6KA1	5.64E-06	-0.49599	0	0.133	0.084342	Undiff-G	RPS6KA1
SPEN	5.68E-06	-0.27514	0.015	0.161	0.084942	Undiff-G	SPEN
BMS1	5.78E-06	-0.20704	0.007	0.147	0.086309	Undiff-G	BMS1
HSDL1	5.78E-06	-0.36934	0	0.132	0.086379	Undiff-G	HSDL1
WSCD1	5.83E-06	-0.33632	0	0.132	0.087068	Undiff-G	WSCD1
AIFM1	5.83E-06	-0.12681	0.007	0.147	0.087082	Undiff-G	AIFM1
TMX3	5.84E-06	-0.2896	0.015	0.159	0.087241	Undiff-G	TMX3
WBSCR16	5.87E-06	-0.14526	0.007	0.147	0.087759	Undiff-G	WBSCR16
RTKN	5.89E-06	-0.35529	0.022	0.173	0.088015	Undiff-G	RTKN
FAHD2A	5.90E-06	-0.12962	0.022	0.174	0.088145	Undiff-G	FAHD2A

OPA1	5.92E-06	-0.33168	0	0.132	0.088464	Undiff-G	OPA1
CCDC94	5.95E-06	-0.31419	0.015	0.16	0.088849	Undiff-G	CCDC94
FUBP1	5.95E-06	0.250547	0.051	0.226	0.088938	Undiff-G	FUBP1
CHRAC1	5.96E-06	-0.38433	0.007	0.146	0.089132	Undiff-G	CHRAC1
ANKRD36	5.98E-06	-0.22185	0.029	0.187	0.089373	Undiff-G	ANKRD36
XPC	5.99E-06	-0.22804	0.015	0.16	0.089554	Undiff-G	XPC
GOSR2	6.00E-06	0.130785	0.029	0.187	0.089668	Undiff-G	GOSR2
NSUN5	6.01E-06	0.174322	0.051	0.225	0.089839	Undiff-G	NSUN5
ZFYVE16	6.02E-06	-0.32053	0.022	0.173	0.089918	Undiff-G	ZFYVE16
FCGR2B	6.08E-06	-0.63826	0.007	0.146	0.090876	Undiff-G	FCGR2B
DUS3L	6.11E-06	-0.33619	0.007	0.145	0.091273	Undiff-G	DUS3L
KAT6A	6.12E-06	-0.22793	0.015	0.16	0.091531	Undiff-G	KAT6A
AGPAT3	6.16E-06	-0.33621	0	0.132	0.092049	Undiff-G	AGPAT3
PIM1	6.16E-06	-0.44869	0	0.132	0.092049	Undiff-G	PIM1
TNIK	6.16E-06	-0.30362	0.007	0.146	0.092087	Undiff-G	TNIK
CWF19L2	6.17E-06	-0.2692	0.022	0.173	0.092131	Undiff-G	CWF19L2
DDX19A	6.17E-06	-0.1138	0.015	0.16	0.092262	Undiff-G	DDX19A
CCDC43	6.18E-06	0.12261	0.037	0.201	0.09231	Undiff-G	CCDC43
KIF3B	6.21E-06	-0.28398	0	0.132	0.092783	Undiff-G	KIF3B
SNCA	6.25E-06	-0.5735	0.007	0.146	0.093342	Undiff-G	SNCA
CNIH2	6.26E-06	-0.30453	0	0.131	0.093522	Undiff-G	CNIH2
CNTFR	6.26E-06	-0.34282	0	0.131	0.093522	Undiff-G	CNTFR
LETM1	6.33E-06	-0.1932	0.029	0.187	0.094601	Undiff-G	LETM1
HMGCS1	6.33E-06	-0.2216	0.029	0.185	0.094608	Undiff-G	HMGCS1
RAB40B	6.36E-06	-0.3835	0	0.131	0.095019	Undiff-G	RAB40B
PHF1	6.39E-06	-0.32672	0.022	0.171	0.09545	Undiff-G	PHF1
TPD52	6.39E-06	0.304093	0.059	0.238	0.095548	Undiff-G	TPD52
SETD2	6.40E-06	-0.32273	0.007	0.145	0.095654	Undiff-G	SETD2
DSTN	6.44E-06	0.558181	0.257	0.622	0.09629	Undiff-G	DSTN
RNMTL1	6.46E-06	-0.32143	0	0.131	0.096539	Undiff-G	RNMTL1
CLN6	6.46E-06	-0.37019	0	0.131	0.096539	Undiff-G	CLN6
VASH1	6.46E-06	-0.43831	0	0.131	0.096539	Undiff-G	VASH1
KCNQ1OT1	6.47E-06	0.344083	0.081	0.28	0.096639	Undiff-G	KCNQ1OT1
CTNND2	6.47E-06	-0.28949	0.015	0.159	0.096664	Undiff-G	CTNND2
HDAC5	6.50E-06	-0.28625	0.022	0.171	0.097087	Undiff-G	HDAC5
CCDC58	6.51E-06	-0.10802	0.007	0.145	0.097269	Undiff-G	CCDC58
NAPG	6.52E-06	-0.17546	0.015	0.16	0.097398	Undiff-G	NAPG
SKIV2L2	6.56E-06	-0.22135	0.015	0.158	0.098053	Undiff-G	SKIV2L2
TMEM161A	6.59E-06	-0.14174	0.022	0.173	0.09849	Undiff-G	TMEM161A
PTEN	6.62E-06	-0.37139	0	0.131	0.098865	Undiff-G	PTEN
NEIL2	6.67E-06	0.309747	0.037	0.2	0.099622	Undiff-G	NEIL2
DLL3	6.67E-06	-0.39729	0.007	0.145	0.099648	Undiff-G	DLL3
GALE	6.67E-06	-0.3352	0.015	0.157	0.09975	Undiff-G	GALE
SMARCA1	6.68E-06	-0.16601	0.022	0.173	0.099851	Undiff-G	SMARCA1
GOLT1B	6.71E-06	0.352032	0.059	0.24	0.100328	Undiff-G	GOLT1B
ENDOD1	6.71E-06	-0.20878	0.007	0.145	0.100341	Undiff-G	ENDOD1
POP4	6.72E-06	0.513083	0.074	0.267	0.100356	Undiff-G	POP4
CD84	6.72E-06	-0.55079	0	0.131	0.100445	Undiff-G	CD84
PAK3	6.73E-06	-0.41816	0.007	0.145	0.100526	Undiff-G	PAK3
PARP9	6.76E-06	-0.132	0.022	0.172	0.101017	Undiff-G	PARP9
S100A11	6.80E-06	1.058217	0.551	0.658	0.101557	Undiff-G	S100A11

ZCCHC7	6.87E-06	-0.32524	0.007	0.145	0.102663	Undiff-G	ZCCHC7
TBC1D17	6.91E-06	-0.34856	0.015	0.157	0.103221	Undiff-G	TBC1D17
PHF6	6.91E-06	-0.3108	0.022	0.172	0.103316	Undiff-G	PHF6
MED8	6.91E-06	0.330229	0.051	0.226	0.103334	Undiff-G	MED8
HPS3	6.92E-06	-0.36543	0.007	0.144	0.103436	Undiff-G	HPS3
B4GALNT1	6.93E-06	-0.38831	0.007	0.145	0.10361	Undiff-G	B4GALNT1
NUMA1	6.95E-06	-0.16303	0.022	0.172	0.103803	Undiff-G	NUMA1
RALB	6.95E-06	-0.25665	0.015	0.159	0.103844	Undiff-G	RALB
TRIP6	6.96E-06	0.17454	0.066	0.251	0.103993	Undiff-G	TRIP6
THSD7A	6.96E-06	-0.47423	0.007	0.144	0.103997	Undiff-G	THSD7A
NUP62	6.96E-06	0.471106	0.059	0.239	0.104082	Undiff-G	NUP62
LINC00094	6.99E-06	-0.29299	0	0.13	0.104507	Undiff-G	LINC00094
CSNK1G2	7.10E-06	-0.27864	0.007	0.144	0.10611	Undiff-G	CSNK1G2
CHAMP1	7.16E-06	-0.29332	0	0.13	0.10702	Undiff-G	CHAMP1
TSTD1	7.20E-06	0.153979	0.037	0.199	0.107557	Undiff-G	TSTD1
FBXO44	7.20E-06	-0.36518	0.007	0.143	0.1076	Undiff-G	FBXO44
WDR12	7.25E-06	-0.26836	0.007	0.144	0.10839	Undiff-G	WDR12
SSBP3	7.28E-06	-0.33714	0	0.13	0.108729	Undiff-G	SSBP3
ARHGEF40	7.28E-06	-0.37899	0	0.13	0.108729	Undiff-G	ARHGEF40
IREB2	7.29E-06	-0.11025	0.015	0.157	0.108931	Undiff-G	IREB2
REEP4	7.31E-06	-0.36616	0.022	0.169	0.109299	Undiff-G	REEP4
MED15	7.32E-06	-0.24698	0.007	0.144	0.109443	Undiff-G	MED15
C3orf17	7.32E-06	-0.19027	0.007	0.144	0.109465	Undiff-G	C3orf17
ZNF394	7.33E-06	-0.39292	0.007	0.144	0.109475	Undiff-G	ZNF394
MAD2L1	7.33E-06	-0.43307	0	0.13	0.109594	Undiff-G	MAD2L1
GNPTAB	7.35E-06	-0.33886	0.015	0.158	0.109794	Undiff-G	GNPTAB
C19orf12	7.38E-06	-0.17964	0.007	0.144	0.11029	Undiff-G	C19orf12
LRRC40	7.44E-06	-0.25959	0.007	0.144	0.111122	Undiff-G	LRRC40
ADCK3	7.45E-06	-0.35444	0	0.13	0.111343	Undiff-G	ADCK3
RRS1	7.50E-06	-0.21172	0.007	0.144	0.112113	Undiff-G	RRS1
TULP4	7.57E-06	-0.21267	0.007	0.144	0.113069	Undiff-G	TULP4
RRP1	7.58E-06	0.225174	0.044	0.212	0.113229	Undiff-G	RRP1
SACS	7.65E-06	-0.28447	0.015	0.156	0.114352	Undiff-G	SACS
CDC26	7.66E-06	0.470214	0.074	0.264	0.114545	Undiff-G	CDC26
RCBTB2	7.68E-06	-0.37623	0.007	0.144	0.114763	Undiff-G	RCBTB2
DNAL4	7.69E-06	-0.31806	0	0.129	0.114923	Undiff-G	DNAL4
NUP88	7.69E-06	-0.33015	0	0.129	0.114923	Undiff-G	NUP88
SEPN1	7.78E-06	0.225006	0.044	0.211	0.116316	Undiff-G	SEPN1
RNF180	7.86E-06	-0.2396	0.022	0.169	0.117453	Undiff-G	RNF180
ORAI3	7.87E-06	-0.36661	0.007	0.143	0.117659	Undiff-G	ORAI3
C16orf72	7.87E-06	-0.33934	0	0.129	0.117683	Undiff-G	C16orf72
PSMD5-AS1	7.88E-06	-0.27836	0.029	0.184	0.117723	Undiff-G	PSMD5-AS1
APH1B	7.94E-06	-0.42723	0	0.129	0.118617	Undiff-G	APH1B
IST1	7.97E-06	0.298717	0.074	0.262	0.119167	Undiff-G	IST1
EXTL2	7.98E-06	-0.28553	0.022	0.169	0.119253	Undiff-G	EXTL2
REPS1	8.00E-06	-0.29833	0	0.129	0.119559	Undiff-G	REPS1
SDC3	8.04E-06	0.184091	0.037	0.198	0.120084	Undiff-G	SDC3
PRDX1	8.06E-06	0.442511	0.346	0.814	0.120481	Undiff-G	PRDX1
TCF7	8.10E-06	-0.38128	0.007	0.141	0.121028	Undiff-G	TCF7
NPTXR	8.13E-06	-0.29535	0	0.129	0.121464	Undiff-G	NPTXR
HCFC1	8.15E-06	-0.27868	0.007	0.143	0.12176	Undiff-G	HCFC1

FAM129A	8.15E-06	-0.36966	0.007	0.141	0.121865	Undiff-G	FAM129A
TMEM55A	8.26E-06	0.306119	0.059	0.235	0.123371	Undiff-G	TMEM55A
UBE2T	8.26E-06	-0.42019	0	0.128	0.123399	Undiff-G	UBE2T
NFKBID	8.26E-06	-0.52877	0.007	0.142	0.123402	Undiff-G	NFKBID
SOX6	8.30E-06	-0.29499	0.007	0.143	0.124084	Undiff-G	SOX6
RP11-452F	8.32E-06	-0.46705	0	0.128	0.124378	Undiff-G	RP11-452F19.3
SMYD2	8.33E-06	-0.32248	0.007	0.142	0.124545	Undiff-G	SMYD2
SMIM3	8.35E-06	1.140906	0.103	0.035	0.124743	Undiff-G	SMIM3
PMM1	8.35E-06	0.401968	0.059	0.235	0.124845	Undiff-G	PMM1
RHNO1	8.37E-06	0.124197	0.037	0.197	0.125083	Undiff-G	RHNO1
DCUN1D1	8.39E-06	-0.24074	0.015	0.157	0.125346	Undiff-G	DCUN1D1
EPDR1	8.46E-06	-0.30146	0	0.128	0.126358	Undiff-G	EPDR1
MFNG	8.46E-06	-0.51314	0	0.128	0.126358	Undiff-G	MFNG
CCDC22	8.52E-06	-0.34844	0	0.128	0.12736	Undiff-G	CCDC22
DVL3	8.53E-06	-0.28529	0.015	0.155	0.127493	Undiff-G	DVL3
WHSC1	8.58E-06	-0.30318	0.015	0.155	0.128227	Undiff-G	WHSC1
SPIN1	8.61E-06	-0.11982	0.022	0.168	0.128702	Undiff-G	SPIN1
MICAL1	8.65E-06	-0.23195	0.007	0.142	0.129276	Undiff-G	MICAL1
EIF4EBP2	8.65E-06	-0.23501	0.007	0.142	0.129302	Undiff-G	EIF4EBP2
C16orf91	8.69E-06	0.282106	0.037	0.197	0.129816	Undiff-G	C16orf91
FOPNL	8.69E-06	0.191551	0.037	0.195	0.129906	Undiff-G	FOPNL
MYH10	8.69E-06	-0.21899	0.015	0.155	0.129918	Undiff-G	MYH10
STK19	8.73E-06	-0.31447	0	0.128	0.130413	Undiff-G	STK19
CERS6	8.73E-06	-0.32562	0	0.128	0.130413	Undiff-G	CERS6
TMEM173	8.73E-06	-0.5528	0	0.128	0.130413	Undiff-G	TMEM173
LINC0093€	8.80E-06	-0.39293	0.007	0.142	0.131439	Undiff-G	LINC00936
PDP1	8.80E-06	-0.34567	0	0.128	0.131446	Undiff-G	PDP1
PPP2R5B	8.83E-06	-0.27139	0.022	0.168	0.131988	Undiff-G	PPP2R5B
SHPRH	8.87E-06	-0.33013	0	0.128	0.132488	Undiff-G	SHPRH
CENPH	8.87E-06	-0.34232	0	0.128	0.132488	Undiff-G	CENPH
DPH7	8.90E-06	-0.27426	0.022	0.169	0.133004	Undiff-G	DPH7
HMOX1	8.95E-06	0.129851	0.096	0.297	0.133783	Undiff-G	HMOX1
SRR	8.99E-06	-0.29427	0.015	0.155	0.134382	Undiff-G	SRR
DPF1	8.99E-06	-0.21858	0.007	0.142	0.134392	Undiff-G	DPF1
DOCK10	9.08E-06	-0.39901	0	0.127	0.135661	Undiff-G	DOCK10
SHD	9.08E-06	-0.45222	0	0.127	0.135661	Undiff-G	SHD
TATDN3	9.15E-06	-0.19065	0.015	0.155	0.136756	Undiff-G	TATDN3
NAB1	9.22E-06	-0.31487	0	0.127	0.137818	Undiff-G	NAB1
NCOA3	9.22E-06	-0.32903	0	0.127	0.137818	Undiff-G	NCOA3
RNF115	9.34E-06	0.182531	0.066	0.245	0.139642	Undiff-G	RNF115
MLLT10	9.37E-06	-0.33899	0	0.127	0.140009	Undiff-G	MLLT10
CDK19	9.37E-06	-0.36362	0	0.127	0.140009	Undiff-G	CDK19
TUBB	9.37E-06	0.461019	0.331	0.776	0.140013	Undiff-G	TUBB
IFI35	9.39E-06	0.216652	0.059	0.233	0.140376	Undiff-G	IFI35
SLC25A46	9.49E-06	-0.31247	0.015	0.155	0.141763	Undiff-G	SLC25A46
NELFA	9.51E-06	-0.21307	0.007	0.141	0.142088	Undiff-G	NELFA
TPPP3	9.52E-06	-0.3053	0.015	0.155	0.142315	Undiff-G	TPPP3
SLC25A25	9.57E-06	-0.19254	0.022	0.167	0.143022	Undiff-G	SLC25A25
GPATCH11	9.64E-06	-0.34061	0.007	0.14	0.143994	Undiff-G	GPATCH11
SLC2A8	9.67E-06	-0.32537	0.007	0.141	0.144556	Undiff-G	SLC2A8
NDEL1	9.73E-06	-0.15251	0.015	0.155	0.145376	Undiff-G	NDEL1

ATXN7L3B	9.74E-06	0.177683	0.029	0.183	0.145495	Undiff-G	ATXN7L3B
IFI16	9.78E-06	0.538435	0.147	0.396	0.146086	Undiff-G	IFI16
NADK	9.81E-06	-0.14854	0.022	0.169	0.146564	Undiff-G	NADK
ELOVL1	9.86E-06	0.22512	0.059	0.233	0.14733	Undiff-G	ELOVL1
TMEM158	9.88E-06	-0.22209	0.007	0.141	0.147672	Undiff-G	TMEM158
ASIC4	9.89E-06	-0.41141	0.007	0.141	0.147759	Undiff-G	ASIC4
SEZ6	9.93E-06	-0.38041	0.007	0.14	0.148367	Undiff-G	SEZ6
NMRK1	9.94E-06	-0.33635	0.007	0.141	0.148576	Undiff-G	NMRK1
SIPA1	9.97E-06	-0.28866	0.007	0.141	0.149012	Undiff-G	SIPA1
ORAI1	9.98E-06	-0.28954	0	0.126	0.149118	Undiff-G	ORAI1
SHOC2	1.00E-05	-0.33775	0.015	0.153	0.149385	Undiff-G	SHOC2
CLASP1	1.00E-05	-0.33025	0.007	0.139	0.149675	Undiff-G	CLASP1
PHKB	1.00E-05	-0.26166	0.037	0.194	0.149818	Undiff-G	PHKB
NSMCE4A	1.00E-05	-0.14456	0.015	0.154	0.14992	Undiff-G	NSMCE4A
NKAIN3	1.01E-05	-0.32162	0	0.126	0.151485	Undiff-G	NKAIN3
GALM	1.01E-05	-0.43313	0	0.126	0.151485	Undiff-G	GALM
FAM98C	1.01E-05	-0.3528	0.007	0.139	0.151627	Undiff-G	FAM98C
BHLHE40	1.01E-05	0.236514	0.037	0.195	0.151634	Undiff-G	BHLHE40
UBE4A	1.02E-05	-0.24075	0.015	0.154	0.151734	Undiff-G	UBE4A
MARK2	1.02E-05	-0.31614	0	0.126	0.152682	Undiff-G	MARK2
PAN3	1.02E-05	-0.3671	0	0.126	0.152682	Undiff-G	PAN3
MEG3	1.03E-05	-0.11703	0.103	0.307	0.153717	Undiff-G	MEG3
CCND3	1.03E-05	0.504508	0.074	0.262	0.15388	Undiff-G	CCND3
PNMA2	1.03E-05	-0.25114	0.007	0.14	0.154651	Undiff-G	PNMA2
RABGAP1	1.04E-05	-0.32963	0	0.126	0.155105	Undiff-G	RABGAP1
LANCL2	1.04E-05	-0.29841	0.015	0.154	0.156071	Undiff-G	LANCL2
ZMIZ1	1.05E-05	-0.15358	0.007	0.14	0.156206	Undiff-G	ZMIZ1
TMEM19	1.05E-05	-0.21894	0.015	0.154	0.15658	Undiff-G	TMEM19
GNL2	1.05E-05	0.271037	0.044	0.208	0.156859	Undiff-G	GNL2
IRF1	1.05E-05	0.12993	0.044	0.208	0.156963	Undiff-G	IRF1
ZNF576	1.05E-05	-0.35626	0.007	0.139	0.157418	Undiff-G	ZNF576
THAP5	1.06E-05	-0.12776	0.029	0.181	0.158206	Undiff-G	THAP5
PRPF18	1.06E-05	-0.3562	0.007	0.139	0.158377	Undiff-G	PRPF18
FKBP15	1.06E-05	-0.37281	0.007	0.14	0.158613	Undiff-G	FKBP15
ABHD13	1.06E-05	-0.28877	0.007	0.14	0.159088	Undiff-G	ABHD13
AL592183.	1.07E-05	-0.34617	0.022	0.167	0.159672	Undiff-G	AL592183.1
ANXA2R	1.07E-05	-0.25037	0.015	0.154	0.160114	Undiff-G	ANXA2R
KCTD13	1.08E-05	-0.29787	0.007	0.14	0.160913	Undiff-G	KCTD13
FMNL1	1.08E-05	-0.49655	0	0.125	0.161327	Undiff-G	FMNL1
CDK12	1.08E-05	-0.19118	0.022	0.167	0.161618	Undiff-G	CDK12
BTBD3	1.08E-05	-0.30087	0.007	0.139	0.162087	Undiff-G	BTBD3
GK	1.09E-05	-0.16974	0.007	0.14	0.162461	Undiff-G	GK
MT1G	1.09E-05	1.459658	0.228	0.127	0.162568	Undiff-G	MT1G
C15orf57	1.09E-05	-0.38397	0	0.125	0.162601	Undiff-G	C15orf57
NABP1	1.09E-05	-0.50767	0	0.125	0.162601	Undiff-G	NABP1
IPO9	1.09E-05	0.172682	0.059	0.233	0.163074	Undiff-G	IPO9
LIMCH1	1.09E-05	-0.15375	0.015	0.154	0.163455	Undiff-G	LIMCH1
PWP2	1.10E-05	-0.3655	0	0.125	0.163884	Undiff-G	PWP2
AASDH	1.10E-05	-0.27821	0.022	0.165	0.164086	Undiff-G	AASDH
ERF	1.10E-05	-0.24657	0.029	0.179	0.164251	Undiff-G	ERF
FAR1	1.10E-05	-0.36688	0.007	0.139	0.164939	Undiff-G	FAR1

PTGER4	1.11E-05	-0.52779	0	0.125	0.165178	Undiff-G	PTGER4
STK40	1.11E-05	-0.30061	0	0.125	0.166481	Undiff-G	STK40
MESDC1	1.11E-05	-0.33004	0	0.125	0.166481	Undiff-G	MESDC1
SENP5	1.12E-05	-0.27511	0.007	0.139	0.167974	Undiff-G	SENP5
LRCH4	1.14E-05	-0.19367	0.037	0.19	0.17103	Undiff-G	LRCH4
CCZ1B	1.15E-05	-0.27823	0.015	0.153	0.171451	Undiff-G	CCZ1B
LYST	1.15E-05	-0.12995	0.015	0.152	0.171566	Undiff-G	LYST
NCF1	1.15E-05	-0.49853	0	0.125	0.171797	Undiff-G	NCF1
MGMT	1.16E-05	-0.14766	0.015	0.153	0.172861	Undiff-G	MGMT
STRN4	1.16E-05	-0.29853	0	0.125	0.173152	Undiff-G	STRN4
ZNF704	1.16E-05	-0.33246	0	0.125	0.173152	Undiff-G	ZNF704
MT3	1.16E-05	1.232347	0.485	0.49	0.1739	Undiff-G	MT3
MED25	1.17E-05	-0.31299	0	0.125	0.174517	Undiff-G	MED25
PPP6R2	1.17E-05	-0.31336	0.015	0.151	0.175338	Undiff-G	PPP6R2
PITPNC1	1.18E-05	-0.28664	0	0.125	0.175893	Undiff-G	PITPNC1
AOAH	1.19E-05	-0.55993	0	0.124	0.17728	Undiff-G	AOAH
AKAP11	1.19E-05	-0.25757	0.015	0.151	0.17757	Undiff-G	AKAP11
ALG14	1.19E-05	-0.15242	0.015	0.153	0.177668	Undiff-G	ALG14
GLCCI1	1.19E-05	-0.3382	0.015	0.153	0.178421	Undiff-G	GLCCI1
PRKAG2	1.19E-05	-0.22947	0.007	0.139	0.178492	Undiff-G	PRKAG2
NCLN	1.20E-05	-0.1448	0.022	0.166	0.178901	Undiff-G	NCLN
CADM1	1.20E-05	0.609517	0.11	0.327	0.179864	Undiff-G	CADM1
EVA1B	1.21E-05	-0.25668	0	0.124	0.180085	Undiff-G	EVA1B
METTL13	1.21E-05	-0.31281	0	0.124	0.180085	Undiff-G	METTL13
CLEC2B	1.21E-05	0.10262	0.044	0.207	0.180273	Undiff-G	CLEC2B
ATM	1.21E-05	-0.20053	0.037	0.191	0.180781	Undiff-G	ATM
MBD3	1.21E-05	-0.2645	0.022	0.165	0.181066	Undiff-G	MBD3
NSF	1.21E-05	-0.16228	0.007	0.139	0.181276	Undiff-G	NSF
CARD16	1.21E-05	0.179899	0.059	0.231	0.181328	Undiff-G	CARD16
NRIP1	1.21E-05	-0.29488	0	0.124	0.181504	Undiff-G	NRIP1
ITGA7	1.22E-05	-0.27859	0.007	0.138	0.181683	Undiff-G	ITGA7
SMAD1	1.22E-05	-0.15863	0.029	0.179	0.18214	Undiff-G	SMAD1
RPS6KA3	1.22E-05	-0.23694	0.007	0.139	0.182594	Undiff-G	RPS6KA3
LINC00685	1.22E-05	-0.29814	0.015	0.151	0.182813	Undiff-G	LINC00685
GUCD1	1.22E-05	-0.31476	0	0.124	0.182934	Undiff-G	GUCD1
TBC1D1	1.22E-05	-0.34374	0	0.124	0.182934	Undiff-G	TBC1D1
AC004540.	1.22E-05	-0.43918	0	0.124	0.182934	Undiff-G	AC004540.4
SUMF1	1.23E-05	-0.28346	0.007	0.138	0.18317	Undiff-G	SUMF1
RMND5B	1.23E-05	-0.25705	0.022	0.166	0.183824	Undiff-G	RMND5B
AC093673.	1.23E-05	0.239964	0.029	0.18	0.183882	Undiff-G	AC093673.5
CTTNBP2	1.23E-05	-0.39445	0.007	0.138	0.184302	Undiff-G	CTTNBP2
MOB3A	1.24E-05	-0.22718	0.022	0.164	0.184602	Undiff-G	MOB3A
PDK2	1.24E-05	-0.10129	0.022	0.166	0.185526	Undiff-G	PDK2
ZNF544	1.24E-05	-0.33266	0	0.124	0.185827	Undiff-G	ZNF544
CNOT10	1.24E-05	-0.3561	0	0.124	0.185827	Undiff-G	CNOT10
DSCR3	1.25E-05	0.204738	0.022	0.166	0.187174	Undiff-G	DSCR3
ASMTL	1.25E-05	0.2192	0.022	0.166	0.187242	Undiff-G	ASMTL
OST4	1.25E-05	0.501482	0.331	0.785	0.187412	Undiff-G	OST4
ENHO	1.26E-05	-0.26791	0.015	0.152	0.188068	Undiff-G	ENHO
UROD	1.26E-05	0.566122	0.132	0.362	0.188609	Undiff-G	UROD
OLFM1	1.27E-05	-0.21149	0.015	0.152	0.189406	Undiff-G	OLFM1

FADS2	1.27E-05	-0.29736	0.007	0.138	0.189931	Undiff-G	FADS2
MACROD1	1.27E-05	-0.28502	0	0.124	0.190251	Undiff-G	MACROD1
AGPS	1.27E-05	-0.31949	0	0.124	0.190251	Undiff-G	AGPS
ZNF639	1.28E-05	-0.28328	0.007	0.138	0.191651	Undiff-G	ZNF639
FAM107B	1.28E-05	-0.44201	0	0.124	0.191749	Undiff-G	FAM107B
UCHL5	1.28E-05	-0.12643	0.015	0.15	0.191872	Undiff-G	UCHL5
DVL2	1.29E-05	-0.25098	0.029	0.178	0.192402	Undiff-G	DVL2
ZFAND2A	1.30E-05	0.481448	0.088	0.285	0.194308	Undiff-G	ZFAND2A
MIB2	1.30E-05	-0.10076	0.015	0.151	0.194661	Undiff-G	MIB2
NAT9	1.30E-05	-0.11535	0.044	0.205	0.194692	Undiff-G	NAT9
FADD	1.30E-05	-0.27302	0.007	0.138	0.195015	Undiff-G	FADD
ZC3H7A	1.31E-05	-0.31246	0.015	0.151	0.195142	Undiff-G	ZC3H7A
GAR1	1.31E-05	0.191312	0.037	0.192	0.195221	Undiff-G	GAR1
ANKRD36C	1.31E-05	-0.33874	0.022	0.164	0.195342	Undiff-G	ANKRD36C
CHST2	1.31E-05	-0.28131	0.007	0.137	0.195976	Undiff-G	CHST2
FEN1	1.31E-05	-0.11251	0.007	0.138	0.196036	Undiff-G	FEN1
RP11-71N1	1.31E-05	-0.33389	0.007	0.137	0.196205	Undiff-G	RP11-71N10.1
CNOT11	1.31E-05	-0.29243	0	0.123	0.196312	Undiff-G	CNOT11
MIOS	1.31E-05	-0.34385	0	0.123	0.196312	Undiff-G	MIOS
DCTN5	1.31E-05	-0.34688	0	0.123	0.196312	Undiff-G	DCTN5
TOMM34	1.32E-05	-0.25859	0.007	0.138	0.196615	Undiff-G	TOMM34
NCSTN	1.32E-05	0.304041	0.074	0.256	0.197138	Undiff-G	NCSTN
ZYG11B	1.33E-05	-0.19301	0.015	0.15	0.198872	Undiff-G	ZYG11B
ELP2	1.33E-05	0.148017	0.044	0.205	0.199168	Undiff-G	ELP2
SMAD4	1.33E-05	-0.27795	0	0.123	0.199413	Undiff-G	SMAD4
C16orf62	1.35E-05	-0.2252	0.015	0.15	0.201069	Undiff-G	C16orf62
ARRDC1	1.35E-05	-0.38626	0.007	0.136	0.201075	Undiff-G	ARRDC1
CRKL	1.36E-05	-0.3068	0	0.123	0.202562	Undiff-G	CRKL
RPS6KA4	1.36E-05	-0.34465	0	0.123	0.202562	Undiff-G	RPS6KA4
RIOK2	1.36E-05	-0.36204	0	0.123	0.202562	Undiff-G	RIOK2
LLPH	1.36E-05	0.485116	0.081	0.27	0.202649	Undiff-G	LLPH
CCNG1	1.36E-05	0.440231	0.081	0.272	0.203691	Undiff-G	CCNG1
EHD1	1.37E-05	-0.31945	0	0.123	0.204154	Undiff-G	EHD1
KIAA0141	1.37E-05	-0.3635	0	0.123	0.204154	Undiff-G	KIAA0141
ING1	1.37E-05	-0.13704	0.007	0.137	0.205412	Undiff-G	ING1
TAZ	1.38E-05	-0.36489	0.007	0.136	0.205484	Undiff-G	TAZ
KANSL2	1.38E-05	-0.17183	0.015	0.151	0.206417	Undiff-G	KANSL2
SNAPC3	1.38E-05	-0.2152	0.007	0.137	0.206863	Undiff-G	SNAPC3
BTN3A2	1.39E-05	-0.44348	0.015	0.148	0.208251	Undiff-G	BTN3A2
SLC30A7	1.40E-05	-0.13674	0.022	0.163	0.209773	Undiff-G	SLC30A7
EXOSC9	1.40E-05	-0.11342	0.015	0.151	0.209845	Undiff-G	EXOSC9
UTP23	1.40E-05	-0.19276	0.015	0.151	0.209909	Undiff-G	UTP23
LRRCC1	1.40E-05	-0.22563	0.007	0.137	0.209916	Undiff-G	LRRCC1
SEZ6L	1.41E-05	-0.37282	0.007	0.137	0.210137	Undiff-G	SEZ6L
KLC2	1.41E-05	-0.28509	0	0.123	0.210649	Undiff-G	KLC2
WDYHV1	1.41E-05	-0.3517	0	0.123	0.210649	Undiff-G	WDYHV1
ZCCHC6	1.42E-05	-0.36434	0.007	0.137	0.211983	Undiff-G	ZCCHC6
PUSL1	1.42E-05	-0.28323	0	0.122	0.212305	Undiff-G	PUSL1
TRIM22	1.42E-05	-0.44435	0.007	0.136	0.212906	Undiff-G	TRIM22
NUP160	1.44E-05	-0.30264	0.022	0.164	0.214685	Undiff-G	NUP160
FBXO18	1.44E-05	-0.29491	0.007	0.136	0.215415	Undiff-G	FBXO18

DOCK8	1.44E-05	-0.50724	0	0.122	0.215654	Undiff-G	DOCK8
PACS2	1.45E-05	-0.36096	0.007	0.136	0.216382	Undiff-G	PACS2
MUS81	1.45E-05	-0.26736	0.029	0.176	0.216461	Undiff-G	MUS81
NAV1	1.45E-05	-0.21986	0.022	0.164	0.216925	Undiff-G	NAV1
SCYL2	1.45E-05	-0.26923	0.007	0.137	0.21722	Undiff-G	SCYL2
ARMCX6	1.47E-05	0.423975	0.066	0.244	0.220265	Undiff-G	ARMCX6
ATP6V1G2	1.48E-05	-0.1308	0.015	0.151	0.220444	Undiff-G	ATP6V1G2
NSG1	1.48E-05	-0.16313	0.029	0.177	0.220588	Undiff-G	NSG1
RASGEF1B	1.48E-05	-0.51807	0	0.122	0.220774	Undiff-G	RASGEF1B
GATAD1	1.48E-05	-0.14258	0.044	0.203	0.221204	Undiff-G	GATAD1
TBC1D9B	1.49E-05	-0.32448	0.022	0.162	0.222064	Undiff-G	TBC1D9B
HERC2	1.50E-05	-0.21647	0.037	0.19	0.224084	Undiff-G	HERC2
GCFC2	1.50E-05	-0.31618	0	0.122	0.224255	Undiff-G	GCFC2
TIMM10B	1.50E-05	0.149349	0.029	0.177	0.224551	Undiff-G	TIMM10B
PARN	1.51E-05	-0.3394	0	0.122	0.226015	Undiff-G	PARN
FAHD1	1.52E-05	0.200053	0.029	0.177	0.226902	Undiff-G	FAHD1
C3	1.52E-05	0.272902	0.154	0.404	0.227095	Undiff-G	C3
FTSJ3	1.52E-05	-0.11395	0.015	0.15	0.227186	Undiff-G	FTSJ3
TSPAN12	1.52E-05	-0.32889	0	0.122	0.227789	Undiff-G	TSPAN12
B4GALT1	1.52E-05	-0.48928	0	0.122	0.227789	Undiff-G	B4GALT1
PDCD4	1.53E-05	-0.21722	0.015	0.15	0.227967	Undiff-G	PDCD4
WDR26	1.53E-05	-0.29461	0.022	0.162	0.228133	Undiff-G	WDR26
GPM6B	1.53E-05	1.01384	0.537	0.62	0.228863	Undiff-G	GPM6B
ATP5S	1.54E-05	0.108555	0.029	0.177	0.230033	Undiff-G	ATP5S
RNF168	1.54E-05	-0.11877	0.015	0.15	0.230369	Undiff-G	RNF168
RP11-294J	1.54E-05	-0.32196	0.007	0.136	0.230809	Undiff-G	RP11-294J22.6
DMTF1	1.55E-05	-0.28678	0.015	0.15	0.231081	Undiff-G	DMTF1
TTC9B	1.55E-05	-0.46611	0	0.122	0.231377	Undiff-G	TTC9B
IKBKG	1.56E-05	-0.30668	0.007	0.135	0.232463	Undiff-G	IKBKG
ZNF322	1.56E-05	-0.29048	0.007	0.135	0.232472	Undiff-G	ZNF322
C11orf96	1.56E-05	-0.38159	0.007	0.136	0.232799	Undiff-G	C11orf96
DPP6	1.56E-05	-0.24631	0.022	0.163	0.233161	Undiff-G	DPP6
RAB30-AS1	1.56E-05	-0.17215	0.015	0.15	0.233224	Undiff-G	RAB30-AS1
GEMIN6	1.56E-05	0.110427	0.022	0.164	0.233397	Undiff-G	GEMIN6
FGD4	1.57E-05	-0.45834	0.015	0.148	0.235046	Undiff-G	FGD4
RSAD1	1.57E-05	-0.2314	0.015	0.15	0.235304	Undiff-G	RSAD1
SMARCA2	1.58E-05	-0.18811	0.007	0.136	0.236263	Undiff-G	SMARCA2
SNRPN	1.58E-05	1.472872	0.191	0.097	0.236657	Undiff-G	SNRPN
ZBTB44	1.59E-05	-0.32036	0	0.121	0.236865	Undiff-G	ZBTB44
TOM1L2	1.59E-05	-0.32353	0	0.121	0.236865	Undiff-G	TOM1L2
CD33	1.59E-05	-0.5506	0	0.121	0.236865	Undiff-G	CD33
LENG1	1.59E-05	0.307017	0.044	0.203	0.237378	Undiff-G	LENG1
NUP107	1.59E-05	-0.19153	0.059	0.225	0.237625	Undiff-G	NUP107
MICU1	1.59E-05	-0.34786	0.007	0.135	0.238215	Undiff-G	MICU1
NFKBIE	1.60E-05	-0.32269	0.007	0.135	0.238401	Undiff-G	NFKBIE
ARHGEF9	1.60E-05	-0.34142	0	0.121	0.238722	Undiff-G	ARHGEF9
RP11-552D	1.60E-05	-0.6415	0	0.121	0.238722	Undiff-G	RP11-552D4.1
CBR4	1.60E-05	0.144809	0.037	0.19	0.239508	Undiff-G	CBR4
LINC0111€	1.60E-05	-0.34192	0.007	0.134	0.239528	Undiff-G	LINC01116
CCAR2	1.61E-05	-0.19935	0.015	0.148	0.240811	Undiff-G	CCAR2
ZNF444	1.61E-05	-0.18028	0.022	0.162	0.240967	Undiff-G	ZNF444

RNF220	1.61E-05	-0.2108	0.015	0.149	0.24115	Undiff-G	RNF220
REXO2	1.62E-05	0.45809	0.11	0.321	0.242198	Undiff-G	REXO2
LHFPL3	1.64E-05	-0.35893	0	0.121	0.244381	Undiff-G	LHFPL3
HLTF	1.64E-05	0.164207	0.029	0.175	0.244869	Undiff-G	HLTF
CALCOCO1	1.64E-05	-0.22127	0.015	0.149	0.24574	Undiff-G	CALCOCO1
THOC1	1.65E-05	-0.18628	0.015	0.148	0.246314	Undiff-G	THOC1
PACSIN2	1.66E-05	-0.10471	0.029	0.176	0.248423	Undiff-G	PACSIN2
RASD1	1.66E-05	-0.46798	0.007	0.135	0.248591	Undiff-G	RASD1
RHOQ	1.66E-05	0.119793	0.022	0.163	0.24877	Undiff-G	RHOQ
PQLC2	1.67E-05	-0.28567	0	0.121	0.250171	Undiff-G	PQLC2
MED13L	1.67E-05	-0.34747	0	0.121	0.250171	Undiff-G	MED13L
CDKN2AIP	1.68E-05	-0.16832	0.015	0.149	0.251067	Undiff-G	CDKN2AIP
KAT2A	1.68E-05	-0.28827	0.022	0.162	0.251125	Undiff-G	KAT2A
ZNF771	1.69E-05	-0.25791	0.015	0.148	0.251906	Undiff-G	ZNF771
WDR5	1.69E-05	-0.29821	0	0.121	0.252131	Undiff-G	WDR5
IL17RA	1.69E-05	-0.39651	0	0.121	0.252131	Undiff-G	IL17RA
WWTR1	1.70E-05	-0.15197	0.022	0.162	0.253693	Undiff-G	WWTR1
GPR108	1.70E-05	0.275571	0.081	0.268	0.253737	Undiff-G	GPR108
RP11-390F	1.70E-05	-0.31137	0.007	0.134	0.254419	Undiff-G	RP11-390P2.4
MTG2	1.70E-05	-0.20921	0.015	0.149	0.254675	Undiff-G	MTG2
NFATC3	1.71E-05	-0.25578	0.015	0.147	0.255566	Undiff-G	NFATC3
RMDN1	1.71E-05	0.299171	0.037	0.19	0.255793	Undiff-G	RMDN1
EFEMP1	1.71E-05	1.353128	0.316	0.22	0.255826	Undiff-G	EFEMP1
C2orf80	1.71E-05	-0.35127	0	0.12	0.256096	Undiff-G	C2orf80
ST8SIA4	1.71E-05	-0.43201	0	0.12	0.256096	Undiff-G	ST8SIA4
RAC2	1.71E-05	-0.53784	0	0.12	0.256096	Undiff-G	RAC2
DCAF5	1.72E-05	-0.30581	0.007	0.135	0.257558	Undiff-G	DCAF5
TOM1	1.73E-05	-0.19144	0.022	0.162	0.259137	Undiff-G	TOM1
TMEM51	1.74E-05	-0.36194	0	0.12	0.260123	Undiff-G	TMEM51
TMEM42	1.74E-05	0.116355	0.022	0.162	0.26021	Undiff-G	TMEM42
C1R	1.75E-05	1.39382	0.309	0.214	0.261316	Undiff-G	C1R
GMCL1	1.75E-05	-0.30762	0.022	0.16	0.262146	Undiff-G	GMCL1
PDDC1	1.76E-05	-0.22207	0.015	0.147	0.26326	Undiff-G	PDDC1
MPI	1.76E-05	-0.31247	0.007	0.134	0.263434	Undiff-G	MPI
MCCC1	1.77E-05	-0.23068	0.015	0.148	0.263879	Undiff-G	MCCC1
KBTBD6	1.77E-05	-0.33465	0.007	0.133	0.264291	Undiff-G	KBTBD6
FAM118A	1.77E-05	-0.37593	0.007	0.134	0.265081	Undiff-G	FAM118A
IFFO1	1.78E-05	-0.35173	0	0.12	0.266279	Undiff-G	IFFO1
POLD2	1.80E-05	0.654288	0.169	0.424	0.268567	Undiff-G	POLD2
HSPA1B	1.80E-05	0.388906	0.162	0.413	0.268635	Undiff-G	HSPA1B
KLHL42	1.81E-05	-0.21713	0.022	0.16	0.269932	Undiff-G	KLHL42
FRMD4B	1.81E-05	-0.2133	0.022	0.161	0.27045	Undiff-G	FRMD4B
MED1	1.81E-05	-0.2944	0	0.12	0.270463	Undiff-G	MED1
NDUFB2	1.82E-05	0.514024	0.338	0.814	0.271504	Undiff-G	NDUFB2
PLEKHB1	1.82E-05	0.144351	0.051	0.215	0.272279	Undiff-G	PLEKHB1
FAM214B	1.82E-05	-0.32308	0	0.12	0.272579	Undiff-G	FAM214B
CNTRL	1.82E-05	-0.35593	0	0.12	0.272579	Undiff-G	CNTRL
IKBIP	1.83E-05	0.330642	0.059	0.228	0.273091	Undiff-G	IKBIP
TXNRD2	1.84E-05	-0.28953	0	0.12	0.274711	Undiff-G	TXNRD2
ZNF559	1.85E-05	-0.30814	0.015	0.146	0.276218	Undiff-G	ZNF559
BACE1	1.88E-05	-0.33074	0	0.119	0.281207	Undiff-G	BACE1

TMEM131	1.88E-05	-0.23811	0.015	0.148	0.281283	Undiff-G	TMEM131
PBX3	1.90E-05	-0.30736	0	0.119	0.283405	Undiff-G	PBX3
TUBB4A	1.90E-05	-0.54582	0	0.119	0.283405	Undiff-G	TUBB4A
PEX11B	1.91E-05	-0.17911	0.007	0.134	0.284732	Undiff-G	PEX11B
CD55	1.91E-05	-0.5128	0	0.119	0.285621	Undiff-G	CD55
UBR4	1.91E-05	-0.31762	0.015	0.147	0.28589	Undiff-G	UBR4
DPF2	1.92E-05	0.191819	0.059	0.226	0.286277	Undiff-G	DPF2
PNPT1	1.93E-05	-0.10238	0.044	0.198	0.288916	Undiff-G	PNPT1
SRP14	1.94E-05	0.455224	0.375	0.872	0.289176	Undiff-G	SRP14
RAB11B-AS	1.94E-05	-0.16072	0.007	0.133	0.289359	Undiff-G	RAB11B-AS1
CTC-524C5	1.94E-05	-0.28325	0	0.119	0.290103	Undiff-G	CTC-524C5.2
GEMIN7	1.95E-05	0.105152	0.022	0.161	0.291842	Undiff-G	GEMIN7
SLC6A8	1.96E-05	-0.34686	0	0.119	0.29237	Undiff-G	SLC6A8
GBA	1.96E-05	-0.37618	0.007	0.132	0.292437	Undiff-G	GBA
NPRL2	1.97E-05	-0.21226	0.022	0.159	0.294394	Undiff-G	NPRL2
RP11-849I	1.98E-05	-0.298	0.007	0.133	0.295835	Undiff-G	RP11-849I19.1
COPG2	1.99E-05	-0.30003	0	0.119	0.296956	Undiff-G	COPG2
SAYSD1	1.99E-05	-0.33067	0	0.119	0.296956	Undiff-G	SAYSD1
MGLL	2.01E-05	-0.31402	0.015	0.147	0.300112	Undiff-G	MGLL
NARFL	2.01E-05	-0.27928	0.015	0.147	0.301032	Undiff-G	NARFL
SBF2	2.02E-05	-0.22824	0.037	0.184	0.302122	Undiff-G	SBF2
ITPR2	2.03E-05	-0.22576	0.007	0.133	0.303218	Undiff-G	ITPR2
MAPK6	2.03E-05	0.134417	0.029	0.174	0.303323	Undiff-G	MAPK6
NPAS3	2.03E-05	-0.26898	0.022	0.16	0.303793	Undiff-G	NPAS3
CEP290	2.03E-05	-0.11104	0.022	0.159	0.303824	Undiff-G	CEP290
TMEM214	2.03E-05	-0.29908	0	0.118	0.303968	Undiff-G	TMEM214
MAML1	2.03E-05	-0.39098	0	0.118	0.303968	Undiff-G	MAML1
FBXO17	2.04E-05	-0.29499	0.015	0.145	0.304769	Undiff-G	FBXO17
TRAPPC2P1	2.04E-05	0.145106	0.059	0.226	0.305439	Undiff-G	TRAPPC2P1
FOXN2	2.06E-05	-0.35826	0.007	0.132	0.307953	Undiff-G	FOXN2
ARHGEF7	2.06E-05	-0.30745	0.022	0.16	0.30845	Undiff-G	ARHGEF7
TGFBR2	2.07E-05	-0.40273	0	0.118	0.308733	Undiff-G	TGFBR2
KLHDC10	2.07E-05	-0.28939	0.007	0.132	0.309752	Undiff-G	KLHDC10
LMNB2	2.08E-05	-0.27367	0	0.118	0.311142	Undiff-G	LMNB2
N4BP2	2.08E-05	-0.2946	0	0.118	0.311142	Undiff-G	N4BP2
INSM1	2.08E-05	-0.33397	0	0.118	0.311142	Undiff-G	INSM1
LANCL1	2.08E-05	-0.3651	0	0.118	0.311142	Undiff-G	LANCL1
SLC35E2B	2.08E-05	-0.23235	0.015	0.146	0.311411	Undiff-G	SLC35E2B
PTGR1	2.09E-05	0.242445	0.037	0.187	0.31244	Undiff-G	PTGR1
XPNPEP1	2.14E-05	-0.333	0.015	0.144	0.320087	Undiff-G	XPNPEP1
DCTN4	2.15E-05	0.215735	0.037	0.187	0.320786	Undiff-G	DCTN4
SREBF1	2.15E-05	-0.35868	0	0.118	0.320968	Undiff-G	SREBF1
RP1-39G22	2.16E-05	-0.17657	0.007	0.132	0.322631	Undiff-G	RP1-39G22.7
PPP1R8	2.17E-05	0.450352	0.059	0.225	0.324219	Undiff-G	PPP1R8
PLP1	2.18E-05	-0.26725	0.029	0.173	0.325519	Undiff-G	PLP1
GAS1	2.18E-05	-0.29123	0.015	0.146	0.326001	Undiff-G	GAS1
PCIF1	2.19E-05	-0.31903	0.015	0.144	0.327517	Undiff-G	PCIF1
SERPINH1	2.19E-05	0.370554	0.051	0.211	0.327658	Undiff-G	SERPINH1
MYD88	2.20E-05	-0.3245	0.007	0.132	0.328362	Undiff-G	MYD88
HKR1	2.20E-05	-0.31377	0	0.118	0.328537	Undiff-G	HKR1
SPG11	2.20E-05	-0.32974	0	0.118	0.328537	Undiff-G	SPG11

SRRD	2.22E-05	-0.25344	0	0.118	0.331099	Undiff-G	SRRD
AP006222.	2.23E-05	0.29988	0.051	0.213	0.333323	Undiff-G	AP006222.2
TRAFD1	2.25E-05	-0.30843	0.007	0.131	0.336279	Undiff-G	TRAFD1
ZNRF1	2.25E-05	-0.25776	0	0.117	0.336281	Undiff-G	ZNRF1
BCL10	2.26E-05	-0.14065	0.007	0.132	0.338118	Undiff-G	BCL10
GNG4	2.27E-05	-0.30867	0	0.117	0.338902	Undiff-G	GNG4
ZNF738	2.28E-05	-0.10731	0.015	0.145	0.340145	Undiff-G	ZNF738
CEP89	2.28E-05	-0.16222	0.007	0.132	0.340417	Undiff-G	CEP89
MSH2	2.29E-05	-0.30325	0	0.117	0.341543	Undiff-G	MSH2
GLS	2.29E-05	-0.34015	0.007	0.13	0.342126	Undiff-G	GLS
NUP93	2.29E-05	-0.26257	0.037	0.183	0.34244	Undiff-G	NUP93
ZSWIM6	2.29E-05	-0.31608	0.007	0.13	0.342525	Undiff-G	ZSWIM6
ZNF770	2.29E-05	-0.26291	0.007	0.131	0.342861	Undiff-G	ZNF770
RNPEPL1	2.30E-05	-0.14264	0.007	0.132	0.343396	Undiff-G	RNPEPL1
PAK4	2.30E-05	-0.28382	0	0.117	0.344204	Undiff-G	PAK4
CD99L2	2.31E-05	-0.28223	0.007	0.131	0.344927	Undiff-G	CD99L2
9-Mar	2.31E-05	-0.37676	0.022	0.156	0.344988	Undiff-G	9-Mar
YDJC	2.32E-05	-0.2292	0.015	0.145	0.346447	Undiff-G	YDJC
4-Sep	2.32E-05	-0.42551	0.007	0.131	0.346798	Undiff-G	4-Sep
SNX12	2.34E-05	-0.29198	0.007	0.131	0.349496	Undiff-G	SNX12
ARHGAP4	2.34E-05	-0.46327	0	0.117	0.349588	Undiff-G	ARHGAP4
PPAT	2.35E-05	-0.20834	0.015	0.145	0.3514	Undiff-G	PPAT
SAMD4B	2.36E-05	0.2805	0.059	0.225	0.352376	Undiff-G	SAMD4B
SYNJ2BP	2.36E-05	-0.17373	0.015	0.145	0.353321	Undiff-G	SYNJ2BP
TCF3	2.38E-05	-0.26143	0.007	0.131	0.355143	Undiff-G	TCF3
POGK	2.38E-05	-0.27884	0.007	0.131	0.355905	Undiff-G	POGK
LHX2	2.39E-05	-0.22532	0.007	0.131	0.356673	Undiff-G	LHX2
TTYH2	2.39E-05	-0.35603	0	0.117	0.35782	Undiff-G	TTYH2
PSPH	2.41E-05	0.111403	0.022	0.159	0.36069	Undiff-G	PSPH
LGALS3BP	2.42E-05	0.51194	0.184	0.452	0.3615	Undiff-G	LGALS3BP
ERCC4	2.42E-05	-0.29976	0.007	0.131	0.361596	Undiff-G	ERCC4
ATG13	2.42E-05	-0.17606	0.007	0.131	0.362376	Undiff-G	ATG13
CBX4	2.43E-05	-0.31187	0	0.117	0.363413	Undiff-G	CBX4
ADAM10	2.43E-05	0.336262	0.037	0.186	0.363467	Undiff-G	ADAM10
EFR3A	2.44E-05	-0.25185	0.015	0.144	0.363984	Undiff-G	EFR3A
RRAGD	2.44E-05	-0.14632	0.007	0.131	0.365261	Undiff-G	RRAGD
COPB1	2.46E-05	0.512541	0.096	0.288	0.367891	Undiff-G	COPB1
PHF19	2.47E-05	-0.32267	0.007	0.131	0.368373	Undiff-G	PHF19
TMEM198	2.47E-05	-0.26552	0	0.116	0.369092	Undiff-G	TMEM198
SLC37A3	2.48E-05	-0.10657	0.007	0.131	0.370955	Undiff-G	SLC37A3
STAG1	2.49E-05	-0.32016	0.007	0.129	0.371939	Undiff-G	STAG1
POSTN	2.51E-05	-0.3655	0.007	0.131	0.374629	Undiff-G	POSTN
CDYL	2.51E-05	-0.25933	0	0.116	0.374858	Undiff-G	CDYL
ANXA4	2.53E-05	0.254427	0.044	0.196	0.378149	Undiff-G	ANXA4
DPP9	2.56E-05	-0.28648	0.007	0.13	0.382588	Undiff-G	DPP9
MCAT	2.60E-05	-0.16191	0.007	0.13	0.388641	Undiff-G	MCAT
EML3	2.63E-05	-0.2857	0	0.116	0.392693	Undiff-G	EML3
STX11	2.63E-05	-0.49966	0	0.116	0.392693	Undiff-G	STX11
C12orf73	2.63E-05	-0.11211	0.022	0.156	0.392915	Undiff-G	C12orf73
PITPNA-AS	2.64E-05	0.335802	0.044	0.197	0.394987	Undiff-G	PITPNA-AS1
AMER2	2.65E-05	-0.46236	0	0.116	0.395745	Undiff-G	AMER2

MCM3	2.65E-05	-0.24903	0.015	0.144	0.396472	Undiff-G	MCM3
CSTA	2.68E-05	-0.38114	0.007	0.13	0.399909	Undiff-G	CSTA
HPS5	2.69E-05	-0.3288	0	0.115	0.401921	Undiff-G	HPS5
ELMO2	2.70E-05	-0.2624	0.007	0.13	0.402922	Undiff-G	ELMO2
ZNF579	2.71E-05	-0.24856	0	0.115	0.405044	Undiff-G	ZNF579
WDR73	2.71E-05	-0.30226	0.015	0.143	0.40532	Undiff-G	WDR73
SLC11A2	2.74E-05	0.117439	0.029	0.17	0.410134	Undiff-G	SLC11A2
ZNF263	2.76E-05	-0.29612	0.007	0.129	0.411877	Undiff-G	ZNF263
RNF44	2.82E-05	-0.32338	0	0.115	0.42102	Undiff-G	RNF44
PLEKHF2	2.83E-05	-0.14728	0.007	0.129	0.423093	Undiff-G	PLEKHF2
FBXW7	2.85E-05	-0.27843	0.007	0.129	0.426268	Undiff-G	FBXW7
GPR89A	2.85E-05	-0.2912	0.007	0.129	0.426364	Undiff-G	GPR89A
SP100	2.86E-05	0.475523	0.096	0.289	0.427702	Undiff-G	SP100
ARID1B	2.87E-05	-0.28952	0.007	0.129	0.428987	Undiff-G	ARID1B
FAM222B	2.88E-05	-0.24869	0	0.115	0.430901	Undiff-G	FAM222B
HEXDC	2.88E-05	-0.28671	0	0.115	0.430901	Undiff-G	HEXDC
STARD4-AS	2.89E-05	-0.29851	0.007	0.128	0.431581	Undiff-G	STARD4-AS1
PRKAR1B	2.89E-05	-0.25971	0.007	0.129	0.432163	Undiff-G	PRKAR1B
TMEM99	2.89E-05	-0.21174	0.007	0.129	0.432413	Undiff-G	TMEM99
FBRS	2.91E-05	-0.29827	0	0.115	0.434245	Undiff-G	FBRS
R3HDM2	2.91E-05	-0.23985	0.029	0.169	0.435371	Undiff-G	R3HDM2
CCDC93	2.93E-05	-0.27789	0.007	0.129	0.437773	Undiff-G	CCDC93
PCDHB16	2.96E-05	-0.16246	0.015	0.141	0.442338	Undiff-G	PCDHB16
TREX1	2.97E-05	-0.31031	0	0.114	0.444432	Undiff-G	TREX1
TNK2	2.98E-05	-0.27589	0.007	0.129	0.445011	Undiff-G	TNK2
MSH6	2.99E-05	-0.29663	0.007	0.128	0.447513	Undiff-G	MSH6
USP53	2.99E-05	-0.21278	0.015	0.143	0.447539	Undiff-G	USP53
ZNF883	3.00E-05	-0.26161	0.015	0.142	0.447719	Undiff-G	ZNF883
SEPHS1	3.00E-05	-0.28435	0	0.114	0.447879	Undiff-G	SEPHS1
MFSD5	3.00E-05	-0.28723	0	0.114	0.447879	Undiff-G	MFSD5
PPIP5K2	3.00E-05	-0.25959	0.007	0.129	0.448022	Undiff-G	PPIP5K2
SMURF2	3.01E-05	-0.30212	0.007	0.127	0.449093	Undiff-G	SMURF2
WDR43	3.03E-05	0.387603	0.044	0.196	0.453017	Undiff-G	WDR43
PTK2B	3.05E-05	-0.32499	0.007	0.128	0.455061	Undiff-G	PTK2B
HCG18	3.07E-05	-0.28501	0	0.114	0.45838	Undiff-G	HCG18
BRI3BP	3.07E-05	-0.29762	0	0.114	0.45838	Undiff-G	BRI3BP
APBB2	3.07E-05	-0.30672	0	0.114	0.45838	Undiff-G	APBB2
FAM208A	3.12E-05	-0.26812	0.015	0.141	0.465629	Undiff-G	FAM208A
RUFY1	3.13E-05	-0.16698	0.022	0.154	0.468268	Undiff-G	RUFY1
BIN2	3.14E-05	-0.48282	0	0.114	0.469122	Undiff-G	BIN2
PLEKHA4	3.14E-05	-0.20398	0.007	0.128	0.469581	Undiff-G	PLEKHA4
SERGEF	3.14E-05	0.264491	0.029	0.169	0.46991	Undiff-G	SERGEF
RP11-14N7	3.16E-05	0.172266	0.051	0.206	0.472498	Undiff-G	RP11-14N7.2
HEPACAM	3.16E-05	-0.20285	0.037	0.179	0.47263	Undiff-G	HEPACAM
ORMDL3	3.17E-05	-0.22578	0.007	0.128	0.474125	Undiff-G	ORMDL3
GIT2	3.19E-05	-0.24538	0.022	0.155	0.476113	Undiff-G	GIT2
ZMYM4	3.19E-05	-0.26009	0.007	0.127	0.476331	Undiff-G	ZMYM4
FAM131A	3.20E-05	-0.27888	0.007	0.128	0.477759	Undiff-G	FAM131A
NUDT11	3.21E-05	-0.31809	0.007	0.128	0.479607	Undiff-G	NUDT11
ATP9A	3.21E-05	-0.28405	0	0.113	0.480112	Undiff-G	ATP9A
STYXL1	3.22E-05	-0.24645	0.007	0.128	0.48176	Undiff-G	STYXL1

TMEM68	3.24E-05	0.187534	0.029	0.169	0.48356	Undiff-G	TMEM68
CTSO	3.24E-05	-0.30101	0	0.113	0.483831	Undiff-G	CTSO
CYTH4	3.24E-05	-0.48393	0	0.113	0.483831	Undiff-G	CYTH4
TGFB1I1	3.24E-05	-0.22025	0.007	0.128	0.483987	Undiff-G	TGFB1I1
PTAFR	3.25E-05	-0.50124	0.007	0.127	0.484982	Undiff-G	PTAFR
ABHD17B	3.25E-05	-0.34525	0.007	0.126	0.485244	Undiff-G	ABHD17B
TCIRG1	3.26E-05	-0.42954	0.007	0.127	0.486932	Undiff-G	TCIRG1
UBTD1	3.26E-05	-0.27165	0	0.113	0.487579	Undiff-G	UBTD1
LRP6	3.26E-05	-0.28301	0	0.113	0.487579	Undiff-G	LRP6
IFITM10	3.27E-05	-0.40486	0.007	0.127	0.488008	Undiff-G	IFITM10
EPS8	3.27E-05	-0.27767	0.007	0.128	0.488951	Undiff-G	EPS8
DOLK	3.28E-05	-0.10197	0.007	0.128	0.489706	Undiff-G	DOLK
XXYLT1	3.28E-05	-0.1383	0.007	0.128	0.489706	Undiff-G	XXYLT1
LMO2	3.28E-05	-0.21509	0.029	0.166	0.490597	Undiff-G	LMO2
SH3BP1	3.29E-05	-0.36272	0	0.113	0.491355	Undiff-G	SH3BP1
DIAPH1	3.33E-05	-0.21593	0.015	0.14	0.497052	Undiff-G	DIAPH1
SRPX	3.33E-05	-0.11123	0.037	0.178	0.497097	Undiff-G	SRPX
DLAT	3.33E-05	-0.12053	0.007	0.127	0.497108	Undiff-G	DLAT
POGLUT1	3.36E-05	-0.28683	0	0.113	0.502856	Undiff-G	POGLUT1
PRKX	3.36E-05	-0.33081	0	0.113	0.502856	Undiff-G	PRKX
PIP4K2A	3.38E-05	-0.40219	0.015	0.138	0.504409	Undiff-G	PIP4K2A
INPP1	3.38E-05	-0.2939	0.022	0.153	0.505256	Undiff-G	INPP1
DLG1	3.38E-05	-0.16489	0.029	0.167	0.505579	Undiff-G	DLG1
SLC35F1	3.40E-05	-0.10303	0.007	0.127	0.508811	Undiff-G	SLC35F1
CEP68	3.41E-05	-0.14502	0.022	0.153	0.50947	Undiff-G	CEP68
DNAJB4	3.42E-05	-0.3336	0.007	0.127	0.510558	Undiff-G	DNAJB4
ACSS3	3.42E-05	-0.24212	0	0.113	0.510671	Undiff-G	ACSS3
ST3GAL6	3.42E-05	-0.44031	0	0.113	0.510671	Undiff-G	ST3GAL6
HMGXB3	3.43E-05	-0.26046	0.007	0.127	0.512221	Undiff-G	HMGXB3
KIAA0232	3.45E-05	-0.17603	0.022	0.153	0.516224	Undiff-G	KIAA0232
TXLNA	3.46E-05	-0.20354	0.015	0.141	0.517759	Undiff-G	TXLNA
USP36	3.46E-05	-0.33014	0.015	0.14	0.517778	Undiff-G	USP36
MTIF2	3.46E-05	0.10473	0.015	0.141	0.517787	Undiff-G	MTIF2
C5orf56	3.47E-05	-0.32979	0	0.113	0.518604	Undiff-G	C5orf56
FAM126B	3.47E-05	-0.26188	0.015	0.141	0.519097	Undiff-G	FAM126B
CXorf40B	3.48E-05	0.166046	0.022	0.155	0.520693	Undiff-G	CXorf40B
GGA2	3.49E-05	-0.32535	0.015	0.14	0.522053	Undiff-G	GGA2
GLI4	3.50E-05	-0.28204	0	0.112	0.522616	Undiff-G	GLI4
CAMK1D	3.50E-05	-0.34345	0	0.112	0.522616	Undiff-G	CAMK1D
TMC6	3.50E-05	-0.44595	0	0.112	0.522616	Undiff-G	TMC6
HYOU1	3.50E-05	-0.22732	0.015	0.141	0.523074	Undiff-G	HYOU1
PRKAG2-AS	3.52E-05	-0.45762	0.007	0.126	0.525765	Undiff-G	PRKAG2-AS1
TULP3	3.52E-05	-0.23651	0.007	0.127	0.526006	Undiff-G	TULP3
KIAA0196	3.52E-05	-0.29565	0	0.112	0.526659	Undiff-G	KIAA0196
ATG7	3.52E-05	-0.34987	0	0.112	0.526659	Undiff-G	ATG7
TLR2	3.52E-05	-0.50063	0	0.112	0.526659	Undiff-G	TLR2
DHX40	3.53E-05	-0.18184	0.015	0.141	0.528013	Undiff-G	DHX40
DHCR7	3.54E-05	0.354785	0.037	0.179	0.528794	Undiff-G	DHCR7
RGS2	3.55E-05	0.46579	0.184	0.448	0.53089	Undiff-G	RGS2
XPR1	3.56E-05	-0.21836	0.007	0.127	0.531701	Undiff-G	XPR1
CUTC	3.56E-05	-0.25716	0.007	0.127	0.531701	Undiff-G	CUTC

SRPK1	3.57E-05	0.14719	0.029	0.168	0.533604	Undiff-G	SRPK1
EPS15	3.57E-05	-0.33777	0.022	0.152	0.534084	Undiff-G	EPS15
CHL1	3.59E-05	0.147934	0.022	0.154	0.535917	Undiff-G	CHL1
TMEM41A	3.59E-05	-0.2008	0.007	0.127	0.536421	Undiff-G	TMEM41A
C17orf49	3.60E-05	-0.25373	0.015	0.139	0.53821	Undiff-G	C17orf49
FAM171B	3.61E-05	-0.2917	0	0.112	0.538972	Undiff-G	FAM171B
UGGT1	3.61E-05	-0.30701	0	0.112	0.538972	Undiff-G	UGGT1
SHMT2	3.61E-05	0.410547	0.066	0.233	0.539707	Undiff-G	SHMT2
COLGALT2	3.61E-05	-0.1065	0.015	0.14	0.540179	Undiff-G	COLGALT2
SART3	3.62E-05	-0.10667	0.007	0.127	0.541183	Undiff-G	SART3
STAT2	3.64E-05	-0.13854	0.015	0.14	0.543552	Undiff-G	STAT2
NOL3	3.66E-05	0.109432	0.029	0.167	0.546874	Undiff-G	NOL3
RALGAPA2	3.66E-05	-0.25936	0	0.112	0.547338	Undiff-G	RALGAPA2
CEP57L1	3.66E-05	-0.29537	0	0.112	0.547338	Undiff-G	CEP57L1
AMPD2	3.66E-05	-0.13577	0.022	0.153	0.547622	Undiff-G	AMPD2
STRIP1	3.67E-05	-0.29976	0.007	0.126	0.548018	Undiff-G	STRIP1
TPT1-AS1	3.67E-05	-0.12177	0.015	0.14	0.548758	Undiff-G	TPT1-AS1
SLC35B3	3.68E-05	-0.10763	0.007	0.126	0.549561	Undiff-G	SLC35B3
LPIN1	3.68E-05	-0.3264	0.015	0.138	0.549564	Undiff-G	LPIN1
WDR48	3.68E-05	-0.24916	0.015	0.14	0.550078	Undiff-G	WDR48
NOTCH1	3.69E-05	-0.27339	0	0.112	0.551568	Undiff-G	NOTCH1
RNF135	3.69E-05	-0.35885	0	0.112	0.551568	Undiff-G	RNF135
ANK2	3.70E-05	-0.19373	0.029	0.165	0.552485	Undiff-G	ANK2
PPAN	3.74E-05	-0.16728	0.029	0.166	0.559318	Undiff-G	PPAN
BIVM	3.75E-05	-0.26753	0	0.112	0.560126	Undiff-G	BIVM
PRPS2	3.75E-05	-0.26235	0.007	0.126	0.560314	Undiff-G	PRPS2
NLGN1	3.76E-05	-0.25682	0.015	0.139	0.56212	Undiff-G	NLGN1
LRRC41	3.76E-05	0.312865	0.074	0.243	0.562426	Undiff-G	LRRC41
ENOSF1	3.77E-05	-0.27086	0.007	0.126	0.563188	Undiff-G	ENOSF1
PI3	3.77E-05	1.445171	0.125	0.052	0.563392	Undiff-G	PI3
RALGAPB	3.78E-05	-0.24023	0	0.112	0.564453	Undiff-G	RALGAPB
GTPBP8	3.78E-05	-0.31588	0	0.112	0.564453	Undiff-G	GTPBP8
CCP110	3.78E-05	-0.33809	0.007	0.125	0.565615	Undiff-G	CCP110
ST6GAL1	3.81E-05	-0.44118	0	0.112	0.568814	Undiff-G	ST6GAL1
TMEM175	3.81E-05	-0.24418	0.022	0.153	0.569118	Undiff-G	TMEM175
SH3BP2	3.82E-05	-0.27719	0.015	0.14	0.570354	Undiff-G	SH3BP2
DCAF15	3.84E-05	-0.26418	0	0.111	0.573208	Undiff-G	DCAF15
MTA1	3.85E-05	-0.17443	0.044	0.192	0.575515	Undiff-G	MTA1
FASTKD5	3.87E-05	-0.28627	0	0.111	0.577635	Undiff-G	FASTKD5
RP11-390E	3.88E-05	-0.20134	0.007	0.126	0.579118	Undiff-G	RP11-390E23.6
MERTK	3.88E-05	-0.32017	0.007	0.126	0.57923	Undiff-G	MERTK
FUT8	3.88E-05	-0.20113	0.007	0.126	0.579676	Undiff-G	FUT8
DDAH1	3.88E-05	-0.16137	0.007	0.126	0.579787	Undiff-G	DDAH1
KLHL12	3.88E-05	-0.10691	0.007	0.126	0.579899	Undiff-G	KLHL12
TCAIM	3.90E-05	-0.16895	0.015	0.138	0.582138	Undiff-G	TCAIM
RPP21	3.90E-05	-0.26747	0.007	0.126	0.582232	Undiff-G	RPP21
MGST3	3.90E-05	0.697724	0.294	0.698	0.583516	Undiff-G	MGST3
ZFC3H1	3.91E-05	-0.28284	0.007	0.125	0.584989	Undiff-G	ZFC3H1
CD200	3.93E-05	-0.33025	0	0.111	0.58659	Undiff-G	CD200
LAPTM4A	3.93E-05	0.545913	0.331	0.778	0.587602	Undiff-G	LAPTM4A
PXN	3.93E-05	-0.2146	0.007	0.126	0.587959	Undiff-G	PXN

HIRIP3	3.95E-05	0.186084	0.029	0.167	0.590667	Undiff-G	HIRIP3
GNB5	3.96E-05	-0.26856	0	0.111	0.591118	Undiff-G	GNB5
TMEM43	3.96E-05	0.332105	0.044	0.193	0.59149	Undiff-G	TMEM43
FAM91A1	3.97E-05	-0.19422	0.007	0.125	0.592601	Undiff-G	FAM91A1
RAB3GAP1	3.97E-05	-0.19776	0.015	0.139	0.593257	Undiff-G	RAB3GAP1
PDE12	3.99E-05	-0.29725	0	0.111	0.595681	Undiff-G	PDE12
ZNF12	3.99E-05	-0.24543	0.007	0.125	0.595998	Undiff-G	ZNF12
UTRN	4.02E-05	-0.30029	0	0.111	0.600279	Undiff-G	UTRN
KLRC2	4.02E-05	-0.40053	0	0.111	0.600279	Undiff-G	KLRC2
JADE1	4.02E-05	-0.2559	0.007	0.125	0.600834	Undiff-G	JADE1
WEE1	4.03E-05	-0.24093	0.022	0.151	0.602492	Undiff-G	WEE1
ZNF451	4.04E-05	-0.11097	0.029	0.164	0.604088	Undiff-G	ZNF451
OPA3	4.05E-05	-0.28891	0	0.111	0.604911	Undiff-G	OPA3
CYB561A3	4.06E-05	-0.25282	0.022	0.152	0.606268	Undiff-G	CYB561A3
USP42	4.07E-05	-0.29835	0.007	0.124	0.607751	Undiff-G	USP42
TLK2	4.09E-05	-0.25098	0.007	0.125	0.611941	Undiff-G	TLK2
NFAT5	4.10E-05	-0.26168	0.015	0.138	0.612155	Undiff-G	NFAT5
AMDHD2	4.11E-05	-0.30799	0.007	0.124	0.613672	Undiff-G	AMDHD2
RFTN1	4.11E-05	-0.26403	0	0.111	0.614282	Undiff-G	RFTN1
MOSPD2	4.11E-05	-0.31681	0	0.111	0.614282	Undiff-G	MOSPD2
HDDC3	4.11E-05	-0.13187	0.022	0.151	0.614725	Undiff-G	HDDC3
ZNF830	4.13E-05	0.382289	0.044	0.193	0.61676	Undiff-G	ZNF830
SALL1	4.14E-05	-0.18887	0.015	0.139	0.618448	Undiff-G	SALL1
CREB3L2	4.14E-05	-0.29853	0.007	0.125	0.618828	Undiff-G	CREB3L2
ZZZ3	4.14E-05	-0.2812	0	0.111	0.61902	Undiff-G	ZZZ3
C6orf136	4.14E-05	-0.29448	0	0.111	0.61902	Undiff-G	C6orf136
ZNF512	4.17E-05	-0.25917	0.007	0.125	0.623229	Undiff-G	ZNF512
ARFGEF1	4.18E-05	-0.19446	0.007	0.125	0.625149	Undiff-G	ARFGEF1
JAKMIP2	4.19E-05	0.125691	0.022	0.152	0.625815	Undiff-G	JAKMIP2
PRDX2	4.19E-05	0.432061	0.316	0.706	0.626863	Undiff-G	PRDX2
XRCC1	4.20E-05	-0.2527	0.007	0.125	0.627057	Undiff-G	XRCC1
METTL16	4.21E-05	-0.26979	0	0.11	0.628606	Undiff-G	METTL16
OXSR1	4.21E-05	-0.29347	0	0.11	0.628606	Undiff-G	OXSR1
PIP4K2C	4.21E-05	-0.34251	0	0.11	0.628606	Undiff-G	PIP4K2C
CLPX	4.21E-05	-0.15507	0.022	0.151	0.629735	Undiff-G	CLPX
CTTN	4.23E-05	0.462827	0.066	0.231	0.632497	Undiff-G	CTTN
RUFY2	4.26E-05	-0.22451	0.022	0.151	0.636354	Undiff-G	RUFY2
BEND5	4.26E-05	-0.12392	0.015	0.138	0.636986	Undiff-G	BEND5
CHST7	4.27E-05	-0.23897	0	0.11	0.638337	Undiff-G	CHST7
COL6A1	4.27E-05	0.175136	0.066	0.231	0.638584	Undiff-G	COL6A1
KCNF1	4.28E-05	-0.25829	0.007	0.125	0.639683	Undiff-G	KCNF1
TAF3	4.30E-05	-0.30951	0.007	0.123	0.643122	Undiff-G	TAF3
PRKRIR	4.30E-05	-0.24835	0	0.11	0.643258	Undiff-G	PRKRIR
PPP3CB	4.30E-05	-0.29962	0	0.11	0.643258	Undiff-G	PPP3CB
PELP1	4.31E-05	0.131889	0.022	0.152	0.643553	Undiff-G	PELP1
LACTB2	4.33E-05	0.141422	0.022	0.152	0.647002	Undiff-G	LACTB2
ZNF721	4.34E-05	-0.26551	0.022	0.15	0.648863	Undiff-G	ZNF721
PLK2	4.34E-05	-0.10589	0.015	0.138	0.649176	Undiff-G	PLK2
CCNY	4.37E-05	-0.29873	0	0.11	0.653212	Undiff-G	CCNY
SLC27A3	4.39E-05	-0.3075	0.007	0.124	0.656192	Undiff-G	SLC27A3
SH2B3	4.40E-05	-0.39906	0	0.11	0.658246	Undiff-G	SH2B3

PRPS1	4.41E-05	0.344619	0.044	0.191	0.658898	Undiff-G	PRPS1
PLOD2	4.42E-05	-0.1708	0.029	0.163	0.660887	Undiff-G	PLOD2
CLASRP	4.43E-05	-0.17512	0.022	0.15	0.66257	Undiff-G	CLASRP
SH2B2	4.44E-05	-0.2648	0	0.11	0.663318	Undiff-G	SH2B2
TPP2	4.45E-05	-0.11727	0.007	0.124	0.66567	Undiff-G	TPP2
CNOT6L	4.47E-05	-0.30807	0	0.11	0.668428	Undiff-G	CNOT6L
HMHA1	4.47E-05	-0.45688	0	0.11	0.668428	Undiff-G	HMHA1
TBL3	4.49E-05	-0.28916	0.007	0.124	0.6711	Undiff-G	TBL3
MSI1	4.50E-05	-0.26633	0.015	0.137	0.672866	Undiff-G	MSI1
LINC00847	4.51E-05	-0.30792	0	0.11	0.673577	Undiff-G	LINC00847
TBC1D22A	4.52E-05	-0.13991	0.007	0.124	0.676067	Undiff-G	TBC1D22A
YTHDF3	4.53E-05	-0.26451	0.015	0.138	0.676538	Undiff-G	YTHDF3
DLX2	4.54E-05	-0.46095	0	0.11	0.678765	Undiff-G	DLX2
C20orf194	4.59E-05	-0.30154	0.015	0.136	0.68563	Undiff-G	C20orf194
MFN2	4.61E-05	-0.28739	0	0.109	0.689259	Undiff-G	MFN2
FADS3	4.62E-05	-0.17703	0.022	0.15	0.691025	Undiff-G	FADS3
NXPH1	4.64E-05	-0.28837	0.015	0.137	0.693359	Undiff-G	NXPH1
STXBP1	4.65E-05	-0.29239	0.007	0.122	0.694321	Undiff-G	STXBP1
SMG1	4.65E-05	-0.15566	0.029	0.164	0.694589	Undiff-G	SMG1
FAM126A	4.66E-05	0.131925	0.015	0.138	0.696769	Undiff-G	FAM126A
ZNF528	4.68E-05	-0.2575	0	0.109	0.699913	Undiff-G	ZNF528
ZNF439	4.71E-05	-0.25148	0.022	0.149	0.704008	Undiff-G	ZNF439
USE1	4.75E-05	0.537267	0.125	0.332	0.709647	Undiff-G	USE1
ALDOA	4.78E-05	0.931123	0.596	0.848	0.71369	Undiff-G	ALDOA
BCL2L13	4.78E-05	0.125067	0.022	0.151	0.714198	Undiff-G	BCL2L13
SLC35F2	4.79E-05	-0.24665	0	0.109	0.716197	Undiff-G	SLC35F2
ANKFY1	4.80E-05	-0.21643	0.015	0.137	0.716742	Undiff-G	ANKFY1
AKAP1	4.80E-05	-0.26014	0.007	0.123	0.716877	Undiff-G	AKAP1
WBP5	4.81E-05	0.732208	0.213	0.506	0.719274	Undiff-G	WBP5
KAT6B	4.82E-05	-0.27321	0.007	0.123	0.720743	Undiff-G	KAT6B
STX2	4.83E-05	-0.25562	0	0.109	0.721708	Undiff-G	STX2
FAM199X	4.83E-05	-0.25872	0	0.109	0.721708	Undiff-G	FAM199X
GOLGA5	4.84E-05	0.135128	0.022	0.149	0.722928	Undiff-G	GOLGA5
NCKAP1L	4.84E-05	-0.31136	0.007	0.123	0.723264	Undiff-G	NCKAP1L
POU2F1	4.85E-05	-0.18907	0.029	0.162	0.725453	Undiff-G	POU2F1
ARFGAP1	4.86E-05	-0.22517	0.022	0.149	0.725615	Undiff-G	ARFGAP1
CASP6	4.86E-05	0.147359	0.029	0.164	0.72576	Undiff-G	CASP6
SUGP1	4.87E-05	-0.23057	0.015	0.137	0.727195	Undiff-G	SUGP1
FAM216A	4.88E-05	-0.11012	0.007	0.123	0.729652	Undiff-G	FAM216A
RASSF5	4.90E-05	-0.42191	0.007	0.121	0.731636	Undiff-G	RASSF5
PCGF2	4.92E-05	0.290273	0.029	0.164	0.735149	Undiff-G	PCGF2
PPP1R37	4.96E-05	-0.24851	0.015	0.136	0.741323	Undiff-G	PPP1R37
CERK	4.98E-05	-0.2876	0	0.108	0.74417	Undiff-G	CERK
ANAPC4	4.98E-05	-0.29885	0	0.108	0.74417	Undiff-G	ANAPC4
IPO13	4.98E-05	-0.30518	0	0.108	0.74417	Undiff-G	IPO13
TBC1D12	4.98E-05	-0.33121	0	0.108	0.74417	Undiff-G	TBC1D12
CCDC57	4.98E-05	-0.36422	0	0.108	0.74417	Undiff-G	CCDC57
ADSS	5.00E-05	0.180141	0.044	0.188	0.74651	Undiff-G	ADSS
NAA16	5.00E-05	-0.27977	0.007	0.123	0.747021	Undiff-G	NAA16
USP7	5.02E-05	-0.17322	0.015	0.137	0.75055	Undiff-G	USP7
SELPLG	5.05E-05	-0.43868	0.007	0.123	0.755303	Undiff-G	SELPLG

RAN	5.06E-05	0.62373	0.331	0.762	0.75633	Undiff-G	RAN
HBA2	5.07E-05	0.338016	0.051	0.199	0.758336	Undiff-G	HBA2
SMARCAD1	5.10E-05	-0.2557	0	0.108	0.761466	Undiff-G	SMARCAD1
GPR82	5.12E-05	-0.2672	0.015	0.136	0.764732	Undiff-G	GPR82
CHRNB1	5.15E-05	0.202598	0.022	0.15	0.76987	Undiff-G	CHRNB1
TTC9C	5.18E-05	0.354006	0.037	0.177	0.774184	Undiff-G	TTC9C
MAF	5.20E-05	-0.20284	0.015	0.136	0.777427	Undiff-G	MAF
TMEM138	5.23E-05	0.127967	0.037	0.174	0.781319	Undiff-G	TMEM138
LYAR	5.23E-05	0.268452	0.037	0.176	0.781765	Undiff-G	LYAR
WDR11	5.25E-05	-0.3358	0	0.108	0.785144	Undiff-G	WDR11
PHTF1	5.26E-05	-0.25685	0.007	0.122	0.7864	Undiff-G	PHTF1
SLC37A4	5.27E-05	-0.17365	0.007	0.122	0.787254	Undiff-G	SLC37A4
PAAF1	5.28E-05	0.128659	0.051	0.201	0.789448	Undiff-G	PAAF1
MFSD3	5.28E-05	-0.26899	0.007	0.121	0.78972	Undiff-G	MFSD3
ATP1A3	5.30E-05	-0.38568	0.007	0.122	0.792423	Undiff-G	ATP1A3
MANBA	5.33E-05	-0.36865	0.007	0.122	0.796692	Undiff-G	MANBA
SH3RF1	5.33E-05	-0.25208	0	0.108	0.797253	Undiff-G	SH3RF1
SPHK1	5.33E-05	-0.29625	0	0.108	0.797253	Undiff-G	SPHK1
BTN2A1	5.36E-05	-0.30064	0.007	0.121	0.800964	Undiff-G	BTN2A1
PILRB	5.36E-05	-0.18821	0.029	0.163	0.8016	Undiff-G	PILRB
RGS3	5.38E-05	-0.26838	0	0.108	0.803376	Undiff-G	RGS3
BRCC3	5.38E-05	-0.28116	0	0.108	0.803376	Undiff-G	BRCC3
NAP1L5	5.38E-05	-0.24043	0.015	0.136	0.804495	Undiff-G	NAP1L5
AGAP1	5.40E-05	-0.29529	0.007	0.121	0.80638	Undiff-G	AGAP1
BPNT1	5.42E-05	-0.29659	0	0.108	0.809546	Undiff-G	BPNT1
ISYNA1	5.43E-05	-0.15657	0.015	0.135	0.810951	Undiff-G	ISYNA1
ID1	5.43E-05	-0.23195	0.007	0.122	0.811805	Undiff-G	ID1
ESYT1	5.44E-05	-0.31336	0.007	0.122	0.813178	Undiff-G	ESYT1
SERTAD2	5.45E-05	-0.25276	0.007	0.122	0.814579	Undiff-G	SERTAD2
NFS1	5.46E-05	-0.14262	0.015	0.135	0.815771	Undiff-G	NFS1
VPS53	5.48E-05	-0.14418	0.029	0.161	0.819272	Undiff-G	VPS53
NME7	5.50E-05	0.310156	0.029	0.163	0.821329	Undiff-G	NME7
SOBP	5.50E-05	-0.19537	0.015	0.136	0.82241	Undiff-G	SOBP
NBN	5.52E-05	0.144574	0.022	0.149	0.825139	Undiff-G	NBN
LRRN1	5.54E-05	-0.30764	0	0.107	0.828335	Undiff-G	LRRN1
MAN2C1	5.55E-05	-0.3363	0.015	0.135	0.829243	Undiff-G	MAN2C1
IRF2BP1	5.59E-05	-0.23236	0	0.107	0.834692	Undiff-G	IRF2BP1
MAPKAPK2	5.59E-05	-0.15134	0.015	0.134	0.835105	Undiff-G	MAPKAPK2
SLC7A6OS	5.61E-05	-0.21024	0.015	0.135	0.838927	Undiff-G	SLC7A6OS
TECPR1	5.61E-05	-0.24714	0.007	0.122	0.839001	Undiff-G	TECPR1
ZCCHC3	5.63E-05	-0.23444	0	0.107	0.841098	Undiff-G	ZCCHC3
SYK	5.63E-05	-0.43637	0	0.107	0.841098	Undiff-G	SYK
BAG5	5.63E-05	0.218799	0.044	0.186	0.8415	Undiff-G	BAG5
CTPS1	5.67E-05	-0.28408	0	0.107	0.847552	Undiff-G	CTPS1
SNX29	5.67E-05	-0.34404	0	0.107	0.847552	Undiff-G	SNX29
AKAP12	5.68E-05	0.155792	0.044	0.189	0.848593	Undiff-G	AKAP12
FAM134C	5.70E-05	-0.28804	0.007	0.121	0.851932	Undiff-G	FAM134C
PEMT	5.74E-05	0.472447	0.059	0.214	0.857495	Undiff-G	PEMT
SEH1L	5.77E-05	-0.16456	0.007	0.121	0.86236	Undiff-G	SEH1L
GIT1	5.80E-05	-0.25172	0	0.107	0.867208	Undiff-G	GIT1
TMEM121	5.80E-05	-0.25868	0	0.107	0.867208	Undiff-G	TMEM121

ICMT	5.81E-05	-0.13589	0.015	0.134	0.86793	Undiff-G	ICMT
KATNB1	5.82E-05	-0.23188	0.007	0.121	0.870044	Undiff-G	KATNB1
DIMT1	5.83E-05	0.133436	0.044	0.188	0.871205	Undiff-G	DIMT1
ZDHHC16	5.85E-05	-0.25622	0	0.107	0.87386	Undiff-G	ZDHHC16
UBTD2	5.85E-05	-0.26142	0	0.107	0.87386	Undiff-G	UBTD2
RCHY1	5.85E-05	0.502511	0.051	0.202	0.87426	Undiff-G	RCHY1
INPPL1	5.86E-05	-0.12829	0.007	0.121	0.875936	Undiff-G	INPPL1
ALDH16A1	5.87E-05	-0.29141	0.007	0.121	0.87745	Undiff-G	ALDH16A1
ASCC3	5.88E-05	-0.2329	0.015	0.135	0.878837	Undiff-G	ASCC3
MCCC2	5.88E-05	-0.16291	0.015	0.135	0.879319	Undiff-G	MCCC2
NDE1	5.89E-05	-0.28226	0	0.107	0.880561	Undiff-G	NDE1
STK17B	5.90E-05	-0.2576	0.015	0.134	0.881826	Undiff-G	STK17B
DUS1L	5.90E-05	-0.23628	0.015	0.134	0.881994	Undiff-G	DUS1L
CA2	5.92E-05	0.41065	0.066	0.227	0.885229	Undiff-G	CA2
OTUD6B	5.98E-05	-0.2429	0.007	0.121	0.893227	Undiff-G	OTUD6B
CIC	5.98E-05	-0.26091	0.007	0.121	0.893355	Undiff-G	CIC
SLC26A11	5.98E-05	-0.24967	0	0.106	0.894115	Undiff-G	SLC26A11
NBAS	5.98E-05	-0.27418	0	0.106	0.894115	Undiff-G	NBAS
GNAI1	5.98E-05	-0.33626	0	0.106	0.894115	Undiff-G	GNAI1
LHPP	5.98E-05	-0.49502	0	0.106	0.894115	Undiff-G	LHPP
ODF2	6.01E-05	-0.17285	0.015	0.133	0.898322	Undiff-G	ODF2
CENPB	6.03E-05	-0.2676	0	0.106	0.900969	Undiff-G	CENPB
EPB41L3	6.03E-05	-0.43804	0	0.106	0.900969	Undiff-G	EPB41L3
NBPF10	6.07E-05	-0.1509	0.022	0.147	0.906397	Undiff-G	NBPF10
KTI12	6.08E-05	-0.26709	0	0.106	0.907875	Undiff-G	KTI12
DNAJC24	6.08E-05	-0.28403	0	0.106	0.907875	Undiff-G	DNAJC24
TBCD	6.10E-05	-0.23389	0.022	0.147	0.911831	Undiff-G	TBCD
SNX8	6.10E-05	-0.18267	0.022	0.148	0.912197	Undiff-G	SNX8
TMEM119	6.12E-05	-0.50964	0	0.106	0.914832	Undiff-G	TMEM119
NECAP1	6.15E-05	0.229206	0.051	0.2	0.918945	Undiff-G	NECAP1
ANAPC2	6.17E-05	-0.17159	0.015	0.133	0.921635	Undiff-G	ANAPC2
HDAC7	6.17E-05	-0.27773	0	0.106	0.921843	Undiff-G	HDAC7
EEFSEC	6.17E-05	-0.28458	0	0.106	0.921843	Undiff-G	EEFSEC
HUS1	6.17E-05	-0.35116	0.007	0.119	0.922113	Undiff-G	HUS1
QSER1	6.23E-05	-0.24156	0.007	0.119	0.930869	Undiff-G	QSER1
CDC42BPB	6.33E-05	-0.2323	0.007	0.12	0.945717	Undiff-G	CDC42BPB
SPATA33	6.34E-05	-0.13533	0.022	0.147	0.947942	Undiff-G	SPATA33
KIF22	6.36E-05	0.30642	0.051	0.198	0.95016	Undiff-G	KIF22
BIRC5	6.36E-05	-0.44339	0	0.106	0.950415	Undiff-G	BIRC5
GSDMD	6.37E-05	0.190474	0.044	0.186	0.951373	Undiff-G	GSDMD
RGMB	6.38E-05	-0.25845	0.015	0.133	0.953647	Undiff-G	RGMB
PHACTR1	6.39E-05	-0.50577	0.007	0.119	0.954788	Undiff-G	PHACTR1
ABR	6.40E-05	-0.11384	0.022	0.147	0.956519	Undiff-G	ABR
SPATA20	6.40E-05	-0.1926	0.015	0.132	0.956812	Undiff-G	SPATA20
ELMOD2	6.41E-05	-0.2977	0	0.106	0.957693	Undiff-G	ELMOD2
NUSAP1	6.41E-05	-0.44131	0	0.106	0.957693	Undiff-G	NUSAP1
C5orf28	6.42E-05	-0.19693	0.007	0.12	0.959341	Undiff-G	C5orf28
PRKAA1	6.44E-05	-0.21905	0.022	0.147	0.962096	Undiff-G	PRKAA1
SCG2	6.45E-05	0.250833	0.088	0.264	0.963161	Undiff-G	SCG2
ITSN1	6.45E-05	0.105121	0.022	0.147	0.963971	Undiff-G	ITSN1
SENP2	6.46E-05	0.125656	0.015	0.134	0.965099	Undiff-G	SENP2

IQCE	6.46E-05	-0.24417	0.007	0.12	0.965509	Undiff-G	IQCE
GALNS	6.47E-05	-0.23038	0.015	0.133	0.966369	Undiff-G	GALNS
RP11-262H	6.48E-05	-0.25646	0.007	0.12	0.968977	Undiff-G	RP11-262H14.4
THAP9-AS1	6.49E-05	0.338014	0.066	0.223	0.969476	Undiff-G	THAP9-AS1
ZNF652	6.49E-05	-0.24766	0.022	0.146	0.970427	Undiff-G	ZNF652
ZNF44	6.51E-05	-0.2833	0	0.105	0.972414	Undiff-G	ZNF44
FAM179B	6.53E-05	-0.24277	0.007	0.12	0.975114	Undiff-G	FAM179B
PSMA3	6.54E-05	0.799517	0.184	0.444	0.978074	Undiff-G	PSMA3
PARP14	6.55E-05	-0.2836	0.029	0.158	0.97845	Undiff-G	PARP14
GTF3C2	6.56E-05	-0.28783	0	0.105	0.979858	Undiff-G	GTF3C2
SPTY2D1	6.56E-05	-0.22364	0.007	0.12	0.980163	Undiff-G	SPTY2D1
DDX50	6.56E-05	0.256968	0.029	0.161	0.980687	Undiff-G	DDX50
DZIP3	6.57E-05	-0.24994	0.015	0.132	0.98176	Undiff-G	DZIP3
CAPRIN2	6.61E-05	-0.23254	0	0.105	0.987358	Undiff-G	CAPRIN2
CEBPA	6.61E-05	-0.41353	0	0.105	0.987358	Undiff-G	CEBPA
MGAT4B	6.66E-05	-0.22379	0	0.105	0.994914	Undiff-G	MGAT4B
ARL9	6.66E-05	-0.24533	0	0.105	0.994914	Undiff-G	ARL9
SLC25A38	6.69E-05	0.153065	0.022	0.147	1	Undiff-G	SLC25A38
SH3GLB2	6.70E-05	-0.21664	0.007	0.12	1	Undiff-G	SH3GLB2
WDR47	6.71E-05	-0.27669	0	0.105	1	Undiff-G	WDR47
SEMA5A	6.71E-05	-0.28988	0	0.105	1	Undiff-G	SEMA5A
IKBKB	6.76E-05	-0.2694	0.007	0.119	1	Undiff-G	IKBKB
H2AFY2	6.76E-05	-0.23264	0	0.105	1	Undiff-G	H2AFY2
ESRRA	6.76E-05	-0.26663	0	0.105	1	Undiff-G	ESRRA
CTR9	6.78E-05	0.290803	0.037	0.174	1	Undiff-G	CTR9
RRM1	6.79E-05	0.375746	0.044	0.187	1	Undiff-G	RRM1
DNAJB9	6.79E-05	0.845206	0.154	0.388	1	Undiff-G	DNAJB9
CDK2AP1	6.81E-05	-0.24232	0	0.105	1	Undiff-G	CDK2AP1
APOBEC3G	6.81E-05	-0.2805	0.007	0.119	1	Undiff-G	APOBEC3G
SLC8A1	6.83E-05	-0.18495	0.022	0.146	1	Undiff-G	SLC8A1
ING3	6.84E-05	0.381081	0.044	0.186	1	Undiff-G	ING3
SHMT1	6.88E-05	-0.15028	0.007	0.119	1	Undiff-G	SHMT1
LEO1	6.91E-05	-0.10996	0.022	0.145	1	Undiff-G	LEO1
SATB1	6.91E-05	-0.25574	0.015	0.131	1	Undiff-G	SATB1
UBFD1	6.92E-05	-0.25177	0	0.105	1	Undiff-G	UBFD1
LPAR5	6.92E-05	-0.44385	0	0.105	1	Undiff-G	LPAR5
ACADS	6.92E-05	-0.21321	0.007	0.119	1	Undiff-G	ACADS
KIAA0101	6.98E-05	-0.39571	0.007	0.119	1	Undiff-G	KIAA0101
C11orf74	7.01E-05	0.431172	0.037	0.173	1	Undiff-G	C11orf74
AK2	7.02E-05	0.728475	0.162	0.393	1	Undiff-G	AK2
TMEM209	7.03E-05	-0.14674	0.007	0.119	1	Undiff-G	TMEM209
CENPW	7.04E-05	-0.29224	0.007	0.119	1	Undiff-G	CENPW
FUNDC1	7.06E-05	0.486139	0.081	0.248	1	Undiff-G	FUNDC1
GZF1	7.08E-05	-0.27803	0	0.105	1	Undiff-G	GZF1
POLRMT	7.10E-05	-0.22088	0.015	0.132	1	Undiff-G	POLRMT
EIF2B5	7.11E-05	0.188801	0.044	0.185	1	Undiff-G	EIF2B5
RRP9	7.17E-05	0.156646	0.029	0.158	1	Undiff-G	RRP9
SPATA2L	7.18E-05	-0.26148	0	0.104	1	Undiff-G	SPATA2L
RALGAPA1	7.26E-05	-0.28669	0.015	0.131	1	Undiff-G	RALGAPA1
10-Sep	7.31E-05	-0.15302	0.007	0.119	1	Undiff-G	10-Sep
KIAA0907	7.33E-05	0.26136	0.051	0.196	1	Undiff-G	KIAA0907

IL17RC	7.34E-05	-0.23782	0.007	0.118	1	Undiff-G	IL17RC
PIP4K2B	7.35E-05	-0.25037	0	0.104	1	Undiff-G	PIP4K2B
CBL	7.35E-05	-0.26411	0	0.104	1	Undiff-G	CBL
RILP	7.35E-05	-0.297	0	0.104	1	Undiff-G	RILP
ANO6	7.41E-05	-0.2653	0	0.104	1	Undiff-G	ANO6
CARKD	7.41E-05	-0.21134	0.015	0.131	1	Undiff-G	CARKD
E2F6	7.45E-05	-0.13907	0.022	0.146	1	Undiff-G	E2F6
PPIL2	7.46E-05	-0.21857	0.007	0.118	1	Undiff-G	PPIL2
FBP1	7.46E-05	-0.40194	0.007	0.118	1	Undiff-G	FBP1
SRCAP	7.46E-05	-0.27737	0	0.104	1	Undiff-G	SRCAP
MORC3	7.46E-05	-0.28101	0	0.104	1	Undiff-G	MORC3
PCED1A	7.48E-05	-0.21093	0.015	0.132	1	Undiff-G	PCED1A
ATG10	7.48E-05	-0.11719	0.022	0.144	1	Undiff-G	ATG10
PRR3	7.52E-05	-0.27297	0	0.104	1	Undiff-G	PRR3
KCNMB1	7.52E-05	-0.48705	0	0.104	1	Undiff-G	KCNMB1
SERPING1	7.53E-05	1.22101	0.316	0.233	1	Undiff-G	SERPING1
NUP50	7.56E-05	-0.22189	0.007	0.118	1	Undiff-G	NUP50
UBXN7	7.57E-05	-0.26403	0.007	0.118	1	Undiff-G	UBXN7
SH3PXD2B	7.59E-05	-0.11861	0.007	0.118	1	Undiff-G	SH3PXD2B
SNHG15	7.60E-05	0.404723	0.066	0.224	1	Undiff-G	SNHG15
S100A10	7.62E-05	1.192475	0.485	0.545	1	Undiff-G	S100A10
QPRT	7.63E-05	-0.39187	0.007	0.116	1	Undiff-G	QPRT
ZNF268	7.65E-05	-0.15512	0.007	0.118	1	Undiff-G	ZNF268
UBQLN4	7.69E-05	-0.25588	0	0.104	1	Undiff-G	UBQLN4
CDKN2A	7.69E-05	-0.44631	0	0.104	1	Undiff-G	CDKN2A
NSMF	7.70E-05	-0.23378	0.007	0.118	1	Undiff-G	NSMF
ATG9A	7.71E-05	-0.24948	0.007	0.118	1	Undiff-G	ATG9A
ENO2	7.72E-05	0.609941	0.103	0.286	1	Undiff-G	ENO2
RBM47	7.72E-05	-0.30737	0.007	0.118	1	Undiff-G	RBM47
TMEM255A	7.75E-05	-0.25166	0	0.103	1	Undiff-G	TMEM255A
TMOD3	7.78E-05	0.111193	0.022	0.145	1	Undiff-G	TMOD3
RCE1	7.78E-05	-0.26744	0.015	0.13	1	Undiff-G	RCE1
METTL21A	7.81E-05	0.266253	0.044	0.185	1	Undiff-G	METTL21A
RNASE1	7.85E-05	-0.20415	0.037	0.171	1	Undiff-G	RNASE1
LGR4	7.87E-05	-0.20635	0	0.103	1	Undiff-G	LGR4
DENND6B	7.87E-05	-0.34956	0	0.103	1	Undiff-G	DENND6B
PLEKHO2	7.87E-05	-0.36057	0	0.103	1	Undiff-G	PLEKHO2
TFE3	7.88E-05	-0.12119	0.007	0.118	1	Undiff-G	TFE3
FHIT	7.93E-05	-0.27357	0.007	0.118	1	Undiff-G	FHIT
CEPT1	7.94E-05	-0.11939	0.029	0.157	1	Undiff-G	CEPT1
CDK17	7.94E-05	-0.21729	0.007	0.118	1	Undiff-G	CDK17
NT5C2	7.94E-05	-0.31776	0.015	0.131	1	Undiff-G	NT5C2
NSMAF	7.96E-05	-0.2521	0.007	0.118	1	Undiff-G	NSMAF
GPN2	7.97E-05	-0.29788	0.007	0.116	1	Undiff-G	GPN2
SMNDC1	7.98E-05	-0.28093	0.007	0.117	1	Undiff-G	SMNDC1
MDM4	7.99E-05	0.225826	0.081	0.248	1	Undiff-G	MDM4
HDAC8	7.99E-05	-0.2907	0	0.103	1	Undiff-G	HDAC8
GTF2IRD2	8.00E-05	-0.26974	0.007	0.117	1	Undiff-G	GTF2IRD2
DPH2	8.00E-05	-0.2492	0.007	0.117	1	Undiff-G	DPH2
PRRC1	8.01E-05	-0.13179	0.007	0.118	1	Undiff-G	PRRC1
ZSCAN21	8.05E-05	-0.27514	0	0.103	1	Undiff-G	ZSCAN21

NAP1L3	8.05E-05	-0.31032	0	0.103	1	Undiff-G	NAP1L3
NOM1	8.06E-05	-0.25555	0.007	0.117	1	Undiff-G	NOM1
RBL2	8.07E-05	-0.25521	0.015	0.131	1	Undiff-G	RBL2
NCAPH2	8.08E-05	-0.17038	0.015	0.131	1	Undiff-G	NCAPH2
PALD1	8.11E-05	-0.42635	0	0.103	1	Undiff-G	PALD1
TRIM16	8.15E-05	-0.21788	0.015	0.131	1	Undiff-G	TRIM16
DLX6-AS1	8.16E-05	-0.45606	0.015	0.131	1	Undiff-G	DLX6-AS1
SEC16A	8.17E-05	-0.25634	0.007	0.117	1	Undiff-G	SEC16A
KBTBD2	8.17E-05	-0.28463	0.007	0.116	1	Undiff-G	KBTBD2
ZSWIM2	8.18E-05	0.302351	0.007	0	1	Undiff-G	ZSWIM2
ELSPBP1	8.18E-05	0.258194	0.007	0	1	Undiff-G	ELSPBP1
CRTC3	8.20E-05	-0.26648	0.007	0.117	1	Undiff-G	CRTC3
SEC14L2	8.23E-05	-0.26525	0.007	0.116	1	Undiff-G	SEC14L2
SAV1	8.24E-05	-0.26926	0	0.103	1	Undiff-G	SAV1
RTN1	8.29E-05	-0.11968	0.022	0.145	1	Undiff-G	RTN1
SERP2	8.30E-05	-0.29849	0	0.103	1	Undiff-G	SERP2
HIBCH	8.36E-05	0.406965	0.081	0.246	1	Undiff-G	HIBCH
PML	8.36E-05	-0.27564	0	0.103	1	Undiff-G	PML
NCF2	8.36E-05	-0.42844	0	0.103	1	Undiff-G	NCF2
SPHK2	8.38E-05	-0.26596	0.015	0.129	1	Undiff-G	SPHK2
DOLPP1	8.43E-05	-0.27751	0	0.103	1	Undiff-G	DOLPP1
DNAJC11	8.43E-05	-0.30475	0	0.103	1	Undiff-G	DNAJC11
BAK1	8.43E-05	-0.17592	0.007	0.117	1	Undiff-G	BAK1
CCDC126	8.48E-05	-0.26007	0.007	0.117	1	Undiff-G	CCDC126
NAB2	8.48E-05	-0.13027	0.022	0.143	1	Undiff-G	NAB2
HOXA7	8.49E-05	-0.35866	0	0.102	1	Undiff-G	HOXA7
C1orf216	8.50E-05	-0.24731	0.015	0.129	1	Undiff-G	C1orf216
DHX38	8.52E-05	-0.25321	0.007	0.117	1	Undiff-G	DHX38
ABCB8	8.54E-05	-0.21503	0.022	0.143	1	Undiff-G	ABCB8
CX3CR1	8.56E-05	-0.49289	0	0.102	1	Undiff-G	CX3CR1
RRP15	8.60E-05	0.424358	0.066	0.222	1	Undiff-G	RRP15
PLEKHA2	8.61E-05	-0.29776	0.007	0.115	1	Undiff-G	PLEKHA2
JMY	8.62E-05	-0.24716	0	0.102	1	Undiff-G	JMY
CENPF	8.62E-05	-0.4139	0	0.102	1	Undiff-G	CENPF
ACAD9	8.65E-05	-0.23842	0.007	0.117	1	Undiff-G	ACAD9
CDC42EP1	8.67E-05	-0.27341	0.015	0.129	1	Undiff-G	CDC42EP1
YLPM1	8.67E-05	-0.26122	0.007	0.116	1	Undiff-G	YLPM1
TMEM184C	8.69E-05	-0.25217	0	0.102	1	Undiff-G	TMEM184C
MYLIP	8.74E-05	-0.20702	0.015	0.13	1	Undiff-G	MYLIP
NOMO1	8.75E-05	-0.25548	0	0.102	1	Undiff-G	NOMO1
NKAIN4	8.75E-05	-0.42027	0	0.102	1	Undiff-G	NKAIN4
KRBOX4	8.76E-05	-0.19745	0.007	0.117	1	Undiff-G	KRBOX4
MBD6	8.77E-05	-0.36092	0.022	0.14	1	Undiff-G	MBD6
ATMIN	8.77E-05	-0.10343	0.007	0.117	1	Undiff-G	ATMIN
RIN3	8.78E-05	-0.36117	0.007	0.116	1	Undiff-G	RIN3
SNAP25	8.78E-05	-0.15149	0.022	0.144	1	Undiff-G	SNAP25
KIAA1586	8.79E-05	-0.26626	0.007	0.116	1	Undiff-G	KIAA1586
INPP4A	8.82E-05	-0.28753	0	0.102	1	Undiff-G	INPP4A
ZNF33A	8.82E-05	-0.31622	0	0.102	1	Undiff-G	ZNF33A
C10orf11	8.82E-05	-0.38229	0	0.102	1	Undiff-G	C10orf11
TNFRSF14	8.83E-05	-0.19417	0.007	0.116	1	Undiff-G	TNFRSF14

IFT43	8.92E-05	0.69303	0.088	0.259	1	Undiff-G	IFT43
NUDCD1	8.96E-05	-0.26985	0	0.102	1	Undiff-G	NUDCD1
AAGAB	8.96E-05	-0.14179	0.007	0.116	1	Undiff-G	AAGAB
NUFIP1	8.97E-05	-0.12347	0.007	0.116	1	Undiff-G	NUFIP1
MAP1B	8.99E-05	0.418545	0.243	0.537	1	Undiff-G	MAP1B
RYBP	9.03E-05	-0.21946	0.007	0.116	1	Undiff-G	RYBP
RP11-25K1	9.16E-05	-0.24667	0	0.102	1	Undiff-G	RP11-25K19.1
DENND3	9.16E-05	-0.42497	0	0.102	1	Undiff-G	DENND3
LCP2	9.17E-05	-0.32772	0.007	0.116	1	Undiff-G	LCP2
RIN2	9.21E-05	-0.3861	0.015	0.127	1	Undiff-G	RIN2
SLC38A1	9.22E-05	-0.27054	0.022	0.143	1	Undiff-G	SLC38A1
PRKCZ	9.23E-05	-0.26693	0	0.102	1	Undiff-G	PRKCZ
PCMTD1	9.28E-05	-0.12178	0.029	0.154	1	Undiff-G	PCMTD1
CUEDC1	9.28E-05	-0.2229	0.007	0.116	1	Undiff-G	CUEDC1
FAM189B	9.30E-05	-0.25187	0	0.101	1	Undiff-G	FAM189B
C3orf70	9.30E-05	-0.26653	0	0.101	1	Undiff-G	C3orf70
MTFMT	9.30E-05	-0.27239	0	0.101	1	Undiff-G	MTFMT
C1QL1	9.33E-05	-0.25111	0.007	0.116	1	Undiff-G	C1QL1
BCL6	9.36E-05	-0.23731	0.015	0.129	1	Undiff-G	BCL6
SIK1	9.37E-05	-0.40305	0	0.101	1	Undiff-G	SIK1
NOL10	9.38E-05	0.220273	0.029	0.156	1	Undiff-G	NOL10
VWA9	9.41E-05	0.137981	0.015	0.13	1	Undiff-G	VWA9
ABHD14B	9.41E-05	0.274146	0.044	0.181	1	Undiff-G	ABHD14B
PCGF3	9.43E-05	-0.21765	0.007	0.116	1	Undiff-G	PCGF3
DBT	9.44E-05	-0.27584	0	0.101	1	Undiff-G	DBT
HMBOX1	9.47E-05	0.111862	0.051	0.194	1	Undiff-G	HMBOX1
FDXR	9.50E-05	-0.27358	0.007	0.115	1	Undiff-G	FDXR
FGFBP3	9.52E-05	-0.23745	0	0.101	1	Undiff-G	FGFBP3
SLMAP	9.55E-05	-0.19819	0.015	0.128	1	Undiff-G	SLMAP
PRDM1	9.59E-05	-0.41669	0	0.101	1	Undiff-G	PRDM1
RBM4B	9.62E-05	0.396887	0.051	0.193	1	Undiff-G	RBM4B
LPGAT1	9.64E-05	-0.12127	0.015	0.129	1	Undiff-G	LPGAT1
SYT1	9.66E-05	-0.40829	0	0.101	1	Undiff-G	SYT1
PIK3C2A	9.67E-05	-0.11513	0.022	0.141	1	Undiff-G	PIK3C2A
CTB-3102C	9.67E-05	-0.23242	0.007	0.115	1	Undiff-G	CTB-31020.2
FOXK1	9.67E-05	-0.27116	0.015	0.128	1	Undiff-G	FOXK1
TBC1D10B	9.74E-05	-0.25411	0	0.101	1	Undiff-G	TBC1D10B
EFNB2	9.88E-05	-0.20159	0	0.101	1	Undiff-G	EFNB2
CMTM5	9.88E-05	-0.50644	0	0.101	1	Undiff-G	CMTM5
F3	9.92E-05	-0.12868	0.051	0.193	1	Undiff-G	F3
ANKRD26	9.94E-05	-0.24189	0.007	0.115	1	Undiff-G	ANKRD26
JAZF1	9.95E-05	-0.24528	0.007	0.115	1	Undiff-G	JAZF1
COG5	9.96E-05	-0.26986	0	0.101	1	Undiff-G	COG5
DNAJC14	9.96E-05	-0.27891	0	0.101	1	Undiff-G	DNAJC14
CDKN1C	9.97E-05	-0.14749	0.007	0.115	1	Undiff-G	CDKN1C
SLC35F5	9.97E-05	-0.10226	0.022	0.141	1	Undiff-G	SLC35F5
CEP78	0.0001	-0.22546	0.007	0.115	1	Undiff-G	CEP78
ZDHHC9	0.0001	0.390102	0.037	0.169	1	Undiff-G	ZDHHC9
GINS2	0.0001	-0.30341	0	0.101	1	Undiff-G	GINS2
GADD45A	0.000101	0.447245	0.103	0.281	1	Undiff-G	GADD45A
CASC15	0.000101	-0.3069	0	0.1	1	Undiff-G	CASC15

PCDH9	0.000101	0.25199	0.088	0.257	1	Undiff-G	PCDH9
EFHC1	0.000101	0.267571	0.059	0.207	1	Undiff-G	EFHC1
HDAC6	0.000101	-0.2192	0.007	0.115	1	Undiff-G	HDAC6
C18orf8	0.000102	-0.19586	0.007	0.115	1	Undiff-G	C18orf8
FBXO28	0.000102	-0.2598	0	0.1	1	Undiff-G	FBXO28
DDX59	0.000102	-0.26537	0	0.1	1	Undiff-G	DDX59
STAM	0.000102	-0.27415	0	0.1	1	Undiff-G	STAM
TCN2	0.000102	-0.35482	0	0.1	1	Undiff-G	TCN2
PEX26	0.000102	-0.26723	0.007	0.114	1	Undiff-G	PEX26
OTUD4	0.000103	-0.21384	0	0.1	1	Undiff-G	OTUD4
FAM168A	0.000103	-0.10654	0.015	0.128	1	Undiff-G	FAM168A
CYFIP2	0.000103	-0.1532	0.007	0.115	1	Undiff-G	CYFIP2
SNX24	0.000103	-0.18564	0.015	0.127	1	Undiff-G	SNX24
ERI1	0.000103	-0.27938	0.007	0.114	1	Undiff-G	ERI1
TMCC1	0.000103	-0.20501	0.007	0.115	1	Undiff-G	TMCC1
ALG1	0.000103	-0.25112	0.007	0.115	1	Undiff-G	ALG1
POM121C	0.000104	-0.13945	0.022	0.142	1	Undiff-G	POM121C
BCL2L12	0.000104	0.112884	0.015	0.128	1	Undiff-G	BCL2L12
CEP85L	0.000104	-0.24828	0.007	0.114	1	Undiff-G	CEP85L
POLR3D	0.000104	-0.25632	0.015	0.127	1	Undiff-G	POLR3D
KLHL13	0.000104	-0.23039	0	0.1	1	Undiff-G	KLHL13
PGGT1B	0.000104	-0.29148	0	0.1	1	Undiff-G	PGGT1B
TRMT6	0.000104	0.400731	0.044	0.18	1	Undiff-G	TRMT6
ADO	0.000105	-0.1269	0.015	0.127	1	Undiff-G	ADO
KIAA0355	0.000105	-0.13888	0.029	0.153	1	Undiff-G	KIAA0355
LRRC37B	0.000105	-0.24041	0.007	0.114	1	Undiff-G	LRRC37B
SPRED2	0.000105	-0.17913	0.007	0.114	1	Undiff-G	SPRED2
MAPK8	0.000105	-0.25705	0	0.1	1	Undiff-G	MAPK8
PYGL	0.000105	0.242597	0.051	0.194	1	Undiff-G	PYGL
CWC22	0.000105	0.106221	0.015	0.128	1	Undiff-G	CWC22
SLC1A5	0.000106	-0.31745	0.022	0.139	1	Undiff-G	SLC1A5
PWWP2A	0.000106	-0.21643	0.015	0.128	1	Undiff-G	PWWP2A
CSRNP3	0.000106	-0.31866	0	0.1	1	Undiff-G	CSRNP3
ID3	0.000106	0.382645	0.096	0.266	1	Undiff-G	ID3
ZDHHC21	0.000107	-0.25182	0	0.1	1	Undiff-G	ZDHHC21
RNF170	0.000107	0.141275	0.037	0.166	1	Undiff-G	RNF170
CENPQ	0.000107	-0.25462	0	0.1	1	Undiff-G	CENPQ
SLC25A24	0.000107	-0.26836	0	0.1	1	Undiff-G	SLC25A24
MILR1	0.000107	-0.47717	0	0.1	1	Undiff-G	MILR1
NUP54	0.000108	0.437222	0.044	0.179	1	Undiff-G	NUP54
GPX3	0.000108	-0.15867	0.029	0.154	1	Undiff-G	GPX3
SLC33A1	0.000108	-0.1237	0.015	0.128	1	Undiff-G	SLC33A1
STRBP	0.000108	-0.18332	0.015	0.128	1	Undiff-G	STRBP
WWOX	0.000108	-0.28713	0	0.1	1	Undiff-G	WWOX
ASXL2	0.000109	-0.27179	0	0.1	1	Undiff-G	ASXL2
RBMXL1	0.000111	0.307872	0.029	0.155	1	Undiff-G	RBMXL1
FXN	0.000111	-0.26228	0.007	0.114	1	Undiff-G	FXN
NEDD4L	0.000111	-0.25273	0.007	0.114	1	Undiff-G	NEDD4L
PLXND1	0.000112	-0.35228	0	0.099	1	Undiff-G	PLXND1
POLR2D	0.000112	0.149783	0.022	0.14	1	Undiff-G	POLR2D
OTUD1	0.000112	-0.40069	0	0.099	1	Undiff-G	OTUD1

POLR1E	0.000113	-0.25105	0.007	0.113	1	Undiff-G	POLR1E
SLC2A4RG	0.000113	-0.21586	0.015	0.127	1	Undiff-G	SLC2A4RG
KIF1A	0.000113	-0.19565	0.015	0.127	1	Undiff-G	KIF1A
TCHP	0.000114	-0.24934	0.007	0.113	1	Undiff-G	TCHP
TOMM70A	0.000114	-0.18805	0.015	0.126	1	Undiff-G	TOMM70A
NECAB3	0.000115	-0.20403	0	0.099	1	Undiff-G	NECAB3
LONRF1	0.000115	-0.21781	0	0.099	1	Undiff-G	LONRF1
ATG4C	0.000115	-0.1107	0.022	0.14	1	Undiff-G	ATG4C
PSEN2	0.000115	-0.24833	0.007	0.113	1	Undiff-G	PSEN2
INO80C	0.000116	0.309396	0.037	0.167	1	Undiff-G	INO80C
SFRP1	0.000116	-0.22584	0	0.099	1	Undiff-G	SFRP1
KLF13	0.000116	-0.24595	0.015	0.127	1	Undiff-G	KLF13
SPAST	0.000116	-0.22804	0.007	0.113	1	Undiff-G	SPAST
DGCR2	0.000116	0.11609	0.029	0.154	1	Undiff-G	DGCR2
MAP2K4	0.000117	-0.22572	0	0.099	1	Undiff-G	MAP2K4
IER5L	0.000117	-0.24407	0	0.099	1	Undiff-G	IER5L
QRSL1	0.000117	-0.11552	0.015	0.127	1	Undiff-G	QRSL1
FRS2	0.000118	-0.31128	0.007	0.113	1	Undiff-G	FRS2
ELOVL2	0.000118	-0.22566	0	0.099	1	Undiff-G	ELOVL2
ARNT2	0.000118	-0.2352	0.007	0.112	1	Undiff-G	ARNT2
RP4-665J2	0.000118	-0.16354	0.015	0.127	1	Undiff-G	RP4-665J23.1
ZNF708	0.000119	-0.25785	0	0.099	1	Undiff-G	ZNF708
CEP70	0.000119	-0.26681	0	0.099	1	Undiff-G	CEP70
ZNF703	0.000119	-0.2866	0	0.099	1	Undiff-G	ZNF703
SLC8B1	0.000119	-0.30795	0	0.099	1	Undiff-G	SLC8B1
NUP35	0.000119	-0.10386	0.007	0.113	1	Undiff-G	NUP35
MAN2A1	0.000119	-0.3368	0.007	0.112	1	Undiff-G	MAN2A1
PHF12	0.000119	-0.25524	0.007	0.112	1	Undiff-G	PHF12
PEX14	0.000119	-0.2518	0	0.099	1	Undiff-G	PEX14
NOL9	0.000119	-0.26115	0	0.099	1	Undiff-G	NOL9
LAS1L	0.00012	-0.1826	0.022	0.139	1	Undiff-G	LAS1L
SYNGR1	0.00012	-0.30787	0.007	0.113	1	Undiff-G	SYNGR1
VPS8	0.00012	-0.21924	0.007	0.113	1	Undiff-G	VPS8
LAMA5	0.00012	-0.20844	0.007	0.113	1	Undiff-G	LAMA5
CNEP1R1	0.000121	-0.29399	0.007	0.112	1	Undiff-G	CNEP1R1
ZNF302	0.000121	0.488872	0.125	0.317	1	Undiff-G	ZNF302
MAP3K1	0.000121	-0.25156	0	0.098	1	Undiff-G	MAP3K1
HLA-DQB2	0.000122	-0.63212	0	0.098	1	Undiff-G	HLA-DQB2
SRSF10	0.000122	0.459249	0.081	0.242	1	Undiff-G	SRSF10
ATP11A	0.000123	-0.26131	0	0.098	1	Undiff-G	ATP11A
KATNA1	0.000124	0.104826	0.022	0.14	1	Undiff-G	KATNA1
TRIM11	0.000124	-0.2613	0	0.098	1	Undiff-G	TRIM11
PDGFA	0.000124	-0.30823	0	0.098	1	Undiff-G	PDGFA
ASB3	0.000124	0.568942	0.029	0.005	1	Undiff-G	ASB3
TMEM168	0.000125	-0.26479	0.007	0.112	1	Undiff-G	TMEM168
EPSTI1	0.000125	-0.26035	0.015	0.126	1	Undiff-G	EPSTI1
ACO1	0.000125	-0.2629	0	0.098	1	Undiff-G	ACO1
ARHGAP15	0.000125	-0.4367	0	0.098	1	Undiff-G	ARHGAP15
FOXJ3	0.000125	-0.14205	0.007	0.112	1	Undiff-G	FOXJ3
FAM221A	0.000125	0.426644	0.044	0.179	1	Undiff-G	FAM221A
ZNF160	0.000126	-0.23328	0.007	0.112	1	Undiff-G	ZNF160

ARSJ	0.000126	-0.21839	0.015	0.126	1	Undiff-G	ARSJ
MAPK7	0.000126	0.14397	0.015	0.126	1	Undiff-G	MAPK7
DCLK1	0.000126	0.10766	0.029	0.153	1	Undiff-G	DCLK1
NPM1	0.000127	0.478299	0.382	0.874	1	Undiff-G	NPM1
GLDN	0.000127	-0.41538	0	0.098	1	Undiff-G	GLDN
FTSJ1	0.000127	-0.193	0.022	0.138	1	Undiff-G	FTSJ1
NCALD	0.000128	-0.19447	0.015	0.126	1	Undiff-G	NCALD
PREP	0.000128	-0.26415	0	0.098	1	Undiff-G	PREP
EID2	0.000128	-0.19649	0.007	0.112	1	Undiff-G	EID2
SLC35A3	0.000128	-0.26931	0.015	0.124	1	Undiff-G	SLC35A3
ZNF385D	0.000128	-0.24527	0.007	0.112	1	Undiff-G	ZNF385D
CPT2	0.000129	-0.21858	0.007	0.112	1	Undiff-G	CPT2
PIGQ	0.000129	-0.23226	0.007	0.112	1	Undiff-G	PIGQ
PRMT5	0.00013	0.139785	0.029	0.152	1	Undiff-G	PRMT5
PACS1	0.00013	-0.25493	0	0.098	1	Undiff-G	PACS1
TFB1M	0.00013	-0.26797	0	0.098	1	Undiff-G	TFB1M
ZNF48	0.000131	-0.224	0	0.098	1	Undiff-G	ZNF48
CPXM1	0.000131	-0.19392	0.015	0.125	1	Undiff-G	CPXM1
RC3H2	0.000132	-0.24199	0	0.097	1	Undiff-G	RC3H2
IGF2R	0.000132	-0.24548	0	0.097	1	Undiff-G	IGF2R
ATL3	0.000132	0.151681	0.022	0.139	1	Undiff-G	ATL3
ASUN	0.000133	-0.23277	0.007	0.112	1	Undiff-G	ASUN
MAP6	0.000133	-0.21808	0	0.097	1	Undiff-G	MAP6
DUSP2	0.000133	-0.43522	0.007	0.112	1	Undiff-G	DUSP2
CDC42EP3	0.000134	-0.1979	0.015	0.125	1	Undiff-G	CDC42EP3
ARIH1	0.000134	-0.28255	0	0.097	1	Undiff-G	ARIH1
ZRSR2	0.000134	-0.25672	0.015	0.125	1	Undiff-G	ZRSR2
LNPEP	0.000134	-0.22434	0.015	0.125	1	Undiff-G	LNPEP
SFXN5	0.000135	0.22749	0.051	0.19	1	Undiff-G	SFXN5
WDR20	0.000135	0.194209	0.022	0.139	1	Undiff-G	WDR20
ZNF280D	0.000135	-0.18715	0.015	0.125	1	Undiff-G	ZNF280D
FAM219B	0.000136	-0.17014	0.029	0.151	1	Undiff-G	FAM219B
SPRYD7	0.000136	0.335557	0.044	0.178	1	Undiff-G	SPRYD7
RABGAP1L	0.000136	-0.33887	0.007	0.111	1	Undiff-G	RABGAP1L
CHFR	0.000136	-0.24702	0.015	0.124	1	Undiff-G	CHFR
PPOX	0.000136	-0.27588	0.007	0.11	1	Undiff-G	PPOX
TMOD2	0.000136	-0.1902	0.007	0.111	1	Undiff-G	TMOD2
LAMP5	0.000137	-0.25718	0.007	0.11	1	Undiff-G	LAMP5
PNMAL1	0.000137	-0.23995	0	0.097	1	Undiff-G	PNMAL1
LPXN	0.000137	-0.34957	0.007	0.111	1	Undiff-G	LPXN
KPNA1	0.000137	0.231643	0.037	0.165	1	Undiff-G	KPNA1
GTF2IRD2E	0.000137	-0.23201	0.022	0.138	1	Undiff-G	GTF2IRD2B
NAF1	0.000138	-0.31907	0.007	0.111	1	Undiff-G	NAF1
SMG7	0.000138	-0.24206	0	0.097	1	Undiff-G	SMG7
SCAMP5	0.000138	-0.24322	0	0.097	1	Undiff-G	SCAMP5
TARSL2	0.000138	-0.23049	0.007	0.111	1	Undiff-G	TARSL2
TNKS2	0.000138	-0.25013	0.007	0.111	1	Undiff-G	TNKS2
NPHP3	0.000139	-0.26435	0.007	0.11	1	Undiff-G	NPHP3
EFNB1	0.000139	-0.26475	0	0.097	1	Undiff-G	EFNB1
TYRO3	0.000139	-0.25838	0.007	0.11	1	Undiff-G	TYRO3
DIS3	0.00014	0.214756	0.029	0.152	1	Undiff-G	DIS3

PARVA	0.00014	-0.20913	0	0.097	1	Undiff-G	PARVA
DENND5B	0.00014	-0.24388	0	0.097	1	Undiff-G	DENND5B
DTNB	0.00014	-0.26121	0	0.097	1	Undiff-G	DTNB
ZBTB43	0.00014	0.13105	0.029	0.151	1	Undiff-G	ZBTB43
RBM12	0.000141	-0.19727	0.015	0.124	1	Undiff-G	RBM12
RELB	0.000141	-0.23932	0	0.097	1	Undiff-G	RELB
MCF2L	0.000141	-0.30194	0	0.097	1	Undiff-G	MCF2L
MBNL2	0.000141	-0.32173	0	0.097	1	Undiff-G	MBNL2
C9orf9	0.000142	-0.11502	0.007	0.111	1	Undiff-G	C9orf9
LRRC4C	0.000143	-0.3029	0	0.097	1	Undiff-G	LRRC4C
KIF5A	0.000144	-0.27599	0.015	0.123	1	Undiff-G	KIF5A
COG1	0.000145	-0.16666	0.007	0.111	1	Undiff-G	COG1
FAM98B	0.000145	-0.23655	0	0.096	1	Undiff-G	FAM98B
RFXAP	0.000145	-0.24626	0	0.096	1	Undiff-G	RFXAP
SUSD3	0.000145	-0.44	0	0.096	1	Undiff-G	SUSD3
DDX39B	0.000146	-0.21696	0.007	0.111	1	Undiff-G	DDX39B
CIRBP	0.000146	0.391506	0.382	0.871	1	Undiff-G	CIRBP
ASAP2	0.000146	-0.19699	0	0.096	1	Undiff-G	ASAP2
TTC28	0.000146	-0.23502	0	0.096	1	Undiff-G	TTC28
SEMA4C	0.000146	-0.26536	0	0.096	1	Undiff-G	SEMA4C
RMND5A	0.000147	-0.2123	0.015	0.124	1	Undiff-G	RMND5A
PPM1D	0.000147	-0.25724	0	0.096	1	Undiff-G	PPM1D
KDM3B	0.000148	-0.27228	0.007	0.11	1	Undiff-G	KDM3B
WBP1L	0.000148	-0.25508	0.007	0.11	1	Undiff-G	WBP1L
TSC2	0.000148	-0.23078	0.022	0.137	1	Undiff-G	TSC2
DHX8	0.000149	-0.10627	0.007	0.11	1	Undiff-G	DHX8
TSPAN6	0.000149	0.109787	0.029	0.151	1	Undiff-G	TSPAN6
AC004158.	0.000149	-0.25616	0.015	0.124	1	Undiff-G	AC004158.3
NUP85	0.000149	0.109116	0.015	0.124	1	Undiff-G	NUP85
EPC2	0.00015	-0.24252	0	0.096	1	Undiff-G	EPC2
DIXDC1	0.00015	-0.25805	0	0.096	1	Undiff-G	DIXDC1
GNA12	0.00015	0.259415	0.022	0.138	1	Undiff-G	GNA12
GNA15	0.000151	-0.23946	0.007	0.11	1	Undiff-G	GNA15
KLHL9	0.000151	-0.18403	0.007	0.11	1	Undiff-G	KLHL9
TP53INP2	0.000151	-0.25808	0	0.096	1	Undiff-G	TP53INP2
C17orf75	0.000151	-0.27529	0	0.096	1	Undiff-G	C17orf75
HIP1R	0.000151	-0.27728	0.007	0.11	1	Undiff-G	HIP1R
RENBP	0.000151	-0.15035	0.022	0.137	1	Undiff-G	RENBP
ITCH	0.000152	-0.25205	0.007	0.11	1	Undiff-G	ITCH
MAP2K5	0.000152	-0.24167	0	0.096	1	Undiff-G	MAP2K5
CA12	0.000152	0.122282	0.022	0.137	1	Undiff-G	CA12
HNRNPL	0.000153	0.396281	0.096	0.266	1	Undiff-G	HNRNPL
PARD6A	0.000153	-0.22484	0	0.096	1	Undiff-G	PARD6A
AP3M1	0.000153	-0.26637	0	0.096	1	Undiff-G	AP3M1
ZNF350	0.000153	-0.29227	0	0.096	1	Undiff-G	ZNF350
TNFAIP8L2	0.000153	-0.41087	0	0.096	1	Undiff-G	TNFAIP8L2
UIMC1	0.000154	-0.12394	0.007	0.11	1	Undiff-G	UIMC1
ZNF768	0.000154	-0.22308	0	0.096	1	Undiff-G	ZNF768
RNF38	0.000154	-0.24647	0	0.096	1	Undiff-G	RNF38
MCC	0.000154	-0.21318	0.015	0.123	1	Undiff-G	MCC
HMGCR	0.000155	-0.2134	0.007	0.11	1	Undiff-G	HMGCR

ZNF775	0.000155	-0.22812	0	0.096	1	Undiff-G	ZNF775
ST3GAL4	0.000156	-0.12438	0.015	0.124	1	Undiff-G	ST3GAL4
WDR59	0.000156	-0.2752	0.007	0.109	1	Undiff-G	WDR59
NVL	0.000157	-0.25174	0.007	0.11	1	Undiff-G	NVL
TANC1	0.000157	-0.21867	0.007	0.109	1	Undiff-G	TANC1
PLBD1	0.000158	-0.35977	0	0.095	1	Undiff-G	PLBD1
ARHGAP30	0.000158	-0.39294	0	0.095	1	Undiff-G	ARHGAP30
CSPG5	0.000158	-0.31825	0.007	0.109	1	Undiff-G	CSPG5
AKAP8	0.000159	-0.17398	0.022	0.137	1	Undiff-G	AKAP8
SLC1A2	0.000159	-0.16815	0.022	0.137	1	Undiff-G	SLC1A2
THBD	0.000159	-0.42774	0	0.095	1	Undiff-G	THBD
RBBP9	0.00016	-0.1986	0	0.095	1	Undiff-G	RBBP9
CBWD5	0.000161	-0.15725	0.015	0.123	1	Undiff-G	CBWD5
ZNF506	0.000161	-0.14351	0.015	0.122	1	Undiff-G	ZNF506
ROBO2	0.000161	-0.22394	0.007	0.109	1	Undiff-G	ROBO2
ZNF362	0.000161	-0.23592	0	0.095	1	Undiff-G	ZNF362
SLC23A2	0.000162	-0.25378	0.007	0.108	1	Undiff-G	SLC23A2
SLC25A44	0.000163	-0.25957	0	0.095	1	Undiff-G	SLC25A44
STK32C	0.000163	-0.26516	0	0.095	1	Undiff-G	STK32C
ODF3B	0.000163	-0.34135	0	0.095	1	Undiff-G	ODF3B
CPD	0.000163	0.269363	0.037	0.161	1	Undiff-G	CPD
LRIG1	0.000163	-0.21414	0.007	0.109	1	Undiff-G	LRIG1
LRRC8D	0.000164	-0.24994	0	0.095	1	Undiff-G	LRRC8D
RASA3	0.000164	-0.26948	0	0.095	1	Undiff-G	RASA3
ALAD	0.000165	-0.27179	0.007	0.109	1	Undiff-G	ALAD
MLLT3	0.000165	-0.25559	0	0.095	1	Undiff-G	MLLT3
KIAA1549	0.000165	-0.25633	0	0.095	1	Undiff-G	KIAA1549
PLAA	0.000165	-0.26208	0	0.095	1	Undiff-G	PLAA
ZMAT1	0.000165	-0.28359	0	0.095	1	Undiff-G	ZMAT1
AVPI1	0.000165	-0.37486	0	0.095	1	Undiff-G	AVPI1
MIER2	0.000165	-0.21392	0.007	0.109	1	Undiff-G	MIER2
PIP5K1C	0.000165	-0.20203	0.007	0.109	1	Undiff-G	PIP5K1C
DDX6	0.000166	0.449345	0.059	0.199	1	Undiff-G	DDX6
FAM161A	0.000167	-0.17892	0.007	0.109	1	Undiff-G	FAM161A
IFIT3	0.000167	-0.20589	0.007	0.109	1	Undiff-G	IFIT3
BAG3	0.000167	-0.23054	0.007	0.109	1	Undiff-G	BAG3
BACH1	0.000167	-0.27969	0.007	0.109	1	Undiff-G	BACH1
POGZ	0.000167	0.306905	0.066	0.212	1	Undiff-G	POGZ
RECQL	0.000168	0.110046	0.044	0.174	1	Undiff-G	RECQL
ARAP1	0.000169	-0.1763	0.007	0.109	1	Undiff-G	ARAP1
RHOU	0.000169	-0.14104	0.022	0.136	1	Undiff-G	RHOU
CEP104	0.00017	-0.17092	0.015	0.123	1	Undiff-G	CEP104
EXOSC10	0.00017	-0.11825	0.029	0.148	1	Undiff-G	EXOSC10
ENTPD4	0.00017	-0.25292	0	0.095	1	Undiff-G	ENTPD4
CSGALNACT	0.00017	-0.26909	0	0.095	1	Undiff-G	CSGALNACT2
UBE3B	0.000171	-0.20299	0.007	0.109	1	Undiff-G	UBE3B
DNAJA3	0.000171	0.167515	0.044	0.175	1	Undiff-G	DNAJA3
PGPEP1	0.000171	-0.13919	0.022	0.135	1	Undiff-G	PGPEP1
NRBP2	0.000172	-0.20315	0.015	0.122	1	Undiff-G	NRBP2
DUSP15	0.000173	-0.19682	0	0.094	1	Undiff-G	DUSP15
PKIA	0.000173	-0.31237	0	0.094	1	Undiff-G	PKIA

METTL8	0.000173	-0.2639	0.015	0.121	1	Undiff-G	METTL8
CDKAL1	0.000174	-0.26557	0	0.094	1	Undiff-G	CDKAL1
C9orf69	0.000175	-0.21512	0	0.094	1	Undiff-G	C9orf69
DAGLB	0.000175	-0.31372	0.015	0.122	1	Undiff-G	DAGLB
SYP	0.000176	-0.2496	0.022	0.135	1	Undiff-G	SYP
HACL1	0.000176	-0.19749	0.007	0.108	1	Undiff-G	HACL1
MGME1	0.000176	0.237566	0.029	0.149	1	Undiff-G	MGME1
CDK5R1	0.000177	-0.27508	0	0.094	1	Undiff-G	CDK5R1
RP11-472N	0.000177	-0.26905	0.007	0.108	1	Undiff-G	RP11-472N13.3
GPALPP1	0.000177	-0.233	0.015	0.122	1	Undiff-G	GPALPP1
YAE1D1	0.000178	0.214778	0.037	0.161	1	Undiff-G	YAE1D1
HDAC10	0.000179	-0.15837	0.007	0.108	1	Undiff-G	HDAC10
SORBS1	0.000179	-0.20615	0	0.094	1	Undiff-G	SORBS1
ANKZF1	0.000179	-0.18116	0.022	0.135	1	Undiff-G	ANKZF1
DSTYK	0.00018	-0.19219	0.015	0.122	1	Undiff-G	DSTYK
ADA	0.000181	-0.22699	0	0.094	1	Undiff-G	ADA
SNHG12	0.000181	-0.10724	0.022	0.135	1	Undiff-G	SNHG12
METTL1	0.000181	0.337815	0.029	0.149	1	Undiff-G	METTL1
LRP12	0.000182	-0.233	0	0.094	1	Undiff-G	LRP12
SIGLEC9	0.000182	-0.4055	0	0.094	1	Undiff-G	SIGLEC9
TOPBP1	0.000183	-0.22888	0	0.094	1	Undiff-G	TOPBP1
ZWINT	0.000183	-0.30987	0	0.094	1	Undiff-G	ZWINT
ATCAY	0.000184	-0.21949	0.015	0.122	1	Undiff-G	ATCAY
PEX3	0.000184	-0.14411	0.007	0.108	1	Undiff-G	PEX3
KPNA5	0.000185	-0.25311	0	0.094	1	Undiff-G	KPNA5
1-Sep	0.000185	-0.23366	0.007	0.108	1	Undiff-G	1-Sep
HTT	0.000185	-0.10961	0.015	0.122	1	Undiff-G	HTT
CELSR2	0.000186	-0.2315	0	0.094	1	Undiff-G	CELSR2
ASB16-AS1	0.000186	-0.23478	0	0.094	1	Undiff-G	ASB16-AS1
MINPP1	0.000187	-0.15887	0.007	0.108	1	Undiff-G	MINPP1
METTL14	0.000188	-0.24587	0	0.093	1	Undiff-G	METTL14
WIPF3	0.000188	-0.27869	0	0.093	1	Undiff-G	WIPF3
ADRBK1	0.000188	-0.30803	0	0.093	1	Undiff-G	ADRBK1
MXI1	0.000188	-0.23559	0.022	0.134	1	Undiff-G	MXI1
LARP1B	0.000188	-0.19657	0.015	0.121	1	Undiff-G	LARP1B
MNT	0.000189	-0.24829	0	0.093	1	Undiff-G	MNT
ELMSAN1	0.000189	-0.16505	0.022	0.135	1	Undiff-G	ELMSAN1
MT1A	0.000189	0.973258	0.081	0.029	1	Undiff-G	MT1A
PTRF	0.000189	0.426995	0.051	0.187	1	Undiff-G	PTRF
DIP2B	0.00019	-0.21593	0.015	0.12	1	Undiff-G	DIP2B
MAK16	0.00019	-0.22053	0	0.093	1	Undiff-G	MAK16
TRIM14	0.00019	-0.25445	0	0.093	1	Undiff-G	TRIM14
STOX2	0.00019	-0.25832	0	0.093	1	Undiff-G	STOX2
FAM57B	0.00019	-0.2898	0	0.093	1	Undiff-G	FAM57B
TP53TG1	0.000191	0.269227	0.059	0.199	1	Undiff-G	TP53TG1
SLC12A5	0.000192	-0.34181	0	0.093	1	Undiff-G	SLC12A5
HSD17B14	0.000193	-0.37504	0	0.093	1	Undiff-G	HSD17B14
DDHD2	0.000194	-0.24749	0.007	0.107	1	Undiff-G	DDHD2
CRTC1	0.000195	-0.23457	0	0.093	1	Undiff-G	CRTC1
RCSD1	0.000195	-0.28002	0.015	0.121	1	Undiff-G	RCSD1
IL10	0.000196	-0.27502	0.007	0.107	1	Undiff-G	IL10

ZNF609	0.000196	-0.23068	0	0.093	1	Undiff-G	ZNF609
ELP4	0.000196	-0.2856	0	0.093	1	Undiff-G	ELP4
YTHDC2	0.000196	-0.21792	0.015	0.12	1	Undiff-G	YTHDC2
COQ6	0.000197	-0.25583	0.007	0.107	1	Undiff-G	COQ6
RAB33A	0.000197	-0.17881	0.007	0.107	1	Undiff-G	RAB33A
SSPN	0.000197	-0.28086	0.007	0.107	1	Undiff-G	SSPN
TMEM178A	0.000197	-0.24241	0.015	0.12	1	Undiff-G	TMEM178A
SMCR5	0.000198	-0.23673	0	0.093	1	Undiff-G	SMCR5
SORD	0.000198	-0.25274	0	0.093	1	Undiff-G	SORD
SLC17A5	0.000199	-0.2624	0.007	0.107	1	Undiff-G	SLC17A5
SMIM8	0.000199	-0.25292	0.007	0.107	1	Undiff-G	SMIM8
PTN	0.000199	0.909912	0.515	0.617	1	Undiff-G	PTN
TMEM35	0.000199	-0.2874	0	0.093	1	Undiff-G	TMEM35
LILRB1	0.000199	-0.39291	0	0.093	1	Undiff-G	LILRB1
CSPP1	0.0002	-0.18515	0.007	0.107	1	Undiff-G	CSPP1
BEND6	0.000201	-0.18091	0.015	0.12	1	Undiff-G	BEND6
OAS1	0.000201	-0.22809	0.022	0.134	1	Undiff-G	OAS1
A2M	0.000202	0.501045	0.206	0.467	1	Undiff-G	A2M
AUTS2	0.000202	-0.20569	0.015	0.12	1	Undiff-G	AUTS2
RASA1	0.000202	-0.2066	0.007	0.107	1	Undiff-G	RASA1
ARHGAP26	0.000202	-0.33426	0	0.093	1	Undiff-G	ARHGAP26
TUBGCP3	0.000203	-0.26467	0.007	0.106	1	Undiff-G	TUBGCP3
AP3M2	0.000204	-0.23258	0	0.092	1	Undiff-G	AP3M2
MLLT6	0.000204	-0.15891	0.022	0.133	1	Undiff-G	MLLT6
PHTF2	0.000205	-0.27502	0	0.092	1	Undiff-G	PHTF2
TYW1	0.000205	-0.18155	0.015	0.12	1	Undiff-G	TYW1
G0S2	0.000206	0.186386	0.051	0.186	1	Undiff-G	G0S2
SMCO4	0.000207	0.100045	0.022	0.134	1	Undiff-G	SMCO4
UPF1	0.000207	-0.23532	0	0.092	1	Undiff-G	UPF1
KCNE4	0.000207	-0.23068	0.007	0.106	1	Undiff-G	KCNE4
NLGN4X	0.000208	-0.20202	0.007	0.107	1	Undiff-G	NLGN4X
DEF6	0.000208	-0.36381	0	0.092	1	Undiff-G	DEF6
INSR	0.00021	-0.12772	0.029	0.146	1	Undiff-G	INSR
KIAA0430	0.00021	-0.24526	0	0.092	1	Undiff-G	KIAA0430
ZNF117	0.00021	-0.24182	0.007	0.106	1	Undiff-G	ZNF117
MED22	0.000211	-0.18757	0.007	0.106	1	Undiff-G	MED22
SCRIB	0.000211	-0.2187	0	0.092	1	Undiff-G	SCRIB
KBTBD3	0.000212	-0.22001	0.007	0.106	1	Undiff-G	KBTBD3
ZDHHC17	0.000213	-0.11403	0.007	0.106	1	Undiff-G	ZDHHC17
HAGHL	0.000213	-0.21871	0.007	0.106	1	Undiff-G	HAGHL
FAM219A	0.000213	-0.22844	0	0.092	1	Undiff-G	FAM219A
PEX5	0.000213	-0.26288	0	0.092	1	Undiff-G	PEX5
ITGAX	0.000213	-0.4195	0	0.092	1	Undiff-G	ITGAX
MAGI2	0.000213	-0.24799	0.007	0.106	1	Undiff-G	MAGI2
FASTKD2	0.000214	-0.26602	0.007	0.105	1	Undiff-G	FASTKD2
MPND	0.000215	-0.18838	0	0.092	1	Undiff-G	MPND
ZFX	0.000215	-0.19742	0.015	0.12	1	Undiff-G	ZFX
TP53BP2	0.000216	-0.25229	0.007	0.105	1	Undiff-G	TP53BP2
PRRT2	0.000216	-0.22334	0	0.092	1	Undiff-G	PRRT2
CNKSR3	0.000217	-0.21354	0.015	0.119	1	Undiff-G	CNKSR3
CENPU	0.000217	-0.12161	0.007	0.106	1	Undiff-G	CENPU

FAM188A	0.000218	-0.23708	0	0.092	1	Undiff-G	FAM188A
SAA2	0.000218	1.047951	0.118	0.051	1	Undiff-G	SAA2
TICAM1	0.000219	-0.24574	0	0.092	1	Undiff-G	TICAM1
FBXO34	0.000219	-0.24784	0	0.092	1	Undiff-G	FBXO34
GNAO1	0.000219	-0.3286	0	0.092	1	Undiff-G	GNAO1
EGR3	0.00022	-0.37695	0.007	0.105	1	Undiff-G	EGR3
FAM84A	0.00022	-0.1315	0.029	0.146	1	Undiff-G	FAM84A
ANTXR1	0.000221	-0.23046	0	0.092	1	Undiff-G	ANTXR1
UBAP2	0.000221	-0.2506	0	0.092	1	Undiff-G	UBAP2
DYM	0.000221	-0.25957	0	0.092	1	Undiff-G	DYM
CCNB1	0.000221	-0.35646	0	0.092	1	Undiff-G	CCNB1
RLF	0.000221	-0.21289	0.007	0.106	1	Undiff-G	RLF
C17orf10C	0.000223	-0.19913	0	0.091	1	Undiff-G	C17orf100
RFX5	0.000224	-0.26109	0.007	0.105	1	Undiff-G	RFX5
C8orf76	0.000225	0.188769	0.037	0.159	1	Undiff-G	C8orf76
DYNC1I1	0.000225	-0.34778	0.007	0.105	1	Undiff-G	DYNC1I1
EMC1	0.000226	0.160039	0.022	0.133	1	Undiff-G	EMC1
PDCD2L	0.000226	-0.10023	0.007	0.106	1	Undiff-G	PDCD2L
ANKS3	0.000226	-0.11301	0.044	0.17	1	Undiff-G	ANKS3
KLHDC8B	0.000228	-0.25686	0.007	0.104	1	Undiff-G	KLHDC8B
TOP3A	0.000228	-0.22764	0	0.091	1	Undiff-G	TOP3A
SLC39A4	0.000228	-0.35065	0	0.091	1	Undiff-G	SLC39A4
PPP1R15B	0.00023	-0.16735	0.015	0.119	1	Undiff-G	PPP1R15B
COG2	0.000231	-0.11229	0.015	0.119	1	Undiff-G	COG2
VWA5A	0.000231	-0.28124	0	0.091	1	Undiff-G	VWA5A
METTL17	0.000232	-0.16423	0.015	0.119	1	Undiff-G	METTL17
GSTZ1	0.000232	0.446763	0.037	0.159	1	Undiff-G	GSTZ1
OSER1-AS1	0.000233	0.216595	0.022	0.133	1	Undiff-G	OSER1-AS1
MINK1	0.000233	-0.21737	0.007	0.105	1	Undiff-G	MINK1
PALM	0.000234	-0.19141	0.007	0.105	1	Undiff-G	PALM
LRRC42	0.000234	0.144516	0.029	0.146	1	Undiff-G	LRRC42
CRISPLD1	0.000235	-0.24557	0	0.091	1	Undiff-G	CRISPLD1
CYTIP	0.000235	-0.42128	0.007	0.105	1	Undiff-G	CYTIP
UBE2O	0.000235	-0.20434	0.015	0.118	1	Undiff-G	UBE2O
B3GAT1	0.000237	-0.28883	0	0.091	1	Undiff-G	B3GAT1
SLC26A2	0.000237	-0.22579	0.007	0.105	1	Undiff-G	SLC26A2
ATXN3	0.000237	0.110533	0.037	0.158	1	Undiff-G	ATXN3
NSMCE2	0.000237	0.279818	0.037	0.158	1	Undiff-G	NSMCE2
ZNF649	0.000238	-0.21079	0	0.091	1	Undiff-G	ZNF649
PPP2R2D	0.000238	-0.22168	0	0.091	1	Undiff-G	PPP2R2D
AGO1	0.000238	-0.23255	0	0.091	1	Undiff-G	AGO1
GKAP1	0.000238	-0.23587	0	0.091	1	Undiff-G	GKAP1
IFI44L	0.000239	-0.29421	0.029	0.143	1	Undiff-G	IFI44L
FRMD8	0.000239	-0.24108	0.015	0.117	1	Undiff-G	FRMD8
NFKB1	0.00024	-0.12093	0.007	0.105	1	Undiff-G	NFKB1
PIGH	0.00024	0.480614	0.066	0.21	1	Undiff-G	PIGH
GALNT7	0.00024	-0.23278	0	0.091	1	Undiff-G	GALNT7
CCNT2	0.000241	-0.25542	0.007	0.104	1	Undiff-G	CCNT2
PPFIA3	0.000242	-0.19898	0	0.09	1	Undiff-G	PPFIA3
CAAP1	0.000242	-0.22125	0	0.09	1	Undiff-G	CAAP1
MYC	0.000242	0.237217	0.044	0.171	1	Undiff-G	MYC

C4orf3	0.000243	0.776099	0.294	0.65	1	Undiff-G	C4orf3
SACM1L	0.000243	-0.22502	0.022	0.13	1	Undiff-G	SACM1L
MPHOSPH9	0.000244	-0.19462	0.015	0.118	1	Undiff-G	MPHOSPH9
FASN	0.000244	-0.18984	0.015	0.118	1	Undiff-G	FASN
EID2B	0.000244	-0.23244	0	0.09	1	Undiff-G	EID2B
PID1	0.000245	-0.21446	0.015	0.118	1	Undiff-G	PID1
ZNF503	0.000245	-0.11338	0.007	0.105	1	Undiff-G	ZNF503
PRR14L	0.000246	-0.21855	0	0.09	1	Undiff-G	PRR14L
HIPK3	0.000246	-0.24989	0	0.09	1	Undiff-G	HIPK3
NUP37	0.000246	0.271155	0.022	0.132	1	Undiff-G	NUP37
RNF185	0.000247	-0.10329	0.007	0.105	1	Undiff-G	RNF185
GMDS	0.000247	-0.22095	0	0.09	1	Undiff-G	GMDS
UVRAG	0.000247	-0.25528	0	0.09	1	Undiff-G	UVRAG
ESYT2	0.000247	-0.26474	0	0.09	1	Undiff-G	ESYT2
HPGDS	0.000251	-0.44058	0	0.09	1	Undiff-G	HPGDS
L3MBTL2	0.000251	-0.25739	0.007	0.103	1	Undiff-G	L3MBTL2
CSTF1	0.000252	0.134721	0.022	0.132	1	Undiff-G	CSTF1
SLC30A1	0.000253	-0.22216	0	0.09	1	Undiff-G	SLC30A1
HAUS6	0.000253	-0.23575	0	0.09	1	Undiff-G	HAUS6
PPP2R2B	0.000253	0.466478	0.074	0.221	1	Undiff-G	PPP2R2B
DIS3L	0.000253	-0.20671	0.007	0.104	1	Undiff-G	DIS3L
RRM2B	0.000254	-0.21355	0.007	0.104	1	Undiff-G	RRM2B
NR1D2	0.000257	-0.23143	0	0.09	1	Undiff-G	NR1D2
CCRL2	0.000257	-0.40893	0	0.09	1	Undiff-G	CCRL2
AP5Z1	0.000257	-0.10868	0.022	0.13	1	Undiff-G	AP5Z1
EXOG	0.000257	0.232344	0.037	0.157	1	Undiff-G	EXOG
EP400	0.000258	-0.14083	0.015	0.118	1	Undiff-G	EP400
AGPAT4	0.00026	-0.23916	0.007	0.104	1	Undiff-G	AGPAT4
BRF1	0.000261	-0.17655	0.007	0.104	1	Undiff-G	BRF1
SYVN1	0.000262	0.161902	0.007	0.104	1	Undiff-G	SYVN1
GPR162	0.000262	-0.23947	0.007	0.103	1	Undiff-G	GPR162
SOX5	0.000263	-0.23514	0	0.09	1	Undiff-G	SOX5
RPS6KB1	0.000263	0.143706	0.022	0.13	1	Undiff-G	RPS6KB1
NACC2	0.000263	-0.20036	0.007	0.104	1	Undiff-G	NACC2
CLOCK	0.000264	-0.21002	0.022	0.129	1	Undiff-G	CLOCK
N4BP1	0.000265	-0.22158	0	0.089	1	Undiff-G	N4BP1
RAD17	0.000265	0.226182	0.022	0.131	1	Undiff-G	RAD17
TMEM132B	0.000266	-0.21729	0.007	0.103	1	Undiff-G	TMEM132B
XRRA1	0.000266	-0.16634	0.022	0.13	1	Undiff-G	XRRA1
PTPRF	0.000266	-0.17971	0.015	0.116	1	Undiff-G	PTPRF
MAPK8IP3	0.000267	-0.23853	0	0.089	1	Undiff-G	MAPK8IP3
CCDC92	0.000267	-0.23584	0.007	0.103	1	Undiff-G	CCDC92
ING5	0.000267	-0.1706	0.015	0.117	1	Undiff-G	ING5
LAMC1	0.000268	-0.16805	0.007	0.104	1	Undiff-G	LAMC1
XPO6	0.000268	-0.21288	0.015	0.116	1	Undiff-G	XPO6
ZDHHC6	0.000269	0.267719	0.044	0.169	1	Undiff-G	ZDHHC6
RANGAP1	0.000269	-0.20249	0.007	0.104	1	Undiff-G	RANGAP1
LRRTM2	0.000269	-0.17759	0.007	0.104	1	Undiff-G	LRRTM2
DHRS11	0.00027	-0.12238	0.007	0.104	1	Undiff-G	DHRS11
DZIP1	0.00027	-0.16935	0.007	0.103	1	Undiff-G	DZIP1
ARL13B	0.00027	0.406622	0.029	0.144	1	Undiff-G	ARL13B

RNF103	0.000272	-0.17842	0.007	0.103	1	Undiff-G	RNF103
SCFD2	0.000273	-0.24511	0	0.089	1	Undiff-G	SCFD2
PCDHB10	0.000273	-0.26083	0.007	0.103	1	Undiff-G	PCDHB10
PEG10	0.000274	-0.15007	0.007	0.103	1	Undiff-G	PEG10
KIF3C	0.000274	-0.11593	0.007	0.103	1	Undiff-G	KIF3C
RP11-284F	0.000274	0.581511	0.037	0.008	1	Undiff-G	RP11-284F21.8
EPB41L1	0.000275	-0.20893	0	0.089	1	Undiff-G	EPB41L1
GNPNAT1	0.000278	-0.23207	0.007	0.102	1	Undiff-G	GNPNAT1
ZNF226	0.000278	0.227658	0.066	0.205	1	Undiff-G	ZNF226
LDOC1	0.000279	-0.33693	0	0.089	1	Undiff-G	LDOC1
HS3ST1	0.000279	-0.37238	0	0.089	1	Undiff-G	HS3ST1
TSEN54	0.000279	-0.22025	0.015	0.116	1	Undiff-G	TSEN54
BIN3	0.000279	0.302002	0.037	0.156	1	Undiff-G	BIN3
DMWD	0.00028	-0.15277	0.029	0.143	1	Undiff-G	DMWD
SCRN3	0.000281	-0.23554	0.007	0.102	1	Undiff-G	SCRN3
SGTB	0.000281	-0.21662	0	0.089	1	Undiff-G	SGTB
C9orf114	0.000282	0.230438	0.029	0.143	1	Undiff-G	C9orf114
RP11-108M	0.000282	-0.15472	0.007	0.103	1	Undiff-G	RP11-108M9.4
POFUT2	0.000283	-0.22954	0	0.089	1	Undiff-G	POFUT2
FIGN	0.000285	-0.201	0	0.089	1	Undiff-G	FIGN
RFC3	0.000285	-0.2402	0	0.089	1	Undiff-G	RFC3
BSG	0.000287	0.561913	0.338	0.746	1	Undiff-G	BSG
TRIM25	0.000287	-0.21543	0	0.089	1	Undiff-G	TRIM25
UEVLD	0.000287	-0.22407	0	0.089	1	Undiff-G	UEVLD
MDFI	0.000287	-0.24506	0	0.089	1	Undiff-G	MDFI
NLRP3	0.000287	-0.39602	0	0.089	1	Undiff-G	NLRP3
MBIP	0.000288	0.428515	0.051	0.181	1	Undiff-G	MBIP
NCOA7	0.000288	-0.22733	0.015	0.116	1	Undiff-G	NCOA7
SESN1	0.000288	-0.17415	0.007	0.103	1	Undiff-G	SESN1
ARL5B	0.000289	-0.23368	0	0.088	1	Undiff-G	ARL5B
PHLDB1	0.000292	0.158995	0.029	0.143	1	Undiff-G	PHLDB1
HSPA14	0.000292	0.116509	0.029	0.143	1	Undiff-G	HSPA14
STK39	0.000292	-0.15192	0.007	0.103	1	Undiff-G	STK39
DLX1	0.000292	-0.29229	0.015	0.116	1	Undiff-G	DLX1
SFXN4	0.000293	-0.15591	0.015	0.115	1	Undiff-G	SFXN4
NINJ2	0.000294	-0.43047	0	0.088	1	Undiff-G	NINJ2
FAM98A	0.000294	0.139724	0.029	0.143	1	Undiff-G	FAM98A
KIF20B	0.000296	-0.30915	0	0.088	1	Undiff-G	KIF20B
ASB1	0.000296	-0.25972	0.007	0.101	1	Undiff-G	ASB1
TOX	0.000298	-0.23823	0	0.088	1	Undiff-G	TOX
KIAA1551	0.000298	-0.2767	0	0.088	1	Undiff-G	KIAA1551
SLC16A10	0.000298	-0.43071	0	0.088	1	Undiff-G	SLC16A10
C6orf203	0.000299	0.325645	0.044	0.167	1	Undiff-G	C6orf203
CAPN7	0.000299	-0.14208	0.015	0.115	1	Undiff-G	CAPN7
MXRA7	0.0003	0.293166	0.044	0.168	1	Undiff-G	MXRA7
ZNF217	0.0003	-0.20584	0	0.088	1	Undiff-G	ZNF217
FAM210A	0.000301	0.229199	0.029	0.143	1	Undiff-G	FAM210A
LSM14B	0.000302	-0.20573	0.007	0.102	1	Undiff-G	LSM14B
MIR181A1H	0.000302	-0.31168	0.015	0.114	1	Undiff-G	MIR181A1HG
NDST1	0.000303	-0.21439	0	0.088	1	Undiff-G	NDST1
ZNF574	0.000303	-0.22555	0	0.088	1	Undiff-G	ZNF574

NCKAP5L	0.000304	-0.23444	0.015	0.115	1	Undiff-G	NCKAP5L
MEIS1	0.000305	-0.20776	0	0.088	1	Undiff-G	MEIS1
FBXL17	0.000305	-0.22429	0	0.088	1	Undiff-G	FBXL17
CDH4	0.000307	-0.20549	0	0.088	1	Undiff-G	CDH4
SYBU	0.000307	-0.22393	0	0.088	1	Undiff-G	SYBU
KBTBD7	0.000307	-0.25145	0	0.088	1	Undiff-G	KBTBD7
GRAMD3	0.000307	-0.14627	0.029	0.141	1	Undiff-G	GRAMD3
ZNF827	0.000307	-0.1734	0.015	0.116	1	Undiff-G	ZNF827
FEM1B	0.000308	-0.22948	0.015	0.115	1	Undiff-G	FEM1B
ICAM3	0.00031	-0.15881	0.007	0.102	1	Undiff-G	ICAM3
NIN	0.000311	-0.16987	0.022	0.129	1	Undiff-G	NIN
LLGL1	0.000311	-0.23045	0.007	0.101	1	Undiff-G	LLGL1
ANGEL2	0.000311	-0.24327	0.015	0.114	1	Undiff-G	ANGEL2
LIMK2	0.000312	-0.22921	0	0.088	1	Undiff-G	LIMK2
MEPCE	0.000312	0.120772	0.022	0.129	1	Undiff-G	MEPCE
THOC5	0.000314	-0.249	0	0.087	1	Undiff-G	THOC5
AEN	0.000314	-0.27033	0	0.087	1	Undiff-G	AEN
GAD1	0.000314	-0.15105	0.015	0.114	1	Undiff-G	GAD1
SNX18	0.000314	-0.22836	0.015	0.114	1	Undiff-G	SNX18
RC3H1	0.000315	-0.15	0.007	0.102	1	Undiff-G	RC3H1
CPSF1	0.000316	-0.24959	0	0.087	1	Undiff-G	CPSF1
RP11-51J9	0.000316	0.212498	0.029	0.142	1	Undiff-G	RP11-51J9.5
TAGAP	0.000317	-0.30624	0.007	0.102	1	Undiff-G	TAGAP
GDAP1L1	0.000317	-0.27486	0.007	0.102	1	Undiff-G	GDAP1L1
HDAC9	0.000318	-0.14406	0.022	0.127	1	Undiff-G	HDAC9
NIT2	0.000319	0.598662	0.081	0.23	1	Undiff-G	NIT2
STK24	0.000319	-0.19178	0.007	0.102	1	Undiff-G	STK24
PRMT7	0.000319	-0.2367	0.007	0.101	1	Undiff-G	PRMT7
SLC25A22	0.000319	-0.20121	0.015	0.114	1	Undiff-G	SLC25A22
EDEM3	0.00032	-0.21157	0.015	0.114	1	Undiff-G	EDEM3
PPP3R1	0.00032	-0.21341	0.015	0.114	1	Undiff-G	PPP3R1
ZNF462	0.000321	0.128013	0.051	0.178	1	Undiff-G	ZNF462
UQCC1	0.000322	0.183288	0.044	0.167	1	Undiff-G	UQCC1
FLYWCH1	0.000322	-0.12701	0.007	0.102	1	Undiff-G	FLYWCH1
EIF3J-AS1	0.000322	-0.10407	0.022	0.128	1	Undiff-G	EIF3J-AS1
HELZ2	0.000323	-0.22694	0.007	0.101	1	Undiff-G	HELZ2
NUDT19	0.000323	-0.18475	0.007	0.101	1	Undiff-G	NUDT19
POMZP3	0.000323	-0.21737	0	0.087	1	Undiff-G	POMZP3
JMJD8	0.000323	-0.27444	0	0.087	1	Undiff-G	JMJD8
ARRDC2	0.000323	-0.33567	0	0.087	1	Undiff-G	ARRDC2
GPR75-ASE	0.000326	-0.22869	0	0.087	1	Undiff-G	GPR75-ASB3
PRC1	0.000326	-0.2938	0	0.087	1	Undiff-G	PRC1
DIP2A	0.000327	-0.11923	0.015	0.115	1	Undiff-G	DIP2A
ANKRD54	0.000328	0.106025	0.029	0.141	1	Undiff-G	ANKRD54
ELL	0.000328	-0.26575	0.007	0.101	1	Undiff-G	ELL
ZCCHC8	0.000328	-0.24378	0	0.087	1	Undiff-G	ZCCHC8
ARHGAP9	0.000328	-0.34834	0	0.087	1	Undiff-G	ARHGAP9
ST3GAL2	0.000328	-0.21625	0.007	0.1	1	Undiff-G	ST3GAL2
SMIM13	0.000331	-0.20798	0	0.087	1	Undiff-G	SMIM13
FNTB	0.000331	-0.26251	0	0.087	1	Undiff-G	FNTB
FKBP9	0.000331	0.33993	0.051	0.178	1	Undiff-G	FKBP9

ELMO1	0.000331	0.171265	0.059	0.191	1	Undiff-G	ELMO1
TEFM	0.000333	-0.22187	0.015	0.114	1	Undiff-G	TEFM
RDH10	0.000333	-0.2684	0	0.087	1	Undiff-G	RDH10
IL6R	0.000333	-0.34741	0	0.087	1	Undiff-G	IL6R
BTK	0.000333	-0.35928	0	0.087	1	Undiff-G	BTK
ASCC1	0.000334	-0.20863	0.015	0.115	1	Undiff-G	ASCC1
FAM160A2	0.000334	-0.16303	0.015	0.115	1	Undiff-G	FAM160A2
ARV1	0.000337	0.48809	0.051	0.18	1	Undiff-G	ARV1
DSN1	0.000337	-0.23154	0.007	0.101	1	Undiff-G	DSN1
FAXC	0.000338	-0.23999	0	0.087	1	Undiff-G	FAXC
MVB12B	0.000338	-0.21726	0.007	0.101	1	Undiff-G	MVB12B
DCUN1D4	0.00034	-0.21771	0.007	0.101	1	Undiff-G	DCUN1D4
GNB1L	0.000341	-0.21819	0	0.087	1	Undiff-G	GNB1L
SNCAIP	0.000343	-0.21088	0	0.086	1	Undiff-G	SNCAIP
ZZEF1	0.000343	-0.21689	0	0.086	1	Undiff-G	ZZEF1
ARAP2	0.000343	-0.25469	0	0.086	1	Undiff-G	ARAP2
INF2	0.000343	-0.26842	0	0.086	1	Undiff-G	INF2
METTL12	0.000344	0.164351	0.044	0.166	1	Undiff-G	METTL12
KDM4A	0.000344	-0.20464	0.015	0.114	1	Undiff-G	KDM4A
LRRC27	0.000346	-0.21452	0	0.086	1	Undiff-G	LRRC27
TAF1B	0.000346	-0.24376	0	0.086	1	Undiff-G	TAF1B
TANGO2	0.000346	-0.27579	0	0.086	1	Undiff-G	TANGO2
AGK	0.000347	-0.1373	0.007	0.101	1	Undiff-G	AGK
FGF14	0.000348	-0.21285	0	0.086	1	Undiff-G	FGF14
HOXC9	0.000348	-0.29849	0	0.086	1	Undiff-G	HOXC9
FBLN1	0.000348	-0.32852	0	0.086	1	Undiff-G	FBLN1
RABEP2	0.000348	-0.20844	0.007	0.1	1	Undiff-G	RABEP2
CLEC5A	0.000349	-0.3368	0.007	0.101	1	Undiff-G	CLEC5A
TMBIM4	0.00035	1.022442	0.125	0.058	1	Undiff-G	TMBIM4
SLC20A2	0.00035	-0.23907	0.015	0.114	1	Undiff-G	SLC20A2
PPP2R5A	0.000351	-0.18595	0.015	0.114	1	Undiff-G	PPP2R5A
CUL4B	0.000351	-0.21914	0	0.086	1	Undiff-G	CUL4B
ALDH18A1	0.000351	-0.22364	0	0.086	1	Undiff-G	ALDH18A1
DIRAS1	0.000352	-0.13627	0.015	0.114	1	Undiff-G	DIRAS1
VCL	0.000352	-0.20679	0.007	0.1	1	Undiff-G	VCL
MYO5A	0.000353	-0.17808	0.015	0.113	1	Undiff-G	MYO5A
SNTA1	0.000354	-0.17472	0	0.086	1	Undiff-G	SNTA1
PLXNA1	0.000354	-0.20647	0	0.086	1	Undiff-G	PLXNA1
KLHL2	0.000354	-0.23018	0	0.086	1	Undiff-G	KLHL2
ZUFSP	0.000354	-0.23676	0	0.086	1	Undiff-G	ZUFSP
INPP5D	0.000356	-0.39114	0	0.086	1	Undiff-G	INPP5D
BCAT2	0.00036	-0.10888	0.029	0.14	1	Undiff-G	BCAT2
C14orf159	0.000361	-0.22201	0.022	0.127	1	Undiff-G	C14orf159
ZNF85	0.000362	-0.228	0	0.086	1	Undiff-G	ZNF85
ZMIZ2	0.000364	-0.18936	0.007	0.1	1	Undiff-G	ZMIZ2
SRBD1	0.000364	-0.24382	0	0.086	1	Undiff-G	SRBD1
SNRK	0.000364	-0.2483	0	0.086	1	Undiff-G	SNRK
PPP4R1	0.000366	-0.18422	0.015	0.113	1	Undiff-G	PPP4R1
SPRTN	0.000367	0.285654	0.022	0.127	1	Undiff-G	SPRTN
HK2	0.000367	-0.27783	0	0.086	1	Undiff-G	HK2
WDTC1	0.000368	-0.23209	0.007	0.1	1	Undiff-G	WDTC1

PITX1	0.00037	-0.15933	0	0.086	1	Undiff-G	PITX1
DNAJC25	0.00037	-0.2224	0	0.086	1	Undiff-G	DNAJC25
DIP2C	0.00037	-0.24282	0	0.086	1	Undiff-G	DIP2C
CYTL1	0.00037	-0.35766	0.015	0.111	1	Undiff-G	CYTL1
ZNF227	0.000373	-0.16652	0.007	0.1	1	Undiff-G	ZNF227
KCTD10	0.000373	-0.23337	0	0.085	1	Undiff-G	KCTD10
P2RY13	0.000373	-0.45584	0	0.085	1	Undiff-G	P2RY13
XPNPEP3	0.000373	-0.26377	0.007	0.099	1	Undiff-G	XPNPEP3
PSMD5	0.000373	-0.21241	0.015	0.113	1	Undiff-G	PSMD5
ATF7IP	0.000374	-0.17942	0.015	0.113	1	Undiff-G	ATF7IP
STX17	0.000374	-0.12025	0.015	0.113	1	Undiff-G	STX17
HSD17B8	0.000375	-0.27148	0.007	0.099	1	Undiff-G	HSD17B8
ZNF430	0.000375	0.385903	0.059	0.191	1	Undiff-G	ZNF430
ITGA3	0.000375	-0.15795	0.007	0.1	1	Undiff-G	ITGA3
PLEKHA1	0.000376	-0.14111	0.007	0.1	1	Undiff-G	PLEKHA1
ARHGEF26	0.000378	-0.15166	0.022	0.126	1	Undiff-G	ARHGEF26
TRIM8	0.000378	-0.21236	0.007	0.1	1	Undiff-G	TRIM8
NTM	0.000379	-0.2983	0.015	0.113	1	Undiff-G	NTM
CHN2	0.00038	-0.32027	0.007	0.099	1	Undiff-G	CHN2
TRIM41	0.00038	-0.22919	0.007	0.099	1	Undiff-G	TRIM41
HOXB2	0.000381	-0.29748	0	0.085	1	Undiff-G	HOXB2
TK2	0.000382	-0.132	0.022	0.125	1	Undiff-G	TK2
ENTPD8	0.000382	0.259258	0.007	0	1	Undiff-G	ENTPD8
MAP1LC3B2	0.000384	-0.32298	0	0.085	1	Undiff-G	MAP1LC3B2
KYNU	0.000384	-0.37778	0	0.085	1	Undiff-G	KYNU
RPS6KA2	0.000384	-0.23014	0.015	0.113	1	Undiff-G	RPS6KA2
NRM	0.000387	-0.23099	0	0.085	1	Undiff-G	NRM
CITED1	0.000387	0.370709	0.037	0.152	1	Undiff-G	CITED1
KCNIP1	0.000388	-0.15167	0.007	0.099	1	Undiff-G	KCNIP1
PEX7	0.000389	-0.19553	0	0.085	1	Undiff-G	PEX7
RBM19	0.000389	-0.21116	0	0.085	1	Undiff-G	RBM19
SLC22A18	0.000392	-0.19369	0.022	0.125	1	Undiff-G	SLC22A18
LILRA2	0.000392	-0.4059	0	0.085	1	Undiff-G	LILRA2
SLC9A3R2	0.000395	0.148248	0.015	0.113	1	Undiff-G	SLC9A3R2
S100B	0.000395	0.492476	0.25	0.531	1	Undiff-G	S100B
PATZ1	0.000395	-0.21824	0	0.085	1	Undiff-G	PATZ1
INO80D	0.000395	-0.23512	0	0.085	1	Undiff-G	INO80D
CDK2	0.000395	-0.23552	0	0.085	1	Undiff-G	CDK2
IRAK4	0.000396	-0.24723	0.007	0.099	1	Undiff-G	IRAK4
LSS	0.000396	-0.13022	0.007	0.099	1	Undiff-G	LSS
L3HYPDH	0.000396	-0.13875	0.022	0.126	1	Undiff-G	L3HYPDH
HEMK1	0.000396	-0.18259	0.015	0.112	1	Undiff-G	HEMK1
B4GALT4	0.000399	0.270442	0.029	0.139	1	Undiff-G	B4GALT4
UBE4B	0.000399	-0.26982	0.007	0.098	1	Undiff-G	UBE4B
WDR25	0.000399	-0.10496	0.007	0.099	1	Undiff-G	WDR25
NMT2	0.0004	-0.18668	0.007	0.099	1	Undiff-G	NMT2
EPHB2	0.000401	-0.23377	0	0.085	1	Undiff-G	EPHB2
UNC5B	0.000401	-0.31624	0	0.085	1	Undiff-G	UNC5B
CLK4	0.000401	0.121554	0.044	0.164	1	Undiff-G	CLK4
KCNMB4	0.000403	-0.14199	0.022	0.124	1	Undiff-G	KCNMB4
INTS6	0.000403	0.173838	0.029	0.139	1	Undiff-G	INTS6

DHRS13	0.000404	-0.16841	0.007	0.099	1	Undiff-G	DHRS13
AGBL5	0.000404	-0.17905	0	0.085	1	Undiff-G	AGBL5
ZNF561	0.000407	-0.20906	0.015	0.112	1	Undiff-G	ZNF561
ARHGAP17	0.000407	-0.21923	0	0.084	1	Undiff-G	ARHGAP17
KIF1C	0.00041	-0.22073	0	0.084	1	Undiff-G	KIF1C
PHRF1	0.00041	-0.22094	0	0.084	1	Undiff-G	PHRF1
ACSL4	0.00041	-0.24844	0	0.084	1	Undiff-G	ACSL4
PMAIP1	0.00041	-0.44277	0	0.084	1	Undiff-G	PMAIP1
PTBP3	0.000412	-0.18501	0.015	0.112	1	Undiff-G	PTBP3
AHSA2	0.000413	-0.19244	0.022	0.125	1	Undiff-G	AHSA2
BTBD2	0.000413	-0.1907	0.007	0.099	1	Undiff-G	BTBD2
KLC4	0.000413	-0.21925	0	0.084	1	Undiff-G	KLC4
GCLC	0.000416	-0.25295	0.007	0.098	1	Undiff-G	GCLC
ZNF253	0.000416	-0.21981	0	0.084	1	Undiff-G	ZNF253
UBA6-AS1	0.000416	-0.23692	0	0.084	1	Undiff-G	UBA6-AS1
AP1G1	0.000417	0.212292	0.022	0.126	1	Undiff-G	AP1G1
BRMS1L	0.000417	-0.1329	0.007	0.099	1	Undiff-G	BRMS1L
FN3K	0.000419	-0.23299	0	0.084	1	Undiff-G	FN3K
NFIL3	0.000419	-0.30967	0	0.084	1	Undiff-G	NFIL3
FKBP10	0.000422	0.191244	0.074	0.214	1	Undiff-G	FKBP10
GPR161	0.000423	-0.20758	0	0.084	1	Undiff-G	GPR161
HAUS8	0.000423	-0.24988	0	0.084	1	Undiff-G	HAUS8
INTS3	0.000423	-0.2078	0.015	0.111	1	Undiff-G	INTS3
RBBP8	0.000426	-0.19668	0	0.084	1	Undiff-G	RBBP8
NXT2	0.000426	-0.2038	0	0.084	1	Undiff-G	NXT2
PHF10	0.000426	-0.21069	0	0.084	1	Undiff-G	PHF10
CCDC159	0.000428	0.24902	0.037	0.15	1	Undiff-G	CCDC159
CCDC71	0.000428	-0.16705	0.007	0.098	1	Undiff-G	CCDC71
SOS2	0.000429	-0.20943	0	0.084	1	Undiff-G	SOS2
DYRK1A	0.000429	-0.20994	0	0.084	1	Undiff-G	DYRK1A
ORAOV1	0.000429	-0.22083	0	0.084	1	Undiff-G	ORAOV1
RP11-660L	0.000429	0.137966	0.022	0.125	1	Undiff-G	RP11-660L16.2
EAF1	0.00043	0.103407	0.022	0.125	1	Undiff-G	EAF1
ZNF668	0.000432	-0.20141	0	0.084	1	Undiff-G	ZNF668
PHACTR2	0.000432	-0.20394	0	0.084	1	Undiff-G	PHACTR2
METRNL	0.000432	-0.32614	0	0.084	1	Undiff-G	METRNL
SCIMP	0.000432	-0.36234	0	0.084	1	Undiff-G	SCIMP
RP11-242D	0.000433	0.467059	0.029	0.006	1	Undiff-G	RP11-242D8.1
MECR	0.000434	0.268375	0.044	0.162	1	Undiff-G	MECR
NCBP1	0.000435	-0.21277	0	0.084	1	Undiff-G	NCBP1
ZNF266	0.000435	-0.22019	0	0.084	1	Undiff-G	ZNF266
ARNTL	0.000436	-0.22763	0.007	0.097	1	Undiff-G	ARNTL
FOXRED2	0.000439	-0.196	0	0.084	1	Undiff-G	FOXRED2
RGS17	0.00044	0.287534	0.044	0.164	1	Undiff-G	RGS17
SLC10A4	0.000441	-0.17435	0.007	0.098	1	Undiff-G	SLC10A4
DGCR8	0.000442	-0.20674	0	0.084	1	Undiff-G	DGCR8
FAM193A	0.000442	-0.21686	0	0.084	1	Undiff-G	FAM193A
CNOT1	0.000444	0.27487	0.037	0.151	1	Undiff-G	CNOT1
TRAK1	0.000444	-0.21348	0.007	0.098	1	Undiff-G	TRAK1
BICD2	0.000445	-0.231	0	0.083	1	Undiff-G	BICD2
RXRA	0.000445	-0.26996	0.007	0.097	1	Undiff-G	RXRA

MCM4	0.000446	-0.23658	0.007	0.097	1	Undiff-G	MCM4
TRAPPC2	0.000447	-0.23282	0.007	0.097	1	Undiff-G	TRAPPC2
TUBGCP4	0.000447	-0.15552	0.022	0.124	1	Undiff-G	TUBGCP4
SIN3B	0.000448	-0.22391	0.007	0.097	1	Undiff-G	SIN3B
FAHD2B	0.000448	-0.22457	0	0.083	1	Undiff-G	FAHD2B
SLC10A3	0.000448	-0.23616	0	0.083	1	Undiff-G	SLC10A3
RP11-834C	0.000449	-0.24486	0.007	0.098	1	Undiff-G	RP11-834C11.4
CDCA7	0.00045	-0.13601	0.022	0.124	1	Undiff-G	CDCA7
METTL10	0.000451	-0.14673	0.015	0.11	1	Undiff-G	METTL10
MAPK14	0.000451	-0.19964	0.015	0.111	1	Undiff-G	MAPK14
TERF2	0.000452	-0.20953	0	0.083	1	Undiff-G	TERF2
MAP6D1	0.000452	-0.23784	0	0.083	1	Undiff-G	MAP6D1
SSX2IP	0.000454	-0.16642	0.007	0.097	1	Undiff-G	SSX2IP
UBE3C	0.000456	0.106066	0.015	0.111	1	Undiff-G	UBE3C
POLG	0.000457	-0.22364	0.007	0.097	1	Undiff-G	POLG
C9orf85	0.000458	-0.16644	0.007	0.097	1	Undiff-G	C9orf85
GOLGA1	0.000458	-0.2102	0	0.083	1	Undiff-G	GOLGA1
NCOA5	0.000458	-0.21323	0	0.083	1	Undiff-G	NCOA5
LMBR1	0.000459	-0.11059	0.015	0.111	1	Undiff-G	LMBR1
TMED10	0.00046	0.656313	0.287	0.617	1	Undiff-G	TMED10
BBS7	0.00046	-0.17737	0.007	0.097	1	Undiff-G	BBS7
MRC2	0.00046	0.250118	0.051	0.175	1	Undiff-G	MRC2
ERLIN2	0.000461	0.161339	0.022	0.124	1	Undiff-G	ERLIN2
RIN1	0.000462	-0.17561	0	0.083	1	Undiff-G	RIN1
IFIT2	0.000462	-0.3342	0	0.083	1	Undiff-G	IFIT2
MUTYH	0.000462	-0.15437	0.007	0.097	1	Undiff-G	MUTYH
HVCN1	0.000463	-0.12525	0.007	0.097	1	Undiff-G	HVCN1
POLM	0.000464	-0.19775	0.007	0.097	1	Undiff-G	POLM
BBS2	0.000465	0.245077	0.051	0.174	1	Undiff-G	BBS2
UTP14C	0.000465	-0.18511	0	0.083	1	Undiff-G	UTP14C
PIANP	0.000465	-0.18545	0	0.083	1	Undiff-G	PIANP
GMPR	0.000465	-0.21793	0	0.083	1	Undiff-G	GMPR
BEX5	0.000466	-0.22584	0.007	0.097	1	Undiff-G	BEX5
EBI3	0.000466	-0.2466	0.007	0.097	1	Undiff-G	EBI3
FAM111A	0.000466	-0.22401	0.015	0.11	1	Undiff-G	FAM111A
GPATCH2	0.000468	0.472692	0.044	0.163	1	Undiff-G	GPATCH2
PPARD	0.000468	-0.22518	0.015	0.109	1	Undiff-G	PPARD
RNF6	0.000468	0.107036	0.029	0.137	1	Undiff-G	RNF6
ARHGAP33	0.000469	-0.22002	0	0.083	1	Undiff-G	ARHGAP33
LEAP2	0.000469	-0.26817	0	0.083	1	Undiff-G	LEAP2
CCR1	0.000469	-0.35537	0	0.083	1	Undiff-G	CCR1
RP11-539L	0.000472	-0.14855	0.007	0.097	1	Undiff-G	RP11-539L10.3
MCM3AP	0.000475	-0.23186	0.015	0.109	1	Undiff-G	MCM3AP
MYBBP1A	0.000476	-0.19584	0	0.083	1	Undiff-G	MYBBP1A
UBA7	0.000476	-0.32912	0	0.083	1	Undiff-G	UBA7
VPS11	0.000479	-0.16121	0.007	0.097	1	Undiff-G	VPS11
RP11-400F	0.000479	-0.18218	0	0.083	1	Undiff-G	RP11-400F19.6
CD2AP	0.000479	-0.21378	0	0.083	1	Undiff-G	CD2AP
ZNF669	0.00048	-0.17093	0.007	0.097	1	Undiff-G	ZNF669
FOXK2	0.000483	-0.21026	0	0.082	1	Undiff-G	FOXK2
LPCAT3	0.000484	0.291012	0.037	0.15	1	Undiff-G	LPCAT3

MYL5	0.000485	-0.1843	0.007	0.097	1	Undiff-G	MYL5
SCRN2	0.000485	0.389025	0.051	0.174	1	Undiff-G	SCRN2
GALNT10	0.000486	-0.26906	0.007	0.096	1	Undiff-G	GALNT10
ACSS2	0.000486	-0.19629	0	0.082	1	Undiff-G	ACSS2
AC093323.	0.000487	-0.12797	0.007	0.097	1	Undiff-G	AC093323.3
ZNF692	0.000488	-0.15264	0.015	0.11	1	Undiff-G	ZNF692
PSENEN	0.000489	0.772531	0.228	0.495	1	Undiff-G	PSENEN
DONSON	0.00049	-0.18501	0	0.082	1	Undiff-G	DONSON
KATNAL1	0.00049	-0.19414	0	0.082	1	Undiff-G	KATNAL1
RP11-421L	0.00049	-0.23777	0	0.082	1	Undiff-G	RP11-421L21.3
ZNF606	0.000494	-0.21018	0	0.082	1	Undiff-G	ZNF606
LYL1	0.000494	-0.34383	0	0.082	1	Undiff-G	LYL1
TAPBPL	0.000494	0.115537	0.022	0.123	1	Undiff-G	TAPBPL
SDCCAG3	0.000496	-0.15506	0.007	0.096	1	Undiff-G	SDCCAG3
GPATCH3	0.000497	-0.23221	0	0.082	1	Undiff-G	GPATCH3
NAIP	0.000498	-0.1857	0.015	0.11	1	Undiff-G	NAIP
SERPINA3	0.000499	0.608856	0.029	0.006	1	Undiff-G	SERPINA3
ENTHD2	0.000501	-0.2214	0	0.082	1	Undiff-G	ENTHD2
RAB2B	0.000501	-0.23415	0	0.082	1	Undiff-G	RAB2B
BCAM	0.000502	0.141564	0.015	0.11	1	Undiff-G	BCAM
AGAP2-AS1	0.000502	-0.12565	0.007	0.096	1	Undiff-G	AGAP2-AS1
DHX32	0.000505	-0.19364	0	0.082	1	Undiff-G	DHX32
FCHSD2	0.000505	-0.27017	0	0.082	1	Undiff-G	FCHSD2
GAP43	0.000505	1.106998	0.441	0.467	1	Undiff-G	GAP43
LTBR	0.000507	-0.28273	0.007	0.096	1	Undiff-G	LTBR
SLC4A4	0.000507	-0.16278	0.015	0.11	1	Undiff-G	SLC4A4
TESK1	0.000508	-0.19984	0	0.082	1	Undiff-G	TESK1
DIRC2	0.000508	-0.20543	0	0.082	1	Undiff-G	DIRC2
TAF1C	0.000508	-0.21963	0	0.082	1	Undiff-G	TAF1C
OTUD5	0.00051	0.122607	0.029	0.136	1	Undiff-G	OTUD5
PI4KA	0.000511	-0.21979	0.007	0.096	1	Undiff-G	PI4KA
REEP1	0.000512	-0.22918	0	0.082	1	Undiff-G	REEP1
ZNF250	0.000512	-0.24614	0	0.082	1	Undiff-G	ZNF250
AC009506.	0.000512	-0.25539	0	0.082	1	Undiff-G	AC009506.1
D2HGDH	0.000515	-0.1031	0.015	0.11	1	Undiff-G	D2HGDH
SNTB2	0.000516	-0.13904	0.007	0.096	1	Undiff-G	SNTB2
CDK8	0.000516	-0.20988	0	0.082	1	Undiff-G	CDK8
FAM175B	0.000516	-0.23774	0	0.082	1	Undiff-G	FAM175B
NQO1	0.000516	0.20946	0.015	0.11	1	Undiff-G	NQO1
TBCK	0.00052	-0.20564	0	0.082	1	Undiff-G	TBCK
GABPB1	0.000523	-0.18527	0.015	0.108	1	Undiff-G	GABPB1
TMEM185A	0.000524	-0.1898	0	0.082	1	Undiff-G	TMEM185A
TNFAIP8L1	0.000524	-0.23751	0	0.082	1	Undiff-G	TNFAIP8L1
THAP3	0.000527	0.390565	0.029	0.136	1	Undiff-G	THAP3
AHCTF1	0.000528	-0.19914	0	0.081	1	Undiff-G	AHCTF1
EMILIN2	0.000528	-0.2446	0	0.081	1	Undiff-G	EMILIN2
ADSSL1	0.000528	-0.25204	0	0.081	1	Undiff-G	ADSSL1
GTF2A1	0.000528	-0.18041	0.015	0.109	1	Undiff-G	GTF2A1
H6PD	0.000531	-0.14516	0.007	0.096	1	Undiff-G	H6PD
AC091729.	0.000531	-0.19744	0	0.081	1	Undiff-G	AC091729.9
SEMA4D	0.000531	-0.34301	0	0.081	1	Undiff-G	SEMA4D

STK11	0.000534	-0.17052	0.007	0.095	1	Undiff-G	STK11
PRKD2	0.000535	-0.20936	0	0.081	1	Undiff-G	PRKD2
JMJD4	0.000535	-0.21586	0	0.081	1	Undiff-G	JMJD4
VPS39	0.000539	-0.21848	0	0.081	1	Undiff-G	VPS39
PRKCD	0.000539	-0.30271	0	0.081	1	Undiff-G	PRKCD
OBSL1	0.000541	0.145109	0.037	0.147	1	Undiff-G	OBSL1
ANKS1B	0.000543	-0.27711	0.015	0.109	1	Undiff-G	ANKS1B
MYO9A	0.000543	-0.20738	0	0.081	1	Undiff-G	MYO9A
RP11-140K	0.000543	-0.23903	0	0.081	1	Undiff-G	RP11-140K17.3
LCOR	0.000543	-0.25893	0	0.081	1	Undiff-G	LCOR
SCML1	0.000544	-0.29995	0.007	0.095	1	Undiff-G	SCML1
CETN3	0.000548	0.253059	0.037	0.148	1	Undiff-G	CETN3
MEX3C	0.000548	-0.16668	0.007	0.095	1	Undiff-G	MEX3C
FAM57A	0.000549	-0.14987	0.007	0.095	1	Undiff-G	FAM57A
ILDR2	0.000551	-0.15824	0	0.081	1	Undiff-G	ILDR2
BANP	0.000551	-0.22015	0	0.081	1	Undiff-G	BANP
DNAJB5	0.000551	-0.23604	0	0.081	1	Undiff-G	DNAJB5
AP001055.	0.000551	-0.36832	0	0.081	1	Undiff-G	AP001055.6
NOC3L	0.000552	-0.12207	0.007	0.095	1	Undiff-G	NOC3L
DBF4	0.000554	0.149613	0.022	0.122	1	Undiff-G	DBF4
MGARP	0.000556	-0.16585	0	0.081	1	Undiff-G	MGARP
ZNF232	0.000556	-0.20284	0	0.081	1	Undiff-G	ZNF232
SH3TC1	0.000556	-0.35848	0	0.081	1	Undiff-G	SH3TC1
ERCC2	0.000558	-0.17324	0.007	0.095	1	Undiff-G	ERCC2
TRAF3IP2-	0.00056	-0.21809	0	0.081	1	Undiff-G	TRAF3IP2-AS1
ACAP1	0.00056	-0.27447	0	0.081	1	Undiff-G	ACAP1
DDX23	0.000562	0.342646	0.044	0.16	1	Undiff-G	DDX23
C5orf42	0.000563	-0.13641	0.007	0.095	1	Undiff-G	C5orf42
SCARB1	0.000564	0.156762	0.015	0.109	1	Undiff-G	SCARB1
VAX2	0.000564	-0.19939	0	0.081	1	Undiff-G	VAX2
ZC3H3	0.000564	-0.20666	0	0.081	1	Undiff-G	ZC3H3
GGA3	0.000564	-0.216	0	0.081	1	Undiff-G	GGA3
PIGG	0.000565	-0.20743	0.007	0.095	1	Undiff-G	PIGG
MOB3B	0.000566	-0.22855	0.007	0.095	1	Undiff-G	MOB3B
TCF7L2	0.000568	-0.19393	0.007	0.095	1	Undiff-G	TCF7L2
ELOVL4	0.000568	-0.20584	0	0.081	1	Undiff-G	ELOVL4
NTRK2	0.000571	-0.12825	0.022	0.122	1	Undiff-G	NTRK2
CBLL1	0.000571	0.107685	0.015	0.108	1	Undiff-G	CBLL1
TLN2	0.000572	-0.22318	0.007	0.095	1	Undiff-G	TLN2
LZTS2	0.000572	-0.20521	0	0.08	1	Undiff-G	LZTS2
ADAM19	0.000576	-0.19807	0	0.08	1	Undiff-G	ADAM19
ZNF789	0.000576	-0.22208	0	0.08	1	Undiff-G	ZNF789
AC004540.	0.000576	-0.25652	0	0.08	1	Undiff-G	AC004540.5
ELAVL2	0.000576	-0.27898	0	0.08	1	Undiff-G	ELAVL2
NME1-NME2	0.000577	-0.14149	0.007	0.095	1	Undiff-G	NME1-NME2
LINC00844	0.000577	-0.33273	0.015	0.107	1	Undiff-G	LINC00844
ZNF354A	0.00058	0.274855	0.022	0.122	1	Undiff-G	ZNF354A
RAB40C	0.000581	-0.19028	0	0.08	1	Undiff-G	RAB40C
ZNF133	0.000581	-0.19644	0	0.08	1	Undiff-G	ZNF133
DUS4L	0.000581	-0.20639	0	0.08	1	Undiff-G	DUS4L
KPTN	0.000581	-0.22509	0	0.08	1	Undiff-G	KPTN

FES	0.000581	-0.32809	0	0.08	1	Undiff-G	FES
NUP133	0.000585	-0.13061	0.007	0.095	1	Undiff-G	NUP133
ORC3	0.000587	-0.11415	0.015	0.107	1	Undiff-G	ORC3
ACACA	0.000588	-0.13962	0.007	0.094	1	Undiff-G	ACACA
DTX3L	0.000589	-0.21976	0	0.08	1	Undiff-G	DTX3L
GABPA	0.000589	-0.22439	0	0.08	1	Undiff-G	GABPA
KNSTRN	0.000589	-0.25976	0	0.08	1	Undiff-G	KNSTRN
PCNX	0.000592	-0.16415	0.007	0.094	1	Undiff-G	PCNX
NOS1AP	0.000594	-0.19403	0	0.08	1	Undiff-G	NOS1AP
EIF5AL1	0.000596	0.136121	0.022	0.121	1	Undiff-G	EIF5AL1
RARRES3	0.000596	0.385985	0.096	0.245	1	Undiff-G	RARRES3
GSAP	0.000598	-0.27886	0	0.08	1	Undiff-G	GSAP
LIN7C	0.0006	0.191355	0.022	0.121	1	Undiff-G	LIN7C
TMEM69	0.000601	0.332639	0.051	0.17	1	Undiff-G	TMEM69
PIAS1	0.000602	0.528982	0.044	0.16	1	Undiff-G	PIAS1
CBLN4	0.000603	-0.23539	0	0.08	1	Undiff-G	CBLN4
ZNF420	0.000606	0.166038	0.015	0.108	1	Undiff-G	ZNF420
TASP1	0.000607	-0.21945	0.007	0.093	1	Undiff-G	TASP1
GABBR1	0.000607	-0.21311	0	0.08	1	Undiff-G	GABBR1
SGPP1	0.000607	-0.24409	0	0.08	1	Undiff-G	SGPP1
RIC8B	0.000608	-0.20841	0.007	0.094	1	Undiff-G	RIC8B
WNK3	0.00061	0.212024	0.022	0.121	1	Undiff-G	WNK3
PTPN13	0.000611	-0.12207	0.007	0.094	1	Undiff-G	PTPN13
ELOVL6	0.000611	-0.18149	0.007	0.094	1	Undiff-G	ELOVL6
PNPO	0.000611	-0.25483	0	0.08	1	Undiff-G	PNPO
PAGR1	0.000614	0.780534	0.037	0.009	1	Undiff-G	PAGR1
CELF4	0.000614	-0.32019	0.007	0.094	1	Undiff-G	CELF4
CREBZF	0.000616	-0.12558	0.029	0.132	1	Undiff-G	CREBZF
XKR8	0.000621	-0.22654	0	0.08	1	Undiff-G	XKR8
DFNA5	0.000621	0.412687	0.066	0.197	1	Undiff-G	DFNA5
PRMT6	0.000622	-0.21989	0.007	0.093	1	Undiff-G	PRMT6
PAX6	0.000625	-0.17896	0	0.079	1	Undiff-G	PAX6
PPTC7	0.000625	-0.20196	0	0.079	1	Undiff-G	PPTC7
MEOX2	0.000625	-0.22164	0	0.079	1	Undiff-G	MEOX2
CBR3	0.000627	-0.16408	0.015	0.106	1	Undiff-G	CBR3
EAF2	0.00063	-0.17121	0.007	0.094	1	Undiff-G	EAF2
TMEM177	0.00063	-0.20469	0	0.079	1	Undiff-G	TMEM177
TUSC1	0.00063	-0.22941	0	0.079	1	Undiff-G	TUSC1
FANCL	0.00063	0.111339	0.037	0.144	1	Undiff-G	FANCL
TRAPPC10	0.000631	-0.21148	0.007	0.093	1	Undiff-G	TRAPPC10
C5orf45	0.000631	0.167358	0.051	0.169	1	Undiff-G	C5orf45
ALKBH6	0.000638	-0.16526	0.015	0.106	1	Undiff-G	ALKBH6
BAG2	0.000639	-0.21047	0	0.079	1	Undiff-G	BAG2
BLOC1S5	0.000639	-0.22561	0	0.079	1	Undiff-G	BLOC1S5
PTPRO	0.000639	-0.27331	0	0.079	1	Undiff-G	PTPRO
CROT	0.00064	0.200209	0.029	0.133	1	Undiff-G	CROT
DCAF16	0.000641	0.30995	0.022	0.12	1	Undiff-G	DCAF16
MAST3	0.000644	-0.23576	0	0.079	1	Undiff-G	MAST3
CD48	0.000644	-0.38033	0	0.079	1	Undiff-G	CD48
KLHL28	0.000647	0.147577	0.022	0.12	1	Undiff-G	KLHL28
ERCC3	0.000648	0.196268	0.037	0.146	1	Undiff-G	ERCC3

RRAS2	0.000649	-0.16536	0	0.079	1	Undiff-G	RRAS2
ETV4	0.000649	-0.18859	0	0.079	1	Undiff-G	ETV4
SASH3	0.000649	-0.3331	0	0.079	1	Undiff-G	SASH3
ZNF577	0.000652	-0.16123	0.007	0.093	1	Undiff-G	ZNF577
LRRC25	0.000653	-0.18507	0.007	0.093	1	Undiff-G	LRRC25
SAP30L	0.000657	-0.11508	0.007	0.093	1	Undiff-G	SAP30L
CHST14	0.000658	-0.17162	0	0.079	1	Undiff-G	CHST14
CHSY1	0.000659	-0.22509	0.007	0.092	1	Undiff-G	CHSY1
FXYD7	0.000663	-0.34023	0	0.079	1	Undiff-G	FXYD7
ALG12	0.000665	-0.16265	0.007	0.093	1	Undiff-G	ALG12
NPEPL1	0.000668	-0.13714	0.015	0.106	1	Undiff-G	NPEPL1
IFT74	0.000668	-0.21948	0	0.079	1	Undiff-G	IFT74
ZSWIM8	0.000668	-0.23301	0	0.079	1	Undiff-G	ZSWIM8
NLGN2	0.000669	-0.17788	0.007	0.093	1	Undiff-G	NLGN2
EYA2	0.000673	-0.18287	0	0.079	1	Undiff-G	EYA2
ALDOC	0.000676	0.129833	0.044	0.157	1	Undiff-G	ALDOC
PMS1	0.000676	-0.17905	0.022	0.118	1	Undiff-G	PMS1
LOXL1	0.000678	-0.15409	0	0.079	1	Undiff-G	LOXL1
PHACTR3	0.000678	-0.27455	0	0.079	1	Undiff-G	PHACTR3
TMEFF2	0.000678	-0.28478	0	0.079	1	Undiff-G	TMEFF2
ZNF224	0.00068	-0.22983	0.015	0.105	1	Undiff-G	ZNF224
SMYD4	0.000681	-0.20137	0.022	0.118	1	Undiff-G	SMYD4
CIB2	0.000683	-0.15062	0	0.078	1	Undiff-G	CIB2
F12	0.000683	-0.17262	0	0.078	1	Undiff-G	F12
TST	0.000683	-0.21897	0	0.078	1	Undiff-G	TST
SIGLEC8	0.000683	-0.36512	0	0.078	1	Undiff-G	SIGLEC8
FIG4	0.000689	-0.13641	0.015	0.106	1	Undiff-G	FIG4
PAPOLG	0.00069	-0.26704	0.007	0.092	1	Undiff-G	PAPOLG
CAHM	0.000698	-0.20177	0.007	0.092	1	Undiff-G	CAHM
CNPY1	0.000698	-0.22613	0	0.078	1	Undiff-G	CNPY1
RP2	0.000698	-0.25383	0	0.078	1	Undiff-G	RP2
DDX55	0.000702	-0.18084	0.022	0.119	1	Undiff-G	DDX55
DOCK2	0.000703	-0.337	0	0.078	1	Undiff-G	DOCK2
SESN2	0.000705	-0.25133	0.015	0.105	1	Undiff-G	SESN2
LINC00632	0.000707	-0.2314	0.007	0.092	1	Undiff-G	LINC00632
PYGO2	0.000708	-0.19363	0	0.078	1	Undiff-G	PYGO2
HOXA10	0.000708	-0.26396	0	0.078	1	Undiff-G	HOXA10
EHD4	0.000711	-0.21082	0.007	0.092	1	Undiff-G	EHD4
SETD6	0.000712	-0.21132	0.007	0.091	1	Undiff-G	SETD6
GABARAP	0.000717	0.695817	0.213	0.464	1	Undiff-G	GABARAP
FAM69C	0.000719	-0.16555	0	0.078	1	Undiff-G	FAM69C
MKL2	0.000719	-0.19566	0	0.078	1	Undiff-G	MKL2
ZNF25	0.000719	-0.20159	0	0.078	1	Undiff-G	ZNF25
TACC3	0.000719	-0.25933	0	0.078	1	Undiff-G	TACC3
C1orf198	0.000721	0.11289	0.015	0.106	1	Undiff-G	C1orf198
TAF1	0.000724	-0.20495	0.007	0.092	1	Undiff-G	TAF1
MANEA	0.000724	-0.18335	0	0.078	1	Undiff-G	MANEA
ITGA6	0.000724	-0.22606	0	0.078	1	Undiff-G	ITGA6
RIOK1	0.000724	0.425612	0.044	0.157	1	Undiff-G	RIOK1
ICOSLG	0.000729	-0.26482	0.007	0.092	1	Undiff-G	ICOSLG
OSGEP	0.000729	0.315205	0.074	0.204	1	Undiff-G	OSGEP

RNF169	0.00073	-0.18265	0	0.078	1	Undiff-G	RNF169
ADNP2	0.00073	-0.186	0	0.078	1	Undiff-G	ADNP2
SH3BP4	0.00073	-0.20119	0	0.078	1	Undiff-G	SH3BP4
STAT5B	0.00073	-0.20835	0	0.078	1	Undiff-G	STAT5B
CELF5	0.00073	-0.27192	0.007	0.092	1	Undiff-G	CELF5
H1F0	0.000731	0.437418	0.11	0.265	1	Undiff-G	H1F0
ARL14EP	0.000737	0.499709	0.059	0.182	1	Undiff-G	ARL14EP
HSF2	0.000737	0.10566	0.022	0.119	1	Undiff-G	HSF2
GRIA4	0.000737	-0.12296	0.029	0.13	1	Undiff-G	GRIA4
ABTB1	0.000739	-0.23003	0.015	0.105	1	Undiff-G	ABTB1
CEP19	0.00074	-0.21743	0	0.077	1	Undiff-G	CEP19
WDR55	0.000743	0.136313	0.022	0.118	1	Undiff-G	WDR55
KCNAB2	0.000746	-0.29025	0	0.077	1	Undiff-G	KCNAB2
ZNF562	0.000748	0.119414	0.037	0.142	1	Undiff-G	ZNF562
POFUT1	0.000749	0.106408	0.022	0.118	1	Undiff-G	POFUT1
FAM171A1	0.000751	-0.19003	0	0.077	1	Undiff-G	FAM171A1
CBLB	0.000751	-0.23063	0	0.077	1	Undiff-G	CBLB
TEX10	0.000757	-0.22069	0	0.077	1	Undiff-G	TEX10
ETV3	0.000757	-0.26428	0	0.077	1	Undiff-G	ETV3
PARP6	0.000761	-0.18453	0.015	0.104	1	Undiff-G	PARP6
RP11-395A	0.000763	-0.16654	0	0.077	1	Undiff-G	RP11-395A13.2
PANX1	0.000763	-0.23245	0	0.077	1	Undiff-G	PANX1
ATP2B4	0.000763	0.150505	0.015	0.105	1	Undiff-G	ATP2B4
UBR7	0.000765	0.186042	0.022	0.118	1	Undiff-G	UBR7
GTF2H3	0.000766	0.19185	0.037	0.143	1	Undiff-G	GTF2H3
TRIM65	0.000768	-0.18244	0	0.077	1	Undiff-G	TRIM65
CXorf21	0.000768	-0.36257	0	0.077	1	Undiff-G	CXorf21
C9orf116	0.000774	-0.1702	0	0.077	1	Undiff-G	C9orf116
CDPF1	0.000774	-0.2278	0	0.077	1	Undiff-G	CDPF1
RIT2	0.000774	-0.3273	0	0.077	1	Undiff-G	RIT2
RBM48	0.000774	-0.21834	0.007	0.091	1	Undiff-G	RBM48
NRXN2	0.000778	-0.17642	0.022	0.118	1	Undiff-G	NRXN2
SIRT3	0.000779	-0.1448	0.015	0.104	1	Undiff-G	SIRT3
BOK	0.00078	-0.21597	0	0.077	1	Undiff-G	BOK
NUMBL	0.000781	-0.18872	0.007	0.09	1	Undiff-G	NUMBL
PTPN4	0.000783	-0.21468	0.015	0.103	1	Undiff-G	PTPN4
RYK	0.000784	-0.11169	0.007	0.091	1	Undiff-G	RYK
C12orf4	0.000785	-0.23868	0.007	0.09	1	Undiff-G	C12orf4
RP11-398K	0.000785	-0.2031	0	0.077	1	Undiff-G	RP11-398K22.12
RASA2	0.000785	-0.22904	0	0.077	1	Undiff-G	RASA2
C12orf29	0.000786	0.391357	0.044	0.155	1	Undiff-G	C12orf29
BRF2	0.000788	-0.20723	0.007	0.091	1	Undiff-G	BRF2
SAMD1	0.000789	-0.13714	0.015	0.104	1	Undiff-G	SAMD1
CCHCR1	0.000791	-0.18177	0	0.077	1	Undiff-G	CCHCR1
TSTD2	0.000791	-0.20592	0	0.077	1	Undiff-G	TSTD2
TEAD2	0.000794	-0.13254	0.007	0.091	1	Undiff-G	TEAD2
METTL22	0.000794	-0.27311	0.007	0.09	1	Undiff-G	METTL22
RBM7	0.000796	0.774481	0.096	0.244	1	Undiff-G	RBM7
SLC35C1	0.000797	-0.19018	0	0.077	1	Undiff-G	SLC35C1
PMP22	0.000798	0.615723	0.206	0.43	1	Undiff-G	PMP22
ZBTB8A	0.000798	-0.11674	0.015	0.104	1	Undiff-G	ZBTB8A

UBE2C	0.000802	0.136397	0.022	0.118	1	Undiff-G	UBE2C
GUF1	0.000803	-0.19627	0.007	0.09	1	Undiff-G	GUF1
ABHD10	0.000803	-0.20247	0	0.077	1	Undiff-G	ABHD10
MAN2A2	0.000803	-0.23281	0	0.077	1	Undiff-G	MAN2A2
ITGAM	0.000803	-0.33097	0	0.077	1	Undiff-G	ITGAM
ZBTB4	0.000805	0.416012	0.022	0.118	1	Undiff-G	ZBTB4
LRRC45	0.000806	-0.18319	0.007	0.09	1	Undiff-G	LRRC45
VCPIP1	0.000807	-0.12083	0.007	0.091	1	Undiff-G	VCPIP1
TEX2	0.000809	-0.17154	0	0.076	1	Undiff-G	TEX2
THG1L	0.000809	-0.22837	0	0.076	1	Undiff-G	THG1L
ATP5E	0.000811	0.337668	0.419	0.92	1	Undiff-G	ATP5E
CBFA2T2	0.000811	-0.13184	0.029	0.13	1	Undiff-G	CBFA2T2
ADAM15	0.000814	-0.19444	0.022	0.117	1	Undiff-G	ADAM15
CHPF2	0.000815	0.250662	0.044	0.154	1	Undiff-G	CHPF2
ZNF276	0.000816	-0.18046	0.007	0.091	1	Undiff-G	ZNF276
CHAF1A	0.000816	-0.20669	0.007	0.09	1	Undiff-G	CHAF1A
ATXN8OS	0.000816	0.948176	0.147	0.079	1	Undiff-G	ATXN8OS
C1orf27	0.000817	0.60135	0.059	0.181	1	Undiff-G	C1orf27
RAB30	0.000821	-0.18895	0	0.076	1	Undiff-G	RAB30
TMEM8A	0.000821	-0.19488	0	0.076	1	Undiff-G	TMEM8A
C5orf30	0.000821	-0.21904	0	0.076	1	Undiff-G	C5orf30
PLXNB1	0.000821	-0.2243	0	0.076	1	Undiff-G	PLXNB1
LSG1	0.000827	0.202366	0.029	0.129	1	Undiff-G	LSG1
GEMIN8	0.000833	-0.24136	0	0.076	1	Undiff-G	GEMIN8
PRDX5	0.000836	0.646095	0.338	0.747	1	Undiff-G	PRDX5
C2	0.000836	-0.2176	0.022	0.116	1	Undiff-G	C2
ATG2A	0.000839	-0.24745	0	0.076	1	Undiff-G	ATG2A
STX3	0.000839	-0.24966	0	0.076	1	Undiff-G	STX3
RDH5	0.000841	-0.17507	0.015	0.103	1	Undiff-G	RDH5
MYO1C	0.000841	-0.12223	0.015	0.103	1	Undiff-G	MYO1C
PLP2	0.000844	0.789666	0.154	0.346	1	Undiff-G	PLP2
UBASH3B	0.000845	-0.19328	0	0.076	1	Undiff-G	UBASH3B
IL1RN	0.000845	-0.35893	0	0.076	1	Undiff-G	IL1RN
GPR107	0.000851	0.217149	0.015	0.104	1	Undiff-G	GPR107
MFI2-AS1	0.000851	-0.17401	0	0.076	1	Undiff-G	MFI2-AS1
INO80	0.000851	-0.18766	0	0.076	1	Undiff-G	INO80
ARMCX5	0.000851	-0.22025	0	0.076	1	Undiff-G	ARMCX5
CAMKMT	0.000851	-0.23782	0	0.076	1	Undiff-G	CAMKMT
ITPRIP	0.000851	-0.2564	0	0.076	1	Undiff-G	ITPRIP
EMB	0.000851	-0.32972	0	0.076	1	Undiff-G	EMB
LMAN1	0.000855	0.80668	0.147	0.331	1	Undiff-G	LMAN1
COL9A2	0.000861	-0.23257	0.007	0.09	1	Undiff-G	COL9A2
PPM1M	0.000864	-0.24484	0	0.076	1	Undiff-G	PPM1M
PIK3CA	0.000867	-0.15051	0.007	0.09	1	Undiff-G	PIK3CA
FAM160B2	0.00087	-0.20053	0	0.076	1	Undiff-G	FAM160B2
ECD	0.00087	-0.23719	0	0.076	1	Undiff-G	ECD
TNFAIP2	0.00087	-0.2944	0	0.076	1	Undiff-G	TNFAIP2
CYP20A1	0.000872	0.184695	0.051	0.166	1	Undiff-G	CYP20A1
SLC25A32	0.000874	-0.22537	0.015	0.102	1	Undiff-G	SLC25A32
LMO1	0.000877	-0.27091	0	0.075	1	Undiff-G	LMO1
TTC7A	0.000877	-0.27493	0	0.075	1	Undiff-G	TTC7A

ZNF595	0.000883	-0.22153	0	0.075	1	Undiff-G	ZNF595
PARP12	0.000883	-0.22454	0	0.075	1	Undiff-G	PARP12
SPATA7	0.000883	-0.24721	0	0.075	1	Undiff-G	SPATA7
TMEM100	0.000887	-0.21008	0.007	0.09	1	Undiff-G	TMEM100
FAM213B	0.000888	-0.10322	0.007	0.09	1	Undiff-G	FAM213B
SEC24A	0.000896	-0.18677	0	0.075	1	Undiff-G	SEC24A
ARHGAP22	0.000896	-0.3067	0	0.075	1	Undiff-G	ARHGAP22
PMS2	0.000901	0.155072	0.029	0.129	1	Undiff-G	PMS2
RP11-332H	0.000901	0.144081	0.029	0.128	1	Undiff-G	RP11-332H14.2
DCBLD1	0.000907	-0.18113	0.007	0.089	1	Undiff-G	DCBLD1
ZNF566	0.000907	0.235099	0.022	0.116	1	Undiff-G	ZNF566
ENOX2	0.000909	-0.21511	0	0.075	1	Undiff-G	ENOX2
FCHO2	0.000909	-0.23053	0	0.075	1	Undiff-G	FCHO2
TET2	0.000911	-0.2222	0.007	0.089	1	Undiff-G	TET2
EXOC4	0.000912	0.372335	0.051	0.165	1	Undiff-G	EXOC4
RFX3	0.000916	-0.18423	0	0.075	1	Undiff-G	RFX3
STYX	0.000916	-0.19258	0	0.075	1	Undiff-G	STYX
SIGLEC7	0.000916	-0.32219	0	0.075	1	Undiff-G	SIGLEC7
RFTN2	0.000917	0.161396	0.029	0.129	1	Undiff-G	RFTN2
SETBP1	0.000923	-0.18852	0	0.075	1	Undiff-G	SETBP1
KIF13B	0.000923	-0.21843	0	0.075	1	Undiff-G	KIF13B
AHDC1	0.000929	-0.1547	0.015	0.102	1	Undiff-G	AHDC1
PROSER1	0.00093	-0.18872	0	0.075	1	Undiff-G	PROSER1
GMIP	0.00093	-0.25567	0	0.075	1	Undiff-G	GMIP
DGAT1	0.000935	-0.17474	0.007	0.089	1	Undiff-G	DGAT1
TSHZ1	0.000937	-0.20549	0	0.075	1	Undiff-G	TSHZ1
CENPM	0.000937	-0.23425	0	0.075	1	Undiff-G	CENPM
PTPRN	0.000937	-0.26683	0	0.075	1	Undiff-G	PTPRN
RPS6KL1	0.000943	-0.19345	0.015	0.101	1	Undiff-G	RPS6KL1
EFNB3	0.000943	-0.17467	0	0.075	1	Undiff-G	EFNB3
VAV1	0.000943	-0.30676	0	0.075	1	Undiff-G	VAV1
TMEM136	0.000945	-0.14062	0.007	0.089	1	Undiff-G	TMEM136
MDP1	0.000945	0.12868	0.022	0.116	1	Undiff-G	MDP1
RP11-192H	0.00095	-0.15676	0.022	0.115	1	Undiff-G	RP11-192H23.6
ZNF513	0.00095	-0.18747	0	0.075	1	Undiff-G	ZNF513
ST5	0.000954	0.11805	0.044	0.152	1	Undiff-G	ST5
BCKDHB	0.000954	-0.14174	0.007	0.089	1	Undiff-G	BCKDHB
GPR84	0.000957	-0.3353	0	0.074	1	Undiff-G	GPR84
CACNA2D4	0.000957	-0.34028	0	0.074	1	Undiff-G	CACNA2D4
UCK2	0.000961	-0.19912	0.007	0.088	1	Undiff-G	UCK2
PDLIM1	0.000964	-0.19484	0	0.074	1	Undiff-G	PDLIM1
FAM110A	0.000964	-0.22554	0	0.074	1	Undiff-G	FAM110A
IRAK3	0.000964	-0.31655	0	0.074	1	Undiff-G	IRAK3
MGA	0.000965	-0.1398	0.015	0.102	1	Undiff-G	MGA
DUSP26	0.000966	-0.26521	0.007	0.089	1	Undiff-G	DUSP26
TOP1MT	0.000972	-0.18292	0	0.074	1	Undiff-G	TOP1MT
FAM129B	0.000972	-0.2006	0	0.074	1	Undiff-G	FAM129B
CNTROB	0.000972	-0.20487	0	0.074	1	Undiff-G	CNTROB
GNG3	0.000972	-0.30882	0	0.074	1	Undiff-G	GNG3
RICTOR	0.000973	0.1893	0.066	0.187	1	Undiff-G	RICTOR
ZBTB25	0.000975	-0.14079	0.007	0.089	1	Undiff-G	ZBTB25

KIAA0100	0.000977	-0.16941	0.007	0.088	1	Undiff-G	KIAA0100
DNAJC17	0.000977	-0.22609	0.007	0.088	1	Undiff-G	DNAJC17
CNR1	0.000979	-0.16602	0	0.074	1	Undiff-G	CNR1
ITPRIPL2	0.000979	-0.19444	0	0.074	1	Undiff-G	ITPRIPL2
ZNF689	0.000979	-0.20275	0	0.074	1	Undiff-G	ZNF689
DRAM1	0.000979	-0.22167	0	0.074	1	Undiff-G	DRAM1
TNS3	0.000979	-0.27707	0	0.074	1	Undiff-G	TNS3
PBK	0.000979	-0.31175	0	0.074	1	Undiff-G	PBK
RASSF8	0.00098	-0.1554	0.007	0.088	1	Undiff-G	RASSF8
TOP2A	0.000981	-0.35106	0.007	0.087	1	Undiff-G	TOP2A
PUS3	0.000982	0.19986	0.022	0.115	1	Undiff-G	PUS3
ZNF76	0.000982	0.158089	0.015	0.102	1	Undiff-G	ZNF76
LRFN4	0.000986	-0.14903	0	0.074	1	Undiff-G	LRFN4
ZFHX4	0.000986	0.108508	0.044	0.153	1	Undiff-G	ZFHX4
WDFY3	0.000987	-0.16852	0.007	0.088	1	Undiff-G	WDFY3
EYA3	0.000987	-0.22052	0.007	0.087	1	Undiff-G	EYA3
NAA30	0.000991	-0.20783	0.007	0.087	1	Undiff-G	NAA30
CAPN10	0.000992	-0.21283	0.007	0.087	1	Undiff-G	CAPN10
DUSP28	0.000996	-0.1983	0.007	0.088	1	Undiff-G	DUSP28
CHGB	0.000998	-0.11692	0.007	0.088	1	Undiff-G	CHGB
TRIM16L	0.001	-0.17472	0	0.074	1	Undiff-G	TRIM16L
CNOT6	0.001	-0.17998	0	0.074	1	Undiff-G	CNOT6
DROSHA	0.001	-0.18877	0	0.074	1	Undiff-G	DROSHA
SLC39A11	0.001	-0.20935	0	0.074	1	Undiff-G	SLC39A11
GIMAP1	0.001	-0.31567	0	0.074	1	Undiff-G	GIMAP1
CADPS	0.001001	1.020162	0.096	0.042	1	Undiff-G	CADPS
CACFD1	0.001006	-0.16615	0.007	0.088	1	Undiff-G	CACFD1
AIF1L	0.001007	-0.10631	0.022	0.114	1	Undiff-G	AIF1L
EPHA3	0.001008	-0.13376	0	0.074	1	Undiff-G	EPHA3
MPP6	0.001008	-0.17237	0	0.074	1	Undiff-G	MPP6
ZC3H4	0.001008	-0.18959	0.007	0.088	1	Undiff-G	ZC3H4
WDR83OS	0.001008	0.686002	0.353	0.748	1	Undiff-G	WDR83OS
TP53	0.001014	0.240463	0.022	0.115	1	Undiff-G	TP53
DDX20	0.001015	-0.19723	0	0.074	1	Undiff-G	DDX20
CSRNP2	0.001016	-0.20326	0.007	0.087	1	Undiff-G	CSRNP2
RP11-138A	0.001018	-0.25141	0.029	0.126	1	Undiff-G	RP11-138A9.1
ABCA5	0.001021	-0.18123	0.007	0.088	1	Undiff-G	ABCA5
SRSF12	0.001023	-0.18106	0	0.074	1	Undiff-G	SRSF12
USP19	0.001023	-0.18937	0	0.074	1	Undiff-G	USP19
ASB6	0.001023	-0.19366	0	0.074	1	Undiff-G	ASB6
RFNG	0.001028	-0.19458	0.015	0.1	1	Undiff-G	RFNG
FTX	0.001028	-0.20659	0.015	0.101	1	Undiff-G	FTX
FAM84B	0.001029	-0.18604	0.007	0.088	1	Undiff-G	FAM84B
TP53I11	0.00103	-0.25346	0	0.074	1	Undiff-G	TP53I11
ST14	0.00103	-0.29844	0	0.074	1	Undiff-G	ST14
DBP	0.001033	-0.22588	0.007	0.088	1	Undiff-G	DBP
ZNF584	0.001037	-0.16773	0.007	0.087	1	Undiff-G	ZNF584
VANGL2	0.001038	-0.18739	0	0.074	1	Undiff-G	VANGL2
HAUS2	0.00104	0.194249	0.015	0.101	1	Undiff-G	HAUS2
ACSS1	0.00104	-0.18235	0.015	0.101	1	Undiff-G	ACSS1
PHF21A	0.001044	-0.13107	0.022	0.113	1	Undiff-G	PHF21A

CRLF3	0.001045	0.189673	0.022	0.114	1	Undiff-G	CRLF3
VPS52	0.001045	-0.16656	0.007	0.088	1	Undiff-G	VPS52
MARK4	0.001045	-0.17403	0	0.073	1	Undiff-G	MARK4
ZNF335	0.001045	-0.20232	0	0.073	1	Undiff-G	ZNF335
BTN3A1	0.001045	-0.23399	0	0.073	1	Undiff-G	BTN3A1
TMEM91	0.001045	-0.26122	0	0.073	1	Undiff-G	TMEM91
MON1A	0.001046	-0.18887	0.007	0.087	1	Undiff-G	MON1A
BOC	0.001048	-0.14767	0.007	0.088	1	Undiff-G	BOC
PCTP	0.001053	-0.24596	0	0.073	1	Undiff-G	PCTP
OSCAR	0.001053	-0.28682	0	0.073	1	Undiff-G	OSCAR
ST7L	0.001055	0.139464	0.022	0.114	1	Undiff-G	ST7L
LDOC1L	0.001057	-0.10179	0.007	0.088	1	Undiff-G	LDOC1L
USP38	0.001057	-0.12708	0.007	0.088	1	Undiff-G	USP38
BOLA1	0.001058	0.432836	0.044	0.152	1	Undiff-G	BOLA1
BRINP2	0.001061	-0.17965	0	0.073	1	Undiff-G	BRINP2
FUT11	0.001061	-0.19847	0	0.073	1	Undiff-G	FUT11
ZBTB11	0.001061	-0.20039	0	0.073	1	Undiff-G	ZBTB11
DNASE1L1	0.001061	-0.27304	0	0.073	1	Undiff-G	DNASE1L1
LRRC8B	0.001061	-0.1555	0.007	0.087	1	Undiff-G	LRRC8B
ERCC6L2	0.001065	-0.19028	0.007	0.087	1	Undiff-G	ERCC6L2
CASP7	0.001065	-0.19065	0.007	0.087	1	Undiff-G	CASP7
RGN	0.001069	-0.24425	0	0.073	1	Undiff-G	RGN
CDCA7L	0.001069	0.155729	0.044	0.15	1	Undiff-G	CDCA7L
GBE1	0.001071	0.425617	0.066	0.187	1	Undiff-G	GBE1
SKIV2L	0.001072	-0.12491	0.007	0.087	1	Undiff-G	SKIV2L
UBXN2B	0.001074	0.512712	0.044	0.152	1	Undiff-G	UBXN2B
EMID1	0.001076	-0.16985	0	0.073	1	Undiff-G	EMID1
CMTR1	0.001076	-0.18404	0	0.073	1	Undiff-G	CMTR1
STK38	0.001076	-0.19507	0	0.073	1	Undiff-G	STK38
KLHDC4	0.001076	-0.21931	0	0.073	1	Undiff-G	KLHDC4
RBM41	0.001078	-0.20527	0.007	0.087	1	Undiff-G	RBM41
ST3GAL3	0.001081	0.118935	0.007	0.087	1	Undiff-G	ST3GAL3
GBA2	0.001083	-0.20906	0.007	0.086	1	Undiff-G	GBA2
PEX11G	0.001084	-0.18146	0	0.073	1	Undiff-G	PEX11G
CCL4L2	0.001085	-0.35894	0.007	0.087	1	Undiff-G	CCL4L2
FAM212B	0.001088	-0.18461	0.015	0.1	1	Undiff-G	FAM212B
ZNF7	0.001089	0.134245	0.051	0.162	1	Undiff-G	ZNF7
PRDM4	0.00109	-0.19051	0.007	0.086	1	Undiff-G	PRDM4
RGL1	0.00109	-0.20592	0.007	0.087	1	Undiff-G	RGL1
WDR91	0.001091	-0.24336	0.007	0.087	1	Undiff-G	WDR91
SRRM3	0.001092	-0.26216	0	0.073	1	Undiff-G	SRRM3
BBOX1	0.001098	0.120971	0.037	0.138	1	Undiff-G	BBOX1
USP13	0.0011	-0.16723	0	0.073	1	Undiff-G	USP13
FASTKD3	0.0011	-0.18811	0	0.073	1	Undiff-G	FASTKD3
ZBTB2	0.0011	-0.20295	0	0.073	1	Undiff-G	ZBTB2
OPN3	0.0011	-0.2061	0	0.073	1	Undiff-G	OPN3
SCN3B	0.0011	-0.26551	0	0.073	1	Undiff-G	SCN3B
PI4K2B	0.001101	-0.16668	0.007	0.087	1	Undiff-G	PI4K2B
PTRH2	0.001107	0.329843	0.037	0.139	1	Undiff-G	PTRH2
RP11-379H	0.001108	-0.17484	0	0.073	1	Undiff-G	RP11-379H18.1
FAM135A	0.001113	-0.11087	0.007	0.087	1	Undiff-G	FAM135A

KAT2B	0.001117	-0.2088	0	0.073	1	Undiff-G	KAT2B
SCN3A	0.001117	-0.22696	0	0.073	1	Undiff-G	SCN3A
SCARA3	0.001122	0.137002	0.037	0.139	1	Undiff-G	SCARA3
RCBTB1	0.001125	-0.20845	0	0.073	1	Undiff-G	RCBTB1
COL6A2	0.001125	0.296471	0.051	0.164	1	Undiff-G	COL6A2
CNPY4	0.001127	0.178118	0.022	0.113	1	Undiff-G	CNPY4
E2F3	0.001128	-0.16844	0.007	0.087	1	Undiff-G	E2F3
PLD2	0.00113	-0.21323	0.007	0.087	1	Undiff-G	PLD2
TAMM41	0.001133	-0.22312	0	0.072	1	Undiff-G	TAMM41
FURIN	0.001133	-0.23819	0	0.072	1	Undiff-G	FURIN
POMT1	0.001137	0.167839	0.037	0.138	1	Undiff-G	POMT1
ASPHD1	0.001141	0.226537	0.037	0.139	1	Undiff-G	ASPHD1
SYCE1L	0.001141	-0.15794	0	0.072	1	Undiff-G	SYCE1L
SBK1	0.001141	-0.22078	0	0.072	1	Undiff-G	SBK1
RREB1	0.001141	-0.2423	0	0.072	1	Undiff-G	RREB1
LINC00869	0.001146	0.803815	0.051	0.016	1	Undiff-G	LINC00869
MEGF8	0.001146	0.144203	0.007	0.087	1	Undiff-G	MEGF8
KAZN	0.00115	-0.19097	0	0.072	1	Undiff-G	KAZN
DUS2	0.00115	-0.20254	0	0.072	1	Undiff-G	DUS2
DPCD	0.00115	0.423141	0.051	0.162	1	Undiff-G	DPCD
C2orf47	0.001152	0.102379	0.029	0.125	1	Undiff-G	C2orf47
U2AF1L4	0.001155	0.32368	0.074	0.198	1	Undiff-G	U2AF1L4
LY6H	0.001156	-0.29169	0.007	0.086	1	Undiff-G	LY6H
LYRM9	0.001159	0.333191	0.059	0.174	1	Undiff-G	LYRM9
RP11-378E	0.001162	0.167846	0.007	0.001	1	Undiff-G	RP11-378E13.4
SMAGP	0.001167	-0.14267	0.007	0.086	1	Undiff-G	SMAGP
LMCD1	0.001171	-0.10635	0.007	0.086	1	Undiff-G	LMCD1
RETSAT	0.001171	-0.2135	0.007	0.085	1	Undiff-G	RETSAT
TBC1D24	0.001175	-0.16816	0	0.072	1	Undiff-G	TBC1D24
NUP153	0.001175	-0.21055	0	0.072	1	Undiff-G	NUP153
POLR3F	0.001175	0.121438	0.037	0.138	1	Undiff-G	POLR3F
FAM13B	0.001176	-0.16349	0.007	0.086	1	Undiff-G	FAM13B
HNRNPU-AS	0.001183	-0.20537	0.015	0.099	1	Undiff-G	HNRNPU-AS1
RP11-798M	0.001186	0.244041	0.044	0.15	1	Undiff-G	RP11-798M19.6
ICK	0.001187	-0.17671	0.007	0.086	1	Undiff-G	ICK
MAU2	0.001195	-0.13272	0.029	0.124	1	Undiff-G	MAU2
PM20D2	0.001196	-0.17601	0.007	0.085	1	Undiff-G	PM20D2
CTDP1	0.001201	-0.18883	0	0.072	1	Undiff-G	CTDP1
CECR1	0.001201	-0.31029	0	0.072	1	Undiff-G	CECR1
KLF12	0.001207	-0.18161	0.007	0.086	1	Undiff-G	KLF12
PGBD1	0.00121	-0.16455	0	0.072	1	Undiff-G	PGBD1
NUAK1	0.00121	-0.21304	0	0.072	1	Undiff-G	NUAK1
PPWD1	0.001211	0.195468	0.022	0.113	1	Undiff-G	PPWD1
DCHS1	0.001217	-0.17203	0.007	0.085	1	Undiff-G	DCHS1
NUDT8	0.001219	-0.1529	0	0.072	1	Undiff-G	NUDT8
DTD2	0.001219	-0.19133	0	0.072	1	Undiff-G	DTD2
IL4R	0.001219	-0.28976	0	0.072	1	Undiff-G	IL4R
MRI1	0.001222	-0.12053	0.022	0.111	1	Undiff-G	MRI1
AGO2	0.001228	-0.19082	0	0.072	1	Undiff-G	AGO2
WWP2	0.001228	-0.20903	0	0.072	1	Undiff-G	WWP2
CD93	0.001228	-0.31132	0	0.072	1	Undiff-G	CD93

PHLPP1	0.001228	-0.19391	0.007	0.086	1	Undiff-G	PHLPP1
ITFG2	0.001229	-0.19174	0.022	0.111	1	Undiff-G	ITFG2
DUSP22	0.001236	0.231376	0.022	0.112	1	Undiff-G	DUSP22
ZNF598	0.001237	-0.16419	0	0.071	1	Undiff-G	ZNF598
ALDH1B1	0.001237	-0.17616	0	0.071	1	Undiff-G	ALDH1B1
HECA	0.001237	-0.20968	0	0.071	1	Undiff-G	HECA
EHBP1L1	0.001237	-0.21005	0	0.071	1	Undiff-G	EHBP1L1
RRAGB	0.001238	0.140978	0.022	0.112	1	Undiff-G	RRAGB
SLC2A6	0.001246	-0.16499	0	0.071	1	Undiff-G	SLC2A6
RASSF7	0.001246	-0.20223	0	0.071	1	Undiff-G	RASSF7
CENPBD1	0.001255	-0.19973	0	0.071	1	Undiff-G	CENPBD1
SCN1B	0.001255	-0.24526	0	0.071	1	Undiff-G	SCN1B
TK1	0.001255	-0.26156	0	0.071	1	Undiff-G	TK1
MTAP	0.001255	-0.26253	0	0.071	1	Undiff-G	MTAP
JDP2	0.001255	-0.30361	0	0.071	1	Undiff-G	JDP2
DMD	0.001256	-0.17698	0.007	0.085	1	Undiff-G	DMD
AEBP2	0.001257	-0.15599	0.007	0.085	1	Undiff-G	AEBP2
ZNF71	0.001263	-0.16461	0.007	0.085	1	Undiff-G	ZNF71
PSMC3IP	0.001264	-0.19743	0	0.071	1	Undiff-G	PSMC3IP
GCC1	0.001264	-0.19989	0	0.071	1	Undiff-G	GCC1
CC2D2A	0.001264	-0.2197	0	0.071	1	Undiff-G	CC2D2A
VCPKMT	0.001264	-0.22199	0	0.071	1	Undiff-G	VCPKMT
CTD-3184A	0.001267	-0.16496	0.015	0.098	1	Undiff-G	CTD-3184A7.4
SLC27A5	0.00127	0.156673	0.037	0.137	1	Undiff-G	SLC27A5
SOS1	0.001275	-0.18454	0.007	0.085	1	Undiff-G	SOS1
CASP9	0.001278	-0.2316	0.007	0.085	1	Undiff-G	CASP9
IER3IP1	0.001279	0.847918	0.088	0.038	1	Undiff-G	IER3IP1
FAM208B	0.001279	-0.14369	0.007	0.085	1	Undiff-G	FAM208B
ZNF846	0.001283	-0.28394	0	0.071	1	Undiff-G	ZNF846
NKX2-2	0.001286	0.113651	0.044	0.149	1	Undiff-G	NKX2-2
GPR37L1	0.00129	-0.2565	0.007	0.085	1	Undiff-G	GPR37L1
PAQR8	0.001291	-0.27988	0.015	0.097	1	Undiff-G	PAQR8
MDGA1	0.001292	-0.12904	0	0.071	1	Undiff-G	MDGA1
INO80B	0.001292	-0.17217	0	0.071	1	Undiff-G	INO80B
BLVRB	0.001297	0.794173	0.176	0.377	1	Undiff-G	BLVRB
ZNF174	0.001302	-0.16404	0	0.071	1	Undiff-G	ZNF174
RP3-428L1	0.001302	-0.17447	0	0.071	1	Undiff-G	RP3-428L16.2
CACNG7	0.001303	-0.14011	0.007	0.085	1	Undiff-G	CACNG7
C17orf80	0.001311	-0.12796	0.007	0.085	1	Undiff-G	C17orf80
PODXL	0.001311	-0.1654	0	0.071	1	Undiff-G	PODXL
IPO8	0.001311	-0.16849	0	0.071	1	Undiff-G	IPO8
DHRS3	0.001312	0.363776	0.059	0.172	1	Undiff-G	DHRS3
CAMSAP1	0.001312	-0.15256	0.007	0.085	1	Undiff-G	CAMSAP1
NR2C1	0.001316	-0.18086	0.015	0.098	1	Undiff-G	NR2C1
ADM	0.001319	1.455816	0.25	0.184	1	Undiff-G	ADM
ZNF121	0.001321	0.122399	0.022	0.112	1	Undiff-G	ZNF121
COX15	0.001321	-0.21042	0	0.071	1	Undiff-G	COX15
RUNDC3A	0.001325	-0.11014	0.015	0.098	1	Undiff-G	RUNDC3A
SCAF8	0.001331	-0.18704	0	0.071	1	Undiff-G	SCAF8
SLC19A1	0.00134	-0.18278	0	0.07	1	Undiff-G	SLC19A1
PLEKHH3	0.001343	-0.15127	0.007	0.085	1	Undiff-G	PLEKHH3

LSAMP	0.001348	0.164731	0.059	0.173	1	Undiff-G	LSAMP
MAPK12	0.00135	-0.1378	0	0.07	1	Undiff-G	MAPK12
REXO1	0.00135	-0.16021	0	0.07	1	Undiff-G	REXO1
PACRGL	0.00135	-0.20195	0	0.07	1	Undiff-G	PACRGL
THEM6	0.00135	-0.18949	0.007	0.084	1	Undiff-G	THEM6
FGR	0.001351	-0.31162	0.007	0.084	1	Undiff-G	FGR
ATG14	0.00136	-0.18449	0	0.07	1	Undiff-G	ATG14
ARMC9	0.00136	-0.18714	0	0.07	1	Undiff-G	ARMC9
YPEL2	0.00136	-0.24768	0	0.07	1	Undiff-G	YPEL2
GPRC5C	0.00136	-0.25827	0	0.07	1	Undiff-G	GPRC5C
SRD5A1	0.001362	0.273288	0.022	0.111	1	Undiff-G	SRD5A1
BAMBI	0.00137	-0.1803	0	0.07	1	Undiff-G	BAMBI
RAB11FIP2	0.00137	-0.18425	0	0.07	1	Undiff-G	RAB11FIP2
PIEZO1	0.00137	-0.2075	0	0.07	1	Undiff-G	PIEZO1
ABCA2	0.001376	-0.3048	0.015	0.097	1	Undiff-G	ABCA2
KIAA0586	0.00138	-0.17829	0	0.07	1	Undiff-G	KIAA0586
CLUH	0.00138	-0.18138	0	0.07	1	Undiff-G	CLUH
NUDT6	0.00138	-0.20176	0	0.07	1	Undiff-G	NUDT6
PSTK	0.00138	-0.20927	0	0.07	1	Undiff-G	PSTK
NFX1	0.00138	-0.18767	0.015	0.098	1	Undiff-G	NFX1
MTRR	0.001385	-0.16405	0.007	0.084	1	Undiff-G	MTRR
ZNF300	0.00139	-0.19413	0	0.07	1	Undiff-G	ZNF300
PCK2	0.00139	-0.22646	0	0.07	1	Undiff-G	PCK2
TMEM186	0.001393	0.121944	0.015	0.098	1	Undiff-G	TMEM186
USP6NL	0.001394	-0.2142	0.007	0.084	1	Undiff-G	USP6NL
PACSIN3	0.0014	-0.14977	0	0.07	1	Undiff-G	PACSIN3
NCKIPSD	0.0014	-0.17709	0	0.07	1	Undiff-G	NCKIPSD
ACVR1	0.0014	-0.17856	0	0.07	1	Undiff-G	ACVR1
KMT2B	0.0014	-0.19001	0	0.07	1	Undiff-G	KMT2B
C7orf43	0.0014	-0.19013	0	0.07	1	Undiff-G	C7orf43
SFMBT1	0.0014	-0.20479	0	0.07	1	Undiff-G	SFMBT1
TMEM220	0.0014	-0.21632	0	0.07	1	Undiff-G	TMEM220
C15orf48	0.0014	-0.35112	0	0.07	1	Undiff-G	C15orf48
IQCB1	0.001403	0.167298	0.029	0.123	1	Undiff-G	IQCB1
ZNF493	0.00142	0.236607	0.051	0.159	1	Undiff-G	ZNF493
CCNT1	0.00142	-0.137	0.007	0.084	1	Undiff-G	CCNT1
RPS6KC1	0.001421	-0.17771	0	0.07	1	Undiff-G	RPS6KC1
ZNF569	0.001421	-0.19011	0	0.07	1	Undiff-G	ZNF569
EML2	0.001421	-0.21663	0.007	0.084	1	Undiff-G	EML2
MRRF	0.001429	-0.23932	0.015	0.096	1	Undiff-G	MRRF
VIPR2	0.001439	-0.27612	0.007	0.083	1	Undiff-G	VIPR2
NUP43	0.001442	-0.12387	0.007	0.084	1	Undiff-G	NUP43
PPCDC	0.001442	-0.19834	0	0.07	1	Undiff-G	PPCDC
OSBPL2	0.00145	0.279654	0.037	0.135	1	Undiff-G	OSBPL2
MTOR	0.001452	-0.17696	0	0.07	1	Undiff-G	MTOR
DENND4B	0.001452	-0.19859	0	0.07	1	Undiff-G	DENND4B
RAPGEF2	0.001452	-0.2043	0	0.07	1	Undiff-G	RAPGEF2
CARD9	0.001452	-0.26677	0	0.07	1	Undiff-G	CARD9
ZFP82	0.001453	0.168041	0.022	0.11	1	Undiff-G	ZFP82
CAPN5	0.001463	-0.15945	0	0.069	1	Undiff-G	CAPN5
ZNF500	0.001463	-0.19072	0	0.069	1	Undiff-G	ZNF500

PRKAR2B	0.001463	-0.21139	0	0.069	1	Undiff-G	PRKAR2B
PPP1R3E	0.001463	-0.23941	0	0.069	1	Undiff-G	PPP1R3E
MTFR1	0.001465	0.139006	0.015	0.097	1	Undiff-G	MTFR1
TIMM21	0.001474	0.516311	0.044	0.148	1	Undiff-G	TIMM21
MOB1B	0.001474	-0.1906	0	0.069	1	Undiff-G	MOB1B
TRMT5	0.001474	-0.20883	0	0.069	1	Undiff-G	TRMT5
PFDN5	0.00148	0.493682	0.404	0.889	1	Undiff-G	PFDN5
C16orf52	0.001481	-0.11789	0.015	0.097	1	Undiff-G	C16orf52
LRRC24	0.001485	-0.17416	0	0.069	1	Undiff-G	LRRC24
TET1	0.001498	-0.13172	0.007	0.083	1	Undiff-G	TET1
GORASP1	0.001506	-0.11272	0.007	0.083	1	Undiff-G	GORASP1
GAS2L3	0.001506	-0.17151	0	0.069	1	Undiff-G	GAS2L3
PDSS2	0.001513	-0.17215	0.007	0.083	1	Undiff-G	PDSS2
TRAPPC8	0.001517	-0.17531	0	0.069	1	Undiff-G	TRAPPC8
TMEM254	0.001517	-0.21776	0	0.069	1	Undiff-G	TMEM254
PDE7A	0.001527	-0.15454	0.007	0.083	1	Undiff-G	PDE7A
ARFGEF2	0.001528	-0.15624	0	0.069	1	Undiff-G	ARFGEF2
RP11-977G	0.001528	-0.17309	0	0.069	1	Undiff-G	RP11-977G19.11
GYS1	0.001528	-0.17886	0	0.069	1	Undiff-G	GYS1
MPP5	0.00153	0.137757	0.015	0.097	1	Undiff-G	MPP5
DMXL2	0.001535	-0.22293	0.007	0.082	1	Undiff-G	DMXL2
SCLT1	0.00154	-0.19634	0	0.069	1	Undiff-G	SCLT1
SFR1	0.00154	-0.27135	0	0.069	1	Undiff-G	SFR1
OLFML2B	0.00154	-0.32388	0	0.069	1	Undiff-G	OLFML2B
RP11-111M	0.00154	0.254888	0.015	0.097	1	Undiff-G	RP11-111M22.3
ARRDC4	0.001551	-0.18328	0	0.069	1	Undiff-G	ARRDC4
PLLP	0.001551	-0.34986	0	0.069	1	Undiff-G	PLLP
MAPK9	0.001559	-0.13886	0.007	0.083	1	Undiff-G	MAPK9
DBNDD1	0.001562	-0.16034	0	0.069	1	Undiff-G	DBNDD1
DHODH	0.001572	-0.13442	0.007	0.083	1	Undiff-G	DHODH
AC007405.	0.001574	-0.15648	0	0.069	1	Undiff-G	AC007405.6
PARP8	0.001574	-0.22309	0	0.069	1	Undiff-G	PARP8
CD300C	0.001574	-0.30413	0	0.069	1	Undiff-G	CD300C
FREM2	0.001585	-0.12489	0	0.069	1	Undiff-G	FREM2
BMPR1A	0.001585	-0.1717	0	0.069	1	Undiff-G	BMPR1A
TLR1	0.001585	-0.29428	0	0.069	1	Undiff-G	TLR1
PRIM1	0.001592	-0.19632	0.007	0.082	1	Undiff-G	PRIM1
UHRF1BP1L	0.001597	-0.19808	0	0.068	1	Undiff-G	UHRF1BP1L
DOPEY2	0.001597	-0.22298	0	0.068	1	Undiff-G	DOPEY2
CPPED1	0.001597	-0.27083	0	0.068	1	Undiff-G	CPPED1
TBP	0.001599	0.104774	0.007	0.083	1	Undiff-G	TBP
DHRS4	0.001618	-0.19055	0.007	0.082	1	Undiff-G	DHRS4
CHST8	0.00162	-0.15517	0	0.068	1	Undiff-G	CHST8
RNF165	0.00162	-0.18457	0	0.068	1	Undiff-G	RNF165
RAD54L2	0.001624	0.105465	0.022	0.108	1	Undiff-G	RAD54L2
GFOD1	0.001632	-0.19302	0	0.068	1	Undiff-G	GFOD1
3-Sep	0.001632	-0.2079	0	0.068	1	Undiff-G	3-Sep
HEPN1	0.001632	0.421128	0.081	0.206	1	Undiff-G	HEPN1
IDNK	0.00164	0.111081	0.015	0.096	1	Undiff-G	IDNK
IGF2BP3	0.001649	0.303744	0.022	0.109	1	Undiff-G	IGF2BP3
RP11-277F	0.001656	-0.22359	0	0.068	1	Undiff-G	RP11-277P12.20

SGK3	0.001656	-0.22854	0	0.068	1	Undiff-G	SGK3
NCAM2	0.001656	-0.23913	0	0.068	1	Undiff-G	NCAM2
TOX3	0.001656	-0.25062	0	0.068	1	Undiff-G	TOX3
ZNHIT6	0.001659	-0.17238	0.015	0.095	1	Undiff-G	ZNHIT6
HEXIM2	0.00166	-0.19106	0.007	0.082	1	Undiff-G	HEXIM2
BBC3	0.001664	-0.18902	0.007	0.082	1	Undiff-G	BBC3
MTSS1L	0.001666	-0.17492	0.015	0.095	1	Undiff-G	MTSS1L
RNF111	0.001668	-0.15518	0.007	0.082	1	Undiff-G	RNF111
OAF	0.001668	-0.13336	0	0.068	1	Undiff-G	OAF
ZNF746	0.001668	-0.18581	0	0.068	1	Undiff-G	ZNF746
C5orf63	0.001668	-0.20527	0	0.068	1	Undiff-G	C5orf63
INADL	0.001668	-0.22021	0	0.068	1	Undiff-G	INADL
BLNK	0.001668	-0.34075	0	0.068	1	Undiff-G	BLNK
TTC39C	0.00167	-0.18878	0.007	0.081	1	Undiff-G	TTC39C
SPOCK1	0.001676	-0.26653	0.007	0.082	1	Undiff-G	SPOCK1
UBB	0.001677	0.363246	0.426	0.916	1	Undiff-G	UBB
TLCD1	0.001679	-0.14103	0.007	0.082	1	Undiff-G	TLCD1
RAI1	0.00168	-0.16141	0	0.068	1	Undiff-G	RAI1
C16orf70	0.001693	-0.19388	0	0.068	1	Undiff-G	C16orf70
CD40	0.001693	-0.2792	0	0.068	1	Undiff-G	CD40
MYPOP	0.001701	-0.17015	0.007	0.081	1	Undiff-G	MYPOP
YPEL1	0.001702	-0.16087	0.015	0.094	1	Undiff-G	YPEL1
BYSL	0.001707	0.341152	0.022	0.108	1	Undiff-G	BYSL
CNN3	0.001708	0.604189	0.228	0.471	1	Undiff-G	CNN3
IGF2BP2	0.001708	-0.16989	0.007	0.081	1	Undiff-G	IGF2BP2
GDPD1	0.001717	-0.16588	0	0.068	1	Undiff-G	GDPD1
SIK3	0.001718	-0.14101	0.022	0.107	1	Undiff-G	SIK3
PAPD5	0.001729	-0.21446	0.007	0.081	1	Undiff-G	PAPD5
ANO10	0.00173	-0.17929	0	0.067	1	Undiff-G	ANO10
STIM1	0.00173	-0.1801	0	0.067	1	Undiff-G	STIM1
GPS2	0.001732	0.325276	0.044	0.146	1	Undiff-G	GPS2
BNIP1	0.001741	0.332808	0.029	0.121	1	Undiff-G	BNIP1
CBX2	0.001743	-0.16052	0	0.067	1	Undiff-G	CBX2
ZFP1	0.001743	-0.16636	0	0.067	1	Undiff-G	ZFP1
SBF1	0.001743	-0.19126	0	0.067	1	Undiff-G	SBF1
PPM1F	0.001743	-0.20127	0	0.067	1	Undiff-G	PPM1F
MDK	0.001748	0.584506	0.243	0.494	1	Undiff-G	MDK
HEATR5A	0.001751	-0.14194	0.007	0.082	1	Undiff-G	HEATR5A
GTF3C4	0.001755	-0.15014	0	0.067	1	Undiff-G	GTF3C4
TMEM87B	0.001755	-0.18494	0	0.067	1	Undiff-G	TMEM87B
RPRM	0.001755	-0.27051	0	0.067	1	Undiff-G	RPRM
MYCBP	0.001763	-0.13921	0.015	0.094	1	Undiff-G	MYCBP
DDB2	0.001763	0.131794	0.015	0.095	1	Undiff-G	DDB2
LRRC28	0.001763	0.147076	0.015	0.095	1	Undiff-G	LRRC28
NOVA2	0.001765	-0.16062	0.007	0.081	1	Undiff-G	NOVA2
RNF123	0.001768	-0.18822	0	0.067	1	Undiff-G	RNF123
FBXO38	0.001768	-0.19721	0	0.067	1	Undiff-G	FBXO38
NOP2	0.00177	0.151993	0.022	0.107	1	Undiff-G	NOP2
AMOTL1	0.001781	-0.14754	0	0.067	1	Undiff-G	AMOTL1
DYRK1B	0.001781	-0.16406	0	0.067	1	Undiff-G	DYRK1B
MTA2	0.001781	-0.17405	0	0.067	1	Undiff-G	MTA2

USP37	0.001781	-0.18825	0	0.067	1	Undiff-G	USP37
RP11-386I	0.001781	-0.30119	0	0.067	1	Undiff-G	RP11-386I14.4
B3GNT2	0.001794	-0.18314	0	0.067	1	Undiff-G	B3GNT2
ZNF197	0.001794	-0.18633	0	0.067	1	Undiff-G	ZNF197
KCNH2	0.001794	-0.211	0	0.067	1	Undiff-G	KCNH2
EZH1	0.001805	-0.20689	0.007	0.081	1	Undiff-G	EZH1
THBS1	0.001806	-0.23973	0.007	0.081	1	Undiff-G	THBS1
APAF1	0.001807	-0.2001	0	0.067	1	Undiff-G	APAF1
PLXNC1	0.001807	-0.23351	0	0.067	1	Undiff-G	PLXNC1
APEX2	0.001812	0.11814	0.015	0.095	1	Undiff-G	APEX2
ZNF532	0.001819	-0.16091	0.015	0.094	1	Undiff-G	ZNF532
LINC0052€	0.001826	-0.18693	0.007	0.08	1	Undiff-G	LINC00526
LIMD1	0.001827	-0.16491	0.007	0.08	1	Undiff-G	LIMD1
IRF9	0.001829	-0.22906	0.015	0.094	1	Undiff-G	IRF9
MYSM1	0.001831	-0.13207	0.007	0.081	1	Undiff-G	MYSM1
TRMT1L	0.001834	-0.18714	0	0.067	1	Undiff-G	TRMT1L
TMEM108	0.001835	0.114099	0.007	0.081	1	Undiff-G	TMEM108
MYADM	0.001839	0.656251	0.074	0.194	1	Undiff-G	MYADM
OMG	0.001843	-0.31721	0.007	0.08	1	Undiff-G	OMG
TPI1	0.001846	0.900702	0.574	0.869	1	Undiff-G	TPI1
CDADC1	0.001852	0.201679	0.022	0.107	1	Undiff-G	CDADC1
PLCB1	0.001854	-0.14205	0.007	0.081	1	Undiff-G	PLCB1
MAP4K3	0.001859	-0.14234	0.007	0.081	1	Undiff-G	MAP4K3
KLF9	0.001859	0.116449	0.037	0.132	1	Undiff-G	KLF9
CELF3	0.001861	-0.23031	0	0.067	1	Undiff-G	CELF3
NGRN	0.001864	-0.15488	0.007	0.081	1	Undiff-G	NGRN
RP11-69E1	0.001874	-0.20563	0	0.067	1	Undiff-G	RP11-69E11.4
DDX28	0.001877	-0.16597	0.007	0.081	1	Undiff-G	DDX28
PCDHB2	0.001879	-0.16251	0.007	0.08	1	Undiff-G	PCDHB2
RLIM	0.00188	0.298856	0.022	0.107	1	Undiff-G	RLIM
EML1	0.001884	-0.11411	0.007	0.081	1	Undiff-G	EML1
GOLPH3L	0.001887	-0.20317	0.007	0.08	1	Undiff-G	GOLPH3L
RRNAD1	0.00189	0.171373	0.022	0.107	1	Undiff-G	RRNAD1
GHDC	0.001893	-0.10309	0.015	0.094	1	Undiff-G	GHDC
ZKSCAN5	0.001902	-0.17013	0	0.066	1	Undiff-G	ZKSCAN5
ZNF189	0.001902	-0.19257	0	0.066	1	Undiff-G	ZNF189
NUP155	0.001916	-0.18314	0	0.066	1	Undiff-G	NUP155
ACSF2	0.001916	-0.2026	0	0.066	1	Undiff-G	ACSF2
OSBPL11	0.00192	0.181958	0.015	0.094	1	Undiff-G	OSBPL11
MIS18A	0.001921	0.34284	0.022	0.107	1	Undiff-G	MIS18A
CDK7	0.001933	0.128344	0.015	0.094	1	Undiff-G	CDK7
PISD	0.001935	0.104199	0.022	0.106	1	Undiff-G	PISD
CRADD	0.001939	0.162388	0.022	0.106	1	Undiff-G	CRADD
VTI1A	0.001944	-0.23173	0	0.066	1	Undiff-G	VTI1A
WDR92	0.001947	-0.17536	0.007	0.08	1	Undiff-G	WDR92
E2F5	0.001958	-0.1202	0	0.066	1	Undiff-G	E2F5
C14orf80	0.001958	-0.17068	0	0.066	1	Undiff-G	C14orf80
MED9	0.001959	-0.10709	0.015	0.094	1	Undiff-G	MED9
MIB1	0.00197	-0.14779	0.015	0.093	1	Undiff-G	MIB1
COX10	0.001972	-0.1611	0	0.066	1	Undiff-G	COX10
FKBPL	0.001972	-0.18478	0	0.066	1	Undiff-G	FKBPL

MBD2	0.001972	-0.18553	0	0.066	1	Undiff-G	MBD2
DIAPH2	0.001972	-0.23303	0	0.066	1	Undiff-G	DIAPH2
STRN3	0.001973	0.303265	0.037	0.131	1	Undiff-G	STRN3
AUH	0.001985	-0.11002	0.007	0.08	1	Undiff-G	AUH
DMPK	0.001986	-0.14332	0	0.066	1	Undiff-G	DMPK
KCTD18	0.001986	-0.1866	0	0.066	1	Undiff-G	KCTD18
PUS1	0.001991	-0.17043	0.007	0.08	1	Undiff-G	PUS1
STAC3	0.001999	-0.23221	0.007	0.08	1	Undiff-G	STAC3
DPY19L1	0.002	-0.14836	0.022	0.105	1	Undiff-G	DPY19L1
PLCE1	0.002001	-0.16453	0	0.066	1	Undiff-G	PLCE1
SP140L	0.002007	-0.27968	0.007	0.079	1	Undiff-G	SP140L
TIAM2	0.002009	-0.16583	0.007	0.08	1	Undiff-G	TIAM2
SLC35G2	0.002014	0.174269	0.029	0.118	1	Undiff-G	SLC35G2
SPDL1	0.002016	-0.1615	0	0.066	1	Undiff-G	SPDL1
IL4I1	0.002016	-0.27641	0	0.066	1	Undiff-G	IL4I1
MT1F	0.00202	0.648439	0.14	0.307	1	Undiff-G	MT1F
RNF121	0.002031	0.405117	0.022	0.106	1	Undiff-G	RNF121
FBXO32	0.002034	0.253483	0.051	0.155	1	Undiff-G	FBXO32
PDGFD	0.002038	-0.12726	0.007	0.08	1	Undiff-G	PDGFD
DOT1L	0.002044	-0.14694	0.007	0.08	1	Undiff-G	DOT1L
SOCS5	0.002045	-0.16451	0	0.065	1	Undiff-G	SOCS5
GXYLT1	0.002045	-0.18004	0	0.065	1	Undiff-G	GXYLT1
METTL21B	0.002045	-0.18439	0	0.065	1	Undiff-G	METTL21B
PEG3	0.002045	-0.19302	0	0.065	1	Undiff-G	PEG3
RPRD2	0.002053	0.189337	0.037	0.131	1	Undiff-G	RPRD2
TMED8	0.00206	-0.16107	0	0.065	1	Undiff-G	TMED8
NLE1	0.00206	-0.16107	0	0.065	1	Undiff-G	NLE1
RAB6B	0.00206	-0.16349	0	0.065	1	Undiff-G	RAB6B
GAN	0.00207	0.183008	0.044	0.143	1	Undiff-G	GAN
KIAA0895L	0.00207	-0.17072	0.015	0.092	1	Undiff-G	KIAA0895L
BATF3	0.002074	0.114215	0.015	0.093	1	Undiff-G	BATF3
CCDC136	0.002075	-0.15204	0.015	0.092	1	Undiff-G	CCDC136
SELO	0.002075	-0.16618	0	0.065	1	Undiff-G	SELO
PIK3AP1	0.002075	-0.2513	0	0.065	1	Undiff-G	PIK3AP1
IKZF1	0.002075	-0.28354	0	0.065	1	Undiff-G	IKZF1
CTHRC1	0.00209	-0.13498	0	0.065	1	Undiff-G	CTHRC1
TAF8	0.002103	-0.15673	0.015	0.092	1	Undiff-G	TAF8
DBX2	0.002105	-0.1388	0	0.065	1	Undiff-G	DBX2
SOX21-AS1	0.002105	-0.1395	0	0.065	1	Undiff-G	SOX21-AS1
CACTIN	0.002105	-0.13978	0	0.065	1	Undiff-G	CACTIN
KHDC1	0.002105	-0.19227	0	0.065	1	Undiff-G	KHDC1
HDAC11	0.002105	-0.22044	0	0.065	1	Undiff-G	HDAC11
TMEM2	0.002105	-0.22722	0	0.065	1	Undiff-G	TMEM2
DBR1	0.002111	-0.18616	0.007	0.079	1	Undiff-G	DBR1
ZNF436	0.002113	-0.14152	0.007	0.079	1	Undiff-G	ZNF436
FKBP11	0.002115	0.274957	0.029	0.117	1	Undiff-G	FKBP11
KCTD1	0.002121	-0.15282	0	0.065	1	Undiff-G	KCTD1
TTLL3	0.002121	-0.17005	0	0.065	1	Undiff-G	TTLL3
ORC2	0.002121	-0.18244	0	0.065	1	Undiff-G	ORC2
SYTL3	0.002121	-0.31885	0	0.065	1	Undiff-G	SYTL3
HILPDA	0.002123	1.554594	0.279	0.226	1	Undiff-G	HILPDA

DENND1B	0.002124	-0.156	0.015	0.092	1	Undiff-G	DENND1B
C3orf62	0.002129	-0.13607	0.007	0.079	1	Undiff-G	C3orf62
CNST	0.002136	-0.18198	0	0.065	1	Undiff-G	CNST
FAM175A	0.002136	-0.20893	0	0.065	1	Undiff-G	FAM175A
ZMYM3	0.002138	-0.13939	0.007	0.079	1	Undiff-G	ZMYM3
PTPN7	0.002148	-0.20673	0.007	0.079	1	Undiff-G	PTPN7
C14orf132	0.002151	-0.1972	0.015	0.092	1	Undiff-G	C14orf132
KANSL3	0.002152	-0.18219	0	0.065	1	Undiff-G	KANSL3
LRRC57	0.002152	-0.18692	0	0.065	1	Undiff-G	LRRC57
RP5-827C2	0.002152	-0.19782	0	0.065	1	Undiff-G	RP5-827C21.4
ZNF720	0.002158	0.487317	0.037	0.13	1	Undiff-G	ZNF720
EMP1	0.002158	0.491911	0.066	0.18	1	Undiff-G	EMP1
TMEM64	0.002168	-0.15831	0	0.065	1	Undiff-G	TMEM64
BDNF-AS	0.002168	-0.16474	0	0.065	1	Undiff-G	BDNF-AS
AFF1	0.002168	-0.18221	0	0.065	1	Undiff-G	AFF1
NIM1K	0.002168	-0.19031	0	0.065	1	Undiff-G	NIM1K
LRRC61	0.002168	-0.22118	0	0.065	1	Undiff-G	LRRC61
SLC9A9	0.002168	-0.22381	0.007	0.079	1	Undiff-G	SLC9A9
CXXC4	0.002168	-0.18266	0.007	0.079	1	Undiff-G	CXXC4
CAMTA2	0.002172	-0.13716	0.007	0.079	1	Undiff-G	CAMTA2
LIPT1	0.002173	-0.14113	0.007	0.079	1	Undiff-G	LIPT1
CHST3	0.002183	-0.12803	0	0.065	1	Undiff-G	CHST3
MFSD6	0.002183	-0.17796	0	0.065	1	Undiff-G	MFSD6
ZNF384	0.002183	-0.18268	0	0.065	1	Undiff-G	ZNF384
NEK3	0.002183	-0.18874	0	0.065	1	Undiff-G	NEK3
MAP3K3	0.002183	-0.20054	0	0.065	1	Undiff-G	MAP3K3
MX2	0.002183	-0.28918	0	0.065	1	Undiff-G	MX2
FLVCR1	0.002189	-0.14026	0.007	0.079	1	Undiff-G	FLVCR1
BCL3	0.002198	-0.18914	0.007	0.078	1	Undiff-G	BCL3
SLC35E4	0.002199	-0.1356	0	0.065	1	Undiff-G	SLC35E4
ENKD1	0.002199	-0.1453	0	0.065	1	Undiff-G	ENKD1
PHC1	0.002214	-0.15073	0.007	0.079	1	Undiff-G	PHC1
ERAP1	0.002215	-0.18293	0	0.065	1	Undiff-G	ERAP1
HELQ	0.002215	-0.19648	0	0.065	1	Undiff-G	HELQ
ORC6	0.00222	-0.10836	0.015	0.092	1	Undiff-G	ORC6
GJC1	0.002231	-0.1274	0	0.064	1	Undiff-G	GJC1
SMN1	0.002231	-0.18088	0	0.064	1	Undiff-G	SMN1
PLEKHA5	0.002231	-0.18582	0	0.064	1	Undiff-G	PLEKHA5
TMEM144	0.002232	-0.37874	0.007	0.078	1	Undiff-G	TMEM144
ALG6	0.002236	0.197741	0.022	0.104	1	Undiff-G	ALG6
MAP3K4	0.002238	-0.14076	0.015	0.091	1	Undiff-G	MAP3K4
ZNF558	0.00224	-0.1701	0.007	0.078	1	Undiff-G	ZNF558
GSE1	0.002245	-0.14596	0.015	0.092	1	Undiff-G	GSE1
RHOH	0.00225	-0.34247	0.007	0.078	1	Undiff-G	RHOH
METTL15	0.00226	0.188585	0.037	0.129	1	Undiff-G	METTL15
ARID2	0.002261	-0.12626	0.022	0.104	1	Undiff-G	ARID2
TIPIN	0.002262	0.173703	0.044	0.142	1	Undiff-G	TIPIN
ATXN7L3	0.002264	-0.15793	0	0.064	1	Undiff-G	ATXN7L3
TFEB	0.00227	-0.17894	0.007	0.078	1	Undiff-G	TFEB
UBR1	0.002277	-0.1548	0.007	0.078	1	Undiff-G	UBR1
B3GALNT2	0.002292	-0.1219	0.015	0.092	1	Undiff-G	B3GALNT2

SHOX2	0.002297	-0.13308	0	0.064	1	Undiff-G	SHOX2
ULK2	0.002297	-0.15862	0	0.064	1	Undiff-G	ULK2
STRN	0.002297	-0.16658	0	0.064	1	Undiff-G	STRN
DUOX1	0.002297	-0.19459	0	0.064	1	Undiff-G	DUOX1
MMS22L	0.002297	-0.19837	0	0.064	1	Undiff-G	MMS22L
SLAMF8	0.002297	-0.26983	0	0.064	1	Undiff-G	SLAMF8
MLYCD	0.002298	-0.16195	0.007	0.078	1	Undiff-G	MLYCD
AURKA	0.002301	-0.18365	0.007	0.078	1	Undiff-G	AURKA
REC8	0.002309	-0.20626	0.007	0.078	1	Undiff-G	REC8
TLE2	0.002309	-0.16284	0.015	0.091	1	Undiff-G	TLE2
TTLL12	0.002314	-0.15339	0	0.064	1	Undiff-G	TTLL12
SGSH	0.002314	-0.16104	0	0.064	1	Undiff-G	SGSH
ZMYM6	0.002314	-0.18637	0	0.064	1	Undiff-G	ZMYM6
CCNB2	0.002314	-0.24737	0	0.064	1	Undiff-G	CCNB2
HIST2H2AA	0.002314	-0.25748	0	0.064	1	Undiff-G	HIST2H2AA3
ADCY7	0.002314	-0.26805	0	0.064	1	Undiff-G	ADCY7
GLMN	0.002316	-0.14093	0.015	0.091	1	Undiff-G	GLMN
ARMC8	0.002319	0.629186	0.081	0.202	1	Undiff-G	ARMC8
CXorf56	0.002322	-0.16158	0.007	0.078	1	Undiff-G	CXorf56
IL1RL1	0.002324	0.306524	0.015	0.002	1	Undiff-G	IL1RL1
SFMBT2	0.002324	-0.22131	0.007	0.078	1	Undiff-G	SFMBT2
PIBF1	0.002325	-0.16557	0.015	0.091	1	Undiff-G	PIBF1
MMP15	0.002331	-0.13718	0	0.064	1	Undiff-G	MMP15
CRIM1	0.002331	-0.14615	0	0.064	1	Undiff-G	CRIM1
C22orf29	0.002331	-0.16012	0	0.064	1	Undiff-G	C22orf29
ZRANB1	0.002331	-0.18266	0	0.064	1	Undiff-G	ZRANB1
NEU4	0.002331	-0.27074	0	0.064	1	Undiff-G	NEU4
PAQR3	0.00234	-0.16515	0.007	0.077	1	Undiff-G	PAQR3
PIK3CB	0.002348	-0.17298	0	0.064	1	Undiff-G	PIK3CB
FMNL3	0.002361	-0.20193	0.007	0.078	1	Undiff-G	FMNL3
RBM28	0.002363	0.11487	0.059	0.164	1	Undiff-G	RBM28
NCAPG2	0.002365	-0.17335	0	0.064	1	Undiff-G	NCAPG2
SETD7	0.002381	0.121067	0.022	0.104	1	Undiff-G	SETD7
HEATR6	0.002382	-0.16414	0	0.064	1	Undiff-G	HEATR6
ADAM22	0.002382	-0.18547	0	0.064	1	Undiff-G	ADAM22
CTU1	0.002408	-0.12625	0.007	0.078	1	Undiff-G	CTU1
DACT3	0.00241	-0.11794	0.007	0.078	1	Undiff-G	DACT3
GIMAP2	0.00241	-0.17267	0.007	0.078	1	Undiff-G	GIMAP2
CASD1	0.002411	-0.16556	0.007	0.078	1	Undiff-G	CASD1
NXPH4	0.002417	-0.21135	0	0.064	1	Undiff-G	NXPH4
TMEM37	0.002417	-0.28581	0	0.064	1	Undiff-G	TMEM37
THUMPD2	0.00242	-0.12389	0.015	0.091	1	Undiff-G	THUMPD2
CCDC18	0.002429	-0.1884	0.007	0.077	1	Undiff-G	CCDC18
USP12	0.002435	-0.16939	0	0.063	1	Undiff-G	USP12
RAB39B	0.002442	0.225926	0.015	0.091	1	Undiff-G	RAB39B
ADCK4	0.002447	0.208942	0.037	0.128	1	Undiff-G	ADCK4
UNC119B	0.002452	-0.14434	0	0.063	1	Undiff-G	UNC119B
PITRM1	0.002452	-0.1761	0	0.063	1	Undiff-G	PITRM1
MEX3B	0.002452	-0.18922	0	0.063	1	Undiff-G	MEX3B
HRK	0.002452	-0.20007	0	0.063	1	Undiff-G	HRK
AHR	0.002452	-0.23699	0	0.063	1	Undiff-G	AHR

MKI67	0.002452	-0.24425	0	0.063	1	Undiff-G	MKI67
RRM2	0.002452	-0.25999	0	0.063	1	Undiff-G	RRM2
FAM131B	0.002453	-0.18378	0.007	0.077	1	Undiff-G	FAM131B
HHLA3	0.002455	0.219719	0.044	0.141	1	Undiff-G	HHLA3
ZFP64	0.002462	-0.12627	0.007	0.077	1	Undiff-G	ZFP64
CALR	0.002464	0.662866	0.331	0.696	1	Undiff-G	CALR
ZNF582-AS	0.002469	-0.22005	0.007	0.077	1	Undiff-G	ZNF582-AS1
FAM76A	0.002469	-0.12077	0.015	0.09	1	Undiff-G	FAM76A
FBXO45	0.00247	-0.14547	0	0.063	1	Undiff-G	FBXO45
SLC25A40	0.00247	-0.19876	0	0.063	1	Undiff-G	SLC25A40
NANOS3	0.002488	-0.14104	0	0.063	1	Undiff-G	NANOS3
MFHAS1	0.002488	-0.1564	0	0.063	1	Undiff-G	MFHAS1
LGALSL	0.002488	-0.16115	0	0.063	1	Undiff-G	LGALSL
ACTL6B	0.002492	-0.22522	0.007	0.077	1	Undiff-G	ACTL6B
FARS2	0.002494	0.337507	0.029	0.116	1	Undiff-G	FARS2
SARM1	0.002506	-0.15288	0	0.063	1	Undiff-G	SARM1
NICN1	0.002506	-0.18675	0	0.063	1	Undiff-G	NICN1
DTX2	0.002506	-0.19829	0	0.063	1	Undiff-G	DTX2
LMBRD2	0.002513	-0.15958	0.007	0.077	1	Undiff-G	LMBRD2
PDE4A	0.002514	-0.18858	0.015	0.09	1	Undiff-G	PDE4A
NLK	0.002522	-0.13778	0.007	0.077	1	Undiff-G	NLK
ITGA2	0.002525	-0.14531	0	0.063	1	Undiff-G	ITGA2
TECPR2	0.002525	-0.18036	0	0.063	1	Undiff-G	TECPR2
PTPN23	0.002529	-0.11991	0.007	0.077	1	Undiff-G	PTPN23
THEM4	0.00253	-0.13375	0.007	0.077	1	Undiff-G	THEM4
ANKHD1	0.002532	-0.14646	0.007	0.077	1	Undiff-G	ANKHD1
HIST1H1C	0.002533	0.654665	0.074	0.19	1	Undiff-G	HIST1H1C
AGL	0.002536	-0.16224	0.015	0.09	1	Undiff-G	AGL
PANK3	0.002537	0.161301	0.029	0.115	1	Undiff-G	PANK3
RGAG4	0.002543	-0.1288	0	0.063	1	Undiff-G	RGAG4
SYNDIG1	0.002548	-0.19563	0.015	0.09	1	Undiff-G	SYNDIG1
AKAP6	0.002557	-0.12271	0.022	0.103	1	Undiff-G	AKAP6
GAB1	0.002558	0.10132	0.022	0.102	1	Undiff-G	GAB1
ABCA3	0.002559	-0.13042	0.007	0.077	1	Undiff-G	ABCA3
MIEF2	0.002561	-0.15524	0	0.063	1	Undiff-G	MIEF2
RAB24	0.002561	-0.16087	0	0.063	1	Undiff-G	RAB24
ZDHHC5	0.002561	-0.17792	0	0.063	1	Undiff-G	ZDHHC5
PRMT3	0.002569	-0.1142	0.015	0.09	1	Undiff-G	PRMT3
MEX3D	0.00258	-0.14178	0	0.063	1	Undiff-G	MEX3D
TTI2	0.00258	-0.19053	0	0.063	1	Undiff-G	TTI2
CLSPN	0.00258	-0.20068	0	0.063	1	Undiff-G	CLSPN
NUF2	0.00258	-0.22381	0	0.063	1	Undiff-G	NUF2
KBTBD4	0.002582	0.158044	0.015	0.09	1	Undiff-G	KBTBD4
FASTKD1	0.002598	-0.19805	0.007	0.076	1	Undiff-G	FASTKD1
EXOC6B	0.002599	-0.1711	0	0.063	1	Undiff-G	EXOC6B
NOTCH4	0.002599	-0.18457	0	0.063	1	Undiff-G	NOTCH4
TOMM40L	0.002599	-0.18947	0	0.063	1	Undiff-G	TOMM40L
TMEM163	0.002599	-0.20893	0	0.063	1	Undiff-G	TMEM163
SARS2	0.002608	-0.18642	0.007	0.076	1	Undiff-G	SARS2
AVIL	0.002609	-0.15704	0.015	0.09	1	Undiff-G	AVIL
NACAD	0.002613	-0.13492	0.015	0.09	1	Undiff-G	NACAD

USP30	0.002618	-0.16111	0	0.063	1	Undiff-G	USP30
DNAJA4	0.002618	-0.2511	0	0.063	1	Undiff-G	DNAJA4
CHCHD4	0.002633	0.226962	0.029	0.115	1	Undiff-G	CHCHD4
CEP97	0.002656	-0.14753	0.007	0.077	1	Undiff-G	CEP97
PIGW	0.002656	-0.14986	0	0.062	1	Undiff-G	PIGW
SNX30	0.002656	-0.20506	0	0.062	1	Undiff-G	SNX30
PSMA6	0.002674	0.552338	0.096	0.225	1	Undiff-G	PSMA6
CLYBL	0.002675	-0.21217	0	0.062	1	Undiff-G	CLYBL
ZNF408	0.002682	-0.16507	0.007	0.076	1	Undiff-G	ZNF408
RP11-264E	0.002696	0.340558	0.007	0.001	1	Undiff-G	RP11-264B17.4
C4orf46	0.002702	-0.12542	0.007	0.076	1	Undiff-G	C4orf46
TRAPPC13	0.002712	0.135598	0.029	0.114	1	Undiff-G	TRAPPC13
VPS33B	0.002714	-0.15896	0	0.062	1	Undiff-G	VPS33B
PEX6	0.002714	-0.17273	0	0.062	1	Undiff-G	PEX6
RP11-138A	0.002714	-0.29105	0.022	0.1	1	Undiff-G	RP11-138A9.2
XPO5	0.002718	-0.16311	0.007	0.076	1	Undiff-G	XPO5
TVP23B	0.002735	0.32955	0.022	0.103	1	Undiff-G	TVP23B
CLP1	0.002737	-0.20818	0.007	0.075	1	Undiff-G	CLP1
NUP205	0.002754	-0.16185	0	0.062	1	Undiff-G	NUP205
TGFBRAP1	0.002754	-0.1661	0	0.062	1	Undiff-G	TGFBRAP1
TMEM120B	0.002754	-0.17222	0	0.062	1	Undiff-G	TMEM120B
ZW10	0.002754	-0.17408	0	0.062	1	Undiff-G	ZW10
DNMT3A	0.002765	-0.10534	0.022	0.101	1	Undiff-G	DNMT3A
TMEM104	0.002774	-0.15849	0	0.062	1	Undiff-G	TMEM104
AP006621.	0.002774	-0.15922	0	0.062	1	Undiff-G	AP006621.5
ZBED3	0.002777	-0.1294	0.015	0.089	1	Undiff-G	ZBED3
GS1-124K5	0.002786	0.212764	0.029	0.115	1	Undiff-G	GS1-124K5.4
MICAL3	0.002792	-0.15238	0.007	0.076	1	Undiff-G	MICAL3
TSEN2	0.002792	-0.12234	0.015	0.089	1	Undiff-G	TSEN2
PVRL1	0.002794	-0.18905	0	0.062	1	Undiff-G	PVRL1
HOXB7	0.002794	-0.2501	0	0.062	1	Undiff-G	HOXB7
CAMK2B	0.002797	0.165235	0.029	0.115	1	Undiff-G	CAMK2B
PUS7	0.002815	-0.15197	0	0.062	1	Undiff-G	PUS7
PLCB2	0.002815	-0.2654	0	0.062	1	Undiff-G	PLCB2
AC074289.	0.002815	-0.29393	0	0.062	1	Undiff-G	AC074289.1
ZNF844	0.002816	-0.16357	0.007	0.076	1	Undiff-G	ZNF844
HSBP1L1	0.002819	-0.22171	0.007	0.075	1	Undiff-G	HSBP1L1
GFM2	0.002832	0.147502	0.015	0.089	1	Undiff-G	GFM2
GTPBP3	0.002832	0.166692	0.029	0.113	1	Undiff-G	GTPBP3
SLC46A1	0.002835	-0.16717	0	0.062	1	Undiff-G	SLC46A1
SIRT1	0.002835	-0.17593	0	0.062	1	Undiff-G	SIRT1
SMIM18	0.002835	-0.2122	0	0.062	1	Undiff-G	SMIM18
CLDN12	0.002843	0.321705	0.044	0.139	1	Undiff-G	CLDN12
PPP2R5D	0.002845	-0.16501	0.007	0.075	1	Undiff-G	PPP2R5D
NFKB2	0.002856	-0.21579	0	0.062	1	Undiff-G	NFKB2
FAM198B	0.002856	-0.25767	0	0.062	1	Undiff-G	FAM198B
PICK1	0.002858	-0.15104	0.015	0.089	1	Undiff-G	PICK1
GORAB	0.002861	-0.10117	0.007	0.076	1	Undiff-G	GORAB
FAM220A	0.002864	-0.10475	0.007	0.076	1	Undiff-G	FAM220A
DNM3	0.002869	-0.10552	0.015	0.089	1	Undiff-G	DNM3
SLC2A13	0.002874	-0.14905	0.015	0.088	1	Undiff-G	SLC2A13

MYO1G	0.002877	-0.24572	0	0.061	1	Undiff-G	MYO1G
APBA3	0.002884	-0.12755	0.015	0.089	1	Undiff-G	APBA3
MTRNR2L1C	0.002885	0.948312	0.132	0.073	1	Undiff-G	MTRNR2L10
PTGR2	0.002891	-0.17057	0.007	0.075	1	Undiff-G	PTGR2
MORC4	0.002897	-0.13043	0	0.061	1	Undiff-G	MORC4
ZDHHC15	0.002897	-0.14154	0	0.061	1	Undiff-G	ZDHHC15
ZBTB33	0.002897	-0.15612	0	0.061	1	Undiff-G	ZBTB33
GRAMD4	0.002897	-0.18974	0	0.061	1	Undiff-G	GRAMD4
SCAPER	0.002898	-0.10494	0.029	0.114	1	Undiff-G	SCAPER
TNRC18	0.002901	-0.16732	0.007	0.075	1	Undiff-G	TNRC18
SLC35D2	0.002911	0.322562	0.022	0.102	1	Undiff-G	SLC35D2
MNS1	0.002915	-0.14235	0.007	0.075	1	Undiff-G	MNS1
PPP1R14C	0.002919	-0.13489	0	0.061	1	Undiff-G	PPP1R14C
NCS1	0.002919	-0.14721	0	0.061	1	Undiff-G	NCS1
CACNB3	0.002919	-0.15045	0	0.061	1	Undiff-G	CACNB3
B3GNTL1	0.002919	-0.16967	0	0.061	1	Undiff-G	B3GNTL1
CAMK2G	0.00292	-0.11621	0.022	0.101	1	Undiff-G	CAMK2G
CSGALNACT	0.00292	-0.20485	0.007	0.075	1	Undiff-G	CSGALNACT1
PIGO	0.002922	-0.19112	0.007	0.075	1	Undiff-G	PIGO
KLHL22	0.002931	-0.1096	0.015	0.089	1	Undiff-G	KLHL22
CDCA4	0.002931	0.109672	0.015	0.089	1	Undiff-G	CDCA4
CXorf40A	0.002934	0.158705	0.015	0.089	1	Undiff-G	CXorf40A
TCTA	0.002937	0.315591	0.059	0.162	1	Undiff-G	TCTA
CFI	0.00294	-0.20168	0	0.061	1	Undiff-G	CFI
SGMS1	0.002961	-0.17081	0	0.061	1	Undiff-G	SGMS1
LRRK2	0.002961	-0.22186	0	0.061	1	Undiff-G	LRRK2
CCDC80	0.002971	0.372468	0.029	0.114	1	Undiff-G	CCDC80
EFCAB11	0.002983	-0.16367	0	0.061	1	Undiff-G	EFCAB11
PDK3	0.002983	-0.17909	0	0.061	1	Undiff-G	PDK3
HOXA5	0.002983	-0.21112	0	0.061	1	Undiff-G	HOXA5
BEST3	0.002991	-0.10704	0.015	0.088	1	Undiff-G	BEST3
NIP7	0.002992	0.495929	0.066	0.173	1	Undiff-G	NIP7
TAB1	0.003005	-0.14011	0.007	0.075	1	Undiff-G	TAB1
TAGLN2	0.003013	0.59715	0.257	0.517	1	Undiff-G	TAGLN2
ZNF512B	0.003015	-0.13044	0.007	0.075	1	Undiff-G	ZNF512B
INTS1	0.003024	-0.14343	0.015	0.088	1	Undiff-G	INTS1
NCOA2	0.003026	-0.16024	0	0.061	1	Undiff-G	NCOA2
SENP7	0.003039	-0.14187	0.015	0.087	1	Undiff-G	SENP7
LZTS1	0.003048	-0.14081	0	0.061	1	Undiff-G	LZTS1
SH3BP5L	0.003048	-0.15148	0	0.061	1	Undiff-G	SH3BP5L
ANKS1A	0.003048	-0.1649	0	0.061	1	Undiff-G	ANKS1A
ASF1B	0.003048	-0.18967	0	0.061	1	Undiff-G	ASF1B
ANKRD28	0.003053	0.298626	0.029	0.114	1	Undiff-G	ANKRD28
SLC2A1	0.003056	0.448561	0.044	0.138	1	Undiff-G	SLC2A1
NXPE3	0.003059	0.160651	0.015	0.088	1	Undiff-G	NXPE3
CXorf23	0.00307	-0.14671	0	0.061	1	Undiff-G	CXorf23
VPS37D	0.00307	-0.15873	0	0.061	1	Undiff-G	VPS37D
GAPT	0.00307	-0.2777	0	0.061	1	Undiff-G	GAPT
LRRC17	0.003072	-0.13125	0.007	0.075	1	Undiff-G	LRRC17
MALT1	0.003075	-0.12007	0.007	0.075	1	Undiff-G	MALT1
DDX3Y	0.003081	-0.13726	0.015	0.087	1	Undiff-G	DDX3Y

PBXIP1	0.003089	0.247261	0.044	0.136	1	Undiff-G	PBXIP1
CALHM2	0.003093	-0.21302	0	0.061	1	Undiff-G	CALHM2
RDH13	0.003094	-0.12829	0.007	0.075	1	Undiff-G	RDH13
SMG9	0.003095	0.414862	0.037	0.126	1	Undiff-G	SMG9
TRIB3	0.003099	-0.12609	0.007	0.075	1	Undiff-G	TRIB3
VIPAS39	0.003115	-0.16518	0.007	0.075	1	Undiff-G	VIPAS39
TM4SF1	0.003115	0.334042	0.029	0.113	1	Undiff-G	TM4SF1
MADD	0.003115	-0.18031	0	0.06	1	Undiff-G	MADD
IMPA2	0.003115	-0.18921	0	0.06	1	Undiff-G	IMPA2
MSANTD3	0.003126	0.297078	0.015	0.088	1	Undiff-G	MSANTD3
CTB-113I2	0.003138	-0.13315	0	0.06	1	Undiff-G	CTB-113I20.2
FAM193B	0.003138	-0.15875	0	0.06	1	Undiff-G	FAM193B
NRSN1	0.003138	-0.17023	0	0.06	1	Undiff-G	NRSN1
RMI1	0.003138	-0.17799	0	0.06	1	Undiff-G	RMI1
ZNF124	0.003138	-0.22183	0	0.06	1	Undiff-G	ZNF124
TTBK2	0.003138	-0.1206	0.007	0.075	1	Undiff-G	TTBK2
COX7C	0.003148	0.5246	0.404	0.891	1	Undiff-G	COX7C
HERC5	0.00315	-0.18911	0.007	0.074	1	Undiff-G	HERC5
MPDZ	0.003159	-0.14234	0.015	0.087	1	Undiff-G	MPDZ
SMOX	0.00316	0.767361	0.11	0.249	1	Undiff-G	SMOX
TF	0.003174	-0.42415	0.007	0.074	1	Undiff-G	TF
MAVS	0.003178	-0.12337	0.007	0.074	1	Undiff-G	MAVS
FAM122B	0.003181	0.15348	0.022	0.1	1	Undiff-G	FAM122B
MORC2	0.003184	-0.14526	0	0.06	1	Undiff-G	MORC2
YTHDF3-AS	0.003184	-0.15125	0	0.06	1	Undiff-G	YTHDF3-AS1
FAM69A	0.003184	-0.15959	0	0.06	1	Undiff-G	FAM69A
ATAD2B	0.003184	-0.1633	0	0.06	1	Undiff-G	ATAD2B
UTP15	0.003184	-0.16439	0	0.06	1	Undiff-G	UTP15
SLC9A6	0.003184	-0.16862	0	0.06	1	Undiff-G	SLC9A6
TDP1	0.003184	-0.1694	0	0.06	1	Undiff-G	TDP1
TRIQK	0.003184	-0.21537	0	0.06	1	Undiff-G	TRIQK
FAM20A	0.003184	-0.23844	0	0.06	1	Undiff-G	FAM20A
C19orf53	0.003205	0.640252	0.338	0.687	1	Undiff-G	C19orf53
MPP2	0.003207	-0.15354	0	0.06	1	Undiff-G	MPP2
TBC1D10A	0.003211	-0.10497	0.007	0.074	1	Undiff-G	TBC1D10A
SLC38A6	0.003226	0.302617	0.022	0.1	1	Undiff-G	SLC38A6
SLC27A4	0.00323	-0.1513	0	0.06	1	Undiff-G	SLC27A4
ZNF333	0.003237	-0.14104	0.015	0.087	1	Undiff-G	ZNF333
TMA7	0.003245	0.486267	0.397	0.856	1	Undiff-G	TMA7
LINC00909	0.003253	0.379743	0.029	0.113	1	Undiff-G	LINC00909
PARD3	0.003254	-0.11195	0	0.06	1	Undiff-G	PARD3
GLRB	0.003254	-0.14058	0	0.06	1	Undiff-G	GLRB
RBM12B	0.003261	-0.13534	0.007	0.074	1	Undiff-G	RBM12B
FLCN	0.00327	0.248849	0.044	0.137	1	Undiff-G	FLCN
GAL3ST3	0.003277	-0.13751	0	0.06	1	Undiff-G	GAL3ST3
ZNF530	0.003277	-0.15108	0	0.06	1	Undiff-G	ZNF530
ZCWPW1	0.003301	-0.19078	0	0.06	1	Undiff-G	ZCWPW1
ARNT	0.003322	-0.13351	0.007	0.074	1	Undiff-G	ARNT
RCAN3	0.003325	-0.17344	0	0.06	1	Undiff-G	RCAN3
LRRC8C	0.003325	-0.1953	0	0.06	1	Undiff-G	LRRC8C
LRR1	0.003326	0.137742	0.015	0.087	1	Undiff-G	LRR1

ASGR1	0.003337	-0.20378	0.007	0.074	1	Undiff-G	ASGR1
UBP1	0.003338	-0.16547	0.007	0.073	1	Undiff-G	UBP1
MED26	0.003341	-0.14097	0.007	0.074	1	Undiff-G	MED26
GRB10	0.003356	-0.15599	0.007	0.074	1	Undiff-G	GRB10
ZNF274	0.003357	0.203297	0.029	0.112	1	Undiff-G	ZNF274
1-Mar	0.003373	-0.13315	0	0.06	1	Undiff-G	1-Mar
CCNYL1	0.003373	-0.14537	0	0.06	1	Undiff-G	CCNYL1
KDM1B	0.003373	-0.15094	0	0.06	1	Undiff-G	KDM1B
ZNF286A	0.003373	-0.16099	0	0.06	1	Undiff-G	ZNF286A
PHF2	0.003373	-0.16537	0	0.06	1	Undiff-G	PHF2
ZNF529	0.003373	-0.17381	0	0.06	1	Undiff-G	ZNF529
KIFC1	0.003373	-0.19705	0	0.06	1	Undiff-G	KIFC1
ZMYND19	0.003398	-0.12443	0	0.059	1	Undiff-G	ZMYND19
SPSB4	0.003398	-0.12707	0	0.059	1	Undiff-G	SPSB4
KCTD7	0.003398	-0.17221	0	0.059	1	Undiff-G	KCTD7
IRF5	0.003398	-0.255	0	0.059	1	Undiff-G	IRF5
GPSM1	0.003403	-0.10017	0.007	0.074	1	Undiff-G	GPSM1
NEO1	0.003405	-0.15805	0.007	0.073	1	Undiff-G	NEO1
ASPHD2	0.003423	-0.11154	0	0.059	1	Undiff-G	ASPHD2
MCHR1	0.003423	-0.12215	0	0.059	1	Undiff-G	MCHR1
ZNF496	0.003423	-0.14374	0	0.059	1	Undiff-G	ZNF496
LRP4	0.003423	-0.17153	0	0.059	1	Undiff-G	LRP4
MYT1L	0.003423	-0.22913	0	0.059	1	Undiff-G	MYT1L
TRPV2	0.003423	-0.24262	0	0.059	1	Undiff-G	TRPV2
SDS	0.003423	-0.33333	0	0.059	1	Undiff-G	SDS
PTPRD	0.00343	-0.21137	0.007	0.073	1	Undiff-G	PTPRD
KIF13A	0.003448	-0.14552	0	0.059	1	Undiff-G	KIF13A
CCDC117	0.003448	-0.15262	0	0.059	1	Undiff-G	CCDC117
BRPF1	0.003448	-0.15456	0	0.059	1	Undiff-G	BRPF1
WARS2	0.003448	-0.16855	0	0.059	1	Undiff-G	WARS2
PDLIM4	0.003449	-0.18745	0.007	0.073	1	Undiff-G	PDLIM4
PAQR6	0.003452	-0.21005	0.007	0.073	1	Undiff-G	PAQR6
TUBG2	0.003458	0.13999	0.029	0.111	1	Undiff-G	TUBG2
SLC1A4	0.003469	-0.14019	0.007	0.073	1	Undiff-G	SLC1A4
SH3BGR	0.003472	0.291833	0.037	0.124	1	Undiff-G	SH3BGR
ATR	0.003473	-0.15466	0	0.059	1	Undiff-G	ATR
SWSAP1	0.003473	-0.19559	0	0.059	1	Undiff-G	SWSAP1
ZNHIT2	0.003491	-0.1402	0.015	0.086	1	Undiff-G	ZNHIT2
ZNF318	0.003498	-0.14606	0	0.059	1	Undiff-G	ZNF318
ZNF687	0.003498	-0.16428	0	0.059	1	Undiff-G	ZNF687
LYSMD3	0.003498	-0.18655	0	0.059	1	Undiff-G	LYSMD3
WNT5A	0.003498	-0.21416	0	0.059	1	Undiff-G	WNT5A
ZBTB48	0.003498	0.243802	0.015	0.087	1	Undiff-G	ZBTB48
CBX8	0.00352	-0.16088	0.007	0.073	1	Undiff-G	CBX8
CLSTN3	0.003523	-0.16195	0	0.059	1	Undiff-G	CLSTN3
ERBB4	0.003523	-0.19854	0	0.059	1	Undiff-G	ERBB4
SLC39A8	0.003523	-0.21126	0	0.059	1	Undiff-G	SLC39A8
GPRIN3	0.003523	-0.24059	0	0.059	1	Undiff-G	GPRIN3
GMPPA	0.003527	0.584308	0.059	0.161	1	Undiff-G	GMPPA
AP5S1	0.003535	-0.1016	0.022	0.099	1	Undiff-G	AP5S1
AEBP1	0.003548	0.180295	0.037	0.122	1	Undiff-G	AEBP1

FAM69B	0.003549	-0.11312	0	0.059	1	Undiff-G	FAM69B
PLAGL2	0.003549	-0.1414	0	0.059	1	Undiff-G	PLAGL2
ZBTB45	0.003549	-0.14221	0	0.059	1	Undiff-G	ZBTB45
ADCY3	0.003549	-0.18028	0	0.059	1	Undiff-G	ADCY3
HPSE	0.003549	-0.26429	0	0.059	1	Undiff-G	HPSE
DNAJC13	0.003568	-0.15561	0.007	0.073	1	Undiff-G	DNAJC13
SPATA5L1	0.003575	-0.13872	0	0.059	1	Undiff-G	SPATA5L1
GRPEL2	0.003575	-0.16732	0	0.059	1	Undiff-G	GRPEL2
PDE4D	0.003584	0.145727	0.022	0.099	1	Undiff-G	PDE4D
MTHFSD	0.003601	-0.15637	0	0.059	1	Undiff-G	MTHFSD
PDIK1L	0.003601	-0.15724	0	0.059	1	Undiff-G	PDIK1L
RP11-698N	0.003601	-0.16525	0	0.059	1	Undiff-G	RP11-698N11.2
GLRX	0.003604	0.761651	0.191	0.391	1	Undiff-G	GLRX
HOXA2	0.003605	-0.153	0.007	0.073	1	Undiff-G	HOXA2
OMA1	0.003616	-0.17003	0.007	0.073	1	Undiff-G	OMA1
WRN	0.003627	-0.14774	0	0.059	1	Undiff-G	WRN
TMEM169	0.003627	-0.15179	0	0.059	1	Undiff-G	TMEM169
ZBTB24	0.003627	-0.16892	0	0.059	1	Undiff-G	ZBTB24
TARS2	0.003635	0.423817	0.029	0.112	1	Undiff-G	TARS2
COX6B1	0.00364	0.57487	0.397	0.851	1	Undiff-G	COX6B1
RFWD3	0.003653	-0.15417	0	0.059	1	Undiff-G	RFWD3
KCND3	0.00368	-0.13863	0	0.059	1	Undiff-G	KCND3
ZNF35	0.00368	-0.14739	0	0.059	1	Undiff-G	ZNF35
CTNNA2	0.00368	-0.20224	0	0.059	1	Undiff-G	CTNNA2
CAPS	0.003691	-0.18679	0.007	0.072	1	Undiff-G	CAPS
HEG1	0.003691	-0.11318	0.007	0.073	1	Undiff-G	HEG1
SYNM	0.003693	-0.13004	0.007	0.072	1	Undiff-G	SYNM
TIPARP	0.003696	0.539349	0.066	0.17	1	Undiff-G	TIPARP
STK10	0.003713	-0.17919	0.007	0.072	1	Undiff-G	STK10
EEPD1	0.00372	-0.18948	0.007	0.072	1	Undiff-G	EEPD1
WDFY2	0.003728	-0.18428	0.015	0.085	1	Undiff-G	WDFY2
ZNF74	0.003733	-0.14035	0	0.058	1	Undiff-G	ZNF74
PPARA	0.003733	-0.15809	0	0.058	1	Undiff-G	PPARA
CCL26	0.003743	0.487735	0.015	0.002	1	Undiff-G	CCL26
EVA1C	0.003748	-0.13796	0.007	0.072	1	Undiff-G	EVA1C
PIGU	0.003749	0.569481	0.066	0.172	1	Undiff-G	PIGU
CHM	0.003752	0.142292	0.015	0.086	1	Undiff-G	CHM
FLRT3	0.00376	-0.14652	0	0.058	1	Undiff-G	FLRT3
MFAP3	0.003788	-0.1391	0	0.058	1	Undiff-G	MFAP3
ZNF398	0.003788	-0.15575	0	0.058	1	Undiff-G	ZNF398
FAXDC2	0.003788	-0.18572	0	0.058	1	Undiff-G	FAXDC2
ABHD15	0.003788	-0.20378	0	0.058	1	Undiff-G	ABHD15
SLC46A3	0.003788	-0.22135	0	0.058	1	Undiff-G	SLC46A3
HHEX	0.003788	-0.266	0	0.058	1	Undiff-G	HHEX
ZNF786	0.00379	-0.13609	0.007	0.072	1	Undiff-G	ZNF786
ZNF236	0.003807	-0.13654	0.007	0.072	1	Undiff-G	ZNF236
POLG2	0.003808	-0.14747	0.015	0.085	1	Undiff-G	POLG2
FRMD5	0.003815	-0.11434	0	0.058	1	Undiff-G	FRMD5
SHB	0.003815	-0.13386	0	0.058	1	Undiff-G	SHB
KLF16	0.003815	-0.14942	0	0.058	1	Undiff-G	KLF16
CTD-2017D	0.003815	-0.15508	0	0.058	1	Undiff-G	CTD-2017D11.1

MANSC1	0.003815	-0.1617	0	0.058	1	Undiff-G	MANSC1
IPO11	0.003815	-0.17379	0	0.058	1	Undiff-G	IPO11
TOM1L1	0.003843	-0.11331	0	0.058	1	Undiff-G	TOM1L1
TCF20	0.003843	-0.16373	0	0.058	1	Undiff-G	TCF20
TPRG1L	0.003843	-0.16692	0	0.058	1	Undiff-G	TPRG1L
BTN2A2	0.003863	-0.16677	0.007	0.072	1	Undiff-G	BTN2A2
PPP1R9A	0.003865	-0.17281	0.007	0.072	1	Undiff-G	PPP1R9A
USPL1	0.003874	-0.1603	0.007	0.072	1	Undiff-G	USPL1
VPS13D	0.003875	-0.17516	0.007	0.072	1	Undiff-G	VPS13D
ETFDH	0.003877	0.322672	0.037	0.123	1	Undiff-G	ETFDH
DUSP18	0.003884	-0.12765	0.015	0.085	1	Undiff-G	DUSP18
CPEB2	0.003886	-0.16401	0.007	0.072	1	Undiff-G	CPEB2
CACNA1A	0.003886	-0.16263	0.022	0.096	1	Undiff-G	CACNA1A
EYA4	0.003899	-0.10283	0	0.058	1	Undiff-G	EYA4
RAD18	0.003899	-0.14875	0	0.058	1	Undiff-G	RAD18
PLBD2	0.003899	-0.17746	0	0.058	1	Undiff-G	PLBD2
CBX7	0.003917	-0.20054	0.007	0.072	1	Undiff-G	CBX7
F11R	0.003927	-0.23217	0	0.058	1	Undiff-G	F11R
RASAL3	0.003927	-0.24286	0	0.058	1	Undiff-G	RASAL3
AQR	0.003938	-0.16736	0.007	0.072	1	Undiff-G	AQR
OPHN1	0.00395	-0.13855	0.007	0.072	1	Undiff-G	OPHN1
ZP3	0.003954	-0.10281	0.007	0.072	1	Undiff-G	ZP3
PDSS1	0.003956	-0.13539	0	0.058	1	Undiff-G	PDSS1
SIK2	0.003956	-0.16074	0	0.058	1	Undiff-G	SIK2
LDLRAP1	0.003956	-0.1734	0	0.058	1	Undiff-G	LDLRAP1
HAUS3	0.003958	-0.14928	0.015	0.085	1	Undiff-G	HAUS3
RTN2	0.003965	-0.16995	0.015	0.085	1	Undiff-G	RTN2
ZNF273	0.003966	0.104865	0.015	0.085	1	Undiff-G	ZNF273
AMH	0.003984	-0.13374	0	0.058	1	Undiff-G	AMH
ASB13	0.003984	-0.13693	0	0.058	1	Undiff-G	ASB13
SIX1	0.003984	-0.15371	0	0.058	1	Undiff-G	SIX1
POLD1	0.003984	-0.16131	0	0.058	1	Undiff-G	POLD1
PDZRN3	0.003984	-0.16557	0	0.058	1	Undiff-G	PDZRN3
GHRL	0.003984	-0.20808	0	0.058	1	Undiff-G	GHRL
ZNF8	0.003986	0.454055	0.022	0.005	1	Undiff-G	ZNF8
PARP4	0.003991	-0.15476	0.015	0.085	1	Undiff-G	PARP4
PCDHB7	0.003994	-0.16673	0.007	0.072	1	Undiff-G	PCDHB7
POLR1C	0.004003	0.334998	0.037	0.122	1	Undiff-G	POLR1C
TARBP1	0.004008	-0.13936	0.007	0.072	1	Undiff-G	TARBP1
DLX6	0.004013	-0.2217	0	0.057	1	Undiff-G	DLX6
COX18	0.004018	-0.15459	0.007	0.071	1	Undiff-G	COX18
ALS2	0.004042	-0.1363	0	0.057	1	Undiff-G	ALS2
CHUK	0.004042	-0.15649	0	0.057	1	Undiff-G	CHUK
VEGFA	0.004062	0.606214	0.096	0.217	1	Undiff-G	VEGFA
SCAI	0.004063	-0.12092	0.015	0.084	1	Undiff-G	SCAI
RCCD1	0.004072	-0.15415	0	0.057	1	Undiff-G	RCCD1
FAM184B	0.004078	0.264277	0.029	0.109	1	Undiff-G	FAM184B
IFIT5	0.004092	-0.15515	0.015	0.084	1	Undiff-G	IFIT5
VPS54	0.004101	-0.14732	0	0.057	1	Undiff-G	VPS54
POC1A	0.004101	-0.15413	0	0.057	1	Undiff-G	POC1A
CMPK2	0.004101	-0.19937	0	0.057	1	Undiff-G	CMPK2

IKBKE	0.004101	-0.21704	0	0.057	1	Undiff-G	IKBKE
ZNF514	0.004107	0.191109	0.015	0.085	1	Undiff-G	ZNF514
IFT80	0.00411	-0.13019	0.007	0.071	1	Undiff-G	IFT80
GDF15	0.004116	-0.288	0.007	0.071	1	Undiff-G	GDF15
CPOX	0.004119	-0.14378	0.007	0.071	1	Undiff-G	CPOX
LAYN	0.004131	-0.12442	0	0.057	1	Undiff-G	LAYN
RBM45	0.004146	-0.19928	0.007	0.07	1	Undiff-G	RBM45
SMAD9	0.004187	-0.12617	0.007	0.071	1	Undiff-G	SMAD9
MBTD1	0.004191	-0.14488	0	0.057	1	Undiff-G	MBTD1
TMEM178B	0.004191	-0.14642	0	0.057	1	Undiff-G	TMEM178B
ZNF275	0.004191	-0.14918	0	0.057	1	Undiff-G	ZNF275
CDK1	0.004214	0.310196	0.022	0.097	1	Undiff-G	CDK1
CCNA2	0.004221	-0.19089	0	0.057	1	Undiff-G	CCNA2
FPR3	0.004221	-0.23026	0	0.057	1	Undiff-G	FPR3
HLA-DRB1	0.004234	0.419659	0.272	0.536	1	Undiff-G	HLA-DRB1
CAMKK2	0.004239	-0.12661	0.007	0.071	1	Undiff-G	CAMKK2
PITPNM1	0.004252	-0.1361	0	0.057	1	Undiff-G	PITPNM1
FHOD1	0.004252	-0.19999	0	0.057	1	Undiff-G	FHOD1
NADSYN1	0.004253	0.346912	0.037	0.121	1	Undiff-G	NADSYN1
TBC1D13	0.004258	-0.17107	0.007	0.07	1	Undiff-G	TBC1D13
LRRC1	0.004279	-0.15763	0.007	0.071	1	Undiff-G	LRRC1
TRNT1	0.00428	0.278704	0.044	0.133	1	Undiff-G	TRNT1
SAPCD2	0.004283	-0.15777	0	0.057	1	Undiff-G	SAPCD2
C10orf76	0.004283	-0.1889	0	0.057	1	Undiff-G	C10orf76
AK4	0.004308	0.468184	0.074	0.181	1	Undiff-G	AK4
HSD17B1	0.004314	-0.11423	0	0.057	1	Undiff-G	HSD17B1
MTM1	0.004314	-0.15311	0	0.057	1	Undiff-G	MTM1
TMEM156	0.004314	-0.27591	0	0.057	1	Undiff-G	TMEM156
B3GAT2	0.004326	-0.14794	0.015	0.084	1	Undiff-G	B3GAT2
ALDH5A1	0.004345	-0.12499	0	0.057	1	Undiff-G	ALDH5A1
HECTD3	0.004345	-0.14323	0	0.057	1	Undiff-G	HECTD3
VPS13B	0.004345	-0.15335	0	0.057	1	Undiff-G	VPS13B
HOXA3	0.004345	-0.18676	0	0.057	1	Undiff-G	HOXA3
TFEC	0.004345	-0.23978	0	0.057	1	Undiff-G	TFEC
PIKFYVE	0.004351	-0.11172	0.015	0.083	1	Undiff-G	PIKFYVE
KDM5C	0.004354	0.304393	0.029	0.109	1	Undiff-G	KDM5C
DYRK2	0.004377	-0.13909	0	0.056	1	Undiff-G	DYRK2
RP6-91H8.	0.004377	-0.29536	0	0.056	1	Undiff-G	RP6-91H8.3
MED14	0.004406	-0.16251	0.007	0.07	1	Undiff-G	MED14
SBNO2	0.004409	-0.13938	0	0.056	1	Undiff-G	SBNO2
AGAP2	0.004409	-0.17933	0	0.056	1	Undiff-G	AGAP2
UPP1	0.004423	0.886597	0.118	0.256	1	Undiff-G	UPP1
POC1B	0.004424	0.100802	0.015	0.084	1	Undiff-G	POC1B
ARMC5	0.004426	-0.13204	0.007	0.07	1	Undiff-G	ARMC5
EARS2	0.004441	-0.13659	0	0.056	1	Undiff-G	EARS2
FAM214A	0.004441	-0.15609	0	0.056	1	Undiff-G	FAM214A
ANTXR2	0.004441	-0.16135	0	0.056	1	Undiff-G	ANTXR2
TLR7	0.004441	-0.27707	0	0.056	1	Undiff-G	TLR7
FKRP	0.004473	-0.12723	0	0.056	1	Undiff-G	FKRP
SLC30A6	0.004473	-0.14898	0	0.056	1	Undiff-G	SLC30A6
AKT3	0.004473	-0.16438	0	0.056	1	Undiff-G	AKT3

TXNDC16	0.004473	-0.167	0	0.056	1	Undiff-G	TXNDC16
PEAK1	0.004485	-0.15373	0.007	0.07	1	Undiff-G	PEAK1
B9D2	0.004502	0.187471	0.015	0.084	1	Undiff-G	B9D2
SYNJ1	0.004505	-0.13526	0	0.056	1	Undiff-G	SYNJ1
SETMAR	0.004505	-0.16476	0	0.056	1	Undiff-G	SETMAR
C3orf33	0.004505	-0.16532	0	0.056	1	Undiff-G	C3orf33
SPN	0.004505	-0.2431	0	0.056	1	Undiff-G	SPN
RP5-1085F	0.004515	-0.10674	0.022	0.095	1	Undiff-G	RP5-1085F17.3
ZNF324	0.004518	-0.1391	0.015	0.083	1	Undiff-G	ZNF324
CXorf38	0.004518	-0.15519	0.007	0.07	1	Undiff-G	CXorf38
TMEM145	0.004526	-0.12329	0.007	0.07	1	Undiff-G	TMEM145
TMEM187	0.004527	0.146029	0.015	0.083	1	Undiff-G	TMEM187
TXNL4B	0.004528	0.163477	0.022	0.096	1	Undiff-G	TXNL4B
TTC13	0.00453	-0.11126	0.007	0.07	1	Undiff-G	TTC13
PGAM5	0.004538	-0.12757	0	0.056	1	Undiff-G	PGAM5
KIAA1211L	0.004538	-0.13542	0	0.056	1	Undiff-G	KIAA1211L
MTG1	0.004538	-0.1494	0	0.056	1	Undiff-G	MTG1
LPCAT4	0.004542	-0.11729	0.007	0.07	1	Undiff-G	LPCAT4
SERTAD1	0.004546	0.803751	0.154	0.318	1	Undiff-G	SERTAD1
RP11-386G	0.004556	0.349835	0.051	0.145	1	Undiff-G	RP11-386G11.10
NR2E1	0.004571	-0.11308	0	0.056	1	Undiff-G	NR2E1
C17orf96	0.004571	-0.12359	0	0.056	1	Undiff-G	C17orf96
MED18	0.004571	-0.1464	0	0.056	1	Undiff-G	MED18
TTTY15	0.004571	-0.15587	0	0.056	1	Undiff-G	TTTY15
DDX60L	0.004571	-0.18684	0	0.056	1	Undiff-G	DDX60L
ARL17B	0.004604	-0.14397	0	0.056	1	Undiff-G	ARL17B
DCLRE1B	0.004604	-0.14901	0	0.056	1	Undiff-G	DCLRE1B
SUSD1	0.004604	-0.17095	0	0.056	1	Undiff-G	SUSD1
USF1	0.004604	-0.20726	0	0.056	1	Undiff-G	USF1
DOCK5	0.004604	-0.26335	0	0.056	1	Undiff-G	DOCK5
ITM2A	0.004604	-0.27156	0	0.056	1	Undiff-G	ITM2A
MICU3	0.004605	-0.12015	0.015	0.083	1	Undiff-G	MICU3
BDH1	0.004614	-0.16057	0.007	0.07	1	Undiff-G	BDH1
SLC15A3	0.004621	-0.23367	0.007	0.07	1	Undiff-G	SLC15A3
RASGRP1	0.004622	-0.1281	0.007	0.07	1	Undiff-G	RASGRP1
RP11-182L	0.004623	-0.13746	0.007	0.07	1	Undiff-G	RP11-182L21.6
RAI14	0.004626	-0.11361	0.007	0.07	1	Undiff-G	RAI14
SPSB2	0.00463	-0.17906	0.007	0.069	1	Undiff-G	SPSB2
STK32A	0.004637	-0.11638	0	0.056	1	Undiff-G	STK32A
GEMIN4	0.004637	-0.12976	0	0.056	1	Undiff-G	GEMIN4
TMEM255B	0.004637	-0.28323	0	0.056	1	Undiff-G	TMEM255B
NPAT	0.004656	-0.1615	0.015	0.082	1	Undiff-G	NPAT
FAM64A	0.004665	-0.10548	0.007	0.07	1	Undiff-G	FAM64A
ARVCF	0.004671	-0.1258	0	0.056	1	Undiff-G	ARVCF
KCTD9	0.004671	-0.15517	0	0.056	1	Undiff-G	KCTD9
CENPE	0.004671	-0.20795	0	0.056	1	Undiff-G	CENPE
CCER2	0.004671	-0.21139	0	0.056	1	Undiff-G	CCER2
ARHGAP27	0.004671	-0.2314	0	0.056	1	Undiff-G	ARHGAP27
VGF	0.004675	-0.18135	0.007	0.07	1	Undiff-G	VGF
MKL1	0.004681	-0.12616	0.007	0.07	1	Undiff-G	MKL1
PTPN14	0.004701	-0.15666	0.007	0.069	1	Undiff-G	PTPN14

IGHMBP2	0.004705	-0.14077	0	0.056	1	Undiff-G	IGHMBP2
CA8	0.004705	-0.2261	0	0.056	1	Undiff-G	CA8
PROCR	0.00473	-0.10431	0.007	0.07	1	Undiff-G	PROCR
CREBRF	0.004739	0.520088	0.044	0.132	1	Undiff-G	CREBRF
TMEM17	0.004739	-0.1371	0	0.055	1	Undiff-G	TMEM17
RPTOR	0.004739	-0.13934	0	0.055	1	Undiff-G	RPTOR
ZNF747	0.004739	-0.14371	0	0.055	1	Undiff-G	ZNF747
RP11-556E	0.004739	-0.26918	0	0.055	1	Undiff-G	RP11-556E13.1
ELMOD1	0.004743	0.101236	0.015	0.083	1	Undiff-G	ELMOD1
SETDB1	0.004743	-0.13152	0.007	0.07	1	Undiff-G	SETDB1
MGAT5	0.004766	-0.1959	0.007	0.069	1	Undiff-G	MGAT5
MFAP3L	0.004773	-0.13303	0	0.055	1	Undiff-G	MFAP3L
TNR	0.004773	-0.23332	0	0.055	1	Undiff-G	TNR
IL13RA2	0.004773	-0.25919	0	0.055	1	Undiff-G	IL13RA2
SNX19	0.004777	0.193849	0.022	0.095	1	Undiff-G	SNX19
MIS12	0.004777	0.379204	0.051	0.143	1	Undiff-G	MIS12
RANBP6	0.004799	-0.15125	0.007	0.069	1	Undiff-G	RANBP6
COPZ2	0.004808	-0.14109	0	0.055	1	Undiff-G	COPZ2
RNFT2	0.004829	-0.12095	0.007	0.069	1	Undiff-G	RNFT2
ZNF667	0.004836	-0.13726	0.015	0.082	1	Undiff-G	ZNF667
CLCN4	0.004843	-0.16107	0	0.055	1	Undiff-G	CLCN4
ERN1	0.004843	-0.165	0	0.055	1	Undiff-G	ERN1
SS18	0.004856	0.135606	0.015	0.083	1	Undiff-G	SS18
RWDD2B	0.004863	-0.13609	0.007	0.069	1	Undiff-G	RWDD2B
ZFYVE19	0.004869	0.48269	0.029	0.108	1	Undiff-G	ZFYVE19
VRK2	0.004878	-0.21504	0	0.055	1	Undiff-G	VRK2
BRAF	0.004891	-0.15813	0.007	0.069	1	Undiff-G	BRAF
GINS1	0.004913	-0.12782	0	0.055	1	Undiff-G	GINS1
CCDC157	0.004913	-0.13659	0	0.055	1	Undiff-G	CCDC157
WDR76	0.004913	-0.15085	0	0.055	1	Undiff-G	WDR76
CYP4V2	0.004913	-0.17402	0	0.055	1	Undiff-G	CYP4V2
GALT	0.004926	-0.13078	0.015	0.082	1	Undiff-G	GALT
C1orf174	0.004935	0.565546	0.059	0.155	1	Undiff-G	C1orf174
IL16	0.004936	-0.14602	0.007	0.069	1	Undiff-G	IL16
SYNGAP1	0.004949	-0.14082	0	0.055	1	Undiff-G	SYNGAP1
DNAJC12	0.004956	0.103927	0.015	0.082	1	Undiff-G	DNAJC12
WWP1	0.004968	-0.15081	0.007	0.068	1	Undiff-G	WWP1
BACE2	0.004985	-0.16053	0	0.055	1	Undiff-G	BACE2
PELO	0.004989	0.326638	0.029	0.107	1	Undiff-G	PELO
RPP40	0.005	0.31892	0.037	0.119	1	Undiff-G	RPP40
THAP6	0.005	0.107746	0.015	0.082	1	Undiff-G	THAP6
SPIN2B	0.005005	0.293748	0.029	0.107	1	Undiff-G	SPIN2B
MRE11A	0.005021	-0.17392	0	0.055	1	Undiff-G	MRE11A
FAM120B	0.005029	0.139536	0.015	0.082	1	Undiff-G	FAM120B
RWDD2A	0.005032	-0.10156	0.007	0.069	1	Undiff-G	RWDD2A
SNX16	0.005057	-0.16861	0	0.055	1	Undiff-G	SNX16
IQSEC2	0.005057	-0.18474	0	0.055	1	Undiff-G	IQSEC2
RP11-11N9	0.005057	-0.22292	0	0.055	1	Undiff-G	RP11-11N9.4
APOBEC3A	0.005077	0.477002	0.022	0.005	1	Undiff-G	APOBEC3A
PPP2R3B	0.005079	-0.13493	0.007	0.068	1	Undiff-G	PPP2R3B
APOL6	0.005086	-0.14359	0.022	0.094	1	Undiff-G	APOL6

PKP4	0.005094	-0.1457	0	0.055	1	Undiff-G	PKP4
DOCK11	0.005094	-0.21068	0	0.055	1	Undiff-G	DOCK11
RASL11B	0.005094	-0.23672	0	0.055	1	Undiff-G	RASL11B
ATL2	0.005099	-0.1647	0.007	0.069	1	Undiff-G	ATL2
RP11-488C	0.005115	-0.17316	0.007	0.068	1	Undiff-G	RP11-488C13.5
KCNJ16	0.005131	-0.12808	0	0.055	1	Undiff-G	KCNJ16
C17orf51	0.005138	-0.12567	0.007	0.069	1	Undiff-G	C17orf51
COQ3	0.005143	-0.14539	0.007	0.069	1	Undiff-G	COQ3
PCDH10	0.005147	-0.12265	0.007	0.069	1	Undiff-G	PCDH10
PCCA	0.005158	0.100411	0.015	0.082	1	Undiff-G	PCCA
RP5-940J5	0.005165	0.140977	0.066	0.163	1	Undiff-G	RP5-940J5.9
RALGPS1	0.005168	-0.15719	0	0.054	1	Undiff-G	RALGPS1
GANC	0.005168	-0.15902	0	0.054	1	Undiff-G	GANC
HEATR5B	0.005168	-0.18544	0	0.054	1	Undiff-G	HEATR5B
BBS10	0.005205	-0.1374	0	0.054	1	Undiff-G	BBS10
ASTE1	0.005205	-0.15903	0	0.054	1	Undiff-G	ASTE1
GOLGA6L1C	0.00522	0.278986	0.007	0.001	1	Undiff-G	GOLGA6L10
CNN2	0.005226	-0.22262	0.007	0.067	1	Undiff-G	CNN2
PVR	0.005232	-0.11045	0.007	0.068	1	Undiff-G	PVR
HLA-DRA	0.005235	0.823333	0.5	0.635	1	Undiff-G	HLA-DRA
DPY19L4	0.005237	0.271825	0.022	0.094	1	Undiff-G	DPY19L4
NANP	0.005243	-0.13662	0	0.054	1	Undiff-G	NANP
TBC1D9	0.005243	-0.17732	0	0.054	1	Undiff-G	TBC1D9
HS1BP3	0.00525	0.149243	0.022	0.093	1	Undiff-G	HS1BP3
CEP135	0.005254	-0.14925	0.015	0.081	1	Undiff-G	CEP135
TMEM170B	0.005281	-0.13479	0	0.054	1	Undiff-G	TMEM170B
SPOPL	0.005281	-0.15551	0	0.054	1	Undiff-G	SPOPL
STX1A	0.005295	-0.178	0.007	0.068	1	Undiff-G	STX1A
SOWAHC	0.005319	-0.1076	0.007	0.068	1	Undiff-G	SOWAHC
SLC25A51	0.005319	-0.15599	0	0.054	1	Undiff-G	SLC25A51
AC009948.	0.005319	-0.18202	0	0.054	1	Undiff-G	AC009948.5
COBL	0.005332	-0.10881	0.007	0.068	1	Undiff-G	COBL
EXOSC2	0.005333	0.123757	0.022	0.094	1	Undiff-G	EXOSC2
YEATS2	0.005357	-0.11746	0.007	0.068	1	Undiff-G	YEATS2
GBF1	0.005358	-0.15025	0	0.054	1	Undiff-G	GBF1
SLC37A1	0.005358	-0.17064	0	0.054	1	Undiff-G	SLC37A1
LILRB2	0.005358	-0.25242	0	0.054	1	Undiff-G	LILRB2
IL7R	0.005358	-0.2776	0	0.054	1	Undiff-G	IL7R
ZNF583	0.005362	0.293266	0.029	0.106	1	Undiff-G	ZNF583
SPA17	0.005395	0.179821	0.015	0.081	1	Undiff-G	SPA17
INTS2	0.005397	-0.12562	0	0.054	1	Undiff-G	INTS2
TJP2	0.005397	-0.17026	0	0.054	1	Undiff-G	TJP2
PIM2	0.005397	-0.18166	0	0.054	1	Undiff-G	PIM2
ATOH8	0.005397	-0.19691	0	0.054	1	Undiff-G	ATOH8
SHC1	0.005401	0.401623	0.044	0.13	1	Undiff-G	SHC1
MAN2B1	0.005425	-0.16101	0.007	0.068	1	Undiff-G	MAN2B1
AFMID	0.00543	0.240658	0.029	0.106	1	Undiff-G	AFMID
TNFRSF19	0.005437	0.231735	0.029	0.106	1	Undiff-G	TNFRSF19
CYB561	0.00544	-0.14374	0.015	0.08	1	Undiff-G	CYB561
FAM109A	0.005475	-0.13952	0	0.054	1	Undiff-G	FAM109A
PRAM1	0.005475	-0.24455	0	0.054	1	Undiff-G	PRAM1

LCLAT1	0.005515	-0.12094	0	0.054	1	Undiff-G	LCLAT1
KLHL25	0.005515	-0.12329	0	0.054	1	Undiff-G	KLHL25
SHCBP1	0.005515	-0.15955	0	0.054	1	Undiff-G	SHCBP1
ABLIM1	0.005555	-0.13956	0	0.054	1	Undiff-G	ABLIM1
ZNF222	0.005555	-0.15036	0	0.054	1	Undiff-G	ZNF222
SERPINI1	0.005555	-0.19443	0	0.054	1	Undiff-G	SERPINI1
UACA	0.005571	-0.14858	0.007	0.067	1	Undiff-G	UACA
SERINC5	0.00559	-0.14354	0.007	0.067	1	Undiff-G	SERINC5
NAT6	0.005595	-0.14037	0	0.054	1	Undiff-G	NAT6
UHRF2	0.005616	-0.13531	0.007	0.067	1	Undiff-G	UHRF2
ZNF429	0.005619	0.454997	0.044	0.13	1	Undiff-G	ZNF429
ACOX3	0.005628	0.139175	0.022	0.093	1	Undiff-G	ACOX3
STK11IP	0.005636	-0.15122	0	0.053	1	Undiff-G	STK11IP
ADRBK2	0.005636	-0.19387	0	0.053	1	Undiff-G	ADRBK2
ARIH2OS	0.005649	-0.14482	0.007	0.067	1	Undiff-G	ARIH2OS
IZUMO4	0.005675	-0.14582	0.007	0.067	1	Undiff-G	IZUMO4
COL18A1	0.005677	-0.14358	0	0.053	1	Undiff-G	COL18A1
SOCS1	0.005677	-0.22129	0	0.053	1	Undiff-G	SOCS1
LINC0091C	0.005677	-0.14643	0.015	0.08	1	Undiff-G	LINC00910
SEC14L4	0.005677	0.501809	0.015	0.002	1	Undiff-G	SEC14L4
TGFB2	0.005687	-0.10232	0.007	0.067	1	Undiff-G	TGFB2
EIF2AK3	0.005718	-0.15289	0	0.053	1	Undiff-G	EIF2AK3
SLC7A6	0.005725	-0.16538	0.007	0.067	1	Undiff-G	SLC7A6
PAOX	0.005759	-0.1421	0	0.053	1	Undiff-G	PAOX
COG8	0.005765	0.122291	0.022	0.093	1	Undiff-G	COG8
ACOX2	0.005801	-0.11927	0	0.053	1	Undiff-G	ACOX2
CYP2U1	0.005801	-0.12998	0	0.053	1	Undiff-G	CYP2U1
CYTH3	0.005801	-0.15901	0	0.053	1	Undiff-G	CYTH3
PEX12	0.005843	-0.13412	0	0.053	1	Undiff-G	PEX12
RGS18	0.005843	-0.23979	0	0.053	1	Undiff-G	RGS18
PCYOX1L	0.005876	-0.14967	0.007	0.066	1	Undiff-G	PCYOX1L
MDN1	0.005885	-0.14162	0	0.053	1	Undiff-G	MDN1
ZNF345	0.005885	-0.15749	0	0.053	1	Undiff-G	ZNF345
PIGV	0.005885	-0.17582	0	0.053	1	Undiff-G	PIGV
NFATC1	0.005885	-0.19062	0	0.053	1	Undiff-G	NFATC1
GNGT2	0.005885	-0.23369	0	0.053	1	Undiff-G	GNGT2
TSLP	0.005901	0.871243	0.051	0.019	1	Undiff-G	TSLP
FAT1	0.005906	0.111814	0.007	0.067	1	Undiff-G	FAT1
SLC12A4	0.005927	-0.12717	0.007	0.067	1	Undiff-G	SLC12A4
HID1	0.005928	-0.14339	0	0.053	1	Undiff-G	HID1
ZC4H2	0.005928	-0.16651	0	0.053	1	Undiff-G	ZC4H2
TRAF3IP3	0.005928	-0.26122	0	0.053	1	Undiff-G	TRAF3IP3
RAD51AP1	0.005948	0.144678	0.007	0.067	1	Undiff-G	RAD51AP1
MED7	0.005957	0.143372	0.037	0.116	1	Undiff-G	MED7
TEAD4	0.005971	-0.10132	0	0.053	1	Undiff-G	TEAD4
SPECC1L	0.005971	-0.13787	0	0.053	1	Undiff-G	SPECC1L
TYSND1	0.005971	-0.14171	0	0.053	1	Undiff-G	TYSND1
CENPL	0.005971	-0.15264	0	0.053	1	Undiff-G	CENPL
TRIM39	0.005971	-0.15588	0	0.053	1	Undiff-G	TRIM39
LONRF2	0.005992	0.189619	0.022	0.093	1	Undiff-G	LONRF2
ANKRD16	0.006014	-0.10798	0	0.053	1	Undiff-G	ANKRD16

DNAJC6	0.006014	-0.1563	0	0.053	1	Undiff-G	DNAJC6
RNF19B	0.006014	-0.17819	0	0.053	1	Undiff-G	RNF19B
STAT6	0.006014	-0.23698	0	0.053	1	Undiff-G	STAT6
FANCF	0.006042	0.265076	0.022	0.093	1	Undiff-G	FANCF
PRKAA2	0.006058	-0.12136	0	0.053	1	Undiff-G	PRKAA2
POLA2	0.006058	-0.14875	0	0.053	1	Undiff-G	POLA2
BTBD17	0.006058	-0.18471	0	0.053	1	Undiff-G	BTBD17
FLI1	0.006058	-0.23742	0	0.053	1	Undiff-G	FLI1
TMEM185B	0.006101	-0.16167	0	0.052	1	Undiff-G	TMEM185B
CLCN6	0.006116	-0.15684	0.007	0.066	1	Undiff-G	CLCN6
SEC23IP	0.006124	-0.10653	0.015	0.079	1	Undiff-G	SEC23IP
XRN1	0.006143	0.128985	0.022	0.092	1	Undiff-G	XRN1
DOCK1	0.006146	-0.12912	0	0.052	1	Undiff-G	DOCK1
STAT5A	0.006146	-0.18572	0	0.052	1	Undiff-G	STAT5A
FABP5	0.006162	1.141423	0.382	0.392	1	Undiff-G	FABP5
LRRTM3	0.00619	-0.10389	0	0.052	1	Undiff-G	LRRTM3
AADAT	0.00619	-0.11967	0	0.052	1	Undiff-G	AADAT
MORC2-AS1	0.00619	-0.14271	0	0.052	1	Undiff-G	MORC2-AS1
ZBTB18	0.00619	-0.15368	0	0.052	1	Undiff-G	ZBTB18
KCNJ10	0.00619	-0.19236	0	0.052	1	Undiff-G	KCNJ10
TBC1D2B	0.00619	-0.19966	0	0.052	1	Undiff-G	TBC1D2B
TRPM2	0.00619	-0.22555	0	0.052	1	Undiff-G	TRPM2
MAFG-AS1	0.006193	-0.10251	0.007	0.066	1	Undiff-G	MAFG-AS1
AKAP10	0.006198	0.208191	0.022	0.092	1	Undiff-G	AKAP10
RECK	0.006201	-0.13958	0.007	0.066	1	Undiff-G	RECK
IGFBP6	0.006202	-0.10074	0.007	0.066	1	Undiff-G	IGFBP6
OSCP1	0.006208	0.189112	0.007	0.066	1	Undiff-G	OSCP1
CMTR2	0.00621	0.256177	0.051	0.14	1	Undiff-G	CMTR2
CEP44	0.006226	-0.13893	0.007	0.066	1	Undiff-G	CEP44
C1orf159	0.006235	-0.12828	0	0.052	1	Undiff-G	C1orf159
SNIP1	0.006235	-0.13956	0	0.052	1	Undiff-G	SNIP1
ZSCAN5A	0.006235	-0.17151	0	0.052	1	Undiff-G	ZSCAN5A
RASGRP2	0.006235	-0.19607	0	0.052	1	Undiff-G	RASGRP2
SMO	0.00628	-0.10693	0	0.052	1	Undiff-G	SMO
PLCXD1	0.00628	-0.13914	0	0.052	1	Undiff-G	PLCXD1
ZIK1	0.00628	-0.15447	0	0.052	1	Undiff-G	ZIK1
ACTA2	0.00628	-0.1939	0	0.052	1	Undiff-G	ACTA2
GRASP	0.00628	-0.24698	0	0.052	1	Undiff-G	GRASP
ADAMTS6	0.00629	-0.10538	0.007	0.066	1	Undiff-G	ADAMTS6
ZBED6	0.006316	-0.10383	0.015	0.079	1	Undiff-G	ZBED6
RP11-796E	0.006336	-0.21488	0.007	0.066	1	Undiff-G	RP11-796E2.4
DOK3	0.006337	-0.13595	0.007	0.066	1	Undiff-G	DOK3
USP31	0.006371	-0.1399	0	0.052	1	Undiff-G	USP31
ARHGEF25	0.00638	-0.12433	0.007	0.066	1	Undiff-G	ARHGEF25
NOL4	0.006385	-0.14116	0.007	0.066	1	Undiff-G	NOL4
RFX1	0.006392	0.152107	0.022	0.091	1	Undiff-G	RFX1
GSTP1	0.006394	0.541825	0.404	0.827	1	Undiff-G	GSTP1
VAV3	0.006401	-0.12273	0.007	0.066	1	Undiff-G	VAV3
EXOC2	0.006442	-0.13747	0.007	0.066	1	Undiff-G	EXOC2
HES1	0.006462	0.527664	0.088	0.198	1	Undiff-G	HES1
ALDH4A1	0.006464	-0.1196	0	0.052	1	Undiff-G	ALDH4A1

PDE6B	0.006464	-0.16961	0	0.052	1	Undiff-G	PDE6B
AP1G2	0.006464	-0.21032	0	0.052	1	Undiff-G	AP1G2
PI4K2A	0.006476	-0.16053	0.007	0.066	1	Undiff-G	PI4K2A
PTX3	0.006484	0.156955	0.037	0.116	1	Undiff-G	PTX3
HAUS5	0.006495	-0.1564	0.007	0.065	1	Undiff-G	HAUS5
ACBD4	0.006511	-0.13343	0	0.052	1	Undiff-G	ACBD4
FAM53B	0.006511	-0.19248	0	0.052	1	Undiff-G	FAM53B
PLA2G7	0.006511	-0.21375	0	0.052	1	Undiff-G	PLA2G7
LIAS	0.006511	0.25337	0.029	0.104	1	Undiff-G	LIAS
GPRASP2	0.00653	-0.12922	0.007	0.066	1	Undiff-G	GPRASP2
LRP5	0.006558	-0.11314	0	0.052	1	Undiff-G	LRP5
FAM184A	0.006558	-0.11872	0	0.052	1	Undiff-G	FAM184A
EFNA2	0.006558	-0.12085	0	0.052	1	Undiff-G	EFNA2
FLNB	0.006558	-0.13499	0	0.052	1	Undiff-G	FLNB
ST3GAL1	0.006562	0.160172	0.029	0.103	1	Undiff-G	ST3GAL1
TSPYL4	0.006579	0.330545	0.029	0.104	1	Undiff-G	TSPYL4
EMP3	0.006599	0.816643	0.228	0.451	1	Undiff-G	EMP3
RAB27A	0.006605	0.180853	0.015	0.079	1	Undiff-G	RAB27A
INPP5E	0.006605	-0.12315	0	0.052	1	Undiff-G	INPP5E
MTRF1	0.006605	-0.13577	0	0.052	1	Undiff-G	MTRF1
CA14	0.006605	-0.19577	0	0.052	1	Undiff-G	CA14
FAM206A	0.00661	-0.14451	0.007	0.065	1	Undiff-G	FAM206A
ANKRD22	0.006617	-0.16508	0.007	0.066	1	Undiff-G	ANKRD22
MSL2	0.006659	-0.14477	0.007	0.065	1	Undiff-G	MSL2
AREG	0.006661	-0.14288	0.007	0.065	1	Undiff-G	AREG
PDE8A	0.006681	-0.1436	0.022	0.09	1	Undiff-G	PDE8A
ARAP3	0.006689	-0.11629	0.007	0.065	1	Undiff-G	ARAP3
PHF13	0.006689	-0.12659	0.007	0.065	1	Undiff-G	PHF13
SIN3A	0.006705	0.219888	0.022	0.09	1	Undiff-G	SIN3A
NKX2-5	0.006739	-0.18115	0.007	0.065	1	Undiff-G	NKX2-5
FAM173B	0.00674	-0.15073	0.007	0.065	1	Undiff-G	FAM173B
MORN4	0.00675	-0.12645	0	0.051	1	Undiff-G	MORN4
PSMG3-AS1	0.00675	-0.13967	0	0.051	1	Undiff-G	PSMG3-AS1
ZDHHC2	0.006793	-0.18873	0.007	0.064	1	Undiff-G	ZDHHC2
TMTC2	0.006799	-0.1699	0	0.051	1	Undiff-G	TMTC2
TTF2	0.006832	-0.11253	0.007	0.065	1	Undiff-G	TTF2
RP11-347F	0.006832	-0.25803	0.007	0.065	1	Undiff-G	RP11-347P5.1
FSD1L	0.006848	-0.14026	0	0.051	1	Undiff-G	FSD1L
AGTPBP1	0.006848	-0.16488	0	0.051	1	Undiff-G	AGTPBP1
SORBS2	0.006848	-0.17897	0	0.051	1	Undiff-G	SORBS2
GIMAP5	0.006848	-0.23042	0	0.051	1	Undiff-G	GIMAP5
ZXDC	0.006893	-0.12006	0.007	0.065	1	Undiff-G	ZXDC
LTBP1	0.006898	-0.11044	0	0.051	1	Undiff-G	LTBP1
TLDC1	0.006898	-0.15146	0	0.051	1	Undiff-G	TLDC1
PHLDA2	0.006898	-0.15834	0	0.051	1	Undiff-G	PHLDA2
ATE1	0.006898	-0.16605	0	0.051	1	Undiff-G	ATE1
FGF9	0.006898	-0.18461	0	0.051	1	Undiff-G	FGF9
PLEKHG2	0.006936	-0.11926	0.007	0.065	1	Undiff-G	PLEKHG2
CDR2L	0.006943	-0.13414	0.007	0.064	1	Undiff-G	CDR2L
ZNF248	0.006948	-0.13286	0	0.051	1	Undiff-G	ZNF248
ZNF615	0.006948	-0.13508	0	0.051	1	Undiff-G	ZNF615

KLHL6	0.006948	-0.25143	0	0.051	1	Undiff-G	KLHL6
SLC45A1	0.006998	-0.12387	0	0.051	1	Undiff-G	SLC45A1
TMEM38A	0.006998	-0.14889	0	0.051	1	Undiff-G	TMEM38A
SYT6	0.006998	-0.16791	0	0.051	1	Undiff-G	SYT6
EXOC6	0.007012	-0.18242	0.007	0.064	1	Undiff-G	EXOC6
PDLIM3	0.007027	0.297495	0.037	0.115	1	Undiff-G	PDLIM3
SLC2A10	0.00703	-0.10063	0.007	0.065	1	Undiff-G	SLC2A10
VAV2	0.007049	-0.12045	0	0.051	1	Undiff-G	VAV2
RP11-545E	0.007049	-0.1408	0	0.051	1	Undiff-G	RP11-545E17.3
CTSK	0.007049	-0.16641	0	0.051	1	Undiff-G	CTSK
CDC23	0.007054	0.36653	0.044	0.126	1	Undiff-G	CDC23
CLDN15	0.007077	-0.17556	0.007	0.064	1	Undiff-G	CLDN15
NKIRAS1	0.007085	0.201164	0.029	0.103	1	Undiff-G	NKIRAS1
TPCN1	0.00709	0.120615	0.022	0.09	1	Undiff-G	TPCN1
SEPP1	0.007099	0.485535	0.169	0.33	1	Undiff-G	SEPP1
POC5	0.0071	-0.15321	0	0.051	1	Undiff-G	POC5
ARPP21	0.0071	-0.16224	0	0.051	1	Undiff-G	ARPP21
ACP6	0.007139	-0.14593	0.007	0.064	1	Undiff-G	ACP6
COX4I1	0.007142	0.506154	0.426	0.916	1	Undiff-G	COX4I1
SAP130	0.007151	-0.12393	0	0.051	1	Undiff-G	SAP130
PYCRL	0.007151	-0.13235	0	0.051	1	Undiff-G	PYCRL
HIRA	0.007203	-0.12854	0	0.05	1	Undiff-G	HIRA
PIK3R2	0.007203	-0.14169	0	0.05	1	Undiff-G	PIK3R2
ATP11C	0.007203	-0.14403	0	0.05	1	Undiff-G	ATP11C
GBGT1	0.007203	-0.19531	0	0.05	1	Undiff-G	GBGT1
STEAP3	0.007204	0.681449	0.066	0.163	1	Undiff-G	STEAP3
RAB13	0.007217	0.950898	0.191	0.374	1	Undiff-G	RAB13
GTF2E1	0.007255	-0.14329	0	0.05	1	Undiff-G	GTF2E1
SEC22A	0.007255	0.426944	0.029	0.103	1	Undiff-G	SEC22A
ZNF445	0.007279	-0.13411	0.007	0.064	1	Undiff-G	ZNF445
UBIAD1	0.007283	0.337452	0.029	0.102	1	Undiff-G	UBIAD1
ENTPD5	0.007307	-0.12299	0	0.05	1	Undiff-G	ENTPD5
NAT8L	0.007307	-0.12447	0	0.05	1	Undiff-G	NAT8L
HELLS	0.007308	-0.15274	0.007	0.064	1	Undiff-G	HELLS
BMP1	0.007358	-0.11032	0.007	0.064	1	Undiff-G	BMP1
URB1	0.00736	-0.12406	0	0.05	1	Undiff-G	URB1
ZCCHC14	0.00736	-0.14468	0	0.05	1	Undiff-G	ZCCHC14
FERMT1	0.00736	-0.18977	0	0.05	1	Undiff-G	FERMT1
DLEU7	0.00736	-0.23213	0	0.05	1	Undiff-G	DLEU7
CARF	0.007382	-0.10525	0.015	0.077	1	Undiff-G	CARF
GPX4	0.007404	0.574116	0.404	0.847	1	Undiff-G	GPX4
ZNF213	0.007414	-0.11507	0	0.05	1	Undiff-G	ZNF213
ECT2	0.007414	-0.14235	0	0.05	1	Undiff-G	ECT2
GIN1	0.007414	-0.14723	0	0.05	1	Undiff-G	GIN1
ZNF184	0.007414	-0.15716	0	0.05	1	Undiff-G	ZNF184
ANKRD13B	0.007467	-0.12468	0	0.05	1	Undiff-G	ANKRD13B
KIAA1524	0.007467	-0.13681	0	0.05	1	Undiff-G	KIAA1524
SULT1A1	0.007467	-0.22342	0	0.05	1	Undiff-G	SULT1A1
UGT8	0.007467	-0.25028	0	0.05	1	Undiff-G	UGT8
LRRN3	0.007488	0.141757	0.037	0.114	1	Undiff-G	LRRN3
HOXD11	0.007502	0.158806	0.015	0.077	1	Undiff-G	HOXD11

TRMT12	0.007521	-0.13813	0	0.05	1	Undiff-G	TRMT12
CMKLR1	0.007521	-0.21371	0	0.05	1	Undiff-G	CMKLR1
GCHFR	0.007538	0.149386	0.015	0.077	1	Undiff-G	GCHFR
ZSCAN16	0.007542	-0.15589	0.007	0.064	1	Undiff-G	ZSCAN16
FITM2	0.007576	-0.11687	0	0.05	1	Undiff-G	FITM2
RBL1	0.007576	-0.12307	0	0.05	1	Undiff-G	RBL1
SLC9A8	0.007576	-0.14741	0	0.05	1	Undiff-G	SLC9A8
SLC38A9	0.007599	-0.15706	0.007	0.064	1	Undiff-G	SLC38A9
HLA-DRB5	0.007612	0.431769	0.213	0.402	1	Undiff-G	HLA-DRB5
SLC6A6	0.007613	-0.15043	0.007	0.064	1	Undiff-G	SLC6A6
BCR	0.007616	-0.14616	0.007	0.064	1	Undiff-G	BCR
RAB23	0.007631	-0.10845	0	0.05	1	Undiff-G	RAB23
FIZ1	0.007631	-0.11753	0	0.05	1	Undiff-G	FIZ1
RAB42	0.007631	-0.15987	0	0.05	1	Undiff-G	RAB42
SEMA4A	0.007631	-0.18597	0	0.05	1	Undiff-G	SEMA4A
ZC3H12A	0.007639	0.143594	0.015	0.077	1	Undiff-G	ZC3H12A
HEATR1	0.007657	-0.14573	0.007	0.063	1	Undiff-G	HEATR1
NCAPD2	0.007678	-0.11246	0.007	0.064	1	Undiff-G	NCAPD2
PLEKHA8	0.007686	0.106159	0.007	0.064	1	Undiff-G	PLEKHA8
JRKL	0.007691	0.157206	0.015	0.077	1	Undiff-G	JRKL
HSPA5	0.007705	0.898998	0.331	0.661	1	Undiff-G	HSPA5
STOML1	0.007706	0.331108	0.029	0.102	1	Undiff-G	STOML1
TRAF3	0.007714	-0.11762	0.007	0.064	1	Undiff-G	TRAF3
ATAD5	0.007724	-0.13084	0.015	0.076	1	Undiff-G	ATAD5
DIRAS2	0.007727	-0.12506	0.007	0.064	1	Undiff-G	DIRAS2
XPO4	0.007733	-0.1263	0.007	0.064	1	Undiff-G	XPO4
ADAM23	0.007742	-0.10877	0	0.05	1	Undiff-G	ADAM23
RHPN1	0.007742	-0.11294	0	0.05	1	Undiff-G	RHPN1
ANKRD50	0.007742	-0.12471	0	0.05	1	Undiff-G	ANKRD50
ARSB	0.007742	-0.13082	0	0.05	1	Undiff-G	ARSB
CENPA	0.007742	-0.17386	0	0.05	1	Undiff-G	CENPA
BCL2	0.007742	-0.19556	0	0.05	1	Undiff-G	BCL2
PNPLA4	0.007752	-0.23758	0.007	0.063	1	Undiff-G	PNPLA4
YJEFN3	0.007773	-0.10241	0.007	0.064	1	Undiff-G	YJEFN3
MXD3	0.007797	-0.15701	0.007	0.063	1	Undiff-G	MXD3
CCDC138	0.007798	-0.14857	0	0.05	1	Undiff-G	CCDC138
FBXO30	0.007807	-0.11104	0.007	0.064	1	Undiff-G	FBXO30
DNALI1	0.00781	0.454031	0.037	0.114	1	Undiff-G	DNALI1
MED23	0.007827	-0.15588	0.007	0.063	1	Undiff-G	MED23
ELMOD3	0.007831	0.337965	0.051	0.136	1	Undiff-G	ELMOD3
C16orf46	0.007854	-0.11286	0	0.049	1	Undiff-G	C16orf46
ITPKC	0.007854	-0.1193	0	0.049	1	Undiff-G	ITPKC
EPB41L5	0.007854	-0.13336	0	0.049	1	Undiff-G	EPB41L5
SPC24	0.007854	-0.15191	0	0.049	1	Undiff-G	SPC24
MIPEP	0.007859	-0.12572	0.007	0.063	1	Undiff-G	MIPEP
ATAD2	0.007911	-0.10854	0.015	0.076	1	Undiff-G	ATAD2
ZMYM1	0.007911	-0.11571	0	0.049	1	Undiff-G	ZMYM1
PRR12	0.007911	-0.11945	0	0.049	1	Undiff-G	PRR12
LPAR1	0.007911	-0.15894	0	0.049	1	Undiff-G	LPAR1
WDSUB1	0.007913	0.177732	0.015	0.077	1	Undiff-G	WDSUB1
FAM73B	0.007918	-0.16071	0.007	0.063	1	Undiff-G	FAM73B

IGDCC3	0.007924	-0.11146	0.007	0.063	1	Undiff-G	IGDCC3
UTP20	0.00794	-0.11397	0.007	0.063	1	Undiff-G	UTP20
LHFP	0.007965	0.750832	0.11	0.233	1	Undiff-G	LHFP
TAF4	0.007968	-0.11441	0	0.049	1	Undiff-G	TAF4
RNF182	0.007968	-0.12697	0	0.049	1	Undiff-G	RNF182
AURKB	0.007968	-0.18863	0	0.049	1	Undiff-G	AURKB
FAM212A	0.007968	-0.23648	0	0.049	1	Undiff-G	FAM212A
SH3D19	0.007999	-0.10544	0.007	0.063	1	Undiff-G	SH3D19
IL11RA	0.008008	-0.12025	0.007	0.063	1	Undiff-G	IL11RA
PARP3	0.008026	-0.13666	0	0.049	1	Undiff-G	PARP3
PLXNA2	0.008026	-0.1466	0	0.049	1	Undiff-G	PLXNA2
STARD13	0.008026	-0.15402	0	0.049	1	Undiff-G	STARD13
APOLD1	0.008026	-0.15745	0	0.049	1	Undiff-G	APOLD1
COL5A2	0.008045	-0.10305	0.007	0.063	1	Undiff-G	COL5A2
SMURF1	0.008084	-0.13588	0	0.049	1	Undiff-G	SMURF1
IDUA	0.008084	-0.1365	0	0.049	1	Undiff-G	IDUA
CENPP	0.008084	-0.15111	0	0.049	1	Undiff-G	CENPP
FAM114A1	0.008106	0.22328	0.037	0.113	1	Undiff-G	FAM114A1
ZFP14	0.008141	0.231352	0.037	0.112	1	Undiff-G	ZFP14
UNC13D	0.008142	-0.17896	0	0.049	1	Undiff-G	UNC13D
FABP3	0.008142	-0.23665	0	0.049	1	Undiff-G	FABP3
PYROXD1	0.008144	-0.16208	0.007	0.063	1	Undiff-G	PYROXD1
ATG16L2	0.008195	-0.15083	0.022	0.088	1	Undiff-G	ATG16L2
CCDC74A	0.008201	-0.13004	0	0.049	1	Undiff-G	CCDC74A
CRY2	0.008201	-0.14995	0	0.049	1	Undiff-G	CRY2
STK32B	0.008259	-0.12877	0.007	0.062	1	Undiff-G	STK32B
EPG5	0.008261	-0.12838	0	0.049	1	Undiff-G	EPG5
ZWILCH	0.008308	0.192371	0.015	0.076	1	Undiff-G	ZWILCH
VLDLR	0.008321	-0.11158	0	0.049	1	Undiff-G	VLDLR
HRASLS	0.008321	-0.11647	0	0.049	1	Undiff-G	HRASLS
RP11-159D	0.008321	-0.11668	0	0.049	1	Undiff-G	RP11-159D12.2
BCL2L11	0.008321	-0.17648	0	0.049	1	Undiff-G	BCL2L11
CHEK2	0.008321	-0.19203	0	0.049	1	Undiff-G	CHEK2
CH25H	0.008338	0.378406	0.059	0.148	1	Undiff-G	CH25H
LIG3	0.008354	-0.12364	0.007	0.062	1	Undiff-G	LIG3
LEMD3	0.00838	-0.11265	0.007	0.063	1	Undiff-G	LEMD3
DGKG	0.008381	-0.13303	0	0.049	1	Undiff-G	DGKG
GPD1	0.008381	-0.21684	0	0.049	1	Undiff-G	GPD1
RNGTT	0.008402	0.137681	0.015	0.076	1	Undiff-G	RNGTT
CNKSR2	0.008418	-0.11486	0.007	0.062	1	Undiff-G	CNKSR2
WDR44	0.008441	-0.13131	0	0.049	1	Undiff-G	WDR44
ANKRD9	0.008447	0.154243	0.022	0.088	1	Undiff-G	ANKRD9
SDC1	0.008503	-0.10529	0	0.049	1	Undiff-G	SDC1
TMEM246	0.008503	-0.16174	0	0.049	1	Undiff-G	TMEM246
TBC1D4	0.008503	-0.19076	0	0.049	1	Undiff-G	TBC1D4
PGAM2	0.008503	-0.22647	0	0.049	1	Undiff-G	PGAM2
TRO	0.008525	-0.11143	0.007	0.062	1	Undiff-G	TRO
CLEC4A	0.008549	-0.24134	0.007	0.062	1	Undiff-G	CLEC4A
ZNF550	0.008564	-0.14899	0	0.048	1	Undiff-G	ZNF550
RP11-480C	0.008564	-0.27515	0	0.048	1	Undiff-G	RP11-480C22.1
PLIN2	0.00858	0.680034	0.176	0.344	1	Undiff-G	PLIN2

N4BP2L1	0.008595	0.309123	0.066	0.158	1	Undiff-G	N4BP2L1
ADCYAP1R1	0.008626	-0.13079	0	0.048	1	Undiff-G	ADCYAP1R1
C19orf38	0.008626	-0.23292	0	0.048	1	Undiff-G	C19orf38
SPC25	0.008632	-0.1709	0.007	0.062	1	Undiff-G	SPC25
ORC5	0.008672	0.144221	0.029	0.1	1	Undiff-G	ORC5
TRAM2	0.008689	-0.10667	0	0.048	1	Undiff-G	TRAM2
INPP5F	0.008689	-0.12809	0	0.048	1	Undiff-G	INPP5F
TTC26	0.008689	-0.14851	0	0.048	1	Undiff-G	TTC26
DENND4C	0.00871	0.222976	0.022	0.088	1	Undiff-G	DENND4C
HAUS4	0.008737	-0.22658	0.007	0.062	1	Undiff-G	HAUS4
ZNF853	0.008751	-0.10379	0	0.048	1	Undiff-G	ZNF853
LRP11	0.008751	-0.10758	0	0.048	1	Undiff-G	LRP11
WWC3	0.008751	-0.12303	0	0.048	1	Undiff-G	WWC3
ZNF614	0.008751	-0.12377	0	0.048	1	Undiff-G	ZNF614
ANKRD6	0.008751	-0.12885	0	0.048	1	Undiff-G	ANKRD6
PPIL6	0.008751	-0.13767	0	0.048	1	Undiff-G	PPIL6
IGLON5	0.008751	-0.16764	0	0.048	1	Undiff-G	IGLON5
ACP5	0.008751	-0.21544	0	0.048	1	Undiff-G	ACP5
HSPA6	0.008809	0.208953	0.029	0.1	1	Undiff-G	HSPA6
MED12L	0.008811	-0.11123	0.007	0.062	1	Undiff-G	MED12L
CTPS2	0.008815	-0.12793	0	0.048	1	Undiff-G	CTPS2
PLCD1	0.008815	-0.15186	0	0.048	1	Undiff-G	PLCD1
LRRC16A	0.008859	-0.10525	0.015	0.075	1	Undiff-G	LRRC16A
SLC16A9	0.008879	-0.10694	0	0.048	1	Undiff-G	SLC16A9
IKZF4	0.008879	-0.11088	0	0.048	1	Undiff-G	IKZF4
ZNF618	0.008879	-0.16181	0	0.048	1	Undiff-G	ZNF618
GPATCH1	0.008896	0.143388	0.015	0.075	1	Undiff-G	GPATCH1
AC005477.	0.008899	0.16933	0.007	0.001	1	Undiff-G	AC005477.1
PTGS2	0.00892	-0.13757	0.007	0.062	1	Undiff-G	PTGS2
C9orf40	0.008943	-0.10111	0	0.048	1	Undiff-G	C9orf40
TRMT2B	0.008943	-0.13404	0	0.048	1	Undiff-G	TRMT2B
FAM19A5	0.008943	-0.14018	0	0.048	1	Undiff-G	FAM19A5
USP54	0.008943	-0.1727	0	0.048	1	Undiff-G	USP54
CASP8	0.008943	-0.19009	0	0.048	1	Undiff-G	CASP8
CLECL1	0.008943	-0.19481	0	0.048	1	Undiff-G	CLECL1
SLC15A4	0.008948	-0.11572	0.015	0.075	1	Undiff-G	SLC15A4
CRTC2	0.008967	0.116222	0.015	0.075	1	Undiff-G	CRTC2
ARHGEF10L	0.008979	-0.13595	0.007	0.062	1	Undiff-G	ARHGEF10L
PPM1L	0.009008	-0.11981	0	0.048	1	Undiff-G	PPM1L
SHQ1	0.009008	-0.12395	0	0.048	1	Undiff-G	SHQ1
IFIH1	0.009008	-0.13299	0	0.048	1	Undiff-G	IFIH1
PIK3R5	0.009008	-0.20082	0	0.048	1	Undiff-G	PIK3R5
NRDE2	0.009017	0.126331	0.029	0.099	1	Undiff-G	NRDE2
METTL4	0.009018	0.140508	0.015	0.075	1	Undiff-G	METTL4
ZNF611	0.009044	-0.12083	0.007	0.062	1	Undiff-G	ZNF611
MFSD2A	0.009056	0.218601	0.029	0.099	1	Undiff-G	MFSD2A
ALKBH8	0.009073	-0.10273	0	0.048	1	Undiff-G	ALKBH8
ZNF454	0.009073	-0.11892	0	0.048	1	Undiff-G	ZNF454
EP400NL	0.009073	-0.12822	0	0.048	1	Undiff-G	EP400NL
CCDC149	0.009073	-0.1332	0	0.048	1	Undiff-G	CCDC149
WDR19	0.009073	-0.13746	0	0.048	1	Undiff-G	WDR19

NFRKB	0.00908	0.315075	0.029	0.1	1	Undiff-G	NFRKB
TMEM25	0.009093	0.416934	0.044	0.123	1	Undiff-G	TMEM25
TMEM234	0.009121	0.32685	0.022	0.087	1	Undiff-G	TMEM234
MDC1	0.00913	-0.11587	0.007	0.061	1	Undiff-G	MDC1
PDZD4	0.009138	-0.13204	0	0.048	1	Undiff-G	PDZD4
WDR53	0.009138	-0.13578	0	0.048	1	Undiff-G	WDR53
TMEM52B	0.009138	-0.23	0	0.048	1	Undiff-G	TMEM52B
PLEKHM1	0.009149	0.128186	0.015	0.075	1	Undiff-G	PLEKHM1
PPP5D1	0.009205	-0.1423	0	0.048	1	Undiff-G	PPP5D1
POMC	0.009205	-0.21341	0	0.048	1	Undiff-G	POMC
CCL20	0.009206	-0.21836	0.007	0.062	1	Undiff-G	CCL20
ZNF599	0.009211	0.127791	0.015	0.075	1	Undiff-G	ZNF599
AP001372.	0.009271	-0.12168	0	0.047	1	Undiff-G	AP001372.2
GPD1L	0.009271	-0.12499	0	0.047	1	Undiff-G	GPD1L
SUOX	0.009271	-0.1367	0	0.047	1	Undiff-G	SUOX
ZNF713	0.009271	-0.13851	0	0.047	1	Undiff-G	ZNF713
ZNF736	0.009271	-0.1396	0	0.047	1	Undiff-G	ZNF736
ZGLP1	0.009271	-0.16195	0	0.047	1	Undiff-G	ZGLP1
UAP1L1	0.009272	-0.10263	0.007	0.061	1	Undiff-G	UAP1L1
GABPB2	0.009277	-0.10091	0.007	0.061	1	Undiff-G	GABPB2
COMTD1	0.009306	-0.16999	0.007	0.061	1	Undiff-G	COMTD1
DENND6A	0.009317	-0.15571	0.007	0.061	1	Undiff-G	DENND6A
HSPA4L	0.009318	-0.15906	0.015	0.074	1	Undiff-G	HSPA4L
PRR5	0.009338	-0.18061	0	0.047	1	Undiff-G	PRR5
QPCT	0.009338	-0.19353	0	0.047	1	Undiff-G	QPCT
CAB39L	0.009341	0.16417	0.015	0.074	1	Undiff-G	CAB39L
TRIM56	0.009344	0.218101	0.015	0.074	1	Undiff-G	TRIM56
GNAZ	0.009377	-0.13425	0.007	0.061	1	Undiff-G	GNAZ
IQCG	0.009381	0.444029	0.044	0.123	1	Undiff-G	IQCG
PARG	0.009406	-0.11442	0	0.047	1	Undiff-G	PARG
CUL9	0.009406	-0.12231	0	0.047	1	Undiff-G	CUL9
TRMT10A	0.009406	-0.13429	0	0.047	1	Undiff-G	TRMT10A
ASPM	0.009406	-0.17196	0	0.047	1	Undiff-G	ASPM
FGD2	0.00943	-0.16554	0.007	0.061	1	Undiff-G	FGD2
RP11-127E	0.009474	-0.11805	0	0.047	1	Undiff-G	RP11-127B20.2
HDAC4	0.009474	-0.14773	0	0.047	1	Undiff-G	HDAC4
POLR3C	0.009477	0.119295	0.022	0.087	1	Undiff-G	POLR3C
PPP2R3A	0.009521	-0.11125	0.007	0.061	1	Undiff-G	PPP2R3A
USP24	0.009526	-0.12552	0.015	0.074	1	Undiff-G	USP24
GSR	0.009612	-0.11513	0	0.047	1	Undiff-G	GSR
FIGNL1	0.009612	-0.1437	0	0.047	1	Undiff-G	FIGNL1
ADCK1	0.009612	-0.14719	0	0.047	1	Undiff-G	ADCK1
WDR81	0.009612	-0.14843	0	0.047	1	Undiff-G	WDR81
SMARCD2	0.009627	0.165103	0.022	0.086	1	Undiff-G	SMARCD2
FOSL1	0.009681	-0.11505	0	0.047	1	Undiff-G	FOSL1
ABCD1	0.009681	-0.14544	0	0.047	1	Undiff-G	ABCD1
PAK7	0.009681	-0.15637	0	0.047	1	Undiff-G	PAK7
TMEM86A	0.009681	-0.18564	0	0.047	1	Undiff-G	TMEM86A
CEP112	0.009751	-0.10019	0	0.047	1	Undiff-G	CEP112
TROAP	0.009751	-0.14512	0	0.047	1	Undiff-G	TROAP
ATAD3B	0.009764	-0.12336	0.007	0.061	1	Undiff-G	ATAD3B

DSCC1	0.009818	-0.13292	0.007	0.06	1	Undiff-G	DSCC1
EPHB3	0.009822	-0.11567	0	0.047	1	Undiff-G	EPHB3
FANCI	0.009822	-0.1271	0	0.047	1	Undiff-G	FANCI
TCP11L1	0.009822	-0.13991	0	0.047	1	Undiff-G	TCP11L1
RP11-1275	0.009825	-0.12035	0.007	0.061	1	Undiff-G	RP11-1275H24.1
TMCC3	0.009854	-0.11608	0.015	0.074	1	Undiff-G	TMCC3
ANK3	0.009884	-0.16743	0.007	0.061	1	Undiff-G	ANK3
FHL2	0.009893	-0.1412	0	0.047	1	Undiff-G	FHL2
PPP1R12B	0.009945	-0.15464	0.007	0.06	1	Undiff-G	PPP1R12B
DOK4	0.009965	-0.11957	0	0.047	1	Undiff-G	DOK4
TES	0.009965	-0.2258	0	0.047	1	Undiff-G	TES
SAA1.1	0	1.945196	0.433	0.058	0	AC-G	SAA1
LTF.1	0	1.726356	0.496	0.084	0	AC-G	LTF
SLPI.1	0	1.723881	0.488	0.084	0	AC-G	SLPI
MGST1.1	0	1.716853	0.577	0.06	0	AC-G	MGST1
CHI3L2.1	0	1.710796	0.592	0.1	0	AC-G	CHI3L2
MGP.1	0	1.675959	0.423	0.041	0	AC-G	MGP
C8orf4	0	1.552945	0.614	0.153	0	AC-G	C8orf4
SAA2.1	0	1.534099	0.23	0.017	0	AC-G	SAA2
EFEMP1.1	0	1.521288	0.577	0.152	0	AC-G	EFEMP1
C1R.1	0	1.494219	0.582	0.144	0	AC-G	C1R
CHI3L1.1	0	1.471343	0.845	0.462	0	AC-G	CHI3L1
CAV1.1	0	1.447132	0.489	0.056	0	AC-G	CAV1
C1S	0	1.392277	0.483	0.099	0	AC-G	C1S
SERPING1.	0	1.344355	0.548	0.173	0	AC-G	SERPING1
RARRES2	0	1.285152	0.461	0.089	0	AC-G	RARRES2
CRYAB.1	0	1.216062	0.831	0.435	0	AC-G	CRYAB
MT1X.1	0	1.166703	0.844	0.525	0	AC-G	MT1X
LGALS3.1	0	1.123973	0.786	0.421	0	AC-G	LGALS3
TIMP1.1	0	1.068728	0.858	0.574	0	AC-G	TIMP1
CLU.1	0	1.05073	0.969	0.746	0	AC-G	CLU
SEC61G.1	0	0.944665	0.959	0.768	0	AC-G	SEC61G
MT2A.1	0	0.923151	0.948	0.752	0	AC-G	MT2A
GAPDH.1	0	0.550311	0.986	0.985	0	AC-G	GAPDH
FTL.1	0	0.510835	0.99	0.995	0	AC-G	FTL
CYR61	########	1.101631	0.433	0.116	########	AC-G	CYR61
ACTG1.1	########	0.556899	0.975	0.943	########	AC-G	ACTG1
S100A6.1	########	0.939019	0.84	0.605	########	AC-G	S100A6
BGN	########	0.726882	0.246	0.029	########	AC-G	BGN
CADM3	########	0.861249	0.316	0.058	########	AC-G	CADM3
SOD2	########	1.039437	0.754	0.493	########	AC-G	SOD2
ACTB.1	########	0.487173	0.992	0.992	########	AC-G	ACTB
MT1E.1	########	0.974895	0.645	0.328	########	AC-G	MT1E
NNMT	########	1.190439	0.392	0.112	########	AC-G	NNMT
IL13RA2.1	########	0.713363	0.212	0.024	########	AC-G	IL13RA2
SPOCD1	########	1.096368	0.474	0.167	########	AC-G	SPOCD1
CA9	########	0.542641	0.175	0.014	########	AC-G	CA9
OCIAD2	########	1.179359	0.645	0.337	########	AC-G	OCIAD2
PRSS23	########	0.761303	0.257	0.043	########	AC-G	PRSS23
CD44	########	0.972719	0.64	0.332	########	AC-G	CD44
NTRK2.1	########	0.815746	0.333	0.079	########	AC-G	NTRK2

S100A16	########	0.94998	0.586	0.252	########	AC-G	S100A16
NAMPT.1	########	0.964939	0.683	0.389	########	AC-G	NAMPT
MALAT1.1	########	0.403988	0.996	0.995	########	AC-G	MALAT1
S100A10.1	########	0.838241	0.741	0.506	########	AC-G	S100A10
PIFO	########	0.65452	0.193	0.021	########	AC-G	PIFO
ANXA1	########	0.859717	0.692	0.355	########	AC-G	ANXA1
SYTL2	########	0.678955	0.249	0.042	########	AC-G	SYTL2
C10orf10.	########	0.966883	0.275	0.055	########	AC-G	C10orf10
MARCKS.1	########	-0.78132	0.3	0.655	########	AC-G	MARCKS
YWHAH.1	########	-0.79965	0.245	0.627	########	AC-G	YWHAH
PKM.1	########	0.639652	0.871	0.751	########	AC-G	PKM
GAP43.1	########	0.964588	0.713	0.418	########	AC-G	GAP43
HSPB8	########	0.543558	0.165	0.015	########	AC-G	HSPB8
SPARC	########	0.981813	0.615	0.339	########	AC-G	SPARC
CSRP2	########	1.134832	0.549	0.253	########	AC-G	CSRP2
CP	########	0.512115	0.132	0.008	########	AC-G	CP
TAGLN	########	1.098981	0.228	0.04	########	AC-G	TAGLN
LDHA.1	########	0.636357	0.854	0.749	########	AC-G	LDHA
VSTM2A	########	0.84492	0.296	0.068	########	AC-G	VSTM2A
ALDOA.1	########	0.482173	0.877	0.839	########	AC-G	ALDOA
SPARCL1	########	1.019565	0.573	0.301	########	AC-G	SPARCL1
MT1M	########	0.976046	0.475	0.199	########	AC-G	MT1M
IGFBP5.1	########	1.001125	0.505	0.222	########	AC-G	IGFBP5
RGS10.1	########	-1.23466	0.05	0.399	########	AC-G	RGS10
SLC39A14	########	0.645622	0.268	0.061	########	AC-G	SLC39A14
CD63.1	########	0.535882	0.941	0.921	########	AC-G	CD63
GPM6B.1	########	0.720697	0.823	0.579	########	AC-G	GPM6B
AIF1.1	########	-1.25518	0.138	0.464	########	AC-G	AIF1
FCER1G.1	########	-1.2033	0.172	0.494	########	AC-G	FCER1G
VAMP8.1	########	-1.23532	0.062	0.398	########	AC-G	VAMP8
MT3.1	########	0.79104	0.722	0.445	########	AC-G	MT3
EIF1.1	########	0.382308	0.948	0.979	########	AC-G	EIF1
ARHGDIB.1	########	-1.21071	0.096	0.426	########	AC-G	ARHGDIB
CNN3.1	########	0.715193	0.682	0.426	########	AC-G	CNN3
PRDX6	########	0.783152	0.78	0.594	########	AC-G	PRDX6
SKP1	########	0.491052	0.899	0.858	########	AC-G	SKP1
GALNT15	########	0.581411	0.153	0.017	########	AC-G	GALNT15
CORO1A.1	########	-1.05439	0.097	0.427	########	AC-G	CORO1A
CXCL14	########	0.850501	0.333	0.104	########	AC-G	CXCL14
MS4A6A.1	########	-1.23717	0.075	0.393	########	AC-G	MS4A6A
TYROBP.1	########	-1.08917	0.207	0.509	########	AC-G	TYROBP
TMSB10.1	########	0.469053	0.972	0.963	########	AC-G	TMSB10
CD53.1	########	-1.16859	0.052	0.366	########	AC-G	CD53
AEBP1.1	########	0.762544	0.296	0.087	########	AC-G	AEBP1
DIRAS3	########	0.941369	0.301	0.089	########	AC-G	DIRAS3
C1orf162.	########	-1.19794	0.062	0.373	########	AC-G	C1orf162
LY86.1	########	-1.15318	0.033	0.343	########	AC-G	LY86
ITGB2.1	########	-1.08238	0.064	0.375	########	AC-G	ITGB2
FCGR3A.1	########	-1.19122	0.083	0.392	########	AC-G	FCGR3A
TREM2.1	########	-1.19314	0.092	0.401	########	AC-G	TREM2
AZGP1	########	0.570794	0.187	0.031	########	AC-G	AZGP1

HP	########	0.913928	0.11	0.007	########	AC-G	HP
RAMP1	########	0.962029	0.621	0.371	########	AC-G	RAMP1
GMFG.1	########	-1.17711	0.045	0.347	########	AC-G	GMFG
GJA1	########	0.606367	0.229	0.05	########	AC-G	GJA1
RNASET2.1	########	-0.95819	0.236	0.531	########	AC-G	RNASET2
MS4A7.1	########	-1.14194	0.075	0.382	########	AC-G	MS4A7
ALOX5AP.1	########	-1.1422	0.154	0.447	########	AC-G	ALOX5AP
CD37.1	########	-1.11526	0.047	0.35	########	AC-G	CD37
LAPTM5.1	########	-1.04665	0.197	0.482	########	AC-G	LAPTM5
RNASE6.1	########	-1.15888	0.029	0.323	########	AC-G	RNASE6
HILPDA.1	########	0.93552	0.424	0.189	########	AC-G	HILPDA
COTL1.1	########	-0.72616	0.246	0.561	########	AC-G	COTL1
SH3BGR.1	########	0.771097	0.295	0.09	########	AC-G	SH3BGR
C4orf48.1	########	-0.66133	0.252	0.579	########	AC-G	C4orf48
CYBA.1	########	-1.06347	0.165	0.451	########	AC-G	CYBA
FAM49B.1	########	-0.68739	0.237	0.558	########	AC-G	FAM49B
B2M.1	########	0.409369	0.968	0.971	########	AC-G	B2M
UBC.1	########	0.369425	0.924	0.956	########	AC-G	UBC
VSIG4.1	########	-1.09769	0.121	0.413	########	AC-G	VSIG4
HN1.1	########	-0.51826	0.44	0.721	########	AC-G	HN1
PTN.1	########	0.729352	0.809	0.579	########	AC-G	PTN
PYCARD.1	########	-0.97001	0.039	0.332	########	AC-G	PYCARD
LST1.1	########	-1.13119	0.069	0.36	########	AC-G	LST1
HCST.1	########	-1.12053	0.043	0.331	########	AC-G	HCST
FCGR1A.1	########	-1.14876	0.024	0.304	########	AC-G	FCGR1A
MS4A4A.1	########	-1.1319	0.046	0.333	########	AC-G	MS4A4A
EVL.1	########	-0.83383	0.095	0.402	########	AC-G	EVL
LILRB4.1	########	-1.11554	0.021	0.301	########	AC-G	LILRB4
GPR34.1	########	-1.1388	0.031	0.313	########	AC-G	GPR34
TYMP.1	########	-0.97794	0.065	0.354	########	AC-G	TYMP
FSCN1.1	########	-0.67324	0.246	0.554	########	AC-G	FSCN1
BASP1.1	########	-0.80575	0.121	0.421	########	AC-G	BASP1
VCAM1	########	0.761168	0.206	0.046	########	AC-G	VCAM1
ARRB2.1	########	-0.81599	0.147	0.449	########	AC-G	ARRB2
FCGR2A.1	########	-1.0232	0.052	0.335	########	AC-G	FCGR2A
CREM.1	########	-0.89102	0.154	0.456	########	AC-G	CREM
TPI1.1	########	0.44321	0.878	0.863	########	AC-G	TPI1
RASD1.1	########	0.648681	0.307	0.1	########	AC-G	RASD1
PLA2G2A	########	1.107307	0.136	0.018	########	AC-G	PLA2G2A
CSF1R.1	########	-1.00211	0.036	0.313	########	AC-G	CSF1R
RGS1.1	########	-1.02191	0.228	0.489	########	AC-G	RGS1
GPSM3.1	########	-1.0224	0.034	0.306	########	AC-G	GPSM3
DDIT4L	########	0.30789	0.08	0.003	########	AC-G	DDIT4L
CD9	########	0.630664	0.737	0.532	########	AC-G	CD9
NUPR1	########	0.708715	0.533	0.283	########	AC-G	NUPR1
ADORA3.1	########	-1.03468	0.034	0.305	########	AC-G	ADORA3
HCLS1.1	########	-1.02582	0.061	0.335	########	AC-G	HCLS1
CALD1.1	########	0.728964	0.586	0.387	########	AC-G	CALD1
GRB2.1	########	-0.62157	0.226	0.53	########	AC-G	GRB2
ANGPTL4	########	0.715632	0.254	0.071	########	AC-G	ANGPTL4
FOLR1	########	0.588419	0.128	0.016	########	AC-G	FOLR1

CA3	########	0.760821	0.141	0.02	########	AC-G	CA3
SLC11A1.1	########	-0.96543	0.073	0.35	########	AC-G	SLC11A1
SGCE	########	0.72794	0.323	0.112	########	AC-G	SGCE
TEX26	########	0.265279	0.091	0.006	########	AC-G	TEX26
PDLIM4.1	########	0.634304	0.203	0.047	########	AC-G	PDLIM4
TMEM205.1	########	0.70497	0.657	0.464	########	AC-G	TMEM205
SAMSN1.1	########	-1.03895	0.033	0.294	########	AC-G	SAMSN1
SLN	########	1.252576	0.332	0.128	########	AC-G	SLN
AC009501.	########	0.855997	0.335	0.122	########	AC-G	AC009501.4
UCP2.1	########	-0.94763	0.051	0.315	########	AC-G	UCP2
C10orf54.	########	-1.04022	0.068	0.331	########	AC-G	C10orf54
SPI1.1	########	-0.93509	0.025	0.28	########	AC-G	SPI1
HLA-DMB.1	########	-1.06313	0.1	0.363	########	AC-G	HLA-DMB
TBXAS1.1	########	-0.98655	0.029	0.282	########	AC-G	TBXAS1
APLN	########	0.372938	0.119	0.015	########	AC-G	APLN
SRGN.1	########	-0.86915	0.312	0.535	########	AC-G	SRGN
FPR1.1	########	-1.02467	0.066	0.327	########	AC-G	FPR1
RGS19.1	########	-0.75888	0.055	0.318	########	AC-G	RGS19
HAVCR2.1	########	-0.98773	0.024	0.272	########	AC-G	HAVCR2
IL32	########	0.538792	0.245	0.072	########	AC-G	IL32
FYB.1	########	-0.93831	0.019	0.263	########	AC-G	FYB
VIM	########	0.447559	0.761	0.645	########	AC-G	VIM
LGALS1.1	########	0.53412	0.801	0.685	########	AC-G	LGALS1
ANXA2	########	0.685847	0.696	0.478	########	AC-G	ANXA2
FXYD5.1	########	-0.86797	0.118	0.384	########	AC-G	FXYD5
GNAI2.1	########	-0.59143	0.248	0.528	########	AC-G	GNAI2
CTSS.1	########	-0.8576	0.136	0.396	########	AC-G	CTSS
C3AR1.1	########	-0.96411	0.033	0.278	########	AC-G	C3AR1
REXO2.1	########	0.769224	0.497	0.284	########	AC-G	REXO2
ENO1	########	0.45586	0.853	0.783	########	AC-G	ENO1
TMEM45A	########	0.722675	0.343	0.144	########	AC-G	TMEM45A
STMN1.1	########	-0.58519	0.426	0.671	########	AC-G	STMN1
CLCF1	########	0.43479	0.136	0.023	########	AC-G	CLCF1
CFI.1	########	0.491995	0.173	0.039	########	AC-G	CFI
BIN1.1	########	-0.77482	0.143	0.403	########	AC-G	BIN1
C1QC.1	########	-0.7934	0.313	0.538	########	AC-G	C1QC
NDUFA4	########	0.422602	0.906	0.893	########	AC-G	NDUFA4
RAC1.1	########	-0.4489	0.484	0.793	########	AC-G	RAC1
OLR1.1	########	-0.96761	0.049	0.289	########	AC-G	OLR1
CCL3.1	########	-0.89088	0.219	0.462	########	AC-G	CCL3
TSLP.1	########	0.389547	0.086	0.007	########	AC-G	TSLP
MAOB	########	0.709538	0.234	0.072	########	AC-G	MAOB
GBP1	########	0.646678	0.318	0.122	########	AC-G	GBP1
PI3.1	########	0.943431	0.156	0.032	########	AC-G	PI3
TMEM160.1	########	-0.56184	0.243	0.509	########	AC-G	TMEM160
LIMD2.1	########	-0.6503	0.043	0.289	########	AC-G	LIMD2
ARPC1B.1	########	-0.59855	0.312	0.573	########	AC-G	ARPC1B
PTPRC.1	########	-0.86713	0.013	0.24	########	AC-G	PTPRC
CCL4.1	########	-1.00084	0.118	0.365	########	AC-G	CCL4
CD86.1	########	-0.92724	0.021	0.251	########	AC-G	CD86
GFAP	########	0.941105	0.519	0.317	########	AC-G	GFAP

MT1A.1	########	0.402218	0.109	0.014	########	AC-G	MT1A
SYNGR2.1	########	-0.7445	0.112	0.37	########	AC-G	SYNGR2
BTG1.1	########	-0.4681	0.429	0.739	########	AC-G	BTG1
MYL6.1	########	0.428313	0.921	0.946	########	AC-G	MYL6
SLCO2B1.1	########	-0.91221	0.028	0.258	########	AC-G	SLCO2B1
YBX1.1	########	-0.38873	0.539	0.807	########	AC-G	YBX1
TAGLN2.1	########	0.602884	0.671	0.484	########	AC-G	TAGLN2
PDLIM3.1	########	0.579005	0.258	0.086	########	AC-G	PDLIM3
HIST1H4C.	########	-0.64494	0.203	0.468	########	AC-G	HIST1H4C
CFB	########	0.368088	0.09	0.009	########	AC-G	CFB
SGK1.1	########	-0.77477	0.225	0.478	########	AC-G	SGK1
MYC.1	########	0.672498	0.333	0.138	########	AC-G	MYC
TMBIM6	########	0.389514	0.837	0.838	########	AC-G	TMBIM6
EVI2B.1	########	-0.86506	0.017	0.239	########	AC-G	EVI2B
APOC2.1	########	-1.08364	0.008	0.224	########	AC-G	APOC2
NRN1	########	0.770318	0.416	0.222	########	AC-G	NRN1
CSF2RA.1	########	-0.91817	0.018	0.238	########	AC-G	CSF2RA
CD163.1	########	-0.92959	0.081	0.314	########	AC-G	CD163
ADM.1	########	0.711377	0.348	0.153	########	AC-G	ADM
SOD1	########	0.465836	0.8	0.748	########	AC-G	SOD1
GRN.1	########	-0.52263	0.365	0.61	########	AC-G	GRN
IGFBP6.1	########	0.568825	0.176	0.044	########	AC-G	IGFBP6
DTNA.1	########	0.786517	0.415	0.221	########	AC-G	DTNA
THEMIS2.1	########	-0.79063	0.049	0.279	########	AC-G	THEMIS2
LAIR1.1	########	-0.87222	0.041	0.268	########	AC-G	LAIR1
MEF2C.1	########	-0.74272	0.083	0.322	########	AC-G	MEF2C
CXCL16.1	########	-0.59076	0.235	0.496	########	AC-G	CXCL16
FBXO32.1	########	0.667881	0.305	0.124	########	AC-G	FBXO32
CD14.1	########	-0.90431	0.227	0.444	########	AC-G	CD14
NGFRAP1	########	0.558683	0.78	0.618	########	AC-G	NGFRAP1
C1QA.1	########	-0.75649	0.371	0.569	########	AC-G	C1QA
RB1.1	########	-0.69521	0.099	0.345	1.16E-99	AC-G	RB1
GPR183.1	########	-0.9914	0.099	0.327	1.73E-99	AC-G	GPR183
S100A4.1	########	-0.90083	0.1	0.334	1.92E-99	AC-G	S100A4
SLA.1	########	-0.83357	0.021	0.237	2.76E-99	AC-G	SLA
CPVL.1	########	-0.70737	0.136	0.39	3.08E-99	AC-G	CPVL
ASS1	########	0.420208	0.102	0.014	4.48E-99	AC-G	ASS1
CYSTM1.1	########	0.680436	0.489	0.292	7.59E-99	AC-G	CYSTM1
CELF2.1	########	-0.59855	0.165	0.426	1.72E-98	AC-G	CELF2
MARCKSL1.	########	-0.66222	0.382	0.576	2.01E-98	AC-G	MARCKSL1
ABI3.1	########	-0.82159	0.014	0.224	2.04E-98	AC-G	ABI3
IFI30.1	########	-0.85473	0.034	0.255	4.66E-98	AC-G	IFI30
CD300A.1	########	-0.82203	0.015	0.226	1.55E-97	AC-G	CD300A
FRZB	########	0.516391	0.137	0.027	1.79E-97	AC-G	FRZB
ATP5D.1	########	-0.46112	0.397	0.656	9.20E-97	AC-G	ATP5D
CD99	########	0.583443	0.746	0.616	6.11E-96	AC-G	CD99
PRKCG	########	0.425309	0.082	0.008	1.01E-95	AC-G	PRKCG
FAU.1	########	0.307341	0.912	0.967	1.23E-95	AC-G	FAU
TCF4.1	1.28E-99	-0.53688	0.331	0.56	1.91E-95	AC-G	TCF4
LAT2.1	1.66E-99	-0.8287	0.026	0.237	2.49E-95	AC-G	LAT2
TMSB4X.1	2.71E-99	0.402561	0.992	0.998	4.05E-95	AC-G	TMSB4X

LOX	9.44E-99	0.492185	0.135	0.027	1.41E-94	AC-G	LOX
WBP5.1	1.32E-98	0.575425	0.648	0.474	1.97E-94	AC-G	WBP5
JMJD1C.1	3.06E-98	-0.53943	0.123	0.375	4.57E-94	AC-G	JMJD1C
SPINT2.1	4.26E-98	-0.74925	0.037	0.251	6.37E-94	AC-G	SPINT2
CYBB.1	1.49E-97	-0.8008	0.019	0.226	2.22E-93	AC-G	CYBB
HLA-DQB1.	1.63E-97	-0.85111	0.136	0.362	2.44E-93	AC-G	HLA-DQB1
CETN2.1	2.25E-97	0.724605	0.474	0.292	3.36E-93	AC-G	CETN2
MMP2.1	4.58E-97	-0.55972	0.041	0.263	6.85E-93	AC-G	MMP2
LGALS9.1	5.91E-97	-0.82342	0.056	0.276	8.83E-93	AC-G	LGALS9
H1FX.1	1.53E-96	-0.55178	0.173	0.426	2.29E-92	AC-G	H1FX
EPB41L2.1	6.05E-96	-0.63928	0.115	0.349	9.04E-92	AC-G	EPB41L2
CHCHD10.1	8.83E-96	-0.55528	0.175	0.421	1.32E-91	AC-G	CHCHD10
SERPINA1.	1.22E-95	-0.83237	0.111	0.336	1.82E-91	AC-G	SERPINA1
LPAR6.1	1.32E-95	-0.73279	0.055	0.273	1.98E-91	AC-G	LPAR6
IL18.1	4.64E-95	-0.85813	0.024	0.226	6.93E-91	AC-G	IL18
NINJ1.1	4.82E-95	-0.63618	0.246	0.492	7.20E-91	AC-G	NINJ1
EVI2A.1	4.87E-95	-0.8469	0.044	0.258	7.28E-91	AC-G	EVI2A
TCF12.1	6.06E-95	-0.52049	0.088	0.322	9.06E-91	AC-G	TCF12
S100A13	1.05E-94	0.781105	0.452	0.265	1.57E-90	AC-G	S100A13
CLEC7A.1	1.39E-94	-0.7694	0.011	0.207	2.08E-90	AC-G	CLEC7A
CPM.1	2.46E-93	-0.83371	0.059	0.269	3.67E-89	AC-G	CPM
KCTD12.1	6.70E-93	-0.64869	0.056	0.271	1.00E-88	AC-G	KCTD12
TNFAIP6	1.36E-92	0.728926	0.236	0.085	2.03E-88	AC-G	TNFAIP6
REL.1	1.66E-92	-0.69964	0.111	0.335	2.47E-88	AC-G	REL
H2AFY.1	2.50E-92	-0.4275	0.401	0.647	3.74E-88	AC-G	H2AFY
FCGR1B.1	3.05E-92	-0.86595	0.006	0.194	4.56E-88	AC-G	FCGR1B
EMP3.1	3.29E-92	0.550253	0.606	0.418	4.92E-88	AC-G	EMP3
MGAT4A.1	3.51E-92	-0.74122	0.047	0.254	5.25E-88	AC-G	MGAT4A
MNDA.1	2.03E-91	-0.75929	0.015	0.208	3.04E-87	AC-G	MNDA
ADAP2.1	3.47E-91	-0.74365	0.02	0.216	5.19E-87	AC-G	ADAP2
SERPINF1.	4.59E-91	-0.705	0.048	0.259	6.86E-87	AC-G	SERPINF1
OSTF1.1	4.81E-91	-0.65573	0.104	0.328	7.18E-87	AC-G	OSTF1
RHOG.1	6.47E-91	-0.62736	0.207	0.449	9.66E-87	AC-G	RHOG
DCX.1	9.23E-91	-0.63967	0.034	0.238	1.38E-86	AC-G	DCX
CENPV.1	1.08E-90	-0.56706	0.074	0.29	1.62E-86	AC-G	CENPV
LTC4S.1	1.83E-90	-0.74419	0.01	0.199	2.74E-86	AC-G	LTC4S
MT1G.1	1.97E-90	0.556425	0.266	0.101	2.94E-86	AC-G	MT1G
TNC	2.36E-90	0.788992	0.345	0.173	3.52E-86	AC-G	TNC
CNPY3.1	4.41E-90	-0.60921	0.213	0.451	6.59E-86	AC-G	CNPY3
RASSF2.1	5.67E-90	-0.51559	0.073	0.295	8.47E-86	AC-G	RASSF2
PRSS35	6.01E-90	0.171524	0.059	0.003	8.99E-86	AC-G	PRSS35
MMP7	6.77E-90	0.608901	0.1	0.015	1.01E-85	AC-G	MMP7
WAS.1	1.38E-89	-0.71582	0.015	0.205	2.06E-85	AC-G	WAS
TNFRSF1B.	2.43E-89	-0.71883	0.026	0.22	3.63E-85	AC-G	TNFRSF1B
TGFB1.1	2.67E-89	-0.58386	0.064	0.28	3.98E-85	AC-G	TGFB1
H2AFJ.1	4.86E-89	-0.67822	0.079	0.294	7.26E-85	AC-G	H2AFJ
SAA4	7.51E-89	0.384912	0.05	0.002	1.12E-84	AC-G	SAA4
AP1B1.1	8.50E-89	-0.63894	0.078	0.294	1.27E-84	AC-G	AP1B1
PRDX5.1	9.76E-89	0.45425	0.781	0.735	1.46E-84	AC-G	PRDX5
HNRNPD.1	2.66E-88	-0.48655	0.215	0.453	3.98E-84	AC-G	HNRNPD
RSPH1	2.95E-88	0.289539	0.091	0.013	4.42E-84	AC-G	RSPH1

EMP1.1	3.72E-88	0.583892	0.324	0.15	5.55E-84	AC-G	EMP1
LIPA.1	3.79E-88	-0.63825	0.145	0.371	5.66E-84	AC-G	LIPA
VAMP5	7.49E-88	0.736479	0.546	0.367	1.12E-83	AC-G	VAMP5
CTSZ.1	2.35E-87	-0.78785	0.085	0.289	3.51E-83	AC-G	CTSZ
EFHD2.1	7.04E-87	-0.58231	0.042	0.243	1.05E-82	AC-G	EFHD2
AGT	8.73E-87	0.816913	0.366	0.191	1.31E-82	AC-G	AGT
SRI.1	9.65E-87	0.448671	0.791	0.717	1.44E-82	AC-G	SRI
ABHD12.1	1.06E-86	-0.5169	0.188	0.419	1.58E-82	AC-G	ABHD12
NR4A2.1	1.13E-86	-0.82548	0.083	0.284	1.69E-82	AC-G	NR4A2
COL8A1	1.35E-86	0.326496	0.08	0.009	2.02E-82	AC-G	COL8A1
CD83.1	1.39E-86	-0.76044	0.12	0.329	2.07E-82	AC-G	CD83
TPT1.1	5.26E-86	0.348273	0.924	0.959	7.87E-82	AC-G	TPT1
GLUL	5.94E-86	0.498459	0.812	0.781	8.88E-82	AC-G	GLUL
TIMP4	2.59E-85	0.696973	0.218	0.078	3.87E-81	AC-G	TIMP4
GNG7.1	3.65E-85	-0.57526	0.11	0.336	5.45E-81	AC-G	GNG7
SMAP2.1	5.54E-85	-0.62739	0.065	0.269	8.28E-81	AC-G	SMAP2
PTPN6.1	5.66E-85	-0.67874	0.011	0.191	8.46E-81	AC-G	PTPN6
SBDS.1	8.72E-85	0.450872	0.698	0.593	1.30E-80	AC-G	SBDS
HSPA1A.1	9.43E-85	-0.60084	0.296	0.53	1.41E-80	AC-G	HSPA1A
IGSF6.1	1.75E-84	-0.75258	0.006	0.182	2.61E-80	AC-G	IGSF6
LAPTM4A.1	6.11E-84	0.406304	0.801	0.767	9.14E-80	AC-G	LAPTM4A
PLXDC2.1	1.07E-83	-0.70433	0.045	0.241	1.60E-79	AC-G	PLXDC2
CCDC88A.1	1.39E-83	-0.44019	0.215	0.455	2.08E-79	AC-G	CCDC88A
EGLN3	1.65E-83	0.419653	0.16	0.044	2.46E-79	AC-G	EGLN3
TM6SF1.1	1.87E-83	-0.7694	0.01	0.187	2.79E-79	AC-G	TM6SF1
MYO1F.1	5.53E-83	-0.70842	0.028	0.214	8.27E-79	AC-G	MYO1F
RNASE2.1	9.79E-83	-0.79796	0.011	0.186	1.46E-78	AC-G	RNASE2
FAM105A.1	2.62E-82	-0.69271	0.015	0.191	3.92E-78	AC-G	FAM105A
FRMD4A.1	2.84E-82	-0.60721	0.031	0.218	4.24E-78	AC-G	FRMD4A
DBI.1	3.59E-82	0.486531	0.862	0.84	5.37E-78	AC-G	DBI
LYZ.1	5.20E-82	-0.8032	0.049	0.237	7.77E-78	AC-G	LYZ
ENPP5	9.09E-82	0.285237	0.071	0.008	1.36E-77	AC-G	ENPP5
IRF8.1	1.08E-81	-0.69709	0.013	0.187	1.61E-77	AC-G	IRF8
APOD	1.19E-81	0.473862	0.313	0.131	1.77E-77	AC-G	APOD
YWHAB.1	1.28E-81	-0.30047	0.526	0.737	1.91E-77	AC-G	YWHAB
APBB1IP.1	1.99E-81	-0.67144	0.011	0.185	2.98E-77	AC-G	APBB1IP
PCSK1	2.14E-81	0.419043	0.105	0.02	3.19E-77	AC-G	PCSK1
SLC7A7.1	2.17E-81	-0.69285	0.023	0.204	3.24E-77	AC-G	SLC7A7
FGL2.1	2.33E-81	-0.73643	0.026	0.206	3.49E-77	AC-G	FGL2
RALY.1	2.46E-81	-0.45425	0.265	0.493	3.68E-77	AC-G	RALY
SPTLC2.1	2.47E-81	-0.6447	0.05	0.242	3.68E-77	AC-G	SPTLC2
SLC2A5.1	5.70E-81	-0.80037	0.021	0.199	8.52E-77	AC-G	SLC2A5
PGK1	6.17E-81	0.420127	0.772	0.75	9.23E-77	AC-G	PGK1
QKI.1	7.95E-81	-0.47767	0.285	0.507	1.19E-76	AC-G	QKI
MSR1.1	1.04E-80	-0.7147	0.028	0.21	1.56E-76	AC-G	MSR1
NDRG1	1.28E-80	0.568068	0.312	0.144	1.92E-76	AC-G	NDRG1
C1QB.1	1.41E-80	-0.60003	0.444	0.594	2.10E-76	AC-G	C1QB
RGCC.1	1.43E-80	-0.71909	0.084	0.282	2.14E-76	AC-G	RGCC
TRAF4.1	1.75E-80	-0.5028	0.096	0.303	2.61E-76	AC-G	TRAF4
PLEK.1	2.93E-80	-0.73538	0.026	0.204	4.37E-76	AC-G	PLEK
UBL5	3.71E-80	0.392625	0.846	0.828	5.54E-76	AC-G	UBL5

GEM	7.43E-80	0.507316	0.212	0.074	1.11E-75	AC-G	GEM
IFITM3	7.86E-80	0.711375	0.61	0.482	1.17E-75	AC-G	IFITM3
PREX1.1	8.42E-80	-0.54405	0.055	0.25	1.26E-75	AC-G	PREX1
TOMM7	1.31E-79	0.410198	0.842	0.854	1.96E-75	AC-G	TOMM7
C5AR1.1	1.56E-79	-0.82141	0.077	0.269	2.33E-75	AC-G	C5AR1
FOLR2.1	1.71E-79	-0.86581	0.028	0.207	2.56E-75	AC-G	FOLR2
FAM26F.1	2.03E-79	-0.67611	0.021	0.195	3.03E-75	AC-G	FAM26F
TAF7.1	3.00E-79	0.475787	0.656	0.533	4.49E-75	AC-G	TAF7
NUDT3.1	3.19E-79	-0.46336	0.208	0.437	4.76E-75	AC-G	NUDT3
EIF3L	5.81E-79	-0.33868	0.402	0.646	8.68E-75	AC-G	EIF3L
EPAS1	6.18E-79	0.490374	0.218	0.083	9.24E-75	AC-G	EPAS1
PTGS1.1	1.02E-78	-0.57168	0.014	0.184	1.52E-74	AC-G	PTGS1
CYFIP1.1	1.54E-78	-0.50207	0.106	0.318	2.31E-74	AC-G	CYFIP1
GADD45A.1	2.51E-78	0.611284	0.421	0.251	3.76E-74	AC-G	GADD45A
TSPAN14.1	3.50E-78	-0.51182	0.052	0.244	5.24E-74	AC-G	TSPAN14
SHFM1	4.38E-78	0.432108	0.773	0.713	6.55E-74	AC-G	SHFM1
FAM181B.1	6.61E-78	-0.52323	0.048	0.234	9.87E-74	AC-G	FAM181B
RP11-437L	8.45E-78	0.332877	0.056	0.004	1.26E-73	AC-G	RP11-437L7.1
SIGLEC10.	1.30E-77	-0.68203	0.019	0.19	1.94E-73	AC-G	SIGLEC10
CD81.1	1.54E-77	-0.49931	0.248	0.476	2.31E-73	AC-G	CD81
SH3KBP1.1	1.82E-77	-0.56371	0.078	0.273	2.72E-73	AC-G	SH3KBP1
GIMAP4.1	2.46E-77	-0.68413	0.01	0.177	3.68E-73	AC-G	GIMAP4
RARRES3.1	3.18E-77	0.734498	0.389	0.215	4.75E-73	AC-G	RARRES3
SRGAP2.1	4.04E-77	-0.57552	0.071	0.264	6.04E-73	AC-G	SRGAP2
STXBP2.1	5.89E-77	-0.65857	0.027	0.201	8.80E-73	AC-G	STXBP2
BACE2.1	6.29E-77	0.291192	0.141	0.037	9.39E-73	AC-G	BACE2
MAP3K8.1	7.47E-77	-0.71116	0.036	0.212	1.12E-72	AC-G	MAP3K8
SKIL.1	1.12E-76	-0.55386	0.117	0.319	1.68E-72	AC-G	SKIL
VEGFA.1	1.13E-76	0.621106	0.347	0.19	1.69E-72	AC-G	VEGFA
SLC31A2.1	1.37E-76	-0.7073	0.042	0.222	2.05E-72	AC-G	SLC31A2
HLA-E.1	2.13E-76	-0.48027	0.434	0.628	3.18E-72	AC-G	HLA-E
SRM.1	2.52E-76	-0.4723	0.18	0.401	3.77E-72	AC-G	SRM
CMTM7.1	2.84E-76	-0.57388	0.021	0.192	4.25E-72	AC-G	CMTM7
RNF130.1	2.90E-76	-0.50757	0.275	0.499	4.34E-72	AC-G	RNF130
CHD7.1	3.14E-76	-0.47916	0.114	0.323	4.69E-72	AC-G	CHD7
HCK.1	3.52E-76	-0.67936	0.024	0.195	5.26E-72	AC-G	HCK
CD4.1	5.93E-76	-0.65651	0.016	0.182	8.86E-72	AC-G	CD4
AC015936.	7.51E-76	0.320569	0.074	0.009	1.12E-71	AC-G	AC015936.3
OLFML3.1	9.12E-76	-0.73248	0.068	0.257	1.36E-71	AC-G	OLFML3
ATP1B3.1	9.37E-76	-0.42181	0.353	0.582	1.40E-71	AC-G	ATP1B3
HLA-DMA.1	1.02E-75	-0.63183	0.246	0.44	1.52E-71	AC-G	HLA-DMA
UBE2E3.1	1.65E-75	-0.39604	0.269	0.497	2.46E-71	AC-G	UBE2E3
SAMHD1.1	1.85E-75	-0.59512	0.06	0.245	2.76E-71	AC-G	SAMHD1
ZEB2.1	1.99E-75	-0.51982	0.13	0.337	2.98E-71	AC-G	ZEB2
FMNL2.1	2.23E-75	-0.47292	0.097	0.298	3.33E-71	AC-G	FMNL2
MTSS1.1	7.24E-75	-0.57994	0.059	0.243	1.08E-70	AC-G	MTSS1
AES.1	8.54E-75	-0.44723	0.226	0.446	1.28E-70	AC-G	AES
SOX4.1	8.71E-75	-0.6834	0.292	0.475	1.30E-70	AC-G	SOX4
SET.1	1.34E-74	-0.33818	0.367	0.598	2.00E-70	AC-G	SET
FERMT3.1	1.35E-74	-0.6738	0.026	0.194	2.02E-70	AC-G	FERMT3
PARVG.1	1.37E-74	-0.69162	0.022	0.189	2.04E-70	AC-G	PARVG

IFNGR2.1	1.59E-74	-0.47976	0.206	0.425	2.38E-70	AC-G	IFNGR2
UBE2S.1	1.79E-74	-0.51626	0.166	0.379	2.68E-70	AC-G	UBE2S
C11orf70	1.80E-74	0.235849	0.073	0.009	2.69E-70	AC-G	C11orf70
CSF3R.1	1.92E-74	-0.68139	0.016	0.179	2.87E-70	AC-G	CSF3R
ANP32A.1	2.88E-74	-0.34059	0.232	0.466	4.30E-70	AC-G	ANP32A
SERPINE1	3.15E-74	0.56049	0.212	0.078	4.71E-70	AC-G	SERPINE1
DDX17.1	4.23E-74	-0.45881	0.24	0.46	6.32E-70	AC-G	DDX17
KHDRBS1.1	4.26E-74	-0.42239	0.295	0.523	6.36E-70	AC-G	KHDRBS1
RP11-161M	4.59E-74	-0.52715	0.025	0.194	6.86E-70	AC-G	RP11-161M6.2
SLC16A1.1	6.16E-74	0.570505	0.365	0.208	9.21E-70	AC-G	SLC16A1
GLTSCR2.1	6.86E-74	-0.38936	0.353	0.605	1.03E-69	AC-G	GLTSCR2
MAP1B.1	8.30E-74	0.506063	0.672	0.507	1.24E-69	AC-G	MAP1B
EIF1AY.1	1.10E-73	-0.39538	0.031	0.206	1.64E-69	AC-G	EIF1AY
AP1S2.1	1.21E-73	-0.41016	0.322	0.535	1.81E-69	AC-G	AP1S2
RDH10.1	1.33E-73	0.457072	0.187	0.066	1.99E-69	AC-G	RDH10
ARHGAP29	2.48E-73	0.242176	0.095	0.018	3.70E-69	AC-G	ARHGAP29
LPCAT2.1	2.89E-73	-0.60122	0.014	0.173	4.31E-69	AC-G	LPCAT2
MYCBP2.1	3.28E-73	-0.49958	0.134	0.338	4.90E-69	AC-G	MYCBP2
ZNF331.1	3.29E-73	-0.72484	0.155	0.347	4.92E-69	AC-G	ZNF331
IDH2.1	4.45E-73	-0.44322	0.24	0.463	6.65E-69	AC-G	IDH2
IER3.1	5.05E-73	-0.74885	0.088	0.271	7.55E-69	AC-G	IER3
ACTR2.1	6.83E-73	-0.42752	0.274	0.495	1.02E-68	AC-G	ACTR2
CLIC4	8.11E-73	0.550066	0.472	0.305	1.21E-68	AC-G	CLIC4
LRRC2	1.05E-72	0.21858	0.069	0.009	1.57E-68	AC-G	LRRC2
CREG1.1	1.18E-72	-0.60282	0.135	0.328	1.77E-68	AC-G	CREG1
FOXN3.1	2.04E-72	-0.51336	0.091	0.287	3.05E-68	AC-G	FOXN3
OLIG1.1	2.04E-72	-0.66626	0.036	0.208	3.05E-68	AC-G	OLIG1
S100A9.1	2.30E-72	-0.80822	0.074	0.253	3.44E-68	AC-G	S100A9
HLA-A.1	3.08E-72	0.362848	0.889	0.897	4.60E-68	AC-G	HLA-A
SERPINB9.	3.92E-72	-0.64051	0.014	0.172	5.85E-68	AC-G	SERPINB9
MFSD1.1	5.58E-72	-0.55758	0.173	0.377	8.33E-68	AC-G	MFSD1
UQCRB.1	5.70E-72	-0.29844	0.46	0.721	8.52E-68	AC-G	UQCRB
SFPQ.1	5.94E-72	-0.39168	0.358	0.578	8.88E-68	AC-G	SFPQ
S100A8.1	6.85E-72	-0.80942	0.043	0.213	1.02E-67	AC-G	S100A8
GBE1.1	9.07E-72	0.554073	0.316	0.16	1.36E-67	AC-G	GBE1
LAMTOR5.1	9.83E-72	0.40835	0.74	0.682	1.47E-67	AC-G	LAMTOR5
IL13RA1.1	1.15E-71	-0.61016	0.094	0.279	1.72E-67	AC-G	IL13RA1
GSTP1.1	1.26E-71	0.396784	0.821	0.822	1.88E-67	AC-G	GSTP1
PLOD2.1	1.48E-71	0.566879	0.281	0.139	2.21E-67	AC-G	PLOD2
BDH2	1.61E-71	0.526159	0.262	0.118	2.40E-67	AC-G	BDH2
RNF145.1	1.75E-71	-0.42359	0.197	0.412	2.62E-67	AC-G	RNF145
ARL5A.1	1.90E-71	-0.39723	0.33	0.554	2.84E-67	AC-G	ARL5A
AKAP12.1	2.01E-71	0.635283	0.31	0.163	3.00E-67	AC-G	AKAP12
VASN	2.70E-71	0.214715	0.076	0.011	4.04E-67	AC-G	VASN
DYNLL1	2.84E-71	0.335546	0.848	0.834	4.24E-67	AC-G	DYNLL1
PLD4.1	3.03E-71	-0.70044	0.02	0.179	4.53E-67	AC-G	PLD4
ACSL1.1	4.32E-71	-0.65256	0.077	0.257	6.46E-67	AC-G	ACSL1
HAMP.1	4.98E-71	-0.84045	0.026	0.187	7.44E-67	AC-G	HAMP
BID.1	5.44E-71	-0.40603	0.169	0.393	8.13E-67	AC-G	BID
KLHDC8A.1	7.05E-71	-0.46388	0.091	0.281	1.05E-66	AC-G	KLHDC8A
SRSF9.1	8.32E-71	-0.38614	0.382	0.596	1.24E-66	AC-G	SRSF9

FAS	1.62E-70	0.450774	0.159	0.049	2.43E-66	AC-G	FAS
IL10RA.1	1.78E-70	-0.61681	0.01	0.163	2.66E-66	AC-G	IL10RA
ARHGAP18.	1.86E-70	-0.59144	0.033	0.199	2.78E-66	AC-G	ARHGAP18
LIMS1.1	2.00E-70	-0.43363	0.264	0.474	2.99E-66	AC-G	LIMS1
GAA.1	2.06E-70	-0.48606	0.15	0.353	3.07E-66	AC-G	GAA
WIPF1.1	2.25E-70	-0.53134	0.067	0.249	3.37E-66	AC-G	WIPF1
FTH1.1	2.28E-70	0.187843	0.833	0.952	3.41E-66	AC-G	FTH1
ZNF667-AS	2.47E-70	0.541526	0.515	0.363	3.69E-66	AC-G	ZNF667-AS1
SEPP1.1	2.96E-70	0.575829	0.495	0.296	4.42E-66	AC-G	SEPP1
APOC1.1	3.07E-70	-0.50925	0.372	0.544	4.59E-66	AC-G	APOC1
MARCH1.1	4.14E-70	-0.5444	0.046	0.219	6.18E-66	AC-G	1-Mar
PTPRE.1	4.19E-70	-0.56148	0.108	0.301	6.26E-66	AC-G	PTPRE
SOX8.1	5.27E-70	-0.49987	0.036	0.205	7.88E-66	AC-G	SOX8
CD69.1	6.33E-70	-0.79145	0.021	0.179	9.46E-66	AC-G	CD69
HTRA1.1	8.62E-70	-0.44065	0.27	0.497	1.29E-65	AC-G	HTRA1
PLP2.1	9.42E-70	0.580242	0.475	0.318	1.41E-65	AC-G	PLP2
SCIN.1	1.22E-69	-0.73573	0.02	0.176	1.83E-65	AC-G	SCIN
SLAIN1.1	1.63E-69	-0.52056	0.055	0.232	2.44E-65	AC-G	SLAIN1
TSHZ2	3.91E-69	0.338617	0.129	0.034	5.84E-65	AC-G	TSHZ2
CEP170.1	4.35E-69	-0.44956	0.1	0.293	6.50E-65	AC-G	CEP170
NCF4.1	4.48E-69	-0.65182	0.016	0.171	6.70E-65	AC-G	NCF4
DHRS7.1	4.67E-69	-0.36586	0.363	0.594	6.98E-65	AC-G	DHRS7
FUOM.1	4.89E-69	-0.56691	0.013	0.166	7.31E-65	AC-G	FUOM
SLC16A3.1	8.38E-69	-0.61423	0.162	0.351	1.25E-64	AC-G	SLC16A3
USF2.1	1.17E-68	-0.45002	0.154	0.359	1.75E-64	AC-G	USF2
GPR65.1	1.50E-68	-0.6097	0.009	0.158	2.24E-64	AC-G	GPR65
GNAQ.1	1.69E-68	-0.41098	0.122	0.319	2.53E-64	AC-G	GNAQ
ZFAND5.1	1.71E-68	-0.39166	0.271	0.5	2.55E-64	AC-G	ZFAND5
FAM89B.1	2.02E-68	-0.40865	0.09	0.277	3.03E-64	AC-G	FAM89B
SPOCK2	2.13E-68	0.54157	0.253	0.117	3.18E-64	AC-G	SPOCK2
GNB1.1	3.01E-68	-0.41054	0.236	0.45	4.50E-64	AC-G	GNB1
MEX3A.1	3.41E-68	-0.42213	0.038	0.205	5.10E-64	AC-G	MEX3A
AQP4	7.82E-68	0.66832	0.288	0.142	1.17E-63	AC-G	AQP4
NREP.1	9.55E-68	-0.43595	0.109	0.304	1.43E-63	AC-G	NREP
CA12.1	9.73E-68	0.553412	0.246	0.115	1.45E-63	AC-G	CA12
BCL2A1.1	1.27E-67	-0.67429	0.027	0.182	1.89E-63	AC-G	BCL2A1
ETV5.1	1.52E-67	-0.43153	0.099	0.29	2.26E-63	AC-G	ETV5
C6orf141	1.63E-67	0.374131	0.091	0.018	2.44E-63	AC-G	C6orf141
CTSC.1	6.64E-67	-0.50243	0.219	0.416	9.93E-63	AC-G	CTSC
TXN.1	1.43E-66	0.457218	0.717	0.656	2.14E-62	AC-G	TXN
PTMS.1	1.90E-66	-0.3792	0.402	0.582	2.84E-62	AC-G	PTMS
CSTA.1	1.93E-66	-0.54391	0.008	0.152	2.88E-62	AC-G	CSTA
HMGN1.1	4.97E-66	-0.35132	0.478	0.701	7.43E-62	AC-G	HMGN1
IGSF21.1	6.80E-66	-0.54707	0.03	0.189	1.02E-61	AC-G	IGSF21
REEP3.1	6.81E-66	-0.40707	0.091	0.275	1.02E-61	AC-G	REEP3
PCBP1.1	9.46E-66	-0.33047	0.403	0.624	1.41E-61	AC-G	PCBP1
CD302.1	1.07E-65	-0.59179	0.063	0.232	1.59E-61	AC-G	CD302
MTDH.1	1.16E-65	-0.34982	0.423	0.646	1.74E-61	AC-G	MTDH
MPST.1	1.19E-65	-0.40598	0.138	0.339	1.79E-61	AC-G	MPST
HIF1A	1.72E-65	-0.3621	0.313	0.533	2.57E-61	AC-G	HIF1A
ITM2C.1	1.92E-65	0.633867	0.51	0.399	2.87E-61	AC-G	ITM2C

PODXL2.1	1.93E-65	-0.48656	0.148	0.339	2.89E-61	AC-G	PODXL2
DSTN.1	2.23E-65	0.425968	0.705	0.601	3.33E-61	AC-G	DSTN
FRMD4B.1	2.77E-65	-0.57478	0.029	0.185	4.14E-61	AC-G	FRMD4B
KIF5B.1	3.34E-65	-0.377	0.338	0.545	4.99E-61	AC-G	KIF5B
SRSF2.1	4.42E-65	-0.35142	0.425	0.64	6.60E-61	AC-G	SRSF2
RILPL2.1	8.05E-65	-0.58384	0.109	0.288	1.20E-60	AC-G	RILPL2
AKAP13.1	1.09E-64	-0.44789	0.155	0.358	1.62E-60	AC-G	AKAP13
GPRC5A	1.56E-64	0.180277	0.048	0.004	2.33E-60	AC-G	GPRC5A
ADAM28.1	1.65E-64	-0.63249	0.018	0.165	2.46E-60	AC-G	ADAM28
GBP2	2.71E-64	0.581439	0.341	0.182	4.06E-60	AC-G	GBP2
TRMT112.1	3.08E-64	0.402046	0.748	0.699	4.60E-60	AC-G	TRMT112
CADPS.1	3.65E-64	0.365733	0.114	0.029	5.46E-60	AC-G	CADPS
ARHGAP21.	4.17E-64	-0.44657	0.096	0.28	6.23E-60	AC-G	ARHGAP21
TRABD.1	5.19E-64	-0.45649	0.105	0.289	7.75E-60	AC-G	TRABD
ABCC3	5.40E-64	0.503471	0.2	0.08	8.08E-60	AC-G	ABCC3
PILRA.1	7.12E-64	-0.66863	0.036	0.19	1.06E-59	AC-G	PILRA
GPC2.1	9.65E-64	-0.51024	0.037	0.193	1.44E-59	AC-G	GPC2
HINT1	1.72E-63	0.36961	0.84	0.825	2.57E-59	AC-G	HINT1
VMO1.1	2.83E-63	-0.67645	0.033	0.184	4.23E-59	AC-G	VMO1
HSPA5.1	3.10E-63	0.425902	0.694	0.65	4.63E-59	AC-G	HSPA5
CCNI.1	3.40E-63	-0.2976	0.541	0.738	5.08E-59	AC-G	CCNI
CORO1B.1	4.66E-63	-0.37723	0.192	0.394	6.96E-59	AC-G	CORO1B
FCGR2B.1	5.31E-63	-0.70216	0.022	0.168	7.93E-59	AC-G	FCGR2B
MLIP	8.60E-63	0.150826	0.05	0.005	1.29E-58	AC-G	MLIP
BRI3.1	9.69E-63	-0.3598	0.297	0.501	1.45E-58	AC-G	BRI3
ALOX5.1	1.02E-62	-0.55494	0.018	0.162	1.53E-58	AC-G	ALOX5
RHOC.1	1.09E-62	0.472788	0.631	0.514	1.63E-58	AC-G	RHOC
HNRNPAB.1	1.12E-62	-0.37538	0.225	0.431	1.67E-58	AC-G	HNRNPAB
RHBDF2.1	1.19E-62	-0.60807	0.047	0.203	1.77E-58	AC-G	RHBDF2
TAGLN3.1	1.32E-62	-0.58581	0.055	0.214	1.97E-58	AC-G	TAGLN3
FAM173A.1	1.45E-62	-0.3954	0.127	0.318	2.16E-58	AC-G	FAM173A
KLC1.1	1.56E-62	-0.41425	0.19	0.393	2.33E-58	AC-G	KLC1
MFNG.1	1.67E-62	-0.53598	0.01	0.149	2.50E-58	AC-G	MFNG
PLEKHO1.1	1.69E-62	-0.42393	0.117	0.306	2.52E-58	AC-G	PLEKHO1
NPL.1	2.23E-62	-0.61939	0.064	0.227	3.33E-58	AC-G	NPL
HLA-B.1	2.37E-62	0.354579	0.867	0.849	3.55E-58	AC-G	HLA-B
GYPC.1	2.40E-62	-0.61189	0.158	0.339	3.58E-58	AC-G	GYPC
STAB1.1	2.77E-62	-0.6451	0.034	0.186	4.14E-58	AC-G	STAB1
SLC43A2.1	3.14E-62	-0.49029	0.097	0.274	4.69E-58	AC-G	SLC43A2
CDC42	4.07E-62	-0.20554	0.428	0.658	6.09E-58	AC-G	CDC42
SORL1.1	5.74E-62	-0.54222	0.083	0.255	8.58E-58	AC-G	SORL1
BSG.1	5.76E-62	0.382401	0.776	0.735	8.61E-58	AC-G	BSG
RP11-552D	6.26E-62	-0.69777	0.007	0.142	9.35E-58	AC-G	RP11-552D4.1
DNAH11	7.67E-62	0.185942	0.064	0.009	1.15E-57	AC-G	DNAH11
PRDX2.1	1.16E-61	0.450701	0.774	0.687	1.73E-57	AC-G	PRDX2
KIF5C.1	1.33E-61	-0.45136	0.117	0.297	1.99E-57	AC-G	KIF5C
AP2S1.1	1.43E-61	-0.29841	0.487	0.694	2.14E-57	AC-G	AP2S1
SYPL1.1	1.63E-61	0.488045	0.529	0.382	2.43E-57	AC-G	SYPL1
HES6.1	2.13E-61	-0.58077	0.135	0.315	3.19E-57	AC-G	HES6
MCL1.1	2.22E-61	-0.36075	0.422	0.633	3.31E-57	AC-G	MCL1
ARL6IP4.1	2.41E-61	-0.33263	0.449	0.658	3.60E-57	AC-G	ARL6IP4

RGS12.1	2.93E-61	-0.41757	0.115	0.301	4.37E-57	AC-G	RGS12
KTN1.1	3.10E-61	-0.34608	0.361	0.559	4.64E-57	AC-G	KTN1
PABPC4.1	3.45E-61	-0.44126	0.168	0.36	5.15E-57	AC-G	PABPC4
C12orf75.	3.65E-61	-0.49784	0.059	0.219	5.45E-57	AC-G	C12orf75
TSPO	3.89E-61	-0.31264	0.422	0.628	5.82E-57	AC-G	TSPO
HMGB3.1	3.91E-61	-0.46927	0.126	0.308	5.85E-57	AC-G	HMGB3
SMARCB1.1	4.52E-61	-0.39027	0.244	0.447	6.75E-57	AC-G	SMARCB1
DDX5.1	5.39E-61	-0.18437	0.656	0.848	8.05E-57	AC-G	DDX5
SOCS6.1	6.17E-61	-0.4189	0.084	0.258	9.22E-57	AC-G	SOCS6
LCP1	7.37E-61	-0.56487	0.022	0.165	1.10E-56	AC-G	LCP1
RPS6KA1.1	7.50E-61	-0.50787	0.015	0.154	1.12E-56	AC-G	RPS6KA1
NCKAP1L.1	7.68E-61	-0.5633	0.009	0.144	1.15E-56	AC-G	NCKAP1L
SMARCA4.1	8.21E-61	-0.36873	0.197	0.393	1.23E-56	AC-G	SMARCA4
RCSD1.1	8.33E-61	-0.55713	0.008	0.142	1.24E-56	AC-G	RCSD1
SEZ6L.1	9.11E-61	-0.39686	0.017	0.158	1.36E-56	AC-G	SEZ6L
SRPX2	9.15E-61	0.49067	0.135	0.043	1.37E-56	AC-G	SRPX2
GADD45B	1.03E-60	0.527761	0.618	0.466	1.53E-56	AC-G	GADD45B
CD84.1	1.09E-60	-0.56276	0.014	0.152	1.63E-56	AC-G	CD84
PRTN3	1.12E-60	0.129883	0.032	0.001	1.67E-56	AC-G	PRTN3
COL5A1	1.24E-60	0.149877	0.053	0.006	1.85E-56	AC-G	COL5A1
RAB20.1	1.35E-60	-0.58985	0.094	0.264	2.02E-56	AC-G	RAB20
FOS.1	1.68E-60	0.395717	0.841	0.841	2.52E-56	AC-G	FOS
PMEPA1.1	2.02E-60	-0.41424	0.065	0.228	3.02E-56	AC-G	PMEPA1
BAZ1A.1	2.24E-60	-0.38503	0.158	0.351	3.35E-56	AC-G	BAZ1A
HSPB2	2.97E-60	0.225857	0.061	0.008	4.43E-56	AC-G	HSPB2
CSNK1E.1	3.43E-60	-0.3789	0.146	0.337	5.12E-56	AC-G	CSNK1E
CTDNEP1.1	4.01E-60	-0.39594	0.16	0.343	5.99E-56	AC-G	CTDNEP1
FABP5.1	4.12E-60	1.015851	0.513	0.369	6.16E-56	AC-G	FABP5
HSPB1	4.60E-60	0.431299	0.685	0.648	6.88E-56	AC-G	HSPB1
ARF6.1	4.74E-60	-0.39624	0.18	0.375	7.09E-56	AC-G	ARF6
MBP	8.42E-60	-0.77523	0.1	0.265	1.26E-55	AC-G	MBP
SNCA.1	9.58E-60	-0.58954	0.024	0.167	1.43E-55	AC-G	SNCA
PPP1R18.1	1.12E-59	-0.4594	0.14	0.323	1.67E-55	AC-G	PPP1R18
PRDX1.1	2.62E-59	0.309728	0.795	0.811	3.91E-55	AC-G	PRDX1
AOAH.1	2.62E-59	-0.5755	0.011	0.145	3.91E-55	AC-G	AOAH
CD33.1	3.36E-59	-0.56908	0.009	0.141	5.01E-55	AC-G	CD33
RABL6.1	3.39E-59	-0.38772	0.102	0.275	5.07E-55	AC-G	RABL6
CKLF.1	3.79E-59	-0.3524	0.341	0.554	5.66E-55	AC-G	CKLF
DBN1.1	4.58E-59	-0.42334	0.157	0.341	6.85E-55	AC-G	DBN1
APLNR	4.69E-59	0.202494	0.042	0.003	7.01E-55	AC-G	APLNR
STMN2.1	4.88E-59	-0.81927	0.059	0.211	7.29E-55	AC-G	STMN2
NKD2	5.21E-59	0.103975	0.042	0.003	7.79E-55	AC-G	NKD2
DBNL.1	7.35E-59	-0.34993	0.253	0.459	1.10E-54	AC-G	DBNL
AKR1B1.1	8.43E-59	-0.24823	0.44	0.661	1.26E-54	AC-G	AKR1B1
LIX1	9.59E-59	0.29886	0.068	0.011	1.43E-54	AC-G	LIX1
SMOX.1	1.11E-58	0.539748	0.367	0.223	1.67E-54	AC-G	SMOX
SEPT6.1	1.14E-58	-0.45552	0.041	0.19	1.70E-54	AC-G	6-Sep
PTAFR.1	1.28E-58	-0.56675	0.014	0.147	1.91E-54	AC-G	PTAFR
MPP1.1	1.78E-58	-0.51087	0.069	0.226	2.66E-54	AC-G	MPP1
NACA	1.81E-58	0.241101	0.877	0.934	2.71E-54	AC-G	NACA
MAP2.1	1.84E-58	-0.41116	0.181	0.368	2.76E-54	AC-G	MAP2

PGD.1	1.87E-58	-0.38293	0.136	0.323	2.79E-54	AC-G	PGD
NCF1.1	2.32E-58	-0.52034	0.013	0.145	3.46E-54	AC-G	NCF1
VPS37B.1	2.40E-58	-0.41695	0.108	0.283	3.59E-54	AC-G	VPS37B
CAPZA1.1	3.57E-58	-0.40083	0.23	0.424	5.33E-54	AC-G	CAPZA1
OSR1	6.72E-58	0.41077	0.051	0.006	1.00E-53	AC-G	OSR1
SPCS2.1	6.98E-58	0.357957	0.756	0.725	1.04E-53	AC-G	SPCS2
ATP6AP1.1	7.04E-58	-0.3781	0.323	0.526	1.05E-53	AC-G	ATP6AP1
BLVRB.1	8.15E-58	0.538591	0.498	0.35	1.22E-53	AC-G	BLVRB
BUD31.1	8.43E-58	0.416153	0.695	0.615	1.26E-53	AC-G	BUD31
KISS1R	8.85E-58	0.124092	0.041	0.003	1.32E-53	AC-G	KISS1R
FMNL1.1	8.94E-58	-0.5015	0.014	0.145	1.34E-53	AC-G	FMNL1
ROBO1.1	1.08E-57	-0.36881	0.051	0.203	1.62E-53	AC-G	ROBO1
ENTPD1.1	1.30E-57	-0.59003	0.041	0.186	1.94E-53	AC-G	ENTPD1
MAP7D1.1	1.81E-57	-0.38753	0.189	0.376	2.71E-53	AC-G	MAP7D1
POSTN.1	1.88E-57	-0.437	0.017	0.151	2.81E-53	AC-G	POSTN
LYSMD2.1	1.94E-57	-0.37048	0.061	0.218	2.89E-53	AC-G	LYSMD2
H3F3B.1	2.06E-57	0.239207	0.935	0.978	3.07E-53	AC-G	H3F3B
CHCHD2	2.45E-57	0.293915	0.869	0.882	3.66E-53	AC-G	CHCHD2
GNB2L1.1	3.54E-57	0.251057	0.888	0.953	5.29E-53	AC-G	GNB2L1
IER2.1	3.68E-57	-0.32269	0.426	0.652	5.51E-53	AC-G	IER2
DOCK8.1	4.28E-57	-0.52633	0.013	0.142	6.40E-53	AC-G	DOCK8
TPST1.1	4.47E-57	0.500925	0.351	0.218	6.68E-53	AC-G	TPST1
AKIRIN2.1	7.92E-57	-0.37887	0.216	0.413	1.18E-52	AC-G	AKIRIN2
SRRM2.1	8.22E-57	-0.34152	0.362	0.565	1.23E-52	AC-G	SRRM2
C1orf194	8.80E-57	0.1801	0.054	0.007	1.31E-52	AC-G	C1orf194
PDPN	1.06E-56	0.65787	0.543	0.431	1.58E-52	AC-G	PDPN
DMKN	1.13E-56	0.250048	0.043	0.004	1.69E-52	AC-G	DMKN
HMGA1.1	1.34E-56	-0.36531	0.185	0.376	2.00E-52	AC-G	HMGA1
NR4A3.1	1.37E-56	-0.5941	0.034	0.174	2.05E-52	AC-G	NR4A3
ECM2	1.89E-56	0.235189	0.062	0.009	2.83E-52	AC-G	ECM2
TMEM173.1	2.27E-56	-0.568	0.017	0.148	3.39E-52	AC-G	TMEM173
SPTSSA.1	2.30E-56	0.51486	0.53	0.408	3.44E-52	AC-G	SPTSSA
RBMS1.1	2.42E-56	-0.32819	0.104	0.278	3.61E-52	AC-G	RBMS1
LAMP1.1	2.51E-56	-0.34851	0.341	0.556	3.75E-52	AC-G	LAMP1
GNB4.1	2.53E-56	-0.41531	0.096	0.259	3.79E-52	AC-G	GNB4
PALMD	2.91E-56	0.180861	0.057	0.008	4.34E-52	AC-G	PALMD
IFT57.1	3.37E-56	0.538707	0.363	0.225	5.04E-52	AC-G	IFT57
CTSH.1	3.61E-56	-0.54033	0.153	0.323	5.39E-52	AC-G	CTSH
ENG.1	4.06E-56	-0.54288	0.045	0.188	6.06E-52	AC-G	ENG
PELI1.1	4.71E-56	-0.47877	0.077	0.235	7.04E-52	AC-G	PELI1
CHST11.1	5.33E-56	-0.42794	0.079	0.239	7.97E-52	AC-G	CHST11
LHFPL2.1	6.09E-56	-0.45581	0.04	0.184	9.10E-52	AC-G	LHFPL2
ASCL1.1	7.60E-56	-0.43399	0.057	0.211	1.14E-51	AC-G	ASCL1
CD68.1	8.39E-56	-0.57029	0.317	0.466	1.25E-51	AC-G	CD68
FGD4.1	8.40E-56	-0.4676	0.03	0.169	1.26E-51	AC-G	FGD4
APBA2.1	8.92E-56	-0.33856	0.073	0.233	1.33E-51	AC-G	APBA2
BAZ2B.1	9.39E-56	-0.36075	0.166	0.35	1.40E-51	AC-G	BAZ2B
PLA2G5	1.16E-55	0.662522	0.204	0.09	1.73E-51	AC-G	PLA2G5
SIPA1.1	1.36E-55	-0.43889	0.027	0.161	2.03E-51	AC-G	SIPA1
LRRFIP1.1	1.71E-55	-0.41121	0.194	0.384	2.55E-51	AC-G	LRRFIP1
MIS18BP1.	1.76E-55	-0.44076	0.031	0.169	2.63E-51	AC-G	MIS18BP1

CFD.1	1.83E-55	-0.5954	0.039	0.179	2.74E-51	AC-G	CFD
OSM.1	2.33E-55	-0.67616	0.032	0.167	3.48E-51	AC-G	OSM
PABPC1.1	2.38E-55	-0.28922	0.573	0.778	3.56E-51	AC-G	PABPC1
IFITM2.1	2.50E-55	-0.66236	0.147	0.309	3.74E-51	AC-G	IFITM2
SPRED1.1	2.56E-55	-0.36113	0.069	0.227	3.83E-51	AC-G	SPRED1
LDLRAD4.1	2.79E-55	-0.46185	0.031	0.168	4.17E-51	AC-G	LDLRAD4
NETO2.1	3.76E-55	-0.32258	0.041	0.185	5.62E-51	AC-G	NETO2
PDZK1IP1	3.91E-55	0.198268	0.053	0.007	5.85E-51	AC-G	PDZK1IP1
BIN2.1	5.10E-55	-0.51701	0.009	0.132	7.62E-51	AC-G	BIN2
PKIG.1	5.14E-55	0.582048	0.426	0.288	7.67E-51	AC-G	PKIG
NAGA.1	9.18E-55	-0.50362	0.06	0.209	1.37E-50	AC-G	NAGA
APMAP.1	1.03E-54	-0.34413	0.35	0.551	1.55E-50	AC-G	APMAP
DLL3.1	1.39E-54	-0.55793	0.031	0.166	2.07E-50	AC-G	DLL3
CCND1.1	1.51E-54	-0.43779	0.083	0.245	2.26E-50	AC-G	CCND1
GRINA.1	1.55E-54	-0.38643	0.275	0.468	2.31E-50	AC-G	GRINA
PRDX4.1	1.75E-54	0.499301	0.621	0.532	2.62E-50	AC-G	PRDX4
PARVB.1	1.79E-54	-0.41917	0.044	0.187	2.68E-50	AC-G	PARVB
PSMA2.1	1.96E-54	0.572117	0.14	0.046	2.93E-50	AC-G	PSMA2
CSK.1	2.29E-54	-0.4176	0.032	0.17	3.42E-50	AC-G	CSK
PTGER4.1	2.34E-54	-0.54332	0.018	0.144	3.50E-50	AC-G	PTGER4
TMSB15A.1	2.67E-54	-0.46127	0.056	0.202	3.99E-50	AC-G	TMSB15A
HNRNPU.1	2.75E-54	-0.30368	0.36	0.563	4.11E-50	AC-G	HNRNPU
ALDH1L1	3.71E-54	0.346195	0.143	0.048	5.55E-50	AC-G	ALDH1L1
FAM50A.1	3.80E-54	-0.36539	0.216	0.403	5.68E-50	AC-G	FAM50A
UNC93B1.1	3.95E-54	-0.49677	0.075	0.225	5.90E-50	AC-G	UNC93B1
C4orf47	4.09E-54	0.243303	0.086	0.02	6.12E-50	AC-G	C4orf47
LGMN.1	5.57E-54	-0.48878	0.201	0.38	8.32E-50	AC-G	LGMN
SNRPD2.1	5.74E-54	0.354832	0.763	0.745	8.58E-50	AC-G	SNRPD2
NCK2.1	5.75E-54	-0.33273	0.033	0.17	8.59E-50	AC-G	NCK2
ROGDI.1	5.83E-54	-0.40964	0.092	0.253	8.71E-50	AC-G	ROGDI
AXL.1	6.62E-54	-0.57073	0.06	0.204	9.89E-50	AC-G	AXL
NCOR2.1	6.86E-54	-0.36373	0.094	0.257	1.03E-49	AC-G	NCOR2
HNRNPA3	8.10E-54	-0.22542	0.386	0.587	1.21E-49	AC-G	HNRNPA3
TAF10.1	8.44E-54	-0.34297	0.091	0.252	1.26E-49	AC-G	TAF10
PDE4B.1	1.45E-53	-0.4097	0.129	0.305	2.17E-49	AC-G	PDE4B
CLTA.1	2.05E-53	-0.31936	0.454	0.662	3.06E-49	AC-G	CLTA
CHMP2A.1	2.12E-53	0.431198	0.7	0.646	3.17E-49	AC-G	CHMP2A
C9orf142.	2.13E-53	-0.38837	0.219	0.406	3.18E-49	AC-G	C9orf142
EVA1A	2.29E-53	0.15606	0.055	0.008	3.42E-49	AC-G	EVA1A
SOX11.1	2.35E-53	-0.49738	0.134	0.299	3.51E-49	AC-G	SOX11
TSC22D3	2.44E-53	-0.36702	0.297	0.487	3.65E-49	AC-G	TSC22D3
CYTH4.1	3.24E-53	-0.46539	0.01	0.132	4.84E-49	AC-G	CYTH4
TBL1XR1.1	3.68E-53	-0.34727	0.109	0.275	5.50E-49	AC-G	TBL1XR1
CCDC85B.1	3.87E-53	-0.35221	0.256	0.444	5.78E-49	AC-G	CCDC85B
GGA1.1	4.30E-53	-0.36956	0.143	0.318	6.43E-49	AC-G	GGA1
PON2.1	5.77E-53	0.512247	0.539	0.433	8.62E-49	AC-G	PON2
CYTH1.1	5.79E-53	-0.43368	0.042	0.181	8.66E-49	AC-G	CYTH1
FJX1.1	6.42E-53	-0.37703	0.074	0.226	9.59E-49	AC-G	FJX1
C9orf16.1	7.37E-53	-0.24991	0.409	0.616	1.10E-48	AC-G	C9orf16
SPAG4	8.42E-53	0.348524	0.115	0.034	1.26E-48	AC-G	SPAG4
TGFBR1.1	8.71E-53	-0.45002	0.046	0.184	1.30E-48	AC-G	TGFBR1

BET1	1.04E-52	0.528959	0.335	0.199	1.56E-48	AC-G	BET1
SHD.1	1.28E-52	-0.4356	0.02	0.146	1.91E-48	AC-G	SHD
PTGES	1.34E-52	0.243064	0.064	0.011	2.00E-48	AC-G	PTGES
RTN4.1	1.39E-52	-0.29682	0.518	0.735	2.07E-48	AC-G	RTN4
FAM107A	1.46E-52	0.321761	0.114	0.034	2.18E-48	AC-G	FAM107A
TTYH3.1	1.48E-52	-0.33385	0.106	0.274	2.20E-48	AC-G	TTYH3
CALM3.1	1.75E-52	-0.28415	0.429	0.624	2.62E-48	AC-G	CALM3
LYN.1	2.05E-52	-0.45113	0.056	0.197	3.07E-48	AC-G	LYN
PICALM.1	2.21E-52	-0.42021	0.142	0.308	3.30E-48	AC-G	PICALM
SDC4	2.92E-52	0.45883	0.184	0.079	4.36E-48	AC-G	SDC4
HMHA1.1	3.21E-52	-0.48853	0.01	0.128	4.80E-48	AC-G	HMHA1
OGFRL1.1	3.77E-52	-0.49179	0.062	0.205	5.64E-48	AC-G	OGFRL1
SSH2.1	3.89E-52	-0.41232	0.055	0.2	5.82E-48	AC-G	SSH2
COL9A3.1	4.31E-52	-0.43597	0.063	0.21	6.44E-48	AC-G	COL9A3
KCNF1.1	4.78E-52	-0.32072	0.019	0.144	7.15E-48	AC-G	KCNF1
RBPJ.1	5.15E-52	-0.34174	0.308	0.5	7.69E-48	AC-G	RBPJ
COQ2.1	5.50E-52	-0.39973	0.059	0.203	8.21E-48	AC-G	COQ2
ABRACL.1	5.74E-52	-0.33702	0.157	0.333	8.58E-48	AC-G	ABRACL
NFKBID.1	5.83E-52	-0.50915	0.031	0.162	8.71E-48	AC-G	NFKBID
TSR3.1	5.90E-52	-0.34261	0.135	0.303	8.82E-48	AC-G	TSR3
TGFBR2.1	7.41E-52	-0.3941	0.015	0.137	1.11E-47	AC-G	TGFBR2
ELAVL4.1	9.01E-52	-0.57314	0.061	0.201	1.35E-47	AC-G	ELAVL4
COL6A1.1	9.29E-52	0.494812	0.342	0.206	1.39E-47	AC-G	COL6A1
SYK.1	9.78E-52	-0.45405	0.008	0.125	1.46E-47	AC-G	SYK
DEK.1	1.01E-51	-0.35417	0.311	0.494	1.51E-47	AC-G	DEK
BPTF.1	1.01E-51	-0.35044	0.166	0.339	1.51E-47	AC-G	BPTF
NCF2.1	1.06E-51	-0.43449	0.006	0.12	1.59E-47	AC-G	NCF2
SLC44A3	1.07E-51	0.335582	0.084	0.02	1.61E-47	AC-G	SLC44A3
LYPLA1.1	1.09E-51	-0.34433	0.251	0.437	1.63E-47	AC-G	LYPLA1
LPAR5.1	1.13E-51	-0.47768	0.007	0.122	1.68E-47	AC-G	LPAR5
ARPC2	1.18E-51	-0.23367	0.613	0.805	1.77E-47	AC-G	ARPC2
CORO7.1	1.19E-51	-0.40334	0.043	0.18	1.78E-47	AC-G	CORO7
OLIG2.1	1.30E-51	-0.48456	0.059	0.202	1.95E-47	AC-G	OLIG2
ARL1.1	1.49E-51	0.490279	0.425	0.292	2.22E-47	AC-G	ARL1
GALNT1.1	1.50E-51	-0.32828	0.077	0.231	2.24E-47	AC-G	GALNT1
ABHD17A.1	1.63E-51	-0.34196	0.101	0.261	2.43E-47	AC-G	ABHD17A
PPP1R10.1	2.33E-51	-0.36383	0.142	0.316	3.48E-47	AC-G	PPP1R10
COMMD7.1	2.42E-51	-0.35492	0.247	0.429	3.62E-47	AC-G	COMMD7
CXCR4	2.65E-51	-0.50078	0.244	0.417	3.96E-47	AC-G	CXCR4
DCK.1	3.63E-51	-0.42137	0.128	0.293	5.42E-47	AC-G	DCK
RBM17.1	4.67E-51	-0.35803	0.216	0.398	6.97E-47	AC-G	RBM17
TIMP2.1	5.64E-51	-0.3483	0.169	0.356	8.42E-47	AC-G	TIMP2
FBP1.1	5.76E-51	-0.48762	0.017	0.136	8.60E-47	AC-G	FBP1
GNA13.1	7.27E-51	-0.4343	0.061	0.203	1.09E-46	AC-G	GNA13
CPNE2.1	7.45E-51	-0.3242	0.057	0.201	1.11E-46	AC-G	CPNE2
CD55.1	8.60E-51	-0.49645	0.017	0.137	1.29E-46	AC-G	CD55
VASH1.1	9.06E-51	-0.39049	0.025	0.15	1.35E-46	AC-G	VASH1
CCDC112.1	9.12E-51	-0.35525	0.122	0.288	1.36E-46	AC-G	CCDC112
SRPX.1	1.07E-50	0.407599	0.287	0.155	1.59E-46	AC-G	SRPX
SCAMP2.1	1.16E-50	-0.36283	0.221	0.404	1.74E-46	AC-G	SCAMP2
GNB2.1	1.17E-50	-0.28834	0.398	0.603	1.75E-46	AC-G	GNB2

LSP1.1	1.23E-50	-0.63729	0.053	0.188	1.84E-46	AC-G	LSP1
MARCH2.1	1.34E-50	-0.33024	0.099	0.259	2.00E-46	AC-G	2-Mar
CCDC3	1.59E-50	0.117817	0.042	0.004	2.37E-46	AC-G	CCDC3
MED10.1	1.65E-50	0.452021	0.572	0.466	2.47E-46	AC-G	MED10
VMA21	1.87E-50	-0.38369	0.192	0.369	2.79E-46	AC-G	VMA21
TMEM165.1	1.97E-50	-0.35493	0.3	0.487	2.94E-46	AC-G	TMEM165
UBALD2.1	2.23E-50	-0.35829	0.12	0.287	3.33E-46	AC-G	UBALD2
HNRNPM.1	2.27E-50	-0.33041	0.377	0.576	3.39E-46	AC-G	HNRNPM
UBXN1.1	2.89E-50	-0.2832	0.447	0.654	4.32E-46	AC-G	UBXN1
SERPINB1.	3.39E-50	-0.50495	0.134	0.292	5.07E-46	AC-G	SERPINB1
PARP1.1	3.68E-50	-0.35908	0.313	0.503	5.50E-46	AC-G	PARP1
RCN2.1	4.06E-50	-0.34829	0.322	0.495	6.07E-46	AC-G	RCN2
SKA2	4.68E-50	-0.30239	0.166	0.334	7.00E-46	AC-G	SKA2
GLRX5.1	4.76E-50	-0.35574	0.153	0.324	7.11E-46	AC-G	GLRX5
FN1	5.65E-50	0.49715	0.258	0.134	8.45E-46	AC-G	FN1
HBEGF.1	6.12E-50	-0.57716	0.094	0.239	9.14E-46	AC-G	HBEGF
ARL9.1	6.45E-50	-0.25112	0.009	0.123	9.65E-46	AC-G	ARL9
CNP.1	6.91E-50	-0.50531	0.229	0.406	1.03E-45	AC-G	CNP
KDM6B.1	8.57E-50	-0.46444	0.098	0.247	1.28E-45	AC-G	KDM6B
TMEM38B	9.09E-50	0.506637	0.269	0.146	1.36E-45	AC-G	TMEM38B
MAP2K2.1	9.87E-50	-0.36548	0.158	0.33	1.47E-45	AC-G	MAP2K2
PAK3.1	1.05E-49	-0.38873	0.035	0.164	1.57E-45	AC-G	PAK3
ARF4.1	1.21E-49	0.369252	0.671	0.612	1.81E-45	AC-G	ARF4
TRIP6.1	1.69E-49	0.528387	0.358	0.228	2.53E-45	AC-G	TRIP6
CMTM6.1	2.04E-49	-0.36391	0.273	0.455	3.04E-45	AC-G	CMTM6
C1orf54.1	2.12E-49	-0.47465	0.099	0.25	3.17E-45	AC-G	C1orf54
KIF1B.1	2.91E-49	-0.31031	0.15	0.322	4.35E-45	AC-G	KIF1B
MIDN.1	3.08E-49	-0.34332	0.26	0.444	4.60E-45	AC-G	MIDN
DPYSL5.1	3.53E-49	-0.33801	0.049	0.185	5.28E-45	AC-G	DPYSL5
MCM5.1	3.58E-49	-0.38384	0.04	0.171	5.35E-45	AC-G	MCM5
PGLS.1	5.48E-49	-0.29935	0.348	0.542	8.19E-45	AC-G	PGLS
KCNMA1	6.03E-49	-0.40734	0.035	0.164	9.02E-45	AC-G	KCNMA1
PHACTR1.1	6.56E-49	-0.54384	0.019	0.137	9.81E-45	AC-G	PHACTR1
RASSF5.1	8.20E-49	-0.43037	0.02	0.139	1.23E-44	AC-G	RASSF5
ST6GAL1.1	9.12E-49	-0.45509	0.015	0.129	1.36E-44	AC-G	ST6GAL1
MYL9	9.46E-49	0.333207	0.089	0.023	1.41E-44	AC-G	MYL9
ODC1.1	1.00E-48	-0.37294	0.253	0.421	1.50E-44	AC-G	ODC1
TAGAP.1	1.08E-48	-0.49374	0.008	0.119	1.61E-44	AC-G	TAGAP
TPGS1.1	1.24E-48	-0.34223	0.168	0.337	1.85E-44	AC-G	TPGS1
ID3.1	1.41E-48	0.838019	0.366	0.245	2.11E-44	AC-G	ID3
RP11-472N	1.81E-48	-0.33004	0.012	0.126	2.70E-44	AC-G	RP11-472N13.3
STX11.1	1.91E-48	-0.50433	0.018	0.133	2.86E-44	AC-G	STX11
DGKZ.1	2.00E-48	-0.34303	0.074	0.219	2.99E-44	AC-G	DGKZ
GAL3ST4.1	2.07E-48	-0.51127	0.058	0.193	3.09E-44	AC-G	GAL3ST4
SCN1A	2.14E-48	0.137969	0.066	0.013	3.21E-44	AC-G	SCN1A
REEP4.1	2.41E-48	-0.40985	0.054	0.189	3.61E-44	AC-G	REEP4
ANXA11.1	2.85E-48	-0.47226	0.095	0.239	4.26E-44	AC-G	ANXA11
CX3CR1.1	3.45E-48	-0.51992	0.009	0.119	5.16E-44	AC-G	CX3CR1
CKB.1	3.60E-48	-0.32003	0.455	0.607	5.38E-44	AC-G	CKB
UQCR11.1	3.72E-48	0.672715	0.212	0.096	5.55E-44	AC-G	UQCR11
NFIX.1	3.73E-48	-0.38147	0.193	0.36	5.57E-44	AC-G	NFIX

TGOLN2.1	3.92E-48	-0.34685	0.221	0.402	5.87E-44	AC-G	TGOLN2
PAK2.1	4.16E-48	-0.35284	0.208	0.38	6.22E-44	AC-G	PAK2
FCGBP.1	4.85E-48	-0.7108	0.113	0.252	7.25E-44	AC-G	FCGBP
RBX1.1	5.39E-48	-0.21896	0.502	0.697	8.05E-44	AC-G	RBX1
LCP2.1	5.96E-48	-0.47535	0.018	0.134	8.91E-44	AC-G	LCP2
PPP1R14B.	6.25E-48	-0.33892	0.202	0.372	9.35E-44	AC-G	PPP1R14B
RCAN1.1	6.98E-48	0.778126	0.366	0.249	1.04E-43	AC-G	RCAN1
SLC2A1.1	7.65E-48	0.468863	0.231	0.119	1.14E-43	AC-G	SLC2A1
WDR83OS.1	9.10E-48	0.393345	0.773	0.738	1.36E-43	AC-G	WDR83OS
ARHGAP4.1	9.50E-48	-0.4502	0.019	0.134	1.42E-43	AC-G	ARHGAP4
GNA15.1	1.01E-47	-0.44766	0.015	0.128	1.51E-43	AC-G	GNA15
SUSD3.1	1.24E-47	-0.47474	0.006	0.113	1.86E-43	AC-G	SUSD3
INSM1.1	1.42E-47	-0.31335	0.019	0.136	2.13E-43	AC-G	INSM1
MT1F.1	1.76E-47	0.50215	0.408	0.285	2.63E-43	AC-G	MT1F
HIVEP3.1	1.91E-47	-0.28609	0.043	0.174	2.85E-43	AC-G	HIVEP3
DOCK10.1	1.95E-47	-0.36338	0.026	0.145	2.92E-43	AC-G	DOCK10
CCL2	2.23E-47	0.877182	0.328	0.206	3.34E-43	AC-G	CCL2
PTRF.1	2.29E-47	0.4353	0.285	0.166	3.42E-43	AC-G	PTRF
FBLN5	2.30E-47	0.470254	0.098	0.028	3.43E-43	AC-G	FBLN5
TNFAIP8L2	2.37E-47	-0.4459	0.006	0.112	3.55E-43	AC-G	TNFAIP8L2
PRUNE2	2.42E-47	0.392251	0.212	0.098	3.61E-43	AC-G	PRUNE2
MAF1.1	2.72E-47	-0.32312	0.275	0.458	4.06E-43	AC-G	MAF1
TNRC6B.1	3.26E-47	-0.31242	0.199	0.382	4.87E-43	AC-G	TNRC6B
NUDT14.1	3.31E-47	-0.39025	0.073	0.215	4.95E-43	AC-G	NUDT14
C17orf89.	3.77E-47	-0.33078	0.275	0.45	5.63E-43	AC-G	C17orf89
LZTFL1	3.78E-47	0.324659	0.152	0.06	5.65E-43	AC-G	LZTFL1
STEAP1	4.19E-47	0.284087	0.074	0.017	6.26E-43	AC-G	STEAP1
MYO9B.1	4.21E-47	-0.348	0.133	0.296	6.29E-43	AC-G	MYO9B
MYBPH	4.24E-47	0.193981	0.062	0.012	6.34E-43	AC-G	MYBPH
MICAL1.1	4.58E-47	-0.39097	0.037	0.161	6.85E-43	AC-G	MICAL1
PIK3IP1.1	5.85E-47	-0.49883	0.051	0.182	8.75E-43	AC-G	PIK3IP1
SEZ6.1	5.86E-47	-0.34718	0.034	0.159	8.76E-43	AC-G	SEZ6
CLTB.1	7.53E-47	-0.32193	0.289	0.47	1.12E-42	AC-G	CLTB
LILRB1.1	7.80E-47	-0.43917	0.005	0.108	1.17E-42	AC-G	LILRB1
CLEC5A.1	8.33E-47	-0.51705	0.01	0.117	1.24E-42	AC-G	CLEC5A
GGT5	8.89E-47	0.139533	0.056	0.01	1.33E-42	AC-G	GGT5
MFSD12.1	9.02E-47	-0.3102	0.072	0.215	1.35E-42	AC-G	MFSD12
FXYD6.1	9.94E-47	-0.39144	0.331	0.486	1.48E-42	AC-G	FXYD6
STK4.1	1.00E-46	-0.39174	0.149	0.313	1.49E-42	AC-G	STK4
CNDP2.1	1.02E-46	-0.29141	0.282	0.46	1.52E-42	AC-G	CNDP2
CDK6.1	1.05E-46	-0.33146	0.083	0.229	1.57E-42	AC-G	CDK6
TRIB3.1	1.06E-46	0.444697	0.148	0.06	1.58E-42	AC-G	TRIB3
ARHGAP30.	1.13E-46	-0.42772	0.007	0.111	1.69E-42	AC-G	ARHGAP30
CLN8.1	1.41E-46	-0.41743	0.139	0.294	2.11E-42	AC-G	CLN8
SERPINA3.	1.48E-46	0.222357	0.03	0.002	2.21E-42	AC-G	SERPINA3
CKMT2	1.48E-46	0.132098	0.032	0.002	2.21E-42	AC-G	CKMT2
SYNPO	1.81E-46	0.194544	0.066	0.014	2.71E-42	AC-G	SYNPO
PDK4.1	2.21E-46	-0.60348	0.062	0.19	3.30E-42	AC-G	PDK4
PLAUR.1	2.35E-46	-0.58017	0.251	0.389	3.51E-42	AC-G	PLAUR
IFNGR1.1	2.40E-46	-0.44492	0.31	0.462	3.59E-42	AC-G	IFNGR1
DYNLRB2	2.50E-46	0.172669	0.046	0.006	3.74E-42	AC-G	DYNLRB2

TPM3.1	2.59E-46	-0.28813	0.41	0.587	3.87E-42	AC-G	TPM3
IL1B.1	2.80E-46	-0.63612	0.107	0.244	4.19E-42	AC-G	IL1B
TRIM36.1	3.08E-46	-0.30893	0.066	0.205	4.60E-42	AC-G	TRIM36
ENC1.1	3.34E-46	-0.36417	0.1	0.248	4.99E-42	AC-G	ENC1
CDC42SE1.	3.37E-46	-0.36606	0.166	0.331	5.04E-42	AC-G	CDC42SE1
RP11-108M	3.49E-46	-0.4185	0.012	0.119	5.22E-42	AC-G	RP11-108M9.4
GLA.1	4.44E-46	-0.42228	0.095	0.239	6.64E-42	AC-G	GLA
TMC6.1	4.78E-46	-0.4323	0.018	0.129	7.14E-42	AC-G	TMC6
DUSP2.1	4.80E-46	-0.51867	0.018	0.128	7.17E-42	AC-G	DUSP2
PALD1.1	5.03E-46	-0.4424	0.012	0.119	7.52E-42	AC-G	PALD1
SIGLEC9.1	5.22E-46	-0.42043	0.006	0.11	7.79E-42	AC-G	SIGLEC9
TMEM119.1	5.55E-46	-0.51708	0.014	0.123	8.29E-42	AC-G	TMEM119
ALCAM.1	7.29E-46	-0.35406	0.078	0.219	1.09E-41	AC-G	ALCAM
UBE2L3.1	8.28E-46	-0.23209	0.38	0.565	1.24E-41	AC-G	UBE2L3
EPB41L3.1	8.44E-46	-0.43853	0.014	0.123	1.26E-41	AC-G	EPB41L3
ELF1.1	8.90E-46	-0.49294	0.121	0.268	1.33E-41	AC-G	ELF1
EFNA1	1.15E-45	0.463052	0.22	0.111	1.72E-41	AC-G	EFNA1
DEF6.1	1.20E-45	-0.39135	0.006	0.108	1.80E-41	AC-G	DEF6
HNRNPUL1.	1.39E-45	-0.34435	0.234	0.413	2.08E-41	AC-G	HNRNPUL1
NAV1.1	1.63E-45	-0.30095	0.053	0.184	2.44E-41	AC-G	NAV1
CTNNBIP1.	1.91E-45	-0.30555	0.094	0.238	2.85E-41	AC-G	CTNNBIP1
CKS2.1	2.24E-45	-0.3968	0.228	0.393	3.35E-41	AC-G	CKS2
ROCK1.1	2.29E-45	-0.3527	0.173	0.335	3.42E-41	AC-G	ROCK1
NDUFA7.1	2.32E-45	0.455048	0.103	0.031	3.46E-41	AC-G	NDUFA7
HSD17B11	2.33E-45	-0.4107	0.238	0.404	3.49E-41	AC-G	HSD17B11
PPM1G.1	2.62E-45	-0.31138	0.276	0.446	3.92E-41	AC-G	PPM1G
RFFL.1	2.83E-45	-0.29798	0.056	0.186	4.23E-41	AC-G	RFFL
CHD9.1	3.28E-45	-0.27935	0.313	0.497	4.90E-41	AC-G	CHD9
TACC1.1	3.39E-45	-0.34788	0.178	0.339	5.07E-41	AC-G	TACC1
DOCK4.1	3.62E-45	-0.42561	0.055	0.183	5.41E-41	AC-G	DOCK4
HPGDS.1	3.64E-45	-0.48731	0.005	0.105	5.43E-41	AC-G	HPGDS
SPP1.1	6.75E-45	0.356932	0.856	0.853	1.01E-40	AC-G	SPP1
SCN9A	7.69E-45	0.248111	0.089	0.025	1.15E-40	AC-G	SCN9A
FAM180A	9.13E-45	0.118609	0.03	0.002	1.36E-40	AC-G	FAM180A
USP22.1	9.42E-45	-0.28775	0.179	0.347	1.41E-40	AC-G	USP22
BTK.1	1.02E-44	-0.40056	0.004	0.102	1.53E-40	AC-G	BTK
ACTA2.1	1.03E-44	0.337416	0.115	0.039	1.53E-40	AC-G	ACTA2
CTGF	1.03E-44	0.296981	0.078	0.02	1.54E-40	AC-G	CTGF
ATRX.1	1.06E-44	-0.30731	0.294	0.473	1.59E-40	AC-G	ATRX
SORT1.1	1.09E-44	-0.35118	0.105	0.254	1.63E-40	AC-G	SORT1
NR4A1.1	1.10E-44	-0.53177	0.154	0.296	1.65E-40	AC-G	NR4A1
SEMA6D.1	1.12E-44	-0.25739	0.05	0.178	1.68E-40	AC-G	SEMA6D
FAM210B.1	1.15E-44	-0.35251	0.143	0.295	1.72E-40	AC-G	FAM210B
TNFSF12.1	1.17E-44	-0.37963	0.072	0.208	1.75E-40	AC-G	TNFSF12
SAP30.1	1.23E-44	-0.33521	0.115	0.264	1.83E-40	AC-G	SAP30
DPP7.1	1.25E-44	-0.34589	0.316	0.496	1.87E-40	AC-G	DPP7
RNPEPL1.1	1.29E-44	-0.31256	0.031	0.149	1.93E-40	AC-G	RNPEPL1
SLC25A19.	1.30E-44	-0.44636	0.06	0.19	1.94E-40	AC-G	SLC25A19
FAM181A	1.32E-44	0.476524	0.135	0.052	1.97E-40	AC-G	FAM181A
NINJ2.1	1.68E-44	-0.47468	0.005	0.103	2.50E-40	AC-G	NINJ2
JOSD1.1	1.70E-44	-0.35134	0.051	0.178	2.54E-40	AC-G	JOSD1

P2RY13.1	1.90E-44	-0.50614	0.003	0.1	2.83E-40	AC-G	P2RY13
ACAP3.1	2.20E-44	-0.33664	0.096	0.239	3.29E-40	AC-G	ACAP3
CXADR.1	2.49E-44	-0.3332	0.172	0.334	3.72E-40	AC-G	CXADR
ETV1.1	2.63E-44	-0.33613	0.12	0.269	3.94E-40	AC-G	ETV1
RANBP1.1	3.28E-44	-0.28232	0.418	0.609	4.90E-40	AC-G	RANBP1
NUDT1.1	3.38E-44	-0.33085	0.21	0.378	5.05E-40	AC-G	NUDT1
HMGB2.1	4.02E-44	-0.37199	0.36	0.521	6.01E-40	AC-G	HMGB2
MAGI1.1	4.24E-44	-0.32275	0.079	0.22	6.34E-40	AC-G	MAGI1
HMOX1.1	4.26E-44	-0.49719	0.174	0.318	6.37E-40	AC-G	HMOX1
IL33	4.49E-44	0.295014	0.104	0.033	6.71E-40	AC-G	IL33
GRAMD1A.1	4.63E-44	-0.3413	0.145	0.3	6.92E-40	AC-G	GRAMD1A
NT5DC2.1	4.65E-44	-0.24551	0.054	0.186	6.94E-40	AC-G	NT5DC2
LRRC25.1	4.99E-44	-0.43115	0.008	0.109	7.46E-40	AC-G	LRRC25
PDGFRA.1	5.07E-44	-0.3541	0.045	0.168	7.57E-40	AC-G	PDGFRA
TAOK3.1	5.28E-44	-0.29885	0.239	0.415	7.89E-40	AC-G	TAOK3
RAC2.1	5.50E-44	-0.48159	0.026	0.137	8.22E-40	AC-G	RAC2
CXCL2	6.44E-44	0.48653	0.167	0.071	9.63E-40	AC-G	CXCL2
ARL8A.1	6.74E-44	-0.29573	0.14	0.298	1.01E-39	AC-G	ARL8A
ETS2.1	7.39E-44	-0.51609	0.096	0.229	1.10E-39	AC-G	ETS2
NOP10.1	7.74E-44	-0.24862	0.454	0.636	1.16E-39	AC-G	NOP10
CYB5R4.1	8.83E-44	-0.35998	0.066	0.198	1.32E-39	AC-G	CYB5R4
POU2F2.1	9.28E-44	-0.44013	0.037	0.154	1.39E-39	AC-G	POU2F2
ENHO.1	9.73E-44	-0.25325	0.045	0.171	1.45E-39	AC-G	ENHO
NUP214.1	9.90E-44	-0.49098	0.083	0.217	1.48E-39	AC-G	NUP214
CALU.1	9.97E-44	0.44729	0.488	0.387	1.49E-39	AC-G	CALU
ELAVL3.1	1.02E-43	-0.39148	0.18	0.336	1.52E-39	AC-G	ELAVL3
PTPN18.1	1.11E-43	-0.37968	0.094	0.233	1.66E-39	AC-G	PTPN18
CYTIP.1	1.70E-43	-0.51974	0.016	0.121	2.54E-39	AC-G	CYTIP
SH3BP5.1	1.78E-43	-0.32506	0.084	0.224	2.67E-39	AC-G	SH3BP5
DENND3.1	2.75E-43	-0.43456	0.014	0.117	4.11E-39	AC-G	DENND3
PCSK1N.1	2.86E-43	-0.37742	0.282	0.447	4.27E-39	AC-G	PCSK1N
EIF3F.1	3.30E-43	-0.27686	0.448	0.636	4.93E-39	AC-G	EIF3F
ENOSF1.1	3.40E-43	-0.24216	0.029	0.143	5.09E-39	AC-G	ENOSF1
ROM1	3.56E-43	0.514775	0.196	0.097	5.32E-39	AC-G	ROM1
MORN5	3.59E-43	0.123511	0.043	0.006	5.36E-39	AC-G	MORN5
RNF213.1	3.88E-43	-0.33509	0.133	0.286	5.79E-39	AC-G	RNF213
PRKCA	3.88E-43	-0.2881	0.083	0.221	5.80E-39	AC-G	PRKCA
C12orf57.	4.01E-43	0.447662	0.617	0.521	5.99E-39	AC-G	C12orf57
EMC10.1	4.12E-43	-0.28887	0.298	0.48	6.16E-39	AC-G	EMC10
LYL1.1	5.02E-43	-0.38289	0.003	0.097	7.50E-39	AC-G	LYL1
CEBPB.1	5.62E-43	-0.28975	0.275	0.466	8.39E-39	AC-G	CEBPB
C22orf39.	5.74E-43	-0.31798	0.133	0.284	8.58E-39	AC-G	C22orf39
DLX6-AS1.	6.06E-43	-0.67605	0.036	0.148	9.06E-39	AC-G	DLX6-AS1
TANC2.1	6.74E-43	-0.26847	0.075	0.211	1.01E-38	AC-G	TANC2
RERE.1	7.53E-43	-0.27314	0.091	0.234	1.13E-38	AC-G	RERE
MKNK1.1	8.50E-43	-0.44498	0.064	0.191	1.27E-38	AC-G	MKNK1
ABL2.1	9.02E-43	-0.38005	0.141	0.293	1.35E-38	AC-G	ABL2
RBFOX2.1	1.15E-42	-0.33293	0.098	0.239	1.72E-38	AC-G	RBFOX2
POLD2.1	1.57E-42	0.48657	0.509	0.404	2.35E-38	AC-G	POLD2
ADPGK.1	1.68E-42	-0.37132	0.136	0.286	2.51E-38	AC-G	ADPGK
TCIRG1.1	1.75E-42	-0.43337	0.032	0.144	2.62E-38	AC-G	TCIRG1

PGP.1	1.95E-42	-0.31106	0.1	0.242	2.92E-38	AC-G	PGP
PPP1R3C	2.23E-42	0.226831	0.072	0.018	3.33E-38	AC-G	PPP1R3C
RAMP3	2.28E-42	0.246995	0.072	0.017	3.40E-38	AC-G	RAMP3
CCRL2.1	2.47E-42	-0.43414	0.008	0.105	3.69E-38	AC-G	CCRL2
TMEM51.1	2.76E-42	-0.34122	0.027	0.137	4.13E-38	AC-G	TMEM51
CLN6.1	2.77E-42	-0.31481	0.034	0.148	4.14E-38	AC-G	CLN6
ARHGAP15.	3.00E-42	-0.4524	0.013	0.113	4.48E-38	AC-G	ARHGAP15
CNIH2.1	3.31E-42	-0.2434	0.034	0.148	4.94E-38	AC-G	CNIH2
CTSK.1	4.38E-42	0.297827	0.111	0.038	6.55E-38	AC-G	CTSK
NDUFA5	4.40E-42	0.364521	0.649	0.563	6.58E-38	AC-G	NDUFA5
SLC10A4.1	4.41E-42	-0.20457	0.013	0.113	6.58E-38	AC-G	SLC10A4
DLX5.1	4.41E-42	-0.59576	0.046	0.163	6.60E-38	AC-G	DLX5
STMN4.1	4.79E-42	-0.54913	0.186	0.329	7.16E-38	AC-G	STMN4
ARL4C.1	6.04E-42	-0.32208	0.237	0.406	9.03E-38	AC-G	ARL4C
MERTK.1	6.69E-42	-0.45903	0.032	0.142	1.00E-37	AC-G	MERTK
CFL1	7.88E-42	0.249257	0.908	0.958	1.18E-37	AC-G	CFL1
SEC14L1.1	9.03E-42	-0.26605	0.159	0.317	1.35E-37	AC-G	SEC14L1
POU3F2.1	9.46E-42	-0.33014	0.102	0.238	1.41E-37	AC-G	POU3F2
DAD1.1	1.07E-41	0.328061	0.746	0.716	1.60E-37	AC-G	DAD1
PTOV1.1	1.15E-41	-0.29489	0.304	0.465	1.72E-37	AC-G	PTOV1
C19orf60.	1.17E-41	-0.26732	0.346	0.533	1.75E-37	AC-G	C19orf60
SIGIRR.1	1.54E-41	-0.3491	0.085	0.218	2.30E-37	AC-G	SIGIRR
GTF3A.1	1.55E-41	-0.2743	0.35	0.535	2.31E-37	AC-G	GTF3A
RIN3.1	1.56E-41	-0.39856	0.026	0.133	2.33E-37	AC-G	RIN3
CCNH.1	1.60E-41	-0.34374	0.272	0.439	2.39E-37	AC-G	CCNH
SIRPA.1	1.65E-41	-0.38334	0.125	0.266	2.47E-37	AC-G	SIRPA
DDAH2.1	1.99E-41	-0.21335	0.336	0.519	2.97E-37	AC-G	DDAH2
TTC9B.1	2.20E-41	-0.41517	0.029	0.138	3.28E-37	AC-G	TTC9B
SH3BGRL	2.41E-41	-0.16617	0.476	0.682	3.60E-37	AC-G	SH3BGRL
NT5C.1	2.45E-41	-0.3217	0.187	0.346	3.66E-37	AC-G	NT5C
H2AFY2.1	2.74E-41	-0.21452	0.018	0.121	4.10E-37	AC-G	H2AFY2
LEPROTL1.	2.86E-41	-0.27841	0.247	0.415	4.27E-37	AC-G	LEPROTL1
PAQR4.1	3.17E-41	-0.32344	0.06	0.184	4.74E-37	AC-G	PAQR4
MFSD2A.1	3.35E-41	0.388897	0.177	0.083	5.01E-37	AC-G	MFSD2A
PNRC1.1	3.49E-41	-0.25821	0.372	0.573	5.22E-37	AC-G	PNRC1
NLRP3.1	3.62E-41	-0.42412	0.008	0.103	5.42E-37	AC-G	NLRP3
EVA1B.1	3.89E-41	-0.22402	0.031	0.141	5.82E-37	AC-G	EVA1B
ZFHX3.1	4.42E-41	-0.34024	0.083	0.217	6.60E-37	AC-G	ZFHX3
ACTR1A.1	4.60E-41	-0.28578	0.15	0.303	6.88E-37	AC-G	ACTR1A
SH2B3.1	4.68E-41	-0.39409	0.022	0.125	6.99E-37	AC-G	SH2B3
CD151.1	4.96E-41	0.509715	0.566	0.466	7.41E-37	AC-G	CD151
GNAS.1	5.28E-41	-0.23309	0.518	0.672	7.90E-37	AC-G	GNAS
SLC25A11.	5.41E-41	-0.24093	0.274	0.448	8.09E-37	AC-G	SLC25A11
PACRG	6.43E-41	0.323607	0.088	0.027	9.61E-37	AC-G	PACRG
KLF13.1	6.44E-41	-0.31031	0.033	0.143	9.62E-37	AC-G	KLF13
NAIP.1	6.48E-41	-0.35865	0.021	0.125	9.68E-37	AC-G	NAIP
RASGEF1B.	7.63E-41	-0.47043	0.031	0.138	1.14E-36	AC-G	RASGEF1B
PEBP1.1	8.34E-41	0.273492	0.801	0.804	1.25E-36	AC-G	PEBP1
MXRA8	8.82E-41	0.243373	0.086	0.025	1.32E-36	AC-G	MXRA8
POLR2I.1	9.21E-41	0.404108	0.624	0.539	1.38E-36	AC-G	POLR2I
PTP4A1.1	1.13E-40	-0.28847	0.261	0.435	1.69E-36	AC-G	PTP4A1

H2AFV.1	1.27E-40	-0.26111	0.381	0.55	1.89E-36	AC-G	H2AFV
PLBD1.1	1.35E-40	-0.36945	0.013	0.11	2.02E-36	AC-G	PLBD1
RCN1.1	1.57E-40	0.372787	0.434	0.338	2.34E-36	AC-G	RCN1
EPC1.1	1.73E-40	-0.3238	0.163	0.316	2.58E-36	AC-G	EPC1
DUSP1.1	1.73E-40	-0.29938	0.417	0.605	2.58E-36	AC-G	DUSP1
TMEM206.1	1.83E-40	-0.31339	0.113	0.254	2.74E-36	AC-G	TMEM206
FRMD3	1.91E-40	0.304836	0.118	0.043	2.86E-36	AC-G	FRMD3
EMP2	1.97E-40	0.443419	0.19	0.092	2.95E-36	AC-G	EMP2
CAPZB	2.13E-40	-0.1805	0.551	0.731	3.19E-36	AC-G	CAPZB
TUBA4A	2.32E-40	0.19246	0.117	0.042	3.47E-36	AC-G	TUBA4A
CD48.1	2.43E-40	-0.42585	0.004	0.093	3.63E-36	AC-G	CD48
B3GNT5.1	2.43E-40	-0.37764	0.045	0.16	3.64E-36	AC-G	B3GNT5
ASAH1.1	2.62E-40	-0.2324	0.498	0.681	3.92E-36	AC-G	ASAH1
HEXA	2.73E-40	-0.25175	0.249	0.417	4.07E-36	AC-G	HEXA
MSRB2.1	3.11E-40	-0.33766	0.134	0.278	4.65E-36	AC-G	MSRB2
CDO1.1	3.13E-40	0.516966	0.334	0.215	4.68E-36	AC-G	CDO1
CHD1.1	3.32E-40	-0.33422	0.168	0.317	4.96E-36	AC-G	CHD1
TXN2.1	3.43E-40	-0.25975	0.289	0.462	5.13E-36	AC-G	TXN2
ZNHIT1	3.45E-40	0.344992	0.691	0.644	5.16E-36	AC-G	ZNHIT1
IL10.1	3.68E-40	-0.51816	0.021	0.123	5.50E-36	AC-G	IL10
IGFBP7-AS	4.01E-40	0.251025	0.076	0.02	5.99E-36	AC-G	IGFBP7-AS1
ARHGAP9.1	4.39E-40	-0.36491	0.008	0.101	6.56E-36	AC-G	ARHGAP9
C1RL	4.77E-40	0.303995	0.12	0.044	7.13E-36	AC-G	C1RL
RAB31.1	4.86E-40	-0.26026	0.309	0.489	7.26E-36	AC-G	RAB31
MAPK1.1	4.86E-40	-0.299	0.122	0.266	7.26E-36	AC-G	MAPK1
NCOA4.1	5.45E-40	-0.33094	0.139	0.287	8.15E-36	AC-G	NCOA4
FKBP10.1	5.83E-40	0.472365	0.304	0.194	8.72E-36	AC-G	FKBP10
SSBP4.1	6.72E-40	-0.29249	0.146	0.294	1.00E-35	AC-G	SSBP4
ABI1.1	6.80E-40	-0.3228	0.12	0.264	1.02E-35	AC-G	ABI1
AC114803.	7.04E-40	0.162657	0.037	0.004	1.05E-35	AC-G	AC114803.3
RTCB.1	7.34E-40	-0.31391	0.199	0.36	1.10E-35	AC-G	RTCB
TUBB6	7.56E-40	0.55935	0.274	0.166	1.13E-35	AC-G	TUBB6
ARID4A	7.66E-40	-0.30736	0.15	0.299	1.15E-35	AC-G	ARID4A
PADI2.1	8.44E-40	-0.47013	0.042	0.153	1.26E-35	AC-G	PADI2
TUBB4A.1	8.83E-40	-0.49279	0.03	0.135	1.32E-35	AC-G	TUBB4A
CCL4L2.1	8.95E-40	-0.61393	0.009	0.101	1.34E-35	AC-G	CCL4L2
SLC8A1.1	1.03E-39	-0.33196	0.048	0.164	1.54E-35	AC-G	SLC8A1
CXorf21.1	1.18E-39	-0.40661	0.003	0.09	1.76E-35	AC-G	CXorf21
FGR.1	1.25E-39	-0.38157	0.007	0.098	1.86E-35	AC-G	FGR
F13A1	1.27E-39	0.625391	0.254	0.14	1.91E-35	AC-G	F13A1
ABCG1.1	1.29E-39	-0.39867	0.04	0.149	1.93E-35	AC-G	ABCG1
DSE.1	1.40E-39	-0.42947	0.045	0.156	2.09E-35	AC-G	DSE
TMEM132B.	1.40E-39	-0.2126	0.018	0.118	2.09E-35	AC-G	TMEM132B
PABPN1.1	1.46E-39	-0.20952	0.213	0.383	2.18E-35	AC-G	PABPN1
MACF1	1.51E-39	-0.27766	0.192	0.347	2.26E-35	AC-G	MACF1
SCAND1.1	1.56E-39	-0.26781	0.436	0.597	2.33E-35	AC-G	SCAND1
TMPO.1	1.65E-39	-0.3404	0.086	0.215	2.46E-35	AC-G	TMPO
LITAF.1	1.66E-39	-0.36517	0.336	0.483	2.47E-35	AC-G	LITAF
MBNL1.1	1.79E-39	-0.34696	0.11	0.246	2.67E-35	AC-G	MBNL1
FNIP2.1	1.79E-39	-0.3927	0.087	0.217	2.68E-35	AC-G	FNIP2
PCBP4.1	1.84E-39	-0.31704	0.136	0.279	2.75E-35	AC-G	PCBP4

WHSC1L1.1	2.02E-39	-0.32383	0.241	0.398	3.02E-35	AC-G	WHSC1L1
RNF166.1	2.08E-39	-0.39785	0.082	0.205	3.11E-35	AC-G	RNF166
MGAT1.1	2.19E-39	-0.4051	0.234	0.382	3.28E-35	AC-G	MGAT1
CAPG.1	2.25E-39	-0.38653	0.304	0.436	3.36E-35	AC-G	CAPG
CLDN10	2.61E-39	0.596202	0.115	0.043	3.90E-35	AC-G	CLDN10
RBM25.1	2.70E-39	-0.2628	0.296	0.468	4.04E-35	AC-G	RBM25
SLC25A39	2.75E-39	-0.30379	0.268	0.432	4.12E-35	AC-G	SLC25A39
MEF2A.1	2.82E-39	-0.38568	0.104	0.237	4.21E-35	AC-G	MEF2A
SSR3.1	2.83E-39	0.458432	0.587	0.514	4.24E-35	AC-G	SSR3
ST14.1	2.89E-39	-0.34023	0.002	0.087	4.32E-35	AC-G	ST14
VAV1.1	3.00E-39	-0.34542	0.002	0.088	4.49E-35	AC-G	VAV1
PDCD5.1	3.22E-39	0.437573	0.601	0.527	4.81E-35	AC-G	PDCD5
SMDT1	3.43E-39	-0.16277	0.404	0.589	5.13E-35	AC-G	SMDT1
GTF2I.1	3.93E-39	-0.30606	0.292	0.453	5.87E-35	AC-G	GTF2I
BMP2K	4.51E-39	-0.33609	0.029	0.134	6.74E-35	AC-G	BMP2K
CUX1.1	5.33E-39	-0.26018	0.12	0.264	7.97E-35	AC-G	CUX1
MUM1.1	5.39E-39	-0.23445	0.227	0.39	8.06E-35	AC-G	MUM1
TFDP1.1	6.11E-39	-0.28286	0.084	0.212	9.14E-35	AC-G	TFDP1
UBE2R2.1	6.36E-39	-0.32024	0.164	0.31	9.50E-35	AC-G	UBE2R2
PTGDS	6.42E-39	0.469603	0.271	0.15	9.60E-35	AC-G	PTGDS
LGR4.1	6.67E-39	-0.18207	0.019	0.118	9.97E-35	AC-G	LGR4
SMCO4.1	7.09E-39	-0.403	0.041	0.15	1.06E-34	AC-G	SMCO4
IQCA1	7.12E-39	0.140142	0.045	0.007	1.06E-34	AC-G	IQCA1
PTPN1.1	7.20E-39	-0.3025	0.115	0.255	1.08E-34	AC-G	PTPN1
TLR2.1	7.94E-39	-0.44409	0.026	0.128	1.19E-34	AC-G	TLR2
FEZ2.1	8.39E-39	-0.29061	0.302	0.477	1.25E-34	AC-G	FEZ2
GRK6.1	8.93E-39	-0.26522	0.042	0.153	1.33E-34	AC-G	GRK6
ANP32B.1	9.25E-39	-0.29469	0.313	0.476	1.38E-34	AC-G	ANP32B
MZT2B.1	1.04E-38	-0.25231	0.455	0.62	1.55E-34	AC-G	MZT2B
DNM2.1	1.11E-38	-0.2885	0.123	0.266	1.67E-34	AC-G	DNM2
FAM114A1.	1.33E-38	0.329435	0.194	0.096	1.98E-34	AC-G	FAM114A1
MXD4.1	1.49E-38	-0.30447	0.082	0.209	2.23E-34	AC-G	MXD4
IL17RA.1	1.76E-38	-0.35199	0.032	0.136	2.63E-34	AC-G	IL17RA
IL1R1	1.82E-38	0.177493	0.052	0.01	2.72E-34	AC-G	IL1R1
WSCD1.1	1.83E-38	-0.24394	0.038	0.149	2.73E-34	AC-G	WSCD1
CLASP2.1	1.87E-38	-0.35863	0.09	0.218	2.80E-34	AC-G	CLASP2
DOCK2.1	1.91E-38	-0.35871	0.005	0.091	2.85E-34	AC-G	DOCK2
SCARB2.1	2.10E-38	-0.31068	0.246	0.408	3.14E-34	AC-G	SCARB2
PFKFB3.1	2.10E-38	-0.40029	0.13	0.266	3.14E-34	AC-G	PFKFB3
SAP18	2.49E-38	0.285949	0.764	0.767	3.72E-34	AC-G	SAP18
FAM183A	2.91E-38	0.126006	0.028	0.002	4.34E-34	AC-G	FAM183A
GPCPD1.1	3.28E-38	-0.27986	0.09	0.219	4.90E-34	AC-G	GPCPD1
CORO1C.1	3.36E-38	-0.2217	0.238	0.399	5.03E-34	AC-G	CORO1C
CDKN1C.1	3.36E-38	-0.35592	0.028	0.13	5.03E-34	AC-G	CDKN1C
STX6.1	3.45E-38	-0.32431	0.09	0.218	5.15E-34	AC-G	STX6
ACTN1.1	3.66E-38	0.464172	0.346	0.237	5.47E-34	AC-G	ACTN1
PPP2R5C	3.77E-38	-0.25886	0.134	0.281	5.63E-34	AC-G	PPP2R5C
TLN1.1	4.02E-38	-0.3429	0.164	0.308	6.01E-34	AC-G	TLN1
ITGAM.1	4.24E-38	-0.35691	0.004	0.09	6.34E-34	AC-G	ITGAM
C19orf53.	4.29E-38	0.389193	0.722	0.676	6.42E-34	AC-G	C19orf53
NCOR1.1	4.57E-38	-0.29649	0.335	0.489	6.84E-34	AC-G	NCOR1

RPS6KL1.1	4.95E-38	-0.26886	0.019	0.116	7.39E-34	AC-G	RPS6KL1
PIK3R1.1	5.34E-38	-0.36189	0.187	0.331	7.98E-34	AC-G	PIK3R1
SEP15.1	6.92E-38	0.333687	0.694	0.649	1.03E-33	AC-G	15-Sep
UROS.1	7.19E-38	-0.2995	0.133	0.275	1.07E-33	AC-G	UROS
LILRA2.1	7.36E-38	-0.40327	0.009	0.098	1.10E-33	AC-G	LILRA2
SNF8.1	7.43E-38	-0.25543	0.319	0.487	1.11E-33	AC-G	SNF8
GPS1.1	7.46E-38	-0.24194	0.194	0.349	1.11E-33	AC-G	GPS1
TSPAN5.1	7.73E-38	-0.33911	0.149	0.284	1.16E-33	AC-G	TSPAN5
NAA10.1	7.95E-38	-0.25415	0.286	0.453	1.19E-33	AC-G	NAA10
SUN2.1	8.10E-38	-0.3827	0.109	0.24	1.21E-33	AC-G	SUN2
MARCH9.1	8.44E-38	-0.32191	0.056	0.173	1.26E-33	AC-G	9-Mar
LHX2.1	8.45E-38	-0.24937	0.04	0.147	1.26E-33	AC-G	LHX2
LMNB1.1	9.20E-38	-0.32639	0.055	0.168	1.37E-33	AC-G	LMNB1
ECI1.1	1.04E-37	-0.27908	0.102	0.235	1.55E-33	AC-G	ECI1
DNMT1.1	1.09E-37	-0.26561	0.161	0.31	1.62E-33	AC-G	DNMT1
C17orf62	1.23E-37	-0.1895	0.235	0.394	1.84E-33	AC-G	C17orf62
NXPH1.1	1.26E-37	-0.28558	0.044	0.154	1.89E-33	AC-G	NXPH1
BRD7.1	1.35E-37	-0.29082	0.136	0.277	2.01E-33	AC-G	BRD7
PLEC.1	1.58E-37	-0.3096	0.082	0.207	2.37E-33	AC-G	PLEC
DLX1.1	1.63E-37	-0.37118	0.03	0.131	2.43E-33	AC-G	DLX1
SELPLG.1	1.75E-37	-0.4566	0.034	0.138	2.62E-33	AC-G	SELPLG
HLA-DQB2.	1.84E-37	-0.61531	0.019	0.112	2.75E-33	AC-G	HLA-DQB2
OTUD1.1	1.86E-37	-0.39322	0.019	0.113	2.78E-33	AC-G	OTUD1
CHN2.1	1.87E-37	-0.31724	0.018	0.113	2.80E-33	AC-G	CHN2
OGFR.1	2.02E-37	-0.2622	0.161	0.311	3.02E-33	AC-G	OGFR
PRDM1.1	2.07E-37	-0.40137	0.02	0.115	3.10E-33	AC-G	PRDM1
ZNF704.1	2.12E-37	-0.25094	0.035	0.14	3.17E-33	AC-G	ZNF704
AGPAT2.1	2.16E-37	-0.31752	0.076	0.199	3.22E-33	AC-G	AGPAT2
SHARPIN.1	2.20E-37	-0.29354	0.177	0.326	3.29E-33	AC-G	SHARPIN
MIR4458HG	2.26E-37	0.351488	0.258	0.141	3.37E-33	AC-G	MIR4458HG
ATXN10	2.36E-37	-0.23234	0.281	0.448	3.53E-33	AC-G	ATXN10
SEPT7.1	2.80E-37	0.290673	0.724	0.699	4.18E-33	AC-G	7-Sep
NPTXR.1	3.66E-37	-0.203	0.037	0.145	5.46E-33	AC-G	NPTXR
CDCA7.1	3.67E-37	-0.27027	0.035	0.14	5.49E-33	AC-G	CDCA7
USP53.1	3.92E-37	-0.48306	0.051	0.159	5.86E-33	AC-G	USP53
BRD3.1	3.99E-37	-0.30431	0.156	0.298	5.96E-33	AC-G	BRD3
SH3TC1.1	4.05E-37	-0.37776	0.008	0.094	6.05E-33	AC-G	SH3TC1
TOP2B.1	4.63E-37	-0.25901	0.121	0.26	6.91E-33	AC-G	TOP2B
EPN1.1	5.02E-37	-0.2896	0.208	0.359	7.50E-33	AC-G	EPN1
EBPL.1	5.11E-37	-0.31401	0.179	0.326	7.64E-33	AC-G	EBPL
ITGB1	5.27E-37	-0.25365	0.259	0.41	7.87E-33	AC-G	ITGB1
ORAI1.1	5.41E-37	-0.23958	0.037	0.142	8.09E-33	AC-G	ORAI1
TMCO1.1	5.87E-37	0.379825	0.629	0.565	8.78E-33	AC-G	TMCO1
KIAA0930.	5.93E-37	-0.33746	0.052	0.163	8.86E-33	AC-G	KIAA0930
VASP.1	6.04E-37	-0.35073	0.129	0.261	9.03E-33	AC-G	VASP
S100A11.1	6.21E-37	0.440494	0.691	0.651	9.28E-33	AC-G	S100A11
TMSB15B.1	6.27E-37	-0.30892	0.08	0.201	9.37E-33	AC-G	TMSB15B
DGCR6L.1	6.69E-37	-0.24294	0.203	0.361	9.99E-33	AC-G	DGCR6L
HSPB6	7.08E-37	0.249005	0.122	0.048	1.06E-32	AC-G	HSPB6
MAP3K7CL	7.30E-37	0.27109	0.095	0.032	1.09E-32	AC-G	MAP3K7CL
OSBPL8.1	7.79E-37	-0.25347	0.183	0.336	1.16E-32	AC-G	OSBPL8

OGFOD3.1	8.32E-37	-0.25113	0.109	0.243	1.24E-32	AC-G	OGFOD3
EIF3A.1	8.60E-37	-0.28863	0.206	0.357	1.28E-32	AC-G	EIF3A
SPCS3.1	8.77E-37	-0.27489	0.199	0.35	1.31E-32	AC-G	SPCS3
RP11-195F	8.98E-37	0.329059	0.164	0.076	1.34E-32	AC-G	RP11-195F19.5
NASP.1	1.01E-36	-0.29296	0.276	0.435	1.51E-32	AC-G	NASP
MUC12	1.06E-36	0.322612	0.095	0.032	1.59E-32	AC-G	MUC12
LY96.1	1.08E-36	-0.40434	0.118	0.243	1.61E-32	AC-G	LY96
GDF15.1	1.13E-36	0.440148	0.135	0.057	1.68E-32	AC-G	GDF15
CPNE4	1.19E-36	-0.15132	0.016	0.108	1.77E-32	AC-G	CPNE4
ZNF511.1	1.24E-36	-0.27649	0.09	0.216	1.85E-32	AC-G	ZNF511
TRIB2.1	1.33E-36	-0.27078	0.146	0.285	1.99E-32	AC-G	TRIB2
HOOK3.1	1.37E-36	-0.27878	0.161	0.307	2.04E-32	AC-G	HOOK3
PHIP.1	1.98E-36	-0.27804	0.203	0.357	2.96E-32	AC-G	PHIP
CPXM1.1	2.01E-36	-0.19845	0.036	0.141	3.00E-32	AC-G	CPXM1
HGSNAT.1	2.09E-36	-0.30164	0.1	0.227	3.13E-32	AC-G	HGSNAT
GFPT2	2.13E-36	0.269409	0.11	0.041	3.18E-32	AC-G	GFPT2
TBC1D22A.	2.24E-36	-0.3057	0.037	0.139	3.35E-32	AC-G	TBC1D22A
TMED9.1	2.27E-36	0.308562	0.7	0.687	3.39E-32	AC-G	TMED9
CCDC181	2.29E-36	0.309465	0.148	0.066	3.43E-32	AC-G	CCDC181
SRGAP3.1	2.34E-36	-0.30486	0.053	0.163	3.50E-32	AC-G	SRGAP3
PHLDA1.1	2.35E-36	-0.28902	0.27	0.42	3.51E-32	AC-G	PHLDA1
DNAJC5.1	2.69E-36	-0.28949	0.103	0.233	4.02E-32	AC-G	DNAJC5
AP001055.	2.69E-36	-0.39104	0.009	0.094	4.03E-32	AC-G	AP001055.6
SHKBP1.1	2.82E-36	-0.28617	0.123	0.259	4.21E-32	AC-G	SHKBP1
COPS8.1	2.90E-36	0.406253	0.541	0.456	4.33E-32	AC-G	COPS8
OAZ1	3.10E-36	0.182449	0.876	0.936	4.63E-32	AC-G	OAZ1
RAC3.1	3.35E-36	-0.24579	0.081	0.203	5.01E-32	AC-G	RAC3
ME2	3.91E-36	-0.32006	0.116	0.245	5.84E-32	AC-G	ME2
EBI3.1	3.95E-36	-0.39861	0.019	0.111	5.90E-32	AC-G	EBI3
BIRC5.1	4.19E-36	-0.39614	0.025	0.12	6.26E-32	AC-G	BIRC5
ASIC4.1	4.20E-36	-0.42215	0.05	0.157	6.27E-32	AC-G	ASIC4
NIN.1	4.25E-36	-0.25642	0.04	0.145	6.35E-32	AC-G	NIN
NDUFS5.1	4.45E-36	0.245592	0.789	0.815	6.65E-32	AC-G	NDUFS5
TOMM22.1	4.51E-36	-0.20725	0.331	0.496	6.75E-32	AC-G	TOMM22
PROX1.1	4.65E-36	-0.26748	0.066	0.183	6.95E-32	AC-G	PROX1
MCF2L.1	4.90E-36	-0.29151	0.019	0.111	7.32E-32	AC-G	MCF2L
GSN	5.02E-36	-0.30921	0.391	0.533	7.50E-32	AC-G	GSN
SASH3.1	5.23E-36	-0.34934	0.008	0.092	7.82E-32	AC-G	SASH3
BAX.1	5.37E-36	-0.22623	0.416	0.595	8.02E-32	AC-G	BAX
TFDP2	6.59E-36	-0.3064	0.106	0.233	9.85E-32	AC-G	TFDP2
MIAT.1	6.68E-36	-0.31258	0.069	0.187	9.98E-32	AC-G	MIAT
SSBP2.1	7.37E-36	0.541847	0.358	0.253	1.10E-31	AC-G	SSBP2
ANXA5.1	7.56E-36	0.287479	0.753	0.721	1.13E-31	AC-G	ANXA5
SIGLEC8.1	7.72E-36	-0.39048	0.008	0.091	1.15E-31	AC-G	SIGLEC8
SPAG9.1	7.86E-36	-0.28394	0.228	0.377	1.17E-31	AC-G	SPAG9
MGST2.1	7.97E-36	-0.29159	0.139	0.276	1.19E-31	AC-G	MGST2
NFIB.1	8.04E-36	-0.39509	0.263	0.4	1.20E-31	AC-G	NFIB
FGFBP2	8.48E-36	0.14459	0.036	0.005	1.27E-31	AC-G	FGFBP2
ATP6V1B2.	9.15E-36	-0.2914	0.279	0.434	1.37E-31	AC-G	ATP6V1B2
GLCCI1.1	9.22E-36	-0.32603	0.058	0.169	1.38E-31	AC-G	GLCCI1
PSD3.1	9.97E-36	-0.22462	0.089	0.215	1.49E-31	AC-G	PSD3

PMAIP1.1	1.03E-35	-0.44136	0.011	0.097	1.54E-31	AC-G	PMAIP1
YWHAZ	1.05E-35	-0.18945	0.397	0.562	1.57E-31	AC-G	YWHAZ
MOB1A.1	1.06E-35	-0.29211	0.216	0.364	1.59E-31	AC-G	MOB1A
KYNU.1	1.06E-35	-0.38357	0.012	0.098	1.59E-31	AC-G	KYNU
HNRNPA0.1	1.14E-35	-0.22667	0.491	0.671	1.70E-31	AC-G	HNRNPA0
PHACTR2.1	1.17E-35	-0.20897	0.011	0.097	1.75E-31	AC-G	PHACTR2
PKN1.1	1.24E-35	-0.26738	0.129	0.265	1.85E-31	AC-G	PKN1
COLGALT1.	1.26E-35	-0.32509	0.09	0.212	1.88E-31	AC-G	COLGALT1
WAC.1	1.28E-35	-0.27418	0.147	0.286	1.91E-31	AC-G	WAC
PAFAH1B3.	1.32E-35	-0.26559	0.294	0.449	1.98E-31	AC-G	PAFAH1B3
ARHGAP12.	1.39E-35	-0.28493	0.087	0.209	2.07E-31	AC-G	ARHGAP12
DTD1.1	1.40E-35	-0.27285	0.263	0.416	2.10E-31	AC-G	DTD1
SURF1.1	1.61E-35	-0.26641	0.223	0.377	2.41E-31	AC-G	SURF1
KLHL35	1.66E-35	-0.35549	0.018	0.109	2.48E-31	AC-G	KLHL35
ATP2B1.1	1.78E-35	-0.28096	0.229	0.378	2.66E-31	AC-G	ATP2B1
CDKN2A.1	1.79E-35	-0.35353	0.023	0.118	2.68E-31	AC-G	CDKN2A
KDM1A	2.01E-35	-0.24311	0.097	0.224	3.00E-31	AC-G	KDM1A
SCIMP.1	2.10E-35	-0.37115	0.011	0.097	3.14E-31	AC-G	SCIMP
IMMP2L	2.30E-35	0.393891	0.228	0.128	3.44E-31	AC-G	IMMP2L
RHOB.1	2.51E-35	-0.23765	0.438	0.642	3.75E-31	AC-G	RHOB
PAG1.1	2.55E-35	-0.3388	0.073	0.188	3.80E-31	AC-G	PAG1
CAMK1D.1	2.63E-35	-0.27921	0.03	0.127	3.94E-31	AC-G	CAMK1D
HNRNPA1L2	2.67E-35	-0.30248	0.097	0.221	3.99E-31	AC-G	HNRNPA1L2
KDM2B	2.75E-35	-0.25888	0.046	0.152	4.10E-31	AC-G	KDM2B
KIAA0101.	2.77E-35	-0.37126	0.034	0.134	4.13E-31	AC-G	KIAA0101
MESDC1.1	2.77E-35	-0.26401	0.039	0.14	4.14E-31	AC-G	MESDC1
SLC39A8.1	2.78E-35	0.206806	0.119	0.047	4.15E-31	AC-G	SLC39A8
RANBP2.1	2.98E-35	-0.31358	0.104	0.229	4.46E-31	AC-G	RANBP2
THBD.1	2.99E-35	-0.39615	0.019	0.109	4.47E-31	AC-G	THBD
GRIA2.1	3.17E-35	-0.29013	0.106	0.232	4.74E-31	AC-G	GRIA2
HPCAL1.1	3.25E-35	-0.32414	0.106	0.23	4.86E-31	AC-G	HPCAL1
AK4.1	3.49E-35	0.426776	0.262	0.163	5.21E-31	AC-G	AK4
DDX3Y.1	3.97E-35	-0.22413	0.014	0.101	5.94E-31	AC-G	DDX3Y
COMT.1	4.06E-35	-0.17838	0.409	0.574	6.06E-31	AC-G	COMT
GDI2.1	4.20E-35	-0.26559	0.352	0.515	6.28E-31	AC-G	GDI2
RP11-92G1	4.31E-35	0.175045	0.041	0.007	6.44E-31	AC-G	RP11-92G12.3
LINC01003	4.40E-35	-0.27236	0.09	0.212	6.58E-31	AC-G	LINC01003
IKZF1.1	4.40E-35	-0.32399	0.001	0.077	6.58E-31	AC-G	IKZF1
DRAXIN.1	4.73E-35	-0.22218	0.057	0.171	7.07E-31	AC-G	DRAXIN
DAB2.1	4.96E-35	-0.49054	0.062	0.167	7.41E-31	AC-G	DAB2
RNF149.1	5.25E-35	-0.47302	0.17	0.29	7.85E-31	AC-G	RNF149
NBPF1.1	6.10E-35	-0.25995	0.125	0.257	9.11E-31	AC-G	NBPF1
BLNK.1	6.35E-35	-0.38025	0.003	0.08	9.48E-31	AC-G	BLNK
SH3BP1.1	6.78E-35	-0.32263	0.031	0.128	1.01E-30	AC-G	SH3BP1
CACNA2D4.	6.78E-35	-0.36059	0.006	0.087	1.01E-30	AC-G	CACNA2D4
COMMD3.1	8.04E-35	-0.24459	0.224	0.38	1.20E-30	AC-G	COMMD3
ATP6V0D1.	8.26E-35	-0.20215	0.364	0.539	1.23E-30	AC-G	ATP6V0D1
MDK.1	8.45E-35	0.560776	0.583	0.473	1.26E-30	AC-G	MDK
TFPI	8.74E-35	0.181783	0.111	0.042	1.31E-30	AC-G	TFPI
CTXN1.1	9.12E-35	-0.22373	0.065	0.178	1.36E-30	AC-G	CTXN1
OSMR	9.23E-35	0.332233	0.151	0.072	1.38E-30	AC-G	OSMR

SIGLEC7.1	1.08E-34	-0.33908	0.007	0.087	1.61E-30	AC-G	SIGLEC7
NAGK	1.13E-34	-0.27027	0.252	0.406	1.68E-30	AC-G	NAGK
CDK2AP1.1	1.13E-34	-0.20407	0.025	0.119	1.69E-30	AC-G	CDK2AP1
STUB1.1	1.14E-34	-0.25575	0.325	0.487	1.70E-30	AC-G	STUB1
GJB2	1.19E-34	0.37504	0.103	0.039	1.78E-30	AC-G	GJB2
NPAS3.1	1.20E-34	-0.22801	0.065	0.177	1.80E-30	AC-G	NPAS3
IFI16.1	1.26E-34	0.430877	0.489	0.375	1.88E-30	AC-G	IFI16
EIF3D.1	1.38E-34	-0.25714	0.339	0.503	2.06E-30	AC-G	EIF3D
CASC4.1	1.38E-34	-0.2489	0.115	0.245	2.06E-30	AC-G	CASC4
PTPRU	1.39E-34	0.163508	0.04	0.007	2.07E-30	AC-G	PTPRU
RAB34	1.41E-34	-0.2108	0.158	0.307	2.11E-30	AC-G	RAB34
GLDN.1	1.42E-34	-0.31351	0.021	0.112	2.12E-30	AC-G	GLDN
ATP1A3.1	1.50E-34	-0.35881	0.038	0.137	2.25E-30	AC-G	ATP1A3
AKAP7.1	1.60E-34	-0.22459	0.091	0.215	2.39E-30	AC-G	AKAP7
GOLIM4.1	1.77E-34	-0.24628	0.201	0.351	2.64E-30	AC-G	GOLIM4
IRAK3.1	1.81E-34	-0.33848	0.007	0.087	2.70E-30	AC-G	IRAK3
ARID1A.1	1.86E-34	-0.26545	0.131	0.263	2.78E-30	AC-G	ARID1A
SLC12A9.1	1.94E-34	-0.33379	0.1	0.221	2.90E-30	AC-G	SLC12A9
SOGA1.1	2.01E-34	-0.24177	0.061	0.173	3.00E-30	AC-G	SOGA1
TIMP3	2.02E-34	0.355824	0.203	0.11	3.02E-30	AC-G	TIMP3
MAFB.1	2.12E-34	-0.3635	0.203	0.336	3.16E-30	AC-G	MAFB
CERK.1	2.21E-34	-0.25211	0.028	0.123	3.31E-30	AC-G	CERK
CCNG2.1	2.51E-34	-0.3177	0.084	0.2	3.76E-30	AC-G	CCNG2
CAMP	2.54E-34	0.204019	0.021	0.001	3.79E-30	AC-G	CAMP
FRYL.1	2.69E-34	-0.26289	0.087	0.207	4.02E-30	AC-G	FRYL
GIMAP1.1	2.78E-34	-0.33933	0.007	0.086	4.15E-30	AC-G	GIMAP1
LACTB.1	3.00E-34	-0.30595	0.072	0.184	4.49E-30	AC-G	LACTB
CD74	3.14E-34	0.335501	0.805	0.741	4.70E-30	AC-G	CD74
HOMER1.1	3.27E-34	-0.21859	0.064	0.175	4.88E-30	AC-G	HOMER1
COLGALT2.	3.31E-34	-0.20645	0.049	0.156	4.95E-30	AC-G	COLGALT2
MMP24-AS1	3.45E-34	-0.28572	0.195	0.334	5.16E-30	AC-G	MMP24-AS1
CMTM3.1	3.61E-34	-0.27875	0.202	0.347	5.40E-30	AC-G	CMTM3
NAAA.1	3.70E-34	-0.3335	0.048	0.15	5.52E-30	AC-G	NAAA
SLC39A3.1	3.84E-34	-0.27575	0.218	0.361	5.74E-30	AC-G	SLC39A3
SMARCC1.1	3.88E-34	-0.26183	0.143	0.276	5.80E-30	AC-G	SMARCC1
APLP1.1	3.92E-34	-0.42574	0.237	0.374	5.86E-30	AC-G	APLP1
STAC3.1	4.01E-34	-0.30407	0.01	0.092	5.99E-30	AC-G	STAC3
PPIC	4.52E-34	0.583641	0.266	0.167	6.75E-30	AC-G	PPIC
DYNC1I1.1	4.81E-34	-0.36382	0.028	0.119	7.19E-30	AC-G	DYNC1I1
PTPRS.1	4.94E-34	-0.22528	0.144	0.28	7.38E-30	AC-G	PTPRS
HAGH.1	4.98E-34	-0.25228	0.225	0.378	7.44E-30	AC-G	HAGH
IMPDH2.1	5.25E-34	0.406835	0.492	0.402	7.85E-30	AC-G	IMPDH2
LINC01088	5.27E-34	0.176746	0.031	0.004	7.88E-30	AC-G	LINC01088
FAM49A	5.44E-34	-0.31435	0.075	0.189	8.13E-30	AC-G	FAM49A
SACS.1	6.11E-34	-0.21633	0.062	0.173	9.14E-30	AC-G	SACS
DUT.1	6.39E-34	-0.25711	0.325	0.48	9.55E-30	AC-G	DUT
CHD4.1	6.77E-34	-0.22367	0.199	0.346	1.01E-29	AC-G	CHD4
SLC35B2	7.26E-34	-0.26352	0.183	0.323	1.08E-29	AC-G	SLC35B2
GRHPR.1	8.19E-34	-0.28068	0.265	0.417	1.22E-29	AC-G	GRHPR
DYNC1H1.1	8.69E-34	-0.24966	0.212	0.362	1.30E-29	AC-G	DYNC1H1
MAP3K1.1	9.26E-34	-0.21676	0.022	0.112	1.38E-29	AC-G	MAP3K1

GSTM5	1.01E-33	0.168535	0.036	0.005	1.51E-29	AC-G	GSTM5
AFF4.1	1.04E-33	-0.29815	0.219	0.364	1.56E-29	AC-G	AFF4
EMB.1	1.05E-33	-0.33892	0.008	0.088	1.57E-29	AC-G	EMB
MAML2	1.08E-33	-0.21171	0.095	0.218	1.62E-29	AC-G	MAML2
CD300C.1	1.12E-33	-0.31377	0.004	0.08	1.68E-29	AC-G	CD300C
RTF1.1	1.13E-33	-0.24107	0.297	0.456	1.69E-29	AC-G	RTF1
YPEL3.1	1.18E-33	-0.3077	0.135	0.266	1.76E-29	AC-G	YPEL3
DUS1L.1	1.20E-33	-0.18867	0.046	0.149	1.80E-29	AC-G	DUS1L
ITGAX.1	1.21E-33	-0.38664	0.018	0.105	1.80E-29	AC-G	ITGAX
PDCD2.1	1.30E-33	-0.28343	0.178	0.317	1.94E-29	AC-G	PDCD2
F8A1.1	1.31E-33	-0.24589	0.052	0.157	1.95E-29	AC-G	F8A1
CBX1.1	1.32E-33	-0.24677	0.243	0.391	1.97E-29	AC-G	CBX1
KIF2A.1	1.35E-33	-0.25601	0.127	0.256	2.02E-29	AC-G	KIF2A
RP11-479J	1.47E-33	0.236472	0.048	0.01	2.19E-29	AC-G	RP11-479J7.2
IL6	1.48E-33	0.294665	0.089	0.031	2.21E-29	AC-G	IL6
TSPAN15	1.52E-33	-0.34567	0.01	0.091	2.27E-29	AC-G	TSPAN15
EML4.1	1.58E-33	-0.25992	0.09	0.21	2.36E-29	AC-G	EML4
ARHGDIA.1	1.63E-33	-0.20435	0.43	0.591	2.43E-29	AC-G	ARHGDIA
MIF4GD.1	1.66E-33	-0.27596	0.139	0.274	2.48E-29	AC-G	MIF4GD
LOXL2	1.78E-33	0.219801	0.076	0.024	2.66E-29	AC-G	LOXL2
GALC	1.82E-33	-0.2095	0.025	0.117	2.72E-29	AC-G	GALC
KLHL5.1	2.17E-33	-0.27957	0.075	0.188	3.24E-29	AC-G	KLHL5
PPP1CA	2.52E-33	-0.16486	0.433	0.598	3.76E-29	AC-G	PPP1CA
KIF21A.1	2.59E-33	-0.3309	0.191	0.324	3.87E-29	AC-G	KIF21A
ACER3.1	2.62E-33	-0.29656	0.089	0.204	3.92E-29	AC-G	ACER3
TUBGCP2.1	2.86E-33	-0.25252	0.108	0.233	4.28E-29	AC-G	TUBGCP2
ROBO2.1	2.99E-33	-0.1926	0.029	0.123	4.47E-29	AC-G	ROBO2
GNPTAB.1	3.24E-33	-0.29132	0.065	0.174	4.84E-29	AC-G	GNPTAB
RNASEH2B.	3.28E-33	-0.3149	0.132	0.259	4.90E-29	AC-G	RNASEH2B
TAOK1.1	3.71E-33	-0.26471	0.148	0.281	5.55E-29	AC-G	TAOK1
CARD8	4.42E-33	-0.31558	0.06	0.166	6.61E-29	AC-G	CARD8
NEBL	5.36E-33	0.106822	0.052	0.012	8.01E-29	AC-G	NEBL
UCHL1	5.55E-33	0.404831	0.534	0.417	8.30E-29	AC-G	UCHL1
GPR82.1	5.58E-33	-0.31393	0.05	0.151	8.34E-29	AC-G	GPR82
AKR1A1	5.66E-33	-0.25657	0.363	0.524	8.46E-29	AC-G	AKR1A1
BBOX1.1	5.67E-33	0.675089	0.211	0.123	8.47E-29	AC-G	BBOX1
SLC25A1.1	5.69E-33	-0.25461	0.094	0.214	8.50E-29	AC-G	SLC25A1
RCC2.1	5.83E-33	-0.28216	0.079	0.193	8.71E-29	AC-G	RCC2
APOL2	6.13E-33	0.557217	0.171	0.085	9.16E-29	AC-G	APOL2
ZSWIM6.1	6.16E-33	-0.24901	0.045	0.145	9.21E-29	AC-G	ZSWIM6
BMPR2.1	6.42E-33	-0.24143	0.081	0.196	9.59E-29	AC-G	BMPR2
ACIN1.1	6.48E-33	-0.2315	0.15	0.287	9.68E-29	AC-G	ACIN1
HMGN3.1	7.71E-33	-0.16165	0.462	0.638	1.15E-28	AC-G	HMGN3
HRH1	7.81E-33	0.398207	0.164	0.083	1.17E-28	AC-G	HRH1
CNBP.1	7.94E-33	0.279044	0.751	0.748	1.19E-28	AC-G	CNBP
WIPF3.1	8.46E-33	-0.26272	0.02	0.106	1.26E-28	AC-G	WIPF3
JKAMP.1	8.51E-33	-0.21317	0.18	0.323	1.27E-28	AC-G	JKAMP
P2RX4.1	8.56E-33	-0.34252	0.083	0.195	1.28E-28	AC-G	P2RX4
KCNMB1.1	8.76E-33	-0.37004	0.027	0.117	1.31E-28	AC-G	KCNMB1
PMP2.1	9.53E-33	0.579568	0.434	0.334	1.42E-28	AC-G	PMP2
RP11-390F	1.01E-32	-0.20748	0.047	0.15	1.50E-28	AC-G	RP11-390P2.4

ZWINT.1	1.06E-32	-0.29721	0.021	0.107	1.58E-28	AC-G	ZWINT
SATB1.1	1.23E-32	-0.28969	0.047	0.146	1.85E-28	AC-G	SATB1
OAS1.1	1.26E-32	-0.42507	0.05	0.148	1.88E-28	AC-G	OAS1
CMYA5	1.33E-32	0.148647	0.072	0.021	1.99E-28	AC-G	CMYA5
CCR1.1	1.38E-32	-0.3573	0.014	0.095	2.06E-28	AC-G	CCR1
SERP1.1	1.38E-32	-0.22287	0.451	0.633	2.06E-28	AC-G	SERP1
SMIM3.1	1.38E-32	0.420099	0.082	0.027	2.07E-28	AC-G	SMIM3
ATAT1.1	1.43E-32	-0.27046	0.179	0.31	2.13E-28	AC-G	ATAT1
ITPK1	1.43E-32	-0.25307	0.053	0.155	2.14E-28	AC-G	ITPK1
ARHGEF6.1	1.63E-32	-0.20949	0.054	0.159	2.43E-28	AC-G	ARHGEF6
GAPT.1	1.74E-32	-0.31742	0.001	0.071	2.60E-28	AC-G	GAPT
BTBD3.1	1.80E-32	-0.17395	0.051	0.154	2.68E-28	AC-G	BTBD3
TUBA1A	1.89E-32	0.340913	0.859	0.798	2.82E-28	AC-G	TUBA1A
CLTC.1	1.92E-32	-0.26099	0.229	0.372	2.87E-28	AC-G	CLTC
PLEKHO2.1	2.03E-32	-0.32108	0.028	0.117	3.03E-28	AC-G	PLEKHO2
MORF4L2	2.17E-32	0.258315	0.655	0.619	3.25E-28	AC-G	MORF4L2
USMG5.1	2.39E-32	-0.16635	0.476	0.648	3.57E-28	AC-G	USMG5
CAPRIN1.1	2.41E-32	-0.25952	0.203	0.343	3.60E-28	AC-G	CAPRIN1
STX7.1	2.70E-32	-0.31092	0.159	0.289	4.04E-28	AC-G	STX7
TWF2.1	2.74E-32	-0.24489	0.222	0.364	4.09E-28	AC-G	TWF2
CCND2.1	2.75E-32	-0.32367	0.213	0.349	4.11E-28	AC-G	CCND2
VPS51.1	3.00E-32	-0.26595	0.153	0.285	4.48E-28	AC-G	VPS51
CYTL1.1	3.38E-32	-0.4528	0.034	0.125	5.05E-28	AC-G	CYTL1
SETD5.1	3.44E-32	-0.25481	0.172	0.311	5.13E-28	AC-G	SETD5
NIPBL.1	3.58E-32	-0.26366	0.131	0.26	5.36E-28	AC-G	NIPBL
TMEM259.1	3.66E-32	-0.22944	0.208	0.36	5.48E-28	AC-G	TMEM259
HP1BP3.1	3.77E-32	-0.2319	0.316	0.466	5.63E-28	AC-G	HP1BP3
RNASE1.1	4.11E-32	-0.64296	0.082	0.186	6.14E-28	AC-G	RNASE1
DDIT4.1	4.12E-32	-0.33713	0.286	0.433	6.15E-28	AC-G	DDIT4
PAPPA2	4.21E-32	0.115995	0.03	0.004	6.29E-28	AC-G	PAPPA2
ANKRD11.1	5.55E-32	-0.25241	0.195	0.34	8.29E-28	AC-G	ANKRD11
SPECC1.1	6.05E-32	-0.19643	0.182	0.318	9.03E-28	AC-G	SPECC1
UBE3A.1	6.14E-32	-0.2392	0.172	0.309	9.18E-28	AC-G	UBE3A
PIK3AP1.1	6.39E-32	-0.27574	0.004	0.076	9.54E-28	AC-G	PIK3AP1
NDUFA13	6.85E-32	0.365367	0.769	0.773	1.02E-27	AC-G	NDUFA13
UNC119.1	6.88E-32	-0.24985	0.114	0.237	1.03E-27	AC-G	UNC119
SELENBP1	6.94E-32	0.160217	0.053	0.013	1.04E-27	AC-G	SELENBP1
GNG11	7.09E-32	0.254966	0.084	0.028	1.06E-27	AC-G	GNG11
C1QTNF1	7.17E-32	0.187864	0.073	0.022	1.07E-27	AC-G	C1QTNF1
SLC12A5.1	7.84E-32	-0.33349	0.022	0.106	1.17E-27	AC-G	SLC12A5
KLF4.1	8.15E-32	-0.45901	0.115	0.224	1.22E-27	AC-G	KLF4
MMP16	8.54E-32	-0.20021	0.028	0.118	1.28E-27	AC-G	MMP16
SNAP29.1	8.88E-32	-0.25713	0.12	0.245	1.33E-27	AC-G	SNAP29
GMIP.1	9.05E-32	-0.2636	0.01	0.086	1.35E-27	AC-G	GMIP
ZNF821.1	9.50E-32	-0.24209	0.072	0.181	1.42E-27	AC-G	ZNF821
NRROS	9.58E-32	-0.32887	0.006	0.08	1.43E-27	AC-G	NRROS
CDK1.1	9.63E-32	-0.34951	0.025	0.11	1.44E-27	AC-G	CDK1
SKAP2	1.00E-31	-0.35509	0.168	0.29	1.50E-27	AC-G	SKAP2
ACSL3.1	1.01E-31	-0.28332	0.225	0.364	1.50E-27	AC-G	ACSL3
AIF1L.1	1.18E-31	-0.33754	0.036	0.128	1.76E-27	AC-G	AIF1L
HDAC2.1	1.28E-31	-0.27207	0.325	0.472	1.91E-27	AC-G	HDAC2

PTPRA.1	1.36E-31	-0.27965	0.243	0.376	2.03E-27	AC-G	PTPRA
COX4I1.1	1.41E-31	0.238062	0.861	0.92	2.10E-27	AC-G	COX4I1
IER3IP1.1	1.42E-31	0.345134	0.086	0.029	2.12E-27	AC-G	IER3IP1
MAML1.1	1.43E-31	-0.30051	0.039	0.132	2.13E-27	AC-G	MAML1
AREG.1	1.54E-31	-0.46148	0.005	0.077	2.30E-27	AC-G	AREG
ATP6V0B.1	1.58E-31	-0.16677	0.659	0.825	2.35E-27	AC-G	ATP6V0B
ST3GAL6.1	1.63E-31	-0.35766	0.036	0.126	2.43E-27	AC-G	ST3GAL6
IL4R.1	1.65E-31	-0.30892	0.008	0.083	2.47E-27	AC-G	IL4R
HLA-DPB1.	1.68E-31	-0.34985	0.413	0.51	2.51E-27	AC-G	HLA-DPB1
RAB13.1	1.71E-31	0.537095	0.449	0.357	2.56E-27	AC-G	RAB13
AC004540.	1.74E-31	-0.28105	0.043	0.138	2.61E-27	AC-G	AC004540.4
CELF5.1	1.87E-31	-0.29081	0.021	0.104	2.79E-27	AC-G	CELF5
EIF4A3.1	1.87E-31	-0.26348	0.32	0.475	2.80E-27	AC-G	EIF4A3
ZNF414.1	1.89E-31	-0.21572	0.089	0.204	2.82E-27	AC-G	ZNF414
FGFBP3.1	1.89E-31	-0.19959	0.027	0.114	2.82E-27	AC-G	FGFBP3
THSD7A.1	1.90E-31	-0.39432	0.06	0.159	2.84E-27	AC-G	THSD7A
LRRTM2.1	1.97E-31	-0.18287	0.028	0.117	2.94E-27	AC-G	LRRTM2
MYO5A.1	2.01E-31	-0.2034	0.034	0.127	3.00E-27	AC-G	MYO5A
MSRB3	2.11E-31	0.14463	0.046	0.01	3.16E-27	AC-G	MSRB3
FBXO7.1	2.23E-31	-0.2672	0.193	0.332	3.33E-27	AC-G	FBXO7
FAM107B.1	2.24E-31	-0.38159	0.044	0.137	3.35E-27	AC-G	FAM107B
PLAC8	2.28E-31	-0.36277	0.007	0.08	3.41E-27	AC-G	PLAC8
CLSTN1.1	2.29E-31	-0.14945	0.165	0.305	3.43E-27	AC-G	CLSTN1
CDC34.1	2.45E-31	-0.23348	0.127	0.256	3.66E-27	AC-G	CDC34
C1orf61.1	2.49E-31	0.341317	0.606	0.521	3.73E-27	AC-G	C1orf61
TPPP3.1	2.50E-31	0.334187	0.233	0.138	3.73E-27	AC-G	TPPP3
EWSR1	2.56E-31	-0.18491	0.332	0.49	3.82E-27	AC-G	EWSR1
TMEM59.1	2.61E-31	0.258305	0.764	0.79	3.90E-27	AC-G	TMEM59
RAPGEF1	2.78E-31	-0.27939	0.039	0.132	4.16E-27	AC-G	RAPGEF1
FNBP1L.1	2.81E-31	-0.27387	0.091	0.203	4.20E-27	AC-G	FNBP1L
CACUL1.1	2.97E-31	-0.30688	0.109	0.225	4.43E-27	AC-G	CACUL1
PDGFRL	3.00E-31	0.134011	0.038	0.007	4.48E-27	AC-G	PDGFRL
GDPD2	3.09E-31	0.223992	0.075	0.025	4.61E-27	AC-G	GDPD2
SPCS1.1	3.19E-31	0.293445	0.728	0.738	4.77E-27	AC-G	SPCS1
DEF8.1	3.20E-31	-0.23557	0.157	0.292	4.79E-27	AC-G	DEF8
PQLC1.1	3.23E-31	-0.23214	0.051	0.15	4.83E-27	AC-G	PQLC1
UBE2C.1	3.27E-31	-0.40395	0.039	0.132	4.88E-27	AC-G	UBE2C
LDHB	3.32E-31	0.267729	0.768	0.781	4.96E-27	AC-G	LDHB
ENO2.1	3.32E-31	0.403525	0.366	0.268	4.97E-27	AC-G	ENO2
PRKX.1	3.36E-31	-0.28154	0.036	0.126	5.02E-27	AC-G	PRKX
GDE1.1	3.37E-31	-0.26232	0.185	0.321	5.04E-27	AC-G	GDE1
RABEP1.1	3.44E-31	-0.2589	0.146	0.274	5.13E-27	AC-G	RABEP1
MPND.1	3.70E-31	-0.16619	0.021	0.104	5.53E-27	AC-G	MPND
RRP36.1	3.86E-31	-0.18792	0.059	0.162	5.76E-27	AC-G	RRP36
APC2.1	3.94E-31	-0.21249	0.06	0.165	5.89E-27	AC-G	APC2
PNMA1.1	4.14E-31	-0.24548	0.173	0.305	6.19E-27	AC-G	PNMA1
TBC1D12.1	4.33E-31	-0.2837	0.033	0.122	6.48E-27	AC-G	TBC1D12
VTI1B.1	4.52E-31	-0.21479	0.226	0.37	6.76E-27	AC-G	VTI1B
ADCK2.1	4.57E-31	-0.26224	0.056	0.156	6.84E-27	AC-G	ADCK2
KLF2.1	4.68E-31	-0.43681	0.052	0.147	7.00E-27	AC-G	KLF2
NBL1.1	4.71E-31	-0.29872	0.086	0.196	7.04E-27	AC-G	NBL1

CENPU.1	4.77E-31	-0.30067	0.031	0.119	7.12E-27	AC-G	CENPU
ZBTB7A	4.83E-31	-0.24354	0.084	0.197	7.22E-27	AC-G	ZBTB7A
NIM1K.1	5.55E-31	0.270649	0.122	0.053	8.29E-27	AC-G	NIM1K
PTPN7.1	6.10E-31	-0.34846	0.014	0.091	9.11E-27	AC-G	PTPN7
MAOA	6.68E-31	0.234133	0.073	0.023	9.98E-27	AC-G	MAOA
FPR3.1	6.69E-31	-0.26492	0.001	0.067	1.00E-26	AC-G	FPR3
SLC47A2	7.08E-31	0.239279	0.063	0.018	1.06E-26	AC-G	SLC47A2
C6orf62.1	7.24E-31	-0.21888	0.316	0.478	1.08E-26	AC-G	C6orf62
TNFRSF12A	7.25E-31	0.371309	0.379	0.269	1.08E-26	AC-G	TNFRSF12A
EPHA3.1	7.53E-31	-0.12312	0.01	0.085	1.13E-26	AC-G	EPHA3
IQGAP2.1	7.68E-31	-0.22507	0.062	0.166	1.15E-26	AC-G	IQGAP2
ACAA1.1	7.92E-31	-0.26427	0.229	0.372	1.18E-26	AC-G	ACAA1
MTA1.1	8.42E-31	-0.2305	0.094	0.209	1.26E-26	AC-G	MTA1
KLHDC3.1	8.60E-31	-0.19007	0.241	0.386	1.29E-26	AC-G	KLHDC3
ARID4B.1	9.02E-31	-0.23656	0.281	0.43	1.35E-26	AC-G	ARID4B
DNAJB9.1	9.10E-31	0.412114	0.467	0.369	1.36E-26	AC-G	DNAJB9
PPP2R5E.1	9.14E-31	-0.22773	0.14	0.268	1.37E-26	AC-G	PPP2R5E
FARP1	9.72E-31	-0.2328	0.089	0.204	1.45E-26	AC-G	FARP1
NUSAP1.1	1.02E-30	-0.39542	0.032	0.119	1.53E-26	AC-G	NUSAP1
SOX12	1.03E-30	-0.15103	0.038	0.131	1.54E-26	AC-G	SOX12
PGAM2.1	1.03E-30	0.392463	0.098	0.038	1.54E-26	AC-G	PGAM2
IL16.1	1.08E-30	-0.33851	0.008	0.08	1.61E-26	AC-G	IL16
RHOH.1	1.14E-30	-0.38342	0.013	0.089	1.71E-26	AC-G	RHOH
PRPF38B.1	1.17E-30	-0.26132	0.307	0.46	1.74E-26	AC-G	PRPF38B
PPIF.1	1.20E-30	-0.30963	0.124	0.24	1.80E-26	AC-G	PPIF
ELOVL2.1	1.22E-30	-0.14567	0.025	0.112	1.82E-26	AC-G	ELOVL2
SNAP23.1	1.22E-30	-0.27969	0.141	0.264	1.83E-26	AC-G	SNAP23
ANKRD22.1	1.28E-30	-0.33242	0.006	0.076	1.92E-26	AC-G	ANKRD22
RTN2.1	1.29E-30	-0.26239	0.017	0.097	1.92E-26	AC-G	RTN2
RP11-499E	1.40E-30	0.197171	0.073	0.023	2.10E-26	AC-G	RP11-499E18.1
WSB2.1	1.43E-30	-0.23547	0.122	0.243	2.14E-26	AC-G	WSB2
RP5-940J5	1.61E-30	0.351332	0.247	0.145	2.41E-26	AC-G	RP5-940J5.9
SEMA4D.1	1.69E-30	-0.3433	0.016	0.093	2.52E-26	AC-G	SEMA4D
STARD4-AS	1.71E-30	-0.21975	0.046	0.143	2.55E-26	AC-G	STARD4-AS1
TALDO1.1	1.78E-30	-0.18806	0.459	0.624	2.67E-26	AC-G	TALDO1
CKAP2.1	1.86E-30	-0.28475	0.079	0.186	2.79E-26	AC-G	CKAP2
SWAP70.1	1.94E-30	-0.29644	0.123	0.241	2.91E-26	AC-G	SWAP70
HTATIP2.1	2.00E-30	-0.33832	0.063	0.163	2.99E-26	AC-G	HTATIP2
DAZAP1.1	2.01E-30	-0.24361	0.129	0.252	3.01E-26	AC-G	DAZAP1
CD93.1	2.28E-30	-0.31633	0.009	0.083	3.41E-26	AC-G	CD93
RAP1A.1	2.29E-30	-0.24822	0.397	0.539	3.42E-26	AC-G	RAP1A
CRELD2.1	2.32E-30	-0.24268	0.171	0.305	3.46E-26	AC-G	CRELD2
COX6A1.1	2.32E-30	0.245507	0.827	0.848	3.46E-26	AC-G	COX6A1
CD59.1	2.37E-30	0.422079	0.585	0.547	3.54E-26	AC-G	CD59
PPP6R2.1	2.39E-30	-0.24138	0.064	0.166	3.57E-26	AC-G	PPP6R2
FNBP1.1	2.73E-30	-0.31176	0.157	0.284	4.08E-26	AC-G	FNBP1
ZNF580.1	2.81E-30	-0.26204	0.182	0.312	4.20E-26	AC-G	ZNF580
GK.1	3.03E-30	-0.31763	0.057	0.154	4.53E-26	AC-G	GK
PPIL6.1	3.31E-30	0.201568	0.098	0.038	4.94E-26	AC-G	PPIL6
VWA1.1	3.58E-30	-0.24847	0.06	0.16	5.35E-26	AC-G	VWA1
SCO2.1	3.63E-30	-0.2234	0.112	0.231	5.42E-26	AC-G	SCO2

DHRSX.1	3.90E-30	-0.22453	0.128	0.253	5.82E-26	AC-G	DHRSX
LDLRAD3.1	4.27E-30	-0.11935	0.082	0.194	6.39E-26	AC-G	LDLRAD3
MAD2L2	4.54E-30	-0.15783	0.268	0.417	6.78E-26	AC-G	MAD2L2
ZNF106.1	4.81E-30	-0.2442	0.15	0.278	7.19E-26	AC-G	ZNF106
CENPW.1	4.94E-30	-0.24727	0.041	0.133	7.38E-26	AC-G	CENPW
ZNF280D.1	4.99E-30	-0.24553	0.045	0.139	7.46E-26	AC-G	ZNF280D
PNOC	5.00E-30	0.101006	0.028	0.004	7.47E-26	AC-G	PNOC
PTPRN.1	5.65E-30	0.22921	0.136	0.062	8.45E-26	AC-G	PTPRN
MYH9.1	5.90E-30	-0.25598	0.094	0.206	8.82E-26	AC-G	MYH9
PAK1.1	6.01E-30	-0.37412	0.083	0.187	8.98E-26	AC-G	PAK1
HHEX.1	6.05E-30	-0.29304	0.002	0.068	9.04E-26	AC-G	HHEX
TNFSF13B.	6.20E-30	-0.44277	0.115	0.221	9.26E-26	AC-G	TNFSF13B
ELAVL1.1	6.23E-30	-0.25413	0.227	0.367	9.31E-26	AC-G	ELAVL1
CTTNBP2.1	7.06E-30	-0.3098	0.056	0.152	1.05E-25	AC-G	CTTNBP2
ABHD2.1	8.36E-30	-0.17777	0.123	0.25	1.25E-25	AC-G	ABHD2
WNK1.1	8.48E-30	-0.20715	0.168	0.3	1.27E-25	AC-G	WNK1
MYO1G.1	8.48E-30	-0.27056	0.004	0.072	1.27E-25	AC-G	MYO1G
UBAC1.1	9.16E-30	-0.23205	0.12	0.237	1.37E-25	AC-G	UBAC1
BNIP2.1	9.79E-30	-0.28692	0.159	0.284	1.46E-25	AC-G	BNIP2
CARD9.1	1.01E-29	-0.27027	0.009	0.08	1.51E-25	AC-G	CARD9
CMIP.1	1.06E-29	-0.30486	0.085	0.19	1.58E-25	AC-G	CMIP
FES.1	1.10E-29	-0.30035	0.016	0.092	1.64E-25	AC-G	FES
CECR5.1	1.11E-29	-0.18392	0.145	0.272	1.65E-25	AC-G	CECR5
ASAP1.1	1.11E-29	-0.20669	0.126	0.247	1.66E-25	AC-G	ASAP1
TSPAN7	1.13E-29	0.434311	0.395	0.294	1.69E-25	AC-G	TSPAN7
RNPEP.1	1.14E-29	-0.27971	0.125	0.243	1.71E-25	AC-G	RNPEP
EIF4G3.1	1.14E-29	-0.23058	0.084	0.192	1.71E-25	AC-G	EIF4G3
RALGAPA2.	1.18E-29	-0.18359	0.036	0.125	1.77E-25	AC-G	RALGAPA2
TMEM181.1	1.22E-29	-0.18936	0.075	0.181	1.82E-25	AC-G	TMEM181
EMILIN2.1	1.26E-29	-0.2109	0.016	0.093	1.88E-25	AC-G	EMILIN2
ANKRD9.1	1.29E-29	-0.18664	0.02	0.101	1.92E-25	AC-G	ANKRD9
NSD1.1	1.33E-29	-0.24464	0.131	0.252	1.98E-25	AC-G	NSD1
ITGAV.1	1.36E-29	-0.23779	0.172	0.299	2.04E-25	AC-G	ITGAV
EEF1B2.1	1.77E-29	-0.10345	0.642	0.803	2.65E-25	AC-G	EEF1B2
NENF.1	1.78E-29	-0.21576	0.358	0.52	2.67E-25	AC-G	NENF
TOP2A.1	1.81E-29	-0.35456	0.02	0.099	2.71E-25	AC-G	TOP2A
KLF3.1	1.83E-29	-0.26913	0.118	0.233	2.74E-25	AC-G	KLF3
HIPK2.1	1.92E-29	-0.25626	0.122	0.24	2.87E-25	AC-G	HIPK2
LRCH4.1	2.25E-29	-0.31756	0.098	0.206	3.36E-25	AC-G	LRCH4
PURA.1	2.30E-29	-0.25973	0.112	0.227	3.44E-25	AC-G	PURA
PBRM1	2.41E-29	-0.23941	0.137	0.26	3.60E-25	AC-G	PBRM1
GPR84.1	2.63E-29	-0.33849	0.013	0.085	3.93E-25	AC-G	GPR84
SDF4.1	2.98E-29	-0.26624	0.288	0.426	4.45E-25	AC-G	SDF4
LMBR1L.1	3.12E-29	-0.27027	0.094	0.202	4.66E-25	AC-G	LMBR1L
DPYSL4.1	3.22E-29	-0.21919	0.069	0.171	4.82E-25	AC-G	DPYSL4
FAM69C.1	3.59E-29	-0.1442	0.014	0.089	5.37E-25	AC-G	FAM69C
RNFT1.1	3.87E-29	-0.20678	0.107	0.221	5.79E-25	AC-G	RNFT1
TFEC.1	3.94E-29	-0.27069	0.002	0.066	5.88E-25	AC-G	TFEC
EPN2.1	4.07E-29	-0.24862	0.138	0.256	6.08E-25	AC-G	EPN2
SPSB4.1	4.15E-29	-0.13528	0.004	0.07	6.20E-25	AC-G	SPSB4
STK25.1	4.17E-29	-0.22619	0.192	0.324	6.23E-25	AC-G	STK25

CLEC4E	4.26E-29	-0.36953	0.003	0.068	6.37E-25	AC-G	CLEC4E
FAM64A.1	4.36E-29	-0.27203	0.01	0.081	6.52E-25	AC-G	FAM64A
PRKCD.1	4.40E-29	-0.30382	0.017	0.093	6.58E-25	AC-G	PRKCD
UBL3.1	4.61E-29	-0.23648	0.239	0.376	6.89E-25	AC-G	UBL3
CXXC5.1	4.64E-29	-0.25174	0.268	0.402	6.93E-25	AC-G	CXXC5
ZNF609.1	4.67E-29	-0.1966	0.024	0.105	6.98E-25	AC-G	ZNF609
TMED10.1	4.68E-29	0.386348	0.655	0.605	6.99E-25	AC-G	TMED10
ICA1	4.77E-29	-0.2836	0.04	0.128	7.12E-25	AC-G	ICA1
NOV	5.16E-29	0.129066	0.043	0.009	7.71E-25	AC-G	NOV
SPG21.1	5.42E-29	-0.22977	0.266	0.408	8.09E-25	AC-G	SPG21
ATP5C1.1	5.48E-29	-0.11688	0.458	0.612	8.20E-25	AC-G	ATP5C1
ALYREF.1	5.50E-29	-0.1656	0.062	0.162	8.22E-25	AC-G	ALYREF
PIP4K2A.1	5.81E-29	-0.32901	0.057	0.153	8.69E-25	AC-G	PIP4K2A
WDR82.1	5.91E-29	-0.25117	0.151	0.274	8.83E-25	AC-G	WDR82
MIR181A1H	5.96E-29	-0.24863	0.039	0.127	8.91E-25	AC-G	MIR181A1HG
RP11-326C	6.19E-29	0.150976	0.038	0.007	9.25E-25	AC-G	RP11-326C3.12
ZNF295-AS	6.33E-29	0.109124	0.025	0.003	9.46E-25	AC-G	ZNF295-AS1
PLEKHB2.1	6.36E-29	-0.2073	0.279	0.423	9.51E-25	AC-G	PLEKHB2
ARHGAP35.	6.49E-29	-0.18835	0.063	0.163	9.70E-25	AC-G	ARHGAP35
BCL7C	6.54E-29	-0.21518	0.15	0.275	9.77E-25	AC-G	BCL7C
CLEC2B.1	6.79E-29	-0.22148	0.108	0.224	1.02E-24	AC-G	CLEC2B
KATNBL1.1	7.06E-29	-0.21616	0.147	0.271	1.06E-24	AC-G	KATNBL1
TRIM24.1	7.20E-29	-0.2196	0.099	0.21	1.08E-24	AC-G	TRIM24
GSK3B.1	7.26E-29	-0.22915	0.103	0.216	1.08E-24	AC-G	GSK3B
CTSD.1	7.48E-29	-0.17522	0.511	0.664	1.12E-24	AC-G	CTSD
PDCD7	7.52E-29	-0.22315	0.123	0.24	1.12E-24	AC-G	PDCD7
CFH	8.27E-29	0.117556	0.042	0.009	1.24E-24	AC-G	CFH
FREM2.1	8.30E-29	-0.11832	0.009	0.079	1.24E-24	AC-G	FREM2
DHRS11.1	9.19E-29	-0.12058	0.031	0.116	1.37E-24	AC-G	DHRS11
BTF3	9.36E-29	0.21247	0.854	0.906	1.40E-24	AC-G	BTF3
RHOQ.1	9.40E-29	-0.23034	0.075	0.178	1.40E-24	AC-G	RHOQ
CAB39.1	1.02E-28	-0.26877	0.094	0.201	1.52E-24	AC-G	CAB39
LPXN.1	1.04E-28	-0.35699	0.038	0.124	1.55E-24	AC-G	LPXN
TMEM131.1	1.06E-28	-0.1569	0.063	0.162	1.58E-24	AC-G	TMEM131
METRNL.1	1.08E-28	-0.3192	0.019	0.095	1.62E-24	AC-G	METRNL
PRRC2B.1	1.14E-28	-0.21995	0.135	0.256	1.71E-24	AC-G	PRRC2B
ZNF428.1	1.16E-28	-0.23854	0.449	0.563	1.73E-24	AC-G	ZNF428
SPPL2A.1	1.30E-28	-0.28175	0.096	0.201	1.94E-24	AC-G	SPPL2A
HAP1	1.32E-28	0.272747	0.124	0.057	1.97E-24	AC-G	HAP1
TACC3.1	1.34E-28	-0.23189	0.015	0.089	2.00E-24	AC-G	TACC3
GLS.1	1.44E-28	-0.21406	0.051	0.144	2.16E-24	AC-G	GLS
RP11-556E	1.56E-28	-0.30152	0.002	0.065	2.34E-24	AC-G	RP11-556E13.1
TMEM38A.1	1.57E-28	0.180574	0.1	0.041	2.35E-24	AC-G	TMEM38A
MAST3.1	1.63E-28	-0.19653	0.016	0.09	2.44E-24	AC-G	MAST3
TMBIM4.1	1.77E-28	0.395033	0.115	0.048	2.64E-24	AC-G	TMBIM4
MAPKAPK3	1.91E-28	-0.29459	0.085	0.187	2.85E-24	AC-G	MAPKAPK3
XXYLT1.1	2.00E-28	-0.19598	0.049	0.141	2.99E-24	AC-G	XXYLT1
WIPI2.1	2.03E-28	-0.23774	0.276	0.421	3.04E-24	AC-G	WIPI2
TGFBI.1	2.06E-28	-0.46978	0.122	0.222	3.07E-24	AC-G	TGFBI
NDUFB2.1	2.13E-28	0.293394	0.786	0.813	3.18E-24	AC-G	NDUFB2
FAM110B.1	2.21E-28	-0.21177	0.095	0.204	3.30E-24	AC-G	FAM110B

LCA5L	2.28E-28	0.120262	0.045	0.011	3.41E-24	AC-G	LCA5L
FKBP5.1	2.31E-28	-0.35776	0.218	0.336	3.46E-24	AC-G	FKBP5
SRGAP1.1	2.33E-28	-0.23524	0.128	0.244	3.48E-24	AC-G	SRGAP1
IDH1.1	2.35E-28	-0.20496	0.26	0.404	3.51E-24	AC-G	IDH1
SNHG18	2.35E-28	0.144863	0.056	0.016	3.52E-24	AC-G	SNHG18
SPPL3.1	2.41E-28	-0.22822	0.102	0.216	3.60E-24	AC-G	SPPL3
FAM162A.1	2.49E-28	0.447786	0.473	0.387	3.73E-24	AC-G	FAM162A
CLEC11A.1	2.50E-28	-0.27794	0.136	0.25	3.74E-24	AC-G	CLEC11A
ARRDC1.1	2.52E-28	-0.2922	0.056	0.149	3.77E-24	AC-G	ARRDC1
EHBP1L1.1	2.65E-28	-0.2055	0.011	0.082	3.96E-24	AC-G	EHBP1L1
COPE.1	2.66E-28	0.250163	0.759	0.773	3.97E-24	AC-G	COPE
GIMAP7	2.74E-28	-0.31391	0.002	0.065	4.10E-24	AC-G	GIMAP7
ZMIZ1.1	2.83E-28	-0.25007	0.059	0.154	4.23E-24	AC-G	ZMIZ1
CCDC50.1	2.90E-28	-0.24588	0.192	0.321	4.33E-24	AC-G	CCDC50
RASAL3.1	2.97E-28	-0.26705	0.004	0.067	4.45E-24	AC-G	RASAL3
DENND6B.1	3.09E-28	-0.26052	0.032	0.116	4.61E-24	AC-G	DENND6B
WSB1	3.09E-28	-0.15247	0.505	0.68	4.62E-24	AC-G	WSB1
PRRC2A.1	3.19E-28	-0.20684	0.141	0.263	4.76E-24	AC-G	PRRC2A
DLX2.1	3.35E-28	-0.37545	0.038	0.122	5.01E-24	AC-G	DLX2
CHP1.1	3.50E-28	-0.23556	0.134	0.254	5.23E-24	AC-G	CHP1
CTDSP1.1	3.51E-28	-0.29109	0.125	0.24	5.25E-24	AC-G	CTDSP1
NLGN3	3.77E-28	-0.19464	0.04	0.128	5.64E-24	AC-G	NLGN3
SNN.1	3.93E-28	-0.26181	0.13	0.244	5.87E-24	AC-G	SNN
ETV6.1	4.02E-28	-0.21911	0.066	0.164	6.01E-24	AC-G	ETV6
PLK3.1	4.23E-28	-0.33765	0.148	0.259	6.32E-24	AC-G	PLK3
CTDSP2.1	4.39E-28	-0.1976	0.169	0.298	6.56E-24	AC-G	CTDSP2
SRP9	4.49E-28	0.245435	0.708	0.676	6.71E-24	AC-G	SRP9
ARSA.1	4.54E-28	-0.24301	0.056	0.15	6.78E-24	AC-G	ARSA
ADAM10.1	4.54E-28	-0.22605	0.093	0.202	6.79E-24	AC-G	ADAM10
FHL1.1	5.04E-28	0.345899	0.436	0.341	7.53E-24	AC-G	FHL1
FUCA1.1	5.16E-28	-0.3018	0.128	0.238	7.72E-24	AC-G	FUCA1
NPM1.1	5.18E-28	0.232424	0.821	0.878	7.73E-24	AC-G	NPM1
CYC1.1	5.93E-28	-0.21968	0.406	0.565	8.86E-24	AC-G	CYC1
UBTF.1	6.25E-28	-0.20016	0.126	0.246	9.34E-24	AC-G	UBTF
SLC16A10.	6.37E-28	-0.38672	0.023	0.1	9.51E-24	AC-G	SLC16A10
RAB8B.1	6.47E-28	-0.17385	0.069	0.171	9.67E-24	AC-G	RAB8B
MKI67.1	6.69E-28	-0.26213	0.007	0.073	9.99E-24	AC-G	MKI67
CDH4.1	6.75E-28	-0.16149	0.022	0.099	1.01E-23	AC-G	CDH4
MAP4K4	7.02E-28	-0.23309	0.146	0.268	1.05E-23	AC-G	MAP4K4
PSMD8.1	7.24E-28	0.349694	0.689	0.67	1.08E-23	AC-G	PSMD8
CORO2B	7.53E-28	-0.15297	0.031	0.114	1.13E-23	AC-G	CORO2B
GSTO1	8.34E-28	-0.26207	0.341	0.478	1.25E-23	AC-G	GSTO1
PANK2.1	9.53E-28	-0.19458	0.114	0.227	1.42E-23	AC-G	PANK2
CLPTM1L.1	9.61E-28	-0.19275	0.068	0.168	1.44E-23	AC-G	CLPTM1L
DESI1.1	9.76E-28	-0.21633	0.086	0.193	1.46E-23	AC-G	DESI1
GAS2L3.1	1.01E-27	-0.15514	0.01	0.08	1.51E-23	AC-G	GAS2L3
RGS18.1	1.03E-27	-0.27239	0.002	0.062	1.55E-23	AC-G	RGS18
SUPT6H	1.04E-27	-0.21392	0.084	0.189	1.55E-23	AC-G	SUPT6H
TTYH2.1	1.05E-27	-0.24051	0.042	0.13	1.57E-23	AC-G	TTYH2
RNF157	1.07E-27	-0.15657	0.054	0.149	1.59E-23	AC-G	RNF157
HSPA8	1.09E-27	0.230403	0.834	0.872	1.63E-23	AC-G	HSPA8

SSNA1.1	1.12E-27	-0.2052	0.322	0.47	1.67E-23	AC-G	SSNA1
TRPV2.1	1.13E-27	-0.26588	0.005	0.069	1.69E-23	AC-G	TRPV2
PKIB.1	1.17E-27	-0.12942	0.17	0.306	1.74E-23	AC-G	PKIB
MAP2K3.1	1.21E-27	-0.28063	0.1	0.206	1.81E-23	AC-G	MAP2K3
TP53I13.1	1.23E-27	-0.25136	0.147	0.264	1.84E-23	AC-G	TP53I13
GCA.1	1.25E-27	-0.38002	0.201	0.318	1.87E-23	AC-G	GCA
VPS26B.1	1.34E-27	-0.20664	0.134	0.25	2.00E-23	AC-G	VPS26B
PTX3.1	1.35E-27	0.512073	0.178	0.103	2.02E-23	AC-G	PTX3
COX5A.1	1.36E-27	-0.19677	0.467	0.624	2.04E-23	AC-G	COX5A
KCNAB2.1	1.52E-27	-0.27481	0.016	0.088	2.27E-23	AC-G	KCNAB2
IER5.1	1.67E-27	-0.27025	0.111	0.22	2.50E-23	AC-G	IER5
TLR1.1	1.80E-27	-0.30375	0.011	0.079	2.70E-23	AC-G	TLR1
ELAVL2.1	1.81E-27	-0.23344	0.018	0.091	2.71E-23	AC-G	ELAVL2
SUMO3.1	1.90E-27	-0.19763	0.319	0.463	2.84E-23	AC-G	SUMO3
FAM212B.1	1.97E-27	-0.21168	0.031	0.112	2.95E-23	AC-G	FAM212B
CCDC28B	2.18E-27	-0.14662	0.12	0.238	3.25E-23	AC-G	CCDC28B
DYNLL2.1	2.22E-27	-0.22888	0.113	0.224	3.32E-23	AC-G	DYNLL2
IGFBP3	2.33E-27	0.529364	0.269	0.18	3.48E-23	AC-G	IGFBP3
ADNP	2.35E-27	-0.18756	0.1	0.211	3.51E-23	AC-G	ADNP
RPS6KA4.1	2.43E-27	-0.25865	0.048	0.136	3.63E-23	AC-G	RPS6KA4
RBM26	2.55E-27	-0.2029	0.117	0.231	3.81E-23	AC-G	RBM26
VCP.1	2.57E-27	-0.23224	0.305	0.457	3.84E-23	AC-G	VCP
KRAS.1	2.62E-27	-0.25728	0.155	0.274	3.92E-23	AC-G	KRAS
POU3F3.1	2.66E-27	-0.18874	0.071	0.17	3.97E-23	AC-G	POU3F3
ZMAT5.1	2.67E-27	-0.15274	0.134	0.255	3.99E-23	AC-G	ZMAT5
FAM57B.1	2.72E-27	-0.2267	0.027	0.105	4.07E-23	AC-G	FAM57B
EZH2.1	2.82E-27	-0.23044	0.072	0.169	4.22E-23	AC-G	EZH2
PRKACA.1	2.96E-27	-0.18951	0.067	0.166	4.42E-23	AC-G	PRKACA
SLC29A1.1	2.97E-27	-0.35662	0.061	0.152	4.44E-23	AC-G	SLC29A1
LINC00936	3.11E-27	-0.29771	0.062	0.156	4.65E-23	AC-G	LINC00936
SLC4A7.1	3.20E-27	-0.26913	0.121	0.232	4.78E-23	AC-G	SLC4A7
LASP1.1	3.44E-27	-0.2078	0.117	0.227	5.14E-23	AC-G	LASP1
FAM217B.1	3.65E-27	-0.22171	0.072	0.171	5.46E-23	AC-G	FAM217B
CFL2	3.80E-27	0.373462	0.406	0.312	5.68E-23	AC-G	CFL2
CYCS.1	3.84E-27	0.275084	0.738	0.733	5.74E-23	AC-G	CYCS
HDAC10.1	3.85E-27	-0.2318	0.038	0.121	5.75E-23	AC-G	HDAC10
ZNF582-AS	4.02E-27	0.29539	0.135	0.065	6.01E-23	AC-G	ZNF582-AS1
CALM1.1	4.09E-27	-0.10552	0.656	0.794	6.11E-23	AC-G	CALM1
NUDT2.1	4.14E-27	-0.23064	0.087	0.192	6.18E-23	AC-G	NUDT2
KMT2C.1	4.21E-27	-0.23137	0.159	0.281	6.28E-23	AC-G	KMT2C
EXOC7.1	4.31E-27	-0.18408	0.241	0.374	6.45E-23	AC-G	EXOC7
MLX.1	4.31E-27	-0.24824	0.192	0.319	6.45E-23	AC-G	MLX
CUEDC1.1	4.33E-27	-0.13321	0.042	0.129	6.47E-23	AC-G	CUEDC1
EPSTI1.1	4.35E-27	-0.38001	0.052	0.139	6.50E-23	AC-G	EPSTI1
CRMP1.1	4.52E-27	-0.32581	0.172	0.283	6.75E-23	AC-G	CRMP1
ALDOC.1	4.55E-27	0.300089	0.236	0.14	6.80E-23	AC-G	ALDOC
FMNL3.1	4.85E-27	-0.26698	0.017	0.089	7.25E-23	AC-G	FMNL3
FAM168A.1	5.24E-27	-0.18951	0.051	0.142	7.84E-23	AC-G	FAM168A
GATM.1	5.28E-27	-0.24459	0.242	0.374	7.89E-23	AC-G	GATM
INPP5D.1	5.41E-27	-0.32696	0.022	0.097	8.08E-23	AC-G	INPP5D
ARHGEF1.1	5.43E-27	-0.278	0.107	0.214	8.12E-23	AC-G	ARHGEF1

CYTH2.1	5.73E-27	-0.22636	0.231	0.363	8.56E-23	AC-G	CYTH2
SERTAD1.1	5.85E-27	0.405556	0.399	0.3	8.74E-23	AC-G	SERTAD1
CENPM.1	6.18E-27	-0.22044	0.015	0.085	9.24E-23	AC-G	CENPM
PCMTD2.1	6.32E-27	-0.22158	0.132	0.246	9.45E-23	AC-G	PCMTD2
SGOL1	6.37E-27	-0.22215	0.005	0.068	9.52E-23	AC-G	SGOL1
MPPED2	6.49E-27	-0.20197	0.026	0.102	9.70E-23	AC-G	MPPED2
KCNQ2.1	6.55E-27	-0.13533	0.121	0.233	9.79E-23	AC-G	KCNQ2
WDR26.1	6.67E-27	-0.21715	0.077	0.176	9.97E-23	AC-G	WDR26
NOL12	6.86E-27	-0.16243	0.123	0.239	1.03E-22	AC-G	NOL12
VPS13C.1	6.89E-27	-0.19592	0.087	0.191	1.03E-22	AC-G	VPS13C
CHD3	6.94E-27	-0.25491	0.044	0.129	1.04E-22	AC-G	CHD3
SLC7A11	8.18E-27	0.121722	0.058	0.017	1.22E-22	AC-G	SLC7A11
KIAA2013.	8.22E-27	-0.1869	0.068	0.167	1.23E-22	AC-G	KIAA2013
ADCY7.1	8.43E-27	-0.2769	0.009	0.074	1.26E-22	AC-G	ADCY7
PHLDB1.1	8.61E-27	-0.22394	0.062	0.157	1.29E-22	AC-G	PHLDB1
PTP4A2.1	8.86E-27	-0.20979	0.335	0.476	1.32E-22	AC-G	PTP4A2
RAB32.1	9.28E-27	-0.27237	0.143	0.257	1.39E-22	AC-G	RAB32
TTC3.1	9.34E-27	-0.23622	0.405	0.538	1.40E-22	AC-G	TTC3
TCOF1.1	9.77E-27	-0.30854	0.108	0.212	1.46E-22	AC-G	TCOF1
EIF4G2.1	9.83E-27	-0.15038	0.552	0.68	1.47E-22	AC-G	EIF4G2
IL10RB.1	1.02E-26	-0.24943	0.147	0.262	1.52E-22	AC-G	IL10RB
CDV3	1.05E-26	-0.27035	0.175	0.295	1.56E-22	AC-G	CDV3
TYMS.1	1.05E-26	-0.27444	0.079	0.177	1.57E-22	AC-G	TYMS
CD40.1	1.06E-26	-0.29116	0.011	0.078	1.59E-22	AC-G	CD40
SUZ12.1	1.08E-26	-0.21153	0.104	0.212	1.61E-22	AC-G	SUZ12
ENAH.1	1.12E-26	-0.21988	0.164	0.284	1.67E-22	AC-G	ENAH
GBP3	1.19E-26	0.247426	0.145	0.071	1.78E-22	AC-G	GBP3
RP11-71N1	1.23E-26	-0.14996	0.056	0.151	1.84E-22	AC-G	RP11-71N10.1
C1QBP.1	1.24E-26	-0.20119	0.425	0.58	1.85E-22	AC-G	C1QBP
DOK1.1	1.29E-26	-0.26508	0.075	0.17	1.93E-22	AC-G	DOK1
SFRP4	1.32E-26	0.363438	0.107	0.047	1.97E-22	AC-G	SFRP4
FOXN2.1	1.34E-26	-0.21098	0.055	0.146	2.01E-22	AC-G	FOXN2
M6PR.1	1.37E-26	-0.27486	0.276	0.403	2.05E-22	AC-G	M6PR
B4GALT1.1	1.39E-26	-0.34855	0.049	0.134	2.08E-22	AC-G	B4GALT1
DOK5	1.53E-26	0.433721	0.253	0.169	2.29E-22	AC-G	DOK5
POLR2J.1	1.55E-26	0.344522	0.664	0.64	2.32E-22	AC-G	POLR2J
GFER.1	1.58E-26	-0.23037	0.117	0.227	2.36E-22	AC-G	GFER
LAPTM4B.1	1.68E-26	-0.27555	0.229	0.351	2.51E-22	AC-G	LAPTM4B
GAS1.1	1.78E-26	-0.22818	0.065	0.16	2.65E-22	AC-G	GAS1
TMCC3.1	1.79E-26	-0.24395	0.015	0.084	2.67E-22	AC-G	TMCC3
XRCC5.1	1.79E-26	-0.13578	0.435	0.585	2.68E-22	AC-G	XRCC5
SEMA6A.1	1.79E-26	-0.1947	0.109	0.217	2.68E-22	AC-G	SEMA6A
NISCH.1	1.80E-26	-0.25972	0.09	0.193	2.69E-22	AC-G	NISCH
ITSN2.1	1.86E-26	-0.28332	0.097	0.198	2.79E-22	AC-G	ITSN2
IDI1.1	1.88E-26	-0.25749	0.22	0.344	2.81E-22	AC-G	IDI1
TK1.1	1.92E-26	-0.25377	0.014	0.082	2.87E-22	AC-G	TK1
DEAF1.1	1.93E-26	-0.18938	0.118	0.23	2.89E-22	AC-G	DEAF1
JUND.1	2.13E-26	-0.25938	0.286	0.421	3.19E-22	AC-G	JUND
CENPF.1	2.14E-26	-0.32456	0.034	0.114	3.20E-22	AC-G	CENPF
GTPBP6.1	2.15E-26	-0.20413	0.119	0.23	3.21E-22	AC-G	GTPBP6
RBM15B.1	2.18E-26	-0.2116	0.087	0.187	3.26E-22	AC-G	RBM15B

PRDM2	2.22E-26	-0.20478	0.068	0.164	3.31E-22	AC-G	PRDM2
CCDC170	2.26E-26	0.115334	0.092	0.036	3.37E-22	AC-G	CCDC170
UBE2Q2.1	2.44E-26	-0.17745	0.112	0.223	3.65E-22	AC-G	UBE2Q2
ATF6B.1	2.46E-26	-0.2158	0.272	0.409	3.68E-22	AC-G	ATF6B
ARHGEF40.	2.58E-26	-0.21396	0.053	0.143	3.86E-22	AC-G	ARHGEF40
PRRC2C.1	2.73E-26	-0.17108	0.361	0.506	4.09E-22	AC-G	PRRC2C
ATXN80S.1	2.84E-26	0.320112	0.142	0.067	4.25E-22	AC-G	ATXN80S
YPEL1.1	3.01E-26	-0.15817	0.029	0.106	4.50E-22	AC-G	YPEL1
UBE2T.1	3.02E-26	-0.27459	0.054	0.141	4.51E-22	AC-G	UBE2T
CITED2.1	3.04E-26	-0.24808	0.242	0.374	4.54E-22	AC-G	CITED2
ZNF32.1	3.09E-26	-0.20674	0.127	0.241	4.62E-22	AC-G	ZNF32
BCL7A.1	3.09E-26	-0.21538	0.069	0.165	4.62E-22	AC-G	BCL7A
PTGES2.1	3.14E-26	-0.2363	0.168	0.286	4.70E-22	AC-G	PTGES2
CLIP2.1	3.18E-26	-0.22104	0.091	0.193	4.75E-22	AC-G	CLIP2
LIMK1.1	3.20E-26	-0.23862	0.062	0.153	4.78E-22	AC-G	LIMK1
RBM6	3.43E-26	-0.22572	0.206	0.334	5.13E-22	AC-G	RBM6
OLFML2B.1	3.50E-26	-0.32363	0.013	0.079	5.23E-22	AC-G	OLFML2B
METRN.1	3.69E-26	-0.30169	0.203	0.312	5.51E-22	AC-G	METRN
RASSF4.1	3.72E-26	-0.27307	0.224	0.35	5.56E-22	AC-G	RASSF4
CNTFR.1	3.87E-26	-0.20846	0.056	0.144	5.78E-22	AC-G	CNTFR
TRIM56.1	4.02E-26	0.241704	0.13	0.063	6.01E-22	AC-G	TRIM56
TRAF3IP3.	4.64E-26	-0.2896	0.003	0.062	6.93E-22	AC-G	TRAF3IP3
SH3GLB2.1	4.75E-26	-0.18378	0.047	0.132	7.10E-22	AC-G	SH3GLB2
NRIP1.1	4.84E-26	-0.16317	0.05	0.137	7.23E-22	AC-G	NRIP1
ERICH1.1	4.87E-26	-0.23902	0.174	0.292	7.28E-22	AC-G	ERICH1
GNGT2.1	4.98E-26	-0.25496	0.003	0.062	7.44E-22	AC-G	GNGT2
BTG2.1	5.19E-26	-0.14729	0.335	0.493	7.75E-22	AC-G	BTG2
HLA-DRA.1	5.24E-26	0.369931	0.702	0.62	7.84E-22	AC-G	HLA-DRA
AUTS2.1	5.50E-26	-0.18797	0.047	0.132	8.21E-22	AC-G	AUTS2
CSF1	5.54E-26	0.264163	0.154	0.083	8.28E-22	AC-G	CSF1
B3GALT6.1	5.57E-26	-0.17308	0.061	0.153	8.32E-22	AC-G	B3GALT6
TMEM121.1	5.85E-26	-0.18561	0.038	0.119	8.75E-22	AC-G	TMEM121
GPR155.1	5.87E-26	-0.28622	0.101	0.203	8.78E-22	AC-G	GPR155
DNAJC7.1	6.07E-26	-0.13292	0.339	0.489	9.07E-22	AC-G	DNAJC7
HEBP2.1	6.14E-26	-0.1913	0.127	0.237	9.18E-22	AC-G	HEBP2
ZNF358.1	6.27E-26	-0.17949	0.081	0.181	9.36E-22	AC-G	ZNF358
PDIA6.1	6.42E-26	0.293289	0.659	0.646	9.59E-22	AC-G	PDIA6
SLAMF8.1	6.53E-26	-0.27583	0.01	0.074	9.76E-22	AC-G	SLAMF8
EZR.1	6.56E-26	-0.22852	0.237	0.363	9.80E-22	AC-G	EZR
ZDHHC24.1	6.58E-26	-0.2302	0.102	0.204	9.83E-22	AC-G	ZDHHC24
DIAPH1.1	6.67E-26	-0.19301	0.062	0.154	9.97E-22	AC-G	DIAPH1
RRM2.1	6.77E-26	-0.26586	0.009	0.073	1.01E-21	AC-G	RRM2
ANAPC15.1	6.84E-26	-0.11347	0.156	0.282	1.02E-21	AC-G	ANAPC15
ANKRD17.1	7.45E-26	-0.21479	0.124	0.235	1.11E-21	AC-G	ANKRD17
ZDHHC12.1	7.85E-26	-0.21714	0.159	0.28	1.17E-21	AC-G	ZDHHC12
FDX1.1	8.08E-26	-0.20811	0.2	0.326	1.21E-21	AC-G	FDX1
HYAL2.1	8.17E-26	-0.18118	0.128	0.241	1.22E-21	AC-G	HYAL2
CEP350	8.17E-26	-0.2261	0.104	0.21	1.22E-21	AC-G	CEP350
PFN2.1	8.18E-26	0.389477	0.509	0.419	1.22E-21	AC-G	PFN2
NUFIP2.1	8.37E-26	-0.19323	0.139	0.255	1.25E-21	AC-G	NUFIP2
ABHD14A.1	8.58E-26	-0.20823	0.129	0.24	1.28E-21	AC-G	ABHD14A

IQCG.1	8.90E-26	0.371566	0.188	0.109	1.33E-21	AC-G	IQCG
TRPM2.1	9.17E-26	-0.24848	0.003	0.061	1.37E-21	AC-G	TRPM2
RAB3A.1	9.37E-26	-0.26162	0.082	0.18	1.40E-21	AC-G	RAB3A
ANKRD13D.	9.84E-26	-0.22293	0.084	0.184	1.47E-21	AC-G	ANKRD13D
RBM47.1	9.90E-26	-0.28567	0.047	0.13	1.48E-21	AC-G	RBM47
KIDINS22C	1.01E-25	-0.23106	0.168	0.288	1.51E-21	AC-G	KIDINS220
NEK11	1.05E-25	0.206002	0.093	0.038	1.57E-21	AC-G	NEK11
PRKACB.1	1.05E-25	-0.22825	0.123	0.233	1.57E-21	AC-G	PRKACB
LTBR.1	1.10E-25	-0.31873	0.031	0.108	1.65E-21	AC-G	LTBR
OSCAR.1	1.11E-25	-0.28058	0.016	0.083	1.66E-21	AC-G	OSCAR
NDUFA4L2	1.16E-25	0.209109	0.083	0.032	1.73E-21	AC-G	NDUFA4L2
FST	1.19E-25	0.140704	0.038	0.008	1.78E-21	AC-G	FST
RP11-806H	1.23E-25	0.162144	0.051	0.014	1.84E-21	AC-G	RP11-806H10.4
PBK.1	1.30E-25	-0.30651	0.017	0.084	1.94E-21	AC-G	PBK
TOP1.1	1.30E-25	-0.21066	0.277	0.411	1.95E-21	AC-G	TOP1
PLEKHA2.1	1.34E-25	-0.22678	0.045	0.128	2.00E-21	AC-G	PLEKHA2
TEAD2.1	1.43E-25	-0.13848	0.027	0.102	2.14E-21	AC-G	TEAD2
PPT1	1.51E-25	-0.20952	0.433	0.58	2.25E-21	AC-G	PPT1
PRR7.1	1.54E-25	-0.19768	0.102	0.205	2.31E-21	AC-G	PRR7
PAIP1.1	1.61E-25	-0.23624	0.285	0.417	2.41E-21	AC-G	PAIP1
ARAP1.1	1.65E-25	-0.24974	0.04	0.121	2.46E-21	AC-G	ARAP1
ZEB1.1	1.67E-25	-0.22891	0.105	0.21	2.50E-21	AC-G	ZEB1
CBX3.1	1.69E-25	-0.18479	0.448	0.574	2.53E-21	AC-G	CBX3
NNAT.1	1.79E-25	-0.64975	0.123	0.21	2.67E-21	AC-G	NNAT
PSMB2.1	1.82E-25	0.401888	0.609	0.567	2.73E-21	AC-G	PSMB2
ARHGAP22.	1.87E-25	-0.30611	0.018	0.085	2.80E-21	AC-G	ARHGAP22
RAP2B.1	1.88E-25	-0.25663	0.086	0.18	2.81E-21	AC-G	RAP2B
MBD6.1	1.94E-25	-0.25465	0.063	0.154	2.89E-21	AC-G	MBD6
EGR3.1	1.97E-25	-0.33109	0.039	0.117	2.94E-21	AC-G	EGR3
FLI1.1	2.10E-25	-0.26148	0.004	0.061	3.13E-21	AC-G	FLI1
RELT	2.10E-25	-0.21985	0.024	0.096	3.14E-21	AC-G	RELT
CLECL1.1	2.16E-25	-0.21551	0.001	0.056	3.23E-21	AC-G	CLECL1
PLAU.1	2.25E-25	-0.25963	0.082	0.179	3.36E-21	AC-G	PLAU
ILDR2.1	2.47E-25	-0.11528	0.02	0.092	3.69E-21	AC-G	ILDR2
MYLIP.1	2.58E-25	-0.36699	0.059	0.143	3.85E-21	AC-G	MYLIP
UBE2D1.1	2.59E-25	-0.2245	0.169	0.286	3.87E-21	AC-G	UBE2D1
CEBPA.1	2.67E-25	-0.31257	0.039	0.117	3.99E-21	AC-G	CEBPA
SLC15A3.1	2.73E-25	-0.23998	0.014	0.079	4.08E-21	AC-G	SLC15A3
ATP6V0E1.	3.16E-25	0.256566	0.746	0.763	4.72E-21	AC-G	ATP6V0E1
PIPOX	3.17E-25	-0.15625	0.057	0.147	4.73E-21	AC-G	PIPOX
CALM2	3.17E-25	0.203891	0.888	0.941	4.74E-21	AC-G	CALM2
PITPNA.1	3.23E-25	-0.1812	0.096	0.198	4.82E-21	AC-G	PITPNA
SYNGR1.1	3.24E-25	-0.2463	0.043	0.125	4.85E-21	AC-G	SYNGR1
DOK2	3.44E-25	-0.25119	0.006	0.065	5.14E-21	AC-G	DOK2
NPDC1.1	3.54E-25	-0.26146	0.225	0.342	5.29E-21	AC-G	NPDC1
SALL1.1	3.59E-25	-0.18736	0.062	0.152	5.36E-21	AC-G	SALL1
ADRBK1.1	3.63E-25	-0.25023	0.03	0.105	5.42E-21	AC-G	ADRBK1
CHIT1	3.68E-25	0.300837	0.039	0.009	5.50E-21	AC-G	CHIT1
KLHL23.1	3.87E-25	-0.24733	0.082	0.176	5.78E-21	AC-G	KLHL23
YY1.1	4.06E-25	-0.17403	0.23	0.361	6.07E-21	AC-G	YY1
ADO.1	4.09E-25	-0.17541	0.053	0.14	6.12E-21	AC-G	ADO

TVP23A	4.56E-25	0.173808	0.074	0.027	6.82E-21	AC-G	TVP23A
ILF3.1	4.77E-25	-0.18384	0.3	0.433	7.13E-21	AC-G	ILF3
POLDIP2.1	4.92E-25	-0.22168	0.243	0.367	7.36E-21	AC-G	POLDIP2
SNRNP70.1	4.93E-25	-0.19554	0.346	0.5	7.36E-21	AC-G	SNRNP70
SIK3.1	4.97E-25	-0.175	0.039	0.119	7.43E-21	AC-G	SIK3
AK2.1	5.07E-25	0.397315	0.457	0.378	7.58E-21	AC-G	AK2
UBE2F	5.13E-25	-0.23252	0.223	0.35	7.67E-21	AC-G	UBE2F
TRA2B.1	5.87E-25	-0.14658	0.414	0.564	8.77E-21	AC-G	TRA2B
PERP	5.92E-25	0.156864	0.076	0.028	8.84E-21	AC-G	PERP
MAP3K2.1	5.94E-25	-0.24801	0.114	0.217	8.87E-21	AC-G	MAP3K2
RNF11.1	6.13E-25	-0.19179	0.202	0.328	9.17E-21	AC-G	RNF11
GABARAPL1	6.16E-25	-0.25701	0.2	0.317	9.21E-21	AC-G	GABARAPL1
SNX6.1	6.42E-25	-0.17312	0.309	0.453	9.60E-21	AC-G	SNX6
ASXL1.1	6.69E-25	-0.18034	0.092	0.193	1.00E-20	AC-G	ASXL1
TWSG1	6.95E-25	0.406417	0.231	0.149	1.04E-20	AC-G	TWSG1
CXXC4.1	7.13E-25	-0.17429	0.02	0.089	1.07E-20	AC-G	CXXC4
CLEC4A.1	7.25E-25	-0.29128	0.01	0.071	1.08E-20	AC-G	CLEC4A
CCDC57.1	7.93E-25	-0.27321	0.041	0.12	1.18E-20	AC-G	CCDC57
LRRC4B.1	7.98E-25	-0.15958	0.059	0.147	1.19E-20	AC-G	LRRC4B
GINS2.1	7.98E-25	-0.24444	0.036	0.112	1.19E-20	AC-G	GINS2
G3BP2.1	8.17E-25	-0.24044	0.176	0.295	1.22E-20	AC-G	G3BP2
SMARCD1.1	8.19E-25	-0.1517	0.123	0.232	1.22E-20	AC-G	SMARCD1
FCGRT	8.36E-25	-0.25069	0.452	0.569	1.25E-20	AC-G	FCGRT
VEZF1.1	8.51E-25	-0.21554	0.144	0.256	1.27E-20	AC-G	VEZF1
PAFAH1B1.	9.15E-25	-0.20523	0.258	0.389	1.37E-20	AC-G	PAFAH1B1
NOTCH1.1	9.33E-25	-0.17042	0.042	0.124	1.39E-20	AC-G	NOTCH1
SNX8.1	9.49E-25	-0.18223	0.069	0.162	1.42E-20	AC-G	SNX8
COL1A2	9.59E-25	0.119724	0.043	0.011	1.43E-20	AC-G	COL1A2
RP11-480C	9.66E-25	-0.31314	0.002	0.057	1.44E-20	AC-G	RP11-480C22.1
IFT27.1	1.05E-24	-0.16383	0.157	0.276	1.57E-20	AC-G	IFT27
MCOLN1.1	1.05E-24	-0.22603	0.149	0.261	1.57E-20	AC-G	MCOLN1
KMT2A.1	1.08E-24	-0.18785	0.142	0.254	1.62E-20	AC-G	KMT2A
ITM2B.1	1.09E-24	-0.17126	0.613	0.835	1.63E-20	AC-G	ITM2B
AAK1.1	1.16E-24	-0.27146	0.181	0.295	1.74E-20	AC-G	AAK1
TMEM9.1	1.18E-24	0.399393	0.428	0.355	1.77E-20	AC-G	TMEM9
GSPT1.1	1.25E-24	-0.20583	0.241	0.369	1.87E-20	AC-G	GSPT1
DHFR	1.28E-24	-0.19873	0.073	0.166	1.91E-20	AC-G	DHFR
GRASP.1	1.30E-24	-0.26304	0.004	0.061	1.94E-20	AC-G	GRASP
ADSL.1	1.32E-24	-0.15762	0.158	0.276	1.98E-20	AC-G	ADSL
ATP2A2.1	1.33E-24	-0.19184	0.177	0.299	1.98E-20	AC-G	ATP2A2
ANKRD44	1.34E-24	-0.28049	0.033	0.108	2.00E-20	AC-G	ANKRD44
DOK3.1	1.39E-24	-0.2294	0.013	0.076	2.07E-20	AC-G	DOK3
MAN2A1.1	1.40E-24	-0.27611	0.044	0.123	2.09E-20	AC-G	MAN2A1
AC004158.	1.42E-24	-0.10816	0.051	0.136	2.12E-20	AC-G	AC004158.3
NF1.1	1.46E-24	-0.19177	0.132	0.241	2.18E-20	AC-G	NF1
MED13	1.49E-24	-0.16156	0.08	0.177	2.22E-20	AC-G	MED13
ZMYND11.1	1.50E-24	-0.15462	0.069	0.161	2.24E-20	AC-G	ZMYND11
CISD3.1	1.50E-24	-0.18399	0.156	0.273	2.25E-20	AC-G	CISD3
FGD2.1	1.52E-24	-0.23859	0.009	0.071	2.28E-20	AC-G	FGD2
ACOT7.1	1.56E-24	-0.17455	0.134	0.245	2.33E-20	AC-G	ACOT7
PRAM1.1	1.59E-24	-0.2599	0.005	0.062	2.38E-20	AC-G	PRAM1

DST.1	1.60E-24	-0.23604	0.275	0.396	2.40E-20	AC-G	DST
SGSM3	1.63E-24	-0.18999	0.104	0.207	2.44E-20	AC-G	SGSM3
PRKDC	1.76E-24	-0.20928	0.191	0.312	2.63E-20	AC-G	PRKDC
PLA2G7.1	1.84E-24	-0.23383	0.004	0.06	2.74E-20	AC-G	PLA2G7
GPX1.1	1.84E-24	-0.10076	0.69	0.838	2.74E-20	AC-G	GPX1
ARHGEF2.1	1.85E-24	-0.21815	0.185	0.309	2.77E-20	AC-G	ARHGEF2
ATP5J2.1	1.86E-24	0.2643	0.781	0.813	2.79E-20	AC-G	ATP5J2
UAP1	1.88E-24	0.429665	0.254	0.171	2.81E-20	AC-G	UAP1
RP3-525N1	1.92E-24	-0.20162	0.072	0.165	2.86E-20	AC-G	RP3-525N10.2
OXR1.1	1.95E-24	-0.1838	0.157	0.27	2.91E-20	AC-G	OXR1
PHC2.1	2.14E-24	-0.1963	0.176	0.296	3.19E-20	AC-G	PHC2
TBCD.1	2.19E-24	-0.19103	0.069	0.16	3.28E-20	AC-G	TBCD
MTHFD1L.1	2.49E-24	-0.21698	0.063	0.15	3.72E-20	AC-G	MTHFD1L
IGF1	2.52E-24	-0.282	0.01	0.071	3.77E-20	AC-G	IGF1
RHOT1.1	2.53E-24	-0.20359	0.155	0.267	3.79E-20	AC-G	RHOT1
MTF2.1	2.61E-24	-0.2145	0.157	0.273	3.90E-20	AC-G	MTF2
LPIN2.1	2.61E-24	-0.18599	0.073	0.164	3.91E-20	AC-G	LPIN2
FAM120A.1	2.69E-24	-0.18842	0.122	0.227	4.02E-20	AC-G	FAM120A
TRPM3	2.77E-24	-0.12188	0.015	0.08	4.14E-20	AC-G	TRPM3
PPM1M.1	2.78E-24	-0.2105	0.019	0.086	4.15E-20	AC-G	PPM1M
ZNF362.1	2.89E-24	-0.14937	0.031	0.106	4.32E-20	AC-G	ZNF362
TIA1.1	2.90E-24	-0.19537	0.152	0.267	4.33E-20	AC-G	TIA1
TPP1.1	3.05E-24	-0.21803	0.218	0.343	4.56E-20	AC-G	TPP1
QARS.1	3.06E-24	-0.16967	0.183	0.301	4.58E-20	AC-G	QARS
EEF1A1.1	3.08E-24	0.330487	0.803	0.83	4.61E-20	AC-G	EEF1A1
PQLC3.1	3.20E-24	-0.22509	0.1	0.201	4.79E-20	AC-G	PQLC3
FBXO11.1	3.22E-24	-0.22692	0.12	0.223	4.81E-20	AC-G	FBXO11
SF3B1.1	3.79E-24	-0.21416	0.322	0.458	5.67E-20	AC-G	SF3B1
SLC35D2.1	3.79E-24	-0.1599	0.037	0.113	5.67E-20	AC-G	SLC35D2
MX2.1	3.85E-24	-0.29884	0.013	0.074	5.76E-20	AC-G	MX2
CCSAP	3.90E-24	-0.20622	0.036	0.112	5.82E-20	AC-G	CCSAP
B4GALNT1.	4.04E-24	-0.29061	0.07	0.157	6.04E-20	AC-G	B4GALNT1
SESN3.1	4.09E-24	-0.16102	0.073	0.165	6.10E-20	AC-G	SESN3
DAGLB.1	4.16E-24	-0.30547	0.053	0.134	6.22E-20	AC-G	DAGLB
NUDT16.1	4.25E-24	-0.22927	0.13	0.237	6.35E-20	AC-G	NUDT16
TMEM203.1	4.25E-24	-0.20782	0.159	0.27	6.35E-20	AC-G	TMEM203
EXTL3	4.32E-24	-0.1275	0.058	0.146	6.46E-20	AC-G	EXTL3
MTMR11	4.33E-24	0.307363	0.122	0.061	6.48E-20	AC-G	MTMR11
FBXL15.1	4.41E-24	-0.26043	0.112	0.211	6.59E-20	AC-G	FBXL15
PPM1A.1	4.55E-24	-0.22845	0.1	0.198	6.80E-20	AC-G	PPM1A
SNX9.1	4.59E-24	-0.26402	0.107	0.205	6.86E-20	AC-G	SNX9
RASGRP3	4.79E-24	-0.29046	0.015	0.079	7.15E-20	AC-G	RASGRP3
UGDH.1	4.98E-24	0.353293	0.227	0.145	7.45E-20	AC-G	UGDH
TMEM19.1	5.32E-24	-0.23067	0.076	0.167	7.95E-20	AC-G	TMEM19
CDC123.1	5.45E-24	-0.17029	0.232	0.357	8.15E-20	AC-G	CDC123
LRMP	5.56E-24	-0.22795	0.001	0.053	8.31E-20	AC-G	LRMP
AGAP2.1	5.61E-24	-0.18277	0.007	0.065	8.39E-20	AC-G	AGAP2
TMEM178A.	5.75E-24	-0.18032	0.05	0.132	8.59E-20	AC-G	TMEM178A
PSMB10.1	5.76E-24	-0.25321	0.166	0.272	8.61E-20	AC-G	PSMB10
CCL3L3	5.83E-24	-0.64071	0.006	0.063	8.71E-20	AC-G	CCL3L3
MVB12B.1	6.10E-24	-0.19606	0.037	0.112	9.11E-20	AC-G	MVB12B

GAD1.1	6.18E-24	-0.12643	0.044	0.127	9.24E-20	AC-G	GAD1
C15orf48.	6.40E-24	-0.34444	0.016	0.079	9.57E-20	AC-G	C15orf48
RP11-263K	6.41E-24	0.281636	0.114	0.056	9.58E-20	AC-G	RP11-263K19.4
GNPTG.1	6.42E-24	-0.22938	0.163	0.276	9.59E-20	AC-G	GNPTG
CDC42SE2.	6.48E-24	-0.231	0.131	0.235	9.69E-20	AC-G	CDC42SE2
IFI44.1	6.52E-24	-0.26174	0.103	0.2	9.74E-20	AC-G	IFI44
HNRNPL.1	6.55E-24	-0.2044	0.166	0.283	9.79E-20	AC-G	HNRNPL
TOX.1	6.98E-24	-0.15429	0.027	0.099	1.04E-19	AC-G	TOX
NRBF2.1	7.12E-24	-0.16235	0.173	0.291	1.06E-19	AC-G	NRBF2
TLR7.1	7.16E-24	-0.27845	0.007	0.065	1.07E-19	AC-G	TLR7
NAB1.1	7.26E-24	-0.17015	0.055	0.139	1.09E-19	AC-G	NAB1
MAPK8IP1.	7.27E-24	-0.22154	0.107	0.208	1.09E-19	AC-G	MAPK8IP1
SPC25.1	7.59E-24	-0.18634	0.011	0.071	1.13E-19	AC-G	SPC25
C3orf70.1	7.64E-24	-0.18223	0.037	0.113	1.14E-19	AC-G	C3orf70
GRIA4.1	8.15E-24	-0.20656	0.058	0.143	1.22E-19	AC-G	GRIA4
DHX36.1	8.21E-24	-0.19729	0.315	0.447	1.23E-19	AC-G	DHX36
F2R.1	8.31E-24	-0.16505	0.088	0.184	1.24E-19	AC-G	F2R
GAK.1	8.31E-24	-0.22085	0.08	0.173	1.24E-19	AC-G	GAK
CTCF.1	8.37E-24	-0.19666	0.085	0.179	1.25E-19	AC-G	CTCF
CADM4.1	8.49E-24	-0.21204	0.167	0.282	1.27E-19	AC-G	CADM4
ARID1B.1	8.99E-24	-0.19492	0.056	0.141	1.34E-19	AC-G	ARID1B
LRRN1.1	9.32E-24	-0.19888	0.042	0.119	1.39E-19	AC-G	LRRN1
TMEM219.1	9.56E-24	-0.14185	0.436	0.584	1.43E-19	AC-G	TMEM219
FLNC	9.57E-24	0.141915	0.059	0.019	1.43E-19	AC-G	FLNC
EPHB2.1	9.73E-24	-0.19601	0.026	0.095	1.45E-19	AC-G	EPHB2
CCNA2.1	9.88E-24	-0.19126	0.008	0.066	1.48E-19	AC-G	CCNA2
CRLF3.1	9.98E-24	-0.19924	0.046	0.126	1.49E-19	AC-G	CRLF3
TRIM13.1	1.04E-23	-0.27106	0.158	0.267	1.56E-19	AC-G	TRIM13
DDR2	1.05E-23	0.23874	0.103	0.048	1.56E-19	AC-G	DDR2
SMIM18.1	1.05E-23	-0.21342	0.011	0.071	1.57E-19	AC-G	SMIM18
ZNF524.1	1.05E-23	-0.17563	0.103	0.204	1.57E-19	AC-G	ZNF524
DLL1	1.08E-23	-0.23638	0.017	0.082	1.61E-19	AC-G	DLL1
DCHS1.1	1.13E-23	-0.13721	0.026	0.096	1.68E-19	AC-G	DCHS1
TMEM156.1	1.14E-23	-0.28288	0.008	0.065	1.70E-19	AC-G	TMEM156
CDK5R1.1	1.15E-23	-0.19182	0.032	0.105	1.72E-19	AC-G	CDK5R1
BIRC3	1.16E-23	0.290876	0.115	0.054	1.74E-19	AC-G	BIRC3
PACS2.1	1.19E-23	-0.26195	0.063	0.149	1.78E-19	AC-G	PACS2
PLXNC1.1	1.20E-23	-0.20173	0.014	0.076	1.80E-19	AC-G	PLXNC1
ZNF33A.1	1.21E-23	-0.21497	0.038	0.113	1.81E-19	AC-G	ZNF33A
SCPEP1	1.28E-23	-0.28269	0.257	0.364	1.91E-19	AC-G	SCPEP1
GNG2.1	1.34E-23	-0.21513	0.098	0.195	2.00E-19	AC-G	GNG2
ZMYND8.1	1.36E-23	-0.20174	0.12	0.225	2.03E-19	AC-G	ZMYND8
TBC1D14.1	1.37E-23	-0.24854	0.093	0.186	2.05E-19	AC-G	TBC1D14
MAP3K11.1	1.37E-23	-0.22296	0.102	0.198	2.05E-19	AC-G	MAP3K11
SNRPN.1	1.40E-23	0.453166	0.158	0.087	2.09E-19	AC-G	SNRPN
TGFB2.1	1.42E-23	0.228318	0.117	0.057	2.13E-19	AC-G	TGFB2
PTEN.1	1.48E-23	-0.23921	0.06	0.143	2.21E-19	AC-G	PTEN
EIF4B.1	1.50E-23	-0.2028	0.27	0.399	2.24E-19	AC-G	EIF4B
SPATC1L.1	1.57E-23	-0.20927	0.112	0.21	2.34E-19	AC-G	SPATC1L
CSNK1A1.1	1.62E-23	-0.18387	0.428	0.583	2.42E-19	AC-G	CSNK1A1
RTN1.1	1.67E-23	-0.29906	0.069	0.157	2.49E-19	AC-G	RTN1

TBC1D1.1	1.68E-23	-0.22673	0.054	0.136	2.51E-19	AC-G	TBC1D1
G6PD	1.75E-23	-0.17074	0.138	0.246	2.62E-19	AC-G	G6PD
TBC1D7.1	1.83E-23	-0.18281	0.084	0.178	2.74E-19	AC-G	TBC1D7
C8orf82.1	1.96E-23	-0.18536	0.091	0.187	2.94E-19	AC-G	C8orf82
SCN1B.1	2.11E-23	-0.22882	0.017	0.081	3.15E-19	AC-G	SCN1B
LINC01114	2.41E-23	0.165648	0.07	0.026	3.60E-19	AC-G	LINC01114
TMCC1.1	2.45E-23	-0.159	0.047	0.126	3.66E-19	AC-G	TMCC1
C16orf89	2.57E-23	0.183486	0.046	0.013	3.84E-19	AC-G	C16orf89
SPN.1	2.68E-23	-0.24214	0.008	0.065	4.00E-19	AC-G	SPN
SMIM7.1	2.69E-23	0.316676	0.556	0.495	4.01E-19	AC-G	SMIM7
CCDC167	2.70E-23	-0.13044	0.271	0.404	4.04E-19	AC-G	CCDC167
TMC8	2.72E-23	-0.21339	0.001	0.051	4.06E-19	AC-G	TMC8
SMURF2.1	2.74E-23	-0.18827	0.056	0.139	4.10E-19	AC-G	SMURF2
NUDT5	2.74E-23	-0.19585	0.206	0.325	4.10E-19	AC-G	NUDT5
CHMP3	2.78E-23	-0.13834	0.268	0.4	4.16E-19	AC-G	CHMP3
TNFRSF21.	2.84E-23	-0.21214	0.085	0.178	4.25E-19	AC-G	TNFRSF21
DNAH9	3.02E-23	0.124005	0.062	0.021	4.52E-19	AC-G	DNAH9
IMPAD1	3.09E-23	-0.16158	0.102	0.203	4.62E-19	AC-G	IMPAD1
DHX9.1	3.11E-23	-0.17194	0.18	0.297	4.64E-19	AC-G	DHX9
SFMBT2.1	3.14E-23	-0.32446	0.022	0.088	4.70E-19	AC-G	SFMBT2
NFIA.1	3.18E-23	-0.17647	0.276	0.399	4.76E-19	AC-G	NFIA
C1orf167	3.23E-23	0.12336	0.026	0.004	4.82E-19	AC-G	C1orf167
ALDH16A1.	3.37E-23	-0.2183	0.052	0.133	5.03E-19	AC-G	ALDH16A1
R3HDM1	3.42E-23	-0.1476	0.057	0.141	5.11E-19	AC-G	R3HDM1
CDC20	3.46E-23	-0.25557	0.016	0.078	5.17E-19	AC-G	CDC20
BOK.1	3.47E-23	-0.18032	0.021	0.087	5.19E-19	AC-G	BOK
NUF2.1	3.52E-23	-0.20994	0.012	0.072	5.26E-19	AC-G	NUF2
SLTM.1	3.54E-23	-0.17237	0.239	0.371	5.29E-19	AC-G	SLTM
DIP2B.1	3.65E-23	-0.17612	0.051	0.132	5.45E-19	AC-G	DIP2B
TGIF2	3.72E-23	-0.15195	0.026	0.095	5.56E-19	AC-G	TGIF2
CCNB2.1	3.75E-23	-0.24062	0.013	0.073	5.61E-19	AC-G	CCNB2
HEXDC.1	3.79E-23	-0.17771	0.047	0.126	5.67E-19	AC-G	HEXDC
MOB3B.1	3.86E-23	-0.21958	0.034	0.106	5.77E-19	AC-G	MOB3B
TRAK1.1	3.88E-23	-0.16683	0.035	0.108	5.81E-19	AC-G	TRAK1
CSNK1G2.1	3.89E-23	-0.16868	0.069	0.157	5.82E-19	AC-G	CSNK1G2
LDOC1.1	3.92E-23	0.294091	0.147	0.076	5.85E-19	AC-G	LDOC1
USP3.1	3.94E-23	-0.18147	0.086	0.181	5.89E-19	AC-G	USP3
UBASH3B.1	4.20E-23	-0.13756	0.02	0.086	6.28E-19	AC-G	UBASH3B
DYNLT3	4.20E-23	0.328802	0.365	0.281	6.28E-19	AC-G	DYNLT3
MYEF2.1	4.33E-23	-0.20088	0.111	0.209	6.47E-19	AC-G	MYEF2
MFAP4	4.45E-23	0.308929	0.1	0.045	6.65E-19	AC-G	MFAP4
NDRG3.1	4.49E-23	-0.15463	0.097	0.193	6.71E-19	AC-G	NDRG3
NCOA6.1	4.62E-23	-0.15334	0.063	0.15	6.91E-19	AC-G	NCOA6
MPEG1	4.64E-23	-0.20231	0.001	0.05	6.94E-19	AC-G	MPEG1
GGA2.1	4.71E-23	-0.28643	0.069	0.153	7.04E-19	AC-G	GGA2
PIK3R3	4.80E-23	-0.10959	0.06	0.146	7.17E-19	AC-G	PIK3R3
DTNB.1	4.84E-23	-0.20414	0.035	0.107	7.23E-19	AC-G	DTNB
TAF11	5.05E-23	-0.17375	0.178	0.295	7.54E-19	AC-G	TAF11
PFDN4.1	5.08E-23	-0.17452	0.254	0.38	7.59E-19	AC-G	PFDN4
SRRD.1	5.30E-23	-0.14423	0.049	0.129	7.92E-19	AC-G	SRRD
MOB3A.1	5.39E-23	-0.21135	0.086	0.178	8.06E-19	AC-G	MOB3A

PPIB	5.53E-23	0.295183	0.709	0.727	8.26E-19	AC-G	PPIB
TMEM35.1	5.58E-23	-0.21449	0.032	0.103	8.34E-19	AC-G	TMEM35
KLRC2.1	5.75E-23	-0.30759	0.047	0.122	8.60E-19	AC-G	KLRC2
EHMT2.1	6.51E-23	-0.19894	0.116	0.217	9.73E-19	AC-G	EHMT2
INSIG1.1	6.56E-23	-0.21705	0.137	0.244	9.81E-19	AC-G	INSIG1
INF2.1	6.61E-23	-0.2262	0.028	0.097	9.88E-19	AC-G	INF2
CLK1.1	6.63E-23	-0.22286	0.324	0.442	9.90E-19	AC-G	CLK1
SNX20	6.78E-23	-0.20603	0.002	0.053	1.01E-18	AC-G	SNX20
TECR.1	7.11E-23	0.3131	0.579	0.535	1.06E-18	AC-G	TECR
CD300LF	7.15E-23	-0.2126	0.003	0.054	1.07E-18	AC-G	CD300LF
PGAP1.1	7.18E-23	-0.16913	0.101	0.199	1.07E-18	AC-G	PGAP1
AURKA.1	7.33E-23	-0.22288	0.022	0.088	1.09E-18	AC-G	AURKA
ADA.1	7.33E-23	-0.16197	0.033	0.104	1.10E-18	AC-G	ADA
ZMYM4.1	7.49E-23	-0.15151	0.056	0.139	1.12E-18	AC-G	ZMYM4
ADIPOR1.1	7.72E-23	-0.22487	0.272	0.401	1.15E-18	AC-G	ADIPOR1
ALDH2.1	7.76E-23	-0.26442	0.275	0.389	1.16E-18	AC-G	ALDH2
ZNF395	7.84E-23	0.212991	0.113	0.055	1.17E-18	AC-G	ZNF395
GIT2.1	7.99E-23	-0.21141	0.078	0.168	1.19E-18	AC-G	GIT2
FYN	8.58E-23	-0.20494	0.233	0.344	1.28E-18	AC-G	FYN
ZNF579.1	8.85E-23	-0.12864	0.048	0.127	1.32E-18	AC-G	ZNF579
LMO2.1	9.98E-23	-0.19625	0.089	0.179	1.49E-18	AC-G	LMO2
RAB33A.1	1.00E-22	-0.27824	0.043	0.118	1.49E-18	AC-G	RAB33A
GRSF1.1	1.02E-22	-0.18293	0.224	0.342	1.52E-18	AC-G	GRSF1
DLEU7.1	1.07E-22	-0.252	0.005	0.058	1.60E-18	AC-G	DLEU7
FAM110A.1	1.09E-22	-0.20265	0.02	0.084	1.63E-18	AC-G	FAM110A
PLLP.1	1.14E-22	-0.33318	0.017	0.078	1.70E-18	AC-G	PLLP
RP1-17K7.	1.15E-22	0.273368	0.102	0.045	1.72E-18	AC-G	RP1-17K7.2
SAMM50.1	1.16E-22	-0.14981	0.126	0.233	1.73E-18	AC-G	SAMM50
PLCB2.1	1.18E-22	-0.25035	0.012	0.071	1.76E-18	AC-G	PLCB2
KANSL1.1	1.19E-22	-0.19516	0.088	0.181	1.78E-18	AC-G	KANSL1
AMER2.1	1.21E-22	-0.33564	0.05	0.127	1.80E-18	AC-G	AMER2
KIAA1211L	1.21E-22	-0.12704	0.008	0.065	1.81E-18	AC-G	KIAA1211L
DRAM2.1	1.24E-22	-0.32637	0.248	0.353	1.85E-18	AC-G	DRAM2
SDF2L1.1	1.28E-22	-0.19886	0.25	0.381	1.91E-18	AC-G	SDF2L1
CELF3.1	1.29E-22	-0.20132	0.015	0.076	1.92E-18	AC-G	CELF3
IDH3G.1	1.30E-22	-0.11357	0.288	0.425	1.94E-18	AC-G	IDH3G
PCIF1.1	1.30E-22	-0.10836	0.069	0.157	1.94E-18	AC-G	PCIF1
USP6NL.1	1.31E-22	-0.13936	0.026	0.094	1.95E-18	AC-G	USP6NL
ATP6V0A1.	1.34E-22	-0.19347	0.189	0.304	2.00E-18	AC-G	ATP6V0A1
NCAM1.1	1.39E-22	-0.21868	0.239	0.354	2.07E-18	AC-G	NCAM1
ATPAF2.1	1.42E-22	-0.17031	0.126	0.23	2.12E-18	AC-G	ATPAF2
ATP8B4	1.44E-22	-0.21085	0.002	0.051	2.15E-18	AC-G	ATP8B4
YDJC.1	1.50E-22	-0.19052	0.071	0.158	2.25E-18	AC-G	YDJC
SYNDIG1.1	1.66E-22	-0.22869	0.031	0.101	2.48E-18	AC-G	SYNDIG1
FAM3C.1	1.67E-22	0.302392	0.483	0.428	2.49E-18	AC-G	FAM3C
NUBP1	1.67E-22	-0.18909	0.131	0.235	2.50E-18	AC-G	NUBP1
PRKD3.1	1.68E-22	-0.18262	0.073	0.161	2.52E-18	AC-G	PRKD3
SCG3.1	1.70E-22	-0.23609	0.177	0.284	2.54E-18	AC-G	SCG3
EXOC5	1.70E-22	-0.17246	0.099	0.195	2.55E-18	AC-G	EXOC5
ZMYM2.1	1.73E-22	-0.23656	0.121	0.221	2.58E-18	AC-G	ZMYM2
IKBKG.1	1.75E-22	-0.18201	0.064	0.147	2.62E-18	AC-G	IKBKG

DNASE2.1	1.83E-22	-0.19914	0.284	0.403	2.73E-18	AC-G	DNASE2
LRRC45.1	1.83E-22	-0.15561	0.031	0.1	2.73E-18	AC-G	LRRC45
CTBP2	1.86E-22	-0.20393	0.072	0.158	2.78E-18	AC-G	CTBP2
ACTL6B.1	1.96E-22	-0.23278	0.022	0.087	2.93E-18	AC-G	ACTL6B
KLHL6.1	2.01E-22	-0.24532	0.006	0.059	3.01E-18	AC-G	KLHL6
EBP.1	2.02E-22	-0.10308	0.156	0.27	3.01E-18	AC-G	EBP
BLOC1S6	2.03E-22	-0.23545	0.169	0.278	3.03E-18	AC-G	BLOC1S6
MGST3.1	2.04E-22	0.271752	0.684	0.695	3.05E-18	AC-G	MGST3
B3GAT1.1	2.11E-22	-0.21896	0.032	0.101	3.16E-18	AC-G	B3GAT1
NLGN1.1	2.14E-22	-0.16088	0.066	0.152	3.19E-18	AC-G	NLGN1
ATP13A2.1	2.17E-22	-0.18877	0.069	0.154	3.24E-18	AC-G	ATP13A2
MILR1.1	2.20E-22	-0.29352	0.038	0.11	3.28E-18	AC-G	MILR1
QSER1.1	2.41E-22	-0.13287	0.052	0.131	3.60E-18	AC-G	QSER1
CHMP4B.1	2.41E-22	-0.18257	0.348	0.475	3.60E-18	AC-G	CHMP4B
RAB22A.1	2.44E-22	-0.17932	0.149	0.256	3.64E-18	AC-G	RAB22A
KIAA1549.	2.45E-22	-0.18181	0.034	0.105	3.67E-18	AC-G	KIAA1549
DYRK3	2.46E-22	0.118009	0.053	0.017	3.68E-18	AC-G	DYRK3
LGALS3BP.	2.56E-22	0.39514	0.509	0.438	3.83E-18	AC-G	LGALS3BP
CAHM.1	2.63E-22	-0.17574	0.032	0.103	3.93E-18	AC-G	CAHM
ERH.1	2.71E-22	0.306223	0.726	0.711	4.05E-18	AC-G	ERH
KIAA0040	2.78E-22	0.14708	0.088	0.037	4.16E-18	AC-G	KIAA0040
IRAK1.1	2.79E-22	-0.23725	0.084	0.173	4.17E-18	AC-G	IRAK1
C14orf80.	2.81E-22	-0.14738	0.015	0.075	4.20E-18	AC-G	C14orf80
FBXW5.1	2.87E-22	-0.24813	0.196	0.306	4.30E-18	AC-G	FBXW5
KLHL13.1	3.08E-22	-0.14004	0.038	0.111	4.60E-18	AC-G	KLHL13
CENPK	3.20E-22	-0.20342	0.039	0.111	4.78E-18	AC-G	CENPK
USP15.1	3.24E-22	-0.2637	0.156	0.26	4.83E-18	AC-G	USP15
ADAM19.1	3.25E-22	-0.15755	0.025	0.09	4.85E-18	AC-G	ADAM19
VDAC1.1	3.31E-22	0.284865	0.652	0.627	4.95E-18	AC-G	VDAC1
NIPSNAP1.	3.34E-22	-0.16531	0.091	0.182	4.98E-18	AC-G	NIPSNAP1
HELZ.1	3.36E-22	-0.18651	0.113	0.212	5.01E-18	AC-G	HELZ
POLR2B.1	3.47E-22	-0.1921	0.17	0.28	5.19E-18	AC-G	POLR2B
DNAJB12.1	3.48E-22	-0.23007	0.076	0.162	5.20E-18	AC-G	DNAJB12
HOMER3.1	3.59E-22	-0.19137	0.153	0.259	5.36E-18	AC-G	HOMER3
RP6-91H8.	3.68E-22	-0.29047	0.009	0.065	5.50E-18	AC-G	RP6-91H8.3
LILRB2.1	3.69E-22	-0.22346	0.008	0.062	5.52E-18	AC-G	LILRB2
LHFPL3.1	3.71E-22	-0.21225	0.053	0.132	5.55E-18	AC-G	LHFPL3
SFRP1.1	3.75E-22	-0.10332	0.036	0.11	5.60E-18	AC-G	SFRP1
DGAT1.1	3.75E-22	-0.16467	0.03	0.099	5.60E-18	AC-G	DGAT1
CTSA.1	3.76E-22	-0.17038	0.295	0.419	5.61E-18	AC-G	CTSA
TES.1	3.83E-22	-0.24403	0.004	0.054	5.72E-18	AC-G	TES
TTTY15.1	4.01E-22	-0.14003	0.009	0.064	5.99E-18	AC-G	TTTY15
HNRNPUL2.	4.28E-22	-0.18137	0.087	0.178	6.40E-18	AC-G	HNRNPUL2
TCEB2.1	4.34E-22	0.232904	0.795	0.829	6.48E-18	AC-G	TCEB2
IL1RN.1	4.43E-22	-0.29908	0.022	0.085	6.62E-18	AC-G	IL1RN
HIP1R.1	4.48E-22	-0.21161	0.046	0.121	6.69E-18	AC-G	HIP1R
STAT1	4.50E-22	-0.2104	0.143	0.246	6.72E-18	AC-G	STAT1
TEN1.1	4.54E-22	-0.15154	0.098	0.194	6.78E-18	AC-G	TEN1
CTBP1.1	4.66E-22	-0.19406	0.136	0.239	6.97E-18	AC-G	CTBP1
CERS6.1	4.75E-22	-0.16295	0.059	0.14	7.10E-18	AC-G	CERS6
KIF9.1	4.99E-22	0.399035	0.239	0.162	7.45E-18	AC-G	KIF9

STIM2	5.01E-22	-0.14001	0.086	0.177	7.49E-18	AC-G	STIM2
PLP1.1	5.02E-22	-0.55693	0.097	0.185	7.50E-18	AC-G	PLP1
WHSC1.1	5.08E-22	-0.18639	0.08	0.167	7.60E-18	AC-G	WHSC1
TBC1D9B.1	5.25E-22	-0.18209	0.084	0.175	7.84E-18	AC-G	TBC1D9B
HOXA2.1	5.26E-22	-0.21784	0.02	0.082	7.86E-18	AC-G	HOXA2
RILP.1	5.69E-22	-0.21127	0.042	0.115	8.50E-18	AC-G	RILP
GGCT.1	5.77E-22	0.464763	0.44	0.376	8.62E-18	AC-G	GGCT
NF2.1	5.83E-22	-0.14955	0.077	0.165	8.71E-18	AC-G	NF2
ARF3.1	6.08E-22	-0.1497	0.268	0.399	9.08E-18	AC-G	ARF3
CMKLR1.1	6.10E-22	-0.21924	0.006	0.058	9.12E-18	AC-G	CMKLR1
RTKN.1	6.16E-22	-0.1363	0.093	0.186	9.21E-18	AC-G	RTKN
AP2A2.1	6.41E-22	-0.27209	0.167	0.268	9.58E-18	AC-G	AP2A2
SECISBP2L	6.48E-22	-0.22367	0.096	0.189	9.68E-18	AC-G	SECISBP2L
PHKB.1	6.56E-22	-0.20539	0.112	0.208	9.80E-18	AC-G	PHKB
WBP1L.1	6.58E-22	-0.25419	0.047	0.121	9.84E-18	AC-G	WBP1L
IGDCC3.1	6.60E-22	-0.11362	0.014	0.072	9.86E-18	AC-G	IGDCC3
ACLY.1	6.71E-22	-0.1406	0.129	0.233	1.00E-17	AC-G	ACLY
CDC42BPB.	6.86E-22	-0.15608	0.053	0.132	1.03E-17	AC-G	CDC42BPB
ZNF292.1	6.91E-22	-0.20565	0.173	0.281	1.03E-17	AC-G	ZNF292
FOXM1	7.23E-22	-0.15259	0.007	0.061	1.08E-17	AC-G	FOXM1
TIMELESS	7.36E-22	-0.12472	0.014	0.072	1.10E-17	AC-G	TIMELESS
SNX27.1	7.38E-22	-0.21701	0.156	0.262	1.10E-17	AC-G	SNX27
CRTAP.1	7.50E-22	-0.22503	0.15	0.253	1.12E-17	AC-G	CRTAP
GIMAP6	7.53E-22	-0.2213	0.002	0.051	1.12E-17	AC-G	GIMAP6
IGFBP7.1	7.96E-22	0.318174	0.345	0.254	1.19E-17	AC-G	IGFBP7
TNKS2.1	8.06E-22	-0.20259	0.047	0.122	1.20E-17	AC-G	TNKS2
MAF.1	8.31E-22	-0.27974	0.068	0.148	1.24E-17	AC-G	MAF
RAD51AP1.	8.66E-22	-0.18355	0.016	0.076	1.29E-17	AC-G	RAD51AP1
SIGLEC14	8.69E-22	-0.22342	0.003	0.053	1.30E-17	AC-G	SIGLEC14
LRRC8D.1	8.71E-22	-0.17112	0.035	0.105	1.30E-17	AC-G	LRRC8D
ZFAND3.1	8.73E-22	-0.1938	0.181	0.29	1.30E-17	AC-G	ZFAND3
NOL3.1	8.88E-22	0.343039	0.23	0.153	1.33E-17	AC-G	NOL3
KCTD20.1	9.03E-22	-0.19325	0.108	0.203	1.35E-17	AC-G	KCTD20
SIAH1.1	9.91E-22	-0.19673	0.142	0.246	1.48E-17	AC-G	SIAH1
HLA-C	1.01E-21	0.234173	0.809	0.802	1.52E-17	AC-G	HLA-C
HMGXB4.1	1.04E-21	-0.19446	0.135	0.239	1.56E-17	AC-G	HMGXB4
HECTD1.1	1.06E-21	-0.18425	0.14	0.245	1.58E-17	AC-G	HECTD1
JDP2.1	1.09E-21	-0.24491	0.019	0.08	1.63E-17	AC-G	JDP2
MAPRE1.1	1.12E-21	-0.1423	0.362	0.5	1.68E-17	AC-G	MAPRE1
LIX1L.1	1.15E-21	-0.16353	0.069	0.153	1.72E-17	AC-G	LIX1L
SFT2D1.1	1.17E-21	-0.17493	0.391	0.523	1.74E-17	AC-G	SFT2D1
GATAD2B.1	1.18E-21	-0.19317	0.086	0.175	1.76E-17	AC-G	GATAD2B
PTPRO.1	1.25E-21	-0.23177	0.025	0.089	1.87E-17	AC-G	PTPRO
PCOLCE2	1.27E-21	0.26885	0.134	0.072	1.90E-17	AC-G	PCOLCE2
SPTSSB	1.34E-21	0.317794	0.073	0.03	2.01E-17	AC-G	SPTSSB
SPRY2.1	1.37E-21	-0.17694	0.134	0.234	2.05E-17	AC-G	SPRY2
SPSB1	1.44E-21	0.248829	0.118	0.059	2.15E-17	AC-G	SPSB1
UROD.1	1.45E-21	0.386827	0.425	0.346	2.17E-17	AC-G	UROD
KHSRP.1	1.47E-21	-0.15333	0.147	0.256	2.20E-17	AC-G	KHSRP
CDIPT-AS1	1.53E-21	0.130492	0.027	0.005	2.28E-17	AC-G	CDIPT-AS1
ACBD5.1	1.58E-21	-0.16731	0.091	0.182	2.36E-17	AC-G	ACBD5

NRP1.1	1.64E-21	-0.18025	0.089	0.18	2.44E-17	AC-G	NRP1
SQSTM1.1	1.76E-21	0.245001	0.555	0.517	2.63E-17	AC-G	SQSTM1
VWA5A.1	1.78E-21	0.259837	0.145	0.079	2.66E-17	AC-G	VWA5A
SOCS2.1	1.85E-21	-0.16352	0.082	0.171	2.76E-17	AC-G	SOCS2
PSPC1.1	2.06E-21	-0.20346	0.129	0.227	3.09E-17	AC-G	PSPC1
BCAN.1	2.08E-21	-0.30257	0.187	0.293	3.12E-17	AC-G	BCAN
ZBTB1.1	2.12E-21	-0.22478	0.091	0.179	3.16E-17	AC-G	ZBTB1
NCL.1	2.13E-21	-0.15935	0.434	0.578	3.19E-17	AC-G	NCL
ANLN	2.22E-21	-0.24493	0.015	0.072	3.31E-17	AC-G	ANLN
MMP15.1	2.24E-21	-0.10972	0.015	0.073	3.34E-17	AC-G	MMP15
NSA2.1	2.25E-21	-0.13596	0.293	0.419	3.36E-17	AC-G	NSA2
IL10RB-AS	2.29E-21	-0.21896	0.004	0.053	3.42E-17	AC-G	IL10RB-AS1
KCTD17.1	2.30E-21	-0.15978	0.066	0.148	3.44E-17	AC-G	KCTD17
AC074289.	2.33E-21	-0.24591	0.014	0.07	3.48E-17	AC-G	AC074289.1
ABAT.1	2.35E-21	-0.19335	0.086	0.174	3.51E-17	AC-G	ABAT
CCDC109B.	2.36E-21	-0.2123	0.253	0.367	3.52E-17	AC-G	CCDC109B
LINC00869	2.39E-21	0.194859	0.042	0.012	3.58E-17	AC-G	LINC00869
SKI.1	2.43E-21	-0.1974	0.079	0.165	3.63E-17	AC-G	SKI
KIF3C.1	2.54E-21	-0.14257	0.042	0.114	3.80E-17	AC-G	KIF3C
PAPOLG.1	2.55E-21	-0.21693	0.034	0.103	3.81E-17	AC-G	PAPOLG
NKG7	2.55E-21	-0.28914	0.007	0.058	3.81E-17	AC-G	NKG7
MIR497HG	2.60E-21	0.281213	0.115	0.058	3.89E-17	AC-G	MIR497HG
MEX3B.1	2.61E-21	-0.16527	0.015	0.072	3.90E-17	AC-G	MEX3B
HSPA9.1	2.69E-21	0.346889	0.517	0.482	4.01E-17	AC-G	HSPA9
TBCC.1	2.70E-21	-0.2372	0.138	0.238	4.03E-17	AC-G	TBCC
TMEM127	2.78E-21	-0.20004	0.078	0.163	4.15E-17	AC-G	TMEM127
EGOT	2.89E-21	0.110947	0.022	0.003	4.32E-17	AC-G	EGOT
WASF2.1	2.92E-21	-0.22454	0.333	0.459	4.36E-17	AC-G	WASF2
KCNQ1	3.01E-21	-0.1937	0.001	0.046	4.50E-17	AC-G	KCNQ1
CREBBP	3.05E-21	-0.19266	0.079	0.165	4.56E-17	AC-G	CREBBP
BCKDK.1	3.07E-21	-0.16355	0.216	0.333	4.58E-17	AC-G	BCKDK
R3HDM2.1	3.09E-21	-0.12592	0.092	0.182	4.62E-17	AC-G	R3HDM2
MAP6D1.1	3.17E-21	-0.20392	0.028	0.093	4.74E-17	AC-G	MAP6D1
CHPT1.1	3.26E-21	-0.21476	0.213	0.32	4.88E-17	AC-G	CHPT1
POMC.1	3.30E-21	-0.22671	0.005	0.055	4.93E-17	AC-G	POMC
C20orf27.	3.41E-21	-0.14978	0.262	0.379	5.10E-17	AC-G	C20orf27
LINC01094	3.49E-21	-0.24561	0.084	0.167	5.21E-17	AC-G	LINC01094
POGZ.1	3.60E-21	-0.18881	0.128	0.227	5.38E-17	AC-G	POGZ
SIK1.1	3.64E-21	-0.30962	0.042	0.111	5.45E-17	AC-G	SIK1
ST8SIA4.1	3.83E-21	-0.24102	0.055	0.131	5.73E-17	AC-G	ST8SIA4
SREBF2	3.84E-21	-0.11242	0.054	0.133	5.74E-17	AC-G	SREBF2
EGLN2.1	3.88E-21	-0.18549	0.191	0.301	5.80E-17	AC-G	EGLN2
GSS.1	4.19E-21	-0.12577	0.151	0.257	6.27E-17	AC-G	GSS
HPS4.1	4.32E-21	-0.15419	0.102	0.198	6.46E-17	AC-G	HPS4
TM9SF3.1	4.39E-21	-0.22698	0.137	0.236	6.57E-17	AC-G	TM9SF3
IRF2BPL.1	4.40E-21	-0.19928	0.154	0.259	6.58E-17	AC-G	IRF2BPL
ABCD4.1	4.44E-21	-0.15247	0.164	0.274	6.64E-17	AC-G	ABCD4
OS9.1	4.45E-21	-0.17082	0.425	0.557	6.65E-17	AC-G	OS9
HLA-DQA1.	4.48E-21	-0.1767	0.213	0.324	6.69E-17	AC-G	HLA-DQA1
THAP11.1	4.49E-21	-0.18086	0.099	0.191	6.71E-17	AC-G	THAP11
EFCAB14.1	4.70E-21	-0.21971	0.123	0.219	7.02E-17	AC-G	EFCAB14

RPS6KA3.1	4.86E-21	-0.1688	0.068	0.15	7.27E-17	AC-G	RPS6KA3
PACSIN2.1	4.92E-21	-0.18196	0.097	0.189	7.35E-17	AC-G	PACSIN2
CECR1.1	5.26E-21	-0.25087	0.02	0.081	7.87E-17	AC-G	CECR1
PDGFB	5.58E-21	-0.24225	0.005	0.054	8.33E-17	AC-G	PDGFB
PCBP2.1	5.67E-21	-0.145	0.545	0.686	8.47E-17	AC-G	PCBP2
TRAF7.1	5.80E-21	-0.15225	0.111	0.205	8.66E-17	AC-G	TRAF7
RPAIN	5.83E-21	-0.158	0.379	0.506	8.72E-17	AC-G	RPAIN
FPGS.1	5.89E-21	-0.18111	0.079	0.164	8.81E-17	AC-G	FPGS
UBE2Q1	5.96E-21	-0.11922	0.086	0.176	8.91E-17	AC-G	UBE2Q1
HUWE1.1	5.97E-21	-0.18882	0.096	0.187	8.92E-17	AC-G	HUWE1
ALG12.1	6.28E-21	-0.11811	0.034	0.103	9.38E-17	AC-G	ALG12
BMI1.1	6.32E-21	-0.12645	0.07	0.153	9.45E-17	AC-G	BMI1
FSD1.1	6.39E-21	-0.15255	0.082	0.169	9.55E-17	AC-G	FSD1
TUBA1C.1	6.54E-21	0.390504	0.443	0.367	9.77E-17	AC-G	TUBA1C
DCTN1.1	6.74E-21	-0.20389	0.133	0.232	1.01E-16	AC-G	DCTN1
FAM222B.1	7.01E-21	-0.10609	0.05	0.126	1.05E-16	AC-G	FAM222B
TBX15	7.24E-21	0.103102	0.052	0.017	1.08E-16	AC-G	TBX15
IQGAP1.1	7.46E-21	-0.21703	0.165	0.27	1.11E-16	AC-G	IQGAP1
ZNF618.1	7.49E-21	-0.15938	0.006	0.056	1.12E-16	AC-G	ZNF618
NCOA3.1	7.56E-21	-0.2048	0.061	0.138	1.13E-16	AC-G	NCOA3
BCLAF1.1	7.71E-21	-0.15476	0.281	0.405	1.15E-16	AC-G	BCLAF1
FHL3.1	7.93E-21	-0.18864	0.086	0.173	1.18E-16	AC-G	FHL3
CBL.1	8.13E-21	-0.1488	0.043	0.115	1.21E-16	AC-G	CBL
TERF2IP.1	8.17E-21	-0.21061	0.406	0.539	1.22E-16	AC-G	TERF2IP
FAM171A1.	8.22E-21	-0.15499	0.025	0.087	1.23E-16	AC-G	FAM171A1
PCM1.1	8.28E-21	-0.19097	0.226	0.343	1.24E-16	AC-G	PCM1
DYNC2LI1.	8.84E-21	0.361207	0.228	0.152	1.32E-16	AC-G	DYNC2LI1
CD52	8.86E-21	-0.39194	0.009	0.06	1.32E-16	AC-G	CD52
FASN.1	8.99E-21	-0.14496	0.053	0.129	1.34E-16	AC-G	FASN
DDX46.1	9.20E-21	-0.142	0.334	0.468	1.38E-16	AC-G	DDX46
STT3B.1	9.25E-21	-0.1722	0.123	0.22	1.38E-16	AC-G	STT3B
SMARCC2.1	9.26E-21	-0.1608	0.172	0.281	1.38E-16	AC-G	SMARCC2
RCAN2	9.34E-21	0.1854	0.084	0.036	1.40E-16	AC-G	RCAN2
NCAN	9.41E-21	-0.21242	0.027	0.09	1.41E-16	AC-G	NCAN
EIF3I.1	9.79E-21	0.28856	0.627	0.597	1.46E-16	AC-G	EIF3I
DMWD.1	9.81E-21	-0.13475	0.072	0.155	1.47E-16	AC-G	DMWD
PPP1R9B	9.92E-21	-0.14278	0.041	0.112	1.48E-16	AC-G	PPP1R9B
AURKB.1	1.02E-20	-0.19777	0.007	0.057	1.52E-16	AC-G	AURKB
MXD3.1	1.06E-20	-0.1892	0.016	0.072	1.59E-16	AC-G	MXD3
CUL3.1	1.08E-20	-0.17561	0.119	0.216	1.61E-16	AC-G	CUL3
DCLK2.1	1.08E-20	-0.15198	0.117	0.212	1.62E-16	AC-G	DCLK2
MAPK10.1	1.08E-20	-0.13946	0.115	0.21	1.62E-16	AC-G	MAPK10
PPP2CB.1	1.10E-20	-0.15444	0.205	0.314	1.64E-16	AC-G	PPP2CB
B3GNT2.1	1.11E-20	-0.15793	0.018	0.076	1.65E-16	AC-G	B3GNT2
RCAN3.1	1.15E-20	-0.15594	0.013	0.068	1.71E-16	AC-G	RCAN3
C7orf73.1	1.17E-20	0.388375	0.581	0.549	1.74E-16	AC-G	C7orf73
BRD2.1	1.26E-20	-0.14109	0.332	0.477	1.88E-16	AC-G	BRD2
SHCBP1.1	1.29E-20	-0.15289	0.009	0.062	1.92E-16	AC-G	SHCBP1
APC.1	1.31E-20	-0.17611	0.13	0.229	1.95E-16	AC-G	APC
NRXN1.1	1.31E-20	-0.32414	0.096	0.176	1.96E-16	AC-G	NRXN1
SERPINE2.	1.32E-20	0.350693	0.354	0.281	1.98E-16	AC-G	SERPINE2

MYBPC1	1.35E-20	0.261234	0.084	0.036	2.02E-16	AC-G	MYBPC1
SOX15	1.46E-20	0.239897	0.119	0.061	2.19E-16	AC-G	SOX15
RUNX1.1	1.47E-20	-0.21076	0.113	0.205	2.20E-16	AC-G	RUNX1
HSD11B1	1.48E-20	0.231865	0.054	0.018	2.21E-16	AC-G	HSD11B1
TEX29	1.49E-20	0.126125	0.024	0.004	2.23E-16	AC-G	TEX29
UBE2J2.1	1.54E-20	-0.19076	0.236	0.353	2.30E-16	AC-G	UBE2J2
KPNB1.1	1.55E-20	-0.13315	0.338	0.469	2.31E-16	AC-G	KPNB1
LHPP.1	1.56E-20	-0.37155	0.046	0.117	2.33E-16	AC-G	LHPP
SF1	1.61E-20	-0.15577	0.388	0.53	2.40E-16	AC-G	SF1
TUG1.1	1.65E-20	-0.16801	0.099	0.19	2.47E-16	AC-G	TUG1
TNF	1.66E-20	-0.24531	0.004	0.051	2.47E-16	AC-G	TNF
ECHS1	1.71E-20	-0.14011	0.325	0.456	2.56E-16	AC-G	ECHS1
SBK1.1	1.72E-20	-0.16151	0.021	0.081	2.56E-16	AC-G	SBK1
CHKA.1	1.73E-20	-0.2516	0.112	0.202	2.59E-16	AC-G	CHKA
MTMR4	1.73E-20	-0.14537	0.087	0.175	2.59E-16	AC-G	MTMR4
NECAB3.1	1.75E-20	-0.12337	0.04	0.109	2.61E-16	AC-G	NECAB3
SSH1	1.75E-20	-0.20314	0.03	0.094	2.62E-16	AC-G	SSH1
CRLS1.1	1.78E-20	-0.20933	0.195	0.301	2.66E-16	AC-G	CRLS1
GBGT1.1	1.86E-20	-0.20061	0.008	0.058	2.78E-16	AC-G	GBGT1
TMEM86A.1	1.87E-20	-0.17959	0.006	0.054	2.79E-16	AC-G	TMEM86A
SYNCRIP.1	1.90E-20	-0.16287	0.277	0.404	2.85E-16	AC-G	SYNCRIP
RAB11B-AS	1.91E-20	-0.14687	0.065	0.145	2.86E-16	AC-G	RAB11B-AS1
CACNA1A.1	1.93E-20	-0.16893	0.038	0.107	2.88E-16	AC-G	CACNA1A
RASGRP1.1	1.98E-20	-0.10158	0.019	0.078	2.96E-16	AC-G	RASGRP1
NONO.1	2.01E-20	-0.1668	0.347	0.476	3.00E-16	AC-G	NONO
RNF220.1	2.03E-20	-0.17318	0.078	0.162	3.03E-16	AC-G	RNF220
IFI44L.1	2.09E-20	-0.37658	0.076	0.154	3.13E-16	AC-G	IFI44L
TRIM8.1	2.20E-20	-0.19143	0.041	0.11	3.30E-16	AC-G	TRIM8
GALM.1	2.21E-20	-0.27798	0.062	0.137	3.30E-16	AC-G	GALM
PPM1B.1	2.22E-20	-0.21515	0.209	0.318	3.31E-16	AC-G	PPM1B
CELF4.1	2.33E-20	-0.27868	0.038	0.103	3.49E-16	AC-G	CELF4
POU2F1.1	2.36E-20	-0.18359	0.089	0.175	3.53E-16	AC-G	POU2F1
ASAP2.1	2.45E-20	-0.10456	0.038	0.106	3.66E-16	AC-G	ASAP2
ARNT2.1	2.47E-20	-0.10376	0.049	0.123	3.70E-16	AC-G	ARNT2
HPSE.1	2.48E-20	-0.24589	0.013	0.067	3.71E-16	AC-G	HPSE
THSD1	2.52E-20	-0.10978	0.037	0.105	3.77E-16	AC-G	THSD1
AP3B1.1	2.55E-20	-0.14476	0.096	0.185	3.81E-16	AC-G	AP3B1
DYNC1I2.1	2.59E-20	-0.14582	0.362	0.49	3.87E-16	AC-G	DYNC1I2
PDE4A.1	2.73E-20	-0.17555	0.034	0.1	4.07E-16	AC-G	PDE4A
UBE2O.1	2.77E-20	-0.13734	0.053	0.129	4.15E-16	AC-G	UBE2O
RFX4	2.88E-20	0.397985	0.209	0.139	4.30E-16	AC-G	RFX4
PRR11	2.89E-20	-0.14658	0.017	0.073	4.32E-16	AC-G	PRR11
MAN1A1	2.93E-20	-0.20341	0.007	0.056	4.37E-16	AC-G	MAN1A1
ZNF655.1	2.96E-20	-0.16483	0.154	0.256	4.42E-16	AC-G	ZNF655
RHOU.1	2.98E-20	-0.2707	0.07	0.147	4.45E-16	AC-G	RHOU
NAP1L4.1	3.01E-20	-0.14898	0.303	0.423	4.50E-16	AC-G	NAP1L4
HNRNPH3.1	3.03E-20	-0.15377	0.336	0.467	4.53E-16	AC-G	HNRNPH3
FAM60A.1	3.05E-20	-0.17709	0.1	0.189	4.55E-16	AC-G	FAM60A
CDCA3	3.10E-20	-0.16828	0.009	0.061	4.64E-16	AC-G	CDCA3
RBP7	3.12E-20	-0.243	0.005	0.053	4.67E-16	AC-G	RBP7
IVNS1ABP.	3.16E-20	-0.21363	0.155	0.254	4.72E-16	AC-G	IVNS1ABP

TMEM52B.1	3.20E-20	-0.22236	0.006	0.055	4.78E-16	AC-G	TMEM52B
UGT8.1	3.24E-20	-0.25413	0.008	0.058	4.85E-16	AC-G	UGT8
SYNRG	3.48E-20	-0.1266	0.11	0.204	5.20E-16	AC-G	SYNRG
ZFYVE16.1	3.49E-20	-0.22031	0.099	0.186	5.22E-16	AC-G	ZFYVE16
VIPR2.1	3.60E-20	-0.25007	0.03	0.093	5.38E-16	AC-G	VIPR2
NKAIN3.1	3.60E-20	-0.13381	0.06	0.137	5.38E-16	AC-G	NKAIN3
RSF1	3.64E-20	-0.15918	0.312	0.438	5.44E-16	AC-G	RSF1
PHF14.1	3.78E-20	-0.20515	0.322	0.442	5.66E-16	AC-G	PHF14
PLA2G15.1	3.80E-20	-0.26871	0.073	0.151	5.68E-16	AC-G	PLA2G15
TBC1D16	3.85E-20	-0.19779	0.068	0.147	5.76E-16	AC-G	TBC1D16
ACP5.1	3.92E-20	-0.22532	0.007	0.056	5.85E-16	AC-G	ACP5
TET2.1	4.12E-20	-0.20145	0.034	0.099	6.16E-16	AC-G	TET2
TMEM184B	4.17E-20	-0.19214	0.072	0.151	6.23E-16	AC-G	TMEM184B
RENBP.1	4.25E-20	-0.29139	0.071	0.148	6.35E-16	AC-G	RENBP
TFPT.1	4.28E-20	-0.12761	0.212	0.327	6.40E-16	AC-G	TFPT
PCDH19	4.31E-20	-0.10393	0.014	0.069	6.45E-16	AC-G	PCDH19
ZNF148	4.32E-20	-0.14106	0.16	0.266	6.46E-16	AC-G	ZNF148
FUCA2.1	4.33E-20	-0.18385	0.157	0.26	6.48E-16	AC-G	FUCA2
TM7SF3.1	4.34E-20	-0.22999	0.168	0.267	6.49E-16	AC-G	TM7SF3
RBM4.1	4.35E-20	-0.15854	0.137	0.236	6.50E-16	AC-G	RBM4
HRAS.1	4.38E-20	-0.17495	0.204	0.311	6.54E-16	AC-G	HRAS
TMEM189.1	4.39E-20	-0.19038	0.111	0.202	6.56E-16	AC-G	TMEM189
SPG11.1	4.39E-20	-0.18871	0.055	0.128	6.56E-16	AC-G	SPG11
SMARCA5.1	4.55E-20	-0.19957	0.251	0.367	6.81E-16	AC-G	SMARCA5
TRIP12.1	4.59E-20	-0.10641	0.092	0.18	6.86E-16	AC-G	TRIP12
EHD2	5.10E-20	0.234453	0.13	0.072	7.62E-16	AC-G	EHD2
NFKBIE.1	5.22E-20	-0.23146	0.07	0.146	7.79E-16	AC-G	NFKBIE
DNMT3A.1	5.32E-20	-0.14911	0.042	0.112	7.95E-16	AC-G	DNMT3A
SLC38A10.	5.37E-20	-0.20484	0.121	0.214	8.02E-16	AC-G	SLC38A10
SAE1.1	5.72E-20	-0.20806	0.184	0.29	8.55E-16	AC-G	SAE1
RNF144B	5.75E-20	-0.31473	0.042	0.109	8.60E-16	AC-G	RNF144B
RP11-553L	5.84E-20	-0.15381	0.141	0.243	8.72E-16	AC-G	RP11-553L6.5
CSNK1D.1	5.93E-20	-0.17556	0.259	0.379	8.87E-16	AC-G	CSNK1D
MAT2A	5.99E-20	-0.16284	0.283	0.404	8.95E-16	AC-G	MAT2A
VARS.1	6.05E-20	-0.17381	0.087	0.172	9.04E-16	AC-G	VARS
RNF139.1	6.10E-20	-0.20244	0.172	0.271	9.12E-16	AC-G	RNF139
FOXO3.1	6.17E-20	-0.21521	0.187	0.289	9.22E-16	AC-G	FOXO3
TTC7A.1	6.46E-20	-0.22818	0.025	0.084	9.66E-16	AC-G	TTC7A
KMT2E	6.58E-20	-0.15894	0.432	0.558	9.83E-16	AC-G	KMT2E
ASCL2	6.59E-20	-0.17457	0.001	0.044	9.85E-16	AC-G	ASCL2
PARL.1	6.65E-20	-0.17406	0.243	0.356	9.94E-16	AC-G	PARL
IVD.1	6.67E-20	-0.19282	0.121	0.214	9.97E-16	AC-G	IVD
LSM14B.1	6.99E-20	-0.13304	0.042	0.112	1.04E-15	AC-G	LSM14B
DUSP4.1	7.01E-20	-0.1902	0.103	0.194	1.05E-15	AC-G	DUSP4
PPM1F.1	7.16E-20	-0.15162	0.019	0.076	1.07E-15	AC-G	PPM1F
SFT2D2	7.24E-20	-0.22323	0.079	0.161	1.08E-15	AC-G	SFT2D2
PPP1R35.1	7.35E-20	-0.19583	0.088	0.171	1.10E-15	AC-G	PPP1R35
SAP30L.1	7.37E-20	-0.13075	0.037	0.103	1.10E-15	AC-G	SAP30L
CISD1.1	7.54E-20	0.466844	0.393	0.326	1.13E-15	AC-G	CISD1
PIK3R5.1	7.81E-20	-0.19926	0.007	0.055	1.17E-15	AC-G	PIK3R5
C20orf24.	7.84E-20	-0.16247	0.364	0.487	1.17E-15	AC-G	C20orf24

CSRNP3.1	7.96E-20	-0.20937	0.043	0.11	1.19E-15	AC-G	CSRNP3
SOD3	7.97E-20	0.146862	0.076	0.032	1.19E-15	AC-G	SOD3
PIM3.1	8.28E-20	-0.21085	0.116	0.205	1.24E-15	AC-G	PIM3
ZCCHC3.1	8.31E-20	-0.13538	0.046	0.117	1.24E-15	AC-G	ZCCHC3
OPA1.1	8.43E-20	-0.15932	0.065	0.143	1.26E-15	AC-G	OPA1
CS.1	8.70E-20	-0.16528	0.089	0.175	1.30E-15	AC-G	CS
WDR18.1	9.00E-20	-0.18232	0.196	0.303	1.34E-15	AC-G	WDR18
FAM96B.1	9.22E-20	0.251026	0.667	0.656	1.38E-15	AC-G	FAM96B
CDYL.1	9.29E-20	-0.14263	0.053	0.127	1.39E-15	AC-G	CDYL
HCFC1.1	9.34E-20	-0.14392	0.074	0.155	1.40E-15	AC-G	HCFC1
SLC39A10	9.37E-20	-0.16458	0.086	0.171	1.40E-15	AC-G	SLC39A10
ZIC1	9.40E-20	-0.11687	0.052	0.126	1.40E-15	AC-G	ZIC1
SLC14A1	9.51E-20	0.134678	0.027	0.005	1.42E-15	AC-G	SLC14A1
TFEB.1	9.81E-20	-0.20751	0.027	0.087	1.47E-15	AC-G	TFEB
ATP6V0E2.	9.83E-20	-0.14849	0.357	0.471	1.47E-15	AC-G	ATP6V0E2
ETFA.1	1.02E-19	-0.15798	0.272	0.392	1.52E-15	AC-G	ETFA
CMPK1.1	1.04E-19	-0.17224	0.308	0.437	1.56E-15	AC-G	CMPK1
TDG.1	1.05E-19	-0.12374	0.18	0.291	1.57E-15	AC-G	TDG
XPO1.1	1.12E-19	-0.16089	0.17	0.276	1.67E-15	AC-G	XPO1
KIFC1.1	1.13E-19	-0.15276	0.014	0.068	1.68E-15	AC-G	KIFC1
APOBR	1.15E-19	-0.18383	0.002	0.045	1.72E-15	AC-G	APOBR
RAD21.1	1.20E-19	-0.16409	0.27	0.385	1.79E-15	AC-G	RAD21
ZMYND15	1.21E-19	-0.16093	0.013	0.065	1.80E-15	AC-G	ZMYND15
SPAST.1	1.21E-19	-0.17077	0.052	0.123	1.81E-15	AC-G	SPAST
TRIM33.1	1.22E-19	-0.17182	0.107	0.198	1.82E-15	AC-G	TRIM33
FAM96A	1.24E-19	-0.13041	0.257	0.376	1.85E-15	AC-G	FAM96A
ID4.1	1.26E-19	-0.21126	0.138	0.23	1.89E-15	AC-G	ID4
RCOR1	1.26E-19	-0.14146	0.022	0.081	1.89E-15	AC-G	RCOR1
GPATCH2L.	1.29E-19	-0.15158	0.139	0.239	1.92E-15	AC-G	GPATCH2L
F3.1	1.29E-19	0.314011	0.254	0.179	1.93E-15	AC-G	F3
PRC1.1	1.30E-19	-0.20713	0.033	0.096	1.94E-15	AC-G	PRC1
RP11-277F	1.36E-19	-0.15485	0.02	0.076	2.04E-15	AC-G	RP11-277P12.20
NTAN1.1	1.39E-19	-0.15778	0.123	0.215	2.08E-15	AC-G	NTAN1
ODF3B.1	1.42E-19	-0.24164	0.039	0.105	2.12E-15	AC-G	ODF3B
BLOC1S4.1	1.43E-19	-0.18557	0.161	0.259	2.13E-15	AC-G	BLOC1S4
LRRC8C.1	1.44E-19	-0.18341	0.015	0.068	2.15E-15	AC-G	LRRC8C
ZNF711.1	1.44E-19	-0.21	0.076	0.155	2.16E-15	AC-G	ZNF711
MAFG.1	1.45E-19	-0.17399	0.109	0.199	2.17E-15	AC-G	MAFG
ICMT.1	1.50E-19	-0.17785	0.068	0.145	2.24E-15	AC-G	ICMT
PPP2R3B.1	1.53E-19	-0.11978	0.02	0.077	2.29E-15	AC-G	PPP2R3B
PRPSAP1.1	1.53E-19	-0.17656	0.216	0.324	2.29E-15	AC-G	PRPSAP1
EMID1.1	1.53E-19	-0.10626	0.023	0.082	2.29E-15	AC-G	EMID1
LINC00882	1.55E-19	0.213784	0.062	0.024	2.32E-15	AC-G	LINC00882
RUNX3	1.57E-19	-0.19806	0.002	0.045	2.34E-15	AC-G	RUNX3
PPP3CB.1	1.57E-19	-0.16981	0.05	0.12	2.35E-15	AC-G	PPP3CB
ARFGEF1.1	1.58E-19	-0.15515	0.061	0.136	2.37E-15	AC-G	ARFGEF1
ALKBH7.1	1.59E-19	-0.15281	0.367	0.492	2.38E-15	AC-G	ALKBH7
PLCG2	1.59E-19	-0.21006	0.007	0.056	2.38E-15	AC-G	PLCG2
RAF1.1	1.65E-19	-0.16645	0.188	0.294	2.47E-15	AC-G	RAF1
NUDC.1	1.67E-19	0.265743	0.597	0.576	2.50E-15	AC-G	NUDC
CEP68.1	1.69E-19	-0.15458	0.083	0.165	2.53E-15	AC-G	CEP68

NSMCE4A.1	1.71E-19	-0.2076	0.085	0.166	2.55E-15	AC-G	NSMCE4A
GPC4	1.71E-19	0.155315	0.092	0.044	2.55E-15	AC-G	GPC4
NCAPH2.1	1.72E-19	-0.16806	0.066	0.142	2.57E-15	AC-G	NCAPH2
UBE2M.1	1.74E-19	-0.11002	0.145	0.247	2.60E-15	AC-G	UBE2M
CALR.1	1.75E-19	0.263578	0.683	0.694	2.62E-15	AC-G	CALR
PTGES3	1.76E-19	0.21118	0.707	0.721	2.63E-15	AC-G	PTGES3
CA14.1	1.76E-19	-0.18553	0.009	0.059	2.63E-15	AC-G	CA14
TRIM38	1.84E-19	-0.23083	0.042	0.109	2.74E-15	AC-G	TRIM38
NEMF	1.90E-19	-0.17478	0.23	0.346	2.85E-15	AC-G	NEMF
HERC2.1	1.93E-19	-0.17192	0.111	0.203	2.89E-15	AC-G	HERC2
BATF	2.05E-19	-0.18543	0.002	0.045	3.07E-15	AC-G	BATF
REST.1	2.06E-19	-0.18529	0.122	0.213	3.08E-15	AC-G	REST
CCAR1.1	2.08E-19	-0.19018	0.166	0.266	3.11E-15	AC-G	CCAR1
SMC4.1	2.09E-19	-0.27209	0.136	0.226	3.13E-15	AC-G	SMC4
PHPT1.1	2.11E-19	0.258439	0.643	0.653	3.16E-15	AC-G	PHPT1
MANBA.1	2.16E-19	-0.28871	0.061	0.132	3.23E-15	AC-G	MANBA
TROAP.1	2.22E-19	-0.14752	0.007	0.054	3.32E-15	AC-G	TROAP
PSTPIP2	2.29E-19	-0.14622	0.006	0.052	3.42E-15	AC-G	PSTPIP2
AC109826.	2.34E-19	-0.20202	0.002	0.044	3.49E-15	AC-G	AC109826.1
SRSF6.1	2.36E-19	-0.16555	0.26	0.378	3.52E-15	AC-G	SRSF6
UFD1L.1	2.36E-19	-0.17217	0.235	0.349	3.53E-15	AC-G	UFD1L
JAG1	2.37E-19	-0.1219	0.086	0.173	3.54E-15	AC-G	JAG1
GAMT.1	2.38E-19	-0.18291	0.21	0.317	3.56E-15	AC-G	GAMT
ST3GAL4.1	2.45E-19	-0.16478	0.06	0.134	3.67E-15	AC-G	ST3GAL4
PLSCR4	2.54E-19	0.219222	0.085	0.039	3.80E-15	AC-G	PLSCR4
CAMSAP1.1	2.54E-19	-0.17502	0.032	0.094	3.80E-15	AC-G	CAMSAP1
SP3.1	2.55E-19	-0.18915	0.132	0.225	3.81E-15	AC-G	SP3
CDK19.1	2.60E-19	-0.1964	0.063	0.138	3.88E-15	AC-G	CDK19
HAS2	2.60E-19	0.279612	0.092	0.045	3.89E-15	AC-G	HAS2
ATF7IP2	2.63E-19	-0.21864	0.004	0.049	3.94E-15	AC-G	ATF7IP2
RAB4B.1	2.68E-19	-0.16559	0.176	0.279	4.01E-15	AC-G	RAB4B
C21orf58	2.71E-19	-0.13112	0.011	0.061	4.05E-15	AC-G	C21orf58
SYT17	2.72E-19	-0.13926	0.088	0.173	4.07E-15	AC-G	SYT17
POLR2F.1	2.82E-19	-0.14156	0.438	0.562	4.22E-15	AC-G	POLR2F
CXCL3	2.92E-19	0.316261	0.121	0.063	4.36E-15	AC-G	CXCL3
ZKSCAN1.1	3.01E-19	-0.15852	0.224	0.335	4.49E-15	AC-G	ZKSCAN1
RCBTB2.1	3.02E-19	-0.23271	0.077	0.155	4.52E-15	AC-G	RCBTB2
SGK223	3.12E-19	-0.13014	0.024	0.082	4.66E-15	AC-G	SGK223
KIAA0141.	3.12E-19	-0.21099	0.06	0.133	4.67E-15	AC-G	KIAA0141
PRKRIR.1	3.16E-19	-0.11141	0.05	0.12	4.73E-15	AC-G	PRKRIR
KIF2C	3.33E-19	-0.14631	0.005	0.051	4.98E-15	AC-G	KIF2C
NIPA2.1	3.34E-19	-0.16106	0.176	0.282	4.99E-15	AC-G	NIPA2
IL4I1.1	3.38E-19	-0.25507	0.019	0.074	5.05E-15	AC-G	IL4I1
CTNND1.1	3.54E-19	-0.18621	0.136	0.23	5.29E-15	AC-G	CTNND1
ZDHHC9.1	3.56E-19	0.324278	0.228	0.156	5.32E-15	AC-G	ZDHHC9
CRYBB1	3.60E-19	-0.21952	0.004	0.049	5.38E-15	AC-G	CRYBB1
C1orf56.1	3.61E-19	-0.2066	0.126	0.217	5.40E-15	AC-G	C1orf56
PDE8A.1	3.68E-19	-0.16783	0.036	0.1	5.50E-15	AC-G	PDE8A
ENPP2	3.69E-19	-0.30692	0.006	0.052	5.51E-15	AC-G	ENPP2
MKRN1.1	3.72E-19	-0.18368	0.309	0.427	5.56E-15	AC-G	MKRN1
POLR2A	3.76E-19	-0.17578	0.131	0.227	5.62E-15	AC-G	POLR2A

STC2	3.76E-19	0.143595	0.079	0.034	5.62E-15	AC-G	STC2
DCP2.1	3.80E-19	-0.19992	0.083	0.164	5.68E-15	AC-G	DCP2
PSMB1.1	3.83E-19	0.273308	0.713	0.727	5.72E-15	AC-G	PSMB1
CRABP2	3.87E-19	0.172721	0.055	0.02	5.78E-15	AC-G	CRABP2
SHROOM2	4.08E-19	-0.11454	0.015	0.067	6.09E-15	AC-G	SHROOM2
NDUFC1.1	4.10E-19	0.316546	0.591	0.545	6.13E-15	AC-G	NDUFC1
TNK2.1	4.13E-19	-0.19711	0.065	0.139	6.18E-15	AC-G	TNK2
AGPAT3.1	4.18E-19	-0.15612	0.066	0.143	6.25E-15	AC-G	AGPAT3
NUP62.1	4.27E-19	-0.16629	0.155	0.253	6.38E-15	AC-G	NUP62
EIF4EBP2.	4.55E-19	-0.20268	0.076	0.153	6.80E-15	AC-G	EIF4EBP2
TPX2	4.57E-19	-0.23553	0.032	0.092	6.83E-15	AC-G	TPX2
CD180	4.67E-19	-0.1676	0.002	0.043	6.97E-15	AC-G	CD180
PCNP.1	4.74E-19	0.276138	0.566	0.531	7.09E-15	AC-G	PCNP
FGD3	4.80E-19	-0.18383	0.003	0.046	7.17E-15	AC-G	FGD3
SDCBP2	4.80E-19	0.168053	0.082	0.037	7.17E-15	AC-G	SDCBP2
COL6A2.1	4.82E-19	0.393752	0.22	0.151	7.20E-15	AC-G	COL6A2
CREB1.1	4.82E-19	-0.17805	0.16	0.257	7.21E-15	AC-G	CREB1
TCF25.1	4.87E-19	-0.12743	0.439	0.573	7.28E-15	AC-G	TCF25
TSPAN13.1	4.92E-19	-0.2547	0.222	0.314	7.35E-15	AC-G	TSPAN13
ARHGEF7.1	5.10E-19	-0.21448	0.09	0.171	7.62E-15	AC-G	ARHGEF7
PVRL2.1	5.23E-19	-0.18954	0.161	0.259	7.82E-15	AC-G	PVRL2
PRKCB	5.26E-19	-0.17442	0.005	0.049	7.85E-15	AC-G	PRKCB
PSEN1.1	5.26E-19	-0.19052	0.106	0.193	7.86E-15	AC-G	PSEN1
FBLN1.1	5.27E-19	-0.19888	0.033	0.095	7.88E-15	AC-G	FBLN1
STOX2.1	5.32E-19	-0.16453	0.038	0.103	7.96E-15	AC-G	STOX2
ENKUR	5.47E-19	0.357045	0.117	0.063	8.17E-15	AC-G	ENKUR
CPSF6.1	5.51E-19	-0.12885	0.236	0.348	8.24E-15	AC-G	CPSF6
PTPN9	5.57E-19	-0.12655	0.062	0.137	8.32E-15	AC-G	PTPN9
CNOT6L.1	5.64E-19	-0.17474	0.05	0.12	8.44E-15	AC-G	CNOT6L
STEAP3.1	5.73E-19	0.416588	0.22	0.15	8.56E-15	AC-G	STEAP3
RP11-76E1	6.17E-19	-0.23756	0.002	0.043	9.22E-15	AC-G	RP11-76E17.3
CREB3L2.1	6.40E-19	-0.18476	0.062	0.136	9.57E-15	AC-G	CREB3L2
CTB-61M7.	6.60E-19	-0.22208	0.003	0.045	9.86E-15	AC-G	CTB-61M7.2
ACAT2.1	6.69E-19	-0.18718	0.19	0.295	9.99E-15	AC-G	ACAT2
PNN.1	6.83E-19	-0.14611	0.301	0.421	1.02E-14	AC-G	PNN
COX6C.1	6.84E-19	0.253247	0.795	0.838	1.02E-14	AC-G	COX6C
LINC01102	6.85E-19	-0.15336	0.023	0.08	1.02E-14	AC-G	LINC01102
RAP1GDS1.	6.96E-19	-0.15694	0.102	0.188	1.04E-14	AC-G	RAP1GDS1
ATCAY.1	7.22E-19	-0.21038	0.061	0.132	1.08E-14	AC-G	ATCAY
RP11-262H	7.54E-19	-0.13871	0.057	0.13	1.13E-14	AC-G	RP11-262H14.4
PTMA.1	7.61E-19	0.16034	0.911	0.978	1.14E-14	AC-G	PTMA
RRBP1.1	7.62E-19	-0.22217	0.211	0.314	1.14E-14	AC-G	RRBP1
ADAMTS9-A	7.70E-19	0.136525	0.042	0.013	1.15E-14	AC-G	ADAMTS9-AS2
ELOVL1.1	7.84E-19	-0.1581	0.149	0.247	1.17E-14	AC-G	ELOVL1
CCM2.1	8.30E-19	-0.1587	0.193	0.301	1.24E-14	AC-G	CCM2
ZFP36L2.1	8.50E-19	-0.19624	0.391	0.54	1.27E-14	AC-G	ZFP36L2
SNRPD3.1	8.57E-19	-0.11748	0.311	0.434	1.28E-14	AC-G	SNRPD3
MBOAT7.1	8.67E-19	-0.17843	0.16	0.259	1.30E-14	AC-G	MBOAT7
EFEMP2	8.78E-19	0.422564	0.27	0.203	1.31E-14	AC-G	EFEMP2
RBM38.1	8.80E-19	-0.12841	0.07	0.148	1.32E-14	AC-G	RBM38
PHACTR4.1	8.83E-19	-0.16783	0.124	0.217	1.32E-14	AC-G	PHACTR4

MTUS1.1	8.87E-19	-0.22422	0.082	0.16	1.33E-14	AC-G	MTUS1
COX6B1.1	8.95E-19	0.213088	0.805	0.854	1.34E-14	AC-G	COX6B1
ADAMDEC1	8.98E-19	-0.18556	0.002	0.044	1.34E-14	AC-G	ADAMDEC1
GTSE1	9.00E-19	-0.17097	0.015	0.066	1.34E-14	AC-G	GTSE1
FXYD7.1	9.01E-19	-0.25911	0.029	0.087	1.35E-14	AC-G	FXYD7
TXNL1	9.07E-19	0.348021	0.509	0.464	1.36E-14	AC-G	TXNL1
ARGLU1.1	9.09E-19	-0.18301	0.361	0.489	1.36E-14	AC-G	ARGLU1
NLRP1.1	9.21E-19	-0.14603	0.248	0.356	1.38E-14	AC-G	NLRP1
CENPE.1	9.23E-19	-0.20129	0.013	0.063	1.38E-14	AC-G	CENPE
BBS2.1	9.38E-19	0.350202	0.234	0.16	1.40E-14	AC-G	BBS2
BAG1.1	9.64E-19	-0.21478	0.26	0.37	1.44E-14	AC-G	BAG1
ANK2.1	9.89E-19	-0.12416	0.092	0.178	1.48E-14	AC-G	ANK2
PLEKHG2.1	1.00E-18	-0.16575	0.019	0.073	1.50E-14	AC-G	PLEKHG2
GM2A.1	1.02E-18	-0.27245	0.08	0.156	1.53E-14	AC-G	GM2A
IL7R.1	1.03E-18	-0.26246	0.012	0.061	1.54E-14	AC-G	IL7R
ARHGAP26.	1.04E-18	-0.24498	0.039	0.102	1.56E-14	AC-G	ARHGAP26
TOR2A.1	1.06E-18	-0.18638	0.082	0.16	1.58E-14	AC-G	TOR2A
ARPC4.1	1.06E-18	-0.10841	0.402	0.535	1.58E-14	AC-G	ARPC4
POMGNT1.1	1.09E-18	-0.13679	0.173	0.275	1.62E-14	AC-G	POMGNT1
INA	1.09E-18	-0.15975	0.005	0.049	1.64E-14	AC-G	INA
APOE.1	1.10E-18	-0.10701	0.528	0.716	1.64E-14	AC-G	APOE
SYT11.1	1.11E-18	-0.17399	0.297	0.401	1.66E-14	AC-G	SYT11
YIF1A.1	1.12E-18	0.346902	0.47	0.415	1.67E-14	AC-G	YIF1A
TP53I11.1	1.14E-18	-0.16873	0.025	0.082	1.70E-14	AC-G	TP53I11
AGTRAP.1	1.16E-18	0.494652	0.34	0.27	1.74E-14	AC-G	AGTRAP
RP11-25K1	1.18E-18	-0.14301	0.044	0.111	1.76E-14	AC-G	RP11-25K19.1
RMDN3.1	1.18E-18	-0.16168	0.158	0.256	1.77E-14	AC-G	RMDN3
NUP50.1	1.22E-18	-0.15628	0.057	0.129	1.82E-14	AC-G	NUP50
ASGR1.1	1.23E-18	-0.13208	0.025	0.082	1.84E-14	AC-G	ASGR1
SNRPB	1.26E-18	-0.12433	0.481	0.596	1.88E-14	AC-G	SNRPB
PPP2CA.1	1.36E-18	-0.1497	0.368	0.501	2.03E-14	AC-G	PPP2CA
RUFY3.1	1.36E-18	-0.19878	0.217	0.323	2.03E-14	AC-G	RUFY3
PDE6D.1	1.37E-18	-0.15047	0.114	0.203	2.05E-14	AC-G	PDE6D
SDCCAG8.1	1.39E-18	-0.18203	0.239	0.347	2.07E-14	AC-G	SDCCAG8
TRIOBP	1.40E-18	-0.129	0.036	0.099	2.09E-14	AC-G	TRIOBP
COX5B.1	1.41E-18	0.194636	0.812	0.855	2.10E-14	AC-G	COX5B
DR1.1	1.42E-18	-0.18015	0.154	0.25	2.12E-14	AC-G	DR1
UBE2H.1	1.43E-18	-0.17981	0.175	0.275	2.13E-14	AC-G	UBE2H
ATP8A1	1.43E-18	-0.17248	0.006	0.05	2.13E-14	AC-G	ATP8A1
RNFT2.1	1.43E-18	-0.12182	0.022	0.078	2.14E-14	AC-G	RNFT2
ST18	1.44E-18	-0.18279	0.004	0.046	2.15E-14	AC-G	ST18
CDS2.1	1.46E-18	-0.14468	0.163	0.264	2.18E-14	AC-G	CDS2
ATRN	1.46E-18	-0.12828	0.062	0.136	2.19E-14	AC-G	ATRN
STK17B.1	1.52E-18	-0.34043	0.073	0.144	2.26E-14	AC-G	STK17B
RP3-428L1	1.53E-18	-0.12498	0.023	0.079	2.28E-14	AC-G	RP3-428L16.2
MYT1L.1	1.55E-18	-0.19785	0.016	0.067	2.32E-14	AC-G	MYT1L
HJURP	1.55E-18	-0.14769	0.004	0.046	2.32E-14	AC-G	HJURP
TXNL4A.1	1.55E-18	-0.12054	0.315	0.429	2.32E-14	AC-G	TXNL4A
JTB.1	1.57E-18	-0.16214	0.491	0.632	2.34E-14	AC-G	JTB
MYBL2	1.60E-18	-0.11295	0.007	0.052	2.40E-14	AC-G	MYBL2
DVL3.1	1.60E-18	-0.12102	0.085	0.167	2.40E-14	AC-G	DVL3

SNX5	1.61E-18	-0.16557	0.305	0.412	2.40E-14	AC-G	SNX5
SAC3D1.1	1.61E-18	-0.15416	0.099	0.183	2.41E-14	AC-G	SAC3D1
ASRGL1.1	1.62E-18	-0.22286	0.129	0.215	2.43E-14	AC-G	ASRGL1
RBM5.1	1.63E-18	-0.1577	0.19	0.296	2.44E-14	AC-G	RBM5
GIMAP5.1	1.67E-18	-0.22618	0.01	0.058	2.49E-14	AC-G	GIMAP5
PES1.1	1.68E-18	-0.15795	0.117	0.206	2.51E-14	AC-G	PES1
SDC2.1	1.72E-18	0.267319	0.257	0.18	2.57E-14	AC-G	SDC2
SMARCAD1.	1.74E-18	-0.15289	0.05	0.118	2.61E-14	AC-G	SMARCAD1
PHF20L1.1	1.75E-18	-0.17968	0.216	0.326	2.62E-14	AC-G	PHF20L1
CSAD	1.75E-18	-0.13602	0.097	0.182	2.62E-14	AC-G	CSAD
ATP9A.1	1.79E-18	-0.13124	0.053	0.124	2.67E-14	AC-G	ATP9A
MARK3.1	1.93E-18	-0.15767	0.194	0.305	2.88E-14	AC-G	MARK3
PSMB3.1	1.93E-18	0.320557	0.701	0.704	2.89E-14	AC-G	PSMB3
TK2.1	1.98E-18	-0.18652	0.063	0.136	2.96E-14	AC-G	TK2
SETX.1	1.98E-18	-0.20731	0.098	0.179	2.97E-14	AC-G	SETX
TP53INP2.	1.99E-18	-0.15478	0.041	0.105	2.98E-14	AC-G	TP53INP2
BCAT1.1	2.03E-18	-0.15992	0.149	0.247	3.04E-14	AC-G	BCAT1
PSENEN.1	2.07E-18	0.383592	0.525	0.486	3.10E-14	AC-G	PSENEN
RIN2.1	2.12E-18	-0.29019	0.067	0.137	3.16E-14	AC-G	RIN2
FAM53B.1	2.13E-18	-0.19157	0.011	0.059	3.19E-14	AC-G	FAM53B
APPL1.1	2.23E-18	-0.19055	0.207	0.312	3.34E-14	AC-G	APPL1
GAB1.1	2.28E-18	-0.15961	0.046	0.112	3.40E-14	AC-G	GAB1
YWHAG.1	2.28E-18	-0.13335	0.29	0.41	3.41E-14	AC-G	YWHAG
GPC6	2.31E-18	0.238015	0.076	0.034	3.45E-14	AC-G	GPC6
MAG	2.33E-18	-0.37916	0.003	0.044	3.49E-14	AC-G	MAG
NDC80	2.34E-18	-0.16161	0.009	0.055	3.50E-14	AC-G	NDC80
MITF	2.38E-18	-0.24784	0.041	0.104	3.56E-14	AC-G	MITF
ARFGAP2.1	2.40E-18	-0.16652	0.205	0.309	3.58E-14	AC-G	ARFGAP2
RDH11.1	2.44E-18	-0.14712	0.288	0.407	3.64E-14	AC-G	RDH11
ISG15.1	2.47E-18	-0.21854	0.264	0.375	3.69E-14	AC-G	ISG15
EIF3E	2.48E-18	-0.12376	0.523	0.671	3.71E-14	AC-G	EIF3E
UBXN2B.1	2.56E-18	-0.15916	0.084	0.164	3.82E-14	AC-G	UBXN2B
ATRAID.1	2.60E-18	0.326382	0.621	0.609	3.89E-14	AC-G	ATRAID
IFNAR2.1	2.64E-18	-0.17428	0.124	0.214	3.95E-14	AC-G	IFNAR2
TAL1	2.65E-18	-0.19718	0.001	0.041	3.97E-14	AC-G	TAL1
ARL11	2.70E-18	-0.1896	0.004	0.046	4.03E-14	AC-G	ARL11
MAP3K3.1	2.71E-18	-0.16956	0.019	0.073	4.04E-14	AC-G	MAP3K3
CAPNS1	2.75E-18	-0.12178	0.242	0.358	4.11E-14	AC-G	CAPNS1
SSB.1	2.85E-18	0.278801	0.611	0.588	4.26E-14	AC-G	SSB
MLF2.1	2.89E-18	0.244289	0.639	0.603	4.32E-14	AC-G	MLF2
NME3.1	2.96E-18	-0.13226	0.315	0.437	4.43E-14	AC-G	NME3
RP11-126K	2.98E-18	-0.12658	0.038	0.102	4.45E-14	AC-G	RP11-126K1.6
ETNK1.1	3.00E-18	-0.18647	0.146	0.238	4.48E-14	AC-G	ETNK1
ATM.1	3.03E-18	-0.24048	0.118	0.203	4.53E-14	AC-G	ATM
ACAP2.1	3.08E-18	-0.17252	0.107	0.192	4.61E-14	AC-G	ACAP2
YTHDC1.1	3.10E-18	-0.17791	0.212	0.32	4.63E-14	AC-G	YTHDC1
CBX6.1	3.13E-18	-0.13867	0.157	0.256	4.67E-14	AC-G	CBX6
TAB2	3.17E-18	-0.18328	0.065	0.138	4.73E-14	AC-G	TAB2
MAPK4	3.22E-18	0.144041	0.048	0.017	4.82E-14	AC-G	MAPK4
TCEA2.1	3.32E-18	-0.19751	0.287	0.384	4.96E-14	AC-G	TCEA2
TOM1.1	3.37E-18	-0.2159	0.094	0.173	5.03E-14	AC-G	TOM1

ARHGAP24.	3.46E-18	-0.35113	0.087	0.16	5.17E-14	AC-G	ARHGAP24
ZNF513.1	3.51E-18	-0.10508	0.026	0.083	5.24E-14	AC-G	ZNF513
ZNF827.1	3.54E-18	-0.12628	0.056	0.126	5.28E-14	AC-G	ZNF827
MINK1.1	3.57E-18	-0.16877	0.048	0.115	5.33E-14	AC-G	MINK1
SRA1.1	3.64E-18	-0.12772	0.301	0.417	5.44E-14	AC-G	SRA1
ASPSCR1.1	3.66E-18	-0.15147	0.154	0.252	5.48E-14	AC-G	ASPSCR1
ARRB1	3.83E-18	-0.19144	0.005	0.048	5.72E-14	AC-G	ARRB1
BRD9.1	3.94E-18	-0.18753	0.121	0.208	5.89E-14	AC-G	BRD9
C16orf72.	3.97E-18	-0.12295	0.065	0.14	5.93E-14	AC-G	C16orf72
ADAM17.1	4.05E-18	-0.21267	0.175	0.271	6.06E-14	AC-G	ADAM17
ICOSLG.1	4.09E-18	-0.28052	0.04	0.101	6.11E-14	AC-G	ICOSLG
ID2	4.09E-18	-0.14647	0.539	0.676	6.12E-14	AC-G	ID2
NCKAP5L.1	4.21E-18	-0.20663	0.056	0.126	6.29E-14	AC-G	NCKAP5L
GDI1.1	4.22E-18	-0.14854	0.388	0.505	6.31E-14	AC-G	GDI1
PMP22.1	4.28E-18	0.250346	0.509	0.412	6.40E-14	AC-G	PMP22
SLC22A17.	4.32E-18	-0.15166	0.196	0.301	6.46E-14	AC-G	SLC22A17
EDARADD	4.38E-18	0.147241	0.057	0.022	6.54E-14	AC-G	EDARADD
CKAP2L	4.42E-18	-0.15264	0.008	0.053	6.61E-14	AC-G	CKAP2L
METTL9.1	4.45E-18	-0.17488	0.361	0.489	6.65E-14	AC-G	METTL9
KDM5B.1	4.59E-18	-0.13343	0.093	0.175	6.86E-14	AC-G	KDM5B
PRKCH	4.66E-18	-0.20544	0.007	0.051	6.96E-14	AC-G	PRKCH
ZWILCH.1	4.69E-18	-0.13348	0.027	0.085	7.01E-14	AC-G	ZWILCH
PSIP1.1	4.70E-18	-0.19804	0.284	0.403	7.03E-14	AC-G	PSIP1
PAK7.1	4.72E-18	-0.14912	0.008	0.054	7.06E-14	AC-G	PAK7
CHCHD1.1	4.86E-18	-0.11221	0.184	0.287	7.27E-14	AC-G	CHCHD1
RABAC1.1	4.86E-18	0.270015	0.659	0.665	7.27E-14	AC-G	RABAC1
GPRIN3.1	4.87E-18	-0.2225	0.016	0.067	7.28E-14	AC-G	GPRIN3
PAFAH1B2.	5.02E-18	-0.18155	0.234	0.344	7.51E-14	AC-G	PAFAH1B2
TMTC4	5.08E-18	-0.18426	0.044	0.109	7.59E-14	AC-G	TMTC4
MFSD3.1	5.18E-18	-0.13219	0.061	0.131	7.75E-14	AC-G	MFSD3
KIF20B.1	5.20E-18	-0.20473	0.036	0.097	7.77E-14	AC-G	KIF20B
PLXND1.1	5.24E-18	-0.22871	0.045	0.108	7.83E-14	AC-G	PLXND1
DDX52	5.31E-18	-0.13315	0.161	0.26	7.94E-14	AC-G	DDX52
ACP2.1	5.41E-18	-0.14071	0.162	0.259	8.08E-14	AC-G	ACP2
RABGAP1L.	5.43E-18	-0.22278	0.054	0.121	8.11E-14	AC-G	RABGAP1L
CLEC9A	5.44E-18	-0.24459	0.001	0.039	8.13E-14	AC-G	CLEC9A
STRN4.1	5.44E-18	-0.12826	0.063	0.135	8.13E-14	AC-G	STRN4
MDH2	5.45E-18	0.281249	0.657	0.661	8.15E-14	AC-G	MDH2
IGF2BP3.1	5.48E-18	-0.10727	0.051	0.119	8.19E-14	AC-G	IGF2BP3
CDKN2D.1	5.56E-18	-0.19324	0.094	0.172	8.31E-14	AC-G	CDKN2D
FAM78A	5.57E-18	-0.18017	0.002	0.042	8.32E-14	AC-G	FAM78A
DCAF7.1	5.94E-18	-0.14702	0.23	0.338	8.87E-14	AC-G	DCAF7
CBR4.1	6.08E-18	-0.20098	0.116	0.202	9.08E-14	AC-G	CBR4
NCAPD2.1	6.13E-18	-0.10581	0.019	0.072	9.16E-14	AC-G	NCAPD2
ZFP36	6.22E-18	0.32584	0.631	0.562	9.30E-14	AC-G	ZFP36
SGPP1.1	6.26E-18	-0.1961	0.031	0.088	9.36E-14	AC-G	SGPP1
AGFG1	6.49E-18	-0.14422	0.07	0.143	9.69E-14	AC-G	AGFG1
NRM.1	6.93E-18	-0.16167	0.034	0.094	1.04E-13	AC-G	NRM
RNF111.1	7.07E-18	-0.14038	0.032	0.091	1.06E-13	AC-G	RNF111
NES.1	7.11E-18	-0.22256	0.206	0.289	1.06E-13	AC-G	NES
SGSM2	7.12E-18	-0.11698	0.047	0.114	1.06E-13	AC-G	SGSM2

CPE	7.18E-18	0.354404	0.435	0.381	1.07E-13	AC-G	CPE
TSC22D2.1	7.18E-18	-0.23559	0.092	0.169	1.07E-13	AC-G	TSC22D2
NAGPA	7.19E-18	-0.18901	0.053	0.12	1.07E-13	AC-G	NAGPA
MTRNR2L1	7.22E-18	-0.24969	0.126	0.205	1.08E-13	AC-G	MTRNR2L1
SPATA13.1	7.25E-18	-0.22227	0.098	0.178	1.08E-13	AC-G	SPATA13
STAT6.1	7.33E-18	-0.18667	0.012	0.06	1.10E-13	AC-G	STAT6
DOCK5.1	7.35E-18	-0.23009	0.014	0.063	1.10E-13	AC-G	DOCK5
CDK5.1	7.57E-18	-0.10135	0.156	0.254	1.13E-13	AC-G	CDK5
PRR14L.1	8.03E-18	-0.12525	0.038	0.099	1.20E-13	AC-G	PRR14L
PCF11	8.27E-18	-0.11976	0.129	0.218	1.24E-13	AC-G	PCF11
CCDC107.1	8.28E-18	-0.17884	0.295	0.409	1.24E-13	AC-G	CCDC107
AGFG2	8.43E-18	0.137746	0.075	0.033	1.26E-13	AC-G	AGFG2
ASPM.1	8.47E-18	-0.16925	0.009	0.054	1.27E-13	AC-G	ASPM
ARHGAP27.	8.65E-18	-0.18715	0.014	0.063	1.29E-13	AC-G	ARHGAP27
SOS2.1	8.71E-18	-0.12787	0.033	0.093	1.30E-13	AC-G	SOS2
CDKN3	8.81E-18	-0.2235	0.04	0.102	1.32E-13	AC-G	CDKN3
TRAPPC12.	9.17E-18	-0.18682	0.125	0.212	1.37E-13	AC-G	TRAPPC12
TTL	9.42E-18	-0.15639	0.059	0.129	1.41E-13	AC-G	TTL
C16orf87	9.44E-18	-0.19357	0.13	0.219	1.41E-13	AC-G	C16orf87
SEPT3.1	9.46E-18	-0.1616	0.022	0.076	1.41E-13	AC-G	3-Sep
DIRC2.1	9.52E-18	-0.1441	0.032	0.09	1.42E-13	AC-G	DIRC2
KDM5D	1.00E-17	-0.10548	0.008	0.052	1.49E-13	AC-G	KDM5D
MATR3.1	1.04E-17	-0.16258	0.379	0.507	1.55E-13	AC-G	MATR3
NLK.1	1.06E-17	-0.11422	0.028	0.086	1.58E-13	AC-G	NLK
MPDU1	1.07E-17	-0.15325	0.226	0.335	1.60E-13	AC-G	MPDU1
AP1S1.1	1.09E-17	0.359896	0.501	0.461	1.63E-13	AC-G	AP1S1
ITGB4	1.09E-17	0.175228	0.064	0.027	1.64E-13	AC-G	ITGB4
GPAA1.1	1.10E-17	-0.12592	0.288	0.414	1.65E-13	AC-G	GPAA1
CRB2	1.12E-17	0.349601	0.079	0.037	1.68E-13	AC-G	CRB2
RINT1	1.13E-17	0.186857	0.147	0.085	1.69E-13	AC-G	RINT1
MTMR14.1	1.14E-17	-0.19157	0.145	0.236	1.70E-13	AC-G	MTMR14
PBX3.1	1.14E-17	-0.15436	0.06	0.129	1.71E-13	AC-G	PBX3
PHYHD1	1.15E-17	0.13691	0.053	0.02	1.72E-13	AC-G	PHYHD1
E2F1	1.16E-17	-0.12562	0.014	0.063	1.73E-13	AC-G	E2F1
ZNF652.1	1.17E-17	-0.18428	0.081	0.156	1.75E-13	AC-G	ZNF652
TPST2.1	1.19E-17	-0.17288	0.098	0.181	1.77E-13	AC-G	TPST2
FAM58A.1	1.19E-17	-0.16582	0.089	0.167	1.78E-13	AC-G	FAM58A
USP13.1	1.19E-17	-0.11989	0.026	0.081	1.78E-13	AC-G	USP13
TTC19.1	1.21E-17	-0.17071	0.261	0.374	1.81E-13	AC-G	TTC19
PRELID1.1	1.23E-17	-0.15457	0.433	0.584	1.84E-13	AC-G	PRELID1
STRBP.1	1.23E-17	-0.16504	0.067	0.138	1.84E-13	AC-G	STRBP
ANKIB1.1	1.25E-17	-0.17639	0.134	0.223	1.87E-13	AC-G	ANKIB1
PDS5B.1	1.26E-17	-0.1468	0.098	0.181	1.88E-13	AC-G	PDS5B
SPRED2.1	1.26E-17	-0.10736	0.055	0.125	1.89E-13	AC-G	SPRED2
ZNRF1.1	1.27E-17	-0.11771	0.058	0.127	1.89E-13	AC-G	ZNRF1
SEPT8.1	1.28E-17	-0.21181	0.122	0.21	1.91E-13	AC-G	8-Sep
NELFB.1	1.30E-17	-0.12316	0.083	0.162	1.94E-13	AC-G	NELFB
CTNNAL1.1	1.40E-17	0.265418	0.203	0.136	2.09E-13	AC-G	CTNNAL1
HTT.1	1.40E-17	-0.13148	0.061	0.132	2.10E-13	AC-G	HTT
IKBKB.1	1.41E-17	-0.14528	0.06	0.129	2.10E-13	AC-G	IKBKB
LIPE-AS1	1.42E-17	-0.13519	0.057	0.127	2.12E-13	AC-G	LIPE-AS1

CELSR2.1	1.44E-17	-0.12741	0.04	0.103	2.16E-13	AC-G	CELSR2
OSGIN1	1.45E-17	0.120472	0.057	0.022	2.17E-13	AC-G	OSGIN1
PTBP3.1	1.48E-17	-0.18995	0.055	0.122	2.21E-13	AC-G	PTBP3
MPHOSPH9.	1.49E-17	-0.1196	0.059	0.129	2.23E-13	AC-G	MPHOSPH9
BRI3BP.1	1.51E-17	-0.15312	0.056	0.124	2.26E-13	AC-G	BRI3BP
OSGEP.1	1.51E-17	-0.11678	0.127	0.217	2.26E-13	AC-G	OSGEP
PLXNA4	1.53E-17	-0.14825	0.029	0.086	2.29E-13	AC-G	PLXNA4
RP11-745C	1.55E-17	0.155202	0.028	0.007	2.31E-13	AC-G	RP11-745C15.2
MGEA5.1	1.55E-17	-0.19786	0.155	0.247	2.32E-13	AC-G	MGEA5
JARID2	1.59E-17	-0.18892	0.064	0.134	2.38E-13	AC-G	JARID2
FBXO18.1	1.66E-17	-0.14329	0.073	0.147	2.48E-13	AC-G	FBXO18
SGPL1	1.67E-17	-0.16641	0.071	0.142	2.49E-13	AC-G	SGPL1
SOX6.1	1.69E-17	-0.20586	0.079	0.153	2.52E-13	AC-G	SOX6
SOBP.1	1.70E-17	-0.13445	0.072	0.146	2.54E-13	AC-G	SOBP
SPC24.1	1.70E-17	-0.13846	0.01	0.056	2.54E-13	AC-G	SPC24
SRD5A3.1	1.73E-17	-0.16163	0.095	0.175	2.58E-13	AC-G	SRD5A3
RRP7A.1	1.81E-17	-0.15357	0.203	0.304	2.71E-13	AC-G	RRP7A
UQCRFS1.1	1.83E-17	-0.16774	0.437	0.565	2.74E-13	AC-G	UQCRFS1
PRPF8.1	1.83E-17	-0.15641	0.172	0.273	2.74E-13	AC-G	PRPF8
PLXNB2.1	1.89E-17	-0.1589	0.09	0.17	2.83E-13	AC-G	PLXNB2
CSDE1.1	1.90E-17	-0.13687	0.493	0.622	2.85E-13	AC-G	CSDE1
ZBTB4.1	1.91E-17	-0.14974	0.059	0.128	2.85E-13	AC-G	ZBTB4
ATP11A.1	1.92E-17	-0.10799	0.044	0.107	2.87E-13	AC-G	ATP11A
SNX29.1	1.93E-17	-0.22365	0.052	0.116	2.89E-13	AC-G	SNX29
SMARCD2.1	1.96E-17	-0.12009	0.036	0.095	2.93E-13	AC-G	SMARCD2
OTOA	2.14E-17	-0.15801	0.001	0.038	3.19E-13	AC-G	OTOA
NADK.1	2.14E-17	-0.11668	0.099	0.18	3.20E-13	AC-G	NADK
COX7C.1	2.16E-17	0.198545	0.834	0.896	3.22E-13	AC-G	COX7C
SSR4.1	2.23E-17	0.268173	0.724	0.77	3.33E-13	AC-G	SSR4
MSN.1	2.25E-17	-0.1731	0.285	0.393	3.36E-13	AC-G	MSN
GCAT.1	2.27E-17	-0.12464	0.094	0.174	3.40E-13	AC-G	GCAT
C1QTNF4	2.34E-17	-0.16407	0.016	0.064	3.49E-13	AC-G	C1QTNF4
BTBD1.1	2.34E-17	-0.13242	0.083	0.161	3.49E-13	AC-G	BTBD1
SPAG16.1	2.38E-17	0.323273	0.351	0.285	3.56E-13	AC-G	SPAG16
FOXG1.1	2.45E-17	-0.10133	0.086	0.166	3.66E-13	AC-G	FOXG1
ERICH2	2.45E-17	0.160681	0.063	0.026	3.67E-13	AC-G	ERICH2
MLXIP.1	2.46E-17	-0.20593	0.114	0.196	3.68E-13	AC-G	MLXIP
SOAT1.1	2.48E-17	-0.25649	0.13	0.211	3.71E-13	AC-G	SOAT1
USP34.1	2.49E-17	-0.14357	0.135	0.224	3.72E-13	AC-G	USP34
DCLRE1C	2.50E-17	-0.15208	0.069	0.142	3.74E-13	AC-G	DCLRE1C
RNF165.1	2.54E-17	-0.12706	0.023	0.076	3.79E-13	AC-G	RNF165
ADAM8	2.56E-17	-0.19517	0.007	0.05	3.82E-13	AC-G	ADAM8
ITFG1.1	2.56E-17	-0.12836	0.255	0.366	3.83E-13	AC-G	ITFG1
RASL11B.1	2.57E-17	-0.20851	0.014	0.062	3.84E-13	AC-G	RASL11B
CST7	2.57E-17	-0.22885	0.003	0.042	3.84E-13	AC-G	CST7
CENPN	2.67E-17	-0.18789	0.038	0.097	3.99E-13	AC-G	CENPN
SEMA4A.1	2.67E-17	-0.16796	0.011	0.057	4.00E-13	AC-G	SEMA4A
E2F3.1	2.70E-17	-0.16104	0.037	0.095	4.04E-13	AC-G	E2F3
ASIC1	2.73E-17	-0.13327	0.013	0.06	4.07E-13	AC-G	ASIC1
ANKH.1	2.81E-17	-0.26181	0.089	0.16	4.20E-13	AC-G	ANKH
DLGAP5	2.81E-17	-0.12317	0.003	0.042	4.21E-13	AC-G	DLGAP5

LBR.1	2.82E-17	-0.2251	0.158	0.247	4.22E-13	AC-G	LBR
YME1L1	2.95E-17	-0.19087	0.179	0.276	4.42E-13	AC-G	YME1L1
LGALS8	3.01E-17	-0.19371	0.087	0.164	4.50E-13	AC-G	LGALS8
FILIP1L.1	3.06E-17	-0.24185	0.088	0.161	4.58E-13	AC-G	FILIP1L
KCTD5.1	3.07E-17	-0.18219	0.134	0.222	4.58E-13	AC-G	KCTD5
TMOD2.1	3.11E-17	-0.11638	0.054	0.121	4.65E-13	AC-G	TMOD2
TM7SF2.1	3.24E-17	-0.15278	0.152	0.246	4.84E-13	AC-G	TM7SF2
CUEDC2.1	3.29E-17	-0.11418	0.301	0.42	4.92E-13	AC-G	CUEDC2
COMMD9	3.36E-17	-0.15208	0.222	0.327	5.03E-13	AC-G	COMMD9
GADD45G.1	3.37E-17	-0.24894	0.18	0.27	5.03E-13	AC-G	GADD45G
KLF7.1	3.43E-17	-0.2209	0.136	0.219	5.13E-13	AC-G	KLF7
MSL1.1	3.46E-17	-0.15644	0.092	0.171	5.17E-13	AC-G	MSL1
IL12RB1	3.61E-17	-0.17392	0.001	0.037	5.40E-13	AC-G	IL12RB1
DSEL.1	3.64E-17	-0.19155	0.152	0.239	5.44E-13	AC-G	DSEL
ITPR2.1	3.66E-17	-0.22981	0.073	0.143	5.47E-13	AC-G	ITPR2
LDOC1L.1	3.67E-17	-0.10102	0.037	0.096	5.48E-13	AC-G	LDOC1L
KCTD13.1	3.80E-17	-0.1796	0.077	0.15	5.67E-13	AC-G	KCTD13
TGM2	3.80E-17	0.167015	0.071	0.031	5.67E-13	AC-G	TGM2
CLASP1.1	3.85E-17	-0.10109	0.075	0.15	5.75E-13	AC-G	CLASP1
TRMT1.1	3.87E-17	-0.20384	0.176	0.273	5.78E-13	AC-G	TRMT1
ATG3.1	3.88E-17	-0.15341	0.282	0.395	5.80E-13	AC-G	ATG3
CARS	3.94E-17	0.31146	0.277	0.208	5.89E-13	AC-G	CARS
ZDHHC22	3.98E-17	-0.11207	0.007	0.05	5.94E-13	AC-G	ZDHHC22
N4BP2.1	4.01E-17	-0.14704	0.06	0.128	5.99E-13	AC-G	N4BP2
KAT7	4.02E-17	-0.15019	0.084	0.16	6.01E-13	AC-G	KAT7
IGF2R.1	4.08E-17	-0.1393	0.044	0.107	6.10E-13	AC-G	IGF2R
ARPC1A.1	4.10E-17	0.299889	0.592	0.551	6.13E-13	AC-G	ARPC1A
PATZ1.1	4.13E-17	-0.12463	0.035	0.093	6.18E-13	AC-G	PATZ1
SPATA6.1	4.55E-17	-0.13961	0.091	0.168	6.80E-13	AC-G	SPATA6
HDAC9.1	4.60E-17	-0.16988	0.067	0.137	6.87E-13	AC-G	HDAC9
SPIN1.1	4.60E-17	-0.14996	0.099	0.18	6.88E-13	AC-G	SPIN1
MOBP	4.61E-17	-0.3468	0.003	0.042	6.89E-13	AC-G	MOBP
ZBED3.1	4.62E-17	-0.1157	0.038	0.098	6.90E-13	AC-G	ZBED3
HLA-F.1	4.66E-17	-0.31389	0.088	0.157	6.97E-13	AC-G	HLA-F
PIP5K1C.1	4.67E-17	-0.12029	0.053	0.119	6.98E-13	AC-G	PIP5K1C
STK39.1	4.69E-17	-0.1083	0.048	0.112	7.01E-13	AC-G	STK39
RNF168.1	4.72E-17	-0.18066	0.086	0.16	7.06E-13	AC-G	RNF168
MORN2.1	4.74E-17	0.387348	0.315	0.252	7.08E-13	AC-G	MORN2
CTNNB1.1	4.81E-17	-0.12609	0.262	0.373	7.18E-13	AC-G	CTNNB1
SDS.1	4.81E-17	-0.2982	0.018	0.067	7.20E-13	AC-G	SDS
BMP7.1	4.96E-17	-0.16204	0.099	0.179	7.42E-13	AC-G	BMP7
KDM3B.1	5.16E-17	-0.15604	0.054	0.12	7.70E-13	AC-G	KDM3B
SLC27A4.1	5.16E-17	-0.11763	0.018	0.067	7.71E-13	AC-G	SLC27A4
BCL7B.1	5.18E-17	-0.1604	0.237	0.339	7.74E-13	AC-G	BCL7B
SYTL3.1	5.28E-17	-0.26392	0.022	0.073	7.88E-13	AC-G	SYTL3
FGF9.1	5.36E-17	-0.15505	0.012	0.058	8.01E-13	AC-G	FGF9
FGD5-AS1.	5.37E-17	-0.17236	0.224	0.33	8.03E-13	AC-G	FGD5-AS1
C19orf38.	5.38E-17	-0.20804	0.01	0.055	8.04E-13	AC-G	C19orf38
SYT6.1	5.39E-17	-0.15001	0.012	0.058	8.05E-13	AC-G	SYT6
SF3A1.1	5.50E-17	-0.15542	0.106	0.187	8.22E-13	AC-G	SF3A1
ACAT1.1	5.54E-17	0.300301	0.424	0.355	8.28E-13	AC-G	ACAT1

CTH	5.57E-17	0.205405	0.094	0.048	8.33E-13	AC-G	CTH
ZC3H13.1	5.65E-17	-0.13425	0.242	0.352	8.45E-13	AC-G	ZC3H13
MLEC.1	5.74E-17	-0.14237	0.305	0.419	8.58E-13	AC-G	MLEC
RNF126.1	5.78E-17	-0.13611	0.203	0.308	8.63E-13	AC-G	RNF126
RP11-395E	5.81E-17	0.193137	0.052	0.02	8.68E-13	AC-G	RP11-395B7.4
PPP1R15A.	5.84E-17	-0.15505	0.364	0.487	8.72E-13	AC-G	PPP1R15A
EFTUD2.1	5.86E-17	-0.10248	0.139	0.23	8.76E-13	AC-G	EFTUD2
DENND1C	5.89E-17	-0.17663	0.002	0.04	8.80E-13	AC-G	DENND1C
SYP.1	5.90E-17	-0.18309	0.074	0.146	8.82E-13	AC-G	SYP
HIST2H2AC	6.06E-17	-0.1122	0.017	0.066	9.05E-13	AC-G	HIST2H2AC
AHCYL1.1	6.09E-17	0.45289	0.377	0.318	9.10E-13	AC-G	AHCYL1
SAT2.1	6.18E-17	0.297773	0.615	0.58	9.24E-13	AC-G	SAT2
RP11-386I	6.23E-17	-0.27027	0.024	0.075	9.31E-13	AC-G	RP11-386I14.4
AGPS.1	6.33E-17	-0.17283	0.065	0.133	9.46E-13	AC-G	AGPS
TOMM40.1	6.43E-17	-0.16854	0.235	0.346	9.61E-13	AC-G	TOMM40
IGLON5.1	6.47E-17	-0.1556	0.01	0.055	9.67E-13	AC-G	IGLON5
LRRC37B.1	6.69E-17	-0.1365	0.056	0.123	9.99E-13	AC-G	LRRC37B
ERCC1.1	6.72E-17	-0.14334	0.37	0.492	1.00E-12	AC-G	ERCC1
RBMX.1	6.86E-17	-0.10932	0.462	0.587	1.03E-12	AC-G	RBMX
STK32C.1	7.02E-17	-0.17282	0.043	0.104	1.05E-12	AC-G	STK32C
DLX6.1	7.04E-17	-0.19346	0.017	0.065	1.05E-12	AC-G	DLX6
ACSF3	7.44E-17	-0.14893	0.057	0.124	1.11E-12	AC-G	ACSF3
SETD3.1	7.77E-17	-0.16495	0.091	0.169	1.16E-12	AC-G	SETD3
RP11-1094	7.93E-17	-0.10482	0.154	0.25	1.18E-12	AC-G	RP11-1094M14.11
HENMT1	8.13E-17	-0.17308	0.002	0.038	1.21E-12	AC-G	HENMT1
CCDC106.1	8.19E-17	-0.14101	0.157	0.247	1.22E-12	AC-G	CCDC106
HLA-DOA	8.26E-17	-0.27374	0.044	0.104	1.23E-12	AC-G	HLA-DOA
RYBP.1	8.31E-17	-0.17809	0.06	0.126	1.24E-12	AC-G	RYBP
CAPS.1	8.46E-17	0.20243	0.114	0.063	1.26E-12	AC-G	CAPS
C21orf2.1	8.48E-17	-0.15526	0.123	0.208	1.27E-12	AC-G	C21orf2
YTHDF3.1	8.60E-17	-0.12832	0.076	0.148	1.29E-12	AC-G	YTHDF3
TCTEX1D1	9.31E-17	0.111378	0.038	0.012	1.39E-12	AC-G	TCTEX1D1
RAB21.1	9.36E-17	-0.13485	0.13	0.218	1.40E-12	AC-G	RAB21
ARHGAP25	9.41E-17	-0.20247	0.023	0.074	1.41E-12	AC-G	ARHGAP25
ZBTB18.1	9.45E-17	-0.121	0.013	0.059	1.41E-12	AC-G	ZBTB18
GPNMB.1	9.68E-17	-0.25443	0.132	0.214	1.45E-12	AC-G	GPNMB
PHF19.1	9.74E-17	-0.13501	0.069	0.141	1.46E-12	AC-G	PHF19
NCAPG	9.85E-17	-0.11631	0.007	0.048	1.47E-12	AC-G	NCAPG
TLN2.1	9.97E-17	-0.18253	0.043	0.104	1.49E-12	AC-G	TLN2
AKT1.1	1.04E-16	-0.13522	0.083	0.159	1.56E-12	AC-G	AKT1
UBE2D2.1	1.07E-16	-0.12627	0.473	0.61	1.60E-12	AC-G	UBE2D2
MAPK8.1	1.08E-16	-0.13593	0.046	0.109	1.62E-12	AC-G	MAPK8
KAT6A.1	1.10E-16	-0.18955	0.095	0.171	1.64E-12	AC-G	KAT6A
SEMA6B	1.10E-16	-0.16189	0.011	0.056	1.65E-12	AC-G	SEMA6B
SSBP1.1	1.12E-16	0.30615	0.636	0.639	1.68E-12	AC-G	SSBP1
JAK1.1	1.13E-16	-0.1817	0.175	0.268	1.68E-12	AC-G	JAK1
MAD1L1.1	1.13E-16	-0.17475	0.093	0.169	1.69E-12	AC-G	MAD1L1
ZNF649.1	1.14E-16	-0.13377	0.04	0.099	1.70E-12	AC-G	ZNF649
EPHX2	1.16E-16	0.201971	0.072	0.032	1.73E-12	AC-G	EPHX2
LSM4	1.19E-16	-0.11403	0.467	0.578	1.78E-12	AC-G	LSM4
ANKRD39.1	1.19E-16	-0.13883	0.101	0.18	1.78E-12	AC-G	ANKRD39

BCL11A	1.19E-16	-0.10297	0.011	0.056	1.78E-12	AC-G	BCL11A
AAED1.1	1.22E-16	-0.19443	0.116	0.194	1.83E-12	AC-G	AAED1
ARL5B.1	1.27E-16	-0.15953	0.039	0.097	1.90E-12	AC-G	ARL5B
REV1.1	1.31E-16	-0.1443	0.101	0.18	1.95E-12	AC-G	REV1
CBX4.1	1.34E-16	-0.1579	0.06	0.126	2.00E-12	AC-G	CBX4
LMF2.1	1.35E-16	-0.13297	0.13	0.215	2.01E-12	AC-G	LMF2
ATF6.1	1.39E-16	-0.22082	0.172	0.263	2.08E-12	AC-G	ATF6
PRKAG2.1	1.41E-16	-0.23795	0.079	0.149	2.11E-12	AC-G	PRKAG2
CBLN4.1	1.42E-16	-0.10725	0.031	0.088	2.12E-12	AC-G	CBLN4
IFITM10.1	1.43E-16	-0.25438	0.071	0.136	2.14E-12	AC-G	IFITM10
CDCA5	1.47E-16	-0.12019	0.009	0.052	2.20E-12	AC-G	CDCA5
MVD.1	1.49E-16	-0.14329	0.11	0.192	2.22E-12	AC-G	MVD
KLK6	1.50E-16	-0.29956	0.001	0.037	2.24E-12	AC-G	KLK6
PUM1.1	1.52E-16	-0.14894	0.16	0.252	2.26E-12	AC-G	PUM1
FABP6	1.56E-16	-0.15444	0.005	0.045	2.33E-12	AC-G	FABP6
C20orf194	1.57E-16	-0.15416	0.075	0.146	2.35E-12	AC-G	C20orf194
PHACTR3.1	1.57E-16	-0.1694	0.031	0.087	2.35E-12	AC-G	PHACTR3
MEIS1.1	1.57E-16	-0.11249	0.038	0.096	2.35E-12	AC-G	MEIS1
MMD.1	1.59E-16	-0.1175	0.167	0.26	2.37E-12	AC-G	MMD
UNC13D.1	1.67E-16	-0.16311	0.011	0.056	2.50E-12	AC-G	UNC13D
GCHFR.1	1.68E-16	-0.25704	0.031	0.085	2.51E-12	AC-G	GCHFR
FAM192A.1	1.72E-16	-0.11548	0.264	0.376	2.57E-12	AC-G	FAM192A
SLC35G2.1	1.73E-16	0.342144	0.167	0.107	2.59E-12	AC-G	SLC35G2
MAPT.1	1.74E-16	-0.1589	0.206	0.3	2.60E-12	AC-G	MAPT
RHOBTB3.1	1.86E-16	-0.16312	0.321	0.425	2.78E-12	AC-G	RHOBTB3
ZC3HAV1.1	1.89E-16	-0.21422	0.111	0.189	2.82E-12	AC-G	ZC3HAV1
CHADL	1.89E-16	-0.19193	0.007	0.048	2.82E-12	AC-G	CHADL
TULP4.1	1.92E-16	-0.15888	0.081	0.154	2.88E-12	AC-G	TULP4
ZNF536	1.93E-16	-0.14517	0.008	0.049	2.88E-12	AC-G	ZNF536
TBC1D10B.	1.94E-16	-0.13843	0.048	0.11	2.90E-12	AC-G	TBC1D10B
SMCR5.1	1.94E-16	-0.11199	0.041	0.102	2.90E-12	AC-G	SMCR5
NCAPH	1.96E-16	-0.11158	0.004	0.042	2.94E-12	AC-G	NCAPH
BHLHE41.1	2.04E-16	-0.28547	0.141	0.221	3.05E-12	AC-G	BHLHE41
SUPT20H.1	2.05E-16	-0.1256	0.134	0.221	3.06E-12	AC-G	SUPT20H
LRRC4	2.09E-16	-0.13785	0.009	0.052	3.12E-12	AC-G	LRRC4
ADRB2	2.10E-16	-0.22243	0.01	0.053	3.14E-12	AC-G	ADRB2
FHOD1.1	2.12E-16	-0.16864	0.017	0.064	3.16E-12	AC-G	FHOD1
CPPED1.1	2.17E-16	-0.2139	0.025	0.076	3.24E-12	AC-G	CPPED1
IL2RG	2.18E-16	-0.25914	0.008	0.05	3.25E-12	AC-G	IL2RG
LMNB2.1	2.18E-16	-0.13605	0.061	0.128	3.26E-12	AC-G	LMNB2
ENDOG.1	2.25E-16	-0.12771	0.085	0.159	3.36E-12	AC-G	ENDOG
FUT8.1	2.26E-16	-0.11719	0.066	0.136	3.37E-12	AC-G	FUT8
DCAF11.1	2.27E-16	-0.10392	0.119	0.204	3.39E-12	AC-G	DCAF11
PARP14.1	2.27E-16	-0.20896	0.094	0.168	3.40E-12	AC-G	PARP14
LMO1.1	2.32E-16	-0.19719	0.03	0.083	3.47E-12	AC-G	LMO1
SLC25A24.	2.33E-16	-0.16594	0.048	0.108	3.48E-12	AC-G	SLC25A24
ABR.1	2.35E-16	-0.16084	0.084	0.157	3.51E-12	AC-G	ABR
GNG5.1	2.44E-16	0.272994	0.677	0.638	3.65E-12	AC-G	GNG5
TMEM109.1	2.47E-16	-0.22137	0.139	0.217	3.69E-12	AC-G	TMEM109
XRN2.1	2.51E-16	-0.13146	0.361	0.476	3.74E-12	AC-G	XRN2
HEPN1.1	2.53E-16	0.395823	0.262	0.193	3.79E-12	AC-G	HEPN1

LARP4B	2.58E-16	-0.1359	0.041	0.099	3.85E-12	AC-G	LARP4B
GTPBP1	2.66E-16	-0.15929	0.049	0.111	3.97E-12	AC-G	GTPBP1
ASCC2	2.76E-16	-0.18502	0.134	0.219	4.13E-12	AC-G	ASCC2
BMS1.1	2.77E-16	-0.13028	0.083	0.157	4.14E-12	AC-G	BMS1
SSBP3.1	2.78E-16	-0.13547	0.07	0.14	4.16E-12	AC-G	SSBP3
SIGMAR1.1	2.85E-16	-0.15432	0.106	0.185	4.27E-12	AC-G	SIGMAR1
LAMP5.1	2.86E-16	-0.12799	0.055	0.12	4.27E-12	AC-G	LAMP5
CHMP1B.1	2.89E-16	-0.1978	0.35	0.455	4.32E-12	AC-G	CHMP1B
GRAMD3.1	2.90E-16	0.291721	0.193	0.13	4.34E-12	AC-G	GRAMD3
ZNF281.1	2.91E-16	-0.20648	0.12	0.198	4.35E-12	AC-G	ZNF281
DARS.1	3.05E-16	0.286024	0.448	0.393	4.55E-12	AC-G	DARS
ENTPD6.1	3.17E-16	-0.11414	0.127	0.212	4.74E-12	AC-G	ENTPD6
MARK2.1	3.26E-16	-0.15547	0.068	0.136	4.87E-12	AC-G	MARK2
GSK3A.1	3.28E-16	-0.12248	0.119	0.204	4.90E-12	AC-G	GSK3A
OIP5	3.28E-16	-0.1071	0.007	0.048	4.91E-12	AC-G	OIP5
SUSD1.1	3.36E-16	-0.12953	0.016	0.063	5.01E-12	AC-G	SUSD1
FAM212A.1	3.41E-16	-0.20859	0.012	0.056	5.09E-12	AC-G	FAM212A
PNKP.1	3.45E-16	-0.13997	0.109	0.191	5.16E-12	AC-G	PNKP
TRPC4AP.1	3.50E-16	-0.15916	0.173	0.266	5.23E-12	AC-G	TRPC4AP
ARHGAP33.	3.53E-16	-0.12296	0.034	0.091	5.27E-12	AC-G	ARHGAP33
SQRDL.1	3.54E-16	-0.264	0.101	0.172	5.29E-12	AC-G	SQRDL
TMED1	3.60E-16	0.415003	0.344	0.281	5.38E-12	AC-G	TMED1
PPP2R2D.1	3.63E-16	-0.1175	0.04	0.099	5.42E-12	AC-G	PPP2R2D
TBC1D10C	3.74E-16	-0.17367	0.001	0.036	5.59E-12	AC-G	TBC1D10C
CSPG5.1	3.77E-16	-0.21293	0.056	0.118	5.64E-12	AC-G	CSPG5
ATP6V1G2.	4.00E-16	-0.14353	0.087	0.161	5.97E-12	AC-G	ATP6V1G2
MGRN1.1	4.03E-16	-0.1572	0.137	0.222	6.02E-12	AC-G	MGRN1
TRIM27.1	4.04E-16	-0.14867	0.137	0.224	6.04E-12	AC-G	TRIM27
CD58.1	4.14E-16	-0.21182	0.155	0.241	6.18E-12	AC-G	CD58
PI4KA.1	4.14E-16	-0.16133	0.045	0.105	6.18E-12	AC-G	PI4KA
CHKB	4.15E-16	-0.13575	0.017	0.064	6.21E-12	AC-G	CHKB
EGR2.1	4.16E-16	-0.23302	0.125	0.205	6.22E-12	AC-G	EGR2
RP3-47704	4.25E-16	-0.16762	0	0.034	6.35E-12	AC-G	RP3-47704.14
PCYOX1L.1	4.34E-16	-0.12632	0.024	0.074	6.48E-12	AC-G	PCYOX1L
CA4	4.36E-16	0.180845	0.018	0.003	6.51E-12	AC-G	CA4
CLEC18B	4.36E-16	0.139343	0.033	0.01	6.52E-12	AC-G	CLEC18B
HAS2-AS1	4.38E-16	0.112485	0.037	0.012	6.54E-12	AC-G	HAS2-AS1
RREB1.1	4.50E-16	-0.15649	0.028	0.08	6.72E-12	AC-G	RREB1
PDE7A.1	4.54E-16	-0.16198	0.036	0.091	6.78E-12	AC-G	PDE7A
RASSF3	4.56E-16	-0.15592	0.008	0.048	6.82E-12	AC-G	RASSF3
BTBD7.1	4.57E-16	-0.12998	0.078	0.151	6.83E-12	AC-G	BTBD7
NUP210	4.64E-16	-0.12281	0.021	0.071	6.94E-12	AC-G	NUP210
B4GALT7.1	4.75E-16	-0.11628	0.137	0.223	7.09E-12	AC-G	B4GALT7
ANGPT2	4.83E-16	0.264284	0.079	0.039	7.22E-12	AC-G	ANGPT2
RBM12.1	5.01E-16	-0.13765	0.066	0.134	7.48E-12	AC-G	RBM12
EID2.1	5.01E-16	-0.11755	0.057	0.121	7.49E-12	AC-G	EID2
NIPAL3	5.02E-16	-0.16087	0.034	0.09	7.50E-12	AC-G	NIPAL3
CHD6.1	5.07E-16	-0.13046	0.168	0.26	7.57E-12	AC-G	CHD6
FAM159A	5.12E-16	-0.17643	0.01	0.052	7.65E-12	AC-G	FAM159A
GOLPH3.1	5.13E-16	-0.1523	0.117	0.198	7.67E-12	AC-G	GOLPH3
PITPNC1.1	5.16E-16	-0.10776	0.066	0.134	7.71E-12	AC-G	PITPNC1

XIST.1	5.21E-16	0.135856	0.357	0.248	7.79E-12	AC-G	XIST
FAXC.1	5.25E-16	-0.15009	0.038	0.095	7.84E-12	AC-G	FAXC
FSTL1	5.30E-16	0.287462	0.16	0.105	7.92E-12	AC-G	FSTL1
OSBPL1A.1	5.44E-16	-0.18935	0.124	0.204	8.13E-12	AC-G	OSBPL1A
PKMYT1	5.53E-16	-0.11333	0.008	0.049	8.27E-12	AC-G	PKMYT1
ABHD15.1	5.61E-16	-0.18566	0.018	0.065	8.38E-12	AC-G	ABHD15
P2RY11	5.75E-16	-0.21275	0.04	0.096	8.59E-12	AC-G	P2RY11
NFASC	5.93E-16	0.219499	0.152	0.093	8.86E-12	AC-G	NFASC
PDXK.1	5.93E-16	-0.12456	0.206	0.3	8.87E-12	AC-G	PDXK
NCLN.1	6.03E-16	-0.14665	0.1	0.177	9.02E-12	AC-G	NCLN
ZNF462.1	6.67E-16	-0.10872	0.112	0.189	9.96E-12	AC-G	ZNF462
CTTNBP2NL	6.72E-16	-0.15059	0.092	0.165	1.00E-11	AC-G	CTTNBP2NL
MPHOSPH6.	6.74E-16	-0.15396	0.21	0.306	1.01E-11	AC-G	MPHOSPH6
STK24.1	6.93E-16	-0.13895	0.049	0.111	1.04E-11	AC-G	STK24
MEIS3	6.99E-16	-0.10066	0.072	0.141	1.04E-11	AC-G	MEIS3
GDAP1.1	7.04E-16	-0.16565	0.091	0.164	1.05E-11	AC-G	GDAP1
ST6GAL2	7.45E-16	-0.12144	0.005	0.043	1.11E-11	AC-G	ST6GAL2
MEST.1	7.49E-16	-0.19393	0.31	0.401	1.12E-11	AC-G	MEST
KRT7	7.70E-16	0.306498	0.07	0.033	1.15E-11	AC-G	KRT7
PXDC1.1	8.08E-16	-0.14813	0.09	0.164	1.21E-11	AC-G	PXDC1
IBTK.1	8.27E-16	-0.14491	0.103	0.18	1.24E-11	AC-G	IBTK
CHAF1A.1	8.28E-16	-0.12471	0.04	0.098	1.24E-11	AC-G	CHAF1A
LAMA2	8.31E-16	0.174849	0.046	0.017	1.24E-11	AC-G	LAMA2
SLC17A9	8.31E-16	-0.13974	0.001	0.035	1.24E-11	AC-G	SLC17A9
MAN2B1.1	8.32E-16	-0.15696	0.025	0.075	1.24E-11	AC-G	MAN2B1
UBXN6.1	8.42E-16	-0.14663	0.216	0.315	1.26E-11	AC-G	UBXN6
PLB1	8.44E-16	-0.14877	0.008	0.049	1.26E-11	AC-G	PLB1
DNAAF2.1	8.63E-16	-0.1207	0.106	0.186	1.29E-11	AC-G	DNAAF2
CREBL2.1	8.95E-16	-0.19684	0.123	0.202	1.34E-11	AC-G	CREBL2
SLAIN2.1	8.99E-16	-0.12253	0.079	0.15	1.34E-11	AC-G	SLAIN2
INSIG2	9.08E-16	0.297857	0.261	0.191	1.36E-11	AC-G	INSIG2
OSR2	9.24E-16	0.186014	0.043	0.015	1.38E-11	AC-G	OSR2
SNX30.1	9.30E-16	-0.16512	0.021	0.07	1.39E-11	AC-G	SNX30
GDAP1L1.1	9.34E-16	-0.16594	0.05	0.111	1.40E-11	AC-G	GDAP1L1
FAM195A.1	9.54E-16	-0.17348	0.179	0.269	1.43E-11	AC-G	FAM195A
JAZF1.1	9.71E-16	-0.16899	0.061	0.124	1.45E-11	AC-G	JAZF1
ATG16L2.1	9.98E-16	-0.23086	0.041	0.096	1.49E-11	AC-G	ATG16L2
WARS.1	1.00E-15	0.303	0.255	0.191	1.50E-11	AC-G	WARS
PNPLA6.1	1.00E-15	-0.1612	0.102	0.179	1.50E-11	AC-G	PNPLA6
CEP78.1	1.01E-15	-0.13169	0.06	0.124	1.51E-11	AC-G	CEP78
RP3-460G2	1.01E-15	0.364959	0.098	0.052	1.51E-11	AC-G	RP3-460G2.2
MTMR10	1.03E-15	-0.18334	0.025	0.074	1.54E-11	AC-G	MTMR10
TAZ.1	1.03E-15	-0.18786	0.077	0.145	1.54E-11	AC-G	TAZ
FDFT1.1	1.04E-15	-0.12863	0.223	0.322	1.56E-11	AC-G	FDFT1
SIAH2.1	1.05E-15	-0.18469	0.141	0.224	1.57E-11	AC-G	SIAH2
NRP2.1	1.06E-15	-0.11747	0.147	0.236	1.58E-11	AC-G	NRP2
AP3D1.1	1.06E-15	-0.16337	0.223	0.325	1.58E-11	AC-G	AP3D1
MDM4.1	1.06E-15	-0.18956	0.171	0.261	1.59E-11	AC-G	MDM4
MRFAP1.1	1.07E-15	0.225383	0.681	0.669	1.60E-11	AC-G	MRFAP1
DNM3.1	1.07E-15	-0.16215	0.041	0.097	1.61E-11	AC-G	DNM3
DPP8.1	1.11E-15	-0.14196	0.095	0.17	1.66E-11	AC-G	DPP8

C9orf69.1	1.11E-15	-0.11976	0.044	0.103	1.66E-11	AC-G	C9orf69
FANCI.1	1.12E-15	-0.11174	0.011	0.053	1.68E-11	AC-G	FANCI
ZNF90	1.13E-15	0.32233	0.44	0.368	1.69E-11	AC-G	ZNF90
CNDP1	1.15E-15	-0.29043	0.004	0.04	1.72E-11	AC-G	CNDP1
RNASE3	1.17E-15	-0.20575	0.003	0.039	1.74E-11	AC-G	RNASE3
FSTL3	1.17E-15	0.154868	0.08	0.039	1.75E-11	AC-G	FSTL3
FCHO1	1.21E-15	-0.15858	0.007	0.045	1.80E-11	AC-G	FCHO1
AKR7A2.1	1.22E-15	-0.17013	0.229	0.331	1.82E-11	AC-G	AKR7A2
SLCO4A1	1.30E-15	0.132334	0.066	0.029	1.94E-11	AC-G	SLCO4A1
FER	1.30E-15	-0.12648	0.044	0.103	1.95E-11	AC-G	FER
C19orf43.	1.31E-15	-0.10462	0.566	0.693	1.96E-11	AC-G	C19orf43
MIIP	1.32E-15	-0.16174	0.126	0.211	1.97E-11	AC-G	MIIP
C19orf24.	1.32E-15	-0.1299	0.278	0.382	1.98E-11	AC-G	C19orf24
MTHFR	1.33E-15	-0.16742	0.033	0.087	1.99E-11	AC-G	MTHFR
ARHGAP11A	1.38E-15	-0.10737	0.006	0.045	2.06E-11	AC-G	ARHGAP11A
CCSER2.1	1.38E-15	-0.13685	0.155	0.243	2.06E-11	AC-G	CCSER2
EIF2AK2.1	1.39E-15	-0.1538	0.221	0.318	2.07E-11	AC-G	EIF2AK2
CCDC74A.1	1.40E-15	0.142646	0.084	0.042	2.09E-11	AC-G	CCDC74A
SPATS2L.1	1.43E-15	-0.16605	0.253	0.347	2.13E-11	AC-G	SPATS2L
NNT	1.43E-15	-0.15581	0.078	0.148	2.14E-11	AC-G	NNT
MCUR1.1	1.49E-15	-0.15658	0.185	0.277	2.23E-11	AC-G	MCUR1
ABCA2.1	1.50E-15	-0.28511	0.047	0.105	2.25E-11	AC-G	ABCA2
ABHD6	1.56E-15	-0.13254	0.051	0.112	2.33E-11	AC-G	ABHD6
GRAMD4.1	1.57E-15	-0.15481	0.021	0.068	2.34E-11	AC-G	GRAMD4
LYVE1	1.59E-15	-0.31657	0.006	0.044	2.37E-11	AC-G	LYVE1
PGM3.1	1.60E-15	0.2564	0.226	0.164	2.38E-11	AC-G	PGM3
TMOD1	1.60E-15	0.20872	0.214	0.146	2.39E-11	AC-G	TMOD1
SCAF11.1	1.61E-15	-0.13896	0.303	0.415	2.41E-11	AC-G	SCAF11
DUSP10	1.62E-15	-0.18554	0.114	0.192	2.42E-11	AC-G	DUSP10
HMMR	1.66E-15	-0.12589	0.007	0.046	2.48E-11	AC-G	HMMR
QSOX1.1	1.68E-15	-0.17604	0.163	0.25	2.51E-11	AC-G	QSOX1
MYRF	1.69E-15	-0.20706	0.002	0.036	2.53E-11	AC-G	MYRF
GPD1.1	1.72E-15	-0.16998	0.013	0.055	2.57E-11	AC-G	GPD1
FAM98B.1	1.73E-15	-0.1114	0.045	0.105	2.58E-11	AC-G	FAM98B
SOX9.1	1.77E-15	-0.2377	0.207	0.284	2.65E-11	AC-G	SOX9
GBA2.1	1.84E-15	-0.1289	0.039	0.095	2.74E-11	AC-G	GBA2
PFKL.1	1.84E-15	-0.16399	0.277	0.38	2.75E-11	AC-G	PFKL
KIAA0232.	1.85E-15	-0.15338	0.091	0.163	2.76E-11	AC-G	KIAA0232
CDC45	1.85E-15	-0.10489	0.005	0.041	2.76E-11	AC-G	CDC45
GANAB.1	1.87E-15	-0.14084	0.192	0.288	2.80E-11	AC-G	GANAB
SAPCD2.1	1.90E-15	-0.12704	0.018	0.064	2.83E-11	AC-G	SAPCD2
SPNS1.1	1.92E-15	-0.14373	0.209	0.306	2.86E-11	AC-G	SPNS1
FLOT1.1	1.92E-15	-0.10799	0.398	0.523	2.87E-11	AC-G	FLOT1
ZFHX4.1	1.99E-15	-0.11665	0.09	0.164	2.97E-11	AC-G	ZFHX4
RALGDS.1	2.02E-15	-0.23514	0.095	0.163	3.02E-11	AC-G	RALGDS
SEPHS1.1	2.10E-15	-0.10606	0.059	0.123	3.13E-11	AC-G	SEPHS1
PDPK1.1	2.10E-15	-0.15818	0.121	0.2	3.14E-11	AC-G	PDPK1
FAM72A	2.10E-15	-0.15956	0.008	0.048	3.14E-11	AC-G	FAM72A
RHPN2	2.13E-15	0.192582	0.069	0.032	3.19E-11	AC-G	RHPN2
PVRL1.1	2.16E-15	-0.1004	0.021	0.069	3.22E-11	AC-G	PVRL1
DOPEY2.1	2.33E-15	-0.14769	0.026	0.076	3.49E-11	AC-G	DOPEY2

PKIA.1	2.38E-15	-0.19864	0.046	0.102	3.55E-11	AC-G	PKIA
APAF1.1	2.38E-15	-0.14954	0.025	0.074	3.56E-11	AC-G	APAF1
PRKCDBP	2.39E-15	0.129958	0.061	0.027	3.58E-11	AC-G	PRKCDBP
DPEP2	2.41E-15	-0.16227	0.004	0.039	3.61E-11	AC-G	DPEP2
NUCB1.1	2.42E-15	-0.13971	0.349	0.464	3.62E-11	AC-G	NUCB1
DMXL2.1	2.44E-15	-0.16043	0.036	0.09	3.64E-11	AC-G	DMXL2
TNPO1.1	2.46E-15	-0.13554	0.198	0.293	3.67E-11	AC-G	TNPO1
SYNE2.1	2.47E-15	-0.11603	0.109	0.186	3.69E-11	AC-G	SYNE2
SLC3A2.1	2.52E-15	0.294535	0.583	0.588	3.76E-11	AC-G	SLC3A2
CNOT6.1	2.52E-15	-0.11435	0.03	0.082	3.77E-11	AC-G	CNOT6
ABHD3.1	2.55E-15	-0.1827	0.118	0.195	3.81E-11	AC-G	ABHD3
HSCB.1	2.59E-15	-0.10939	0.107	0.184	3.87E-11	AC-G	HSCB
RP11-488C	2.60E-15	-0.13323	0.026	0.075	3.88E-11	AC-G	RP11-488C13.5
FLOT2.1	2.71E-15	-0.12092	0.117	0.199	4.05E-11	AC-G	FLOT2
RASA1.1	2.74E-15	-0.12504	0.054	0.116	4.10E-11	AC-G	RASA1
TRAPPC2P1	2.75E-15	0.334438	0.28	0.213	4.11E-11	AC-G	TRAPPC2P1
SMCHD1.1	2.75E-15	-0.16838	0.121	0.2	4.11E-11	AC-G	SMCHD1
KCNN3	2.75E-15	0.135668	0.06	0.026	4.11E-11	AC-G	KCNN3
ARHGEF9.1	2.78E-15	-0.12886	0.065	0.13	4.15E-11	AC-G	ARHGEF9
NEU4.1	2.79E-15	-0.22257	0.024	0.071	4.17E-11	AC-G	NEU4
ZMYND12	2.88E-15	0.120223	0.027	0.007	4.30E-11	AC-G	ZMYND12
PCNX.1	3.00E-15	-0.1436	0.045	0.103	4.48E-11	AC-G	PCNX
PHF12.1	3.07E-15	-0.11948	0.058	0.121	4.59E-11	AC-G	PHF12
TCF3.1	3.13E-15	-0.11104	0.073	0.14	4.68E-11	AC-G	TCF3
RGL1.1	3.17E-15	-0.13242	0.039	0.095	4.74E-11	AC-G	RGL1
NEDD4L.1	3.24E-15	-0.11101	0.06	0.123	4.84E-11	AC-G	NEDD4L
MLLT4.1	3.27E-15	-0.17435	0.101	0.174	4.88E-11	AC-G	MLLT4
BUB1	3.34E-15	-0.12043	0.008	0.046	4.99E-11	AC-G	BUB1
C4orf3.1	3.57E-15	0.278617	0.647	0.646	5.33E-11	AC-G	C4orf3
MND1	3.62E-15	-0.11854	0.01	0.051	5.41E-11	AC-G	MND1
ZNF738.1	3.63E-15	-0.10889	0.084	0.156	5.42E-11	AC-G	ZNF738
IMPDH1.1	3.67E-15	-0.14318	0.096	0.169	5.48E-11	AC-G	IMPDH1
DNASE1L1.	3.68E-15	-0.1763	0.03	0.081	5.49E-11	AC-G	DNASE1L1
CADM2	3.68E-15	-0.21467	0.125	0.201	5.50E-11	AC-G	CADM2
GNG4.1	3.69E-15	-0.13109	0.062	0.126	5.51E-11	AC-G	GNG4
BTBD17.1	3.69E-15	-0.15972	0.016	0.059	5.52E-11	AC-G	BTBD17
GLIPR1L2	3.79E-15	0.136475	0.069	0.032	5.67E-11	AC-G	GLIPR1L2
ACSL4.1	3.82E-15	-0.178	0.039	0.092	5.71E-11	AC-G	ACSL4
CCNDBP1.1	3.85E-15	-0.20201	0.137	0.218	5.76E-11	AC-G	CCNDBP1
WDR78	3.86E-15	0.122908	0.042	0.015	5.76E-11	AC-G	WDR78
ZC3H18.1	3.92E-15	-0.11718	0.096	0.169	5.86E-11	AC-G	ZC3H18
ITGA4	3.96E-15	-0.15582	0.009	0.049	5.91E-11	AC-G	ITGA4
PRR4.1	3.97E-15	-0.12773	0.146	0.23	5.93E-11	AC-G	PRR4
USP9X.1	4.05E-15	-0.14699	0.133	0.215	6.05E-11	AC-G	USP9X
MOG	4.06E-15	-0.25614	0.003	0.037	6.07E-11	AC-G	MOG
CKAP5.1	4.18E-15	-0.11283	0.095	0.168	6.25E-11	AC-G	CKAP5
PEAK1.1	4.24E-15	-0.11372	0.027	0.077	6.34E-11	AC-G	PEAK1
FAM127B.1	4.44E-15	0.314073	0.354	0.29	6.64E-11	AC-G	FAM127B
MARCH6.1	4.47E-15	-0.10157	0.271	0.374	6.68E-11	AC-G	6-Mar
HMG20B.1	4.51E-15	-0.16418	0.254	0.352	6.75E-11	AC-G	HMG20B
PPP1R14A	4.69E-15	-0.34891	0.057	0.117	7.00E-11	AC-G	PPP1R14A

LETM1.1	4.77E-15	-0.12333	0.12	0.198	7.12E-11	AC-G	LETM1
TLE3	4.80E-15	-0.23191	0.083	0.148	7.18E-11	AC-G	TLE3
KCNQ3	4.82E-15	-0.17189	0.009	0.048	7.21E-11	AC-G	KCNQ3
BANF1.1	4.83E-15	0.250662	0.677	0.659	7.22E-11	AC-G	BANF1
LZTS2.1	4.85E-15	-0.13015	0.035	0.088	7.25E-11	AC-G	LZTS2
PAM.1	4.86E-15	0.293967	0.214	0.155	7.26E-11	AC-G	PAM
VRK1	4.86E-15	-0.13662	0.051	0.111	7.26E-11	AC-G	VRK1
SSPN.1	4.88E-15	0.217182	0.154	0.096	7.29E-11	AC-G	SSPN
MYT1	5.09E-15	-0.13123	0.007	0.045	7.61E-11	AC-G	MYT1
DCUN1D1.1	5.10E-15	-0.13976	0.096	0.167	7.62E-11	AC-G	DCUN1D1
SRSF4.1	5.12E-15	-0.12535	0.27	0.377	7.66E-11	AC-G	SRSF4
EGFR.1	5.20E-15	-0.23236	0.23	0.314	7.77E-11	AC-G	EGFR
TBC1D2B.1	5.24E-15	-0.16721	0.016	0.059	7.83E-11	AC-G	TBC1D2B
TLR5	5.28E-15	-0.20609	0.011	0.052	7.90E-11	AC-G	TLR5
KIF23	5.46E-15	-0.11379	0.008	0.046	8.15E-11	AC-G	KIF23
RMI2	5.59E-15	-0.11211	0.007	0.045	8.35E-11	AC-G	RMI2
NCOA1.1	5.70E-15	-0.11035	0.099	0.173	8.52E-11	AC-G	NCOA1
SYT1.1	5.81E-15	-0.24623	0.053	0.109	8.68E-11	AC-G	SYT1
RPUSD1.1	5.81E-15	-0.1489	0.078	0.145	8.69E-11	AC-G	RPUSD1
MAPK9.1	5.94E-15	-0.12653	0.037	0.091	8.87E-11	AC-G	MAPK9
TTLL7.1	6.02E-15	-0.22925	0.167	0.251	8.99E-11	AC-G	TTLL7
BEST3.1	6.03E-15	-0.19752	0.041	0.096	9.02E-11	AC-G	BEST3
TTYH1.1	6.09E-15	-0.24871	0.196	0.282	9.10E-11	AC-G	TTYH1
CSNK1G3.1	6.11E-15	-0.13539	0.092	0.165	9.12E-11	AC-G	CSNK1G3
FAM20C.1	6.12E-15	0.333509	0.274	0.213	9.15E-11	AC-G	FAM20C
MED1.1	6.16E-15	-0.12216	0.065	0.129	9.21E-11	AC-G	MED1
ALDH9A1.1	6.20E-15	0.278155	0.386	0.329	9.27E-11	AC-G	ALDH9A1
TRMT11	6.21E-15	0.233268	0.165	0.107	9.28E-11	AC-G	TRMT11
PRPF4B.1	6.22E-15	-0.1064	0.195	0.289	9.29E-11	AC-G	PRPF4B
FCHSD2.1	6.30E-15	-0.18802	0.037	0.09	9.41E-11	AC-G	FCHSD2
UBE2L6.1	6.32E-15	0.410907	0.497	0.469	9.44E-11	AC-G	UBE2L6
APOBEC3C	6.33E-15	-0.14226	0.074	0.139	9.46E-11	AC-G	APOBEC3C
FKBP2.1	6.37E-15	0.303229	0.599	0.588	9.51E-11	AC-G	FKBP2
C21orf62	6.52E-15	0.34246	0.209	0.148	9.74E-11	AC-G	C21orf62
AC015987.	6.74E-15	0.102291	0.023	0.005	1.01E-10	AC-G	AC015987.1
GAS6.1	6.89E-15	-0.28865	0.146	0.216	1.03E-10	AC-G	GAS6
CMPK2.1	6.89E-15	-0.17865	0.019	0.064	1.03E-10	AC-G	CMPK2
POLG.1	7.09E-15	-0.1528	0.048	0.106	1.06E-10	AC-G	POLG
AGRN	7.20E-15	-0.11605	0.046	0.104	1.08E-10	AC-G	AGRN
IRF5.1	7.25E-15	-0.21843	0.021	0.066	1.08E-10	AC-G	IRF5
BRF1.1	7.25E-15	-0.12621	0.053	0.113	1.08E-10	AC-G	BRF1
GPR132	7.44E-15	-0.1596	0.003	0.036	1.11E-10	AC-G	GPR132
YIPF2	7.62E-15	0.348289	0.167	0.109	1.14E-10	AC-G	YIPF2
STARD7.1	7.65E-15	-0.17922	0.236	0.332	1.14E-10	AC-G	STARD7
IRGQ	7.81E-15	-0.12595	0.041	0.096	1.17E-10	AC-G	IRGQ
HPRT1.1	7.98E-15	-0.22008	0.163	0.244	1.19E-10	AC-G	HPRT1
CSRNP1.1	8.07E-15	-0.28701	0.133	0.202	1.21E-10	AC-G	CSRNP1
RANGAP1.1	8.07E-15	-0.13984	0.053	0.112	1.21E-10	AC-G	RANGAP1
VEZT.1	8.16E-15	-0.12532	0.155	0.241	1.22E-10	AC-G	VEZT
UBE2G1.1	8.25E-15	-0.12644	0.181	0.273	1.23E-10	AC-G	UBE2G1
CCER2.1	8.32E-15	-0.18033	0.018	0.062	1.24E-10	AC-G	CCER2

MTMR9.1	8.39E-15	-0.13566	0.124	0.202	1.25E-10	AC-G	MTMR9
ZNF131.1	8.69E-15	-0.15148	0.161	0.246	1.30E-10	AC-G	ZNF131
PTDSS2.1	8.92E-15	-0.13339	0.092	0.164	1.33E-10	AC-G	PTDSS2
HEXB.1	9.16E-15	-0.18936	0.319	0.422	1.37E-10	AC-G	HEXB
ASF1A.1	9.35E-15	-0.13197	0.162	0.248	1.40E-10	AC-G	ASF1A
CDT1	9.36E-15	-0.13071	0.022	0.068	1.40E-10	AC-G	CDT1
MELK	9.61E-15	-0.10537	0.012	0.053	1.44E-10	AC-G	MELK
CUL2	9.65E-15	-0.15988	0.068	0.132	1.44E-10	AC-G	CUL2
PSMA1.1	9.76E-15	0.28921	0.627	0.608	1.46E-10	AC-G	PSMA1
SMPDL3A	9.79E-15	-0.2097	0.072	0.135	1.46E-10	AC-G	SMPDL3A
FCHO2.1	9.88E-15	-0.16261	0.032	0.082	1.48E-10	AC-G	FCHO2
RP11-452F	1.01E-14	-0.20251	0.073	0.137	1.51E-10	AC-G	RP11-452F19.3
EIF4A2.1	1.02E-14	0.191918	0.704	0.722	1.52E-10	AC-G	EIF4A2
RP5-1177M	1.02E-14	-0.17204	0.011	0.051	1.53E-10	AC-G	RP5-1177M21.1
SP110.1	1.04E-14	-0.23917	0.135	0.21	1.55E-10	AC-G	SP110
BEX2.1	1.05E-14	0.224001	0.329	0.243	1.57E-10	AC-G	BEX2
PDE9A	1.05E-14	-0.12209	0.029	0.079	1.57E-10	AC-G	PDE9A
ESCO2	1.07E-14	-0.10042	0.005	0.04	1.61E-10	AC-G	ESCO2
NDUFAF2.1	1.09E-14	0.294769	0.324	0.262	1.62E-10	AC-G	NDUFAF2
PDE3B	1.11E-14	-0.14151	0.008	0.045	1.65E-10	AC-G	PDE3B
CYB561A3.	1.11E-14	-0.18263	0.093	0.162	1.66E-10	AC-G	CYB561A3
FAM134A.1	1.12E-14	-0.16747	0.22	0.313	1.68E-10	AC-G	FAM134A
CRIP1	1.14E-14	-0.21369	0.008	0.045	1.70E-10	AC-G	CRIP1
MB21D2	1.20E-14	-0.15624	0.017	0.061	1.79E-10	AC-G	MB21D2
ZNF467	1.20E-14	-0.13636	0.005	0.039	1.79E-10	AC-G	ZNF467
PAOX.1	1.22E-14	-0.12419	0.017	0.06	1.82E-10	AC-G	PAOX
ANKS1B.1	1.22E-14	-0.22752	0.059	0.117	1.82E-10	AC-G	ANKS1B
DICER1.1	1.23E-14	-0.18048	0.112	0.187	1.84E-10	AC-G	DICER1
PPP2R1A.1	1.24E-14	-0.12099	0.418	0.534	1.86E-10	AC-G	PPP2R1A
DOCK11.1	1.25E-14	-0.18001	0.018	0.061	1.87E-10	AC-G	DOCK11
ABCD1.1	1.26E-14	-0.13293	0.013	0.053	1.88E-10	AC-G	ABCD1
GPR161.1	1.27E-14	-0.13475	0.039	0.092	1.90E-10	AC-G	GPR161
PFN1.1	1.28E-14	-0.131	0.662	0.837	1.91E-10	AC-G	PFN1
ATMIN.1	1.28E-14	-0.11616	0.063	0.125	1.92E-10	AC-G	ATMIN
ZNF326.1	1.29E-14	-0.10878	0.172	0.262	1.93E-10	AC-G	ZNF326
BRWD1.1	1.30E-14	-0.17679	0.172	0.255	1.94E-10	AC-G	BRWD1
COL9A2.1	1.31E-14	-0.15718	0.043	0.098	1.96E-10	AC-G	COL9A2
GTF2E2.1	1.31E-14	-0.13212	0.122	0.201	1.96E-10	AC-G	GTF2E2
ADD1	1.34E-14	-0.16413	0.228	0.323	2.00E-10	AC-G	ADD1
PRKAG2-AS	1.35E-14	-0.27803	0.073	0.135	2.02E-10	AC-G	PRKAG2-AS1
GCLC.1	1.36E-14	-0.1551	0.05	0.106	2.03E-10	AC-G	GCLC
NUAK1.1	1.36E-14	-0.16128	0.03	0.079	2.03E-10	AC-G	NUAK1
UBE2J1.1	1.36E-14	-0.15658	0.205	0.295	2.04E-10	AC-G	UBE2J1
ANK3.1	1.44E-14	-0.1565	0.022	0.067	2.15E-10	AC-G	ANK3
EDIL3	1.46E-14	-0.22942	0.004	0.037	2.17E-10	AC-G	EDIL3
WDFY3.1	1.46E-14	-0.12607	0.042	0.096	2.18E-10	AC-G	WDFY3
EFS	1.47E-14	-0.10557	0.055	0.116	2.19E-10	AC-G	EFS
MASTL	1.49E-14	-0.10237	0.007	0.043	2.23E-10	AC-G	MASTL
SLC8B1.1	1.53E-14	-0.17159	0.05	0.107	2.28E-10	AC-G	SLC8B1
SMC1A.1	1.54E-14	-0.18763	0.15	0.23	2.31E-10	AC-G	SMC1A
RPP38	1.55E-14	-0.11247	0.077	0.143	2.31E-10	AC-G	RPP38

RAN.1	1.58E-14	0.199542	0.74	0.76	2.36E-10	AC-G	RAN
UBA7.1	1.59E-14	-0.25197	0.039	0.09	2.38E-10	AC-G	UBA7
IPO5.1	1.61E-14	-0.1114	0.146	0.229	2.41E-10	AC-G	IPO5
HECA.1	1.62E-14	-0.15695	0.03	0.079	2.43E-10	AC-G	HECA
AKAP11.1	1.63E-14	-0.13741	0.091	0.161	2.43E-10	AC-G	AKAP11
DUSP26.1	1.67E-14	-0.20396	0.042	0.096	2.49E-10	AC-G	DUSP26
ITPRIPL2.	1.72E-14	-0.14806	0.032	0.081	2.57E-10	AC-G	ITPRIPL2
RP3-522D1	1.73E-14	-0.14525	0.007	0.043	2.59E-10	AC-G	RP3-522D1.1
HHLA3.1	1.75E-14	0.328422	0.189	0.13	2.61E-10	AC-G	HHLA3
BRINP2.1	1.80E-14	-0.10749	0.031	0.081	2.69E-10	AC-G	BRINP2
TECPR1.1	1.81E-14	-0.13152	0.067	0.13	2.71E-10	AC-G	TECPR1
ZNF302.1	1.82E-14	0.339576	0.369	0.304	2.72E-10	AC-G	ZNF302
AKNA	1.82E-14	-0.24055	0.031	0.08	2.72E-10	AC-G	AKNA
NKD1	1.84E-14	-0.11394	0.005	0.04	2.75E-10	AC-G	NKD1
POLDIP3	1.85E-14	-0.11296	0.12	0.199	2.77E-10	AC-G	POLDIP3
SPINT1	1.88E-14	-0.14034	0.005	0.039	2.80E-10	AC-G	SPINT1
CDPF1.1	1.89E-14	-0.10192	0.033	0.084	2.82E-10	AC-G	CDPF1
KAT6B.1	1.90E-14	-0.12928	0.068	0.132	2.83E-10	AC-G	KAT6B
ZNF267.1	1.92E-14	-0.18831	0.078	0.143	2.87E-10	AC-G	ZNF267
FKBP15.1	1.94E-14	-0.1737	0.083	0.149	2.89E-10	AC-G	FKBP15
DSC2	1.94E-14	-0.12552	0.004	0.038	2.90E-10	AC-G	DSC2
CDCA8	1.95E-14	-0.13666	0.008	0.045	2.91E-10	AC-G	CDCA8
UBN1.1	1.98E-14	-0.14388	0.106	0.178	2.96E-10	AC-G	UBN1
EIF2AK4.1	2.02E-14	-0.14032	0.118	0.193	3.02E-10	AC-G	EIF2AK4
CA10	2.03E-14	-0.11037	0.004	0.037	3.03E-10	AC-G	CA10
GPX4.1	2.04E-14	0.235019	0.803	0.85	3.05E-10	AC-G	GPX4
CENPA.1	2.06E-14	-0.15171	0.015	0.056	3.07E-10	AC-G	CENPA
IRF7.1	2.16E-14	-0.18667	0.102	0.172	3.23E-10	AC-G	IRF7
TAPBP.1	2.24E-14	-0.14683	0.258	0.359	3.34E-10	AC-G	TAPBP
DNAJA4.1	2.24E-14	-0.16399	0.024	0.069	3.35E-10	AC-G	DNAJA4
RIC3	2.30E-14	0.319712	0.288	0.22	3.43E-10	AC-G	RIC3
EHD4.1	2.32E-14	-0.19146	0.046	0.1	3.47E-10	AC-G	EHD4
XKR4	2.33E-14	-0.12288	0.02	0.064	3.48E-10	AC-G	XKR4
FAR2	2.33E-14	-0.14004	0.019	0.062	3.48E-10	AC-G	FAR2
ARHGAP11E	2.34E-14	-0.10469	0.008	0.044	3.49E-10	AC-G	ARHGAP11B
TBC1D4.1	2.40E-14	-0.16695	0.014	0.055	3.58E-10	AC-G	TBC1D4
RQCD1	2.40E-14	-0.11639	0.122	0.2	3.59E-10	AC-G	RQCD1
YLPM1.1	2.43E-14	-0.11003	0.063	0.125	3.63E-10	AC-G	YLPM1
VEGFB	2.49E-14	-0.15352	0.087	0.156	3.72E-10	AC-G	VEGFB
TOX3.1	2.53E-14	-0.18617	0.028	0.075	3.78E-10	AC-G	TOX3
CAMK2N1.1	2.64E-14	-0.21093	0.248	0.33	3.95E-10	AC-G	CAMK2N1
A2M.1	2.64E-14	0.458795	0.479	0.461	3.95E-10	AC-G	A2M
MLLT6.1	2.68E-14	-0.12691	0.076	0.142	4.00E-10	AC-G	MLLT6
ZNF672.1	2.70E-14	-0.14888	0.084	0.151	4.03E-10	AC-G	ZNF672
PAQR8.1	2.87E-14	-0.21522	0.05	0.105	4.29E-10	AC-G	PAQR8
TSPAN17.1	2.88E-14	-0.13533	0.09	0.159	4.31E-10	AC-G	TSPAN17
TYRO3.1	2.89E-14	-0.10192	0.058	0.119	4.32E-10	AC-G	TYRO3
ATXN2	2.94E-14	-0.12675	0.152	0.235	4.39E-10	AC-G	ATXN2
EP300	3.13E-14	-0.12598	0.034	0.084	4.68E-10	AC-G	EP300
HSPE1.1	3.14E-14	0.210445	0.714	0.732	4.69E-10	AC-G	HSPE1
PDIA3.1	3.17E-14	-0.13722	0.463	0.606	4.73E-10	AC-G	PDIA3

FAM219B.1	3.26E-14	-0.13591	0.091	0.161	4.87E-10	AC-G	FAM219B
CCDC88C	3.27E-14	-0.10478	0.01	0.049	4.89E-10	AC-G	CCDC88C
AC009506.	3.28E-14	-0.10518	0.037	0.089	4.91E-10	AC-G	AC009506.1
REEP1.1	3.29E-14	-0.1584	0.038	0.089	4.91E-10	AC-G	REEP1
ATP6V1A.1	3.35E-14	-0.13584	0.134	0.211	5.01E-10	AC-G	ATP6V1A
ANKS1A.1	3.41E-14	-0.12989	0.022	0.067	5.10E-10	AC-G	ANKS1A
CCNE2	3.43E-14	-0.16788	0.021	0.066	5.13E-10	AC-G	CCNE2
CNOT11.1	3.46E-14	-0.13299	0.069	0.132	5.18E-10	AC-G	CNOT11
TNKS.1	3.47E-14	-0.1182	0.09	0.16	5.18E-10	AC-G	TNKS
GALNT6	3.48E-14	-0.13681	0.003	0.035	5.20E-10	AC-G	GALNT6
NR1D1	3.50E-14	0.187033	0.082	0.043	5.24E-10	AC-G	NR1D1
NDE1.1	3.54E-14	-0.15002	0.056	0.115	5.29E-10	AC-G	NDE1
RANBP3.1	3.55E-14	-0.13617	0.152	0.236	5.31E-10	AC-G	RANBP3
SLC32A1	3.59E-14	-0.14791	0.007	0.043	5.37E-10	AC-G	SLC32A1
SESTD1	3.60E-14	-0.15265	0.101	0.17	5.39E-10	AC-G	SESTD1
EFR3A.1	3.62E-14	-0.15944	0.086	0.153	5.41E-10	AC-G	EFR3A
NARS	3.76E-14	0.289258	0.467	0.432	5.62E-10	AC-G	NARS
TPMT	3.81E-14	-0.19672	0.13	0.204	5.70E-10	AC-G	TPMT
NUCB2	3.83E-14	0.327552	0.39	0.338	5.72E-10	AC-G	NUCB2
U2AF1.1	3.84E-14	-0.12356	0.517	0.653	5.73E-10	AC-G	U2AF1
CCNY.1	3.85E-14	-0.15296	0.059	0.118	5.76E-10	AC-G	CCNY
IFNAR1.1	3.98E-14	-0.10119	0.211	0.305	5.95E-10	AC-G	IFNAR1
NDST1.1	3.99E-14	-0.11605	0.042	0.096	5.96E-10	AC-G	NDST1
KCNMB4.1	4.07E-14	-0.15703	0.07	0.134	6.09E-10	AC-G	KCNMB4
CNTN2	4.10E-14	-0.27583	0.01	0.047	6.12E-10	AC-G	CNTN2
AC006946.	4.13E-14	0.1032	0.017	0.003	6.18E-10	AC-G	AC006946.16
SNX24.1	4.15E-14	-0.1278	0.072	0.137	6.20E-10	AC-G	SNX24
RASSF7.1	4.18E-14	-0.1205	0.03	0.078	6.25E-10	AC-G	RASSF7
DIDO1	4.18E-14	-0.10724	0.089	0.16	6.25E-10	AC-G	DIDO1
MTMR6.1	4.21E-14	-0.1353	0.088	0.156	6.29E-10	AC-G	MTMR6
EAF2.1	4.23E-14	-0.23337	0.048	0.101	6.32E-10	AC-G	EAF2
SPRY1.1	4.23E-14	-0.17981	0.164	0.246	6.32E-10	AC-G	SPRY1
EMILIN3	4.24E-14	-0.10172	0.004	0.037	6.33E-10	AC-G	EMILIN3
IL2RA	4.31E-14	-0.25111	0.004	0.037	6.44E-10	AC-G	IL2RA
ZNF480	4.32E-14	-0.16929	0.057	0.115	6.46E-10	AC-G	ZNF480
GLG1.1	4.52E-14	-0.12944	0.19	0.281	6.76E-10	AC-G	GLG1
TXNRD2.1	4.56E-14	-0.11378	0.066	0.128	6.81E-10	AC-G	TXNRD2
ECHDC3	4.57E-14	-0.14678	0.005	0.038	6.82E-10	AC-G	ECHDC3
NUTM2A-AS	4.59E-14	-0.17005	0.075	0.138	6.86E-10	AC-G	NUTM2A-AS1
PLK1	4.62E-14	-0.16648	0.019	0.062	6.90E-10	AC-G	PLK1
NRBP2.1	4.63E-14	-0.10603	0.068	0.131	6.91E-10	AC-G	NRBP2
NKX6-2	4.63E-14	-0.20182	0	0.029	6.92E-10	AC-G	NKX6-2
AC147651.	4.71E-14	-0.16143	0.007	0.042	7.03E-10	AC-G	AC147651.4
RP11-489C	4.71E-14	-0.17246	0.002	0.033	7.04E-10	AC-G	RP11-489O18.1
SAR1A.1	4.73E-14	-0.11754	0.222	0.313	7.07E-10	AC-G	SAR1A
C10orf11.	4.77E-14	-0.2325	0.055	0.11	7.13E-10	AC-G	C10orf11
ZADH2	4.81E-14	-0.13177	0.055	0.113	7.18E-10	AC-G	ZADH2
CD7	4.82E-14	-0.2699	0.011	0.05	7.20E-10	AC-G	CD7
P2RX7	4.90E-14	-0.18177	0.04	0.091	7.32E-10	AC-G	P2RX7
HPS1	4.91E-14	-0.23723	0.062	0.12	7.34E-10	AC-G	HPS1
EIF3J.1	4.95E-14	-0.11887	0.29	0.395	7.40E-10	AC-G	EIF3J

NCKAP5	5.08E-14	-0.13654	0.026	0.073	7.59E-10	AC-G	NCKAP5
APLP2.1	5.10E-14	-0.12615	0.489	0.613	7.62E-10	AC-G	APLP2
SIRT7	5.17E-14	-0.13387	0.139	0.219	7.72E-10	AC-G	SIRT7
OSTC.1	5.23E-14	0.320713	0.53	0.498	7.82E-10	AC-G	OSTC
EHMT1.1	5.26E-14	-0.14185	0.09	0.157	7.85E-10	AC-G	EHMT1
CNTNAP3B	5.30E-14	0.157729	0.062	0.029	7.92E-10	AC-G	CNTNAP3B
ADRBK2.1	5.42E-14	-0.15054	0.018	0.06	8.10E-10	AC-G	ADRBK2
RNF44.1	5.45E-14	-0.14828	0.064	0.123	8.14E-10	AC-G	RNF44
C14orf37	5.52E-14	-0.15854	0.062	0.121	8.24E-10	AC-G	C14orf37
SCAMP4.1	5.60E-14	-0.10628	0.195	0.283	8.36E-10	AC-G	SCAMP4
PDP1.1	5.66E-14	-0.11301	0.073	0.137	8.46E-10	AC-G	PDP1
AP005530.	5.66E-14	-0.16215	0.003	0.034	8.46E-10	AC-G	AP005530.2
ZCCHC11	5.76E-14	-0.11308	0.167	0.253	8.61E-10	AC-G	ZCCHC11
GLI4.1	5.95E-14	-0.15702	0.062	0.121	8.89E-10	AC-G	GLI4
CPEB2.1	6.00E-14	-0.10745	0.03	0.079	8.96E-10	AC-G	CPEB2
HSD11B1L.	6.10E-14	-0.13929	0.14	0.217	9.11E-10	AC-G	HSD11B1L
HSBP1.1	6.14E-14	0.221924	0.699	0.718	9.17E-10	AC-G	HSBP1
PIK3C2A.1	6.22E-14	-0.13154	0.084	0.151	9.29E-10	AC-G	PIK3C2A
NR1H2.1	6.34E-14	-0.16383	0.226	0.318	9.47E-10	AC-G	NR1H2
NEK2	6.55E-14	-0.11196	0.005	0.039	9.78E-10	AC-G	NEK2
C5orf28.1	6.61E-14	0.213353	0.166	0.11	9.88E-10	AC-G	C5orf28
PDIA4.1	6.64E-14	0.321741	0.483	0.457	9.92E-10	AC-G	PDIA4
MEGF9	6.71E-14	-0.13835	0.047	0.101	1.00E-09	AC-G	MEGF9
ARSB.1	6.76E-14	-0.10505	0.015	0.056	1.01E-09	AC-G	ARSB
INPPL1.1	6.83E-14	-0.12011	0.068	0.13	1.02E-09	AC-G	INPPL1
ZBED1.1	6.84E-14	-0.15346	0.085	0.15	1.02E-09	AC-G	ZBED1
MGMT.1	6.84E-14	-0.24453	0.098	0.162	1.02E-09	AC-G	MGMT
AKAP17A	6.90E-14	-0.17209	0.109	0.179	1.03E-09	AC-G	AKAP17A
PCED1B	6.99E-14	-0.15042	0.007	0.042	1.05E-09	AC-G	PCED1B
PI4K2A.1	7.00E-14	-0.17089	0.027	0.072	1.05E-09	AC-G	PI4K2A
ATF5.1	7.10E-14	-0.21649	0.132	0.206	1.06E-09	AC-G	ATF5
PEX26.1	7.12E-14	-0.10639	0.062	0.123	1.06E-09	AC-G	PEX26
CARD11	7.16E-14	-0.12347	0.002	0.033	1.07E-09	AC-G	CARD11
SLC40A1.1	7.23E-14	-0.16797	0.085	0.149	1.08E-09	AC-G	SLC40A1
ADAR	7.26E-14	-0.14833	0.228	0.323	1.08E-09	AC-G	ADAR
DDX39B.1	7.28E-14	-0.12307	0.06	0.119	1.09E-09	AC-G	DDX39B
TFRC.1	7.30E-14	-0.22957	0.147	0.221	1.09E-09	AC-G	TFRC
RP11-742E	7.37E-14	-0.16311	0.004	0.036	1.10E-09	AC-G	RP11-742B18.1
SCAF1.1	7.51E-14	-0.12262	0.095	0.165	1.12E-09	AC-G	SCAF1
ADAP1	7.53E-14	-0.16014	0.01	0.047	1.12E-09	AC-G	ADAP1
SDCCAG3.1	7.74E-14	-0.12996	0.049	0.104	1.16E-09	AC-G	SDCCAG3
ZNF775.1	7.89E-14	-0.1117	0.049	0.103	1.18E-09	AC-G	ZNF775
DENND5B.1	7.95E-14	-0.10397	0.049	0.105	1.19E-09	AC-G	DENND5B
MAPK8IP3.	8.17E-14	-0.11651	0.043	0.097	1.22E-09	AC-G	MAPK8IP3
RP11-212F	8.30E-14	-0.11854	0.045	0.1	1.24E-09	AC-G	RP11-212P7.2
GAS2L1.1	8.33E-14	-0.11255	0.093	0.163	1.24E-09	AC-G	GAS2L1
CAMTA1.1	8.44E-14	-0.11328	0.364	0.466	1.26E-09	AC-G	CAMTA1
LINC00665	8.66E-14	0.279664	0.236	0.176	1.29E-09	AC-G	LINC00665
NFATC2	8.71E-14	-0.17809	0.014	0.053	1.30E-09	AC-G	NFATC2
FURIN.1	8.86E-14	-0.15102	0.031	0.079	1.32E-09	AC-G	FURIN
ZNF439.1	9.16E-14	-0.1067	0.09	0.159	1.37E-09	AC-G	ZNF439

RHBDD2.1	9.26E-14	0.280465	0.481	0.455	1.38E-09	AC-G	RHBDD2
PPARG	9.33E-14	-0.12615	0.001	0.029	1.39E-09	AC-G	PPARG
HNRNPLL.1	9.34E-14	-0.17084	0.129	0.203	1.40E-09	AC-G	HNRNPLL
DAPP1	9.47E-14	-0.14481	0.002	0.033	1.41E-09	AC-G	DAPP1
CNTRL.1	9.47E-14	-0.19691	0.068	0.128	1.41E-09	AC-G	CNTRL
MOAP1.1	9.64E-14	-0.13007	0.149	0.231	1.44E-09	AC-G	MOAP1
YEATS4.1	9.73E-14	-0.19643	0.183	0.265	1.45E-09	AC-G	YEATS4
MASP1	9.91E-14	0.174914	0.07	0.034	1.48E-09	AC-G	MASP1
UBE2W	9.99E-14	-0.11138	0.146	0.226	1.49E-09	AC-G	UBE2W
NFAM1	1.01E-13	-0.14809	0.006	0.039	1.51E-09	AC-G	NFAM1
DLG1.1	1.03E-13	-0.14027	0.106	0.177	1.53E-09	AC-G	DLG1
TMEM33.1	1.06E-13	-0.12264	0.21	0.3	1.58E-09	AC-G	TMEM33
GJB1	1.06E-13	-0.19912	0.001	0.029	1.58E-09	AC-G	GJB1
TUBB2A.1	1.07E-13	-0.17329	0.416	0.49	1.59E-09	AC-G	TUBB2A
HECTD4	1.09E-13	-0.11309	0.045	0.099	1.62E-09	AC-G	HECTD4
CCR5	1.10E-13	-0.12267	0.001	0.03	1.65E-09	AC-G	CCR5
DCTN4.1	1.13E-13	-0.14829	0.122	0.198	1.68E-09	AC-G	DCTN4
TM4SF1.1	1.17E-13	0.37492	0.155	0.104	1.74E-09	AC-G	TM4SF1
FADS3.1	1.17E-13	0.199153	0.197	0.14	1.75E-09	AC-G	FADS3
CLSPN.1	1.19E-13	-0.13875	0.025	0.069	1.77E-09	AC-G	CLSPN
DENR.1	1.22E-13	-0.1123	0.24	0.336	1.82E-09	AC-G	DENR
ENTPD4.1	1.23E-13	-0.1187	0.048	0.102	1.84E-09	AC-G	ENTPD4
TBCA.1	1.25E-13	0.240653	0.727	0.707	1.87E-09	AC-G	TBCA
CTD-2090I	1.25E-13	0.185847	0.089	0.046	1.87E-09	AC-G	CTD-2090I13.1
IFFO1.1	1.26E-13	-0.12831	0.068	0.128	1.88E-09	AC-G	IFFO1
RP11-676J	1.27E-13	-0.10488	0.004	0.036	1.89E-09	AC-G	RP11-676J15.1
PIF1	1.27E-13	-0.11963	0.017	0.057	1.90E-09	AC-G	PIF1
HSP90AA1.	1.27E-13	0.123957	0.829	0.9	1.90E-09	AC-G	HSP90AA1
PHF6.1	1.29E-13	-0.1563	0.111	0.181	1.93E-09	AC-G	PHF6
LCORL	1.31E-13	-0.11424	0.051	0.107	1.96E-09	AC-G	LCORL
AMDHD2.1	1.37E-13	-0.16661	0.072	0.133	2.05E-09	AC-G	AMDHD2
ABHD8.1	1.39E-13	-0.13796	0.089	0.156	2.08E-09	AC-G	ABHD8
STAT5A.1	1.40E-13	-0.15828	0.018	0.058	2.09E-09	AC-G	STAT5A
KIF3B.1	1.43E-13	-0.1046	0.076	0.14	2.14E-09	AC-G	KIF3B
CTTN.1	1.43E-13	0.300364	0.279	0.22	2.14E-09	AC-G	CTTN
ERF.1	1.44E-13	-0.12114	0.114	0.189	2.15E-09	AC-G	ERF
PTPRD.1	1.48E-13	-0.17977	0.033	0.08	2.21E-09	AC-G	PTPRD
TRIM14.1	1.49E-13	-0.13898	0.048	0.101	2.23E-09	AC-G	TRIM14
RAP2A	1.51E-13	-0.12411	0.078	0.142	2.26E-09	AC-G	RAP2A
PCSK7.1	1.52E-13	-0.15523	0.206	0.296	2.27E-09	AC-G	PCSK7
SRCIN1	1.53E-13	-0.14591	0.004	0.036	2.29E-09	AC-G	SRCIN1
GTDC1	1.54E-13	-0.12985	0.053	0.108	2.30E-09	AC-G	GTDC1
C19orf25.	1.54E-13	-0.13716	0.221	0.314	2.30E-09	AC-G	C19orf25
LTB	1.55E-13	-0.20176	0.005	0.038	2.31E-09	AC-G	LTB
DNALI1.1	1.55E-13	0.318365	0.155	0.105	2.32E-09	AC-G	DNALI1
HAPLN2	1.56E-13	-0.17514	0.005	0.038	2.33E-09	AC-G	HAPLN2
CDC27.1	1.63E-13	-0.12517	0.104	0.174	2.44E-09	AC-G	CDC27
LNPEP.1	1.66E-13	-0.16405	0.073	0.134	2.49E-09	AC-G	LNPEP
KLF5	1.68E-13	0.112985	0.051	0.022	2.51E-09	AC-G	KLF5
KPNA3.1	1.71E-13	-0.16876	0.186	0.269	2.56E-09	AC-G	KPNA3
MLF1	1.73E-13	0.281839	0.29	0.232	2.59E-09	AC-G	MLF1

ALOX15B	1.74E-13	-0.15368	0.002	0.031	2.61E-09	AC-G	ALOX15B
PCMT1.1	1.77E-13	0.241929	0.545	0.54	2.64E-09	AC-G	PCMT1
STAU2.1	1.77E-13	-0.11172	0.113	0.185	2.64E-09	AC-G	STAU2
SEPT11.1	1.78E-13	-0.13435	0.229	0.317	2.65E-09	AC-G	11-Sep
LILRB3	1.79E-13	-0.16829	0.01	0.046	2.67E-09	AC-G	LILRB3
TNS3.1	1.79E-13	-0.20642	0.034	0.081	2.68E-09	AC-G	TNS3
MCM2	1.80E-13	-0.12454	0.027	0.073	2.69E-09	AC-G	MCM2
RAB4A.1	1.84E-13	-0.13725	0.262	0.356	2.76E-09	AC-G	RAB4A
ZNF639.1	1.89E-13	-0.10882	0.082	0.147	2.83E-09	AC-G	ZNF639
NAA30.1	1.92E-13	-0.11478	0.043	0.095	2.87E-09	AC-G	NAA30
SUGP2	1.95E-13	-0.11087	0.173	0.26	2.91E-09	AC-G	SUGP2
ABI2	1.96E-13	-0.12603	0.283	0.377	2.93E-09	AC-G	ABI2
LMO4.1	1.97E-13	-0.11028	0.396	0.494	2.94E-09	AC-G	LMO4
NUP107.1	2.02E-13	-0.19208	0.159	0.236	3.02E-09	AC-G	NUP107
D2HGDH.1	2.03E-13	-0.10858	0.06	0.118	3.03E-09	AC-G	D2HGDH
TMEM63A	2.04E-13	-0.15858	0.031	0.078	3.05E-09	AC-G	TMEM63A
PLEKHA1.1	2.09E-13	-0.16342	0.053	0.108	3.12E-09	AC-G	PLEKHA1
USP46.1	2.11E-13	-0.12874	0.084	0.148	3.15E-09	AC-G	USP46
COPS6.1	2.14E-13	0.265474	0.608	0.603	3.20E-09	AC-G	COPS6
EIF4EBP3	2.14E-13	0.103964	0.023	0.006	3.20E-09	AC-G	EIF4EBP3
SUSD4	2.17E-13	0.148428	0.071	0.035	3.24E-09	AC-G	SUSD4
RASGRP2.1	2.18E-13	-0.15855	0.018	0.058	3.26E-09	AC-G	RASGRP2
CASP1.1	2.19E-13	-0.17725	0.164	0.238	3.27E-09	AC-G	CASP1
DHTKD1	2.27E-13	-0.14133	0.074	0.135	3.39E-09	AC-G	DHTKD1
GPSM1.1	2.32E-13	-0.11806	0.033	0.081	3.47E-09	AC-G	GPSM1
RP11-403I	2.35E-13	-0.19639	0.006	0.039	3.51E-09	AC-G	RP11-403I13.5
MAP2K1.1	2.35E-13	-0.11648	0.195	0.283	3.51E-09	AC-G	MAP2K1
NDUFB4.1	2.41E-13	0.244797	0.673	0.681	3.59E-09	AC-G	NDUFB4
ZNF195.1	2.41E-13	-0.14801	0.105	0.173	3.60E-09	AC-G	ZNF195
GNS.1	2.42E-13	-0.13792	0.135	0.212	3.61E-09	AC-G	GNS
IKBKE.1	2.51E-13	-0.15848	0.021	0.063	3.74E-09	AC-G	IKBKE
NDUFB7.1	2.53E-13	0.248367	0.724	0.719	3.78E-09	AC-G	NDUFB7
NKIRAS2	2.53E-13	-0.11821	0.191	0.277	3.79E-09	AC-G	NKIRAS2
SESN2.1	2.53E-13	0.17421	0.149	0.095	3.79E-09	AC-G	SESN2
STAG2	2.61E-13	-0.17077	0.174	0.256	3.90E-09	AC-G	STAG2
EGR1	2.66E-13	-0.12772	0.414	0.512	3.98E-09	AC-G	EGR1
AP000439.	2.68E-13	-0.16803	0.002	0.032	4.01E-09	AC-G	AP000439.1
CEACAM4	2.70E-13	-0.11924	0.001	0.028	4.04E-09	AC-G	CEACAM4
RP11-212I	2.71E-13	-0.13587	0	0.027	4.05E-09	AC-G	RP11-212I21.4
CAMSAP2	2.81E-13	-0.10313	0.093	0.16	4.19E-09	AC-G	CAMSAP2
FFAR4	2.82E-13	-0.14499	0.004	0.035	4.22E-09	AC-G	FFAR4
XPO6.1	2.88E-13	-0.10678	0.065	0.125	4.30E-09	AC-G	XPO6
KLHL2.1	2.92E-13	-0.10198	0.042	0.093	4.36E-09	AC-G	KLHL2
GOT2	2.94E-13	-0.10295	0.133	0.209	4.39E-09	AC-G	GOT2
KBTBD2.1	2.96E-13	-0.11503	0.066	0.124	4.42E-09	AC-G	KBTBD2
KCNK6	3.02E-13	-0.1611	0.01	0.045	4.52E-09	AC-G	KCNK6
ICK.1	3.14E-13	-0.12203	0.042	0.093	4.69E-09	AC-G	ICK
SPG7.1	3.14E-13	-0.17692	0.173	0.254	4.69E-09	AC-G	SPG7
ARHGAP23	3.15E-13	-0.10852	0.018	0.059	4.70E-09	AC-G	ARHGAP23
KLF12.1	3.18E-13	-0.10219	0.042	0.093	4.75E-09	AC-G	KLF12
CNOT1.1	3.30E-13	-0.15564	0.094	0.16	4.93E-09	AC-G	CNOT1

TOR1A.1	3.30E-13	-0.10681	0.191	0.275	4.93E-09	AC-G	TOR1A
ULK3	3.34E-13	-0.1469	0.092	0.156	5.00E-09	AC-G	ULK3
SARS2.1	3.40E-13	-0.10056	0.034	0.083	5.08E-09	AC-G	SARS2
SRRM3.1	3.46E-13	-0.16815	0.033	0.08	5.17E-09	AC-G	SRRM3
TACO1.1	3.47E-13	-0.11056	0.114	0.186	5.19E-09	AC-G	TACO1
GRIPAP1.1	3.49E-13	-0.12416	0.13	0.205	5.21E-09	AC-G	GRIPAP1
ICAM2	3.54E-13	-0.17769	0.007	0.04	5.28E-09	AC-G	ICAM2
GAB3	3.72E-13	-0.13886	0.006	0.038	5.55E-09	AC-G	GAB3
EMX2	3.72E-13	0.150282	0.053	0.024	5.56E-09	AC-G	EMX2
RP11-536K	3.80E-13	-0.20062	0.008	0.043	5.67E-09	AC-G	RP11-536K7.3
NFKB1.1	3.83E-13	-0.21782	0.059	0.113	5.72E-09	AC-G	NFKB1
TTC26.1	3.83E-13	0.165749	0.079	0.042	5.72E-09	AC-G	TTC26
SCLT1.1	3.91E-13	-0.12947	0.03	0.076	5.85E-09	AC-G	SCLT1
RFTN2.1	3.98E-13	-0.15489	0.077	0.137	5.95E-09	AC-G	RFTN2
VCPIP1.1	3.99E-13	-0.11795	0.046	0.098	5.97E-09	AC-G	VCPIP1
ILKAP.1	4.06E-13	-0.14646	0.162	0.242	6.07E-09	AC-G	ILKAP
KDM2A	4.17E-13	-0.12254	0.075	0.137	6.23E-09	AC-G	KDM2A
SSR1	4.29E-13	-0.12274	0.374	0.487	6.41E-09	AC-G	SSR1
TLR4	4.31E-13	-0.14202	0.011	0.047	6.44E-09	AC-G	TLR4
KCNE3	4.33E-13	-0.11796	0.002	0.031	6.47E-09	AC-G	KCNE3
FBRSL1	4.37E-13	-0.10986	0.02	0.062	6.52E-09	AC-G	FBRSL1
UBR4.1	4.42E-13	-0.10775	0.089	0.156	6.61E-09	AC-G	UBR4
ZNF628	4.48E-13	-0.10315	0.018	0.058	6.69E-09	AC-G	ZNF628
NUMB.1	4.50E-13	-0.17246	0.113	0.182	6.72E-09	AC-G	NUMB
ATP6V0A2	4.51E-13	-0.10272	0.051	0.104	6.74E-09	AC-G	ATP6V0A2
KATNB1.1	4.53E-13	-0.11693	0.07	0.129	6.77E-09	AC-G	KATNB1
MEF2D	4.53E-13	-0.16712	0.034	0.081	6.77E-09	AC-G	MEF2D
RAB3IP	4.62E-13	-0.22835	0.183	0.259	6.91E-09	AC-G	RAB3IP
ASF1B.1	4.87E-13	-0.12448	0.024	0.067	7.28E-09	AC-G	ASF1B
TLE2.1	4.91E-13	-0.12206	0.047	0.099	7.34E-09	AC-G	TLE2
TMEM116	5.01E-13	0.197376	0.099	0.056	7.48E-09	AC-G	TMEM116
SPAG5	5.04E-13	-0.10732	0.013	0.05	7.53E-09	AC-G	SPAG5
SURF2.1	5.06E-13	-0.10334	0.164	0.245	7.56E-09	AC-G	SURF2
SCYL2.1	5.09E-13	-0.11904	0.082	0.145	7.61E-09	AC-G	SCYL2
SLC38A7	5.09E-13	-0.13211	0.061	0.117	7.61E-09	AC-G	SLC38A7
PTDSS1.1	5.13E-13	-0.13594	0.139	0.214	7.66E-09	AC-G	PTDSS1
RC3H1.1	5.16E-13	-0.13125	0.054	0.11	7.71E-09	AC-G	RC3H1
PDZRN4	5.17E-13	-0.14127	0.009	0.044	7.73E-09	AC-G	PDZRN4
SLC18B1.1	5.18E-13	-0.12258	0.119	0.19	7.74E-09	AC-G	SLC18B1
FANCA	5.30E-13	-0.10772	0.023	0.065	7.92E-09	AC-G	FANCA
TRAPPC11	5.33E-13	-0.10491	0.042	0.093	7.96E-09	AC-G	TRAPPC11
RP11-514C	5.37E-13	-0.1239	0.003	0.032	8.03E-09	AC-G	RP11-514O12.4
ISCU.1	5.38E-13	-0.10389	0.469	0.585	8.04E-09	AC-G	ISCU
DIP2C.1	5.44E-13	-0.13693	0.042	0.093	8.13E-09	AC-G	DIP2C
CD72	5.47E-13	-0.17998	0.018	0.057	8.17E-09	AC-G	CD72
C19orf48.	5.52E-13	-0.11248	0.161	0.24	8.25E-09	AC-G	C19orf48
RFWD2.1	5.56E-13	-0.14754	0.108	0.176	8.30E-09	AC-G	RFWD2
SIRT2.1	5.73E-13	-0.14279	0.332	0.432	8.56E-09	AC-G	SIRT2
DCAF13	5.76E-13	0.270722	0.365	0.308	8.60E-09	AC-G	DCAF13
XBP1.1	5.91E-13	-0.14414	0.29	0.387	8.83E-09	AC-G	XBP1
PACS1.1	5.94E-13	-0.11025	0.051	0.105	8.88E-09	AC-G	PACS1

ATF7IP.1	5.96E-13	-0.15201	0.065	0.121	8.90E-09	AC-G	ATF7IP
NADSYN1.1	6.04E-13	-0.10093	0.07	0.13	9.02E-09	AC-G	NADSYN1
PTCRA	6.04E-13	-0.12941	0.001	0.028	9.03E-09	AC-G	PTCRA
VAX2.1	6.09E-13	-0.12443	0.039	0.088	9.10E-09	AC-G	VAX2
LILRA4	6.15E-13	-0.14523	0.001	0.028	9.20E-09	AC-G	LILRA4
TNFAIP3.1	6.32E-13	-0.3042	0.112	0.173	9.44E-09	AC-G	TNFAIP3
BNIP3	6.41E-13	0.332704	0.263	0.205	9.57E-09	AC-G	BNIP3
GMFB	6.44E-13	-0.12059	0.144	0.221	9.63E-09	AC-G	GMFB
RUNDC3A.1	6.54E-13	-0.1577	0.053	0.106	9.77E-09	AC-G	RUNDC3A
USP7.1	6.54E-13	-0.10995	0.083	0.145	9.78E-09	AC-G	USP7
INPP4A.1	6.64E-13	-0.12395	0.055	0.11	9.93E-09	AC-G	INPP4A
PIK3CD	6.76E-13	-0.13268	0.005	0.036	1.01E-08	AC-G	PIK3CD
SUPT16H.1	6.78E-13	-0.12099	0.178	0.262	1.01E-08	AC-G	SUPT16H
TMEM125	6.89E-13	-0.1676	0	0.026	1.03E-08	AC-G	TMEM125
ZNF37A.1	7.02E-13	-0.11587	0.086	0.149	1.05E-08	AC-G	ZNF37A
MICU1.1	7.04E-13	-0.11873	0.082	0.144	1.05E-08	AC-G	MICU1
VPS18	7.16E-13	-0.10752	0.038	0.087	1.07E-08	AC-G	VPS18
AGAP3	7.18E-13	-0.15531	0.055	0.108	1.07E-08	AC-G	AGAP3
TSPAN6.1	7.32E-13	0.335935	0.194	0.141	1.09E-08	AC-G	TSPAN6
TMEM134.1	7.49E-13	-0.10859	0.235	0.324	1.12E-08	AC-G	TMEM134
TUBB3	7.51E-13	0.110389	0.008	0.001	1.12E-08	AC-G	TUBB3
SP9	7.64E-13	-0.1329	0.011	0.046	1.14E-08	AC-G	SP9
SNAI1	7.75E-13	-0.19052	0.014	0.051	1.16E-08	AC-G	SNAI1
AKIRIN1.1	7.75E-13	-0.11329	0.282	0.385	1.16E-08	AC-G	AKIRIN1
FICD	7.91E-13	0.178324	0.116	0.071	1.18E-08	AC-G	FICD
BROX.1	8.00E-13	-0.14829	0.167	0.248	1.20E-08	AC-G	BROX
IRF2BP2.1	8.11E-13	-0.13882	0.238	0.331	1.21E-08	AC-G	IRF2BP2
DAP.1	8.25E-13	-0.10648	0.203	0.291	1.23E-08	AC-G	DAP
TMOD3.1	8.46E-13	-0.11263	0.089	0.155	1.26E-08	AC-G	TMOD3
MICU3.1	8.53E-13	-0.16339	0.042	0.09	1.27E-08	AC-G	MICU3
MGAT5.1	8.59E-13	-0.14202	0.03	0.075	1.28E-08	AC-G	MGAT5
RP11-445F	8.70E-13	-0.12246	0.004	0.033	1.30E-08	AC-G	RP11-445F12.1
DERL2.1	8.74E-13	0.314722	0.396	0.343	1.31E-08	AC-G	DERL2
PSTPIP1	8.74E-13	-0.16151	0.011	0.047	1.31E-08	AC-G	PSTPIP1
PLEKHF2.1	8.82E-13	-0.14895	0.078	0.138	1.32E-08	AC-G	PLEKHF2
RAB42.1	8.88E-13	-0.14614	0.017	0.055	1.33E-08	AC-G	RAB42
CASD1.1	8.94E-13	-0.12745	0.037	0.085	1.34E-08	AC-G	CASD1
WDR91.1	9.07E-13	-0.17608	0.044	0.094	1.36E-08	AC-G	WDR91
UQCRQ.1	9.10E-13	0.209811	0.768	0.805	1.36E-08	AC-G	UQCRQ
TRIM44.1	9.14E-13	-0.12727	0.212	0.301	1.37E-08	AC-G	TRIM44
OTUD5.1	9.30E-13	-0.1283	0.082	0.145	1.39E-08	AC-G	OTUD5
BCL3.1	9.36E-13	-0.12134	0.037	0.085	1.40E-08	AC-G	BCL3
SLC25A33.	9.37E-13	-0.11257	0.151	0.231	1.40E-08	AC-G	SLC25A33
SLC46A1.1	9.47E-13	-0.10857	0.025	0.068	1.41E-08	AC-G	SLC46A1
OPN3.1	9.55E-13	-0.1267	0.033	0.079	1.43E-08	AC-G	OPN3
SBF1.1	9.58E-13	-0.11633	0.029	0.074	1.43E-08	AC-G	SBF1
MLXIPL	9.78E-13	-0.17874	0.01	0.045	1.46E-08	AC-G	MLXIPL
TESK1.1	9.82E-13	-0.10859	0.04	0.089	1.47E-08	AC-G	TESK1
LPGAT1.1	9.90E-13	-0.12972	0.078	0.138	1.48E-08	AC-G	LPGAT1
PHF11.1	9.92E-13	-0.1549	0.089	0.152	1.48E-08	AC-G	PHF11
TRIM22.1	9.95E-13	-0.23079	0.085	0.144	1.49E-08	AC-G	TRIM22

MAML3	1.01E-12	-0.13495	0.008	0.041	1.51E-08	AC-G	MAML3
SCAF4	1.04E-12	-0.1269	0.087	0.151	1.56E-08	AC-G	SCAF4
CTA-384D8	1.09E-12	-0.11761	0.001	0.028	1.63E-08	AC-G	CTA-384D8.34
VPS36.1	1.10E-12	-0.11455	0.138	0.213	1.65E-08	AC-G	VPS36
PIGS.1	1.11E-12	-0.10436	0.122	0.194	1.65E-08	AC-G	PIGS
GALNT10.1	1.12E-12	-0.12329	0.051	0.103	1.67E-08	AC-G	GALNT10
H2AFX.1	1.12E-12	-0.21699	0.162	0.231	1.67E-08	AC-G	H2AFX
TSPAN33	1.14E-12	-0.14623	0.013	0.048	1.70E-08	AC-G	TSPAN33
CARNS1	1.14E-12	-0.21884	0.004	0.033	1.70E-08	AC-G	CARNS1
NPPA	1.14E-12	-0.14889	0.004	0.033	1.71E-08	AC-G	NPPA
MAZ.1	1.17E-12	-0.11793	0.181	0.264	1.74E-08	AC-G	MAZ
CD2AP.1	1.18E-12	-0.11447	0.041	0.09	1.76E-08	AC-G	CD2AP
DUSP14.1	1.18E-12	0.348352	0.224	0.17	1.77E-08	AC-G	DUSP14
NHSL2	1.20E-12	0.124485	0.086	0.045	1.79E-08	AC-G	NHSL2
PICK1.1	1.25E-12	-0.17129	0.046	0.096	1.87E-08	AC-G	PICK1
LTB4R	1.25E-12	-0.1178	0.007	0.039	1.87E-08	AC-G	LTB4R
RP11-353N	1.28E-12	-0.1241	0	0.026	1.92E-08	AC-G	RP11-353N4.5
APOBEC3G.	1.31E-12	-0.18498	0.071	0.127	1.96E-08	AC-G	APOBEC3G
MAN1A2.1	1.32E-12	-0.11191	0.141	0.216	1.97E-08	AC-G	MAN1A2
SART1.1	1.34E-12	-0.13519	0.137	0.21	2.00E-08	AC-G	SART1
ZNF550.1	1.34E-12	0.158058	0.079	0.042	2.01E-08	AC-G	ZNF550
PIAS1.1	1.35E-12	-0.17186	0.104	0.169	2.02E-08	AC-G	PIAS1
LHFP.1	1.38E-12	0.391133	0.281	0.222	2.07E-08	AC-G	LHFP
MFSD7	1.42E-12	-0.11237	0.006	0.037	2.12E-08	AC-G	MFSD7
RALGAPA1.	1.47E-12	-0.16018	0.08	0.14	2.19E-08	AC-G	RALGAPA1
LINC01152	1.48E-12	0.202753	0.042	0.017	2.21E-08	AC-G	LINC01152
LRCH3.1	1.50E-12	-0.10863	0.085	0.148	2.24E-08	AC-G	LRCH3
CAP1	1.51E-12	-0.12371	0.497	0.611	2.26E-08	AC-G	CAP1
TMEM151A	1.52E-12	-0.14035	0.013	0.048	2.27E-08	AC-G	TMEM151A
FBXW2.1	1.53E-12	-0.12844	0.13	0.205	2.29E-08	AC-G	FBXW2
MED24.1	1.57E-12	-0.11699	0.111	0.179	2.35E-08	AC-G	MED24
PHGDH.1	1.58E-12	0.233835	0.25	0.19	2.36E-08	AC-G	PHGDH
TRAPPC10.	1.61E-12	-0.136	0.05	0.101	2.40E-08	AC-G	TRAPPC10
AATK	1.65E-12	-0.19325	0.007	0.038	2.46E-08	AC-G	AATK
SELO.1	1.65E-12	-0.11457	0.028	0.072	2.47E-08	AC-G	SELO
WDFY4	1.67E-12	-0.13057	0.003	0.031	2.50E-08	AC-G	WDFY4
TMEM144.1	1.69E-12	-0.32919	0.039	0.085	2.53E-08	AC-G	TMEM144
DNAJC13.1	1.71E-12	-0.13317	0.034	0.08	2.56E-08	AC-G	DNAJC13
PNPLA2.1	1.72E-12	-0.14511	0.149	0.224	2.56E-08	AC-G	PNPLA2
ZYX.1	1.77E-12	-0.13928	0.325	0.417	2.64E-08	AC-G	ZYX
SULF2.1	1.78E-12	-0.14023	0.105	0.17	2.66E-08	AC-G	SULF2
R3HDM4.1	1.79E-12	-0.11114	0.138	0.212	2.68E-08	AC-G	R3HDM4
ZYG11B.1	1.79E-12	-0.12475	0.095	0.159	2.68E-08	AC-G	ZYG11B
AP2B1	1.82E-12	-0.10467	0.264	0.355	2.71E-08	AC-G	AP2B1
TCTN1	1.90E-12	0.363869	0.255	0.203	2.84E-08	AC-G	TCTN1
SYAP1	1.92E-12	-0.14713	0.308	0.402	2.87E-08	AC-G	SYAP1
PLVAP	1.93E-12	-0.15068	0.005	0.035	2.89E-08	AC-G	PLVAP
TMEM87B.1	1.95E-12	-0.12403	0.03	0.074	2.91E-08	AC-G	TMEM87B
MAPK13	1.97E-12	-0.11922	0.005	0.036	2.94E-08	AC-G	MAPK13
FLII.1	1.98E-12	-0.12799	0.128	0.198	2.95E-08	AC-G	FLII
AGO1.1	2.02E-12	-0.12844	0.048	0.098	3.01E-08	AC-G	AGO1

RNF13.1	2.03E-12	-0.1446	0.4	0.496	3.03E-08	AC-G	RNF13
RP11-597D	2.03E-12	-0.15048	0.001	0.027	3.03E-08	AC-G	RP11-597D13.8
RNF5	2.03E-12	-0.11712	0.308	0.406	3.04E-08	AC-G	RNF5
CC2D1A	2.04E-12	-0.12852	0.126	0.198	3.05E-08	AC-G	CC2D1A
CEP135.1	2.05E-12	-0.12688	0.04	0.088	3.07E-08	AC-G	CEP135
FAR1.1	2.08E-12	-0.11926	0.085	0.147	3.10E-08	AC-G	FAR1
TMEM50B.1	2.08E-12	0.291449	0.347	0.294	3.11E-08	AC-G	TMEM50B
XIAP.1	2.09E-12	-0.13434	0.149	0.226	3.12E-08	AC-G	XIAP
TMEM57.1	2.12E-12	-0.15895	0.118	0.186	3.17E-08	AC-G	TMEM57
TNRC6C.1	2.14E-12	-0.10867	0.114	0.183	3.20E-08	AC-G	TNRC6C
MAST4	2.14E-12	0.169816	0.084	0.046	3.20E-08	AC-G	MAST4
RUFY1.1	2.16E-12	-0.10368	0.098	0.163	3.23E-08	AC-G	RUFY1
SLCO5A1	2.17E-12	-0.12251	0.012	0.047	3.24E-08	AC-G	SLCO5A1
CHPF.1	2.19E-12	0.278112	0.299	0.251	3.27E-08	AC-G	CHPF
FAM122A.1	2.24E-12	-0.14224	0.091	0.154	3.35E-08	AC-G	FAM122A
CROT.1	2.26E-12	0.311604	0.176	0.124	3.37E-08	AC-G	CROT
FAM222A	2.31E-12	-0.11069	0.005	0.034	3.45E-08	AC-G	FAM222A
MOGS	2.31E-12	-0.12048	0.074	0.133	3.46E-08	AC-G	MOGS
TIAM1	2.32E-12	-0.12719	0.039	0.087	3.47E-08	AC-G	TIAM1
SESN1.1	2.32E-12	-0.22265	0.059	0.11	3.47E-08	AC-G	SESN1
FAM20A.1	2.34E-12	-0.20278	0.026	0.066	3.49E-08	AC-G	FAM20A
CCDC6	2.36E-12	-0.10991	0.063	0.12	3.53E-08	AC-G	CCDC6
KCNQ1OT1.	2.40E-12	-0.19477	0.213	0.291	3.59E-08	AC-G	KCNQ1OT1
DTNBP1.1	2.47E-12	-0.13988	0.144	0.219	3.68E-08	AC-G	DTNBP1
PTBP1.1	2.54E-12	-0.10432	0.243	0.339	3.80E-08	AC-G	PTBP1
NCEH1	2.55E-12	-0.12736	0.031	0.075	3.81E-08	AC-G	NCEH1
TNRC18.1	2.61E-12	-0.12803	0.036	0.082	3.89E-08	AC-G	TNRC18
COPA.1	2.62E-12	-0.11653	0.237	0.328	3.92E-08	AC-G	COPA
ISG20L2	2.62E-12	-0.14036	0.161	0.237	3.92E-08	AC-G	ISG20L2
PWP2.1	2.65E-12	-0.19496	0.076	0.133	3.96E-08	AC-G	PWP2
ACOT9.1	2.67E-12	-0.12153	0.124	0.193	3.99E-08	AC-G	ACOT9
RP11-347F	2.69E-12	-0.26845	0.029	0.071	4.01E-08	AC-G	RP11-347P5.1
BAK1.1	2.73E-12	-0.13557	0.069	0.125	4.07E-08	AC-G	BAK1
IL27RA	2.78E-12	-0.11703	0.022	0.062	4.16E-08	AC-G	IL27RA
DUSP8	2.86E-12	-0.12353	0.019	0.057	4.28E-08	AC-G	DUSP8
MPZL1.1	2.94E-12	-0.15816	0.184	0.261	4.40E-08	AC-G	MPZL1
ASNS.1	2.97E-12	0.237841	0.226	0.167	4.43E-08	AC-G	ASNS
GOPC.1	2.98E-12	-0.11274	0.188	0.271	4.45E-08	AC-G	GOPC
SMAP1.1	3.00E-12	-0.12431	0.225	0.312	4.48E-08	AC-G	SMAP1
CPOX.1	3.01E-12	-0.10485	0.033	0.078	4.50E-08	AC-G	CPOX
CHCHD7	3.01E-12	-0.13567	0.22	0.299	4.50E-08	AC-G	CHCHD7
REEP5.1	3.03E-12	0.223447	0.596	0.582	4.53E-08	AC-G	REEP5
PAPD5.1	3.19E-12	-0.11204	0.04	0.088	4.77E-08	AC-G	PAPD5
PTPN4.1	3.19E-12	-0.1193	0.057	0.111	4.77E-08	AC-G	PTPN4
RIF1	3.25E-12	-0.11688	0.158	0.232	4.86E-08	AC-G	RIF1
PPP1R16A	3.26E-12	0.244072	0.214	0.16	4.88E-08	AC-G	PPP1R16A
MPG.1	3.28E-12	-0.10371	0.265	0.361	4.90E-08	AC-G	MPG
DLD.1	3.35E-12	0.319384	0.386	0.336	5.00E-08	AC-G	DLD
PIEZO1.1	3.39E-12	-0.10733	0.032	0.077	5.06E-08	AC-G	PIEZO1
ZDHHC3.1	3.45E-12	-0.11666	0.139	0.211	5.15E-08	AC-G	ZDHHC3
KDM4B.1	3.48E-12	-0.12374	0.082	0.142	5.20E-08	AC-G	KDM4B

ANTXR1.1	3.60E-12	-0.12124	0.049	0.099	5.38E-08	AC-G	ANTXR1
SCARF1	3.62E-12	-0.10969	0.003	0.031	5.41E-08	AC-G	SCARF1
RAPGEF5	3.62E-12	-0.19449	0.013	0.048	5.41E-08	AC-G	RAPGEF5
PLAGL1	3.64E-12	-0.13341	0.012	0.046	5.44E-08	AC-G	PLAGL1
KB-1507C5	3.67E-12	-0.12346	0.002	0.028	5.49E-08	AC-G	KB-1507C5.4
KAT2B.1	3.70E-12	-0.10015	0.034	0.079	5.54E-08	AC-G	KAT2B
HRCT1	3.75E-12	0.175415	0.052	0.024	5.61E-08	AC-G	HRCT1
EPS15.1	3.78E-12	-0.18704	0.099	0.16	5.65E-08	AC-G	EPS15
RASA3.1	3.81E-12	-0.15341	0.052	0.102	5.70E-08	AC-G	RASA3
TNFRSF11A	3.90E-12	-0.11121	0.002	0.029	5.82E-08	AC-G	TNFRSF11A
ERBB4.1	3.90E-12	-0.12793	0.025	0.065	5.83E-08	AC-G	ERBB4
FAM149A	4.02E-12	-0.13448	0.007	0.037	6.01E-08	AC-G	FAM149A
ADIPOR2.1	4.04E-12	-0.19995	0.099	0.16	6.04E-08	AC-G	ADIPOR2
CSTB.1	4.04E-12	0.197876	0.672	0.693	6.04E-08	AC-G	CSTB
GUCD1.1	4.04E-12	-0.11403	0.074	0.132	6.04E-08	AC-G	GUCD1
NME7.1	4.05E-12	0.277084	0.206	0.152	6.05E-08	AC-G	NME7
KPNA4.1	4.08E-12	-0.11298	0.122	0.191	6.10E-08	AC-G	KPNA4
CELF1.1	4.11E-12	-0.11023	0.233	0.322	6.14E-08	AC-G	CELF1
NDRG4.1	4.14E-12	-0.11951	0.106	0.171	6.18E-08	AC-G	NDRG4
FAM84B.1	4.24E-12	-0.13303	0.046	0.095	6.34E-08	AC-G	FAM84B
WTAP.1	4.30E-12	0.300874	0.428	0.419	6.42E-08	AC-G	WTAP
TMEM100.1	4.35E-12	0.225349	0.13	0.081	6.50E-08	AC-G	TMEM100
MDM2.1	4.43E-12	-0.17039	0.147	0.218	6.62E-08	AC-G	MDM2
EIF4E.1	4.49E-12	-0.10753	0.403	0.505	6.71E-08	AC-G	EIF4E
TMEM208.1	4.61E-12	0.269184	0.491	0.462	6.89E-08	AC-G	TMEM208
TMA7.1	4.74E-12	0.186992	0.815	0.858	7.08E-08	AC-G	TMA7
SIGLEC1	4.81E-12	-0.14184	0.005	0.034	7.19E-08	AC-G	SIGLEC1
TOR4A	4.81E-12	-0.10429	0.003	0.03	7.19E-08	AC-G	TOR4A
SUMF2	4.83E-12	-0.11881	0.265	0.358	7.21E-08	AC-G	SUMF2
GALNT7.1	4.83E-12	-0.10947	0.048	0.098	7.22E-08	AC-G	GALNT7
ATP9B	4.87E-12	-0.11689	0.039	0.086	7.28E-08	AC-G	ATP9B
PHLPP1.1	4.94E-12	-0.1125	0.044	0.093	7.39E-08	AC-G	PHLPP1
PNO1	5.00E-12	0.236068	0.251	0.193	7.47E-08	AC-G	PNO1
DCAF5.1	5.08E-12	-0.11729	0.083	0.143	7.59E-08	AC-G	DCAF5
GLUD1.1	5.27E-12	-0.18594	0.187	0.26	7.88E-08	AC-G	GLUD1
ELMO1.1	5.43E-12	-0.20573	0.136	0.2	8.12E-08	AC-G	ELMO1
CERS4.1	5.69E-12	-0.11859	0.139	0.211	8.50E-08	AC-G	CERS4
RCOR2	5.73E-12	-0.10442	0.016	0.052	8.56E-08	AC-G	RCOR2
MIER1.1	5.79E-12	-0.15309	0.118	0.185	8.65E-08	AC-G	MIER1
PWWP2B	5.81E-12	-0.10955	0.008	0.039	8.69E-08	AC-G	PWWP2B
HSPA4L.1	5.95E-12	0.157448	0.109	0.066	8.89E-08	AC-G	HSPA4L
ASIP	6.01E-12	0.112005	0.043	0.018	8.98E-08	AC-G	ASIP
DNM1L.1	6.05E-12	-0.1146	0.169	0.244	9.04E-08	AC-G	DNM1L
DISC1	6.18E-12	-0.11242	0.016	0.052	9.23E-08	AC-G	DISC1
TRAF5	6.27E-12	-0.11078	0.023	0.063	9.37E-08	AC-G	TRAF5
RP11-557H	6.45E-12	-0.15258	0.004	0.031	9.64E-08	AC-G	RP11-557H15.4
RP11-367J	6.50E-12	0.107108	0.027	0.009	9.71E-08	AC-G	RP11-367J11.3
SAMD8.1	6.57E-12	-0.10381	0.091	0.154	9.82E-08	AC-G	SAMD8
C7orf43.1	6.66E-12	-0.10778	0.033	0.076	9.95E-08	AC-G	C7orf43
FBXO34.1	6.70E-12	-0.1305	0.049	0.099	1.00E-07	AC-G	FBXO34
HINT2.1	6.81E-12	-0.10528	0.27	0.361	1.02E-07	AC-G	HINT2

PARP8.1	6.91E-12	-0.14791	0.032	0.075	1.03E-07	AC-G	PARP8
ZC3H4.1	6.94E-12	-0.11042	0.046	0.095	1.04E-07	AC-G	ZC3H4
KIF21B	6.98E-12	-0.11238	0.004	0.032	1.04E-07	AC-G	KIF21B
BAZ1B.1	7.01E-12	-0.11103	0.225	0.312	1.05E-07	AC-G	BAZ1B
TPK1	7.18E-12	-0.12975	0.016	0.051	1.07E-07	AC-G	TPK1
CLCN7.1	7.32E-12	-0.11663	0.101	0.165	1.09E-07	AC-G	CLCN7
TGDS	7.54E-12	0.145553	0.111	0.067	1.13E-07	AC-G	TGDS
IPO7.1	7.56E-12	-0.10115	0.148	0.224	1.13E-07	AC-G	IPO7
DACT3.1	7.62E-12	-0.12051	0.039	0.084	1.14E-07	AC-G	DACT3
STK17A.1	8.20E-12	-0.12237	0.222	0.307	1.23E-07	AC-G	STK17A
MIOS.1	8.21E-12	-0.15042	0.076	0.131	1.23E-07	AC-G	MIOS
LRRK2.1	8.22E-12	-0.14333	0.027	0.067	1.23E-07	AC-G	LRRK2
GRID2	8.27E-12	-0.11728	0.016	0.052	1.24E-07	AC-G	GRID2
HNMT.1	8.35E-12	-0.14622	0.163	0.229	1.25E-07	AC-G	HNMT
SLC7A6.1	8.41E-12	-0.1375	0.031	0.073	1.26E-07	AC-G	SLC7A6
FBXO9.1	8.53E-12	-0.1235	0.145	0.218	1.27E-07	AC-G	FBXO9
THRAP3.1	8.55E-12	-0.10192	0.348	0.455	1.28E-07	AC-G	THRAP3
ANG	8.63E-12	0.148073	0.124	0.076	1.29E-07	AC-G	ANG
REC8.1	8.66E-12	-0.14589	0.039	0.085	1.29E-07	AC-G	REC8
PIK3CG	8.69E-12	-0.1065	0.001	0.026	1.30E-07	AC-G	PIK3CG
YIPF4.1	8.69E-12	-0.13575	0.295	0.39	1.30E-07	AC-G	YIPF4
TMEM220.1	8.76E-12	-0.13212	0.033	0.076	1.31E-07	AC-G	TMEM220
PHRF1.1	8.80E-12	-0.10102	0.043	0.091	1.31E-07	AC-G	PHRF1
WWP2.1	8.89E-12	-0.10557	0.034	0.078	1.33E-07	AC-G	WWP2
AQR.1	8.92E-12	-0.10852	0.034	0.078	1.33E-07	AC-G	AQR
SMIM10	8.94E-12	0.194451	0.077	0.042	1.34E-07	AC-G	SMIM10
ARRDC2.1	8.95E-12	-0.22417	0.047	0.094	1.34E-07	AC-G	ARRDC2
SNRPA	8.96E-12	-0.11868	0.184	0.266	1.34E-07	AC-G	SNRPA
CHFR.1	9.00E-12	-0.13237	0.075	0.132	1.34E-07	AC-G	CHFR
SNX2.1	9.05E-12	-0.10698	0.332	0.425	1.35E-07	AC-G	SNX2
TMEM106A	9.05E-12	-0.16486	0.029	0.07	1.35E-07	AC-G	TMEM106A
ITGA6.1	9.06E-12	-0.13616	0.039	0.084	1.35E-07	AC-G	ITGA6
ZNFX1	9.15E-12	-0.13604	0.089	0.151	1.37E-07	AC-G	ZNFX1
IFNLR1	9.34E-12	-0.12523	0.002	0.028	1.40E-07	AC-G	IFNLR1
CASK	9.57E-12	-0.10792	0.075	0.133	1.43E-07	AC-G	CASK
MAP4K5.1	9.69E-12	-0.13	0.101	0.165	1.45E-07	AC-G	MAP4K5
CYFIP2.1	9.80E-12	-0.12395	0.068	0.122	1.46E-07	AC-G	CYFIP2
PRIM1.1	9.93E-12	-0.11766	0.042	0.089	1.48E-07	AC-G	PRIM1
PLEKHA3.1	9.99E-12	-0.13464	0.168	0.244	1.49E-07	AC-G	PLEKHA3
CDH2.1	1.01E-11	-0.11339	0.22	0.297	1.51E-07	AC-G	CDH2
KCNJ10.1	1.02E-11	-0.15775	0.02	0.058	1.53E-07	AC-G	KCNJ10
DUOX1.1	1.03E-11	-0.12087	0.029	0.07	1.54E-07	AC-G	DUOX1
LANCL1.1	1.04E-11	-0.12361	0.071	0.126	1.56E-07	AC-G	LANCL1
ZDHHC7.1	1.05E-11	-0.15906	0.097	0.157	1.56E-07	AC-G	ZDHHC7
XAF1	1.05E-11	-0.2239	0.065	0.117	1.57E-07	AC-G	XAF1
TMEM141.1	1.08E-11	-0.10544	0.185	0.265	1.62E-07	AC-G	TMEM141
SEL1L	1.12E-11	-0.12785	0.117	0.184	1.68E-07	AC-G	SEL1L
SRPK1.1	1.14E-11	-0.12769	0.111	0.177	1.70E-07	AC-G	SRPK1
ADCY3.1	1.14E-11	-0.12676	0.025	0.065	1.71E-07	AC-G	ADCY3
EPOR	1.15E-11	-0.11916	0.033	0.076	1.72E-07	AC-G	EPOR
CDC42EP1.	1.16E-11	-0.12735	0.079	0.137	1.73E-07	AC-G	CDC42EP1

ZNF100	1.16E-11	-0.11203	0.031	0.074	1.73E-07	AC-G	ZNF100
ITGB2-AS1	1.16E-11	-0.13603	0.005	0.033	1.74E-07	AC-G	ITGB2-AS1
TMEM2.1	1.17E-11	-0.14914	0.03	0.071	1.74E-07	AC-G	TMEM2
UBXN11	1.20E-11	0.217711	0.181	0.127	1.80E-07	AC-G	UBXN11
PPIL2.1	1.22E-11	-0.10663	0.071	0.126	1.82E-07	AC-G	PPIL2
MAL	1.22E-11	-0.24426	0.012	0.045	1.83E-07	AC-G	MAL
ST3GAL1.1	1.22E-11	-0.15903	0.06	0.111	1.83E-07	AC-G	ST3GAL1
TP53TG1.1	1.24E-11	0.361007	0.242	0.188	1.85E-07	AC-G	TP53TG1
CLEC12A	1.25E-11	-0.1095	0	0.024	1.87E-07	AC-G	CLEC12A
SEC61B.1	1.29E-11	0.177466	0.708	0.742	1.93E-07	AC-G	SEC61B
KHDRBS3.1	1.31E-11	-0.10475	0.155	0.227	1.96E-07	AC-G	KHDRBS3
SETD2.1	1.31E-11	-0.10349	0.092	0.154	1.96E-07	AC-G	SETD2
RP11-796E	1.33E-11	-0.20675	0.031	0.072	1.98E-07	AC-G	RP11-796E2.4
CHMP7.1	1.36E-11	-0.108	0.121	0.188	2.03E-07	AC-G	CHMP7
POLR2C.1	1.39E-11	0.272934	0.385	0.343	2.08E-07	AC-G	POLR2C
INTS10.1	1.39E-11	-0.1273	0.233	0.315	2.08E-07	AC-G	INTS10
CASP9.1	1.40E-11	-0.11824	0.044	0.092	2.09E-07	AC-G	CASP9
ZNF226.1	1.40E-11	0.363454	0.246	0.195	2.09E-07	AC-G	ZNF226
DIS3L.1	1.40E-11	-0.11985	0.06	0.112	2.09E-07	AC-G	DIS3L
COG4.1	1.41E-11	-0.13153	0.133	0.202	2.11E-07	AC-G	COG4
AMPD3	1.45E-11	-0.16411	0.021	0.058	2.17E-07	AC-G	AMPD3
KIAA0196.	1.45E-11	-0.10907	0.066	0.12	2.17E-07	AC-G	KIAA0196
CSF2RB	1.50E-11	-0.10524	0.003	0.029	2.25E-07	AC-G	CSF2RB
MYO10	1.57E-11	-0.1047	0.136	0.205	2.34E-07	AC-G	MYO10
TAF3.1	1.57E-11	-0.13164	0.075	0.131	2.35E-07	AC-G	TAF3
TLE4.1	1.58E-11	-0.12723	0.109	0.174	2.37E-07	AC-G	TLE4
DHX33	1.59E-11	-0.10519	0.074	0.13	2.37E-07	AC-G	DHX33
ECE1	1.63E-11	0.201724	0.115	0.073	2.43E-07	AC-G	ECE1
MOB1B.1	1.64E-11	-0.10943	0.033	0.076	2.45E-07	AC-G	MOB1B
AVPI1.1	1.64E-11	-0.26576	0.054	0.101	2.46E-07	AC-G	AVPI1
CCNK	1.66E-11	-0.10424	0.076	0.133	2.48E-07	AC-G	CCNK
EIF1B.1	1.67E-11	0.275107	0.597	0.586	2.50E-07	AC-G	EIF1B
PDCD4.1	1.68E-11	-0.21229	0.1	0.158	2.51E-07	AC-G	PDCD4
CCDC80.1	1.70E-11	0.228941	0.154	0.105	2.54E-07	AC-G	CCDC80
NEUROD1	1.71E-11	-0.10551	0.011	0.043	2.55E-07	AC-G	NEUROD1
RRP1B.1	1.71E-11	-0.10264	0.103	0.166	2.55E-07	AC-G	RRP1B
PLSCR1.1	1.73E-11	-0.13174	0.293	0.372	2.58E-07	AC-G	PLSCR1
SPOCK3	1.76E-11	-0.20438	0.008	0.038	2.63E-07	AC-G	SPOCK3
ICAM3.1	1.77E-11	-0.13944	0.058	0.109	2.65E-07	AC-G	ICAM3
RBM7.1	1.80E-11	0.230329	0.289	0.234	2.69E-07	AC-G	RBM7
BCL11B	1.81E-11	-0.11557	0.008	0.038	2.71E-07	AC-G	BCL11B
SMG1.1	1.83E-11	-0.11543	0.108	0.173	2.73E-07	AC-G	SMG1
WRB	1.86E-11	0.300136	0.451	0.414	2.78E-07	AC-G	WRB
PLEKHH1	1.93E-11	-0.13645	0.007	0.037	2.89E-07	AC-G	PLEKHH1
STK10.1	1.97E-11	-0.17537	0.036	0.079	2.94E-07	AC-G	STK10
TMEM70.1	2.00E-11	-0.16726	0.322	0.409	2.99E-07	AC-G	TMEM70
CHD2.1	2.01E-11	-0.10604	0.219	0.306	3.00E-07	AC-G	CHD2
HIST1H2AC	2.02E-11	0.345698	0.212	0.158	3.02E-07	AC-G	HIST1H2AC
SIDT1	2.07E-11	-0.14345	0.002	0.027	3.09E-07	AC-G	SIDT1
HSD17B10	2.10E-11	0.329324	0.514	0.492	3.14E-07	AC-G	HSD17B10
CACNA2D3	2.11E-11	-0.114	0.007	0.036	3.16E-07	AC-G	CACNA2D3

SLC39A11.	2.14E-11	-0.11557	0.037	0.08	3.20E-07	AC-G	SLC39A11
HRSP12.1	2.16E-11	0.291308	0.243	0.189	3.23E-07	AC-G	HRSP12
ARFRP1	2.18E-11	-0.10892	0.182	0.26	3.25E-07	AC-G	ARFRP1
PLEKHG3	2.18E-11	-0.10834	0.005	0.033	3.25E-07	AC-G	PLEKHG3
ERMN	2.21E-11	-0.35927	0.011	0.043	3.30E-07	AC-G	ERMN
PTBP2	2.24E-11	-0.13094	0.063	0.114	3.35E-07	AC-G	PTBP2
CALB1	2.24E-11	0.218424	0.03	0.011	3.35E-07	AC-G	CALB1
CNPY1.1	2.28E-11	-0.11233	0.04	0.085	3.41E-07	AC-G	CNPY1
L3MBTL2.1	2.29E-11	-0.11984	0.06	0.111	3.42E-07	AC-G	L3MBTL2
OAS2	2.35E-11	-0.15065	0.013	0.045	3.50E-07	AC-G	OAS2
GNAO1.1	2.35E-11	-0.17496	0.051	0.098	3.51E-07	AC-G	GNAO1
CCDC18.1	2.42E-11	-0.15302	0.04	0.084	3.62E-07	AC-G	CCDC18
BEND7	2.43E-11	-0.15886	0.026	0.064	3.63E-07	AC-G	BEND7
LIMA1.1	2.48E-11	-0.13519	0.253	0.33	3.71E-07	AC-G	LIMA1
TTC37.1	2.51E-11	-0.11227	0.128	0.194	3.75E-07	AC-G	TTC37
ELF2	2.51E-11	-0.16364	0.126	0.192	3.75E-07	AC-G	ELF2
RIPK3	2.58E-11	-0.10079	0	0.023	3.86E-07	AC-G	RIPK3
ALDH1A1	2.58E-11	-0.13917	0.006	0.034	3.86E-07	AC-G	ALDH1A1
SNX14.1	2.62E-11	-0.13109	0.152	0.222	3.92E-07	AC-G	SNX14
RGP1	2.64E-11	-0.12503	0.051	0.098	3.95E-07	AC-G	RGP1
HYI	2.66E-11	-0.11772	0.089	0.149	3.97E-07	AC-G	HYI
KXD1.1	2.76E-11	0.300155	0.479	0.443	4.12E-07	AC-G	KXD1
LRRC34	2.76E-11	0.10902	0.068	0.036	4.13E-07	AC-G	LRRC34
ARID2.1	2.77E-11	-0.12472	0.06	0.111	4.14E-07	AC-G	ARID2
MALT1.1	2.77E-11	-0.11311	0.038	0.081	4.14E-07	AC-G	MALT1
PRKAR2B.1	2.85E-11	-0.13949	0.034	0.075	4.27E-07	AC-G	PRKAR2B
SCG5.1	2.88E-11	0.382998	0.369	0.32	4.30E-07	AC-G	SCG5
NFATC3.1	2.92E-11	-0.14581	0.096	0.156	4.36E-07	AC-G	NFATC3
PNPLA8.1	2.97E-11	0.282633	0.383	0.34	4.43E-07	AC-G	PNPLA8
SPEN.1	3.01E-11	-0.1031	0.106	0.169	4.50E-07	AC-G	SPEN
PLEK2	3.05E-11	-0.10834	0.003	0.028	4.56E-07	AC-G	PLEK2
DCAF6.1	3.06E-11	-0.14814	0.114	0.176	4.57E-07	AC-G	DCAF6
GSTM4.1	3.09E-11	-0.10904	0.145	0.216	4.61E-07	AC-G	GSTM4
NFYC.1	3.10E-11	-0.10218	0.159	0.233	4.64E-07	AC-G	NFYC
DUSP28.1	3.11E-11	-0.1072	0.048	0.095	4.65E-07	AC-G	DUSP28
GLIS3	3.12E-11	0.170793	0.112	0.071	4.67E-07	AC-G	GLIS3
PPP1CC.1	3.14E-11	-0.10952	0.361	0.461	4.69E-07	AC-G	PPP1CC
SOX10	3.17E-11	-0.15478	0.006	0.033	4.73E-07	AC-G	SOX10
ZC3H11A.1	3.26E-11	-0.10855	0.141	0.211	4.87E-07	AC-G	ZC3H11A
ARHGEF25.	3.27E-11	-0.10175	0.031	0.072	4.88E-07	AC-G	ARHGEF25
RACGAP1	3.29E-11	-0.12407	0.045	0.09	4.92E-07	AC-G	RACGAP1
SEMA4C.1	3.35E-11	-0.13529	0.055	0.103	5.01E-07	AC-G	SEMA4C
LPCAT4.1	3.67E-11	-0.11369	0.034	0.076	5.48E-07	AC-G	LPCAT4
RP2.1	3.82E-11	-0.10497	0.04	0.084	5.70E-07	AC-G	RP2
GHRL.1	3.82E-11	-0.15346	0.025	0.063	5.71E-07	AC-G	GHRL
WAC-AS1	3.86E-11	-0.10623	0.09	0.15	5.77E-07	AC-G	WAC-AS1
C10orf128	3.88E-11	-0.14798	0.007	0.035	5.79E-07	AC-G	C10orf128
KLF11	3.90E-11	-0.11226	0.013	0.045	5.83E-07	AC-G	KLF11
CUL5.1	3.90E-11	-0.1297	0.162	0.234	5.83E-07	AC-G	CUL5
LGI3	3.94E-11	-0.14425	0.001	0.025	5.88E-07	AC-G	LGI3
QRICH1.1	4.01E-11	-0.11132	0.098	0.159	5.99E-07	AC-G	QRICH1

KDM5A.1	4.11E-11	-0.16277	0.185	0.26	6.14E-07	AC-G	KDM5A
LIMK2.1	4.12E-11	-0.1397	0.048	0.094	6.16E-07	AC-G	LIMK2
MLLT10.1	4.14E-11	-0.10092	0.079	0.135	6.19E-07	AC-G	MLLT10
SMC6.1	4.17E-11	-0.11034	0.106	0.167	6.22E-07	AC-G	SMC6
FAM177A1	4.19E-11	-0.1064	0.304	0.404	6.26E-07	AC-G	FAM177A1
GCSH.1	4.22E-11	-0.13645	0.276	0.361	6.31E-07	AC-G	GCSH
CYBRD1.1	4.26E-11	0.273261	0.22	0.168	6.37E-07	AC-G	CYBRD1
NUMA1.1	4.34E-11	-0.12589	0.115	0.18	6.48E-07	AC-G	NUMA1
CHAMP1.1	4.34E-11	-0.10506	0.081	0.138	6.49E-07	AC-G	CHAMP1
RCBTB1.1	4.50E-11	-0.12635	0.036	0.079	6.72E-07	AC-G	RCBTB1
GCNT2	4.51E-11	-0.11942	0.015	0.048	6.75E-07	AC-G	GCNT2
ZNF385A.1	4.63E-11	-0.12716	0.088	0.146	6.93E-07	AC-G	ZNF385A
ABHD17B.1	4.67E-11	-0.17308	0.08	0.133	6.97E-07	AC-G	ABHD17B
LEMD2.1	4.71E-11	-0.12132	0.098	0.158	7.04E-07	AC-G	LEMD2
SLBP.1	4.76E-11	-0.13033	0.241	0.324	7.11E-07	AC-G	SLBP
DHRS9	4.81E-11	-0.15649	0.002	0.026	7.19E-07	AC-G	DHRS9
LRP6.1	4.94E-11	-0.10851	0.068	0.12	7.39E-07	AC-G	LRP6
OSTM1.1	4.96E-11	-0.14303	0.168	0.24	7.41E-07	AC-G	OSTM1
FGFR2	4.98E-11	-0.17269	0.018	0.053	7.44E-07	AC-G	FGFR2
GSE1.1	4.98E-11	-0.14847	0.052	0.099	7.44E-07	AC-G	GSE1
TREML1	5.04E-11	-0.12334	0.002	0.025	7.54E-07	AC-G	TREML1
SENP6.1	5.15E-11	-0.14811	0.159	0.23	7.69E-07	AC-G	SENP6
SCD5.1	5.19E-11	-0.14066	0.32	0.409	7.75E-07	AC-G	SCD5
MAP4K1	5.26E-11	-0.14328	0.006	0.034	7.86E-07	AC-G	MAP4K1
REPIN1.1	5.27E-11	-0.11094	0.207	0.287	7.87E-07	AC-G	REPIN1
KIAA1109	5.32E-11	-0.13471	0.075	0.129	7.95E-07	AC-G	KIAA1109
ITGAL	5.40E-11	-0.12738	0.003	0.028	8.07E-07	AC-G	ITGAL
ATP5F1.1	5.50E-11	0.234986	0.639	0.645	8.22E-07	AC-G	ATP5F1
ADAM22.1	5.54E-11	-0.12359	0.03	0.069	8.28E-07	AC-G	ADAM22
FAM53C.1	5.56E-11	-0.10803	0.179	0.254	8.32E-07	AC-G	FAM53C
PPARD.1	5.66E-11	-0.15387	0.066	0.117	8.46E-07	AC-G	PPARD
TFG	5.75E-11	0.242669	0.438	0.402	8.60E-07	AC-G	TFG
PIGX.1	5.80E-11	-0.12172	0.189	0.265	8.66E-07	AC-G	PIGX
LRRC61.1	5.81E-11	0.11054	0.098	0.057	8.68E-07	AC-G	LRRC61
POMZP3.1	5.91E-11	-0.12411	0.048	0.094	8.83E-07	AC-G	POMZP3
PHF3	5.92E-11	-0.12852	0.138	0.205	8.84E-07	AC-G	PHF3
PSME4	6.16E-11	-0.10401	0.088	0.145	9.21E-07	AC-G	PSME4
ZNF276.1	6.32E-11	-0.14546	0.051	0.097	9.45E-07	AC-G	ZNF276
CCDC69	6.37E-11	-0.12052	0.01	0.041	9.52E-07	AC-G	CCDC69
ADCY2	6.43E-11	0.143969	0.064	0.034	9.61E-07	AC-G	ADCY2
SENCR	6.44E-11	-0.10765	0.001	0.023	9.62E-07	AC-G	SENCR
HVCN1.1	6.85E-11	-0.1611	0.056	0.104	1.02E-06	AC-G	HVCN1
DENND4B.1	6.89E-11	-0.12705	0.034	0.075	1.03E-06	AC-G	DENND4B
MAPRE2	7.04E-11	-0.1617	0.132	0.197	1.05E-06	AC-G	MAPRE2
SGOL2	7.19E-11	-0.16588	0.052	0.097	1.07E-06	AC-G	SGOL2
SP140	7.32E-11	-0.13588	0.007	0.035	1.09E-06	AC-G	SP140
USP4	7.36E-11	-0.13925	0.122	0.185	1.10E-06	AC-G	USP4
OTUD4.1	7.65E-11	-0.10742	0.059	0.107	1.14E-06	AC-G	OTUD4
WIPI1.1	7.66E-11	-0.18929	0.132	0.196	1.14E-06	AC-G	WIPI1
GAS7	7.67E-11	-0.1634	0.067	0.118	1.15E-06	AC-G	GAS7
ORC6.1	7.86E-11	-0.13528	0.052	0.099	1.17E-06	AC-G	ORC6

PTPN22	7.95E-11	-0.11954	0.005	0.031	1.19E-06	AC-G	PTPN22
MSC	7.96E-11	0.122487	0.018	0.005	1.19E-06	AC-G	MSC
WNT5A.1	8.21E-11	-0.1671	0.027	0.065	1.23E-06	AC-G	WNT5A
HSPB11.1	8.33E-11	-0.10881	0.254	0.343	1.25E-06	AC-G	HSPB11
ZFP91.1	8.59E-11	-0.11398	0.087	0.145	1.28E-06	AC-G	ZFP91
CCDC74B	8.59E-11	0.111802	0.059	0.03	1.28E-06	AC-G	CCDC74B
CEACAM21	8.61E-11	-0.10912	0.001	0.024	1.29E-06	AC-G	CEACAM21
IL17RB	8.69E-11	0.200884	0.073	0.04	1.30E-06	AC-G	IL17RB
TANGO2.1	8.81E-11	-0.10062	0.048	0.093	1.32E-06	AC-G	TANGO2
TMEM129.1	8.82E-11	-0.10562	0.119	0.183	1.32E-06	AC-G	TMEM129
FAM120AOS	8.86E-11	-0.11216	0.13	0.196	1.32E-06	AC-G	FAM120AOS
SLC7A5.1	9.14E-11	-0.26095	0.096	0.147	1.37E-06	AC-G	SLC7A5
DIP2A.1	9.29E-11	-0.12304	0.07	0.122	1.39E-06	AC-G	DIP2A
RUFY2.1	9.33E-11	-0.12179	0.099	0.159	1.39E-06	AC-G	RUFY2
RAPGEF6	9.38E-11	-0.11148	0.019	0.054	1.40E-06	AC-G	RAPGEF6
ATOH8.1	9.49E-11	-0.13545	0.023	0.059	1.42E-06	AC-G	ATOH8
COQ7.1	9.74E-11	-0.11579	0.132	0.198	1.46E-06	AC-G	COQ7
NFATC1.1	9.85E-11	-0.14757	0.022	0.058	1.47E-06	AC-G	NFATC1
RPH3A	1.02E-10	-0.10126	0.008	0.037	1.52E-06	AC-G	RPH3A
AFMID.1	1.02E-10	-0.1575	0.065	0.113	1.53E-06	AC-G	AFMID
TIMM17A.1	1.03E-10	0.328617	0.406	0.37	1.54E-06	AC-G	TIMM17A
CDC5L.1	1.03E-10	-0.10342	0.214	0.294	1.54E-06	AC-G	CDC5L
CDK5R2	1.05E-10	-0.11073	0.009	0.038	1.56E-06	AC-G	CDK5R2
HES4.1	1.05E-10	-0.1331	0.134	0.197	1.57E-06	AC-G	HES4
HPS5.1	1.05E-10	-0.11895	0.071	0.123	1.58E-06	AC-G	HPS5
NDUFAF6.1	1.06E-10	-0.10348	0.126	0.191	1.58E-06	AC-G	NDUFAF6
FA2H	1.07E-10	-0.14637	0.005	0.031	1.60E-06	AC-G	FA2H
OPHN1.1	1.09E-10	-0.1133	0.036	0.078	1.62E-06	AC-G	OPHN1
IPCEF1	1.10E-10	-0.10844	0.002	0.026	1.65E-06	AC-G	IPCEF1
HLTF.1	1.11E-10	-0.10339	0.12	0.184	1.66E-06	AC-G	HLTF
HSP90B1.1	1.14E-10	0.221888	0.616	0.666	1.71E-06	AC-G	HSP90B1
DENND1B.1	1.15E-10	-0.1515	0.053	0.099	1.72E-06	AC-G	DENND1B
NELL2	1.15E-10	-0.13823	0.064	0.114	1.73E-06	AC-G	NELL2
PPP3R1.1	1.16E-10	-0.14517	0.071	0.121	1.73E-06	AC-G	PPP3R1
RGL2.1	1.16E-10	-0.12708	0.147	0.215	1.74E-06	AC-G	RGL2
CATSPER1	1.17E-10	-0.12068	0.005	0.03	1.75E-06	AC-G	CATSPER1
YWHAQ.1	1.17E-10	-0.10972	0.575	0.657	1.75E-06	AC-G	YWHAQ
KNSTRN.1	1.18E-10	-0.12708	0.043	0.086	1.76E-06	AC-G	KNSTRN
CDK20	1.18E-10	0.126567	0.089	0.052	1.76E-06	AC-G	CDK20
TBC1D5.1	1.18E-10	-0.14689	0.094	0.151	1.76E-06	AC-G	TBC1D5
DNAJC21	1.20E-10	-0.11299	0.077	0.131	1.80E-06	AC-G	DNAJC21
LINC00461	1.21E-10	-0.10169	0.254	0.333	1.81E-06	AC-G	LINC00461
VNN2	1.22E-10	-0.11478	0.004	0.029	1.82E-06	AC-G	VNN2
GABARAPL2	1.22E-10	0.204355	0.723	0.747	1.82E-06	AC-G	GABARAPL2
ARL4D.1	1.23E-10	-0.14303	0.119	0.179	1.84E-06	AC-G	ARL4D
EP400.1	1.26E-10	-0.10711	0.073	0.125	1.88E-06	AC-G	EP400
FAM118A.1	1.26E-10	-0.2051	0.089	0.141	1.89E-06	AC-G	FAM118A
FAM204A.1	1.26E-10	-0.12027	0.195	0.269	1.89E-06	AC-G	FAM204A
FLT1	1.27E-10	-0.10475	0.001	0.023	1.89E-06	AC-G	FLT1
MTAP.1	1.27E-10	-0.11477	0.036	0.077	1.90E-06	AC-G	MTAP
PUM2.1	1.28E-10	-0.1031	0.103	0.164	1.92E-06	AC-G	PUM2

C9orf72.1	1.30E-10	-0.2225	0.104	0.159	1.94E-06	AC-G	C9orf72
STX2.1	1.31E-10	-0.11261	0.065	0.116	1.95E-06	AC-G	STX2
IGSF8.1	1.31E-10	-0.11555	0.157	0.224	1.96E-06	AC-G	IGSF8
ACSL5	1.34E-10	-0.13419	0.01	0.039	2.00E-06	AC-G	ACSL5
HPCA	1.40E-10	-0.1144	0.028	0.066	2.10E-06	AC-G	HPCA
KCNK13	1.45E-10	-0.10009	0.003	0.027	2.17E-06	AC-G	KCNK13
EIF4A1.1	1.51E-10	0.138968	0.795	0.874	2.26E-06	AC-G	EIF4A1
PNMAL1.1	1.51E-10	-0.10011	0.056	0.104	2.26E-06	AC-G	PNMAL1
ELOF1	1.54E-10	0.301023	0.45	0.418	2.30E-06	AC-G	ELOF1
RETN	1.57E-10	-0.12052	0.001	0.023	2.34E-06	AC-G	RETN
ZC2HC1A.1	1.58E-10	-0.13126	0.156	0.221	2.36E-06	AC-G	ZC2HC1A
PNP	1.61E-10	-0.16363	0.063	0.111	2.40E-06	AC-G	PNP
CCNG1.1	1.64E-10	0.222352	0.317	0.26	2.45E-06	AC-G	CCNG1
COMMD8.1	1.65E-10	-0.1246	0.167	0.235	2.47E-06	AC-G	COMMD8
C2orf69.1	1.72E-10	-0.11438	0.139	0.205	2.56E-06	AC-G	C2orf69
LRAT	1.72E-10	0.145363	0.028	0.01	2.57E-06	AC-G	LRAT
ZFP90.1	1.74E-10	-0.11803	0.085	0.14	2.61E-06	AC-G	ZFP90
CYLD	1.78E-10	-0.15908	0.115	0.174	2.66E-06	AC-G	CYLD
RP11-84C1	1.78E-10	-0.12485	0.001	0.022	2.67E-06	AC-G	RP11-84C10.2
C16orf52.	1.81E-10	-0.10147	0.056	0.104	2.71E-06	AC-G	C16orf52
NRXN2.1	1.82E-10	-0.15602	0.074	0.125	2.71E-06	AC-G	NRXN2
ADPRH	1.85E-10	-0.11649	0.009	0.038	2.76E-06	AC-G	ADPRH
AFF3	1.86E-10	-0.12724	0.023	0.058	2.78E-06	AC-G	AFF3
TNR.1	1.87E-10	-0.13901	0.025	0.061	2.79E-06	AC-G	TNR
SNX11	1.89E-10	-0.11355	0.059	0.107	2.83E-06	AC-G	SNX11
CDK5RAP1.	1.90E-10	-0.10666	0.108	0.168	2.83E-06	AC-G	CDK5RAP1
TARBP1.1	1.92E-10	-0.10748	0.037	0.077	2.86E-06	AC-G	TARBP1
BIRC6.1	1.94E-10	-0.10117	0.13	0.194	2.89E-06	AC-G	BIRC6
TPCN1.1	1.97E-10	-0.1781	0.052	0.097	2.95E-06	AC-G	TPCN1
FGF7	1.97E-10	0.157312	0.047	0.022	2.95E-06	AC-G	FGF7
SMYD4.1	1.97E-10	-0.10581	0.073	0.126	2.95E-06	AC-G	SMYD4
NDUFS8.1	1.99E-10	0.294168	0.606	0.609	2.97E-06	AC-G	NDUFS8
NXN	2.04E-10	-0.1081	0.011	0.04	3.05E-06	AC-G	NXN
KCNIP1.1	2.05E-10	-0.12344	0.059	0.106	3.07E-06	AC-G	KCNIP1
USF1.1	2.06E-10	-0.14361	0.025	0.061	3.07E-06	AC-G	USF1
CTDSPL2	2.07E-10	-0.10407	0.066	0.117	3.10E-06	AC-G	CTDSPL2
FAM177B	2.08E-10	-0.12917	0.008	0.035	3.10E-06	AC-G	FAM177B
MAPK14.1	2.08E-10	-0.10998	0.067	0.118	3.11E-06	AC-G	MAPK14
NEAT1	2.11E-10	0.159521	0.741	0.767	3.15E-06	AC-G	NEAT1
C19orf70.	2.11E-10	0.246957	0.584	0.577	3.15E-06	AC-G	C19orf70
ZNF638.1	2.12E-10	-0.10208	0.188	0.264	3.17E-06	AC-G	ZNF638
RPA3.1	2.13E-10	0.278856	0.382	0.335	3.18E-06	AC-G	RPA3
GTF2IRD2E	2.14E-10	-0.10857	0.09	0.146	3.19E-06	AC-G	GTF2IRD2B
PRRG4	2.18E-10	-0.16682	0.018	0.05	3.26E-06	AC-G	PRRG4
TOMM70A.1	2.23E-10	-0.1093	0.08	0.134	3.34E-06	AC-G	TOMM70A
PKP4.1	2.27E-10	-0.10106	0.024	0.06	3.39E-06	AC-G	PKP4
XPNPEP1.1	2.30E-10	-0.1162	0.096	0.152	3.44E-06	AC-G	XPNPEP1
SELL	2.36E-10	-0.15545	0.012	0.042	3.53E-06	AC-G	SELL
KLHL36	2.39E-10	-0.10812	0.055	0.102	3.58E-06	AC-G	KLHL36
HIST2H2AA	2.41E-10	-0.16331	0.031	0.07	3.59E-06	AC-G	HIST2H2AA3
ABTB1.1	2.42E-10	-0.20193	0.065	0.112	3.62E-06	AC-G	ABTB1

BCOR	2.43E-10	-0.11619	0.024	0.06	3.63E-06	AC-G	BCOR
JAM2.1	2.43E-10	0.311725	0.292	0.245	3.63E-06	AC-G	JAM2
AGA.1	2.49E-10	0.24398	0.196	0.145	3.71E-06	AC-G	AGA
YPEL2.1	2.51E-10	-0.15352	0.036	0.076	3.75E-06	AC-G	YPEL2
ALKBH6.1	2.68E-10	-0.11661	0.064	0.113	4.00E-06	AC-G	ALKBH6
PINLYP	2.68E-10	0.196656	0.083	0.048	4.00E-06	AC-G	PINLYP
SAT1	2.68E-10	0.22712	0.781	0.807	4.01E-06	AC-G	SAT1
CRYZ	2.70E-10	0.298906	0.161	0.116	4.03E-06	AC-G	CRYZ
SEC24B	2.74E-10	-0.11393	0.042	0.084	4.09E-06	AC-G	SEC24B
SHOC2.1	2.75E-10	-0.13977	0.103	0.161	4.11E-06	AC-G	SHOC2
QTRT1.1	2.79E-10	0.320674	0.367	0.324	4.17E-06	AC-G	QTRT1
CAMLG.1	2.82E-10	0.234718	0.508	0.491	4.22E-06	AC-G	CAMLG
RNASEK	2.83E-10	-0.15142	0.043	0.086	4.23E-06	AC-G	RNASEK
CNIH4	2.90E-10	0.266112	0.409	0.367	4.34E-06	AC-G	CNIH4
CPEB4.1	2.96E-10	-0.13567	0.188	0.258	4.43E-06	AC-G	CPEB4
FOLH1	2.98E-10	-0.11342	0.001	0.021	4.45E-06	AC-G	FOLH1
MAEA	3.11E-10	-0.10571	0.176	0.246	4.65E-06	AC-G	MAEA
RP11-783K	3.12E-10	0.291854	0.075	0.043	4.66E-06	AC-G	RP11-783K16.5
ABCC4	3.15E-10	-0.112	0.018	0.05	4.71E-06	AC-G	ABCC4
PER1	3.16E-10	-0.24118	0.137	0.194	4.72E-06	AC-G	PER1
LIPE	3.16E-10	-0.10781	0.029	0.067	4.72E-06	AC-G	LIPE
NET1	3.22E-10	-0.12825	0.015	0.045	4.80E-06	AC-G	NET1
MORC3.1	3.26E-10	-0.12929	0.063	0.111	4.87E-06	AC-G	MORC3
IL6R.1	3.26E-10	-0.23044	0.051	0.093	4.87E-06	AC-G	IL6R
LYPLA2.1	3.33E-10	-0.10688	0.162	0.232	4.98E-06	AC-G	LYPLA2
CCK	3.38E-10	0.101713	0.019	0.005	5.05E-06	AC-G	CCK
ZNF124.1	3.45E-10	-0.12137	0.029	0.066	5.16E-06	AC-G	ZNF124
GIMAP2.1	3.52E-10	-0.1806	0.042	0.084	5.26E-06	AC-G	GIMAP2
ADD3	3.55E-10	0.258971	0.173	0.125	5.31E-06	AC-G	ADD3
HAUS4.1	3.57E-10	-0.11158	0.03	0.067	5.34E-06	AC-G	HAUS4
YTHDC2.1	3.57E-10	-0.13487	0.076	0.127	5.34E-06	AC-G	YTHDC2
ARMC1.1	3.62E-10	-0.11268	0.201	0.276	5.41E-06	AC-G	ARMC1
ATPAF1.1	3.62E-10	-0.15621	0.181	0.251	5.41E-06	AC-G	ATPAF1
TLR10	3.68E-10	-0.11508	0.004	0.028	5.50E-06	AC-G	TLR10
RARA	3.73E-10	-0.1062	0.057	0.103	5.57E-06	AC-G	RARA
LCOR.1	3.75E-10	-0.11121	0.044	0.087	5.61E-06	AC-G	LCOR
BNIP3L.1	3.76E-10	0.213173	0.59	0.601	5.62E-06	AC-G	BNIP3L
RAP2C	3.96E-10	-0.12003	0.081	0.134	5.91E-06	AC-G	RAP2C
PGM2L1	3.97E-10	0.325947	0.298	0.247	5.93E-06	AC-G	PGM2L1
GALT.1	4.06E-10	-0.17884	0.046	0.088	6.07E-06	AC-G	GALT
IL6ST.1	4.09E-10	-0.10479	0.191	0.266	6.11E-06	AC-G	IL6ST
SCN3A.1	4.10E-10	-0.13031	0.039	0.078	6.13E-06	AC-G	SCN3A
TOMM5.1	4.56E-10	-0.11878	0.154	0.22	6.82E-06	AC-G	TOMM5
EPDR1.1	4.59E-10	0.250714	0.164	0.119	6.86E-06	AC-G	EPDR1
MED15.1	4.61E-10	-0.12515	0.095	0.152	6.89E-06	AC-G	MED15
RAB11FIP1	4.62E-10	-0.10437	0.006	0.031	6.90E-06	AC-G	RAB11FIP1
TATDN3.1	4.69E-10	-0.12078	0.107	0.163	7.02E-06	AC-G	TATDN3
LACC1	4.70E-10	-0.1177	0.01	0.039	7.03E-06	AC-G	LACC1
PPP3CA.1	4.75E-10	-0.13255	0.187	0.258	7.10E-06	AC-G	PPP3CA
SLC16A4	4.78E-10	0.180082	0.091	0.056	7.15E-06	AC-G	SLC16A4
SLC12A2	4.80E-10	-0.16223	0.085	0.138	7.18E-06	AC-G	SLC12A2

NFATC2IP.	4.81E-10	-0.11304	0.113	0.172	7.19E-06	AC-G	NFATC2IP
SUDS3.1	4.82E-10	-0.10388	0.134	0.199	7.21E-06	AC-G	SUDS3
TMIGD2	4.87E-10	-0.14831	0.002	0.023	7.27E-06	AC-G	TMIGD2
NEDD9.1	4.97E-10	-0.12665	0.162	0.232	7.42E-06	AC-G	NEDD9
TUBGCP3.1	5.00E-10	-0.12634	0.065	0.112	7.47E-06	AC-G	TUBGCP3
PRKCZ.1	5.03E-10	-0.10353	0.061	0.108	7.52E-06	AC-G	PRKCZ
AC007228.	5.12E-10	0.112738	0.039	0.017	7.65E-06	AC-G	AC007228.11
AP2A1.1	5.38E-10	-0.11434	0.177	0.248	8.04E-06	AC-G	AP2A1
ASXL2.1	5.38E-10	-0.11694	0.059	0.106	8.05E-06	AC-G	ASXL2
UBQLN2.1	5.39E-10	-0.12962	0.165	0.231	8.05E-06	AC-G	UBQLN2
RFX5.1	5.70E-10	-0.10309	0.064	0.112	8.51E-06	AC-G	RFX5
LPAR2	5.78E-10	-0.10179	0.01	0.037	8.63E-06	AC-G	LPAR2
SLC29A3	5.80E-10	-0.14664	0.018	0.049	8.67E-06	AC-G	SLC29A3
NHSL1	5.87E-10	-0.11546	0.063	0.111	8.77E-06	AC-G	NHSL1
TMEM30A.1	5.96E-10	-0.10676	0.265	0.347	8.91E-06	AC-G	TMEM30A
ARX	5.96E-10	-0.11812	0.015	0.045	8.91E-06	AC-G	ARX
RNF141.1	6.13E-10	-0.13062	0.157	0.225	9.17E-06	AC-G	RNF141
SORBS3.1	6.17E-10	-0.1013	0.12	0.183	9.22E-06	AC-G	SORBS3
E2F4.1	6.19E-10	-0.1368	0.158	0.225	9.25E-06	AC-G	E2F4
RHOF	6.19E-10	-0.11241	0.023	0.057	9.26E-06	AC-G	RHOF
RNF125	6.21E-10	-0.11754	0.004	0.028	9.28E-06	AC-G	RNF125
HNRNPC.1	6.31E-10	0.203911	0.677	0.697	9.43E-06	AC-G	HNRNPC
BLVRA.1	6.60E-10	0.392237	0.372	0.333	9.86E-06	AC-G	BLVRA
KLHL8.1	6.62E-10	-0.11727	0.088	0.142	9.89E-06	AC-G	KLHL8
KLHL42.1	6.67E-10	-0.10049	0.109	0.169	9.96E-06	AC-G	KLHL42
BCL2.1	6.67E-10	-0.15458	0.021	0.054	9.97E-06	AC-G	BCL2
CDCP1	6.74E-10	-0.13123	0.01	0.038	1.01E-05	AC-G	CDCP1
EXOC6.1	6.80E-10	-0.11201	0.032	0.07	1.02E-05	AC-G	EXOC6
WDR81.1	6.91E-10	-0.10246	0.019	0.052	1.03E-05	AC-G	WDR81
C12orf29.	7.02E-10	0.255015	0.193	0.146	1.05E-05	AC-G	C12orf29
LDLRAP1.1	7.04E-10	-0.11644	0.027	0.063	1.05E-05	AC-G	LDLRAP1
PLCL1	7.38E-10	-0.10761	0.009	0.036	1.10E-05	AC-G	PLCL1
PHYKPL.1	7.66E-10	-0.10754	0.149	0.212	1.15E-05	AC-G	PHYKPL
MLKL	7.70E-10	-0.12803	0.007	0.032	1.15E-05	AC-G	MLKL
AGO3	7.79E-10	-0.11848	0.138	0.203	1.16E-05	AC-G	AGO3
USP36.1	7.86E-10	-0.15317	0.094	0.147	1.17E-05	AC-G	USP36
RCC1	7.97E-10	-0.10403	0.061	0.107	1.19E-05	AC-G	RCC1
CTD-2336C	8.14E-10	-0.10959	0.166	0.235	1.22E-05	AC-G	CTD-233602.1
SLC35F6.1	8.15E-10	-0.10196	0.154	0.22	1.22E-05	AC-G	SLC35F6
TNNI2	8.22E-10	-0.11161	0.002	0.023	1.23E-05	AC-G	TNNI2
LINC00657	8.37E-10	-0.12938	0.264	0.345	1.25E-05	AC-G	LINC00657
FADS1.1	8.50E-10	-0.11202	0.16	0.229	1.27E-05	AC-G	FADS1
SLC27A1.1	8.64E-10	-0.10974	0.111	0.17	1.29E-05	AC-G	SLC27A1
QDPR.1	8.65E-10	-0.12639	0.29	0.374	1.29E-05	AC-G	QDPR
GLYR1	8.79E-10	-0.11397	0.154	0.22	1.31E-05	AC-G	GLYR1
PRCD	8.80E-10	-0.1312	0.01	0.038	1.32E-05	AC-G	PRCD
TSPYL1.1	9.02E-10	-0.11495	0.182	0.249	1.35E-05	AC-G	TSPYL1
ANKRD13A	9.13E-10	-0.10132	0.059	0.104	1.36E-05	AC-G	ANKRD13A
EDEM1	9.18E-10	-0.12178	0.044	0.086	1.37E-05	AC-G	EDEM1
MEMO1	9.27E-10	-0.13295	0.063	0.109	1.39E-05	AC-G	MEMO1
SRRM4	9.32E-10	-0.10199	0.009	0.035	1.39E-05	AC-G	SRRM4

SLFN11	9.54E-10	-0.10651	0.017	0.048	1.43E-05	AC-G	SLFN11
RICTOR.1	9.59E-10	-0.18249	0.137	0.196	1.43E-05	AC-G	RICTOR
ZC3H7A.1	9.89E-10	-0.1423	0.103	0.158	1.48E-05	AC-G	ZC3H7A
NME1-NME2	9.90E-10	-0.11403	0.056	0.101	1.48E-05	AC-G	NME1-NME2
PSAT1	9.90E-10	0.181321	0.244	0.195	1.48E-05	AC-G	PSAT1
ZMIZ2.1	9.92E-10	-0.10524	0.061	0.106	1.48E-05	AC-G	ZMIZ2
SERPINI1.	1.01E-09	-0.12791	0.025	0.059	1.51E-05	AC-G	SERPINI1
SCP2.1	1.01E-09	0.258498	0.58	0.584	1.52E-05	AC-G	SCP2
YIPF5.1	1.02E-09	0.24175	0.301	0.254	1.52E-05	AC-G	YIPF5
SHPRH.1	1.05E-09	-0.1187	0.083	0.135	1.57E-05	AC-G	SHPRH
PKN2.1	1.06E-09	-0.10472	0.154	0.222	1.58E-05	AC-G	PKN2
PARP6.1	1.06E-09	-0.11104	0.064	0.111	1.58E-05	AC-G	PARP6
TBC1D9.1	1.08E-09	-0.12212	0.025	0.059	1.61E-05	AC-G	TBC1D9
L1CAM	1.10E-09	-0.11479	0.011	0.039	1.65E-05	AC-G	L1CAM
PEX5L	1.16E-09	-0.13056	0.004	0.026	1.73E-05	AC-G	PEX5L
TMEM37.1	1.17E-09	0.361998	0.091	0.057	1.74E-05	AC-G	TMEM37
EPB41	1.17E-09	-0.12505	0.03	0.066	1.75E-05	AC-G	EPB41
CPED1	1.18E-09	-0.11952	0.01	0.037	1.77E-05	AC-G	CPED1
IFRD1.1	1.25E-09	0.206685	0.512	0.482	1.86E-05	AC-G	IFRD1
TREM1	1.27E-09	0.339604	0.208	0.155	1.90E-05	AC-G	TREM1
FMN1	1.29E-09	-0.1144	0.01	0.037	1.92E-05	AC-G	FMN1
TIPARP.1	1.30E-09	0.206483	0.21	0.161	1.94E-05	AC-G	TIPARP
ENOX2.1	1.33E-09	-0.10839	0.041	0.081	1.99E-05	AC-G	ENOX2
FPR2	1.38E-09	-0.10727	0.002	0.022	2.06E-05	AC-G	FPR2
STRIP1.1	1.39E-09	-0.13177	0.083	0.133	2.07E-05	AC-G	STRIP1
FKBP9.1	1.42E-09	0.244182	0.213	0.17	2.12E-05	AC-G	FKBP9
AMPD2.1	1.44E-09	-0.15651	0.107	0.161	2.15E-05	AC-G	AMPD2
NOA1.1	1.46E-09	-0.1167	0.098	0.152	2.18E-05	AC-G	NOA1
P2RY6	1.46E-09	-0.1012	0.01	0.037	2.19E-05	AC-G	P2RY6
PTAR1.1	1.51E-09	-0.10198	0.091	0.145	2.26E-05	AC-G	PTAR1
KLHL18	1.54E-09	-0.1132	0.077	0.127	2.30E-05	AC-G	KLHL18
STK38L	1.54E-09	-0.13128	0.105	0.159	2.31E-05	AC-G	STK38L
PDXDC1	1.57E-09	-0.12128	0.121	0.181	2.35E-05	AC-G	PDXDC1
HS3ST1.1	1.62E-09	-0.16314	0.052	0.095	2.42E-05	AC-G	HS3ST1
EXOSC3.1	1.63E-09	-0.10755	0.112	0.169	2.43E-05	AC-G	EXOSC3
FGF20	1.66E-09	-0.12009	0.001	0.021	2.47E-05	AC-G	FGF20
FRAT2	1.70E-09	-0.10165	0.019	0.051	2.54E-05	AC-G	FRAT2
PRR5.1	1.71E-09	-0.13568	0.02	0.052	2.55E-05	AC-G	PRR5
TTC38	1.75E-09	-0.11336	0.052	0.096	2.62E-05	AC-G	TTC38
ZDHHC21.1	1.79E-09	-0.12239	0.061	0.106	2.67E-05	AC-G	ZDHHC21
SNX3.1	1.83E-09	0.116795	0.672	0.725	2.73E-05	AC-G	SNX3
TRIM47.1	1.89E-09	0.3065	0.216	0.172	2.82E-05	AC-G	TRIM47
NDFIP1.1	1.90E-09	-0.10572	0.491	0.623	2.84E-05	AC-G	NDFIP1
MS4A14	1.91E-09	-0.10627	0.004	0.026	2.86E-05	AC-G	MS4A14
RPF2	2.05E-09	0.320441	0.266	0.219	3.06E-05	AC-G	RPF2
PTPN2	2.06E-09	-0.11549	0.193	0.259	3.08E-05	AC-G	PTPN2
GRM3	2.07E-09	-0.13083	0.004	0.026	3.09E-05	AC-G	GRM3
STEAP2	2.14E-09	0.13263	0.034	0.015	3.20E-05	AC-G	STEAP2
PTPRJ	2.14E-09	-0.111	0.008	0.034	3.20E-05	AC-G	PTPRJ
SRBD1.1	2.21E-09	-0.1208	0.05	0.092	3.30E-05	AC-G	SRBD1
AL592183.	2.24E-09	-0.18139	0.119	0.174	3.35E-05	AC-G	AL592183.1

SPHK2.1	2.27E-09	-0.10363	0.085	0.136	3.39E-05	AC-G	SPHK2
POLB	2.31E-09	-0.10366	0.05	0.091	3.44E-05	AC-G	POLB
MEIS2.1	2.31E-09	-0.17162	0.156	0.213	3.45E-05	AC-G	MEIS2
CREB5	2.35E-09	-0.10855	0.178	0.246	3.51E-05	AC-G	CREB5
LACTB2.1	2.37E-09	0.187085	0.192	0.143	3.54E-05	AC-G	LACTB2
KCNE1	2.38E-09	-0.1064	0.004	0.026	3.55E-05	AC-G	KCNE1
UPP1.1	2.42E-09	0.265537	0.296	0.246	3.61E-05	AC-G	UPP1
ERBB3	2.44E-09	-0.13557	0.014	0.042	3.65E-05	AC-G	ERBB3
AKAP9.1	2.50E-09	-0.10099	0.372	0.465	3.74E-05	AC-G	AKAP9
RNF150	2.52E-09	-0.10352	0.016	0.046	3.76E-05	AC-G	RNF150
SPHK1.1	2.53E-09	-0.10138	0.067	0.114	3.78E-05	AC-G	SPHK1
NPTN	2.53E-09	-0.10421	0.032	0.068	3.78E-05	AC-G	NPTN
SWI5.1	2.54E-09	0.347555	0.282	0.238	3.80E-05	AC-G	SWI5
THOC6	2.59E-09	-0.11101	0.121	0.179	3.87E-05	AC-G	THOC6
C1orf228	2.61E-09	-0.11739	0.01	0.036	3.90E-05	AC-G	C1orf228
ZBED5.1	2.63E-09	-0.10419	0.129	0.188	3.93E-05	AC-G	ZBED5
GAB2	2.64E-09	-0.12304	0.035	0.072	3.94E-05	AC-G	GAB2
MGAT2.1	2.66E-09	-0.10239	0.135	0.197	3.97E-05	AC-G	MGAT2
SMC2	2.71E-09	-0.15779	0.151	0.209	4.04E-05	AC-G	SMC2
TIAM2.1	2.99E-09	-0.12498	0.046	0.085	4.47E-05	AC-G	TIAM2
GLO1.1	3.04E-09	0.232155	0.435	0.406	4.55E-05	AC-G	GLO1
GCC2	3.05E-09	-0.13355	0.215	0.283	4.56E-05	AC-G	GCC2
RP11-862L	3.07E-09	-0.15042	0.001	0.02	4.58E-05	AC-G	RP11-862L9.3
FBXO17.1	3.10E-09	0.170766	0.181	0.136	4.64E-05	AC-G	FBXO17
CIDEB	3.13E-09	-0.10211	0.006	0.029	4.68E-05	AC-G	CIDEB
CDKN1B.1	3.14E-09	-0.15677	0.204	0.27	4.69E-05	AC-G	CDKN1B
RAB35.1	3.21E-09	-0.10051	0.118	0.175	4.79E-05	AC-G	RAB35
GZF1.1	3.23E-09	-0.10486	0.065	0.111	4.82E-05	AC-G	GZF1
AFG3L2.1	3.26E-09	-0.1009	0.153	0.218	4.87E-05	AC-G	AFG3L2
SERPINF2	3.30E-09	-0.12143	0.006	0.03	4.94E-05	AC-G	SERPINF2
GRK4	3.33E-09	-0.10658	0.061	0.105	4.97E-05	AC-G	GRK4
WDR54.1	3.41E-09	0.286911	0.268	0.227	5.09E-05	AC-G	WDR54
CASC15.1	3.48E-09	-0.11853	0.062	0.107	5.20E-05	AC-G	CASC15
HINT3.1	3.50E-09	-0.11399	0.147	0.209	5.24E-05	AC-G	HINT3
C11orf1.1	3.75E-09	0.29755	0.182	0.137	5.60E-05	AC-G	C11orf1
NEK3.1	3.78E-09	-0.12399	0.034	0.07	5.65E-05	AC-G	NEK3
TPTEP1	4.18E-09	-0.12624	0.009	0.035	6.25E-05	AC-G	TPTEP1
PREP.1	4.19E-09	-0.11914	0.061	0.104	6.26E-05	AC-G	PREP
MIR22HG	4.30E-09	0.26316	0.15	0.106	6.43E-05	AC-G	MIR22HG
BCL6.1	4.75E-09	0.135238	0.167	0.12	7.11E-05	AC-G	BCL6
GPR137B.1	4.83E-09	-0.1117	0.175	0.24	7.22E-05	AC-G	GPR137B
ZCCHC24	5.00E-09	-0.12786	0.063	0.107	7.48E-05	AC-G	ZCCHC24
C9orf85.1	5.05E-09	-0.1262	0.06	0.103	7.55E-05	AC-G	C9orf85
PTPRN2.1	5.28E-09	-0.13246	0.164	0.221	7.88E-05	AC-G	PTPRN2
PRRX1.1	5.32E-09	0.333836	0.206	0.164	7.95E-05	AC-G	PRRX1
TRPM4	5.52E-09	-0.11251	0.016	0.045	8.25E-05	AC-G	TRPM4
TMEM258.1	5.52E-09	0.185479	0.696	0.735	8.25E-05	AC-G	TMEM258
ASNA1.1	5.54E-09	0.234616	0.538	0.516	8.28E-05	AC-G	ASNA1
GAR1.1	5.57E-09	0.291363	0.228	0.183	8.32E-05	AC-G	GAR1
TMEM255B.	5.59E-09	-0.16664	0.028	0.06	8.35E-05	AC-G	TMEM255B
CTSW	5.62E-09	-0.19523	0.009	0.033	8.41E-05	AC-G	CTSW

SMAD7	5.85E-09	-0.11685	0.012	0.038	8.75E-05	AC-G	SMAD7
ATP1B1.1	5.93E-09	-0.18387	0.189	0.243	8.86E-05	AC-G	ATP1B1
PINK1.1	6.07E-09	-0.13794	0.156	0.217	9.07E-05	AC-G	PINK1
FAM13B.1	6.09E-09	-0.10481	0.051	0.092	9.10E-05	AC-G	FAM13B
ITGB7	6.10E-09	-0.10271	0.006	0.029	9.11E-05	AC-G	ITGB7
MYO7A	6.30E-09	-0.13421	0.011	0.037	9.42E-05	AC-G	MYO7A
SLC44A1.1	6.40E-09	-0.1408	0.248	0.326	9.57E-05	AC-G	SLC44A1
KIF5A.1	6.66E-09	-0.15552	0.083	0.129	9.96E-05	AC-G	KIF5A
CHRNE	6.67E-09	-0.12605	0.007	0.03	9.96E-05	AC-G	CHRNE
TSEN54.1	6.86E-09	-0.11113	0.076	0.123	0.000103	AC-G	TSEN54
AC104653.	6.88E-09	0.107865	0.018	0.005	0.000103	AC-G	AC104653.1
PCNXL2	7.09E-09	-0.10759	0.05	0.09	0.000106	AC-G	PCNXL2
SLCO3A1	7.16E-09	-0.10082	0.01	0.036	0.000107	AC-G	SLCO3A1
HOTAIRM1.	7.20E-09	-0.14055	0.137	0.191	0.000108	AC-G	HOTAIRM1
RP13-516M	7.33E-09	-0.12907	0.01	0.036	0.00011	AC-G	RP13-516M14.4
DENND6A.1	7.56E-09	-0.10287	0.031	0.066	0.000113	AC-G	DENND6A
FEM1A	7.66E-09	-0.10358	0.083	0.134	0.000114	AC-G	FEM1A
TMEM91.1	7.96E-09	-0.12201	0.041	0.079	0.000119	AC-G	TMEM91
HHIP	7.99E-09	-0.13871	0.007	0.03	0.000119	AC-G	HHIP
POFUT2.1	8.11E-09	0.133542	0.12	0.081	0.000121	AC-G	POFUT2
VTI1A.1	8.85E-09	-0.12377	0.036	0.071	0.000132	AC-G	VTI1A
BRIX1.1	8.92E-09	0.248267	0.288	0.246	0.000133	AC-G	BRIX1
MAFF.1	9.03E-09	-0.22786	0.131	0.179	0.000135	AC-G	MAFF
NDN.1	9.21E-09	-0.1135	0.146	0.206	0.000138	AC-G	NDN
LAMP2.1	9.27E-09	0.21617	0.501	0.465	0.000139	AC-G	LAMP2
FZR1.1	9.65E-09	-0.10265	0.11	0.164	0.000144	AC-G	FZR1
NRIP3	9.99E-09	-0.10815	0.014	0.041	0.000149	AC-G	NRIP3
RP11-345J	1.01E-08	-0.11784	0.18	0.245	0.000152	AC-G	RP11-345J4.5
ZNF277	1.05E-08	-0.10977	0.126	0.181	0.000157	AC-G	ZNF277
CBFA2T2.1	1.06E-08	-0.10597	0.087	0.136	0.000158	AC-G	CBFA2T2
FAM214B.1	1.07E-08	-0.12854	0.079	0.126	0.00016	AC-G	FAM214B
TRAPPC4.1	1.09E-08	0.23797	0.463	0.441	0.000163	AC-G	TRAPPC4
KIAA0895L	1.13E-08	-0.10345	0.057	0.098	0.000169	AC-G	KIAA0895L
NEUROD2	1.14E-08	-0.11636	0.009	0.033	0.000171	AC-G	NEUROD2
C6orf15	1.15E-08	-0.15511	0.008	0.032	0.000171	AC-G	C6orf15
ZEB2-AS1	1.15E-08	-0.13003	0.013	0.038	0.000172	AC-G	ZEB2-AS1
C16orf54	1.17E-08	-0.10201	0.001	0.019	0.000175	AC-G	C16orf54
PCSK6	1.18E-08	-0.11005	0.002	0.02	0.000176	AC-G	PCSK6
PLXNB1.1	1.18E-08	-0.11846	0.044	0.082	0.000176	AC-G	PLXNB1
LINC00599	1.19E-08	-0.10009	0.013	0.039	0.000177	AC-G	LINC00599
NXPE3.1	1.21E-08	-0.13137	0.054	0.094	0.000181	AC-G	NXPE3
NKAIN4.1	1.25E-08	-0.14624	0.066	0.108	0.000187	AC-G	NKAIN4
RBBP7.1	1.27E-08	0.308664	0.446	0.42	0.00019	AC-G	RBBP7
CLN3	1.27E-08	0.14705	0.022	0.008	0.00019	AC-G	CLN3
MCM3.1	1.29E-08	-0.14053	0.101	0.15	0.000193	AC-G	MCM3
SHMT1.1	1.37E-08	-0.18156	0.08	0.125	0.000205	AC-G	SHMT1
UNC5B.1	1.38E-08	-0.18961	0.052	0.09	0.000206	AC-G	UNC5B
CD3E	1.39E-08	-0.17828	0.002	0.021	0.000207	AC-G	CD3E
YAP1	1.39E-08	0.133761	0.065	0.038	0.000208	AC-G	YAP1
ARV1.1	1.43E-08	0.214485	0.217	0.171	0.000214	AC-G	ARV1
GOLT1B.1	1.50E-08	0.237373	0.273	0.231	0.000225	AC-G	GOLT1B

LANCL2.1	1.51E-08	-0.13864	0.11	0.16	0.000226	AC-G	LANCL2
LRRC8B.1	1.53E-08	-0.10246	0.053	0.093	0.000228	AC-G	LRRC8B
KPNA6.1	1.56E-08	-0.10699	0.18	0.245	0.000233	AC-G	KPNA6
TMEM106C	1.58E-08	0.314702	0.299	0.26	0.000237	AC-G	TMEM106C
FAM91A1.1	1.63E-08	-0.11574	0.085	0.132	0.000244	AC-G	FAM91A1
CD200.1	1.65E-08	-0.14166	0.074	0.117	0.000246	AC-G	CD200
UQCRH.1	1.67E-08	0.131106	0.739	0.789	0.000249	AC-G	UQCRH
TRADD.1	1.71E-08	-0.12124	0.122	0.177	0.000256	AC-G	TRADD
ZNF703.1	1.72E-08	-0.13456	0.063	0.104	0.000257	AC-G	ZNF703
FXYD1	1.74E-08	0.122604	0.053	0.028	0.00026	AC-G	FXYD1
C8orf46	1.78E-08	-0.1321	0.099	0.147	0.000266	AC-G	C8orf46
TM2D2	1.83E-08	0.305405	0.372	0.334	0.000274	AC-G	TM2D2
GNL3.1	1.87E-08	0.27893	0.37	0.341	0.00028	AC-G	GNL3
HAAO	1.89E-08	-0.11728	0.005	0.026	0.000282	AC-G	HAAO
XPR1.1	1.93E-08	-0.10731	0.086	0.133	0.000289	AC-G	XPR1
SLC2A9	2.06E-08	-0.10921	0.007	0.029	0.000308	AC-G	SLC2A9
PDCL	2.06E-08	-0.13058	0.146	0.203	0.000308	AC-G	PDCL
SLAMF9	2.07E-08	-0.12773	0.003	0.022	0.00031	AC-G	SLAMF9
TSHZ1.1	2.22E-08	-0.10982	0.043	0.08	0.000331	AC-G	TSHZ1
GOLPH3L.1	2.28E-08	0.144306	0.11	0.074	0.000341	AC-G	GOLPH3L
SELK.1	2.28E-08	0.176124	0.683	0.704	0.000341	AC-G	SELK
ESYT1.1	2.30E-08	-0.12713	0.082	0.128	0.000343	AC-G	ESYT1
PAPSS2	2.31E-08	-0.10487	0.005	0.026	0.000345	AC-G	PAPSS2
KIAA1147	2.33E-08	-0.12549	0.041	0.077	0.000348	AC-G	KIAA1147
C9orf116.	2.38E-08	0.196397	0.104	0.071	0.000355	AC-G	C9orf116
PLA2G16.1	2.51E-08	0.206423	0.437	0.398	0.000376	AC-G	PLA2G16
SRP54.1	2.74E-08	0.270175	0.305	0.267	0.00041	AC-G	SRP54
PARK7.1	2.82E-08	0.246813	0.745	0.77	0.000421	AC-G	PARK7
EMG1	2.84E-08	0.287064	0.27	0.228	0.000425	AC-G	EMG1
TXNDC15	2.95E-08	0.266395	0.326	0.287	0.00044	AC-G	TXNDC15
BOD1L1.1	2.96E-08	-0.11231	0.115	0.168	0.000443	AC-G	BOD1L1
NHLH1	3.03E-08	-0.11204	0.007	0.028	0.000453	AC-G	NHLH1
NKAIN2	3.14E-08	-0.10594	0.002	0.02	0.000469	AC-G	NKAIN2
NPHP1	3.17E-08	0.211048	0.096	0.064	0.000474	AC-G	NPHP1
FAM134C.1	3.18E-08	-0.12485	0.083	0.127	0.000475	AC-G	FAM134C
C7orf26.1	3.23E-08	-0.10655	0.115	0.166	0.000482	AC-G	C7orf26
DEPTOR	3.23E-08	-0.12566	0.02	0.049	0.000482	AC-G	DEPTOR
PISD.1	3.30E-08	-0.12055	0.069	0.112	0.000493	AC-G	PISD
ITPRIP.1	3.34E-08	-0.14309	0.045	0.081	0.0005	AC-G	ITPRIP
PPP1R3B	3.36E-08	-0.10684	0.038	0.072	0.000501	AC-G	PPP1R3B
FAM111A.1	3.45E-08	-0.16156	0.074	0.116	0.000515	AC-G	FAM111A
CASP8.1	3.49E-08	-0.10603	0.023	0.052	0.000522	AC-G	CASP8
ATP5G1.1	3.50E-08	0.229016	0.605	0.62	0.000523	AC-G	ATP5G1
HK1.1	3.62E-08	-0.13077	0.148	0.204	0.000542	AC-G	HK1
UVRAG.1	3.72E-08	-0.11838	0.056	0.096	0.000556	AC-G	UVRAG
SNRK.1	3.90E-08	-0.10715	0.053	0.091	0.000583	AC-G	SNRK
OPALIN	3.91E-08	-0.18889	0	0.016	0.000585	AC-G	OPALIN
AC004540.	4.02E-08	-0.11679	0.049	0.085	0.000601	AC-G	AC004540.5
AKTIP	4.06E-08	0.233093	0.204	0.161	0.000607	AC-G	AKTIP
SLC1A5.1	4.11E-08	-0.17265	0.099	0.146	0.000614	AC-G	SLC1A5
SCD.1	4.16E-08	-0.1471	0.16	0.222	0.000621	AC-G	SCD

TMEM14A	4.22E-08	0.313368	0.349	0.319	0.000631	AC-G	TMEM14A
TOR3A.1	4.57E-08	-0.13058	0.162	0.219	0.000683	AC-G	TOR3A
TMEM147	4.59E-08	0.223725	0.586	0.597	0.000686	AC-G	TMEM147
NANS.1	4.69E-08	-0.14478	0.306	0.37	0.000701	AC-G	NANS
ANKRD28.1	4.71E-08	0.176671	0.146	0.106	0.000703	AC-G	ANKRD28
HES1.1	4.76E-08	0.22685	0.236	0.189	0.000711	AC-G	HES1
DNAJC19.1	4.76E-08	0.317617	0.408	0.385	0.000711	AC-G	DNAJC19
PPIA	4.76E-08	0.170652	0.815	0.887	0.000712	AC-G	PPIA
TCTEX1D2	4.88E-08	0.324812	0.259	0.221	0.000729	AC-G	TCTEX1D2
TUBA1B	4.92E-08	0.205882	0.842	0.906	0.000735	AC-G	TUBA1B
CAP2	5.04E-08	0.138525	0.083	0.052	0.000753	AC-G	CAP2
GRB10.1	5.16E-08	0.196716	0.1	0.068	0.000772	AC-G	GRB10
TRIO.1	5.38E-08	-0.10044	0.126	0.181	0.000804	AC-G	TRIO
EMC8.1	5.50E-08	-0.10111	0.218	0.282	0.000822	AC-G	EMC8
SGK3.1	5.59E-08	-0.13656	0.039	0.073	0.000835	AC-G	SGK3
CD2	5.70E-08	-0.18328	0.004	0.022	0.000852	AC-G	CD2
RGMB.1	5.73E-08	-0.19895	0.096	0.139	0.000857	AC-G	RGMB
KIAA1033.	5.92E-08	-0.10163	0.189	0.248	0.000884	AC-G	KIAA1033
SDHD	6.01E-08	0.228034	0.426	0.39	0.000898	AC-G	SDHD
PLBD2.1	6.06E-08	-0.10093	0.031	0.062	0.000905	AC-G	PLBD2
HSD3B7	6.08E-08	0.142136	0.104	0.07	0.000908	AC-G	HSD3B7
SDHC.1	6.18E-08	0.248634	0.558	0.557	0.000923	AC-G	SDHC
CCL20.1	6.18E-08	-0.17072	0.034	0.066	0.000924	AC-G	CCL20
SPNS3	6.68E-08	-0.1123	0.006	0.026	0.000998	AC-G	SPNS3
CCND2-AS1	6.80E-08	0.108255	0.042	0.022	0.001017	AC-G	CCND2-AS1
KIAA1551.	6.91E-08	-0.13061	0.055	0.093	0.001032	AC-G	KIAA1551
SLC9A3R1.	7.04E-08	-0.12962	0.109	0.158	0.001051	AC-G	SLC9A3R1
CD1D	7.20E-08	-0.13537	0.016	0.042	0.001077	AC-G	CD1D
NAA60	7.21E-08	0.130092	0.025	0.01	0.001077	AC-G	NAA60
ATP6AP1L	7.31E-08	0.141683	0.068	0.041	0.001092	AC-G	ATP6AP1L
PGM2	7.68E-08	-0.10516	0.108	0.156	0.001147	AC-G	PGM2
FRS2.1	7.77E-08	-0.10364	0.076	0.118	0.001161	AC-G	FRS2
C9orf3	7.83E-08	0.161946	0.138	0.098	0.00117	AC-G	C9orf3
LIG1	7.94E-08	-0.11707	0.043	0.077	0.001187	AC-G	LIG1
PHF1.1	7.98E-08	0.168804	0.205	0.162	0.001192	AC-G	PHF1
BAD	8.05E-08	0.231534	0.44	0.413	0.001203	AC-G	BAD
TPP2.1	8.13E-08	-0.12176	0.085	0.13	0.001215	AC-G	TPP2
SLC31A1.1	8.20E-08	-0.15368	0.126	0.176	0.001225	AC-G	SLC31A1
SCOC.1	8.63E-08	0.212521	0.436	0.41	0.001289	AC-G	SCOC
UBE2E1	8.66E-08	0.250214	0.459	0.449	0.001294	AC-G	UBE2E1
ITSN1.1	9.11E-08	-0.1574	0.107	0.153	0.001361	AC-G	ITSN1
GLTP.1	9.62E-08	-0.12156	0.215	0.279	0.001437	AC-G	GLTP
LEAP2.1	9.77E-08	-0.10928	0.051	0.088	0.001459	AC-G	LEAP2
ARID5B.1	9.80E-08	0.273212	0.38	0.35	0.001465	AC-G	ARID5B
FUBP1.1	1.00E-07	-0.1011	0.176	0.234	0.001501	AC-G	FUBP1
NDUFC2.1	1.01E-07	0.208281	0.715	0.76	0.001503	AC-G	NDUFC2
LONP2	1.03E-07	-0.11377	0.196	0.258	0.001545	AC-G	LONP2
BMPR1B	1.05E-07	0.100377	0.038	0.019	0.001563	AC-G	BMPR1B
CCL5	1.06E-07	-0.29285	0.031	0.06	0.00159	AC-G	CCL5
TUBE1	1.08E-07	0.142339	0.124	0.088	0.001614	AC-G	TUBE1
CIRBP.1	1.10E-07	0.135769	0.811	0.876	0.001646	AC-G	CIRBP

TMED4.1	1.11E-07	0.272016	0.449	0.436	0.001655	AC-G	TMED4
CAPN3	1.17E-07	-0.22376	0.021	0.047	0.001743	AC-G	CAPN3
SERGEF.1	1.17E-07	0.224063	0.201	0.161	0.001748	AC-G	SERGEF
XPOT.1	1.20E-07	0.187611	0.2	0.161	0.0018	AC-G	XPOT
DPYD	1.22E-07	0.109253	0.135	0.094	0.00182	AC-G	DPYD
TSPYL2	1.22E-07	0.142969	0.146	0.106	0.001828	AC-G	TSPYL2
GCLM	1.27E-07	0.177613	0.191	0.152	0.001899	AC-G	GCLM
ARID3A	1.28E-07	-0.11328	0.078	0.122	0.001909	AC-G	ARID3A
SVIP.1	1.28E-07	-0.24819	0.131	0.177	0.001915	AC-G	SVIP
C1QTNF3	1.30E-07	-0.10089	0.017	0.043	0.001937	AC-G	C1QTNF3
MCM4.1	1.31E-07	-0.10751	0.063	0.102	0.001953	AC-G	MCM4
RGN.1	1.40E-07	0.154565	0.1	0.067	0.002099	AC-G	RGN
ZSWIM8.1	1.41E-07	-0.10371	0.048	0.084	0.002102	AC-G	ZSWIM8
CCDC151	1.42E-07	0.153646	0.089	0.058	0.002129	AC-G	CCDC151
AGTPBP1.1	1.45E-07	-0.10653	0.027	0.055	0.002168	AC-G	AGTPBP1
PCGF1	1.46E-07	0.170804	0.125	0.089	0.002182	AC-G	PCGF1
NDUFA6.1	1.47E-07	0.266328	0.571	0.572	0.002192	AC-G	NDUFA6
ZNF804A	1.49E-07	-0.10093	0.017	0.042	0.002223	AC-G	ZNF804A
CLOCK.1	1.51E-07	-0.10384	0.091	0.135	0.002251	AC-G	CLOCK
MANF.1	1.52E-07	0.24962	0.46	0.449	0.002275	AC-G	MANF
ETV3.1	1.59E-07	-0.13248	0.048	0.082	0.002382	AC-G	ETV3
PSMC2.1	1.60E-07	0.259915	0.474	0.456	0.002384	AC-G	PSMC2
PAK1IP1.1	1.66E-07	0.228454	0.25	0.209	0.002476	AC-G	PAK1IP1
TNFAIP8	1.70E-07	-0.11983	0.042	0.075	0.002534	AC-G	TNFAIP8
SPOCK1.1	1.74E-07	-0.14664	0.052	0.087	0.002593	AC-G	SPOCK1
TNFRSF14.	1.75E-07	-0.18399	0.082	0.122	0.00262	AC-G	TNFRSF14
PLEKHA5.1	1.78E-07	-0.10404	0.037	0.069	0.002657	AC-G	PLEKHA5
MCM7.1	1.79E-07	-0.14382	0.207	0.263	0.002677	AC-G	MCM7
HOXC10	1.87E-07	0.132691	0.078	0.049	0.002794	AC-G	HOXC10
C1orf109	1.96E-07	0.201532	0.163	0.126	0.002936	AC-G	C1orf109
CA2.1	1.99E-07	0.274375	0.263	0.218	0.002975	AC-G	CA2
SH2B2.1	2.03E-07	-0.10564	0.075	0.115	0.003031	AC-G	SH2B2
AC093673.	2.05E-07	0.221233	0.211	0.172	0.003061	AC-G	AC093673.5
ARG2	2.05E-07	-0.13264	0.067	0.106	0.003063	AC-G	ARG2
STK3	2.12E-07	0.185118	0.139	0.103	0.00317	AC-G	STK3
HOXC9.1	2.19E-07	-0.13644	0.056	0.091	0.003269	AC-G	HOXC9
KBTBD8	2.21E-07	-0.10623	0.011	0.033	0.003309	AC-G	KBTBD8
ZNF419	2.22E-07	0.159234	0.087	0.057	0.003314	AC-G	ZNF419
OCIAD1.1	2.23E-07	0.23201	0.621	0.639	0.003337	AC-G	OCIAD1
ANAPC11.1	2.24E-07	0.196357	0.717	0.728	0.003342	AC-G	ANAPC11
C10orf76.	2.31E-07	-0.1132	0.031	0.061	0.003455	AC-G	C10orf76
MITD1.1	2.33E-07	0.229336	0.269	0.227	0.00348	AC-G	MITD1
PSMB7.1	2.36E-07	0.208318	0.629	0.642	0.003531	AC-G	PSMB7
FAM195B	2.43E-07	0.194828	0.095	0.063	0.003629	AC-G	FAM195B
SERPINH1.	2.47E-07	0.235646	0.24	0.203	0.003685	AC-G	SERPINH1
LINC00623	2.62E-07	0.145393	0.022	0.009	0.00392	AC-G	LINC00623
ARL13B.1	2.71E-07	0.180968	0.176	0.136	0.004056	AC-G	ARL13B
ITGB5	2.80E-07	-0.13893	0.032	0.061	0.004182	AC-G	ITGB5
SPAG1	2.86E-07	0.107592	0.067	0.042	0.00428	AC-G	SPAG1
NRARP	2.90E-07	-0.10763	0.016	0.039	0.004327	AC-G	NRARP
ELL2.1	2.94E-07	-0.19391	0.157	0.2	0.0044	AC-G	ELL2

SPG20	3.00E-07	0.169966	0.272	0.231	0.004483	AC-G	SPG20
APOL4	3.04E-07	0.118904	0.064	0.039	0.004537	AC-G	APOL4
MDH1	3.04E-07	0.219791	0.552	0.55	0.004548	AC-G	MDH1
CALHM2.1	3.05E-07	-0.11629	0.034	0.065	0.004557	AC-G	CALHM2
NQO1.1	3.23E-07	0.316397	0.136	0.103	0.004822	AC-G	NQO1
MLLT11	3.23E-07	-0.1073	0.411	0.462	0.004828	AC-G	MLLT11
ZDHHC14	3.24E-07	-0.12814	0.049	0.083	0.004848	AC-G	ZDHHC14
NT5C2.1	3.39E-07	-0.11624	0.094	0.136	0.005061	AC-G	NT5C2
TMX2	3.47E-07	0.249597	0.416	0.395	0.005184	AC-G	TMX2
COPZ1	3.54E-07	0.261555	0.496	0.483	0.005285	AC-G	COPZ1
CYSLTR1	3.54E-07	-0.10131	0.008	0.028	0.005295	AC-G	CYSLTR1
SAR1B.1	3.58E-07	0.210046	0.347	0.317	0.005345	AC-G	SAR1B
FCER1A	3.70E-07	-0.12391	0.008	0.027	0.005532	AC-G	FCER1A
DDIT3.1	3.87E-07	0.255739	0.44	0.426	0.00579	AC-G	DDIT3
KRBOX1	3.89E-07	0.109599	0.013	0.004	0.005813	AC-G	KRBOX1
GPBP1L1.1	4.04E-07	-0.10148	0.16	0.215	0.006043	AC-G	GPBP1L1
SMOC1	4.10E-07	-0.11584	0.015	0.038	0.00613	AC-G	SMOC1
C14orf1	4.22E-07	0.110951	0.229	0.305	0.006303	AC-G	C14orf1
ANKRD36.1	4.33E-07	-0.15361	0.145	0.193	0.00647	AC-G	ANKRD36
NACAD.1	4.37E-07	-0.10112	0.059	0.095	0.006538	AC-G	NACAD
PMM1.1	4.41E-07	0.313143	0.26	0.228	0.006593	AC-G	PMM1
FBXO8	4.61E-07	0.196464	0.176	0.138	0.006892	AC-G	FBXO8
KIAA2026.	4.76E-07	-0.12747	0.107	0.15	0.007112	AC-G	KIAA2026
SMIM14.1	4.81E-07	0.253483	0.359	0.325	0.007193	AC-G	SMIM14
CUTA.1	5.09E-07	0.138039	0.652	0.688	0.007607	AC-G	CUTA
S100A2	5.12E-07	0.136687	0.052	0.03	0.007645	AC-G	S100A2
RIPK2.1	5.16E-07	-0.14474	0.13	0.175	0.007718	AC-G	RIPK2
ATG4C.1	5.20E-07	-0.12207	0.102	0.146	0.007776	AC-G	ATG4C
SFXN5.1	5.30E-07	0.274388	0.218	0.182	0.007917	AC-G	SFXN5
HOXB2.1	5.51E-07	-0.11363	0.056	0.09	0.008232	AC-G	HOXB2
NR3C1.1	5.72E-07	-0.11602	0.2	0.258	0.008553	AC-G	NR3C1
MOCS2.1	5.77E-07	0.24961	0.293	0.257	0.008616	AC-G	MOCS2
SCML1.1	5.85E-07	0.135437	0.124	0.088	0.008747	AC-G	SCML1
KHDC1.1	6.03E-07	0.1002	0.09	0.059	0.009008	AC-G	KHDC1
SHMT2.1	6.67E-07	0.2697	0.26	0.226	0.009966	AC-G	SHMT2
PDLIM2	6.72E-07	0.28511	0.269	0.234	0.010048	AC-G	PDLIM2
NME5	7.05E-07	0.157005	0.07	0.045	0.010532	AC-G	NME5
DPM1.1	7.07E-07	0.283486	0.394	0.375	0.010564	AC-G	DPM1
METTL22.1	7.49E-07	-0.12233	0.06	0.095	0.01119	AC-G	METTL22
CTSL.1	7.96E-07	0.164913	0.561	0.516	0.011889	AC-G	CTSL
CHRAC1.1	8.22E-07	-0.10686	0.107	0.152	0.012283	AC-G	CHRAC1
ELN	8.33E-07	0.259885	0.138	0.102	0.012446	AC-G	ELN
YARS	8.36E-07	0.211096	0.246	0.212	0.012495	AC-G	YARS
PPP1R9A.1	8.39E-07	-0.14216	0.046	0.076	0.012534	AC-G	PPP1R9A
HOPX	8.73E-07	0.147522	0.347	0.27	0.013051	AC-G	HOPX
C1QTNF9B-	8.74E-07	0.134863	0.041	0.022	0.013059	AC-G	C1QTNF9B-AS1
FIBIN.1	8.78E-07	0.270616	0.262	0.22	0.013116	AC-G	FIBIN
BLZF1	9.14E-07	0.142722	0.147	0.111	0.013664	AC-G	BLZF1
PDLIM1.1	9.17E-07	0.219121	0.098	0.069	0.013701	AC-G	PDLIM1
SNX18.1	9.24E-07	-0.12127	0.08	0.119	0.013807	AC-G	SNX18
IP6K2.1	9.32E-07	0.242327	0.452	0.431	0.01392	AC-G	IP6K2

VPS26A	9.70E-07	-0.13594	0.2	0.251	0.014489	AC-G	VPS26A
CYB5B.1	9.70E-07	0.22364	0.363	0.332	0.014503	AC-G	CYB5B
BACE1.1	9.78E-07	-0.12187	0.085	0.124	0.01461	AC-G	BACE1
3-Mar	9.97E-07	0.142308	0.135	0.099	0.014901	AC-G	3-Mar
CNPY4.1	9.97E-07	0.202856	0.141	0.107	0.014905	AC-G	CNPY4
ATP5J	1.00E-06	0.200513	0.716	0.758	0.015009	AC-G	ATP5J
PSMB6	1.01E-06	0.212956	0.672	0.701	0.015099	AC-G	PSMB6
POR	1.02E-06	0.210582	0.295	0.259	0.015225	AC-G	POR
ATE1.1	1.02E-06	-0.10869	0.028	0.055	0.015312	AC-G	ATE1
NDUFA1.1	1.03E-06	0.150921	0.688	0.731	0.015356	AC-G	NDUFA1
IFIT2.1	1.03E-06	-0.13493	0.054	0.087	0.015398	AC-G	IFIT2
GPRC5C.1	1.05E-06	-0.1349	0.044	0.074	0.015758	AC-G	GPRC5C
GBP4	1.06E-06	-0.12942	0.042	0.072	0.015867	AC-G	GBP4
TULP3.1	1.08E-06	0.193796	0.154	0.12	0.016139	AC-G	TULP3
ZSCAN16.1	1.08E-06	-0.11359	0.039	0.068	0.016153	AC-G	ZSCAN16
C9orf9.1	1.12E-06	0.203989	0.137	0.104	0.016703	AC-G	C9orf9
PCP4	1.14E-06	0.175754	0.059	0.035	0.016962	AC-G	PCP4
CA8.1	1.17E-06	-0.12969	0.032	0.059	0.017454	AC-G	CA8
RAPH1	1.17E-06	-0.10045	0.041	0.071	0.017506	AC-G	RAPH1
SLC1A4.1	1.19E-06	-0.12122	0.046	0.078	0.017853	AC-G	SLC1A4
SPA17.1	1.20E-06	0.206977	0.106	0.076	0.017906	AC-G	SPA17
KLHL24.1	1.23E-06	-0.11363	0.14	0.186	0.018378	AC-G	KLHL24
IDNK.1	1.39E-06	0.166607	0.123	0.089	0.020821	AC-G	IDNK
NPRL2.1	1.53E-06	0.19403	0.189	0.152	0.02285	AC-G	NPRL2
CYB561.1	1.57E-06	0.167808	0.104	0.075	0.023479	AC-G	CYB561
TMEM230	1.58E-06	0.216523	0.61	0.618	0.023544	AC-G	TMEM230
CAPN2	1.61E-06	0.193229	0.279	0.247	0.024046	AC-G	CAPN2
ANGPTL2	1.61E-06	0.171808	0.127	0.094	0.024077	AC-G	ANGPTL2
SUN1.1	1.61E-06	0.206453	0.239	0.202	0.024088	AC-G	SUN1
TCEAL2	1.62E-06	0.118762	0.222	0.171	0.024136	AC-G	TCEAL2
SCAMP1	1.66E-06	-0.10432	0.172	0.222	0.024823	AC-G	SCAMP1
COPB2.1	1.67E-06	0.287507	0.327	0.302	0.024885	AC-G	COPB2
PCDHB10.1	1.72E-06	-0.13143	0.073	0.108	0.025646	AC-G	PCDHB10
ATP6V1F.1	1.73E-06	0.163097	0.744	0.813	0.025851	AC-G	ATP6V1F
AAMDC.1	1.74E-06	0.237778	0.236	0.2	0.025994	AC-G	AAMDC
UPF2	1.85E-06	-0.10997	0.174	0.226	0.027646	AC-G	UPF2
VPS4B.1	1.97E-06	-0.10386	0.2	0.258	0.029409	AC-G	VPS4B
KRTCAP2	1.99E-06	0.199356	0.559	0.556	0.0297	AC-G	KRTCAP2
GARS.1	2.05E-06	0.21737	0.316	0.289	0.030575	AC-G	GARS
AAMP.1	2.07E-06	0.224402	0.438	0.425	0.030869	AC-G	AAMP
WNT3	2.19E-06	0.13144	0.045	0.026	0.032788	AC-G	WNT3
IFI35.1	2.28E-06	0.255345	0.261	0.225	0.034114	AC-G	IFI35
C8orf48	2.35E-06	0.107615	0.019	0.008	0.035054	AC-G	C8orf48
GPX8	2.41E-06	0.213011	0.084	0.059	0.036041	AC-G	GPX8
CLUAP1	2.41E-06	0.257399	0.197	0.162	0.036079	AC-G	CLUAP1
PFDN5.1	2.47E-06	0.125189	0.813	0.898	0.036977	AC-G	PFDN5
ECHDC2.1	2.51E-06	0.230821	0.218	0.182	0.03758	AC-G	ECHDC2
MOK	2.54E-06	0.125448	0.089	0.061	0.038009	AC-G	MOK
GNLY	2.56E-06	-0.17654	0.013	0.033	0.038279	AC-G	GNLY
RP11-166C	2.57E-06	-0.1172	0.033	0.06	0.038377	AC-G	RP11-16604.6
CD3G	2.63E-06	-0.10013	0.001	0.013	0.039276	AC-G	CD3G

RAB40B.1	2.63E-06	-0.1215	0.096	0.136	0.03932	AC-G	RAB40B
PLD2.1	2.64E-06	-0.10506	0.058	0.091	0.039479	AC-G	PLD2
METTL21B.	2.65E-06	0.132174	0.088	0.06	0.039536	AC-G	METTL21B
PSORS1C1	2.69E-06	0.181571	0.054	0.033	0.040213	AC-G	PSORS1C1
PTGR1.1	2.72E-06	0.299148	0.212	0.18	0.040617	AC-G	PTGR1
MTRNR2L12	2.99E-06	0.371199	0.228	0.19	0.044665	AC-G	MTRNR2L12
CCP110.1	2.99E-06	-0.11721	0.092	0.13	0.044716	AC-G	CCP110
ACP1.1	3.13E-06	0.200992	0.505	0.497	0.046726	AC-G	ACP1
INCA1	3.20E-06	0.119662	0.031	0.016	0.047787	AC-G	INCA1
SEPT4.1	3.32E-06	-0.18397	0.097	0.136	0.049576	AC-G	4-Sep
CX3CL1	3.33E-06	0.133373	0.057	0.036	0.049814	AC-G	CX3CL1
CMBL	3.42E-06	0.177275	0.154	0.121	0.051069	AC-G	CMBL
ECH1.1	3.53E-06	0.227777	0.555	0.559	0.052797	AC-G	ECH1
TFB2M	3.58E-06	0.165767	0.111	0.08	0.053489	AC-G	TFB2M
CD3D	3.63E-06	-0.21191	0.006	0.021	0.054263	AC-G	CD3D
NFKB2.1	3.66E-06	-0.13053	0.038	0.065	0.054703	AC-G	NFKB2
PEX12.1	3.72E-06	0.161775	0.073	0.048	0.055561	AC-G	PEX12
TRAM1.1	3.76E-06	0.162126	0.45	0.43	0.056162	AC-G	TRAM1
EFHC1.1	3.98E-06	0.298509	0.231	0.2	0.059403	AC-G	EFHC1
PARP10	4.16E-06	-0.12907	0.06	0.092	0.062228	AC-G	PARP10
NKX2-2.1	4.23E-06	-0.11796	0.114	0.154	0.063145	AC-G	NKX2-2
ISG20	4.23E-06	0.199799	0.151	0.116	0.063146	AC-G	ISG20
AC002456.	4.23E-06	0.205615	0.063	0.04	0.063178	AC-G	AC002456.2
DNAJC25.1	4.34E-06	0.132024	0.109	0.08	0.064797	AC-G	DNAJC25
RP11-798M	4.35E-06	0.19155	0.178	0.143	0.065009	AC-G	RP11-798M19.6
COMTD1.1	4.49E-06	-0.10593	0.038	0.064	0.067147	AC-G	COMTD1
CDC42EP2	4.50E-06	-0.10614	0.019	0.04	0.067306	AC-G	CDC42EP2
FAM229B	4.63E-06	0.213361	0.316	0.287	0.069168	AC-G	FAM229B
RAB3IL1	4.67E-06	-0.10328	0.016	0.036	0.069743	AC-G	RAB3IL1
RP11-620J	4.70E-06	0.218887	0.302	0.263	0.070216	AC-G	RP11-620J15.3
LCK	4.75E-06	-0.11898	0.003	0.017	0.071056	AC-G	LCK
MCMBP.1	4.81E-06	-0.10755	0.11	0.151	0.071855	AC-G	MCMBP
PTPRF.1	4.83E-06	0.163264	0.142	0.11	0.072225	AC-G	PTPRF
PCID2	5.25E-06	0.23404	0.207	0.174	0.078529	AC-G	PCID2
IBSP	5.35E-06	-0.15067	0.009	0.027	0.080017	AC-G	IBSP
C5orf56.1	5.36E-06	0.127789	0.139	0.106	0.080041	AC-G	C5orf56
POLR1C.1	5.56E-06	0.173604	0.149	0.116	0.083137	AC-G	POLR1C
NR2F2	5.92E-06	0.104048	0.064	0.041	0.088397	AC-G	NR2F2
HSD17B6	5.97E-06	0.106366	0.032	0.017	0.089184	AC-G	HSD17B6
ISOC1	6.21E-06	0.149452	0.195	0.158	0.092757	AC-G	ISOC1
CMTM5.1	6.33E-06	-0.25709	0.074	0.105	0.094609	AC-G	CMTM5
PSMA7.1	6.60E-06	0.169393	0.758	0.809	0.098619	AC-G	PSMA7
NT5E	6.72E-06	0.141843	0.083	0.058	0.100406	AC-G	NT5E
ABCA8	6.78E-06	0.177837	0.097	0.07	0.101332	AC-G	ABCA8
OAT	6.85E-06	0.156124	0.273	0.23	0.102298	AC-G	OAT
VRK2.1	6.93E-06	-0.1543	0.034	0.059	0.103578	AC-G	VRK2
PTTG1.1	7.04E-06	-0.16668	0.192	0.238	0.105203	AC-G	PTTG1
WDR19.1	7.15E-06	0.118343	0.066	0.043	0.106789	AC-G	WDR19
SEC62.1	7.47E-06	0.167428	0.65	0.699	0.111596	AC-G	SEC62
DGUOK	7.75E-06	0.259317	0.515	0.516	0.115841	AC-G	DGUOK
LMAN2.1	7.79E-06	0.213157	0.564	0.576	0.11645	AC-G	LMAN2

MAD2L1.1	8.18E-06	-0.11032	0.098	0.134	0.1223	AC-G	MAD2L1
SARS.1	8.34E-06	0.198892	0.448	0.442	0.124589	AC-G	SARS
RP11-1143	8.99E-06	-0.14394	0.031	0.056	0.134371	AC-G	RP11-1143G9.4
PELO.1	9.07E-06	0.13111	0.133	0.101	0.135615	AC-G	PELO
SELM.1	9.10E-06	0.114172	0.222	0.182	0.135948	AC-G	SELM
GSTZ1.1	9.38E-06	0.112727	0.117	0.165	0.140231	AC-G	GSTZ1
QPCT.1	9.46E-06	-0.1449	0.028	0.051	0.141432	AC-G	QPCT
HDHD3	9.74E-06	0.208216	0.135	0.104	0.145485	AC-G	HDHD3
ZDHHC4.1	1.03E-05	0.252408	0.373	0.351	0.15348	AC-G	ZDHHC4
SERF2	1.06E-05	0.160261	0.837	0.907	0.157763	AC-G	SERF2
OMG.1	1.09E-05	-0.13129	0.055	0.084	0.162944	AC-G	OMG
TMEM43.1	1.12E-05	0.141894	0.22	0.186	0.167084	AC-G	TMEM43
GFPT1	1.15E-05	0.117164	0.121	0.09	0.171529	AC-G	GFPT1
CCT8	1.17E-05	0.158747	0.541	0.534	0.175375	AC-G	CCT8
NDUFB9.1	1.20E-05	0.174026	0.665	0.694	0.179501	AC-G	NDUFB9
SMS.1	1.21E-05	0.217349	0.565	0.574	0.180375	AC-G	SMS
NOP16.1	1.23E-05	0.286829	0.224	0.194	0.183356	AC-G	NOP16
HSPD1.1	1.26E-05	0.161916	0.582	0.621	0.188088	AC-G	HSPD1
DNAJB1	1.26E-05	0.252018	0.608	0.61	0.188852	AC-G	DNAJB1
NIFK	1.27E-05	0.252296	0.366	0.345	0.189574	AC-G	NIFK
LRRCC1.1	1.29E-05	0.246646	0.159	0.131	0.192952	AC-G	LRRCC1
CLDN12.1	1.29E-05	0.167397	0.165	0.133	0.193105	AC-G	CLDN12
FOSL1.1	1.32E-05	0.113982	0.065	0.043	0.197443	AC-G	FOSL1
ST5.1	1.33E-05	0.19517	0.177	0.146	0.199415	AC-G	ST5
NDUFS4	1.36E-05	0.237125	0.503	0.505	0.203696	AC-G	NDUFS4
HS2ST1.1	1.41E-05	0.275453	0.198	0.17	0.211111	AC-G	HS2ST1
TXLNG	1.45E-05	0.155511	0.16	0.128	0.21735	AC-G	TXLNG
MTURN	1.51E-05	-0.1116	0.055	0.084	0.225233	AC-G	MTURN
VBP1.1	1.56E-05	0.212364	0.495	0.485	0.233576	AC-G	VBP1
TXNDC12.1	1.57E-05	0.144757	0.448	0.429	0.234542	AC-G	TXNDC12
CCDC25.1	1.59E-05	0.222404	0.318	0.293	0.237134	AC-G	CCDC25
TPD52.1	1.63E-05	0.208187	0.262	0.231	0.242857	AC-G	TPD52
SLC46A3.1	1.64E-05	-0.13341	0.037	0.061	0.245673	AC-G	SLC46A3
DPH3	1.72E-05	0.269163	0.239	0.208	0.256486	AC-G	DPH3
PTPRCAP	1.75E-05	-0.196	0.012	0.029	0.261488	AC-G	PTPRCAP
ANPEP	1.77E-05	-0.10194	0.016	0.035	0.263854	AC-G	ANPEP
ORC5.1	1.77E-05	0.183563	0.123	0.095	0.264924	AC-G	ORC5
SOX2.1	1.81E-05	-0.11065	0.396	0.436	0.270547	AC-G	SOX2
DPCD.1	1.82E-05	0.205916	0.187	0.156	0.272096	AC-G	DPCD
ATP5B.1	1.85E-05	0.165797	0.734	0.784	0.277047	AC-G	ATP5B
ZFAS1.1	1.95E-05	0.202434	0.664	0.704	0.291313	AC-G	ZFAS1
PARP12.1	1.99E-05	-0.1095	0.052	0.079	0.297005	AC-G	PARP12
PRNP	2.00E-05	0.175827	0.471	0.464	0.298519	AC-G	PRNP
S100A1	2.05E-05	-0.21096	0.021	0.041	0.306069	AC-G	S100A1
NFIL3.1	2.14E-05	-0.16931	0.06	0.088	0.320118	AC-G	NFIL3
WWTR1.1	2.18E-05	0.192994	0.187	0.155	0.325744	AC-G	WWTR1
LBH.1	2.20E-05	-0.12486	0.123	0.159	0.328147	AC-G	LBH
PPP1R15B.	2.22E-05	-0.10267	0.089	0.123	0.332381	AC-G	PPP1R15B
NMD3	2.23E-05	0.172266	0.242	0.21	0.333442	AC-G	NMD3
CCL7	2.38E-05	0.121028	0.023	0.011	0.355319	AC-G	CCL7
CTC-479C5	2.43E-05	-0.11037	0.032	0.055	0.363393	AC-G	CTC-479C5.12

TF.1	2.44E-05	-0.1219	0.098	0.068	0.364462	AC-G	TF
DUSP22.1	2.47E-05	-0.13493	0.085	0.117	0.368776	AC-G	DUSP22
TLR3	2.50E-05	-0.10776	0.017	0.036	0.373367	AC-G	TLR3
PTGS2.1	2.52E-05	-0.13458	0.041	0.065	0.376778	AC-G	PTGS2
ARMCX2	2.55E-05	0.245076	0.172	0.141	0.380479	AC-G	ARMCX2
CYB5A.1	2.59E-05	0.314065	0.355	0.339	0.387327	AC-G	CYB5A
SLC17A5.1	2.64E-05	-0.13213	0.08	0.111	0.394087	AC-G	SLC17A5
RAB6B.1	2.67E-05	0.10043	0.085	0.061	0.399241	AC-G	RAB6B
MBNL2.1	2.76E-05	-0.11468	0.071	0.1	0.412041	AC-G	MBNL2
PDIA5	2.77E-05	0.187871	0.09	0.066	0.414521	AC-G	PDIA5
JAGN1.1	2.91E-05	0.271199	0.341	0.316	0.4351	AC-G	JAGN1
PIR	2.93E-05	0.132587	0.076	0.053	0.437383	AC-G	PIR
RNF135.1	2.97E-05	-0.14291	0.085	0.116	0.443423	AC-G	RNF135
IFI6.1	3.02E-05	0.204931	0.421	0.404	0.451074	AC-G	IFI6
DNAAF1	3.14E-05	-0.12665	0.013	0.03	0.469634	AC-G	DNAAF1
CTD-2015H	3.17E-05	0.119401	0.059	0.039	0.474196	AC-G	CTD-2015H6.3
TUBG2.1	3.37E-05	0.177301	0.134	0.106	0.503021	AC-G	TUBG2
GTF2H2	3.40E-05	0.212924	0.21	0.18	0.508758	AC-G	GTF2H2
NOP58.1	3.74E-05	0.206797	0.312	0.288	0.558713	AC-G	NOP58
INSR.1	3.85E-05	-0.11854	0.115	0.151	0.575192	AC-G	INSR
TMEM99.1	3.90E-05	0.166129	0.152	0.123	0.582897	AC-G	TMEM99
NR2C2AP	4.32E-05	0.234027	0.189	0.161	0.645292	AC-G	NR2C2AP
NECAB1	4.40E-05	-0.11642	0.021	0.04	0.657522	AC-G	NECAB1
DNAJC1.1	4.41E-05	-0.10253	0.15	0.192	0.658387	AC-G	DNAJC1
EVA1C.1	4.56E-05	0.155911	0.091	0.068	0.680787	AC-G	EVA1C
SLC25A4.1	4.68E-05	0.211424	0.44	0.424	0.699539	AC-G	SLC25A4
TAF1D.1	4.88E-05	0.220794	0.354	0.341	0.729949	AC-G	TAF1D
MTRR.1	5.10E-05	0.128412	0.105	0.079	0.762741	AC-G	MTRR
TMED5.1	5.21E-05	0.170761	0.33	0.301	0.778655	AC-G	TMED5
EML2.1	5.26E-05	-0.11629	0.06	0.087	0.786464	AC-G	EML2
MEAF6.1	5.45E-05	0.195987	0.478	0.477	0.81399	AC-G	MEAF6
MYL12B.1	5.53E-05	0.203357	0.693	0.734	0.826839	AC-G	MYL12B
DDX39A.1	5.55E-05	0.235357	0.404	0.39	0.82901	AC-G	DDX39A
POT1	5.81E-05	0.120148	0.103	0.077	0.868367	AC-G	POT1
UNC50	5.88E-05	0.189169	0.369	0.352	0.878092	AC-G	UNC50
ZFP36L1	5.93E-05	0.200128	0.561	0.559	0.886535	AC-G	ZFP36L1
SLC25A18	6.80E-05	0.305002	0.107	0.083	1	AC-G	SLC25A18
RP11-629G	6.86E-05	0.11658	0.033	0.019	1	AC-G	RP11-629G13.1
FOSL2.1	6.98E-05	-0.11268	0.153	0.192	1	AC-G	FOSL2
CDC37L1	7.05E-05	0.142962	0.164	0.134	1	AC-G	CDC37L1
TMEM231	7.16E-05	0.120812	0.081	0.059	1	AC-G	TMEM231
KDM7A	7.30E-05	-0.10653	0.043	0.066	1	AC-G	KDM7A
GZMA	7.59E-05	-0.15417	0.005	0.016	1	AC-G	GZMA
NFKBIZ.1	7.82E-05	0.145601	0.204	0.169	1	AC-G	NFKBIZ
TIMMDC1	7.83E-05	0.202793	0.397	0.385	1	AC-G	TIMMDC1
MKL2.1	8.02E-05	0.12628	0.097	0.073	1	AC-G	MKL2
MTRNR2L8	8.18E-05	0.21333	0.221	0.185	1	AC-G	MTRNR2L8
MXD1	8.39E-05	-0.14764	0.103	0.133	1	AC-G	MXD1
SLC35E3	8.48E-05	-0.13968	0.156	0.193	1	AC-G	SLC35E3
EEF1D.1	8.81E-05	0.128834	0.782	0.857	1	AC-G	EEF1D
NAF1.1	8.86E-05	-0.11187	0.085	0.114	1	AC-G	NAF1

FLNA.1	8.95E-05	0.184974	0.273	0.246	1	AC-G	FLNA
MATN2	9.02E-05	0.137078	0.085	0.062	1	AC-G	MATN2
MIR210HG	9.26E-05	0.160382	0.112	0.087	1	AC-G	MIR210HG
PTPRZ1.1	9.33E-05	-0.1016	0.363	0.388	1	AC-G	PTPRZ1
TMEM216	9.44E-05	0.209495	0.101	0.076	1	AC-G	TMEM216
LSM5	9.59E-05	0.200673	0.503	0.508	1	AC-G	LSM5
C3.1	9.95E-05	0.167269	0.438	0.394	1	AC-G	C3
TROVE2	0.0001	0.169332	0.433	0.433	1	AC-G	TROVE2
BZW1.1	0.000101	0.166921	0.521	0.541	1	AC-G	BZW1
YIPF3.1	0.000107	0.225492	0.48	0.484	1	AC-G	YIPF3
DYNC2H1	0.00011	0.112667	0.068	0.048	1	AC-G	DYNC2H1
PIN4.1	0.00011	0.237783	0.422	0.417	1	AC-G	PIN4
HIBADH.1	0.000111	0.219507	0.25	0.223	1	AC-G	HIBADH
TXNDC17.1	0.000112	0.250435	0.525	0.525	1	AC-G	TXNDC17
COMMD6.1	0.000112	0.164745	0.726	0.786	1	AC-G	COMMD6
ZCRB1.1	0.000119	0.264853	0.48	0.493	1	AC-G	ZCRB1
NRCAM	0.00012	0.242264	0.289	0.266	1	AC-G	NRCAM
PLS3.1	0.000121	0.28494	0.245	0.216	1	AC-G	PLS3
CALCOCO2.	0.000125	0.123163	0.219	0.188	1	AC-G	CALCOCO2
OSCP1.1	0.000127	0.109584	0.084	0.062	1	AC-G	OSCP1
ATP6V1G1.	0.00013	0.1692	0.718	0.761	1	AC-G	ATP6V1G1
CBR1	0.000133	0.175981	0.476	0.476	1	AC-G	CBR1
IFIT3.1	0.000135	-0.1217	0.084	0.113	1	AC-G	IFIT3
CD320	0.000138	0.15547	0.339	0.314	1	AC-G	CD320
PLRG1.1	0.000142	0.210132	0.259	0.233	1	AC-G	PLRG1
TMEM47	0.000142	0.124299	0.099	0.076	1	AC-G	TMEM47
ATF3.1	0.000142	0.114156	0.334	0.296	1	AC-G	ATF3
SEC11C.1	0.000147	0.198685	0.483	0.5	1	AC-G	SEC11C
CDK7.1	0.000148	0.160675	0.114	0.089	1	AC-G	CDK7
TMEM176B.	0.000156	-0.2242	0.335	0.355	1	AC-G	TMEM176B
CEBPZ.1	0.000157	0.201405	0.289	0.268	1	AC-G	CEBPZ
NEIL2.1	0.000169	0.245321	0.217	0.195	1	AC-G	NEIL2
ZNF230	0.000172	0.104348	0.067	0.048	1	AC-G	ZNF230
AGPAT5	0.000177	0.259037	0.228	0.206	1	AC-G	AGPAT5
ZNF408.1	0.000179	0.104189	0.095	0.072	1	AC-G	ZNF408
RAD23A.1	0.000181	0.129455	0.569	0.566	1	AC-G	RAD23A
AARS	0.000184	0.150169	0.251	0.226	1	AC-G	AARS
GOLGA7	0.000184	0.16346	0.467	0.463	1	AC-G	GOLGA7
LRTOMT	0.000184	0.156255	0.089	0.068	1	AC-G	LRTOMT
CCDC47.1	0.000185	0.120487	0.412	0.403	1	AC-G	CCDC47
PGM1.1	0.000186	0.21563	0.271	0.247	1	AC-G	PGM1
ZNF568	0.000189	0.137817	0.119	0.094	1	AC-G	ZNF568
RPRM.1	0.000192	-0.12712	0.048	0.07	1	AC-G	RPRM
OST4.1	0.000195	0.186117	0.734	0.789	1	AC-G	OST4
MX1	0.000197	-0.17745	0.097	0.124	1	AC-G	MX1
TMEFF2.1	0.000214	-0.11631	0.058	0.081	1	AC-G	TMEFF2
CRY1	0.000215	0.208311	0.157	0.132	1	AC-G	CRY1
GNG12	0.000216	0.213526	0.157	0.134	1	AC-G	GNG12
SLC9A9.1	0.000216	-0.14822	0.058	0.082	1	AC-G	SLC9A9
PTHLH	0.00022	0.100434	0.046	0.031	1	AC-G	PTHLH
FERMT2.1	0.000229	0.175409	0.221	0.199	1	AC-G	FERMT2

VMP1.1	0.000231	0.17156	0.472	0.472	1	AC-G	VMP1
ZCCHC6.1	0.000248	-0.10823	0.109	0.14	1	AC-G	ZCCHC6
ATOX1.1	0.000255	0.231185	0.591	0.607	1	AC-G	ATOX1
IRF1.1	0.000256	0.162379	0.232	0.201	1	AC-G	IRF1
TAX1BP1	0.000272	0.143841	0.493	0.516	1	AC-G	TAX1BP1
RIT2.1	0.000278	0.12621	0.096	0.072	1	AC-G	RIT2
ATP1B2.1	0.000287	0.17917	0.257	0.228	1	AC-G	ATP1B2
TEX10.1	0.000289	0.107269	0.096	0.073	1	AC-G	TEX10
ABCF2.1	0.000293	0.224488	0.236	0.212	1	AC-G	ABCF2
LMAN1.1	0.000294	0.235462	0.338	0.327	1	AC-G	LMAN1
PRAF2.1	0.000296	0.15814	0.311	0.3	1	AC-G	PRAF2
ANXA4.1	0.000301	0.174144	0.218	0.189	1	AC-G	ANXA4
ANAPC13.1	0.000303	0.168874	0.369	0.351	1	AC-G	ANAPC13
COA3	0.000307	0.246274	0.466	0.479	1	AC-G	COA3
NSG1.1	0.000309	-0.19118	0.155	0.18	1	AC-G	NSG1
HEPACAM.1	0.000321	0.234289	0.201	0.173	1	AC-G	HEPACAM
CASP4.1	0.000324	0.184275	0.255	0.222	1	AC-G	CASP4
NEXN	0.000347	0.103962	0.045	0.03	1	AC-G	NEXN
C1orf52.1	0.000359	0.209396	0.322	0.304	1	AC-G	C1orf52
CRISPLD1.	0.00036	0.136598	0.109	0.086	1	AC-G	CRISPLD1
ROMO1.1	0.000362	0.187793	0.579	0.598	1	AC-G	ROMO1
CIB1.1	0.000364	0.161685	0.5	0.491	1	AC-G	CIB1
IARS	0.000368	0.179701	0.227	0.207	1	AC-G	IARS
FBL.1	0.000374	0.211398	0.387	0.376	1	AC-G	FBL
LOXL3	0.000384	-0.10143	0.054	0.077	1	AC-G	LOXL3
NMB.1	0.000385	0.193961	0.222	0.198	1	AC-G	NMB
VAMP4	0.00039	0.132157	0.139	0.114	1	AC-G	VAMP4
GMNN.1	0.000399	0.21198	0.196	0.172	1	AC-G	GMNN
NDUFV2.1	0.000425	0.193067	0.576	0.594	1	AC-G	NDUFV2
TVP23B.1	0.000438	0.106275	0.122	0.098	1	AC-G	TVP23B
SNRPG.1	0.000449	0.156122	0.62	0.643	1	AC-G	SNRPG
PCDH1	0.000471	0.116871	0.075	0.056	1	AC-G	PCDH1
TNKS1BP1	0.000474	0.136272	0.09	0.07	1	AC-G	TNKS1BP1
EGFL7	0.000488	-0.10588	0.023	0.039	1	AC-G	EGFL7
USE1.1	0.000493	0.279485	0.341	0.328	1	AC-G	USE1
OTUD7B	0.000505	0.130429	0.12	0.097	1	AC-G	OTUD7B
GS1-124K5	0.000523	0.194648	0.134	0.11	1	AC-G	GS1-124K5.4
SIVA1.1	0.000524	0.176313	0.485	0.486	1	AC-G	SIVA1
LINC00116	0.000527	0.253357	0.368	0.364	1	AC-G	LINC00116
TMEM222.1	0.00054	0.169127	0.297	0.276	1	AC-G	TMEM222
RWDD1.1	0.000548	0.170823	0.516	0.529	1	AC-G	RWDD1
TRIP10	0.000553	0.152594	0.117	0.095	1	AC-G	TRIP10
PODXL.1	0.000559	0.155197	0.086	0.067	1	AC-G	PODXL
GNAI1.1	0.000586	-0.14095	0.086	0.109	1	AC-G	GNAI1
TIMM44.1	0.00059	0.201901	0.274	0.26	1	AC-G	TIMM44
PSMA6.1	0.000591	0.191979	0.245	0.219	1	AC-G	PSMA6
B9D1	0.000591	0.297602	0.118	0.097	1	AC-G	B9D1
GZMK	0.000603	-0.11521	0.003	0.012	1	AC-G	GZMK
RNU12	0.00061	-0.11433	0.024	0.04	1	AC-G	RNU12
THYN1.1	0.000613	0.250455	0.41	0.406	1	AC-G	THYN1
SH3GLB1.1	0.000616	0.207677	0.431	0.43	1	AC-G	SH3GLB1

HNRNPA2B1	0.000616	0.15006	0.79	0.856	1	AC-G	HNRNPA2B1
MORF4L1.1	0.00063	0.110979	0.711	0.751	1	AC-G	MORF4L1
MIF.1	0.00064	0.14544	0.615	0.681	1	AC-G	MIF
ZNF181	0.000676	0.164122	0.115	0.093	1	AC-G	ZNF181
AIMP2.1	0.000678	0.203287	0.259	0.237	1	AC-G	AIMP2
HSD17B12.	0.000711	0.144349	0.417	0.413	1	AC-G	HSD17B12
TRNT1.1	0.000712	0.234986	0.15	0.128	1	AC-G	TRNT1
MAGED2.1	0.000715	0.158322	0.503	0.51	1	AC-G	MAGED2
FAM221A.1	0.000718	0.1577	0.202	0.173	1	AC-G	FAM221A
MUT.1	0.00073	0.107782	0.178	0.153	1	AC-G	MUT
CLDN11	0.000731	-0.14652	0.031	0.048	1	AC-G	CLDN11
LYRM1.1	0.000733	0.269899	0.203	0.179	1	AC-G	LYRM1
DNAJC2.1	0.00074	0.156765	0.312	0.295	1	AC-G	DNAJC2
APIP.1	0.000754	-0.10034	0.202	0.239	1	AC-G	APIP
TOPORS.1	0.000765	-0.11353	0.152	0.184	1	AC-G	TOPORS
ZNF136	0.00077	0.126146	0.115	0.094	1	AC-G	ZNF136
CCT3.1	0.000788	0.175123	0.563	0.58	1	AC-G	CCT3
FHIT.1	0.000793	-0.18193	0.096	0.12	1	AC-G	FHIT
LINC00493	0.000796	0.192837	0.522	0.532	1	AC-G	LINC00493
DCAKD.1	0.000837	0.102297	0.112	0.144	1	AC-G	DCAKD
AHR.1	0.000843	-0.14204	0.047	0.066	1	AC-G	AHR
CDC42EP4.	0.000856	0.114769	0.225	0.196	1	AC-G	CDC42EP4
EIF2S2.1	0.000915	0.154725	0.564	0.593	1	AC-G	EIF2S2
HOXA10.1	0.000934	-0.10585	0.06	0.08	1	AC-G	HOXA10
TMEM50A.1	0.000936	0.219531	0.567	0.597	1	AC-G	TMEM50A
PCOLCE	0.001014	0.277359	0.097	0.078	1	AC-G	PCOLCE
ADAMTS4	0.001071	-0.10272	0.02	0.034	1	AC-G	ADAMTS4
IFT46	0.001075	0.157776	0.127	0.106	1	AC-G	IFT46
ALKBH2.1	0.001079	0.225008	0.212	0.192	1	AC-G	ALKBH2
CHMP5	0.001083	0.144788	0.523	0.534	1	AC-G	CHMP5
IQCK	0.001087	0.208843	0.143	0.123	1	AC-G	IQCK
DYNLRB1	0.001088	0.173339	0.647	0.669	1	AC-G	DYNLRB1
RP11-11N9	0.001199	0.108604	0.069	0.051	1	AC-G	RP11-11N9.4
GALK1.1	0.001217	0.106246	0.169	0.211	1	AC-G	GALK1
C1orf53	0.001237	0.223796	0.099	0.08	1	AC-G	C1orf53
FOXP1	0.001253	0.276006	0.254	0.226	1	AC-G	FOXP1
ZFAND2A.1	0.001287	0.254226	0.299	0.28	1	AC-G	ZFAND2A
TSC22D4.1	0.001287	0.187315	0.476	0.469	1	AC-G	TSC22D4
MICALL2	0.001289	0.10539	0.085	0.066	1	AC-G	MICALL2
CAPZA2.1	0.001352	0.174737	0.611	0.662	1	AC-G	CAPZA2
ABLIM1.1	0.001355	0.102235	0.067	0.05	1	AC-G	ABLIM1
WBSCR22.1	0.001439	0.203187	0.483	0.495	1	AC-G	WBSCR22
TMEM55A.1	0.001474	0.124198	0.251	0.229	1	AC-G	TMEM55A
STK33	0.001478	0.11171	0.083	0.065	1	AC-G	STK33
TMEM186.1	0.001529	0.128497	0.114	0.094	1	AC-G	TMEM186
PTRH2.1	0.00153	0.155856	0.156	0.134	1	AC-G	PTRH2
SNTG1	0.001555	0.364537	0.168	0.15	1	AC-G	SNTG1
MAP9	0.001581	0.181936	0.206	0.185	1	AC-G	MAP9
PHOSPHO2	0.001587	0.103855	0.057	0.042	1	AC-G	PHOSPHO2
CDK18	0.001633	-0.11584	0.061	0.081	1	AC-G	CDK18
PDK1	0.001735	0.105797	0.101	0.081	1	AC-G	PDK1

AMD1	0.00177	0.1503	0.39	0.382	1	AC-G	AMD1
TGFB1I1.1	0.001782	0.271062	0.141	0.123	1	AC-G	TGFB1I1
SEC61A1	0.001784	0.150247	0.368	0.365	1	AC-G	SEC61A1
CCT4	0.001804	0.170062	0.543	0.564	1	AC-G	CCT4
NIP7.1	0.00181	0.149862	0.191	0.168	1	AC-G	NIP7
ZNF211	0.001822	0.12726	0.081	0.063	1	AC-G	ZNF211
TAPBPL.1	0.001858	0.156201	0.143	0.118	1	AC-G	TAPBPL
MAP7	0.001917	-0.12122	0.027	0.041	1	AC-G	MAP7
PRPS1.1	0.001919	0.120013	0.21	0.186	1	AC-G	PRPS1
ISOC2.1	0.001936	0.175529	0.359	0.347	1	AC-G	ISOC2
NUDCD1.1	0.002001	0.109598	0.118	0.097	1	AC-G	NUDCD1
SMYD3.1	0.002183	0.18297	0.157	0.135	1	AC-G	SMYD3
GPX7	0.002194	0.124007	0.145	0.183	1	AC-G	GPX7
EAPP.1	0.002216	0.258867	0.356	0.354	1	AC-G	EAPP
ZNF503.1	0.002339	-0.10764	0.085	0.107	1	AC-G	ZNF503
RP11-421L	0.002343	0.146508	0.097	0.078	1	AC-G	RP11-421L21.3
EIF5B	0.002406	0.201629	0.51	0.524	1	AC-G	EIF5B
LRRC8A	0.002456	0.150053	0.184	0.165	1	AC-G	LRRC8A
MAP3K13.1	0.002533	0.184681	0.395	0.393	1	AC-G	MAP3K13
RNF19A.1	0.002546	0.154002	0.219	0.199	1	AC-G	RNF19A
TBCE	0.00261	0.128421	0.124	0.104	1	AC-G	TBCE
ASL	0.002633	0.20048	0.189	0.167	1	AC-G	ASL
DNAJB4.1	0.002648	0.10432	0.145	0.122	1	AC-G	DNAJB4
TMEM60.1	0.002777	0.194147	0.262	0.244	1	AC-G	TMEM60
UBE2D3	0.002865	0.122272	0.678	0.737	1	AC-G	UBE2D3
TAF12.1	0.002928	0.205013	0.318	0.307	1	AC-G	TAF12
PPP2R2B.1	0.002949	0.245296	0.234	0.216	1	AC-G	PPP2R2B
EIF3M.1	0.003057	0.157232	0.513	0.528	1	AC-G	EIF3M
PDE4DIP	0.003085	0.124227	0.287	0.259	1	AC-G	PDE4DIP
SNHG12.1	0.003096	0.125975	0.154	0.13	1	AC-G	SNHG12
TAF9.1	0.003297	0.14664	0.48	0.504	1	AC-G	TAF9
MAGOH.1	0.003371	0.195899	0.411	0.415	1	AC-G	MAGOH
RIOK2.1	0.003383	0.117758	0.14	0.118	1	AC-G	RIOK2
TOM1L1.1	0.003408	0.121803	0.07	0.055	1	AC-G	TOM1L1
GORAB.1	0.003412	0.104095	0.089	0.072	1	AC-G	GORAB
PRMT5.1	0.003504	0.165574	0.168	0.147	1	AC-G	PRMT5
PSMB5.1	0.003541	0.199609	0.578	0.598	1	AC-G	PSMB5
FOPNL.1	0.003573	0.162822	0.21	0.19	1	AC-G	FOPNL
NFYB	0.003575	0.199129	0.197	0.181	1	AC-G	NFYB
GPI	0.003618	0.175506	0.361	0.355	1	AC-G	GPI
NOVA1.1	0.003685	-0.11233	0.495	0.476	1	AC-G	NOVA1
KIAA1191	0.003833	0.213423	0.276	0.262	1	AC-G	KIAA1191
C1orf21.1	0.003876	0.233595	0.213	0.198	1	AC-G	C1orf21
KLHDC9	0.00393	0.112153	0.087	0.07	1	AC-G	KLHDC9
CHD1L.1	0.004094	0.146742	0.156	0.134	1	AC-G	CHD1L
TTC1.1	0.004102	0.181523	0.374	0.373	1	AC-G	TTC1
TIMM21.1	0.004166	0.169338	0.162	0.144	1	AC-G	TIMM21
PBXIP1.1	0.004242	0.179834	0.152	0.132	1	AC-G	PBXIP1
POLR2G.1	0.004277	0.232585	0.544	0.572	1	AC-G	POLR2G
ASNSD1.1	0.004503	0.161337	0.283	0.269	1	AC-G	ASNSD1
RAB39B.1	0.004524	0.110504	0.106	0.087	1	AC-G	RAB39B

ANKRA2	0.004531	0.157332	0.164	0.144	1	AC-G	ANKRA2
RPA2	0.004571	0.197322	0.328	0.318	1	AC-G	RPA2
SDCBP	0.004602	0.108211	0.684	0.737	1	AC-G	SDCBP
EPRS.1	0.004657	0.162249	0.303	0.292	1	AC-G	EPRS
SLC24A2	0.004742	-0.11288	0.021	0.033	1	AC-G	SLC24A2
PNMA2.1	0.004749	0.141546	0.156	0.136	1	AC-G	PNMA2
ARL6IP1.1	0.004829	0.121638	0.699	0.75	1	AC-G	ARL6IP1
LAMTOR3	0.004991	0.174982	0.272	0.258	1	AC-G	LAMTOR3
MANBAL.1	0.005075	0.214304	0.343	0.333	1	AC-G	MANBAL
DLGAP1-AS	0.005094	0.178623	0.106	0.089	1	AC-G	DLGAP1-AS1
KIAA0907.	0.005135	0.135013	0.208	0.191	1	AC-G	KIAA0907
SCRG1.1	0.005223	0.269388	0.344	0.346	1	AC-G	SCRG1
TTC32	0.005244	0.172865	0.166	0.146	1	AC-G	TTC32
SPRED3	0.005267	0.115903	0.026	0.017	1	AC-G	SPRED3
ATP5EP2.1	0.005749	0.125311	0.383	0.459	1	AC-G	ATP5EP2
ARL2.1	0.005773	0.182004	0.435	0.439	1	AC-G	ARL2
UBXN4.1	0.005775	0.135151	0.513	0.55	1	AC-G	UBXN4
LINC00152	0.005865	0.270435	0.27	0.256	1	AC-G	LINC00152
PGRMC2.1	0.006334	0.107802	0.24	0.223	1	AC-G	PGRMC2
NHP2.1	0.006529	0.158138	0.561	0.584	1	AC-G	NHP2
RCHY1.1	0.006675	0.124754	0.217	0.197	1	AC-G	RCHY1
C1orf27.1	0.00669	0.13623	0.195	0.176	1	AC-G	C1orf27
AIMP1.1	0.006699	0.222537	0.37	0.371	1	AC-G	AIMP1
C3orf14	0.006772	0.143857	0.279	0.262	1	AC-G	C3orf14
NPC2	0.006956	0.131922	0.683	0.693	1	AC-G	NPC2
ARHGAP5-A	0.007262	0.124825	0.042	0.031	1	AC-G	ARHGAP5-AS1
TCEAL1.1	0.007283	0.205272	0.266	0.253	1	AC-G	TCEAL1
FKBP11.1	0.007565	0.160533	0.131	0.113	1	AC-G	FKBP11
IFIT1	0.007664	-0.10191	0.059	0.076	1	AC-G	IFIT1
FKBP1A.1	0.007764	0.211478	0.648	0.71	1	AC-G	FKBP1A
PSMD6.1	0.0079	0.223722	0.358	0.349	1	AC-G	PSMD6
CCBL2	0.008025	0.115686	0.142	0.123	1	AC-G	CCBL2
SON.1	0.008231	0.158958	0.651	0.722	1	AC-G	SON
PDE8B	0.008295	0.102755	0.059	0.046	1	AC-G	PDE8B
SUGT1.1	0.008342	0.15426	0.351	0.343	1	AC-G	SUGT1
PSMA3.1	0.008347	0.23633	0.435	0.442	1	AC-G	PSMA3
ALG2.1	0.008426	0.106936	0.179	0.16	1	AC-G	ALG2
STYXL1.1	0.008486	0.124928	0.143	0.123	1	AC-G	STYXL1
BIN3.1	0.008635	0.176686	0.17	0.152	1	AC-G	BIN3
NBEAL1	0.008691	0.165028	0.648	0.668	1	AC-G	NBEAL1
BAALC.1	0.008947	0.110581	0.356	0.343	1	AC-G	BAALC
FKBP8	0.008964	0.158929	0.62	0.651	1	AC-G	FKBP8
C6orf1.1	0.00907	0.186208	0.363	0.36	1	AC-G	C6orf1
ARFIP2.1	0.009133	0.155766	0.255	0.242	1	AC-G	ARFIP2
C10orf107	0.009152	0.110888	0.048	0.036	1	AC-G	C10orf107
OXSM	0.009185	0.134958	0.094	0.079	1	AC-G	OXSM
HMCES	0.009842	0.144765	0.184	0.166	1	AC-G	HMCES
GPC1.1	0.00991	0.11504	0.262	0.232	1	AC-G	GPC1
MALAT1.2	########	0.531237	0.991	0.996	########	CSCL	MALAT1
GAPDH.2	########	0.582499	0.991	0.985	########	CSCL	GAPDH
PTN.2	########	0.936858	0.827	0.605	########	CSCL	PTN

MT3.2	########	1.205963	0.752	0.477	########	CSCL	MT3
GNB2L1.2	########	0.532251	0.916	0.943	########	CSCL	GNB2L1
DBI.2	########	0.73164	0.867	0.842	########	CSCL	DBI
TMSB4X.2	########	0.396188	0.977	0.998	########	CSCL	TMSB4X
ACTG1.2	########	0.416111	0.92	0.95	########	CSCL	ACTG1
B2M.2	########	0.506641	0.946	0.972	########	CSCL	B2M
MT2A.2	########	0.800887	0.848	0.781	########	CSCL	MT2A
H3F3B.2	########	0.388256	0.911	0.974	########	CSCL	H3F3B
TUBA1A.1	########	0.548885	0.864	0.805	########	CSCL	TUBA1A
FTL.2	########	0.399136	0.956	0.996	########	CSCL	FTL
GPM6B.2	########	0.721635	0.794	0.61	########	CSCL	GPM6B
CLU.2	########	0.69222	0.838	0.779	1.99E-97	CSCL	CLU
SEC61G.2	2.35E-97	0.768328	0.845	0.797	3.51E-93	CSCL	SEC61G
FAU.2	2.79E-95	0.459097	0.892	0.961	4.17E-91	CSCL	FAU
NDUFA4.1	3.18E-94	0.501622	0.857	0.897	4.75E-90	CSCL	NDUFA4
NGFRAP1.1	1.41E-91	0.760332	0.771	0.638	2.10E-87	CSCL	NGFRAP1
PTMA.2	4.94E-88	0.373547	0.895	0.97	7.39E-84	CSCL	PTMA
CD63.2	3.87E-87	0.517373	0.866	0.927	5.78E-83	CSCL	CD63
ACTB.2	3.50E-86	0.278353	0.934	0.995	5.22E-82	CSCL	ACTB
EEF1A1.2	4.75E-86	0.580968	0.82	0.826	7.10E-82	CSCL	EEF1A1
RNASET2.2	1.06E-85	-1.1528	0.051	0.505	1.59E-81	CSCL	RNASET2
EIF1.2	9.91E-83	0.361683	0.899	0.978	1.48E-78	CSCL	EIF1
SRI.2	2.74E-79	0.709852	0.757	0.727	4.09E-75	CSCL	SRI
UBC.2	1.05E-77	0.379336	0.862	0.955	1.57E-73	CSCL	UBC
TMSB10.2	3.84E-76	0.394808	0.899	0.968	5.75E-72	CSCL	TMSB10
ARPC1B.2	3.94E-73	-0.8413	0.101	0.553	5.88E-69	CSCL	ARPC1B
TPT1.2	4.14E-73	0.405813	0.873	0.957	6.19E-69	CSCL	TPT1
COTL1.2	2.18E-72	-0.68998	0.084	0.531	3.25E-68	CSCL	COTL1
CYBA.2	4.55E-71	-1.22483	0.028	0.424	6.80E-67	CSCL	CYBA
FCER1G.2	1.91E-70	-1.26457	0.061	0.461	2.85E-66	CSCL	FCER1G
LAPTM5.2	3.29E-69	-1.20279	0.058	0.455	4.92E-65	CSCL	LAPTM5
RGS1.2	5.35E-69	-1.22378	0.07	0.466	8.00E-65	CSCL	RGS1
RAMP1.1	7.38E-68	0.916881	0.613	0.402	1.10E-63	CSCL	RAMP1
CXCL16.2	1.59E-67	-0.79509	0.061	0.473	2.38E-63	CSCL	CXCL16
AIF1.2	1.79E-67	-1.29248	0.045	0.429	2.67E-63	CSCL	AIF1
SKP1.1	1.44E-66	0.500326	0.798	0.868	2.15E-62	CSCL	SKP1
C1QC.2	1.62E-66	-1.0666	0.117	0.521	2.42E-62	CSCL	C1QC
ATP1B3.2	2.26E-66	-0.6101	0.119	0.566	3.37E-62	CSCL	ATP1B3
LDHB.1	6.64E-66	0.65865	0.75	0.78	9.93E-62	CSCL	LDHB
FAM49B.2	7.59E-66	-0.62528	0.098	0.527	1.13E-61	CSCL	FAM49B
NINJ1.2	8.50E-66	-0.78567	0.065	0.472	1.27E-61	CSCL	NINJ1
C1orf61.2	1.59E-65	0.895509	0.658	0.529	2.38E-61	CSCL	C1orf61
CFL1.1	3.33E-65	0.311685	0.857	0.954	4.98E-61	CSCL	CFL1
YWHAH.2	1.81E-64	-0.58228	0.136	0.587	2.71E-60	CSCL	YWHAH
GRB2.2	3.16E-64	-0.61243	0.082	0.501	4.72E-60	CSCL	GRB2
HSP90AA1.	1.39E-63	0.42003	0.799	0.893	2.08E-59	CSCL	HSP90AA1
RNF130.2	9.47E-63	-0.7273	0.08	0.482	1.42E-58	CSCL	RNF130
TYROBP.2	1.15E-61	-1.09568	0.098	0.478	1.72E-57	CSCL	TYROBP
CD14.2	1.24E-61	-1.14489	0.056	0.426	1.85E-57	CSCL	CD14
VSIG4.2	8.11E-61	-1.27284	0.028	0.382	1.21E-56	CSCL	VSIG4
ALOX5AP.2	1.74E-60	-1.19019	0.056	0.417	2.61E-56	CSCL	ALOX5AP

RGS10.2	2.92E-60	-1.21883	0.016	0.359	4.37E-56	CSCL	RGS10
ARHGDIB.2	3.28E-60	-1.18723	0.035	0.389	4.89E-56	CSCL	ARHGDIB
FCGR3A.2	5.17E-60	-1.3121	0.016	0.358	7.73E-56	CSCL	FCGR3A
SRGN.2	6.75E-59	-0.98812	0.136	0.518	1.01E-54	CSCL	SRGN
ITGB2.2	2.16E-58	-1.22567	0.009	0.34	3.23E-54	CSCL	ITGB2
IFNGR1.2	2.25E-58	-0.76079	0.075	0.456	3.36E-54	CSCL	IFNGR1
RHOG.2	3.04E-58	-0.77793	0.058	0.427	4.55E-54	CSCL	RHOG
VAMP8.2	3.30E-58	-1.20011	0.021	0.36	4.93E-54	CSCL	VAMP8
C1QA.2	4.46E-58	-0.8621	0.155	0.557	6.67E-54	CSCL	C1QA
PPIA.1	1.19E-57	0.448283	0.791	0.879	1.78E-53	CSCL	PPIA
CORO1A.2	1.70E-57	-1.00712	0.04	0.39	2.54E-53	CSCL	CORO1A
C4orf48.2	1.99E-57	-0.51283	0.126	0.546	2.98E-53	CSCL	C4orf48
PRDX2.2	4.03E-57	0.640399	0.716	0.7	6.02E-53	CSCL	PRDX2
C1orf162.	2.67E-56	-1.24162	0.012	0.338	4.00E-52	CSCL	C1orf162
MGAT1.2	2.79E-56	-0.77395	0.033	0.374	4.17E-52	CSCL	MGAT1
BASP1.2	3.51E-56	-0.78282	0.042	0.389	5.24E-52	CSCL	BASP1
MS4A6A.2	9.75E-56	-1.20796	0.026	0.357	1.46E-51	CSCL	MS4A6A
UQCR11.2	2.79E-55	0.845115	0.312	0.105	4.17E-51	CSCL	UQCR11
GSN.1	5.11E-55	-0.59737	0.124	0.529	7.63E-51	CSCL	GSN
CD53.2	2.43E-54	-1.1931	0.014	0.331	3.63E-50	CSCL	CD53
CAPG.2	6.61E-54	-0.86334	0.077	0.432	9.87E-50	CSCL	CAPG
SLC16A3.2	1.01E-53	-0.95371	0.016	0.336	1.51E-49	CSCL	SLC16A3
C20orf24.	2.34E-53	-0.4957	0.101	0.486	3.49E-49	CSCL	C20orf24
MYL12A.1	3.33E-53	-0.47389	0.143	0.564	4.98E-49	CSCL	MYL12A
TREM2.2	3.62E-53	-1.12952	0.038	0.367	5.41E-49	CSCL	TREM2
RALY.2	4.15E-53	-0.52124	0.094	0.475	6.20E-49	CSCL	RALY
ARRB2.2	6.27E-53	-0.70608	0.066	0.417	9.37E-49	CSCL	ARRB2
GRN.2	7.94E-53	-0.53487	0.175	0.59	1.19E-48	CSCL	GRN
CTSD.2	1.14E-52	-0.4882	0.218	0.661	1.70E-48	CSCL	CTSD
SYNGR2.2	2.40E-52	-0.8531	0.024	0.344	3.58E-48	CSCL	SYNGR2
BIN1.2	2.89E-52	-0.76848	0.045	0.377	4.32E-48	CSCL	BIN1
CD68.2	8.69E-52	-0.80197	0.099	0.459	1.30E-47	CSCL	CD68
CAP1.1	8.75E-52	-0.38684	0.175	0.614	1.31E-47	CSCL	CAP1
ACTR2.2	1.52E-51	-0.48674	0.099	0.477	2.27E-47	CSCL	ACTR2
MS4A7.2	1.78E-51	-1.10813	0.031	0.347	2.66E-47	CSCL	MS4A7
CD9.1	2.09E-51	0.762353	0.66	0.561	3.12E-47	CSCL	CD9
CNPY3.2	4.05E-51	-0.62907	0.077	0.429	6.06E-47	CSCL	CNPY3
CTSS.2	4.98E-51	-0.93532	0.042	0.37	7.44E-47	CSCL	CTSS
APOC1.2	8.94E-51	-0.73965	0.159	0.534	1.34E-46	CSCL	APOC1
GMFG.2	9.54E-51	-1.12127	0.012	0.312	1.43E-46	CSCL	GMFG
LST1.2	1.12E-50	-1.07776	0.021	0.328	1.68E-46	CSCL	LST1
SERP1.2	1.16E-50	-0.39764	0.19	0.625	1.73E-46	CSCL	SERP1
TPI1.2	2.27E-50	0.459396	0.766	0.871	3.39E-46	CSCL	TPI1
ADIPOR1.2	2.29E-50	-0.516	0.058	0.396	3.41E-46	CSCL	ADIPOR1
MAP1LC3B.	4.20E-50	-0.4022	0.166	0.586	6.27E-46	CSCL	MAP1LC3B
KDELR2.1	7.03E-50	-0.34349	0.164	0.589	1.05E-45	CSCL	KDELR2
GTF3A.2	7.58E-50	-0.35515	0.127	0.524	1.13E-45	CSCL	GTF3A
TYMP.2	1.00E-49	-0.94347	0.019	0.321	1.50E-45	CSCL	TYMP
CCL3.2	1.02E-49	-0.90689	0.101	0.439	1.53E-45	CSCL	CCL3
BTF3.1	1.10E-49	0.405236	0.78	0.904	1.64E-45	CSCL	BTF3
CEBPB.2	1.16E-49	-0.57265	0.094	0.452	1.73E-45	CSCL	CEBPB

HLA-A.2	1.23E-49	0.396144	0.789	0.901	1.84E-45	CSCL	HLA-A
ATP6V1B2.	2.14E-49	-0.54867	0.077	0.426	3.20E-45	CSCL	ATP6V1B2
SGK1.2	3.16E-49	-0.66018	0.099	0.454	4.73E-45	CSCL	SGK1
MTCH1.1	3.58E-49	-0.35188	0.15	0.554	5.35E-45	CSCL	MTCH1
ZNF331.2	4.35E-49	-0.91575	0.028	0.33	6.50E-45	CSCL	ZNF331
PPP4C.1	7.97E-49	-0.36102	0.152	0.563	1.19E-44	CSCL	PPP4C
HERPUD1.1	9.41E-49	-0.49885	0.234	0.663	1.41E-44	CSCL	HERPUD1
HLA-DMB.2	9.59E-49	-1.06728	0.033	0.335	1.43E-44	CSCL	HLA-DMB
C6orf62.2	9.63E-49	-0.45094	0.103	0.47	1.44E-44	CSCL	C6orf62
COX7C.2	1.89E-48	0.425668	0.77	0.892	2.83E-44	CSCL	COX7C
C10orf54.	2.67E-48	-1.04253	0.012	0.302	3.99E-44	CSCL	C10orf54
ISCU.2	3.03E-48	-0.36633	0.171	0.586	4.53E-44	CSCL	ISCU
RER1.1	3.89E-48	-0.38609	0.129	0.516	5.81E-44	CSCL	RER1
TMEM160.2	4.93E-48	-0.37534	0.112	0.484	7.37E-44	CSCL	TMEM160
RNASE6.2	5.13E-48	-1.12771	0.005	0.289	7.67E-44	CSCL	RNASE6
HEXB.2	9.46E-48	-0.45706	0.079	0.422	1.41E-43	CSCL	HEXB
RAP1A.2	9.72E-48	-0.40524	0.143	0.535	1.45E-43	CSCL	RAP1A
EIF4A3.2	1.18E-47	-0.35748	0.103	0.468	1.76E-43	CSCL	EIF4A3
CD37.2	1.49E-47	-1.06356	0.023	0.315	2.23E-43	CSCL	CD37
MS4A4A.2	1.93E-47	-1.14624	0.014	0.3	2.88E-43	CSCL	MS4A4A
LIMS1.2	2.73E-47	-0.52338	0.101	0.457	4.09E-43	CSCL	LIMS1
LY86.2	3.25E-47	-1.08762	0.017	0.306	4.86E-43	CSCL	LY86
LAMP1.2	3.28E-47	-0.39612	0.148	0.54	4.91E-43	CSCL	LAMP1
HLA-DPB1.	4.94E-47	-0.7473	0.155	0.512	7.38E-43	CSCL	HLA-DPB1
S100B.1	5.45E-47	0.884743	0.613	0.523	8.14E-43	CSCL	S100B
CTSC.2	8.70E-47	-0.76293	0.073	0.4	1.30E-42	CSCL	CTSC
PNRC1.2	1.77E-46	-0.39231	0.164	0.559	2.64E-42	CSCL	PNRC1
CPVL.2	1.91E-46	-0.85162	0.054	0.364	2.85E-42	CSCL	CPVL
PYCARD.2	2.63E-46	-0.94121	0.016	0.298	3.94E-42	CSCL	PYCARD
HLA-DQB1.	2.73E-46	-1.00811	0.042	0.34	4.07E-42	CSCL	HLA-DQB1
NANS.2	3.94E-46	-0.61886	0.058	0.375	5.89E-42	CSCL	NANS
IFNGR2.2	4.66E-46	-0.41276	0.073	0.406	6.97E-42	CSCL	IFNGR2
CAPZA1.2	5.15E-46	-0.45795	0.075	0.409	7.70E-42	CSCL	CAPZA1
XBP1.2	5.92E-46	-0.57943	0.066	0.387	8.85E-42	CSCL	XBP1
RGS19.2	6.30E-46	-0.75791	0.01	0.289	9.41E-42	CSCL	RGS19
DEK.2	7.24E-46	-0.33334	0.113	0.482	1.08E-41	CSCL	DEK
HCLS1.2	8.23E-46	-1.01521	0.021	0.304	1.23E-41	CSCL	HCLS1
ATP6V0D1.	1.18E-45	-0.39409	0.145	0.529	1.77E-41	CSCL	ATP6V0D1
AKR1B1.2	1.33E-45	-0.37281	0.215	0.646	1.99E-41	CSCL	AKR1B1
GNAI2.2	1.39E-45	-0.38235	0.129	0.5	2.08E-41	CSCL	GNAI2
LIPA.2	2.17E-45	-0.70565	0.045	0.349	3.25E-41	CSCL	LIPA
CXCR4.1	2.24E-45	-0.76137	0.082	0.404	3.35E-41	CSCL	CXCR4
PLAUR.2	2.59E-45	-0.80519	0.068	0.382	3.88E-41	CSCL	PLAUR
NAA10.2	2.65E-45	-0.33437	0.094	0.443	3.97E-41	CSCL	NAA10
NDUFA7.2	3.05E-45	0.726736	0.157	0.037	4.55E-41	CSCL	NDUFA7
CALM2.1	3.09E-45	0.290614	0.815	0.938	4.62E-41	CSCL	CALM2
DDAH2.2	4.45E-45	-0.37557	0.134	0.507	6.65E-41	CSCL	DDAH2
ALDOA.2	4.97E-45	0.454374	0.742	0.851	7.43E-41	CSCL	ALDOA
CREG1.2	6.00E-45	-0.71262	0.024	0.311	8.97E-41	CSCL	CREG1
ANP32B.2	6.22E-45	-0.31709	0.11	0.467	9.29E-41	CSCL	ANP32B
CREM.2	6.45E-45	-0.55891	0.089	0.423	9.64E-41	CSCL	CREM

DPP7.2	6.47E-45	-0.39467	0.122	0.485	9.67E-41	CSCL	DPP7
FCGR2A.2	1.10E-44	-0.9522	0.021	0.303	1.64E-40	CSCL	FCGR2A
CKLF.2	1.41E-44	-0.3306	0.152	0.538	2.11E-40	CSCL	CKLF
S100A4.2	1.63E-44	-0.99352	0.026	0.309	2.44E-40	CSCL	S100A4
GPR183.2	2.81E-44	-1.07654	0.026	0.303	4.20E-40	CSCL	GPR183
SERPINA1.	2.83E-44	-0.9347	0.03	0.313	4.23E-40	CSCL	SERPINA1
EPB41L2.2	3.07E-44	-0.64537	0.035	0.325	4.59E-40	CSCL	EPB41L2
HCST.2	4.45E-44	-1.02795	0.021	0.298	6.65E-40	CSCL	HCST
CDC42.1	4.72E-44	-0.29124	0.209	0.642	7.06E-40	CSCL	CDC42
NDUFS5.2	4.94E-44	0.528172	0.724	0.815	7.39E-40	CSCL	NDUFS5
TMEM70.2	6.28E-44	-0.44558	0.084	0.411	9.38E-40	CSCL	TMEM70
FXYD5.2	7.76E-44	-0.81784	0.054	0.355	1.16E-39	CSCL	FXYD5
OSTF1.2	9.22E-44	-0.73317	0.024	0.305	1.38E-39	CSCL	OSTF1
CALM3.2	1.01E-43	-0.31323	0.195	0.613	1.51E-39	CSCL	CALM3
SSNA1.2	1.17E-43	-0.3207	0.11	0.463	1.75E-39	CSCL	SSNA1
FPR1.2	1.79E-43	-1.02366	0.023	0.298	2.68E-39	CSCL	FPR1
GPR34.2	1.80E-43	-1.04966	0.012	0.28	2.70E-39	CSCL	GPR34
JTB.2	1.96E-43	-0.2622	0.206	0.63	2.93E-39	CSCL	JTB
SFT2D1.2	2.15E-43	-0.34473	0.145	0.52	3.22E-39	CSCL	SFT2D1
NACA.1	2.43E-43	0.345396	0.785	0.932	3.62E-39	CSCL	NACA
CHCHD2.1	3.69E-43	0.332548	0.77	0.886	5.51E-39	CSCL	CHCHD2
MCL1.2	3.74E-43	-0.3483	0.204	0.619	5.59E-39	CSCL	MCL1
LILRB4.2	4.42E-43	-1.05518	0.007	0.268	6.61E-39	CSCL	LILRB4
CD83.2	4.44E-43	-0.85495	0.03	0.308	6.64E-39	CSCL	CD83
CMTM6.2	4.67E-43	-0.44603	0.105	0.442	6.97E-39	CSCL	CMTM6
TSC22D3.1	4.85E-43	-0.49198	0.127	0.473	7.25E-39	CSCL	TSC22D3
GPSM3.2	5.19E-43	-0.96888	0.01	0.275	7.76E-39	CSCL	GPSM3
ARL6IP4.2	5.48E-43	-0.31898	0.216	0.645	8.18E-39	CSCL	ARL6IP4
CCDC109B.	6.59E-43	-0.48142	0.058	0.364	9.84E-39	CSCL	CCDC109B
MYL6.2	6.66E-43	0.296818	0.815	0.948	9.95E-39	CSCL	MYL6
SAMSN1.2	1.09E-42	-1.00903	0.005	0.264	1.63E-38	CSCL	SAMSN1
MFSD1.2	1.37E-42	-0.63254	0.059	0.358	2.05E-38	CSCL	MFSD1
RB1.2	1.54E-42	-0.67041	0.037	0.319	2.30E-38	CSCL	RB1
CD163.2	1.91E-42	-0.98636	0.021	0.289	2.85E-38	CSCL	CD163
TACC1.2	1.94E-42	-0.51084	0.04	0.327	2.90E-38	CSCL	TACC1
TNFRSF1A.	2.29E-42	-0.45795	0.073	0.387	3.43E-38	CSCL	TNFRSF1A
APMAP.2	2.94E-42	-0.331	0.157	0.536	4.39E-38	CSCL	APMAP
RAB10.1	3.11E-42	-0.23966	0.138	0.514	4.64E-38	CSCL	RAB10
PTP4A1.2	3.43E-42	-0.37678	0.094	0.423	5.13E-38	CSCL	PTP4A1
UCP2.2	4.07E-42	-0.87232	0.019	0.285	6.09E-38	CSCL	UCP2
SCAMP2.2	4.20E-42	-0.48916	0.077	0.39	6.28E-38	CSCL	SCAMP2
AP1S2.2	5.57E-42	-0.27303	0.147	0.518	8.33E-38	CSCL	AP1S2
MAFB.2	5.77E-42	-0.72379	0.044	0.328	8.62E-38	CSCL	MAFB
SPG21.2	9.41E-42	-0.29932	0.08	0.4	1.41E-37	CSCL	SPG21
ABHD12.2	1.66E-41	-0.38485	0.082	0.397	2.49E-37	CSCL	ABHD12
DUT.2	1.97E-41	-0.27402	0.12	0.472	2.94E-37	CSCL	DUT
PPP1R18.2	1.97E-41	-0.58753	0.031	0.306	2.94E-37	CSCL	PPP1R18
IL13RA1.2	2.31E-41	-0.72199	0.007	0.261	3.45E-37	CSCL	IL13RA1
CHMP1B.2	2.53E-41	-0.39946	0.117	0.454	3.78E-37	CSCL	CHMP1B
HSPA8.1	3.10E-41	0.37277	0.742	0.872	4.64E-37	CSCL	HSPA8
CIRBP.2	3.57E-41	0.386062	0.742	0.872	5.33E-37	CSCL	CIRBP

DNAJB11.1	3.62E-41	-0.32525	0.112	0.45	5.41E-37	CSCL	DNAJB11
CTSZ.2	3.83E-41	-0.84405	0.012	0.269	5.73E-37	CSCL	CTSZ
CSF1R.2	3.88E-41	-0.93013	0.019	0.281	5.80E-37	CSCL	CSF1R
LRRC59.1	5.24E-41	-0.40854	0.068	0.373	7.83E-37	CSCL	LRRC59
ASAH1.2	5.40E-41	-0.37024	0.248	0.672	8.08E-37	CSCL	ASAH1
FCGR1A.2	5.99E-41	-0.98652	0.014	0.271	8.95E-37	CSCL	FCGR1A
COX4I1.2	6.66E-41	0.3725	0.766	0.918	9.95E-37	CSCL	COX4I1
SDF4.2	7.67E-41	-0.31884	0.092	0.419	1.15E-36	CSCL	SDF4
PRELID1.2	1.27E-40	-0.22439	0.183	0.579	1.90E-36	CSCL	PRELID1
CAPZB.1	1.40E-40	-0.33459	0.272	0.724	2.09E-36	CSCL	CAPZB
PPT1.1	1.45E-40	-0.33257	0.187	0.575	2.16E-36	CSCL	PPT1
SLC25A39.	1.45E-40	-0.29263	0.094	0.421	2.17E-36	CSCL	SLC25A39
C19orf24.	1.70E-40	-0.31716	0.072	0.38	2.54E-36	CSCL	C19orf24
HLA-DMA.2	1.81E-40	-0.72305	0.112	0.423	2.71E-36	CSCL	HLA-DMA
ITGB1.1	1.94E-40	-0.41246	0.086	0.401	2.90E-36	CSCL	ITGB1
SERPINB6.	2.02E-40	-0.29595	0.138	0.495	3.02E-36	CSCL	SERPINB6
CORO1B.2	2.34E-40	-0.37691	0.072	0.376	3.50E-36	CSCL	CORO1B
CHCHD10.2	2.75E-40	-0.37708	0.084	0.396	4.11E-36	CSCL	CHCHD10
GLIPR1.1	2.85E-40	-0.56456	0.058	0.349	4.26E-36	CSCL	GLIPR1
PDIA3.2	2.86E-40	-0.26447	0.199	0.603	4.27E-36	CSCL	PDIA3
RNF149.2	2.89E-40	-0.69937	0.023	0.283	4.32E-36	CSCL	RNF149
SLC11A1.2	3.12E-40	-0.7711	0.042	0.319	4.67E-36	CSCL	SLC11A1
SURF1.2	3.14E-40	-0.35726	0.066	0.367	4.69E-36	CSCL	SURF1
C9orf142.	4.10E-40	-0.31266	0.08	0.391	6.12E-36	CSCL	C9orf142
RILPL2.2	4.11E-40	-0.75899	0.016	0.271	6.14E-36	CSCL	RILPL2
PGLS.2	4.62E-40	-0.24067	0.154	0.529	6.91E-36	CSCL	PGLS
FCGRT.1	4.95E-40	-0.42346	0.194	0.568	7.39E-36	CSCL	FCGRT
CLTB.2	5.36E-40	-0.24864	0.117	0.458	8.01E-36	CSCL	CLTB
ARPC2.1	5.78E-40	-0.32198	0.323	0.797	8.63E-36	CSCL	ARPC2
BRD2.2	6.64E-40	-0.32257	0.126	0.47	9.93E-36	CSCL	BRD2
OLR1.2	6.98E-40	-0.98712	0.014	0.262	1.04E-35	CSCL	OLR1
CTSH.2	6.98E-40	-0.78837	0.038	0.309	1.04E-35	CSCL	CTSH
CSNK1D.2	7.20E-40	-0.38085	0.072	0.374	1.08E-35	CSCL	CSNK1D
ABRACL.2	7.53E-40	-0.4971	0.042	0.318	1.13E-35	CSCL	ABRACL
RNF114.1	8.11E-40	-0.30322	0.103	0.431	1.21E-35	CSCL	RNF114
WIPI2.2	8.33E-40	-0.31887	0.094	0.413	1.25E-35	CSCL	WIPI2
EVL.2	8.41E-40	-0.5954	0.073	0.366	1.26E-35	CSCL	EVL
COMMD7.2	8.44E-40	-0.29601	0.094	0.415	1.26E-35	CSCL	COMMD7
C17orf62.	9.15E-40	-0.4287	0.079	0.383	1.37E-35	CSCL	C17orf62
SYAP1.1	9.81E-40	-0.33211	0.087	0.402	1.47E-35	CSCL	SYAP1
PPM1G.2	9.83E-40	-0.31819	0.106	0.434	1.47E-35	CSCL	PPM1G
ACTR3	1.22E-39	-0.27984	0.188	0.578	1.83E-35	CSCL	ACTR3
GDI2.2	1.25E-39	-0.21175	0.143	0.506	1.87E-35	CSCL	GDI2
PABPC4.2	1.33E-39	-0.4165	0.056	0.343	1.98E-35	CSCL	PABPC4
ADORA3.2	1.39E-39	-0.92373	0.019	0.273	2.08E-35	CSCL	ADORA3
CMPK1.2	1.70E-39	-0.28335	0.105	0.432	2.54E-35	CSCL	CMPK1
AKIRIN1.2	1.70E-39	-0.26058	0.077	0.383	2.54E-35	CSCL	AKIRIN1
H2AFJ.2	2.06E-39	-0.63731	0.017	0.271	3.08E-35	CSCL	H2AFJ
SH3GLB1.2	2.06E-39	-0.31846	0.113	0.446	3.08E-35	CSCL	SH3GLB1
TPM4.1	2.14E-39	-0.32641	0.155	0.515	3.19E-35	CSCL	TPM4
AKIRIN2.2	2.28E-39	-0.34692	0.087	0.396	3.41E-35	CSCL	AKIRIN2

TBXAS1.2	2.36E-39	-0.9387	0.009	0.253	3.53E-35	CSCL	TBXAS1
SRSF2.2	2.46E-39	-0.26501	0.215	0.625	3.68E-35	CSCL	SRSF2
HNRNPM.2	2.82E-39	-0.2342	0.175	0.562	4.22E-35	CSCL	HNRNPM
LRRFIP1.2	2.86E-39	-0.37243	0.072	0.368	4.27E-35	CSCL	LRRFIP1
HMGA1.2	2.93E-39	-0.37093	0.066	0.359	4.38E-35	CSCL	HMGA1
KTN1.2	5.02E-39	-0.2875	0.171	0.545	7.50E-35	CSCL	KTN1
ARHGDIA.2	5.02E-39	-0.25353	0.188	0.584	7.50E-35	CSCL	ARHGDIA
UBB.1	5.66E-39	0.309611	0.768	0.918	8.46E-35	CSCL	UBB
UBE2I.1	6.26E-39	-0.24897	0.197	0.596	9.36E-35	CSCL	UBE2I
ATP6AP1.2	7.55E-39	-0.24377	0.152	0.51	1.13E-34	CSCL	ATP6AP1
HIF1A.1	7.81E-39	-0.3391	0.157	0.514	1.17E-34	CSCL	HIF1A
MSN.2	8.22E-39	-0.37608	0.087	0.39	1.23E-34	CSCL	MSN
HMOX1.2	9.40E-39	-0.74162	0.042	0.308	1.41E-34	CSCL	HMOX1
C3AR1.2	1.02E-38	-0.96402	0.009	0.25	1.53E-34	CSCL	C3AR1
MAF1.2	1.12E-38	-0.26364	0.115	0.444	1.67E-34	CSCL	MAF1
RBM17.2	1.15E-38	-0.34385	0.08	0.383	1.71E-34	CSCL	RBM17
CMTM3.2	1.18E-38	-0.43527	0.056	0.337	1.76E-34	CSCL	CMTM3
H2AFY.2	1.57E-38	-0.27558	0.215	0.627	2.35E-34	CSCL	H2AFY
C19orf60.	1.64E-38	-0.23855	0.157	0.52	2.45E-34	CSCL	C19orf60
ARPC5L.1	1.73E-38	-0.31306	0.08	0.384	2.59E-34	CSCL	ARPC5L
CLTA.2	1.74E-38	-0.16505	0.225	0.649	2.60E-34	CSCL	CLTA
SCPEP1.1	1.81E-38	-0.52883	0.07	0.36	2.71E-34	CSCL	SCPEP1
HAGH.2	1.87E-38	-0.33549	0.072	0.368	2.80E-34	CSCL	HAGH
SPI1.2	1.91E-38	-0.86488	0.01	0.251	2.85E-34	CSCL	SPI1
MAP7D1.2	2.04E-38	-0.34813	0.068	0.36	3.05E-34	CSCL	MAP7D1
ALKBH5.1	2.23E-38	-0.40316	0.056	0.337	3.33E-34	CSCL	ALKBH5
ARPC4.2	2.46E-38	-0.22469	0.161	0.532	3.67E-34	CSCL	ARPC4
PICALM.2	2.58E-38	-0.49582	0.033	0.294	3.85E-34	CSCL	PICALM
RGS2.1	3.10E-38	-0.53025	0.136	0.461	4.63E-34	CSCL	RGS2
GYPC.2	3.23E-38	-0.54435	0.049	0.323	4.83E-34	CSCL	GYPC
IDH2.2	3.98E-38	-0.29568	0.115	0.442	5.95E-34	CSCL	IDH2
RTFDC1.1	5.56E-38	-0.18844	0.18	0.557	8.32E-34	CSCL	RTFDC1
HAVCR2.2	6.51E-38	-0.93814	0.007	0.243	9.73E-34	CSCL	HAVCR2
CHMP4B.2	6.90E-38	-0.2499	0.129	0.471	1.03E-33	CSCL	CHMP4B
PSMD3.1	7.53E-38	-0.26095	0.103	0.42	1.13E-33	CSCL	PSMD3
SUMO3.2	8.17E-38	-0.29892	0.124	0.455	1.22E-33	CSCL	SUMO3
DCK.2	9.82E-38	-0.54609	0.026	0.278	1.47E-33	CSCL	DCK
NR4A2.2	1.07E-37	-0.86638	0.021	0.263	1.60E-33	CSCL	NR4A2
NFKBIA.1	1.12E-37	-0.371	0.168	0.517	1.67E-33	CSCL	NFKBIA
REL.2	1.20E-37	-0.64228	0.047	0.311	1.79E-33	CSCL	REL
SPCS3.2	1.26E-37	-0.43911	0.059	0.339	1.88E-33	CSCL	SPCS3
UBE2J2.2	1.47E-37	-0.25835	0.063	0.348	2.20E-33	CSCL	UBE2J2
MTDH.2	1.62E-37	-0.29018	0.223	0.63	2.41E-33	CSCL	MTDH
MOB1A.2	1.63E-37	-0.40079	0.068	0.354	2.43E-33	CSCL	MOB1A
FNTA.1	1.65E-37	-0.31109	0.094	0.4	2.46E-33	CSCL	FNTA
FLOT1.2	1.67E-37	-0.23952	0.159	0.52	2.50E-33	CSCL	FLOT1
EIF3F.2	1.70E-37	-0.19987	0.215	0.625	2.55E-33	CSCL	EIF3F
ATG3.2	1.80E-37	-0.23109	0.089	0.392	2.69E-33	CSCL	ATG3
CTSA.2	1.96E-37	-0.26594	0.101	0.414	2.92E-33	CSCL	CTSA
GNAI3.1	3.73E-37	-0.26583	0.098	0.408	5.58E-33	CSCL	GNAI3
ERP44	3.83E-37	-0.35151	0.101	0.411	5.72E-33	CSCL	ERP44

HLA-DQA1.	4.22E-37	-0.82278	0.054	0.319	6.30E-33	CSCL	HLA-DQA1
HNRNPA1.1	4.24E-37	0.360135	0.742	0.884	6.34E-33	CSCL	HNRNPA1
POLD4	4.79E-37	-0.37724	0.117	0.436	7.16E-33	CSCL	POLD4
TGOLN2.2	5.45E-37	-0.38504	0.089	0.387	8.14E-33	CSCL	TGOLN2
PITHD1.1	6.52E-37	-0.34236	0.073	0.36	9.75E-33	CSCL	PITHD1
GAA.2	6.95E-37	-0.43341	0.059	0.333	1.04E-32	CSCL	GAA
ANP32A.2	7.49E-37	-0.33088	0.122	0.444	1.12E-32	CSCL	ANP32A
STAU1.1	7.52E-37	-0.2705	0.084	0.381	1.12E-32	CSCL	STAU1
HNRNPA2B1	7.53E-37	0.362928	0.717	0.852	1.13E-32	CSCL	HNRNPA2B1
SNX2.2	8.60E-37	-0.31373	0.11	0.426	1.29E-32	CSCL	SNX2
PAFAH1B1.	1.11E-36	-0.34811	0.086	0.382	1.65E-32	CSCL	PAFAH1B1
TMEM183A.	1.20E-36	-0.28153	0.042	0.305	1.79E-32	CSCL	TMEM183A
CCDC50.2	1.48E-36	-0.31192	0.047	0.313	2.21E-32	CSCL	CCDC50
AKR1A1.1	1.69E-36	-0.25147	0.161	0.515	2.53E-32	CSCL	AKR1A1
IER3.2	1.81E-36	-0.85869	0.017	0.253	2.70E-32	CSCL	IER3
TSPO.1	1.89E-36	-0.29678	0.223	0.614	2.83E-32	CSCL	TSPO
CKB.2	2.00E-36	0.66548	0.607	0.581	2.99E-32	CSCL	CKB
RBCK1.1	2.02E-36	-0.33092	0.07	0.351	3.02E-32	CSCL	RBCK1
SIRPA.2	2.31E-36	-0.54772	0.017	0.255	3.46E-32	CSCL	SIRPA
COX6C.2	2.49E-36	0.435763	0.712	0.837	3.72E-32	CSCL	COX6C
HNRNPAB.2	2.53E-36	-0.23562	0.101	0.412	3.78E-32	CSCL	HNRNPAB
PPP1CA.1	2.80E-36	-0.23509	0.202	0.59	4.19E-32	CSCL	PPP1CA
TLN1.2	3.15E-36	-0.40589	0.04	0.297	4.71E-32	CSCL	TLN1
CCDC107.2	3.18E-36	-0.24605	0.099	0.405	4.75E-32	CSCL	CCDC107
KIF5B.2	3.18E-36	-0.20957	0.169	0.529	4.75E-32	CSCL	KIF5B
TGFB1.2	3.18E-36	-0.60355	0.019	0.256	4.76E-32	CSCL	TGFB1
THEMIS2.2	3.44E-36	-0.74796	0.017	0.253	5.14E-32	CSCL	THEMIS2
AUP1.1	4.92E-36	-0.24921	0.178	0.54	7.35E-32	CSCL	AUP1
HDAC2.2	5.26E-36	-0.24761	0.133	0.464	7.87E-32	CSCL	HDAC2
MEF2C.2	5.46E-36	-0.61795	0.042	0.295	8.16E-32	CSCL	MEF2C
IFITM2.2	5.62E-36	-0.6956	0.044	0.295	8.40E-32	CSCL	IFITM2
NUTF2.1	5.94E-36	-0.14523	0.133	0.466	8.88E-32	CSCL	NUTF2
FEZ2.2	5.99E-36	-0.25274	0.134	0.465	8.95E-32	CSCL	FEZ2
UBE2L3.2	6.25E-36	-0.1971	0.182	0.553	9.34E-32	CSCL	UBE2L3
GNB2.2	6.84E-36	-0.24184	0.202	0.588	1.02E-31	CSCL	GNB2
PTTG1IP.1	8.37E-36	-0.20619	0.138	0.472	1.25E-31	CSCL	PTTG1IP
CSDE1.2	8.44E-36	-0.23171	0.225	0.621	1.26E-31	CSCL	CSDE1
GNB1.2	8.49E-36	-0.21259	0.113	0.431	1.27E-31	CSCL	GNB1
SLCO2B1.2	8.50E-36	-0.89375	0.007	0.231	1.27E-31	CSCL	SLCO2B1
PCBP1.2	8.81E-36	-0.19583	0.213	0.607	1.32E-31	CSCL	PCBP1
SSR1.1	8.86E-36	-0.27407	0.148	0.485	1.32E-31	CSCL	SSR1
M6PR.2	9.01E-36	-0.29056	0.096	0.397	1.35E-31	CSCL	M6PR
C1QB.2	9.79E-36	-0.57684	0.25	0.586	1.46E-31	CSCL	C1QB
MTPN.1	1.06E-35	-0.13427	0.122	0.448	1.58E-31	CSCL	MTPN
UBE2D2.2	1.16E-35	-0.24329	0.216	0.607	1.73E-31	CSCL	UBE2D2
MLEC.2	1.43E-35	-0.18639	0.105	0.416	2.14E-31	CSCL	MLEC
TPM3.2	1.72E-35	-0.2156	0.197	0.577	2.56E-31	CSCL	TPM3
ALDH2.2	1.77E-35	-0.42454	0.094	0.385	2.64E-31	CSCL	ALDH2
SKIL.2	2.14E-35	-0.4027	0.044	0.298	3.20E-31	CSCL	SKIL
SPINT2.2	2.22E-35	-0.74933	0.005	0.227	3.32E-31	CSCL	SPINT2
BRI3.2	2.64E-35	-0.20022	0.143	0.484	3.94E-31	CSCL	BRI3

PLEKHB2.2	3.31E-35	-0.2563	0.108	0.414	4.95E-31	CSCL	PLEKHB2
ZFAND5.2	3.60E-35	-0.25781	0.145	0.479	5.38E-31	CSCL	ZFAND5
SERPINB1.	4.15E-35	-0.69219	0.035	0.279	6.20E-31	CSCL	SERPINB1
MAPRE1.2	4.23E-35	-0.1735	0.152	0.494	6.32E-31	CSCL	MAPRE1
LGALS9.2	4.27E-35	-0.75587	0.019	0.251	6.38E-31	CSCL	LGALS9
CRELD2.2	4.54E-35	-0.38937	0.042	0.295	6.78E-31	CSCL	CRELD2
ELF1.2	4.58E-35	-0.60555	0.021	0.255	6.85E-31	CSCL	ELF1
UBL3.2	4.98E-35	-0.26924	0.082	0.368	7.44E-31	CSCL	UBL3
TWF2.2	5.37E-35	-0.37509	0.077	0.354	8.02E-31	CSCL	TWF2
SPNS1.2	6.14E-35	-0.34278	0.047	0.303	9.18E-31	CSCL	SPNS1
TIMP2.2	6.83E-35	-0.381	0.068	0.339	1.02E-30	CSCL	TIMP2
CCDC85B.2	7.18E-35	-0.22763	0.115	0.429	1.07E-30	CSCL	CCDC85B
RNH1.1	8.33E-35	-0.28158	0.192	0.555	1.25E-30	CSCL	RNH1
DHRS7.2	8.38E-35	-0.33204	0.208	0.574	1.25E-30	CSCL	DHRS7
LSM12.1	1.02E-34	-0.35415	0.03	0.271	1.52E-30	CSCL	LSM12
C20orf27.	1.08E-34	-0.25504	0.086	0.374	1.61E-30	CSCL	C20orf27
HNRNPF.1	1.13E-34	-0.18336	0.171	0.525	1.69E-30	CSCL	HNRNPF
PAK2.2	1.42E-34	-0.29958	0.082	0.366	2.12E-30	CSCL	PAK2
HSPH1.1	1.52E-34	-0.2609	0.087	0.375	2.27E-30	CSCL	HSPH1
PFDN4.2	1.52E-34	-0.19078	0.086	0.373	2.28E-30	CSCL	PFDN4
RAP1B.1	1.57E-34	-0.23292	0.129	0.45	2.34E-30	CSCL	RAP1B
EIF3A.2	1.65E-34	-0.26901	0.072	0.346	2.46E-30	CSCL	EIF3A
ACSL1.2	1.85E-34	-0.69288	0.014	0.238	2.77E-30	CSCL	ACSL1
OSBPL8.2	1.90E-34	-0.31082	0.059	0.324	2.83E-30	CSCL	OSBPL8
VPS4A.1	1.98E-34	-0.266	0.058	0.321	2.96E-30	CSCL	VPS4A
C5AR1.2	2.11E-34	-0.81707	0.021	0.249	3.16E-30	CSCL	C5AR1
DAP.2	2.19E-34	-0.34298	0.04	0.288	3.27E-30	CSCL	DAP
LIMD2.2	2.30E-34	-0.50628	0.026	0.26	3.44E-30	CSCL	LIMD2
TMEM123.1	2.54E-34	-0.27415	0.108	0.411	3.79E-30	CSCL	TMEM123
OLFML3.2	2.79E-34	-0.79598	0.014	0.237	4.16E-30	CSCL	OLFML3
VMA21.1	2.92E-34	-0.31644	0.077	0.354	4.36E-30	CSCL	VMA21
MEG3.1	3.06E-34	0.875201	0.445	0.297	4.57E-30	CSCL	MEG3
CNDP2.2	3.51E-34	-0.1793	0.127	0.446	5.25E-30	CSCL	CNDP2
CCL4.2	3.90E-34	-0.79959	0.084	0.337	5.83E-30	CSCL	CCL4
ROCK1.2	4.21E-34	-0.3199	0.059	0.321	6.29E-30	CSCL	ROCK1
APLP2.2	4.42E-34	-0.22998	0.223	0.612	6.60E-30	CSCL	APLP2
MMD.2	4.43E-34	-0.492	0.024	0.256	6.63E-30	CSCL	MMD
FYB.2	4.48E-34	-0.8444	0.014	0.234	6.69E-30	CSCL	FYB
SEC63.1	4.67E-34	-0.20205	0.105	0.402	6.98E-30	CSCL	SEC63
PKN1.2	4.77E-34	-0.3523	0.023	0.254	7.13E-30	CSCL	PKN1
AKAP13.2	4.82E-34	-0.41843	0.072	0.338	7.20E-30	CSCL	AKAP13
OGFR.2	4.92E-34	-0.3316	0.047	0.299	7.36E-30	CSCL	OGFR
FBXO7.2	5.12E-34	-0.31341	0.061	0.322	7.65E-30	CSCL	FBXO7
PPP1R15A.	5.31E-34	-0.30576	0.155	0.483	7.93E-30	CSCL	PPP1R15A
FAM50A.2	5.72E-34	-0.26667	0.096	0.387	8.55E-30	CSCL	FAM50A
HRAS.2	6.52E-34	-0.2871	0.051	0.306	9.75E-30	CSCL	HRAS
PAIP1.2	7.22E-34	-0.20958	0.108	0.41	1.08E-29	CSCL	PAIP1
PTPMT1.1	7.48E-34	-0.25296	0.084	0.366	1.12E-29	CSCL	PTPMT1
ACAA1.2	8.68E-34	-0.22771	0.082	0.363	1.30E-29	CSCL	ACAA1
AMD1.1	9.08E-34	-0.2957	0.103	0.398	1.36E-29	CSCL	AMD1
NUDT3.2	9.52E-34	-0.26756	0.113	0.414	1.42E-29	CSCL	NUDT3

ADI1.1	1.03E-33	-0.25222	0.117	0.421	1.54E-29	CSCL	ADI1
KPNA2.1	1.08E-33	-0.37514	0.089	0.368	1.61E-29	CSCL	KPNA2
C16orf13.	1.10E-33	-0.16327	0.145	0.476	1.65E-29	CSCL	C16orf13
H3F3A	1.19E-33	0.437924	0.684	0.751	1.78E-29	CSCL	H3F3A
EIF3J.2	1.29E-33	-0.23644	0.099	0.392	1.93E-29	CSCL	EIF3J
RBPJ.2	1.37E-33	-0.18249	0.15	0.485	2.05E-29	CSCL	RBPJ
CD86.2	1.50E-33	-0.84952	0.009	0.224	2.23E-29	CSCL	CD86
HMGN1.2	1.52E-33	-0.26391	0.267	0.685	2.28E-29	CSCL	HMGN1
TOP1.2	1.88E-33	-0.21227	0.105	0.404	2.81E-29	CSCL	TOP1
MMP24-AS1	2.02E-33	-0.34333	0.063	0.324	3.02E-29	CSCL	MMP24-AS1
IFI30.2	2.12E-33	-0.81576	0.012	0.23	3.17E-29	CSCL	IFI30
LEPROTL1.	2.15E-33	-0.26434	0.106	0.402	3.22E-29	CSCL	LEPROTL1
CITED2.2	2.19E-33	-0.39476	0.089	0.366	3.28E-29	CSCL	CITED2
BCL7C.1	2.50E-33	-0.39773	0.031	0.266	3.74E-29	CSCL	BCL7C
LYZ.2	2.76E-33	-0.81671	0.005	0.216	4.13E-29	CSCL	LYZ
SLBP.2	2.83E-33	-0.26592	0.063	0.324	4.23E-29	CSCL	SLBP
PRPF40A.1	3.13E-33	-0.166	0.141	0.466	4.68E-29	CSCL	PRPF40A
RAB32.2	3.16E-33	-0.40129	0.023	0.25	4.72E-29	CSCL	RAB32
PDXK.2	3.50E-33	-0.3434	0.049	0.297	5.24E-29	CSCL	PDXK
CTDNEP1.2	3.55E-33	-0.28586	0.065	0.326	5.31E-29	CSCL	CTDNEP1
VAMP3.1	3.61E-33	-0.25079	0.084	0.362	5.39E-29	CSCL	VAMP3
RAB5C	4.31E-33	-0.21206	0.255	0.673	6.44E-29	CSCL	RAB5C
DNAJC3	4.64E-33	-0.29861	0.052	0.304	6.94E-29	CSCL	DNAJC3
HLA-E.2	4.80E-33	-0.26908	0.236	0.615	7.17E-29	CSCL	HLA-E
PSMD11.1	5.02E-33	-0.16774	0.117	0.422	7.51E-29	CSCL	PSMD11
ARL5A.2	5.26E-33	-0.18146	0.182	0.534	7.87E-29	CSCL	ARL5A
FDX1.2	5.52E-33	-0.3216	0.061	0.318	8.25E-29	CSCL	FDX1
BCKDK.2	5.57E-33	-0.34563	0.066	0.327	8.32E-29	CSCL	BCKDK
ECHS1.1	5.75E-33	-0.17672	0.133	0.45	8.60E-29	CSCL	ECHS1
JMJD6.1	6.87E-33	-0.40015	0.049	0.295	1.03E-28	CSCL	JMJD6
NXT1.1	7.02E-33	-0.30173	0.068	0.329	1.05E-28	CSCL	NXT1
PIGT.1	7.14E-33	-0.21768	0.113	0.412	1.07E-28	CSCL	PIGT
GPAA1.2	7.18E-33	-0.29366	0.112	0.408	1.07E-28	CSCL	GPAA1
SEPHS2.1	7.50E-33	-0.23779	0.065	0.326	1.12E-28	CSCL	SEPHS2
KHDRBS1.2	8.19E-33	-0.20013	0.164	0.502	1.22E-28	CSCL	KHDRBS1
SMARCA5.2	8.59E-33	-0.24836	0.086	0.362	1.28E-28	CSCL	SMARCA5
CHD1.2	9.04E-33	-0.40713	0.056	0.305	1.35E-28	CSCL	CHD1
TMEM165.2	9.84E-33	-0.23421	0.148	0.472	1.47E-28	CSCL	TMEM165
RNF126.2	9.85E-33	-0.29426	0.052	0.303	1.47E-28	CSCL	RNF126
ENO1.1	9.96E-33	0.48059	0.696	0.799	1.49E-28	CSCL	ENO1
ANXA11.2	1.03E-32	-0.6024	0.012	0.226	1.54E-28	CSCL	ANXA11
GAMT.2	1.09E-32	-0.27506	0.058	0.312	1.63E-28	CSCL	GAMT
GSTO1.1	1.10E-32	-0.23355	0.15	0.472	1.64E-28	CSCL	GSTO1
TRABD.2	1.11E-32	-0.45734	0.037	0.27	1.65E-28	CSCL	TRABD
BTG1.2	1.11E-32	-0.30985	0.295	0.709	1.66E-28	CSCL	BTG1
SMAP2.2	1.12E-32	-0.57286	0.024	0.247	1.67E-28	CSCL	SMAP2
PTP4A2.2	1.12E-32	-0.16539	0.143	0.469	1.68E-28	CSCL	PTP4A2
SSR3.2	1.22E-32	-0.20121	0.188	0.544	1.83E-28	CSCL	SSR3
FBXW5.2	1.23E-32	-0.32423	0.052	0.3	1.83E-28	CSCL	FBXW5
EVI2B.2	1.24E-32	-0.80776	0.005	0.213	1.86E-28	CSCL	EVI2B
TRAM1.2	1.26E-32	-0.26413	0.136	0.449	1.88E-28	CSCL	TRAM1

RRP7A.2	1.36E-32	-0.41659	0.052	0.299	2.03E-28	CSCL		RRP7A
PPP1R2	1.43E-32	-0.25767	0.098	0.382	2.14E-28	CSCL		PPP1R2
CCNL1.1	1.57E-32	-0.26084	0.194	0.542	2.34E-28	CSCL		CCNL1
NDUFAF3	1.63E-32	-0.15905	0.183	0.534	2.43E-28	CSCL		NDUFAF3
RAB20.2	1.78E-32	-0.63199	0.024	0.247	2.66E-28	CSCL		RAB20
SYNCRIP.2	1.83E-32	-0.23724	0.108	0.398	2.74E-28	CSCL		SYNCRIP
DRAM2.2	1.96E-32	-0.41444	0.084	0.349	2.93E-28	CSCL		DRAM2
CSNK1A1.2	2.02E-32	-0.2229	0.204	0.576	3.02E-28	CSCL		CSNK1A1
CDV3.1	2.07E-32	-0.3607	0.045	0.287	3.09E-28	CSCL		CDV3
UBE2D1.2	2.20E-32	-0.32581	0.04	0.278	3.29E-28	CSCL		UBE2D1
GDE1.2	2.25E-32	-0.31467	0.059	0.311	3.37E-28	CSCL		GDE1
WHSC1L1.2	2.30E-32	-0.27506	0.101	0.387	3.43E-28	CSCL		WHSC1L1
METTL9.2	2.41E-32	-0.11098	0.152	0.485	3.60E-28	CSCL		METTL9
USF2.2	2.62E-32	-0.29188	0.075	0.339	3.92E-28	CSCL		USF2
PFKL.2	2.81E-32	-0.24098	0.096	0.377	4.20E-28	CSCL		PFKL
PTPRC.2	3.08E-32	-0.79132	0.007	0.213	4.61E-28	CSCL		PTPRC
PLXDC2.2	3.36E-32	-0.76423	0.01	0.219	5.02E-28	CSCL		PLXDC2
S100A9.2	3.41E-32	-0.89994	0.019	0.234	5.09E-28	CSCL		S100A9
MKRN1.2	3.50E-32	-0.20883	0.122	0.423	5.24E-28	CSCL		MKRN1
FKBP4.1	3.81E-32	-0.19866	0.072	0.334	5.70E-28	CSCL		FKBP4
TM9SF2.1	3.82E-32	-0.30388	0.084	0.352	5.71E-28	CSCL		TM9SF2
FNBP1.2	4.14E-32	-0.41724	0.04	0.274	6.19E-28	CSCL		FNBP1
HP1BP3.2	4.51E-32	-0.21796	0.141	0.457	6.74E-28	CSCL		HP1BP3
SH3KBP1.2	4.69E-32	-0.44595	0.028	0.252	7.01E-28	CSCL		SH3KBP1
GBAS.1	4.82E-32	-0.12867	0.117	0.414	7.20E-28	CSCL		GBAS
STK4.2	4.82E-32	-0.35089	0.054	0.299	7.21E-28	CSCL		STK4
VSTM2A.1	4.91E-32	0.850316	0.237	0.099	7.34E-28	CSCL		VSTM2A
SLC25A11.	5.19E-32	-0.14851	0.127	0.434	7.76E-28	CSCL		SLC25A11
WDR1.1	5.33E-32	-0.2274	0.127	0.429	7.96E-28	CSCL		WDR1
PLEKHO1.2	5.84E-32	-0.35163	0.047	0.287	8.73E-28	CSCL		PLEKHO1
GSPT1.2	6.63E-32	-0.18774	0.087	0.362	9.90E-28	CSCL		GSPT1
EIF2AK1.1	7.29E-32	-0.24234	0.079	0.345	1.09E-27	CSCL		EIF2AK1
MPST.2	7.55E-32	-0.28548	0.065	0.319	1.13E-27	CSCL		MPST
JAK1.2	8.44E-32	-0.3414	0.035	0.264	1.26E-27	CSCL		JAK1
AP1B1.2	8.74E-32	-0.51184	0.04	0.27	1.31E-27	CSCL		AP1B1
THRAP3.2	9.27E-32	-0.1387	0.14	0.453	1.39E-27	CSCL		THRAP3
TGIF1.1	9.70E-32	-0.345	0.096	0.371	1.45E-27	CSCL		TGIF1
HIST1H4C.	9.82E-32	-0.30321	0.136	0.439	1.47E-27	CSCL		HIST1H4C
DBNL.2	1.03E-31	-0.22756	0.136	0.44	1.53E-27	CSCL		DBNL
P4HB.1	1.11E-31	-0.19778	0.218	0.59	1.66E-27	CSCL		P4HB
SAP30.2	1.20E-31	-0.3951	0.028	0.251	1.79E-27	CSCL		SAP30
LAT2.2	1.25E-31	-0.77402	0.009	0.213	1.87E-27	CSCL		LAT2
CNOT7.1	1.25E-31	-0.19413	0.099	0.382	1.87E-27	CSCL		CNOT7
DPYSL2.1	1.25E-31	-0.21277	0.134	0.438	1.87E-27	CSCL		DPYSL2
CD164.1	1.32E-31	-0.25161	0.175	0.507	1.97E-27	CSCL		CD164
COPRS.1	1.48E-31	-0.23082	0.08	0.347	2.21E-27	CSCL		COPRS
LY96.2	1.49E-31	-0.50601	0.019	0.233	2.23E-27	CSCL		LY96
DDX21	1.56E-31	-0.29681	0.063	0.312	2.34E-27	CSCL		DDX21
NOL7.1	1.59E-31	-0.15728	0.141	0.454	2.38E-27	CSCL		NOL7
LITAF.2	1.67E-31	-0.30258	0.159	0.474	2.49E-27	CSCL		LITAF
HMGB2.2	1.78E-31	-0.32048	0.187	0.51	2.67E-27	CSCL		HMGB2

ABI3.2	1.80E-31	-0.79454	0.002	0.199	2.70E-27	CSCL	ABI3
DDT	1.84E-31	-0.20041	0.19	0.539	2.74E-27	CSCL	DDT
RTCB.2	2.05E-31	-0.29814	0.082	0.346	3.07E-27	CSCL	RTCB
PGP.2	2.07E-31	-0.41259	0.017	0.23	3.10E-27	CSCL	PGP
PRKAR1A.1	2.21E-31	-0.21426	0.162	0.488	3.30E-27	CSCL	PRKAR1A
APOC2.2	2.26E-31	-1.01408	0.002	0.199	3.37E-27	CSCL	APOC2
CD300A.2	2.50E-31	-0.79637	0.003	0.201	3.74E-27	CSCL	CD300A
ARL2.2	2.51E-31	-0.13913	0.141	0.454	3.75E-27	CSCL	ARL2
PNPLA8.2	2.72E-31	-0.31916	0.091	0.36	4.07E-27	CSCL	PNPLA8
VPS35	2.76E-31	-0.2148	0.14	0.445	4.13E-27	CSCL	VPS35
MATR3.2	2.93E-31	-0.15872	0.169	0.503	4.38E-27	CSCL	MATR3
EZR.2	3.27E-31	-0.28329	0.087	0.355	4.89E-27	CSCL	EZR
C17orf89.	3.29E-31	-0.13717	0.129	0.436	4.92E-27	CSCL	C17orf89
UBE2S.2	3.35E-31	-0.24133	0.089	0.357	5.01E-27	CSCL	UBE2S
CD47.1	3.54E-31	-0.21435	0.112	0.398	5.30E-27	CSCL	CD47
SERINC3.1	4.96E-31	-0.23744	0.101	0.379	7.41E-27	CSCL	SERINC3
MCUR1.2	5.18E-31	-0.2538	0.042	0.273	7.74E-27	CSCL	MCUR1
TPRKB.1	5.29E-31	-0.17904	0.099	0.377	7.90E-27	CSCL	TPRKB
VASP.2	5.32E-31	-0.36368	0.03	0.251	7.94E-27	CSCL	VASP
CXADR.2	5.40E-31	-0.3853	0.07	0.319	8.08E-27	CSCL	CXADR
THOC7	5.42E-31	-0.16317	0.113	0.403	8.11E-27	CSCL	THOC7
SNX5.1	5.48E-31	-0.25853	0.119	0.408	8.19E-27	CSCL	SNX5
ADAP2.2	5.58E-31	-0.75939	0	0.193	8.33E-27	CSCL	ADAP2
EGLN2.2	5.66E-31	-0.30736	0.054	0.295	8.46E-27	CSCL	EGLN2
NPC2.1	6.00E-31	-0.31542	0.311	0.711	8.97E-27	CSCL	NPC2
CISD3.2	6.43E-31	-0.35506	0.038	0.265	9.61E-27	CSCL	CISD3
NAGK.1	7.08E-31	-0.29539	0.113	0.395	1.06E-26	CSCL	NAGK
NR1H2.2	7.17E-31	-0.2739	0.066	0.315	1.07E-26	CSCL	NR1H2
GRINA.2	7.28E-31	-0.19839	0.143	0.452	1.09E-26	CSCL	GRINA
SNX10.1	7.69E-31	-0.4538	0.024	0.239	1.15E-26	CSCL	SNX10
MPC2	8.70E-31	-0.18824	0.225	0.6	1.30E-26	CSCL	MPC2
SF1.1	9.07E-31	-0.20818	0.185	0.524	1.35E-26	CSCL	SF1
MYCBP2.2	9.07E-31	-0.30954	0.068	0.317	1.35E-26	CSCL	MYCBP2
STUB1.2	9.78E-31	-0.14664	0.154	0.477	1.46E-26	CSCL	STUB1
G3BP2.2	9.78E-31	-0.31481	0.051	0.287	1.46E-26	CSCL	G3BP2
BNIP2.2	1.03E-30	-0.41258	0.045	0.275	1.54E-26	CSCL	BNIP2
STK25.2	1.08E-30	-0.31662	0.066	0.314	1.62E-26	CSCL	STK25
EIF4B.2	1.12E-30	-0.12064	0.108	0.392	1.67E-26	CSCL	EIF4B
TERF2IP.2	1.14E-30	-0.22797	0.197	0.534	1.71E-26	CSCL	TERF2IP
TAPBP.2	1.18E-30	-0.23478	0.089	0.356	1.77E-26	CSCL	TAPBP
BROX.2	1.21E-30	-0.39305	0.028	0.245	1.81E-26	CSCL	BROX
PTPRE.2	1.26E-30	-0.39082	0.049	0.281	1.89E-26	CSCL	PTPRE
BRMS1.1	1.34E-30	-0.27254	0.072	0.323	2.00E-26	CSCL	BRMS1
RAB8A	1.44E-30	-0.15005	0.096	0.369	2.16E-26	CSCL	RAB8A
RALA.1	1.46E-30	-0.22932	0.099	0.373	2.18E-26	CSCL	RALA
EMD	1.48E-30	-0.32624	0.056	0.295	2.22E-26	CSCL	EMD
SLC39A1	1.52E-30	-0.24192	0.115	0.401	2.26E-26	CSCL	SLC39A1
SHKBP1.2	1.54E-30	-0.38495	0.03	0.248	2.31E-26	CSCL	SHKBP1
HLA-DRB5.	1.56E-30	-0.59816	0.138	0.413	2.34E-26	CSCL	HLA-DRB5
ADPGK.2	1.65E-30	-0.353	0.044	0.272	2.46E-26	CSCL	ADPGK
GLTSCR2.2	1.69E-30	-0.10253	0.216	0.582	2.53E-26	CSCL	GLTSCR2

ABL2.2	1.76E-30	-0.46945	0.049	0.279	2.63E-26	CSCL	ABL2
CD81.2	1.82E-30	-0.24463	0.15	0.453	2.73E-26	CSCL	CD81
PLIN2.1	1.86E-30	-0.51421	0.096	0.355	2.78E-26	CSCL	PLIN2
MARCH2.2	2.18E-30	-0.36857	0.028	0.243	3.25E-26	CSCL	2-Mar
FAM210B.2	2.22E-30	-0.35337	0.049	0.281	3.31E-26	CSCL	FAM210B
DYNC1I2.2	2.28E-30	-0.17112	0.161	0.485	3.40E-26	CSCL	DYNC1I2
COX16.1	2.32E-30	-0.14036	0.178	0.517	3.46E-26	CSCL	COX16
LSM2	2.33E-30	-0.12097	0.147	0.463	3.48E-26	CSCL	LSM2
CHMP2B.1	2.35E-30	-0.25919	0.07	0.319	3.51E-26	CSCL	CHMP2B
SLA.2	2.37E-30	-0.75541	0.012	0.212	3.55E-26	CSCL	SLA
APPL1.2	2.49E-30	-0.25968	0.063	0.307	3.73E-26	CSCL	APPL1
FXYD6.2	2.58E-30	0.802169	0.525	0.457	3.85E-26	CSCL	FXYD6
COMMD9.1	2.67E-30	-0.26683	0.072	0.322	3.99E-26	CSCL	COMMD9
GNAQ.2	2.73E-30	-0.24792	0.058	0.298	4.08E-26	CSCL	GNAQ
IDI1.2	2.83E-30	-0.18847	0.079	0.337	4.23E-26	CSCL	IDI1
MLX.2	2.87E-30	-0.32329	0.066	0.311	4.29E-26	CSCL	MLX
LAIR1.2	2.91E-30	-0.74481	0.03	0.241	4.34E-26	CSCL	LAIR1
TALDO1.2	3.20E-30	-0.16373	0.239	0.616	4.78E-26	CSCL	TALDO1
NT5C3B.1	3.22E-30	-0.25895	0.084	0.344	4.82E-26	CSCL	NT5C3B
C12orf75.	3.36E-30	-0.53865	0.007	0.202	5.02E-26	CSCL	C12orf75
METTL23.1	3.60E-30	-0.237	0.077	0.331	5.38E-26	CSCL	METTL23
ELP5.1	3.95E-30	-0.2836	0.047	0.278	5.91E-26	CSCL	ELP5
UBE2R2.2	4.02E-30	-0.25119	0.058	0.298	6.01E-26	CSCL	UBE2R2
H2AFV.2	4.61E-30	-0.12621	0.192	0.539	6.89E-26	CSCL	H2AFV
TOMM22.2	4.89E-30	-0.11791	0.161	0.485	7.31E-26	CSCL	TOMM22
SMARCB1.2	5.22E-30	-0.18561	0.131	0.428	7.80E-26	CSCL	SMARCB1
HEBP2.2	5.30E-30	-0.34643	0.021	0.229	7.93E-26	CSCL	HEBP2
KPNB1.2	5.95E-30	-0.1867	0.152	0.463	8.90E-26	CSCL	KPNB1
PPA1.1	6.05E-30	-0.12173	0.195	0.546	9.05E-26	CSCL	PPA1
SMAP1.2	6.19E-30	-0.17276	0.065	0.309	9.25E-26	CSCL	SMAP1
KLHDC3.2	6.21E-30	-0.13467	0.101	0.375	9.28E-26	CSCL	KLHDC3
ALKBH7.2	6.30E-30	-0.1119	0.164	0.487	9.42E-26	CSCL	ALKBH7
SNRNP70.2	6.34E-30	-0.17788	0.166	0.491	9.48E-26	CSCL	SNRNP70
PVRL2.2	6.40E-30	-0.327	0.035	0.254	9.56E-26	CSCL	PVRL2
ETS2.2	6.48E-30	-0.61998	0.016	0.217	9.69E-26	CSCL	ETS2
TUSC2.1	6.73E-30	-0.17919	0.087	0.348	1.01E-25	CSCL	TUSC2
PEBP1.2	6.92E-30	0.41668	0.688	0.81	1.03E-25	CSCL	PEBP1
FUCA1.2	7.54E-30	-0.4792	0.023	0.23	1.13E-25	CSCL	FUCA1
FAM173A.2	7.81E-30	-0.26073	0.059	0.299	1.17E-25	CSCL	FAM173A
SMARCE1.1	8.64E-30	-0.191	0.112	0.392	1.29E-25	CSCL	SMARCE1
CELF2.2	9.08E-30	-0.29755	0.119	0.397	1.36E-25	CSCL	CELF2
PRR13	9.55E-30	-0.13439	0.206	0.56	1.43E-25	CSCL	PRR13
ARPC5.1	9.61E-30	-0.18799	0.281	0.692	1.44E-25	CSCL	ARPC5
CCNH.2	1.00E-29	-0.23573	0.134	0.426	1.50E-25	CSCL	CCNH
PSMD12.1	1.19E-29	-0.22906	0.092	0.356	1.77E-25	CSCL	PSMD12
TMEM176B.	1.21E-29	-0.51139	0.105	0.365	1.81E-25	CSCL	TMEM176B
TGFBI.2	1.21E-29	-0.73336	0.016	0.215	1.81E-25	CSCL	TGFBI
UBAC2.1	1.27E-29	-0.3498	0.052	0.282	1.89E-25	CSCL	UBAC2
WTAP.2	1.27E-29	-0.17117	0.138	0.435	1.89E-25	CSCL	WTAP
CYBB.2	1.31E-29	-0.74521	0.009	0.201	1.96E-25	CSCL	CYBB
UBQLN1.1	1.37E-29	-0.25915	0.058	0.293	2.05E-25	CSCL	UBQLN1

BAG6.1	1.43E-29	-0.22132	0.086	0.344	2.14E-25	CSCL	BAG6
WNK1.2	1.60E-29	-0.26632	0.056	0.29	2.38E-25	CSCL	WNK1
EMC6.1	1.63E-29	-0.1794	0.143	0.449	2.43E-25	CSCL	EMC6
ZYX.2	1.66E-29	-0.17429	0.127	0.416	2.48E-25	CSCL	ZYX
ARL4C.2	1.68E-29	-0.28146	0.113	0.392	2.51E-25	CSCL	ARL4C
SPTLC2.2	1.73E-29	-0.61248	0.019	0.22	2.59E-25	CSCL	SPTLC2
PTPN1.2	1.74E-29	-0.32282	0.03	0.242	2.60E-25	CSCL	PTPN1
JOSD2.1	1.76E-29	-0.24543	0.089	0.347	2.62E-25	CSCL	JOSD2
MTIF3.1	1.89E-29	-0.11694	0.092	0.359	2.83E-25	CSCL	MTIF3
FGFR1OP2.	2.12E-29	-0.23437	0.079	0.33	3.16E-25	CSCL	FGFR1OP2
TMEM9B.1	2.16E-29	-0.18283	0.206	0.553	3.22E-25	CSCL	TMEM9B
NCOA4.2	2.21E-29	-0.27467	0.047	0.274	3.31E-25	CSCL	NCOA4
SAFB.1	2.51E-29	-0.25831	0.063	0.301	3.75E-25	CSCL	SAFB
IL18.2	2.64E-29	-0.77063	0.01	0.202	3.95E-25	CSCL	IL18
QSOX1.2	2.74E-29	-0.35399	0.033	0.247	4.09E-25	CSCL	QSOX1
MCOLN1.2	2.77E-29	-0.35667	0.037	0.254	4.14E-25	CSCL	MCOLN1
CAPRIN1.2	2.84E-29	-0.14151	0.079	0.332	4.25E-25	CSCL	CAPRIN1
POP7.1	2.96E-29	-0.16411	0.122	0.408	4.43E-25	CSCL	POP7
SQSTM1.2	3.12E-29	-0.14059	0.202	0.54	4.66E-25	CSCL	SQSTM1
UROS.2	3.14E-29	-0.29414	0.042	0.263	4.69E-25	CSCL	UROS
KCTD12.2	3.17E-29	-0.40221	0.033	0.247	4.74E-25	CSCL	KCTD12
CYFIP1.2	3.41E-29	-0.33609	0.061	0.294	5.10E-25	CSCL	CYFIP1
BAX.2	3.66E-29	-0.12564	0.222	0.583	5.48E-25	CSCL	BAX
TMEM259.2	3.68E-29	-0.18724	0.089	0.348	5.50E-25	CSCL	TMEM259
TPD52L2.1	3.80E-29	-0.1263	0.082	0.336	5.68E-25	CSCL	TPD52L2
CSF2RA.2	3.81E-29	-0.75826	0.016	0.212	5.69E-25	CSCL	CSF2RA
DDRGK1.1	4.31E-29	-0.15307	0.136	0.432	6.45E-25	CSCL	DDRGK1
ANAPC16	4.37E-29	-0.13481	0.201	0.542	6.54E-25	CSCL	ANAPC16
CCDC112.2	4.39E-29	-0.30821	0.047	0.272	6.56E-25	CSCL	CCDC112
CLEC2B.2	4.42E-29	-0.69551	0.017	0.214	6.60E-25	CSCL	CLEC2B
EPC1.2	4.44E-29	-0.27184	0.065	0.303	6.64E-25	CSCL	EPC1
HEXA.1	4.79E-29	-0.2752	0.124	0.403	7.15E-25	CSCL	HEXA
SLTM.2	5.02E-29	-0.13011	0.098	0.362	7.51E-25	CSCL	SLTM
SRSF4.2	5.19E-29	-0.12144	0.103	0.373	7.76E-25	CSCL	SRSF4
H1FX.2	5.25E-29	-0.2691	0.122	0.398	7.84E-25	CSCL	H1FX
WDR45B.1	5.27E-29	-0.19791	0.129	0.417	7.88E-25	CSCL	WDR45B
STX7.2	5.61E-29	-0.35031	0.052	0.279	8.38E-25	CSCL	STX7
PAFAH1B2.	5.70E-29	-0.19039	0.084	0.338	8.52E-25	CSCL	PAFAH1B2
IFNAR1.2	6.39E-29	-0.26652	0.065	0.301	9.55E-25	CSCL	IFNAR1
CAST.1	6.72E-29	-0.16749	0.08	0.33	1.00E-24	CSCL	CAST
STIP1	7.87E-29	-0.11378	0.11	0.385	1.18E-24	CSCL	STIP1
RNF213.2	8.34E-29	-0.32794	0.049	0.272	1.25E-24	CSCL	RNF213
PPIF.2	8.46E-29	-0.40758	0.026	0.231	1.26E-24	CSCL	PPIF
ARF6.2	8.53E-29	-0.20224	0.096	0.356	1.27E-24	CSCL	ARF6
ARGLU1.2	8.60E-29	-0.1736	0.166	0.484	1.29E-24	CSCL	ARGLU1
NRBF2.2	9.17E-29	-0.25156	0.054	0.283	1.37E-24	CSCL	NRBF2
NUDT14.2	9.70E-29	-0.50837	0.01	0.201	1.45E-24	CSCL	NUDT14
SNX17	9.88E-29	-0.16252	0.157	0.465	1.48E-24	CSCL	SNX17
RNF145.2	1.04E-28	-0.23425	0.115	0.391	1.55E-24	CSCL	RNF145
FCGBP.2	1.10E-28	-0.80423	0.035	0.239	1.65E-24	CSCL	FCGBP
MPP1.2	1.15E-28	-0.52131	0.016	0.21	1.72E-24	CSCL	MPP1

IQGAP1.2	1.18E-28	-0.26312	0.044	0.263	1.77E-24	CSCL	IQGAP1
RHOT1.2	1.20E-28	-0.27659	0.042	0.259	1.79E-24	CSCL	RHOT1
DNASE2.2	1.22E-28	-0.22183	0.12	0.397	1.82E-24	CSCL	DNASE2
UFD1L.2	1.37E-28	-0.13594	0.087	0.343	2.04E-24	CSCL	UFD1L
GLRX3.1	1.37E-28	-0.24862	0.073	0.316	2.05E-24	CSCL	GLRX3
SCARB2.2	1.45E-28	-0.18775	0.119	0.395	2.16E-24	CSCL	SCARB2
NOP10.2	1.47E-28	-0.18083	0.248	0.625	2.20E-24	CSCL	NOP10
WAS.2	1.48E-28	-0.702	0.002	0.183	2.21E-24	CSCL	WAS
C1orf54.2	1.50E-28	-0.4956	0.03	0.236	2.25E-24	CSCL	C1orf54
SLC7A7.2	1.50E-28	-0.74076	0.002	0.183	2.25E-24	CSCL	SLC7A7
HMGB1	1.51E-28	0.330759	0.728	0.868	2.25E-24	CSCL	HMGB1
TMEM50A.2	1.55E-28	-0.13166	0.237	0.61	2.32E-24	CSCL	TMEM50A
ACADVL.1	1.59E-28	-0.10343	0.155	0.466	2.37E-24	CSCL	ACADVL
SNF8.2	1.63E-28	-0.14538	0.162	0.475	2.43E-24	CSCL	SNF8
ARID5A	1.70E-28	-0.29384	0.066	0.301	2.54E-24	CSCL	ARID5A
PFKFB3.2	1.70E-28	-0.45677	0.042	0.254	2.54E-24	CSCL	PFKFB3
MGAT4A.2	1.71E-28	-0.58985	0.028	0.23	2.56E-24	CSCL	MGAT4A
BAZ1B.2	1.72E-28	-0.23988	0.07	0.31	2.57E-24	CSCL	BAZ1B
PRRC2C.2	1.93E-28	-0.11739	0.175	0.498	2.89E-24	CSCL	PRRC2C
RRBP1.2	1.96E-28	-0.26097	0.07	0.308	2.93E-24	CSCL	RRBP1
BLOC1S2.1	1.97E-28	-0.148	0.11	0.382	2.95E-24	CSCL	BLOC1S2
CCDC88A.2	2.08E-28	-0.17581	0.14	0.43	3.11E-24	CSCL	CCDC88A
MANF.2	2.08E-28	-0.19837	0.161	0.466	3.11E-24	CSCL	MANF
MAP3K8.2	2.13E-28	-0.68611	0.007	0.192	3.19E-24	CSCL	MAP3K8
GGNBP2	2.19E-28	-0.2013	0.126	0.404	3.28E-24	CSCL	GGNBP2
FSCN1.2	2.25E-28	-0.19735	0.192	0.52	3.37E-24	CSCL	FSCN1
VOPP1.1	2.52E-28	-0.29949	0.068	0.302	3.76E-24	CSCL	VOPP1
TRPC4AP.2	2.82E-28	-0.26543	0.044	0.261	4.21E-24	CSCL	TRPC4AP
EWSR1.1	2.85E-28	-0.13989	0.168	0.48	4.26E-24	CSCL	EWSR1
RABEP1.2	2.88E-28	-0.2751	0.045	0.263	4.30E-24	CSCL	RABEP1
FAM195A.2	2.93E-28	-0.23001	0.045	0.265	4.37E-24	CSCL	FAM195A
SAMHD1.2	2.94E-28	-0.48505	0.024	0.224	4.40E-24	CSCL	SAMHD1
NARF	3.14E-28	-0.238	0.089	0.342	4.70E-24	CSCL	NARF
MBOAT7.2	3.53E-28	-0.24035	0.04	0.253	5.27E-24	CSCL	MBOAT7
SHARPIN.2	3.59E-28	-0.20219	0.073	0.313	5.37E-24	CSCL	SHARPIN
GLRX.1	3.77E-28	-0.29328	0.127	0.402	5.63E-24	CSCL	GLRX
PARL.2	3.78E-28	-0.19494	0.094	0.35	5.65E-24	CSCL	PARL
ATP6V1A.2	3.82E-28	-0.35631	0.016	0.208	5.71E-24	CSCL	ATP6V1A
ARRDC3	4.03E-28	-0.27747	0.073	0.313	6.02E-24	CSCL	ARRDC3
FMNL2.2	4.16E-28	-0.31203	0.052	0.276	6.22E-24	CSCL	FMNL2
RNPEP.2	4.29E-28	-0.35809	0.03	0.234	6.40E-24	CSCL	RNPEP
UBXN1.2	4.49E-28	-0.13273	0.257	0.639	6.70E-24	CSCL	UBXN1
RP11-345J	4.82E-28	-0.20575	0.035	0.245	7.20E-24	CSCL	RP11-345J4.5
DNMT1.2	4.89E-28	-0.18117	0.065	0.297	7.30E-24	CSCL	DNMT1
USB1.1	4.93E-28	-0.3357	0.033	0.24	7.36E-24	CSCL	USB1
PDCD10.1	5.04E-28	-0.21609	0.063	0.294	7.53E-24	CSCL	PDCD10
ELOVL5	5.15E-28	-0.25488	0.087	0.336	7.70E-24	CSCL	ELOVL5
RRAGA.1	5.22E-28	-0.18626	0.126	0.402	7.80E-24	CSCL	RRAGA
PLK3.2	6.14E-28	-0.48813	0.042	0.251	9.17E-24	CSCL	PLK3
NFE2L2.1	6.23E-28	-0.13804	0.171	0.483	9.31E-24	CSCL	NFE2L2
YWHAG.2	6.39E-28	-0.11114	0.124	0.404	9.55E-24	CSCL	YWHAG

TMX4.1	6.72E-28	-0.26971	0.042	0.256	1.00E-23	CSCL	TMX4
NSA2.2	7.21E-28	-0.11807	0.129	0.412	1.08E-23	CSCL	NSA2
CSRP1.1	7.29E-28	-0.34137	0.038	0.248	1.09E-23	CSCL	CSRP1
S100A8.2	7.54E-28	-0.88532	0.01	0.194	1.13E-23	CSCL	S100A8
TAF10.2	7.99E-28	-0.30782	0.031	0.236	1.19E-23	CSCL	TAF10
CCDC47.2	8.07E-28	-0.15024	0.133	0.419	1.21E-23	CSCL	CCDC47
AP2B1.1	8.31E-28	-0.2161	0.096	0.353	1.24E-23	CSCL	AP2B1
PPP1R7	8.64E-28	-0.13217	0.103	0.366	1.29E-23	CSCL	PPP1R7
HOMER3.2	9.02E-28	-0.25555	0.04	0.252	1.35E-23	CSCL	HOMER3
MNDA.2	9.10E-28	-0.71781	0.005	0.186	1.36E-23	CSCL	MNDA
STARD3	9.69E-28	-0.30714	0.049	0.266	1.45E-23	CSCL	STARD3
WIPF1.2	1.05E-27	-0.45381	0.03	0.229	1.57E-23	CSCL	WIPF1
CEP170.2	1.05E-27	-0.27451	0.052	0.272	1.57E-23	CSCL	CEP170
NRP2.2	1.08E-27	-0.3193	0.03	0.231	1.61E-23	CSCL	NRP2
AGPAT2.2	1.13E-27	-0.46762	0.007	0.188	1.70E-23	CSCL	AGPAT2
COX6B1.2	1.16E-27	0.43803	0.688	0.854	1.73E-23	CSCL	COX6B1
WASF2.2	1.22E-27	-0.10518	0.155	0.453	1.82E-23	CSCL	WASF2
POLDIP2.2	1.24E-27	-0.14689	0.099	0.36	1.86E-23	CSCL	POLDIP2
NDFIP1.2	1.25E-27	-0.18098	0.243	0.62	1.86E-23	CSCL	NDFIP1
NEK6.1	1.25E-27	-0.23115	0.068	0.301	1.87E-23	CSCL	NEK6
RTN4.2	1.27E-27	-0.25211	0.309	0.72	1.89E-23	CSCL	RTN4
ETF1.1	1.35E-27	-0.21341	0.101	0.358	2.01E-23	CSCL	ETF1
CISD2.1	1.46E-27	-0.13665	0.134	0.418	2.19E-23	CSCL	CISD2
ADRM1	1.51E-27	-0.12584	0.195	0.525	2.25E-23	CSCL	ADRM1
FYTTD1.1	1.57E-27	-0.19689	0.068	0.3	2.34E-23	CSCL	FYTTD1
XRCC5.2	1.57E-27	-0.15532	0.227	0.578	2.35E-23	CSCL	XRCC5
CYTH2.2	1.64E-27	-0.14866	0.098	0.354	2.46E-23	CSCL	CYTH2
POLR1D.1	1.67E-27	-0.10079	0.14	0.429	2.50E-23	CSCL	POLR1D
PDCD6IP	1.68E-27	-0.23024	0.068	0.3	2.51E-23	CSCL	PDCD6IP
HINT1.1	1.69E-27	0.400173	0.686	0.835	2.53E-23	CSCL	HINT1
FGD5-AS1.	1.69E-27	-0.20179	0.082	0.324	2.53E-23	CSCL	FGD5-AS1
HSD17B11.	1.79E-27	-0.3294	0.124	0.39	2.68E-23	CSCL	HSD17B11
CTSB	1.85E-27	-0.31727	0.37	0.776	2.77E-23	CSCL	CTSB
NRBP1	1.90E-27	-0.17259	0.098	0.353	2.83E-23	CSCL	NRBP1
ACAT2.2	1.91E-27	-0.16255	0.061	0.289	2.86E-23	CSCL	ACAT2
GPR137B.2	1.97E-27	-0.27989	0.035	0.239	2.94E-23	CSCL	GPR137B
BECN1.1	2.02E-27	-0.27798	0.035	0.24	3.02E-23	CSCL	BECN1
RTF1.2	2.03E-27	-0.1074	0.148	0.445	3.04E-23	CSCL	RTF1
FOLR2.2	2.16E-27	-0.89059	0.007	0.186	3.23E-23	CSCL	FOLR2
SH3BP5.2	2.18E-27	-0.39854	0.019	0.21	3.26E-23	CSCL	SH3BP5
UNC93B1.2	2.21E-27	-0.4041	0.019	0.21	3.31E-23	CSCL	UNC93B1
THOP1.1	2.22E-27	-0.26383	0.019	0.211	3.32E-23	CSCL	THOP1
SLC43A2.2	2.31E-27	-0.42462	0.045	0.256	3.46E-23	CSCL	SLC43A2
LTC4S.2	2.32E-27	-0.67641	0.002	0.177	3.47E-23	CSCL	LTC4S
USP14.1	2.36E-27	-0.22998	0.051	0.268	3.53E-23	CSCL	USP14
RAB14	2.48E-27	-0.14785	0.141	0.431	3.70E-23	CSCL	RAB14
PTS.1	2.60E-27	-0.11782	0.089	0.339	3.89E-23	CSCL	PTS
CRYL1	2.66E-27	-0.27263	0.082	0.321	3.97E-23	CSCL	CRYL1
BTG3.1	2.67E-27	-0.21466	0.119	0.384	3.99E-23	CSCL	BTG3
CELF1.2	2.74E-27	-0.21131	0.08	0.319	4.10E-23	CSCL	CELF1
PTBP1.2	2.98E-27	-0.12019	0.087	0.335	4.46E-23	CSCL	PTBP1

ZDHHC12.2	3.15E-27	-0.24959	0.052	0.271	4.71E-23	CSCL	ZDHHC12
IFNAR2.2	3.18E-27	-0.36971	0.019	0.209	4.75E-23	CSCL	IFNAR2
TP53I13.2	3.33E-27	-0.28058	0.044	0.255	4.98E-23	CSCL	TP53I13
UBE2F.1	3.36E-27	-0.18962	0.092	0.342	5.01E-23	CSCL	UBE2F
BIRC2.1	3.39E-27	-0.2003	0.065	0.292	5.06E-23	CSCL	BIRC2
TMUB1.1	3.40E-27	-0.22144	0.056	0.277	5.08E-23	CSCL	TMUB1
MSR1.2	3.53E-27	-0.69308	0.009	0.189	5.27E-23	CSCL	MSR1
PDCD2.2	3.57E-27	-0.14841	0.072	0.306	5.34E-23	CSCL	PDCD2
SLC35B2.1	3.62E-27	-0.2204	0.075	0.312	5.41E-23	CSCL	SLC35B2
MFSD10.1	3.91E-27	-0.22166	0.077	0.313	5.84E-23	CSCL	MFSD10
GNA13.2	4.00E-27	-0.49608	0.009	0.189	5.98E-23	CSCL	GNA13
TCP1	4.19E-27	-0.15101	0.204	0.539	6.25E-23	CSCL	TCP1
CLEC7A.2	4.19E-27	-0.71298	0.007	0.184	6.26E-23	CSCL	CLEC7A
OSTM1.2	4.19E-27	-0.25299	0.035	0.238	6.26E-23	CSCL	OSTM1
FABP5.2	4.20E-27	0.949157	0.485	0.387	6.28E-23	CSCL	FABP5
KARS.1	4.33E-27	-0.19032	0.119	0.387	6.47E-23	CSCL	KARS
TOR1A.2	4.50E-27	-0.2656	0.054	0.272	6.73E-23	CSCL	TOR1A
GOLGB1.1	4.60E-27	-0.32934	0.042	0.25	6.87E-23	CSCL	GOLGB1
CD302.2	4.60E-27	-0.54039	0.023	0.214	6.88E-23	CSCL	CD302
PDCD6.1	4.66E-27	-0.11494	0.208	0.551	6.97E-23	CSCL	PDCD6
SIGIRR.2	4.68E-27	-0.38369	0.017	0.205	7.00E-23	CSCL	SIGIRR
CTNNB1.2	4.76E-27	-0.26517	0.112	0.367	7.11E-23	CSCL	CTNNB1
TMEM248.1	4.82E-27	-0.18463	0.073	0.307	7.21E-23	CSCL	TMEM248
HNRNPH3.2	5.29E-27	-0.12293	0.159	0.46	7.91E-23	CSCL	HNRNPH3
SVIP.2	5.37E-27	-0.58999	0.003	0.178	8.03E-23	CSCL	SVIP
HNRNPA0.2	5.38E-27	-0.14439	0.271	0.661	8.04E-23	CSCL	HNRNPA0
NR4A1.2	5.46E-27	-0.50125	0.065	0.283	8.16E-23	CSCL	NR4A1
SLC52A2.1	5.56E-27	-0.22722	0.065	0.291	8.30E-23	CSCL	SLC52A2
CLIC1	5.59E-27	-0.24286	0.271	0.641	8.35E-23	CSCL	CLIC1
TIMP1.2	6.00E-27	0.584599	0.62	0.62	8.96E-23	CSCL	TIMP1
PLEK.2	6.18E-27	-0.72546	0.007	0.184	9.23E-23	CSCL	PLEK
SCO2.2	6.72E-27	-0.3189	0.026	0.221	1.00E-22	CSCL	SCO2
HPCAL1.2	6.89E-27	-0.34792	0.026	0.219	1.03E-22	CSCL	HPCAL1
PTPN6.2	7.19E-27	-0.67486	0	0.17	1.07E-22	CSCL	PTPN6
TAX1BP3.1	7.25E-27	-0.13758	0.126	0.395	1.08E-22	CSCL	TAX1BP3
MZT2A.1	7.34E-27	-0.10798	0.141	0.428	1.10E-22	CSCL	MZT2A
HNRNPD.2	7.58E-27	-0.15221	0.141	0.428	1.13E-22	CSCL	HNRNPD
NIPA2.2	7.73E-27	-0.18267	0.056	0.275	1.15E-22	CSCL	NIPA2
NME3.2	7.73E-27	-0.16325	0.145	0.431	1.16E-22	CSCL	NME3
TMED2.1	8.18E-27	-0.14813	0.257	0.63	1.22E-22	CSCL	TMED2
VIMP.1	8.38E-27	-0.14465	0.168	0.471	1.25E-22	CSCL	VIMP
EIF4EBP1.	8.50E-27	-0.1546	0.15	0.441	1.27E-22	CSCL	EIF4EBP1
ITFG1.2	8.69E-27	-0.20692	0.105	0.361	1.30E-22	CSCL	ITFG1
TPP1.2	8.98E-27	-0.28619	0.092	0.335	1.34E-22	CSCL	TPP1
IVNS1ABP.	9.12E-27	-0.37855	0.042	0.248	1.36E-22	CSCL	IVNS1ABP
GPX1.2	9.72E-27	-0.29228	0.396	0.835	1.45E-22	CSCL	GPX1
GHITM.1	9.72E-27	-0.12057	0.208	0.539	1.45E-22	CSCL	GHITM
UBL5.1	1.10E-26	0.419513	0.684	0.839	1.65E-22	CSCL	UBL5
IFT27.2	1.17E-26	-0.25804	0.052	0.267	1.74E-22	CSCL	IFT27
GINM1	1.22E-26	-0.16868	0.075	0.308	1.82E-22	CSCL	GINM1
SCAF11.2	1.22E-26	-0.15122	0.133	0.41	1.82E-22	CSCL	SCAF11

YIF1B	1.22E-26	-0.16345	0.096	0.345	1.83E-22	CSCL	YIF1B
IL10RB.2	1.27E-26	-0.36057	0.045	0.253	1.90E-22	CSCL	IL10RB
SLC31A2.2	1.29E-26	-0.56428	0.017	0.201	1.92E-22	CSCL	SLC31A2
C1orf35.1	1.30E-26	-0.20347	0.056	0.274	1.94E-22	CSCL	C1orf35
SSRP1.1	1.31E-26	-0.12503	0.108	0.367	1.95E-22	CSCL	SSRP1
HCK.2	1.34E-26	-0.65836	0.003	0.176	2.01E-22	CSCL	HCK
NT5C.2	1.38E-26	-0.18386	0.089	0.332	2.06E-22	CSCL	NT5C
ARMC10.1	1.43E-26	-0.13824	0.075	0.308	2.13E-22	CSCL	ARMC10
CHD2.2	1.51E-26	-0.22835	0.073	0.303	2.25E-22	CSCL	CHD2
MGST2.2	1.51E-26	-0.44212	0.052	0.264	2.26E-22	CSCL	MGST2
UBE3A.2	1.52E-26	-0.21076	0.07	0.298	2.26E-22	CSCL	UBE3A
HBEGF.2	1.52E-26	-0.54856	0.031	0.225	2.28E-22	CSCL	HBEGF
MAP2K3.2	1.56E-26	-0.41616	0.016	0.198	2.34E-22	CSCL	MAP2K3
FAM26F.2	1.63E-26	-0.63951	0.003	0.175	2.44E-22	CSCL	FAM26F
DDX17.2	1.65E-26	-0.15706	0.15	0.438	2.46E-22	CSCL	DDX17
SRSF6.2	1.66E-26	-0.16385	0.112	0.372	2.48E-22	CSCL	SRSF6
MINOS1.1	1.71E-26	-0.12253	0.271	0.658	2.56E-22	CSCL	MINOS1
STXBP2.2	1.82E-26	-0.62965	0.007	0.181	2.72E-22	CSCL	STXBP2
RBBP6.1	1.88E-26	-0.18446	0.086	0.325	2.81E-22	CSCL	RBBP6
FAM89B.2	1.95E-26	-0.24553	0.047	0.257	2.92E-22	CSCL	FAM89B
BRD4.1	1.98E-26	-0.12666	0.092	0.339	2.96E-22	CSCL	BRD4
FERMT3.2	2.03E-26	-0.63809	0.003	0.175	3.03E-22	CSCL	FERMT3
UBE2J1.2	2.04E-26	-0.18894	0.066	0.292	3.05E-22	CSCL	UBE2J1
IKBIP.1	2.06E-26	-0.38864	0.037	0.236	3.08E-22	CSCL	IKBIP
C5orf24.1	2.14E-26	-0.16614	0.075	0.307	3.20E-22	CSCL	C5orf24
FAM105A.2	2.17E-26	-0.66768	0.002	0.171	3.24E-22	CSCL	FAM105A
OGFRL1.2	2.20E-26	-0.52223	0.012	0.19	3.29E-22	CSCL	OGFRL1
UBE2K	2.26E-26	-0.13728	0.077	0.31	3.38E-22	CSCL	UBE2K
SCAND1.2	2.28E-26	-0.15476	0.234	0.588	3.40E-22	CSCL	SCAND1
DNAJA1	2.37E-26	-0.1447	0.267	0.647	3.54E-22	CSCL	DNAJA1
RDX.1	2.45E-26	-0.16161	0.19	0.506	3.67E-22	CSCL	RDX
MEF2A.2	2.46E-26	-0.46003	0.031	0.225	3.68E-22	CSCL	MEF2A
ATF6.2	2.51E-26	-0.2699	0.049	0.259	3.75E-22	CSCL	ATF6
ITGAV.2	2.57E-26	-0.21957	0.066	0.289	3.84E-22	CSCL	ITGAV
CDIPT.1	2.69E-26	-0.15763	0.07	0.297	4.02E-22	CSCL	CDIPT
TM7SF3.2	3.48E-26	-0.21059	0.051	0.261	5.20E-22	CSCL	TM7SF3
NQO2.1	3.48E-26	-0.27587	0.052	0.263	5.21E-22	CSCL	NQO2
EPN1.2	3.64E-26	-0.1284	0.098	0.347	5.43E-22	CSCL	EPN1
ORMDL2	3.87E-26	-0.3329	0.065	0.283	5.78E-22	CSCL	ORMDL2
CHMP3.1	3.97E-26	-0.12858	0.124	0.392	5.93E-22	CSCL	CHMP3
ROGDI.2	4.04E-26	-0.3113	0.038	0.237	6.03E-22	CSCL	ROGDI
RHBDF2.2	4.20E-26	-0.60629	0.01	0.186	6.27E-22	CSCL	RHBDF2
TIMM10.1	4.24E-26	-0.10606	0.11	0.366	6.34E-22	CSCL	TIMM10
RPN1.1	4.43E-26	-0.14597	0.143	0.423	6.61E-22	CSCL	RPN1
ZNF207	4.43E-26	-0.14626	0.169	0.47	6.62E-22	CSCL	ZNF207
LPAR6.2	4.44E-26	-0.47207	0.045	0.248	6.63E-22	CSCL	LPAR6
TUBB4B.1	4.45E-26	-0.17206	0.192	0.507	6.66E-22	CSCL	TUBB4B
CMTM7.2	4.49E-26	-0.56921	0.003	0.172	6.71E-22	CSCL	CMTM7
CAPNS1.1	4.49E-26	-0.15109	0.101	0.352	6.71E-22	CSCL	CAPNS1
NUDT5.1	4.65E-26	-0.11049	0.082	0.318	6.94E-22	CSCL	NUDT5
SAFB2.1	4.65E-26	-0.20114	0.098	0.343	6.95E-22	CSCL	SAFB2

BLOC1S6.1	4.65E-26	-0.2206	0.056	0.27	6.95E-22	CSCL	BLOC1S6
RAB7A.1	4.79E-26	-0.11425	0.279	0.678	7.16E-22	CSCL	RAB7A
FGL2.2	4.80E-26	-0.67676	0.01	0.185	7.18E-22	CSCL	FGL2
CFLAR.1	4.90E-26	-0.25266	0.092	0.33	7.32E-22	CSCL	CFLAR
CD58.2	4.90E-26	-0.33435	0.038	0.237	7.32E-22	CSCL	CD58
TNFRSF1B.	5.00E-26	-0.57661	0.017	0.197	7.47E-22	CSCL	TNFRSF1B
RNF10.1	5.23E-26	-0.10933	0.098	0.344	7.82E-22	CSCL	RNF10
UPP1.2	5.30E-26	-0.30621	0.054	0.265	7.92E-22	CSCL	UPP1
TMEM33.2	5.44E-26	-0.25083	0.072	0.296	8.13E-22	CSCL	TMEM33
DTD1.2	5.50E-26	-0.12312	0.133	0.405	8.23E-22	CSCL	DTD1
YME1L1.1	5.64E-26	-0.22864	0.058	0.271	8.42E-22	CSCL	YME1L1
NTAN1.2	5.82E-26	-0.32065	0.023	0.209	8.70E-22	CSCL	NTAN1
MBNL1.2	6.26E-26	-0.35669	0.037	0.233	9.36E-22	CSCL	MBNL1
PDK4.2	6.53E-26	-0.69563	0.007	0.178	9.76E-22	CSCL	PDK4
CCM2.2	6.56E-26	-0.16751	0.07	0.295	9.80E-22	CSCL	CCM2
DYNLL1.1	6.87E-26	0.344622	0.695	0.843	1.03E-21	CSCL	DYNLL1
DNM2.2	7.32E-26	-0.25717	0.047	0.252	1.09E-21	CSCL	DNM2
GALNT1.2	7.38E-26	-0.32163	0.026	0.215	1.10E-21	CSCL	GALNT1
PPP2R5E.2	7.43E-26	-0.2183	0.049	0.257	1.11E-21	CSCL	PPP2R5E
ATG4B	7.88E-26	-0.22697	0.066	0.287	1.18E-21	CSCL	ATG4B
DDX41.1	8.19E-26	-0.22514	0.047	0.253	1.22E-21	CSCL	DDX41
MVP	8.37E-26	-0.34686	0.038	0.235	1.25E-21	CSCL	MVP
RBM42.1	8.51E-26	-0.11398	0.129	0.399	1.27E-21	CSCL	RBM42
BCL7B.2	8.62E-26	-0.1658	0.092	0.334	1.29E-21	CSCL	BCL7B
E2F4.2	8.90E-26	-0.27527	0.031	0.223	1.33E-21	CSCL	E2F4
AATF	8.94E-26	-0.18172	0.075	0.302	1.34E-21	CSCL	AATF
NCOR1.2	9.07E-26	-0.11084	0.175	0.479	1.35E-21	CSCL	NCOR1
NUFIP2.2	9.14E-26	-0.21454	0.044	0.246	1.37E-21	CSCL	NUFIP2
LGMN.2	9.24E-26	-0.23285	0.113	0.363	1.38E-21	CSCL	LGMN
RNASEH2B.	9.68E-26	-0.23891	0.045	0.249	1.45E-21	CSCL	RNASEH2B
EIF2S1	9.78E-26	-0.12438	0.091	0.331	1.46E-21	CSCL	EIF2S1
LLPH.1	1.02E-25	-0.1632	0.061	0.279	1.52E-21	CSCL	LLPH
GPCPD1.2	1.03E-25	-0.35124	0.023	0.207	1.55E-21	CSCL	GPCPD1
LARP7.1	1.04E-25	-0.15133	0.115	0.372	1.55E-21	CSCL	LARP7
TMED3.1	1.04E-25	-0.18264	0.086	0.319	1.55E-21	CSCL	TMED3
PLTP	1.08E-25	-0.39801	0.194	0.484	1.62E-21	CSCL	PLTP
CKS2.2	1.13E-25	-0.2959	0.126	0.379	1.68E-21	CSCL	CKS2
UBA2.1	1.13E-25	-0.10854	0.113	0.371	1.69E-21	CSCL	UBA2
WAC.2	1.17E-25	-0.20519	0.059	0.274	1.75E-21	CSCL	WAC
CTNNBIP1.	1.20E-25	-0.19693	0.031	0.224	1.79E-21	CSCL	CTNNBIP1
DDX27.1	1.22E-25	-0.1626	0.07	0.293	1.82E-21	CSCL	DDX27
PARVG.2	1.22E-25	-0.67716	0.003	0.17	1.83E-21	CSCL	PARVG
IRF8.2	1.25E-25	-0.6649	0.002	0.166	1.87E-21	CSCL	IRF8
ADAM17.2	1.30E-25	-0.31557	0.056	0.266	1.94E-21	CSCL	ADAM17
DOHH.1	1.35E-25	-0.33563	0.021	0.203	2.01E-21	CSCL	DOHH
GID8	1.37E-25	-0.20959	0.051	0.257	2.05E-21	CSCL	GID8
CDC5L.2	1.41E-25	-0.19797	0.07	0.292	2.10E-21	CSCL	CDC5L
RAD21.2	1.41E-25	-0.13275	0.119	0.379	2.11E-21	CSCL	RAD21
ABCF1.1	1.48E-25	-0.15741	0.072	0.295	2.21E-21	CSCL	ABCF1
PDLIM7.1	1.50E-25	-0.25129	0.045	0.248	2.24E-21	CSCL	PDLIM7
SF3B1.2	1.51E-25	-0.12707	0.161	0.45	2.25E-21	CSCL	SF3B1

PTGES2.2	1.54E-25	-0.14697	0.061	0.278	2.30E-21	CSCL	PTGES2
SRSF9.2	1.57E-25	-0.11003	0.229	0.578	2.35E-21	CSCL	SRSF9
RANBP2.2	1.65E-25	-0.31392	0.03	0.218	2.46E-21	CSCL	RANBP2
MTMR14.2	1.72E-25	-0.29322	0.037	0.231	2.57E-21	CSCL	MTMR14
MAPKAPK3.	1.74E-25	-0.45544	0.009	0.179	2.60E-21	CSCL	MAPKAPK3
COPS3	1.79E-25	-0.11237	0.12	0.381	2.67E-21	CSCL	COPS3
NEU1.1	1.92E-25	-0.24359	0.079	0.303	2.87E-21	CSCL	NEU1
IMMT.1	1.95E-25	-0.12494	0.082	0.313	2.92E-21	CSCL	IMMT
MAZ.2	1.99E-25	-0.19122	0.052	0.26	2.98E-21	CSCL	MAZ
CHMP4A.1	2.07E-25	-0.12107	0.18	0.483	3.09E-21	CSCL	CHMP4A
DEGS1	2.12E-25	-0.12875	0.175	0.474	3.16E-21	CSCL	DEGS1
PRPF38B.2	2.12E-25	-0.12816	0.161	0.449	3.17E-21	CSCL	PRPF38B
IFRD1.2	2.18E-25	-0.19021	0.195	0.502	3.25E-21	CSCL	IFRD1
G6PD.1	2.18E-25	-0.1837	0.04	0.238	3.25E-21	CSCL	G6PD
CRTAP.2	2.25E-25	-0.26072	0.045	0.246	3.37E-21	CSCL	CRTAP
ZDHHC3.2	2.32E-25	-0.2744	0.024	0.208	3.46E-21	CSCL	ZDHHC3
SMARCC1.2	2.39E-25	-0.23632	0.056	0.265	3.57E-21	CSCL	SMARCC1
SEC23B.1	2.43E-25	-0.15779	0.065	0.281	3.63E-21	CSCL	SEC23B
C19orf25.	2.45E-25	-0.13058	0.08	0.31	3.66E-21	CSCL	C19orf25
IL1B.2	2.46E-25	-0.63448	0.04	0.231	3.68E-21	CSCL	IL1B
CTSL.2	2.62E-25	-0.20883	0.222	0.539	3.91E-21	CSCL	CTSL
SNAP23.2	2.65E-25	-0.32081	0.051	0.254	3.97E-21	CSCL	SNAP23
FTH1.2	2.77E-25	0.243423	0.806	0.939	4.14E-21	CSCL	FTH1
UTP18.1	2.86E-25	-0.16606	0.061	0.274	4.27E-21	CSCL	UTP18
CHCHD1.2	2.93E-25	-0.11652	0.065	0.281	4.38E-21	CSCL	CHCHD1
SS18L2	3.05E-25	-0.13863	0.087	0.322	4.56E-21	CSCL	SS18L2
ME2.1	3.44E-25	-0.26081	0.038	0.233	5.14E-21	CSCL	ME2
MFSD12.2	3.65E-25	-0.32886	0.021	0.2	5.45E-21	CSCL	MFSD12
BID.2	3.65E-25	-0.11133	0.115	0.369	5.46E-21	CSCL	BID
ECHDC1.1	3.69E-25	-0.14375	0.098	0.338	5.52E-21	CSCL	ECHDC1
ADAM9.1	3.85E-25	-0.16889	0.099	0.342	5.75E-21	CSCL	ADAM9
DEF8.2	3.89E-25	-0.12161	0.065	0.281	5.82E-21	CSCL	DEF8
CCDC66.1	3.97E-25	-0.26796	0.042	0.239	5.93E-21	CSCL	CCDC66
KDM6B.2	4.00E-25	-0.41018	0.04	0.232	5.97E-21	CSCL	KDM6B
PCM1.2	4.21E-25	-0.13856	0.096	0.335	6.30E-21	CSCL	PCM1
TUBGCP2.2	4.26E-25	-0.26569	0.033	0.222	6.36E-21	CSCL	TUBGCP2
VTI1B.2	4.37E-25	-0.12971	0.108	0.359	6.53E-21	CSCL	VTI1B
RAB31.2	4.38E-25	-0.14676	0.178	0.474	6.55E-21	CSCL	RAB31
RBM27.1	4.47E-25	-0.2682	0.04	0.235	6.69E-21	CSCL	RBM27
FOXO3.2	4.56E-25	-0.2114	0.066	0.282	6.81E-21	CSCL	FOXO3
CAPN1.1	4.85E-25	-0.24647	0.059	0.269	7.25E-21	CSCL	CAPN1
PPP2CB.2	5.12E-25	-0.19322	0.08	0.308	7.65E-21	CSCL	PPP2CB
ZNF524.2	5.23E-25	-0.26724	0.019	0.196	7.82E-21	CSCL	ZNF524
SWAP70.2	5.36E-25	-0.27366	0.038	0.231	8.01E-21	CSCL	SWAP70
COX6A1.2	5.58E-25	0.347532	0.681	0.853	8.34E-21	CSCL	COX6A1
FAM120A.2	5.58E-25	-0.23738	0.031	0.219	8.34E-21	CSCL	FAM120A
SND1	5.73E-25	-0.23642	0.058	0.266	8.57E-21	CSCL	SND1
CHCHD7.1	5.75E-25	-0.30369	0.077	0.297	8.59E-21	CSCL	CHCHD7
DHX29.1	5.86E-25	-0.16319	0.089	0.321	8.75E-21	CSCL	DHX29
ENOPH1.1	5.94E-25	-0.10341	0.133	0.402	8.88E-21	CSCL	ENOPH1
RRAGC.1	6.16E-25	-0.18806	0.052	0.257	9.20E-21	CSCL	RRAGC

RABGGTB.1	6.18E-25	-0.18744	0.079	0.302	9.23E-21	CSCL	RABGGTB
COMT.2	6.27E-25	-0.11207	0.223	0.564	9.36E-21	CSCL	COMT
ID2.1	6.78E-25	-0.17412	0.3	0.672	1.01E-20	CSCL	ID2
TOMM7.1	6.84E-25	0.382771	0.695	0.86	1.02E-20	CSCL	TOMM7
ANXA7.1	6.93E-25	-0.17678	0.106	0.35	1.04E-20	CSCL	ANXA7
RB1CC1.1	6.98E-25	-0.25766	0.07	0.287	1.04E-20	CSCL	RB1CC1
PNPLA2.2	7.11E-25	-0.28287	0.033	0.221	1.06E-20	CSCL	PNPLA2
BLOC1S4.2	7.14E-25	-0.20378	0.051	0.253	1.07E-20	CSCL	BLOC1S4
MAD2L2.1	7.26E-25	-0.12714	0.136	0.405	1.09E-20	CSCL	MAD2L2
BCL2A1.2	7.81E-25	-0.65379	0.003	0.165	1.17E-20	CSCL	BCL2A1
NCBP2-AS2	7.91E-25	-0.14977	0.07	0.287	1.18E-20	CSCL	NCBP2-AS2
APOE.2	8.29E-25	-0.18412	0.33	0.704	1.24E-20	CSCL	APOE
TMED5.2	8.54E-25	-0.14553	0.087	0.317	1.28E-20	CSCL	TMED5
MTRNR2L10	8.69E-25	0.558755	0.182	0.069	1.30E-20	CSCL	MTRNR2L10
STX4.1	9.02E-25	-0.22111	0.092	0.325	1.35E-20	CSCL	STX4
ZC3H13.2	9.56E-25	-0.11181	0.103	0.346	1.43E-20	CSCL	ZC3H13
MID1IP1.1	9.64E-25	-0.32726	0.063	0.272	1.44E-20	CSCL	MID1IP1
PLGRKT.1	9.64E-25	-0.18812	0.065	0.278	1.44E-20	CSCL	PLGRKT
PTPN18.2	1.01E-24	-0.34267	0.033	0.22	1.51E-20	CSCL	PTPN18
AKIP1.1	1.02E-24	-0.20684	0.077	0.297	1.52E-20	CSCL	AKIP1
TNFSF13B.	1.03E-24	-0.51997	0.03	0.212	1.54E-20	CSCL	TNFSF13B
SCYL1.1	1.05E-24	-0.25578	0.042	0.236	1.57E-20	CSCL	SCYL1
IST1.1	1.09E-24	-0.28853	0.061	0.27	1.63E-20	CSCL	IST1
FCGR1B.2	1.12E-24	-0.70809	0.007	0.171	1.67E-20	CSCL	FCGR1B
FAM204A.2	1.16E-24	-0.22348	0.059	0.267	1.74E-20	CSCL	FAM204A
C9orf16.2	1.17E-24	-0.12301	0.246	0.599	1.75E-20	CSCL	C9orf16
UNC119.2	1.18E-24	-0.2744	0.037	0.226	1.76E-20	CSCL	UNC119
CLN8.2	1.18E-24	-0.33399	0.068	0.279	1.77E-20	CSCL	CLN8
KRT10.1	1.18E-24	-0.10158	0.23	0.575	1.77E-20	CSCL	KRT10
PLSCR1.2	1.19E-24	-0.22854	0.12	0.371	1.78E-20	CSCL	PLSCR1
TUBA1B.1	1.27E-24	0.260814	0.735	0.904	1.90E-20	CSCL	TUBA1B
SLC25A33.	1.27E-24	-0.18058	0.037	0.227	1.90E-20	CSCL	SLC25A33
MYH9.2	1.30E-24	-0.42262	0.021	0.197	1.94E-20	CSCL	MYH9
KRAS.2	1.31E-24	-0.18095	0.058	0.264	1.96E-20	CSCL	KRAS
SEPT6.2	1.36E-24	-0.4588	0.009	0.174	2.04E-20	CSCL	6-Sep
YTHDC1.2	1.40E-24	-0.13112	0.086	0.313	2.09E-20	CSCL	YTHDC1
COX5B.2	1.42E-24	0.323184	0.696	0.856	2.12E-20	CSCL	COX5B
AXL.2	1.43E-24	-0.60306	0.017	0.189	2.14E-20	CSCL	AXL
CTD-2336C	1.48E-24	-0.22864	0.04	0.233	2.22E-20	CSCL	CTD-233602.1
NBL1.2	1.51E-24	-0.38936	0.016	0.187	2.26E-20	CSCL	NBL1
ENG.2	1.52E-24	-0.57684	0.009	0.173	2.27E-20	CSCL	ENG
DAZAP2.1	1.53E-24	-0.10512	0.223	0.552	2.28E-20	CSCL	DAZAP2
GNB4.2	1.59E-24	-0.18505	0.045	0.242	2.37E-20	CSCL	GNB4
MAP2K1.2	1.60E-24	-0.19767	0.066	0.279	2.39E-20	CSCL	MAP2K1
ANKRD11.2	1.60E-24	-0.13814	0.094	0.327	2.39E-20	CSCL	ANKRD11
PIM3.2	1.62E-24	-0.40002	0.023	0.199	2.42E-20	CSCL	PIM3
PPM1A.2	1.76E-24	-0.33441	0.017	0.19	2.63E-20	CSCL	PPM1A
RAP2B.2	1.83E-24	-0.41815	0.009	0.173	2.73E-20	CSCL	RAP2B
TSTA3.1	1.84E-24	-0.14177	0.066	0.28	2.74E-20	CSCL	TSTA3
RNF11.2	1.87E-24	-0.10357	0.089	0.319	2.80E-20	CSCL	RNF11
PCBD1	1.88E-24	-0.13067	0.103	0.342	2.80E-20	CSCL	PCBD1

LSP1.2	1.92E-24	-0.71382	0.01	0.174	2.87E-20	CSCL	LSP1
JUND.2	2.09E-24	-0.18765	0.147	0.412	3.12E-20	CSCL	JUND
KHSRP.2	2.12E-24	-0.17613	0.049	0.248	3.17E-20	CSCL	KHSRP
SRGAP2.2	2.14E-24	-0.30493	0.047	0.242	3.19E-20	CSCL	SRGAP2
CYB5R3.1	2.19E-24	-0.13372	0.12	0.373	3.27E-20	CSCL	CYB5R3
DNTTIP2.1	2.22E-24	-0.22344	0.079	0.297	3.31E-20	CSCL	DNTTIP2
DNAJC5.2	2.26E-24	-0.30547	0.035	0.221	3.38E-20	CSCL	DNAJC5
ETV5.2	2.26E-24	-0.18707	0.061	0.269	3.38E-20	CSCL	ETV5
A1BG.1	2.46E-24	-0.20034	0.084	0.31	3.68E-20	CSCL	A1BG
PSMA2.2	2.46E-24	0.554617	0.159	0.056	3.68E-20	CSCL	PSMA2
STX5.1	2.49E-24	-0.18639	0.068	0.279	3.73E-20	CSCL	STX5
OGFOD3.2	2.54E-24	-0.22976	0.04	0.231	3.79E-20	CSCL	OGFOD3
RNASE2.2	2.57E-24	-0.71872	0.005	0.165	3.84E-20	CSCL	RNASE2
EBAG9.1	2.67E-24	-0.19296	0.058	0.262	3.99E-20	CSCL	EBAG9
RAB6A.1	2.67E-24	-0.12291	0.14	0.407	4.00E-20	CSCL	RAB6A
SFPQ.2	2.68E-24	-0.13549	0.225	0.558	4.00E-20	CSCL	SFPQ
CHD4.2	2.74E-24	-0.14963	0.098	0.334	4.09E-20	CSCL	CHD4
RNFT1.2	2.74E-24	-0.308	0.03	0.211	4.10E-20	CSCL	RNFT1
MPZL1.2	3.16E-24	-0.21634	0.056	0.258	4.72E-20	CSCL	MPZL1
REEP4.2	3.19E-24	-0.42426	0.01	0.175	4.76E-20	CSCL	REEP4
PHF20L1.2	3.20E-24	-0.13997	0.089	0.319	4.78E-20	CSCL	PHF20L1
FUCA2.2	3.21E-24	-0.20939	0.052	0.253	4.80E-20	CSCL	FUCA2
PARVB.2	3.44E-24	-0.37509	0.009	0.172	5.14E-20	CSCL	PARVB
CDK2AP2.1	3.46E-24	-0.10966	0.101	0.341	5.17E-20	CSCL	CDK2AP2
GTF2B.1	3.47E-24	-0.22129	0.086	0.311	5.18E-20	CSCL	GTF2B
WBP11.1	3.62E-24	-0.13436	0.08	0.302	5.41E-20	CSCL	WBP11
SAR1A.2	3.63E-24	-0.18369	0.086	0.309	5.43E-20	CSCL	SAR1A
COPB2.2	3.64E-24	-0.11415	0.089	0.317	5.44E-20	CSCL	COPB2
CHMP1A.1	3.66E-24	-0.1438	0.07	0.284	5.47E-20	CSCL	CHMP1A
GIMAP4.2	3.76E-24	-0.69992	0.002	0.157	5.61E-20	CSCL	GIMAP4
BOD1.1	3.88E-24	-0.14836	0.087	0.314	5.81E-20	CSCL	BOD1
ZFHX3.2	4.12E-24	-0.33121	0.026	0.204	6.15E-20	CSCL	ZFHX3
PRPSAP1.2	4.18E-24	-0.13662	0.089	0.317	6.24E-20	CSCL	PRPSAP1
FAM134A.2	4.27E-24	-0.17486	0.086	0.309	6.38E-20	CSCL	FAM134A
DYNLT3.1	4.33E-24	-0.13696	0.082	0.305	6.47E-20	CSCL	DYNLT3
CFD.2	4.45E-24	-0.63861	0.005	0.164	6.64E-20	CSCL	CFD
UBN1.2	4.47E-24	-0.32356	0.01	0.174	6.68E-20	CSCL	UBN1
GNPTG.2	4.57E-24	-0.16365	0.061	0.268	6.82E-20	CSCL	GNPTG
REEP3.2	4.63E-24	-0.17738	0.054	0.254	6.92E-20	CSCL	REEP3
ARID5B.2	4.66E-24	-0.14405	0.12	0.367	6.96E-20	CSCL	ARID5B
UBE2M.2	4.73E-24	-0.15686	0.045	0.24	7.07E-20	CSCL	UBE2M
RTCA.1	4.90E-24	-0.14355	0.068	0.28	7.32E-20	CSCL	RTCA
NBPF1.2	5.01E-24	-0.16515	0.049	0.245	7.49E-20	CSCL	NBPF1
ABI1.2	5.02E-24	-0.24192	0.052	0.25	7.50E-20	CSCL	ABI1
TOLLIP.1	5.05E-24	-0.18153	0.066	0.276	7.54E-20	CSCL	TOLLIP
SERPINF1.	5.05E-24	-0.52544	0.045	0.233	7.55E-20	CSCL	SERPINF1
CIR1.1	5.22E-24	-0.14612	0.103	0.341	7.79E-20	CSCL	CIR1
PJA2.1	5.27E-24	-0.11067	0.106	0.347	7.88E-20	CSCL	PJA2
WDR46.1	5.31E-24	-0.21731	0.049	0.245	7.93E-20	CSCL	WDR46
SMIM19.1	5.38E-24	-0.12777	0.099	0.335	8.04E-20	CSCL	SMIM19
AIP	5.90E-24	-0.13143	0.089	0.315	8.82E-20	CSCL	AIP

TM6SF1.2	5.99E-24	-0.68162	0.007	0.166	8.96E-20	CSCL	TM6SF1
PHF20.1	6.13E-24	-0.1333	0.098	0.332	9.17E-20	CSCL	PHF20
ROBO1.2	6.35E-24	-0.33434	0.017	0.186	9.48E-20	CSCL	ROBO1
CRYAB.2	6.36E-24	0.613515	0.56	0.496	9.51E-20	CSCL	CRYAB
CAMK1.1	6.55E-24	-0.19225	0.044	0.235	9.79E-20	CSCL	CAMK1
KLF4.2	6.69E-24	-0.52556	0.035	0.215	1.00E-19	CSCL	KLF4
PTTG1.2	6.73E-24	-0.304	0.047	0.24	1.01E-19	CSCL	PTTG1
GTF2E2.2	6.74E-24	-0.28591	0.023	0.196	1.01E-19	CSCL	GTF2E2
ADPRHL2.1	6.86E-24	-0.16578	0.065	0.271	1.02E-19	CSCL	ADPRHL2
CD4.2	6.92E-24	-0.61358	0.005	0.163	1.03E-19	CSCL	CD4
GGA1.2	6.92E-24	-0.24465	0.082	0.3	1.03E-19	CSCL	GGA1
BSG.2	7.27E-24	0.433635	0.635	0.747	1.09E-19	CSCL	BSG
HYAL2.2	7.29E-24	-0.20568	0.042	0.232	1.09E-19	CSCL	HYAL2
SF3A3.1	7.36E-24	-0.14622	0.079	0.296	1.10E-19	CSCL	SF3A3
ABHD17A.2	7.55E-24	-0.15921	0.049	0.245	1.13E-19	CSCL	ABHD17A
BHLHE41.2	7.58E-24	-0.40135	0.035	0.217	1.13E-19	CSCL	BHLHE41
NUBP1.1	7.83E-24	-0.32873	0.04	0.227	1.17E-19	CSCL	NUBP1
BHLHE40.1	7.92E-24	-0.32365	0.026	0.202	1.18E-19	CSCL	BHLHE40
AC010642.	7.94E-24	-0.18604	0.042	0.232	1.19E-19	CSCL	AC010642.1
DTYMK.1	8.19E-24	-0.10355	0.103	0.341	1.22E-19	CSCL	DTYMK
TEN1.2	8.38E-24	-0.26104	0.017	0.186	1.25E-19	CSCL	TEN1
JMJD1C.2	8.44E-24	-0.20067	0.108	0.346	1.26E-19	CSCL	JMJD1C
SLC2A5.2	8.76E-24	-0.68325	0.014	0.178	1.31E-19	CSCL	SLC2A5
NMT1.1	8.90E-24	-0.18463	0.068	0.278	1.33E-19	CSCL	NMT1
DYNLL2.2	8.91E-24	-0.24305	0.033	0.215	1.33E-19	CSCL	DYNLL2
TMEM109.2	8.96E-24	-0.40155	0.033	0.213	1.34E-19	CSCL	TMEM109
CERS2.1	9.19E-24	-0.1827	0.047	0.241	1.37E-19	CSCL	CERS2
PBRM1.1	1.02E-23	-0.22984	0.052	0.249	1.52E-19	CSCL	PBRM1
LPCAT2.2	1.02E-23	-0.57993	0.002	0.155	1.53E-19	CSCL	LPCAT2
MAPK1.2	1.03E-23	-0.22983	0.054	0.252	1.53E-19	CSCL	MAPK1
GORASP2.1	1.05E-23	-0.12269	0.082	0.303	1.56E-19	CSCL	GORASP2
C19orf43.	1.07E-23	-0.10709	0.297	0.692	1.60E-19	CSCL	C19orf43
BCLAF1.2	1.08E-23	-0.16216	0.138	0.398	1.62E-19	CSCL	BCLAF1
MAP1LC3A.	1.14E-23	-0.11302	0.124	0.377	1.70E-19	CSCL	MAP1LC3A
HSPA4.1	1.14E-23	-0.1277	0.073	0.286	1.70E-19	CSCL	HSPA4
TSPAN14.2	1.14E-23	-0.36987	0.038	0.221	1.70E-19	CSCL	TSPAN14
TNIP1	1.14E-23	-0.24897	0.035	0.217	1.70E-19	CSCL	TNIP1
KATNBL1.2	1.15E-23	-0.19906	0.059	0.261	1.72E-19	CSCL	KATNBL1
BAZ1A.2	1.16E-23	-0.1699	0.099	0.331	1.74E-19	CSCL	BAZ1A
TMBIM1	1.18E-23	-0.281	0.056	0.254	1.76E-19	CSCL	TMBIM1
NUDT16.2	1.18E-23	-0.30151	0.042	0.228	1.76E-19	CSCL	NUDT16
APBB1IP.2	1.32E-23	-0.57708	0.007	0.164	1.97E-19	CSCL	APBB1IP
PLD3.1	1.34E-23	-0.10629	0.22	0.545	2.00E-19	CSCL	PLD3
HAMP.2	1.35E-23	-0.80578	0.01	0.168	2.02E-19	CSCL	HAMP
LSM4.1	1.36E-23	-0.11184	0.239	0.577	2.04E-19	CSCL	LSM4
STOM.1	1.39E-23	-0.35188	0.061	0.261	2.08E-19	CSCL	STOM
TBL1XR1.2	1.41E-23	-0.19508	0.058	0.258	2.10E-19	CSCL	TBL1XR1
CENPV.2	1.42E-23	-0.32947	0.063	0.265	2.13E-19	CSCL	CENPV
IGSF6.2	1.43E-23	-0.64656	0.005	0.161	2.14E-19	CSCL	IGSF6
UBE2D3.1	1.43E-23	-0.18559	0.335	0.748	2.14E-19	CSCL	UBE2D3
TUBG1	1.46E-23	-0.12692	0.049	0.243	2.18E-19	CSCL	TUBG1

TRAP1.1	1.47E-23	-0.12766	0.063	0.268	2.19E-19	CSCL	TRAP1
SLC25A36	1.51E-23	-0.15588	0.07	0.279	2.26E-19	CSCL	SLC25A36
NECAP2.1	1.51E-23	-0.15692	0.084	0.302	2.26E-19	CSCL	NECAP2
RNF139.2	1.53E-23	-0.23184	0.063	0.265	2.29E-19	CSCL	RNF139
SUN2.2	1.54E-23	-0.36145	0.042	0.228	2.31E-19	CSCL	SUN2
COQ2.2	1.62E-23	-0.30481	0.019	0.188	2.42E-19	CSCL	COQ2
ZFP36L2.2	1.65E-23	-0.20487	0.223	0.531	2.47E-19	CSCL	ZFP36L2
TINF2.1	1.76E-23	-0.15877	0.058	0.258	2.63E-19	CSCL	TINF2
CMC2	1.78E-23	-0.11347	0.091	0.316	2.66E-19	CSCL	CMC2
RNASEH1.1	1.86E-23	-0.2182	0.047	0.238	2.78E-19	CSCL	RNASEH1
EVI2A.2	1.87E-23	-0.52692	0.045	0.232	2.80E-19	CSCL	EVI2A
STRADB	1.89E-23	-0.1666	0.042	0.229	2.83E-19	CSCL	STRADB
CSF3R.2	1.95E-23	-0.64151	0.005	0.16	2.91E-19	CSCL	CSF3R
STK17A.2	2.09E-23	-0.11103	0.084	0.304	3.13E-19	CSCL	STK17A
SORT1.2	2.10E-23	-0.14367	0.047	0.239	3.14E-19	CSCL	SORT1
NCF4.2	2.10E-23	-0.6666	0.002	0.153	3.14E-19	CSCL	NCF4
AFF4.2	2.12E-23	-0.18516	0.113	0.352	3.17E-19	CSCL	AFF4
ATF5.2	2.19E-23	-0.37815	0.028	0.202	3.27E-19	CSCL	ATF5
TFPT.2	2.23E-23	-0.12437	0.092	0.319	3.33E-19	CSCL	TFPT
PILRA.2	2.25E-23	-0.64682	0.012	0.173	3.37E-19	CSCL	PILRA
PSIP1.2	2.28E-23	-0.13393	0.14	0.396	3.41E-19	CSCL	PSIP1
GAS6.2	2.30E-23	-0.39154	0.035	0.213	3.44E-19	CSCL	GAS6
ERICH1.2	2.34E-23	-0.19783	0.073	0.283	3.49E-19	CSCL	ERICH1
PPIL3.1	2.38E-23	-0.25108	0.038	0.222	3.55E-19	CSCL	PPIL3
SNX27.2	2.40E-23	-0.14647	0.056	0.254	3.58E-19	CSCL	SNX27
RAF1.2	2.60E-23	-0.1792	0.075	0.287	3.89E-19	CSCL	RAF1
PRNP.1	2.65E-23	-0.101	0.185	0.48	3.96E-19	CSCL	PRNP
CECR5.2	2.70E-23	-0.21239	0.061	0.261	4.04E-19	CSCL	CECR5
TXNRD1.1	2.75E-23	-0.18512	0.052	0.247	4.11E-19	CSCL	TXNRD1
SMC3.1	2.77E-23	-0.15919	0.08	0.296	4.14E-19	CSCL	SMC3
MYO1F.2	2.84E-23	-0.46698	0.023	0.192	4.24E-19	CSCL	MYO1F
KLC1.2	3.00E-23	-0.11174	0.124	0.372	4.48E-19	CSCL	KLC1
HSPA1A.2	3.01E-23	-0.1761	0.209	0.506	4.50E-19	CSCL	HSPA1A
CD69.2	3.11E-23	-0.76046	0.007	0.161	4.65E-19	CSCL	CD69
ORAI2.1	3.22E-23	-0.18849	0.075	0.287	4.81E-19	CSCL	ORAI2
OXLD1.1	3.29E-23	-0.15283	0.061	0.262	4.91E-19	CSCL	OXLD1
HPRT1.2	3.34E-23	-0.27794	0.049	0.24	4.98E-19	CSCL	HPRT1
TMEM189.2	3.36E-23	-0.25942	0.024	0.195	5.02E-19	CSCL	TMEM189
ARID4B.2	3.36E-23	-0.11121	0.15	0.419	5.03E-19	CSCL	ARID4B
NDN.2	3.48E-23	-0.18025	0.03	0.205	5.19E-19	CSCL	NDN
AZI2.1	3.68E-23	-0.20948	0.092	0.312	5.50E-19	CSCL	AZI2
CCNC	3.72E-23	-0.12563	0.087	0.306	5.56E-19	CSCL	CCNC
HLA-DPA1.	3.78E-23	-0.3661	0.257	0.547	5.65E-19	CSCL	HLA-DPA1
HLA-DRB1.	3.85E-23	-0.41791	0.265	0.547	5.76E-19	CSCL	HLA-DRB1
MYO9B.2	3.89E-23	-0.217	0.072	0.279	5.81E-19	CSCL	MYO9B
ACTN1.2	3.99E-23	-0.1979	0.063	0.265	5.96E-19	CSCL	ACTN1
GFER.2	4.12E-23	-0.20502	0.037	0.218	6.15E-19	CSCL	GFER
ADAR.1	4.20E-23	-0.11149	0.094	0.318	6.28E-19	CSCL	ADAR
SMPD1.1	4.21E-23	-0.20903	0.052	0.246	6.30E-19	CSCL	SMPD1
IL6ST.2	4.37E-23	-0.1822	0.063	0.264	6.53E-19	CSCL	IL6ST
PREX1.2	4.46E-23	-0.17157	0.042	0.227	6.67E-19	CSCL	PREX1

TTYH3.2	4.48E-23	-0.23485	0.059	0.257	6.70E-19	CSCL	TTYH3
MAT2A.1	4.70E-23	-0.11597	0.138	0.396	7.03E-19	CSCL	MAT2A
UBAC1.2	4.71E-23	-0.17716	0.042	0.227	7.04E-19	CSCL	UBAC1
UBE2Q2.2	4.74E-23	-0.23436	0.035	0.214	7.09E-19	CSCL	UBE2Q2
LMBRD1.1	4.78E-23	-0.31696	0.042	0.225	7.14E-19	CSCL	LMBRD1
COLGALT1.	4.85E-23	-0.27607	0.028	0.2	7.26E-19	CSCL	COLGALT1
KIAA1143	4.95E-23	-0.22405	0.038	0.219	7.40E-19	CSCL	KIAA1143
TAF15.1	5.03E-23	-0.13063	0.073	0.284	7.51E-19	CSCL	TAF15
PHC2.2	5.24E-23	-0.18374	0.077	0.287	7.83E-19	CSCL	PHC2
C22orf39.	5.29E-23	-0.12151	0.066	0.269	7.90E-19	CSCL	C22orf39
DNAJB2.1	5.38E-23	-0.10357	0.098	0.325	8.04E-19	CSCL	DNAJB2
AZIN1.1	5.50E-23	-0.1004	0.106	0.341	8.22E-19	CSCL	AZIN1
VAT1.1	5.53E-23	-0.26247	0.033	0.21	8.26E-19	CSCL	VAT1
BUB3.1	5.75E-23	-0.2055	0.096	0.318	8.59E-19	CSCL	BUB3
SIGLEC10.	6.35E-23	-0.57522	0.012	0.17	9.49E-19	CSCL	SIGLEC10
DAPK3.1	6.69E-23	-0.14401	0.054	0.248	9.99E-19	CSCL	DAPK3
SPRED1.2	6.75E-23	-0.2531	0.033	0.21	1.01E-18	CSCL	SPRED1
HDAC3.1	6.85E-23	-0.11261	0.068	0.273	1.02E-18	CSCL	HDAC3
RAB22A.2	7.20E-23	-0.12904	0.054	0.248	1.08E-18	CSCL	RAB22A
CTDSP1.2	7.70E-23	-0.25227	0.045	0.231	1.15E-18	CSCL	CTDSP1
PTPN2.1	7.71E-23	-0.27969	0.061	0.258	1.15E-18	CSCL	PTPN2
PROSC.1	7.86E-23	-0.15975	0.063	0.261	1.17E-18	CSCL	PROSC
DHRS4L2	8.05E-23	-0.20149	0.068	0.272	1.20E-18	CSCL	DHRS4L2
JKAMP.2	8.14E-23	-0.19866	0.091	0.31	1.22E-18	CSCL	JKAMP
RUNX1.2	8.38E-23	-0.33503	0.028	0.199	1.25E-18	CSCL	RUNX1
APRT	9.06E-23	-0.10834	0.225	0.547	1.35E-18	CSCL	APRT
LRRFIP2.1	9.28E-23	-0.19434	0.026	0.196	1.39E-18	CSCL	LRRFIP2
COPA.2	9.82E-23	-0.12494	0.098	0.324	1.47E-18	CSCL	COPA
UBE2G1.2	1.03E-22	-0.14842	0.066	0.268	1.55E-18	CSCL	UBE2G1
STAB1.2	1.05E-22	-0.5888	0.012	0.169	1.57E-18	CSCL	STAB1
MAP3K2.2	1.06E-22	-0.25469	0.033	0.209	1.59E-18	CSCL	MAP3K2
TAF11.1	1.08E-22	-0.12677	0.077	0.287	1.62E-18	CSCL	TAF11
EFCAB14.2	1.09E-22	-0.23283	0.035	0.212	1.63E-18	CSCL	EFCAB14
ATP6V1H	1.11E-22	-0.10623	0.105	0.334	1.66E-18	CSCL	ATP6V1H
RHOQ.2	1.12E-22	-0.35255	0.012	0.169	1.67E-18	CSCL	RHOQ
ERBB2IP.1	1.14E-22	-0.1954	0.045	0.23	1.70E-18	CSCL	ERBB2IP
GMPS.1	1.15E-22	-0.2587	0.024	0.192	1.71E-18	CSCL	GMPS
PQLC3.2	1.15E-22	-0.26895	0.024	0.193	1.72E-18	CSCL	PQLC3
PRKCA.1	1.15E-22	-0.29428	0.033	0.207	1.73E-18	CSCL	PRKCA
VAPB.1	1.16E-22	-0.18309	0.031	0.206	1.73E-18	CSCL	VAPB
ANP32E.1	1.21E-22	-0.11132	0.072	0.278	1.81E-18	CSCL	ANP32E
GCA.2	1.21E-22	-0.22152	0.091	0.31	1.81E-18	CSCL	GCA
SKAP2.1	1.23E-22	-0.38366	0.077	0.28	1.83E-18	CSCL	SKAP2
TSSC4.1	1.32E-22	-0.11281	0.098	0.323	1.97E-18	CSCL	TSSC4
UBALD2.2	1.36E-22	-0.17043	0.068	0.269	2.03E-18	CSCL	UBALD2
SOCS3.1	1.36E-22	-0.1716	0.124	0.36	2.04E-18	CSCL	SOCS3
VMO1.2	1.37E-22	-0.65139	0.012	0.167	2.05E-18	CSCL	VMO1
FOXN3.2	1.39E-22	-0.31274	0.068	0.265	2.07E-18	CSCL	FOXN3
PHIP.2	1.41E-22	-0.12984	0.11	0.343	2.11E-18	CSCL	PHIP
MCRS1	1.45E-22	-0.1138	0.073	0.28	2.16E-18	CSCL	MCRS1
LYN.2	1.46E-22	-0.41088	0.019	0.182	2.18E-18	CSCL	LYN

ZEB2.2	1.47E-22	-0.245	0.096	0.314	2.19E-18	CSCL	ZEB2
MTRF1L.1	1.48E-22	-0.23541	0.028	0.198	2.21E-18	CSCL	MTRF1L
EEA1.1	1.50E-22	-0.12339	0.059	0.255	2.24E-18	CSCL	EEA1
RNF166.2	1.50E-22	-0.37113	0.026	0.193	2.25E-18	CSCL	RNF166
CASC4.2	1.55E-22	-0.18737	0.047	0.233	2.31E-18	CSCL	CASC4
PLD4.2	1.56E-22	-0.62807	0.009	0.161	2.33E-18	CSCL	PLD4
USP22.2	1.56E-22	-0.13295	0.103	0.33	2.33E-18	CSCL	USP22
FUOM.2	1.66E-22	-0.54909	0.002	0.148	2.48E-18	CSCL	FUOM
FAM104A	1.67E-22	-0.13413	0.056	0.249	2.49E-18	CSCL	FAM104A
SORL1.2	1.68E-22	-0.43905	0.052	0.236	2.51E-18	CSCL	SORL1
HOOK3.2	1.68E-22	-0.12068	0.082	0.294	2.52E-18	CSCL	HOOK3
ASH1L	1.73E-22	-0.10146	0.098	0.321	2.59E-18	CSCL	ASH1L
TRMT1.2	1.75E-22	-0.24159	0.068	0.267	2.62E-18	CSCL	TRMT1
H2AFX.2	1.76E-22	-0.24642	0.045	0.228	2.63E-18	CSCL	H2AFX
DUSP1.2	1.81E-22	-0.1994	0.271	0.59	2.70E-18	CSCL	DUSP1
UBE2W.1	1.85E-22	-0.27537	0.042	0.222	2.77E-18	CSCL	UBE2W
ZNF511.2	1.86E-22	-0.20336	0.031	0.204	2.79E-18	CSCL	ZNF511
HSF1.1	1.92E-22	-0.13747	0.079	0.288	2.87E-18	CSCL	HSF1
POLE4.1	1.96E-22	-0.16396	0.084	0.295	2.93E-18	CSCL	POLE4
TXNDC9	2.08E-22	-0.1162	0.068	0.27	3.11E-18	CSCL	TXNDC9
WSB2.2	2.11E-22	-0.1383	0.047	0.233	3.15E-18	CSCL	WSB2
RIT1.1	2.13E-22	-0.17997	0.042	0.223	3.18E-18	CSCL	RIT1
PLA2G16.2	2.20E-22	-0.17894	0.159	0.417	3.29E-18	CSCL	PLA2G16
CCDC106.2	2.20E-22	-0.15744	0.052	0.241	3.29E-18	CSCL	CCDC106
HTATIP2.2	2.24E-22	-0.44192	0.005	0.154	3.35E-18	CSCL	HTATIP2
SERPINB9.	2.25E-22	-0.56997	0.005	0.154	3.36E-18	CSCL	SERPINB9
U2AF2.1	2.38E-22	-0.18028	0.068	0.267	3.55E-18	CSCL	U2AF2
DAZAP1.2	2.39E-22	-0.14797	0.052	0.241	3.57E-18	CSCL	DAZAP1
REPIN1.2	2.48E-22	-0.14004	0.077	0.284	3.71E-18	CSCL	REPIN1
MSRB2.2	2.53E-22	-0.17624	0.066	0.264	3.78E-18	CSCL	MSRB2
STX6.2	2.56E-22	-0.27426	0.033	0.206	3.82E-18	CSCL	STX6
A2M.2	2.61E-22	-0.15709	0.19	0.478	3.90E-18	CSCL	A2M
SNX4.1	2.65E-22	-0.15763	0.063	0.259	3.96E-18	CSCL	SNX4
CHP1.2	2.65E-22	-0.15851	0.054	0.244	3.96E-18	CSCL	CHP1
ATP6V1C1	2.66E-22	-0.22847	0.059	0.253	3.98E-18	CSCL	ATP6V1C1
CHST11.2	2.78E-22	-0.25985	0.042	0.221	4.16E-18	CSCL	CHST11
EPHX1.1	2.81E-22	-0.26922	0.054	0.241	4.20E-18	CSCL	EPHX1
TMF1.1	2.81E-22	-0.21273	0.045	0.228	4.20E-18	CSCL	TMF1
SART1.2	2.88E-22	-0.16975	0.033	0.206	4.30E-18	CSCL	SART1
SRPRB.1	2.90E-22	-0.16974	0.066	0.265	4.33E-18	CSCL	SRPRB
CGGBP1.1	3.04E-22	-0.14643	0.075	0.279	4.54E-18	CSCL	CGGBP1
IER2.2	3.10E-22	-0.12121	0.277	0.632	4.63E-18	CSCL	IER2
TFDP1.2	3.16E-22	-0.16547	0.03	0.2	4.73E-18	CSCL	TFDP1
PRR7.2	3.17E-22	-0.25393	0.028	0.196	4.73E-18	CSCL	PRR7
SLC35F6.2	3.19E-22	-0.24112	0.04	0.218	4.77E-18	CSCL	SLC35F6
ELOVL1.2	3.29E-22	-0.22567	0.052	0.24	4.92E-18	CSCL	ELOVL1
C2orf69.2	3.31E-22	-0.23269	0.031	0.202	4.94E-18	CSCL	C2orf69
GPR108.1	3.34E-22	-0.15299	0.073	0.275	4.99E-18	CSCL	GPR108
CDKN1B.2	3.39E-22	-0.20761	0.07	0.269	5.07E-18	CSCL	CDKN1B
CKS1B	3.42E-22	-0.15399	0.068	0.269	5.10E-18	CSCL	CKS1B
NAGA.2	3.42E-22	-0.35661	0.026	0.193	5.11E-18	CSCL	NAGA

VPS37B.2	3.45E-22	-0.19082	0.066	0.264	5.16E-18	CSCL	VPS37B
PITPNA.2	3.46E-22	-0.23342	0.024	0.19	5.17E-18	CSCL	PITPNA
FOSL2.2	3.56E-22	-0.35463	0.028	0.194	5.32E-18	CSCL	FOSL2
NUDT22.1	3.65E-22	-0.11141	0.075	0.28	5.45E-18	CSCL	NUDT22
CCNL2	3.74E-22	-0.13897	0.087	0.301	5.60E-18	CSCL	CCNL2
TMEM60.2	3.76E-22	-0.20324	0.063	0.257	5.62E-18	CSCL	TMEM60
B4GALT7.2	3.80E-22	-0.20043	0.04	0.218	5.68E-18	CSCL	B4GALT7
TMEM30A.2	3.96E-22	-0.1381	0.113	0.345	5.91E-18	CSCL	TMEM30A
RSBN1.1	3.98E-22	-0.27298	0.045	0.226	5.95E-18	CSCL	RSBN1
SCIN.2	3.98E-22	-0.69434	0.009	0.158	5.95E-18	CSCL	SCIN
LYSMD2.2	4.15E-22	-0.21818	0.031	0.201	6.19E-18	CSCL	LYSMD2
GPNMB.2	4.25E-22	-0.50144	0.037	0.209	6.35E-18	CSCL	GPNMB
TSPAN4	4.42E-22	-0.32175	0.024	0.189	6.60E-18	CSCL	TSPAN4
MTFR1L.1	4.50E-22	-0.13036	0.087	0.301	6.72E-18	CSCL	MTFR1L
GLG1.2	4.51E-22	-0.12302	0.073	0.276	6.75E-18	CSCL	GLG1
MAP1S.1	4.57E-22	-0.28029	0.019	0.179	6.83E-18	CSCL	MAP1S
SIAH2.2	4.58E-22	-0.27418	0.042	0.219	6.85E-18	CSCL	SIAH2
HIST1H2BK	4.62E-22	-0.2342	0.04	0.217	6.91E-18	CSCL	HIST1H2BK
LHFPL2.2	5.23E-22	-0.4139	0.014	0.168	7.82E-18	CSCL	LHFPL2
BRWD1.2	5.28E-22	-0.17854	0.059	0.251	7.89E-18	CSCL	BRWD1
FBXL15.2	5.43E-22	-0.2591	0.033	0.204	8.12E-18	CSCL	FBXL15
CYB5R4.2	5.68E-22	-0.3262	0.023	0.184	8.48E-18	CSCL	CYB5R4
DDX19B	5.68E-22	-0.27008	0.037	0.21	8.49E-18	CSCL	DDX19B
SLC4A7.2	5.69E-22	-0.232	0.044	0.223	8.50E-18	CSCL	SLC4A7
ENTPD1.2	5.72E-22	-0.55692	0.016	0.17	8.55E-18	CSCL	ENTPD1
SF3A2.1	5.76E-22	-0.19346	0.07	0.269	8.61E-18	CSCL	SF3A2
KIF2A.2	5.92E-22	-0.16516	0.056	0.244	8.85E-18	CSCL	KIF2A
SPECC1.2	5.93E-22	-0.14474	0.091	0.306	8.85E-18	CSCL	SPECC1
KLHDC2.1	6.01E-22	-0.10033	0.094	0.313	8.97E-18	CSCL	KLHDC2
DPH3.1	6.02E-22	-0.26154	0.044	0.222	9.00E-18	CSCL	DPH3
ARID1A.2	6.17E-22	-0.15156	0.059	0.251	9.22E-18	CSCL	ARID1A
PANK2.2	6.20E-22	-0.16705	0.04	0.217	9.26E-18	CSCL	PANK2
SBNO1.1	6.23E-22	-0.1446	0.073	0.275	9.31E-18	CSCL	SBNO1
KLHL5.2	6.26E-22	-0.28573	0.019	0.178	9.35E-18	CSCL	KLHL5
ABHD3.2	6.56E-22	-0.30656	0.026	0.19	9.80E-18	CSCL	ABHD3
ABCA1.1	6.59E-22	-0.28995	0.059	0.248	9.84E-18	CSCL	ABCA1
ATP5E.1	6.66E-22	0.246896	0.729	0.923	9.95E-18	CSCL	ATP5E
ENC1.2	6.80E-22	-0.29228	0.051	0.233	1.02E-17	CSCL	ENC1
C1orf52.2	7.09E-22	-0.1148	0.098	0.317	1.06E-17	CSCL	C1orf52
RBMS1.2	7.51E-22	-0.15119	0.065	0.26	1.12E-17	CSCL	RBMS1
TGFBR1.2	7.60E-22	-0.42727	0.016	0.169	1.14E-17	CSCL	TGFBR1
MBD4.1	7.86E-22	-0.16988	0.073	0.273	1.17E-17	CSCL	MBD4
FABP7.1	8.04E-22	0.775365	0.461	0.379	1.20E-17	CSCL	FABP7
MTX2	8.07E-22	-0.10455	0.106	0.332	1.21E-17	CSCL	MTX2
ITSN2.2	8.21E-22	-0.28184	0.026	0.189	1.23E-17	CSCL	ITSN2
CEP350.1	8.32E-22	-0.15762	0.031	0.201	1.24E-17	CSCL	CEP350
FAM207A.1	8.37E-22	-0.16078	0.038	0.213	1.25E-17	CSCL	FAM207A
TMEM134.2	8.39E-22	-0.11202	0.099	0.32	1.25E-17	CSCL	TMEM134
CCNI.2	8.83E-22	-0.15358	0.328	0.725	1.32E-17	CSCL	CCNI
TRIM36.2	9.11E-22	-0.29039	0.026	0.19	1.36E-17	CSCL	TRIM36
FAM3A	9.42E-22	-0.23996	0.049	0.23	1.41E-17	CSCL	FAM3A

RELA.1	9.46E-22	-0.16541	0.038	0.212	1.41E-17	CSCL	RELA
PPP4R2	9.69E-22	-0.11482	0.072	0.271	1.45E-17	CSCL	PPP4R2
ARCN1	9.73E-22	-0.25172	0.023	0.183	1.45E-17	CSCL	ARCN1
DNAJC4.1	1.01E-21	-0.16715	0.052	0.237	1.51E-17	CSCL	DNAJC4
MIF4GD.2	1.02E-21	-0.15105	0.066	0.261	1.52E-17	CSCL	MIF4GD
CDKN1A.1	1.02E-21	-0.26442	0.103	0.318	1.52E-17	CSCL	CDKN1A
ARHGAP18.	1.03E-21	-0.42607	0.021	0.18	1.54E-17	CSCL	ARHGAP18
VPS26B.2	1.07E-21	-0.15758	0.054	0.24	1.59E-17	CSCL	VPS26B
SLC30A9.1	1.07E-21	-0.13641	0.066	0.261	1.59E-17	CSCL	SLC30A9
SIPA1.2	1.09E-21	-0.43351	0.003	0.146	1.63E-17	CSCL	SIPA1
FOSB.1	1.15E-21	-0.24501	0.157	0.404	1.72E-17	CSCL	FOSB
SIRT7.1	1.16E-21	-0.19959	0.04	0.215	1.73E-17	CSCL	SIRT7
KDSR	1.20E-21	-0.18232	0.068	0.263	1.79E-17	CSCL	KDSR
UBLCP1	1.21E-21	-0.21044	0.035	0.206	1.81E-17	CSCL	UBLCP1
CPM.2	1.21E-21	-0.4602	0.059	0.244	1.81E-17	CSCL	CPM
NRP1.2	1.25E-21	-0.28841	0.017	0.173	1.87E-17	CSCL	NRP1
RABL6.2	1.27E-21	-0.11334	0.063	0.256	1.90E-17	CSCL	RABL6
GLA.2	1.29E-21	-0.3247	0.047	0.224	1.92E-17	CSCL	GLA
USP1.1	1.33E-21	-0.10533	0.07	0.268	1.99E-17	CSCL	USP1
SMCHD1.2	1.34E-21	-0.21071	0.03	0.195	2.00E-17	CSCL	SMCHD1
CAT.1	1.38E-21	-0.20023	0.099	0.315	2.06E-17	CSCL	CAT
NIPSNAP3A	1.39E-21	-0.20385	0.037	0.208	2.08E-17	CSCL	NIPSNAP3A
ALG3	1.40E-21	-0.19146	0.056	0.241	2.09E-17	CSCL	ALG3
IL10RA.2	1.41E-21	-0.57387	0.003	0.145	2.11E-17	CSCL	IL10RA
RARS2	1.51E-21	-0.19493	0.031	0.199	2.26E-17	CSCL	RARS2
EIF4G1.1	1.55E-21	-0.10258	0.082	0.287	2.31E-17	CSCL	EIF4G1
TMEM199	1.55E-21	-0.10815	0.061	0.252	2.32E-17	CSCL	TMEM199
CHIC2.1	1.63E-21	-0.1909	0.063	0.253	2.44E-17	CSCL	CHIC2
NR4A3.2	1.65E-21	-0.5589	0.01	0.158	2.47E-17	CSCL	NR4A3
CEBPG.1	1.68E-21	-0.19405	0.066	0.258	2.50E-17	CSCL	CEBPG
RAB4B.2	1.70E-21	-0.11759	0.073	0.272	2.53E-17	CSCL	RAB4B
GALNT2.1	1.75E-21	-0.18036	0.063	0.253	2.62E-17	CSCL	GALNT2
TNFSF12.2	1.79E-21	-0.28839	0.03	0.194	2.68E-17	CSCL	TNFSF12
P2RX4.2	1.82E-21	-0.35581	0.024	0.184	2.72E-17	CSCL	P2RX4
HNMT.2	1.82E-21	-0.36888	0.049	0.227	2.72E-17	CSCL	HNMT
CBFB.1	1.91E-21	-0.11351	0.056	0.242	2.85E-17	CSCL	CBFB
WIPI1.2	1.96E-21	-0.32876	0.03	0.193	2.93E-17	CSCL	WIPI1
LINC01094	1.97E-21	-0.56274	0.012	0.161	2.95E-17	CSCL	LINC01094
GPR137.1	2.10E-21	-0.14025	0.042	0.217	3.14E-17	CSCL	GPR137
PMEPA1.2	2.18E-21	-0.24787	0.038	0.209	3.26E-17	CSCL	PMEPA1
TMEM128.1	2.20E-21	-0.14435	0.091	0.302	3.29E-17	CSCL	TMEM128
SLAIN1.2	2.38E-21	-0.30022	0.04	0.211	3.56E-17	CSCL	SLAIN1
R3HDM4.2	2.55E-21	-0.20428	0.038	0.209	3.81E-17	CSCL	R3HDM4
SQRDL.2	2.60E-21	-0.4586	0.016	0.168	3.88E-17	CSCL	SQRDL
ABCE1	2.78E-21	-0.13423	0.066	0.259	4.15E-17	CSCL	ABCE1
CDC42SE2.	2.90E-21	-0.22157	0.049	0.227	4.33E-17	CSCL	CDC42SE2
VPS4B.2	2.92E-21	-0.10492	0.066	0.258	4.36E-17	CSCL	VPS4B
DERL1.1	3.06E-21	-0.12065	0.105	0.323	4.58E-17	CSCL	DERL1
OSM.2	3.11E-21	-0.67521	0.009	0.152	4.64E-17	CSCL	OSM
KIFAP3.1	3.30E-21	-0.26287	0.04	0.211	4.92E-17	CSCL	KIFAP3
CCND3.1	3.30E-21	-0.17462	0.073	0.269	4.93E-17	CSCL	CCND3

GRAMD1A.2	3.35E-21	-0.14401	0.082	0.285	5.00E-17	CSCL	GRAMD1A
GPBP1L1.2	3.36E-21	-0.22634	0.042	0.214	5.02E-17	CSCL	GPBP1L1
FNBP1L.2	3.42E-21	-0.23042	0.03	0.193	5.11E-17	CSCL	FNBP1L
CUL5.2	3.45E-21	-0.13567	0.051	0.231	5.15E-17	CSCL	CUL5
IER5.2	3.49E-21	-0.23794	0.04	0.211	5.22E-17	CSCL	IER5
LMF2.2	3.51E-21	-0.28606	0.04	0.209	5.25E-17	CSCL	LMF2
SLC25A19.	3.61E-21	-0.38283	0.021	0.176	5.40E-17	CSCL	SLC25A19
TMED7.1	3.64E-21	-0.15123	0.08	0.281	5.44E-17	CSCL	TMED7
PPP2R5C.1	3.66E-21	-0.11611	0.072	0.267	5.46E-17	CSCL	PPP2R5C
CDC16.1	3.74E-21	-0.20404	0.037	0.204	5.59E-17	CSCL	CDC16
TMEM251.1	3.76E-21	-0.13742	0.063	0.252	5.62E-17	CSCL	TMEM251
ANXA5.2	3.78E-21	0.502776	0.621	0.732	5.65E-17	CSCL	ANXA5
PSMB10.2	3.83E-21	-0.24211	0.072	0.264	5.72E-17	CSCL	PSMB10
MT1X.2	3.86E-21	0.66092	0.576	0.578	5.77E-17	CSCL	MT1X
PRRC2B.2	3.97E-21	-0.17255	0.059	0.245	5.94E-17	CSCL	PRRC2B
CIAO1.1	3.99E-21	-0.11492	0.063	0.252	5.96E-17	CSCL	CIAO1
SRD5A3.2	4.08E-21	-0.28155	0.017	0.17	6.09E-17	CSCL	SRD5A3
PELI1.2	4.12E-21	-0.38122	0.045	0.218	6.16E-17	CSCL	PELI1
TOMM5.2	4.15E-21	-0.1573	0.044	0.218	6.20E-17	CSCL	TOMM5
ARL8B.1	4.19E-21	-0.1939	0.042	0.214	6.26E-17	CSCL	ARL8B
SNX9.2	4.26E-21	-0.2731	0.033	0.197	6.37E-17	CSCL	SNX9
FBXO9.2	4.37E-21	-0.11219	0.042	0.215	6.53E-17	CSCL	FBXO9
QARS.2	4.38E-21	-0.13518	0.087	0.292	6.55E-17	CSCL	QARS
GABARAPL1	4.45E-21	-0.20921	0.098	0.308	6.65E-17	CSCL	GABARAPL1
ASCC2.1	4.72E-21	-0.2154	0.042	0.214	7.06E-17	CSCL	ASCC2
RNF19A.2	4.84E-21	-0.25135	0.04	0.21	7.23E-17	CSCL	RNF19A
ORC4.1	5.24E-21	-0.20562	0.059	0.244	7.83E-17	CSCL	ORC4
ADAM28.2	5.29E-21	-0.61126	0.007	0.148	7.90E-17	CSCL	ADAM28
SOAT1.2	5.53E-21	-0.28716	0.038	0.206	8.26E-17	CSCL	SOAT1
HES4.2	5.60E-21	-0.20553	0.031	0.195	8.37E-17	CSCL	HES4
HK1.2	5.65E-21	-0.22625	0.037	0.203	8.45E-17	CSCL	HK1
ALOX5.2	5.84E-21	-0.53547	0.005	0.145	8.72E-17	CSCL	ALOX5
CSTA.2	5.86E-21	-0.55002	0	0.135	8.76E-17	CSCL	CSTA
IPO7.2	5.95E-21	-0.13633	0.045	0.22	8.90E-17	CSCL	IPO7
PADI2.2	6.01E-21	-0.55726	0.003	0.142	8.99E-17	CSCL	PADI2
IRF7.2	6.13E-21	-0.34229	0.017	0.168	9.16E-17	CSCL	IRF7
PLAU.2	6.55E-21	-0.39622	0.019	0.171	9.79E-17	CSCL	PLAU
B3GNT5.2	6.71E-21	-0.39202	0.007	0.148	1.00E-16	CSCL	B3GNT5
PAK1.2	6.72E-21	-0.40894	0.023	0.177	1.00E-16	CSCL	PAK1
HLA-B.2	6.84E-21	0.322661	0.716	0.859	1.02E-16	CSCL	HLA-B
AP2A2.2	6.92E-21	-0.16493	0.07	0.261	1.03E-16	CSCL	AP2A2
FCGR2B.2	7.04E-21	-0.63434	0.009	0.151	1.05E-16	CSCL	FCGR2B
SNX1.1	7.05E-21	-0.2243	0.052	0.231	1.05E-16	CSCL	SNX1
IMPDH1.2	7.18E-21	-0.27987	0.016	0.165	1.07E-16	CSCL	IMPDH1
CAMK2N1.2	7.34E-21	-0.11893	0.108	0.327	1.10E-16	CSCL	CAMK2N1
NPL.2	7.41E-21	-0.48504	0.042	0.209	1.11E-16	CSCL	NPL
CCSER2.2	7.43E-21	-0.15256	0.056	0.237	1.11E-16	CSCL	CCSER2
GOSR1.1	7.58E-21	-0.10155	0.079	0.277	1.13E-16	CSCL	GOSR1
ZNF106.2	7.76E-21	-0.14257	0.073	0.267	1.16E-16	CSCL	ZNF106
USP53.2	7.97E-21	-0.51296	0.007	0.148	1.19E-16	CSCL	USP53
DAB2.2	8.33E-21	-0.53403	0.012	0.157	1.24E-16	CSCL	DAB2

ABHD14B.1	8.37E-21	-0.29621	0.028	0.187	1.25E-16	CSCL	ABHD14B
PPP3CA.2	8.60E-21	-0.12641	0.066	0.255	1.28E-16	CSCL	PPP3CA
INO80E.1	8.74E-21	-0.18422	0.054	0.234	1.31E-16	CSCL	INO80E
SEL1L.1	8.78E-21	-0.21794	0.024	0.181	1.31E-16	CSCL	SEL1L
SNCA.2	8.87E-21	-0.57734	0.009	0.151	1.33E-16	CSCL	SNCA
CCNDBP1.2	9.31E-21	-0.31045	0.044	0.213	1.39E-16	CSCL	CCNDBP1
TBC1D20.1	9.33E-21	-0.12253	0.03	0.191	1.39E-16	CSCL	TBC1D20
CHMP6.1	9.70E-21	-0.13937	0.037	0.203	1.45E-16	CSCL	CHMP6
MAN1B1	1.01E-20	-0.13133	0.052	0.23	1.51E-16	CSCL	MAN1B1
TMEM141.2	1.02E-20	-0.10112	0.07	0.262	1.52E-16	CSCL	TMEM141
PTGS1.2	1.02E-20	-0.41265	0.016	0.164	1.53E-16	CSCL	PTGS1
COQ10B.1	1.03E-20	-0.14907	0.077	0.272	1.53E-16	CSCL	COQ10B
ATP5G2.1	1.03E-20	0.338203	0.691	0.878	1.54E-16	CSCL	ATP5G2
MIS18BP1.	1.03E-20	-0.40898	0.01	0.154	1.54E-16	CSCL	MIS18BP1
GCC2.1	1.07E-20	-0.14559	0.082	0.282	1.60E-16	CSCL	GCC2
PRADC1.1	1.12E-20	-0.15991	0.049	0.225	1.67E-16	CSCL	PRADC1
NUB1	1.13E-20	-0.15985	0.072	0.262	1.69E-16	CSCL	NUB1
GPR65.2	1.15E-20	-0.55474	0.003	0.14	1.72E-16	CSCL	GPR65
ZBTB7A.1	1.18E-20	-0.2345	0.028	0.186	1.77E-16	CSCL	ZBTB7A
ARL8A.2	1.19E-20	-0.10581	0.082	0.282	1.77E-16	CSCL	ARL8A
CDC42SE1.	1.20E-20	-0.12169	0.101	0.314	1.80E-16	CSCL	CDC42SE1
WDR43.1	1.20E-20	-0.1783	0.037	0.202	1.80E-16	CSCL	WDR43
SDCCAG8.2	1.24E-20	-0.13399	0.119	0.34	1.86E-16	CSCL	SDCCAG8
ZC3H11A.2	1.30E-20	-0.20369	0.04	0.207	1.94E-16	CSCL	ZC3H11A
MAX	1.31E-20	-0.10632	0.096	0.304	1.96E-16	CSCL	MAX
UBL7	1.34E-20	-0.1674	0.058	0.239	2.00E-16	CSCL	UBL7
SMCO4.2	1.34E-20	-0.48833	0.003	0.139	2.00E-16	CSCL	SMCO4
CDC27.2	1.35E-20	-0.24522	0.019	0.17	2.01E-16	CSCL	CDC27
DHX30.1	1.38E-20	-0.1051	0.073	0.267	2.07E-16	CSCL	DHX30
UHMK1.1	1.39E-20	-0.15961	0.042	0.211	2.07E-16	CSCL	UHMK1
TSPAN13.2	1.39E-20	-0.28483	0.103	0.309	2.08E-16	CSCL	TSPAN13
TRIP12.2	1.40E-20	-0.20434	0.021	0.173	2.10E-16	CSCL	TRIP12
AAED1.2	1.44E-20	-0.25668	0.03	0.189	2.15E-16	CSCL	AAED1
SNN.2	1.45E-20	-0.20238	0.056	0.234	2.16E-16	CSCL	SNN
ATG5	1.51E-20	-0.20879	0.045	0.217	2.26E-16	CSCL	ATG5
RPS6KB2.1	1.52E-20	-0.16111	0.056	0.236	2.28E-16	CSCL	RPS6KB2
ZDHHC24.2	1.54E-20	-0.12968	0.033	0.196	2.29E-16	CSCL	ZDHHC24
TSSC1	1.55E-20	-0.12093	0.075	0.269	2.32E-16	CSCL	TSSC1
TP53RK.1	1.55E-20	-0.1205	0.035	0.199	2.32E-16	CSCL	TP53RK
PRPF8.2	1.57E-20	-0.10172	0.073	0.266	2.35E-16	CSCL	PRPF8
TSPYL1.2	1.58E-20	-0.1973	0.063	0.247	2.36E-16	CSCL	TSPYL1
MKNK1.2	1.64E-20	-0.41773	0.024	0.178	2.45E-16	CSCL	MKNK1
PRKRIP1.1	1.65E-20	-0.13479	0.065	0.25	2.47E-16	CSCL	PRKRIP1
GUSB.1	1.69E-20	-0.17057	0.084	0.282	2.53E-16	CSCL	GUSB
TBRG1.1	1.75E-20	-0.11856	0.066	0.254	2.61E-16	CSCL	TBRG1
AIDA	1.82E-20	-0.11583	0.061	0.245	2.72E-16	CSCL	AIDA
GNL1	1.87E-20	-0.13664	0.065	0.251	2.80E-16	CSCL	GNL1
SPSB3.1	2.03E-20	-0.12179	0.103	0.315	3.03E-16	CSCL	SPSB3
GOLT1B.2	2.05E-20	-0.15044	0.063	0.247	3.06E-16	CSCL	GOLT1B
SLC38A2.1	2.08E-20	-0.18573	0.089	0.289	3.10E-16	CSCL	SLC38A2
TMEM55B.1	2.14E-20	-0.22103	0.052	0.227	3.20E-16	CSCL	TMEM55B

BFAR.1	2.17E-20	-0.17014	0.058	0.237	3.24E-16	CSCL	BFAR
CCDC137.1	2.19E-20	-0.15949	0.042	0.21	3.27E-16	CSCL	CCDC137
TACO1.2	2.22E-20	-0.15806	0.026	0.182	3.31E-16	CSCL	TACO1
TOR3A.2	2.37E-20	-0.1671	0.047	0.219	3.54E-16	CSCL	TOR3A
PKN2.2	2.39E-20	-0.1678	0.047	0.219	3.58E-16	CSCL	PKN2
GALK1.2	2.41E-20	-0.17747	0.044	0.212	3.60E-16	CSCL	GALK1
KPNA4.2	2.56E-20	-0.17992	0.03	0.188	3.83E-16	CSCL	KPNA4
RNF14.1	2.61E-20	-0.20204	0.038	0.203	3.90E-16	CSCL	RNF14
TBCC.2	2.79E-20	-0.21791	0.054	0.23	4.16E-16	CSCL	TBCC
ATPAF2.2	2.84E-20	-0.13085	0.049	0.222	4.25E-16	CSCL	ATPAF2
FNDC4	3.04E-20	-0.15828	0.04	0.206	4.54E-16	CSCL	FNDC4
GLRX2.1	3.08E-20	-0.27218	0.021	0.17	4.61E-16	CSCL	GLRX2
COPS2.1	3.15E-20	-0.17518	0.047	0.218	4.70E-16	CSCL	COPS2
TMEM218.1	3.22E-20	-0.17575	0.054	0.23	4.82E-16	CSCL	TMEM218
ACER3.2	3.38E-20	-0.26376	0.033	0.193	5.05E-16	CSCL	ACER3
MTF2.2	3.52E-20	-0.14603	0.073	0.263	5.27E-16	CSCL	MTF2
TOB1	3.62E-20	-0.19109	0.075	0.264	5.41E-16	CSCL	TOB1
FLNA.2	3.78E-20	-0.15557	0.072	0.26	5.65E-16	CSCL	FLNA
CBX3.2	3.85E-20	-0.10036	0.248	0.569	5.75E-16	CSCL	CBX3
DESI1.2	3.87E-20	-0.24158	0.028	0.183	5.79E-16	CSCL	DESI1
MAFG.2	3.91E-20	-0.18311	0.033	0.192	5.84E-16	CSCL	MAFG
USO1.1	3.92E-20	-0.16756	0.051	0.224	5.86E-16	CSCL	USO1
DCAF6.2	3.96E-20	-0.2749	0.023	0.173	5.92E-16	CSCL	DCAF6
OSER1	4.15E-20	-0.12023	0.103	0.312	6.21E-16	CSCL	OSER1
LACTB.2	4.17E-20	-0.27736	0.023	0.173	6.24E-16	CSCL	LACTB
FGD4.2	4.18E-20	-0.41646	0.012	0.153	6.24E-16	CSCL	FGD4
CTBS.1	4.20E-20	-0.31969	0.017	0.163	6.27E-16	CSCL	CTBS
DESI2.1	4.27E-20	-0.10583	0.038	0.202	6.38E-16	CSCL	DESI2
CAB39.2	4.31E-20	-0.24366	0.033	0.191	6.43E-16	CSCL	CAB39
LCP1.1	4.33E-20	-0.51773	0.01	0.149	6.47E-16	CSCL	LCP1
APLP1.2	4.34E-20	-0.25846	0.14	0.362	6.48E-16	CSCL	APLP1
NUDT2.2	4.37E-20	-0.24337	0.028	0.182	6.53E-16	CSCL	NUDT2
GNS.2	4.52E-20	-0.20331	0.042	0.207	6.76E-16	CSCL	GNS
FNDC3A.1	4.71E-20	-0.15783	0.051	0.223	7.04E-16	CSCL	FNDC3A
UBQLN2.2	4.88E-20	-0.18031	0.054	0.229	7.29E-16	CSCL	UBQLN2
GARS.2	4.89E-20	-0.13387	0.098	0.303	7.31E-16	CSCL	GARS
TMEM242.1	5.06E-20	-0.16256	0.066	0.25	7.55E-16	CSCL	TMEM242
ATP1B1.2	5.14E-20	-0.30255	0.065	0.243	7.68E-16	CSCL	ATP1B1
ACOT7.2	5.14E-20	-0.10826	0.058	0.236	7.69E-16	CSCL	ACOT7
FBXW11.1	5.15E-20	-0.21126	0.031	0.188	7.69E-16	CSCL	FBXW11
RNF115.1	5.16E-20	-0.1686	0.068	0.252	7.71E-16	CSCL	RNF115
SMC2.1	5.24E-20	-0.16999	0.042	0.208	7.84E-16	CSCL	SMC2
TM9SF3.2	5.57E-20	-0.16119	0.054	0.228	8.32E-16	CSCL	TM9SF3
DDX54.1	5.69E-20	-0.16635	0.042	0.207	8.51E-16	CSCL	DDX54
DALRD3	5.72E-20	-0.21277	0.023	0.173	8.54E-16	CSCL	DALRD3
EGLN1.1	5.73E-20	-0.15616	0.035	0.195	8.56E-16	CSCL	EGLN1
RP11-395G	5.93E-20	-0.2374	0.03	0.185	8.86E-16	CSCL	RP11-395G23.3
AKAP17A.1	5.97E-20	-0.28033	0.024	0.175	8.92E-16	CSCL	AKAP17A
GTPBP6.2	5.98E-20	-0.10174	0.049	0.22	8.94E-16	CSCL	GTPBP6
ENTPD6.2	6.11E-20	-0.19547	0.042	0.207	9.13E-16	CSCL	ENTPD6
SLC12A9.2	6.14E-20	-0.21958	0.044	0.209	9.18E-16	CSCL	SLC12A9

CSE1L.1	6.22E-20	-0.11617	0.056	0.231	9.30E-16	CSCL	CSE1L
SYNRG.1	6.25E-20	-0.18631	0.037	0.197	9.34E-16	CSCL	SYNRG
C6orf89	6.52E-20	-0.16391	0.054	0.228	9.74E-16	CSCL	C6orf89
KIAA1715.	6.52E-20	-0.12836	0.075	0.264	9.75E-16	CSCL	KIAA1715
ALG13.1	6.84E-20	-0.20093	0.054	0.228	1.02E-15	CSCL	ALG13
DGKZ.2	7.00E-20	-0.14406	0.04	0.204	1.05E-15	CSCL	DGKZ
TMEM223.1	7.09E-20	-0.15431	0.058	0.233	1.06E-15	CSCL	TMEM223
SMARCD1.2	7.28E-20	-0.11462	0.051	0.223	1.09E-15	CSCL	SMARCD1
MTHFD1L.2	7.38E-20	-0.32382	0.007	0.142	1.10E-15	CSCL	MTHFD1L
USP15.2	7.67E-20	-0.27008	0.07	0.251	1.15E-15	CSCL	USP15
IRF2BP2.2	7.79E-20	-0.11951	0.112	0.326	1.16E-15	CSCL	IRF2BP2
TRADD.2	7.83E-20	-0.17798	0.024	0.175	1.17E-15	CSCL	TRADD
IER3IP1.2	8.11E-20	0.537239	0.108	0.035	1.21E-15	CSCL	IER3IP1
SRGAP1.2	8.37E-20	-0.199	0.058	0.234	1.25E-15	CSCL	SRGAP1
ZC3HAV1.2	8.93E-20	-0.22884	0.03	0.184	1.33E-15	CSCL	ZC3HAV1
DNAJC1.2	9.19E-20	-0.1996	0.035	0.193	1.37E-15	CSCL	DNAJC1
C17orf58.	9.41E-20	-0.20793	0.035	0.194	1.41E-15	CSCL	C17orf58
PAPD4.1	9.42E-20	-0.23781	0.031	0.187	1.41E-15	CSCL	PAPD4
RNF13.2	9.44E-20	-0.1131	0.213	0.494	1.41E-15	CSCL	RNF13
RPS6KA1.2	9.57E-20	-0.47729	0.005	0.137	1.43E-15	CSCL	RPS6KA1
PUM1.2	9.63E-20	-0.12362	0.065	0.246	1.44E-15	CSCL	PUM1
PIK3IP1.2	9.75E-20	-0.51184	0.023	0.168	1.46E-15	CSCL	PIK3IP1
PES1.2	9.84E-20	-0.12119	0.038	0.2	1.47E-15	CSCL	PES1
PRDX1.2	9.98E-20	0.415244	0.665	0.815	1.49E-15	CSCL	PRDX1
PCGF5	1.01E-19	-0.24437	0.031	0.187	1.51E-15	CSCL	PCGF5
THOC3.1	1.02E-19	-0.2167	0.026	0.177	1.52E-15	CSCL	THOC3
TMPO.2	1.07E-19	-0.24828	0.04	0.201	1.60E-15	CSCL	TMPO
DR1.2	1.09E-19	-0.1553	0.065	0.243	1.62E-15	CSCL	DR1
CTTNBP2NL	1.11E-19	-0.30861	0.017	0.16	1.66E-15	CSCL	CTTNBP2NL
PDE4B.2	1.12E-19	-0.14925	0.089	0.286	1.68E-15	CSCL	PDE4B
ETNK1.2	1.15E-19	-0.19661	0.058	0.232	1.72E-15	CSCL	ETNK1
CACUL1.2	1.15E-19	-0.22439	0.047	0.214	1.72E-15	CSCL	CACUL1
CKAP2.2	1.16E-19	-0.25767	0.026	0.176	1.73E-15	CSCL	CKAP2
VPS37A	1.19E-19	-0.16335	0.047	0.214	1.79E-15	CSCL	VPS37A
CSK.2	1.20E-19	-0.33376	0.014	0.154	1.79E-15	CSCL	CSK
DTNBP1.2	1.21E-19	-0.15377	0.047	0.215	1.81E-15	CSCL	DTNBP1
SP100.1	1.23E-19	-0.28787	0.098	0.296	1.84E-15	CSCL	SP100
PRKAR2A	1.23E-19	-0.1117	0.072	0.258	1.85E-15	CSCL	PRKAR2A
KLF10.1	1.25E-19	-0.29034	0.065	0.242	1.86E-15	CSCL	KLF10
FAM228B.1	1.26E-19	-0.12394	0.052	0.224	1.89E-15	CSCL	FAM228B
ANKRD40.1	1.33E-19	-0.19192	0.03	0.182	1.98E-15	CSCL	ANKRD40
NCF1.2	1.37E-19	-0.50684	0.002	0.13	2.04E-15	CSCL	NCF1
CSRNP1.2	1.39E-19	-0.32122	0.04	0.198	2.07E-15	CSCL	CSRNP1
SLC20A1	1.40E-19	-0.23429	0.031	0.186	2.09E-15	CSCL	SLC20A1
MRGBP.1	1.42E-19	-0.21621	0.014	0.154	2.13E-15	CSCL	MRGBP
CTXN1.2	1.49E-19	-0.22804	0.021	0.167	2.22E-15	CSCL	CTXN1
MARCH1.2	1.53E-19	-0.39043	0.04	0.198	2.28E-15	CSCL	1-Mar
ARFIP2.2	1.55E-19	-0.14749	0.07	0.253	2.31E-15	CSCL	ARFIP2
SOCS6.2	1.55E-19	-0.10786	0.061	0.238	2.32E-15	CSCL	SOCS6
FLII.2	1.56E-19	-0.20852	0.037	0.195	2.34E-15	CSCL	FLII
PDCL3.1	1.59E-19	-0.10636	0.099	0.303	2.38E-15	CSCL	PDCL3

EIF2AK4.2	1.60E-19	-0.17799	0.033	0.189	2.38E-15	CSCL	EIF2AK4
CES2.1	1.60E-19	-0.17555	0.035	0.192	2.39E-15	CSCL	CES2
TMEM87A.1	1.60E-19	-0.10935	0.087	0.283	2.39E-15	CSCL	TMEM87A
UBAP1.1	1.61E-19	-0.17478	0.035	0.192	2.40E-15	CSCL	UBAP1
BTBD10.1	1.74E-19	-0.19702	0.033	0.189	2.61E-15	CSCL	BTBD10
SELM.2	1.75E-19	-0.32899	0.038	0.196	2.61E-15	CSCL	SELM
ADCK2.2	1.77E-19	-0.28356	0.01	0.146	2.64E-15	CSCL	ADCK2
AOAH.2	1.80E-19	-0.56213	0.002	0.129	2.69E-15	CSCL	AOAH
SUCLG2	1.81E-19	-0.16247	0.072	0.254	2.71E-15	CSCL	SUCLG2
TCERG1.1	1.87E-19	-0.17313	0.047	0.212	2.79E-15	CSCL	TCERG1
SNHG15.1	1.87E-19	-0.18025	0.058	0.23	2.80E-15	CSCL	SNHG15
SIL1.1	1.96E-19	-0.11726	0.077	0.264	2.93E-15	CSCL	SIL1
EVA1B.2	2.03E-19	-0.23738	0.002	0.129	3.03E-15	CSCL	EVA1B
CD84.2	2.06E-19	-0.52715	0.005	0.135	3.09E-15	CSCL	CD84
C21orf2.2	2.08E-19	-0.1965	0.042	0.202	3.11E-15	CSCL	C21orf2
HEXIM1.1	2.08E-19	-0.20891	0.056	0.227	3.11E-15	CSCL	HEXIM1
JOSD1.2	2.09E-19	-0.29779	0.021	0.165	3.12E-15	CSCL	JOSD1
FBXW2.2	2.18E-19	-0.14874	0.04	0.2	3.25E-15	CSCL	FBXW2
LASP1.2	2.21E-19	-0.18572	0.051	0.218	3.30E-15	CSCL	LASP1
KIF5C.2	2.21E-19	-0.19279	0.086	0.277	3.30E-15	CSCL	KIF5C
TRAPPC12.	2.21E-19	-0.18594	0.044	0.205	3.31E-15	CSCL	TRAPPC12
LRRC47.1	2.22E-19	-0.13274	0.054	0.225	3.32E-15	CSCL	LRRC47
TOB2.1	2.23E-19	-0.26017	0.044	0.205	3.33E-15	CSCL	TOB2
C7orf49.1	2.27E-19	-0.11867	0.049	0.216	3.39E-15	CSCL	C7orf49
GLYR1.1	2.29E-19	-0.17427	0.051	0.218	3.42E-15	CSCL	GLYR1
LRRC58.1	2.49E-19	-0.17047	0.049	0.214	3.73E-15	CSCL	LRRC58
USP34.2	2.51E-19	-0.14319	0.051	0.218	3.76E-15	CSCL	USP34
NUP62.2	2.54E-19	-0.11096	0.066	0.246	3.79E-15	CSCL	NUP62
TRAF7.2	2.59E-19	-0.14519	0.038	0.197	3.86E-15	CSCL	TRAF7
PHLDA1.2	2.61E-19	-0.10022	0.164	0.407	3.90E-15	CSCL	PHLDA1
RIF1.1	2.63E-19	-0.11044	0.056	0.228	3.94E-15	CSCL	RIF1
SNAP29.2	2.70E-19	-0.11487	0.059	0.233	4.04E-15	CSCL	SNAP29
FAM120AOS	2.72E-19	-0.14008	0.037	0.193	4.06E-15	CSCL	FAM120AOS
FAM58A.2	2.77E-19	-0.23973	0.019	0.161	4.14E-15	CSCL	FAM58A
ATAT1.2	2.77E-19	-0.11713	0.098	0.298	4.15E-15	CSCL	ATAT1
CCPG1.1	2.79E-19	-0.14368	0.052	0.221	4.17E-15	CSCL	CCPG1
NCKAP1L.2	2.83E-19	-0.52657	0.002	0.128	4.23E-15	CSCL	NCKAP1L
RRP36.2	2.86E-19	-0.18868	0.014	0.152	4.28E-15	CSCL	RRP36
MTSS1.2	2.91E-19	-0.27893	0.054	0.221	4.35E-15	CSCL	MTSS1
SPIN1.2	2.96E-19	-0.25314	0.026	0.174	4.43E-15	CSCL	SPIN1
ZNF414.2	3.00E-19	-0.15317	0.037	0.193	4.48E-15	CSCL	ZNF414
R3HDM2.2	3.15E-19	-0.21459	0.026	0.174	4.71E-15	CSCL	R3HDM2
ATP6AP2.1	3.20E-19	-0.14068	0.311	0.674	4.79E-15	CSCL	ATP6AP2
TCOF1.2	3.27E-19	-0.27903	0.044	0.203	4.89E-15	CSCL	TCOF1
NGLY1.1	3.43E-19	-0.14979	0.054	0.223	5.13E-15	CSCL	NGLY1
ARF1.1	3.65E-19	-0.11236	0.33	0.713	5.45E-15	CSCL	ARF1
SEMA6D.2	3.71E-19	-0.23502	0.021	0.164	5.55E-15	CSCL	SEMA6D
PPP2R5B.1	3.72E-19	-0.19564	0.026	0.173	5.56E-15	CSCL	PPP2R5B
CDKN2D.2	3.77E-19	-0.23729	0.023	0.167	5.64E-15	CSCL	CDKN2D
RPP14.1	3.79E-19	-0.19362	0.038	0.195	5.67E-15	CSCL	RPP14
KIAA1033.	3.80E-19	-0.12405	0.068	0.247	5.68E-15	CSCL	KIAA1033

SEPN1.1	3.83E-19	-0.1258	0.051	0.218	5.73E-15	CSCL	SEPN1
ATF3.2	3.90E-19	-0.11153	0.108	0.312	5.83E-15	CSCL	ATF3
RPIA.1	3.91E-19	-0.1864	0.038	0.195	5.84E-15	CSCL	RPIA
EIF4G2.2	3.91E-19	-0.16009	0.318	0.677	5.84E-15	CSCL	EIF4G2
UXS1.1	3.92E-19	-0.15179	0.047	0.211	5.85E-15	CSCL	UXS1
DEDD2.1	4.04E-19	-0.23447	0.03	0.179	6.04E-15	CSCL	DEDD2
NFE2L1.1	4.05E-19	-0.16502	0.038	0.195	6.05E-15	CSCL	NFE2L1
ZHX1.1	4.27E-19	-0.11891	0.051	0.217	6.39E-15	CSCL	ZHX1
MAEA.1	4.34E-19	-0.15604	0.066	0.243	6.48E-15	CSCL	MAEA
KMT2C.2	4.38E-19	-0.13716	0.082	0.271	6.55E-15	CSCL	KMT2C
PCYT2.1	4.46E-19	-0.17954	0.023	0.167	6.67E-15	CSCL	PCYT2
SDSL.1	4.63E-19	-0.19288	0.038	0.195	6.92E-15	CSCL	SDSL
HERPUD2.1	4.90E-19	-0.2218	0.031	0.182	7.32E-15	CSCL	HERPUD2
USP39.1	4.92E-19	-0.12252	0.037	0.192	7.35E-15	CSCL	USP39
CLEC11A.2	5.07E-19	-0.12111	0.065	0.24	7.58E-15	CSCL	CLEC11A
INSIG1.2	5.12E-19	-0.20605	0.063	0.235	7.66E-15	CSCL	INSIG1
LPIN2.2	5.19E-19	-0.27566	0.017	0.156	7.75E-15	CSCL	LPIN2
PSPC1.2	5.20E-19	-0.14708	0.052	0.219	7.78E-15	CSCL	PSPC1
MAPKAPK5.	5.43E-19	-0.20722	0.037	0.191	8.12E-15	CSCL	MAPKAPK5
FNBP4.1	5.46E-19	-0.14487	0.077	0.261	8.15E-15	CSCL	FNBP4
CYLD.1	5.63E-19	-0.26981	0.026	0.172	8.41E-15	CSCL	CYLD
MOB2	5.71E-19	-0.17516	0.038	0.195	8.53E-15	CSCL	MOB2
PCBP4.2	5.76E-19	-0.10703	0.079	0.265	8.61E-15	CSCL	PCBP4
ANKRD39.2	6.01E-19	-0.2948	0.028	0.174	8.97E-15	CSCL	ANKRD39
PSME3.1	6.01E-19	-0.11619	0.061	0.235	8.98E-15	CSCL	PSME3
VEZF1.2	6.18E-19	-0.11102	0.068	0.247	9.23E-15	CSCL	VEZF1
DIMT1.1	6.35E-19	-0.21486	0.038	0.194	9.48E-15	CSCL	DIMT1
SDC2.2	6.37E-19	-0.21513	0.042	0.2	9.51E-15	CSCL	SDC2
DDX52.1	6.65E-19	-0.16393	0.072	0.253	9.94E-15	CSCL	DDX52
RUFY2.2	6.75E-19	-0.24657	0.017	0.156	1.01E-14	CSCL	RUFY2
TOR1AIP2	6.91E-19	-0.14195	0.077	0.26	1.03E-14	CSCL	TOR1AIP2
HNRNPK.1	6.96E-19	0.361021	0.634	0.797	1.04E-14	CSCL	HNRNPK
ASH2L.1	7.07E-19	-0.19122	0.033	0.184	1.06E-14	CSCL	ASH2L
SNX14.2	7.12E-19	-0.12034	0.052	0.219	1.06E-14	CSCL	SNX14
MARCH5.1	7.17E-19	-0.20011	0.042	0.199	1.07E-14	CSCL	5-Mar
PFDN5.2	7.23E-19	0.301735	0.695	0.894	1.08E-14	CSCL	PFDN5
CERS4.2	7.26E-19	-0.11407	0.045	0.207	1.08E-14	CSCL	CERS4
RASSF5.2	7.27E-19	-0.43396	0.002	0.126	1.09E-14	CSCL	RASSF5
BRD3.2	7.32E-19	-0.11968	0.091	0.284	1.09E-14	CSCL	BRD3
TFRC.2	7.50E-19	-0.18719	0.052	0.217	1.12E-14	CSCL	TFRC
WIPF2	7.57E-19	-0.13384	0.047	0.209	1.13E-14	CSCL	WIPF2
REST.2	7.60E-19	-0.16097	0.045	0.206	1.14E-14	CSCL	REST
PTAFR.2	7.62E-19	-0.51286	0.005	0.131	1.14E-14	CSCL	PTAFR
UCHL5.1	7.62E-19	-0.26752	0.017	0.156	1.14E-14	CSCL	UCHL5
SKI.2	7.91E-19	-0.2771	0.019	0.158	1.18E-14	CSCL	SKI
DOK1.2	8.04E-19	-0.31686	0.021	0.161	1.20E-14	CSCL	DOK1
KLF6.1	8.06E-19	-0.14726	0.251	0.546	1.20E-14	CSCL	KLF6
MMP2.2	8.22E-19	-0.1635	0.063	0.235	1.23E-14	CSCL	MMP2
TOP2B.2	8.33E-19	-0.11033	0.068	0.246	1.24E-14	CSCL	TOP2B
DPH7.1	8.38E-19	-0.19008	0.028	0.174	1.25E-14	CSCL	DPH7
GK.2	8.49E-19	-0.39311	0.012	0.145	1.27E-14	CSCL	GK

CYB561D2	8.54E-19	-0.19223	0.063	0.234	1.28E-14	CSCL	CYB561D2
RAC2.2	8.62E-19	-0.53547	0.002	0.125	1.29E-14	CSCL	RAC2
IARS2	8.72E-19	-0.14356	0.049	0.212	1.30E-14	CSCL	IARS2
ATP2C1.1	8.83E-19	-0.16159	0.061	0.232	1.32E-14	CSCL	ATP2C1
SFSWAP.1	9.23E-19	-0.14217	0.033	0.184	1.38E-14	CSCL	SFSWAP
KIAA0930.	9.44E-19	-0.33198	0.016	0.151	1.41E-14	CSCL	KIAA0930
CMIP.2	9.48E-19	-0.21062	0.031	0.18	1.42E-14	CSCL	CMIP
SLC1A5.2	9.56E-19	-0.37187	0.012	0.144	1.43E-14	CSCL	SLC1A5
ELL2.2	9.61E-19	-0.33775	0.044	0.2	1.44E-14	CSCL	ELL2
C19orf52.	9.65E-19	-0.13426	0.031	0.181	1.44E-14	CSCL	C19orf52
NADK.2	9.83E-19	-0.13829	0.028	0.174	1.47E-14	CSCL	NADK
MED30.1	9.83E-19	-0.11945	0.079	0.262	1.47E-14	CSCL	MED30
TRIM24.2	1.00E-18	-0.13433	0.042	0.199	1.50E-14	CSCL	TRIM24
LEMD2.2	1.01E-18	-0.20457	0.017	0.155	1.50E-14	CSCL	LEMD2
THAP11.2	1.03E-18	-0.17196	0.033	0.183	1.54E-14	CSCL	THAP11
RAB21.2	1.05E-18	-0.11006	0.049	0.212	1.57E-14	CSCL	RAB21
TSC22D2.2	1.09E-18	-0.28623	0.023	0.163	1.63E-14	CSCL	TSC22D2
APIP.2	1.11E-18	-0.12483	0.066	0.242	1.67E-14	CSCL	APIP
SLC4A1AP	1.14E-18	-0.15083	0.033	0.183	1.70E-14	CSCL	SLC4A1AP
FAM45A.1	1.14E-18	-0.13563	0.042	0.199	1.70E-14	CSCL	FAM45A
NCK2.2	1.15E-18	-0.23936	0.017	0.154	1.71E-14	CSCL	NCK2
KMT2A.2	1.17E-18	-0.10348	0.068	0.245	1.75E-14	CSCL	KMT2A
OSBPL1A.2	1.20E-18	-0.14974	0.042	0.198	1.79E-14	CSCL	OSBPL1A
TCEB3.1	1.22E-18	-0.1675	0.033	0.183	1.82E-14	CSCL	TCEB3
EXOSC3.2	1.22E-18	-0.21534	0.024	0.167	1.83E-14	CSCL	EXOSC3
UBL4A.1	1.24E-18	-0.13087	0.037	0.19	1.86E-14	CSCL	UBL4A
TTC9C.1	1.27E-18	-0.24509	0.033	0.182	1.89E-14	CSCL	TTC9C
CASP3.1	1.29E-18	-0.16243	0.045	0.205	1.93E-14	CSCL	CASP3
CHTF8	1.32E-18	-0.16787	0.035	0.186	1.97E-14	CSCL	CHTF8
PTGER4.2	1.33E-18	-0.49885	0.005	0.13	1.99E-14	CSCL	PTGER4
USP3.2	1.35E-18	-0.22862	0.028	0.173	2.02E-14	CSCL	USP3
HLA-DQA2.	1.38E-18	-0.61079	0.033	0.179	2.07E-14	CSCL	HLA-DQA2
FMNL1.2	1.41E-18	-0.46803	0.005	0.13	2.10E-14	CSCL	FMNL1
HINT3.2	1.41E-18	-0.16541	0.047	0.206	2.11E-14	CSCL	HINT3
MVD.2	1.43E-18	-0.13355	0.035	0.186	2.13E-14	CSCL	MVD
GTF2H5.1	1.44E-18	0.111964	0.164	0.417	2.15E-14	CSCL	GTF2H5
MOB3A.2	1.45E-18	-0.20707	0.026	0.17	2.17E-14	CSCL	MOB3A
EVI5.1	1.48E-18	-0.10835	0.038	0.192	2.21E-14	CSCL	EVI5
GYG1	1.51E-18	-0.13217	0.08	0.263	2.26E-14	CSCL	GYG1
CNOT8.1	1.53E-18	-0.12825	0.037	0.189	2.29E-14	CSCL	CNOT8
DCAF11.2	1.56E-18	-0.14264	0.042	0.198	2.33E-14	CSCL	DCAF11
EDEM2.1	1.59E-18	-0.10451	0.072	0.249	2.37E-14	CSCL	EDEM2
RASGEF1B.	1.66E-18	-0.49206	0.003	0.127	2.48E-14	CSCL	RASGEF1B
MMP14.1	1.67E-18	-0.15692	0.061	0.23	2.50E-14	CSCL	MMP14
THOC6.1	1.68E-18	-0.15083	0.03	0.176	2.51E-14	CSCL	THOC6
GAR1.2	1.72E-18	-0.12601	0.042	0.198	2.57E-14	CSCL	GAR1
USP9X.2	1.78E-18	-0.14506	0.049	0.209	2.65E-14	CSCL	USP9X
ATM.2	1.82E-18	-0.24567	0.042	0.196	2.73E-14	CSCL	ATM
RP11-552D	1.86E-18	-0.63713	0.003	0.126	2.78E-14	CSCL	RP11-552D4.1
ADSS.1	1.92E-18	-0.12875	0.04	0.194	2.87E-14	CSCL	ADSS
FBP1.2	1.94E-18	-0.47423	0.002	0.123	2.90E-14	CSCL	FBP1

CKAP5.2	1.96E-18	-0.17503	0.023	0.163	2.93E-14	CSCL	CKAP5
LONP2.1	2.06E-18	-0.10823	0.077	0.257	3.08E-14	CSCL	LONP2
SSH2.2	2.15E-18	-0.30493	0.035	0.183	3.22E-14	CSCL	SSH2
DBN1.2	2.16E-18	-0.14772	0.117	0.321	3.22E-14	CSCL	DBN1
TNIP2.1	2.18E-18	-0.14098	0.028	0.172	3.26E-14	CSCL	TNIP2
TMEM173.2	2.21E-18	-0.49229	0.007	0.132	3.30E-14	CSCL	TMEM173
HIVEP3.2	2.25E-18	-0.18466	0.021	0.159	3.37E-14	CSCL	HIVEP3
MGRN1.2	2.27E-18	-0.22255	0.054	0.216	3.40E-14	CSCL	MGRN1
TRIB1.1	2.40E-18	-0.36303	0.024	0.164	3.59E-14	CSCL	TRIB1
LRPPRC.1	2.45E-18	-0.21459	0.03	0.175	3.66E-14	CSCL	LRPPRC
MAP3K11.2	2.47E-18	-0.19271	0.038	0.19	3.69E-14	CSCL	MAP3K11
UBE2Q1.1	2.55E-18	-0.18103	0.026	0.168	3.81E-14	CSCL	UBE2Q1
TMSB15A.2	2.55E-18	-0.27678	0.037	0.186	3.81E-14	CSCL	TMSB15A
HPS3.1	2.56E-18	-0.28426	0.016	0.149	3.82E-14	CSCL	HPS3
ANKLE2	2.58E-18	-0.21374	0.037	0.187	3.86E-14	CSCL	ANKLE2
GAK.2	2.76E-18	-0.20816	0.024	0.165	4.12E-14	CSCL	GAK
CASP4.2	2.80E-18	-0.273	0.066	0.236	4.19E-14	CSCL	CASP4
SUZ12.2	2.84E-18	-0.11013	0.045	0.202	4.24E-14	CSCL	SUZ12
EFR3A.2	2.85E-18	-0.2246	0.016	0.149	4.27E-14	CSCL	EFR3A
POU2F2.2	2.86E-18	-0.40871	0.012	0.142	4.27E-14	CSCL	POU2F2
CLCN7.2	2.92E-18	-0.2371	0.023	0.161	4.37E-14	CSCL	CLCN7
CHST2.1	2.93E-18	-0.16611	0.012	0.142	4.38E-14	CSCL	CHST2
RRP1.1	2.93E-18	-0.10457	0.054	0.218	4.38E-14	CSCL	RRP1
ZNF800.1	2.98E-18	-0.15607	0.038	0.189	4.45E-14	CSCL	ZNF800
ALG2.2	2.99E-18	-0.25645	0.028	0.17	4.47E-14	CSCL	ALG2
BIN2.2	3.01E-18	-0.4977	0	0.118	4.50E-14	CSCL	BIN2
GSDMD.1	3.13E-18	-0.22732	0.04	0.192	4.67E-14	CSCL	GSDMD
PPIL4.1	3.15E-18	-0.1161	0.077	0.255	4.70E-14	CSCL	PPIL4
TCIRG1.2	3.25E-18	-0.39186	0.007	0.132	4.86E-14	CSCL	TCIRG1
UTP6.1	3.41E-18	-0.13043	0.054	0.217	5.09E-14	CSCL	UTP6
LAMP2.2	3.49E-18	-0.11816	0.213	0.484	5.21E-14	CSCL	LAMP2
UAP1.1	3.49E-18	-0.18927	0.04	0.192	5.22E-14	CSCL	UAP1
SNAPC1.1	3.55E-18	-0.13287	0.042	0.196	5.30E-14	CSCL	SNAPC1
NOTCH2NL.	3.60E-18	-0.186	0.03	0.173	5.39E-14	CSCL	NOTCH2NL
KIAA2013.	3.61E-18	-0.24062	0.021	0.157	5.40E-14	CSCL	KIAA2013
SACS.2	3.64E-18	-0.17108	0.023	0.161	5.44E-14	CSCL	SACS
CDK11A.1	3.71E-18	-0.23157	0.047	0.204	5.54E-14	CSCL	CDK11A
DSE.2	3.81E-18	-0.38878	0.014	0.144	5.69E-14	CSCL	DSE
ACOX1.1	3.81E-18	-0.16257	0.03	0.173	5.70E-14	CSCL	ACOX1
TOR2A.2	3.83E-18	-0.27865	0.019	0.154	5.72E-14	CSCL	TOR2A
NUP214.2	3.91E-18	-0.23361	0.047	0.203	5.85E-14	CSCL	NUP214
EGR2.2	3.99E-18	-0.2565	0.045	0.199	5.96E-14	CSCL	EGR2
ZMYM2.2	4.01E-18	-0.16756	0.052	0.213	5.99E-14	CSCL	ZMYM2
PDPK1.2	4.08E-18	-0.14767	0.042	0.195	6.10E-14	CSCL	PDPK1
LCP2.2	4.14E-18	-0.48553	0.002	0.121	6.19E-14	CSCL	LCP2
FBXO11.2	4.15E-18	-0.13681	0.052	0.214	6.20E-14	CSCL	FBXO11
DOCK4.2	4.21E-18	-0.32395	0.028	0.169	6.30E-14	CSCL	DOCK4
RHOT2.1	4.22E-18	-0.14018	0.08	0.26	6.30E-14	CSCL	RHOT2
SLC38A10.	4.25E-18	-0.15467	0.049	0.206	6.35E-14	CSCL	SLC38A10
ACAP2.2	4.32E-18	-0.18433	0.037	0.185	6.46E-14	CSCL	ACAP2
MZT1.1	4.42E-18	-0.12013	0.084	0.264	6.60E-14	CSCL	MZT1

KCMF1.1	4.49E-18	-0.10561	0.044	0.199	6.71E-14	CSCL	KCMF1
NFKBID.2	4.54E-18	-0.44284	0.016	0.147	6.79E-14	CSCL	NFKBID
YDJC.2	4.92E-18	-0.24041	0.017	0.15	7.36E-14	CSCL	YDJC
STX11.2	4.98E-18	-0.50255	0.002	0.12	7.45E-14	CSCL	STX11
CYTH1.2	5.18E-18	-0.33027	0.026	0.165	7.75E-14	CSCL	CYTH1
VPS26A.1	5.42E-18	-0.13955	0.075	0.251	8.11E-14	CSCL	VPS26A
LY6E.1	5.44E-18	-0.10247	0.157	0.387	8.13E-14	CSCL	LY6E
FAM122A.2	5.49E-18	-0.22521	0.017	0.15	8.20E-14	CSCL	FAM122A
HGSNAT.2	5.53E-18	-0.17081	0.054	0.214	8.26E-14	CSCL	HGSNAT
THAP4.1	5.63E-18	-0.20811	0.026	0.166	8.42E-14	CSCL	THAP4
KANSL1-AS	5.76E-18	-0.12829	0.045	0.201	8.61E-14	CSCL	KANSL1-AS1
SLC31A1.2	5.86E-18	-0.20953	0.031	0.175	8.75E-14	CSCL	SLC31A1
HLA-F.2	5.98E-18	-0.37958	0.019	0.153	8.94E-14	CSCL	HLA-F
PKM.2	6.04E-18	0.381697	0.642	0.777	9.02E-14	CSCL	PKM
PDXDC1.1	6.12E-18	-0.1551	0.033	0.178	9.15E-14	CSCL	PDXDC1
F8A1.2	6.28E-18	-0.24792	0.016	0.146	9.39E-14	CSCL	F8A1
ADNP.1	6.29E-18	-0.1421	0.045	0.2	9.40E-14	CSCL	ADNP
MIA3.1	6.37E-18	-0.15163	0.056	0.217	9.52E-14	CSCL	MIA3
CEP57.1	6.52E-18	-0.13435	0.068	0.238	9.74E-14	CSCL	CEP57
ISG20L2.1	6.57E-18	-0.11135	0.065	0.233	9.82E-14	CSCL	ISG20L2
POLR2A.1	6.70E-18	-0.1527	0.058	0.219	1.00E-13	CSCL	POLR2A
ACLY.2	6.71E-18	-0.11599	0.059	0.224	1.00E-13	CSCL	ACLY
ZFAND2B.1	6.74E-18	-0.13877	0.054	0.214	1.01E-13	CSCL	ZFAND2B
FNDC3B.1	6.91E-18	-0.15514	0.035	0.181	1.03E-13	CSCL	FNDC3B
RRP1B.2	6.95E-18	-0.18162	0.024	0.163	1.04E-13	CSCL	RRP1B
SDAD1.1	7.27E-18	-0.10952	0.061	0.227	1.09E-13	CSCL	SDAD1
CD33.2	7.32E-18	-0.52112	0.005	0.126	1.09E-13	CSCL	CD33
GGA2.2	7.51E-18	-0.33151	0.016	0.145	1.12E-13	CSCL	GGA2
MXD4.2	7.64E-18	-0.19743	0.044	0.196	1.14E-13	CSCL	MXD4
NELFB.2	7.69E-18	-0.18428	0.021	0.155	1.15E-13	CSCL	NELFB
SERGEF.2	8.11E-18	-0.1253	0.031	0.175	1.21E-13	CSCL	SERGEF
OXA1L.1	8.28E-18	-0.10809	0.113	0.311	1.24E-13	CSCL	OXA1L
VPS25	8.34E-18	0.10267	0.126	0.34	1.25E-13	CSCL	VPS25
PREB.1	8.44E-18	-0.11589	0.056	0.218	1.26E-13	CSCL	PREB
RNF168.2	8.73E-18	-0.23165	0.021	0.155	1.30E-13	CSCL	RNF168
TIMM17B	8.96E-18	0.104978	0.133	0.353	1.34E-13	CSCL	TIMM17B
APC.2	9.23E-18	-0.10468	0.058	0.221	1.38E-13	CSCL	APC
UBE2Z.1	9.42E-18	-0.15976	0.037	0.183	1.41E-13	CSCL	UBE2Z
CS.2	9.59E-18	-0.12018	0.028	0.168	1.43E-13	CSCL	CS
CWC25	1.03E-17	-0.2141	0.052	0.209	1.54E-13	CSCL	CWC25
AHI1.1	1.03E-17	-0.15752	0.054	0.213	1.54E-13	CSCL	AHI1
DUSP3.1	1.04E-17	-0.19288	0.037	0.183	1.56E-13	CSCL	DUSP3
CUX1.2	1.05E-17	-0.10269	0.075	0.249	1.56E-13	CSCL	CUX1
SPPL2A.2	1.08E-17	-0.19574	0.042	0.191	1.61E-13	CSCL	SPPL2A
FRMD4A.2	1.19E-17	-0.31608	0.045	0.195	1.78E-13	CSCL	FRMD4A
SAC3D1.2	1.22E-17	-0.11531	0.033	0.177	1.82E-13	CSCL	SAC3D1
SLC25A37.	1.22E-17	-0.14497	0.119	0.32	1.83E-13	CSCL	SLC25A37
CHKA.2	1.23E-17	-0.16132	0.044	0.195	1.84E-13	CSCL	CHKA
RASSF4.2	1.26E-17	-0.11191	0.131	0.339	1.88E-13	CSCL	RASSF4
KLF3.2	1.26E-17	-0.1109	0.059	0.222	1.89E-13	CSCL	KLF3
RNASE1.2	1.27E-17	-0.6648	0.037	0.176	1.90E-13	CSCL	RNASE1

KPNA1.1	1.28E-17	-0.11046	0.03	0.171	1.92E-13	CSCL	KPNA1
YPEL3.2	1.31E-17	-0.13641	0.079	0.253	1.96E-13	CSCL	YPEL3
BCAN.2	1.31E-17	0.923939	0.354	0.272	1.96E-13	CSCL	BCAN
DENND5A.1	1.32E-17	-0.12925	0.038	0.186	1.97E-13	CSCL	DENND5A
HSPB11.2	1.35E-17	0.139746	0.126	0.339	2.01E-13	CSCL	HSPB11
CEP63.1	1.36E-17	-0.14478	0.038	0.185	2.03E-13	CSCL	CEP63
ATP13A3.1	1.37E-17	-0.20074	0.023	0.157	2.05E-13	CSCL	ATP13A3
TNFRSF21.	1.40E-17	-0.15154	0.03	0.17	2.10E-13	CSCL	TNFRSF21
PIP4K2A.2	1.41E-17	-0.34478	0.016	0.143	2.10E-13	CSCL	PIP4K2A
SLC36A4.1	1.45E-17	-0.10886	0.047	0.201	2.16E-13	CSCL	SLC36A4
RECQL.1	1.45E-17	-0.11886	0.035	0.18	2.16E-13	CSCL	RECQL
SPATA13.2	1.48E-17	-0.23755	0.031	0.172	2.21E-13	CSCL	SPATA13
NDUFB2.2	1.48E-17	0.389981	0.639	0.817	2.22E-13	CSCL	NDUFB2
VAC14.1	1.49E-17	-0.18887	0.017	0.147	2.23E-13	CSCL	VAC14
SAMM50.2	1.51E-17	-0.16056	0.061	0.223	2.26E-13	CSCL	SAMM50
PACSIN2.2	1.52E-17	-0.21525	0.037	0.181	2.28E-13	CSCL	PACSIN2
ZNF672.2	1.55E-17	-0.24958	0.017	0.147	2.32E-13	CSCL	ZNF672
LBR.2	1.75E-17	-0.1914	0.072	0.241	2.61E-13	CSCL	LBR
DOCK8.2	1.76E-17	-0.45697	0.007	0.127	2.62E-13	CSCL	DOCK8
HGS.1	1.77E-17	-0.10421	0.061	0.224	2.65E-13	CSCL	HGS
TMEM176A.	1.78E-17	-0.33812	0.07	0.235	2.65E-13	CSCL	TMEM176A
FAF2.1	1.78E-17	-0.17216	0.045	0.197	2.67E-13	CSCL	FAF2
TMA7.2	1.80E-17	0.32132	0.674	0.86	2.68E-13	CSCL	TMA7
ENOSF1.2	1.85E-17	-0.2016	0.009	0.13	2.76E-13	CSCL	ENOSF1
PHF3.1	1.88E-17	-0.13477	0.049	0.202	2.81E-13	CSCL	PHF3
RGL2.2	1.89E-17	-0.14487	0.054	0.212	2.83E-13	CSCL	RGL2
NAAA.2	1.90E-17	-0.31078	0.014	0.14	2.83E-13	CSCL	NAAA
NCOR2.2	1.94E-17	-0.11341	0.07	0.239	2.90E-13	CSCL	NCOR2
NISCH.2	1.96E-17	-0.21924	0.038	0.183	2.93E-13	CSCL	NISCH
CORO7.2	2.00E-17	-0.29812	0.028	0.165	2.99E-13	CSCL	CORO7
ELAVL3.2	2.04E-17	-0.13132	0.12	0.32	3.05E-13	CSCL	ELAVL3
NFKBIE.2	2.11E-17	-0.20304	0.014	0.14	3.15E-13	CSCL	NFKBIE
HN1L.1	2.13E-17	-0.21185	0.024	0.159	3.18E-13	CSCL	HN1L
DCTN1.2	2.15E-17	-0.10903	0.061	0.224	3.21E-13	CSCL	DCTN1
AGPAT1.1	2.20E-17	-0.13197	0.068	0.235	3.29E-13	CSCL	AGPAT1
SLC25A29	2.20E-17	-0.2025	0.024	0.159	3.29E-13	CSCL	SLC25A29
UBE2T.2	2.21E-17	-0.33462	0.01	0.133	3.30E-13	CSCL	UBE2T
BCL2L12.1	2.26E-17	-0.21239	0.01	0.133	3.38E-13	CSCL	BCL2L12
GNL2.1	2.28E-17	-0.12587	0.056	0.214	3.41E-13	CSCL	GNL2
ARFGEF1.2	2.32E-17	-0.23943	0.009	0.129	3.47E-13	CSCL	ARFGEF1
RBM47.2	2.36E-17	-0.37063	0.005	0.122	3.52E-13	CSCL	RBM47
DCBLD2.1	2.40E-17	-0.21098	0.019	0.149	3.58E-13	CSCL	DCBLD2
LRCH4.2	2.45E-17	-0.29603	0.047	0.196	3.66E-13	CSCL	LRCH4
CLINT1.1	2.47E-17	-0.13832	0.052	0.208	3.69E-13	CSCL	CLINT1
NPM1.2	2.56E-17	0.279914	0.686	0.878	3.82E-13	CSCL	NPM1
FAM129A.1	2.57E-17	-0.20421	0.017	0.146	3.84E-13	CSCL	FAM129A
PTPN12.1	2.57E-17	-0.10608	0.065	0.23	3.84E-13	CSCL	PTPN12
VPS41.1	2.59E-17	-0.10026	0.052	0.209	3.87E-13	CSCL	VPS41
PFN1.2	2.60E-17	-0.16135	0.417	0.829	3.89E-13	CSCL	PFN1
IRAK1.2	2.61E-17	-0.15415	0.028	0.165	3.90E-13	CSCL	IRAK1
PHF1.2	2.62E-17	-0.17935	0.035	0.176	3.92E-13	CSCL	PHF1

PREPL	2.67E-17	-0.12781	0.045	0.196	3.98E-13	CSCL	PREPL
FNIP2.2	2.67E-17	-0.26909	0.051	0.203	3.99E-13	CSCL	FNIP2
NRAS	2.69E-17	-0.16163	0.044	0.192	4.01E-13	CSCL	NRAS
SUB1.1	2.70E-17	0.379623	0.62	0.763	4.03E-13	CSCL	SUB1
NIPSNAP1.	2.79E-17	-0.1594	0.033	0.174	4.17E-13	CSCL	NIPSNAP1
NR3C1.2	2.82E-17	-0.11725	0.082	0.257	4.21E-13	CSCL	NR3C1
SUDS3.2	2.84E-17	-0.12035	0.045	0.195	4.24E-13	CSCL	SUDS3
CSNK1G2.2	2.84E-17	-0.13085	0.019	0.149	4.24E-13	CSCL	CSNK1G2
GSK3B.2	2.90E-17	-0.12086	0.051	0.205	4.34E-13	CSCL	GSK3B
ACBD5.2	2.94E-17	-0.14373	0.033	0.174	4.39E-13	CSCL	ACBD5
RCSD1.2	2.94E-17	-0.46136	0.007	0.126	4.39E-13	CSCL	RCSD1
PDS5A.1	2.94E-17	-0.18557	0.028	0.165	4.39E-13	CSCL	PDS5A
PSAP.1	2.94E-17	-0.21464	0.438	0.838	4.40E-13	CSCL	PSAP
C11orf24.	3.04E-17	-0.2067	0.019	0.149	4.54E-13	CSCL	C11orf24
RNF8.1	3.18E-17	-0.12971	0.033	0.174	4.75E-13	CSCL	RNF8
KIDINS220	3.22E-17	-0.10439	0.094	0.278	4.81E-13	CSCL	KIDINS220
DUSP23.1	3.26E-17	-0.25172	0.045	0.195	4.87E-13	CSCL	DUSP23
FRMD4B.2	3.27E-17	-0.35659	0.03	0.166	4.89E-13	CSCL	FRMD4B
NRIP1.2	3.40E-17	-0.21241	0.009	0.129	5.08E-13	CSCL	NRIP1
EML4.2	3.51E-17	-0.11343	0.047	0.198	5.24E-13	CSCL	EML4
ABHD8.2	3.62E-17	-0.1877	0.021	0.151	5.41E-13	CSCL	ABHD8
COX19.1	3.65E-17	-0.13519	0.072	0.239	5.46E-13	CSCL	COX19
ABHD11.1	3.67E-17	-0.23883	0.035	0.176	5.49E-13	CSCL	ABHD11
B3GALNT1.	3.76E-17	-0.17333	0.044	0.191	5.62E-13	CSCL	B3GALNT1
PLA2G15.2	3.77E-17	-0.28198	0.017	0.145	5.63E-13	CSCL	PLA2G15
SLC29A1.2	4.11E-17	-0.37389	0.017	0.144	6.14E-13	CSCL	SLC29A1
IL1RAP.1	4.24E-17	-0.23136	0.03	0.166	6.34E-13	CSCL	IL1RAP
BLMH.1	4.25E-17	-0.14375	0.038	0.182	6.35E-13	CSCL	BLMH
SMPDL3A.1	4.26E-17	-0.34457	0.01	0.131	6.37E-13	CSCL	SMPDL3A
GRK6.2	4.32E-17	-0.16895	0.016	0.141	6.45E-13	CSCL	GRK6
PHKB.2	4.54E-17	-0.14254	0.049	0.2	6.78E-13	CSCL	PHKB
RAP2C.1	4.68E-17	-0.258	0.01	0.131	7.00E-13	CSCL	RAP2C
RABIF.1	4.72E-17	-0.10805	0.042	0.188	7.06E-13	CSCL	RABIF
HMHA1.2	4.82E-17	-0.45535	0.002	0.114	7.21E-13	CSCL	HMHA1
RIOK3.1	5.00E-17	-0.10567	0.049	0.2	7.47E-13	CSCL	RIOK3
FILIP1L.2	5.24E-17	-0.30389	0.024	0.156	7.84E-13	CSCL	FILIP1L
MERTK.2	5.30E-17	-0.40411	0.01	0.13	7.92E-13	CSCL	MERTK
INO80C.1	5.40E-17	-0.13608	0.033	0.172	8.07E-13	CSCL	INO80C
PRKACA.2	5.41E-17	-0.1885	0.024	0.156	8.09E-13	CSCL	PRKACA
GSTP1.2	5.44E-17	0.369395	0.653	0.83	8.13E-13	CSCL	GSTP1
C6orf47.1	5.68E-17	-0.11731	0.019	0.147	8.49E-13	CSCL	C6orf47
TFDP2.1	5.69E-17	-0.14488	0.061	0.22	8.50E-13	CSCL	TFDP2
NDUFB8	5.84E-17	0.484052	0.134	0.052	8.73E-13	CSCL	NDUFB8
NOC4L.1	5.93E-17	-0.1765	0.028	0.163	8.87E-13	CSCL	NOC4L
NDUFA13.1	6.03E-17	0.380168	0.632	0.779	9.00E-13	CSCL	NDUFA13
CNOT3.1	6.03E-17	-0.13675	0.031	0.169	9.01E-13	CSCL	CNOT3
CREBL2.2	6.03E-17	-0.16544	0.047	0.196	9.02E-13	CSCL	CREBL2
CASC3	6.36E-17	-0.13462	0.045	0.193	9.50E-13	CSCL	CASC3
MED16.1	6.43E-17	-0.14817	0.03	0.165	9.61E-13	CSCL	MED16
PRR14.1	6.58E-17	-0.13891	0.054	0.208	9.83E-13	CSCL	PRR14
CNOT1.2	6.64E-17	-0.19892	0.024	0.156	9.92E-13	CSCL	CNOT1

SP3.2	6.65E-17	-0.10508	0.059	0.218	9.94E-13	CSCL	SP3
ZDHHC6.1	6.77E-17	-0.17813	0.035	0.175	1.01E-12	CSCL	ZDHHC6
BMP2K.1	6.81E-17	-0.37692	0.007	0.122	1.02E-12	CSCL	BMP2K
CEBPA.2	6.90E-17	-0.4267	0	0.109	1.03E-12	CSCL	CEBPA
ZMYND11.2	6.96E-17	-0.13473	0.023	0.153	1.04E-12	CSCL	ZMYND11
POLDIP3.1	7.06E-17	-0.1113	0.045	0.193	1.05E-12	CSCL	POLDIP3
C8orf82.2	7.30E-17	-0.11771	0.037	0.178	1.09E-12	CSCL	C8orf82
SERINC2	7.42E-17	-0.25273	0.009	0.126	1.11E-12	CSCL	SERINC2
SEPT4.2	7.49E-17	-0.38213	0.014	0.136	1.12E-12	CSCL	4-Sep
TGFBR2.2	7.64E-17	-0.34749	0.007	0.123	1.14E-12	CSCL	TGFBR2
IBTK.2	7.66E-17	-0.15907	0.035	0.174	1.14E-12	CSCL	IBTK
DVL2.1	7.95E-17	-0.15248	0.04	0.183	1.19E-12	CSCL	DVL2
TMEM131.2	7.96E-17	-0.16085	0.023	0.152	1.19E-12	CSCL	TMEM131
FAM208A.1	8.11E-17	-0.19521	0.019	0.146	1.21E-12	CSCL	FAM208A
DNAJC10.1	8.32E-17	-0.13372	0.052	0.204	1.24E-12	CSCL	DNAJC10
B4GALT1.2	8.36E-17	-0.39108	0.009	0.126	1.25E-12	CSCL	B4GALT1
TRIM13.2	8.82E-17	-0.14141	0.084	0.258	1.32E-12	CSCL	TRIM13
RFFL.2	8.92E-17	-0.13573	0.033	0.171	1.33E-12	CSCL	RFFL
TPST2.2	9.02E-17	-0.1056	0.035	0.174	1.35E-12	CSCL	TPST2
CDK5RAP1.	9.23E-17	-0.16803	0.03	0.165	1.38E-12	CSCL	CDK5RAP1
NDEL1.1	9.42E-17	-0.28309	0.028	0.16	1.41E-12	CSCL	NDEL1
FAM104B.1	9.76E-17	-0.12884	0.049	0.198	1.46E-12	CSCL	FAM104B
AAK1.2	9.82E-17	-0.12116	0.101	0.286	1.47E-12	CSCL	AAK1
CTR9.1	1.00E-16	-0.18236	0.038	0.179	1.50E-12	CSCL	CTR9
TMEM181.2	1.05E-16	-0.10212	0.033	0.17	1.57E-12	CSCL	TMEM181
ARHGAP4.2	1.06E-16	-0.43234	0.007	0.121	1.58E-12	CSCL	ARHGAP4
FAH.1	1.09E-16	-0.15235	0.03	0.164	1.62E-12	CSCL	FAH
SLAIN2.2	1.11E-16	-0.20318	0.019	0.145	1.66E-12	CSCL	SLAIN2
NXF1.1	1.14E-16	-0.14095	0.054	0.205	1.70E-12	CSCL	NXF1
MLXIP.2	1.16E-16	-0.2059	0.045	0.19	1.74E-12	CSCL	MLXIP
RIN3.2	1.18E-16	-0.42408	0.007	0.121	1.76E-12	CSCL	RIN3
ST8SIA4.2	1.19E-16	-0.37783	0.009	0.125	1.77E-12	CSCL	ST8SIA4
GNA15.2	1.22E-16	-0.42763	0.003	0.115	1.83E-12	CSCL	GNA15
EPS15.2	1.23E-16	-0.24256	0.026	0.156	1.84E-12	CSCL	EPS15
GM2A.2	1.23E-16	-0.34639	0.023	0.15	1.84E-12	CSCL	GM2A
EFHD2.2	1.23E-16	-0.10602	0.061	0.218	1.84E-12	CSCL	EFHD2
ZC3H18.2	1.28E-16	-0.17991	0.03	0.163	1.92E-12	CSCL	ZC3H18
CANT1.1	1.30E-16	-0.12769	0.04	0.182	1.95E-12	CSCL	CANT1
MAPK3	1.32E-16	-0.11013	0.059	0.214	1.98E-12	CSCL	MAPK3
SCAP.1	1.34E-16	-0.14693	0.03	0.164	2.00E-12	CSCL	SCAP
ATXN2L	1.41E-16	-0.14923	0.042	0.184	2.10E-12	CSCL	ATXN2L
SLC50A1.1	1.47E-16	-0.24104	0.037	0.174	2.20E-12	CSCL	SLC50A1
FKBP1B	1.50E-16	-0.17397	0.01	0.128	2.24E-12	CSCL	FKBP1B
MCM5.2	1.50E-16	-0.26956	0.026	0.156	2.24E-12	CSCL	MCM5
DUSP11	1.52E-16	-0.12841	0.045	0.19	2.27E-12	CSCL	DUSP11
E2F6.1	1.54E-16	-0.14432	0.023	0.15	2.30E-12	CSCL	E2F6
WWTR1.2	1.56E-16	-0.10027	0.031	0.167	2.33E-12	CSCL	WWTR1
SH3BP1.2	1.57E-16	-0.32557	0.005	0.117	2.34E-12	CSCL	SH3BP1
RAB35.2	1.59E-16	-0.10511	0.035	0.172	2.37E-12	CSCL	RAB35
TMOD3.2	1.62E-16	-0.15855	0.023	0.15	2.41E-12	CSCL	TMOD3
PODXL2.2	1.67E-16	-0.10043	0.12	0.317	2.49E-12	CSCL	PODXL2

COMMD8.2	1.68E-16	-0.11134	0.07	0.232	2.51E-12	CSCL	COMMD8
MTG2.1	1.70E-16	-0.15634	0.024	0.153	2.55E-12	CSCL	MTG2
FAM214B.2	1.71E-16	-0.26772	0.009	0.124	2.55E-12	CSCL	FAM214B
VPS53.1	1.74E-16	-0.15367	0.031	0.166	2.60E-12	CSCL	VPS53
NR2F6.1	1.76E-16	-0.12226	0.021	0.147	2.63E-12	CSCL	NR2F6
PDHX.1	1.77E-16	-0.11268	0.038	0.178	2.65E-12	CSCL	PDHX
DUSP4.2	1.79E-16	-0.15218	0.044	0.186	2.67E-12	CSCL	DUSP4
FAM49A.1	1.82E-16	-0.18173	0.038	0.177	2.73E-12	CSCL	FAM49A
C6orf120.	1.88E-16	-0.13818	0.019	0.144	2.81E-12	CSCL	C6orf120
TOM1.2	1.90E-16	-0.25114	0.033	0.167	2.84E-12	CSCL	TOM1
CCDC28A.1	1.91E-16	-0.2417	0.031	0.165	2.85E-12	CSCL	CCDC28A
EIF2B4.1	1.91E-16	-0.1348	0.049	0.195	2.86E-12	CSCL	EIF2B4
TPRA1.1	1.94E-16	-0.13013	0.033	0.168	2.90E-12	CSCL	TPRA1
EXT2.1	1.94E-16	-0.11824	0.038	0.178	2.90E-12	CSCL	EXT2
PARK7.2	1.94E-16	0.446544	0.611	0.774	2.90E-12	CSCL	PARK7
FZR1.2	1.95E-16	-0.16708	0.03	0.162	2.92E-12	CSCL	FZR1
RNF138	2.05E-16	-0.10163	0.044	0.187	3.06E-12	CSCL	RNF138
KCNMA1.1	2.11E-16	-0.24866	0.023	0.149	3.15E-12	CSCL	KCNMA1
RBM15B.2	2.18E-16	-0.10605	0.038	0.178	3.26E-12	CSCL	RBM15B
FAM168A.2	2.22E-16	-0.21258	0.014	0.133	3.32E-12	CSCL	FAM168A
NSRP1	2.25E-16	0.106858	0.147	0.367	3.37E-12	CSCL	NSRP1
ICAM3.2	2.32E-16	-0.30956	0	0.106	3.47E-12	CSCL	ICAM3
ARHGAP35.	2.35E-16	-0.13817	0.024	0.153	3.51E-12	CSCL	ARHGAP35
ACOT9.2	2.38E-16	-0.12452	0.045	0.189	3.55E-12	CSCL	ACOT9
ZNF385A.2	2.49E-16	-0.27839	0.019	0.143	3.71E-12	CSCL	ZNF385A
MAP3K13.2	2.57E-16	0.124815	0.168	0.405	3.84E-12	CSCL	MAP3K13
NRSN2.1	2.78E-16	-0.10669	0.035	0.171	4.15E-12	CSCL	NRSN2
ZYG11B.2	2.85E-16	-0.17111	0.026	0.155	4.26E-12	CSCL	ZYG11B
SLC40A1.2	2.86E-16	-0.25463	0.021	0.145	4.27E-12	CSCL	SLC40A1
MAPK6.1	2.90E-16	-0.15678	0.04	0.179	4.33E-12	CSCL	MAPK6
ZCCHC6.2	2.91E-16	-0.28603	0.019	0.141	4.35E-12	CSCL	ZCCHC6
PAQR4.2	2.96E-16	-0.17672	0.035	0.171	4.42E-12	CSCL	PAQR4
LMBR1L.2	2.97E-16	-0.20463	0.047	0.191	4.44E-12	CSCL	LMBR1L
TBCD.2	2.99E-16	-0.14722	0.024	0.151	4.46E-12	CSCL	TBCD
MYADM.1	3.01E-16	-0.16112	0.052	0.2	4.50E-12	CSCL	MYADM
LPAR5.2	3.04E-16	-0.43241	0.002	0.109	4.54E-12	CSCL	LPAR5
CTA-29F11	3.05E-16	-0.18023	0.058	0.208	4.56E-12	CSCL	CTA-29F11.1
LGALS3.2	3.12E-16	0.634026	0.501	0.48	4.67E-12	CSCL	LGALS3
NUP93.1	3.19E-16	-0.10047	0.045	0.188	4.76E-12	CSCL	NUP93
ZNF330	3.21E-16	-0.11866	0.066	0.223	4.79E-12	CSCL	ZNF330
UBXN2A.1	3.25E-16	-0.11917	0.049	0.195	4.86E-12	CSCL	UBXN2A
RIN2.2	3.27E-16	-0.33324	0.014	0.132	4.88E-12	CSCL	RIN2
CYC1.2	3.32E-16	0.106518	0.248	0.554	4.96E-12	CSCL	CYC1
PNPLA6.2	3.32E-16	-0.13661	0.037	0.173	4.96E-12	CSCL	PNPLA6
LARP6.1	3.32E-16	-0.10196	0.052	0.2	4.97E-12	CSCL	LARP6
P4HA1	3.35E-16	-0.10276	0.072	0.232	5.01E-12	CSCL	P4HA1
MICAL1.2	3.46E-16	-0.31733	0.023	0.147	5.17E-12	CSCL	MICAL1
ALDH16A1.	3.50E-16	-0.26473	0.01	0.125	5.23E-12	CSCL	ALDH16A1
SPHK1.2	3.51E-16	-0.27094	0.003	0.112	5.25E-12	CSCL	SPHK1
PLEC.2	3.52E-16	-0.13335	0.049	0.194	5.26E-12	CSCL	PLEC
C14orf119	3.53E-16	0.126398	0.122	0.323	5.28E-12	CSCL	C14orf119

C6orf226.	3.59E-16	-0.10219	0.051	0.197	5.36E-12	CSCL	C6orf226
FKBP5.2	3.61E-16	-0.12465	0.131	0.326	5.39E-12	CSCL	FKBP5
RENBP.2	3.67E-16	-0.31701	0.019	0.141	5.49E-12	CSCL	RENBP
PHF11.2	3.70E-16	-0.1916	0.023	0.148	5.53E-12	CSCL	PHF11
B3GALT6.2	3.95E-16	-0.13732	0.021	0.144	5.90E-12	CSCL	B3GALT6
SETX.2	3.99E-16	-0.10903	0.037	0.173	5.96E-12	CSCL	SETX
ZNF608	4.01E-16	-0.12534	0.031	0.164	5.99E-12	CSCL	ZNF608
VAMP7.1	4.05E-16	-0.14495	0.037	0.173	6.05E-12	CSCL	VAMP7
DPYSL5.2	4.12E-16	-0.13511	0.035	0.169	6.16E-12	CSCL	DPYSL5
LINC00936	4.15E-16	-0.30594	0.023	0.147	6.20E-12	CSCL	LINC00936
LDLRAD4.2	4.18E-16	-0.33946	0.026	0.152	6.25E-12	CSCL	LDLRAD4
KANSL1.2	4.21E-16	-0.16196	0.037	0.172	6.28E-12	CSCL	KANSL1
PELP1.1	4.27E-16	-0.13671	0.028	0.157	6.38E-12	CSCL	PELP1
LTA4H.1	4.41E-16	-0.11325	0.075	0.237	6.59E-12	CSCL	LTA4H
FAM168B.1	4.47E-16	-0.11999	0.035	0.169	6.68E-12	CSCL	FAM168B
SLC2A3.1	4.52E-16	-0.20687	0.092	0.263	6.76E-12	CSCL	SLC2A3
NDRG3.2	4.62E-16	-0.11298	0.044	0.184	6.90E-12	CSCL	NDRG3
CASP6.1	4.64E-16	-0.14634	0.035	0.169	6.93E-12	CSCL	CASP6
ATL3.1	4.71E-16	-0.18161	0.021	0.144	7.04E-12	CSCL	ATL3
OCEL1.1	4.72E-16	-0.1625	0.031	0.163	7.06E-12	CSCL	OCEL1
COL4A3BP.	4.74E-16	-0.17329	0.024	0.15	7.08E-12	CSCL	COL4A3BP
UBE4A.1	4.75E-16	-0.19178	0.03	0.159	7.10E-12	CSCL	UBE4A
PARP14.2	4.79E-16	-0.14873	0.031	0.163	7.16E-12	CSCL	PARP14
SEPT7.2	4.87E-16	0.462455	0.595	0.708	7.28E-12	CSCL	7-Sep
CYTH4.2	4.89E-16	-0.41908	0.007	0.117	7.30E-12	CSCL	CYTH4
TAF3.2	4.89E-16	-0.23971	0.012	0.128	7.31E-12	CSCL	TAF3
SETD3.2	4.90E-16	-0.18662	0.031	0.162	7.32E-12	CSCL	SETD3
XAB2	4.91E-16	-0.13134	0.044	0.184	7.33E-12	CSCL	XAB2
AFTPH.1	4.95E-16	-0.17924	0.03	0.159	7.40E-12	CSCL	AFTPH
ARHGEF1.2	5.00E-16	-0.18706	0.056	0.204	7.47E-12	CSCL	ARHGEF1
HIPK2.2	5.03E-16	-0.1367	0.07	0.229	7.52E-12	CSCL	HIPK2
PQBP1.1	5.13E-16	0.1082	0.141	0.355	7.67E-12	CSCL	PQBP1
PLEKHO2.2	5.24E-16	-0.33736	0.002	0.107	7.82E-12	CSCL	PLEKHO2
LINC00493	5.36E-16	0.115725	0.244	0.545	8.01E-12	CSCL	LINC00493
LYST.1	5.36E-16	-0.13161	0.028	0.156	8.01E-12	CSCL	LYST
PHACTR1.2	5.49E-16	-0.48467	0.01	0.123	8.20E-12	CSCL	PHACTR1
POLR2M.1	5.55E-16	-0.10798	0.033	0.166	8.30E-12	CSCL	POLR2M
CD55.2	5.61E-16	-0.42879	0.01	0.123	8.38E-12	CSCL	CD55
IREB2.1	5.63E-16	-0.2193	0.031	0.161	8.41E-12	CSCL	IREB2
ANKH.2	5.79E-16	-0.24488	0.028	0.155	8.65E-12	CSCL	ANKH
ATP13A1.1	6.19E-16	-0.15792	0.035	0.168	9.24E-12	CSCL	ATP13A1
NSMCE4A.2	6.42E-16	-0.11237	0.03	0.159	9.60E-12	CSCL	NSMCE4A
SLC35A2	6.46E-16	-0.20308	0.038	0.173	9.66E-12	CSCL	SLC35A2
GSPT2	6.68E-16	-0.1823	0.028	0.155	9.98E-12	CSCL	GSPT2
PHYKPL.2	6.73E-16	-0.13181	0.059	0.209	1.01E-11	CSCL	PHYKPL
KLF2.2	7.09E-16	-0.41272	0.019	0.137	1.06E-11	CSCL	KLF2
PDCD4.2	7.15E-16	-0.26619	0.028	0.155	1.07E-11	CSCL	PDCD4
DCTPP1.1	7.42E-16	0.132552	0.134	0.34	1.11E-11	CSCL	DCTPP1
SNRPD2.2	7.92E-16	0.481447	0.606	0.755	1.18E-11	CSCL	SNRPD2
MESDC1.2	8.19E-16	-0.21207	0.014	0.129	1.22E-11	CSCL	MESDC1
FBXO25.1	8.33E-16	-0.19572	0.03	0.158	1.24E-11	CSCL	FBXO25

PRKAB1.1	8.59E-16	-0.17955	0.033	0.164	1.28E-11	CSCL	PRKAB1
LPXN.2	8.66E-16	-0.40914	0.007	0.115	1.29E-11	CSCL	LPXN
RCBTB2.2	8.76E-16	-0.27208	0.024	0.148	1.31E-11	CSCL	RCBTB2
MED13.1	8.82E-16	-0.10015	0.035	0.167	1.32E-11	CSCL	MED13
RP11-452F	8.92E-16	-0.2906	0.016	0.133	1.33E-11	CSCL	RP11-452F19.3
NOC2L.1	9.15E-16	0.109897	0.124	0.322	1.37E-11	CSCL	NOC2L
LHX2.2	9.40E-16	-0.16683	0.017	0.135	1.41E-11	CSCL	LHX2
UBE2C.2	9.42E-16	-0.4156	0.01	0.122	1.41E-11	CSCL	UBE2C
RAB40B.2	9.43E-16	-0.22358	0.017	0.135	1.41E-11	CSCL	RAB40B
FADD.1	9.48E-16	-0.14506	0.021	0.142	1.42E-11	CSCL	FADD
TULP4.2	9.59E-16	-0.16363	0.024	0.148	1.43E-11	CSCL	TULP4
PLXNB2.2	9.60E-16	-0.17563	0.033	0.163	1.43E-11	CSCL	PLXNB2
TBC1D12.2	9.65E-16	-0.28455	0.005	0.112	1.44E-11	CSCL	TBC1D12
UBE2N.1	9.68E-16	0.102846	0.244	0.536	1.45E-11	CSCL	UBE2N
C1orf174.	9.95E-16	-0.15498	0.031	0.16	1.49E-11	CSCL	C1orf174
SCO1	1.01E-15	-0.10969	0.038	0.173	1.50E-11	CSCL	SCO1
TMEM101.1	1.01E-15	-0.10196	0.063	0.214	1.51E-11	CSCL	TMEM101
C1GALT1.1	1.01E-15	-0.18219	0.037	0.169	1.51E-11	CSCL	C1GALT1
RRAGD.1	1.03E-15	-0.16012	0.017	0.135	1.54E-11	CSCL	RRAGD
TAB2.1	1.07E-15	-0.19948	0.016	0.132	1.60E-11	CSCL	TAB2
TEX261.1	1.07E-15	-0.13286	0.038	0.173	1.60E-11	CSCL	TEX261
PCID2.1	1.09E-15	-0.15404	0.047	0.187	1.63E-11	CSCL	PCID2
AIFM1.1	1.11E-15	-0.12873	0.026	0.151	1.66E-11	CSCL	AIFM1
TBC1D22A.	1.15E-15	-0.23918	0.014	0.128	1.71E-11	CSCL	TBC1D22A
APTX.1	1.16E-15	-0.18448	0.023	0.144	1.74E-11	CSCL	APTX
WBSCR16.1	1.17E-15	-0.10152	0.026	0.151	1.75E-11	CSCL	WBSCR16
NFATC2IP.	1.17E-15	-0.14068	0.037	0.169	1.75E-11	CSCL	NFATC2IP
GIT2.2	1.18E-15	-0.23493	0.031	0.159	1.77E-11	CSCL	GIT2
KDM5B.2	1.21E-15	-0.17249	0.037	0.168	1.80E-11	CSCL	KDM5B
SRFBP1.1	1.21E-15	-0.10225	0.047	0.188	1.82E-11	CSCL	SRFBP1
ARL4D.2	1.22E-15	-0.25654	0.042	0.175	1.83E-11	CSCL	ARL4D
ARSA.2	1.23E-15	-0.27797	0.021	0.141	1.84E-11	CSCL	ARSA
MTMR6.2	1.24E-15	-0.18752	0.026	0.151	1.86E-11	CSCL	MTMR6
SUCO	1.25E-15	-0.1332	0.031	0.16	1.87E-11	CSCL	SUCO
ZSWIM6.2	1.28E-15	-0.22785	0.017	0.134	1.91E-11	CSCL	ZSWIM6
SFT2D2.1	1.34E-15	-0.17318	0.028	0.154	2.00E-11	CSCL	SFT2D2
PITPNB.1	1.34E-15	-0.10844	0.042	0.178	2.01E-11	CSCL	PITPNB
TNFAIP3.2	1.36E-15	-0.31252	0.038	0.17	2.03E-11	CSCL	TNFAIP3
KLHL24.2	1.36E-15	-0.22448	0.047	0.186	2.04E-11	CSCL	KLHL24
VWA1.2	1.41E-15	-0.15786	0.026	0.15	2.11E-11	CSCL	VWA1
MAPRE2.1	1.47E-15	-0.10433	0.051	0.193	2.19E-11	CSCL	MAPRE2
UBALD1.1	1.58E-15	-0.1424	0.04	0.174	2.36E-11	CSCL	UBALD1
SNX8.2	1.58E-15	-0.20672	0.028	0.153	2.36E-11	CSCL	SNX8
IMPACT.1	1.59E-15	-0.10017	0.037	0.169	2.37E-11	CSCL	IMPACT
NUMB.2	1.60E-15	-0.16234	0.042	0.177	2.39E-11	CSCL	NUMB
KCTD17.2	1.60E-15	-0.10518	0.021	0.141	2.39E-11	CSCL	KCTD17
RUSC1.1	1.64E-15	-0.12722	0.023	0.144	2.44E-11	CSCL	RUSC1
CCNG2.2	1.64E-15	-0.21524	0.049	0.188	2.45E-11	CSCL	CCNG2
RPS6KA4.2	1.68E-15	-0.24726	0.014	0.127	2.50E-11	CSCL	RPS6KA4
BEND6.1	1.73E-15	-0.16294	0.012	0.124	2.58E-11	CSCL	BEND6
MPHOSPH8.	1.78E-15	0.111747	0.178	0.41	2.66E-11	CSCL	MPHOSPH8

SETD2.2	1.79E-15	-0.12874	0.026	0.15	2.68E-11	CSCL	SETD2
NOTCH2.1	1.82E-15	-0.13652	0.028	0.153	2.71E-11	CSCL	NOTCH2
SUSD3.2	1.88E-15	-0.45383	0	0.1	2.81E-11	CSCL	SUSD3
SH3GLB2.2	1.89E-15	-0.23144	0.012	0.124	2.82E-11	CSCL	SH3GLB2
TLR2.2	1.98E-15	-0.45132	0.009	0.116	2.96E-11	CSCL	TLR2
PRPF18.1	1.98E-15	-0.17028	0.023	0.143	2.96E-11	CSCL	PRPF18
TUG1.2	1.98E-15	-0.12508	0.045	0.182	2.97E-11	CSCL	TUG1
EPB41L3.2	1.99E-15	-0.40585	0.005	0.11	2.98E-11	CSCL	EPB41L3
FRYL.2	2.02E-15	-0.13703	0.052	0.195	3.02E-11	CSCL	FRYL
RNPEPL1.2	2.03E-15	-0.25843	0.019	0.136	3.03E-11	CSCL	RNPEPL1
ZC3H7A.2	2.03E-15	-0.16062	0.03	0.155	3.04E-11	CSCL	ZC3H7A
DPM1.2	2.04E-15	0.110874	0.164	0.389	3.04E-11	CSCL	DPM1
TMC6.2	2.14E-15	-0.39419	0.009	0.116	3.20E-11	CSCL	TMC6
OPA1.2	2.27E-15	-0.15414	0.019	0.136	3.39E-11	CSCL	OPA1
FAM217B.2	2.28E-15	-0.14705	0.033	0.161	3.40E-11	CSCL	FAM217B
TNFAIP8L2	2.34E-15	-0.42397	0	0.099	3.50E-11	CSCL	TNFAIP8L2
CPQ	2.35E-15	-0.20632	0.047	0.184	3.51E-11	CSCL	CPQ
METTL21A.	2.37E-15	-0.16316	0.051	0.19	3.55E-11	CSCL	METTL21A
KLC2.1	2.37E-15	-0.18338	0.014	0.127	3.55E-11	CSCL	KLC2
REPS1.1	2.41E-15	-0.15585	0.017	0.133	3.60E-11	CSCL	REPS1
CENPW.2	2.44E-15	-0.261	0.012	0.123	3.64E-11	CSCL	CENPW
PYGL.1	2.45E-15	-0.16327	0.056	0.199	3.66E-11	CSCL	PYGL
MIB2.1	2.45E-15	-0.12753	0.03	0.155	3.66E-11	CSCL	MIB2
TMEM41B	2.46E-15	-0.12866	0.045	0.182	3.68E-11	CSCL	TMEM41B
IKBKG.2	2.48E-15	-0.17228	0.021	0.139	3.71E-11	CSCL	IKBKG
RALB.1	2.49E-15	-0.17046	0.035	0.163	3.72E-11	CSCL	RALB
DUSP2.2	2.55E-15	-0.46232	0.009	0.116	3.81E-11	CSCL	DUSP2
CTC-524C5	2.58E-15	-0.16654	0.012	0.123	3.86E-11	CSCL	CTC-524C5.2
KIF3B.2	2.62E-15	-0.15621	0.019	0.136	3.92E-11	CSCL	KIF3B
SOD1.1	2.63E-15	0.441838	0.604	0.764	3.94E-11	CSCL	SOD1
TRIAP1	2.69E-15	0.130231	0.126	0.323	4.02E-11	CSCL	TRIAP1
ACAA2.1	2.69E-15	-0.12703	0.066	0.217	4.02E-11	CSCL	ACAA2
ALYREF.2	2.72E-15	-0.10376	0.028	0.152	4.06E-11	CSCL	ALYREF
TYMS.2	2.76E-15	-0.12301	0.037	0.167	4.12E-11	CSCL	TYMS
UBXN2B.2	2.77E-15	-0.20974	0.031	0.157	4.14E-11	CSCL	UBXN2B
MTMR4.1	2.78E-15	-0.10401	0.037	0.167	4.15E-11	CSCL	MTMR4
RNF41	2.83E-15	-0.11192	0.04	0.173	4.23E-11	CSCL	RNF41
GAL3ST4.2	2.85E-15	-0.37399	0.045	0.178	4.26E-11	CSCL	GAL3ST4
NF2.2	2.86E-15	-0.10679	0.031	0.157	4.27E-11	CSCL	NF2
PCF11.1	2.86E-15	-0.11908	0.063	0.211	4.28E-11	CSCL	PCF11
PPP1R35.2	2.87E-15	-0.13498	0.035	0.164	4.29E-11	CSCL	PPP1R35
UBE2E2.1	2.91E-15	-0.141	0.086	0.248	4.34E-11	CSCL	UBE2E2
CLK3.1	2.93E-15	-0.13638	0.038	0.17	4.38E-11	CSCL	CLK3
DHX40.1	2.98E-15	-0.16862	0.024	0.145	4.46E-11	CSCL	DHX40
ODF3B.2	3.01E-15	-0.3526	0	0.099	4.50E-11	CSCL	ODF3B
DENND3.2	3.01E-15	-0.41809	0.003	0.105	4.51E-11	CSCL	DENND3
TNKS.2	3.02E-15	-0.11827	0.03	0.155	4.51E-11	CSCL	TNKS
POLR2H.1	3.07E-15	0.115526	0.133	0.332	4.59E-11	CSCL	POLR2H
DIDO1.1	3.10E-15	-0.14725	0.03	0.154	4.63E-11	CSCL	DIDO1
TMEM57.2	3.13E-15	-0.13667	0.045	0.181	4.67E-11	CSCL	TMEM57
AMDHD2.2	3.15E-15	-0.2139	0.016	0.129	4.71E-11	CSCL	AMDHD2

HDAC10.2	3.18E-15	-0.25272	0.007	0.112	4.76E-11	CSCL	HDAC10
ATXN8OS.2	3.19E-15	0.4095	0.166	0.075	4.76E-11	CSCL	ATXN8OS
POGK.1	3.21E-15	-0.15369	0.019	0.135	4.79E-11	CSCL	POGK
ANXA4.2	3.44E-15	-0.13627	0.058	0.201	5.13E-11	CSCL	ANXA4
EEF2	3.44E-15	0.346036	0.644	0.839	5.14E-11	CSCL	EEF2
ORAI3.1	3.55E-15	-0.17595	0.026	0.147	5.30E-11	CSCL	ORAI3
SH3BP2.1	3.62E-15	-0.20571	0.024	0.144	5.41E-11	CSCL	SH3BP2
MFSD3.2	3.63E-15	-0.15678	0.014	0.125	5.42E-11	CSCL	MFSD3
CTSF.1	3.69E-15	0.111939	0.101	0.278	5.52E-11	CSCL	CTSF
TMEM106C.	3.77E-15	0.109535	0.099	0.275	5.64E-11	CSCL	TMEM106C
CENPH.1	3.78E-15	-0.18813	0.017	0.132	5.64E-11	CSCL	CENPH
GLDN.2	3.81E-15	-0.404	0.002	0.102	5.69E-11	CSCL	GLDN
TXNDC11	4.01E-15	-0.23561	0.012	0.122	5.99E-11	CSCL	TXNDC11
PMF1.1	4.12E-15	0.104607	0.133	0.33	6.15E-11	CSCL	PMF1
STK38L.1	4.20E-15	-0.16427	0.031	0.156	6.28E-11	CSCL	STK38L
UQCRQ.2	4.22E-15	0.361778	0.63	0.808	6.31E-11	CSCL	UQCRQ
ARRDC1.2	4.24E-15	-0.21577	0.023	0.14	6.33E-11	CSCL	ARRDC1
LIN7B	4.25E-15	-0.20245	0.016	0.128	6.35E-11	CSCL	LIN7B
YY1AP1	4.29E-15	-0.12945	0.044	0.177	6.41E-11	CSCL	YY1AP1
CARD8.1	4.46E-15	-0.25034	0.031	0.155	6.67E-11	CSCL	CARD8
GTF2H1	4.46E-15	-0.10743	0.047	0.183	6.67E-11	CSCL	GTF2H1
NFKBIZ.2	4.47E-15	-0.24606	0.047	0.182	6.68E-11	CSCL	NFKBIZ
PIGS.2	4.52E-15	-0.10134	0.051	0.189	6.76E-11	CSCL	PIGS
ST6GALNAC	4.54E-15	-0.1825	0.017	0.131	6.78E-11	CSCL	ST6GALNAC4
UTRN.1	4.59E-15	-0.20648	0.009	0.115	6.86E-11	CSCL	UTRN
IL17RA.2	4.65E-15	-0.26185	0.014	0.125	6.94E-11	CSCL	IL17RA
EBI3.2	4.66E-15	-0.43785	0.002	0.101	6.96E-11	CSCL	EBI3
PPP6R2.2	4.67E-15	-0.14166	0.031	0.156	6.98E-11	CSCL	PPP6R2
STK17B.2	4.69E-15	-0.39649	0.023	0.138	7.01E-11	CSCL	STK17B
TMEM19.2	4.71E-15	-0.20597	0.033	0.159	7.03E-11	CSCL	TMEM19
CLEC5A.2	4.75E-15	-0.45931	0.003	0.104	7.10E-11	CSCL	CLEC5A
ANKRD13D.	4.82E-15	-0.10742	0.042	0.174	7.21E-11	CSCL	ANKRD13D
FUBP1.2	4.89E-15	-0.11387	0.077	0.232	7.31E-11	CSCL	FUBP1
EIF4EBP2.	4.94E-15	-0.13887	0.026	0.147	7.38E-11	CSCL	EIF4EBP2
HAX1	4.96E-15	0.143397	0.204	0.458	7.41E-11	CSCL	HAX1
NOVA1.2	5.06E-15	0.503279	0.482	0.479	7.56E-11	CSCL	NOVA1
SLC35B4.1	5.09E-15	-0.10759	0.056	0.198	7.60E-11	CSCL	SLC35B4
PTPRZ1.2	5.12E-15	0.688181	0.421	0.382	7.66E-11	CSCL	PTPRZ1
ARPC3	5.13E-15	-0.15121	0.419	0.824	7.66E-11	CSCL	ARPC3
ITPR2.2	5.15E-15	-0.13977	0.021	0.137	7.69E-11	CSCL	ITPR2
TBC1D16.1	5.20E-15	-0.21733	0.023	0.139	7.77E-11	CSCL	TBC1D16
SYK.2	5.21E-15	-0.36613	0.007	0.111	7.79E-11	CSCL	SYK
TTYH2.2	5.30E-15	-0.26173	0.012	0.121	7.92E-11	CSCL	TTYH2
RBM33	5.39E-15	-0.1962	0.031	0.155	8.05E-11	CSCL	RBM33
OAZ1.1	5.40E-15	0.17235	0.736	0.936	8.07E-11	CSCL	OAZ1
RFWD2.2	5.42E-15	-0.10017	0.04	0.171	8.10E-11	CSCL	RFWD2
GALNS.1	5.47E-15	-0.14271	0.021	0.137	8.18E-11	CSCL	GALNS
APH1B.1	5.50E-15	-0.25038	0.019	0.133	8.22E-11	CSCL	APH1B
DHRS11.2	5.55E-15	-0.17785	0.005	0.107	8.30E-11	CSCL	DHRS11
ASRGL1.2	5.61E-15	-0.13386	0.063	0.208	8.38E-11	CSCL	ASRGL1
CHD1L.2	5.79E-15	-0.11684	0.024	0.143	8.65E-11	CSCL	CHD1L

RELB.1	5.90E-15	-0.23303	0.002	0.1	8.81E-11	CSCL	RELB
CWC15.1	5.98E-15	0.113099	0.227	0.5	8.94E-11	CSCL	CWC15
TIMMDC1.1	6.11E-15	0.104966	0.171	0.398	9.13E-11	CSCL	TIMMDC1
DARS.2	6.14E-15	0.105492	0.182	0.414	9.17E-11	CSCL	DARS
ZNF771.1	6.18E-15	-0.11244	0.03	0.152	9.23E-11	CSCL	ZNF771
ANK2.2	6.32E-15	-0.117	0.04	0.17	9.44E-11	CSCL	ANK2
ZCCHC17.1	6.38E-15	0.13686	0.15	0.362	9.53E-11	CSCL	ZCCHC17
TLE4.2	6.57E-15	-0.13952	0.04	0.17	9.81E-11	CSCL	TLE4
SLC30A7.1	6.57E-15	-0.11973	0.038	0.167	9.82E-11	CSCL	SLC30A7
ABR.2	6.62E-15	-0.2246	0.03	0.151	9.89E-11	CSCL	ABR
BAP1.1	6.65E-15	-0.16541	0.026	0.146	9.94E-11	CSCL	BAP1
ANAPC5.1	6.65E-15	0.116301	0.176	0.407	9.94E-11	CSCL	ANAPC5
UCKL1	6.79E-15	-0.10111	0.037	0.164	1.01E-10	CSCL	UCKL1
KLHL12.1	6.83E-15	-0.18696	0.017	0.13	1.02E-10	CSCL	KLHL12
PRKX.2	7.13E-15	-0.24197	0.01	0.117	1.07E-10	CSCL	PRKX
C3.2	7.58E-15	-0.16556	0.194	0.412	1.13E-10	CSCL	C3
BTBD1.2	7.60E-15	-0.15637	0.031	0.154	1.14E-10	CSCL	BTBD1
RPP30.1	7.62E-15	-0.1747	0.044	0.175	1.14E-10	CSCL	RPP30
PUSL1.1	7.64E-15	-0.14115	0.016	0.126	1.14E-10	CSCL	PUSL1
USP4.1	7.74E-15	-0.104	0.047	0.181	1.16E-10	CSCL	USP4
NCF2.2	7.74E-15	-0.38583	0.005	0.106	1.16E-10	CSCL	NCF2
CD99L2.1	7.82E-15	-0.13761	0.021	0.136	1.17E-10	CSCL	CD99L2
LRRC4B.2	8.07E-15	-0.14752	0.023	0.139	1.21E-10	CSCL	LRRC4B
CUEDC1.2	8.07E-15	-0.17156	0.012	0.12	1.21E-10	CSCL	CUEDC1
FAM118A.2	8.11E-15	-0.25607	0.023	0.138	1.21E-10	CSCL	FAM118A
EML3.1	8.17E-15	-0.17469	0.012	0.12	1.22E-10	CSCL	EML3
PFDN6.1	8.23E-15	0.102598	0.152	0.363	1.23E-10	CSCL	PFDN6
DEF6.2	8.48E-15	-0.37567	0	0.096	1.27E-10	CSCL	DEF6
CENPF.2	8.60E-15	-0.36346	0.005	0.106	1.29E-10	CSCL	CENPF
CX3CR1.2	8.60E-15	-0.44693	0.005	0.106	1.29E-10	CSCL	CX3CR1
C9orf72.2	8.64E-15	-0.25064	0.033	0.156	1.29E-10	CSCL	C9orf72
DHRS3.1	8.71E-15	-0.20534	0.045	0.177	1.30E-10	CSCL	DHRS3
LYAR.1	8.72E-15	-0.11368	0.047	0.181	1.30E-10	CSCL	LYAR
FEN1.1	8.80E-15	-0.14708	0.024	0.142	1.32E-10	CSCL	FEN1
VHL.1	8.88E-15	-0.12683	0.04	0.169	1.33E-10	CSCL	VHL
CLN6.2	9.07E-15	-0.20315	0.021	0.135	1.36E-10	CSCL	CLN6
CNIH2.2	9.11E-15	-0.15293	0.021	0.135	1.36E-10	CSCL	CNIH2
SERF2.1	9.14E-15	0.225209	0.703	0.905	1.37E-10	CSCL	SERF2
TATDN3.2	9.24E-15	-0.13447	0.035	0.16	1.38E-10	CSCL	TATDN3
RAB3A.2	9.24E-15	-0.23453	0.042	0.17	1.38E-10	CSCL	RAB3A
FOS.2	9.27E-15	0.327552	0.667	0.85	1.39E-10	CSCL	FOS
WDR55.1	9.38E-15	-0.18769	0.014	0.123	1.40E-10	CSCL	WDR55
TSC22D4.2	9.40E-15	0.687583	0.475	0.47	1.40E-10	CSCL	TSC22D4
CERCAM	9.53E-15	-0.19114	0.021	0.135	1.42E-10	CSCL	CERCAM
HELZ.2	9.58E-15	-0.11501	0.061	0.203	1.43E-10	CSCL	HELZ
RNMTL1.1	9.63E-15	-0.15442	0.021	0.135	1.44E-10	CSCL	RNMTL1
EBNA1BP2.	9.66E-15	0.189177	0.152	0.364	1.44E-10	CSCL	EBNA1BP2
THBD.2	9.69E-15	-0.41956	0.002	0.099	1.45E-10	CSCL	THBD
S100A11.2	9.70E-15	-0.10333	0.34	0.673	1.45E-10	CSCL	S100A11
CERK.2	9.98E-15	-0.2201	0.009	0.112	1.49E-10	CSCL	CERK
MAN2A1.2	1.00E-14	-0.27426	0.01	0.115	1.49E-10	CSCL	MAN2A1

MTHFD2.1	1.00E-14	-0.10704	0.119	0.297	1.50E-10	CSCL	MTHFD2
TAGAP.2	1.02E-14	-0.46092	0.005	0.105	1.52E-10	CSCL	TAGAP
MOGS.1	1.02E-14	-0.19112	0.017	0.129	1.53E-10	CSCL	MOGS
RIPK2.2	1.02E-14	-0.16194	0.044	0.174	1.53E-10	CSCL	RIPK2
DUS1L.2	1.03E-14	-0.10923	0.023	0.138	1.54E-10	CSCL	DUS1L
STRN4.2	1.03E-14	-0.14279	0.017	0.129	1.55E-10	CSCL	STRN4
ASL.1	1.06E-14	-0.15991	0.045	0.177	1.58E-10	CSCL	ASL
FKBP15.2	1.07E-14	-0.18181	0.026	0.144	1.61E-10	CSCL	FKBP15
KCNF1.2	1.08E-14	-0.15559	0.017	0.129	1.61E-10	CSCL	KCNF1
LMNB2.2	1.08E-14	-0.17884	0.014	0.122	1.61E-10	CSCL	LMNB2
GMCL1.1	1.11E-14	-0.18481	0.038	0.165	1.66E-10	CSCL	GMCL1
HCFC1.2	1.11E-14	-0.16066	0.028	0.147	1.66E-10	CSCL	HCFC1
CXorf40B.	1.11E-14	-0.11347	0.035	0.159	1.66E-10	CSCL	CXorf40B
DLX6-AS1.	1.12E-14	-0.51307	0.023	0.135	1.68E-10	CSCL	DLX6-AS1
NCOA6.2	1.13E-14	-0.10822	0.024	0.141	1.69E-10	CSCL	NCOA6
INPPL1.2	1.15E-14	-0.16985	0.016	0.125	1.72E-10	CSCL	INPPL1
GPX3.1	1.18E-14	-0.21952	0.035	0.159	1.76E-10	CSCL	GPX3
SLC25A46.	1.18E-14	-0.12539	0.035	0.159	1.77E-10	CSCL	SLC25A46
RBMXL1.1	1.19E-14	-0.15251	0.035	0.159	1.78E-10	CSCL	RBMXL1
ALCAM.2	1.20E-14	-0.13941	0.061	0.203	1.80E-10	CSCL	ALCAM
ARHGAP15.	1.21E-14	-0.41155	0.003	0.102	1.82E-10	CSCL	ARHGAP15
PER1.1	1.24E-14	-0.24811	0.056	0.191	1.85E-10	CSCL	PER1
SPATA20.1	1.28E-14	-0.22925	0.023	0.137	1.91E-10	CSCL	SPATA20
SCD.2	1.40E-14	-0.10098	0.072	0.219	2.09E-10	CSCL	SCD
DYNC1LI2.	1.41E-14	0.10159	0.148	0.354	2.11E-10	CSCL	DYNC1LI2
PLEKHF2.2	1.42E-14	-0.18106	0.021	0.133	2.12E-10	CSCL	PLEKHF2
SELPLG.2	1.47E-14	-0.42394	0.017	0.127	2.19E-10	CSCL	SELPLG
SESTD1.1	1.51E-14	-0.12232	0.038	0.165	2.25E-10	CSCL	SESTD1
KDM2B.1	1.51E-14	-0.14367	0.024	0.14	2.26E-10	CSCL	KDM2B
MBTPS1	1.51E-14	-0.10181	0.047	0.18	2.26E-10	CSCL	MBTPS1
LMNB1.2	1.54E-14	-0.16697	0.033	0.155	2.30E-10	CSCL	LMNB1
TOPORS.2	1.55E-14	-0.1211	0.051	0.185	2.31E-10	CSCL	TOPORS
ZNRF1.2	1.59E-14	-0.11064	0.014	0.121	2.37E-10	CSCL	ZNRF1
DCX.2	1.61E-14	-0.21977	0.068	0.211	2.41E-10	CSCL	DCX
NABP1.1	1.61E-14	-0.37458	0.019	0.129	2.41E-10	CSCL	NABP1
FAM118B.1	1.62E-14	-0.10598	0.031	0.152	2.42E-10	CSCL	FAM118B
SLC7A5.2	1.64E-14	-0.32215	0.028	0.144	2.45E-10	CSCL	SLC7A5
PBDC1	1.66E-14	0.116619	0.099	0.269	2.48E-10	CSCL	PBDC1
EHMT1.2	1.68E-14	-0.13554	0.031	0.152	2.51E-10	CSCL	EHMT1
RPS6KA3.2	1.73E-14	-0.15359	0.026	0.143	2.58E-10	CSCL	RPS6KA3
LRRC25.2	1.83E-14	-0.40702	0.002	0.097	2.73E-10	CSCL	LRRC25
UTP23.1	1.84E-14	-0.13028	0.033	0.155	2.74E-10	CSCL	UTP23
CCDC91.1	1.84E-14	-0.1262	0.063	0.204	2.75E-10	CSCL	CCDC91
ORAI1.2	1.87E-14	-0.11773	0.019	0.13	2.79E-10	CSCL	ORAI1
TXNRD2.2	1.88E-14	-0.18603	0.016	0.123	2.81E-10	CSCL	TXNRD2
C8orf76.1	1.88E-14	-0.12335	0.038	0.164	2.81E-10	CSCL	C8orf76
ICAM1.1	1.92E-14	-0.18016	0.042	0.17	2.88E-10	CSCL	ICAM1
UGCG.1	1.93E-14	-0.11951	0.038	0.164	2.88E-10	CSCL	UGCG
TAF13	1.94E-14	-0.11304	0.026	0.143	2.90E-10	CSCL	TAF13
C7orf26.2	1.97E-14	-0.13867	0.038	0.164	2.94E-10	CSCL	C7orf26
FAM91A1.2	2.01E-14	-0.18646	0.019	0.129	3.00E-10	CSCL	FAM91A1

CCRL2.2	2.03E-14	-0.42198	0	0.093	3.04E-10	CSCL	CCRL2
SLC2A8.1	2.04E-14	-0.17516	0.028	0.145	3.05E-10	CSCL	SLC2A8
DENND6B.2	2.10E-14	-0.28196	0.007	0.107	3.14E-10	CSCL	DENND6B
NMRK1.1	2.11E-14	-0.22214	0.028	0.145	3.16E-10	CSCL	NMRK1
TRIM8.2	2.12E-14	-0.2655	0.005	0.103	3.16E-10	CSCL	TRIM8
NUP54.1	2.12E-14	-0.12837	0.051	0.184	3.17E-10	CSCL	NUP54
NAA35	2.13E-14	-0.13934	0.026	0.142	3.18E-10	CSCL	NAA35
ARID1B.2	2.14E-14	-0.16987	0.021	0.133	3.20E-10	CSCL	ARID1B
PHKG2.1	2.25E-14	-0.13114	0.037	0.161	3.36E-10	CSCL	PHKG2
PALD1.2	2.27E-14	-0.34798	0.007	0.107	3.39E-10	CSCL	PALD1
RAB11A	2.27E-14	0.147699	0.218	0.479	3.39E-10	CSCL	RAB11A
TUBB2B.1	2.33E-14	0.392196	0.553	0.555	3.49E-10	CSCL	TUBB2B
DBF4.1	2.34E-14	-0.22746	0.017	0.126	3.50E-10	CSCL	DBF4
PHTF1.1	2.36E-14	-0.18057	0.017	0.126	3.53E-10	CSCL	PHTF1
RANBP9.1	2.37E-14	-0.12912	0.035	0.157	3.55E-10	CSCL	RANBP9
TK2.2	2.38E-14	-0.14274	0.019	0.129	3.56E-10	CSCL	TK2
ZNF267.2	2.39E-14	-0.25534	0.024	0.138	3.58E-10	CSCL	ZNF267
PRKAG2.2	2.47E-14	-0.29207	0.028	0.143	3.69E-10	CSCL	PRKAG2
CYB561A3.	2.59E-14	-0.19441	0.035	0.156	3.88E-10	CSCL	CYB561A3
DPYSL4.2	2.64E-14	-0.13056	0.037	0.16	3.95E-10	CSCL	DPYSL4
FECH	2.65E-14	-0.13504	0.017	0.126	3.96E-10	CSCL	FECH
CRNKL1.1	2.69E-14	-0.10063	0.035	0.157	4.03E-10	CSCL	CRNKL1
SLC11A2.1	2.73E-14	-0.12468	0.045	0.175	4.08E-10	CSCL	SLC11A2
PTEN.2	2.75E-14	-0.18439	0.023	0.135	4.10E-10	CSCL	PTEN
KIAA0101.	2.78E-14	-0.27239	0.016	0.123	4.16E-10	CSCL	KIAA0101
DCP2.2	2.82E-14	-0.11725	0.035	0.156	4.21E-10	CSCL	DCP2
SLC48A1.1	2.91E-14	-0.19765	0.024	0.138	4.35E-10	CSCL	SLC48A1
TMEM14A.1	2.91E-14	0.104087	0.138	0.333	4.35E-10	CSCL	TMEM14A
MAPKAPK2.	2.96E-14	-0.11068	0.024	0.138	4.42E-10	CSCL	MAPKAPK2
CDKN2AIP.	2.99E-14	-0.15479	0.033	0.153	4.47E-10	CSCL	CDKN2AIP
MOSPD1	2.99E-14	-0.13027	0.023	0.135	4.47E-10	CSCL	MOSPD1
ATG4C.2	3.07E-14	-0.15615	0.028	0.144	4.59E-10	CSCL	ATG4C
ICA1.1	3.09E-14	-0.26451	0.014	0.118	4.61E-10	CSCL	ICA1
ATPAF1.2	3.12E-14	0.134268	0.087	0.248	4.67E-10	CSCL	ATPAF1
SLC35E2B.	3.23E-14	-0.13781	0.031	0.15	4.83E-10	CSCL	SLC35E2B
NLRP3.2	3.24E-14	-0.40874	0	0.092	4.84E-10	CSCL	NLRP3
ARHGAP30.	3.24E-14	-0.36038	0.003	0.099	4.85E-10	CSCL	ARHGAP30
DNAJC9.1	3.28E-14	-0.13104	0.042	0.168	4.91E-10	CSCL	DNAJC9
NDUFV1.1	3.31E-14	0.119149	0.227	0.492	4.94E-10	CSCL	NDUFV1
APOD.1	3.38E-14	0.612872	0.255	0.156	5.05E-10	CSCL	APOD
SH3PXD2B.	3.39E-14	-0.11819	0.016	0.122	5.07E-10	CSCL	SH3PXD2B
RRM1.1	3.41E-14	-0.10507	0.056	0.192	5.10E-10	CSCL	RRM1
C18orf8.1	3.48E-14	-0.15188	0.014	0.119	5.20E-10	CSCL	C18orf8
SYNE2.2	3.52E-14	-0.12246	0.049	0.18	5.26E-10	CSCL	SYNE2
MCAT.1	3.58E-14	-0.16957	0.023	0.134	5.36E-10	CSCL	MCAT
ATP6V0B.2	3.61E-14	-0.10946	0.421	0.817	5.39E-10	CSCL	ATP6V0B
FAM107B.2	3.64E-14	-0.25568	0.019	0.127	5.44E-10	CSCL	FAM107B
RASSF1.1	3.68E-14	-0.11341	0.04	0.165	5.50E-10	CSCL	RASSF1
SIK1.2	3.69E-14	-0.32934	0.007	0.105	5.51E-10	CSCL	SIK1
C12orf10	3.69E-14	0.142825	0.134	0.327	5.52E-10	CSCL	C12orf10
G0S2.1	3.71E-14	-0.27227	0.058	0.191	5.55E-10	CSCL	G0S2

CBFA2T2.2	3.72E-14	-0.1754	0.023	0.134	5.57E-10	CSCL	CBFA2T2
ZDHHC7.2	3.75E-14	-0.14867	0.033	0.153	5.60E-10	CSCL	ZDHHC7
KAT8.1	3.76E-14	-0.14593	0.051	0.183	5.61E-10	CSCL	KAT8
SNHG12.2	3.76E-14	-0.28274	0.026	0.14	5.62E-10	CSCL	SNHG12
ZDHHC9.2	3.77E-14	-0.10318	0.045	0.174	5.64E-10	CSCL	ZDHHC9
RCE1.1	3.79E-14	-0.16977	0.023	0.134	5.66E-10	CSCL	RCE1
OPTN	3.83E-14	-0.10465	0.026	0.141	5.72E-10	CSCL	OPTN
DCAF15.1	3.88E-14	-0.15875	0.012	0.115	5.79E-10	CSCL	DCAF15
ABCG1.2	3.90E-14	-0.30208	0.024	0.137	5.83E-10	CSCL	ABCG1
APBB1.1	3.92E-14	-0.13778	0.059	0.196	5.86E-10	CSCL	APBB1
OTUD5.2	3.93E-14	-0.16422	0.026	0.14	5.87E-10	CSCL	OTUD5
ANXA2.1	4.07E-14	-0.12008	0.265	0.526	6.08E-10	CSCL	ANXA2
PRDM1.2	4.07E-14	-0.38166	0.007	0.105	6.08E-10	CSCL	PRDM1
RPS6KB1.1	4.14E-14	-0.11907	0.023	0.134	6.18E-10	CSCL	RPS6KB1
DDX49.1	4.16E-14	0.1033	0.108	0.283	6.22E-10	CSCL	DDX49
KLF7.2	4.20E-14	-0.11298	0.07	0.213	6.27E-10	CSCL	KLF7
CD200.2	4.20E-14	-0.20792	0.012	0.115	6.27E-10	CSCL	CD200
ERGIC2	4.28E-14	0.116053	0.155	0.362	6.40E-10	CSCL	ERGIC2
HMOX2.1	4.33E-14	0.113462	0.122	0.304	6.47E-10	CSCL	HMOX2
ATP13A2.2	4.34E-14	-0.12227	0.03	0.146	6.48E-10	CSCL	ATP13A2
SIGLEC9.2	4.54E-14	-0.40552	0.003	0.097	6.79E-10	CSCL	SIGLEC9
ZCRB1.2	4.55E-14	0.167036	0.234	0.504	6.80E-10	CSCL	ZCRB1
HSDL1.1	4.57E-14	-0.16313	0.024	0.136	6.83E-10	CSCL	HSDL1
ABCF3	4.60E-14	-0.1337	0.031	0.149	6.87E-10	CSCL	ABCF3
SH2B1.1	4.62E-14	-0.1253	0.035	0.155	6.90E-10	CSCL	SH2B1
BARD1.1	4.65E-14	-0.11485	0.033	0.152	6.95E-10	CSCL	BARD1
AGFG1.1	4.75E-14	-0.12493	0.024	0.137	7.10E-10	CSCL	AGFG1
CNIH4.1	4.77E-14	0.12087	0.168	0.384	7.13E-10	CSCL	CNIH4
LPGAT1.2	4.87E-14	-0.11809	0.023	0.133	7.28E-10	CSCL	LPGAT1
CTDSPL2.1	5.01E-14	-0.17067	0.012	0.114	7.48E-10	CSCL	CTDSPL2
ATXN1.1	5.05E-14	-0.12525	0.044	0.169	7.55E-10	CSCL	ATXN1
CDC42BPB.	5.09E-14	-0.13406	0.017	0.124	7.61E-10	CSCL	CDC42BPB
PQLC1.2	5.10E-14	-0.12266	0.026	0.14	7.63E-10	CSCL	PQLC1
CCZ1B.1	5.14E-14	-0.16194	0.037	0.157	7.69E-10	CSCL	CCZ1B
NSUN2.1	5.15E-14	-0.14598	0.026	0.14	7.69E-10	CSCL	NSUN2
MSH3	5.17E-14	-0.18848	0.01	0.111	7.72E-10	CSCL	MSH3
CUL4A.1	5.46E-14	-0.12226	0.033	0.151	8.16E-10	CSCL	CUL4A
SOX12.1	5.52E-14	-0.12624	0.016	0.121	8.26E-10	CSCL	SOX12
ELMSAN1.1	5.66E-14	-0.18116	0.026	0.139	8.46E-10	CSCL	ELMSAN1
TP53I3.1	5.73E-14	-0.16078	0.033	0.151	8.56E-10	CSCL	TP53I3
NUP88.1	5.83E-14	-0.13381	0.023	0.133	8.72E-10	CSCL	NUP88
RANGAP1.2	5.84E-14	-0.19826	0.009	0.107	8.73E-10	CSCL	RANGAP1
HPGDS.2	5.87E-14	-0.44577	0.002	0.093	8.77E-10	CSCL	HPGDS
HYOU1.1	5.93E-14	-0.12316	0.03	0.145	8.86E-10	CSCL	HYOU1
BTK.2	6.03E-14	-0.37102	0	0.09	9.01E-10	CSCL	BTK
RND3.1	6.21E-14	-0.18914	0.096	0.25	9.28E-10	CSCL	RND3
FAM32A.1	6.21E-14	0.145593	0.213	0.464	9.28E-10	CSCL	FAM32A
USP36.2	6.31E-14	-0.17104	0.03	0.144	9.43E-10	CSCL	USP36
NAV1.2	6.38E-14	-0.1117	0.044	0.169	9.53E-10	CSCL	NAV1
ANKRD37	6.40E-14	-0.2013	0.031	0.147	9.56E-10	CSCL	ANKRD37
ZFC3H1.1	6.70E-14	-0.13384	0.021	0.129	1.00E-09	CSCL	ZFC3H1

QSER1.2	6.71E-14	-0.12056	0.017	0.123	1.00E-09	CSCL	QSER1
WIPF3.2	6.72E-14	-0.23761	0.003	0.097	1.00E-09	CSCL	WIPF3
TBC1D14.2	6.82E-14	-0.10421	0.049	0.177	1.02E-09	CSCL	TBC1D14
OSBPL2.1	6.88E-14	-0.10513	0.026	0.139	1.03E-09	CSCL	OSBPL2
ZDHHC16.1	6.93E-14	-0.12516	0.01	0.11	1.03E-09	CSCL	ZDHHC16
SNRPN.2	7.07E-14	0.659855	0.18	0.094	1.06E-09	CSCL	SNRPN
SMG1.2	7.21E-14	-0.1342	0.044	0.168	1.08E-09	CSCL	SMG1
CDYL.2	7.22E-14	-0.12498	0.016	0.12	1.08E-09	CSCL	CDYL
DUSP22.2	7.36E-14	-0.24265	0.014	0.116	1.10E-09	CSCL	DUSP22
MAML1.2	7.38E-14	-0.25567	0.017	0.122	1.10E-09	CSCL	MAML1
NCAPH2.2	7.52E-14	-0.13684	0.024	0.135	1.12E-09	CSCL	NCAPH2
THAP2	7.62E-14	-0.2048	0.023	0.132	1.14E-09	CSCL	THAP2
CYB5B.2	7.93E-14	0.154336	0.148	0.347	1.18E-09	CSCL	CYB5B
TBK1.1	8.05E-14	-0.13119	0.037	0.156	1.20E-09	CSCL	TBK1
GNPAT.1	8.05E-14	0.107202	0.089	0.247	1.20E-09	CSCL	GNPAT
ATP5SL.1	8.09E-14	0.123439	0.084	0.238	1.21E-09	CSCL	ATP5SL
LILRB1.2	8.33E-14	-0.37015	0.003	0.096	1.24E-09	CSCL	LILRB1
ZBTB43.1	8.38E-14	-0.14123	0.037	0.156	1.25E-09	CSCL	ZBTB43
NDUFA10.1	8.38E-14	0.121558	0.206	0.45	1.25E-09	CSCL	NDUFA10
ARAP1.2	8.46E-14	-0.26901	0.012	0.113	1.26E-09	CSCL	ARAP1
CDKN3.1	8.58E-14	-0.2763	0.003	0.096	1.28E-09	CSCL	CDKN3
NUSAP1.2	8.60E-14	-0.34232	0.01	0.109	1.29E-09	CSCL	NUSAP1
VEGFB.1	8.62E-14	-0.11894	0.033	0.15	1.29E-09	CSCL	VEGFB
FAM210A.1	9.23E-14	-0.13528	0.031	0.147	1.38E-09	CSCL	FAM210A
STRBP.2	9.43E-14	-0.10993	0.023	0.132	1.41E-09	CSCL	STRBP
CCDC57.2	9.48E-14	-0.2563	0.012	0.112	1.42E-09	CSCL	CCDC57
DOCK10.2	9.85E-14	-0.19763	0.023	0.131	1.47E-09	CSCL	DOCK10
POP5.1	9.96E-14	0.136862	0.124	0.305	1.49E-09	CSCL	POP5
HLA-DQB2.	9.97E-14	-0.56479	0.007	0.102	1.49E-09	CSCL	HLA-DQB2
CHN2.2	9.97E-14	-0.30877	0.007	0.102	1.49E-09	CSCL	CHN2
PSMG3.1	1.01E-13	0.120333	0.143	0.336	1.51E-09	CSCL	PSMG3
ABCB8.1	1.01E-13	-0.11278	0.031	0.147	1.52E-09	CSCL	ABCB8
CEP78.2	1.02E-13	-0.10928	0.016	0.119	1.53E-09	CSCL	CEP78
PCYT1A	1.03E-13	-0.10315	0.024	0.135	1.54E-09	CSCL	PCYT1A
ADAM10.2	1.05E-13	-0.11433	0.058	0.19	1.57E-09	CSCL	ADAM10
S100A6.2	1.06E-13	0.504829	0.574	0.647	1.59E-09	CSCL	S100A6
RNF135.2	1.08E-13	-0.20231	0.014	0.116	1.62E-09	CSCL	RNF135
TBC1D1.2	1.11E-13	-0.16575	0.021	0.128	1.66E-09	CSCL	TBC1D1
UQCC2	1.14E-13	0.140132	0.201	0.438	1.70E-09	CSCL	UQCC2
C11orf73.	1.15E-13	0.133116	0.195	0.432	1.72E-09	CSCL	C11orf73
CCDC25.2	1.16E-13	0.173514	0.124	0.306	1.73E-09	CSCL	CCDC25
RNF2.1	1.17E-13	-0.11265	0.035	0.152	1.75E-09	CSCL	RNF2
SENP5.1	1.18E-13	-0.11416	0.03	0.143	1.76E-09	CSCL	SENP5
TMEM119.2	1.20E-13	-0.44931	0.012	0.11	1.79E-09	CSCL	TMEM119
NDUFA8	1.27E-13	0.152898	0.234	0.504	1.89E-09	CSCL	NDUFA8
DGUOK.1	1.27E-13	0.108443	0.25	0.529	1.90E-09	CSCL	DGUOK
CTD-3184A	1.28E-13	-0.15645	0.007	0.102	1.91E-09	CSCL	CTD-3184A7.4
IL10.2	1.30E-13	-0.44269	0.012	0.111	1.95E-09	CSCL	IL10
PMPCA.1	1.31E-13	0.102768	0.094	0.254	1.96E-09	CSCL	PMPCA
POMT1.1	1.34E-13	-0.11128	0.03	0.143	2.01E-09	CSCL	POMT1
PTDSS2.2	1.37E-13	-0.10827	0.038	0.158	2.04E-09	CSCL	PTDSS2

PXK.1	1.38E-13	-0.15046	0.031	0.146	2.07E-09	CSCL	PXK
NONO.2	1.42E-13	0.167505	0.215	0.467	2.12E-09	CSCL	NONO
PPP3R1.2	1.42E-13	-0.1361	0.016	0.118	2.13E-09	CSCL	PPP3R1
KLHL23.2	1.45E-13	-0.12322	0.044	0.167	2.16E-09	CSCL	KLHL23
MFSD5.1	1.48E-13	-0.13536	0.016	0.118	2.21E-09	CSCL	MFSD5
SIRT2.2	1.49E-13	0.122118	0.197	0.427	2.23E-09	CSCL	SIRT2
ANAPC13.2	1.50E-13	0.164444	0.159	0.364	2.24E-09	CSCL	ANAPC13
FBXW7.1	1.52E-13	-0.17461	0.024	0.133	2.27E-09	CSCL	FBXW7
FAM111A.2	1.52E-13	-0.21176	0.014	0.114	2.27E-09	CSCL	FAM111A
LGALS8.1	1.58E-13	-0.1073	0.038	0.157	2.35E-09	CSCL	LGALS8
RILP.2	1.59E-13	-0.20866	0.01	0.108	2.37E-09	CSCL	RILP
BRI3BP.2	1.60E-13	-0.13911	0.016	0.118	2.40E-09	CSCL	BRI3BP
CREB3L2.2	1.62E-13	-0.26744	0.023	0.129	2.42E-09	CSCL	CREB3L2
NCOA7.1	1.66E-13	-0.24267	0.017	0.12	2.49E-09	CSCL	NCOA7
MARK2.2	1.67E-13	-0.13148	0.023	0.13	2.49E-09	CSCL	MARK2
TOMM70A.2	1.67E-13	-0.1189	0.023	0.13	2.50E-09	CSCL	TOMM70A
RC3H2.1	1.70E-13	-0.197	0.007	0.101	2.55E-09	CSCL	RC3H2
PTAR1.2	1.71E-13	-0.10148	0.03	0.142	2.55E-09	CSCL	PTAR1
TCAIM.1	1.73E-13	-0.10957	0.03	0.142	2.59E-09	CSCL	TCAIM
PRPS2.1	1.74E-13	-0.11897	0.023	0.13	2.60E-09	CSCL	PRPS2
NAGPA.1	1.74E-13	-0.19214	0.014	0.114	2.61E-09	CSCL	NAGPA
SMAD4.1	1.75E-13	-0.10859	0.021	0.127	2.61E-09	CSCL	SMAD4
CSTF1.1	1.75E-13	-0.1117	0.026	0.136	2.62E-09	CSCL	CSTF1
CNTRL.2	1.79E-13	-0.16425	0.019	0.123	2.67E-09	CSCL	CNTRL
NBPF10.1	1.81E-13	-0.1444	0.035	0.151	2.70E-09	CSCL	NBPF10
CEP104.1	1.86E-13	-0.1286	0.021	0.127	2.78E-09	CSCL	CEP104
ZWINT.2	1.87E-13	-0.28287	0.005	0.097	2.79E-09	CSCL	ZWINT
ARHGAP9.2	1.88E-13	-0.34922	0.002	0.09	2.81E-09	CSCL	ARHGAP9
KRBOX1.1	1.90E-13	0.246798	0.026	0.004	2.84E-09	CSCL	KRBOX1
TRAPPC10.	1.93E-13	-0.23862	0.005	0.097	2.89E-09	CSCL	TRAPPC10
SH2B3.2	2.01E-13	-0.25861	0.014	0.113	3.01E-09	CSCL	SH2B3
TTL.1	2.07E-13	-0.18838	0.019	0.123	3.09E-09	CSCL	TTL
RABL2B	2.07E-13	-0.13871	0.024	0.132	3.10E-09	CSCL	RABL2B
PBX3.2	2.22E-13	-0.13697	0.019	0.123	3.31E-09	CSCL	PBX3
PAXBP1.1	2.22E-13	-0.10873	0.044	0.165	3.32E-09	CSCL	PAXBP1
LGR4.2	2.26E-13	-0.12747	0.01	0.107	3.38E-09	CSCL	LGR4
SLC30A1.1	2.27E-13	-0.1838	0.003	0.093	3.39E-09	CSCL	SLC30A1
SLC25A24.	2.28E-13	-0.21645	0.009	0.103	3.41E-09	CSCL	SLC25A24
TSPAN5.2	2.31E-13	0.101335	0.105	0.27	3.45E-09	CSCL	TSPAN5
PIAS1.2	2.32E-13	-0.15944	0.044	0.164	3.47E-09	CSCL	PIAS1
CYTL1.2	2.35E-13	-0.4319	0.016	0.115	3.51E-09	CSCL	CYTL1
SLC22A18.	2.44E-13	-0.12487	0.023	0.129	3.65E-09	CSCL	SLC22A18
KXD1.2	2.44E-13	0.112835	0.215	0.461	3.65E-09	CSCL	KXD1
HPS5.2	2.52E-13	-0.16201	0.017	0.119	3.76E-09	CSCL	HPS5
CENPU.2	2.52E-13	-0.21256	0.012	0.11	3.76E-09	CSCL	CENPU
SEPT10.1	2.54E-13	-0.13844	0.019	0.122	3.79E-09	CSCL	10-Sep
PAG1.2	2.56E-13	-0.18267	0.051	0.175	3.83E-09	CSCL	PAG1
CYTIP.2	2.62E-13	-0.42143	0.012	0.109	3.91E-09	CSCL	CYTIP
KCNMB4.2	2.62E-13	-0.17748	0.023	0.128	3.92E-09	CSCL	KCNMB4
CERS6.2	2.65E-13	-0.10179	0.024	0.132	3.96E-09	CSCL	CERS6
PTBP3.2	2.70E-13	-0.18998	0.016	0.116	4.03E-09	CSCL	PTBP3

PSMA4	2.73E-13	0.150929	0.26	0.552	4.07E-09	CSCL	PSMA4
EXOC3	2.84E-13	-0.10472	0.026	0.135	4.24E-09	CSCL	EXOC3
RAB3GAP1.	2.86E-13	-0.16694	0.031	0.143	4.27E-09	CSCL	RAB3GAP1
GLS.2	2.89E-13	-0.1754	0.026	0.134	4.32E-09	CSCL	GLS
CEP57L1.1	2.93E-13	-0.19753	0.016	0.116	4.37E-09	CSCL	CEP57L1
SLC35F2.1	2.95E-13	-0.16383	0.014	0.112	4.41E-09	CSCL	SLC35F2
NUP50.2	2.96E-13	-0.10834	0.019	0.122	4.42E-09	CSCL	NUP50
CDK9.1	2.96E-13	-0.10776	0.033	0.146	4.43E-09	CSCL	CDK9
INPP5K	2.99E-13	-0.13161	0.021	0.125	4.46E-09	CSCL	INPP5K
SNRPD1	3.00E-13	0.107327	0.277	0.578	4.48E-09	CSCL	SNRPD1
GPT2	3.02E-13	-0.12241	0.037	0.153	4.52E-09	CSCL	GPT2
COA4	3.05E-13	0.167493	0.178	0.397	4.56E-09	CSCL	COA4
S100A16.1	3.08E-13	0.733624	0.363	0.304	4.60E-09	CSCL	S100A16
CDK19.2	3.13E-13	-0.18082	0.024	0.131	4.67E-09	CSCL	CDK19
PLBD1.2	3.30E-13	-0.30802	0.007	0.099	4.93E-09	CSCL	PLBD1
MINK1.2	3.36E-13	-0.1723	0.012	0.109	5.02E-09	CSCL	MINK1
NINJ2.2	3.42E-13	-0.42826	0.003	0.092	5.12E-09	CSCL	NINJ2
ITGAX.2	3.45E-13	-0.38485	0.005	0.095	5.16E-09	CSCL	ITGAX
RP11-1094	3.49E-13	0.123861	0.089	0.242	5.21E-09	CSCL	RP11-1094M14.11
SEPT11.2	3.57E-13	0.173	0.129	0.312	5.33E-09	CSCL	11-Sep
SRM.2	3.59E-13	0.170037	0.166	0.375	5.37E-09	CSCL	SRM
CHCHD5.1	3.60E-13	0.138383	0.171	0.383	5.38E-09	CSCL	CHCHD5
FAM103A1.	3.64E-13	0.174097	0.094	0.25	5.45E-09	CSCL	FAM103A1
GAD1.2	3.65E-13	-0.18075	0.017	0.118	5.46E-09	CSCL	GAD1
POM121C.1	3.68E-13	-0.11522	0.033	0.146	5.51E-09	CSCL	POM121C
KYNU.2	3.70E-13	-0.37416	0.002	0.088	5.53E-09	CSCL	KYNU
FASN.2	3.71E-13	-0.12159	0.019	0.121	5.54E-09	CSCL	FASN
H2AFY2.2	3.87E-13	-0.12701	0.012	0.109	5.79E-09	CSCL	H2AFY2
ANKRD9.2	3.88E-13	-0.17698	0.003	0.092	5.79E-09	CSCL	ANKRD9
GCSH.2	3.91E-13	0.211959	0.154	0.357	5.85E-09	CSCL	GCSH
THOC1.1	3.92E-13	-0.10223	0.037	0.152	5.86E-09	CSCL	THOC1
AGPS.2	4.04E-13	-0.10475	0.023	0.127	6.04E-09	CSCL	AGPS
PLAA.1	4.10E-13	-0.21256	0.007	0.098	6.12E-09	CSCL	PLAA
POLRMT.1	4.12E-13	-0.10044	0.028	0.136	6.16E-09	CSCL	POLRMT
NUTM2A-AS	4.14E-13	-0.15462	0.026	0.133	6.19E-09	CSCL	NUTM2A-AS1
PMAIP1.2	4.16E-13	-0.45309	0.002	0.088	6.22E-09	CSCL	PMAIP1
CYSTM1.2	4.46E-13	0.116275	0.145	0.334	6.66E-09	CSCL	CYSTM1
TMEM39B	4.47E-13	-0.16201	0.026	0.133	6.67E-09	CSCL	TMEM39B
ADAM15.1	4.51E-13	-0.15615	0.019	0.121	6.74E-09	CSCL	ADAM15
FXN.1	4.53E-13	-0.15528	0.017	0.117	6.78E-09	CSCL	FXN
ZNF821.2	4.54E-13	-0.11064	0.047	0.169	6.79E-09	CSCL	ZNF821
MYLIP.2	4.68E-13	-0.31774	0.028	0.134	7.00E-09	CSCL	MYLIP
RFTN1.1	4.78E-13	-0.12797	0.016	0.114	7.15E-09	CSCL	RFTN1
KLHL9.1	4.94E-13	-0.20156	0.016	0.114	7.38E-09	CSCL	KLHL9
LINC00869	4.97E-13	0.371356	0.054	0.015	7.43E-09	CSCL	LINC00869
PXN.1	5.01E-13	-0.15238	0.024	0.129	7.48E-09	CSCL	PXN
HYI.1	5.03E-13	-0.14084	0.033	0.144	7.51E-09	CSCL	HYI
WRNIP1	5.07E-13	-0.13487	0.012	0.108	7.58E-09	CSCL	WRNIP1
CSTF2T	5.12E-13	-0.16636	0.023	0.126	7.65E-09	CSCL	CSTF2T
DCLRE1C.1	5.15E-13	-0.13528	0.028	0.135	7.70E-09	CSCL	DCLRE1C
MED1.2	5.18E-13	-0.10008	0.021	0.123	7.73E-09	CSCL	MED1

DUSP15.1	5.24E-13	-0.13669	0.007	0.098	7.83E-09	CSCL	DUSP15
AP1G1.1	5.25E-13	-0.13906	0.024	0.129	7.85E-09	CSCL	AP1G1
UNC50.1	5.32E-13	0.124143	0.162	0.364	7.95E-09	CSCL	UNC50
DEDD	5.33E-13	-0.14999	0.021	0.123	7.96E-09	CSCL	DEDD
AGPAT3.2	5.47E-13	-0.11737	0.028	0.135	8.17E-09	CSCL	AGPAT3
MYO5A.2	5.59E-13	-0.16007	0.017	0.117	8.35E-09	CSCL	MYO5A
EFNB2.1	5.62E-13	-0.11942	0.01	0.104	8.40E-09	CSCL	EFNB2
PSMA3.2	5.63E-13	0.115233	0.213	0.452	8.41E-09	CSCL	PSMA3
ERCC1.2	5.64E-13	0.136004	0.232	0.484	8.42E-09	CSCL	ERCC1
RALGAPA1.	5.74E-13	-0.12381	0.028	0.135	8.57E-09	CSCL	RALGAPA1
CNIH1.1	5.74E-13	0.100697	0.175	0.383	8.58E-09	CSCL	CNIH1
DAGLB.2	5.91E-13	-0.26504	0.023	0.125	8.83E-09	CSCL	DAGLB
UBTD1.1	5.91E-13	-0.12874	0.017	0.117	8.83E-09	CSCL	UBTD1
OTUD4.2	5.93E-13	-0.15946	0.01	0.104	8.86E-09	CSCL	OTUD4
HNRNPH2.1	6.03E-13	0.10137	0.19	0.411	9.02E-09	CSCL	HNRNPH2
ESYT1.2	6.07E-13	-0.19091	0.023	0.125	9.07E-09	CSCL	ESYT1
PHACTR2.2	6.16E-13	-0.19261	0.002	0.087	9.20E-09	CSCL	PHACTR2
NDUFB7.2	6.22E-13	0.448182	0.581	0.727	9.30E-09	CSCL	NDUFB7
CDC37L1.1	6.32E-13	-0.11294	0.033	0.144	9.45E-09	CSCL	CDC37L1
MILR1.2	6.33E-13	-0.36277	0.01	0.103	9.46E-09	CSCL	MILR1
TMEM209.1	6.36E-13	-0.13493	0.021	0.123	9.50E-09	CSCL	TMEM209
EAF2.2	6.48E-13	-0.29863	0.007	0.097	9.68E-09	CSCL	EAF2
PARN.1	6.51E-13	-0.17462	0.023	0.125	9.74E-09	CSCL	PARN
DTNB.2	6.54E-13	-0.21409	0.009	0.1	9.77E-09	CSCL	DTNB
APPBP2.1	6.54E-13	0.110072	0.075	0.216	9.77E-09	CSCL	APPBP2
ZMIZ1.2	6.60E-13	-0.10179	0.033	0.144	9.86E-09	CSCL	ZMIZ1
MAPK8.2	6.74E-13	-0.17593	0.01	0.103	1.01E-08	CSCL	MAPK8
CENPK.1	6.75E-13	-0.19063	0.01	0.104	1.01E-08	CSCL	CENPK
PTK2B.1	6.85E-13	-0.10383	0.026	0.132	1.02E-08	CSCL	PTK2B
APOBEC3C.	6.90E-13	-0.23997	0.028	0.133	1.03E-08	CSCL	APOBEC3C
CDKN1C.2	7.07E-13	-0.26451	0.019	0.119	1.06E-08	CSCL	CDKN1C
OTUD1.2	7.22E-13	-0.33775	0.01	0.103	1.08E-08	CSCL	OTUD1
EMG1.1	7.59E-13	0.117382	0.091	0.242	1.13E-08	CSCL	EMG1
DHCR7.1	7.72E-13	0.126466	0.056	0.184	1.15E-08	CSCL	DHCR7
CENPC	7.79E-13	-0.2026	0.016	0.113	1.16E-08	CSCL	CENPC
HDHD2.1	7.83E-13	0.134664	0.105	0.265	1.17E-08	CSCL	HDHD2
HPS1.1	7.91E-13	-0.28289	0.017	0.115	1.18E-08	CSCL	HPS1
KIAA1109.	7.92E-13	-0.11884	0.023	0.125	1.18E-08	CSCL	KIAA1109
CPT2.1	8.07E-13	-0.17223	0.017	0.116	1.21E-08	CSCL	CPT2
BCL7A.2	8.36E-13	-0.11314	0.04	0.155	1.25E-08	CSCL	BCL7A
NADSYN1.2	8.39E-13	-0.11201	0.023	0.125	1.25E-08	CSCL	NADSYN1
PIK3C3	8.43E-13	-0.13339	0.023	0.125	1.26E-08	CSCL	PIK3C3
WRAP73.1	8.46E-13	-0.11742	0.038	0.152	1.26E-08	CSCL	WRAP73
TUBGCP4.1	8.58E-13	-0.12725	0.024	0.128	1.28E-08	CSCL	TUBGCP4
TLE3.1	8.60E-13	-0.19938	0.033	0.142	1.28E-08	CSCL	TLE3
CCR1.2	8.62E-13	-0.33646	0.002	0.086	1.29E-08	CSCL	CCR1
COX17	8.73E-13	0.147647	0.255	0.531	1.30E-08	CSCL	COX17
ODF2.1	8.73E-13	-0.11474	0.03	0.137	1.30E-08	CSCL	ODF2
GLIPR2.1	8.94E-13	0.119994	0.113	0.277	1.34E-08	CSCL	GLIPR2
GUCD1.2	9.03E-13	-0.11835	0.024	0.128	1.35E-08	CSCL	GUCD1
IMMP1L.1	9.03E-13	0.106592	0.089	0.239	1.35E-08	CSCL	IMMP1L

CD48.2	9.32E-13	-0.39263	0	0.082	1.39E-08	CSCL	CD48
SMARCA2.1	9.35E-13	-0.18648	0.031	0.14	1.40E-08	CSCL	SMARCA2
EMC1.1	9.45E-13	-0.11548	0.03	0.137	1.41E-08	CSCL	EMC1
DENND1B.2	9.49E-13	-0.20181	0.007	0.096	1.42E-08	CSCL	DENND1B
EPN2.2	9.57E-13	0.173439	0.092	0.244	1.43E-08	CSCL	EPN2
SLC25A32.	9.61E-13	-0.1971	0.012	0.105	1.44E-08	CSCL	SLC25A32
KNOP1.1	9.79E-13	0.134804	0.106	0.267	1.46E-08	CSCL	KNOP1
APOO.1	9.87E-13	0.176319	0.087	0.236	1.48E-08	CSCL	APOO
MEAF6.2	9.92E-13	0.140674	0.234	0.489	1.48E-08	CSCL	MEAF6
KIFC3	1.04E-12	-0.18301	0.014	0.109	1.56E-08	CSCL	KIFC3
SPG11.2	1.06E-12	-0.14363	0.021	0.121	1.58E-08	CSCL	SPG11
RALGAPB.1	1.10E-12	-0.10442	0.017	0.115	1.64E-08	CSCL	RALGAPB
MFNG.2	1.13E-12	-0.294	0.028	0.132	1.68E-08	CSCL	MFNG
NSMAF.1	1.13E-12	-0.11125	0.021	0.121	1.69E-08	CSCL	NSMAF
MAPK14.2	1.13E-12	-0.15097	0.017	0.114	1.69E-08	CSCL	MAPK14
RPUSD1.2	1.15E-12	-0.10582	0.031	0.139	1.71E-08	CSCL	RPUSD1
WBP1L.2	1.18E-12	-0.23541	0.017	0.114	1.76E-08	CSCL	WBP1L
MIOS.2	1.19E-12	-0.11363	0.024	0.127	1.77E-08	CSCL	MIOS
SNX18.2	1.20E-12	-0.13325	0.019	0.118	1.79E-08	CSCL	SNX18
NOM1.1	1.20E-12	-0.1497	0.021	0.121	1.79E-08	CSCL	NOM1
NAF1.2	1.20E-12	-0.21213	0.017	0.114	1.80E-08	CSCL	NAF1
IFFO1.2	1.21E-12	-0.21124	0.023	0.123	1.81E-08	CSCL	IFFO1
UBE2V2.1	1.23E-12	0.116433	0.244	0.508	1.84E-08	CSCL	UBE2V2
DHX16	1.25E-12	-0.16321	0.016	0.111	1.87E-08	CSCL	DHX16
GNB5.1	1.26E-12	-0.12186	0.017	0.115	1.88E-08	CSCL	GNB5
ATG16L2.2	1.26E-12	-0.27705	0.005	0.091	1.89E-08	CSCL	ATG16L2
SLC25A44.	1.27E-12	-0.1781	0.009	0.098	1.90E-08	CSCL	SLC25A44
ELK1	1.28E-12	-0.13563	0.012	0.105	1.91E-08	CSCL	ELK1
VIM.1	1.28E-12	0.424132	0.588	0.667	1.92E-08	CSCL	VIM
YTHDC2.2	1.31E-12	-0.10436	0.023	0.123	1.96E-08	CSCL	YTHDC2
SGPL1.1	1.33E-12	-0.12867	0.03	0.136	1.99E-08	CSCL	SGPL1
DOCK2.2	1.34E-12	-0.34813	0	0.081	2.01E-08	CSCL	DOCK2
UBR4.2	1.35E-12	-0.12186	0.038	0.15	2.01E-08	CSCL	UBR4
CCDC93.1	1.36E-12	-0.12616	0.028	0.132	2.03E-08	CSCL	CCDC93
MITF.1	1.37E-12	-0.25961	0.009	0.098	2.05E-08	CSCL	MITF
SHMT1.2	1.37E-12	-0.20464	0.023	0.123	2.05E-08	CSCL	SHMT1
NABP2.1	1.39E-12	0.163682	0.117	0.284	2.08E-08	CSCL	NABP2
PPIP5K2.1	1.39E-12	-0.10502	0.028	0.132	2.08E-08	CSCL	PPIP5K2
KCNMB1.2	1.41E-12	-0.37833	0.014	0.107	2.10E-08	CSCL	KCNMB1
BCL10.1	1.42E-12	-0.12503	0.03	0.135	2.12E-08	CSCL	BCL10
KIAA1551.	1.42E-12	-0.24089	0.005	0.091	2.12E-08	CSCL	KIAA1551
COG1.1	1.42E-12	-0.12155	0.017	0.114	2.13E-08	CSCL	COG1
RABGAP1.1	1.46E-12	-0.12075	0.026	0.129	2.19E-08	CSCL	RABGAP1
SLC12A2.1	1.47E-12	-0.16928	0.03	0.135	2.19E-08	CSCL	SLC12A2
DSN1.1	1.49E-12	-0.19494	0.012	0.104	2.23E-08	CSCL	DSN1
MAD1L1.2	1.50E-12	-0.10866	0.045	0.162	2.24E-08	CSCL	MAD1L1
CHRAC1.2	1.52E-12	-0.11141	0.038	0.15	2.27E-08	CSCL	CHRAC1
IRF2BP1.1	1.52E-12	-0.15497	0.016	0.111	2.27E-08	CSCL	IRF2BP1
SLC39A13	1.54E-12	-0.12834	0.033	0.141	2.30E-08	CSCL	SLC39A13
CDK1.2	1.55E-12	-0.27247	0.01	0.101	2.31E-08	CSCL	CDK1
ESD.1	1.57E-12	0.181169	0.239	0.503	2.35E-08	CSCL	ESD

ACAD9.1	1.60E-12	-0.11954	0.021	0.12	2.39E-08	CSCL	ACAD9
SNRPA1.1	1.60E-12	0.116219	0.129	0.303	2.40E-08	CSCL	SNRPA1
EAF1.1	1.60E-12	-0.16068	0.026	0.129	2.40E-08	CSCL	EAF1
CCDC22.1	1.65E-12	-0.15097	0.028	0.132	2.47E-08	CSCL	CCDC22
AHNAK	1.66E-12	-0.13968	0.023	0.123	2.48E-08	CSCL	AHNAK
CEPT1.1	1.69E-12	-0.11575	0.045	0.161	2.53E-08	CSCL	CEPT1
PGS1.1	1.69E-12	-0.13255	0.033	0.141	2.53E-08	CSCL	PGS1
RYBP.2	1.70E-12	-0.14129	0.021	0.12	2.54E-08	CSCL	RYBP
PRKRIR.2	1.71E-12	-0.12638	0.017	0.114	2.56E-08	CSCL	PRKRIR
SH3TC1.2	1.74E-12	-0.3445	0.002	0.084	2.60E-08	CSCL	SH3TC1
MSH6.1	1.76E-12	-0.12527	0.028	0.132	2.63E-08	CSCL	MSH6
PCMTD1.1	1.77E-12	-0.14103	0.044	0.158	2.65E-08	CSCL	PCMTD1
HSF2.1	1.78E-12	-0.14963	0.023	0.122	2.67E-08	CSCL	HSF2
LARP4B.1	1.80E-12	-0.18214	0.007	0.094	2.69E-08	CSCL	LARP4B
CNOT6L.2	1.80E-12	-0.18699	0.017	0.113	2.69E-08	CSCL	CNOT6L
CHPF2.1	1.80E-12	-0.11921	0.044	0.159	2.70E-08	CSCL	CHPF2
MAFF.2	1.81E-12	-0.17231	0.056	0.177	2.71E-08	CSCL	MAFF
MXD1.1	1.83E-12	-0.24098	0.03	0.134	2.73E-08	CSCL	MXD1
PPP3CC.1	1.85E-12	-0.15092	0.031	0.138	2.77E-08	CSCL	PPP3CC
SCIMP.2	1.96E-12	-0.34563	0.003	0.087	2.93E-08	CSCL	SCIMP
STAMBP	1.98E-12	0.151565	0.112	0.274	2.96E-08	CSCL	STAMBP
DCTN6.1	2.02E-12	0.133749	0.216	0.455	3.01E-08	CSCL	DCTN6
ADA.2	2.02E-12	-0.11419	0.009	0.097	3.02E-08	CSCL	ADA
SEH1L.1	2.03E-12	-0.15031	0.024	0.125	3.03E-08	CSCL	SEH1L
MGMT.2	2.07E-12	-0.21817	0.044	0.157	3.10E-08	CSCL	MGMT
ST6GAL1.2	2.26E-12	-0.3102	0.019	0.115	3.38E-08	CSCL	ST6GAL1
LARP1B.1	2.28E-12	-0.11623	0.024	0.125	3.40E-08	CSCL	LARP1B
LINC0011€	2.29E-12	0.134199	0.171	0.374	3.42E-08	CSCL	LINC00116
ALAD.1	2.29E-12	-0.13694	0.017	0.112	3.43E-08	CSCL	ALAD
ZC3H6.1	2.30E-12	-0.11515	0.04	0.152	3.44E-08	CSCL	ZC3H6
ITGAM.2	2.33E-12	-0.34193	0	0.08	3.48E-08	CSCL	ITGAM
SREBF1.1	2.33E-12	-0.21906	0.023	0.121	3.48E-08	CSCL	SREBF1
EGR3.2	2.33E-12	-0.3126	0.016	0.109	3.49E-08	CSCL	EGR3
ELMOD3.1	2.34E-12	-0.1018	0.033	0.14	3.49E-08	CSCL	ELMOD3
MPC1.1	2.34E-12	0.103112	0.244	0.5	3.49E-08	CSCL	MPC1
WBP4.1	2.36E-12	0.19744	0.136	0.314	3.53E-08	CSCL	WBP4
SUMO2	2.38E-12	0.301941	0.642	0.83	3.55E-08	CSCL	SUMO2
CARHSP1.1	2.41E-12	0.104259	0.206	0.434	3.60E-08	CSCL	CARHSP1
WBP2.1	2.46E-12	0.121149	0.223	0.464	3.68E-08	CSCL	WBP2
MMGT1	2.49E-12	-0.12361	0.019	0.116	3.72E-08	CSCL	MMGT1
PGM1.2	2.50E-12	0.136762	0.103	0.258	3.73E-08	CSCL	PGM1
PLXND1.2	2.51E-12	-0.2596	0.012	0.103	3.75E-08	CSCL	PLXND1
QRSL1.1	2.53E-12	-0.1228	0.028	0.13	3.78E-08	CSCL	QRSL1
TRIOBP.1	2.56E-12	-0.16936	0.007	0.093	3.83E-08	CSCL	TRIOBP
SNAPIN	2.59E-12	0.142383	0.178	0.387	3.87E-08	CSCL	SNAPIN
BLVRB.2	2.61E-12	0.108631	0.178	0.384	3.89E-08	CSCL	BLVRB
RP11-472N	2.64E-12	-0.18656	0.017	0.112	3.95E-08	CSCL	RP11-472N13.3
INPP5D.2	2.74E-12	-0.36369	0.005	0.089	4.09E-08	CSCL	INPP5D
JMY.1	2.75E-12	-0.15889	0.014	0.106	4.11E-08	CSCL	JMY
TRIM4.1	2.76E-12	-0.10114	0.04	0.151	4.12E-08	CSCL	TRIM4
SLC8B1.2	2.80E-12	-0.23082	0.012	0.102	4.19E-08	CSCL	SLC8B1

SASH3.2	2.81E-12	-0.34053	0.002	0.082	4.20E-08	CSCL	SASH3
GTPBP1.1	2.82E-12	-0.1882	0.014	0.106	4.22E-08	CSCL	GTPBP1
PAPOLG.2	2.91E-12	-0.25902	0.009	0.096	4.34E-08	CSCL	PAPOLG
C10orf11.	2.91E-12	-0.23012	0.014	0.105	4.35E-08	CSCL	C10orf11
USP42.1	2.96E-12	-0.13025	0.026	0.127	4.43E-08	CSCL	USP42
CREBRF.1	2.97E-12	-0.1226	0.031	0.136	4.44E-08	CSCL	CREBRF
PRDM2.1	2.99E-12	-0.1025	0.042	0.154	4.46E-08	CSCL	PRDM2
MLLT6.2	3.01E-12	-0.12248	0.031	0.136	4.50E-08	CSCL	MLLT6
USP46.2	3.06E-12	-0.10641	0.035	0.143	4.57E-08	CSCL	USP46
TP53INP2.	3.08E-12	-0.13126	0.01	0.099	4.61E-08	CSCL	TP53INP2
MAD2L1.2	3.09E-12	-0.13696	0.03	0.133	4.62E-08	CSCL	MAD2L1
FRMD8.1	3.11E-12	-0.12581	0.023	0.121	4.65E-08	CSCL	FRMD8
ICOSLG.2	3.13E-12	-0.30063	0.009	0.095	4.67E-08	CSCL	ICOSLG
HLA-DOA.1	3.17E-12	-0.33161	0.01	0.098	4.74E-08	CSCL	HLA-DOA
HBB.1	3.21E-12	0.171555	0.202	0.409	4.79E-08	CSCL	HBB
FOXN2.2	3.21E-12	-0.12235	0.031	0.136	4.80E-08	CSCL	FOXN2
DIP2B.2	3.22E-12	-0.12843	0.024	0.124	4.81E-08	CSCL	DIP2B
DHRS13.1	3.25E-12	-0.10845	0.012	0.102	4.86E-08	CSCL	DHRS13
CCDC90B.1	3.27E-12	0.104414	0.201	0.42	4.89E-08	CSCL	CCDC90B
HDDC2	3.29E-12	0.15565	0.194	0.411	4.91E-08	CSCL	HDDC2
LYL1.2	3.31E-12	-0.31832	0.003	0.085	4.95E-08	CSCL	LYL1
RNF38.1	3.35E-12	-0.13657	0.01	0.099	5.00E-08	CSCL	RNF38
STK11.1	3.41E-12	-0.13487	0.01	0.099	5.09E-08	CSCL	STK11
GGPS1.1	3.48E-12	0.144677	0.145	0.326	5.20E-08	CSCL	GGPS1
TELO2	3.49E-12	-0.13305	0.031	0.135	5.21E-08	CSCL	TELO2
GPATCH3.1	3.55E-12	-0.22126	0.003	0.085	5.31E-08	CSCL	GPATCH3
MANBA.2	3.64E-12	-0.23395	0.026	0.125	5.43E-08	CSCL	MANBA
STX8	3.64E-12	0.129441	0.171	0.37	5.44E-08	CSCL	STX8
PPP1R21	3.64E-12	-0.12864	0.021	0.117	5.45E-08	CSCL	PPP1R21
RELT.1	3.66E-12	-0.22465	0.005	0.088	5.47E-08	CSCL	RELT
SNX29.2	3.76E-12	-0.22541	0.017	0.11	5.62E-08	CSCL	SNX29
MSH2.1	3.79E-12	-0.12335	0.023	0.121	5.67E-08	CSCL	MSH2
BAK1.2	3.80E-12	-0.10125	0.023	0.12	5.68E-08	CSCL	BAK1
AVPI1.2	3.84E-12	-0.28183	0.01	0.098	5.74E-08	CSCL	AVPI1
PSMB4	3.86E-12	0.197314	0.201	0.428	5.76E-08	CSCL	PSMB4
CHD3.1	3.91E-12	-0.15442	0.023	0.12	5.84E-08	CSCL	CHD3
COPE.2	3.93E-12	0.381378	0.6	0.779	5.87E-08	CSCL	COPE
KLHL36.1	3.94E-12	-0.14944	0.01	0.098	5.89E-08	CSCL	KLHL36
ZFAND6	3.96E-12	0.153734	0.22	0.462	5.92E-08	CSCL	ZFAND6
HAUS2.1	4.02E-12	-0.11905	0.014	0.105	6.01E-08	CSCL	HAUS2
ATXN1L	4.05E-12	-0.17918	0.003	0.085	6.05E-08	CSCL	ATXN1L
SIGLEC8.2	4.07E-12	-0.34499	0.002	0.081	6.09E-08	CSCL	SIGLEC8
DHX38.1	4.14E-12	-0.11691	0.023	0.12	6.19E-08	CSCL	DHX38
RTN2.2	4.14E-12	-0.28861	0.005	0.088	6.19E-08	CSCL	RTN2
ATP11A.2	4.15E-12	-0.13116	0.012	0.101	6.19E-08	CSCL	ATP11A
ARL5B.2	4.15E-12	-0.1691	0.007	0.092	6.20E-08	CSCL	ARL5B
SUGP2.1	4.15E-12	0.141323	0.101	0.254	6.21E-08	CSCL	SUGP2
DHX8.1	4.19E-12	-0.13305	0.019	0.114	6.27E-08	CSCL	DHX8
AUTS2.2	4.24E-12	-0.11969	0.024	0.123	6.33E-08	CSCL	AUTS2
KLHDC8B.1	4.25E-12	-0.10774	0.016	0.108	6.35E-08	CSCL	KLHDC8B
PPP3CB.2	4.25E-12	-0.16585	0.019	0.113	6.36E-08	CSCL	PPP3CB

CENPN.1	4.35E-12	-0.18264	0.007	0.091	6.49E-08	CSCL	CENPN
PPP1R15B.	4.40E-12	-0.1918	0.024	0.122	6.58E-08	CSCL	PPP1R15B
ST3GAL6.2	4.46E-12	-0.26832	0.021	0.116	6.67E-08	CSCL	ST3GAL6
C15orf57.	4.51E-12	-0.12884	0.028	0.129	6.74E-08	CSCL	C15orf57
MOB4.1	4.66E-12	0.11929	0.176	0.382	6.96E-08	CSCL	MOB4
CCNY.2	4.77E-12	-0.14887	0.019	0.113	7.13E-08	CSCL	CCNY
ATP5J2.2	4.78E-12	0.327462	0.621	0.817	7.14E-08	CSCL	ATP5J2
RP11-108M	4.78E-12	-0.28295	0.016	0.106	7.15E-08	CSCL	RP11-108M9.4
ARID3A.1	4.81E-12	-0.23246	0.023	0.119	7.18E-08	CSCL	ARID3A
GPR84.2	4.82E-12	-0.34639	0	0.077	7.21E-08	CSCL	GPR84
PRKCD.2	4.88E-12	-0.27755	0.003	0.084	7.30E-08	CSCL	PRKCD
IRAK3.2	4.97E-12	-0.32711	0	0.077	7.43E-08	CSCL	IRAK3
SAT1.1	5.00E-12	-0.11059	0.461	0.82	7.48E-08	CSCL	SAT1
BAG3.1	5.08E-12	-0.2093	0.019	0.112	7.59E-08	CSCL	BAG3
USP7.2	5.35E-12	-0.10394	0.035	0.14	7.99E-08	CSCL	USP7
ULK3.1	5.44E-12	-0.10089	0.042	0.151	8.13E-08	CSCL	ULK3
FDX1L	5.45E-12	0.124835	0.11	0.267	8.15E-08	CSCL	FDX1L
PIGF.1	5.47E-12	0.136181	0.127	0.296	8.18E-08	CSCL	PIGF
HTT.2	5.51E-12	-0.10202	0.026	0.125	8.24E-08	CSCL	HTT
SH2B2.2	5.62E-12	-0.14796	0.019	0.113	8.40E-08	CSCL	SH2B2
LIMK2.2	5.63E-12	-0.14785	0.007	0.091	8.42E-08	CSCL	LIMK2
VCPIP1.2	5.74E-12	-0.15854	0.009	0.094	8.58E-08	CSCL	VCPIP1
OBFC1	5.77E-12	-0.18009	0.007	0.091	8.62E-08	CSCL	OBFC1
GIMAP1.2	5.78E-12	-0.32621	0	0.077	8.64E-08	CSCL	GIMAP1
MAGEF1.1	5.79E-12	0.128829	0.173	0.373	8.66E-08	CSCL	MAGEF1
HERC4	5.87E-12	-0.15621	0.023	0.119	8.77E-08	CSCL	HERC4
IRF3	5.89E-12	0.105571	0.101	0.251	8.80E-08	CSCL	IRF3
ITM2B.2	5.91E-12	-0.1046	0.429	0.818	8.84E-08	CSCL	ITM2B
CYHR1.1	5.94E-12	0.10513	0.122	0.286	8.88E-08	CSCL	CYHR1
EPHA3.2	5.96E-12	-0.13865	0	0.077	8.91E-08	CSCL	EPHA3
CXorf21.2	6.04E-12	-0.3665	0.002	0.08	9.02E-08	CSCL	CXorf21
ALKBH6.2	6.05E-12	-0.13236	0.017	0.11	9.04E-08	CSCL	ALKBH6
NCOA3.2	6.10E-12	-0.14268	0.03	0.131	9.12E-08	CSCL	NCOA3
MSTO1	6.48E-12	-0.14473	0.007	0.09	9.68E-08	CSCL	MSTO1
TOP2A.2	6.56E-12	-0.3032	0.007	0.09	9.81E-08	CSCL	TOP2A
NFRKB.1	6.61E-12	-0.16878	0.014	0.103	9.87E-08	CSCL	NFRKB
RNF7.1	6.79E-12	0.147543	0.265	0.546	1.01E-07	CSCL	RNF7
ARL3.1	6.80E-12	0.165438	0.162	0.356	1.02E-07	CSCL	ARL3
METRNL.2	6.80E-12	-0.24809	0.005	0.087	1.02E-07	CSCL	METRNL
BOK.2	7.02E-12	-0.20394	0.002	0.08	1.05E-07	CSCL	BOK
OSCAR.2	7.15E-12	-0.29653	0	0.076	1.07E-07	CSCL	OSCAR
LRRC45.2	7.15E-12	-0.14064	0.009	0.093	1.07E-07	CSCL	LRRC45
NME2	7.15E-12	-0.17469	0.007	0.09	1.07E-07	CSCL	NME2
RNF144B.1	7.21E-12	-0.29798	0.014	0.103	1.08E-07	CSCL	RNF144B
DLX5.2	7.22E-12	-0.38348	0.044	0.149	1.08E-07	CSCL	DLX5
SCARB1.1	7.22E-12	-0.1543	0.019	0.112	1.08E-07	CSCL	SCARB1
BACH1.1	7.29E-12	-0.17573	0.019	0.112	1.09E-07	CSCL	BACH1
MIR22HG.1	7.36E-12	-0.2346	0.023	0.118	1.10E-07	CSCL	MIR22HG
SDHD.1	7.43E-12	0.116647	0.192	0.406	1.11E-07	CSCL	SDHD
FAM199X.1	7.46E-12	-0.12115	0.019	0.112	1.12E-07	CSCL	FAM199X
KLF13.2	7.59E-12	-0.10888	0.03	0.13	1.13E-07	CSCL	KLF13

TAF12.2	7.66E-12	0.137922	0.141	0.317	1.14E-07	CSCL	TAF12
FAM160A2.	7.70E-12	-0.14664	0.023	0.118	1.15E-07	CSCL	FAM160A2
GTF2F2	7.84E-12	0.180911	0.134	0.308	1.17E-07	CSCL	GTF2F2
CHAF1A.2	7.85E-12	-0.16824	0.009	0.093	1.17E-07	CSCL	CHAF1A
TARSL2.1	7.90E-12	-0.18598	0.021	0.115	1.18E-07	CSCL	TARSL2
GABPB1.1	7.94E-12	-0.15477	0.019	0.112	1.19E-07	CSCL	GABPB1
SEC16A.1	8.45E-12	-0.15562	0.024	0.121	1.26E-07	CSCL	SEC16A
PAN3.1	8.64E-12	-0.17691	0.03	0.129	1.29E-07	CSCL	PAN3
ZNF394.1	8.93E-12	-0.12119	0.04	0.147	1.33E-07	CSCL	ZNF394
PIM1.1	9.19E-12	-0.11094	0.033	0.135	1.37E-07	CSCL	PIM1
ZNF703.2	9.32E-12	-0.17343	0.014	0.102	1.39E-07	CSCL	ZNF703
NEDD4L.2	9.44E-12	-0.17834	0.023	0.117	1.41E-07	CSCL	NEDD4L
TNKS2.2	9.49E-12	-0.12423	0.021	0.114	1.42E-07	CSCL	TNKS2
WHAMM	9.70E-12	-0.11755	0.019	0.111	1.45E-07	CSCL	WHAMM
PTPRO.2	9.89E-12	-0.25764	0.003	0.082	1.48E-07	CSCL	PTPRO
CUEDC2.2	1.01E-11	0.13764	0.195	0.411	1.51E-07	CSCL	CUEDC2
SCAMP3.1	1.01E-11	0.150112	0.131	0.298	1.51E-07	CSCL	SCAMP3
FUT8.2	1.01E-11	-0.1277	0.03	0.129	1.51E-07	CSCL	FUT8
PHTF2.1	1.03E-11	-0.16898	0.01	0.095	1.54E-07	CSCL	PHTF2
MFF	1.03E-11	0.115394	0.244	0.502	1.54E-07	CSCL	MFF
ING1.1	1.04E-11	-0.12748	0.037	0.141	1.56E-07	CSCL	ING1
ABHD17B.2	1.04E-11	-0.13044	0.03	0.129	1.56E-07	CSCL	ABHD17B
P2RY13.2	1.04E-11	-0.3914	0.007	0.088	1.56E-07	CSCL	P2RY13
MON1B	1.05E-11	-0.12463	0.019	0.111	1.57E-07	CSCL	MON1B
MAST3.2	1.05E-11	-0.21295	0.003	0.082	1.57E-07	CSCL	MAST3
SFXN1	1.09E-11	0.109273	0.086	0.224	1.63E-07	CSCL	SFXN1
SPTY2D1.1	1.10E-11	-0.10662	0.026	0.123	1.64E-07	CSCL	SPTY2D1
RPRD1B	1.10E-11	-0.14048	0.014	0.101	1.65E-07	CSCL	RPRD1B
RP11-25K1	1.11E-11	-0.1247	0.016	0.105	1.66E-07	CSCL	RP11-25K19.1
ERC1	1.13E-11	-0.10782	0.028	0.126	1.69E-07	CSCL	ERC1
BEX1.1	1.13E-11	0.734242	0.384	0.349	1.69E-07	CSCL	BEX1
PSME4.1	1.15E-11	-0.10655	0.037	0.141	1.71E-07	CSCL	PSME4
PITX1.1	1.16E-11	-0.10782	0.007	0.089	1.73E-07	CSCL	PITX1
SIGLEC7.2	1.19E-11	-0.32843	0.002	0.078	1.77E-07	CSCL	SIGLEC7
ARHGAP22.	1.20E-11	-0.30695	0.002	0.078	1.79E-07	CSCL	ARHGAP22
CECR1.2	1.23E-11	-0.32067	0	0.075	1.84E-07	CSCL	CECR1
PRPF31.1	1.24E-11	0.147135	0.19	0.402	1.85E-07	CSCL	PRPF31
CCDC71.1	1.25E-11	-0.11393	0.014	0.101	1.86E-07	CSCL	CCDC71
SIK3.2	1.26E-11	-0.11877	0.019	0.111	1.89E-07	CSCL	SIK3
HMG20A	1.26E-11	-0.13842	0.019	0.11	1.89E-07	CSCL	HMG20A
SLC17A5.2	1.27E-11	-0.18393	0.019	0.11	1.90E-07	CSCL	SLC17A5
RBBP4	1.30E-11	0.133375	0.213	0.442	1.94E-07	CSCL	RBBP4
CCNB1.1	1.31E-11	-0.23321	0.01	0.095	1.96E-07	CSCL	CCNB1
PWP2.2	1.36E-11	-0.10783	0.03	0.129	2.03E-07	CSCL	PWP2
LILRA2.2	1.36E-11	-0.34979	0.007	0.088	2.04E-07	CSCL	LILRA2
LLGL1.1	1.38E-11	-0.12149	0.016	0.104	2.06E-07	CSCL	LLGL1
AES.2	1.39E-11	0.148333	0.202	0.421	2.07E-07	CSCL	AES
TOX.2	1.40E-11	-0.15742	0.009	0.091	2.09E-07	CSCL	TOX
CBL.2	1.40E-11	-0.10448	0.017	0.107	2.09E-07	CSCL	CBL
UPF3A.1	1.46E-11	0.189769	0.162	0.354	2.18E-07	CSCL	UPF3A
AK2.2	1.46E-11	0.189764	0.19	0.401	2.18E-07	CSCL	AK2

BCL2L1.1	1.46E-11	0.121202	0.115	0.272	2.18E-07	CSCL	BCL2L1
CAMK1D.2	1.49E-11	-0.16749	0.023	0.116	2.23E-07	CSCL	CAMK1D
USP11.1	1.50E-11	0.143459	0.169	0.362	2.24E-07	CSCL	USP11
VAV1.2	1.53E-11	-0.30068	0.002	0.077	2.28E-07	CSCL	VAV1
JAZF1.2	1.54E-11	-0.17149	0.024	0.118	2.29E-07	CSCL	JAZF1
P2RY11.1	1.55E-11	-0.20798	0.009	0.091	2.31E-07	CSCL	P2RY11
YARS2	1.59E-11	-0.13389	0.019	0.11	2.37E-07	CSCL	YARS2
MTF1	1.59E-11	-0.10526	0.021	0.113	2.38E-07	CSCL	MTF1
RABEP2.1	1.60E-11	-0.10833	0.016	0.104	2.39E-07	CSCL	RABEP2
PCGF3.1	1.60E-11	-0.11702	0.024	0.119	2.39E-07	CSCL	PCGF3
AFMID.2	1.60E-11	-0.17713	0.019	0.11	2.40E-07	CSCL	AFMID
CNKSR3.1	1.61E-11	-0.10521	0.026	0.122	2.40E-07	CSCL	CNKSR3
SAYSD1.1	1.61E-11	-0.10015	0.026	0.122	2.41E-07	CSCL	SAYSD1
VRK3.1	1.62E-11	0.113695	0.087	0.225	2.41E-07	CSCL	VRK3
CST3.1	1.62E-11	0.449114	0.616	0.795	2.42E-07	CSCL	CST3
L3MBTL2.2	1.62E-11	-0.13295	0.017	0.107	2.42E-07	CSCL	L3MBTL2
SGOL2.1	1.62E-11	-0.19456	0.01	0.094	2.43E-07	CSCL	SGOL2
CACNA2D4.	1.63E-11	-0.32599	0.002	0.077	2.44E-07	CSCL	CACNA2D4
DHRS12	1.64E-11	-0.19406	0.012	0.097	2.44E-07	CSCL	DHRS12
SESN1.2	1.64E-11	-0.26175	0.017	0.106	2.45E-07	CSCL	SESN1
NCBP2	1.68E-11	0.139251	0.218	0.452	2.51E-07	CSCL	NCBP2
FAM20C.2	1.72E-11	0.100545	0.091	0.23	2.58E-07	CSCL	FAM20C
ARNTL.1	1.73E-11	-0.10094	0.014	0.1	2.58E-07	CSCL	ARNTL
PPP2R1A.2	1.74E-11	0.162032	0.26	0.528	2.60E-07	CSCL	PPP2R1A
PPP2R2D.2	1.74E-11	-0.13725	0.01	0.094	2.60E-07	CSCL	PPP2R2D
KIF3C.2	1.75E-11	-0.14675	0.017	0.107	2.61E-07	CSCL	KIF3C
TAOK2	1.76E-11	-0.14773	0.012	0.097	2.63E-07	CSCL	TAOK2
PPP1R12C	1.79E-11	-0.16054	0.007	0.087	2.68E-07	CSCL	PPP1R12C
KTI12.1	1.80E-11	-0.12341	0.019	0.109	2.69E-07	CSCL	KTI12
PLK2.1	1.81E-11	-0.16435	0.038	0.142	2.71E-07	CSCL	PLK2
RRAGB.1	1.83E-11	-0.11017	0.023	0.116	2.73E-07	CSCL	RRAGB
PDLIM2.1	1.84E-11	0.114778	0.101	0.247	2.74E-07	CSCL	PDLIM2
PRKAG2-AS	1.86E-11	-0.23207	0.031	0.129	2.78E-07	CSCL	PRKAG2-AS1
ARRDC2.2	1.90E-11	-0.26564	0.009	0.09	2.84E-07	CSCL	ARRDC2
FAM133B.1	1.99E-11	0.131316	0.237	0.483	2.97E-07	CSCL	FAM133B
ASXL2.2	2.01E-11	-0.14771	0.016	0.103	3.00E-07	CSCL	ASXL2
TOPBP1.1	2.03E-11	-0.10586	0.012	0.097	3.03E-07	CSCL	TOPBP1
MED28.1	2.03E-11	0.17779	0.161	0.348	3.04E-07	CSCL	MED28
SMIM13.1	2.04E-11	-0.17028	0.009	0.09	3.05E-07	CSCL	SMIM13
ZDHHC21.2	2.07E-11	-0.10296	0.016	0.103	3.09E-07	CSCL	ZDHHC21
MCM4.2	2.07E-11	-0.15392	0.014	0.1	3.10E-07	CSCL	MCM4
CRADD.1	2.07E-11	-0.1039	0.019	0.109	3.10E-07	CSCL	CRADD
TPD52.2	2.10E-11	0.103587	0.099	0.243	3.15E-07	CSCL	TPD52
TRIM14.2	2.19E-11	-0.15049	0.012	0.096	3.27E-07	CSCL	TRIM14
FES.2	2.21E-11	-0.28787	0.005	0.083	3.30E-07	CSCL	FES
ST14.2	2.22E-11	-0.28003	0.002	0.076	3.31E-07	CSCL	ST14
E2F3.2	2.30E-11	-0.15513	0.009	0.09	3.43E-07	CSCL	E2F3
HAUS6.1	2.31E-11	-0.15937	0.01	0.093	3.45E-07	CSCL	HAUS6
CNEP1R1.1	2.38E-11	-0.11097	0.023	0.115	3.56E-07	CSCL	CNEP1R1
ELK4	2.39E-11	-0.12037	0.021	0.112	3.57E-07	CSCL	ELK4
TET1.1	2.45E-11	-0.12025	0.007	0.086	3.66E-07	CSCL	TET1

VASH1.2	2.50E-11	-0.1904	0.035	0.134	3.73E-07	CSCL	VASH1
SGPP1.2	2.50E-11	-0.21238	0.005	0.083	3.73E-07	CSCL	SGPP1
EHD1.1	2.51E-11	-0.11138	0.03	0.126	3.75E-07	CSCL	EHD1
DCPS.1	2.53E-11	0.126012	0.059	0.177	3.78E-07	CSCL	DCPS
MSMO1.1	2.56E-11	0.122951	0.077	0.207	3.82E-07	CSCL	MSMO1
CBX5.1	2.58E-11	0.129885	0.166	0.353	3.85E-07	CSCL	CBX5
CC2D1A.1	2.59E-11	0.101573	0.068	0.192	3.88E-07	CSCL	CC2D1A
ATAD2.1	2.62E-11	-0.17432	0.003	0.079	3.91E-07	CSCL	ATAD2
TSPYL2.1	2.65E-11	-0.14897	0.024	0.117	3.96E-07	CSCL	TSPYL2
NFKB1.2	2.65E-11	-0.15644	0.019	0.108	3.96E-07	CSCL	NFKB1
TANGO2.2	2.66E-11	-0.17749	0.009	0.089	3.97E-07	CSCL	TANGO2
RMND1	2.71E-11	-0.12666	0.017	0.105	4.05E-07	CSCL	RMND1
HOXC9.2	2.72E-11	-0.22509	0.009	0.089	4.06E-07	CSCL	HOXC9
NPM3.1	2.74E-11	0.112541	0.087	0.223	4.09E-07	CSCL	NPM3
NMRAL1	2.79E-11	0.109559	0.101	0.246	4.17E-07	CSCL	NMRAL1
ELK3	2.80E-11	-0.15466	0.01	0.092	4.19E-07	CSCL	ELK3
POLR2E.1	2.81E-11	0.131672	0.272	0.548	4.20E-07	CSCL	POLR2E
KLHDC8A.2	2.82E-11	0.131331	0.108	0.258	4.21E-07	CSCL	KLHDC8A
HERC1	2.91E-11	-0.13664	0.016	0.102	4.35E-07	CSCL	HERC1
PTPN7.2	3.09E-11	-0.31246	0.005	0.082	4.61E-07	CSCL	PTPN7
UBASH3B.2	3.12E-11	-0.18033	0.003	0.079	4.66E-07	CSCL	UBASH3B
SWI5.2	3.13E-11	0.241409	0.105	0.252	4.67E-07	CSCL	SWI5
C12orf76.	3.19E-11	0.136669	0.119	0.274	4.77E-07	CSCL	C12orf76
CARKD.1	3.22E-11	-0.1166	0.035	0.134	4.81E-07	CSCL	CARKD
EPB41L1.1	3.26E-11	-0.1249	0.01	0.092	4.87E-07	CSCL	EPB41L1
EMB.2	3.27E-11	-0.30606	0.003	0.079	4.88E-07	CSCL	EMB
CD2AP.2	3.30E-11	-0.13547	0.007	0.085	4.93E-07	CSCL	CD2AP
HK2.1	3.33E-11	-0.21608	0.009	0.089	4.98E-07	CSCL	HK2
DIS3L.2	3.34E-11	-0.11476	0.019	0.107	4.99E-07	CSCL	DIS3L
MORN2.2	3.37E-11	0.225104	0.115	0.269	5.04E-07	CSCL	MORN2
STXBP1.1	3.47E-11	-0.10205	0.03	0.126	5.19E-07	CSCL	STXBP1
GCLC.2	3.48E-11	-0.14674	0.016	0.101	5.20E-07	CSCL	GCLC
DLX1.2	3.56E-11	-0.22697	0.026	0.119	5.32E-07	CSCL	DLX1
DONSON.1	3.56E-11	-0.11764	0.007	0.085	5.32E-07	CSCL	DONSON
CH25H.1	3.58E-11	-0.33536	0.047	0.152	5.35E-07	CSCL	CH25H
TRIP4	3.62E-11	-0.14079	0.024	0.116	5.42E-07	CSCL	TRIP4
TBRG4.1	3.64E-11	0.143826	0.101	0.245	5.44E-07	CSCL	TBRG4
G6PC3.1	3.66E-11	0.117181	0.169	0.356	5.47E-07	CSCL	G6PC3
BIRC5.2	3.69E-11	-0.28444	0.021	0.109	5.51E-07	CSCL	BIRC5
ASNA1.2	3.74E-11	0.184417	0.262	0.533	5.58E-07	CSCL	ASNA1
LBH.2	3.86E-11	-0.18977	0.051	0.159	5.77E-07	CSCL	LBH
TLR1.2	3.87E-11	-0.30421	0	0.071	5.78E-07	CSCL	TLR1
DHX32.1	3.90E-11	-0.1581	0.007	0.085	5.83E-07	CSCL	DHX32
RASA1.2	4.00E-11	-0.12635	0.021	0.11	5.98E-07	CSCL	RASA1
DDX56.1	4.03E-11	0.117198	0.113	0.264	6.03E-07	CSCL	DDX56
PPARD.2	4.08E-11	-0.1171	0.023	0.113	6.10E-07	CSCL	PPARD
DDHD2.1	4.12E-11	-0.1292	0.021	0.11	6.16E-07	CSCL	DDHD2
DPP4	4.13E-11	-0.10776	0.005	0.081	6.17E-07	CSCL	DPP4
SLC35A3.1	4.15E-11	-0.10073	0.031	0.128	6.20E-07	CSCL	SLC35A3
TGIF2.1	4.19E-11	-0.14418	0.009	0.088	6.26E-07	CSCL	TGIF2
ZNF480.1	4.23E-11	-0.11522	0.021	0.11	6.32E-07	CSCL	ZNF480

SFMBT2.2	4.24E-11	-0.32261	0.005	0.081	6.33E-07	CSCL	SFMBT2
MED10.2	4.25E-11	0.146154	0.246	0.496	6.35E-07	CSCL	MED10
HLA-C.1	4.32E-11	0.28744	0.64	0.811	6.46E-07	CSCL	HLA-C
RPP25L	4.35E-11	0.121514	0.058	0.173	6.50E-07	CSCL	RPP25L
IL4R.2	4.39E-11	-0.27996	0.002	0.074	6.56E-07	CSCL	IL4R
NOB1.1	4.40E-11	0.174111	0.073	0.199	6.58E-07	CSCL	NOB1
CD93.2	4.44E-11	-0.30883	0.002	0.074	6.64E-07	CSCL	CD93
CDK17.1	4.46E-11	-0.15865	0.028	0.121	6.66E-07	CSCL	CDK17
UNC5B.2	4.50E-11	-0.24148	0.009	0.087	6.73E-07	CSCL	UNC5B
ANKRD49	4.53E-11	-0.10905	0.023	0.113	6.77E-07	CSCL	ANKRD49
NDST1.2	4.59E-11	-0.1369	0.01	0.091	6.86E-07	CSCL	NDST1
POLR3GL	4.67E-11	0.189072	0.171	0.36	6.98E-07	CSCL	POLR3GL
AP001055.	4.68E-11	-0.33859	0.007	0.084	6.99E-07	CSCL	AP001055.6
RMND5A.1	4.69E-11	-0.14061	0.031	0.128	7.00E-07	CSCL	RMND5A
POLG.2	4.69E-11	-0.14467	0.016	0.1	7.00E-07	CSCL	POLG
RHOH.2	4.74E-11	-0.35011	0.005	0.081	7.08E-07	CSCL	RHOH
NDUFAF2.2	4.75E-11	0.189592	0.122	0.28	7.09E-07	CSCL	NDUFAF2
SLC26A11.	4.79E-11	-0.10937	0.021	0.11	7.16E-07	CSCL	SLC26A11
ZNF512B.1	4.90E-11	-0.12862	0.003	0.077	7.32E-07	CSCL	ZNF512B
COMMD1	4.93E-11	0.143218	0.175	0.366	7.36E-07	CSCL	COMMD1
SLC35B1	4.93E-11	0.126147	0.192	0.401	7.36E-07	CSCL	SLC35B1
VCL.1	5.06E-11	-0.10428	0.017	0.103	7.56E-07	CSCL	VCL
CD40.2	5.06E-11	-0.28869	0	0.07	7.57E-07	CSCL	CD40
MYO1C.1	5.11E-11	-0.13904	0.019	0.106	7.64E-07	CSCL	MYO1C
KRIT1.1	5.15E-11	0.116222	0.089	0.223	7.70E-07	CSCL	KRIT1
ACSL4.2	5.37E-11	-0.17093	0.009	0.087	8.02E-07	CSCL	ACSL4
DYNC1I1.2	5.42E-11	-0.26467	0.021	0.108	8.10E-07	CSCL	DYNC1I1
RP3-428L1	5.62E-11	-0.17352	0.002	0.074	8.39E-07	CSCL	RP3-428L16.2
HBA2.1	5.64E-11	0.278541	0.079	0.203	8.43E-07	CSCL	HBA2
LRR1.1	5.73E-11	-0.13136	0.01	0.09	8.56E-07	CSCL	LRR1
TRIM38.1	5.73E-11	-0.23375	0.017	0.102	8.57E-07	CSCL	TRIM38
GATM.2	5.74E-11	0.115196	0.173	0.361	8.57E-07	CSCL	GATM
ITPRIPL2.	5.78E-11	-0.17486	0.003	0.077	8.64E-07	CSCL	ITPRIPL2
TMTC4.1	5.80E-11	-0.1457	0.017	0.103	8.67E-07	CSCL	TMTC4
PSMC6.1	5.88E-11	0.12618	0.192	0.397	8.79E-07	CSCL	PSMC6
NOL11.1	5.94E-11	0.157836	0.072	0.195	8.87E-07	CSCL	NOL11
TMEM5	6.02E-11	0.108096	0.084	0.215	9.00E-07	CSCL	TMEM5
SLC39A9	6.08E-11	-0.11655	0.016	0.099	9.09E-07	CSCL	SLC39A9
ALG1.1	6.17E-11	-0.11943	0.026	0.118	9.22E-07	CSCL	ALG1
TMEM208.2	6.30E-11	0.182276	0.237	0.479	9.41E-07	CSCL	TMEM208
SLC12A5.2	6.49E-11	-0.2173	0.014	0.096	9.69E-07	CSCL	SLC12A5
TMEM222.2	6.60E-11	0.185394	0.127	0.287	9.87E-07	CSCL	TMEM222
PI4KB.1	6.67E-11	0.136254	0.086	0.216	9.97E-07	CSCL	PI4KB
CLNS1A.1	6.68E-11	0.176678	0.25	0.504	9.99E-07	CSCL	CLNS1A
EDEM1.1	6.82E-11	-0.21585	0.007	0.083	1.02E-06	CSCL	EDEM1
UBE3C.1	6.94E-11	-0.11642	0.024	0.114	1.04E-06	CSCL	UBE3C
STAC3.2	6.98E-11	-0.27397	0.007	0.083	1.04E-06	CSCL	STAC3
TAF6L	7.07E-11	-0.13892	0.012	0.093	1.06E-06	CSCL	TAF6L
ZFYVE21.1	7.13E-11	0.151734	0.12	0.274	1.06E-06	CSCL	ZFYVE21
ACTR10.1	7.18E-11	0.175182	0.176	0.369	1.07E-06	CSCL	ACTR10
PROS1	7.43E-11	-0.10233	0.014	0.096	1.11E-06	CSCL	PROS1

TPX2.1	7.43E-11	-0.20837	0.009	0.086	1.11E-06	CSCL	TPX2
TFCP2	7.55E-11	-0.1035	0.026	0.117	1.13E-06	CSCL	TFCP2
RANBP1.2	7.67E-11	0.195063	0.298	0.592	1.15E-06	CSCL	RANBP1
PRKD3.2	7.98E-11	-0.10767	0.047	0.152	1.19E-06	CSCL	PRKD3
SH3YL1.1	7.98E-11	0.117745	0.068	0.187	1.19E-06	CSCL	SH3YL1
NSL1.1	8.06E-11	0.151296	0.169	0.356	1.20E-06	CSCL	NSL1
MOSPD2.1	8.09E-11	-0.13193	0.024	0.114	1.21E-06	CSCL	MOSPD2
ACTN4.1	8.15E-11	0.173694	0.161	0.341	1.22E-06	CSCL	ACTN4
CDKL3	8.19E-11	-0.13521	0.024	0.114	1.22E-06	CSCL	CDKL3
N4BP1.1	8.21E-11	-0.13317	0.012	0.092	1.23E-06	CSCL	N4BP1
FARSB	8.23E-11	0.101833	0.099	0.239	1.23E-06	CSCL	FARSB
RRM2B.1	8.27E-11	-0.17255	0.021	0.107	1.24E-06	CSCL	RRM2B
TMEM39A	8.29E-11	-0.20304	0.014	0.095	1.24E-06	CSCL	TMEM39A
LCORL.1	8.55E-11	-0.11262	0.017	0.102	1.28E-06	CSCL	LCORL
CARD9.2	8.63E-11	-0.26728	0.002	0.072	1.29E-06	CSCL	CARD9
RLIM.1	8.69E-11	-0.12117	0.023	0.111	1.30E-06	CSCL	RLIM
EED	8.91E-11	-0.15297	0.017	0.101	1.33E-06	CSCL	EED
KLHL2.2	9.03E-11	-0.11583	0.01	0.089	1.35E-06	CSCL	KLHL2
MTFMT.1	9.07E-11	-0.12061	0.019	0.104	1.36E-06	CSCL	MTFMT
CTD-2369F	9.08E-11	0.136117	0.014	0.001	1.36E-06	CSCL	CTD-2369P2.10
WDR45	9.31E-11	0.137428	0.112	0.258	1.39E-06	CSCL	WDR45
CHN1.1	9.51E-11	0.197119	0.089	0.222	1.42E-06	CSCL	CHN1
MOB1B.2	9.56E-11	-0.18436	0.002	0.072	1.43E-06	CSCL	MOB1B
PRKCZ.2	9.72E-11	-0.11538	0.019	0.105	1.45E-06	CSCL	PRKCZ
FAM76B	9.79E-11	-0.10629	0.026	0.116	1.46E-06	CSCL	FAM76B
SRCAP.1	9.88E-11	-0.12733	0.021	0.107	1.48E-06	CSCL	SRCAP
PPP1R9B.1	9.88E-11	-0.10886	0.019	0.104	1.48E-06	CSCL	PPP1R9B
DMXL1	1.01E-10	-0.15061	0.012	0.092	1.51E-06	CSCL	DMXL1
FAM65A	1.02E-10	-0.12824	0.019	0.104	1.52E-06	CSCL	FAM65A
IL16.2	1.02E-10	-0.29842	0.002	0.072	1.53E-06	CSCL	IL16
ANKRD22.2	1.04E-10	-0.31899	0	0.068	1.55E-06	CSCL	ANKRD22
KIF1A.1	1.04E-10	-0.12748	0.035	0.131	1.56E-06	CSCL	KIF1A
GAS2L3.2	1.05E-10	-0.16373	0.002	0.072	1.57E-06	CSCL	GAS2L3
RPF1.1	1.05E-10	0.1517	0.108	0.252	1.57E-06	CSCL	RPF1
NAIP.2	1.06E-10	-0.19755	0.024	0.113	1.59E-06	CSCL	NAIP
SREK1.1	1.08E-10	0.188381	0.155	0.331	1.62E-06	CSCL	SREK1
SLC38A7.1	1.09E-10	-0.14303	0.024	0.113	1.64E-06	CSCL	SLC38A7
RFC3.1	1.10E-10	-0.11918	0.012	0.091	1.64E-06	CSCL	RFC3
TREM1.1	1.11E-10	-0.18457	0.059	0.169	1.66E-06	CSCL	TREM1
OLFML2B.2	1.11E-10	-0.32251	0.002	0.071	1.66E-06	CSCL	OLFML2B
APEX2.1	1.12E-10	-0.11133	0.016	0.098	1.67E-06	CSCL	APEX2
D2HGDH.2	1.12E-10	-0.12248	0.024	0.113	1.67E-06	CSCL	D2HGDH
PREP.2	1.13E-10	-0.14088	0.017	0.101	1.68E-06	CSCL	PREP
TCEB2.2	1.15E-10	0.299976	0.623	0.834	1.72E-06	CSCL	TCEB2
TSPAN15.1	1.16E-10	-0.28051	0.007	0.081	1.73E-06	CSCL	TSPAN15
NRROS.1	1.17E-10	-0.30908	0.002	0.071	1.74E-06	CSCL	NRROS
SMARCD3.1	1.17E-10	0.135118	0.099	0.238	1.75E-06	CSCL	SMARCD3
PIK3AP1.2	1.17E-10	-0.25995	0	0.068	1.75E-06	CSCL	PIK3AP1
IKZF1.2	1.17E-10	-0.29316	0	0.068	1.75E-06	CSCL	IKZF1
SEMA4D.2	1.18E-10	-0.29143	0.009	0.084	1.76E-06	CSCL	SEMA4D
KDM5C.1	1.18E-10	-0.13217	0.024	0.112	1.77E-06	CSCL	KDM5C

MGAT5.2	1.18E-10	-0.20203	0.002	0.071	1.77E-06	CSCL	MGAT5
YPEL1.2	1.21E-10	-0.11028	0.016	0.098	1.80E-06	CSCL	YPEL1
GALC.1	1.22E-10	-0.12923	0.021	0.106	1.83E-06	CSCL	GALC
BCAP29.1	1.23E-10	0.124207	0.154	0.325	1.84E-06	CSCL	BCAP29
C1orf123	1.24E-10	0.206037	0.166	0.35	1.85E-06	CSCL	C1orf123
CSGALNACT	1.24E-10	-0.13807	0.016	0.097	1.85E-06	CSCL	CSGALNACT2
CCL4L2.2	1.25E-10	-0.52181	0.012	0.09	1.87E-06	CSCL	CCL4L2
UBE2E1.1	1.26E-10	0.176329	0.23	0.462	1.89E-06	CSCL	UBE2E1
MX1.1	1.27E-10	-0.15577	0.031	0.124	1.89E-06	CSCL	MX1
EXOSC8.1	1.27E-10	0.150621	0.136	0.297	1.90E-06	CSCL	EXOSC8
SS18L1	1.29E-10	-0.11692	0.014	0.094	1.93E-06	CSCL	SS18L1
BBS7.1	1.30E-10	-0.11457	0.017	0.1	1.95E-06	CSCL	BBS7
CDK5R1.2	1.34E-10	-0.12347	0.016	0.097	2.01E-06	CSCL	CDK5R1
RYK.1	1.35E-10	-0.11208	0.014	0.094	2.01E-06	CSCL	RYK
MARCH3.1	1.38E-10	-0.17972	0.023	0.109	2.06E-06	CSCL	3-Mar
NUAK1.2	1.40E-10	-0.18652	0.003	0.074	2.08E-06	CSCL	NUAK1
CLOCK.2	1.40E-10	-0.11263	0.037	0.133	2.09E-06	CSCL	CLOCK
GMIP.2	1.41E-10	-0.23508	0.005	0.077	2.10E-06	CSCL	GMIP
FIG4.1	1.41E-10	-0.11597	0.023	0.109	2.11E-06	CSCL	FIG4
SPATA2	1.42E-10	-0.15757	0.002	0.071	2.12E-06	CSCL	SPATA2
PLEKHM1.1	1.43E-10	-0.16841	0.005	0.078	2.13E-06	CSCL	PLEKHM1
PISD.2	1.43E-10	-0.12569	0.023	0.109	2.14E-06	CSCL	PISD
MUM1.2	1.45E-10	0.133302	0.18	0.373	2.17E-06	CSCL	MUM1
EHBP1L1.2	1.48E-10	-0.17897	0.003	0.074	2.22E-06	CSCL	EHBP1L1
HSBP1L1.1	1.49E-10	-0.15351	0.005	0.078	2.23E-06	CSCL	HSBP1L1
UXT.1	1.50E-10	0.14189	0.279	0.551	2.23E-06	CSCL	UXT
FGR.2	1.50E-10	-0.29004	0.01	0.087	2.24E-06	CSCL	FGR
SCN1B.2	1.56E-10	-0.23175	0.003	0.074	2.33E-06	CSCL	SCN1B
IGFBP2.1	1.57E-10	0.73086	0.394	0.373	2.35E-06	CSCL	IGFBP2
COQ9.1	1.58E-10	0.183434	0.106	0.249	2.36E-06	CSCL	COQ9
SYNDIG1.2	1.63E-10	-0.2408	0.014	0.093	2.43E-06	CSCL	SYNDIG1
TK1.2	1.63E-10	-0.21241	0.003	0.074	2.43E-06	CSCL	TK1
PSMD14.1	1.64E-10	0.220644	0.237	0.479	2.46E-06	CSCL	PSMD14
GALNT7.2	1.65E-10	-0.1368	0.014	0.093	2.47E-06	CSCL	GALNT7
NNAT.2	1.68E-10	-0.31075	0.086	0.201	2.50E-06	CSCL	NNAT
TIMM9	1.72E-10	0.192557	0.129	0.284	2.57E-06	CSCL	TIMM9
ASB16-AS1	1.77E-10	-0.11202	0.016	0.096	2.65E-06	CSCL	ASB16-AS1
SLAMF8.2	1.78E-10	-0.27904	0	0.067	2.66E-06	CSCL	SLAMF8
RABEPK.1	1.79E-10	0.191206	0.08	0.205	2.68E-06	CSCL	RABEPK
GFPT1.1	1.79E-10	-0.1598	0.017	0.099	2.68E-06	CSCL	GFPT1
HIPK3.1	1.80E-10	-0.1277	0.014	0.093	2.69E-06	CSCL	HIPK3
C2.1	1.80E-10	-0.25616	0.03	0.119	2.70E-06	CSCL	C2
SMPD4	1.81E-10	-0.10021	0.023	0.108	2.71E-06	CSCL	SMPD4
FERMT2.2	1.82E-10	0.171444	0.082	0.208	2.72E-06	CSCL	FERMT2
YIPF6.1	1.83E-10	0.136633	0.152	0.321	2.73E-06	CSCL	YIPF6
CCNB2.2	1.83E-10	-0.25591	0	0.067	2.74E-06	CSCL	CCNB2
PRC1.2	1.87E-10	-0.18124	0.012	0.09	2.79E-06	CSCL	PRC1
FNIP1	1.88E-10	-0.14045	0.023	0.108	2.81E-06	CSCL	FNIP1
ALG12.2	1.90E-10	-0.1275	0.016	0.096	2.85E-06	CSCL	ALG12
APOBEC3G.	1.91E-10	-0.14983	0.031	0.123	2.85E-06	CSCL	APOBEC3G
GCHFR.2	1.93E-10	-0.25159	0.007	0.08	2.88E-06	CSCL	GCHFR

RAD54L2.1	1.93E-10	-0.10041	0.024	0.111	2.88E-06	CSCL	RAD54L2
DLX2.2	1.93E-10	-0.27316	0.026	0.112	2.89E-06	CSCL	DLX2
LYPLAL1	2.00E-10	0.106488	0.105	0.243	2.99E-06	CSCL	LYPLAL1
PTPN23.1	2.04E-10	-0.11256	0.007	0.08	3.05E-06	CSCL	PTPN23
CFDP1.1	2.05E-10	0.215222	0.211	0.426	3.06E-06	CSCL	CFDP1
SEPT1.1	2.07E-10	-0.11452	0.024	0.111	3.09E-06	CSCL	1-Sep
ZNF33A.2	2.11E-10	-0.12602	0.021	0.105	3.15E-06	CSCL	ZNF33A
ARSG	2.13E-10	-0.16826	0.012	0.089	3.18E-06	CSCL	ARSG
PRDM4.1	2.13E-10	-0.10724	0.012	0.089	3.19E-06	CSCL	PRDM4
RP11-395A	2.15E-10	-0.11125	0.007	0.08	3.21E-06	CSCL	RP11-395A13.2
GSAP.1	2.22E-10	-0.21667	0.009	0.083	3.32E-06	CSCL	GSAP
CCNG1.2	2.23E-10	0.116482	0.126	0.277	3.33E-06	CSCL	CCNG1
CBX4.2	2.28E-10	-0.10672	0.03	0.12	3.40E-06	CSCL	CBX4
VPS18.1	2.29E-10	-0.16478	0.009	0.083	3.42E-06	CSCL	VPS18
TP53I11.2	2.29E-10	-0.20616	0.005	0.076	3.43E-06	CSCL	TP53I11
SGK223.1	2.40E-10	-0.16214	0.005	0.076	3.58E-06	CSCL	SGK223
ACVR1B	2.42E-10	-0.16498	0.012	0.089	3.62E-06	CSCL	ACVR1B
CHSY1.1	2.45E-10	-0.11777	0.016	0.095	3.66E-06	CSCL	CHSY1
PCK2.1	2.45E-10	-0.19346	0.003	0.073	3.66E-06	CSCL	PCK2
ARHGAP25.	2.50E-10	-0.23355	0.002	0.069	3.73E-06	CSCL	ARHGAP25
MVB12B.2	2.50E-10	-0.1649	0.021	0.104	3.74E-06	CSCL	MVB12B
SLC16A10.	2.53E-10	-0.33984	0.014	0.091	3.78E-06	CSCL	SLC16A10
DNASE1L1.	2.55E-10	-0.21969	0.005	0.076	3.81E-06	CSCL	DNASE1L1
BBIP1.1	2.56E-10	0.11638	0.068	0.183	3.83E-06	CSCL	BBIP1
TAGLN2.2	2.61E-10	0.593224	0.471	0.517	3.90E-06	CSCL	TAGLN2
SERTAD2.1	2.65E-10	-0.1069	0.033	0.125	3.96E-06	CSCL	SERTAD2
CDCA4.1	2.74E-10	-0.13622	0.014	0.092	4.09E-06	CSCL	CDCA4
LRIF1.1	2.80E-10	0.158896	0.08	0.203	4.19E-06	CSCL	LRIF1
PSMC5	2.82E-10	0.145774	0.286	0.568	4.21E-06	CSCL	PSMC5
NUF2.2	2.86E-10	-0.23163	0	0.065	4.28E-06	CSCL	NUF2
STAT1.1	2.88E-10	0.199258	0.099	0.236	4.30E-06	CSCL	STAT1
PAQR8.2	2.92E-10	-0.20411	0.019	0.1	4.36E-06	CSCL	PAQR8
IL1RN.2	2.93E-10	-0.28833	0.007	0.079	4.37E-06	CSCL	IL1RN
RP11-400F	2.97E-10	-0.11248	0.01	0.085	4.44E-06	CSCL	RP11-400F19.6
GSTK1	3.00E-10	0.129754	0.281	0.543	4.48E-06	CSCL	GSTK1
ZMYND8.2	3.01E-10	0.106724	0.087	0.214	4.49E-06	CSCL	ZMYND8
SGTB.1	3.02E-10	-0.11166	0.014	0.092	4.51E-06	CSCL	SGTB
RETSAT.1	3.07E-10	-0.13907	0.012	0.088	4.58E-06	CSCL	RETSAT
WDR41	3.16E-10	0.217769	0.098	0.232	4.73E-06	CSCL	WDR41
INSM1.2	3.21E-10	-0.12248	0.031	0.121	4.79E-06	CSCL	INSM1
PLAC8.1	3.24E-10	-0.34912	0.003	0.071	4.85E-06	CSCL	PLAC8
RASAL2	3.25E-10	-0.17169	0.009	0.081	4.86E-06	CSCL	RASAL2
FAM213A.1	3.34E-10	0.117704	0.195	0.392	4.99E-06	CSCL	FAM213A
IGF1.1	3.42E-10	-0.30991	0	0.065	5.11E-06	CSCL	IGF1
RNF111.2	3.43E-10	-0.11546	0.01	0.085	5.12E-06	CSCL	RNF111
ARG2.1	3.43E-10	-0.19666	0.021	0.103	5.12E-06	CSCL	ARG2
CHD7.2	3.46E-10	0.107688	0.14	0.296	5.18E-06	CSCL	CHD7
CHST14.1	3.46E-10	-0.12925	0.009	0.081	5.18E-06	CSCL	CHST14
AREG.2	3.48E-10	-0.44117	0.002	0.068	5.20E-06	CSCL	AREG
RFX1.1	3.48E-10	-0.12138	0.016	0.094	5.21E-06	CSCL	RFX1
SYCE1L.1	3.49E-10	-0.11145	0.005	0.075	5.21E-06	CSCL	SYCE1L

NIPAL3.1	3.55E-10	-0.18374	0.01	0.085	5.30E-06	CSCL	NIPAL3
SPARC.1	3.55E-10	0.62124	0.403	0.383	5.31E-06	CSCL	SPARC
NFYC.2	3.63E-10	0.143749	0.096	0.227	5.42E-06	CSCL	NFYC
DENND4C.1	3.63E-10	-0.14874	0.014	0.091	5.42E-06	CSCL	DENND4C
MED13L.1	3.65E-10	-0.13316	0.033	0.124	5.46E-06	CSCL	MED13L
DHPS.1	3.69E-10	0.153757	0.232	0.458	5.52E-06	CSCL	DHPS
NSMCE1.1	3.71E-10	0.191982	0.206	0.415	5.54E-06	CSCL	NSMCE1
LMF1	3.71E-10	-0.10574	0.023	0.106	5.54E-06	CSCL	LMF1
CBWD5.1	3.71E-10	-0.13755	0.035	0.126	5.55E-06	CSCL	CBWD5
PPIL1.1	3.71E-10	0.221873	0.079	0.2	5.55E-06	CSCL	PPIL1
PTRF.2	3.74E-10	0.150999	0.073	0.191	5.59E-06	CSCL	PTRF
RCL1	3.89E-10	-0.16745	0.009	0.081	5.82E-06	CSCL	RCL1
SELO.2	3.90E-10	-0.14976	0.002	0.068	5.83E-06	CSCL	SELO
ADSSL1.1	3.95E-10	-0.15913	0.01	0.084	5.90E-06	CSCL	ADSSL1
DYRK4.1	3.97E-10	0.126197	0.094	0.223	5.93E-06	CSCL	DYRK4
FUNDC1.1	3.98E-10	0.147091	0.112	0.253	5.95E-06	CSCL	FUNDC1
AURKA.2	4.01E-10	-0.18751	0.009	0.081	6.00E-06	CSCL	AURKA
TIA1.2	4.03E-10	0.127385	0.113	0.255	6.02E-06	CSCL	TIA1
OARD1.1	4.09E-10	0.163428	0.103	0.239	6.11E-06	CSCL	OARD1
MYCBP.1	4.11E-10	-0.10111	0.017	0.097	6.14E-06	CSCL	MYCBP
ABTB1.2	4.12E-10	-0.16087	0.024	0.108	6.15E-06	CSCL	ABTB1
GPANK1.1	4.18E-10	0.156423	0.079	0.199	6.25E-06	CSCL	GPANK1
ZNF787	4.23E-10	-0.11273	0.007	0.078	6.32E-06	CSCL	ZNF787
SYTL3.2	4.23E-10	-0.30769	0.002	0.068	6.33E-06	CSCL	SYTL3
C3orf58	4.28E-10	-0.15967	0.016	0.093	6.40E-06	CSCL	C3orf58
TM2D3.1	4.30E-10	0.207178	0.164	0.339	6.42E-06	CSCL	TM2D3
FBXO21	4.32E-10	0.106065	0.15	0.316	6.45E-06	CSCL	FBXO21
WDTC1.1	4.32E-10	-0.10694	0.021	0.103	6.46E-06	CSCL	WDTC1
WWP2.2	4.39E-10	-0.18912	0.005	0.074	6.55E-06	CSCL	WWP2
ADRBK1.2	4.40E-10	-0.15624	0.017	0.096	6.57E-06	CSCL	ADRBK1
CCT7.1	4.40E-10	0.18139	0.276	0.543	6.57E-06	CSCL	CCT7
JADE1.1	4.43E-10	-0.13246	0.037	0.128	6.62E-06	CSCL	JADE1
RGS12.2	4.46E-10	0.14286	0.127	0.278	6.67E-06	CSCL	RGS12
ARHGAP21.	4.48E-10	0.113912	0.115	0.257	6.70E-06	CSCL	ARHGAP21
GKAP1.1	4.50E-10	-0.10789	0.016	0.093	6.72E-06	CSCL	GKAP1
AGO1.2	4.50E-10	-0.12882	0.016	0.093	6.73E-06	CSCL	AGO1
MX2.2	4.51E-10	-0.29044	0.002	0.067	6.74E-06	CSCL	MX2
MALT1.2	4.52E-10	-0.14725	0.007	0.077	6.75E-06	CSCL	MALT1
THOC5.1	4.53E-10	-0.13171	0.014	0.09	6.77E-06	CSCL	THOC5
RP2.2	4.53E-10	-0.16482	0.009	0.081	6.77E-06	CSCL	RP2
CISD1.2	4.54E-10	0.170278	0.168	0.345	6.78E-06	CSCL	CISD1
BCL3.2	4.58E-10	-0.10213	0.009	0.081	6.84E-06	CSCL	BCL3
UFC1.1	4.61E-10	0.151144	0.297	0.575	6.88E-06	CSCL	UFC1
NIFK.1	4.63E-10	0.230001	0.173	0.357	6.91E-06	CSCL	NIFK
CENPM.2	4.66E-10	-0.1802	0.007	0.077	6.97E-06	CSCL	CENPM
RABGAP1L.	4.67E-10	-0.12731	0.028	0.114	6.98E-06	CSCL	RABGAP1L
HECA.2	4.68E-10	-0.17862	0.005	0.074	6.99E-06	CSCL	HECA
TDRD3	4.71E-10	-0.10454	0.017	0.096	7.04E-06	CSCL	TDRD3
PCBD2	4.72E-10	-0.13735	0.012	0.087	7.06E-06	CSCL	PCBD2
PYGO2.1	4.73E-10	-0.1201	0.009	0.081	7.07E-06	CSCL	PYGO2
POMZP3.2	4.75E-10	-0.13003	0.014	0.09	7.11E-06	CSCL	POMZP3

BLNK.2	4.77E-10	-0.32765	0.003	0.07	7.14E-06	CSCL	BLNK
SSBP2.2	4.87E-10	0.129068	0.127	0.277	7.28E-06	CSCL	SSBP2
TACC3.2	4.89E-10	-0.16819	0.009	0.08	7.31E-06	CSCL	TACC3
GMDS.1	4.95E-10	-0.10154	0.016	0.093	7.40E-06	CSCL	GMDS
GPR161.2	4.99E-10	-0.1491	0.012	0.087	7.45E-06	CSCL	GPR161
INADL.1	5.04E-10	-0.2036	0.003	0.07	7.53E-06	CSCL	INADL
PI4K2B.1	5.13E-10	-0.11238	0.014	0.09	7.66E-06	CSCL	PI4K2B
ITGA6.2	5.27E-10	-0.10693	0.009	0.08	7.88E-06	CSCL	ITGA6
RACGAP1.1	5.28E-10	-0.16032	0.012	0.087	7.89E-06	CSCL	RACGAP1
SEC24D	5.40E-10	-0.10053	0.017	0.096	8.07E-06	CSCL	SEC24D
EPC2.1	5.45E-10	-0.12799	0.019	0.099	8.14E-06	CSCL	EPC2
NCBP1.1	5.70E-10	-0.11086	0.012	0.086	8.51E-06	CSCL	NCBP1
PRPF38A.1	5.90E-10	0.209953	0.14	0.298	8.82E-06	CSCL	PRPF38A
DIXDC1.1	5.98E-10	-0.11893	0.019	0.099	8.94E-06	CSCL	DIXDC1
PAK3.2	6.01E-10	-0.12266	0.049	0.148	8.99E-06	CSCL	PAK3
NDUFB5	6.08E-10	0.125389	0.295	0.575	9.09E-06	CSCL	NDUFB5
THEM4.1	6.08E-10	-0.15906	0.009	0.08	9.09E-06	CSCL	THEM4
SLC39A11.	6.10E-10	-0.14918	0.007	0.076	9.12E-06	CSCL	SLC39A11
PLD2.2	6.14E-10	-0.11698	0.014	0.089	9.17E-06	CSCL	PLD2
SNRPD3.2	6.28E-10	0.2299	0.211	0.424	9.39E-06	CSCL	SNRPD3
SYNGR1.2	6.41E-10	-0.11702	0.03	0.116	9.57E-06	CSCL	SYNGR1
AKAP10.1	6.41E-10	-0.12241	0.017	0.095	9.57E-06	CSCL	AKAP10
ZZEF1.1	6.42E-10	-0.13237	0.014	0.089	9.60E-06	CSCL	ZZEF1
RP11-386I	6.45E-10	-0.28317	0.003	0.07	9.63E-06	CSCL	RP11-386I14.4
RAB5B.1	6.49E-10	0.10513	0.092	0.22	9.71E-06	CSCL	RAB5B
AUH.1	6.76E-10	-0.13187	0.01	0.083	1.01E-05	CSCL	AUH
FAM20A.2	6.77E-10	-0.24671	0	0.063	1.01E-05	CSCL	FAM20A
BRIX1.2	6.81E-10	0.183894	0.117	0.26	1.02E-05	CSCL	BRIX1
PUS1.1	6.93E-10	-0.13487	0.01	0.083	1.04E-05	CSCL	PUS1
VPS8.1	6.93E-10	-0.10304	0.03	0.116	1.04E-05	CSCL	VPS8
TRIM11.1	6.93E-10	-0.10217	0.021	0.101	1.04E-05	CSCL	TRIM11
UQCRB.2	7.01E-10	0.106749	0.363	0.694	1.05E-05	CSCL	UQCRB
MAML2.1	7.04E-10	0.105309	0.082	0.204	1.05E-05	CSCL	MAML2
RAD51AP1.	7.09E-10	-0.17641	0.003	0.069	1.06E-05	CSCL	RAD51AP1
C15orf48.	7.22E-10	-0.31525	0.005	0.072	1.08E-05	CSCL	C15orf48
TMEM220.2	7.31E-10	-0.18204	0.005	0.072	1.09E-05	CSCL	TMEM220
FAM64A.2	7.32E-10	-0.2386	0.005	0.072	1.09E-05	CSCL	FAM64A
RRM2.2	7.35E-10	-0.26019	0.002	0.066	1.10E-05	CSCL	RRM2
SETD5.2	7.46E-10	0.179792	0.138	0.296	1.11E-05	CSCL	SETD5
TBCA.2	7.62E-10	0.416112	0.562	0.718	1.14E-05	CSCL	TBCA
UBA7.2	7.70E-10	-0.20466	0.012	0.085	1.15E-05	CSCL	UBA7
CCDC12.1	7.82E-10	0.141847	0.225	0.441	1.17E-05	CSCL	CCDC12
C7orf43.2	7.92E-10	-0.15436	0.005	0.072	1.18E-05	CSCL	C7orf43
AGAP3.1	7.94E-10	-0.13002	0.023	0.104	1.19E-05	CSCL	AGAP3
GPM6A.1	8.01E-10	0.465673	0.471	0.506	1.20E-05	CSCL	GPM6A
AKNA.1	8.04E-10	-0.25008	0.007	0.075	1.20E-05	CSCL	AKNA
RCAN3.2	8.08E-10	-0.17965	0	0.062	1.21E-05	CSCL	RCAN3
UBE4B.1	8.09E-10	-0.10661	0.021	0.101	1.21E-05	CSCL	UBE4B
PDHB.1	8.18E-10	0.205558	0.185	0.372	1.22E-05	CSCL	PDHB
NUP133.1	8.50E-10	-0.11919	0.019	0.097	1.27E-05	CSCL	NUP133
GNAI1.2	8.62E-10	-0.12925	0.026	0.109	1.29E-05	CSCL	GNAI1

SLC15A3.2	8.63E-10	-0.24181	0.005	0.072	1.29E-05	CSCL	SLC15A3
FUNDC2.1	8.69E-10	0.211786	0.208	0.413	1.30E-05	CSCL	FUNDC2
UPF3B.1	8.80E-10	0.129202	0.117	0.258	1.32E-05	CSCL	UPF3B
ACVR2A	8.81E-10	-0.13694	0.005	0.072	1.32E-05	CSCL	ACVR2A
IRF5.2	8.83E-10	-0.26377	0	0.062	1.32E-05	CSCL	IRF5
MSRA.1	8.84E-10	0.128071	0.12	0.263	1.32E-05	CSCL	MSRA
APOPT1	8.87E-10	0.218582	0.194	0.391	1.33E-05	CSCL	APOPT1
CALM1.2	8.90E-10	0.382553	0.597	0.781	1.33E-05	CSCL	CALM1
BBC3.1	8.98E-10	-0.14567	0.012	0.085	1.34E-05	CSCL	BBC3
RXRA.1	9.02E-10	-0.12407	0.021	0.1	1.35E-05	CSCL	RXRA
PRRX1.2	9.15E-10	0.123684	0.066	0.176	1.37E-05	CSCL	PRRX1
OPN3.2	9.15E-10	-0.14459	0.007	0.075	1.37E-05	CSCL	OPN3
DOK3.2	9.23E-10	-0.22131	0.003	0.069	1.38E-05	CSCL	DOK3
KIAA0319L	9.39E-10	0.102477	0.075	0.19	1.40E-05	CSCL	KIAA0319L
ARMCX3.1	9.54E-10	0.172477	0.222	0.44	1.43E-05	CSCL	ARMCX3
AK4.2	9.54E-10	0.165835	0.072	0.185	1.43E-05	CSCL	AK4
SERPINB8	9.59E-10	-0.1802	0.01	0.081	1.43E-05	CSCL	SERPINB8
HTRA1.2	9.63E-10	0.133263	0.244	0.471	1.44E-05	CSCL	HTRA1
MNAT1.1	9.69E-10	0.172915	0.08	0.199	1.45E-05	CSCL	MNAT1
ELP6	9.77E-10	0.142966	0.094	0.221	1.46E-05	CSCL	ELP6
TRUB2	9.78E-10	0.145811	0.092	0.219	1.46E-05	CSCL	TRUB2
EEPD1.1	1.00E-09	-0.1929	0.007	0.075	1.50E-05	CSCL	EEPD1
PPM1M.2	1.01E-09	-0.16948	0.009	0.078	1.51E-05	CSCL	PPM1M
TUBB6.1	1.03E-09	0.100805	0.075	0.19	1.53E-05	CSCL	TUBB6
CDC20.1	1.03E-09	-0.23121	0.005	0.071	1.53E-05	CSCL	CDC20
CDC42BPA	1.03E-09	0.123459	0.072	0.184	1.55E-05	CSCL	CDC42BPA
CELF4.2	1.07E-09	-0.21023	0.019	0.096	1.60E-05	CSCL	CELF4
CDK8.1	1.09E-09	-0.10476	0.012	0.084	1.63E-05	CSCL	CDK8
IL4I1.2	1.09E-09	-0.25262	0.003	0.068	1.64E-05	CSCL	IL4I1
PARP10.1	1.11E-09	-0.17694	0.016	0.09	1.66E-05	CSCL	PARP10
CD300C.2	1.12E-09	-0.28353	0.005	0.071	1.68E-05	CSCL	CD300C
CLP1.1	1.13E-09	-0.1463	0.009	0.078	1.69E-05	CSCL	CLP1
EPHB2.2	1.14E-09	-0.13614	0.014	0.087	1.70E-05	CSCL	EPHB2
FCHO2.2	1.15E-09	-0.18987	0.009	0.078	1.72E-05	CSCL	FCHO2
RFX5.2	1.17E-09	-0.11772	0.026	0.108	1.75E-05	CSCL	RFX5
ZNF22.1	1.19E-09	0.17553	0.133	0.283	1.77E-05	CSCL	ZNF22
GPR82.2	1.21E-09	-0.13118	0.045	0.139	1.81E-05	CSCL	GPR82
TNFRSF14.	1.21E-09	-0.15189	0.033	0.119	1.81E-05	CSCL	TNFRSF14
TADA2A	1.22E-09	-0.11055	0.009	0.078	1.82E-05	CSCL	TADA2A
PPP2R5D.1	1.22E-09	-0.12669	0.009	0.078	1.83E-05	CSCL	PPP2R5D
PARP8.2	1.24E-09	-0.18848	0.005	0.071	1.85E-05	CSCL	PARP8
TMEM158.1	1.25E-09	0.108153	0.047	0.144	1.86E-05	CSCL	TMEM158
MEF2D.1	1.29E-09	-0.20664	0.009	0.077	1.92E-05	CSCL	MEF2D
PHRF1.2	1.30E-09	-0.12779	0.014	0.087	1.94E-05	CSCL	PHRF1
UVRAG.2	1.31E-09	-0.14746	0.017	0.093	1.96E-05	CSCL	UVRAG
EPS8.1	1.32E-09	-0.13864	0.04	0.131	1.97E-05	CSCL	EPS8
ECSIT.1	1.33E-09	0.201544	0.127	0.275	1.99E-05	CSCL	ECSIT
ATP5B.2	1.34E-09	0.343556	0.586	0.785	2.00E-05	CSCL	ATP5B
PRR4.2	1.35E-09	0.133623	0.096	0.223	2.02E-05	CSCL	PRR4
ARHGAP24.	1.37E-09	-0.22323	0.056	0.153	2.04E-05	CSCL	ARHGAP24
CCL20.2	1.37E-09	-0.31365	0.002	0.064	2.05E-05	CSCL	CCL20

TMEM42.1	1.39E-09	0.106299	0.061	0.166	2.07E-05	CSCL	TMEM42
GCAT.2	1.40E-09	0.123741	0.061	0.166	2.09E-05	CSCL	GCAT
BANP.1	1.41E-09	-0.13734	0.012	0.083	2.10E-05	CSCL	BANP
RASSF7.2	1.41E-09	-0.17154	0.007	0.074	2.11E-05	CSCL	RASSF7
NFKB2.2	1.44E-09	-0.19191	0.002	0.064	2.14E-05	CSCL	NFKB2
ERF.2	1.48E-09	0.140406	0.072	0.183	2.21E-05	CSCL	ERF
PAPD5.2	1.49E-09	-0.14929	0.012	0.083	2.22E-05	CSCL	PAPD5
MAP3K3.2	1.49E-09	-0.18155	0.003	0.067	2.23E-05	CSCL	MAP3K3
ZNF598.1	1.49E-09	-0.10099	0.007	0.074	2.23E-05	CSCL	ZNF598
PRR11.1	1.49E-09	-0.1398	0.003	0.067	2.23E-05	CSCL	PRR11
IQSEC1	1.52E-09	-0.1414	0.005	0.07	2.28E-05	CSCL	IQSEC1
LSM6	1.54E-09	0.227228	0.166	0.339	2.30E-05	CSCL	LSM6
RAN.2	1.54E-09	0.379364	0.579	0.766	2.30E-05	CSCL	RAN
TRMT61B	1.56E-09	0.108622	0.063	0.168	2.33E-05	CSCL	TRMT61B
ATP5L.1	1.57E-09	0.274058	0.63	0.871	2.34E-05	CSCL	ATP5L
IGSF21.2	1.57E-09	-0.11739	0.065	0.168	2.35E-05	CSCL	IGSF21
BCAT1.2	1.60E-09	0.196943	0.105	0.238	2.39E-05	CSCL	BCAT1
SGMS1.1	1.62E-09	-0.16911	0.002	0.063	2.42E-05	CSCL	SGMS1
HIGD1A.1	1.64E-09	0.198486	0.222	0.438	2.45E-05	CSCL	HIGD1A
ST3GAL1.2	1.65E-09	-0.20217	0.026	0.106	2.46E-05	CSCL	ST3GAL1
YAF2.1	1.69E-09	0.168235	0.176	0.355	2.53E-05	CSCL	YAF2
DOK5.1	1.72E-09	0.120286	0.075	0.188	2.56E-05	CSCL	DOK5
SARS.2	1.75E-09	0.221631	0.23	0.454	2.62E-05	CSCL	SARS
HDAC9.2	1.75E-09	-0.10897	0.04	0.13	2.62E-05	CSCL	HDAC9
IFT43.1	1.76E-09	0.188978	0.122	0.264	2.63E-05	CSCL	IFT43
ARHGEF12	1.77E-09	0.114086	0.061	0.165	2.65E-05	CSCL	ARHGEF12
EHD4.2	1.77E-09	-0.12306	0.019	0.095	2.65E-05	CSCL	EHD4
AGPAT5.1	1.77E-09	0.120175	0.092	0.215	2.65E-05	CSCL	AGPAT5
ADD2	1.80E-09	-0.15185	0.017	0.092	2.69E-05	CSCL	ADD2
ZNF24	1.81E-09	0.224645	0.148	0.308	2.70E-05	CSCL	ZNF24
BLVRA.2	1.82E-09	0.162964	0.173	0.348	2.73E-05	CSCL	BLVRA
GAPT.2	1.88E-09	-0.27479	0.002	0.063	2.81E-05	CSCL	GAPT
TNFRSF10E	1.94E-09	-0.15751	0.026	0.106	2.90E-05	CSCL	TNFRSF10B
ASGR1.2	1.94E-09	-0.1929	0.009	0.076	2.90E-05	CSCL	ASGR1
SCAF8.1	1.95E-09	-0.14024	0.007	0.073	2.91E-05	CSCL	SCAF8
AFAP1	1.98E-09	-0.11927	0.016	0.089	2.97E-05	CSCL	AFAP1
PSMB9.1	1.98E-09	0.206203	0.211	0.415	2.97E-05	CSCL	PSMB9
PLEKHA8.1	1.99E-09	-0.14804	0.003	0.066	2.97E-05	CSCL	PLEKHA8
TLN2.2	2.01E-09	-0.14236	0.021	0.097	3.01E-05	CSCL	TLN2
CLUAP1.1	2.02E-09	0.105723	0.066	0.173	3.02E-05	CSCL	CLUAP1
CRLF3.2	2.04E-09	-0.14203	0.033	0.117	3.05E-05	CSCL	CRLF3
SLC19A1.1	2.06E-09	-0.11153	0.007	0.073	3.07E-05	CSCL	SLC19A1
SEC23IP.1	2.06E-09	-0.15083	0.012	0.082	3.08E-05	CSCL	SEC23IP
YPEL2.2	2.06E-09	-0.20086	0.007	0.073	3.08E-05	CSCL	YPEL2
ORC6.2	2.07E-09	-0.13174	0.019	0.095	3.09E-05	CSCL	ORC6
NCAPD2.2	2.13E-09	-0.12819	0.003	0.066	3.18E-05	CSCL	NCAPD2
FPR3.2	2.14E-09	-0.23828	0	0.059	3.20E-05	CSCL	FPR3
DOT1L.1	2.17E-09	-0.1065	0.012	0.082	3.24E-05	CSCL	DOT1L
SLC1A4.2	2.18E-09	-0.1361	0.009	0.076	3.25E-05	CSCL	SLC1A4
RFNG.1	2.22E-09	-0.10262	0.024	0.103	3.32E-05	CSCL	RFNG
EPOR.1	2.24E-09	-0.17505	0.007	0.072	3.35E-05	CSCL	EPOR

SUPT16H.2	2.25E-09	0.206678	0.117	0.255	3.36E-05	CSCL	SUPT16H
PRKCSH.1	2.27E-09	0.130771	0.229	0.441	3.39E-05	CSCL	PRKCSH
ANKRD13A.	2.30E-09	-0.12704	0.023	0.1	3.44E-05	CSCL	ANKRD13A
ZBTB25.1	2.39E-09	-0.11347	0.017	0.091	3.57E-05	CSCL	ZBTB25
TIMM10B.1	2.41E-09	0.105594	0.072	0.181	3.60E-05	CSCL	TIMM10B
EAPP.2	2.43E-09	0.19542	0.183	0.363	3.62E-05	CSCL	EAPP
KIF20B.2	2.45E-09	-0.17674	0.017	0.091	3.66E-05	CSCL	KIF20B
MKI67.2	2.47E-09	-0.19719	0.003	0.066	3.69E-05	CSCL	MKI67
PCNP.2	2.49E-09	0.133913	0.283	0.55	3.72E-05	CSCL	PCNP
MFAP1.1	2.50E-09	0.138256	0.07	0.178	3.74E-05	CSCL	MFAP1
ZNF629	2.51E-09	-0.10557	0.007	0.072	3.74E-05	CSCL	ZNF629
STK10.2	2.52E-09	-0.21065	0.009	0.075	3.77E-05	CSCL	STK10
COMMD10.1	2.55E-09	0.17034	0.103	0.231	3.81E-05	CSCL	COMMD10
GPC2.2	2.55E-09	-0.14466	0.07	0.173	3.82E-05	CSCL	GPC2
LSM1	2.55E-09	0.19504	0.241	0.467	3.82E-05	CSCL	LSM1
DYNC2LI1.	2.62E-09	0.124765	0.065	0.169	3.91E-05	CSCL	DYNC2LI1
CAMTA2.1	2.71E-09	-0.10498	0.012	0.081	4.04E-05	CSCL	CAMTA2
GHDC.1	2.71E-09	-0.10022	0.021	0.097	4.06E-05	CSCL	GHDC
DENND4B.2	2.73E-09	-0.14936	0.007	0.072	4.08E-05	CSCL	DENND4B
ZC3H14.1	2.73E-09	0.184124	0.089	0.209	4.08E-05	CSCL	ZC3H14
MBNL2.2	2.77E-09	-0.13255	0.023	0.099	4.14E-05	CSCL	MBNL2
REXO2.2	2.80E-09	0.138725	0.162	0.327	4.18E-05	CSCL	REXO2
CCNYL1.1	2.81E-09	-0.13805	0.002	0.062	4.20E-05	CSCL	CCNYL1
TRPV2.2	2.82E-09	-0.24394	0.002	0.062	4.22E-05	CSCL	TRPV2
PSMC1.1	2.84E-09	0.225301	0.199	0.394	4.25E-05	CSCL	PSMC1
PIGZ	2.86E-09	-0.10416	0.003	0.065	4.27E-05	CSCL	PIGZ
SCP2.2	2.93E-09	0.103468	0.314	0.597	4.37E-05	CSCL	SCP2
MIR181A1H	2.95E-09	-0.13391	0.033	0.117	4.41E-05	CSCL	MIR181A1HG
MYT1L.2	3.00E-09	-0.21973	0.002	0.062	4.48E-05	CSCL	MYT1L
CTNS	3.07E-09	-0.10848	0.016	0.087	4.59E-05	CSCL	CTNS
LRP1.1	3.08E-09	0.160384	0.182	0.359	4.61E-05	CSCL	LRP1
TPCN1.2	3.09E-09	-0.14756	0.019	0.093	4.61E-05	CSCL	TPCN1
RREB1.2	3.11E-09	-0.1508	0.009	0.075	4.65E-05	CSCL	RREB1
SRP14.1	3.27E-09	0.215219	0.64	0.878	4.89E-05	CSCL	SRP14
BRSK1	3.29E-09	-0.11729	0.016	0.087	4.91E-05	CSCL	BRSK1
SEC24A.1	3.29E-09	-0.10118	0.01	0.078	4.92E-05	CSCL	SEC24A
UPRT	3.32E-09	-0.11283	0.017	0.09	4.96E-05	CSCL	UPRT
SYT1.2	3.32E-09	-0.25074	0.026	0.104	4.97E-05	CSCL	SYT1
PRMT3.1	3.35E-09	-0.1056	0.019	0.093	5.01E-05	CSCL	PRMT3
EMILIN2.2	3.37E-09	-0.1148	0.014	0.084	5.04E-05	CSCL	EMILIN2
IL6R.2	3.41E-09	-0.23792	0.017	0.089	5.09E-05	CSCL	IL6R
DRG2.1	3.41E-09	0.173964	0.087	0.206	5.09E-05	CSCL	DRG2
RP11-556E	3.43E-09	-0.27838	0	0.058	5.13E-05	CSCL	RP11-556E13.1
CCDC28B.1	3.48E-09	0.207874	0.099	0.225	5.20E-05	CSCL	CCDC28B
NTPCR.1	3.51E-09	0.205561	0.134	0.28	5.25E-05	CSCL	NTPCR
FMNL3.2	3.61E-09	-0.18174	0.012	0.08	5.39E-05	CSCL	FMNL3
EML2.2	3.62E-09	-0.13906	0.016	0.086	5.40E-05	CSCL	EML2
PRPS1.2	3.63E-09	0.114019	0.082	0.195	5.42E-05	CSCL	PRPS1
CFL2.1	3.65E-09	0.199471	0.168	0.336	5.46E-05	CSCL	CFL2
NLE1.1	3.74E-09	-0.105	0.005	0.068	5.60E-05	CSCL	NLE1
FYN.1	3.82E-09	0.174605	0.166	0.334	5.71E-05	CSCL	FYN

ACAP1.1	3.87E-09	-0.1348	0.014	0.083	5.78E-05	CSCL	ACAP1
PROSER1.1	4.00E-09	-0.1228	0.01	0.077	5.98E-05	CSCL	PROSER1
CTHRC1.1	4.00E-09	-0.10985	0.005	0.068	5.98E-05	CSCL	CTHRC1
GIMAP7.1	4.09E-09	-0.28791	0	0.057	6.12E-05	CSCL	GIMAP7
CPPED1.2	4.10E-09	-0.18916	0.007	0.071	6.13E-05	CSCL	CPPED1
CLEC4E.1	4.11E-09	-0.33056	0.002	0.061	6.15E-05	CSCL	CLEC4E
TCEAL1.2	4.11E-09	0.112668	0.124	0.262	6.15E-05	CSCL	TCEAL1
TRIM26	4.15E-09	-0.12499	0.024	0.101	6.21E-05	CSCL	TRIM26
HHEX.2	4.22E-09	-0.26745	0.002	0.06	6.31E-05	CSCL	HHEX
ZC3H3.1	4.23E-09	-0.12199	0.014	0.083	6.31E-05	CSCL	ZC3H3
MBD6.2	4.23E-09	-0.10305	0.051	0.144	6.32E-05	CSCL	MBD6
KCNAB2.2	4.23E-09	-0.20685	0.012	0.08	6.32E-05	CSCL	KCNAB2
GTSE1.1	4.28E-09	-0.19546	0.002	0.06	6.39E-05	CSCL	GTSE1
SOS2.2	4.33E-09	-0.10386	0.016	0.086	6.46E-05	CSCL	SOS2
ATG7.1	4.39E-09	-0.12631	0.033	0.115	6.56E-05	CSCL	ATG7
ABHD15.2	4.39E-09	-0.20134	0.002	0.06	6.56E-05	CSCL	ABHD15
TRIM47.2	4.48E-09	0.143576	0.075	0.185	6.70E-05	CSCL	TRIM47
SGOL1.1	4.52E-09	-0.20264	0.002	0.06	6.75E-05	CSCL	SGOL1
PDE4DIP.1	4.53E-09	0.103998	0.131	0.271	6.77E-05	CSCL	PDE4DIP
MOCS2.2	4.54E-09	0.211352	0.127	0.27	6.78E-05	CSCL	MOCS2
INF2.2	4.56E-09	-0.14799	0.017	0.089	6.82E-05	CSCL	INF2
ETV3.2	4.61E-09	-0.15341	0.012	0.08	6.89E-05	CSCL	ETV3
PAQR3.1	4.63E-09	-0.10548	0.012	0.08	6.92E-05	CSCL	PAQR3
MYO1G.2	4.66E-09	-0.22047	0.003	0.064	6.96E-05	CSCL	MYO1G
DRAM1.1	4.80E-09	-0.12463	0.01	0.077	7.17E-05	CSCL	DRAM1
LUC7L3	4.90E-09	0.139903	0.276	0.522	7.32E-05	CSCL	LUC7L3
SMAD5.1	4.92E-09	0.138404	0.07	0.176	7.35E-05	CSCL	SMAD5
RASAL3.2	4.94E-09	-0.24319	0.002	0.06	7.39E-05	CSCL	RASAL3
B3GNTL1.1	4.99E-09	-0.11895	0.003	0.063	7.46E-05	CSCL	B3GNTL1
KNSTRN.2	5.08E-09	-0.14876	0.014	0.083	7.59E-05	CSCL	KNSTRN
CNN2.1	5.15E-09	-0.18641	0.007	0.07	7.70E-05	CSCL	CNN2
LDLRAP1.2	5.24E-09	-0.17146	0.002	0.06	7.83E-05	CSCL	LDLRAP1
PDK3.1	5.33E-09	-0.16285	0.003	0.063	7.96E-05	CSCL	PDK3
CLIP3.1	5.34E-09	0.166493	0.087	0.203	7.98E-05	CSCL	CLIP3
PSRC1	5.39E-09	0.145715	0.129	0.271	8.06E-05	CSCL	PSRC1
HVCN1.2	5.44E-09	-0.14151	0.024	0.1	8.13E-05	CSCL	HVCN1
TMCC3.2	5.44E-09	-0.16753	0.01	0.076	8.13E-05	CSCL	TMCC3
ARNT.1	5.45E-09	-0.10172	0.01	0.076	8.15E-05	CSCL	ARNT
ADCY7.2	5.55E-09	-0.24507	0.005	0.066	8.29E-05	CSCL	ADCY7
SFXN5.2	5.60E-09	0.102655	0.082	0.194	8.37E-05	CSCL	SFXN5
SCFD1.1	5.67E-09	0.151823	0.122	0.259	8.48E-05	CSCL	SCFD1
FBXO34.2	5.68E-09	-0.12255	0.021	0.094	8.49E-05	CSCL	FBXO34
ZMYND15.1	5.72E-09	-0.15964	0.002	0.06	8.54E-05	CSCL	ZMYND15
DPYSL3.1	5.73E-09	0.127822	0.166	0.33	8.56E-05	CSCL	DPYSL3
ELOF1.1	5.79E-09	0.21868	0.223	0.434	8.65E-05	CSCL	ELOF1
RASA2.1	5.80E-09	-0.11037	0.012	0.079	8.67E-05	CSCL	RASA2
HIST2H2AA	5.88E-09	-0.20555	0.005	0.066	8.79E-05	CSCL	HIST2H2AA3
WDR6.1	5.91E-09	0.106357	0.11	0.238	8.83E-05	CSCL	WDR6
TROVE2.1	6.03E-09	0.235691	0.23	0.443	9.01E-05	CSCL	TROVE2
PRAM1.2	6.17E-09	-0.253	0	0.056	9.23E-05	CSCL	PRAM1
TNFAIP8.1	6.18E-09	-0.23344	0.009	0.073	9.23E-05	CSCL	TNFAIP8

WDSUB1.1	6.18E-09	-0.11431	0.012	0.079	9.23E-05	CSCL	WDSUB1
LYRM5	6.25E-09	0.218447	0.176	0.349	9.35E-05	CSCL	LYRM5
MAN2A2.1	6.34E-09	-0.10198	0.012	0.079	9.47E-05	CSCL	MAN2A2
SPARCL1.1	6.40E-09	0.593504	0.373	0.344	9.56E-05	CSCL	SPARCL1
PIEZO1.2	6.40E-09	-0.11677	0.009	0.073	9.56E-05	CSCL	PIEZO1
LPCAT4.2	6.40E-09	-0.12144	0.009	0.072	9.57E-05	CSCL	LPCAT4
RP11-138A	6.42E-09	-0.20082	0.044	0.129	9.59E-05	CSCL	RP11-138A9.1
B3GNT2.2	6.47E-09	-0.13516	0.007	0.069	9.68E-05	CSCL	B3GNT2
TUSC1.1	6.60E-09	-0.12252	0.014	0.082	9.86E-05	CSCL	TUSC1
STMN2.2	6.62E-09	-0.30542	0.089	0.192	9.90E-05	CSCL	STMN2
PTPRS.2	6.72E-09	0.109619	0.127	0.265	0.0001	CSCL	PTPRS
KLHDC4.1	6.74E-09	-0.15705	0.01	0.075	0.000101	CSCL	KLHDC4
SLC9A9.2	6.79E-09	-0.19005	0.014	0.081	0.000102	CSCL	SLC9A9
ARHGAP20	6.82E-09	-0.11023	0.003	0.063	0.000102	CSCL	ARHGAP20
GGCT.2	6.82E-09	0.222729	0.202	0.396	0.000102	CSCL	GGCT
ZMYM3.1	7.01E-09	-0.11862	0.014	0.082	0.000105	CSCL	ZMYM3
NMI.1	7.12E-09	0.128907	0.079	0.188	0.000106	CSCL	NMI
ATAD5.1	7.15E-09	-0.10836	0.012	0.079	0.000107	CSCL	ATAD5
FHOD1.2	7.20E-09	-0.18832	0.002	0.059	0.000108	CSCL	FHOD1
ITPRIP.2	7.23E-09	-0.17178	0.012	0.078	0.000108	CSCL	ITPRIP
TMEM156.2	7.27E-09	-0.27623	0.002	0.059	0.000109	CSCL	TMEM156
MAP1A.1	7.40E-09	0.131656	0.11	0.236	0.000111	CSCL	MAP1A
HEATR3	7.49E-09	-0.1604	0.002	0.059	0.000112	CSCL	HEATR3
EIF1AX.1	7.57E-09	0.130523	0.297	0.573	0.000113	CSCL	EIF1AX
PAOX.2	7.58E-09	-0.14727	0	0.055	0.000113	CSCL	PAOX
TTC9B.2	7.60E-09	-0.16407	0.04	0.124	0.000114	CSCL	TTC9B
ZNF669.1	7.73E-09	-0.11062	0.024	0.099	0.000116	CSCL	ZNF669
ITGA5	7.97E-09	-0.11106	0.017	0.087	0.000119	CSCL	ITGA5
ZNF37A.2	8.04E-09	0.103448	0.051	0.143	0.00012	CSCL	ZNF37A
TMEM144.2	8.06E-09	-0.30119	0.014	0.081	0.00012	CSCL	TMEM144
LTBR.2	8.07E-09	-0.18795	0.024	0.099	0.000121	CSCL	LTBR
TCTEX1D2.	8.09E-09	0.247876	0.106	0.233	0.000121	CSCL	TCTEX1D2
GNGT2.2	8.28E-09	-0.24181	0	0.055	0.000124	CSCL	GNGT2
PARP12.2	8.33E-09	-0.12846	0.012	0.078	0.000125	CSCL	PARP12
LRRC8C.2	8.39E-09	-0.16642	0.003	0.062	0.000125	CSCL	LRRC8C
DOK2.1	8.42E-09	-0.2611	0.002	0.058	0.000126	CSCL	DOK2
FURIN.2	8.46E-09	-0.15415	0.01	0.075	0.000126	CSCL	FURIN
STRAP.1	8.46E-09	0.106775	0.3	0.566	0.000126	CSCL	STRAP
SEZ6L2.1	8.62E-09	0.139795	0.136	0.28	0.000129	CSCL	SEZ6L2
ALDH3B1	8.73E-09	-0.16584	0.01	0.075	0.00013	CSCL	ALDH3B1
GCDH	8.95E-09	0.127205	0.089	0.203	0.000134	CSCL	GCDH
MEX3D.1	8.99E-09	-0.12043	0.005	0.065	0.000134	CSCL	MEX3D
S100A10.2	9.02E-09	0.514263	0.492	0.547	0.000135	CSCL	S100A10
PGAM5.1	9.05E-09	-0.12773	0.002	0.058	0.000135	CSCL	PGAM5
DAPK1	9.12E-09	-0.16014	0.003	0.062	0.000136	CSCL	DAPK1
MAP2.2	9.12E-09	0.131171	0.176	0.346	0.000136	CSCL	MAP2
UBR1.1	9.23E-09	-0.12126	0.014	0.081	0.000138	CSCL	UBR1
WDR91.2	9.27E-09	-0.12485	0.019	0.089	0.000139	CSCL	WDR91
FLI1.2	9.30E-09	-0.24566	0	0.055	0.000139	CSCL	FLI1
ZDHHC4.2	9.39E-09	0.225735	0.185	0.363	0.00014	CSCL	ZDHHC4
TRAPPC8.1	9.39E-09	-0.11735	0.009	0.071	0.00014	CSCL	TRAPPC8

WDR36	9.42E-09	-0.10427	0.014	0.081	0.000141	CSCL	WDR36
CRTC2.1	9.47E-09	-0.10308	0.012	0.077	0.000142	CSCL	CRTC2
NFIL3.2	9.49E-09	-0.1923	0.017	0.087	0.000142	CSCL	NFIL3
IL27RA.1	9.69E-09	-0.13201	0.002	0.058	0.000145	CSCL	IL27RA
PI4K2A.2	9.72E-09	-0.13697	0.007	0.068	0.000145	CSCL	PI4K2A
STOX2.2	9.73E-09	-0.1071	0.023	0.096	0.000145	CSCL	STOX2
PEMT.1	9.78E-09	0.185267	0.098	0.218	0.000146	CSCL	PEMT
DNAJC19.2	9.82E-09	0.222995	0.206	0.398	0.000147	CSCL	DNAJC19
LHFP.2	9.98E-09	0.172126	0.11	0.238	0.000149	CSCL	LHFP
FAM195B.1	1.00E-08	0.575865	0.122	0.065	0.000149	CSCL	FAM195B
ZNF75A.1	1.03E-08	0.141697	0.099	0.22	0.000153	CSCL	ZNF75A
SPDL1.1	1.03E-08	-0.11748	0.007	0.068	0.000155	CSCL	SPDL1
NSG1.2	1.04E-08	-0.1383	0.079	0.181	0.000155	CSCL	NSG1
HPSE.2	1.04E-08	-0.25152	0.003	0.061	0.000155	CSCL	HPSE
TRIP6.2	1.04E-08	0.140451	0.122	0.255	0.000156	CSCL	TRIP6
TUBB4A.2	1.05E-08	-0.1943	0.04	0.122	0.000156	CSCL	TUBB4A
PET100.1	1.05E-08	0.130614	0.283	0.537	0.000157	CSCL	PET100
THAP3.1	1.05E-08	0.117145	0.049	0.139	0.000158	CSCL	THAP3
ATP5H.1	1.06E-08	0.120784	0.353	0.664	0.000159	CSCL	ATP5H
PCSK7.2	1.11E-08	0.121813	0.143	0.289	0.000165	CSCL	PCSK7
MR1	1.12E-08	-0.12578	0.021	0.092	0.000167	CSCL	MR1
CLEC4A.2	1.13E-08	-0.22061	0.005	0.064	0.000169	CSCL	CLEC4A
VRK2.2	1.18E-08	-0.21679	0.002	0.057	0.000176	CSCL	VRK2
ZFAND2A.2	1.22E-08	0.182372	0.145	0.29	0.000183	CSCL	ZFAND2A
ABCA2.2	1.25E-08	-0.22205	0.026	0.099	0.000187	CSCL	ABCA2
DECR1.1	1.27E-08	0.152088	0.222	0.422	0.00019	CSCL	DECR1
FAM13B.2	1.28E-08	-0.12273	0.019	0.089	0.000191	CSCL	FAM13B
AKT2	1.28E-08	0.123147	0.082	0.191	0.000191	CSCL	AKT2
TMBIM6.1	1.30E-08	0.222361	0.623	0.849	0.000194	CSCL	TMBIM6
HIST2H2AC	1.30E-08	-0.11301	0.003	0.061	0.000195	CSCL	HIST2H2AC
KCTD2	1.31E-08	-0.11451	0.007	0.067	0.000196	CSCL	KCTD2
KIAA1549.	1.32E-08	-0.11593	0.024	0.097	0.000198	CSCL	KIAA1549
ATG16L1	1.33E-08	-0.11309	0.009	0.07	0.000198	CSCL	ATG16L1
PDE7A.2	1.33E-08	-0.10997	0.017	0.086	0.000199	CSCL	PDE7A
MTURN.1	1.38E-08	-0.14564	0.016	0.082	0.000207	CSCL	MTURN
ZFYVE27	1.38E-08	-0.1289	0.016	0.082	0.000207	CSCL	ZFYVE27
FGD2.2	1.39E-08	-0.27368	0.005	0.063	0.000208	CSCL	FGD2
RP11-284F	1.40E-08	0.19621	0.03	0.007	0.000209	CSCL	RP11-284F21.8
NEK3.2	1.41E-08	-0.12714	0.007	0.067	0.000211	CSCL	NEK3
C15orf40	1.41E-08	0.127888	0.066	0.166	0.000211	CSCL	C15orf40
TNS3.2	1.42E-08	-0.1271	0.012	0.076	0.000213	CSCL	TNS3
PANX1.1	1.44E-08	-0.11434	0.014	0.079	0.000215	CSCL	PANX1
GSE1.2	1.45E-08	-0.10271	0.023	0.095	0.000217	CSCL	GSE1
NDUFA9	1.45E-08	0.21509	0.204	0.393	0.000217	CSCL	NDUFA9
CXXC5.2	1.50E-08	0.182265	0.202	0.389	0.000223	CSCL	CXXC5
MAGEH1.1	1.50E-08	0.126046	0.168	0.325	0.000224	CSCL	MAGEH1
SLC25A6.1	1.50E-08	0.19963	0.635	0.869	0.000224	CSCL	SLC25A6
METAP2.1	1.56E-08	0.220961	0.253	0.481	0.000234	CSCL	METAP2
TVP23B.2	1.59E-08	-0.10423	0.03	0.105	0.000238	CSCL	TVP23B
TTLL12.1	1.62E-08	-0.12786	0.007	0.066	0.000242	CSCL	TTLL12
ANG.1	1.62E-08	-0.20304	0.019	0.087	0.000242	CSCL	ANG

DDX20.1	1.62E-08	-0.10842	0.012	0.076	0.000242	CSCL	DDX20
FAM120B.1	1.63E-08	-0.13215	0.017	0.085	0.000244	CSCL	FAM120B
ATXN7L3.1	1.63E-08	-0.10081	0.007	0.066	0.000244	CSCL	ATXN7L3
TBC1D13.1	1.68E-08	-0.12091	0.01	0.073	0.000252	CSCL	TBC1D13
REXO1.1	1.70E-08	-0.10667	0.01	0.073	0.000253	CSCL	REXO1
CPNE2.2	1.70E-08	0.238848	0.077	0.183	0.000254	CSCL	CPNE2
TMEM91.2	1.74E-08	-0.14092	0.012	0.076	0.000261	CSCL	TMEM91
USP37.1	1.79E-08	-0.13153	0.009	0.069	0.000267	CSCL	USP37
YIF1A.2	1.80E-08	0.193845	0.227	0.434	0.000269	CSCL	YIF1A
LILRB2.2	1.81E-08	-0.24653	0.002	0.056	0.00027	CSCL	LILRB2
IL7R.2	1.83E-08	-0.26355	0.002	0.056	0.000273	CSCL	IL7R
TTC13.1	1.84E-08	-0.12522	0.01	0.072	0.000274	CSCL	TTC13
ZRANB1.1	1.86E-08	-0.11828	0.007	0.066	0.000277	CSCL	ZRANB1
GBGT1.2	1.88E-08	-0.20223	0	0.052	0.00028	CSCL	GBGT1
MTHFR.1	1.92E-08	-0.14391	0.016	0.081	0.000287	CSCL	MTHFR
IKBKE.2	1.93E-08	-0.18244	0.003	0.059	0.000289	CSCL	IKBKE
LAYN.1	1.95E-08	-0.10335	0.003	0.059	0.000292	CSCL	LAYN
SETD9	2.02E-08	0.102821	0.049	0.137	0.000303	CSCL	SETD9
PFDN2.1	2.04E-08	0.126031	0.332	0.625	0.000305	CSCL	PFDN2
ZWILCH.2	2.05E-08	-0.11246	0.014	0.078	0.000306	CSCL	ZWILCH
LAPTM4B.2	2.05E-08	0.218083	0.173	0.339	0.000306	CSCL	LAPTM4B
SHCBP1.2	2.07E-08	-0.14322	0.002	0.056	0.000309	CSCL	SHCBP1
PPP5C.1	2.08E-08	0.18531	0.077	0.182	0.00031	CSCL	PPP5C
RNGTT.1	2.08E-08	-0.12624	0.014	0.078	0.000311	CSCL	RNGTT
LYRM9.1	2.10E-08	0.101689	0.075	0.178	0.000314	CSCL	LYRM9
MXD3.2	2.13E-08	-0.10335	0.007	0.066	0.000318	CSCL	MXD3
FDPS.1	2.15E-08	0.274128	0.222	0.426	0.000322	CSCL	FDPS
REEP1.2	2.18E-08	-0.11883	0.017	0.084	0.000325	CSCL	REEP1
PITPNM1.1	2.18E-08	-0.12698	0.003	0.059	0.000325	CSCL	PITPNM1
GAB2.1	2.28E-08	-0.14514	0.009	0.069	0.000341	CSCL	GAB2
TTC7A.2	2.29E-08	-0.16874	0.014	0.078	0.000342	CSCL	TTC7A
SUPV3L1	2.30E-08	-0.15272	0.009	0.069	0.000344	CSCL	SUPV3L1
SRR.1	2.31E-08	0.107377	0.063	0.159	0.000345	CSCL	SRR
ECD.1	2.32E-08	-0.11464	0.014	0.078	0.000346	CSCL	ECD
IGDCC3.2	2.32E-08	-0.10764	0.007	0.065	0.000347	CSCL	IGDCC3
ACSF2.1	2.36E-08	-0.13046	0.009	0.068	0.000352	CSCL	ACSF2
FGFR1OP	2.38E-08	-0.10162	0.017	0.084	0.000356	CSCL	FGFR1OP
TFEC.2	2.39E-08	-0.22345	0.003	0.059	0.000358	CSCL	TFEC
TFEB.2	2.40E-08	-0.1071	0.016	0.081	0.000359	CSCL	TFEB
ANLN.1	2.44E-08	-0.18785	0.007	0.065	0.000364	CSCL	ANLN
MYLK	2.46E-08	-0.18817	0.005	0.062	0.000368	CSCL	MYLK
FBXL6	2.47E-08	-0.11353	0.01	0.071	0.00037	CSCL	FBXL6
RABL3	2.49E-08	-0.12096	0.009	0.068	0.000372	CSCL	RABL3
LINC00667	2.50E-08	0.19968	0.12	0.25	0.000374	CSCL	LINC00667
AKAP7.2	2.51E-08	0.129997	0.089	0.2	0.000375	CSCL	AKAP7
ARL13B.2	2.55E-08	0.142766	0.056	0.147	0.000381	CSCL	ARL13B
RGS18.2	2.56E-08	-0.23641	0.002	0.055	0.000382	CSCL	RGS18
ARHGAP23.	2.60E-08	-0.14503	0.002	0.055	0.000389	CSCL	ARHGAP23
TRAF3IP3.	2.62E-08	-0.26146	0.002	0.055	0.000392	CSCL	TRAF3IP3
RUNDC3A.2	2.65E-08	-0.10615	0.028	0.101	0.000397	CSCL	RUNDC3A
KDM1B.1	2.68E-08	-0.12629	0.005	0.062	0.0004	CSCL	KDM1B

CDCA3.1	2.69E-08	-0.15147	0.002	0.055	0.000402	CSCL	CDCA3
SEC11A.1	2.73E-08	0.16489	0.319	0.605	0.000407	CSCL	SEC11A
SPN.2	2.74E-08	-0.2284	0.003	0.058	0.000409	CSCL	SPN
CLSPN.2	2.74E-08	-0.14411	0.007	0.065	0.00041	CSCL	CLSPN
DCLK2.2	2.77E-08	0.14967	0.091	0.201	0.000415	CSCL	DCLK2
GOLM1.1	2.82E-08	0.224358	0.164	0.324	0.000422	CSCL	GOLM1
SEL1L3	2.90E-08	-0.10841	0.012	0.074	0.000434	CSCL	SEL1L3
ANKRD16.1	2.92E-08	-0.10259	0.002	0.055	0.000436	CSCL	ANKRD16
MICU3.2	2.99E-08	-0.12691	0.019	0.086	0.000447	CSCL	MICU3
UNC45A	3.04E-08	0.110968	0.068	0.165	0.000455	CSCL	UNC45A
GBP2.1	3.10E-08	0.105242	0.099	0.214	0.000464	CSCL	GBP2
ELAVL2.2	3.11E-08	-0.15888	0.017	0.083	0.000464	CSCL	ELAVL2
NUDC.2	3.12E-08	0.184104	0.314	0.593	0.000466	CSCL	NUDC
LRCH1	3.13E-08	-0.13965	0.005	0.061	0.000468	CSCL	LRCH1
SNRNP35.1	3.16E-08	0.155945	0.091	0.201	0.000473	CSCL	SNRNP35
CENPE.2	3.16E-08	-0.18246	0.003	0.058	0.000473	CSCL	CENPE
ZNF226.2	3.27E-08	0.176089	0.096	0.209	0.000488	CSCL	ZNF226
SLC25A4.2	3.31E-08	0.161514	0.232	0.436	0.000494	CSCL	SLC25A4
UHRF1BP1L	3.32E-08	-0.1171	0.01	0.071	0.000496	CSCL	UHRF1BP1L
RASGRP2.2	3.36E-08	-0.18841	0.002	0.054	0.000502	CSCL	RASGRP2
IFT57.2	3.37E-08	0.172171	0.124	0.254	0.000504	CSCL	IFT57
CELF5.2	3.43E-08	-0.15434	0.024	0.094	0.000513	CSCL	CELF5
HOPX.1	3.46E-08	0.66021	0.321	0.28	0.000517	CSCL	HOPX
TCF19	3.46E-08	-0.13965	0	0.051	0.000517	CSCL	TCF19
ARMC6	3.51E-08	0.13748	0.068	0.165	0.000524	CSCL	ARMC6
GCC1.1	3.56E-08	-0.12443	0.012	0.073	0.000532	CSCL	GCC1
PSMC3IP.1	3.57E-08	-0.10174	0.012	0.073	0.000534	CSCL	PSMC3IP
MAPT.2	3.58E-08	0.100847	0.15	0.292	0.000534	CSCL	MAPT
SGK3.2	3.65E-08	-0.14378	0.01	0.07	0.000545	CSCL	SGK3
RFWD3.1	3.66E-08	-0.12888	0.005	0.061	0.000546	CSCL	RFWD3
KIF16B	3.67E-08	-0.10997	0.007	0.064	0.000548	CSCL	KIF16B
TBC1D4.2	3.67E-08	-0.19754	0	0.05	0.000548	CSCL	TBC1D4
FAM84A.1	3.68E-08	0.134038	0.058	0.149	0.000549	CSCL	FAM84A
RP11-480C	3.78E-08	-0.28452	0	0.05	0.000565	CSCL	RP11-480C22.1
LINC00152	3.84E-08	0.190789	0.131	0.265	0.000574	CSCL	LINC00152
TCTN1.1	3.85E-08	0.162209	0.101	0.218	0.000575	CSCL	TCTN1
C19orf38.	3.89E-08	-0.24102	0	0.05	0.000581	CSCL	C19orf38
HOXB2.2	3.92E-08	-0.17357	0.021	0.087	0.000586	CSCL	HOXB2
MAN1A1.1	4.00E-08	-0.19551	0	0.05	0.000598	CSCL	MAN1A1
PLA2G7.2	4.04E-08	-0.20297	0.002	0.054	0.000603	CSCL	PLA2G7
TSC22D1.1	4.04E-08	0.458933	0.459	0.514	0.000604	CSCL	TSC22D1
ILF3-AS1.	4.06E-08	0.213519	0.162	0.317	0.000607	CSCL	ILF3-AS1
RGS16.1	4.09E-08	0.182771	0.143	0.282	0.000612	CSCL	RGS16
CLIC4.1	4.11E-08	0.148983	0.178	0.34	0.000614	CSCL	CLIC4
ACP5.2	4.12E-08	-0.223	0	0.05	0.000616	CSCL	ACP5
RBX1.2	4.13E-08	0.106806	0.36	0.681	0.000617	CSCL	RBX1
FAM13A	4.15E-08	-0.13851	0.03	0.102	0.000621	CSCL	FAM13A
WDR37	4.18E-08	-0.13572	0.005	0.06	0.000625	CSCL	WDR37
ANKRD44.1	4.22E-08	-0.14724	0.028	0.099	0.000631	CSCL	ANKRD44
SMOX.2	4.29E-08	0.235183	0.124	0.253	0.000642	CSCL	SMOX
DHRS4.1	4.31E-08	-0.11064	0.019	0.085	0.000645	CSCL	DHRS4

CD59.2	4.34E-08	0.133715	0.304	0.566	0.000649	CSCL	CD59
ENPP4	4.35E-08	-0.18651	0.003	0.057	0.000651	CSCL	ENPP4
CLECL1.2	4.50E-08	-0.20171	0	0.05	0.000672	CSCL	CLECL1
SNUPN.1	4.51E-08	0.15959	0.07	0.167	0.000674	CSCL	SNUPN
CDT1.1	4.61E-08	-0.12404	0.007	0.063	0.000689	CSCL	CDT1
PDDC1.1	4.65E-08	0.10274	0.059	0.15	0.000694	CSCL	PDDC1
MORN4.1	4.67E-08	-0.10295	0.002	0.053	0.000698	CSCL	MORN4
METTL22.2	4.69E-08	-0.10941	0.024	0.093	0.000701	CSCL	METTL22
ALKBH2.2	4.70E-08	0.207462	0.091	0.2	0.000702	CSCL	ALKBH2
TUBD1	4.71E-08	-0.11921	0.021	0.087	0.000704	CSCL	TUBD1
ARMC8.1	4.79E-08	0.106154	0.094	0.206	0.000715	CSCL	ARMC8
GIMAP5.2	4.90E-08	-0.22235	0.002	0.053	0.000732	CSCL	GIMAP5
TMEM52B.2	4.91E-08	-0.23801	0	0.05	0.000734	CSCL	TMEM52B
ZNF574.1	4.94E-08	-0.10334	0.023	0.09	0.000739	CSCL	ZNF574
PXMP4	5.01E-08	-0.10131	0.009	0.066	0.000749	CSCL	PXMP4
WDR54.2	5.04E-08	0.216492	0.115	0.24	0.000753	CSCL	WDR54
KLHL35.1	5.05E-08	-0.20131	0.028	0.097	0.000754	CSCL	KLHL35
POMC.2	5.05E-08	-0.2209	0	0.049	0.000755	CSCL	POMC
RAB2B.1	5.12E-08	-0.10261	0.019	0.084	0.000765	CSCL	RAB2B
KLHL6.2	5.15E-08	-0.24551	0.002	0.053	0.00077	CSCL	KLHL6
ZNF300.1	5.16E-08	-0.11353	0.012	0.072	0.000772	CSCL	ZNF300
ATG2A.1	5.25E-08	-0.14148	0.016	0.078	0.000784	CSCL	ATG2A
APAF1.2	5.25E-08	-0.1388	0.01	0.069	0.000785	CSCL	APAF1
MAGI1.2	5.26E-08	0.150799	0.092	0.203	0.000787	CSCL	MAGI1
ZSWIM8.2	5.27E-08	-0.12069	0.017	0.081	0.000788	CSCL	ZSWIM8
TRMT1L.1	5.29E-08	-0.12881	0.01	0.069	0.000791	CSCL	TRMT1L
RAB27A.1	5.30E-08	-0.11862	0.017	0.081	0.000792	CSCL	RAB27A
PLEKHA4.1	5.31E-08	0.147244	0.047	0.131	0.000793	CSCL	PLEKHA4
SPOPL.1	5.34E-08	-0.13302	0.003	0.056	0.000797	CSCL	SPOPL
CELF3.2	5.36E-08	-0.17661	0.01	0.069	0.0008	CSCL	CELF3
PRR5.2	5.36E-08	-0.18706	0	0.049	0.0008	CSCL	PRR5
SLFN5	5.54E-08	-0.15399	0.007	0.063	0.000828	CSCL	SLFN5
RITA1	5.57E-08	0.149566	0.051	0.136	0.000833	CSCL	RITA1
YJEFN3.1	5.58E-08	-0.11167	0.009	0.066	0.000833	CSCL	YJEFN3
C14orf166	5.70E-08	0.180471	0.316	0.598	0.000851	CSCL	C14orf166
NKG7.1	5.76E-08	-0.28442	0.002	0.052	0.000861	CSCL	NKG7
RASGRP3.1	5.78E-08	-0.25482	0.012	0.071	0.000864	CSCL	RASGRP3
HSPBAP1	5.81E-08	-0.17345	0.014	0.075	0.000868	CSCL	HSPBAP1
HSP90AB1.	5.89E-08	0.24591	0.602	0.832	0.00088	CSCL	HSP90AB1
PDZD11.1	5.89E-08	0.295126	0.19	0.367	0.00088	CSCL	PDZD11
POC1A.1	5.90E-08	-0.12342	0.005	0.059	0.000882	CSCL	POC1A
HOOK2	5.93E-08	0.219867	0.117	0.24	0.000886	CSCL	HOOK2
PROCR.1	6.00E-08	-0.13483	0.012	0.072	0.000897	CSCL	PROCR
AHR.2	6.01E-08	-0.1527	0.009	0.065	0.000898	CSCL	AHR
TNFAIP8L1	6.05E-08	-0.11063	0.019	0.084	0.000904	CSCL	TNFAIP8L1
MORC2.1	6.13E-08	-0.10333	0.007	0.062	0.000916	CSCL	MORC2
BEX4.1	6.15E-08	0.11098	0.269	0.487	0.000918	CSCL	BEX4
NNT-AS1.1	6.18E-08	0.254393	0.108	0.227	0.000924	CSCL	NNT-AS1
IFI27L1	6.23E-08	0.194765	0.086	0.191	0.000931	CSCL	IFI27L1
SLC9A6.1	6.23E-08	-0.10884	0.007	0.062	0.000932	CSCL	SLC9A6
THBS1.1	6.30E-08	-0.23902	0.019	0.083	0.000941	CSCL	THBS1

C21orf58.	6.34E-08	-0.10759	0.003	0.056	0.000947	CSCL	C21orf58
COX7A2L	6.42E-08	0.127137	0.337	0.627	0.000959	CSCL	COX7A2L
PAXIP1-AS	6.43E-08	0.121407	0.068	0.163	0.00096	CSCL	PAXIP1-AS1
TIMM8B.1	6.43E-08	0.190972	0.295	0.557	0.000961	CSCL	TIMM8B
MLH1	6.50E-08	0.135274	0.065	0.157	0.000972	CSCL	MLH1
MAP3K5	6.53E-08	-0.1433	0.002	0.052	0.000976	CSCL	MAP3K5
TNFAIP2.1	6.59E-08	-0.1127	0.016	0.078	0.000984	CSCL	TNFAIP2
CCNA2.2	6.59E-08	-0.16055	0.005	0.059	0.000985	CSCL	CCNA2
LY6H.1	6.61E-08	-0.18679	0.023	0.089	0.000988	CSCL	LY6H
DLEU7.2	6.61E-08	-0.21242	0.002	0.052	0.000988	CSCL	DLEU7
AKAP12.2	6.79E-08	0.133683	0.087	0.192	0.001014	CSCL	AKAP12
ECT2.1	6.82E-08	-0.11283	0.002	0.052	0.001019	CSCL	ECT2
C10orf76.	6.89E-08	-0.15639	0.005	0.059	0.00103	CSCL	C10orf76
BRCA2	6.96E-08	-0.13729	0	0.048	0.00104	CSCL	BRCA2
LCOR.2	7.08E-08	-0.12191	0.019	0.083	0.001058	CSCL	LCOR
CMKLR1.2	7.10E-08	-0.20923	0.002	0.052	0.00106	CSCL	CMKLR1
SLC22A17.	7.12E-08	0.157271	0.148	0.291	0.001064	CSCL	SLC22A17
CCDC18.2	7.22E-08	-0.13922	0.017	0.08	0.001079	CSCL	CCDC18
EIF2B2	7.25E-08	0.10096	0.079	0.178	0.001083	CSCL	EIF2B2
NCEH1.1	7.31E-08	-0.10083	0.012	0.071	0.001092	CSCL	NCEH1
TIMELESS.	7.33E-08	-0.1077	0.009	0.065	0.001095	CSCL	TIMELESS
MDH1.1	7.34E-08	0.156945	0.3	0.563	0.001097	CSCL	MDH1
C6orf15.1	7.48E-08	0.57167	0.063	0.026	0.001117	CSCL	C6orf15
TAP1	7.70E-08	0.258061	0.129	0.26	0.001151	CSCL	TAP1
CENPA.2	7.77E-08	-0.17312	0.002	0.051	0.001162	CSCL	CENPA
RASSF8-AS	7.91E-08	0.100936	0.07	0.164	0.001182	CSCL	RASSF8-AS1
TSFM	7.98E-08	0.138818	0.145	0.283	0.001193	CSCL	TSFM
PDGFB.1	8.04E-08	-0.23276	0	0.048	0.001202	CSCL	PDGFB
DPY30.1	8.10E-08	0.244367	0.271	0.507	0.001211	CSCL	DPY30
AAMDC.2	8.18E-08	0.20899	0.099	0.212	0.001223	CSCL	AAMDC
F2R.2	8.73E-08	0.170424	0.075	0.173	0.001305	CSCL	F2R
JDP2.2	8.77E-08	-0.18714	0.014	0.073	0.001311	CSCL	JDP2
DPM3.1	8.79E-08	0.250256	0.188	0.361	0.001314	CSCL	DPM3
WNT5A.2	8.85E-08	-0.15713	0.007	0.061	0.001323	CSCL	WNT5A
PLXNA4.1	8.86E-08	-0.10461	0.017	0.079	0.001324	CSCL	PLXNA4
ALDH1L1.1	9.10E-08	0.544299	0.112	0.061	0.001359	CSCL	ALDH1L1
PTGS2.2	9.20E-08	-0.20095	0.009	0.064	0.001375	CSCL	PTGS2
AP5B1	9.51E-08	-0.14602	0.003	0.054	0.001421	CSCL	AP5B1
PBK.2	9.56E-08	-0.12087	0.016	0.076	0.001429	CSCL	PBK
AMPD3.1	9.60E-08	-0.16103	0.003	0.054	0.001434	CSCL	AMPD3
MCF2L.2	9.65E-08	-0.12858	0.03	0.099	0.001442	CSCL	MCF2L
RP11-792A	9.66E-08	0.147314	0.103	0.216	0.001444	CSCL	RP11-792A8.4
PSPH.1	9.92E-08	0.151747	0.068	0.162	0.001482	CSCL	PSPH
ZC3H12A.1	9.92E-08	-0.12277	0.017	0.079	0.001483	CSCL	ZC3H12A
ZNF846.1	9.94E-08	-0.15571	0.014	0.073	0.001485	CSCL	ZNF846
BLOC1S1.1	9.95E-08	0.100852	0.36	0.663	0.001487	CSCL	BLOC1S1
ZFHX4.2	1.01E-07	0.12095	0.065	0.156	0.00151	CSCL	ZFHX4
TRPM2.2	1.02E-07	-0.1859	0.003	0.054	0.001525	CSCL	TRPM2
TSKU	1.02E-07	0.107042	0.023	0.088	0.001531	CSCL	TSKU
PDK1.1	1.04E-07	-0.12155	0.023	0.088	0.001549	CSCL	PDK1
BET1.1	1.05E-07	0.130283	0.11	0.227	0.001562	CSCL	BET1

PLCB2.2	1.06E-07	-0.19631	0.009	0.064	0.001585	CSCL	PLCB2
LRMP.1	1.11E-07	-0.20871	0	0.047	0.001654	CSCL	LRMP
ENPP2.1	1.11E-07	-0.28812	0	0.047	0.001654	CSCL	ENPP2
ATP5D.2	1.13E-07	0.178379	0.332	0.628	0.001691	CSCL	ATP5D
SPATA33.1	1.13E-07	0.181251	0.061	0.15	0.001691	CSCL	SPATA33
SIRT1.1	1.14E-07	-0.1104	0.009	0.064	0.001698	CSCL	SIRT1
USP31.1	1.15E-07	-0.10205	0.003	0.054	0.00172	CSCL	USP31
NPAS3.2	1.16E-07	0.154711	0.07	0.163	0.001734	CSCL	NPAS3
SNX20.1	1.17E-07	-0.18825	0	0.047	0.001753	CSCL	SNX20
TMEM140	1.19E-07	-0.18796	0.002	0.05	0.001778	CSCL	TMEM140
OXCT1	1.19E-07	0.155914	0.063	0.152	0.001781	CSCL	OXCT1
ZNF626	1.20E-07	0.132022	0.059	0.147	0.001791	CSCL	ZNF626
C4orf33	1.22E-07	-0.11332	0.009	0.063	0.001816	CSCL	C4orf33
FAM96B.2	1.22E-07	0.127218	0.36	0.673	0.001828	CSCL	FAM96B
RBBP7.2	1.23E-07	0.245265	0.232	0.434	0.001842	CSCL	RBBP7
RP11-553L	1.24E-07	0.123679	0.113	0.232	0.00185	CSCL	RP11-553L6.5
ZNF791	1.26E-07	0.132137	0.143	0.277	0.001876	CSCL	ZNF791
MMS19	1.27E-07	-0.10655	0.009	0.063	0.001898	CSCL	MMS19
DVL1	1.27E-07	-0.10339	0.003	0.053	0.001899	CSCL	DVL1
HSD3B7.1	1.28E-07	-0.12162	0.017	0.078	0.001911	CSCL	HSD3B7
ARL6IP5	1.29E-07	0.118183	0.361	0.665	0.001925	CSCL	ARL6IP5
VWA9.1	1.29E-07	0.117738	0.051	0.132	0.001928	CSCL	VWA9
ULK2.1	1.31E-07	-0.10842	0.01	0.066	0.001955	CSCL	ULK2
ARMCX1.1	1.32E-07	0.108552	0.106	0.219	0.001972	CSCL	ARMCX1
IGFBP5.2	1.35E-07	0.740082	0.307	0.266	0.002015	CSCL	IGFBP5
SPAG5.1	1.36E-07	-0.13419	0	0.046	0.002026	CSCL	SPAG5
PIF1.1	1.36E-07	-0.13815	0.003	0.053	0.002038	CSCL	PIF1
APBA2.2	1.37E-07	0.16229	0.101	0.212	0.002046	CSCL	APBA2
NUP85.1	1.39E-07	0.105656	0.047	0.127	0.00208	CSCL	NUP85
PLBD2.2	1.40E-07	-0.1161	0.007	0.06	0.00209	CSCL	PLBD2
SLC16A1.2	1.43E-07	0.173845	0.119	0.239	0.00213	CSCL	SLC16A1
PLXNC1.2	1.44E-07	-0.14594	0.012	0.069	0.002149	CSCL	PLXNC1
COMTD1.2	1.45E-07	-0.12825	0.009	0.063	0.002174	CSCL	COMTD1
AGTPBP1.2	1.46E-07	-0.15439	0.003	0.053	0.002175	CSCL	AGTPBP1
RNF157.1	1.47E-07	0.143054	0.054	0.138	0.002201	CSCL	RNF157
ANKS1A.2	1.48E-07	-0.11476	0.009	0.063	0.002213	CSCL	ANKS1A
WARS.2	1.48E-07	0.247748	0.098	0.207	0.002217	CSCL	WARS
GHRL.2	1.49E-07	-0.16015	0.007	0.059	0.002223	CSCL	GHRL
ASTE1.1	1.49E-07	-0.13297	0.005	0.056	0.002229	CSCL	ASTE1
PRKCH.1	1.52E-07	-0.21866	0	0.046	0.002275	CSCL	PRKCH
SCRN1.1	1.54E-07	0.182843	0.084	0.184	0.002306	CSCL	SCRN1
DLX6.2	1.55E-07	-0.16208	0.007	0.059	0.002318	CSCL	DLX6
RCBTB1.2	1.56E-07	-0.10254	0.016	0.075	0.002334	CSCL	RCBTB1
SMPD2	1.56E-07	-0.12304	0.002	0.049	0.002337	CSCL	SMPD2
MKLN1	1.62E-07	0.173879	0.117	0.235	0.002417	CSCL	MKLN1
PGAP2	1.67E-07	0.109002	0.044	0.121	0.002492	CSCL	PGAP2
LYPD1	1.67E-07	0.220457	0.094	0.201	0.002498	CSCL	LYPD1
SYNJ2BP.1	1.67E-07	0.104512	0.061	0.148	0.002501	CSCL	SYNJ2BP
CYB5D1	1.71E-07	-0.14462	0.005	0.056	0.002559	CSCL	CYB5D1
MPP2.1	1.75E-07	-0.10534	0.009	0.062	0.002619	CSCL	MPP2
ASPM.2	1.77E-07	-0.15629	0.002	0.049	0.002643	CSCL	ASPM

PIGU.1	1.77E-07	0.193141	0.079	0.176	0.002651	CSCL	PIGU
NDRG4.2	1.80E-07	0.158628	0.072	0.165	0.002695	CSCL	NDRG4
ATP11C.1	1.84E-07	-0.12344	0.003	0.052	0.002742	CSCL	ATP11C
MOXD1	1.84E-07	-0.11096	0.005	0.056	0.002745	CSCL	MOXD1
ADAM8.1	1.86E-07	-0.19791	0	0.045	0.002786	CSCL	ADAM8
DTX2.1	1.87E-07	-0.12357	0.01	0.065	0.002789	CSCL	DTX2
ACYP2.1	1.88E-07	0.229346	0.168	0.32	0.002815	CSCL	ACYP2
SERPINI1.	1.92E-07	-0.15888	0.005	0.055	0.002875	CSCL	SERPINI1
SLC25A28	1.98E-07	-0.10899	0.005	0.055	0.002955	CSCL	SLC25A28
SUCLA2.1	2.00E-07	0.214966	0.154	0.294	0.002982	CSCL	SUCLA2
UFSP2	2.00E-07	0.268332	0.108	0.222	0.002983	CSCL	UFSP2
RP6-91H8.	2.02E-07	-0.24979	0.007	0.058	0.003024	CSCL	RP6-91H8.3
PEX11B.1	2.03E-07	0.156573	0.054	0.136	0.003027	CSCL	PEX11B
SFR1.1	2.11E-07	-0.12709	0.014	0.071	0.003154	CSCL	SFR1
DPH5	2.12E-07	0.168828	0.075	0.169	0.003161	CSCL	DPH5
RHOJ	2.13E-07	0.137675	0.033	0.103	0.00318	CSCL	RHOJ
NUPR1.1	2.13E-07	0.177665	0.182	0.331	0.003189	CSCL	NUPR1
BNIP3.1	2.15E-07	0.207236	0.108	0.22	0.003208	CSCL	BNIP3
SYMPK.1	2.16E-07	0.249037	0.112	0.229	0.003227	CSCL	SYMPK
SBNO2.1	2.17E-07	-0.10389	0.007	0.058	0.003246	CSCL	SBNO2
RP11-660L	2.18E-07	0.138598	0.049	0.128	0.003255	CSCL	RP11-660L16.2
TMEM163.1	2.18E-07	-0.14286	0.01	0.065	0.003262	CSCL	TMEM163
TES.2	2.23E-07	-0.20791	0.002	0.048	0.003327	CSCL	TES
TMEM63A.1	2.23E-07	-0.14036	0.016	0.073	0.003331	CSCL	TMEM63A
PUF60.1	2.24E-07	0.124018	0.298	0.554	0.003343	CSCL	PUF60
TLR7.2	2.25E-07	-0.22077	0.007	0.058	0.003365	CSCL	TLR7
SDS.2	2.26E-07	-0.22677	0.009	0.061	0.003374	CSCL	SDS
MARCO	2.26E-07	-0.27577	0.009	0.061	0.003375	CSCL	MARCO
NDUFV2.2	2.26E-07	0.152976	0.325	0.605	0.003379	CSCL	NDUFV2
CBR4.2	2.27E-07	0.115947	0.091	0.193	0.003386	CSCL	CBR4
NPTN.1	2.30E-07	-0.10731	0.01	0.064	0.003433	CSCL	NPTN
DOPEY2.2	2.33E-07	-0.1218	0.014	0.07	0.003477	CSCL	DOPEY2
RFXANK	2.36E-07	0.321222	0.175	0.332	0.003532	CSCL	RFXANK
CBR1.1	2.37E-07	0.13426	0.267	0.487	0.003537	CSCL	CBR1
RAB42.2	2.37E-07	-0.11536	0.003	0.052	0.003543	CSCL	RAB42
TF.2	2.39E-07	-0.30573	0.017	0.076	0.003575	CSCL	TF
ADRB2.1	2.41E-07	-0.23049	0.002	0.048	0.003597	CSCL	ADRB2
RPP40.1	2.47E-07	0.11229	0.045	0.122	0.003684	CSCL	RPP40
SPC25.2	2.52E-07	-0.10776	0.01	0.064	0.003768	CSCL	SPC25
TSPAN33.1	2.56E-07	-0.15675	0	0.044	0.003829	CSCL	TSPAN33
AURKB.2	2.58E-07	-0.17956	0.003	0.051	0.003851	CSCL	AURKB
SPC24.2	2.64E-07	-0.12794	0.003	0.051	0.003947	CSCL	SPC24
NT5DC1	2.65E-07	-0.17096	0.005	0.054	0.003957	CSCL	NT5DC1
CD300LF.1	2.65E-07	-0.18866	0.002	0.048	0.003958	CSCL	CD300LF
MAN2B1.2	2.66E-07	-0.14075	0.014	0.07	0.003972	CSCL	MAN2B1
SUSD1.2	2.71E-07	-0.1052	0.007	0.058	0.004047	CSCL	SUSD1
FAM212A.2	2.71E-07	-0.21526	0.003	0.051	0.004056	CSCL	FAM212A
FOXM1.1	2.73E-07	-0.10968	0.005	0.054	0.004075	CSCL	FOXM1
CKAP2L.1	2.74E-07	-0.14308	0.002	0.048	0.004092	CSCL	CKAP2L
MPEG1.1	2.79E-07	-0.18066	0	0.044	0.004176	CSCL	MPEG1
ARHGAP27.	2.85E-07	-0.16395	0.007	0.057	0.00426	CSCL	ARHGAP27

AC074289.	2.86E-07	-0.18702	0.01	0.064	0.004278	CSCL	AC074289.1
KIF21A.2	2.88E-07	0.300741	0.162	0.309	0.004303	CSCL	KIF21A
CCDC170.1	3.04E-07	-0.14612	0.002	0.047	0.004542	CSCL	CCDC170
GRASP.2	3.08E-07	-0.20905	0.005	0.054	0.004597	CSCL	GRASP
NAA38.1	3.09E-07	0.239837	0.265	0.482	0.004624	CSCL	NAA38
SPRY1.2	3.11E-07	0.148436	0.12	0.238	0.004654	CSCL	SPRY1
C5orf45.1	3.13E-07	0.160391	0.079	0.172	0.004684	CSCL	C5orf45
RASSF3.1	3.14E-07	-0.15765	0	0.044	0.004687	CSCL	RASSF3
ATF7IP2.1	3.14E-07	-0.20853	0	0.044	0.004687	CSCL	ATF7IP2
FAM159A.1	3.14E-07	-0.18514	0.002	0.047	0.004688	CSCL	FAM159A
PRCP.1	3.17E-07	0.204761	0.197	0.363	0.004731	CSCL	PRCP
AKTIP.1	3.17E-07	0.165646	0.079	0.173	0.004734	CSCL	AKTIP
NIPA1	3.19E-07	-0.12213	0.005	0.054	0.004766	CSCL	NIPA1
USP28	3.20E-07	-0.10757	0.007	0.057	0.004784	CSCL	USP28
PLK1.1	3.21E-07	-0.12839	0.007	0.057	0.004794	CSCL	PLK1
BCL11A.1	3.21E-07	-0.12784	0.003	0.051	0.004795	CSCL	BCL11A
TIFA	3.22E-07	-0.12799	0.009	0.06	0.004812	CSCL	TIFA
CCL3L3.1	3.24E-07	-0.59656	0.007	0.056	0.004835	CSCL	CCL3L3
ABCC4.1	3.25E-07	-0.12386	0.002	0.047	0.004851	CSCL	ABCC4
CLCN4.1	3.28E-07	-0.11204	0.007	0.057	0.004895	CSCL	CLCN4
NCAPG.1	3.32E-07	-0.12656	0	0.044	0.004966	CSCL	NCAPG
MYBL2.1	3.34E-07	-0.1036	0.002	0.047	0.004997	CSCL	MYBL2
DUOX1.2	3.51E-07	-0.12562	0.012	0.066	0.005248	CSCL	DUOX1
SLC46A3.2	3.53E-07	-0.12834	0.009	0.06	0.005274	CSCL	SLC46A3
ZNF738.2	3.53E-07	0.109789	0.063	0.148	0.005281	CSCL	ZNF738
C16orf59	3.64E-07	-0.10229	0.002	0.047	0.005433	CSCL	C16orf59
NDC80.1	3.71E-07	-0.14194	0.003	0.05	0.005537	CSCL	NDC80
KHDRBS3.2	3.72E-07	0.253993	0.108	0.22	0.00556	CSCL	KHDRBS3
PSTPIP2.1	3.73E-07	-0.13483	0.002	0.047	0.005572	CSCL	PSTPIP2
CDK6.2	3.76E-07	0.151232	0.103	0.211	0.005612	CSCL	CDK6
PSTPIP1.1	3.84E-07	-0.18603	0	0.043	0.005737	CSCL	PSTPIP1
SIGLEC14.	3.84E-07	-0.19474	0.002	0.047	0.005744	CSCL	SIGLEC14
PKIA.2	4.15E-07	-0.11666	0.031	0.096	0.006196	CSCL	PKIA
PRKAR2B.2	4.26E-07	-0.10976	0.016	0.071	0.006367	CSCL	PRKAR2B
SNRPB2.1	4.29E-07	0.199345	0.309	0.577	0.00641	CSCL	SNRPB2
GPSM2.1	4.42E-07	0.101784	0.086	0.181	0.006606	CSCL	GPSM2
PLCG1	4.43E-07	0.107168	0.035	0.103	0.006621	CSCL	PLCG1
WIZ	4.45E-07	0.114562	0.042	0.114	0.006648	CSCL	WIZ
SLC29A3.1	4.45E-07	-0.16812	0.002	0.046	0.006648	CSCL	SLC29A3
SHQ1.1	4.47E-07	-0.11188	0.003	0.05	0.00668	CSCL	SHQ1
PPM1L.1	4.47E-07	-0.10545	0.003	0.05	0.006681	CSCL	PPM1L
IPMK	4.53E-07	-0.12515	0.002	0.046	0.006773	CSCL	IPMK
TBC1D9.2	4.57E-07	-0.10786	0.007	0.056	0.006836	CSCL	TBC1D9
DDR1.1	4.69E-07	0.213585	0.182	0.339	0.00701	CSCL	DDR1
ATPIF1	4.69E-07	0.150435	0.347	0.637	0.007015	CSCL	ATPIF1
PIM2.1	4.73E-07	-0.1457	0.007	0.056	0.007071	CSCL	PIM2
SLC37A1.1	4.83E-07	-0.11218	0.007	0.056	0.007217	CSCL	SLC37A1
PHF19.2	4.93E-07	0.139414	0.054	0.133	0.007371	CSCL	PHF19
ITGB5.1	4.95E-07	-0.14498	0.009	0.059	0.007392	CSCL	ITGB5
INTS12	5.00E-07	0.178173	0.077	0.169	0.007479	CSCL	INTS12
PEF1.1	5.06E-07	0.317316	0.175	0.327	0.007557	CSCL	PEF1

SYT11.2	5.17E-07	0.177524	0.216	0.392	0.007728	CSCL	SYT11
QPCT.2	5.18E-07	-0.17	0.003	0.049	0.007742	CSCL	QPCT
DZIP1.1	5.24E-07	0.165819	0.037	0.106	0.007826	CSCL	DZIP1
BUB1.1	5.27E-07	-0.12486	0	0.042	0.007879	CSCL	BUB1
DOLK.1	5.28E-07	0.100212	0.052	0.13	0.007886	CSCL	DOLK
CD7.1	5.37E-07	-0.27675	0.002	0.045	0.008028	CSCL	CD7
DNAJA4.2	5.37E-07	-0.17567	0.012	0.064	0.008031	CSCL	DNAJA4
ANKHD1.1	5.38E-07	-0.11246	0.021	0.079	0.008036	CSCL	ANKHD1
CCS.1	5.44E-07	0.225892	0.14	0.266	0.008136	CSCL	CCS
RP11-841C	5.69E-07	-0.13042	0.009	0.058	0.008498	CSCL	RP11-841020.2
SNRPF.1	5.73E-07	0.223058	0.304	0.559	0.008558	CSCL	SNRPF
ATP5J.1	5.74E-07	0.362042	0.558	0.761	0.008573	CSCL	ATP5J
TOMM40L.1	5.81E-07	-0.10867	0.012	0.064	0.008688	CSCL	TOMM40L
ADRBK2.2	5.89E-07	-0.13781	0.007	0.055	0.008805	CSCL	ADRBK2
IL2RG.1	5.93E-07	-0.25086	0.002	0.045	0.008864	CSCL	IL2RG
BTAF1	5.95E-07	-0.11609	0.005	0.052	0.008892	CSCL	BTAF1
PHLDB1.2	5.96E-07	0.116583	0.063	0.146	0.008906	CSCL	PHLDB1
NFATC2.1	5.99E-07	-0.19357	0.003	0.048	0.008958	CSCL	NFATC2
SETD4	6.04E-07	0.144972	0.052	0.129	0.009027	CSCL	SETD4
ATP8B4.1	6.12E-07	-0.16703	0.002	0.045	0.009146	CSCL	ATP8B4
PLIN3.1	6.13E-07	0.229559	0.199	0.366	0.009159	CSCL	PLIN3
TROAP.2	6.28E-07	-0.10891	0.003	0.049	0.009379	CSCL	TROAP
WRB.1	6.41E-07	0.22823	0.236	0.429	0.009575	CSCL	WRB
RIN1.1	6.48E-07	0.100377	0.024	0.085	0.009691	CSCL	RIN1
EIF4A1.2	6.52E-07	0.163955	0.628	0.873	0.009745	CSCL	EIF4A1
HSPA13.1	6.55E-07	0.142433	0.101	0.205	0.009785	CSCL	HSPA13
NR2C2AP.1	6.82E-07	0.22301	0.079	0.17	0.010186	CSCL	NR2C2AP
HJURP.1	6.83E-07	-0.14157	0	0.041	0.010211	CSCL	HJURP
NFATC1.2	6.85E-07	-0.1435	0.007	0.055	0.010242	CSCL	NFATC1
CD52.1	6.98E-07	-0.29382	0.007	0.054	0.010435	CSCL	CD52
SEMA4A.2	7.02E-07	-0.13394	0.005	0.051	0.010483	CSCL	SEMA4A
PARVA.1	7.07E-07	0.10926	0.033	0.099	0.010562	CSCL	PARVA
ZNF468	7.09E-07	-0.10744	0.003	0.048	0.010594	CSCL	ZNF468
WDR61	7.11E-07	0.284294	0.194	0.356	0.010627	CSCL	WDR61
MMP19	7.13E-07	-0.16227	0.01	0.061	0.010657	CSCL	MMP19
CDCA7L.1	7.17E-07	0.12825	0.068	0.153	0.010719	CSCL	CDCA7L
ITGA4.1	7.20E-07	-0.16519	0.002	0.045	0.010756	CSCL	ITGA4
KANSL3.1	7.20E-07	-0.10191	0.014	0.067	0.010761	CSCL	KANSL3
UMPS	7.23E-07	0.116925	0.058	0.137	0.010804	CSCL	UMPS
PARPBP	7.28E-07	-0.11199	0.003	0.048	0.010882	CSCL	PARPBP
GPRIN3.2	7.28E-07	-0.1364	0.01	0.061	0.010886	CSCL	GPRIN3
IFI27L2.1	7.28E-07	0.161177	0.271	0.481	0.010887	CSCL	IFI27L2
COA3.1	7.42E-07	0.237023	0.269	0.487	0.011082	CSCL	COA3
STAT6.2	7.45E-07	-0.17198	0.007	0.054	0.011139	CSCL	STAT6
TMC8.1	7.70E-07	-0.16211	0.002	0.045	0.011502	CSCL	TMC8
ENO2.2	7.90E-07	0.23743	0.157	0.29	0.0118	CSCL	ENO2
LPAR1.1	8.02E-07	-0.10502	0.005	0.051	0.011987	CSCL	LPAR1
SUCLG1.1	8.09E-07	0.289197	0.244	0.446	0.012091	CSCL	SUCLG1
METTL8.1	8.10E-07	0.137864	0.049	0.123	0.0121	CSCL	METTL8
ZNF883.1	8.13E-07	0.146899	0.063	0.145	0.012143	CSCL	ZNF883
NRCAM.1	8.13E-07	0.149522	0.147	0.276	0.012147	CSCL	NRCAM

MBOAT2.1	8.30E-07	0.262097	0.089	0.186	0.012409	CSCL	MBOAT2
ISOC2.2	8.36E-07	0.281415	0.194	0.357	0.012486	CSCL	ISOC2
PLB1.1	8.58E-07	-0.16351	0.002	0.044	0.012817	CSCL	PLB1
PRKCB.1	8.64E-07	-0.14465	0.002	0.044	0.012917	CSCL	PRKCB
FAM181B.2	8.71E-07	0.11251	0.105	0.208	0.013012	CSCL	FAM181B
SEPT8.2	8.73E-07	0.185034	0.099	0.201	0.013044	CSCL	8-Sep
MORF4L2.1	8.89E-07	0.104251	0.353	0.639	0.013292	CSCL	MORF4L2
SPAG16.2	9.01E-07	0.214259	0.164	0.303	0.013464	CSCL	SPAG16
HS3ST1.2	9.20E-07	-0.15351	0.03	0.091	0.013745	CSCL	HS3ST1
RUNX3.1	9.38E-07	-0.18378	0	0.04	0.014015	CSCL	RUNX3
SBK1.2	9.69E-07	-0.10363	0.019	0.074	0.014479	CSCL	SBK1
PRRG4.1	9.73E-07	-0.16796	0.003	0.047	0.01454	CSCL	PRRG4
CYB5D2	9.74E-07	0.198437	0.124	0.238	0.014555	CSCL	CYB5D2
SLC41A2	9.79E-07	-0.10935	0.003	0.047	0.014637	CSCL	SLC41A2
TCF12.2	9.84E-07	0.279297	0.154	0.29	0.014699	CSCL	TCF12
ARPC1A.2	1.01E-06	0.144316	0.316	0.571	0.015125	CSCL	ARPC1A
SLC38A9.1	1.02E-06	-0.10742	0.014	0.066	0.015312	CSCL	SLC38A9
CRYBB1.1	1.05E-06	-0.19954	0.002	0.044	0.015634	CSCL	CRYBB1
PRKCG.1	1.05E-06	0.358275	0.047	0.019	0.015646	CSCL	PRKCG
SNAI1.1	1.05E-06	-0.16779	0.003	0.047	0.015677	CSCL	SNAI1
TTLL7.2	1.05E-06	0.11259	0.129	0.243	0.015717	CSCL	TTLL7
LYVE1.1	1.08E-06	-0.30814	0	0.04	0.016184	CSCL	LYVE1
ARRB1.1	1.09E-06	-0.1648	0.002	0.043	0.016233	CSCL	ARRB1
IL10RB-AS	1.10E-06	-0.17929	0.003	0.047	0.016458	CSCL	IL10RB-AS1
PLA2G5.1	1.10E-06	0.782665	0.157	0.106	0.016494	CSCL	PLA2G5
HOXA10.2	1.12E-06	-0.11049	0.023	0.08	0.016789	CSCL	HOXA10
CCDC151.1	1.13E-06	-0.11172	0.014	0.065	0.016876	CSCL	CCDC151
RCN1.2	1.14E-06	0.260087	0.197	0.362	0.017099	CSCL	RCN1
KIF9.2	1.15E-06	0.237991	0.086	0.179	0.017152	CSCL	KIF9
OIP5.1	1.15E-06	-0.10418	0.002	0.043	0.01716	CSCL	OIP5
ZNF397	1.17E-06	0.109881	0.079	0.167	0.017537	CSCL	ZNF397
SHISA4.1	1.18E-06	0.289268	0.131	0.252	0.017618	CSCL	SHISA4
APOBR.1	1.18E-06	-0.17057	0	0.04	0.017642	CSCL	APOBR
PMPCB.1	1.20E-06	0.254394	0.194	0.351	0.017911	CSCL	PMPCB
ZNF618.2	1.21E-06	-0.141	0.005	0.05	0.018057	CSCL	ZNF618
ACAP3.2	1.21E-06	0.141039	0.113	0.221	0.018146	CSCL	ACAP3
LHPP.2	1.21E-06	-0.13662	0.042	0.108	0.018155	CSCL	LHPP
CD72.1	1.22E-06	-0.16246	0.007	0.053	0.018244	CSCL	CD72
LIMCH1.1	1.23E-06	0.138128	0.072	0.156	0.01834	CSCL	LIMCH1
ZC3H8	1.23E-06	0.135948	0.068	0.151	0.018403	CSCL	ZC3H8
MB21D2.1	1.24E-06	-0.12712	0.009	0.056	0.018523	CSCL	MB21D2
MGLL.1	1.24E-06	0.221589	0.066	0.149	0.018529	CSCL	MGLL
UQCRH.2	1.25E-06	0.346216	0.569	0.792	0.018623	CSCL	UQCRH
GAP43.2	1.26E-06	0.395031	0.447	0.467	0.018785	CSCL	GAP43
C4orf3.2	1.26E-06	0.140247	0.36	0.661	0.018806	CSCL	C4orf3
RBM8A.1	1.27E-06	0.175463	0.344	0.627	0.019014	CSCL	RBM8A
AC009948.	1.29E-06	-0.12759	0.009	0.056	0.019219	CSCL	AC009948.5
KMT2E.1	1.29E-06	0.139098	0.307	0.549	0.019259	CSCL	KMT2E
CASP8.2	1.30E-06	-0.13051	0.005	0.05	0.019389	CSCL	CASP8
PLAGL1.1	1.30E-06	-0.1608	0.002	0.043	0.019394	CSCL	PLAGL1
EIF3K.1	1.31E-06	0.348673	0.571	0.768	0.019513	CSCL	EIF3K

SLC25A51.	1.32E-06	-0.10146	0.009	0.056	0.019673	CSCL	SLC25A51
ADAP1.1	1.34E-06	-0.16332	0.002	0.043	0.020048	CSCL	ADAP1
PIK3R5.2	1.34E-06	-0.14742	0.005	0.049	0.020054	CSCL	PIK3R5
EXOSC5	1.36E-06	0.178728	0.091	0.185	0.020368	CSCL	EXOSC5
ANKRD46	1.37E-06	0.128919	0.075	0.161	0.020472	CSCL	ANKRD46
GSTM3	1.39E-06	0.293035	0.147	0.274	0.020742	CSCL	GSTM3
MASTL.1	1.40E-06	-0.10798	0	0.039	0.020963	CSCL	MASTL
SLC32A1.1	1.40E-06	-0.15731	0	0.039	0.020963	CSCL	SLC32A1
ASCL2.1	1.44E-06	-0.1566	0	0.039	0.021574	CSCL	ASCL2
AC109826.	1.44E-06	-0.18576	0	0.039	0.021574	CSCL	AC109826.1
NHSL2.1	1.45E-06	0.312663	0.094	0.049	0.021643	CSCL	NHSL2
HNRNPC.2	1.48E-06	0.399634	0.532	0.702	0.022072	CSCL	HNRNPC
CHML	1.48E-06	-0.10662	0.002	0.042	0.022142	CSCL	CHML
DHTKD1.1	1.50E-06	0.110675	0.054	0.129	0.022449	CSCL	DHTKD1
RP11-536K	1.53E-06	-0.21985	0	0.039	0.02285	CSCL	RP11-536K7.3
KCNQ1OT1.	1.54E-06	0.149737	0.155	0.284	0.023074	CSCL	KCNQ1OT1
TXNDC17.2	1.60E-06	0.217735	0.295	0.537	0.02389	CSCL	TXNDC17
FEZ1.1	1.60E-06	0.133935	0.25	0.439	0.02392	CSCL	FEZ1
JAM2.2	1.61E-06	0.196086	0.138	0.258	0.024016	CSCL	JAM2
SELL.1	1.62E-06	-0.20567	0	0.039	0.024201	CSCL	SELL
IQCG.2	1.64E-06	0.143977	0.052	0.126	0.024557	CSCL	IQCG
MT1E.2	1.66E-06	0.591272	0.38	0.38	0.024822	CSCL	MT1E
ZCCHC14.1	1.71E-06	-0.10127	0.007	0.052	0.025606	CSCL	ZCCHC14
CD180.1	1.72E-06	-0.1566	0	0.039	0.025632	CSCL	CD180
KIF2C.1	1.73E-06	-0.11793	0.003	0.045	0.02583	CSCL	KIF2C
CGRRF1.1	1.73E-06	0.145404	0.08	0.168	0.025874	CSCL	CGRRF1
HSPA1B.1	1.73E-06	0.124013	0.239	0.419	0.025913	CSCL	HSPA1B
HAUS4.2	1.74E-06	-0.14849	0.014	0.064	0.026046	CSCL	HAUS4
MYEOV2.1	1.75E-06	0.117594	0.377	0.689	0.026206	CSCL	MYEOV2
SULT1A1.1	1.77E-06	-0.16176	0.007	0.052	0.026414	CSCL	SULT1A1
TESC	1.80E-06	-0.11838	0.002	0.042	0.026935	CSCL	TESC
NME7.2	1.81E-06	0.167387	0.079	0.165	0.027028	CSCL	NME7
CDK10.1	1.85E-06	0.133449	0.08	0.168	0.027621	CSCL	CDK10
TNF.1	1.86E-06	-0.20864	0.003	0.045	0.027799	CSCL	TNF
MARVELD1	1.87E-06	-0.1053	0.002	0.042	0.027934	CSCL	MARVELD1
DOCK5.2	1.88E-06	-0.18011	0.01	0.057	0.028112	CSCL	DOCK5
PRIM2	1.89E-06	-0.10111	0.01	0.058	0.028233	CSCL	PRIM2
RP11-76E1	1.92E-06	-0.21492	0	0.038	0.028752	CSCL	RP11-76E17.3
SRSF10.1	1.95E-06	0.210202	0.131	0.246	0.029186	CSCL	SRSF10
TP53TG1.2	1.99E-06	0.174471	0.103	0.202	0.029812	CSCL	TP53TG1
TMEM255B.	2.04E-06	-0.1503	0.01	0.057	0.030474	CSCL	TMEM255B
CDCA8.1	2.07E-06	-0.13432	0.002	0.041	0.030952	CSCL	CDCA8
GIMAP6.1	2.10E-06	-0.17696	0.003	0.045	0.031377	CSCL	GIMAP6
PKMYT1.1	2.12E-06	-0.11424	0.003	0.045	0.031652	CSCL	PKMYT1
TMEFF2.2	2.12E-06	-0.11346	0.024	0.08	0.031659	CSCL	TMEFF2
AP1S1.2	2.12E-06	0.252897	0.265	0.478	0.031721	CSCL	AP1S1
PSMC3.1	2.13E-06	0.201546	0.302	0.542	0.031784	CSCL	PSMC3
KLF11.1	2.13E-06	-0.12805	0.002	0.041	0.031882	CSCL	KLF11
NDUFB3.1	2.15E-06	0.262173	0.305	0.566	0.032183	CSCL	NDUFB3
FGD3.1	2.16E-06	-0.15635	0.002	0.041	0.032226	CSCL	FGD3
SC5D.1	2.16E-06	0.180697	0.087	0.178	0.032229	CSCL	SC5D

GRAMD4.2	2.22E-06	-0.10239	0.014	0.063	0.033228	CSCL	GRAMD4
ID3.2	2.27E-06	0.129059	0.15	0.27	0.033957	CSCL	ID3
TPK1.1	2.28E-06	-0.15159	0.005	0.048	0.034119	CSCL	TPK1
SMURF1.1	2.32E-06	-0.10793	0.007	0.051	0.034743	CSCL	SMURF1
JAG1.1	2.37E-06	0.249571	0.077	0.163	0.035471	CSCL	JAG1
INA.1	2.40E-06	-0.12885	0.003	0.044	0.035838	CSCL	INA
RHOF.1	2.40E-06	-0.13962	0.009	0.054	0.035921	CSCL	RHOF
TCF20.1	2.42E-06	-0.10151	0.012	0.06	0.036116	CSCL	TCF20
C21orf62.	2.42E-06	0.181169	0.077	0.162	0.036235	CSCL	C21orf62
TNFSF10	2.45E-06	-0.13319	0.023	0.077	0.036582	CSCL	TNFSF10
MAP7.1	2.46E-06	-0.20125	0.002	0.041	0.036784	CSCL	MAP7
NGDN.1	2.47E-06	0.200002	0.147	0.269	0.036939	CSCL	NGDN
FAM78A.1	2.49E-06	-0.16678	0	0.037	0.037227	CSCL	FAM78A
RBP7.1	2.49E-06	-0.16604	0.005	0.047	0.037232	CSCL	RBP7
TRMT13	2.53E-06	0.140427	0.063	0.14	0.037805	CSCL	TRMT13
CDC45.1	2.56E-06	-0.10534	0	0.037	0.038311	CSCL	CDC45
C11orf1.2	2.62E-06	0.22441	0.068	0.148	0.039209	CSCL	C11orf1
KCNQ1.1	2.68E-06	-0.16252	0.002	0.041	0.040039	CSCL	KCNQ1
NRF1	2.72E-06	-0.10315	0	0.037	0.040573	CSCL	NRF1
SLC27A3.1	2.74E-06	0.134571	0.054	0.126	0.040926	CSCL	SLC27A3
DEPDC1	2.79E-06	-0.1024	0	0.037	0.041754	CSCL	DEPDC1
DLGAP5.1	2.79E-06	-0.11676	0	0.037	0.041754	CSCL	DLGAP5
CST7.1	2.79E-06	-0.21171	0	0.037	0.041754	CSCL	CST7
MTRNR2L8.	2.81E-06	0.485706	0.243	0.188	0.042043	CSCL	MTRNR2L8
AP1G2.1	2.82E-06	-0.12331	0.009	0.053	0.042105	CSCL	AP1G2
TMEM69.1	2.83E-06	0.122675	0.086	0.173	0.042361	CSCL	TMEM69
ASNS.2	2.85E-06	0.135426	0.091	0.181	0.042518	CSCL	ASNS
ZNF544.1	2.88E-06	0.143333	0.054	0.126	0.043097	CSCL	ZNF544
MAP1LC3B2	2.91E-06	-0.12353	0.03	0.087	0.043447	CSCL	MAP1LC3B2
DOCK11.2	2.91E-06	-0.11537	0.01	0.056	0.043448	CSCL	DOCK11
DENND2D	3.06E-06	-0.12654	0.005	0.047	0.045743	CSCL	DENND2D
NUDT18	3.09E-06	-0.11226	0.002	0.04	0.046106	CSCL	NUDT18
PDZRN4.1	3.11E-06	-0.14413	0.002	0.04	0.046482	CSCL	PDZRN4
BATF.1	3.12E-06	-0.16058	0.002	0.04	0.046603	CSCL	BATF
CTB-61M7.	3.18E-06	-0.19429	0.002	0.04	0.047488	CSCL	CTB-61M7.2
HS3ST3B1	3.21E-06	-0.12122	0.003	0.043	0.047957	CSCL	HS3ST3B1
CDK4	3.24E-06	0.203483	0.279	0.494	0.048483	CSCL	CDK4
NDUFS6.1	3.26E-06	0.136882	0.351	0.642	0.048712	CSCL	NDUFS6
ANAPC11.2	3.27E-06	0.367077	0.55	0.736	0.048844	CSCL	ANAPC11
FLVCR2	3.32E-06	-0.14014	0	0.036	0.049593	CSCL	FLVCR2
CCND2.2	3.40E-06	0.243684	0.187	0.334	0.050827	CSCL	CCND2
POLR2F.2	3.53E-06	0.221244	0.311	0.554	0.052723	CSCL	POLR2F
ICAM2.1	3.62E-06	-0.17888	0	0.036	0.054047	CSCL	ICAM2
EIF1B.2	3.65E-06	0.170425	0.339	0.601	0.054538	CSCL	EIF1B
MANBAL.2	3.65E-06	0.294073	0.188	0.342	0.054546	CSCL	MANBAL
CITED1.1	3.67E-06	0.235224	0.073	0.155	0.0549	CSCL	CITED1
ZC3HC1	3.73E-06	0.200084	0.056	0.128	0.055708	CSCL	ZC3HC1
ITGB3BP	3.79E-06	0.239483	0.068	0.147	0.05668	CSCL	ITGB3BP
ACSL5.1	3.83E-06	-0.14913	0	0.036	0.057235	CSCL	ACSL5
ZNF462.2	3.86E-06	0.166496	0.091	0.181	0.057725	CSCL	ZNF462
TGM2.1	3.88E-06	-0.13918	0.002	0.039	0.057963	CSCL	TGM2

LMO4.2	3.88E-06	0.161207	0.283	0.488	0.057985	CSCL	LMO4
CD1D.1	3.92E-06	-0.18441	0.002	0.039	0.058642	CSCL	CD1D
DPH2.1	3.95E-06	0.132428	0.051	0.119	0.059078	CSCL	DPH2
HOXA3.1	4.02E-06	-0.10818	0.012	0.058	0.06003	CSCL	HOXA3
DENND1A	4.05E-06	-0.10966	0.01	0.055	0.060586	CSCL	DENND1A
MYOF	4.08E-06	-0.13223	0.005	0.046	0.060958	CSCL	MYOF
LRIG1.1	4.20E-06	0.119201	0.045	0.111	0.062728	CSCL	LRIG1
CLIC2	4.26E-06	-0.14143	0.005	0.046	0.063689	CSCL	CLIC2
TMEM59L.1	4.28E-06	0.193859	0.145	0.264	0.063994	CSCL	TMEM59L
MAG.1	4.29E-06	-0.36341	0.002	0.039	0.064182	CSCL	MAG
F3.2	4.32E-06	0.189864	0.101	0.196	0.064505	CSCL	F3
FBL.2	4.38E-06	0.26163	0.218	0.386	0.0654	CSCL	FBL
RP11-386G	4.38E-06	0.100464	0.07	0.147	0.065408	CSCL	RP11-386G11.10
ZNF467.1	4.42E-06	-0.13477	0	0.036	0.066052	CSCL	ZNF467
TRIM44.2	4.45E-06	0.309255	0.161	0.293	0.066505	CSCL	TRIM44
ADAMDEC1.	4.45E-06	-0.18176	0.002	0.039	0.066547	CSCL	ADAMDEC1
ERV3-1.1	4.48E-06	0.172927	0.147	0.266	0.066928	CSCL	ERV3-1
ITM2C.2	4.49E-06	0.130842	0.248	0.426	0.06705	CSCL	ITM2C
TRIB2.2	4.50E-06	0.323597	0.145	0.269	0.067188	CSCL	TRIB2
PIN4.2	4.50E-06	0.2735	0.241	0.427	0.067202	CSCL	PIN4
NET1.1	4.53E-06	-0.12953	0.003	0.042	0.067717	CSCL	NET1
IMPDH2.2	4.60E-06	0.319081	0.239	0.426	0.068789	CSCL	IMPDH2
DENND1C.1	4.68E-06	-0.16683	0	0.035	0.069947	CSCL	DENND1C
TMEM86A.2	4.73E-06	-0.14633	0.007	0.048	0.070683	CSCL	TMEM86A
CDCP1.1	4.82E-06	-0.14926	0	0.035	0.07198	CSCL	CDCP1
CHID1.1	4.89E-06	0.277748	0.251	0.446	0.07314	CSCL	CHID1
TSTD1.1	4.92E-06	0.251945	0.105	0.202	0.073556	CSCL	TSTD1
DPEP2.1	4.96E-06	-0.17879	0	0.035	0.074071	CSCL	DPEP2
EIF5B.1	5.01E-06	0.233504	0.3	0.533	0.074832	CSCL	EIF5B
ABHD16A	5.04E-06	-0.12417	0.002	0.039	0.07536	CSCL	ABHD16A
BNIP1.1	5.09E-06	0.131717	0.054	0.123	0.076111	CSCL	BNIP1
C19orf53.	5.16E-06	0.412485	0.534	0.691	0.077096	CSCL	C19orf53
PAFAH1B3.	5.22E-06	0.307342	0.243	0.433	0.078036	CSCL	PAFAH1B3
NYAP1	5.27E-06	-0.11293	0.002	0.038	0.078797	CSCL	NYAP1
ARHGAP12.	5.52E-06	0.310528	0.099	0.194	0.082499	CSCL	ARHGAP12
GPI.1	5.53E-06	0.263481	0.206	0.364	0.082701	CSCL	GPI
ATP8A1.1	5.54E-06	-0.11681	0.005	0.045	0.082759	CSCL	ATP8A1
RAB3IL1.1	5.56E-06	-0.14221	0	0.035	0.083062	CSCL	RAB3IL1
ARX.1	5.59E-06	-0.121	0.003	0.042	0.08357	CSCL	ARX
MBIP.1	5.65E-06	0.15643	0.094	0.184	0.08449	CSCL	MBIP
ATP1A2	5.70E-06	0.650921	0.101	0.06	0.085136	CSCL	ATP1A2
P2RY6.1	5.72E-06	-0.14184	0	0.035	0.085474	CSCL	P2RY6
NOL3.2	5.72E-06	0.130218	0.086	0.17	0.085502	CSCL	NOL3
KIF23.1	5.74E-06	-0.10348	0.003	0.042	0.085811	CSCL	KIF23
RBKS	5.77E-06	-0.12425	0.003	0.041	0.086275	CSCL	RBKS
HRH2	5.87E-06	-0.14443	0.003	0.041	0.08777	CSCL	HRH2
STAT5A.2	5.90E-06	-0.11374	0.01	0.054	0.088096	CSCL	STAT5A
MAL.1	5.94E-06	-0.24827	0.003	0.041	0.088703	CSCL	MAL
CERS1	6.07E-06	0.181512	0.059	0.131	0.09075	CSCL	CERS1
TBC1D2B.2	6.16E-06	-0.10478	0.01	0.054	0.092026	CSCL	TBC1D2B
KCNN4	6.23E-06	-0.12666	0	0.034	0.09314	CSCL	KCNN4

FAM50B	6.27E-06	-0.13431	0.005	0.044	0.093766	CSCL	FAM50B
ARHGEF26.	6.36E-06	0.179544	0.058	0.128	0.095017	CSCL	ARHGEF26
RP11-796E	6.38E-06	-0.14296	0.019	0.068	0.095349	CSCL	RP11-796E2.4
ST18.1	6.39E-06	-0.15148	0.003	0.041	0.095477	CSCL	ST18
ECHDC3.1	6.41E-06	-0.14539	0	0.034	0.095845	CSCL	ECHDC3
TMEM151A.	6.56E-06	-0.14606	0.005	0.044	0.09803	CSCL	TMEM151A
CLEC9A.1	6.60E-06	-0.21889	0	0.034	0.098628	CSCL	CLEC9A
ZNF814	6.62E-06	0.193271	0.056	0.126	0.098886	CSCL	ZNF814
TLR5.1	6.68E-06	-0.16902	0.007	0.047	0.099816	CSCL	TLR5
GSTA4.1	6.71E-06	0.252206	0.166	0.296	0.100256	CSCL	GSTA4
LTB.1	6.79E-06	-0.19935	0	0.034	0.101492	CSCL	LTB
FCHO1.1	6.84E-06	-0.11276	0.003	0.041	0.102154	CSCL	FCHO1
FKBP10.2	6.89E-06	0.240044	0.115	0.217	0.102902	CSCL	FKBP10
METTL1.1	6.97E-06	0.10843	0.073	0.151	0.104103	CSCL	METTL1
PCED1B.1	7.00E-06	-0.1303	0.002	0.038	0.104563	CSCL	PCED1B
GPR37L1.1	7.01E-06	0.578454	0.127	0.082	0.104799	CSCL	GPR37L1
F11R.1	7.07E-06	-0.10645	0.014	0.059	0.105709	CSCL	F11R
RAPGEF5.1	7.11E-06	-0.18304	0.005	0.044	0.106238	CSCL	RAPGEF5
HENMT1.1	7.19E-06	-0.15762	0	0.034	0.107471	CSCL	HENMT1
SCARA3.1	7.21E-06	0.17615	0.066	0.141	0.107784	CSCL	SCARA3
FAM53B.2	7.24E-06	-0.12009	0.01	0.053	0.108264	CSCL	FAM53B
ARL11.1	7.37E-06	-0.14811	0.003	0.041	0.110141	CSCL	ARL11
NELFA.1	7.41E-06	0.139279	0.068	0.143	0.110775	CSCL	NELFA
ZNF124.2	7.46E-06	-0.10618	0.016	0.062	0.111489	CSCL	ZNF124
GNG10	7.53E-06	-0.10072	0.024	0.076	0.112471	CSCL	GNG10
ADORA1	7.56E-06	0.162389	0.056	0.125	0.113027	CSCL	ADORA1
C1R.2	7.66E-06	0.567867	0.257	0.213	0.114507	CSCL	C1R
PSME1.1	7.72E-06	0.219079	0.333	0.596	0.115369	CSCL	PSME1
N6AMT2	7.85E-06	0.262669	0.063	0.136	0.117263	CSCL	N6AMT2
C2orf80.1	7.86E-06	0.118291	0.054	0.122	0.117509	CSCL	C2orf80
IFT88	8.01E-06	0.111557	0.047	0.111	0.119627	CSCL	IFT88
MGST1.2	8.05E-06	0.486228	0.195	0.142	0.120283	CSCL	MGST1
ZBTB7B	8.06E-06	-0.10151	0.005	0.044	0.120462	CSCL	ZBTB7B
ARNT2.2	8.16E-06	0.123487	0.049	0.114	0.121951	CSCL	ARNT2
FAM83D	8.19E-06	-0.1036	0.002	0.037	0.122418	CSCL	FAM83D
C16orf86	8.27E-06	-0.11339	0.003	0.04	0.123515	CSCL	C16orf86
IL15RA	8.54E-06	-0.10148	0	0.033	0.1276	CSCL	IL15RA
IL2RA.1	8.54E-06	-0.24155	0	0.033	0.1276	CSCL	IL2RA
COPS8.2	8.56E-06	0.250458	0.272	0.48	0.127911	CSCL	COPS8
PIPOX.1	8.64E-06	0.261545	0.063	0.136	0.129168	CSCL	PIPOX
BMP7.2	8.67E-06	0.219286	0.086	0.17	0.129531	CSCL	BMP7
LRRC8A.1	8.73E-06	0.2079	0.087	0.172	0.130481	CSCL	LRRC8A
ZNF577.1	8.82E-06	0.10131	0.037	0.095	0.131817	CSCL	ZNF577
MFSD7.1	9.04E-06	-0.12278	0	0.033	0.135113	CSCL	MFSD7
ANPEP.1	9.04E-06	-0.14711	0	0.033	0.135113	CSCL	ANPEP
CREBZF.1	9.14E-06	0.12753	0.063	0.135	0.136598	CSCL	CREBZF
ATOX1.2	9.22E-06	0.186664	0.344	0.618	0.137843	CSCL	ATOX1
PAICS.1	9.27E-06	0.319237	0.176	0.315	0.138548	CSCL	PAICS
ZNF562.1	9.40E-06	0.138765	0.07	0.145	0.140543	CSCL	ZNF562
CCL5.1	9.43E-06	-0.25902	0.014	0.058	0.140944	CSCL	CCL5
OTOA.1	9.57E-06	-0.14096	0	0.033	0.143068	CSCL	OTOA

TSPAN31.1	9.60E-06	0.174026	0.173	0.301	0.143392	CSCL	TSPAN31
TM4SF1.2	9.67E-06	0.110073	0.051	0.116	0.144468	CSCL	TM4SF1
ZBTB20.1	9.73E-06	0.203452	0.225	0.394	0.145333	CSCL	ZBTB20
RP1-39G22	9.87E-06	0.187277	0.063	0.134	0.147428	CSCL	RP1-39G22.7
CSGALNACT	9.94E-06	-0.12124	0.026	0.077	0.148572	CSCL	CSGALNACT1
CNTN2.1	1.01E-05	-0.2056	0.005	0.043	0.151192	CSCL	CNTN2
ISG15.2	1.02E-05	0.362795	0.208	0.364	0.151728	CSCL	ISG15
TMEM53	1.04E-05	0.134275	0.045	0.108	0.155933	CSCL	TMEM53
TLR4.1	1.08E-05	-0.12924	0.005	0.043	0.161973	CSCL	TLR4
CNBP.2	1.09E-05	0.333856	0.553	0.758	0.162323	CSCL	CNBP
HIBCH.1	1.10E-05	0.238148	0.138	0.249	0.165106	CSCL	HIBCH
SEC14L2.1	1.11E-05	0.220039	0.052	0.118	0.165359	CSCL	SEC14L2
AC004158.	1.13E-05	0.121706	0.058	0.126	0.168197	CSCL	AC004158.3
VEGFA.2	1.16E-05	0.205818	0.12	0.221	0.173432	CSCL	VEGFA
PSD4	1.17E-05	-0.11703	0	0.033	0.174774	CSCL	PSD4
TAL1.1	1.18E-05	-0.17068	0.002	0.036	0.17586	CSCL	TAL1
CBS.1	1.18E-05	0.229971	0.08	0.161	0.176525	CSCL	CBS
HLX	1.20E-05	-0.11749	0.005	0.042	0.178969	CSCL	HLX
SYT5	1.20E-05	-0.11217	0.007	0.045	0.178978	CSCL	SYT5
TMEM106A.	1.24E-05	-0.11296	0.019	0.066	0.185483	CSCL	TMEM106A
SMIM3.2	1.25E-05	0.292177	0.068	0.034	0.186979	CSCL	SMIM3
CDO1.2	1.26E-05	0.257769	0.133	0.24	0.188594	CSCL	CDO1
LACC1.1	1.28E-05	-0.13254	0.002	0.036	0.191038	CSCL	LACC1
ZNF428.2	1.29E-05	0.194959	0.318	0.556	0.192353	CSCL	ZNF428
SP9.1	1.29E-05	-0.11486	0.005	0.042	0.192782	CSCL	SP9
RP11-100C	1.31E-05	-0.12599	0.005	0.042	0.195708	CSCL	RP11-1000B6.3
SMAD7.1	1.35E-05	-0.11742	0.002	0.036	0.201397	CSCL	SMAD7
RP11-403I	1.35E-05	-0.17603	0.002	0.036	0.201471	CSCL	RP11-403I13.5
RP11-742E	1.35E-05	-0.15462	0	0.032	0.20163	CSCL	RP11-742B18.1
CAPN3.1	1.36E-05	-0.23244	0.007	0.045	0.203188	CSCL	CAPN3
NDUFS7	1.38E-05	0.154978	0.358	0.631	0.205525	CSCL	NDUFS7
MLC1.1	1.40E-05	0.214168	0.075	0.152	0.209125	CSCL	MLC1
IL15	1.40E-05	-0.14382	0.003	0.039	0.209724	CSCL	IL15
RP11-89N1	1.44E-05	0.121099	0.007	0.001	0.214913	CSCL	RP11-89N17.4
SLPI.2	1.47E-05	0.508555	0.202	0.147	0.219611	CSCL	SLPI
CDIPT-AS1	1.48E-05	0.25309	0.024	0.008	0.22116	CSCL	CDIPT-AS1
MKL2.2	1.50E-05	0.107113	0.028	0.079	0.22404	CSCL	MKL2
DISC1.1	1.50E-05	-0.10474	0.009	0.048	0.224869	CSCL	DISC1
TBC1D10C.	1.51E-05	-0.15573	0	0.032	0.22605	CSCL	TBC1D10C
RTKN.2	1.52E-05	0.222791	0.091	0.175	0.227244	CSCL	RTKN
NEDD8.1	1.54E-05	0.342217	0.557	0.755	0.230001	CSCL	NEDD8
LTB4R.1	1.55E-05	-0.11649	0.002	0.035	0.231822	CSCL	LTB4R
SPINT1.1	1.58E-05	-0.12379	0.002	0.035	0.236559	CSCL	SPINT1
NEK2.1	1.59E-05	-0.10528	0.002	0.035	0.237343	CSCL	NEK2
CDC42EP2.	1.60E-05	-0.10337	0.003	0.038	0.239475	CSCL	CDC42EP2
KALRN	1.60E-05	0.140226	0.04	0.098	0.239637	CSCL	KALRN
CAMLG.2	1.61E-05	0.282455	0.288	0.504	0.239989	CSCL	CAMLG
SELK.2	1.69E-05	0.109368	0.4	0.716	0.252024	CSCL	SELK
MYO7A.1	1.69E-05	-0.13408	0.002	0.035	0.253072	CSCL	MYO7A
HMBOX1.1	1.71E-05	0.217656	0.106	0.197	0.255988	CSCL	HMBOX1
ZNF536.1	1.72E-05	-0.12248	0.007	0.044	0.256669	CSCL	ZNF536

AC069363.	1.73E-05	-0.18255	0.012	0.053	0.258859	CSCL	AC069363.1
VCAN.1	1.75E-05	0.27919	0.141	0.251	0.261104	CSCL	VCAN
LAP3	1.76E-05	0.214745	0.288	0.502	0.262838	CSCL	LAP3
PLVAP.1	1.80E-05	-0.15491	0	0.031	0.268327	CSCL	PLVAP
GPX8.1	1.83E-05	0.113675	0.019	0.065	0.272898	CSCL	GPX8
MLXIPL.1	1.84E-05	-0.14159	0.005	0.041	0.274245	CSCL	MLXIPL
CHEK2.1	1.84E-05	-0.12052	0.01	0.05	0.274456	CSCL	CHEK2
ERGIC3.1	1.84E-05	0.159318	0.333	0.588	0.274782	CSCL	ERGIC3
SLC17A9.1	1.85E-05	-0.12945	0	0.031	0.276104	CSCL	SLC17A9
PAAF1.1	1.85E-05	0.270286	0.11	0.204	0.276254	CSCL	PAAF1
AC147651.	1.86E-05	-0.1358	0.003	0.038	0.277213	CSCL	AC147651.4
MYL6B.1	1.87E-05	0.246444	0.258	0.452	0.278933	CSCL	MYL6B
GRIA3.1	1.88E-05	0.250372	0.089	0.172	0.281105	CSCL	GRIA3
TSPAN3.1	1.89E-05	0.262761	0.265	0.47	0.282209	CSCL	TSPAN3
CEND1	1.98E-05	0.175303	0.07	0.142	0.295409	CSCL	CEND1
GAS1.2	1.99E-05	0.193916	0.073	0.148	0.296759	CSCL	GAS1
KIAA0907.	2.04E-05	0.142532	0.108	0.198	0.30496	CSCL	KIAA0907
DSC2.1	2.05E-05	-0.13187	0.002	0.034	0.30644	CSCL	DSC2
ALDH1A1.1	2.07E-05	-0.15236	0	0.031	0.309529	CSCL	ALDH1A1
EIF3M.2	2.08E-05	0.241535	0.309	0.537	0.310512	CSCL	EIF3M
C14orf1.1	2.10E-05	0.321492	0.169	0.299	0.3141	CSCL	C14orf1
ST8SIA1	2.16E-05	0.100105	0.038	0.094	0.322355	CSCL	ST8SIA1
WLS	2.16E-05	0.263936	0.12	0.219	0.323391	CSCL	WLS
CCDC69.1	2.20E-05	-0.1051	0.003	0.037	0.32816	CSCL	CCDC69
HSD17B7	2.24E-05	0.124053	0.056	0.121	0.334259	CSCL	HSD17B7
CHST8.1	2.25E-05	0.101211	0.023	0.07	0.335816	CSCL	CHST8
CACNG8	2.29E-05	0.14802	0.026	0.075	0.341613	CSCL	CACNG8
RPRM.2	2.29E-05	-0.10227	0.023	0.069	0.342004	CSCL	RPRM
PID1.1	2.30E-05	0.101966	0.056	0.12	0.343003	CSCL	PID1
FMN1.1	2.30E-05	-0.12573	0.002	0.034	0.343234	CSCL	FMN1
FAM162A.2	2.31E-05	0.277221	0.237	0.409	0.344502	CSCL	FAM162A
SIGLEC1.1	2.32E-05	-0.1423	0	0.03	0.346993	CSCL	SIGLEC1
AP005530.	2.32E-05	-0.14937	0	0.03	0.346993	CSCL	AP005530.2
NXN.1	2.33E-05	-0.10594	0.003	0.037	0.347465	CSCL	NXN
MAML3.1	2.37E-05	-0.1113	0.003	0.037	0.353798	CSCL	MAML3
PMS2.1	2.38E-05	0.176188	0.063	0.131	0.355174	CSCL	PMS2
FAIM2.1	2.42E-05	0.205884	0.106	0.195	0.361901	CSCL	FAIM2
CHI3L1.2	2.53E-05	0.477446	0.475	0.527	0.37856	CSCL	CHI3L1
SOCS1.1	2.55E-05	-0.12273	0.014	0.055	0.381104	CSCL	SOCS1
NAA60.1	2.58E-05	0.224092	0.031	0.012	0.38585	CSCL	NAA60
PSME2.1	2.59E-05	0.241028	0.325	0.569	0.38676	CSCL	PSME2
ZNF576.1	2.60E-05	0.230599	0.07	0.141	0.387932	CSCL	ZNF576
FABP6.1	2.61E-05	-0.11974	0.005	0.04	0.389552	CSCL	FABP6
ZEB1.2	2.68E-05	0.295962	0.106	0.197	0.399807	CSCL	ZEB1
GORAB.2	2.72E-05	0.101958	0.028	0.077	0.406711	CSCL	GORAB
AP4M1	2.75E-05	0.204309	0.108	0.198	0.410719	CSCL	AP4M1
GNAS.2	2.77E-05	0.472157	0.497	0.655	0.413451	CSCL	GNAS
PNN.2	2.81E-05	0.285424	0.237	0.41	0.42034	CSCL	PNN
SOD2.1	2.98E-05	0.191263	0.33	0.547	0.445618	CSCL	SOD2
RP11-489C	3.00E-05	-0.15802	0	0.03	0.448685	CSCL	RP11-489018.1
LSS.1	3.05E-05	0.127339	0.044	0.101	0.455721	CSCL	LSS

UBXN11.1	3.25E-05	0.11225	0.07	0.139	0.485489	CSCL	UBXN11
CARD11.1	3.27E-05	-0.11824	0	0.029	0.488812	CSCL	CARD11
ATXN3.1	3.29E-05	0.187391	0.084	0.16	0.491025	CSCL	ATXN3
ELOVL2.2	3.33E-05	0.14386	0.044	0.1	0.497705	CSCL	ELOVL2
IL12RB1.1	3.37E-05	-0.14582	0.002	0.033	0.503968	CSCL	IL12RB1
WSB1.1	3.41E-05	0.103849	0.377	0.666	0.509213	CSCL	WSB1
CACNA2D3.	3.44E-05	-0.11679	0.002	0.033	0.513769	CSCL	CACNA2D3
PBX1.1	3.46E-05	0.212517	0.122	0.219	0.517727	CSCL	PBX1
NECAB1.1	3.47E-05	-0.14059	0.005	0.039	0.518953	CSCL	NECAB1
ZNF680	3.48E-05	0.150655	0.096	0.178	0.520425	CSCL	ZNF680
KLK6.1	3.59E-05	-0.25614	0.002	0.032	0.535843	CSCL	KLK6
CNPY2.1	3.60E-05	0.159474	0.328	0.581	0.537458	CSCL	CNPY2
LILRB3.1	3.60E-05	-0.14837	0.007	0.042	0.537914	CSCL	LILRB3
HSPD1.2	3.60E-05	0.185408	0.36	0.628	0.538717	CSCL	HSPD1
DNAJC24.1	3.61E-05	0.115216	0.049	0.108	0.539356	CSCL	DNAJC24
RAMP2.1	3.62E-05	0.154206	0.07	0.139	0.540848	CSCL	RAMP2
SLC38A6.1	3.65E-05	0.107401	0.045	0.102	0.545193	CSCL	SLC38A6
PMP2.2	3.69E-05	0.509492	0.344	0.35	0.551036	CSCL	PMP2
AQP4.1	3.73E-05	0.683468	0.202	0.164	0.557039	CSCL	AQP4
NFAM1.1	3.73E-05	-0.13884	0.003	0.035	0.557428	CSCL	NFAM1
SP140.1	3.76E-05	-0.11897	0.002	0.032	0.56141	CSCL	SP140
GLDC	3.81E-05	0.114768	0.024	0.071	0.569927	CSCL	GLDC
ST5.2	3.82E-05	0.264971	0.08	0.155	0.571404	CSCL	ST5
PEA15.1	3.85E-05	0.329902	0.26	0.452	0.57472	CSCL	PEA15
RP11-347F	3.96E-05	-0.16647	0.023	0.066	0.591216	CSCL	RP11-347P5.1
PRMT5.2	4.06E-05	0.21321	0.08	0.154	0.606078	CSCL	PRMT5
MAPK13.1	4.08E-05	-0.11138	0.002	0.032	0.60954	CSCL	MAPK13
GPR132.1	4.10E-05	-0.11033	0.002	0.032	0.612338	CSCL	GPR132
RP11-514C	4.23E-05	-0.11873	0	0.028	0.632013	CSCL	RP11-514O12.4
FGF1	4.29E-05	0.145324	0.042	0.097	0.64181	CSCL	FGF1
AC007405.	4.53E-05	0.119455	0.024	0.07	0.677386	CSCL	AC007405.6
C6orf48.1	4.55E-05	0.1441	0.375	0.661	0.680182	CSCL	C6orf48
RP11-557H	4.61E-05	-0.14768	0	0.028	0.68852	CSCL	RP11-557H15.4
PGGT1B.1	4.70E-05	0.121976	0.045	0.102	0.701831	CSCL	PGGT1B
ZNF521	4.74E-05	0.150858	0.035	0.086	0.708449	CSCL	ZNF521
PSAT1.1	4.81E-05	0.261929	0.117	0.208	0.719476	CSCL	PSAT1
DNAJC11.1	4.84E-05	0.15581	0.047	0.104	0.723756	CSCL	DNAJC11
PVR.1	4.85E-05	0.133155	0.024	0.07	0.724949	CSCL	PVR
RNASE3.1	4.88E-05	-0.17966	0.003	0.035	0.729556	CSCL	RNASE3
AATK.1	4.89E-05	-0.17635	0.003	0.035	0.730448	CSCL	AATK
VAMP5.1	5.01E-05	0.233706	0.241	0.405	0.748176	CSCL	VAMP5
HRH1.1	5.01E-05	0.173608	0.044	0.099	0.748509	CSCL	HRH1
SPA17.2	5.07E-05	0.103873	0.033	0.083	0.757209	CSCL	SPA17
PTPN22.1	5.16E-05	-0.12124	0	0.028	0.771793	CSCL	PTPN22
SCARF1.1	5.16E-05	-0.12413	0	0.028	0.771793	CSCL	SCARF1
KCNE3.1	5.31E-05	-0.11632	0	0.028	0.794139	CSCL	KCNE3
C8orf4.1	5.42E-05	0.632031	0.262	0.226	0.809992	CSCL	C8orf4
CHRNB1.1	5.44E-05	0.197915	0.08	0.152	0.813056	CSCL	CHRNB1
CATSPER1.	5.63E-05	-0.14308	0	0.028	0.84079	CSCL	CATSPER1
MOBP.1	5.70E-05	-0.27064	0.005	0.037	0.851386	CSCL	MOBP
WDFY4.1	5.79E-05	-0.12467	0	0.027	0.865133	CSCL	WDFY4

FFAR4.1	5.85E-05	-0.13223	0.002	0.031	0.874744	CSCL	FFAR4
HAPLN2.1	5.93E-05	-0.15096	0.003	0.034	0.886024	CSCL	HAPLN2
AC018647.	6.07E-05	-0.13153	0.009	0.043	0.907116	CSCL	AC018647.3
MAP4K1.1	6.09E-05	-0.12047	0.002	0.031	0.910749	CSCL	MAP4K1
LAMB2	6.31E-05	0.152286	0.044	0.098	0.943118	CSCL	LAMB2
PLA2G4A	6.38E-05	-0.1101	0.003	0.034	0.953096	CSCL	PLA2G4A
PEAK1.2	6.44E-05	0.129516	0.026	0.071	0.962183	CSCL	PEAK1
LINC00665	6.49E-05	0.352898	0.105	0.19	0.969743	CSCL	LINC00665
NAP1L1	6.84E-05	0.134059	0.387	0.689	1	CSCL	NAP1L1
NXPH1.2	6.91E-05	0.113124	0.072	0.139	1	CSCL	NXPH1
CCR5.1	7.07E-05	-0.11253	0	0.027	1	CSCL	CCR5
SKIV2L2.1	7.12E-05	0.189423	0.086	0.16	1	CSCL	SKIV2L2
SLC12A4.1	7.16E-05	0.106457	0.024	0.068	1	CSCL	SLC12A4
EPAS1.1	7.19E-05	0.131154	0.051	0.107	1	CSCL	EPAS1
TSHZ1.2	7.25E-05	0.100889	0.03	0.076	1	CSCL	TSHZ1
ERMN.1	7.32E-05	-0.2748	0.007	0.039	1	CSCL	ERMN
RWDD1.2	7.33E-05	0.291644	0.309	0.538	1	CSCL	RWDD1
KANK1	7.36E-05	0.10209	0.042	0.095	1	CSCL	KANK1
OCIAD2.1	7.43E-05	0.586128	0.365	0.388	1	CSCL	OCIAD2
SNRPE.1	7.50E-05	0.264207	0.339	0.592	1	CSCL	SNRPE
PLEKHH1.1	7.55E-05	-0.11353	0.003	0.033	1	CSCL	PLEKHH1
DENND2A	7.55E-05	0.142932	0.044	0.097	1	CSCL	DENND2A
TOR4A.1	7.70E-05	-0.10101	0	0.027	1	CSCL	TOR4A
ITGB7.1	7.70E-05	-0.11119	0	0.027	1	CSCL	ITGB7
MEIS1.2	7.71E-05	0.122399	0.038	0.089	1	CSCL	MEIS1
EDIL3.1	7.74E-05	-0.16508	0.003	0.033	1	CSCL	EDIL3
TMEM25.1	7.79E-05	0.172622	0.063	0.125	1	CSCL	TMEM25
NOS1AP.1	7.79E-05	0.224079	0.033	0.081	1	CSCL	NOS1AP
AQP9	7.86E-05	-0.11061	0.002	0.03	1	CSCL	AQP9
VNN2.1	7.92E-05	-0.10998	0	0.027	1	CSCL	VNN2
METRN.2	8.14E-05	0.245117	0.178	0.3	1	CSCL	METRN
CPE.1	8.16E-05	0.205169	0.239	0.397	1	CSCL	CPE
RGMA.1	8.28E-05	0.324943	0.115	0.205	1	CSCL	RGMA
FUS.1	8.48E-05	0.153179	0.373	0.657	1	CSCL	FUS
FKBP7	8.52E-05	0.103313	0.042	0.094	1	CSCL	FKBP7
HK3	8.63E-05	-0.10141	0	0.026	1	CSCL	HK3
DLC1	8.77E-05	0.159134	0.038	0.089	1	CSCL	DLC1
CARNS1.1	8.79E-05	-0.18954	0.002	0.03	1	CSCL	CARNS1
CYSLTR1.1	8.88E-05	-0.11854	0	0.026	1	CSCL	CYSLTR1
TMEM177.1	9.06E-05	0.100253	0.033	0.081	1	CSCL	TMEM177
LDHA.2	9.13E-05	0.282277	0.579	0.776	1	CSCL	LDHA
PPARG.1	9.40E-05	-0.11336	0	0.026	1	CSCL	PPARG
FAM175B.1	9.44E-05	0.115186	0.035	0.083	1	CSCL	FAM175B
ARMCX2.1	9.46E-05	0.184471	0.08	0.15	1	CSCL	ARMCX2
LAT	9.59E-05	-0.11648	0.005	0.036	1	CSCL	LAT
NFIB.2	9.82E-05	0.144421	0.239	0.385	1	CSCL	NFIB
SUN1.2	9.85E-05	0.243656	0.122	0.212	1	CSCL	SUN1
SERPINE2.	9.86E-05	0.232449	0.18	0.299	1	CSCL	SERPINE2
C16orf45.	9.89E-05	0.382567	0.19	0.325	1	CSCL	C16orf45
WDR83OS.2	9.89E-05	0.381234	0.545	0.754	1	CSCL	WDR83OS
TNFRSF11A	9.96E-05	-0.105	0	0.026	1	CSCL	TNFRSF11A

TMEM168.1	1.00E-04	0.149227	0.056	0.114	1	CSCL	TMEM168
AL592528.	0.0001	0.147273	0.012	0.003	1	CSCL	AL592528.1
TIPIN.1	0.000103	0.226656	0.077	0.144	1	CSCL	TIPIN
CSF2RB.1	0.000105	-0.10276	0	0.026	1	CSCL	CSF2RB
TLR10.1	0.000105	-0.1187	0	0.026	1	CSCL	TLR10
CETN2.2	0.000107	0.335053	0.195	0.329	1	CSCL	CETN2
IFITM1	0.000107	-0.16025	0.007	0.038	1	CSCL	IFITM1
GEMIN8.1	0.000109	0.111613	0.031	0.077	1	CSCL	GEMIN8
NAMPT.2	0.000109	0.149838	0.276	0.446	1	CSCL	NAMPT
CRYZ.1	0.000111	0.186183	0.065	0.126	1	CSCL	CRYZ
DAPP1.1	0.000111	-0.10565	0.002	0.029	1	CSCL	DAPP1
METTL7A	0.000111	0.196947	0.133	0.225	1	CSCL	METTL7A
FAM177B.1	0.000112	-0.13221	0.003	0.032	1	CSCL	FAM177B
OLFM1.1	0.000114	0.167712	0.084	0.154	1	CSCL	OLFM1
RP11-620J	0.000115	0.253806	0.164	0.275	1	CSCL	RP11-620J15.3
CA12.2	0.000116	0.263083	0.073	0.139	1	CSCL	CA12
SOX8.2	0.000117	0.163863	0.103	0.181	1	CSCL	SOX8
MAGED2.2	0.000117	0.210999	0.305	0.519	1	CSCL	MAGED2
MTRNR2L12	0.000119	0.405487	0.237	0.194	1	CSCL	MTRNR2L12
RNF125.1	0.000122	-0.12152	0	0.025	1	CSCL	RNF125
FBXO17.2	0.000122	0.297404	0.079	0.147	1	CSCL	FBXO17
TRIT1	0.000123	0.11442	0.04	0.09	1	CSCL	TRIT1
CEACAM4.1	0.000125	-0.10843	0	0.025	1	CSCL	CEACAM4
IFNLR1.1	0.000125	-0.12592	0	0.025	1	CSCL	IFNLR1
CTNND2.1	0.000128	0.23141	0.089	0.161	1	CSCL	CTNND2
PLEK2.1	0.000129	-0.10464	0	0.025	1	CSCL	PLEK2
CXCL12	0.000129	-0.1084	0.01	0.044	1	CSCL	CXCL12
DHODH.1	0.000131	0.111162	0.037	0.084	1	CSCL	DHODH
CTA-384D8	0.000132	-0.10827	0	0.025	1	CSCL	CTA-384D8.34
SBDS.2	0.000135	0.135269	0.366	0.622	1	CSCL	SBDS
ITM2A.1	0.000137	-0.12659	0.019	0.057	1	CSCL	ITM2A
CCDC181.1	0.000138	0.134312	0.035	0.082	1	CSCL	CCDC181
RAB11FIP1	0.000141	-0.11058	0.002	0.028	1	CSCL	RAB11FIP1
NR2F2-AS1	0.000143	0.185984	0.028	0.011	1	CSCL	NR2F2-AS1
ARHGAP32	0.000144	0.1218	0.038	0.087	1	CSCL	ARHGAP32
NUP107.2	0.000145	0.232181	0.134	0.228	1	CSCL	NUP107
HELZ2.1	0.000147	0.155671	0.049	0.102	1	CSCL	HELZ2
EEF1D.2	0.000151	0.228193	0.611	0.857	1	CSCL	EEF1D
MTOR.1	0.000151	0.107389	0.028	0.071	1	CSCL	MTOR
CUTA.2	0.000151	0.117148	0.398	0.696	1	CSCL	CUTA
TTYH1.2	0.000152	0.746315	0.277	0.267	1	CSCL	TTYH1
CBLN4.2	0.000152	0.123077	0.035	0.081	1	CSCL	CBLN4
TENM1	0.000153	0.113154	0.03	0.074	1	CSCL	TENM1
2-Sep	0.000154	0.203408	0.344	0.593	1	CSCL	2-Sep
FAM229B.1	0.000155	0.389223	0.176	0.298	1	CSCL	FAM229B
PTCRA.1	0.000157	-0.11633	0	0.024	1	CSCL	PTCRA
SPNS3.1	0.000157	-0.12223	0	0.024	1	CSCL	SPNS3
RP11-597D	0.000157	-0.1385	0	0.024	1	CSCL	RP11-597D13.8
SIDT1.1	0.000157	-0.13947	0	0.024	1	CSCL	SIDT1
IRF1.2	0.000159	0.183056	0.124	0.21	1	CSCL	IRF1
TMEM132B.	0.000165	0.118739	0.051	0.104	1	CSCL	TMEM132B

CDH6	0.000165	0.112933	0.028	0.071	1	CSCL	CDH6
RP11-495F	0.00017	0.121808	0.009	0.002	1	CSCL	RP11-495P10.1
NME6	0.000175	0.155867	0.056	0.111	1	CSCL	NME6
NDRG2	0.000178	0.603702	0.342	0.36	1	CSCL	NDRG2
FAM149A.1	0.000178	-0.10485	0.005	0.034	1	CSCL	FAM149A
ALOX15B.1	0.000179	-0.11879	0.002	0.027	1	CSCL	ALOX15B
HEY1.1	0.00018	0.317638	0.148	0.252	1	CSCL	HEY1
BPNT1.1	0.00018	0.16627	0.054	0.109	1	CSCL	BPNT1
DPF1.1	0.000183	0.165395	0.079	0.144	1	CSCL	DPF1
VDAC1.2	0.000183	0.157682	0.372	0.644	1	CSCL	VDAC1
CHRNE.1	0.000185	-0.12191	0.002	0.027	1	CSCL	CHRNE
GPR89A.1	0.000185	0.10247	0.07	0.131	1	CSCL	GPR89A
CNN3.2	0.000186	0.44221	0.417	0.471	1	CSCL	CNN3
PSMC4.1	0.000186	0.275482	0.297	0.504	1	CSCL	PSMC4
B3GALNT2.	0.000187	0.102836	0.044	0.093	1	CSCL	B3GALNT2
NLGN3.1	0.00019	0.212402	0.059	0.116	1	CSCL	NLGN3
ALDOC.2	0.000193	0.620421	0.188	0.154	1	CSCL	ALDOC
ALDH1L1-A	0.000196	0.178902	0.026	0.01	1	CSCL	ALDH1L1-AS2
DHRS9.1	0.000198	-0.16901	0	0.024	1	CSCL	DHRS9
LZTS1.1	0.000198	0.121792	0.023	0.062	1	CSCL	LZTS1
PPP1R14A.	0.000201	-0.1171	0.058	0.109	1	CSCL	PPP1R14A
VAMP2.1	0.000202	0.103892	0.375	0.635	1	CSCL	VAMP2
EIF4EBP3.	0.000204	0.111622	0.023	0.008	1	CSCL	EIF4EBP3
PNKD	0.000205	0.298196	0.279	0.466	1	CSCL	PNKD
ETV4.1	0.000206	0.202512	0.035	0.08	1	CSCL	ETV4
MIR7-3HG	0.00021	0.509528	0.059	0.032	1	CSCL	MIR7-3HG
IFI6.2	0.000212	0.346222	0.251	0.414	1	CSCL	IFI6
NMT2.1	0.000216	0.111771	0.049	0.1	1	CSCL	NMT2
AIM1	0.000216	-0.10703	0.007	0.036	1	CSCL	AIM1
SNCAIP.1	0.00022	0.127375	0.04	0.088	1	CSCL	SNCAIP
MS4A14.1	0.000221	-0.11292	0	0.023	1	CSCL	MS4A14
SDR39U1	0.000222	0.379242	0.168	0.28	1	CSCL	SDR39U1
DCAF13.1	0.000223	0.341719	0.194	0.324	1	CSCL	DCAF13
EID1.1	0.000226	0.323745	0.532	0.716	1	CSCL	EID1
HIP1	0.000232	0.236894	0.098	0.171	1	CSCL	HIP1
FGF14.1	0.000232	0.153967	0.04	0.088	1	CSCL	FGF14
BTN3A2.1	0.000237	0.210814	0.084	0.15	1	CSCL	BTN3A2
GFAP.1	0.000238	0.507029	0.346	0.35	1	CSCL	GFAP
ADM.2	0.000244	0.183763	0.112	0.189	1	CSCL	ADM
SLC24A2.1	0.000244	-0.11335	0.005	0.033	1	CSCL	SLC24A2
FBXO32.2	0.000245	0.186516	0.089	0.157	1	CSCL	FBXO32
IPCEF1.1	0.000248	-0.10698	0	0.023	1	CSCL	IPCEF1
ANXA2R.1	0.000252	0.224034	0.087	0.155	1	CSCL	ANXA2R
PSENEN.2	0.000253	0.259375	0.3	0.502	1	CSCL	PSENEN
CEP250	0.000257	0.122308	0.03	0.071	1	CSCL	CEP250
SYP.2	0.000258	0.130834	0.075	0.137	1	CSCL	SYP
ITGB2-AS1	0.000263	-0.11139	0.003	0.029	1	CSCL	ITGB2-AS1
FHL1.2	0.000263	0.285335	0.22	0.364	1	CSCL	FHL1
MOG.1	0.000264	-0.19255	0.005	0.032	1	CSCL	MOG
TTC8	0.000265	0.153453	0.042	0.089	1	CSCL	TTC8
WBSCR22.2	0.000267	0.252156	0.297	0.503	1	CSCL	WBSCR22

GABARAPL2	0.000268	0.30237	0.541	0.753	1	CSCL	GABARAPL2
MLF2.2	0.000269	0.160484	0.36	0.622	1	CSCL	MLF2
RP3-47704	0.00027	-0.10824	0.003	0.029	1	CSCL	RP3-47704.14
RP11-353N	0.00027	-0.10998	0	0.023	1	CSCL	RP11-353N4.5
FAM46A	0.000271	0.304835	0.117	0.2	1	CSCL	FAM46A
LINC00662	0.000272	0.283204	0.108	0.187	1	CSCL	LINC00662
KPTN.1	0.000274	0.145599	0.037	0.082	1	CSCL	KPTN
NDUFS2.1	0.000277	0.240878	0.326	0.562	1	CSCL	NDUFS2
TREML1.1	0.000278	-0.11426	0	0.023	1	CSCL	TREML1
CNDP1.1	0.000283	-0.161	0.007	0.035	1	CSCL	CNDP1
MYRF.1	0.000285	-0.15603	0.005	0.032	1	CSCL	MYRF
TRPM3.1	0.000289	0.176687	0.03	0.071	1	CSCL	TRPM3
C1QL1.1	0.00029	0.20103	0.061	0.117	1	CSCL	C1QL1
GJB1.1	0.000293	-0.1701	0.002	0.026	1	CSCL	GJB1
ZHX3	0.000294	0.208982	0.065	0.122	1	CSCL	ZHX3
LUC7L2	0.000295	0.159088	0.021	0.007	1	CSCL	LUC7L2
GS1-124K5	0.000295	0.167518	0.061	0.117	1	CSCL	GS1-124K5.4
AKAP6.1	0.000295	0.208267	0.052	0.104	1	CSCL	AKAP6
NCAM1.2	0.000296	0.23602	0.208	0.342	1	CSCL	NCAM1
SRCIN1.1	0.000296	-0.10317	0.005	0.032	1	CSCL	SRCIN1
OR4N2	0.000302	0.225645	0.028	0.011	1	CSCL	OR4N2
ZNF90.1	0.000306	0.419196	0.372	0.38	1	CSCL	ZNF90
RNF180.1	0.00031	0.286894	0.098	0.171	1	CSCL	RNF180
CADM2.1	0.000321	0.159215	0.113	0.192	1	CSCL	CADM2
BCL2L15	0.000323	0.138868	0.026	0.01	1	CSCL	BCL2L15
NDUFS8.2	0.000324	0.212305	0.358	0.621	1	CSCL	NDUFS8
ROM1.1	0.000327	0.178856	0.061	0.116	1	CSCL	ROM1
SPTSSA.2	0.000331	0.301136	0.264	0.436	1	CSCL	SPTSSA
DPY19L3	0.000334	0.141445	0.044	0.091	1	CSCL	DPY19L3
NKX6-2.1	0.000335	-0.15483	0.002	0.025	1	CSCL	NKX6-2
OBSL1.1	0.000335	0.172177	0.084	0.149	1	CSCL	OBSL1
ZIC1.1	0.000335	0.165376	0.061	0.117	1	CSCL	ZIC1
NRDE2.1	0.000342	0.156905	0.051	0.101	1	CSCL	NRDE2
GPR89B	0.000349	0.143112	0.017	0.006	1	CSCL	GPR89B
CRIM1.1	0.000349	0.10368	0.026	0.065	1	CSCL	CRIM1
TSEN2.1	0.000353	0.117242	0.044	0.091	1	CSCL	TSEN2
PRDX4.2	0.000358	0.220435	0.326	0.558	1	CSCL	PRDX4
LGI3.1	0.00036	-0.1304	0	0.022	1	CSCL	LGI3
FCER1A.1	0.000362	-0.12334	0.002	0.025	1	CSCL	FCER1A
TAC3	0.000364	-0.10717	0.002	0.025	1	CSCL	TAC3
TEKT1	0.000366	0.116786	0.012	0.003	1	CSCL	TEKT1
FA2H.1	0.000367	-0.13069	0.003	0.028	1	CSCL	FA2H
POLR2J3.1	0.000375	0.30163	0.209	0.345	1	CSCL	POLR2J3
DNAAF1.1	0.000377	-0.15229	0.003	0.028	1	CSCL	DNAAF1
BGN.1	0.000387	0.431624	0.096	0.063	1	CSCL	BGN
PKD1	0.000395	0.200669	0.065	0.121	1	CSCL	PKD1
OLIG1.2	0.0004	0.580498	0.209	0.178	1	CSCL	OLIG1
GRAMD3.2	0.000401	0.21574	0.08	0.143	1	CSCL	GRAMD3
IPP	0.000408	0.119101	0.024	0.062	1	CSCL	IPP
ACSM5	0.000417	-0.10564	0.007	0.034	1	CSCL	ACSM5
RPGRIP1L	0.000419	0.112266	0.023	0.059	1	CSCL	RPGRIP1L

RND2.1	0.00042	0.292817	0.096	0.167	1	CSCL	RND2
AP000439.	0.000421	-0.11805	0.003	0.028	1	CSCL	AP000439.1
SHROOM3	0.000422	0.10051	0.012	0.043	1	CSCL	SHROOM3
HSPE1.2	0.000422	0.295149	0.538	0.739	1	CSCL	HSPE1
VPS28.1	0.000426	0.196437	0.379	0.649	1	CSCL	VPS28
CEACAM21.	0.00044	-0.10395	0	0.021	1	CSCL	CEACAM21
SPRY4	0.000442	0.134942	0.03	0.069	1	CSCL	SPRY4
POLR2G.2	0.000445	0.224093	0.344	0.579	1	CSCL	POLR2G
RP11-14N7	0.000455	0.351045	0.124	0.209	1	CSCL	RP11-14N7.2
CTSW.1	0.000462	-0.16814	0.005	0.031	1	CSCL	CTSW
MT1A.2	0.000469	0.337768	0.052	0.028	1	CSCL	MT1A
PGM2L1.1	0.000478	0.281114	0.159	0.26	1	CSCL	PGM2L1
ZNF429.1	0.000506	0.232984	0.073	0.132	1	CSCL	ZNF429
PTPRCAP.1	0.000511	-0.16132	0.003	0.027	1	CSCL	PTPRCAP
RETN.1	0.000522	-0.11224	0	0.021	1	CSCL	RETN
RHBDF1	0.000522	0.143421	0.019	0.053	1	CSCL	RHBDF1
NRXN2.2	0.000529	0.126393	0.065	0.119	1	CSCL	NRXN2
QTRT1.2	0.000536	0.368824	0.206	0.337	1	CSCL	QTRT1
P2RY12	0.000537	-0.11789	0	0.021	1	CSCL	P2RY12
IL1R2	0.000538	-0.13697	0.005	0.03	1	CSCL	IL1R2
RP11-212I	0.000541	-0.10602	0.002	0.024	1	CSCL	RP11-212I21.4
MT1F.2	0.000548	0.299232	0.192	0.311	1	CSCL	MT1F
HAAO.1	0.000554	-0.11631	0.002	0.024	1	CSCL	HAAO
PCOLCE.1	0.000561	0.105519	0.04	0.083	1	CSCL	PCOLCE
CARF.1	0.000563	0.106736	0.037	0.078	1	CSCL	CARF
FIS1.1	0.000564	0.161886	0.382	0.651	1	CSCL	FIS1
SDHC.2	0.000566	0.286692	0.332	0.569	1	CSCL	SDHC
CLEC10A	0.000569	-0.1135	0	0.02	1	CSCL	CLEC10A
POLR1C.2	0.000576	0.26095	0.068	0.124	1	CSCL	POLR1C
GADD45B.1	0.000582	0.410489	0.433	0.493	1	CSCL	GADD45B
CHMP2A.2	0.000586	0.455347	0.49	0.663	1	CSCL	CHMP2A
RP11-421L	0.000597	0.171091	0.04	0.083	1	CSCL	RP11-421L21.3
BCAT2.1	0.0006	0.314841	0.08	0.141	1	CSCL	BCAT2
TNNI2.1	0.000603	-0.10441	0	0.02	1	CSCL	TNNI2
APP.1	0.000607	0.171955	0.319	0.535	1	CSCL	APP
ASB3.1	0.000609	0.126042	0.016	0.005	1	CSCL	ASB3
UBE2L6.2	0.00061	0.303977	0.291	0.482	1	CSCL	UBE2L6
RHOBTB3.2	0.000611	0.293222	0.253	0.416	1	CSCL	RHOBTB3
PSMA1.2	0.000621	0.205522	0.368	0.624	1	CSCL	PSMA1
TRMT5.1	0.000632	0.107447	0.031	0.07	1	CSCL	TRMT5
CLDN12.2	0.000634	0.206903	0.08	0.141	1	CSCL	CLDN12
LANCL2.2	0.000639	0.247411	0.091	0.155	1	CSCL	LANCL2
GTPBP10	0.000647	0.140996	0.051	0.098	1	CSCL	GTPBP10
CAV1.2	0.000688	0.441159	0.162	0.125	1	CSCL	CAV1
SLC20A2.1	0.000693	0.210019	0.063	0.115	1	CSCL	SLC20A2
FPR2.1	0.000695	-0.10058	0	0.02	1	CSCL	FPR2
CASK.1	0.000696	0.11256	0.072	0.127	1	CSCL	CASK
CDH11	0.000702	0.187356	0.065	0.118	1	CSCL	CDH11
RFTN2.2	0.000707	0.185497	0.073	0.13	1	CSCL	RFTN2
HIST1H2BC	0.000734	-0.12183	0.002	0.023	1	CSCL	HIST1H2BC
AK5	0.000752	-0.10942	0.003	0.026	1	CSCL	AK5

SIDT2	0.000771	0.104656	0.037	0.077	1	CSCL	SIDT2
CCNB1IP1	0.000783	0.268186	0.099	0.168	1	CSCL	CCNB1IP1
HSPA6.1	0.000792	0.160992	0.054	0.101	1	CSCL	HSPA6
SCG3.2	0.000793	0.642654	0.272	0.266	1	CSCL	SCG3
GADD45A.2	0.000794	0.327976	0.176	0.284	1	CSCL	GADD45A
PRDX6.1	0.000806	0.368019	0.489	0.631	1	CSCL	PRDX6
HNRNPDL.1	0.000813	0.219409	0.565	0.809	1	CSCL	HNRNPDL
NES.2	0.000815	0.289727	0.175	0.281	1	CSCL	NES
RP11-51J9	0.000828	0.198657	0.084	0.144	1	CSCL	RP11-51J9.5
PHLPP1.2	0.000836	0.132532	0.044	0.087	1	CSCL	PHLPP1
RP11-1143	0.000839	-0.10653	0.021	0.053	1	CSCL	RP11-1143G9.4
SPOCK3.1	0.000844	-0.18831	0.009	0.034	1	CSCL	SPOCK3
USE1.2	0.000852	0.35177	0.209	0.336	1	CSCL	USE1
MAGED1.1	0.000868	0.352012	0.215	0.351	1	CSCL	MAGED1
PKIG.2	0.000878	0.366403	0.195	0.316	1	CSCL	PKIG
KLHL4	0.000891	0.299334	0.073	0.13	1	CSCL	KLHL4
PRKAA2.1	0.00091	0.11623	0.021	0.054	1	CSCL	PRKAA2
SLC35E3.1	0.000913	0.16494	0.117	0.19	1	CSCL	SLC35E3
NUCKS1.1	0.000935	0.197282	0.382	0.649	1	CSCL	NUCKS1
CTTNBP2.2	0.000963	0.101048	0.082	0.139	1	CSCL	CTTNBP2
CD3E.1	0.000981	-0.16538	0	0.019	1	CSCL	CD3E
ZNF395.1	0.000984	0.106613	0.03	0.066	1	CSCL	ZNF395
DSTN.2	0.001006	0.103758	0.379	0.63	1	CSCL	DSTN
FGF20.1	0.00101	-0.11081	0	0.019	1	CSCL	FGF20
DBX2.1	0.001012	0.152431	0.03	0.066	1	CSCL	DBX2
ZNF16	0.001022	0.139205	0.031	0.069	1	CSCL	ZNF16
RP11-539L	0.001023	0.205458	0.052	0.098	1	CSCL	RP11-539L10.3
RNPC3	0.001035	0.104408	0.049	0.093	1	CSCL	RNPC3
C7orf73.2	0.001046	0.228217	0.337	0.566	1	CSCL	C7orf73
BBS2.2	0.001051	0.24015	0.106	0.176	1	CSCL	BBS2
ZNF667.1	0.00106	0.139513	0.042	0.084	1	CSCL	ZNF667
BDNF-AS.1	0.001061	0.101086	0.03	0.066	1	CSCL	BDNF-AS
TRIM9.1	0.001068	0.31654	0.147	0.237	1	CSCL	TRIM9
SLITRK4	0.001076	0.109187	0.012	0.003	1	CSCL	SLITRK4
TCF4.2	0.001077	0.140669	0.333	0.533	1	CSCL	TCF4
CDC42EP4.	0.001078	0.402541	0.126	0.205	1	CSCL	CDC42EP4
ACSBG1	0.001166	0.178207	0.037	0.076	1	CSCL	ACSBG1
LHFPL3.2	0.001172	0.152382	0.07	0.122	1	CSCL	LHFPL3
ABAT.2	0.001175	0.213689	0.098	0.163	1	CSCL	ABAT
ACSS1.1	0.001195	0.10977	0.056	0.102	1	CSCL	ACSS1
C14orf2.1	0.001201	0.1824	0.389	0.668	1	CSCL	C14orf2
RNU12.1	0.00122	-0.12233	0.012	0.039	1	CSCL	RNU12
RCAN1.2	0.001225	0.301676	0.173	0.273	1	CSCL	RCAN1
HINFP	0.001228	0.158153	0.051	0.095	1	CSCL	HINFP
MAGI2.1	0.001228	0.100138	0.059	0.107	1	CSCL	MAGI2
C7orf55.1	0.001261	0.320927	0.264	0.432	1	CSCL	C7orf55
APBB2.1	0.001286	0.241701	0.065	0.115	1	CSCL	APBB2
SLC35G2.2	0.001305	0.170593	0.068	0.119	1	CSCL	SLC35G2
NCALD.1	0.001334	0.179606	0.073	0.127	1	CSCL	NCALD
TSHR	0.001348	0.115042	0.012	0.004	1	CSCL	TSHR
BBS4	0.001365	0.151127	0.045	0.087	1	CSCL	BBS4

C1orf21.2	0.001385	0.36325	0.126	0.204	1	CSCL	C1orf21
NPPA.1	0.001393	0.330165	0.049	0.027	1	CSCL	NPPA
SPEG	0.001425	0.115058	0.035	0.072	1	CSCL	SPEG
TMEM67	0.001457	0.273995	0.052	0.097	1	CSCL	TMEM67
NEAT1.1	0.00146	0.18747	0.581	0.772	1	CSCL	NEAT1
GTF2IRD2.	0.00147	0.126769	0.068	0.119	1	CSCL	GTF2IRD2
HSD17B10.	0.001478	0.369423	0.302	0.505	1	CSCL	HSD17B10
SIAE	0.001483	0.152405	0.049	0.092	1	CSCL	SIAE
BOLA2B	0.001527	0.155766	0.037	0.074	1	CSCL	BOLA2B
CREB5.1	0.00159	0.300983	0.15	0.239	1	CSCL	CREB5
PRDX5.2	0.001622	0.296952	0.532	0.753	1	CSCL	PRDX5
SLC1A3.1	0.001646	0.245574	0.3	0.486	1	CSCL	SLC1A3
ZMAT1.1	0.001671	0.137046	0.052	0.096	1	CSCL	ZMAT1
ZBTB8A.1	0.001674	0.140372	0.059	0.106	1	CSCL	ZBTB8A
CASP9.2	0.001676	0.109385	0.045	0.086	1	CSCL	CASP9
SS18.1	0.001677	0.136522	0.044	0.084	1	CSCL	SS18
IGSF11	0.001686	0.135774	0.033	0.069	1	CSCL	IGSF11
MSX1	0.001709	0.118965	0.031	0.066	1	CSCL	MSX1
CLN3.1	0.001742	0.158692	0.023	0.009	1	CSCL	CLN3
PCDH17	0.001767	0.392209	0.12	0.195	1	CSCL	PCDH17
SEZ6.2	0.001785	0.156067	0.086	0.142	1	CSCL	SEZ6
SNAP25.1	0.001795	0.200495	0.087	0.145	1	CSCL	SNAP25
CAMK2B.1	0.001805	0.291474	0.066	0.116	1	CSCL	CAMK2B
ISG20.1	0.00182	0.161199	0.073	0.124	1	CSCL	ISG20
PCDH15	0.001824	0.26606	0.03	0.014	1	CSCL	PCDH15
CD2.1	0.001853	-0.14223	0.002	0.02	1	CSCL	CD2
BDH1.1	0.001862	0.136495	0.035	0.071	1	CSCL	BDH1
NDUFA12.1	0.001871	0.336543	0.337	0.569	1	CSCL	NDUFA12
RBM41.1	0.001871	0.110977	0.047	0.088	1	CSCL	RBM41
EFHC1.2	0.00188	0.33802	0.131	0.209	1	CSCL	EFHC1
PTPRF.2	0.001897	0.222794	0.068	0.118	1	CSCL	PTPRF
SETDB1.1	0.001941	0.101102	0.035	0.071	1	CSCL	SETDB1
RP11-390E	0.001948	0.195543	0.075	0.127	1	CSCL	RP11-390E23.6
BCAS4	0.001948	0.179875	0.047	0.088	1	CSCL	BCAS4
SAT2.2	0.001953	0.254875	0.36	0.597	1	CSCL	SAT2
COX7A2	0.001966	0.202598	0.567	0.823	1	CSCL	COX7A2
HAPLN1	0.002	0.136995	0.021	0.009	1	CSCL	HAPLN1
IGF1R	0.002001	0.175471	0.035	0.071	1	CSCL	IGF1R
ZNF559.1	0.002025	0.267734	0.089	0.148	1	CSCL	ZNF559
TNC.1	0.002058	0.311271	0.129	0.205	1	CSCL	TNC
CD3D.1	0.002074	-0.17635	0.002	0.02	1	CSCL	CD3D
ETV1.2	0.002095	0.375635	0.157	0.249	1	CSCL	ETV1
NDUFA3.1	0.002155	0.143044	0.391	0.662	1	CSCL	NDUFA3
FAT3	0.002179	0.113002	0.026	0.058	1	CSCL	FAT3
FMN2	0.002207	0.143851	0.035	0.07	1	CSCL	FMN2
MYO10.1	0.002272	0.346849	0.124	0.198	1	CSCL	MYO10
LAMA4	0.002276	0.188326	0.037	0.073	1	CSCL	LAMA4
FABP3.1	0.002315	0.136561	0.021	0.05	1	CSCL	FABP3
XIST.2	0.002326	0.21309	0.175	0.271	1	CSCL	XIST
COL9A3.2	0.002335	0.345052	0.119	0.189	1	CSCL	COL9A3
EZH1.1	0.00234	0.124362	0.044	0.082	1	CSCL	EZH1

RGS6	0.002358	0.272522	0.051	0.092	1	CSCL	RGS6
TCEAL5	0.002393	0.157552	0.073	0.123	1	CSCL	TCEAL5
CPNE5	0.00242	0.113666	0.023	0.052	1	CSCL	CPNE5
THBS4	0.002436	0.102538	0.023	0.052	1	CSCL	THBS4
LRRC69	0.00247	-0.12011	0.01	0.034	1	CSCL	LRRC69
PPP1R1C	0.002548	0.35648	0.045	0.026	1	CSCL	PPP1R1C
DHRS4-AS1	0.002561	0.101898	0.016	0.006	1	CSCL	DHRS4-AS1
STK33.1	0.002565	0.210015	0.035	0.07	1	CSCL	STK33
NSUN6	0.002588	0.102763	0.04	0.077	1	CSCL	NSUN6
SOX2.2	0.002594	0.451143	0.365	0.433	1	CSCL	SOX2
HRASLS.1	0.002597	0.109294	0.021	0.05	1	CSCL	HRASLS
PSMA7.2	0.002598	0.2028	0.572	0.813	1	CSCL	PSMA7
CD82	0.002729	0.369553	0.155	0.244	1	CSCL	CD82
GDAP1.2	0.002814	0.261672	0.096	0.155	1	CSCL	GDAP1
B3GALT1	0.002814	0.109641	0.023	0.052	1	CSCL	B3GALT1
RP11-299L	0.00283	0.122737	0.017	0.007	1	CSCL	RP11-299L17.3
KCND3.1	0.00285	0.142621	0.028	0.059	1	CSCL	KCND3
ZFHX4-AS1	0.002855	0.109119	0.023	0.052	1	CSCL	ZFHX4-AS1
RAB13.2	0.002912	0.353413	0.243	0.378	1	CSCL	RAB13
TRIM32	0.002988	0.113135	0.028	0.059	1	CSCL	TRIM32
UBE3D	0.003007	0.112423	0.028	0.059	1	CSCL	UBE3D
RP11-1114	0.003018	0.10689	0.047	0.086	1	CSCL	RP11-1114A5.4
STK32A.1	0.003063	0.190199	0.026	0.057	1	CSCL	STK32A
HHLA3.2	0.003078	0.298964	0.087	0.142	1	CSCL	HHLA3
CPT1C	0.003144	0.148518	0.063	0.107	1	CSCL	CPT1C
ZNF783	0.003155	0.128368	0.028	0.059	1	CSCL	ZNF783
TMEM100.2	0.003163	0.473561	0.115	0.087	1	CSCL	TMEM100
HSPB1.1	0.003342	0.173026	0.403	0.667	1	CSCL	HSPB1
ANGPTL1	0.003368	0.112792	0.016	0.041	1	CSCL	ANGPTL1
COL16A1	0.003494	0.173714	0.04	0.076	1	CSCL	COL16A1
TSGA10	0.003581	0.108348	0.031	0.063	1	CSCL	TSGA10
C2orf74	0.003622	0.301173	0.044	0.025	1	CSCL	C2orf74
TMED1.1	0.003669	0.433549	0.19	0.296	1	CSCL	TMED1
CTD-2090I	0.003685	0.185615	0.079	0.052	1	CSCL	CTD-2090I13.1
CLYBL.1	0.003699	0.161866	0.031	0.063	1	CSCL	CLYBL
DDAH1.1	0.00382	0.296866	0.077	0.127	1	CSCL	DDAH1
CD74.1	0.003842	0.146223	0.621	0.758	1	CSCL	CD74
RARRES2.1	0.003873	0.451637	0.176	0.148	1	CSCL	RARRES2
PHB.1	0.003908	0.258623	0.34	0.565	1	CSCL	PHB
MPV17L	0.003912	0.145975	0.04	0.075	1	CSCL	MPV17L
WASF3	0.003918	0.208889	0.042	0.078	1	CSCL	WASF3
SHROOM2.1	0.003985	0.129937	0.03	0.06	1	CSCL	SHROOM2
PDCD5.2	0.004006	0.288533	0.337	0.55	1	CSCL	PDCD5
U2AF1L4.1	0.004024	0.347673	0.129	0.2	1	CSCL	U2AF1L4
HKR1.1	0.004027	0.217729	0.072	0.119	1	CSCL	HKR1
ATP6V1G1.	0.00405	0.285185	0.534	0.765	1	CSCL	ATP6V1G1
KCNJ16.1	0.004108	0.126455	0.026	0.055	1	CSCL	KCNJ16
SSR2.1	0.004108	0.11159	0.431	0.728	1	CSCL	SSR2
BST2.1	0.00416	0.468827	0.403	0.49	1	CSCL	BST2
NDP	0.004191	0.276554	0.068	0.114	1	CSCL	NDP
HRSP12.2	0.004239	0.359724	0.129	0.202	1	CSCL	HRSP12

CD99.1	0.004252	0.312195	0.483	0.645	1	CSCL	CD99
MDFI.1	0.004316	0.216505	0.051	0.089	1	CSCL	MDFI
ELP4.1	0.004476	0.223089	0.054	0.094	1	CSCL	ELP4
FAM221A.2	0.004483	0.216321	0.117	0.181	1	CSCL	FAM221A
AHCYL1.2	0.004586	0.372388	0.215	0.333	1	CSCL	AHCYL1
COQ3.1	0.004687	0.113455	0.037	0.069	1	CSCL	COQ3
SSBP1.2	0.004724	0.223707	0.387	0.651	1	CSCL	SSBP1
POLR2J.2	0.004791	0.435467	0.475	0.652	1	CSCL	POLR2J
ZNF273.1	0.004802	0.129111	0.049	0.086	1	CSCL	ZNF273
SCG2.1	0.004802	0.32902	0.175	0.266	1	CSCL	SCG2
ADAMTS9	0.005025	0.264023	0.044	0.079	1	CSCL	ADAMTS9
ABLIM1.2	0.005066	0.110274	0.026	0.054	1	CSCL	ABLIM1
TMEM9.2	0.005084	0.386675	0.239	0.374	1	CSCL	TMEM9
HSPB6.1	0.005184	0.137685	0.031	0.062	1	CSCL	HSPB6
RP3-525N1	0.005307	0.203739	0.098	0.153	1	CSCL	RP3-525N10.2
FGGY	0.005326	0.220882	0.051	0.088	1	CSCL	FGGY
GABBR1.1	0.005346	0.120684	0.045	0.081	1	CSCL	GABBR1
TMBIM4.2	0.005391	0.123772	0.086	0.058	1	CSCL	TMBIM4
DYNLRB1.1	0.005445	0.18149	0.405	0.678	1	CSCL	DYNLRB1
SHFM1.1	0.005483	0.31066	0.515	0.734	1	CSCL	SHFM1
CCDC8	0.005505	0.148802	0.023	0.049	1	CSCL	CCDC8
ZNF398.1	0.005516	0.122337	0.03	0.059	1	CSCL	ZNF398
LGALS1.2	0.005538	0.239162	0.539	0.712	1	CSCL	LGALS1
FLRT3.1	0.005552	0.133903	0.03	0.059	1	CSCL	FLRT3
UQCR10.1	0.005619	0.118045	0.443	0.757	1	CSCL	UQCR10
NMB.2	0.005725	0.500905	0.133	0.206	1	CSCL	NMB
TMEM231.1	0.005765	0.135219	0.033	0.064	1	CSCL	TMEM231
PON2.2	0.005834	0.51161	0.382	0.454	1	CSCL	PON2
SETD1A	0.005979	0.112106	0.021	0.046	1	CSCL	SETD1A
EXOG.1	0.006035	0.34635	0.101	0.159	1	CSCL	EXOG
TPPP3.2	0.006243	0.278731	0.101	0.156	1	CSCL	TPPP3
YWHAE.1	0.006335	0.188672	0.55	0.763	1	CSCL	YWHAE
THY1.1	0.006383	0.317957	0.171	0.26	1	CSCL	THY1
HIGD2A.1	0.006566	0.243045	0.379	0.631	1	CSCL	HIGD2A
PPP5D1.1	0.006718	0.101241	0.023	0.048	1	CSCL	PPP5D1
AHDC1.1	0.00672	0.195446	0.063	0.103	1	CSCL	AHDC1
METTL3	0.006856	0.218506	0.079	0.125	1	CSCL	METTL3
ATP6V0E2.	0.006885	0.304677	0.29	0.461	1	CSCL	ATP6V0E2
CCBE1	0.006947	0.109691	0.021	0.009	1	CSCL	CCBE1
IFIT3.2	0.006997	0.213124	0.068	0.11	1	CSCL	IFIT3
IBSP.1	0.007147	-0.11892	0.007	0.025	1	CSCL	IBSP
LRRN3.1	0.007183	0.222314	0.072	0.115	1	CSCL	LRRN3
SERPINE1.	0.007188	0.116576	0.063	0.102	1	CSCL	SERPINE1
SLIRP	0.007206	0.207688	0.384	0.633	1	CSCL	SLIRP
NR1D1.1	0.007231	0.126104	0.024	0.05	1	CSCL	NR1D1
GLRA3	0.007241	0.140353	0.021	0.01	1	CSCL	GLRA3
SLC25A3.1	0.007304	0.108486	0.45	0.761	1	CSCL	SLC25A3
SNTG1.1	0.007456	0.616596	0.171	0.152	1	CSCL	SNTG1
FKBP3.1	0.007535	0.336731	0.302	0.486	1	CSCL	FKBP3
DIRAS3.1	0.007539	0.5443	0.147	0.122	1	CSCL	DIRAS3
BUD31.2	0.007547	0.20812	0.386	0.64	1	CSCL	BUD31

EPSTI1.2	0.007601	0.300046	0.08	0.127	1	CSCL	EPSTI1
MGP.2	0.007669	0.410064	0.131	0.102	1	CSCL	MGP
CC2D2A.1	0.007709	0.177529	0.04	0.072	1	CSCL	CC2D2A
CCDC30	0.007709	0.122659	0.024	0.05	1	CSCL	CCDC30
EIF3E.1	0.007728	0.263206	0.394	0.66	1	CSCL	EIF3E
ZNF714	0.007728	0.151162	0.065	0.105	1	CSCL	ZNF714
DMD.1	0.007815	0.215014	0.051	0.086	1	CSCL	DMD
METAP1D	0.007948	0.181016	0.08	0.126	1	CSCL	METAP1D
CLDN11.1	0.008011	-0.1205	0.023	0.047	1	CSCL	CLDN11
AASS	0.008019	0.160983	0.073	0.117	1	CSCL	AASS
LPL	0.008036	0.293969	0.105	0.161	1	CSCL	LPL
RP11-263K	0.008155	0.169227	0.037	0.067	1	CSCL	RP11-263K19.4
CRIP2	0.008263	0.326813	0.209	0.319	1	CSCL	CRIP2
CCDC102B	0.008305	0.263897	0.063	0.103	1	CSCL	CCDC102B
COA7	0.008631	0.133801	0.035	0.064	1	CSCL	COA7
SEC62.2	0.008642	0.139651	0.419	0.705	1	CSCL	SEC62
GZMA.1	0.00872	-0.12462	0.002	0.015	1	CSCL	GZMA
ATP1B2.2	0.008997	0.318074	0.157	0.237	1	CSCL	ATP1B2
OAS3	0.009155	0.148079	0.028	0.054	1	CSCL	OAS3
APOL2.1	0.009472	0.203583	0.063	0.101	1	CSCL	APOL2
DSEL.2	0.009515	0.302251	0.152	0.229	1	CSCL	DSEL
ERH.2	0.009813	0.333346	0.504	0.724	1	CSCL	ERH
FCER1A.2	0	1.633094	0.615	0.014	0	DC	FCER1A
AREG.3	0	1.517188	0.749	0.054	0	DC	AREG
CLEC10A.1	0	1.314866	0.471	0.012	0	DC	CLEC10A
CD1C	0	1.283364	0.369	0.002	0	DC	CD1C
CD1E	0	1.195941	0.332	0.002	0	DC	CD1E
CD1A	########	0.60682	0.123	0.001	########	DC	CD1A
EREG	########	1.055164	0.348	0.019	########	DC	EREG
IL1R2.1	########	1.09421	0.358	0.024	########	DC	IL1R2
CD1D.2	########	1.040838	0.412	0.032	########	DC	CD1D
IL18R1	########	0.663384	0.209	0.008	########	DC	IL18R1
LSP1.3	########	1.200771	0.824	0.155	########	DC	LSP1
AKR1C3	########	0.893188	0.23	0.013	########	DC	AKR1C3
FCGR2B.3	########	1.114918	0.727	0.135	########	DC	FCGR2B
CYTIP.3	########	1.087507	0.599	0.096	########	DC	CYTIP
C15orf48.	########	1.261493	0.471	0.063	########	DC	C15orf48
CLEC5A.3	########	1.060313	0.578	0.092	########	DC	CLEC5A
LYZ.3	########	1.149797	0.802	0.196	########	DC	LYZ
CLEC4E.2	########	0.94503	0.428	0.052	########	DC	CLEC4E
CST7.2	3.22E-99	0.999589	0.316	0.031	4.82E-95	DC	CST7
S100A4.3	1.02E-97	1.031391	0.963	0.285	1.52E-93	DC	S100A4
HLA-DQA2.	1.78E-95	1.013786	0.749	0.163	2.66E-91	DC	HLA-DQA2
STK17B.3	3.40E-93	0.993996	0.626	0.125	5.08E-89	DC	STK17B
HLA-DQB2.	7.00E-93	1.022505	0.545	0.09	1.05E-88	DC	HLA-DQB2
HLA-DQB1.	2.90E-90	0.956046	0.989	0.315	4.33E-86	DC	HLA-DQB1
FGR.3	5.10E-89	0.913558	0.481	0.077	7.61E-85	DC	FGR
TACSTD2	2.44E-88	0.355951	0.091	0.002	3.65E-84	DC	TACSTD2
CFP	6.86E-86	0.700315	0.193	0.013	1.02E-81	DC	CFP
SDPR	8.54E-84	0.345576	0.075	0.001	1.28E-79	DC	SDPR
PLAC8.2	4.20E-83	0.927015	0.422	0.062	6.27E-79	DC	PLAC8

IL2RG.2	3.50E-82	0.816138	0.326	0.038	5.23E-78	DC	IL2RG
C1orf162.	3.63E-82	0.921348	0.973	0.312	5.42E-78	DC	C1orf162
CEACAM4.2	6.49E-82	0.665463	0.235	0.02	9.70E-78	DC	CEACAM4
HLA-DQA1.	1.61E-80	0.877927	0.936	0.296	2.41E-76	DC	HLA-DQA1
LCP1.2	2.07E-79	0.898558	0.615	0.134	3.10E-75	DC	LCP1
RP11-404C	2.58E-79	0.280921	0.053	0.001	3.86E-75	DC	RP11-404013.5
EVI2B.3	4.77E-79	0.879305	0.759	0.194	7.12E-75	DC	EVI2B
GPR183.3	6.76E-75	0.893229	0.888	0.28	1.01E-70	DC	GPR183
CSTA.3	4.12E-74	0.879915	0.561	0.121	6.16E-70	DC	CSTA
PHACTR1.3	2.06E-73	0.906313	0.54	0.111	3.08E-69	DC	PHACTR1
CD207	3.98E-73	0.295832	0.048	0.001	5.94E-69	DC	CD207
GAPT.3	7.89E-73	0.840802	0.369	0.055	1.18E-68	DC	GAPT
RNASE6.3	8.59E-73	0.873716	0.877	0.265	1.28E-68	DC	RNASE6
LST1.3	1.12E-72	0.861019	0.925	0.303	1.67E-68	DC	LST1
TES.3	1.84E-72	0.659986	0.321	0.042	2.75E-68	DC	TES
CD55.3	2.83E-72	0.883066	0.535	0.111	4.23E-68	DC	CD55
HCLS1.3	1.64E-71	0.840118	0.893	0.281	2.46E-67	DC	HCLS1
CD52.2	3.30E-71	0.790845	0.342	0.047	4.93E-67	DC	CD52
ALOX5.3	1.19E-70	0.865721	0.583	0.131	1.78E-66	DC	ALOX5
MAP3K8.3	1.80E-70	0.913313	0.674	0.175	2.69E-66	DC	MAP3K8
CTSH.3	2.85E-68	0.848962	0.861	0.286	4.26E-64	DC	CTSH
CD86.3	2.34E-67	0.82856	0.738	0.205	3.49E-63	DC	CD86
MNDA.3	5.10E-67	0.859964	0.658	0.169	7.63E-63	DC	MNDA
IFI30.3	1.13E-66	0.830701	0.754	0.211	1.69E-62	DC	IFI30
NLRP3.3	1.71E-66	0.84779	0.439	0.082	2.55E-62	DC	NLRP3
GMFG.3	2.82E-66	0.819048	0.893	0.288	4.21E-62	DC	GMFG
FXYD5.3	2.05E-64	0.810928	0.925	0.331	3.06E-60	DC	FXYD5
MS4A6A.3	2.46E-64	0.773493	0.952	0.331	3.68E-60	DC	MS4A6A
CRIP1.1	5.09E-64	0.69431	0.278	0.035	7.61E-60	DC	CRIP1
QPCT.3	2.97E-63	0.806632	0.299	0.043	4.44E-59	DC	QPCT
FAM26F.3	4.39E-62	0.868907	0.61	0.16	6.57E-58	DC	FAM26F
CD48.3	9.23E-62	0.78205	0.401	0.073	1.38E-57	DC	CD48
HPGD	1.09E-61	0.323203	0.091	0.004	1.62E-57	DC	HPGD
ICAM3.3	1.56E-61	0.947963	0.439	0.095	2.33E-57	DC	ICAM3
CNN2.2	2.53E-61	0.801117	0.358	0.062	3.78E-57	DC	CNN2
SAMSN1.3	4.23E-61	0.820156	0.786	0.243	6.32E-57	DC	SAMSN1
DUSP2.3	1.64E-60	0.868991	0.481	0.104	2.45E-56	DC	DUSP2
ARHGDIB.3	2.61E-60	0.759098	0.973	0.362	3.90E-56	DC	ARHGDIB
ST14.3	5.95E-60	0.739169	0.38	0.068	8.89E-56	DC	ST14
ITGB2.3	6.93E-60	0.765632	0.909	0.314	1.04E-55	DC	ITGB2
NR4A2.3	2.11E-59	0.829081	0.77	0.243	3.16E-55	DC	NR4A2
CXCR4.2	1.65E-58	0.750859	0.914	0.38	2.46E-54	DC	CXCR4
PLBD1.3	5.22E-58	0.816988	0.428	0.089	7.80E-54	DC	PLBD1
CLEC7A.3	6.64E-58	0.752869	0.626	0.168	9.92E-54	DC	CLEC7A
ADAM8.2	2.40E-57	0.674845	0.278	0.039	3.59E-53	DC	ADAM8
GPSM3.3	2.90E-57	0.806089	0.786	0.254	4.33E-53	DC	GPSM3
HLA-DOB	4.59E-57	0.409718	0.107	0.006	6.86E-53	DC	HLA-DOB
FGL2.3	1.36E-56	0.780211	0.626	0.169	2.03E-52	DC	FGL2
TYMP.3	3.92E-56	0.814215	0.818	0.298	5.86E-52	DC	TYMP
CD83.3	9.34E-56	0.799962	0.807	0.286	1.40E-51	DC	CD83
FCGR2A.3	4.83E-55	0.746955	0.834	0.28	7.21E-51	DC	FCGR2A

RHOH.3	3.74E-54	0.699835	0.38	0.072	5.58E-50	DC	RHOH
NR4A3.3	2.53E-53	0.786097	0.556	0.144	3.78E-49	DC	NR4A3
CSF2RA.3	4.29E-53	0.745929	0.663	0.195	6.41E-49	DC	CSF2RA
CTSS.3	4.41E-53	0.694581	0.914	0.345	6.60E-49	DC	CTSS
PKIB.2	1.29E-52	0.831909	0.717	0.277	1.93E-48	DC	PKIB
CORO1A.3	1.94E-52	0.719788	0.898	0.364	2.90E-48	DC	CORO1A
MALAT1.3	1.96E-52	-0.61708	1	0.995	2.93E-48	DC	MALAT1
PLAUR.3	2.37E-52	0.731666	0.893	0.358	3.54E-48	DC	PLAUR
RIPK2.3	3.18E-52	0.832131	0.551	0.162	4.75E-48	DC	RIPK2
FBP1.3	4.78E-52	0.840215	0.465	0.111	7.14E-48	DC	FBP1
DOK2.2	1.08E-51	0.740776	0.305	0.051	1.61E-47	DC	DOK2
THBD.3	1.14E-51	0.836252	0.412	0.089	1.71E-47	DC	THBD
IL13RA1.3	2.26E-51	0.829228	0.679	0.242	3.37E-47	DC	IL13RA1
HLA-DMA.3	2.92E-51	0.66058	0.968	0.399	4.36E-47	DC	HLA-DMA
PTPRC.3	5.08E-51	0.772069	0.652	0.196	7.59E-47	DC	PTPRC
SPINT2.3	5.59E-51	0.795118	0.647	0.209	8.35E-47	DC	SPINT2
IRF4	5.95E-51	0.370172	0.086	0.004	8.89E-47	DC	IRF4
SERPINA1.	1.44E-50	0.750318	0.813	0.291	2.15E-46	DC	SERPINA1
HLA-DRB5.	2.14E-50	0.626061	0.952	0.391	3.20E-46	DC	HLA-DRB5
PLEK2.2	3.48E-50	0.627334	0.187	0.021	5.21E-46	DC	PLEK2
S100A12	1.16E-49	0.366848	0.096	0.006	1.73E-45	DC	S100A12
VAMP8.3	1.50E-49	0.689992	0.898	0.334	2.25E-45	DC	VAMP8
RNF144B.2	1.82E-49	0.740371	0.417	0.093	2.72E-45	DC	RNF144B
NCF2.3	2.02E-49	0.755854	0.422	0.096	3.02E-45	DC	NCF2
HLA-DPB1.	2.08E-49	0.642446	1	0.486	3.11E-45	DC	HLA-DPB1
SPI1.3	1.93E-48	0.765469	0.711	0.231	2.89E-44	DC	SPI1
SNAI1.2	3.25E-48	0.70883	0.262	0.041	4.86E-44	DC	SNAI1
HLA-DMB.3	3.73E-48	0.646563	0.872	0.311	5.57E-44	DC	HLA-DMB
PTGER4.3	6.26E-48	0.726302	0.476	0.118	9.36E-44	DC	PTGER4
PTPN6.3	9.30E-48	0.760495	0.551	0.156	1.39E-43	DC	PTPN6
SERPINB9.	1.48E-47	0.708296	0.524	0.14	2.22E-43	DC	SERPINB9
RAC2.3	2.15E-47	0.7784	0.46	0.113	3.21E-43	DC	RAC2
IFITM2.3	2.52E-47	0.767527	0.749	0.275	3.77E-43	DC	IFITM2
AIF1.3	6.73E-47	0.63532	0.984	0.401	1.01E-42	DC	AIF1
PARVG.3	1.02E-46	0.725703	0.551	0.155	1.53E-42	DC	PARVG
SPIB	1.04E-46	0.408407	0.128	0.011	1.56E-42	DC	SPIB
FMNL1.3	5.47E-46	0.686695	0.465	0.118	8.17E-42	DC	FMNL1
LY86.3	7.04E-46	0.654476	0.818	0.284	1.05E-41	DC	LY86
HMHA1.3	7.58E-46	0.668759	0.433	0.103	1.13E-41	DC	HMHA1
ALOX5AP.3	1.07E-45	0.664725	0.947	0.39	1.59E-41	DC	ALOX5AP
GPR132.2	1.11E-45	0.50165	0.209	0.028	1.65E-41	DC	GPR132
HAS1	1.44E-45	0.487301	0.134	0.012	2.16E-41	DC	HAS1
GPR65.3	3.29E-45	0.727323	0.487	0.128	4.92E-41	DC	GPR65
TBXAS1.3	3.41E-45	0.6765	0.701	0.234	5.09E-41	DC	TBXAS1
PYCARD.3	4.76E-45	0.709296	0.759	0.277	7.11E-41	DC	PYCARD
CREM.3	5.30E-45	0.697462	0.84	0.399	7.93E-41	DC	CREM
OGFRL1.3	6.65E-45	0.815345	0.545	0.176	9.94E-41	DC	OGFRL1
CTLA4	1.30E-44	0.424341	0.112	0.008	1.94E-40	DC	CTLA4
CD53.3	1.55E-44	0.653441	0.84	0.307	2.32E-40	DC	CD53
TGFBI.3	4.46E-44	0.750701	0.62	0.199	6.67E-40	DC	TGFBI
FPR1.3	4.92E-44	0.626906	0.786	0.276	7.35E-40	DC	FPR1

LGALS2	5.13E-44	0.247351	0.053	0.002	7.66E-40	DC	LGALS2
KYNU.3	5.45E-44	0.676797	0.364	0.08	8.14E-40	DC	KYNU
TYROBP.3	6.60E-44	0.605502	1	0.451	9.87E-40	DC	TYROBP
DAPP1.2	6.82E-44	0.500109	0.193	0.025	1.02E-39	DC	DAPP1
THEMIS2.3	7.19E-44	0.704839	0.674	0.235	1.07E-39	DC	THEMIS2
IL18.3	8.28E-44	0.661854	0.604	0.186	1.24E-39	DC	IL18
BCL2A1.3	2.40E-43	0.727064	0.524	0.151	3.59E-39	DC	BCL2A1
ETS2.3	3.00E-43	0.752163	0.604	0.201	4.49E-39	DC	ETS2
RGS1.3	3.23E-43	0.583894	0.973	0.438	4.82E-39	DC	RGS1
RILPL2.3	3.48E-43	0.722422	0.674	0.252	5.20E-39	DC	RILPL2
CD37.3	3.55E-43	0.63248	0.807	0.293	5.30E-39	DC	CD37
PLEK.3	3.74E-43	0.733279	0.556	0.169	5.59E-39	DC	PLEK
PTPRE.3	4.69E-43	0.832654	0.647	0.264	7.01E-39	DC	PTPRE
HCST.3	8.60E-43	0.657153	0.77	0.276	1.29E-38	DC	HCST
RGS2.2	9.16E-43	0.578859	0.92	0.438	1.37E-38	DC	RGS2
CYP2S1	1.83E-42	0.384173	0.139	0.014	2.74E-38	DC	CYP2S1
RGS10.3	2.35E-42	0.622727	0.856	0.334	3.51E-38	DC	RGS10
MYO1G.3	9.81E-42	0.61718	0.294	0.057	1.47E-37	DC	MYO1G
MYL12A.2	1.35E-41	0.550327	0.936	0.537	2.02E-37	DC	MYL12A
SRGN.3	3.06E-41	0.56436	0.995	0.491	4.57E-37	DC	SRGN
ARPC1B.3	3.16E-41	0.575069	0.947	0.524	4.72E-37	DC	ARPC1B
S1PR4	3.32E-41	0.37384	0.112	0.009	4.96E-37	DC	S1PR4
MYO1F.3	4.84E-41	0.654125	0.567	0.177	7.23E-37	DC	MYO1F
PSTPIP2.2	5.91E-41	0.597534	0.241	0.041	8.84E-37	DC	PSTPIP2
LAPTM5.3	8.97E-41	0.595096	0.963	0.427	1.34E-36	DC	LAPTM5
WAS.3	9.40E-41	0.686334	0.545	0.168	1.40E-36	DC	WAS
OLR1.3	1.35E-40	0.678419	0.695	0.243	2.02E-36	DC	OLR1
FCER1G.3	1.43E-40	0.58382	0.968	0.433	2.14E-36	DC	FCER1G
CAPG.3	1.59E-40	0.636753	0.893	0.407	2.37E-36	DC	CAPG
GLIPR1.2	2.21E-40	0.651376	0.749	0.328	3.30E-36	DC	GLIPR1
KLF4.3	2.86E-40	0.743469	0.583	0.2	4.27E-36	DC	KLF4
RIN3.3	3.92E-40	0.739779	0.417	0.11	5.86E-36	DC	RIN3
SMAP2.3	6.00E-40	0.756582	0.61	0.23	8.96E-36	DC	SMAP2
SAMHD1.3	6.58E-40	0.71698	0.583	0.209	9.83E-36	DC	SAMHD1
ITGA4.2	9.38E-40	0.561196	0.235	0.039	1.40E-35	DC	ITGA4
HS3ST3B1.	1.12E-39	0.535096	0.23	0.038	1.67E-35	DC	HS3ST3B1
LAT2.3	1.49E-39	0.680147	0.599	0.196	2.23E-35	DC	LAT2
CLEC4A.3	1.91E-39	0.68307	0.289	0.058	2.86E-35	DC	CLEC4A
SPN.3	3.44E-39	0.597743	0.273	0.052	5.15E-35	DC	SPN
STK38L.2	3.62E-39	0.768886	0.465	0.145	5.41E-35	DC	STK38L
BTK.3	5.05E-39	0.589745	0.353	0.081	7.54E-35	DC	BTK
IRAK3.3	8.85E-39	0.584399	0.321	0.07	1.32E-34	DC	IRAK3
CYBA.3	2.08E-38	0.651878	0.893	0.397	3.11E-34	DC	CYBA
CCDC69.2	2.86E-38	0.509604	0.209	0.033	4.27E-34	DC	CCDC69
COTL1.3	3.67E-38	0.628468	0.85	0.504	5.48E-34	DC	COTL1
MAP4K1.2	4.96E-38	0.510662	0.187	0.027	7.42E-34	DC	MAP4K1
SERPINB1.	1.05E-37	0.629994	0.701	0.26	1.56E-33	DC	SERPINB1
CKLF.3	1.33E-37	0.596032	0.872	0.514	1.98E-33	DC	CKLF
REL.3	1.70E-37	0.728673	0.684	0.292	2.55E-33	DC	REL
CSF3R.3	2.43E-37	0.660098	0.492	0.147	3.63E-33	DC	CSF3R
CD300LB	2.61E-37	0.312448	0.102	0.008	3.91E-33	DC	CD300LB

AHR.3	4.51E-37	0.617481	0.283	0.059	6.74E-33	DC	AHR
AIM1.1	4.89E-37	0.522938	0.203	0.032	7.31E-33	DC	AIM1
FAM105A.3	6.81E-37	0.655039	0.508	0.157	1.02E-32	DC	FAM105A
STXBP2.3	9.66E-37	0.678976	0.519	0.167	1.44E-32	DC	STXBP2
RGS18.3	1.59E-36	0.564482	0.257	0.049	2.37E-32	DC	RGS18
IL10RA.3	1.68E-36	0.637869	0.46	0.133	2.51E-32	DC	IL10RA
RASSF5.3	1.71E-36	0.660892	0.412	0.115	2.55E-32	DC	RASSF5
HCK.3	1.91E-36	0.651532	0.519	0.162	2.85E-32	DC	HCK
HLA-DPA1.	2.32E-36	0.501271	0.995	0.526	3.47E-32	DC	HLA-DPA1
STX11.3	2.76E-36	0.674211	0.406	0.11	4.12E-32	DC	STX11
LILRA2.3	3.21E-36	0.579911	0.342	0.08	4.79E-32	DC	LILRA2
GNA15.3	9.25E-36	0.656985	0.39	0.105	1.38E-31	DC	GNA15
GRN.3	1.13E-35	0.511188	0.92	0.564	1.69E-31	DC	GRN
NR4A1.3	1.56E-35	0.641469	0.679	0.266	2.33E-31	DC	NR4A1
OSM.3	2.16E-35	0.652188	0.476	0.14	3.23E-31	DC	OSM
IGSF6.3	2.60E-35	0.648043	0.487	0.148	3.89E-31	DC	IGSF6
RP6-91H8.	7.46E-35	0.618445	0.262	0.052	1.12E-30	DC	RP6-91H8.3
PTN.3	7.67E-35	-1.41099	0.16	0.624	1.15E-30	DC	PTN
CCL22	1.19E-34	0.203169	0.059	0.003	1.78E-30	DC	CCL22
HLA-DRB1.	2.48E-34	0.488381	1	0.525	3.71E-30	DC	HLA-DRB1
LGALS9.3	3.41E-34	0.597987	0.642	0.234	5.10E-30	DC	LGALS9
PPT1.2	4.28E-34	0.533359	0.877	0.55	6.39E-30	DC	PPT1
ZNF331.3	4.91E-34	0.640957	0.706	0.309	7.34E-30	DC	ZNF331
MEF2C.3	5.27E-34	0.630281	0.663	0.277	7.87E-30	DC	MEF2C
AXL.3	5.28E-34	0.641438	0.519	0.175	7.88E-30	DC	AXL
DEF6.3	6.26E-34	0.573402	0.348	0.087	9.36E-30	DC	DEF6
GSN.2	7.90E-34	0.533075	0.914	0.503	1.18E-29	DC	GSN
CMTM7.3	1.16E-33	0.600006	0.487	0.159	1.73E-29	DC	CMTM7
EMP3.2	1.28E-33	0.565245	0.829	0.443	1.92E-29	DC	EMP3
GRASP.3	1.45E-33	0.62102	0.246	0.048	2.17E-29	DC	GRASP
MS4A7.3	1.63E-33	0.56339	0.797	0.324	2.43E-29	DC	MS4A7
NOVA1.3	2.15E-33	-1.51093	0.016	0.486	3.21E-29	DC	NOVA1
PLXDC2.3	2.47E-33	0.599945	0.578	0.203	3.69E-29	DC	PLXDC2
TCIRG1.3	3.76E-33	0.576441	0.417	0.121	5.62E-29	DC	TCIRG1
LITAF.3	4.88E-33	0.5426	0.84	0.453	7.30E-29	DC	LITAF
TNFAIP8.2	5.18E-33	0.580341	0.289	0.066	7.74E-29	DC	TNFAIP8
LILRB3.2	6.40E-33	0.56177	0.209	0.037	9.56E-29	DC	LILRB3
CXCL16.3	6.52E-33	0.560956	0.84	0.447	9.74E-29	DC	CXCL16
ANXA11.3	6.54E-33	0.650448	0.556	0.21	9.78E-29	DC	ANXA11
PLSCR1.3	7.07E-33	0.606526	0.733	0.353	1.06E-28	DC	PLSCR1
LPCAT2.3	8.82E-33	0.600958	0.46	0.142	1.32E-28	DC	LPCAT2
SGK1.3	8.84E-33	0.584667	0.834	0.43	1.32E-28	DC	SGK1
RNASET2.3	1.03E-32	0.575392	0.898	0.476	1.54E-28	DC	RNASET2
TPSAB1	1.08E-32	0.451899	0.15	0.02	1.61E-28	DC	TPSAB1
C10orf54.	1.92E-32	0.604457	0.701	0.281	2.88E-28	DC	C10orf54
RAB11FIP1	2.04E-32	0.417795	0.166	0.025	3.05E-28	DC	RAB11FIP1
FLT1.1	3.09E-32	0.397261	0.139	0.018	4.62E-28	DC	FLT1
CLEC2B.3	3.31E-32	0.583797	0.556	0.199	4.94E-28	DC	CLEC2B
HLA-DOA.2	3.73E-32	0.548279	0.348	0.09	5.57E-28	DC	HLA-DOA
ANXA1.1	3.84E-32	0.450937	0.882	0.402	5.74E-28	DC	ANXA1
CD300A.3	3.85E-32	0.599778	0.545	0.186	5.75E-28	DC	CD300A

HBEGF.3	3.89E-32	0.709006	0.551	0.21	5.81E-28	DC	HBEGF
MAP2K1.3	4.17E-32	0.73845	0.578	0.264	6.23E-28	DC	MAP2K1
NGFRAP1.2	4.18E-32	-1.10342	0.193	0.651	6.25E-28	DC	NGFRAP1
DSE.3	4.43E-32	0.644969	0.428	0.133	6.62E-28	DC	DSE
ENTPD1.3	4.86E-32	0.623812	0.481	0.157	7.26E-28	DC	ENTPD1
VASP.3	5.06E-32	0.728974	0.551	0.235	7.56E-28	DC	VASP
TMSB4X.3	5.79E-32	-0.44348	1	0.997	8.66E-28	DC	TMSB4X
LYN.3	5.93E-32	0.619295	0.492	0.169	8.86E-28	DC	LYN
BANK1	1.13E-31	0.290081	0.07	0.005	1.68E-27	DC	BANK1
DDIT4.2	1.60E-31	0.575714	0.791	0.402	2.39E-27	DC	DDIT4
GPM6B.3	1.63E-31	-1.24384	0.23	0.625	2.44E-27	DC	GPM6B
CAT.2	1.80E-31	0.647935	0.642	0.299	2.70E-27	DC	CAT
CASP4.3	1.84E-31	0.598345	0.567	0.222	2.75E-27	DC	CASP4
ISG20.2	3.10E-31	0.677538	0.39	0.117	4.63E-27	DC	ISG20
CD163.3	3.19E-31	0.567145	0.679	0.269	4.77E-27	DC	CD163
CKB.3	3.37E-31	-1.16686	0.171	0.589	5.03E-27	DC	CKB
RP11-1143	4.82E-31	0.626791	0.235	0.049	7.21E-27	DC	RP11-1143G9.4
CSF1R.3	9.60E-31	0.528704	0.69	0.261	1.43E-26	DC	CSF1R
IFNGR1.3	9.93E-31	0.579773	0.786	0.431	1.48E-26	DC	IFNGR1
ATF7IP2.2	1.55E-30	0.533408	0.209	0.039	2.32E-26	DC	ATF7IP2
TNFRSF1B.	2.04E-30	0.606624	0.519	0.183	3.05E-26	DC	TNFRSF1B
THBS1.2	2.20E-30	0.697698	0.305	0.077	3.29E-26	DC	THBS1
NCKAP1L.3	2.93E-30	0.541653	0.401	0.117	4.38E-26	DC	NCKAP1L
PRKCH.2	3.51E-30	0.471847	0.214	0.041	5.24E-26	DC	PRKCH
TUBB2B.2	4.40E-30	-1.33144	0.176	0.561	6.58E-26	DC	TUBB2B
ITGB2-AS1	5.29E-30	0.383585	0.166	0.026	7.90E-26	DC	ITGB2-AS1
FXYD6.3	1.19E-29	-1.23648	0.027	0.468	1.77E-25	DC	FXYD6
CLEC12A.1	1.22E-29	0.396416	0.134	0.018	1.83E-25	DC	CLEC12A
ADAM28.3	1.82E-29	0.562949	0.433	0.137	2.72E-25	DC	ADAM28
SMCO4.3	1.99E-29	0.63672	0.401	0.128	2.97E-25	DC	SMCO4
OSTF1.3	2.00E-29	0.571205	0.652	0.285	2.99E-25	DC	OSTF1
HERPUD1.2	2.39E-29	0.438738	0.936	0.637	3.57E-25	DC	HERPUD1
RNF149.3	2.43E-29	0.605386	0.62	0.265	3.64E-25	DC	RNF149
PAK1.3	2.94E-29	0.670957	0.455	0.165	4.39E-25	DC	PAK1
ACTB.3	2.96E-29	-0.40283	1	0.992	4.43E-25	DC	ACTB
ASGR2	3.67E-29	0.380071	0.091	0.009	5.49E-25	DC	ASGR2
NET1.2	4.21E-29	0.502862	0.198	0.038	6.30E-25	DC	NET1
PLP2.2	4.93E-29	0.591939	0.668	0.339	7.36E-25	DC	PLP2
SEPT6.3	5.59E-29	0.679277	0.444	0.161	8.35E-25	DC	6-Sep
TLR2.3	6.93E-29	0.556678	0.369	0.107	1.04E-24	DC	TLR2
AVPI1.3	1.09E-28	0.654867	0.321	0.09	1.63E-24	DC	AVPI1
SQRDL.3	1.18E-28	0.581831	0.455	0.155	1.76E-24	DC	SQRDL
FERMT3.3	1.44E-28	0.612945	0.471	0.161	2.15E-24	DC	FERMT3
VNN1	1.83E-28	0.295457	0.096	0.01	2.73E-24	DC	VNN1
GK.3	2.13E-28	0.621252	0.401	0.134	3.18E-24	DC	GK
RP11-347F	2.26E-28	0.55295	0.262	0.061	3.38E-24	DC	RP11-347P5.1
SYTL3.3	2.84E-28	0.507712	0.262	0.061	4.24E-24	DC	SYTL3
LILRA5	2.93E-28	0.348291	0.102	0.011	4.38E-24	DC	LILRA5
SPINT1.2	3.82E-28	0.402435	0.176	0.031	5.71E-24	DC	SPINT1
RP11-76E1	3.87E-28	0.419433	0.187	0.034	5.78E-24	DC	RP11-76E17.3
ARHGAP30.	4.05E-28	0.594874	0.326	0.091	6.05E-24	DC	ARHGAP30

EFHD2.3	5.05E-28	0.655563	0.508	0.205	7.55E-24	DC	EFHD2
ITGB7.2	5.92E-28	0.431076	0.15	0.023	8.85E-24	DC	ITGB7
ZC3H12A.2	7.74E-28	0.557799	0.283	0.073	1.16E-23	DC	ZC3H12A
TREM1.2	8.09E-28	0.534276	0.471	0.159	1.21E-23	DC	TREM1
LDLRAD4.3	1.33E-27	0.675056	0.412	0.142	1.99E-23	DC	LDLRAD4
MAP1B.2	1.73E-27	-1.1829	0.139	0.54	2.59E-23	DC	MAP1B
CASP1.2	1.80E-27	0.563749	0.545	0.221	2.70E-23	DC	CASP1
RHOG.3	2.14E-27	0.558846	0.759	0.404	3.19E-23	DC	RHOG
RETN.2	2.82E-27	0.422386	0.128	0.018	4.21E-23	DC	RETN
C1orf61.3	3.18E-27	-1.18294	0.144	0.541	4.75E-23	DC	C1orf61
ARHGAP15.	3.50E-27	0.533438	0.332	0.093	5.23E-23	DC	ARHGAP15
PTEN.3	5.85E-27	0.639627	0.374	0.125	8.74E-23	DC	PTEN
GPRIN3.3	6.12E-27	0.484209	0.241	0.055	9.14E-23	DC	GPRIN3
IL1B.3	6.40E-27	0.699298	0.54	0.216	9.56E-23	DC	IL1B
ARRB2.3	6.59E-27	0.5583	0.749	0.394	9.84E-23	DC	ARRB2
CLIC1.1	7.32E-27	0.39364	0.973	0.617	1.09E-22	DC	CLIC1
AKAP13.3	8.07E-27	0.587508	0.636	0.32	1.21E-22	DC	AKAP13
CXorf21.3	8.44E-27	0.531803	0.283	0.073	1.26E-22	DC	CXorf21
FILIP1L.3	8.44E-27	0.640667	0.406	0.145	1.26E-22	DC	FILIP1L
SERP1.3	8.75E-27	0.469139	0.856	0.6	1.31E-22	DC	SERP1
BIN2.3	9.32E-27	0.477113	0.364	0.108	1.39E-22	DC	BIN2
RP11-138A	1.03E-26	0.609665	0.374	0.121	1.54E-22	DC	RP11-138A9.1
CCR2	1.04E-26	0.189271	0.059	0.004	1.55E-22	DC	CCR2
ALDH2.3	1.28E-26	0.604861	0.674	0.366	1.92E-22	DC	ALDH2
SYK.3	1.35E-26	0.484657	0.348	0.102	2.02E-22	DC	SYK
FCGRT.2	1.46E-26	0.434567	0.925	0.544	2.19E-22	DC	FCGRT
SIGLEC10.	1.87E-26	0.542923	0.455	0.157	2.80E-22	DC	SIGLEC10
OPN3.3	2.79E-26	0.616183	0.262	0.069	4.18E-22	DC	OPN3
LRRFIP1.3	3.97E-26	0.612689	0.647	0.349	5.93E-22	DC	LRRFIP1
CPVL.3	5.52E-26	0.522969	0.706	0.343	8.25E-22	DC	CPVL
SDS.3	5.71E-26	0.723086	0.235	0.056	8.54E-22	DC	SDS
MFSD1.3	6.25E-26	0.516285	0.684	0.338	9.35E-22	DC	MFSD1
METRNL.3	9.61E-26	0.548905	0.289	0.079	1.44E-21	DC	METRNL
CTSZ.3	9.85E-26	0.524106	0.62	0.25	1.47E-21	DC	CTSZ
CD300C.3	1.49E-25	0.575184	0.257	0.065	2.23E-21	DC	CD300C
TNFAIP3.3	2.23E-25	0.610149	0.439	0.159	3.33E-21	DC	TNFAIP3
CALD1.2	2.28E-25	-1.07703	0.027	0.426	3.40E-21	DC	CALD1
SOX2.3	3.36E-25	-1.17054	0.053	0.435	5.02E-21	DC	SOX2
VSIG4.3	3.41E-25	0.451741	0.781	0.358	5.09E-21	DC	VSIG4
TSC22D1.2	6.20E-25	-1.02112	0.139	0.518	9.27E-21	DC	TSC22D1
RPS6KA1.3	6.55E-25	0.54936	0.38	0.127	9.79E-21	DC	RPS6KA1
CD58.3	8.28E-25	0.630069	0.513	0.223	1.24E-20	DC	CD58
CD302.3	9.14E-25	0.539158	0.497	0.2	1.37E-20	DC	CD302
SIK1.3	1.14E-24	0.587306	0.321	0.097	1.71E-20	DC	SIK1
PABPC1.2	1.28E-24	0.344018	0.952	0.741	1.91E-20	DC	PABPC1
GPM6A.2	1.53E-24	-1.05118	0.128	0.51	2.28E-20	DC	GPM6A
EIF3L.1	1.87E-24	0.441772	0.872	0.602	2.80E-20	DC	EIF3L
HAVCR2.3	1.98E-24	0.480008	0.578	0.226	2.96E-20	DC	HAVCR2
GDI2.3	2.65E-24	0.500455	0.743	0.484	3.96E-20	DC	GDI2
ARHGAP9.3	2.96E-24	0.470434	0.294	0.083	4.43E-20	DC	ARHGAP9
PFN2.2	3.44E-24	-1.0828	0.059	0.44	5.14E-20	DC	PFN2

TSC22D3.2	3.50E-24	0.455637	0.791	0.451	5.22E-20	DC	TSC22D3
CD33.3	3.56E-24	0.496371	0.364	0.116	5.32E-20	DC	CD33
CLECL1.3	3.60E-24	0.430132	0.203	0.045	5.38E-20	DC	CLECL1
TUBA1A.2	4.23E-24	-0.67532	0.754	0.808	6.33E-20	DC	TUBA1A
UCP2.3	5.10E-24	0.563208	0.594	0.267	7.62E-20	DC	UCP2
MSN.3	6.92E-24	0.533375	0.647	0.371	1.03E-19	DC	MSN
HLA-DRA.2	9.39E-24	0.331902	1	0.627	1.40E-19	DC	HLA-DRA
PLIN2.2	1.18E-23	0.444592	0.717	0.336	1.76E-19	DC	PLIN2
NKG7.2	1.25E-23	0.460811	0.209	0.047	1.86E-19	DC	NKG7
SLC16A3.3	1.25E-23	0.519842	0.658	0.315	1.86E-19	DC	SLC16A3
ARHGAP18.	1.30E-23	0.525661	0.449	0.168	1.95E-19	DC	ARHGAP18
GAP43.3	1.33E-23	-1.27523	0.096	0.472	1.99E-19	DC	GAP43
CAP1.2	1.57E-23	0.394019	0.834	0.588	2.34E-19	DC	CAP1
PRR13.1	1.69E-23	0.414459	0.829	0.538	2.52E-19	DC	PRR13
CYBB.3	1.72E-23	0.504517	0.497	0.187	2.57E-19	DC	CYBB
MCL1.3	2.54E-23	0.435076	0.856	0.594	3.80E-19	DC	MCL1
CHMP1B.3	2.66E-23	0.540502	0.717	0.433	3.97E-19	DC	CHMP1B
FKBP5.3	2.67E-23	0.51646	0.626	0.312	3.99E-19	DC	FKBP5
B3GNT5.3	2.79E-23	0.563774	0.38	0.138	4.17E-19	DC	B3GNT5
ST3GAL1.3	3.02E-23	0.523456	0.316	0.099	4.51E-19	DC	ST3GAL1
TUBB2A.2	3.23E-23	-0.94	0.112	0.483	4.83E-19	DC	TUBB2A
SASH3.3	3.75E-23	0.435588	0.273	0.075	5.60E-19	DC	SASH3
UCHL1.1	4.30E-23	-1.09839	0.07	0.442	6.42E-19	DC	UCHL1
SLC16A10.	5.25E-23	0.558622	0.289	0.084	7.85E-19	DC	SLC16A10
AOAH.3	6.43E-23	0.498615	0.364	0.119	9.60E-19	DC	AOAH
RBP1.1	8.52E-23	-1.10669	0.107	0.465	1.27E-18	DC	RBP1
GAPDH.3	9.13E-23	-0.40339	1	0.985	1.37E-18	DC	GAPDH
SLC25A4.3	9.30E-23	-0.91196	0.059	0.432	1.39E-18	DC	SLC25A4
IRF8.3	1.09E-22	0.516847	0.428	0.154	1.63E-18	DC	IRF8
FEZ1.2	1.46E-22	-0.95119	0.064	0.436	2.18E-18	DC	FEZ1
PEBP1.3	1.48E-22	-0.58069	0.652	0.806	2.21E-18	DC	PEBP1
B4GALT1.3	1.55E-22	0.524899	0.348	0.117	2.32E-18	DC	B4GALT1
FAM49B.3	1.59E-22	0.47869	0.781	0.502	2.38E-18	DC	FAM49B
CNPY3.3	1.65E-22	0.482298	0.727	0.407	2.46E-18	DC	CNPY3
MGAT1.3	1.86E-22	0.534858	0.647	0.353	2.78E-18	DC	MGAT1
CSRNP1.3	1.99E-22	0.586702	0.455	0.186	2.97E-18	DC	CSRNP1
IKZF1.3	2.02E-22	0.460136	0.241	0.062	3.02E-18	DC	IKZF1
PCSK1N.2	2.29E-22	-1.09541	0.08	0.426	3.42E-18	DC	PCSK1N
FSCN1.3	2.97E-22	-0.85839	0.15	0.51	4.45E-18	DC	FSCN1
ANPEP.2	3.00E-22	0.35908	0.155	0.03	4.49E-18	DC	ANPEP
LRRK2.2	3.35E-22	0.472631	0.225	0.058	5.00E-18	DC	LRRK2
FPR3.3	3.36E-22	0.437324	0.219	0.054	5.02E-18	DC	FPR3
WBP5.2	3.51E-22	-0.91407	0.128	0.509	5.24E-18	DC	WBP5
CST6	3.71E-22	0.339303	0.102	0.014	5.55E-18	DC	CST6
EEF1B2.2	3.73E-22	0.267195	0.968	0.773	5.58E-18	DC	EEF1B2
SKIL.3	4.66E-22	0.543246	0.561	0.281	6.96E-18	DC	SKIL
LIMS1.3	4.73E-22	0.484701	0.722	0.435	7.07E-18	DC	LIMS1
APOBR.2	4.82E-22	0.429683	0.171	0.036	7.20E-18	DC	APOBR
SH3KBP1.3	5.68E-22	0.541375	0.513	0.237	8.49E-18	DC	SH3KBP1
ACTG1.3	7.74E-22	-0.37805	0.973	0.948	1.16E-17	DC	ACTG1
SCD5.2	8.08E-22	-0.9207	0.037	0.4	1.21E-17	DC	SCD5

MX2.3	8.54E-22	0.443061	0.235	0.061	1.28E-17	DC	MX2
CARD16.1	8.88E-22	0.4688	0.524	0.225	1.33E-17	DC	CARD16
NFKBID.3	1.07E-21	0.469076	0.39	0.136	1.60E-17	DC	NFKBID
PDPN.1	1.30E-21	-1.03342	0.086	0.455	1.94E-17	DC	PDPN
USP53.3	1.46E-21	0.507638	0.385	0.137	2.19E-17	DC	USP53
CNN3.3	1.47E-21	-1.01938	0.123	0.474	2.20E-17	DC	CNN3
S100A6.3	1.79E-21	0.23234	0.968	0.638	2.68E-17	DC	S100A6
LTB.2	1.83E-21	0.392206	0.155	0.031	2.74E-17	DC	LTB
PMAIP1.3	2.03E-21	0.479956	0.278	0.08	3.03E-17	DC	PMAIP1
TMEM109.3	2.03E-21	0.539193	0.465	0.2	3.04E-17	DC	TMEM109
EVI2A.3	2.04E-21	0.415742	0.535	0.218	3.04E-17	DC	EVI2A
RASAL3.3	2.09E-21	0.390276	0.219	0.054	3.13E-17	DC	RASAL3
HMGB2.3	2.21E-21	0.442872	0.802	0.489	3.30E-17	DC	HMGB2
DOCK5.3	2.22E-21	0.420126	0.214	0.053	3.32E-17	DC	DOCK5
HEXB.3	2.34E-21	0.504734	0.674	0.401	3.50E-17	DC	HEXB
ZNF385A.3	2.72E-21	0.536627	0.364	0.133	4.07E-17	DC	ZNF385A
PPA1.2	3.14E-21	0.438853	0.775	0.525	4.69E-17	DC	PPA1
GCA.3	3.62E-21	0.549829	0.588	0.294	5.40E-17	DC	GCA
CLEC9A.2	3.74E-21	0.437766	0.155	0.031	5.59E-17	DC	CLEC9A
FABP7.2	4.99E-21	-1.29597	0.053	0.389	7.46E-17	DC	FABP7
PARVB.3	5.05E-21	0.600859	0.396	0.16	7.54E-17	DC	PARVB
RHOBTB3.3	5.23E-21	-0.86642	0.059	0.414	7.81E-17	DC	RHOBTB3
ITGAM.3	6.11E-21	0.501907	0.257	0.073	9.14E-17	DC	ITGAM
ARHGAP4.3	6.41E-21	0.494899	0.337	0.112	9.58E-17	DC	ARHGAP4
CIB1.2	6.44E-21	0.440573	0.765	0.488	9.63E-17	DC	CIB1
PRDX2.3	6.93E-21	-0.71043	0.422	0.706	1.04E-16	DC	PRDX2
NFIB.3	6.96E-21	-1.01201	0.037	0.383	1.04E-16	DC	NFIB
RASSF3.2	6.96E-21	0.379526	0.176	0.039	1.04E-16	DC	RASSF3
PSTPIP1.2	7.00E-21	0.387259	0.176	0.039	1.05E-16	DC	PSTPIP1
TPM3.3	9.21E-21	0.386858	0.797	0.554	1.38E-16	DC	TPM3
FCN1	9.75E-21	0.375185	0.075	0.008	1.46E-16	DC	FCN1
MCM5.3	1.04E-20	0.548902	0.38	0.146	1.56E-16	DC	MCM5
RP11-386I	1.07E-20	0.439259	0.235	0.064	1.59E-16	DC	RP11-386I14.4
KCNE1.1	1.21E-20	0.393511	0.123	0.021	1.81E-16	DC	KCNE1
TLR10.2	1.43E-20	0.359503	0.128	0.023	2.13E-16	DC	TLR10
MDK.2	1.45E-20	-0.98872	0.155	0.496	2.17E-16	DC	MDK
ATP1B3.3	1.51E-20	0.452254	0.77	0.541	2.26E-16	DC	ATP1B3
RGS19.3	1.69E-20	0.548853	0.551	0.271	2.52E-16	DC	RGS19
PLB1.2	1.73E-20	0.431046	0.176	0.04	2.59E-16	DC	PLB1
RNF130.3	1.79E-20	0.49134	0.722	0.458	2.67E-16	DC	RNF130
LPXN.3	1.92E-20	0.470871	0.321	0.107	2.88E-16	DC	LPXN
SLA.3	2.03E-20	0.474022	0.492	0.197	3.04E-16	DC	SLA
FES.3	2.05E-20	0.439851	0.262	0.076	3.06E-16	DC	FES
KCNK6.1	2.32E-20	0.371546	0.171	0.037	3.47E-16	DC	KCNK6
CATSPER1.	2.88E-20	0.389922	0.134	0.024	4.30E-16	DC	CATSPER1
CEBPD.1	2.97E-20	0.465954	0.759	0.454	4.43E-16	DC	CEBPD
SYNGR2.3	3.00E-20	0.473289	0.626	0.323	4.48E-16	DC	SYNGR2
PTPN22.2	3.68E-20	0.338255	0.134	0.025	5.51E-16	DC	PTPN22
HSH2D	3.79E-20	0.243166	0.07	0.007	5.66E-16	DC	HSH2D
SLC2A3.2	4.45E-20	0.532039	0.529	0.25	6.66E-16	DC	SLC2A3
TRPV2.3	4.80E-20	0.475451	0.214	0.056	7.17E-16	DC	TRPV2

CAMK2N1.3	5.21E-20	-0.91849	0	0.322	7.79E-16	DC	CAMK2N1
TAGAP.3	5.84E-20	0.472405	0.305	0.097	8.73E-16	DC	TAGAP
CD14.3	7.30E-20	0.411758	0.781	0.402	1.09E-15	DC	CD14
PLD4.3	7.68E-20	0.490416	0.406	0.149	1.15E-15	DC	PLD4
RPPH1	7.92E-20	0.428167	0.144	0.029	1.18E-15	DC	RPPH1
DUSP5	8.03E-20	0.608164	0.235	0.07	1.20E-15	DC	DUSP5
CD68.3	1.07E-19	0.389855	0.834	0.435	1.61E-15	DC	CD68
CACNA2D4.	1.07E-19	0.465105	0.246	0.071	1.61E-15	DC	CACNA2D4
SCG5.2	1.17E-19	-0.97584	0.011	0.333	1.75E-15	DC	SCG5
CACNA2D3.	1.21E-19	0.434007	0.144	0.029	1.80E-15	DC	CACNA2D3
MEST.2	1.30E-19	-0.87655	0.053	0.392	1.94E-15	DC	MEST
MLLT11.1	1.30E-19	-0.96038	0.123	0.459	1.94E-15	DC	MLLT11
ARPC3.1	1.34E-19	0.233635	0.984	0.801	2.00E-15	DC	ARPC3
SYAP1.2	1.44E-19	0.549947	0.62	0.383	2.15E-15	DC	SYAP1
H2AFY.3	1.56E-19	0.377578	0.813	0.604	2.33E-15	DC	H2AFY
PRKAG2-AS	1.57E-19	0.557538	0.337	0.121	2.35E-15	DC	PRKAG2-AS1
SLC11A1.3	2.02E-19	0.468325	0.626	0.3	3.02E-15	DC	SLC11A1
TM6SF1.3	2.16E-19	0.47489	0.406	0.154	3.22E-15	DC	TM6SF1
MAP2.3	2.26E-19	-0.84442	0.021	0.343	3.38E-15	DC	MAP2
SP100.2	2.30E-19	0.435929	0.578	0.282	3.44E-15	DC	SP100
LACTB.3	2.39E-19	0.553123	0.39	0.162	3.57E-15	DC	LACTB
ACTR2.3	3.14E-19	0.470271	0.69	0.455	4.68E-15	DC	ACTR2
NFIX.2	3.22E-19	-0.81479	0.016	0.337	4.81E-15	DC	NFIX
NAAA.3	3.46E-19	0.504856	0.342	0.13	5.18E-15	DC	NAAA
ARPC5.2	3.76E-19	0.296027	0.882	0.669	5.62E-15	DC	ARPC5
RASGEF1B.	4.32E-19	0.430265	0.337	0.117	6.46E-15	DC	RASGEF1B
CTSC.3	4.51E-19	0.394169	0.738	0.378	6.74E-15	DC	CTSC
GPX1.3	5.62E-19	0.224357	0.995	0.811	8.40E-15	DC	GPX1
MARCKS.2	5.90E-19	-0.65715	0.337	0.602	8.82E-15	DC	MARCKS
CXXC5.3	6.11E-19	-0.83256	0.059	0.385	9.13E-15	DC	CXXC5
TMEM173.3	6.16E-19	0.460301	0.348	0.123	9.21E-15	DC	TMEM173
DUSP23.2	7.96E-19	0.489689	0.444	0.184	1.19E-14	DC	DUSP23
CDKN1A.2	8.13E-19	0.474206	0.594	0.303	1.22E-14	DC	CDKN1A
MARCKSL1.	8.83E-19	-0.9476	0.316	0.548	1.32E-14	DC	MARCKSL1
IGFBP2.2	8.98E-19	-1.09642	0.07	0.379	1.34E-14	DC	IGFBP2
SLC7A7.3	9.28E-19	0.455161	0.428	0.17	1.39E-14	DC	SLC7A7
ACSL6	1.07E-18	0.404014	0.182	0.045	1.59E-14	DC	ACSL6
NFKBIA.2	1.18E-18	0.438593	0.775	0.495	1.76E-14	DC	NFKBIA
CCNH.3	1.25E-18	0.476702	0.663	0.407	1.87E-14	DC	CCNH
ETV3.3	1.27E-18	0.526925	0.241	0.074	1.90E-14	DC	ETV3
TRABD.3	1.52E-18	0.528951	0.497	0.255	2.27E-14	DC	TRABD
ANAPC16.1	1.72E-18	0.360165	0.781	0.521	2.58E-14	DC	ANAPC16
SEC11A.2	1.79E-18	0.305638	0.829	0.587	2.67E-14	DC	SEC11A
C10orf128	1.82E-18	0.437019	0.139	0.029	2.73E-14	DC	C10orf128
MOB1A.3	2.31E-18	0.469559	0.594	0.336	3.45E-14	DC	MOB1A
GSTO1.2	2.34E-18	0.440634	0.722	0.452	3.50E-14	DC	GSTO1
STAT6.3	3.37E-18	0.397672	0.193	0.05	5.04E-14	DC	STAT6
FGD2.3	3.65E-18	0.33273	0.214	0.058	5.45E-14	DC	FGD2
ELF1.3	3.89E-18	0.45808	0.508	0.24	5.81E-14	DC	ELF1
SPTLC2.3	5.06E-18	0.463131	0.465	0.206	7.56E-14	DC	SPTLC2
ZNF667-AS	5.44E-18	-0.79994	0.07	0.393	8.13E-14	DC	ZNF667-AS1

SH3BP1.3	6.09E-18	0.560712	0.299	0.109	9.10E-14	DC	SH3BP1
ETHE1	6.11E-18	0.510166	0.529	0.285	9.13E-14	DC	ETHE1
WRB.2	7.49E-18	-0.74597	0.091	0.425	1.12E-13	DC	WRB
CELF2.3	7.88E-18	0.445896	0.636	0.379	1.18E-13	DC	CELF2
RGCC.2	7.89E-18	0.499067	0.519	0.246	1.18E-13	DC	RGCC
GOLM1.2	8.07E-18	-0.73873	0.016	0.321	1.21E-13	DC	GOLM1
ANXA2.2	8.40E-18	0.333064	0.84	0.508	1.26E-13	DC	ANXA2
ELAVL3.3	8.56E-18	-0.81511	0.016	0.315	1.28E-13	DC	ELAVL3
CD38	9.28E-18	0.372845	0.107	0.019	1.39E-13	DC	CD38
TSPO.2	9.90E-18	0.36544	0.84	0.591	1.48E-13	DC	TSPO
P2RY13.3	1.06E-17	0.393283	0.262	0.082	1.58E-13	DC	P2RY13
IER3.3	1.12E-17	0.455041	0.524	0.237	1.68E-13	DC	IER3
ETF1.2	1.14E-17	0.485869	0.578	0.342	1.70E-13	DC	ETF1
SLC25A5	1.22E-17	0.271862	0.92	0.718	1.82E-13	DC	SLC25A5
HHEX.3	1.36E-17	0.388374	0.203	0.055	2.03E-13	DC	HHEX
CMTM6.3	1.96E-17	0.437286	0.652	0.422	2.94E-13	DC	CMTM6
BAALC.2	1.97E-17	-0.84656	0.048	0.35	2.94E-13	DC	BAALC
MEG3.2	2.05E-17	-1.07313	0.016	0.309	3.06E-13	DC	MEG3
PMP2.3	2.24E-17	-1.0228	0.053	0.355	3.34E-13	DC	PMP2
VRK2.3	2.45E-17	0.415885	0.193	0.052	3.65E-13	DC	VRK2
NFKB1.3	2.53E-17	0.507301	0.283	0.101	3.78E-13	DC	NFKB1
PTGS2.3	2.85E-17	0.45797	0.209	0.059	4.26E-13	DC	PTGS2
NCF4.3	2.89E-17	0.440305	0.369	0.142	4.32E-13	DC	NCF4
SH3BGRL.1	2.93E-17	0.312911	0.872	0.645	4.38E-13	DC	SH3BGRL
PTPRZ1.3	3.18E-17	-0.90032	0.086	0.389	4.76E-13	DC	PTPRZ1
NPC2.2	3.22E-17	0.267366	0.989	0.687	4.81E-13	DC	NPC2
CCND3.2	3.29E-17	0.508142	0.492	0.256	4.92E-13	DC	CCND3
SERPINF2.	3.77E-17	0.350179	0.123	0.024	5.64E-13	DC	SERPINF2
ARPC2.2	4.51E-17	0.256642	0.952	0.771	6.74E-13	DC	ARPC2
C19orf33	4.56E-17	0.152138	0.032	0.002	6.82E-13	DC	C19orf33
DRAM2.3	5.23E-17	0.441446	0.594	0.332	7.82E-13	DC	DRAM2
HBB.2	5.34E-17	0.383054	0.668	0.394	7.99E-13	DC	HBB
FHL1.3	5.41E-17	-0.77049	0.059	0.361	8.09E-13	DC	FHL1
FAM49A.2	5.49E-17	0.524794	0.38	0.167	8.20E-13	DC	FAM49A
ACSL1.3	5.53E-17	0.484075	0.476	0.223	8.27E-13	DC	ACSL1
RCSD1.3	6.01E-17	0.447623	0.321	0.117	8.99E-13	DC	RCSD1
S100A10.3	6.12E-17	0.282951	0.845	0.54	9.15E-13	DC	S100A10
CHRNE.2	6.26E-17	0.28065	0.123	0.024	9.35E-13	DC	CHRNE
STMN3.1	6.26E-17	-0.72208	0.021	0.311	9.35E-13	DC	STMN3
NCAM1.3	6.66E-17	-0.78316	0.043	0.34	9.95E-13	DC	NCAM1
PABPC4.3	7.75E-17	0.527417	0.551	0.325	1.16E-12	DC	PABPC4
CCL17	7.78E-17	0.194762	0.048	0.004	1.16E-12	DC	CCL17
ICAM2.2	7.84E-17	0.412001	0.144	0.033	1.17E-12	DC	ICAM2
ABI3.3	9.54E-17	0.40379	0.444	0.186	1.42E-12	DC	ABI3
RHBDF2.3	1.00E-16	0.460023	0.412	0.174	1.50E-12	DC	RHBDF2
C3AR1.3	1.17E-16	0.337058	0.529	0.233	1.75E-12	DC	C3AR1
ODF3B.3	1.20E-16	0.43836	0.267	0.091	1.79E-12	DC	ODF3B
STMN1.2	1.20E-16	-0.7254	0.353	0.635	1.80E-12	DC	STMN1
LINC00461	1.27E-16	-0.76449	0.032	0.325	1.90E-12	DC	LINC00461
EAF2.3	1.32E-16	0.39428	0.267	0.09	1.97E-12	DC	EAF2
NFIA.2	1.36E-16	-0.80648	0.08	0.384	2.04E-12	DC	NFIA

MT3.3	1.37E-16	-1.12174	0.219	0.495	2.04E-12	DC	MT3
LIMD2.3	1.42E-16	0.572726	0.465	0.245	2.12E-12	DC	LIMD2
FTL.3	1.56E-16	-0.35645	1	0.994	2.34E-12	DC	FTL
MAPT.3	1.63E-16	-0.79037	0.011	0.289	2.44E-12	DC	MAPT
CARD9.3	1.68E-16	0.381659	0.219	0.066	2.50E-12	DC	CARD9
KIF21A.3	1.68E-16	-0.72181	0.021	0.307	2.52E-12	DC	KIF21A
TRIM38.2	1.72E-16	0.45444	0.273	0.095	2.57E-12	DC	TRIM38
ACAP1.2	1.75E-16	0.4642	0.235	0.077	2.61E-12	DC	ACAP1
CD44.1	1.84E-16	0.294357	0.684	0.378	2.75E-12	DC	CD44
YWHAH.3	1.90E-16	0.409706	0.759	0.562	2.84E-12	DC	YWHAH
NPDC1.2	2.01E-16	-0.7435	0.043	0.328	3.00E-12	DC	NPDC1
SLC1A5.3	2.12E-16	0.515137	0.337	0.135	3.17E-12	DC	SLC1A5
DDX21.1	2.16E-16	0.487112	0.524	0.296	3.23E-12	DC	DDX21
HLA-E.3	2.26E-16	0.299675	0.898	0.591	3.38E-12	DC	HLA-E
ZBTB20.2	2.33E-16	-0.71784	0.08	0.39	3.49E-12	DC	ZBTB20
PODXL2.3	2.35E-16	-0.72575	0.027	0.312	3.51E-12	DC	PODXL2
EIF3F.3	2.40E-16	0.333589	0.786	0.602	3.59E-12	DC	EIF3F
CDH2.2	2.53E-16	-0.67788	0.011	0.289	3.78E-12	DC	CDH2
BCL2.2	2.65E-16	0.387173	0.176	0.047	3.96E-12	DC	BCL2
LTC4S.3	2.76E-16	0.422017	0.406	0.164	4.13E-12	DC	LTC4S
CRIP2.1	2.81E-16	-0.70033	0.027	0.318	4.20E-12	DC	CRIP2
MS4A4A.3	2.86E-16	0.324303	0.61	0.281	4.27E-12	DC	MS4A4A
BEX1.2	2.89E-16	-0.91551	0.064	0.355	4.31E-12	DC	BEX1
CEBPB.3	2.91E-16	0.415776	0.695	0.431	4.35E-12	DC	CEBPB
CD2.2	2.97E-16	0.209448	0.102	0.018	4.44E-12	DC	CD2
SOX4.2	3.08E-16	-0.98379	0.166	0.45	4.61E-12	DC	SOX4
PRKCB.2	3.17E-16	0.348391	0.16	0.04	4.74E-12	DC	PRKCB
S100A11.3	3.44E-16	0.231562	0.989	0.652	5.14E-12	DC	S100A11
APRT.1	3.75E-16	0.318522	0.765	0.527	5.61E-12	DC	APRT
RP11-586D	3.79E-16	0.215527	0.059	0.007	5.67E-12	DC	RP11-586D19.1
GSTA4.2	4.01E-16	-0.76063	0.016	0.294	5.99E-12	DC	GSTA4
DSTN.3	4.14E-16	-0.61895	0.385	0.622	6.19E-12	DC	DSTN
H3F3B.3	4.28E-16	-0.2913	0.995	0.97	6.40E-12	DC	H3F3B
ATP6V0E2.	4.53E-16	-0.71181	0.16	0.457	6.76E-12	DC	ATP6V0E2
KCNAB2.3	4.78E-16	0.436272	0.23	0.074	7.14E-12	DC	KCNAB2
LAPTM4B.3	5.02E-16	-0.70406	0.048	0.336	7.50E-12	DC	LAPTM4B
FOSL2.3	5.04E-16	0.555558	0.39	0.183	7.53E-12	DC	FOSL2
CCR7	5.07E-16	0.405566	0.07	0.009	7.58E-12	DC	CCR7
NT5DC1.1	5.28E-16	0.40497	0.182	0.05	7.89E-12	DC	NT5DC1
CPPED1.3	5.30E-16	0.418337	0.214	0.065	7.92E-12	DC	CPPED1
LILRB4.3	6.12E-16	0.328604	0.561	0.25	9.15E-12	DC	LILRB4
MYL6B.2	6.31E-16	-0.70978	0.139	0.447	9.42E-12	DC	MYL6B
SERPINF1.	6.59E-16	0.45613	0.471	0.22	9.86E-12	DC	SERPINF1
SEC61G.3	6.61E-16	-0.75129	0.743	0.8	9.87E-12	DC	SEC61G
NABP1.2	6.82E-16	0.447187	0.316	0.121	1.02E-11	DC	NABP1
QARS.3	7.44E-16	0.48479	0.503	0.279	1.11E-11	DC	QARS
FAM229B.2	7.47E-16	-0.73797	0.021	0.296	1.12E-11	DC	FAM229B
SLC31A2.3	7.64E-16	0.453918	0.422	0.189	1.14E-11	DC	SLC31A2
GBGT1.3	7.74E-16	0.356942	0.176	0.048	1.16E-11	DC	GBGT1
STMN4.2	8.62E-16	-1.07988	0.037	0.31	1.29E-11	DC	STMN4
CD74.2	8.82E-16	0.221203	1	0.747	1.32E-11	DC	CD74

APLP1.3	9.07E-16	-0.80643	0.064	0.356	1.36E-11	DC	APLP1
KIF5C.3	9.49E-16	-0.70826	0.005	0.272	1.42E-11	DC	KIF5C
CAPZA1.3	9.98E-16	0.439482	0.599	0.389	1.49E-11	DC	CAPZA1
ZNF428.3	1.06E-15	-0.61837	0.278	0.549	1.59E-11	DC	ZNF428
GPR34.3	1.24E-15	0.322712	0.572	0.262	1.85E-11	DC	GPR34
NES.3	1.42E-15	-0.86406	0.016	0.28	2.12E-11	DC	NES
TSPAN7.1	1.46E-15	-0.81381	0.037	0.314	2.19E-11	DC	TSPAN7
RUNX3.2	1.51E-15	0.338131	0.15	0.037	2.25E-11	DC	RUNX3
DPYSL3.2	1.61E-15	-0.728	0.043	0.326	2.41E-11	DC	DPYSL3
GSTK1.1	1.63E-15	0.330783	0.765	0.526	2.44E-11	DC	GSTK1
IGFLR1	1.77E-15	0.363285	0.187	0.053	2.64E-11	DC	IGFLR1
TGIF1.2	1.83E-15	0.500371	0.578	0.354	2.73E-11	DC	TGIF1
TWF2.3	2.39E-15	0.517583	0.551	0.337	3.57E-11	DC	TWF2
LYL1.3	2.43E-15	0.358181	0.241	0.079	3.64E-11	DC	LYL1
CD320.1	2.47E-15	-0.68714	0.043	0.322	3.69E-11	DC	CD320
C7orf55.2	2.86E-15	-0.68822	0.123	0.428	4.27E-11	DC	C7orf55
ARF6.3	3.11E-15	0.492157	0.551	0.34	4.65E-11	DC	ARF6
YPEL5.1	3.22E-15	0.416083	0.663	0.449	4.81E-11	DC	YPEL5
RP11-24F1	3.22E-15	0.227762	0.086	0.014	4.81E-11	DC	RP11-24F11.2
PILRA.3	3.34E-15	0.44409	0.385	0.161	4.98E-11	DC	PILRA
PRAM1.3	3.53E-15	0.361376	0.182	0.051	5.27E-11	DC	PRAM1
TCEAL7.1	3.61E-15	-0.76048	0.032	0.307	5.40E-11	DC	TCEAL7
MT1E.3	3.75E-15	-1.01674	0.102	0.384	5.60E-11	DC	MT1E
SERPINE2.	3.78E-15	-0.74259	0.027	0.297	5.65E-11	DC	SERPINE2
DDR1.2	4.11E-15	-0.68175	0.053	0.336	6.14E-11	DC	DDR1
OSTC.2	4.12E-15	0.348136	0.711	0.5	6.16E-11	DC	OSTC
SCG3.3	4.13E-15	-0.83855	0.011	0.271	6.18E-11	DC	SCG3
SEZ6L2.2	4.26E-15	-0.69997	0.016	0.278	6.36E-11	DC	SEZ6L2
SOX11.2	4.29E-15	-0.84476	0.016	0.277	6.42E-11	DC	SOX11
SELL.2	4.31E-15	0.320737	0.144	0.035	6.44E-11	DC	SELL
TMSB10.3	4.54E-15	-0.33231	1	0.964	6.79E-11	DC	TMSB10
LINC00937	4.77E-15	0.338568	0.134	0.031	7.13E-11	DC	LINC00937
KDM6B.3	4.79E-15	0.486165	0.444	0.219	7.16E-11	DC	KDM6B
IFNGR2.3	4.92E-15	0.459857	0.594	0.386	7.35E-11	DC	IFNGR2
PRDM1.3	5.12E-15	0.403612	0.273	0.097	7.64E-11	DC	PRDM1
VMO1.3	5.36E-15	0.485638	0.369	0.156	8.01E-11	DC	VMO1
ATAT1.3	5.48E-15	-0.69337	0.027	0.293	8.18E-11	DC	ATAT1
NBEAL1.1	6.20E-15	0.231535	0.898	0.661	9.27E-11	DC	NBEAL1
CAMK1D.3	6.36E-15	0.511008	0.278	0.108	9.51E-11	DC	CAMK1D
TSPAN32	6.60E-15	0.202369	0.064	0.009	9.86E-11	DC	TSPAN32
CCRL2.3	7.10E-15	0.478298	0.251	0.086	1.06E-10	DC	CCRL2
SERPINB8.	7.18E-15	0.431733	0.225	0.076	1.07E-10	DC	SERPINB8
CFL2.2	7.70E-15	-0.74568	0.053	0.332	1.15E-10	DC	CFL2
PPP1R15A.	7.85E-15	0.400602	0.706	0.463	1.17E-10	DC	PPP1R15A
PKIG.3	7.86E-15	-0.71201	0.043	0.315	1.18E-10	DC	PKIG
ACRBP	8.22E-15	0.242672	0.075	0.011	1.23E-10	DC	ACRBP
ADAP2.3	8.25E-15	0.374443	0.422	0.18	1.23E-10	DC	ADAP2
SAT1.2	8.32E-15	0.179651	0.989	0.8	1.24E-10	DC	SAT1
ARID5A.1	8.67E-15	0.453084	0.508	0.286	1.30E-10	DC	ARID5A
KCNMB1.3	9.37E-15	0.391156	0.278	0.1	1.40E-10	DC	KCNMB1
DOCK8.3	9.77E-15	0.378159	0.31	0.118	1.46E-10	DC	DOCK8

S100A16.2	9.93E-15	-0.99795	0.043	0.311	1.48E-10	DC	S100A16
XBP1.3	1.02E-14	0.451996	0.599	0.367	1.53E-10	DC	XBP1
GLRX.2	1.07E-14	0.337863	0.652	0.384	1.60E-10	DC	GLRX
OXA1L.2	1.09E-14	0.432115	0.529	0.297	1.63E-10	DC	OXA1L
INSIG1.3	1.09E-14	0.556024	0.428	0.224	1.63E-10	DC	INSIG1
SNX20.2	1.12E-14	0.33172	0.16	0.043	1.67E-10	DC	SNX20
LRRK1	1.22E-14	0.270612	0.086	0.015	1.83E-10	DC	LRRK1
RP11-356I	1.23E-14	0.255164	0.102	0.02	1.84E-10	DC	RP11-356I2.4
TMEM71	1.26E-14	0.312439	0.112	0.024	1.88E-10	DC	TMEM71
ENG.3	1.31E-14	0.414606	0.374	0.162	1.95E-10	DC	ENG
DNER.1	1.35E-14	-0.80323	0.027	0.284	2.01E-10	DC	DNER
HOPX.2	1.36E-14	-1.10112	0.032	0.286	2.03E-10	DC	HOPX
M6PR.3	1.39E-14	0.410297	0.599	0.379	2.07E-10	DC	M6PR
NCOA4.3	1.39E-14	0.502918	0.471	0.259	2.08E-10	DC	NCOA4
PALD1.3	1.45E-14	0.389782	0.273	0.099	2.17E-10	DC	PALD1
CRMP1.2	1.48E-14	-0.75211	0.016	0.269	2.21E-10	DC	CRMP1
RGS13	1.51E-14	0.218439	0.075	0.012	2.26E-10	DC	RGS13
HIGD2A.2	1.59E-14	0.276107	0.824	0.615	2.37E-10	DC	HIGD2A
MTHFD2.2	1.81E-14	0.420882	0.519	0.285	2.70E-10	DC	MTHFD2
SH3TC1.3	1.85E-14	0.330281	0.235	0.077	2.76E-10	DC	SH3TC1
SNAP23.3	1.91E-14	0.456201	0.455	0.241	2.85E-10	DC	SNAP23
SPTBN1.1	1.95E-14	-0.63914	0.021	0.279	2.92E-10	DC	SPTBN1
FAM159A.2	2.15E-14	0.332874	0.16	0.043	3.21E-10	DC	FAM159A
UNC13D.2	2.28E-14	0.418684	0.166	0.047	3.40E-10	DC	UNC13D
DOCK2.3	2.52E-14	0.423709	0.225	0.075	3.77E-10	DC	DOCK2
ROCK1.3	2.82E-14	0.479761	0.508	0.305	4.22E-10	DC	ROCK1
FYB.3	2.87E-14	0.403237	0.476	0.219	4.28E-10	DC	FYB
LCP2.3	3.16E-14	0.402091	0.294	0.112	4.72E-10	DC	LCP2
LIMA1.2	3.22E-14	-0.71443	0.059	0.322	4.82E-10	DC	LIMA1
APBB1IP.3	3.25E-14	0.302327	0.38	0.153	4.86E-10	DC	APBB1IP
FOSB.2	3.33E-14	0.363135	0.668	0.387	4.97E-10	DC	FOSB
IMPA2.1	3.38E-14	0.346651	0.187	0.058	5.05E-10	DC	IMPA2
GNAI2.3	3.63E-14	0.39715	0.679	0.479	5.42E-10	DC	GNAI2
IL16.3	3.77E-14	0.369515	0.209	0.066	5.63E-10	DC	IL16
MAGED1.2	4.04E-14	-0.66154	0.075	0.349	6.04E-10	DC	MAGED1
FZD3	4.35E-14	-0.61527	0.027	0.28	6.50E-10	DC	FZD3
KCTD12.3	4.70E-14	0.437515	0.455	0.233	7.02E-10	DC	KCTD12
LILRB2.3	4.89E-14	0.378791	0.176	0.051	7.31E-10	DC	LILRB2
CXADR.3	4.94E-14	-0.68498	0.048	0.311	7.39E-10	DC	CXADR
ACSL5.2	5.17E-14	0.272894	0.134	0.033	7.73E-10	DC	ACSL5
GNA13.3	5.46E-14	0.51003	0.374	0.177	8.15E-10	DC	GNA13
PRKAG2.3	5.47E-14	0.439435	0.321	0.134	8.17E-10	DC	PRKAG2
DBNL.3	6.08E-14	0.429277	0.62	0.422	9.09E-10	DC	DBNL
HSD17B11.	6.15E-14	0.355482	0.626	0.373	9.19E-10	DC	HSD17B11
CD300E	6.34E-14	0.162655	0.064	0.009	9.47E-10	DC	CD300E
ZFP36.1	6.50E-14	0.236207	0.909	0.568	9.72E-10	DC	ZFP36
NCF1.3	6.80E-14	0.380268	0.31	0.12	1.02E-09	DC	NCF1
KRT18	7.08E-14	0.184147	0.053	0.007	1.06E-09	DC	KRT18
MILR1.3	7.21E-14	0.400757	0.262	0.096	1.08E-09	DC	MILR1
AP1S2.3	7.31E-14	0.39059	0.701	0.497	1.09E-09	DC	AP1S2
SCG2.2	8.17E-14	-0.78869	0.021	0.266	1.22E-09	DC	SCG2

KCNN4.1	8.27E-14	0.306158	0.128	0.031	1.24E-09	DC	KCNN4
MYL12B.2	8.71E-14	0.20568	0.904	0.724	1.30E-09	DC	MYL12B
LY96.3	9.00E-14	0.404345	0.439	0.219	1.34E-09	DC	LY96
EIF3M.3	9.28E-14	0.334243	0.722	0.523	1.39E-09	DC	EIF3M
EIF3K.2	9.38E-14	0.198809	0.952	0.755	1.40E-09	DC	EIF3K
RAP1A.3	1.03E-13	0.322056	0.727	0.512	1.54E-09	DC	RAP1A
SOX9.2	1.04E-13	-0.6844	0.027	0.276	1.55E-09	DC	SOX9
NRROS.2	1.18E-13	0.389075	0.203	0.066	1.76E-09	DC	NRROS
ANP32B.3	1.20E-13	0.390607	0.652	0.446	1.79E-09	DC	ANP32B
DENND3.3	1.24E-13	0.332514	0.267	0.098	1.85E-09	DC	DENND3
CADM4.2	1.24E-13	-0.59944	0.021	0.267	1.86E-09	DC	CADM4
SKAP2.2	1.34E-13	0.36274	0.513	0.266	2.00E-09	DC	SKAP2
ITGAX.3	1.36E-13	0.403594	0.246	0.088	2.03E-09	DC	ITGAX
ATP6V1B2.	1.52E-13	0.420179	0.615	0.405	2.28E-09	DC	ATP6V1B2
CENPV.3	1.54E-13	-0.66385	0.021	0.259	2.30E-09	DC	CENPV
PELI1.3	1.69E-13	0.517387	0.406	0.206	2.52E-09	DC	PELI1
RNPEP.3	1.78E-13	0.449803	0.417	0.221	2.66E-09	DC	RNPEP
CTD-2006K	1.81E-13	0.190338	0.027	0.002	2.71E-09	DC	CTD-2006K23.1
TKT.1	1.87E-13	0.345981	0.695	0.506	2.79E-09	DC	TKT
TNFSF13B.	1.89E-13	0.379946	0.428	0.2	2.82E-09	DC	TNFSF13B
NFE2L2.2	1.92E-13	0.361243	0.658	0.465	2.87E-09	DC	NFE2L2
UBB.2	2.21E-13	-0.28097	0.936	0.91	3.31E-09	DC	UBB
PER1.2	2.31E-13	0.40203	0.385	0.181	3.45E-09	DC	PER1
KLHDC8A.3	2.41E-13	-0.62005	0.016	0.254	3.60E-09	DC	KLHDC8A
CHST11.3	2.52E-13	0.45203	0.406	0.21	3.77E-09	DC	CHST11
CCR6	2.77E-13	0.228504	0.053	0.007	4.14E-09	DC	CCR6
MIS18BP1.	2.78E-13	0.410818	0.326	0.144	4.15E-09	DC	MIS18BP1
CD4.3	2.97E-13	0.413151	0.353	0.152	4.44E-09	DC	CD4
C16orf45.	3.01E-13	-0.56733	0.059	0.322	4.50E-09	DC	C16orf45
MYCL	3.02E-13	0.272014	0.112	0.026	4.52E-09	DC	MYCL
PIK3R5.3	3.02E-13	0.296941	0.16	0.045	4.52E-09	DC	PIK3R5
APOBEC3A.	3.18E-13	0.199268	0.043	0.005	4.75E-09	DC	APOBEC3A
SNHG8.1	3.33E-13	0.337668	0.679	0.465	4.97E-09	DC	SNHG8
SGCB	3.41E-13	-0.66132	0.07	0.33	5.10E-09	DC	SGCB
ETV1.3	3.43E-13	-0.69778	0.016	0.248	5.12E-09	DC	ETV1
CD300LF.2	3.44E-13	0.361777	0.155	0.044	5.14E-09	DC	CD300LF
EIF3E.2	3.49E-13	0.23865	0.813	0.644	5.21E-09	DC	EIF3E
CYSLTR1.2	3.86E-13	0.285307	0.107	0.024	5.77E-09	DC	CYSLTR1
UBE2D3.2	3.92E-13	0.219559	0.888	0.725	5.86E-09	DC	UBE2D3
PCBP4.3	4.06E-13	-0.60664	0.021	0.259	6.06E-09	DC	PCBP4
RHOF.2	4.24E-13	0.370223	0.166	0.05	6.33E-09	DC	RHOF
DPEP2.2	4.26E-13	0.304503	0.128	0.032	6.36E-09	DC	DPEP2
MT1M.1	4.36E-13	-0.87151	0.016	0.247	6.52E-09	DC	MT1M
QSOX1.3	4.38E-13	0.456299	0.422	0.233	6.54E-09	DC	QSOX1
ENAH.2	4.67E-13	-0.55676	0.027	0.268	6.98E-09	DC	ENAH
NFAM1.2	5.00E-13	0.324546	0.128	0.032	7.47E-09	DC	NFAM1
ZNF467.2	5.07E-13	0.302002	0.128	0.032	7.57E-09	DC	ZNF467
VDR	5.36E-13	0.292519	0.08	0.015	8.00E-09	DC	VDR
LINC00528	5.37E-13	0.139201	0.048	0.006	8.02E-09	DC	LINC00528
PCDH9.1	5.47E-13	-0.71144	0.021	0.259	8.17E-09	DC	PCDH9
SDCBP.1	5.52E-13	0.21902	0.92	0.725	8.25E-09	DC	SDCBP

RP11-796E	5.53E-13	0.498147	0.193	0.063	8.26E-09	DC	RP11-796E2.4
FTH1.3	5.55E-13	-0.19185	0.989	0.932	8.30E-09	DC	FTH1
PPP1CA.2	5.59E-13	0.329682	0.754	0.568	8.35E-09	DC	PPP1CA
CDO1.3	5.87E-13	-0.69881	0.011	0.238	8.78E-09	DC	CDO1
TSPAN5.3	6.01E-13	-0.56377	0.027	0.266	8.98E-09	DC	TSPAN5
GHITM.2	6.50E-13	0.304443	0.711	0.52	9.71E-09	DC	GHITM
DENND1C.2	6.50E-13	0.240173	0.128	0.032	9.71E-09	DC	DENND1C
SPNS3.2	7.10E-13	0.278886	0.102	0.022	1.06E-08	DC	SPNS3
STK4.3	7.44E-13	0.447541	0.481	0.284	1.11E-08	DC	STK4
TCF4.3	8.13E-13	-0.61505	0.283	0.527	1.21E-08	DC	TCF4
RND3.2	8.18E-13	-0.7227	0.016	0.246	1.22E-08	DC	RND3
CHPF.2	8.36E-13	-0.55823	0.027	0.262	1.25E-08	DC	CHPF
RIC3.1	8.57E-13	-0.70668	0.011	0.234	1.28E-08	DC	RIC3
SRI.3	9.10E-13	-0.50135	0.578	0.731	1.36E-08	DC	SRI
EDEM1.2	9.12E-13	0.411802	0.214	0.077	1.36E-08	DC	EDEM1
C12orf57.	9.35E-13	-0.59181	0.267	0.541	1.40E-08	DC	C12orf57
HEY1.2	1.03E-12	-0.63693	0.021	0.25	1.53E-08	DC	HEY1
SUSD3.3	1.03E-12	0.34817	0.251	0.093	1.54E-08	DC	SUSD3
CPE.2	1.04E-12	-0.69489	0.134	0.394	1.55E-08	DC	CPE
SNX10.2	1.05E-12	0.449984	0.417	0.225	1.57E-08	DC	SNX10
TMEM59L.2	1.13E-12	-0.5677	0.027	0.262	1.68E-08	DC	TMEM59L
GPC1.2	1.13E-12	-0.69314	0.016	0.24	1.68E-08	DC	GPC1
CAPZB.2	1.26E-12	0.200293	0.898	0.698	1.88E-08	DC	CAPZB
EGFR.2	1.29E-12	-0.71017	0.059	0.304	1.93E-08	DC	EGFR
CTTN.2	1.30E-12	-0.57189	0.011	0.233	1.94E-08	DC	CTTN
DBN1.3	1.32E-12	-0.60173	0.07	0.315	1.98E-08	DC	DBN1
MANBAL.3	1.34E-12	-0.56387	0.08	0.339	2.00E-08	DC	MANBAL
HPS1.2	1.37E-12	0.359939	0.273	0.108	2.05E-08	DC	HPS1
HSP90AA1.	1.42E-12	-0.29678	0.877	0.888	2.13E-08	DC	HSP90AA1
ATP5C1.2	1.53E-12	0.269062	0.759	0.584	2.28E-08	DC	ATP5C1
IL17RA.3	1.54E-12	0.439219	0.278	0.117	2.30E-08	DC	IL17RA
PGRMC1.1	1.62E-12	-0.53	0.182	0.451	2.42E-08	DC	PGRMC1
SH2B3.3	1.64E-12	0.477945	0.262	0.106	2.45E-08	DC	SH2B3
RAP1B.2	1.64E-12	0.33131	0.62	0.431	2.45E-08	DC	RAP1B
CECR1.3	1.69E-12	0.321329	0.203	0.069	2.52E-08	DC	CECR1
METRN.3	1.75E-12	-0.68058	0.064	0.298	2.61E-08	DC	METRN
NREP.2	1.91E-12	-0.6235	0.043	0.276	2.86E-08	DC	NREP
CSRP2.1	1.92E-12	-0.90429	0.064	0.305	2.87E-08	DC	CSRP2
PTPRS.3	1.95E-12	-0.6279	0.032	0.262	2.92E-08	DC	PTPRS
ZNF302.2	1.96E-12	-0.57077	0.064	0.319	2.93E-08	DC	ZNF302
PTPN1.3	1.97E-12	0.524492	0.401	0.229	2.95E-08	DC	PTPN1
CDC42SE2.	2.01E-12	0.484819	0.396	0.216	3.01E-08	DC	CDC42SE2
MIR22HG.2	2.11E-12	0.436394	0.267	0.111	3.15E-08	DC	MIR22HG
GTF2B.2	2.11E-12	0.431775	0.503	0.297	3.15E-08	DC	GTF2B
POU3F2.2	2.27E-12	-0.59774	0.005	0.219	3.40E-08	DC	POU3F2
POLD4.1	2.30E-12	0.324796	0.642	0.417	3.44E-08	DC	POLD4
ITSN2.3	2.32E-12	0.411864	0.358	0.179	3.46E-08	DC	ITSN2
MT1F.3	2.41E-12	-0.66185	0.064	0.309	3.61E-08	DC	MT1F
OTUD1.3	2.51E-12	0.357858	0.251	0.096	3.75E-08	DC	OTUD1
HNRNPF.2	2.54E-12	0.314012	0.684	0.505	3.79E-08	DC	HNRNPF
TNFAIP8L2	2.71E-12	0.344594	0.246	0.092	4.04E-08	DC	TNFAIP8L2

DNAJC15.1	2.76E-12	0.383737	0.578	0.387	4.13E-08	DC	DNAJC15
PTPN7.3	2.91E-12	0.407078	0.214	0.076	4.35E-08	DC	PTPN7
LRRC25.3	3.03E-12	0.367031	0.241	0.09	4.53E-08	DC	LRRC25
CHN1.2	3.09E-12	-0.52537	0.005	0.219	4.62E-08	DC	CHN1
LRCH4.3	3.12E-12	0.459986	0.369	0.185	4.67E-08	DC	LRCH4
KCNQ2.2	3.20E-12	-0.57333	0.005	0.218	4.78E-08	DC	KCNQ2
EPN2.3	3.24E-12	-0.4715	0.016	0.241	4.84E-08	DC	EPN2
TRIM2.1	3.36E-12	-0.55503	0.011	0.227	5.01E-08	DC	TRIM2
FAM127B.2	3.52E-12	-0.64997	0.064	0.304	5.26E-08	DC	FAM127B
LMO4.3	3.62E-12	-0.57133	0.23	0.482	5.41E-08	DC	LMO4
ALOX15B.2	3.76E-12	0.254674	0.107	0.025	5.62E-08	DC	ALOX15B
CHPT1.2	3.77E-12	0.501285	0.476	0.3	5.64E-08	DC	CHPT1
OSCAR.3	3.78E-12	0.369719	0.203	0.07	5.64E-08	DC	OSCAR
INHBA	4.17E-12	0.366915	0.091	0.019	6.23E-08	DC	INHBA
TMEM161B-	4.36E-12	-0.6689	0.059	0.293	6.51E-08	DC	TMEM161B-AS1
TRIB2.3	4.37E-12	-0.59111	0.037	0.266	6.53E-08	DC	TRIB2
GRIA2.2	4.39E-12	-0.58197	0.005	0.215	6.56E-08	DC	GRIA2
CYTH4.3	4.46E-12	0.313013	0.278	0.109	6.66E-08	DC	CYTH4
IL7R.3	4.70E-12	0.374024	0.166	0.052	7.02E-08	DC	IL7R
TSPAN13.3	4.82E-12	-0.63619	0.064	0.303	7.20E-08	DC	TSPAN13
AC016831.	5.10E-12	0.341816	0.198	0.069	7.63E-08	DC	AC016831.7
FAM181B.3	5.11E-12	-0.60466	0	0.207	7.64E-08	DC	FAM181B
SP140.2	5.15E-12	0.249052	0.118	0.029	7.70E-08	DC	SP140
POLR1D.2	5.39E-12	0.416981	0.594	0.412	8.05E-08	DC	POLR1D
WIPF1.3	5.49E-12	0.41917	0.406	0.217	8.20E-08	DC	WIPF1
ADPGK.3	5.72E-12	0.40496	0.455	0.258	8.54E-08	DC	ADPGK
NRCAM.2	5.80E-12	-0.57743	0.043	0.273	8.67E-08	DC	NRCAM
SLC22A17.	5.81E-12	-0.57093	0.053	0.288	8.68E-08	DC	SLC22A17
LTA4H.2	6.01E-12	0.498859	0.412	0.226	8.98E-08	DC	LTA4H
ERP44.1	6.13E-12	0.321548	0.599	0.392	9.16E-08	DC	ERP44
FKBP10.3	6.33E-12	-0.53754	0.005	0.215	9.46E-08	DC	FKBP10
BNIP2.3	6.61E-12	0.449561	0.444	0.261	9.88E-08	DC	BNIP2
FJX1.2	6.63E-12	-0.58189	0	0.205	9.90E-08	DC	FJX1
CHP1.3	6.98E-12	0.475628	0.406	0.232	1.04E-07	DC	CHP1
MT2A.3	6.98E-12	-0.6852	0.727	0.785	1.04E-07	DC	MT2A
ATP6V0D1.	7.22E-12	0.355892	0.69	0.507	1.08E-07	DC	ATP6V0D1
TMEM98.1	7.26E-12	-0.60358	0.021	0.238	1.09E-07	DC	TMEM98
NDUFA4.2	7.56E-12	-0.27561	0.941	0.894	1.13E-07	DC	NDUFA4
LY75	7.86E-12	0.174281	0.08	0.016	1.18E-07	DC	LY75
NLRP1.2	8.59E-12	-0.55311	0.091	0.343	1.28E-07	DC	NLRP1
TBC1D22A.	8.60E-12	0.461845	0.273	0.12	1.29E-07	DC	TBC1D22A
FIBIN.2	8.75E-12	-0.65208	0.016	0.23	1.31E-07	DC	FIBIN
GPER1	8.82E-12	0.179442	0.048	0.006	1.32E-07	DC	GPER1
CFLAR.2	9.36E-12	0.415963	0.503	0.316	1.40E-07	DC	CFLAR
AP1G2.2	9.42E-12	0.268245	0.16	0.049	1.41E-07	DC	AP1G2
GCSH.3	9.44E-12	-0.55127	0.107	0.351	1.41E-07	DC	GCSH
RBFOX2.2	1.01E-11	-0.56253	0.011	0.219	1.50E-07	DC	RBFOX2
CYB5R4.3	1.02E-11	0.476625	0.342	0.173	1.52E-07	DC	CYB5R4
TTYH1.3	1.04E-11	-0.72651	0.048	0.271	1.56E-07	DC	TTYH1
RBM47.3	1.06E-11	0.434171	0.267	0.114	1.59E-07	DC	RBM47
FNDC4.1	1.07E-11	-0.48081	0	0.201	1.60E-07	DC	FNDC4

ATG16L2.3	1.10E-11	0.329932	0.225	0.085	1.64E-07	DC	ATG16L2
EMILIN2.3	1.14E-11	0.385748	0.209	0.078	1.71E-07	DC	EMILIN2
PBX1.2	1.35E-11	-0.5149	0.011	0.218	2.02E-07	DC	PBX1
DCX.3	1.39E-11	-0.6732	0.005	0.208	2.08E-07	DC	DCX
HES6.2	1.48E-11	-0.72912	0.064	0.289	2.21E-07	DC	HES6
DUSP1.3	1.48E-11	0.310005	0.84	0.57	2.22E-07	DC	DUSP1
NAE1.1	1.49E-11	-0.53936	0.096	0.344	2.23E-07	DC	NAE1
DEPTOR.1	1.49E-11	0.31066	0.144	0.042	2.23E-07	DC	DEPTOR
FGD4.3	1.53E-11	0.434947	0.316	0.144	2.29E-07	DC	FGD4
PTGS1.3	1.63E-11	0.393318	0.332	0.153	2.44E-07	DC	PTGS1
CBFA2T3	1.78E-11	0.130213	0.043	0.005	2.66E-07	DC	CBFA2T3
MEIS2.2	1.82E-11	-0.64181	0.005	0.207	2.72E-07	DC	MEIS2
REEP2.1	1.94E-11	-0.46186	0.005	0.205	2.89E-07	DC	REEP2
PTRHD1	2.02E-11	0.418423	0.492	0.316	3.02E-07	DC	PTRHD1
CBR1.2	2.03E-11	-0.58076	0.225	0.48	3.04E-07	DC	CBR1
LAIR1.3	2.09E-11	0.321222	0.455	0.227	3.12E-07	DC	LAIR1
MAP1LC3A.	2.11E-11	-0.56125	0.128	0.369	3.15E-07	DC	MAP1LC3A
PHF14.2	2.15E-11	-0.50468	0.166	0.427	3.21E-07	DC	PHF14
AP2S1.2	2.18E-11	0.254252	0.824	0.658	3.25E-07	DC	AP2S1
NLRP2	2.29E-11	0.113048	0.048	0.006	3.42E-07	DC	NLRP2
CACUL1.3	2.33E-11	0.461107	0.374	0.203	3.49E-07	DC	CACUL1
CTSW.2	2.37E-11	0.218487	0.112	0.028	3.55E-07	DC	CTSW
PDCL3.2	2.38E-11	0.451503	0.465	0.29	3.55E-07	DC	PDCL3
SYT11.3	2.40E-11	-0.55073	0.139	0.388	3.59E-07	DC	SYT11
TNNI2.2	2.42E-11	0.288992	0.086	0.018	3.62E-07	DC	TNNI2
SLC43A2.3	2.59E-11	0.43704	0.428	0.243	3.87E-07	DC	SLC43A2
HVCN1.3	2.64E-11	0.460242	0.235	0.094	3.95E-07	DC	HVCN1
COL6A1.2	2.68E-11	-0.56458	0.021	0.232	4.01E-07	DC	COL6A1
CKAP4.1	2.72E-11	-0.46178	0.011	0.213	4.06E-07	DC	CKAP4
MB21D2.2	2.73E-11	0.344885	0.16	0.052	4.08E-07	DC	MB21D2
MSI2.1	2.75E-11	-0.60653	0.096	0.325	4.11E-07	DC	MSI2
PGM2L1.2	2.97E-11	-0.57862	0.043	0.259	4.44E-07	DC	PGM2L1
SHKBP1.3	3.22E-11	0.423845	0.406	0.234	4.81E-07	DC	SHKBP1
RAB3IP.1	3.37E-11	-0.622	0.037	0.25	5.04E-07	DC	RAB3IP
POMC.3	3.42E-11	0.324017	0.15	0.045	5.11E-07	DC	POMC
SEMA6A.2	3.49E-11	-0.48338	0.005	0.203	5.21E-07	DC	SEMA6A
CHD7.3	3.82E-11	-0.57628	0.07	0.292	5.70E-07	DC	CHD7
TBC1D10C.	3.96E-11	0.263275	0.112	0.029	5.91E-07	DC	TBC1D10C
CTA-384D8	4.06E-11	0.270146	0.096	0.023	6.06E-07	DC	CTA-384D8.34
EBI3.3	4.21E-11	0.320719	0.241	0.094	6.30E-07	DC	EBI3
NR1H2.3	4.24E-11	0.422621	0.471	0.301	6.34E-07	DC	NR1H2
NCKAP1.1	4.34E-11	-0.44063	0	0.191	6.48E-07	DC	NCKAP1
FMNL2.3	4.34E-11	-0.51054	0.048	0.269	6.49E-07	DC	FMNL2
CD79B	4.36E-11	0.175111	0.043	0.005	6.51E-07	DC	CD79B
RHOA.1	4.39E-11	0.180209	0.92	0.752	6.57E-07	DC	RHOA
MT1X.3	4.55E-11	-0.90276	0.364	0.581	6.80E-07	DC	MT1X
TMEM54.1	4.86E-11	-0.46275	0.005	0.198	7.26E-07	DC	TMEM54
S100Z	5.12E-11	0.208774	0.064	0.011	7.65E-07	DC	S100Z
HPRT1.3	5.19E-11	0.375466	0.417	0.227	7.76E-07	DC	HPRT1
SLAIN1.3	5.29E-11	-0.59417	0.011	0.206	7.91E-07	DC	SLAIN1
SCOC.2	5.32E-11	-0.52564	0.171	0.418	7.95E-07	DC	SCOC

C1orf122	5.46E-11	-0.54577	0.251	0.492	8.16E-07	DC	C1orf122
PRKCD.3	5.55E-11	0.340873	0.209	0.078	8.30E-07	DC	PRKCD
MSR1.3	5.90E-11	0.351703	0.374	0.177	8.82E-07	DC	MSR1
FOXN3.3	5.99E-11	0.378953	0.439	0.252	8.95E-07	DC	FOXN3
ASCL1.2	6.37E-11	-0.61918	0	0.189	9.53E-07	DC	ASCL1
WBP1L.3	6.54E-11	0.377371	0.251	0.107	9.78E-07	DC	WBP1L
RUFY3.2	6.57E-11	-0.51694	0.08	0.309	9.82E-07	DC	RUFY3
KLHL6.3	6.67E-11	0.271698	0.155	0.049	9.97E-07	DC	KLHL6
NAALADL1	6.82E-11	0.284256	0.08	0.017	1.02E-06	DC	NAALADL1
KIF1B.2	7.30E-11	-0.53935	0.075	0.297	1.09E-06	DC	KIF1B
TMEM106B	7.32E-11	-0.55963	0.102	0.334	1.09E-06	DC	TMEM106B
TNFSF14	7.38E-11	0.197915	0.059	0.01	1.10E-06	DC	TNFSF14
ANKRD44.2	7.61E-11	0.265414	0.235	0.093	1.14E-06	DC	ANKRD44
SOCS3.2	7.87E-11	0.360296	0.556	0.345	1.18E-06	DC	SOCS3
KIAA0513	8.29E-11	0.37195	0.139	0.043	1.24E-06	DC	KIAA0513
NIPAL2	8.34E-11	0.249587	0.07	0.013	1.25E-06	DC	NIPAL2
MXD1.2	8.38E-11	0.416789	0.278	0.126	1.25E-06	DC	MXD1
PSME1.2	8.61E-11	0.203661	0.781	0.58	1.29E-06	DC	PSME1
GALNT3	8.84E-11	0.170154	0.043	0.006	1.32E-06	DC	GALNT3
F3.3	9.12E-11	-0.5585	0.005	0.194	1.36E-06	DC	F3
PICK1.2	9.25E-11	0.404289	0.214	0.086	1.38E-06	DC	PICK1
S100A9.3	9.37E-11	0.397255	0.417	0.221	1.40E-06	DC	S100A9
NDFIP2.1	9.51E-11	-0.48657	0.005	0.195	1.42E-06	DC	NDFIP2
PNRC1.3	9.96E-11	0.318687	0.706	0.537	1.49E-06	DC	PNRC1
PSRC1.1	9.97E-11	-0.6052	0.053	0.267	1.49E-06	DC	PSRC1
MYD88.1	1.01E-10	0.460848	0.273	0.128	1.52E-06	DC	MYD88
PABPC3	1.02E-10	0.250548	0.096	0.023	1.53E-06	DC	PABPC3
ID4.2	1.03E-10	-0.62491	0.021	0.218	1.53E-06	DC	ID4
RP11-620J	1.07E-10	-0.64613	0.059	0.273	1.60E-06	DC	RP11-620J15.3
RP11-553L	1.09E-10	-0.47322	0.027	0.229	1.63E-06	DC	RP11-553L6.5
C1QA.3	1.09E-10	0.224199	0.824	0.533	1.63E-06	DC	C1QA
MARCH1.3	1.10E-10	0.367676	0.374	0.187	1.64E-06	DC	1-Mar
NFKBIZ.3	1.11E-10	0.402809	0.342	0.172	1.66E-06	DC	NFKBIZ
SEMA4A.3	1.12E-10	0.329533	0.15	0.048	1.67E-06	DC	SEMA4A
PSMB10.3	1.12E-10	0.414196	0.444	0.252	1.67E-06	DC	PSMB10
DKK3.1	1.12E-10	-0.47449	0.011	0.203	1.68E-06	DC	DKK3
RDX.2	1.14E-10	-0.48645	0.251	0.494	1.70E-06	DC	RDX
AP001055.	1.15E-10	0.318455	0.209	0.078	1.72E-06	DC	AP001055.6
JAM2.3	1.15E-10	-0.54386	0.043	0.256	1.72E-06	DC	JAM2
MRFAP1.2	1.15E-10	-0.46016	0.481	0.674	1.72E-06	DC	MRFAP1
PCDH17.1	1.20E-10	-0.47531	0.005	0.194	1.79E-06	DC	PCDH17
MAPK8IP1.	1.21E-10	-0.49417	0.005	0.194	1.80E-06	DC	MAPK8IP1
SIGLEC7.3	1.21E-10	0.329679	0.198	0.072	1.81E-06	DC	SIGLEC7
CSF2RB.2	1.24E-10	0.220316	0.096	0.023	1.85E-06	DC	CSF2RB
RGMA.2	1.28E-10	-0.50216	0.011	0.203	1.92E-06	DC	RGMA
BCAN.3	1.40E-10	-0.78164	0.07	0.279	2.10E-06	DC	BCAN
HAUS4.3	1.43E-10	0.389621	0.171	0.059	2.14E-06	DC	HAUS4
MYO6.1	1.44E-10	-0.49759	0.027	0.228	2.15E-06	DC	MYO6
GAMT.3	1.45E-10	-0.46512	0.075	0.303	2.17E-06	DC	GAMT
TAGLN3.2	1.47E-10	-0.65207	0.005	0.191	2.20E-06	DC	TAGLN3
TTLL7.3	1.48E-10	-0.45715	0.032	0.241	2.21E-06	DC	TTLL7

MAGEF1.2	1.50E-10	-0.4873	0.128	0.367	2.25E-06	DC	MAGEF1
MAGI1.3	1.51E-10	-0.52145	0.011	0.2	2.26E-06	DC	MAGI1
HBA2.2	1.64E-10	0.312234	0.369	0.195	2.45E-06	DC	HBA2
CXCL2.1	1.66E-10	0.342139	0.219	0.084	2.48E-06	DC	CXCL2
DDOST.1	1.66E-10	0.313004	0.631	0.462	2.48E-06	DC	DDOST
FUOM.3	1.67E-10	0.383427	0.299	0.138	2.50E-06	DC	FUOM
HEPN1.2	1.71E-10	-0.64999	0.016	0.207	2.56E-06	DC	HEPN1
H2AFJ.3	1.74E-10	0.400672	0.439	0.256	2.60E-06	DC	H2AFJ
RP11-442H	1.83E-10	0.249679	0.059	0.01	2.73E-06	DC	RP11-442H21.2
MEX3A.2	1.84E-10	-0.49438	0	0.181	2.75E-06	DC	MEX3A
PICALM.3	1.84E-10	0.434819	0.455	0.278	2.76E-06	DC	PICALM
WASF2.3	1.85E-10	0.290684	0.626	0.435	2.77E-06	DC	WASF2
TET2.2	1.87E-10	0.387387	0.214	0.086	2.79E-06	DC	TET2
GNGT2.3	1.89E-10	0.30727	0.155	0.051	2.82E-06	DC	GNGT2
DEXI.1	1.89E-10	-0.4249	0.016	0.21	2.82E-06	DC	DEXI
PSAT1.2	1.91E-10	-0.57548	0.016	0.206	2.85E-06	DC	PSAT1
FMNL3.3	1.95E-10	0.3811	0.198	0.075	2.91E-06	DC	FMNL3
KNOP1.2	2.14E-10	-0.50264	0.053	0.263	3.20E-06	DC	KNOP1
EFEMP2.1	2.24E-10	-0.47557	0.021	0.217	3.35E-06	DC	EFEMP2
PIP4K2A.3	2.24E-10	0.378191	0.289	0.135	3.35E-06	DC	PIP4K2A
MGAT4A.3	2.47E-10	0.336258	0.417	0.217	3.70E-06	DC	MGAT4A
TM7SF2.2	2.65E-10	-0.44325	0.032	0.234	3.96E-06	DC	TM7SF2
CD7.2	2.65E-10	0.194681	0.139	0.042	3.96E-06	DC	CD7
TRAF4.2	2.71E-10	-0.44401	0.059	0.272	4.05E-06	DC	TRAF4
CLU.3	2.72E-10	-0.62948	0.69	0.784	4.07E-06	DC	CLU
VGLL4.1	2.77E-10	-0.47715	0.096	0.321	4.13E-06	DC	VGLL4
S100A8.3	2.80E-10	0.372642	0.364	0.182	4.18E-06	DC	S100A8
NAMPT.3	2.83E-10	0.145918	0.674	0.434	4.22E-06	DC	NAMPT
PPIC.1	2.88E-10	-0.50654	0.005	0.186	4.30E-06	DC	PPIC
RAMP1.2	2.95E-10	-0.81032	0.193	0.415	4.40E-06	DC	RAMP1
PAFAH1B3.	3.06E-10	-0.46775	0.187	0.428	4.57E-06	DC	PAFAH1B3
CHD1.3	3.18E-10	0.471152	0.444	0.29	4.75E-06	DC	CHD1
SV2A.1	3.25E-10	-0.42486	0.016	0.205	4.85E-06	DC	SV2A
AK1.1	3.37E-10	-0.51035	0.107	0.339	5.04E-06	DC	AK1
IL15.1	3.54E-10	0.261129	0.123	0.036	5.29E-06	DC	IL15
CRYAB.3	3.55E-10	-0.95487	0.289	0.503	5.31E-06	DC	CRYAB
KMO	3.59E-10	0.2157	0.08	0.018	5.36E-06	DC	KMO
CCDC57.3	3.67E-10	0.424186	0.241	0.105	5.49E-06	DC	CCDC57
CMTM3.3	3.78E-10	0.400051	0.487	0.321	5.65E-06	DC	CMTM3
NRN1.1	3.91E-10	-0.54447	0.048	0.257	5.84E-06	DC	NRN1
PPP2R2B.2	4.07E-10	-0.53098	0.027	0.222	6.09E-06	DC	PPP2R2B
HRAS.3	4.11E-10	-0.4584	0.08	0.297	6.14E-06	DC	HRAS
PVRL2.3	4.14E-10	0.458441	0.401	0.241	6.19E-06	DC	PVRL2
MYADM.2	4.22E-10	0.371177	0.353	0.19	6.31E-06	DC	MYADM
RP11-108M	4.31E-10	0.374172	0.235	0.1	6.45E-06	DC	RP11-108M9.4
ZFAND5.3	4.61E-10	0.358144	0.604	0.46	6.89E-06	DC	ZFAND5
DSEL.3	4.74E-10	-0.50474	0.032	0.228	7.08E-06	DC	DSEL
TGFB1.3	4.80E-10	0.371715	0.417	0.242	7.17E-06	DC	TGFB1
PSIP1.3	4.84E-10	-0.4752	0.155	0.387	7.23E-06	DC	PSIP1
EIF3H.1	4.88E-10	0.223895	0.813	0.666	7.29E-06	DC	EIF3H
EIF3D.2	4.93E-10	0.300091	0.636	0.474	7.37E-06	DC	EIF3D

KBTBD8.1	4.96E-10	0.24967	0.107	0.029	7.41E-06	DC	KBTBD8
GTF3A.3	4.97E-10	0.317336	0.652	0.502	7.43E-06	DC	GTF3A
TAX1BP3.2	4.98E-10	0.374594	0.545	0.379	7.45E-06	DC	TAX1BP3
POP7.2	5.05E-10	-0.48137	0.16	0.398	7.55E-06	DC	POP7
MAP4K4.1	5.14E-10	-0.46317	0.048	0.251	7.67E-06	DC	MAP4K4
ITGB8.1	5.28E-10	-0.43667	0.043	0.243	7.89E-06	DC	ITGB8
NANS.3	5.32E-10	0.316099	0.551	0.357	7.95E-06	DC	NANS
CSDE1.3	5.41E-10	0.247336	0.749	0.599	8.08E-06	DC	CSDE1
CD69.3	5.52E-10	0.248845	0.332	0.15	8.24E-06	DC	CD69
GLIPR2.2	5.67E-10	0.425886	0.433	0.266	8.47E-06	DC	GLIPR2
KHDRBS3.3	5.76E-10	-0.47139	0.027	0.218	8.61E-06	DC	KHDRBS3
PPIAL4G	5.81E-10	0.123438	0.043	0.006	8.68E-06	DC	PPIAL4G
TCEA2.2	5.84E-10	-0.51319	0.144	0.372	8.72E-06	DC	TCEA2
APC.3	5.94E-10	-0.4556	0.027	0.216	8.87E-06	DC	APC
DCK.3	5.99E-10	0.387488	0.433	0.263	8.96E-06	DC	DCK
DYNLL1.2	6.02E-10	-0.30595	0.824	0.836	9.00E-06	DC	DYNLL1
PJA2.2	6.12E-10	-0.48	0.118	0.339	9.15E-06	DC	PJA2
TRIM9.2	6.28E-10	-0.42947	0.037	0.236	9.38E-06	DC	TRIM9
ANXA7.2	6.36E-10	0.365976	0.508	0.336	9.50E-06	DC	ANXA7
HSD11B1L.	6.52E-10	-0.417	0.021	0.208	9.74E-06	DC	HSD11B1L
OLIG2.2	6.59E-10	-0.57873	0.005	0.181	9.85E-06	DC	OLIG2
SLC7A5.3	6.60E-10	0.405079	0.289	0.136	9.86E-06	DC	SLC7A5
PLS3.2	6.88E-10	-0.51839	0.032	0.224	1.03E-05	DC	PLS3
C10orf11.	6.95E-10	0.325884	0.235	0.099	1.04E-05	DC	C10orf11
EMB.3	7.00E-10	0.362092	0.193	0.073	1.05E-05	DC	EMB
GGA2.3	7.14E-10	0.334955	0.289	0.137	1.07E-05	DC	GGA2
SP110.2	7.36E-10	0.330733	0.364	0.195	1.10E-05	DC	SP110
SDC3.1	7.98E-10	-0.50087	0.016	0.199	1.19E-05	DC	SDC3
SOX8.3	8.05E-10	-0.53047	0.005	0.18	1.20E-05	DC	SOX8
SCRG1.2	8.12E-10	-0.66874	0.139	0.349	1.21E-05	DC	SCRG1
TMEM39A.1	8.26E-10	0.361618	0.214	0.089	1.23E-05	DC	TMEM39A
CNDP2.3	8.69E-10	0.310794	0.588	0.428	1.30E-05	DC	CNDP2
FARP1.1	8.76E-10	-0.43473	0.011	0.189	1.31E-05	DC	FARP1
RNF24	8.77E-10	-0.42673	0.048	0.252	1.31E-05	DC	RNF24
ALCAM.3	8.78E-10	0.409019	0.353	0.193	1.31E-05	DC	ALCAM
ACY3	8.79E-10	0.26383	0.08	0.018	1.31E-05	DC	ACY3
FCGR3A.3	8.81E-10	0.255161	0.61	0.337	1.32E-05	DC	FCGR3A
MYO10.2	8.92E-10	-0.47165	0.016	0.197	1.33E-05	DC	MYO10
SPAG16.3	9.00E-10	-0.49747	0.086	0.299	1.34E-05	DC	SPAG16
RP3-47704	9.10E-10	0.277673	0.102	0.027	1.36E-05	DC	RP3-47704.14
IP6K2.2	9.39E-10	-0.53335	0.203	0.438	1.40E-05	DC	IP6K2
MAPK10.2	9.62E-10	-0.45435	0.016	0.198	1.44E-05	DC	MAPK10
GIT2.3	9.80E-10	0.37857	0.299	0.151	1.46E-05	DC	GIT2
RP11-110C	9.82E-10	0.19893	0.07	0.014	1.47E-05	DC	RP11-1100L3.8
BMP7.3	9.90E-10	-0.43038	0	0.168	1.48E-05	DC	BMP7
PTPRA.2	1.02E-09	-0.45786	0.134	0.358	1.52E-05	DC	PTPRA
HNRNPA1L2	1.04E-09	0.410194	0.353	0.198	1.55E-05	DC	HNRNPA1L2
PTRF.3	1.09E-09	-0.45377	0.011	0.188	1.63E-05	DC	PTRF
PNMA1.2	1.11E-09	-0.43683	0.075	0.287	1.65E-05	DC	PNMA1
ATG3.3	1.12E-09	0.337392	0.524	0.375	1.67E-05	DC	ATG3
IL1RN.3	1.13E-09	0.346127	0.193	0.073	1.69E-05	DC	IL1RN

LARP6.2	1.15E-09	-0.48184	0.016	0.196	1.71E-05	DC	LARP6
PPP2CB.3	1.15E-09	-0.46748	0.091	0.3	1.72E-05	DC	PPP2CB
PGAP1.2	1.21E-09	-0.42233	0.011	0.186	1.80E-05	DC	PGAP1
JOSD1.3	1.24E-09	0.462465	0.299	0.155	1.85E-05	DC	JOSD1
HMGA1.3	1.27E-09	0.389129	0.497	0.343	1.90E-05	DC	HMGA1
EIF4A3.3	1.28E-09	0.370201	0.594	0.447	1.91E-05	DC	EIF4A3
NME1-NME2	1.33E-09	0.364419	0.214	0.092	1.99E-05	DC	NME1-NME2
BST2.2	1.35E-09	0.1911	0.781	0.481	2.01E-05	DC	BST2
SYNE2.3	1.35E-09	-0.43096	0.005	0.177	2.02E-05	DC	SYNE2
RAC3.2	1.35E-09	-0.3885	0.011	0.186	2.02E-05	DC	RAC3
MS4A4E	1.39E-09	0.118477	0.021	0.002	2.08E-05	DC	MS4A4E
WLS.1	1.39E-09	-0.45561	0.032	0.217	2.08E-05	DC	WLS
DNAJB2.2	1.39E-09	-0.42746	0.096	0.318	2.08E-05	DC	DNAJB2
STMN2.3	1.40E-09	-0.94044	0.016	0.189	2.09E-05	DC	STMN2
VPS26A.2	1.41E-09	0.407135	0.401	0.24	2.10E-05	DC	VPS26A
MFNG.3	1.42E-09	0.271462	0.278	0.124	2.13E-05	DC	MFNG
SLC8A1.2	1.53E-09	0.360108	0.289	0.142	2.28E-05	DC	SLC8A1
LYPD1.1	1.60E-09	-0.52634	0.021	0.198	2.39E-05	DC	LYPD1
FAM92A1.1	1.60E-09	-0.40729	0.021	0.204	2.40E-05	DC	FAM92A1
WDR1.2	1.61E-09	0.338847	0.567	0.412	2.40E-05	DC	WDR1
EZR.3	1.62E-09	0.356388	0.503	0.34	2.43E-05	DC	EZR
PRRX1.3	1.73E-09	-0.44713	0.005	0.174	2.58E-05	DC	PRRX1
TAOK3.2	1.73E-09	0.336674	0.54	0.384	2.58E-05	DC	TAOK3
ENC1.3	1.76E-09	-0.50159	0.037	0.227	2.62E-05	DC	ENC1
LRRC8C.3	1.76E-09	0.275735	0.16	0.057	2.63E-05	DC	LRRC8C
AP2B1.2	1.77E-09	-0.40342	0.118	0.344	2.64E-05	DC	AP2B1
TRIM47.3	1.78E-09	-0.44017	0.011	0.182	2.65E-05	DC	TRIM47
RTN3.1	1.85E-09	-0.41898	0.465	0.658	2.76E-05	DC	RTN3
KLHL23.3	1.89E-09	-0.44206	0	0.164	2.82E-05	DC	KLHL23
NAT14	1.90E-09	-0.38616	0.011	0.182	2.84E-05	DC	NAT14
CFD.3	1.91E-09	0.33062	0.321	0.153	2.85E-05	DC	CFD
FDX1L.1	1.92E-09	-0.4305	0.059	0.262	2.88E-05	DC	FDX1L
CADM2.2	1.97E-09	-0.51036	0.016	0.191	2.95E-05	DC	CADM2
CDC42.2	1.99E-09	0.197999	0.77	0.618	2.97E-05	DC	CDC42
FLII.3	2.00E-09	0.447441	0.332	0.185	2.99E-05	DC	FLII
SH3BGRL3	2.01E-09	0.149016	0.936	0.775	3.01E-05	DC	SH3BGRL3
NIPSNAP3E	2.02E-09	0.157806	0.07	0.015	3.02E-05	DC	NIPSNAP3B
TJP1.1	2.02E-09	-0.38089	0.011	0.183	3.02E-05	DC	TJP1
CRNDE	2.05E-09	-0.41134	0.011	0.182	3.07E-05	DC	CRNDE
TRAM1.3	2.08E-09	0.305027	0.599	0.431	3.10E-05	DC	TRAM1
F2R.3	2.08E-09	-0.40433	0.005	0.171	3.11E-05	DC	F2R
FLI1.3	2.09E-09	0.222401	0.15	0.05	3.12E-05	DC	FLI1
OCIAD2.2	2.16E-09	-0.90305	0.209	0.39	3.23E-05	DC	OCIAD2
PCBP2.2	2.23E-09	0.238202	0.813	0.66	3.33E-05	DC	PCBP2
APBA2.3	2.25E-09	-0.40497	0.027	0.21	3.36E-05	DC	APBA2
BEX2.2	2.28E-09	-0.67629	0.07	0.26	3.40E-05	DC	BEX2
GADD45G.2	2.31E-09	-0.58716	0.064	0.258	3.45E-05	DC	GADD45G
MAPRE3.1	2.43E-09	-0.40208	0.011	0.182	3.63E-05	DC	MAPRE3
KIF3A.1	2.44E-09	-0.4899	0.048	0.237	3.64E-05	DC	KIF3A
MAGED2.3	2.45E-09	-0.46026	0.278	0.512	3.67E-05	DC	MAGED2
BCHE.1	2.45E-09	-0.47395	0.016	0.189	3.67E-05	DC	BCHE

IGFBP5.3	2.50E-09	-0.78239	0.075	0.271	3.74E-05	DC	IGFBP5
CAMK2D.1	2.56E-09	-0.46913	0.053	0.247	3.83E-05	DC	CAMK2D
DCLK2.3	2.58E-09	-0.41532	0.021	0.199	3.85E-05	DC	DCLK2
RP11-138A	2.65E-09	0.325285	0.225	0.097	3.95E-05	DC	RP11-138A9.2
RBPJ.3	2.65E-09	0.339885	0.615	0.467	3.96E-05	DC	RBPJ
GRIA3.2	2.71E-09	-0.39099	0.005	0.171	4.04E-05	DC	GRIA3
ATP6AP1.3	2.77E-09	0.270884	0.642	0.49	4.13E-05	DC	ATP6AP1
CD72.2	2.86E-09	0.308408	0.144	0.049	4.27E-05	DC	CD72
GHRL.3	2.90E-09	0.278773	0.155	0.055	4.33E-05	DC	GHRL
SPARCL1.2	2.92E-09	-0.78764	0.15	0.349	4.36E-05	DC	SPARCL1
RNF180.2	2.93E-09	-0.40351	0.005	0.17	4.37E-05	DC	RNF180
RP11-161M	2.95E-09	-0.51959	0.005	0.169	4.41E-05	DC	RP11-161M6.2
ZFP36L2.3	2.98E-09	0.26655	0.69	0.513	4.45E-05	DC	ZFP36L2
ARRDC1.3	3.00E-09	0.43143	0.267	0.132	4.48E-05	DC	ARRDC1
RNASE2.3	3.00E-09	0.342974	0.321	0.155	4.48E-05	DC	RNASE2
RCN2.2	3.06E-09	-0.43916	0.262	0.47	4.58E-05	DC	RCN2
FAIM2.2	3.07E-09	-0.55197	0.021	0.194	4.58E-05	DC	FAIM2
RAB31.3	3.07E-09	0.315069	0.615	0.457	4.59E-05	DC	RAB31
TMEM9.3	3.08E-09	-0.48817	0.144	0.371	4.60E-05	DC	TMEM9
CBS.2	3.08E-09	-0.38192	0	0.16	4.61E-05	DC	CBS
KLHL7.1	3.14E-09	-0.45407	0.08	0.282	4.70E-05	DC	KLHL7
TMEM14A.2	3.24E-09	-0.49889	0.112	0.327	4.85E-05	DC	TMEM14A
IDH2.3	3.27E-09	0.321015	0.583	0.424	4.89E-05	DC	IDH2
SNCA.3	3.32E-09	0.235212	0.305	0.141	4.96E-05	DC	SNCA
EIF2S3.1	3.35E-09	0.409588	0.444	0.292	5.01E-05	DC	EIF2S3
C3orf14.1	3.38E-09	-0.49846	0.07	0.268	5.05E-05	DC	C3orf14
MGST2.3	3.39E-09	0.239582	0.449	0.25	5.07E-05	DC	MGST2
TUBB.1	3.44E-09	-0.34581	0.722	0.771	5.14E-05	DC	TUBB
MORN2.3	3.46E-09	-0.45415	0.064	0.265	5.18E-05	DC	MORN2
MYH9.3	3.48E-09	0.492543	0.337	0.186	5.21E-05	DC	MYH9
SOCS2.2	3.49E-09	-0.42008	0	0.159	5.22E-05	DC	SOCS2
FOXP1.1	3.58E-09	0.209898	0.412	0.227	5.35E-05	DC	FOXP1
SLC39A6.1	3.65E-09	-0.45557	0.118	0.332	5.45E-05	DC	SLC39A6
MAPKAPK3.	3.73E-09	0.406009	0.316	0.168	5.57E-05	DC	MAPKAPK3
COMMD8.3	3.74E-09	0.372655	0.38	0.222	5.59E-05	DC	COMMD8
CD93.3	3.81E-09	0.319937	0.182	0.069	5.70E-05	DC	CD93
PHGDH.2	3.87E-09	-0.56185	0.027	0.202	5.78E-05	DC	PHGDH
FAM213A.2	3.88E-09	-0.48471	0.16	0.386	5.80E-05	DC	FAM213A
POLR2I.2	3.91E-09	-0.4724	0.326	0.556	5.85E-05	DC	POLR2I
GPRC5B.1	3.94E-09	-0.48337	0.037	0.222	5.89E-05	DC	GPRC5B
MVP.1	3.95E-09	0.389578	0.38	0.223	5.90E-05	DC	MVP
SH3BP5.3	3.95E-09	0.446274	0.342	0.199	5.90E-05	DC	SH3BP5
C8orf4.2	4.10E-09	-0.87445	0.048	0.231	6.12E-05	DC	C8orf4
ACP5.3	4.17E-09	0.251422	0.139	0.046	6.23E-05	DC	ACP5
NRXN1.2	4.20E-09	-0.58528	0.005	0.165	6.28E-05	DC	NRXN1
PROX1.2	4.21E-09	-0.43114	0.005	0.167	6.28E-05	DC	PROX1
RAPGEF1.1	4.23E-09	0.447857	0.246	0.115	6.33E-05	DC	RAPGEF1
TCEAL2.1	4.28E-09	-0.6276	0.016	0.182	6.39E-05	DC	TCEAL2
C16orf54.	4.29E-09	0.196033	0.07	0.015	6.41E-05	DC	C16orf54
SAP30.3	4.29E-09	0.45421	0.38	0.238	6.42E-05	DC	SAP30
STAC3.3	4.45E-09	0.380499	0.193	0.077	6.65E-05	DC	STAC3

NRSN2.2	4.47E-09	-0.35826	0.005	0.167	6.68E-05	DC	NRSN2
MED31	4.50E-09	-0.45184	0.037	0.219	6.72E-05	DC	MED31
PROCR.2	4.51E-09	0.291517	0.176	0.067	6.74E-05	DC	PROCR
C1orf21.3	4.54E-09	-0.44395	0.027	0.203	6.78E-05	DC	C1orf21
TMEM156.3	4.65E-09	0.242615	0.155	0.054	6.95E-05	DC	TMEM156
POU3F3.2	4.69E-09	-0.38889	0	0.157	7.01E-05	DC	POU3F3
WDR34.1	4.70E-09	-0.41808	0.032	0.21	7.02E-05	DC	WDR34
SLCO4A1.1	4.97E-09	0.258062	0.112	0.034	7.43E-05	DC	SLCO4A1
OLIG1.3	4.98E-09	-0.63616	0.016	0.183	7.44E-05	DC	OLIG1
PGK1.1	5.01E-09	0.143726	0.914	0.751	7.48E-05	DC	PGK1
TOM1.3	5.01E-09	0.449206	0.299	0.158	7.49E-05	DC	TOM1
LHFP.3	5.02E-09	-0.51995	0.048	0.235	7.50E-05	DC	LHFP
TNRC6C.2	5.17E-09	-0.41217	0.011	0.174	7.72E-05	DC	TNRC6C
NINJ2.3	5.22E-09	0.257447	0.209	0.085	7.80E-05	DC	NINJ2
TMSB15B.2	5.30E-09	-0.41821	0.016	0.184	7.92E-05	DC	TMSB15B
FOXG1.2	5.38E-09	-0.35166	0	0.156	8.03E-05	DC	FOXG1
ANKRD22.3	5.57E-09	0.245925	0.171	0.063	8.33E-05	DC	ANKRD22
PCGF2.1	5.61E-09	-0.36523	0.005	0.165	8.38E-05	DC	PCGF2
PLEKHB1.1	5.80E-09	-0.56107	0.037	0.216	8.66E-05	DC	PLEKHB1
RP11-395G	5.90E-09	-0.45395	0.016	0.18	8.82E-05	DC	RP11-395G23.3
MAGEH1.2	5.97E-09	-0.48703	0.112	0.321	8.93E-05	DC	MAGEH1
GRB2.3	6.05E-09	0.299352	0.636	0.478	9.03E-05	DC	GRB2
ICAM1.2	6.12E-09	0.343966	0.316	0.161	9.14E-05	DC	ICAM1
ENSA.1	6.19E-09	0.246228	0.652	0.498	9.25E-05	DC	ENSA
CD164.2	6.22E-09	0.263112	0.652	0.488	9.30E-05	DC	CD164
CFL1.2	6.33E-09	-0.18744	0.995	0.949	9.46E-05	DC	CFL1
ENO2.3	6.34E-09	-0.4581	0.086	0.287	9.48E-05	DC	ENO2
IMMP1L.2	6.40E-09	-0.4443	0.048	0.235	9.56E-05	DC	IMMP1L
TNFSF8	6.41E-09	0.154861	0.048	0.008	9.57E-05	DC	TNFSF8
ZFHX4.3	6.44E-09	-0.35841	0	0.154	9.62E-05	DC	ZFHX4
MAP9.1	6.44E-09	-0.40992	0.021	0.191	9.63E-05	DC	MAP9
COL9A3.3	6.48E-09	-0.53304	0.021	0.189	9.68E-05	DC	COL9A3
THY1.2	6.60E-09	-0.48155	0.064	0.259	9.86E-05	DC	THY1
NSG1.3	6.61E-09	-0.6771	0.016	0.178	9.88E-05	DC	NSG1
GUSB.2	6.75E-09	0.331562	0.433	0.269	0.000101	DC	GUSB
DPP6.1	6.80E-09	-0.41006	0.005	0.164	0.000102	DC	DPP6
NBL1.3	6.94E-09	-0.44596	0.016	0.181	0.000104	DC	NBL1
DCLK1.1	6.97E-09	-0.40581	0	0.154	0.000104	DC	DCLK1
JAKMIP2.1	7.21E-09	-0.37885	0	0.153	0.000108	DC	JAKMIP2
BASP1.3	7.36E-09	0.394568	0.54	0.37	0.00011	DC	BASP1
GLTSCR2.3	7.37E-09	0.254578	0.69	0.562	0.00011	DC	GLTSCR2
ECHDC3.2	7.38E-09	0.263743	0.107	0.032	0.00011	DC	ECHDC3
GMIP.3	7.39E-09	0.324206	0.182	0.072	0.000111	DC	GMIP
SCPEP1.2	7.46E-09	0.273887	0.545	0.343	0.000111	DC	SCPEP1
NPAS3.3	7.70E-09	-0.39243	0.005	0.161	0.000115	DC	NPAS3
TCTEX1D2.	7.82E-09	-0.48264	0.048	0.23	0.000117	DC	TCTEX1D2
SLC41A2.1	7.93E-09	0.300939	0.128	0.044	0.000118	DC	SLC41A2
CTSF.2	8.00E-09	-0.43443	0.08	0.273	0.00012	DC	CTSF
G0S2.2	8.10E-09	0.482576	0.332	0.182	0.000121	DC	G0S2
EIF1.3	8.11E-09	-0.25169	1	0.974	0.000121	DC	EIF1
RP3-525N1	8.25E-09	-0.46415	0	0.152	0.000123	DC	RP3-525N10.2

ATP8B4.2	8.41E-09	0.252113	0.128	0.042	0.000126	DC	ATP8B4
CTXN1.3	8.52E-09	-0.35045	0.005	0.162	0.000127	DC	CTXN1
AKAP12.3	8.52E-09	-0.44037	0.021	0.19	0.000127	DC	AKAP12
C9orf72.3	8.55E-09	0.383925	0.289	0.148	0.000128	DC	C9orf72
HEXA.2	8.57E-09	0.298558	0.551	0.387	0.000128	DC	HEXA
PRR7.3	8.58E-09	-0.35939	0.021	0.191	0.000128	DC	PRR7
GPNMB.3	9.06E-09	0.296019	0.374	0.198	0.000135	DC	GPNMB
TRIB1.2	9.28E-09	0.370499	0.299	0.154	0.000139	DC	TRIB1
MXRA7.1	9.38E-09	-0.39232	0.011	0.17	0.00014	DC	MXRA7
ERP29	9.78E-09	0.210009	0.754	0.609	0.000146	DC	ERP29
ARPC1A.3	9.78E-09	-0.43967	0.348	0.562	0.000146	DC	ARPC1A
OLFM2.1	9.87E-09	-0.50684	0.027	0.194	0.000147	DC	OLFM2
ZDHHC9.3	1.03E-08	-0.46354	0.011	0.17	0.000155	DC	ZDHHC9
RP11-443E	1.05E-08	0.122033	0.059	0.012	0.000157	DC	RP11-443B7.1
ZNF462.3	1.07E-08	-0.41591	0.016	0.179	0.00016	DC	ZNF462
MICAL1.3	1.08E-08	0.367475	0.278	0.139	0.000161	DC	MICAL1
SEMA6D.3	1.09E-08	-0.4127	0.005	0.159	0.000163	DC	SEMA6D
FNBP1L.3	1.11E-08	-0.40871	0.021	0.187	0.000166	DC	FNBP1L
ODC1.2	1.11E-08	-0.4831	0.198	0.397	0.000166	DC	ODC1
NNT-AS1.2	1.12E-08	-0.40049	0.043	0.224	0.000167	DC	NNT-AS1
PSD3.2	1.13E-08	-0.39188	0.027	0.197	0.000169	DC	PSD3
CDC42EP4.	1.14E-08	-0.46332	0.032	0.203	0.00017	DC	CDC42EP4
MCTP1	1.14E-08	0.203296	0.08	0.02	0.000171	DC	MCTP1
CARD11.2	1.14E-08	0.203048	0.096	0.027	0.000171	DC	CARD11
RP11-325F	1.19E-08	0.129953	0.027	0.003	0.000177	DC	RP11-325F22.2
GLB1	1.21E-08	0.357435	0.396	0.242	0.00018	DC	GLB1
CBX1.2	1.21E-08	-0.37737	0.155	0.37	0.000181	DC	CBX1
ST3GAL6.3	1.22E-08	0.289413	0.241	0.109	0.000182	DC	ST3GAL6
C1R.3	1.24E-08	-0.77011	0.043	0.218	0.000185	DC	C1R
SOCS1.2	1.24E-08	0.273276	0.144	0.051	0.000186	DC	SOCS1
SUPT4H1	1.25E-08	0.280489	0.636	0.492	0.000186	DC	SUPT4H1
OSBPL6	1.28E-08	-0.3345	0.005	0.159	0.000192	DC	OSBPL6
ARPC4.3	1.34E-08	0.255327	0.631	0.512	0.0002	DC	ARPC4
ACSL4.3	1.39E-08	0.334481	0.193	0.082	0.000208	DC	ACSL4
C4orf32	1.40E-08	0.17998	0.07	0.016	0.000209	DC	C4orf32
BHLHE40.2	1.42E-08	0.376376	0.337	0.191	0.000212	DC	BHLHE40
DPYSL4.3	1.44E-08	-0.36665	0.005	0.157	0.000216	DC	DPYSL4
FN1.1	1.45E-08	0.512734	0.289	0.152	0.000217	DC	FN1
ATF3.3	1.47E-08	0.308526	0.487	0.299	0.00022	DC	ATF3
CD63.3	1.48E-08	-0.31964	0.947	0.924	0.000221	DC	CD63
C12orf76.	1.49E-08	-0.43509	0.075	0.269	0.000222	DC	C12orf76
IL23A	1.51E-08	0.16509	0.048	0.008	0.000225	DC	IL23A
CCNDBP1.3	1.52E-08	0.374158	0.353	0.202	0.000227	DC	CCNDBP1
APBB1.2	1.52E-08	-0.43793	0.027	0.192	0.000227	DC	APBB1
RP11-214C	1.54E-08	0.136465	0.048	0.008	0.000231	DC	RP11-214O1.3
PTMS.2	1.56E-08	-0.4154	0.439	0.555	0.000233	DC	PTMS
APOL1	1.62E-08	0.296977	0.118	0.038	0.000242	DC	APOL1
KIAA1191.	1.62E-08	-0.42863	0.075	0.267	0.000243	DC	KIAA1191
CDH26	1.68E-08	0.149481	0.053	0.01	0.000251	DC	CDH26
COL6A2.2	1.69E-08	-0.48382	0.011	0.165	0.000253	DC	COL6A2
NFIC.1	1.72E-08	-0.46807	0.235	0.437	0.000257	DC	NFIC

CRTAP.3	1.75E-08	0.37402	0.38	0.234	0.000261	DC	CRTAP
AGT.1	1.84E-08	-0.61479	0.048	0.223	0.000275	DC	AGT
FBXO17.3	1.84E-08	-0.361	0	0.146	0.000275	DC	FBXO17
DRAXIN.2	1.85E-08	-0.39594	0.005	0.155	0.000277	DC	DRAXIN
SATB1.2	1.92E-08	0.402686	0.257	0.128	0.000286	DC	SATB1
LIMCH1.2	1.95E-08	-0.4276	0.005	0.154	0.000291	DC	LIMCH1
SNTG1.2	1.98E-08	-0.48645	0.005	0.156	0.000295	DC	SNTG1
LSAMP.1	2.01E-08	-0.46094	0.016	0.175	0.0003	DC	LSAMP
NDN.3	2.05E-08	-0.39279	0.032	0.199	0.000306	DC	NDN
RTKN.3	2.13E-08	-0.4009	0.016	0.173	0.000318	DC	RTKN
RASGRP3.2	2.14E-08	0.339927	0.171	0.067	0.00032	DC	RASGRP3
SPPL2A.3	2.16E-08	0.376224	0.321	0.182	0.000323	DC	SPPL2A
TSPAN33.2	2.17E-08	0.256837	0.123	0.041	0.000324	DC	TSPAN33
C21orf59	2.19E-08	-0.40236	0.112	0.319	0.000327	DC	C21orf59
IL27RA.2	2.24E-08	0.315409	0.144	0.054	0.000335	DC	IL27RA
CLIP3.2	2.25E-08	-0.4022	0.032	0.2	0.000337	DC	CLIP3
MEF2A.3	2.29E-08	0.38863	0.369	0.213	0.000342	DC	MEF2A
ZKSCAN1.2	2.32E-08	-0.36826	0.112	0.32	0.000347	DC	ZKSCAN1
RGS17.1	2.32E-08	-0.37844	0.011	0.165	0.000347	DC	RGS17
YWHAQ.2	2.36E-08	-0.36461	0.535	0.645	0.000353	DC	YWHAQ
OLFM1.2	2.37E-08	-0.41956	0.005	0.153	0.000354	DC	OLFM1
C1QB.3	2.40E-08	0.169963	0.818	0.566	0.000358	DC	C1QB
IL12RB1.2	2.45E-08	0.207299	0.102	0.03	0.000366	DC	IL12RB1
RNH1.2	2.46E-08	0.214174	0.684	0.535	0.000368	DC	RNH1
AFF3.1	2.49E-08	0.364864	0.139	0.051	0.000372	DC	AFF3
CD180.2	2.53E-08	0.244734	0.112	0.035	0.000378	DC	CD180
GPC2.3	2.55E-08	-0.50328	0.016	0.17	0.000381	DC	GPC2
SOX6.2	2.57E-08	-0.40265	0	0.144	0.000384	DC	SOX6
SLAMF9.1	2.58E-08	0.211385	0.075	0.018	0.000386	DC	SLAMF9
YWHAE.2	2.61E-08	-0.2354	0.743	0.752	0.00039	DC	YWHAE
SIPA1.3	2.63E-08	0.333573	0.273	0.137	0.000393	DC	SIPA1
GPATCH8.1	2.65E-08	-0.40968	0.107	0.307	0.000397	DC	GPATCH8
UPP1.3	2.73E-08	0.382596	0.401	0.252	0.000408	DC	UPP1
CHCHD2.2	2.77E-08	-0.21981	0.909	0.88	0.000413	DC	CHCHD2
MACC1	2.78E-08	0.177948	0.07	0.016	0.000416	DC	MACC1
SMARCD3.2	2.82E-08	-0.34615	0.053	0.234	0.000421	DC	SMARCD3
PSMA1.3	2.84E-08	0.214229	0.743	0.609	0.000425	DC	PSMA1
PJA1.1	2.85E-08	-0.41957	0.032	0.195	0.000426	DC	PJA1
RPAIN.1	2.85E-08	-0.4405	0.278	0.489	0.000426	DC	RPAIN
BEST1.1	2.89E-08	0.373275	0.428	0.272	0.000432	DC	BEST1
RASGRP2.3	2.95E-08	0.346724	0.139	0.05	0.000441	DC	RASGRP2
TNFRSF14.	2.98E-08	0.31834	0.241	0.113	0.000446	DC	TNFRSF14
RFX4.1	2.99E-08	-0.41852	0.005	0.153	0.000447	DC	RFX4
BICD1.1	3.04E-08	-0.30343	0.037	0.207	0.000454	DC	BICD1
DENND6B.3	3.07E-08	0.347736	0.219	0.1	0.000459	DC	DENND6B
MPEG1.2	3.10E-08	0.227077	0.123	0.041	0.000463	DC	MPEG1
PCDHB16.1	3.10E-08	-0.34521	0	0.142	0.000464	DC	PCDHB16
DAPK1.1	3.12E-08	0.322192	0.15	0.057	0.000467	DC	DAPK1
NDRG4.3	3.14E-08	-0.3605	0.011	0.162	0.00047	DC	NDRG4
DAGLB.3	3.16E-08	0.324327	0.246	0.118	0.000472	DC	DAGLB
COX5A.2	3.18E-08	0.255835	0.727	0.596	0.000475	DC	COX5A

BPGM	3.22E-08	-0.42054	0.059	0.237	0.000481	DC	BPGM
C8orf46.1	3.24E-08	-0.44539	0	0.142	0.000484	DC	C8orf46
JAM3	3.34E-08	-0.36816	0.005	0.151	0.000499	DC	JAM3
MLF1.1	3.36E-08	-0.43015	0.064	0.245	0.000502	DC	MLF1
DUSP22.3	3.42E-08	0.364042	0.23	0.109	0.000511	DC	DUSP22
CALM1.3	3.42E-08	-0.2762	0.738	0.772	0.000511	DC	CALM1
FAM96A.1	3.46E-08	0.323495	0.508	0.354	0.000517	DC	FAM96A
C19orf38.	3.49E-08	0.223601	0.134	0.046	0.000521	DC	C19orf38
ZNF608.1	3.57E-08	-0.34029	0.011	0.16	0.000534	DC	ZNF608
RGS16.2	3.59E-08	-0.52035	0.091	0.278	0.000536	DC	RGS16
PRAF2.2	3.59E-08	-0.38904	0.107	0.305	0.000536	DC	PRAF2
ACSF2.2	3.61E-08	0.364668	0.16	0.064	0.000539	DC	ACSF2
MYEF2.2	3.68E-08	-0.32526	0.032	0.196	0.00055	DC	MYEF2
GDI1.2	3.71E-08	-0.43444	0.278	0.489	0.000554	DC	GDI1
MIR181A1H	3.94E-08	0.419466	0.23	0.111	0.000588	DC	MIR181A1HG
NDUFS5.3	3.95E-08	-0.27016	0.791	0.811	0.00059	DC	NDUFS5
PPP1R14B.	3.96E-08	-0.39393	0.15	0.348	0.000591	DC	PPP1R14B
NLGN1.2	4.00E-08	-0.32797	0	0.14	0.000597	DC	NLGN1
THRA.1	4.00E-08	-0.40042	0.064	0.242	0.000598	DC	THRA
CPQ.1	4.11E-08	0.36356	0.321	0.175	0.000614	DC	CPQ
UPK3A	4.13E-08	0.107477	0.043	0.007	0.000617	DC	UPK3A
MIF4GD.3	4.15E-08	0.411693	0.39	0.25	0.00062	DC	MIF4GD
APP.2	4.19E-08	-0.45118	0.353	0.527	0.000626	DC	APP
S100A13.1	4.27E-08	-0.618	0.112	0.299	0.000638	DC	S100A13
LINC00954	4.29E-08	0.209862	0.048	0.009	0.000641	DC	LINC00954
ITPR1	4.34E-08	0.214419	0.107	0.033	0.000649	DC	ITPR1
IL10RB.3	4.34E-08	0.397209	0.385	0.241	0.000649	DC	IL10RB
PTPN2.2	4.38E-08	0.319012	0.396	0.246	0.000654	DC	PTPN2
ARC.1	4.39E-08	-0.57106	0.048	0.21	0.000656	DC	ARC
TMEM106C.	4.46E-08	-0.49791	0.086	0.269	0.000667	DC	TMEM106C
CYP27A1.1	4.50E-08	-0.39972	0.016	0.167	0.000673	DC	CYP27A1
PPFIBP1.1	4.66E-08	-0.3537	0.011	0.158	0.000696	DC	PPFIBP1
RABEPK.2	4.67E-08	-0.41664	0.037	0.202	0.000698	DC	RABEPK
IGFBP3.1	4.68E-08	-0.64094	0.037	0.197	0.0007	DC	IGFBP3
POLR2J3.2	4.73E-08	-0.42889	0.139	0.342	0.000707	DC	POLR2J3
TPM2	4.82E-08	-0.44801	0.016	0.165	0.00072	DC	TPM2
SRPK2.1	4.85E-08	-0.42661	0.203	0.414	0.000725	DC	SRPK2
COA3.2	4.91E-08	-0.42311	0.262	0.48	0.000733	DC	COA3
WDR60	4.94E-08	-0.34522	0.059	0.235	0.000738	DC	WDR60
SIRPB2	5.07E-08	0.234369	0.064	0.015	0.000757	DC	SIRPB2
DPF3	5.09E-08	-0.33877	0	0.138	0.00076	DC	DPF3
SIGLEC9.3	5.14E-08	0.294388	0.209	0.091	0.000768	DC	SIGLEC9
DPYSL5.3	5.16E-08	-0.3767	0.016	0.165	0.000771	DC	DPYSL5
GGT7.1	5.23E-08	-0.31936	0.016	0.168	0.000782	DC	GGT7
VAMP2.2	5.24E-08	-0.41252	0.471	0.625	0.000784	DC	VAMP2
SPTAN1.1	5.29E-08	-0.39587	0.08	0.263	0.00079	DC	SPTAN1
SPG21.3	5.37E-08	0.327322	0.519	0.383	0.000803	DC	SPG21
DAG1.1	5.42E-08	-0.32708	0.021	0.176	0.000809	DC	DAG1
ZYX.3	5.43E-08	0.330056	0.545	0.399	0.000811	DC	ZYX
EIF3A.3	5.44E-08	0.375881	0.46	0.33	0.000813	DC	EIF3A
SLC1A2.1	5.55E-08	-0.40536	0	0.137	0.00083	DC	SLC1A2

GSTM3.1	5.72E-08	-0.44216	0.086	0.271	0.000854	DC	GSTM3
C5orf24.2	5.76E-08	-0.35207	0.102	0.299	0.00086	DC	C5orf24
ELMO1.2	5.82E-08	0.301438	0.348	0.187	0.00087	DC	ELMO1
GAS1.3	5.92E-08	-0.38236	0.005	0.147	0.000884	DC	GAS1
PDIA3.3	5.92E-08	0.218069	0.711	0.581	0.000884	DC	PDIA3
FMN1.2	5.96E-08	0.219064	0.102	0.031	0.000891	DC	FMN1
SMARCA1.1	6.02E-08	-0.33587	0.021	0.174	0.0009	DC	SMARCA1
APOD.2	6.07E-08	-0.88774	0.016	0.163	0.000906	DC	APOD
FSD1.2	6.10E-08	-0.31606	0.011	0.157	0.000911	DC	FSD1
ZNF75A.2	6.12E-08	-0.3961	0.048	0.217	0.000914	DC	ZNF75A
USP3.3	6.13E-08	0.334137	0.294	0.164	0.000916	DC	USP3
TMEM132A.	6.17E-08	-0.30466	0.005	0.147	0.000922	DC	TMEM132A
SSR1.2	6.22E-08	0.276129	0.604	0.466	0.00093	DC	SSR1
RNF187.1	6.43E-08	-0.41738	0.171	0.381	0.000961	DC	RNF187
DCBLD2.2	6.53E-08	-0.34108	0.005	0.145	0.000976	DC	DCBLD2
HILPDA.2	6.55E-08	-0.60292	0.059	0.23	0.000979	DC	HILPDA
JAK3	6.63E-08	0.179184	0.091	0.026	0.000992	DC	JAK3
BIRC3.1	6.66E-08	0.239811	0.16	0.063	0.000995	DC	BIRC3
ARL4D.3	6.68E-08	-0.46611	0.021	0.171	0.000998	DC	ARL4D
NUDT14.3	6.89E-08	0.350347	0.337	0.19	0.001029	DC	NUDT14
IDH1.2	6.94E-08	-0.43497	0.176	0.383	0.001037	DC	IDH1
SNAI3	7.01E-08	0.127927	0.064	0.015	0.001047	DC	SNAI3
TAGLN2.3	7.27E-08	0.17484	0.701	0.511	0.001086	DC	TAGLN2
ROBO1.3	7.27E-08	-0.35949	0.027	0.18	0.001086	DC	ROBO1
CSK.3	7.33E-08	0.351603	0.278	0.145	0.001096	DC	CSK
ELAVL4.2	7.36E-08	-0.63457	0.032	0.181	0.0011	DC	ELAVL4
LRRC4B.3	7.38E-08	-0.29959	0	0.135	0.001102	DC	LRRC4B
CDKN2C.1	7.44E-08	-0.48232	0.102	0.285	0.001112	DC	CDKN2C
CNP.2	7.70E-08	-0.5053	0.176	0.38	0.001151	DC	CNP
HYAL2.3	7.72E-08	-0.32132	0.053	0.225	0.001153	DC	HYAL2
ZFAND1	7.78E-08	0.378956	0.31	0.175	0.001163	DC	ZFAND1
PLAGL1.2	8.01E-08	0.198742	0.118	0.04	0.001196	DC	PLAGL1
KIFAP3.2	8.02E-08	-0.39128	0.043	0.205	0.001199	DC	KIFAP3
BCAR1.1	8.04E-08	-0.3199	0.021	0.174	0.001202	DC	BCAR1
TRAF3IP3.	8.24E-08	0.255675	0.139	0.051	0.001232	DC	TRAF3IP3
HIF1A.2	8.49E-08	0.292231	0.631	0.495	0.001268	DC	HIF1A
C11orf49.	8.57E-08	-0.35073	0.064	0.24	0.00128	DC	C11orf49
CD82.1	8.69E-08	-0.53178	0.075	0.242	0.001299	DC	CD82
DPF1.2	8.72E-08	-0.39443	0.005	0.143	0.001303	DC	DPF1
CITED1.2	8.84E-08	-0.43921	0.011	0.153	0.001321	DC	CITED1
CASS4	8.85E-08	0.155773	0.064	0.015	0.001323	DC	CASS4
JUN	8.94E-08	-0.36058	0.561	0.674	0.001336	DC	JUN
GCAT.3	8.97E-08	-0.27138	0.016	0.163	0.00134	DC	GCAT
SUMO3.3	9.00E-08	0.276695	0.583	0.437	0.001345	DC	SUMO3
PHYHIPL.1	9.00E-08	-0.49542	0.032	0.188	0.001345	DC	PHYHIPL
ST5.3	9.17E-08	-0.35791	0.011	0.153	0.00137	DC	ST5
SLC1A3.2	9.33E-08	-0.4598	0.283	0.48	0.001394	DC	SLC1A3
ARHGAP27.	9.53E-08	0.213483	0.144	0.054	0.001424	DC	ARHGAP27
PTPN18.3	9.57E-08	0.366491	0.348	0.208	0.00143	DC	PTPN18
PCBP1.3	9.61E-08	0.220341	0.701	0.586	0.001436	DC	PCBP1
IQGAP1.3	9.62E-08	0.367702	0.385	0.25	0.001438	DC	IQGAP1

ZNF90.2	9.68E-08	0.250924	0.567	0.377	0.001446	DC	ZNF90
TMEM45A.1	9.94E-08	-0.49702	0.027	0.179	0.001486	DC	TMEM45A
GPSM2.2	1.01E-07	-0.34119	0.027	0.179	0.001504	DC	GPSM2
FKBP9.2	1.01E-07	-0.33411	0.027	0.179	0.001509	DC	FKBP9
RELT.2	1.01E-07	0.36575	0.187	0.083	0.001515	DC	RELT
PTOV1.2	1.02E-07	-0.395	0.235	0.442	0.001524	DC	PTOV1
LINC00665	1.04E-07	-0.36533	0.032	0.188	0.001549	DC	LINC00665
YES1	1.04E-07	-0.29553	0	0.132	0.001561	DC	YES1
ATP6V1G2.	1.06E-07	-0.37531	0.011	0.151	0.001589	DC	ATP6V1G2
EPHX1.2	1.08E-07	-0.45679	0.064	0.235	0.001621	DC	EPHX1
NKX2-2.2	1.09E-07	-0.4596	0.011	0.15	0.001622	DC	NKX2-2
HOMER3.3	1.10E-07	-0.37452	0.07	0.244	0.001642	DC	HOMER3
CNIH2.3	1.10E-07	-0.30568	0	0.132	0.001648	DC	CNIH2
CNTFR.2	1.10E-07	-0.3441	0	0.132	0.001648	DC	CNTFR
TMEM219.2	1.12E-07	0.203651	0.701	0.558	0.001679	DC	TMEM219
LHX2.3	1.13E-07	-0.31736	0	0.132	0.001684	DC	LHX2
KLF6.2	1.16E-07	0.187315	0.727	0.528	0.001733	DC	KLF6
LPAR6.3	1.16E-07	0.289119	0.396	0.235	0.001741	DC	LPAR6
CTNND2.2	1.17E-07	-0.35975	0.016	0.16	0.001743	DC	CTNND2
METTL7B	1.17E-07	-0.64412	0.053	0.215	0.001754	DC	METTL7B
NOL3.3	1.20E-07	-0.40773	0.021	0.168	0.00179	DC	NOL3
TMEM158.2	1.21E-07	-0.33771	0.005	0.142	0.001806	DC	TMEM158
POSTN.2	1.22E-07	-0.44367	0	0.131	0.001817	DC	POSTN
LAMTOR1.1	1.24E-07	0.159891	0.802	0.652	0.001858	DC	LAMTOR1
ATP1B2.3	1.25E-07	-0.36138	0.064	0.236	0.001873	DC	ATP1B2
APC2.2	1.26E-07	-0.3598	0.011	0.15	0.00188	DC	APC2
PYCR1	1.28E-07	-0.3453	0.032	0.187	0.001913	DC	PYCR1
ARHGAP26.	1.29E-07	0.350293	0.198	0.09	0.001935	DC	ARHGAP26
COL4A2	1.31E-07	-0.29279	0	0.131	0.00196	DC	COL4A2
TMSB15A.3	1.32E-07	-0.47234	0.032	0.181	0.001974	DC	TMSB15A
RP11-14N7	1.34E-07	-0.39775	0.048	0.207	0.002009	DC	RP11-14N7.2
CEND1.1	1.35E-07	-0.33375	0.005	0.141	0.002015	DC	CEND1
PMP22.2	1.35E-07	-0.50462	0.235	0.431	0.00202	DC	PMP22
TNC.2	1.37E-07	-0.44941	0.043	0.203	0.00205	DC	TNC
SGCE.1	1.38E-07	-0.57567	0.011	0.148	0.002056	DC	SGCE
IL10.3	1.38E-07	0.341955	0.225	0.104	0.002059	DC	IL10
CERS1.1	1.41E-07	-0.31536	0	0.13	0.002114	DC	CERS1
DST.2	1.43E-07	-0.37612	0.171	0.38	0.002138	DC	DST
FCHO1.2	1.43E-07	0.281614	0.112	0.038	0.002141	DC	FCHO1
AHNAK.1	1.45E-07	0.398757	0.23	0.116	0.00217	DC	AHNAK
PTMA.3	1.46E-07	-0.2359	1	0.966	0.002187	DC	PTMA
ASPHD1.1	1.47E-07	-0.36997	0.005	0.14	0.002195	DC	ASPHD1
ABRACL.3	1.51E-07	0.38108	0.439	0.302	0.002252	DC	ABRACL
COX6C.3	1.52E-07	-0.26815	0.802	0.831	0.002269	DC	COX6C
DOK5.2	1.54E-07	-0.37726	0.032	0.185	0.002307	DC	DOK5
KIAA1715.	1.55E-07	-0.36748	0.08	0.258	0.002324	DC	KIAA1715
PSME2.2	1.57E-07	0.171871	0.711	0.555	0.00235	DC	PSME2
CCDC28B.2	1.59E-07	-0.27772	0.053	0.222	0.002369	DC	CCDC28B
PLXNC1.3	1.60E-07	0.235492	0.16	0.065	0.002385	DC	PLXNC1
ARRDC2.3	1.60E-07	0.244409	0.193	0.084	0.002387	DC	ARRDC2
LTB4R.2	1.61E-07	0.22527	0.102	0.032	0.002406	DC	LTB4R

STARD4-AS	1.61E-07	-0.33045	0	0.129	0.002407	DC	STARD4-AS1
KLHL4.1	1.63E-07	-0.32449	0	0.129	0.002433	DC	KLHL4
MAFK	1.63E-07	0.212781	0.112	0.038	0.002438	DC	MAFK
CDC34.2	1.65E-07	-0.37171	0.07	0.237	0.002473	DC	CDC34
EIF4B.3	1.66E-07	0.3079	0.508	0.376	0.002486	DC	EIF4B
SRGAP1.3	1.67E-07	0.34852	0.358	0.223	0.0025	DC	SRGAP1
ZNF771.2	1.73E-07	-0.2811	0.011	0.148	0.002587	DC	ZNF771
IVNS1ABP.	1.73E-07	0.31822	0.374	0.235	0.002593	DC	IVNS1ABP
PLEKHO2.3	1.74E-07	0.328449	0.214	0.1	0.002597	DC	PLEKHO2
RP11-71N1	1.78E-07	-0.36072	0.005	0.138	0.002667	DC	RP11-71N10.1
CLN8.3	1.79E-07	0.330442	0.417	0.266	0.00268	DC	CLN8
MSI1.1	1.81E-07	-0.33184	0.005	0.137	0.002705	DC	MSI1
CDC25B.1	1.81E-07	-0.38928	0.043	0.196	0.002706	DC	CDC25B
MBNL1.3	1.83E-07	0.351296	0.358	0.222	0.002738	DC	MBNL1
CA12.3	1.85E-07	-0.36373	0.005	0.138	0.002763	DC	CA12
LIPA.3	1.88E-07	0.253247	0.513	0.331	0.002814	DC	LIPA
SLFN12	1.89E-07	0.169957	0.086	0.025	0.002824	DC	SLFN12
TCOF1.3	1.92E-07	0.42678	0.321	0.193	0.002862	DC	TCOF1
ALKBH4.1	1.94E-07	-0.28901	0.043	0.201	0.002895	DC	ALKBH4
EEF1D.3	1.95E-07	0.102307	0.989	0.843	0.002917	DC	EEF1D
SACS.3	1.95E-07	-0.28974	0.016	0.157	0.002919	DC	SACS
TWF1.1	2.01E-07	-0.3398	0.086	0.266	0.003004	DC	TWF1
SLC25A3.2	2.02E-07	0.126346	0.872	0.744	0.003016	DC	SLC25A3
ENDOV	2.02E-07	-0.34706	0.059	0.223	0.003026	DC	ENDOV
OPTN.1	2.05E-07	-0.32612	0.005	0.137	0.003062	DC	OPTN
PGM2.1	2.06E-07	0.263658	0.273	0.146	0.003076	DC	PGM2
MGMT.3	2.09E-07	0.257439	0.289	0.149	0.003122	DC	MGMT
SYP.3	2.09E-07	-0.4224	0.005	0.136	0.00313	DC	SYP
ARSJ.1	2.13E-07	-0.30244	0	0.127	0.003184	DC	ARSJ
NKAIN3.2	2.13E-07	-0.32283	0	0.127	0.003184	DC	NKAIN3
RPS6KA4.3	2.13E-07	0.353783	0.235	0.12	0.003187	DC	RPS6KA4
SPRY1.3	2.15E-07	-0.45634	0.07	0.235	0.003207	DC	SPRY1
FCGR1A.3	2.15E-07	0.207566	0.46	0.255	0.003211	DC	FCGR1A
P4HTM.1	2.18E-07	-0.325	0.086	0.264	0.003261	DC	P4HTM
PRDX1.3	2.19E-07	-0.33163	0.711	0.81	0.003276	DC	PRDX1
TNIK.1	2.27E-07	-0.29308	0.011	0.146	0.003393	DC	TNIK
SLC39A8.2	2.33E-07	0.283403	0.144	0.057	0.003477	DC	SLC39A8
GADD45GIF	2.33E-07	-0.35633	0.433	0.565	0.003481	DC	GADD45GIP1
STX6.3	2.34E-07	0.365581	0.326	0.195	0.003492	DC	STX6
ALDH3B1.1	2.34E-07	0.292037	0.166	0.07	0.003496	DC	ALDH3B1
RP11-660L	2.35E-07	-0.3387	0	0.126	0.003507	DC	RP11-660L16.2
MYH10.1	2.37E-07	-0.32621	0.016	0.155	0.003538	DC	MYH10
HRH2.1	2.38E-07	0.241703	0.112	0.038	0.003562	DC	HRH2
FAM84A.2	2.40E-07	-0.2829	0.011	0.146	0.003591	DC	FAM84A
JARID2.1	2.41E-07	0.349898	0.235	0.12	0.003597	DC	JARID2
SNX8.3	2.44E-07	0.38436	0.267	0.145	0.003645	DC	SNX8
ZNF711.2	2.46E-07	-0.36171	0.011	0.144	0.003673	DC	ZNF711
TCTN1.2	2.48E-07	-0.36201	0.053	0.214	0.003711	DC	TCTN1
SPATS2.1	2.49E-07	-0.37068	0.086	0.261	0.003727	DC	SPATS2
CHST12.1	2.52E-07	-0.34209	0.075	0.249	0.003767	DC	CHST12
RNF157.2	2.52E-07	-0.27816	0.005	0.136	0.00377	DC	RNF157

SMAD1.1	2.55E-07	-0.31392	0.032	0.18	0.003804	DC	SMAD1
KCNF1.3	2.56E-07	-0.33169	0	0.125	0.003821	DC	KCNF1
CDC42BPA.	2.56E-07	-0.31075	0.032	0.181	0.003822	DC	CDC42BPA
PLCG2.1	2.57E-07	0.172604	0.128	0.046	0.003834	DC	PLCG2
MIAT.2	2.58E-07	-0.43799	0.027	0.17	0.003855	DC	MIAT
SNAP25.2	2.59E-07	-0.45077	0.011	0.145	0.003864	DC	SNAP25
NDRG2.1	2.60E-07	0.178613	0.551	0.356	0.003893	DC	NDRG2
EDNRB.1	2.61E-07	-0.38125	0.037	0.189	0.003902	DC	EDNRB
TPP1.3	2.62E-07	0.29712	0.471	0.32	0.003916	DC	TPP1
TMEM237.1	2.64E-07	-0.29764	0.037	0.189	0.003942	DC	TMEM237
CNIH3	2.70E-07	-0.34318	0.027	0.171	0.004037	DC	CNIH3
NUPR1.2	2.72E-07	-0.57001	0.144	0.327	0.004058	DC	NUPR1
HEPACAM.2	2.75E-07	-0.44191	0.032	0.18	0.004103	DC	HEPACAM
AC004540.	2.76E-07	-0.44075	0	0.125	0.004119	DC	AC004540.4
STAT5A.3	2.80E-07	0.231333	0.134	0.05	0.004181	DC	STAT5A
NDUFB7.3	2.80E-07	-0.33055	0.631	0.722	0.004191	DC	NDUFB7
SEPT8.3	2.82E-07	-0.36978	0.043	0.198	0.004216	DC	8-Sep
NAGA.3	2.91E-07	0.307558	0.321	0.182	0.004347	DC	NAGA
FBL.3	2.97E-07	0.244128	0.508	0.375	0.004435	DC	FBL
PDRG1.1	3.04E-07	-0.3831	0.096	0.271	0.004547	DC	PDRG1
PDE6G	3.11E-07	0.148773	0.053	0.012	0.004648	DC	PDE6G
NR2F6.2	3.16E-07	-0.28324	0.011	0.143	0.004717	DC	NR2F6
PSMB5.2	3.17E-07	-0.39818	0.406	0.598	0.004734	DC	PSMB5
FGFR1	3.17E-07	-0.29399	0.027	0.173	0.00474	DC	FGFR1
ZNF710	3.19E-07	0.249205	0.102	0.034	0.00476	DC	ZNF710
ZNF91	3.20E-07	-0.33751	0.053	0.212	0.004782	DC	ZNF91
TLE1.1	3.21E-07	-0.28984	0.027	0.172	0.004803	DC	TLE1
ENHO.2	3.23E-07	-0.29911	0.016	0.153	0.004829	DC	ENHO
RNASEH1-A	3.25E-07	-0.28742	0.032	0.18	0.004853	DC	RNASEH1-AS1
VPS29.1	3.25E-07	0.225018	0.674	0.551	0.004856	DC	VPS29
HADHA.1	3.26E-07	0.26941	0.567	0.439	0.004877	DC	HADHA
HCAR3	3.27E-07	0.136645	0.032	0.005	0.004885	DC	HCAR3
OSER1-AS1	3.28E-07	-0.33918	0.005	0.133	0.004894	DC	OSER1-AS1
CADM1.1	3.28E-07	-0.34423	0.134	0.328	0.004899	DC	CADM1
RIMKLB.1	3.29E-07	-0.3291	0.016	0.153	0.004913	DC	RIMKLB
TIMM22	3.29E-07	-0.32084	0.075	0.245	0.004923	DC	TIMM22
GNG5.2	3.36E-07	0.160935	0.856	0.641	0.005023	DC	GNG5
HSPBP1	3.43E-07	-0.29625	0.107	0.29	0.005122	DC	HSPBP1
SRPX.2	3.51E-07	-0.39544	0.032	0.179	0.005252	DC	SRPX
EXTL2.1	3.55E-07	-0.27045	0.027	0.17	0.005311	DC	EXTL2
PLCB2.3	3.67E-07	0.2307	0.15	0.06	0.005482	DC	PLCB2
WSCD1.2	3.70E-07	-0.3041	0.005	0.133	0.005536	DC	WSCD1
TSPAN12.1	3.72E-07	-0.33013	0	0.122	0.005556	DC	TSPAN12
ASCL2.2	3.76E-07	0.207938	0.107	0.036	0.005615	DC	ASCL2
HS2ST1.2	3.88E-07	-0.34941	0.032	0.177	0.005795	DC	HS2ST1
NDUFA5.1	3.91E-07	-0.40647	0.39	0.58	0.005848	DC	NDUFA5
GSPT2.1	3.92E-07	-0.31493	0.016	0.151	0.005864	DC	GSPT2
FAM3C.2	3.96E-07	-0.34639	0.246	0.44	0.005913	DC	FAM3C
C1S.1	4.02E-07	-0.70444	0.027	0.164	0.006007	DC	C1S
C21orf62.	4.08E-07	-0.38322	0.021	0.16	0.006095	DC	C21orf62
GRIA4.2	4.10E-07	-0.34715	0.005	0.131	0.00612	DC	GRIA4

FERMT2.3	4.11E-07	-0.31571	0.048	0.205	0.006136	DC	FERMT2
DLX5.3	4.17E-07	-0.6825	0.016	0.146	0.006237	DC	DLX5
ATP6V0B.3	4.20E-07	0.129071	0.93	0.796	0.006278	DC	ATP6V0B
TREM2.3	4.24E-07	0.208714	0.578	0.347	0.006343	DC	TREM2
PNMA2.2	4.27E-07	-0.3248	0.011	0.141	0.006381	DC	PNMA2
HGSNAT.3	4.31E-07	0.407726	0.326	0.204	0.006442	DC	HGSNAT
MEIS3.1	4.39E-07	-0.28191	0.005	0.131	0.006559	DC	MEIS3
RAB7A.2	4.41E-07	0.17871	0.759	0.657	0.006587	DC	RAB7A
KRIT1.2	4.59E-07	-0.33215	0.059	0.219	0.006859	DC	KRIT1
NIPSNAP3A	4.72E-07	0.349232	0.326	0.198	0.007049	DC	NIPSNAP3A
RCN1.3	4.77E-07	-0.32609	0.166	0.357	0.007125	DC	RCN1
MAFF.3	4.80E-07	0.317292	0.299	0.169	0.007179	DC	MAFF
NR2F1	4.82E-07	-0.29393	0.027	0.168	0.007205	DC	NR2F1
GNG12.1	5.02E-07	-0.27951	0.011	0.14	0.007498	DC	GNG12
VAPA.1	5.03E-07	0.209928	0.775	0.631	0.007522	DC	VAPA
RAB32.3	5.13E-07	0.39055	0.364	0.236	0.007663	DC	RAB32
DLL3.2	5.16E-07	-0.53476	0.016	0.146	0.007718	DC	DLL3
GTF2I.2	5.22E-07	-0.4236	0.246	0.43	0.007798	DC	GTF2I
THSD7A.2	5.31E-07	-0.4838	0.016	0.145	0.007933	DC	THSD7A
CETN2.3	5.32E-07	-0.42027	0.144	0.325	0.007955	DC	CETN2
NUDT11.1	5.34E-07	-0.3588	0.005	0.128	0.007986	DC	NUDT11
HLTF.2	5.35E-07	-0.29687	0.032	0.176	0.007988	DC	HLTF
ZMAT3.1	5.35E-07	-0.38162	0.053	0.205	0.008002	DC	ZMAT3
TNFSF12.3	5.36E-07	0.310193	0.316	0.183	0.008013	DC	TNFSF12
SENCR.1	5.37E-07	0.139265	0.07	0.019	0.008019	DC	SENCR
INSR.2	5.53E-07	0.440499	0.257	0.143	0.008263	DC	INSR
PDE4A.2	5.54E-07	0.311783	0.187	0.088	0.008283	DC	PDE4A
BBOX1.2	5.59E-07	-0.37567	0.011	0.139	0.008357	DC	BBOX1
EIF4E2.1	5.69E-07	0.364975	0.449	0.331	0.008499	DC	EIF4E2
ACOT9.3	5.70E-07	0.336374	0.305	0.18	0.008511	DC	ACOT9
PHAX.1	5.71E-07	-0.35236	0.123	0.308	0.008529	DC	PHAX
AKNA.2	5.76E-07	0.259629	0.166	0.071	0.008611	DC	AKNA
PDE4B.3	5.78E-07	0.401565	0.401	0.274	0.00863	DC	PDE4B
CYB5D2.1	5.79E-07	-0.4261	0.075	0.235	0.00865	DC	CYB5D2
PLOD2.2	5.80E-07	-0.34962	0.027	0.164	0.008673	DC	PLOD2
ATXN2.1	5.81E-07	-0.33847	0.064	0.224	0.008687	DC	ATXN2
PXMP2.1	5.98E-07	-0.30243	0.064	0.226	0.008935	DC	PXMP2
SMARCA4.2	6.04E-07	-0.3854	0.176	0.364	0.009021	DC	SMARCA4
SUGP2.2	6.07E-07	-0.30195	0.08	0.249	0.009074	DC	SUGP2
PITPNA-AS	6.21E-07	-0.33955	0.048	0.198	0.009281	DC	PITPNA-AS1
ARV1.2	6.37E-07	-0.38537	0.037	0.181	0.009526	DC	ARV1
GNG4.2	6.39E-07	-0.30984	0	0.118	0.009552	DC	GNG4
DUSP14.2	6.49E-07	-0.3194	0.037	0.182	0.009705	DC	DUSP14
OBSL1.2	6.50E-07	-0.28365	0.016	0.148	0.009716	DC	OBSL1
LRWD1	6.57E-07	-0.29049	0.032	0.172	0.009816	DC	LRWD1
DTX3	6.57E-07	-0.47591	0.096	0.258	0.009824	DC	DTX3
RND2.2	6.58E-07	-0.34461	0.027	0.165	0.009833	DC	RND2
RPS6KA3.3	6.62E-07	0.387892	0.246	0.135	0.009891	DC	RPS6KA3
SOBP.2	6.67E-07	-0.3225	0.011	0.136	0.009966	DC	SOBP
TAF9B	6.67E-07	-0.27326	0.016	0.147	0.00997	DC	TAF9B
POLD2.2	6.74E-07	-0.3742	0.225	0.424	0.010065	DC	POLD2

CARHSP1.2	6.81E-07	-0.39202	0.23	0.426	0.010179	DC	CARHSP1
SIRPA.3	6.83E-07	0.300325	0.38	0.241	0.010203	DC	SIRPA
PPDPF	6.86E-07	-0.28686	0.69	0.711	0.01025	DC	PPDPF
PLA2G4A.1	6.94E-07	0.196398	0.096	0.031	0.010366	DC	PLA2G4A
ASNS.3	6.99E-07	-0.35423	0.037	0.179	0.010451	DC	ASNS
DNAJB12.2	7.05E-07	0.29295	0.267	0.146	0.010533	DC	DNAJB12
TEAD1.1	7.07E-07	-0.25453	0.016	0.147	0.010567	DC	TEAD1
PAK3.3	7.08E-07	-0.39643	0.016	0.145	0.010585	DC	PAK3
PTAFR.3	7.15E-07	0.223132	0.251	0.123	0.010681	DC	PTAFR
TMEM198.1	7.18E-07	-0.26654	0	0.117	0.010731	DC	TMEM198
SNRPB.1	7.22E-07	0.25203	0.727	0.575	0.010788	DC	SNRPB
NPM3.2	7.30E-07	0.408319	0.337	0.215	0.01091	DC	NPM3
SFXN5.3	7.38E-07	-0.33925	0.043	0.191	0.011032	DC	SFXN5
PCYOX1	7.48E-07	-0.25614	0.037	0.183	0.011171	DC	PCYOX1
ABHD15.3	7.50E-07	0.264993	0.139	0.056	0.011208	DC	ABHD15
PRR4.3	7.64E-07	-0.33645	0.064	0.219	0.011422	DC	PRR4
C1QL1.2	7.65E-07	-0.32241	0	0.116	0.011433	DC	C1QL1
DOK3.3	7.72E-07	0.224688	0.155	0.064	0.011529	DC	DOK3
RSPRY1.1	7.72E-07	-0.34369	0.086	0.251	0.011536	DC	RSPRY1
LINC00152	7.78E-07	0.375709	0.385	0.256	0.011623	DC	LINC00152
RASA2.2	7.83E-07	0.309165	0.166	0.074	0.011697	DC	RASA2
FAM20C.3	7.89E-07	-0.3996	0.07	0.226	0.011798	DC	FAM20C
PFKL.3	7.90E-07	0.32774	0.481	0.361	0.011803	DC	PFKL
NBPF1.3	7.91E-07	-0.27507	0.075	0.238	0.011816	DC	NBPF1
DDAH1.2	8.23E-07	-0.24203	0.005	0.126	0.012292	DC	DDAH1
ZNF704.2	8.24E-07	-0.3195	0.005	0.125	0.012318	DC	ZNF704
ANXA4.3	8.36E-07	0.333443	0.316	0.192	0.012493	DC	ANXA4
CALU.2	8.39E-07	-0.38465	0.209	0.406	0.012532	DC	CALU
CDCA7.2	8.47E-07	-0.3882	0.005	0.125	0.012651	DC	CDCA7
USP15.3	8.50E-07	0.327506	0.374	0.24	0.012703	DC	USP15
ANXA2R.2	8.78E-07	-0.33348	0.021	0.154	0.013126	DC	ANXA2R
ASIC4.2	8.84E-07	-0.49882	0.016	0.141	0.013204	DC	ASIC4
PDCD5.3	8.88E-07	-0.39901	0.348	0.542	0.013265	DC	PDCD5
BOD1.2	8.89E-07	-0.32754	0.128	0.306	0.013281	DC	BOD1
IRF7.3	8.89E-07	0.342838	0.278	0.159	0.013281	DC	IRF7
GATM.3	9.11E-07	-0.43172	0.176	0.355	0.013611	DC	GATM
HENMT1.2	9.15E-07	0.166646	0.096	0.031	0.013678	DC	HENMT1
ST8SIA4.3	9.18E-07	0.347158	0.23	0.117	0.013724	DC	ST8SIA4
AC004158.	9.29E-07	-0.32856	0.005	0.124	0.01388	DC	AC004158.3
NUMB.3	9.40E-07	0.361583	0.289	0.169	0.014049	DC	NUMB
SSR3.3	9.43E-07	0.179079	0.652	0.524	0.014097	DC	SSR3
ZNF3.1	9.54E-07	-0.37612	0.059	0.208	0.014263	DC	ZNF3
WWTR1.3	9.57E-07	-0.32282	0.027	0.162	0.014306	DC	WWTR1
ZNF664.1	9.62E-07	-0.31062	0.021	0.151	0.01438	DC	ZNF664
AC092484.	9.74E-07	0.138937	0.053	0.012	0.01455	DC	AC092484.1
RP11-849I	9.76E-07	-0.34063	0.011	0.134	0.014578	DC	RP11-849I19.1
GADD45A.3	9.80E-07	-0.45041	0.112	0.281	0.014651	DC	GADD45A
INHBA-AS1	9.84E-07	0.106181	0.027	0.004	0.014702	DC	INHBA-AS1
GBP2.2	9.93E-07	0.269079	0.342	0.206	0.014837	DC	GBP2
SMOX.3	9.96E-07	-0.39649	0.086	0.25	0.014889	DC	SMOX
ZNF883.2	9.99E-07	-0.3066	0.016	0.143	0.014925	DC	ZNF883

CIDEB.1	9.99E-07	0.227691	0.08	0.024	0.014925	DC	CIDEB
MTX2.1	1.01E-06	-0.39337	0.144	0.324	0.015161	DC	MTX2
ACAD8	1.02E-06	-0.29625	0.043	0.185	0.01521	DC	ACAD8
PIK3R3.1	1.02E-06	-0.37638	0.011	0.134	0.015289	DC	PIK3R3
PPP2R5B.2	1.04E-06	-0.30582	0.032	0.168	0.015506	DC	PPP2R5B
IL3RA	1.05E-06	0.173862	0.053	0.013	0.01571	DC	IL3RA
QDPR.2	1.07E-06	-0.40363	0.176	0.363	0.015937	DC	QDPR
IL10RB-AS	1.10E-06	0.247559	0.118	0.043	0.016415	DC	IL10RB-AS1
SFT2D1.3	1.10E-06	0.203132	0.626	0.499	0.016504	DC	SFT2D1
PTK2B.2	1.10E-06	0.305826	0.235	0.125	0.016513	DC	PTK2B
COMMD6.2	1.11E-06	0.129705	0.909	0.774	0.016552	DC	COMMD6
NTM.1	1.12E-06	-0.44613	0	0.113	0.016706	DC	NTM
GABARAPL1	1.13E-06	0.325801	0.428	0.295	0.016817	DC	GABARAPL1
FAM216A.1	1.13E-06	-0.25334	0.005	0.124	0.016822	DC	FAM216A
ERCC1.3	1.13E-06	0.238413	0.604	0.47	0.016879	DC	ERCC1
TMEM88	1.13E-06	0.191127	0.075	0.022	0.016938	DC	TMEM88
TCEAL5.1	1.14E-06	-0.4325	0.005	0.122	0.01706	DC	TCEAL5
TTC9B.3	1.14E-06	-0.4492	0.005	0.122	0.017091	DC	TTC9B
DMWD.2	1.15E-06	-0.21421	0.016	0.143	0.017161	DC	DMWD
LRMP.2	1.15E-06	0.244274	0.118	0.044	0.017229	DC	LRMP
VMA21.2	1.18E-06	0.349897	0.455	0.339	0.017628	DC	VMA21
TPPP3.3	1.19E-06	-0.52576	0.027	0.156	0.017768	DC	TPPP3
NRBP2.2	1.20E-06	-0.26946	0.005	0.123	0.017871	DC	NRBP2
PGM3.2	1.22E-06	-0.30025	0.037	0.176	0.018291	DC	PGM3
TSPAN6.2	1.22E-06	-0.31394	0.021	0.152	0.018299	DC	TSPAN6
COL4A1	1.23E-06	-0.23983	0	0.112	0.018362	DC	COL4A1
ARF3.2	1.24E-06	0.275801	0.492	0.376	0.018501	DC	ARF3
TCF3.2	1.24E-06	-0.26736	0.011	0.131	0.018541	DC	TCF3
CD84.3	1.24E-06	0.202186	0.257	0.127	0.018584	DC	CD84
SCO2.3	1.24E-06	0.354805	0.332	0.209	0.018589	DC	SCO2
FNBP1.3	1.26E-06	0.337734	0.385	0.261	0.018857	DC	FNBP1
IFT81.1	1.27E-06	-0.22428	0.027	0.161	0.01891	DC	IFT81
PNKD.1	1.27E-06	-0.37048	0.262	0.46	0.018942	DC	PNKD
WNK3.1	1.28E-06	-0.28384	0.005	0.122	0.019123	DC	WNK3
AK4.3	1.29E-06	-0.36535	0.043	0.182	0.019323	DC	AK4
SLC37A3.1	1.29E-06	-0.31767	0.011	0.131	0.019343	DC	SLC37A3
WEE1.1	1.30E-06	-0.23635	0.021	0.152	0.019445	DC	WEE1
ATN1.1	1.31E-06	-0.28467	0.043	0.186	0.019584	DC	ATN1
HLA-F.3	1.32E-06	0.26181	0.273	0.144	0.019682	DC	HLA-F
LHFPL3.3	1.32E-06	-0.34373	0.005	0.121	0.019694	DC	LHFPL3
ZNF439.2	1.34E-06	-0.27512	0.021	0.15	0.019951	DC	ZNF439
RAB20.3	1.36E-06	0.328476	0.38	0.234	0.020303	DC	RAB20
TRIM36.3	1.37E-06	-0.316	0.043	0.184	0.020499	DC	TRIM36
GPT2.1	1.38E-06	-0.30857	0.021	0.149	0.020603	DC	GPT2
ATP5A1.1	1.38E-06	0.138333	0.802	0.672	0.02065	DC	ATP5A1
CLDN12.3	1.42E-06	-0.28427	0.016	0.14	0.021214	DC	CLDN12
C2orf80.2	1.43E-06	-0.33494	0.005	0.121	0.021398	DC	C2orf80
EFNA1.1	1.46E-06	-0.40968	0.011	0.13	0.021763	DC	EFNA1
UNC93B1.3	1.46E-06	0.280449	0.332	0.199	0.021833	DC	UNC93B1
TCF12.3	1.48E-06	-0.34508	0.118	0.286	0.02216	DC	TCF12
RP11-345F	1.50E-06	-0.2926	0.032	0.166	0.022377	DC	RP11-345P4.9

ZNF821.3	1.50E-06	-0.33317	0.032	0.165	0.022476	DC	ZNF821
SCD.3	1.51E-06	-0.43552	0.064	0.215	0.022501	DC	SCD
CES1	1.51E-06	0.150458	0.048	0.011	0.022516	DC	CES1
BCAM.1	1.52E-06	-0.25659	0	0.111	0.022644	DC	BCAM
XRRA1.1	1.52E-06	-0.27	0.011	0.131	0.022766	DC	XRRA1
FAM221A.3	1.53E-06	-0.4169	0.043	0.18	0.022938	DC	FAM221A
NXPH1.3	1.54E-06	-0.34293	0.016	0.138	0.022941	DC	NXPH1
ITGA7.1	1.54E-06	-0.24687	0.016	0.139	0.023018	DC	ITGA7
RHBDD2.2	1.55E-06	-0.38136	0.267	0.462	0.0231	DC	RHBDD2
LSM6.1	1.55E-06	0.302422	0.455	0.329	0.023119	DC	LSM6
MFGE8	1.56E-06	-0.27767	0.048	0.193	0.023376	DC	MFGE8
TMEM178A.	1.57E-06	-0.28929	0.005	0.12	0.023526	DC	TMEM178A
GLCCI1.2	1.58E-06	-0.42987	0.027	0.153	0.02365	DC	GLCCI1
SLC4A4.1	1.60E-06	-0.31755	0	0.11	0.02386	DC	SLC4A4
BLOC1S6.2	1.60E-06	0.368843	0.374	0.258	0.023885	DC	BLOC1S6
KIF1A.2	1.61E-06	-0.39985	0.011	0.128	0.024049	DC	KIF1A
BRD8.1	1.63E-06	-0.33729	0.032	0.164	0.024311	DC	BRD8
CSPG5.2	1.65E-06	-0.37059	0	0.11	0.024621	DC	CSPG5
B2M.3	1.65E-06	-0.27471	1	0.97	0.024633	DC	B2M
C1QC.3	1.66E-06	0.133485	0.738	0.497	0.024793	DC	C1QC
KDM1A.1	1.67E-06	-0.32926	0.059	0.206	0.025004	DC	KDM1A
SIRPB1	1.69E-06	0.132147	0.048	0.011	0.025287	DC	SIRPB1
NPTXR.2	1.70E-06	-0.25655	0.011	0.129	0.0254	DC	NPTXR
PPP1R3B.1	1.72E-06	0.244803	0.15	0.065	0.025639	DC	PPP1R3B
FAM161A.1	1.72E-06	-0.28904	0	0.11	0.025672	DC	FAM161A
MSMO1.2	1.74E-06	-0.36492	0.059	0.203	0.026017	DC	MSMO1
SCARA3.2	1.75E-06	-0.22217	0.016	0.14	0.026112	DC	SCARA3
INSM1.3	1.77E-06	-0.32246	0.005	0.119	0.026407	DC	INSM1
MGLL.2	1.77E-06	-0.33809	0.021	0.147	0.026473	DC	MGLL
DEK.3	1.77E-06	0.274109	0.588	0.462	0.02648	DC	DEK
PIK3AP1.3	1.77E-06	0.241678	0.15	0.063	0.026484	DC	PIK3AP1
PELI2	1.78E-06	0.20331	0.08	0.025	0.026586	DC	PELI2
SEPT10.2	1.81E-06	-0.27568	0.005	0.119	0.026996	DC	10-Sep
CPNE2.3	1.81E-06	-0.3541	0.043	0.18	0.027113	DC	CPNE2
NFIL3.3	1.83E-06	0.28554	0.176	0.082	0.027327	DC	NFIL3
CNRIP1.1	1.83E-06	-0.34917	0.059	0.207	0.027372	DC	CNRIP1
AKAP7.3	1.85E-06	-0.30035	0.053	0.197	0.027661	DC	AKAP7
ATP5F1.2	1.89E-06	0.127495	0.754	0.642	0.028314	DC	ATP5F1
ZNF358.2	1.91E-06	-0.22301	0.032	0.167	0.028576	DC	ZNF358
RYR1	1.92E-06	0.146639	0.059	0.015	0.028665	DC	RYR1
TXNIP.1	1.92E-06	0.217319	0.594	0.444	0.028692	DC	TXNIP
ICOSLG.3	1.98E-06	0.338999	0.187	0.089	0.029613	DC	ICOSLG
AQP4.2	1.98E-06	-0.49587	0.037	0.168	0.029635	DC	AQP4
PPAT.1	1.99E-06	-0.28455	0.021	0.146	0.029799	DC	PPAT
RFK.1	2.00E-06	-0.33354	0.086	0.24	0.029849	DC	RFK
SPATC1L.2	2.00E-06	-0.23883	0.053	0.197	0.029912	DC	SPATC1L
KIDINS220	2.01E-06	-0.32916	0.107	0.271	0.029971	DC	KIDINS220
MED19	2.02E-06	-0.33206	0.112	0.284	0.030137	DC	MED19
GNAQ.3	2.02E-06	0.373703	0.401	0.285	0.030248	DC	GNAQ
RSL1D1.1	2.05E-06	0.218603	0.61	0.495	0.030673	DC	RSL1D1
IQCK.1	2.06E-06	-0.28609	0.011	0.128	0.03072	DC	IQCK

ANK2.3	2.06E-06	-0.24973	0.032	0.166	0.030791	DC	ANK2
CDK4.1	2.07E-06	-0.42584	0.305	0.487	0.03095	DC	CDK4
GNA11	2.07E-06	-0.24579	0	0.108	0.030977	DC	GNA11
TMEM181.3	2.08E-06	-0.20495	0.032	0.166	0.031034	DC	TMEM181
RP4-665J2	2.13E-06	-0.28913	0.011	0.127	0.031863	DC	RP4-665J23.1
BAK1.3	2.13E-06	0.359947	0.214	0.114	0.031894	DC	BAK1
PRTFDC1	2.15E-06	-0.31189	0.016	0.137	0.032182	DC	PRTFDC1
NNMT.1	2.17E-06	-0.65273	0.032	0.16	0.032416	DC	NNMT
VWA1.3	2.20E-06	-0.28815	0.021	0.146	0.032834	DC	VWA1
SDC2.3	2.20E-06	0.456633	0.305	0.19	0.032933	DC	SDC2
KIF2A.3	2.22E-06	-0.35114	0.086	0.238	0.033104	DC	KIF2A
ATP1A1.1	2.22E-06	0.21597	0.594	0.471	0.03321	DC	ATP1A1
C11orf96.	2.23E-06	-0.35993	0.016	0.136	0.033389	DC	C11orf96
SLC16A1.3	2.24E-06	-0.3406	0.08	0.236	0.033501	DC	SLC16A1
MAP1A.2	2.25E-06	-0.30387	0.075	0.232	0.033585	DC	MAP1A
SOX12.2	2.27E-06	-0.24268	0.005	0.117	0.033982	DC	SOX12
EFS.1	2.28E-06	-0.30416	0	0.107	0.034021	DC	EFS
SLC35F1.1	2.31E-06	-0.22833	0.011	0.128	0.034452	DC	SLC35F1
MAT2A.2	2.31E-06	0.331597	0.497	0.382	0.034552	DC	MAT2A
UQCRB.3	2.31E-06	0.135428	0.781	0.676	0.034575	DC	UQCRB
GIPC1.1	2.32E-06	-0.33548	0.107	0.27	0.034706	DC	GIPC1
TMEM255B.	2.32E-06	0.243093	0.134	0.054	0.034706	DC	TMEM255B
DLX1.3	2.33E-06	-0.41426	0.005	0.117	0.034751	DC	DLX1
SEMA4D.3	2.33E-06	0.196354	0.176	0.079	0.034871	DC	SEMA4D
MSANTD4	2.34E-06	-0.27918	0.011	0.127	0.034913	DC	MSANTD4
RARRES2.2	2.34E-06	-0.66096	0.027	0.151	0.034944	DC	RARRES2
PTX3.2	2.35E-06	-0.33122	0.005	0.117	0.0351	DC	PTX3
ACYP1	2.35E-06	-0.33104	0.043	0.179	0.035122	DC	ACYP1
CARD8.2	2.37E-06	0.272063	0.262	0.147	0.035492	DC	CARD8
PNPLA2.3	2.38E-06	0.329088	0.326	0.21	0.035558	DC	PNPLA2
TNPO2.1	2.38E-06	-0.25437	0.043	0.182	0.035608	DC	TNPO2
RASSF8-AS	2.39E-06	-0.32864	0.032	0.162	0.035647	DC	RASSF8-AS1
TNFRSF19.	2.50E-06	-0.22251	0	0.107	0.03736	DC	TNFRSF19
SPARC.2	2.50E-06	-0.53253	0.214	0.387	0.03743	DC	SPARC
AMER2.2	2.54E-06	-0.44222	0.005	0.116	0.037903	DC	AMER2
SIGIRR.3	2.54E-06	0.394815	0.31	0.194	0.037905	DC	SIGIRR
DAAM1.1	2.56E-06	-0.38449	0.091	0.243	0.038261	DC	DAAM1
ROCK2	2.58E-06	-0.25401	0.027	0.154	0.03851	DC	ROCK2
KCNE4.1	2.58E-06	-0.25139	0	0.106	0.038544	DC	KCNE4
CPXM1.2	2.59E-06	-0.27043	0.011	0.126	0.038732	DC	CPXM1
ZSCAN18.1	2.63E-06	-0.26718	0.08	0.234	0.03927	DC	ZSCAN18
FRMD4B.3	2.63E-06	0.254436	0.283	0.157	0.039346	DC	FRMD4B
CHL1.1	2.65E-06	-0.2843	0.027	0.155	0.039552	DC	CHL1
DTNA.2	2.65E-06	-0.38926	0.096	0.255	0.039666	DC	DTNA
CLIC2.1	2.67E-06	0.321797	0.112	0.043	0.039889	DC	CLIC2
ZNF827.2	2.73E-06	-0.24368	0.005	0.116	0.040722	DC	ZNF827
CTC-428H1	2.73E-06	0.199268	0.07	0.02	0.040767	DC	CTC-428H11.2
ALDH7A1	2.73E-06	-0.27753	0.112	0.278	0.040862	DC	ALDH7A1
FLOT1.3	2.75E-06	0.2329	0.615	0.501	0.041053	DC	FLOT1
PDLIM3.2	2.77E-06	-0.40056	0.005	0.116	0.041326	DC	PDLIM3
NT5C3B.2	2.77E-06	-0.33851	0.16	0.334	0.041386	DC	NT5C3B

PALM.1	2.77E-06	-0.23822	0	0.106	0.041451	DC	PALM
ARL9.2	2.77E-06	-0.24629	0	0.106	0.041451	DC	ARL9
TSPAN3.2	2.78E-06	-0.31508	0.283	0.462	0.041563	DC	TSPAN3
SEPP1.2	2.79E-06	-0.67186	0.176	0.331	0.041677	DC	SEPP1
SLC25A23.	2.80E-06	-0.22468	0.016	0.135	0.041916	DC	SLC25A23
PDGFRA.2	2.84E-06	-0.37217	0.027	0.15	0.042378	DC	PDGFRA
SMDT1.1	2.84E-06	0.176357	0.668	0.557	0.042403	DC	SMDT1
EHMT2.2	2.85E-06	-0.30014	0.059	0.203	0.042571	DC	EHMT2
AHCYL1.3	2.87E-06	-0.39663	0.155	0.33	0.042874	DC	AHCYL1
ABAT.3	2.87E-06	-0.28501	0.032	0.162	0.042955	DC	ABAT
MTDH.3	2.90E-06	0.195028	0.727	0.608	0.04332	DC	MTDH
PPP1R18.3	2.90E-06	0.328278	0.417	0.291	0.043344	DC	PPP1R18
HMGB3.2	2.93E-06	-0.33591	0.118	0.281	0.04376	DC	HMGB3
DNALI1.2	2.94E-06	-0.2905	0.005	0.115	0.043888	DC	DNALI1
ID3.3	2.95E-06	0.138827	0.412	0.262	0.044063	DC	ID3
CAMK1.2	2.95E-06	0.414728	0.337	0.224	0.044147	DC	CAMK1
RPAP2.1	2.95E-06	-0.28434	0.048	0.187	0.044159	DC	RPAP2
ZBTB8A.2	2.98E-06	-0.30009	0	0.105	0.044574	DC	ZBTB8A
RP11-792A	3.00E-06	-0.37067	0.07	0.212	0.044782	DC	RP11-792A8.4
UCHL3.1	3.03E-06	0.336123	0.369	0.245	0.045262	DC	UCHL3
LGALS3BP.	3.04E-06	-0.46571	0.273	0.452	0.045386	DC	LGALS3BP
PIPOX.2	3.06E-06	-0.27687	0.016	0.134	0.045722	DC	PIPOX
IKBIP.2	3.10E-06	0.234206	0.353	0.224	0.046383	DC	IKBIP
CBR4.3	3.12E-06	-0.35425	0.053	0.19	0.046638	DC	CBR4
MACF1.1	3.13E-06	-0.25857	0.144	0.325	0.046803	DC	MACF1
SPATS2L.2	3.14E-06	-0.33541	0.155	0.335	0.046935	DC	SPATS2L
LTBP3.1	3.16E-06	-0.32041	0.07	0.212	0.047234	DC	LTBP3
CAMSAP2.1	3.16E-06	-0.26924	0.027	0.151	0.04727	DC	CAMSAP2
TLN1.3	3.20E-06	0.298981	0.406	0.283	0.047894	DC	TLN1
JMJD1C.3	3.25E-06	0.357317	0.444	0.332	0.048507	DC	JMJD1C
KLHL42.2	3.26E-06	-0.23596	0.032	0.161	0.048684	DC	KLHL42
EIF4EBP2.	3.26E-06	0.327638	0.246	0.139	0.048751	DC	EIF4EBP2
LYRM9.2	3.27E-06	-0.3585	0.043	0.175	0.048869	DC	LYRM9
MESP1	3.29E-06	-0.24784	0.016	0.133	0.049129	DC	MESP1
DIRAS1.1	3.30E-06	-0.24232	0.005	0.115	0.049322	DC	DIRAS1
LAMC1.1	3.34E-06	-0.20932	0	0.104	0.049958	DC	LAMC1
ADORA1.1	3.36E-06	-0.28969	0.011	0.124	0.050263	DC	ADORA1
ATP9A.2	3.41E-06	-0.26884	0.005	0.114	0.050912	DC	ATP9A
SHISA4.2	3.42E-06	-0.28593	0.091	0.249	0.051089	DC	SHISA4
ANKIB1.2	3.44E-06	-0.2794	0.064	0.211	0.051451	DC	ANKIB1
TRIM24.3	3.47E-06	-0.26552	0.053	0.194	0.0518	DC	TRIM24
GTF2H2.1	3.52E-06	-0.25348	0.048	0.187	0.052569	DC	GTF2H2
MTUS1.2	3.53E-06	-0.37833	0.027	0.149	0.052729	DC	MTUS1
FRYL.3	3.55E-06	-0.24791	0.053	0.19	0.053001	DC	FRYL
DBI.3	3.60E-06	-0.32755	0.861	0.843	0.053822	DC	DBI
GPR84.3	3.60E-06	0.32339	0.16	0.072	0.053867	DC	GPR84
TMEM132B.	3.63E-06	-0.23795	0	0.103	0.054271	DC	TMEM132B
AKAP6.2	3.63E-06	-0.28472	0	0.103	0.054271	DC	AKAP6
SUN1.3	3.72E-06	-0.30188	0.064	0.21	0.055591	DC	SUN1
ATP1A3.2	3.73E-06	-0.38456	0.011	0.122	0.055762	DC	ATP1A3
SERP2.1	3.75E-06	-0.29963	0	0.103	0.055982	DC	SERP2

ACTR3.1	3.76E-06	0.187412	0.642	0.558	0.056186	DC	ACTR3
PFKFB3.3	3.77E-06	0.388718	0.364	0.242	0.056302	DC	PFKFB3
SCIMP.3	3.77E-06	0.246293	0.176	0.081	0.056363	DC	SCIMP
MUM1.3	3.78E-06	-0.33993	0.187	0.367	0.056485	DC	MUM1
NDP.1	3.79E-06	-0.30538	0.005	0.113	0.056578	DC	NDP
ID1.1	3.81E-06	-0.34089	0.011	0.122	0.056869	DC	ID1
MARCH9.2	3.84E-06	-0.3742	0.032	0.156	0.057391	DC	9-Mar
PSMA4.1	3.85E-06	0.177803	0.652	0.536	0.057473	DC	PSMA4
NT5DC2.2	3.85E-06	-0.27076	0.037	0.166	0.057473	DC	NT5DC2
HOXA7.1	3.86E-06	-0.35999	0	0.103	0.057745	DC	HOXA7
ITGB1.2	3.87E-06	0.241967	0.524	0.383	0.057862	DC	ITGB1
DAZAP2.2	3.89E-06	0.221827	0.642	0.535	0.058196	DC	DAZAP2
LAMA5.1	3.98E-06	-0.21101	0.005	0.113	0.059492	DC	LAMA5
PXDN	3.99E-06	-0.23897	0	0.103	0.059564	DC	PXDN
H1FX.3	4.00E-06	-0.36687	0.214	0.387	0.059807	DC	H1FX
ARHGAP21.	4.06E-06	-0.34447	0.096	0.253	0.060697	DC	ARHGAP21
C5AR1.3	4.08E-06	0.227611	0.39	0.235	0.060929	DC	C5AR1
DTYMK.2	4.08E-06	-0.35651	0.16	0.332	0.061045	DC	DTYMK
NAP1L1.1	4.12E-06	0.133517	0.786	0.672	0.061571	DC	NAP1L1
CD200.3	4.16E-06	-0.31838	0.005	0.112	0.062183	DC	CD200
ARHGAP12.	4.17E-06	-0.30162	0.053	0.191	0.062304	DC	ARHGAP12
SNAPC3.1	4.19E-06	-0.31522	0.021	0.138	0.062648	DC	SNAPC3
RENBP.3	4.29E-06	0.25539	0.251	0.134	0.064141	DC	RENBP
COLGALT2.	4.40E-06	-0.26233	0.021	0.141	0.06577	DC	COLGALT2
LINC00094	4.44E-06	-0.24176	0.016	0.131	0.066369	DC	LINC00094
HSPA5.2	4.46E-06	0.137285	0.807	0.654	0.066716	DC	HSPA5
PLCG1.1	4.47E-06	-0.23047	0	0.102	0.066729	DC	PLCG1
MAP4.1	4.47E-06	-0.30847	0.16	0.338	0.066818	DC	MAP4
YWHAG.3	4.52E-06	-0.34404	0.214	0.393	0.067483	DC	YWHAG
SHD.2	4.52E-06	-0.40507	0.016	0.128	0.067493	DC	SHD
ZNF677.1	4.54E-06	-0.26557	0.059	0.199	0.067861	DC	ZNF677
CLCN3.1	4.58E-06	-0.29077	0.096	0.251	0.068507	DC	CLCN3
B3GNT2.3	4.59E-06	0.291705	0.144	0.065	0.06859	DC	B3GNT2
TGFBR1.3	4.60E-06	0.300819	0.278	0.16	0.068668	DC	TGFBR1
RNF125.2	4.61E-06	0.1545	0.075	0.023	0.068849	DC	RNF125
EFHC1.3	4.61E-06	-0.32337	0.064	0.207	0.068964	DC	EFHC1
EEF1A1.3	4.62E-06	-0.28125	0.898	0.824	0.068986	DC	EEF1A1
SOGA1.2	4.68E-06	-0.28135	0.032	0.157	0.069952	DC	SOGA1
JUNB.1	4.68E-06	0.153873	0.85	0.722	0.069995	DC	JUNB
EFNB2.2	4.75E-06	-0.2024	0	0.101	0.070989	DC	EFNB2
NUP160.1	4.76E-06	-0.3228	0.037	0.164	0.071074	DC	NUP160
SRSF10.2	4.77E-06	-0.37807	0.091	0.242	0.071274	DC	SRSF10
SLAMF8.3	4.78E-06	0.234691	0.144	0.062	0.071367	DC	SLAMF8
GPR108.2	4.79E-06	0.340035	0.374	0.264	0.071581	DC	GPR108
AGAP1.1	4.82E-06	-0.25497	0.011	0.121	0.071983	DC	AGAP1
IGSF10	4.85E-06	-0.21717	0	0.101	0.072468	DC	IGSF10
TRAPPC6A.	4.86E-06	0.295147	0.385	0.257	0.07262	DC	TRAPPC6A
MORF4L2.2	4.86E-06	-0.32592	0.449	0.628	0.07267	DC	MORF4L2
PHLDA3	4.87E-06	-0.2579	0.043	0.173	0.072722	DC	PHLDA3
SKP1.2	4.87E-06	-0.25979	0.877	0.864	0.072766	DC	SKP1
RFTN2.3	4.94E-06	-0.34333	0.016	0.129	0.073854	DC	RFTN2

PSMB8.1	5.00E-06	0.225628	0.54	0.413	0.074648	DC	PSMB8
AUTS2.3	5.05E-06	-0.27202	0.011	0.12	0.075469	DC	AUTS2
KLHL13.2	5.11E-06	-0.2313	0	0.101	0.076299	DC	KLHL13
COPS8.3	5.12E-06	-0.43584	0.299	0.472	0.076538	DC	COPS8
RP11-262H	5.14E-06	-0.26891	0.011	0.12	0.07685	DC	RP11-262H14.4
FBXO3.1	5.15E-06	-0.29804	0.043	0.172	0.076997	DC	FBXO3
PAM.2	5.15E-06	-0.20987	0.037	0.167	0.077005	DC	PAM
MAML3.2	5.21E-06	0.183164	0.096	0.034	0.077885	DC	MAML3
FBXO32.3	5.22E-06	-0.37963	0.032	0.156	0.077958	DC	FBXO32
ANKS1B.2	5.28E-06	-0.41474	0.005	0.109	0.078886	DC	ANKS1B
PLA2G5.2	5.31E-06	-0.4739	0.005	0.11	0.079395	DC	PLA2G5
CDC42EP1.	5.32E-06	-0.2812	0.016	0.13	0.079575	DC	CDC42EP1
ASRGL1.3	5.47E-06	-0.3002	0.064	0.203	0.081796	DC	ASRGL1
TMEM51.2	5.50E-06	0.279075	0.219	0.117	0.082176	DC	TMEM51
PDCD4.3	5.51E-06	0.234792	0.262	0.147	0.082355	DC	PDCD4
SEZ6L.2	5.54E-06	-0.33365	0.021	0.137	0.082827	DC	SEZ6L
RP11-47L3	5.54E-06	0.126237	0.043	0.009	0.082858	DC	RP11-47L3.1
ANKRD35	5.63E-06	-0.23268	0.021	0.139	0.084156	DC	ANKRD35
TMEM99.2	5.64E-06	-0.25258	0.016	0.129	0.084286	DC	TMEM99
RIPK3.1	5.64E-06	0.129805	0.064	0.018	0.08434	DC	RIPK3
LANCL2.3	5.70E-06	-0.29953	0.032	0.154	0.085208	DC	LANCL2
GDAP1.3	5.70E-06	-0.33846	0.032	0.154	0.085208	DC	GDAP1
GABARAP.1	5.74E-06	0.217267	0.583	0.459	0.085735	DC	GABARAP
WHSC1.2	5.82E-06	-0.25849	0.032	0.155	0.086977	DC	WHSC1
ZEB1.3	5.83E-06	-0.26803	0.059	0.195	0.087188	DC	ZEB1
DCTN4.2	5.85E-06	-0.30659	0.053	0.187	0.087446	DC	DCTN4
ELN.1	5.89E-06	-0.32778	0.005	0.11	0.08809	DC	ELN
FAM78A.2	5.90E-06	0.189776	0.096	0.035	0.088138	DC	FAM78A
ACAP3.3	5.93E-06	-0.31062	0.075	0.218	0.08857	DC	ACAP3
PRDX3.1	5.94E-06	0.197696	0.588	0.465	0.088708	DC	PRDX3
JDP2.3	6.01E-06	0.223165	0.155	0.069	0.089847	DC	JDP2
THAP5.1	6.03E-06	-0.28175	0.048	0.181	0.090134	DC	THAP5
GRK4.1	6.08E-06	-0.26398	0	0.099	0.090884	DC	GRK4
CEP70.1	6.08E-06	-0.26784	0	0.099	0.090884	DC	CEP70
TMEM52B.3	6.12E-06	0.167107	0.118	0.046	0.091403	DC	TMEM52B
PLEKHA4.2	6.18E-06	-0.2404	0.016	0.128	0.092416	DC	PLEKHA4
ANP32A.3	6.18E-06	0.227681	0.54	0.426	0.092417	DC	ANP32A
VEZF1.3	6.18E-06	-0.31418	0.091	0.24	0.092427	DC	VEZF1
UQCRC2	6.19E-06	0.184999	0.62	0.511	0.092561	DC	UQCRC2
FZD7	6.21E-06	-0.21418	0	0.099	0.092771	DC	FZD7
PREPL.1	6.24E-06	-0.25787	0.053	0.191	0.093183	DC	PREPL
JAG1.2	6.24E-06	-0.28791	0.037	0.161	0.093289	DC	JAG1
USMG5.2	6.28E-06	0.140239	0.733	0.618	0.093898	DC	USMG5
RP11-514C	6.33E-06	0.180019	0.08	0.026	0.094656	DC	RP11-514O12.4
SPCS3.3	6.34E-06	0.320822	0.433	0.324	0.094683	DC	SPCS3
CBY1.1	6.35E-06	-0.30274	0.037	0.161	0.09488	DC	CBY1
C19orf12.	6.40E-06	-0.28936	0.027	0.144	0.095626	DC	C19orf12
LRRN1.2	6.43E-06	-0.29968	0.005	0.108	0.096105	DC	LRRN1
CYR61.1	6.52E-06	-0.43655	0.043	0.17	0.097448	DC	CYR61
POU2F2.3	6.56E-06	0.281937	0.246	0.133	0.097982	DC	POU2F2
POU2F1.2	6.62E-06	-0.26659	0.037	0.163	0.098907	DC	POU2F1

ZNF420.1	6.64E-06	-0.28539	0.005	0.108	0.099269	DC	ZNF420
ARRB1.2	6.65E-06	0.151115	0.107	0.04	0.099367	DC	ARRB1
CDKN2D.3	6.66E-06	-0.27917	0.037	0.162	0.099477	DC	CDKN2D
SFMBT2.3	6.66E-06	0.247426	0.166	0.076	0.09957	DC	SFMBT2
RASSF7.3	6.67E-06	0.295298	0.15	0.069	0.099683	DC	RASSF7
SLC10A4.2	6.81E-06	-0.20591	0	0.098	0.101753	DC	SLC10A4
SELK.3	6.83E-06	0.121347	0.802	0.699	0.102142	DC	SELK
UXT.2	6.84E-06	0.195606	0.647	0.536	0.102191	DC	UXT
LBH.3	6.85E-06	-0.44365	0.037	0.155	0.102373	DC	LBH
NAIP.3	6.96E-06	0.247495	0.209	0.107	0.103941	DC	NAIP
NELL2.1	7.05E-06	-0.29603	0.005	0.108	0.10532	DC	NELL2
SOD1.2	7.05E-06	-0.3246	0.684	0.758	0.105342	DC	SOD1
MAP6.1	7.09E-06	-0.21895	0	0.098	0.106016	DC	MAP6
CTSA.3	7.10E-06	0.300013	0.508	0.397	0.106071	DC	CTSA
CTD-2020K	7.10E-06	0.133855	0.075	0.023	0.106159	DC	CTD-2020K17.1
TGFB1I1.2	7.12E-06	-0.21168	0.016	0.128	0.106469	DC	TGFB1I1
ENDOD1.1	7.16E-06	-0.2449	0.027	0.145	0.106951	DC	ENDOD1
ORMDL2.1	7.22E-06	0.291022	0.401	0.27	0.107845	DC	ORMDL2
ATL1	7.29E-06	-0.29503	0.011	0.118	0.108876	DC	ATL1
NUFIP1.1	7.37E-06	-0.28889	0.011	0.117	0.110123	DC	NUFIP1
GGPS1.2	7.56E-06	-0.30312	0.15	0.32	0.112954	DC	GGPS1
ZNRF1.3	7.56E-06	-0.20035	0.011	0.118	0.112994	DC	ZNRF1
NLN.1	7.56E-06	-0.25599	0.053	0.187	0.113016	DC	NLN
RAB9A.1	7.64E-06	-0.3702	0.144	0.306	0.1141	DC	RAB9A
TMEM243.1	7.64E-06	0.322029	0.321	0.2	0.114145	DC	TMEM243
CCDC34.1	7.72E-06	-0.29842	0.043	0.169	0.115359	DC	CCDC34
CDH11.1	7.74E-06	-0.26126	0.011	0.117	0.11561	DC	CDH11
DIRAS3.2	7.79E-06	-0.50775	0.016	0.125	0.116444	DC	DIRAS3
P2RY6.2	7.84E-06	0.184781	0.091	0.032	0.117235	DC	P2RY6
LRRC4C.1	7.86E-06	-0.30406	0	0.097	0.11746	DC	LRRC4C
GATAD1.1	7.90E-06	-0.24588	0.064	0.203	0.118117	DC	GATAD1
TMEM121.2	7.91E-06	-0.23513	0.005	0.107	0.118133	DC	TMEM121
LINC01003	8.02E-06	-0.26499	0.059	0.194	0.119904	DC	LINC01003
AASS.1	8.11E-06	-0.26229	0.011	0.117	0.121237	DC	AASS
DYNC1I1.3	8.23E-06	-0.38015	0.005	0.106	0.122964	DC	DYNC1I1
SQLE.1	8.24E-06	-0.31064	0.102	0.251	0.123132	DC	SQLE
TMEM25.2	8.26E-06	-0.28011	0.016	0.124	0.123494	DC	TMEM25
MANBA.3	8.30E-06	0.35794	0.219	0.119	0.124016	DC	MANBA
MMAB.1	8.30E-06	-0.29866	0.064	0.199	0.124076	DC	MMAB
FAM210B.3	8.36E-06	-0.28477	0.118	0.272	0.124863	DC	FAM210B
ATP5L2	8.38E-06	0.188838	0.187	0.091	0.125245	DC	ATP5L2
DNASE1L1.	8.42E-06	0.253072	0.155	0.071	0.125775	DC	DNASE1L1
TBCB.1	8.43E-06	-0.32508	0.535	0.66	0.126043	DC	TBCB
HTRA1.3	8.44E-06	-0.34358	0.278	0.463	0.12616	DC	HTRA1
SEC14L2.2	8.48E-06	-0.23076	0.011	0.117	0.126662	DC	SEC14L2
SRRM1	8.49E-06	0.221442	0.615	0.516	0.126874	DC	SRRM1
ANKS3.1	8.51E-06	-0.23214	0.043	0.17	0.127117	DC	ANKS3
CPT1C.1	8.56E-06	-0.25605	0.005	0.107	0.127952	DC	CPT1C
CBX5.2	8.60E-06	-0.34982	0.176	0.347	0.128528	DC	CBX5
CSNK1E.2	8.70E-06	-0.28144	0.144	0.309	0.129987	DC	CSNK1E
ZNF254	8.70E-06	-0.28186	0.027	0.143	0.130078	DC	ZNF254

INPP5D.3	8.75E-06	0.235078	0.176	0.084	0.130803	DC		INPP5D
LINC00657	8.78E-06	-0.31852	0.16	0.334	0.131141	DC		LINC00657
CSF1.1	8.89E-06	-0.24669	0	0.096	0.132812	DC		CSF1
SH3YL1.2	8.89E-06	-0.32239	0.053	0.184	0.132889	DC		SH3YL1
FGD3.2	8.98E-06	0.219192	0.102	0.038	0.134181	DC		FGD3
TRAPPC1	9.00E-06	0.135894	0.733	0.613	0.134537	DC		TRAPPC1
TTC3.2	9.03E-06	-0.36262	0.38	0.518	0.134891	DC		TTC3
NFE2L3	9.12E-06	0.269583	0.086	0.03	0.136262	DC		NFE2L3
ZNF579.2	9.12E-06	-0.21258	0.011	0.116	0.136309	DC		ZNF579
THSD1.1	9.16E-06	-0.20041	0	0.096	0.136949	DC		THSD1
FGF1.1	9.35E-06	-0.30926	0	0.095	0.139778	DC		FGF1
ABCA7	9.38E-06	0.139937	0.053	0.014	0.140202	DC		ABCA7
TRIM22.2	9.39E-06	0.244588	0.241	0.133	0.140308	DC		TRIM22
ADRBK2.3	9.39E-06	0.275335	0.123	0.052	0.140356	DC		ADRBK2
TM4SF1.3	9.40E-06	-0.3245	0.011	0.114	0.140464	DC		TM4SF1
IPO9.1	9.46E-06	-0.20716	0.086	0.234	0.14143	DC		IPO9
NLGN3.2	9.49E-06	-0.26941	0.011	0.115	0.141858	DC		NLGN3
TMEM14C.1	9.50E-06	0.145964	0.695	0.589	0.141978	DC		TMEM14C
KIAA1549.	9.55E-06	-0.25733	0	0.095	0.142664	DC		KIAA1549
SECISBP2L	9.59E-06	-0.29874	0.048	0.176	0.143371	DC		SECISBP2L
MTSS1L.1	9.64E-06	-0.23525	0	0.095	0.14413	DC		MTSS1L
PTRH1.1	9.66E-06	-0.11772	0.053	0.189	0.14438	DC		PTRH1
CAMK2B.2	9.68E-06	-0.26389	0.011	0.115	0.14461	DC		CAMK2B
TSPYL5	9.69E-06	0.103821	0.043	0.01	0.144823	DC		TSPYL5
COPS7B	9.70E-06	-0.30647	0.064	0.199	0.145019	DC		COPS7B
CORO1B.3	9.74E-06	0.243979	0.471	0.359	0.145577	DC		CORO1B
NACA2	9.76E-06	0.286682	0.283	0.166	0.145914	DC		NACA2
COX7B.1	9.80E-06	0.131086	0.781	0.64	0.146498	DC		COX7B
NTRK2.2	1.00E-05	-0.4653	0.016	0.122	0.150005	DC		NTRK2
LRRC58.2	1.00E-05	-0.21115	0.07	0.209	0.150161	DC		LRRC58
FYN.2	1.01E-05	-0.3308	0.16	0.329	0.150747	DC		FYN
PIM2.2	1.01E-05	0.288841	0.123	0.052	0.151074	DC		PIM2
ATXN7L3B.	1.03E-05	-0.24369	0.053	0.183	0.153402	DC		ATXN7L3B
MMP16.1	1.03E-05	-0.23065	0.005	0.105	0.153998	DC		MMP16
PFKM	1.03E-05	-0.25992	0.053	0.186	0.15444	DC		PFKM
VPS35.1	1.03E-05	0.220262	0.529	0.429	0.154535	DC		VPS35
GAD1.3	1.03E-05	-0.26219	0.011	0.115	0.154603	DC		GAD1
CCDC80.2	1.04E-05	-0.32444	0.011	0.114	0.154874	DC		CCDC80
KIF3B.3	1.04E-05	-0.2333	0.021	0.132	0.155154	DC		KIF3B
CCL3.3	1.04E-05	0.104212	0.61	0.419	0.155634	DC		CCL3
CDK5R1.3	1.05E-05	-0.27614	0	0.095	0.156399	DC		CDK5R1
SMARCD1.3	1.05E-05	-0.21151	0.075	0.217	0.156731	DC		SMARCD1
SOX21	1.06E-05	-0.19876	0	0.094	0.158003	DC		SOX21
POLR2F.3	1.08E-05	-0.35078	0.39	0.544	0.161442	DC		POLR2F
DYNLL2.3	1.08E-05	-0.23859	0.07	0.208	0.162025	DC		DYNLL2
CPNE4.1	1.09E-05	-0.19588	0	0.094	0.162915	DC		CPNE4
SEPT4.3	1.09E-05	-0.42082	0.021	0.132	0.162963	DC		4-Sep
APBB2.2	1.11E-05	-0.25263	0.011	0.114	0.166322	DC		APBB2
PCDHB10.2	1.15E-05	-0.28194	0.005	0.104	0.171128	DC		PCDHB10
RP11-742E	1.15E-05	0.175499	0.086	0.03	0.171215	DC		RP11-742B18.1
KIF9.3	1.15E-05	-0.27569	0.048	0.176	0.171669	DC		KIF9

FAM57B.2	1.16E-05	-0.29091	0	0.094	0.173194	DC	FAM57B
CERCAM.1	1.17E-05	-0.27867	0.021	0.131	0.174561	DC	CERCAM
DZIP1.2	1.17E-05	-0.19355	0.005	0.104	0.174877	DC	DZIP1
NRP2.3	1.17E-05	-0.33045	0.086	0.223	0.175143	DC	NRP2
LRRTM2.2	1.18E-05	-0.19875	0.005	0.104	0.175956	DC	LRRTM2
CHCHD6.1	1.18E-05	-0.23805	0.091	0.238	0.1763	DC	CHCHD6
RSU1	1.19E-05	0.312272	0.374	0.267	0.17722	DC	RSU1
LGR4.3	1.19E-05	-0.1895	0.005	0.104	0.17815	DC	LGR4
CASP3.2	1.20E-05	-0.27873	0.064	0.199	0.178771	DC	CASP3
GALK1.3	1.20E-05	0.296802	0.31	0.202	0.178779	DC	GALK1
SMAD5.2	1.20E-05	-0.29237	0.048	0.173	0.178957	DC	SMAD5
NME7.3	1.21E-05	-0.31554	0.043	0.163	0.180383	DC	NME7
MCC.1	1.21E-05	-0.19906	0.016	0.123	0.180812	DC	MCC
TAPBP.3	1.21E-05	0.259251	0.449	0.341	0.181289	DC	TAPBP
FAM127A	1.21E-05	-0.28207	0.273	0.462	0.181504	DC	FAM127A
GDAP1L1.2	1.22E-05	-0.3623	0.005	0.102	0.18225	DC	GDAP1L1
SFXN1.1	1.23E-05	-0.29698	0.08	0.219	0.183079	DC	SFXN1
TUBB4A.3	1.25E-05	-0.49366	0.016	0.12	0.186327	DC	TUBB4A
FAM171B.1	1.25E-05	-0.2528	0.011	0.112	0.187268	DC	FAM171B
ST8SIA1.1	1.26E-05	-0.21144	0	0.093	0.187902	DC	ST8SIA1
PEMT.2	1.26E-05	-0.28875	0.075	0.215	0.188355	DC	PEMT
SCCPDH.1	1.26E-05	-0.36628	0.209	0.38	0.188986	DC	SCCPDH
FOXJ3.1	1.27E-05	-0.23291	0.011	0.113	0.1891	DC	FOXJ3
ACSS3.1	1.28E-05	-0.204	0.011	0.113	0.190941	DC	ACSS3
ARNT2.3	1.28E-05	-0.21967	0.011	0.113	0.191695	DC	ARNT2
LINC00632	1.28E-05	-0.34164	0	0.093	0.191768	DC	LINC00632
LAMB1	1.28E-05	-0.2175	0.005	0.103	0.191773	DC	LAMB1
DCAKD.2	1.29E-05	-0.24534	0.027	0.14	0.192033	DC	DCAKD
USP46.3	1.30E-05	-0.25566	0.027	0.139	0.193543	DC	USP46
ADORA3.3	1.30E-05	0.174991	0.439	0.258	0.193826	DC	ADORA3
SEPT7.3	1.30E-05	-0.32819	0.631	0.704	0.193907	DC	7-Sep
NEIL2.2	1.30E-05	-0.22565	0.064	0.201	0.194402	DC	NEIL2
SYT1.3	1.30E-05	-0.40076	0.005	0.101	0.195011	DC	SYT1
ALKBH2.3	1.31E-05	-0.24693	0.064	0.197	0.196164	DC	ALKBH2
COMMD9.2	1.32E-05	0.400019	0.406	0.308	0.197238	DC	COMMD9
STK25.3	1.32E-05	-0.27791	0.144	0.305	0.1973	DC	STK25
DNAL4.1	1.33E-05	-0.26519	0.021	0.129	0.198134	DC	DNAL4
B4GALNT1.	1.33E-05	-0.3873	0.032	0.145	0.198315	DC	B4GALNT1
TUBG1.1	1.34E-05	-0.2629	0.091	0.236	0.200402	DC	TUBG1
SLC2A9.1	1.34E-05	0.124049	0.075	0.024	0.200727	DC	SLC2A9
C20orf27.	1.34E-05	0.252009	0.465	0.358	0.200968	DC	C20orf27
RBM4B.1	1.35E-05	-0.30947	0.064	0.194	0.202297	DC	RBM4B
KLC1.3	1.36E-05	-0.29519	0.193	0.362	0.203045	DC	KLC1
CCDC102B.	1.37E-05	-0.26046	0.005	0.102	0.204245	DC	CCDC102B
TNFAIP8L3	1.37E-05	0.224497	0.08	0.028	0.204612	DC	TNFAIP8L3
ZHX3.1	1.37E-05	-0.19103	0.016	0.121	0.205314	DC	ZHX3
CADM3.1	1.38E-05	-0.4351	0.005	0.102	0.206248	DC	CADM3
NKAIN4.2	1.38E-05	-0.37792	0.005	0.102	0.206428	DC	NKAIN4
ZNF385D.1	1.38E-05	-0.22922	0.011	0.112	0.206538	DC	ZNF385D
BIVM.1	1.38E-05	-0.22902	0.011	0.112	0.206754	DC	BIVM
VDAC2	1.38E-05	0.129077	0.759	0.637	0.206787	DC	VDAC2

MAN2B1.3	1.39E-05	0.217343	0.144	0.066	0.207128	DC	MAN2B1
ITPRIP.3	1.39E-05	0.262066	0.155	0.074	0.20713	DC	ITPRIP
LIFR	1.40E-05	-0.25423	0.011	0.111	0.209049	DC	LIFR
CD59.3	1.40E-05	-0.41342	0.39	0.556	0.209365	DC	CD59
YAE1D1.1	1.41E-05	-0.30792	0.043	0.162	0.210959	DC	YAE1D1
RAB3A.3	1.41E-05	-0.38292	0.048	0.166	0.21121	DC	RAB3A
CLASP1.2	1.42E-05	-0.24094	0.027	0.14	0.211747	DC	CLASP1
TOR4A.2	1.42E-05	0.140662	0.075	0.024	0.212001	DC	TOR4A
TLE2.2	1.42E-05	-0.24562	0	0.092	0.212306	DC	TLE2
TNFAIP6.1	1.43E-05	-0.43597	0.011	0.111	0.213923	DC	TNFAIP6
B9D1.1	1.45E-05	-0.25851	0.005	0.102	0.217292	DC	B9D1
LINC00511	1.46E-05	-0.25064	0.016	0.121	0.217645	DC	LINC00511
ZNF146.1	1.46E-05	-0.2253	0.086	0.23	0.21793	DC	ZNF146
UBXN1.3	1.46E-05	0.174858	0.717	0.619	0.218252	DC	UBXN1
ZNF559.2	1.47E-05	-0.29147	0.032	0.147	0.219109	DC	ZNF559
MBD3.1	1.49E-05	-0.21283	0.043	0.166	0.222212	DC	MBD3
PTS.2	1.49E-05	-0.2938	0.166	0.329	0.22337	DC	PTS
CMTM5.2	1.50E-05	-0.47998	0.005	0.101	0.223845	DC	CMTM5
TEAD2.2	1.51E-05	-0.1924	0	0.091	0.225657	DC	TEAD2
LINC0093€	1.52E-05	0.339753	0.241	0.139	0.226838	DC	LINC00936
DNAJC4.2	1.52E-05	0.281244	0.337	0.227	0.227065	DC	DNAJC4
LAMP5.2	1.52E-05	-0.23926	0.011	0.111	0.227493	DC	LAMP5
GSAP.2	1.54E-05	0.25984	0.16	0.078	0.229497	DC	GSAP
LRRC8A.2	1.54E-05	-0.29014	0.048	0.17	0.230613	DC	LRRC8A
ARL6IP4.3	1.55E-05	0.182251	0.722	0.623	0.231368	DC	ARL6IP4
SSBP3.2	1.55E-05	-0.23777	0.021	0.13	0.232062	DC	SSBP3
IFI16.2	1.55E-05	0.145217	0.54	0.391	0.232247	DC	IFI16
STRBP.3	1.56E-05	-0.24837	0.021	0.128	0.233403	DC	STRBP
TMED1.2	1.57E-05	-0.34798	0.139	0.294	0.233994	DC	TMED1
IFT57.3	1.57E-05	-0.31792	0.102	0.25	0.234096	DC	IFT57
SCAMP2.3	1.57E-05	0.264961	0.492	0.372	0.235199	DC	SCAMP2
TPM1.1	1.57E-05	-0.28156	0.107	0.255	0.235364	DC	TPM1
TAF7.2	1.58E-05	-0.36041	0.401	0.556	0.236446	DC	TAF7
ADIPOR1.3	1.58E-05	0.284442	0.481	0.378	0.236449	DC	ADIPOR1
SLC6A8.1	1.59E-05	-0.29618	0.016	0.119	0.237699	DC	SLC6A8
EIF3J-AS1	1.59E-05	-0.29287	0.021	0.129	0.237975	DC	EIF3J-AS1
PTK2.1	1.60E-05	-0.22587	0.053	0.18	0.238783	DC	PTK2
SNRNP27.1	1.60E-05	-0.31657	0.187	0.355	0.239429	DC	SNRNP27
PPFIA3.1	1.60E-05	-0.19978	0	0.091	0.239835	DC	PPFIA3
RAD51C.1	1.61E-05	-0.2215	0.112	0.269	0.240362	DC	RAD51C
RALB.2	1.61E-05	0.301266	0.257	0.155	0.241224	DC	RALB
CMPK1.3	1.62E-05	0.238698	0.508	0.415	0.241926	DC	CMPK1
LAP3.1	1.63E-05	0.131346	0.631	0.489	0.242852	DC	LAP3
TMX1.1	1.64E-05	0.260151	0.508	0.401	0.24506	DC	TMX1
DLX6-AS1.	1.64E-05	-0.65292	0.027	0.132	0.24535	DC	DLX6-AS1
CHST7.1	1.66E-05	-0.20667	0.011	0.111	0.247389	DC	CHST7
SNX21	1.66E-05	-0.21512	0.016	0.12	0.248776	DC	SNX21
SEC22C.1	1.68E-05	-0.26601	0.086	0.23	0.25127	DC	SEC22C
GSK3A.2	1.70E-05	-0.27126	0.064	0.192	0.25342	DC	GSK3A
NRXN2.3	1.70E-05	-0.30106	0.016	0.118	0.254217	DC	NRXN2
NAPEPLD	1.70E-05	-0.2554	0.005	0.1	0.25472	DC	NAPEPLD

PIM3.3	1.72E-05	0.321923	0.294	0.189	0.256644	DC	PIM3
ARL10	1.76E-05	-0.21038	0	0.09	0.262769	DC	ARL10
CSRNP3.2	1.76E-05	-0.29115	0.005	0.1	0.263076	DC	CSRNP3
NME1.1	1.80E-05	-0.2982	0.422	0.57	0.268937	DC	NME1
MTSS1.3	1.80E-05	-0.39243	0.086	0.215	0.269455	DC	MTSS1
TANC1.1	1.81E-05	-0.21059	0.011	0.11	0.27085	DC	TANC1
KCNIP1.2	1.81E-05	-0.27738	0.005	0.1	0.271037	DC	KCNIP1
SESN1.3	1.82E-05	0.256519	0.193	0.1	0.272192	DC	SESN1
USP4.2	1.83E-05	0.343306	0.273	0.173	0.273876	DC	USP4
CNKSR3.2	1.83E-05	-0.19379	0.016	0.119	0.274198	DC	CNKSR3
ARF1.2	1.84E-05	0.10962	0.802	0.692	0.275657	DC	ARF1
DHCR7.2	1.85E-05	-0.25916	0.053	0.18	0.275965	DC	DHCR7
NAP1L5.1	1.87E-05	-0.26444	0.027	0.136	0.278721	DC	NAP1L5
ADD2.1	1.87E-05	-0.23394	0	0.09	0.279248	DC	ADD2
TSPAN15.2	1.88E-05	0.203838	0.16	0.076	0.281466	DC	TSPAN15
LRIG1.2	1.88E-05	-0.22535	0.011	0.11	0.281609	DC	LRIG1
XIST.3	1.89E-05	0.196618	0.417	0.263	0.282	DC	XIST
XRCC6	1.90E-05	0.14521	0.647	0.556	0.283647	DC	XRCC6
BAZ1B.3	1.90E-05	-0.21861	0.144	0.3	0.284408	DC	BAZ1B
BPHL.1	1.91E-05	-0.28269	0.053	0.176	0.284783	DC	BPHL
YTHDF1	1.91E-05	-0.27003	0.059	0.185	0.286002	DC	YTHDF1
DTD1.3	1.92E-05	-0.25533	0.23	0.394	0.286981	DC	DTD1
ROBO2.2	1.92E-05	-0.21185	0.011	0.109	0.28743	DC	ROBO2
LDOC1.2	1.95E-05	-0.33819	0	0.089	0.290796	DC	LDOC1
TULP3.2	1.96E-05	-0.23269	0.021	0.127	0.292891	DC	TULP3
PCYT2.2	1.97E-05	-0.21876	0.043	0.162	0.29415	DC	PCYT2
LTBR.3	1.98E-05	0.181563	0.187	0.094	0.295739	DC	LTBR
UPF3A.2	1.98E-05	-0.32505	0.182	0.347	0.29633	DC	UPF3A
DUSP26.2	1.99E-05	-0.34762	0	0.089	0.296746	DC	DUSP26
REEP5.2	1.99E-05	0.130411	0.706	0.583	0.29692	DC	REEP5
APAF1.3	2.00E-05	0.281837	0.139	0.065	0.2992	DC	APAF1
NAP1L4.2	2.00E-05	-0.33115	0.235	0.406	0.299362	DC	NAP1L4
EEF1G	2.02E-05	0.24389	0.193	0.101	0.301428	DC	EEF1G
SFRP1.2	2.02E-05	-0.20341	0.005	0.099	0.301754	DC	SFRP1
GDE1.3	2.03E-05	-0.26445	0.144	0.302	0.302661	DC	GDE1
ADO.2	2.03E-05	-0.18316	0.021	0.128	0.303227	DC	ADO
HK1.3	2.04E-05	0.294392	0.299	0.194	0.304373	DC	HK1
ELOVL2.3	2.04E-05	-0.20754	0.005	0.099	0.304669	DC	ELOVL2
ADAP1.2	2.05E-05	0.214211	0.102	0.04	0.306137	DC	ADAP1
BAD.1	2.06E-05	-0.30718	0.246	0.42	0.307841	DC	BAD
EIF3C	2.06E-05	0.1922	0.086	0.031	0.308471	DC	EIF3C
MKKS.1	2.07E-05	0.242616	0.508	0.402	0.308651	DC	MKKS
ARHGEF12.	2.08E-05	-0.18448	0.043	0.162	0.311113	DC	ARHGEF12
ACPP	2.09E-05	0.117838	0.048	0.013	0.311685	DC	ACPP
MYL6.3	2.10E-05	-0.19334	0.984	0.941	0.313845	DC	MYL6
MSANTD3.1	2.11E-05	-0.21475	0	0.089	0.315327	DC	MSANTD3
RIF1.2	2.11E-05	-0.2769	0.086	0.222	0.31593	DC	RIF1
HKR1.2	2.12E-05	-0.21349	0.016	0.118	0.316231	DC	HKR1
ERRFI1.1	2.12E-05	-0.25792	0.037	0.153	0.316986	DC	ERRFI1
PLA2G12A.	2.13E-05	-0.26671	0.096	0.236	0.317792	DC	PLA2G12A
BTG1.3	2.13E-05	0.163375	0.834	0.686	0.318726	DC	BTG1

CIITA	2.14E-05	0.188035	0.096	0.037	0.319433	DC		CIITA
ZIC1.2	2.15E-05	-0.26084	0.016	0.116	0.320663	DC		ZIC1
CTU2	2.15E-05	-0.24981	0.027	0.135	0.321055	DC		CTU2
CHRAC1.3	2.16E-05	0.355839	0.241	0.143	0.322098	DC		CHRAC1
TMEM68.1	2.16E-05	-0.22302	0.048	0.169	0.322576	DC		TMEM68
FAF2.2	2.16E-05	-0.2827	0.064	0.191	0.322703	DC		FAF2
FAM200A	2.16E-05	-0.25731	0.005	0.098	0.323329	DC		FAM200A
ENY2.1	2.17E-05	0.128005	0.711	0.602	0.32418	DC		ENY2
ZNF322.1	2.17E-05	-0.23178	0.027	0.135	0.324592	DC		ZNF322
LINC00662	2.17E-05	-0.24668	0.059	0.185	0.324778	DC		LINC00662
FKBP1A.2	2.17E-05	0.112755	0.834	0.697	0.324822	DC		FKBP1A
ARHGEF26.	2.18E-05	-0.23427	0.021	0.127	0.325519	DC		ARHGEF26
C4orf27.1	2.18E-05	-0.26698	0.155	0.321	0.325529	DC		C4orf27
PLAU.3	2.18E-05	0.310026	0.267	0.161	0.326262	DC		PLAU
SLC12A9.3	2.19E-05	0.294962	0.305	0.2	0.326606	DC		SLC12A9
TRIAP1.1	2.19E-05	-0.26425	0.15	0.316	0.327614	DC		TRIAP1
ERLEC1.1	2.21E-05	-0.29035	0.198	0.364	0.330848	DC		ERLEC1
CDH4.2	2.22E-05	-0.20631	0	0.088	0.331686	DC		CDH4
C1orf198.	2.23E-05	-0.3257	0.011	0.106	0.333221	DC		C1orf198
LDOC1L.2	2.24E-05	-0.19352	0	0.088	0.335057	DC		LDOC1L
ZNF566.1	2.25E-05	-0.28112	0.016	0.117	0.336717	DC		ZNF566
AIF1L.2	2.26E-05	-0.38974	0.016	0.115	0.337577	DC		AIF1L
ENPP4.1	2.28E-05	0.21678	0.123	0.053	0.341135	DC		ENPP4
FUCA2.3	2.29E-05	0.272673	0.353	0.241	0.34175	DC		FUCA2
PRPSAP1.3	2.29E-05	-0.28891	0.15	0.309	0.341854	DC		PRPSAP1
MAGI2.2	2.29E-05	-0.27153	0.011	0.107	0.342889	DC		MAGI2
ATPAF1.3	2.30E-05	-0.29581	0.102	0.242	0.344317	DC		ATPAF1
GABPB1.2	2.31E-05	0.396577	0.193	0.106	0.345837	DC		GABPB1
MTMR14.3	2.36E-05	0.358348	0.321	0.22	0.352516	DC		MTMR14
PLEKHB2.3	2.36E-05	0.2731	0.492	0.398	0.35339	DC		PLEKHB2
MRFAP1L1.	2.37E-05	-0.2981	0.086	0.222	0.35386	DC		MRFAP1L1
NCALD.2	2.37E-05	-0.22237	0.021	0.126	0.35445	DC		NCALD
TUBA1B.2	2.37E-05	-0.19534	0.957	0.894	0.35456	DC		TUBA1B
RGS3.1	2.38E-05	-0.19427	0.011	0.108	0.356303	DC		RGS3
TMEM53.1	2.39E-05	-0.27397	0.011	0.106	0.357219	DC		TMEM53
CCDC90B.2	2.40E-05	-0.3205	0.241	0.412	0.359035	DC		CCDC90B
C9orf3.1	2.40E-05	-0.35733	0.011	0.106	0.359311	DC		C9orf3
RNF19A.3	2.41E-05	-0.29748	0.075	0.204	0.360563	DC		RNF19A
SORL1.3	2.41E-05	0.278815	0.353	0.225	0.360589	DC		SORL1
SMIM7.2	2.42E-05	-0.36876	0.337	0.508	0.361233	DC		SMIM7
MOCS2.3	2.44E-05	-0.27589	0.118	0.266	0.365232	DC		MOCS2
SH3BGR.2	2.45E-05	-0.36702	0.021	0.125	0.365666	DC		SH3BGR
TOB1.1	2.46E-05	0.349091	0.348	0.253	0.368128	DC		TOB1
RABEP1.3	2.47E-05	-0.25174	0.107	0.255	0.368535	DC		RABEP1
ENOPH1.2	2.49E-05	-0.31012	0.225	0.392	0.371505	DC		ENOPH1
NUBP2.1	2.49E-05	-0.29516	0.209	0.384	0.372777	DC		NUBP2
GFOD2	2.50E-05	-0.25113	0.027	0.133	0.373225	DC		GFOD2
SIPA1L2	2.51E-05	-0.28801	0.021	0.123	0.374712	DC		SIPA1L2
PLP1.2	2.55E-05	-0.79389	0.059	0.173	0.380383	DC		PLP1
NLGN4X.1	2.56E-05	-0.20825	0.011	0.107	0.381949	DC		NLGN4X
KCNMB4.3	2.57E-05	-0.27868	0.021	0.125	0.383899	DC		KCNMB4

CYTH1.3	2.58E-05	0.20579	0.267	0.156	0.386265	DC	CYTH1
ASAP2.2	2.59E-05	-0.18665	0.005	0.097	0.387597	DC	ASAP2
COX11.1	2.60E-05	-0.28103	0.16	0.321	0.387912	DC	COX11
SLC39A14.	2.61E-05	-0.2947	0.005	0.096	0.390722	DC	SLC39A14
MAN1A1.2	2.62E-05	0.203861	0.112	0.047	0.390916	DC	MAN1A1
METTL2A.1	2.62E-05	-0.19545	0.043	0.16	0.391274	DC	METTL2A
KALRN.1	2.62E-05	-0.2204	0.005	0.097	0.391724	DC	KALRN
PARVA.2	2.62E-05	-0.17913	0.005	0.097	0.392008	DC	PARVA
USP1.2	2.63E-05	-0.25063	0.112	0.261	0.393045	DC	USP1
ANKRD37.1	2.63E-05	0.281297	0.241	0.14	0.393184	DC	ANKRD37
SRSF2.3	2.63E-05	0.1896	0.722	0.603	0.39349	DC	SRSF2
AGPAT2.3	2.63E-05	0.326361	0.278	0.178	0.393588	DC	AGPAT2
FGF14.2	2.64E-05	-0.2137	0	0.087	0.39383	DC	FGF14
HDAC1.1	2.64E-05	0.301325	0.321	0.224	0.39499	DC	HDAC1
RUFY2.3	2.66E-05	-0.24113	0.037	0.151	0.396786	DC	RUFY2
TSKU.1	2.66E-05	-0.18057	0	0.087	0.397824	DC	TSKU
DHCR24	2.66E-05	-0.19503	0.005	0.097	0.397842	DC	DHCR24
CPEB4.2	2.68E-05	-0.2838	0.107	0.249	0.400373	DC	CPEB4
PQBP1.2	2.68E-05	0.283444	0.444	0.343	0.400955	DC	PQBP1
DALRD3.1	2.69E-05	-0.2018	0.048	0.167	0.401599	DC	DALRD3
SNTA1.1	2.69E-05	-0.17542	0	0.086	0.401858	DC	SNTA1
AP4M1.1	2.73E-05	-0.31271	0.07	0.195	0.408626	DC	AP4M1
TMEM67.1	2.74E-05	-0.22343	0.005	0.097	0.408889	DC	TMEM67
OSMR.1	2.74E-05	-0.1998	0	0.086	0.410048	DC	OSMR
ABI2.1	2.78E-05	-0.28218	0.198	0.364	0.416004	DC	ABI2
DUSP4.3	2.79E-05	0.417259	0.273	0.178	0.417479	DC	DUSP4
PSMB9.2	2.80E-05	0.173278	0.529	0.403	0.418812	DC	PSMB9
CRYZ.2	2.82E-05	-0.23308	0.021	0.125	0.421735	DC	CRYZ
ADRBK1.3	2.83E-05	0.27355	0.176	0.091	0.42229	DC	ADRBK1
CHST10	2.84E-05	-0.22575	0.032	0.141	0.425029	DC	CHST10
IRF2BPL.2	2.87E-05	-0.24909	0.102	0.244	0.428561	DC	IRF2BPL
LRRN3.2	2.88E-05	-0.29867	0.016	0.115	0.429801	DC	LRRN3
RSBN1L.1	2.90E-05	-0.33721	0.203	0.357	0.433621	DC	RSBN1L
DDT.1	2.91E-05	0.167123	0.636	0.52	0.434199	DC	DDT
CYTL1.3	2.91E-05	-0.50129	0.016	0.112	0.434601	DC	CYTL1
PRELID1.3	2.91E-05	0.237137	0.647	0.558	0.435407	DC	PRELID1
KCNE3.2	2.92E-05	0.156955	0.075	0.026	0.435716	DC	KCNE3
CKAP5.3	2.92E-05	-0.23946	0.043	0.158	0.436883	DC	CKAP5
DENND2A.1	2.93E-05	-0.21352	0.005	0.096	0.438263	DC	DENND2A
IL1RL2	2.94E-05	0.113445	0.027	0.005	0.438838	DC	IL1RL2
DCHS1.2	2.94E-05	-0.19254	0	0.086	0.440033	DC	DCHS1
AKAP8L.1	2.96E-05	-0.24863	0.107	0.251	0.441881	DC	AKAP8L
PDZD11.2	2.96E-05	-0.32229	0.193	0.361	0.442656	DC	PDZD11
MBOAT2.2	3.00E-05	-0.24973	0.059	0.184	0.447835	DC	MBOAT2
ZNF426	3.03E-05	-0.25249	0.016	0.114	0.452091	DC	ZNF426
IL6ST.3	3.03E-05	-0.29671	0.112	0.256	0.453221	DC	IL6ST
EPM2AIP1.	3.03E-05	-0.18375	0.07	0.2	0.45328	DC	EPM2AIP1
CACNG7.1	3.03E-05	-0.18129	0	0.085	0.453538	DC	CACNG7
MMP7.1	3.05E-05	0.206524	0.08	0.028	0.455574	DC	MMP7
PPP1R10.2	3.06E-05	0.272312	0.396	0.286	0.457051	DC	PPP1R10
WASF1	3.06E-05	-0.22849	0.005	0.096	0.457065	DC	WASF1

CAPRIN2.1	3.08E-05	-0.17636	0.011	0.106	0.460389	DC	CAPRIN2
STXBP1.2	3.09E-05	-0.2372	0.021	0.123	0.46184	DC	STXBP1
CTNNAL1.2	3.10E-05	-0.2527	0.037	0.149	0.463819	DC	CTNNAL1
SPOCD1.1	3.11E-05	-0.44319	0.086	0.22	0.464135	DC	SPOCD1
FSTL1.1	3.11E-05	-0.18682	0.016	0.115	0.464626	DC	FSTL1
CLASP2.2	3.13E-05	-0.32527	0.075	0.199	0.467439	DC	CLASP2
FAM222B.2	3.13E-05	-0.17581	0.016	0.115	0.468368	DC	FAM222B
PTPN4.2	3.14E-05	-0.25103	0.011	0.104	0.469776	DC	PTPN4
SLC17A9.2	3.18E-05	0.237104	0.08	0.029	0.474692	DC	SLC17A9
VOPP1.2	3.18E-05	0.256397	0.401	0.289	0.474698	DC	VOPP1
FAU.3	3.18E-05	-0.19842	0.995	0.957	0.475835	DC	FAU
BRSK1.1	3.19E-05	-0.21837	0	0.085	0.476961	DC	BRSK1
DPYSL2.2	3.21E-05	-0.29878	0.251	0.426	0.480166	DC	DPYSL2
PSMG3.2	3.22E-05	-0.28299	0.171	0.33	0.481083	DC	PSMG3
NFASC.1	3.22E-05	-0.32528	0.011	0.104	0.481589	DC	NFASC
ZNF521.1	3.22E-05	-0.19048	0	0.085	0.481788	DC	ZNF521
CCDC106.3	3.26E-05	-0.22963	0.096	0.234	0.48663	DC	CCDC106
CHI3L1.3	3.26E-05	-0.75984	0.39	0.527	0.486722	DC	CHI3L1
STK17A.3	3.27E-05	0.301243	0.396	0.292	0.488559	DC	STK17A
TMEM255A.	3.29E-05	-0.22689	0.011	0.104	0.491343	DC	TMEM255A
DUSP15.2	3.31E-05	-0.18368	0.005	0.095	0.495317	DC	DUSP15
PRKAR1B.1	3.32E-05	-0.27626	0.027	0.129	0.495443	DC	PRKAR1B
PACSIN2.3	3.33E-05	0.310529	0.273	0.173	0.498105	DC	PACSIN2
APOBEC3C.	3.33E-05	0.229106	0.225	0.127	0.498264	DC	APOBEC3C
DNAJC3.1	3.34E-05	0.250247	0.396	0.29	0.498665	DC	DNAJC3
PTGFRN	3.34E-05	-0.15081	0.011	0.105	0.499207	DC	PTGFRN
ERV3-1.2	3.35E-05	-0.25416	0.118	0.263	0.50015	DC	ERV3-1
RAP2B.3	3.36E-05	0.292493	0.262	0.164	0.501649	DC	RAP2B
PRCC	3.36E-05	-0.31169	0.15	0.306	0.501917	DC	PRCC
LAMTOR2.1	3.36E-05	0.141047	0.701	0.585	0.502621	DC	LAMTOR2
WIPF3.3	3.38E-05	-0.27063	0.005	0.094	0.504439	DC	WIPF3
KIF3C.3	3.38E-05	-0.23046	0.011	0.104	0.504931	DC	KIF3C
HIST2H2BE	3.38E-05	-0.28581	0.037	0.149	0.505759	DC	HIST2H2BE
FAM217B.3	3.39E-05	-0.23348	0.043	0.157	0.506673	DC	FAM217B
SCAMP1.1	3.40E-05	-0.2274	0.08	0.216	0.508299	DC	SCAMP1
TOP2B.3	3.44E-05	-0.26491	0.102	0.239	0.51427	DC	TOP2B
PKIA.3	3.44E-05	-0.27976	0.005	0.095	0.514703	DC	PKIA
SLC25A36.	3.49E-05	-0.24403	0.123	0.271	0.521526	DC	SLC25A36
PTDSS1.2	3.57E-05	0.334142	0.299	0.2	0.533705	DC	PTDSS1
PYGB.1	3.57E-05	-0.21777	0.08	0.213	0.533706	DC	PYGB
BTBD3.2	3.58E-05	-0.2224	0.032	0.139	0.535124	DC	BTBD3
LMO3	3.60E-05	-0.32587	0	0.084	0.538167	DC	LMO3
SREK1.2	3.62E-05	-0.31269	0.171	0.325	0.540524	DC	SREK1
PARP4.1	3.63E-05	0.330296	0.16	0.082	0.541814	DC	PARP4
GAB3.1	3.65E-05	0.19787	0.086	0.032	0.546073	DC	GAB3
TET1.2	3.67E-05	-0.17288	0	0.084	0.549096	DC	TET1
ITM2C.3	3.68E-05	-0.36415	0.251	0.42	0.55043	DC	ITM2C
KLRC2.2	3.69E-05	-0.34258	0.016	0.111	0.551748	DC	KLRC2
SLC29A4	3.71E-05	-0.21724	0	0.084	0.554643	DC	SLC29A4
B3GALT6.3	3.72E-05	-0.21246	0.032	0.14	0.555956	DC	B3GALT6
PSMA6.2	3.72E-05	0.217179	0.337	0.222	0.556481	DC	PSMA6

CELSR2.2	3.75E-05	-0.20926	0.005	0.094	0.560485	DC	CELSR2
CTDSP1.3	3.80E-05	0.274317	0.326	0.22	0.567535	DC	CTDSP1
IARS.1	3.80E-05	-0.22122	0.08	0.212	0.56801	DC	IARS
ZNF429.2	3.81E-05	-0.23435	0.027	0.131	0.568818	DC	ZNF429
CALM2.2	3.81E-05	-0.18925	0.979	0.932	0.569201	DC	CALM2
PCOLCE2.1	3.83E-05	-0.26103	0	0.083	0.571618	DC	PCOLCE2
SCRN3.1	3.83E-05	-0.22962	0.011	0.102	0.572744	DC	SCRN3
TMEM35.2	3.84E-05	-0.27164	0.005	0.093	0.573931	DC	TMEM35
AKTIP.2	3.86E-05	-0.29402	0.053	0.17	0.577016	DC	AKTIP
AHDC1.2	3.88E-05	-0.2495	0.011	0.103	0.579915	DC	AHDC1
RHOU.2	3.89E-05	-0.32207	0.032	0.136	0.580837	DC	RHOU
CHD6.2	3.90E-05	-0.2142	0.107	0.247	0.583186	DC	CHD6
PIANP.1	3.90E-05	-0.1862	0	0.083	0.583218	DC	PIANP
C3orf70.2	3.93E-05	-0.24466	0.011	0.102	0.587674	DC	C3orf70
OXR1.2	3.93E-05	-0.2731	0.112	0.254	0.587812	DC	OXR1
RAB8A.1	3.97E-05	0.192983	0.455	0.354	0.593605	DC	RAB8A
EFEMP1.2	3.98E-05	-0.64547	0.096	0.223	0.594855	DC	EFEMP1
STOX2.3	3.98E-05	-0.21586	0.005	0.094	0.595314	DC	STOX2
ASTN1	4.04E-05	-0.19506	0.005	0.093	0.60305	DC	ASTN1
IFI27L1.1	4.05E-05	-0.2316	0.064	0.187	0.60506	DC	IFI27L1
C1orf52.3	4.08E-05	-0.30023	0.155	0.309	0.609401	DC	C1orf52
FAM129A.2	4.09E-05	0.303948	0.23	0.138	0.611635	DC	FAM129A
ORAI3.2	4.11E-05	0.271845	0.235	0.14	0.614049	DC	ORAI3
C12orf73.	4.12E-05	-0.20317	0.043	0.156	0.615664	DC	C12orf73
HIP1R.2	4.18E-05	-0.27343	0.016	0.11	0.624643	DC	HIP1R
DYNC1LI2.	4.18E-05	-0.25214	0.187	0.347	0.625131	DC	DYNC1LI2
TMEM63B	4.24E-05	-0.21787	0.032	0.137	0.633121	DC	TMEM63B
WASL.1	4.24E-05	-0.24452	0.064	0.185	0.634221	DC	WASL
TMEM206.2	4.24E-05	-0.21999	0.096	0.233	0.634326	DC	TMEM206
SLC25A45	4.25E-05	0.211621	0.107	0.045	0.635435	DC	SLC25A45
BID.3	4.25E-05	0.298064	0.46	0.355	0.635846	DC	BID
LILRB1.3	4.26E-05	0.174127	0.182	0.09	0.636504	DC	LILRB1
IQCE.1	4.26E-05	-0.22741	0.021	0.12	0.636684	DC	IQCE
MED10.3	4.27E-05	-0.317	0.31	0.486	0.638322	DC	MED10
COQ7.2	4.28E-05	-0.3334	0.07	0.189	0.638868	DC	COQ7
CELF5.3	4.33E-05	-0.30156	0.005	0.092	0.647735	DC	CELF5
DENND1B.3	4.34E-05	0.296345	0.171	0.09	0.648029	DC	DENND1B
HIVEP3.3	4.34E-05	-0.22216	0.043	0.154	0.649181	DC	HIVEP3
ZNF846.2	4.37E-05	0.213253	0.144	0.069	0.653144	DC	ZNF846
NDUFAF5	4.37E-05	-0.27173	0.032	0.135	0.65344	DC	NDUFAF5
MNAT1.2	4.38E-05	-0.22645	0.07	0.195	0.654975	DC	MNAT1
CORO2B.1	4.40E-05	-0.20603	0.011	0.102	0.656926	DC	CORO2B
UBC.3	4.40E-05	-0.22256	0.973	0.95	0.657712	DC	UBC
ALDH6A1.1	4.44E-05	-0.2692	0.08	0.209	0.663219	DC	ALDH6A1
TCEAL4.1	4.44E-05	-0.32079	0.401	0.574	0.66387	DC	TCEAL4
REEP1.3	4.45E-05	-0.23008	0	0.082	0.664503	DC	REEP1
MIR4458HG	4.46E-05	-0.42128	0.053	0.162	0.666826	DC	MIR4458HG
CKS2.3	4.47E-05	0.205342	0.497	0.364	0.668358	DC	CKS2
TALDO1.3	4.49E-05	0.139888	0.695	0.596	0.671519	DC	TALDO1
SLC25A51.	4.52E-05	0.230392	0.118	0.052	0.675811	DC	SLC25A51
FAM110B.2	4.53E-05	-0.22457	0.064	0.189	0.677624	DC	FAM110B

ING4.1	4.61E-05	-0.30509	0.128	0.269	0.689022	DC	ING4
NDUFC1.2	4.61E-05	-0.34093	0.38	0.555	0.689135	DC	NDUFC1
TRPM4.1	4.62E-05	0.302441	0.096	0.039	0.690185	DC	TRPM4
SYNC.1	4.64E-05	-0.22176	0.059	0.178	0.693245	DC	SYNC
ELP6.1	4.66E-05	-0.25338	0.086	0.217	0.696587	DC	ELP6
ADPRH.1	4.71E-05	0.186012	0.086	0.032	0.703476	DC	ADPRH
RP11-556E	4.71E-05	0.144544	0.123	0.054	0.704214	DC	RP11-556E13.1
RHOJ.1	4.71E-05	-0.19034	0.011	0.101	0.704404	DC	RHOJ
MPPED2.1	4.72E-05	-0.23467	0.005	0.091	0.705961	DC	MPPED2
ZFP82.1	4.73E-05	-0.22881	0.016	0.111	0.706325	DC	ZFP82
UBE2D4.1	4.73E-05	-0.23745	0.07	0.191	0.70757	DC	UBE2D4
PTDSS2.3	4.79E-05	-0.21288	0.043	0.154	0.716105	DC	PTDSS2
RGS6.1	4.80E-05	-0.21114	0.005	0.092	0.71729	DC	RGS6
C14orf1.2	4.81E-05	-0.2726	0.144	0.295	0.719399	DC	C14orf1
ISYNA1.1	4.87E-05	-0.24671	0.032	0.135	0.727174	DC	ISYNA1
RAMP2.2	4.92E-05	-0.24382	0.032	0.137	0.734531	DC	RAMP2
ILDR2.2	4.92E-05	-0.15888	0	0.081	0.734561	DC	ILDR2
ARL11.2	4.92E-05	0.190896	0.096	0.038	0.735556	DC	ARL11
RP11-332H	4.92E-05	-0.21959	0.027	0.128	0.735732	DC	RP11-332H14.2
ECE1.1	4.96E-05	-0.22994	0	0.081	0.741956	DC	ECE1
HTATIP2.3	4.98E-05	0.317953	0.241	0.145	0.744877	DC	HTATIP2
ANKRD46.1	4.99E-05	-0.3144	0.048	0.159	0.745441	DC	ANKRD46
STX7.3	5.04E-05	0.275654	0.374	0.266	0.752646	DC	STX7
JAK2	5.04E-05	0.152033	0.086	0.032	0.753113	DC	JAK2
ORMDL3.1	5.04E-05	-0.23424	0.027	0.128	0.753373	DC	ORMDL3
DFNA5.1	5.06E-05	-0.30164	0.075	0.197	0.756368	DC	DFNA5
VAX2.2	5.07E-05	-0.20019	0	0.081	0.756967	DC	VAX2
TIMP3.1	5.09E-05	-0.27078	0.027	0.127	0.760454	DC	TIMP3
ELOVL4.1	5.12E-05	-0.20666	0	0.081	0.764585	DC	ELOVL4
ODF2L.1	5.13E-05	-0.27155	0.059	0.177	0.766966	DC	ODF2L
ZNF593.1	5.13E-05	-0.19289	0.144	0.304	0.767098	DC	ZNF593
FASN.3	5.14E-05	-0.15075	0.021	0.118	0.768016	DC	FASN
B3GAT1.2	5.15E-05	-0.26166	0.005	0.091	0.769255	DC	B3GAT1
CCDC130.1	5.16E-05	-0.28366	0.096	0.23	0.770895	DC	CCDC130
IGF2BP3.2	5.16E-05	-0.197	0.016	0.109	0.771472	DC	IGF2BP3
CMBL.1	5.18E-05	-0.21526	0.027	0.128	0.773985	DC	CMBL
RHOC.2	5.21E-05	0.145379	0.695	0.53	0.779048	DC	RHOC
CCDC181.2	5.22E-05	-0.23549	0	0.081	0.780049	DC	CCDC181
NAV2	5.26E-05	-0.23168	0.021	0.119	0.786527	DC	NAV2
NUMBL.1	5.28E-05	-0.18929	0.005	0.091	0.789039	DC	NUMBL
RNF135.3	5.30E-05	0.248456	0.198	0.109	0.792587	DC	RNF135
TMEM100.3	5.32E-05	-0.34958	0.005	0.09	0.795034	DC	TMEM100
DOLK.2	5.33E-05	-0.21241	0.027	0.128	0.797052	DC	DOLK
EPDR1.2	5.34E-05	-0.201	0.027	0.128	0.797671	DC	EPDR1
AASDH.1	5.35E-05	-0.30329	0.053	0.166	0.799548	DC	AASDH
SNHG15.2	5.35E-05	0.260779	0.321	0.22	0.800186	DC	SNHG15
PCDHB2.1	5.38E-05	-0.18299	0	0.08	0.803826	DC	PCDHB2
HPS4.2	5.42E-05	-0.2624	0.064	0.185	0.809529	DC	HPS4
NQO1.2	5.43E-05	-0.21789	0.016	0.11	0.812082	DC	NQO1
PFDN6.2	5.46E-05	-0.27313	0.193	0.355	0.815728	DC	PFDN6
NUP107.3	5.48E-05	-0.34086	0.096	0.225	0.819065	DC	NUP107

LDLR	5.49E-05	-0.23204	0.011	0.1	0.819682	DC	LDLR
CBLN4.3	5.54E-05	-0.23631	0	0.08	0.82832	DC	CBLN4
BSDC1.1	5.62E-05	-0.2462	0.144	0.293	0.839157	DC	BSDC1
EHBP1.1	5.64E-05	-0.22164	0.053	0.169	0.842456	DC	EHBP1
PDGFD.1	5.65E-05	-0.15866	0	0.08	0.845058	DC	PDGFD
TRABD2A	5.66E-05	0.143232	0.043	0.011	0.846243	DC	TRABD2A
PSPH.2	5.73E-05	-0.22155	0.048	0.159	0.855545	DC	PSPH
SMPD1.2	5.73E-05	-0.23334	0.102	0.239	0.856138	DC	SMPD1
PPARD.3	5.80E-05	0.266025	0.193	0.107	0.866567	DC	PPARD
PLD3.2	5.80E-05	-0.33126	0.369	0.532	0.867332	DC	PLD3
GPR137.2	5.85E-05	-0.22069	0.08	0.211	0.873914	DC	GPR137
ATP6V1E1.	5.89E-05	0.219974	0.524	0.431	0.879534	DC	ATP6V1E1
ZDHHC4.3	5.95E-05	-0.328	0.198	0.357	0.88933	DC	ZDHHC4
CACNB4	5.99E-05	-0.20702	0.005	0.09	0.89546	DC	CACNB4
MECR.1	6.00E-05	-0.14534	0.048	0.163	0.896174	DC	MECR
PTP4A3	6.02E-05	-0.21332	0.005	0.089	0.898897	DC	PTP4A3
FASTK.1	6.02E-05	-0.26107	0.176	0.34	0.899411	DC	FASTK
AP1S1.3	6.06E-05	-0.34992	0.31	0.47	0.905312	DC	AP1S1
CEP290.1	6.06E-05	-0.2302	0.048	0.159	0.906252	DC	CEP290
UTRN.2	6.07E-05	0.271635	0.193	0.108	0.907049	DC	UTRN
ALG13.2	6.08E-05	0.292071	0.321	0.218	0.909283	DC	ALG13
GLUD1.2	6.12E-05	0.195088	0.364	0.246	0.914978	DC	GLUD1
SOX5.1	6.12E-05	-0.1882	0.005	0.09	0.915141	DC	SOX5
ETV4.2	6.13E-05	-0.18935	0	0.079	0.915417	DC	ETV4
KIF5A.2	6.15E-05	-0.31388	0.027	0.123	0.919242	DC	KIF5A
RHBDD3.1	6.18E-05	-0.22642	0.043	0.151	0.923934	DC	RHBDD3
PIGF.2	6.23E-05	-0.31473	0.144	0.29	0.930596	DC	PIGF
USE1.3	6.23E-05	-0.33944	0.176	0.332	0.931037	DC	USE1
PAXBP1.2	6.23E-05	-0.1891	0.048	0.161	0.93131	DC	PAXBP1
EPB41L3.3	6.26E-05	0.181099	0.198	0.104	0.935342	DC	EPB41L3
C20orf96.	6.33E-05	-0.15489	0.043	0.152	0.946166	DC	C20orf96
TRIO.2	6.34E-05	-0.27839	0.059	0.174	0.947972	DC	TRIO
IPMK.1	6.39E-05	0.243338	0.102	0.043	0.955248	DC	IPMK
MDFI.2	6.41E-05	-0.23074	0.005	0.089	0.957432	DC	MDFI
PPME1.1	6.47E-05	-0.25159	0.064	0.183	0.966484	DC	PPME1
SLC2A4RG.	6.48E-05	-0.17041	0.027	0.127	0.967886	DC	SLC2A4RG
NUBP1.2	6.50E-05	0.300212	0.316	0.216	0.971028	DC	NUBP1
LOXL1.1	6.50E-05	-0.15472	0	0.079	0.971959	DC	LOXL1
PPP1R37.1	6.51E-05	-0.17429	0.032	0.136	0.973251	DC	PPP1R37
CSAD.1	6.53E-05	-0.20371	0.053	0.17	0.976525	DC	CSAD
FIGN.1	6.55E-05	-0.18524	0.005	0.089	0.979167	DC	FIGN
CIB2.1	6.57E-05	-0.15123	0	0.079	0.981713	DC	CIB2
QKI.2	6.58E-05	-0.32665	0.337	0.473	0.982738	DC	QKI
PDGFA.1	6.62E-05	-0.25947	0.011	0.098	0.98955	DC	PDGFA
XXbac-B13	6.65E-05	0.127225	0.059	0.019	0.994215	DC	XXbac-B135H6.15
PTPRF.3	6.69E-05	-0.21252	0.021	0.117	0.999671	DC	PTPRF
ZNF512.1	6.70E-05	-0.2222	0.027	0.125	1	DC	ZNF512
SPIN2B.1	6.72E-05	-0.21978	0.016	0.108	1	DC	SPIN2B
HNRNPM.3	6.72E-05	0.199876	0.631	0.542	1	DC	HNRNPM
UPF2.1	6.75E-05	0.269023	0.316	0.216	1	DC	UPF2
CNPY1.2	6.77E-05	-0.22702	0	0.079	1	DC	CNPY1

KDM4C	6.77E-05	0.258745	0.096	0.04	1	DC	KDM4C
SRSF5	6.77E-05	0.116097	0.791	0.692	1	DC	SRSF5
OAT.1	6.77E-05	0.274857	0.342	0.236	1	DC	OAT
TIA1.3	6.79E-05	-0.25277	0.112	0.25	1	DC	TIA1
RUNDC3A.3	6.81E-05	-0.24082	0.011	0.099	1	DC	RUNDC3A
TRIM28.1	6.85E-05	-0.24025	0.112	0.252	1	DC	TRIM28
RNF166.3	6.88E-05	0.221505	0.289	0.183	1	DC	RNF166
FAM84B.2	6.89E-05	-0.23928	0.005	0.088	1	DC	FAM84B
UBTF.2	6.89E-05	-0.21744	0.096	0.229	1	DC	UBTF
SYBU.1	6.91E-05	-0.21204	0.005	0.088	1	DC	SYBU
ADD1.1	6.93E-05	-0.2671	0.16	0.31	1	DC	ADD1
COPRS.2	6.98E-05	-0.22861	0.187	0.336	1	DC	COPRS
TMEM205.2	6.98E-05	-0.35465	0.337	0.498	1	DC	TMEM205
ADCY7.3	7.04E-05	0.17724	0.134	0.062	1	DC	ADCY7
FAM69C.2	7.04E-05	-0.16622	0	0.078	1	DC	FAM69C
MEIS1.3	7.08E-05	-0.19329	0.005	0.088	1	DC	MEIS1
POMZP3.3	7.16E-05	-0.2092	0.005	0.087	1	DC	POMZP3
CYLD.2	7.19E-05	0.281114	0.262	0.163	1	DC	CYLD
POMGNT2.1	7.21E-05	-0.19581	0.037	0.142	1	DC	POMGNT2
ELOF1.2	7.21E-05	-0.31269	0.262	0.426	1	DC	ELOF1
CNPY2.2	7.22E-05	-0.27933	0.406	0.571	1	DC	CNPY2
SMIM15	7.29E-05	-0.27368	0.176	0.335	1	DC	SMIM15
VTI1A.2	7.36E-05	0.280513	0.134	0.064	1	DC	VTI1A
CTTNBP2.3	7.37E-05	-0.31608	0.037	0.138	1	DC	CTTNBP2
LINC01116	7.43E-05	-0.20826	0.032	0.134	1	DC	LINC01116
YIPF6.2	7.45E-05	-0.33513	0.166	0.316	1	DC	YIPF6
HOXD11.1	7.48E-05	-0.16115	0	0.078	1	DC	HOXD11
FAM171A1.	7.48E-05	-0.19079	0	0.078	1	DC	FAM171A1
ZNF582-AS	7.48E-05	-0.27212	0	0.078	1	DC	ZNF582-AS1
DIXDC1.2	7.48E-05	-0.23727	0.011	0.096	1	DC	DIXDC1
TMEM5.1	7.54E-05	-0.26368	0.086	0.21	1	DC	TMEM5
LONP2.2	7.54E-05	0.305427	0.342	0.246	1	DC	LONP2
RMND5B.1	7.60E-05	-0.21673	0.053	0.166	1	DC	RMND5B
HOXC9.3	7.63E-05	-0.28891	0.005	0.087	1	DC	HOXC9
NMI.2	7.63E-05	0.258476	0.283	0.181	1	DC	NMI
DMRTA2	7.63E-05	-0.16491	0	0.077	1	DC	DMRTA2
SESN2.2	7.63E-05	0.283033	0.187	0.103	1	DC	SESN2
MLC1.2	7.67E-05	-0.23142	0.043	0.15	1	DC	MLC1
IFT43.2	7.69E-05	-0.28423	0.118	0.259	1	DC	IFT43
ZFP90.2	7.70E-05	-0.21627	0.032	0.133	1	DC	ZFP90
TTC28.1	7.72E-05	-0.21106	0.011	0.097	1	DC	TTC28
PXK.2	7.74E-05	0.279743	0.23	0.139	1	DC	PXK
DLC1.1	7.74E-05	-0.18702	0.005	0.088	1	DC	DLC1
WDR83.1	7.74E-05	-0.2004	0.059	0.173	1	DC	WDR83
KLF9.1	7.75E-05	0.275325	0.219	0.129	1	DC	KLF9
MKLN1.1	7.82E-05	-0.25134	0.102	0.232	1	DC	MKLN1
HSBP1.2	7.85E-05	-0.25475	0.636	0.716	1	DC	HSBP1
EZH2.2	7.85E-05	-0.2589	0.048	0.155	1	DC	EZH2
BOK.3	7.86E-05	-0.21683	0	0.077	1	DC	BOK
TSR1.1	7.91E-05	-0.20199	0.07	0.191	1	DC	TSR1
MAPKAPK5-	7.92E-05	-0.31505	0.096	0.225	1	DC	MAPKAPK5-AS1

BEND5.1	7.98E-05	-0.26981	0.037	0.138	1	DC	BEND5
DNAJC18	8.00E-05	-0.21197	0.027	0.124	1	DC	DNAJC18
EXOC7.2	8.01E-05	-0.28986	0.198	0.354	1	DC	EXOC7
ITGA5.1	8.01E-05	0.212072	0.16	0.082	1	DC	ITGA5
DPP7.3	8.02E-05	0.17313	0.572	0.465	1	DC	DPP7
C17orf58.	8.03E-05	-0.21866	0.07	0.188	1	DC	C17orf58
FAXC.2	8.11E-05	-0.22124	0.005	0.087	1	DC	FAXC
SNCAIP.2	8.12E-05	-0.19892	0.005	0.087	1	DC	SNCAIP
ATCAY.2	8.13E-05	-0.27935	0.027	0.122	1	DC	ATCAY
CACNA1A.2	8.14E-05	-0.27447	0.011	0.097	1	DC	CACNA1A
AGPAT5.2	8.15E-05	-0.28019	0.086	0.211	1	DC	AGPAT5
SEPW1.1	8.23E-05	-0.29095	0.578	0.674	1	DC	SEPW1
LAMB2.1	8.26E-05	-0.20578	0.011	0.097	1	DC	LAMB2
ATP2B1.2	8.29E-05	0.25289	0.449	0.353	1	DC	ATP2B1
PSMG2.1	8.31E-05	0.197638	0.503	0.411	1	DC	PSMG2
VASH1.3	8.33E-05	0.327562	0.219	0.128	1	DC	VASH1
PEG10.1	8.33E-05	-0.32693	0.016	0.104	1	DC	PEG10
POMGNT1.2	8.38E-05	-0.23282	0.118	0.26	1	DC	POMGNT1
HES4.3	8.45E-05	-0.21847	0.07	0.189	1	DC	HES4
PHLDA1.3	8.48E-05	-0.34985	0.273	0.397	1	DC	PHLDA1
MS4A14.2	8.48E-05	0.147395	0.064	0.021	1	DC	MS4A14
LXN	8.54E-05	0.217392	0.07	0.024	1	DC	LXN
EIF2AK4.3	8.61E-05	0.358487	0.267	0.18	1	DC	EIF2AK4
SHOC2.2	8.62E-05	0.260129	0.241	0.15	1	DC	SHOC2
LMAN2L	8.69E-05	-0.18307	0.021	0.115	1	DC	LMAN2L
SEMA5A.1	8.78E-05	-0.22827	0.016	0.105	1	DC	SEMA5A
DHX40.2	8.83E-05	-0.18447	0.037	0.141	1	DC	DHX40
TSTD1.2	8.90E-05	0.209867	0.305	0.195	1	DC	TSTD1
GORAB.3	8.95E-05	-0.19087	0	0.076	1	DC	GORAB
C17orf107	8.95E-05	0.191878	0.048	0.014	1	DC	C17orf107
MAP1LC3B.	8.95E-05	0.208908	0.647	0.565	1	DC	MAP1LC3B
SUMO2.1	8.99E-05	-0.14963	0.85	0.821	1	DC	SUMO2
THOC6.2	9.03E-05	0.31828	0.262	0.168	1	DC	THOC6
CNPY4.2	9.03E-05	-0.22792	0.021	0.114	1	DC	CNPY4
SEPT9.1	9.04E-05	-0.29899	0.198	0.343	1	DC	9-Sep
ZNF775.2	9.07E-05	-0.18521	0.011	0.096	1	DC	ZNF775
PRKAR1A.2	9.08E-05	0.177216	0.561	0.47	1	DC	PRKAR1A
GGH.1	9.13E-05	-0.23592	0.128	0.265	1	DC	GGH
LMO1.2	9.22E-05	-0.27196	0	0.076	1	DC	LMO1
RP1-39G22	9.25E-05	-0.16784	0.032	0.132	1	DC	RP1-39G22.7
SCARB2.3	9.27E-05	-0.27508	0.225	0.384	1	DC	SCARB2
RBX1.3	9.33E-05	0.120264	0.765	0.664	1	DC	RBX1
SPRY2.2	9.35E-05	-0.28743	0.096	0.22	1	DC	SPRY2
GPR37L1.2	9.40E-05	-0.3742	0.005	0.086	1	DC	GPR37L1
SPATA33.2	9.45E-05	-0.23805	0.043	0.147	1	DC	SPATA33
MLST8.1	9.47E-05	-0.15114	0.102	0.238	1	DC	MLST8
FAM57A.1	9.48E-05	-0.14269	0.011	0.096	1	DC	FAM57A
SURF2.2	9.52E-05	-0.2404	0.102	0.234	1	DC	SURF2
KLC2.2	9.55E-05	-0.17427	0.027	0.123	1	DC	KLC2
DSCAM	9.59E-05	-0.24212	0	0.075	1	DC	DSCAM
KRT8	9.61E-05	0.15086	0.048	0.014	1	DC	KRT8

PITX1.2	9.62E-05	-0.13735	0.005	0.086	1	DC	PITX1
MLLT3.1	9.63E-05	-0.21149	0.011	0.095	1	DC	MLLT3
CENPT.1	9.64E-05	-0.2729	0.075	0.192	1	DC	CENPT
NAP1L3.1	9.67E-05	-0.25739	0.016	0.103	1	DC	NAP1L3
AZI2.2	9.67E-05	0.323786	0.39	0.299	1	DC	AZI2
DOCK10.3	9.71E-05	0.300763	0.214	0.124	1	DC	DOCK10
MAP2K2.2	9.72E-05	-0.2907	0.166	0.304	1	DC	MAP2K2
ZMYND11.3	9.73E-05	-0.183	0.043	0.148	1	DC	ZMYND11
ZNF639.2	9.78E-05	-0.21446	0.037	0.138	1	DC	ZNF639
POLR2B.2	9.83E-05	-0.28791	0.128	0.264	1	DC	POLR2B
THAP7.1	9.86E-05	-0.19804	0.086	0.211	1	DC	THAP7
EI24.1	9.86E-05	-0.26852	0.273	0.436	1	DC	EI24
LRRC17.1	9.88E-05	-0.22287	0	0.075	1	DC	LRRC17
COL16A1.1	9.98E-05	-0.19034	0	0.075	1	DC	COL16A1
SAMD1.1	0.000101	-0.20206	0.016	0.104	1	DC	SAMD1
PTPRN.2	0.000101	-0.26786	0	0.075	1	DC	PTPRN
MAP2K3.3	0.000101	0.29833	0.283	0.188	1	DC	MAP2K3
ZFP14.1	0.000102	-0.24654	0.021	0.113	1	DC	ZFP14
CAB39L.1	0.000102	-0.20215	0	0.075	1	DC	CAB39L
FADS2.1	0.000103	-0.20838	0.037	0.138	1	DC	FADS2
RUVBL1	0.000103	-0.24123	0.128	0.268	1	DC	RUVBL1
NNAT.3	0.000104	-0.69295	0.096	0.197	1	DC	NNAT
CELF4.3	0.000104	-0.31287	0.011	0.094	1	DC	CELF4
VIPR2.2	0.000104	-0.31009	0.005	0.084	1	DC	VIPR2
DDX1.1	0.000106	-0.26997	0.203	0.362	1	DC	DDX1
SDHAF1.1	0.000106	-0.19708	0.064	0.178	1	DC	SDHAF1
TLR1.3	0.000106	0.190028	0.139	0.067	1	DC	TLR1
DLX2.3	0.000108	-0.37679	0.021	0.11	1	DC	DLX2
NSMAF.2	0.000108	0.27383	0.198	0.115	1	DC	NSMAF
KEAP1.1	0.000109	-0.23036	0.134	0.274	1	DC	KEAP1
KLHDC9.1	0.000109	-0.19762	0	0.074	1	DC	KLHDC9
C14orf37.	0.00011	-0.15917	0.021	0.113	1	DC	C14orf37
SALL1.2	0.00011	-0.1711	0.037	0.139	1	DC	SALL1
CLUAP1.2	0.00011	-0.25177	0.059	0.17	1	DC	CLUAP1
SORBS1.1	0.00011	-0.17486	0.011	0.094	1	DC	SORBS1
GCHFR.3	0.000111	0.200416	0.15	0.075	1	DC	GCHFR
RARA.1	0.000111	0.290098	0.171	0.095	1	DC	RARA
MPP6.1	0.000111	-0.17306	0	0.074	1	DC	MPP6
PRDX4.3	0.000111	-0.31614	0.385	0.549	1	DC	PRDX4
USP32	0.000112	0.315334	0.176	0.099	1	DC	USP32
PRRC2A.2	0.000112	-0.12449	0.107	0.245	1	DC	PRRC2A
CCDC92.1	0.000112	-0.18175	0.016	0.103	1	DC	CCDC92
CUTA.3	0.000112	-0.2755	0.583	0.683	1	DC	CUTA
DNM1	0.000112	-0.14902	0	0.074	1	DC	DNM1
ELOVL6.1	0.000113	-0.17072	0.011	0.094	1	DC	ELOVL6
DIAPH1.2	0.000114	0.38046	0.219	0.137	1	DC	DIAPH1
HSPA4L.2	0.000114	-0.22979	0	0.074	1	DC	HSPA4L
ARHGEF1.3	0.000114	0.318765	0.289	0.195	1	DC	ARHGEF1
MARCH2.3	0.000114	0.282724	0.326	0.231	1	DC	2-Mar
EIF2AK3.1	0.000115	0.252412	0.112	0.052	1	DC	EIF2AK3
SATB2	0.000115	-0.15286	0	0.074	1	DC	SATB2

AC069363.	0.000115	0.206077	0.112	0.05	1	DC	AC069363.1
TBL2.1	0.000115	-0.24172	0.075	0.191	1	DC	TBL2
AKIRIN2.3	0.000115	0.259482	0.46	0.38	1	DC	AKIRIN2
KANK1.1	0.000116	-0.20508	0.011	0.093	1	DC	KANK1
HNRNPA3.1	0.000116	0.132107	0.642	0.553	1	DC	HNRNPA3
GIMAP4.3	0.000116	0.104174	0.262	0.148	1	DC	GIMAP4
RPS6KL1.2	0.000116	-0.23571	0.016	0.102	1	DC	RPS6KL1
MAP6D1.2	0.000116	-0.22654	0.005	0.084	1	DC	MAP6D1
KMT2A.3	0.000117	-0.22435	0.107	0.238	1	DC	KMT2A
CTSL.3	0.000117	-0.38788	0.38	0.526	1	DC	CTSL
SLC2A5.3	0.000118	-0.53085	0.07	0.172	1	DC	SLC2A5
ZNF577.2	0.000118	-0.17278	0.011	0.093	1	DC	ZNF577
PRKRIP1.2	0.000119	-0.18453	0.112	0.243	1	DC	PRKRIP1
USP22.3	0.000119	-0.24278	0.171	0.322	1	DC	USP22
TXNDC11.1	0.000119	0.267165	0.198	0.115	1	DC	TXNDC11
SERINC2.1	0.000119	-0.15144	0.027	0.122	1	DC	SERINC2
RP13-516M	0.000119	0.169421	0.08	0.031	1	DC	RP13-516M14.4
MFSD7.2	0.000119	0.159164	0.08	0.031	1	DC	MFSD7
MAD2L2.2	0.00012	-0.33607	0.257	0.394	1	DC	MAD2L2
KHSRP.3	0.00012	-0.22278	0.107	0.24	1	DC	KHSRP
FAM198B.1	0.000121	0.132771	0.128	0.06	1	DC	FAM198B
RAB5C.1	0.000122	0.109703	0.759	0.651	1	DC	RAB5C
EMID1.2	0.000122	-0.17053	0	0.073	1	DC	EMID1
B4GALT5.1	0.000122	-0.22737	0.08	0.198	1	DC	B4GALT5
FAM102B	0.000122	0.140152	0.075	0.028	1	DC	FAM102B
BOLA1.1	0.000122	-0.24239	0.048	0.153	1	DC	BOLA1
DLG1.2	0.000122	-0.24239	0.059	0.167	1	DC	DLG1
JAZF1.3	0.000123	0.220563	0.198	0.112	1	DC	JAZF1
HABP4	0.000123	-0.16139	0.027	0.121	1	DC	HABP4
CTC-444N2	0.000123	-0.21087	0.037	0.137	1	DC	CTC-444N24.11
HAAO.2	0.000123	0.190216	0.064	0.022	1	DC	HAAO
KIAA0895L	0.000124	-0.20687	0.011	0.093	1	DC	KIAA0895L
DPH7.2	0.000124	-0.21891	0.059	0.169	1	DC	DPH7
ENKUR.1	0.000124	-0.23307	0	0.073	1	DC	ENKUR
SRRM3.2	0.000124	-0.26317	0	0.073	1	DC	SRRM3
CASP10	0.000124	0.150578	0.07	0.025	1	DC	CASP10
ADPRHL2.2	0.000124	-0.20449	0.123	0.263	1	DC	ADPRHL2
ZNF267.3	0.000124	0.264985	0.219	0.131	1	DC	ZNF267
OTUD7B.1	0.000125	-0.20602	0.016	0.102	1	DC	OTUD7B
C14orf132	0.000125	-0.27888	0.011	0.093	1	DC	C14orf132
SCN3B.1	0.000125	-0.26654	0	0.073	1	DC	SCN3B
CCDC136.1	0.000126	-0.23403	0.011	0.092	1	DC	CCDC136
LONRF2.1	0.000126	-0.18452	0.011	0.093	1	DC	LONRF2
AMOTL2	0.000127	-0.25645	0.005	0.082	1	DC	AMOTL2
CBX8.1	0.000128	-0.18136	0	0.073	1	DC	CBX8
KCNQ1OT1.	0.000128	-0.33242	0.15	0.28	1	DC	KCNQ1OT1
CYHR1.2	0.000128	-0.17987	0.139	0.281	1	DC	CYHR1
DDX55.1	0.000128	-0.21881	0.027	0.119	1	DC	DDX55
FAM168A.3	0.000129	-0.19119	0.032	0.129	1	DC	FAM168A
NTN1	0.000129	-0.1367	0.005	0.083	1	DC	NTN1
HSPE1.3	0.000129	-0.23869	0.69	0.73	1	DC	HSPE1

DDX42.1	0.000129	-0.24562	0.096	0.22	1	DC	DDX42
TYRO3.2	0.00013	-0.20922	0.021	0.111	1	DC	TYRO3
THAP3.2	0.00013	-0.23425	0.037	0.136	1	DC	THAP3
CASC15.2	0.000131	-0.2576	0.016	0.101	1	DC	CASC15
LATS2	0.000131	0.207756	0.091	0.038	1	DC	LATS2
STAMBP.1	0.000131	-0.21711	0.128	0.268	1	DC	STAMBP
RASGRP4	0.000132	0.146639	0.059	0.019	1	DC	RASGRP4
TNFSF10.1	0.000132	0.145369	0.15	0.073	1	DC	TNFSF10
SBK1.3	0.000132	-0.22166	0	0.073	1	DC	SBK1
GCLM.1	0.000132	-0.18731	0.053	0.16	1	DC	GCLM
CCDC43.1	0.000132	-0.24103	0.08	0.201	1	DC	CCDC43
ZNF786.1	0.000133	-0.18272	0	0.073	1	DC	ZNF786
TIPARP.2	0.000133	0.253539	0.262	0.167	1	DC	TIPARP
ID2.2	0.000133	0.123834	0.802	0.652	1	DC	ID2
SWAP70.3	0.000134	0.223451	0.321	0.22	1	DC	SWAP70
HSPA8.2	0.000134	-0.21777	0.861	0.866	1	DC	HSPA8
SETD4.1	0.000134	-0.23888	0.032	0.127	1	DC	SETD4
CASP2	0.000135	-0.20619	0.016	0.101	1	DC	CASP2
ABTB1.3	0.000135	0.196256	0.187	0.103	1	DC	ABTB1
LMNB2.3	0.000135	-0.1982	0.027	0.118	1	DC	LMNB2
NUDCD3.1	0.000136	-0.22596	0.08	0.2	1	DC	NUDCD3
PPP1R9A.2	0.000136	-0.2439	0	0.072	1	DC	PPP1R9A
CHTOP.1	0.000136	-0.3153	0.219	0.369	1	DC	CHTOP
CREB1.2	0.000137	-0.30988	0.118	0.243	1	DC	CREB1
FAM188A.1	0.000137	-0.2042	0.011	0.092	1	DC	FAM188A
PGAM1.1	0.000138	0.158652	0.578	0.5	1	DC	PGAM1
ZNF667.2	0.000138	-0.17937	0.005	0.083	1	DC	ZNF667
ORC4.2	0.000139	-0.22012	0.107	0.237	1	DC	ORC4
TSPAN31.2	0.000139	-0.2715	0.16	0.297	1	DC	TSPAN31
RGMB.2	0.000139	-0.29906	0.037	0.133	1	DC	RGMB
PAXIP1-AS	0.000139	-0.15599	0.053	0.16	1	DC	PAXIP1-AS1
CEP41	0.00014	-0.21508	0.048	0.153	1	DC	CEP41
RGS14	0.00014	0.180976	0.086	0.034	1	DC	RGS14
CNOT4	0.00014	-0.22416	0.139	0.274	1	DC	CNOT4
KANK2	0.00014	-0.16369	0.011	0.092	1	DC	KANK2
PPM1M.3	0.000141	0.252896	0.144	0.074	1	DC	PPM1M
MAML2.2	0.000141	-0.21758	0.08	0.2	1	DC	MAML2
FAM162A.3	0.000141	0.160785	0.503	0.399	1	DC	FAM162A
HMGCR.1	0.000142	-0.18807	0.021	0.11	1	DC	HMGCR
NRARP.1	0.000142	0.204346	0.086	0.034	1	DC	NRARP
TPM4.2	0.000142	0.165863	0.604	0.495	1	DC	TPM4
CRISPLD1.	0.000142	-0.21827	0.011	0.091	1	DC	CRISPLD1
LLGL1.2	0.000143	-0.16536	0.016	0.101	1	DC	LLGL1
C17orf10C	0.000144	-0.16942	0.011	0.092	1	DC	C17orf100
BEND6.2	0.000144	-0.11955	0.027	0.12	1	DC	BEND6
MYLIP.3	0.000145	0.159781	0.225	0.127	1	DC	MYLIP
TLCD1.1	0.000145	-0.1472	0.005	0.082	1	DC	TLCD1
ARHGAP25.	0.000145	0.179558	0.134	0.065	1	DC	ARHGAP25
WNT5A.3	0.000146	0.171045	0.123	0.057	1	DC	WNT5A
HEXIM2.1	0.000146	-0.19476	0.005	0.082	1	DC	HEXIM2
ANKRD17.2	0.000147	-0.20904	0.096	0.219	1	DC	ANKRD17

RP9.1	0.000147	-0.15124	0.07	0.181	1	DC	RP9
UBL5.2	0.000148	-0.21119	0.834	0.831	1	DC	UBL5
PRRT2.1	0.000149	-0.14071	0.011	0.092	1	DC	PRRT2
CHCHD10.3	0.00015	0.187285	0.487	0.379	1	DC	CHCHD10
CEACAM21.	0.00015	0.118485	0.059	0.019	1	DC	CEACAM21
TMEM108.1	0.000151	-0.17626	0.005	0.081	1	DC	TMEM108
LINC01102	0.000151	-0.2558	0	0.071	1	DC	LINC01102
PLTP.1	0.000151	-0.41567	0.321	0.472	1	DC	PLTP
DYNC1H1.2	0.000152	-0.2677	0.193	0.34	1	DC	DYNC1H1
ZNF260	0.000152	-0.17896	0.016	0.101	1	DC	ZNF260
ELAVL2.3	0.000152	-0.26783	0.005	0.081	1	DC	ELAVL2
UBE2O.2	0.000152	-0.18301	0.027	0.118	1	DC	UBE2O
POLR2C.2	0.000153	-0.23577	0.198	0.352	1	DC	POLR2C
PTBP2.1	0.000154	-0.26082	0.021	0.107	1	DC	PTBP2
GRAMD3.3	0.000155	-0.25464	0.043	0.142	1	DC	GRAMD3
NACC1	0.000155	-0.18873	0.048	0.152	1	DC	NACC1
TAF6	0.000156	-0.21573	0.107	0.238	1	DC	TAF6
AC091729.	0.000156	-0.1616	0.005	0.082	1	DC	AC091729.9
METAP1D.1	0.000157	-0.21411	0.032	0.126	1	DC	METAP1D
CHFR.2	0.000157	0.275394	0.203	0.121	1	DC	CHFR
SYDE1	0.000158	-0.13882	0	0.071	1	DC	SYDE1
LRRC1.1	0.000158	-0.20952	0	0.071	1	DC	LRRC1
HELZ2.2	0.000158	-0.15563	0.016	0.101	1	DC	HELZ2
SLC4A3	0.000159	-0.15807	0.011	0.091	1	DC	SLC4A3
MBOAT7.3	0.000159	0.304398	0.321	0.242	1	DC	MBOAT7
RAB27A.2	0.000159	0.182179	0.15	0.077	1	DC	RAB27A
TMEM47.1	0.000159	-0.16463	0.005	0.081	1	DC	TMEM47
AC004540.	0.00016	-0.24153	0.005	0.081	1	DC	AC004540.5
SLC35G2.3	0.000161	-0.18795	0.027	0.118	1	DC	SLC35G2
MUT.2	0.000161	-0.20157	0.053	0.159	1	DC	MUT
CSNK2B	0.000162	0.147248	0.684	0.607	1	DC	CSNK2B
PAK4.1	0.000163	-0.1968	0.027	0.117	1	DC	PAK4
BIK	0.000165	0.133363	0.043	0.012	1	DC	BIK
TPK1.2	0.000165	0.184765	0.102	0.045	1	DC	TPK1
HMGB1.1	0.000165	-0.17922	0.92	0.86	1	DC	HMGB1
PDE1B	0.000165	0.118016	0.059	0.02	1	DC	PDE1B
SPOCK2.1	0.000165	-0.26231	0.043	0.141	1	DC	SPOCK2
MAPK12.1	0.000165	-0.13836	0	0.071	1	DC	MAPK12
TMEM55A.2	0.000166	-0.22724	0.107	0.235	1	DC	TMEM55A
C8orf33	0.000166	-0.22892	0.171	0.321	1	DC	C8orf33
FBXO44.1	0.000167	-0.21723	0.043	0.143	1	DC	FBXO44
GPR171	0.000167	0.193735	0.059	0.02	1	DC	GPR171
CCDC167.1	0.000167	-0.20706	0.235	0.385	1	DC	CCDC167
DPY19L3.1	0.00017	-0.19457	0.011	0.09	1	DC	DPY19L3
HMGN1.3	0.000171	0.147941	0.791	0.663	1	DC	HMGN1
ACTR3B	0.000172	-0.16529	0.021	0.109	1	DC	ACTR3B
CDADC1.1	0.000172	-0.23861	0.021	0.108	1	DC	CDADC1
THAP8	0.000172	-0.21778	0.005	0.08	1	DC	THAP8
NOS1AP.2	0.000173	-0.18058	0.005	0.08	1	DC	NOS1AP
FKBP4.2	0.000174	-0.25043	0.182	0.323	1	DC	FKBP4
PACSIN3.1	0.000174	-0.15038	0	0.07	1	DC	PACSIN3

S100B.2	0.000174	-0.37077	0.38	0.53	1	DC	S100B
IGF1R.1	0.000176	-0.19499	0	0.07	1	DC	IGF1R
FAM64A.3	0.000176	-0.25967	0	0.07	1	DC	FAM64A
SNX9.3	0.000177	0.329427	0.278	0.188	1	DC	SNX9
RNF219	0.000177	-0.2338	0.027	0.116	1	DC	RNF219
SLC30A9.2	0.000177	-0.2049	0.123	0.254	1	DC	SLC30A9
CSNK1D.3	0.000177	0.220543	0.449	0.358	1	DC	CSNK1D
CUEDC1.3	0.000179	-0.19171	0.027	0.116	1	DC	CUEDC1
CAV1.3	0.000179	-0.7007	0.037	0.128	1	DC	CAV1
POM121C.2	0.00018	-0.20361	0.043	0.142	1	DC	POM121C
IRF2BP1.2	0.00018	-0.16604	0.021	0.107	1	DC	IRF2BP1
B3GAT3.1	0.00018	-0.27291	0.23	0.381	1	DC	B3GAT3
LINC00685	0.000181	-0.19356	0.048	0.151	1	DC	LINC00685
TLR5.2	0.000181	0.180482	0.102	0.044	1	DC	TLR5
RASGRP1.2	0.000181	-0.14846	0	0.07	1	DC	RASGRP1
CHCHD7.2	0.000182	0.202816	0.401	0.284	1	DC	CHCHD7
FMN2.1	0.000183	-0.17298	0	0.07	1	DC	FMN2
LNX1	0.000183	-0.22275	0	0.07	1	DC	LNX1
DHRS7.3	0.000183	0.127372	0.674	0.554	1	DC	DHRS7
SH3RF1.1	0.000183	-0.17345	0.021	0.108	1	DC	SH3RF1
SIDT1.2	0.000183	0.167802	0.064	0.023	1	DC	SIDT1
ZNF397.1	0.000184	-0.22981	0.059	0.164	1	DC	ZNF397
SRRD.2	0.000184	-0.11054	0.027	0.118	1	DC	SRRD
MEOX2.1	0.000184	-0.20661	0.005	0.08	1	DC	MEOX2
MGST3.2	0.000185	-0.31534	0.567	0.695	1	DC	MGST3
RRP1B.3	0.000186	-0.19229	0.053	0.157	1	DC	RRP1B
L3HYPDH.1	0.000187	-0.16732	0.032	0.126	1	DC	L3HYPDH
CDH6.1	0.000188	-0.14869	0	0.07	1	DC	CDH6
CARKD.2	0.000189	-0.2471	0.037	0.131	1	DC	CARKD
BIRC5.3	0.000189	-0.33733	0.021	0.106	1	DC	BIRC5
TMEM177.2	0.000189	-0.18827	0.005	0.08	1	DC	TMEM177
ATMIN.2	0.000189	-0.17049	0.027	0.117	1	DC	ATMIN
ING2.1	0.000189	-0.2015	0.091	0.208	1	DC	ING2
GLDC.1	0.00019	-0.16362	0	0.07	1	DC	GLDC
RSAD1.1	0.00019	-0.16699	0.048	0.15	1	DC	RSAD1
EPAS1.2	0.00019	-0.26768	0.021	0.106	1	DC	EPAS1
PLEKHA3.2	0.000191	-0.20494	0.107	0.233	1	DC	PLEKHA3
PMM1.2	0.000191	-0.32885	0.112	0.235	1	DC	PMM1
NDUFS3.1	0.000192	-0.24294	0.321	0.492	1	DC	NDUFS3
MYC.2	0.000193	-0.28647	0.064	0.171	1	DC	MYC
RP11-597D	0.000194	0.121695	0.064	0.023	1	DC	RP11-597D13.8
PAX6.1	0.000195	-0.14104	0.005	0.08	1	DC	PAX6
TMEM136.1	0.000195	-0.17308	0.011	0.089	1	DC	TMEM136
TCEAL1.3	0.000195	-0.24167	0.128	0.257	1	DC	TCEAL1
FAM134B.1	0.000195	-0.17899	0.059	0.167	1	DC	FAM134B
NFKB2.3	0.000196	0.255848	0.123	0.06	1	DC	NFKB2
BCL2L1.2	0.000197	-0.22565	0.134	0.266	1	DC	BCL2L1
ZNF436.1	0.000198	-0.14879	0.005	0.079	1	DC	ZNF436
HHLA3.3	0.000198	-0.16689	0.043	0.141	1	DC	HHLA3
CLHC1	0.0002	-0.18017	0	0.069	1	DC	CLHC1
PLLP.2	0.0002	-0.35117	0	0.069	1	DC	PLLP

WDFY4.2	0.0002	0.129792	0.07	0.025	1	DC	WDFY4
CPED1.1	0.0002	0.140617	0.08	0.032	1	DC	CPED1
GNL1.1	0.0002	-0.17703	0.112	0.244	1	DC	GNL1
PHACTR3.2	0.0002	-0.25686	0.005	0.079	1	DC	PHACTR3
DBNDD1.1	0.000202	-0.16099	0	0.069	1	DC	DBNDD1
ADAMTS9.1	0.000202	-0.17208	0.005	0.079	1	DC	ADAMTS9
ZNF583.1	0.000203	-0.20591	0.021	0.107	1	DC	ZNF583
AC007405.	0.000204	-0.15712	0	0.069	1	DC	AC007405.6
EXT2.2	0.000204	-0.19065	0.064	0.173	1	DC	EXT2
ERCC4.1	0.000205	-0.23895	0.037	0.131	1	DC	ERCC4
FAM228B.2	0.000205	-0.23846	0.096	0.217	1	DC	FAM228B
FREM2.2	0.000206	-0.12541	0	0.069	1	DC	FREM2
PNP.1	0.000206	0.241664	0.182	0.102	1	DC	PNP
CXXC4.2	0.000206	-0.19186	0.005	0.079	1	DC	CXXC4
OSBPL1A.3	0.000207	-0.26236	0.08	0.193	1	DC	OSBPL1A
N4BP2L1.1	0.000207	0.267572	0.246	0.156	1	DC	N4BP2L1
LY6H.2	0.000207	-0.3282	0.011	0.087	1	DC	LY6H
SSFA2.1	0.000207	-0.22319	0.075	0.188	1	DC	SSFA2
HRH1.2	0.000207	-0.22551	0.016	0.097	1	DC	HRH1
EPSTI1.3	0.000207	0.156503	0.214	0.124	1	DC	EPSTI1
VSTM2A.2	0.000208	-0.40595	0.021	0.107	1	DC	VSTM2A
SDHD.2	0.000208	0.207084	0.476	0.394	1	DC	SDHD
LRP10.1	0.000208	0.250905	0.348	0.25	1	DC	LRP10
BARD1.2	0.000209	-0.21691	0.048	0.148	1	DC	BARD1
PDE4DIP.2	0.000209	-0.19378	0.134	0.266	1	DC	PDE4DIP
B3GALNT1.	0.00021	-0.24322	0.075	0.185	1	DC	B3GALNT1
ASXL1.2	0.00021	-0.20906	0.07	0.179	1	DC	ASXL1
KCTD13.2	0.00021	-0.20311	0.043	0.14	1	DC	KCTD13
RBM28.1	0.000211	-0.19698	0.059	0.165	1	DC	RBM28
CHST8.2	0.000212	-0.1558	0	0.069	1	DC	CHST8
SOWAHC.1	0.000212	-0.16572	0	0.069	1	DC	SOWAHC
RNF165.2	0.000212	-0.18531	0	0.069	1	DC	RNF165
UBE2L3.3	0.000212	0.189978	0.594	0.534	1	DC	UBE2L3
PBRM1.2	0.000212	-0.25746	0.118	0.242	1	DC	PBRM1
C16orf52.	0.000212	-0.17044	0.016	0.097	1	DC	C16orf52
LIN7B.1	0.000213	-0.1919	0.032	0.124	1	DC	LIN7B
TNKS.3	0.000213	-0.14618	0.048	0.15	1	DC	TNKS
FHL3.2	0.000214	0.29187	0.241	0.158	1	DC	FHL3
ATF4.1	0.000214	0.162923	0.61	0.515	1	DC	ATF4
FHOD1.3	0.000215	0.157403	0.118	0.055	1	DC	FHOD1
PPA2.1	0.000215	0.226928	0.401	0.314	1	DC	PPA2
MED22.1	0.000215	-0.19342	0.021	0.107	1	DC	MED22
DPY19L1.1	0.000215	-0.1922	0.021	0.106	1	DC	DPY19L1
FAM168B.2	0.000215	-0.19822	0.059	0.165	1	DC	FAM168B
MPP1.3	0.000215	0.211961	0.305	0.199	1	DC	MPP1
MIR497HG.	0.000216	-0.2481	0	0.068	1	DC	MIR497HG
CCDC14.1	0.000216	-0.26744	0.107	0.229	1	DC	CCDC14
SCAMP5.1	0.000217	-0.19079	0.016	0.097	1	DC	SCAMP5
BOC.1	0.000217	-0.15438	0.011	0.088	1	DC	BOC
LRRC47.2	0.000217	-0.20628	0.096	0.218	1	DC	LRRC47
CDKN2A.2	0.000218	-0.35723	0.021	0.104	1	DC	CDKN2A

IGSF11.1	0.000218	-0.17422	0	0.068	1	DC	IGSF11
RP11-277F	0.000218	-0.22447	0	0.068	1	DC	RP11-277P12.20
NCAM2.1	0.000218	-0.24007	0	0.068	1	DC	NCAM2
TOX3.2	0.000218	-0.2516	0	0.068	1	DC	TOX3
GTF2H2C	0.000219	-0.20443	0.011	0.088	1	DC	GTF2H2C
PNMAL1.2	0.000219	-0.17797	0.016	0.097	1	DC	PNMAL1
PLEKHJ1.1	0.00022	-0.20919	0.214	0.369	1	DC	PLEKHJ1
MARK1	0.00022	-0.13962	0	0.068	1	DC	MARK1
INADL.2	0.00022	-0.22109	0	0.068	1	DC	INADL
CSGALNACT	0.000222	0.240993	0.166	0.092	1	DC	CSGALNACT2
UFC1.2	0.000222	0.140066	0.631	0.56	1	DC	UFC1
KCNQ1.2	0.000222	0.121543	0.091	0.038	1	DC	KCNQ1
ZC3H8.1	0.000224	-0.17745	0.048	0.149	1	DC	ZC3H8
AEBP1.2	0.000224	-0.25362	0.032	0.123	1	DC	AEBP1
CEP89.1	0.000225	-0.18595	0.037	0.132	1	DC	CEP89
UBE2M.3	0.000227	-0.15972	0.107	0.232	1	DC	UBE2M
SEC11C.2	0.000228	-0.29115	0.348	0.499	1	DC	SEC11C
CCDC124	0.000229	-0.29332	0.321	0.473	1	DC	CCDC124
SLC12A2.2	0.00023	-0.22794	0.037	0.131	1	DC	SLC12A2
CLNS1A.2	0.00023	0.196568	0.567	0.49	1	DC	CLNS1A
RP11-66N1	0.00023	-0.18958	0.011	0.088	1	DC	RP11-66N11.8
MPP5.1	0.000231	-0.12729	0.016	0.097	1	DC	MPP5
IL6R.3	0.000231	0.225568	0.16	0.085	1	DC	IL6R
FAM127C	0.000231	-0.22746	0.021	0.105	1	DC	FAM127C
LRP6.2	0.000231	-0.20331	0.027	0.113	1	DC	LRP6
PLCD3	0.000231	-0.13258	0	0.068	1	DC	PLCD3
SLC22A23	0.000231	-0.17022	0	0.068	1	DC	SLC22A23
SLC44A2.1	0.000232	-0.17785	0.086	0.204	1	DC	SLC44A2
PLXNA4.2	0.000232	-0.2139	0.005	0.077	1	DC	PLXNA4
LPL.1	0.000232	-0.33188	0.059	0.16	1	DC	LPL
TPT1-AS1.	0.000233	-0.18068	0.043	0.14	1	DC	TPT1-AS1
CLIP2.2	0.000234	-0.21431	0.07	0.178	1	DC	CLIP2
MCMBP.2	0.000234	0.280716	0.225	0.143	1	DC	MCMBP
SDR39U1.1	0.000235	-0.25659	0.144	0.277	1	DC	SDR39U1
MPDZ.1	0.000236	-0.16409	0.011	0.087	1	DC	MPDZ
ZNF43.1	0.000236	-0.19504	0.043	0.139	1	DC	ZNF43
YIPF3.2	0.000236	-0.29491	0.326	0.486	1	DC	YIPF3
SERPINH1.	0.000237	-0.14176	0.091	0.211	1	DC	SERPINH1
TBC1D9.3	0.000238	0.18858	0.112	0.053	1	DC	TBC1D9
FBLN1.2	0.000239	-0.28518	0.011	0.086	1	DC	FBLN1
SAMD4A	0.000243	-0.18467	0.011	0.087	1	DC	SAMD4A
TRIM52	0.000244	-0.19278	0.005	0.077	1	DC	TRIM52
ZNF682	0.000244	-0.18497	0.005	0.077	1	DC	ZNF682
SCAF1.2	0.000244	-0.16398	0.053	0.155	1	DC	SCAF1
AGPAT4.1	0.000246	-0.2279	0.021	0.104	1	DC	AGPAT4
NUDT3.3	0.000246	0.217443	0.476	0.399	1	DC	NUDT3
DENND5B.2	0.000246	-0.13107	0.016	0.097	1	DC	DENND5B
MUL1	0.000246	-0.2046	0.059	0.162	1	DC	MUL1
POLR3F.1	0.000246	-0.19189	0.043	0.138	1	DC	POLR3F
PLAT	0.000247	-0.24733	0.032	0.121	1	DC	PLAT
CCDC82	0.000248	-0.16884	0.134	0.274	1	DC	CCDC82

ANAPC11.3	0.000249	-0.21315	0.668	0.728	1	DC	ANAPC11
TRIM69.1	0.00025	-0.2489	0.086	0.199	1	DC	TRIM69
FKBP14	0.000252	-0.17021	0.016	0.096	1	DC	FKBP14
SRR.2	0.000252	-0.19679	0.053	0.155	1	DC	SRR
FAM131B.1	0.000253	-0.18556	0.005	0.077	1	DC	FAM131B
UBE2S.3	0.000254	-0.27688	0.209	0.346	1	DC	UBE2S
JRKL.1	0.000254	-0.16437	0.005	0.077	1	DC	JRKL
PDDC1.2	0.000255	-0.18513	0.048	0.147	1	DC	PDDC1
CELF3.3	0.000255	-0.23122	0	0.067	1	DC	CELF3
SYTL2.1	0.000256	-0.27348	0.005	0.077	1	DC	SYTL2
HNRNPUL1.	0.000256	0.228197	0.46	0.382	1	DC	HNRNPUL1
GCLC.3	0.000257	0.243128	0.171	0.096	1	DC	GCLC
FAM181A.1	0.000258	-0.24408	0	0.067	1	DC	FAM181A
KLHDC8B.2	0.000259	-0.19515	0.021	0.105	1	DC	KLHDC8B
RP11-115C	0.000261	-0.16253	0.011	0.087	1	DC	RP11-115C21.2
SSBP2.3	0.000262	-0.20748	0.134	0.272	1	DC	SSBP2
MED28.2	0.000262	0.216566	0.422	0.337	1	DC	MED28
FRS2.2	0.000263	-0.22876	0.027	0.113	1	DC	FRS2
ZNF506.1	0.000263	-0.16811	0.032	0.123	1	DC	ZNF506
SWI5.3	0.000263	-0.23803	0.118	0.247	1	DC	SWI5
FBXW9	0.000263	-0.17388	0.027	0.113	1	DC	FBXW9
ZSCAN21.1	0.000264	-0.21803	0.021	0.103	1	DC	ZSCAN21
MED24.2	0.000266	-0.17259	0.064	0.17	1	DC	MED24
AFAP1.1	0.000266	-0.16159	0.011	0.086	1	DC	AFAP1
DNAAF1.2	0.000267	0.277225	0.07	0.026	1	DC	DNAAF1
TOMM5.3	0.000269	0.369409	0.289	0.208	1	DC	TOMM5
PEX10.1	0.00027	-0.18393	0.08	0.194	1	DC	PEX10
VAV1.3	0.00027	0.152007	0.144	0.073	1	DC	VAV1
NOL4.1	0.000274	-0.18497	0	0.066	1	DC	NOL4
RAB8B.2	0.000275	0.283524	0.235	0.153	1	DC	RAB8B
DIP2C.2	0.000275	-0.20608	0.011	0.086	1	DC	DIP2C
PLXNB1.2	0.000275	-0.19644	0.005	0.077	1	DC	PLXNB1
GTF2IRD2E	0.000276	-0.19814	0.043	0.138	1	DC	GTF2IRD2B
CMSS1	0.000276	-0.23668	0.155	0.292	1	DC	CMSS1
XPOT.2	0.000277	-0.20012	0.064	0.169	1	DC	XPOT
C5orf42.1	0.000278	-0.15606	0.016	0.095	1	DC	C5orf42
PDCL.1	0.00028	-0.23491	0.086	0.196	1	DC	PDCL
GPRASP2.1	0.000282	-0.17583	0	0.066	1	DC	GPRASP2
RPRD1A.1	0.000282	-0.14165	0.091	0.209	1	DC	RPRD1A
PIK3IP1.3	0.000282	0.142369	0.262	0.159	1	DC	PIK3IP1
CCL2.1	0.000283	-0.58316	0.118	0.227	1	DC	CCL2
SNHG10	0.000283	-0.17081	0.016	0.095	1	DC	SNHG10
GNB2L1.3	0.000283	-0.17492	0.989	0.941	1	DC	GNB2L1
RP11-1114	0.000284	-0.19383	0.011	0.085	1	DC	RP11-1114A5.4
ARHGAP32.	0.000284	-0.1786	0.011	0.086	1	DC	ARHGAP32
PDGFB.2	0.000284	0.117156	0.102	0.045	1	DC	PDGFB
PDGFC	0.000286	-0.17168	0.032	0.121	1	DC	PDGFC
C1orf54.3	0.000287	0.203099	0.332	0.224	1	DC	C1orf54
VIM.2	0.000289	0.133243	0.941	0.659	1	DC	VIM
TSC2.1	0.000289	-0.19945	0.043	0.137	1	DC	TSC2
DNAAF2.2	0.000289	-0.19098	0.07	0.174	1	DC	DNAAF2

TLE3.2	0.00029	0.19968	0.225	0.136	1	DC	TLE3
PEG3.1	0.00029	-0.1938	0	0.066	1	DC	PEG3
HLA-A.3	0.000291	-0.22453	0.963	0.895	1	DC	HLA-A
FEZ2.3	0.000292	-0.22915	0.294	0.451	1	DC	FEZ2
DOCK11.3	0.000293	0.252357	0.112	0.053	1	DC	DOCK11
RAB6B.2	0.000293	-0.16416	0	0.066	1	DC	RAB6B
UQCRQ.3	0.000294	-0.20624	0.786	0.799	1	DC	UQCRQ
ZNF680.1	0.000295	-0.23761	0.07	0.176	1	DC	ZNF680
DERA.1	0.000296	0.215223	0.342	0.25	1	DC	DERA
RORA	0.000297	-0.19872	0.005	0.076	1	DC	RORA
ABCA8.1	0.000298	-0.2829	0.005	0.075	1	DC	ABCA8
RP11-410L	0.000299	-0.17596	0.037	0.13	1	DC	RP11-410L14.2
CTHRC1.2	0.000299	-0.13554	0	0.066	1	DC	CTHRC1
BNIP3.2	0.000299	-0.33073	0.102	0.217	1	DC	BNIP3
NACC2.1	0.0003	-0.14999	0.021	0.104	1	DC	NACC2
C17orf80.	0.000301	-0.16498	0.011	0.085	1	DC	C17orf80
PTK7	0.000301	-0.12924	0.016	0.095	1	DC	PTK7
GNAS.3	0.000301	-0.24107	0.61	0.648	1	DC	GNAS
SOX21-AS1	0.000302	-0.14007	0	0.065	1	DC	SOX21-AS1
KHDC1.2	0.000302	-0.19304	0	0.065	1	DC	KHDC1
SLC46A3.3	0.000302	0.169762	0.118	0.057	1	DC	SLC46A3
VBP1.2	0.000302	-0.23162	0.321	0.49	1	DC	VBP1
PGLS.3	0.000302	0.167924	0.61	0.509	1	DC	PGLS
GLT8D1	0.000304	-0.2123	0.144	0.278	1	DC	GLT8D1
KCTD1.1	0.000305	-0.15344	0	0.065	1	DC	KCTD1
FBXO21.1	0.000306	-0.23569	0.171	0.31	1	DC	FBXO21
SCYL2.2	0.000306	-0.17728	0.043	0.137	1	DC	SCYL2
RMDN3.2	0.000307	-0.21673	0.118	0.242	1	DC	RMDN3
ZDHHC12.3	0.000307	0.321654	0.348	0.259	1	DC	ZDHHC12
NAGPA.2	0.000307	0.288914	0.182	0.108	1	DC	NAGPA
ABL1	0.000308	-0.19983	0.016	0.094	1	DC	ABL1
RDH5.1	0.000308	-0.16756	0.021	0.104	1	DC	RDH5
PLK2.2	0.000308	0.294412	0.219	0.136	1	DC	PLK2
ZNF793	0.000309	-0.21019	0.027	0.112	1	DC	ZNF793
FAM110A.2	0.000311	0.214827	0.139	0.072	1	DC	FAM110A
BLCAP	0.000311	-0.23185	0.16	0.292	1	DC	BLCAP
BTG3.2	0.000312	-0.22959	0.225	0.373	1	DC	BTG3
POLR3D.1	0.000313	-0.20165	0.037	0.127	1	DC	POLR3D
TUBG2.2	0.000314	-0.20608	0.027	0.112	1	DC	TUBG2
SLC2A10.1	0.000314	-0.13193	0	0.065	1	DC	SLC2A10
SERPINE1.	0.000316	-0.40905	0.021	0.101	1	DC	SERPINE1
YDJC.3	0.000316	0.322648	0.219	0.142	1	DC	YDJC
RALGAPA2.	0.000318	-0.14042	0.027	0.112	1	DC	RALGAPA2
ASB8	0.000318	-0.22162	0.102	0.219	1	DC	ASB8
UBA6-AS1.	0.000318	-0.17117	0.011	0.084	1	DC	UBA6-AS1
TRIB3.2	0.000319	-0.21011	0.005	0.075	1	DC	TRIB3
LANCL1.2	0.000319	-0.22327	0.032	0.118	1	DC	LANCL1
FAM179B.1	0.000319	-0.2051	0.032	0.12	1	DC	FAM179B
CDC42BPB.	0.000319	-0.16519	0.032	0.12	1	DC	CDC42BPB
ZNF444.1	0.000321	-0.16077	0.059	0.162	1	DC	ZNF444
NVL.1	0.000325	-0.22609	0.027	0.11	1	DC	NVL

GNG3.1	0.000325	-0.28693	0.005	0.075	1	DC	GNG3
ANGPTL2.1	0.000325	-0.26675	0.021	0.101	1	DC	ANGPTL2
FTX.1	0.000326	-0.2487	0.021	0.101	1	DC	FTX
GJC1.1	0.000327	-0.12793	0	0.065	1	DC	GJC1
PLEKHH3.1	0.000327	-0.11512	0.011	0.085	1	DC	PLEKHH3
PBK.3	0.000328	-0.29184	0.005	0.074	1	DC	PBK
PLA2G16.3	0.000332	-0.32139	0.251	0.407	1	DC	PLA2G16
CIRBP.3	0.000335	-0.18127	0.888	0.865	1	DC	CIRBP
GPR37	0.000337	-0.32191	0.005	0.074	1	DC	GPR37
ESF1.1	0.000339	-0.2168	0.203	0.348	1	DC	ESF1
SHOX2.1	0.00034	-0.13363	0	0.064	1	DC	SHOX2
ICA1L	0.000341	-0.22583	0.021	0.101	1	DC	ICA1L
TIMP4.1	0.000342	-0.30952	0.021	0.102	1	DC	TIMP4
PRUNE2.1	0.000342	-0.29325	0.032	0.118	1	DC	PRUNE2
RP11-448A	0.000343	-0.14581	0.016	0.093	1	DC	RP11-448A19.1
SEZ6.3	0.000343	-0.26424	0.048	0.14	1	DC	SEZ6
TRIM33.2	0.000346	-0.26688	0.08	0.184	1	DC	TRIM33
PPIF.3	0.000346	0.266938	0.316	0.219	1	DC	PPIF
MMP15.2	0.000346	-0.13774	0	0.064	1	DC	MMP15
NLGN2.1	0.000346	-0.15917	0.016	0.093	1	DC	NLGN2
GPR161.3	0.000348	-0.15699	0.011	0.084	1	DC	GPR161
IFT46.1	0.000349	-0.19114	0.027	0.111	1	DC	IFT46
ABHD17C	0.000351	-0.12497	0.011	0.084	1	DC	ABHD17C
CAST.2	0.000351	0.273727	0.401	0.317	1	DC	CAST
RBM26.1	0.000354	-0.16689	0.096	0.215	1	DC	RBM26
PTPRD.2	0.000354	-0.2458	0.005	0.074	1	DC	PTPRD
MTHFR.2	0.000354	0.251778	0.144	0.077	1	DC	MTHFR
CNR1.1	0.000355	-0.10526	0.005	0.074	1	DC	CNR1
ABCD3.1	0.000356	-0.18367	0.086	0.197	1	DC	ABCD3
DIRAS2.1	0.000357	-0.16882	0	0.064	1	DC	DIRAS2
ADAM22.2	0.000357	-0.18622	0	0.064	1	DC	ADAM22
SLC25A37.	0.000358	0.213631	0.401	0.308	1	DC	SLC25A37
TPX2.2	0.000358	-0.232	0.011	0.084	1	DC	TPX2
MIB1.1	0.00036	-0.14569	0.016	0.093	1	DC	MIB1
PIK3CD.1	0.000362	0.14888	0.075	0.03	1	DC	PIK3CD
NXPH4.1	0.000364	-0.21219	0	0.064	1	DC	NXPH4
CLIC4.2	0.000365	-0.29619	0.198	0.334	1	DC	CLIC4
SRSF12.1	0.000366	-0.1605	0.005	0.074	1	DC	SRSF12
PGPEP1.1	0.000367	-0.18965	0.043	0.135	1	DC	PGPEP1
TM2D2.1	0.000368	-0.26364	0.198	0.343	1	DC	TM2D2
COX7A2L.1	0.000368	0.106261	0.695	0.611	1	DC	COX7A2L
GRB10.2	0.000369	-0.17124	0.005	0.074	1	DC	GRB10
CACNG8.1	0.00037	-0.13871	0.005	0.074	1	DC	CACNG8
GATS	0.000371	-0.19015	0	0.064	1	DC	GATS
SDHB	0.000372	0.156073	0.503	0.423	1	DC	SDHB
ZCCHC24.1	0.000373	-0.23661	0.021	0.101	1	DC	ZCCHC24
MT1G.2	0.000375	-0.58465	0.043	0.129	1	DC	MT1G
SCN3A.2	0.000376	-0.2176	0.005	0.073	1	DC	SCN3A
TRMT11.1	0.000377	-0.18857	0.032	0.118	1	DC	TRMT11
DMXL2.2	0.000378	-0.21375	0.011	0.082	1	DC	DMXL2
SMPDL3A.2	0.000378	-0.2447	0.037	0.126	1	DC	SMPDL3A

NDEL1.2	0.000378	0.257267	0.235	0.152	1 DC	NDEL1
NANOS3.1	0.000378	-0.14162	0	0.063	1 DC	NANOS3
PTPRG	0.000379	-0.16306	0.005	0.074	1 DC	PTPRG
RNF220.2	0.000381	-0.21901	0.053	0.149	1 DC	RNF220
MICU3.3	0.000381	-0.18588	0.011	0.084	1 DC	MICU3
ZNF334	0.000384	-0.1576	0.005	0.073	1 DC	ZNF334
VANGL2.1	0.000384	-0.12671	0.005	0.074	1 DC	VANGL2
COL5A2.1	0.000386	-0.12331	0	0.063	1 DC	COL5A2
AGAP3.2	0.000387	-0.15863	0.021	0.101	1 DC	AGAP3
HOXA2.2	0.000387	-0.22838	0.005	0.073	1 DC	HOXA2
RB1.3	0.00039	0.223796	0.406	0.303	1 DC	RB1
LINC00667	0.00039	-0.26351	0.123	0.246	1 DC	LINC00667
IFT88.1	0.00039	-0.16408	0.027	0.11	1 DC	IFT88
FAM98A.1	0.00039	-0.18407	0.048	0.143	1 DC	FAM98A
PWAR6	0.000391	-0.15314	0.005	0.073	1 DC	PWAR6
ZNF138.1	0.000393	-0.23448	0.053	0.147	1 DC	ZNF138
AP1G1.2	0.000394	-0.20743	0.037	0.126	1 DC	AP1G1
CREG1.3	0.000395	0.173991	0.417	0.295	1 DC	CREG1
GKAP1.2	0.000395	-0.19922	0.016	0.091	1 DC	GKAP1
PPP1R2.1	0.000397	0.206102	0.455	0.367	1 DC	PPP1R2
FUBP3.1	0.000401	-0.12747	0.118	0.242	1 DC	FUBP3
TMTC4.2	0.000402	-0.21931	0.021	0.1	1 DC	TMTC4
ELMOD1.1	0.000403	-0.12287	0.011	0.083	1 DC	ELMOD1
CREB3L1	0.000405	-0.12223	0	0.063	1 DC	CREB3L1
GIMAP8	0.000405	0.134633	0.053	0.018	1 DC	GIMAP8
CYB5A.2	0.000408	-0.32353	0.209	0.344	1 DC	CYB5A
CD151.2	0.000409	-0.31506	0.337	0.484	1 DC	CD151
RP13-516M	0.00041	0.113135	0.043	0.013	1 DC	RP13-516M14.1
SLC50A1.2	0.000411	0.248586	0.251	0.166	1 DC	SLC50A1
GNPDA2	0.000413	-0.13628	0.037	0.126	1 DC	GNPDA2
CNKSR2.1	0.000413	-0.13517	0	0.063	1 DC	CNKSR2
EVA1C.2	0.000414	-0.166	0.005	0.073	1 DC	EVA1C
PCDHB7.1	0.000416	-0.2095	0.005	0.072	1 DC	PCDHB7
UNG	0.000417	-0.24643	0.037	0.123	1 DC	UNG
GPD2	0.000417	-0.17782	0.016	0.091	1 DC	GPD2
TBC1D8	0.000418	0.218618	0.096	0.044	1 DC	TBC1D8
TRUB2.1	0.000419	-0.1688	0.096	0.214	1 DC	TRUB2
NDUFB3.2	0.000421	0.123389	0.631	0.552	1 DC	NDUFB3
ZNF528.1	0.000421	-0.16239	0.027	0.109	1 DC	ZNF528
PIAS4	0.000423	-0.20085	0.043	0.133	1 DC	PIAS4
GTF2IRD2.	0.000424	-0.19975	0.032	0.118	1 DC	GTF2IRD2
EPHA4	0.000425	-0.15115	0.011	0.082	1 DC	EPHA4
MOB3B.2	0.000427	0.199587	0.166	0.093	1 DC	MOB3B
NACAD.2	0.000427	-0.22954	0.016	0.09	1 DC	NACAD
EHD2.1	0.000427	-0.13766	0.011	0.082	1 DC	EHD2
SPA17.3	0.000428	-0.20793	0.011	0.082	1 DC	SPA17
SLC25A14	0.000429	-0.18293	0.016	0.091	1 DC	SLC25A14
GPR160	0.000431	0.154531	0.064	0.024	1 DC	GPR160
ABCA1.2	0.000432	-0.25111	0.123	0.241	1 DC	ABCA1
CEP95	0.000433	-0.18237	0.037	0.125	1 DC	CEP95
SYNM.1	0.000433	-0.12958	0.005	0.073	1 DC	SYNM

ITGA3.1	0.000434	-0.17073	0.021	0.1	1	DC	ITGA3
HYOU1.2	0.000435	-0.21057	0.048	0.141	1	DC	HYOU1
CD47.2	0.000437	0.225677	0.465	0.383	1	DC	CD47
HOMER1.2	0.000439	-0.17538	0.059	0.158	1	DC	HOMER1
TENM1.1	0.000439	-0.14774	0.005	0.072	1	DC	TENM1
C1orf109.	0.00044	-0.17059	0.043	0.134	1	DC	C1orf109
HSDL1.2	0.00044	-0.16584	0.043	0.132	1	DC	HSDL1
AVIL.1	0.000444	-0.22582	0.016	0.09	1	DC	AVIL
LSS.2	0.000444	-0.19472	0.021	0.099	1	DC	LSS
EXTL3.1	0.000445	-0.18212	0.043	0.133	1	DC	EXTL3
EEA1.2	0.000447	-0.20374	0.123	0.247	1	DC	EEA1
FAM102A	0.000447	-0.22257	0.021	0.099	1	DC	FAM102A
CNTRL.3	0.000448	0.264197	0.193	0.117	1	DC	CNTRL
EID2B.1	0.000449	-0.17348	0.016	0.091	1	DC	EID2B
SRSF6.3	0.00045	0.255955	0.433	0.358	1	DC	SRSF6
RAB3GAP2.	0.00045	-0.2032	0.07	0.172	1	DC	RAB3GAP2
SMIM18.2	0.000451	-0.21305	0	0.062	1	DC	SMIM18
AC004951.	0.000452	-0.1675	0.059	0.158	1	DC	AC004951.6
HRSP12.3	0.000453	-0.23628	0.091	0.2	1	DC	HRSP12
NOVA2.1	0.000454	-0.15832	0.011	0.082	1	DC	NOVA2
ZNF232.1	0.000456	-0.17621	0.011	0.081	1	DC	ZNF232
UBE2K.1	0.000461	0.280099	0.374	0.297	1	DC	UBE2K
PLK3.3	0.000463	0.234276	0.337	0.239	1	DC	PLK3
MIS18A.1	0.000465	-0.20708	0.027	0.107	1	DC	MIS18A
ZDHHC15.1	0.000465	-0.14212	0	0.062	1	DC	ZDHHC15
CRLF3.3	0.000465	0.261522	0.187	0.112	1	DC	CRLF3
SPON2	0.000466	0.184494	0.059	0.021	1	DC	SPON2
LAMA4.1	0.000469	-0.14261	0.005	0.072	1	DC	LAMA4
PPP1R14C.	0.000469	-0.13545	0	0.062	1	DC	PPP1R14C
NELFA.2	0.000471	-0.18882	0.048	0.141	1	DC	NELFA
SMKR1	0.000471	-0.16951	0.005	0.072	1	DC	SMKR1
PIK3CG.1	0.000474	0.168746	0.059	0.021	1	DC	PIK3CG
YWHAZ.1	0.000474	0.148785	0.626	0.534	1	DC	YWHAZ
CTC-479C5	0.000474	0.14288	0.107	0.05	1	DC	CTC-479C5.12
SMAD9.1	0.000477	-0.18868	0.005	0.071	1	DC	SMAD9
SOX15.1	0.000478	-0.23021	0.005	0.071	1	DC	SOX15
ZNF337	0.000479	-0.14837	0.005	0.072	1	DC	ZNF337
YBEY	0.000479	-0.20048	0.107	0.223	1	DC	YBEY
HSPA13.2	0.000481	-0.19822	0.091	0.201	1	DC	HSPA13
AP006222.	0.000482	-0.22064	0.096	0.213	1	DC	AP006222.2
TRIM37.1	0.000485	-0.13072	0.053	0.151	1	DC	TRIM37
ROM1.2	0.000485	-0.26058	0.032	0.115	1	DC	ROM1
SLC25A19.	0.000488	0.274502	0.257	0.167	1	DC	SLC25A19
CETN3.1	0.000488	-0.21444	0.053	0.148	1	DC	CETN3
NOTCH3	0.000488	-0.12381	0	0.061	1	DC	NOTCH3
KBTBD6.1	0.000489	-0.1854	0.043	0.133	1	DC	KBTBD6
LRRC45.3	0.00049	-0.14506	0.016	0.09	1	DC	LRRC45
REEP4.3	0.000491	0.201484	0.257	0.166	1	DC	REEP4
CFDP1.2	0.000492	-0.30399	0.267	0.418	1	DC	CFDP1
MTHFD2L.1	0.000493	-0.214	0.096	0.208	1	DC	MTHFD2L
GDF15.2	0.000493	-0.30485	0.005	0.071	1	DC	GDF15

NELFCD.1	0.000494	-0.26324	0.203	0.339	1	DC	NELFCD
NCBP2-AS2	0.000495	-0.159	0.15	0.279	1	DC	NCBP2-AS2
GLRX3.2	0.000496	0.236102	0.385	0.303	1	DC	GLRX3
STARD8	0.000497	0.140452	0.037	0.011	1	DC	STARD8
PELO.2	0.000497	-0.14521	0.027	0.108	1	DC	PELO
PCDH19.1	0.000498	-0.13168	0	0.061	1	DC	PCDH19
IPP.1	0.000498	-0.1486	0	0.061	1	DC	IPP
TIAM2.2	0.000499	-0.20393	0.011	0.08	1	DC	TIAM2
WDR48.1	0.000501	-0.19078	0.048	0.14	1	DC	WDR48
CD226	0.000502	0.129442	0.043	0.013	1	DC	CD226
WDR82.2	0.000502	-0.19154	0.134	0.256	1	DC	WDR82
VPS37D.1	0.000503	-0.15938	0	0.061	1	DC	VPS37D
PLCB1.1	0.000503	-0.13486	0.011	0.081	1	DC	PLCB1
KAT2A.1	0.000506	-0.18627	0.064	0.162	1	DC	KAT2A
ZNF124.3	0.000507	0.205749	0.118	0.059	1	DC	ZNF124
CCNL2.1	0.000508	-0.13244	0.16	0.292	1	DC	CCNL2
HP1BP3.3	0.00051	-0.24824	0.299	0.444	1	DC	HP1BP3
RP3-428L1	0.000511	-0.14809	0.005	0.071	1	DC	RP3-428L16.2
MDGA1.1	0.000512	-0.10395	0.005	0.071	1	DC	MDGA1
STK10.3	0.000513	0.253881	0.134	0.071	1	DC	STK10
TP53BP1	0.000514	-0.17095	0.021	0.098	1	DC	TP53BP1
CRKL.1	0.000515	-0.13838	0.037	0.123	1	DC	CRKL
CTB-113I2	0.000518	-0.1337	0	0.061	1	DC	CTB-113I20.2
PAICS.2	0.00052	-0.22078	0.176	0.31	1	DC	PAICS
SIGLEC14.	0.00052	0.20114	0.096	0.044	1	DC	SIGLEC14
TMED4.2	0.000522	-0.29456	0.294	0.44	1	DC	TMED4
UBE2H.2	0.000523	-0.19803	0.134	0.261	1	DC	UBE2H
ARHGEF19	0.000525	0.158188	0.048	0.016	1	DC	ARHGEF19
SNAP47.1	0.000527	-0.18315	0.091	0.204	1	DC	SNAP47
PCDH1.1	0.000528	-0.14044	0	0.061	1	DC	PCDH1
HPCA.1	0.000528	-0.20328	0	0.061	1	DC	HPCA
POLR3H.1	0.000529	-0.17473	0.102	0.216	1	DC	POLR3H
SBDS.3	0.000529	-0.31111	0.492	0.612	1	DC	SBDS
TCF25.2	0.000531	0.11953	0.626	0.55	1	DC	TCF25
MED17	0.000532	-0.20711	0.086	0.191	1	DC	MED17
PRKCA.2	0.000533	-0.20463	0.091	0.2	1	DC	PRKCA
NIP7.2	0.000533	-0.24102	0.075	0.174	1	DC	NIP7
MPP2.2	0.000533	-0.15417	0	0.06	1	DC	MPP2
ZNF37A.3	0.000535	-0.1707	0.048	0.14	1	DC	ZNF37A
MITF.2	0.000535	0.180058	0.166	0.093	1	DC	MITF
NT5C3A.1	0.000538	-0.2517	0.171	0.296	1	DC	NT5C3A
BAMBI.1	0.000538	-0.16143	0.005	0.07	1	DC	BAMBI
ZNF300.2	0.00054	-0.17628	0.005	0.07	1	DC	ZNF300
CTC-250I1	0.00054	0.177041	0.064	0.025	1	DC	CTC-250I14.6
ZNF629.1	0.000541	-0.15118	0.005	0.07	1	DC	ZNF629
ZNF181.1	0.000542	-0.16585	0.021	0.098	1	DC	ZNF181
GLRB.1	0.000544	-0.14116	0	0.06	1	DC	GLRB
SAC3D1.3	0.000549	-0.16198	0.07	0.171	1	DC	SAC3D1
GAL3ST3.1	0.000549	-0.13808	0	0.06	1	DC	GAL3ST3
KIAA0319L	0.000552	-0.17718	0.08	0.186	1	DC	KIAA0319L
NPRL3.1	0.000554	-0.18512	0.086	0.192	1	DC	NPRL3

TTI1	0.000554	-0.13825	0.021	0.098	1	DC	TTI1
BAX.3	0.000554	0.149436	0.642	0.564	1	DC	BAX
PTRH2.2	0.000554	-0.14312	0.048	0.139	1	DC	PTRH2
ABHD14A.2	0.000557	-0.17437	0.107	0.224	1	DC	ABHD14A
RAI14.1	0.000558	-0.17126	0.005	0.07	1	DC	RAI14
SAT2.3	0.00056	-0.25252	0.449	0.588	1	DC	SAT2
TGS1	0.00056	-0.22913	0.064	0.162	1	DC	TGS1
FXYD7.2	0.000562	-0.28473	0.011	0.079	1	DC	FXYD7
ZNF48.1	0.000565	-0.15452	0.021	0.098	1	DC	ZNF48
USP11.2	0.000566	-0.20958	0.209	0.355	1	DC	USP11
BAG5.1	0.000568	-0.20942	0.08	0.187	1	DC	BAG5
LRRCC1.2	0.00057	-0.22215	0.048	0.137	1	DC	LRRCC1
PAPOLA.1	0.00057	0.160493	0.556	0.483	1	DC	PAPOLA
SHROOM2.2	0.000571	-0.14419	0	0.06	1	DC	SHROOM2
GBP4.1	0.000573	0.189258	0.128	0.066	1	DC	GBP4
HSPA2	0.000574	-0.44277	0.043	0.126	1	DC	HSPA2
EGR1.1	0.000575	-0.34086	0.396	0.497	1	DC	EGR1
IRF5.3	0.000575	0.139013	0.118	0.058	1	DC	IRF5
TGOLN2.3	0.000576	0.255307	0.449	0.371	1	DC	TGOLN2
SPSB4.2	0.000577	-0.1276	0	0.06	1	DC	SPSB4
TMEM60.3	0.000577	-0.20836	0.128	0.249	1	DC	TMEM60
ZBTB1.2	0.000578	0.34705	0.241	0.163	1	DC	ZBTB1
TMCC1.2	0.000579	-0.16075	0.032	0.115	1	DC	TMCC1
PAN3.2	0.000579	0.254374	0.198	0.123	1	DC	PAN3
CACFD1.1	0.00058	-0.16407	0.016	0.088	1	DC	CACFD1
RRAS2.1	0.00058	-0.12527	0.011	0.079	1	DC	RRAS2
MRAS.1	0.000581	-0.1749	0.07	0.169	1	DC	MRAS
FAM200B.1	0.000581	-0.24493	0.23	0.368	1	DC	FAM200B
ALDH3A2.1	0.000582	-0.22487	0.118	0.234	1	DC	ALDH3A2
MCHR1.1	0.000582	-0.12265	0	0.06	1	DC	MCHR1
MICALL2.1	0.000584	-0.13171	0.005	0.07	1	DC	MICALL2
CRCP.1	0.000587	-0.21353	0.08	0.183	1	DC	CRCP
COQ5.1	0.000588	-0.2105	0.091	0.198	1	DC	COQ5
BUD31.3	0.00059	-0.27239	0.513	0.63	1	DC	BUD31
MOSPD1.1	0.000591	-0.14954	0.043	0.131	1	DC	MOSPD1
DNAJC30.1	0.000592	-0.13708	0.059	0.155	1	DC	DNAJC30
PLEKHH2	0.000592	-0.1408	0.005	0.07	1	DC	PLEKHH2
CAPN10.1	0.000596	-0.18696	0.016	0.088	1	DC	CAPN10
MACROD1.1	0.000596	-0.13784	0.037	0.124	1	DC	MACROD1
TRPM2.3	0.000597	0.185919	0.107	0.051	1	DC	TRPM2
NRP1.3	0.000598	-0.17537	0.07	0.167	1	DC	NRP1
TUSC2.2	0.0006	-0.1643	0.193	0.337	1	DC	TUSC2
F12.1	0.0006	-0.14664	0.011	0.079	1	DC	F12
SCIN.3	0.000601	-0.46113	0.064	0.152	1	DC	SCIN
PTPRN2.2	0.000602	-0.35484	0.112	0.213	1	DC	PTPRN2
COQ4.1	0.000602	-0.22055	0.144	0.269	1	DC	COQ4
GSDMD.2	0.000603	0.188322	0.273	0.183	1	DC	GSDMD
AAMDC.3	0.000603	-0.34572	0.102	0.208	1	DC	AAMDC
LONP1.1	0.000603	-0.14903	0.096	0.206	1	DC	LONP1
UBE2E2.2	0.000603	0.19086	0.337	0.239	1	DC	UBE2E2
CDK20.1	0.000605	-0.16179	0	0.059	1	DC	CDK20

POMT1.2	0.000607	-0.14649	0.048	0.139	1	DC	POMT1
SLC25A22.	0.000607	-0.14987	0.032	0.114	1	DC	SLC25A22
DEF8.3	0.000608	-0.179	0.144	0.272	1	DC	DEF8
EIF1AY.2	0.000609	-0.16098	0.075	0.179	1	DC	EIF1AY
RNFT2.2	0.00061	-0.12991	0.005	0.07	1	DC	RNFT2
FAM69B.1	0.000611	-0.11359	0	0.059	1	DC	FAM69B
LRRC37B.2	0.000613	-0.16008	0.032	0.114	1	DC	LRRC37B
SNX2.3	0.000613	0.143255	0.497	0.409	1	DC	SNX2
ZC2HC1A.2	0.000613	-0.27808	0.107	0.212	1	DC	ZC2HC1A
PAPOLG.3	0.000615	0.227799	0.16	0.09	1	DC	PAPOLG
ITGAV.3	0.000616	-0.18823	0.155	0.28	1	DC	ITGAV
RAP1GDS1.	0.000616	0.289072	0.251	0.172	1	DC	RAP1GDS1
RDH10.2	0.000616	-0.22243	0.016	0.087	1	DC	RDH10
ALYREF.3	0.000617	-0.15688	0.053	0.147	1	DC	ALYREF
C12orf29.	0.00062	-0.19665	0.059	0.155	1	DC	C12orf29
GIMAP2.2	0.000622	0.146306	0.144	0.076	1	DC	GIMAP2
KRTCAP2.1	0.000622	0.122112	0.642	0.555	1	DC	KRTCAP2
RP11-698N	0.000623	-0.16592	0	0.059	1	DC	RP11-698N11.2
UBR4.3	0.000629	-0.18885	0.053	0.146	1	DC	UBR4
TMEM169.1	0.00063	-0.15241	0	0.059	1	DC	TMEM169
STK33.2	0.000633	-0.13424	0.005	0.069	1	DC	STK33
ARHGEF9.2	0.000633	-0.20829	0.037	0.121	1	DC	ARHGEF9
PDCD2L.1	0.000634	-0.12347	0.027	0.106	1	DC	PDCD2L
ACTL6B.2	0.000634	-0.2585	0.011	0.078	1	DC	ACTL6B
HOXA10.3	0.000634	-0.21954	0.011	0.078	1	DC	HOXA10
KBTBD7.1	0.000635	-0.18205	0.016	0.088	1	DC	KBTBD7
C5orf56.2	0.000639	-0.17035	0.032	0.113	1	DC	C5orf56
KCND3.2	0.000642	-0.1392	0	0.059	1	DC	KCND3
PSMG4.1	0.000642	-0.18038	0.091	0.2	1	DC	PSMG4
VPS72.1	0.000643	-0.19152	0.15	0.278	1	DC	VPS72
SLC35E2B.	0.000643	-0.16857	0.053	0.146	1	DC	SLC35E2B
LSG1.1	0.000643	-0.1799	0.043	0.129	1	DC	LSG1
DPP4.1	0.000643	-0.127	0.011	0.079	1	DC	DPP4
BACE1.2	0.000645	-0.22976	0.037	0.119	1	DC	BACE1
ARL13B.3	0.000645	-0.24474	0.053	0.144	1	DC	ARL13B
TFEC.3	0.000647	0.183277	0.112	0.055	1	DC	TFEC
ALG10B	0.000647	0.136943	0.037	0.011	1	DC	ALG10B
SYT17.1	0.000649	-0.18517	0.064	0.161	1	DC	SYT17
PINK1.2	0.000649	-0.22851	0.102	0.209	1	DC	PINK1
COMT.3	0.000649	0.118407	0.647	0.546	1	DC	COMT
TMEM11	0.000651	-0.19408	0.187	0.321	1	DC	TMEM11
GPR75-ASE	0.000653	-0.17002	0.016	0.087	1	DC	GPR75-ASB3
HIRIP3.1	0.000654	-0.23764	0.07	0.167	1	DC	HIRIP3
ZNF584.1	0.000655	-0.13105	0.016	0.087	1	DC	ZNF584
PCDH10.1	0.000655	-0.14607	0.005	0.069	1	DC	PCDH10
GAS2L1.2	0.000659	-0.14539	0.059	0.153	1	DC	GAS2L1
CAPN1.2	0.00066	0.246968	0.342	0.257	1	DC	CAPN1
CRB1	0.000661	-0.20843	0.005	0.068	1	DC	CRB1
FLRT3.2	0.000661	-0.14712	0	0.059	1	DC	FLRT3
ABI1.3	0.000662	0.285624	0.321	0.239	1	DC	ABI1
RRP1.2	0.000663	-0.17743	0.102	0.212	1	DC	RRP1

UTP23.2	0.000666	-0.21616	0.059	0.151	1	DC	UTP23
FAM98B.2	0.000667	-0.14657	0.021	0.096	1	DC	FAM98B
CEP19.1	0.000668	-0.18652	0.011	0.078	1	DC	CEP19
RIT2.2	0.000669	-0.28862	0.011	0.077	1	DC	RIT2
TTC37.2	0.000672	-0.16638	0.08	0.185	1	DC	TTC37
SHFM1.2	0.000674	-0.30411	0.668	0.724	1	DC	SHFM1
YKT6.1	0.000681	-0.16963	0.123	0.24	1	DC	YKT6
PRRG4.2	0.000681	0.144913	0.096	0.044	1	DC	PRRG4
PCMTD2.2	0.000685	-0.16871	0.112	0.229	1	DC	PCMTD2
LUC7L3.1	0.000686	-0.26445	0.385	0.512	1	DC	LUC7L3
CSGALNACT	0.000686	0.184751	0.139	0.074	1	DC	CSGALNACT1
CARF.2	0.000687	-0.18357	0.011	0.077	1	DC	CARF
SHC4	0.000687	-0.14131	0	0.058	1	DC	SHC4
GAR1.3	0.000689	-0.17896	0.086	0.192	1	DC	GAR1
AGRN.1	0.000689	-0.15502	0.021	0.095	1	DC	AGRN
SGSM2.1	0.000692	-0.15423	0.027	0.104	1	DC	SGSM2
EYA4.1	0.000694	-0.10327	0	0.058	1	DC	EYA4
LRRC27.1	0.000696	-0.16671	0.016	0.086	1	DC	LRRC27
B4GALT2	0.000699	-0.1335	0.011	0.078	1	DC	B4GALT2
CTU1.1	0.0007	-0.12753	0.011	0.078	1	DC	CTU1
C10orf35	0.000701	-0.21323	0	0.058	1	DC	C10orf35
DOK1.3	0.000706	0.191103	0.235	0.153	1	DC	DOK1
COPA.3	0.000707	-0.21034	0.182	0.315	1	DC	COPA
HNRNPL.2	0.000708	-0.24527	0.144	0.266	1	DC	HNRNPL
FARSB.1	0.00071	-0.14771	0.118	0.234	1	DC	FARSB
BCAS4.1	0.000712	-0.13469	0.016	0.087	1	DC	BCAS4
JMJD6.2	0.000714	0.21069	0.369	0.281	1	DC	JMJD6
AMH.1	0.000715	-0.13429	0	0.058	1	DC	AMH
SIX1.1	0.000715	-0.15433	0	0.058	1	DC	SIX1
CDKN3.2	0.000715	-0.28476	0.021	0.093	1	DC	CDKN3
NDUFS1.1	0.000716	-0.1859	0.134	0.257	1	DC	NDUFS1
FAM53C.2	0.000717	0.171868	0.332	0.24	1	DC	FAM53C
CEP57L1.2	0.000717	-0.18701	0.032	0.112	1	DC	CEP57L1
PHF23.1	0.000718	-0.14469	0.096	0.209	1	DC	PHF23
SLX1A	0.00072	0.124979	0.043	0.014	1	DC	SLX1A
CTA-445C9	0.000722	-0.14852	0	0.058	1	DC	CTA-445C9.14
DLX6.3	0.000722	-0.22258	0	0.058	1	DC	DLX6
PTPRK	0.000722	-0.22614	0	0.058	1	DC	PTPRK
PAPSS2.1	0.000722	0.142151	0.059	0.022	1	DC	PAPSS2
LDLRAD3.2	0.000723	-0.13327	0.075	0.177	1	DC	LDLRAD3
GDPD1.1	0.000724	-0.14835	0.005	0.068	1	DC	GDPD1
PSMA5.1	0.000726	0.184206	0.513	0.441	1	DC	PSMA5
RP11-83A2	0.000727	-0.16171	0.048	0.137	1	DC	RP11-83A24.2
PKD1.1	0.000728	-0.18685	0.037	0.119	1	DC	PKD1
ASNSD1.2	0.000729	-0.24283	0.15	0.273	1	DC	ASNSD1
NIPSNAP1.	0.000731	-0.16308	0.07	0.169	1	DC	NIPSNAP1
ZNF16.1	0.000731	-0.1736	0.005	0.068	1	DC	ZNF16
NCBP2.1	0.000732	-0.2132	0.305	0.443	1	DC	NCBP2
PPP2R2A	0.000733	-0.231	0.171	0.302	1	DC	PPP2R2A
CBX2.1	0.000733	-0.14546	0.005	0.068	1	DC	CBX2
UBE2C.3	0.000735	-0.33426	0.037	0.118	1	DC	UBE2C

ABCA3.1	0.000735	-0.14683	0.011	0.077	1	DC	ABCA3
GPHN	0.000738	-0.16526	0.005	0.068	1	DC	GPHN
RP11-1094	0.000739	-0.16018	0.118	0.236	1	DC	RP11-1094M14.11
ZNF768.1	0.000745	-0.11533	0.021	0.096	1	DC	ZNF768
DLG5	0.000745	-0.14824	0.011	0.077	1	DC	DLG5
YPEL1.3	0.000745	-0.1433	0.021	0.095	1	DC	YPEL1
RUNX2	0.000745	0.100348	0.07	0.028	1	DC	RUNX2
ZFYVE21.2	0.000748	-0.22467	0.144	0.268	1	DC	ZFYVE21
KLF12.2	0.000748	-0.16152	0.016	0.086	1	DC	KLF12
FLCN.1	0.00075	-0.16278	0.048	0.137	1	DC	FLCN
LAYN.2	0.00075	-0.12494	0	0.057	1	DC	LAYN
PDAP1.1	0.000757	-0.2453	0.401	0.538	1	DC	PDAP1
FAT3.1	0.000758	-0.12743	0	0.057	1	DC	FAT3
ANKRD52	0.00076	-0.11359	0.011	0.077	1	DC	ANKRD52
PIGX.2	0.000762	-0.18868	0.134	0.255	1	DC	PIGX
NCOA6.3	0.000763	-0.13791	0.048	0.137	1	DC	NCOA6
RCC2.2	0.000763	0.311304	0.251	0.173	1	DC	RCC2
SLC25A39.	0.000766	0.227998	0.465	0.404	1	DC	SLC25A39
MPHOSPH1C	0.000768	-0.16989	0.123	0.24	1	DC	MPHOSPH10
CEP97.1	0.000768	-0.15959	0.011	0.077	1	DC	CEP97
KCNH2.1	0.00077	-0.1884	0.005	0.067	1	DC	KCNH2
GLA.3	0.000771	0.237244	0.305	0.214	1	DC	GLA
ZBTB16	0.000777	0.13626	0.246	0.154	1	DC	ZBTB16
ZNF493.1	0.000778	-0.17614	0.064	0.159	1	DC	ZNF493
NTRK3	0.00078	-0.17636	0.005	0.067	1	DC	NTRK3
DHFR.1	0.000781	-0.13045	0.059	0.152	1	DC	DHFR
STARD5	0.000782	0.112531	0.048	0.016	1	DC	STARD5
CDC42SE1.	0.000782	0.250679	0.385	0.303	1	DC	CDC42SE1
SP3.3	0.000784	-0.16763	0.102	0.212	1	DC	SP3
ASUN.1	0.000786	-0.14706	0.032	0.112	1	DC	ASUN
FAT1.1	0.000787	-0.11279	0.005	0.067	1	DC	FAT1
WASF3.1	0.000787	-0.14741	0.011	0.077	1	DC	WASF3
AMOTL1.1	0.000787	-0.10554	0.005	0.067	1	DC	AMOTL1
SAPCD2.2	0.000788	-0.15841	0	0.057	1	DC	SAPCD2
NEDD1	0.000789	-0.13903	0.016	0.086	1	DC	NEDD1
LMBR1.1	0.000791	-0.15223	0.032	0.111	1	DC	LMBR1
SEC61A1.1	0.000791	-0.25344	0.225	0.368	1	DC	SEC61A1
DPY19L4.1	0.000795	-0.1189	0.021	0.095	1	DC	DPY19L4
HSD17B1.1	0.000796	-0.11471	0	0.057	1	DC	HSD17B1
GLYCTK	0.000796	0.110068	0.07	0.028	1	DC	GLYCTK
IL1R1.1	0.000797	0.121968	0.048	0.016	1	DC	IL1R1
RGPD5	0.000797	0.113997	0.053	0.019	1	DC	RGPD5
NME4.1	0.000797	-0.1875	0.241	0.384	1	DC	NME4
DNPH1.1	0.000797	-0.24447	0.246	0.379	1	DC	DNPH1
RBMX.2	0.000799	0.125939	0.663	0.565	1	DC	RBMX
TRMT61B.1	0.000802	-0.22355	0.07	0.164	1	DC	TRMT61B
PM20D2.1	0.000803	-0.12703	0.016	0.086	1	DC	PM20D2
HOXA3.2	0.000804	-0.18751	0	0.057	1	DC	HOXA3
ILF3-AS1.	0.000805	-0.18506	0.176	0.312	1	DC	ILF3-AS1
TMEM41B.1	0.000806	-0.12568	0.075	0.177	1	DC	TMEM41B
TMC6.3	0.000809	0.138565	0.193	0.11	1	DC	TMC6

ACLY.3	0.000812	-0.18786	0.107	0.218	1	DC	ACLY
FER.1	0.000813	-0.13692	0.021	0.095	1	DC	FER
RMND1.1	0.000814	-0.19273	0.027	0.102	1	DC	RMND1
DDX27.2	0.000819	0.244027	0.358	0.281	1	DC	DDX27
ABCD4.2	0.00082	-0.21291	0.139	0.258	1	DC	ABCD4
MAGOH.2	0.000822	0.190121	0.492	0.413	1	DC	MAGOH
C9orf9.2	0.000824	-0.17334	0.032	0.111	1	DC	C9orf9
REPIN1.3	0.000826	-0.14579	0.15	0.276	1	DC	REPIN1
MOK.1	0.000826	-0.15532	0.005	0.067	1	DC	MOK
TTYH3.3	0.000829	-0.18242	0.128	0.249	1	DC	TTYH3
TMEM128.2	0.000829	-0.2104	0.166	0.293	1	DC	TMEM128
MAFG-AS1.	0.00083	-0.11374	0.005	0.066	1	DC	MAFG-AS1
VAMP7.2	0.000831	0.260261	0.241	0.165	1	DC	VAMP7
PPP1R35.3	0.000831	-0.16219	0.064	0.159	1	DC	PPP1R35
ZNF844.1	0.000834	-0.17615	0.011	0.076	1	DC	ZNF844
GPX4.2	0.000835	-0.19006	0.861	0.842	1	DC	GPX4
MAF.2	0.000835	-0.3543	0.053	0.136	1	DC	MAF
ARHGEF25.	0.000837	-0.17633	0.005	0.066	1	DC	ARHGEF25
ZNF532.1	0.000837	-0.1435	0.021	0.094	1	DC	ZNF532
MFI2-AS1.	0.00084	-0.14026	0.011	0.076	1	DC	MFI2-AS1
C19orf52.	0.000841	-0.14658	0.075	0.175	1	DC	C19orf52
RABGAP1L.	0.000841	0.268176	0.182	0.109	1	DC	RABGAP1L
OSCP1.2	0.000842	-0.13717	0.005	0.067	1	DC	OSCP1
TNFAIP1.1	0.000849	-0.13194	0.075	0.177	1	DC	TNFAIP1
COPG2.1	0.000853	-0.17471	0.037	0.119	1	DC	COPG2
SMYD5	0.000856	-0.1308	0.005	0.067	1	DC	SMYD5
ZKSCAN5.1	0.000858	-0.13217	0.005	0.067	1	DC	ZKSCAN5
STT3B.2	0.000858	0.287086	0.278	0.203	1	DC	STT3B
PAIP1.3	0.000858	-0.1929	0.251	0.398	1	DC	PAIP1
AKAP11.2	0.00086	-0.1506	0.059	0.151	1	DC	AKAP11
STOML1.1	0.00086	-0.18607	0.027	0.102	1	DC	STOML1
SSB.2	0.000864	-0.27084	0.46	0.594	1	DC	SSB
HSPA1B.2	0.000865	-0.36826	0.299	0.412	1	DC	HSPA1B
MAN2A1.3	0.000866	0.190603	0.182	0.109	1	DC	MAN2A1
METTL16.1	0.000868	-0.16945	0.032	0.11	1	DC	METTL16
ITM2A.2	0.000869	-0.2726	0	0.056	1	DC	ITM2A
RP11-263K	0.000871	-0.13296	0.005	0.066	1	DC	RP11-263K19.4
STK32A.2	0.000877	-0.11686	0	0.056	1	DC	STK32A
EBF4	0.000877	-0.11841	0	0.056	1	DC	EBF4
EPC1.3	0.00088	0.305639	0.364	0.29	1	DC	EPC1
ZNF253.1	0.00088	-0.16922	0.016	0.084	1	DC	ZNF253
DUT.3	0.000883	0.211995	0.529	0.454	1	DC	DUT
ECI2.1	0.000885	-0.30535	0.198	0.325	1	DC	ECI2
MAN1C1	0.000889	-0.13575	0.016	0.085	1	DC	MAN1C1
HMOX1.3	0.00089	0.100929	0.422	0.292	1	DC	HMOX1
TMEM222.3	0.00089	-0.15631	0.155	0.282	1	DC	TMEM222
RSRC1.1	0.000891	-0.20189	0.176	0.307	1	DC	RSRC1
ZNF599.1	0.000892	-0.16638	0.011	0.075	1	DC	ZNF599
SLC48A1.2	0.000892	-0.24199	0.048	0.134	1	DC	SLC48A1
MNS1.1	0.000897	-0.14438	0.011	0.076	1	DC	MNS1
PFN1.3	0.000897	0.128954	0.93	0.807	1	DC	PFN1

RABGAP1.2	0.000897	-0.19159	0.043	0.126	1	DC	RABGAP1
STOM.2	0.000908	0.15233	0.353	0.25	1	DC	STOM
MAPK11	0.000912	-0.10475	0	0.056	1	DC	MAPK11
IL13RA2.2	0.000912	-0.26019	0	0.056	1	DC	IL13RA2
ANKH.3	0.000918	0.216244	0.23	0.147	1	DC	ANKH
DYNLRB1.2	0.00092	-0.22407	0.594	0.666	1	DC	DYNLRB1
USP36.3	0.000921	0.223693	0.214	0.137	1	DC	USP36
UQCC1.1	0.000923	-0.14494	0.07	0.167	1	DC	UQCC1
MSX1.1	0.000925	-0.13833	0.005	0.066	1	DC	MSX1
WNK1.3	0.000926	0.212988	0.358	0.277	1	DC	WNK1
KCTD3	0.000932	-0.10733	0.053	0.144	1	DC	KCTD3
CCDC88B	0.000934	0.235232	0.144	0.08	1	DC	CCDC88B
ZNF205	0.000935	-0.15294	0.032	0.11	1	DC	ZNF205
ZNF12.1	0.000935	-0.16152	0.043	0.125	1	DC	ZNF12
RP11-563K	0.000938	-0.16295	0.005	0.065	1	DC	RP11-563K23.1
PRPF6.1	0.000939	-0.2231	0.219	0.355	1	DC	PRPF6
PEX5.1	0.000939	-0.19858	0.021	0.092	1	DC	PEX5
HES1.2	0.000939	-0.25829	0.096	0.198	1	DC	HES1
FOXO1	0.000941	-0.18317	0.011	0.075	1	DC	FOXO1
TCF7.1	0.000942	-0.20206	0.053	0.141	1	DC	TCF7
XAB2.1	0.000947	-0.20899	0.08	0.178	1	DC	XAB2
DLG4	0.000949	-0.16878	0.011	0.074	1	DC	DLG4
QTRT1.3	0.000951	-0.30105	0.203	0.333	1	DC	QTRT1
GON4L.1	0.000953	-0.14791	0.107	0.216	1	DC	GON4L
ELK3.1	0.000956	0.276534	0.15	0.087	1	DC	ELK3
ACYP2.2	0.000956	-0.23235	0.187	0.314	1	DC	ACYP2
VAPB.2	0.000957	-0.20458	0.096	0.199	1	DC	VAPB
GAPVD1	0.000958	-0.18205	0.08	0.177	1	DC	GAPVD1
R3HCC1.1	0.000959	-0.1765	0.16	0.288	1	DC	R3HCC1
RAB11B-AS	0.000959	-0.16985	0.048	0.133	1	DC	RAB11B-AS1
ARFGAP1.1	0.000961	-0.17118	0.059	0.149	1	DC	ARFGAP1
DBX2.2	0.000963	-0.12335	0.005	0.065	1	DC	DBX2
CTDP1.1	0.000964	0.220884	0.128	0.07	1	DC	CTDP1
FAM212B.2	0.000965	-0.15889	0.027	0.1	1	DC	FAM212B
NKX2-5.1	0.000965	-0.19111	0.005	0.065	1	DC	NKX2-5
ISOC2.3	0.000965	-0.18511	0.209	0.351	1	DC	ISOC2
NIM1K.2	0.000966	-0.17685	0.005	0.065	1	DC	NIM1K
LYRM1.2	0.000971	-0.21347	0.086	0.185	1	DC	LYRM1
SAMD14	0.000972	-0.15536	0.005	0.065	1	DC	SAMD14
RP11-25K1	0.000975	-0.1345	0.027	0.102	1	DC	RP11-25K19.1
MCM7.2	0.000976	-0.26302	0.144	0.256	1	DC	MCM7
CHST3.1	0.000977	-0.11952	0.005	0.065	1	DC	CHST3
ATG9A.1	0.00098	-0.16457	0.037	0.118	1	DC	ATG9A
ALDH1L1.2	0.00098	-0.27985	0.005	0.064	1	DC	ALDH1L1
ILK.1	0.000981	0.200098	0.444	0.364	1	DC	ILK
E4F1	0.000985	-0.1882	0.037	0.116	1	DC	E4F1
FAM173B.1	0.000986	-0.15193	0.005	0.065	1	DC	FAM173B
DGCR6	0.000987	-0.17739	0.064	0.155	1	DC	DGCR6
FAM126B.1	0.000989	-0.17757	0.053	0.141	1	DC	FAM126B
TMED3.2	0.000991	0.236125	0.38	0.307	1	DC	TMED3
SLC23A2.1	0.000991	-0.177	0.032	0.108	1	DC	SLC23A2

TSFM.1	0.000992	-0.2484	0.16	0.278	1	DC	TSFM
ZNF512B.2	0.000993	-0.10791	0.011	0.075	1	DC	ZNF512B
CAHM.2	0.000994	-0.16168	0.021	0.092	1	DC	CAHM
RASL11B.2	0.000996	-0.23765	0	0.055	1	DC	RASL11B
PPM1F.2	0.000999	0.211522	0.123	0.066	1	DC	PPM1F
AP3M2.1	0.001001	-0.11639	0.021	0.093	1	DC	AP3M2
MALT1.3	0.001001	0.193397	0.134	0.073	1	DC	MALT1
KCNJ16.2	0.001005	-0.12861	0	0.055	1	DC	KCNJ16
POFUT1.1	0.001007	-0.15671	0.037	0.118	1	DC	POFUT1
SPPL3.2	0.001008	-0.20297	0.096	0.199	1	DC	SPPL3
SEL1L3.1	0.001011	0.232277	0.128	0.07	1	DC	SEL1L3
HMGN3.2	0.001012	0.116191	0.69	0.608	1	DC	HMGN3
SLC16A2	0.001015	-0.11242	0	0.055	1	DC	SLC16A2
SCAF11.3	0.001016	0.201129	0.46	0.395	1	DC	SCAF11
EFNB3.1	0.001018	-0.11547	0.011	0.075	1	DC	EFNB3
TCEB2.3	0.001019	-0.14307	0.856	0.823	1	DC	TCEB2
ZC3HC1.1	0.00102	-0.16129	0.043	0.126	1	DC	ZC3HC1
GLG1.3	0.00102	-0.1843	0.15	0.268	1	DC	GLG1
TTYH2.3	0.001023	-0.22512	0.037	0.117	1	DC	TTYH2
MAEA.2	0.001026	-0.22308	0.123	0.237	1	DC	MAEA
ORC5.2	0.001026	-0.18707	0.027	0.1	1	DC	ORC5
MIB2.2	0.001029	-0.13193	0.059	0.151	1	DC	MIB2
HOXC10.1	0.001035	-0.18086	0	0.055	1	DC	HOXC10
SNRPN.3	0.001037	-0.41547	0.027	0.1	1	DC	SNRPN
VKORC1L1	0.001038	-0.11515	0.048	0.133	1	DC	VKORC1L1
NFE2L1.2	0.001038	-0.12748	0.086	0.189	1	DC	NFE2L1
ZNF226.3	0.00104	-0.27939	0.102	0.205	1	DC	ZNF226
SORBS3.2	0.001045	-0.11926	0.075	0.174	1	DC	SORBS3
FAIM.1	0.001045	-0.20331	0.059	0.148	1	DC	FAIM
NCOA5.1	0.001047	-0.15233	0.016	0.083	1	DC	NCOA5
BRINP2.2	0.001052	-0.15491	0.011	0.073	1	DC	BRINP2
PPP5C.2	0.001053	-0.18482	0.08	0.178	1	DC	PPP5C
RUNX1.3	0.001054	0.281729	0.267	0.189	1	DC	RUNX1
LRPAP1.1	0.001054	0.105205	0.626	0.546	1	DC	LRPAP1
CLDN15.1	0.001056	-0.17842	0.005	0.064	1	DC	CLDN15
GPSM1.2	0.001057	-0.1556	0.011	0.074	1	DC	GPSM1
DMAP1.1	0.00106	-0.18378	0.134	0.253	1	DC	DMAP1
THOP1.2	0.001063	-0.14177	0.096	0.203	1	DC	THOP1
NUDT16.3	0.001064	0.245333	0.299	0.218	1	DC	NUDT16
ACD.1	0.001069	-0.20019	0.075	0.169	1	DC	ACD
CSTF3.1	0.001077	-0.13872	0.15	0.269	1	DC	CSTF3
NADK2	0.001079	-0.12895	0.027	0.101	1	DC	NADK2
CRIM1.2	0.00108	-0.13396	0.005	0.064	1	DC	CRIM1
DSC2.2	0.001085	0.11074	0.075	0.032	1	DC	DSC2
CCDC144A	0.001087	-0.1481	0.016	0.083	1	DC	CCDC144A
MAOB.1	0.001087	-0.26974	0.027	0.1	1	DC	MAOB
GOLIM4.2	0.001087	-0.21276	0.203	0.329	1	DC	GOLIM4
PPIE.1	0.001088	0.253225	0.358	0.282	1	DC	PPIE
C22orf29.	0.001089	-0.14509	0.005	0.064	1	DC	C22orf29
TFEB.3	0.001092	0.167226	0.139	0.077	1	DC	TFEB
FAM174A.1	0.001092	-0.16885	0.112	0.224	1	DC	FAM174A

KBTBD4.1	0.001092	-0.19899	0.021	0.091	1	DC	KBTBD4
IER5L.1	0.001095	-0.17467	0.027	0.099	1	DC	IER5L
CCND2.3	0.001099	-0.2965	0.209	0.329	1	DC	CCND2
GTF3C1	0.0011	-0.16859	0.032	0.108	1	DC	GTF3C1
TMEM203.2	0.001107	-0.10552	0.134	0.254	1	DC	TMEM203
MOXD1.1	0.001108	-0.13852	0	0.054	1	DC	MOXD1
RAB39B.2	0.001109	-0.15458	0.021	0.091	1	DC	RAB39B
CTNNB1.3	0.001111	0.205491	0.439	0.354	1	DC	CTNNB1
NFKBIE.3	0.001113	0.260411	0.203	0.133	1	DC	NFKBIE
NOP16.2	0.001114	-0.18922	0.096	0.2	1	DC	NOP16
ANKRD54.1	0.001116	-0.14906	0.053	0.141	1	DC	ANKRD54
FGFBP3.2	0.001116	-0.1031	0.027	0.101	1	DC	FGFBP3
SLC37A2	0.001119	0.140435	0.053	0.02	1	DC	SLC37A2
SERPINI1.	0.001119	-0.19521	0	0.054	1	DC	SERPINI1
PREX1.3	0.00112	0.228919	0.299	0.217	1	DC	PREX1
C16orf58	0.001123	-0.17597	0.037	0.116	1	DC	C16orf58
FBXO8.1	0.001127	-0.19695	0.059	0.146	1	DC	FBXO8
LGALSL.1	0.001133	-0.15277	0.005	0.063	1	DC	LGALSL
GPX8.2	0.001134	-0.13981	0.005	0.064	1	DC	GPX8
DLL1.1	0.001135	-0.24509	0.011	0.072	1	DC	DLL1
BRF2.1	0.001137	-0.1766	0.021	0.091	1	DC	BRF2
ZNF606.1	0.001138	-0.15467	0.016	0.082	1	DC	ZNF606
KLHDC10.1	0.001139	-0.16176	0.048	0.131	1	DC	KLHDC10
THAP6.1	0.00114	-0.15646	0.016	0.082	1	DC	THAP6
IGDCC3.3	0.001142	-0.13253	0.005	0.063	1	DC	IGDCC3
CUX1.3	0.001145	-0.19839	0.128	0.243	1	DC	CUX1
PAQR6.1	0.001146	-0.19249	0.011	0.073	1	DC	PAQR6
AGAP2-AS1	0.001147	-0.29951	0.027	0.096	1	DC	AGAP2-AS1
HS6ST2	0.001148	-0.1078	0.005	0.063	1	DC	HS6ST2
UTP20.1	0.00115	-0.14192	0.005	0.064	1	DC	UTP20
HRK.1	0.001151	-0.17974	0.005	0.064	1	DC	HRK
SPOCK1.2	0.001151	-0.21767	0.016	0.082	1	DC	SPOCK1
AAED1.3	0.001151	0.240905	0.257	0.18	1	DC	AAED1
ANLN.2	0.001152	-0.26024	0.005	0.063	1	DC	ANLN
SDSL.2	0.001154	0.277705	0.262	0.186	1	DC	SDSL
ETAA1	0.001163	-0.13209	0	0.053	1	DC	ETAA1
ISCA1.1	0.001165	-0.15977	0.166	0.299	1	DC	ISCA1
ENDOG.2	0.001165	-0.10714	0.059	0.149	1	DC	ENDOG
SH3BP2.2	0.00117	0.166265	0.214	0.137	1	DC	SH3BP2
RIMKLA	0.001175	-0.10819	0	0.053	1	DC	RIMKLA
TMEM18	0.001177	-0.28307	0.107	0.209	1	DC	TMEM18
ALG14.1	0.00118	-0.22597	0.064	0.153	1	DC	ALG14
PEX11G.1	0.00118	-0.13565	0.011	0.073	1	DC	PEX11G
C11orf68	0.001181	-0.13832	0.032	0.108	1	DC	C11orf68
NUF2.3	0.001182	-0.21077	0.005	0.063	1	DC	NUF2
SPIRE1	0.001191	-0.13911	0.027	0.099	1	DC	SPIRE1
ASF1A.2	0.0012	-0.15423	0.123	0.236	1	DC	ASF1A
UBE2T.3	0.001201	-0.25111	0.048	0.128	1	DC	UBE2T
ABCA2.3	0.001202	-0.32289	0.027	0.097	1	DC	ABCA2
SGTA.1	0.001205	-0.20317	0.187	0.318	1	DC	SGTA
CWC27.1	0.001206	-0.18215	0.144	0.266	1	DC	CWC27

ZNF649.2	0.001208	-0.12383	0.021	0.091	1	DC	ZNF649
GYPC.3	0.001208	-0.32124	0.203	0.311	1	DC	GYPC
ARHGAP23.	0.00121	-0.14734	0	0.053	1	DC	ARHGAP23
PSMD5-AS1	0.001211	-0.21822	0.086	0.183	1	DC	PSMD5-AS1
CCPG1.2	0.001213	0.331933	0.283	0.212	1	DC	CCPG1
TBCK.1	0.001215	-0.15086	0.016	0.082	1	DC	TBCK
MEX3D.2	0.001216	-0.1321	0.005	0.063	1	DC	MEX3D
NMT2.2	0.001218	-0.11281	0.027	0.099	1	DC	NMT2
TRIM16.1	0.001224	-0.16552	0.048	0.131	1	DC	TRIM16
COQ9.2	0.001229	-0.20892	0.128	0.244	1	DC	COQ9
TEAD4.1	0.001233	-0.10174	0	0.053	1	DC	TEAD4
BEST3.2	0.001235	-0.28403	0.021	0.088	1	DC	BEST3
MYSM1.1	0.001237	-0.16782	0.016	0.081	1	DC	MYSM1
TNFRSF10E	0.001237	0.171043	0.171	0.101	1	DC	TNFRSF10B
SCARF1.2	0.001237	0.106751	0.064	0.026	1	DC	SCARF1
ITGA2.1	0.001251	-0.11233	0.005	0.063	1	DC	ITGA2
RAB6A.2	0.001251	-0.16354	0.251	0.396	1	DC	RAB6A
PIGZ.1	0.001253	-0.11	0.005	0.063	1	DC	PIGZ
DDX60L.1	0.001254	0.195218	0.107	0.054	1	DC	DDX60L
PRKAA2.2	0.001258	-0.12186	0	0.053	1	DC	PRKAA2
KAZN.1	0.001258	-0.14913	0.011	0.072	1	DC	KAZN
DCP2.3	0.001258	0.323019	0.219	0.149	1	DC	DCP2
TXLNA.1	0.001261	-0.13114	0.053	0.141	1	DC	TXLNA
RIC8A	0.001262	-0.20308	0.166	0.288	1	DC	RIC8A
CD99L2.2	0.001264	-0.16912	0.048	0.131	1	DC	CD99L2
WDR92.1	0.001265	-0.18573	0.016	0.08	1	DC	WDR92
UBE2V2.2	0.001265	-0.24788	0.358	0.497	1	DC	UBE2V2
ZNF277.1	0.001266	0.216831	0.251	0.171	1	DC	ZNF277
ASIC1.1	0.00127	-0.15957	0	0.053	1	DC	ASIC1
ACOT13.1	0.001271	-0.22142	0.112	0.219	1	DC	ACOT13
LRTOMT.1	0.001276	-0.15946	0.011	0.072	1	DC	LRTOMT
KLHL35.2	0.001277	-0.31204	0.027	0.095	1	DC	KLHL35
LPCAT1.1	0.001279	-0.17856	0.086	0.183	1	DC	LPCAT1
SEMA3E	0.001282	-0.12435	0	0.053	1	DC	SEMA3E
LRRTM3.1	0.001295	-0.10433	0	0.053	1	DC	LRRTM3
ERBB2IP.2	0.001296	-0.18518	0.118	0.223	1	DC	ERBB2IP
GRWD1	0.001297	-0.149	0.064	0.156	1	DC	GRWD1
DUSP6	0.001298	-0.23848	0.134	0.246	1	DC	DUSP6
POLR1C.3	0.001302	-0.18646	0.043	0.123	1	DC	POLR1C
HIP1.1	0.001305	-0.17816	0.075	0.169	1	DC	HIP1
TRIM23	0.001306	-0.1508	0.016	0.081	1	DC	TRIM23
NOP10.3	0.001307	0.126707	0.695	0.605	1	DC	NOP10
SPC25.3	0.001308	-0.19767	0.005	0.062	1	DC	SPC25
MTFR1L.2	0.00131	-0.22138	0.171	0.292	1	DC	MTFR1L
UNC50.2	0.00131	-0.25104	0.225	0.357	1	DC	UNC50
ITGB3BP.1	0.001315	-0.21375	0.059	0.144	1	DC	ITGB3BP
STK32B.1	0.001316	-0.12214	0.005	0.063	1	DC	STK32B
NHLRC3.1	0.001317	-0.21232	0.155	0.272	1	DC	NHLRC3
UROD.2	0.001319	-0.27232	0.23	0.361	1	DC	UROD
SKA2.1	0.00132	-0.23006	0.187	0.309	1	DC	SKA2
SMO.1	0.00132	-0.10737	0	0.052	1	DC	SMO

NAA16.1	0.001322	-0.16853	0.043	0.122	1	DC	NAA16
TSEN2.2	0.001323	-0.15716	0.021	0.089	1	DC	TSEN2
TRAPPC4.2	0.001327	-0.28754	0.316	0.447	1	DC	TRAPPC4
TBC1D24.1	0.001331	-0.1215	0.011	0.072	1	DC	TBC1D24
PRRT1	0.001333	-0.13724	0	0.052	1	DC	PRRT1
C1orf43	0.001333	0.127882	0.679	0.595	1	DC	C1orf43
CTD-3184A	0.001335	-0.13001	0.027	0.098	1	DC	CTD-3184A7.4
C6orf120.	0.001336	-0.13552	0.053	0.139	1	DC	C6orf120
CDCA7L.2	0.001344	-0.22296	0.064	0.15	1	DC	CDCA7L
FGF14-AS2	0.001359	-0.13352	0	0.052	1	DC	FGF14-AS2
MRC2.1	0.001359	-0.17396	0.08	0.175	1	DC	MRC2
KCNMA1.2	0.001362	-0.22782	0.059	0.144	1	DC	KCNMA1
FAM126A.1	0.001363	-0.11368	0.053	0.138	1	DC	FAM126A
UBE2R2.3	0.001364	0.219151	0.358	0.285	1	DC	UBE2R2
RNF8.2	0.001365	-0.1682	0.075	0.169	1	DC	RNF8
JAK1.3	0.001367	0.264716	0.321	0.252	1	DC	JAK1
BSG.3	0.001368	-0.21029	0.733	0.742	1	DC	BSG
NFYB.1	0.00137	-0.15294	0.086	0.185	1	DC	NFYB
XPA.1	0.001371	-0.20084	0.118	0.223	1	DC	XPA
PGBD1.1	0.001372	-0.12136	0.011	0.072	1	DC	PGBD1
NR3C1.3	0.001376	0.266273	0.321	0.247	1	DC	NR3C1
TUSC1.2	0.001379	-0.18662	0.016	0.079	1	DC	TUSC1
ZNF703.3	0.00138	-0.14408	0.027	0.099	1	DC	ZNF703
PAQR4.3	0.00138	-0.20225	0.075	0.165	1	DC	PAQR4
ROMO1.2	0.001381	-0.24454	0.487	0.597	1	DC	ROMO1
ADH5.1	0.001382	-0.24521	0.257	0.391	1	DC	ADH5
CENPB.1	0.001383	-0.15145	0.032	0.106	1	DC	CENPB
SOAT1.3	0.001384	0.149766	0.283	0.196	1	DC	SOAT1
RP1-79C4.	0.001386	-0.10739	0	0.052	1	DC	RP1-79C4.4
MCAT.2	0.00139	-0.12624	0.048	0.13	1	DC	MCAT
ERGIC1.1	0.001393	0.226214	0.358	0.288	1	DC	ERGIC1
TRAF1	0.001397	0.159326	0.059	0.023	1	DC	TRAF1
EFNA2.1	0.001399	-0.12136	0	0.052	1	DC	EFNA2
CSNK2A1.1	0.001403	-0.18675	0.193	0.318	1	DC	CSNK2A1
DDX23.1	0.001404	-0.18058	0.07	0.16	1	DC	DDX23
AMPD2.2	0.001405	0.218174	0.225	0.151	1	DC	AMPD2
C1orf216.	0.001408	-0.15691	0.048	0.129	1	DC	C1orf216
FAM73A	0.001408	-0.14334	0.021	0.089	1	DC	FAM73A
MED12L.1	0.001409	-0.10872	0.005	0.062	1	DC	MED12L
CPNE5.1	0.001413	-0.11071	0	0.052	1	DC	CPNE5
THBS4.1	0.001413	-0.12216	0	0.052	1	DC	THBS4
CA14.2	0.001413	-0.19655	0	0.052	1	DC	CA14
FAM134A.3	0.001414	-0.21368	0.176	0.3	1	DC	FAM134A
WHSC1L1.3	0.001415	-0.22435	0.246	0.375	1	DC	WHSC1L1
PCNA.1	0.001418	-0.23644	0.193	0.317	1	DC	PCNA
C1orf35.2	0.001419	-0.15875	0.15	0.265	1	DC	C1orf35
PDE9A.1	0.001421	-0.11584	0.011	0.072	1	DC	PDE9A
SLC46A1.2	0.001422	-0.14768	0.005	0.062	1	DC	SLC46A1
POLDIP2.3	0.001422	-0.1747	0.219	0.349	1	DC	POLDIP2
CACNB3.1	0.001424	-0.14033	0.005	0.061	1	DC	CACNB3
HOOK2.1	0.001425	-0.17198	0.123	0.236	1	DC	HOOK2

PRKD1	0.001427	-0.11082	0	0.052	1	DC	PRKD1
C19orf53.	0.001428	-0.23172	0.615	0.684	1	DC	C19orf53
YTHDF2.1	0.001431	-0.22787	0.299	0.433	1	DC	YTHDF2
EXOC6.2	0.001436	0.194707	0.118	0.063	1	DC	EXOC6
CCT8.1	0.001438	0.129185	0.61	0.534	1	DC	CCT8
GABBR1.2	0.001442	-0.1502	0.016	0.08	1	DC	GABBR1
UBA7.3	0.001444	0.179513	0.144	0.081	1	DC	UBA7
AARS.1	0.001445	-0.15937	0.123	0.232	1	DC	AARS
PSMD7	0.001447	0.180131	0.545	0.489	1	DC	PSMD7
MORN4.2	0.001455	-0.12698	0	0.052	1	DC	MORN4
DUSP8.1	0.001455	-0.15165	0	0.052	1	DC	DUSP8
COG6	0.001463	-0.18884	0.027	0.096	1	DC	COG6
BAZ1A.3	0.001464	0.168955	0.401	0.319	1	DC	BAZ1A
EHD1.2	0.001471	-0.129	0.043	0.123	1	DC	EHD1
MORC4.1	0.001472	-0.11482	0.005	0.062	1	DC	MORC4
FIBP.1	0.001478	0.16304	0.556	0.481	1	DC	FIBP
BMF	0.001478	0.137307	0.053	0.02	1	DC	BMF
ZNF618.3	0.00148	0.128922	0.096	0.047	1	DC	ZNF618
SORBS2.1	0.001484	-0.17969	0	0.051	1	DC	SORBS2
ZFAND2A.3	0.001484	-0.24983	0.166	0.285	1	DC	ZFAND2A
SASH1	0.001484	-0.14699	0.011	0.071	1	DC	SASH1
SPEG.1	0.001492	-0.12973	0.011	0.071	1	DC	SPEG
RBM39.1	0.001494	0.104357	0.77	0.652	1	DC	RBM39
B3GALT1.1	0.001498	-0.10283	0	0.051	1	DC	B3GALT1
LTBP1.1	0.001498	-0.1109	0	0.051	1	DC	LTBP1
ZNF14	0.001498	-0.13841	0	0.051	1	DC	ZNF14
FGF9.2	0.001498	-0.18535	0	0.051	1	DC	FGF9
CARS.1	0.001502	-0.24386	0.118	0.221	1	DC	CARS
UBE2E3.2	0.001503	-0.20996	0.337	0.462	1	DC	UBE2E3
PRSS23.1	0.001515	-0.25856	0.016	0.079	1	DC	PRSS23
NCS1.1	0.001518	-0.11005	0.005	0.061	1	DC	NCS1
TOP2A.3	0.001519	-0.2146	0.021	0.087	1	DC	TOP2A
EFNA3	0.001521	-0.18611	0.005	0.061	1	DC	EFNA3
AC093323.	0.001521	-0.13419	0.027	0.097	1	DC	AC093323.3
TMEM123.2	0.001523	0.226037	0.46	0.395	1	DC	TMEM123
VGF.1	0.001525	-0.2129	0.011	0.07	1	DC	VGF
LCA5	0.001527	-0.12331	0	0.051	1	DC	LCA5
TMEM38A.2	0.001527	-0.1495	0	0.051	1	DC	TMEM38A
ARMC9.1	0.00153	-0.15484	0.011	0.07	1	DC	ARMC9
RP11-305L	0.001537	0.112554	0.032	0.009	1	DC	RP11-305L7.3
BST1	0.001539	0.17251	0.08	0.037	1	DC	BST1
CLTC.2	0.001541	-0.17979	0.219	0.351	1	DC	CLTC
MVK	0.001543	-0.15324	0.016	0.079	1	DC	MVK
C8orf44	0.001545	-0.13401	0.005	0.061	1	DC	C8orf44
TTC7A.3	0.001546	0.185532	0.134	0.074	1	DC	TTC7A
ARHGEF10	0.001553	-0.20364	0.011	0.07	1	DC	ARHGEF10
COA5.1	0.001555	-0.18431	0.182	0.308	1	DC	COA5
TMEFF2.3	0.001557	-0.22301	0.016	0.079	1	DC	TMEFF2
ARPP21.1	0.001557	-0.1629	0	0.051	1	DC	ARPP21
LZTS1.2	0.001565	-0.12571	0.005	0.061	1	DC	LZTS1
NUDT10	0.001573	-0.1542	0	0.051	1	DC	NUDT10

ZCCHC6.3	0.001578	0.192444	0.209	0.134	1	DC	ZCCHC6
PDXK.3	0.001581	0.176988	0.364	0.283	1	DC	PDXK
PPCS	0.001584	0.126173	0.406	0.319	1	DC	PPCS
CCDC58.1	0.001586	-0.12677	0.059	0.145	1	DC	CCDC58
SGIP1	0.001588	-0.18386	0	0.051	1	DC	SGIP1
HNRNPH2.2	0.001588	0.150888	0.476	0.399	1	DC	HNRNPH2
DGKA	0.001588	0.122503	0.08	0.037	1	DC	DGKA
NAA30.2	0.00159	-0.16188	0.021	0.088	1	DC	NAA30
DYNC2LI1.	0.001591	-0.21623	0.075	0.166	1	DC	DYNC2LI1
FAM104A.1	0.001594	-0.1487	0.128	0.242	1	DC	FAM104A
COMMD2.1	0.001597	-0.21253	0.171	0.291	1	DC	COMMD2
PADI2.3	0.001598	0.119432	0.219	0.133	1	DC	PADI2
NSA2.3	0.001602	0.22731	0.455	0.397	1	DC	NSA2
PPP4C.2	0.001609	0.175309	0.599	0.542	1	DC	PPP4C
PHACTR2.3	0.001611	0.277755	0.139	0.082	1	DC	PHACTR2
TTC8.1	0.001612	-0.14553	0.021	0.088	1	DC	TTC8
ARHGEF7.2	0.00162	-0.148	0.07	0.159	1	DC	ARHGEF7
C16orf91.	0.001621	-0.16644	0.096	0.197	1	DC	C16orf91
NPHP1.1	0.00163	-0.14125	0.011	0.07	1	DC	NPHP1
ZNF660	0.001635	-0.11913	0	0.05	1	DC	ZNF660
WDR73.1	0.001637	-0.18748	0.059	0.143	1	DC	WDR73
COX5B.3	0.001641	-0.14111	0.904	0.847	1	DC	COX5B
PSEN2.1	0.001645	-0.15389	0.037	0.113	1	DC	PSEN2
BROX.3	0.001646	0.272782	0.299	0.234	1	DC	BROX
ACTL6A.1	0.001646	-0.25108	0.15	0.259	1	DC	ACTL6A
FNTB.1	0.001649	-0.19212	0.021	0.087	1	DC	FNTB
INTU	0.00165	-0.143	0.011	0.07	1	DC	INTU
SLC25A18.	0.00165	-0.11719	0.021	0.088	1	DC	SLC25A18
DNAL1	0.001655	-0.10289	0.032	0.105	1	DC	DNAL1
SRD5A3.3	0.001656	0.23221	0.235	0.161	1	DC	SRD5A3
RINT1.1	0.001656	-0.16771	0.027	0.096	1	DC	RINT1
MRPS23.1	0.001661	-0.2352	0.235	0.369	1	DC	MRPS23
ZNF620	0.001662	-0.1486	0.005	0.06	1	DC	ZNF620
KRI1	0.001662	-0.11436	0.032	0.105	1	DC	KRI1
ZNF625	0.001667	-0.12203	0	0.05	1	DC	ZNF625
ANKRD13B.	0.001667	-0.1252	0	0.05	1	DC	ANKRD13B
CACNG4	0.001667	-0.13511	0	0.05	1	DC	CACNG4
GNAI3.2	0.00167	0.172485	0.46	0.392	1	DC	GNAI3
IL32.1	0.00167	-0.41071	0.032	0.101	1	DC	IL32
CYP3A5	0.00167	-0.13835	0.005	0.06	1	DC	CYP3A5
PIH1D1.1	0.001675	-0.23981	0.257	0.392	1	DC	PIH1D1
AC109826.	0.001679	0.168836	0.08	0.036	1	DC	AC109826.1
GYG1.1	0.001679	0.250874	0.326	0.253	1	DC	GYG1
ST3GAL3.1	0.00168	-0.14531	0.021	0.087	1	DC	ST3GAL3
C19orf25.	0.001681	-0.16668	0.176	0.301	1	DC	C19orf25
TPT1.3	0.001682	-0.17833	0.995	0.952	1	DC	TPT1
SLC25A17.	0.001684	-0.17875	0.07	0.158	1	DC	SLC25A17
PARD3.1	0.001691	-0.10214	0.005	0.06	1	DC	PARD3
DHX33.1	0.001694	-0.10449	0.043	0.122	1	DC	DHX33
MBIP.2	0.001695	-0.19473	0.086	0.181	1	DC	MBIP
CAPNS1.2	0.001702	0.205207	0.406	0.338	1	DC	CAPNS1

FIZ1.1	0.001716	-0.11802	0	0.05	1	DC	FIZ1
ZNF212	0.00172	-0.16357	0.011	0.069	1	DC	ZNF212
ZNF530.1	0.001723	-0.13415	0.005	0.06	1	DC	ZNF530
BCKDK.3	0.001724	0.175337	0.39	0.313	1	DC	BCKDK
CTC-338M1	0.001725	-0.177	0.011	0.07	1	DC	CTC-338M12.5
STARD10	0.001733	-0.14148	0.016	0.079	1	DC	STARD10
RFC4.1	0.001735	-0.18092	0.07	0.158	1	DC	RFC4
TMEM97.1	0.001735	-0.16385	0.08	0.173	1	DC	TMEM97
REXO2.3	0.001741	-0.34175	0.203	0.321	1	DC	REXO2
CCZ1B.2	0.001741	-0.12701	0.064	0.153	1	DC	CCZ1B
WDR5.1	0.001741	-0.13285	0.043	0.12	1	DC	WDR5
SNW1	0.001745	0.204665	0.417	0.352	1	DC	SNW1
ANKRD50.1	0.00175	-0.12522	0	0.05	1	DC	ANKRD50
ABHD14B.2	0.001753	0.161869	0.257	0.178	1	DC	ABHD14B
PRADC1.2	0.001756	-0.16476	0.112	0.218	1	DC	PRADC1
B3GNT7	0.001757	0.183833	0.064	0.027	1	DC	B3GNT7
ESYT1.3	0.001758	0.213275	0.187	0.119	1	DC	ESYT1
GSG1L	0.001761	-0.18444	0.005	0.06	1	DC	GSG1L
ZNF529.1	0.001762	-0.15828	0.005	0.06	1	DC	ZNF529
CENPF.3	0.001762	-0.25705	0.032	0.102	1	DC	CENPF
PHLDB1.3	0.001764	-0.1651	0.059	0.143	1	DC	PHLDB1
ERCC6L2.1	0.00178	-0.10145	0.021	0.087	1	DC	ERCC6L2
ADNP.2	0.001784	-0.11377	0.096	0.194	1	DC	ADNP
EPB41L5.1	0.001784	-0.13391	0	0.05	1	DC	EPB41L5
SPC24.3	0.001784	-0.15253	0	0.05	1	DC	SPC24
ARPP19.1	0.001793	-0.25848	0.289	0.428	1	DC	ARPP19
PURA.2	0.001794	-0.17114	0.107	0.21	1	DC	PURA
MED16.2	0.001794	-0.11781	0.07	0.16	1	DC	MED16
RGL1.2	0.001795	-0.16014	0.021	0.087	1	DC	RGL1
PGRMC2.2	0.001796	-0.17419	0.123	0.227	1	DC	PGRMC2
COBL.1	0.001798	-0.18079	0.011	0.068	1	DC	COBL
NUP62.3	0.001798	-0.22201	0.134	0.239	1	DC	NUP62
ZNF776	0.0018	-0.12236	0.005	0.06	1	DC	ZNF776
MARC1.1	0.001806	-0.11475	0.005	0.06	1	DC	1-Mar
TMEM161A.	0.001808	-0.15617	0.08	0.173	1	DC	TMEM161A
RNF25	0.001811	-0.16364	0.032	0.103	1	DC	RNF25
RNF26.1	0.001812	-0.12347	0.07	0.159	1	DC	RNF26
PRR3.1	0.001816	-0.14474	0.032	0.104	1	DC	PRR3
RNF182.1	0.001819	-0.1275	0	0.05	1	DC	RNF182
GPR137C	0.001819	-0.13608	0	0.05	1	DC	GPR137C
KCTD7.1	0.001822	-0.14806	0.005	0.06	1	DC	KCTD7
EMP2.1	0.001823	-0.2751	0.037	0.109	1	DC	EMP2
ADCK4.1	0.001824	-0.13113	0.048	0.128	1	DC	ADCK4
GOSR2.1	0.001825	-0.18346	0.091	0.187	1	DC	GOSR2
MON1A.1	0.001826	-0.11731	0.021	0.087	1	DC	MON1A
SIRT3.1	0.001827	-0.1146	0.032	0.104	1	DC	SIRT3
GPR162.1	0.001827	-0.16203	0.032	0.103	1	DC	GPR162
CD40.3	0.001829	0.208758	0.123	0.066	1	DC	CD40
IL4R.3	0.001832	0.195119	0.128	0.07	1	DC	IL4R
PPIL1.2	0.001834	-0.16901	0.096	0.196	1	DC	PPIL1
IFIT3.3	0.001838	-0.23504	0.037	0.109	1	DC	IFIT3

AC004069.	0.001841	0.125937	0.053	0.021	1	DC	AC004069.2
ABHD17B.3	0.001842	-0.19778	0.048	0.126	1	DC	ABHD17B
C17orf51.	0.001851	-0.12421	0.011	0.069	1	DC	C17orf51
SLC35B2.2	0.001854	-0.11573	0.176	0.302	1	DC	SLC35B2
RP11-488L	0.001855	0.170894	0.102	0.052	1	DC	RP11-488L18.10
TTL.2	0.001856	-0.17237	0.043	0.119	1	DC	TTL
CREB5.2	0.001869	-0.18406	0.128	0.237	1	DC	CREB5
GARS.3	0.001871	-0.13393	0.176	0.295	1	DC	GARS
TSC22D4.3	0.001873	-0.43173	0.385	0.471	1	DC	TSC22D4
MYT1L.3	0.001883	-0.16864	0.005	0.06	1	DC	MYT1L
SEPN1.2	0.001885	-0.14029	0.107	0.211	1	DC	SEPN1
ZNF268.1	0.001898	-0.18563	0.043	0.118	1	DC	ZNF268
SEPT3.2	0.001899	-0.17075	0.011	0.068	1	DC	3-Sep
POLDIP3.2	0.001901	-0.15249	0.091	0.187	1	DC	POLDIP3
EFHD1	0.001903	-0.26176	0.005	0.059	1	DC	EFHD1
PRRC2C.3	0.001903	0.134249	0.545	0.481	1	DC	PRRC2C
TDP2.1	0.001904	-0.21796	0.091	0.184	1	DC	TDP2
TANGO2.3	0.001905	0.192202	0.144	0.084	1	DC	TANGO2
CD36	0.001911	0.136056	0.043	0.015	1	DC	CD36
BMPR1A.1	0.001913	-0.13372	0.011	0.069	1	DC	BMPR1A
FOLR2.3	0.001928	-0.605	0.102	0.179	1	DC	FOLR2
LMLN	0.001928	-0.11276	0	0.049	1	DC	LMLN
SPRY4.1	0.001929	-0.12188	0.011	0.068	1	DC	SPRY4
SEC13.1	0.00193	0.246177	0.428	0.367	1	DC	SEC13
HMGCS1.1	0.00194	-0.19525	0.091	0.184	1	DC	HMGCS1
POLE4.2	0.001944	0.234798	0.364	0.283	1	DC	POLE4
SEPT11.3	0.001945	-0.20937	0.187	0.305	1	DC	11-Sep
GPD1.2	0.001947	-0.2177	0	0.049	1	DC	GPD1
TCF7L2.1	0.001951	-0.14262	0.027	0.095	1	DC	TCF7L2
NEDD9.2	0.001953	0.225909	0.294	0.219	1	DC	NEDD9
LINC00844	0.001954	-0.32094	0.037	0.108	1	DC	LINC00844
RBBP9.1	0.001955	-0.11173	0.027	0.095	1	DC	RBBP9
IGBP1.1	0.001955	0.208282	0.417	0.344	1	DC	IGBP1
OGFOD1	0.001956	-0.15542	0.064	0.152	1	DC	OGFOD1
IMPAD1.1	0.001956	-0.16325	0.091	0.188	1	DC	IMPAD1
RPRM.3	0.001958	-0.23492	0.011	0.067	1	DC	RPRM
EID2.2	0.001961	-0.10461	0.037	0.112	1	DC	EID2
SETD5.3	0.001965	-0.15047	0.171	0.29	1	DC	SETD5
DEAF1.2	0.001966	-0.13047	0.107	0.214	1	DC	DEAF1
LASP1.3	0.001969	0.199809	0.283	0.208	1	DC	LASP1
ZNF692.1	0.001971	-0.16954	0.037	0.11	1	DC	ZNF692
ZNF414.3	0.001977	-0.15096	0.091	0.187	1	DC	ZNF414
PRRC2B.3	0.00198	-0.16352	0.128	0.238	1	DC	PRRC2B
WSB2.3	0.001982	-0.18825	0.123	0.225	1	DC	WSB2
OSTM1.3	0.001983	0.300126	0.294	0.227	1	DC	OSTM1
LENG1.1	0.001984	-0.15899	0.102	0.203	1	DC	LENG1
TMEM246.1	0.001985	-0.1624	0	0.049	1	DC	TMEM246
CTNNA2.1	0.001994	-0.18021	0.005	0.059	1	DC	CTNNA2
NLK.2	0.001996	-0.12086	0.016	0.077	1	DC	NLK
PLXNA1.1	0.001997	-0.10685	0.021	0.086	1	DC	PLXNA1
SLC41A1	0.002003	-0.13704	0.011	0.068	1	DC	SLC41A1

RP11-480C	0.002005	-0.27621	0	0.049	1	DC	RP11-480C22.1
PEX11A	0.002011	-0.14863	0.005	0.059	1	DC	PEX11A
ASGR1.3	0.002021	0.201054	0.128	0.072	1	DC	ASGR1
DLG2	0.002024	-0.16006	0	0.049	1	DC	DLG2
FGF12	0.002024	-0.19631	0	0.049	1	DC	FGF12
CD109	0.002026	0.125872	0.059	0.024	1	DC	CD109
GGA1.3	0.002026	0.276936	0.358	0.288	1	DC	GGA1
TLR4.2	0.002028	0.119993	0.086	0.04	1	DC	TLR4
ZNF341	0.002029	0.162094	0.048	0.018	1	DC	ZNF341
COG1.2	0.002033	-0.13794	0.037	0.111	1	DC	COG1
ZNF638.2	0.002038	-0.17858	0.144	0.253	1	DC	ZNF638
TTC26.2	0.002044	-0.14912	0	0.048	1	DC	TTC26
RP11-398K	0.002047	-0.13354	0.016	0.077	1	DC	RP11-398K22.12
ZYG11B.3	0.002047	-0.12529	0.064	0.15	1	DC	ZYG11B
SEC14L1.2	0.00205	0.30995	0.358	0.291	1	DC	SEC14L1
CROT.2	0.002058	-0.16274	0.053	0.133	1	DC	CROT
ZNF398.2	0.002061	-0.14067	0.005	0.058	1	DC	ZNF398
RBM18.1	0.002062	-0.14021	0.107	0.208	1	DC	RBM18
ZNF853.1	0.002064	-0.10423	0	0.048	1	DC	ZNF853
WWC3.1	0.002064	-0.12354	0	0.048	1	DC	WWC3
EMC9	0.002066	-0.20501	0.086	0.178	1	DC	EMC9
NAT9.1	0.002079	-0.22893	0.107	0.204	1	DC	NAT9
UBE2G1.3	0.002081	-0.12966	0.144	0.26	1	DC	UBE2G1
WBP4.2	0.002089	-0.16562	0.182	0.307	1	DC	WBP4
ACOX3.1	0.002094	-0.15955	0.027	0.093	1	DC	ACOX3
SLC12A5.3	0.002104	-0.18913	0.027	0.093	1	DC	SLC12A5
SLC16A9.1	0.002104	-0.10739	0	0.048	1	DC	SLC16A9
RPGRIP1L.	0.002107	-0.13789	0.005	0.058	1	DC	RPGRIP1L
ZBED5.2	0.002109	-0.14216	0.086	0.18	1	DC	ZBED5
C8orf59.1	0.002116	0.159708	0.455	0.392	1	DC	C8orf59
DHRS7B	0.002122	-0.1372	0.091	0.188	1	DC	DHRS7B
NEK11.1	0.002125	-0.13759	0	0.048	1	DC	NEK11
CCDC77	0.002125	-0.14681	0.005	0.058	1	DC	CCDC77
TPMT.1	0.002133	0.200381	0.267	0.19	1	DC	TPMT
MAPK7.1	0.00214	-0.1285	0.048	0.126	1	DC	MAPK7
ZNF273.2	0.002141	-0.17709	0.021	0.085	1	DC	ZNF273
FGFR2.1	0.002145	-0.20274	0	0.048	1	DC	FGFR2
ACTR6	0.002146	-0.15684	0.128	0.236	1	DC	ACTR6
ZNF514.1	0.002155	-0.16353	0.021	0.085	1	DC	ZNF514
PRKCZ.3	0.002161	-0.15	0.032	0.101	1	DC	PRKCZ
C5orf30.1	0.002162	-0.15757	0.016	0.076	1	DC	C5orf30
ZNF454.1	0.002166	-0.11942	0	0.048	1	DC	ZNF454
RAB30.1	0.002172	-0.13283	0.016	0.076	1	DC	RAB30
LUC7L.1	0.002174	-0.20917	0.139	0.247	1	DC	LUC7L
TSEN15.1	0.002187	-0.1409	0.128	0.235	1	DC	TSEN15
PDZD4.1	0.002187	-0.13259	0	0.048	1	DC	PDZD4
GTF3C4.1	0.002188	-0.11512	0.011	0.067	1	DC	GTF3C4
RP11-195F	0.002189	-0.21988	0.027	0.092	1	DC	RP11-195F19.5
KLHL20	0.002194	-0.16886	0.059	0.14	1	DC	KLHL20
PTPN13.1	0.002196	-0.11805	0.027	0.094	1	DC	PTPN13
URGCP	0.002198	-0.17548	0.043	0.117	1	DC	URGCP

TDRD3.1	0.0022	-0.13176	0.027	0.093	1	DC	TDRD3
SRGAP3.2	0.002219	-0.19193	0.064	0.147	1	DC	SRGAP3
COQ10A	0.002221	-0.11333	0.032	0.102	1	DC	COQ10A
RAF1.3	0.002224	-0.14758	0.16	0.279	1	DC	RAF1
SAMD10	0.00223	-0.1009	0.005	0.058	1	DC	SAMD10
SESTD1.2	0.00223	-0.19153	0.075	0.16	1	DC	SESTD1
BLOC1S2.2	0.002236	0.179601	0.439	0.367	1	DC	BLOC1S2
FUNDC1.2	0.002258	-0.2019	0.139	0.247	1	DC	FUNDC1
DMTF1.1	0.002262	-0.14153	0.064	0.149	1	DC	DMTF1
ASCC2.2	0.002266	0.294041	0.273	0.204	1	DC	ASCC2
METTL14.1	0.002269	-0.13678	0.027	0.093	1	DC	METTL14
XKR4.1	0.002272	-0.1473	0.005	0.058	1	DC	XKR4
RITA1.1	0.002288	-0.15515	0.053	0.133	1	DC	RITA1
MLLT4.2	0.002289	-0.11259	0.075	0.163	1	DC	MLLT4
THEM6.1	0.002296	-0.14881	0.021	0.084	1	DC	THEM6
FIS1.2	0.0023	-0.24326	0.54	0.639	1	DC	FIS1
ESCO1	0.002301	-0.15075	0.048	0.125	1	DC	ESCO1
FAHD1.1	0.00231	-0.16484	0.086	0.177	1	DC	FAHD1
CREBZF.2	0.00231	-0.16103	0.053	0.132	1	DC	CREBZF
RANBP2.3	0.002311	0.241307	0.283	0.208	1	DC	RANBP2
ARL5B.3	0.002318	0.224359	0.144	0.086	1	DC	ARL5B
CHPF2.2	0.002327	-0.17939	0.07	0.154	1	DC	CHPF2
TMCC3.3	0.00233	0.169832	0.128	0.072	1	DC	TMCC3
LINC00116	0.002336	-0.1884	0.23	0.367	1	DC	LINC00116
IFIT5.1	0.002338	-0.15145	0.021	0.084	1	DC	IFIT5
TYW5	0.00236	-0.1902	0.027	0.093	1	DC	TYW5
PAK7.2	0.002364	-0.15701	0	0.047	1	DC	PAK7
RLF.1	0.002366	0.217314	0.166	0.104	1	DC	RLF
CCDC66.2	0.002367	-0.13249	0.123	0.231	1	DC	CCDC66
TMEM178B.	0.002368	-0.13131	0.005	0.057	1	DC	TMEM178B
RP11-390E	0.00237	-0.10326	0.048	0.126	1	DC	RP11-390E23.6
ZNF580.2	0.002371	-0.16059	0.176	0.293	1	DC	ZNF580
BEX5.1	0.002381	-0.31488	0.032	0.097	1	DC	BEX5
PIR.1	0.00239	-0.16071	0.005	0.057	1	DC	PIR
FDXR.1	0.002392	-0.18115	0.043	0.115	1	DC	FDXR
CDKN1C.3	0.002399	-0.21778	0.043	0.115	1	DC	CDKN1C
NUCB1.2	0.002399	0.147256	0.513	0.444	1	DC	NUCB1
CASP6.2	0.00241	-0.1495	0.075	0.164	1	DC	CASP6
CORO7.3	0.002416	0.246712	0.23	0.157	1	DC	CORO7
IPO13.1	0.002419	-0.17409	0.037	0.108	1	DC	IPO13
SNRPA1.2	0.002422	-0.20067	0.176	0.297	1	DC	SNRPA1
ZNF140	0.002433	-0.1357	0.037	0.108	1	DC	ZNF140
SLC35F6.3	0.002434	-0.18738	0.112	0.211	1	DC	SLC35F6
GPATCH2.1	0.002441	-0.17121	0.075	0.163	1	DC	GPATCH2
RAPH1.1	0.002444	0.178493	0.118	0.065	1	DC	RAPH1
PLXNA3	0.002445	-0.1087	0.011	0.066	1	DC	PLXNA3
SAP30L.2	0.002452	-0.10765	0.027	0.093	1	DC	SAP30L
RER1.2	0.002453	0.169395	0.561	0.496	1	DC	RER1
DOK4.1	0.002457	-0.12007	0	0.047	1	DC	DOK4
LEMD1	0.002457	-0.14384	0	0.047	1	DC	LEMD1
TRIP6.3	0.002463	-0.16079	0.139	0.251	1	DC	TRIP6

IMMP2L.1	0.002464	-0.21627	0.064	0.146	1	DC	IMMP2L
TROVE2.2	0.002465	-0.22891	0.294	0.435	1	DC	TROVE2
RDH11.2	0.002467	-0.24505	0.267	0.39	1	DC	RDH11
TTLL1	0.002469	-0.15144	0.027	0.092	1	DC	TTLL1
RERE.2	0.002471	-0.18023	0.112	0.212	1	DC	RERE
AKT1S1.1	0.002477	-0.1136	0.134	0.242	1	DC	AKT1S1
GRID2.1	0.002481	-0.13776	0	0.047	1	DC	GRID2
MED21	0.002481	-0.21019	0.112	0.212	1	DC	MED21
PLCE1.1	0.002487	-0.13632	0.011	0.066	1	DC	PLCE1
TF.3	0.002503	-0.40299	0.016	0.074	1	DC	TF
AC009501.	0.002506	-0.41191	0.075	0.158	1	DC	AC009501.4
ADAR.2	0.002508	-0.158	0.187	0.309	1	DC	ADAR
CDK2AP2.2	0.002518	0.239679	0.39	0.328	1	DC	CDK2AP2
PRPF38B.3	0.002529	0.221073	0.492	0.434	1	DC	PRPF38B
CD276.1	0.00254	-0.17721	0.123	0.224	1	DC	CD276
COG5.1	0.002548	-0.12176	0.032	0.101	1	DC	COG5
RP11-212F	0.002551	-0.13315	0.027	0.092	1	DC	RP11-212P7.2
IL17RB.1	0.002555	-0.15274	0	0.046	1	DC	IL17RB
ARHGEF2.2	0.002557	-0.25032	0.182	0.29	1	DC	ARHGEF2
TBC1D12.3	0.002561	0.183644	0.171	0.106	1	DC	TBC1D12
RAE1.1	0.002564	-0.17672	0.155	0.269	1	DC	RAE1
SETBP1.1	0.002568	-0.12628	0.016	0.075	1	DC	SETBP1
GNPAT.2	0.002577	-0.16574	0.134	0.241	1	DC	GNPAT
BOLA3.1	0.002577	-0.24868	0.214	0.337	1	DC	BOLA3
RP11-486G	0.00258	-0.12869	0	0.046	1	DC	RP11-486G15.2
WDR3	0.002603	-0.13126	0.032	0.101	1	DC	WDR3
ABL2.3	0.002603	0.227325	0.348	0.267	1	DC	ABL2
AIFM1.2	0.002608	0.224682	0.209	0.144	1	DC	AIFM1
NECAP1.1	0.002611	-0.23406	0.107	0.2	1	DC	NECAP1
GALM.2	0.002613	0.203018	0.193	0.124	1	DC	GALM
ZNF131.2	0.002613	-0.15877	0.128	0.234	1	DC	ZNF131
VPS53.2	0.002614	-0.19257	0.075	0.16	1	DC	VPS53
FAM172A.1	0.002616	-0.17705	0.102	0.198	1	DC	FAM172A
INTS5	0.00262	-0.10628	0.021	0.084	1	DC	INTS5
FAM199X.2	0.002621	-0.11884	0.037	0.109	1	DC	FAM199X
RPIA.2	0.002634	0.263984	0.257	0.187	1	DC	RPIA
MAGI3	0.002639	-0.11097	0.005	0.056	1	DC	MAGI3
CHORDC1.1	0.002647	-0.22653	0.118	0.214	1	DC	CHORDC1
NDFIP1.3	0.002657	-0.17649	0.535	0.602	1	DC	NDFIP1
FKRP.1	0.002658	-0.10329	0.005	0.056	1	DC	FKRP
ZNF433	0.002664	-0.12702	0.005	0.056	1	DC	ZNF433
ADAM9.2	0.002667	-0.20232	0.214	0.332	1	DC	ADAM9
PELP1.2	0.002674	-0.17336	0.07	0.152	1	DC	PELP1
CLDN10.1	0.002676	-0.28614	0.005	0.056	1	DC	CLDN10
FRMD3.1	0.002677	-0.12009	0.005	0.056	1	DC	FRMD3
C2orf69.3	0.002678	-0.16304	0.102	0.196	1	DC	C2orf69
DLGAP1	0.002682	-0.12741	0	0.046	1	DC	DLGAP1
CDCA5.1	0.002682	-0.13194	0	0.046	1	DC	CDCA5
ATP6AP1L.	0.002682	-0.14621	0	0.046	1	DC	ATP6AP1L
CYSTM1.3	0.002687	-0.30565	0.214	0.326	1	DC	CYSTM1
PIN1.1	0.002697	-0.21872	0.433	0.538	1	DC	PIN1

SPIN1.3	0.002701	-0.15243	0.08	0.168	1	DC	SPIN1
SMARCAD1.	0.002708	-0.11258	0.037	0.108	1	DC	SMARCAD1
MAML1.3	0.002717	0.211121	0.182	0.116	1	DC	MAML1
ZNF324.1	0.002718	-0.13442	0.021	0.083	1	DC	ZNF324
FBXO6	0.00272	0.18467	0.128	0.074	1	DC	FBXO6
PUS3.1	0.00272	-0.12764	0.043	0.115	1	DC	PUS3
CSPP1.1	0.002721	-0.14887	0.037	0.107	1	DC	CSPP1
RYK.2	0.002736	-0.14216	0.027	0.091	1	DC	RYK
C1orf50.1	0.002738	-0.19925	0.091	0.181	1	DC	C1orf50
RFX1.2	0.002741	-0.11568	0.027	0.091	1	DC	RFX1
HK3.1	0.002745	0.106807	0.059	0.024	1	DC	HK3
SIL1.2	0.002756	0.209689	0.326	0.253	1	DC	SIL1
RAB34.1	0.002767	0.213616	0.369	0.282	1	DC	RAB34
HIST1H4C.	0.002769	-0.31113	0.342	0.426	1	DC	HIST1H4C
SYNDIG1.3	0.002774	-0.20937	0.027	0.09	1	DC	SYNDIG1
RIN1.2	0.002776	-0.11531	0.021	0.083	1	DC	RIN1
FAM229A	0.002788	-0.1205	0	0.046	1	DC	FAM229A
CYFIP2.2	0.002791	-0.16321	0.043	0.115	1	DC	CYFIP2
UBE4A.2	0.002795	-0.13568	0.07	0.154	1	DC	UBE4A
ZNF830.1	0.002804	-0.15607	0.096	0.192	1	DC	ZNF830
NEU4.2	0.002809	-0.23972	0.011	0.064	1	DC	NEU4
ARVCF.1	0.00281	-0.10311	0.005	0.056	1	DC	ARVCF
CCER2.2	0.002811	-0.1765	0.005	0.056	1	DC	CCER2
EIF2B2.1	0.002813	-0.15	0.086	0.175	1	DC	EIF2B2
B4GALNT4	0.002815	-0.11263	0	0.046	1	DC	B4GALNT4
RP5-1177M	0.002815	-0.2005	0	0.046	1	DC	RP5-1177M21.1
LOX.1	0.002815	-0.21765	0	0.046	1	DC	LOX
TRMT10C.1	0.002815	-0.19396	0.144	0.251	1	DC	TRMT10C
CLCN4.2	0.002828	-0.1475	0.005	0.055	1	DC	CLCN4
FTSJ2.1	0.002836	-0.18644	0.134	0.239	1	DC	FTSJ2
GRIK3	0.002842	-0.11273	0	0.046	1	DC	GRIK3
ZNF117.1	0.002844	-0.16431	0.037	0.106	1	DC	ZNF117
MFAP3L.1	0.002844	-0.1179	0.005	0.056	1	DC	MFAP3L
DNASE1	0.002845	-0.14709	0.021	0.083	1	DC	DNASE1
LARP1.1	0.002851	-0.15778	0.144	0.254	1	DC	LARP1
PGD.2	0.002861	0.229577	0.358	0.292	1	DC	PGD
MCF2L.3	0.002864	-0.21156	0.032	0.097	1	DC	MCF2L
UBL4A.2	0.002867	-0.14179	0.091	0.184	1	DC	UBL4A
CASKIN2	0.00287	-0.1107	0	0.045	1	DC	CASKIN2
TIMM8A	0.002882	-0.16667	0.032	0.099	1	DC	TIMM8A
ENKD1.1	0.002888	-0.11798	0.011	0.065	1	DC	ENKD1
PIK3R1.2	0.002888	-0.27396	0.209	0.309	1	DC	PIK3R1
C16orf59.	0.002898	-0.11005	0	0.045	1	DC	C16orf59
NEO1.1	0.002904	-0.13981	0.016	0.073	1	DC	NEO1
LRRC61.2	0.002913	-0.1662	0.011	0.065	1	DC	LRRC61
IER5.3	0.00292	0.222248	0.273	0.201	1	DC	IER5
TMEM87B.2	0.00292	0.163928	0.118	0.066	1	DC	TMEM87B
TMEM199.1	0.002932	-0.14305	0.139	0.244	1	DC	TMEM199
COL1A2.1	0.002934	0.122931	0.043	0.015	1	DC	COL1A2
FBXO31	0.002936	-0.10892	0.005	0.055	1	DC	FBXO31
NUDT16L1.	0.002937	-0.14913	0.166	0.283	1	DC	NUDT16L1

AP1B1.3	0.002944	0.158988	0.348	0.257	1	DC	AP1B1
FAM114A2	0.002944	-0.19577	0.053	0.128	1	DC	FAM114A2
DPYD.1	0.002945	0.115938	0.166	0.099	1	DC	DPYD
ZNF136.1	0.002949	-0.17723	0.032	0.098	1	DC	ZNF136
HSPB1.2	0.002955	-0.24477	0.61	0.655	1	DC	HSPB1
MAFG.3	0.002964	-0.19218	0.096	0.186	1	DC	MAFG
ANAPC13.3	0.002965	-0.23096	0.235	0.356	1	DC	ANAPC13
MARK4.1	0.002969	-0.12376	0.016	0.073	1	DC	MARK4
CCDC157.1	0.00298	-0.1181	0.005	0.055	1	DC	CCDC157
CDYL.3	0.00298	-0.1007	0.043	0.116	1	DC	CDYL
TECPR1.2	0.002983	-0.13533	0.048	0.121	1	DC	TECPR1
ZNF573	0.002986	-0.13974	0.011	0.065	1	DC	ZNF573
FAM120A.3	0.002991	0.248456	0.273	0.209	1	DC	FAM120A
COX6A1.3	0.002992	-0.15849	0.866	0.844	1	DC	COX6A1
BNIP1.2	0.002995	-0.19643	0.048	0.121	1	DC	BNIP1
RBM48.1	0.003007	-0.13015	0.027	0.091	1	DC	RBM48
MKRN3	0.003012	-0.13397	0	0.045	1	DC	MKRN3
SYNGAP1.1	0.003018	-0.1196	0.005	0.055	1	DC	SYNGAP1
BRAT1.1	0.00302	-0.18601	0.096	0.185	1	DC	BRAT1
GPX3.2	0.003026	0.222797	0.225	0.152	1	DC	GPX3
EN1	0.003028	-0.13224	0.011	0.065	1	DC	EN1
RNF214	0.003032	-0.13812	0.037	0.107	1	DC	RNF214
PCDHB14	0.003039	-0.10055	0.005	0.055	1	DC	PCDHB14
TMEM186.2	0.003043	-0.17062	0.032	0.098	1	DC	TMEM186
RGN.2	0.003046	-0.13633	0.016	0.073	1	DC	RGN
STEAP3.2	0.003051	-0.24826	0.08	0.163	1	DC	STEAP3
RCOR3	0.003052	-0.17126	0.053	0.129	1	DC	RCOR3
RUVBL2	0.003055	-0.20221	0.198	0.32	1	DC	RUVBL2
C11orf74.	0.003056	-0.16338	0.086	0.173	1	DC	C11orf74
HN1.2	0.003059	-0.21011	0.647	0.675	1	DC	HN1
NR2C2AP.2	0.003061	-0.18635	0.08	0.167	1	DC	NR2C2AP
MFAP4.1	0.003064	-0.17627	0.005	0.055	1	DC	MFAP4
BRD7.2	0.00307	-0.13524	0.144	0.256	1	DC	BRD7
GPR155.2	0.003076	-0.16786	0.096	0.188	1	DC	GPR155
PKP4.2	0.003077	-0.13062	0.005	0.055	1	DC	PKP4
SREK1IP1.	0.00308	-0.24204	0.219	0.34	1	DC	SREK1IP1
BCR.1	0.00309	-0.15387	0.011	0.064	1	DC	BCR
PITHD1.2	0.003091	-0.21328	0.23	0.348	1	DC	PITHD1
ABCF1.2	0.003093	-0.1243	0.171	0.286	1	DC	ABCF1
ARHGEF4	0.003098	-0.11864	0.005	0.055	1	DC	ARHGEF4
BACE2.2	0.003099	-0.10215	0.005	0.055	1	DC	BACE2
ZNF781	0.003101	-0.12561	0	0.045	1	DC	ZNF781
DUOX1.3	0.003102	-0.14735	0.011	0.064	1	DC	DUOX1
VCAM1.1	0.003106	-0.22788	0.016	0.073	1	DC	VCAM1
PHACTR4.2	0.003109	-0.12926	0.107	0.204	1	DC	PHACTR4
NDUFAF6.2	0.003115	-0.11249	0.091	0.182	1	DC	NDUFAF6
PINLYP.1	0.003121	-0.1865	0.005	0.054	1	DC	PINLYP
CRB2.1	0.003131	-0.1655	0	0.045	1	DC	CRB2
TNIP1.1	0.003134	0.248696	0.273	0.207	1	DC	TNIP1
FBXL16	0.003162	-0.10715	0	0.045	1	DC	FBXL16
REPS2	0.003165	0.108164	0.043	0.016	1	DC	REPS2

ZNF654	0.003171	-0.10586	0.005	0.055	1	DC	ZNF654
GALNT13	0.003193	-0.16652	0	0.044	1	DC	GALNT13
GALNT11.1	0.003196	-0.17305	0.128	0.228	1	DC	GALNT11
PRR14L.2	0.003196	-0.10546	0.027	0.09	1	DC	PRR14L
BTBD2.1	0.003204	-0.11103	0.032	0.099	1	DC	BTBD2
RGS12.3	0.003217	-0.20395	0.166	0.272	1	DC	RGS12
BBS2.3	0.003223	-0.17679	0.086	0.174	1	DC	BBS2
FKTN	0.003229	-0.11346	0.011	0.064	1	DC	FKTN
PMEPA1.3	0.003231	-0.21993	0.112	0.202	1	DC	PMEPA1
PYGL.2	0.003231	0.129226	0.273	0.191	1	DC	PYGL
STX3.1	0.003233	0.215982	0.128	0.074	1	DC	STX3
RP11-379H	0.003237	-0.11517	0.016	0.073	1	DC	RP11-379H18.1
SRA1.2	0.003239	0.211196	0.449	0.398	1	DC	SRA1
KLHL28.1	0.003241	-0.18452	0.048	0.12	1	DC	KLHL28
KDM2B.2	0.003245	0.208677	0.198	0.134	1	DC	KDM2B
NCK2.3	0.003248	-0.19058	0.07	0.149	1	DC	NCK2
SYT5.1	0.003255	-0.15182	0	0.044	1	DC	SYT5
HSD17B4.1	0.003257	0.163593	0.316	0.24	1	DC	HSD17B4
GOT2.1	0.003257	-0.11376	0.102	0.198	1	DC	GOT2
SSH2.3	0.00326	0.219876	0.246	0.175	1	DC	SSH2
METTL1.2	0.003263	-0.20608	0.07	0.149	1	DC	METTL1
IGF2BP2.1	0.003265	-0.11811	0.021	0.081	1	DC	IGF2BP2
HEG1.1	0.00327	-0.10059	0.016	0.073	1	DC	HEG1
BLOC1S4.3	0.003283	-0.162	0.139	0.245	1	DC	BLOC1S4
NDUFB1.1	0.003283	0.100796	0.668	0.592	1	DC	NDUFB1
PON2.3	0.003285	-0.30874	0.353	0.452	1	DC	PON2
PLA2G4C	0.003287	-0.13051	0	0.044	1	DC	PLA2G4C
ANKMY2	0.003288	-0.20268	0.048	0.119	1	DC	ANKMY2
TESC.1	0.003292	0.222926	0.08	0.039	1	DC	TESC
NR2C1.1	0.003302	-0.12849	0.032	0.098	1	DC	NR2C1
NUAK1.3	0.003307	-0.16454	0.016	0.072	1	DC	NUAK1
ZP3.1	0.003307	-0.16081	0.016	0.072	1	DC	ZP3
PIGU.2	0.003314	-0.17456	0.086	0.172	1	DC	PIGU
N6AMT1	0.003314	-0.10152	0.011	0.064	1	DC	N6AMT1
SLC35E3.2	0.003317	-0.21569	0.102	0.188	1	DC	SLC35E3
PMS2.2	0.003328	-0.13806	0.053	0.129	1	DC	PMS2
SYMPK.2	0.00333	-0.1733	0.123	0.224	1	DC	SYMPK
ONECUT2	0.003351	-0.14472	0	0.044	1	DC	ONECUT2
SLC27A5.1	0.003351	-0.13424	0.059	0.137	1	DC	SLC27A5
ZFR.1	0.003359	-0.14944	0.187	0.307	1	DC	ZFR
ZNF24.1	0.003394	-0.1232	0.182	0.302	1	DC	ZNF24
CDH13	0.003395	-0.20428	0.005	0.054	1	DC	CDH13
ATP5E.2	0.003401	-0.12384	0.979	0.913	1	DC	ATP5E
ATP1A2.1	0.003407	-0.26187	0.011	0.063	1	DC	ATP1A2
NGRN.1	0.00341	-0.15295	0.021	0.081	1	DC	NGRN
PLEKHF1	0.003417	-0.10024	0	0.044	1	DC	PLEKHF1
ZDHHC22.1	0.003417	-0.12909	0	0.044	1	DC	ZDHHC22
RP11-22P6	0.003417	-0.1647	0	0.044	1	DC	RP11-22P6.2
BRIX1.3	0.003417	-0.17117	0.15	0.255	1	DC	BRIX1
MIEF2.1	0.003421	-0.13108	0.011	0.063	1	DC	MIEF2
GTF3C5.1	0.003442	-0.16821	0.096	0.187	1	DC	GTF3C5

ZBTB17	0.003464	-0.15356	0.043	0.113	1	DC	ZBTB17
RAB40C.1	0.00347	-0.12759	0.021	0.08	1	DC	RAB40C
NMD3.1	0.003472	-0.15731	0.118	0.216	1	DC	NMD3
KLHL25.1	0.003476	-0.1069	0.005	0.054	1	DC	KLHL25
RAB9B	0.003485	-0.1569	0.005	0.053	1	DC	RAB9B
SND1.1	0.003486	0.173759	0.326	0.254	1	DC	SND1
YY1.2	0.003495	0.222454	0.396	0.339	1	DC	YY1
RILP.3	0.003495	0.289881	0.16	0.102	1	DC	RILP
HOXB7.1	0.003498	-0.22666	0.011	0.062	1	DC	HOXB7
TMEM115.1	0.003514	-0.16505	0.112	0.209	1	DC	TMEM115
TXN.2	0.003514	-0.28363	0.61	0.667	1	DC	TXN
BCKDHB.1	0.003519	-0.12996	0.027	0.089	1	DC	BCKDHB
CWC15.2	0.003525	-0.28504	0.353	0.489	1	DC	CWC15
B3GALT4	0.003534	0.13775	0.086	0.042	1	DC	B3GALT4
ARMCX1.2	0.00355	-0.188	0.118	0.215	1	DC	ARMCX1
CCDC28A.2	0.003558	0.228886	0.225	0.157	1	DC	CCDC28A
CIC.1	0.003559	-0.12972	0.048	0.12	1	DC	CIC
RSF1.1	0.003561	-0.18309	0.294	0.419	1	DC	RSF1
ERI3.1	0.003578	-0.10865	0.241	0.371	1	DC	ERI3
ZBTB41	0.003586	-0.10487	0	0.043	1	DC	ZBTB41
SHF	0.003586	-0.1177	0	0.043	1	DC	SHF
SDCCAG3.2	0.003586	-0.13299	0.032	0.096	1	DC	SDCCAG3
HDAC2.3	0.00359	-0.23327	0.332	0.45	1	DC	HDAC2
PARP2	0.003603	-0.1794	0.043	0.112	1	DC	PARP2
PIP4K2C.1	0.003603	-0.20336	0.043	0.11	1	DC	PIP4K2C
KAT8.2	0.003605	0.212978	0.246	0.175	1	DC	KAT8
ZNF567	0.00361	-0.13371	0.032	0.097	1	DC	ZNF567
SART3.1	0.003624	-0.18006	0.053	0.126	1	DC	SART3
KLHDC2.2	0.003627	-0.17902	0.187	0.304	1	DC	KLHDC2
SPECC1.3	0.003633	-0.14068	0.182	0.298	1	DC	SPECC1
RAB11B.1	0.003635	-0.18798	0.369	0.506	1	DC	RAB11B
UBL3.3	0.003637	0.238626	0.406	0.353	1	DC	UBL3
ST8SIA5	0.003657	-0.12626	0	0.043	1	DC	ST8SIA5
AC005618.	0.003657	-0.14447	0	0.043	1	DC	AC005618.6
MUC12.1	0.003657	-0.1935	0	0.043	1	DC	MUC12
RREB1.3	0.003664	0.193265	0.123	0.071	1	DC	RREB1
TMEM57.3	0.003674	-0.18174	0.091	0.176	1	DC	TMEM57
IGSF21.3	0.003682	-0.28691	0.086	0.165	1	DC	IGSF21
CYB561.2	0.003686	-0.11075	0.021	0.081	1	DC	CYB561
LINC00526	0.003689	-0.11256	0.021	0.08	1	DC	LINC00526
RNF144A	0.003692	-0.13628	0	0.043	1	DC	RNF144A
BCAS1	0.003692	-0.22802	0	0.043	1	DC	BCAS1
PARP1.2	0.003697	-0.22151	0.353	0.474	1	DC	PARP1
ZC4H2.1	0.003707	-0.15127	0.005	0.053	1	DC	ZC4H2
GFPT1.2	0.00371	-0.15269	0.032	0.096	1	DC	GFPT1
VPS52.1	0.003712	-0.16265	0.027	0.088	1	DC	VPS52
CDCA3.2	0.003712	-0.16893	0.005	0.053	1	DC	CDCA3
REPS1.2	0.003717	-0.11264	0.053	0.128	1	DC	REPS1
ACP2.2	0.003727	-0.22083	0.144	0.245	1	DC	ACP2
GMNN.2	0.003729	-0.18021	0.091	0.178	1	DC	GMNN
ZNF561.1	0.003739	-0.1522	0.043	0.112	1	DC	ZNF561

SERINC1	0.003744	-0.21614	0.294	0.42	1	DC	SERINC1
SLC25A1.2	0.003746	-0.12142	0.102	0.196	1	DC	SLC25A1
SERPINB6.	0.003756	0.153748	0.545	0.477	1	DC	SERPINB6
RNF170.1	0.003761	-0.1307	0.08	0.166	1	DC	RNF170
MAPK8IP2	0.003764	-0.12443	0	0.043	1	DC	MAPK8IP2
SUV39H2	0.003772	-0.14589	0.011	0.062	1	DC	SUV39H2
IMPDH2.3	0.003777	0.107234	0.481	0.415	1	DC	IMPDH2
TMOD1.1	0.003782	-0.26755	0.08	0.158	1	DC	TMOD1
BTBD17.2	0.003793	-0.16857	0.005	0.053	1	DC	BTBD17
ANKFY1.1	0.003795	-0.1125	0.059	0.137	1	DC	ANKFY1
INTS12.1	0.003809	-0.15614	0.08	0.166	1	DC	INTS12
STK38.1	0.003814	0.163433	0.123	0.071	1	DC	STK38
IRGQ.1	0.003839	-0.1133	0.027	0.088	1	DC	IRGQ
BEX4.2	0.003854	0.129151	0.561	0.475	1	DC	BEX4
PHF6.2	0.003857	-0.13687	0.086	0.171	1	DC	PHF6
TXLNG.1	0.003861	-0.15971	0.059	0.135	1	DC	TXLNG
PDK3.2	0.003864	0.247312	0.107	0.06	1	DC	PDK3
TOX.3	0.003869	-0.12448	0.027	0.088	1	DC	TOX
MCAM	0.003876	-0.1266	0	0.043	1	DC	MCAM
CKAP2.3	0.003877	-0.17217	0.086	0.17	1	DC	CKAP2
COX14.1	0.003878	0.179822	0.513	0.459	1	DC	COX14
MYLPF	0.003886	0.121493	0.043	0.016	1	DC	MYLPF
EXOG.2	0.003903	-0.13051	0.075	0.157	1	DC	EXOG
PUS7L	0.003906	-0.16342	0.08	0.163	1	DC	PUS7L
MTHFS.1	0.003913	0.25834	0.257	0.188	1	DC	MTHFS
TERF2IP.3	0.00392	-0.21734	0.412	0.519	1	DC	TERF2IP
VTA1	0.00394	-0.14186	0.096	0.185	1	DC	VTA1
CPLX1	0.003951	-0.15558	0	0.043	1	DC	CPLX1
ZNF174.1	0.003954	-0.11194	0.016	0.071	1	DC	ZNF174
RBM6.1	0.003956	-0.17338	0.198	0.315	1	DC	RBM6
SENP2.1	0.003961	-0.12677	0.059	0.134	1	DC	SENP2
SLC39A4.1	0.003967	0.136243	0.15	0.089	1	DC	SLC39A4
SPG20.1	0.003967	-0.19338	0.139	0.239	1	DC	SPG20
PVRL1.2	0.003973	-0.1428	0.011	0.062	1	DC	PVRL1
ZMAT1.2	0.003999	-0.13802	0.032	0.095	1	DC	ZMAT1
YIF1B.1	0.004003	0.243768	0.385	0.332	1	DC	YIF1B
GPBP1L1.3	0.004006	0.328668	0.267	0.205	1	DC	GPBP1L1
UBN2	0.004012	-0.15754	0.043	0.11	1	DC	UBN2
ARAP2.1	0.004027	-0.16972	0.027	0.086	1	DC	ARAP2
NINJ1.3	0.00403	0.117233	0.551	0.45	1	DC	NINJ1
COIL.1	0.004039	-0.14116	0.096	0.187	1	DC	COIL
BCYRN1	0.004041	-0.14138	0.005	0.053	1	DC	BCYRN1
CD99.2	0.004054	-0.29785	0.604	0.638	1	DC	CD99
RSPH3	0.004055	-0.1254	0.021	0.079	1	DC	RSPH3
FICD.1	0.004067	-0.14151	0.021	0.079	1	DC	FICD
AC018647.	0.004068	-0.22761	0	0.042	1	DC	AC018647.3
TMEM145.1	0.004095	-0.14037	0.016	0.07	1	DC	TMEM145
USP31.2	0.004102	-0.12654	0.005	0.052	1	DC	USP31
RPP40.2	0.004106	-0.12666	0.048	0.119	1	DC	RPP40
LIN7A	0.004108	-0.10107	0	0.042	1	DC	LIN7A
GNAZ.1	0.004108	-0.13311	0.011	0.061	1	DC	GNAZ

CHERP	0.004109	-0.1716	0.086	0.17	1	DC	CHERP
CHURC1.1	0.004112	0.149547	0.46	0.4	1	DC	CHURC1
C15orf41	0.004118	-0.13325	0.005	0.052	1	DC	C15orf41
NUDT9.1	0.004122	-0.10823	0.123	0.224	1	DC	NUDT9
HBS1L.1	0.004124	-0.12896	0.102	0.193	1	DC	HBS1L
SDF2	0.004127	-0.16866	0.241	0.358	1	DC	SDF2
PCMTD1.2	0.004135	0.216714	0.219	0.151	1	DC	PCMTD1
GMPR2.1	0.004142	0.304712	0.278	0.214	1	DC	GMPR2
DOLPP1.1	0.004155	-0.16717	0.037	0.102	1	DC	DOLPP1
HSPBAP1.1	0.004163	0.204839	0.123	0.071	1	DC	HSPBAP1
RALGDS.2	0.004177	-0.20151	0.075	0.153	1	DC	RALGDS
PC	0.004179	-0.13207	0.016	0.071	1	DC	PC
MTHFD1L.3	0.004186	0.209878	0.198	0.135	1	DC	MTHFD1L
UMPS.1	0.004189	-0.15841	0.059	0.134	1	DC	UMPS
PTCD3.1	0.00419	-0.18173	0.134	0.231	1	DC	PTCD3
ARMCX2.2	0.004192	-0.20735	0.07	0.148	1	DC	ARMCX2
CSRNP2.1	0.004194	-0.12298	0.027	0.087	1	DC	CSRNP2
PARP6.2	0.004195	-0.12401	0.037	0.104	1	DC	PARP6
NOC2L.2	0.004216	-0.12433	0.193	0.315	1	DC	NOC2L
MAP7D3	0.004216	-0.16491	0.048	0.118	1	DC	MAP7D3
KMT2B.1	0.004225	-0.13942	0.016	0.07	1	DC	KMT2B
SEMA7A	0.004226	0.142787	0.08	0.04	1	DC	SEMA7A
EFCAB7	0.004235	-0.13157	0.016	0.07	1	DC	EFCAB7
RFFL.3	0.004238	-0.14311	0.08	0.166	1	DC	RFFL
TARSL2.2	0.004245	-0.12569	0.043	0.111	1	DC	TARSL2
ZFP37	0.004249	-0.13213	0.005	0.052	1	DC	ZFP37
ANK3.2	0.004253	-0.18356	0.011	0.061	1	DC	ANK3
DHPS.2	0.004259	-0.18326	0.316	0.449	1	DC	DHPS
ZNF92.1	0.004265	-0.17542	0.07	0.147	1	DC	ZNF92
CHMP7.2	0.004268	-0.11428	0.091	0.178	1	DC	CHMP7
GNG7.2	0.00427	-0.25769	0.198	0.301	1	DC	GNG7
CLCF1.1	0.00427	-0.1631	0	0.042	1	DC	CLCF1
ACVR1B.1	0.004277	-0.15543	0.027	0.086	1	DC	ACVR1B
WDR54.3	0.004282	-0.15083	0.134	0.235	1	DC	WDR54
KAT7.1	0.004287	-0.1301	0.07	0.149	1	DC	KAT7
RPS6KB2.2	0.004292	0.200115	0.289	0.226	1	DC	RPS6KB2
ST6GALNAC	0.004306	-0.15486	0.075	0.155	1	DC	ST6GALNAC6
SLCO5A1.1	0.004311	-0.14582	0	0.042	1	DC	SLCO5A1
RNF213.3	0.004329	0.208031	0.326	0.26	1	DC	RNF213
LRP3	0.004346	-0.12009	0.011	0.061	1	DC	LRP3
PSD2	0.004353	-0.14077	0	0.042	1	DC	PSD2
GLRX5.2	0.004355	-0.17214	0.182	0.298	1	DC	GLRX5
SLC2A1.2	0.004357	-0.2053	0.064	0.139	1	DC	SLC2A1
SECTM1	0.00436	0.185942	0.048	0.019	1	DC	SECTM1
MRI1.1	0.004368	-0.10693	0.043	0.111	1	DC	MRI1
SRP9.1	0.00437	-0.17879	0.599	0.682	1	DC	SRP9
TBC1D1.3	0.004371	0.267143	0.182	0.122	1	DC	TBC1D1
TCEAL3.1	0.004377	-0.21105	0.225	0.344	1	DC	TCEAL3
LINC00672	0.004397	-0.15842	0.005	0.052	1	DC	LINC00672
MAZ.3	0.004412	-0.10848	0.144	0.252	1	DC	MAZ
NDUFV3.1	0.004428	-0.17485	0.278	0.412	1	DC	NDUFV3

ZSWIM4	0.004429	-0.14325	0.005	0.052	1	DC	ZSWIM4
ANAPC7.1	0.004433	-0.12843	0.086	0.173	1	DC	ANAPC7
PQLC3.3	0.004437	0.270246	0.246	0.183	1	DC	PQLC3
NMNAT1	0.004438	-0.15553	0.021	0.078	1	DC	NMNAT1
SALL3	0.004439	-0.10206	0	0.042	1	DC	SALL3
CKMT2-AS1	0.004439	-0.12549	0	0.042	1	DC	CKMT2-AS1
RAPGEF2.1	0.004443	-0.1482	0.016	0.07	1	DC	RAPGEF2
TM9SF2.2	0.004449	0.179165	0.396	0.338	1	DC	TM9SF2
GOSR1.2	0.00445	-0.11059	0.16	0.269	1	DC	GOSR1
HSPB6.2	0.004462	-0.12906	0.011	0.061	1	DC	HSPB6
EVI5L	0.004474	-0.12696	0.011	0.06	1	DC	EVI5L
SAA1.2	0.004475	-0.87009	0.053	0.12	1	DC	SAA1
RNF150.1	0.004482	-0.13779	0	0.041	1	DC	RNF150
DPH3.2	0.004482	-0.14296	0.118	0.215	1	DC	DPH3
TAPBPL.2	0.004496	0.124586	0.187	0.121	1	DC	TAPBPL
RIC8B.1	0.004499	-0.14841	0.032	0.094	1	DC	RIC8B
ZNF585A	0.004504	-0.12013	0.011	0.061	1	DC	ZNF585A
IDH3G.2	0.004512	0.112324	0.465	0.402	1	DC	IDH3G
PCGF5.1	0.004516	0.233327	0.246	0.178	1	DC	PCGF5
ZNF471	0.004526	-0.1047	0	0.041	1	DC	ZNF471
USP2	0.004526	-0.11889	0	0.041	1	DC	USP2
TRPM3.2	0.00453	-0.10646	0.016	0.07	1	DC	TRPM3
ARSD	0.004543	0.224875	0.144	0.09	1	DC	ARSD
AC018816.	0.004557	0.100361	0.037	0.013	1	DC	AC018816.3
ZNF71.1	0.004558	-0.15552	0.027	0.085	1	DC	ZNF71
METTL15.1	0.004563	0.24477	0.187	0.127	1	DC	METTL15
LINC-PINT	0.004564	0.221047	0.166	0.105	1	DC	LINC-PINT
ZNF627	0.004573	-0.10208	0.021	0.078	1	DC	ZNF627
FAM193B.1	0.00458	-0.12541	0.011	0.061	1	DC	FAM193B
RFC1.1	0.004607	-0.18784	0.171	0.278	1	DC	RFC1
SNN.3	0.004618	-0.1795	0.134	0.227	1	DC	SNN
SUCLA2.2	0.004624	-0.19888	0.182	0.289	1	DC	SUCLA2
MSANTD2	0.00463	-0.10474	0.011	0.061	1	DC	MSANTD2
PTCH1	0.004632	-0.11736	0.005	0.051	1	DC	PTCH1
LMCD1.1	0.004641	-0.11912	0.027	0.086	1	DC	LMCD1
POLR2E.2	0.004652	0.12816	0.594	0.534	1	DC	POLR2E
PRPF18.2	0.004656	0.255754	0.198	0.136	1	DC	PRPF18
SP9.2	0.004659	-0.15472	0	0.041	1	DC	SP9
NRSN1.1	0.004664	-0.12923	0.011	0.061	1	DC	NRSN1
PTPN14.1	0.00467	-0.10705	0.016	0.069	1	DC	PTPN14
JPH4	0.004676	-0.12482	0.011	0.061	1	DC	JPH4
LRRC24.1	0.00468	-0.1141	0.016	0.069	1	DC	LRRC24
ZNF180	0.004704	-0.10731	0	0.041	1	DC	ZNF180
RNF169.1	0.004706	-0.11234	0.021	0.078	1	DC	RNF169
POLR3A	0.004734	-0.11571	0.011	0.06	1	DC	POLR3A
GAS8	0.004741	-0.11759	0.016	0.069	1	DC	GAS8
TBC1D14.3	0.004753	-0.19139	0.091	0.173	1	DC	TBC1D14
MTMR4.2	0.004762	-0.14835	0.08	0.162	1	DC	MTMR4
VRK3.2	0.004768	-0.1296	0.123	0.22	1	DC	VRK3
YTHDF3-AS	0.004778	-0.11054	0.011	0.06	1	DC	YTHDF3-AS1
SH3PXD2B.	0.004791	-0.11878	0.048	0.118	1	DC	SH3PXD2B

N6AMT2.1	0.004792	-0.13587	0.059	0.134	1	DC	N6AMT2
C19orf68	0.004796	-0.10634	0	0.041	1	DC	C19orf68
CD163L1	0.004796	-0.1465	0	0.041	1	DC	CD163L1
MARCH6.2	0.0048	-0.21918	0.241	0.359	1	DC	6-Mar
ARHGAP35.	0.004803	-0.12822	0.07	0.147	1	DC	ARHGAP35
STX1A.1	0.004821	-0.16969	0.016	0.068	1	DC	STX1A
EIF5A.1	0.004827	0.104257	0.588	0.526	1	DC	EIF5A
COL8A2	0.004832	0.108192	0.053	0.023	1	DC	COL8A2
AGFG2.1	0.004843	-0.10925	0	0.041	1	DC	AGFG2
OBFC1.1	0.004855	0.227545	0.139	0.086	1	DC	OBFC1
LYPLA1.2	0.004859	0.199013	0.46	0.406	1	DC	LYPLA1
PCF11.2	0.004863	0.2194	0.267	0.203	1	DC	PCF11
HPGDS.3	0.004886	0.109819	0.15	0.088	1	DC	HPGDS
NRG2	0.00489	-0.10546	0	0.041	1	DC	NRG2
NDUFA4L2.	0.00489	-0.17465	0	0.041	1	DC	NDUFA4L2
MED7.1	0.004891	-0.1651	0.048	0.116	1	DC	MED7
PIK3R2.1	0.004898	-0.12539	0.005	0.051	1	DC	PIK3R2
ATP5J2.3	0.004918	-0.19454	0.791	0.808	1	DC	ATP5J2
FERMT1.1	0.004921	-0.17358	0.005	0.05	1	DC	FERMT1
PHLPP1.3	0.004922	-0.13281	0.027	0.086	1	DC	PHLPP1
VPS51.2	0.004933	0.24171	0.321	0.262	1	DC	VPS51
FAM13C	0.004938	-0.13161	0	0.041	1	DC	FAM13C
CA9.1	0.004938	-0.17962	0	0.041	1	DC	CA9
EIF2B3	0.004946	-0.21293	0.091	0.174	1	DC	EIF2B3
FEM1C	0.004952	-0.13042	0.064	0.141	1	DC	FEM1C
UGT8.2	0.004963	-0.23161	0.005	0.05	1	DC	UGT8
PYCRL.1	0.004964	-0.10445	0.005	0.051	1	DC	PYCRL
AEBP2.1	0.004966	-0.11778	0.027	0.085	1	DC	AEBP2
C6orf89.1	0.004968	-0.14591	0.123	0.221	1	DC	C6orf89
FAM129B.1	0.004977	0.154922	0.123	0.073	1	DC	FAM129B
RPUSD2	0.004984	-0.11812	0.016	0.069	1	DC	RPUSD2
PHTF1.2	0.00499	0.270228	0.176	0.12	1	DC	PHTF1
HADHB.1	0.005018	0.131837	0.524	0.46	1	DC	HADHB
TNFRSF12A	0.005025	-0.28282	0.203	0.288	1	DC	TNFRSF12A
ENTPD5.1	0.005031	-0.1071	0.005	0.05	1	DC	ENTPD5
ARX.2	0.005035	-0.14436	0	0.04	1	DC	ARX
PRRT3	0.005062	-0.11397	0.016	0.069	1	DC	PRRT3
EIF4G3.2	0.005065	-0.14521	0.091	0.176	1	DC	EIF4G3
RBM43	0.005073	-0.14025	0.005	0.05	1	DC	RBM43
GIN1.1	0.005075	-0.13094	0.005	0.05	1	DC	GIN1
SLC38A7.2	0.005075	-0.13022	0.043	0.109	1	DC	SLC38A7
MID1	0.00508	-0.10315	0.005	0.051	1	DC	MID1
RABL2A	0.005107	-0.10479	0.021	0.077	1	DC	RABL2A
BECN1.2	0.005119	0.252424	0.289	0.229	1	DC	BECN1
SNAI2	0.005133	-0.10032	0	0.04	1	DC	SNAI2
SLC1A4.3	0.005142	0.181581	0.123	0.072	1	DC	SLC1A4
CHKB.1	0.005149	0.159265	0.102	0.056	1	DC	CHKB
RBM22.1	0.005169	-0.17047	0.193	0.304	1	DC	RBM22
TMEM119.3	0.005169	-0.25573	0.043	0.106	1	DC	TMEM119
KIFC2	0.005171	-0.1042	0.016	0.068	1	DC	KIFC2
SLC8B1.3	0.005173	0.162479	0.155	0.097	1	DC	SLC8B1

RTN1.2	0.005175	0.252568	0.203	0.142	1	DC	RTN1
ZNF501	0.005183	-0.12007	0	0.04	1	DC	ZNF501
HDAC3.2	0.005195	0.246141	0.321	0.262	1	DC	HDAC3
RDH14	0.005196	-0.13865	0.102	0.189	1	DC	RDH14
PIFO.1	0.005198	-0.21824	0.005	0.05	1	DC	PIFO
EIF4E.2	0.005201	0.125667	0.529	0.488	1	DC	EIF4E
KDM5C.2	0.00521	-0.1332	0.043	0.109	1	DC	KDM5C
BOLA3-AS1	0.005234	-0.10813	0	0.04	1	DC	BOLA3-AS1
MAL.2	0.005234	-0.26721	0	0.04	1	DC	MAL
TSHZ2.1	0.005256	-0.16693	0.005	0.05	1	DC	TSHZ2
ZNF446	0.005265	-0.12776	0.021	0.076	1	DC	ZNF446
TARBP2.1	0.005278	-0.10535	0.07	0.148	1	DC	TARBP2
SOD3.1	0.005285	-0.10471	0	0.04	1	DC	SOD3
BRSK2	0.005285	-0.10995	0	0.04	1	DC	BRSK2
RALA.2	0.005298	0.27506	0.406	0.359	1	DC	RALA
CBX4.3	0.005323	-0.13438	0.048	0.116	1	DC	CBX4
SYNE1	0.005326	-0.14889	0.053	0.124	1	DC	SYNE1
ERBB4.2	0.00533	-0.15221	0.011	0.059	1	DC	ERBB4
HOTAIRM1.	0.005334	0.228575	0.257	0.181	1	DC	HOTAIRM1
ADAM19.2	0.005342	0.230161	0.128	0.079	1	DC	ADAM19
MLH3	0.005352	-0.18175	0.043	0.107	1	DC	MLH3
APTX.2	0.005354	-0.11526	0.064	0.14	1	DC	APTX
CENPA.3	0.005365	-0.15303	0.005	0.05	1	DC	CENPA
HIST2H2AA	0.005367	0.125481	0.112	0.062	1	DC	HIST2H2AA3
NR1D1.2	0.005375	-0.13555	0.005	0.05	1	DC	NR1D1
WDYHV1.1	0.005385	-0.19562	0.053	0.122	1	DC	WDYHV1
DDX25	0.005388	-0.12169	0	0.04	1	DC	DDX25
C15orf61.	0.005405	-0.14016	0.091	0.175	1	DC	C15orf61
NEDD4L.3	0.005406	-0.19348	0.048	0.114	1	DC	NEDD4L
SPATA5L1.	0.005417	-0.10621	0.011	0.059	1	DC	SPATA5L1
CHRNA9	0.005421	-0.11875	0.005	0.05	1	DC	CHRNA9
ST3GAL5.1	0.005478	-0.21183	0.107	0.192	1	DC	ST3GAL5
MYT1.1	0.005494	-0.14155	0	0.04	1	DC	MYT1
RNF34.1	0.005497	-0.11215	0.107	0.196	1	DC	RNF34
STX2.2	0.005498	-0.10898	0.043	0.109	1	DC	STX2
SARNP	0.005505	0.212661	0.182	0.121	1	DC	SARNP
PIGH.1	0.005516	-0.18263	0.118	0.209	1	DC	PIGH
ZSCAN32	0.005519	-0.11805	0.016	0.068	1	DC	ZSCAN32
PANK3.1	0.005521	-0.14658	0.048	0.115	1	DC	PANK3
ZNF106.3	0.005532	0.248661	0.316	0.256	1	DC	ZNF106
NXF1.2	0.005539	0.241485	0.262	0.197	1	DC	NXF1
SYNJ2BP.2	0.005552	-0.18549	0.07	0.145	1	DC	SYNJ2BP
NME5.1	0.005576	-0.10391	0.005	0.05	1	DC	NME5
NFATC1.3	0.005577	0.131901	0.096	0.052	1	DC	NFATC1
CDK2.1	0.005578	-0.12799	0.027	0.085	1	DC	CDK2
PCP4.1	0.005601	-0.213	0	0.039	1	DC	PCP4
RASSF4.3	0.005613	0.131436	0.412	0.328	1	DC	RASSF4
ZHX1.2	0.00563	-0.15294	0.118	0.211	1	DC	ZHX1
CHST13	0.005633	0.112843	0.043	0.017	1	DC	CHST13
ADCY2.1	0.005656	-0.10912	0	0.039	1	DC	ADCY2
GS1-124K5	0.005666	-0.1828	0.048	0.115	1	DC	GS1-124K5.4

FANCL.1	0.005671	-0.1016	0.07	0.144	1	DC	FANCL
ANKRD36C.	0.005674	-0.18883	0.086	0.163	1	DC	ANKRD36C
SLC12A7	0.005721	0.159224	0.08	0.041	1	DC	SLC12A7
GAS6.3	0.005724	0.125413	0.289	0.203	1	DC	GAS6
EIF5AL1.1	0.005735	0.233476	0.176	0.119	1	DC	EIF5AL1
STARD13.1	0.005747	-0.12684	0.005	0.049	1	DC	STARD13
WBSCR16.2	0.005747	-0.11821	0.07	0.146	1	DC	WBSCR16
ZCCHC7.1	0.005753	-0.14199	0.07	0.144	1	DC	ZCCHC7
LUZP2	0.00576	-0.15173	0.005	0.049	1	DC	LUZP2
FAM63B	0.005761	-0.11213	0.112	0.206	1	DC	FAM63B
FDXACB1	0.005766	-0.12489	0	0.039	1	DC	FDXACB1
FANCF.1	0.005776	-0.11555	0.032	0.093	1	DC	FANCF
BCS1L.1	0.005778	-0.10467	0.086	0.167	1	DC	BCS1L
TGFB2.2	0.005781	-0.11175	0.016	0.068	1	DC	TGFB2
MLF2.3	0.005799	-0.22088	0.524	0.61	1	DC	MLF2
GOLGA4.1	0.005805	-0.21193	0.241	0.348	1	DC	GOLGA4
FAM8A1	0.005806	-0.11254	0.027	0.084	1	DC	FAM8A1
ATOX1.3	0.005812	-0.216	0.513	0.606	1	DC	ATOX1
CHRNB1.2	0.005846	-0.20965	0.075	0.15	1	DC	CHRNB1
ELK4.1	0.005848	-0.10557	0.043	0.108	1	DC	ELK4
PGAM2.2	0.005856	-0.21602	0.005	0.049	1	DC	PGAM2
TBCC.3	0.005865	-0.19769	0.134	0.223	1	DC	TBCC
ZNF93	0.005926	-0.1214	0.011	0.058	1	DC	ZNF93
C1orf53.1	0.005926	-0.12847	0.027	0.084	1	DC	C1orf53
EIF5A2	0.005927	-0.1172	0.011	0.058	1	DC	EIF5A2
ARHGAP11A	0.005937	-0.1079	0	0.039	1	DC	ARHGAP11A
ZNF595.1	0.005937	-0.14382	0.021	0.075	1	DC	ZNF595
STAM.1	0.00594	-0.11538	0.037	0.1	1	DC	STAM
TOMM22.3	0.005948	0.150196	0.513	0.468	1	DC	TOMM22
JAGN1.2	0.005978	-0.1932	0.203	0.322	1	DC	JAGN1
ZFR2	0.005994	-0.13183	0	0.039	1	DC	ZFR2
FABP6.2	0.005994	-0.15129	0	0.039	1	DC	FABP6
SRPX2.1	0.005997	-0.14088	0.011	0.058	1	DC	SRPX2
IZUMO4.1	0.006011	-0.1103	0.016	0.067	1	DC	IZUMO4
DHFRL1	0.006013	-0.10489	0.016	0.067	1	DC	DHFRL1
TUBGCP2.3	0.006014	0.205756	0.273	0.211	1	DC	TUBGCP2
SHB.1	0.006036	-0.10103	0.011	0.058	1	DC	SHB
ARMC1.2	0.006057	-0.11429	0.16	0.266	1	DC	ARMC1
DHODH.2	0.006081	-0.17322	0.027	0.083	1	DC	DHODH
FOXO3.3	0.006105	0.235081	0.332	0.271	1	DC	FOXO3
LZTFL1.1	0.006107	-0.12366	0.021	0.075	1	DC	LZTFL1
LRRC42.1	0.00611	-0.13956	0.07	0.145	1	DC	LRRC42
LYRM5.1	0.006111	-0.19388	0.23	0.343	1	DC	LYRM5
CDK16.1	0.006117	-0.14657	0.139	0.237	1	DC	CDK16
PDZRN3.1	0.00612	-0.13387	0.011	0.058	1	DC	PDZRN3
DGKG.1	0.006122	-0.10468	0.005	0.049	1	DC	DGKG
LYRM7.1	0.006131	-0.16417	0.112	0.203	1	DC	LYRM7
AZGP1.1	0.006133	-0.24154	0.011	0.057	1	DC	AZGP1
RP11-635N	0.006162	-0.1224	0.011	0.058	1	DC	RP11-635N19.1
FAH.2	0.006173	0.278841	0.219	0.157	1	DC	FAH
CHKA.3	0.006195	0.232796	0.246	0.186	1	DC	CHKA

ZNF738.3	0.006202	-0.14137	0.07	0.145	1	DC	ZNF738
FAM169A	0.00622	-0.10768	0.005	0.049	1	DC	FAM169A
ZCCHC3.2	0.006237	-0.11029	0.043	0.107	1	DC	ZCCHC3
UBN1.3	0.006239	-0.10572	0.086	0.168	1	DC	UBN1
TBC1D17.1	0.006253	-0.14801	0.08	0.156	1	DC	TBC1D17
MSRA.2	0.006256	0.244824	0.321	0.255	1	DC	MSRA
USP54.1	0.00628	-0.16425	0.005	0.048	1	DC	USP54
CLDND1.1	0.006282	-0.28647	0.273	0.395	1	DC	CLDND1
CTD-2139E	0.006287	0.107138	0.037	0.014	1	DC	CTD-2139B15.2
RP2.3	0.006291	0.134669	0.128	0.076	1	DC	RP2
AC093627.	0.006293	0.103502	0.043	0.017	1	DC	AC093627.10
BGN.2	0.006327	-0.27104	0.016	0.065	1	DC	BGN
ZC3H7B	0.006347	-0.13381	0.043	0.107	1	DC	ZC3H7B
C3orf18	0.006353	-0.10196	0	0.038	1	DC	C3orf18
RORB	0.006353	-0.14241	0	0.038	1	DC	RORB
ERBB3.1	0.006353	-0.17821	0	0.038	1	DC	ERBB3
DLGAP4	0.006354	-0.12021	0.182	0.295	1	DC	DLGAP4
TACC2	0.006361	-0.10529	0.011	0.058	1	DC	TACC2
SETD6.1	0.006364	-0.11877	0.032	0.091	1	DC	SETD6
MIS12.1	0.00637	-0.16836	0.07	0.143	1	DC	MIS12
ZDHHC17.1	0.006372	-0.13226	0.043	0.106	1	DC	ZDHHC17
PLOD3.1	0.006395	-0.15116	0.128	0.219	1	DC	PLOD3
PPIL6.2	0.006404	-0.11694	0.005	0.048	1	DC	PPIL6
NCAN.1	0.006411	-0.20617	0.027	0.081	1	DC	NCAN
PMVK.1	0.006432	-0.21763	0.31	0.439	1	DC	PMVK
DDR2.1	0.006436	-0.12004	0.011	0.058	1	DC	DDR2
HECTD2	0.00645	-0.11904	0.005	0.048	1	DC	HECTD2
DIP2B.3	0.006451	-0.16446	0.053	0.12	1	DC	DIP2B
DNAJC19.3	0.006454	-0.15487	0.262	0.391	1	DC	DNAJC19
PROSER1.2	0.006461	-0.11338	0.021	0.075	1	DC	PROSER1
BCL7A.3	0.006463	-0.13951	0.075	0.151	1	DC	BCL7A
VWA3B	0.006478	-0.10244	0	0.038	1	DC	VWA3B
BTBD6	0.006478	-0.10397	0	0.038	1	DC	BTBD6
THBS2	0.006478	-0.12837	0	0.038	1	DC	THBS2
THYN1.2	0.006504	-0.24642	0.294	0.408	1	DC	THYN1
NCDN	0.006517	-0.10992	0.032	0.091	1	DC	NCDN
RCAN1.3	0.006538	-0.28099	0.171	0.27	1	DC	RCAN1
SCHIP1	0.006541	-0.10648	0	0.038	1	DC	SCHIP1
PIK3R4	0.006541	-0.1093	0	0.038	1	DC	PIK3R4
TOPORS-AS	0.006549	-0.19361	0.075	0.148	1	DC	TOPORS-AS1
ZC3H13.3	0.006552	-0.16115	0.225	0.336	1	DC	ZC3H13
C6orf203.	0.006556	-0.165	0.086	0.167	1	DC	C6orf203
PCOLCE.2	0.006561	-0.19624	0.027	0.082	1	DC	PCOLCE
COA6.1	0.006567	-0.10898	0.171	0.278	1	DC	COA6
ZNF275.1	0.006572	-0.12066	0.011	0.057	1	DC	ZNF275
CTBP1.2	0.006576	-0.11879	0.128	0.224	1	DC	CTBP1
NKTR.1	0.006581	-0.21291	0.246	0.359	1	DC	NKTR
RTN4.3	0.006605	-0.13641	0.738	0.699	1	DC	RTN4
COQ6.1	0.006611	-0.12765	0.043	0.107	1	DC	COQ6
C17orf97	0.006614	-0.10685	0.011	0.057	1	DC	C17orf97
PRIM1.2	0.006616	-0.12076	0.027	0.082	1	DC	PRIM1

SLC7A6OS.	0.006623	-0.1628	0.064	0.135	1 DC	SLC7A6OS
TMEM256.1	0.006641	0.118435	0.497	0.429	1 DC	TMEM256
BAG1.2	0.006642	0.181464	0.412	0.351	1 DC	BAG1
ASL.2	0.006653	0.193451	0.235	0.17	1 DC	ASL
ARHGAP33.	0.006659	-0.10779	0.027	0.083	1 DC	ARHGAP33
TNK2.2	0.006664	-0.12518	0.059	0.128	1 DC	TNK2
CDC37	0.006665	0.107354	0.583	0.543	1 DC	CDC37
NECAB1.2	0.006669	-0.1759	0	0.038	1 DC	NECAB1
F11R.2	0.006675	0.129159	0.102	0.056	1 DC	F11R
SLC39A3.2	0.006701	-0.15478	0.225	0.34	1 DC	SLC39A3
SIAE.1	0.006709	-0.11017	0.032	0.091	1 DC	SIAE
FNDC3A.2	0.006714	-0.13009	0.123	0.216	1 DC	FNDC3A
PRKAR2A.1	0.006725	-0.10523	0.15	0.25	1 DC	PRKAR2A
FAM124A	0.006734	-0.1296	0	0.038	1 DC	FAM124A
WDR19.2	0.006746	-0.11517	0.005	0.048	1 DC	WDR19
DSTYK.1	0.00676	-0.12198	0.053	0.122	1 DC	DSTYK
RGS11	0.00677	-0.10082	0.005	0.048	1 DC	RGS11
THUMPD3	0.006788	-0.12541	0.107	0.195	1 DC	THUMPD3
AARSD1	0.006789	-0.12822	0.043	0.106	1 DC	AARSD1
UBAP2L	0.006792	-0.10121	0.123	0.218	1 DC	UBAP2L
HIST1H2AC	0.006801	-0.2489	0.091	0.168	1 DC	HIST1H2AC
ZNF568.1	0.006809	-0.12507	0.037	0.099	1 DC	ZNF568
GIT1.1	0.006824	-0.10442	0.043	0.107	1 DC	GIT1
APITD1	0.006829	-0.10951	0.027	0.083	1 DC	APITD1
RND1	0.006836	-0.18215	0.016	0.065	1 DC	RND1
WDR12.1	0.006839	-0.13462	0.07	0.144	1 DC	WDR12
USB1.2	0.006842	0.310013	0.283	0.229	1 DC	USB1
TMEM79	0.006843	-0.12354	0.011	0.057	1 DC	TMEM79
ZDHHC7.3	0.006844	0.240918	0.203	0.146	1 DC	ZDHHC7
C1QTNF4.1	0.006844	-0.13385	0.011	0.057	1 DC	C1QTNF4
PRNP.2	0.006849	-0.22878	0.342	0.467	1 DC	PRNP
HOXA-AS2	0.006866	-0.14067	0	0.038	1 DC	HOXA-AS2
UBQLN2.3	0.006868	-0.10328	0.128	0.222	1 DC	UBQLN2
PPP5D1.2	0.006886	-0.11738	0.005	0.048	1 DC	PPP5D1
SIRT2.3	0.006894	-0.2435	0.294	0.418	1 DC	SIRT2
ASPM.3	0.006895	-0.15363	0.005	0.047	1 DC	ASPM
ETFA.2	0.006897	0.193809	0.417	0.371	1 DC	ETFA
NPEPL1.1	0.006902	-0.11794	0.043	0.106	1 DC	NPEPL1
CTC-459F4	0.006911	-0.12126	0.016	0.066	1 DC	CTC-459F4.3
ST6GAL2.1	0.006933	-0.11553	0	0.038	1 DC	ST6GAL2
APLP2.3	0.006982	0.101311	0.658	0.592	1 DC	APLP2
SPAG9.2	0.007014	-0.10381	0.235	0.355	1 DC	SPAG9
APOO.2	0.007021	-0.13222	0.134	0.23	1 DC	APOO
GANAB.2	0.007021	-0.17042	0.171	0.274	1 DC	GANAB
CYP46A1	0.007044	-0.11253	0.005	0.047	1 DC	CYP46A1
GPALPP1.1	0.007044	-0.12869	0.053	0.121	1 DC	GPALPP1
ZSCAN16-A	0.007056	-0.18598	0.16	0.259	1 DC	ZSCAN16-AS1
RFC5	0.007066	-0.11597	0.032	0.091	1 DC	RFC5
TIGD1	0.007111	-0.12747	0.016	0.065	1 DC	TIGD1
TSN.1	0.007127	-0.17718	0.262	0.379	1 DC	TSN
SH3D21	0.007136	0.131075	0.053	0.023	1 DC	SH3D21

MTFR1.1	0.007155	-0.14185	0.037	0.097	1	DC	MTFR1
PET117	0.007159	-0.11459	0.096	0.181	1	DC	PET117
SLC39A7	0.007162	-0.178	0.198	0.307	1	DC	SLC39A7
TXNDC16.1	0.00717	-0.13074	0.011	0.056	1	DC	TXNDC16
CALCOCO2.	0.007177	0.209834	0.251	0.192	1	DC	CALCOCO2
AR	0.00718	-0.10719	0.005	0.047	1	DC	AR
HPCAL1.3	0.007181	0.287394	0.267	0.209	1	DC	HPCAL1
METTL21B.	0.007188	-0.10451	0.016	0.066	1	DC	METTL21B
FBXO5	0.007191	-0.13155	0.037	0.097	1	DC	FBXO5
PXN-AS1	0.007193	-0.12466	0.027	0.082	1	DC	PXN-AS1
DISC1.2	0.007204	0.14634	0.086	0.045	1	DC	DISC1
CCNF	0.007207	-0.10238	0	0.037	1	DC	CCNF
NYAP1.1	0.007207	-0.11625	0	0.037	1	DC	NYAP1
CGRRF1.2	0.00721	-0.1007	0.086	0.165	1	DC	CGRRF1
PRKD2.1	0.007228	-0.13379	0.027	0.081	1	DC	PRKD2
HEATR5A.1	0.00724	-0.1452	0.027	0.082	1	DC	HEATR5A
DEGS1.1	0.007257	0.138931	0.508	0.459	1	DC	DEGS1
AP2A2.3	0.007258	-0.18615	0.16	0.253	1	DC	AP2A2
MED20	0.007258	-0.10965	0.011	0.056	1	DC	MED20
TFAP2A	0.007278	-0.12883	0	0.037	1	DC	TFAP2A
SLC16A1-A	0.007298	-0.16464	0.005	0.047	1	DC	SLC16A1-AS1
BDNF-AS.2	0.007309	-0.12232	0.016	0.065	1	DC	BDNF-AS
TMPO.3	0.007312	0.27493	0.257	0.193	1	DC	TMPO
WFS1	0.00733	-0.12753	0.011	0.056	1	DC	WFS1
RAD50	0.007332	-0.12498	0.16	0.267	1	DC	RAD50
NGFR	0.007349	-0.1309	0	0.037	1	DC	NGFR
MAP3K2.3	0.00735	0.241562	0.257	0.199	1	DC	MAP3K2
ZNF22.2	0.007396	-0.17127	0.176	0.277	1	DC	ZNF22
CA8.2	0.007409	-0.18442	0.011	0.056	1	DC	CA8
BRMS1.2	0.007409	0.258684	0.358	0.31	1	DC	BRMS1
THRAP3.3	0.007424	0.158556	0.497	0.437	1	DC	THRAP3
GTPBP10.1	0.007445	-0.15249	0.037	0.097	1	DC	GTPBP10
APH1A.1	0.007458	0.122211	0.54	0.484	1	DC	APH1A
SAR1A.3	0.007465	0.190967	0.364	0.297	1	DC	SAR1A
NEK1	0.007492	-0.13954	0.059	0.127	1	DC	NEK1
PSORS1C1.	0.007492	-0.137	0	0.037	1	DC	PSORS1C1
CYB5R3.2	0.007499	0.207771	0.406	0.36	1	DC	CYB5R3
C12orf45.	0.007511	0.253092	0.235	0.177	1	DC	C12orf45
SPRTN.1	0.007517	-0.12849	0.059	0.127	1	DC	SPRTN
FGD5-AS1.	0.00755	-0.12628	0.198	0.314	1	DC	FGD5-AS1
SNRPD3.3	0.007587	0.149535	0.46	0.413	1	DC	SNRPD3
SLC25A40.	0.007601	0.181772	0.107	0.062	1	DC	SLC25A40
FBLIM1	0.007602	-0.15378	0.032	0.09	1	DC	FBLIM1
NUDT5.2	0.007651	0.222627	0.358	0.305	1	DC	NUDT5
CISD1.3	0.007652	-0.24657	0.23	0.338	1	DC	CISD1
CCDC97	0.007662	-0.103	0.043	0.105	1	DC	CCDC97
RRP7A.3	0.007672	0.143839	0.353	0.286	1	DC	RRP7A
NABP2.2	0.007683	-0.1244	0.171	0.278	1	DC	NABP2
ZNF44.1	0.007701	-0.11068	0.043	0.105	1	DC	ZNF44
TNR.2	0.007704	-0.18612	0.011	0.055	1	DC	TNR
CSNK1A1.3	0.007705	0.117948	0.631	0.557	1	DC	CSNK1A1

ANKZF1.1	0.007713	-0.12911	0.064	0.135	1 DC	ANKZF1
RCOR2.1	0.007734	-0.12236	0.005	0.046	1 DC	RCOR2
TTC21B	0.0078	-0.10189	0.005	0.047	1 DC	TTC21B
EXOSC8.2	0.00782	-0.20705	0.187	0.291	1 DC	EXOSC8
AC084219.	0.007865	-0.10713	0	0.036	1 DC	AC084219.4
COCH	0.007865	-0.1425	0	0.036	1 DC	COCH
RECQL.2	0.007866	0.216841	0.23	0.172	1 DC	RECQL
CSE1L.2	0.007883	-0.16514	0.134	0.224	1 DC	CSE1L
OTUD6B.1	0.007906	-0.1048	0.053	0.12	1 DC	OTUD6B
SLC41A3	0.007937	-0.15323	0.075	0.148	1 DC	SLC41A3
LINGO1	0.007942	-0.11482	0	0.036	1 DC	LINGO1
C1orf123.	0.007956	-0.2309	0.23	0.343	1 DC	C1orf123
EPHB1	0.007969	-0.11308	0.005	0.046	1 DC	EPHB1
C11orf57	0.008	-0.15566	0.08	0.155	1 DC	C11orf57
KCND2	0.008019	-0.10964	0	0.036	1 DC	KCND2
TCEAL6	0.008019	-0.15598	0	0.036	1 DC	TCEAL6
CC2D1A.2	0.008031	-0.1107	0.102	0.188	1 DC	CC2D1A
EHD4.3	0.008061	0.13903	0.144	0.09	1 DC	EHD4
TMC7	0.008072	-0.10517	0.005	0.046	1 DC	TMC7
TMOD2.2	0.008082	-0.13729	0.048	0.111	1 DC	TMOD2
KCNN2	0.008097	-0.10034	0	0.036	1 DC	KCNN2
MOBP.2	0.008097	-0.31577	0	0.036	1 DC	MOBP
ANGPT2.1	0.008098	-0.15794	0.005	0.046	1 DC	ANGPT2
CDC14B	0.008115	-0.10283	0.016	0.064	1 DC	CDC14B
MMS22L.1	0.008117	-0.13143	0.016	0.064	1 DC	MMS22L
GAN.1	0.008132	-0.10328	0.07	0.143	1 DC	GAN
SMARCC2.2	0.008143	-0.1685	0.166	0.265	1 DC	SMARCC2
ZNF236.1	0.008164	-0.12692	0.021	0.072	1 DC	ZNF236
TMEM63A.2	0.008166	-0.19112	0.021	0.071	1 DC	TMEM63A
ABCE1.1	0.008222	-0.1695	0.155	0.252	1 DC	ABCE1
CHD2.3	0.008248	0.269793	0.342	0.291	1 DC	CHD2
MAF1.3	0.008275	0.135549	0.476	0.427	1 DC	MAF1
SFSWAP.2	0.00829	-0.13446	0.096	0.178	1 DC	SFSWAP
RCAN3.3	0.008292	0.166663	0.102	0.058	1 DC	RCAN3
VPS36.2	0.008319	0.198855	0.262	0.2	1 DC	VPS36
TTLL5	0.008339	-0.10467	0.011	0.055	1 DC	TTLL5
ERN1.1	0.008347	0.18221	0.096	0.054	1 DC	ERN1
DZIP3.1	0.008353	-0.16612	0.064	0.132	1 DC	DZIP3
CKAP2L.2	0.00836	-0.11001	0.005	0.046	1 DC	CKAP2L
NAPA.1	0.008365	0.143435	0.444	0.393	1 DC	NAPA
APBA3.1	0.008388	-0.1095	0.032	0.089	1 DC	APBA3
TRAF3IP2-	0.0084	-0.1121	0.027	0.081	1 DC	TRAF3IP2-AS1
ALG6.1	0.008402	-0.11866	0.043	0.104	1 DC	ALG6
ULK3.2	0.008404	-0.13093	0.075	0.147	1 DC	ULK3
HMBOX1.2	0.008434	-0.18123	0.112	0.194	1 DC	HMBOX1
MVB12A.1	0.008472	-0.19175	0.198	0.304	1 DC	MVB12A
DHX57	0.008486	-0.12713	0.037	0.096	1 DC	DHX57
ARL6IP1.2	0.008487	-0.19113	0.701	0.743	1 DC	ARL6IP1
TRPT1.1	0.008492	-0.19525	0.134	0.225	1 DC	TRPT1
COA7.1	0.0085	-0.14226	0.016	0.064	1 DC	COA7
DLGAP5.2	0.0085	-0.11307	0	0.036	1 DC	DLGAP5

RP11-315A	0.0085	-0.11325	0	0.036	1	DC	RP11-315A16.1
PPFIA1	0.008523	-0.14117	0.091	0.168	1	DC	PPFIA1
PNPT1.1	0.00855	-0.15294	0.112	0.198	1	DC	PNPT1
GPR19	0.008578	-0.15026	0.005	0.046	1	DC	GPR19
KIAA1147.	0.008588	-0.12574	0.021	0.072	1	DC	KIAA1147
IGF1.2	0.008607	-0.24375	0.016	0.062	1	DC	IGF1
CCP110.2	0.008613	-0.1447	0.059	0.125	1	DC	CCP110
SENP7.1	0.008626	-0.13237	0.032	0.087	1	DC	SENP7
BRD4.2	0.008628	-0.15815	0.219	0.329	1	DC	BRD4
DYNLT1.1	0.008643	-0.1801	0.545	0.63	1	DC	DYNLT1
TTC9C.2	0.008648	-0.16585	0.096	0.176	1	DC	TTC9C
MYBL2.2	0.008658	-0.10449	0.005	0.046	1	DC	MYBL2
TMCO1.2	0.008734	-0.2286	0.455	0.577	1	DC	TMCO1
SLC2A8.2	0.008741	-0.13441	0.07	0.14	1	DC	SLC2A8
LINC00599	0.008751	-0.13581	0	0.035	1	DC	LINC00599
MFHAS1.1	0.008753	-0.10715	0.016	0.063	1	DC	MFHAS1
MAX.1	0.008755	0.191995	0.353	0.293	1	DC	MAX
VPS13A	0.00877	-0.10741	0.027	0.08	1	DC	VPS13A
IL33.1	0.008771	-0.11201	0.005	0.046	1	DC	IL33
UBAC2.2	0.008809	0.237599	0.326	0.27	1	DC	UBAC2
NR2F2.1	0.008813	-0.13279	0.005	0.045	1	DC	NR2F2
ZNF790	0.008819	-0.12052	0.037	0.096	1	DC	ZNF790
TTLL4	0.008825	-0.118	0.021	0.072	1	DC	TTLL4
NR1H3	0.008837	-0.15934	0.011	0.054	1	DC	NR1H3
ZNF574.2	0.008839	-0.11385	0.032	0.088	1	DC	ZNF574
NFS1.1	0.008844	-0.10555	0.064	0.135	1	DC	NFS1
TNFRSF11E	0.008853	-0.1652	0.011	0.054	1	DC	TNFRSF11B
DNAJB9.2	0.008879	0.102645	0.46	0.384	1	DC	DNAJB9
TIMELESS.	0.008908	-0.12036	0.016	0.063	1	DC	TIMELESS
POLR2K	0.008927	-0.17263	0.364	0.491	1	DC	POLR2K
LARP4B.2	0.008932	0.161091	0.139	0.089	1	DC	LARP4B
SUCLG2.1	0.008943	0.18991	0.305	0.244	1	DC	SUCLG2
GCNT1	0.00896	0.112276	0.053	0.024	1	DC	GCNT1
HAMP.3	0.008973	-0.42353	0.096	0.162	1	DC	HAMP
IL11RA.1	0.008994	-0.10692	0.016	0.063	1	DC	IL11RA
TSGA10.1	0.009056	-0.13064	0.016	0.063	1	DC	TSGA10
NPEPPS	0.00907	-0.13194	0.134	0.226	1	DC	NPEPPS
NUDT22.2	0.009079	0.171941	0.326	0.269	1	DC	NUDT22
ZC3H14.2	0.009087	-0.14187	0.118	0.204	1	DC	ZC3H14
DHRS1	0.009125	-0.15283	0.08	0.156	1	DC	DHRS1
CDC23.1	0.009151	-0.14271	0.059	0.126	1	DC	CDC23
APOPT1.1	0.009223	-0.22376	0.267	0.383	1	DC	APOPT1
FAR2.1	0.00923	0.236522	0.096	0.055	1	DC	FAR2
NAA15.1	0.009241	-0.15863	0.128	0.217	1	DC	NAA15
CELSR3	0.009277	-0.1001	0	0.035	1	DC	CELSR3
MR1.1	0.009303	0.196487	0.139	0.088	1	DC	MR1
PSMC3IP.2	0.009341	-0.11206	0.021	0.071	1	DC	PSMC3IP
ZBTB37	0.009361	-0.10276	0.011	0.054	1	DC	ZBTB37
MAST2	0.009379	-0.124	0.021	0.07	1	DC	MAST2
ALKBH5.2	0.009382	-0.15293	0.219	0.325	1	DC	ALKBH5
C21orf58.	0.009392	-0.10658	0.011	0.054	1	DC	C21orf58

TIMM21.2	0.009397	-0.14592	0.075	0.148	1	DC	TIMM21
ENOSF1.3	0.009417	-0.12561	0.059	0.126	1	DC	ENOSF1
BLOC1S5.1	0.009429	-0.13042	0.027	0.079	1	DC	BLOC1S5
CAD	0.009449	-0.10904	0.011	0.054	1	DC	CAD
FBXL15.3	0.009461	0.209709	0.257	0.194	1	DC	FBXL15
SLC25A44.	0.009489	-0.11234	0.037	0.095	1	DC	SLC25A44
NBPF12	0.009551	-0.10116	0	0.035	1	DC	NBPF12
RUNX1T1	0.009551	-0.10128	0	0.035	1	DC	RUNX1T1
ABCG1.3	0.009555	0.120382	0.198	0.13	1	DC	ABCG1
CHMP4A.2	0.009624	0.108759	0.524	0.467	1	DC	CHMP4A
SLITRK2	0.009644	-0.10904	0	0.035	1	DC	SLITRK2
FAM45A.2	0.009659	-0.16774	0.112	0.192	1	DC	FAM45A
RAB5B.2	0.009675	-0.12538	0.128	0.215	1	DC	RAB5B
EXOC6B.1	0.009685	-0.10284	0.016	0.063	1	DC	EXOC6B
MYOM2	0.009712	-0.11401	0.005	0.045	1	DC	MYOM2
MARVELD1.	0.009751	0.161187	0.075	0.039	1	DC	MARVELD1
ZNF222.1	0.009769	-0.11079	0.011	0.054	1	DC	ZNF222
HGS.2	0.009779	-0.10363	0.128	0.218	1	DC	HGS
PDE3B.1	0.009802	0.156392	0.075	0.039	1	DC	PDE3B
DNMT3A.2	0.009812	-0.13789	0.043	0.101	1	DC	DNMT3A
SCRN1.2	0.009812	-0.15542	0.102	0.181	1	DC	SCRN1
LINC00237	0.009834	-0.10297	0	0.034	1	DC	LINC00237
NDUFAF4.1	0.009886	-0.13069	0.134	0.223	1	DC	NDUFAF4
COQ10B.2	0.009902	0.253626	0.316	0.262	1	DC	COQ10B
RP11-102N	0.009929	0.1037	0.043	0.018	1	DC	RP11-102N12.3
PPHLN1.1	0.009932	0.225406	0.406	0.366	1	DC	PPHLN1
ACHE	0.009967	-0.1194	0.005	0.044	1	DC	ACHE
SASS6	0.009968	-0.12225	0.016	0.062	1	DC	SASS6
OSBPL8.3	0.00997	0.202751	0.358	0.311	1	DC	OSBPL8
LGALS8.2	0.009986	0.192419	0.209	0.151	1	DC	LGALS8
PHC1.1	0.009991	-0.10489	0.027	0.079	1	DC	PHC1
RAB40B.3	0.009998	-0.11355	0.064	0.131	1	DC	RAB40B
CD83.4	0	1.37207	0.819	0.189	0	M1	CD83
IL1B.4	0	1.318572	0.645	0.136	0	M1	IL1B
OLR1.4	0	1.31801	0.762	0.147	0	M1	OLR1
GPR183.4	0	1.30168	0.81	0.185	0	M1	GPR183
NR4A2.4	0	1.264021	0.727	0.156	0	M1	NR4A2
HLA-DMB.4	0	1.245278	0.899	0.204	0	M1	HLA-DMB
C5AR1.4	0	1.236866	0.702	0.145	0	M1	C5AR1
MS4A7.4	0	1.236615	0.912	0.216	0	M1	MS4A7
IER3.4	0	1.217332	0.68	0.153	0	M1	IER3
OSM.4	0	1.209506	0.495	0.075	0	M1	OSM
GPR34.4	0	1.205934	0.785	0.163	0	M1	GPR34
LY86.4	0	1.199875	0.832	0.184	0	M1	LY86
FPR1.4	0	1.183846	0.798	0.181	0	M1	FPR1
C3AR1.4	0	1.181988	0.711	0.143	0	M1	C3AR1
FCGR1A.4	0	1.180792	0.748	0.16	0	M1	FCGR1A
C10orf54.	0	1.179503	0.794	0.186	0	M1	C10orf54
TREM2.4	0	1.17036	0.918	0.237	0	M1	TREM2
HLA-DQB1.	0	1.170179	0.865	0.217	0	M1	HLA-DQB1
LILRB4.4	0	1.169458	0.743	0.157	0	M1	LILRB4

NR4A1.4	0	1.163448	0.701	0.187	0	M1	NR4A1
NR4A3.4	0	1.152534	0.507	0.079	0	M1	NR4A3
CSF2RA.4	0	1.151605	0.627	0.117	0	M1	CSF2RA
SLC11A1.4	0	1.145752	0.776	0.211	0	M1	SLC11A1
FCGR3A.4	0	1.144157	0.898	0.23	0	M1	FCGR3A
APOC2.3	0	1.141357	0.6	0.107	0	M1	APOC2
TBXAS1.4	0	1.140559	0.69	0.151	0	M1	TBXAS1
CCL4.3	0	1.134266	0.719	0.245	0	M1	CCL4
MS4A6A.4	0	1.132866	0.899	0.229	0	M1	MS4A6A
FCGR2A.4	0	1.132486	0.785	0.19	0	M1	FCGR2A
ZNF331.4	0	1.13123	0.717	0.235	0	M1	ZNF331
CD69.4	0	1.131226	0.529	0.078	0	M1	CD69
CCL3.4	0	1.126151	0.883	0.33	0	M1	CCL3
RNASE6.4	0	1.119904	0.756	0.178	0	M1	RNASE6
PLD4.4	0	1.118331	0.543	0.075	0	M1	PLD4
CD37.4	0	1.118037	0.814	0.198	0	M1	CD37
LST1.4	0	1.116422	0.818	0.211	0	M1	LST1
CSF1R.4	0	1.112176	0.741	0.173	0	M1	CSF1R
RGS10.4	0	1.111036	0.854	0.239	0	M1	RGS10
KLF4.4	0	1.110277	0.566	0.134	0	M1	KLF4
IFI30.4	0	1.107808	0.625	0.138	0	M1	IFI30
TM6SF1.4	0	1.107443	0.518	0.086	0	M1	TM6SF1
HAVCR2.4	0	1.106527	0.662	0.145	0	M1	HAVCR2
NFKBID.4	0	1.101156	0.479	0.072	0	M1	NFKBID
ADORA3.4	0	1.100751	0.707	0.171	0	M1	ADORA3
SGK1.4	0	1.094535	0.897	0.344	0	M1	SGK1
HLA-DMA.4	0	1.088697	0.949	0.3	0	M1	HLA-DMA
PLAUR.4	0	1.085636	0.825	0.275	0	M1	PLAUR
HBEGF.4	0	1.083828	0.546	0.149	0	M1	HBEGF
RP11-552D	0	1.0808	0.44	0.056	0	M1	RP11-552D4.1
PDK4.3	0	1.075032	0.497	0.104	0	M1	PDK4
VSIG4.4	0	1.074237	0.912	0.255	0	M1	VSIG4
RGS1.4	0	1.073837	0.99	0.338	0	M1	RGS1
HCLS1.4	0	1.071294	0.773	0.193	0	M1	HCLS1
IRF8.4	0	1.07068	0.507	0.088	0	M1	IRF8
CH25H.2	0	1.066501	0.457	0.084	0	M1	CH25H
CSF3R.4	0	1.065897	0.496	0.084	0	M1	CSF3R
RAB20.4	0	1.062993	0.584	0.166	0	M1	RAB20
C1orf162.	0	1.062981	0.833	0.219	0	M1	C1orf162
MS4A4A.4	0	1.059997	0.771	0.189	0	M1	MS4A4A
CCL4L2.3	0	1.059143	0.343	0.035	0	M1	CCL4L2
SAMSN1.4	0	1.055739	0.682	0.165	0	M1	SAMSN1
IL18.4	0	1.049604	0.562	0.119	0	M1	IL18
CXCR4.3	0	1.046349	0.814	0.303	0	M1	CXCR4
HAMP.4	0	1.044528	0.471	0.098	0	M1	HAMP
FCGR1B.3	0	1.043699	0.511	0.093	0	M1	FCGR1B
CD86.4	0	1.038836	0.594	0.137	0	M1	CD86
CTSS.4	0	1.037257	0.85	0.254	0	M1	CTSS
HLA-DQA1.	0	1.035227	0.801	0.207	0	M1	HLA-DQA1
CD14.4	0	1.033837	0.93	0.304	0	M1	CD14
AIF1.4	0	1.032337	0.954	0.302	0	M1	AIF1

OLFML3.3	0	1.030327	0.603	0.151	0	M1	OLFML3
PLXDC2.4	0	1.027422	0.586	0.134	0	M1	PLXDC2
CD53.4	0	1.027017	0.81	0.216	0	M1	CD53
CLEC7A.4	0	1.026848	0.516	0.107	0	M1	CLEC7A
LAT2.4	0	1.026796	0.575	0.128	0	M1	LAT2
FGL2.4	0	1.025592	0.517	0.108	0	M1	FGL2
SERPINA1.	0	1.02434	0.766	0.206	0	M1	SERPINA1
RNASET2.4	0	1.024333	0.888	0.401	0	M1	RNASET2
FCER1G.4	0	1.022679	0.975	0.334	0	M1	FCER1G
FYB.4	0	1.021633	0.606	0.146	0	M1	FYB
SERPINB9.	0	1.021334	0.464	0.083	0	M1	SERPINB9
SLCO2B1.3	0	1.018497	0.617	0.141	0	M1	SLCO2B1
CPVL.4	0	1.015892	0.772	0.264	0	M1	CPVL
SCIN.4	0	1.01426	0.475	0.086	0	M1	SCIN
KLF2.3	0	1.01404	0.385	0.081	0	M1	KLF2
REL.4	0	1.01358	0.637	0.23	0	M1	REL
RILPL2.4	0	1.012831	0.579	0.194	0	M1	RILPL2
TYROBP.4	0	1.011159	0.992	0.353	0	M1	TYROBP
CYBA.4	0	1.008976	0.869	0.312	0	M1	CYBA
ALOX5AP.4	0	1.006244	0.918	0.295	0	M1	ALOX5AP
PLEK.4	0	1.001843	0.512	0.107	0	M1	PLEK
MAP3K8.4	0	0.992766	0.505	0.119	0	M1	MAP3K8
SLC2A5.4	0	0.992589	0.511	0.102	0	M1	SLC2A5
BCL2A1.4	0	0.990598	0.457	0.097	0	M1	BCL2A1
LAPTM5.4	0	0.988502	0.966	0.329	0	M1	LAPTM5
CXCL16.4	0	0.986414	0.841	0.376	0	M1	CXCL16
ARHGDIB.4	0	0.981478	0.886	0.268	0	M1	ARHGDIB
HCST.4	0	0.981446	0.717	0.198	0	M1	HCST
LTC4S.4	0	0.978477	0.504	0.101	0	M1	LTC4S
C1QC.4	0	0.977927	0.995	0.402	0	M1	C1QC
PHACTR1.4	0	0.977867	0.365	0.068	0	M1	PHACTR1
ITGB2.4	0	0.976042	0.792	0.23	0	M1	ITGB2
STX11.4	0	0.973273	0.381	0.061	0	M1	STX11
EVI2B.4	0	0.964641	0.56	0.131	0	M1	EVI2B
AXL.4	0	0.964626	0.495	0.117	0	M1	AXL
LAIR1.4	0	0.959135	0.596	0.157	0	M1	LAIR1
GPSM3.4	0	0.956254	0.672	0.18	0	M1	GPSM3
IGSF6.4	0	0.955896	0.452	0.094	0	M1	IGSF6
ADAM28.4	0	0.955197	0.431	0.083	0	M1	ADAM28
RHBDF2.4	0	0.955085	0.498	0.113	0	M1	RHBDF2
PILRA.4	0	0.954131	0.477	0.102	0	M1	PILRA
THEMIS2.4	0	0.949542	0.581	0.174	0	M1	THEMIS2
GMFG.4	0	0.948853	0.738	0.209	0	M1	GMFG
TYMP.4	0	0.94714	0.678	0.232	0	M1	TYMP
CD300A.4	0	0.946274	0.531	0.123	0	M1	CD300A
C1QA.4	0	0.94299	0.995	0.445	0	M1	C1QA
ABI3.4	0	0.941002	0.534	0.121	0	M1	ABI3
CREM.4	0	0.939332	0.716	0.344	0	M1	CREM
CD68.4	0	0.938658	0.919	0.346	0	M1	CD68
CYBB.4	0	0.936858	0.53	0.124	0	M1	CYBB
SPI1.4	0	0.934809	0.62	0.162	0	M1	SPI1

FCGBP.3	0	0.933391	0.569	0.161	0	M1	FCGBP
SRGN.4	0	0.926415	0.989	0.401	0	M1	SRGN
HLA-DQA2.	0	0.926158	0.473	0.112	0	M1	HLA-DQA2
LGALS9.4	0	0.926141	0.598	0.168	0	M1	LGALS9
PYCARD.4	0	0.926092	0.666	0.208	0	M1	PYCARD
HLA-DPB1.	0	0.924532	0.975	0.398	0	M1	HLA-DPB1
CTSH.4	0	0.9239	0.681	0.218	0	M1	CTSH
VAMP8.4	0	0.922596	0.813	0.249	0	M1	VAMP8
APBB1IP.4	0	0.918095	0.459	0.096	0	M1	APBB1IP
TMEM119.4	0	0.91669	0.36	0.054	0	M1	TMEM119
MSR1.4	0	0.915857	0.507	0.114	0	M1	MSR1
SIGLEC10.	0	0.914896	0.461	0.102	0	M1	SIGLEC10
SERPINF1.	0	0.908229	0.549	0.159	0	M1	SERPINF1
PADI2.4	0	0.905408	0.4	0.082	0	M1	PADI2
FXYD5.4	0	0.904576	0.76	0.256	0	M1	FXYD5
C3.3	0	0.903742	0.864	0.308	0	M1	C3
CPM.3	0	0.903519	0.546	0.172	0	M1	CPM
APOC1.3	0	0.901985	0.951	0.428	0	M1	APOC1
CD163.4	0	0.899559	0.653	0.2	0	M1	CD163
PTGER4.4	0	0.89929	0.391	0.07	0	M1	PTGER4
TAGAP.4	0	0.899094	0.341	0.052	0	M1	TAGAP
PTPRC.4	0	0.898774	0.537	0.136	0	M1	PTPRC
ADAP2.4	0	0.89731	0.495	0.122	0	M1	ADAP2
FOLR2.4	0	0.896704	0.49	0.115	0	M1	FOLR2
CORO1A.4	0	0.896323	0.782	0.291	0	M1	CORO1A
C1QB.4	0	0.895246	0.997	0.484	0	M1	C1QB
FAM105A.4	0	0.893774	0.452	0.104	0	M1	FAM105A
CTSC.4	0	0.888834	0.803	0.299	0	M1	CTSC
ENTPD1.4	0	0.883206	0.442	0.106	0	M1	ENTPD1
MNDA.4	0	0.883018	0.476	0.117	0	M1	MNDA
SLA.4	0	0.881022	0.528	0.136	0	M1	SLA
AOAH.4	0	0.880709	0.381	0.071	0	M1	AOAH
LPCAT2.4	0	0.872712	0.422	0.092	0	M1	LPCAT2
TNFRSF1B.	0	0.869995	0.483	0.129	0	M1	TNFRSF1B
CX3CR1.3	0	0.867193	0.355	0.05	0	M1	CX3CR1
IFNGR1.4	0	0.860774	0.739	0.376	0	M1	IFNGR1
IFITM2.4	0	0.851726	0.624	0.214	0	M1	IFITM2
HLA-DRB5.	0	0.848189	0.844	0.31	0	M1	HLA-DRB5
CNPY3.4	0	0.847632	0.71	0.352	0	M1	CNPY3
TMEM176B.	0	0.843993	0.71	0.28	0	M1	TMEM176B
SCPEP1.3	0	0.840862	0.65	0.285	0	M1	SCPEP1
PLIN2.3	0	0.84084	0.709	0.269	0	M1	PLIN2
CAPG.4	0	0.835944	0.799	0.337	0	M1	CAPG
SLC7A7.4	0	0.832685	0.463	0.116	0	M1	SLC7A7
HLA-DPA1.	0	0.818187	0.992	0.441	0	M1	HLA-DPA1
RGS2.3	0	0.806329	0.846	0.365	0	M1	RGS2
FCGRT.3	0	0.801225	0.929	0.474	0	M1	FCGRT
ARPC1B.4	0	0.79582	0.881	0.461	0	M1	ARPC1B
HMOX1.4	0	0.793797	0.631	0.227	0	M1	HMOX1
SERPINB1.	0	0.791356	0.611	0.198	0	M1	SERPINB1
HLA-DRB1.	0	0.787473	0.995	0.44	0	M1	HLA-DRB1

KLF6.3	0	0.787058	0.853	0.467	0	M1	KLF6
FOSB.3	0	0.784978	0.709	0.328	0	M1	FOSB
HLA-E.4	0	0.747926	0.94	0.527	0	M1	HLA-E
DUSP1.4	0	0.718372	0.864	0.517	0	M1	DUSP1
GSN.3	0	0.704783	0.836	0.444	0	M1	GSN
MCL1.4	0	0.7018	0.82	0.554	0	M1	MCL1
HERPUD1.3	0	0.662616	0.899	0.59	0	M1	HERPUD1
GRN.4	0	0.656047	0.836	0.517	0	M1	GRN
ZFP36.2	0	0.622479	0.944	0.499	0	M1	ZFP36
ID2.3	0	0.604165	0.914	0.602	0	M1	ID2
HLA-DRA.3	0	0.574742	0.997	0.56	0	M1	HLA-DRA
NPC2.3	0	0.564639	0.977	0.634	0	M1	NPC2
SAT1.3	0	0.477781	0.997	0.764	0	M1	SAT1
CD74.3	0	0.465858	1	0.701	0	M1	CD74
CTSB.1	0	0.459235	0.978	0.712	0	M1	CTSB
GPX1.4	0	0.451631	0.972	0.782	0	M1	GPX1
MALAT1.4	0	-0.49706	0.999	0.995	0	M1	MALAT1
TUBA1A.3	0	-0.92837	0.573	0.855	0	M1	TUBA1A
NGFRAP1.3	0	-1.25461	0.197	0.734	0	M1	NGFRAP1
MAP1B.3	0	-1.28509	0.104	0.62	0	M1	MAP1B
GPM6B.4	0	-1.36134	0.174	0.708	0	M1	GPM6B
TUBB2B.3	0	-1.43756	0.145	0.637	0	M1	TUBB2B
PTN.4	0	-1.45181	0.167	0.706	0	M1	PTN
NOVA1.4	0	-1.51347	0.039	0.567	0	M1	NOVA1
PSAP.2	########	0.400544	0.963	0.79	########	M1	PSAP
IL10.4	########	0.942985	0.338	0.06	########	M1	IL10
EBI3.4	########	0.843091	0.318	0.052	########	M1	EBI3
CCRL2.4	########	0.863601	0.301	0.046	########	M1	CCRL2
ARRB2.4	########	0.799266	0.694	0.341	########	M1	ARRB2
RBP1.2	########	-1.2788	0.068	0.538	########	M1	RBP1
SOX2.4	########	-1.27826	0.045	0.506	########	M1	SOX2
RASGEF1B.	########	0.865268	0.36	0.073	########	M1	RASGEF1B
A2M.3	########	0.610341	0.796	0.397	########	M1	A2M
SLC16A3.4	########	0.804026	0.63	0.258	########	M1	SLC16A3
MEF2C.4	########	0.865758	0.572	0.225	########	M1	MEF2C
C1orf61.4	########	-1.1977	0.16	0.61	########	M1	C1orf61
ASAH1.3	########	0.575831	0.851	0.611	########	M1	ASAH1
PARVG.4	########	0.832878	0.429	0.108	########	M1	PARVG
MARCKSL1.	########	-1.20333	0.199	0.614	########	M1	MARCKSL1
CNN3.4	########	-1.19148	0.077	0.546	########	M1	CNN3
LPAR5.3	########	0.754839	0.327	0.059	########	M1	LPAR5
TSC22D1.3	########	-1.0744	0.124	0.59	########	M1	TSC22D1
GPR65.4	########	0.794644	0.379	0.084	########	M1	GPR65
HCK.4	########	0.801487	0.436	0.113	########	M1	HCK
GYPC.4	########	0.814115	0.606	0.25	########	M1	GYPC
CFD.4	########	0.856271	0.416	0.104	########	M1	CFD
NINJ1.4	########	0.754077	0.71	0.4	########	M1	NINJ1
LITAF.4	########	0.738652	0.728	0.405	########	M1	LITAF
GIMAP4.4	########	0.77113	0.409	0.098	########	M1	GIMAP4
CALD1.3	########	-1.14864	0.041	0.496	########	M1	CALD1
TNFSF13B.	########	0.805077	0.485	0.147	########	M1	TNFSF13B

PALD1.4	########	0.799746	0.317	0.059	########	M1	PALD1
RCSD1.4	########	0.775374	0.354	0.073	########	M1	RCSD1
WAS.4	########	0.809714	0.442	0.121	########	M1	WAS
MYO1F.4	########	0.838096	0.453	0.13	########	M1	MYO1F
GAP43.4	########	-1.33693	0.088	0.542	########	M1	GAP43
OTUD1.4	########	0.853564	0.305	0.057	########	M1	OTUD1
MAFB.3	########	0.80097	0.606	0.256	########	M1	MAFB
FEZ1.3	########	-1.08018	0.053	0.505	########	M1	FEZ1
DUSP2.4	########	0.764357	0.333	0.066	########	M1	DUSP2
SORL1.4	########	0.86038	0.5	0.172	########	M1	SORL1
TMEM173.4	########	0.814785	0.361	0.079	########	M1	TMEM173
PFN2.3	########	-1.10896	0.059	0.509	########	M1	PFN2
CTSZ.4	########	0.788492	0.554	0.196	########	M1	CTSZ
FAM26F.4	########	0.759862	0.428	0.114	########	M1	FAM26F
S100A11.4	########	0.413267	0.953	0.598	########	M1	S100A11
APOE.3	########	0.51594	0.857	0.651	########	M1	APOE
IL10RA.4	########	0.787206	0.378	0.09	########	M1	IL10RA
FXYD6.4	########	-1.10378	0.094	0.534	########	M1	FXYD6
UCHL1.2	########	-1.15639	0.071	0.509	########	M1	UCHL1
CDKN1A.3	########	0.811412	0.591	0.25	########	M1	CDKN1A
PPP1R15A.	########	0.7351	0.706	0.419	########	M1	PPP1R15A
UCP2.4	########	0.780758	0.559	0.215	########	M1	UCP2
MFSD1.4	########	0.797382	0.615	0.289	########	M1	MFSD1
LPAR6.4	########	0.865478	0.507	0.184	########	M1	LPAR6
ACSL1.4	########	0.850047	0.491	0.174	########	M1	ACSL1
CEBPB.4	########	0.733009	0.681	0.385	########	M1	CEBPB
MGAT4A.4	########	0.854016	0.485	0.167	########	M1	MGAT4A
PCSK1N.3	########	-1.15912	0.071	0.49	########	M1	PCSK1N
OSTF1.4	########	0.804879	0.57	0.235	########	M1	OSTF1
P2RY13.4	########	0.809932	0.277	0.046	########	M1	P2RY13
MDK.3	########	-1.10734	0.126	0.564	########	M1	MDK
HLA-DQB2.	########	0.937212	0.301	0.056	########	M1	HLA-DQB2
SEC61G.4	########	-0.98033	0.58	0.843	########	M1	SEC61G
ELF1.4	########	0.859514	0.502	0.192	########	M1	ELF1
CYTL1.4	########	0.855383	0.322	0.068	########	M1	CYTL1
MGST2.4	########	0.797032	0.524	0.199	########	M1	MGST2
USP53.4	########	0.863909	0.37	0.095	########	M1	USP53
ETS2.4	########	0.803856	0.468	0.155	########	M1	ETS2
PABPC1.3	########	0.437485	0.886	0.716	########	M1	PABPC1
NFIB.4	########	-1.1322	0.033	0.447	########	M1	NFIB
NLRP3.4	########	0.780824	0.278	0.049	########	M1	NLRP3
DHRS7.4	########	0.646822	0.767	0.514	########	M1	DHRS7
CD84.4	########	0.761703	0.354	0.084	########	M1	CD84
GPM6A.3	########	-0.96002	0.136	0.578	########	M1	GPM6A
NFKBIA.3	########	0.64873	0.737	0.452	########	M1	NFKBIA
RNF149.4	########	0.814959	0.532	0.218	########	M1	RNF149
CD302.4	########	0.851357	0.453	0.155	########	M1	CD302
IL13RA1.4	########	0.828531	0.502	0.198	########	M1	IL13RA1
CTSD.3	########	0.504797	0.831	0.601	########	M1	CTSD
SUSD3.4	########	0.751412	0.292	0.056	########	M1	SUSD3
BST2.3	########	0.55261	0.803	0.422	########	M1	BST2

SH3TC1.4	########	0.729492	0.26	0.044	########	M1	SH3TC1
ZNF667-AS	########	-0.96379	0.042	0.458	########	M1	ZNF667-AS1
RHOG.4	########	0.711978	0.675	0.356	########	M1	RHOG
CD4.4	########	0.729299	0.394	0.107	########	M1	CD4
IGFBP2.3	########	-1.23644	0.042	0.441	########	M1	IGFBP2
ATF3.4	########	0.808714	0.559	0.251	########	M1	ATF3
CAPZB.3	########	0.44995	0.85	0.672	########	M1	CAPZB
PPT1.3	########	0.600515	0.75	0.517	########	M1	PPT1
PMAIP1.4	########	0.796069	0.265	0.047	########	M1	PMAIP1
SNCA.4	########	0.766426	0.373	0.098	########	M1	SNCA
PTPN6.4	########	0.723526	0.403	0.114	########	M1	PTPN6
TSPO.3	########	0.534075	0.793	0.555	########	M1	TSPO
PRDX2.4	########	-0.76191	0.385	0.765	########	M1	PRDX2
HPGDS.4	########	0.682766	0.277	0.051	########	M1	HPGDS
FABP7.3	########	-1.31865	0.061	0.448	########	M1	FABP7
LINC01094	########	0.773559	0.385	0.107	########	M1	LINC01094
ARHGAP24.	########	0.77799	0.377	0.102	########	M1	ARHGAP24
EVI2A.4	########	0.694566	0.492	0.169	########	M1	EVI2A
AP001055.	########	0.736175	0.255	0.045	########	M1	AP001055.6
NFIX.3	########	-0.92762	0.015	0.396	########	M1	NFIX
PTAFR.4	########	0.753782	0.337	0.083	########	M1	PTAFR
CEBPD.2	########	0.656655	0.699	0.411	########	M1	CEBPD
RNASE2.4	########	0.742318	0.395	0.11	########	M1	RNASE2
SPTLC2.4	########	0.825964	0.448	0.162	########	M1	SPTLC2
FHL1.4	########	-0.91454	0.032	0.422	########	M1	FHL1
SIGLEC8.3	########	0.689953	0.248	0.043	########	M1	SIGLEC8
RASSF5.4	########	0.747503	0.32	0.079	########	M1	RASSF5
VMO1.4	########	0.804752	0.388	0.114	########	M1	VMO1
SOX4.3	########	-1.15338	0.126	0.509	########	M1	SOX4
CSRNP1.4	########	0.80104	0.418	0.145	########	M1	CSRNP1
MGAT1.4	########	0.773797	0.59	0.311	########	M1	MGAT1
EVL.3	########	0.777142	0.596	0.303	########	M1	EVL
CPE.3	########	-0.96278	0.064	0.455	########	M1	CPE
EPB41L2.3	########	0.805021	0.539	0.265	########	M1	EPB41L2
FYN.3	########	-0.87183	0.017	0.388	########	M1	FYN
PLSCR1.4	########	0.72383	0.605	0.31	########	M1	PLSCR1
BEX1.3	########	-1.08361	0.039	0.413	########	M1	BEX1
FAM49B.4	########	0.62976	0.698	0.467	########	M1	FAM49B
S100A4.4	########	0.615149	0.586	0.237	########	M1	S100A4
MAP2.4	########	-0.89778	0.03	0.4	########	M1	MAP2
GADD45B.2	########	0.486622	0.771	0.434	########	M1	GADD45B
SCG5.3	########	-0.98889	0.022	0.389	########	M1	SCG5
ELAVL3.4	########	-0.90838	0.012	0.371	########	M1	ELAVL3
CAMK2N1.4	########	-0.94165	0.016	0.377	########	M1	CAMK2N1
NPDC1.3	########	-0.86964	0.021	0.384	########	M1	NPDC1
ARPC3.2	########	0.326025	0.915	0.782	########	M1	ARPC3
MLLT11.2	########	-0.94888	0.127	0.519	########	M1	MLLT11
LILRB1.4	########	0.670433	0.269	0.056	########	M1	LILRB1
ALOX5.4	########	0.680644	0.35	0.096	########	M1	ALOX5
LINC00461	########	-0.91613	0.021	0.38	########	M1	LINC00461
APLP1.4	########	-0.95262	0.043	0.413	########	M1	APLP1

KIF21A.4	########	-0.86649	0.01	0.361	########	M1	KIF21A
KCNMB1.4	########	0.738356	0.288	0.065	########	M1	KCNMB1
SCD5.3	########	-0.88057	0.075	0.458	########	M1	SCD5
APP.3	########	-0.73923	0.183	0.593	########	M1	APP
BLNK.3	########	0.680086	0.221	0.036	########	M1	BLNK
ACTG1.4	########	-0.42689	0.894	0.959	########	M1	ACTG1
CKB.4	########	-0.82532	0.309	0.637	########	M1	CKB
TUBB2A.3	########	-0.85555	0.157	0.542	########	M1	TUBB2A
ELL2.3	########	0.735502	0.418	0.147	########	M1	ELL2
DOCK8.4	########	0.7164	0.318	0.081	########	M1	DOCK8
LAPTM4B.4	########	-0.81632	0.029	0.392	########	M1	LAPTM4B
HLA-DOA.3	########	0.642173	0.272	0.058	########	M1	HLA-DOA
SDCBP.2	########	0.402987	0.861	0.702	########	M1	SDCBP
CD33.4	########	0.724212	0.317	0.08	########	M1	CD33
TLR2.4	########	0.723816	0.301	0.073	########	M1	TLR2
BIN1.3	########	0.69304	0.589	0.315	########	M1	BIN1
CEBPA.3	########	0.676846	0.287	0.067	########	M1	CEBPA
STXBP2.4	########	0.701683	0.398	0.127	########	M1	STXBP2
CXorf21.4	########	0.743865	0.236	0.044	########	M1	CXorf21
ST6GAL1.3	########	0.694274	0.297	0.073	########	M1	ST6GAL1
ITGAX.4	########	0.739571	0.263	0.056	########	M1	ITGAX
TRIB1.3	########	0.812281	0.36	0.116	########	M1	TRIB1
AKR1B1.3	########	0.505658	0.776	0.595	########	M1	AKR1B1
CLEC2B.4	########	0.664415	0.444	0.157	########	M1	CLEC2B
SMAP2.4	########	0.802376	0.457	0.192	########	M1	SMAP2
WBP5.3	########	-0.76554	0.16	0.571	########	M1	WBP5
LIMS1.4	########	0.653114	0.642	0.399	########	M1	LIMS1
PPDPF.1	########	-0.62616	0.462	0.761	########	M1	PPDPF
ANKRD22.4	########	0.644749	0.213	0.035	########	M1	ANKRD22
RP11-480C	########	0.579301	0.178	0.022	########	M1	RP11-480C22.1
RGS19.4	########	0.771364	0.499	0.231	########	M1	RGS19
HSD17B11.	########	0.690691	0.611	0.33	########	M1	HSD17B11
FERMT3.4	########	0.652556	0.388	0.122	########	M1	FERMT3
C16orf45.	########	-0.82722	0.026	0.377	########	M1	C16orf45
NCF4.4	########	0.706711	0.357	0.104	########	M1	NCF4
STMN3.2	########	-0.78946	0.019	0.364	########	M1	STMN3
LCP2.4	########	0.713522	0.303	0.077	########	M1	LCP2
DPYSL3.3	########	-0.82745	0.03	0.381	########	M1	DPYSL3
KCTD12.4	########	0.723427	0.46	0.191	########	M1	KCTD12
LIPA.4	########	0.690159	0.566	0.288	########	M1	LIPA
NCAM1.4	########	-0.8164	0.039	0.395	########	M1	NCAM1
SIK1.4	########	0.704708	0.275	0.065	########	M1	SIK1
PTPRZ1.4	########	-0.93811	0.087	0.444	########	M1	PTPRZ1
PTGS1.4	########	0.741492	0.359	0.116	########	M1	PTGS1
ATP6V0E2.	########	-0.7746	0.139	0.515	########	M1	ATP6V0E2
SYNGR2.4	########	0.702447	0.558	0.282	########	M1	SYNGR2
DENND3.4	########	0.661834	0.277	0.065	########	M1	DENND3
CDH2.3	########	-0.79555	0.009	0.34	########	M1	CDH2
PODXL2.4	########	-0.80804	0.024	0.365	########	M1	PODXL2
CYTH4.4	########	0.665573	0.298	0.075	########	M1	CYTH4
BIN2.4	########	0.661306	0.299	0.075	########	M1	BIN2

NEAT1.2	########	0.364709	0.98	0.719	########	M1	NEAT1
GOLM1.3	########	-0.77864	0.029	0.373	########	M1	GOLM1
CD320.2	########	-0.77625	0.03	0.376	########	M1	CD320
MYL12A.3	########	0.473077	0.755	0.501	########	M1	MYL12A
CLIC1.2	########	0.425668	0.844	0.578	########	M1	CLIC1
RB1.4	########	0.749637	0.524	0.261	########	M1	RB1
CXXC5.4	########	-0.79164	0.076	0.441	########	M1	CXXC5
NRROS.3	########	0.664571	0.215	0.038	########	M1	NRROS
KDM6B.4	########	0.787288	0.435	0.18	########	M1	KDM6B
PLK3.4	########	0.779928	0.453	0.198	########	M1	PLK3
NPL.3	########	0.660922	0.427	0.155	########	M1	NPL
RP6-91H8.	########	0.6831	0.191	0.029	########	M1	RP6-91H8.3
MERTK.3	########	0.697314	0.312	0.087	########	M1	MERTK
SGCB.1	########	-0.81387	0.039	0.383	########	M1	SGCB
DBN1.4	########	-0.8001	0.031	0.367	########	M1	DBN1
LYN.4	########	0.678818	0.384	0.132	########	M1	LYN
ZFP36L1.1	########	0.473851	0.759	0.52	########	M1	ZFP36L1
SLC31A2.4	########	0.724529	0.411	0.149	########	M1	SLC31A2
LILRA2.4	########	0.635021	0.246	0.051	########	M1	LILRA2
SELPLG.3	########	0.75353	0.308	0.084	########	M1	SELPLG
TMEM176A.	########	0.699593	0.455	0.181	########	M1	TMEM176A
OCIAD2.3	########	-1.12533	0.092	0.446	########	M1	OCIAD2
ABL2.4	########	0.817081	0.476	0.226	########	M1	ABL2
TSC22D3.3	########	0.601463	0.666	0.414	########	M1	TSC22D3
TCEAL7.2	########	-0.8715	0.026	0.358	########	M1	TCEAL7
CREG1.4	########	0.682513	0.52	0.252	########	M1	CREG1
HMHA1.4	########	0.64657	0.287	0.073	########	M1	HMHA1
DDR1.3	########	-0.77238	0.046	0.388	########	M1	DDR1
COTL1.4	########	0.554837	0.686	0.474	########	M1	COTL1
EGR2.3	########	0.861939	0.388	0.152	########	M1	EGR2
MT3.4	########	-1.13406	0.208	0.547	########	M1	MT3
PMP2.4	########	-1.04094	0.068	0.406	########	M1	PMP2
GRASP.4	########	0.57487	0.179	0.026	########	M1	GRASP
EGR3.3	########	0.744952	0.274	0.07	########	M1	EGR3
RHOBTB3.4	########	-0.73125	0.103	0.47	########	M1	RHOBTB3
BAALC.3	########	-0.84069	0.06	0.403	########	M1	BAALC
PTGS2.4	########	0.574076	0.199	0.034	########	M1	PTGS2
JUNB.2	########	0.386551	0.856	0.697	########	M1	JUNB
TNFAIP3.4	########	0.708773	0.368	0.122	########	M1	TNFAIP3
NES.4	########	-0.93768	0.013	0.329	########	M1	NES
ZBTB20.3	########	-0.71357	0.084	0.446	########	M1	ZBTB20
TSPAN7.2	########	-0.86175	0.034	0.366	########	M1	TSPAN7
CRIP2.2	########	-0.78052	0.034	0.37	########	M1	CRIP2
PDGFB.3	########	0.612048	0.165	0.022	########	M1	PDGFB
MAPT.4	########	-0.81872	0.018	0.338	########	M1	MAPT
GAS6.4	########	0.704327	0.411	0.163	########	M1	GAS6
SP100.3	########	0.623082	0.519	0.24	########	M1	SP100
SEZ6L2.3	########	-0.76304	0.011	0.326	########	M1	SEZ6L2
CMTM7.4	########	0.689117	0.363	0.124	########	M1	CMTM7
SKIL.4	########	0.763215	0.485	0.246	########	M1	SKIL
CSRP2.2	########	-1.05494	0.03	0.356	########	M1	CSRP2

CMTM6.4	########	0.66099	0.608	0.389	########	M1	CMTM6
RNF130.4	########	0.599959	0.647	0.426	########	M1	RNF130
ZFP36L2.4	########	0.542692	0.7	0.478	########	M1	ZFP36L2
CD55.4	########	0.676239	0.298	0.082	########	M1	CD55
XBP1.4	########	0.684723	0.57	0.331	########	M1	XBP1
SCAMP2.4	########	0.677932	0.578	0.333	########	M1	SCAMP2
S100A16.3	########	-1.09315	0.036	0.361	########	M1	S100A16
DRAM2.4	########	0.744446	0.543	0.295	########	M1	DRAM2
SPINT2.4	########	0.66503	0.438	0.172	########	M1	SPINT2
LYZ.4	########	0.649437	0.433	0.161	########	M1	LYZ
SERPINE2.	########	-0.79574	0.026	0.347	########	M1	SERPINE2
SOX11.3	########	-0.92595	0.017	0.324	########	M1	SOX11
TMEM156.4	########	0.574555	0.185	0.03	########	M1	TMEM156
GAL3ST4.3	########	0.735407	0.371	0.131	########	M1	GAL3ST4
MYL6B.3	########	-0.67283	0.132	0.504	########	M1	MYL6B
KIF5C.4	########	-0.74347	0.013	0.319	########	M1	KIF5C
FKBP5.4	########	0.672337	0.532	0.273	########	M1	FKBP5
C1orf54.4	########	0.689453	0.436	0.183	########	M1	C1orf54
SH3BGRL.2	########	0.44044	0.778	0.622	########	M1	SH3BGRL
SLC29A1.3	########	0.710322	0.321	0.101	########	M1	SLC29A1
RASSF4.4	########	0.679602	0.537	0.287	########	M1	RASSF4
SYT11.4	########	-0.68376	0.091	0.443	########	M1	SYT11
OGFRL1.4	########	0.759479	0.373	0.143	########	M1	OGFRL1
SCG3.4	########	-0.88797	0.014	0.317	########	M1	SCG3
SCG2.3	########	-0.90173	0.012	0.312	########	M1	SCG2
ENAH.3	########	-0.67842	0.013	0.314	########	M1	ENAH
CFL2.3	########	-0.77471	0.055	0.383	########	M1	CFL2
MEST.3	########	-0.76089	0.103	0.443	########	M1	MEST
AKAP13.4	########	0.743367	0.521	0.286	########	M1	AKAP13
DDIT4.3	########	0.594853	0.63	0.364	########	M1	DDIT4
RP11-76E1	########	0.557181	0.14	0.016	########	M1	RP11-76E17.3
MKNK1.3	########	0.718933	0.363	0.132	########	M1	MKNK1
FAM229B.3	########	-0.71955	0.032	0.344	########	M1	FAM229B
EEF1B2.3	########	0.28725	0.87	0.757	########	M1	EEF1B2
MEF2A.4	########	0.742034	0.412	0.176	########	M1	MEF2A
GSTA4.3	########	-0.76182	0.033	0.341	########	M1	GSTA4
CRMP1.3	########	-0.80557	0.017	0.315	########	M1	CRMP1
STMN4.3	########	-1.07235	0.05	0.357	########	M1	STMN4
NLRP1.3	########	-0.71395	0.066	0.393	########	M1	NLRP1
TSPAN5.4	########	-0.69019	0.015	0.312	########	M1	TSPAN5
CELF2.4	########	0.693387	0.569	0.346	########	M1	CELF2
BTG2.2	########	0.565749	0.654	0.43	########	M1	BTG2
MEG3.3	########	-0.99208	0.047	0.356	########	M1	MEG3
GLDN.3	########	0.573385	0.257	0.065	########	M1	GLDN
POP7.3	########	-0.65981	0.104	0.452	########	M1	POP7
BTK.4	########	0.599047	0.236	0.056	########	M1	BTK
ATAT1.4	########	-0.69957	0.033	0.34	########	M1	ATAT1
PCBP4.4	########	-0.69725	0.012	0.304	########	M1	PCBP4
ATP6V0B.4	########	0.327945	0.895	0.779	########	M1	ATP6V0B
CADM4.3	########	-0.67164	0.017	0.313	########	M1	CADM4
STMN1.3	########	-0.73407	0.371	0.683	########	M1	STMN1

TNF.2	########	0.566098	0.153	0.021	########	M1	TNF
STAB1.3	########	0.631679	0.36	0.122	########	M1	STAB1
NREP.3	########	-0.71453	0.024	0.322	########	M1	NREP
LIMA1.3	########	-0.74206	0.057	0.37	########	M1	LIMA1
FZD3.1	########	-0.64902	0.025	0.327	########	M1	FZD3
TRIB2.4	########	-0.72163	0.018	0.312	########	M1	TRIB2
ENO2.4	########	-0.73672	0.031	0.335	########	M1	ENO2
SPTBN1.2	########	-0.65172	0.024	0.325	########	M1	SPTBN1
SOX9.3	########	-0.80549	0.026	0.321	########	M1	SOX9
LGMN.3	########	0.625972	0.547	0.311	########	M1	LGMN
FGD4.4	########	0.725455	0.325	0.111	########	M1	FGD4
ZNF428.4	########	-0.62438	0.265	0.601	########	M1	ZNF428
CENPV.4	########	-0.72056	0.014	0.303	########	M1	CENPV
GPR84.4	########	0.638814	0.212	0.046	########	M1	GPR84
RAP1A.4	########	0.52868	0.677	0.483	########	M1	RAP1A
PCDH9.2	########	-0.82316	0.014	0.303	########	M1	PCDH9
GNA13.4	########	0.743078	0.364	0.143	########	M1	GNA13
KLHDC8A.4	########	-0.73551	0.013	0.298	########	M1	KLHDC8A
PIK3IP1.4	########	0.643044	0.35	0.123	########	M1	PIK3IP1
AP1B1.4	########	0.702817	0.456	0.219	########	M1	AP1B1
DNER.2	########	-0.82751	0.036	0.329	########	M1	DNER
NCKAP1L.4	########	0.608467	0.293	0.087	########	M1	NCKAP1L
ARHGAP4.4	########	0.593673	0.283	0.082	########	M1	ARHGAP4
SCIMP.4	########	0.61678	0.227	0.054	########	M1	SCIMP
SLC25A4.4	########	-0.64055	0.136	0.485	########	M1	SLC25A4
DAB2.3	########	0.625628	0.337	0.113	########	M1	DAB2
PKIG.4	########	-0.72529	0.053	0.362	########	M1	PKIG
TMEM59L.3	########	-0.66118	0.02	0.306	########	M1	TMEM59L
MAGED1.3	########	-0.65198	0.076	0.399	########	M1	MAGED1
CTB-61M7.	########	0.502446	0.14	0.018	########	M1	CTB-61M7.2
CCL3L3.2	########	0.757989	0.175	0.03	########	M1	CCL3L3
CLEC4E.3	########	0.640134	0.18	0.033	########	M1	CLEC4E
THY1.3	########	-0.73797	0.02	0.303	########	M1	THY1
MLXIPL.2	########	0.496204	0.142	0.019	########	M1	MLXIPL
PRDM1.4	########	0.612451	0.254	0.069	########	M1	PRDM1
GAPDH.4	########	-0.38028	0.972	0.987	########	M1	GAPDH
GAA.3	########	0.663342	0.505	0.282	########	M1	GAA
ARHGAP15.	########	0.607071	0.251	0.066	########	M1	ARHGAP15
LYL1.4	########	0.559906	0.223	0.053	########	M1	LYL1
C7orf55.3	########	-0.6365	0.135	0.481	########	M1	C7orf55
TLR5.3	########	0.516905	0.154	0.023	########	M1	TLR5
FRMD4A.3	########	0.709871	0.374	0.15	########	M1	FRMD4A
PLAC8.3	########	0.558227	0.2	0.042	########	M1	PLAC8
INPP5D.4	########	0.602593	0.23	0.056	########	M1	INPP5D
PAFAH1B3.	########	-0.60267	0.143	0.48	########	M1	PAFAH1B3
SPARC.3	########	-0.87274	0.117	0.438	########	M1	SPARC
RNF144B.3	########	0.645652	0.251	0.068	########	M1	RNF144B
PFKFB3.4	########	0.725134	0.43	0.207	########	M1	PFKFB3
HEY1.3	########	-0.69952	0.017	0.293	########	M1	HEY1
NRN1.2	########	-0.78674	0.021	0.3	########	M1	NRN1
RP11-386I	########	0.62465	0.194	0.041	########	M1	RP11-386I14.4

SLC22A17.	########	-0.62711	0.04	0.333	########	M1	SLC22A17
TXN.3	########	-0.59955	0.398	0.72	########	M1	TXN
GPC1.3	########	-0.75997	0.012	0.282	########	M1	GPC1
TLR7.3	########	0.536689	0.174	0.032	########	M1	TLR7
CYFIP1.3	########	0.666846	0.466	0.246	########	M1	CYFIP1
TMEM98.2	########	-0.69968	0.012	0.28	########	M1	TMEM98
NCF1.4	########	0.544768	0.292	0.089	########	M1	NCF1
PRAF2.3	########	-0.59543	0.053	0.352	########	M1	PRAF2
UNC93B1.4	########	0.671791	0.382	0.164	########	M1	UNC93B1
S100B.3	########	-0.80218	0.263	0.581	########	M1	S100B
PSIP1.4	########	-0.61151	0.117	0.437	########	M1	PSIP1
FMNL1.4	########	0.616981	0.288	0.091	########	M1	FMNL1
DNASE2.3	########	0.629285	0.549	0.35	########	M1	DNASE2
CKLF.4	########	0.520789	0.67	0.489	########	M1	CKLF
CSTA.4	########	0.578109	0.298	0.094	########	M1	CSTA
DSE.4	########	0.644847	0.305	0.104	########	M1	DSE
ARHGAP18.	########	0.622086	0.357	0.135	########	M1	ARHGAP18
DSTN.4	########	-0.56377	0.352	0.672	########	M1	DSTN
POLD2.3	########	-0.62202	0.141	0.477	########	M1	POLD2
RHOB.2	########	0.447022	0.742	0.582	########	M1	RHOB
POU3F2.3	########	-0.67344	0.003	0.259	########	M1	POU3F2
CTTN.3	########	-0.62578	0.011	0.273	########	M1	CTTN
CTSL.4	########	0.445322	0.7	0.488	########	M1	CTSL
TTYH1.4	########	-0.84365	0.039	0.314	########	M1	TTYH1
PHF14.3	########	-0.55084	0.143	0.479	########	M1	PHF14
NFIA.3	########	-0.68252	0.12	0.431	########	M1	NFIA
RCN1.4	########	-0.64077	0.097	0.406	########	M1	RCN1
TRIM2.2	########	-0.61828	0.008	0.267	########	M1	TRIM2
ARPC2.3	########	0.274335	0.861	0.756	########	M1	ARPC2
PTOV1.3	########	-0.57265	0.16	0.494	########	M1	PTOV1
RIC3.2	########	-0.73613	0.013	0.275	########	M1	RIC3
TMEM161B-	########	-0.7005	0.052	0.336	########	M1	TMEM161B-AS1
IP6K2.3	########	-0.58538	0.152	0.491	########	M1	IP6K2
PTPRE.4	########	0.772744	0.443	0.235	########	M1	PTPRE
YWHAQ.3	########	-0.51741	0.401	0.692	########	M1	YWHAQ
PLS3.3	########	-0.66556	0.008	0.264	########	M1	PLS3
ATP1B3.4	########	0.532362	0.664	0.521	########	M1	ATP1B3
METRN.4	########	-0.68143	0.056	0.342	########	M1	METRN
NAGA.4	########	0.666804	0.362	0.149	########	M1	NAGA
RUFY3.3	########	-0.574	0.061	0.354	########	M1	RUFY3
FIBIN.3	########	-0.74044	0.012	0.27	########	M1	FIBIN
DEF6.4	########	0.514639	0.234	0.062	########	M1	DEF6
CHMP1B.4	########	0.66214	0.583	0.409	########	M1	CHMP1B
IGFBP5.4	########	-1.01015	0.041	0.314	########	M1	IGFBP5
TNFAIP8L2	########	0.540696	0.24	0.065	########	M1	TNFAIP8L2
ALDH2.4	########	0.629907	0.547	0.336	########	M1	ALDH2
SRPK2.2	########	-0.56399	0.138	0.465	########	M1	SRPK2
PGM2L1.3	########	-0.64898	0.03	0.301	########	M1	PGM2L1
LHFP.4	########	-0.68213	0.016	0.275	########	M1	LHFP
CLEC9A.3	########	0.500596	0.122	0.015	########	M1	CLEC9A
TLR1.4	########	0.552664	0.192	0.043	########	M1	TLR1

NME1.2	########	-0.58404	0.296	0.622	########	M1	NME1
CARHSP1.3	########	-0.59809	0.15	0.478	########	M1	CARHSP1
NRCAM.3	########	-0.57631	0.038	0.316	########	M1	NRCAM
IGF1.3	########	0.542917	0.18	0.038	########	M1	IGF1
BEX2.3	########	-0.80815	0.035	0.302	########	M1	BEX2
SMARCA4.3	########	-0.55402	0.104	0.412	########	M1	SMARCA4
ITM2B.3	########	0.256653	0.871	0.785	########	M1	ITM2B
THBD.4	########	0.600628	0.236	0.066	########	M1	THBD
CHN1.3	########	-0.57181	0.009	0.257	########	M1	CHN1
C12orf57.	########	-0.58004	0.241	0.596	########	M1	C12orf57
CLIC4.3	########	-0.61255	0.082	0.383	########	M1	CLIC4
CDO1.4	########	-0.71633	0.02	0.278	########	M1	CDO1
AP005530.	########	0.363424	0.112	0.012	########	M1	AP005530.2
NT5C3B.3	########	-0.53693	0.083	0.382	########	M1	NT5C3B
TMSB4X.4	########	-0.32756	1	0.996	########	M1	TMSB4X
MT1M.2	########	-0.86897	0.027	0.287	########	M1	MT1M
CLU.4	########	-0.73029	0.62	0.815	########	M1	CLU
TAL1.2	########	0.487872	0.124	0.016	########	M1	TAL1
ACTB.4	########	-0.31832	0.994	0.992	########	M1	ACTB
MAP1LC3A.	########	-0.58565	0.113	0.416	########	M1	MAP1LC3A
ID4.3	########	-0.73824	0.01	0.257	########	M1	ID4
TRAF4.3	########	-0.59728	0.043	0.314	########	M1	TRAF4
B4GALT1.4	########	0.638898	0.274	0.089	########	M1	B4GALT1
SPARCL1.3	########	-0.92409	0.108	0.393	########	M1	SPARCL1
PTPRS.4	########	-0.64797	0.036	0.302	########	M1	PTPRS
HMGB3.3	########	-0.63374	0.05	0.325	########	M1	HMGB3
ATP6V0E1.	########	0.240289	0.856	0.741	########	M1	ATP6V0E1
RBFOX2.3	########	-0.62062	0.011	0.257	########	M1	RBFOX2
COL6A1.3	########	-0.65281	0.018	0.271	########	M1	COL6A1
STOM.3	########	0.575701	0.44	0.213	########	M1	STOM
IKZF1.4	########	0.504137	0.184	0.041	########	M1	IKZF1
EGFR.3	########	-0.73254	0.068	0.347	########	M1	EGFR
SFMBT2.4	########	0.58935	0.206	0.052	########	M1	SFMBT2
KLHL6.4	########	0.531554	0.156	0.029	########	M1	KLHL6
NANS.4	########	0.620178	0.53	0.325	########	M1	NANS
ANXA11.4	########	0.600766	0.396	0.18	########	M1	ANXA11
HLA-F.4	########	0.609733	0.311	0.113	########	M1	HLA-F
VAV1.4	########	0.510926	0.199	0.049	########	M1	VAV1
SKAP2.3	########	0.577467	0.46	0.232	########	M1	SKAP2
GCSH.4	########	-0.60421	0.103	0.396	########	M1	GCSH
RPS6KA1.4	########	0.569375	0.288	0.099	########	M1	RPS6KA1
CHPF.3	########	-0.54461	0.038	0.303	########	M1	CHPF
GLIPR1.3	########	0.575173	0.521	0.297	########	M1	GLIPR1
ETV1.4	########	-0.67019	0.031	0.287	########	M1	ETV1
TGFB1.4	########	0.655856	0.415	0.21	########	M1	TGFB1
MSI2.2	########	-0.58213	0.081	0.37	########	M1	MSI2
PPP2R2B.3	########	-0.64034	0.014	0.26	########	M1	PPP2R2B
FKBP10.4	########	-0.5836	0.011	0.252	########	M1	FKBP10
POLR2I.3	########	-0.58227	0.27	0.609	########	M1	POLR2I
RIN3.4	########	0.558722	0.265	0.085	########	M1	RIN3
LMO4.4	########	-0.57883	0.218	0.53	########	M1	LMO4

FJX1.3	########	-0.63182	0.006	0.241	########	M1	FJX1
SV2A.2	########	-0.51276	0.006	0.241	########	M1	SV2A
NABP1.3	########	0.568466	0.278	0.093	########	M1	NABP1
MILR1.4	########	0.585886	0.24	0.07	########	M1	MILR1
PNMA1.3	########	-0.5273	0.056	0.329	########	M1	PNMA1
KCNQ2.3	########	-0.59155	0.013	0.255	########	M1	KCNQ2
MAP1A.3	########	-0.59231	0.022	0.271	########	M1	MAP1A
FAM181B.4	########	-0.64582	0.008	0.243	########	M1	FAM181B
GRIA2.3	########	-0.60916	0.013	0.252	########	M1	GRIA2
PBX1.3	########	-0.5576	0.014	0.254	########	M1	PBX1
REEP2.2	########	-0.50929	0.007	0.241	########	M1	REEP2
RP11-553L	########	-0.5228	0.021	0.267	########	M1	RP11-553L6.5
PPP1R14B.	########	-0.57965	0.103	0.393	########	M1	PPP1R14B
TRIM9.3	########	-0.53976	0.024	0.274	########	M1	TRIM9
SIGLEC9.4	########	0.514188	0.23	0.065	########	M1	SIGLEC9
TTC3.3	########	-0.5401	0.261	0.567	########	M1	TTC3
YWHAH.4	########	0.440319	0.662	0.546	########	M1	YWHAH
SLC39A6.2	########	-0.49959	0.087	0.376	########	M1	SLC39A6
JDP2.4	########	0.545334	0.19	0.047	########	M1	JDP2
ENG.4	########	0.556274	0.333	0.131	########	M1	ENG
LHFPL2.3	########	0.635866	0.319	0.129	########	M1	LHFPL2
RAB3IP.2	########	-0.61143	0.035	0.289	########	M1	RAB3IP
HLA-B.3	########	0.139776	0.991	0.824	########	M1	HLA-B
APBA2.4	########	-0.51571	0.013	0.246	########	M1	APBA2
IL6R.4	########	0.5674	0.214	0.06	########	M1	IL6R
AES.3	########	-0.52255	0.152	0.462	########	M1	AES
SEMA6A.3	########	-0.52234	0.009	0.238	########	M1	SEMA6A
SPP1.2	########	0.162266	0.938	0.837	########	M1	SPP1
STK17B.4	########	0.5963	0.286	0.102	########	M1	STK17B
TCIRG1.4	########	0.539517	0.276	0.095	########	M1	TCIRG1
CRYAB.4	########	-0.98429	0.261	0.548	########	M1	CRYAB
DOCK2.4	########	0.501099	0.201	0.052	########	M1	DOCK2
FERMT2.4	########	-0.49398	0.011	0.241	########	M1	FERMT2
GLRX.3	########	0.472489	0.576	0.351	########	M1	GLRX
TREM1.3	########	0.482531	0.339	0.129	########	M1	TREM1
RCN2.3	########	-0.5264	0.213	0.518	########	M1	RCN2
PLVAP.2	########	0.373241	0.109	0.014	########	M1	PLVAP
CCDC167.2	########	-0.53683	0.134	0.433	########	M1	CCDC167
MANBAL.4	########	-0.49473	0.096	0.383	########	M1	MANBAL
COPS8.4	########	-0.52988	0.197	0.524	########	M1	COPS8
HEPN1.3	########	-0.71623	0.014	0.243	########	M1	HEPN1
RGMA.3	########	-0.57374	0.011	0.238	########	M1	RGMA
CCND2.4	########	-0.5791	0.095	0.373	########	M1	CCND2
SLC7A5.4	########	0.621372	0.289	0.108	########	M1	SLC7A5
SCRG1.3	########	-0.71214	0.119	0.391	########	M1	SCRG1
FNDC4.2	########	-0.49944	0.01	0.236	########	M1	FNDC4
TCEA2.3	########	-0.50783	0.124	0.417	########	M1	TCEA2
CLN8.4	########	0.674484	0.433	0.236	########	M1	CLN8
GAPT.4	########	0.481446	0.17	0.038	########	M1	GAPT
SEPT7.4	########	-0.51152	0.476	0.748	########	M1	7-Sep
CCNH.4	########	0.637531	0.555	0.383	########	M1	CCNH

CD81.3	########	0.529612	0.592	0.408	########	M1	CD81
LARP6.3	########	-0.56763	0.007	0.23	########	M1	LARP6
FAM177B.2	########	0.3971	0.111	0.015	########	M1	FAM177B
MAPK10.3	########	-0.55353	0.009	0.232	########	M1	MAPK10
HLA-C.2	########	0.177469	0.941	0.775	########	M1	HLA-C
PRAM1.4	########	0.478609	0.156	0.032	########	M1	PRAM1
NBEAL1.2	########	0.318045	0.798	0.638	########	M1	NBEAL1
PCDH17.2	########	-0.53933	0.007	0.228	########	M1	PCDH17
TMEM52B.4	########	0.472936	0.145	0.028	########	M1	TMEM52B
FGD2.4	########	0.507105	0.17	0.039	########	M1	FGD2
TTLL7.4	########	-0.45952	0.033	0.278	########	M1	TTLL7
FBP1.4	########	0.485547	0.265	0.087	########	M1	FBP1
TAF7.3	########	-0.51431	0.278	0.609	########	M1	TAF7
DKK3.2	########	-0.51274	0.012	0.237	########	M1	DKK3
NCKAP1.2	########	-0.45521	0.006	0.225	########	M1	NCKAP1
MEIS2.3	########	-0.67041	0.015	0.241	########	M1	MEIS2
SLAIN1.4	########	-0.62008	0.015	0.241	########	M1	SLAIN1
CASP1.3	########	0.557558	0.4	0.191	########	M1	CASP1
VGLL4.2	########	-0.48519	0.088	0.363	########	M1	VGLL4
DCX.4	########	-0.69281	0.017	0.242	########	M1	DCX
YWHAE.3	########	-0.37389	0.589	0.785	########	M1	YWHAE
IL1RN.4	########	0.544593	0.194	0.051	########	M1	IL1RN
C1orf21.4	########	-0.55118	0.013	0.238	########	M1	C1orf21
COL9A3.4	########	-0.65548	0.006	0.222	########	M1	COL9A3
C10orf11.	########	0.515954	0.234	0.074	########	M1	C10orf11
DYNC1LI2.	########	-0.47255	0.106	0.392	########	M1	DYNC1LI2
WDR54.4	########	-0.52248	0.034	0.274	########	M1	WDR54
GGH.2	########	-0.54645	0.053	0.305	########	M1	GGH
VCAN.2	########	-0.58248	0.043	0.287	########	M1	VCAN
CCDC28B.3	########	-0.48746	0.024	0.258	########	M1	CCDC28B
PRR13.2	########	0.408003	0.675	0.517	########	M1	PRR13
CA2.2	########	-0.82398	0.032	0.264	########	M1	CA2
SRP9.2	########	-0.43691	0.434	0.73	########	M1	SRP9
IL4R.4	########	0.514298	0.185	0.048	########	M1	IL4R
KLHL7.2	########	-0.47383	0.064	0.322	########	M1	KLHL7
BLVRA.3	########	-0.55631	0.107	0.386	########	M1	BLVRA
ACOT7.3	########	-0.54894	0.032	0.266	########	M1	ACOT7
RASAL3.4	########	0.437644	0.162	0.036	########	M1	RASAL3
SYK.4	########	0.504943	0.243	0.078	########	M1	SYK
GPRC5B.2	########	-0.54123	0.026	0.257	########	M1	GPRC5B
THRA.2	########	-0.56378	0.04	0.279	########	M1	THRA
AP1S1.4	########	-0.51212	0.207	0.52	########	M1	AP1S1
BUD31.4	########	-0.49188	0.362	0.681	########	M1	BUD31
PNRC1.4	########	0.43653	0.64	0.52	########	M1	PNRC1
RNF24.1	########	-0.48992	0.045	0.289	########	M1	RNF24
MT1E.4	########	-0.85281	0.156	0.425	########	M1	MT1E
DOCK4.3	########	0.638512	0.311	0.132	########	M1	DOCK4
EFEMP2.2	########	-0.52977	0.025	0.252	########	M1	EFEMP2
DSEL.4	########	-0.5549	0.032	0.264	########	M1	DSEL
POU2F2.4	########	0.586145	0.281	0.106	########	M1	POU2F2
WRB.3	########	-0.47629	0.164	0.471	########	M1	WRB

CREB5.3	########	-0.53005	0.037	0.275	########	M1		CREB5
BOD1.3	########	-0.46132	0.08	0.347	########	M1		BOD1
MEX3A.3	########	-0.54243	0.004	0.213	########	M1		MEX3A
TCTEX1D2.	########	-0.53816	0.033	0.266	########	M1		TCTEX1D2
RGS18.4	########	0.436361	0.152	0.032	########	M1		RGS18
LY96.4	########	0.56677	0.384	0.19	########	M1		LY96
ARHGAP9.4	########	0.495787	0.21	0.061	########	M1		ARHGAP9
SQLE.2	########	-0.46371	0.045	0.289	########	M1		SQLE
ITGB8.2	########	-0.4586	0.04	0.28	########	M1		ITGB8
ATP1B2.4	########	-0.5175	0.035	0.272	########	M1		ATP1B2
SIPA1.4	########	0.587665	0.281	0.111	########	M1		SIPA1
ICOSLG.4	########	0.555018	0.215	0.066	########	M1		ICOSLG
PYCR1.1	########	-0.46648	0.008	0.22	########	M1		PYCR1
FAM92A1.2	########	-0.49909	0.018	0.238	########	M1		FAM92A1
GNA15.4	########	0.501034	0.247	0.082	########	M1		GNA15
SMCO4.4	########	0.542251	0.277	0.104	########	M1		SMCO4
EMB.4	########	0.44187	0.193	0.051	########	M1		EMB
SLC1A3.3	########	0.453529	0.635	0.445	########	M1		SLC1A3
FLNA.3	########	-0.49344	0.048	0.291	########	M1		FLNA
MAGI1.4	########	-0.53256	0.016	0.234	########	M1		MAGI1
HIF1A.3	########	0.478409	0.61	0.474	########	M1		HIF1A
ARC.2	########	-0.69775	0.025	0.245	########	M1		ARC
NELFCD.2	########	-0.46651	0.108	0.383	########	M1		NELFCD
RP11-620J	########	-0.64036	0.066	0.311	########	M1		RP11-620J15.3
RP11-556E	########	0.451313	0.156	0.035	########	M1		RP11-556E13.1
PAICS.3	########	-0.52228	0.088	0.352	########	M1		PAICS
F3.4	########	-0.56041	0.013	0.227	########	M1		F3
FGR.4	########	0.502686	0.204	0.059	########	M1		FGR
ASCL1.3	########	-0.64868	0.011	0.221	########	M1		ASCL1
BCHE.2	########	-0.54855	0.011	0.222	########	M1		BCHE
FBXO21.2	########	-0.46497	0.087	0.352	########	M1		FBXO21
POLD4.2	########	0.449963	0.577	0.39	########	M1		POLD4
CBX1.3	########	-0.50018	0.134	0.414	1.01E-99	M1		CBX1
TCOF1.4	########	0.714088	0.342	0.165	1.14E-99	M1		TCOF1
SPAG16.4	########	-0.4938	0.079	0.34	2.12E-99	M1		SPAG16
CADM2.3	########	-0.60648	0.013	0.223	2.13E-99	M1		CADM2
ZNF302.3	########	-0.41998	0.088	0.36	3.10E-99	M1		ZNF302
DCLK2.4	########	-0.49748	0.017	0.232	3.25E-99	M1		DCLK2
SRI.4	########	-0.49514	0.535	0.768	7.14E-99	M1		SRI
RP11-489C	########	0.363053	0.101	0.013	1.02E-98	M1		RP11-489O18.1
TNC.3	########	-0.61732	0.019	0.237	1.16E-98	M1		TNC
AKR1A1.2	########	0.49553	0.616	0.474	1.53E-98	M1		AKR1A1
LRRC25.4	########	0.503774	0.219	0.067	1.79E-98	M1		LRRC25
PTRF.4	########	-0.50809	0.011	0.22	1.83E-98	M1		PTRF
PPIC.2	########	-0.53464	0.01	0.218	2.14E-98	M1		PPIC
CAPN2.1	########	-0.4569	0.051	0.293	2.71E-98	M1		CAPN2
SYTL3.4	########	0.504018	0.172	0.043	3.35E-98	M1		SYTL3
PLTP.2	########	0.444856	0.61	0.442	4.26E-98	M1		PLTP
NAE1.2	########	-0.47632	0.111	0.386	4.65E-98	M1		NAE1
CSNK1E.3	########	-0.43438	0.087	0.35	9.04E-98	M1		CSNK1E
TCF4.4	########	-0.50522	0.287	0.57	1.85E-97	M1		TCF4

KIF3A.2	########	-0.48637	0.041	0.273	2.74E-97	M1	KIF3A
OLFML2B.3	########	0.541351	0.177	0.046	3.70E-97	M1	OLFML2B
NDUFA5.2	########	-0.4587	0.311	0.63	4.20E-97	M1	NDUFA5
LYPD1.2	########	-0.55674	0.018	0.231	7.34E-97	M1	LYPD1
MYEF2.3	########	-0.43491	0.017	0.229	8.36E-97	M1	MYEF2
SAMHD1.4	########	0.579373	0.373	0.183	9.89E-97	M1	SAMHD1
IL16.4	########	0.459431	0.178	0.046	1.09E-96	M1	IL16
DNAAF1.3	########	0.457891	0.098	0.013	1.35E-96	M1	DNAAF1
PRR7.4	########	-0.43033	0.014	0.223	1.72E-96	M1	PRR7
SPATS2.2	########	-0.42592	0.056	0.299	1.87E-96	M1	SPATS2
HOPX.3	########	-0.90969	0.092	0.321	2.28E-96	M1	HOPX
TBCA.3	########	-0.44573	0.492	0.754	2.41E-96	M1	TBCA
GLUL.1	########	0.154787	0.892	0.765	2.45E-96	M1	GLUL
CLIP3.3	########	-0.4866	0.019	0.233	2.49E-96	M1	CLIP3
TAGLN3.3	########	-0.67882	0.016	0.222	3.56E-96	M1	TAGLN3
PLCB2.4	########	0.463153	0.165	0.04	4.11E-96	M1	PLCB2
LGALS3.3	########	-0.84324	0.271	0.523	4.72E-96	M1	LGALS3
UBA7.4	########	0.546864	0.198	0.058	4.82E-96	M1	UBA7
C1R.4	########	-0.8736	0.032	0.252	4.83E-96	M1	C1R
MAPK8IP1.	########	-0.50241	0.016	0.226	5.83E-96	M1	MAPK8IP1
SRGAP2.3	########	0.706023	0.378	0.203	6.20E-96	M1	SRGAP2
ENC1.4	########	-0.555	0.038	0.262	9.34E-96	M1	ENC1
RND3.3	########	-0.5963	0.049	0.282	9.65E-96	M1	RND3
PTMS.3	########	-0.50378	0.354	0.593	1.09E-95	M1	PTMS
KB-1507C5	########	0.341359	0.09	0.01	1.21E-95	M1	KB-1507C5.4
ISCU.3	########	0.419764	0.652	0.549	1.46E-95	M1	ISCU
CKAP4.2	1.09E-99	-0.42491	0.027	0.246	1.64E-95	M1	CKAP4
BNIP3.3	1.18E-99	-0.59518	0.031	0.252	1.76E-95	M1	BNIP3
SRSF5.1	1.34E-99	0.342752	0.763	0.68	2.00E-95	M1	SRSF5
ST3GAL6.4	1.50E-99	0.610742	0.241	0.085	2.24E-95	M1	ST3GAL6
AK1.2	1.58E-99	-0.43263	0.109	0.381	2.36E-95	M1	AK1
TGIF1.3	1.99E-99	0.631399	0.496	0.33	2.97E-95	M1	TGIF1
CDC42BPA.	2.04E-99	-0.43372	0.01	0.213	3.05E-95	M1	CDC42BPA
SMAD1.2	2.19E-99	-0.45808	0.009	0.211	3.27E-95	M1	SMAD1
SCOC.3	2.72E-99	-0.45273	0.169	0.463	4.07E-95	M1	SCOC
TMSB15A.4	7.05E-99	-0.60145	0.011	0.212	1.05E-94	M1	TMSB15A
MBOAT2.3	7.18E-99	-0.45097	0.012	0.216	1.07E-94	M1	MBOAT2
IGFBP7.2	7.87E-99	-0.73382	0.074	0.308	1.18E-94	M1	IGFBP7
TMC6.4	7.91E-99	0.499331	0.244	0.084	1.18E-94	M1	TMC6
GRIA3.3	1.90E-98	-0.44019	0.005	0.201	2.84E-94	M1	GRIA3
NR2F1.1	2.27E-98	-0.42981	0.003	0.198	3.40E-94	M1	NR2F1
EEF1D.4	2.61E-98	0.190527	0.926	0.829	3.89E-94	M1	EEF1D
RAC3.3	4.74E-98	-0.43666	0.013	0.217	7.09E-94	M1	RAC3
MAGEF1.3	5.02E-98	-0.45225	0.131	0.41	7.51E-94	M1	MAGEF1
ODC1.3	5.42E-98	-0.51193	0.167	0.439	8.09E-94	M1	ODC1
HRAS.4	6.40E-98	-0.42921	0.082	0.336	9.56E-94	M1	HRAS
SKA2.2	7.60E-98	-0.4906	0.095	0.349	1.14E-93	M1	SKA2
HHEX.4	8.22E-98	0.471033	0.157	0.038	1.23E-93	M1	HHEX
AGT.2	8.72E-98	-0.70573	0.037	0.257	1.30E-93	M1	AGT
RHOA.2	1.03E-97	0.212997	0.824	0.741	1.54E-93	M1	RHOA
SIGLEC14.	1.29E-97	0.411822	0.133	0.027	1.93E-93	M1	SIGLEC14

PSAT1.3	1.40E-97	-0.56965	0.026	0.239	2.09E-93	M1	PSAT1
TPM3.4	2.19E-97	0.39767	0.656	0.539	3.27E-93	M1	TPM3
PRRX1.4	2.44E-97	-0.48829	0.007	0.204	3.64E-93	M1	PRRX1
CRNDE.1	4.17E-97	-0.4639	0.012	0.212	6.23E-93	M1	CRNDE
TMEM106B.	4.67E-97	-0.41566	0.107	0.375	6.97E-93	M1	TMEM106B
MFNG.4	6.49E-97	0.509445	0.265	0.099	9.69E-93	M1	MFNG
SEPT9.2	6.89E-97	-0.49801	0.122	0.385	1.03E-92	M1	9-Sep
SHISA4.3	7.24E-97	-0.48415	0.053	0.285	1.08E-92	M1	SHISA4
SIDT1.3	1.02E-96	0.407685	0.088	0.01	1.53E-92	M1	SIDT1
PGRMC1.2	1.27E-96	-0.42251	0.2	0.497	1.90E-92	M1	PGRMC1
AKAP12.4	1.54E-96	-0.54203	0.017	0.221	2.30E-92	M1	AKAP12
CBX5.3	1.57E-96	-0.44077	0.12	0.389	2.34E-92	M1	CBX5
FAIM2.3	1.83E-96	-0.56554	0.019	0.226	2.74E-92	M1	FAIM2
SRPX.3	2.13E-96	-0.59301	0.011	0.21	3.19E-92	M1	SRPX
ICAM1.3	3.42E-96	0.590698	0.309	0.135	5.11E-92	M1	ICAM1
HES6.3	3.49E-96	-0.64163	0.087	0.326	5.22E-92	M1	HES6
PXMP2.2	3.49E-96	-0.41447	0.038	0.26	5.22E-92	M1	PXMP2
CYSTM1.4	4.35E-96	-0.51237	0.109	0.368	6.50E-92	M1	CYSTM1
RGS17.2	4.68E-96	-0.48108	0.003	0.194	6.99E-92	M1	RGS17
PHYHIPL.2	5.01E-96	-0.62875	0.017	0.219	7.48E-92	M1	PHYHIPL
WDR34.2	6.52E-96	-0.41287	0.029	0.243	9.74E-92	M1	WDR34
ROBO1.4	9.29E-96	-0.45095	0.012	0.211	1.39E-91	M1	ROBO1
TCEAL2.2	9.59E-96	-0.69058	0.014	0.213	1.43E-91	M1	TCEAL2
DHPS.3	9.81E-96	-0.45606	0.199	0.497	1.47E-91	M1	DHPS
FUOM.4	1.02E-95	0.586418	0.281	0.113	1.52E-91	M1	FUOM
EPN2.4	1.04E-95	-0.44053	0.047	0.275	1.55E-91	M1	EPN2
TMSB15B.3	1.36E-95	-0.47043	0.014	0.215	2.03E-91	M1	TMSB15B
RGCC.3	1.38E-95	0.516137	0.415	0.217	2.06E-91	M1	RGCC
TIMM22.1	1.45E-95	-0.39608	0.05	0.281	2.17E-91	M1	TIMM22
SMARCD3.3	1.65E-95	-0.42566	0.044	0.269	2.46E-91	M1	SMARCD3
C8orf4.3	1.70E-95	-0.90279	0.045	0.264	2.54E-91	M1	C8orf4
LSP1.4	1.82E-95	0.485399	0.326	0.134	2.72E-91	M1	LSP1
TMEM45A.2	2.30E-95	-0.63235	0.012	0.209	3.43E-91	M1	TMEM45A
NAT14.1	5.89E-95	-0.40545	0.013	0.213	8.80E-91	M1	NAT14
TPST1.2	6.77E-95	-0.42075	0.048	0.278	1.01E-90	M1	TPST1
PLBD1.4	7.94E-95	0.497389	0.214	0.07	1.19E-90	M1	PLBD1
RAMP1.3	7.97E-95	-0.75995	0.194	0.456	1.19E-90	M1	RAMP1
CCR1.3	7.99E-95	0.512355	0.196	0.059	1.19E-90	M1	CCR1
HEXA.3	1.10E-94	0.544134	0.527	0.362	1.65E-90	M1	HEXA
ARHGAP30.	1.23E-94	0.472914	0.217	0.07	1.84E-90	M1	ARHGAP30
PTPN7.4	1.31E-94	0.489951	0.191	0.056	1.96E-90	M1	PTPN7
TMEM14A.3	2.49E-94	-0.46742	0.109	0.367	3.73E-90	M1	TMEM14A
MAD2L2.3	2.63E-94	-0.46393	0.164	0.438	3.93E-90	M1	MAD2L2
BCAR1.2	2.71E-94	-0.43294	0.01	0.204	4.04E-90	M1	BCAR1
CTXN1.4	2.88E-94	-0.40638	0.003	0.191	4.30E-90	M1	CTXN1
CDC42EP4.	3.38E-94	-0.50112	0.027	0.236	5.05E-90	M1	CDC42EP4
IGFBP3.2	4.43E-94	-0.79187	0.026	0.228	6.62E-90	M1	IGFBP3
PHGDH.3	5.45E-94	-0.6151	0.028	0.234	8.14E-90	M1	PHGDH
HSD11B1L.	6.85E-94	-0.41612	0.029	0.24	1.02E-89	M1	HSD11B1L
LRMP.3	7.14E-94	0.404507	0.131	0.027	1.07E-89	M1	LRMP
PDRG1.2	8.50E-94	-0.38495	0.067	0.308	1.27E-89	M1	PDRG1

SOX8.4	1.77E-93	-0.56579	0.014	0.21	2.65E-89	M1	SOX8
SEPT11.4	1.78E-93	-0.4381	0.094	0.345	2.66E-89	M1	11-Sep
PRKCD.4	1.85E-93	0.491976	0.191	0.058	2.77E-89	M1	PRKCD
POLR2J3.3	2.11E-93	-0.38577	0.117	0.383	3.15E-89	M1	POLR2J3
RP11-14N7	2.50E-93	-0.46499	0.03	0.24	3.74E-89	M1	RP11-14N7.2
NDN.4	2.88E-93	-0.44469	0.025	0.231	4.30E-89	M1	NDN
RALA.3	3.29E-93	-0.42898	0.135	0.405	4.92E-89	M1	RALA
MAP3K13.3	3.43E-93	-0.43039	0.157	0.441	5.13E-89	M1	MAP3K13
DTD1.4	4.50E-93	-0.42849	0.159	0.438	6.73E-89	M1	DTD1
SERP1.4	5.40E-93	0.360895	0.672	0.59	8.06E-89	M1	SERP1
NPAS3.4	5.55E-93	-0.43411	0.004	0.19	8.30E-89	M1	NPAS3
MAGEH1.3	6.39E-93	-0.45982	0.106	0.36	9.55E-89	M1	MAGEH1
AK4.4	6.70E-93	-0.48787	0.015	0.212	1.00E-88	M1	AK4
HILPDA.3	7.92E-93	-0.66832	0.045	0.263	1.18E-88	M1	HILPDA
LSAMP.2	9.28E-93	-0.5679	0.012	0.204	1.39E-88	M1	LSAMP
C3orf14.2	1.14E-92	-0.49452	0.071	0.303	1.71E-88	M1	C3orf14
MOB1A.4	1.16E-92	0.53832	0.481	0.312	1.73E-88	M1	MOB1A
F2R.4	1.35E-92	-0.39837	0.009	0.201	2.02E-88	M1	F2R
PER1.3	1.75E-92	0.585659	0.331	0.155	2.62E-88	M1	PER1
ZNF462.4	1.82E-92	-0.47815	0.014	0.209	2.72E-88	M1	ZNF462
IRAK3.4	2.07E-92	0.491623	0.181	0.052	3.10E-88	M1	IRAK3
CALU.3	2.08E-92	-0.44773	0.169	0.45	3.11E-88	M1	CALU
AREG.4	5.01E-92	0.563874	0.167	0.044	7.49E-88	M1	AREG
CNIH3.1	5.39E-92	-0.44839	0.01	0.2	8.05E-88	M1	CNIH3
ADRB2.2	1.04E-91	0.45345	0.133	0.028	1.56E-87	M1	ADRB2
DPP7.4	1.25E-91	0.472559	0.578	0.445	1.87E-87	M1	DPP7
DLEU7.3	1.66E-91	0.424127	0.14	0.032	2.49E-87	M1	DLEU7
NDFIP2.2	1.69E-91	-0.41327	0.023	0.226	2.52E-87	M1	NDFIP2
GPC2.4	2.56E-91	-0.57372	0.011	0.2	3.82E-87	M1	GPC2
C8orf33.1	2.73E-91	-0.3991	0.105	0.361	4.08E-87	M1	C8orf33
SNTG1.3	2.98E-91	-0.57251	0.002	0.184	4.45E-87	M1	SNTG1
EI24.2	2.99E-91	-0.40223	0.19	0.482	4.46E-87	M1	EI24
KLHL23.4	3.66E-91	-0.46627	0.007	0.192	5.47E-87	M1	KLHL23
FAM134B.2	4.66E-91	-0.42062	0.009	0.197	6.96E-87	M1	FAM134B
ZFHX4.4	6.14E-91	-0.40452	0.002	0.182	9.18E-87	M1	ZFHX4
NAP1L4.3	6.31E-91	-0.43538	0.17	0.45	9.42E-87	M1	NAP1L4
CD40.4	6.44E-91	0.421224	0.17	0.046	9.62E-87	M1	CD40
CDC34.3	1.03E-90	-0.39808	0.05	0.272	1.53E-86	M1	CDC34
NRXN1.3	1.04E-90	-0.63366	0.009	0.194	1.55E-86	M1	NRXN1
MYO6.2	1.14E-90	-0.43195	0.045	0.261	1.71E-86	M1	MYO6
TM7SF2.3	1.38E-90	-0.43844	0.048	0.267	2.06E-86	M1	TM7SF2
PEBP1.4	1.57E-90	-0.39464	0.65	0.835	2.35E-86	M1	PEBP1
JAG1.3	1.76E-90	-0.41625	0.006	0.19	2.63E-86	M1	JAG1
FAM127A.1	1.93E-90	-0.42839	0.214	0.508	2.89E-86	M1	FAM127A
PDCD5.4	2.05E-90	-0.47924	0.286	0.59	3.07E-86	M1	PDCD5
BCAN.4	2.27E-90	-0.73508	0.09	0.313	3.39E-86	M1	BCAN
NSG1.4	2.49E-90	-0.73773	0.018	0.207	3.73E-86	M1	NSG1
DYNLL1.3	3.13E-90	-0.37733	0.698	0.864	4.68E-86	M1	DYNLL1
TERF2IP.4	3.34E-90	-0.47979	0.287	0.564	5.00E-86	M1	TERF2IP
TMEM9.4	3.77E-90	-0.43848	0.141	0.412	5.63E-86	M1	TMEM9
CBS.3	4.02E-90	-0.40858	0.005	0.188	6.01E-86	M1	CBS

DLGAP4.1	6.75E-90	-0.41841	0.09	0.334	1.01E-85	M1	DLGAP4
MYO10.3	7.07E-90	-0.45373	0.026	0.228	1.06E-85	M1	MYO10
TMEM54.2	7.58E-90	-0.40146	0.027	0.229	1.13E-85	M1	TMEM54
BAZ1B.4	9.26E-90	-0.40908	0.094	0.339	1.38E-85	M1	BAZ1B
RNF180.3	1.10E-89	-0.42683	0.011	0.199	1.65E-85	M1	RNF180
MAP9.2	1.19E-89	-0.42433	0.023	0.222	1.78E-85	M1	MAP9
VBP1.3	1.38E-89	-0.42283	0.238	0.537	2.06E-85	M1	VBP1
TLE1.2	1.89E-89	-0.38276	0.012	0.201	2.83E-85	M1	TLE1
LINC00665	2.03E-89	-0.38917	0.021	0.219	3.04E-85	M1	LINC00665
TROVE2.3	2.25E-89	-0.42767	0.195	0.481	3.36E-85	M1	TROVE2
GDI1.3	2.43E-89	-0.38474	0.238	0.535	3.63E-85	M1	GDI1
CD72.3	2.61E-89	0.400005	0.14	0.033	3.90E-85	M1	CD72
IFT57.4	4.30E-89	-0.44894	0.06	0.285	6.43E-85	M1	IFT57
NRSN2.3	5.84E-89	-0.37193	0.01	0.195	8.73E-85	M1	NRSN2
SOCS2.3	7.11E-89	-0.44847	0.006	0.187	1.06E-84	M1	SOCS2
DPYSL4.4	9.18E-89	-0.40616	0.005	0.184	1.37E-84	M1	DPYSL4
WDR60.1	9.55E-89	-0.41302	0.051	0.268	1.43E-84	M1	WDR60
S100A9.4	9.76E-89	0.465038	0.393	0.19	1.46E-84	M1	S100A9
DPYSL5.4	9.81E-89	-0.41951	0.009	0.194	1.47E-84	M1	DPYSL5
FOXG1.3	1.78E-88	-0.37546	0.004	0.183	2.66E-84	M1	FOXG1
HDAC2.4	2.31E-88	-0.41188	0.214	0.495	3.46E-84	M1	HDAC2
PLOD2.3	2.96E-88	-0.44219	0.009	0.193	4.42E-84	M1	PLOD2
SFXN1.2	2.97E-88	-0.39855	0.041	0.252	4.43E-84	M1	SFXN1
OLIG2.3	3.26E-88	-0.58355	0.02	0.21	4.87E-84	M1	OLIG2
PGAP1.3	3.63E-88	-0.39877	0.021	0.216	5.42E-84	M1	PGAP1
GSTO1.3	4.87E-88	0.430268	0.6	0.427	7.27E-84	M1	GSTO1
MIAT.3	5.95E-88	-0.55265	0.013	0.199	8.89E-84	M1	MIAT
SH2B3.4	6.49E-88	0.583972	0.228	0.085	9.69E-84	M1	SH2B3
PRR4.4	6.87E-88	-0.41841	0.042	0.251	1.03E-83	M1	PRR4
MCM7.3	7.95E-88	-0.50975	0.07	0.291	1.19E-83	M1	MCM7
COL6A2.3	8.22E-88	-0.52698	0.01	0.193	1.23E-83	M1	COL6A2
DTNA.3	1.17E-87	-0.52662	0.066	0.29	1.75E-83	M1	DTNA
NNT-AS1.3	2.42E-87	-0.40806	0.045	0.257	3.62E-83	M1	NNT-AS1
KIF1B.3	3.10E-87	-0.39486	0.093	0.334	4.64E-83	M1	KIF1B
RHBDD2.3	3.92E-87	-0.40729	0.22	0.507	5.86E-83	M1	RHBDD2
ZKSCAN1.3	5.12E-87	-0.36185	0.107	0.358	7.66E-83	M1	ZKSCAN1
CHL1.2	7.18E-87	-0.44278	0.005	0.182	1.07E-82	M1	CHL1
SPOCD1.2	1.07E-86	-0.6996	0.044	0.252	1.60E-82	M1	SPOCD1
UPF3A.3	1.15E-86	-0.40384	0.129	0.388	1.71E-82	M1	UPF3A
HINT1.2	1.29E-86	-0.37306	0.683	0.857	1.92E-82	M1	HINT1
TJP1.2	1.42E-86	-0.39994	0.021	0.212	2.12E-82	M1	TJP1
NT5DC2.3	1.49E-86	-0.38534	0.012	0.195	2.23E-82	M1	NT5DC2
BMP7.4	1.69E-86	-0.42035	0.013	0.196	2.52E-82	M1	BMP7
PPFIBP1.2	1.74E-86	-0.40836	0.007	0.186	2.61E-82	M1	PPFIBP1
JAKMIP2.2	1.82E-86	-0.40478	0.005	0.18	2.72E-82	M1	JAKMIP2
UBE2V2.3	1.88E-86	-0.429	0.252	0.544	2.81E-82	M1	UBE2V2
POU3F3.3	1.93E-86	-0.41388	0.007	0.184	2.89E-82	M1	POU3F3
ANK2.4	2.06E-86	-0.3841	0.012	0.194	3.07E-82	M1	ANK2
TUBB.2	2.47E-86	-0.39381	0.631	0.799	3.70E-82	M1	TUBB
FFAR4.2	3.08E-86	0.337991	0.098	0.016	4.61E-82	M1	FFAR4
NDRG4.4	3.32E-86	-0.4238	0.01	0.19	4.96E-82	M1	NDRG4

SYNE2.4	3.71E-86	-0.44	0.018	0.205	5.54E-82	M1	SYNE2
FNBP1L.4	4.57E-86	-0.44569	0.025	0.217	6.83E-82	M1	FNBP1L
OLIG1.4	4.85E-86	-0.70317	0.023	0.211	7.25E-82	M1	OLIG1
ATP6V1B2.	7.02E-86	0.525186	0.53	0.384	1.05E-81	M1	ATP6V1B2
MORN2.4	1.01E-85	-0.46773	0.074	0.3	1.52E-81	M1	MORN2
EFEMP1.3	1.06E-85	-0.84214	0.049	0.255	1.58E-81	M1	EFEMP1
PMP22.3	1.10E-85	-0.57133	0.215	0.47	1.64E-81	M1	PMP22
SASH3.4	1.14E-85	0.438263	0.185	0.057	1.71E-81	M1	SASH3
TFRC.3	1.51E-85	0.616859	0.345	0.182	2.25E-81	M1	TFRC
DOK5.3	1.60E-85	-0.43222	0.023	0.215	2.40E-81	M1	DOK5
LUC7L3.2	2.59E-85	-0.40534	0.267	0.559	3.87E-81	M1	LUC7L3
TEAD1.2	2.71E-85	-0.34306	0.002	0.173	4.04E-81	M1	TEAD1
IFNGR2.4	2.97E-85	0.509254	0.502	0.367	4.44E-81	M1	IFNGR2
HNMT.3	4.61E-85	0.489786	0.371	0.188	6.89E-81	M1	HNMT
RENBP.4	5.16E-85	0.514658	0.266	0.109	7.72E-81	M1	RENBP
PHAX.2	7.60E-85	-0.41553	0.103	0.346	1.14E-80	M1	PHAX
RP11-161M	8.44E-85	-0.52754	0.015	0.197	1.26E-80	M1	RP11-161M6.2
HSPBP1.1	9.35E-85	-0.36786	0.09	0.327	1.40E-80	M1	HSPBP1
ASNS.4	1.08E-84	-0.48384	0.021	0.208	1.62E-80	M1	ASNS
WWTR1.4	1.84E-84	-0.40326	0.011	0.19	2.75E-80	M1	WWTR1
GIPC1.2	1.87E-84	-0.3934	0.077	0.306	2.79E-80	M1	GIPC1
WASF2.4	1.99E-84	0.480106	0.549	0.416	2.98E-80	M1	WASF2
BICD1.2	2.23E-84	-0.36278	0.037	0.238	3.34E-80	M1	BICD1
ALDH7A1.1	2.99E-84	-0.43843	0.085	0.314	4.47E-80	M1	ALDH7A1
PITPNA-AS	3.33E-84	-0.41187	0.032	0.228	4.98E-80	M1	PITPNA-AS1
ARL5A.3	3.89E-84	0.421445	0.596	0.501	5.81E-80	M1	ARL5A
SEPT8.4	6.07E-84	-0.45231	0.033	0.228	9.07E-80	M1	8-Sep
APBB1.3	6.59E-84	-0.41391	0.029	0.222	9.85E-80	M1	APBB1
MX2.4	6.83E-84	0.454998	0.16	0.045	1.02E-79	M1	MX2
PTS.3	6.96E-84	-0.39454	0.118	0.368	1.04E-79	M1	PTS
JAM3.1	8.09E-84	-0.41437	0.006	0.178	1.21E-79	M1	JAM3
RND2.3	8.16E-84	-0.40873	0.013	0.193	1.22E-79	M1	RND2
DMWD.3	9.32E-84	-0.3447	0.002	0.169	1.39E-79	M1	DMWD
FAM84A.3	9.61E-84	-0.40479	0.003	0.173	1.44E-79	M1	FAM84A
TMEM219.3	1.17E-83	0.374226	0.651	0.542	1.76E-79	M1	TMEM219
PIGX.3	1.18E-83	-0.34882	0.068	0.29	1.77E-79	M1	PIGX
KIF2A.4	1.26E-83	-0.38318	0.058	0.271	1.89E-79	M1	KIF2A
ENHO.3	1.35E-83	-0.38864	0.007	0.179	2.01E-79	M1	ENHO
ACAP3.4	1.35E-83	-0.43603	0.046	0.25	2.02E-79	M1	ACAP3
CITED1.3	1.37E-83	-0.51624	0.007	0.18	2.05E-79	M1	CITED1
PPP2CB.4	1.55E-83	-0.4014	0.1	0.336	2.31E-79	M1	PPP2CB
CARD9.4	1.92E-83	0.441274	0.167	0.049	2.86E-79	M1	CARD9
AC069363.	2.08E-83	0.45363	0.138	0.034	3.11E-79	M1	AC069363.1
KNOP1.3	2.14E-83	-0.39216	0.074	0.297	3.20E-79	M1	KNOP1
CBR1.3	2.40E-83	-0.47832	0.248	0.522	3.59E-79	M1	CBR1
IFT81.2	2.46E-83	-0.35683	0.011	0.189	3.68E-79	M1	IFT81
TGFBI.4	2.48E-83	0.449367	0.368	0.173	3.70E-79	M1	TGFBI
ELAVL4.3	2.74E-83	-0.68763	0.026	0.209	4.10E-79	M1	ELAVL4
SEC11A.3	3.13E-83	0.330488	0.676	0.574	4.68E-79	M1	SEC11A
METTL7B.1	3.64E-83	-0.69844	0.047	0.245	5.44E-79	M1	METTL7B
GADD45A.4	4.07E-83	-0.51464	0.089	0.317	6.09E-79	M1	GADD45A

FBXO17.4	7.12E-83	-0.39632	0.004	0.172	1.06E-78	M1	FBXO17
MED19.1	7.33E-83	-0.37232	0.088	0.32	1.10E-78	M1	MED19
ACTN4.2	7.84E-83	-0.39192	0.125	0.374	1.17E-78	M1	ACTN4
B3GNT5.4	9.50E-83	0.664143	0.263	0.117	1.42E-78	M1	B3GNT5
DPP6.2	9.96E-83	-0.40638	0.013	0.191	1.49E-78	M1	DPP6
LDLRAD3.3	1.00E-82	-0.38029	0.021	0.207	1.50E-78	M1	LDLRAD3
TMEM132A.	1.10E-82	-0.35324	0.004	0.173	1.64E-78	M1	TMEM132A
CDKN2C.2	1.15E-82	-0.51697	0.095	0.32	1.72E-78	M1	CDKN2C
RDX.3	1.50E-82	-0.42124	0.267	0.535	2.23E-78	M1	RDX
G0S2.3	1.57E-82	0.576406	0.321	0.156	2.35E-78	M1	G0S2
TMEM106C.	1.70E-82	-0.42554	0.079	0.304	2.54E-78	M1	TMEM106C
FOXN3.4	1.73E-82	0.579977	0.392	0.228	2.58E-78	M1	FOXN3
GPSM2.3	1.76E-82	-0.41461	0.022	0.208	2.63E-78	M1	GPSM2
HIST2H2AA	1.98E-82	0.492805	0.157	0.044	2.96E-78	M1	HIST2H2AA3
UBE2S.4	2.51E-82	-0.45054	0.138	0.385	3.76E-78	M1	UBE2S
CAP1.3	3.42E-82	0.334996	0.662	0.578	5.11E-78	M1	CAP1
GCA.4	4.01E-82	0.535028	0.442	0.27	5.99E-78	M1	GCA
GPNMB.4	7.04E-82	0.484001	0.354	0.17	1.05E-77	M1	GPNMB
CCNL1.2	7.15E-82	0.434293	0.616	0.506	1.07E-77	M1	CCNL1
BHLHE41.3	8.74E-82	0.613936	0.342	0.181	1.31E-77	M1	BHLHE41
TRIM47.4	8.91E-82	-0.42269	0.025	0.21	1.33E-77	M1	TRIM47
HPSE.3	9.90E-82	0.404703	0.15	0.04	1.48E-77	M1	HPSE
FAM20A.3	1.55E-81	0.420734	0.151	0.041	2.31E-77	M1	FAM20A
SEMA6D.4	1.86E-81	-0.40333	0.012	0.186	2.79E-77	M1	SEMA6D
BPGM.1	2.02E-81	-0.40063	0.059	0.269	3.01E-77	M1	BPGM
TGFBR1.4	2.21E-81	0.549795	0.291	0.136	3.31E-77	M1	TGFBR1
SSBP2.4	2.27E-81	-0.41131	0.082	0.308	3.39E-77	M1	SSBP2
ACTN1.3	2.70E-81	-0.43361	0.074	0.291	4.04E-77	M1	ACTN1
WEE1.2	2.96E-81	-0.37013	0.008	0.178	4.42E-77	M1	WEE1
BMP2K.2	2.96E-81	0.519133	0.234	0.093	4.42E-77	M1	BMP2K
RFX4.2	3.28E-81	-0.47305	0.009	0.179	4.90E-77	M1	RFX4
NDUFA4.3	3.62E-81	-0.31789	0.824	0.909	5.42E-77	M1	NDUFA4
HEPACAM.3	3.64E-81	-0.53566	0.025	0.208	5.44E-77	M1	HEPACAM
LINC01003	3.73E-81	-0.36756	0.033	0.224	5.57E-77	M1	LINC01003
FAM200B.2	4.32E-81	-0.37257	0.15	0.41	6.46E-77	M1	FAM200B
HTRA1.4	7.99E-81	0.410012	0.58	0.436	1.19E-76	M1	HTRA1
VAMP2.3	8.61E-81	-0.40824	0.399	0.667	1.29E-76	M1	VAMP2
ZNF3.2	1.08E-80	-0.39648	0.042	0.238	1.62E-76	M1	ZNF3
FSD1.3	1.29E-80	-0.35455	0.011	0.183	1.92E-76	M1	FSD1
C11orf49.	1.29E-80	-0.37047	0.061	0.272	1.92E-76	M1	C11orf49
EMP1.2	1.37E-80	-0.505	0.025	0.209	2.05E-76	M1	EMP1
UBE2D3.3	1.41E-80	0.248011	0.772	0.719	2.11E-76	M1	UBE2D3
TMEM158.3	2.10E-80	-0.39106	0.003	0.167	3.14E-76	M1	TMEM158
APC.4	2.27E-80	-0.36019	0.046	0.247	3.39E-76	M1	APC
DDX49.2	2.68E-80	-0.37154	0.086	0.312	4.00E-76	M1	DDX49
MYLIP.4	2.98E-80	0.531121	0.253	0.104	4.45E-76	M1	MYLIP
NKX2-2.3	3.82E-80	-0.51902	0.008	0.175	5.71E-76	M1	NKX2-2
PCDHB16.2	4.15E-80	-0.3724	0.004	0.167	6.21E-76	M1	PCDHB16
CDKN2D.4	4.40E-80	-0.4216	0.015	0.189	6.57E-76	M1	CDKN2D
ABI2.2	4.85E-80	-0.33787	0.147	0.405	7.24E-76	M1	ABI2
TRIM24.4	8.38E-80	-0.33337	0.033	0.223	1.25E-75	M1	TRIM24

RGS16.3	9.01E-80	0.560095	0.41	0.249	1.35E-75	M1	RGS16
SNAP25.3	1.02E-79	-0.56695	0.006	0.17	1.53E-75	M1	SNAP25
FGF20.2	1.26E-79	0.321716	0.069	0.007	1.88E-75	M1	FGF20
CALM1.4	1.68E-79	-0.33248	0.643	0.797	2.51E-75	M1	CALM1
S100A6.4	1.98E-79	-0.64767	0.472	0.678	2.97E-75	M1	S100A6
OLFM1.3	2.07E-79	-0.43038	0.01	0.179	3.09E-75	M1	OLFM1
HEXB.4	2.07E-79	0.479829	0.522	0.382	3.10E-75	M1	HEXB
SGCE.2	2.35E-79	-0.65037	0.008	0.174	3.52E-75	M1	SGCE
PDAP1.2	3.35E-79	-0.38232	0.296	0.584	5.01E-75	M1	PDAP1
TSPAN6.3	5.23E-79	-0.40469	0.01	0.178	7.81E-75	M1	TSPAN6
SUN1.4	5.70E-79	-0.3684	0.044	0.241	8.52E-75	M1	SUN1
NCF2.4	6.44E-79	0.418764	0.218	0.078	9.62E-75	M1	NCF2
B4GALT5.2	7.72E-79	-0.37974	0.038	0.228	1.15E-74	M1	B4GALT5
DRAXIN.3	7.83E-79	-0.36038	0.011	0.181	1.17E-74	M1	DRAXIN
SOAT1.4	1.06E-78	0.566791	0.329	0.171	1.59E-74	M1	SOAT1
IRF5.4	1.37E-78	0.465022	0.148	0.041	2.05E-74	M1	IRF5
CHRNE.3	1.74E-78	0.343239	0.087	0.014	2.59E-74	M1	CHRNE
APC2.3	1.83E-78	-0.39587	0.009	0.176	2.74E-74	M1	APC2
NOL3.4	2.11E-78	-0.43513	0.02	0.195	3.15E-74	M1	NOL3
DCLK1.2	2.14E-78	-0.40489	0.011	0.179	3.20E-74	M1	DCLK1
ANXA6	2.69E-78	-0.42584	0.032	0.215	4.02E-74	M1	ANXA6
ARPC1A.4	3.03E-78	-0.3972	0.317	0.607	4.53E-74	M1	ARPC1A
DPF3.1	4.13E-78	-0.36902	0.003	0.163	6.17E-74	M1	DPF3
DPF1.3	4.22E-78	-0.4252	0.006	0.167	6.31E-74	M1	DPF1
FCGR2B.4	4.44E-78	0.5635	0.275	0.118	6.63E-74	M1	FCGR2B
YWHAG.4	4.58E-78	-0.39643	0.18	0.433	6.85E-74	M1	YWHAG
GARS.4	5.24E-78	-0.38409	0.104	0.331	7.82E-74	M1	GARS
CNP.3	5.29E-78	-0.53679	0.169	0.419	7.90E-74	M1	CNP
MYH10.2	5.89E-78	-0.37927	0.013	0.181	8.81E-74	M1	MYH10
LPXN.4	6.82E-78	0.503974	0.226	0.087	1.02E-73	M1	LPXN
KHDRBS3.4	9.96E-78	-0.38836	0.049	0.248	1.49E-73	M1	KHDRBS3
AQP4.3	9.99E-78	-0.61274	0.021	0.194	1.49E-73	M1	AQP4
ZC2HC1A.3	1.04E-77	-0.43064	0.049	0.243	1.55E-73	M1	ZC2HC1A
SLC16A1.4	1.26E-77	-0.43891	0.064	0.267	1.88E-73	M1	SLC16A1
ATP6V1G2.	1.50E-77	-0.41455	0.011	0.177	2.24E-73	M1	ATP6V1G2
RNF187.2	1.55E-77	-0.41293	0.167	0.42	2.32E-73	M1	RNF187
CBX6.2	1.55E-77	-0.37544	0.067	0.275	2.32E-73	M1	CBX6
CCZ1.1	1.79E-77	-0.34461	0.143	0.393	2.68E-73	M1	CCZ1
PABPC4.4	2.03E-77	0.556243	0.445	0.305	3.03E-73	M1	PABPC4
C1orf122.	2.04E-77	-0.44809	0.269	0.532	3.04E-73	M1	C1orf122
KIF9.4	2.22E-77	-0.37982	0.025	0.204	3.31E-73	M1	KIF9
PREPL.2	2.92E-77	-0.38214	0.035	0.219	4.37E-73	M1	PREPL
AC074289.	3.05E-77	0.488004	0.151	0.043	4.55E-73	M1	AC074289.1
PROX1.3	3.36E-77	-0.37125	0.019	0.193	5.03E-73	M1	PROX1
LTBR.4	3.52E-77	0.472006	0.204	0.073	5.26E-73	M1	LTBR
PTCRA.2	4.10E-77	0.303156	0.08	0.012	6.12E-73	M1	PTCRA
PSD3.3	4.56E-77	-0.35923	0.038	0.226	6.82E-73	M1	PSD3
MLF2.4	6.54E-77	-0.38595	0.382	0.654	9.77E-73	M1	MLF2
UBE2E3.3	7.61E-77	-0.40114	0.241	0.504	1.14E-72	M1	UBE2E3
C21orf62.	7.79E-77	-0.43723	0.016	0.186	1.16E-72	M1	C21orf62
ENDOV.1	9.04E-77	-0.32337	0.053	0.254	1.35E-72	M1	ENDOV

TCHH	9.38E-77	0.276139	0.07	0.008	1.40E-72	M1	TCHH
THSD7A.3	1.14E-76	-0.55918	0.009	0.169	1.70E-72	M1	THSD7A
DAG1.2	1.24E-76	-0.32032	0.025	0.203	1.86E-72	M1	DAG1
SMOX.4	1.37E-76	-0.43206	0.072	0.282	2.04E-72	M1	SMOX
PCGF2.2	1.41E-76	-0.32572	0.019	0.191	2.11E-72	M1	PCGF2
TSC22D4.4	1.47E-76	-0.57456	0.268	0.51	2.19E-72	M1	TSC22D4
PCMTD2.3	1.49E-76	-0.33982	0.059	0.261	2.23E-72	M1	PCMTD2
ACSM5.1	1.60E-76	0.334643	0.1	0.019	2.40E-72	M1	ACSM5
CDK4.2	1.61E-76	-0.46304	0.261	0.529	2.40E-72	M1	CDK4
TNFSF10.2	1.88E-76	0.386007	0.174	0.054	2.82E-72	M1	TNFSF10
FAM3C.3	3.23E-76	-0.38447	0.219	0.481	4.83E-72	M1	FAM3C
PICALM.4	3.30E-76	0.561116	0.406	0.256	4.94E-72	M1	PICALM
MIS18BP1.	3.47E-76	0.563426	0.267	0.122	5.19E-72	M1	MIS18BP1
ZSCAN18.2	4.18E-76	-0.35048	0.062	0.265	6.25E-72	M1	ZSCAN18
CEND1.2	4.39E-76	-0.37357	0.006	0.165	6.56E-72	M1	CEND1
TPGS2.1	5.24E-76	-0.36976	0.137	0.379	7.83E-72	M1	TPGS2
NT5C3A.2	5.66E-76	-0.36523	0.106	0.332	8.46E-72	M1	NT5C3A
SQRDL.4	7.46E-76	0.486266	0.292	0.134	1.11E-71	M1	SQRDL
EHBP1.2	8.50E-76	-0.32129	0.022	0.196	1.27E-71	M1	EHBP1
GCAT.4	1.12E-75	-0.30429	0.019	0.19	1.67E-71	M1	GCAT
POSTN.3	1.27E-75	-0.49754	0.002	0.155	1.90E-71	M1	POSTN
MAF.3	1.36E-75	0.486063	0.257	0.11	2.03E-71	M1	MAF
KLC1.4	1.44E-75	-0.35229	0.154	0.401	2.15E-71	M1	KLC1
C5orf24.3	2.13E-75	-0.34131	0.106	0.334	3.18E-71	M1	C5orf24
NFATC2.2	2.22E-75	0.369237	0.125	0.03	3.31E-71	M1	NFATC2
DTX3.1	2.83E-75	-0.50243	0.084	0.289	4.23E-71	M1	DTX3
RBM4B.2	3.45E-75	-0.34673	0.038	0.223	5.15E-71	M1	RBM4B
RP3-525N1	3.54E-75	-0.43672	0.013	0.177	5.29E-71	M1	RP3-525N10.2
SETD5.4	3.83E-75	-0.36652	0.101	0.326	5.72E-71	M1	SETD5
CCT2	4.11E-75	-0.45094	0.322	0.59	6.14E-71	M1	CCT2
LRRC4B.4	4.20E-75	-0.32123	0.004	0.159	6.28E-71	M1	LRRC4B
NLGN1.3	4.21E-75	-0.34272	0.007	0.164	6.29E-71	M1	NLGN1
DCTN3	4.21E-75	-0.38131	0.285	0.563	6.29E-71	M1	DCTN3
TPM2.1	4.68E-75	-0.44009	0.021	0.191	6.99E-71	M1	TPM2
KIAA1191.	4.87E-75	-0.3435	0.085	0.3	7.28E-71	M1	KIAA1191
COLGALT2.	4.98E-75	-0.34611	0.007	0.165	7.44E-71	M1	COLGALT2
ASNSD1.3	5.69E-75	-0.33572	0.09	0.307	8.50E-71	M1	ASNSD1
IPO9.2	5.81E-75	-0.317	0.062	0.265	8.68E-71	M1	IPO9
NUDT16L1.	8.06E-75	-0.32893	0.097	0.318	1.20E-70	M1	NUDT16L1
CSTF3.2	8.80E-75	-0.36825	0.088	0.303	1.31E-70	M1	CSTF3
KCNQ1OT1.	9.01E-75	-0.41874	0.098	0.314	1.35E-70	M1	KCNQ1OT1
CTNNAL1.3	9.54E-75	-0.34363	0.012	0.174	1.43E-70	M1	CTNNAL1
S100A8.4	9.89E-75	0.397596	0.329	0.156	1.48E-70	M1	S100A8
ZNF75A.3	1.02E-74	-0.34293	0.052	0.247	1.52E-70	M1	ZNF75A
ST8SIA4.4	1.04E-74	0.523123	0.233	0.096	1.55E-70	M1	ST8SIA4
TMEM237.2	1.04E-74	-0.34641	0.035	0.217	1.56E-70	M1	TMEM237
LHX2.4	1.13E-74	-0.34858	0.003	0.155	1.69E-70	M1	LHX2
FOS.3	1.17E-74	0.145301	0.959	0.817	1.74E-70	M1	FOS
ELOF1.3	1.25E-74	-0.38894	0.204	0.468	1.87E-70	M1	ELOF1
CFDP1.3	1.27E-74	-0.35925	0.194	0.46	1.89E-70	M1	CFDP1
APMAP.3	1.38E-74	0.438265	0.586	0.504	2.06E-70	M1	APMAP

ZNF608.2	1.60E-74	-0.31666	0.018	0.185	2.39E-70	M1	ZNF608
LIMCH1.3	1.75E-74	-0.41255	0.015	0.179	2.62E-70	M1	LIMCH1
CTNND2.3	1.82E-74	-0.37188	0.018	0.186	2.71E-70	M1	CTNND2
NUCB2.1	1.97E-74	-0.36876	0.147	0.387	2.94E-70	M1	NUCB2
PAK3.4	2.00E-74	-0.45568	0.01	0.17	2.99E-70	M1	PAK3
IFNLR1.2	3.17E-74	0.296552	0.081	0.012	4.74E-70	M1	IFNLR1
ASPHD1.2	3.27E-74	-0.40787	0.007	0.164	4.88E-70	M1	ASPHD1
NXPH1.4	3.66E-74	-0.41912	0.007	0.162	5.47E-70	M1	NXPH1
MXRA7.2	3.78E-74	-0.34499	0.024	0.196	5.66E-70	M1	MXRA7
SCARA3.3	3.90E-74	-0.34167	0.007	0.164	5.82E-70	M1	SCARA3
SSRP1.2	3.97E-74	-0.35948	0.151	0.396	5.94E-70	M1	SSRP1
PIK3R5.4	5.87E-74	0.381352	0.126	0.032	8.77E-70	M1	PIK3R5
BBOX1.3	6.17E-74	-0.4691	0.007	0.163	9.23E-70	M1	BBOX1
PTPRA.3	9.23E-74	-0.38127	0.155	0.394	1.38E-69	M1	PTPRA
TYMS.3	1.11E-73	-0.43499	0.022	0.189	1.66E-69	M1	TYMS
CDC25B.2	1.13E-73	-0.36317	0.041	0.225	1.69E-69	M1	CDC25B
GIMAP1.3	1.15E-73	0.422716	0.169	0.054	1.72E-69	M1	GIMAP1
TNRC6C.3	1.27E-73	-0.36535	0.028	0.201	1.90E-69	M1	TNRC6C
CHST12.2	1.36E-73	-0.30178	0.073	0.28	2.04E-69	M1	CHST12
MLLT4.3	1.45E-73	-0.37192	0.022	0.19	2.16E-69	M1	MLLT4
C19orf38.	1.58E-73	0.431404	0.126	0.032	2.36E-69	M1	C19orf38
SLC2A3.3	1.85E-73	0.534233	0.392	0.227	2.77E-69	M1	SLC2A3
SOBP.3	2.24E-73	-0.3759	0.006	0.16	3.35E-69	M1	SOBP
ITGAM.4	2.37E-73	0.403864	0.173	0.056	3.54E-69	M1	ITGAM
CLCN3.2	2.41E-73	-0.34566	0.077	0.283	3.60E-69	M1	CLCN3
RP11-71N1	3.07E-73	-0.39267	0.007	0.162	4.59E-69	M1	RP11-71N10.1
CHST2.2	3.31E-73	-0.32083	0.007	0.162	4.95E-69	M1	CHST2
SLC25A5.1	3.40E-73	0.222162	0.782	0.709	5.09E-69	M1	SLC25A5
CISD1.4	3.46E-73	-0.4271	0.141	0.376	5.17E-69	M1	CISD1
MPP1.4	4.34E-73	0.507827	0.334	0.174	6.48E-69	M1	MPP1
PELI1.4	4.55E-73	0.590129	0.336	0.184	6.80E-69	M1	PELI1
CBX3.3	4.80E-73	-0.36301	0.328	0.599	7.17E-69	M1	CBX3
EAF2.4	4.86E-73	0.521026	0.196	0.072	7.26E-69	M1	EAF2
IMMP1L.3	4.93E-73	-0.32777	0.065	0.265	7.37E-69	M1	IMMP1L
STMN2.4	5.53E-73	-0.90841	0.046	0.215	8.27E-69	M1	STMN2
NAMPT.4	5.63E-73	0.231571	0.59	0.407	8.42E-69	M1	NAMPT
CHD7.4	6.07E-73	-0.38069	0.107	0.325	9.08E-69	M1	CHD7
SUGP2.3	6.61E-73	-0.30379	0.074	0.281	9.87E-69	M1	SUGP2
CNIH2.4	6.68E-73	-0.33104	0.004	0.155	9.99E-69	M1	CNIH2
MAP4.2	6.81E-73	-0.32974	0.137	0.375	1.02E-68	M1	MAP4
SLC15A3.3	7.02E-73	0.400831	0.161	0.05	1.05E-68	M1	SLC15A3
KEAP1.2	7.44E-73	-0.28927	0.091	0.308	1.11E-68	M1	KEAP1
CERS1.2	8.08E-73	-0.33794	0.003	0.153	1.21E-68	M1	CERS1
RP11-353N	1.14E-72	0.28115	0.075	0.011	1.71E-68	M1	RP11-353N4.5
ZNF711.3	1.25E-72	-0.38953	0.011	0.168	1.87E-68	M1	ZNF711
LINC00662	1.40E-72	-0.38296	0.035	0.213	2.09E-68	M1	LINC00662
GNG12.2	1.64E-72	-0.32092	0.009	0.164	2.45E-68	M1	GNG12
ALKBH4.2	1.65E-72	-0.31321	0.044	0.229	2.47E-68	M1	ALKBH4
C8orf46.2	1.73E-72	-0.45981	0.01	0.165	2.58E-68	M1	C8orf46
GTF2I.3	1.79E-72	-0.39079	0.224	0.467	2.68E-68	M1	GTF2I
KCNF1.4	1.98E-72	-0.378	0.001	0.148	2.96E-68	M1	KCNF1

DTYMK.3	2.00E-72	-0.39182	0.137	0.368	3.00E-68	M1	DTYMK
LCP1.3	2.26E-72	0.39508	0.271	0.116	3.38E-68	M1	LCP1
RSBN1L.2	2.74E-72	-0.30928	0.153	0.396	4.10E-68	M1	RSBN1L
NPTXR.3	2.95E-72	-0.31749	0.003	0.152	4.41E-68	M1	NPTXR
MSI1.2	3.02E-72	-0.35954	0.008	0.161	4.51E-68	M1	MSI1
RAMP2.3	3.12E-72	-0.39084	0.008	0.161	4.66E-68	M1	RAMP2
TGFBR2.3	3.27E-72	0.519929	0.227	0.095	4.89E-68	M1	TGFBR2
IFT52.1	3.83E-72	-0.30912	0.082	0.291	5.72E-68	M1	IFT52
KLHL4.2	3.96E-72	-0.35418	0.003	0.152	5.92E-68	M1	KLHL4
ZNF146.2	4.08E-72	-0.33172	0.063	0.26	6.10E-68	M1	ZNF146
STX4.2	4.29E-72	0.54528	0.433	0.29	6.42E-68	M1	STX4
SLC35F1.2	4.37E-72	-0.30761	0.003	0.151	6.53E-68	M1	SLC35F1
CXADR.4	4.37E-72	-0.40992	0.124	0.344	6.54E-68	M1	CXADR
SMARCD1.4	4.64E-72	-0.32119	0.055	0.246	6.93E-68	M1	SMARCD1
CA12.4	5.20E-72	-0.40097	0.008	0.162	7.76E-68	M1	CA12
CECR1.4	5.42E-72	0.384303	0.165	0.052	8.10E-68	M1	CECR1
PEMT.3	5.53E-72	-0.36413	0.054	0.244	8.27E-68	M1	PEMT
IFT43.3	5.83E-72	-0.36809	0.083	0.292	8.71E-68	M1	IFT43
LRRC8A.3	6.44E-72	-0.37363	0.027	0.197	9.63E-68	M1	LRRC8A
PNMA2.3	7.38E-72	-0.33998	0.01	0.165	1.10E-67	M1	PNMA2
C4orf27.2	7.87E-72	-0.36225	0.127	0.357	1.18E-67	M1	C4orf27
CCDC34.2	8.41E-72	-0.38859	0.026	0.195	1.26E-67	M1	CCDC34
RP11-597D	8.76E-72	0.346029	0.078	0.012	1.31E-67	M1	RP11-597D13.8
PLEKHO2.4	9.53E-72	0.467677	0.208	0.081	1.42E-67	M1	PLEKHO2
RFK.2	9.55E-72	-0.33844	0.071	0.271	1.43E-67	M1	RFK
NIFK.2	1.12E-71	-0.36568	0.15	0.388	1.67E-67	M1	NIFK
AASDHPPT.	1.13E-71	-0.34163	0.104	0.325	1.69E-67	M1	AASDHPPT
CD164.3	1.14E-71	0.406547	0.581	0.472	1.70E-67	M1	CD164
SPATC1L.3	1.22E-71	-0.32486	0.043	0.225	1.82E-67	M1	SPATC1L
KIFAP3.3	1.33E-71	-0.36295	0.049	0.234	1.99E-67	M1	KIFAP3
WSCD1.3	1.34E-71	-0.35226	0.006	0.156	2.00E-67	M1	WSCD1
TNIK.2	1.50E-71	-0.29699	0.013	0.17	2.23E-67	M1	TNIK
SLC25A19.	1.84E-71	0.554201	0.29	0.144	2.76E-67	M1	SLC25A19
POLR2J.3	1.95E-71	-0.39474	0.414	0.689	2.91E-67	M1	POLR2J
C2.2	2.02E-71	0.442472	0.229	0.092	3.02E-67	M1	C2
MEIS3.2	2.10E-71	-0.34769	0.005	0.154	3.13E-67	M1	MEIS3
CLECL1.4	2.41E-71	0.3134	0.125	0.032	3.59E-67	M1	CLECL1
TLR3.1	3.06E-71	0.322129	0.098	0.02	4.57E-67	M1	TLR3
CLUAP1.3	4.57E-71	-0.3608	0.028	0.196	6.83E-67	M1	CLUAP1
C1S.2	4.90E-71	-0.6845	0.024	0.19	7.32E-67	M1	C1S
TLK1.1	5.32E-71	-0.3172	0.065	0.261	7.95E-67	M1	TLK1
WASL.2	6.40E-71	-0.29425	0.036	0.213	9.56E-67	M1	WASL
RNASEH1-A	7.50E-71	-0.33158	0.034	0.207	1.12E-66	M1	RNASEH1-AS1
VDAC3.1	7.65E-71	-0.36701	0.241	0.509	1.14E-66	M1	VDAC3
KYNU.4	8.22E-71	0.461145	0.183	0.064	1.23E-66	M1	KYNU
ING4.2	8.35E-71	-0.3152	0.092	0.302	1.25E-66	M1	ING4
USP1.3	1.09E-70	-0.35764	0.087	0.293	1.63E-66	M1	USP1
C12orf76.	1.12E-70	-0.29028	0.089	0.302	1.67E-66	M1	C12orf76
CCDC14.2	1.15E-70	-0.31126	0.064	0.259	1.72E-66	M1	CCDC14
NETO2.2	1.26E-70	-0.29866	0.023	0.189	1.89E-66	M1	NETO2
EIF3L.2	1.27E-70	0.339607	0.673	0.593	1.89E-66	M1	EIF3L

ITGA7.2	1.40E-70	-0.29941	0.01	0.162	2.10E-66	M1	ITGA7
RPAIN.2	1.71E-70	-0.33594	0.261	0.53	2.56E-66	M1	RPAIN
P4HTM.2	1.78E-70	-0.29322	0.087	0.296	2.67E-66	M1	P4HTM
SPTAN1.2	2.17E-70	-0.31495	0.086	0.295	3.24E-66	M1	SPTAN1
IFI44L.2	2.28E-70	0.495589	0.263	0.117	3.41E-66	M1	IFI44L
ATF7IP2.3	2.29E-70	0.376928	0.114	0.027	3.42E-66	M1	ATF7IP2
SLC43A2.4	2.37E-70	0.576541	0.364	0.222	3.54E-66	M1	SLC43A2
C11orf96.	2.55E-70	-0.44302	0.009	0.159	3.82E-66	M1	C11orf96
NR2F6.3	2.65E-70	-0.29338	0.012	0.167	3.96E-66	M1	NR2F6
YES1.1	2.97E-70	-0.30788	0.007	0.155	4.45E-66	M1	YES1
REEP4.4	3.28E-70	0.534706	0.286	0.143	4.90E-66	M1	REEP4
KDM1A.2	3.45E-70	-0.3226	0.05	0.234	5.16E-66	M1	KDM1A
PJA1.2	3.76E-70	-0.36801	0.044	0.222	5.62E-66	M1	PJA1
C14orf1.3	4.10E-70	-0.37064	0.11	0.329	6.13E-66	M1	C14orf1
RAB3A.4	4.47E-70	-0.41379	0.026	0.191	6.69E-66	M1	RAB3A
COL4A2.1	4.65E-70	-0.2988	0.006	0.153	6.95E-66	M1	COL4A2
LSM5.1	5.16E-70	-0.38358	0.287	0.551	7.71E-66	M1	LSM5
ARHGAP22.	5.17E-70	0.500177	0.167	0.056	7.73E-66	M1	ARHGAP22
SLAMF8.4	6.24E-70	0.411493	0.15	0.046	9.33E-66	M1	SLAMF8
S100A10.4	6.31E-70	-0.59064	0.364	0.581	9.43E-66	M1	S100A10
CLDN12.4	6.56E-70	-0.36557	0.011	0.164	9.81E-66	M1	CLDN12
NNMT.2	6.76E-70	-0.7403	0.024	0.185	1.01E-65	M1	NNMT
ANAPC16.2	8.22E-70	0.363535	0.616	0.507	1.23E-65	M1	ANAPC16
ABCG1.4	8.64E-70	0.478477	0.247	0.108	1.29E-65	M1	ABCG1
GAS1.4	9.52E-70	-0.39349	0.015	0.17	1.42E-65	M1	GAS1
NCBP2.2	9.73E-70	-0.31069	0.219	0.485	1.45E-65	M1	NCBP2
PPP2R1A.3	1.09E-69	-0.35018	0.286	0.561	1.63E-65	M1	PPP2R1A
RP11-345F	1.60E-69	-0.30107	0.026	0.192	2.39E-65	M1	RP11-345P4.9
FAM210B.4	1.60E-69	-0.3732	0.097	0.304	2.39E-65	M1	FAM210B
RP9.2	1.65E-69	-0.33854	0.036	0.208	2.47E-65	M1	RP9
MPLKIP.1	1.78E-69	-0.34037	0.221	0.489	2.66E-65	M1	MPLKIP
ARSJ.2	1.82E-69	-0.32387	0.004	0.149	2.73E-65	M1	ARSJ
GRIA4.3	1.83E-69	-0.38233	0.007	0.154	2.73E-65	M1	GRIA4
SLC1A2.2	2.12E-69	-0.40818	0.01	0.16	3.17E-65	M1	SLC1A2
DCBLD2.3	2.33E-69	-0.3161	0.014	0.169	3.49E-65	M1	DCBLD2
SPOCK2.2	2.81E-69	-0.37659	0.012	0.165	4.19E-65	M1	SPOCK2
RP11-347F	3.00E-69	0.47248	0.152	0.047	4.48E-65	M1	RP11-347P5.1
STARD4-AS	3.32E-69	-0.34956	0.006	0.151	4.96E-65	M1	STARD4-AS1
S100A13.2	3.67E-69	-0.59453	0.122	0.331	5.49E-65	M1	S100A13
KMT2A.4	4.02E-69	-0.31157	0.072	0.269	6.01E-65	M1	KMT2A
BEND5.2	4.37E-69	-0.37911	0.011	0.162	6.53E-65	M1	BEND5
TMEM97.2	4.93E-69	-0.31466	0.032	0.2	7.36E-65	M1	TMEM97
OSBPL6.1	5.05E-69	-0.27339	0.022	0.183	7.54E-65	M1	OSBPL6
PFDN4.3	5.78E-69	-0.3551	0.16	0.399	8.63E-65	M1	PFDN4
TFEC.4	9.73E-69	0.398211	0.138	0.039	1.45E-64	M1	TFEC
TPPP3.4	9.83E-69	-0.55567	0.022	0.18	1.47E-64	M1	TPPP3
MLC1.3	1.04E-68	-0.33947	0.018	0.175	1.55E-64	M1	MLC1
MAFF.4	1.23E-68	0.519522	0.291	0.147	1.84E-64	M1	MAFF
ASIC4.3	1.41E-68	-0.54952	0.014	0.165	2.11E-64	M1	ASIC4
ST14.4	1.42E-68	0.381177	0.165	0.054	2.12E-64	M1	ST14
NABP2.3	1.43E-68	-0.32744	0.099	0.311	2.14E-64	M1	NABP2

SLC44A1.2	2.16E-68	-0.38761	0.129	0.35	3.23E-64	M1	SLC44A1
ZNF292.2	2.17E-68	-0.31391	0.092	0.298	3.24E-64	M1	ZNF292
WHSC1L1.4	2.23E-68	-0.31061	0.171	0.413	3.34E-64	M1	WHSC1L1
LYRM7.2	2.50E-68	-0.30473	0.05	0.231	3.74E-64	M1	LYRM7
QTRT1.4	2.58E-68	-0.35451	0.14	0.369	3.86E-64	M1	QTRT1
LRWD1.1	2.61E-68	-0.29939	0.031	0.198	3.90E-64	M1	LRWD1
TRIAP1.2	3.34E-68	-0.32326	0.125	0.351	5.00E-64	M1	TRIAP1
HSPA1A.3	3.46E-68	0.409939	0.623	0.466	5.16E-64	M1	HSPA1A
CNTFR.3	3.53E-68	-0.35249	0.008	0.154	5.27E-64	M1	CNTFR
STAC3.4	3.84E-68	0.423422	0.173	0.06	5.74E-64	M1	STAC3
TREML1.2	4.77E-68	0.302952	0.073	0.011	7.13E-64	M1	TREML1
AC004158.	4.94E-68	-0.3523	0.004	0.146	7.38E-64	M1	AC004158.3
ARMC1.3	5.89E-68	-0.33025	0.093	0.298	8.80E-64	M1	ARMC1
NKAIN3.3	5.96E-68	-0.34741	0.006	0.149	8.90E-64	M1	NKAIN3
SLC25A23.	6.22E-68	-0.28992	0.01	0.158	9.29E-64	M1	SLC25A23
DLX5.4	6.56E-68	-0.74139	0.019	0.169	9.80E-64	M1	DLX5
GTF2H2.2	7.49E-68	-0.29364	0.04	0.213	1.12E-63	M1	GTF2H2
NAF1.3	7.68E-68	0.545689	0.212	0.089	1.15E-63	M1	NAF1
NUDT11.2	7.80E-68	-0.39554	0.007	0.15	1.17E-63	M1	NUDT11
CHCHD10.4	8.10E-68	0.394124	0.489	0.36	1.21E-63	M1	CHCHD10
DCAF13.2	9.34E-68	-0.3256	0.13	0.355	1.40E-63	M1	DCAF13
C7orf73.3	9.42E-68	-0.38325	0.328	0.6	1.41E-63	M1	C7orf73
PPP1R18.4	1.00E-67	0.540006	0.412	0.269	1.50E-63	M1	PPP1R18
NDUFB7.4	1.04E-67	-0.37353	0.535	0.757	1.55E-63	M1	NDUFB7
MESP1.1	1.25E-67	-0.29608	0.009	0.156	1.87E-63	M1	MESP1
KIF1A.3	1.30E-67	-0.44881	0.007	0.15	1.95E-63	M1	KIF1A
PGM1.3	1.64E-67	-0.34266	0.085	0.284	2.45E-63	M1	PGM1
KRT10.2	1.82E-67	-0.36402	0.347	0.6	2.71E-63	M1	KRT10
SEZ6L.3	2.15E-67	-0.40368	0.012	0.16	3.22E-63	M1	SEZ6L
ZDHHC9.4	2.26E-67	-0.37179	0.031	0.195	3.38E-63	M1	ZDHHC9
DAPK3.2	2.37E-67	-0.30364	0.075	0.271	3.54E-63	M1	DAPK3
BCL7A.4	2.80E-67	-0.32104	0.02	0.176	4.19E-63	M1	BCL7A
GNG7.3	4.65E-67	0.504756	0.421	0.274	6.94E-63	M1	GNG7
HIP1.2	5.77E-67	-0.33296	0.031	0.195	8.62E-63	M1	HIP1
REPIN1.4	7.92E-67	-0.30654	0.1	0.309	1.18E-62	M1	REPIN1
FKBP9.3	8.07E-67	-0.30331	0.036	0.205	1.21E-62	M1	FKBP9
RFTN2.4	8.65E-67	-0.42405	0.008	0.151	1.29E-62	M1	RFTN2
TSN.2	9.32E-67	-0.31939	0.176	0.417	1.39E-62	M1	TSN
NUDT14.4	9.66E-67	0.537679	0.31	0.168	1.44E-62	M1	NUDT14
OLFM2.2	9.99E-67	-0.42995	0.047	0.22	1.49E-62	M1	OLFM2
TUBG1.2	1.38E-66	-0.32432	0.074	0.266	2.06E-62	M1	TUBG1
PAQR4.4	1.61E-66	-0.36336	0.03	0.191	2.40E-62	M1	PAQR4
TMED4.3	1.94E-66	-0.36697	0.229	0.48	2.90E-62	M1	TMED4
DDAH1.3	2.54E-66	-0.32338	0.007	0.148	3.80E-62	M1	DDAH1
MAPRE3.2	2.56E-66	-0.32735	0.039	0.207	3.83E-62	M1	MAPRE3
H1FX.4	2.77E-66	-0.35628	0.186	0.424	4.14E-62	M1	H1FX
VWA1.4	3.14E-66	-0.3287	0.018	0.169	4.69E-62	M1	VWA1
POLR2H.2	3.20E-66	-0.32061	0.136	0.36	4.77E-62	M1	POLR2H
RP11-849I	3.35E-66	-0.39272	0.011	0.156	5.00E-62	M1	RP11-849I19.1
TGFB1I1.3	3.40E-66	-0.30946	0.008	0.15	5.08E-62	M1	TGFB1I1
TBCB.2	3.45E-66	-0.39582	0.452	0.699	5.16E-62	M1	TBCB

C21orf59.	3.51E-66	-0.28012	0.128	0.354	5.24E-62	M1	C21orf59
AP2B1.3	3.73E-66	-0.3006	0.147	0.379	5.57E-62	M1	AP2B1
MFGE8.1	4.04E-66	-0.3117	0.045	0.219	6.03E-62	M1	MFGE8
PDGFRA.3	4.24E-66	-0.42914	0.021	0.174	6.33E-62	M1	PDGFRA
ARID5A.2	4.40E-66	0.5181	0.406	0.267	6.57E-62	M1	ARID5A
ZEB1.4	4.44E-66	-0.30871	0.047	0.222	6.63E-62	M1	ZEB1
ECSIT.2	4.96E-66	-0.32051	0.097	0.302	7.42E-62	M1	ECSIT
MAN1A1.3	5.00E-66	0.376961	0.122	0.033	7.47E-62	M1	MAN1A1
TRPM2.4	6.19E-66	0.368651	0.129	0.036	9.25E-62	M1	TRPM2
SLC37A3.2	6.30E-66	-0.33687	0.01	0.153	9.42E-62	M1	SLC37A3
NCBP2-AS2	6.36E-66	-0.30455	0.102	0.312	9.50E-62	M1	NCBP2-AS2
SMPD1.3	6.61E-66	-0.27418	0.074	0.269	9.87E-62	M1	SMPD1
GSPT2.2	7.08E-66	-0.32482	0.021	0.175	1.06E-61	M1	GSPT2
TNFAIP1.2	7.43E-66	-0.28377	0.036	0.203	1.11E-61	M1	TNFAIP1
FLI1.4	7.44E-66	0.387377	0.129	0.036	1.11E-61	M1	FLI1
BRIX1.4	9.08E-66	-0.30918	0.087	0.287	1.36E-61	M1	BRIX1
PIPOX.3	1.26E-65	-0.31003	0.011	0.156	1.88E-61	M1	PIPOX
DEXI.2	1.29E-65	-0.29958	0.057	0.237	1.93E-61	M1	DEXI
ZNF771.3	1.30E-65	-0.27252	0.019	0.172	1.94E-61	M1	ZNF771
CDC42EP1.	1.39E-65	-0.34666	0.009	0.152	2.08E-61	M1	CDC42EP1
KIAA1715.	1.40E-65	-0.29419	0.089	0.288	2.09E-61	M1	KIAA1715
PLCG2.2	1.42E-65	0.340772	0.122	0.033	2.12E-61	M1	PLCG2
SMARCE1.2	1.42E-65	-0.31372	0.178	0.419	2.12E-61	M1	SMARCE1
TSPAN12.2	1.64E-65	-0.34891	0.005	0.143	2.46E-61	M1	TSPAN12
WSB2.4	1.84E-65	-0.28615	0.067	0.255	2.75E-61	M1	WSB2
EFHC1.4	1.88E-65	-0.32011	0.056	0.235	2.80E-61	M1	EFHC1
LANCL2.4	1.95E-65	-0.40682	0.024	0.178	2.92E-61	M1	LANCL2
KCNQ1.3	1.99E-65	0.325699	0.106	0.025	2.98E-61	M1	KCNQ1
ZCCHC17.2	2.13E-65	-0.3127	0.157	0.391	3.18E-61	M1	ZCCHC17
ZNF439.3	2.15E-65	-0.28557	0.02	0.173	3.22E-61	M1	ZNF439
RP11-701F	2.40E-65	0.259187	0.055	0.006	3.58E-61	M1	RP11-701P16.2
SLC38A1.1	2.77E-65	-0.44368	0.018	0.166	4.13E-61	M1	SLC38A1
ANKIB1.3	3.12E-65	-0.29134	0.058	0.238	4.66E-61	M1	ANKIB1
FRMD4B.4	3.37E-65	0.505537	0.279	0.135	5.04E-61	M1	FRMD4B
SOX6.3	3.60E-65	-0.31458	0.017	0.166	5.38E-61	M1	SOX6
UBE2H.3	3.98E-65	-0.28348	0.091	0.293	5.95E-61	M1	UBE2H
PAM.3	4.11E-65	-0.29537	0.031	0.192	6.13E-61	M1	PAM
MATR3.3	4.29E-65	-0.31797	0.267	0.53	6.41E-61	M1	MATR3
SACS.4	4.65E-65	-0.26569	0.024	0.181	6.95E-61	M1	SACS
AC004540.	5.29E-65	-0.46274	0.007	0.146	7.90E-61	M1	AC004540.4
MED10.4	6.47E-65	-0.33654	0.259	0.529	9.67E-61	M1	MED10
HIST1H4C.	6.71E-65	-0.38138	0.227	0.464	1.00E-60	M1	HIST1H4C
CD58.4	7.61E-65	0.564203	0.346	0.203	1.14E-60	M1	CD58
TMEM109.4	8.18E-65	0.493158	0.324	0.181	1.22E-60	M1	TMEM109
KLHL42.3	8.20E-65	-0.28513	0.028	0.185	1.23E-60	M1	KLHL42
TSPAN14.3	8.46E-65	0.553399	0.328	0.189	1.26E-60	M1	TSPAN14
STRA13	9.20E-65	-0.34273	0.237	0.494	1.38E-60	M1	STRA13
EFNA1.2	9.34E-65	-0.44417	0.01	0.152	1.40E-60	M1	EFNA1
ZNF680.2	1.02E-64	-0.33028	0.037	0.201	1.53E-60	M1	ZNF680
KDM5B.3	1.05E-64	-0.30191	0.03	0.189	1.58E-60	M1	KDM5B
DLG1.3	1.11E-64	-0.30533	0.032	0.192	1.65E-60	M1	DLG1

INSM1.4	1.20E-64	-0.36601	0.004	0.14	1.79E-60	M1	INSM1
MTHFD2L.2	1.31E-64	-0.26807	0.057	0.236	1.96E-60	M1	MTHFD2L
ITGB1BP1.	1.33E-64	-0.34824	0.186	0.427	1.99E-60	M1	ITGB1BP1
EPDR1.3	1.39E-64	-0.28933	0.009	0.15	2.08E-60	M1	EPDR1
CDCA7L.3	1.41E-64	-0.35507	0.022	0.174	2.11E-60	M1	CDCA7L
C7orf55-L	1.66E-64	-0.28544	0.149	0.377	2.48E-60	M1	C7orf55-LUC7L2
RBM10.1	1.81E-64	-0.28223	0.055	0.232	2.70E-60	M1	RBM10
SERPINH1.	1.88E-64	-0.32791	0.06	0.239	2.81E-60	M1	SERPINH1
TTC7A.4	2.41E-64	0.436613	0.163	0.057	3.61E-60	M1	TTC7A
GPT2.2	2.54E-64	-0.30341	0.021	0.172	3.80E-60	M1	GPT2
IARS.2	3.29E-64	-0.27858	0.06	0.241	4.92E-60	M1	IARS
CHCHD7.3	3.68E-64	0.46634	0.407	0.262	5.49E-60	M1	CHCHD7
BAD.2	4.00E-64	-0.32874	0.209	0.459	5.98E-60	M1	BAD
PMM1.3	4.11E-64	-0.33513	0.076	0.265	6.14E-60	M1	PMM1
ENDOD1.2	4.21E-64	-0.31323	0.019	0.168	6.29E-60	M1	ENDOD1
PSPH.3	4.95E-64	-0.30991	0.028	0.183	7.39E-60	M1	PSPH
OPTN.2	5.05E-64	-0.32924	0.015	0.159	7.55E-60	M1	OPTN
ZFR.2	5.54E-64	-0.31316	0.125	0.342	8.28E-60	M1	ZFR
WNK3.2	5.83E-64	-0.30421	0.006	0.143	8.72E-60	M1	WNK3
FADS1.2	7.18E-64	-0.26111	0.065	0.249	1.07E-59	M1	FADS1
ARL16.1	7.75E-64	-0.29694	0.133	0.356	1.16E-59	M1	ARL16
AGAP1.2	8.46E-64	-0.32784	0.006	0.142	1.26E-59	M1	AGAP1
BEND6.3	8.70E-64	-0.26298	0.006	0.141	1.30E-59	M1	BEND6
RNF157.3	8.70E-64	-0.26273	0.014	0.158	1.30E-59	M1	RNF157
ILF3.2	8.87E-64	-0.29916	0.203	0.453	1.33E-59	M1	ILF3
ZNF704.3	9.43E-64	-0.33003	0.008	0.146	1.41E-59	M1	ZNF704
UBTF.3	1.31E-63	-0.30317	0.072	0.258	1.96E-59	M1	UBTF
DUSP23.3	1.44E-63	0.397647	0.319	0.162	2.15E-59	M1	DUSP23
EEF1E1.1	1.59E-63	-0.33661	0.134	0.352	2.38E-59	M1	EEF1E1
RP11-536K	1.63E-63	0.366929	0.102	0.024	2.44E-59	M1	RP11-536K7.3
ARL6IP5.1	1.73E-63	0.268714	0.705	0.639	2.58E-59	M1	ARL6IP5
SOCS3.3	1.80E-63	0.471524	0.461	0.326	2.69E-59	M1	SOCS3
SLC35B2.3	1.86E-63	-0.29792	0.122	0.336	2.78E-59	M1	SLC35B2
OSCAR.4	2.02E-63	0.38258	0.16	0.055	3.02E-59	M1	OSCAR
MSMO1.3	2.21E-63	-0.34287	0.057	0.23	3.30E-59	M1	MSMO1
GNG4.3	2.22E-63	-0.33469	0.005	0.138	3.32E-59	M1	GNG4
RAE1.2	2.24E-63	-0.28328	0.098	0.301	3.35E-59	M1	RAE1
SEC14L2.3	2.24E-63	-0.30722	0.004	0.137	3.35E-59	M1	SEC14L2
ASRGL1.4	2.28E-63	-0.33134	0.056	0.23	3.40E-59	M1	ASRGL1
TMEM198.2	2.37E-63	-0.29063	0.004	0.137	3.55E-59	M1	TMEM198
PSRC1.2	2.86E-63	-0.3579	0.099	0.297	4.27E-59	M1	PSRC1
PIK3AP1.4	3.05E-63	0.367824	0.148	0.048	4.55E-59	M1	PIK3AP1
CSNK2A1.2	3.16E-63	-0.29703	0.134	0.353	4.73E-59	M1	CSNK2A1
RP11-1094	3.39E-63	-0.26427	0.076	0.266	5.07E-59	M1	RP11-1094M14.11
CPXM1.3	3.63E-63	-0.31408	0.009	0.147	5.43E-59	M1	CPXM1
DCAF7.2	4.40E-63	-0.28065	0.135	0.358	6.58E-59	M1	DCAF7
RP13-516M	4.57E-63	0.392463	0.091	0.02	6.83E-59	M1	RP13-516M14.4
CCL20.3	4.61E-63	0.459458	0.142	0.045	6.90E-59	M1	CCL20
RNF19A.4	5.01E-63	-0.3101	0.057	0.231	7.48E-59	M1	RNF19A
TLR10.3	5.39E-63	0.2725	0.077	0.014	8.05E-59	M1	TLR10
WDFY4.3	5.43E-63	0.281293	0.081	0.015	8.12E-59	M1	WDFY4

IL17RA.4	6.56E-63	0.47749	0.222	0.098	9.81E-59	M1	IL17RA
SNRPE.2	8.14E-63	-0.36529	0.364	0.623	1.22E-58	M1	SNRPE
ARL8A.3	9.46E-63	-0.30927	0.104	0.306	1.41E-58	M1	ARL8A
GLA.4	9.99E-63	0.522766	0.332	0.192	1.49E-58	M1	GLA
PPP1R16A.	1.03E-62	-0.3068	0.036	0.196	1.54E-58	M1	PPP1R16A
TCTN1.3	1.04E-62	-0.32558	0.064	0.242	1.56E-58	M1	TCTN1
DAGLB.4	1.06E-62	0.521828	0.223	0.1	1.59E-58	M1	DAGLB
LAPTM4A.2	1.12E-62	0.150099	0.827	0.761	1.67E-58	M1	LAPTM4A
CHST11.4	1.13E-62	0.5164	0.327	0.19	1.68E-58	M1	CHST11
BAX.4	1.21E-62	0.332634	0.625	0.554	1.80E-58	M1	BAX
BZW2.1	1.24E-62	-0.32136	0.104	0.303	1.85E-58	M1	BZW2
CHST10.1	1.64E-62	-0.29287	0.018	0.164	2.45E-58	M1	CHST10
NOC2L.3	1.67E-62	-0.28132	0.131	0.349	2.49E-58	M1	NOC2L
DNALI1.3	1.96E-62	-0.32064	0.004	0.135	2.92E-58	M1	DNALI1
RUVBL1.1	2.09E-62	-0.3224	0.1	0.299	3.12E-58	M1	RUVBL1
C2orf80.3	2.15E-62	-0.3661	0.007	0.141	3.22E-58	M1	C2orf80
LINC00936	2.27E-62	0.607814	0.245	0.12	3.39E-58	M1	LINC00936
FAM89B.3	2.86E-62	-0.29668	0.087	0.278	4.28E-58	M1	FAM89B
SELL.3	2.91E-62	0.287722	0.101	0.024	4.35E-58	M1	SELL
BCAP31.1	3.15E-62	0.288907	0.67	0.608	4.70E-58	M1	BCAP31
IQCK.2	3.47E-62	-0.31842	0.011	0.149	5.18E-58	M1	IQCK
DHRS9.2	3.50E-62	0.313781	0.073	0.012	5.23E-58	M1	DHRS9
B4GALNT1.	4.15E-62	-0.48714	0.023	0.167	6.20E-58	M1	B4GALNT1
PPAT.2	4.92E-62	-0.29685	0.021	0.168	7.35E-58	M1	PPAT
EBNA1BP2.	5.10E-62	-0.30669	0.162	0.392	7.62E-58	M1	EBNA1BP2
TIMM44.2	6.33E-62	-0.28385	0.097	0.295	9.45E-58	M1	TIMM44
MRFAP1L1.	6.38E-62	-0.28793	0.069	0.25	9.54E-58	M1	MRFAP1L1
MTRF1L.2	7.01E-62	-0.26478	0.049	0.218	1.05E-57	M1	MTRF1L
CMKLR1.3	7.83E-62	0.386561	0.122	0.035	1.17E-57	M1	CMKLR1
BRD7.3	8.27E-62	-0.28461	0.091	0.287	1.24E-57	M1	BRD7
FAM32A.2	8.45E-62	-0.3329	0.239	0.495	1.26E-57	M1	FAM32A
RTKN.4	9.06E-62	-0.33219	0.038	0.198	1.35E-57	M1	RTKN
LBH.4	9.16E-62	-0.46769	0.029	0.178	1.37E-57	M1	LBH
CAMK2B.3	9.20E-62	-0.33912	0.005	0.135	1.37E-57	M1	CAMK2B
ZNF827.3	1.14E-61	-0.27808	0.005	0.136	1.71E-57	M1	ZNF827
TMC8.2	1.33E-61	0.323978	0.111	0.029	1.99E-57	M1	TMC8
COIL.2	1.38E-61	-0.267	0.047	0.213	2.06E-57	M1	COIL
CMSS1.1	1.60E-61	-0.32015	0.117	0.324	2.38E-57	M1	CMSS1
SP110.3	1.65E-61	0.497468	0.313	0.175	2.47E-57	M1	SP110
YAE1D1.2	1.66E-61	-0.34158	0.032	0.186	2.48E-57	M1	YAE1D1
PRPF6.2	1.79E-61	-0.2835	0.163	0.391	2.68E-57	M1	PRPF6
PHLDA3.1	1.83E-61	-0.32609	0.039	0.197	2.73E-57	M1	PHLDA3
ZNF493.2	1.92E-61	-0.32341	0.031	0.183	2.87E-57	M1	ZNF493
CDCA7.3	1.94E-61	-0.40021	0.01	0.145	2.90E-57	M1	CDCA7
ENOPH1.3	2.34E-61	-0.29883	0.19	0.429	3.50E-57	M1	ENOPH1
P2RX4.3	2.54E-61	0.491338	0.29	0.154	3.79E-57	M1	P2RX4
BPTF.2	2.55E-61	-0.31059	0.133	0.346	3.82E-57	M1	BPTF
MAP2K2.3	2.73E-61	-0.32506	0.128	0.337	4.08E-57	M1	MAP2K2
CDH11.2	2.73E-61	-0.32323	0.006	0.137	4.08E-57	M1	CDH11
C17orf58.	2.92E-61	-0.28057	0.048	0.213	4.37E-57	M1	C17orf58
USP47	2.93E-61	-0.27861	0.086	0.277	4.38E-57	M1	USP47

C1QL1.3	3.08E-61	-0.34202	0.006	0.136	4.61E-57	M1	C1QL1
FES.4	3.14E-61	0.402393	0.169	0.061	4.70E-57	M1	FES
GDAP1.4	4.12E-61	-0.38267	0.028	0.177	6.16E-57	M1	GDAP1
TMEM203.3	4.57E-61	-0.26672	0.091	0.285	6.83E-57	M1	TMEM203
ADORA1.2	4.83E-61	-0.30455	0.01	0.144	7.22E-57	M1	ADORA1
MRAS.2	4.90E-61	-0.28896	0.037	0.194	7.32E-57	M1	MRAS
TRIM38.3	5.04E-61	0.462609	0.194	0.079	7.53E-57	M1	TRIM38
CSE1L.3	6.17E-61	-0.2883	0.072	0.253	9.22E-57	M1	CSE1L
LONP1.2	6.57E-61	-0.25894	0.06	0.234	9.81E-57	M1	LONP1
LSM7.1	7.15E-61	-0.34497	0.439	0.669	1.07E-56	M1	LSM7
RP11-792A	7.62E-61	-0.31557	0.064	0.239	1.14E-56	M1	RP11-792A8.4
CLEC5A.4	7.79E-61	0.45149	0.2	0.079	1.16E-56	M1	CLEC5A
RARRES2.3	8.07E-61	-0.77249	0.029	0.173	1.21E-56	M1	RARRES2
CD200.4	8.08E-61	-0.35788	0.004	0.131	1.21E-56	M1	CD200
SFT2D1.4	8.22E-61	0.36084	0.574	0.487	1.23E-56	M1	SFT2D1
UBXN2A.2	8.32E-61	-0.23853	0.049	0.215	1.24E-56	M1	UBXN2A
LAMP5.3	8.53E-61	-0.30644	0.003	0.13	1.27E-56	M1	LAMP5
TRAF3IP3.	8.71E-61	0.368256	0.127	0.037	1.30E-56	M1	TRAF3IP3
MED31.1	8.80E-61	-0.25127	0.067	0.246	1.31E-56	M1	MED31
CCDC82.1	9.09E-61	-0.28886	0.105	0.305	1.36E-56	M1	CCDC82
RAB6A.3	9.34E-61	-0.27908	0.194	0.434	1.40E-56	M1	RAB6A
ERI3.2	1.00E-60	-0.28927	0.174	0.408	1.50E-56	M1	ERI3
DHFR.2	1.15E-60	-0.30489	0.027	0.176	1.72E-56	M1	DHFR
ZCRB1.3	1.17E-60	-0.33163	0.275	0.534	1.76E-56	M1	ZCRB1
EDNRB.2	1.23E-60	-0.36729	0.05	0.214	1.84E-56	M1	EDNRB
PYGB.2	1.50E-60	-0.26093	0.064	0.24	2.23E-56	M1	PYGB
IGF2BP3.3	1.50E-60	-0.25957	0.003	0.129	2.25E-56	M1	IGF2BP3
ACYP1.1	1.60E-60	-0.3202	0.043	0.204	2.39E-56	M1	ACYP1
ACAT2.3	1.60E-60	-0.28641	0.11	0.311	2.39E-56	M1	ACAT2
NFYB.2	1.78E-60	-0.3039	0.047	0.211	2.67E-56	M1	NFYB
NDP.2	1.94E-60	-0.34593	0.005	0.133	2.90E-56	M1	NDP
COL4A1.1	2.06E-60	-0.24871	0.004	0.132	3.07E-56	M1	COL4A1
GNAS.4	2.07E-60	-0.34124	0.48	0.681	3.09E-56	M1	GNAS
CNPY2.3	2.22E-60	-0.33615	0.346	0.613	3.32E-56	M1	CNPY2
PRKRIP1.3	2.24E-60	-0.26757	0.085	0.273	3.34E-56	M1	PRKRIP1
ZNF385D.2	2.24E-60	-0.28345	0.004	0.132	3.35E-56	M1	ZNF385D
LYRM1.3	2.33E-60	-0.34307	0.047	0.21	3.48E-56	M1	LYRM1
SLC2A1.3	2.52E-60	-0.38095	0.02	0.161	3.77E-56	M1	SLC2A1
CIAPIN1.1	2.53E-60	-0.29969	0.099	0.292	3.78E-56	M1	CIAPIN1
FAM221A.4	2.72E-60	-0.37857	0.045	0.204	4.07E-56	M1	FAM221A
DHRS3.2	2.85E-60	0.424452	0.288	0.148	4.26E-56	M1	DHRS3
SH3BP1.4	2.89E-60	0.46501	0.21	0.092	4.31E-56	M1	SH3BP1
BSDC1.2	2.90E-60	-0.26546	0.119	0.325	4.34E-56	M1	BSDC1
ATP9A.3	2.98E-60	-0.30053	0.005	0.134	4.46E-56	M1	ATP9A
CLDND1.2	3.03E-60	-0.40221	0.197	0.433	4.53E-56	M1	CLDND1
FBXO32.4	3.26E-60	-0.42505	0.03	0.179	4.88E-56	M1	FBXO32
TMCO3	3.37E-60	-0.25146	0.06	0.233	5.03E-56	M1	TMCO3
C16orf91.	3.40E-60	-0.28414	0.055	0.224	5.09E-56	M1	C16orf91
KRIT1.3	3.62E-60	-0.24849	0.068	0.246	5.41E-56	M1	KRIT1
AKTIP.3	3.67E-60	-0.32843	0.038	0.194	5.48E-56	M1	AKTIP
SOGA1.3	3.86E-60	-0.24756	0.03	0.18	5.76E-56	M1	SOGA1

VEZF1.4	4.00E-60	-0.2713	0.083	0.269	5.98E-56	M1	VEZF1
PLA2G12A.	4.15E-60	-0.26688	0.08	0.265	6.21E-56	M1	PLA2G12A
NTHL1.1	5.66E-60	-0.25946	0.049	0.215	8.45E-56	M1	NTHL1
DEAF1.3	5.80E-60	-0.25182	0.065	0.241	8.66E-56	M1	DEAF1
ADCY7.4	5.98E-60	0.385859	0.144	0.047	8.93E-56	M1	ADCY7
ZNF91.1	6.36E-60	-0.23292	0.064	0.239	9.51E-56	M1	ZNF91
FSTL1.2	6.47E-60	-0.26231	0.006	0.135	9.67E-56	M1	FSTL1
FAM127B.3	6.84E-60	-0.35519	0.131	0.334	1.02E-55	M1	FAM127B
ENDOG.3	7.07E-60	-0.27158	0.025	0.172	1.06E-55	M1	ENDOG
ZIC1.3	7.33E-60	-0.31264	0.007	0.136	1.09E-55	M1	ZIC1
RSPRY1.2	8.49E-60	-0.26464	0.09	0.28	1.27E-55	M1	RSPRY1
GABARAP.2	1.01E-59	0.418624	0.551	0.443	1.51E-55	M1	GABARAP
RBM47.4	1.03E-59	0.465629	0.217	0.097	1.54E-55	M1	RBM47
UBE2M.4	1.05E-59	-0.30234	0.078	0.261	1.57E-55	M1	UBE2M
EHMT2.3	1.12E-59	-0.2886	0.059	0.229	1.67E-55	M1	EHMT2
ACSS3.2	1.21E-59	-0.2375	0.005	0.133	1.80E-55	M1	ACSS3
TRPV2.4	1.21E-59	0.376171	0.136	0.043	1.82E-55	M1	TRPV2
GAD1.4	1.23E-59	-0.32213	0.006	0.134	1.84E-55	M1	GAD1
NRXN2.4	1.32E-59	-0.35707	0.008	0.138	1.97E-55	M1	NRXN2
COQ9.3	1.39E-59	-0.26952	0.086	0.273	2.07E-55	M1	COQ9
MCC.2	1.72E-59	-0.23866	0.011	0.144	2.58E-55	M1	MCC
CHCHD2.3	1.75E-59	-0.25805	0.805	0.895	2.62E-55	M1	CHCHD2
MRFAP1.3	1.76E-59	-0.32537	0.47	0.711	2.62E-55	M1	MRFAP1
DCUN1D5	1.78E-59	-0.27926	0.117	0.321	2.65E-55	M1	DCUN1D5
RSBN1.2	1.91E-59	-0.30939	0.071	0.246	2.85E-55	M1	RSBN1
RUFY2.4	2.00E-59	-0.27532	0.027	0.174	2.99E-55	M1	RUFY2
ARL9.3	2.24E-59	-0.27876	0.002	0.125	3.35E-55	M1	ARL9
FUCA1.3	2.56E-59	0.525327	0.332	0.197	3.82E-55	M1	FUCA1
SYP.4	2.73E-59	-0.37326	0.018	0.157	4.08E-55	M1	SYP
OLA1.1	2.87E-59	-0.30388	0.275	0.531	4.29E-55	M1	OLA1
SYNC.2	3.21E-59	-0.2914	0.044	0.202	4.80E-55	M1	SYNC
MARCH9.3	3.48E-59	-0.38286	0.032	0.179	5.20E-55	M1	9-Mar
PSMB10.4	3.63E-59	0.443158	0.376	0.231	5.43E-55	M1	PSMB10
KCTD3.1	3.67E-59	-0.24865	0.023	0.166	5.49E-55	M1	KCTD3
PAPOLG.4	3.76E-59	0.487946	0.182	0.073	5.62E-55	M1	PAPOLG
KIDINS220	3.85E-59	-0.27788	0.107	0.301	5.76E-55	M1	KIDINS220
ANKS3.2	4.39E-59	-0.27985	0.039	0.194	6.56E-55	M1	ANKS3
MFF.1	4.53E-59	-0.30729	0.273	0.533	6.76E-55	M1	MFF
RPAP2.2	4.87E-59	-0.26433	0.049	0.212	7.28E-55	M1	RPAP2
EXTL3.2	4.90E-59	-0.26344	0.017	0.154	7.32E-55	M1	EXTL3
ZNF677.2	5.39E-59	-0.2293	0.057	0.225	8.06E-55	M1	ZNF677
URI1.1	5.40E-59	-0.26259	0.072	0.251	8.07E-55	M1	URI1
KHSRP.4	5.75E-59	-0.24931	0.084	0.269	8.60E-55	M1	KHSRP
BRD3.3	6.55E-59	-0.26735	0.11	0.308	9.79E-55	M1	BRD3
MTX2.2	6.74E-59	-0.27486	0.144	0.357	1.01E-54	M1	MTX2
RP11-395G	6.87E-59	-0.34055	0.046	0.204	1.03E-54	M1	RP11-395G23.3
TSEN15.2	7.77E-59	-0.27114	0.081	0.263	1.16E-54	M1	TSEN15
PEF1.2	8.13E-59	-0.30306	0.142	0.355	1.21E-54	M1	PEF1
CKS2.4	8.48E-59	0.463083	0.475	0.345	1.27E-54	M1	CKS2
SEPT4.4	8.91E-59	-0.49367	0.017	0.153	1.33E-54	M1	4-Sep
CIB1.3	9.17E-59	0.327997	0.582	0.474	1.37E-54	M1	CIB1

STARD3NL.	1.11E-58	-0.27388	0.2	0.437	1.66E-54	M1	STARD3NL
TMEM25.3	1.25E-58	-0.29823	0.012	0.144	1.87E-54	M1	TMEM25
SLC6A8.2	1.34E-58	-0.34723	0.01	0.139	2.00E-54	M1	SLC6A8
SLC1A5.4	1.44E-58	0.502127	0.241	0.117	2.15E-54	M1	SLC1A5
TCEAL5.2	1.47E-58	-0.44591	0.012	0.142	2.19E-54	M1	TCEAL5
ALYREF.4	1.56E-58	-0.24362	0.025	0.17	2.33E-54	M1	ALYREF
SHD.3	1.72E-58	-0.44142	0.015	0.148	2.58E-54	M1	SHD
CC2D1A.3	1.74E-58	-0.24793	0.05	0.213	2.59E-54	M1	CC2D1A
NFIL3.4	1.76E-58	0.449732	0.171	0.066	2.64E-54	M1	NFIL3
TMEM181.4	1.86E-58	-0.26348	0.037	0.189	2.78E-54	M1	TMEM181
ANP32A.4	2.05E-58	0.417632	0.511	0.412	3.06E-54	M1	ANP32A
NLGN4X.2	2.19E-58	-0.27358	0.003	0.126	3.27E-54	M1	NLGN4X
CSGALNACT	2.23E-58	0.433893	0.158	0.058	3.33E-54	M1	CSGALNACT1
MT1F.4	2.41E-58	-0.39353	0.138	0.339	3.60E-54	M1	MT1F
RRM1.2	2.53E-58	-0.3149	0.051	0.212	3.79E-54	M1	RRM1
DOHH.2	2.55E-58	-0.28712	0.057	0.221	3.81E-54	M1	DOHH
CETN2.4	2.62E-58	-0.39815	0.149	0.357	3.91E-54	M1	CETN2
TIMM13.1	2.82E-58	-0.33574	0.388	0.632	4.22E-54	M1	TIMM13
RP11-452F	2.83E-58	0.496238	0.228	0.106	4.23E-54	M1	RP11-452F19.3
AHCYL1.4	2.90E-58	-0.32597	0.15	0.363	4.33E-54	M1	AHCYL1
CDK16.2	2.95E-58	-0.23054	0.082	0.266	4.41E-54	M1	CDK16
GNLY.1	3.14E-58	0.328026	0.086	0.018	4.69E-54	M1	GNLY
OBSL1.3	3.39E-58	-0.26223	0.026	0.17	5.06E-54	M1	OBSL1
MAN1A2.2	3.80E-58	-0.26103	0.063	0.232	5.67E-54	M1	MAN1A2
NRARP.2	3.87E-58	0.325482	0.095	0.023	5.78E-54	M1	NRARP
ZNF791.1	4.54E-58	-0.25145	0.107	0.303	6.79E-54	M1	ZNF791
CYR61.2	4.63E-58	-0.52798	0.042	0.193	6.92E-54	M1	CYR61
ROBO2.3	5.85E-58	-0.26273	0.005	0.128	8.73E-54	M1	ROBO2
CCDC80.3	6.15E-58	-0.39438	0.008	0.134	9.19E-54	M1	CCDC80
RUVBL2.1	6.20E-58	-0.25344	0.141	0.353	9.26E-54	M1	RUVBL2
SNAP47.2	6.33E-58	-0.25579	0.062	0.231	9.46E-54	M1	SNAP47
HMGCS1.2	6.41E-58	-0.28069	0.049	0.21	9.59E-54	M1	HMGCS1
SRPK1.2	6.61E-58	-0.2952	0.039	0.191	9.87E-54	M1	SRPK1
CLEC4A.4	6.61E-58	0.463334	0.139	0.046	9.88E-54	M1	CLEC4A
TSEN34.1	6.69E-58	-0.27037	0.176	0.407	9.99E-54	M1	TSEN34
DLL3.3	6.91E-58	-0.53458	0.028	0.167	1.03E-53	M1	DLL3
NBPF1.4	7.01E-58	-0.23044	0.083	0.266	1.05E-53	M1	NBPF1
PTK2.2	7.74E-58	-0.28025	0.046	0.204	1.16E-53	M1	PTK2
ODF3B.4	7.94E-58	0.416792	0.186	0.075	1.19E-53	M1	ODF3B
SRR.3	8.77E-58	-0.25762	0.031	0.178	1.31E-53	M1	SRR
PLA2G5.3	9.31E-58	-0.54938	0.006	0.129	1.39E-53	M1	PLA2G5
DIRAS3.3	9.81E-58	-0.53109	0.014	0.145	1.47E-53	M1	DIRAS3
ARPC5L.2	1.14E-57	-0.29102	0.181	0.407	1.70E-53	M1	ARPC5L
EZH2.3	1.18E-57	-0.32769	0.032	0.178	1.77E-53	M1	EZH2
CDKN1B.3	1.21E-57	-0.30108	0.102	0.29	1.80E-53	M1	CDKN1B
SPATA33.3	1.28E-57	-0.27035	0.026	0.17	1.92E-53	M1	SPATA33
TIMM8B.2	1.38E-57	-0.34291	0.338	0.585	2.06E-53	M1	TIMM8B
LINC00511	1.48E-57	-0.26444	0.011	0.141	2.21E-53	M1	LINC00511
APBB2.3	1.51E-57	-0.30008	0.008	0.134	2.26E-53	M1	APBB2
KCNMB4.4	1.54E-57	-0.34766	0.014	0.145	2.30E-53	M1	KCNMB4
AMER2.3	1.56E-57	-0.48409	0.009	0.135	2.33E-53	M1	AMER2

PAXBP1.3	1.56E-57	-0.23482	0.035	0.184	2.34E-53	M1	PAXBP1
SLC4A4.2	1.67E-57	-0.33517	0.006	0.129	2.49E-53	M1	SLC4A4
PALLD.1	1.67E-57	-0.2591	0.06	0.226	2.49E-53	M1	PALLD
ATP1A3.3	1.72E-57	-0.41184	0.013	0.142	2.57E-53	M1	ATP1A3
POLR2K.1	1.83E-57	-0.31561	0.28	0.531	2.73E-53	M1	POLR2K
CCT7.2	1.88E-57	-0.31646	0.316	0.572	2.81E-53	M1	CCT7
SH3BGRL3.	2.03E-57	0.107197	0.849	0.763	3.04E-53	M1	SH3BGRL3
EFS.2	2.08E-57	-0.32663	0.004	0.126	3.10E-53	M1	EFS
NAV2.1	2.21E-57	-0.30092	0.011	0.139	3.31E-53	M1	NAV2
APOD.3	2.39E-57	-0.83248	0.04	0.185	3.57E-53	M1	APOD
TMEM132B.	2.44E-57	-0.26394	0.002	0.122	3.65E-53	M1	TMEM132B
ASF1A.3	2.66E-57	-0.24975	0.084	0.264	3.97E-53	M1	ASF1A
CDK6.3	2.72E-57	-0.3483	0.067	0.233	4.07E-53	M1	CDK6
ATCAY.3	3.04E-57	-0.36669	0.013	0.142	4.55E-53	M1	ATCAY
KCNE4.2	3.06E-57	-0.26677	0.004	0.125	4.58E-53	M1	KCNE4
KLRC2.3	3.12E-57	-0.41965	0.007	0.13	4.66E-53	M1	KLRC2
HS2ST1.3	3.26E-57	-0.28497	0.045	0.2	4.88E-53	M1	HS2ST1
TYRO3.3	3.27E-57	-0.28334	0.006	0.13	4.88E-53	M1	TYRO3
WHSC1.3	3.37E-57	-0.26949	0.032	0.178	5.03E-53	M1	WHSC1
DVL2.2	3.51E-57	-0.24483	0.046	0.203	5.25E-53	M1	DVL2
AKAP8L.2	3.81E-57	-0.26216	0.093	0.28	5.70E-53	M1	AKAP8L
DZIP1.3	3.86E-57	-0.23984	0.003	0.122	5.78E-53	M1	DZIP1
HS3ST1.3	4.12E-57	0.463987	0.177	0.07	6.15E-53	M1	HS3ST1
BRD8.2	4.40E-57	-0.26829	0.037	0.187	6.58E-53	M1	BRD8
GSTK1.2	4.46E-57	0.298886	0.603	0.515	6.66E-53	M1	GSTK1
CD300C.4	4.79E-57	0.397867	0.149	0.052	7.16E-53	M1	CD300C
MPHOSPH1C	5.34E-57	-0.28135	0.087	0.268	7.98E-53	M1	MPHOSPH10
ISCA1.2	5.39E-57	-0.23113	0.127	0.331	8.06E-53	M1	ISCA1
RFC4.2	5.49E-57	-0.32735	0.035	0.181	8.20E-53	M1	RFC4
APOO.3	5.79E-57	-0.28089	0.08	0.259	8.66E-53	M1	APOO
GIMAP6.2	5.97E-57	0.3116	0.109	0.029	8.92E-53	M1	GIMAP6
NTM.2	6.33E-57	-0.46025	0.008	0.132	9.46E-53	M1	NTM
MX1.2	6.38E-57	0.421223	0.221	0.1	9.53E-53	M1	MX1
PSMD14.2	7.18E-57	-0.31083	0.259	0.509	1.07E-52	M1	PSMD14
SNX21.1	8.03E-57	-0.23628	0.011	0.14	1.20E-52	M1	SNX21
RAD21.3	8.28E-57	-0.29848	0.182	0.403	1.24E-52	M1	RAD21
PAPSS1.1	9.21E-57	-0.27426	0.128	0.328	1.38E-52	M1	PAPSS1
ALKBH2.4	9.26E-57	-0.25887	0.058	0.223	1.38E-52	M1	ALKBH2
RB1CC1.2	9.41E-57	-0.26102	0.114	0.309	1.41E-52	M1	RB1CC1
TRIM16.2	9.81E-57	-0.25507	0.018	0.152	1.47E-52	M1	TRIM16
TNPO2.2	1.05E-56	-0.2277	0.048	0.206	1.58E-52	M1	TNPO2
PPP2R5B.3	1.08E-56	-0.26071	0.04	0.191	1.61E-52	M1	PPP2R5B
DPEP2.3	1.16E-56	0.357218	0.092	0.022	1.74E-52	M1	DPEP2
TTC9B.4	1.25E-56	-0.46315	0.014	0.141	1.87E-52	M1	TTC9B
PSMG3.3	1.38E-56	-0.26816	0.15	0.363	2.07E-52	M1	PSMG3
IFI27L1.2	1.45E-56	-0.30833	0.053	0.212	2.16E-52	M1	IFI27L1
ERV3-1.3	1.53E-56	-0.29357	0.104	0.292	2.29E-52	M1	ERV3-1
MGST1.3	1.59E-56	-0.88177	0.03	0.168	2.37E-52	M1	MGST1
RP11-410L	1.60E-56	-0.26157	0.017	0.15	2.38E-52	M1	RP11-410L14.2
ARNT2.4	1.68E-56	-0.23926	0.008	0.132	2.51E-52	M1	ARNT2
TIMP3.2	1.70E-56	-0.35629	0.016	0.147	2.54E-52	M1	TIMP3

GOT2.2	1.78E-56	-0.26448	0.06	0.224	2.65E-52	M1	GOT2
PRPSAP1.4	1.84E-56	-0.21965	0.133	0.341	2.76E-52	M1	PRPSAP1
RBBP4.1	1.87E-56	-0.30197	0.231	0.471	2.79E-52	M1	RBBP4
ARHGAP27.	2.24E-56	0.358639	0.128	0.04	3.34E-52	M1	ARHGAP27
POLR2C.3	2.27E-56	-0.30803	0.169	0.386	3.39E-52	M1	POLR2C
NELL2.2	2.30E-56	-0.32929	0.005	0.126	3.44E-52	M1	NELL2
PTPRF.4	2.33E-56	-0.2959	0.01	0.136	3.48E-52	M1	PTPRF
ATP8B4.3	2.36E-56	0.329492	0.109	0.03	3.52E-52	M1	ATP8B4
PRPSAP2.1	2.51E-56	-0.26217	0.107	0.298	3.75E-52	M1	PRPSAP2
LIFR.1	2.62E-56	-0.28647	0.007	0.13	3.91E-52	M1	LIFR
FAM78A.3	2.71E-56	0.309617	0.095	0.024	4.05E-52	M1	FAM78A
OAS1.2	2.75E-56	0.405813	0.239	0.111	4.10E-52	M1	OAS1
C19orf25.	3.05E-56	-0.22661	0.128	0.334	4.56E-52	M1	C19orf25
NAIP.4	3.08E-56	0.446547	0.203	0.09	4.61E-52	M1	NAIP
DLX1.4	3.18E-56	-0.44162	0.011	0.136	4.75E-52	M1	DLX1
AUTS2.4	3.35E-56	-0.29042	0.012	0.14	5.01E-52	M1	AUTS2
ZNF883.3	3.35E-56	-0.2687	0.025	0.164	5.01E-52	M1	ZNF883
TNFRSF19.	3.46E-56	-0.22705	0.005	0.125	5.17E-52	M1	TNFRSF19
SEZ6.4	3.51E-56	-0.34854	0.024	0.162	5.24E-52	M1	SEZ6
USP11.3	3.80E-56	-0.29644	0.171	0.389	5.68E-52	M1	USP11
TPP1.4	3.83E-56	0.467027	0.43	0.301	5.72E-52	M1	TPP1
TMEM121.3	3.87E-56	-0.27454	0.005	0.126	5.78E-52	M1	TMEM121
SWI5.4	3.99E-56	-0.28566	0.093	0.276	5.96E-52	M1	SWI5
ANKS1B.3	4.73E-56	-0.43207	0.007	0.128	7.07E-52	M1	ANKS1B
SNHG9.1	5.25E-56	-0.2467	0.133	0.339	7.85E-52	M1	SNHG9
GGT7.2	5.38E-56	-0.22484	0.04	0.191	8.04E-52	M1	GGT7
RP1-39G22	5.78E-56	-0.27704	0.019	0.153	8.64E-52	M1	RP1-39G22.7
EPB41L3.4	6.20E-56	0.363646	0.203	0.085	9.26E-52	M1	EPB41L3
ATL1.1	7.19E-56	-0.3305	0.011	0.137	1.07E-51	M1	ATL1
ASPH.1	7.24E-56	-0.27121	0.116	0.309	1.08E-51	M1	ASPH
ANXA2R.3	7.40E-56	-0.32093	0.033	0.176	1.11E-51	M1	ANXA2R
TNFAIP6.2	7.45E-56	-0.50476	0.008	0.13	1.11E-51	M1	TNFAIP6
TMX4.2	8.15E-56	-0.24061	0.093	0.276	1.22E-51	M1	TMX4
MMP16.2	8.22E-56	-0.26843	0.004	0.123	1.23E-51	M1	MMP16
AEBP1.3	8.22E-56	-0.38339	0.014	0.143	1.23E-51	M1	AEBP1
GNGT2.4	8.88E-56	0.375391	0.123	0.038	1.33E-51	M1	GNGT2
MGMT.4	8.97E-56	0.429463	0.262	0.129	1.34E-51	M1	MGMT
ROM1.3	9.56E-56	-0.3414	0.01	0.134	1.43E-51	M1	ROM1
GPATCH8.2	9.69E-56	-0.23105	0.131	0.338	1.45E-51	M1	GPATCH8
PPME1.2	1.20E-55	-0.22172	0.05	0.208	1.80E-51	M1	PPME1
GLT8D1.1	1.21E-55	-0.24596	0.113	0.309	1.81E-51	M1	GLT8D1
GK.4	1.30E-55	0.472927	0.239	0.118	1.95E-51	M1	GK
ADH5.2	1.38E-55	-0.28868	0.199	0.427	2.06E-51	M1	ADH5
ECHDC3.3	1.45E-55	0.297367	0.09	0.021	2.16E-51	M1	ECHDC3
ZNF326.2	1.49E-55	-0.23184	0.094	0.278	2.23E-51	M1	ZNF326
ZNF664.2	1.54E-55	-0.25065	0.031	0.173	2.30E-51	M1	ZNF664
STXBP1.3	1.60E-55	-0.2811	0.014	0.142	2.39E-51	M1	STXBP1
CYFIP2.3	1.74E-55	-0.2982	0.01	0.134	2.60E-51	M1	CYFIP2
DGUOK.2	1.76E-55	-0.32261	0.301	0.559	2.63E-51	M1	DGUOK
TM4SF1.4	1.76E-55	-0.35579	0.01	0.133	2.64E-51	M1	TM4SF1
FKBP4.3	1.93E-55	-0.29158	0.15	0.355	2.88E-51	M1	FKBP4

NHP2.2	1.98E-55	-0.32762	0.366	0.623	2.96E-51	M1	NHP2
TIA1.4	2.05E-55	-0.23315	0.095	0.279	3.07E-51	M1	TIA1
NMB.3	2.10E-55	-0.39558	0.067	0.229	3.14E-51	M1	NMB
RP4-665J2	2.22E-55	-0.2745	0.017	0.147	3.32E-51	M1	RP4-665J23.1
ATN1.2	2.38E-55	-0.22855	0.052	0.21	3.55E-51	M1	ATN1
GLCCI1.3	2.42E-55	-0.39811	0.034	0.175	3.62E-51	M1	GLCCI1
RP11-25K1	2.63E-55	-0.27029	0.003	0.12	3.93E-51	M1	RP11-25K19.1
BTBD3.3	3.02E-55	-0.24033	0.023	0.16	4.51E-51	M1	BTBD3
ABCD3.2	3.07E-55	-0.23869	0.06	0.223	4.59E-51	M1	ABCD3
IER2.3	3.09E-55	0.291454	0.718	0.594	4.62E-51	M1	IER2
RNF166.4	3.19E-55	0.499512	0.289	0.164	4.77E-51	M1	RNF166
NME7.4	3.45E-55	-0.29727	0.039	0.186	5.16E-51	M1	NME7
FZD7.1	3.52E-55	-0.24047	0.002	0.117	5.27E-51	M1	FZD7
PTX3.3	3.70E-55	-0.32792	0.011	0.136	5.52E-51	M1	PTX3
GRB2.4	4.04E-55	0.371441	0.544	0.468	6.04E-51	M1	GRB2
FAM104A.2	4.08E-55	-0.25222	0.089	0.27	6.10E-51	M1	FAM104A
PDLIM3.3	4.19E-55	-0.40435	0.011	0.135	6.26E-51	M1	PDLIM3
DNAJB2.3	4.27E-55	-0.28481	0.144	0.348	6.37E-51	M1	DNAJB2
STRADB.1	4.42E-55	-0.24149	0.076	0.249	6.60E-51	M1	STRADB
FDX1L.2	4.52E-55	-0.29712	0.104	0.29	6.75E-51	M1	FDX1L
GSK3A.3	4.81E-55	-0.22098	0.057	0.217	7.19E-51	M1	GSK3A
M6PR.4	5.30E-55	0.430611	0.47	0.364	7.92E-51	M1	M6PR
KLHL13.3	5.56E-55	-0.25151	0.003	0.118	8.32E-51	M1	KLHL13
SLC25A6.2	5.70E-55	0.130606	0.914	0.846	8.51E-51	M1	SLC25A6
FHIT.2	5.74E-55	0.481057	0.212	0.097	8.58E-51	M1	FHIT
ZHX3.2	5.87E-55	-0.23783	0.014	0.141	8.77E-51	M1	ZHX3
GPR137.3	6.28E-55	-0.24693	0.068	0.237	9.39E-51	M1	GPR137
EPSTI1.4	6.34E-55	0.414831	0.225	0.105	9.48E-51	M1	EPSTI1
ADPGK.4	6.56E-55	0.483053	0.365	0.24	9.80E-51	M1	ADPGK
WLS.2	7.35E-55	-0.22779	0.072	0.243	1.10E-50	M1	WLS
CARD16.2	7.42E-55	0.353629	0.356	0.204	1.11E-50	M1	CARD16
SFRP1.3	7.97E-55	-0.24561	0.002	0.117	1.19E-50	M1	SFRP1
CSPG5.3	8.01E-55	-0.38749	0.008	0.128	1.20E-50	M1	CSPG5
TPM1.2	8.07E-55	-0.26453	0.1	0.283	1.21E-50	M1	TPM1
SESTD1.3	8.31E-55	-0.30791	0.038	0.183	1.24E-50	M1	SESTD1
CORO2B.2	8.37E-55	-0.26073	0.004	0.12	1.25E-50	M1	CORO2B
PRKAG2-AS	8.85E-55	0.462871	0.224	0.105	1.32E-50	M1	PRKAG2-AS1
NFKBIZ.4	8.94E-55	0.479458	0.282	0.154	1.34E-50	M1	NFKBIZ
CKS1B.1	9.92E-55	-0.36748	0.107	0.289	1.48E-50	M1	CKS1B
NAP1L5.2	1.00E-54	-0.28876	0.023	0.157	1.50E-50	M1	NAP1L5
FILIP1L.4	1.07E-54	0.553912	0.247	0.13	1.59E-50	M1	FILIP1L
TRIM22.3	1.16E-54	0.464697	0.234	0.114	1.73E-50	M1	TRIM22
LILRA4.1	1.17E-54	0.304636	0.071	0.014	1.74E-50	M1	LILRA4
GTF2B.3	1.20E-54	0.523137	0.4	0.28	1.79E-50	M1	GTF2B
RHOH.4	1.21E-54	0.437271	0.16	0.06	1.81E-50	M1	RHOH
ZNRF1.4	1.25E-54	-0.24588	0.012	0.137	1.86E-50	M1	ZNRF1
PCYT2.3	1.36E-54	-0.22818	0.038	0.184	2.04E-50	M1	PCYT2
LRRN3.3	1.37E-54	-0.34431	0.011	0.134	2.05E-50	M1	LRRN3
ST3GAL4.2	1.42E-54	-0.2676	0.016	0.144	2.12E-50	M1	ST3GAL4
PCDHB10.3	1.48E-54	-0.31606	0.005	0.122	2.21E-50	M1	PCDHB10
EIF2AK1.2	1.53E-54	-0.26125	0.156	0.367	2.28E-50	M1	EIF2AK1

ZNF414.4	1.54E-54	-0.22919	0.054	0.212	2.30E-50	M1	ZNF414
OXCT1.1	1.56E-54	-0.25762	0.031	0.172	2.33E-50	M1	OXCT1
ARL6IP6	1.57E-54	-0.26259	0.121	0.313	2.35E-50	M1	ARL6IP6
EIF3B.1	1.65E-54	-0.2521	0.095	0.277	2.46E-50	M1	EIF3B
LTBP3.2	1.65E-54	-0.23612	0.07	0.238	2.46E-50	M1	LTBP3
TSPAN3.3	1.71E-54	-0.28743	0.259	0.5	2.56E-50	M1	TSPAN3
ERH.3	1.74E-54	-0.36121	0.525	0.751	2.59E-50	M1	ERH
POLR2F.4	2.04E-54	-0.30075	0.334	0.584	3.04E-50	M1	POLR2F
DLX2.4	2.06E-54	-0.4759	0.009	0.128	3.09E-50	M1	DLX2
CRIPT	2.33E-54	-0.24815	0.152	0.361	3.48E-50	M1	CRIPT
EIF3K.3	2.35E-54	0.160083	0.798	0.75	3.51E-50	M1	EIF3K
STK39.2	2.72E-54	-0.24422	0.005	0.121	4.06E-50	M1	STK39
RABEPK.3	2.77E-54	-0.2567	0.064	0.227	4.13E-50	M1	RABEPK
ZNHIT1.1	2.83E-54	-0.34375	0.44	0.694	4.22E-50	M1	ZNHIT1
RAB11B.2	2.87E-54	-0.27152	0.291	0.547	4.28E-50	M1	RAB11B
SOD2.2	3.05E-54	0.124768	0.66	0.511	4.55E-50	M1	SOD2
FAM171B.2	3.17E-54	-0.27508	0.01	0.131	4.74E-50	M1	FAM171B
ZNF397.2	3.26E-54	-0.23228	0.04	0.187	4.87E-50	M1	ZNF397
NDUFS5.4	3.31E-54	-0.29754	0.683	0.837	4.95E-50	M1	NDUFS5
LAMC1.2	3.49E-54	-0.21987	0.005	0.122	5.22E-50	M1	LAMC1
DOK3.4	3.49E-54	0.331131	0.143	0.05	5.22E-50	M1	DOK3
LINC00094	3.57E-54	-0.22324	0.02	0.151	5.34E-50	M1	LINC00094
C7orf50.1	3.86E-54	-0.30749	0.305	0.548	5.77E-50	M1	C7orf50
AKAP7.4	3.86E-54	-0.27109	0.061	0.221	5.77E-50	M1	AKAP7
SHFM1.3	4.03E-54	-0.35347	0.54	0.76	6.02E-50	M1	SHFM1
USP22.4	4.06E-54	-0.22469	0.145	0.354	6.07E-50	M1	USP22
SMARCA5.3	4.24E-54	-0.24987	0.168	0.385	6.33E-50	M1	SMARCA5
SUB1.2	4.71E-54	-0.26786	0.638	0.78	7.04E-50	M1	SUB1
TAF9B.1	4.91E-54	-0.27205	0.03	0.168	7.33E-50	M1	TAF9B
LRRC4C.2	5.69E-54	-0.33475	0.002	0.114	8.50E-50	M1	LRRC4C
PGM3.3	6.97E-54	-0.25946	0.048	0.2	1.04E-49	M1	PGM3
PXDN.1	7.21E-54	-0.24753	0.005	0.12	1.08E-49	M1	PXDN
TACO1.3	7.39E-54	-0.24089	0.048	0.2	1.10E-49	M1	TACO1
CCDC25.3	7.83E-54	-0.29759	0.133	0.33	1.17E-49	M1	CCDC25
ASAP2.3	8.53E-54	-0.21917	0.002	0.114	1.27E-49	M1	ASAP2
LAMA5.2	8.68E-54	-0.20679	0.01	0.132	1.30E-49	M1	LAMA5
TULP3.3	8.80E-54	-0.26075	0.018	0.147	1.31E-49	M1	TULP3
FBXO3.2	9.24E-54	-0.25626	0.046	0.195	1.38E-49	M1	FBXO3
EXOG.3	9.40E-54	-0.27164	0.037	0.18	1.41E-49	M1	EXOG
PHF23.2	9.53E-54	-0.23772	0.069	0.235	1.42E-49	M1	PHF23
CPD.1	9.70E-54	-0.26917	0.039	0.184	1.45E-49	M1	CPD
SERP2.2	1.03E-53	-0.3149	0.005	0.121	1.54E-49	M1	SERP2
SLC2A4RG.	1.05E-53	-0.22067	0.018	0.147	1.56E-49	M1	SLC2A4RG
PAIP1.4	1.08E-53	-0.265	0.204	0.434	1.61E-49	M1	PAIP1
ORC4.3	1.08E-53	-0.25617	0.089	0.264	1.61E-49	M1	ORC4
SKP1.3	1.12E-53	-0.31753	0.758	0.886	1.67E-49	M1	SKP1
TAF6.1	1.16E-53	-0.229	0.089	0.266	1.73E-49	M1	TAF6
CLIP2.3	1.17E-53	-0.27107	0.05	0.202	1.74E-49	M1	CLIP2
PPIL1.3	1.18E-53	-0.28396	0.062	0.221	1.76E-49	M1	PPIL1
HDGF.1	1.21E-53	-0.23771	0.114	0.305	1.81E-49	M1	HDGF
SOX12.3	1.27E-53	-0.21134	0.013	0.136	1.89E-49	M1	SOX12

STRAP.2	1.29E-53	-0.28895	0.338	0.597	1.93E-49	M1	STRAP
DLD.2	1.32E-53	-0.28113	0.168	0.379	1.98E-49	M1	DLD
CWC27.2	1.52E-53	-0.24294	0.108	0.295	2.27E-49	M1	CWC27
LAP3.2	1.58E-53	0.298116	0.571	0.476	2.36E-49	M1	LAP3
ARL4D.4	1.63E-53	-0.34526	0.046	0.193	2.43E-49	M1	ARL4D
ZNF43.2	1.67E-53	-0.23682	0.025	0.16	2.50E-49	M1	ZNF43
NCALD.3	1.69E-53	-0.26785	0.018	0.146	2.52E-49	M1	NCALD
PFKM.1	1.72E-53	-0.29233	0.055	0.209	2.57E-49	M1	PFKM
SNRPG.2	1.76E-53	-0.32889	0.443	0.679	2.64E-49	M1	SNRPG
OXA1L.3	1.82E-53	0.455081	0.408	0.28	2.72E-49	M1	OXA1L
DHCR7.3	1.95E-53	-0.28519	0.051	0.203	2.92E-49	M1	DHCR7
VAPB.3	2.01E-53	-0.21716	0.063	0.224	3.01E-49	M1	VAPB
LHFPL3.4	2.17E-53	-0.32591	0.016	0.14	3.25E-49	M1	LHFPL3
RP11-660L	2.31E-53	-0.28344	0.018	0.145	3.45E-49	M1	RP11-660L16.2
PLEKHB1.2	2.38E-53	-0.39949	0.076	0.24	3.55E-49	M1	PLEKHB1
TSR1.2	2.44E-53	-0.21495	0.058	0.216	3.64E-49	M1	TSR1
TCF3.3	2.46E-53	-0.22725	0.021	0.151	3.68E-49	M1	TCF3
STIM2.1	2.51E-53	-0.20587	0.04	0.186	3.76E-49	M1	STIM2
MAP6.2	2.56E-53	-0.24109	0.003	0.115	3.82E-49	M1	MAP6
DDX52.2	2.58E-53	-0.20894	0.094	0.274	3.85E-49	M1	DDX52
WBP4.3	2.61E-53	-0.25452	0.137	0.339	3.89E-49	M1	WBP4
MLST8.2	2.71E-53	-0.21339	0.089	0.266	4.05E-49	M1	MLST8
ECI2.2	2.80E-53	-0.30681	0.156	0.356	4.18E-49	M1	ECI2
ALOX15B.3	3.16E-53	0.295775	0.076	0.016	4.72E-49	M1	ALOX15B
C12orf10.	3.23E-53	-0.25303	0.146	0.352	4.83E-49	M1	C12orf10
SERPINB6.	3.35E-53	0.326491	0.548	0.464	5.01E-49	M1	SERPINB6
C17orf107	3.39E-53	0.220344	0.052	0.007	5.06E-49	M1	C17orf107
FIP1L1.1	3.60E-53	-0.2746	0.133	0.329	5.38E-49	M1	FIP1L1
SLC10A4.3	3.89E-53	-0.23205	0.003	0.115	5.82E-49	M1	SLC10A4
AGGF1.1	3.90E-53	-0.21501	0.077	0.248	5.83E-49	M1	AGGF1
COA3.3	4.13E-53	-0.31439	0.273	0.518	6.18E-49	M1	COA3
P2RY12.1	4.17E-53	0.244589	0.063	0.011	6.23E-49	M1	P2RY12
ARAP1.3	4.34E-53	0.44342	0.199	0.089	6.49E-49	M1	ARAP1
RNASE3.2	4.52E-53	0.308553	0.09	0.022	6.75E-49	M1	RNASE3
TWF1.2	4.59E-53	-0.22434	0.107	0.295	6.86E-49	M1	TWF1
PDDC1.3	4.70E-53	-0.22696	0.031	0.169	7.02E-49	M1	PDDC1
CNKSR3.3	4.78E-53	-0.19399	0.014	0.138	7.14E-49	M1	CNKSR3
P2RY11.2	4.92E-53	0.400648	0.171	0.07	7.35E-49	M1	P2RY11
B9D1.2	5.50E-53	-0.30118	0.005	0.12	8.22E-49	M1	B9D1
HIBCH.2	5.87E-53	-0.25018	0.095	0.274	8.77E-49	M1	HIBCH
ASCL2.3	6.41E-53	0.263985	0.096	0.025	9.57E-49	M1	ASCL2
TSPAN13.4	6.76E-53	-0.37285	0.147	0.329	1.01E-48	M1	TSPAN13
YTHDF2.2	7.01E-53	-0.24744	0.231	0.471	1.05E-48	M1	YTHDF2
EPM2AIP1.	7.05E-53	-0.25062	0.065	0.225	1.05E-48	M1	EPM2AIP1
COMMD2.2	7.09E-53	-0.24146	0.127	0.321	1.06E-48	M1	COMMD2
TRIM37.2	7.15E-53	-0.2008	0.033	0.173	1.07E-48	M1	TRIM37
APPBP2.2	9.55E-53	-0.21517	0.071	0.237	1.43E-48	M1	APPBP2
METTL2B	9.63E-53	-0.23417	0.065	0.225	1.44E-48	M1	METTL2B
RFC1.2	9.95E-53	-0.24329	0.118	0.308	1.49E-48	M1	RFC1
RSF1.2	1.01E-52	-0.23258	0.221	0.456	1.51E-48	M1	RSF1
AKAP6.3	1.03E-52	-0.29386	0.006	0.121	1.54E-48	M1	AKAP6

PRKCH.3	1.04E-52	0.305943	0.108	0.031	1.55E-48	M1	PRKCH
HNRNPR	1.05E-52	-0.29242	0.317	0.56	1.56E-48	M1	HNRNPR
FMNL3.4	1.10E-52	0.432861	0.157	0.061	1.65E-48	M1	FMNL3
TSFM.2	1.15E-52	-0.3513	0.125	0.306	1.73E-48	M1	TSFM
PALM.2	1.22E-52	-0.21076	0.007	0.123	1.82E-48	M1	PALM
LRP6.3	1.30E-52	-0.25256	0.012	0.132	1.95E-48	M1	LRP6
SYT1.4	1.35E-52	-0.44591	0.006	0.119	2.01E-48	M1	SYT1
RASGRP3.3	1.36E-52	0.373781	0.147	0.053	2.04E-48	M1	RASGRP3
TOP2B.4	1.38E-52	-0.23765	0.091	0.266	2.06E-48	M1	TOP2B
SCCPDH.2	1.38E-52	-0.27732	0.192	0.414	2.07E-48	M1	SCCPDH
ARHGEF12.	1.48E-52	-0.20369	0.04	0.184	2.21E-48	M1	ARHGEF12
CDKN2AIPN	1.57E-52	-0.2365	0.098	0.279	2.35E-48	M1	CDKN2AIPNL
CBY1.2	1.64E-52	-0.24022	0.04	0.183	2.45E-48	M1	CBY1
TCP1.1	1.83E-52	-0.2835	0.319	0.564	2.73E-48	M1	TCP1
RNF13.3	2.06E-52	0.380516	0.554	0.466	3.08E-48	M1	RNF13
LONRF2.2	2.29E-52	-0.25865	0.001	0.11	3.43E-48	M1	LONRF2
AP000439.	2.31E-52	0.261851	0.077	0.017	3.45E-48	M1	AP000439.1
PPM1G.3	2.32E-52	-0.27547	0.23	0.456	3.47E-48	M1	PPM1G
NAP1L3.2	2.63E-52	-0.32378	0.007	0.121	3.93E-48	M1	NAP1L3
CSAD.2	2.73E-52	-0.20113	0.045	0.192	4.08E-48	M1	CSAD
GDAP1L1.3	3.09E-52	-0.39779	0.006	0.12	4.62E-48	M1	GDAP1L1
MAD2L1.3	3.14E-52	-0.37729	0.022	0.149	4.69E-48	M1	MAD2L1
ZNF254.1	3.16E-52	-0.29085	0.03	0.164	4.72E-48	M1	ZNF254
WDR18.2	3.19E-52	-0.22989	0.124	0.318	4.77E-48	M1	WDR18
OTUD7B.2	3.19E-52	-0.28486	0.006	0.12	4.77E-48	M1	OTUD7B
PLP2.3	3.38E-52	-0.38857	0.177	0.377	5.05E-48	M1	PLP2
C1orf131.	3.42E-52	-0.2514	0.067	0.227	5.11E-48	M1	C1orf131
DNAJC19.4	3.43E-52	-0.29081	0.203	0.426	5.13E-48	M1	DNAJC19
INPP1.1	3.76E-52	-0.29564	0.036	0.174	5.62E-48	M1	INPP1
PLEKHA4.3	4.08E-52	-0.22581	0.02	0.148	6.10E-48	M1	PLEKHA4
NAV1.3	4.27E-52	-0.22875	0.042	0.187	6.38E-48	M1	NAV1
RPRD1A.2	4.33E-52	-0.21936	0.071	0.235	6.48E-48	M1	RPRD1A
ROCK2.1	4.41E-52	-0.19707	0.035	0.175	6.59E-48	M1	ROCK2
CYTH2.3	4.68E-52	-0.223	0.165	0.377	6.99E-48	M1	CYTH2
SERINC2.2	4.85E-52	-0.24345	0.017	0.141	7.25E-48	M1	SERINC2
TRABD.4	4.86E-52	0.497999	0.357	0.239	7.26E-48	M1	TRABD
MTMR4.3	5.05E-52	-0.23388	0.041	0.184	7.55E-48	M1	MTMR4
ZNF821.4	5.11E-52	-0.26296	0.044	0.187	7.63E-48	M1	ZNF821
LRRN1.3	5.88E-52	-0.30351	0.009	0.125	8.79E-48	M1	LRRN1
SBNO1.2	6.16E-52	-0.20744	0.111	0.297	9.21E-48	M1	SBNO1
RHOJ.2	6.97E-52	-0.22424	0.006	0.119	1.04E-47	M1	RHOJ
YEATS4.2	7.13E-52	-0.34274	0.107	0.28	1.07E-47	M1	YEATS4
RP11-332H	7.33E-52	-0.24185	0.021	0.148	1.10E-47	M1	RP11-332H14.2
SOX21.1	7.89E-52	-0.21464	0.002	0.111	1.18E-47	M1	SOX21
UBA2.2	8.99E-52	-0.25595	0.179	0.394	1.34E-47	M1	UBA2
ABAT.4	9.29E-52	-0.27741	0.042	0.183	1.39E-47	M1	ABAT
SWAP70.4	9.61E-52	0.503322	0.32	0.202	1.44E-47	M1	SWAP70
TERF1.1	9.83E-52	-0.25546	0.153	0.358	1.47E-47	M1	TERF1
ELOVL2.4	9.90E-52	-0.23384	0.005	0.116	1.48E-47	M1	ELOVL2
LRRC58.3	9.95E-52	-0.23562	0.071	0.234	1.49E-47	M1	LRRC58
LINC00578	1.04E-51	0.17433	0.039	0.003	1.56E-47	M1	LINC00578

PARVA.3	1.07E-51	-0.2062	0.004	0.114	1.59E-47	M1	PARVA
COA5.2	1.09E-51	-0.25217	0.141	0.339	1.62E-47	M1	COA5
PJA2.3	1.12E-51	-0.27542	0.168	0.369	1.67E-47	M1	PJA2
PPP1R8.1	1.15E-51	-0.25504	0.082	0.251	1.72E-47	M1	PPP1R8
HIPK2.3	1.18E-51	-0.31197	0.085	0.248	1.77E-47	M1	HIPK2
VPS72.2	1.30E-51	-0.22158	0.119	0.308	1.94E-47	M1	VPS72
ZNF738.4	1.38E-51	-0.30058	0.032	0.166	2.06E-47	M1	ZNF738
KALRN.2	1.49E-51	-0.25215	0.004	0.114	2.23E-47	M1	KALRN
PID1.2	1.51E-51	-0.30302	0.016	0.137	2.26E-47	M1	PID1
MTMR9.2	1.57E-51	-0.2216	0.061	0.215	2.34E-47	M1	MTMR9
NRBP2.3	1.63E-51	-0.23005	0.018	0.142	2.43E-47	M1	NRBP2
SNRNP25.1	1.63E-51	-0.2501	0.173	0.385	2.43E-47	M1	SNRNP25
FADS2.2	1.77E-51	-0.23057	0.027	0.158	2.64E-47	M1	FADS2
DDX1.2	1.86E-51	-0.27368	0.183	0.395	2.78E-47	M1	DDX1
HEBP2.3	1.89E-51	-0.25032	0.08	0.247	2.83E-47	M1	HEBP2
FNIP2.3	2.22E-51	0.550668	0.296	0.175	3.31E-47	M1	FNIP2
PRKRA.1	2.23E-51	-0.25968	0.182	0.397	3.34E-47	M1	PRKRA
CFLAR.3	2.69E-51	0.434235	0.416	0.299	4.03E-47	M1	CFLAR
PAXIP1-AS	2.95E-51	-0.25515	0.041	0.182	4.40E-47	M1	PAXIP1-AS1
CPNE3.1	3.05E-51	-0.25451	0.159	0.365	4.55E-47	M1	CPNE3
INHBA.1	3.06E-51	0.281915	0.064	0.012	4.57E-47	M1	INHBA
SDC3.2	3.27E-51	-0.25215	0.065	0.222	4.89E-47	M1	SDC3
TRAPPC4.3	3.32E-51	-0.264	0.249	0.484	4.96E-47	M1	TRAPPC4
ATXN7L3B.	3.37E-51	-0.20978	0.055	0.206	5.03E-47	M1	ATXN7L3B
SIRPA.4	3.41E-51	0.478471	0.344	0.223	5.10E-47	M1	SIRPA
EXTL2.2	3.69E-51	-0.23609	0.046	0.192	5.52E-47	M1	EXTL2
THAP5.2	3.80E-51	-0.24467	0.054	0.204	5.68E-47	M1	THAP5
METTL2A.2	3.92E-51	-0.21575	0.041	0.182	5.86E-47	M1	METTL2A
MOB4.2	4.04E-51	-0.23762	0.19	0.408	6.03E-47	M1	MOB4
ITM2C.4	4.06E-51	-0.34268	0.231	0.455	6.07E-47	M1	ITM2C
RIMKLB.2	4.33E-51	-0.23941	0.036	0.173	6.47E-47	M1	RIMKLB
TRIM28.2	4.61E-51	-0.24467	0.101	0.279	6.89E-47	M1	TRIM28
DAAM1.2	4.70E-51	-0.23016	0.096	0.27	7.02E-47	M1	DAAM1
TBCC.4	5.04E-51	-0.26058	0.084	0.249	7.53E-47	M1	TBCC
RIN2.3	5.05E-51	0.468368	0.219	0.107	7.55E-47	M1	RIN2
LGR4.4	5.23E-51	-0.19754	0.008	0.121	7.81E-47	M1	LGR4
POMGNT2.2	5.34E-51	-0.23162	0.03	0.163	7.97E-47	M1	POMGNT2
CTB-31020	5.41E-51	-0.22797	0.014	0.134	8.09E-47	M1	CTB-31020.2
CTNNBIP1.	6.09E-51	-0.24562	0.078	0.242	9.10E-47	M1	CTNNBIP1
NDUFAF2.3	7.30E-51	-0.28757	0.12	0.303	1.09E-46	M1	NDUFAF2
NLN.2	7.42E-51	-0.24378	0.059	0.211	1.11E-46	M1	NLN
GNA11.1	7.44E-51	-0.21892	0.01	0.126	1.11E-46	M1	GNA11
NLGN3.3	7.67E-51	-0.27275	0.014	0.133	1.15E-46	M1	NLGN3
MANBA.4	7.76E-51	0.472252	0.213	0.102	1.16E-46	M1	MANBA
NINJ2.4	8.10E-51	0.338889	0.173	0.07	1.21E-46	M1	NINJ2
SSBP3.3	8.16E-51	-0.25639	0.023	0.149	1.22E-46	M1	SSBP3
AIF1L.3	8.33E-51	-0.40979	0.015	0.133	1.25E-46	M1	AIF1L
TRIM44.3	8.78E-51	-0.27197	0.13	0.318	1.31E-46	M1	TRIM44
RBBP7.3	1.22E-50	-0.31272	0.232	0.463	1.82E-46	M1	RBBP7
DIRAS1.2	1.23E-50	-0.25132	0.014	0.133	1.84E-46	M1	DIRAS1
ZNF12.2	1.31E-50	-0.22492	0.02	0.145	1.96E-46	M1	ZNF12

NR2C2AP.3	1.34E-50	-0.29454	0.047	0.189	2.01E-46	M1	NR2C2AP
HIGD2A.3	1.36E-50	0.241355	0.669	0.608	2.03E-46	M1	HIGD2A
YTHDF1.1	1.40E-50	-0.22165	0.057	0.208	2.09E-46	M1	YTHDF1
MAZ.4	1.57E-50	-0.2403	0.102	0.28	2.35E-46	M1	MAZ
ATP6V1F.2	1.58E-50	0.133855	0.826	0.796	2.36E-46	M1	ATP6V1F
CAMSAP2.2	1.65E-50	-0.21973	0.036	0.172	2.46E-46	M1	CAMSAP2
CCT6A.1	1.72E-50	-0.29002	0.375	0.615	2.57E-46	M1	CCT6A
NFASC.2	1.72E-50	-0.34316	0.009	0.122	2.57E-46	M1	NFASC
RNF126.3	1.80E-50	-0.21874	0.13	0.323	2.69E-46	M1	RNF126
CTC-444N2	2.20E-50	-0.20824	0.027	0.157	3.29E-46	M1	CTC-444N24.11
ST8SIA1.2	2.23E-50	-0.23295	0.003	0.109	3.33E-46	M1	ST8SIA1
PLA2G15.3	2.36E-50	0.428364	0.234	0.119	3.53E-46	M1	PLA2G15
CAV1.4	2.38E-50	-0.81379	0.024	0.147	3.55E-46	M1	CAV1
SNAI1.3	2.54E-50	0.337554	0.107	0.032	3.79E-46	M1	SNAI1
LINC00632	2.71E-50	-0.35398	0.003	0.109	4.05E-46	M1	LINC00632
RGS6.2	2.78E-50	-0.25548	0.002	0.108	4.15E-46	M1	RGS6
BCAT1.3	2.97E-50	-0.24151	0.09	0.26	4.44E-46	M1	BCAT1
ZNF593.2	3.32E-50	-0.27187	0.14	0.334	4.96E-46	M1	ZNF593
ZFPL1.1	3.39E-50	-0.22237	0.092	0.264	5.07E-46	M1	ZFPL1
COX20	3.67E-50	-0.27673	0.264	0.499	5.48E-46	M1	COX20
DDX5.2	3.91E-50	0.147384	0.88	0.804	5.84E-46	M1	DDX5
ERAL1.1	4.17E-50	-0.21987	0.096	0.27	6.23E-46	M1	ERAL1
ZNF429.3	4.21E-50	-0.26024	0.024	0.15	6.29E-46	M1	ZNF429
LAMB1.1	4.33E-50	-0.21806	0.008	0.12	6.47E-46	M1	LAMB1
SRM.3	4.56E-50	-0.30114	0.191	0.4	6.81E-46	M1	SRM
DYNC1I1.4	4.74E-50	-0.41417	0.011	0.123	7.09E-46	M1	DYNC1I1
SNHG8.2	4.84E-50	0.354365	0.544	0.453	7.23E-46	M1	SNHG8
TOMM40.2	4.86E-50	-0.24628	0.158	0.362	7.26E-46	M1	TOMM40
NUP107.4	4.97E-50	-0.33225	0.088	0.25	7.43E-46	M1	NUP107
FAM159A.3	5.06E-50	0.28578	0.108	0.032	7.56E-46	M1	FAM159A
SEMA5A.2	5.15E-50	-0.26641	0.01	0.123	7.70E-46	M1	SEMA5A
NQO1.3	5.35E-50	-0.27674	0.012	0.128	8.00E-46	M1	NQO1
HOXA7.2	5.64E-50	-0.37429	0.009	0.12	8.42E-46	M1	HOXA7
PLB1.3	5.99E-50	0.33295	0.102	0.03	8.95E-46	M1	PLB1
PABPN1.2	6.18E-50	-0.24345	0.179	0.39	9.23E-46	M1	PABPN1
ANAPC11.4	6.19E-50	-0.3025	0.563	0.76	9.25E-46	M1	ANAPC11
NTRK2.3	7.54E-50	-0.49928	0.021	0.141	1.13E-45	M1	NTRK2
CPNE4.2	7.65E-50	-0.20068	0.004	0.111	1.14E-45	M1	CPNE4
SNRNP27.2	7.83E-50	-0.21933	0.176	0.387	1.17E-45	M1	SNRNP27
RNF34.2	8.05E-50	-0.21572	0.066	0.221	1.20E-45	M1	RNF34
MTA1.2	8.34E-50	-0.21167	0.062	0.216	1.25E-45	M1	MTA1
ACTR3B.1	8.41E-50	-0.23708	0.012	0.126	1.26E-45	M1	ACTR3B
COPRS.3	9.60E-50	-0.26821	0.166	0.367	1.43E-45	M1	COPRS
FBXW9.1	9.84E-50	-0.22552	0.014	0.131	1.47E-45	M1	FBXW9
MDFI.3	9.99E-50	-0.27945	0.001	0.105	1.49E-45	M1	MDFI
DEPTOR.2	1.18E-49	0.32797	0.106	0.032	1.76E-45	M1	DEPTOR
ZNF358.3	1.18E-49	-0.19861	0.046	0.189	1.77E-45	M1	ZNF358
CBR3.1	1.22E-49	-0.23869	0.011	0.124	1.83E-45	M1	CBR3
ZNF92.2	1.23E-49	-0.29158	0.036	0.168	1.83E-45	M1	ZNF92
TMSB10.4	1.27E-49	-0.25504	0.991	0.959	1.90E-45	M1	TMSB10
RP11-403I	1.27E-49	0.366493	0.089	0.023	1.90E-45	M1	RP11-403I13.5

FAM172A.2	1.33E-49	-0.21458	0.066	0.222	1.99E-45	M1	FAM172A
GMNN.3	1.49E-49	-0.26546	0.055	0.201	2.22E-45	M1	GMNN
ADAMTSL2	1.55E-49	0.17803	0.049	0.007	2.31E-45	M1	ADAMTSL2
IFI16.3	1.57E-49	0.296517	0.499	0.372	2.35E-45	M1	IFI16
MT1X.4	1.65E-49	-0.6972	0.425	0.608	2.47E-45	M1	MT1X
CRYBB1.2	1.66E-49	0.290658	0.102	0.029	2.48E-45	M1	CRYBB1
NNAT.4	1.74E-49	-0.76079	0.084	0.218	2.60E-45	M1	NNAT
SAC3D1.4	1.92E-49	-0.22556	0.05	0.194	2.87E-45	M1	SAC3D1
SH3KBP1.4	2.00E-49	0.449647	0.341	0.221	2.99E-45	M1	SH3KBP1
CST3.2	2.02E-49	0.124024	0.878	0.768	3.02E-45	M1	CST3
NKTR.2	2.09E-49	-0.197	0.179	0.392	3.13E-45	M1	NKTR
CANT1.2	2.19E-49	-0.21558	0.053	0.199	3.27E-45	M1	CANT1
PTK7.1	2.20E-49	-0.19173	0.005	0.112	3.29E-45	M1	PTK7
IGSF8.2	2.21E-49	-0.27258	0.079	0.24	3.30E-45	M1	IGSF8
TEAD2.3	2.21E-49	-0.2003	0.003	0.107	3.31E-45	M1	TEAD2
PRCC.1	2.31E-49	-0.24003	0.141	0.336	3.45E-45	M1	PRCC
DHCR24.1	2.31E-49	-0.24804	0.006	0.114	3.45E-45	M1	DHCR24
SNAP23.4	2.56E-49	0.532165	0.34	0.225	3.82E-45	M1	SNAP23
ZNF512.2	2.75E-49	-0.26048	0.022	0.144	4.11E-45	M1	ZNF512
VASH1.4	2.92E-49	0.45651	0.222	0.111	4.36E-45	M1	VASH1
FGFBP3.3	3.07E-49	-0.23494	0.008	0.118	4.59E-45	M1	FGFBP3
GADD45GIF	3.08E-49	-0.30933	0.4	0.596	4.60E-45	M1	GADD45GIP1
ACP1.2	3.10E-49	-0.26713	0.293	0.54	4.63E-45	M1	ACP1
CD300LF.3	3.14E-49	0.296571	0.108	0.033	4.70E-45	M1	CD300LF
BLOC1S4.4	3.70E-49	-0.21506	0.098	0.272	5.53E-45	M1	BLOC1S4
MARCH6.3	3.82E-49	-0.25075	0.182	0.392	5.71E-45	M1	6-Mar
NDUFS1.2	3.84E-49	-0.21496	0.107	0.285	5.73E-45	M1	NDUFS1
BAG5.2	4.01E-49	-0.2435	0.06	0.21	5.99E-45	M1	BAG5
KIF3B.4	4.01E-49	-0.19972	0.025	0.151	5.99E-45	M1	KIF3B
FAM57B.3	4.07E-49	-0.31168	0.004	0.11	6.08E-45	M1	FAM57B
CAPRIN1.3	4.15E-49	-0.24012	0.153	0.354	6.20E-45	M1	CAPRIN1
DCTN2	4.34E-49	-0.35775	0.318	0.545	6.48E-45	M1	DCTN2
BCAM.2	4.43E-49	-0.21517	0.013	0.128	6.62E-45	M1	BCAM
TMEM11.1	4.67E-49	-0.20082	0.152	0.352	6.98E-45	M1	TMEM11
FAM161A.2	4.82E-49	-0.25	0.013	0.127	7.21E-45	M1	FAM161A
YARS.1	4.87E-49	-0.25612	0.083	0.244	7.28E-45	M1	YARS
THSD1.2	5.56E-49	-0.20321	0.005	0.112	8.31E-45	M1	THSD1
SCAMP5.2	5.63E-49	-0.24168	0.006	0.114	8.42E-45	M1	SCAMP5
PTPN13.2	6.12E-49	-0.21402	0.005	0.111	9.14E-45	M1	PTPN13
DIXDC1.3	6.16E-49	-0.25002	0.006	0.113	9.20E-45	M1	DIXDC1
TSSC4.2	6.21E-49	-0.1925	0.146	0.345	9.28E-45	M1	TSSC4
TMEM35.3	6.54E-49	-0.31427	0.004	0.109	9.77E-45	M1	TMEM35
RAB11B-AS	6.66E-49	-0.21648	0.027	0.153	9.95E-45	M1	RAB11B-AS1
TNFSF12.4	6.93E-49	0.468112	0.283	0.166	1.04E-44	M1	TNFSF12
KLHDC10.2	7.23E-49	-0.22037	0.026	0.151	1.08E-44	M1	KLHDC10
KIAA1549.	7.38E-49	-0.25639	0.005	0.112	1.10E-44	M1	KIAA1549
KCTD6	8.13E-49	-0.22386	0.018	0.137	1.22E-44	M1	KCTD6
PAFAH1B2.	9.53E-49	-0.21953	0.159	0.359	1.42E-44	M1	PAFAH1B2
B3GAT1.3	9.77E-49	-0.31245	0.003	0.107	1.46E-44	M1	B3GAT1
PITPNC1.2	9.96E-49	-0.23421	0.022	0.143	1.49E-44	M1	PITPNC1
CCDC124.1	1.08E-48	-0.27209	0.273	0.51	1.61E-44	M1	CCDC124

FASN.4	1.15E-48	-0.20659	0.018	0.136	1.71E-44	M1	FASN
MPPED2.2	1.17E-48	-0.2674	0.003	0.107	1.75E-44	M1	MPPED2
GFOD2.1	1.21E-48	-0.25009	0.028	0.153	1.81E-44	M1	GFOD2
MAGI2.3	1.29E-48	-0.28271	0.012	0.124	1.93E-44	M1	MAGI2
PLCG1.2	1.35E-48	-0.21438	0.009	0.118	2.02E-44	M1	PLCG1
IRF2BPL.3	1.49E-48	-0.2239	0.099	0.27	2.22E-44	M1	IRF2BPL
PAK1IP1.2	1.50E-48	-0.23475	0.081	0.242	2.24E-44	M1	PAK1IP1
PPP2R2A.1	1.51E-48	-0.21514	0.14	0.332	2.26E-44	M1	PPP2R2A
LRRTM2.3	1.55E-48	-0.24136	0.01	0.121	2.31E-44	M1	LRRTM2
DCTPP1.2	1.68E-48	-0.27105	0.165	0.363	2.50E-44	M1	DCTPP1
SUCLA2.3	1.76E-48	-0.23909	0.132	0.318	2.63E-44	M1	SUCLA2
SF3A2.2	1.78E-48	-0.21937	0.111	0.289	2.66E-44	M1	SF3A2
TRMT10C.2	1.88E-48	-0.22091	0.105	0.279	2.81E-44	M1	TRMT10C
C6orf203.	1.90E-48	-0.23301	0.048	0.189	2.84E-44	M1	C6orf203
FGF14.3	2.27E-48	-0.2421	0.001	0.102	3.39E-44	M1	FGF14
CCDC137.2	2.38E-48	-0.21112	0.072	0.228	3.56E-44	M1	CCDC137
TFG.1	2.42E-48	-0.25812	0.225	0.445	3.61E-44	M1	TFG
ELN.2	2.56E-48	-0.3345	0.014	0.127	3.82E-44	M1	ELN
TOX.4	2.71E-48	-0.2662	0.002	0.104	4.04E-44	M1	TOX
GOLGA7.1	2.81E-48	-0.25016	0.266	0.503	4.20E-44	M1	GOLGA7
GTF2F2.1	2.83E-48	-0.24057	0.141	0.331	4.23E-44	M1	GTF2F2
NLGN2.2	2.85E-48	-0.22321	0.005	0.109	4.27E-44	M1	NLGN2
KBTBD8.2	2.93E-48	0.306152	0.08	0.02	4.38E-44	M1	KBTBD8
ARHGAP5.1	3.08E-48	-0.22409	0.067	0.219	4.61E-44	M1	ARHGAP5
SEC11C.3	3.11E-48	-0.28375	0.299	0.537	4.64E-44	M1	SEC11C
LRRC47.3	3.16E-48	-0.22801	0.082	0.243	4.72E-44	M1	LRRC47
PTP4A3.1	3.41E-48	-0.24831	0.003	0.105	5.10E-44	M1	PTP4A3
MTMR2.1	3.43E-48	-0.19815	0.036	0.168	5.13E-44	M1	MTMR2
SYMPK.3	3.81E-48	-0.22762	0.087	0.25	5.70E-44	M1	SYMPK
C12orf29.	4.02E-48	-0.26939	0.041	0.176	6.01E-44	M1	C12orf29
GDI2.4	4.14E-48	0.369712	0.546	0.477	6.18E-44	M1	GDI2
DHX29.2	4.25E-48	-0.21017	0.147	0.342	6.35E-44	M1	DHX29
FLAD1.1	4.25E-48	-0.23313	0.093	0.26	6.36E-44	M1	FLAD1
TLE2.3	4.34E-48	-0.26482	0.004	0.108	6.49E-44	M1	TLE2
FLYWCH2.1	4.47E-48	-0.26819	0.149	0.342	6.68E-44	M1	FLYWCH2
FGFR1.1	4.47E-48	-0.20661	0.052	0.194	6.68E-44	M1	FGFR1
CDH4.3	4.57E-48	-0.22364	0.002	0.104	6.82E-44	M1	CDH4
GON4L.2	4.60E-48	-0.20941	0.08	0.241	6.88E-44	M1	GON4L
NFE2L2.3	4.73E-48	0.364258	0.527	0.456	7.07E-44	M1	NFE2L2
DCAKD.3	4.85E-48	-0.21534	0.032	0.16	7.25E-44	M1	DCAKD
MKLN1.2	5.11E-48	-0.21781	0.092	0.257	7.64E-44	M1	MKLN1
DNAJA1.1	5.30E-48	0.26943	0.667	0.621	7.92E-44	M1	DNAJA1
SOX5.2	5.30E-48	-0.25427	0.003	0.106	7.92E-44	M1	SOX5
ZNF138.2	5.54E-48	-0.2675	0.037	0.167	8.28E-44	M1	ZNF138
R3HCC1.2	5.94E-48	-0.21872	0.131	0.317	8.88E-44	M1	R3HCC1
CARS.2	6.05E-48	-0.24194	0.085	0.246	9.04E-44	M1	CARS
SEPN1.3	6.12E-48	-0.23317	0.078	0.236	9.14E-44	M1	SEPN1
CSRNP3.3	7.04E-48	-0.32455	0.009	0.117	1.05E-43	M1	CSRNP3
ZNF444.2	7.12E-48	-0.20085	0.045	0.183	1.06E-43	M1	ZNF444
SNRNP200	7.22E-48	-0.2152	0.13	0.315	1.08E-43	M1	SNRNP200
C3orf70.3	7.43E-48	-0.25685	0.01	0.118	1.11E-43	M1	C3orf70

AP4M1.2	8.00E-48	-0.24148	0.068	0.219	1.20E-43	M1	AP4M1
MPC2.1	8.92E-48	0.284796	0.61	0.576	1.33E-43	M1	MPC2
USP46.4	9.94E-48	-0.22421	0.032	0.159	1.48E-43	M1	USP46
NPRL3.2	1.00E-47	-0.21673	0.065	0.215	1.50E-43	M1	NPRL3
HTATSF1.1	1.03E-47	-0.26889	0.126	0.304	1.54E-43	M1	HTATSF1
KCTD13.3	1.09E-47	-0.25354	0.033	0.16	1.63E-43	M1	KCTD13
PKIA.4	1.23E-47	-0.32981	0.006	0.111	1.84E-43	M1	PKIA
SLC4A3.1	1.24E-47	-0.21607	0.004	0.107	1.85E-43	M1	SLC4A3
LARS.1	1.28E-47	-0.22082	0.173	0.375	1.91E-43	M1	LARS
C9orf9.3	1.33E-47	-0.24462	0.015	0.129	1.98E-43	M1	C9orf9
SCRN1.3	1.35E-47	-0.22001	0.058	0.204	2.01E-43	M1	SCRN1
NUDT16.4	1.41E-47	0.510257	0.315	0.2	2.11E-43	M1	NUDT16
RAD1	1.41E-47	-0.25809	0.058	0.202	2.11E-43	M1	RAD1
FRYL.4	1.44E-47	-0.23552	0.064	0.212	2.15E-43	M1	FRYL
SELK.4	1.57E-47	0.220071	0.705	0.7	2.34E-43	M1	SELK
RPA3.2	1.61E-47	-0.31414	0.18	0.375	2.41E-43	M1	RPA3
ARL4C.3	1.77E-47	-0.24914	0.206	0.413	2.64E-43	M1	ARL4C
DENND2A.2	1.79E-47	-0.2009	0.007	0.112	2.68E-43	M1	DENND2A
CHCHD3.1	1.81E-47	-0.21594	0.191	0.404	2.71E-43	M1	CHCHD3
LDLRAD4.4	1.84E-47	0.446232	0.241	0.127	2.75E-43	M1	LDLRAD4
CREBL2.3	1.90E-47	0.459078	0.286	0.169	2.84E-43	M1	CREBL2
ZNF559.3	1.91E-47	-0.25365	0.037	0.167	2.86E-43	M1	ZNF559
KLC2.3	1.95E-47	-0.2133	0.022	0.141	2.92E-43	M1	KLC2
ARL2BP.1	1.97E-47	-0.26549	0.223	0.442	2.94E-43	M1	ARL2BP
CPT1C.2	2.01E-47	-0.27928	0.013	0.124	3.00E-43	M1	CPT1C
FAXC.3	2.10E-47	-0.26706	0.002	0.102	3.14E-43	M1	FAXC
SLC25A22.	2.15E-47	-0.22376	0.017	0.132	3.22E-43	M1	SLC25A22
BOC.2	2.20E-47	-0.2022	0.003	0.104	3.29E-43	M1	BOC
FIGN.2	2.27E-47	-0.21228	0.003	0.105	3.40E-43	M1	FIGN
HSPA6.2	2.34E-47	0.421407	0.186	0.082	3.49E-43	M1	HSPA6
CD93.4	2.37E-47	0.36137	0.146	0.056	3.54E-43	M1	CD93
C9orf3.2	2.39E-47	-0.35984	0.013	0.123	3.57E-43	M1	C9orf3
ZNF626.1	2.64E-47	-0.20162	0.035	0.164	3.94E-43	M1	ZNF626
C17orf10C	2.67E-47	-0.19966	0.005	0.108	3.98E-43	M1	C17orf100
SMC4.2	2.82E-47	-0.29223	0.083	0.238	4.21E-43	M1	SMC4
CELF4.4	2.83E-47	-0.38898	0.006	0.11	4.23E-43	M1	CELF4
SMARCA1.2	2.91E-47	-0.21223	0.053	0.195	4.35E-43	M1	SMARCA1
PCYOX1.1	2.93E-47	-0.1951	0.059	0.205	4.38E-43	M1	PCYOX1
MAPK6.2	2.96E-47	-0.16397	0.053	0.196	4.43E-43	M1	MAPK6
COPS7B.1	3.19E-47	-0.19805	0.07	0.222	4.77E-43	M1	COPS7B
RAB39B.3	3.21E-47	-0.23246	0.005	0.107	4.80E-43	M1	RAB39B
NARF.1	3.23E-47	-0.20994	0.164	0.362	4.82E-43	M1	NARF
MAPKAPK5-	3.39E-47	-0.24891	0.089	0.25	5.07E-43	M1	MAPKAPK5-AS1
CD151.3	3.45E-47	-0.367	0.302	0.518	5.15E-43	M1	CD151
CKAP2.4	3.47E-47	-0.27268	0.053	0.192	5.19E-43	M1	CKAP2
SAE1.2	3.82E-47	-0.26625	0.124	0.303	5.71E-43	M1	SAE1
TSSC1.1	3.82E-47	-0.22237	0.114	0.289	5.71E-43	M1	TSSC1
ELOVL6.2	3.85E-47	-0.21888	0.006	0.11	5.75E-43	M1	ELOVL6
EFNB2.3	3.97E-47	-0.17796	0.01	0.118	5.94E-43	M1	EFNB2
TBC1D4.3	3.98E-47	0.320072	0.11	0.035	5.95E-43	M1	TBC1D4
TMEM183A.	4.16E-47	-0.23933	0.138	0.323	6.22E-43	M1	TMEM183A

MTFR1L.3	4.46E-47	-0.20556	0.134	0.322	6.66E-43	M1	MTFR1L
DHX40.3	4.56E-47	-0.20212	0.033	0.16	6.82E-43	M1	DHX40
SDHAF1.2	4.59E-47	-0.22242	0.057	0.2	6.86E-43	M1	SDHAF1
ABHD17A.3	4.61E-47	-0.23323	0.097	0.263	6.89E-43	M1	ABHD17A
SLC35F2.2	4.81E-47	-0.19232	0.014	0.126	7.18E-43	M1	SLC35F2
LRIF1.2	4.81E-47	-0.24091	0.071	0.223	7.18E-43	M1	LRIF1
MNAT1.3	4.87E-47	-0.2396	0.068	0.218	7.27E-43	M1	MNAT1
MEIS1.4	4.96E-47	-0.2303	0.003	0.104	7.41E-43	M1	MEIS1
MSANTD4.1	5.11E-47	-0.24518	0.025	0.145	7.64E-43	M1	MSANTD4
GCLM.2	5.26E-47	-0.22629	0.046	0.181	7.86E-43	M1	GCLM
VSTM2A.3	5.31E-47	-0.56605	0.014	0.124	7.93E-43	M1	VSTM2A
COPS6.2	5.39E-47	-0.2913	0.399	0.645	8.06E-43	M1	COPS6
SPNS3.3	5.53E-47	0.234805	0.068	0.014	8.26E-43	M1	SPNS3
ABCF2.2	5.76E-47	-0.22227	0.084	0.242	8.60E-43	M1	ABCF2
SLC9A3R2.	5.79E-47	-0.20824	0.017	0.131	8.66E-43	M1	SLC9A3R2
CNOT4.1	5.97E-47	-0.21119	0.123	0.302	8.92E-43	M1	CNOT4
ANKRD46.2	6.07E-47	-0.30095	0.046	0.179	9.07E-43	M1	ANKRD46
THAP3.3	6.11E-47	-0.23452	0.031	0.155	9.14E-43	M1	THAP3
GGA2.4	6.60E-47	0.410879	0.232	0.12	9.86E-43	M1	GGA2
CMBL.2	6.74E-47	-0.20695	0.025	0.147	1.01E-42	M1	CMBL
ATXN2.2	7.05E-47	-0.15636	0.087	0.249	1.05E-42	M1	ATXN2
UBE2C.4	7.26E-47	-0.45533	0.021	0.136	1.08E-42	M1	UBE2C
DNAJA3.1	7.30E-47	-0.23961	0.056	0.197	1.09E-42	M1	DNAJA3
POMC.4	7.62E-47	0.311878	0.109	0.035	1.14E-42	M1	POMC
TRMT6.1	7.73E-47	-0.22946	0.059	0.202	1.16E-42	M1	TRMT6
DYNC2LI1.	7.81E-47	-0.26028	0.05	0.187	1.17E-42	M1	DYNC2LI1
CELF5.4	7.93E-47	-0.34831	0.006	0.108	1.18E-42	M1	CELF5
ERLEC1.2	7.97E-47	-0.25675	0.188	0.396	1.19E-42	M1	ERLEC1
MOSPD3.1	8.47E-47	-0.237	0.134	0.318	1.27E-42	M1	MOSPD3
ARMC6.1	8.79E-47	-0.22629	0.047	0.183	1.31E-42	M1	ARMC6
PDLIM2.2	9.01E-47	-0.28124	0.102	0.268	1.35E-42	M1	PDLIM2
C19orf52.	9.25E-47	-0.1832	0.055	0.198	1.38E-42	M1	C19orf52
AKT1S1.2	9.29E-47	-0.22125	0.1	0.268	1.39E-42	M1	AKT1S1
STOX2.4	9.50E-47	-0.25344	0.006	0.109	1.42E-42	M1	STOX2
BARD1.3	9.91E-47	-0.25024	0.039	0.168	1.48E-42	M1	BARD1
CHD6.3	9.91E-47	-0.20688	0.103	0.273	1.48E-42	M1	CHD6
HCAR2	9.94E-47	0.201998	0.043	0.005	1.49E-42	M1	HCAR2
ANKRD54.2	1.00E-46	-0.19293	0.034	0.161	1.50E-42	M1	ANKRD54
PEX10.2	1.08E-46	-0.18974	0.067	0.217	1.61E-42	M1	PEX10
DDX42.2	1.10E-46	-0.19656	0.085	0.245	1.65E-42	M1	DDX42
HBS1L.2	1.12E-46	-0.20709	0.067	0.216	1.67E-42	M1	HBS1L
PRKCZ.4	1.17E-46	-0.24081	0.011	0.118	1.74E-42	M1	PRKCZ
TET1.3	1.19E-46	-0.19492	0.001	0.099	1.78E-42	M1	TET1
DHX33.2	1.21E-46	-0.20935	0.022	0.141	1.81E-42	M1	DHX33
GLTP.2	1.25E-46	-0.29976	0.124	0.297	1.86E-42	M1	GLTP
MLLT3.2	1.31E-46	-0.24174	0.007	0.111	1.96E-42	M1	MLLT3
MPDZ.2	1.41E-46	-0.21812	0.003	0.103	2.11E-42	M1	MPDZ
RP11-212I	1.46E-46	0.263703	0.067	0.014	2.18E-42	M1	RP11-212I21.4
PWP1.1	1.58E-46	-0.22168	0.146	0.334	2.36E-42	M1	PWP1
HLTF.3	1.60E-46	-0.21518	0.056	0.197	2.40E-42	M1	HLTF
OSER1-AS1	1.66E-46	-0.22624	0.029	0.152	2.48E-42	M1	OSER1-AS1

PPP5C.3	1.81E-46	-0.20996	0.058	0.2	2.70E-42	M1	PPP5C
DESI2.2	1.83E-46	-0.18834	0.069	0.219	2.74E-42	M1	DESI2
ABHD17C.1	1.84E-46	-0.18727	0.002	0.1	2.75E-42	M1	ABHD17C
PSMB5.3	1.93E-46	-0.32306	0.389	0.636	2.88E-42	M1	PSMB5
ZNF322.2	1.94E-46	-0.19156	0.03	0.154	2.90E-42	M1	ZNF322
CHTOP.2	2.27E-46	-0.23415	0.191	0.402	3.39E-42	M1	CHTOP
RTN3.2	2.39E-46	-0.27631	0.47	0.692	3.57E-42	M1	RTN3
NUBP2.2	2.41E-46	-0.21886	0.203	0.417	3.60E-42	M1	NUBP2
DAXX	2.73E-46	-0.20592	0.075	0.229	4.08E-42	M1	DAXX
PSMD1.1	2.73E-46	-0.22605	0.16	0.355	4.08E-42	M1	PSMD1
RAB3GAP2.	2.88E-46	-0.19876	0.053	0.194	4.30E-42	M1	RAB3GAP2
ALDH1A1.2	3.04E-46	0.288817	0.078	0.019	4.54E-42	M1	ALDH1A1
LINC01116	3.08E-46	-0.21411	0.03	0.153	4.61E-42	M1	LINC01116
NCOA6.4	3.09E-46	-0.17749	0.032	0.156	4.61E-42	M1	NCOA6
TSR3.2	3.24E-46	-0.22429	0.126	0.306	4.84E-42	M1	TSR3
TRUB2.2	3.53E-46	-0.21587	0.082	0.239	5.28E-42	M1	TRUB2
PITX1.3	3.65E-46	-0.17576	0.003	0.101	5.45E-42	M1	PITX1
CSNK1G3.2	3.96E-46	-0.19468	0.043	0.175	5.92E-42	M1	CSNK1G3
ZNF521.2	4.05E-46	-0.20551	0.002	0.1	6.06E-42	M1	ZNF521
HABP4.1	4.18E-46	-0.18033	0.022	0.139	6.24E-42	M1	HABP4
MAGOHB.1	4.21E-46	-0.24668	0.101	0.265	6.29E-42	M1	MAGOHB
AGPAT5.3	4.27E-46	-0.2341	0.08	0.235	6.38E-42	M1	AGPAT5
CNOT7.2	4.38E-46	-0.21101	0.195	0.403	6.54E-42	M1	CNOT7
SNX20.3	4.40E-46	0.28569	0.104	0.033	6.58E-42	M1	SNX20
POLR2B.3	4.50E-46	-0.24087	0.119	0.291	6.72E-42	M1	POLR2B
CYB5D2.2	4.51E-46	-0.19551	0.096	0.26	6.74E-42	M1	CYB5D2
TLN1.4	4.67E-46	0.456806	0.373	0.267	6.98E-42	M1	TLN1
SBDS.4	5.18E-46	-0.31102	0.405	0.651	7.74E-42	M1	SBDS
GPRIN3.4	5.35E-46	0.322704	0.126	0.045	7.99E-42	M1	GPRIN3
ITGA3.2	5.47E-46	-0.19692	0.01	0.116	8.17E-42	M1	ITGA3
GIMAP2.3	5.51E-46	0.395044	0.152	0.062	8.24E-42	M1	GIMAP2
ZNF793.1	5.93E-46	-0.18193	0.017	0.129	8.86E-42	M1	ZNF793
ANGPTL2.2	6.02E-46	-0.32318	0.011	0.117	9.00E-42	M1	ANGPTL2
VPS26B.3	6.11E-46	-0.22735	0.096	0.258	9.13E-42	M1	VPS26B
CACNG7.2	6.33E-46	-0.2004	0.003	0.1	9.46E-42	M1	CACNG7
TWF2.4	6.47E-46	0.432713	0.425	0.324	9.67E-42	M1	TWF2
NTN1.1	6.49E-46	-0.20167	0.002	0.098	9.69E-42	M1	NTN1
HAS1.1	6.61E-46	0.228587	0.048	0.007	9.88E-42	M1	HAS1
WASF1.1	7.52E-46	-0.24912	0.008	0.112	1.12E-41	M1	WASF1
HARS	7.69E-46	-0.22693	0.13	0.311	1.15E-41	M1	HARS
EEF1A1.4	8.00E-46	-0.29819	0.796	0.832	1.20E-41	M1	EEF1A1
ATG16L2.4	8.03E-46	0.433716	0.165	0.072	1.20E-41	M1	ATG16L2
TRIM36.4	8.25E-46	-0.22987	0.063	0.206	1.23E-41	M1	TRIM36
FGF1.2	8.35E-46	-0.30687	0.008	0.111	1.25E-41	M1	FGF1
SDR39U1.2	8.58E-46	-0.22424	0.126	0.304	1.28E-41	M1	SDR39U1
SYBU.2	9.23E-46	-0.24014	0.004	0.103	1.38E-41	M1	SYBU
MZT1.2	1.02E-45	-0.19912	0.113	0.284	1.53E-41	M1	MZT1
SH3BGR.3	1.05E-45	-0.37944	0.026	0.143	1.57E-41	M1	SH3BGR
ARFIP2.3	1.08E-45	-0.17829	0.104	0.272	1.61E-41	M1	ARFIP2
SLC12A2.3	1.15E-45	-0.24394	0.029	0.15	1.72E-41	M1	SLC12A2
MZT2A.2	1.17E-45	-0.2432	0.233	0.45	1.75E-41	M1	MZT2A

PDLIM7.2	1.28E-45	-0.22373	0.101	0.266	1.92E-41	M1	PDLIM7
CAMK2D.2	1.30E-45	-0.25223	0.106	0.271	1.95E-41	M1	CAMK2D
PDZD11.3	1.36E-45	-0.27698	0.192	0.392	2.03E-41	M1	PDZD11
ZNF22.3	1.54E-45	-0.24828	0.128	0.305	2.31E-41	M1	ZNF22
ETF1.3	1.66E-45	0.452449	0.422	0.33	2.48E-41	M1	ETF1
PDE4B.4	1.86E-45	0.516748	0.365	0.258	2.77E-41	M1	PDE4B
DNAJC2.2	2.15E-45	-0.19647	0.144	0.329	3.21E-41	M1	DNAJC2
CREB3	2.18E-45	-0.22535	0.128	0.307	3.26E-41	M1	CREB3
MTSS1L.2	2.19E-45	-0.21983	0.008	0.111	3.27E-41	M1	MTSS1L
ZNHIT3	2.22E-45	-0.19582	0.206	0.42	3.31E-41	M1	ZNHIT3
CHAMP1.2	2.38E-45	-0.19798	0.029	0.149	3.56E-41	M1	CHAMP1
HMGB1.2	2.39E-45	-0.24807	0.778	0.878	3.57E-41	M1	HMGB1
ARRDC2.4	2.42E-45	0.399981	0.164	0.07	3.62E-41	M1	ARRDC2
TAOK1.2	2.52E-45	-0.21494	0.116	0.288	3.77E-41	M1	TAOK1
SPAG9.3	2.56E-45	-0.20541	0.184	0.387	3.83E-41	M1	SPAG9
NPEPPS.1	2.59E-45	-0.17025	0.091	0.252	3.88E-41	M1	NPEPPS
SRRT.1	2.81E-45	-0.21718	0.087	0.243	4.20E-41	M1	SRRT
TMEM108.2	2.91E-45	-0.20962	0.001	0.096	4.34E-41	M1	TMEM108
CDK5R1.4	3.11E-45	-0.27318	0.008	0.11	4.64E-41	M1	CDK5R1
PRRT2.2	3.26E-45	-0.20144	0.007	0.108	4.87E-41	M1	PRRT2
TMEM115.2	3.54E-45	-0.20155	0.079	0.234	5.29E-41	M1	TMEM115
ILDR2.3	3.62E-45	-0.18015	0.001	0.096	5.41E-41	M1	ILDR2
GAN.2	3.74E-45	-0.18328	0.037	0.162	5.59E-41	M1	GAN
KIF5A.3	4.01E-45	-0.32915	0.026	0.141	6.00E-41	M1	KIF5A
GNB5.2	4.06E-45	-0.18315	0.017	0.128	6.07E-41	M1	GNB5
TCEAL4.2	4.48E-45	-0.30661	0.369	0.612	6.70E-41	M1	TCEAL4
STAMBP.2	4.56E-45	-0.21815	0.12	0.295	6.82E-41	M1	STAMBP
CALM3.3	4.65E-45	-0.30034	0.405	0.63	6.95E-41	M1	CALM3
MMAB.2	4.70E-45	-0.22756	0.073	0.222	7.02E-41	M1	MMAB
PDGFD.2	4.80E-45	-0.18049	0.001	0.095	7.17E-41	M1	PDGFD
TMEM99.3	4.88E-45	-0.21943	0.029	0.147	7.30E-41	M1	TMEM99
KHDRBS1.3	5.02E-45	-0.28043	0.302	0.523	7.50E-41	M1	KHDRBS1
DVL3.2	5.04E-45	-0.18827	0.044	0.175	7.53E-41	M1	DVL3
WIPF1.4	5.05E-45	0.46142	0.315	0.2	7.55E-41	M1	WIPF1
COX11.2	5.08E-45	-0.19473	0.157	0.351	7.59E-41	M1	COX11
DYNLL2.4	5.15E-45	-0.21496	0.079	0.231	7.69E-41	M1	DYNLL2
ZNF667.3	5.63E-45	-0.23072	0.002	0.097	8.41E-41	M1	ZNF667
SLC35B1.1	5.78E-45	-0.22769	0.215	0.426	8.63E-41	M1	SLC35B1
POLR3F.2	5.88E-45	-0.21025	0.034	0.158	8.79E-41	M1	POLR3F
RUNDC3A.4	5.89E-45	-0.30173	0.011	0.115	8.80E-41	M1	RUNDC3A
ACBD6.1	6.23E-45	-0.19153	0.127	0.307	9.31E-41	M1	ACBD6
CREBZF.3	6.37E-45	-0.21638	0.031	0.151	9.52E-41	M1	CREBZF
RWDD4	6.74E-45	-0.17985	0.093	0.254	1.01E-40	M1	RWDD4
ZNF638.3	6.74E-45	-0.18548	0.111	0.28	1.01E-40	M1	ZNF638
ITPRIP.4	7.17E-45	0.387423	0.147	0.06	1.07E-40	M1	ITPRIP
GLRX5.3	7.58E-45	-0.23726	0.142	0.327	1.13E-40	M1	GLRX5
DPH7.3	7.71E-45	-0.19339	0.053	0.19	1.15E-40	M1	DPH7
BSG.4	7.74E-45	-0.28409	0.602	0.769	1.16E-40	M1	BSG
GRAMD3.4	7.79E-45	-0.25423	0.037	0.161	1.16E-40	M1	GRAMD3
SRSF10.3	8.22E-45	-0.21765	0.103	0.267	1.23E-40	M1	SRSF10
ATP5G1.2	8.22E-45	-0.30519	0.421	0.657	1.23E-40	M1	ATP5G1

SLC30A9.3	8.33E-45	-0.18382	0.111	0.28	1.25E-40	M1	SLC30A9
LSM1.1	8.67E-45	-0.20043	0.264	0.494	1.30E-40	M1	LSM1
ZNF775.3	9.06E-45	-0.21273	0.009	0.112	1.35E-40	M1	ZNF775
LYRM2.1	9.22E-45	-0.17302	0.14	0.325	1.38E-40	M1	LYRM2
PTCD3.2	9.57E-45	-0.2049	0.096	0.257	1.43E-40	M1	PTCD3
PET117.1	9.69E-45	-0.20563	0.062	0.203	1.45E-40	M1	PET117
SNTA1.2	1.01E-44	-0.1837	0.004	0.101	1.51E-40	M1	SNTA1
UBE2G1.4	1.05E-44	-0.20731	0.115	0.286	1.58E-40	M1	UBE2G1
PCDHB2.2	1.06E-44	-0.20602	0.001	0.095	1.58E-40	M1	PCDHB2
ROMO1.3	1.09E-44	-0.2905	0.41	0.632	1.64E-40	M1	ROMO1
CPED1.2	1.10E-44	0.258179	0.083	0.022	1.64E-40	M1	CPED1
LRRC42.2	1.12E-44	-0.23116	0.039	0.165	1.67E-40	M1	LRRC42
M1AP	1.14E-44	0.171982	0.039	0.004	1.71E-40	M1	M1AP
RITA1.2	1.15E-44	-0.22623	0.032	0.152	1.72E-40	M1	RITA1
HBB.3	1.17E-44	0.280427	0.503	0.378	1.75E-40	M1	HBB
ESF1.2	1.28E-44	-0.24403	0.181	0.378	1.92E-40	M1	ESF1
TMEM255B.	1.31E-44	0.390968	0.12	0.042	1.96E-40	M1	TMEM255B
FDPS.2	1.31E-44	-0.27741	0.238	0.451	1.96E-40	M1	FDPS
CBLN4.4	1.32E-44	-0.26969	0.001	0.095	1.98E-40	M1	CBLN4
MBD3.2	1.60E-44	-0.18896	0.051	0.186	2.40E-40	M1	MBD3
LRRCC1.3	1.74E-44	-0.25217	0.034	0.156	2.61E-40	M1	LRRCC1
PPP1CC.2	1.77E-44	-0.24173	0.26	0.482	2.64E-40	M1	PPP1CC
IL15.2	1.92E-44	0.281628	0.091	0.026	2.87E-40	M1	IL15
PTTG1.3	1.96E-44	-0.37837	0.103	0.256	2.93E-40	M1	PTTG1
UQCC1.2	2.10E-44	-0.19483	0.053	0.188	3.14E-40	M1	UQCC1
SLN.1	2.21E-44	-0.67777	0.053	0.183	3.30E-40	M1	SLN
QDPR.3	2.23E-44	-0.32796	0.197	0.393	3.33E-40	M1	QDPR
SNCAIP.3	2.28E-44	-0.21659	0.005	0.102	3.40E-40	M1	SNCAIP
WIZ.1	2.44E-44	-0.19671	0.019	0.129	3.64E-40	M1	WIZ
EIF1AY.3	2.45E-44	-0.20522	0.061	0.201	3.65E-40	M1	EIF1AY
TP53RK.2	2.48E-44	-0.16441	0.069	0.215	3.70E-40	M1	TP53RK
GTF2H5.2	2.48E-44	-0.30654	0.23	0.439	3.71E-40	M1	GTF2H5
GAMT.4	2.60E-44	-0.22622	0.146	0.33	3.88E-40	M1	GAMT
MCRS1.1	2.64E-44	-0.22099	0.124	0.299	3.95E-40	M1	MCRS1
TCF12.4	2.64E-44	-0.24613	0.139	0.313	3.95E-40	M1	TCF12
BIRC5.4	2.67E-44	-0.4144	0.016	0.122	3.98E-40	M1	BIRC5
TMX2.1	2.69E-44	-0.23952	0.222	0.433	4.02E-40	M1	TMX2
CAMLG.3	2.88E-44	-0.23865	0.299	0.533	4.31E-40	M1	CAMLG
PDLIM5.1	2.93E-44	-0.19275	0.089	0.246	4.38E-40	M1	PDLIM5
FBXO5.1	3.12E-44	-0.22466	0.011	0.113	4.67E-40	M1	FBXO5
EPHA4.1	3.33E-44	-0.21866	0.003	0.097	4.97E-40	M1	EPHA4
NOP56	3.52E-44	-0.2471	0.233	0.45	5.27E-40	M1	NOP56
EPRS.2	3.58E-44	-0.21518	0.143	0.324	5.35E-40	M1	EPRS
ARMC10.2	3.78E-44	-0.21291	0.144	0.327	5.65E-40	M1	ARMC10
RP11-390E	3.86E-44	-0.27542	0.028	0.144	5.77E-40	M1	RP11-390E23.6
TCEA1	3.99E-44	-0.29214	0.301	0.521	5.97E-40	M1	TCEA1
MACF1.2	4.19E-44	-0.22136	0.164	0.353	6.26E-40	M1	MACF1
RGMB.3	4.29E-44	-0.32969	0.033	0.152	6.41E-40	M1	RGMB
SMARCC2.3	4.31E-44	-0.16079	0.119	0.292	6.44E-40	M1	SMARCC2
ZHX1.3	4.51E-44	-0.21825	0.083	0.234	6.74E-40	M1	ZHX1
DUSP15.3	4.61E-44	-0.18243	0.009	0.11	6.89E-40	M1	DUSP15

ARMCX1.3	4.89E-44	-0.21162	0.087	0.239	7.30E-40	M1	ARMCX1
RCAN1.4	4.99E-44	-0.38758	0.128	0.296	7.45E-40	M1	RCAN1
ARHGEF26.	5.09E-44	-0.18298	0.028	0.144	7.61E-40	M1	ARHGEF26
THOP1.3	5.15E-44	-0.17146	0.077	0.226	7.70E-40	M1	THOP1
SLC39A7.1	5.44E-44	-0.22273	0.151	0.336	8.13E-40	M1	SLC39A7
LRIG1.3	5.53E-44	-0.22	0.018	0.126	8.26E-40	M1	LRIG1
ANAPC7.2	5.64E-44	-0.20669	0.058	0.195	8.43E-40	M1	ANAPC7
DUSP12.1	5.80E-44	-0.196	0.1	0.262	8.67E-40	M1	DUSP12
ELMOD1.2	6.01E-44	-0.16065	0.003	0.098	8.97E-40	M1	ELMOD1
ALDH3A2.2	6.03E-44	-0.20568	0.098	0.259	9.02E-40	M1	ALDH3A2
CERCAM.2	6.04E-44	-0.25395	0.031	0.149	9.03E-40	M1	CERCAM
FAM69C.3	6.08E-44	-0.19218	0.001	0.092	9.09E-40	M1	FAM69C
DNAJC18.1	6.08E-44	-0.2196	0.026	0.142	9.09E-40	M1	DNAJC18
GPX3.3	6.12E-44	0.444612	0.246	0.134	9.15E-40	M1	GPX3
NACC1.1	6.34E-44	-0.15943	0.043	0.172	9.47E-40	M1	NACC1
GNL1.2	6.44E-44	-0.18213	0.104	0.27	9.62E-40	M1	GNL1
KIAA0907.	6.57E-44	-0.19372	0.072	0.218	9.82E-40	M1	KIAA0907
ADD2.2	6.62E-44	-0.23941	0.007	0.105	9.90E-40	M1	ADD2
NAGK.2	6.81E-44	0.376838	0.457	0.366	1.02E-39	M1	NAGK
BRSK1.2	6.81E-44	-0.22805	0.004	0.1	1.02E-39	M1	BRSK1
TCEB2.4	6.99E-44	-0.25425	0.718	0.845	1.04E-39	M1	TCEB2
POLR3H.2	7.07E-44	-0.21622	0.086	0.24	1.06E-39	M1	POLR3H
FAHD1.2	7.20E-44	-0.20954	0.061	0.199	1.08E-39	M1	FAHD1
ARL6IP4.4	7.27E-44	0.234237	0.65	0.619	1.09E-39	M1	ARL6IP4
CMTM5.3	7.74E-44	-0.49185	0.014	0.117	1.16E-39	M1	CMTM5
AP3D1.2	7.92E-44	-0.18327	0.152	0.34	1.18E-39	M1	AP3D1
DUSP26.3	8.35E-44	-0.36468	0.007	0.104	1.25E-39	M1	DUSP26
DPP4.2	8.36E-44	-0.19136	0.001	0.093	1.25E-39	M1	DPP4
IMPAD1.2	8.54E-44	-0.19929	0.067	0.21	1.28E-39	M1	IMPAD1
BIVM.2	8.88E-44	-0.21098	0.019	0.129	1.33E-39	M1	BIVM
CCP110.3	8.98E-44	-0.27944	0.028	0.143	1.34E-39	M1	CCP110
CYHR1.3	9.04E-44	-0.21506	0.131	0.308	1.35E-39	M1	CYHR1
BCS1L.2	9.08E-44	-0.21855	0.055	0.188	1.36E-39	M1	BCS1L
FARSB.2	9.12E-44	-0.21041	0.099	0.259	1.36E-39	M1	FARSB
DHX30.2	9.19E-44	-0.16896	0.115	0.286	1.37E-39	M1	DHX30
NOVA2.2	9.71E-44	-0.22081	0.003	0.096	1.45E-39	M1	NOVA2
LOXL1.2	9.94E-44	-0.17384	0.001	0.093	1.49E-39	M1	LOXL1
PAX6.2	1.02E-43	-0.19802	0.002	0.094	1.52E-39	M1	PAX6
PRKDC.1	1.06E-43	-0.18553	0.141	0.322	1.59E-39	M1	PRKDC
CUL5.3	1.10E-43	-0.17387	0.091	0.249	1.65E-39	M1	CUL5
MSANTD3.2	1.10E-43	-0.212	0.006	0.103	1.65E-39	M1	MSANTD3
PLA2G4A.2	1.16E-43	0.250305	0.083	0.022	1.73E-39	M1	PLA2G4A
CPSF4.1	1.21E-43	-0.1856	0.074	0.22	1.80E-39	M1	CPSF4
CASP4.4	1.26E-43	0.416601	0.329	0.207	1.88E-39	M1	CASP4
RARS2.1	1.30E-43	-0.18836	0.07	0.215	1.94E-39	M1	RARS2
COX6C.4	1.32E-43	-0.26796	0.736	0.85	1.97E-39	M1	COX6C
UBA1.1	1.33E-43	-0.20675	0.177	0.375	1.99E-39	M1	UBA1
MUM1.4	1.44E-43	-0.23016	0.196	0.397	2.16E-39	M1	MUM1
HIP1R.3	1.45E-43	-0.25403	0.019	0.127	2.16E-39	M1	HIP1R
PEG10.2	1.48E-43	-0.36506	0.016	0.12	2.21E-39	M1	PEG10
MARCH1.4	1.49E-43	0.399992	0.292	0.17	2.23E-39	M1	1-Mar

TXNL4A.2	1.54E-43	-0.25376	0.233	0.446	2.30E-39	M1		TXNL4A
MRPS23.2	1.58E-43	-0.24802	0.198	0.4	2.36E-39	M1		MRPS23
RFFL.4	1.69E-43	-0.20032	0.053	0.187	2.52E-39	M1		RFFL
AP3M2.2	1.71E-43	-0.21233	0.009	0.108	2.55E-39	M1		AP3M2
NOS1AP.3	1.73E-43	-0.21518	0.002	0.095	2.59E-39	M1		NOS1AP
MAOB.2	1.80E-43	-0.35649	0.013	0.116	2.70E-39	M1		MAOB
REEP1.4	1.84E-43	-0.2528	0.003	0.096	2.74E-39	M1		REEP1
NELFA.3	1.88E-43	-0.20136	0.038	0.16	2.81E-39	M1		NELFA
DUSP14.3	1.89E-43	-0.17643	0.063	0.203	2.83E-39	M1		DUSP14
PLCB1.2	1.89E-43	-0.21368	0.003	0.096	2.83E-39	M1		PLCB1
SARS.3	1.90E-43	-0.22734	0.256	0.48	2.83E-39	M1		SARS
RNPS1	1.94E-43	-0.24675	0.34	0.573	2.90E-39	M1		RNPS1
COA4.1	2.08E-43	-0.27981	0.217	0.42	3.11E-39	M1		COA4
C12orf73.	2.15E-43	-0.21481	0.047	0.176	3.21E-39	M1		C12orf73
TXNDC17.3	2.23E-43	-0.3228	0.349	0.56	3.33E-39	M1		TXNDC17
GPR162.2	2.24E-43	-0.22567	0.015	0.119	3.34E-39	M1		GPR162
C11orf57.	2.50E-43	-0.22184	0.047	0.175	3.73E-39	M1		C11orf57
TLCD1.2	2.54E-43	-0.16142	0.003	0.096	3.80E-39	M1		TLCD1
NUDCD2.1	2.60E-43	-0.25604	0.225	0.436	3.88E-39	M1		NUDCD2
CARD11.3	2.62E-43	0.222374	0.074	0.019	3.91E-39	M1		CARD11
ELAVL2.4	2.82E-43	-0.31264	0.003	0.095	4.21E-39	M1		ELAVL2
CXXC4.3	2.85E-43	-0.24037	0.002	0.093	4.27E-39	M1		CXXC4
TMEM178A.	2.96E-43	-0.24209	0.025	0.137	4.42E-39	M1		TMEM178A
TNPO3	3.10E-43	-0.25059	0.04	0.163	4.63E-39	M1		TNPO3
UBXN1.4	3.17E-43	0.278994	0.638	0.617	4.74E-39	M1		UBXN1
ELOVL4.2	3.21E-43	-0.21922	0.003	0.095	4.80E-39	M1		ELOVL4
TRIO.3	3.23E-43	-0.24118	0.06	0.195	4.82E-39	M1		TRIO
PHF6.3	3.35E-43	-0.18294	0.057	0.193	5.00E-39	M1		PHF6
CKAP5.4	3.44E-43	-0.20752	0.048	0.178	5.14E-39	M1		CKAP5
SLC25A18.	3.69E-43	-0.26083	0.007	0.103	5.51E-39	M1		SLC25A18
ACTR10.2	4.32E-43	-0.24129	0.194	0.393	6.46E-39	M1		ACTR10
SREK1.3	4.44E-43	-0.20444	0.165	0.354	6.63E-39	M1		SREK1
RBM4.2	4.55E-43	-0.17321	0.091	0.246	6.80E-39	M1		RBM4
CSF1.2	4.58E-43	-0.21924	0.011	0.111	6.84E-39	M1		CSF1
NOP10.4	4.66E-43	0.251273	0.644	0.598	6.96E-39	M1		NOP10
TMEM205.3	4.73E-43	-0.3653	0.319	0.531	7.07E-39	M1		TMEM205
CASC15.3	4.87E-43	-0.2476	0.014	0.116	7.28E-39	M1		CASC15
VNN2.2	4.98E-43	0.241902	0.069	0.016	7.44E-39	M1		VNN2
CRCP.2	5.93E-43	-0.17823	0.065	0.205	8.86E-39	M1		CRCP
HRH1.3	6.16E-43	-0.22271	0.012	0.113	9.20E-39	M1		HRH1
DPF2.1	7.05E-43	-0.17352	0.093	0.25	1.05E-38	M1		DPF2
PIANP.2	7.12E-43	-0.19045	0.004	0.098	1.06E-38	M1		PIANP
AHCY.1	7.30E-43	-0.19513	0.145	0.324	1.09E-38	M1		AHCY
FANCL.2	7.61E-43	-0.26417	0.041	0.163	1.14E-38	M1		FANCL
PRRG4.3	7.87E-43	0.332915	0.102	0.033	1.18E-38	M1		PRRG4
PUF60.2	8.08E-43	-0.24681	0.345	0.581	1.21E-38	M1		PUF60
MED21.1	8.49E-43	-0.21675	0.085	0.235	1.27E-38	M1		MED21
LINC00685	8.56E-43	-0.19026	0.044	0.17	1.28E-38	M1		LINC00685
UBL4A.3	8.63E-43	-0.18571	0.066	0.206	1.29E-38	M1		UBL4A
CMAS.1	8.83E-43	-0.20344	0.125	0.295	1.32E-38	M1		CMAS
VAX2.3	9.92E-43	-0.21828	0.003	0.095	1.48E-38	M1		VAX2

SUDS3.3	1.06E-42	-0.16947	0.069	0.212	1.59E-38	M1	SUDS3
TMEM5.2	1.16E-42	-0.22867	0.085	0.233	1.73E-38	M1	TMEM5
VKORC1L1.	1.17E-42	-0.19721	0.033	0.151	1.75E-38	M1	VKORC1L1
MAGED2.4	1.19E-42	-0.27134	0.322	0.546	1.78E-38	M1	MAGED2
VEZT.2	1.20E-42	-0.16123	0.096	0.253	1.79E-38	M1	VEZT
BOLA3.2	1.21E-42	-0.26528	0.176	0.367	1.81E-38	M1	BOLA3
PTBP2.2	1.23E-42	-0.2687	0.018	0.123	1.84E-38	M1	PTBP2
CCDC181.3	1.26E-42	-0.25674	0.003	0.095	1.89E-38	M1	CCDC181
PLAT.1	1.29E-42	-0.2513	0.026	0.138	1.93E-38	M1	PLAT
TMEM41B.2	1.31E-42	-0.19699	0.062	0.199	1.95E-38	M1	TMEM41B
TMTC4.3	1.34E-42	-0.25306	0.014	0.115	2.00E-38	M1	TMTC4
EPB41L1.2	1.37E-42	-0.20388	0.008	0.104	2.05E-38	M1	EPB41L1
PIK3R3.2	1.42E-42	-0.16299	0.033	0.152	2.12E-38	M1	PIK3R3
CLASP1.3	1.49E-42	-0.19349	0.037	0.158	2.23E-38	M1	CLASP1
F12.2	1.57E-42	-0.19218	0.002	0.093	2.34E-38	M1	F12
MED17.1	1.57E-42	-0.1899	0.071	0.213	2.34E-38	M1	MED17
DHRS11.3	1.61E-42	-0.15696	0.016	0.12	2.40E-38	M1	DHRS11
ATP6AP1.4	1.63E-42	0.345075	0.536	0.484	2.43E-38	M1	ATP6AP1
TIMP4.2	1.64E-42	-0.41895	0.015	0.118	2.45E-38	M1	TIMP4
DLX6-AS1.	1.66E-42	-0.65117	0.037	0.149	2.48E-38	M1	DLX6-AS1
LINC00152	1.71E-42	-0.30927	0.123	0.286	2.56E-38	M1	LINC00152
TMEM136.2	1.72E-42	-0.20511	0.008	0.104	2.57E-38	M1	TMEM136
TPT1-AS1.	1.72E-42	-0.16356	0.038	0.159	2.57E-38	M1	TPT1-AS1
IGF2BP2.2	1.76E-42	-0.20555	0.004	0.096	2.63E-38	M1	IGF2BP2
BLCAP.1	1.77E-42	-0.21776	0.143	0.32	2.64E-38	M1	BLCAP
PIGU.3	1.79E-42	-0.25715	0.06	0.193	2.67E-38	M1	PIGU
LMNB2.4	1.84E-42	-0.18667	0.024	0.135	2.75E-38	M1	LMNB2
PCOLCE2.2	1.84E-42	-0.2702	0.005	0.098	2.75E-38	M1	PCOLCE2
KBTBD6.2	1.86E-42	-0.19784	0.033	0.151	2.79E-38	M1	KBTBD6
USP48	1.89E-42	-0.17806	0.088	0.24	2.83E-38	M1	USP48
IL32.2	2.01E-42	-0.48952	0.015	0.117	3.00E-38	M1	IL32
PIGF.3	2.04E-42	-0.21819	0.141	0.317	3.04E-38	M1	PIGF
TARDBP	2.21E-42	-0.19238	0.145	0.324	3.30E-38	M1	TARDBP
TMEM70.3	2.22E-42	0.434317	0.46	0.382	3.32E-38	M1	TMEM70
HP1BP3.4	2.29E-42	-0.23436	0.259	0.479	3.42E-38	M1	HP1BP3
ZBED5.3	2.29E-42	-0.16918	0.064	0.202	3.42E-38	M1	ZBED5
TMEM199.2	2.29E-42	-0.16242	0.107	0.269	3.43E-38	M1	TMEM199
AMOTL2.1	2.35E-42	-0.28252	0.004	0.096	3.51E-38	M1	AMOTL2
DMRTA2.1	2.36E-42	-0.18411	0.002	0.091	3.52E-38	M1	DMRTA2
LEO1.1	2.59E-42	-0.22491	0.042	0.164	3.88E-38	M1	LEO1
RNF8.3	2.69E-42	-0.19236	0.057	0.189	4.02E-38	M1	RNF8
ATG4D.1	2.73E-42	-0.18002	0.052	0.182	4.08E-38	M1	ATG4D
HDDC2.1	2.75E-42	-0.27271	0.228	0.435	4.10E-38	M1	HDDC2
FAM57A.2	2.76E-42	-0.16318	0.011	0.111	4.13E-38	M1	FAM57A
CCT3.2	2.76E-42	-0.27214	0.376	0.618	4.13E-38	M1	CCT3
PSMC3.2	2.82E-42	-0.25696	0.333	0.57	4.22E-38	M1	PSMC3
ZNF544.2	2.87E-42	-0.19419	0.028	0.141	4.29E-38	M1	ZNF544
HSF2.2	2.91E-42	-0.24739	0.025	0.136	4.35E-38	M1	HSF2
DIDO1.2	2.93E-42	-0.15029	0.044	0.169	4.38E-38	M1	DIDO1
ASTN1.1	3.06E-42	-0.15817	0.01	0.108	4.57E-38	M1	ASTN1
GALNT11.2	3.24E-42	-0.17263	0.097	0.252	4.84E-38	M1	GALNT11

MXI1.1	3.37E-42	-0.29174	0.036	0.153	5.03E-38	M1	MXI1
TMEM100.4	3.42E-42	-0.36601	0.009	0.105	5.12E-38	M1	TMEM100
CLPP.1	3.45E-42	-0.23726	0.224	0.433	5.15E-38	M1	CLPP
FAM63B.1	3.77E-42	-0.19121	0.082	0.229	5.64E-38	M1	FAM63B
PSMB7.2	3.81E-42	-0.28572	0.444	0.68	5.70E-38	M1	PSMB7
ILKAP.2	3.82E-42	-0.19664	0.099	0.255	5.70E-38	M1	ILKAP
HSD17B10.	3.87E-42	-0.2917	0.306	0.533	5.78E-38	M1	HSD17B10
AC004951.	4.11E-42	-0.14788	0.049	0.178	6.14E-38	M1	AC004951.6
CCDC106.4	4.38E-42	-0.17195	0.1	0.258	6.55E-38	M1	CCDC106
HOOK2.2	4.39E-42	-0.15835	0.101	0.261	6.57E-38	M1	HOOK2
THUMPD3.1	4.63E-42	-0.18349	0.074	0.218	6.92E-38	M1	THUMPD3
ATG9A.2	4.95E-42	-0.17069	0.024	0.135	7.39E-38	M1	ATG9A
GLOD4.1	5.21E-42	-0.22319	0.215	0.424	7.79E-38	M1	GLOD4
GPR132.3	5.22E-42	0.267878	0.078	0.021	7.80E-38	M1	GPR132
HKR1.3	5.31E-42	-0.2275	0.025	0.135	7.93E-38	M1	HKR1
TNKS.4	5.49E-42	-0.15546	0.044	0.169	8.21E-38	M1	TNKS
FAM102A.1	5.62E-42	-0.24398	0.014	0.114	8.41E-38	M1	FAM102A
MEOX2.2	5.66E-42	-0.23714	0.003	0.094	8.45E-38	M1	MEOX2
EMC6.2	5.70E-42	-0.23286	0.257	0.469	8.52E-38	M1	EMC6
SLC25A17.	5.80E-42	-0.21161	0.05	0.178	8.67E-38	M1	SLC25A17
ZC3H8.2	5.83E-42	-0.21276	0.044	0.168	8.70E-38	M1	ZC3H8
TBRG4.2	5.83E-42	-0.1899	0.105	0.264	8.71E-38	M1	TBRG4
HYAL2.4	5.98E-42	-0.16591	0.094	0.249	8.93E-38	M1	HYAL2
CTD-3184A	6.00E-42	-0.1782	0.013	0.114	8.96E-38	M1	CTD-3184A7.4
EMC9.1	6.01E-42	-0.22699	0.064	0.2	8.99E-38	M1	EMC9
ING2.2	6.42E-42	-0.1851	0.084	0.231	9.60E-38	M1	ING2
RNF10.2	6.48E-42	-0.17622	0.172	0.365	9.68E-38	M1	RNF10
RRP36.3	6.68E-42	-0.15552	0.042	0.166	9.98E-38	M1	RRP36
TTF1.1	7.16E-42	-0.16835	0.081	0.227	1.07E-37	M1	TTF1
HOXC9.4	7.23E-42	-0.31123	0.007	0.101	1.08E-37	M1	HOXC9
PSMB2.2	7.41E-42	-0.31327	0.374	0.614	1.11E-37	M1	PSMB2
CORO1B.4	7.41E-42	0.400129	0.433	0.347	1.11E-37	M1	CORO1B
MAPK7.2	7.50E-42	-0.19234	0.03	0.144	1.12E-37	M1	MAPK7
YIPF3.3	7.74E-42	-0.22618	0.289	0.522	1.16E-37	M1	YIPF3
PPP1CA.3	8.11E-42	0.260862	0.609	0.564	1.21E-37	M1	PPP1CA
GGPS1.3	8.62E-42	-0.20329	0.162	0.349	1.29E-37	M1	GGPS1
IQCE.2	8.76E-42	-0.20076	0.026	0.137	1.31E-37	M1	IQCE
AC093673.	9.17E-42	-0.19251	0.064	0.201	1.37E-37	M1	AC093673.5
TOM1L2.1	9.27E-42	-0.20681	0.027	0.138	1.39E-37	M1	TOM1L2
OSMR.2	9.66E-42	-0.17531	0.007	0.101	1.44E-37	M1	OSMR
SMC6.2	1.01E-41	-0.19401	0.05	0.178	1.51E-37	M1	SMC6
ETV4.3	1.05E-41	-0.20101	0.003	0.093	1.56E-37	M1	ETV4
NOP16.3	1.05E-41	-0.24993	0.08	0.223	1.57E-37	M1	NOP16
NOL11.2	1.09E-41	-0.20526	0.072	0.212	1.63E-37	M1	NOL11
LMCD1.2	1.12E-41	-0.20688	0.007	0.101	1.67E-37	M1	LMCD1
BOLA1.2	1.12E-41	-0.20438	0.047	0.172	1.67E-37	M1	BOLA1
TMEM47.2	1.13E-41	-0.19058	0.004	0.095	1.69E-37	M1	TMEM47
UBQLN2.4	1.19E-41	-0.17315	0.094	0.246	1.77E-37	M1	UBQLN2
SH3GL1.1	1.20E-41	-0.21646	0.113	0.277	1.80E-37	M1	SH3GL1
TFAM.1	1.28E-41	-0.16595	0.066	0.204	1.91E-37	M1	TFAM
DNM1L.2	1.38E-41	-0.15358	0.1	0.258	2.06E-37	M1	DNM1L

EPHA3.3	1.39E-41	-0.15263	0.001	0.088	2.08E-37	M1	EPHA3
ZNF436.2	1.42E-41	-0.16557	0.003	0.093	2.12E-37	M1	ZNF436
ARHGAP26.	1.50E-41	0.404941	0.167	0.076	2.25E-37	M1	ARHGAP26
DNAL4.2	1.53E-41	-0.17792	0.032	0.147	2.29E-37	M1	DNAL4
SORBS1.2	1.54E-41	-0.16026	0.011	0.109	2.30E-37	M1	SORBS1
TARS.1	1.70E-41	-0.16417	0.097	0.253	2.55E-37	M1	TARS
SPOCK1.3	1.82E-41	-0.34198	0.005	0.096	2.71E-37	M1	SPOCK1
ZNF814.1	1.93E-41	-0.20045	0.029	0.141	2.89E-37	M1	ZNF814
CADM3.2	1.94E-41	-0.41506	0.016	0.117	2.90E-37	M1	CADM3
NDUFAF4.2	1.99E-41	-0.21879	0.095	0.247	2.98E-37	M1	NDUFAF4
ADAM19.3	2.02E-41	-0.20717	0.004	0.095	3.01E-37	M1	ADAM19
VIPR2.3	2.09E-41	-0.34209	0.006	0.098	3.12E-37	M1	VIPR2
LDLR.1	2.38E-41	-0.18902	0.015	0.116	3.56E-37	M1	LDLR
TNFAIP2.2	2.39E-41	0.344341	0.145	0.061	3.57E-37	M1	TNFAIP2
GMCL1.2	2.47E-41	-0.20546	0.052	0.18	3.69E-37	M1	GMCL1
SH3PXD2B.	2.64E-41	-0.16586	0.025	0.135	3.95E-37	M1	SH3PXD2B
HOXD11.2	2.66E-41	-0.17401	0.003	0.091	3.97E-37	M1	HOXD11
ADAMTS9.2	2.70E-41	-0.19171	0.003	0.092	4.04E-37	M1	ADAMTS9
AASS.2	2.71E-41	-0.20373	0.024	0.133	4.05E-37	M1	AASS
NAA35.1	2.80E-41	-0.1819	0.038	0.157	4.19E-37	M1	NAA35
TMED1.3	2.83E-41	-0.24911	0.145	0.321	4.24E-37	M1	TMED1
DOCK7	2.86E-41	-0.15986	0.031	0.145	4.28E-37	M1	DOCK7
CACNA2D4.	3.08E-41	0.365547	0.145	0.059	4.60E-37	M1	CACNA2D4
SNAPC2	3.30E-41	-0.17906	0.082	0.228	4.93E-37	M1	SNAPC2
TANC1.2	3.31E-41	-0.16798	0.02	0.126	4.95E-37	M1	TANC1
TRAP1.2	3.36E-41	-0.19019	0.12	0.286	5.02E-37	M1	TRAP1
ZNF639.3	3.37E-41	-0.17438	0.038	0.156	5.04E-37	M1	ZNF639
STK25.4	3.38E-41	-0.15479	0.15	0.333	5.05E-37	M1	STK25
NKAIN4.3	3.39E-41	-0.38866	0.017	0.118	5.07E-37	M1	NKAIN4
RRAS2.2	3.41E-41	-0.17581	0.004	0.093	5.10E-37	M1	RRAS2
VTA1.1	3.46E-41	-0.19427	0.069	0.207	5.17E-37	M1	VTA1
ZNF48.2	3.62E-41	-0.18356	0.014	0.113	5.41E-37	M1	ZNF48
ANKRD28.2	3.72E-41	-0.28362	0.024	0.131	5.55E-37	M1	ANKRD28
FBLN1.3	3.74E-41	-0.33009	0.008	0.101	5.59E-37	M1	FBLN1
EIF4A3.4	3.89E-41	0.396186	0.492	0.441	5.82E-37	M1	EIF4A3
CCZ1B.3	3.93E-41	-0.17285	0.047	0.172	5.87E-37	M1	CCZ1B
NOP58.2	4.09E-41	-0.2395	0.148	0.321	6.12E-37	M1	NOP58
RBM15B.3	4.19E-41	-0.15534	0.06	0.193	6.27E-37	M1	RBM15B
CHD4.3	4.24E-41	-0.20055	0.168	0.353	6.34E-37	M1	CHD4
COQ4.2	4.28E-41	-0.1738	0.126	0.296	6.40E-37	M1	COQ4
NAPEPLD.1	4.31E-41	-0.21424	0.015	0.116	6.45E-37	M1	NAPEPLD
LSM14A.1	4.32E-41	-0.2098	0.211	0.415	6.46E-37	M1	LSM14A
C14orf132	5.23E-41	-0.30598	0.011	0.107	7.82E-37	M1	C14orf132
SLBP.3	5.29E-41	-0.21929	0.161	0.341	7.90E-37	M1	SLBP
RDH5.2	5.90E-41	-0.19872	0.017	0.12	8.81E-37	M1	RDH5
SLC39A14.	6.17E-41	-0.26832	0.013	0.111	9.22E-37	M1	SLC39A14
KLHL35.3	6.19E-41	-0.37809	0.013	0.11	9.25E-37	M1	KLHL35
ZNF579.3	6.40E-41	-0.14777	0.024	0.132	9.56E-37	M1	ZNF579
TSKU.2	6.81E-41	-0.17839	0.008	0.101	1.02E-36	M1	TSKU
SUCO.1	6.91E-41	-0.16789	0.049	0.175	1.03E-36	M1	SUCO
APOPT1.2	7.02E-41	-0.21416	0.21	0.415	1.05E-36	M1	APOPT1

LILRB2.4	7.07E-41	0.316652	0.115	0.041	1.06E-36	M1	LILRB2
EFNB3.2	7.32E-41	-0.1938	0.002	0.088	1.09E-36	M1	EFNB3
SLC35A5	7.65E-41	-0.18897	0.071	0.21	1.14E-36	M1	SLC35A5
CERS4.3	7.96E-41	-0.17851	0.079	0.223	1.19E-36	M1	CERS4
ITGAE	8.02E-41	-0.19785	0.198	0.398	1.20E-36	M1	ITGAE
SH3RF1.2	8.06E-41	-0.18184	0.02	0.124	1.20E-36	M1	SH3RF1
ASAP1.2	8.07E-41	-0.19271	0.1	0.253	1.21E-36	M1	ASAP1
ARL10.1	8.53E-41	-0.17413	0.01	0.105	1.27E-36	M1	ARL10
ZNF506.2	8.71E-41	-0.15459	0.029	0.14	1.30E-36	M1	ZNF506
RGS3.2	8.80E-41	-0.20062	0.02	0.124	1.31E-36	M1	RGS3
LRRFIP1.4	8.89E-41	0.449425	0.425	0.339	1.33E-36	M1	LRRFIP1
LMNB1.3	8.98E-41	-0.25899	0.048	0.17	1.34E-36	M1	LMNB1
CCT5.1	9.10E-41	-0.25874	0.297	0.519	1.36E-36	M1	CCT5
TIMP1.3	9.14E-41	-0.54408	0.507	0.643	1.37E-36	M1	TIMP1
LCMT1	9.46E-41	-0.18884	0.124	0.29	1.41E-36	M1	LCMT1
FBXO44.2	9.51E-41	-0.22506	0.042	0.161	1.42E-36	M1	FBXO44
CASP10.1	9.97E-41	0.230899	0.069	0.017	1.49E-36	M1	CASP10
CENPH.2	1.01E-40	-0.23522	0.033	0.145	1.51E-36	M1	CENPH
ZNF644.1	1.01E-40	-0.15892	0.116	0.28	1.51E-36	M1	ZNF644
NEIL2.3	1.06E-40	-0.17248	0.079	0.222	1.58E-36	M1	NEIL2
PKD1.2	1.06E-40	-0.19016	0.027	0.136	1.58E-36	M1	PKD1
NGDN.2	1.08E-40	-0.1827	0.125	0.291	1.62E-36	M1	NGDN
CRK.1	1.10E-40	-0.17124	0.161	0.348	1.64E-36	M1	CRK
C11orf73.	1.11E-40	-0.22275	0.241	0.456	1.66E-36	M1	C11orf73
TRMT13.1	1.13E-40	-0.21414	0.039	0.156	1.68E-36	M1	TRMT13
ZNF566.2	1.14E-40	-0.22426	0.025	0.133	1.71E-36	M1	ZNF566
FKBP1B.1	1.15E-40	-0.17598	0.03	0.141	1.71E-36	M1	FKBP1B
BBS2.4	1.17E-40	-0.22781	0.062	0.194	1.75E-36	M1	BBS2
CNPY1.3	1.21E-40	-0.23966	0.004	0.092	1.80E-36	M1	CNPY1
GNPDA2.1	1.21E-40	-0.1914	0.031	0.143	1.81E-36	M1	GNPDA2
TBC1D20.2	1.23E-40	-0.16582	0.068	0.206	1.84E-36	M1	TBC1D20
DLG5.1	1.24E-40	-0.20532	0.003	0.091	1.85E-36	M1	DLG5
BCL2.3	1.30E-40	0.322914	0.107	0.037	1.94E-36	M1	BCL2
CLASP2.3	1.32E-40	-0.25808	0.081	0.22	1.97E-36	M1	CLASP2
PTDSS2.4	1.33E-40	-0.15238	0.048	0.173	1.99E-36	M1	PTDSS2
DDA1	1.42E-40	-0.18809	0.172	0.36	2.12E-36	M1	DDA1
KLHDC9.2	1.43E-40	-0.22416	0.002	0.088	2.13E-36	M1	KLHDC9
SMARCC1.3	1.46E-40	-0.16539	0.119	0.282	2.18E-36	M1	SMARCC1
SCARF1.3	1.48E-40	0.232604	0.07	0.018	2.20E-36	M1	SCARF1
TUBB4A.4	1.48E-40	-0.45584	0.029	0.136	2.21E-36	M1	TUBB4A
METTL1.3	1.50E-40	-0.26804	0.047	0.167	2.24E-36	M1	METTL1
EID2.3	1.53E-40	-0.18604	0.023	0.129	2.29E-36	M1	EID2
LINC00844	1.56E-40	-0.40855	0.021	0.123	2.33E-36	M1	LINC00844
IGFLR1.1	1.56E-40	0.323706	0.117	0.043	2.33E-36	M1	IGFLR1
HSPA13.3	1.63E-40	-0.19549	0.082	0.223	2.44E-36	M1	HSPA13
TMEM222.4	1.64E-40	-0.21629	0.138	0.308	2.45E-36	M1	TMEM222
U2SURP.1	1.66E-40	-0.17939	0.202	0.403	2.48E-36	M1	U2SURP
DST.3	1.70E-40	-0.23599	0.218	0.408	2.54E-36	M1	DST
ACTR6.1	1.73E-40	-0.20164	0.105	0.26	2.58E-36	M1	ACTR6
ECI1.2	1.74E-40	-0.16477	0.088	0.238	2.60E-36	M1	ECI1
RNU12.2	1.75E-40	0.304296	0.089	0.027	2.61E-36	M1	RNU12

TTC14	1.77E-40	-0.13584	0.127	0.298	2.65E-36	M1	TTC14
STAU1.2	1.80E-40	-0.21304	0.202	0.399	2.70E-36	M1	STAU1
UFSP2.1	1.83E-40	-0.20917	0.092	0.241	2.74E-36	M1	UFSP2
NUPL2.1	1.83E-40	-0.15509	0.128	0.296	2.74E-36	M1	NUPL2
HMCES.1	1.86E-40	-0.19296	0.06	0.191	2.77E-36	M1	HMCES
ENKUR.2	1.88E-40	-0.26337	0.001	0.086	2.80E-36	M1	ENKUR
NSFL1C.1	1.90E-40	-0.20102	0.229	0.436	2.84E-36	M1	NSFL1C
RUNDC1	2.00E-40	-0.17205	0.038	0.155	2.99E-36	M1	RUNDC1
ZCCHC11.1	2.19E-40	-0.17142	0.107	0.265	3.27E-36	M1	ZCCHC11
AGRN.2	2.20E-40	-0.18765	0.013	0.11	3.29E-36	M1	AGRN
REXO4.1	2.23E-40	-0.17129	0.074	0.214	3.33E-36	M1	REXO4
GMIP.4	2.24E-40	0.332925	0.143	0.06	3.34E-36	M1	GMIP
RASSF8-AS	2.26E-40	-0.20105	0.054	0.181	3.38E-36	M1	RASSF8-AS1
ARHGEF2.3	2.30E-40	-0.23596	0.148	0.317	3.44E-36	M1	ARHGEF2
GLG1.4	2.52E-40	-0.16731	0.126	0.294	3.77E-36	M1	GLG1
LMAN2L.1	2.55E-40	-0.21238	0.025	0.131	3.81E-36	M1	LMAN2L
SRSF12.2	2.60E-40	-0.19846	0.002	0.087	3.88E-36	M1	SRSF12
STRBP.4	2.60E-40	-0.17257	0.033	0.145	3.88E-36	M1	STRBP
IGSF10.1	2.64E-40	-0.14943	0.016	0.116	3.95E-36	M1	IGSF10
LY6H.3	2.76E-40	-0.37765	0.009	0.101	4.12E-36	M1	LY6H
C19orf48.	2.94E-40	-0.20703	0.101	0.253	4.39E-36	M1	C19orf48
CRY1.1	3.06E-40	-0.22371	0.039	0.156	4.57E-36	M1	CRY1
KIAA0232.	3.11E-40	-0.17563	0.049	0.172	4.65E-36	M1	KIAA0232
RIF1.3	3.22E-40	-0.18211	0.094	0.245	4.81E-36	M1	RIF1
CEBPZ.2	3.30E-40	-0.20037	0.132	0.299	4.93E-36	M1	CEBPZ
FAM171A1.	3.42E-40	-0.20606	0.004	0.091	5.10E-36	M1	FAM171A1
SEC61A1.2	3.43E-40	-0.17518	0.2	0.398	5.13E-36	M1	SEC61A1
CIB2.2	3.45E-40	-0.15169	0.004	0.092	5.15E-36	M1	CIB2
GBGT1.4	3.51E-40	0.265474	0.109	0.038	5.24E-36	M1	GBGT1
PFKP	3.53E-40	-0.23057	0.049	0.172	5.27E-36	M1	PFKP
LSM12.2	3.75E-40	-0.17874	0.122	0.287	5.60E-36	M1	LSM12
SPN.4	3.86E-40	0.311275	0.117	0.043	5.77E-36	M1	SPN
LSS.3	4.01E-40	-0.22492	0.016	0.115	5.99E-36	M1	LSS
USP5	4.13E-40	-0.20478	0.099	0.25	6.17E-36	M1	USP5
SYNE1.1	4.24E-40	-0.23187	0.031	0.141	6.33E-36	M1	SYNE1
MIR4458HG	4.27E-40	-0.3709	0.058	0.181	6.38E-36	M1	MIR4458HG
ALDH6A1.2	4.37E-40	-0.19858	0.086	0.231	6.53E-36	M1	ALDH6A1
DLC1.2	4.66E-40	-0.21685	0.009	0.102	6.96E-36	M1	DLC1
SELT.1	4.75E-40	0.214619	0.67	0.657	7.09E-36	M1	SELT
GLO1.2	4.79E-40	-0.23065	0.237	0.445	7.15E-36	M1	GLO1
TLR4.3	4.79E-40	0.308159	0.094	0.03	7.15E-36	M1	TLR4
CNR1.2	4.83E-40	-0.18356	0.002	0.088	7.21E-36	M1	CNR1
SAMD4B.1	5.32E-40	-0.15836	0.096	0.248	7.95E-36	M1	SAMD4B
PIN1.2	5.81E-40	-0.25131	0.358	0.573	8.69E-36	M1	PIN1
POR.1	5.82E-40	-0.21319	0.128	0.293	8.69E-36	M1	POR
RAB5C.2	6.06E-40	0.191706	0.664	0.651	9.06E-36	M1	RAB5C
LARP1.2	6.10E-40	-0.18307	0.118	0.279	9.11E-36	M1	LARP1
RP11-156K	6.24E-40	0.237877	0.056	0.011	9.33E-36	M1	RP11-156K13.3
FAM20C.4	6.33E-40	-0.23872	0.099	0.248	9.46E-36	M1	FAM20C
ZNF24.2	6.41E-40	-0.17136	0.151	0.33	9.57E-36	M1	ZNF24
C19orf12.	6.58E-40	-0.18489	0.043	0.162	9.83E-36	M1	C19orf12

FAM53C.3	6.68E-40	0.479187	0.326	0.225	9.98E-36	M1	FAM53C
MGARP.1	6.79E-40	-0.15505	0.006	0.095	1.01E-35	M1	MGARP
SUCLG1.2	6.88E-40	-0.20992	0.255	0.473	1.03E-35	M1	SUCLG1
CASS4.1	6.91E-40	0.205052	0.049	0.009	1.03E-35	M1	CASS4
SRRM3.3	6.94E-40	-0.29453	0.002	0.086	1.04E-35	M1	SRRM3
FAM168B.3	6.99E-40	-0.1656	0.057	0.184	1.05E-35	M1	FAM168B
GTF3C5.2	7.17E-40	-0.17707	0.072	0.209	1.07E-35	M1	GTF3C5
SGTA.2	7.31E-40	-0.186	0.163	0.346	1.09E-35	M1	SGTA
SLC29A4.1	7.50E-40	-0.21392	0.007	0.097	1.12E-35	M1	SLC29A4
SUZ12.3	7.66E-40	-0.15549	0.077	0.218	1.14E-35	M1	SUZ12
SLC25A38.	7.95E-40	-0.22228	0.046	0.166	1.19E-35	M1	SLC25A38
CCR5.2	8.00E-40	0.212713	0.068	0.017	1.20E-35	M1	CCR5
CAND1.1	8.14E-40	-0.17321	0.106	0.261	1.22E-35	M1	CAND1
CCDC112.3	8.16E-40	-0.18663	0.125	0.288	1.22E-35	M1	CCDC112
CEP70.2	8.32E-40	-0.2236	0.016	0.114	1.24E-35	M1	CEP70
SOD1.3	8.35E-40	-0.31974	0.605	0.787	1.25E-35	M1	SOD1
AARS.2	8.45E-40	-0.22626	0.104	0.256	1.26E-35	M1	AARS
B4GALT2.1	9.02E-40	-0.18176	0.004	0.091	1.35E-35	M1	B4GALT2
C1orf109.	9.24E-40	-0.17459	0.037	0.151	1.38E-35	M1	C1orf109
TPD52.3	9.35E-40	-0.26082	0.109	0.262	1.40E-35	M1	TPD52
ADNP.3	9.35E-40	-0.16058	0.076	0.216	1.40E-35	M1	ADNP
DYNC1H1.3	9.61E-40	-0.20564	0.181	0.369	1.44E-35	M1	DYNC1H1
CTCF.2	1.01E-39	-0.17431	0.057	0.185	1.51E-35	M1	CTCF
SECISBP2L	1.02E-39	-0.20845	0.065	0.196	1.52E-35	M1	SECISBP2L
LMO3.1	1.04E-39	-0.33219	0.008	0.098	1.56E-35	M1	LMO3
EHD2.2	1.20E-39	-0.16821	0.007	0.096	1.80E-35	M1	EHD2
PLXNA4.3	1.23E-39	-0.2428	0.004	0.091	1.83E-35	M1	PLXNA4
RP11-796E	1.23E-39	0.435837	0.13	0.052	1.84E-35	M1	RP11-796E2.4
RRP1.3	1.23E-39	-0.17627	0.089	0.234	1.84E-35	M1	RRP1
NUMBL.2	1.26E-39	-0.17495	0.011	0.105	1.88E-35	M1	NUMBL
FPR3.4	1.29E-39	0.26233	0.118	0.044	1.93E-35	M1	FPR3
CCDC66.3	1.34E-39	-0.14217	0.101	0.255	2.00E-35	M1	CCDC66
COPS5	1.38E-39	-0.20571	0.236	0.445	2.07E-35	M1	COPS5
ERP44.2	1.52E-39	0.368918	0.467	0.381	2.28E-35	M1	ERP44
PPP1R14A.	1.55E-39	-0.54339	0.023	0.124	2.32E-35	M1	PPP1R14A
NONO.3	1.59E-39	-0.24953	0.278	0.491	2.37E-35	M1	NONO
ALDOC.3	1.70E-39	-0.41537	0.056	0.175	2.54E-35	M1	ALDOC
CRISPLD1.	1.74E-39	-0.22097	0.012	0.105	2.61E-35	M1	CRISPLD1
MDH2.1	1.81E-39	-0.26673	0.477	0.697	2.70E-35	M1	MDH2
EXOC7.3	1.85E-39	-0.15401	0.19	0.385	2.76E-35	M1	EXOC7
FEN1.2	1.93E-39	-0.25438	0.041	0.155	2.88E-35	M1	FEN1
MRGBP.2	1.95E-39	-0.13028	0.046	0.167	2.91E-35	M1	MRGBP
TADA3	1.95E-39	-0.21078	0.221	0.424	2.92E-35	M1	TADA3
ANKMY2.1	2.18E-39	-0.25329	0.029	0.136	3.26E-35	M1	ANKMY2
ATP6V1D.1	2.21E-39	-0.20565	0.206	0.403	3.31E-35	M1	ATP6V1D
CCDC90B.3	2.28E-39	-0.19857	0.235	0.444	3.40E-35	M1	CCDC90B
PIN4.3	2.29E-39	-0.23917	0.245	0.453	3.43E-35	M1	PIN4
PRTFDC1.1	2.31E-39	-0.22443	0.04	0.154	3.45E-35	M1	PRTFDC1
MFSD7.3	2.44E-39	0.236198	0.078	0.022	3.64E-35	M1	MFSD7
RNASEH2A.	2.56E-39	-0.24071	0.092	0.235	3.83E-35	M1	RNASEH2A
CDKN3.3	2.66E-39	-0.30669	0.013	0.107	3.98E-35	M1	CDKN3

IRF2BP1.3	2.68E-39	-0.14489	0.021	0.123	4.01E-35	M1	IRF2BP1
MDGA1.2	2.76E-39	-0.14511	0.001	0.084	4.12E-35	M1	MDGA1
ARFRP1.1	2.96E-39	-0.16785	0.115	0.274	4.42E-35	M1	ARFRP1
POLDIP2.4	3.01E-39	-0.20597	0.191	0.378	4.50E-35	M1	POLDIP2
UNG.1	3.13E-39	-0.24785	0.032	0.14	4.68E-35	M1	UNG
GRB10.3	3.21E-39	-0.21779	0.003	0.087	4.80E-35	M1	GRB10
GRK4.2	3.24E-39	-0.19387	0.016	0.114	4.84E-35	M1	GRK4
PPP1R37.2	3.39E-39	-0.17089	0.039	0.153	5.06E-35	M1	PPP1R37
PPP4R2.1	3.55E-39	-0.15124	0.124	0.289	5.30E-35	M1	PPP4R2
TUBGCP4.2	3.58E-39	-0.14808	0.032	0.141	5.35E-35	M1	TUBGCP4
C20orf96.	3.62E-39	-0.15285	0.049	0.171	5.40E-35	M1	C20orf96
NACAD.3	3.65E-39	-0.25584	0.012	0.105	5.45E-35	M1	NACAD
REXO2.4	3.78E-39	-0.33598	0.174	0.348	5.64E-35	M1	REXO2
LRRC40.1	3.83E-39	-0.20996	0.045	0.162	5.73E-35	M1	LRRC40
SNTB2.1	4.01E-39	-0.16159	0.015	0.111	5.99E-35	M1	SNTB2
MAML2.3	4.08E-39	-0.16479	0.08	0.221	6.10E-35	M1	MAML2
AC007405.	4.16E-39	-0.18319	0	0.081	6.21E-35	M1	AC007405.6
EAPP.3	4.30E-39	-0.23841	0.197	0.386	6.43E-35	M1	EAPP
PFDN6.3	4.40E-39	-0.23007	0.194	0.384	6.57E-35	M1	PFDN6
WDR82.3	4.43E-39	-0.1716	0.121	0.281	6.62E-35	M1	WDR82
ZFP82.2	4.53E-39	-0.18696	0.023	0.126	6.77E-35	M1	ZFP82
FAM217B.4	4.67E-39	-0.16245	0.052	0.176	6.97E-35	M1	FAM217B
SLC35B4.2	4.92E-39	-0.18609	0.077	0.214	7.36E-35	M1	SLC35B4
LDOC1.3	4.98E-39	-0.32823	0.011	0.103	7.44E-35	M1	LDOC1
FUNDC1.3	5.15E-39	-0.23253	0.116	0.272	7.70E-35	M1	FUNDC1
DMTF1.2	5.32E-39	-0.16257	0.048	0.168	7.95E-35	M1	DMTF1
FAIM.2	5.55E-39	-0.2115	0.047	0.166	8.29E-35	M1	FAIM
OGFOD1.1	5.55E-39	-0.18112	0.049	0.17	8.29E-35	M1	OGFOD1
ZC3HC1.2	5.71E-39	-0.1985	0.033	0.143	8.53E-35	M1	ZC3HC1
ZC3H12A.3	5.84E-39	0.32064	0.145	0.062	8.72E-35	M1	ZC3H12A
PRRC2B.4	6.03E-39	-0.18106	0.108	0.262	9.01E-35	M1	PRRC2B
OGG1	6.06E-39	-0.16054	0.049	0.17	9.05E-35	M1	OGG1
GNPAT.3	6.17E-39	-0.13413	0.109	0.266	9.23E-35	M1	GNPAT
ZSCAN21.2	6.20E-39	-0.21204	0.019	0.118	9.26E-35	M1	ZSCAN21
REV3L.1	6.26E-39	-0.1886	0.09	0.233	9.36E-35	M1	REV3L
ADPRHL2.3	6.29E-39	-0.16288	0.125	0.289	9.40E-35	M1	ADPRHL2
CDH6.2	6.47E-39	-0.17079	0.001	0.082	9.67E-35	M1	CDH6
HMBS.1	6.70E-39	-0.14489	0.08	0.22	1.00E-34	M1	HMBS
ACTL6A.2	6.95E-39	-0.20817	0.124	0.284	1.04E-34	M1	ACTL6A
NUP160.2	7.04E-39	-0.18377	0.058	0.183	1.05E-34	M1	NUP160
RGN.3	7.04E-39	-0.26253	0.003	0.086	1.05E-34	M1	RGN
FKBP3.2	7.17E-39	-0.27715	0.298	0.513	1.07E-34	M1	FKBP3
ACD.2	7.22E-39	-0.20048	0.062	0.189	1.08E-34	M1	ACD
SUPT20H.2	7.31E-39	-0.15998	0.087	0.231	1.09E-34	M1	SUPT20H
BOK.4	7.42E-39	-0.22629	0.005	0.09	1.11E-34	M1	BOK
TMEM53.2	7.71E-39	-0.19165	0.021	0.122	1.15E-34	M1	TMEM53
PAAF1.2	7.94E-39	-0.18195	0.083	0.222	1.19E-34	M1	PAAF1
PRKAR1B.2	8.17E-39	-0.24153	0.036	0.146	1.22E-34	M1	PRKAR1B
CLSTN1.2	8.39E-39	-0.18911	0.142	0.311	1.25E-34	M1	CLSTN1
PDCD7.1	8.52E-39	-0.16541	0.096	0.246	1.27E-34	M1	PDCD7
LRRC17.2	8.58E-39	-0.24098	0.004	0.088	1.28E-34	M1	LRRC17

GINS2.2	8.78E-39	-0.2495	0.018	0.116	1.31E-34	M1	GINS2
DDX23.2	9.46E-39	-0.18461	0.056	0.18	1.41E-34	M1	DDX23
CEP290.2	9.58E-39	-0.17739	0.055	0.178	1.43E-34	M1	CEP290
EIF2B1	9.93E-39	-0.18633	0.142	0.311	1.48E-34	M1	EIF2B1
RYR1.1	9.95E-39	0.213605	0.049	0.009	1.49E-34	M1	RYR1
PTGFRN.1	1.03E-38	-0.17648	0.02	0.12	1.54E-34	M1	PTGFRN
CHRNB1.3	1.04E-38	-0.21296	0.049	0.169	1.56E-34	M1	CHRNB1
TMEM67.2	1.08E-38	-0.17245	0.015	0.111	1.61E-34	M1	TMEM67
CCDC136.2	1.08E-38	-0.23912	0.013	0.106	1.62E-34	M1	CCDC136
POMGNT1.3	1.09E-38	-0.14741	0.121	0.285	1.62E-34	M1	POMGNT1
ZNF880	1.10E-38	-0.18858	0.046	0.165	1.65E-34	M1	ZNF880
ARHGAP25.	1.10E-38	0.327543	0.13	0.053	1.65E-34	M1	ARHGAP25
LAMTOR5.2	1.11E-38	-0.30169	0.521	0.726	1.65E-34	M1	LAMTOR5
SLC35G2.4	1.11E-38	-0.20123	0.029	0.135	1.66E-34	M1	SLC35G2
BHLHE40.3	1.13E-38	0.425809	0.279	0.176	1.68E-34	M1	BHLHE40
MPP6.2	1.17E-38	-0.1768	0.003	0.087	1.74E-34	M1	MPP6
PHLPP1.4	1.22E-38	-0.247	0.01	0.1	1.82E-34	M1	PHLPP1
MAML1.4	1.22E-38	0.42712	0.195	0.102	1.82E-34	M1	MAML1
HOXA10.4	1.23E-38	-0.28341	0.006	0.091	1.83E-34	M1	HOXA10
PMPCA.2	1.23E-38	-0.17562	0.116	0.272	1.84E-34	M1	PMPCA
DHRS13.2	1.24E-38	-0.15303	0.017	0.114	1.85E-34	M1	DHRS13
DNAAF2.3	1.24E-38	-0.17477	0.065	0.194	1.85E-34	M1	DNAAF2
ZNF426.1	1.24E-38	-0.19253	0.026	0.13	1.86E-34	M1	ZNF426
ALKBH5.3	1.27E-38	-0.19753	0.174	0.353	1.89E-34	M1	ALKBH5
SLC25A36.	1.28E-38	-0.17477	0.132	0.297	1.91E-34	M1	SLC25A36
GNAO1.2	1.28E-38	-0.30826	0.013	0.106	1.91E-34	M1	GNAO1
DNMT1.3	1.29E-38	-0.18737	0.146	0.314	1.93E-34	M1	DNMT1
TUBG2.3	1.30E-38	-0.19798	0.024	0.127	1.94E-34	M1	TUBG2
CEP89.2	1.30E-38	-0.19256	0.037	0.149	1.94E-34	M1	CEP89
NACC2.2	1.36E-38	-0.16816	0.02	0.119	2.03E-34	M1	NACC2
CTDSP1.4	1.41E-38	0.511761	0.302	0.205	2.11E-34	M1	CTDSP1
THYN1.3	1.42E-38	-0.23859	0.237	0.44	2.13E-34	M1	THYN1
FAM134A.4	1.53E-38	-0.16918	0.152	0.327	2.29E-34	M1	FAM134A
GORASP2.2	1.56E-38	-0.15463	0.149	0.321	2.33E-34	M1	GORASP2
STX2.3	1.58E-38	-0.16146	0.023	0.125	2.36E-34	M1	STX2
PRKCI	1.60E-38	-0.18328	0.02	0.12	2.39E-34	M1	PRKCI
SBK1.4	1.64E-38	-0.24459	0.003	0.085	2.45E-34	M1	SBK1
KPNB1.3	1.75E-38	-0.24005	0.273	0.483	2.62E-34	M1	KPNB1
FAM98A.2	1.77E-38	-0.20899	0.045	0.161	2.65E-34	M1	FAM98A
RP11-262H	1.78E-38	-0.16127	0.03	0.136	2.67E-34	M1	RP11-262H14.4
AASDH.2	1.85E-38	-0.2055	0.06	0.185	2.77E-34	M1	AASDH
C16orf13.	1.86E-38	-0.22764	0.282	0.496	2.77E-34	M1	C16orf13
PDS5B.2	1.91E-38	-0.16992	0.061	0.189	2.86E-34	M1	PDS5B
SMG6	1.97E-38	-0.14274	0.032	0.141	2.95E-34	M1	SMG6
PWWP2A.1	2.01E-38	-0.19022	0.035	0.145	3.01E-34	M1	PWWP2A
RP11-108M	2.09E-38	0.399289	0.178	0.087	3.12E-34	M1	RP11-108M9.4
FXYD7.3	2.09E-38	-0.35168	0.006	0.092	3.13E-34	M1	FXYD7
IPO5.2	2.14E-38	-0.16562	0.094	0.24	3.20E-34	M1	IPO5
TXLNA.2	2.16E-38	-0.17021	0.043	0.159	3.22E-34	M1	TXLNA
HMOX2.2	2.30E-38	-0.23769	0.153	0.323	3.43E-34	M1	HMOX2
TMEM131.3	2.30E-38	-0.14021	0.047	0.166	3.44E-34	M1	TMEM131

ZNF830.2	2.36E-38	-0.16898	0.077	0.214	3.52E-34	M1	ZNF830
TMIGD2.1	2.37E-38	0.229304	0.057	0.012	3.55E-34	M1	TMIGD2
FAM222B.3	2.38E-38	-0.14638	0.026	0.131	3.55E-34	M1	FAM222B
SYNM.2	2.48E-38	-0.15956	0.003	0.085	3.70E-34	M1	SYNM
ANAPC15.2	2.51E-38	-0.19042	0.127	0.288	3.75E-34	M1	ANAPC15
TENM1.2	2.56E-38	-0.18472	0.003	0.085	3.83E-34	M1	TENM1
GLDC.2	2.65E-38	-0.18629	0.001	0.082	3.95E-34	M1	GLDC
ACYP2.3	2.74E-38	-0.25012	0.167	0.341	4.10E-34	M1	ACYP2
PHACTR3.3	2.85E-38	-0.28822	0.006	0.092	4.26E-34	M1	PHACTR3
FUZ.1	3.03E-38	-0.18618	0.058	0.182	4.53E-34	M1	FUZ
FAM131B.2	3.06E-38	-0.21163	0.005	0.09	4.58E-34	M1	FAM131B
RNF11.3	3.11E-38	-0.17094	0.16	0.337	4.64E-34	M1	RNF11
RAD23B.1	3.11E-38	-0.21943	0.214	0.413	4.65E-34	M1	RAD23B
RAB33A.2	3.14E-38	-0.33375	0.023	0.123	4.69E-34	M1	RAB33A
PRRC2A.3	3.39E-38	-0.14694	0.113	0.269	5.07E-34	M1	PRRC2A
N6AMT2.2	3.56E-38	-0.22138	0.039	0.151	5.32E-34	M1	N6AMT2
ANKRD35.1	3.57E-38	-0.18917	0.042	0.156	5.34E-34	M1	ANKRD35
FTSJ2.2	3.61E-38	-0.18208	0.11	0.262	5.40E-34	M1	FTSJ2
TRMT61B.2	3.62E-38	-0.19841	0.06	0.184	5.41E-34	M1	TRMT61B
DZIP3.2	3.72E-38	-0.20013	0.038	0.149	5.57E-34	M1	DZIP3
FADD.2	3.76E-38	-0.1738	0.041	0.155	5.62E-34	M1	FADD
VRK3.3	3.77E-38	-0.15557	0.096	0.243	5.63E-34	M1	VRK3
MED16.3	3.78E-38	-0.13837	0.056	0.18	5.65E-34	M1	MED16
MTHFD1.1	3.83E-38	-0.19552	0.075	0.21	5.72E-34	M1	MTHFD1
ABCA5.1	3.83E-38	-0.19319	0.011	0.102	5.72E-34	M1	ABCA5
ZBTB8A.3	3.86E-38	-0.19905	0.021	0.12	5.77E-34	M1	ZBTB8A
E2F6.2	3.91E-38	-0.12379	0.046	0.164	5.85E-34	M1	E2F6
SPNS1.3	4.10E-38	-0.1555	0.146	0.319	6.13E-34	M1	SPNS1
EFTUD2.2	4.12E-38	-0.18753	0.095	0.239	6.16E-34	M1	EFTUD2
KATNAL1.1	4.20E-38	-0.18871	0.008	0.096	6.28E-34	M1	KATNAL1
FUBP3.2	4.20E-38	-0.16037	0.111	0.266	6.28E-34	M1	FUBP3
ILF3-AS1.	4.24E-38	-0.17098	0.162	0.339	6.34E-34	M1	ILF3-AS1
KCMF1.2	4.49E-38	-0.13469	0.077	0.214	6.71E-34	M1	KCMF1
DENND5B.3	4.49E-38	-0.16237	0.016	0.112	6.72E-34	M1	DENND5B
MRTO4	4.77E-38	-0.18417	0.116	0.272	7.13E-34	M1	MRTO4
XRRA1.2	4.82E-38	-0.16453	0.037	0.148	7.20E-34	M1	XRRA1
LAMA4.2	5.20E-38	-0.2045	0.003	0.085	7.77E-34	M1	LAMA4
PPFIA3.2	5.45E-38	-0.16034	0.013	0.105	8.15E-34	M1	PPFIA3
GNG3.2	5.57E-38	-0.32817	0.004	0.087	8.32E-34	M1	GNG3
ACSBG1.1	5.66E-38	-0.17785	0.004	0.088	8.46E-34	M1	ACSBG1
ATP6AP2.2	5.71E-38	0.207044	0.669	0.654	8.53E-34	M1	ATP6AP2
STIP1.1	5.79E-38	-0.18413	0.209	0.404	8.65E-34	M1	STIP1
HPS1.3	5.93E-38	0.354372	0.19	0.095	8.86E-34	M1	HPS1
NUCKS1.2	5.94E-38	-0.2679	0.472	0.669	8.88E-34	M1	NUCKS1
CENPF.4	5.95E-38	-0.34937	0.021	0.117	8.90E-34	M1	CENPF
POU2F1.3	5.98E-38	-0.2046	0.058	0.181	8.94E-34	M1	POU2F1
C1orf198.	6.28E-38	-0.27701	0.022	0.121	9.38E-34	M1	C1orf198
ANKRD17.3	6.29E-38	-0.14068	0.095	0.242	9.41E-34	M1	ANKRD17
BEST3.3	6.31E-38	-0.34565	0.012	0.102	9.42E-34	M1	BEST3
ZNF181.2	6.62E-38	-0.19971	0.017	0.113	9.90E-34	M1	ZNF181
KLHDC2.3	6.90E-38	-0.19025	0.157	0.331	1.03E-33	M1	KLHDC2

POLDIP3.3	7.33E-38	-0.17724	0.074	0.208	1.10E-33	M1	POLDIP3
DDX56.2	7.74E-38	-0.18593	0.125	0.283	1.16E-33	M1	DDX56
SSX2IP.1	7.84E-38	-0.12756	0.017	0.112	1.17E-33	M1	SSX2IP
CELSR2.3	7.89E-38	-0.18718	0.015	0.108	1.18E-33	M1	CELSR2
HOXA2.3	7.91E-38	-0.27417	0.004	0.086	1.18E-33	M1	HOXA2
PDE6D.2	8.02E-38	-0.17415	0.077	0.211	1.20E-33	M1	PDE6D
PBK.4	8.12E-38	-0.32333	0.004	0.087	1.21E-33	M1	PBK
NOL8.1	8.17E-38	-0.17449	0.074	0.207	1.22E-33	M1	NOL8
TRMT1.3	8.41E-38	0.493856	0.336	0.241	1.26E-33	M1	TRMT1
URGCP.1	8.58E-38	-0.18213	0.029	0.133	1.28E-33	M1	URGCP
SMARCB1.3	8.73E-38	-0.20939	0.246	0.448	1.31E-33	M1	SMARCB1
MIEF1	8.93E-38	-0.16964	0.048	0.165	1.33E-33	M1	MIEF1
KAZN.2	9.23E-38	-0.19892	0.003	0.085	1.38E-33	M1	KAZN
MIR210HG.	9.38E-38	-0.20376	0.014	0.107	1.40E-33	M1	MIR210HG
TMEM55A.3	9.43E-38	-0.19123	0.108	0.258	1.41E-33	M1	TMEM55A
ZMYND11.4	9.64E-38	-0.14012	0.048	0.166	1.44E-33	M1	ZMYND11
TTC19.2	1.03E-37	-0.14703	0.195	0.388	1.54E-33	M1	TTC19
PCNA.2	1.08E-37	-0.23304	0.17	0.344	1.61E-33	M1	PCNA
NCOR1.3	1.12E-37	-0.22363	0.291	0.499	1.67E-33	M1	NCOR1
FEM1C.1	1.13E-37	-0.14775	0.044	0.159	1.69E-33	M1	FEM1C
TNRC6A	1.15E-37	-0.17095	0.094	0.239	1.72E-33	M1	TNRC6A
PNN.3	1.27E-37	-0.21882	0.235	0.435	1.90E-33	M1	PNN
RORA.1	1.27E-37	-0.2499	0.005	0.089	1.90E-33	M1	RORA
ABCF1.3	1.28E-37	-0.12774	0.143	0.312	1.91E-33	M1	ABCF1
HRSP12.4	1.29E-37	-0.24463	0.085	0.221	1.93E-33	M1	HRSP12
SPECC1.4	1.35E-37	-0.17978	0.154	0.324	2.02E-33	M1	SPECC1
IGSF21.4	1.41E-37	0.391085	0.252	0.146	2.11E-33	M1	IGSF21
UBE2T.4	1.42E-37	-0.28905	0.037	0.145	2.12E-33	M1	UBE2T
MKRN1.3	1.46E-37	-0.22072	0.246	0.441	2.18E-33	M1	MKRN1
METAP2.2	1.52E-37	-0.23668	0.288	0.507	2.28E-33	M1	METAP2
PDCD4.4	1.54E-37	0.392835	0.232	0.132	2.30E-33	M1	PDCD4
MARK1.1	1.56E-37	-0.15808	0.001	0.08	2.33E-33	M1	MARK1
PTPRD.3	1.57E-37	-0.28492	0.004	0.086	2.34E-33	M1	PTPRD
PIAS4.1	1.62E-37	-0.15987	0.039	0.15	2.42E-33	M1	PIAS4
CDH26.1	1.68E-37	0.191336	0.038	0.005	2.51E-33	M1	CDH26
GPR161.4	1.72E-37	-0.17054	0.01	0.098	2.56E-33	M1	GPR161
SYDE1.1	1.73E-37	-0.14732	0.003	0.084	2.59E-33	M1	SYDE1
GPR37.1	1.75E-37	-0.35374	0.004	0.086	2.61E-33	M1	GPR37
ASB8.1	1.77E-37	-0.15458	0.097	0.242	2.64E-33	M1	ASB8
SLC17A9.3	1.82E-37	0.23032	0.074	0.021	2.71E-33	M1	SLC17A9
PPP2R5E.3	1.83E-37	-0.15907	0.118	0.273	2.74E-33	M1	PPP2R5E
C1orf216.	1.92E-37	-0.1814	0.037	0.146	2.86E-33	M1	C1orf216
SIRT6.1	1.94E-37	-0.15933	0.08	0.218	2.89E-33	M1	SIRT6
IWS1.1	2.01E-37	-0.14858	0.093	0.237	3.01E-33	M1	IWS1
TEX30.1	2.12E-37	-0.17064	0.061	0.186	3.17E-33	M1	TEX30
TUBB6.2	2.19E-37	-0.25729	0.076	0.206	3.27E-33	M1	TUBB6
HOMER1.3	2.24E-37	-0.14824	0.055	0.177	3.34E-33	M1	HOMER1
ABCA8.2	2.35E-37	-0.31879	0.005	0.088	3.52E-33	M1	ABCA8
SCRN3.2	2.48E-37	-0.19129	0.02	0.117	3.71E-33	M1	SCRN3
UBTD2.1	2.54E-37	-0.17003	0.023	0.122	3.79E-33	M1	UBTD2
PDLIM1.2	2.61E-37	-0.19465	0.005	0.087	3.90E-33	M1	PDLIM1

ZRANB2	2.63E-37	-0.15791	0.176	0.36	3.93E-33	M1	ZRANB2
RANBP2.4	2.73E-37	0.484686	0.287	0.193	4.08E-33	M1	RANBP2
SLC29A3.2	2.82E-37	0.312243	0.097	0.033	4.22E-33	M1	SLC29A3
EXOSC8.3	2.83E-37	-0.20024	0.15	0.317	4.23E-33	M1	EXOSC8
HVCN1.4	2.90E-37	0.401174	0.169	0.082	4.34E-33	M1	HVCN1
TNFRSF1A.	2.94E-37	0.346874	0.441	0.358	4.39E-33	M1	TNFRSF1A
ID1.2	2.95E-37	-0.25401	0.033	0.138	4.41E-33	M1	ID1
DCHS1.3	3.04E-37	-0.17404	0.011	0.099	4.54E-33	M1	DCHS1
PLRG1.2	3.19E-37	-0.15899	0.112	0.263	4.76E-33	M1	PLRG1
IL12RB1.3	3.19E-37	0.238287	0.076	0.022	4.77E-33	M1	IL12RB1
ELP6.2	3.21E-37	-0.17717	0.096	0.239	4.80E-33	M1	ELP6
CNIH4.2	3.25E-37	-0.22145	0.213	0.406	4.86E-33	M1	CNIH4
UGDH.2	3.27E-37	-0.22968	0.058	0.178	4.88E-33	M1	UGDH
MAP6D1.3	3.37E-37	-0.22119	0.01	0.097	5.03E-33	M1	MAP6D1
SPTSSA.3	3.37E-37	-0.28728	0.26	0.462	5.03E-33	M1	SPTSSA
SLC25A1.3	3.48E-37	-0.1478	0.08	0.217	5.20E-33	M1	SLC25A1
DPY19L1.2	3.50E-37	-0.17427	0.022	0.121	5.23E-33	M1	DPY19L1
ZC3H14.3	3.57E-37	-0.1535	0.087	0.226	5.33E-33	M1	ZC3H14
CNRIP1.2	3.57E-37	-0.1798	0.09	0.227	5.33E-33	M1	CNRIP1
TMTC3	3.60E-37	-0.15325	0.014	0.105	5.37E-33	M1	TMTC3
COBL.2	3.61E-37	-0.22973	0.002	0.081	5.40E-33	M1	COBL
RDH10.3	3.63E-37	-0.23033	0.012	0.101	5.42E-33	M1	RDH10
PPARG.2	3.64E-37	0.203252	0.065	0.017	5.44E-33	M1	PPARG
LRCH3.2	3.77E-37	-0.13924	0.043	0.157	5.63E-33	M1	LRCH3
CHST8.3	3.77E-37	-0.17303	0.002	0.081	5.63E-33	M1	CHST8
NAAA.4	3.81E-37	0.473804	0.211	0.118	5.70E-33	M1	NAAA
MARK3.2	3.90E-37	-0.17325	0.147	0.315	5.83E-33	M1	MARK3
MAFG-AS1.	4.06E-37	-0.14168	0.001	0.078	6.06E-33	M1	MAFG-AS1
ACTL6B.3	4.15E-37	-0.30354	0.007	0.091	6.20E-33	M1	ACTL6B
ELMOD3.2	4.17E-37	-0.16831	0.042	0.154	6.23E-33	M1	ELMOD3
CDC5L.3	4.20E-37	-0.172	0.143	0.309	6.27E-33	M1	CDC5L
PRSS23.2	4.48E-37	-0.299	0.007	0.092	6.69E-33	M1	PRSS23
ARID4B.3	4.49E-37	-0.1862	0.237	0.44	6.71E-33	M1	ARID4B
STAT6.4	4.52E-37	0.293051	0.11	0.041	6.75E-33	M1	STAT6
ZNF576.2	4.69E-37	-0.18913	0.043	0.156	7.01E-33	M1	ZNF576
RGS13.1	4.71E-37	0.167383	0.042	0.007	7.03E-33	M1	RGS13
LYRM5.2	4.73E-37	-0.20993	0.19	0.371	7.06E-33	M1	LYRM5
MOCS2.4	4.92E-37	-0.18733	0.131	0.29	7.35E-33	M1	MOCS2
LDOC1L.3	4.94E-37	-0.16698	0.012	0.102	7.38E-33	M1	LDOC1L
CXCL12.1	5.00E-37	0.346937	0.094	0.032	7.48E-33	M1	CXCL12
HNRNPD.3	5.06E-37	-0.20452	0.248	0.448	7.56E-33	M1	HNRNPD
GHITM.3	5.07E-37	0.267335	0.564	0.515	7.58E-33	M1	GHITM
VGF.2	5.22E-37	-0.27645	0.003	0.082	7.80E-33	M1	VGF
SOX15.2	5.51E-37	-0.25499	0.003	0.084	8.23E-33	M1	SOX15
PRKAG2.4	5.90E-37	0.421162	0.219	0.121	8.82E-33	M1	PRKAG2
POGZ.2	5.92E-37	-0.14633	0.092	0.234	8.85E-33	M1	POGZ
PTPN18.4	6.07E-37	0.439531	0.293	0.194	9.08E-33	M1	PTPN18
GTF2IRD2E	6.09E-37	-0.18174	0.043	0.156	9.09E-33	M1	GTF2IRD2B
SPOP.1	6.13E-37	-0.14611	0.16	0.338	9.16E-33	M1	SPOP
SNRNP40.1	6.24E-37	-0.2039	0.179	0.359	9.33E-33	M1	SNRNP40
SRSF1	6.28E-37	-0.18651	0.229	0.431	9.39E-33	M1	SRSF1

MCM4.3	6.59E-37	-0.20985	0.018	0.111	9.85E-33	M1	MCM4
NELFB.3	6.70E-37	-0.16009	0.051	0.169	1.00E-32	M1	NELFB
SATB2.1	7.24E-37	-0.15516	0.005	0.086	1.08E-32	M1	SATB2
WIPF2.1	7.33E-37	-0.15198	0.086	0.225	1.10E-32	M1	WIPF2
GIT1.2	7.68E-37	-0.1352	0.023	0.122	1.15E-32	M1	GIT1
SREBF2.1	7.69E-37	-0.15256	0.032	0.137	1.15E-32	M1	SREBF2
YKT6.2	7.70E-37	-0.16436	0.113	0.264	1.15E-32	M1	YKT6
RAD50.1	7.78E-37	-0.1588	0.131	0.292	1.16E-32	M1	RAD50
ADD1.2	8.25E-37	-0.16561	0.161	0.337	1.23E-32	M1	ADD1
AC147651.	8.26E-37	0.240189	0.085	0.026	1.23E-32	M1	AC147651.4
VPS25.1	8.52E-37	-0.22428	0.18	0.36	1.27E-32	M1	VPS25
BET1.2	8.53E-37	-0.2132	0.102	0.245	1.27E-32	M1	BET1
KIF3C.4	8.98E-37	-0.19836	0.022	0.118	1.34E-32	M1	KIF3C
AFAP1.2	9.42E-37	-0.17898	0.012	0.1	1.41E-32	M1	AFAP1
IL10RB-AS	9.48E-37	0.354682	0.097	0.034	1.42E-32	M1	IL10RB-AS1
ANKRD36C.	9.51E-37	-0.21286	0.061	0.182	1.42E-32	M1	ANKRD36C
SURF2.3	9.60E-37	-0.14927	0.107	0.257	1.44E-32	M1	SURF2
SCD.4	9.72E-37	-0.32146	0.098	0.235	1.45E-32	M1	SCD
TMEFF2.4	9.81E-37	-0.28908	0.008	0.092	1.47E-32	M1	TMEFF2
U2AF2.2	9.89E-37	-0.14096	0.124	0.284	1.48E-32	M1	U2AF2
CENPK.2	1.00E-36	-0.2022	0.02	0.115	1.50E-32	M1	CENPK
GABBR1.3	1.00E-36	-0.19645	0.008	0.093	1.50E-32	M1	GABBR1
MYO7A.2	1.02E-36	0.282552	0.079	0.024	1.53E-32	M1	MYO7A
RALGAPB.2	1.11E-36	-0.14507	0.026	0.127	1.65E-32	M1	RALGAPB
MSLN	1.12E-36	0.15755	0.035	0.004	1.68E-32	M1	MSLN
SIAH1.2	1.18E-36	-0.14689	0.106	0.253	1.76E-32	M1	SIAH1
RBM27.2	1.18E-36	-0.13769	0.103	0.251	1.77E-32	M1	RBM27
ELMO2.1	1.19E-36	-0.15638	0.038	0.146	1.78E-32	M1	ELMO2
RP11-557H	1.21E-36	0.248647	0.069	0.018	1.81E-32	M1	RP11-557H15.4
MPRIP.1	1.22E-36	-0.14667	0.056	0.177	1.82E-32	M1	MPRIP
NUDCD3.2	1.24E-36	-0.19011	0.086	0.221	1.85E-32	M1	NUDCD3
AGAP2-AS1	1.27E-36	-0.34028	0.019	0.111	1.90E-32	M1	AGAP2-AS1
TMEM208.3	1.38E-36	-0.2086	0.287	0.503	2.06E-32	M1	TMEM208
LMO1.3	1.44E-36	-0.27337	0.006	0.088	2.15E-32	M1	LMO1
RBM26.2	1.44E-36	-0.11961	0.094	0.237	2.16E-32	M1	RBM26
CTHRC1.3	1.45E-36	-0.15497	0.001	0.077	2.17E-32	M1	CTHRC1
DCAF16.1	1.46E-36	-0.15105	0.032	0.137	2.18E-32	M1	DCAF16
HN1L.2	1.47E-36	-0.1642	0.054	0.172	2.20E-32	M1	HN1L
CCDC43.2	1.52E-36	-0.15308	0.085	0.222	2.28E-32	M1	CCDC43
ZNF90.3	1.62E-36	0.346318	0.471	0.361	2.42E-32	M1	ZNF90
DNM3.2	1.74E-36	-0.25051	0.014	0.103	2.60E-32	M1	DNM3
CD1D.3	1.92E-36	0.262357	0.087	0.028	2.87E-32	M1	CD1D
MPHOSPH8.	1.98E-36	-0.17611	0.231	0.433	2.95E-32	M1	MPHOSPH8
CHST7.2	2.00E-36	-0.15254	0.026	0.126	2.98E-32	M1	CHST7
STK33.3	2.06E-36	-0.16519	0.003	0.081	3.07E-32	M1	STK33
DEF8.4	2.14E-36	-0.13246	0.135	0.298	3.20E-32	M1	DEF8
GPS1.2	2.18E-36	-0.15502	0.174	0.354	3.26E-32	M1	GPS1
FASTK.2	2.22E-36	-0.18383	0.185	0.368	3.32E-32	M1	FASTK
PDK2.1	2.25E-36	-0.13779	0.062	0.185	3.36E-32	M1	PDK2
MIS18A.2	2.29E-36	-0.20356	0.024	0.122	3.43E-32	M1	MIS18A
RBM18.2	2.37E-36	-0.19248	0.092	0.229	3.54E-32	M1	RBM18

DNM1.1	2.37E-36	-0.12804	0.005	0.087	3.55E-32	M1	DNM1
CACNG8.2	2.41E-36	-0.14443	0.005	0.087	3.61E-32	M1	CACNG8
ICAM2.3	2.49E-36	0.2468	0.082	0.025	3.73E-32	M1	ICAM2
BCL7C.2	2.55E-36	-0.17709	0.125	0.281	3.82E-32	M1	BCL7C
FAM84B.3	2.57E-36	-0.22903	0.013	0.102	3.85E-32	M1	FAM84B
SMAD5.3	2.66E-36	-0.15108	0.066	0.192	3.98E-32	M1	SMAD5
GNG2.2	2.67E-36	-0.20795	0.074	0.2	3.98E-32	M1	GNG2
CRAT.1	2.83E-36	-0.14938	0.059	0.181	4.23E-32	M1	CRAT
ZNF195.2	2.88E-36	-0.16408	0.061	0.183	4.31E-32	M1	ZNF195
RASGRP1.3	2.93E-36	-0.15791	0.003	0.082	4.38E-32	M1	RASGRP1
MITF.3	3.04E-36	0.346178	0.165	0.079	4.54E-32	M1	MITF
FAM200A.1	3.28E-36	-0.21059	0.019	0.112	4.90E-32	M1	FAM200A
RNASEH1.2	3.31E-36	-0.11964	0.106	0.254	4.94E-32	M1	RNASEH1
RPRM.4	3.47E-36	-0.30581	0.002	0.079	5.19E-32	M1	RPRM
ZNF253.2	3.51E-36	-0.18466	0.011	0.098	5.24E-32	M1	ZNF253
FAM103A1.	3.54E-36	-0.22361	0.117	0.268	5.29E-32	M1	FAM103A1
PMS2.3	3.60E-36	-0.17258	0.038	0.145	5.39E-32	M1	PMS2
ORC6.3	3.64E-36	-0.19859	0.016	0.106	5.45E-32	M1	ORC6
DCAF10.1	3.65E-36	-0.14066	0.071	0.199	5.46E-32	M1	DCAF10
NCAN.2	3.65E-36	-0.28926	0.01	0.094	5.46E-32	M1	NCAN
FREM2.3	3.66E-36	-0.13421	0.003	0.081	5.47E-32	M1	FREM2
FAF2.3	3.68E-36	-0.13936	0.079	0.211	5.51E-32	M1	FAF2
RTF1.3	3.70E-36	-0.19471	0.258	0.465	5.53E-32	M1	RTF1
GATAD2B.2	3.75E-36	-0.15013	0.06	0.181	5.60E-32	M1	GATAD2B
UBFD1.1	3.81E-36	-0.15269	0.023	0.12	5.69E-32	M1	UBFD1
ECE1.2	3.83E-36	-0.17659	0.009	0.094	5.72E-32	M1	ECE1
DOLPP1.2	3.95E-36	-0.18358	0.022	0.117	5.90E-32	M1	DOLPP1
CBX2.2	4.20E-36	-0.17855	0.002	0.08	6.27E-32	M1	CBX2
PHLDB1.4	4.20E-36	-0.19468	0.047	0.16	6.28E-32	M1	PHLDB1
CASC3.1	4.23E-36	-0.14595	0.077	0.207	6.33E-32	M1	CASC3
ARL3.2	4.25E-36	-0.20787	0.193	0.377	6.35E-32	M1	ARL3
MAPK12.2	4.29E-36	-0.13912	0.004	0.083	6.41E-32	M1	MAPK12
SETD4.2	4.31E-36	-0.19467	0.037	0.144	6.44E-32	M1	SETD4
VCAM1.2	4.42E-36	-0.34217	0.005	0.086	6.61E-32	M1	VCAM1
ANAPC13.4	4.46E-36	-0.24288	0.205	0.384	6.67E-32	M1	ANAPC13
LRP12.1	4.68E-36	-0.17772	0.017	0.108	7.00E-32	M1	LRP12
ERRFI1.2	4.79E-36	-0.21641	0.055	0.171	7.15E-32	M1	ERRFI1
SIRT7.2	4.89E-36	-0.13954	0.09	0.23	7.31E-32	M1	SIRT7
C18orf21.	4.95E-36	-0.15694	0.063	0.186	7.39E-32	M1	C18orf21
SLC9A9.3	5.07E-36	0.331642	0.145	0.065	7.58E-32	M1	SLC9A9
RALBP1.1	5.15E-36	-0.18094	0.16	0.331	7.70E-32	M1	RALBP1
GAR1.4	5.16E-36	-0.19283	0.08	0.212	7.71E-32	M1	GAR1
MFI2-AS1.	5.18E-36	-0.1628	0.007	0.089	7.74E-32	M1	MFI2-AS1
EID2B.2	5.39E-36	-0.17545	0.015	0.104	8.06E-32	M1	EID2B
TOX3.3	5.42E-36	-0.27895	0.003	0.08	8.11E-32	M1	TOX3
IFT88.2	5.46E-36	-0.20502	0.026	0.125	8.17E-32	M1	IFT88
HMGCR.2	5.47E-36	-0.17158	0.026	0.125	8.17E-32	M1	HMGCR
HMGN2	5.59E-36	-0.33479	0.507	0.656	8.36E-32	M1	HMGN2
AC093323.	5.66E-36	-0.14428	0.018	0.111	8.46E-32	M1	AC093323.3
TRIM33.3	5.67E-36	-0.17113	0.075	0.204	8.47E-32	M1	TRIM33
CACFD1.2	5.78E-36	-0.18207	0.014	0.102	8.63E-32	M1	CACFD1

CHID1.2	5.79E-36	-0.22443	0.265	0.471	8.66E-32	M1	CHID1
ZNF205.1	5.80E-36	-0.17325	0.026	0.125	8.66E-32	M1	ZNF205
PCDHB7.2	5.82E-36	-0.23384	0.005	0.084	8.70E-32	M1	PCDHB7
LSM14B.2	6.28E-36	-0.15479	0.021	0.116	9.39E-32	M1	LSM14B
SOX21-AS1	6.30E-36	-0.15408	0.001	0.077	9.41E-32	M1	SOX21-AS1
TMEM184B.	6.39E-36	-0.17753	0.045	0.157	9.55E-32	M1	TMEM184B
FAM126A.2	6.40E-36	-0.14229	0.044	0.155	9.56E-32	M1	FAM126A
TMEM206.3	6.44E-36	-0.14834	0.109	0.255	9.63E-32	M1	TMEM206
RRP9.1	6.47E-36	-0.19021	0.058	0.176	9.67E-32	M1	RRP9
FAT1.2	6.58E-36	-0.14827	0.002	0.079	9.83E-32	M1	FAT1
C1orf52.4	6.62E-36	-0.13225	0.163	0.336	9.89E-32	M1	C1orf52
SNRPF.2	6.74E-36	-0.2427	0.361	0.584	1.01E-31	M1	SNRPF
FANCF.2	6.75E-36	-0.19457	0.016	0.107	1.01E-31	M1	FANCF
RBM28.2	7.09E-36	-0.17142	0.062	0.183	1.06E-31	M1	RBM28
ZNF568.2	7.12E-36	-0.22416	0.02	0.114	1.06E-31	M1	ZNF568
NOL4.2	7.19E-36	-0.20231	0.002	0.078	1.07E-31	M1	NOL4
FAHD2A.1	7.20E-36	-0.16443	0.068	0.193	1.08E-31	M1	FAHD2A
KCNIP1.3	7.43E-36	-0.2164	0.02	0.114	1.11E-31	M1	KCNIP1
ZNF268.2	7.47E-36	-0.16536	0.032	0.134	1.12E-31	M1	ZNF268
KATNB1.2	7.52E-36	-0.16722	0.034	0.137	1.12E-31	M1	KATNB1
RP3-428L1	7.63E-36	-0.17976	0.004	0.083	1.14E-31	M1	RP3-428L16.2
AGPAT4.2	7.88E-36	-0.18228	0.023	0.119	1.18E-31	M1	AGPAT4
PODXL.2	7.98E-36	-0.17593	0.004	0.083	1.19E-31	M1	PODXL
YRDC.1	8.21E-36	-0.15585	0.06	0.181	1.23E-31	M1	YRDC
PSMD3.2	8.43E-36	-0.16239	0.237	0.438	1.26E-31	M1	PSMD3
GALNS.2	8.45E-36	-0.13581	0.041	0.15	1.26E-31	M1	GALNS
CRLS1.2	8.51E-36	-0.18445	0.146	0.311	1.27E-31	M1	CRLS1
CES2.2	8.68E-36	-0.14341	0.075	0.206	1.30E-31	M1	CES2
ATAD3A.1	9.32E-36	-0.14142	0.054	0.171	1.39E-31	M1	ATAD3A
CHCHD6.2	9.32E-36	-0.16138	0.112	0.26	1.39E-31	M1	CHCHD6
UBE2Q1.2	9.33E-36	-0.16268	0.061	0.182	1.39E-31	M1	UBE2Q1
DGCR6.1	9.85E-36	-0.14889	0.056	0.174	1.47E-31	M1	DGCR6
ETV3.4	9.90E-36	0.360803	0.141	0.063	1.48E-31	M1	ETV3
PNO1.1	1.00E-35	-0.17223	0.089	0.225	1.49E-31	M1	PNO1
PRPF38A.2	1.01E-35	-0.13915	0.15	0.318	1.51E-31	M1	PRPF38A
SMKR1.1	1.03E-35	-0.20185	0.005	0.084	1.54E-31	M1	SMKR1
ARPC5.3	1.06E-35	0.15663	0.689	0.669	1.58E-31	M1	ARPC5
DBNDD1.2	1.07E-35	-0.15962	0.003	0.081	1.60E-31	M1	DBNDD1
HDHD2.2	1.08E-35	-0.17943	0.128	0.283	1.61E-31	M1	HDHD2
UBE2J2.3	1.10E-35	-0.17246	0.183	0.365	1.65E-31	M1	UBE2J2
P4HA2	1.11E-35	-0.20304	0.01	0.095	1.65E-31	M1	P4HA2
CHST3.2	1.12E-35	-0.1407	0.001	0.077	1.67E-31	M1	CHST3
RABEP1.4	1.12E-35	-0.12973	0.123	0.279	1.68E-31	M1	RABEP1
CCDC102B.	1.14E-35	-0.17936	0.022	0.117	1.71E-31	M1	CCDC102B
SNRPD1.1	1.17E-35	-0.25169	0.384	0.6	1.74E-31	M1	SNRPD1
RAD51C.2	1.17E-35	-0.14077	0.133	0.293	1.74E-31	M1	RAD51C
CASC4.3	1.17E-35	-0.1108	0.102	0.248	1.74E-31	M1	CASC4
HIST1H2BK	1.21E-35	0.434879	0.289	0.192	1.80E-31	M1	HIST1H2BK
SMIM15.1	1.25E-35	-0.19973	0.183	0.362	1.87E-31	M1	SMIM15
ACLY.4	1.26E-35	-0.11771	0.097	0.24	1.89E-31	M1	ACLY
CREB3L1.1	1.30E-35	-0.14291	0	0.074	1.94E-31	M1	CREB3L1

TCERG1.2	1.31E-35	-0.12444	0.09	0.227	1.95E-31	M1	TCERG1
ZNF37A.4	1.32E-35	-0.17107	0.046	0.157	1.97E-31	M1	ZNF37A
KAT7.2	1.37E-35	-0.15533	0.051	0.167	2.05E-31	M1	KAT7
TBPL1	1.38E-35	-0.16868	0.1	0.241	2.06E-31	M1	TBPL1
PPP1R35.4	1.42E-35	-0.12108	0.058	0.177	2.12E-31	M1	PPP1R35
METTL7A.1	1.46E-35	0.369696	0.311	0.203	2.18E-31	M1	METTL7A
GNAI2.4	1.49E-35	0.301393	0.52	0.475	2.23E-31	M1	GNAI2
RNF219.1	1.52E-35	-0.22143	0.031	0.131	2.28E-31	M1	RNF219
PAK4.2	1.54E-35	-0.14931	0.031	0.133	2.31E-31	M1	PAK4
SMAD9.2	1.55E-35	-0.21456	0.005	0.084	2.31E-31	M1	SMAD9
SRP68.1	1.58E-35	-0.13861	0.107	0.254	2.37E-31	M1	SRP68
MTHFD2.3	1.66E-35	0.375368	0.376	0.271	2.48E-31	M1	MTHFD2
RPS6KL1.3	1.67E-35	-0.22332	0.022	0.116	2.50E-31	M1	RPS6KL1
PDIA3.4	1.68E-35	0.208297	0.607	0.578	2.51E-31	M1	PDIA3
PCIF1.2	1.71E-35	-0.15549	0.049	0.162	2.55E-31	M1	PCIF1
GOSR2.2	1.75E-35	-0.17194	0.078	0.207	2.62E-31	M1	GOSR2
ZNF281.2	1.77E-35	-0.16706	0.078	0.206	2.64E-31	M1	ZNF281
MORF4L2.3	1.77E-35	-0.2441	0.448	0.661	2.65E-31	M1	MORF4L2
GPR37L1.3	1.78E-35	-0.34156	0.013	0.099	2.66E-31	M1	GPR37L1
RNF165.3	1.78E-35	-0.19807	0.003	0.08	2.66E-31	M1	RNF165
LRFN4.1	1.83E-35	-0.13638	0.006	0.087	2.73E-31	M1	LRFN4
H2AFJ.4	1.91E-35	0.449145	0.34	0.243	2.86E-31	M1	H2AFJ
HSPE1.4	1.92E-35	-0.24796	0.594	0.756	2.87E-31	M1	HSPE1
TPX2.3	1.97E-35	-0.27535	0.012	0.097	2.95E-31	M1	TPX2
RP11-263K	1.98E-35	-0.19244	0.002	0.078	2.96E-31	M1	RP11-263K19.4
SPRY2.3	2.01E-35	-0.16117	0.1	0.242	3.00E-31	M1	SPRY2
HS6ST2.1	2.02E-35	-0.13351	0.001	0.075	3.02E-31	M1	HS6ST2
COL16A1.2	2.06E-35	-0.1846	0.007	0.087	3.08E-31	M1	COL16A1
TIAM2.3	2.07E-35	-0.22486	0.01	0.093	3.09E-31	M1	TIAM2
IL4I1.3	2.09E-35	0.271404	0.127	0.052	3.12E-31	M1	IL4I1
DFFA.1	2.10E-35	-0.12771	0.101	0.243	3.14E-31	M1	DFFA
YIPF5.2	2.19E-35	-0.17368	0.132	0.288	3.28E-31	M1	YIPF5
RIT2.3	2.22E-35	-0.32895	0.008	0.09	3.32E-31	M1	RIT2
MAP2K7.1	2.25E-35	-0.14598	0.085	0.218	3.37E-31	M1	MAP2K7
LRRC1.2	2.27E-35	-0.2186	0.005	0.083	3.39E-31	M1	LRRC1
C16orf87.	2.28E-35	-0.13912	0.091	0.227	3.41E-31	M1	C16orf87
DBX2.3	2.30E-35	-0.15458	0.002	0.077	3.44E-31	M1	DBX2
WDR13.1	2.37E-35	-0.17909	0.149	0.313	3.54E-31	M1	WDR13
ACOT8	2.45E-35	-0.13333	0.091	0.229	3.66E-31	M1	ACOT8
CCDC130.2	2.50E-35	-0.12828	0.106	0.252	3.73E-31	M1	CCDC130
LONRF1.1	2.57E-35	-0.13158	0.02	0.113	3.84E-31	M1	LONRF1
SNRPA1.3	2.63E-35	-0.17906	0.156	0.322	3.93E-31	M1	SNRPA1
NIPSNAP1.	2.69E-35	-0.16762	0.066	0.188	4.02E-31	M1	NIPSNAP1
ITGA2.2	2.80E-35	-0.16791	0.001	0.075	4.18E-31	M1	ITGA2
CCNB1.2	2.92E-35	-0.30895	0.017	0.105	4.37E-31	M1	CCNB1
C1orf123.	2.95E-35	-0.20407	0.191	0.371	4.41E-31	M1	C1orf123
PPID	3.00E-35	-0.15191	0.094	0.233	4.48E-31	M1	PPID
RP11-66N1	3.04E-35	-0.16918	0.014	0.101	4.55E-31	M1	RP11-66N11.8
LLGL1.3	3.06E-35	-0.15559	0.022	0.116	4.57E-31	M1	LLGL1
AKT2.1	3.15E-35	-0.18022	0.078	0.207	4.71E-31	M1	AKT2
MYL12B.3	3.45E-35	0.110242	0.739	0.725	5.15E-31	M1	MYL12B

SIGLEC7.4	3.48E-35	0.289674	0.14	0.061	5.20E-31	M1	SIGLEC7
PAK1.4	3.53E-35	0.405614	0.253	0.153	5.28E-31	M1	PAK1
AVIL.2	3.58E-35	-0.23306	0.016	0.104	5.35E-31	M1	AVIL
WDR6.2	3.60E-35	-0.17912	0.111	0.256	5.38E-31	M1	WDR6
SLC30A5.1	3.61E-35	-0.14073	0.091	0.228	5.39E-31	M1	SLC30A5
MSH2.2	3.71E-35	-0.19006	0.032	0.133	5.54E-31	M1	MSH2
RPS6KA4.4	3.75E-35	0.409546	0.194	0.107	5.60E-31	M1	RPS6KA4
SEC23B.2	3.93E-35	-0.17918	0.138	0.297	5.87E-31	M1	SEC23B
PLAGL1.3	3.96E-35	0.297112	0.09	0.031	5.91E-31	M1	PLAGL1
THAP11.3	3.99E-35	-0.12405	0.071	0.197	5.96E-31	M1	THAP11
NEK1.1	4.00E-35	-0.16935	0.039	0.144	5.98E-31	M1	NEK1
CDC16.2	4.25E-35	-0.15203	0.086	0.218	6.35E-31	M1	CDC16
PPP1R9A.3	4.42E-35	-0.24957	0.006	0.084	6.61E-31	M1	PPP1R9A
SNAI3.1	4.54E-35	0.189066	0.047	0.009	6.79E-31	M1	SNAI3
ABHD15.4	4.61E-35	0.355876	0.114	0.046	6.88E-31	M1	ABHD15
FMN2.2	4.75E-35	-0.16672	0.004	0.082	7.10E-31	M1	FMN2
ZCCHC7.2	4.81E-35	-0.18518	0.05	0.162	7.19E-31	M1	ZCCHC7
AC004540.	4.86E-35	-0.22659	0.01	0.093	7.26E-31	M1	AC004540.5
ABCA3.2	5.30E-35	-0.15659	0.008	0.09	7.92E-31	M1	ABCA3
LINC01102	5.36E-35	-0.26222	0.005	0.083	8.01E-31	M1	LINC01102
PLCD3.1	5.46E-35	-0.13743	0.003	0.079	8.16E-31	M1	PLCD3
EXOC5.1	5.63E-35	-0.16886	0.074	0.201	8.41E-31	M1	EXOC5
ACIN1.2	5.71E-35	-0.15515	0.134	0.291	8.53E-31	M1	ACIN1
DENND1C.3	6.03E-35	0.269275	0.079	0.025	9.02E-31	M1	DENND1C
ZMYND8.3	6.25E-35	-0.15041	0.094	0.23	9.35E-31	M1	ZMYND8
TUBA1C.2	6.29E-35	-0.27139	0.23	0.41	9.39E-31	M1	TUBA1C
SPEG.2	6.90E-35	-0.18387	0.005	0.083	1.03E-30	M1	SPEG
RBM42.2	6.93E-35	-0.17453	0.225	0.419	1.04E-30	M1	RBM42
LANCL1.3	7.10E-35	-0.23664	0.034	0.134	1.06E-30	M1	LANCL1
SEC22C.2	7.18E-35	-0.15724	0.107	0.252	1.07E-30	M1	SEC22C
IGDCC3.4	7.47E-35	-0.16157	0.001	0.075	1.12E-30	M1	IGDCC3
SESN1.4	7.76E-35	0.354584	0.174	0.087	1.16E-30	M1	SESN1
GTF2H3.1	7.81E-35	-0.14118	0.048	0.16	1.17E-30	M1	GTF2H3
ADAMTS6.1	7.89E-35	-0.15423	0.003	0.078	1.18E-30	M1	ADAMTS6
OXLD1.2	8.00E-35	-0.18223	0.126	0.278	1.20E-30	M1	OXLD1
NSD1.2	8.04E-35	-0.13885	0.112	0.257	1.20E-30	M1	NSD1
PSMG4.2	8.06E-35	-0.1716	0.087	0.22	1.20E-30	M1	PSMG4
RNF170.2	8.11E-35	-0.13818	0.064	0.185	1.21E-30	M1	RNF170
C16orf52.	8.26E-35	-0.14292	0.02	0.111	1.24E-30	M1	C16orf52
RAI14.2	8.49E-35	-0.19413	0.005	0.082	1.27E-30	M1	RAI14
SLC48A1.3	8.55E-35	-0.27717	0.044	0.15	1.28E-30	M1	SLC48A1
SLC36A4.2	8.60E-35	-0.14445	0.084	0.215	1.29E-30	M1	SLC36A4
PELP1.3	8.69E-35	-0.18034	0.055	0.17	1.30E-30	M1	PELP1
MECR.2	9.06E-35	-0.145	0.061	0.181	1.35E-30	M1	MECR
C7orf26.3	9.44E-35	-0.16923	0.06	0.177	1.41E-30	M1	C7orf26
COL22A1	9.79E-35	-0.13878	0.006	0.085	1.46E-30	M1	COL22A1
RBM38.2	1.02E-34	-0.14505	0.045	0.154	1.52E-30	M1	RBM38
KIAA1586.	1.02E-34	-0.18506	0.032	0.132	1.52E-30	M1	KIAA1586
SCN3A.3	1.08E-34	-0.21608	0.006	0.085	1.62E-30	M1	SCN3A
RBBP9.2	1.10E-34	-0.12175	0.019	0.109	1.65E-30	M1	RBBP9
SMIM7.3	1.14E-34	-0.22882	0.322	0.542	1.70E-30	M1	SMIM7

SEMA4A.4	1.18E-34	0.246846	0.103	0.038	1.76E-30	M1	SEMA4A
RNF40.1	1.19E-34	-0.14544	0.071	0.196	1.77E-30	M1	RNF40
MBIP.3	1.20E-34	-0.16219	0.074	0.201	1.79E-30	M1	MBIP
CELF3.4	1.23E-34	-0.25257	0.003	0.078	1.83E-30	M1	CELF3
ZCCHC3.3	1.29E-34	-0.13666	0.026	0.122	1.93E-30	M1	ZCCHC3
PSMD8.2	1.32E-34	-0.29493	0.502	0.708	1.97E-30	M1	PSMD8
DMPK.1	1.32E-34	-0.14172	0.003	0.078	1.98E-30	M1	DMPK
SAP30L.3	1.36E-34	-0.14787	0.018	0.107	2.03E-30	M1	SAP30L
SULF2.2	1.40E-34	-0.12215	0.06	0.179	2.08E-30	M1	SULF2
PNPT1.2	1.42E-34	-0.14746	0.087	0.219	2.12E-30	M1	PNPT1
FAM212A.3	1.43E-34	0.337641	0.102	0.038	2.13E-30	M1	FAM212A
XIAP.2	1.43E-34	-0.11417	0.097	0.237	2.14E-30	M1	XIAP
MIB2.3	1.45E-34	-0.12347	0.053	0.168	2.16E-30	M1	MIB2
RP11-196G	1.48E-34	0.172784	0.045	0.009	2.21E-30	M1	RP11-196G11.2
MUT.3	1.54E-34	-0.15464	0.06	0.177	2.29E-30	M1	MUT
DDB1	1.59E-34	-0.16936	0.153	0.317	2.38E-30	M1	DDB1
EID1.2	1.59E-34	-0.22246	0.55	0.739	2.38E-30	M1	EID1
PES1.3	1.63E-34	-0.12784	0.083	0.214	2.44E-30	M1	PES1
HIRIP3.2	1.65E-34	-0.19079	0.066	0.185	2.47E-30	M1	HIRIP3
BRINP2.3	1.69E-34	-0.16936	0.007	0.086	2.52E-30	M1	BRINP2
C9orf116.	1.74E-34	-0.14346	0.009	0.09	2.60E-30	M1	C9orf116
FAM181A.2	1.80E-34	-0.2589	0.003	0.078	2.69E-30	M1	FAM181A
TBC1D8.1	1.82E-34	0.268902	0.095	0.034	2.72E-30	M1	TBC1D8
MSH6.2	1.93E-34	-0.16421	0.04	0.144	2.89E-30	M1	MSH6
ZNF260.1	1.97E-34	-0.14472	0.022	0.115	2.95E-30	M1	ZNF260
BYSL.1	1.98E-34	-0.18182	0.028	0.123	2.96E-30	M1	BYSL
RAB9A.2	2.06E-34	-0.15657	0.162	0.332	3.08E-30	M1	RAB9A
GGCT.3	2.07E-34	-0.23873	0.229	0.418	3.09E-30	M1	GGCT
GJC1.2	2.08E-34	-0.12529	0.002	0.076	3.10E-30	M1	GJC1
PRR5.3	2.09E-34	0.281116	0.099	0.036	3.12E-30	M1	PRR5
GAS2L1.3	2.12E-34	-0.14524	0.056	0.171	3.16E-30	M1	GAS2L1
MED9.1	2.21E-34	-0.16523	0.018	0.107	3.31E-30	M1	MED9
TRIB3.3	2.24E-34	-0.22117	0.008	0.087	3.35E-30	M1	TRIB3
ST5.4	2.27E-34	-0.17103	0.056	0.17	3.40E-30	M1	ST5
WASF3.2	2.28E-34	-0.17802	0.009	0.089	3.41E-30	M1	WASF3
C12orf43	2.41E-34	-0.15558	0.06	0.176	3.60E-30	M1	C12orf43
NDUFAF6.3	2.43E-34	-0.12435	0.075	0.202	3.62E-30	M1	NDUFAF6
CSK.4	2.47E-34	0.3948	0.225	0.132	3.69E-30	M1	CSK
PWAR6.1	2.48E-34	-0.1595	0.007	0.085	3.71E-30	M1	PWAR6
HRK.2	2.56E-34	-0.22074	0.002	0.075	3.83E-30	M1	HRK
TTC32.1	2.58E-34	-0.19493	0.055	0.168	3.85E-30	M1	TTC32
TSPYL4.1	2.65E-34	-0.18699	0.025	0.119	3.97E-30	M1	TSPYL4
CNOT3.2	2.70E-34	-0.11317	0.063	0.182	4.03E-30	M1	CNOT3
PDHA1.1	2.75E-34	-0.16458	0.164	0.331	4.10E-30	M1	PDHA1
MZT2B.2	2.80E-34	-0.25288	0.441	0.624	4.19E-30	M1	MZT2B
TNFRSF14.	2.85E-34	0.403892	0.19	0.1	4.27E-30	M1	TNFRSF14
ADPRH.2	3.00E-34	0.257259	0.077	0.024	4.48E-30	M1	ADPRH
METAP1D.2	3.19E-34	-0.20523	0.039	0.141	4.76E-30	M1	METAP1D
WDR48.2	3.21E-34	-0.17854	0.048	0.157	4.80E-30	M1	WDR48
SHOX2.2	3.26E-34	-0.14326	0.002	0.076	4.87E-30	M1	SHOX2
PCID2.2	3.32E-34	-0.15597	0.076	0.201	4.97E-30	M1	PCID2

GOPC.2	3.33E-34	-0.13012	0.13	0.283	4.98E-30	M1	GOPC
C5orf42.2	3.38E-34	-0.14361	0.019	0.109	5.04E-30	M1	C5orf42
USE1.4	3.39E-34	-0.18388	0.183	0.359	5.06E-30	M1	USE1
BRWD1.3	3.60E-34	-0.16819	0.119	0.266	5.38E-30	M1	BRWD1
PRUNE2.2	3.68E-34	-0.31755	0.035	0.133	5.50E-30	M1	PRUNE2
ICA1L.1	3.79E-34	-0.15759	0.023	0.115	5.66E-30	M1	ICA1L
TIMM21.3	3.85E-34	-0.16717	0.053	0.166	5.75E-30	M1	TIMM21
R3HDM1.1	3.88E-34	-0.16921	0.04	0.144	5.80E-30	M1	R3HDM1
ADO.3	4.13E-34	-0.13362	0.039	0.144	6.17E-30	M1	ADO
MMP15.3	4.16E-34	-0.15117	0.002	0.075	6.21E-30	M1	MMP15
PDE4D.1	4.22E-34	-0.1738	0.022	0.113	6.31E-30	M1	PDE4D
METTL5.1	4.25E-34	-0.15983	0.205	0.391	6.35E-30	M1	METTL5
EMC8.2	4.29E-34	-0.14751	0.139	0.298	6.41E-30	M1	EMC8
CBR4.4	4.59E-34	-0.12646	0.081	0.21	6.85E-30	M1	CBR4
PEG3.2	4.86E-34	-0.21584	0.003	0.077	7.27E-30	M1	PEG3
MAP4K4.2	5.05E-34	-0.20655	0.126	0.272	7.54E-30	M1	MAP4K4
ZNF708.1	5.27E-34	-0.17281	0.022	0.113	7.87E-30	M1	ZNF708
TP53BP1.1	5.41E-34	-0.13882	0.021	0.112	8.08E-30	M1	TP53BP1
SPA17.4	5.49E-34	-0.19984	0.012	0.094	8.20E-30	M1	SPA17
CD7.3	5.54E-34	0.207526	0.095	0.033	8.28E-30	M1	CD7
DHX15.1	5.66E-34	-0.16285	0.122	0.269	8.46E-30	M1	DHX15
PTPN4.3	5.71E-34	-0.18886	0.025	0.118	8.53E-30	M1	PTPN4
SCN3B.2	5.86E-34	-0.26088	0.007	0.085	8.76E-30	M1	SCN3B
NXPH4.2	5.90E-34	-0.23857	0.002	0.075	8.82E-30	M1	NXPH4
EN1.1	6.01E-34	-0.20354	0.003	0.076	8.98E-30	M1	EN1
ADA.3	6.03E-34	-0.14904	0.019	0.108	9.02E-30	M1	ADA
SPPL3.3	6.30E-34	-0.11742	0.087	0.219	9.41E-30	M1	SPPL3
KANSL2.1	6.30E-34	-0.17495	0.056	0.168	9.42E-30	M1	KANSL2
RHBDD3.2	6.36E-34	-0.13355	0.055	0.168	9.50E-30	M1	RHBDD3
NDUFAF5.1	6.50E-34	-0.15317	0.045	0.152	9.72E-30	M1	NDUFAF5
CHERP.1	6.56E-34	-0.15723	0.068	0.189	9.81E-30	M1	CHERP
PTPRN.3	6.95E-34	-0.25359	0.008	0.087	1.04E-29	M1	PTPRN
GLTSCR2.4	6.97E-34	0.222184	0.59	0.559	1.04E-29	M1	GLTSCR2
NAT9.2	7.23E-34	-0.11888	0.091	0.225	1.08E-29	M1	NAT9
SLC26A11.	7.81E-34	-0.12279	0.026	0.121	1.17E-29	M1	SLC26A11
FAM96A.2	8.03E-34	0.372789	0.423	0.343	1.20E-29	M1	FAM96A
PNKD.2	8.04E-34	-0.20912	0.285	0.491	1.20E-29	M1	PNKD
TTC37.3	8.05E-34	-0.13748	0.078	0.205	1.20E-29	M1	TTC37
FTO.1	8.36E-34	-0.14536	0.045	0.152	1.25E-29	M1	FTO
BASP1.4	8.67E-34	0.330496	0.455	0.356	1.30E-29	M1	BASP1
RAC2.4	8.74E-34	0.283063	0.2	0.103	1.31E-29	M1	RAC2
ARHGEF9.3	8.77E-34	-0.1751	0.036	0.137	1.31E-29	M1	ARHGEF9
RP11-514C	8.79E-34	0.225479	0.067	0.019	1.31E-29	M1	RP11-514O12.4
SLC8B1.4	8.79E-34	0.389662	0.165	0.084	1.31E-29	M1	SLC8B1
SMC3.2	8.80E-34	-0.16616	0.153	0.312	1.32E-29	M1	SMC3
MCHR1.2	8.82E-34	-0.1434	0	0.07	1.32E-29	M1	MCHR1
GPSM1.3	8.82E-34	-0.18552	0.008	0.086	1.32E-29	M1	GPSM1
CSRP1.2	8.91E-34	-0.27386	0.12	0.262	1.33E-29	M1	CSRP1
FAF1	9.13E-34	-0.15553	0.066	0.185	1.36E-29	M1	FAF1
RAB5B.3	9.53E-34	-0.14859	0.1	0.237	1.42E-29	M1	RAB5B
ADD3.1	9.60E-34	-0.2395	0.045	0.15	1.43E-29	M1	ADD3

SDC4.1	9.61E-34	-0.24826	0.021	0.111	1.44E-29	M1	SDC4
RSAD1.2	9.73E-34	-0.1338	0.054	0.167	1.45E-29	M1	RSAD1
C8orf82.3	9.81E-34	-0.13212	0.07	0.191	1.47E-29	M1	C8orf82
SLC35C2.1	9.83E-34	-0.16257	0.127	0.279	1.47E-29	M1	SLC35C2
MAD2L1BP	1.01E-33	-0.15157	0.063	0.18	1.51E-29	M1	MAD2L1BP
QSER1.3	1.02E-33	-0.12219	0.035	0.135	1.53E-29	M1	QSER1
HSBP1.3	1.04E-33	-0.26942	0.555	0.747	1.55E-29	M1	HSBP1
GSTM3.2	1.09E-33	-0.13878	0.137	0.295	1.63E-29	M1	GSTM3
GLIS3.1	1.10E-33	-0.13502	0.01	0.091	1.65E-29	M1	GLIS3
MS4A14.3	1.18E-33	0.217785	0.059	0.015	1.76E-29	M1	MS4A14
DYNC1I2.3	1.19E-33	-0.22181	0.302	0.503	1.78E-29	M1	DYNC1I2
SAMD14.1	1.25E-33	-0.19047	0.003	0.077	1.86E-29	M1	SAMD14
GPX7.1	1.25E-33	-0.12483	0.073	0.198	1.86E-29	M1	GPX7
TDG.2	1.26E-33	-0.12772	0.141	0.3	1.88E-29	M1	TDG
MNS1.2	1.27E-33	-0.15834	0.009	0.088	1.90E-29	M1	MNS1
NFKB2.4	1.27E-33	0.339322	0.118	0.049	1.90E-29	M1	NFKB2
NF1.2	1.28E-33	-0.14144	0.106	0.247	1.91E-29	M1	NF1
RAB22A.3	1.28E-33	-0.11968	0.115	0.263	1.91E-29	M1	RAB22A
ST7	1.30E-33	-0.21547	0.028	0.122	1.95E-29	M1	ST7
PSMD5-AS1	1.31E-33	-0.15779	0.078	0.203	1.96E-29	M1	PSMD5-AS1
BRMS1L.1	1.32E-33	-0.18202	0.022	0.113	1.97E-29	M1	BRMS1L
BBIP1.2	1.32E-33	-0.1512	0.075	0.199	1.97E-29	M1	BBIP1
TIMM8A.1	1.33E-33	-0.19367	0.022	0.113	1.99E-29	M1	TIMM8A
DSTYK.2	1.34E-33	-0.15461	0.037	0.137	2.01E-29	M1	DSTYK
ZNF532.2	1.34E-33	-0.15665	0.019	0.107	2.01E-29	M1	ZNF532
RP11-563K	1.36E-33	-0.18632	0.003	0.077	2.03E-29	M1	RP11-563K23.1
TXNRD1.2	1.37E-33	-0.14718	0.118	0.262	2.04E-29	M1	TXNRD1
CHMP2A.3	1.38E-33	-0.30784	0.477	0.691	2.06E-29	M1	CHMP2A
ACAD8.1	1.39E-33	-0.15628	0.078	0.204	2.08E-29	M1	ACAD8
E2F5.1	1.42E-33	-0.1126	0.004	0.078	2.12E-29	M1	E2F5
KTI12.2	1.47E-33	-0.17084	0.027	0.121	2.20E-29	M1	KTI12
WDR83.2	1.52E-33	-0.12764	0.07	0.191	2.27E-29	M1	WDR83
C22orf39.	1.55E-33	-0.14496	0.132	0.285	2.32E-29	M1	C22orf39
FAM69B.2	1.56E-33	-0.13291	0	0.07	2.33E-29	M1	FAM69B
NEO1.2	1.58E-33	-0.18118	0.008	0.086	2.36E-29	M1	NEO1
LMBR1.2	1.58E-33	-0.15862	0.03	0.126	2.36E-29	M1	LMBR1
NUP214.3	1.59E-33	0.488069	0.274	0.179	2.37E-29	M1	NUP214
ISOC2.4	1.60E-33	-0.20288	0.201	0.379	2.39E-29	M1	ISOC2
SFSWAP.3	1.61E-33	-0.10202	0.073	0.197	2.40E-29	M1	SFSWAP
FAM219A.1	1.66E-33	-0.1652	0.018	0.105	2.48E-29	M1	FAM219A
GAL3ST3.2	1.71E-33	-0.15797	0.001	0.071	2.56E-29	M1	GAL3ST3
MAP4K5.2	1.72E-33	-0.13298	0.059	0.174	2.57E-29	M1	MAP4K5
MEN1	1.75E-33	-0.13908	0.034	0.133	2.61E-29	M1	MEN1
EXOC1.1	1.80E-33	-0.19282	0.128	0.273	2.69E-29	M1	EXOC1
PCED1A.1	1.82E-33	-0.12977	0.043	0.148	2.73E-29	M1	PCED1A
AAAS.1	1.85E-33	-0.15151	0.081	0.207	2.76E-29	M1	AAAS
TTC28.2	1.85E-33	-0.14543	0.021	0.11	2.77E-29	M1	TTC28
ODF2.2	1.88E-33	-0.16487	0.044	0.15	2.82E-29	M1	ODF2
RNMTL1.2	1.89E-33	-0.11482	0.042	0.147	2.82E-29	M1	RNMTL1
EVA1C.3	1.92E-33	-0.15959	0.007	0.084	2.87E-29	M1	EVA1C
ENPP1	1.99E-33	0.170423	0.046	0.01	2.97E-29	M1	ENPP1

ACSL3.2	1.99E-33	-0.14334	0.193	0.371	2.97E-29	M1	ACSL3
DGCR2.1	2.04E-33	-0.14439	0.058	0.171	3.05E-29	M1	DGCR2
GART.1	2.09E-33	-0.13035	0.09	0.222	3.12E-29	M1	GART
XPOT.3	2.18E-33	-0.14801	0.068	0.187	3.26E-29	M1	XPOT
DYNLRB1.3	2.20E-33	-0.23405	0.494	0.699	3.28E-29	M1	DYNLRB1
TNFRSF21.	2.20E-33	-0.16686	0.065	0.183	3.29E-29	M1	TNFRSF21
ST20	2.23E-33	-0.16304	0.031	0.127	3.34E-29	M1	ST20
CTU2.1	2.30E-33	-0.12654	0.045	0.15	3.43E-29	M1	CTU2
RP11-111M	2.33E-33	-0.17731	0.021	0.111	3.48E-29	M1	RP11-111M22.3
SEPHS2.2	2.42E-33	-0.17014	0.173	0.341	3.61E-29	M1	SEPHS2
RRP8	2.43E-33	-0.17117	0.041	0.144	3.63E-29	M1	RRP8
TIMM17A.2	2.46E-33	-0.18432	0.22	0.407	3.68E-29	M1	TIMM17A
FAM64A.4	2.47E-33	-0.26773	0.006	0.082	3.69E-29	M1	FAM64A
TMEM9B.2	2.55E-33	0.279167	0.554	0.533	3.81E-29	M1	TMEM9B
CEP95.1	2.56E-33	-0.15082	0.039	0.141	3.82E-29	M1	CEP95
RP11-115C	2.56E-33	-0.19369	0.015	0.099	3.83E-29	M1	RP11-115C21.2
BZW1.2	2.63E-33	-0.23624	0.365	0.573	3.93E-29	M1	BZW1
PLA2G7.3	2.67E-33	0.245881	0.105	0.04	3.99E-29	M1	PLA2G7
HMGB2.4	2.71E-33	0.2606	0.564	0.48	4.05E-29	M1	HMGB2
SSB.3	2.92E-33	-0.24528	0.409	0.629	4.37E-29	M1	SSB
ATMIN.3	3.05E-33	-0.14592	0.034	0.132	4.55E-29	M1	ATMIN
PLXNB1.3	3.06E-33	-0.2063	0.01	0.089	4.58E-29	M1	PLXNB1
ITGB3BP.2	3.08E-33	-0.18144	0.052	0.161	4.60E-29	M1	ITGB3BP
ZNF131.3	3.12E-33	-0.15334	0.114	0.256	4.66E-29	M1	ZNF131
SPRY4.2	3.13E-33	-0.14958	0.005	0.08	4.67E-29	M1	SPRY4
NRSN1.2	3.23E-33	-0.19273	0.001	0.071	4.83E-29	M1	NRSN1
ZZZ3.1	3.35E-33	-0.15029	0.03	0.125	5.00E-29	M1	ZZZ3
CNKSR2.2	3.35E-33	-0.14547	0.002	0.074	5.01E-29	M1	CNKSR2
PIK3CG.2	3.53E-33	0.190941	0.058	0.015	5.28E-29	M1	PIK3CG
DDHD2.2	3.54E-33	-0.16856	0.028	0.121	5.29E-29	M1	DDHD2
PDIA4.2	3.57E-33	-0.2425	0.293	0.495	5.34E-29	M1	PDIA4
KCNH2.2	3.61E-33	-0.21837	0.005	0.079	5.39E-29	M1	KCNH2
UPF3B.2	3.61E-33	-0.12421	0.127	0.276	5.40E-29	M1	UPF3B
PACSIN3.2	3.69E-33	-0.14489	0.006	0.082	5.51E-29	M1	PACSIN3
AGBL5.1	3.78E-33	-0.12209	0.014	0.097	5.64E-29	M1	AGBL5
ABHD17B.4	3.95E-33	-0.2113	0.041	0.141	5.90E-29	M1	ABHD17B
SNX2.4	4.00E-33	0.367328	0.464	0.399	5.97E-29	M1	SNX2
MT2A.4	4.03E-33	-0.51603	0.744	0.792	6.02E-29	M1	MT2A
RRS1.1	4.05E-33	-0.15035	0.051	0.161	6.05E-29	M1	RRS1
C2orf69.4	4.13E-33	-0.14225	0.088	0.215	6.17E-29	M1	C2orf69
CDK14	4.20E-33	-0.15957	0.011	0.092	6.28E-29	M1	CDK14
KMT2E.2	4.26E-33	-0.1946	0.361	0.573	6.36E-29	M1	KMT2E
WDR3.1	4.32E-33	-0.16248	0.024	0.115	6.45E-29	M1	WDR3
MPP2.3	4.32E-33	-0.17588	0.001	0.071	6.45E-29	M1	MPP2
C1orf53.2	4.32E-33	-0.19179	0.014	0.097	6.46E-29	M1	C1orf53
IFT27.3	4.45E-33	-0.13534	0.131	0.282	6.65E-29	M1	IFT27
GNAI1.3	4.47E-33	-0.25459	0.029	0.121	6.68E-29	M1	GNAI1
SPIN2B.2	4.55E-33	-0.13602	0.028	0.122	6.79E-29	M1	SPIN2B
KCTD1.2	4.59E-33	-0.15122	0.004	0.077	6.86E-29	M1	KCTD1
RAB30.2	4.61E-33	-0.15894	0.01	0.089	6.89E-29	M1	RAB30
VANGL2.2	4.78E-33	-0.15849	0.008	0.086	7.15E-29	M1	VANGL2

FRMD5.1	4.84E-33	-0.13433	0	0.069	7.23E-29	M1	FRMD5
CHGB.1	5.00E-33	-0.23695	0.017	0.101	7.47E-29	M1	CHGB
SLC25A29.	5.07E-33	-0.12246	0.058	0.171	7.58E-29	M1	SLC25A29
ZNF226.4	5.55E-33	-0.20854	0.095	0.226	8.30E-29	M1	ZNF226
BCAS4.2	5.62E-33	-0.14414	0.016	0.1	8.40E-29	M1	BCAS4
COL5A2.2	5.64E-33	-0.12725	0.003	0.074	8.42E-29	M1	COL5A2
CDK5.2	5.72E-33	-0.1308	0.118	0.262	8.55E-29	M1	CDK5
UBE3A.3	5.81E-33	-0.14484	0.153	0.314	8.68E-29	M1	UBE3A
LRRC41.1	5.91E-33	-0.16871	0.12	0.265	8.83E-29	M1	LRRC41
SHC4.1	6.07E-33	-0.16497	0	0.069	9.07E-29	M1	SHC4
GPRASP2.2	6.20E-33	-0.17579	0.004	0.077	9.27E-29	M1	GPRASP2
DMAP1.2	6.24E-33	-0.14309	0.127	0.276	9.33E-29	M1	DMAP1
CCDC58.2	6.36E-33	-0.13531	0.052	0.162	9.50E-29	M1	CCDC58
TXNL1.1	6.46E-33	-0.21813	0.298	0.506	9.65E-29	M1	TXNL1
PCDH10.2	6.47E-33	-0.16241	0.006	0.08	9.67E-29	M1	PCDH10
CHEK1	6.80E-33	-0.18454	0.013	0.094	1.02E-28	M1	CHEK1
MCCC2.1	6.81E-33	-0.14173	0.046	0.151	1.02E-28	M1	MCCC2
KANK1.2	6.91E-33	-0.20055	0.02	0.106	1.03E-28	M1	KANK1
LIN7B.2	6.95E-33	-0.15092	0.039	0.139	1.04E-28	M1	LIN7B
GNB4.3	7.29E-33	0.432397	0.304	0.218	1.09E-28	M1	GNB4
UBA5.1	7.54E-33	-0.15233	0.08	0.205	1.13E-28	M1	UBA5
DIRAS2.2	7.79E-33	-0.17868	0.003	0.075	1.16E-28	M1	DIRAS2
HES4.4	7.80E-33	-0.17763	0.083	0.208	1.17E-28	M1	HES4
UNK	7.80E-33	-0.13766	0.041	0.144	1.17E-28	M1	UNK
PCDH19.2	7.85E-33	-0.14013	0.002	0.072	1.17E-28	M1	PCDH19
SLC4A1AP.	7.90E-33	-0.15122	0.074	0.196	1.18E-28	M1	SLC4A1AP
PNMAL1.3	7.90E-33	-0.11991	0.022	0.111	1.18E-28	M1	PNMAL1
MED24.3	8.34E-33	-0.15594	0.07	0.188	1.25E-28	M1	MED24
UQCRQ.4	8.35E-33	-0.2441	0.686	0.822	1.25E-28	M1	UQCRQ
SETBP1.2	8.41E-33	-0.16086	0.009	0.087	1.26E-28	M1	SETBP1
COA1.1	8.43E-33	-0.13795	0.234	0.43	1.26E-28	M1	COA1
TNKS1BP1.	8.58E-33	-0.12811	0.009	0.086	1.28E-28	M1	TNKS1BP1
SRD5A1.1	8.75E-33	-0.16754	0.031	0.126	1.31E-28	M1	SRD5A1
MEX3C.1	8.78E-33	-0.13339	0.021	0.109	1.31E-28	M1	MEX3C
RRAGD.2	8.82E-33	-0.14017	0.043	0.147	1.32E-28	M1	RRAGD
C1QBP.2	8.89E-33	-0.24412	0.397	0.586	1.33E-28	M1	C1QBP
ZFP90.3	9.21E-33	-0.11446	0.044	0.148	1.38E-28	M1	ZFP90
SIKE1.1	9.23E-33	-0.12711	0.083	0.209	1.38E-28	M1	SIKE1
RIC8B.2	9.50E-33	-0.16997	0.02	0.107	1.42E-28	M1	RIC8B
RP11-277F	9.89E-33	-0.23318	0.006	0.08	1.48E-28	M1	RP11-277P12.20
FAM213B.1	1.02E-32	-0.15836	0.018	0.103	1.52E-28	M1	FAM213B
RIN1.3	1.03E-32	-0.12968	0.014	0.096	1.54E-28	M1	RIN1
NOL10.1	1.03E-32	-0.16153	0.061	0.174	1.54E-28	M1	NOL10
GHRL.4	1.06E-32	0.361501	0.111	0.046	1.58E-28	M1	GHRL
SNHG10.1	1.07E-32	-0.1369	0.021	0.109	1.60E-28	M1	SNHG10
UBL5.3	1.11E-32	-0.27342	0.732	0.851	1.66E-28	M1	UBL5
SEC61A2	1.14E-32	-0.16156	0.013	0.093	1.70E-28	M1	SEC61A2
PLEKHJ1.2	1.15E-32	-0.13461	0.212	0.397	1.72E-28	M1	PLEKHJ1
FARSA.1	1.16E-32	-0.15175	0.177	0.348	1.73E-28	M1	FARSA
MIR497HG.	1.17E-32	-0.2412	0.006	0.08	1.74E-28	M1	MIR497HG
RP11-212F	1.18E-32	-0.14464	0.019	0.106	1.76E-28	M1	RP11-212P7.2

UCKL1.1	1.23E-32	-0.12441	0.062	0.177	1.84E-28	M1	UCKL1
NAA15.2	1.25E-32	-0.15981	0.103	0.238	1.87E-28	M1	NAA15
ACTR2.4	1.26E-32	0.3324	0.492	0.452	1.88E-28	M1	ACTR2
PBRM1.3	1.27E-32	-0.12522	0.12	0.264	1.89E-28	M1	PBRM1
FBXO9.3	1.27E-32	-0.12786	0.096	0.228	1.90E-28	M1	FBXO9
NFATC1.4	1.27E-32	0.328241	0.105	0.042	1.90E-28	M1	NFATC1
ZDHHC15.2	1.28E-32	-0.14798	0.002	0.072	1.91E-28	M1	ZDHHC15
FOXRED2.1	1.28E-32	-0.13755	0.014	0.096	1.91E-28	M1	FOXRED2
YBEY.1	1.29E-32	-0.16266	0.107	0.244	1.92E-28	M1	YBEY
CROT.3	1.30E-32	-0.17197	0.046	0.149	1.94E-28	M1	CROT
BDP1.1	1.34E-32	-0.14213	0.124	0.269	2.00E-28	M1	BDP1
PREB.2	1.34E-32	-0.13518	0.098	0.232	2.01E-28	M1	PREB
DNTTIP1.1	1.35E-32	-0.14682	0.185	0.359	2.02E-28	M1	DNTTIP1
ZNF770.1	1.39E-32	-0.12704	0.044	0.147	2.07E-28	M1	ZNF770
SLC52A2.2	1.41E-32	-0.14236	0.148	0.307	2.10E-28	M1	SLC52A2
PDGFC.1	1.42E-32	-0.13104	0.037	0.136	2.12E-28	M1	PDGFC
KIAA0895L	1.43E-32	-0.18301	0.02	0.106	2.13E-28	M1	KIAA0895L
GPANK1.2	1.54E-32	-0.13796	0.087	0.214	2.30E-28	M1	GPANK1
GS1-124K5	1.55E-32	-0.20713	0.034	0.13	2.32E-28	M1	GS1-124K5.4
SMIM14.2	1.55E-32	-0.17915	0.187	0.359	2.32E-28	M1	SMIM14
CIITA.1	1.60E-32	0.246084	0.084	0.028	2.39E-28	M1	CIITA
SLC39A3.3	1.64E-32	-0.15702	0.192	0.367	2.44E-28	M1	SLC39A3
MATN2.1	1.65E-32	-0.19469	0.005	0.078	2.46E-28	M1	MATN2
LPGAT1.3	1.67E-32	-0.16227	0.043	0.145	2.50E-28	M1	LPGAT1
YPEL1.4	1.77E-32	-0.12787	0.021	0.108	2.64E-28	M1	YPEL1
AAR2.1	1.81E-32	-0.13815	0.048	0.154	2.71E-28	M1	AAR2
INTS12.2	1.84E-32	-0.1728	0.068	0.184	2.76E-28	M1	INTS12
HSDL1.3	2.00E-32	-0.18397	0.045	0.148	2.99E-28	M1	HSDL1
DIAPH1.3	2.02E-32	-0.13948	0.05	0.156	3.01E-28	M1	DIAPH1
NIM1K.3	2.03E-32	-0.19755	0.004	0.076	3.03E-28	M1	NIM1K
TNFRSF10E	2.05E-32	0.383499	0.17	0.089	3.06E-28	M1	TNFRSF10B
SCO1.1	2.06E-32	-0.12253	0.068	0.186	3.08E-28	M1	SCO1
MCAT.3	2.09E-32	-0.13557	0.043	0.146	3.13E-28	M1	MCAT
DRG2.2	2.10E-32	-0.13815	0.092	0.222	3.14E-28	M1	DRG2
CCDC93.2	2.15E-32	-0.18131	0.043	0.144	3.21E-28	M1	CCDC93
FMN1.3	2.20E-32	0.2412	0.075	0.024	3.28E-28	M1	FMN1
BDNF-AS.3	2.25E-32	-0.16412	0.004	0.076	3.36E-28	M1	BDNF-AS
ACY3.1	2.25E-32	0.246067	0.052	0.012	3.36E-28	M1	ACY3
AP1M1.1	2.25E-32	-0.12833	0.108	0.246	3.37E-28	M1	AP1M1
OSBPL2.2	2.28E-32	-0.13502	0.046	0.151	3.40E-28	M1	OSBPL2
TBC1D24.2	2.31E-32	-0.164	0.008	0.084	3.45E-28	M1	TBC1D24
PSMC3IP.3	2.33E-32	-0.18221	0.008	0.083	3.49E-28	M1	PSMC3IP
SIAE.2	2.36E-32	-0.17934	0.019	0.104	3.53E-28	M1	SIAE
RP11-698N	2.43E-32	-0.19106	0.001	0.07	3.64E-28	M1	RP11-698N11.2
ITGB2-AS1	2.57E-32	0.259689	0.068	0.02	3.84E-28	M1	ITGB2-AS1
TNFRSF12A	2.57E-32	-0.31501	0.172	0.31	3.84E-28	M1	TNFRSF12A
BBS7.2	2.63E-32	-0.1656	0.023	0.111	3.93E-28	M1	BBS7
TMEM88.1	2.64E-32	0.202143	0.059	0.015	3.95E-28	M1	TMEM88
ASNA1.3	2.71E-32	-0.21434	0.345	0.554	4.05E-28	M1	ASNA1
RNF14.2	2.74E-32	-0.11743	0.088	0.216	4.09E-28	M1	RNF14
MUS81.1	2.81E-32	-0.12046	0.073	0.194	4.20E-28	M1	MUS81

ZNF420.2	2.82E-32	-0.1678	0.03	0.122	4.22E-28	M1	ZNF420
ZMYND15.2	2.84E-32	0.321363	0.111	0.046	4.24E-28	M1	ZMYND15
SYNDIG1.4	3.04E-32	0.371326	0.154	0.076	4.54E-28	M1	SYNDIG1
CD82.2	3.05E-32	-0.26271	0.126	0.262	4.56E-28	M1	CD82
HEY2	3.05E-32	-0.13683	0.004	0.076	4.56E-28	M1	HEY2
WRNIP1.1	3.09E-32	-0.12581	0.027	0.118	4.62E-28	M1	WRNIP1
CBFA2T2.3	3.11E-32	-0.12087	0.043	0.145	4.65E-28	M1	CBFA2T2
EIF3F.4	3.17E-32	0.216039	0.609	0.605	4.74E-28	M1	EIF3F
PDLIM4.2	3.20E-32	-0.30529	0.009	0.085	4.79E-28	M1	PDLIM4
TRIM16L.1	3.23E-32	-0.13754	0.009	0.086	4.82E-28	M1	TRIM16L
ZNF649.3	3.26E-32	-0.13414	0.019	0.104	4.88E-28	M1	ZNF649
YIF1A.3	3.30E-32	-0.22458	0.265	0.456	4.93E-28	M1	YIF1A
RP11-195F	3.30E-32	-0.235	0.02	0.105	4.94E-28	M1	RP11-195F19.5
TAF15.2	3.36E-32	-0.17624	0.145	0.299	5.02E-28	M1	TAF15
GSG1L.1	3.39E-32	-0.22021	0.002	0.07	5.06E-28	M1	GSG1L
RPP40.3	3.41E-32	-0.18194	0.037	0.135	5.09E-28	M1	RPP40
TARBP2.2	3.43E-32	-0.11064	0.055	0.165	5.12E-28	M1	TARBP2
MSX1.2	3.45E-32	-0.14798	0.005	0.077	5.16E-28	M1	MSX1
EBPL.2	3.46E-32	-0.18832	0.167	0.329	5.16E-28	M1	EBPL
XXYLT1.2	3.47E-32	-0.11905	0.042	0.143	5.19E-28	M1	XXYLT1
ZNF714.1	3.64E-32	-0.20242	0.028	0.118	5.44E-28	M1	ZNF714
SNAPC3.2	3.69E-32	-0.14847	0.048	0.153	5.51E-28	M1	SNAPC3
NPRL2.2	3.77E-32	-0.15697	0.064	0.176	5.64E-28	M1	NPRL2
AP2A1.2	3.83E-32	-0.12065	0.117	0.26	5.73E-28	M1	AP2A1
ZNF267.4	3.88E-32	0.447463	0.203	0.118	5.80E-28	M1	ZNF267
PTPN11.1	3.91E-32	-0.14112	0.057	0.167	5.84E-28	M1	PTPN11
NADK2.1	4.00E-32	-0.13251	0.025	0.114	5.97E-28	M1	NADK2
GPX8.3	4.12E-32	-0.14418	0.004	0.075	6.15E-28	M1	GPX8
POM121C.3	4.35E-32	-0.144	0.051	0.158	6.51E-28	M1	POM121C
LYRM9.3	4.48E-32	-0.1759	0.074	0.193	6.70E-28	M1	LYRM9
INO80E.2	4.50E-32	-0.13011	0.111	0.249	6.72E-28	M1	INO80E
RRN3	4.54E-32	-0.18204	0.048	0.151	6.78E-28	M1	RRN3
TTLL1.1	4.56E-32	-0.17326	0.02	0.105	6.82E-28	M1	TTLL1
PCOLCE.3	4.61E-32	-0.24187	0.014	0.095	6.89E-28	M1	PCOLCE
SEC23A.1	4.62E-32	-0.128	0.088	0.214	6.90E-28	M1	SEC23A
TMEM8B	4.68E-32	-0.1374	0.004	0.074	6.99E-28	M1	TMEM8B
CD48.4	4.69E-32	0.284792	0.143	0.065	7.00E-28	M1	CD48
CCAR2.1	4.90E-32	-0.18557	0.056	0.165	7.33E-28	M1	CCAR2
B3GALNT1.	4.97E-32	-0.14433	0.082	0.204	7.43E-28	M1	B3GALNT1
HELZ2.3	5.01E-32	-0.11643	0.025	0.115	7.49E-28	M1	HELZ2
ZNF790.1	5.10E-32	-0.17628	0.022	0.109	7.62E-28	M1	ZNF790
FAM46A.1	5.24E-32	-0.1346	0.09	0.217	7.83E-28	M1	FAM46A
NANOS3.2	5.36E-32	-0.14815	0.004	0.074	8.01E-28	M1	NANOS3
CENPT.2	5.37E-32	-0.10248	0.085	0.211	8.02E-28	M1	CENPT
CSTB.2	5.42E-32	0.135677	0.706	0.687	8.10E-28	M1	CSTB
KANK2.1	5.63E-32	-0.11004	0.02	0.105	8.42E-28	M1	KANK2
ACACA.1	5.64E-32	-0.1299	0.021	0.108	8.43E-28	M1	ACACA
ALG14.2	5.66E-32	-0.15881	0.06	0.17	8.47E-28	M1	ALG14
AC091729.	5.72E-32	-0.16037	0.014	0.094	8.54E-28	M1	AC091729.9
NMT2.3	5.95E-32	-0.1708	0.024	0.113	8.89E-28	M1	NMT2
AP5S1.1	5.96E-32	-0.12965	0.024	0.112	8.91E-28	M1	AP5S1

NKIRAS2.1	6.20E-32	-0.1159	0.137	0.289	9.26E-28	M1	NKIRAS2
OXR1.3	6.20E-32	-0.13461	0.129	0.276	9.26E-28	M1	OXR1
BIK.1	6.21E-32	0.169381	0.039	0.007	9.29E-28	M1	BIK
ERBB2IP.3	6.27E-32	-0.14174	0.107	0.244	9.37E-28	M1	ERBB2IP
CECR5.3	6.64E-32	-0.16621	0.129	0.276	9.92E-28	M1	CECR5
LUC7L.2	6.70E-32	-0.12087	0.124	0.27	1.00E-27	M1	LUC7L
ZFAND2A.4	6.76E-32	-0.15033	0.153	0.309	1.01E-27	M1	ZFAND2A
KIAA0101.	7.02E-32	-0.24585	0.038	0.133	1.05E-27	M1	KIAA0101
GPALPP1.2	7.18E-32	-0.12727	0.039	0.137	1.07E-27	M1	GPALPP1
ARHGAP35.	7.22E-32	-0.10168	0.055	0.165	1.08E-27	M1	ARHGAP35
BMPR2.2	7.27E-32	-0.11578	0.077	0.198	1.09E-27	M1	BMPR2
CEP97.2	7.27E-32	-0.17414	0.011	0.089	1.09E-27	M1	CEP97
TBC1D2B.3	7.41E-32	0.321596	0.103	0.041	1.11E-27	M1	TBC1D2B
NUP35.1	7.41E-32	-0.20041	0.034	0.128	1.11E-27	M1	NUP35
CUEDC1.4	7.45E-32	-0.12593	0.035	0.131	1.11E-27	M1	CUEDC1
PRADC1.3	7.54E-32	-0.15349	0.105	0.239	1.13E-27	M1	PRADC1
VPS4A.2	7.63E-32	-0.11625	0.169	0.336	1.14E-27	M1	VPS4A
PLEKHH2.1	7.72E-32	-0.13388	0.007	0.081	1.15E-27	M1	PLEKHH2
PARD3.2	7.92E-32	-0.12067	0.002	0.071	1.18E-27	M1	PARD3
ZNF528.2	7.98E-32	-0.14356	0.031	0.123	1.19E-27	M1	ZNF528
SRRD.3	8.50E-32	-0.11414	0.036	0.132	1.27E-27	M1	SRRD
EPS15L1	8.52E-32	-0.11356	0.069	0.185	1.27E-27	M1	EPS15L1
YIPF6.3	9.09E-32	-0.11969	0.173	0.341	1.36E-27	M1	YIPF6
TUSC2.3	9.30E-32	-0.14693	0.191	0.364	1.39E-27	M1	TUSC2
PVRL1.3	9.39E-32	-0.19934	0.003	0.073	1.40E-27	M1	PVRL1
RHOU.3	9.66E-32	-0.22986	0.049	0.151	1.44E-27	M1	RHOU
FAM192A.2	9.67E-32	-0.15855	0.207	0.388	1.44E-27	M1	FAM192A
TMEM50B.2	9.74E-32	-0.14969	0.167	0.33	1.46E-27	M1	TMEM50B
IP6K1.1	9.89E-32	-0.13978	0.059	0.17	1.48E-27	M1	IP6K1
ZFP14.2	9.97E-32	-0.1811	0.033	0.127	1.49E-27	M1	ZFP14
TLR6	1.00E-31	0.200629	0.051	0.012	1.50E-27	M1	TLR6
EBAG9.2	1.03E-31	-0.15234	0.131	0.277	1.54E-27	M1	EBAG9
DIS3.1	1.08E-31	-0.14305	0.059	0.169	1.62E-27	M1	DIS3
PRMT6.1	1.12E-31	-0.13799	0.021	0.106	1.67E-27	M1	PRMT6
RAPGEF1.2	1.16E-31	0.394462	0.186	0.103	1.73E-27	M1	RAPGEF1
UBN2.1	1.16E-31	-0.11919	0.032	0.125	1.73E-27	M1	UBN2
CEP78.3	1.16E-31	-0.13924	0.035	0.129	1.74E-27	M1	CEP78
ZNF512B.3	1.17E-31	-0.12469	0.01	0.087	1.75E-27	M1	ZNF512B
HGS.3	1.23E-31	-0.12816	0.104	0.239	1.83E-27	M1	HGS
XPR1.2	1.24E-31	-0.16584	0.042	0.142	1.85E-27	M1	XPR1
C11orf68.	1.25E-31	-0.13954	0.03	0.122	1.86E-27	M1	C11orf68
ZNF577.3	1.27E-31	-0.11537	0.021	0.106	1.89E-27	M1	ZNF577
NELFE	1.31E-31	-0.154	0.222	0.408	1.96E-27	M1	NELFE
ISYNA1.2	1.34E-31	-0.14644	0.048	0.151	2.01E-27	M1	ISYNA1
EXT2.3	1.37E-31	-0.114	0.073	0.191	2.04E-27	M1	EXT2
PDPK1.3	1.41E-31	-0.10491	0.084	0.208	2.11E-27	M1	PDPK1
ABHD12.3	1.46E-31	0.335044	0.431	0.371	2.19E-27	M1	ABHD12
TPRKB.2	1.47E-31	-0.20295	0.216	0.393	2.20E-27	M1	TPRKB
KHDC1.3	1.51E-31	-0.197	0.005	0.076	2.26E-27	M1	KHDC1
TOP2A.4	1.56E-31	-0.32653	0.018	0.1	2.33E-27	M1	TOP2A
SS18L1.1	1.57E-31	-0.13307	0.02	0.104	2.35E-27	M1	SS18L1

TOMM70A.3	1.67E-31	-0.10806	0.042	0.142	2.49E-27	M1	TOMM70A
C6orf48.2	1.71E-31	0.18295	0.657	0.645	2.56E-27	M1	C6orf48
TARSL2.3	1.74E-31	-0.16783	0.033	0.126	2.59E-27	M1	TARSL2
FAM129B.2	1.75E-31	-0.17028	0.01	0.086	2.61E-27	M1	FAM129B
CDCA4.2	1.77E-31	-0.18077	0.019	0.102	2.64E-27	M1	CDCA4
FAM174A.2	1.77E-31	-0.13534	0.109	0.245	2.64E-27	M1	FAM174A
SRPX2.2	1.77E-31	-0.24707	0.002	0.069	2.65E-27	M1	SRPX2
PPP1R14C.	1.81E-31	-0.14338	0.003	0.072	2.71E-27	M1	PPP1R14C
ARHGAP33.	1.84E-31	-0.17743	0.015	0.095	2.75E-27	M1	ARHGAP33
CRNKL1.2	1.84E-31	-0.10698	0.059	0.169	2.76E-27	M1	CRNKL1
GNAZ.2	1.86E-31	-0.18157	0.003	0.072	2.78E-27	M1	GNAZ
TRPM3.3	1.87E-31	-0.13417	0.008	0.081	2.79E-27	M1	TRPM3
ZMYM4.2	1.92E-31	-0.12713	0.042	0.142	2.87E-27	M1	ZMYM4
GOSR1.3	1.97E-31	-0.12507	0.141	0.293	2.94E-27	M1	GOSR1
SIK3.3	2.02E-31	-0.1521	0.03	0.121	3.02E-27	M1	SIK3
DNASE1L1.	2.05E-31	0.330838	0.132	0.06	3.06E-27	M1	DNASE1L1
TMEM177.3	2.05E-31	-0.16882	0.013	0.092	3.07E-27	M1	TMEM177
USP10.1	2.08E-31	-0.12731	0.123	0.267	3.10E-27	M1	USP10
TMEM209.2	2.16E-31	-0.11969	0.037	0.134	3.22E-27	M1	TMEM209
DPY30.2	2.17E-31	-0.17839	0.32	0.53	3.25E-27	M1	DPY30
LENG8.1	2.20E-31	-0.12178	0.11	0.246	3.29E-27	M1	LENG8
TXNDC9.1	2.22E-31	-0.16809	0.138	0.285	3.31E-27	M1	TXNDC9
EIF5B.2	2.22E-31	-0.22774	0.345	0.557	3.32E-27	M1	EIF5B
ERBB4.3	2.25E-31	-0.21985	0.002	0.07	3.36E-27	M1	ERBB4
RAB2A.1	2.25E-31	-0.23378	0.419	0.626	3.36E-27	M1	RAB2A
FAM212B.3	2.30E-31	-0.11971	0.025	0.114	3.44E-27	M1	FAM212B
LAYN.3	2.37E-31	-0.1406	0.001	0.068	3.55E-27	M1	LAYN
STX7.4	2.39E-31	0.443299	0.338	0.253	3.58E-27	M1	STX7
PELO.3	2.42E-31	-0.18328	0.031	0.122	3.62E-27	M1	PELO
PUS3.2	2.46E-31	-0.15559	0.035	0.13	3.68E-27	M1	PUS3
PHF20L1.3	2.49E-31	-0.14082	0.171	0.335	3.72E-27	M1	PHF20L1
RP11-398K	2.55E-31	-0.14017	0.012	0.089	3.81E-27	M1	RP11-398K22.12
GLRB.2	2.62E-31	-0.1517	0.003	0.071	3.92E-27	M1	GLRB
CYTIP.4	2.62E-31	0.280181	0.177	0.089	3.92E-27	M1	CYTIP
CPNE2.4	2.68E-31	-0.17168	0.078	0.197	4.01E-27	M1	CPNE2
ZNF273.3	2.68E-31	-0.19619	0.017	0.098	4.01E-27	M1	ZNF273
SLC2A6.1	2.69E-31	-0.14064	0.009	0.083	4.01E-27	M1	SLC2A6
ARHGAP32.	2.69E-31	-0.15124	0.017	0.098	4.02E-27	M1	ARHGAP32
TSC22D2.3	2.70E-31	0.418005	0.228	0.142	4.04E-27	M1	TSC22D2
RFC3.2	2.74E-31	-0.18164	0.019	0.101	4.09E-27	M1	RFC3
CHI3L1.4	2.77E-31	-0.70333	0.404	0.548	4.14E-27	M1	CHI3L1
ZNF430.1	2.79E-31	-0.16099	0.087	0.21	4.17E-27	M1	ZNF430
UBE2O.3	2.82E-31	-0.10706	0.037	0.133	4.22E-27	M1	UBE2O
COPB2.3	2.89E-31	-0.19454	0.172	0.333	4.32E-27	M1	COPB2
UBALD1.2	2.90E-31	-0.10085	0.07	0.187	4.33E-27	M1	UBALD1
NFAM1.3	2.94E-31	0.221953	0.077	0.025	4.39E-27	M1	NFAM1
RGAG4.1	2.95E-31	-0.12489	0.004	0.074	4.41E-27	M1	RGAG4
STX1A.2	3.00E-31	-0.22076	0.007	0.08	4.48E-27	M1	STX1A
ADAM22.3	3.03E-31	-0.18667	0.005	0.075	4.53E-27	M1	ADAM22
SCAF1.3	3.04E-31	-0.12015	0.061	0.172	4.54E-27	M1	SCAF1
HNRNPLL.2	3.10E-31	-0.15752	0.088	0.211	4.64E-27	M1	HNRNPLL

ZNF300.3	3.15E-31	-0.18289	0.008	0.082	4.71E-27	M1	ZNF300
C1orf35.3	3.20E-31	-0.15081	0.139	0.288	4.78E-27	M1	C1orf35
LRRC8C.4	3.23E-31	0.293713	0.113	0.048	4.83E-27	M1	LRRC8C
DPY19L3.2	3.26E-31	-0.14431	0.019	0.102	4.87E-27	M1	DPY19L3
TCTA.1	3.35E-31	-0.16472	0.067	0.18	5.01E-27	M1	TCTA
VPS37D.2	3.51E-31	-0.16975	0.003	0.071	5.24E-27	M1	VPS37D
TRMU	3.51E-31	-0.13495	0.047	0.149	5.25E-27	M1	TRMU
EYA4.2	3.68E-31	-0.11205	0.002	0.068	5.51E-27	M1	EYA4
SMIM18.3	3.72E-31	-0.22571	0.004	0.072	5.56E-27	M1	SMIM18
TMEM63B.1	3.80E-31	-0.10105	0.049	0.153	5.67E-27	M1	TMEM63B
RABL6.3	4.06E-31	-0.10944	0.125	0.271	6.06E-27	M1	RABL6
AIMP2.2	4.11E-31	-0.16184	0.124	0.264	6.14E-27	M1	AIMP2
POLR2D.1	4.20E-31	-0.13175	0.051	0.156	6.27E-27	M1	POLR2D
NCDN.1	4.23E-31	-0.1411	0.02	0.104	6.32E-27	M1	NCDN
ATRN.1	4.25E-31	-0.12147	0.042	0.141	6.35E-27	M1	ATRN
RAB6B.3	4.34E-31	-0.15335	0.006	0.077	6.49E-27	M1	RAB6B
ARHGEF25.	4.44E-31	-0.19055	0.006	0.077	6.64E-27	M1	ARHGEF25
LNX1.1	4.44E-31	-0.22095	0.008	0.081	6.64E-27	M1	LNX1
ZFHX3.3	4.53E-31	0.444678	0.267	0.181	6.77E-27	M1	ZFHX3
ADAL	4.56E-31	-0.12148	0.035	0.13	6.81E-27	M1	ADAL
OGFOD3.3	4.60E-31	-0.15648	0.11	0.244	6.87E-27	M1	OGFOD3
CBX8.2	4.69E-31	-0.14544	0.01	0.084	7.01E-27	M1	CBX8
MAPKAPK3.	4.79E-31	0.380245	0.245	0.156	7.16E-27	M1	MAPKAPK3
EIF3J-AS1	4.84E-31	-0.11275	0.044	0.144	7.24E-27	M1	EIF3J-AS1
DCK.4	4.91E-31	0.44032	0.334	0.253	7.34E-27	M1	DCK
NFS1.2	5.13E-31	-0.10345	0.048	0.151	7.66E-27	M1	NFS1
CAMK1.3	5.14E-31	-0.14936	0.113	0.248	7.68E-27	M1	CAMK1
TMEM68.2	5.57E-31	-0.13683	0.071	0.186	8.33E-27	M1	TMEM68
PTPRG.1	5.76E-31	-0.14714	0.01	0.085	8.60E-27	M1	PTPRG
CAPRIN2.2	5.82E-31	-0.11518	0.029	0.119	8.69E-27	M1	CAPRIN2
CDYL.4	5.86E-31	-0.1008	0.036	0.131	8.76E-27	M1	CDYL
MFAP1.2	5.94E-31	-0.14845	0.075	0.193	8.87E-27	M1	MFAP1
BTBD10.2	6.09E-31	-0.14311	0.08	0.201	9.10E-27	M1	BTBD10
EIF2B5.1	6.10E-31	-0.12588	0.083	0.204	9.12E-27	M1	EIF2B5
SSFA2.2	6.20E-31	-0.12135	0.084	0.206	9.26E-27	M1	SSFA2
IGSF11.2	6.21E-31	-0.15025	0.007	0.079	9.28E-27	M1	IGSF11
DNAJC30.2	6.28E-31	-0.15763	0.063	0.172	9.38E-27	M1	DNAJC30
MOAP1.2	6.38E-31	-0.13492	0.106	0.24	9.54E-27	M1	MOAP1
AARSD1.1	6.54E-31	-0.14942	0.03	0.12	9.77E-27	M1	AARSD1
KLHL22.1	6.66E-31	-0.16484	0.019	0.101	9.95E-27	M1	KLHL22
ANKRA2.1	6.69E-31	-0.14507	0.058	0.165	9.99E-27	M1	ANKRA2
C2orf76.1	6.75E-31	-0.15048	0.069	0.183	1.01E-26	M1	C2orf76
ZNF786.2	6.78E-31	-0.1617	0.01	0.084	1.01E-26	M1	ZNF786
MED13.2	6.78E-31	-0.1008	0.066	0.18	1.01E-26	M1	MED13
ZNF584.2	6.84E-31	-0.12802	0.018	0.1	1.02E-26	M1	ZNF584
RAN.3	6.86E-31	-0.24699	0.614	0.785	1.03E-26	M1	RAN
GTF3C1.1	6.91E-31	-0.14151	0.031	0.122	1.03E-26	M1	GTF3C1
ARMCX3.2	7.14E-31	-0.15302	0.264	0.463	1.07E-26	M1	ARMCX3
ANTXR1.2	7.42E-31	-0.14345	0.021	0.104	1.11E-26	M1	ANTXR1
SPIRE1.1	7.50E-31	-0.13167	0.025	0.112	1.12E-26	M1	SPIRE1
SLC9A3R1.	7.57E-31	-0.13613	0.06	0.168	1.13E-26	M1	SLC9A3R1

WDR73.2	7.74E-31	-0.10644	0.053	0.159	1.16E-26	M1	WDR73
BPHL.2	7.85E-31	-0.14588	0.076	0.194	1.17E-26	M1	BPHL
MKRN2	7.85E-31	-0.1359	0.061	0.17	1.17E-26	M1	MKRN2
CD99L2.3	7.92E-31	-0.16314	0.046	0.147	1.18E-26	M1	CD99L2
ZNF334.1	8.08E-31	-0.13209	0.01	0.085	1.21E-26	M1	ZNF334
TMEM186.3	8.09E-31	-0.16409	0.025	0.111	1.21E-26	M1	TMEM186
CARKD.3	8.09E-31	-0.16574	0.046	0.146	1.21E-26	M1	CARKD
LRPAP1.2	8.35E-31	0.257914	0.571	0.542	1.25E-26	M1	LRPAP1
ZNF395.2	8.47E-31	-0.18724	0.006	0.076	1.27E-26	M1	ZNF395
LRRFIP2.2	8.74E-31	-0.10289	0.085	0.208	1.31E-26	M1	LRRFIP2
DCTN1.3	9.30E-31	-0.16484	0.107	0.237	1.39E-26	M1	DCTN1
PLEKHG1	9.41E-31	-0.11432	0.001	0.066	1.41E-26	M1	PLEKHG1
DRG1.1	9.82E-31	-0.11421	0.127	0.269	1.47E-26	M1	DRG1
FAT3.2	9.85E-31	-0.1403	0.002	0.067	1.47E-26	M1	FAT3
CBX4.4	9.87E-31	-0.18449	0.037	0.131	1.47E-26	M1	CBX4
RERE.3	1.01E-30	-0.11786	0.102	0.232	1.50E-26	M1	RERE
B4GALT3.1	1.06E-30	-0.13949	0.107	0.24	1.58E-26	M1	B4GALT3
TOM1L1.2	1.07E-30	-0.11768	0.002	0.068	1.59E-26	M1	TOM1L1
MGLL.3	1.07E-30	-0.20287	0.058	0.163	1.60E-26	M1	MGLL
KLF12.3	1.09E-30	-0.16253	0.018	0.098	1.63E-26	M1	KLF12
NOTCH3.1	1.11E-30	-0.10175	0.004	0.072	1.66E-26	M1	NOTCH3
RP13-1032	1.12E-30	-0.13253	0.014	0.092	1.67E-26	M1	RP13-1032I1.7
RRM2.3	1.12E-30	-0.26994	0.005	0.074	1.68E-26	M1	RRM2
PSMD7.1	1.18E-30	-0.19896	0.316	0.525	1.77E-26	M1	PSMD7
SMAD4.2	1.19E-30	-0.10706	0.041	0.138	1.77E-26	M1	SMAD4
GNL3.2	1.22E-30	-0.19106	0.204	0.374	1.82E-26	M1	GNL3
PGRMC2.3	1.24E-30	-0.12805	0.113	0.248	1.85E-26	M1	PGRMC2
DPYSL2.3	1.28E-30	-0.21298	0.27	0.454	1.91E-26	M1	DPYSL2
OSCP1.3	1.28E-30	-0.14512	0.007	0.078	1.91E-26	M1	OSCP1
PRMT1.1	1.29E-30	-0.22221	0.378	0.589	1.93E-26	M1	PRMT1
NPHP3.1	1.31E-30	-0.12148	0.033	0.124	1.96E-26	M1	NPHP3
PTRH1.2	1.33E-30	-0.10961	0.085	0.207	1.99E-26	M1	PTRH1
MKKS.2	1.35E-30	-0.13364	0.247	0.436	2.01E-26	M1	MKKS
TES.4	1.38E-30	0.297092	0.095	0.036	2.06E-26	M1	TES
SAMD10.1	1.41E-30	-0.13345	0.002	0.068	2.11E-26	M1	SAMD10
ORMDL3.2	1.44E-30	-0.1741	0.045	0.143	2.16E-26	M1	ORMDL3
NKX2-5.2	1.51E-30	-0.22854	0.006	0.076	2.26E-26	M1	NKX2-5
FH.1	1.52E-30	-0.16147	0.113	0.248	2.27E-26	M1	FH
C17orf89.	1.56E-30	-0.2183	0.273	0.451	2.33E-26	M1	C17orf89
METTL16.2	1.65E-30	-0.12907	0.033	0.124	2.46E-26	M1	METTL16
INADL.3	1.70E-30	-0.19613	0.008	0.079	2.55E-26	M1	INADL
CNPY4.3	1.74E-30	-0.13854	0.035	0.128	2.59E-26	M1	CNPY4
ACBD3.1	1.74E-30	-0.12237	0.076	0.194	2.59E-26	M1	ACBD3
PTPN2.3	1.77E-30	0.422871	0.319	0.234	2.65E-26	M1	PTPN2
PRPF31.2	1.79E-30	-0.15404	0.236	0.423	2.68E-26	M1	PRPF31
TRMT11.2	1.79E-30	-0.14219	0.038	0.133	2.68E-26	M1	TRMT11
MPHOSPH9.	1.81E-30	-0.15001	0.038	0.133	2.71E-26	M1	MPHOSPH9
PSMB4.1	1.82E-30	-0.19492	0.256	0.45	2.71E-26	M1	PSMB4
TATDN1.1	1.86E-30	-0.12409	0.121	0.259	2.78E-26	M1	TATDN1
ANLN.3	1.95E-30	-0.27641	0.005	0.074	2.91E-26	M1	ANLN
SPSB4.3	2.00E-30	-0.12972	0.003	0.07	2.98E-26	M1	SPSB4

ATP5SL.2	2.00E-30	-0.12776	0.117	0.254	2.99E-26	M1	ATP5SL
KXD1.3	2.00E-30	-0.20348	0.286	0.481	2.99E-26	M1	KXD1
HPCA.2	2.04E-30	-0.21778	0.004	0.071	3.05E-26	M1	HPCA
TXLNG.2	2.07E-30	-0.16022	0.049	0.15	3.09E-26	M1	TXLNG
TUG1.3	2.11E-30	-0.10316	0.077	0.195	3.15E-26	M1	TUG1
RP11-192H	2.12E-30	-0.11772	0.036	0.13	3.17E-26	M1	RP11-192H23.6
SSBP4.2	2.14E-30	-0.14624	0.145	0.295	3.20E-26	M1	SSBP4
SORBS3.3	2.17E-30	-0.11163	0.075	0.192	3.24E-26	M1	SORBS3
MAP7D1.3	2.17E-30	-0.14043	0.201	0.375	3.24E-26	M1	MAP7D1
SCAMP3.2	2.18E-30	-0.14674	0.159	0.316	3.26E-26	M1	SCAMP3
NTRK3.1	2.25E-30	-0.16114	0.007	0.078	3.36E-26	M1	NTRK3
PCDH1.2	2.32E-30	-0.14698	0.004	0.071	3.46E-26	M1	PCDH1
APITD1.1	2.33E-30	-0.1611	0.016	0.095	3.48E-26	M1	APITD1
KCNK13.1	2.34E-30	0.192267	0.059	0.016	3.50E-26	M1	KCNK13
FAM104B.2	2.35E-30	-0.15784	0.089	0.211	3.52E-26	M1	FAM104B
NDUFS4.1	2.37E-30	-0.21307	0.331	0.54	3.54E-26	M1	NDUFS4
EHD1.3	2.43E-30	-0.16633	0.042	0.137	3.64E-26	M1	EHD1
HMBOX1.3	2.45E-30	-0.16025	0.091	0.213	3.66E-26	M1	HMBOX1
SETD6.2	2.47E-30	-0.14125	0.021	0.104	3.69E-26	M1	SETD6
NAPB	2.51E-30	-0.16677	0.014	0.091	3.74E-26	M1	NAPB
BRF2.2	2.52E-30	-0.1798	0.021	0.104	3.76E-26	M1	BRF2
TULP4.3	2.57E-30	-0.16806	0.056	0.16	3.85E-26	M1	TULP4
TAF9.2	2.62E-30	-0.17968	0.323	0.535	3.92E-26	M1	TAF9
RAD17.1	2.67E-30	-0.13336	0.047	0.146	3.99E-26	M1	RAD17
CTU1.2	2.73E-30	-0.12085	0.013	0.09	4.08E-26	M1	CTU1
DHX36.2	2.76E-30	-0.15014	0.263	0.459	4.13E-26	M1	DHX36
THAP7.2	2.85E-30	-0.12252	0.101	0.231	4.25E-26	M1	THAP7
EIF2B2.2	2.85E-30	-0.12932	0.076	0.193	4.25E-26	M1	EIF2B2
CHMP7.3	2.90E-30	-0.15384	0.079	0.196	4.33E-26	M1	CHMP7
LILRA1	2.94E-30	0.155357	0.045	0.01	4.40E-26	M1	LILRA1
PLLP.3	2.95E-30	-0.35277	0.009	0.08	4.42E-26	M1	PLLP
STRN4.3	3.06E-30	-0.11132	0.042	0.14	4.57E-26	M1	STRN4
CAPN5.1	3.14E-30	-0.13252	0.009	0.081	4.69E-26	M1	CAPN5
LRRC24.2	3.16E-30	-0.15768	0.009	0.08	4.72E-26	M1	LRRC24
TTI1.1	3.26E-30	-0.13183	0.025	0.111	4.87E-26	M1	TTI1
IPCEF1.2	3.27E-30	0.183447	0.056	0.015	4.88E-26	M1	IPCEF1
CETN3.2	3.38E-30	-0.16036	0.059	0.164	5.05E-26	M1	CETN3
SLC2A10.2	3.42E-30	-0.11396	0.006	0.076	5.11E-26	M1	SLC2A10
PCDHB14.1	3.43E-30	-0.18356	0.001	0.065	5.13E-26	M1	PCDHB14
UBQLN1.2	3.46E-30	-0.11521	0.153	0.307	5.17E-26	M1	UBQLN1
TMCC1.3	3.56E-30	-0.13886	0.036	0.129	5.32E-26	M1	TMCC1
USP13.2	3.57E-30	-0.14709	0.011	0.084	5.34E-26	M1	USP13
JAGN1.3	3.71E-30	-0.15265	0.181	0.348	5.55E-26	M1	JAGN1
EIF2B4.2	3.84E-30	-0.14139	0.087	0.208	5.74E-26	M1	EIF2B4
AKNA.3	3.91E-30	0.308192	0.131	0.06	5.84E-26	M1	AKNA
SPAST.2	3.94E-30	-0.12661	0.035	0.127	5.89E-26	M1	SPAST
SYAP1.3	3.95E-30	0.417615	0.426	0.379	5.91E-26	M1	SYAP1
JPH4.1	3.96E-30	-0.23199	0.004	0.071	5.91E-26	M1	JPH4
FAM3B	4.02E-30	0.17586	0.034	0.006	6.00E-26	M1	FAM3B
ZNHIT2.1	4.10E-30	-0.13483	0.018	0.098	6.12E-26	M1	ZNHIT2
C5orf30.2	4.10E-30	-0.18108	0.013	0.088	6.13E-26	M1	C5orf30

MYLK.1	4.17E-30	-0.26054	0.004	0.07	6.23E-26	M1	MYLK
CEACAM21.	4.20E-30	0.169903	0.053	0.014	6.28E-26	M1	CEACAM21
CEP57L1.3	4.24E-30	-0.14454	0.035	0.126	6.34E-26	M1	CEP57L1
SUGT1.2	4.44E-30	-0.14362	0.201	0.373	6.63E-26	M1	SUGT1
URM1	4.52E-30	-0.16032	0.212	0.387	6.76E-26	M1	URM1
LZTS1.3	4.58E-30	-0.14227	0.004	0.071	6.85E-26	M1	LZTS1
JAM2.4	4.59E-30	-0.12552	0.133	0.276	6.86E-26	M1	JAM2
IPP.2	4.62E-30	-0.14725	0.004	0.071	6.90E-26	M1	IPP
STK16.1	4.62E-30	-0.12537	0.087	0.208	6.90E-26	M1	STK16
GEMIN2	4.63E-30	-0.15795	0.035	0.126	6.92E-26	M1	GEMIN2
TEX261.2	4.76E-30	-0.11193	0.071	0.185	7.11E-26	M1	TEX261
SPC25.4	4.83E-30	-0.22943	0.005	0.073	7.22E-26	M1	SPC25
ERCC4.2	4.85E-30	-0.13826	0.047	0.146	7.24E-26	M1	ERCC4
RFC5.1	5.12E-30	-0.16353	0.021	0.103	7.65E-26	M1	RFC5
USP33	5.15E-30	-0.11739	0.081	0.199	7.69E-26	M1	USP33
EBF4.1	5.47E-30	-0.12662	0.002	0.066	8.18E-26	M1	EBF4
S100Z.1	5.48E-30	0.138674	0.038	0.007	8.19E-26	M1	S100Z
CCNL2.2	5.55E-30	-0.12579	0.161	0.316	8.30E-26	M1	CCNL2
CYB561.3	5.67E-30	-0.14377	0.015	0.093	8.48E-26	M1	CYB561
RAF1.4	5.70E-30	-0.10458	0.15	0.302	8.51E-26	M1	RAF1
SIX1.2	5.70E-30	-0.16918	0.003	0.068	8.52E-26	M1	SIX1
FAM60A.2	5.82E-30	-0.14553	0.078	0.194	8.69E-26	M1	FAM60A
KBTBD7.2	5.91E-30	-0.16213	0.02	0.1	8.83E-26	M1	KBTBD7
RFXAP.1	5.95E-30	-0.14774	0.025	0.109	8.89E-26	M1	RFXAP
NAP1L1.2	6.03E-30	0.144815	0.679	0.673	9.01E-26	M1	NAP1L1
FAM198B.2	6.14E-30	0.248987	0.116	0.05	9.18E-26	M1	FAM198B
RP11-345J	6.21E-30	-0.12674	0.119	0.258	9.28E-26	M1	RP11-345J4.5
TSC2.2	6.24E-30	-0.10359	0.051	0.152	9.33E-26	M1	TSC2
FAM127C.1	6.26E-30	-0.18245	0.031	0.119	9.36E-26	M1	FAM127C
SHROOM2.3	6.31E-30	-0.14434	0.004	0.07	9.43E-26	M1	SHROOM2
CACYBP.1	6.41E-30	-0.17595	0.334	0.54	9.58E-26	M1	CACYBP
IZUMO4.2	6.54E-30	-0.14221	0.008	0.078	9.77E-26	M1	IZUMO4
ATXN10.1	6.67E-30	-0.16445	0.264	0.452	9.97E-26	M1	ATXN10
DSCAM.1	6.95E-30	-0.18204	0.012	0.087	1.04E-25	M1	DSCAM
CENPU.3	7.33E-30	-0.22666	0.032	0.12	1.10E-25	M1	CENPU
CACNB3.2	7.38E-30	-0.1393	0.005	0.072	1.10E-25	M1	CACNB3
RNF4	7.49E-30	-0.13608	0.08	0.197	1.12E-25	M1	RNF4
POFUT1.2	7.55E-30	-0.10023	0.038	0.132	1.13E-25	M1	POFUT1
CUL3.2	7.59E-30	-0.11395	0.096	0.221	1.13E-25	M1	CUL3
PARP2.1	7.78E-30	-0.18265	0.036	0.126	1.16E-25	M1	PARP2
CRYZ.3	8.08E-30	-0.16591	0.043	0.14	1.21E-25	M1	CRYZ
ASXL1.3	8.11E-30	-0.11806	0.079	0.196	1.21E-25	M1	ASXL1
PPIF.4	8.22E-30	0.40121	0.291	0.207	1.23E-25	M1	PPIF
PDCD2L.2	8.28E-30	-0.16032	0.031	0.119	1.24E-25	M1	PDCD2L
PLOD3.2	8.78E-30	-0.11902	0.108	0.24	1.31E-25	M1	PLOD3
GSDMD.3	9.39E-30	0.392564	0.258	0.17	1.40E-25	M1	GSDMD
IER5.4	9.50E-30	0.471196	0.27	0.189	1.42E-25	M1	IER5
TP53I3.2	9.51E-30	-0.15065	0.059	0.163	1.42E-25	M1	TP53I3
DGCR8.1	9.57E-30	-0.14397	0.017	0.096	1.43E-25	M1	DGCR8
EPAS1.3	9.64E-30	-0.20471	0.032	0.119	1.44E-25	M1	EPAS1
MICAL1.4	9.67E-30	0.380698	0.211	0.127	1.45E-25	M1	MICAL1

CRTC3.1	9.98E-30	-0.13592	0.038	0.131	1.49E-25	M1	CRTC3
CRIM1.3	1.03E-29	-0.13532	0.006	0.075	1.54E-25	M1	CRIM1
TMEM14C.2	1.06E-29	0.213423	0.603	0.588	1.59E-25	M1	TMEM14C
IMMP2L.2	1.07E-29	-0.18226	0.059	0.162	1.60E-25	M1	IMMP2L
ATG12	1.10E-29	-0.10023	0.201	0.374	1.64E-25	M1	ATG12
ZNF451.1	1.12E-29	-0.14745	0.07	0.181	1.67E-25	M1	ZNF451
RAB40B.4	1.16E-29	-0.20165	0.049	0.146	1.74E-25	M1	RAB40B
FAM228B.3	1.17E-29	-0.12707	0.107	0.237	1.74E-25	M1	FAM228B
SDAD1.2	1.18E-29	-0.13235	0.11	0.24	1.77E-25	M1	SDAD1
SCAI.1	1.23E-29	-0.15179	0.018	0.096	1.84E-25	M1	SCAI
FGFRL1	1.24E-29	-0.1157	0.002	0.066	1.85E-25	M1	FGFRL1
ARF1.3	1.25E-29	0.151209	0.695	0.694	1.87E-25	M1	ARF1
PSPC1.3	1.25E-29	-0.11771	0.104	0.232	1.87E-25	M1	PSPC1
BRD4.3	1.29E-29	-0.12993	0.188	0.355	1.93E-25	M1	BRD4
TMEM178B.	1.33E-29	-0.16008	0.003	0.067	1.99E-25	M1	TMEM178B
C1orf50.2	1.35E-29	-0.1226	0.082	0.2	2.01E-25	M1	C1orf50
ZFP1.1	1.36E-29	-0.13794	0.008	0.078	2.03E-25	M1	ZFP1
OARD1.2	1.36E-29	-0.15175	0.119	0.255	2.04E-25	M1	OARD1
IGF1R.2	1.39E-29	-0.17118	0.01	0.081	2.08E-25	M1	IGF1R
MAP3K7.1	1.39E-29	-0.11964	0.1	0.226	2.08E-25	M1	MAP3K7
SLC35E3.3	1.43E-29	-0.2242	0.091	0.206	2.14E-25	M1	SLC35E3
NCAM2.2	1.44E-29	-0.22348	0.009	0.079	2.15E-25	M1	NCAM2
RHBDF1.1	1.45E-29	-0.12576	0	0.062	2.16E-25	M1	RHBDF1
NUFIP1.2	1.45E-29	-0.15304	0.038	0.13	2.16E-25	M1	NUFIP1
PIP4K2B.1	1.58E-29	-0.11239	0.03	0.118	2.36E-25	M1	PIP4K2B
DLL1.2	1.71E-29	-0.25448	0.011	0.083	2.56E-25	M1	DLL1
THAP1	1.75E-29	-0.14742	0.056	0.158	2.62E-25	M1	THAP1
SYTL2.2	1.76E-29	-0.23887	0.014	0.088	2.64E-25	M1	SYTL2
SLC16A4.1	1.79E-29	-0.12861	0.006	0.073	2.67E-25	M1	SLC16A4
AC113189.	1.80E-29	-0.18733	0.008	0.077	2.69E-25	M1	AC113189.5
MEX3B.2	1.84E-29	-0.18679	0.006	0.074	2.75E-25	M1	MEX3B
STK32A.3	1.87E-29	-0.12785	0.002	0.066	2.80E-25	M1	STK32A
SAPCD2.3	1.90E-29	-0.17236	0.003	0.067	2.84E-25	M1	SAPCD2
ZNF567.1	1.98E-29	-0.14765	0.026	0.11	2.97E-25	M1	ZNF567
TEAD4.2	2.00E-29	-0.1133	0.001	0.062	2.99E-25	M1	TEAD4
ADCK4.2	2.01E-29	-0.13512	0.046	0.143	3.01E-25	M1	ADCK4
RP1-79C4.	2.03E-29	-0.12572	0	0.061	3.03E-25	M1	RP1-79C4.4
PHF19.3	2.11E-29	-0.16831	0.048	0.145	3.15E-25	M1	PHF19
CENPM.3	2.11E-29	-0.19908	0.013	0.086	3.15E-25	M1	CENPM
ZDHHC4.4	2.13E-29	-0.15364	0.211	0.384	3.18E-25	M1	ZDHHC4
ARVCF.2	2.14E-29	-0.13445	0.002	0.066	3.20E-25	M1	ARVCF
HNRNPAB.3	2.19E-29	-0.17377	0.248	0.427	3.28E-25	M1	HNRNPAB
DHX9.2	2.23E-29	-0.12643	0.153	0.303	3.33E-25	M1	DHX9
CTA-445C9	2.29E-29	-0.16065	0.003	0.068	3.42E-25	M1	CTA-445C9.14
LRRN2	2.29E-29	-0.1084	0.003	0.067	3.43E-25	M1	LRRN2
SPHK2.2	2.30E-29	-0.1297	0.047	0.144	3.43E-25	M1	SPHK2
TNFRSF10D	2.40E-29	0.140666	0.036	0.007	3.59E-25	M1	TNFRSF10D
GPATCH2.2	2.47E-29	-0.12203	0.069	0.18	3.69E-25	M1	GPATCH2
SMC2.2	2.50E-29	-0.189	0.099	0.22	3.73E-25	M1	SMC2
AMN1	2.50E-29	-0.16601	0.045	0.14	3.73E-25	M1	AMN1
POMT1.3	2.53E-29	-0.11591	0.053	0.154	3.78E-25	M1	POMT1

IFT46.2	2.53E-29	-0.15328	0.035	0.124	3.79E-25	M1	IFT46
FZR1.3	2.56E-29	-0.10035	0.065	0.173	3.82E-25	M1	FZR1
IL13RA2.3	2.60E-29	-0.2893	0.002	0.065	3.88E-25	M1	IL13RA2
SLC16A2.1	2.63E-29	-0.1261	0.002	0.064	3.92E-25	M1	SLC16A2
CTC-338M1	2.75E-29	-0.19504	0.01	0.08	4.10E-25	M1	CTC-338M12.5
ILF2.1	2.77E-29	-0.20351	0.403	0.608	4.13E-25	M1	ILF2
NSMF.1	2.93E-29	-0.12075	0.039	0.132	4.39E-25	M1	NSMF
WIPI1.3	2.95E-29	0.435457	0.255	0.171	4.40E-25	M1	WIPI1
MUL1.1	2.96E-29	-0.12309	0.069	0.179	4.43E-25	M1	MUL1
PLEKHA3.3	3.01E-29	-0.12642	0.119	0.254	4.50E-25	M1	PLEKHA3
SEPT3.3	3.03E-29	-0.19845	0.009	0.079	4.52E-25	M1	3-Sep
C17orf51.	3.23E-29	-0.13227	0.009	0.08	4.82E-25	M1	C17orf51
DALRD3.2	3.25E-29	-0.10729	0.072	0.184	4.85E-25	M1	DALRD3
NEDD9.3	3.26E-29	0.52575	0.286	0.207	4.87E-25	M1	NEDD9
ANKRD36.2	3.29E-29	-0.13359	0.087	0.205	4.92E-25	M1	ANKRD36
ZNF580.3	3.34E-29	-0.15497	0.164	0.317	4.99E-25	M1	ZNF580
FLRT3.3	3.37E-29	-0.14476	0.004	0.068	5.03E-25	M1	FLRT3
MLH3.1	3.38E-29	-0.14706	0.033	0.12	5.05E-25	M1	MLH3
BANF1.2	3.44E-29	-0.26329	0.491	0.696	5.14E-25	M1	BANF1
SENCR.2	3.48E-29	0.210103	0.051	0.013	5.20E-25	M1	SENCR
GPBP1.1	3.75E-29	-0.15274	0.283	0.482	5.60E-25	M1	GPBP1
CIC.2	3.89E-29	-0.10895	0.041	0.135	5.81E-25	M1	CIC
CAHM.3	3.98E-29	-0.13975	0.023	0.105	5.94E-25	M1	CAHM
CRB1.1	3.99E-29	-0.21044	0.009	0.079	5.96E-25	M1	CRB1
RANBP1.3	4.24E-29	-0.24187	0.426	0.608	6.34E-25	M1	RANBP1
C9orf72.4	4.43E-29	0.420509	0.221	0.136	6.61E-25	M1	C9orf72
LINC00526	4.46E-29	-0.15107	0.016	0.092	6.66E-25	M1	LINC00526
PCED1B.2	4.46E-29	0.202779	0.078	0.027	6.67E-25	M1	PCED1B
LSM2.1	4.50E-29	-0.17724	0.286	0.48	6.72E-25	M1	LSM2
SNRPB2.2	4.61E-29	-0.23014	0.4	0.597	6.89E-25	M1	SNRPB2
ACOX2.1	4.62E-29	-0.13535	0.001	0.063	6.90E-25	M1	ACOX2
GM2A.3	4.64E-29	0.388839	0.214	0.129	6.94E-25	M1	GM2A
RP11-448A	4.69E-29	-0.13146	0.024	0.106	7.00E-25	M1	RP11-448A19.1
ABCA2.4	4.80E-29	-0.29099	0.027	0.109	7.17E-25	M1	ABCA2
MBP.1	4.87E-29	0.166609	0.329	0.22	7.27E-25	M1	MBP
ATRX.2	4.90E-29	-0.17951	0.287	0.476	7.32E-25	M1	ATRX
ZNF682.1	4.94E-29	-0.13377	0.014	0.089	7.38E-25	M1	ZNF682
H3F3B.4	5.01E-29	-0.15855	0.988	0.967	7.48E-25	M1	H3F3B
RP11-379H	5.12E-29	-0.11708	0.012	0.084	7.65E-25	M1	RP11-379H18.1
LRCH4.4	5.13E-29	0.40348	0.26	0.174	7.66E-25	M1	LRCH4
SLC12A5.4	5.22E-29	0.364986	0.154	0.08	7.80E-25	M1	SLC12A5
DSC2.3	5.50E-29	0.21868	0.073	0.024	8.21E-25	M1	DSC2
RP11-305L	5.53E-29	0.175901	0.032	0.005	8.26E-25	M1	RP11-305L7.3
NR2E1.1	5.55E-29	-0.1176	0.003	0.066	8.30E-25	M1	NR2E1
PM20D2.2	5.67E-29	-0.11502	0.019	0.098	8.47E-25	M1	PM20D2
EIF4H.1	5.77E-29	-0.22092	0.371	0.574	8.63E-25	M1	EIF4H
CLDN10.2	5.95E-29	-0.33294	0.003	0.066	8.89E-25	M1	CLDN10
NDUFA12.2	6.20E-29	-0.24179	0.385	0.593	9.27E-25	M1	NDUFA12
MYT1L.4	6.21E-29	-0.24102	0.005	0.07	9.27E-25	M1	MYT1L
TTC1.2	6.32E-29	-0.15473	0.226	0.403	9.45E-25	M1	TTC1
RPP14.2	6.44E-29	-0.10848	0.088	0.207	9.63E-25	M1	RPP14

MARC1.2	7.07E-29	-0.1354	0.005	0.07	1.06E-24	M1	1-Mar
ASH1L.1	7.41E-29	-0.10773	0.177	0.336	1.11E-24	M1	ASH1L
BABAM1	7.43E-29	-0.1625	0.25	0.437	1.11E-24	M1	BABAM1
LGALSL.2	7.44E-29	-0.14558	0.007	0.074	1.11E-24	M1	LGALSL
SHISA5	7.53E-29	-0.18072	0.19	0.352	1.13E-24	M1	SHISA5
POLRMT.2	7.77E-29	-0.12024	0.049	0.147	1.16E-24	M1	POLRMT
DYNC1LI1.	7.91E-29	-0.15156	0.148	0.29	1.18E-24	M1	DYNC1LI1
CARD8.3	8.00E-29	0.385859	0.219	0.134	1.20E-24	M1	CARD8
RIC8A.1	8.02E-29	-0.13282	0.159	0.311	1.20E-24	M1	RIC8A
LRRC49	8.14E-29	-0.14703	0.005	0.07	1.22E-24	M1	LRRC49
TASP1.1	8.31E-29	-0.14457	0.024	0.106	1.24E-24	M1	TASP1
AAMP.2	8.39E-29	-0.15476	0.266	0.459	1.25E-24	M1	AAMP
SLC25A37.	8.51E-29	0.406375	0.372	0.297	1.27E-24	M1	SLC25A37
FN1.2	8.61E-29	-0.25801	0.068	0.172	1.29E-24	M1	FN1
ANKRD44.3	8.74E-29	0.381328	0.158	0.083	1.31E-24	M1	ANKRD44
AZGP1.2	8.82E-29	-0.30907	0.004	0.067	1.32E-24	M1	AZGP1
KCNJ16.3	8.98E-29	-0.14082	0.002	0.064	1.34E-24	M1	KCNJ16
GPN1.1	9.15E-29	-0.15271	0.115	0.246	1.37E-24	M1	GPN1
PRKAA2.3	9.25E-29	-0.13628	0.001	0.062	1.38E-24	M1	PRKAA2
DENR.2	9.29E-29	-0.13768	0.185	0.348	1.39E-24	M1	DENR
IQCG.3	9.43E-29	-0.18618	0.044	0.138	1.41E-24	M1	IQCG
INIP.1	9.47E-29	-0.14023	0.096	0.217	1.42E-24	M1	INIP
TMEM161A.	9.58E-29	-0.12519	0.077	0.19	1.43E-24	M1	TMEM161A
MINOS1.2	9.72E-29	0.149225	0.644	0.638	1.45E-24	M1	MINOS1
ZBTB38	9.88E-29	-0.14777	0.089	0.207	1.48E-24	M1	ZBTB38
ELMO1.3	1.01E-28	0.35365	0.27	0.174	1.50E-24	M1	ELMO1
GKAP1.3	1.01E-28	-0.14408	0.023	0.103	1.52E-24	M1	GKAP1
ARV1.3	1.03E-28	-0.14129	0.083	0.198	1.54E-24	M1	ARV1
SYNRG.2	1.05E-28	-0.10025	0.089	0.209	1.57E-24	M1	SYNRG
HOXC10.2	1.07E-28	-0.20256	0.002	0.064	1.60E-24	M1	HOXC10
SERTAD1.2	1.07E-28	0.308101	0.384	0.302	1.60E-24	M1	SERTAD1
ZNF599.2	1.10E-28	-0.12785	0.013	0.086	1.65E-24	M1	ZNF599
MAPK11.1	1.14E-28	-0.10291	0.003	0.065	1.70E-24	M1	MAPK11
MKI67.3	1.16E-28	-0.25103	0.007	0.074	1.73E-24	M1	MKI67
SUPT16H.3	1.16E-28	-0.13985	0.133	0.271	1.73E-24	M1	SUPT16H
SGOL1.2	1.17E-28	-0.21247	0.004	0.068	1.74E-24	M1	SGOL1
PDHX.2	1.23E-28	-0.1191	0.077	0.19	1.83E-24	M1	PDHX
LRRTM3.2	1.24E-28	-0.11445	0.001	0.062	1.86E-24	M1	LRRTM3
NPB	1.32E-28	-0.14843	0.01	0.079	1.97E-24	M1	NPB
NUSAP1.3	1.33E-28	-0.31271	0.034	0.119	1.99E-24	M1	NUSAP1
TPR.1	1.35E-28	-0.15303	0.249	0.436	2.02E-24	M1	TPR
ZNF583.2	1.36E-28	-0.13227	0.033	0.12	2.03E-24	M1	ZNF583
SRP72.1	1.43E-28	-0.1673	0.298	0.49	2.14E-24	M1	SRP72
YJEFN3.2	1.46E-28	-0.13132	0.007	0.074	2.18E-24	M1	YJEFN3
MEX3D.3	1.52E-28	-0.13273	0.007	0.073	2.27E-24	M1	MEX3D
SLU7	1.53E-28	-0.12731	0.181	0.34	2.29E-24	M1	SLU7
ZC3H6.2	1.53E-28	-0.13602	0.061	0.164	2.29E-24	M1	ZC3H6
SYT17.2	1.55E-28	-0.12274	0.069	0.177	2.32E-24	M1	SYT17
RIMKLA.1	1.57E-28	-0.11528	0.002	0.063	2.34E-24	M1	RIMKLA
RUSC1.2	1.57E-28	-0.11515	0.055	0.155	2.35E-24	M1	RUSC1
SLC2A9.2	1.59E-28	0.200717	0.061	0.018	2.37E-24	M1	SLC2A9

VIM.3	1.62E-28	-0.26916	0.659	0.664	2.42E-24	M1	VIM
RASD1.2	1.63E-28	-0.29686	0.054	0.15	2.43E-24	M1	RASD1
TSEN2.3	1.65E-28	-0.15542	0.022	0.102	2.46E-24	M1	TSEN2
HENMT1.3	1.67E-28	0.224733	0.072	0.024	2.50E-24	M1	HENMT1
TMEM43.2	1.68E-28	-0.1176	0.091	0.211	2.51E-24	M1	TMEM43
ZNF846.3	1.69E-28	0.362292	0.125	0.059	2.53E-24	M1	ZNF846
GATS.1	1.70E-28	-0.18138	0.007	0.074	2.55E-24	M1	GATS
[truncated: 451,841 more chars]
